# Comprehensive transcriptomic study on horse gram (*Macrotyloma uniflorum*): *De novo* assembly, functional characterization and comparative analysis in relation to drought stress

**DOI:** 10.1186/1471-2164-14-647

**Published:** 2013-09-23

**Authors:** Jyoti Bhardwaj, Rohit Chauhan, Mohit Kumar Swarnkar, Rakesh Kumar Chahota, Anil Kumar Singh, Ravi Shankar, Sudesh Kumar Yadav

**Affiliations:** 1 Plant Metabolic Engineering Laboratory, Council of Scientific and Industrial Research-Institute of Himalayan Bioresource Technology, Palampur 176061, HP, India; 2 Studio of Computational Biology & Bioinformatics, Council of Scientific and Industrial Research-Institute of Himalayan Bioresource Technology, Palampur 176061, HP, India; 3 Plant Genomics Laboratory, Biotechnology Division, CSIR-Institute of Himalayan Bioresource Technology, Council of Scientific and Industrial Research-Institute of Himalayan Bioresource Technology, Palampur 176061, HP, India; 4 Department of Genetics and Plant Breeding, Choudhary Sarwan Kumar Himachal Pradesh Krishi Vishvavidyalaya, Palampur 176061, HP, India

**Keywords:** Horse gram, Illumina sequencing, *De novo* assembly, Drought responsive genes

## Abstract

**Background:**

Drought tolerance is an attribute maintained in plants by cross-talk between multiple and cascading metabolic pathways. Without a sequenced genome available for horse gram, it is difficult to comprehend such complex networks and intercalated genes associated with drought tolerance of horse gram (*Macrotyloma uniflorum*). Therefore, *de novo* transcriptome discovery and associated analyses was done for this highly drought tolerant yet under exploited legume to decipher its genetic makeup.

**Results:**

Eight samples comprising of shoot and root tissues of two horse gram genotypes (drought-sensitive; M-191 and drought-tolerant; M-249) were used for comparison under control and polyethylene glycol-induced drought stress conditions. Using Illumina sequencing technology, a total of 229,297,896 paired end read pairs were generated and utilized for *de novo* assembly of horse gram. Significant BLAST hits were obtained for 26,045 transcripts while, 3,558 transcripts had no hits but contained important conserved domains. A total of 21,887 unigenes were identified. SSRs containing sequences covered 16.25% of the transcriptome with predominant tri- and mono-nucleotides (43%). The total GC content of the transcriptome was found to be 43.44%. Under Gene Ontology response to stimulus, DNA binding and catalytic activity was highly expressed during drought stress conditions. Serine/threonine protein kinase was found to dominate in Enzyme Classification while pathways belonging to ribosome metabolism followed by plant pathogen interaction and plant hormone signal transduction were predominant in Kyoto Encyclopedia of Genes and Genomes analysis. Independent search on plant metabolic network pathways suggested valine degradation, gluconeogenesis and purine nucleotide degradation to be highly influenced under drought stress in horse gram. Transcription factors belonging to NAC, MYB-related, and WRKY families were found highly represented under drought stress. qRT-PCR validated the expression profile for 9 out of 10 genes analyzed in response to drought stress.

**Conclusions:**

*De novo* transcriptome discovery and analysis has generated enormous information over horse gram genomics. The genes and pathways identified suggest efficient regulation leading to active adaptation as a basal defense response against drought stress by horse gram. The knowledge generated can be further utilized for exploring other underexploited plants for stress responsive genes and improving plant tolerance.

## Background

Scarcity of natural resources such as water leads to calamities like drought, which is considered as a major abiotic stress in the flora and fauna world [1]. Horse gram is a highly drought tolerant legume, whose tolerance capacity is attributed in parts to various pathways like antioxidant and osmolyte biosynthesis, making it sturdy enough to withstand long periods of drought with minimum management [2,3]. Its nutritious composition, medicinal properties and indomitable pest resistance makes it a rich yet cheap source of food, fodder, fuel supplement and green manure [4-7]. It has been identified as potential food source for future by the U.S. National Academy of Sciences [8,9].

However, information over horse gram genetic resources is scarce compared to other plants. There are only 1,025 Expressed Sequence Tags (ESTs) available at NCBI as compared to other legumes like *Glycine max* (1,461,624), *Cicer arietinum* (44,982), *Medicago truncatula* (269,501), *Lotus japonicus* (242,432) and *Pisum sativum* (18,576). No Genome Survey Sequences (GSS) is available for horse gram as compared to the above mentioned legumes. Furthermore, till date there is no data related to global size genomic, transcriptomic or protein biology studies on horse gram. This study is a comprehensive report on transcriptomic analysis of horse gram in response to drought stress.

Transcriptome is coding region of the mRNA set derived from a genome [10]. Massively parallel sequencing includes next generation sequencing techniques like 454 pyrosequencing/Roche, Illumina/Solexa GAIIx, ABI/SOLiD, Pac Biosciences/PacBioRS and Helicos Biosciences/tSMS and DRS [11-14]. These techniques do not require prior knowledge of genomic sequence and are much advanced in terms of time, cost, labor, amount of data produced, data coverage, sensitivity and accuracy as compared to the orthodox sequencing methods [15-17].

The estimated genome size of horse gram is 400 Mb. Genomic-level questions can be addressed through developing informative transcriptome producing huge amounts of data [18,19]. When answering fundamental questions like mechanisms involved or related to a particular trait like drought tolerance, it becomes imperative to draw a conclusion based on a comparative study [20]. Comparison of plant genotypes differing in their sensitivities towards drought is an inevitable approach to discover natural drought tolerance mechanisms [21]. Avoiding this, we could miss out some vital information regarding the common and divergent regulatory networks involved in drought tolerance [22]. Horse gram has inherent drought tolerance trait, still its genotypes display different sensitivities towards drought stress. Therefore, we consider horse gram to be a wonderful source for understanding the genetic basis of responses to drought tolerance.

This study describes transcriptome analysis conducted for eight shoot and root tissues of a drought sensitive (M-191) and a tolerant (M-249) genotype of horse gram under control and drought stress conditions using Illumina GAIIx. The development of genetic resource for horse gram facilitated functional characterization of transcripts responsive to induced drought stress conditions. Creating genetic resources regarding GC content, SSRs markers, genes, pathways and transcription factors associated with depauperate plants like horse gram would augment the relating research programs. Our study would possibly create awareness on global scale for betterment of horse gram and other less exploited plants.

## Methods

### Tissue sampling, cDNA library preparation and Illumina sequencing

The seeds of horse gram were obtained from Department of Plant Breeding and Genetics, CSK HPKV, Palampur, India. The shoot and root tissues were collected from control (Hoagland medium without drought stress) and 48 h stressed (induced drought stress with 18% PEG-6000 in Hoagland medium) samples of horse gram plants M-191 (drought-sensitive genotype) and M-249 (drought-tolerant genotype). Eight samples (V1SHC, V1SHS, V1RC, V1RS, V2SHC, V2SHS, V2RC and V2RS) were taken for the present study. V1 is the sensitive genotype (M-191) while V2 is the tolerant genotype (M-249). Control condition is denoted by ‘C’ while drought stress condition is denoted by ‘S’. SH, stands for shoot and R stands for root tissues. RNA extraction was done using *iRIS* method [23] from three different biological replicates for each tissue. Obtained RNA was checked for quality and quantity on Bioanalyzer (Agilent technologies, USA) and 0.8% formaldehyde agarose gel. The best RNA samples having 5 μg of concentration, good quality on gel and RIN above 7 were chosen for cDNA library preparation. Illumina TruSeq RNA sample preparation kit v2 (Illumina Inc., USA) was used for library preparation. The prepared libraries were quantified on Qubit fluorometer using Qubit dsDNA BR assay kit (Life Technologies, USA). The validation insert size in libraries was done using Bioanalyzer. These libraries were further loaded onto the flow cell for generating clusters on cluster station using TruSeq PE Cluster Kit v5-CS-GA (Illumina Inc., USA). The flow cell containing clonally amplified clusters was loaded onto the Genome Analyser IIx (Illumina) and paired-end (PE) (2×72) was performed.

### *De novo* assembly, sequence clustering and homology search

Using CASAVA package, provided by Illumina, PE sequence reads of length 72 bp each were generated. Quality assessment of reads was done using read quality filtering tool, filteR [24]. *De novo* assembling of high quality reads was performed using assembler SOAPdenovo-trans [25]. In order to assemble the reads to obtain high quality assembly contigs, filtered reads were first split into smaller substrings (k-mers). SOAPdenovo-trans was run for different k-mer lengths ranging from 19–71 bases. K-mer size of 65 and 67 were found to be best in-terms of number of transcripts produced, average length of transcripts, coverage and N50 value. Scaffold sequences were obtained by merging two contigs into a single scaffold sequence, which shares the PE reads separated by an average insert length of 200 bp. GapCloser was used to close the gaps emerging during the scaffolding process by SOAPdenovo-trans. In the first step of hierarchical clustering, clustering and merging was done using Cluster Database at High Identity with Tolerance (CD-HIT) EST with minimum similarity cut-off of 90% [26]. In follow-up, TIGR gene indices clustering tool (TGICL) CAP3 (Contig assembly program version3) clustering was run on 90% identity to get the assembled transcripts without overlaps [27]. Following the hierarchical clustering process, the number of total assembled sequences was reduced. This set of assembled transcript sequences were used to scan against NR protein database using BLASTX (Basic local alignment search tool) with the E-value threshold of 10^-5^[28,29]. The contigs/scaffolds that had no sequence similarity among themselves but may belong to the different regions of a single gene were identified using Dissimilar Sequence (DS) clustering approach [24,30]. The longest sequence with highest bit score from each cluster was taken as the representative sequence. This clustering approach yielded non-inflated representation of total number of unique genes, which would otherwise remain falsely high.

### Assembly validation and similarity search for assembled transcripts

To estimate assembly accuracy, about 1,025 experimentally validated horse gram EST sequences, reported at NCBI were used to comparatively validate the assembled sequences. These EST sequences were searched against the assembled transcripts as the database, using BLASTN with an E-value threshold of 10^-5^.

### Ontology and annotation

Assembled transcripts were searched against UniProt databases and associated GO, KEGG and EC annotations were derived using Annot8r [31,32]. Annotation was performed with an E-value threshold of 10^-1^ and ten maximum hits were allowed. Top hits were considered based on highest bit score and E-value. PlnTFDB (Plant Transcription factor database) provides complete set of transcription factors and other transcription regulators of 20 different plant species [33]. In PlnTFDB version 3.0 different protein models and sequences are further categorized into 84 different gene families. In the current study, data for all 20 plant species which consists of 29,473 transcription factors was downloaded. The assembled transcript sequences (sequences obtained after hierarchical clustering) were searched against this database using BLASTX with an E-value threshold of 10^-5^. Further DS clustering was performed to choose best representatives. AgriGO tool was used to identify the enriched Gene Ontology terms [34]. The singular enrichment analysis was performed at significance level of 0.05 in all the comparative conditions which used complete assembled transcript unigene GO annotations of horse gram as the background reference. The query list contained only the GO terms for transcripts having two fold or above differential expression for the given conditions. Hyper-geometric statistical test was applied with Bonferroni correction method to counterbalance the problem of multiple comparisons.

### Plant metabolic network (PMN) pathways analysis

PlantCyc version 7.0 reference database which hosts more than 800 experimentally validated pathways, their catalytic enzymes and genes was used to study the up regulated pathways in drought stress conditions [35]. Locus IDs of the identified unigenes after DS clustering were looked across NCBI and pathways corresponding to four different plant species namely *Arabidopsis thaliana, Glycine max, Vitis vinifera* and *Populus trichocarpa* were searched in the database.

### Functional domains search for unknown sequences

The assembled transcripts which did not return any homologous sequence hit through BLASTX were searched against conserved domain database (CDD) using RPS-BLAST at an E-value threshold of 10^-5^[36-38]. This way it was possible to functionally characterize even those sequences whose sequence homology might be missing but presence of conserved functional domain could be identified.

### Read mapping and transcript abundance (expression) measurement

To measure the expression of all the assembled transcripts RPKM (Reads per exon kilobase per million) level measurement approach was used [39]. Combination of tools SeqMap and Rseq was used for RPKM measurement [40,41]. The filtered reads from different samples were mapped back individually to the assembled transcripts using SeqMap with two mismatches allowed. Rseq was used for RPKM based expression measurement on each sample separately. Similar transcripts were searched for their RPKM value in each sample. Differential expression was calculated for six different comparative conditions by comparing the RPKM values of similar transcript under different conditions. Only those transcripts were considered as differentially expressed which exhibited two fold or above differential expression.

### GC content analysis and SSRs identification

Emboss GeeCee program was used to calculate the GC content of all the assembled transcripts and simple sequence repeats were found using MISA tool [42,43].

### Quantitative real-time polymerase chain reaction (qRT-PCR)

Total RNA was given DNase I (Fermentas life Sciences, USA) treatment to remove any DNA contamination. First strand cDNA synthesis was conducted with 5 μg of the total RNA using high capacity cDNA reverse transcription kit (Applied Biosystems, USA) according to the manufacturer’s instructions. Gene specific primers for qRT-PCR were designed using primer express 3.0 software. qRT-PCR was performed in three biological replicates on a Step One real-time PCR machine (Applied Biosystems, USA) using SYBR Green qPCR Master Mix (Thermo Scientific, USA). The conditions for qRT-PCR were kept as; 4 min at 94°C, 40 cycles each of 30s at 94°C, 30s at annealing temperature and 72°C for 30s and a final melting curve analysis was performed. Transcript level of all the genes was normalized to an internal reference eukaryotic translation elongation factor gene from horse gram. The relative expression ratio of each gene was calculated using comparative Ct value method [44]. All primers used in this study are listed in (Additional file 1).

## Results and discussion

**Figure 1:**
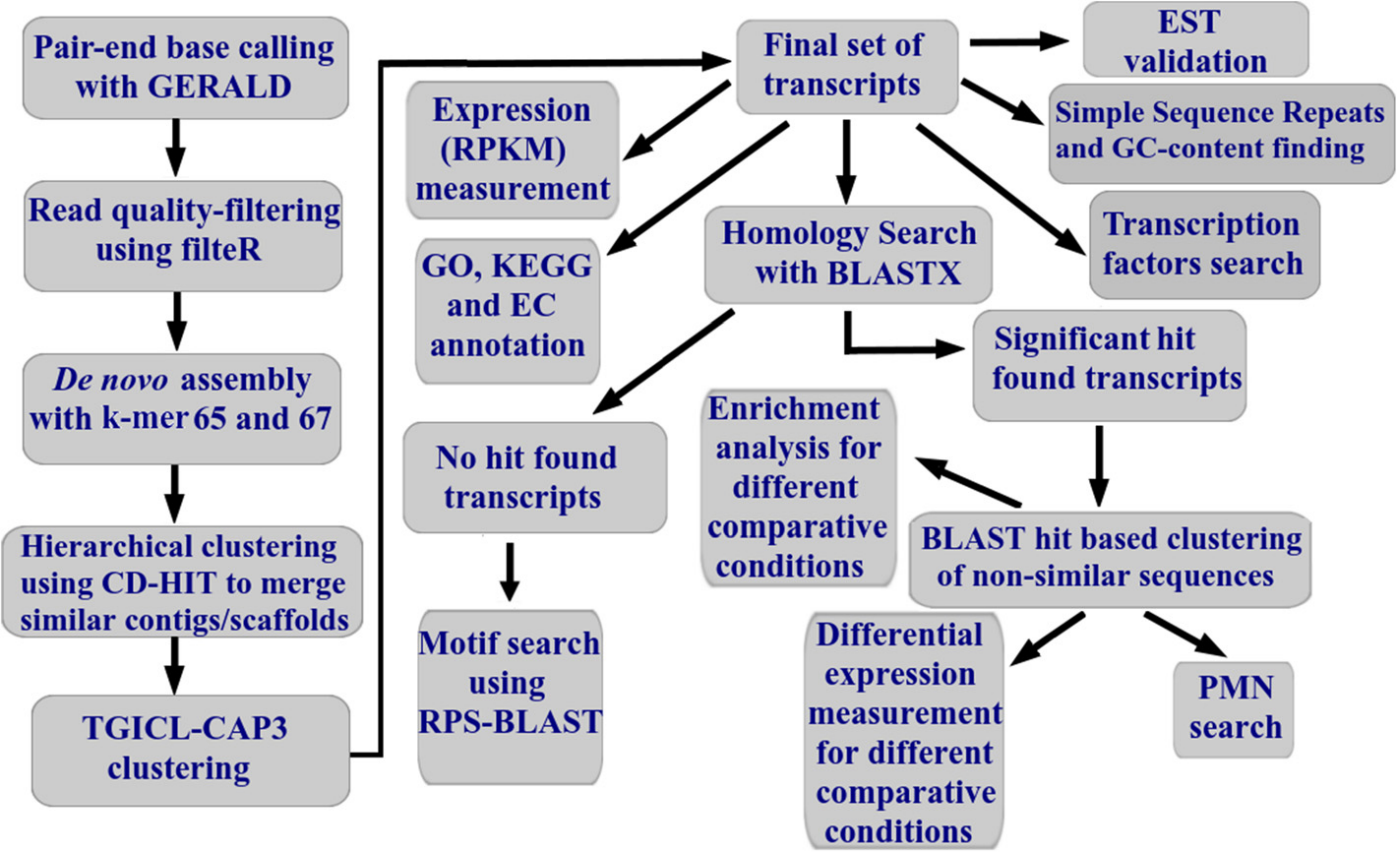
**Work-flow of NGS pipeline.** Work-flow of NGS was developed for *de novo* transcriptome data generation and analysis of horse gram*.*

Comprehensive study on *de novo* transcriptome assembly of horse gram (*Macrotyloma uniflorum*) was conducted using high throughput sequencing on Illumina GAIIx (Figure 1). Shoot and root tissues of two horse gram genotypes, a drought sensitive (M-191) and a drought tolerant (M-249) were used for trancriptomic analysis under control and stressed conditions (48 h of PEG-induced drought stress). Transcripts obtained were computationally annotated and analyzed. Transcripts belonging to drought responsive genes and pathways were identified.

### Reads generation

**Figure 2:**
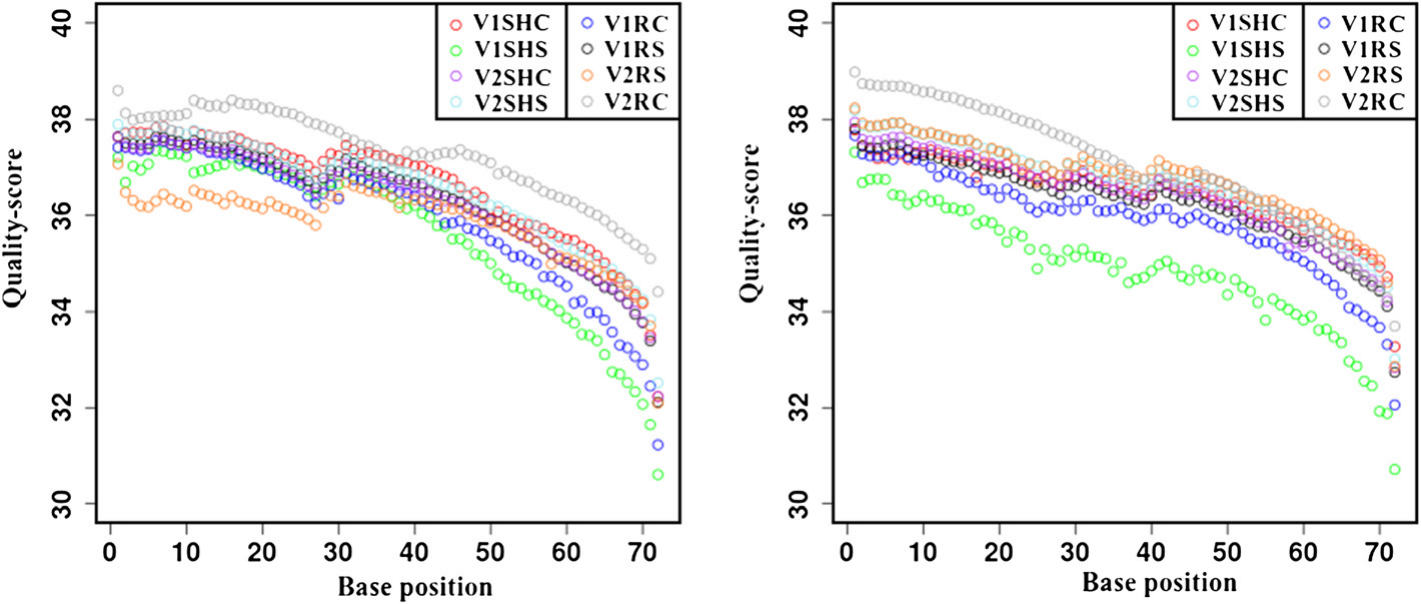
**Read quality-scores.** Plots showing the read quality-score for all the samples in PE data. The quality-score for all the reads generated was observed to be greater than 30 for all samples.

For high-throughput sequencing of horse gram transcriptome, PE run of 2×72 cycles for each sample was performed on Illumina genome analyzer IIx platform (Illumina, USA). CASAVA package was used to convert reads into FASTQ format. The details of total number of reads obtained for eight different samples are given in Table 1. The total number of read pairs obtained before quality filtering were 295, 842,219. No trimming in the read length was done because the average read quality score was found to be greater than 30 for all the samples (Figure 2). This shows that the quality of reads obtained was very good. Only reads with adapter contamination were filtered out using FilteR [24]. This reduced the read pairs from 295, 842,219 to 229,297,896.

**Table 1:** Details of various samples taken for study and read sequence information

**Description**	**Total PE Read Pairs before quality-filtering**	**Total PE Read Pairs after quality-filtering**
Shoot of genotype 1control (V1SHC)	37,723,307	30,618,500
Shoot of genotype 1 stressed (V1SHS)	37,196,581	27,552,147
Root tissue of genotype 1 control (V1RC)	37,291,296	28,898,641
Root tissue of genotype 1 stressed (V1RS)	40,064,602	32,032,375
Shoot tissue of genotype 2 control (V2SHC)	40,543,204	32,311,123
Shoot tissue of genotype 2 stressed (V2SHS)	40,157,434	27,344,755
Root tissue of genotype 2 control (V2RC)	27,586,042	22,571,840
Root tissue of genotype 2 stressed (V2RS)	35,279,753	27,968,515
Total	295,842,219	229,297,896

Different samples taken in transcriptomic study of horse gram and total PE Read Pairs before and after quality-filtering.

### *De novo* assembly, sequence clustering and homology search

For more accurate and sensitive assembly, PE reads with insert length of 200 bp were used. SOAPdenovo-trans [25] was used for *de novo* assembly and was run from k-mer size 19–71 with read length of 72 bp. The evaluation of assembly on different k-mers was done on the basis of chosen parameters which included total number of assembled transcripts, number and percent of transcripts ≥ 1000 bp, N50 value and coverage. K-mer of size 65 and 67 were found to be the best and were taken together for further analysis (Additional file 2). For all the assembled transcripts minimum cut-off length was 100 bp. The total transcript sequences obtained after primary assembly steps were 62,065 wherein 28,110 transcripts had length ≥ 1000 bp. The maximum length of the transcript was found to be 15,501 bp and average transcript length was 1,114.72 bp with N 50 value of 1,728 bp and high coverage of 295X (Table 2). Sequences with gaps were further scrutinized to map the gap regions with gap filler.

**Table 2:** Different assembly steps involved in the transcriptomic study of horse gram

**Assembly steps**	**Total transcripts**	**Number of transcripts (≥1000 bp)**	**Percent of transcripts (≥1000 bp)**	**Maximum transcript length (bp)**	**Average transcript length (bp)**	**N 50 value (bp)**	**Coverage (X)**
**Primary assembly step**	62, 065	28, 110	45.29	15, 501	1, 114.72	1, 728	295
**Hierarchical clustering**	29, 603	14, 855	50.18	15, 764	1, 221.20	1, 770	568
**Total Unique Gene groups**	21, 887	12, 706	58.05	15, 764	1, 364.09	1, 805	667

All the steps performed for the *de novo* assembly of horse gram and calculation of different statistics for each step.

Redundancy in the assembled transcripts was removed using two clustering tools namely, CD-HIT and 90% similarity cut off and TGICL [26,27]. After hierarchical clustering total numbers of assembled transcripts were reduced from 62,065 to 29,603 wherein 14,855 transcripts had lengths above 1000 bp (Additional file 3). Maximum transcript length was found to be 15,764 bp with an average transcript length of 1,221.2 bp. The N50 value was 1,770 bp with coverage of 568X (Table 2).

**Figure 3:**
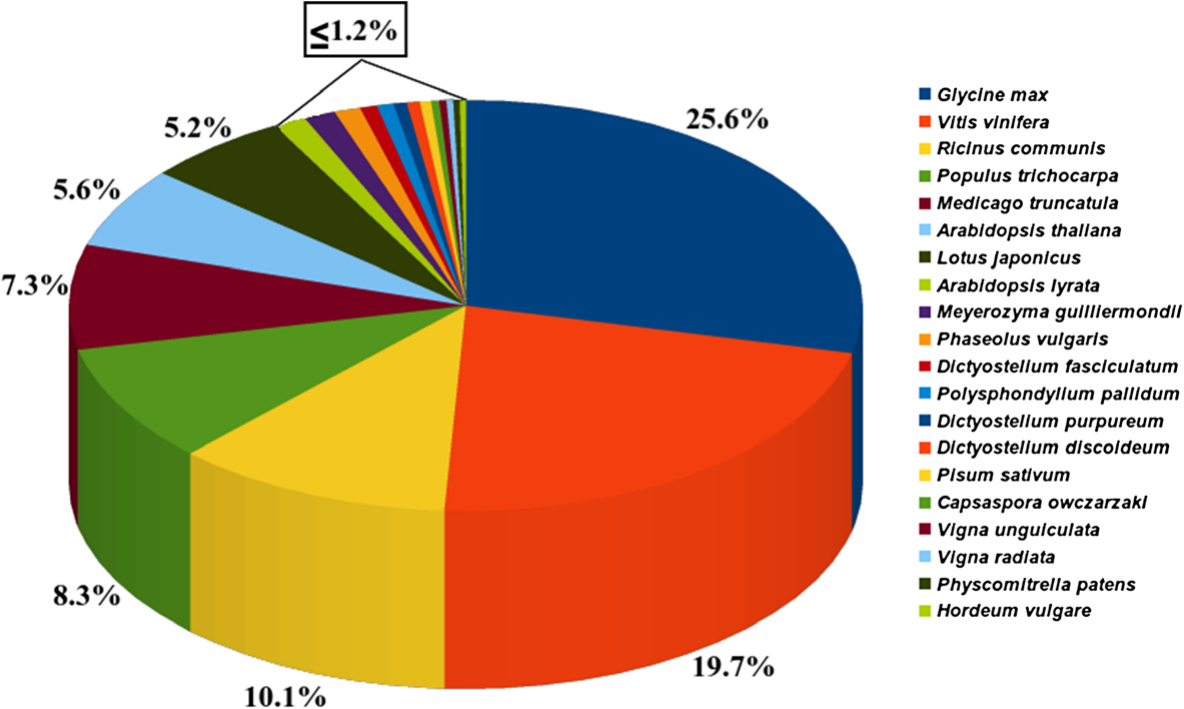
**Homology search.** Representation of the percent of top 20 BLASTX top-hits with different plant species. Maximum homology of horse gram transcripts were observed with *Glycine max*.

Homology search for the sequences obtained after clustering, was done using BLASTX against protein sequences at non-redundant (NR) databases at NCBI with cut off E-value of 10^-5^[28,29]. Significant BLAST hits were obtained for 26,045 sequences while no hits were found for 3,558 sequences. There can be multiple representatives of a single gene or a single gene may have many isoforms so another clustering step known as DS clustering [24,30] was performed, which curtailed inflation in the number of unigenes that could have occurred otherwise. Hence, the total number of transcripts was reduced from 26,045 to 21,887 for best group representation. The details of clustered sequences are available as (Additional files 4 and 5). In the best group representation, 12,706 transcripts were found to be ≥ 1000 bp with a maximum length of 15,764 bp and average transcript length of 1,364.09 bp. The N50 value was 1,805 bp with coverage of 667X (Table 2). The maximum number of BLASTX top hits for best group representatives were found with *Glycine max* (25.6%) followed by *Vitis vinifera* (19.7%), *Ricinus communis* (10.1%), *Populus trichocarpa* (8.3%). However, it also showed homology to a lesser extent with model legume plants, *Medicago truncatula* (7.3%) and *Lotus japonicus* (5.2%) and non-legume model plant *Arabidopsis thaliana* (5.6%) (Figure 3).

### Validation of assembled sequences against EST’s of horse gram

Validation of the assembled sequences of horse gram was conducted using BLASTN analysis performed with an E-value threshold of 10^-05^[28]. Mis-assemblies and alignment conditions were screened using in-house developed scripts. A total of 1,025 ESTs for horse gram were available at NCBI dbEST (Additional file 6) out of which maximum number (>850) of the ESTs were submitted in response to drought stress [45]. Out of 1,025 ESTs significant hits were observed for 843 ESTs (82.24%) while only 181 ESTs (17.65%) had no hits. Maximum width coverage for a single EST was 99.5% and 22 ESTs had coverage greater than 90% while 642 ESTs had coverage greater than 50% validating the precision of the sequence data obtained (Additional file 7). No major number was found reporting chimeric arrangement of assembled transcript sequences. This suggests correct alignment and continuity of the sequences obtained. Significant hits accounting for 83.4% of ESTs in *P. kurrooa*, 87% in *A. thaliana*, 46.60% in *P. vulgaris* and 62.28% in *L. japonicus* have been reported previously [24,46-48]. The percentages of significant hits with ESTs obtained for horse gram are higher than those from other legumes and comparable to those from other non-legumes. This validation confirms that all the transcripts from the present study showing significant hits with the available ESTs of horse gram should belong to drought stress inducible genes.

### GC content and identification of short sequence repeats (SSRs)

GC content is the percentage of guanine and cytosine nucleotides in a genome. Usually, GC content ranges between 25 to 75% and is affected by genome size, environment and temperature. Functional relevance of GC content includes sequence variations in a genome, repeat element distribution, methylation pattern, thermostability and gene density [49-51]. The average GC content for all the assembled transcripts (29,603) was found to be 43.44% (Additional file 8). The average GC content of plants like chickpea, soybean and *Arabidopsis* ranges between 40-42% [52]. However, higher GC content (55%) has been observed in rice [53]. The GC content for horse gram is well within the range.

SSRs or microsatellites are stretches of short nucleotide motifs ranging from 1 to 6 nucleotides in length. These are repeated in tandem and are evenly spread across prokaryotic and eukaryotic genome. Due to high mutation rates affecting the number of repeat units, SSRs show high length polymorphisms which are easily detected through amplified fragment length polymorphism (AFLP) techniques. Thus, SSRs serve as important molecular marker discovery centers for studying linkage maps of plants, genetic analysis for economically important quantitative traits, plant evolution and breeding studies [54,55]. From 29,603 assembled transcripts, a total of 6,195 SSR loci were identified which were found to be highly abundant (16.25%) in the 4,810 sequences. Out of these, the most prevalent SSR type was tri-nucleotides (43.93%), immediately followed by mono-nucleotides (43.43%), then di-nucleotides (36.65%), tetra-nucleotides (2.41%), hexa-nucleotides (1.41%) and penta-nucleotides (0.96%) (Table 3). In earlier studies also tri-nucleotide repeat type of SSRs have been found to occur at the highest frequency as compared to other types [17,24,56-59].

**Table 3:** Simple sequence repeats (SSRs) identified in transcriptome of horse gram

**SSR mining**	
Total number of sequences examined:	29, 603
Total size of examined sequences (bp):	36, 151, 506
Total number of identified SSRs:	6, 195
Number of SSR containing sequences:	4, 810 (16.25%)
Number of sequences containing more than one SSR	1, 024
Number of SSRs present in compound formation:	514
**Distribution of SSRs in different repeat types**	
**Unit size**	**Number of SSRs**
Mononucleotide (1)	2, 089 (43.43%)
Dinucleotide (2)	1, 763 (36.65%)
Trinucleotide (3)	2, 113 (43.93%)
Tetranucleotide (4)	116 (2.41%)
Pentanucleotide (5)	46 (0.96%)
Hexanucleotide (6)	68 (1.41%)

Details of various SSRs found in horse gram along with their type and occurrence.

### Functional annotation and classification

Functional annotation was conducted using Annot8r [31]. All the assembled transcripts were compared against NR protein sequences available at UniProt database using BLASTX algorithm with E-value threshold of 10^-01^[28,32]. The highest scoring hits attained for the sequences were assigned their corresponding functional categories namely GO, EC and KEGG (Additional file 9).

**Figure 4:**
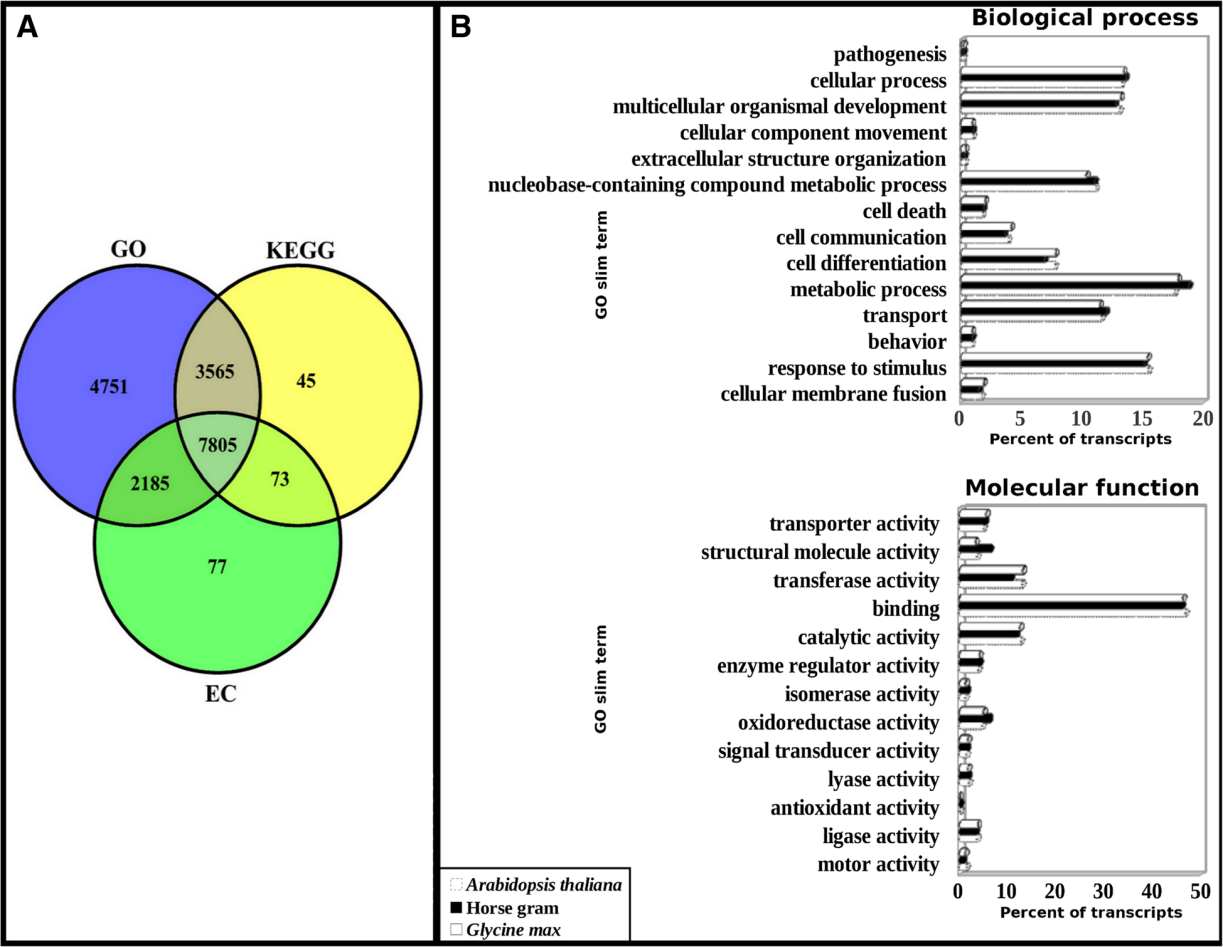
**Annotation details.** Venn-diagram showing the annotation details of unigenes for GO, EC and KEGG analysis **(A)**, and comparative study of horse gram based on GO annotation with *Glycine max* and *Arabidopsis thaliana***(B)***.*

**Figure 5:**
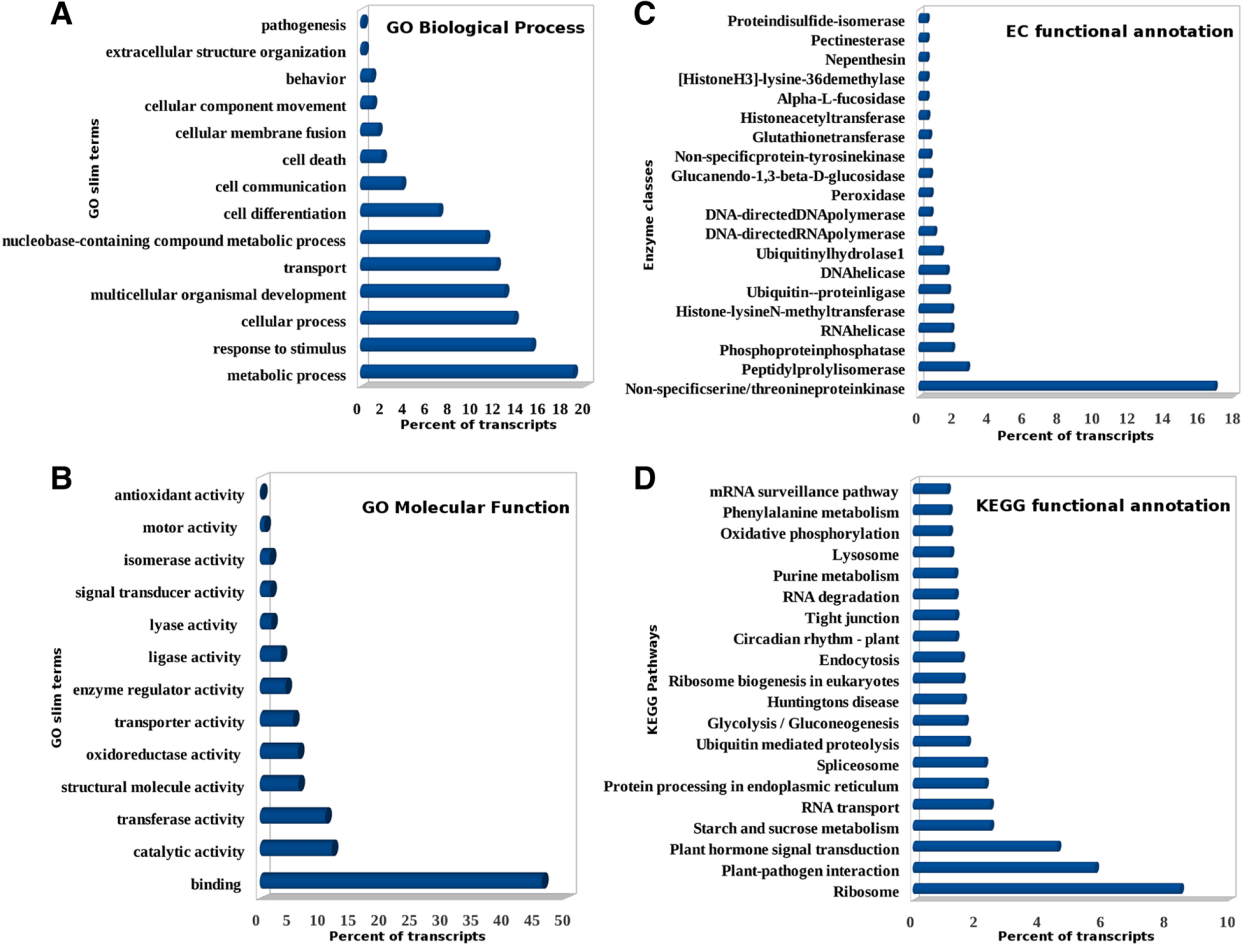
**Gene Ontology based study.** Occurrence of different biological processes based on GO slim categories in horse gram with corresponding percentage **(A)**, occurrence of different molecular functions based on GO slim categories in horse gram with corresponding percentage **(B)**, top-20 enzyme classes with corresponding percent of occurrence found in horse gram based on EC annotations **(C)**, and top-20 KEGG pathways with corresponding percent of occurrence found in horse gram based on KEGG annotations **(D)**.

GO classification was obtained for 18,306 transcripts out of a total of 21,887 transcripts (best representatives of unigenes obtained after dissimilar sequence clustering) taken as input (Figure 4A). GO classification includes biological processes (Figure 5A) and molecular functions (Figure 5B) as sub-categories. The highly represented groups among biological processes category were metabolic processes (18.86%), response to stimulus (15.16%) and cellular processes (13.62%). Under molecular functions category genes for DNA binding (46.06%), catalytic activity (11.73%) and transferase activity (10.67%) were observed to be highly represented. In chickpea, 28.19% of sequences corresponded to metabolic processes, 27.62% to cellular processes, 7.29% to response to stimulus, 46.35% to DNA binding and 37.92% to catalytic activity [60]. About 14-18% of the genes in cassava and 12-17% in peanut have been documented under GO functional categories [56,61]. In pigeonpea out of 2,106 sequences, 571 belonged to metabolic processes followed by 542 sequences for cellular processes and 152 sequences for response to stimulus. While molecular functions like DNA binding activity retained highest number of 594 sequences followed by 513 sequences for catalytic activity in pigeonpea [62]. It is suggested that the genes showing high representation for all these processes are from metabolically active developing tissues and hence are diverse in function. These processes and activities could be involved in gene regulation and basal defense responses via stress signaling pathways which might be abiotic stress activated or pathogen stimulated [17,61,63].

To assess the importance and validity of the identified functions and processes, a GO comparison was drawn for horse gram with a common legume *Glycine max* which showed highest homology with horse gram unigenes in BLASTX results and a non-legume model plant *Arabidopsis thaliana* (Figure 4B). Under biological processes category, higher numbers of transcripts representing metabolic (18.86%) and cellular processes (13.62%) were found in horse gram as compared to other two plants. *Arabidopsis* showed 17.68% for metabolic and 13.28% for cellular processes while *Glycine max* showed 17.93% for metabolic and 13.42% for cellular processes. However, under molecular functions category the representation of transcripts was more or less the same for all three plants except for structural molecular activity (6.29%) and oxidoreductase activity (6.21%) being more represented in horse gram than *Arabidopsis* (3.71% and 4.80%, respectively) and *Glycine max* (3.32% and 5.02%, respectively). In a similar comparison drawn between *P. kurrooa*, *Arabidopsis* and *Medicago*, *P. kurrooa* showed higher number of transcripts representing metabolic processes. However, for DNA binding and catalytic activity, higher number of transcripts belonged to *Arabidopsis* and *Medicago*[24]. Thus, GO analysis suggests that metabolic processes, cellular processes, structural molecular and oxidoreductase activity could be responsible for growth and defense system of horse gram. The above stated highly represented processes and functions remained more or less the same for all the three plants compared, suggesting their indispensable role in over all plant growth, development and defense. This knowledge allows the comparison and transfer of genetic information between plant species.

EC classification was obtained for 10,140 transcripts while KEGG classification was found for 11,488 transcripts out of the 21,887 transcripts taken as input. A total of 1,400 enzyme classes and 248 KEGG pathways were identified in this study. Out of the top 20 EC classes analyzed serine/threonine protein kinase (16.86%) was found to dominate followed by peptidyl prolylisomerase (2.75%) and phosphoprotein phosphatase (1.89%) (Figure 5C). In transcriptomic analysis of chickpea, sequences primarily belonged to transferases (728), hydrolases (671), and oxidoreductases (474) [60]. In transcriptomic profiling studies on pigeonpea, 31% of the unigenes belonged to transferases, followed by hydrolases (28%), oxido-reductases (25%), ligases, lyases and isomerases (5-6%) [62]. Similar categorization of enzyme classes identified for horse gram suggested that 16.24% transcripts for oxidoreductases, 41.9% transcripts for transferases, 27.45% transcripts for hydrolases, 3.65% transcripts for lyases, 5.58% transcripts for isomerases and 5.18% transcripts for ligases. Upon analyzing the top 20 KEGG pathways, dominance was observed for pathways belonging to ribosome (8.43%) followed by plant pathogen interaction (5.75%) and plant hormone signal transduction (4.56%) (Figure 5D). Similar results have been observed for *P. kurrooa* where highest number of the assembled transcripts represented serine/threonine protein kinase (14.6%) followed by plant pathogen interaction (6.13%) [24]. In semi-mangrove plant (*M. Pinnata*), 21.8% of the unigenes identified with 124 KEGG pathways wherein ribosome, plant pathogen interaction, plant hormone biosynthesis and spliceosome pathways were found to be most represented [64]. Besides housekeeping functions these enzymes and pathways are expected to be involved in active cellular responses associated with growth, development, differentiation, defense, inflammation, energy metabolism, photosynthesis and apoptosis leading to efficient environmental adaptation [24,62,65].

### Comparative analysis for identification of drought responsive genes

**Figure 6:**
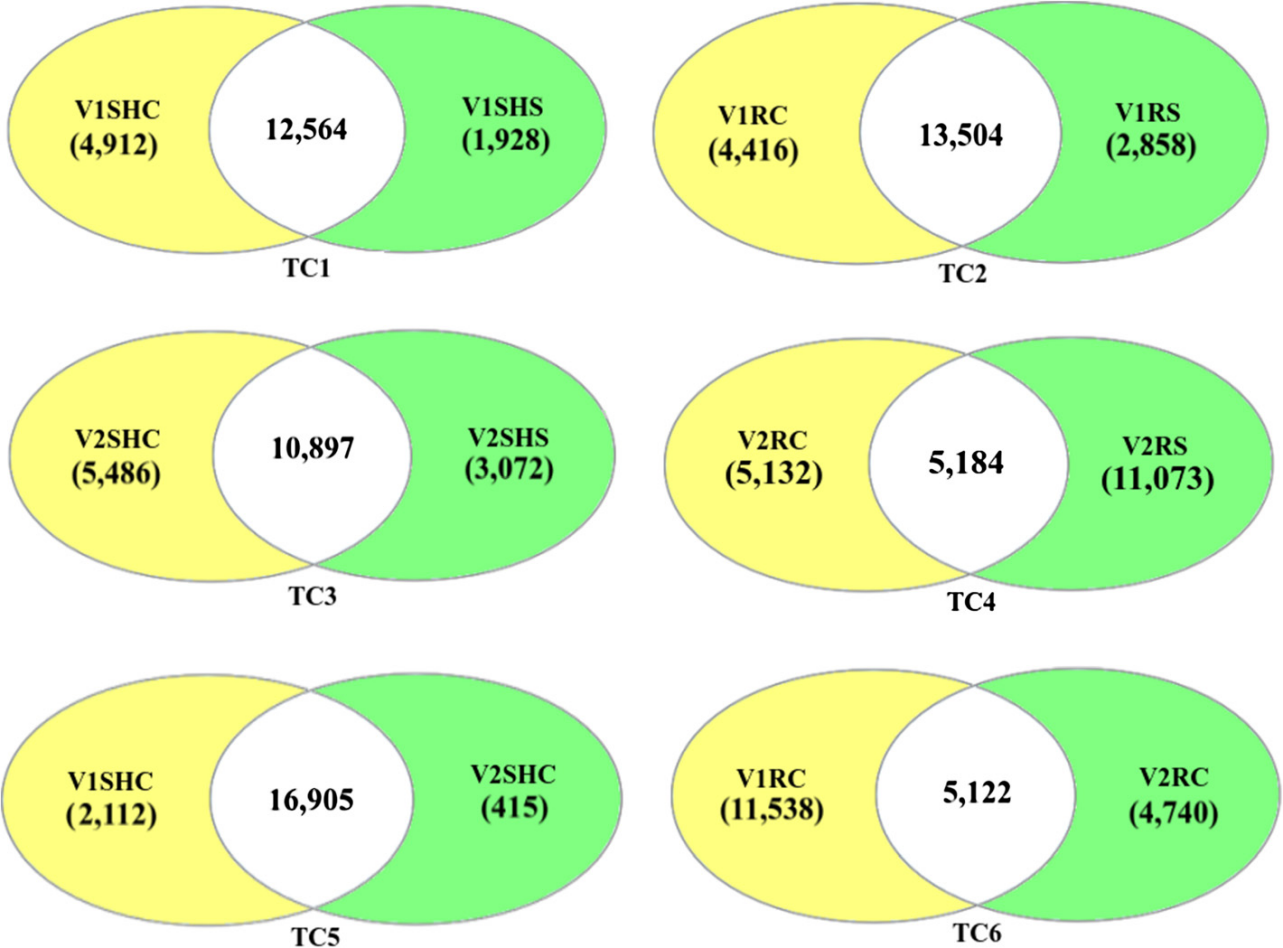
**Differential expression based study for comparative test conditions.** Number of up-regulated transcripts, down-regulated transcripts and transcripts showing no major change among best group representatives found in various comparative test conditions. The figure does not include the cases in which we have very small RPKM value (RPKM value < 2.0) in one condition and have zero RPKM value in the other condition. Transcripts having zero RPKM value in both the conditions were also not considered here.

To generate a resource for mining stress responsive genes, representative unigene transcripts showing two-fold and above differential expression were analyzed under control and drought stress conditions in horse gram (Figure 6). The different test conditions taken into consideration have been abbreviated as TC1: Transcripts up regulated in shoots of genotype M-191 under drought stress condition (V1SHS/V1SHC); TC2: Transcripts up regulated in roots of genotype M-191 under drought stress condition (V1RS/V1RC); TC3: Transcripts up regulated in shoots of genotype M-249 under drought stress condition (V2SHS/V2SHC); TC4: Transcripts up regulated in roots of genotype M-249 under drought stress condition (V2RS/V2RC); TC5: Transcripts up regulated in shoots of genotype M-249 in comparison to genotype M-191 under control conditions (V2SHC/V1SHC); and TC6: Transcripts up regulated in roots of genotype M-249 in comparison to genotype M-191 under control conditions (V2RC/V1RC). The first four test conditions (TC1-TC4) compared the changes in transcript levels of two tissues under stress conditions with that of control conditions within a single genotype. While the last two test conditions (TC5, TC6) compared the changes in native transcript levels of the two tissues between the two genotypes under control conditions. Henceforth, these abbreviated forms will be used for describing the comparative test conditions taken for this study. RPKM expression value and differential expression of all the unigenes under different TCs is given in the (Additional file 10).

In TC1, 1,928 transcripts were found to be up regulated, 4,912 down regulated and 12,564 remained unchanged whereas in TC2, 2,858 transcripts were found to be up regulated, 4,416 down regulated and 13,504 showed no major change under drought stress conditions. In TC3, 3,072 transcripts were observed to be up regulated, 5,486 were down regulated and 10,897 remained without any major change in expression level. Similarly, in TC4, 11,073 transcripts were found to be up regulated, 5,132 were down regulated and 5,184 remained without any major change. To identify the differentially expressed genes in the two genotypes TC5 and TC6 were taken into account. In TC5, 415 transcripts were found up regulated, 2,112 were down regulated and 16,905 showed no major change in the tolerant genotype compared to the sensitive one. Similarly in TC6 4,740 transcripts were up regulated, 11,538 were down regulated and 5,122 remained without major change in the tolerant genotype as compared to the sensitive one. Fusarium wilt and sterility mosaic disease had up regulated 5,000-9,000 unigenes and down regulated 100–4,000 unigenes in five genotypes of pigeonpea differing in their sensitivity towards these biotic stresses [62]. Three genotypes of cassava have also been reported to show up regulation of 169 genes and down regulation of 69 genes in response to drought stress [61]. In *M. pinnata* higher numbers of genes were found to be up regulated (7,491) and down regulated (8,972) in the root tissue as compared to the leaf tissue (5,239 and 4,519, respectively) under salt stress [64]. In horse gram higher numbers of transcripts were observed to be up regulated in the root tissue as compared to the shoot tissue within as well as between the two genotypes. Root tissue of the tolerant genotype showed the highest number of up regulated transcripts under drought stress conditions. Hence, up regulated genes in the root tissue of horse gram could be playing crucial role in its tolerance towards drought.

To find the most represented GO category under drought stress, a comparative analysis was done for different TCs based on GO terms (Additional files 11 and 12). It was observed that under biological processes, glycolysis predominated in three test conditions (TC2, TC3 and TC6) while response to heat, vegetative to reproductive phase transition of meristem and stamen development were found most represented in TC1, TC4 and TC5, respectively. Under molecular functions category, protein binding predominated in TC1, TC4 and TC5. While molecular function of ubiquitin protein ligase binding dominated in TC2 and structural constituent of ribosome was most represented in TC3 and TC6.

To relate the drought tolerance of horse gram to its functionally annotated transcripts enrichment analysis was conducted using AgriGO Singular Enrichment Analysis module to locate the transcripts for highly enriched drought responsive biological processes and molecular functions at significance level of 0.05 for all the TCs [34] (Additional file 13). All the available GO IDs of unigenes were used as reference and those which are differentially expressed (having two-fold or above differential expression) in particular TC were taken in the query list. Out of all the TCs taken into study highly enriched transcripts for either biological processes or molecular function category were found for all the TCs except TC4. For TC1 under molecular function category hydrolase activity (p-value: 0.0101) and ion transmembrane transporter activity (p-value: 0.0328) was found to be enriched. For TC2 among biological processes, response to stimulus (p-value: 2.89e-07) was observed to be highly enriched. For TC3 under molecular function category, substrate specific transmembrane transporter activity (p-value: 0.0454) was found enriched while under biological processes, response to stimulus (p-value: 0.0025) was enriched. For TC5, structural molecule activity (p-value: 1.1e-13) and structural constituent of ribosome (p-value: 1.21e-13) was enriched in shoot of sensitive genotype. While under biological category, metabolic and catabolic processes associated with carbohydrates and alcohol (p-value: 5.04e-10 to 1.03e-08) were found to be highly significant. In TC6, under molecular functions category, structural molecule activity (p-value: 1.89e-49) and structural constituent of ribosome (p-value: 3.36e-50) were found enriched in root of tolerant genotype while biological processes like generation of precursor metabolites, energy (p-value: 7.12e-09), glycolysis (p-value: 5.57e-07), metabolic, catabolic processes associated with carbohydrates, alcohol and small molecules (p-value: 3.75e-12 to 3.8e-08) were found enriched.

In an insectivorous plant *Sarracenia,* hydrolase activity has been found to be highly represented among all the unigenes [66]. Metabolic processes for metabolites and energy generation have been found to be enriched in *M. pinnata* and chickpea unigenes [60,64]. Changes in glycolysis, Kreb’s cycle and electron transport chain have been previously implicated during drought stress in plants [67]. These molecules help in timely response to the stimulus and in the maintenance of membrane integrity which is the first line of defense to stress introduction [24,60,64]. The enrichment of these processes suggests that horse gram derives and maintains its energy requirements during drought stress adaptation through an active demand for metabolites like carbohydrates and alcohol. High representations of these biological processes and molecular functions further suggested active and efficient adaptation by horse gram to the external stimuli.

To assess the important drought responsive enzymes, EC analysis was conducted for all the TCs (Additional file 14). Unambiguously in all the TCs serine/threonine protein kinase was most represented (10-18%) followed by ubiquitin protein ligase and peptidylprolylisomerase (2-4%). However, few other enzymes like histone-lysine N methyl transferase, phosphoproteinphosphatase, peroxidase and RNA helicase were also found commonly represented (1-3%) in all TCs. Similarly, important drought responsive pathways were identified through KEGG analysis conducted for comparison under different TCs (Additional file 15). The pathways found most represented for all the TCs were ribosome (2-23%), plant-pathogen interaction (4-10%) and plant hormone signal transduction (6-10%). These were followed by alpha-linolenic acid metabolism, ribosome, spliceosome, starch, sucrose metabolism and glycolysis. These ranged from 1-3% in abundance. Like horse gram, *P. kurrooa* also showed plant-pathogen interaction (6.13%) to be highly represented [24]. Transcriptomic analysis of *M. pinnata* has also documented maximum transcripts to plant pathogen interaction (2,859) from a total of 2,933 unigenes [64].

### Plant metabolic network associated with drought tolerance in horse gram

A separate PMN search was conducted to identify and associate the drought responsive transcripts of horse gram with plant metabolic networks or pathways. A total of 220 out of 21,887 unigenes were found to be associated with 709 pathways. Important pathways were selected from these 709 pathways only for transcripts showing two fold or above differential expression. These selected pathways were considered to be up regulated in different TCs (Additional file 16A and B). Among these up regulated pathways also, only those showing highest differential expression are mentioned under different conditions. In TC1, pathways found highly influenced were of valine degradation (5.11), γ-glutamyl cycle (3.47) and acetyl-CoA biosynthesis (CO_2_ fixation, 2.59). While in TC2 gluconeogenesis (85.31), sucrose degradation (9.83) and pyridoxal 5-phosphate biosynthesis II (5.37) pathways were found most influenced. Similarly, in TC3 besides valine degradation (9.98), glucosinolate biosynthesis (8.26) and UDP-glucose biosynthesis (5.90) pathways were observed to respond significantly. While in TC4 purine nucleotides degradation I (17.0), flavonol biosynthesis (16.66) and CO_2_ fixation (9.59) were found to be most influenced.

Valine degradation in shoots, gluconeogenesis and purine nucleotide degradation pathways in roots were among highly up regulated pathways under stress conditions suggesting their critical role against drought stress in horse gram. Amino acids like valine, leucine, isoleucine act as a precursor for the synthesis of polyamines which along with proline produce synergistic osmoprotective effect against drought stress [68]. Glucosinolates are the nitrogen and sulphur containing compounds derived from amino acids like valine, leucine, alanine. These compounds have been known to be accumulated in response to drought stress conditions [69,70]. Simultaneously, catabolic enzymes of these branched chain amino acids (BCAA) are also activated under stressed conditions to prevent the rise of these BCAA to a toxic level [71,72].

Gluconeogenesis produces glucose from non-carbohydrate sources. Normally, no change or down regulation of gluconeogenesis process should be an energy saving measure taken by plants under drought stress. Interestingly, up regulation of gluconeogenesis in roots of drought sensitive horse gram genotype only, allows us to hypothesize it as a quick response of this genotype to cope with drought challenge. When shoots of this genotype are unable to maintain a normal homeostasis under stress, roots might perceive the danger and utilize gluconeogenesis pathway to produce enough glucose as energy source for plant. The key enzyme of gluconeogenesis (fructose bis-phosphate aldolase) has been found to be up regulated in drought susceptible genotype of chickpea and down regulated in the leaves of tomato and *Arabidopsis* under drought stress [71,73].

Nucleotide metabolism is known to affect plant growth and development. Under drought stress conditions these otherwise normal processes are challenged. Therefore, nucleotide biosynthesis as well as degradation has been shown to be implicated in drought stress responses in rice, paddy rice and *Arabidopsis* plants [74-76].

Involvement of other pathways showing high representation in horse gram has also been well implicated under drought stress conditions. Under extreme dehydration conditions, some carbohydrates (non-reducing sugars like sucrose, trehalose and oligosaccharides) provide protection to the proteins by forming a hydration shell in lieu of water [77]. In a study on *Arabidopsis,* enzymes of UDP-glucose biosynthesis (UDP-glucose 4-epimerase) have been implicated in shoot and root growth through cell wall carbohydrate biosynthesis under stress conditions [78]. Genes involved in flavonoid biosynthesis, sucrose degradation and gluconeogenesis pathways have been found to be strongly affected in response to drought conditions in *Vitis vinifera* and tomato [73,79,80]. Gamma-glutamyl cycle involves glutathione metabolism which is well known to mitigate the stress levels in plants [81,82]. The variation in phenotypic response to drought stress of these two genotypes of horse gram could be attributed to the influence of various regulatory network pathways mentioned above as has been documented for plants like cowpea and chickpea under biotic and abiotic stresses [22,60].

### Transcription factors

**Figure 7:**
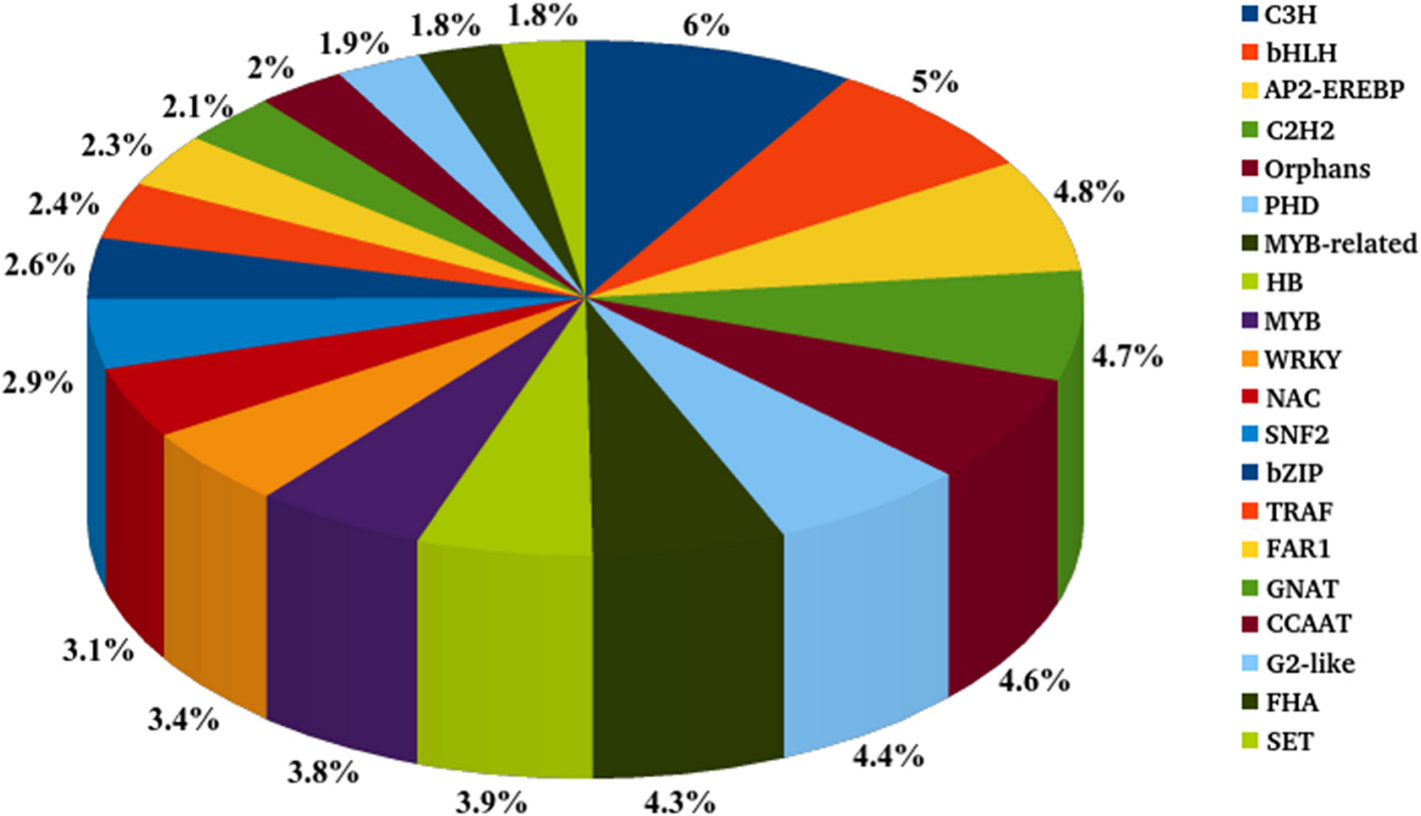
**Identification of transcription factor families and their abundance.** Occurrence of top-20 transcription factor families found in horse gram based on sequence similarity using PlnTFDB (v3.0) database.

Sequences from different plants for various transcription factors are available at PlnTFDB [33]. These sequences are further categorized under 29,474 categories. Around 6,637 transcript sequences exhibited similarity to transcription factor, represented by 2,280 unique transcription factor genes (Additional file 17). The most abundant TF families observed in case of horse gram are C3H (6%), bHLH (5%), AP2-EREBP (4.8%) (Figure 7). A comparative study was also conducted for all the TCs (Additional file 18). The most abundant TF families observed under stressed condition in the shoot tissue of both genotypes were NAC (1-9%), MYB-related (3-9%), G-2 like (1-4%), and WRKY (1-12%). While in case of roots others remaining the same, C3H and PHD (5-6%) families were an addition. However, the order of abundance varied under different conditions. On comparison, AP2-EREBP, MYB-related and bHLH were highly abundant in shoot of M-191 while C3H, GNAT, TIG and G-2 like were high in shoot of M-249. In case of roots, C3H, PHD and bHLH were abundant in M-191 while Orphans, AP2-EREBP and MYB-related were abundant in M-249. All these families ranged from 1-8% in abundance in horse gram.

TFs are key regulators of plant growth, development and response systems. They can control a cascade of metabolic reactions and hence alter important agronomical traits in plants [56]. From the analysis of TFs in horse gram it can be suggested that the most important and responsive players in defense of horse gram against drought stress are NAC, MYB-related, WRKY, C3H, PHD families. These families have been previously shown to act in improving drought tolerance and pest resistance, reducing water loss by regulating stomatal movement, increasing transpiration efficiency, regulating embryogenesis, chromatin mediated transcription and systematic acquired resistance (SAR) in plants like soybean, peanut, chickpea and ground nut [24,56,60,63,83-86].

### Unknown genes

**Figure 8:**
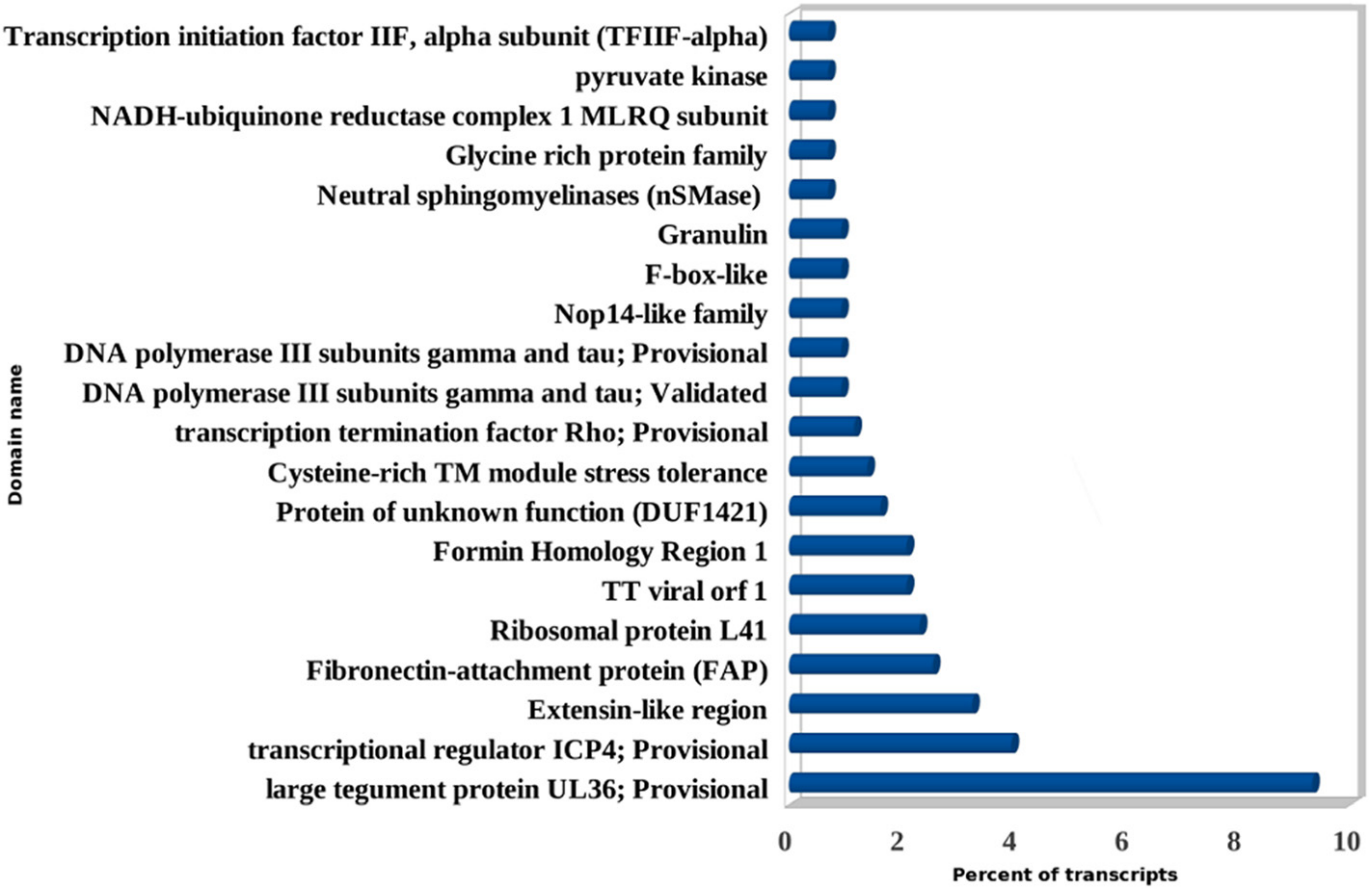
**Conserved domains identification for unknown genes.** Different conserved domains found in horse gram using RPS-BLAST and CDD database on no-hit BLASTX transcripts and their abundance.

No hits were obtained for a total of 3,558 transcripts in the BLASTX search. Therefore, search for these transcripts was conducted against conserved domain database using RPS-BLAST at an E-value threshold of 10^-5^[36-38]. Out of 3,558 sequences, hits were found for 429 transcripts mainly belonging to conserved domain categories (Additional file 19). Highest occurrence was shown by important domains like large tegument protein UL36 (9.32%); transcriptional regulator ICP4 (3.96%); Extensin-like region (3.26%) (Figure 8). Among the 29,622 unknown genes of *P. Kurrooa*, conserved domains were identified for 1,225 transcripts only. In *P. Kurrooa* also, large tegument protein UL36 and Extensin-like region were among the most represented domains [24]. The large tegument protein UL36 is a high molecular weight protein (3,164 amino acids) found on capsid surface of herpes simplex virus (HSV). It is essential for morphogenesis and protein-protein interaction of virus. ICP4 is crucial protein of HSV involved in the regulation of viral gene expression for productive infection [87-89]. Extensin-like regions are similar to hydroxyproline rich glycoproteins (HRGP) known to be associated with pollen tube growth, cell wall self assembly and cell extension [90]. The presence of these conserved domains suggests critical function of their corresponding transcripts in horse gram for cell wall assembly, protein-protein interaction and pollen growth.

### Experimental validation of differential expression data by qRT-PCR

**Figure 9:**
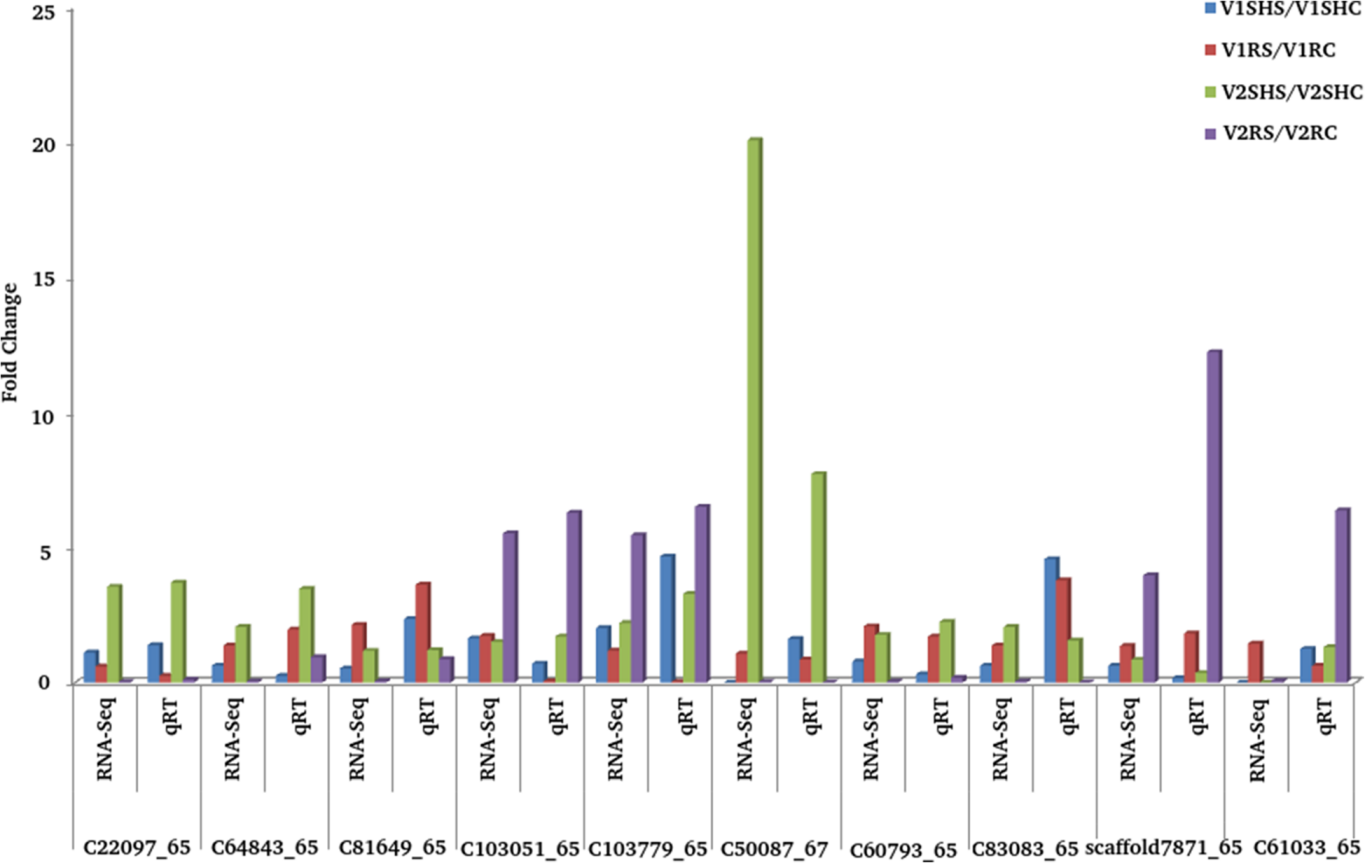
**Validation of expression profile through qRT-PCR.** A total of 10 drought responsive genes were selected and validated using qRT-PCR against their expression profile from RNA-seq.

In order to validate differential gene expression obtained through RNA-seq, a total of 10 genes were selected, out of the transcripts having two-fold and above differential expression under different TCs and their qRT-PCR analysis was performed (Figure 9). Among the chosen transcripts genes encoding heat shock protein (C103051_65), cysteine proteinase inhibitor 4 (C22097_65), 40S ribosomal protein S 19–3 (C64843_65), transcription factor bHLH (Scaffold7871_65), calmodulin binding factor (C103779_65), dehydrin (C83083_65), DEAD-box ATP dependent RNA helicase (C60793_65) have been known to be previously involved in response to abiotic stresses [91,92]. Besides these, qRT-PCR was also performed for transcripts encoding for pod storage protein (C81649_65) and eukaryotic translation initiation factor (C50087_67), which were found to be drought-responsive in horse gram in this study. The expression pattern of most of the genes obtained through qRT-PCR data largely corroborated with RNA-seq data. However, one transcript (C61033_65) coding for ubiquitin 40S ribosomal protein S 27–3 did not exactly match with its RNA-seq value (Figure 9). The qRT-PCR analysis confirms that RNA-seq approach has provided reliable data regarding differential gene expression of horse gram under drought stress.

## Conclusions

In order to reveal the genetic architecture and response towards drought stress, transcriptomic study was conducted in shoot and root tissue of a sensitive (M-191) and tolerant (M-249) genotype of horse gram. High quality reads generated with wide coverage presented a comprehensive overview of horse gram at genetic level. Attaining substantial number of transcripts with high average length and coverage suggested making of good quality *de novo* assembly. Validation of the obtained unigenes against already known drought responsive ESTs of horse gram suggested their prominent role under drought stress conditions. Functional annotation and validation of horse gram transcripts against other plant species showed a number of known pathways, enzymes, metabolic processes and transcription factors up regulated under stressed conditions. However, a few unknown genes were also found to be significantly responsive to drought stress but their annotation and functional validation demands further investigation. SSR markers obtained from this study can be utilized for molecular breeding programs. Identification and characterization of genes responsible for horse gram’s indomitable pest resistance remains a future work to better understand plant-pathogen interactions. This genetic information developed in this study will be very useful for the improvement of horse gram as well as other agricultural important crops.

### Availability of supporting data

The Illumina sequence data from this study have been submitted as BioProject ID [PRJNA216977] to the NCBI Sequence read archive under the accession number [SRP029360]. All the supporting data are included as additional files.

## Abbreviations

CAP3: Contig assembly program version3; CD HIT: Cluster Database at High Identity with Tolerance; CDD: Conserved domain database; DS: Dissimilar sequence; HRGP: Hydroxyproline rich glycoproteins; KEGG: Kyoto encyclopedia of genes and genome; NCBI: National centre for biotechnology information; RIN: RNA integrity number; RPKM: Reads per exon kilobase per million; SAR: Systemtic acquired resistance; TGICL: TIGR gene indices clustering tool.

## Competing interests

The authors declare that they have no competing interests.

## Authors’ contributions

RKC generated and provided the plant material. JB raised the plant material and carried out entire wet lab experiments. JB and MKS prepared cDNA library for Illumina sequencing and performed sequencing run on Illumina. RC performed the entire computational part of this study. RS developed the computational analysis part protocols, tools, algorithms and supervised the entire computational part of the study. JB conducted the qRT-PCR experiments. AKS supervised Illumina sequencing and qRT-PCR, data interpretation and analysis. JB and SKY designed the experiments, interpreted and analyzed the results. SKY conceptualized and mentored the whole study. JB, RC wrote the MS. RS and SKY edited and approved the MS. All authors have read and approved the manuscript.

## Supplementary Material

Additional file 1 Details of the primers used for qRT-PCR. Click here for file

Additional file 2 Calculation of various parameters to select best k-mer size. Click here for file

Additional file 3 Assembled sequences of horse gram. Click here for file

Additional file 4 Dissimilar sequence clustering information. Click here for file

Additional file 5 Unigenes related information. Click here for file

Additional file 6 Experimentally validated ESTs of horse gram. Click here for file

Additional file 7 Assembly validation results. Click here for file

Additional file 8 Guanine-cytosine (GC) content related information. Click here for file

Additional file 9 Functional annotation details. Click here for file

Additional file 10 Differential expression of all the unigenes. Click here for file

Additional file 11 Top-20 biological processes in comparative conditions. Click here for file

Additional file 12 Top-20 molecular functions in comparative conditions. Click here for file

Additional file 13 AgriGO enrichment analysis. Click here for file

Additional file 14 Top-20 enzyme classes in comparative conditions. Click here for file

Additional file 15 Top-20 KEGG pathways in comparative conditions. Click here for file

Additional file 16 A and B: Stress related up-regulated PMN pathways. Click here for file

Additional file 17 Details of Transcription factor families. Click here for file

Additional file 18 Top-20 transcription factor families in comparative conditions. Click here for file

Additional file 19 RPS BLAST results for unknown sequences. Click here for file
