# Supplementary figures and images for: Comprehensive transcriptomic study on horse gram (Macrotyloma uniflorum): De novo assembly, functional characterization and comparative analysis in relation to drought stress (part 1 of 4)
Source: BMC Genomics. 2013 Sep 23;14:647. doi: 10.1186/1471-2164-14-647 (PMC3853109; doi:10.1186/1471-2164-14-647)

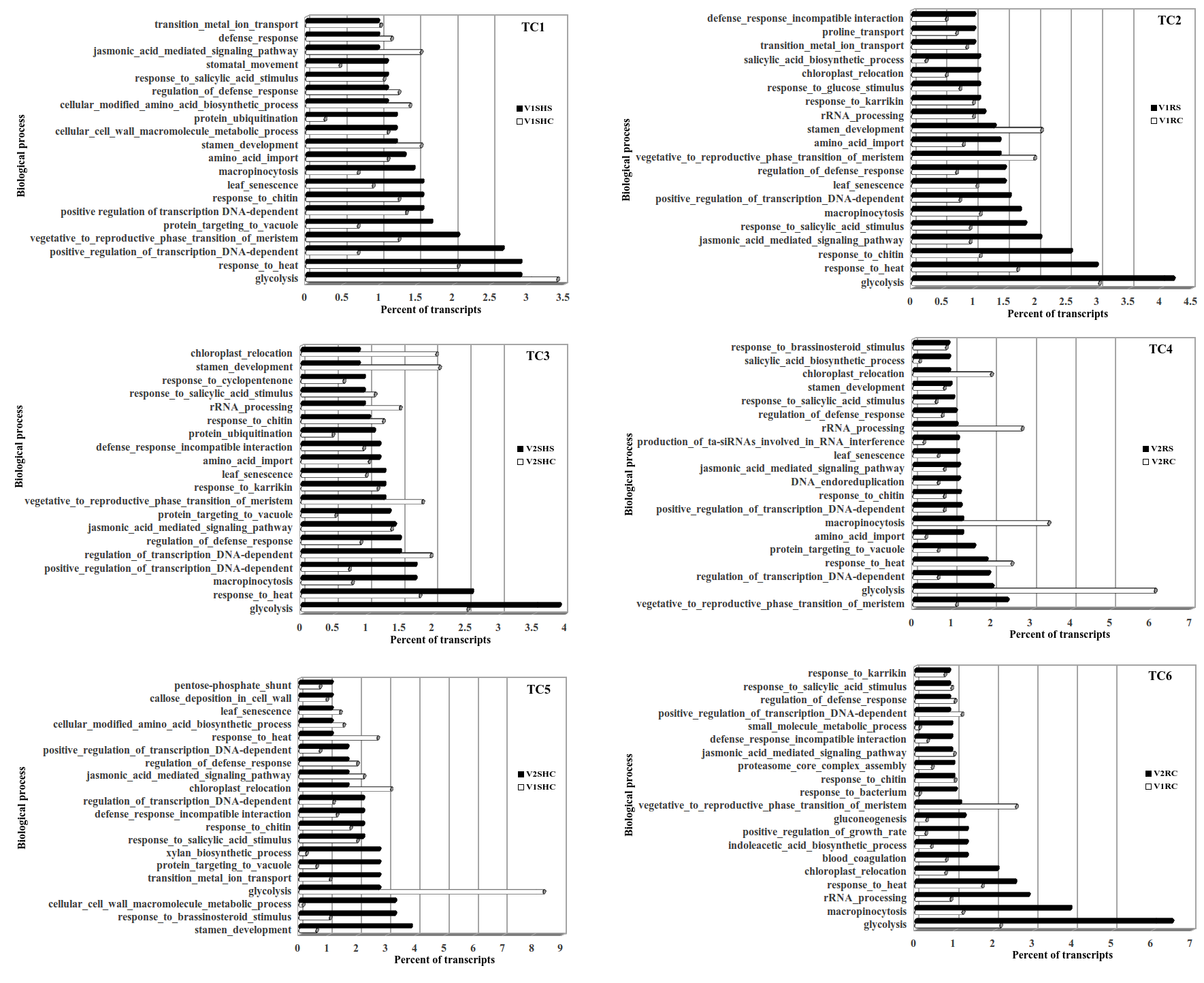

Supplement: Additional file 11 — Top-20 biological processes in comparative conditions. [file 1471-2164-14-647-S11.png]

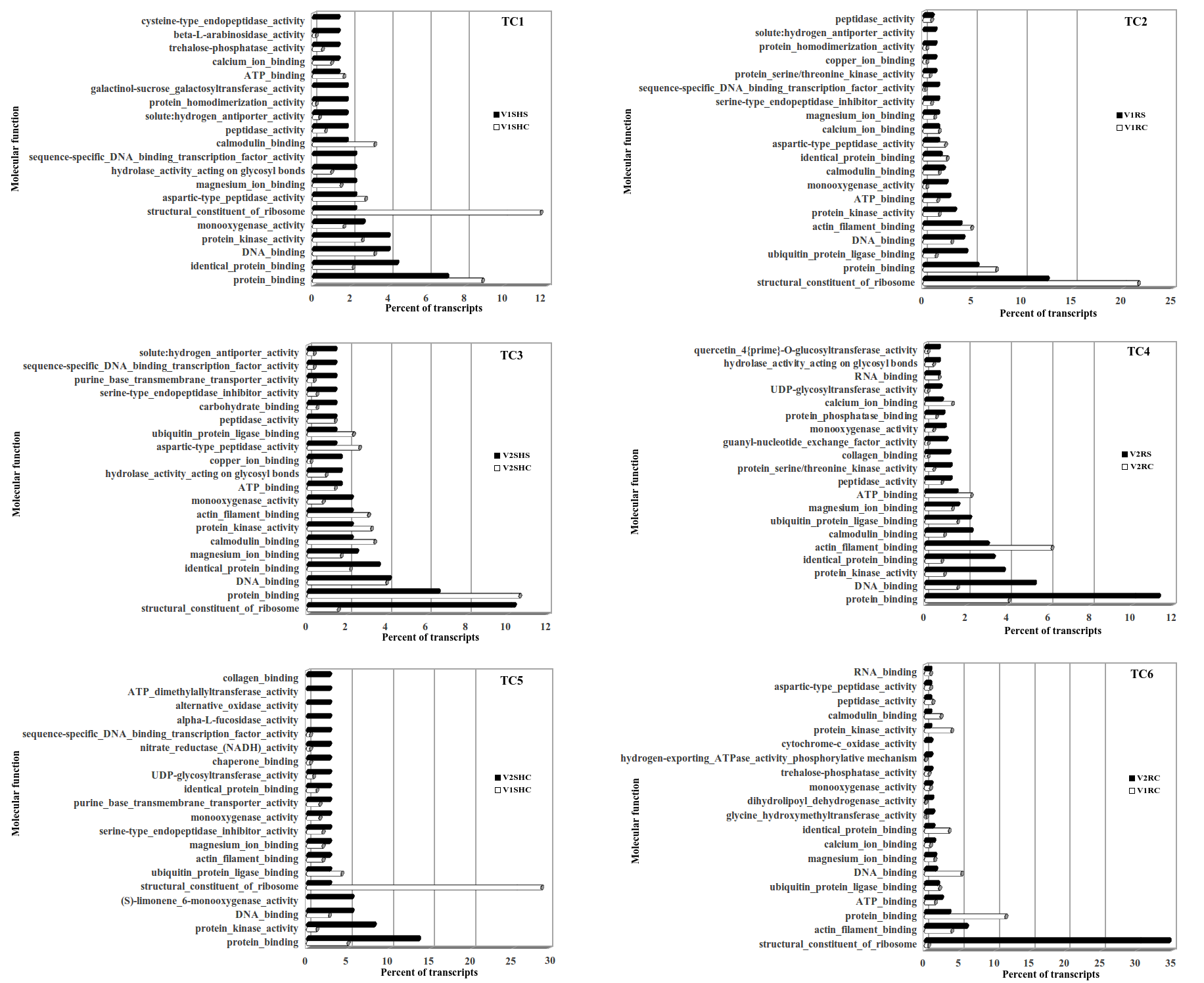

Supplement: Additional file 12 — Top-20 molecular functions in comparative conditions. [file 1471-2164-14-647-S12.png]

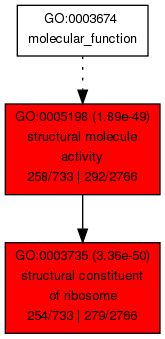

Supplement: Additional file 13 — AgriGO enrichment analysis. [file 1471-2164-14-647-S13.zip › additional_file12_enrichment_images/TC6_V1RC_V2RC/Molecular_function_V2RC_i_V1RC_V2RC.png]

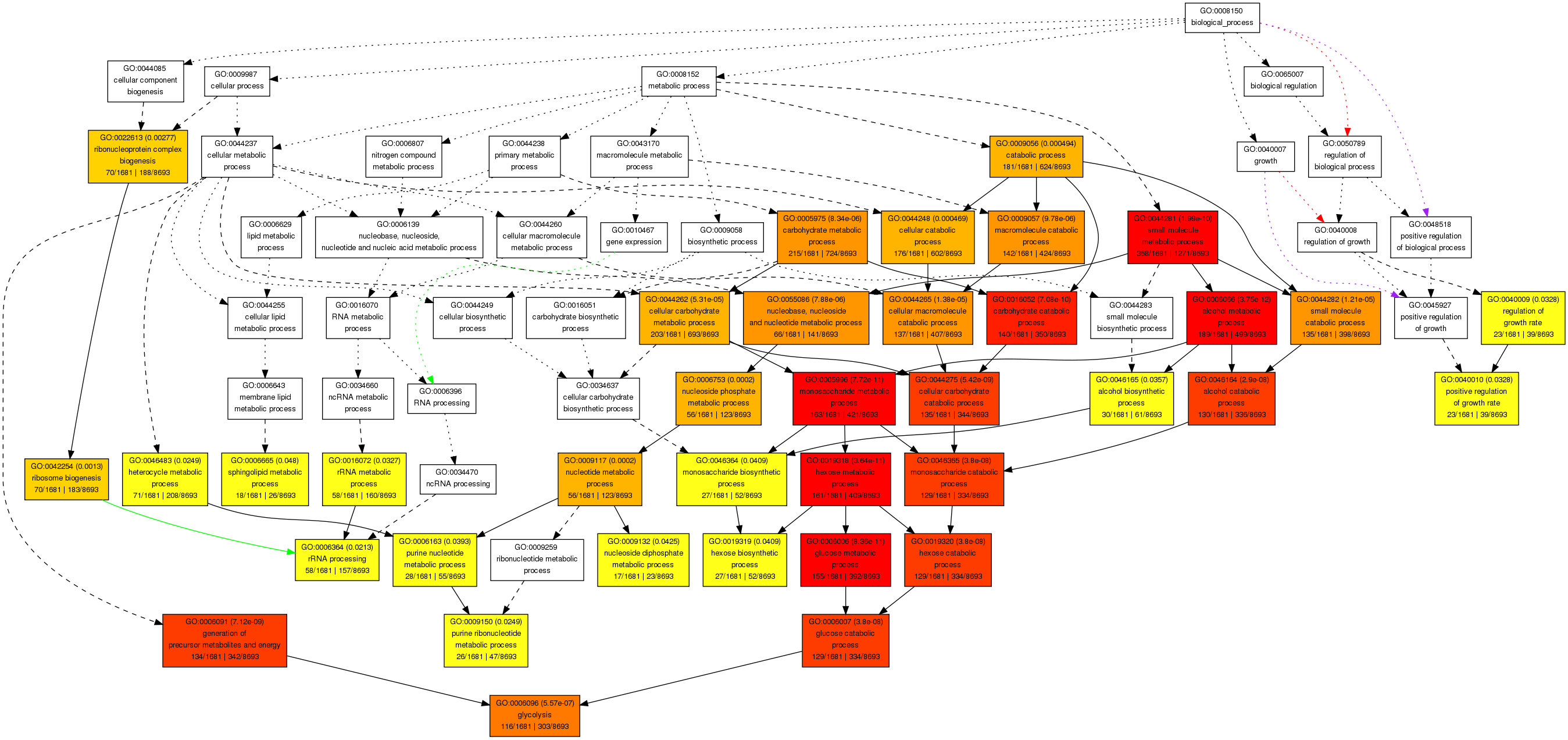

Supplement: Additional file 13 — AgriGO enrichment analysis. [file 1471-2164-14-647-S13.zip › additional_file12_enrichment_images/TC6_V1RC_V2RC/Biological_process_V2RC_i_V1RC_V2RC.png]

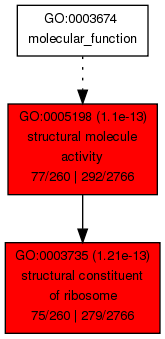

Supplement: Additional file 13 — AgriGO enrichment analysis. [file 1471-2164-14-647-S13.zip › additional_file12_enrichment_images/TC5_V2SHC_V1SHC/Molecular_function_V1SH1C_i_V1SH1C_V2SH1C.png]

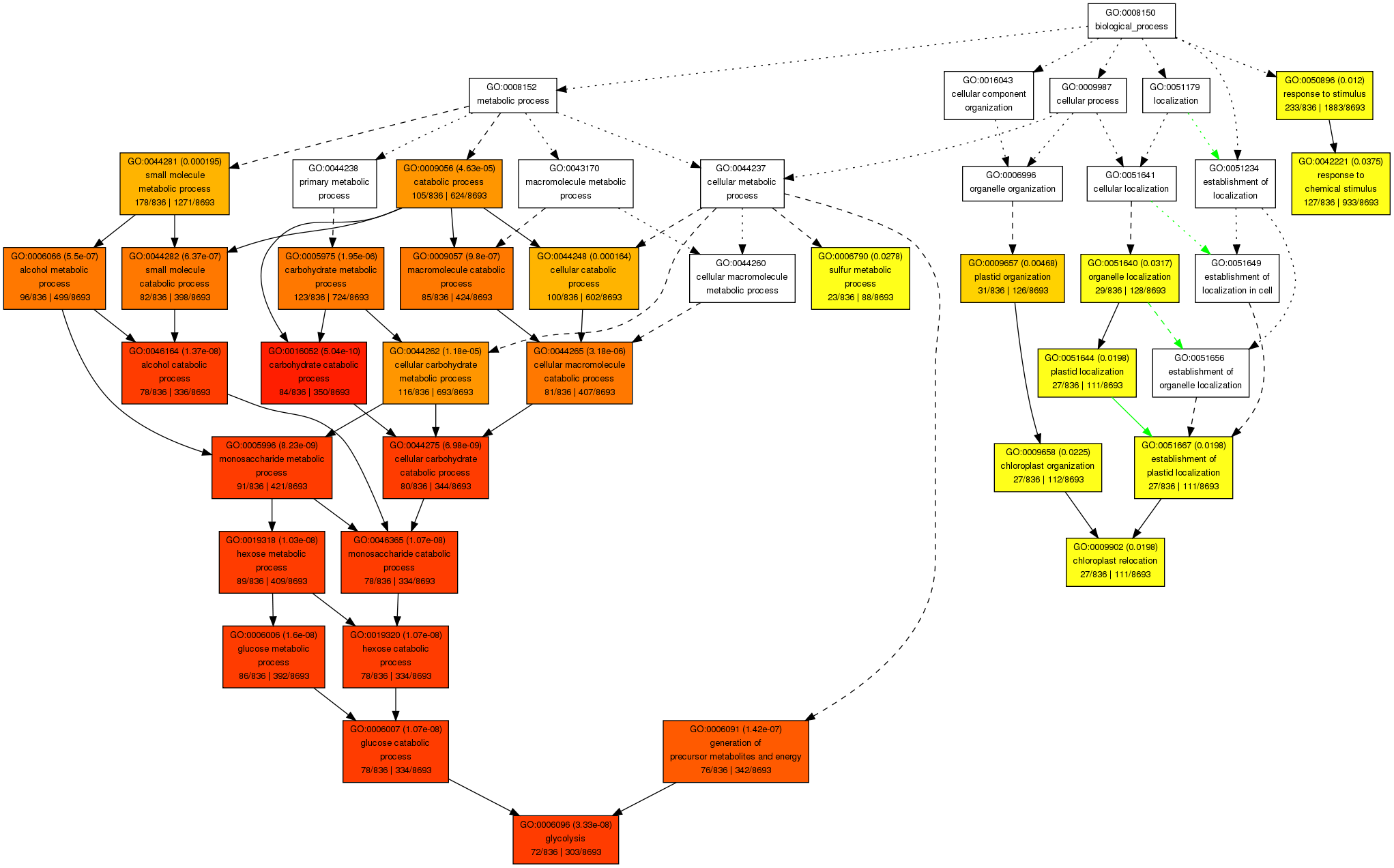

Supplement: Additional file 13 — AgriGO enrichment analysis. [file 1471-2164-14-647-S13.zip › additional_file12_enrichment_images/TC5_V2SHC_V1SHC/Biological_process_V1SH1C_i_V1SH1C_V2SH1C.png]

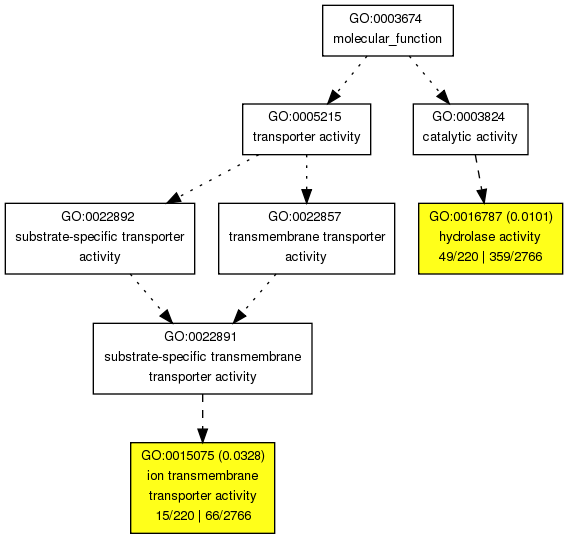

Supplement: Additional file 13 — AgriGO enrichment analysis. [file 1471-2164-14-647-S13.zip › additional_file12_enrichment_images/TC1_Molecular_function_V1SHS.png]

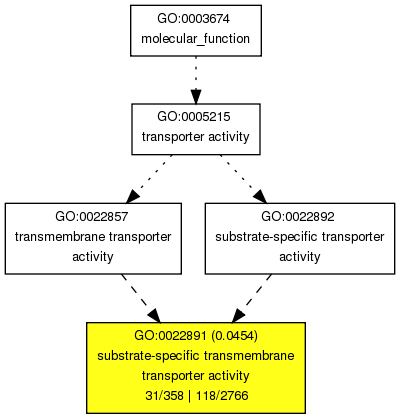

Supplement: Additional file 13 — AgriGO enrichment analysis. [file 1471-2164-14-647-S13.zip › additional_file12_enrichment_images/TC3_Molecular_function_V2SHS.png]

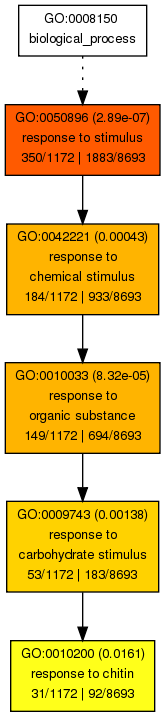

Supplement: Additional file 13 — AgriGO enrichment analysis. [file 1471-2164-14-647-S13.zip › additional_file12_enrichment_images/TC2_Biological_process_V1RS.png]

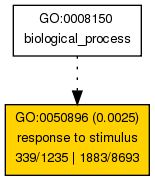

Supplement: Additional file 13 — AgriGO enrichment analysis. [file 1471-2164-14-647-S13.zip › additional_file12_enrichment_images/TC3_Biological_process_V2SHS.png]

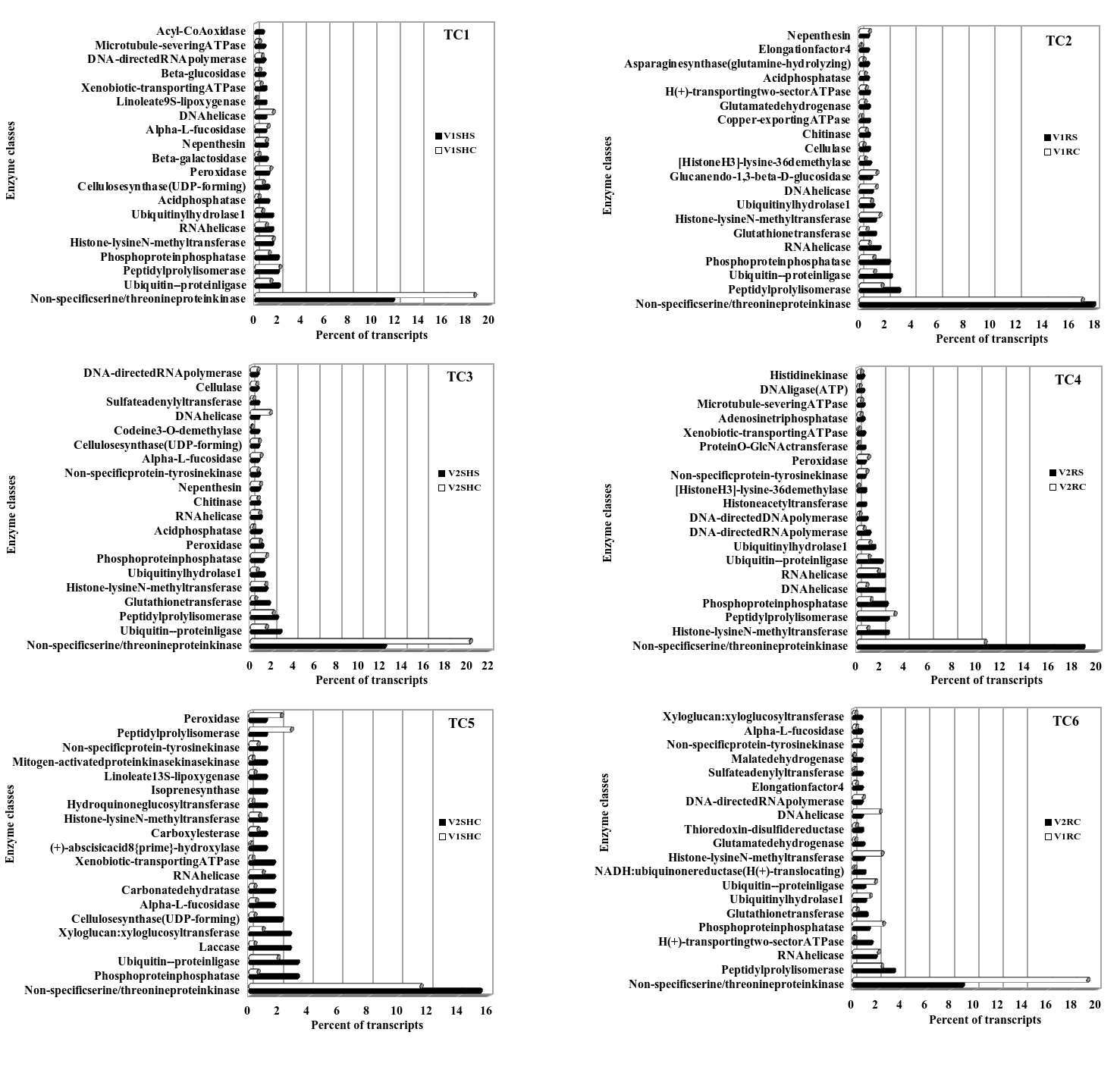

Supplement: Additional file 14 — Top-20 enzyme classes in comparative conditions. [file 1471-2164-14-647-S14.png]

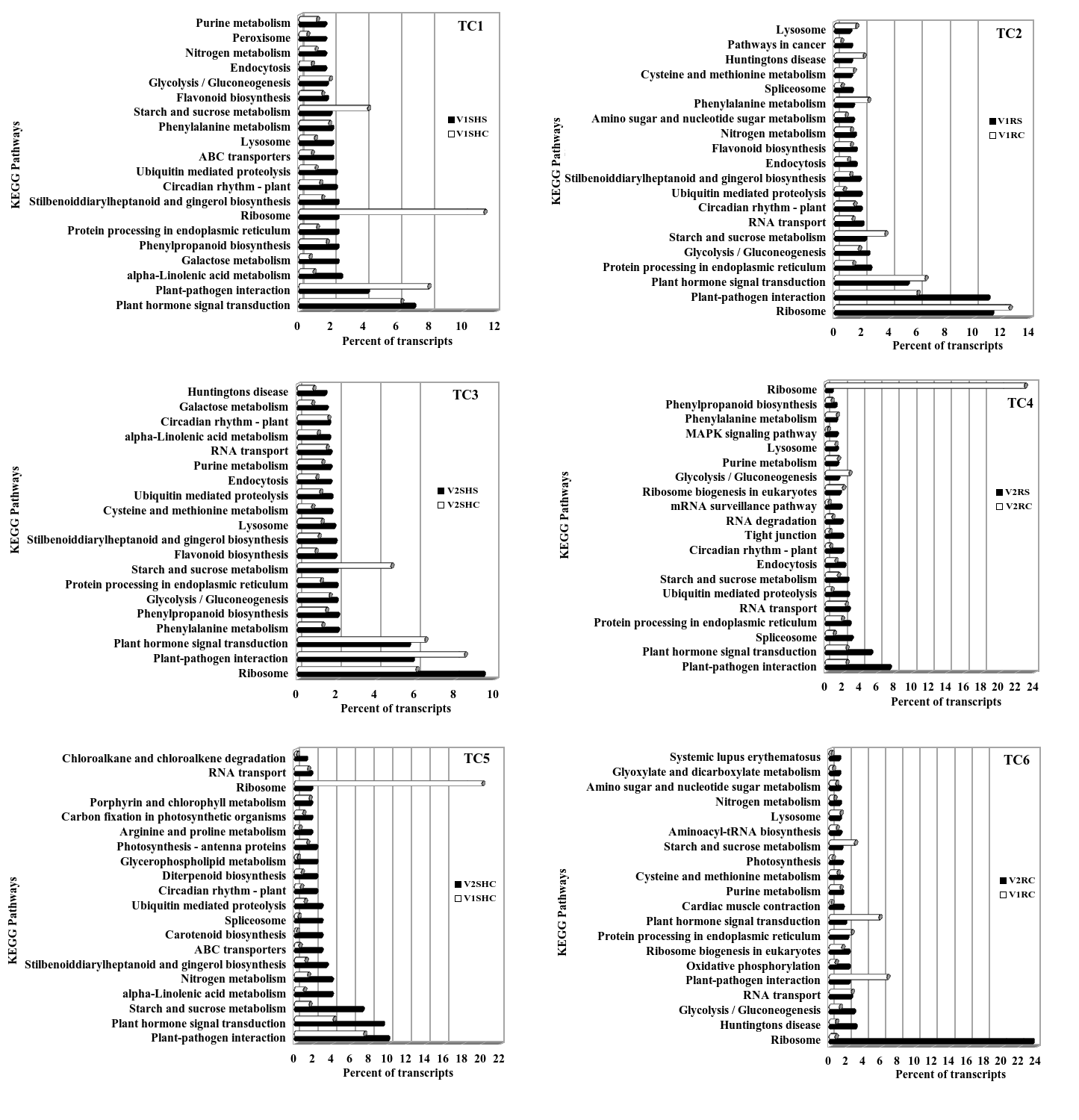

Supplement: Additional file 15 — Top-20 KEGG pathways in comparative conditions. [file 1471-2164-14-647-S15.png]

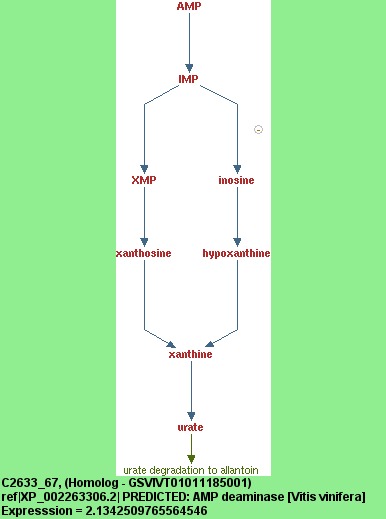

Supplement: Additional file 16 — A and B: Stress related up-regulated PMN pathways. [file 1471-2164-14-647-S16.zip › Additional_file16A_Upregulated_PMN_pathways_in_Shoot/V1SHS/C2633_67_GSVIVT01011185001_1_adenosine_nucleotides_degradation_I.jpg]

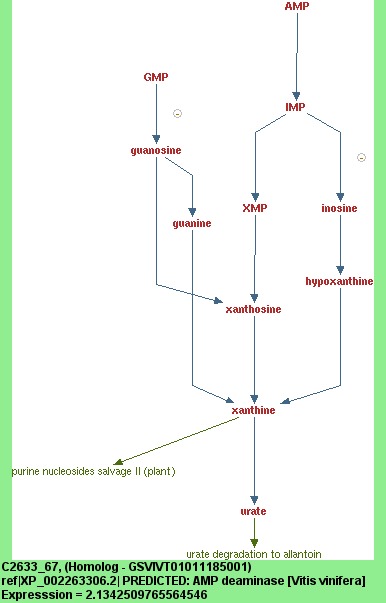

Supplement: Additional file 16 — A and B: Stress related up-regulated PMN pathways. [file 1471-2164-14-647-S16.zip › Additional_file16A_Upregulated_PMN_pathways_in_Shoot/V1SHS/C2633_67_GSVIVT01011185001_2_purine_nucleotides_degradation_I_(plants).jpg]

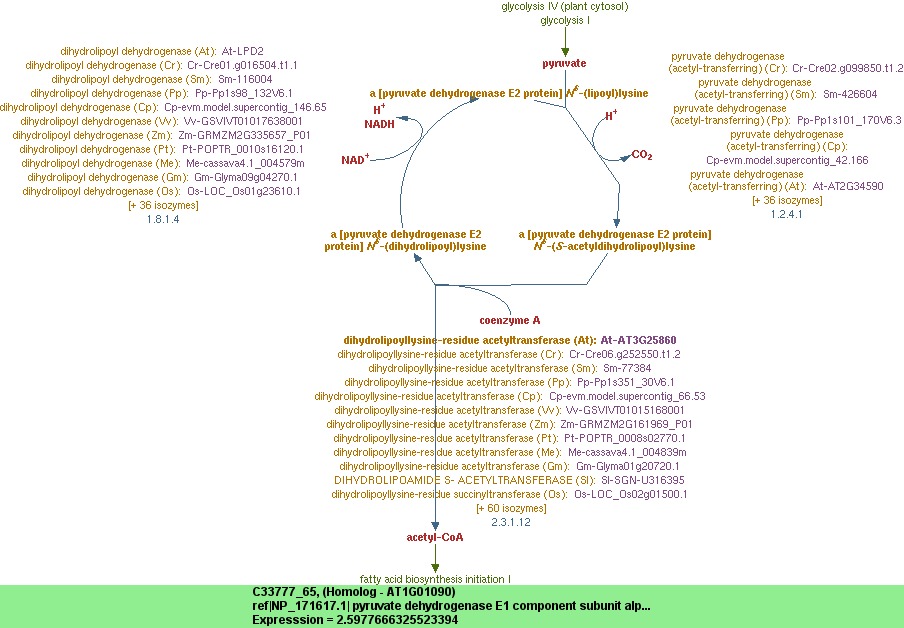

Supplement: Additional file 16 — A and B: Stress related up-regulated PMN pathways. [file 1471-2164-14-647-S16.zip › Additional_file16A_Upregulated_PMN_pathways_in_Shoot/V1SHS/C33777_65_AT1G01090_1_acetyl-CoA_biosynthesis_I_(pyruvate_dehydrogenase_complex).jpg]

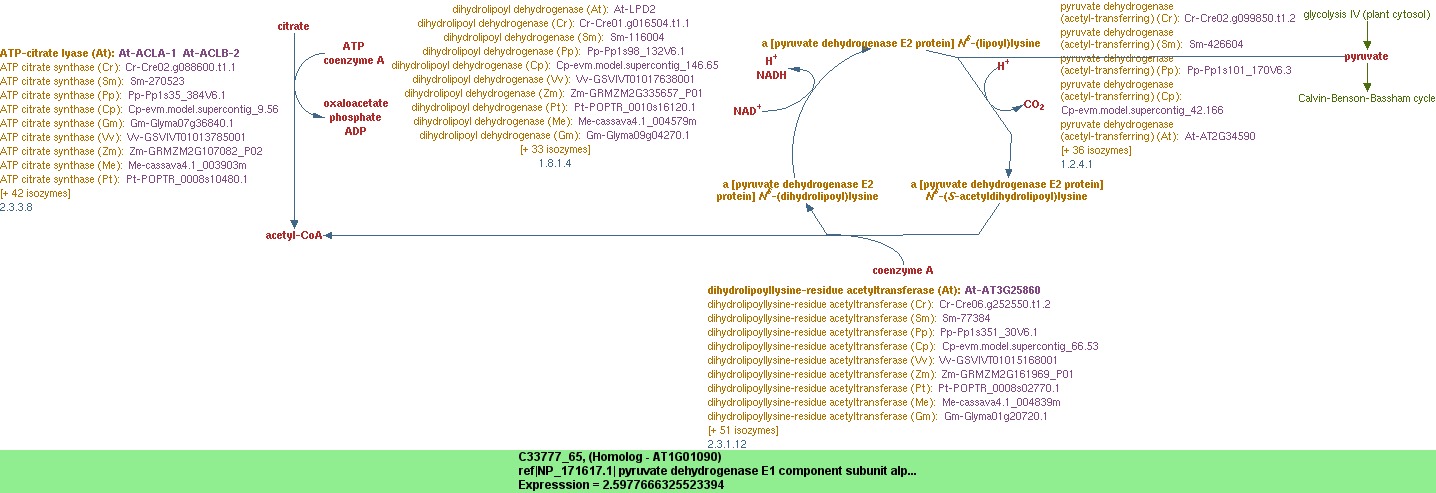

Supplement: Additional file 16 — A and B: Stress related up-regulated PMN pathways. [file 1471-2164-14-647-S16.zip › Additional_file16A_Upregulated_PMN_pathways_in_Shoot/V1SHS/C33777_65_AT1G01090_3_superpathway_of_acetyl-CoA_biosynthesis.jpg]

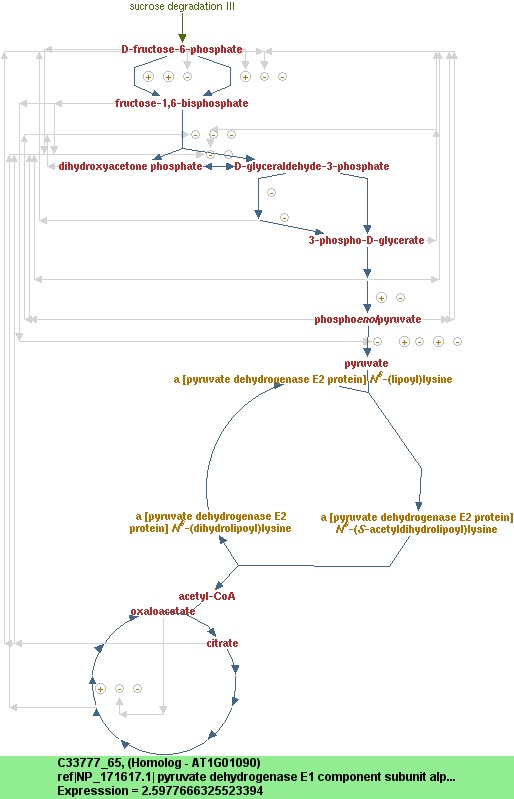

Supplement: Additional file 16 — A and B: Stress related up-regulated PMN pathways. [file 1471-2164-14-647-S16.zip › Additional_file16A_Upregulated_PMN_pathways_in_Shoot/V1SHS/C33777_65_AT1G01090_5_superpathway_of_cytosolic_glycolysis_(plants),_pyruvate_dehydrogenase_and_TCA_cycle.jpg]

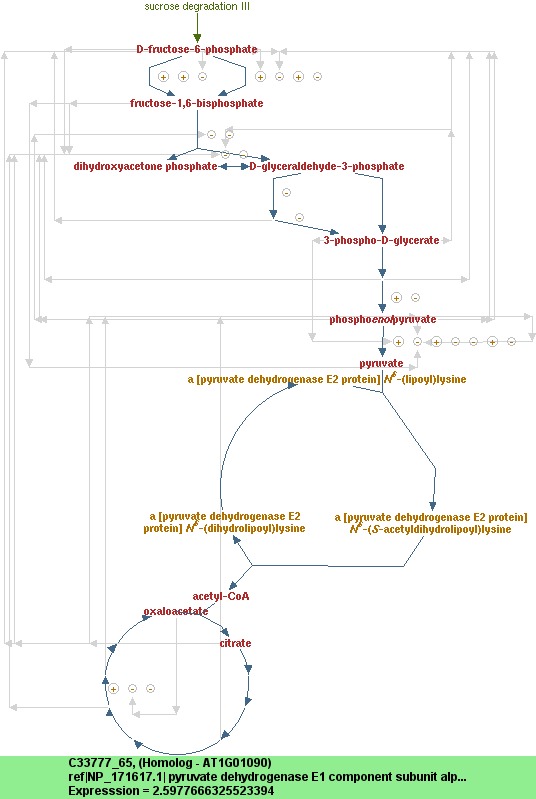

Supplement: Additional file 16 — A and B: Stress related up-regulated PMN pathways. [file 1471-2164-14-647-S16.zip › Additional_file16A_Upregulated_PMN_pathways_in_Shoot/V1SHS/C33777_65_AT1G01090_7_superpathway_of_cytosolic_glycolysis_(plants),_pyruvate_dehydrogenase_and_TCA_cycle.jpg]

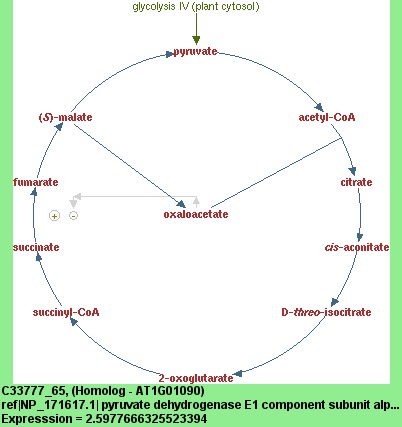

Supplement: Additional file 16 — A and B: Stress related up-regulated PMN pathways. [file 1471-2164-14-647-S16.zip › Additional_file16A_Upregulated_PMN_pathways_in_Shoot/V1SHS/C33777_65_AT1G01090_9_TCA_cycle_variation_V_(plant).jpg]

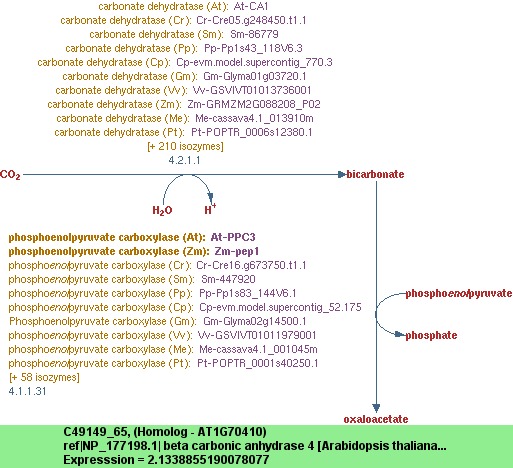

Supplement: Additional file 16 — A and B: Stress related up-regulated PMN pathways. [file 1471-2164-14-647-S16.zip › Additional_file16A_Upregulated_PMN_pathways_in_Shoot/V1SHS/C49149_65_AT1G70410_1_CO_fixation_into_oxaloacetate_(anapleurotic).jpg]

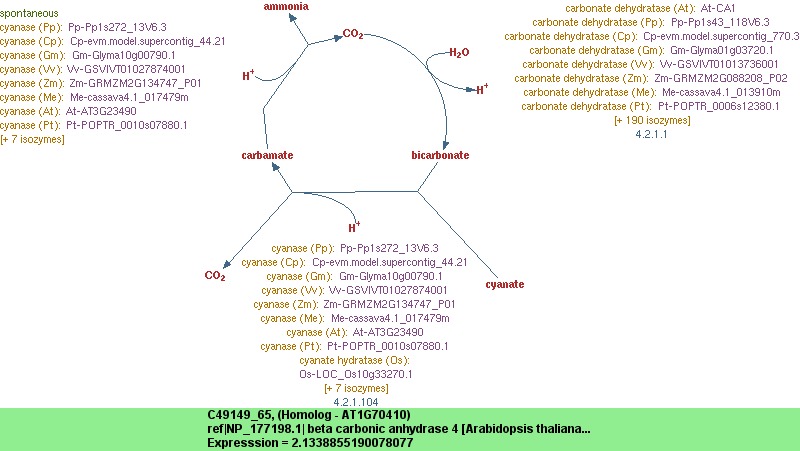

Supplement: Additional file 16 — A and B: Stress related up-regulated PMN pathways. [file 1471-2164-14-647-S16.zip › Additional_file16A_Upregulated_PMN_pathways_in_Shoot/V1SHS/C49149_65_AT1G70410_3_cyanate_degradation.jpg]

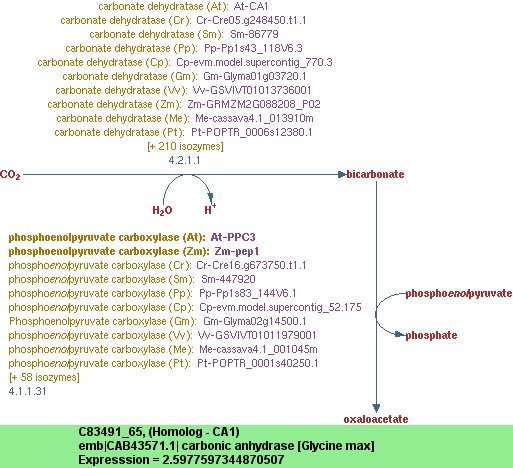

Supplement: Additional file 16 — A and B: Stress related up-regulated PMN pathways. [file 1471-2164-14-647-S16.zip › Additional_file16A_Upregulated_PMN_pathways_in_Shoot/V1SHS/C83491_65_CA1_1_CO_fixation_into_oxaloacetate_(anapleurotic).jpg]

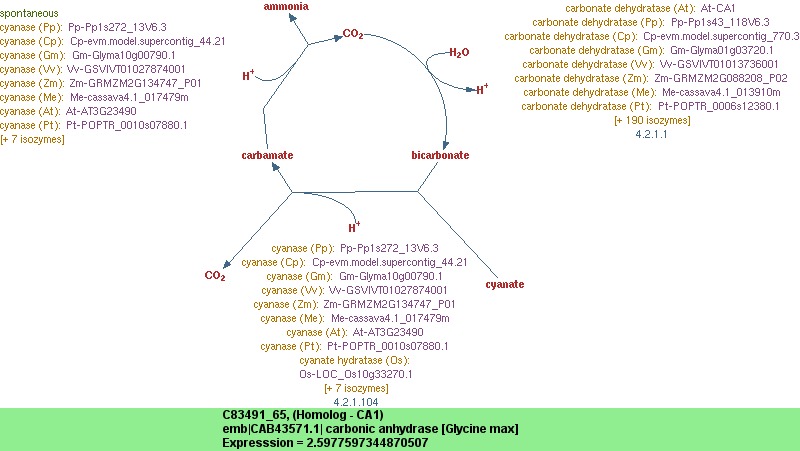

Supplement: Additional file 16 — A and B: Stress related up-regulated PMN pathways. [file 1471-2164-14-647-S16.zip › Additional_file16A_Upregulated_PMN_pathways_in_Shoot/V1SHS/C83491_65_CA1_2_cyanate_degradation.jpg]

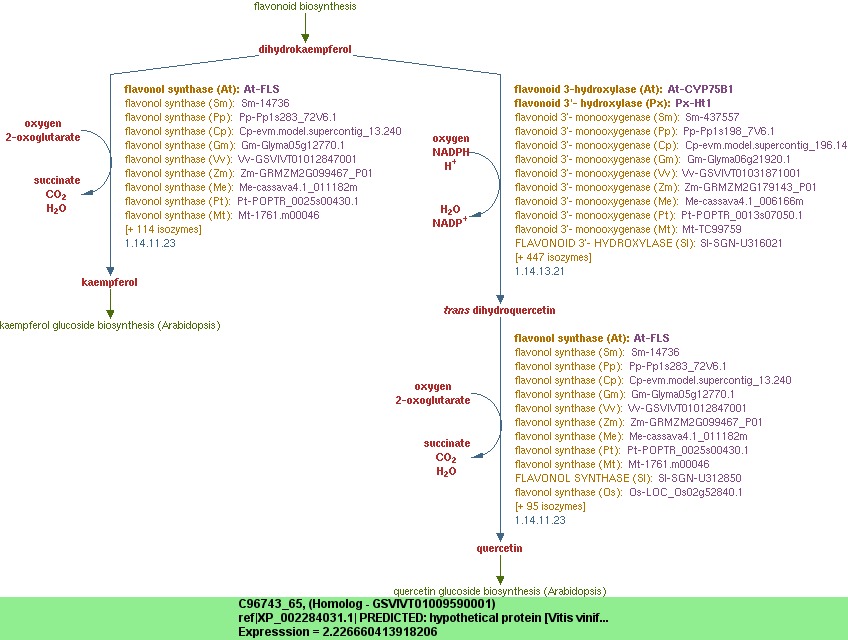

Supplement: Additional file 16 — A and B: Stress related up-regulated PMN pathways. [file 1471-2164-14-647-S16.zip › Additional_file16A_Upregulated_PMN_pathways_in_Shoot/V1SHS/C96743_65_GSVIVT01009590001_1_flavonol_biosynthesis.jpg]

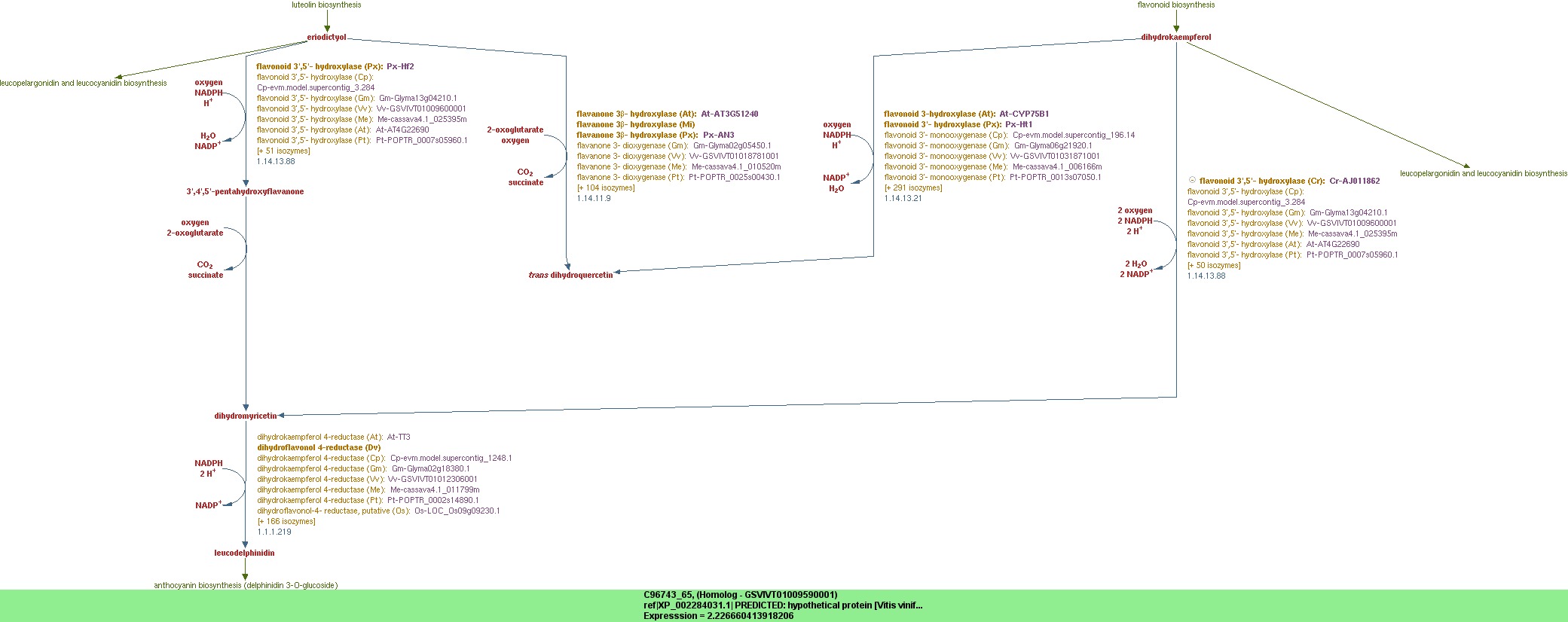

Supplement: Additional file 16 — A and B: Stress related up-regulated PMN pathways. [file 1471-2164-14-647-S16.zip › Additional_file16A_Upregulated_PMN_pathways_in_Shoot/V1SHS/C96743_65_GSVIVT01009590001_2_leucodelphinidin_biosynthesis.jpg]

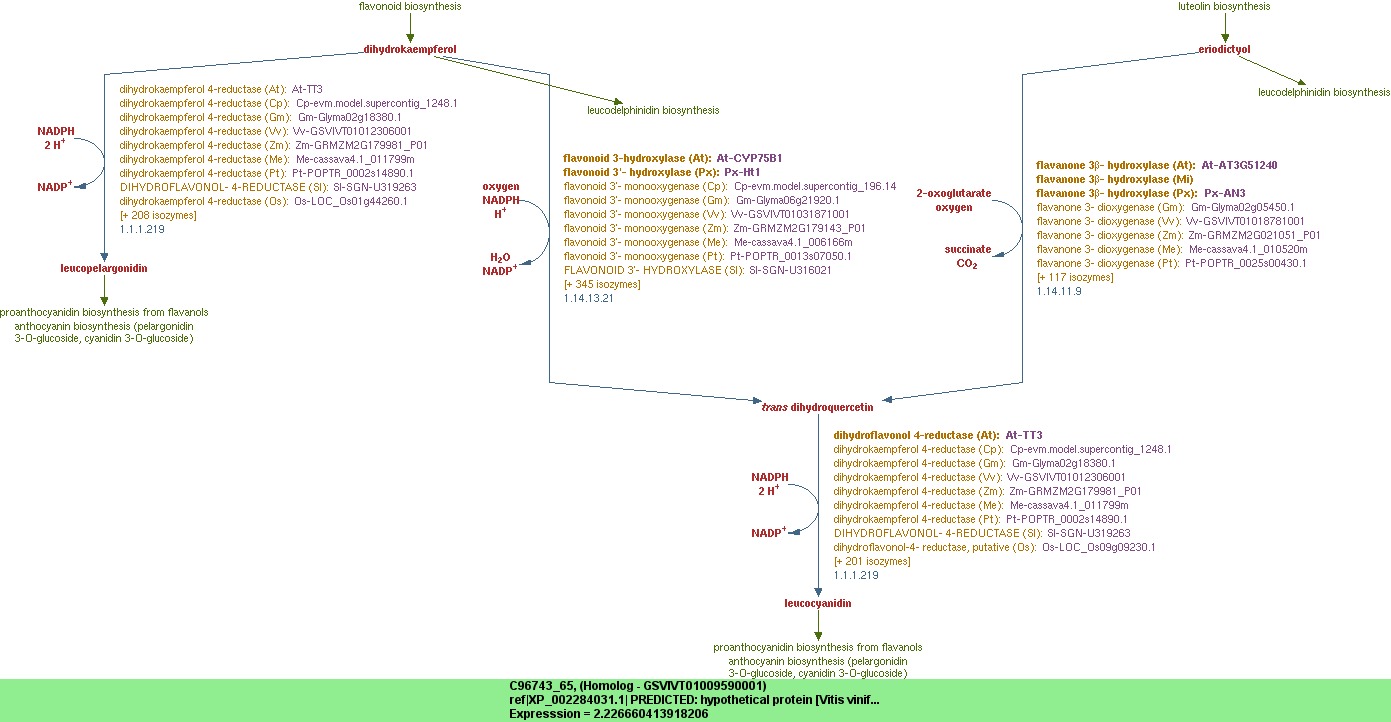

Supplement: Additional file 16 — A and B: Stress related up-regulated PMN pathways. [file 1471-2164-14-647-S16.zip › Additional_file16A_Upregulated_PMN_pathways_in_Shoot/V1SHS/C96743_65_GSVIVT01009590001_3_leucopelargonidin_and_leucocyanidin_biosynthesis.jpg]

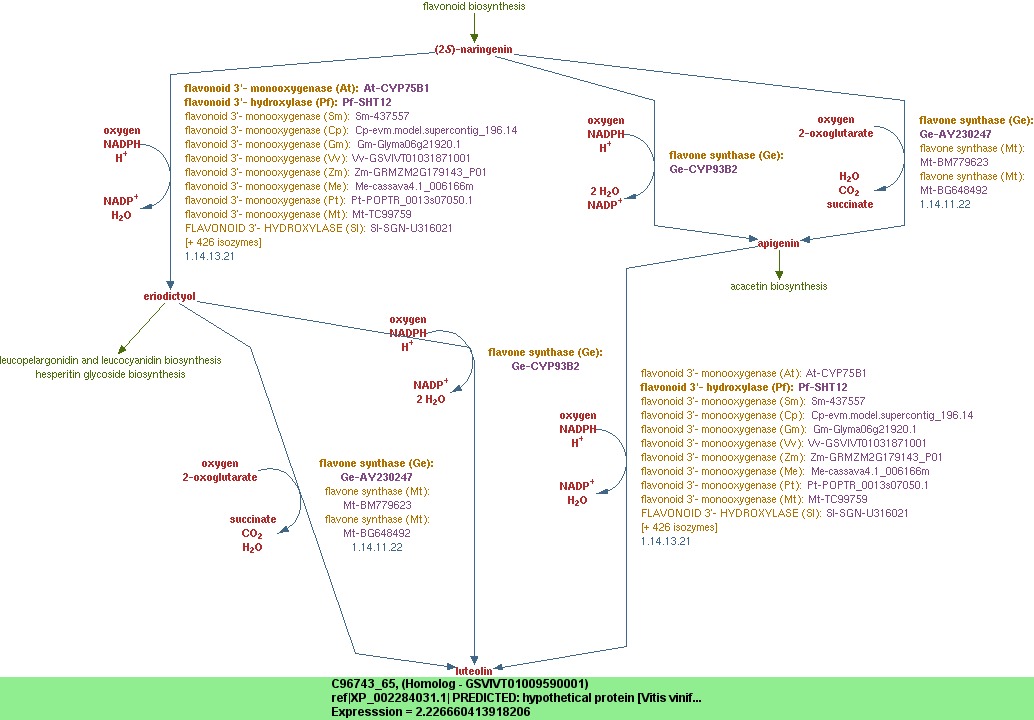

Supplement: Additional file 16 — A and B: Stress related up-regulated PMN pathways. [file 1471-2164-14-647-S16.zip › Additional_file16A_Upregulated_PMN_pathways_in_Shoot/V1SHS/C96743_65_GSVIVT01009590001_4_luteolin_biosynthesis.jpg]

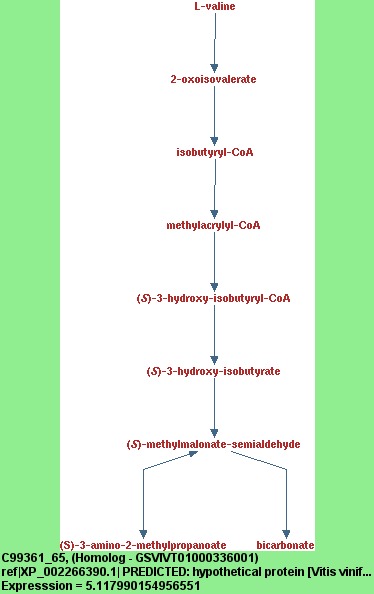

Supplement: Additional file 16 — A and B: Stress related up-regulated PMN pathways. [file 1471-2164-14-647-S16.zip › Additional_file16A_Upregulated_PMN_pathways_in_Shoot/V1SHS/C99361_65_GSVIVT01000336001_1_valine_degradation_I.jpg]

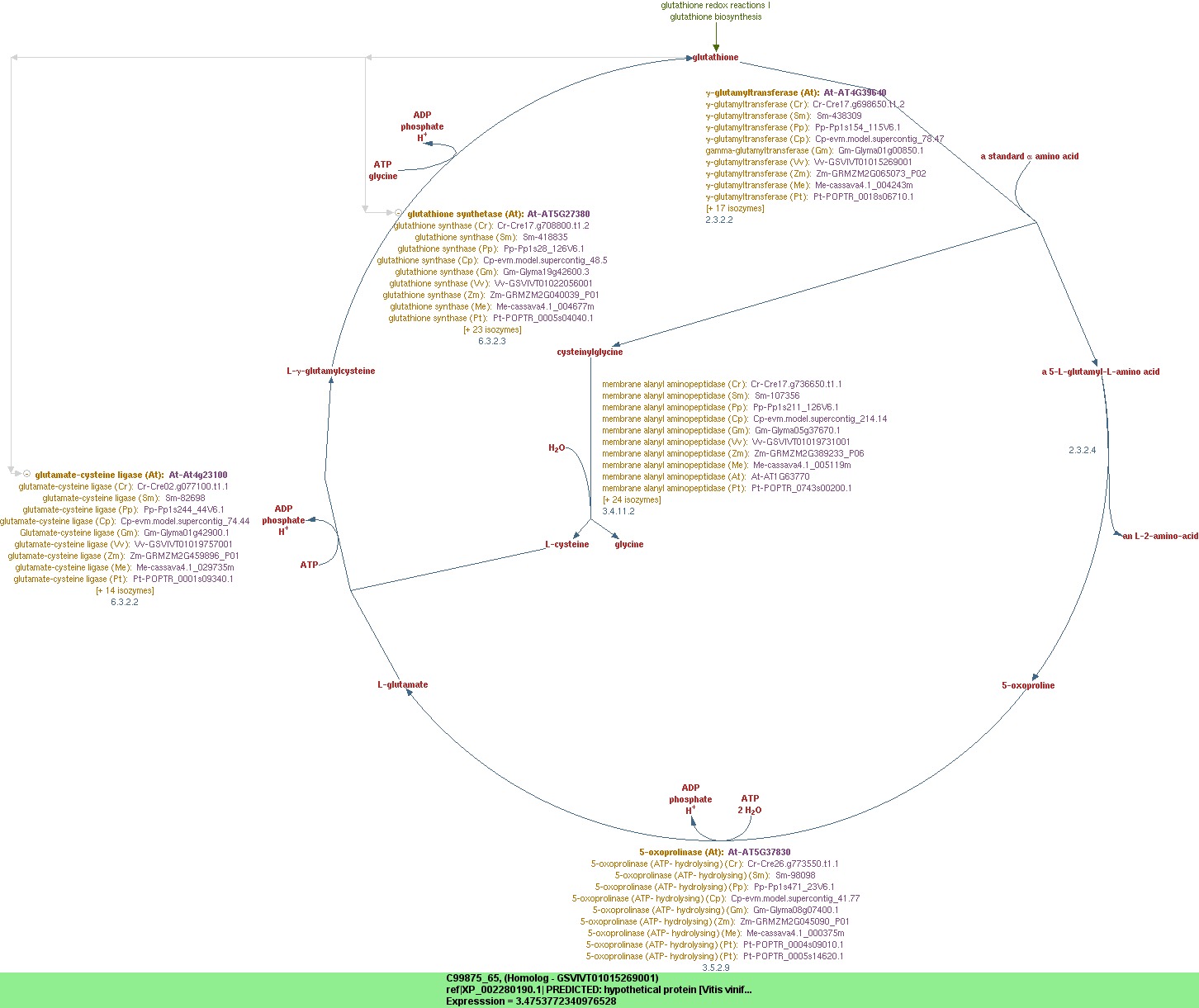

Supplement: Additional file 16 — A and B: Stress related up-regulated PMN pathways. [file 1471-2164-14-647-S16.zip › Additional_file16A_Upregulated_PMN_pathways_in_Shoot/V1SHS/C99875_65_GSVIVT01015269001_1_&gamma-glutamyl_cycle.jpg]

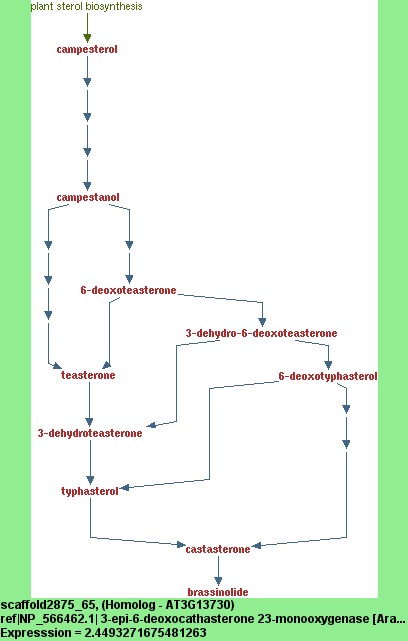

Supplement: Additional file 16 — A and B: Stress related up-regulated PMN pathways. [file 1471-2164-14-647-S16.zip › Additional_file16A_Upregulated_PMN_pathways_in_Shoot/V1SHS/scaffold2875_65_AT3G13730_1_brassinosteroid_biosynthesis_I.jpg]

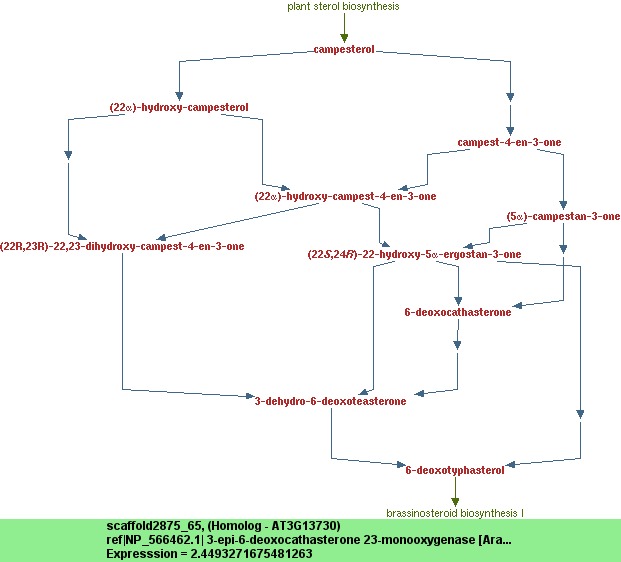

Supplement: Additional file 16 — A and B: Stress related up-regulated PMN pathways. [file 1471-2164-14-647-S16.zip › Additional_file16A_Upregulated_PMN_pathways_in_Shoot/V1SHS/scaffold2875_65_AT3G13730_2_brassinosteroid_biosynthesis_II.jpg]

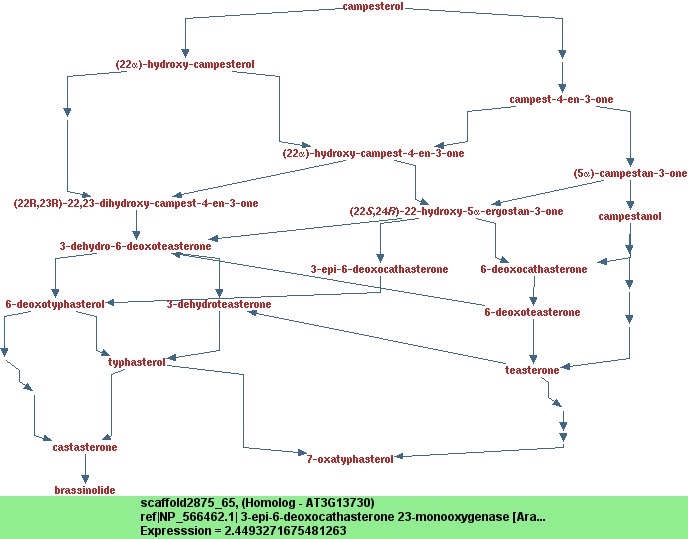

Supplement: Additional file 16 — A and B: Stress related up-regulated PMN pathways. [file 1471-2164-14-647-S16.zip › Additional_file16A_Upregulated_PMN_pathways_in_Shoot/V1SHS/scaffold2875_65_AT3G13730_3_superpathway_of_C28_brassinosteroid_biosynthesis.jpg]

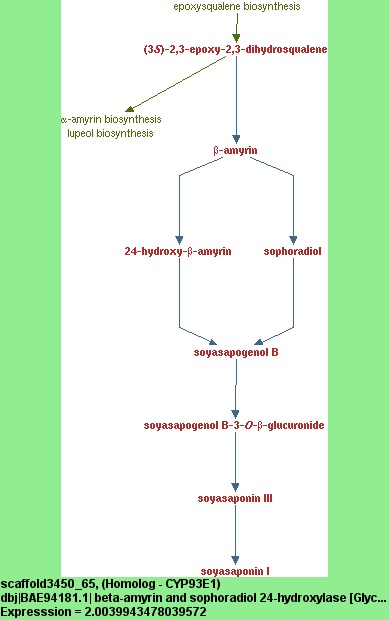

Supplement: Additional file 16 — A and B: Stress related up-regulated PMN pathways. [file 1471-2164-14-647-S16.zip › Additional_file16A_Upregulated_PMN_pathways_in_Shoot/V1SHS/scaffold3450_65_CYP93E1_1_soybean_saponin_I_biosynthesis.jpg]

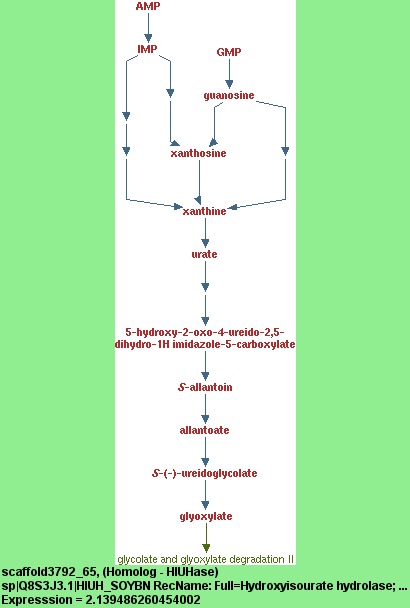

Supplement: Additional file 16 — A and B: Stress related up-regulated PMN pathways. [file 1471-2164-14-647-S16.zip › Additional_file16A_Upregulated_PMN_pathways_in_Shoot/V1SHS/scaffold3792_65_HIUHase_1_superpathway_of_purines_degradation_in_plants.jpg]

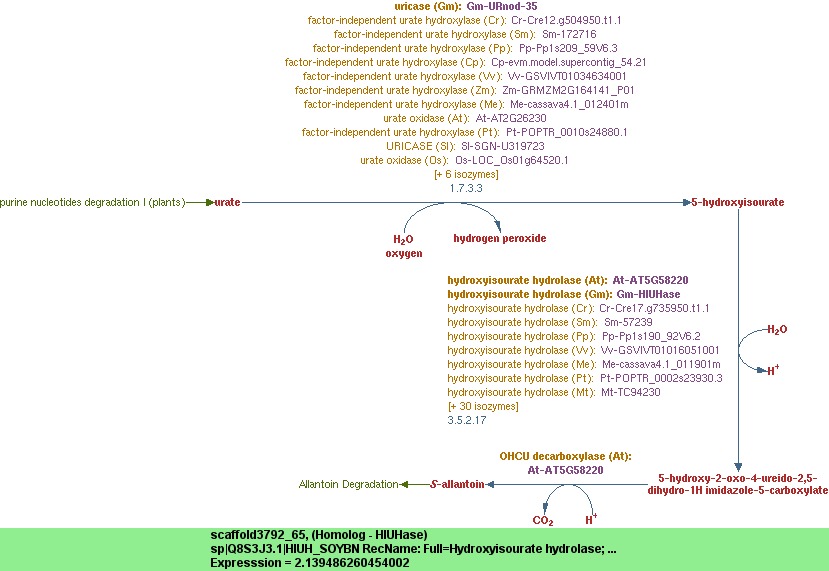

Supplement: Additional file 16 — A and B: Stress related up-regulated PMN pathways. [file 1471-2164-14-647-S16.zip › Additional_file16A_Upregulated_PMN_pathways_in_Shoot/V1SHS/scaffold3792_65_HIUHase_2_urate_degradation_to_allantoin.jpg]

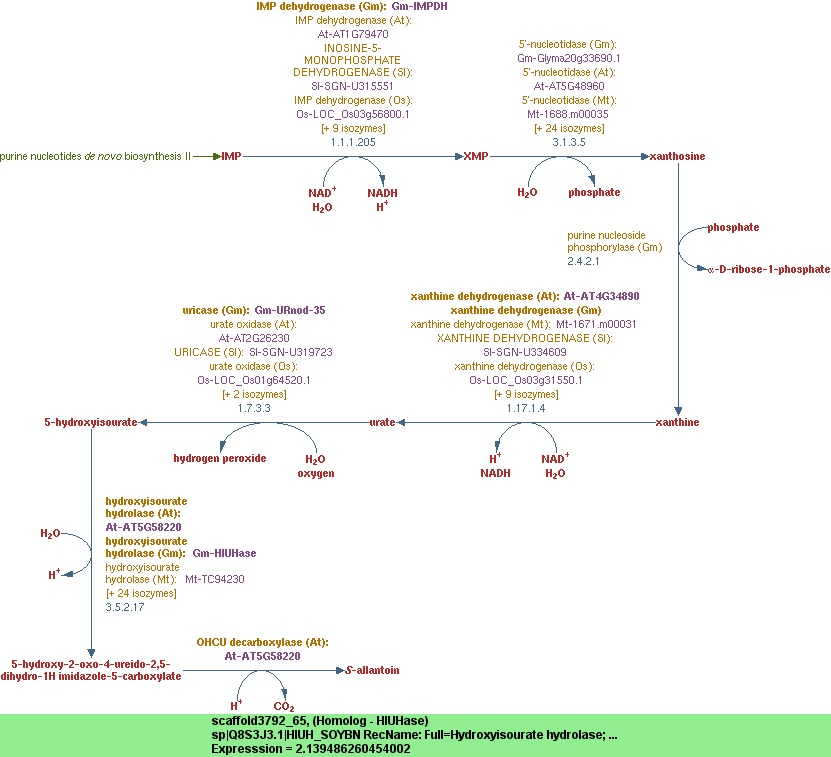

Supplement: Additional file 16 — A and B: Stress related up-regulated PMN pathways. [file 1471-2164-14-647-S16.zip › Additional_file16A_Upregulated_PMN_pathways_in_Shoot/V1SHS/scaffold3792_65_HIUHase_3_ureide_biosynthesis.jpg]

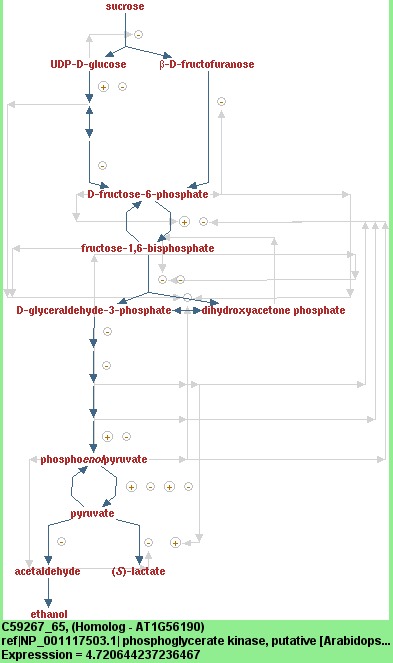

Supplement: Additional file 16 — A and B: Stress related up-regulated PMN pathways. [file 1471-2164-14-647-S16.zip › Additional_file16A_Upregulated_PMN_pathways_in_Shoot/V2SHS/C59267_65_AT1G56190_15_sucrose_degradation_VI_(anaerobic).jpg]

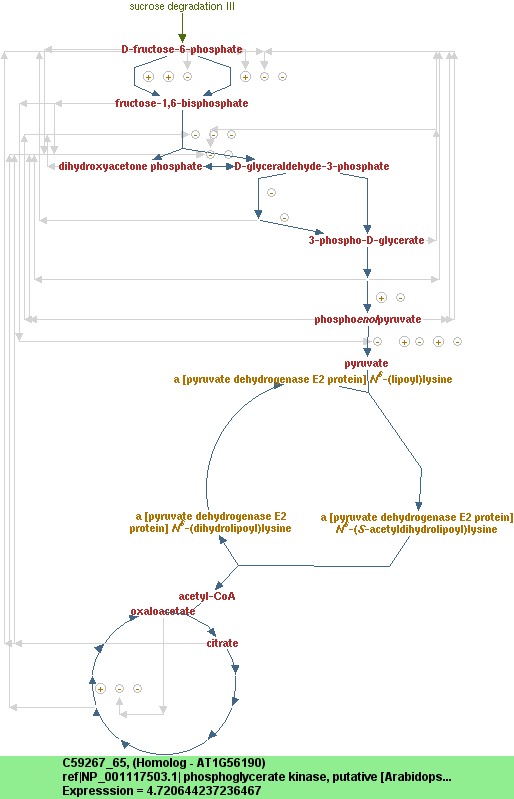

Supplement: Additional file 16 — A and B: Stress related up-regulated PMN pathways. [file 1471-2164-14-647-S16.zip › Additional_file16A_Upregulated_PMN_pathways_in_Shoot/V2SHS/C59267_65_AT1G56190_17_superpathway_of_cytosolic_glycolysis_(plants),_pyruvate_dehydrogenase_and_TCA_cycle.jpg]

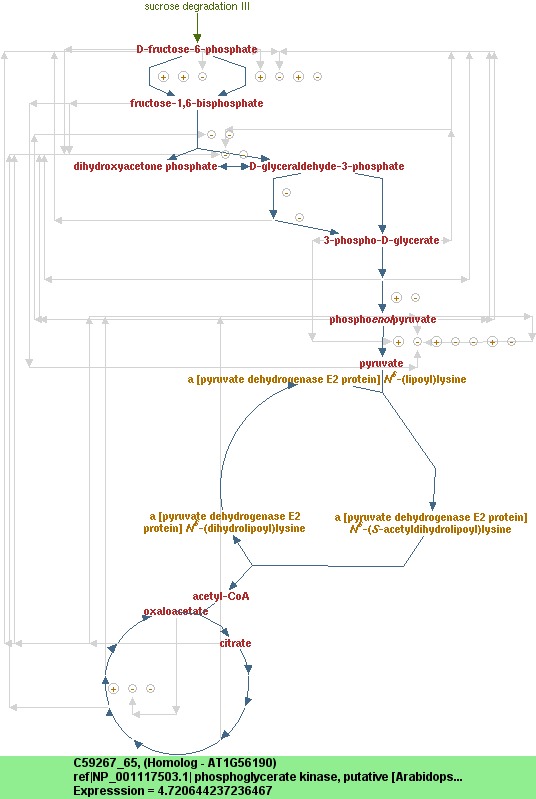

Supplement: Additional file 16 — A and B: Stress related up-regulated PMN pathways. [file 1471-2164-14-647-S16.zip › Additional_file16A_Upregulated_PMN_pathways_in_Shoot/V2SHS/C59267_65_AT1G56190_19_superpathway_of_cytosolic_glycolysis_(plants),_pyruvate_dehydrogenase_and_TCA_cycle.jpg]

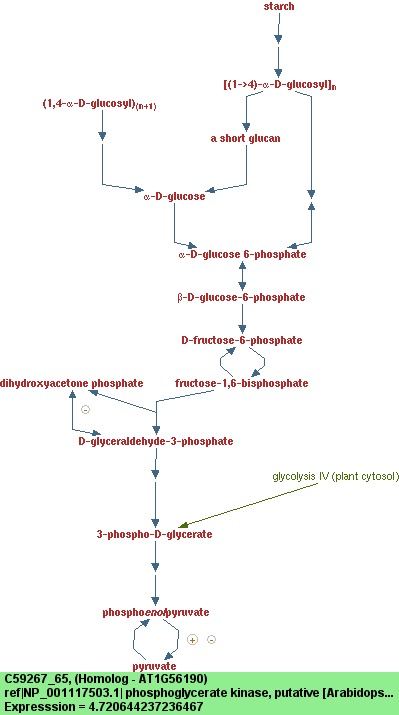

Supplement: Additional file 16 — A and B: Stress related up-regulated PMN pathways. [file 1471-2164-14-647-S16.zip › Additional_file16A_Upregulated_PMN_pathways_in_Shoot/V2SHS/C59267_65_AT1G56190_21_superpathway_of_starch_degradation_to_pyruvate.jpg]

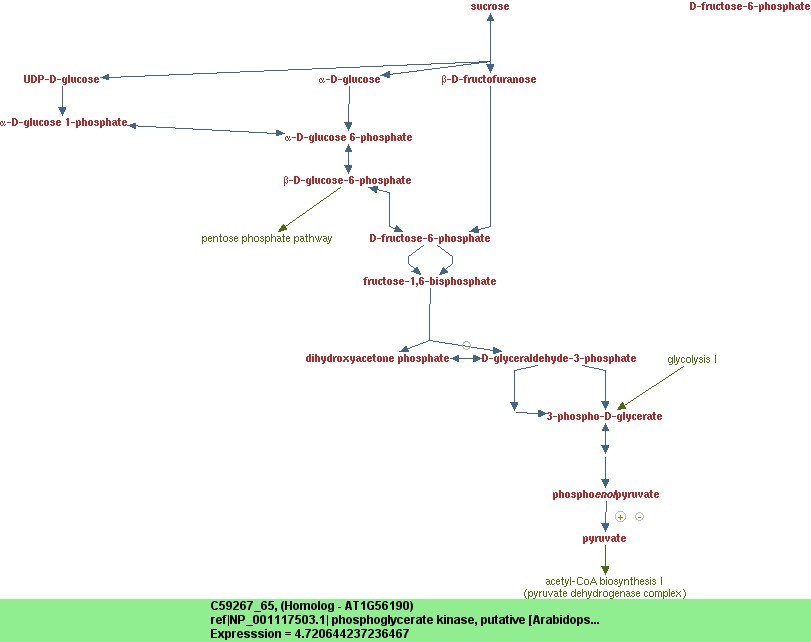

Supplement: Additional file 16 — A and B: Stress related up-regulated PMN pathways. [file 1471-2164-14-647-S16.zip › Additional_file16A_Upregulated_PMN_pathways_in_Shoot/V2SHS/C59267_65_AT1G56190_23_superpathway_of_sucrose_degradation_to_pyruvate.jpg]

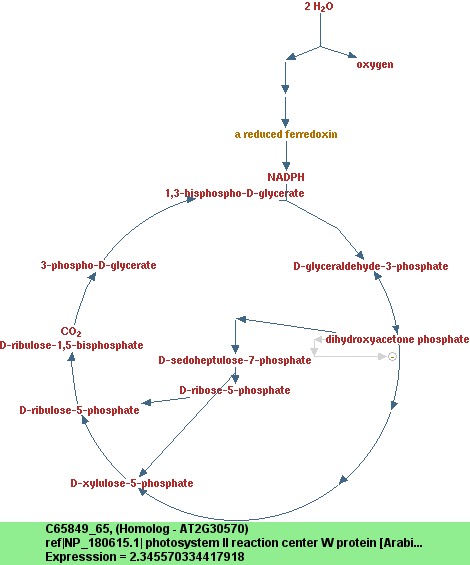

Supplement: Additional file 16 — A and B: Stress related up-regulated PMN pathways. [file 1471-2164-14-647-S16.zip › Additional_file16A_Upregulated_PMN_pathways_in_Shoot/V2SHS/C65849_65_AT2G30570_1_oxygenic_photosynthesis.jpg]

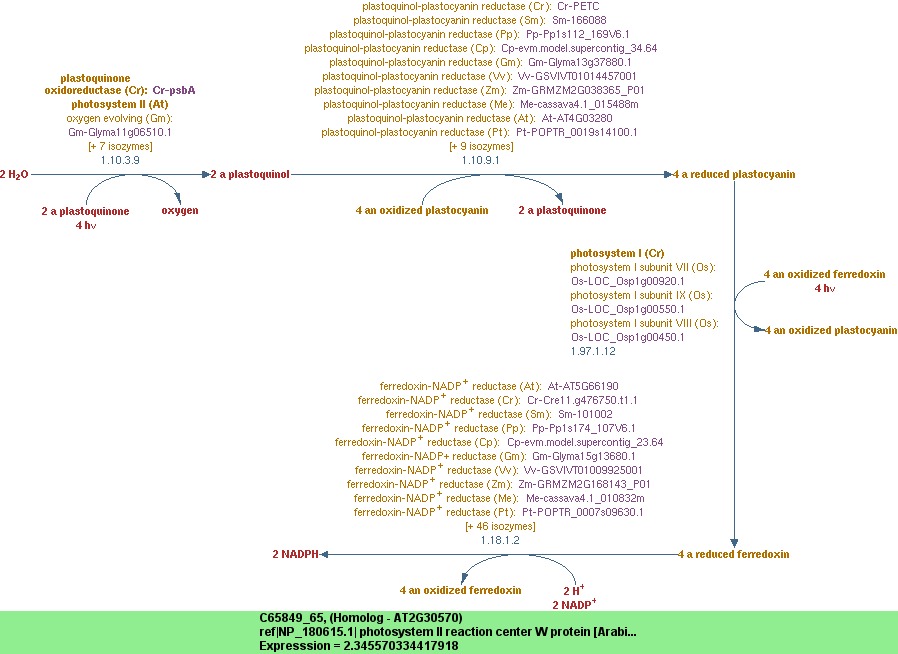

Supplement: Additional file 16 — A and B: Stress related up-regulated PMN pathways. [file 1471-2164-14-647-S16.zip › Additional_file16A_Upregulated_PMN_pathways_in_Shoot/V2SHS/C65849_65_AT2G30570_2_photosynthesis_light_reactions.jpg]

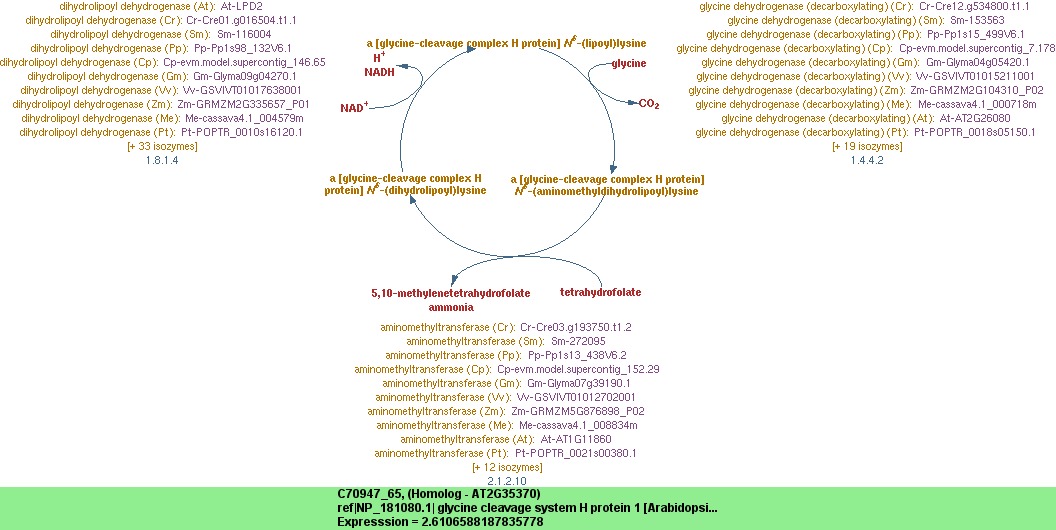

Supplement: Additional file 16 — A and B: Stress related up-regulated PMN pathways. [file 1471-2164-14-647-S16.zip › Additional_file16A_Upregulated_PMN_pathways_in_Shoot/V2SHS/C70947_65_AT2G35370_1_glycine_cleavage_complex.jpg]

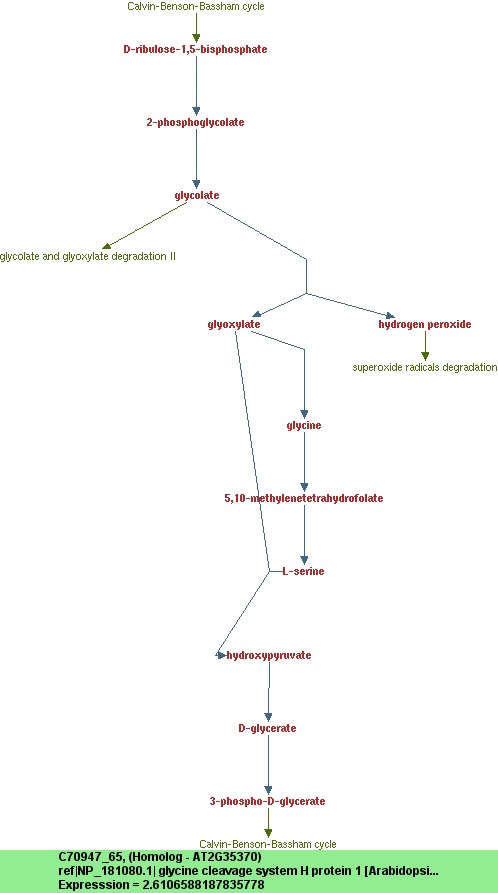

Supplement: Additional file 16 — A and B: Stress related up-regulated PMN pathways. [file 1471-2164-14-647-S16.zip › Additional_file16A_Upregulated_PMN_pathways_in_Shoot/V2SHS/C70947_65_AT2G35370_3_photorespiration.jpg]

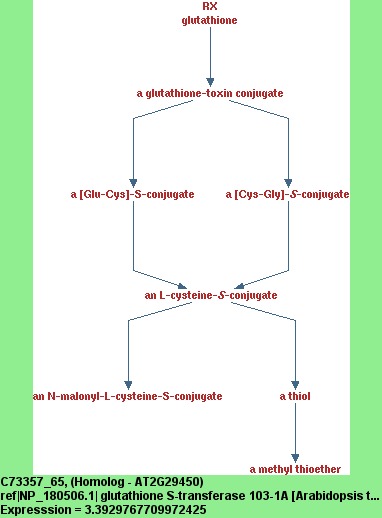

Supplement: Additional file 16 — A and B: Stress related up-regulated PMN pathways. [file 1471-2164-14-647-S16.zip › Additional_file16A_Upregulated_PMN_pathways_in_Shoot/V2SHS/C73357_65_AT2G29450_1_glutathione-mediated_detoxification_II.jpg]

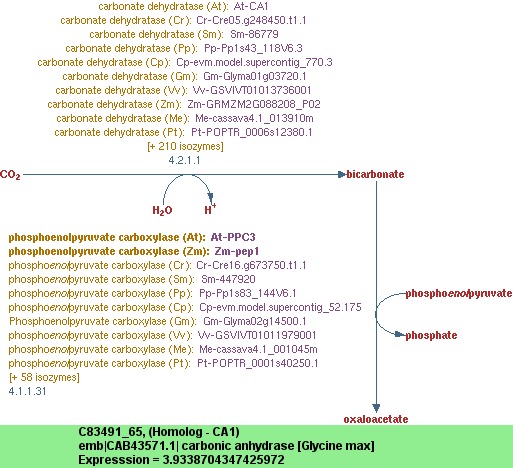

Supplement: Additional file 16 — A and B: Stress related up-regulated PMN pathways. [file 1471-2164-14-647-S16.zip › Additional_file16A_Upregulated_PMN_pathways_in_Shoot/V2SHS/C83491_65_CA1_1_CO_fixation_into_oxaloacetate_(anapleurotic).jpg]

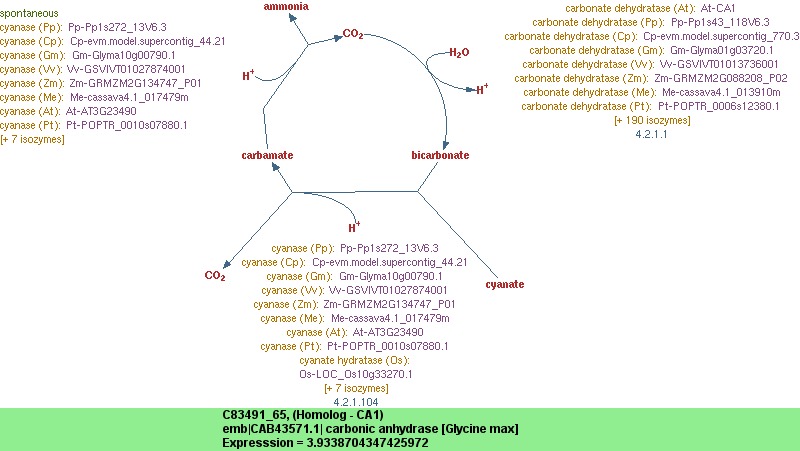

Supplement: Additional file 16 — A and B: Stress related up-regulated PMN pathways. [file 1471-2164-14-647-S16.zip › Additional_file16A_Upregulated_PMN_pathways_in_Shoot/V2SHS/C83491_65_CA1_2_cyanate_degradation.jpg]

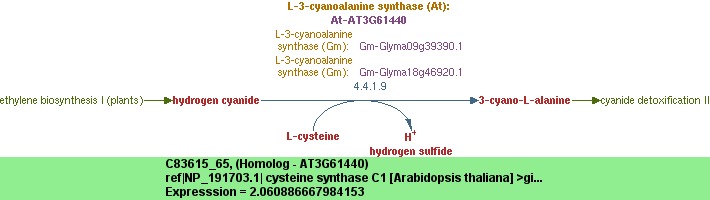

Supplement: Additional file 16 — A and B: Stress related up-regulated PMN pathways. [file 1471-2164-14-647-S16.zip › Additional_file16A_Upregulated_PMN_pathways_in_Shoot/V2SHS/C83615_65_AT3G61440_1_cyanide_degradation.jpg]

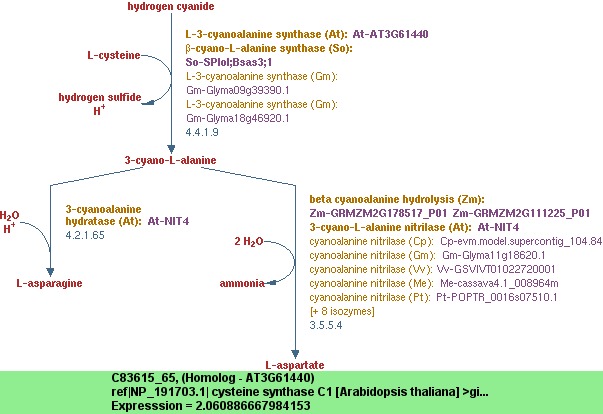

Supplement: Additional file 16 — A and B: Stress related up-regulated PMN pathways. [file 1471-2164-14-647-S16.zip › Additional_file16A_Upregulated_PMN_pathways_in_Shoot/V2SHS/C83615_65_AT3G61440_3_cyanide_detoxification_II.jpg]

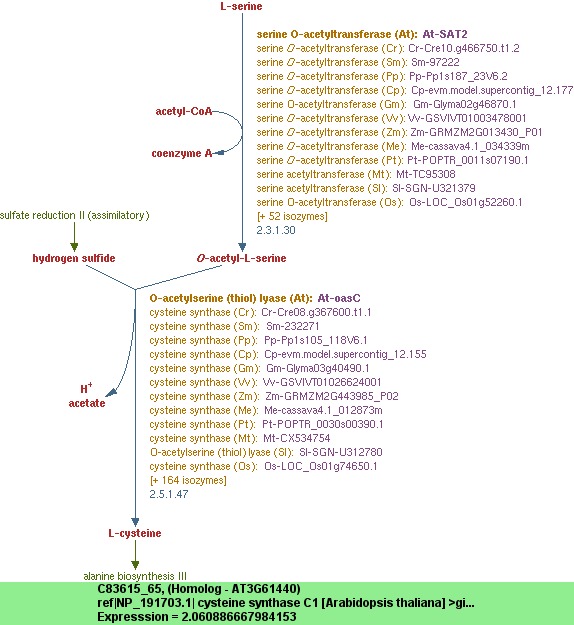

Supplement: Additional file 16 — A and B: Stress related up-regulated PMN pathways. [file 1471-2164-14-647-S16.zip › Additional_file16A_Upregulated_PMN_pathways_in_Shoot/V2SHS/C83615_65_AT3G61440_5_cysteine_biosynthesis_I.jpg]

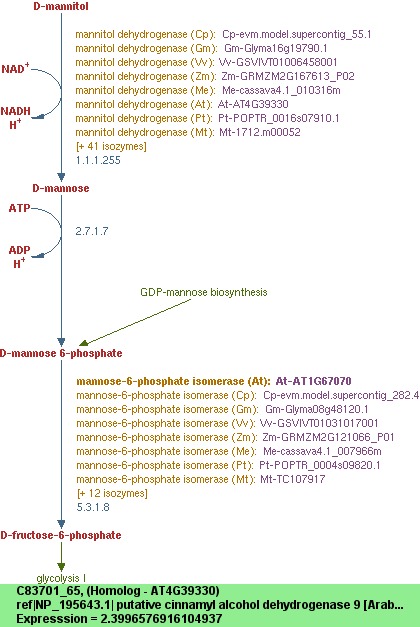

Supplement: Additional file 16 — A and B: Stress related up-regulated PMN pathways. [file 1471-2164-14-647-S16.zip › Additional_file16A_Upregulated_PMN_pathways_in_Shoot/V2SHS/C83701_65_AT4G39330_1_mannitol_degradation_II.jpg]

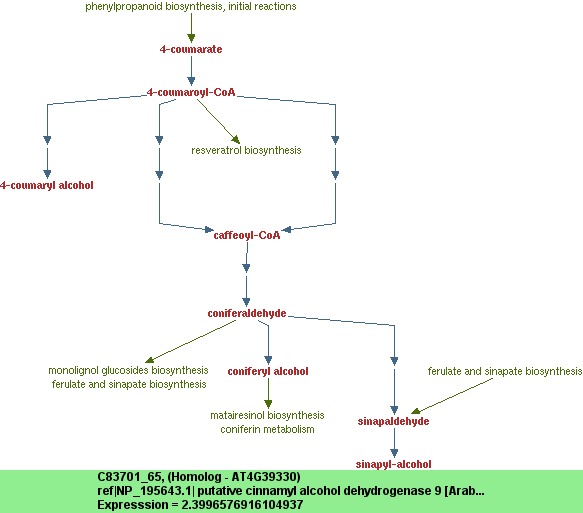

Supplement: Additional file 16 — A and B: Stress related up-regulated PMN pathways. [file 1471-2164-14-647-S16.zip › Additional_file16A_Upregulated_PMN_pathways_in_Shoot/V2SHS/C83701_65_AT4G39330_3_phenylpropanoid_biosynthesis.jpg]

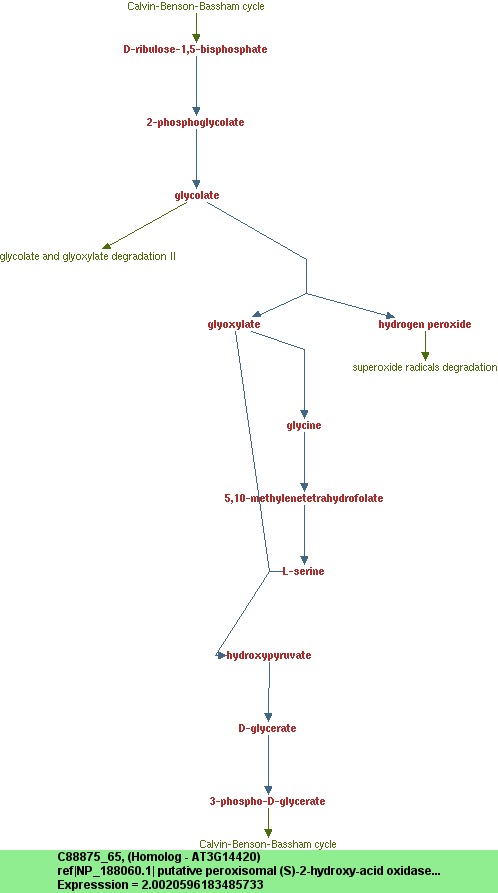

Supplement: Additional file 16 — A and B: Stress related up-regulated PMN pathways. [file 1471-2164-14-647-S16.zip › Additional_file16A_Upregulated_PMN_pathways_in_Shoot/V2SHS/C88875_65_AT3G14420_1_photorespiration.jpg]

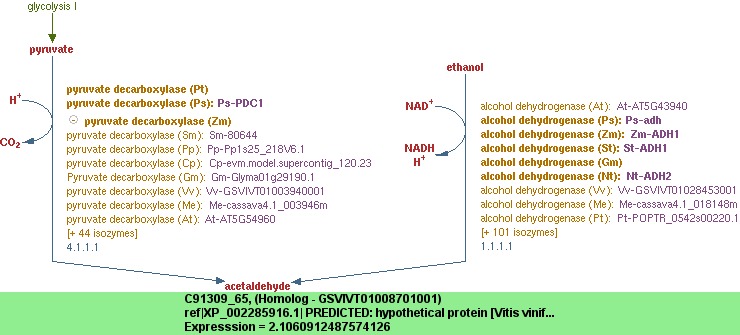

Supplement: Additional file 16 — A and B: Stress related up-regulated PMN pathways. [file 1471-2164-14-647-S16.zip › Additional_file16A_Upregulated_PMN_pathways_in_Shoot/V2SHS/C91309_65_GSVIVT01008701001_1_acetaldehyde_biosynthesis_I.jpg]

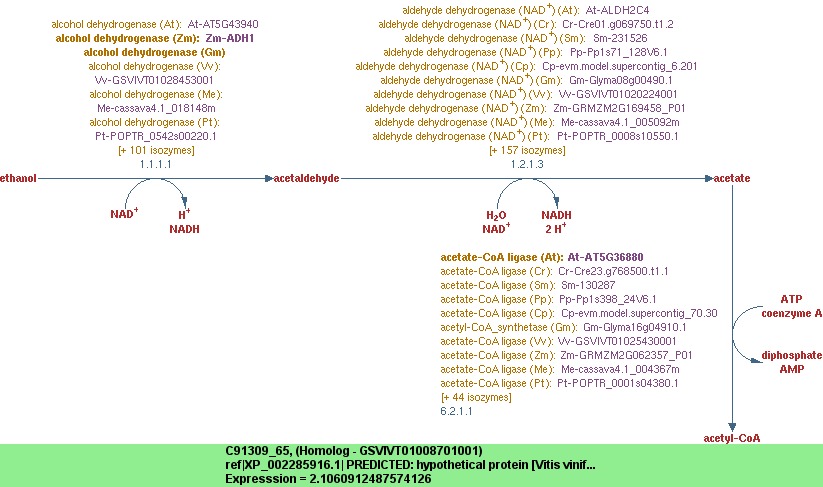

Supplement: Additional file 16 — A and B: Stress related up-regulated PMN pathways. [file 1471-2164-14-647-S16.zip › Additional_file16A_Upregulated_PMN_pathways_in_Shoot/V2SHS/C91309_65_GSVIVT01008701001_2_ethanol_degradation_II.jpg]

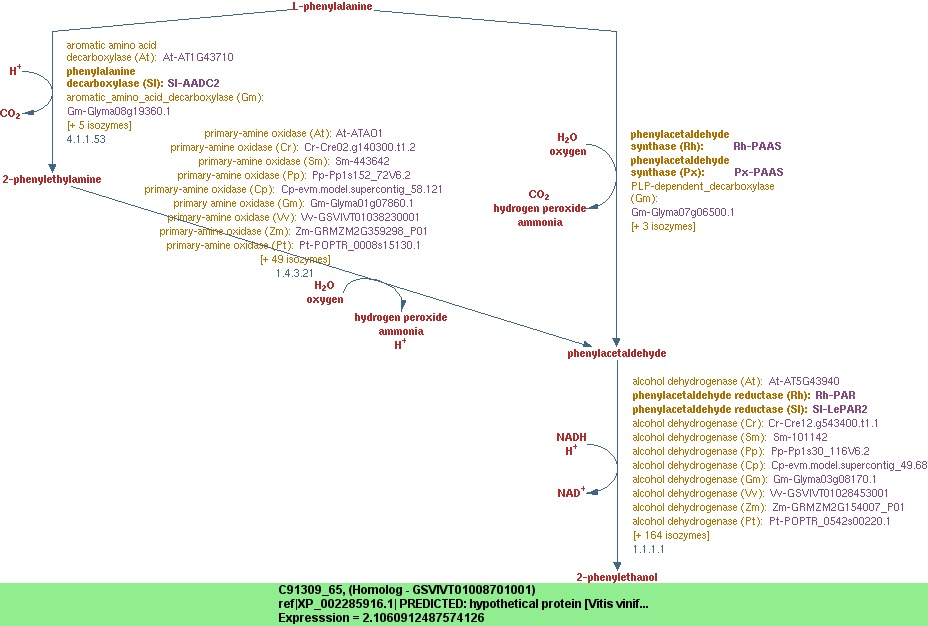

Supplement: Additional file 16 — A and B: Stress related up-regulated PMN pathways. [file 1471-2164-14-647-S16.zip › Additional_file16A_Upregulated_PMN_pathways_in_Shoot/V2SHS/C91309_65_GSVIVT01008701001_3_phenylethanol_biosynthesis.jpg]

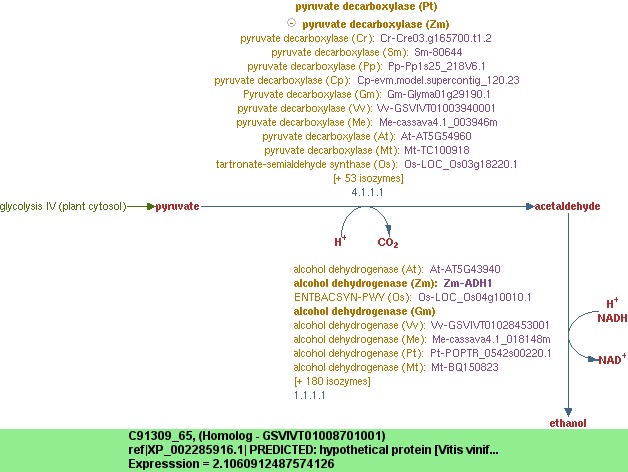

Supplement: Additional file 16 — A and B: Stress related up-regulated PMN pathways. [file 1471-2164-14-647-S16.zip › Additional_file16A_Upregulated_PMN_pathways_in_Shoot/V2SHS/C91309_65_GSVIVT01008701001_4_pyruvate_fermentation_to_ethanol_II.jpg]

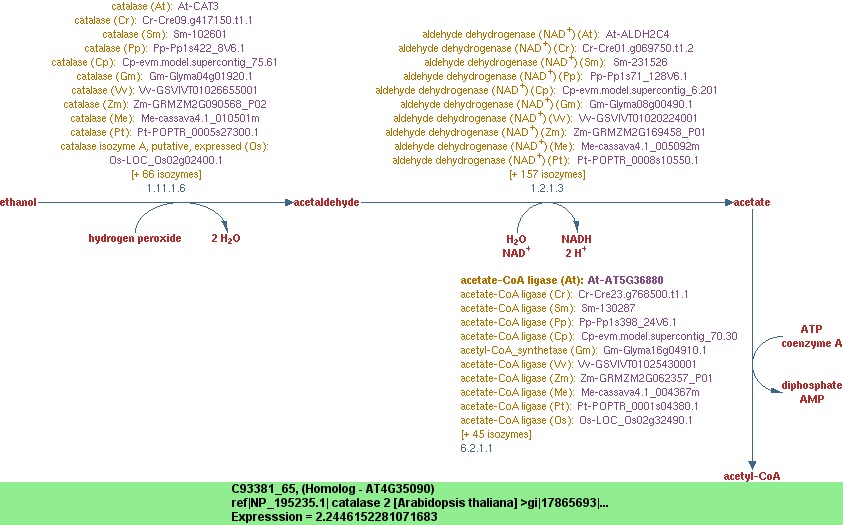

Supplement: Additional file 16 — A and B: Stress related up-regulated PMN pathways. [file 1471-2164-14-647-S16.zip › Additional_file16A_Upregulated_PMN_pathways_in_Shoot/V2SHS/C93381_65_AT4G35090_1_ethanol_degradation_IV.jpg]

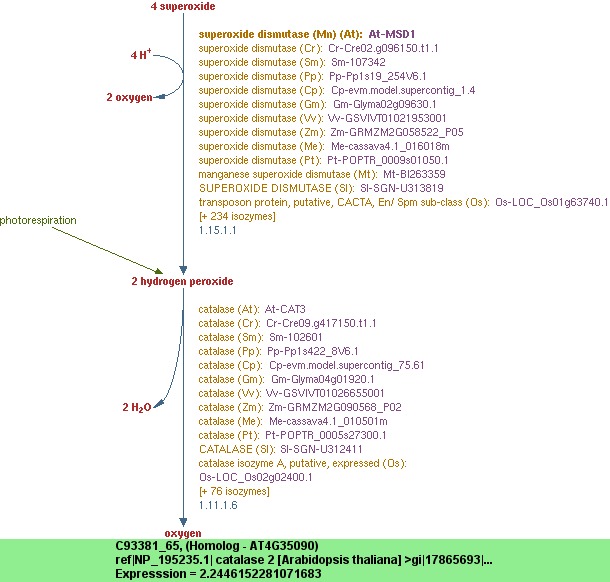

Supplement: Additional file 16 — A and B: Stress related up-regulated PMN pathways. [file 1471-2164-14-647-S16.zip › Additional_file16A_Upregulated_PMN_pathways_in_Shoot/V2SHS/C93381_65_AT4G35090_2_superoxide_radicals_degradation.jpg]

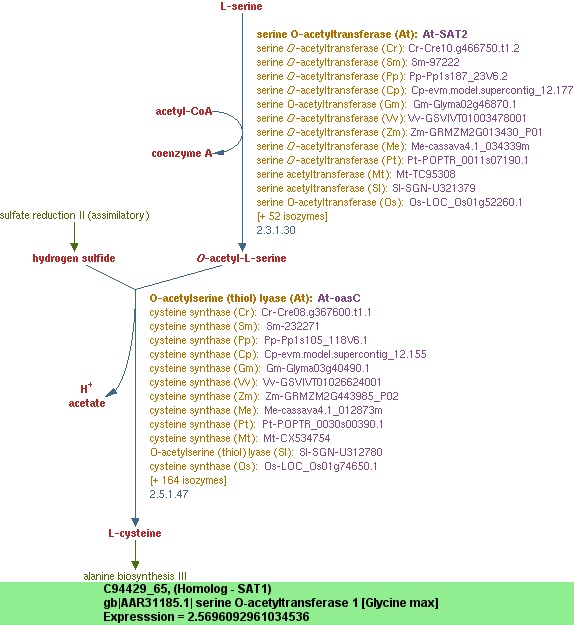

Supplement: Additional file 16 — A and B: Stress related up-regulated PMN pathways. [file 1471-2164-14-647-S16.zip › Additional_file16A_Upregulated_PMN_pathways_in_Shoot/V2SHS/C94429_65_SAT1_1_cysteine_biosynthesis_I.jpg]

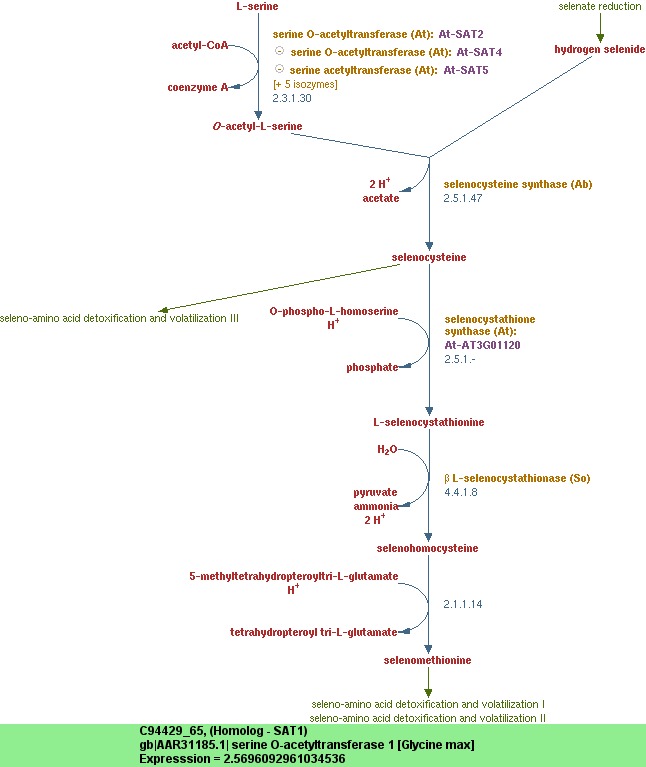

Supplement: Additional file 16 — A and B: Stress related up-regulated PMN pathways. [file 1471-2164-14-647-S16.zip › Additional_file16A_Upregulated_PMN_pathways_in_Shoot/V2SHS/C94429_65_SAT1_2_seleno-amino_acid_biosynthesis.jpg]

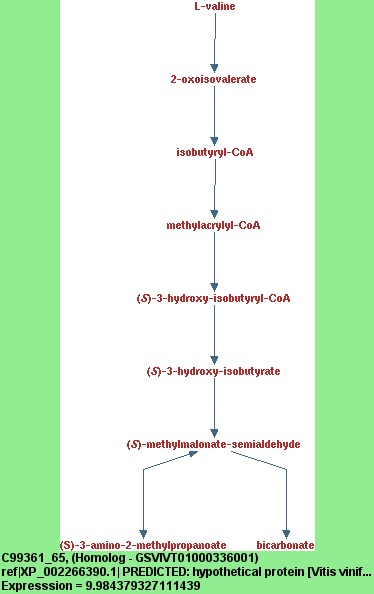

Supplement: Additional file 16 — A and B: Stress related up-regulated PMN pathways. [file 1471-2164-14-647-S16.zip › Additional_file16A_Upregulated_PMN_pathways_in_Shoot/V2SHS/C99361_65_GSVIVT01000336001_1_valine_degradation_I.jpg]

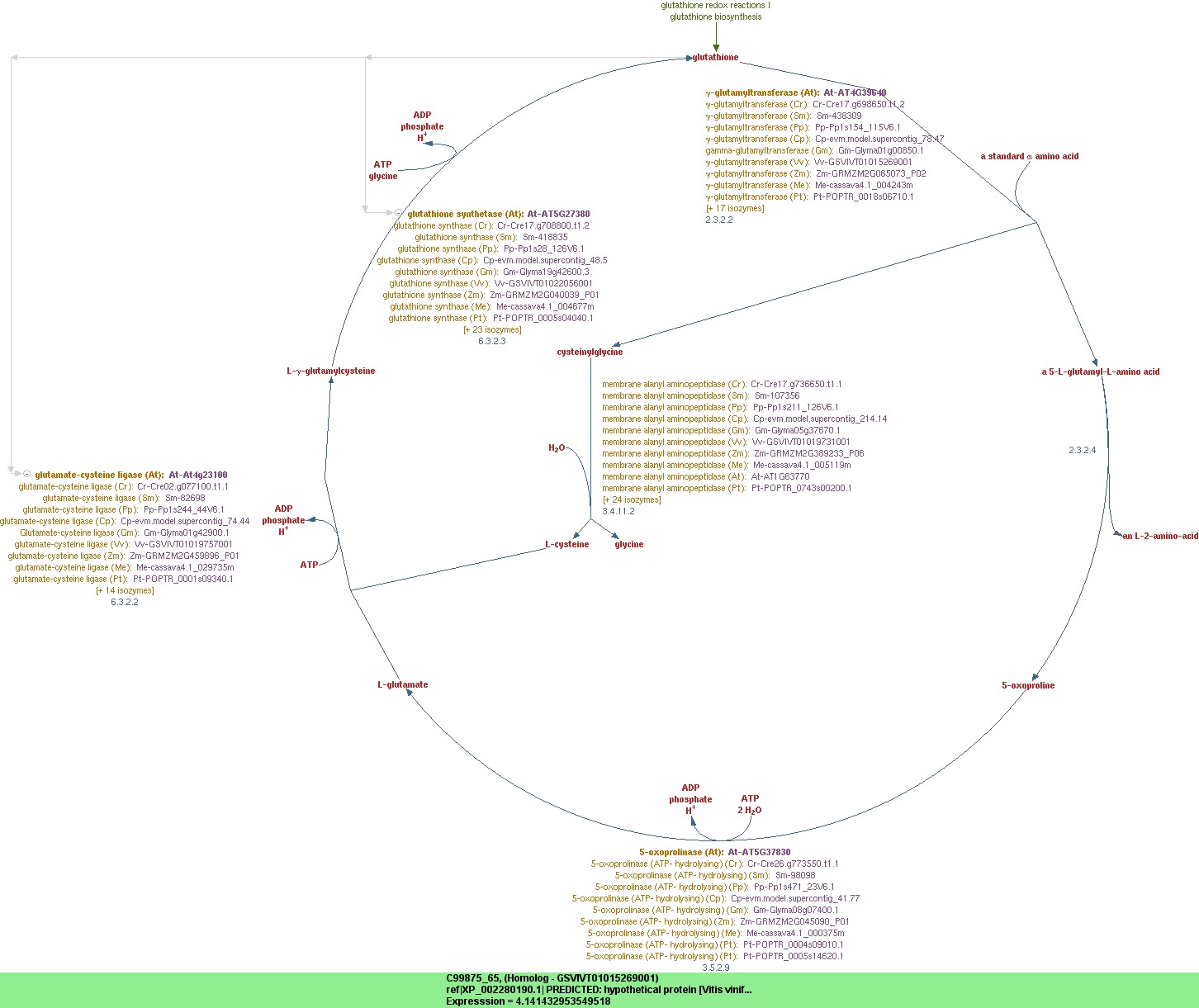

Supplement: Additional file 16 — A and B: Stress related up-regulated PMN pathways. [file 1471-2164-14-647-S16.zip › Additional_file16A_Upregulated_PMN_pathways_in_Shoot/V2SHS/C99875_65_GSVIVT01015269001_1_&gamma-glutamyl_cycle.jpg]

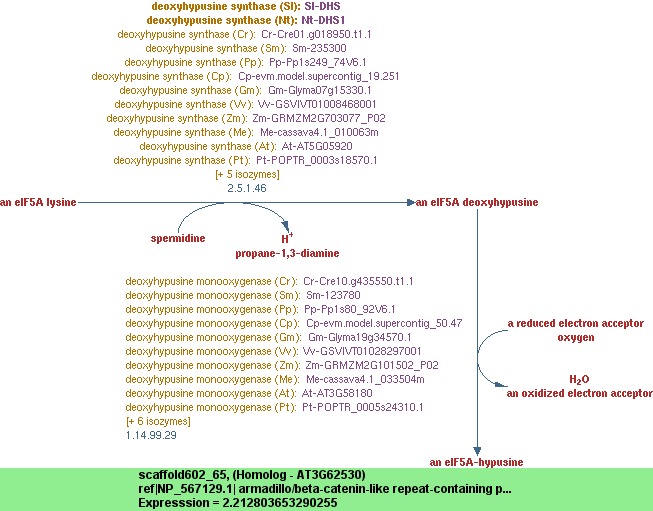

Supplement: Additional file 16 — A and B: Stress related up-regulated PMN pathways. [file 1471-2164-14-647-S16.zip › Additional_file16A_Upregulated_PMN_pathways_in_Shoot/V2SHS/scaffold602_65_AT3G62530_1_hypusine_biosynthesis.jpg]

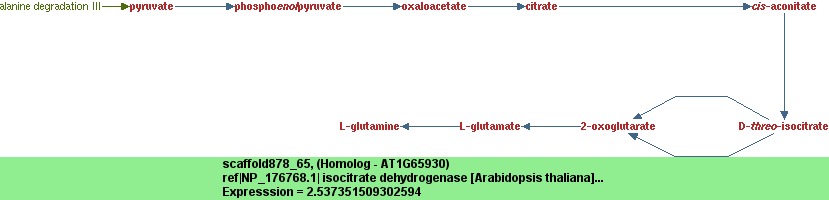

Supplement: Additional file 16 — A and B: Stress related up-regulated PMN pathways. [file 1471-2164-14-647-S16.zip › Additional_file16A_Upregulated_PMN_pathways_in_Shoot/V2SHS/scaffold878_65_AT1G65930_1_glutamine_biosynthesis_III.jpg]

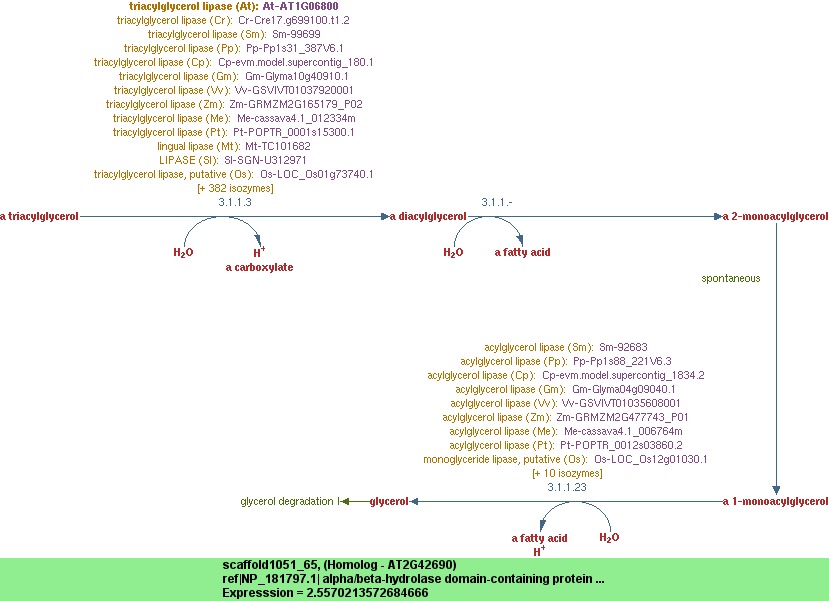

Supplement: Additional file 16 — A and B: Stress related up-regulated PMN pathways. [file 1471-2164-14-647-S16.zip › Additional_file16A_Upregulated_PMN_pathways_in_Shoot/V2SHS/scaffold1051_65_AT2G42690_1_triacylglycerol_degradation.jpg]

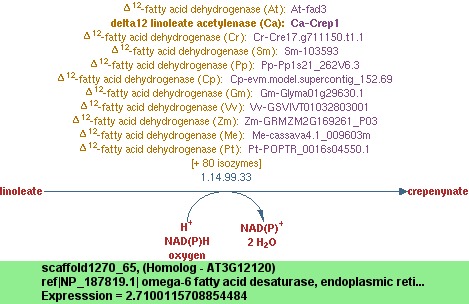

Supplement: Additional file 16 — A and B: Stress related up-regulated PMN pathways. [file 1471-2164-14-647-S16.zip › Additional_file16A_Upregulated_PMN_pathways_in_Shoot/V2SHS/scaffold1270_65_AT3G12120_1_crepenynic_acid_biosynthesis.jpg]

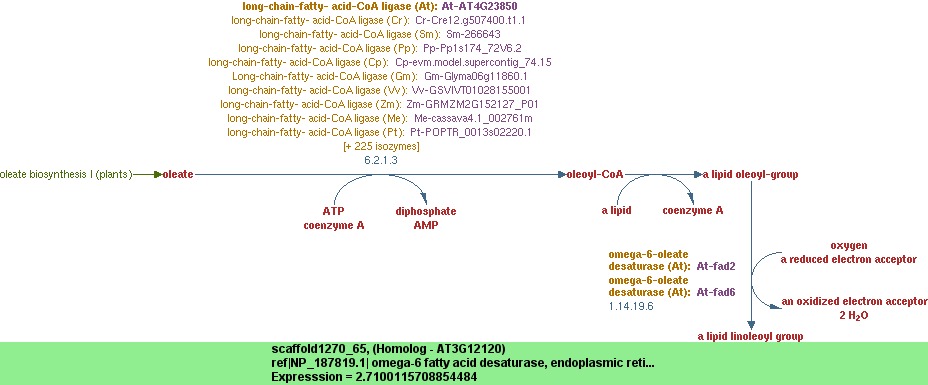

Supplement: Additional file 16 — A and B: Stress related up-regulated PMN pathways. [file 1471-2164-14-647-S16.zip › Additional_file16A_Upregulated_PMN_pathways_in_Shoot/V2SHS/scaffold1270_65_AT3G12120_2_linoleate_biosynthesis_I_(plants).jpg]

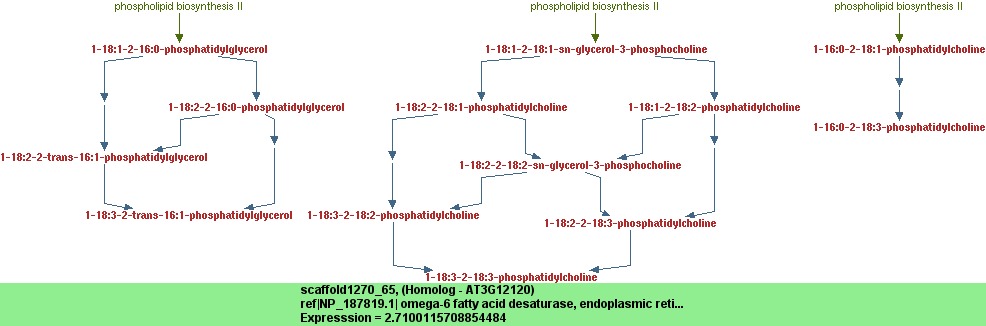

Supplement: Additional file 16 — A and B: Stress related up-regulated PMN pathways. [file 1471-2164-14-647-S16.zip › Additional_file16A_Upregulated_PMN_pathways_in_Shoot/V2SHS/scaffold1270_65_AT3G12120_3_phospholipid_desaturation.jpg]

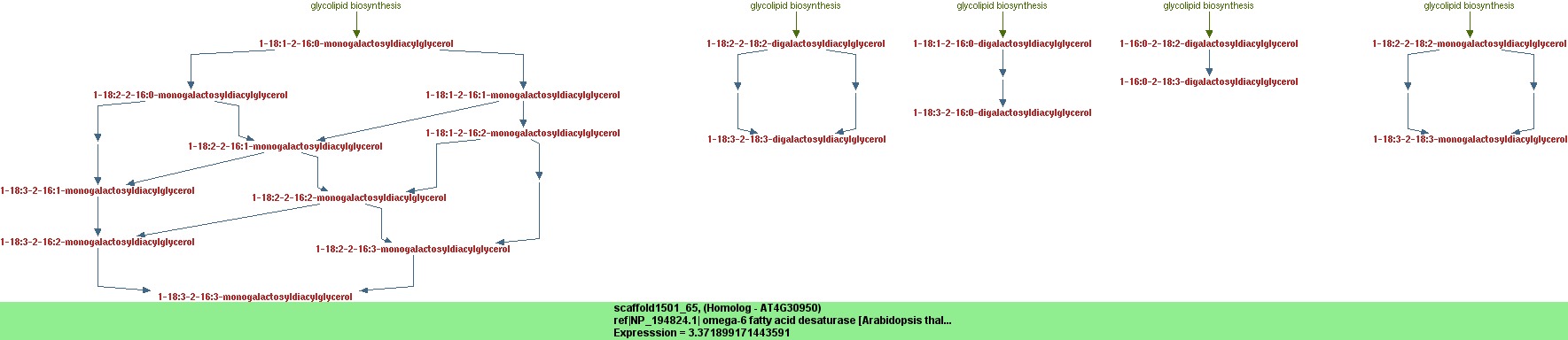

Supplement: Additional file 16 — A and B: Stress related up-regulated PMN pathways. [file 1471-2164-14-647-S16.zip › Additional_file16A_Upregulated_PMN_pathways_in_Shoot/V2SHS/scaffold1501_65_AT4G30950_1_glycolipid_desaturation.jpg]

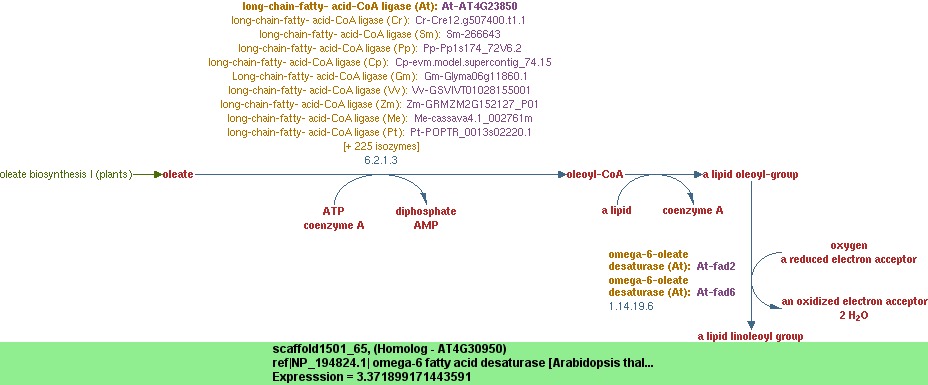

Supplement: Additional file 16 — A and B: Stress related up-regulated PMN pathways. [file 1471-2164-14-647-S16.zip › Additional_file16A_Upregulated_PMN_pathways_in_Shoot/V2SHS/scaffold1501_65_AT4G30950_2_linoleate_biosynthesis_I_(plants).jpg]

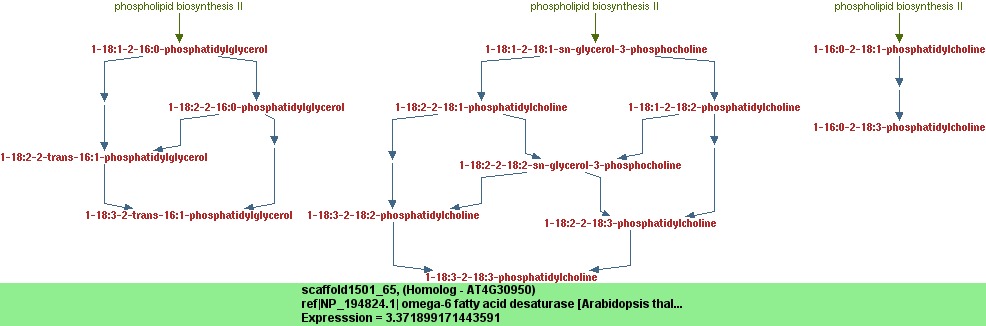

Supplement: Additional file 16 — A and B: Stress related up-regulated PMN pathways. [file 1471-2164-14-647-S16.zip › Additional_file16A_Upregulated_PMN_pathways_in_Shoot/V2SHS/scaffold1501_65_AT4G30950_3_phospholipid_desaturation.jpg]

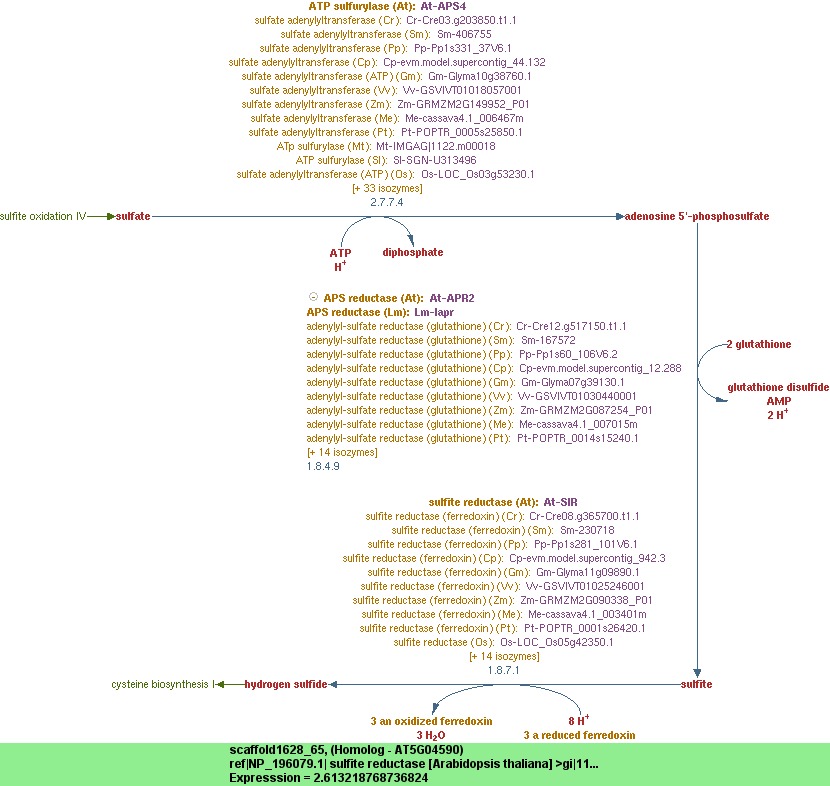

Supplement: Additional file 16 — A and B: Stress related up-regulated PMN pathways. [file 1471-2164-14-647-S16.zip › Additional_file16A_Upregulated_PMN_pathways_in_Shoot/V2SHS/scaffold1628_65_AT5G04590_1_sulfate_reduction_II_(assimilatory).jpg]

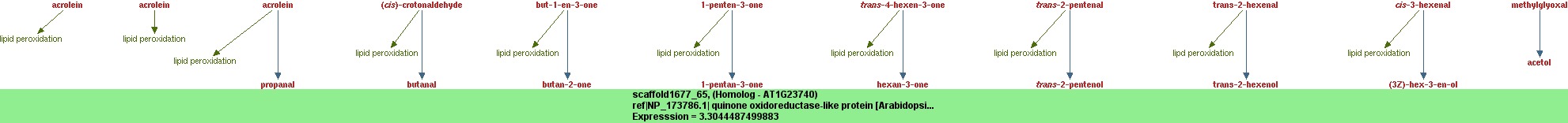

Supplement: Additional file 16 — A and B: Stress related up-regulated PMN pathways. [file 1471-2164-14-647-S16.zip › Additional_file16A_Upregulated_PMN_pathways_in_Shoot/V2SHS/scaffold1677_65_AT1G23740_1_detoxification_of_reactive_carbonyls_in_chloroplasts.jpg]

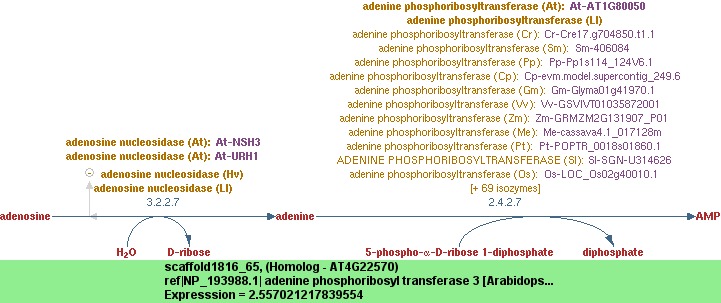

Supplement: Additional file 16 — A and B: Stress related up-regulated PMN pathways. [file 1471-2164-14-647-S16.zip › Additional_file16A_Upregulated_PMN_pathways_in_Shoot/V2SHS/scaffold1816_65_AT4G22570_1_adenine_and_adenosine_salvage_II.jpg]

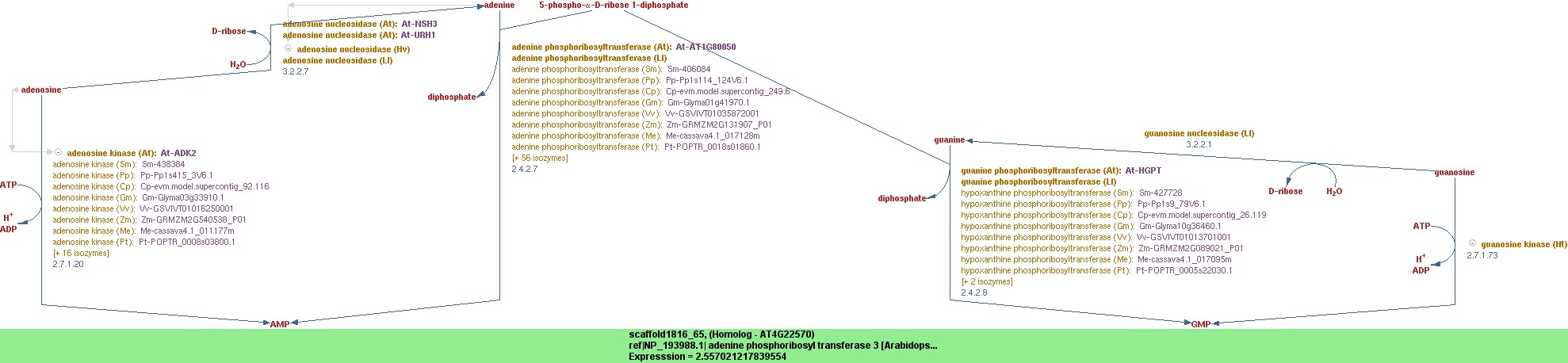

Supplement: Additional file 16 — A and B: Stress related up-regulated PMN pathways. [file 1471-2164-14-647-S16.zip › Additional_file16A_Upregulated_PMN_pathways_in_Shoot/V2SHS/scaffold1816_65_AT4G22570_3_purine_nucleosides_salvage_II_(plant).jpg]

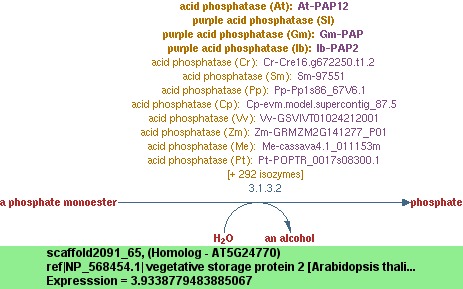

Supplement: Additional file 16 — A and B: Stress related up-regulated PMN pathways. [file 1471-2164-14-647-S16.zip › Additional_file16A_Upregulated_PMN_pathways_in_Shoot/V2SHS/scaffold2091_65_AT5G24770_1_phosphate_acquisition.jpg]

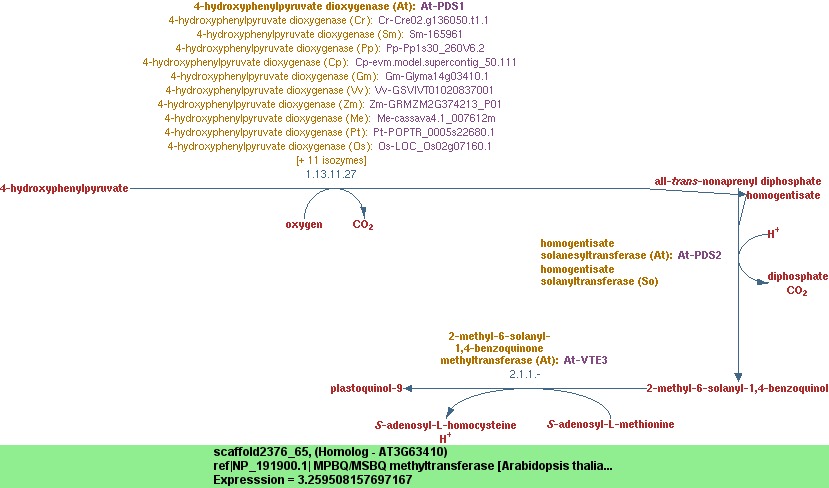

Supplement: Additional file 16 — A and B: Stress related up-regulated PMN pathways. [file 1471-2164-14-647-S16.zip › Additional_file16A_Upregulated_PMN_pathways_in_Shoot/V2SHS/scaffold2376_65_AT3G63410_1_plastoquinol-9_biosynthesis_I.jpg]

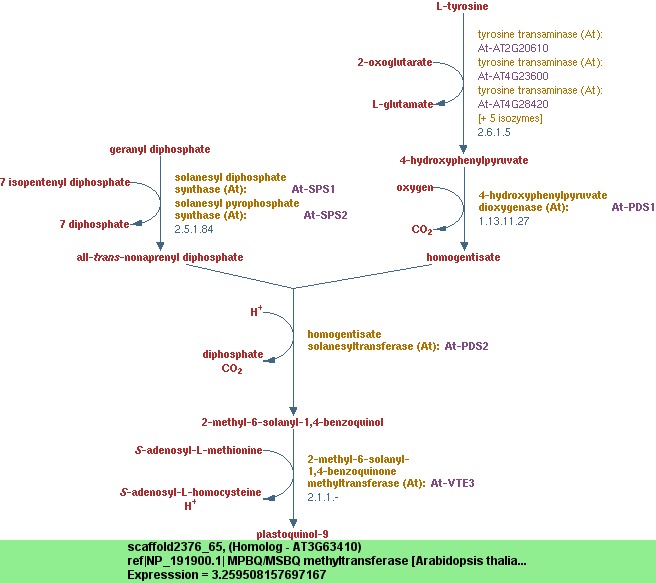

Supplement: Additional file 16 — A and B: Stress related up-regulated PMN pathways. [file 1471-2164-14-647-S16.zip › Additional_file16A_Upregulated_PMN_pathways_in_Shoot/V2SHS/scaffold2376_65_AT3G63410_2_superpathway_of_plastoquinol_biosynthesis.jpg]

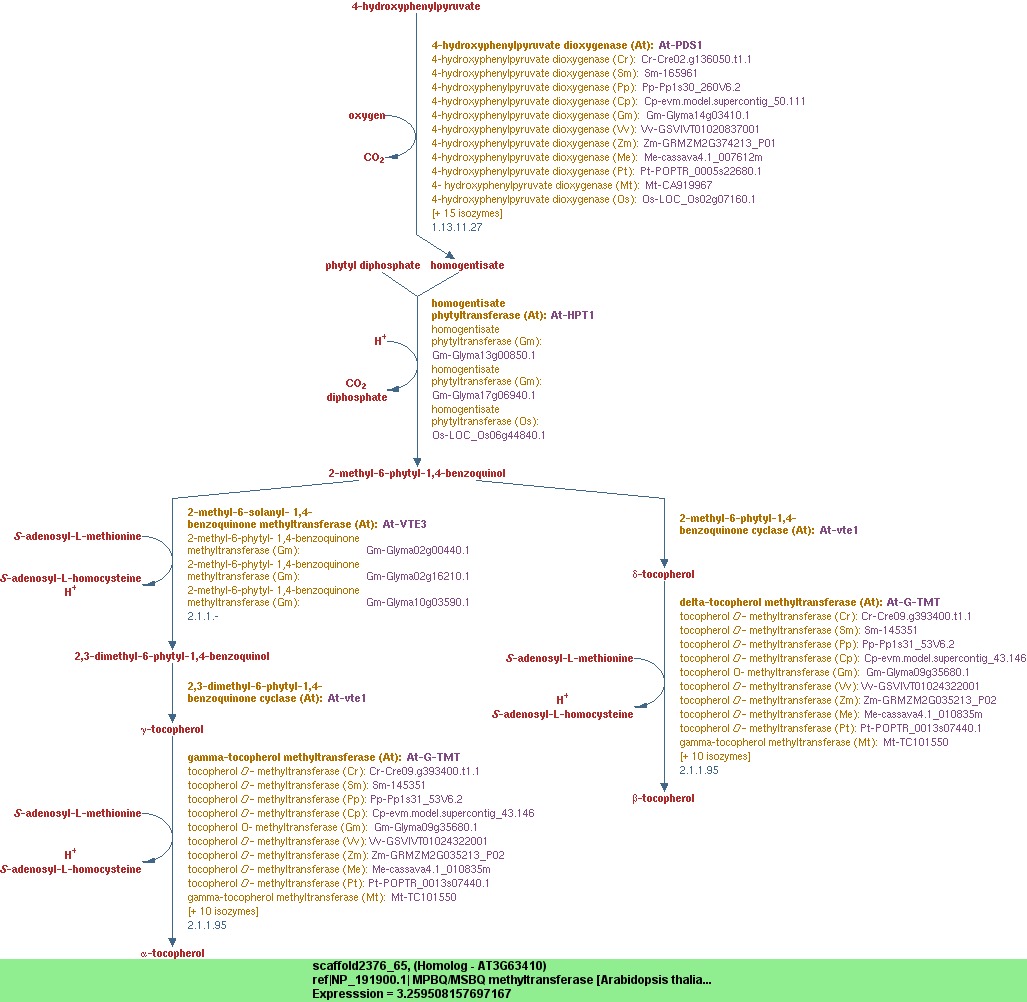

Supplement: Additional file 16 — A and B: Stress related up-regulated PMN pathways. [file 1471-2164-14-647-S16.zip › Additional_file16A_Upregulated_PMN_pathways_in_Shoot/V2SHS/scaffold2376_65_AT3G63410_3_vitamin_E_biosynthesis.jpg]

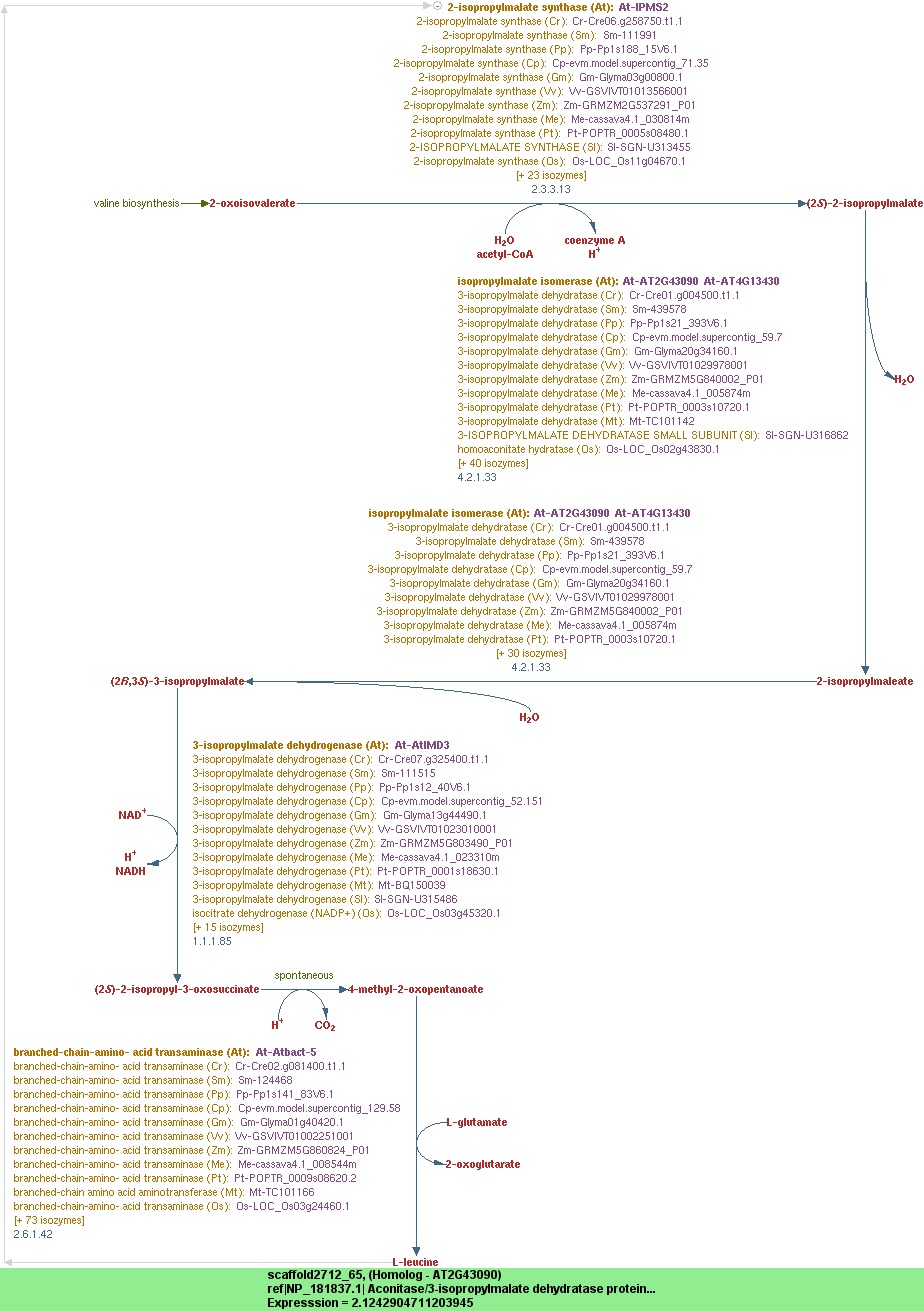

Supplement: Additional file 16 — A and B: Stress related up-regulated PMN pathways. [file 1471-2164-14-647-S16.zip › Additional_file16A_Upregulated_PMN_pathways_in_Shoot/V2SHS/scaffold2712_65_AT2G43090_1_leucine_biosynthesis.jpg]

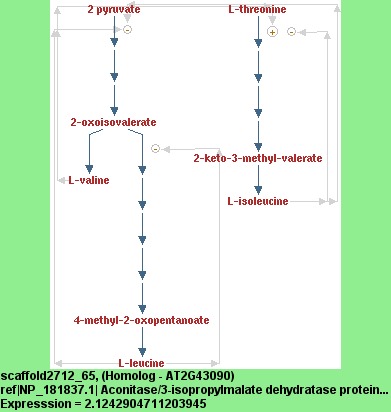

Supplement: Additional file 16 — A and B: Stress related up-regulated PMN pathways. [file 1471-2164-14-647-S16.zip › Additional_file16A_Upregulated_PMN_pathways_in_Shoot/V2SHS/scaffold2712_65_AT2G43090_3_superpathway_of_leucine,_valine,_and_isoleucine_biosynthesis.jpg]

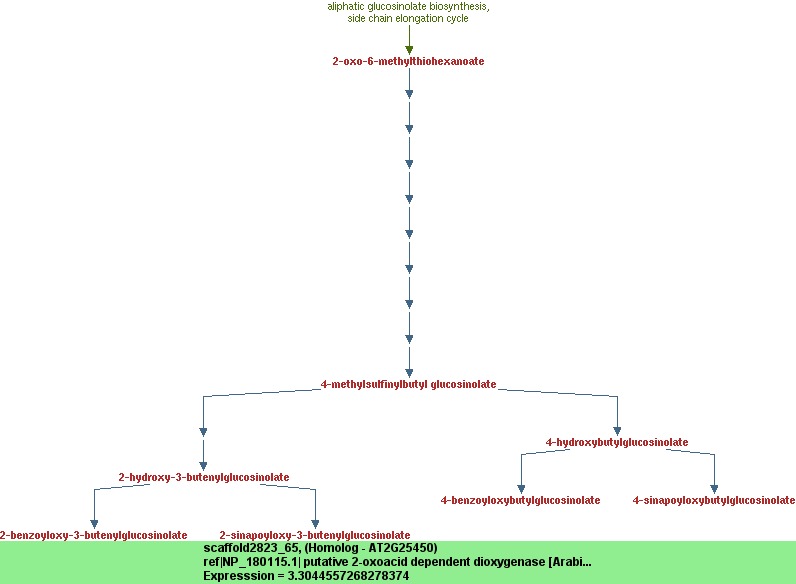

Supplement: Additional file 16 — A and B: Stress related up-regulated PMN pathways. [file 1471-2164-14-647-S16.zip › Additional_file16A_Upregulated_PMN_pathways_in_Shoot/V2SHS/scaffold2823_65_AT2G25450_1_glucosinolate_biosynthesis_from_dihomomethionine.jpg]

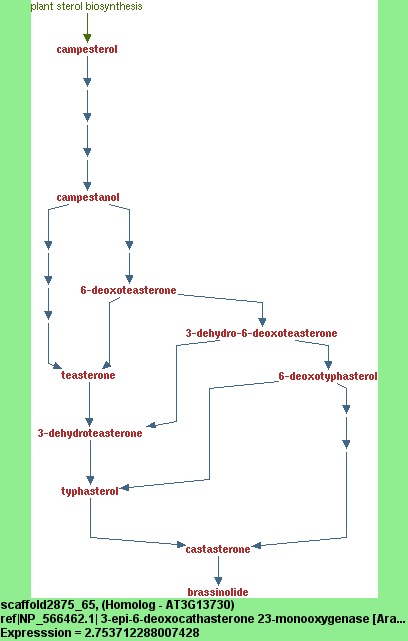

Supplement: Additional file 16 — A and B: Stress related up-regulated PMN pathways. [file 1471-2164-14-647-S16.zip › Additional_file16A_Upregulated_PMN_pathways_in_Shoot/V2SHS/scaffold2875_65_AT3G13730_1_brassinosteroid_biosynthesis_I.jpg]

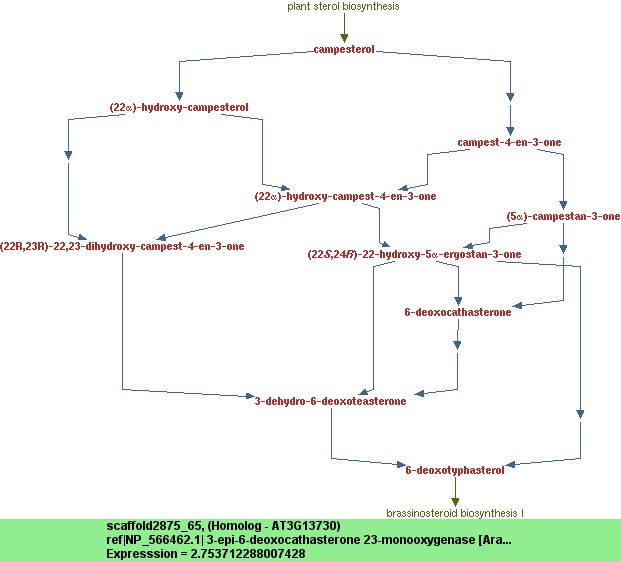

Supplement: Additional file 16 — A and B: Stress related up-regulated PMN pathways. [file 1471-2164-14-647-S16.zip › Additional_file16A_Upregulated_PMN_pathways_in_Shoot/V2SHS/scaffold2875_65_AT3G13730_2_brassinosteroid_biosynthesis_II.jpg]

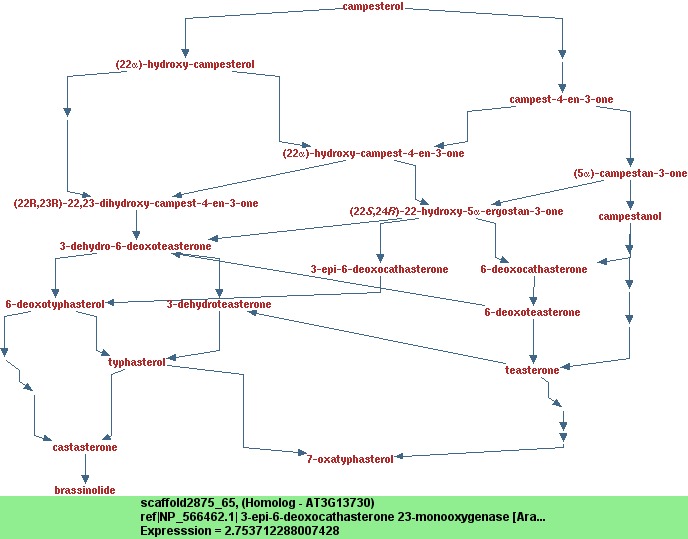

Supplement: Additional file 16 — A and B: Stress related up-regulated PMN pathways. [file 1471-2164-14-647-S16.zip › Additional_file16A_Upregulated_PMN_pathways_in_Shoot/V2SHS/scaffold2875_65_AT3G13730_3_superpathway_of_C28_brassinosteroid_biosynthesis.jpg]

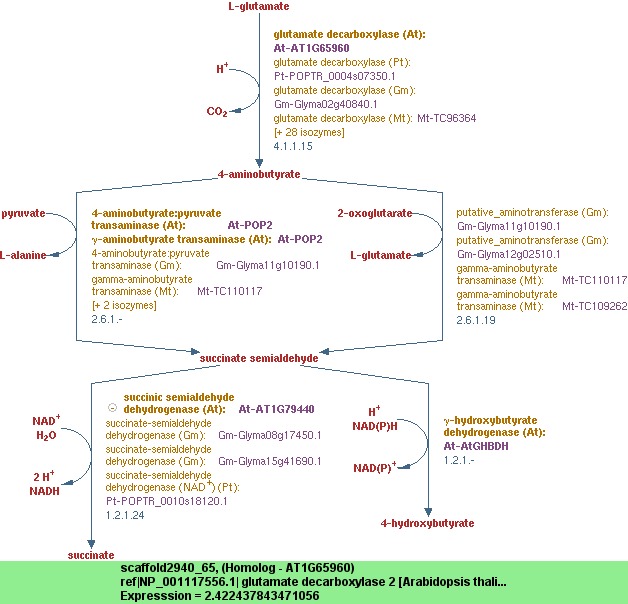

Supplement: Additional file 16 — A and B: Stress related up-regulated PMN pathways. [file 1471-2164-14-647-S16.zip › Additional_file16A_Upregulated_PMN_pathways_in_Shoot/V2SHS/scaffold2940_65_AT1G65960_1_glutamate_degradation_IV.jpg]

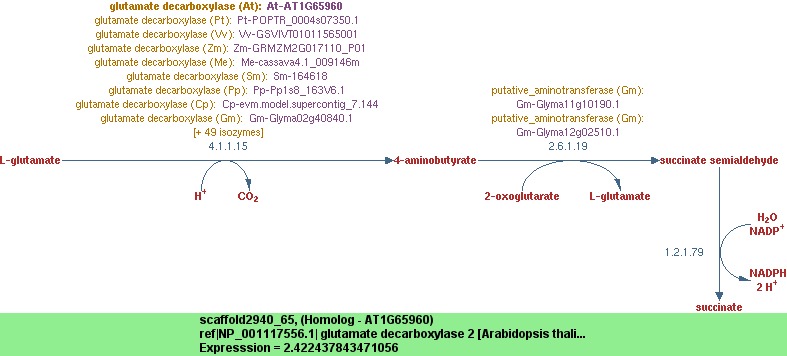

Supplement: Additional file 16 — A and B: Stress related up-regulated PMN pathways. [file 1471-2164-14-647-S16.zip › Additional_file16A_Upregulated_PMN_pathways_in_Shoot/V2SHS/scaffold2940_65_AT1G65960_3_glutamate_degradation_IX_(via_4-aminobutyrate).jpg]

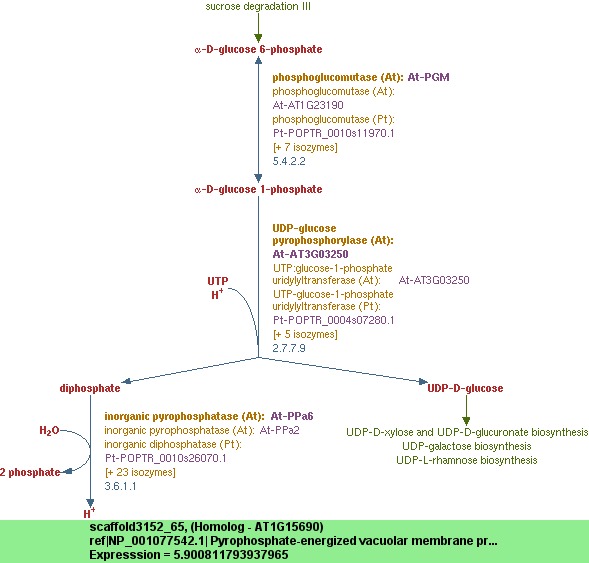

Supplement: Additional file 16 — A and B: Stress related up-regulated PMN pathways. [file 1471-2164-14-647-S16.zip › Additional_file16A_Upregulated_PMN_pathways_in_Shoot/V2SHS/scaffold3152_65_AT1G15690_1_UDP-glucose_biosynthesis_(from_glucose_6-phosphate).jpg]

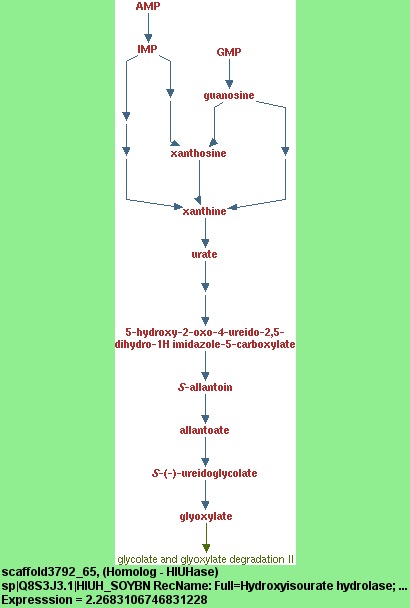

Supplement: Additional file 16 — A and B: Stress related up-regulated PMN pathways. [file 1471-2164-14-647-S16.zip › Additional_file16A_Upregulated_PMN_pathways_in_Shoot/V2SHS/scaffold3792_65_HIUHase_1_superpathway_of_purines_degradation_in_plants.jpg]

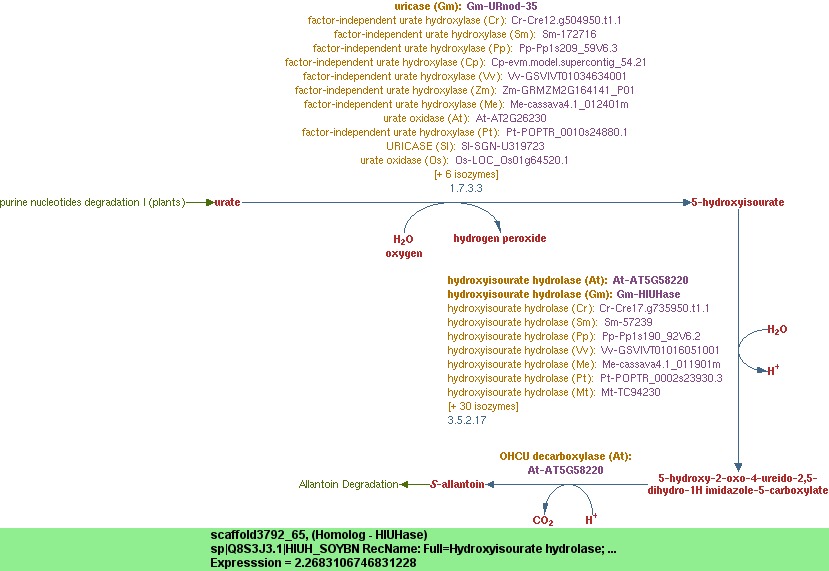

Supplement: Additional file 16 — A and B: Stress related up-regulated PMN pathways. [file 1471-2164-14-647-S16.zip › Additional_file16A_Upregulated_PMN_pathways_in_Shoot/V2SHS/scaffold3792_65_HIUHase_2_urate_degradation_to_allantoin.jpg]

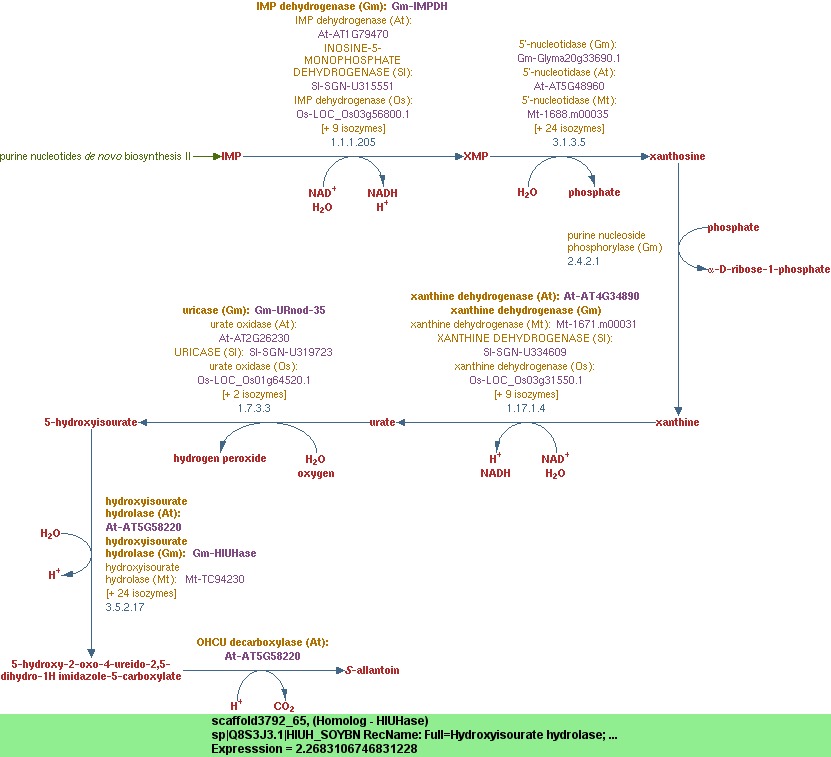

Supplement: Additional file 16 — A and B: Stress related up-regulated PMN pathways. [file 1471-2164-14-647-S16.zip › Additional_file16A_Upregulated_PMN_pathways_in_Shoot/V2SHS/scaffold3792_65_HIUHase_3_ureide_biosynthesis.jpg]

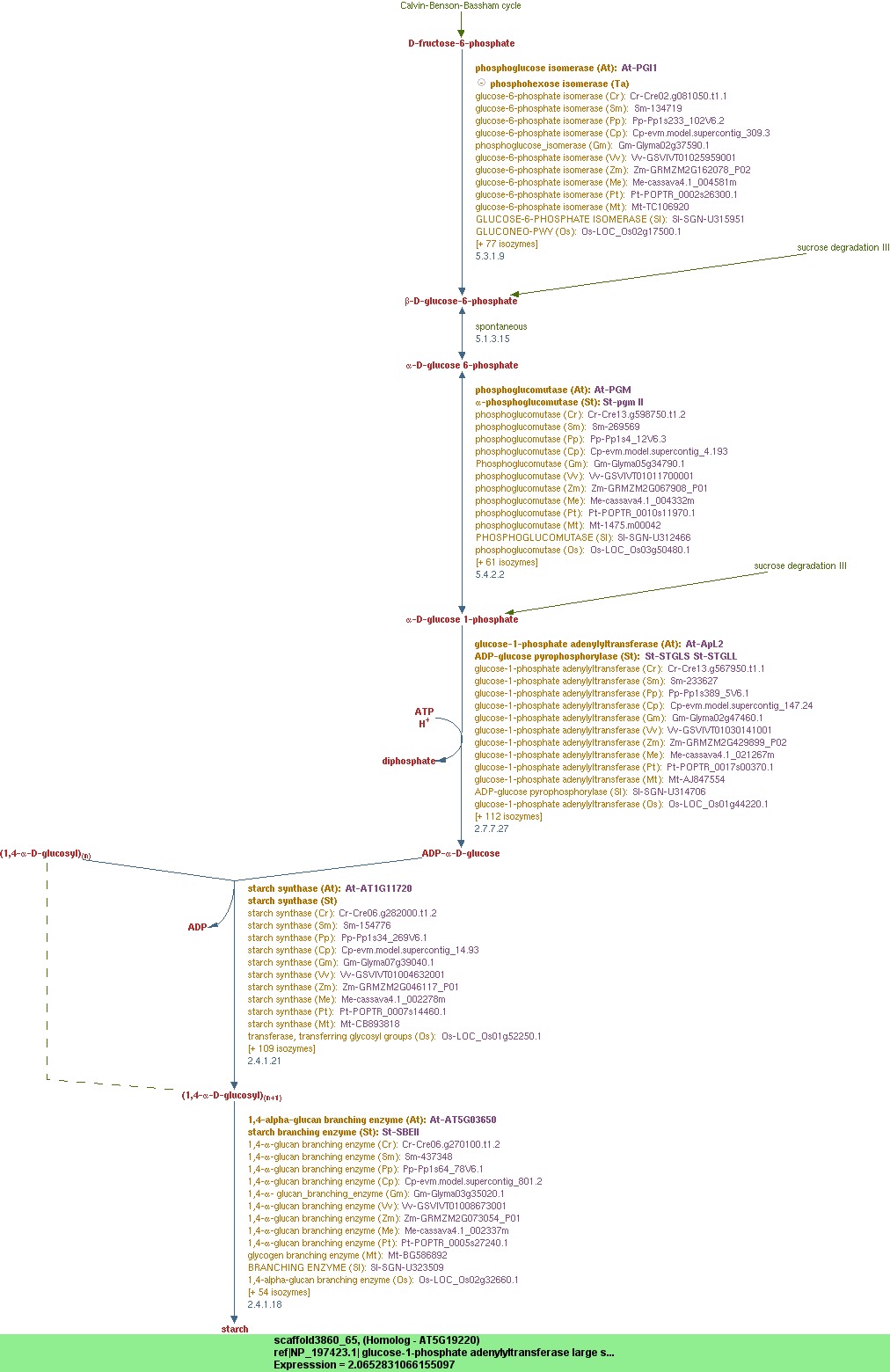

Supplement: Additional file 16 — A and B: Stress related up-regulated PMN pathways. [file 1471-2164-14-647-S16.zip › Additional_file16A_Upregulated_PMN_pathways_in_Shoot/V2SHS/scaffold3860_65_AT5G19220_1_starch_biosynthesis.jpg]

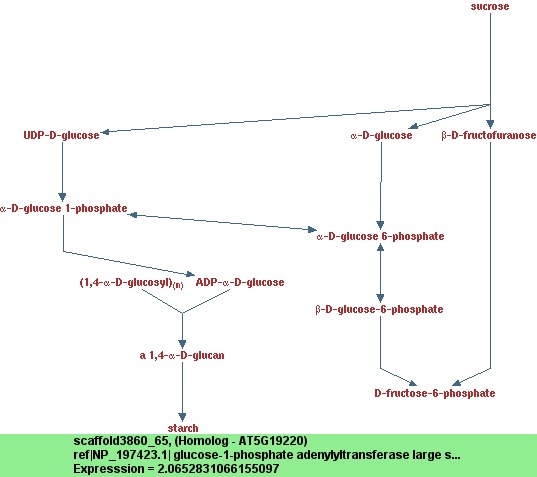

Supplement: Additional file 16 — A and B: Stress related up-regulated PMN pathways. [file 1471-2164-14-647-S16.zip › Additional_file16A_Upregulated_PMN_pathways_in_Shoot/V2SHS/scaffold3860_65_AT5G19220_2_superpathway_of_sucrose_and_starch_metabolism_I_(non-photosynthetic_tissue).jpg]

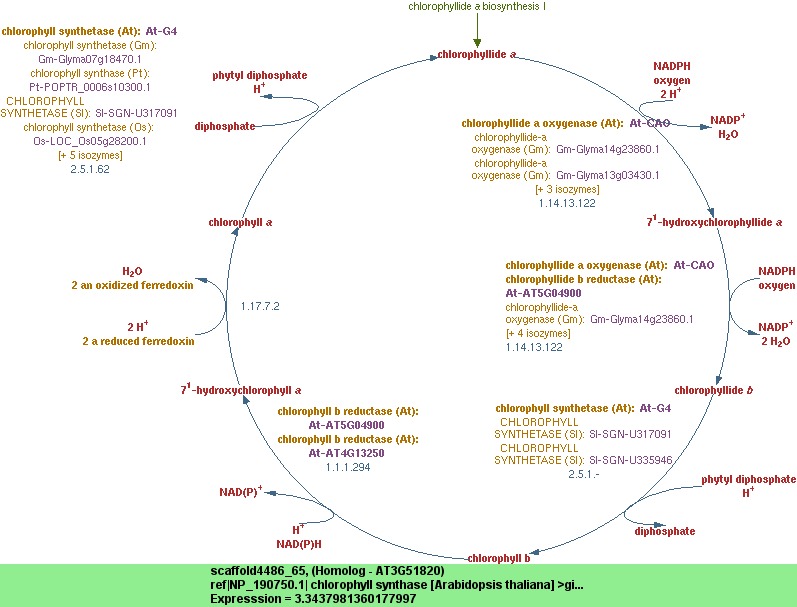

Supplement: Additional file 16 — A and B: Stress related up-regulated PMN pathways. [file 1471-2164-14-647-S16.zip › Additional_file16A_Upregulated_PMN_pathways_in_Shoot/V2SHS/scaffold4486_65_AT3G51820_1_chlorophyll_cycle.jpg]

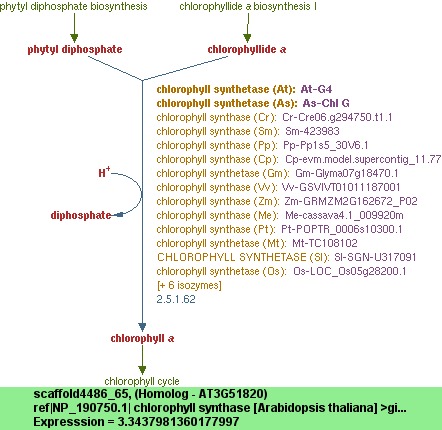

Supplement: Additional file 16 — A and B: Stress related up-regulated PMN pathways. [file 1471-2164-14-647-S16.zip › Additional_file16A_Upregulated_PMN_pathways_in_Shoot/V2SHS/scaffold4486_65_AT3G51820_2_chlorophyll_a_biosynthesis_I.jpg]

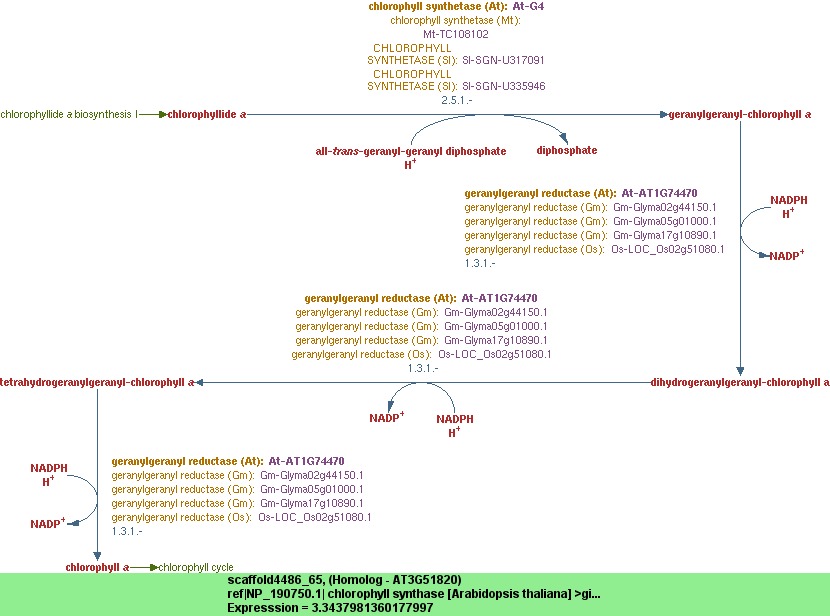

Supplement: Additional file 16 — A and B: Stress related up-regulated PMN pathways. [file 1471-2164-14-647-S16.zip › Additional_file16A_Upregulated_PMN_pathways_in_Shoot/V2SHS/scaffold4486_65_AT3G51820_3_chlorophyll_a_biosynthesis_II.jpg]

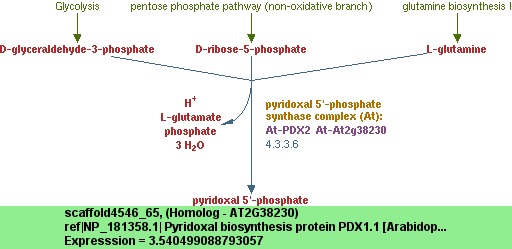

Supplement: Additional file 16 — A and B: Stress related up-regulated PMN pathways. [file 1471-2164-14-647-S16.zip › Additional_file16A_Upregulated_PMN_pathways_in_Shoot/V2SHS/scaffold4546_65_AT2G38230_1_pyridoxal_5'-phosphate_biosynthesis_II.jpg]

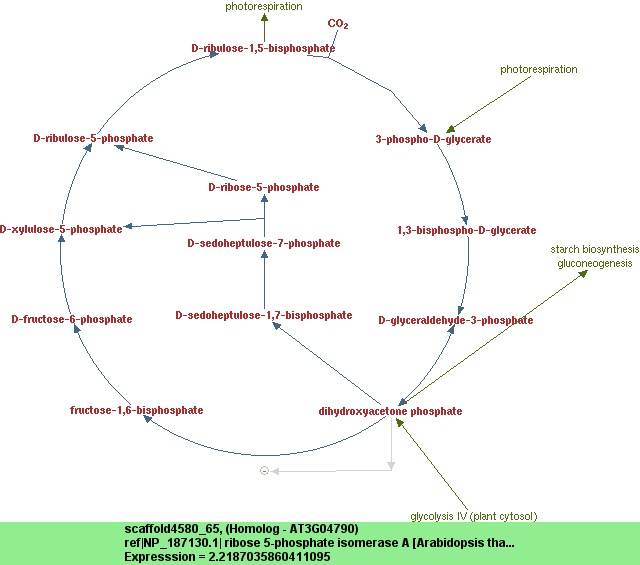

Supplement: Additional file 16 — A and B: Stress related up-regulated PMN pathways. [file 1471-2164-14-647-S16.zip › Additional_file16A_Upregulated_PMN_pathways_in_Shoot/V2SHS/scaffold4580_65_AT3G04790_1_Calvin-Benson-Bassham_cycle.jpg]
