# Supplementary figures and images for: Comprehensive transcriptomic study on horse gram (Macrotyloma uniflorum): De novo assembly, functional characterization and comparative analysis in relation to drought stress (part 3 of 4)
Source: BMC Genomics. 2013 Sep 23;14:647. doi: 10.1186/1471-2164-14-647 (PMC3853109; doi:10.1186/1471-2164-14-647)

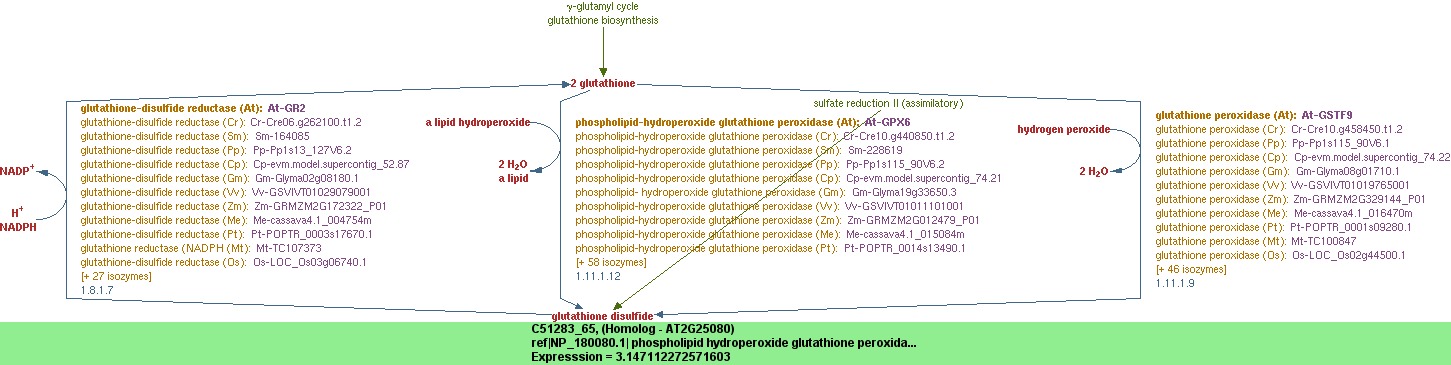

Supplement: Additional file 16 — A and B: Stress related up-regulated PMN pathways. [file 1471-2164-14-647-S16.zip › Additional_file16A_Upregulated_PMN_pathways_in_Shoot/V2SHS/C51283_65_AT2G25080_1_glutathione_redox_reactions_I.jpg]

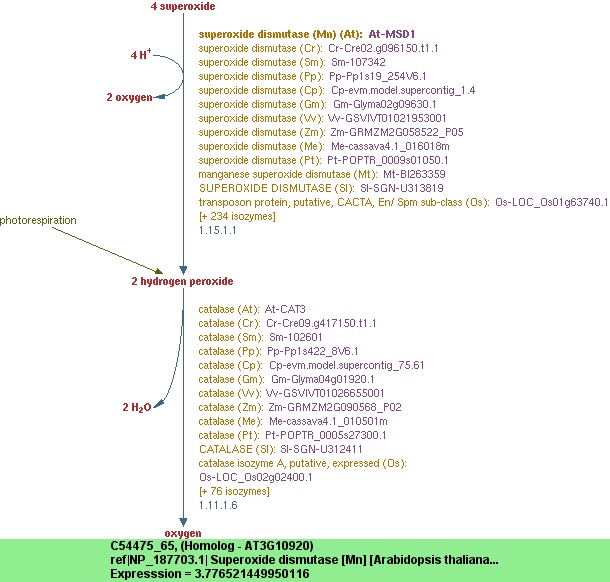

Supplement: Additional file 16 — A and B: Stress related up-regulated PMN pathways. [file 1471-2164-14-647-S16.zip › Additional_file16A_Upregulated_PMN_pathways_in_Shoot/V2SHS/C54475_65_AT3G10920_1_superoxide_radicals_degradation.jpg]

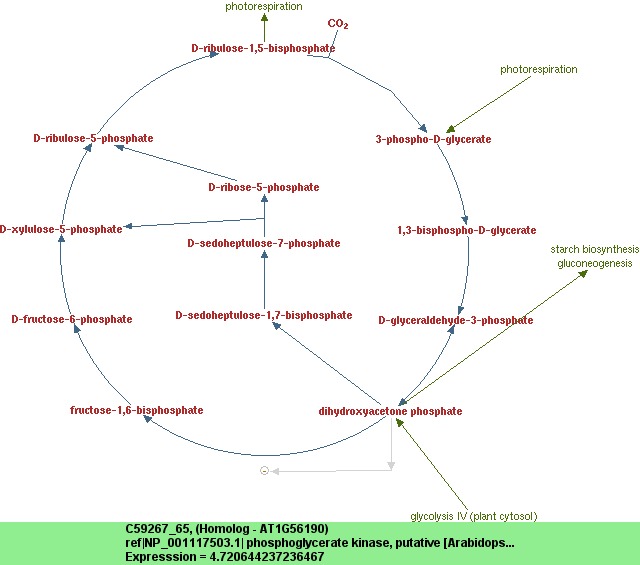

Supplement: Additional file 16 — A and B: Stress related up-regulated PMN pathways. [file 1471-2164-14-647-S16.zip › Additional_file16A_Upregulated_PMN_pathways_in_Shoot/V2SHS/C59267_65_AT1G56190_1_Calvin-Benson-Bassham_cycle.jpg]

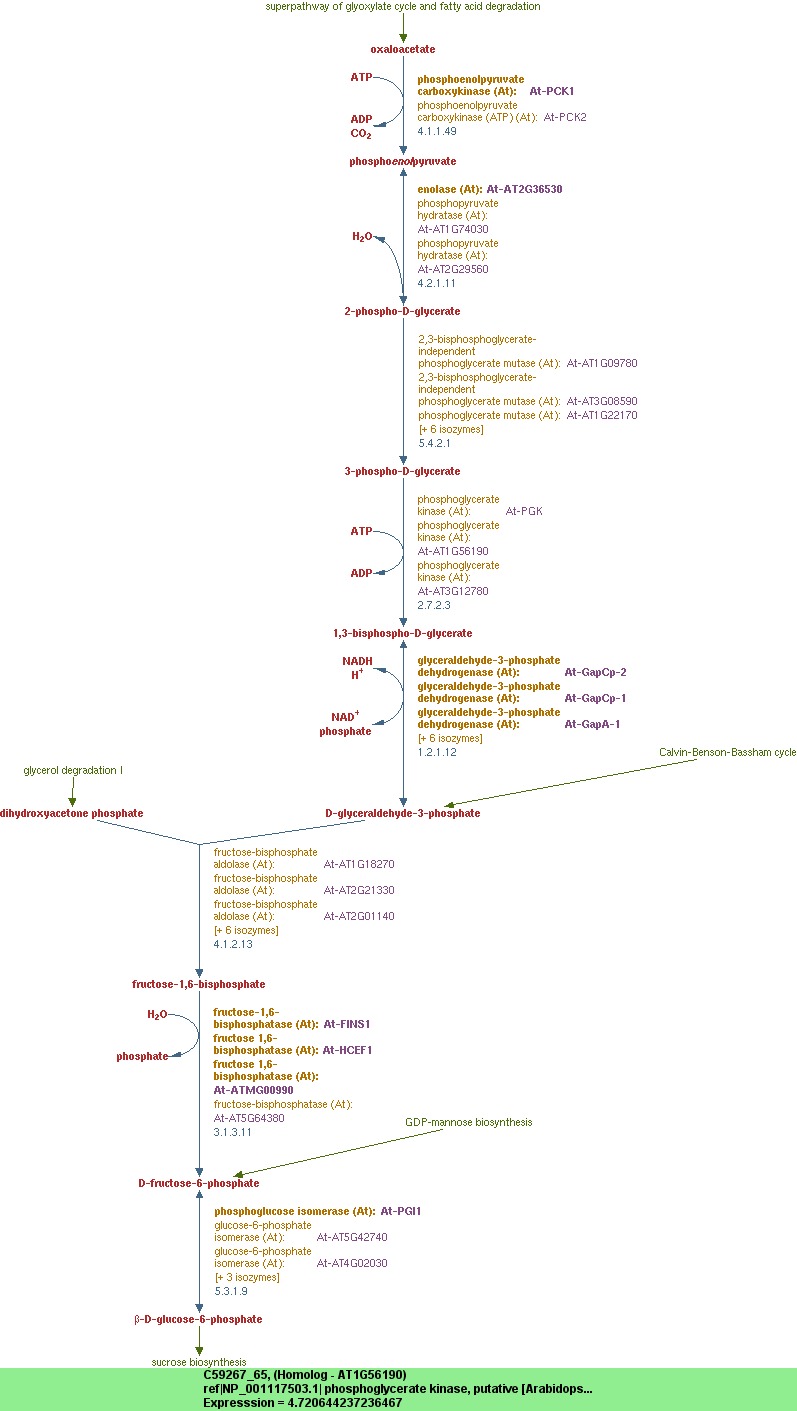

Supplement: Additional file 16 — A and B: Stress related up-regulated PMN pathways. [file 1471-2164-14-647-S16.zip › Additional_file16A_Upregulated_PMN_pathways_in_Shoot/V2SHS/C59267_65_AT1G56190_3_gluconeogenesis.jpg]

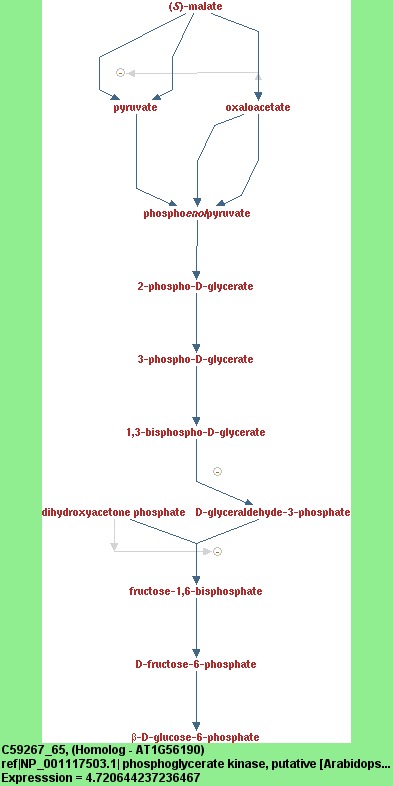

Supplement: Additional file 16 — A and B: Stress related up-regulated PMN pathways. [file 1471-2164-14-647-S16.zip › Additional_file16A_Upregulated_PMN_pathways_in_Shoot/V2SHS/C59267_65_AT1G56190_5_gluconeogenesis_I.jpg]

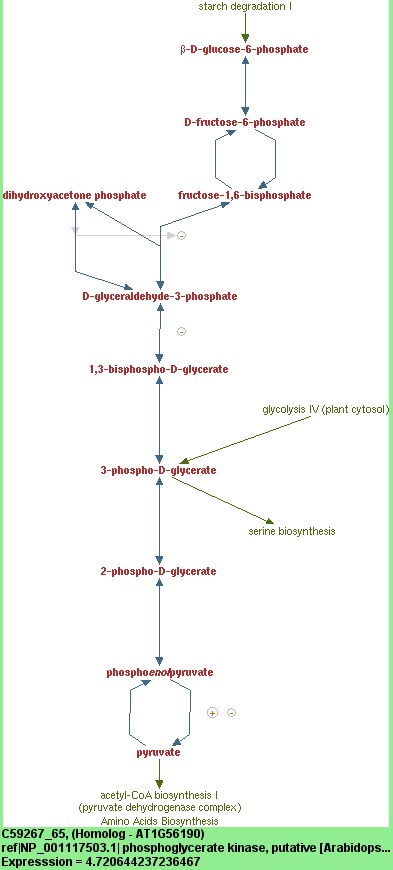

Supplement: Additional file 16 — A and B: Stress related up-regulated PMN pathways. [file 1471-2164-14-647-S16.zip › Additional_file16A_Upregulated_PMN_pathways_in_Shoot/V2SHS/C59267_65_AT1G56190_7_glycolysis_I.jpg]

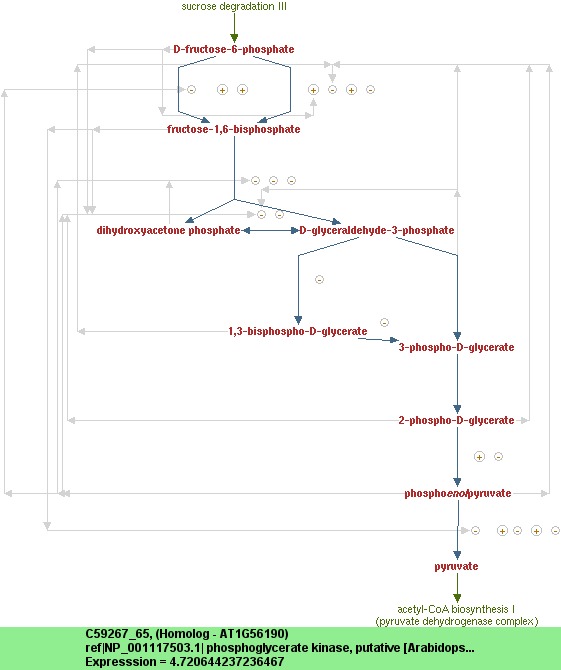

Supplement: Additional file 16 — A and B: Stress related up-regulated PMN pathways. [file 1471-2164-14-647-S16.zip › Additional_file16A_Upregulated_PMN_pathways_in_Shoot/V2SHS/C59267_65_AT1G56190_9_glycolysis_IV_(plant_cytosol).jpg]

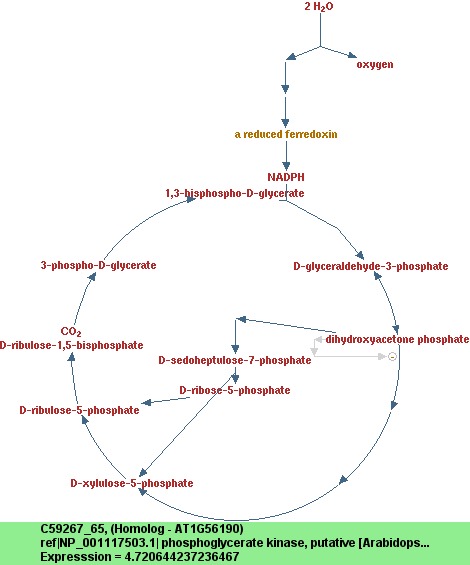

Supplement: Additional file 16 — A and B: Stress related up-regulated PMN pathways. [file 1471-2164-14-647-S16.zip › Additional_file16A_Upregulated_PMN_pathways_in_Shoot/V2SHS/C59267_65_AT1G56190_11_oxygenic_photosynthesis.jpg]

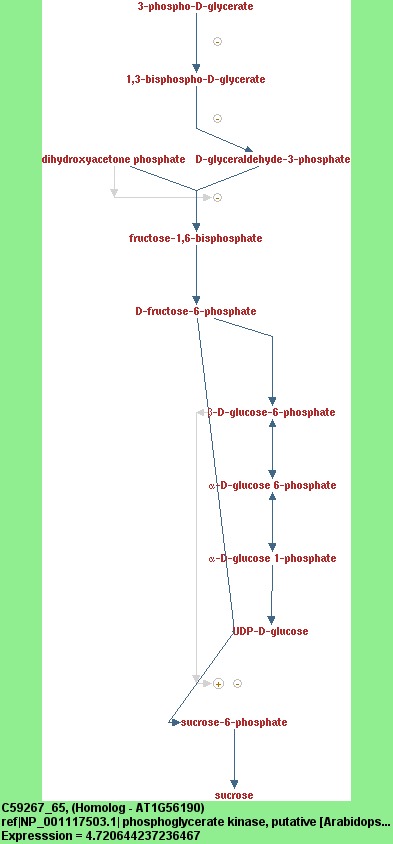

Supplement: Additional file 16 — A and B: Stress related up-regulated PMN pathways. [file 1471-2164-14-647-S16.zip › Additional_file16A_Upregulated_PMN_pathways_in_Shoot/V2SHS/C59267_65_AT1G56190_13_sucrose_biosynthesis.jpg]

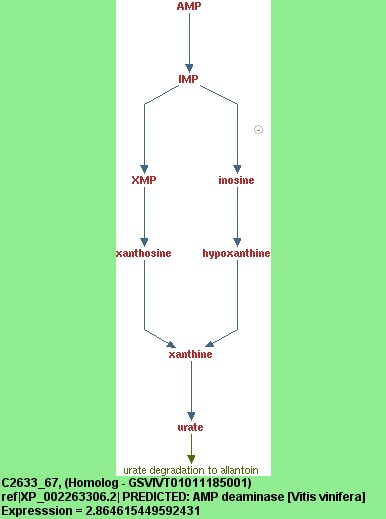

Supplement: Additional file 17 — Details of Transcription factor families. [file 1471-2164-14-647-S17.zip › Additional_file16B_Upregulated_PMN_pathways_in_Root/V1RS/C2633_67_GSVIVT01011185001_1_adenosine_nucleotides_degradation_I.jpg]

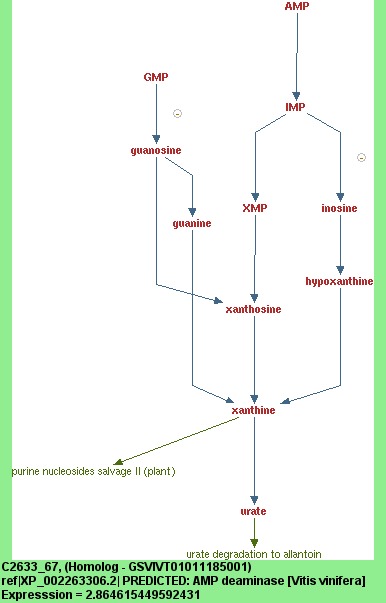

Supplement: Additional file 17 — Details of Transcription factor families. [file 1471-2164-14-647-S17.zip › Additional_file16B_Upregulated_PMN_pathways_in_Root/V1RS/C2633_67_GSVIVT01011185001_2_purine_nucleotides_degradation_I_(plants).jpg]

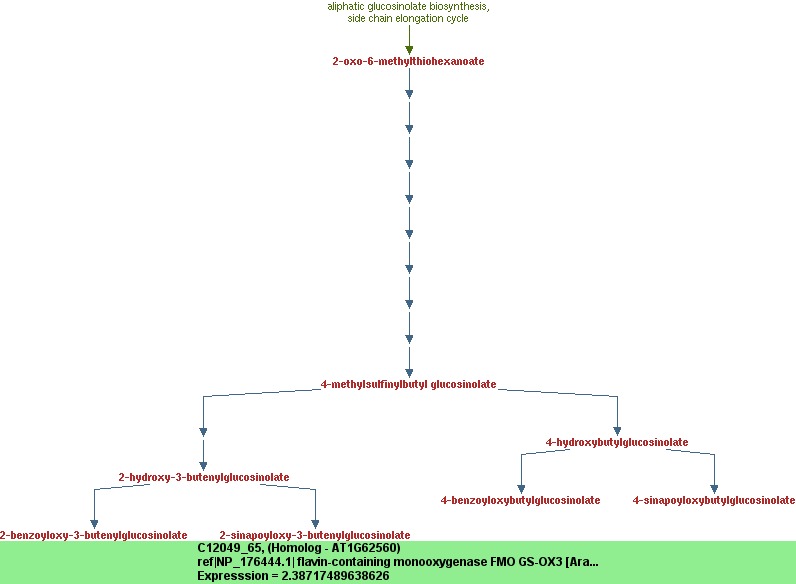

Supplement: Additional file 17 — Details of Transcription factor families. [file 1471-2164-14-647-S17.zip › Additional_file16B_Upregulated_PMN_pathways_in_Root/V1RS/C12049_65_AT1G62560_1_glucosinolate_biosynthesis_from_dihomomethionine.jpg]

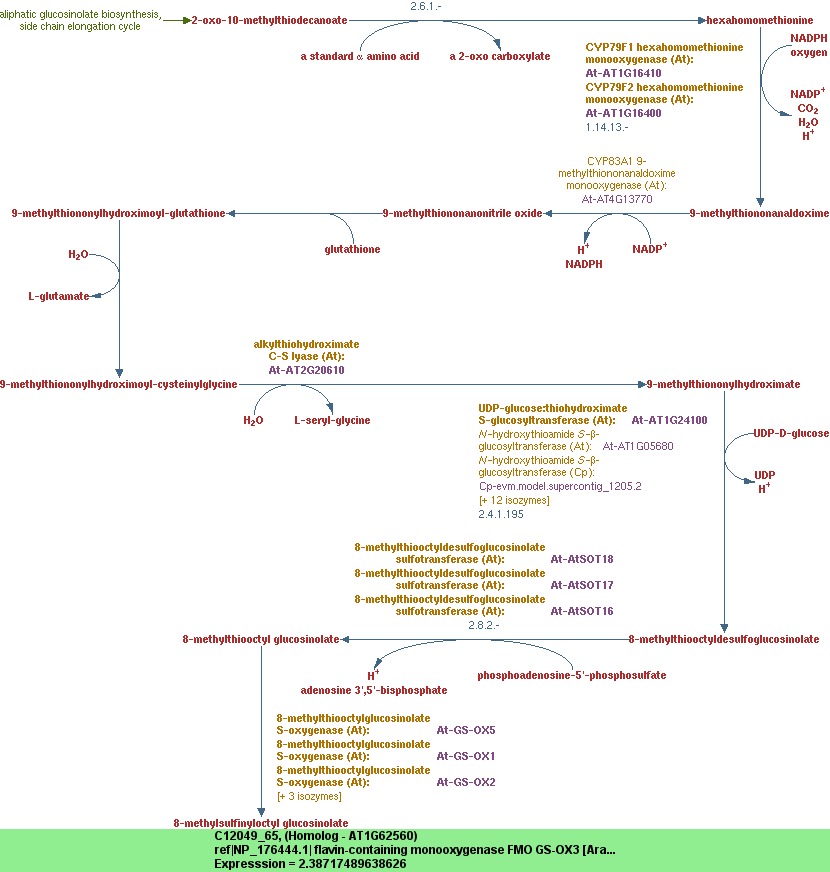

Supplement: Additional file 17 — Details of Transcription factor families. [file 1471-2164-14-647-S17.zip › Additional_file16B_Upregulated_PMN_pathways_in_Root/V1RS/C12049_65_AT1G62560_2_glucosinolate_biosynthesis_from_hexahomomethionine.jpg]

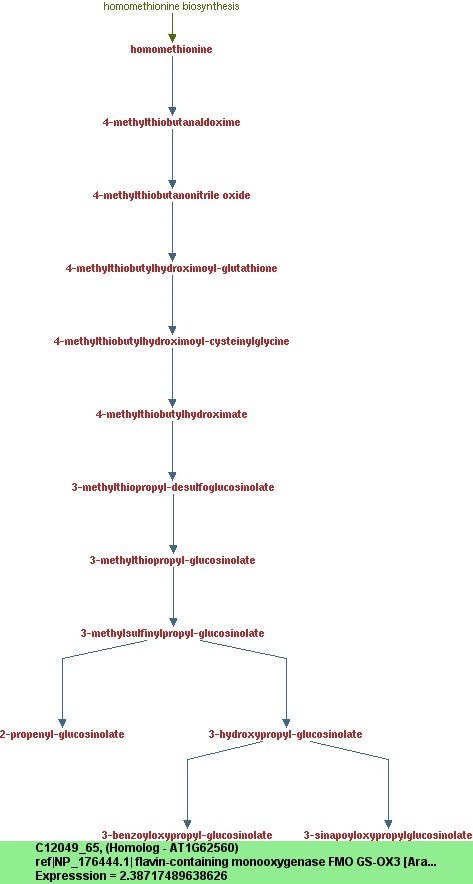

Supplement: Additional file 17 — Details of Transcription factor families. [file 1471-2164-14-647-S17.zip › Additional_file16B_Upregulated_PMN_pathways_in_Root/V1RS/C12049_65_AT1G62560_3_glucosinolate_biosynthesis_from_homomethionine.jpg]

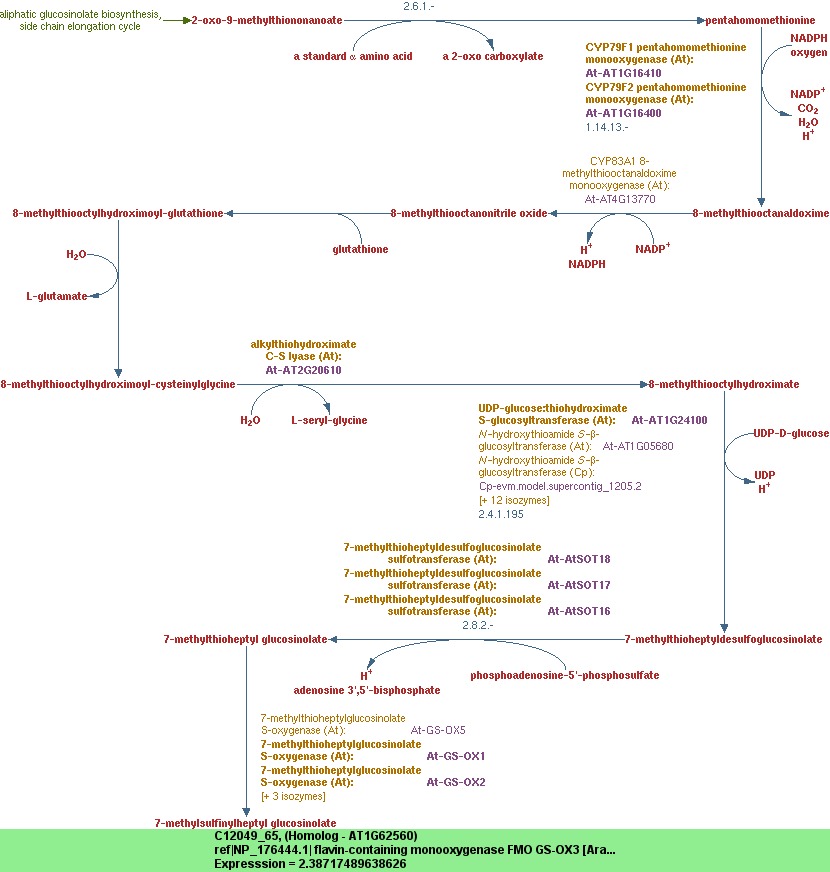

Supplement: Additional file 17 — Details of Transcription factor families. [file 1471-2164-14-647-S17.zip › Additional_file16B_Upregulated_PMN_pathways_in_Root/V1RS/C12049_65_AT1G62560_4_glucosinolate_biosynthesis_from_pentahomomethionine.jpg]

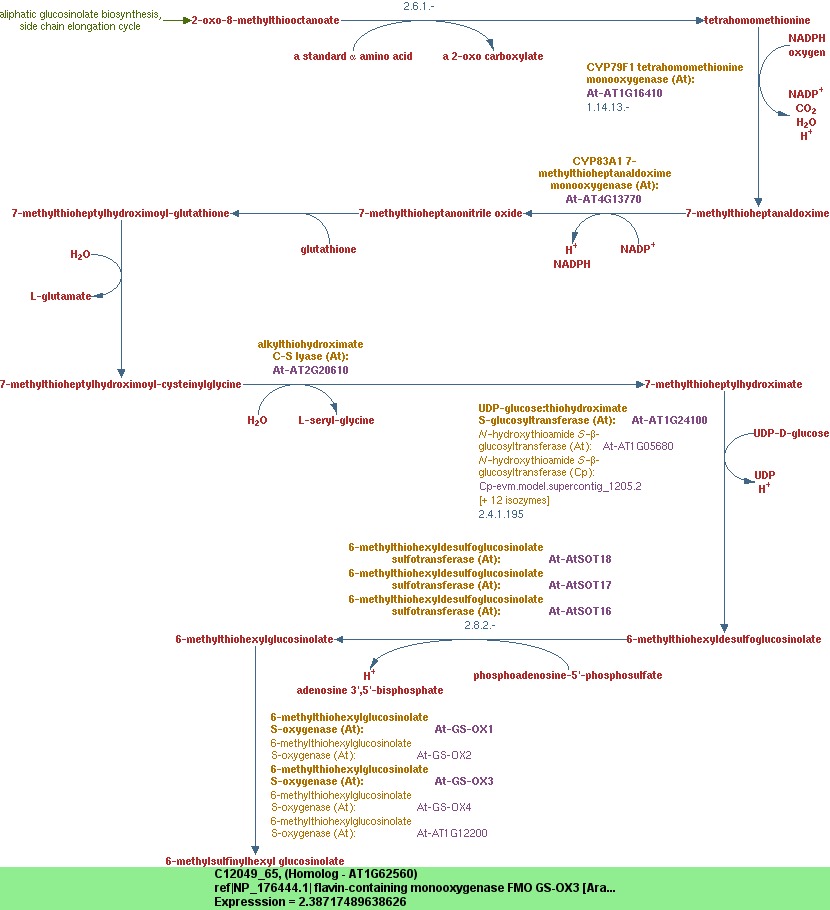

Supplement: Additional file 17 — Details of Transcription factor families. [file 1471-2164-14-647-S17.zip › Additional_file16B_Upregulated_PMN_pathways_in_Root/V1RS/C12049_65_AT1G62560_5_glucosinolate_biosynthesis_from_tetrahomomethionine.jpg]

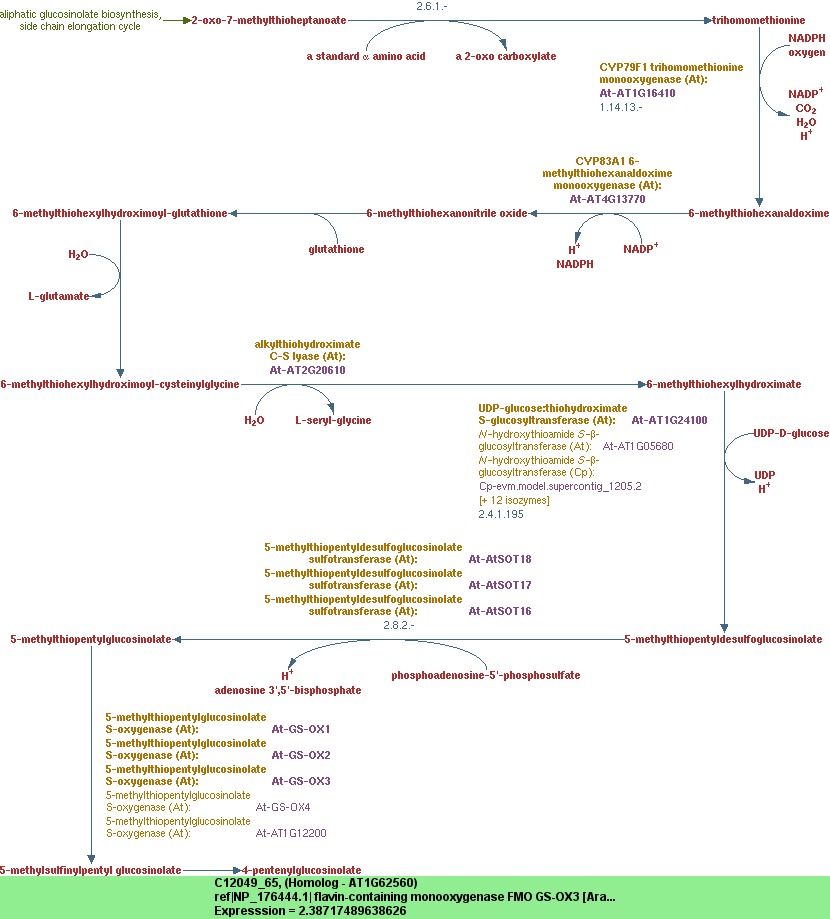

Supplement: Additional file 17 — Details of Transcription factor families. [file 1471-2164-14-647-S17.zip › Additional_file16B_Upregulated_PMN_pathways_in_Root/V1RS/C12049_65_AT1G62560_6_glucosinolate_biosynthesis_from_trihomomethionine.jpg]

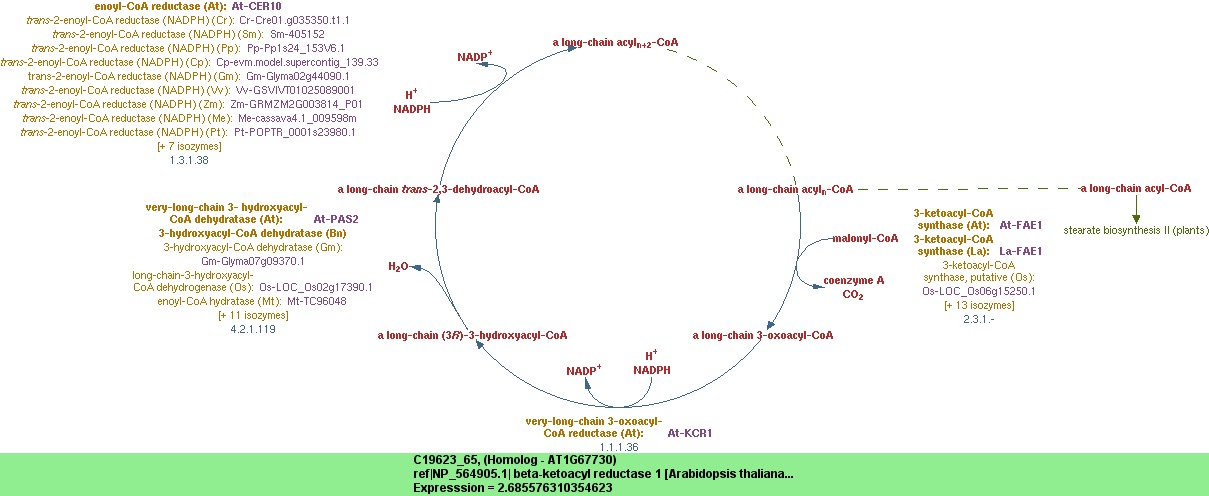

Supplement: Additional file 17 — Details of Transcription factor families. [file 1471-2164-14-647-S17.zip › Additional_file16B_Upregulated_PMN_pathways_in_Root/V1RS/C19623_65_AT1G67730_1_very_long_chain_fatty_acid_biosynthesis.jpg]

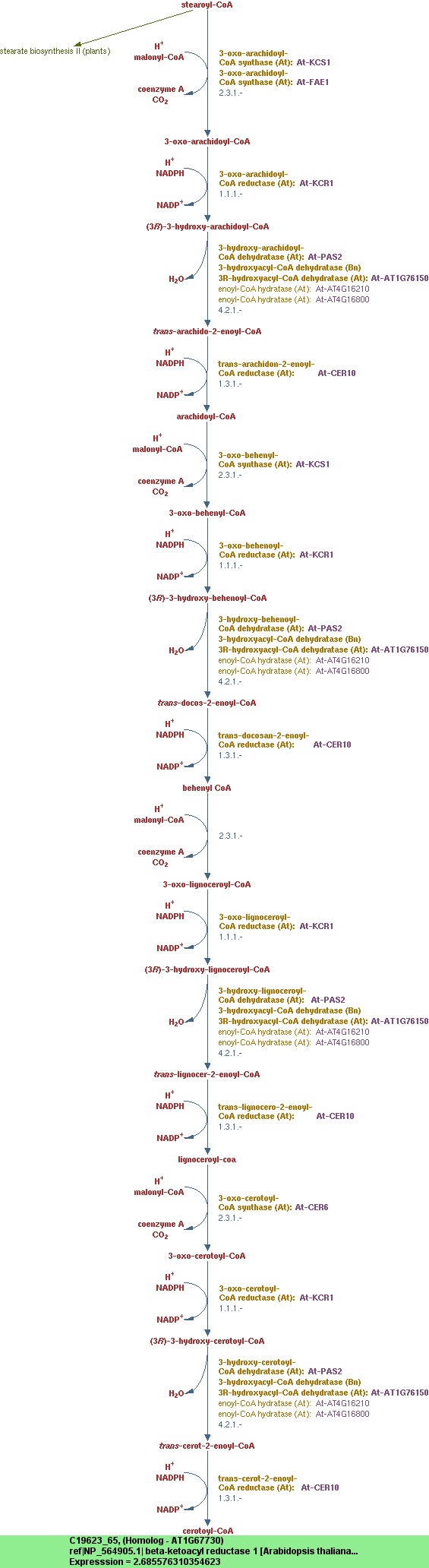

Supplement: Additional file 17 — Details of Transcription factor families. [file 1471-2164-14-647-S17.zip › Additional_file16B_Upregulated_PMN_pathways_in_Root/V1RS/C19623_65_AT1G67730_2_very_long_chain_fatty_acid_biosynthesis_II.jpg]

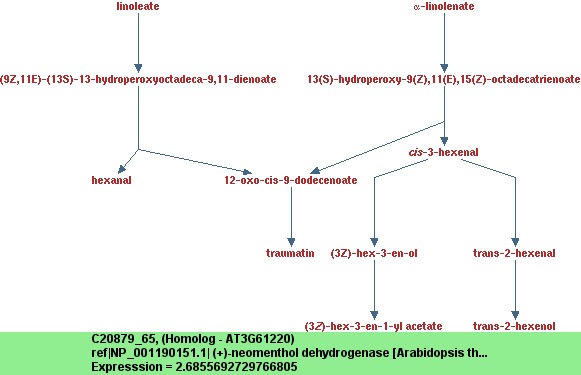

Supplement: Additional file 17 — Details of Transcription factor families. [file 1471-2164-14-647-S17.zip › Additional_file16B_Upregulated_PMN_pathways_in_Root/V1RS/C20879_65_AT3G61220_1_traumatin_and_(Z)-3-hexen-1-yl_acetate_biosynthesis.jpg]

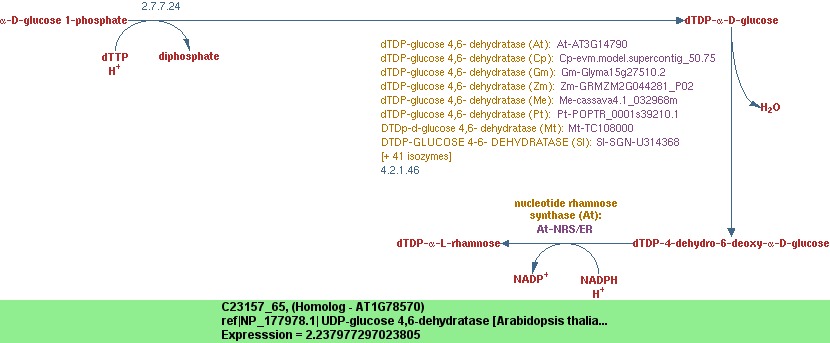

Supplement: Additional file 17 — Details of Transcription factor families. [file 1471-2164-14-647-S17.zip › Additional_file16B_Upregulated_PMN_pathways_in_Root/V1RS/C23157_65_AT1G78570_1_dTDP-L-rhamnose_biosynthesis_II.jpg]

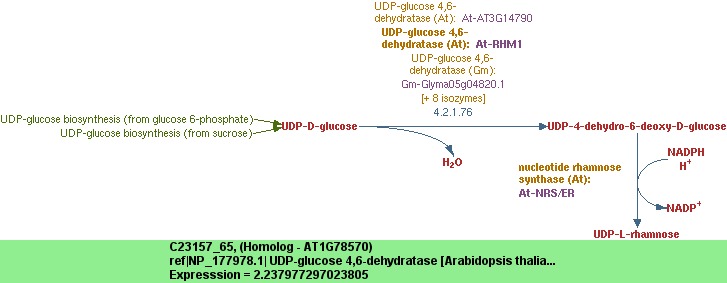

Supplement: Additional file 17 — Details of Transcription factor families. [file 1471-2164-14-647-S17.zip › Additional_file16B_Upregulated_PMN_pathways_in_Root/V1RS/C23157_65_AT1G78570_2_UDP-L-rhamnose_biosynthesis.jpg]

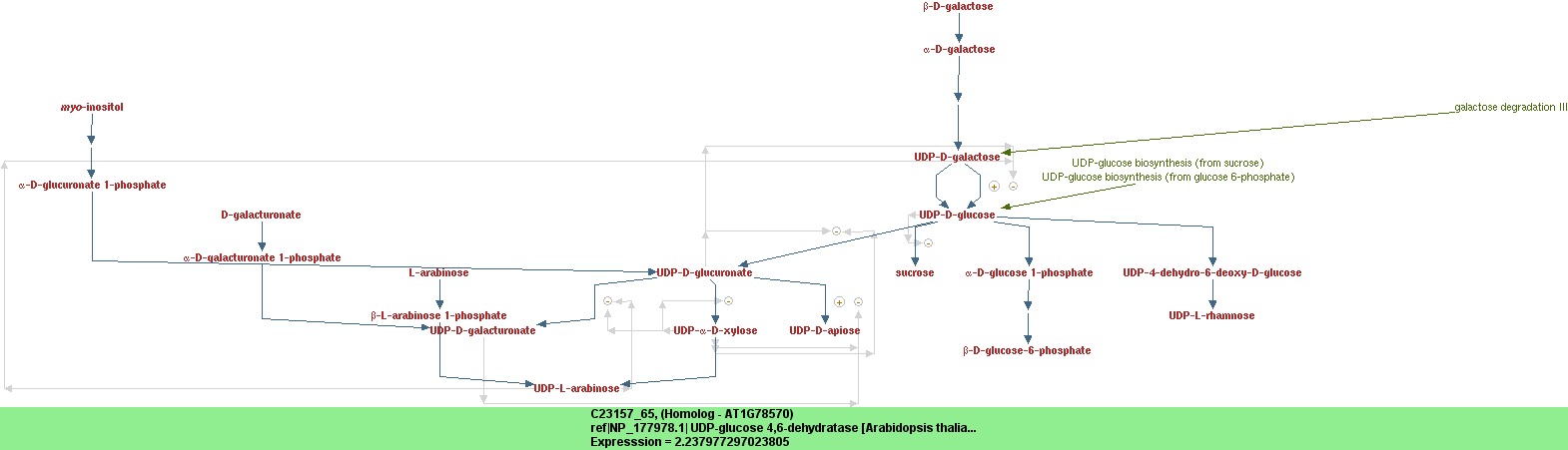

Supplement: Additional file 17 — Details of Transcription factor families. [file 1471-2164-14-647-S17.zip › Additional_file16B_Upregulated_PMN_pathways_in_Root/V1RS/C23157_65_AT1G78570_3_UDP-sugars_interconversion.jpg]

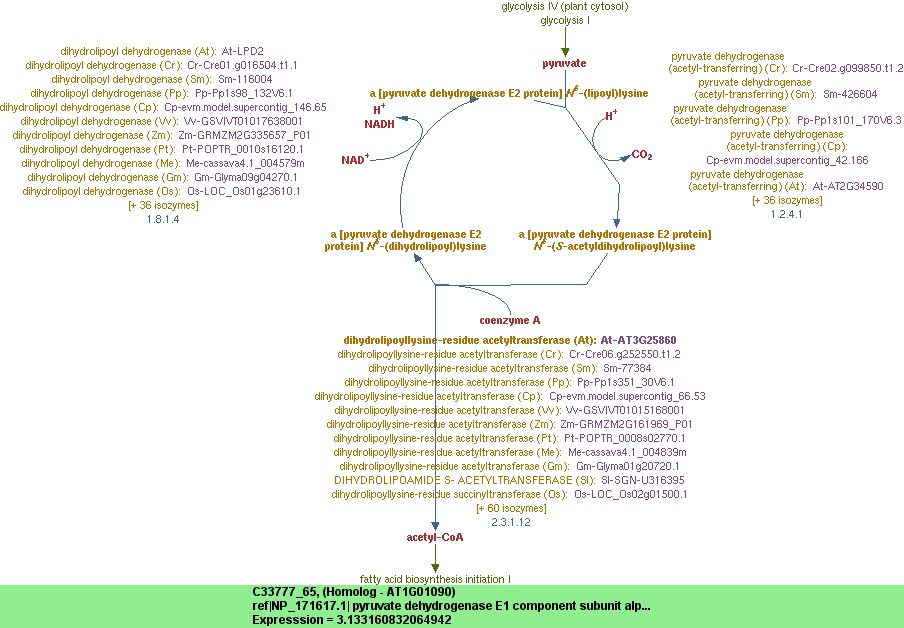

Supplement: Additional file 17 — Details of Transcription factor families. [file 1471-2164-14-647-S17.zip › Additional_file16B_Upregulated_PMN_pathways_in_Root/V1RS/C33777_65_AT1G01090_1_acetyl-CoA_biosynthesis_I_(pyruvate_dehydrogenase_complex).jpg]

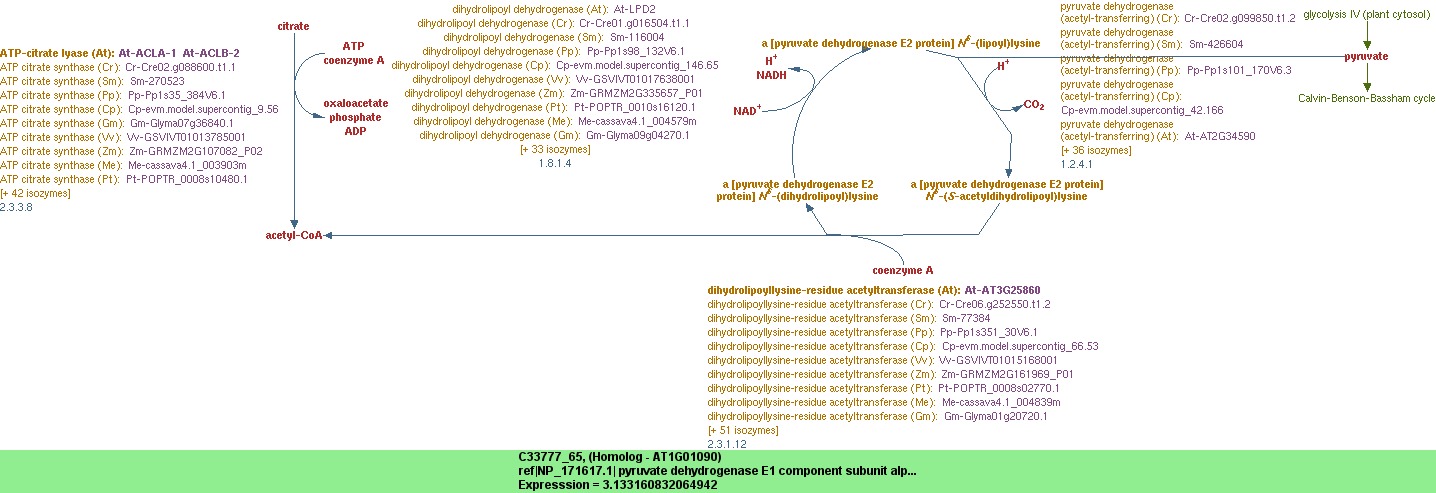

Supplement: Additional file 17 — Details of Transcription factor families. [file 1471-2164-14-647-S17.zip › Additional_file16B_Upregulated_PMN_pathways_in_Root/V1RS/C33777_65_AT1G01090_3_superpathway_of_acetyl-CoA_biosynthesis.jpg]

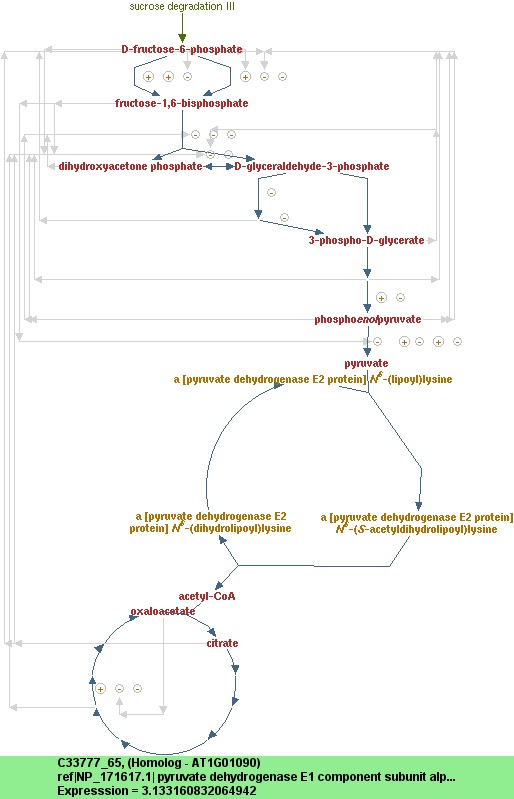

Supplement: Additional file 17 — Details of Transcription factor families. [file 1471-2164-14-647-S17.zip › Additional_file16B_Upregulated_PMN_pathways_in_Root/V1RS/C33777_65_AT1G01090_5_superpathway_of_cytosolic_glycolysis_(plants),_pyruvate_dehydrogenase_and_TCA_cycle.jpg]

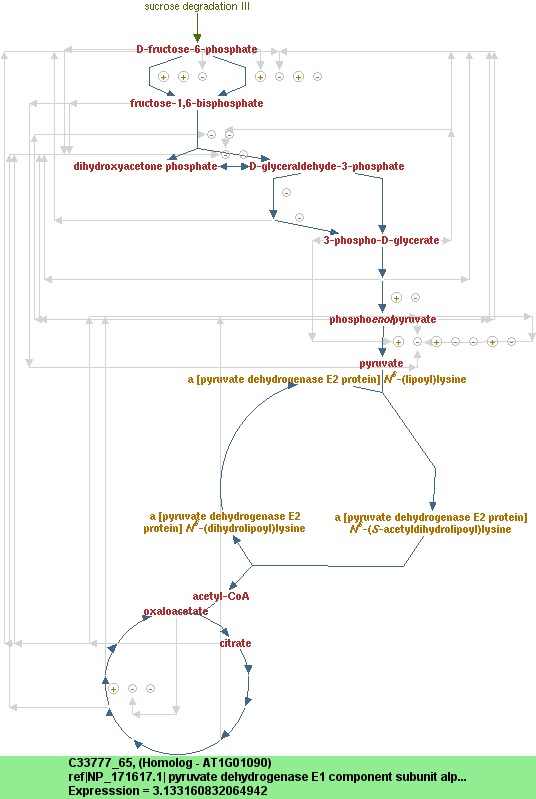

Supplement: Additional file 17 — Details of Transcription factor families. [file 1471-2164-14-647-S17.zip › Additional_file16B_Upregulated_PMN_pathways_in_Root/V1RS/C33777_65_AT1G01090_7_superpathway_of_cytosolic_glycolysis_(plants),_pyruvate_dehydrogenase_and_TCA_cycle.jpg]

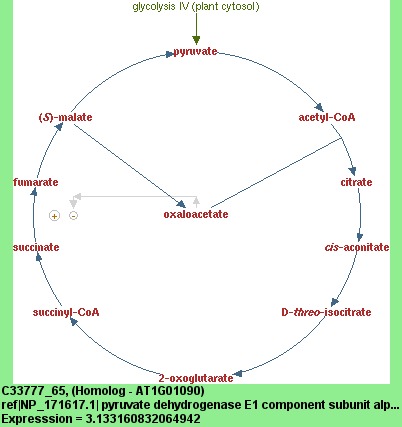

Supplement: Additional file 17 — Details of Transcription factor families. [file 1471-2164-14-647-S17.zip › Additional_file16B_Upregulated_PMN_pathways_in_Root/V1RS/C33777_65_AT1G01090_9_TCA_cycle_variation_V_(plant).jpg]

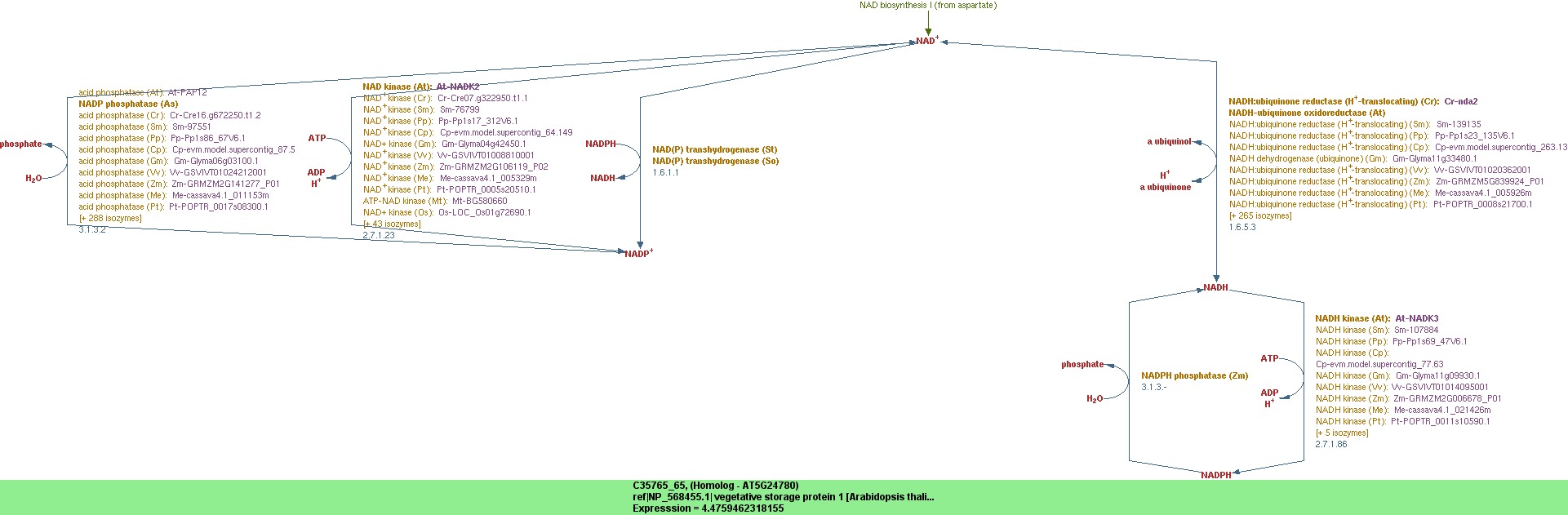

Supplement: Additional file 17 — Details of Transcription factor families. [file 1471-2164-14-647-S17.zip › Additional_file16B_Upregulated_PMN_pathways_in_Root/V1RS/C35765_65_AT5G24780_1_NAD-NADH_phosphorylation_and_dephosphorylation.jpg]

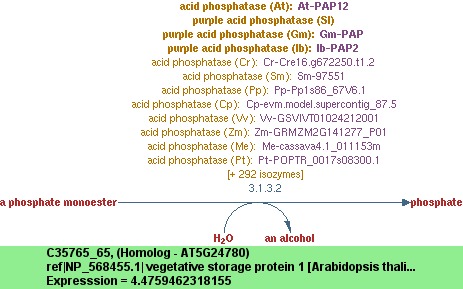

Supplement: Additional file 17 — Details of Transcription factor families. [file 1471-2164-14-647-S17.zip › Additional_file16B_Upregulated_PMN_pathways_in_Root/V1RS/C35765_65_AT5G24780_3_phosphate_acquisition.jpg]

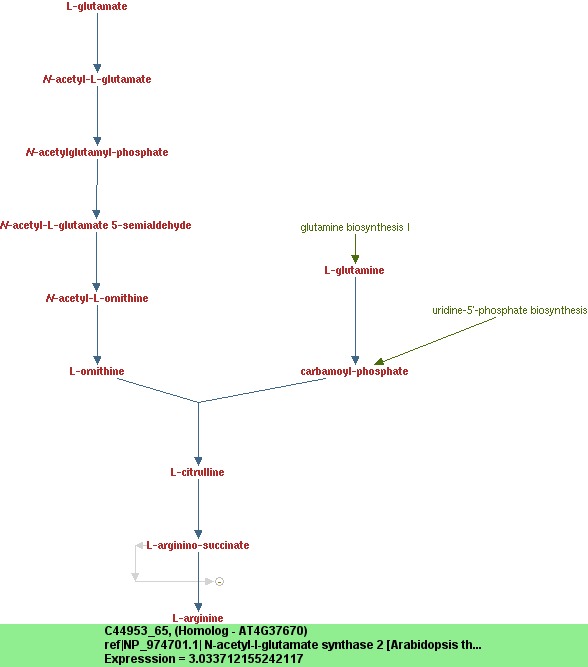

Supplement: Additional file 17 — Details of Transcription factor families. [file 1471-2164-14-647-S17.zip › Additional_file16B_Upregulated_PMN_pathways_in_Root/V1RS/C44953_65_AT4G37670_1_arginine_biosynthesis_I.jpg]

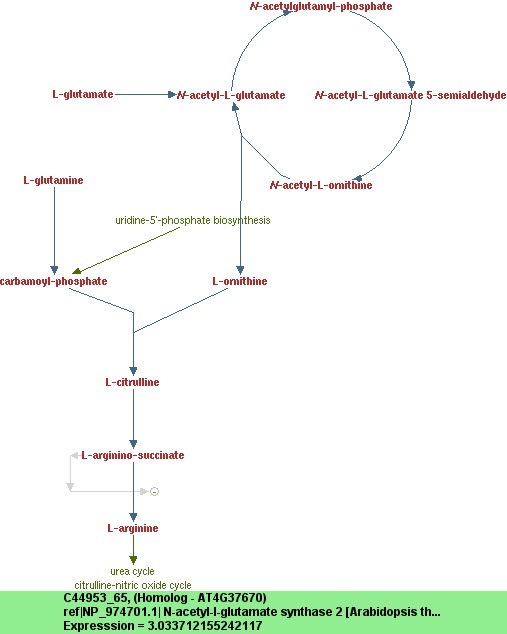

Supplement: Additional file 17 — Details of Transcription factor families. [file 1471-2164-14-647-S17.zip › Additional_file16B_Upregulated_PMN_pathways_in_Root/V1RS/C44953_65_AT4G37670_3_arginine_biosynthesis_II_(acetyl_cycle).jpg]

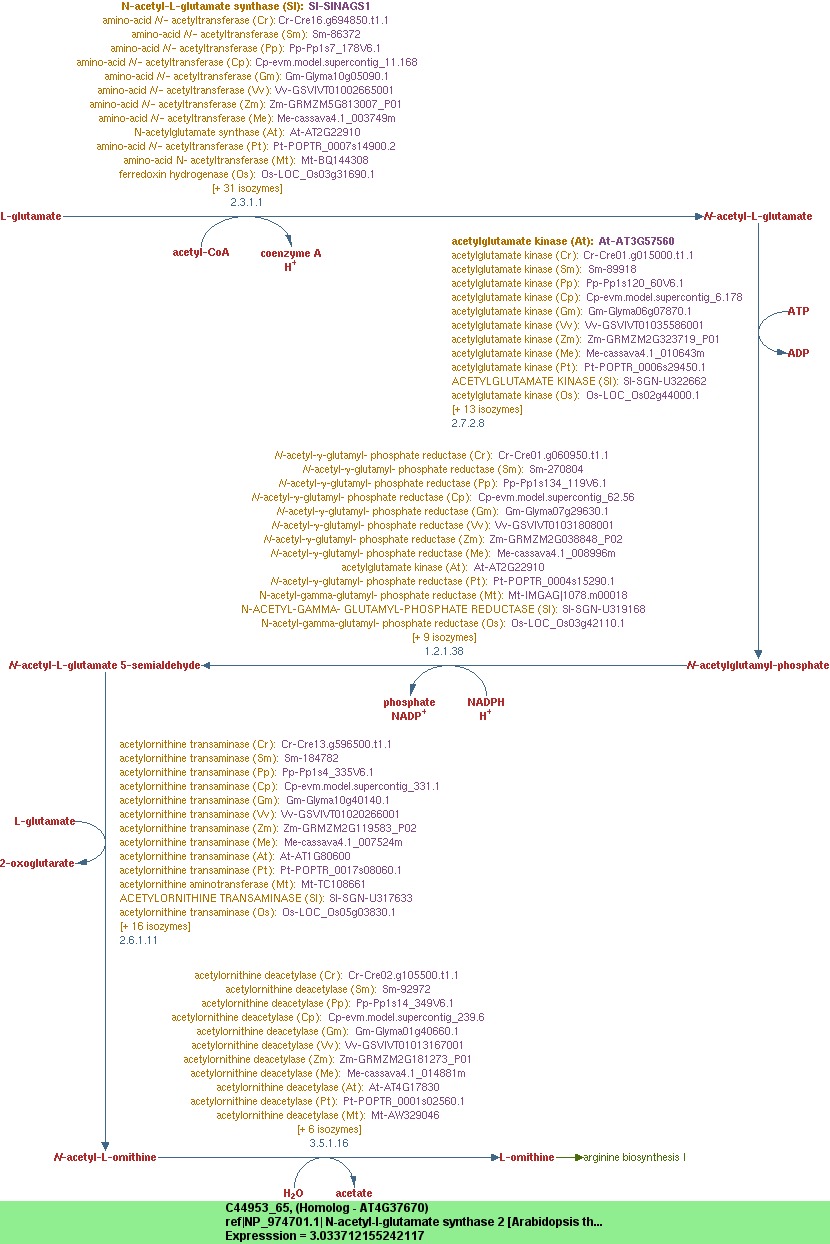

Supplement: Additional file 17 — Details of Transcription factor families. [file 1471-2164-14-647-S17.zip › Additional_file16B_Upregulated_PMN_pathways_in_Root/V1RS/C44953_65_AT4G37670_5_ornithine_biosynthesis.jpg]

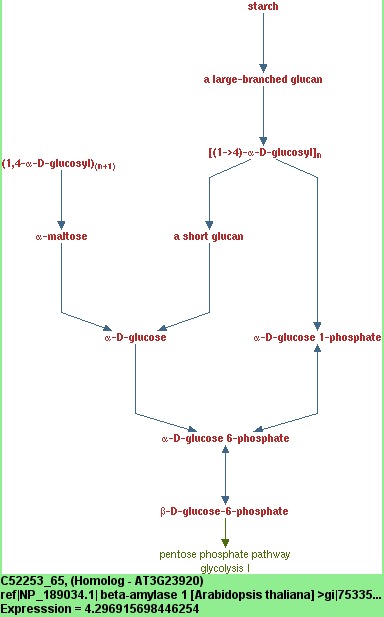

Supplement: Additional file 17 — Details of Transcription factor families. [file 1471-2164-14-647-S17.zip › Additional_file16B_Upregulated_PMN_pathways_in_Root/V1RS/C52253_65_AT3G23920_1_starch_degradation_I.jpg]

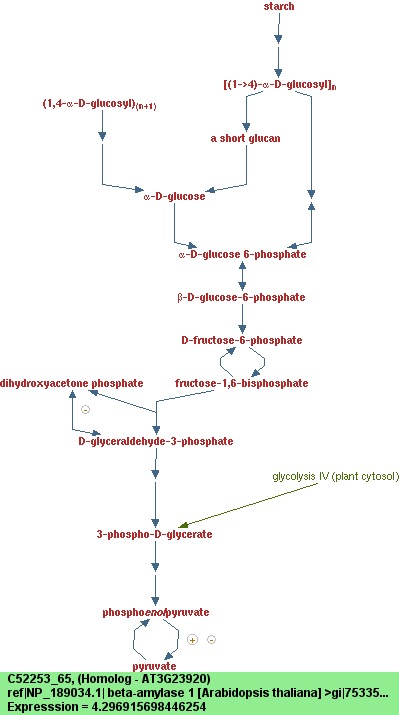

Supplement: Additional file 17 — Details of Transcription factor families. [file 1471-2164-14-647-S17.zip › Additional_file16B_Upregulated_PMN_pathways_in_Root/V1RS/C52253_65_AT3G23920_2_superpathway_of_starch_degradation_to_pyruvate.jpg]

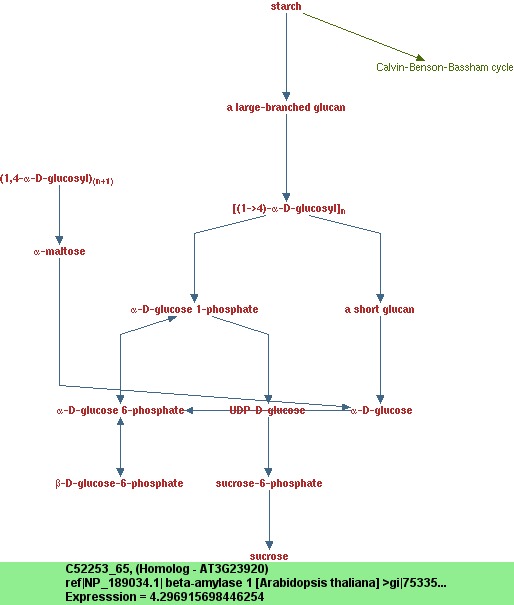

Supplement: Additional file 17 — Details of Transcription factor families. [file 1471-2164-14-647-S17.zip › Additional_file16B_Upregulated_PMN_pathways_in_Root/V1RS/C52253_65_AT3G23920_3_superpathway_of_sucrose_and_starch_metabolism_II_(photosynthetic_tissue).jpg]

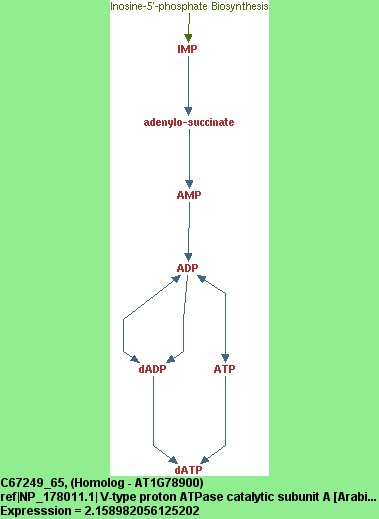

Supplement: Additional file 17 — Details of Transcription factor families. [file 1471-2164-14-647-S17.zip › Additional_file16B_Upregulated_PMN_pathways_in_Root/V1RS/C67249_65_AT1G78900_1_adenosine_nucleotides_de_novo_biosynthesis.jpg]

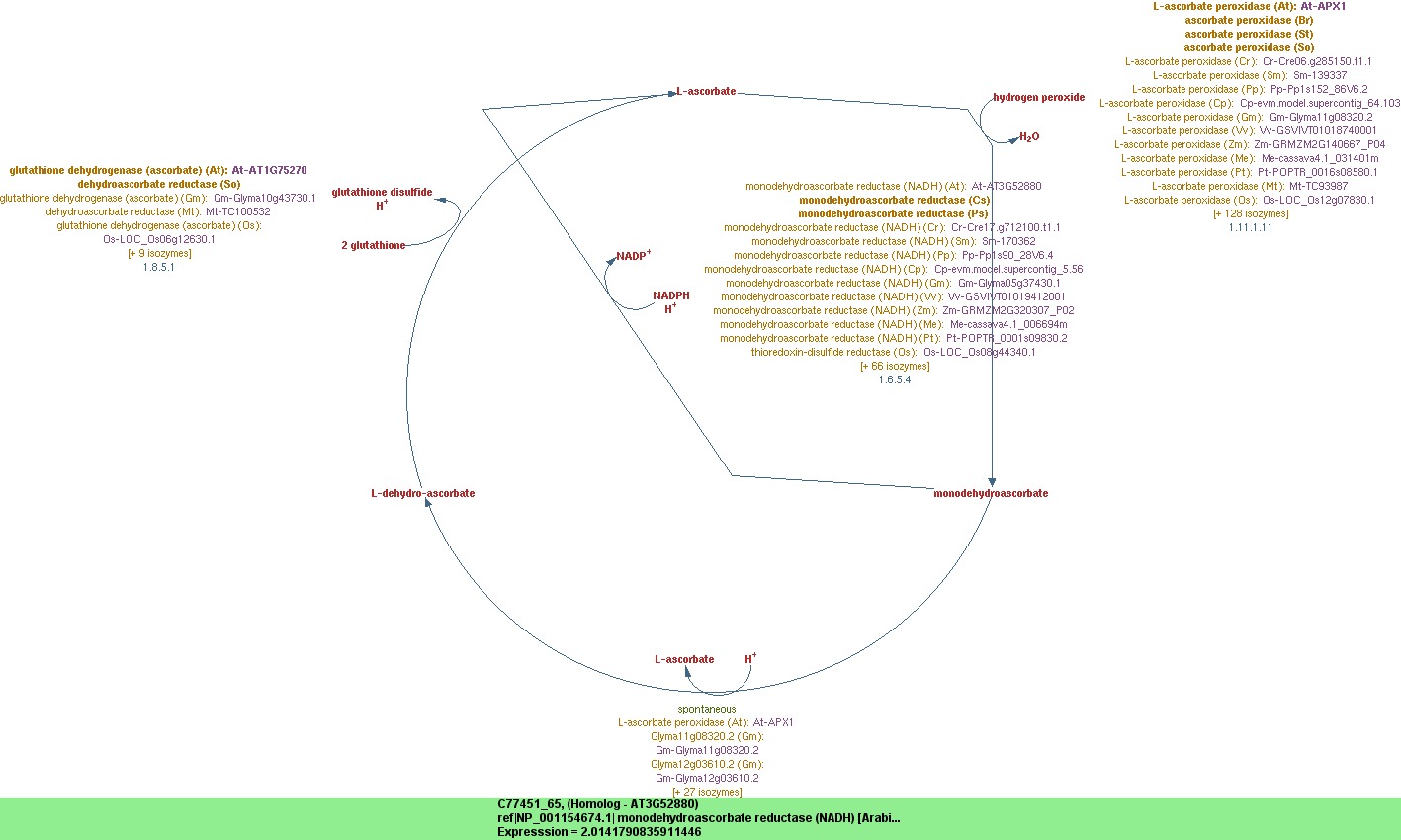

Supplement: Additional file 17 — Details of Transcription factor families. [file 1471-2164-14-647-S17.zip › Additional_file16B_Upregulated_PMN_pathways_in_Root/V1RS/C77451_65_AT3G52880_1_ascorbate_glutathione_cycle.jpg]

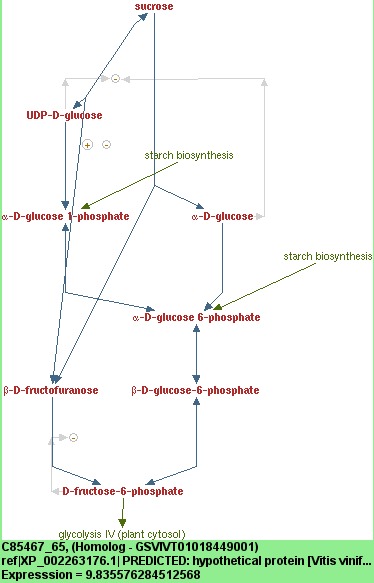

Supplement: Additional file 17 — Details of Transcription factor families. [file 1471-2164-14-647-S17.zip › Additional_file16B_Upregulated_PMN_pathways_in_Root/V1RS/C85467_65_GSVIVT01018449001_1_sucrose_degradation_III.jpg]

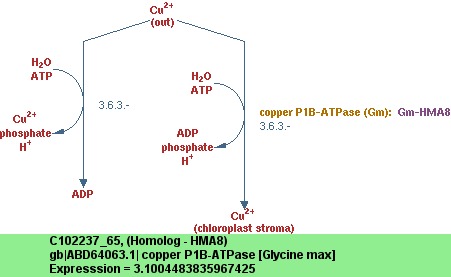

Supplement: Additional file 17 — Details of Transcription factor families. [file 1471-2164-14-647-S17.zip › Additional_file16B_Upregulated_PMN_pathways_in_Root/V1RS/C102237_65_HMA8_1_copper_transport_I.jpg]

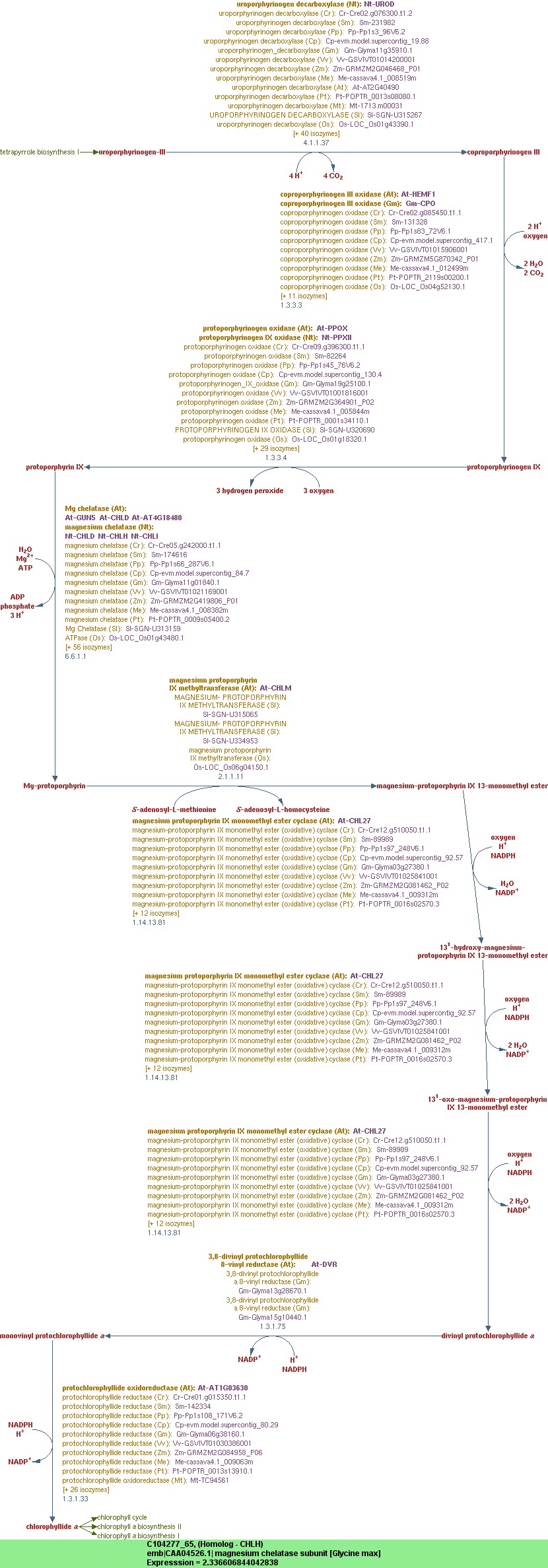

Supplement: Additional file 17 — Details of Transcription factor families. [file 1471-2164-14-647-S17.zip › Additional_file16B_Upregulated_PMN_pathways_in_Root/V1RS/C104277_65_CHLH_1_chlorophyllide_a_biosynthesis_I.jpg]

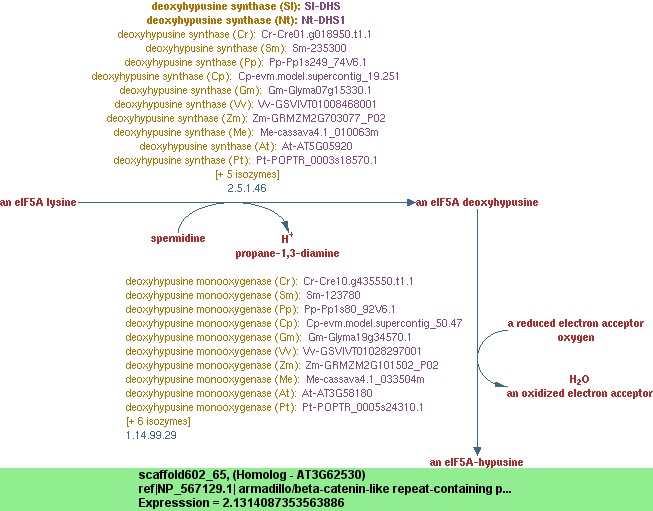

Supplement: Additional file 17 — Details of Transcription factor families. [file 1471-2164-14-647-S17.zip › Additional_file16B_Upregulated_PMN_pathways_in_Root/V1RS/scaffold602_65_AT3G62530_1_hypusine_biosynthesis.jpg]

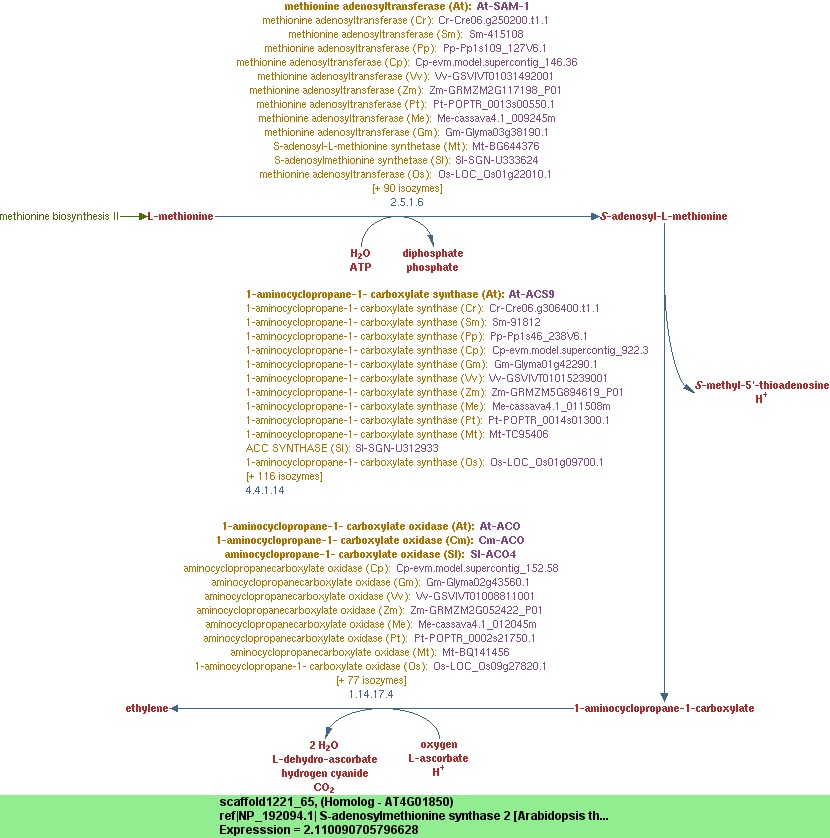

Supplement: Additional file 17 — Details of Transcription factor families. [file 1471-2164-14-647-S17.zip › Additional_file16B_Upregulated_PMN_pathways_in_Root/V1RS/scaffold1221_65_AT4G01850_1_ethylene_biosynthesis_I_(plants).jpg]

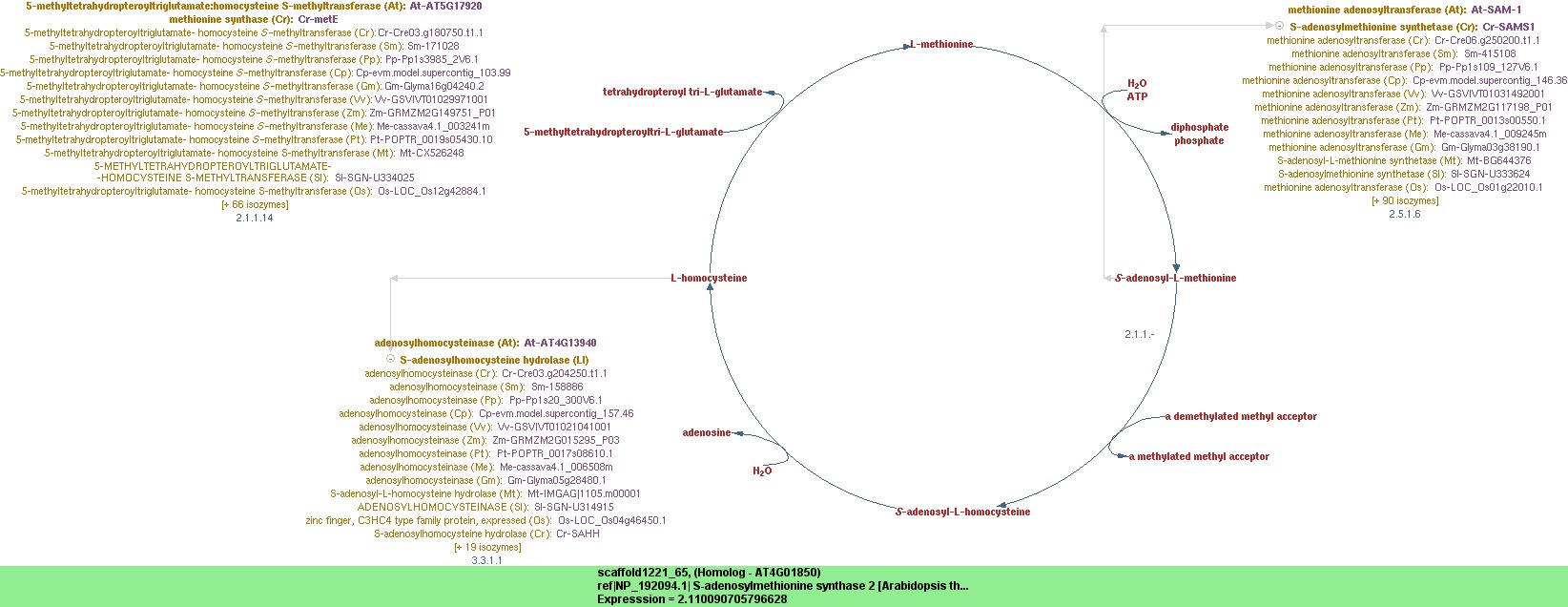

Supplement: Additional file 17 — Details of Transcription factor families. [file 1471-2164-14-647-S17.zip › Additional_file16B_Upregulated_PMN_pathways_in_Root/V1RS/scaffold1221_65_AT4G01850_3_-adenosyl-L-methionine_cycle_II.jpg]

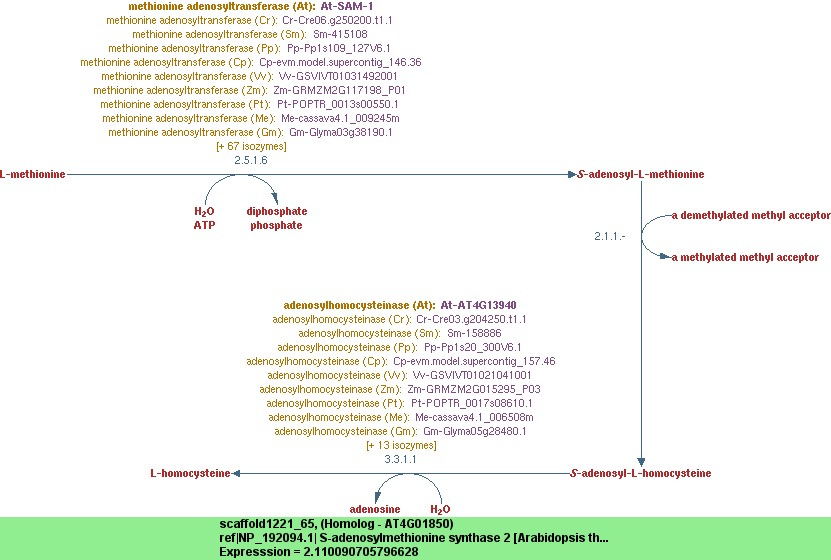

Supplement: Additional file 17 — Details of Transcription factor families. [file 1471-2164-14-647-S17.zip › Additional_file16B_Upregulated_PMN_pathways_in_Root/V1RS/scaffold1221_65_AT4G01850_5_methionine_degradation_I_(to_homocysteine).jpg]

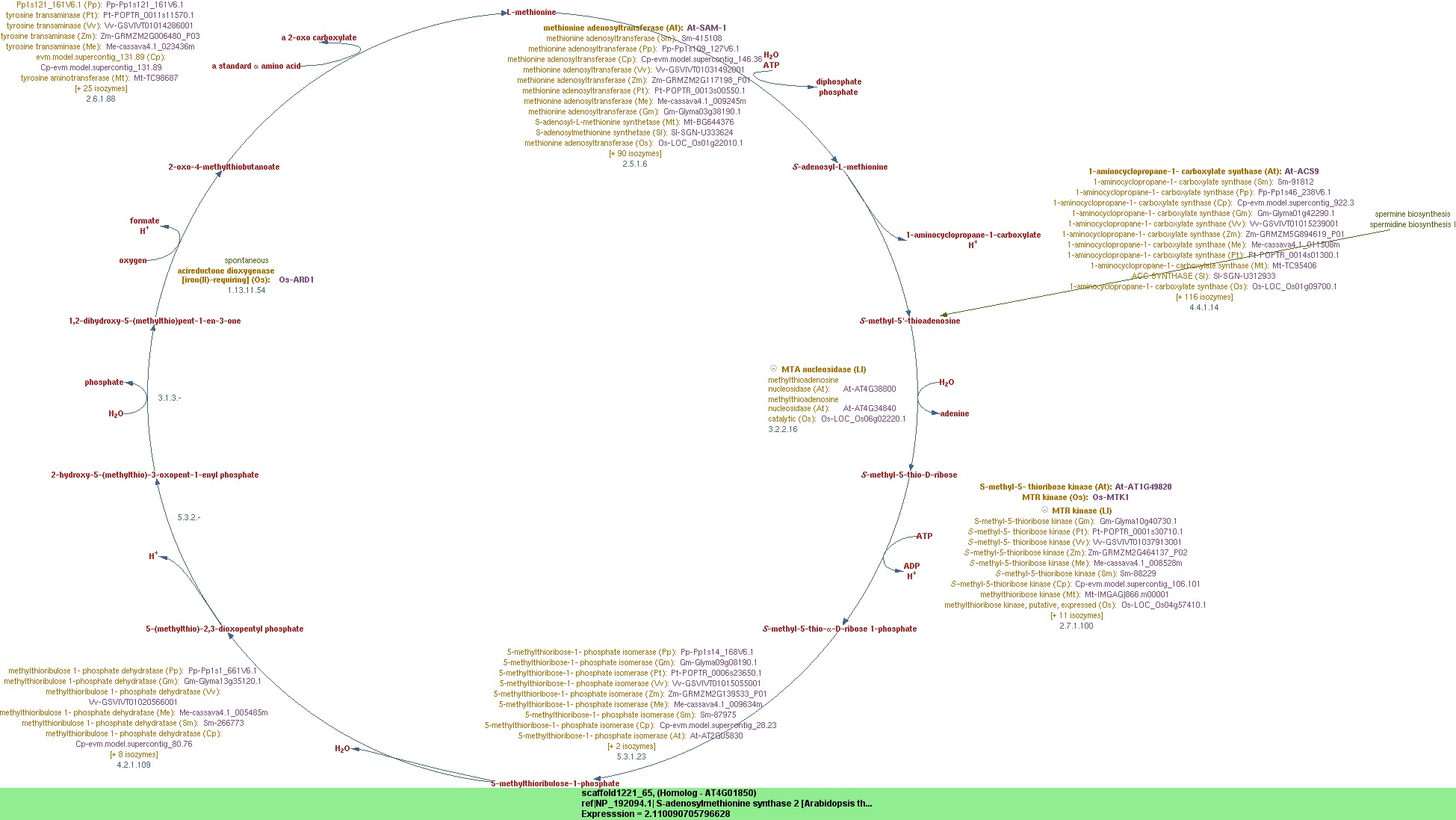

Supplement: Additional file 17 — Details of Transcription factor families. [file 1471-2164-14-647-S17.zip › Additional_file16B_Upregulated_PMN_pathways_in_Root/V1RS/scaffold1221_65_AT4G01850_7_methionine_salvage_pathway.jpg]

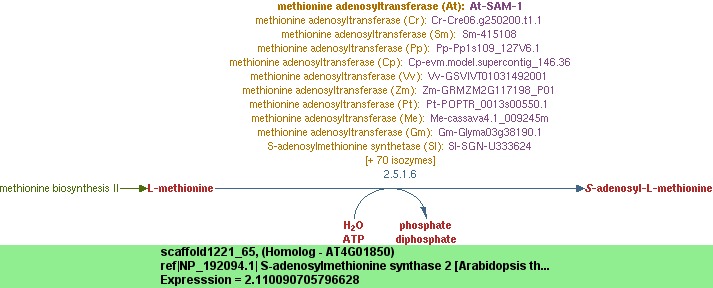

Supplement: Additional file 17 — Details of Transcription factor families. [file 1471-2164-14-647-S17.zip › Additional_file16B_Upregulated_PMN_pathways_in_Root/V1RS/scaffold1221_65_AT4G01850_9_S-adenosyl-L-methionine_biosynthesis.jpg]

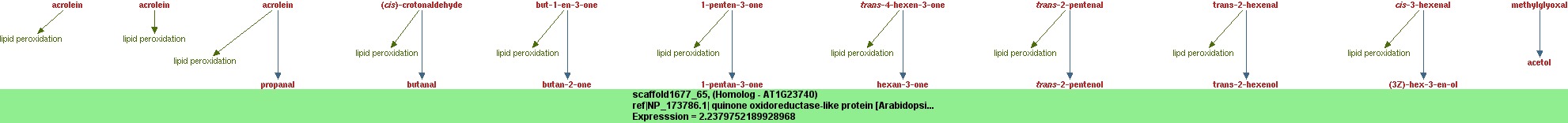

Supplement: Additional file 17 — Details of Transcription factor families. [file 1471-2164-14-647-S17.zip › Additional_file16B_Upregulated_PMN_pathways_in_Root/V1RS/scaffold1677_65_AT1G23740_1_detoxification_of_reactive_carbonyls_in_chloroplasts.jpg]

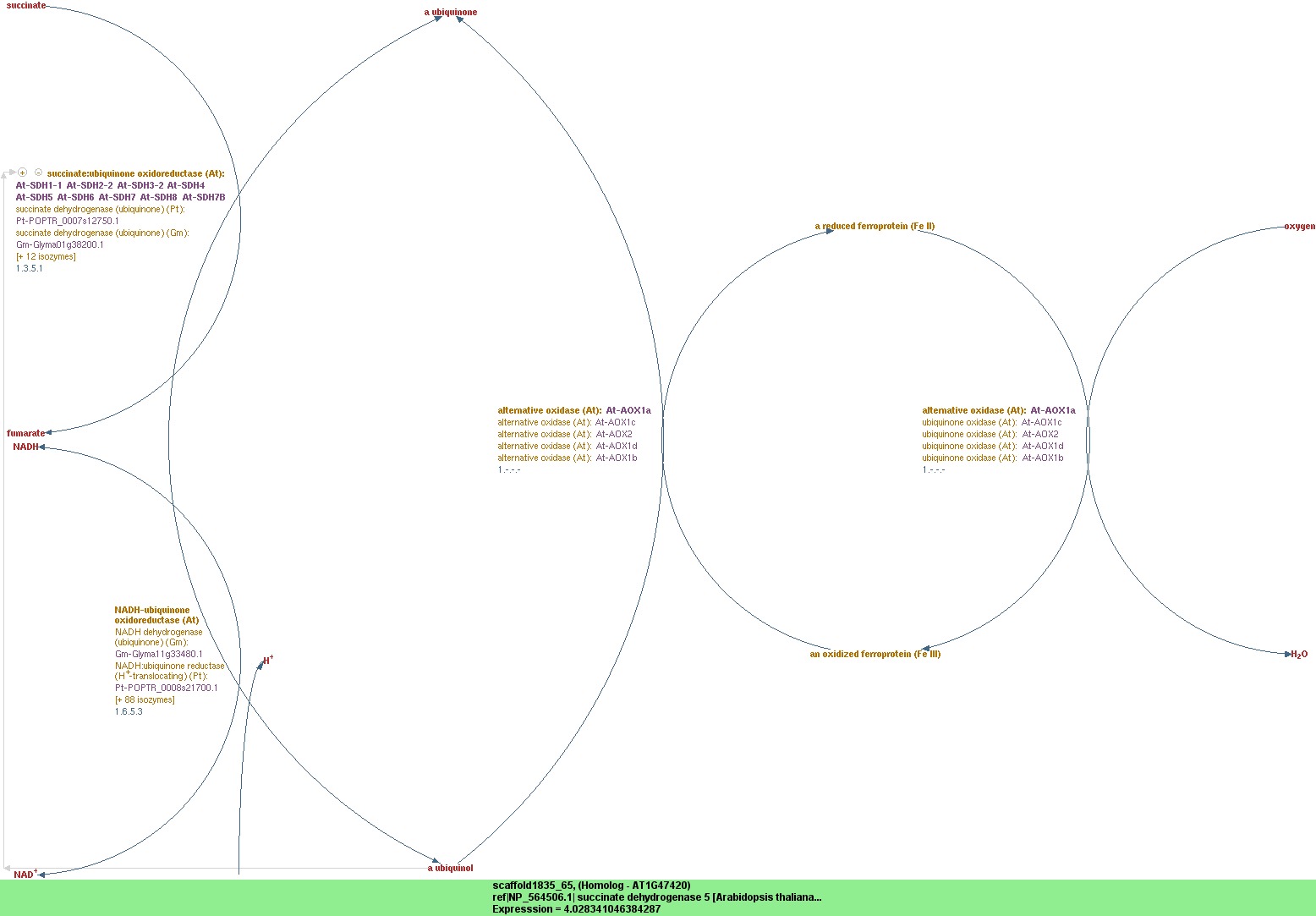

Supplement: Additional file 17 — Details of Transcription factor families. [file 1471-2164-14-647-S17.zip › Additional_file16B_Upregulated_PMN_pathways_in_Root/V1RS/scaffold1835_65_AT1G47420_1_aerobic_respiration_(alternative_oxidase_pathway).jpg]

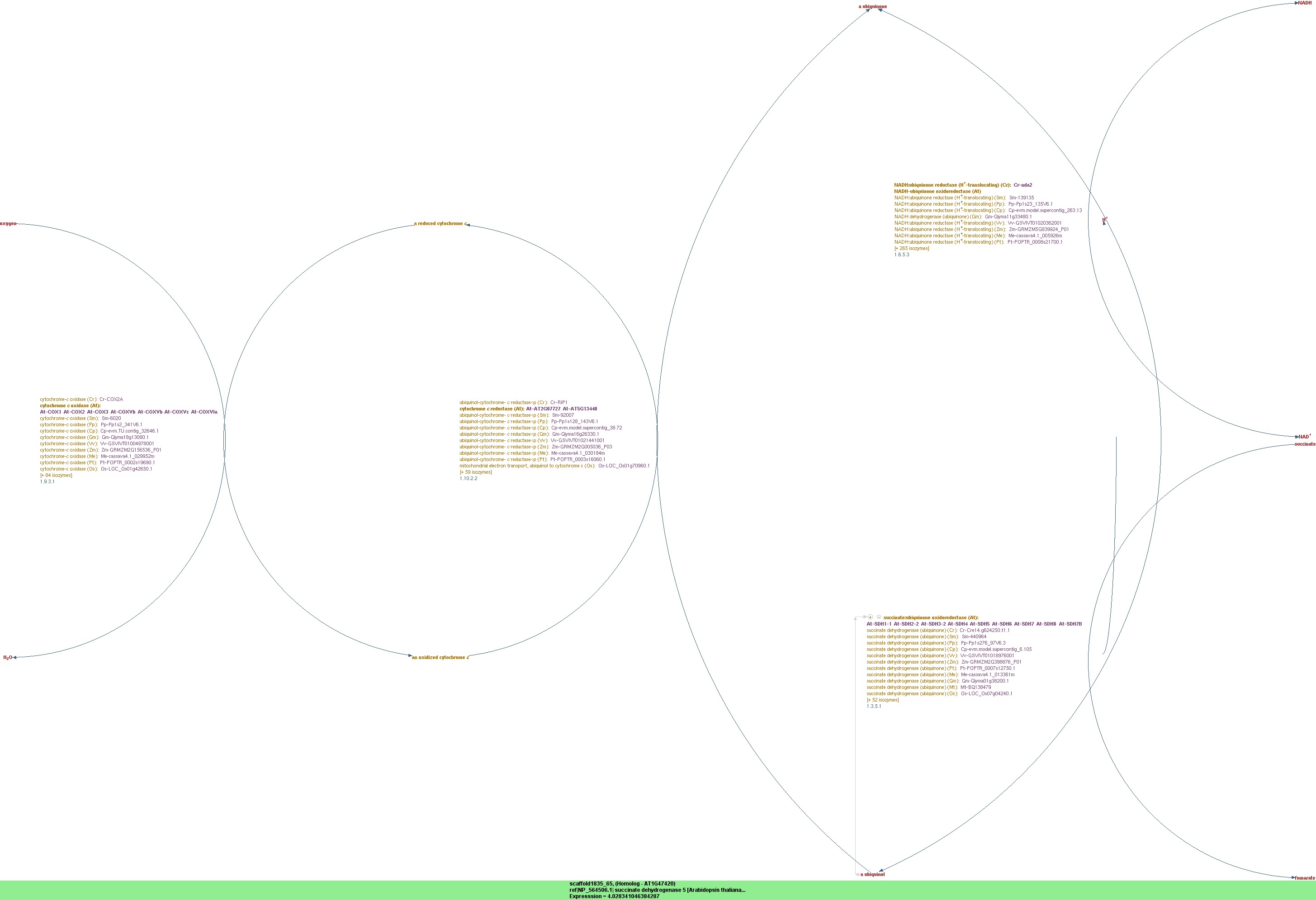

Supplement: Additional file 17 — Details of Transcription factor families. [file 1471-2164-14-647-S17.zip › Additional_file16B_Upregulated_PMN_pathways_in_Root/V1RS/scaffold1835_65_AT1G47420_2_aerobic_respiration_(cytochrome_c).jpg]

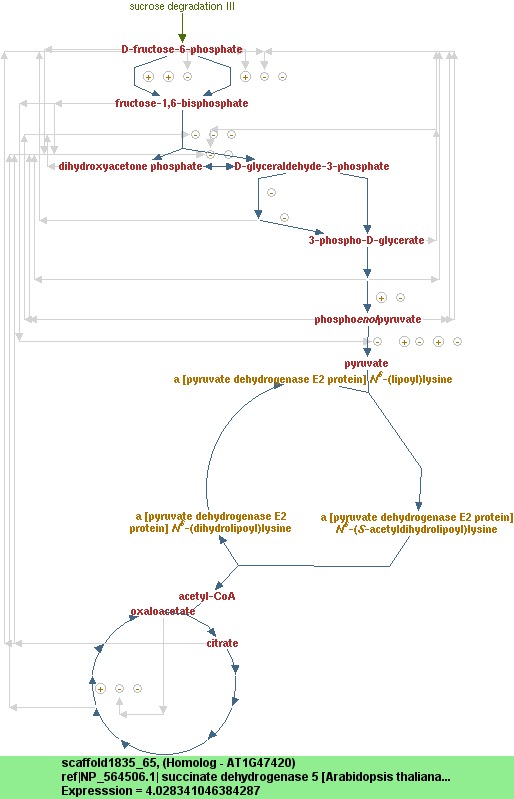

Supplement: Additional file 17 — Details of Transcription factor families. [file 1471-2164-14-647-S17.zip › Additional_file16B_Upregulated_PMN_pathways_in_Root/V1RS/scaffold1835_65_AT1G47420_3_superpathway_of_cytosolic_glycolysis_(plants),_pyruvate_dehydrogenase_and_TCA_cycle.jpg]

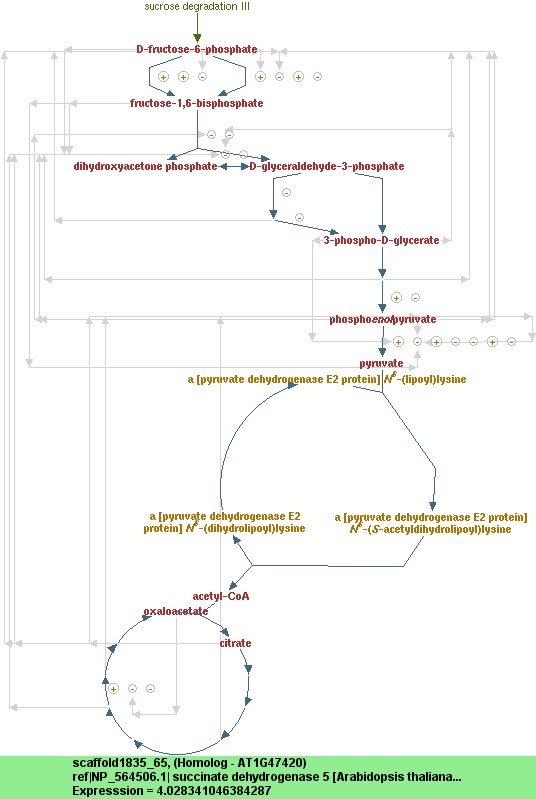

Supplement: Additional file 17 — Details of Transcription factor families. [file 1471-2164-14-647-S17.zip › Additional_file16B_Upregulated_PMN_pathways_in_Root/V1RS/scaffold1835_65_AT1G47420_4_superpathway_of_cytosolic_glycolysis_(plants),_pyruvate_dehydrogenase_and_TCA_cycle.jpg]

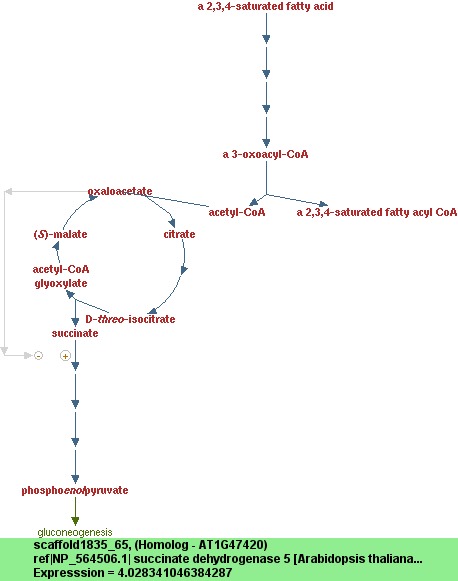

Supplement: Additional file 17 — Details of Transcription factor families. [file 1471-2164-14-647-S17.zip › Additional_file16B_Upregulated_PMN_pathways_in_Root/V1RS/scaffold1835_65_AT1G47420_5_superpathway_of_glyoxylate_cycle_and_fatty_acid_degradation.jpg]

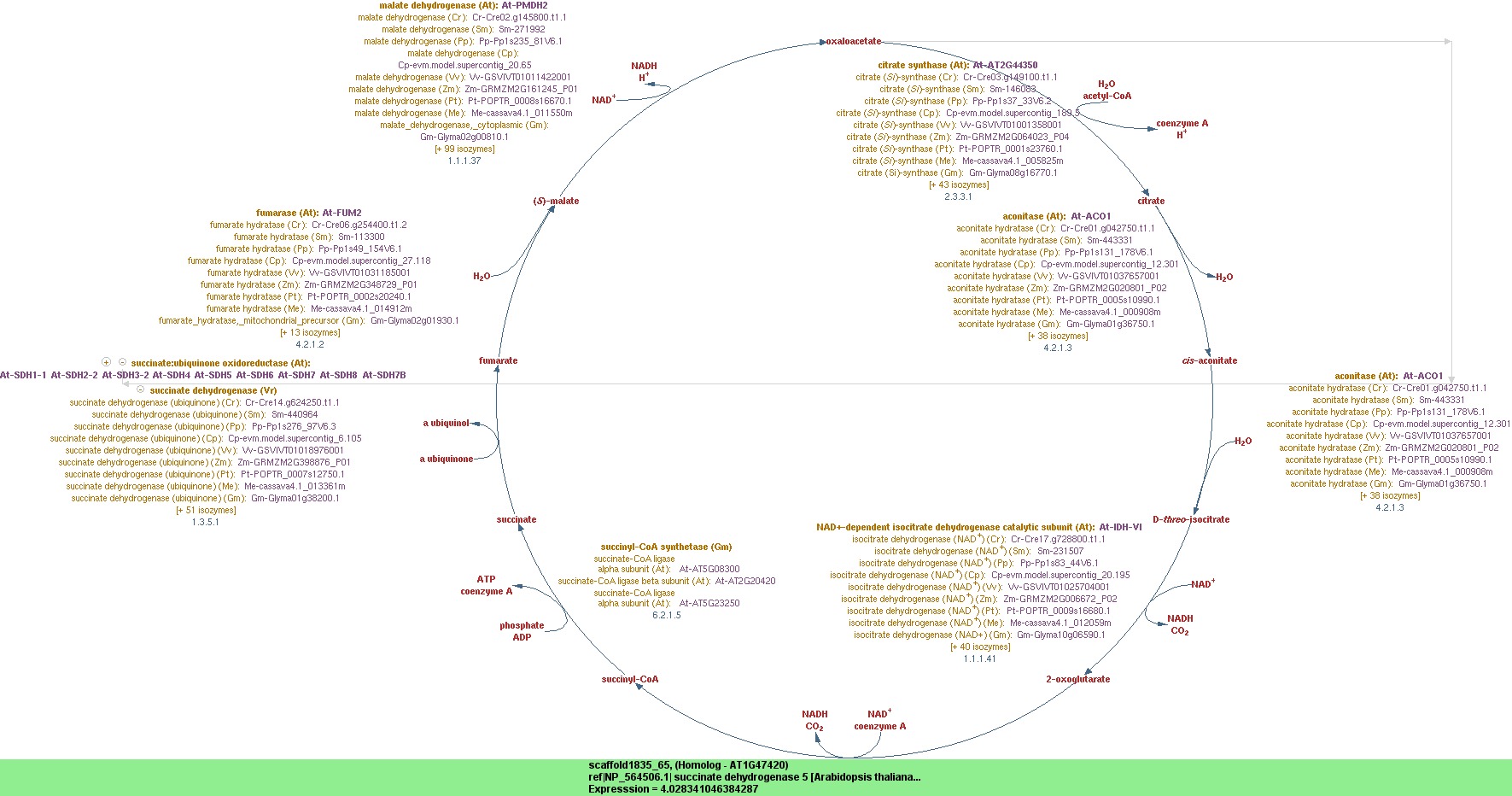

Supplement: Additional file 17 — Details of Transcription factor families. [file 1471-2164-14-647-S17.zip › Additional_file16B_Upregulated_PMN_pathways_in_Root/V1RS/scaffold1835_65_AT1G47420_6_TCA_cycle_II_(eukaryotic).jpg]

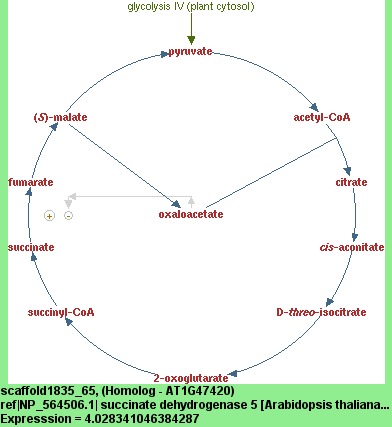

Supplement: Additional file 17 — Details of Transcription factor families. [file 1471-2164-14-647-S17.zip › Additional_file16B_Upregulated_PMN_pathways_in_Root/V1RS/scaffold1835_65_AT1G47420_7_TCA_cycle_variation_V_(plant).jpg]

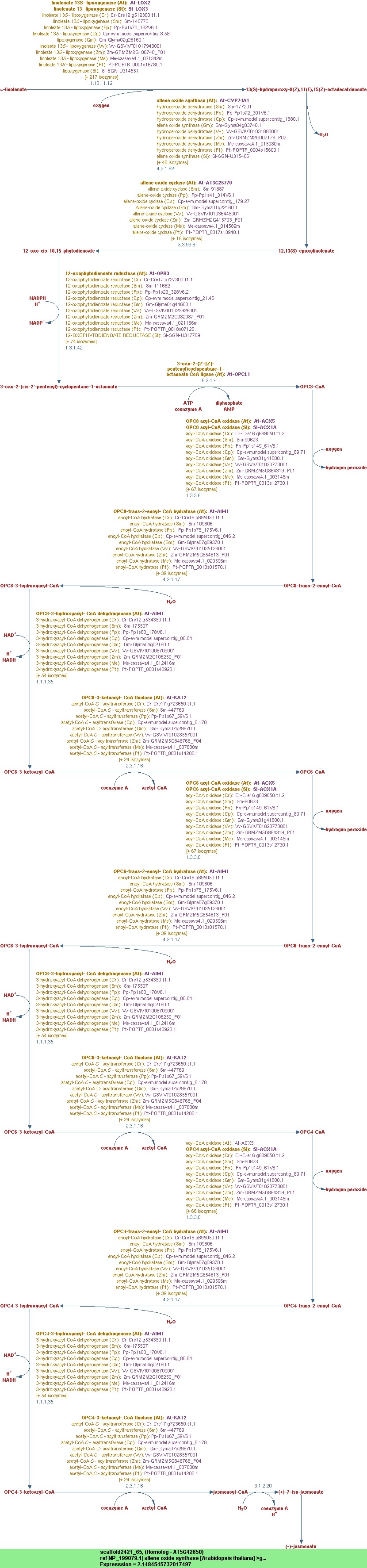

Supplement: Additional file 17 — Details of Transcription factor families. [file 1471-2164-14-647-S17.zip › Additional_file16B_Upregulated_PMN_pathways_in_Root/V1RS/scaffold2421_65_AT5G42650_1_jasmonic_acid_biosynthesis.jpg]

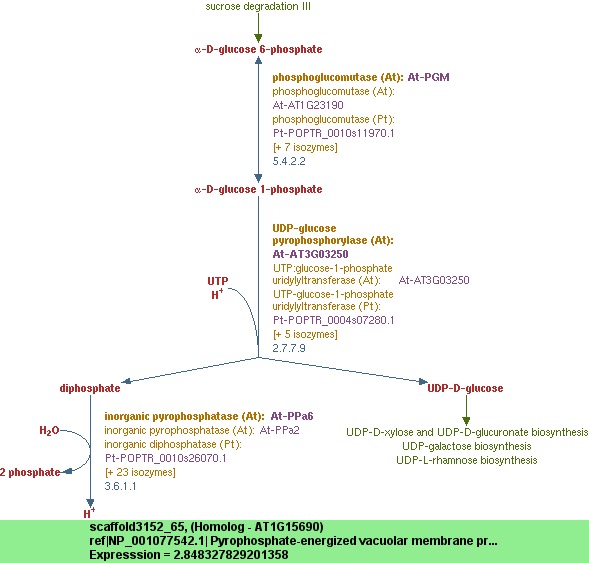

Supplement: Additional file 17 — Details of Transcription factor families. [file 1471-2164-14-647-S17.zip › Additional_file16B_Upregulated_PMN_pathways_in_Root/V1RS/scaffold3152_65_AT1G15690_1_UDP-glucose_biosynthesis_(from_glucose_6-phosphate).jpg]

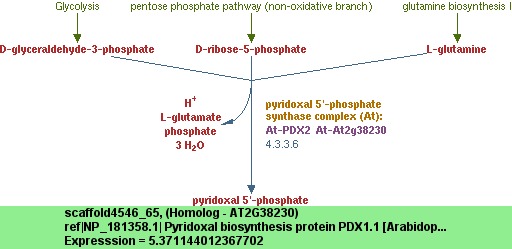

Supplement: Additional file 17 — Details of Transcription factor families. [file 1471-2164-14-647-S17.zip › Additional_file16B_Upregulated_PMN_pathways_in_Root/V1RS/scaffold4546_65_AT2G38230_1_pyridoxal_5'-phosphate_biosynthesis_II.jpg]

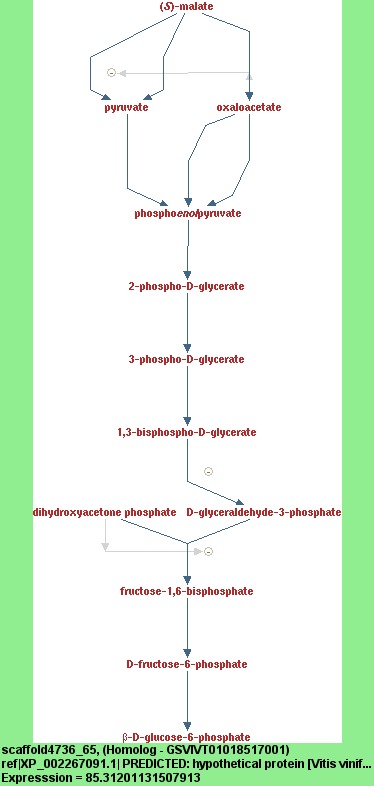

Supplement: Additional file 17 — Details of Transcription factor families. [file 1471-2164-14-647-S17.zip › Additional_file16B_Upregulated_PMN_pathways_in_Root/V1RS/scaffold4736_65_GSVIVT01018517001_1_gluconeogenesis_I.jpg]

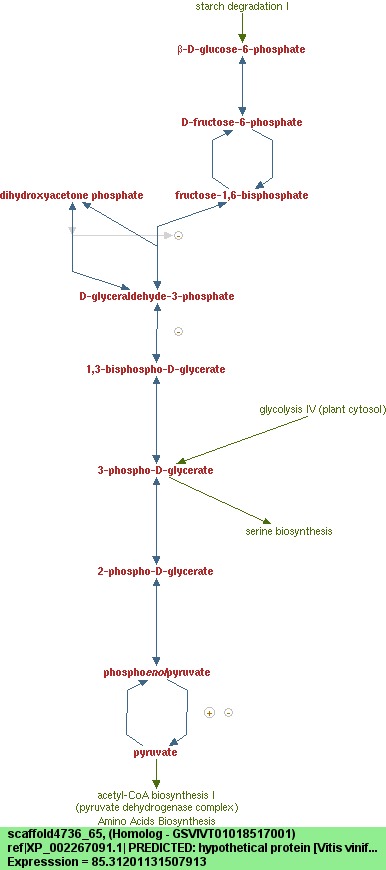

Supplement: Additional file 17 — Details of Transcription factor families. [file 1471-2164-14-647-S17.zip › Additional_file16B_Upregulated_PMN_pathways_in_Root/V1RS/scaffold4736_65_GSVIVT01018517001_2_glycolysis_I.jpg]

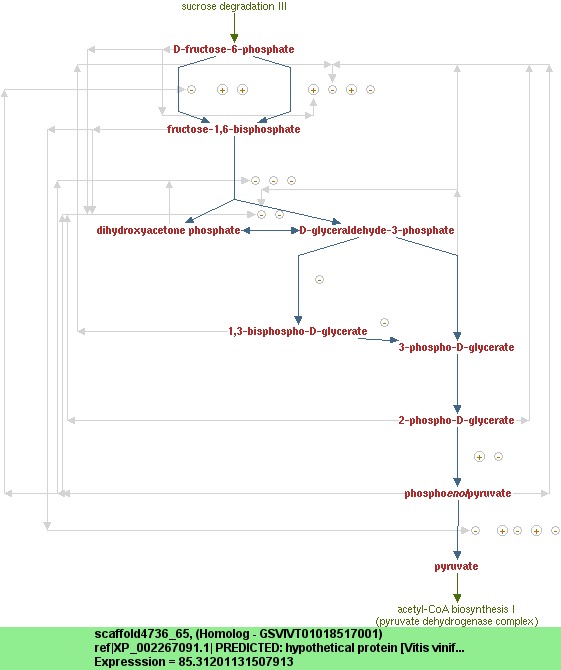

Supplement: Additional file 17 — Details of Transcription factor families. [file 1471-2164-14-647-S17.zip › Additional_file16B_Upregulated_PMN_pathways_in_Root/V1RS/scaffold4736_65_GSVIVT01018517001_3_glycolysis_IV_(plant_cytosol).jpg]

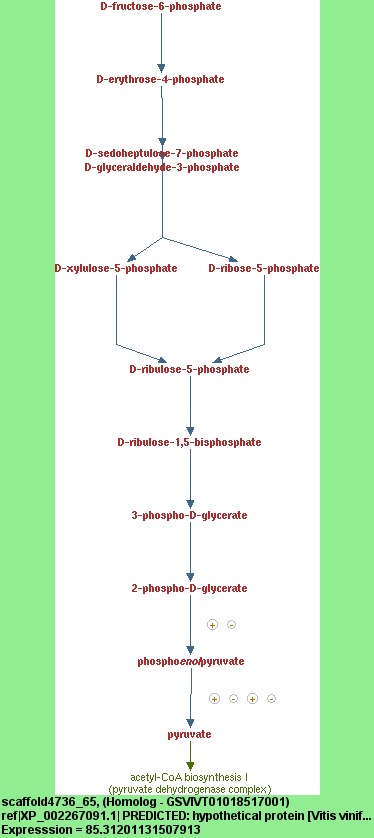

Supplement: Additional file 17 — Details of Transcription factor families. [file 1471-2164-14-647-S17.zip › Additional_file16B_Upregulated_PMN_pathways_in_Root/V1RS/scaffold4736_65_GSVIVT01018517001_4_Rubisco_shunt.jpg]

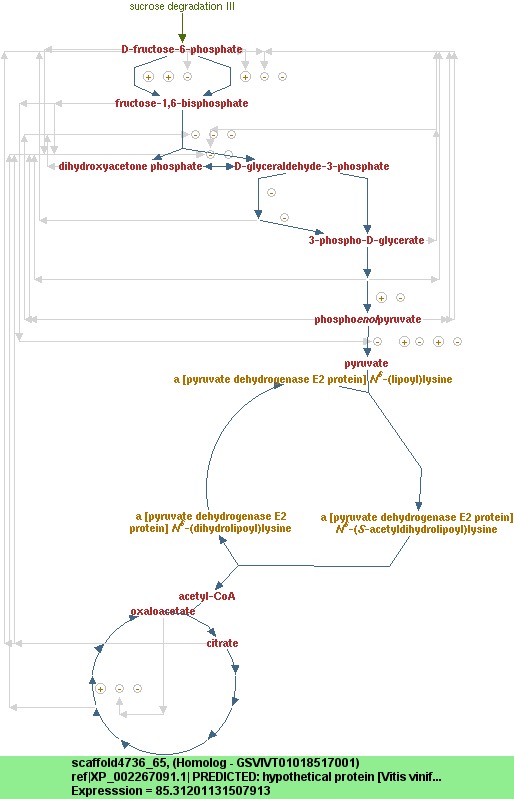

Supplement: Additional file 17 — Details of Transcription factor families. [file 1471-2164-14-647-S17.zip › Additional_file16B_Upregulated_PMN_pathways_in_Root/V1RS/scaffold4736_65_GSVIVT01018517001_5_superpathway_of_cytosolic_glycolysis_(plants),_pyruvate_dehydrogenase_and_TCA_cycle.jpg]

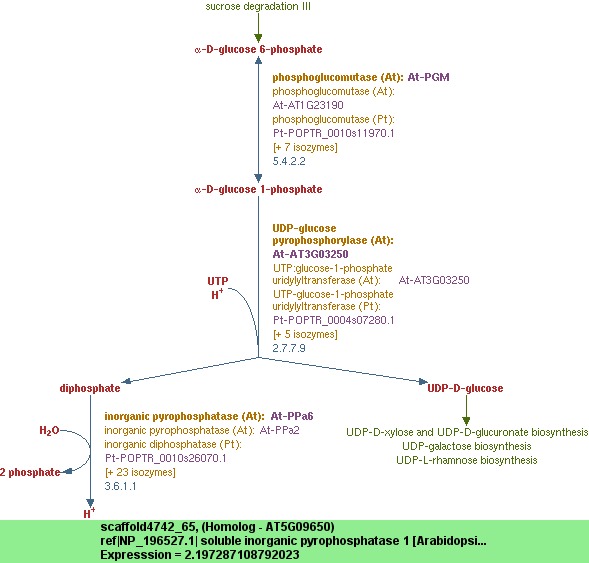

Supplement: Additional file 17 — Details of Transcription factor families. [file 1471-2164-14-647-S17.zip › Additional_file16B_Upregulated_PMN_pathways_in_Root/V1RS/scaffold4742_65_AT5G09650_1_UDP-glucose_biosynthesis_(from_glucose_6-phosphate).jpg]

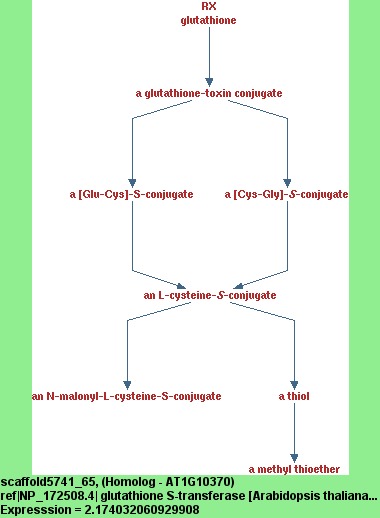

Supplement: Additional file 17 — Details of Transcription factor families. [file 1471-2164-14-647-S17.zip › Additional_file16B_Upregulated_PMN_pathways_in_Root/V1RS/scaffold5741_65_AT1G10370_1_glutathione-mediated_detoxification_II.jpg]

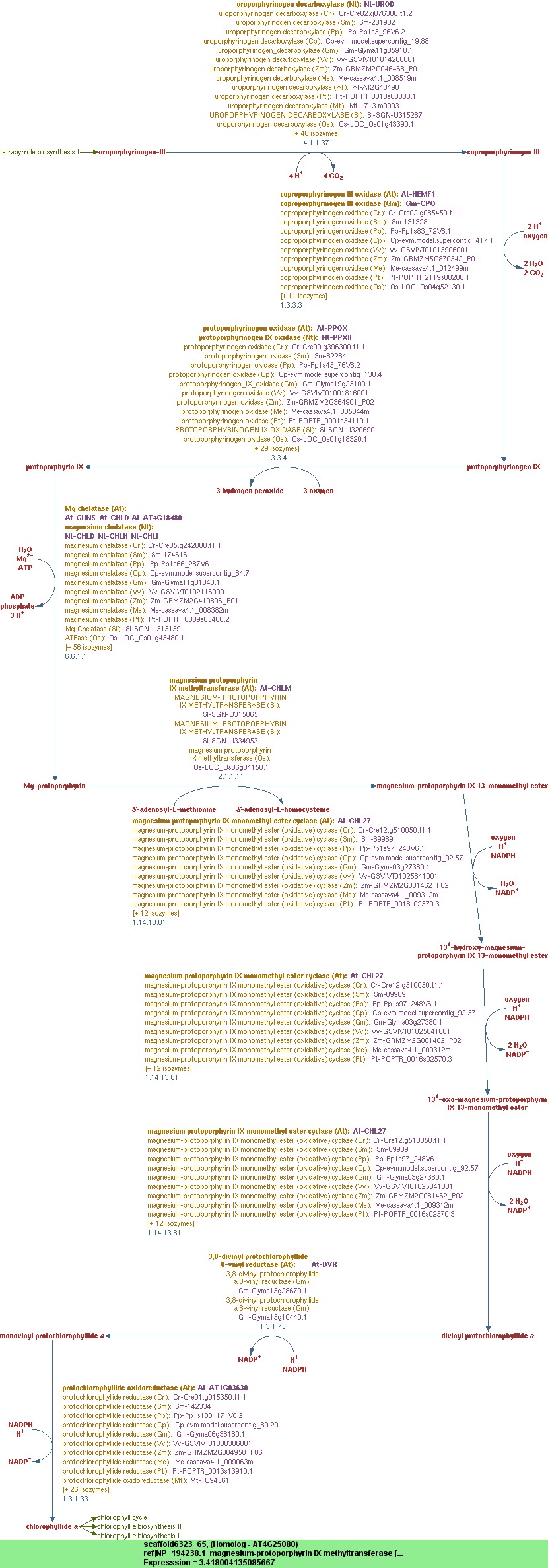

Supplement: Additional file 17 — Details of Transcription factor families. [file 1471-2164-14-647-S17.zip › Additional_file16B_Upregulated_PMN_pathways_in_Root/V1RS/scaffold6323_65_AT4G25080_1_chlorophyllide_a_biosynthesis_I.jpg]

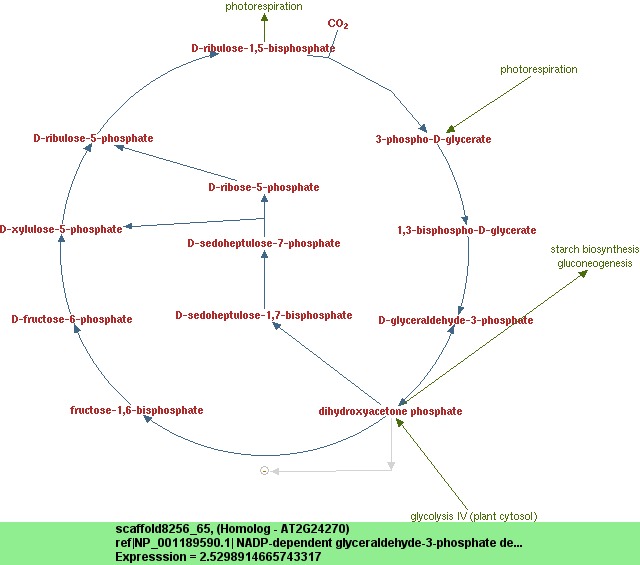

Supplement: Additional file 17 — Details of Transcription factor families. [file 1471-2164-14-647-S17.zip › Additional_file16B_Upregulated_PMN_pathways_in_Root/V1RS/scaffold8256_65_AT2G24270_1_Calvin-Benson-Bassham_cycle.jpg]

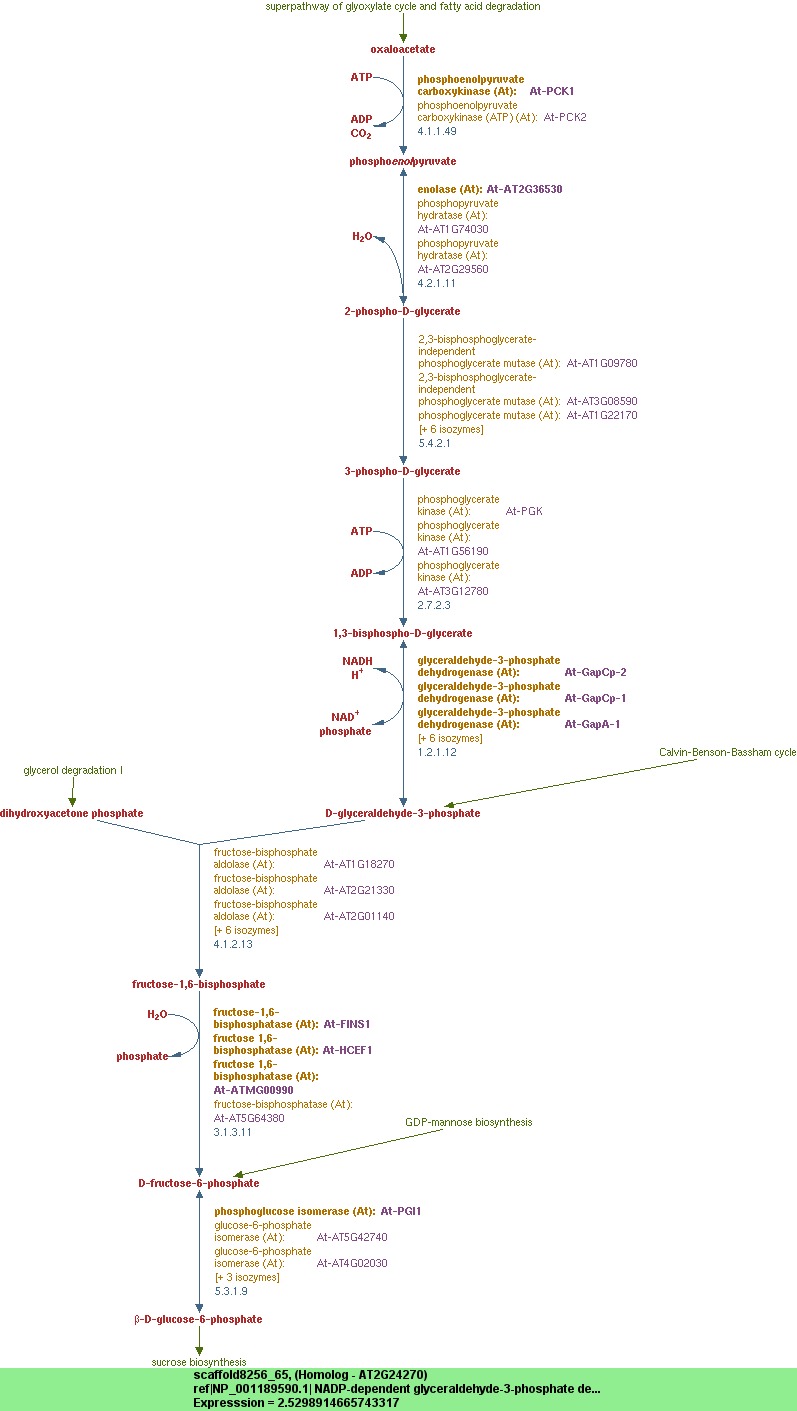

Supplement: Additional file 17 — Details of Transcription factor families. [file 1471-2164-14-647-S17.zip › Additional_file16B_Upregulated_PMN_pathways_in_Root/V1RS/scaffold8256_65_AT2G24270_2_gluconeogenesis.jpg]

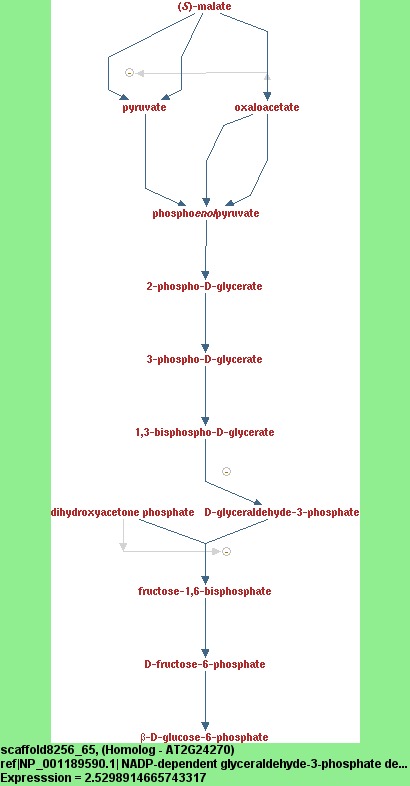

Supplement: Additional file 17 — Details of Transcription factor families. [file 1471-2164-14-647-S17.zip › Additional_file16B_Upregulated_PMN_pathways_in_Root/V1RS/scaffold8256_65_AT2G24270_3_gluconeogenesis_I.jpg]

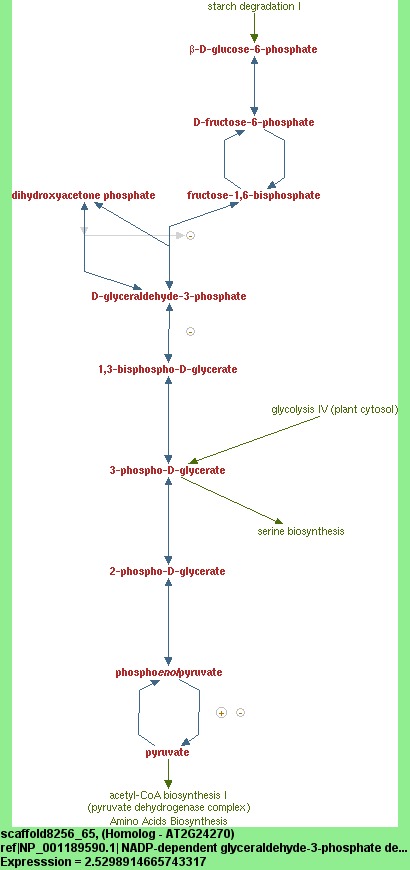

Supplement: Additional file 17 — Details of Transcription factor families. [file 1471-2164-14-647-S17.zip › Additional_file16B_Upregulated_PMN_pathways_in_Root/V1RS/scaffold8256_65_AT2G24270_4_glycolysis_I.jpg]

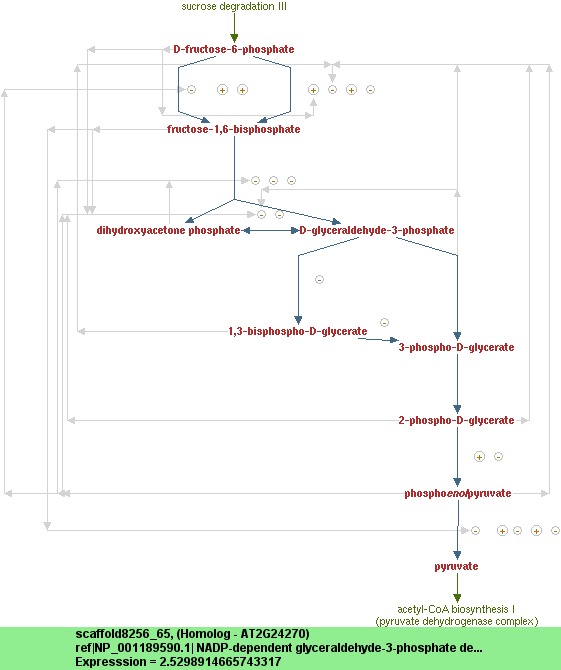

Supplement: Additional file 17 — Details of Transcription factor families. [file 1471-2164-14-647-S17.zip › Additional_file16B_Upregulated_PMN_pathways_in_Root/V1RS/scaffold8256_65_AT2G24270_5_glycolysis_IV_(plant_cytosol).jpg]

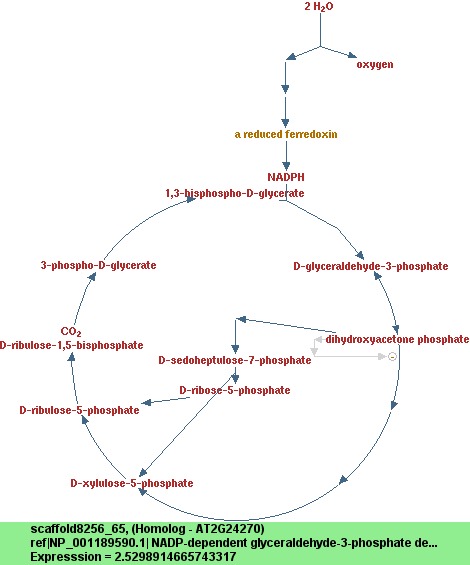

Supplement: Additional file 17 — Details of Transcription factor families. [file 1471-2164-14-647-S17.zip › Additional_file16B_Upregulated_PMN_pathways_in_Root/V1RS/scaffold8256_65_AT2G24270_6_oxygenic_photosynthesis.jpg]

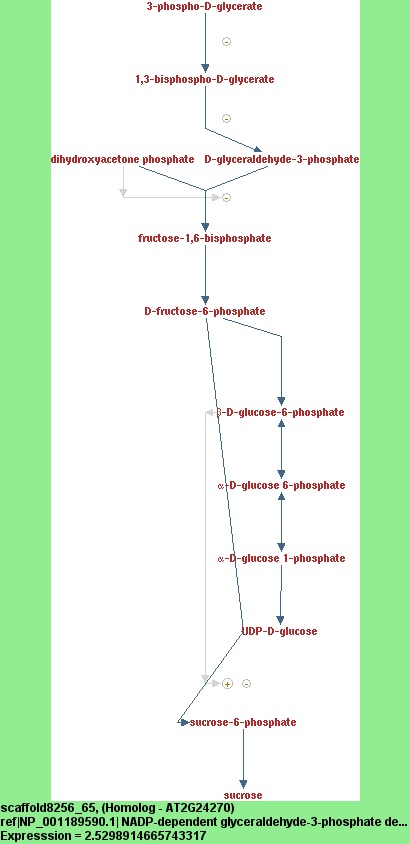

Supplement: Additional file 17 — Details of Transcription factor families. [file 1471-2164-14-647-S17.zip › Additional_file16B_Upregulated_PMN_pathways_in_Root/V1RS/scaffold8256_65_AT2G24270_7_sucrose_biosynthesis.jpg]

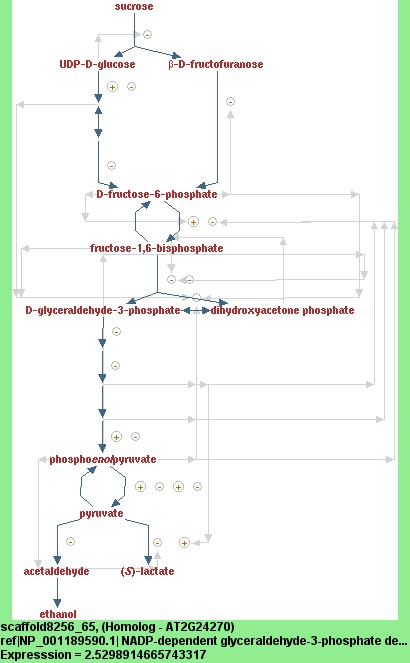

Supplement: Additional file 17 — Details of Transcription factor families. [file 1471-2164-14-647-S17.zip › Additional_file16B_Upregulated_PMN_pathways_in_Root/V1RS/scaffold8256_65_AT2G24270_8_sucrose_degradation_VI_(anaerobic).jpg]

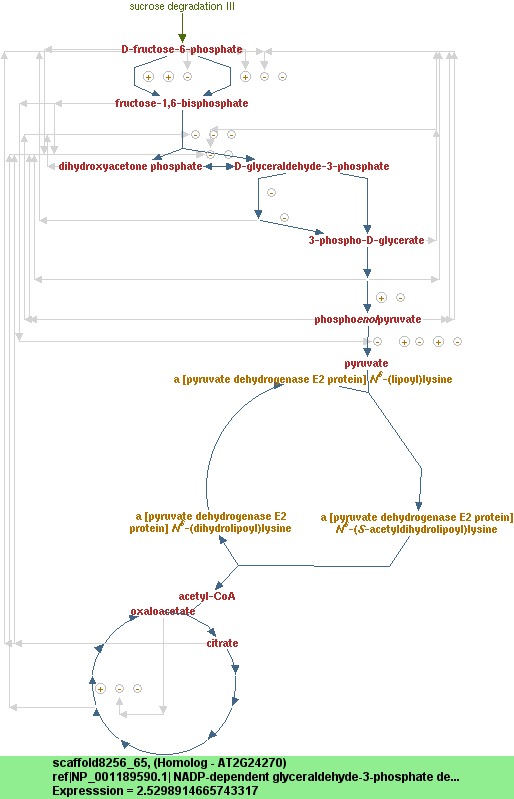

Supplement: Additional file 17 — Details of Transcription factor families. [file 1471-2164-14-647-S17.zip › Additional_file16B_Upregulated_PMN_pathways_in_Root/V1RS/scaffold8256_65_AT2G24270_9_superpathway_of_cytosolic_glycolysis_(plants),_pyruvate_dehydrogenase_and_TCA_cycle.jpg]

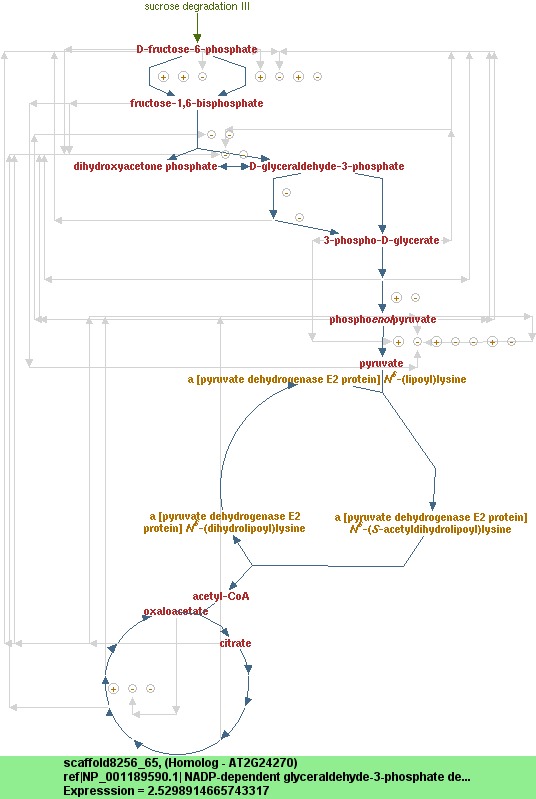

Supplement: Additional file 17 — Details of Transcription factor families. [file 1471-2164-14-647-S17.zip › Additional_file16B_Upregulated_PMN_pathways_in_Root/V1RS/scaffold8256_65_AT2G24270_10_superpathway_of_cytosolic_glycolysis_(plants),_pyruvate_dehydrogenase_and_TCA_cycle.jpg]

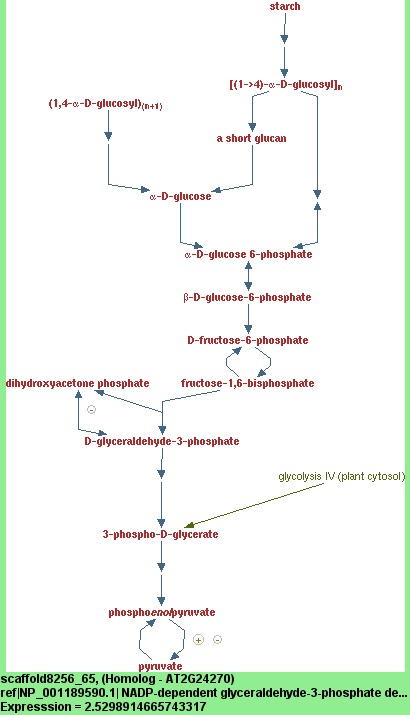

Supplement: Additional file 17 — Details of Transcription factor families. [file 1471-2164-14-647-S17.zip › Additional_file16B_Upregulated_PMN_pathways_in_Root/V1RS/scaffold8256_65_AT2G24270_11_superpathway_of_starch_degradation_to_pyruvate.jpg]

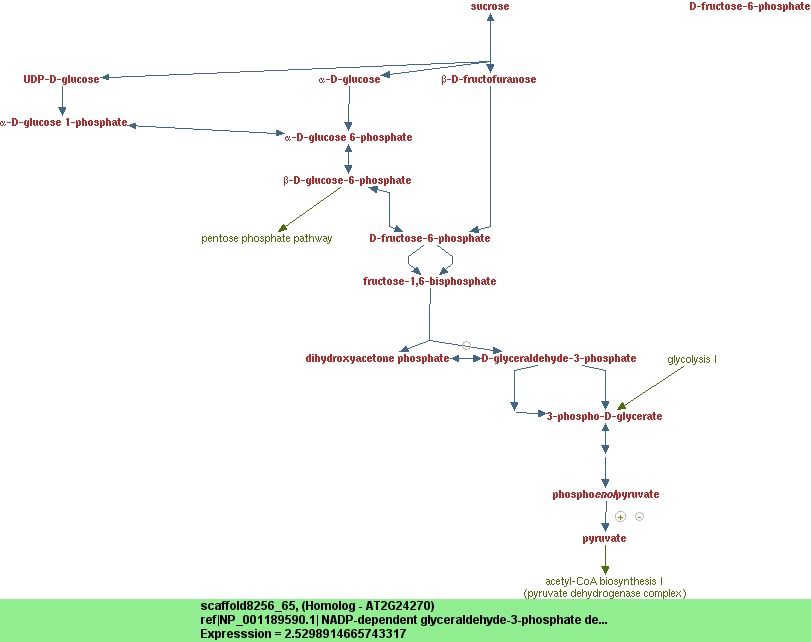

Supplement: Additional file 17 — Details of Transcription factor families. [file 1471-2164-14-647-S17.zip › Additional_file16B_Upregulated_PMN_pathways_in_Root/V1RS/scaffold8256_65_AT2G24270_12_superpathway_of_sucrose_degradation_to_pyruvate.jpg]

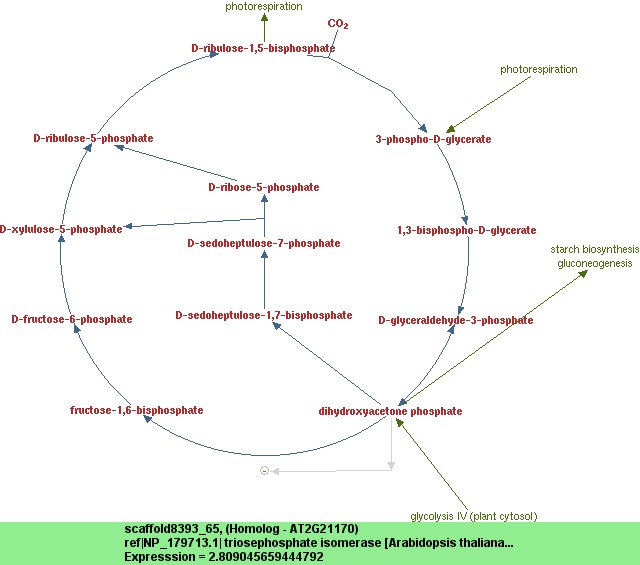

Supplement: Additional file 17 — Details of Transcription factor families. [file 1471-2164-14-647-S17.zip › Additional_file16B_Upregulated_PMN_pathways_in_Root/V1RS/scaffold8393_65_AT2G21170_1_Calvin-Benson-Bassham_cycle.jpg]

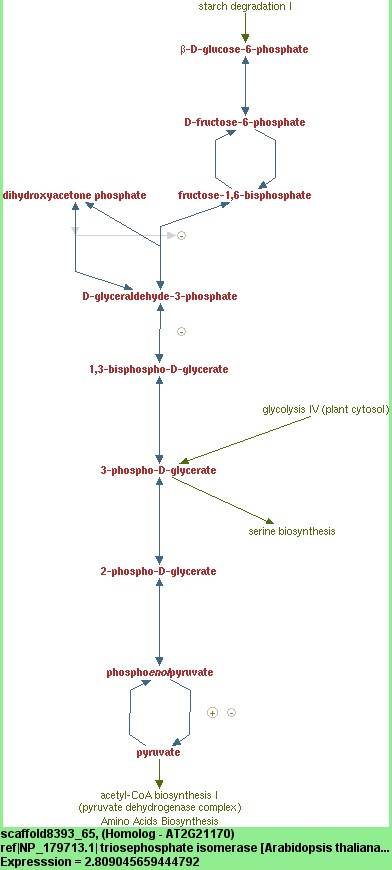

Supplement: Additional file 17 — Details of Transcription factor families. [file 1471-2164-14-647-S17.zip › Additional_file16B_Upregulated_PMN_pathways_in_Root/V1RS/scaffold8393_65_AT2G21170_3_glycolysis_I.jpg]

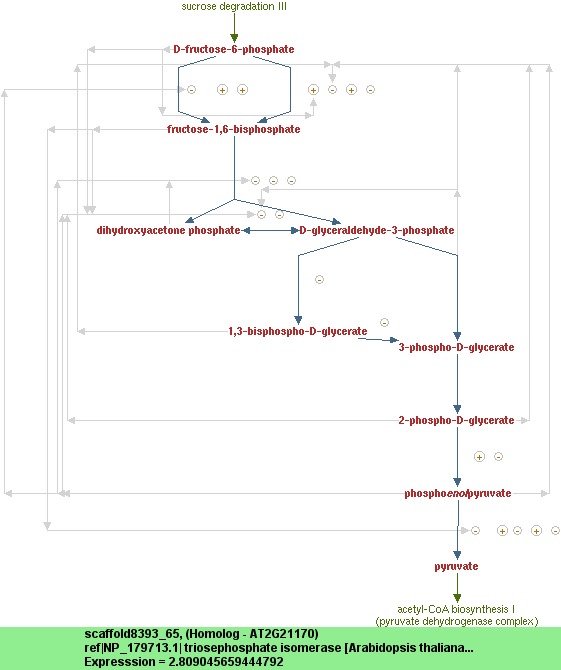

Supplement: Additional file 17 — Details of Transcription factor families. [file 1471-2164-14-647-S17.zip › Additional_file16B_Upregulated_PMN_pathways_in_Root/V1RS/scaffold8393_65_AT2G21170_5_glycolysis_IV_(plant_cytosol).jpg]

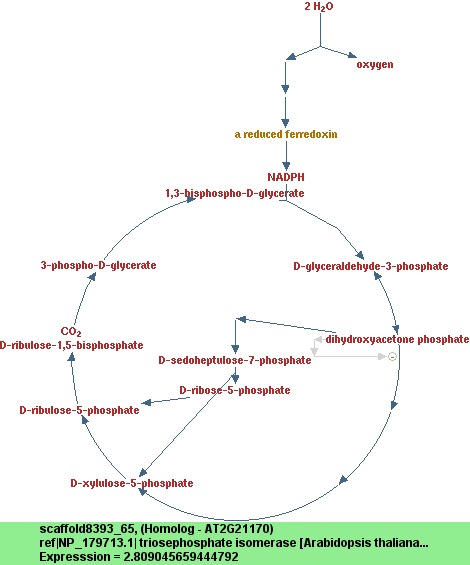

Supplement: Additional file 17 — Details of Transcription factor families. [file 1471-2164-14-647-S17.zip › Additional_file16B_Upregulated_PMN_pathways_in_Root/V1RS/scaffold8393_65_AT2G21170_7_oxygenic_photosynthesis.jpg]

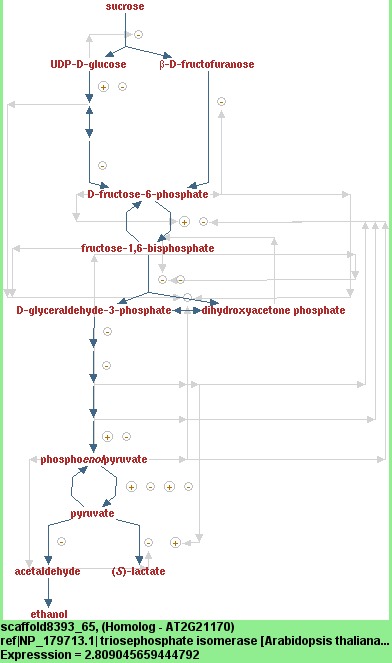

Supplement: Additional file 17 — Details of Transcription factor families. [file 1471-2164-14-647-S17.zip › Additional_file16B_Upregulated_PMN_pathways_in_Root/V1RS/scaffold8393_65_AT2G21170_9_sucrose_degradation_VI_(anaerobic).jpg]

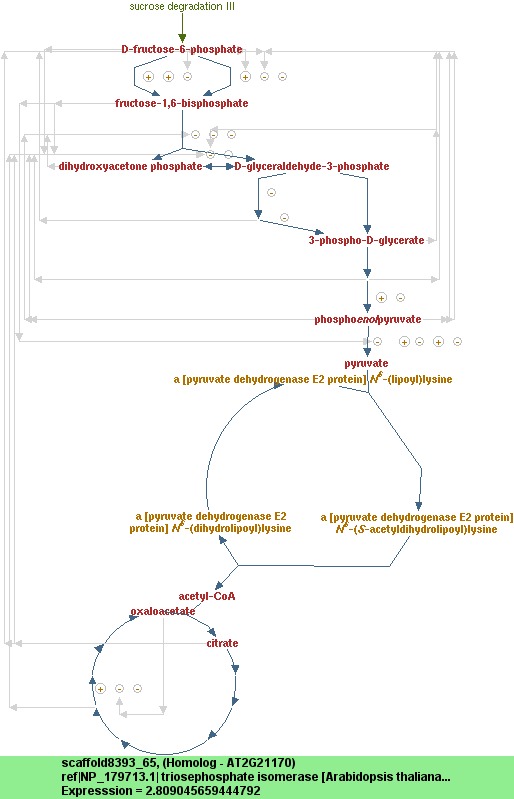

Supplement: Additional file 17 — Details of Transcription factor families. [file 1471-2164-14-647-S17.zip › Additional_file16B_Upregulated_PMN_pathways_in_Root/V1RS/scaffold8393_65_AT2G21170_11_superpathway_of_cytosolic_glycolysis_(plants),_pyruvate_dehydrogenase_and_TCA_cycle.jpg]

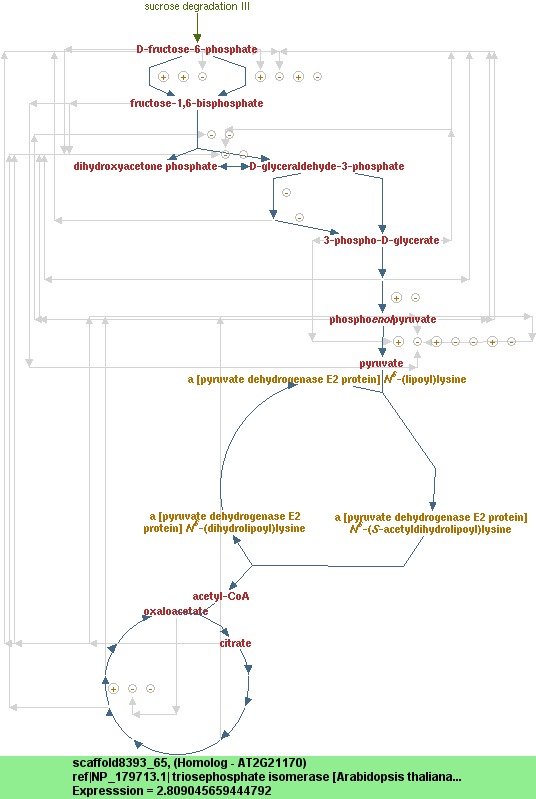

Supplement: Additional file 17 — Details of Transcription factor families. [file 1471-2164-14-647-S17.zip › Additional_file16B_Upregulated_PMN_pathways_in_Root/V1RS/scaffold8393_65_AT2G21170_13_superpathway_of_cytosolic_glycolysis_(plants),_pyruvate_dehydrogenase_and_TCA_cycle.jpg]

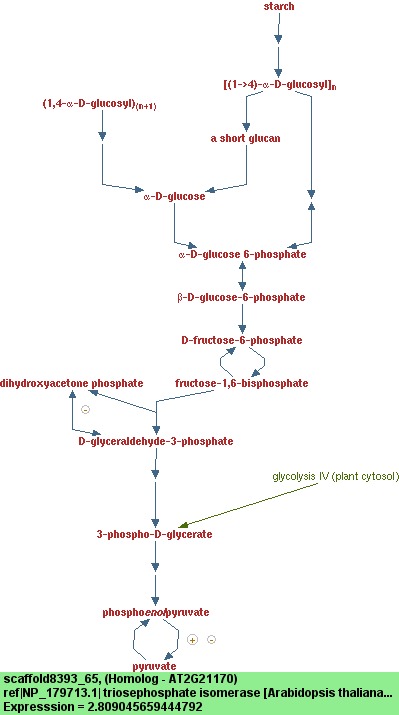

Supplement: Additional file 17 — Details of Transcription factor families. [file 1471-2164-14-647-S17.zip › Additional_file16B_Upregulated_PMN_pathways_in_Root/V1RS/scaffold8393_65_AT2G21170_15_superpathway_of_starch_degradation_to_pyruvate.jpg]

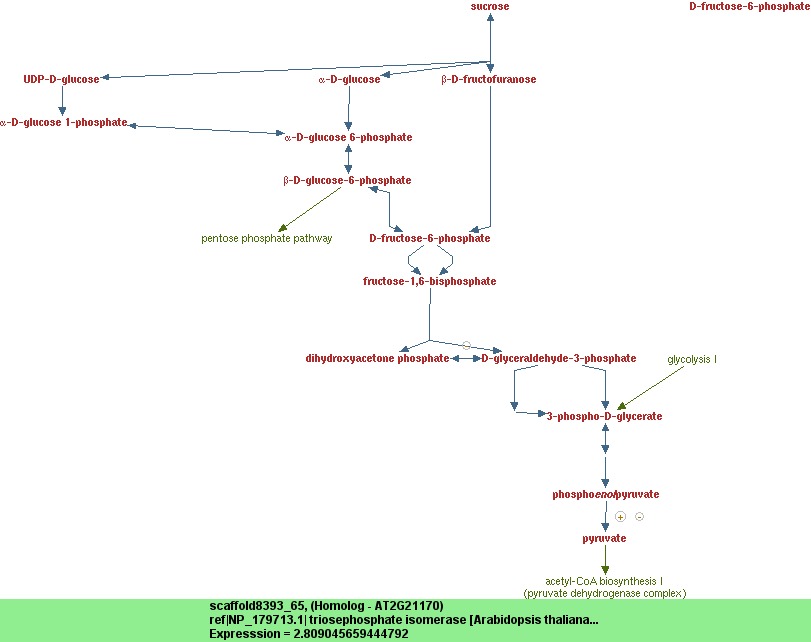

Supplement: Additional file 17 — Details of Transcription factor families. [file 1471-2164-14-647-S17.zip › Additional_file16B_Upregulated_PMN_pathways_in_Root/V1RS/scaffold8393_65_AT2G21170_17_superpathway_of_sucrose_degradation_to_pyruvate.jpg]

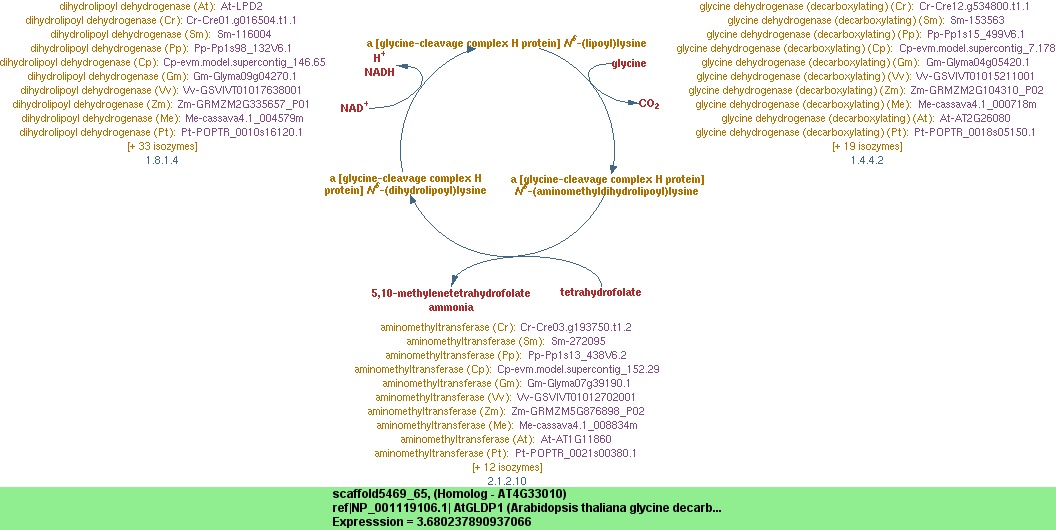

Supplement: Additional file 17 — Details of Transcription factor families. [file 1471-2164-14-647-S17.zip › Additional_file16B_Upregulated_PMN_pathways_in_Root/V1RS/scaffold11049_65_AT4G33010_1_glycine_cleavage_complex.jpg]

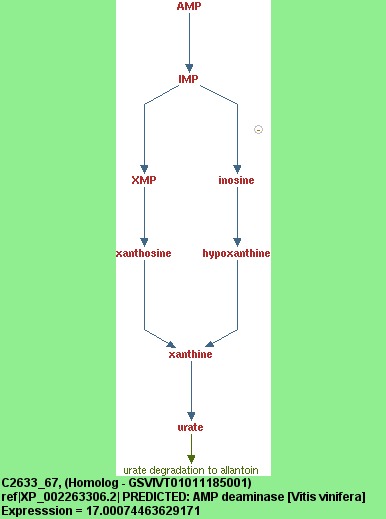

Supplement: Additional file 17 — Details of Transcription factor families. [file 1471-2164-14-647-S17.zip › Additional_file16B_Upregulated_PMN_pathways_in_Root/V2RS/C2633_67_GSVIVT01011185001_1_adenosine_nucleotides_degradation_I.jpg]

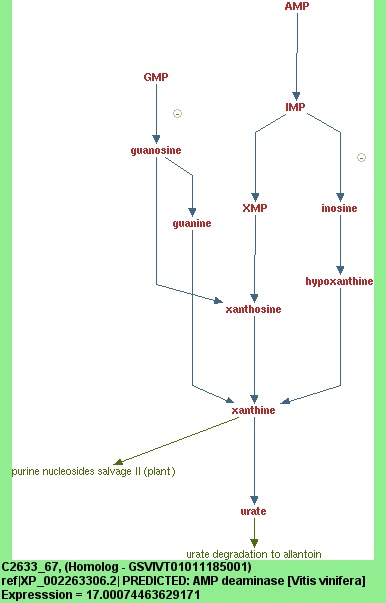

Supplement: Additional file 17 — Details of Transcription factor families. [file 1471-2164-14-647-S17.zip › Additional_file16B_Upregulated_PMN_pathways_in_Root/V2RS/C2633_67_GSVIVT01011185001_2_purine_nucleotides_degradation_I_(plants).jpg]

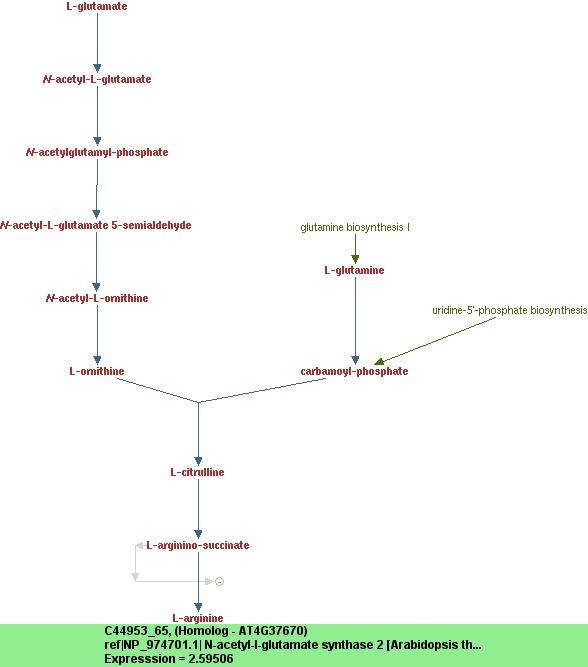

Supplement: Additional file 17 — Details of Transcription factor families. [file 1471-2164-14-647-S17.zip › Additional_file16B_Upregulated_PMN_pathways_in_Root/V2RS/C44953_65_AT4G37670_1_arginine_biosynthesis_I.jpg]

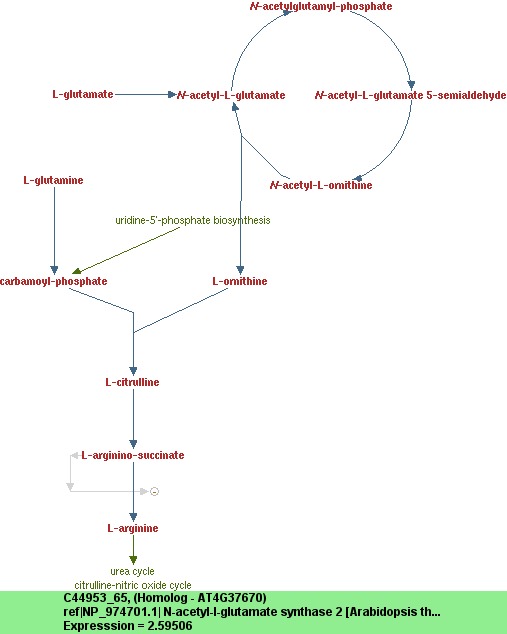

Supplement: Additional file 17 — Details of Transcription factor families. [file 1471-2164-14-647-S17.zip › Additional_file16B_Upregulated_PMN_pathways_in_Root/V2RS/C44953_65_AT4G37670_3_arginine_biosynthesis_II_(acetyl_cycle).jpg]

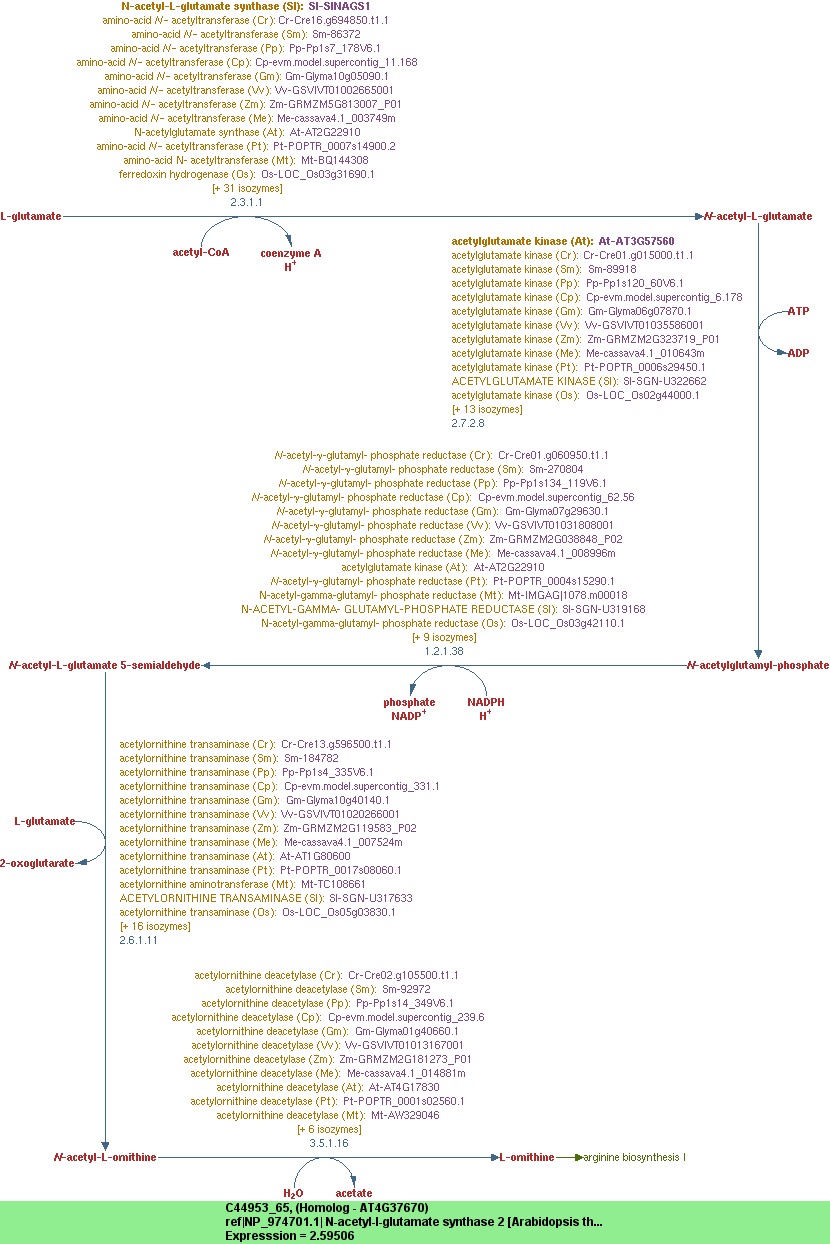

Supplement: Additional file 17 — Details of Transcription factor families. [file 1471-2164-14-647-S17.zip › Additional_file16B_Upregulated_PMN_pathways_in_Root/V2RS/C44953_65_AT4G37670_5_ornithine_biosynthesis.jpg]

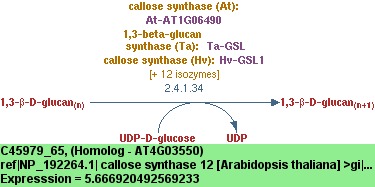

Supplement: Additional file 17 — Details of Transcription factor families. [file 1471-2164-14-647-S17.zip › Additional_file16B_Upregulated_PMN_pathways_in_Root/V2RS/C45979_65_AT4G03550_1_callose_biosynthesis.jpg]

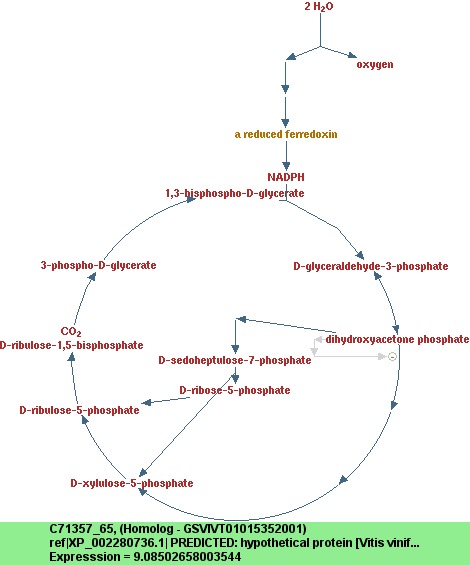

Supplement: Additional file 17 — Details of Transcription factor families. [file 1471-2164-14-647-S17.zip › Additional_file16B_Upregulated_PMN_pathways_in_Root/V2RS/C71357_65_GSVIVT01015352001_1_oxygenic_photosynthesis.jpg]

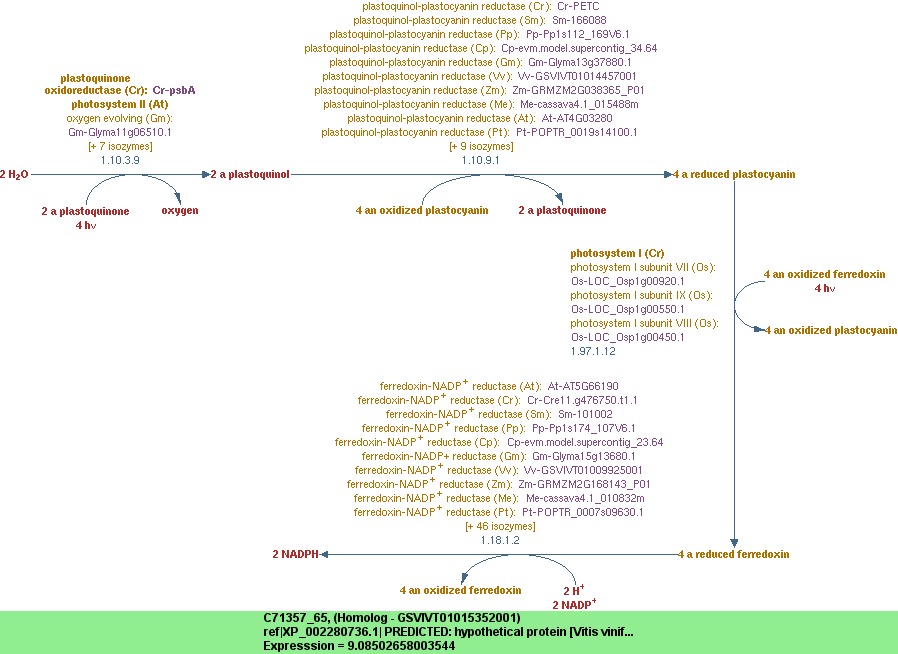

Supplement: Additional file 17 — Details of Transcription factor families. [file 1471-2164-14-647-S17.zip › Additional_file16B_Upregulated_PMN_pathways_in_Root/V2RS/C71357_65_GSVIVT01015352001_2_photosynthesis_light_reactions.jpg]

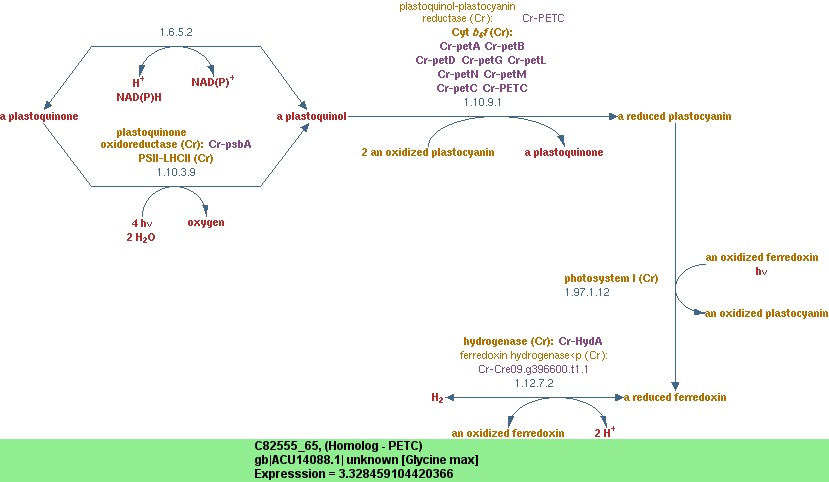

Supplement: Additional file 17 — Details of Transcription factor families. [file 1471-2164-14-647-S17.zip › Additional_file16B_Upregulated_PMN_pathways_in_Root/V2RS/C82555_65_PETC_1_hydrogen_production_VIII.jpg]

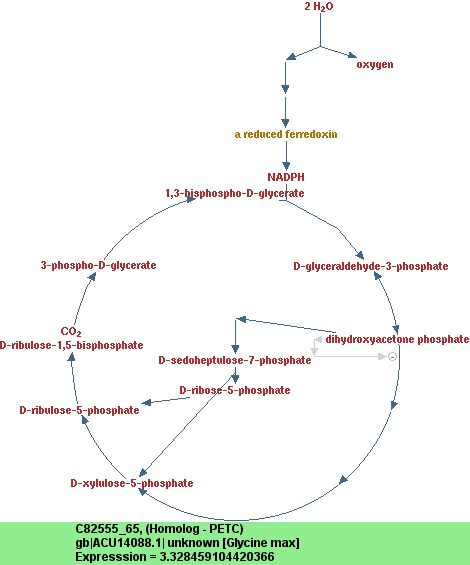

Supplement: Additional file 17 — Details of Transcription factor families. [file 1471-2164-14-647-S17.zip › Additional_file16B_Upregulated_PMN_pathways_in_Root/V2RS/C82555_65_PETC_2_oxygenic_photosynthesis.jpg]

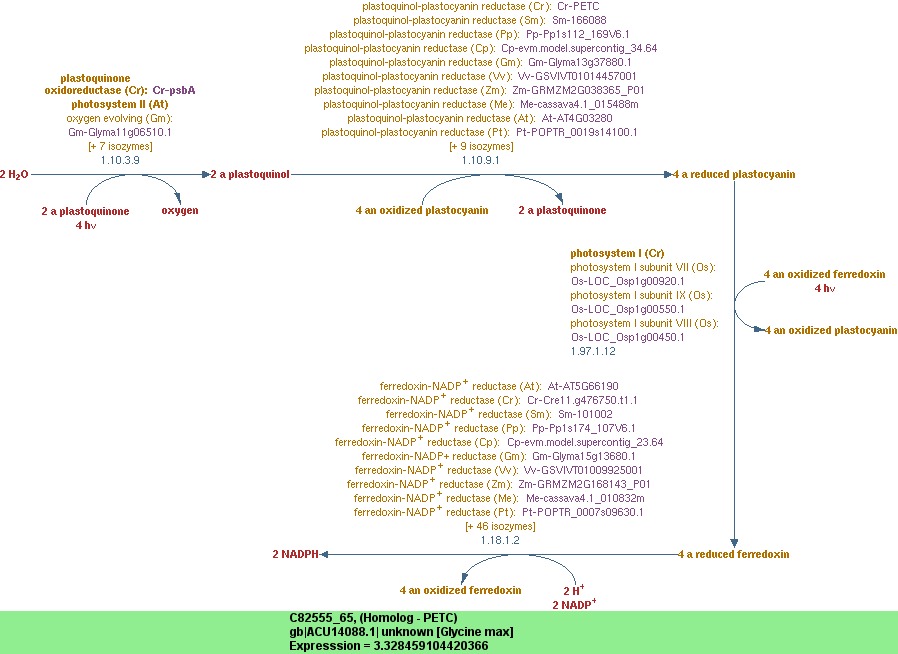

Supplement: Additional file 17 — Details of Transcription factor families. [file 1471-2164-14-647-S17.zip › Additional_file16B_Upregulated_PMN_pathways_in_Root/V2RS/C82555_65_PETC_3_photosynthesis_light_reactions.jpg]

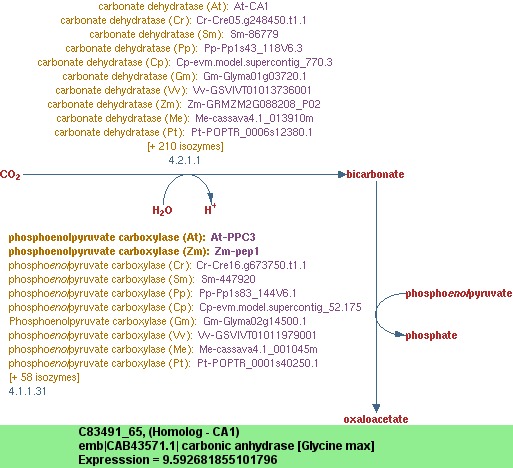

Supplement: Additional file 17 — Details of Transcription factor families. [file 1471-2164-14-647-S17.zip › Additional_file16B_Upregulated_PMN_pathways_in_Root/V2RS/C83491_65_CA1_1_CO_fixation_into_oxaloacetate_(anapleurotic).jpg]
