# Supplementary figures and images for: Comprehensive transcriptomic study on horse gram (Macrotyloma uniflorum): De novo assembly, functional characterization and comparative analysis in relation to drought stress (part 2 of 4)
Source: BMC Genomics. 2013 Sep 23;14:647. doi: 10.1186/1471-2164-14-647 (PMC3853109; doi:10.1186/1471-2164-14-647)

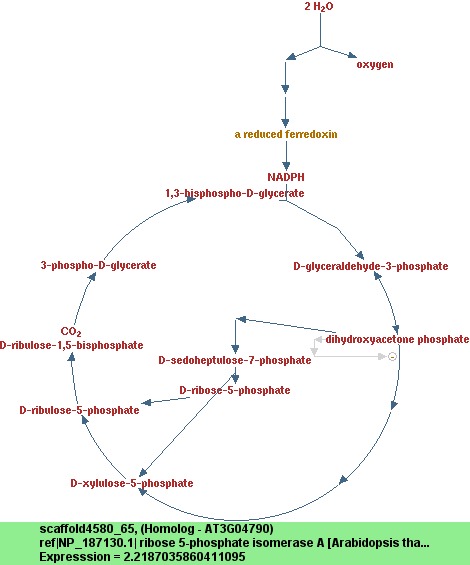

Supplement: Additional file 16 — A and B: Stress related up-regulated PMN pathways. [file 1471-2164-14-647-S16.zip › Additional_file16A_Upregulated_PMN_pathways_in_Shoot/V2SHS/scaffold4580_65_AT3G04790_3_oxygenic_photosynthesis.jpg]

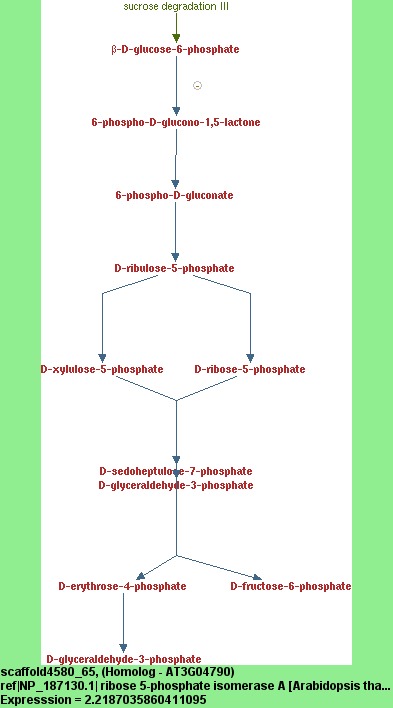

Supplement: Additional file 16 — A and B: Stress related up-regulated PMN pathways. [file 1471-2164-14-647-S16.zip › Additional_file16A_Upregulated_PMN_pathways_in_Shoot/V2SHS/scaffold4580_65_AT3G04790_5_pentose_phosphate_pathway.jpg]

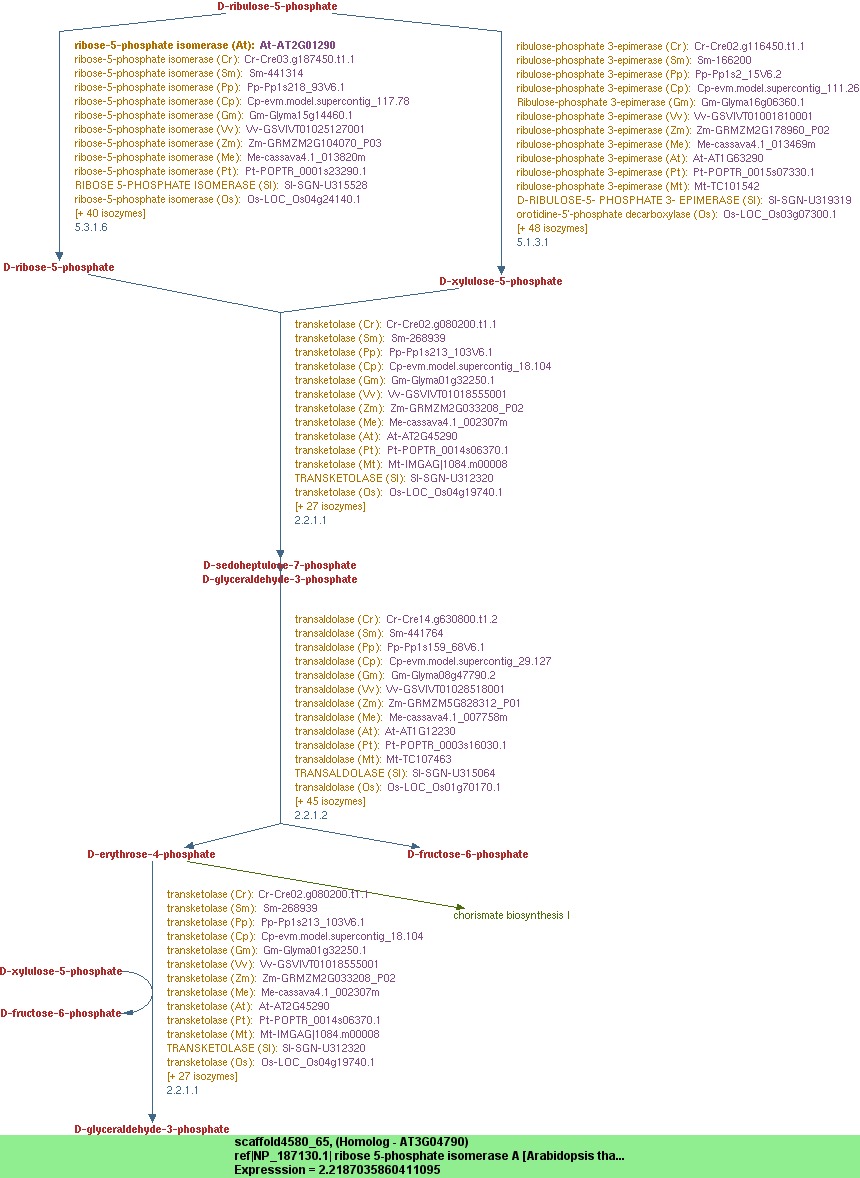

Supplement: Additional file 16 — A and B: Stress related up-regulated PMN pathways. [file 1471-2164-14-647-S16.zip › Additional_file16A_Upregulated_PMN_pathways_in_Shoot/V2SHS/scaffold4580_65_AT3G04790_7_pentose_phosphate_pathway_(non-oxidative_branch).jpg]

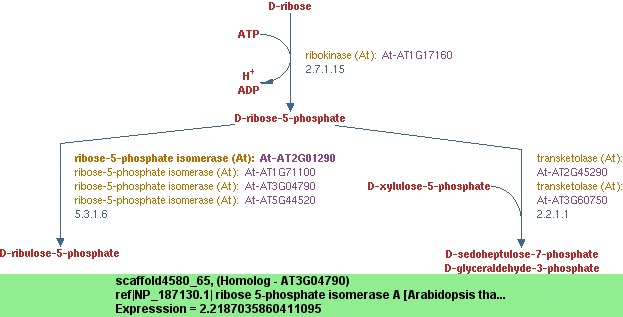

Supplement: Additional file 16 — A and B: Stress related up-regulated PMN pathways. [file 1471-2164-14-647-S16.zip › Additional_file16A_Upregulated_PMN_pathways_in_Shoot/V2SHS/scaffold4580_65_AT3G04790_9_ribose_degradation.jpg]

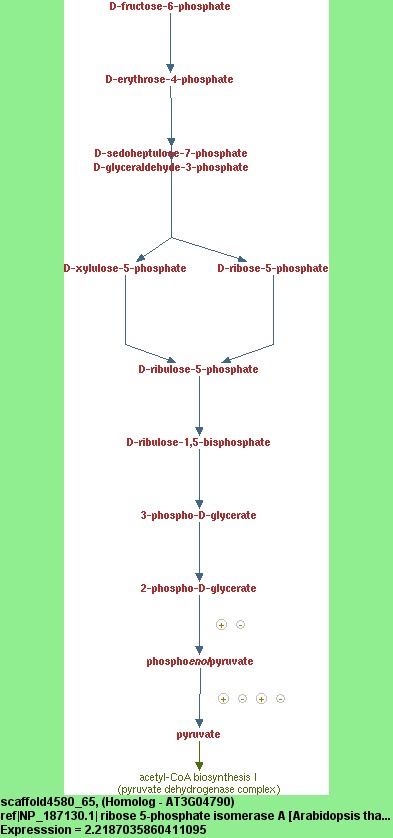

Supplement: Additional file 16 — A and B: Stress related up-regulated PMN pathways. [file 1471-2164-14-647-S16.zip › Additional_file16A_Upregulated_PMN_pathways_in_Shoot/V2SHS/scaffold4580_65_AT3G04790_11_Rubisco_shunt.jpg]

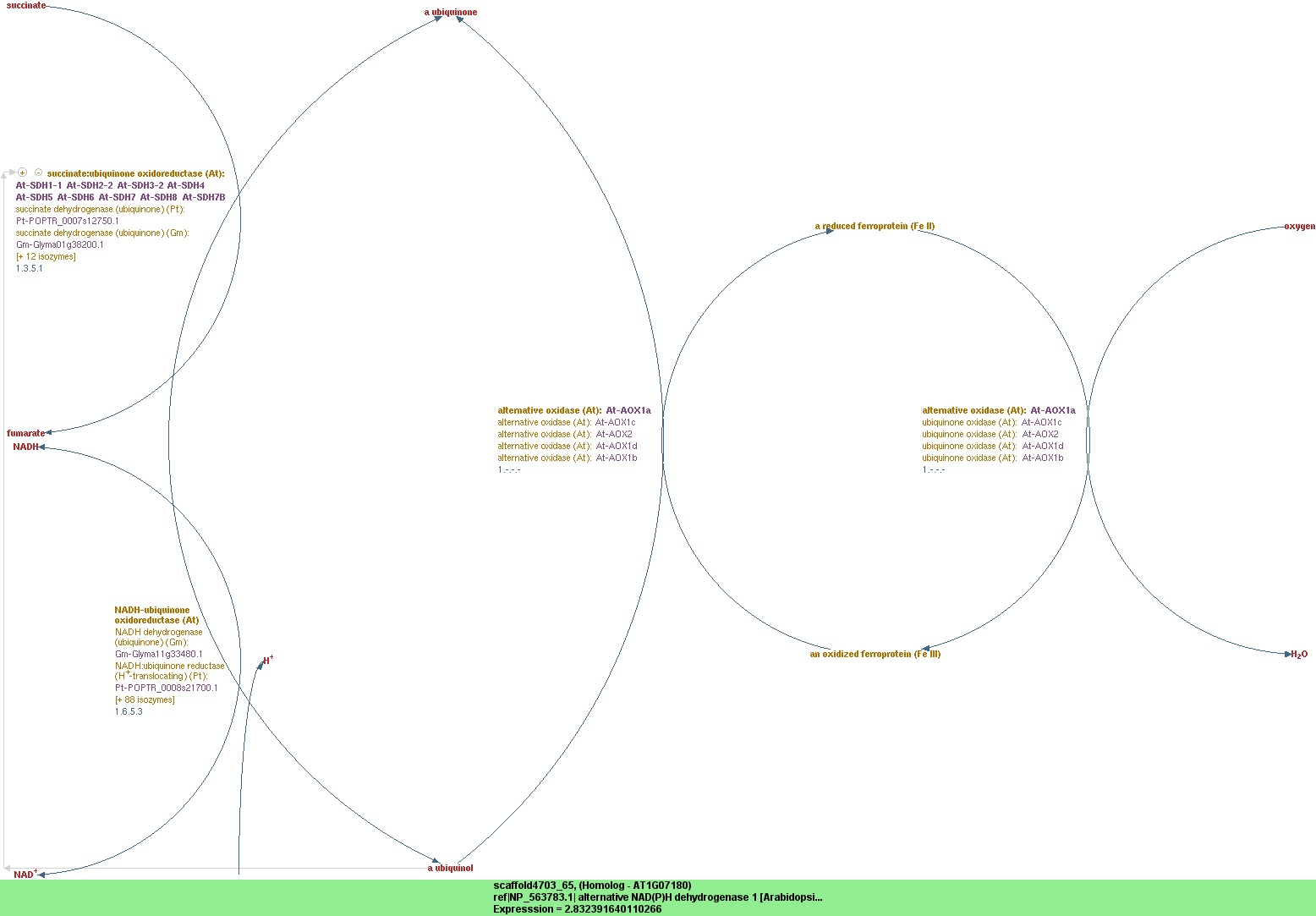

Supplement: Additional file 16 — A and B: Stress related up-regulated PMN pathways. [file 1471-2164-14-647-S16.zip › Additional_file16A_Upregulated_PMN_pathways_in_Shoot/V2SHS/scaffold4703_65_AT1G07180_1_aerobic_respiration_(alternative_oxidase_pathway).jpg]

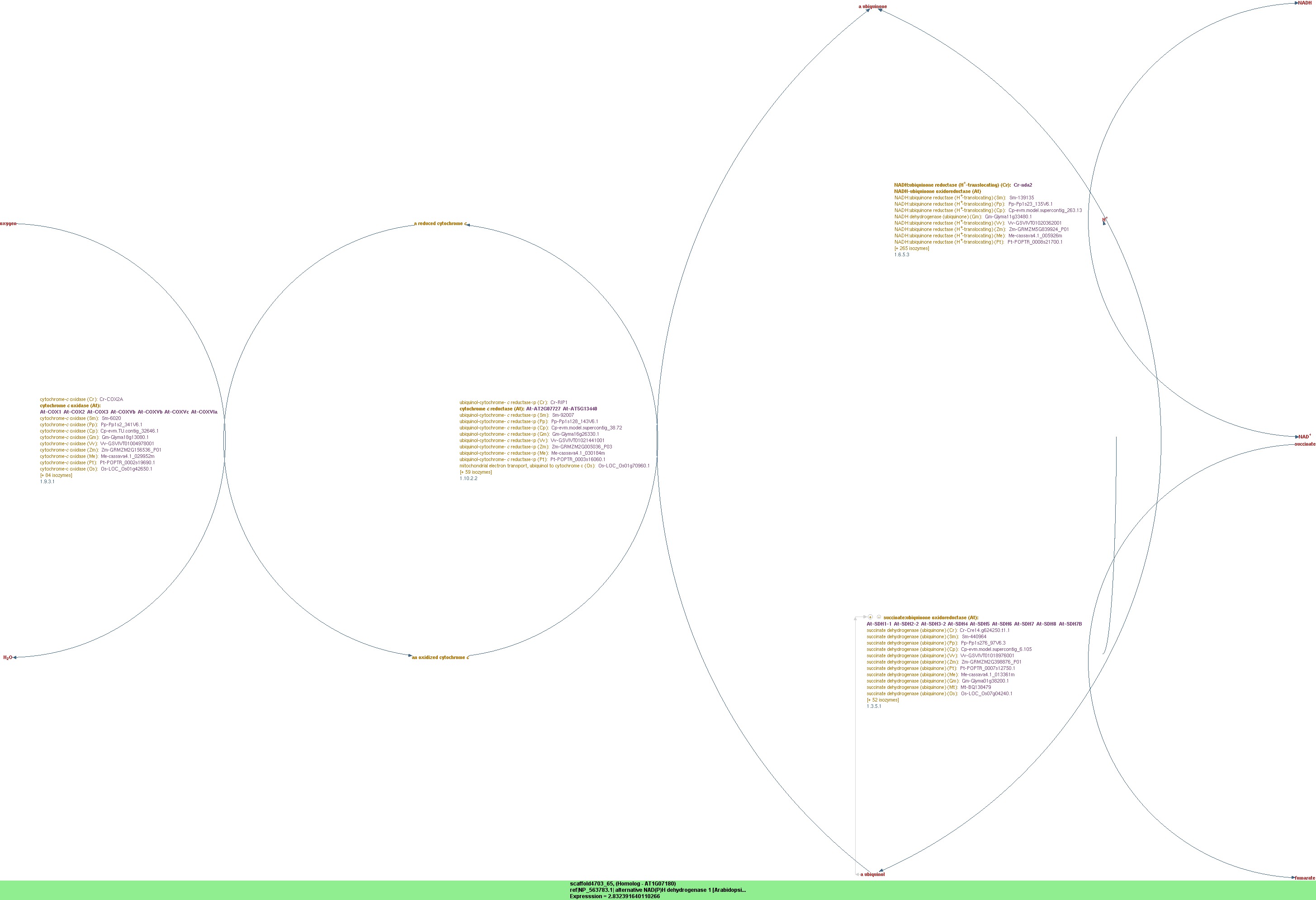

Supplement: Additional file 16 — A and B: Stress related up-regulated PMN pathways. [file 1471-2164-14-647-S16.zip › Additional_file16A_Upregulated_PMN_pathways_in_Shoot/V2SHS/scaffold4703_65_AT1G07180_3_aerobic_respiration_(cytochrome_c).jpg]

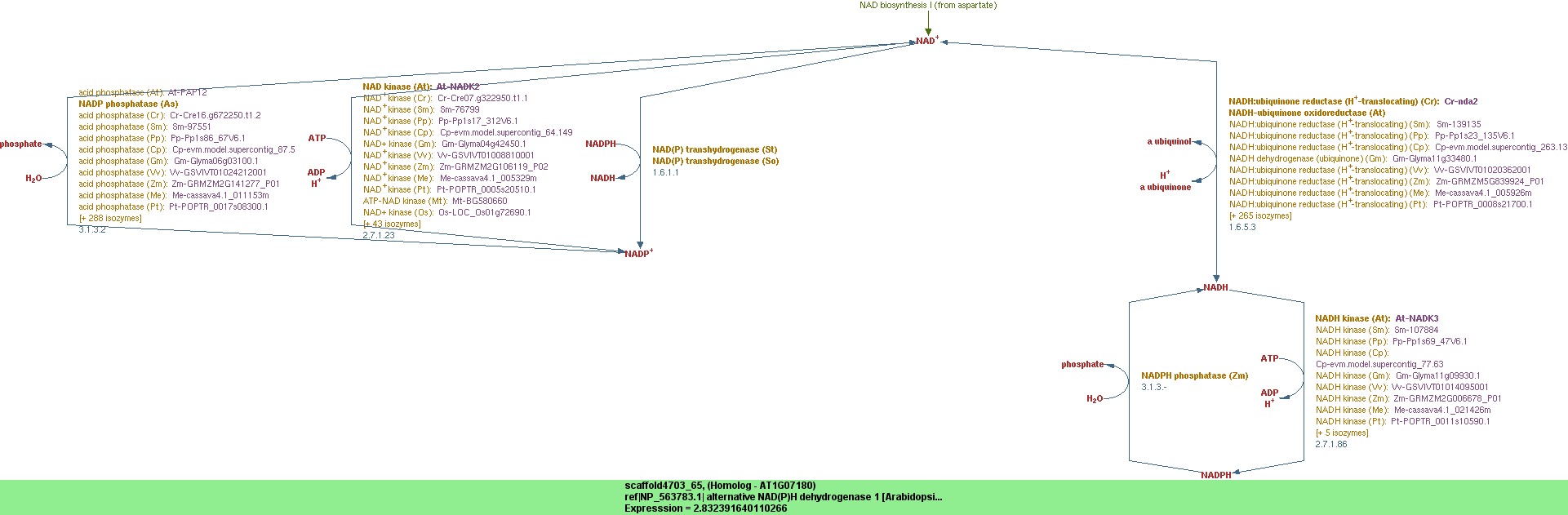

Supplement: Additional file 16 — A and B: Stress related up-regulated PMN pathways. [file 1471-2164-14-647-S16.zip › Additional_file16A_Upregulated_PMN_pathways_in_Shoot/V2SHS/scaffold4703_65_AT1G07180_5_NAD-NADH_phosphorylation_and_dephosphorylation.jpg]

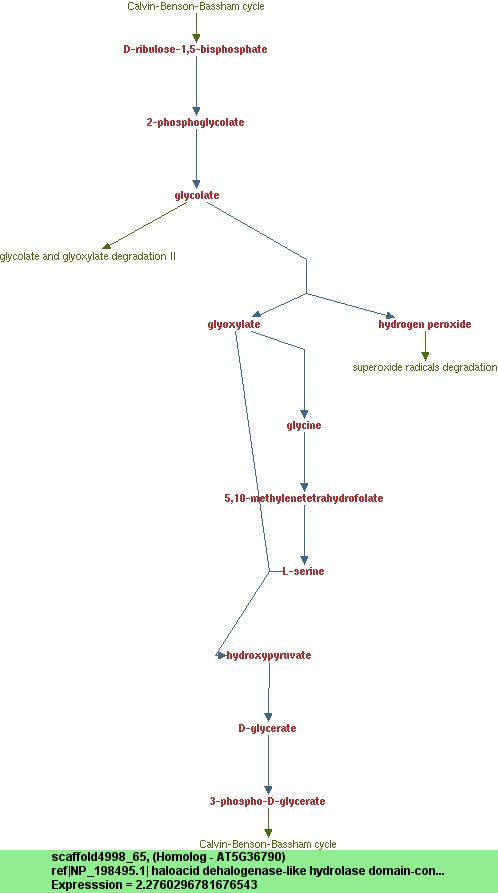

Supplement: Additional file 16 — A and B: Stress related up-regulated PMN pathways. [file 1471-2164-14-647-S16.zip › Additional_file16A_Upregulated_PMN_pathways_in_Shoot/V2SHS/scaffold4998_65_AT5G36790_1_photorespiration.jpg]

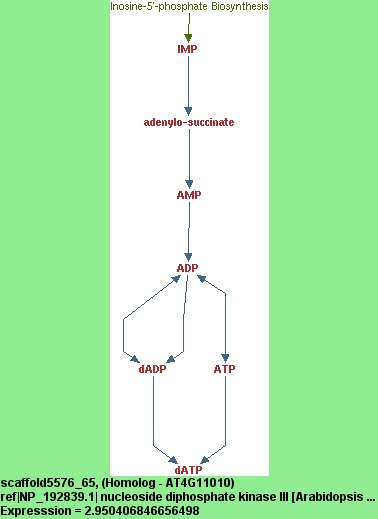

Supplement: Additional file 16 — A and B: Stress related up-regulated PMN pathways. [file 1471-2164-14-647-S16.zip › Additional_file16A_Upregulated_PMN_pathways_in_Shoot/V2SHS/scaffold5576_65_AT4G11010_1_adenosine_nucleotides_de_novo_biosynthesis.jpg]

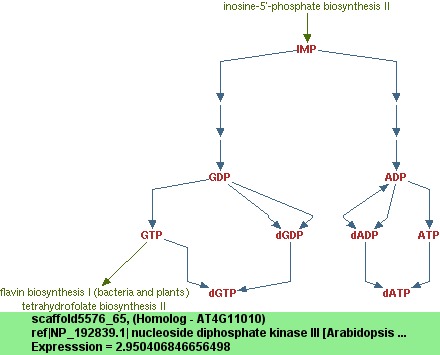

Supplement: Additional file 16 — A and B: Stress related up-regulated PMN pathways. [file 1471-2164-14-647-S16.zip › Additional_file16A_Upregulated_PMN_pathways_in_Shoot/V2SHS/scaffold5576_65_AT4G11010_3_purine_nucleotide_metabolism_(phosphotransfer_and_nucleotide_modification).jpg]

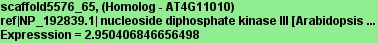

Supplement: Additional file 16 — A and B: Stress related up-regulated PMN pathways. [file 1471-2164-14-647-S16.zip › Additional_file16A_Upregulated_PMN_pathways_in_Shoot/V2SHS/scaffold5576_65_AT4G11010_7_pyrimidine_ribonucleotides_de_novo_biosynthesis.jpg]

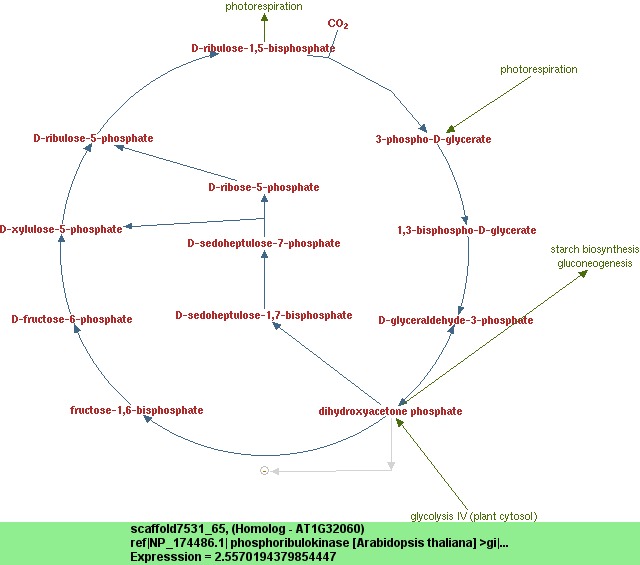

Supplement: Additional file 16 — A and B: Stress related up-regulated PMN pathways. [file 1471-2164-14-647-S16.zip › Additional_file16A_Upregulated_PMN_pathways_in_Shoot/V2SHS/scaffold7531_65_AT1G32060_1_Calvin-Benson-Bassham_cycle.jpg]

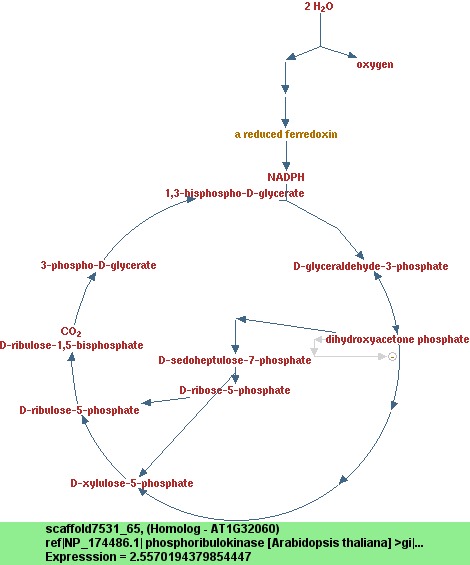

Supplement: Additional file 16 — A and B: Stress related up-regulated PMN pathways. [file 1471-2164-14-647-S16.zip › Additional_file16A_Upregulated_PMN_pathways_in_Shoot/V2SHS/scaffold7531_65_AT1G32060_2_oxygenic_photosynthesis.jpg]

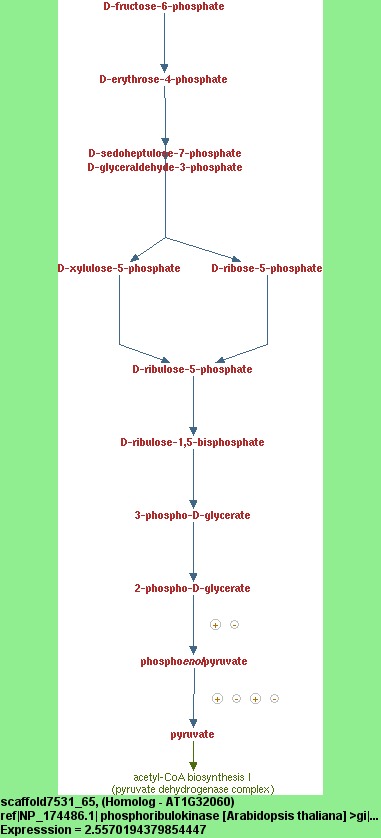

Supplement: Additional file 16 — A and B: Stress related up-regulated PMN pathways. [file 1471-2164-14-647-S16.zip › Additional_file16A_Upregulated_PMN_pathways_in_Shoot/V2SHS/scaffold7531_65_AT1G32060_3_Rubisco_shunt.jpg]

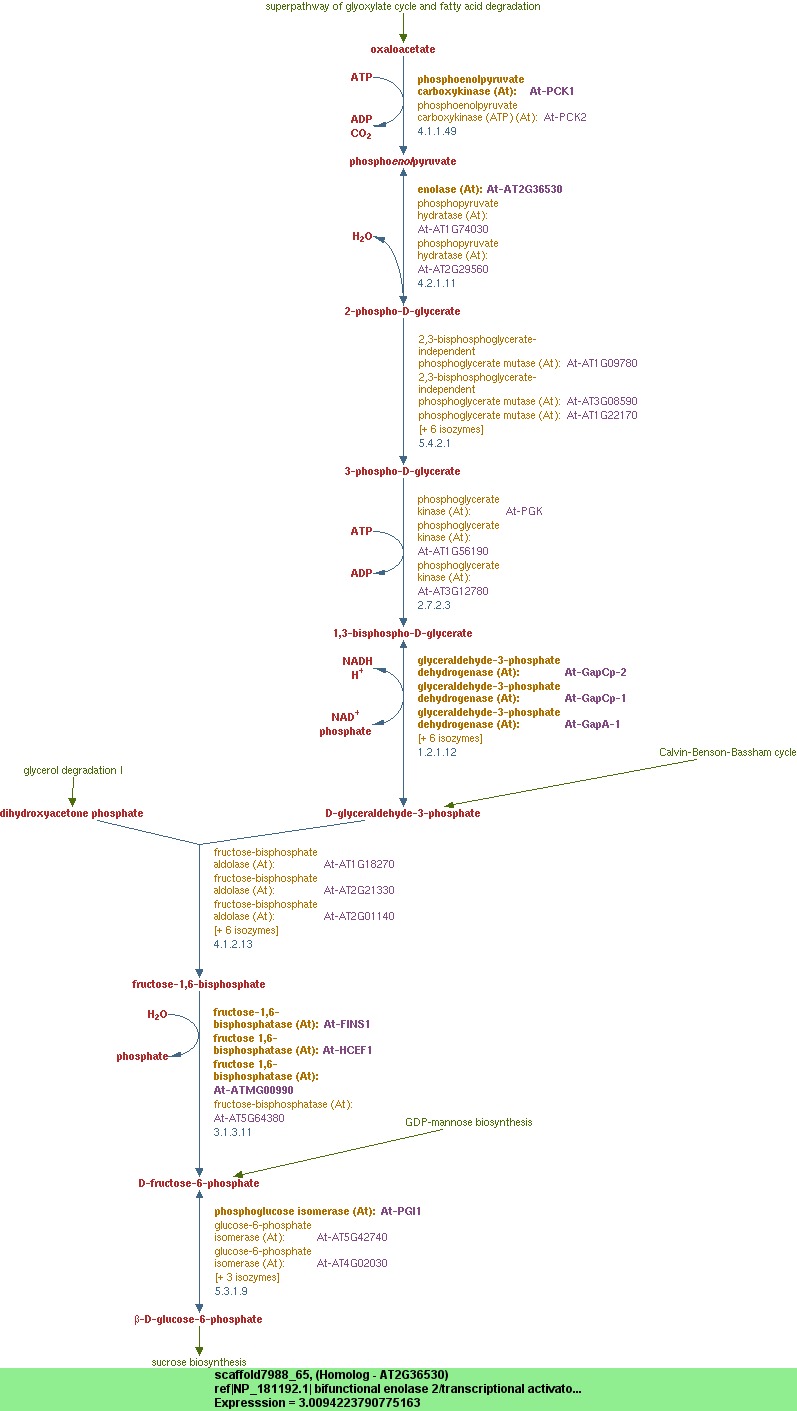

Supplement: Additional file 16 — A and B: Stress related up-regulated PMN pathways. [file 1471-2164-14-647-S16.zip › Additional_file16A_Upregulated_PMN_pathways_in_Shoot/V2SHS/scaffold7988_65_AT2G36530_1_gluconeogenesis.jpg]

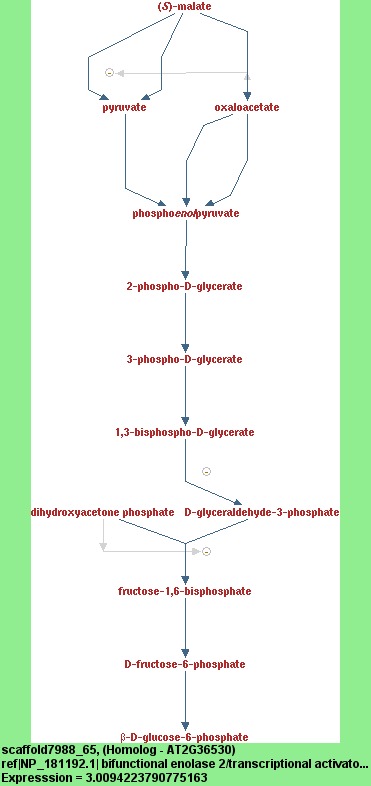

Supplement: Additional file 16 — A and B: Stress related up-regulated PMN pathways. [file 1471-2164-14-647-S16.zip › Additional_file16A_Upregulated_PMN_pathways_in_Shoot/V2SHS/scaffold7988_65_AT2G36530_3_gluconeogenesis_I.jpg]

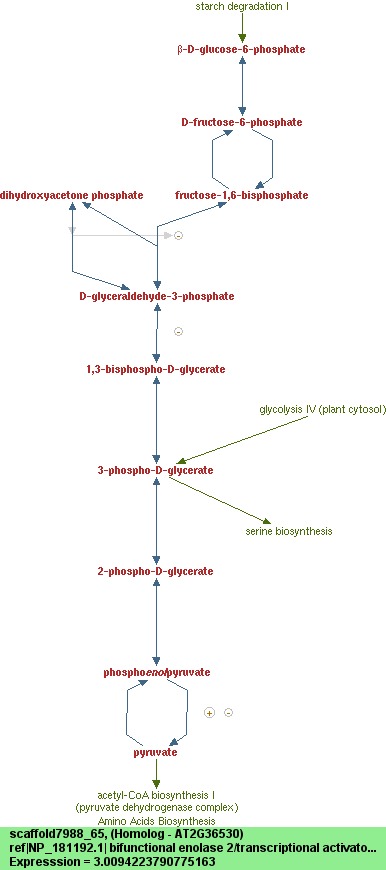

Supplement: Additional file 16 — A and B: Stress related up-regulated PMN pathways. [file 1471-2164-14-647-S16.zip › Additional_file16A_Upregulated_PMN_pathways_in_Shoot/V2SHS/scaffold7988_65_AT2G36530_5_glycolysis_I.jpg]

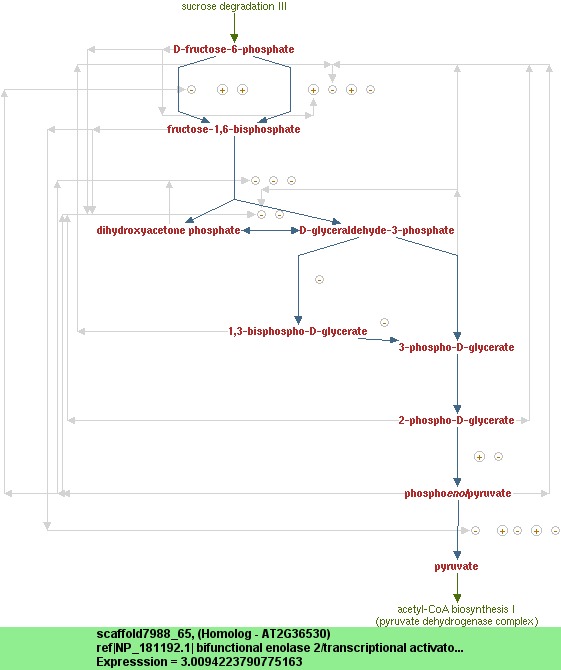

Supplement: Additional file 16 — A and B: Stress related up-regulated PMN pathways. [file 1471-2164-14-647-S16.zip › Additional_file16A_Upregulated_PMN_pathways_in_Shoot/V2SHS/scaffold7988_65_AT2G36530_7_glycolysis_IV_(plant_cytosol).jpg]

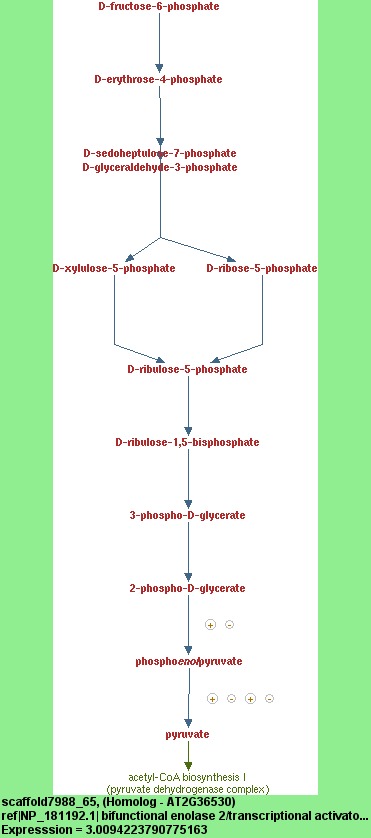

Supplement: Additional file 16 — A and B: Stress related up-regulated PMN pathways. [file 1471-2164-14-647-S16.zip › Additional_file16A_Upregulated_PMN_pathways_in_Shoot/V2SHS/scaffold7988_65_AT2G36530_9_Rubisco_shunt.jpg]

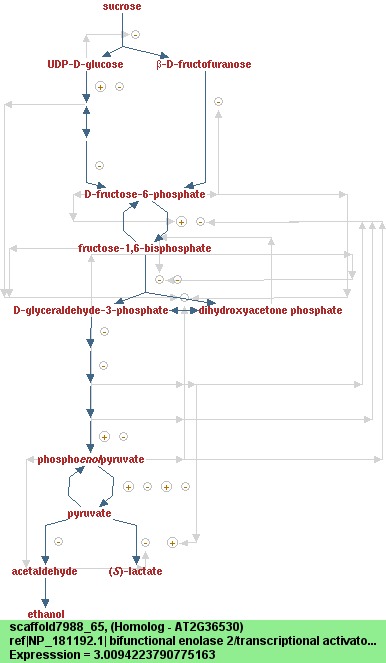

Supplement: Additional file 16 — A and B: Stress related up-regulated PMN pathways. [file 1471-2164-14-647-S16.zip › Additional_file16A_Upregulated_PMN_pathways_in_Shoot/V2SHS/scaffold7988_65_AT2G36530_11_sucrose_degradation_VI_(anaerobic).jpg]

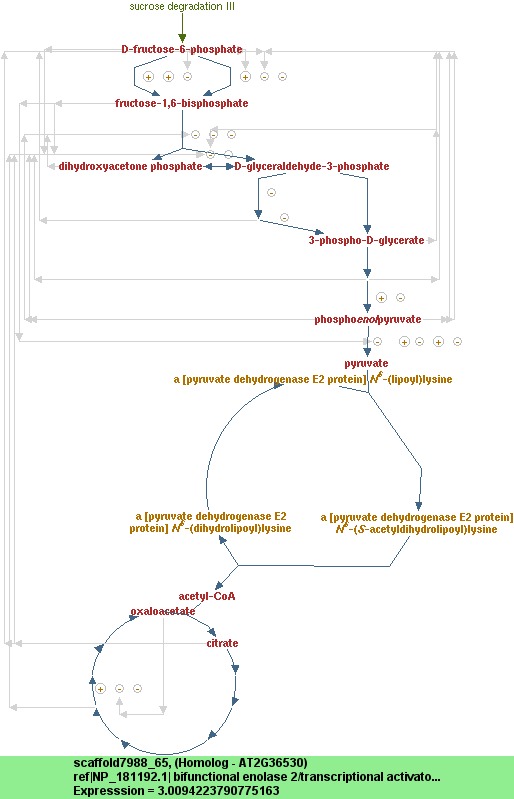

Supplement: Additional file 16 — A and B: Stress related up-regulated PMN pathways. [file 1471-2164-14-647-S16.zip › Additional_file16A_Upregulated_PMN_pathways_in_Shoot/V2SHS/scaffold7988_65_AT2G36530_13_superpathway_of_cytosolic_glycolysis_(plants),_pyruvate_dehydrogenase_and_TCA_cycle.jpg]

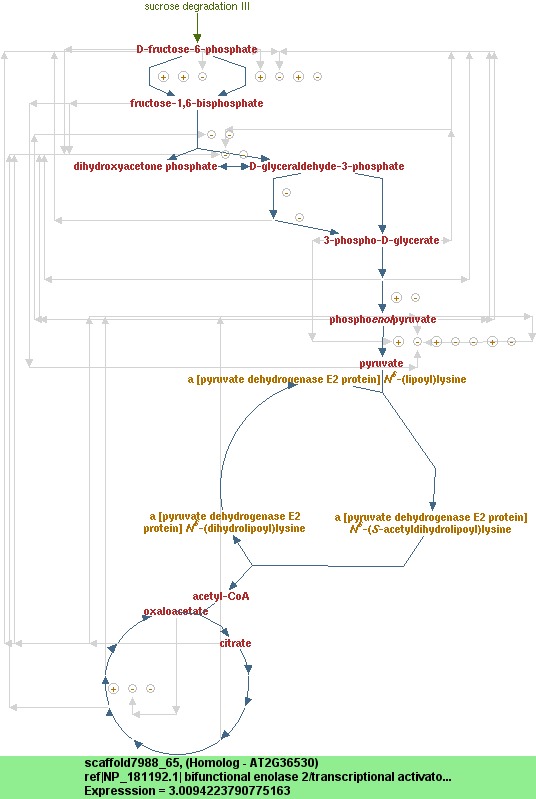

Supplement: Additional file 16 — A and B: Stress related up-regulated PMN pathways. [file 1471-2164-14-647-S16.zip › Additional_file16A_Upregulated_PMN_pathways_in_Shoot/V2SHS/scaffold7988_65_AT2G36530_15_superpathway_of_cytosolic_glycolysis_(plants),_pyruvate_dehydrogenase_and_TCA_cycle.jpg]

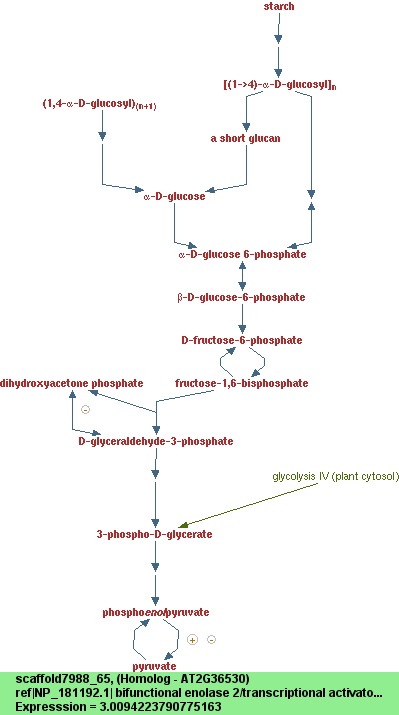

Supplement: Additional file 16 — A and B: Stress related up-regulated PMN pathways. [file 1471-2164-14-647-S16.zip › Additional_file16A_Upregulated_PMN_pathways_in_Shoot/V2SHS/scaffold7988_65_AT2G36530_17_superpathway_of_starch_degradation_to_pyruvate.jpg]

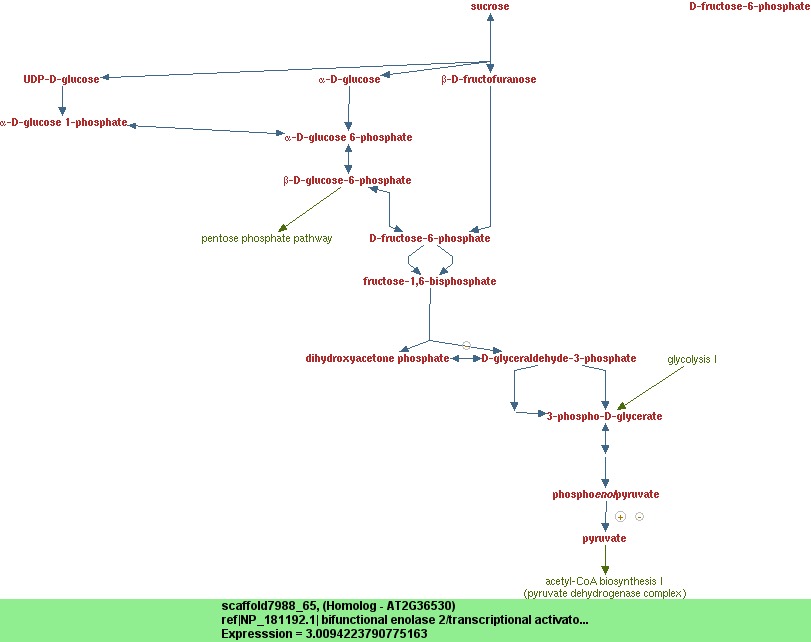

Supplement: Additional file 16 — A and B: Stress related up-regulated PMN pathways. [file 1471-2164-14-647-S16.zip › Additional_file16A_Upregulated_PMN_pathways_in_Shoot/V2SHS/scaffold7988_65_AT2G36530_19_superpathway_of_sucrose_degradation_to_pyruvate.jpg]

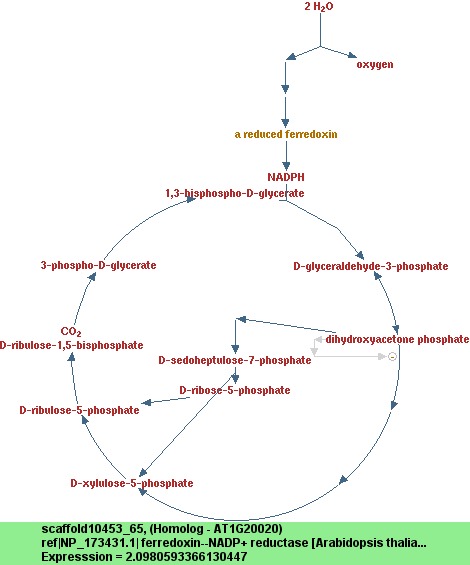

Supplement: Additional file 16 — A and B: Stress related up-regulated PMN pathways. [file 1471-2164-14-647-S16.zip › Additional_file16A_Upregulated_PMN_pathways_in_Shoot/V2SHS/scaffold10453_65_AT1G20020_1_oxygenic_photosynthesis.jpg]

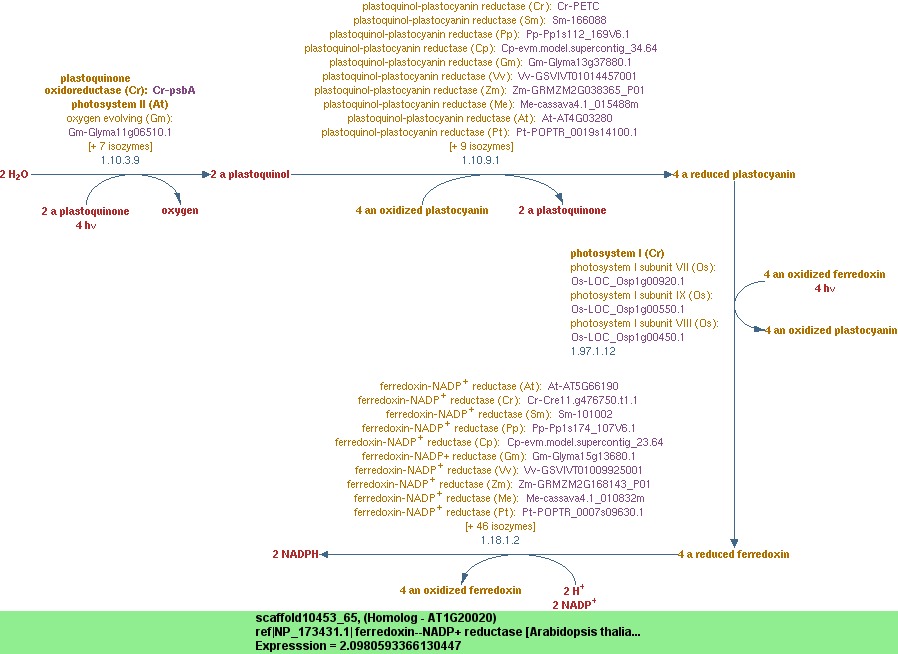

Supplement: Additional file 16 — A and B: Stress related up-regulated PMN pathways. [file 1471-2164-14-647-S16.zip › Additional_file16A_Upregulated_PMN_pathways_in_Shoot/V2SHS/scaffold10453_65_AT1G20020_3_photosynthesis_light_reactions.jpg]

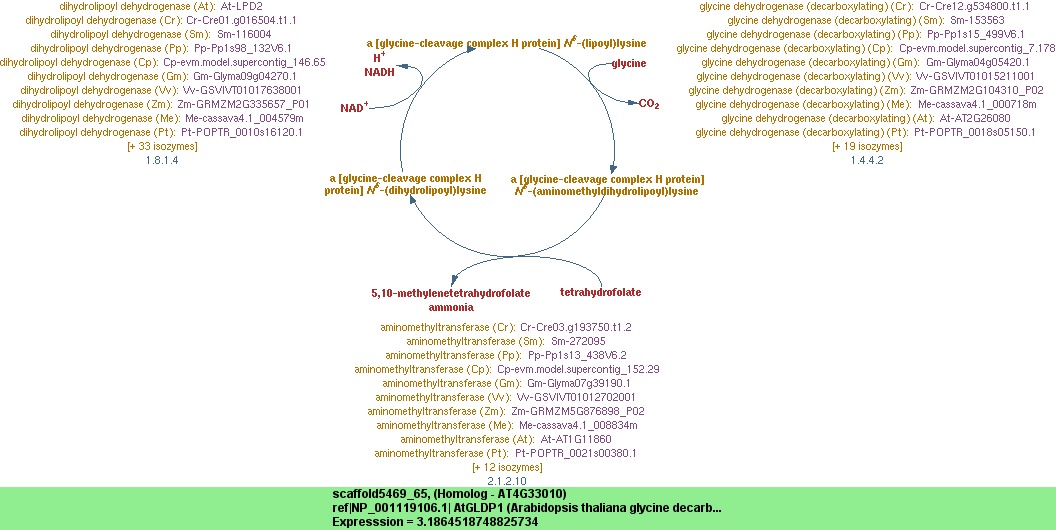

Supplement: Additional file 16 — A and B: Stress related up-regulated PMN pathways. [file 1471-2164-14-647-S16.zip › Additional_file16A_Upregulated_PMN_pathways_in_Shoot/V2SHS/scaffold11049_65_AT4G33010_1_glycine_cleavage_complex.jpg]

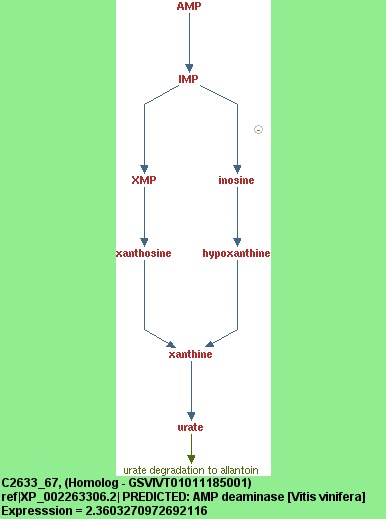

Supplement: Additional file 16 — A and B: Stress related up-regulated PMN pathways. [file 1471-2164-14-647-S16.zip › Additional_file16A_Upregulated_PMN_pathways_in_Shoot/V2SHS/C2633_67_GSVIVT01011185001_1_adenosine_nucleotides_degradation_I.jpg]

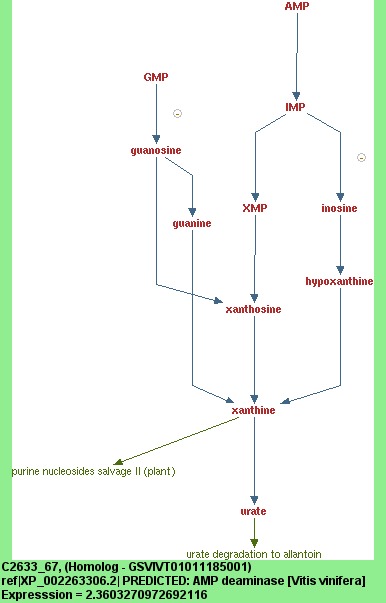

Supplement: Additional file 16 — A and B: Stress related up-regulated PMN pathways. [file 1471-2164-14-647-S16.zip › Additional_file16A_Upregulated_PMN_pathways_in_Shoot/V2SHS/C2633_67_GSVIVT01011185001_2_purine_nucleotides_degradation_I_(plants).jpg]

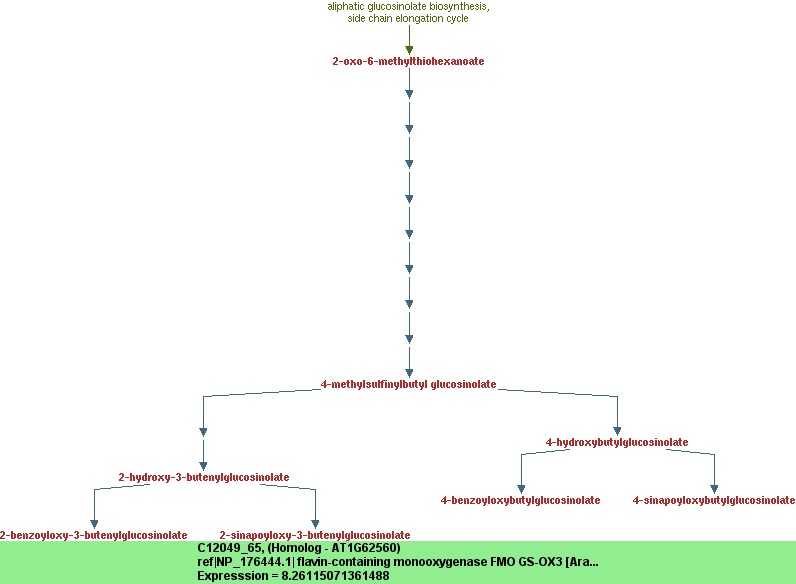

Supplement: Additional file 16 — A and B: Stress related up-regulated PMN pathways. [file 1471-2164-14-647-S16.zip › Additional_file16A_Upregulated_PMN_pathways_in_Shoot/V2SHS/C12049_65_AT1G62560_1_glucosinolate_biosynthesis_from_dihomomethionine.jpg]

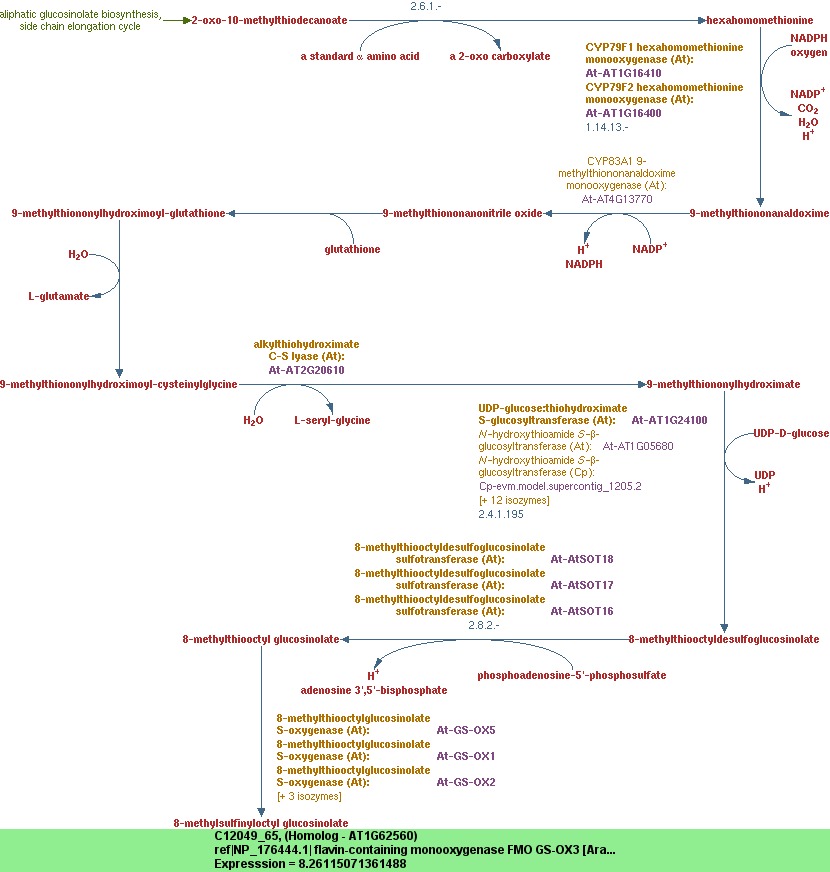

Supplement: Additional file 16 — A and B: Stress related up-regulated PMN pathways. [file 1471-2164-14-647-S16.zip › Additional_file16A_Upregulated_PMN_pathways_in_Shoot/V2SHS/C12049_65_AT1G62560_2_glucosinolate_biosynthesis_from_hexahomomethionine.jpg]

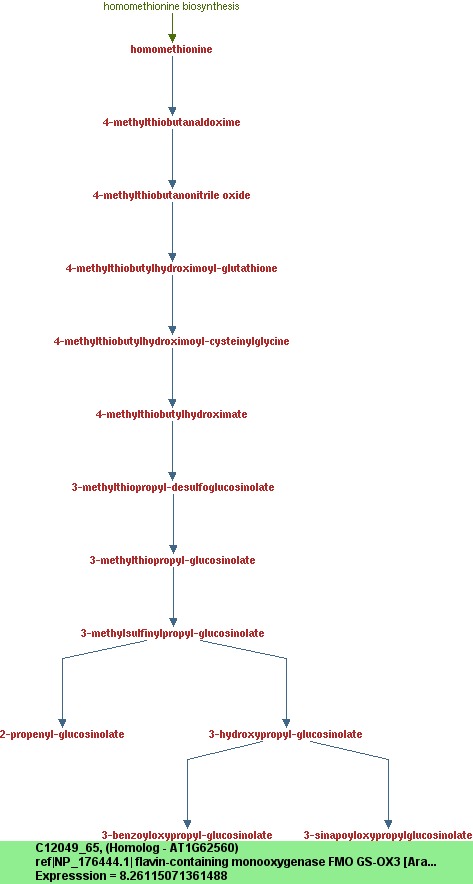

Supplement: Additional file 16 — A and B: Stress related up-regulated PMN pathways. [file 1471-2164-14-647-S16.zip › Additional_file16A_Upregulated_PMN_pathways_in_Shoot/V2SHS/C12049_65_AT1G62560_3_glucosinolate_biosynthesis_from_homomethionine.jpg]

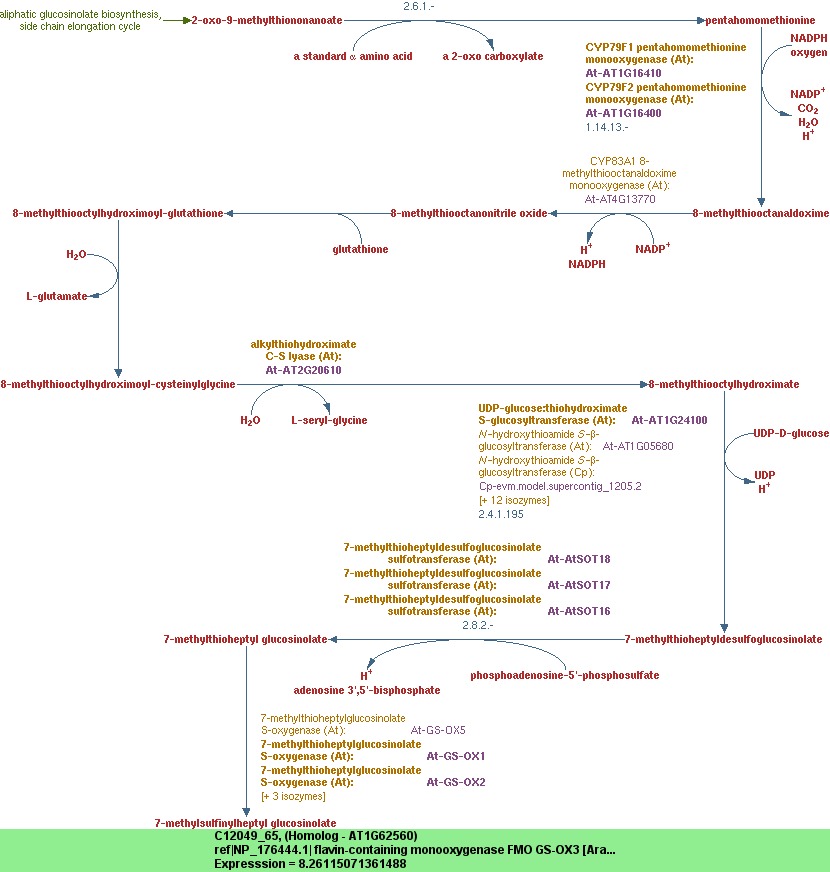

Supplement: Additional file 16 — A and B: Stress related up-regulated PMN pathways. [file 1471-2164-14-647-S16.zip › Additional_file16A_Upregulated_PMN_pathways_in_Shoot/V2SHS/C12049_65_AT1G62560_4_glucosinolate_biosynthesis_from_pentahomomethionine.jpg]

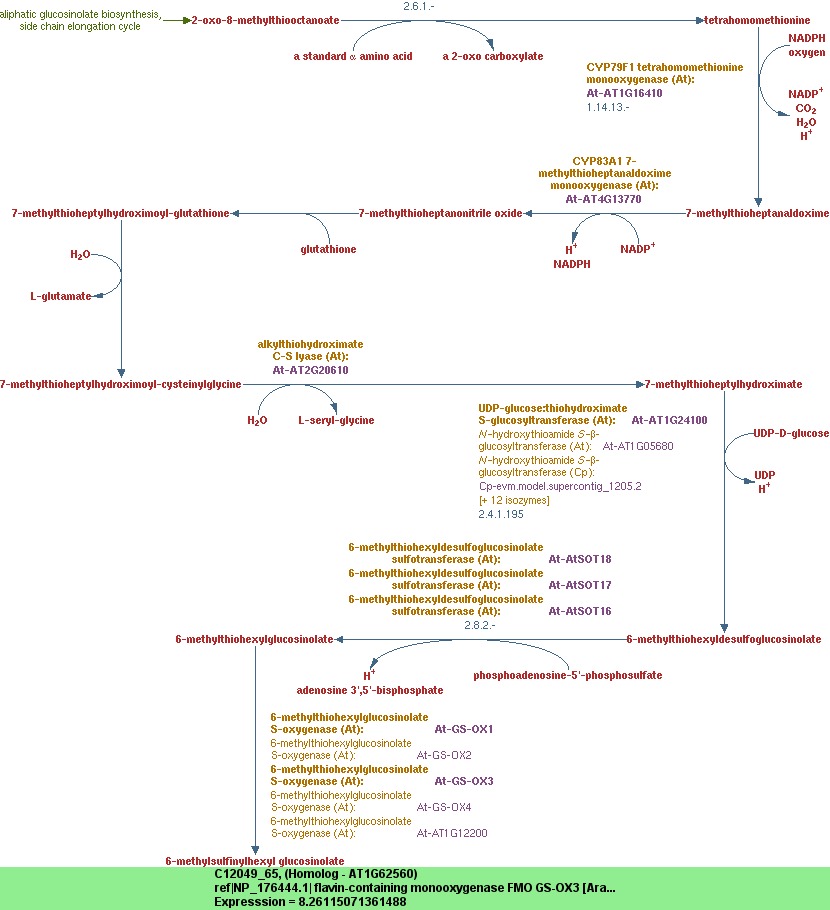

Supplement: Additional file 16 — A and B: Stress related up-regulated PMN pathways. [file 1471-2164-14-647-S16.zip › Additional_file16A_Upregulated_PMN_pathways_in_Shoot/V2SHS/C12049_65_AT1G62560_5_glucosinolate_biosynthesis_from_tetrahomomethionine.jpg]

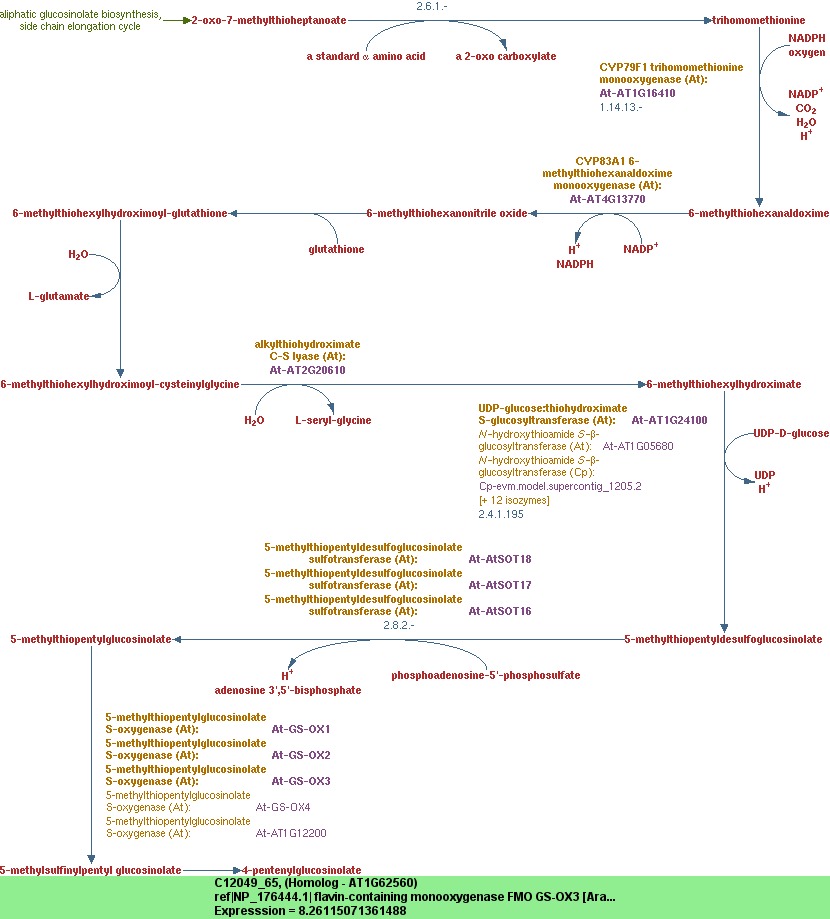

Supplement: Additional file 16 — A and B: Stress related up-regulated PMN pathways. [file 1471-2164-14-647-S16.zip › Additional_file16A_Upregulated_PMN_pathways_in_Shoot/V2SHS/C12049_65_AT1G62560_6_glucosinolate_biosynthesis_from_trihomomethionine.jpg]

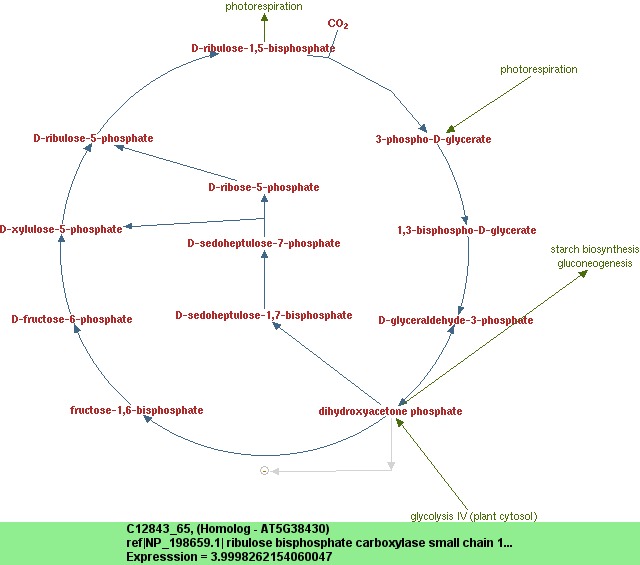

Supplement: Additional file 16 — A and B: Stress related up-regulated PMN pathways. [file 1471-2164-14-647-S16.zip › Additional_file16A_Upregulated_PMN_pathways_in_Shoot/V2SHS/C12843_65_AT5G38430_1_Calvin-Benson-Bassham_cycle.jpg]

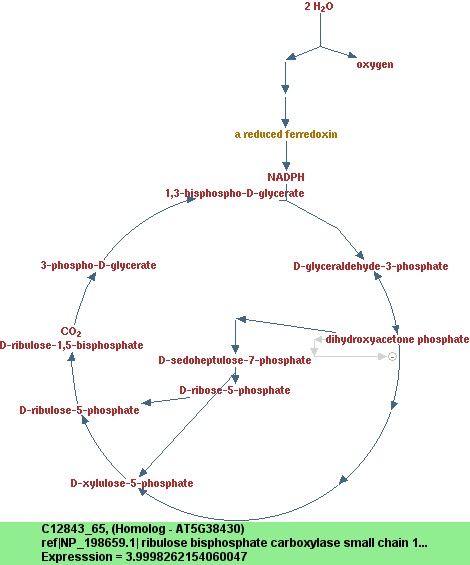

Supplement: Additional file 16 — A and B: Stress related up-regulated PMN pathways. [file 1471-2164-14-647-S16.zip › Additional_file16A_Upregulated_PMN_pathways_in_Shoot/V2SHS/C12843_65_AT5G38430_2_oxygenic_photosynthesis.jpg]

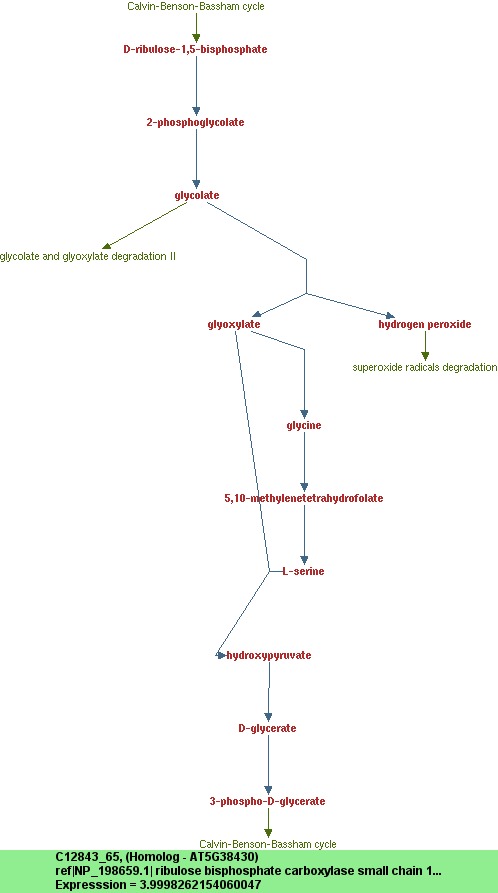

Supplement: Additional file 16 — A and B: Stress related up-regulated PMN pathways. [file 1471-2164-14-647-S16.zip › Additional_file16A_Upregulated_PMN_pathways_in_Shoot/V2SHS/C12843_65_AT5G38430_3_photorespiration.jpg]

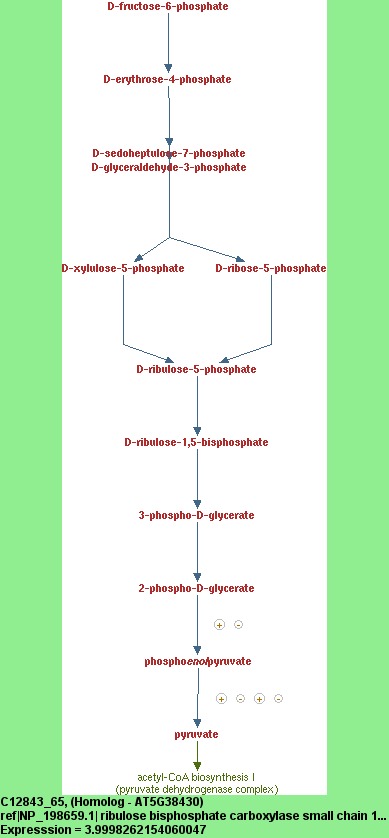

Supplement: Additional file 16 — A and B: Stress related up-regulated PMN pathways. [file 1471-2164-14-647-S16.zip › Additional_file16A_Upregulated_PMN_pathways_in_Shoot/V2SHS/C12843_65_AT5G38430_4_Rubisco_shunt.jpg]

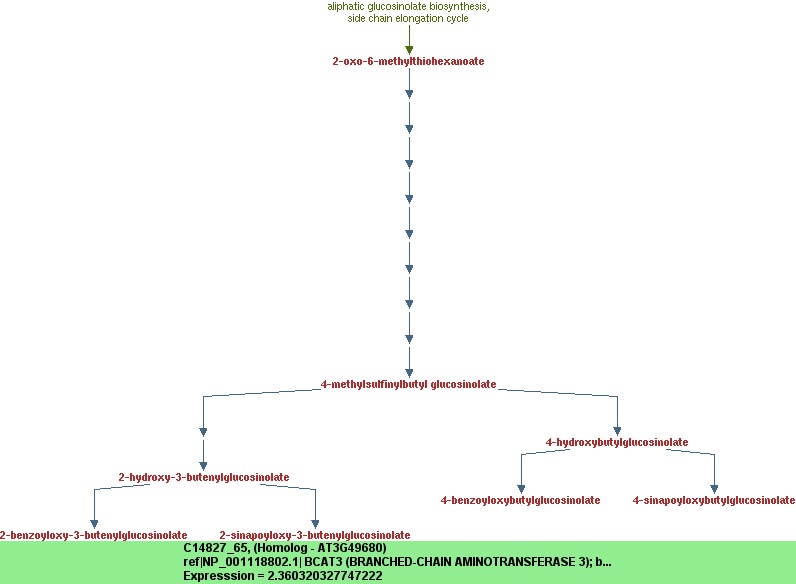

Supplement: Additional file 16 — A and B: Stress related up-regulated PMN pathways. [file 1471-2164-14-647-S16.zip › Additional_file16A_Upregulated_PMN_pathways_in_Shoot/V2SHS/C14827_65_AT3G49680_1_glucosinolate_biosynthesis_from_dihomomethionine.jpg]

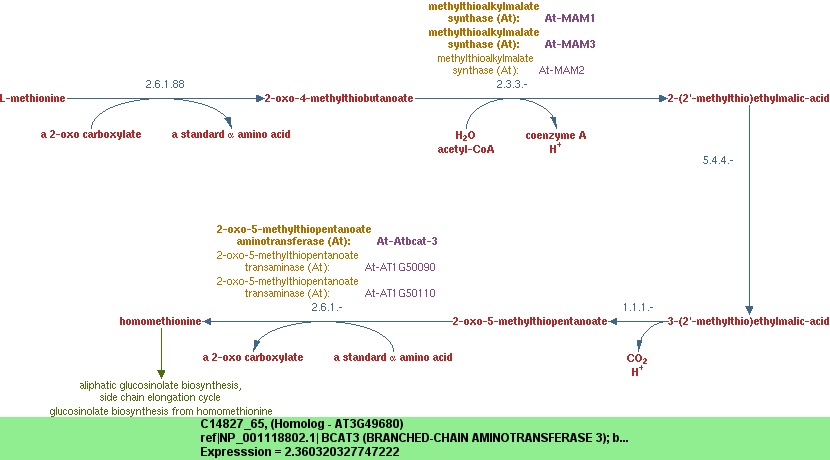

Supplement: Additional file 16 — A and B: Stress related up-regulated PMN pathways. [file 1471-2164-14-647-S16.zip › Additional_file16A_Upregulated_PMN_pathways_in_Shoot/V2SHS/C14827_65_AT3G49680_2_homomethionine_biosynthesis.jpg]

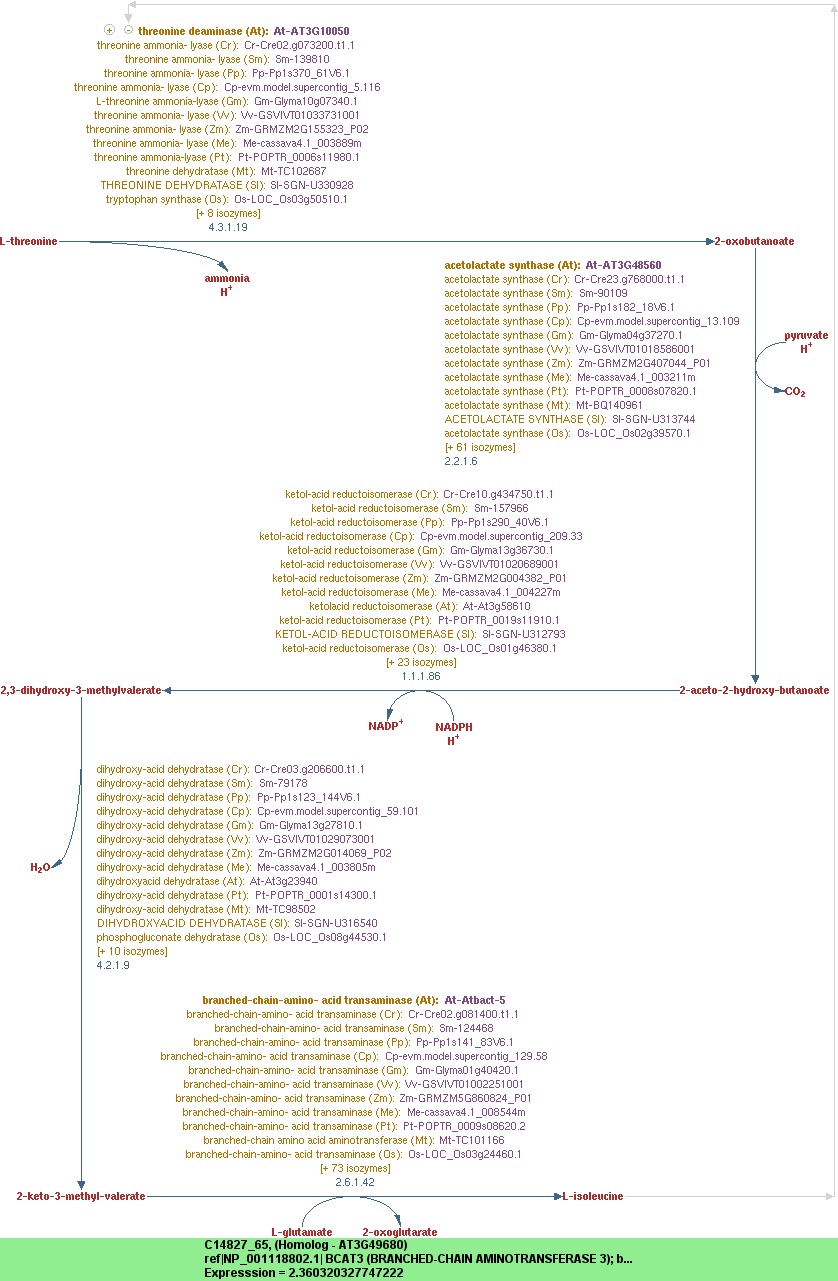

Supplement: Additional file 16 — A and B: Stress related up-regulated PMN pathways. [file 1471-2164-14-647-S16.zip › Additional_file16A_Upregulated_PMN_pathways_in_Shoot/V2SHS/C14827_65_AT3G49680_3_isoleucine_biosynthesis_I_(from_threonine).jpg]

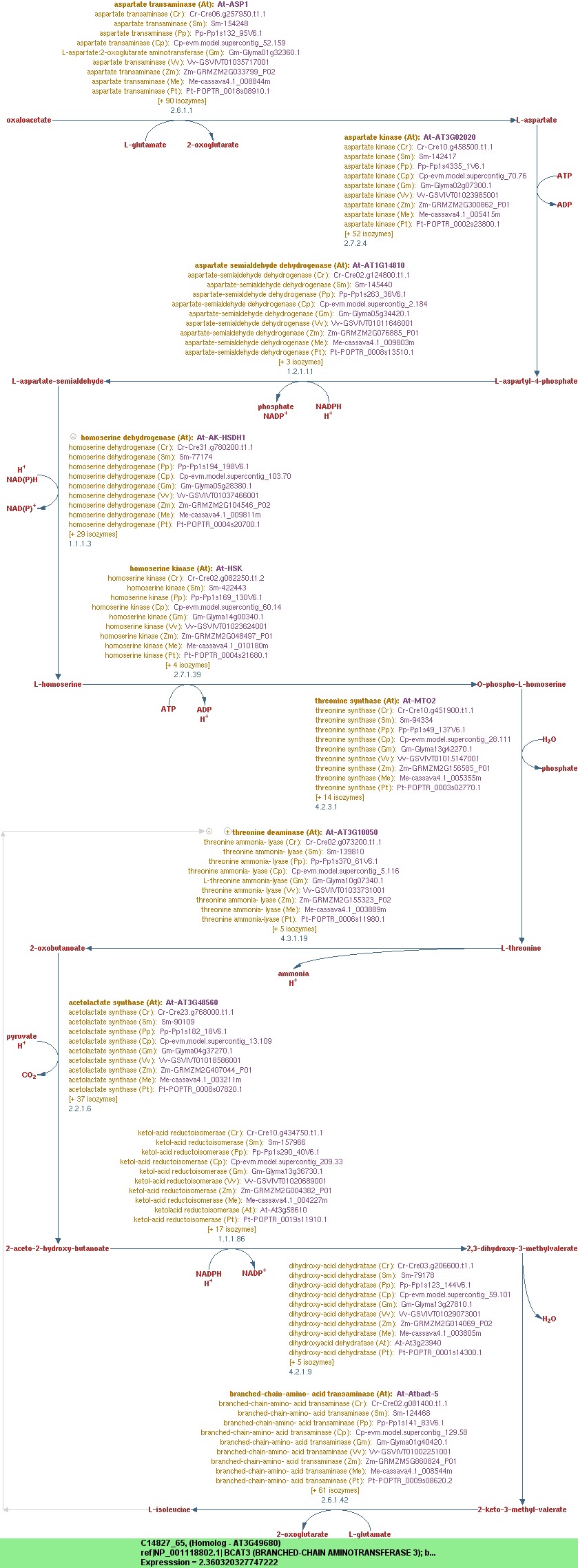

Supplement: Additional file 16 — A and B: Stress related up-regulated PMN pathways. [file 1471-2164-14-647-S16.zip › Additional_file16A_Upregulated_PMN_pathways_in_Shoot/V2SHS/C14827_65_AT3G49680_4_isoleucine_biosynthesis_I.jpg]

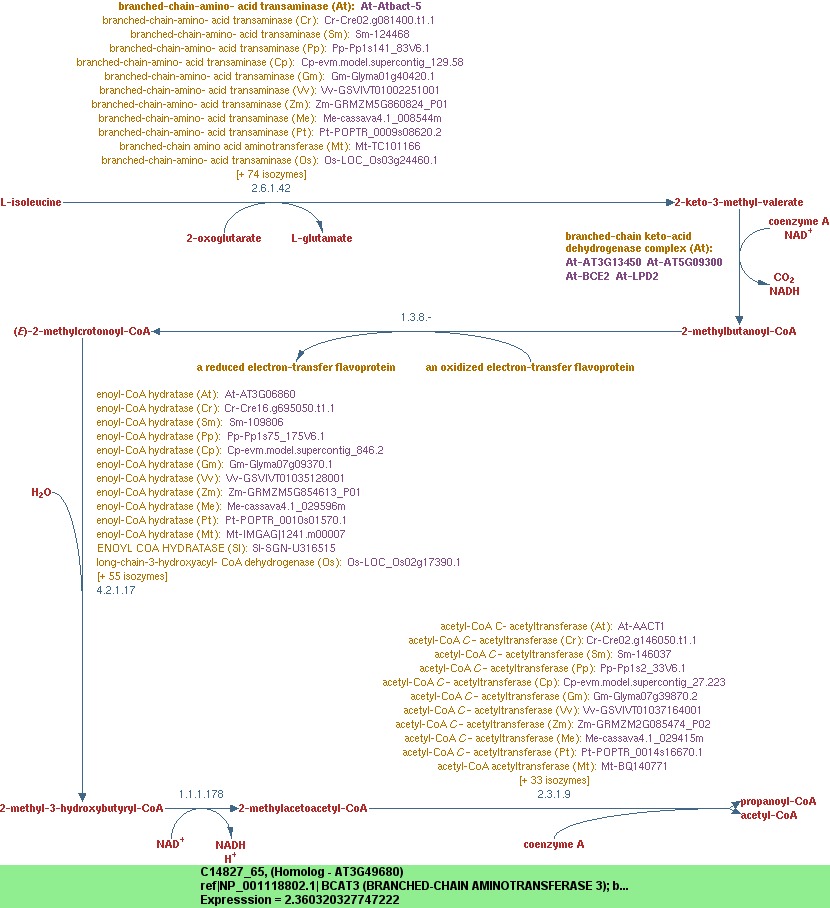

Supplement: Additional file 16 — A and B: Stress related up-regulated PMN pathways. [file 1471-2164-14-647-S16.zip › Additional_file16A_Upregulated_PMN_pathways_in_Shoot/V2SHS/C14827_65_AT3G49680_5_isoleucine_degradation_I.jpg]

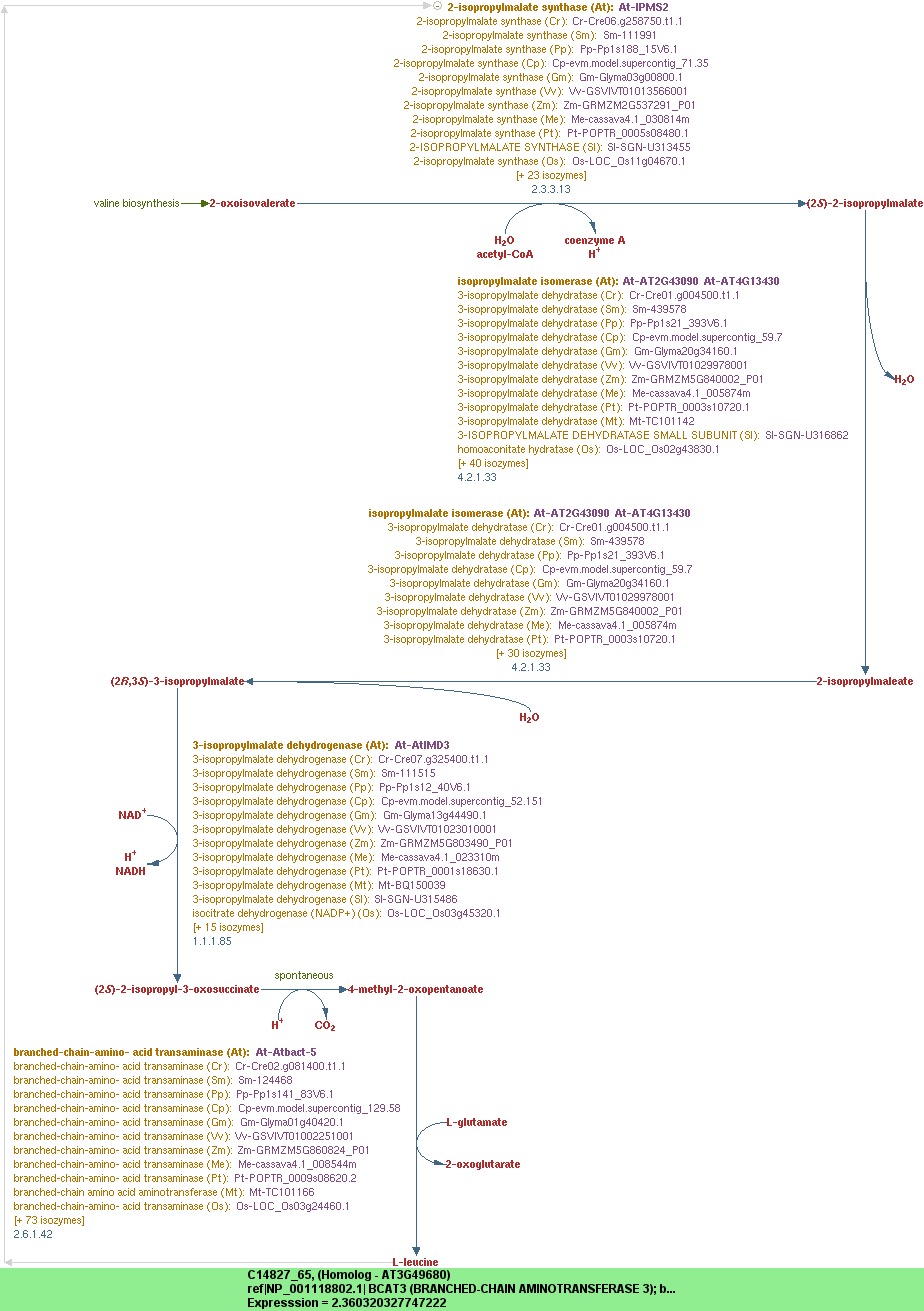

Supplement: Additional file 16 — A and B: Stress related up-regulated PMN pathways. [file 1471-2164-14-647-S16.zip › Additional_file16A_Upregulated_PMN_pathways_in_Shoot/V2SHS/C14827_65_AT3G49680_6_leucine_biosynthesis.jpg]

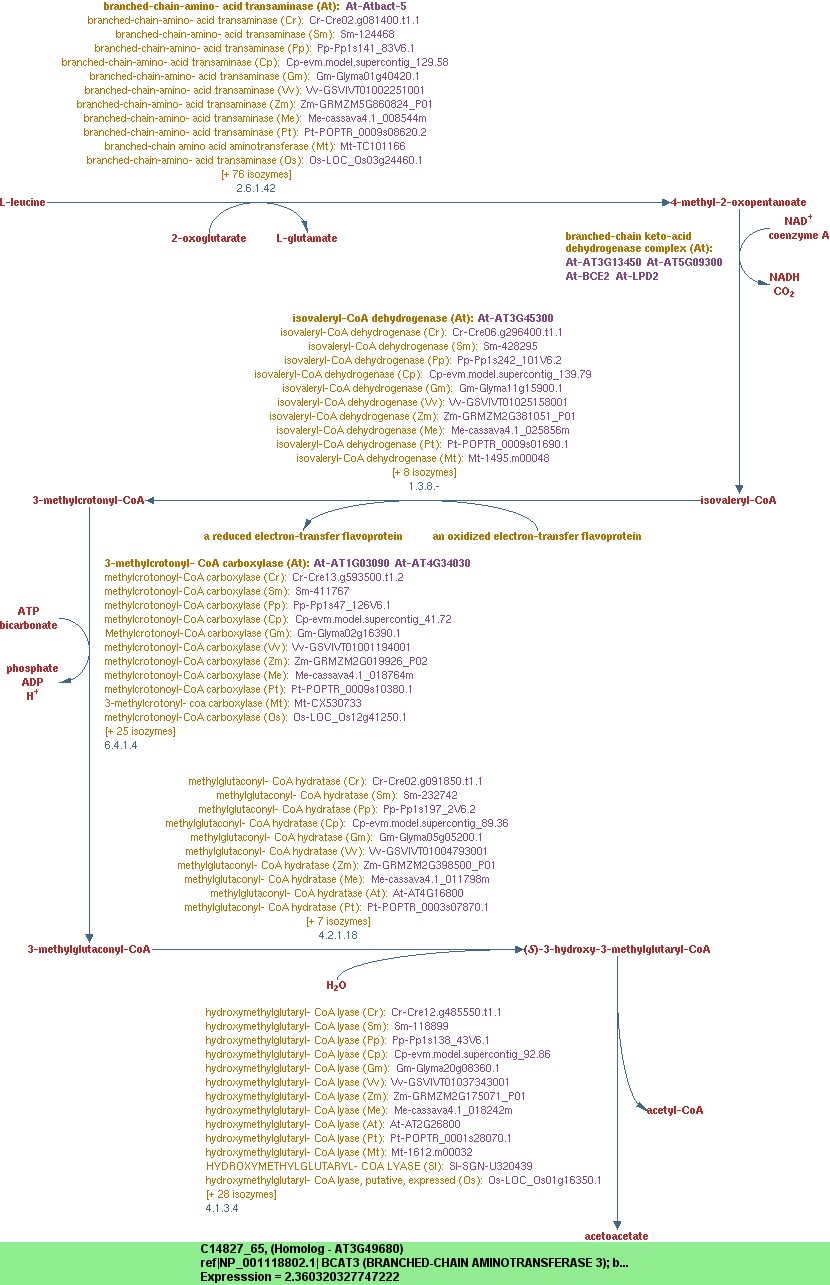

Supplement: Additional file 16 — A and B: Stress related up-regulated PMN pathways. [file 1471-2164-14-647-S16.zip › Additional_file16A_Upregulated_PMN_pathways_in_Shoot/V2SHS/C14827_65_AT3G49680_7_leucine_degradation_I.jpg]

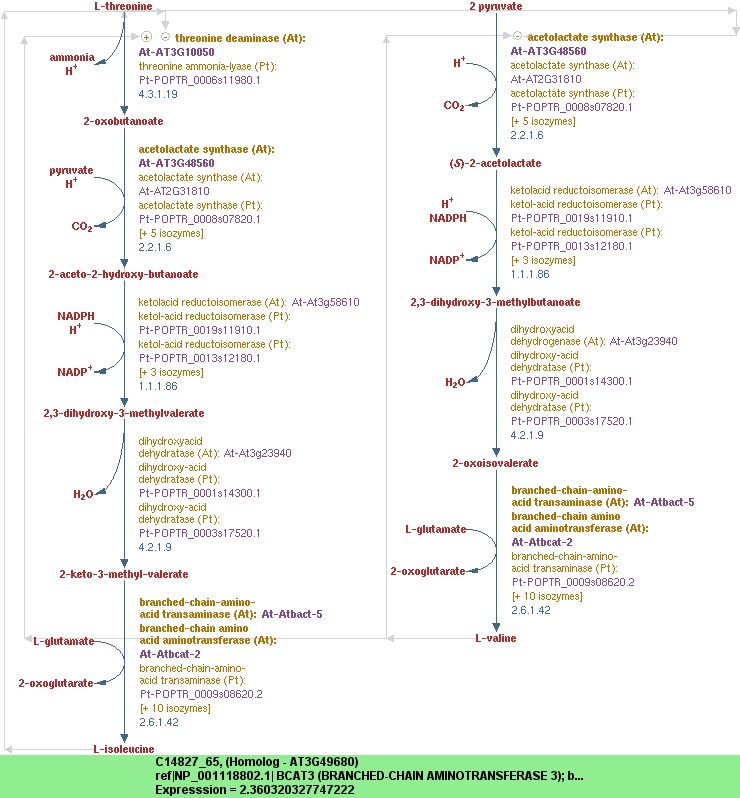

Supplement: Additional file 16 — A and B: Stress related up-regulated PMN pathways. [file 1471-2164-14-647-S16.zip › Additional_file16A_Upregulated_PMN_pathways_in_Shoot/V2SHS/C14827_65_AT3G49680_8_superpathway_of_isoleucine_and_valine_biosynthesis.jpg]

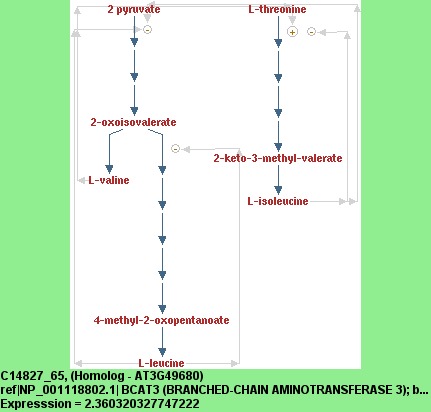

Supplement: Additional file 16 — A and B: Stress related up-regulated PMN pathways. [file 1471-2164-14-647-S16.zip › Additional_file16A_Upregulated_PMN_pathways_in_Shoot/V2SHS/C14827_65_AT3G49680_9_superpathway_of_leucine,_valine,_and_isoleucine_biosynthesis.jpg]

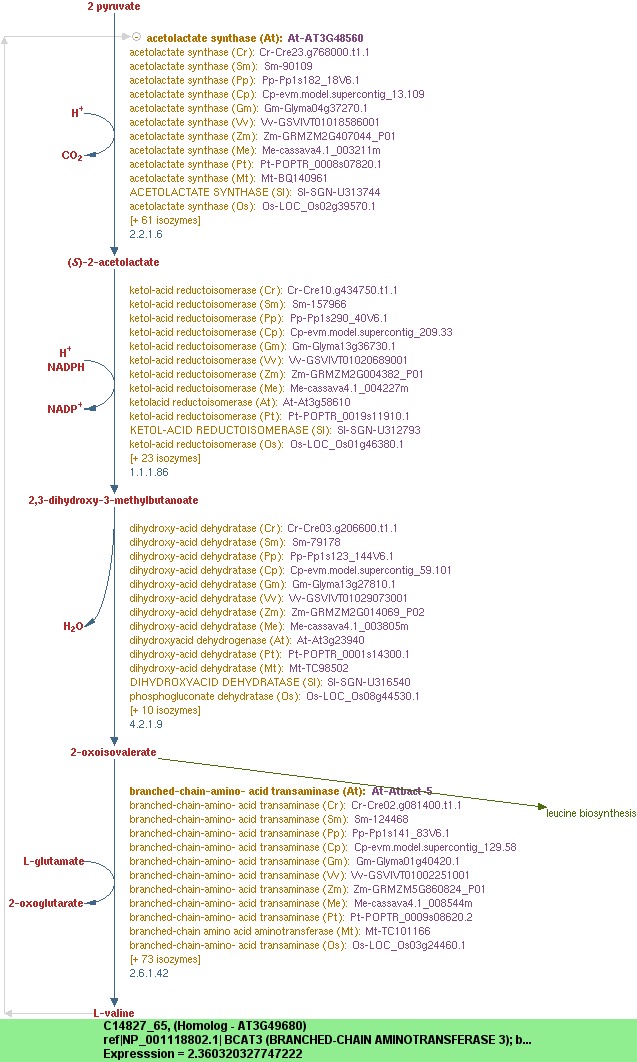

Supplement: Additional file 16 — A and B: Stress related up-regulated PMN pathways. [file 1471-2164-14-647-S16.zip › Additional_file16A_Upregulated_PMN_pathways_in_Shoot/V2SHS/C14827_65_AT3G49680_10_valine_biosynthesis.jpg]

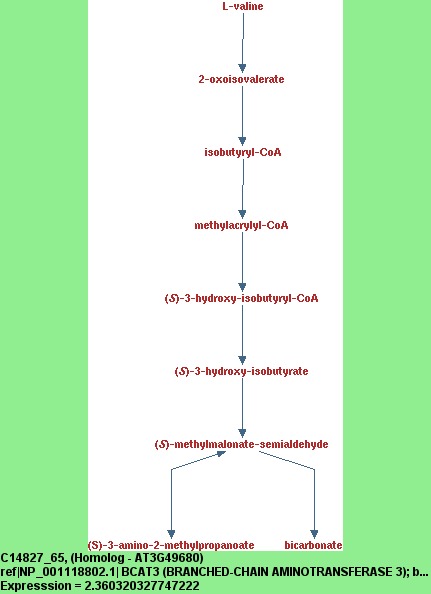

Supplement: Additional file 16 — A and B: Stress related up-regulated PMN pathways. [file 1471-2164-14-647-S16.zip › Additional_file16A_Upregulated_PMN_pathways_in_Shoot/V2SHS/C14827_65_AT3G49680_11_valine_degradation_I.jpg]

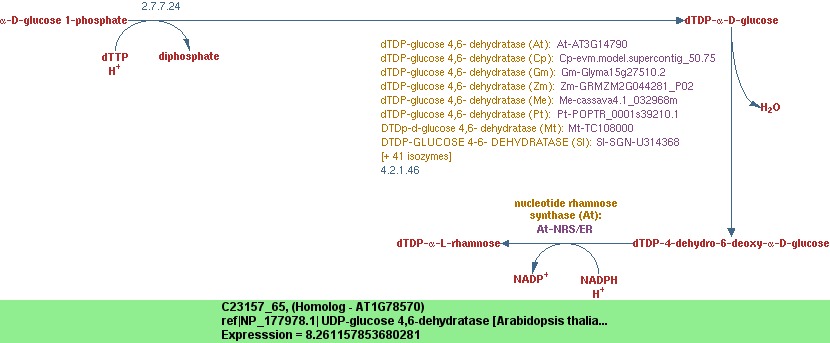

Supplement: Additional file 16 — A and B: Stress related up-regulated PMN pathways. [file 1471-2164-14-647-S16.zip › Additional_file16A_Upregulated_PMN_pathways_in_Shoot/V2SHS/C23157_65_AT1G78570_1_dTDP-L-rhamnose_biosynthesis_II.jpg]

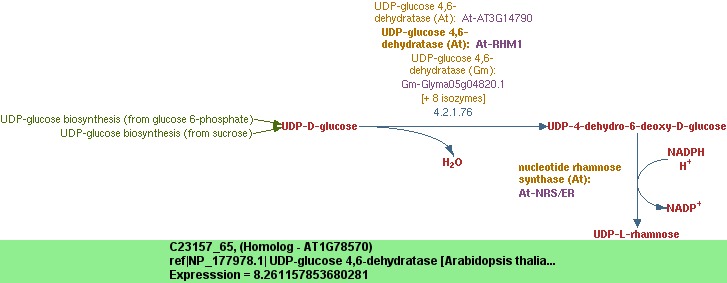

Supplement: Additional file 16 — A and B: Stress related up-regulated PMN pathways. [file 1471-2164-14-647-S16.zip › Additional_file16A_Upregulated_PMN_pathways_in_Shoot/V2SHS/C23157_65_AT1G78570_2_UDP-L-rhamnose_biosynthesis.jpg]

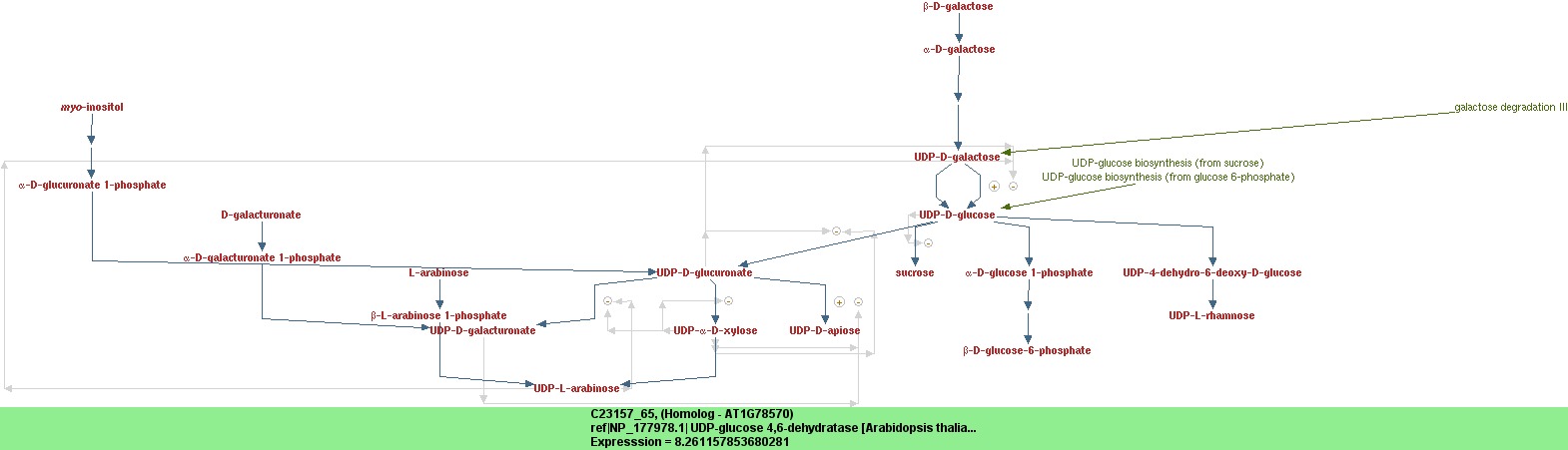

Supplement: Additional file 16 — A and B: Stress related up-regulated PMN pathways. [file 1471-2164-14-647-S16.zip › Additional_file16A_Upregulated_PMN_pathways_in_Shoot/V2SHS/C23157_65_AT1G78570_3_UDP-sugars_interconversion.jpg]

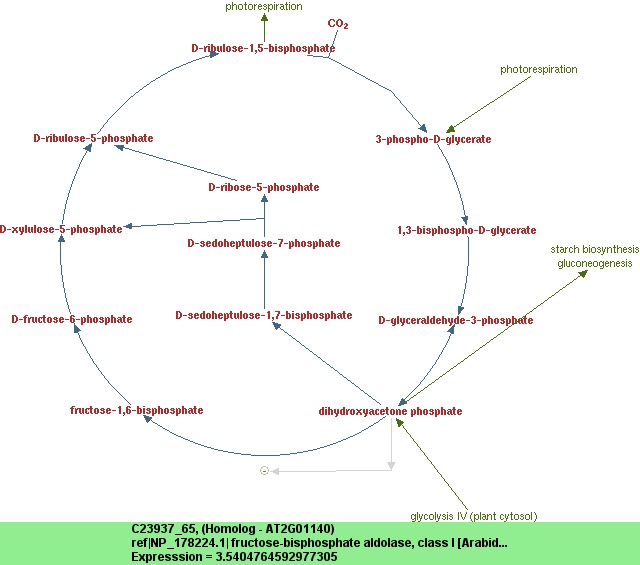

Supplement: Additional file 16 — A and B: Stress related up-regulated PMN pathways. [file 1471-2164-14-647-S16.zip › Additional_file16A_Upregulated_PMN_pathways_in_Shoot/V2SHS/C23937_65_AT2G01140_1_Calvin-Benson-Bassham_cycle.jpg]

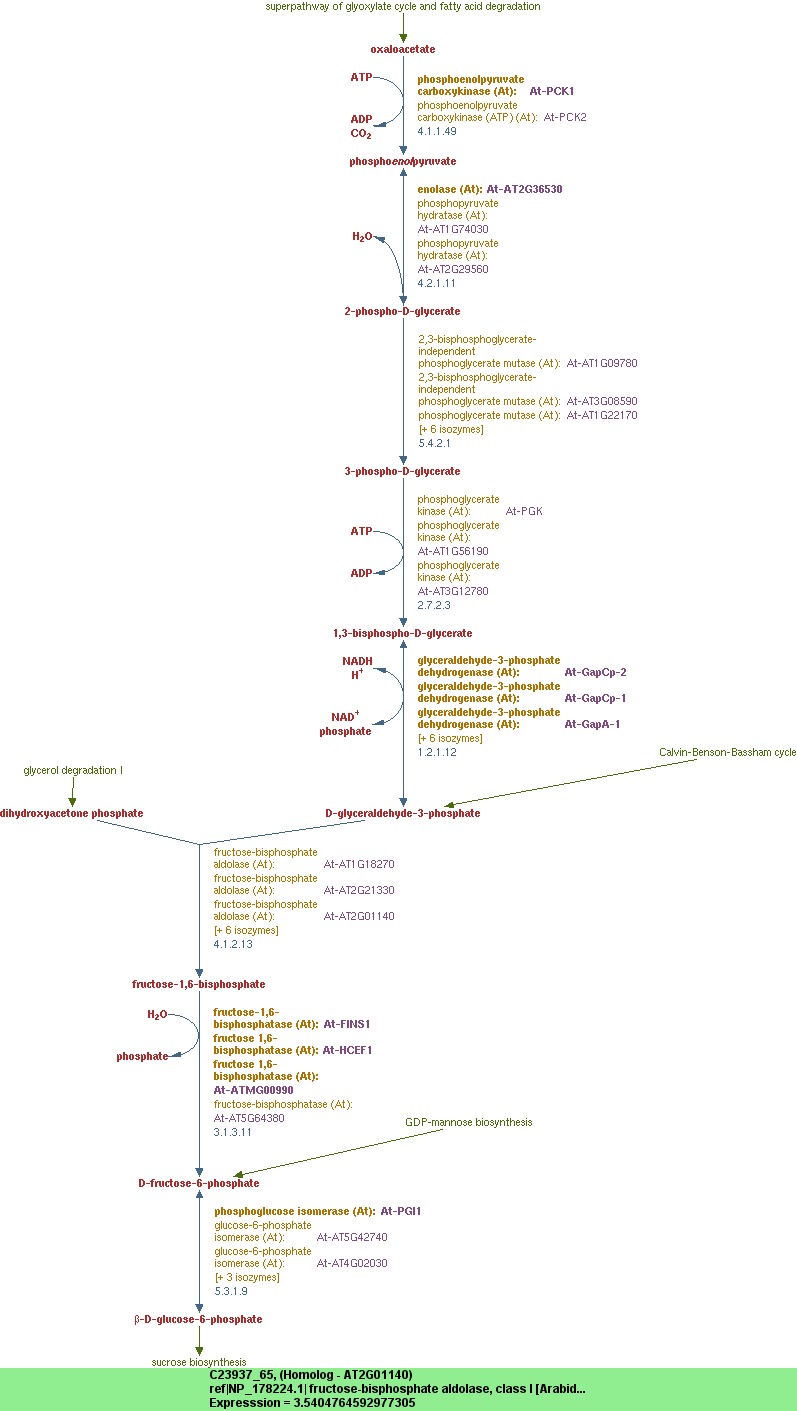

Supplement: Additional file 16 — A and B: Stress related up-regulated PMN pathways. [file 1471-2164-14-647-S16.zip › Additional_file16A_Upregulated_PMN_pathways_in_Shoot/V2SHS/C23937_65_AT2G01140_3_gluconeogenesis.jpg]

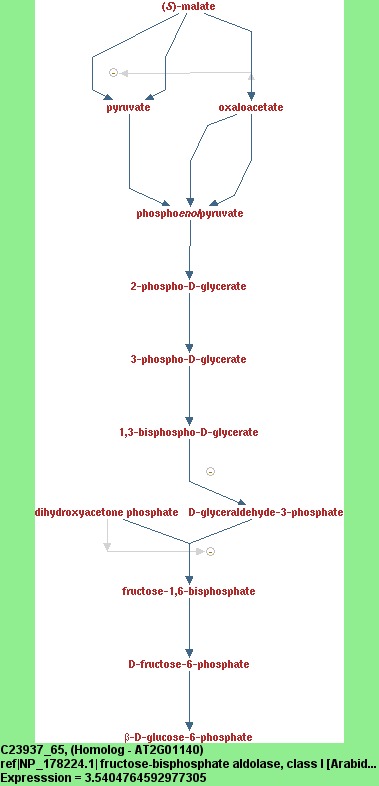

Supplement: Additional file 16 — A and B: Stress related up-regulated PMN pathways. [file 1471-2164-14-647-S16.zip › Additional_file16A_Upregulated_PMN_pathways_in_Shoot/V2SHS/C23937_65_AT2G01140_5_gluconeogenesis_I.jpg]

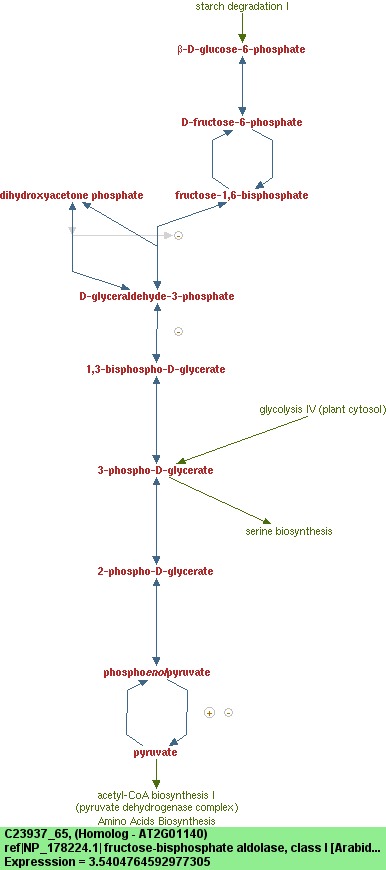

Supplement: Additional file 16 — A and B: Stress related up-regulated PMN pathways. [file 1471-2164-14-647-S16.zip › Additional_file16A_Upregulated_PMN_pathways_in_Shoot/V2SHS/C23937_65_AT2G01140_7_glycolysis_I.jpg]

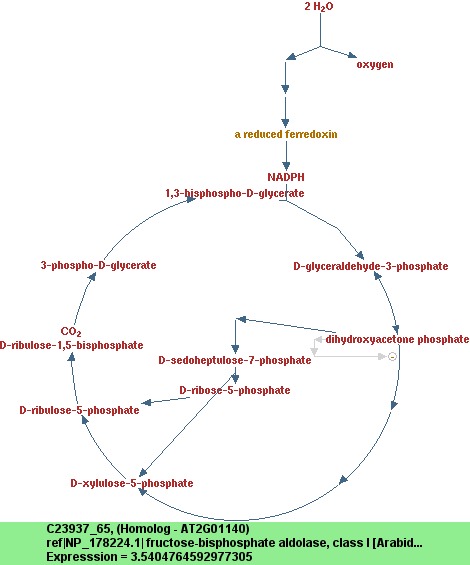

Supplement: Additional file 16 — A and B: Stress related up-regulated PMN pathways. [file 1471-2164-14-647-S16.zip › Additional_file16A_Upregulated_PMN_pathways_in_Shoot/V2SHS/C23937_65_AT2G01140_9_oxygenic_photosynthesis.jpg]

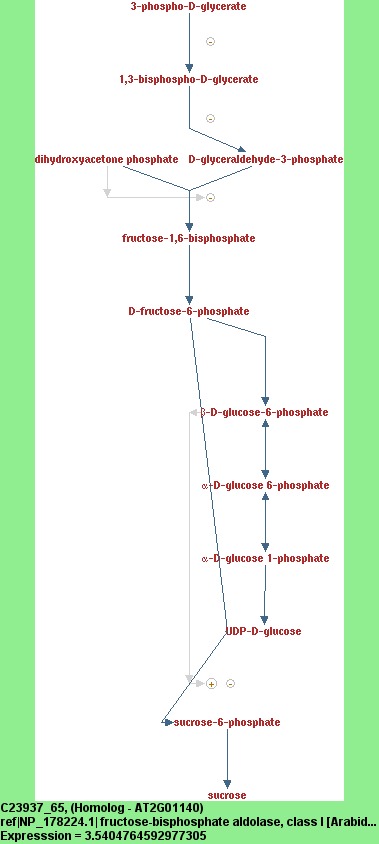

Supplement: Additional file 16 — A and B: Stress related up-regulated PMN pathways. [file 1471-2164-14-647-S16.zip › Additional_file16A_Upregulated_PMN_pathways_in_Shoot/V2SHS/C23937_65_AT2G01140_11_sucrose_biosynthesis.jpg]

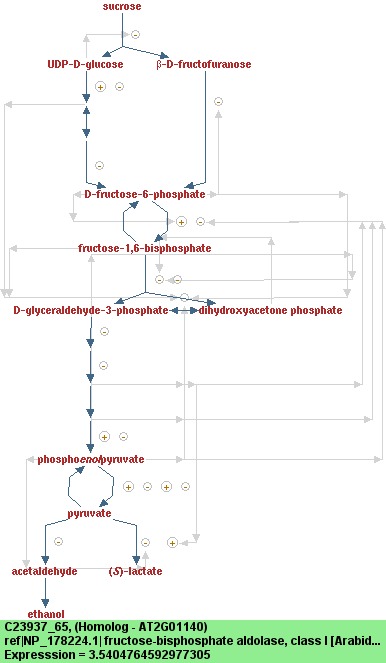

Supplement: Additional file 16 — A and B: Stress related up-regulated PMN pathways. [file 1471-2164-14-647-S16.zip › Additional_file16A_Upregulated_PMN_pathways_in_Shoot/V2SHS/C23937_65_AT2G01140_13_sucrose_degradation_VI_(anaerobic).jpg]

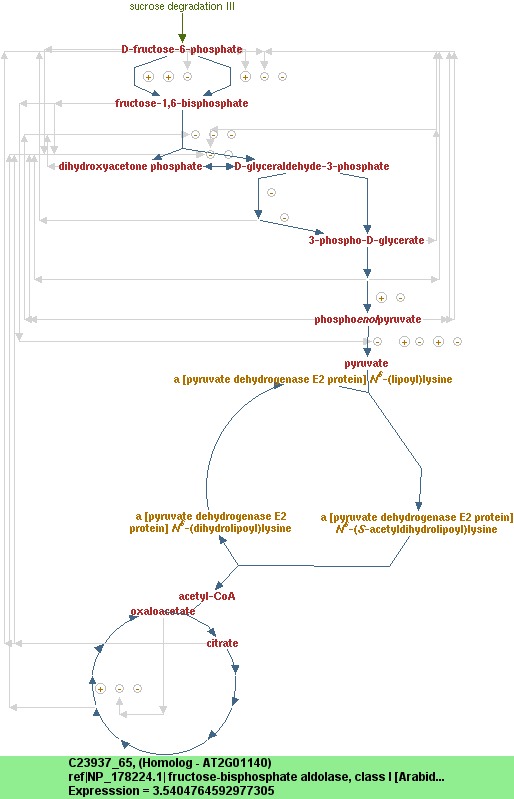

Supplement: Additional file 16 — A and B: Stress related up-regulated PMN pathways. [file 1471-2164-14-647-S16.zip › Additional_file16A_Upregulated_PMN_pathways_in_Shoot/V2SHS/C23937_65_AT2G01140_15_superpathway_of_cytosolic_glycolysis_(plants),_pyruvate_dehydrogenase_and_TCA_cycle.jpg]

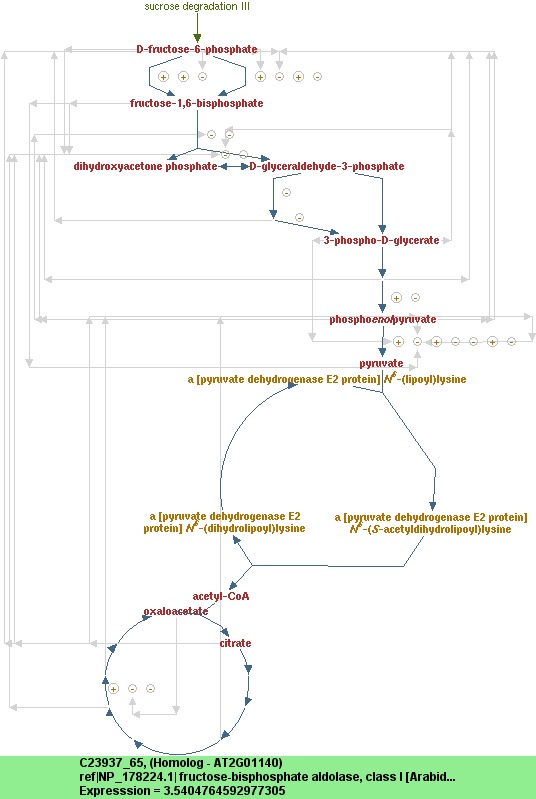

Supplement: Additional file 16 — A and B: Stress related up-regulated PMN pathways. [file 1471-2164-14-647-S16.zip › Additional_file16A_Upregulated_PMN_pathways_in_Shoot/V2SHS/C23937_65_AT2G01140_17_superpathway_of_cytosolic_glycolysis_(plants),_pyruvate_dehydrogenase_and_TCA_cycle.jpg]

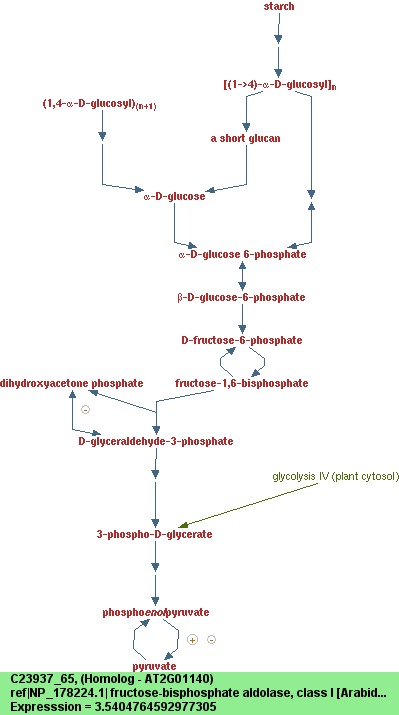

Supplement: Additional file 16 — A and B: Stress related up-regulated PMN pathways. [file 1471-2164-14-647-S16.zip › Additional_file16A_Upregulated_PMN_pathways_in_Shoot/V2SHS/C23937_65_AT2G01140_19_superpathway_of_starch_degradation_to_pyruvate.jpg]

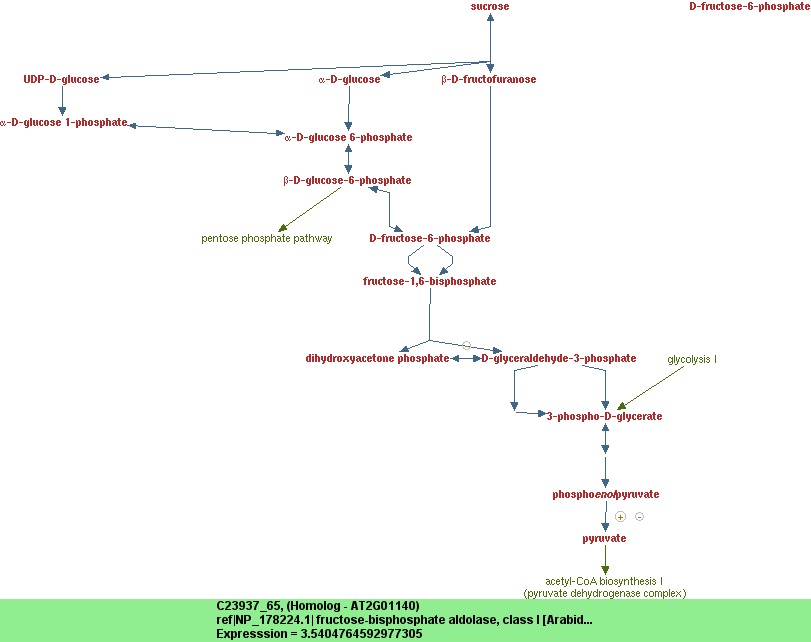

Supplement: Additional file 16 — A and B: Stress related up-regulated PMN pathways. [file 1471-2164-14-647-S16.zip › Additional_file16A_Upregulated_PMN_pathways_in_Shoot/V2SHS/C23937_65_AT2G01140_21_superpathway_of_sucrose_degradation_to_pyruvate.jpg]

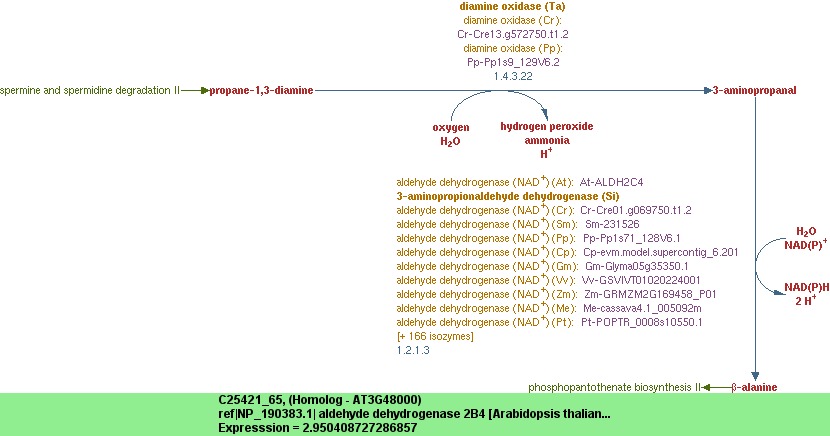

Supplement: Additional file 16 — A and B: Stress related up-regulated PMN pathways. [file 1471-2164-14-647-S16.zip › Additional_file16A_Upregulated_PMN_pathways_in_Shoot/V2SHS/C25421_65_AT3G48000_1_&beta-alanine_biosynthesis_I.jpg]

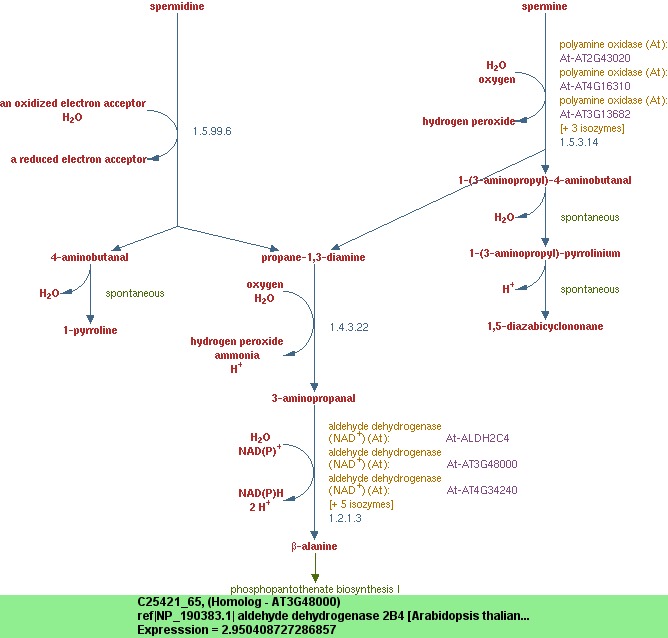

Supplement: Additional file 16 — A and B: Stress related up-regulated PMN pathways. [file 1471-2164-14-647-S16.zip › Additional_file16A_Upregulated_PMN_pathways_in_Shoot/V2SHS/C25421_65_AT3G48000_3_&beta-alanine_biosynthesis_I.jpg]

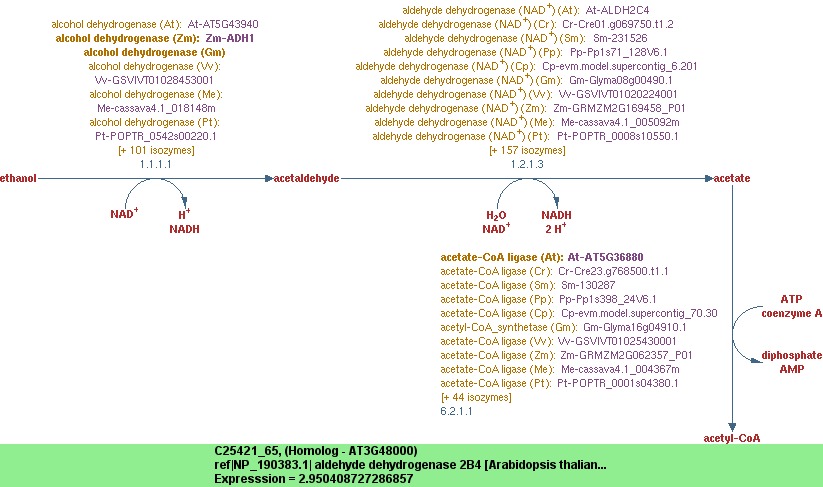

Supplement: Additional file 16 — A and B: Stress related up-regulated PMN pathways. [file 1471-2164-14-647-S16.zip › Additional_file16A_Upregulated_PMN_pathways_in_Shoot/V2SHS/C25421_65_AT3G48000_5_ethanol_degradation_II.jpg]

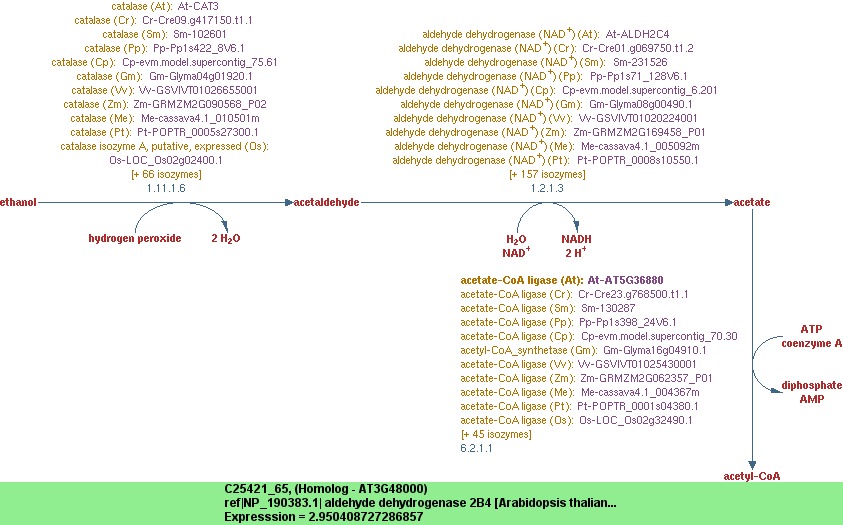

Supplement: Additional file 16 — A and B: Stress related up-regulated PMN pathways. [file 1471-2164-14-647-S16.zip › Additional_file16A_Upregulated_PMN_pathways_in_Shoot/V2SHS/C25421_65_AT3G48000_7_ethanol_degradation_IV.jpg]

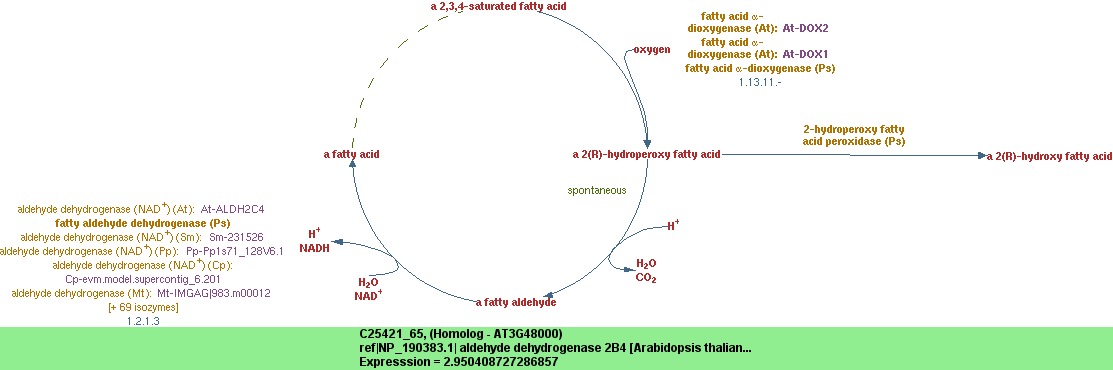

Supplement: Additional file 16 — A and B: Stress related up-regulated PMN pathways. [file 1471-2164-14-647-S16.zip › Additional_file16A_Upregulated_PMN_pathways_in_Shoot/V2SHS/C25421_65_AT3G48000_9_fatty_acid_&alpha-oxidation.jpg]

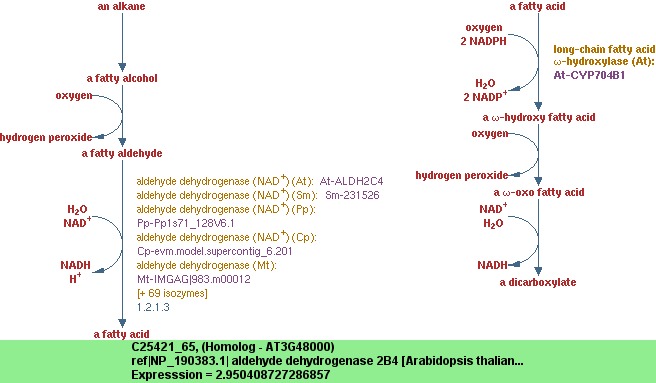

Supplement: Additional file 16 — A and B: Stress related up-regulated PMN pathways. [file 1471-2164-14-647-S16.zip › Additional_file16A_Upregulated_PMN_pathways_in_Shoot/V2SHS/C25421_65_AT3G48000_11_fatty_acid_&omega-oxidation.jpg]

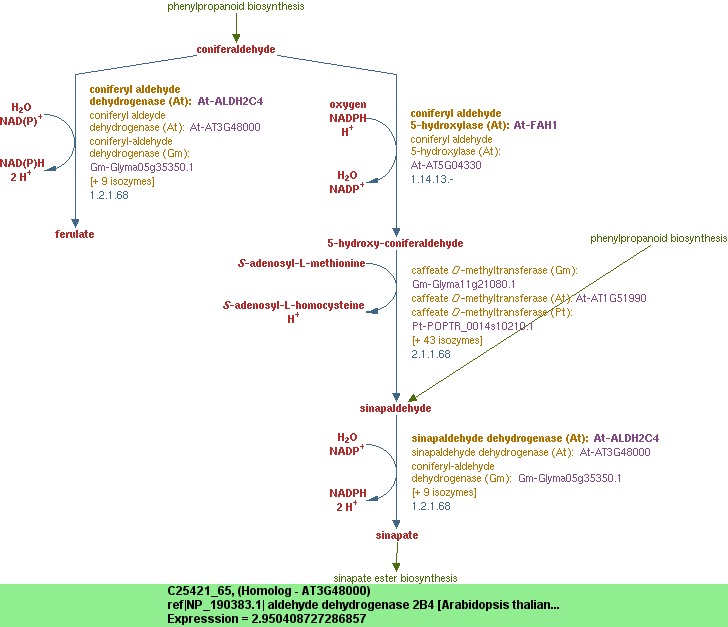

Supplement: Additional file 16 — A and B: Stress related up-regulated PMN pathways. [file 1471-2164-14-647-S16.zip › Additional_file16A_Upregulated_PMN_pathways_in_Shoot/V2SHS/C25421_65_AT3G48000_13_ferulate_and_sinapate_biosynthesis.jpg]

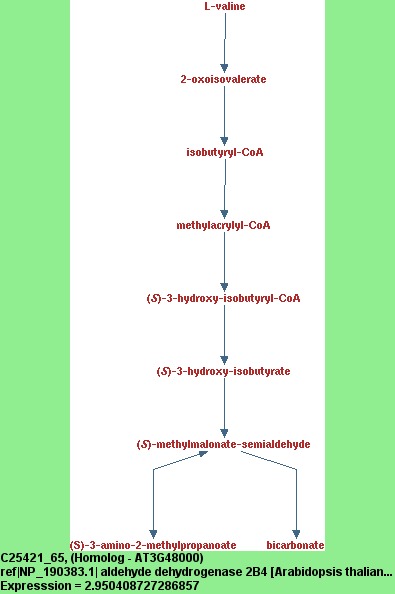

Supplement: Additional file 16 — A and B: Stress related up-regulated PMN pathways. [file 1471-2164-14-647-S16.zip › Additional_file16A_Upregulated_PMN_pathways_in_Shoot/V2SHS/C25421_65_AT3G48000_15_valine_degradation_I.jpg]

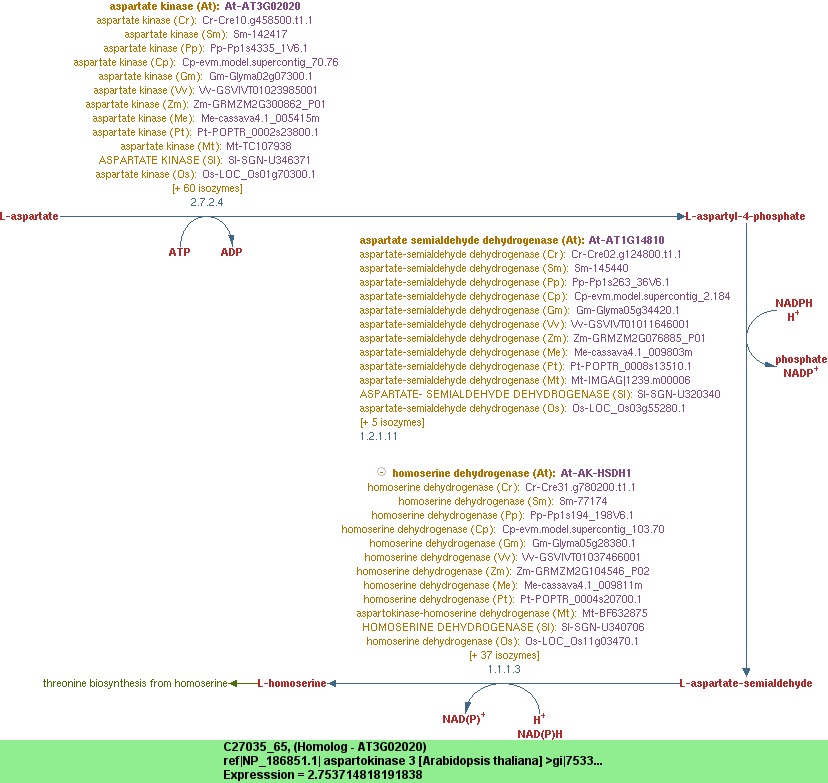

Supplement: Additional file 16 — A and B: Stress related up-regulated PMN pathways. [file 1471-2164-14-647-S16.zip › Additional_file16A_Upregulated_PMN_pathways_in_Shoot/V2SHS/C27035_65_AT3G02020_1_homoserine_biosynthesis.jpg]

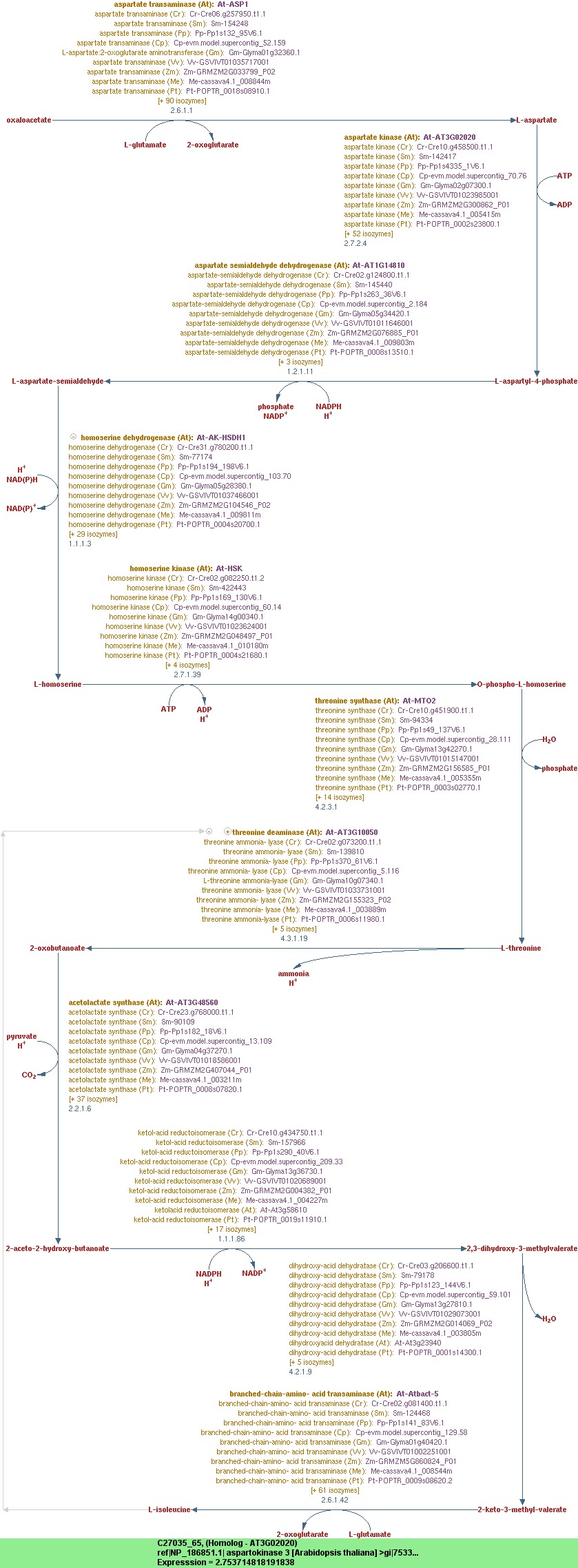

Supplement: Additional file 16 — A and B: Stress related up-regulated PMN pathways. [file 1471-2164-14-647-S16.zip › Additional_file16A_Upregulated_PMN_pathways_in_Shoot/V2SHS/C27035_65_AT3G02020_3_isoleucine_biosynthesis_I.jpg]

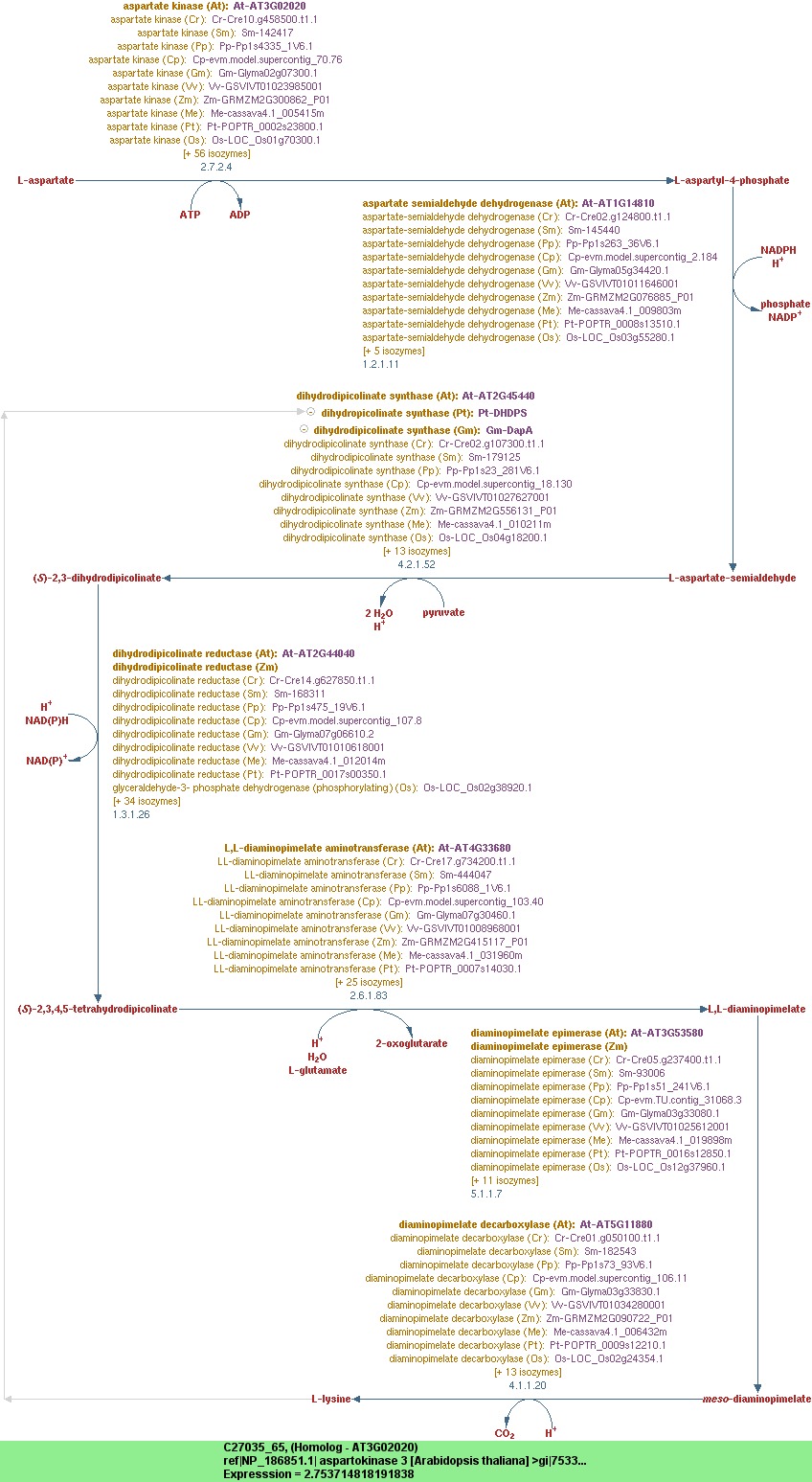

Supplement: Additional file 16 — A and B: Stress related up-regulated PMN pathways. [file 1471-2164-14-647-S16.zip › Additional_file16A_Upregulated_PMN_pathways_in_Shoot/V2SHS/C27035_65_AT3G02020_5_lysine_biosynthesis_VI.jpg]

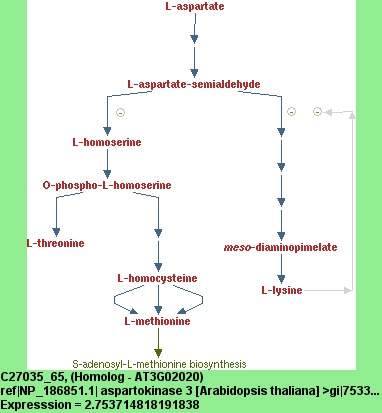

Supplement: Additional file 16 — A and B: Stress related up-regulated PMN pathways. [file 1471-2164-14-647-S16.zip › Additional_file16A_Upregulated_PMN_pathways_in_Shoot/V2SHS/C27035_65_AT3G02020_7_superpathway_of_lysine,_threonine_and_methionine_biosynthesis_II.jpg]

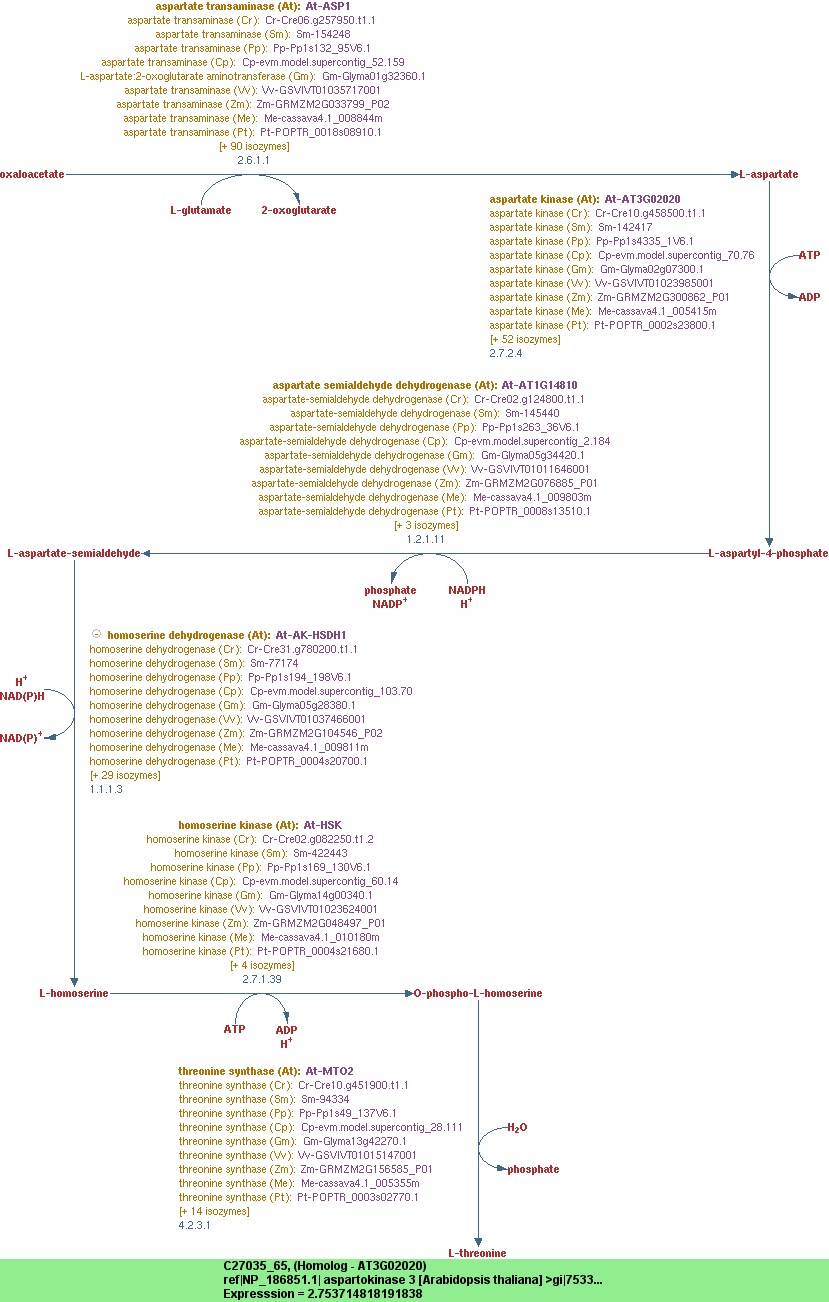

Supplement: Additional file 16 — A and B: Stress related up-regulated PMN pathways. [file 1471-2164-14-647-S16.zip › Additional_file16A_Upregulated_PMN_pathways_in_Shoot/V2SHS/C27035_65_AT3G02020_9_threonine_biosynthesis.jpg]

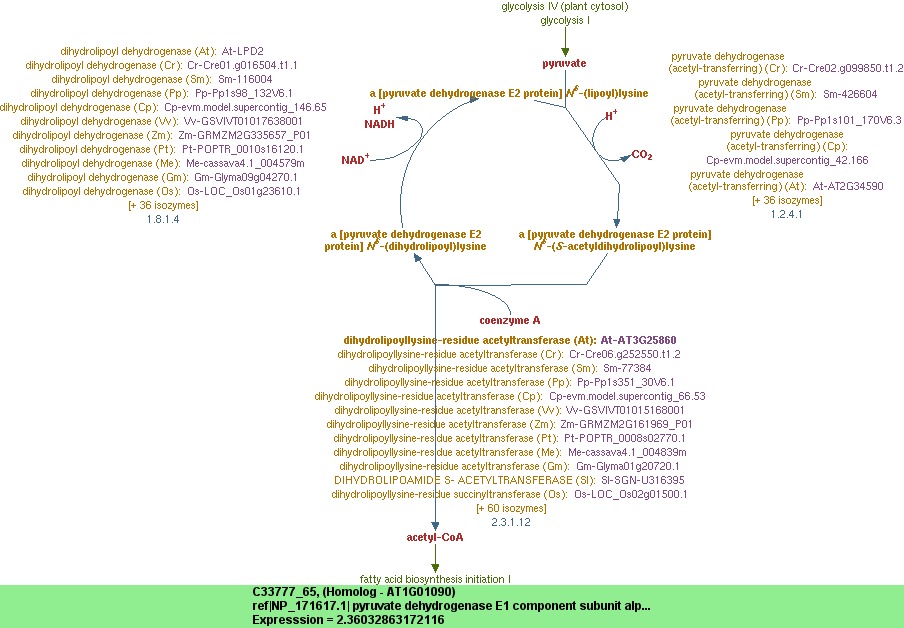

Supplement: Additional file 16 — A and B: Stress related up-regulated PMN pathways. [file 1471-2164-14-647-S16.zip › Additional_file16A_Upregulated_PMN_pathways_in_Shoot/V2SHS/C33777_65_AT1G01090_1_acetyl-CoA_biosynthesis_I_(pyruvate_dehydrogenase_complex).jpg]

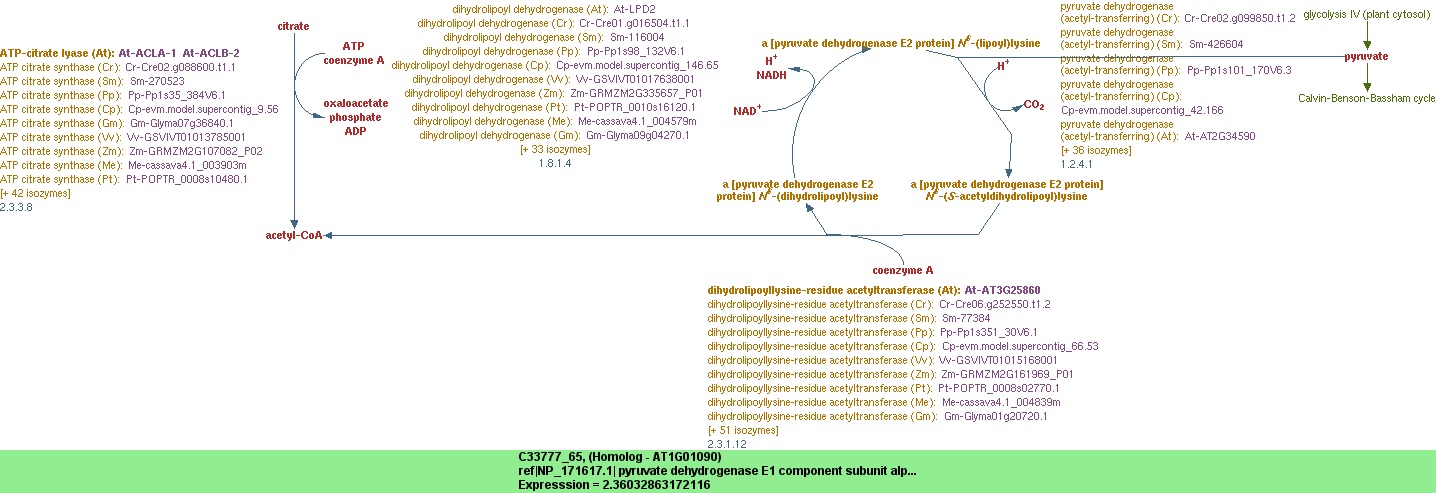

Supplement: Additional file 16 — A and B: Stress related up-regulated PMN pathways. [file 1471-2164-14-647-S16.zip › Additional_file16A_Upregulated_PMN_pathways_in_Shoot/V2SHS/C33777_65_AT1G01090_3_superpathway_of_acetyl-CoA_biosynthesis.jpg]

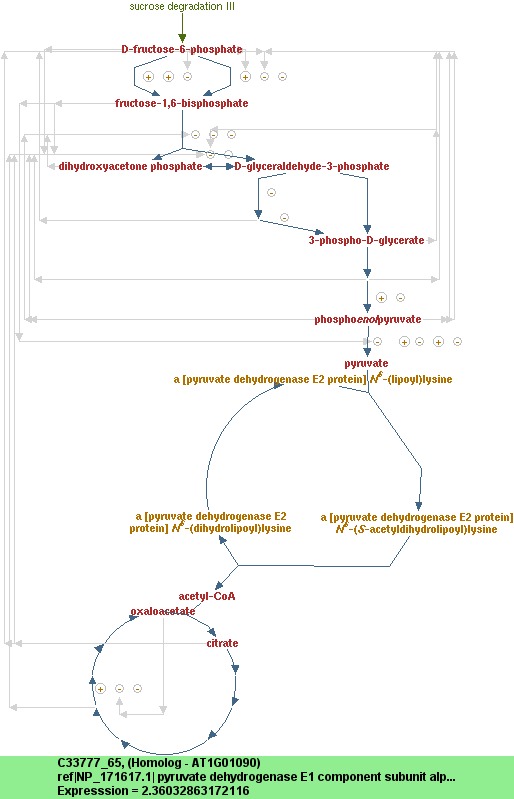

Supplement: Additional file 16 — A and B: Stress related up-regulated PMN pathways. [file 1471-2164-14-647-S16.zip › Additional_file16A_Upregulated_PMN_pathways_in_Shoot/V2SHS/C33777_65_AT1G01090_5_superpathway_of_cytosolic_glycolysis_(plants),_pyruvate_dehydrogenase_and_TCA_cycle.jpg]

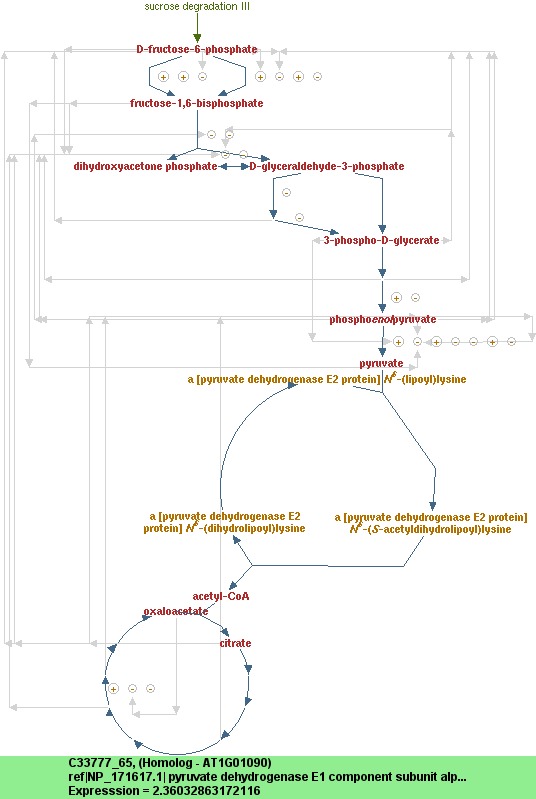

Supplement: Additional file 16 — A and B: Stress related up-regulated PMN pathways. [file 1471-2164-14-647-S16.zip › Additional_file16A_Upregulated_PMN_pathways_in_Shoot/V2SHS/C33777_65_AT1G01090_7_superpathway_of_cytosolic_glycolysis_(plants),_pyruvate_dehydrogenase_and_TCA_cycle.jpg]

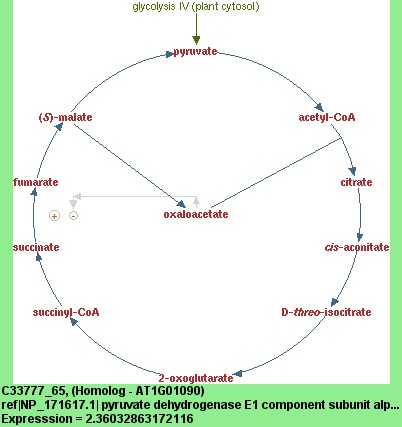

Supplement: Additional file 16 — A and B: Stress related up-regulated PMN pathways. [file 1471-2164-14-647-S16.zip › Additional_file16A_Upregulated_PMN_pathways_in_Shoot/V2SHS/C33777_65_AT1G01090_9_TCA_cycle_variation_V_(plant).jpg]

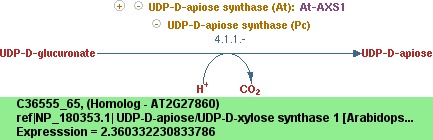

Supplement: Additional file 16 — A and B: Stress related up-regulated PMN pathways. [file 1471-2164-14-647-S16.zip › Additional_file16A_Upregulated_PMN_pathways_in_Shoot/V2SHS/C36555_65_AT2G27860_1_UDP-D-apiose_biosynthesis_(from_UDP-D-glucuronate).jpg]

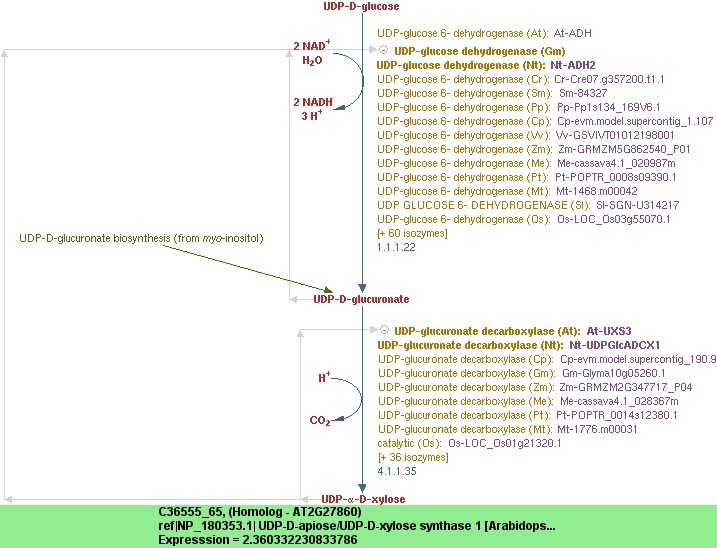

Supplement: Additional file 16 — A and B: Stress related up-regulated PMN pathways. [file 1471-2164-14-647-S16.zip › Additional_file16A_Upregulated_PMN_pathways_in_Shoot/V2SHS/C36555_65_AT2G27860_2_UDP-D-xylose_and_UDP-D-glucuronate_biosynthesis.jpg]

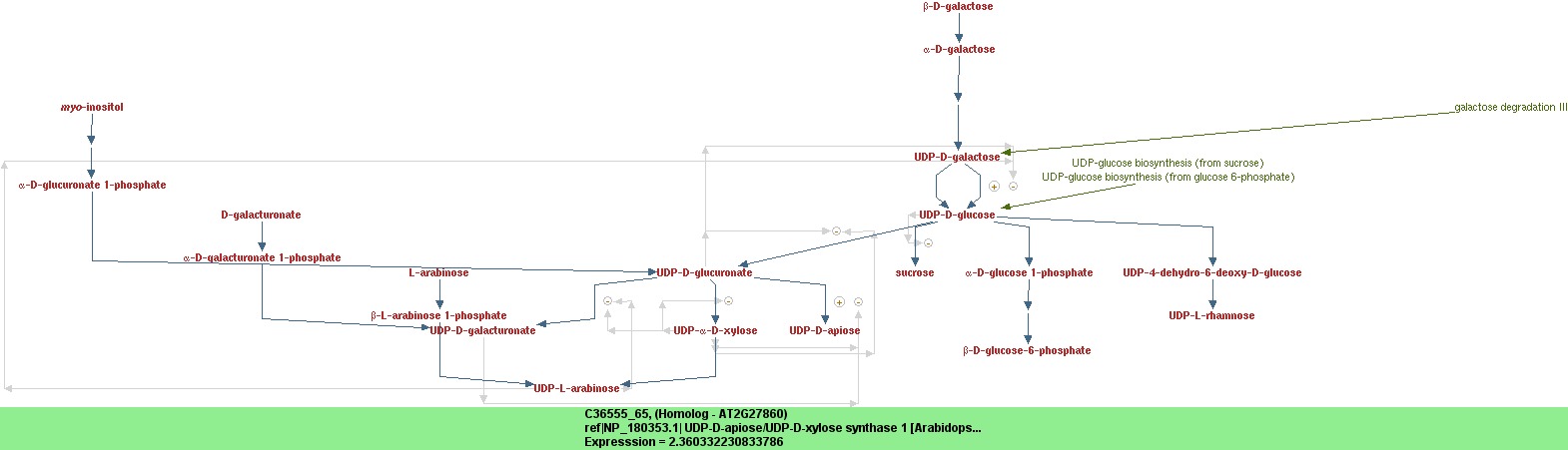

Supplement: Additional file 16 — A and B: Stress related up-regulated PMN pathways. [file 1471-2164-14-647-S16.zip › Additional_file16A_Upregulated_PMN_pathways_in_Shoot/V2SHS/C36555_65_AT2G27860_3_UDP-sugars_interconversion.jpg]

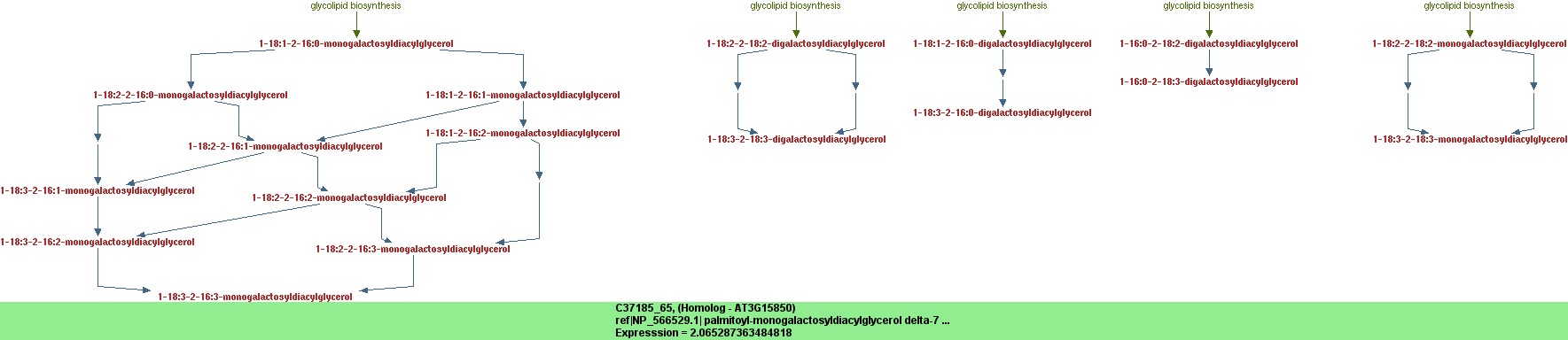

Supplement: Additional file 16 — A and B: Stress related up-regulated PMN pathways. [file 1471-2164-14-647-S16.zip › Additional_file16A_Upregulated_PMN_pathways_in_Shoot/V2SHS/C37185_65_AT3G15850_1_glycolipid_desaturation.jpg]

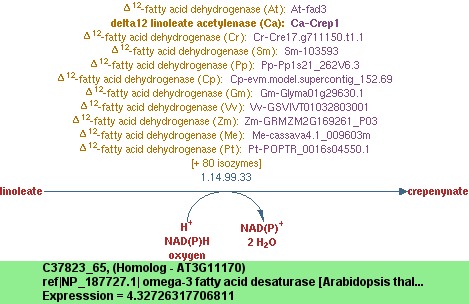

Supplement: Additional file 16 — A and B: Stress related up-regulated PMN pathways. [file 1471-2164-14-647-S16.zip › Additional_file16A_Upregulated_PMN_pathways_in_Shoot/V2SHS/C37823_65_AT3G11170_1_crepenynic_acid_biosynthesis.jpg]

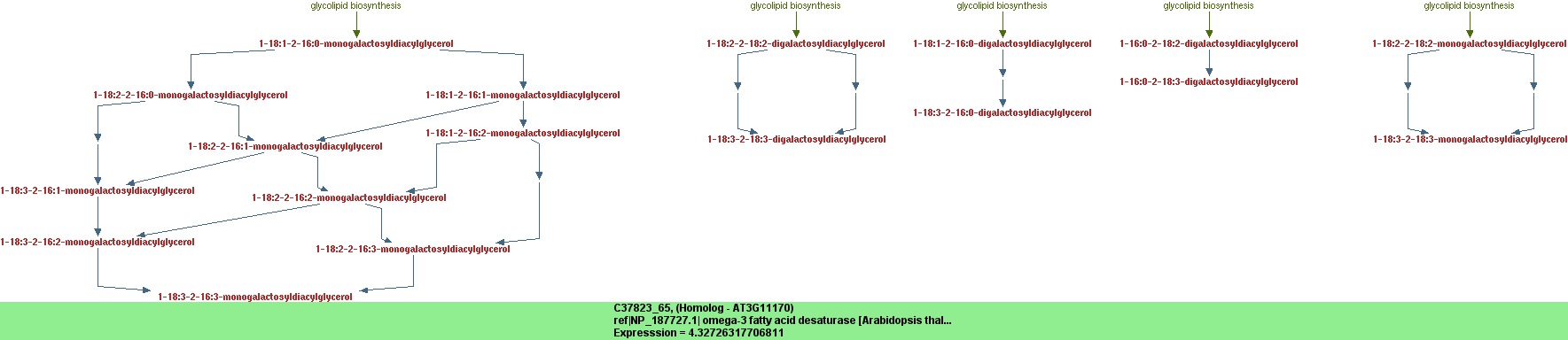

Supplement: Additional file 16 — A and B: Stress related up-regulated PMN pathways. [file 1471-2164-14-647-S16.zip › Additional_file16A_Upregulated_PMN_pathways_in_Shoot/V2SHS/C37823_65_AT3G11170_2_glycolipid_desaturation.jpg]

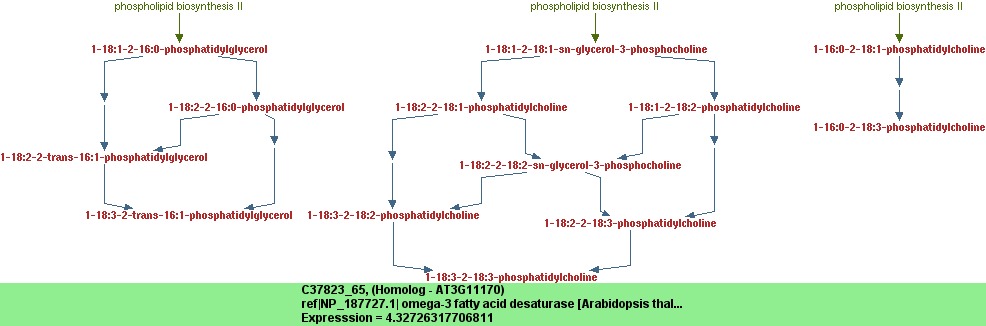

Supplement: Additional file 16 — A and B: Stress related up-regulated PMN pathways. [file 1471-2164-14-647-S16.zip › Additional_file16A_Upregulated_PMN_pathways_in_Shoot/V2SHS/C37823_65_AT3G11170_3_phospholipid_desaturation.jpg]

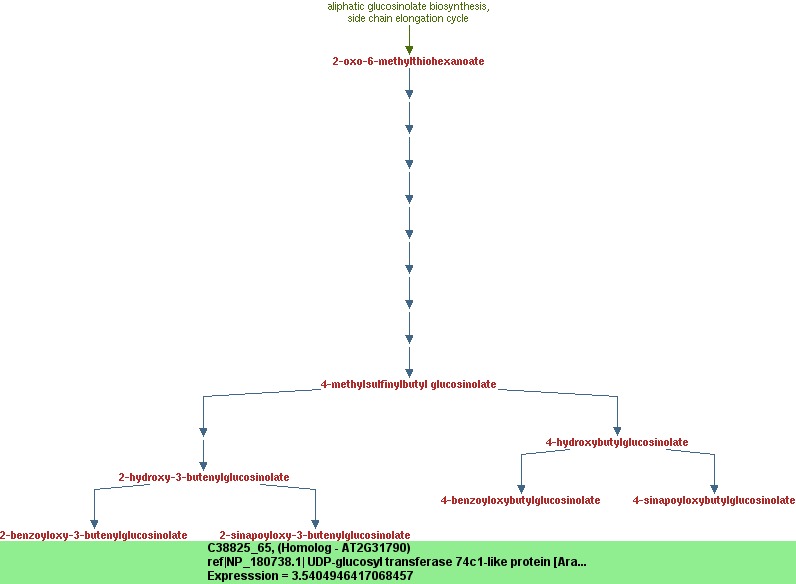

Supplement: Additional file 16 — A and B: Stress related up-regulated PMN pathways. [file 1471-2164-14-647-S16.zip › Additional_file16A_Upregulated_PMN_pathways_in_Shoot/V2SHS/C38825_65_AT2G31790_1_glucosinolate_biosynthesis_from_dihomomethionine.jpg]

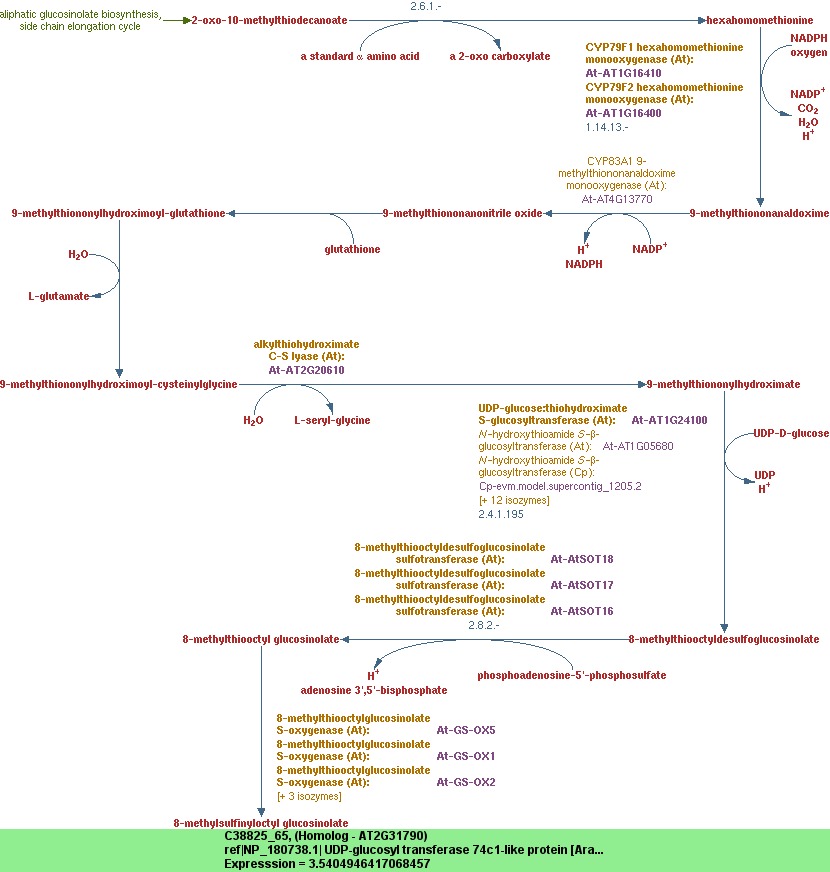

Supplement: Additional file 16 — A and B: Stress related up-regulated PMN pathways. [file 1471-2164-14-647-S16.zip › Additional_file16A_Upregulated_PMN_pathways_in_Shoot/V2SHS/C38825_65_AT2G31790_3_glucosinolate_biosynthesis_from_hexahomomethionine.jpg]

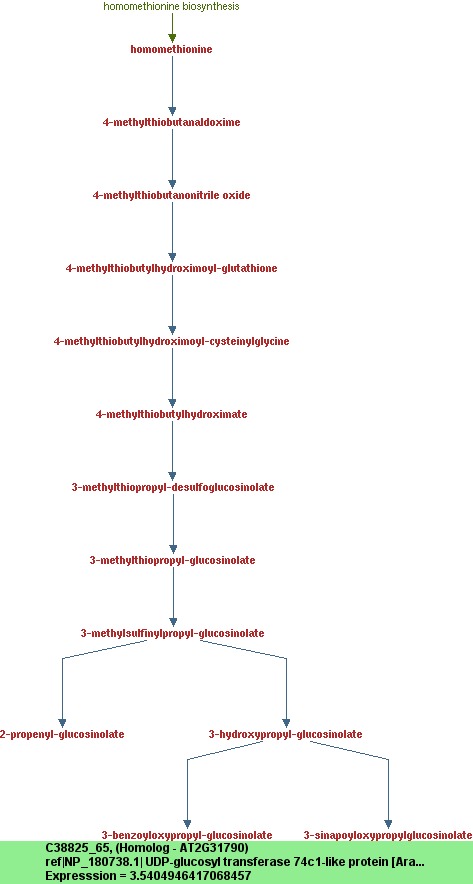

Supplement: Additional file 16 — A and B: Stress related up-regulated PMN pathways. [file 1471-2164-14-647-S16.zip › Additional_file16A_Upregulated_PMN_pathways_in_Shoot/V2SHS/C38825_65_AT2G31790_5_glucosinolate_biosynthesis_from_homomethionine.jpg]

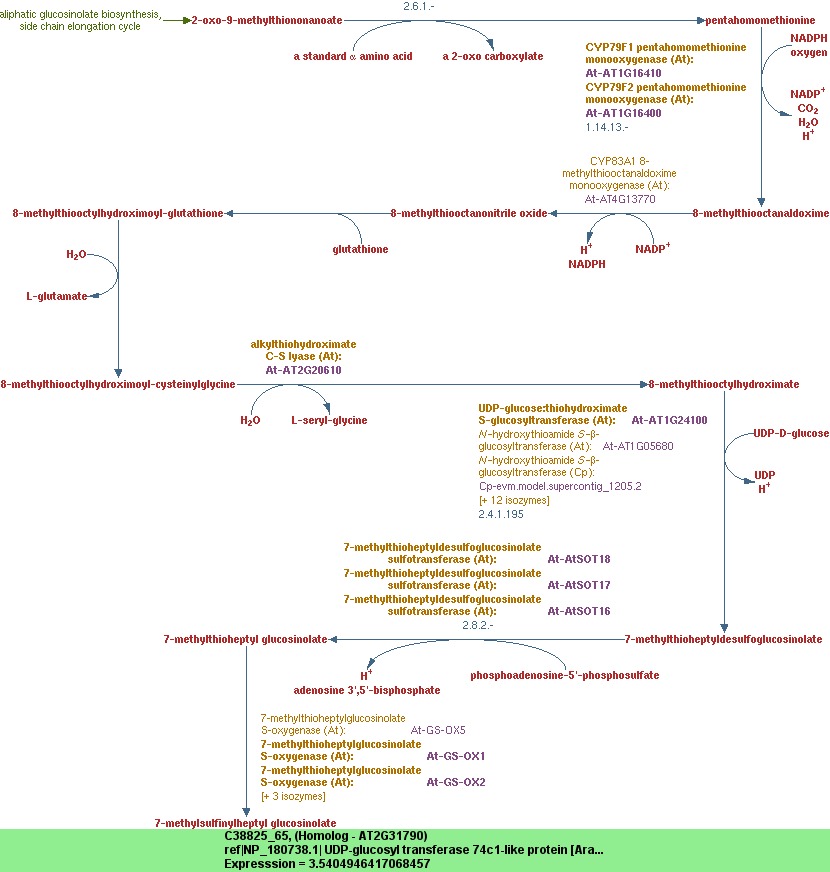

Supplement: Additional file 16 — A and B: Stress related up-regulated PMN pathways. [file 1471-2164-14-647-S16.zip › Additional_file16A_Upregulated_PMN_pathways_in_Shoot/V2SHS/C38825_65_AT2G31790_7_glucosinolate_biosynthesis_from_pentahomomethionine.jpg]

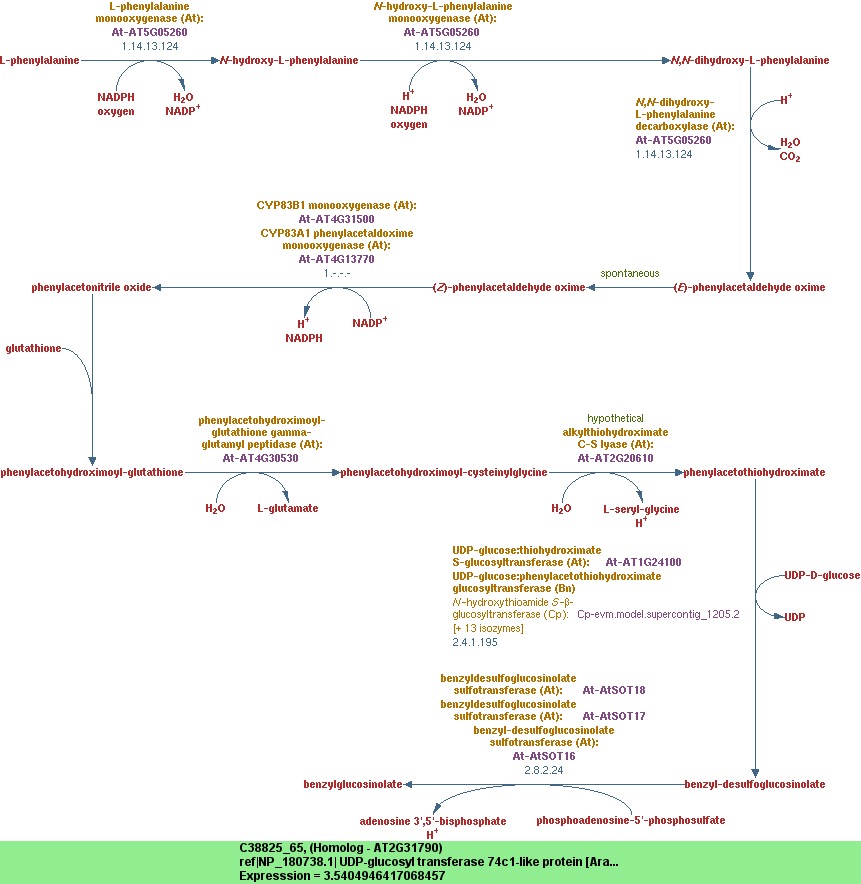

Supplement: Additional file 16 — A and B: Stress related up-regulated PMN pathways. [file 1471-2164-14-647-S16.zip › Additional_file16A_Upregulated_PMN_pathways_in_Shoot/V2SHS/C38825_65_AT2G31790_9_glucosinolate_biosynthesis_from_phenylalanine.jpg]

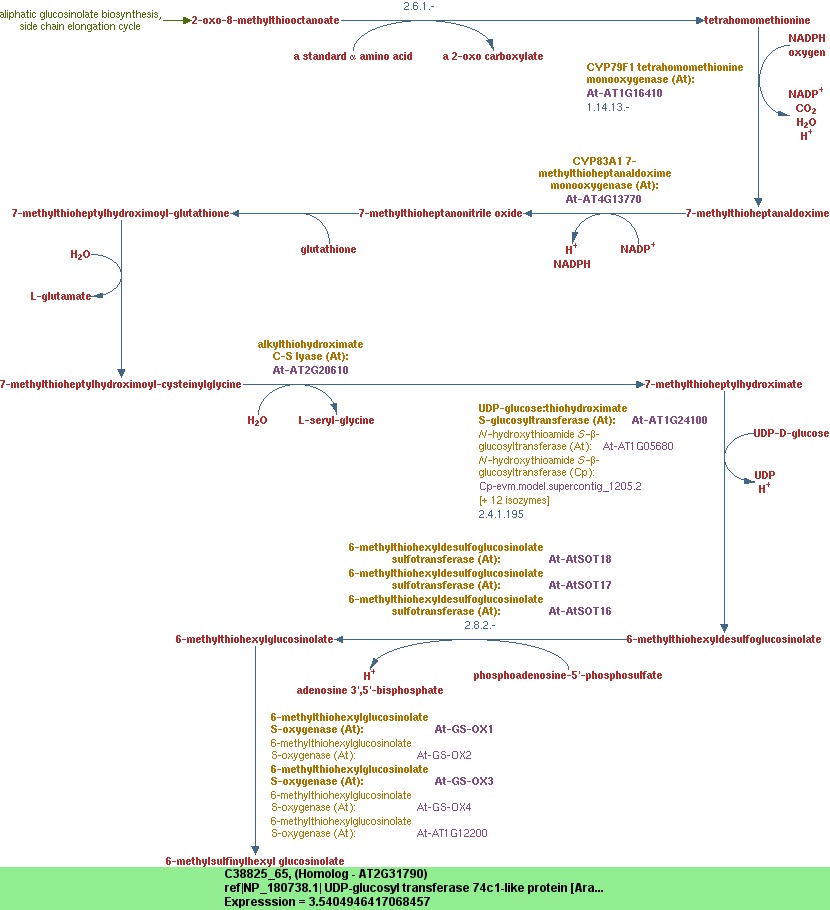

Supplement: Additional file 16 — A and B: Stress related up-regulated PMN pathways. [file 1471-2164-14-647-S16.zip › Additional_file16A_Upregulated_PMN_pathways_in_Shoot/V2SHS/C38825_65_AT2G31790_11_glucosinolate_biosynthesis_from_tetrahomomethionine.jpg]

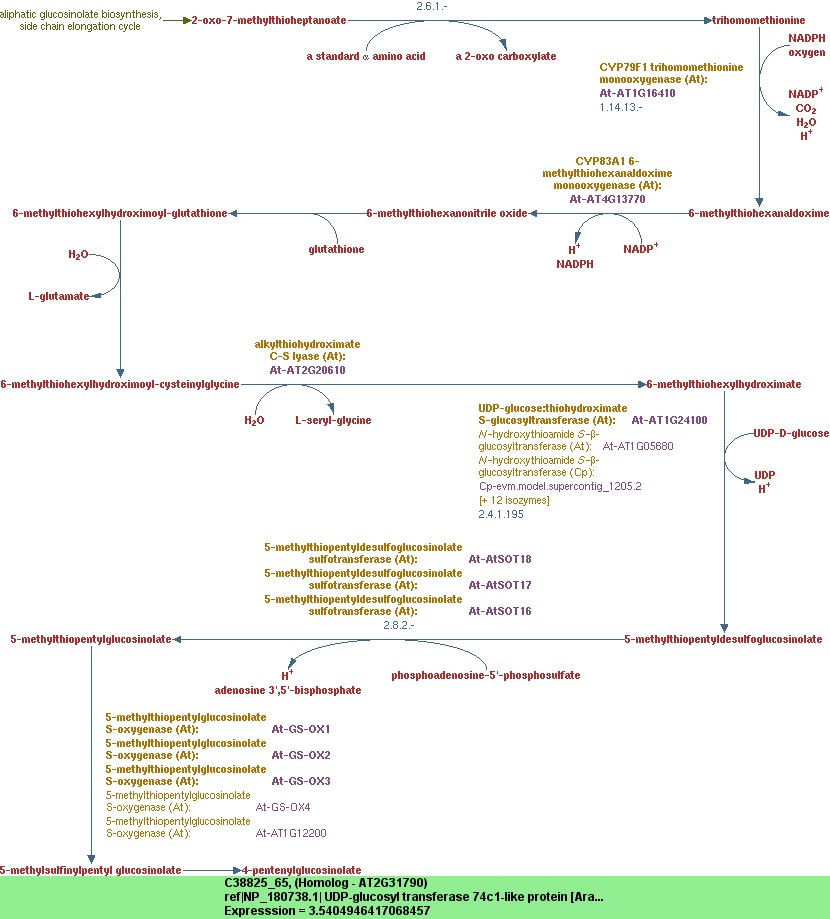

Supplement: Additional file 16 — A and B: Stress related up-regulated PMN pathways. [file 1471-2164-14-647-S16.zip › Additional_file16A_Upregulated_PMN_pathways_in_Shoot/V2SHS/C38825_65_AT2G31790_13_glucosinolate_biosynthesis_from_trihomomethionine.jpg]

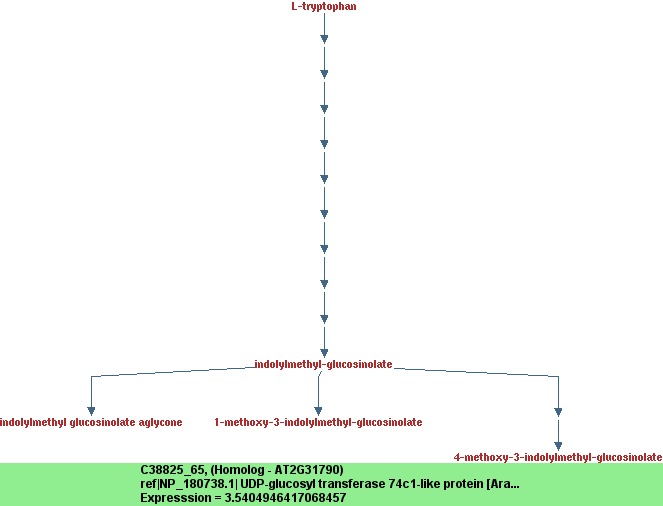

Supplement: Additional file 16 — A and B: Stress related up-regulated PMN pathways. [file 1471-2164-14-647-S16.zip › Additional_file16A_Upregulated_PMN_pathways_in_Shoot/V2SHS/C38825_65_AT2G31790_15_glucosinolate_biosynthesis_from_tryptophan.jpg]

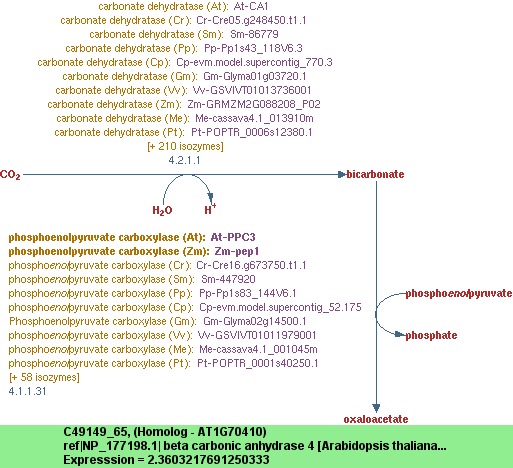

Supplement: Additional file 16 — A and B: Stress related up-regulated PMN pathways. [file 1471-2164-14-647-S16.zip › Additional_file16A_Upregulated_PMN_pathways_in_Shoot/V2SHS/C49149_65_AT1G70410_1_CO_fixation_into_oxaloacetate_(anapleurotic).jpg]

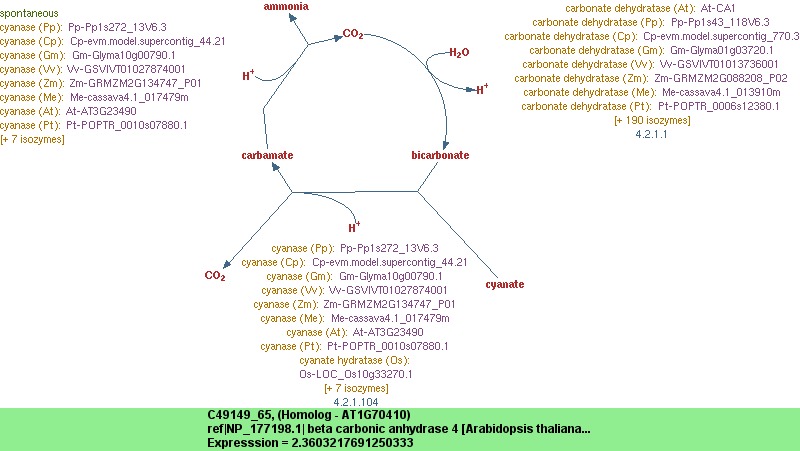

Supplement: Additional file 16 — A and B: Stress related up-regulated PMN pathways. [file 1471-2164-14-647-S16.zip › Additional_file16A_Upregulated_PMN_pathways_in_Shoot/V2SHS/C49149_65_AT1G70410_3_cyanate_degradation.jpg]
