# Supplementary figures and images for: Comprehensive transcriptomic study on horse gram (Macrotyloma uniflorum): De novo assembly, functional characterization and comparative analysis in relation to drought stress (part 4 of 4)
Source: BMC Genomics. 2013 Sep 23;14:647. doi: 10.1186/1471-2164-14-647 (PMC3853109; doi:10.1186/1471-2164-14-647)

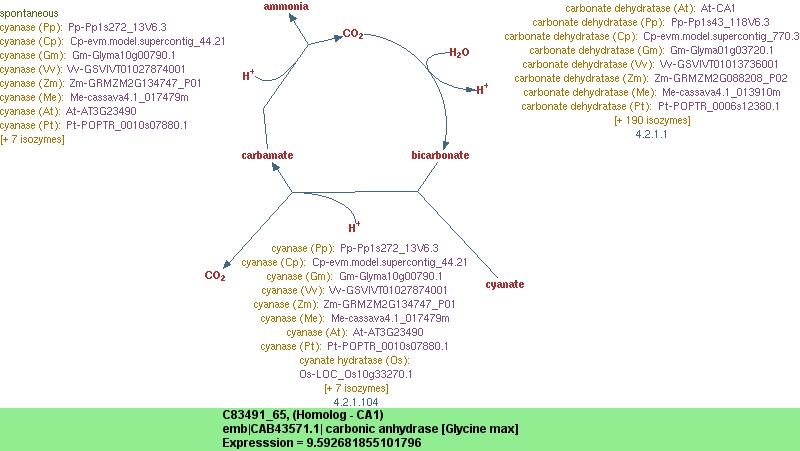

Supplement: Additional file 17 — Details of Transcription factor families. [file 1471-2164-14-647-S17.zip › Additional_file16B_Upregulated_PMN_pathways_in_Root/V2RS/C83491_65_CA1_2_cyanate_degradation.jpg]

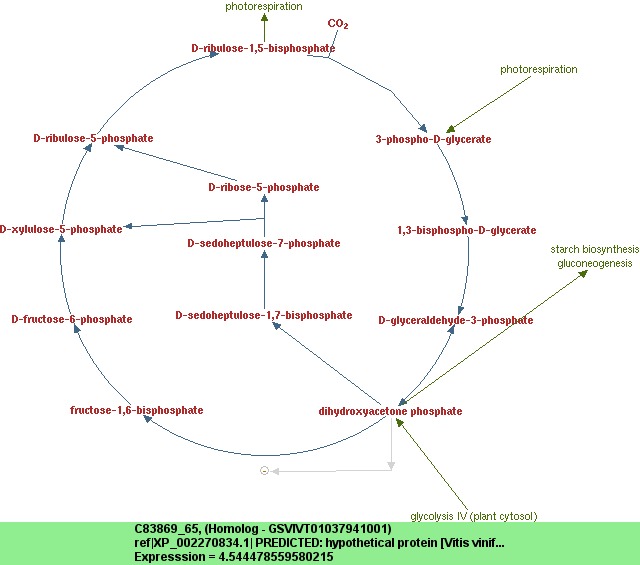

Supplement: Additional file 17 — Details of Transcription factor families. [file 1471-2164-14-647-S17.zip › Additional_file16B_Upregulated_PMN_pathways_in_Root/V2RS/C83869_65_GSVIVT01037941001_1_Calvin-Benson-Bassham_cycle.jpg]

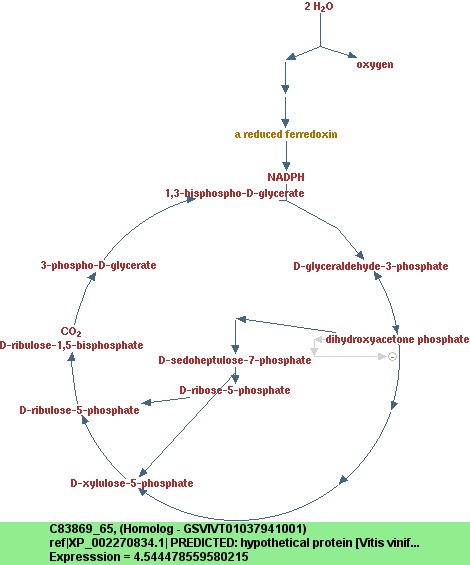

Supplement: Additional file 17 — Details of Transcription factor families. [file 1471-2164-14-647-S17.zip › Additional_file16B_Upregulated_PMN_pathways_in_Root/V2RS/C83869_65_GSVIVT01037941001_2_oxygenic_photosynthesis.jpg]

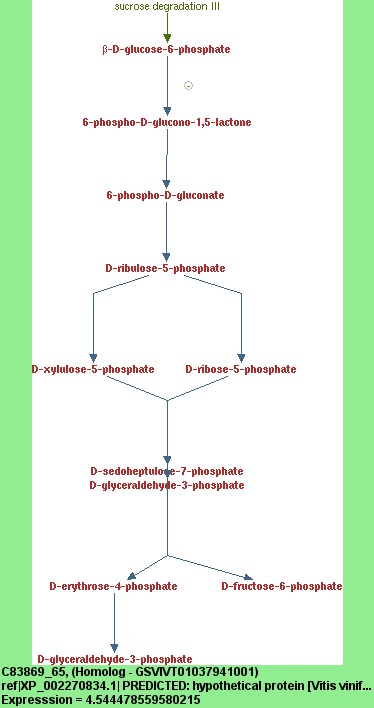

Supplement: Additional file 17 — Details of Transcription factor families. [file 1471-2164-14-647-S17.zip › Additional_file16B_Upregulated_PMN_pathways_in_Root/V2RS/C83869_65_GSVIVT01037941001_3_pentose_phosphate_pathway.jpg]

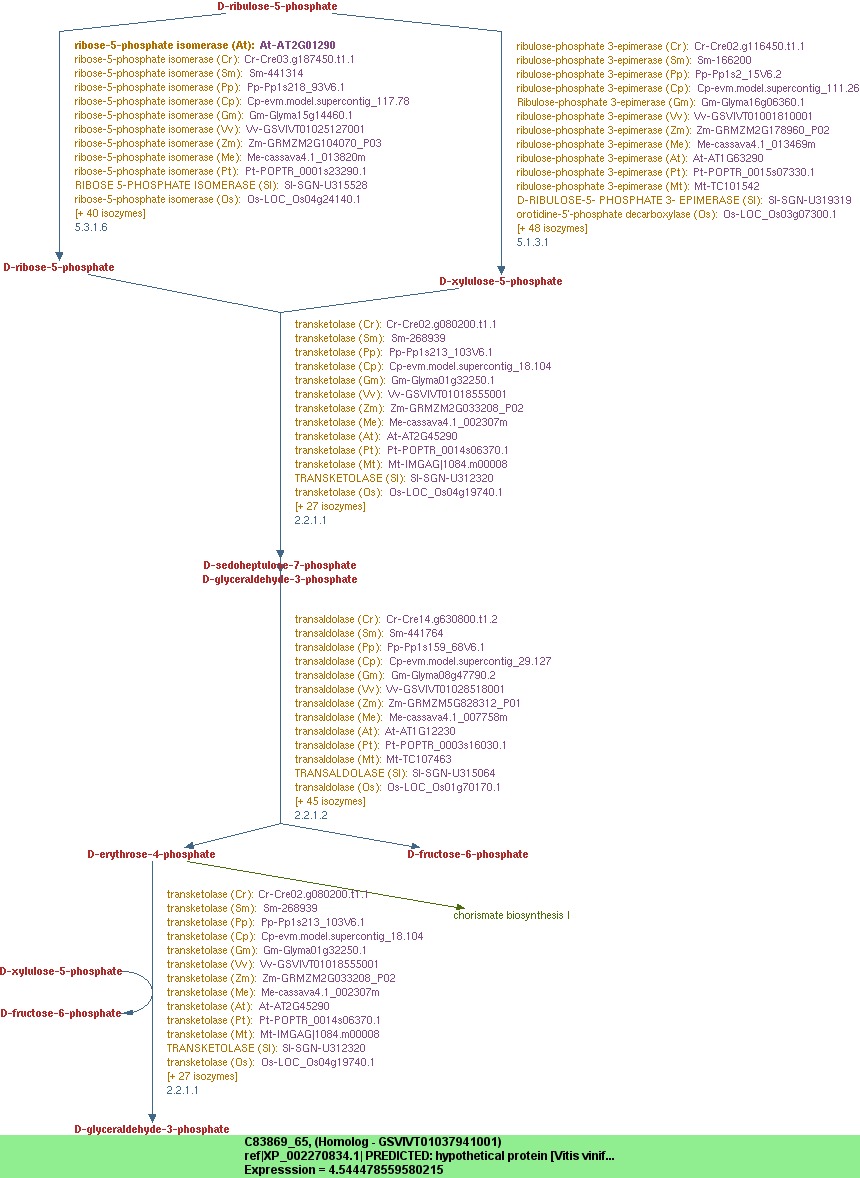

Supplement: Additional file 17 — Details of Transcription factor families. [file 1471-2164-14-647-S17.zip › Additional_file16B_Upregulated_PMN_pathways_in_Root/V2RS/C83869_65_GSVIVT01037941001_4_pentose_phosphate_pathway_(non-oxidative_branch).jpg]

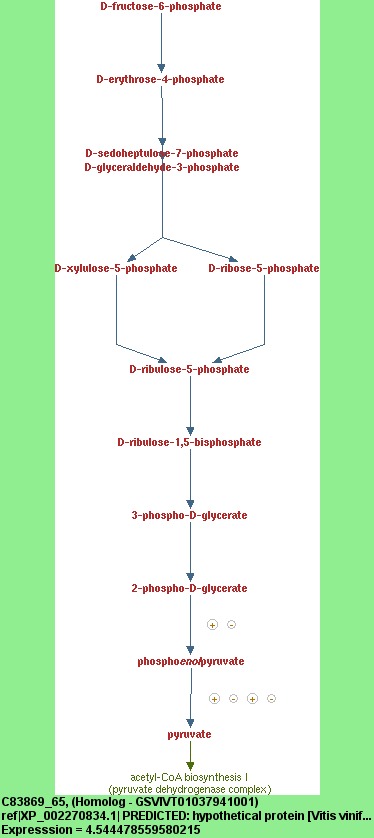

Supplement: Additional file 17 — Details of Transcription factor families. [file 1471-2164-14-647-S17.zip › Additional_file16B_Upregulated_PMN_pathways_in_Root/V2RS/C83869_65_GSVIVT01037941001_5_Rubisco_shunt.jpg]

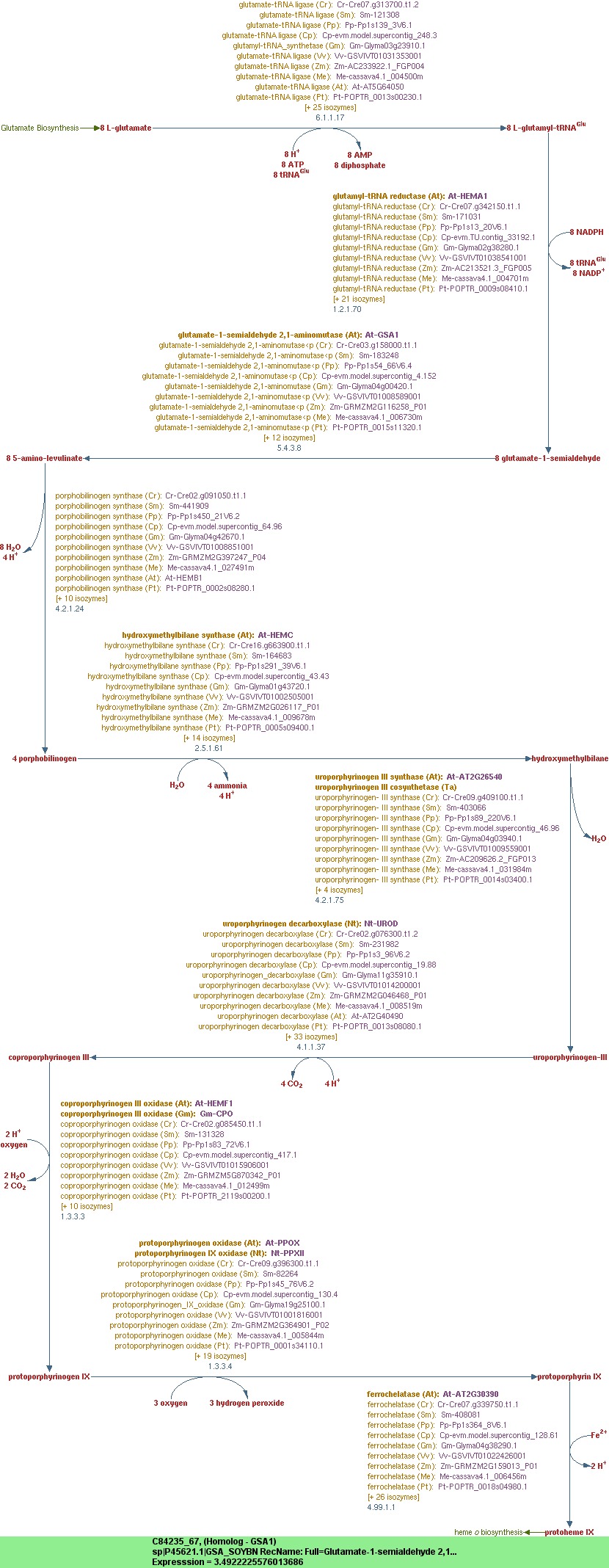

Supplement: Additional file 17 — Details of Transcription factor families. [file 1471-2164-14-647-S17.zip › Additional_file16B_Upregulated_PMN_pathways_in_Root/V2RS/C84235_67_GSA1_1_heme_biosynthesis_I.jpg]

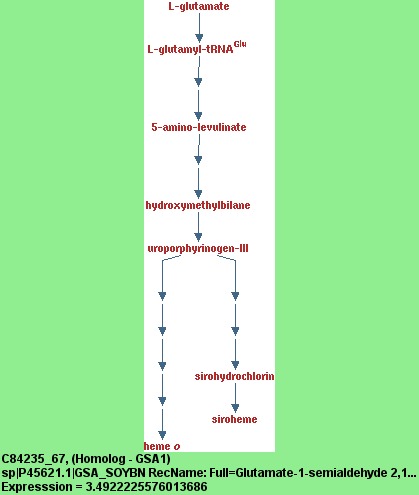

Supplement: Additional file 17 — Details of Transcription factor families. [file 1471-2164-14-647-S17.zip › Additional_file16B_Upregulated_PMN_pathways_in_Root/V2RS/C84235_67_GSA1_2_superpathway_of_proto-_and_siroheme_biosynthesis.jpg]

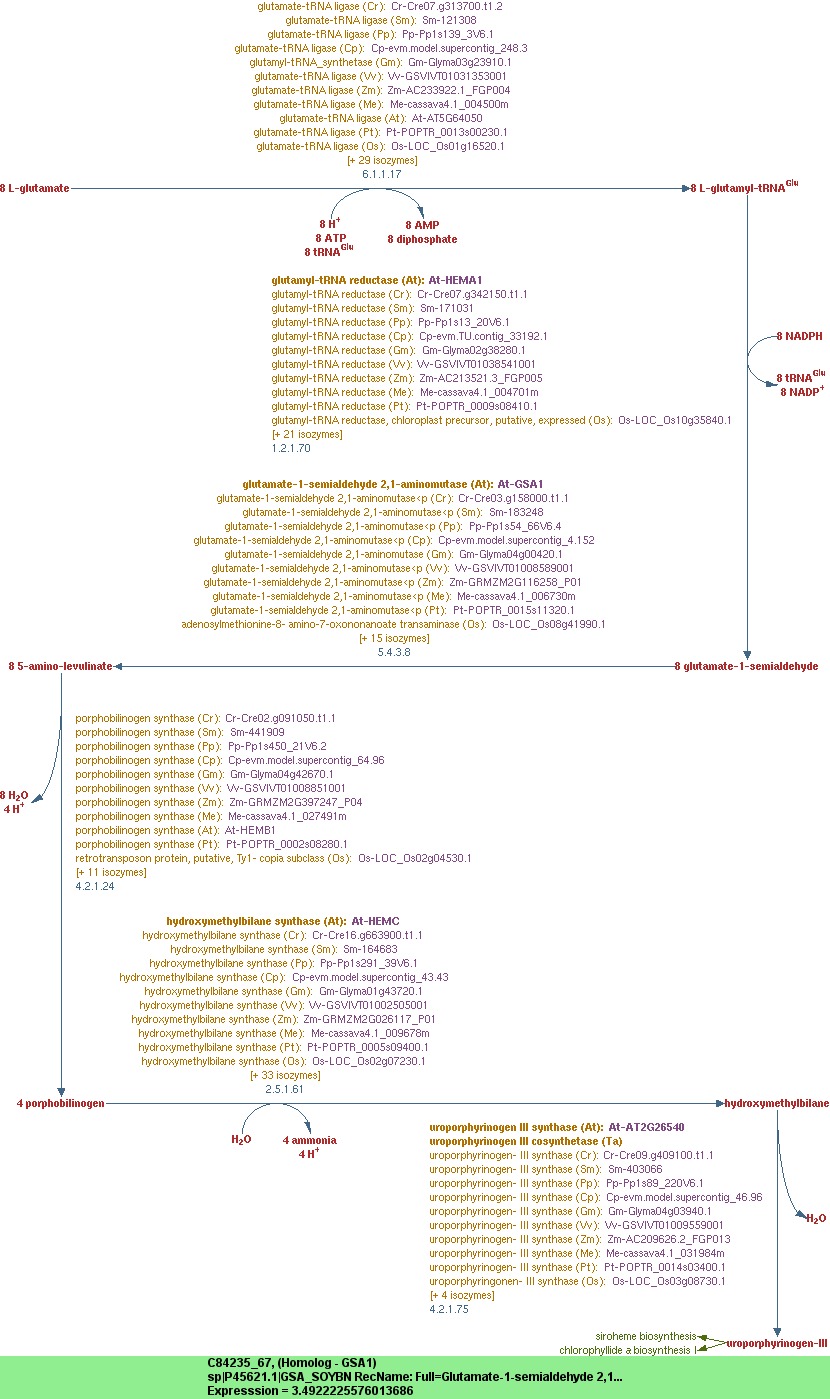

Supplement: Additional file 17 — Details of Transcription factor families. [file 1471-2164-14-647-S17.zip › Additional_file16B_Upregulated_PMN_pathways_in_Root/V2RS/C84235_67_GSA1_3_tetrapyrrole_biosynthesis_I.jpg]

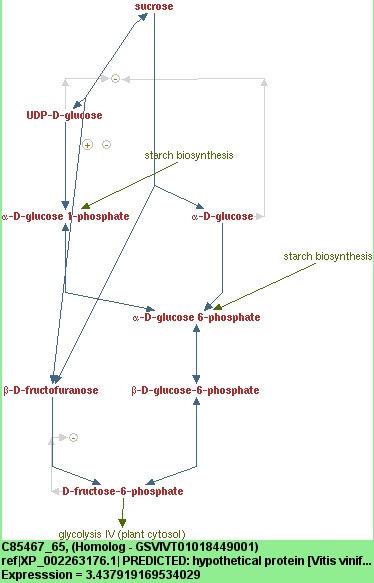

Supplement: Additional file 17 — Details of Transcription factor families. [file 1471-2164-14-647-S17.zip › Additional_file16B_Upregulated_PMN_pathways_in_Root/V2RS/C85467_65_GSVIVT01018449001_1_sucrose_degradation_III.jpg]

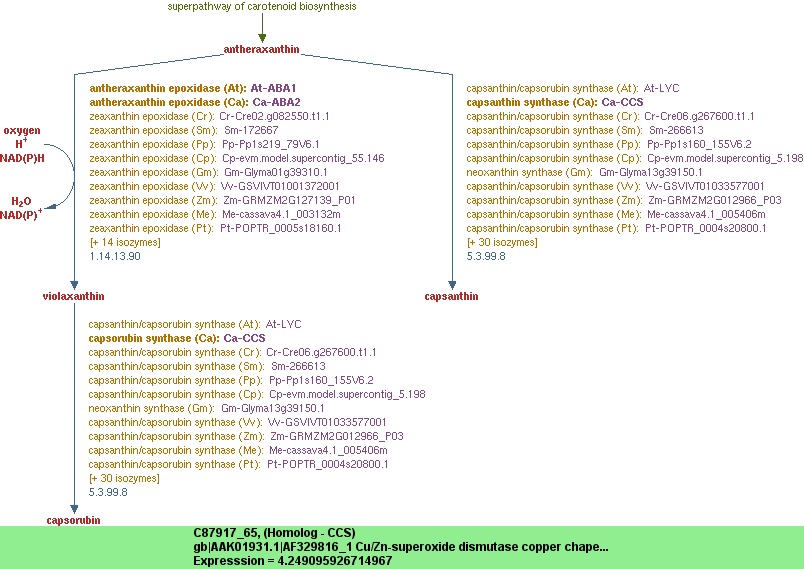

Supplement: Additional file 17 — Details of Transcription factor families. [file 1471-2164-14-647-S17.zip › Additional_file16B_Upregulated_PMN_pathways_in_Root/V2RS/C87917_65_CCS_1_capsanthin_and_capsorubin_biosynthesis.jpg]

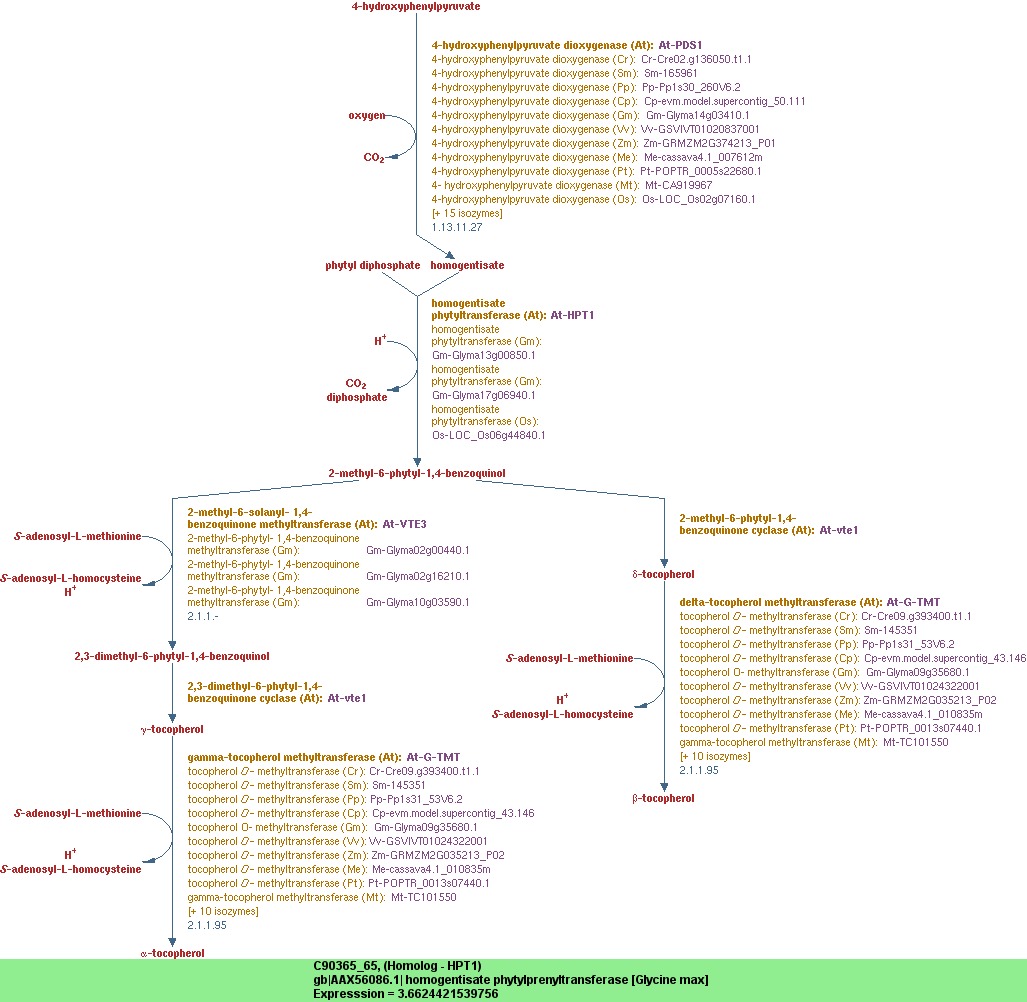

Supplement: Additional file 17 — Details of Transcription factor families. [file 1471-2164-14-647-S17.zip › Additional_file16B_Upregulated_PMN_pathways_in_Root/V2RS/C90365_65_HPT1_1_vitamin_E_biosynthesis.jpg]

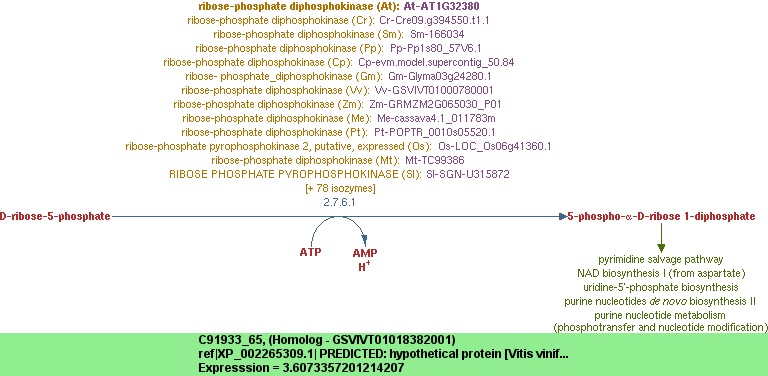

Supplement: Additional file 17 — Details of Transcription factor families. [file 1471-2164-14-647-S17.zip › Additional_file16B_Upregulated_PMN_pathways_in_Root/V2RS/C91933_65_GSVIVT01018382001_1_PRPP_biosynthesis_I.jpg]

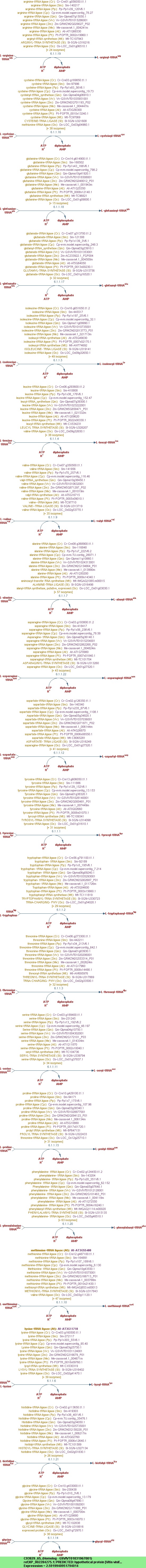

Supplement: Additional file 17 — Details of Transcription factor families. [file 1471-2164-14-647-S17.zip › Additional_file16B_Upregulated_PMN_pathways_in_Root/V2RS/C93829_65_GSVIVT01031967001_1_tRNA_charging.jpg]

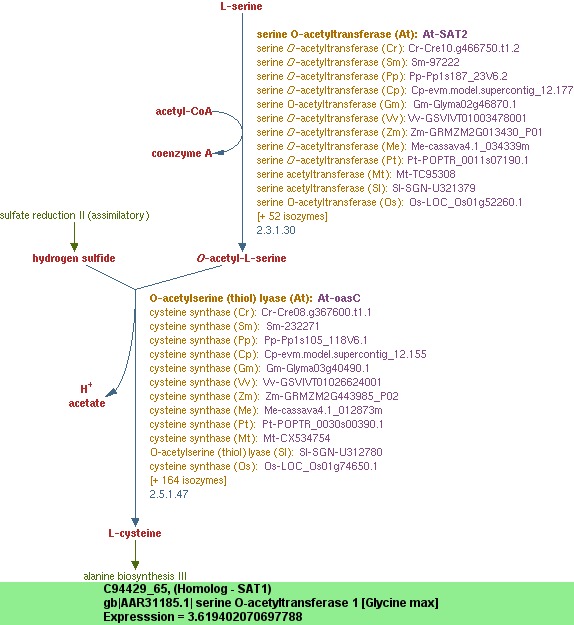

Supplement: Additional file 17 — Details of Transcription factor families. [file 1471-2164-14-647-S17.zip › Additional_file16B_Upregulated_PMN_pathways_in_Root/V2RS/C94429_65_SAT1_1_cysteine_biosynthesis_I.jpg]

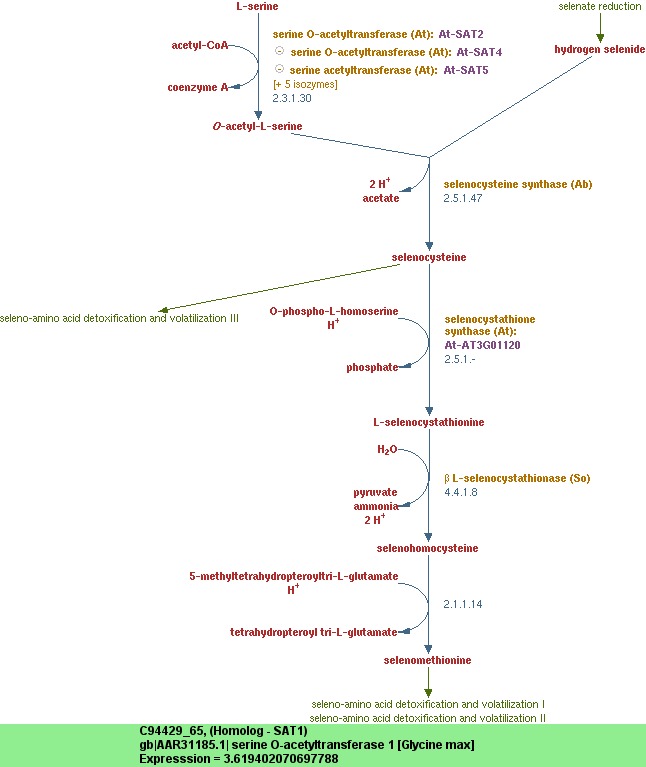

Supplement: Additional file 17 — Details of Transcription factor families. [file 1471-2164-14-647-S17.zip › Additional_file16B_Upregulated_PMN_pathways_in_Root/V2RS/C94429_65_SAT1_2_seleno-amino_acid_biosynthesis.jpg]

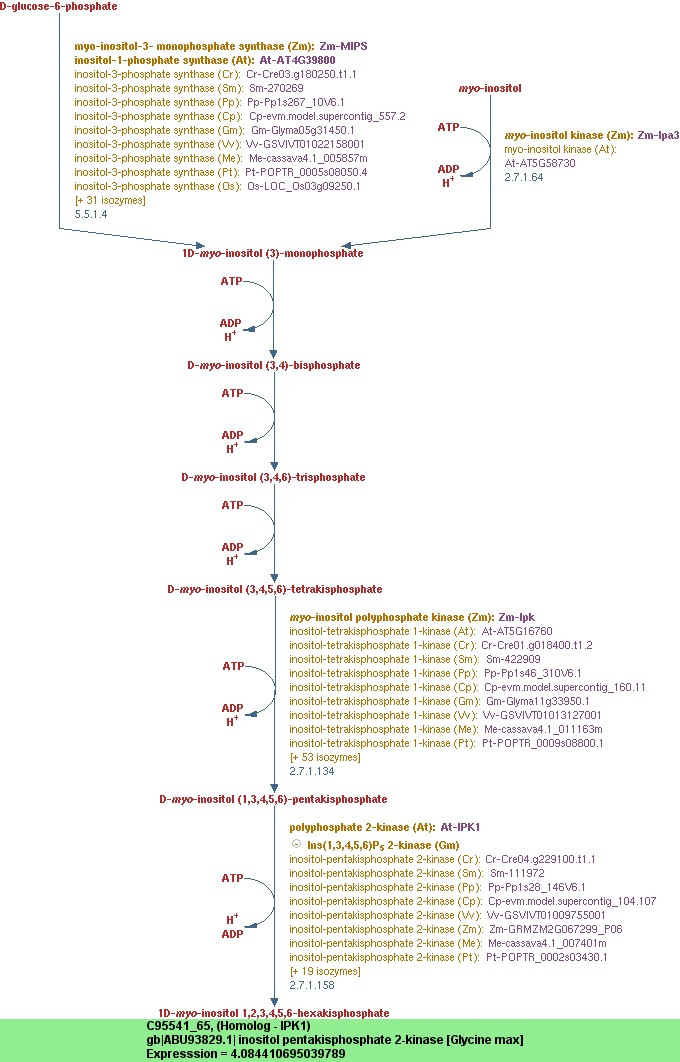

Supplement: Additional file 17 — Details of Transcription factor families. [file 1471-2164-14-647-S17.zip › Additional_file16B_Upregulated_PMN_pathways_in_Root/V2RS/C95541_65_IPK1_1_1D-myo-inositol_hexakisphosphate_biosynthesis_III_(Spirodela_polyrrhiza).jpg]

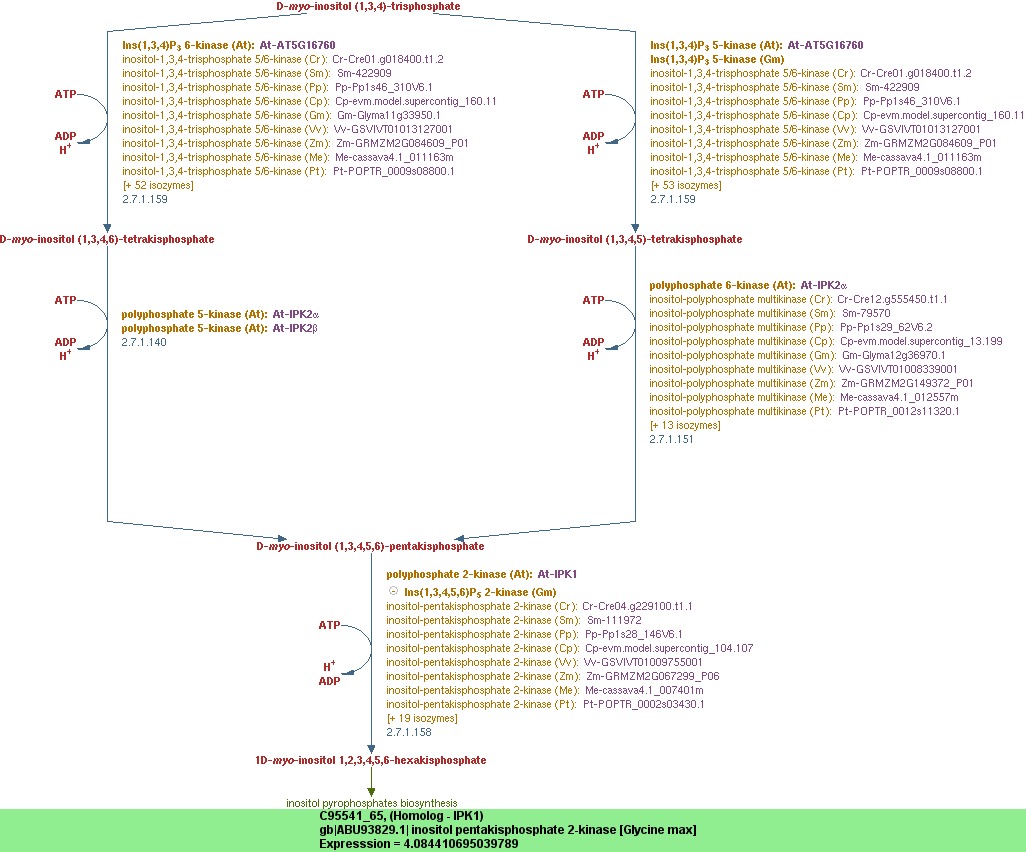

Supplement: Additional file 17 — Details of Transcription factor families. [file 1471-2164-14-647-S17.zip › Additional_file16B_Upregulated_PMN_pathways_in_Root/V2RS/C95541_65_IPK1_2_1D-myo-inositol_hexakisphosphate_biosynthesis_V_(from_Ins(1,3,4)P3).jpg]

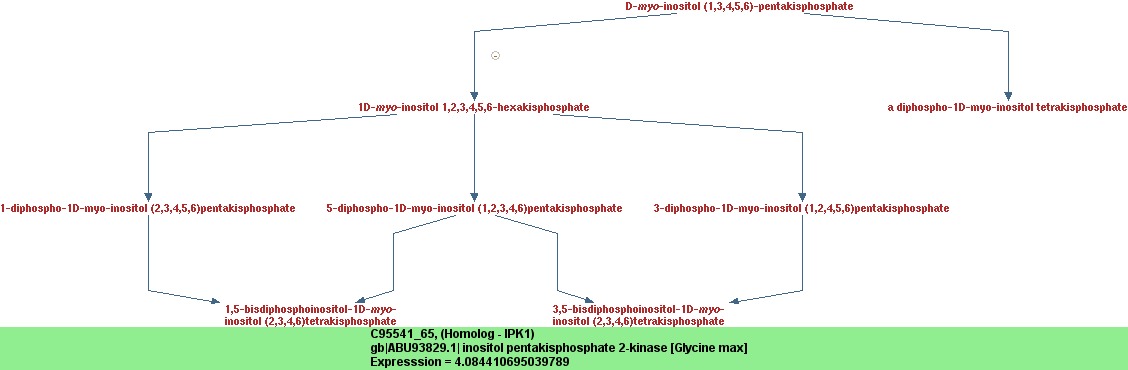

Supplement: Additional file 17 — Details of Transcription factor families. [file 1471-2164-14-647-S17.zip › Additional_file16B_Upregulated_PMN_pathways_in_Root/V2RS/C95541_65_IPK1_3_inositol_pyrophosphates_biosynthesis.jpg]

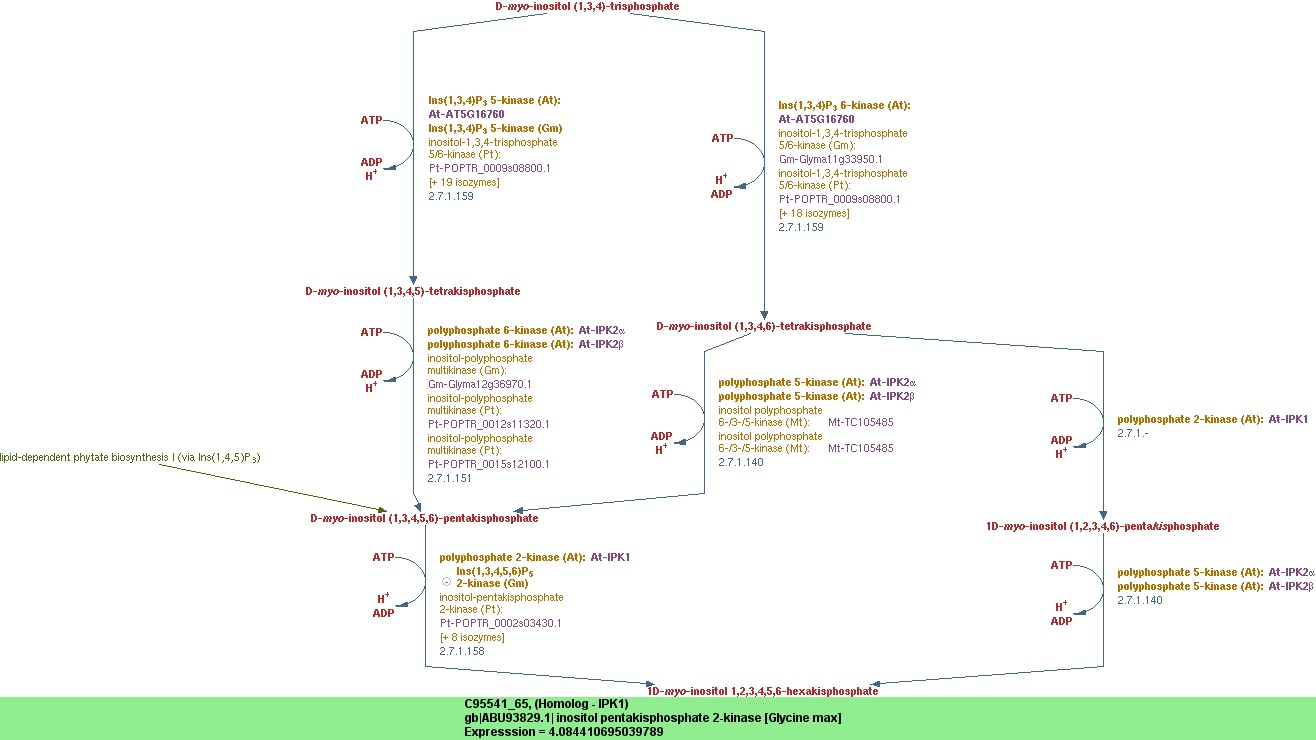

Supplement: Additional file 17 — Details of Transcription factor families. [file 1471-2164-14-647-S17.zip › Additional_file16B_Upregulated_PMN_pathways_in_Root/V2RS/C95541_65_IPK1_4_lipid-dependent_phytate_biosynthesis_II_(via_Ins(1,3,4)P3).jpg]

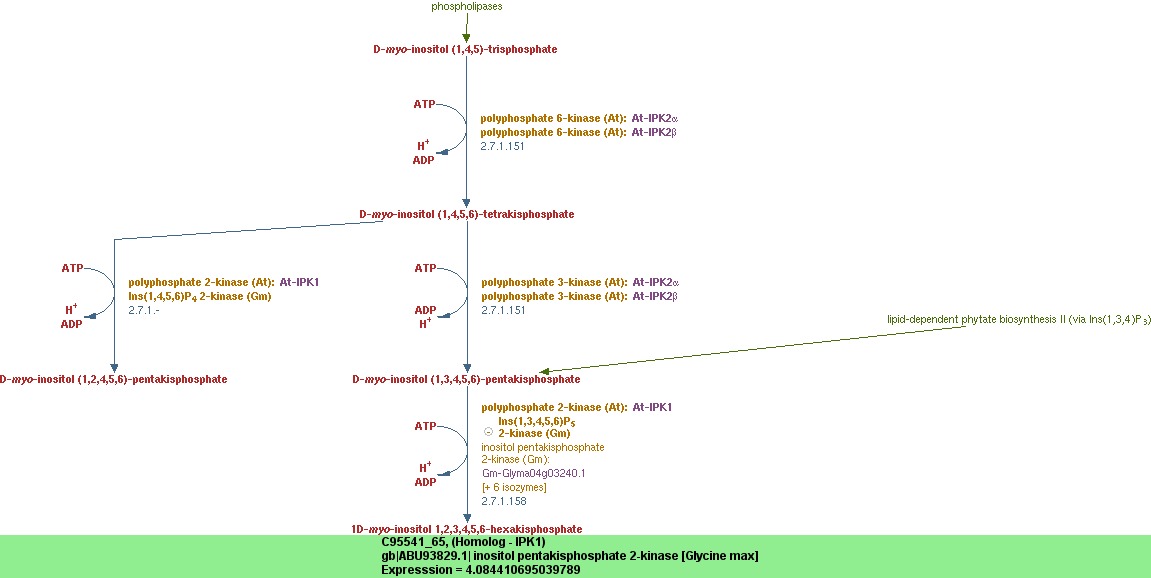

Supplement: Additional file 17 — Details of Transcription factor families. [file 1471-2164-14-647-S17.zip › Additional_file16B_Upregulated_PMN_pathways_in_Root/V2RS/C95541_65_IPK1_5_lipid-dependent_phytate_biosynthesis_I_(via_Ins(1,4,5)P3).jpg]

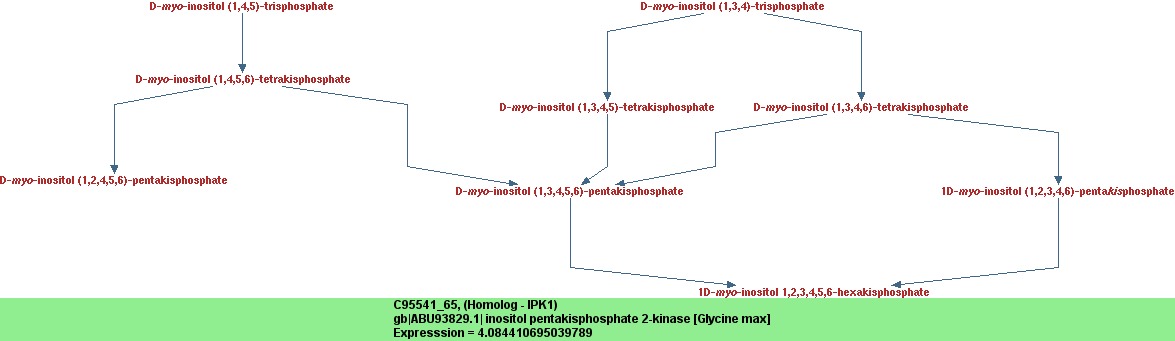

Supplement: Additional file 17 — Details of Transcription factor families. [file 1471-2164-14-647-S17.zip › Additional_file16B_Upregulated_PMN_pathways_in_Root/V2RS/C95541_65_IPK1_6_superpathway_of_lipid-dependent_phytate_biosynthesis.jpg]

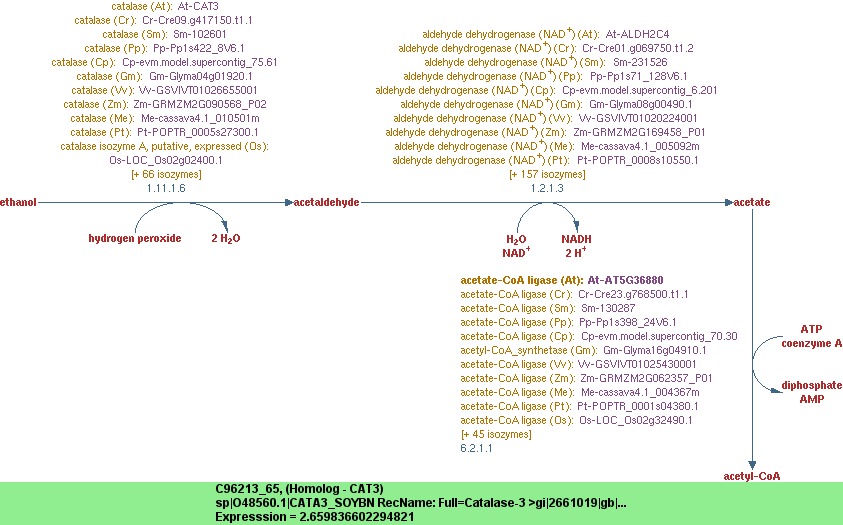

Supplement: Additional file 17 — Details of Transcription factor families. [file 1471-2164-14-647-S17.zip › Additional_file16B_Upregulated_PMN_pathways_in_Root/V2RS/C96213_65_CAT3_1_ethanol_degradation_IV.jpg]

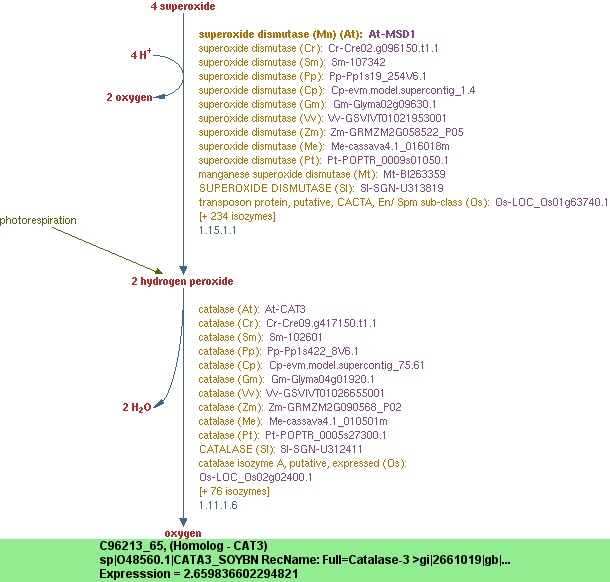

Supplement: Additional file 17 — Details of Transcription factor families. [file 1471-2164-14-647-S17.zip › Additional_file16B_Upregulated_PMN_pathways_in_Root/V2RS/C96213_65_CAT3_2_superoxide_radicals_degradation.jpg]

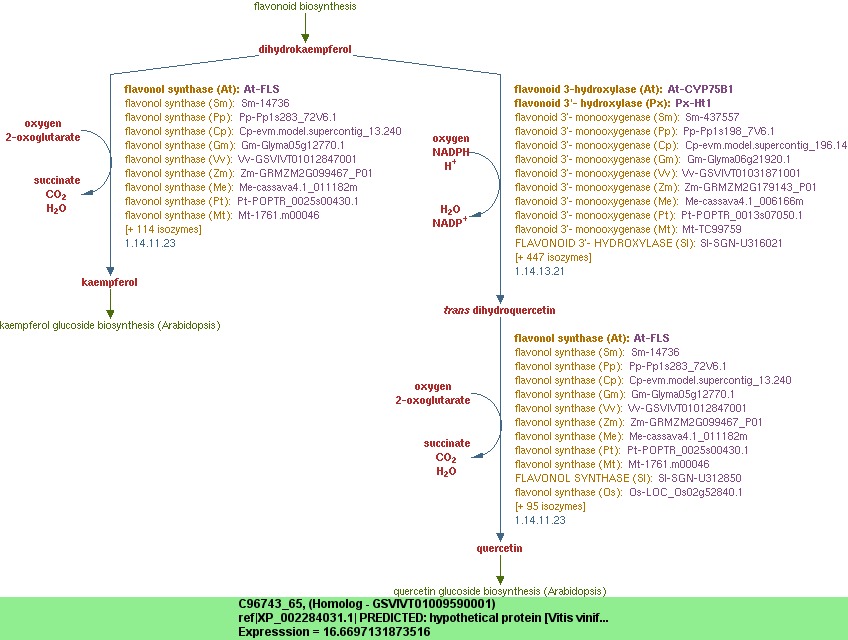

Supplement: Additional file 17 — Details of Transcription factor families. [file 1471-2164-14-647-S17.zip › Additional_file16B_Upregulated_PMN_pathways_in_Root/V2RS/C96743_65_GSVIVT01009590001_1_flavonol_biosynthesis.jpg]

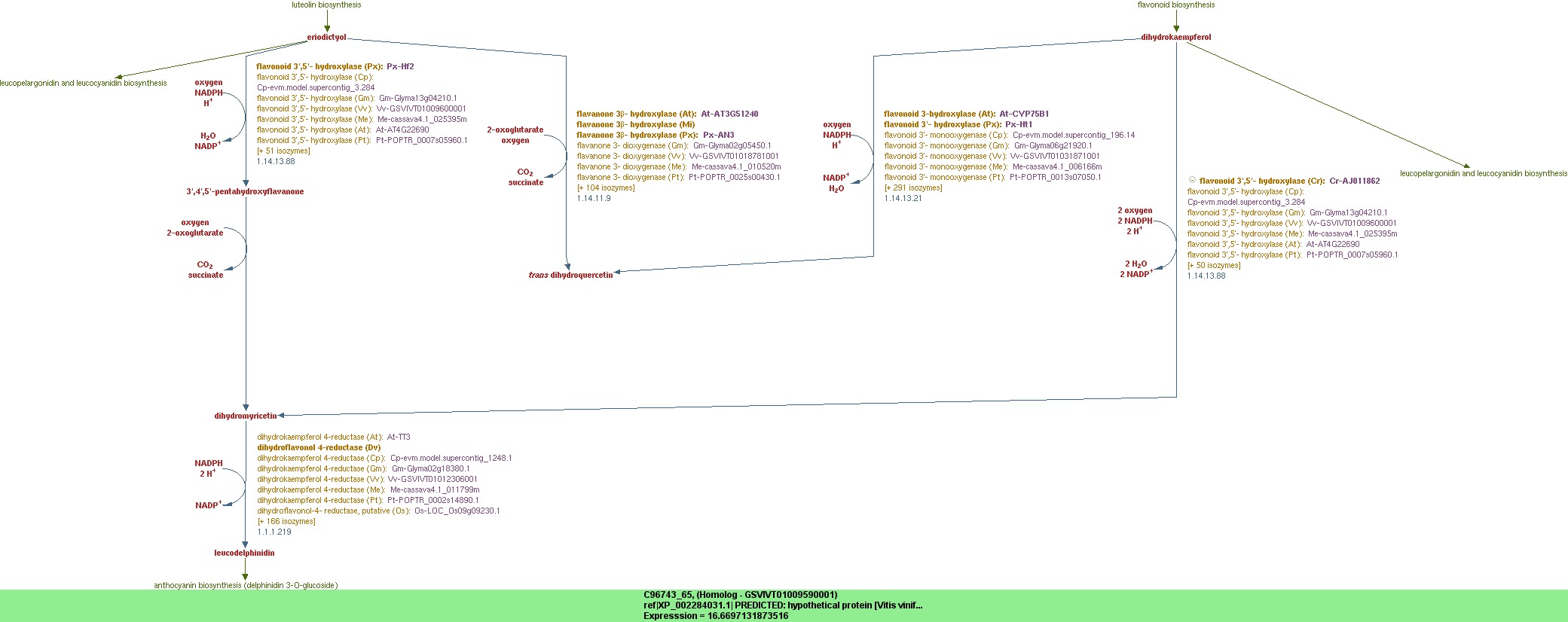

Supplement: Additional file 17 — Details of Transcription factor families. [file 1471-2164-14-647-S17.zip › Additional_file16B_Upregulated_PMN_pathways_in_Root/V2RS/C96743_65_GSVIVT01009590001_2_leucodelphinidin_biosynthesis.jpg]

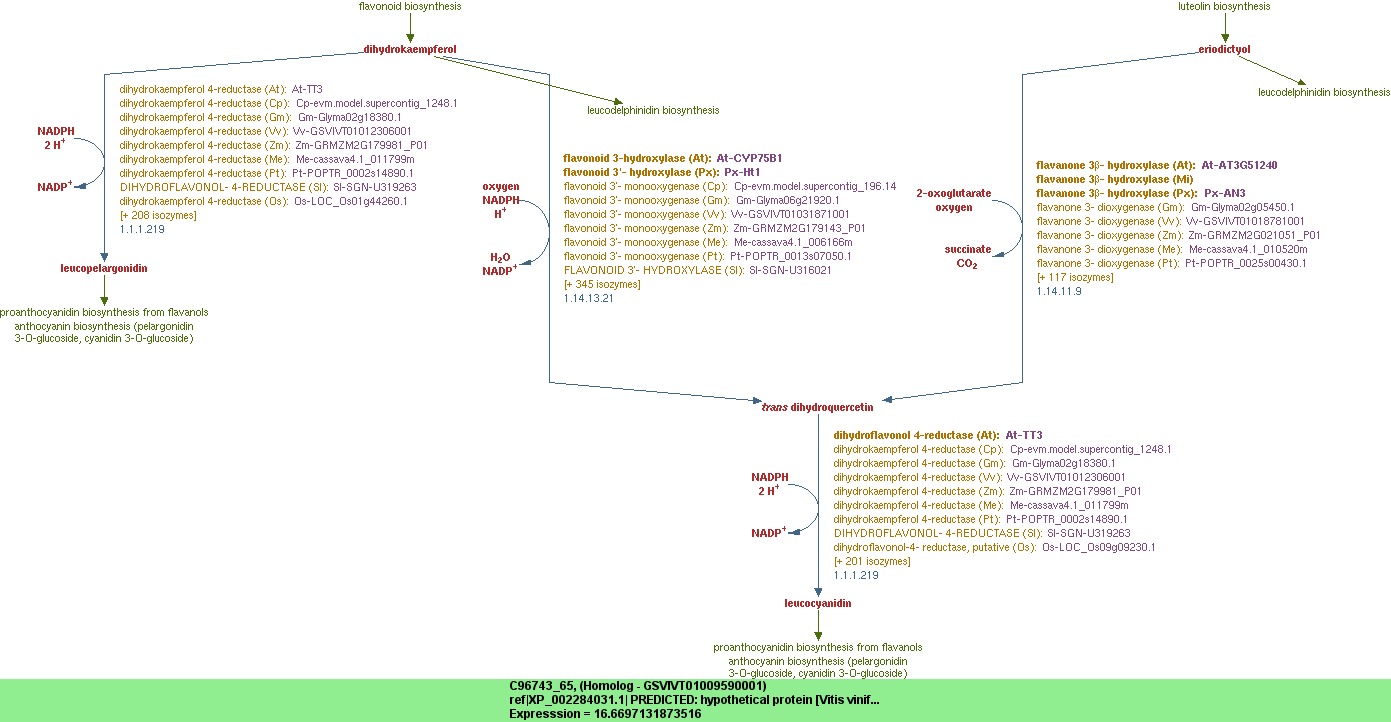

Supplement: Additional file 17 — Details of Transcription factor families. [file 1471-2164-14-647-S17.zip › Additional_file16B_Upregulated_PMN_pathways_in_Root/V2RS/C96743_65_GSVIVT01009590001_3_leucopelargonidin_and_leucocyanidin_biosynthesis.jpg]

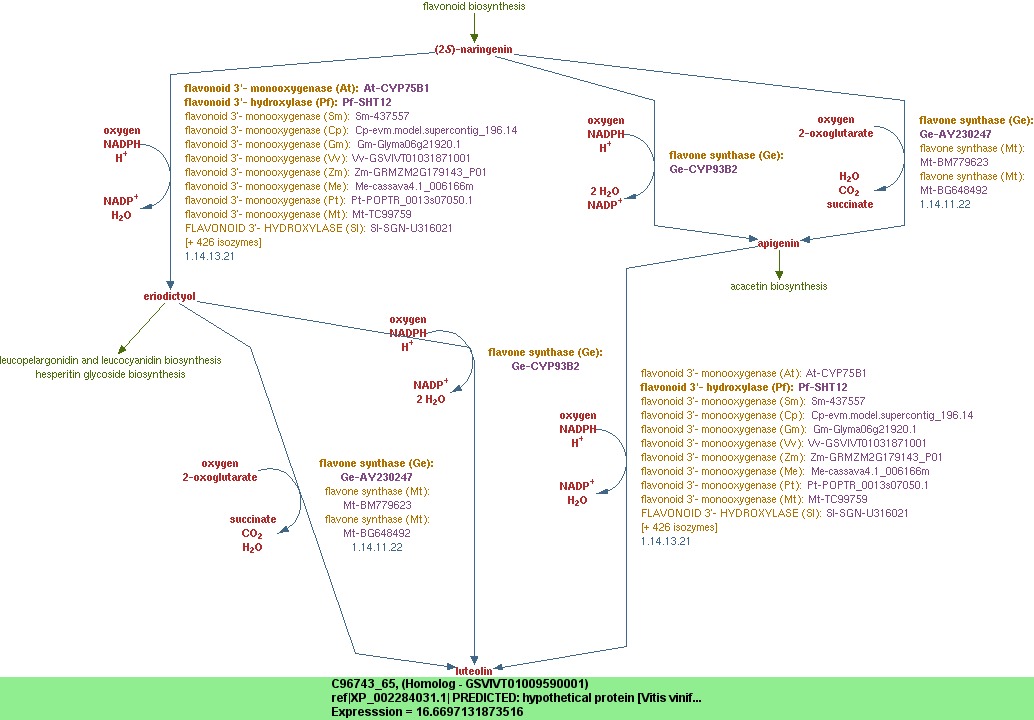

Supplement: Additional file 17 — Details of Transcription factor families. [file 1471-2164-14-647-S17.zip › Additional_file16B_Upregulated_PMN_pathways_in_Root/V2RS/C96743_65_GSVIVT01009590001_4_luteolin_biosynthesis.jpg]

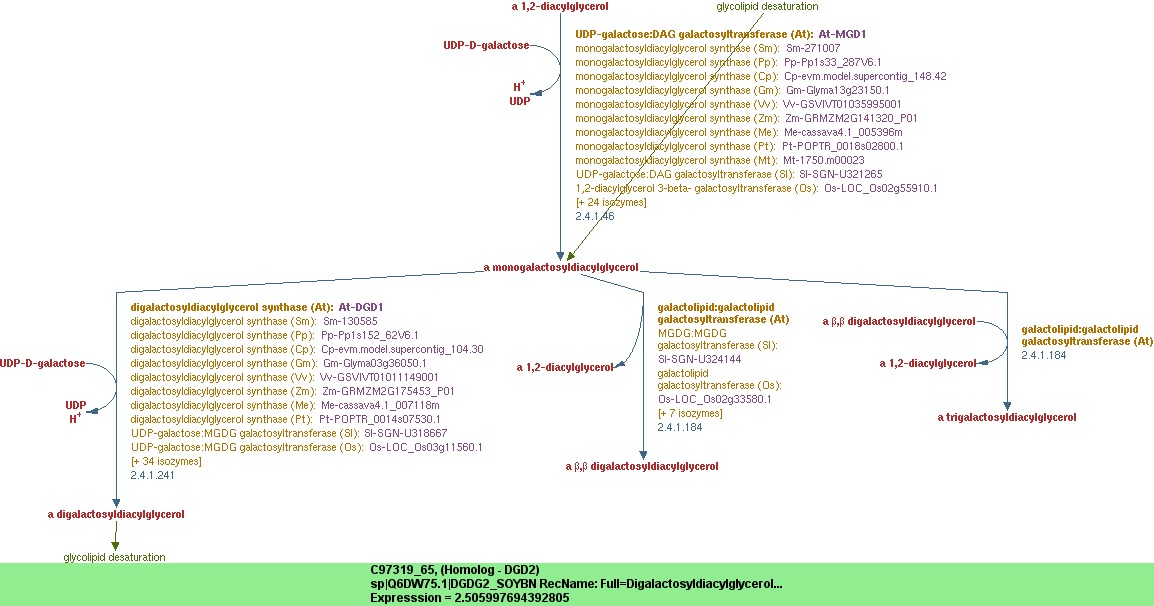

Supplement: Additional file 17 — Details of Transcription factor families. [file 1471-2164-14-647-S17.zip › Additional_file16B_Upregulated_PMN_pathways_in_Root/V2RS/C97319_65_DGD2_1_glycolipid_biosynthesis.jpg]

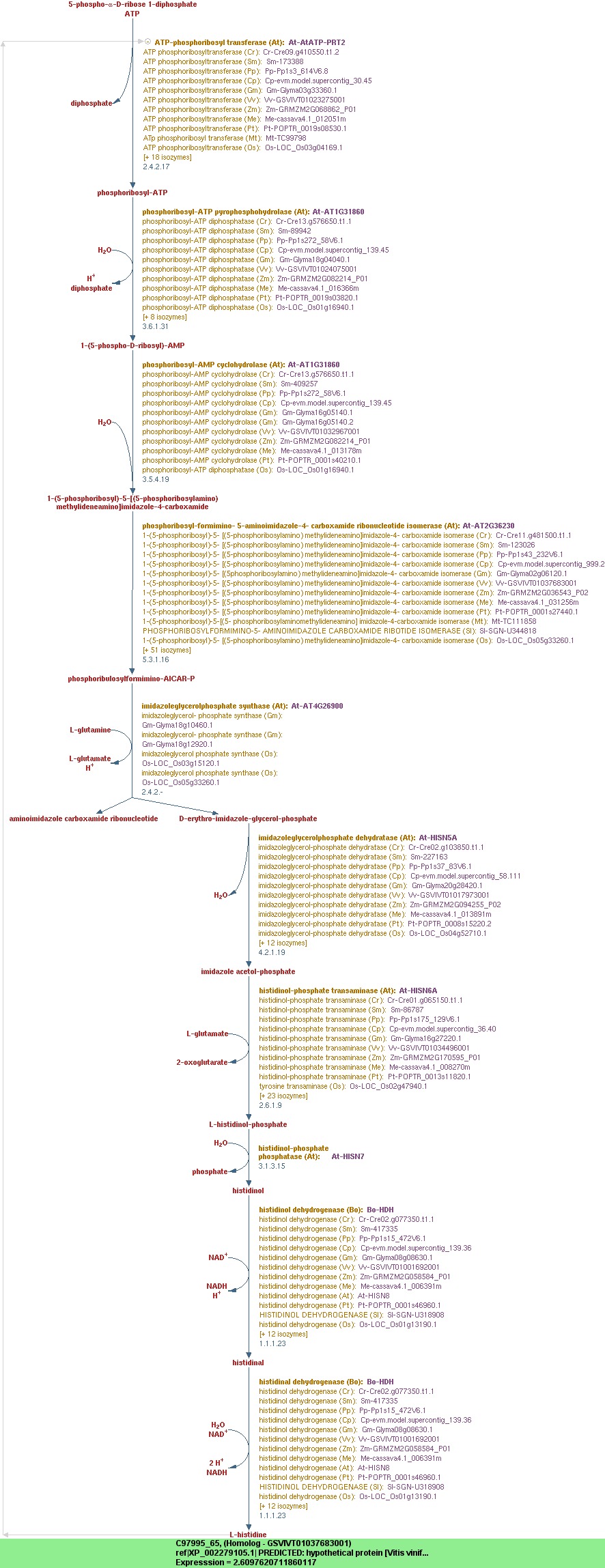

Supplement: Additional file 17 — Details of Transcription factor families. [file 1471-2164-14-647-S17.zip › Additional_file16B_Upregulated_PMN_pathways_in_Root/V2RS/C97995_65_GSVIVT01037683001_1_histidine_biosynthesis.jpg]

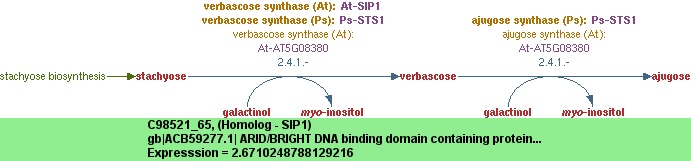

Supplement: Additional file 17 — Details of Transcription factor families. [file 1471-2164-14-647-S17.zip › Additional_file16B_Upregulated_PMN_pathways_in_Root/V2RS/C98521_65_SIP1_1_ajugose_biosynthesis_I_(galactinol-dependent).jpg]

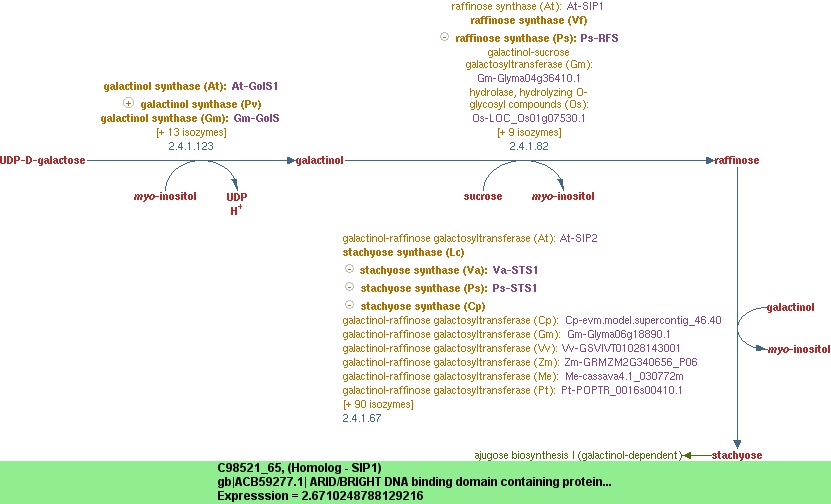

Supplement: Additional file 17 — Details of Transcription factor families. [file 1471-2164-14-647-S17.zip › Additional_file16B_Upregulated_PMN_pathways_in_Root/V2RS/C98521_65_SIP1_2_stachyose_biosynthesis.jpg]

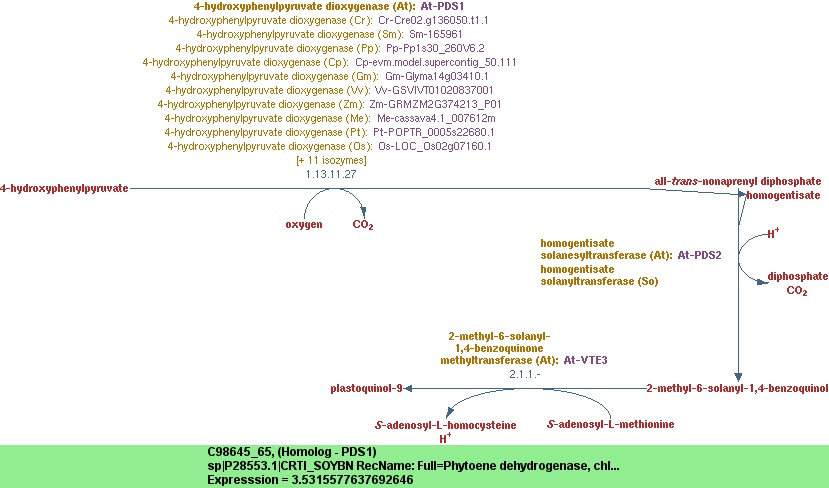

Supplement: Additional file 17 — Details of Transcription factor families. [file 1471-2164-14-647-S17.zip › Additional_file16B_Upregulated_PMN_pathways_in_Root/V2RS/C98645_65_PDS1_1_plastoquinol-9_biosynthesis_I.jpg]

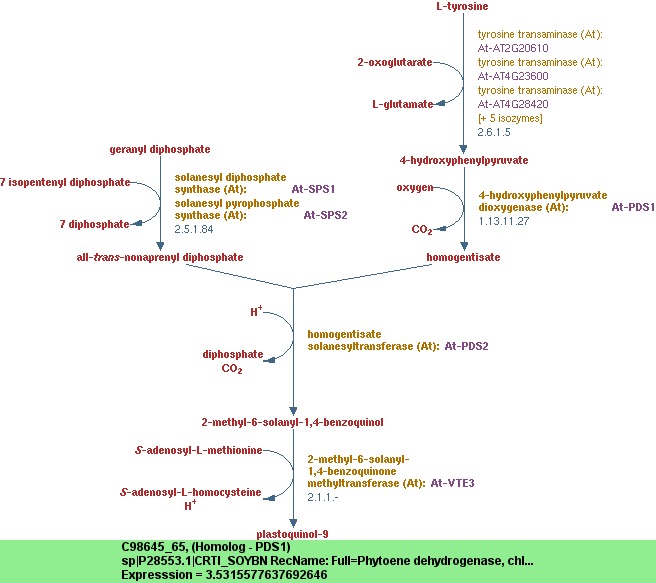

Supplement: Additional file 17 — Details of Transcription factor families. [file 1471-2164-14-647-S17.zip › Additional_file16B_Upregulated_PMN_pathways_in_Root/V2RS/C98645_65_PDS1_2_superpathway_of_plastoquinol_biosynthesis.jpg]

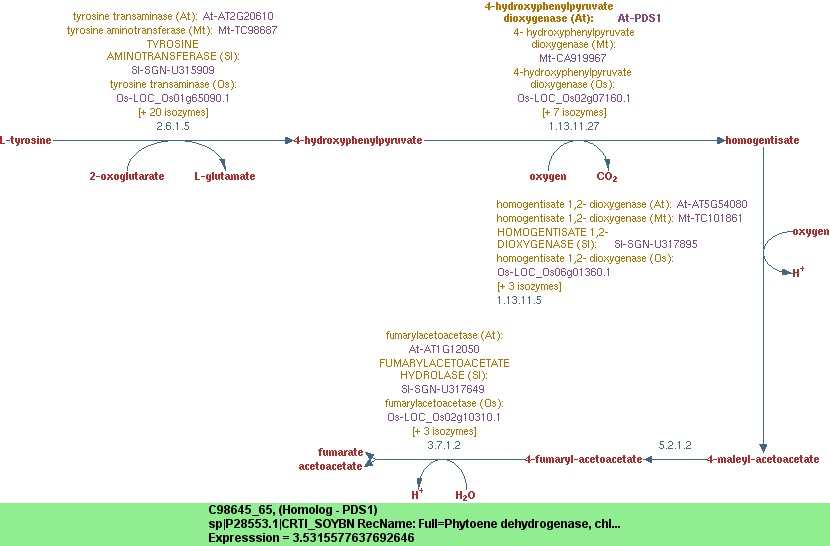

Supplement: Additional file 17 — Details of Transcription factor families. [file 1471-2164-14-647-S17.zip › Additional_file16B_Upregulated_PMN_pathways_in_Root/V2RS/C98645_65_PDS1_3_tyrosine_degradation_I.jpg]

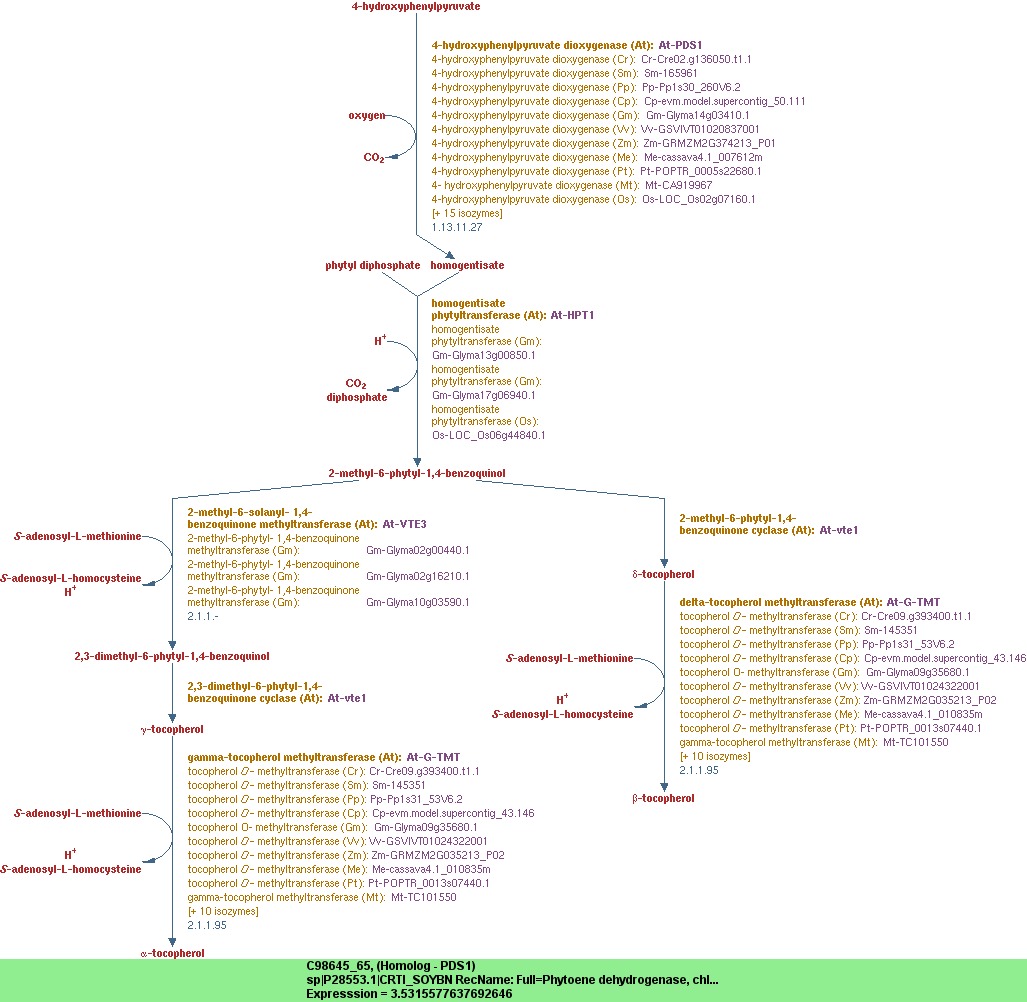

Supplement: Additional file 17 — Details of Transcription factor families. [file 1471-2164-14-647-S17.zip › Additional_file16B_Upregulated_PMN_pathways_in_Root/V2RS/C98645_65_PDS1_4_vitamin_E_biosynthesis.jpg]

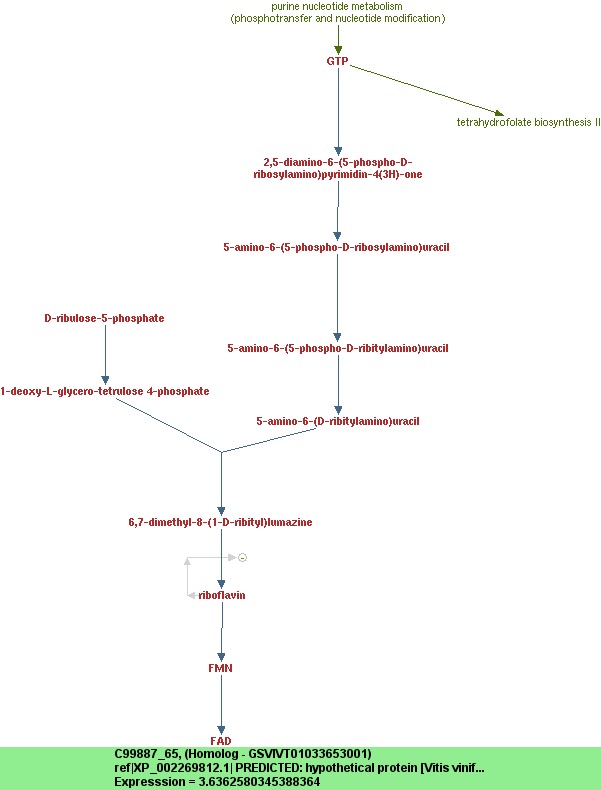

Supplement: Additional file 17 — Details of Transcription factor families. [file 1471-2164-14-647-S17.zip › Additional_file16B_Upregulated_PMN_pathways_in_Root/V2RS/C99887_65_GSVIVT01033653001_1_flavin_biosynthesis_I_(bacteria_and_plants).jpg]

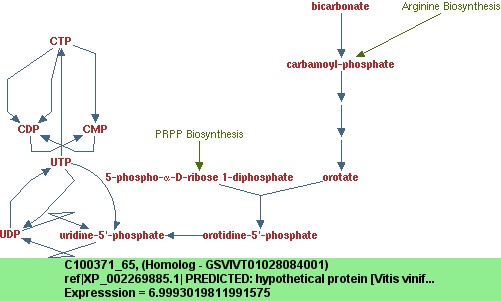

Supplement: Additional file 17 — Details of Transcription factor families. [file 1471-2164-14-647-S17.zip › Additional_file16B_Upregulated_PMN_pathways_in_Root/V2RS/C100371_65_GSVIVT01028084001_1_pyrimidine_ribonucleotides_de_novo_biosynthesis.jpg]

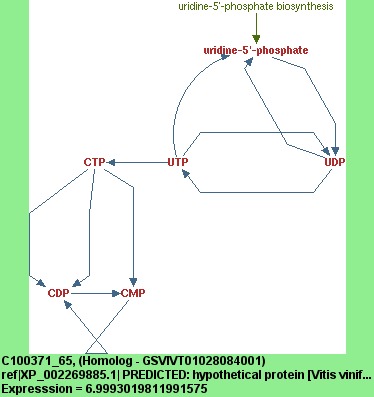

Supplement: Additional file 17 — Details of Transcription factor families. [file 1471-2164-14-647-S17.zip › Additional_file16B_Upregulated_PMN_pathways_in_Root/V2RS/C100371_65_GSVIVT01028084001_2_pyrimidine_ribonucleotides_interconversion.jpg]

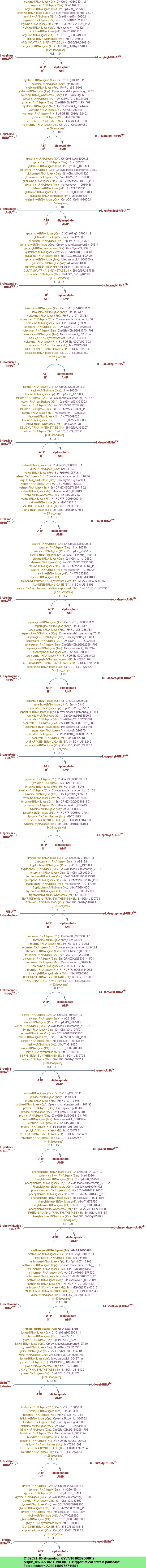

Supplement: Additional file 17 — Details of Transcription factor families. [file 1471-2164-14-647-S17.zip › Additional_file16B_Upregulated_PMN_pathways_in_Root/V2RS/C102031_65_GSVIVT01020206001_1_tRNA_charging.jpg]

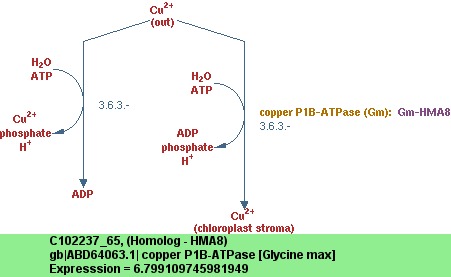

Supplement: Additional file 17 — Details of Transcription factor families. [file 1471-2164-14-647-S17.zip › Additional_file16B_Upregulated_PMN_pathways_in_Root/V2RS/C102237_65_HMA8_1_copper_transport_I.jpg]

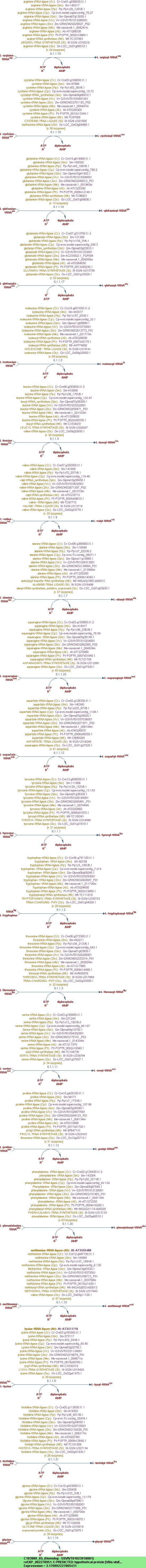

Supplement: Additional file 17 — Details of Transcription factor families. [file 1471-2164-14-647-S17.zip › Additional_file16B_Upregulated_PMN_pathways_in_Root/V2RS/C103889_65_GSVIVT01023918001_1_tRNA_charging.jpg]

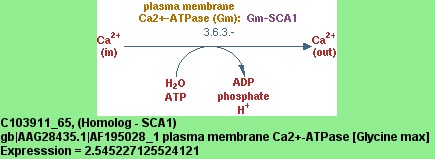

Supplement: Additional file 17 — Details of Transcription factor families. [file 1471-2164-14-647-S17.zip › Additional_file16B_Upregulated_PMN_pathways_in_Root/V2RS/C103911_65_SCA1_1_calmodulin_regulated_calcium_transport.jpg]

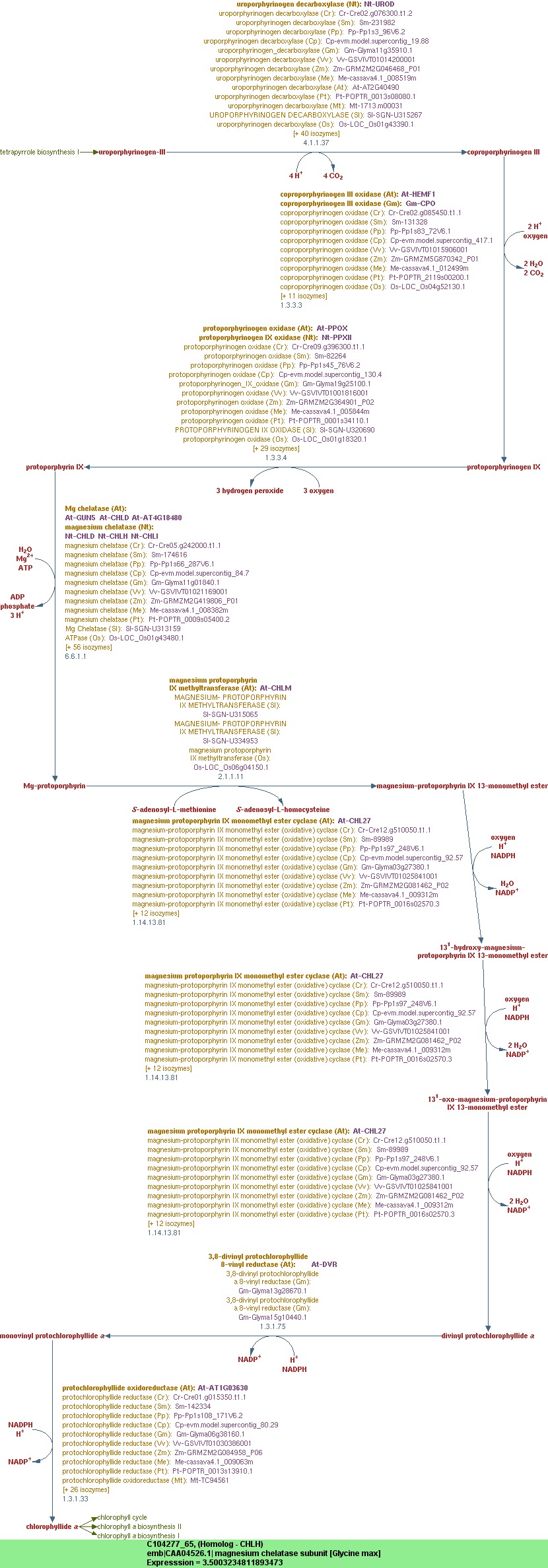

Supplement: Additional file 17 — Details of Transcription factor families. [file 1471-2164-14-647-S17.zip › Additional_file16B_Upregulated_PMN_pathways_in_Root/V2RS/C104277_65_CHLH_1_chlorophyllide_a_biosynthesis_I.jpg]

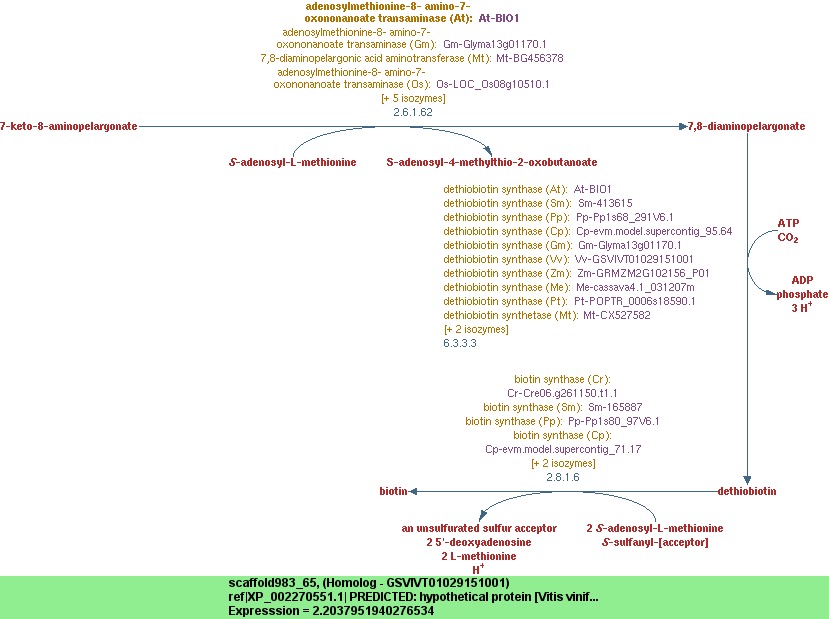

Supplement: Additional file 17 — Details of Transcription factor families. [file 1471-2164-14-647-S17.zip › Additional_file16B_Upregulated_PMN_pathways_in_Root/V2RS/scaffold983_65_GSVIVT01029151001_1_biotin_biosynthesis_from_7-keto-8-aminopelargonate.jpg]

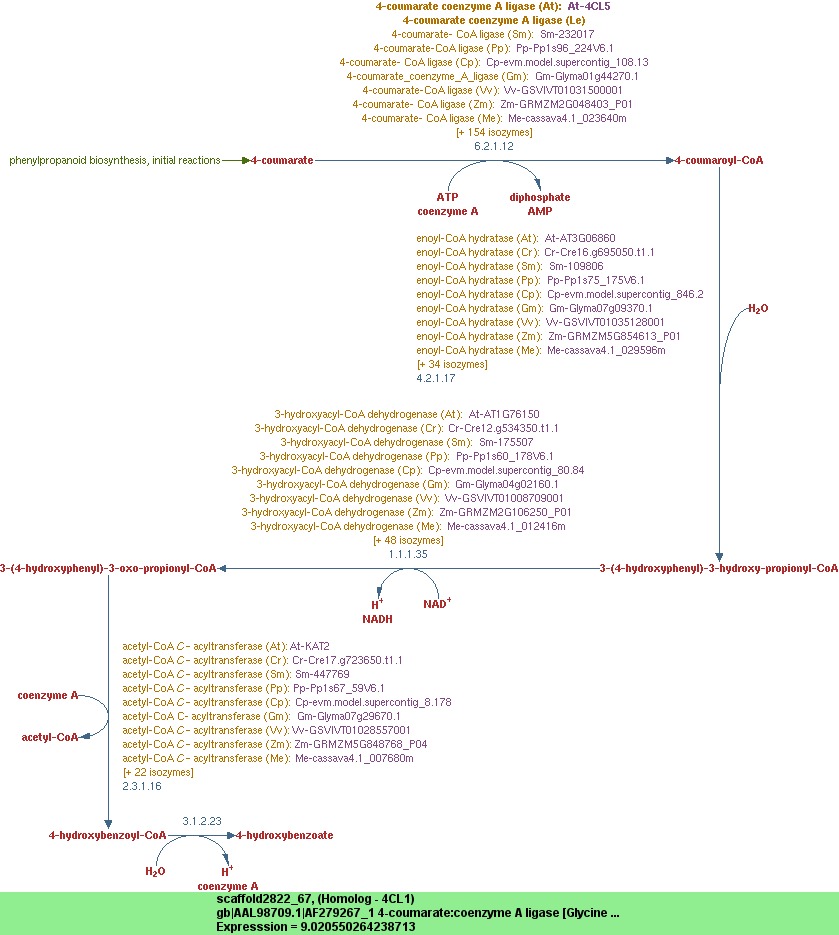

Supplement: Additional file 17 — Details of Transcription factor families. [file 1471-2164-14-647-S17.zip › Additional_file16B_Upregulated_PMN_pathways_in_Root/V2RS/scaffold2822_67_4CL1_1_4-hydroxybenzoate_biosynthesis_V.jpg]

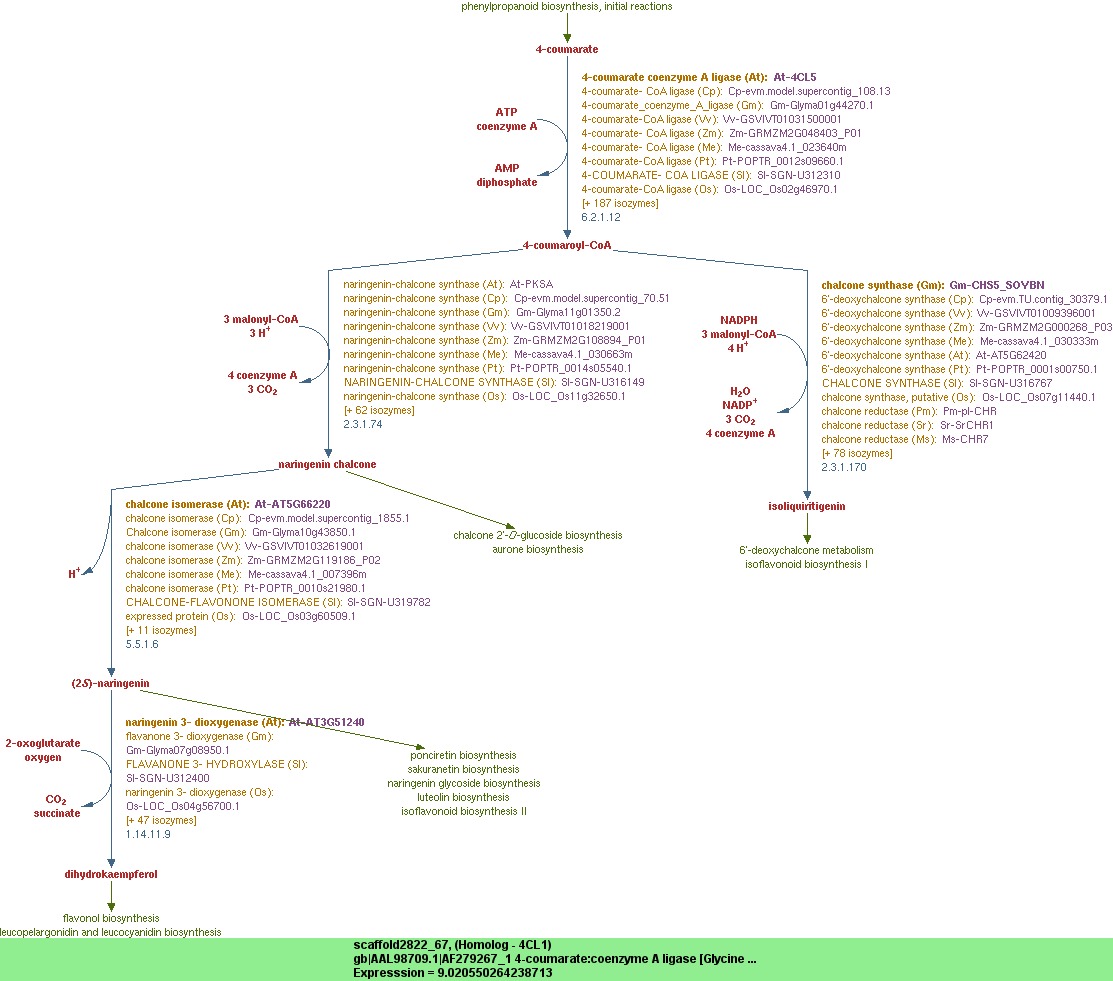

Supplement: Additional file 17 — Details of Transcription factor families. [file 1471-2164-14-647-S17.zip › Additional_file16B_Upregulated_PMN_pathways_in_Root/V2RS/scaffold2822_67_4CL1_2_flavonoid_biosynthesis.jpg]

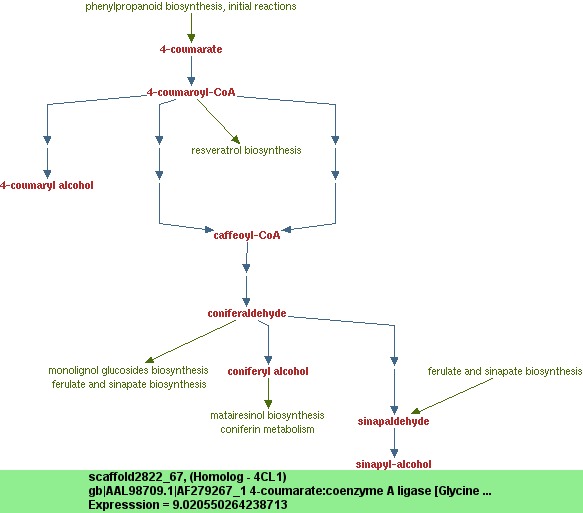

Supplement: Additional file 17 — Details of Transcription factor families. [file 1471-2164-14-647-S17.zip › Additional_file16B_Upregulated_PMN_pathways_in_Root/V2RS/scaffold2822_67_4CL1_3_phenylpropanoid_biosynthesis.jpg]

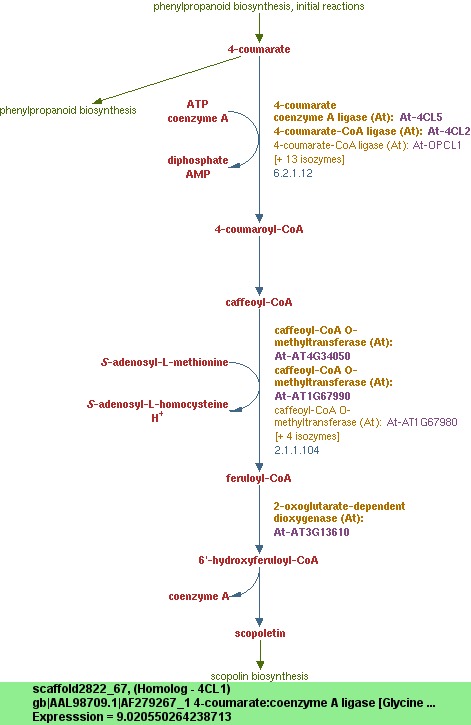

Supplement: Additional file 17 — Details of Transcription factor families. [file 1471-2164-14-647-S17.zip › Additional_file16B_Upregulated_PMN_pathways_in_Root/V2RS/scaffold2822_67_4CL1_4_scopoletin_biosynthesis.jpg]

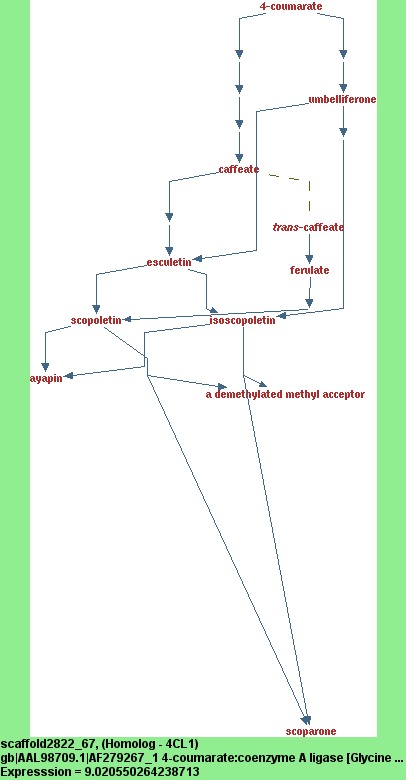

Supplement: Additional file 17 — Details of Transcription factor families. [file 1471-2164-14-647-S17.zip › Additional_file16B_Upregulated_PMN_pathways_in_Root/V2RS/scaffold2822_67_4CL1_5_simplecoumarins_biosynthesis.jpg]

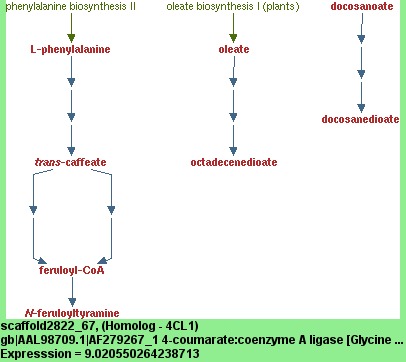

Supplement: Additional file 17 — Details of Transcription factor families. [file 1471-2164-14-647-S17.zip › Additional_file16B_Upregulated_PMN_pathways_in_Root/V2RS/scaffold2822_67_4CL1_6_suberin_biosynthesis.jpg]

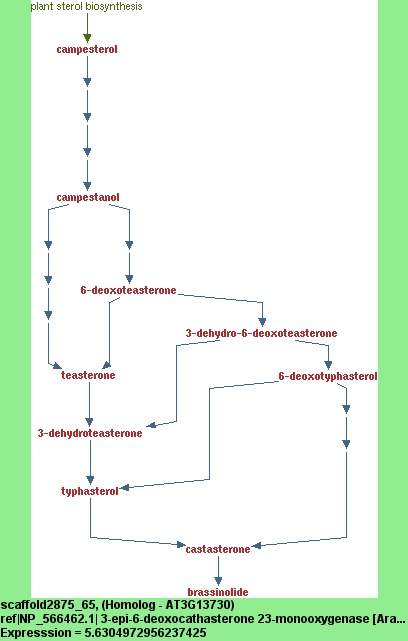

Supplement: Additional file 17 — Details of Transcription factor families. [file 1471-2164-14-647-S17.zip › Additional_file16B_Upregulated_PMN_pathways_in_Root/V2RS/scaffold2875_65_AT3G13730_1_brassinosteroid_biosynthesis_I.jpg]

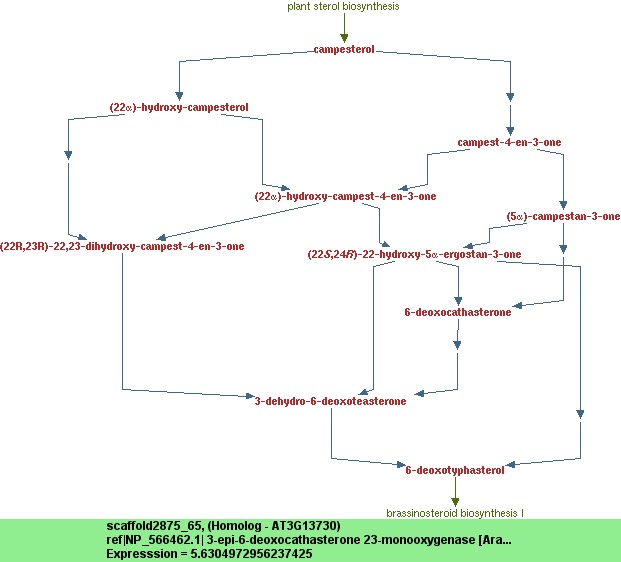

Supplement: Additional file 17 — Details of Transcription factor families. [file 1471-2164-14-647-S17.zip › Additional_file16B_Upregulated_PMN_pathways_in_Root/V2RS/scaffold2875_65_AT3G13730_2_brassinosteroid_biosynthesis_II.jpg]

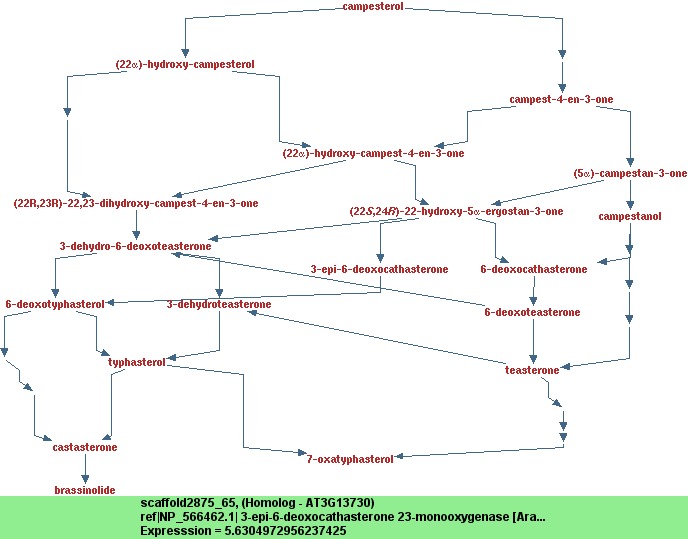

Supplement: Additional file 17 — Details of Transcription factor families. [file 1471-2164-14-647-S17.zip › Additional_file16B_Upregulated_PMN_pathways_in_Root/V2RS/scaffold2875_65_AT3G13730_3_superpathway_of_C28_brassinosteroid_biosynthesis.jpg]

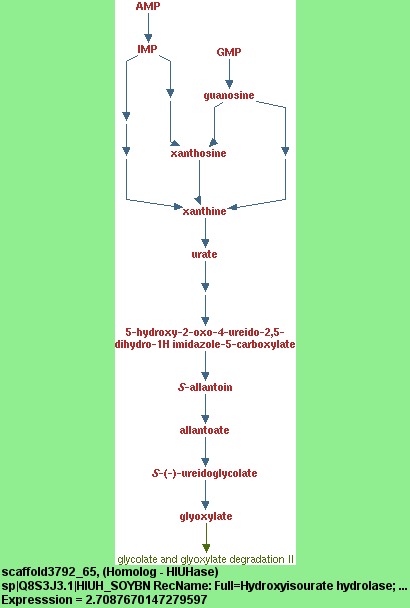

Supplement: Additional file 17 — Details of Transcription factor families. [file 1471-2164-14-647-S17.zip › Additional_file16B_Upregulated_PMN_pathways_in_Root/V2RS/scaffold3792_65_HIUHase_1_superpathway_of_purines_degradation_in_plants.jpg]

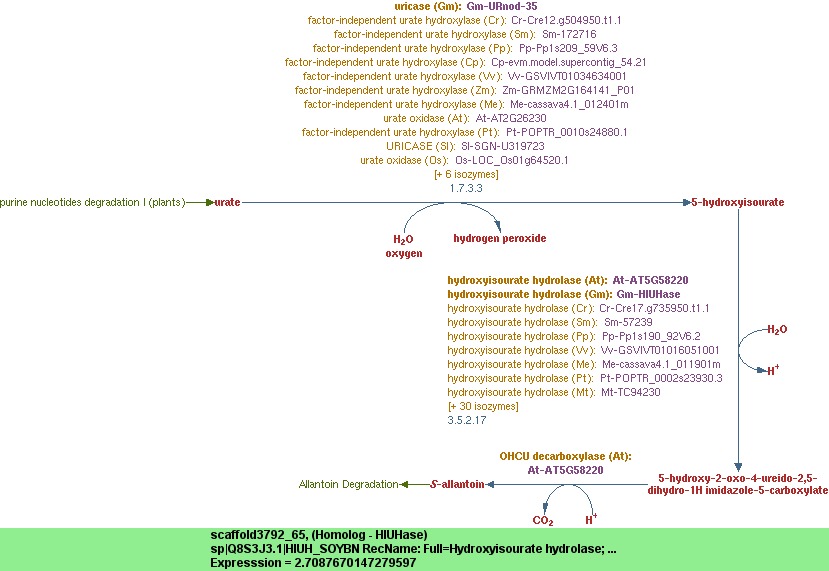

Supplement: Additional file 17 — Details of Transcription factor families. [file 1471-2164-14-647-S17.zip › Additional_file16B_Upregulated_PMN_pathways_in_Root/V2RS/scaffold3792_65_HIUHase_2_urate_degradation_to_allantoin.jpg]

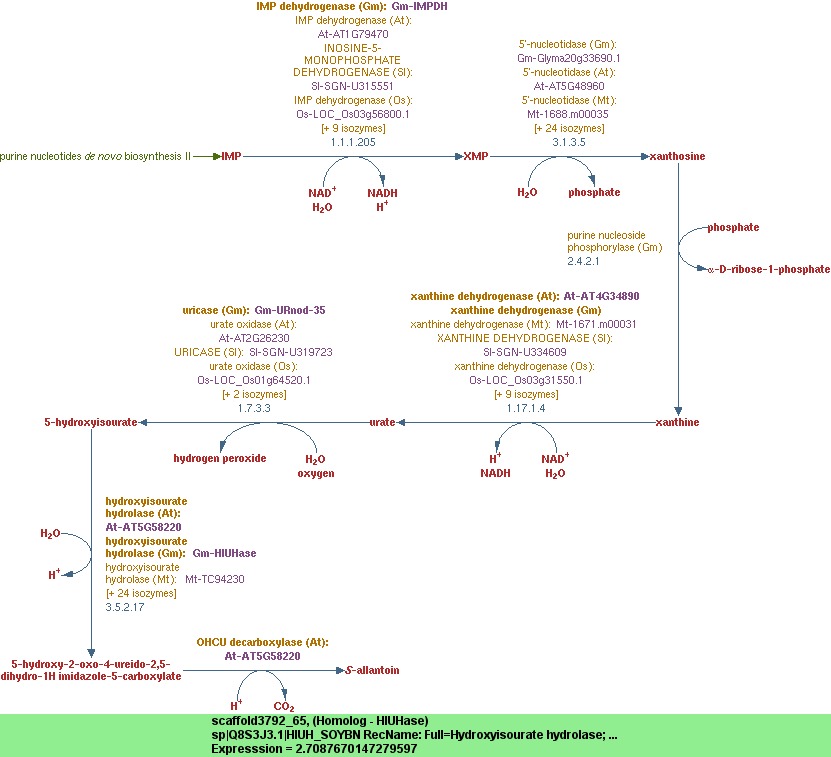

Supplement: Additional file 17 — Details of Transcription factor families. [file 1471-2164-14-647-S17.zip › Additional_file16B_Upregulated_PMN_pathways_in_Root/V2RS/scaffold3792_65_HIUHase_3_ureide_biosynthesis.jpg]

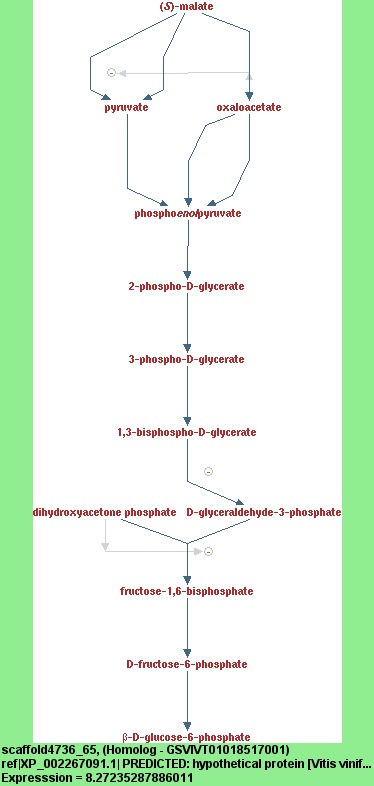

Supplement: Additional file 17 — Details of Transcription factor families. [file 1471-2164-14-647-S17.zip › Additional_file16B_Upregulated_PMN_pathways_in_Root/V2RS/scaffold4736_65_GSVIVT01018517001_1_gluconeogenesis_I.jpg]

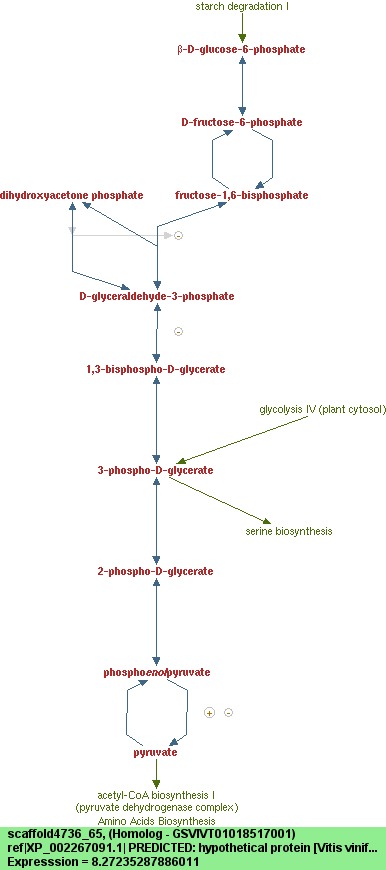

Supplement: Additional file 17 — Details of Transcription factor families. [file 1471-2164-14-647-S17.zip › Additional_file16B_Upregulated_PMN_pathways_in_Root/V2RS/scaffold4736_65_GSVIVT01018517001_2_glycolysis_I.jpg]

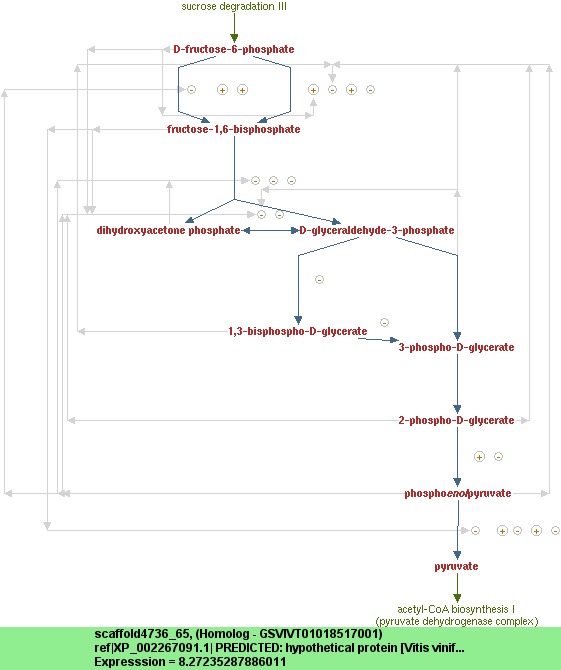

Supplement: Additional file 17 — Details of Transcription factor families. [file 1471-2164-14-647-S17.zip › Additional_file16B_Upregulated_PMN_pathways_in_Root/V2RS/scaffold4736_65_GSVIVT01018517001_3_glycolysis_IV_(plant_cytosol).jpg]

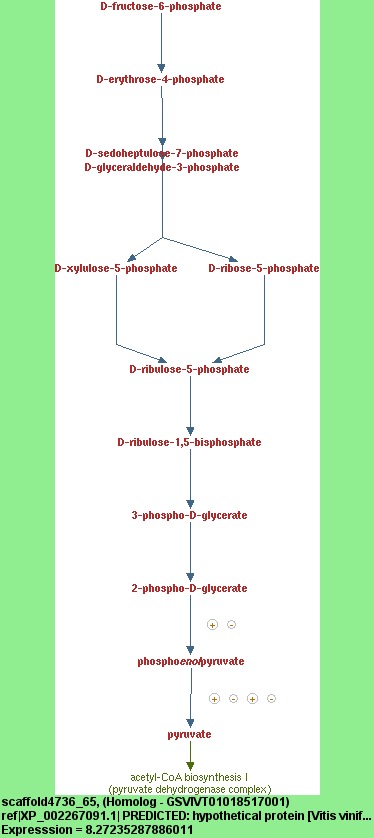

Supplement: Additional file 17 — Details of Transcription factor families. [file 1471-2164-14-647-S17.zip › Additional_file16B_Upregulated_PMN_pathways_in_Root/V2RS/scaffold4736_65_GSVIVT01018517001_4_Rubisco_shunt.jpg]

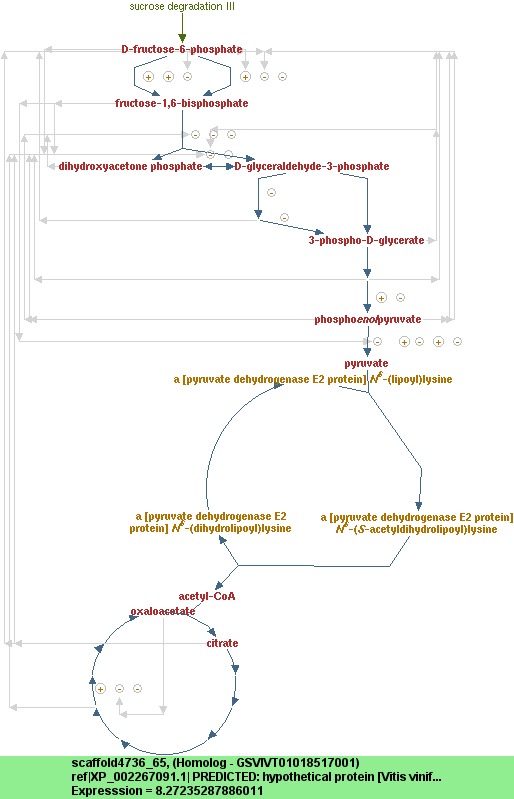

Supplement: Additional file 17 — Details of Transcription factor families. [file 1471-2164-14-647-S17.zip › Additional_file16B_Upregulated_PMN_pathways_in_Root/V2RS/scaffold4736_65_GSVIVT01018517001_5_superpathway_of_cytosolic_glycolysis_(plants),_pyruvate_dehydrogenase_and_TCA_cycle.jpg]

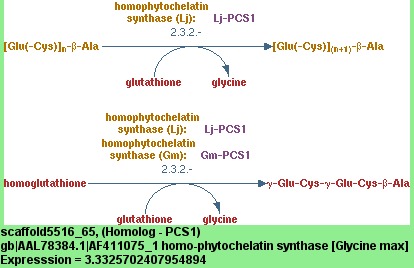

Supplement: Additional file 17 — Details of Transcription factor families. [file 1471-2164-14-647-S17.zip › Additional_file16B_Upregulated_PMN_pathways_in_Root/V2RS/scaffold5516_65_PCS1_1_homophytochelatin_biosynthesis.jpg]

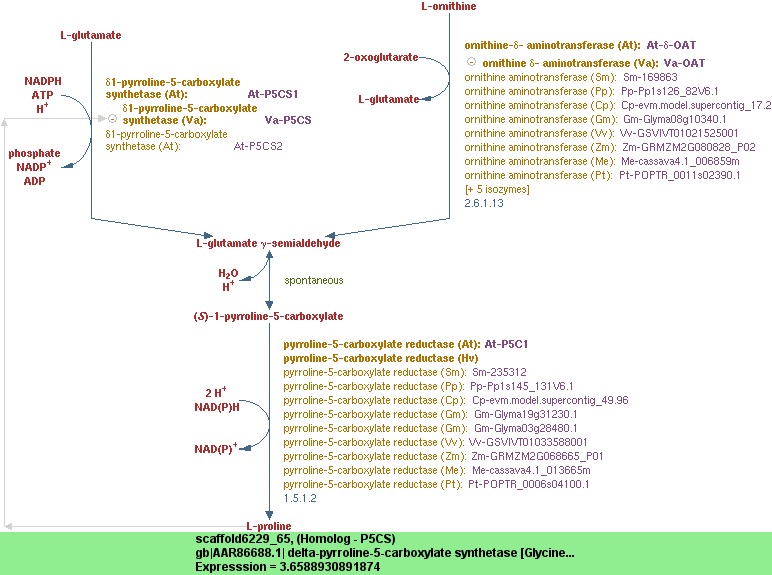

Supplement: Additional file 17 — Details of Transcription factor families. [file 1471-2164-14-647-S17.zip › Additional_file16B_Upregulated_PMN_pathways_in_Root/V2RS/scaffold6229_65_P5CS_1_proline_biosynthesis_III.jpg]

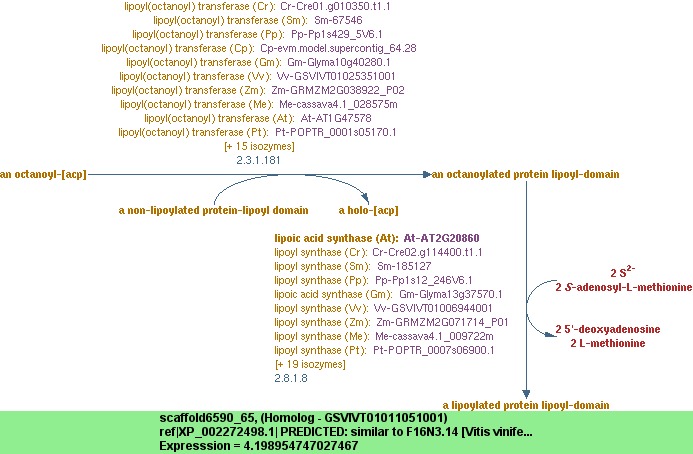

Supplement: Additional file 17 — Details of Transcription factor families. [file 1471-2164-14-647-S17.zip › Additional_file16B_Upregulated_PMN_pathways_in_Root/V2RS/scaffold6590_65_GSVIVT01011051001_1_lipoate_biosynthesis_and_incorporation_I.jpg]

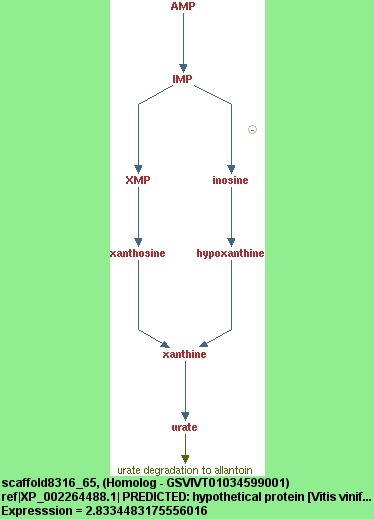

Supplement: Additional file 17 — Details of Transcription factor families. [file 1471-2164-14-647-S17.zip › Additional_file16B_Upregulated_PMN_pathways_in_Root/V2RS/scaffold8316_65_GSVIVT01034599001_1_adenosine_nucleotides_degradation_I.jpg]

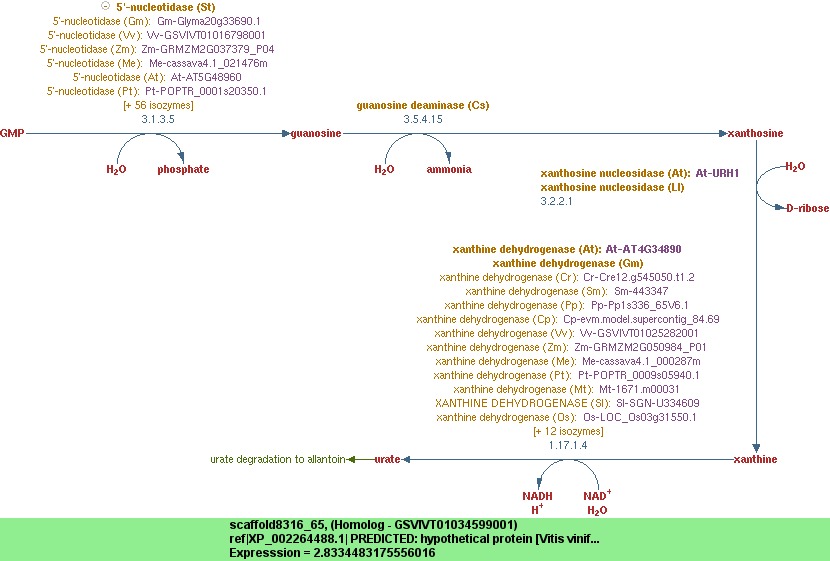

Supplement: Additional file 17 — Details of Transcription factor families. [file 1471-2164-14-647-S17.zip › Additional_file16B_Upregulated_PMN_pathways_in_Root/V2RS/scaffold8316_65_GSVIVT01034599001_2_guanosine_nucleotides_degradation_I.jpg]

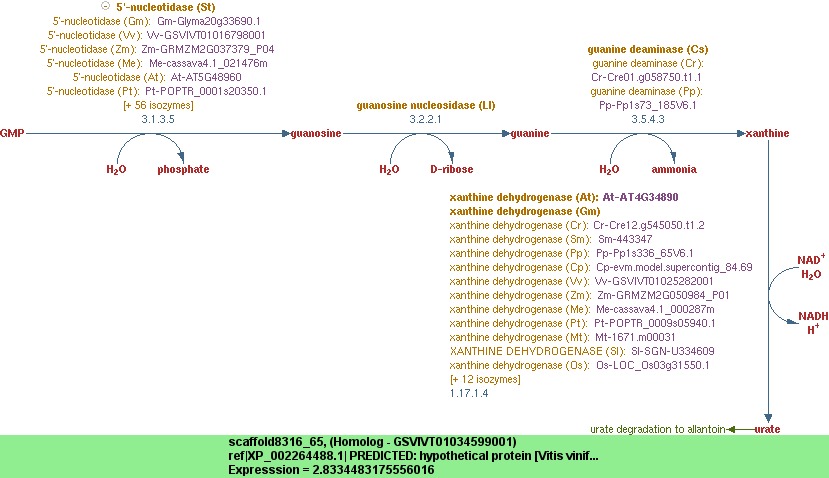

Supplement: Additional file 17 — Details of Transcription factor families. [file 1471-2164-14-647-S17.zip › Additional_file16B_Upregulated_PMN_pathways_in_Root/V2RS/scaffold8316_65_GSVIVT01034599001_3_guanosine_nucleotides_degradation_II.jpg]

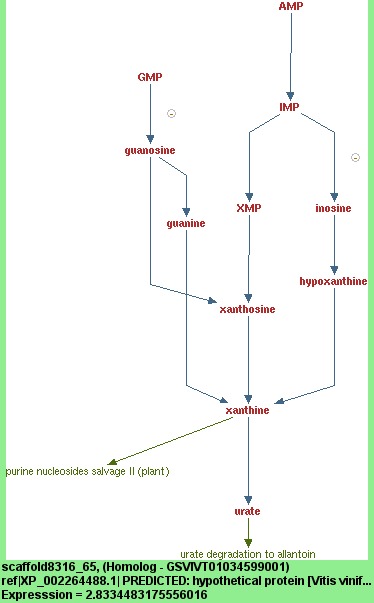

Supplement: Additional file 17 — Details of Transcription factor families. [file 1471-2164-14-647-S17.zip › Additional_file16B_Upregulated_PMN_pathways_in_Root/V2RS/scaffold8316_65_GSVIVT01034599001_4_purine_nucleotides_degradation_I_(plants).jpg]

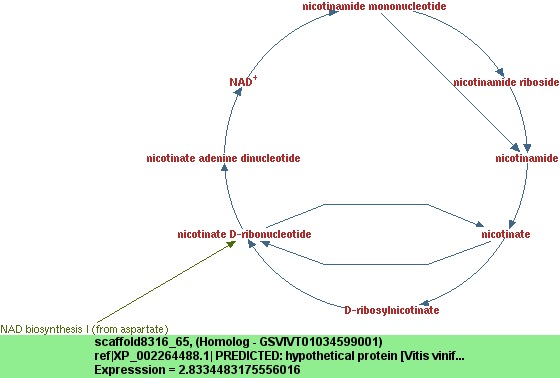

Supplement: Additional file 17 — Details of Transcription factor families. [file 1471-2164-14-647-S17.zip › Additional_file16B_Upregulated_PMN_pathways_in_Root/V2RS/scaffold8316_65_GSVIVT01034599001_5_pyridine_nucleotide_cycling_(plants).jpg]

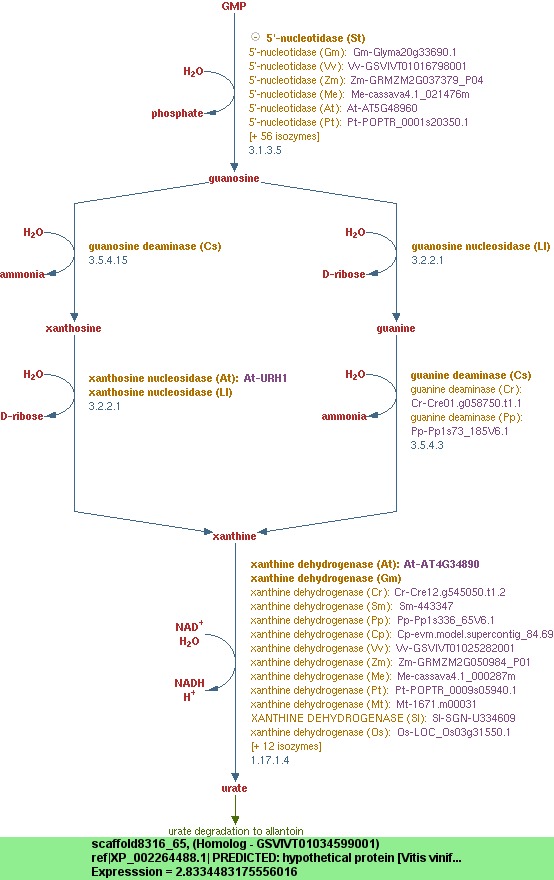

Supplement: Additional file 17 — Details of Transcription factor families. [file 1471-2164-14-647-S17.zip › Additional_file16B_Upregulated_PMN_pathways_in_Root/V2RS/scaffold8316_65_GSVIVT01034599001_6_superpathway_of_guanosine_nucleotides_degradation_(plants).jpg]

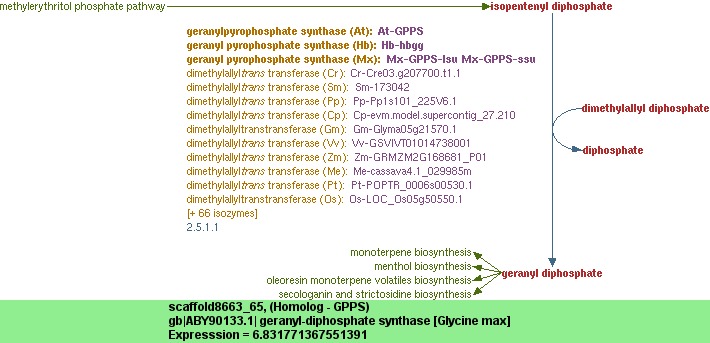

Supplement: Additional file 17 — Details of Transcription factor families. [file 1471-2164-14-647-S17.zip › Additional_file16B_Upregulated_PMN_pathways_in_Root/V2RS/scaffold8663_65_GPPS_1_geranyl_diphosphate_biosynthesis.jpg]

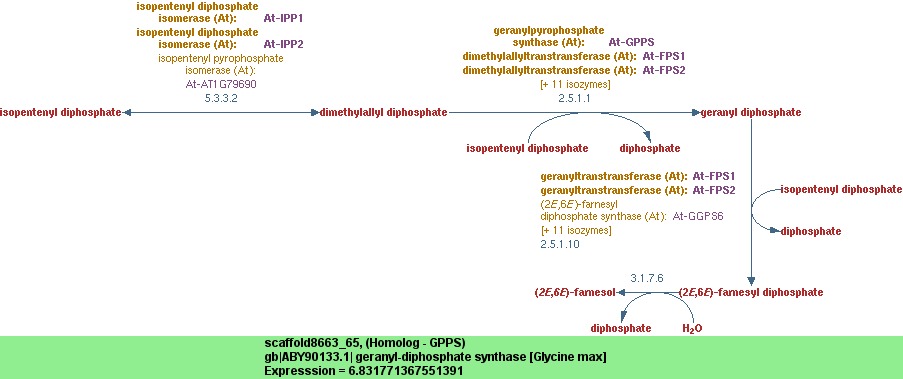

Supplement: Additional file 17 — Details of Transcription factor families. [file 1471-2164-14-647-S17.zip › Additional_file16B_Upregulated_PMN_pathways_in_Root/V2RS/scaffold8663_65_GPPS_2_all-trans-farnesol_biosynthesis.jpg]

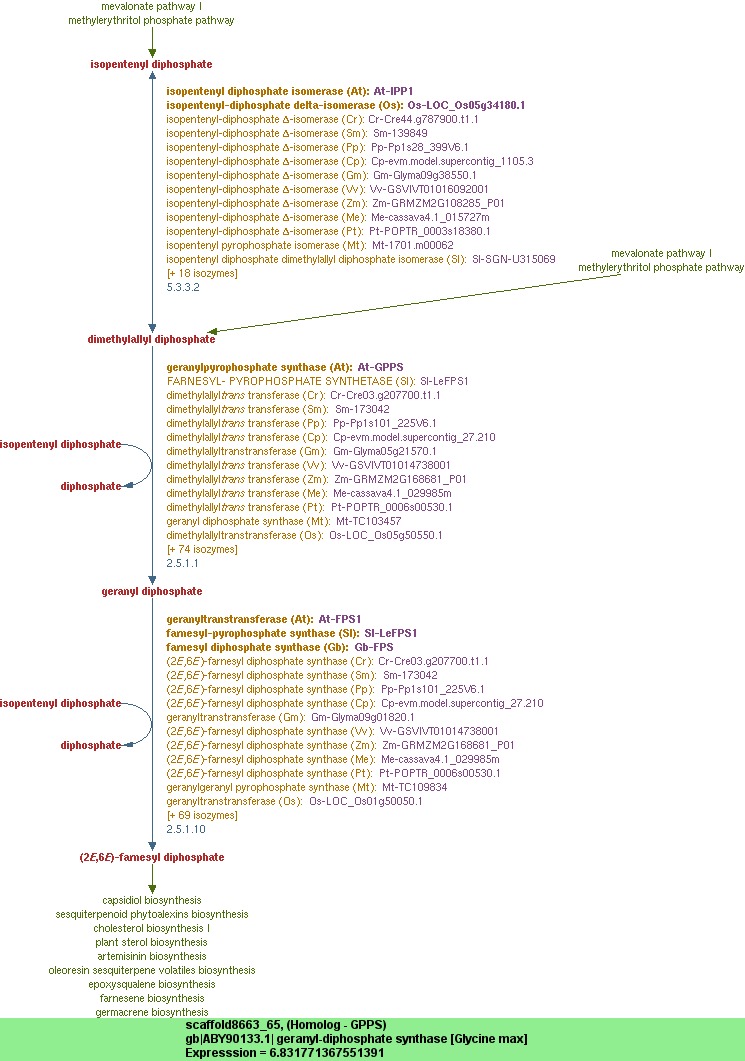

Supplement: Additional file 17 — Details of Transcription factor families. [file 1471-2164-14-647-S17.zip › Additional_file16B_Upregulated_PMN_pathways_in_Root/V2RS/scaffold8663_65_GPPS_3_trans,_trans-farnesyl_diphosphate_biosynthesis.jpg]

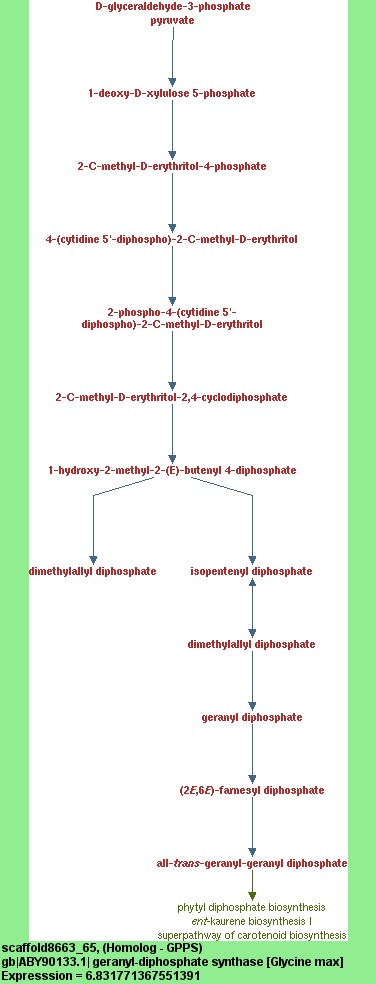

Supplement: Additional file 17 — Details of Transcription factor families. [file 1471-2164-14-647-S17.zip › Additional_file16B_Upregulated_PMN_pathways_in_Root/V2RS/scaffold8663_65_GPPS_4_superpathway_of_geranylgeranyldiphosphate_biosynthesis_II_(via_MEP).jpg]

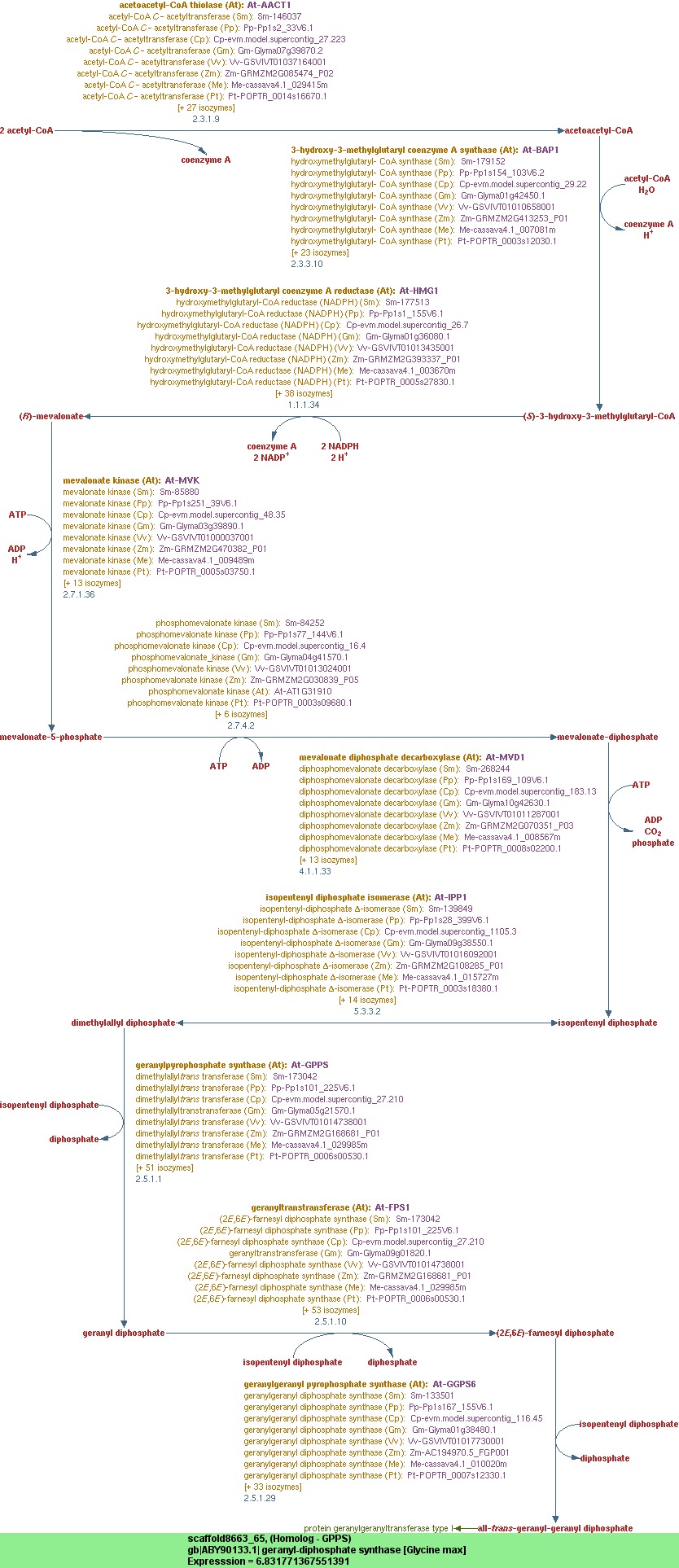

Supplement: Additional file 17 — Details of Transcription factor families. [file 1471-2164-14-647-S17.zip › Additional_file16B_Upregulated_PMN_pathways_in_Root/V2RS/scaffold8663_65_GPPS_5_superpathway_of_geranylgeranyldiphosphate_biosynthesis_I_(via_mevalonate).jpg]

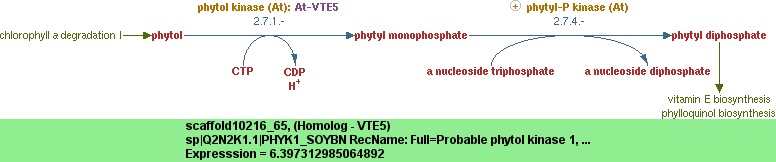

Supplement: Additional file 17 — Details of Transcription factor families. [file 1471-2164-14-647-S17.zip › Additional_file16B_Upregulated_PMN_pathways_in_Root/V2RS/scaffold10216_65_VTE5_1_phytol_salvage_pathway.jpg]

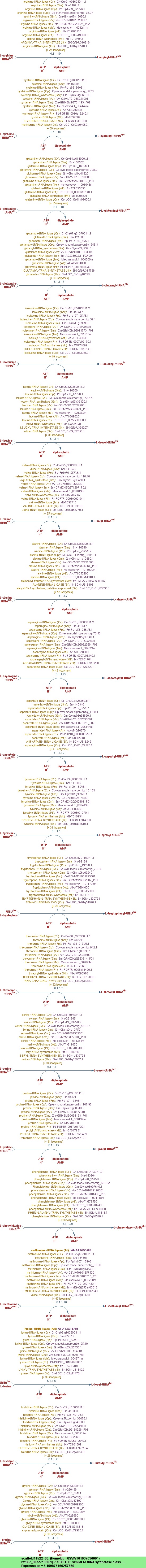

Supplement: Additional file 17 — Details of Transcription factor families. [file 1471-2164-14-647-S17.zip › Additional_file16B_Upregulated_PMN_pathways_in_Root/V2RS/scaffold11522_65_GSVIVT01037036001_1_tRNA_charging.jpg]

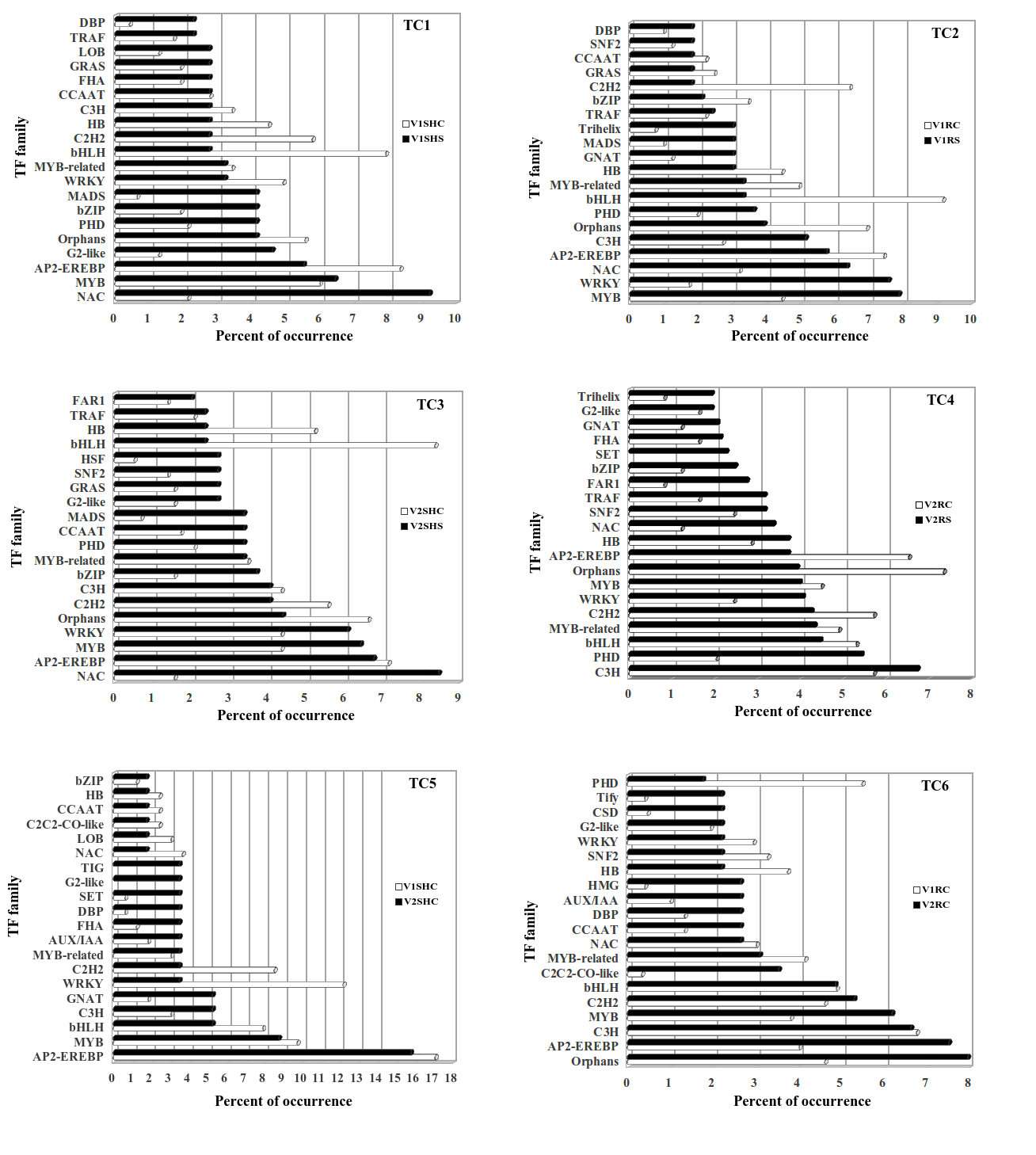

Supplement: Additional file 19 — RPS BLAST results for unknown sequences. [file 1471-2164-14-647-S19.png]
