# Supplementary material for: Comprehensive transcriptomic study on horse gram (Macrotyloma uniflorum): De novo assembly, functional characterization and comparative analysis in relation to drought stress
Source: BMC Genomics. 2013 Sep 23;14:647. doi: 10.1186/1471-2164-14-647 (PMC3853109; doi:10.1186/1471-2164-14-647)
Supplement: Supplementary file 18 [file 1471-2164-14-647-S3.odt]

>C100005_65

ATGCATTTAATAAATAAAGGGATTAATACTTGTAAAAGAAATACTATTCTTTGGAAGAATGCAGTACTAACAGACCATAGTCCAGTATTGCGACTGCAGAACCGCTCATCAAAGTAAAAAACATTCAACTCAGACAATTATTTCCTTTATAGTCTTTATACCATAGTCAACAGCTGCCAAGAAACAGATTACCTTTTTCCTTTTTTTGTTTTCCTTCAGTTCGATCCCAAGATAATAAGCATATAATGTAAACACAGTAAGTCTTTCTACAACTAAACCGAACAGTGGTAGTTGAGTTTCTGATGTTTCTTGGCCGTCCTAGTTACTTTCCACAATCACCTAGTACTATGGGATCAACATTAGAAATACAACTTCTCCCCATGCAGAGCAGGAAGTGGGACAGTAGTTATAATTAACACACAGAACTGCTACCGTTAAACGGTTAGTGATTACAAACAACATTGCGAAAGTAAATTGGATCAAGGTCGTCATATCAGTGGTTACCATCTTCGAGGGTTGTCATTTCTATCGGTAGTAAGCCTGCTGATAAGTGGGTGCCAAGACATTTCCATAGCCACCATATGCAGGATAGTTCATGGGAGCCGCTGGGTAAGACCCTGCAATAGCTGGTGCAGGCTCGGGCGAATAAGCAGCATAAGGATTTGCTGGGGGACTTCTGCTGCCATATATGTGGGGTGGAGAAGGGTATGGTGGAAGAGCAGCTGGGTATTGCCCGTGTGATGGAGACCTAACAAATGTAGGAGCAGCAGGGAAAGATGATACATACGCATTAGTCAAACGCCCAGCTTTGGCTGGTGGCATTGGACCGCCATTGCTATTGCTTGCTCGAGTTCTCTTATTGGCAGGCACAGCCGCCGGTTTCCTTTTCTCGGTCTTCACTGTCTTCACCTTCTCCAACTGTTCAAGTCGCTTCTTCAGATTTTCTGGAGGGAACTCAGCCTCAAGTTTGTATTCTTCAATGCATTTAATCACAGCCCTGAGTGCAGACTGCTCTTTCCTTGCAGCTAGGTACGCAGCTCGGCCTGCATTATTAGGATCTTCCAAAATAGAAGCCGCAACTTTCTTCGCATCCTTGAGAAAAGACTTCAACAAAGGAACAGGAGGGAACTTGTCCACAAGACCCACTTCATATGTAAAATGAACCGCATCAAGCTGCTGCCCTTTGCTGATCAACTCCTCAATCATATCAGGCATTTGCTGAGAAAGACCGAGCGAAAGTGCGAGTTTCGGCATCTGTTTCCTCCAAGCGGAAGCAATAACAAGCTTCCGGTATAAATCCAAGTCCTCGTTCTTGACAATCCCAAAGGTGACCACGTGCTGCAAGAAGGTGTGGACGTCAGGGGTCTTCACGTTTTCCACACCGCCACGCTCCTCCAGGCTGGCCTTCCAGGTCTCCGCTATCTCCGTGGCCTGCTCCTTCACAGTGGGGGTGACCAACAGCCTCGATTTTCCGATGACCGGGTCGACAAAGACCGGAATCAGAGATTCGAGGACAAGGACGCAGGCCCAGCCTAGGTCGTGGCCAGGCTTCTCCCCTCTCTTGTCAACTGGAAAGACCTCAGAGATAGCCTCGAGGACGAATTTGGCTGGATCGACGCATTCGGCGAGAGCGACAGGGACTTCGGCGCGCAAACCGTCGAGCTCCTTCTTCTTGGCGCTGAGGAAGGTCCAGAAGCCGAAGGCGTCCATGCGGAGGCAGAAGGACTTTAGTTTGAGGACGAGGCCGGTGGCGTCGTCGACCTCGCCACAGTCGTCGGCGACGTGATCGTCAAGGAAGGAGGAGAGGGCGGCGGTGCGGCGCGTGTCGAGCGTGCGGAAAGCGATCTGGAGAGTGGTGTCGAGGGAGGTCTCGCGGTGGTCGAGGAGGCGGAGGGAGTCAGAGGTGGCGGTGTCGAGCGTGCGAATCTTGCGTTTAAGGGCTTCGGATTTGTGCTTGAGGTCCTCCTCGAGGGAGGAGAAGTGGTCGGAGAGCTCCTTCCAGAGTAGGGTGCAGCTGGTCATGAGAGAGGTTTGGCGCTGGAACTCGTCGAAGCTCGGCTGAGTCAACTCGCTCAACTCGCCAGGATCGGGGATGGACCCCATGGTCGCGAACTGAAGAGAGAACACACGAAAACCCTACCTCACTGCTTCACTCACTCTCTGCGTTCAATCAATCAGAGAAGAAAGAAAGAAAGAAAGAGCGCAACAAAGAGTAAAGGTGGTGCTGGTAAAATG

>C100009_65

AAAAAAAAAAAAAAAAATAGCAAACTCTACAACAGATGGCACAAAAAAGCCAACTGAATGTTGAACTTCACATACCAGGAGCAGGCTGCATTAATAAAGGTAATGGACATTGATCCACTCACAACCTGTTTACTAACCAGACGCATTCTTCCTTGCAGAATATACCCTGTAAGCTGCCAGCCCAACTACGACCACTGCAGCTCCAACTGAAACAGCCATTAAAGATCGATTAATTAGCCTGTGGTACTGCTTGCGGCTTCTTCCAGCTTCAGTTTCTGGAATGCTTAAATGAGGATGCTCCGCAGCAATTACAATTTTATGGAATAGACTGTTGAAATCTCCTAACTTCACACTGATTGGTATAGGAGCCTCTACTCCCATATCTTGACTAACCCTAGTTGACTCCTGAATAGCCATTGGAAATGAATCCAAATCATCTTTGGCGGCCACTATCAGGCAGGGCACCTCAAAGCCAGTATCTTCTCCATGACTAGCAATTTCAACCAGGAGTTCAGATGATGTTCTCCATGAGGATTCATCAGACCTGTCATGAACAAAAACTGCAATGTCACATGAGGCCAAAGACTCCTTATTGGATAGGAGCTTCCCAACTCCATCTTCTGGAATCTCTCTCAGCACAAGATATTTTTTGTTTTCCATAGAAATATCAACGACATTTACACCATATCGGTCTTCAGTGGTTGGATTGTAACTTTCAGAATATGGCCTTCCAATAAAAGAATTCAATAATGCAGATTTCCCAGCCTTTCTAGGCCCAAAAACAAAGCACTGCAATACATTTCTGTCTGAATGTTGCTTCTTGCGATCCAAACGCCGCCTCCTAGTCACACGGATAGCAGATGATGGATCACCAGGATAACCAATGTAAATCAGATTCTCCACACTAAAAGCTGGGTTTAGAAGTGTCATAAGTGCCCACTCTGACAAAAATGCATCTAGCGATAGTCCTCCAAATGCATTTTTTTCTGCAGCATCCTCAAATGGATTTCCAGTCCAAGGACTTTCAGGGGCAGTAGAAAACAACTCTTCGAGTTCCCGAGATCGTAACATCCCATCCCCATCACCATCAAATGCATCAAAAATTGCCTTCAAAAATTCATATGCTTCATTTGTCAGCTCCACACTCTGATCAGGAGCACGTTTTATAGGTGGAATTAGATCATCAGCAAGCTTGATATCATCATTATATCCAAACTTCCTTAGCACAGTCCATGTTGTCTCGAGACGCCCCTTTTCTATGAAAAGTGCATGAAGAAATAGGAACCCAGTCAAAGTAAGCCCACGTTCATTCACCCCTTCAGACATTTTTTCTTGTACAACCTTCTTTACACCCACAATTTCAGATGGTTGCAAAGGAGCATTGAAACATTTAACCTGAAAATCATTCAGCTCAGCATCACTAAGGGCACCATCTCTGTCATGGTCACAGAGGATAAAAATCCGCTTCAAAGCTCGCACACATCGAGGCTTTAGAGTTTGAGACTCTTGATCAAATAATGGAGCTGTTGGATGAAGAACAGCCTTTTGTGCATAGTAGAAAACTTCGGGGACCTGAATATGCCTAGATGCTGAACACTCAATGCAAGTTTCAATCTCTCTAAACTGTTGCATTATCGGCGACATCACCTGTTCTAGGCTCACCTGCTGATTTTCATCTCTCAAATCAAGCTTACAACCAACTACTATAACTGGAACTTTCACCTCTAATTTGCGAAGATGTGGAAGCCAAAATATACTCAGATTTTCAAGTGTTTCAGGTCGATCACATGCGTATGTAAGGACAACTGTGTCAGCCCGCTGCAATTCTTCAGCAACTTTATCACTATCCTCAGCACGGGAGGAGGTGTCGATGATGGTGATAGGAACGCGATCCGGATAGAGATCCTCAGGCAAGCGCGTGGGAGGCAACACCGGCGGCACATTCACCGGAAAGTTATCGGCGGCGGCGGTGATGATGAGGCTGGATTTTCCGGTGCCTTGGTCTCCGGCAACGACGATACGAACTCCAGTGCGGCTGTGAGGATTGACGGTTCCAGAAGAGGGTTTTGCCATGGTTGGTAGGAGCGGAAAGAGTCCTAACTAGACGGTGCTTGTAGGTGGCCGTCTGGAGCCTTCACTTCTTCCGCGGTTAATATTTGTAGCACCTTTACTTCTCTTCCACCTCAGACTACTGTACCACACTTTCTCACGGAATTCGCTTTTCTCCTTAAAAAC

>C100017_65

CGCTGTTATGATTTGTGAATTCTCCAGAGGATGATGAAGCACATAACATATAACACTTGAAAGAATCACGCAAGCATGTAACTTAATATACAAGGTTTAAATAATGAAATACAGGAAAGTTAGTTTGACAACAAATTGGGGGAAAACCAATCAATTCCGTAATGCCTAATGCCTTTCCTTAATAATAGTATTGAAATGGATAAGAAGTTTATCCCACCTCCTTTCAATCACTTTCAAAGTCTTCATCCCCAAAGGAAAAAACTGAAGCATCTGCTGCAATTGCCTTGATGTCACTTGCCCCTACAGATCCCAAGGTTGTGCTGTTATTTCCTGTATGAATATGTTTATCTGCAGTTGATTCTTCAGAAGAAGAATTGTTTTGCATGCGTTCCTCATGCTGTAGTGACATCCTATCTGTTGTTTGAGTCAACGTCCCTACATGGTCTGTAGTCCCCTCAGATAATTTATGAGGTGCCATAAATTTGTACTCAGTTGCAGCTTGCTTGCTTCGCGTATCTCGGGTCTGCTCTTCATCACTGTCTGAGAATACATCATCTTCTCCATTTCTTGGCAGTTTACTCTCACTTGAATCAGATGTAGTAGTCTTTGGACCAGGAAGGGCATTGCTTGAGTTTCCATCAGATCCTTTGGTAGCAGGATTGACTTTTGTCCTAGCATTATAGTCTATCATGACAACTTCCACCCGGAATCCGGGTGCAGGGATCTTTCTCTTTTCGAAACCATCAAAATCTGAACCATCTAAAATTGTTCGGTTTTCTGTCATTGTTGTATTCATCCAACAGTAGAAATCTCCATGTCTATCATCAAAATGAATTTTGAAGTCTCCTACCAACTCTGCTAGACCAGGCTCTCCCGGTAGTGCAAAAACCACAATTCCTTTCTTCGGTGCACTGATCCAGAAATCTTCTGGCATTAGATCTTTTGTTTTGGGATGCCTTTTTGTGGCAAAAAGAATTCCCTTATGATCAGAAATTGTAATAGACGGTCTAACCCAATAAGGGCACTTGTGAAGCCGAAACCCTCTTAGTATGCACCTTCGTCCAGGCTGGACTTCTCCATTGAATTGTGTTAAAGTCCGTTCAAAGTATTTTACATATCTTATTTGACTAGGGAGGACAAGAGCCTTTCCATCTATACATCTTTTATGGTTAAAGTAATCAATGGCTTCTTCTGCAGTTGGGAAGAACTTAAGAGACAAAAGAAGACAACATATCATCAGTCCAGTCCTCCCCAGACCAGCTTTACAATGAACAACGACCACATTTTGAATATCCTCCTTTAACCATGAATATGCACTTTGACAAAATGATGCTATAAGTTGAATAGGAGGGCAATTATGATCACTAAATGGGAAAGTTGCAACCTTCCCCTGGAATAATGACCCATCATATAACCTTTCTGAACAAAGATTGTATACTTTGTATTTACCCTTATGATGTGTTTCAAAAAACTTGATCACTTCTTCCATGTGATTGCGATAGAACCCCTCAATGTATCCAAAAATTCCAGAGCTCAAATCACCACCCGGAAATCCCATAGCAATTATATTTTCAGTGATATATGTCATATCCAAATCAAATCCATCTTCCTGGTATCTACGTTTATTTTGAGAGACAATATGGCGTGCTTTGACCTGCACTGCTTTTACTGCACTCTTGGAAGAGTCCACCAACCCTTTGGTGAAGGATTCAATCAAATTAGATTGCTCGGTTCCTGAATTGACTGGAGCAGCAGTTTCATTTGACTGCGTTCGCAACCCAAGCCCACCAGTCAGGCGAGCAAAAGCTGACATGCCTGTGTTTTCGGTCTGTGAATTCTGTTGTGCTTGTCCAAGGCCAAGGCCTGGGAATTTCAGATTTCTAGCCCATAATGATATGCCAGAAGCAGGTAACGATGACGGTGAATCATGTGCAATGTTAAACTGTCCAGACACTTTGGTTGAGCGTTGTTCTTCAGTATTAGAAACTGGAGGAGTAGCTTGAGATGAAGAATCAGCTGAAGCTGGTTCCATGAACACTCAAATGCACTTGGTGAGTCGATGGTGATGCACCCTATCCGATCTGTTTTCCAAGGAACAATGGTTTTGTGCAGGGGAAACACACGATTTGCGAGTTTGGGAATTAGATCAATGCAGTTCGATTGGAGTCACTAGCAATTACACAACGGAAATCCAAGGGGAAAATGTGTGAAAAATGAAGATTCATATAAACAGGTAG

>C100019_65

GGGTTCTAGAATTGTTCCCTTCACTCTATCCGCCATGGGTAGGCCATCCTCAACCATCCAAATCCCTCATGAACGCTACCGTCATAACGCTCGTCGCACACCTGACTCCGATGCCTCTGACGAGTATTACCGCCGTCGCAGCCCCAGTTATGACACTTACGACCGCCCCACGGACCGCCGACAGCGCCGTTCGGAATCTCCTACACACAGAAACCCTCGACACACCAATGGACTTCCCAACGAAGACGCTCTTCCGAAGAAATTTGGTCGTCGAAACGGCGCCTATCTTGACCGCGACCGCGGAGATTGGCAGCGTTCCGAGTCGGAATCCGACGAGGAGTTGAAGGGTTTGACCTACGAGGAATACAGAAGGCTCAAGCGCCAGAAGATGAGGAAATCGCTGAAACACTGCATCTGGAACGTCACGCCGAGTCCTCCGCGGCGCGACATCGAAGATCTGGAGGATTATAGCAAACCGGATGAAATCTCTGACCGAGACGACGTCGCTGGGAAGATCGAAAAAGAGGTTAAGCCAAGAGGAAAATTGATATCGGAATCTGATTCCGATTCTGAATCTGATAAATCTATCGGCAGTGAATCAGACGATTCGCGTTCGAAGAAGAGAAGGAAGAGTTCTGCTGGTTCGTCGAGAAGATCGTATAGCGAGAGTGGATCAGAATCTGAATCCGAATCAGAAGAAGCGGGTCGTAGACGAAGGAAGAGTAGGAGGAATAGAAAAAAAAGAAATAACAGGAGCAGCCGGAAGAGAAGATATAGCGATTCTGATGAGAGTGAAGAGAGCGAGAGTGGTGACTCTGACAGCAGCGGGAGAAAAAGGATGAAACGTTCTTCTTCTTCAAGGTCTCGATCGAAGCGTAGCAGAAGAAGCAGAAAAGGAAGAAAGAAAATTTCCGAGACGGAAAGCGAGGGTGCTTATTCGGAGGAGGAGAATGGTTCCGGTTCTGATGATGGTAAGAGTAAAGTTACGATTGATGAGGTGTTGAACTCGGAGATTAATGCGGAGGCTATAAAGTTAAAGGAATTGTTTGAGTCTCAGAAGAAACCCGCATTGGATAACGAACCCTCTGTTGGGCCAATGCCGTTGCCTAGAGCTGAGGGGCATATAAGCTATGGTGGAGCGCTTAGGCCCGGTGAAGGTGATGCCATTGCACAGTATGTTCAACAAGGGAAACGTATTCCGCGAAGAGGAGAAGTGGGTCTTTCTGCGGAGGAGATTCAGAAGTTTGAGAGTCTTGGTTATGTGATGAGTGGTAGTAGGCATCAAAGAATGAATGCCATTCGTATAAGGAAAGAAAACCAGGTTTATAGTGCTGAAGATAAGCGTGCTTTGGCTATGTTTAACTATGAAGAGAAGGCCAAGAGGGAGCATAAGGTTATGGCTGATCTCCAGCGCCTTGTGCAGCGCCACATTGGCCAGGATGTTGGTCCAACTCATGATCCTTTTGCTGCAAAAGCCTCGGATGCTGCTGATGCATGATCCGTAGTACTGATGATTGGTGAGTAATTTCTCTGAAACGCCTGTAGTAAATGCTGCCTTTCGACTCCTACTAGAGAAGGATAAAATCTCAGTGCGTCGTAGCAAGATTGTTTTTTAAATTATAATCCTGTTAAAAGCTACATGTAAGCATTAAAATTCCTTGGTTTTGGCAACCTCAGTAAGATATCCATGTGAAGAATTATTGTAGACATGCTAATCAAATTGCAAAGTTTTTGAAACTGTAGCTCAACAATATCCCTGGTGTTGTCTAGTCGAAGAAGGATTAGTGTAGATTAGCAATAGCAGCAGCCAATGCCTGTTTGTTCATTGTATTTACAAGGCATATAAATATACGAGATTAAATATAAAAAGCTCAATATTCCAGCAACTGTATAGATAGTAGAAACACATAAACACATAAACCTTATTACTACTAGGCTCATGGCTGTGATGATGTCCTCAATATTCTTGTTTGCGGTGGGTGAAGGTCGACAATCTATAGATTTTGGAGTCTGGATCAAGCTTAGGAGTGGAGAGTGTAAGCGGGGTTGTATATTTTGAAAGGAAGCTTTCATATTAATCATGCAACTTGATGGAAGCTTCCACGTAAATGGGCATAACATGGTCATAAATGAGTAAAAGTGGTGTGCATTGCCTGGGCTTTAATCTATTGGACCTGAAGAAATTTGTATTCAAGTTGAATTCTGAATATATCAGCTACTGCATTTTACAGAATGAG

>C100021_65

CCAAAACCCTCCACCACCACTATCTGAACAGACACCATGCAAGAAATCGCTTGAACTCAGTATAAAATACATCGCCCCAAGCTCAATCGCTGTTATCGGTGTCGGAGAGAGTTGAAGCAGCGAAATGCCTCTCGGAGAAAGGTTAGGGGATAAGAGCGAGTCTCGCTACTGCGGTGTGGAGACCGATTTCAACGATGACATGCCACACGTCCTCCATTTCAATCTCTATTATGGAAGCTTTGATTTCGTTGTTGCTCCTCTGATGGATCCTTCTTATCGACCAAGTTTAGTGAAAAAGGATGGTTTGGGGTCTGGTGTCCTGCCATTTGCTGGGTCAGACTTGGTCTTGAGTCCATCCCAATGGAGTAGTCATGTAGTGGGAAAAGTTAGCTCATGGATAGATCTGGATTCTGAGGATGAAACCCTCAGGATAGATTCTGAAGCTACACTTAAACAAGAAATTGCTTGGGCTTCACACCTCTCTTTACAGGCCTGCCTGCTTCCTGCTCCCAAGGGAACTTCTTGTGCTAATTACGCTAGATGTGTTAATCAAATCTTACAGGGTACAAATAATATTCAGTTGTGGCTTAGAACACCATTGATAAAGCCTGATGATGACTCTATGGATGCAAAGTCTGTTACTTTGGATGACTCTTGGGAATCTTGGAATTCATTTCGTCTGCTGTGTGAGCATCATAGTCAATTGTCAGTTGCACTTGATATTATGAGTACTCTTCCTTCCACAAATTCACTAGGACGTTGGTTTGGGGAGCCTGTTAGAGCAGCTATATTAAATACTGATTGCTTCCTAACTAATTCTCATGGCTGTCCATGCCTCTCCAAGCGTCACCAAAGGTTAATTACTGGTTTTTTTAACCATTCTATTCAGATCATTATAACGGGAAGTTCTGTTCATCCAAAAGCAAGTATGGATGCAAATGATAATTCTGATAAAGATTCACAAAGACATCCCTTGCGGCCTTATCTGGACTATGTTGGATTTCTATACCAAAGAATTGATCCTGTTTCTGAGCAAGAACGTTTTGAGCTTGGTTACAGGGATTTTTTGCAGTCACCCTTGCAACCTTTGATGGATAATTTAGAAGCTCAAACTTATGAGACTTTTGAGAAAGATGCAGTCAAATACATTCAGTACCAAAGAGCAGTTAGTAAAGCATTGCTGGACAGGGTTTCTGATGAAGAGGCATCTGTTAAAACAATTGTATTGATGGTTGTTGGGGCAGGGCGTGGGCCTCTTGTGCGGGCATCGTTGCAGGCTGCTGAAGAAACTGGGCGGAAACTCAAAATTTATGCCGTTGAGAAGAATCCAAATGCTGTTGTTACCCTGCATGCATTGGTCAAGTTAGAAGGATGGGAGGGCACTGTCACCATAGTCTCAAGTGACATGCGTCATTGGAATGCTCCTGAAGAAGCTGACATATTGGTTAGTGAATTACTAGGTTCTTTTGGTGACAATGAACTCTCTCCTGAGTGCCTTGATGGAGCCCAGAGGTTTCTAAAGCAAGATGGAATTTCAATACCATCTTCGTACACAAGTTTCCTCCAACCAGTGACAGCTTCAAAGTTATACAATGATGTTAAGGCACATAAAGATCTTGTACATTTTGAAACTGCTTATGTTGTGAAACTACACAACGTAGCCAGACTTGCACCATCTCAACCTGTTTTTACATTTACTCATCCAAAACGCCCTGAACATGAAAGCAATCAGCGATATAAAAAGTTGAATTTTGTGATTCCTAGTGACACTGGGTCAGCAATGGTACACGGATTTGCTGGTTACTTTGATGCAACACTTTACAAGGATGTGCATCTTGGAATTGAACCTTCAACGGCAACACCAAACATGTTCAGTTGGTTTGCAATATTTTTCCCATTAAGGGCACCCGTTTGTGTGGGTCCTGGTTCTACACTTGAAGTGCATTTTTGGCGCTGTTGTGGTTCTACAAAGGTTTGGTATGAGTGGTGTGTTGCTTCTCCTTCGTCGTCCCCAATTCACAATAGTAATGGACGTTCATACTGGGTTGGGCTTTAGAGCCCTGTATTCTCTGGAATATTATGAATACAAAGTTTTTTAGAGGAGGATCCCTTCATTTCGATTCATTATGGGAAATTCTGTTGTACAATAAGCCATTTTATTAGCTAGTATTGTCCTAAATGCATCCATCTATAATGTAGGTAGTTTTATAGTAACCGTATTATCTTAAGTATGTTTC

>C100023_65

CTTGACGTACTTACTAGTCCCTTCTTAGTTCTTACGTTGTTTTCCCGGCAACAAGACCAACTCAAAAACCCTAATTTCCCATCTCCACCGTTCACTGAATTATAGTCTTTCTCTCGCTTCGTCTCTCTCCAATATTTCCTGTCAAATCGATTCATCGTTTTTTAAATTTTCCAATTTGTCTTCCCCAATTTTTTCTTCTTCTGTCACCGTCTCAGAGCGGGTTTGAGTTTGAAGGTCCTTCAATGGCGGAGGACCGGAGTGAGGTGAGTGATTACAGTTCGGAGGACGAAGGAACCGAAGATTACAGGCGCGGAGGGTACCATGCGGTTCGGATTGGTGACACTTTCAAGAATGGGTGCTATGTGGTGCAGAGCAAGCTTGGTTGGGGTCATTTTTCCACTGTGTGGCTCGCTTGGGACACTAACAAATCGCGATATGTTGCCTTAAAAATTCAAAAGAGTGCTCAGCATTACACTGAAGCGGCAATGGATGAAATAAAGATTCTCAAACAAATTGCTGACGGGGATCCAGATGACAAAAAATGTGTTGTAAAGCTTTTGGATCACTTTAAGCATTCAGGGCCTAATGGTCAGCATGTTTGTATGGTTTTTGAATTCCTTGGTGATAATCTTCTCACCCTTATCAAGTATAGTGATTACCGAGGTGTTTCTCTTCCCATGGTGAAGGAAATTTGTTTCCATATTTTGGTGGGTTTGGATTACTTGCATCGGGAGCTTTCTGTAATACACACTGATTTGAAGCCTGAGAATGTCCTGCTTTTATCTCCAATAGATCCATCTAAGGATCCTAGAAAGTCAGGTGTTCCACTTATCCTTCCAAACACAAAGGATAAGGCTGTGACAAAAAATGGGACAATAGCCAATAAGAGTTTGAATGGAGATCTGACCAAGAACCAGAAAAAGAAATTGCGGAAAAAGGCTAAAAAGGCAGCCCAGGGCTGTGTTGGAAGGGAAAATGCCGAGGAAGCTGAGGAGGATTCCAAAGCACCTGATGAGCAAGATGATTGTAGTAATGATGTGAAACCAAGTGTAGATTCTGGTGACCATAAACCTAATAGTCCTGTGAGTAAAGATGAATCAACAAAGACTTCTGAAAATAAGGATACTCCACAAGCAAGTCATGGTCATAGGAGAGGTAGCCGCTCTACAAGAAAGAAGTTGCTTGCATCTGTTGATCTTAAGTGCAAGCTGGTTGATTTTGGTAATGCTTGTTGGACCTATAAACAATTCACAAATGATATTCAGACAAGGCAGTATAGGTGTCCTGAAGTTCTTCTTGGATCTAAATACTCAACTCCAGCAGATCTATGGTCCTTTGCTTGCATTTGCTTTGAGCTTGCCACTGGCGATGTTCTTTTTGATCCTCATAGTGGTGACAACTATGATAGGGATGAGGATCATCTAGCACTGATGATGGAGCTTCTTGGGATGATGCCTCGCAAGATTGCACTTGGTGGCCGCTATTCCCGAGATTTTTTCAATAGATATGGCGATTTGAGACACATACGACGATTGCGCTTCTGGCCTCTTAATAAGGTGATGATGGAGAAGTATGATTTCAGTGAACAAGATGCAAGTAGCATGGCCGACTTCCTTCTTCCATTACTTGATTTTGTCCCTGAAAAGAGGCCGACTGCTGCACAATGCCTTCAGCATCCATGGTTCAGTGCAGGTCCCCGTACTCTTGAGCCCCCAGTGGCTACTGTGCAGCATGCTACTATTGCCGGGGAAATATCTGAAAAAATGCAGAGGGAAAAAGCTGAGCAGGAGGCTGTGGAAGTTGGTATGGGGAATATGGCCATAGATGGAAATCCAAAGCAACTTTAATAGTTCCATTTTGTGAAGCCATCAGAAGGGAAGTTAACTGGCTTGTTTAGGTGGGTATGCCACCTGTTTTTGTTTATGTGGTAAGCCTGGATTGTCCTGGGAGCATGATATTATGGTTAACTGTTATATGTAACAAAGGTCATACACGAGGTTGTCATTTGAGAAGCCCTTCAAGAGGAAGCTAGTCGCCTATTGCAGAATATAATGTCTAAAAGATAGCTTACTTCCTTCACATTCAGTTGAAAGCACACTGTGATATAACCACGGTTTATCTTTTTGGAGAATTTGTTTGAAGCTTCTCCCTAGGTTAGATTTTCAAGCTATATTTGAGGGGTTCGAACTGTACATTCGAATGAGTTATTATTATTATTATTTTTTCTTCTGTATCCG

>C100027_65

TTTTTTTCTTAACTCGATCCTTAGACCTCAGTTTATTAGTCCATTGAGAAAAACAGAGGAAATATTTTAGCAAATCTGAACCCCCATTACCCTGAGAAACTAAGAAACTTGCATTTTCAATAGGTTCATTTATGCATTATTTAAACATACTTTCTATTACACAAGCTGCAAGAACTCATATATAACTCATAGTTTGATAGAATTCATATATATTGTATCTTGGAAACACCATAAGCTAGTTGATTAATTTGTTTATATATGTATAGACCCACAAACTGATTGATTGAGAATCTTATTATGCTAATTAATTAAGAATTGAAGAAAGGGGGTTAATCTTGAATGGCCTAAAGCCATGCTGAAGACTTGCTTGCCATATTTTCTCAACGCTAAACGTGAAATTTCCACATTCGTGAACGTCCCAACCTTCTAGGGCATGCAGTATTCCGGCCACACGCCATGCACTCATCACTCTTCTTGGCAGCCAGTTCTCACAAGAATCCACATTCACTAAAGAAGGGGGAGCTATCATGGCTGGTGTGCTGTGGTAGAAGCAGTCTTTGCGAAGTTTCTTTGGAGGGAATTGAGAAATAGGAATGAACAGTGATCCTTTTGGTGCCTTCTTTTGTTCAGCCTCGTTACATTGGTCTCCTACTAGCCAAATCTTTGCAGTATAGGATCCTGGGAAAACCAAGTTCTTTTTGGACTCGTTAGAGAGTCTTAATTGAAGACTATCATATTCATCCTTATACATAGTTGTAACCTTTGTACCCCTTTCACATAAAGCACTTGCAATGGCATATGCAACCTTATCAGGTTTCCCACAAAGAAGCACTTGACTTGCTTCTTTGGGAATACTGTTAAGAACAATAGCCACAACTAAGCTACTGCCATCCACAATCTTTATCTTAAGCTCAGGAAATCTTTTTATGTAAAGTTCACCATATTTATTCAGTGATTCTCCCTGGTTTGAAAGACCTAGACTTAAAACCTTTGCTTTGCTTGACTCTGCCTGAAGTATTGCTTCTTCTATAAGTCTGTTTAGTGTTTCACTTTGCCATTTGAAAAGGTATTGTACATGGAATCTAGGTATAACCCATGATTGCAAATTGAGCATTTTGAAAGCATTCCTCTCCATGACAAATGTTTGACCATAAAACCAAGTCACAAGAACAGACCAAAAAGTGAAAGGCCACATCAAATAGAGGTACCATGTTGAAGATTGTGGTTTGGAGGCTAAGGAAGCAAATCCCAATCTTAGATGATAAATAGATTCTGGGTTTGTAAGGTGTGTTAGATGAACTACATCAGGTGAGATTTCTTCTCTCTGCAATGAAGTTTCATATGTGGTGTCTGTAGTCTTGTCCATGGTGCCATAAATGTAATCATAAATGGGCATGAAGAGTGAGTAATTGGTCCTAAACTGGGTATGATGCAGTGAGTGGAACGATGGTGTGTACATGAGATACTTTAGGAAGGGAAAGGTTGAGAAAATGGCCTTTGGAATGCACTCGAAATTGCAGTGACCCAAATTGTTCATGAAATCAATGTATGCAAGATAACCAGCATAGGATGCTATAGAAGCTGTTCTTGTAGCCACAGTTGTGTACAATGGAATCGCAAATAACAAAAAGTATGCAATGTGTTCTGCAAATGGGTGGATAACAGACGTAATGGGTTCTGTGACAATGGAAGAGTGGTGGTGAGAATGATATCTAGAGTAGAGAAAGTGATGATGAAGTGCTCTGTGTAACCAATAATACAGAAACTCCACTGGTCCTGCATGTAAAAGAGCAGTGATAACCACACCATCTGTTCTCCACAAGGGCAAGTGACAACTCTCTGGAATTAACATATGACCTACATAAAACACGATTCCATTGAACAAGATCTGGTCATCCCAATTGCTTTCTCGATCAACTTGTTCAAACTCGATGCCCTTGTCAACGATTCTGTTTTTGCCTTTGGCAGTTCTATGGCGAGAAAGTGTGATCCAAATTTGGTCATGCAGCATCCTCACCAACATATAGGGGAAGATGAGGAAGTAGCTAAGGTCTCTCTCCTTTGGATCATTCACTATGAAAGAGTAAGTGCTGTGTGCAATCCATGGTGTCAGAATCACCCACTTGAAACTCCCTAGTGGCTTCCATGGCCAATTGGTCAGTATGCCAGGTTTGGATGCCATGTTTACAAAGGATAAGAAAACGAAGGAACTCCAAATATTTTGTGTGTATGAGATCT

>C100033_65

CAAAAACAGGGGAAAGCTCCCTCTTTCTTGTCTTTTTCATTTGCCGCTGCTACTGTGTCACTGATCTTCGCTCACCAACAGGTAAGCCAAAACAAGATCTGTAATCAATCTCAGATGTTGTCATCAACTTTGGTTCTAATCCCAGTGAGTATCAGCGTATACTTTTATGTTGTGGCTTAGATTTGAACTCAAGGGTATCTGTTCTTATCACAACATATCAAGCTTGTCATGATTATTGCTAGTTAATGCGTTCGCTTATTGCCTATATGCAGATTCTGAGCTTTATTTTGTTTTTGAGATGTTGTGTGAGCAAAAGATTTGGCTTTCCAGTTTTGTGGTGGTGTTAGGCTTAGTCAACTGGTTGTAAATGAACACTGTTCTGTGCACGACCCAATTGAGATTTAAGTTTTCGTTCTAGTCTTTTTTGTGTCAGCATCTTTGAGAATTAGGATTTGTGTTTGTGTTCCAAGAATGAACAGTTCGGGTTTGGGTGGTGGTTTTTTGTCTGGTCCCAGTGGAGGGATTTTGGACTTGGAATCTCCACTTCATAGACACCAACAGACCCAATTAGGTCATTCATCAATACCTGGGCAACAGCACTTGAATATCATAAGTGGTCTTGAAAGTGATCACCCTATTGGCCTAATTGAAGTGAAAAATTTGAATGCGGCGTTGAATTTTGGTAAAGGAAAGGCAATTGCTCCTTCCAATAGTAATGAGTTGAGTGAGGATGATGAGCCTAGCTATGCAGAAGAAGGGAATTGTGAGAACCTGGATGGTGGGAAGAGCAAAAAGGGGTCTCCTTGGCAGCGAATGAAGTGGACAGATAATGTGGTTAGGCTTCTTATAACAGTGGTGAGTTGTGTGGGTGATGATGGCACTATTGGGGGCATGGATGGCCACAAAAGGAAATCTGGGGTTTTACAAAAGAAGGGCAAGTGGAAAACAGTCTCCAAGATAATGATAGGCAAGGGTTGCCATGTGTCGCCACAGCAGTGTGAGGACAAGTTCAATGACTTAAATAAGAGATACAAGAGGTTAAATGACATACTTGGAAGGGGAACTTGTTGTCAAGTGGTTGAGAATCCTGCGCTGATGGATTCAATGCCCAACCTGTCTGCTAAGATGAAGGATGACGTTAGGAAGATCCTGAGCTCAAAACACTTGTTTTATAAGGAGATGTGTGCCTATCATAATGGGCAAAGGATACCAAATTCTCATGAACTTGATTTACAGGGTTATTCTATGGAGCACGGGAGGGACTCTACAAGAGATAATAATGGATCTGAGGATGAAGATGAGGATAACAATGATAGTGAGGATGATGAAATTAATATTAATGCACACGAGGATGGAGGGAGGAGGCAGGAACTCCGTGATAGAAATATATTAAGTGAGGAGGATGGCCATTTTGGCCCACAAACTTCTCGGATGGACAAATTTGAGGTTGAAATGGCAAGAGTTTTTCAAGACCCTGCAAAGTCATTACGTGAACAAAGAGAGTGGATTAAAATCCAGATGTTGCAGCTTCAAGAGCAAAATATCAGCTATCAAGCCCAATCTCTTGAACTTGAGAAACAACGGCTTAAGTGGTTAAGATACTGCAGCAAGAAGGACAAGGAGCTGGAGAAGCTGAGATTGGAGAACAAAAGAATGAAATTAGAAAATGAGCGCAGGATCTTGAAACTGAAGCAGAAAGAGCTAGAGGCAGATTTCAGTACATCTGAGATGTCTTTAGACCCTGCCTCTCTAGGAATCAACCGGCCTCAGGGGAGGGAACATATCAACTTAGGCAGACAACAGTAGAGAGTGCTGTGGCGTTAGCAACTGAGCTGAGATGCGTATTTGGAATATGAATGTTTTGCTCAACTAGTTACGTGCTTTACGTCATTGAATCACTATATATTGGCATGTGGGATTTTTCCAGTATGACAGGATAGAAGGATGTTGCTCCAAGGTTCTTAGCTCTTATTGTTCATCAGTTCATCATTGATGTGGCTTTAACTTCTTTTTTGTTAACTTAATTGTAACTGTTACATGTATCCACAAACCACATGCATATTTTTGGCTTTGCAGCTGTATATTATACACACAAAAAATAATGGTGCAAACTCGGAGGAAAAGGTTACAAGCATCCCTGGGAGTACGTCACCGACTTTGAATTTGTAACTTTTTTTGGTACAGACTTTGTTGTAACTTTGTTACGTTAAGTAGCTAAATGAAGTATGCTTGATTCAT

>C100035_65

GAAACTTTCTCTGCTCATGCGTGTTCTCTCTGAGAAAAATAAAAGCGAGAAAAAGCTATGGGCCACAGCACCTAACATCACAACACAAAACCCTCTGAATTATTCCCTTGATTCTCAAATTCCGCTTCCGTTTATATCTTTATAATTGATCGTTGCTCCTTTCAAGATTTTTTTCCCCTTTCTTTTTTTACTTTCGTCTTCGTTGAGTCAGCATGAGTTCCGTCGAGTGTTATCCCTCGCGCGGGGACGAAGTCGCCGTCGTGTCGCTTGATTCTCTCCCTTTAGGGTTCCGATTCCGACCTACCGATGAGGAACTCATCGATTACTACCTGCGACAGAAGATTAACGGTAATGGTGCCGAGGTTTGGGTTATCCGAGAAATTGATGTCTGCAAATGGGAGCCGTGGGACATGCCTGATTTGTCGGTGATACGGAACAAGGATCCGGAATGGTTCTTCTTCTGTCCCCAGGACAGAAAGTATCCAAATGGGCATCGGTTGAACAGAGCAACGAGTCATGGGTATTGGAAGGCAACTGGGAAGGATCGTAAAATAAAGTCTGGAATGAGTTTGATTGGAATGAAGAAGACTTTGGTGTTCTACACTGGTCGTGCACCCAAAGGGAAGAGGACCAATTGGGTTATGCACGAGTATCGCCCCACCTTGAAGGAGCTTGATGGCACTAATCCTGGACAGAATGCATATGTCCTTTGTCGCTTGTTTAAGAAACAAGATGAGAGCCTTGAAGTTTCACACTGTGATGAGGCAGAACAGACTGCTTCAACTCCAATTGCTGCCAATTACTCTACAGAGGAAATACAGTCTGGCCTGGCTGTTAATGCAGTGTCCCCATCACAGGTTACAGGTGATGATAAGCACCTGGCAATTATCCCTTCGCACTCTGAGGAAGCAATATCCAACGTTATAACTCCGTTTGATTGCCATAGTGATGGATATGATGCTTCTGATGCACAAAATCAATATCTGGCACCAACTGCAGAGGAGTTTCAGCCATTGAACTTTGTTATATGTTCTGACACAAAAAACGAGCTATTGGATGACAAATTATTCTCCCCCGTACTTGCACATAATCAACCAGAGTTTCATTATCAATCAAACAGTGAATCAGATGGTCGATACGGGCTTCAATGTACAAATGAGACAAACATTTCAGACTTTTTGAATTCTGTAGTTAACTGGGACCAACTACCCTACGAGGAGCCAAATTGTCAACAGTGGAGCCACTTTAATGTTCAGAATAACGTATTAGGCAGCGACATAGATTCTGAACTTGCCATTATGCCATATATACAAGCTGGTTACGCTGAGGAGGCAAATGACAGAAAGATTCCTTTGGTGGTAACAGAAGAATTTTGCATCACCTCTGACCTCCCCATTGACTTTGGTGGTGATGAGCAGAAGAACAATGTTGAGTTACTTCAACACAATTCCCAGAGAGCTTATACAGATGCTAATATGGGCCAAGTATACAATGTAGTAAATGATTATCAGCACCAAAGAATCTTTAATACAGTTCCTAGTGGGGATACCGGAATCATAAGGAGGACTCGACTGGTACAAAATGAACAGCTAAGTGCAAGCTCAGCACAAGGTACAGCACACAGGAGAATTCGTTTGCAGATGAAGGAACAAAATGAACTCTCAAATAATATGGTGAAAAATAGAAGTTGTGAACAAGAGCTCAATTCAAAACCAATCATTGCTGTGGAGGAGAAAGCTTCACGAAACCATGCTTCTGAGGAAAGTGCTACTCTTACTAAAGATGGGAATGAACTGCAGAAGACACCTGAGTCAAATGGCAATAGAAAGATTTCCCGACAAGTTACGAAAGCAAGTTCCACCTTGGGGTTAAAAGATTTTTTATTGCTCAGAAGGGTGCCATACATTTCAAAGGCTAACTCCAATCGCGCCAAGTGCTCTTCTGTTTTTGTAGTTTCTGCCGTTTTAGTGATCTCCTTAGTGGTCTTTACTGACATTTGCGGATATCTTAAATTCTAAACTGCAACTGCATCATTCCTTTTCTTTTGTTTTCACCTCTATTTCTTTTTTGGGCTAATTAATAGAGTCGTGTAGGGTACTTAGATTTGTTGATATTTGAATATACATGTGACTGCACATGTAGTTGGTGTATTATATGTATACTCACCATGTACATTAGATATACAGTTTTCCCCTTCCAACTTTTCAGCTCCTCCTTTTAAGTTTGCTGCTTCGCCAA

>C100037_65

GAAGTTTATCCAGATGCAAAAGTAAATAGAGAAACACAATTGAGTGTCTCCCTCTTCATCACTGCACAACTATCTCTCTCTATCTGCACCATAGCAATAATAATAAACCATGCCGTTTTGAGTTTCGCCATCCCACTCTCGCAAAACCCAGGCTTTCGGTGGGAGCTCATGGCTACCATTCATTCCAATCTCGTCCCTAATTTGTCCTTCAATCGCTTCCAGACACCACCATCATCGTACCTTGTTGGTGCTGGTATTGTGCAACGTGGAGTTATTTTGAGCAATTATTGTAGAAGGAGGCTTCCTTGCATAAAGTGCGAAAAGAAAGATGATGAGCACATTGAGCATGTATCGGTTGAGCGTCCACCCTATCACAGTTACTTTGACTCCACCTCTGGCCAGCTCGAGCCTGCCTCCGGAGCCCGAGCGAGTATTCCCAGTCAAGAGTATTGGCCTGAGGGAACTGCTAGTCGTGTGAGGGCTGCAAGGGCTCCTGCTCCAACTGCTGAATCATCAACCTCTCCATCTTATGGAGCCAAGCCTGGAAGTAGGAGGAAGAACTACAAGACATCTGTTCCCGCTGCTGCTGCTTCTGCTTCATCTCAGCCCATTGTTGAATTGACTGGTGCTGATTCTGGTTCTTCTGAGCTTTTGGTTGAATCCCCCGAAGAATCTCAGGATCACTCATCTGAATTTGTTGTTTATCAGTCTGAACCCGAGGAGGAAGAGTTGAGTGAGTATGAGTACTGTAAGAAAGTTGGACTTCCTCATCCCTTTGTTGATCCAAAGGCTAAAAAGCCGATAGAGGGGGTGCTTCCCAATGAGGAGCTGTGGTGGAATTGGAAACAGCCGGAGAAAGAACAATGGTCCAGGTGGCAAAGGAGGAAACCTGATGTTGAAACGGTTTTCCTGAAAGCTATGGCAGAAACTGGACAAATAAAGCTTCATGGAGAACACCCAACATTAACAGAAACTGCCCTTTACCGAGCAAGGCGAGAGATTTATAAAGAAGAAAGGCTTCAGGAGGAGCAAGAGAGATTGGAAAGAGATGGTCCAATTGCATACTATTCAGAATGGGTGAAAGCGTGGAAAAGGGACACATCTCGCGAAGCTATTCAGAAGCATTTTGAAGAGACTGGAGAAGATGAAAATGCTCAACTTATTGAAATGTTTTGCCACCAAACTGACCGAGAGTATCGCATAATGATGGGGACAGATTATCGAATTCAAAGGGACCCTTTAGCAATGCGAATGAGGGAGGATCAGATAAAGCAAATATGGGGTGGAGATCCTGTTTACCCGACTGTGAACTATATTCAAGATCCAAATGAAGTGATTGACTACAGGGGGGCAAATTTTCATGAACCAACGCCAAATATGGTGGCTTATCTGAAGGAGCATGGAAAACTCATTTCAAGGGAGGAATTTGACCAGACTATGGCCAAGGAAAAGACTGAACAAATTGAGATGACGGACATGGATGAAGCTATGGCCAAAGCTGTTGACATTGGGGAAAATGATGATGAAGAGGATAGTGATGTTGAGGAGGTAGAGGGAGAGGTAGAGGGAGAGGGAGAGGGAGAGGGAGAGGAAGAGAAGCTTGGTGATTATTGGAGTGTGTTGAAAAGTACTCCTGAGCTGCGGAAGTCAAAGCCAAAGCCAAAGAAAGATGGTCCAATGTCACTGGACGATGCTATAGACGATTCCGAAAATTTGACTGATTTCCTCATGGACTTTGAAGAAGAATGACATGGCTCCTTTGTTCCACGTCAAGTGGGGCATTGTATTGCATTGTAGACAGAATAGAAGGTTTGGTTGTCAGTCTTCCTTGTCAATCTCTGTACTCTATTAGTAATTCTAGGTAGGCATAGCCGCAGTAGATGATGTTGCCAATGCGATAAAAGTGCAATTCTGATTCAAGGGTTCTAGTTTCTGTTAAGCACTTGTTCCCAACACATATCTCCATAAACAATTTTGATATCCCTCTTCATACGGAACTTAGCAGCAGCTAAATTAACTAGGAAAGTGACCATCCAATATATTAAACACATTGGCGCATGTTGTAATTTTGGTGCAAACTAGATATGTTAAGTCACATATCTTCTTTCTCAAGTAGTTCAAATAACGATGTCCTCGTTCATTGAGATTCACAACCTCCCTCAGACTTTGGCCCAAGGCATGTACAAAGACTTTAGGTTTGGTTGCTTCAATGTATTTGGGTGTTGCACTTTTGTTTTA

>C100041_65

ATCTTTTCATGTTGCAAAGGTTGCATTAATATTATTGTGACATACCACATTCAAATAATCTACGAATACAATGTTACCTAAACCATAGCTAATAGGGAAATTGGTGCTACTTTTAGAGTTCATATACGGATGAATCCACAGATATTCTCTTCACTTCACTTTCTTGCCCATATTTCTCCCCTTTTCATGTGCCAAACAAGATAAAGACAACTTCAAAAACCTTTTGTTCACCAGAAGATAAATAGTAGATCATCGCTTGAGTTGTCTTCTAGCCAAAGCAGGGACTTTATTAGGGATATAGACCTTGTAGCTAACCTGTACCATTTCACCACCCCGCATCACATTCATTGTTTTCTTCTCAAACTGGGAACCCTGGTTGGATTCAACTTGCAATTTGTCTGATTTGTTCTCTACTGATGAAACATCACCATTTCTACCGTTATTAATACTCTGAATTGCAGCATGAGGAGTCTTAGCTGCATTCCTTTCTACATCACCCTCAGTCTCCAGTAACTTTGCATTGGTAGGTTCATGTGAAGCAGATGTTTGAAATGAATCAAGGCACGGGTTTTGTGTTTGTTTGGCCAATTCTAAACTGGTTGTATTGTAATTAGTGGATTCAGTGACTGTTCCACTACGATTGTGTGAATCAATCACCTTCTCTGGTGCACTGGCCAAACTACACTTTGCAGAAGCAGATATAACTTTATCTGTATCCGTGTCTAAAATTCTTTCATTGTTTCTTGGAGAAGCAATTAAAGTTCTCCTCCCAACAGGAAAATTAGTTTCTTCTCCAGGGATTCGCTTATCAGATTTTGTTCCTACAACTGCATTATTGACAACAGACTTACCTTGAACTGGCTCAAGTATATAAGTAATTGGTTGCTTCACCTGGCTAACAATTTTATTTTTCTGACCAGGAGCTTTTGCACTAGACTGCATCTCACCGCGAAATTTTCTCAAGTTTTCCAATGCTCTTTGTCTTAGAATTTTCTCCAAGTCATCATTATTAACATTAGCACCTGAAGTCTCGCTTATTTCCTTAGCAGATTCACTTGTCCCAGCACTATATGCTTTAAGGTTACTGCTGTCATTGTAGCTCCTATCCCTTAGTCCGGGCTCTGCAAAGTTAAGATCAGCATTCACATCTCCTGCCTCATTGTCAATACTCAATGTCTCTTCTGATGCAAGATGTGTATTATGATCCAACTCTCTCTTTGTGCCCCCATCATTACTGTCGTTACTTCTACAAGGGTAATCATGATCATCAACAATCTCCTCCTTGGTTTCATTTCTACATAATTCCCCATATTCTTCCACTTCTTTTGTGACAATAATAACTGATCGGAGCCGCCTGGAATTCTTCTCACCTACATATTTCTCCTTAATAGTTTCAAAACTATTTTCACTACTGCACGAAGAACAACTTCTAGCCCTGTATCTACTACTCCTTTTACTTCCACTTCTTCCTCTTAATCTTCTGTCTTTCTCTTTCCTTTCCGATCTTCCCCTGTGGTTCTTATATCGATTGTCATCACTACCATCCTGACACGTCGAGCAACTCTGTGATCTGCTACTCATTAAATCAACACTTGCTTCTCTCCTAATCTTCTTCTTCTGGAAACGTTTCTTCCTTGGCTCATCACGCTCATTTTTCCTCTTCACCTTTTTTCTCTTCCTAGCATAATGAGAGTCCTCACTGCTGTCACGGCTGTAAGATCGTCCCCTAGCCTTCCTCTTACGACCTTTCACATCTTTTCTTGTGCTAGACCTATCCCTTTTTCTTCTGTTATCTTCAGAACTAGAGGATGCTGATATGTCCAAACTTCTTGAATCATCATAGTCAGAACTCGAAAGAGAGACCTGACGGCGAACCTTCTTGGATTTATTTTTCCTAATCTTCTTACTTCTACTGCTTAACTTGGCCTTGGATACTTTCTTAGAACGTTTCCTCTTGAGTGAAGAGGATGATTTCTCCATTGGAGCGACCAGCAAAATCAAACTTCCAAAAAAATAAAAGAGGAAAAGAAATTGTGGGAGGAAGAAAGAGTGGGAGGGGAAAGAGAGGTGTGAATAGTTGAAGAGGAATTTAGGAAAGGGAAATGTGGAAAAAGCAAAAGGGTGTATGAAAAAATGATGCTTTTTTTTGTTAACGAACAAACCCAATTGTGTCCAATCTCTCTTTTTCTGCAATCGGAACACTGCCAATTTGTGCCCCTTTGAGGTAAACCAACTGTTG

>C100045_65

GGCATTTCCGTTATTTCCTCCACGCGCTGTGTCGTTTATTCAACCTGCATGCGTTCCACTCAATCAAACCCCTTCCCCTGCACCCTATTCACTACACCTTCATCACACTCAAAACAACAACTACAAACACTCTTCAACTCAGCTTAAACCTTCTTCTCCGTTTCCTTTTTCTTCGGTCTACCCAGAAACCATCGTTGCTCGCCGCCGATCGCGACGGCGATTTCCGCTGCTTAGATTTTTGGTTTTTTTTTATTCCATGTATTTTCCGTGCTGTGAAGCCGCCGGAATCCGCCTCAGGTGACGGATGGGTTGCGTGCTCGGGACGCCGGCGGGTGCCGGAGATGGGCGCCGTCGGCGGAGAGAGAAGCGGGACGTTGCTGTCGAAGAAGGTAATAATGCCGTTAGGGTTCGAGATAGGGAGAGGAACCGGCACACCGGGGATTTTCCGGGGACTCTTCCGGCGCCGGAACGGCGGAAACCTAGACTCGATCCGTGCGCCGTCACGCAGCAGGGATGGCCGTCGTGGTTGATGGCTGTCGCCGGTGAAGCAATCGGCGATTGGACTCCTCGTCGCGCCAACACTTTCGAGAAGCTCGCGAAGATTGGGCAAGGGACGTATAGTAATGTGTATAAGGCTCGGGACCTTGTTACGGGGAAGATTGTGGCATTGAAGAAAGTGAGATTTGATAATTTGGAGCCAGAGAGTGTAAAGTTCATGGCAAGAGAAATACTTGTTTTGAGGAGGCTTGATCACCCCAATGTTTTGAAGCTTGAGGGTTTGGTTACTTCTAGAATGTCGTGCAGTCTTTATTTGGTGTTTGAGTATATGGAGCATGATCTTGCAGGGCTTGCAGCTGGCCAAGGGGTTAAGTTCACTGAACCTCAGGTTAAATGCTTTATGAAGCAATTACTCTCTGGTCTTGAGCATTGCCATAGTCGAGGCGTGTTGCACCGGGATATCAAGGGTTCCAATCTGCTTGTAGACAATGAAGGAATCCTTAAAATTGCAGATTTTGGACTGGCAACTTTTTATGATCCTAAGATAAAACAGGCAATGACAAGCAGAGTGGTGACCCTTTGGTATCGTCCTCCCGAGCTTCTTCTTGGAGCTACATTATATGGTGTCGGTATTGACCTTTGGAGTGCTGGTTGCATCTTGGCAGAGCTGCTTGCTGGAAAGCCAATAATGCCTGGCCGAACAGAGGTTGAACAACTGCACAAAATATTTAAGCTGTGTGGCTCTCCATCTGAGGAATATTGGAGGAAGTATAGATTGCCAAACGCTACTATCTTTAAGCCTCAGCAACCATATAAACGTTGCATCTCAGAAACATTCAAAGATTTTCCTCCATCTTCACTACCTCTAATTGAAGCTCTTCTTACAATAGATCCTGATGATCGTGGCACTGCCTCAGCAGCACTAAATAGTGAATTCTTCACCACAGAGCCCTATGCTTGTGAACCATCGAGTTTACCAAAGTATCCTCCCAGCAAAGAATTAGATGTAAAACTGAGAGATGAAGAAGCAAGAAGACAAAAGGCTCTAAATGGAAAAGCTAGTGCAGTTGATGGTGTCAAAAAAGGCAGAGCACGTGAGCGAGGCCGGGCTGTTCCGGCCCCTGAAGCTAATGCAGAGATCCAAACAAACTTAGATAGGTGGAGAGTTGTGACACATGCAAATGCAAAAAGCAAGAGTGAGAAATTTCCACCTCCTCATCAAGATGGAGCTGTTGGGTATCCACAGGATGCATCAAACAGAGGGCCTGTTTCGTTTGGCGCCCCCGACACTTCCTTTTCTTCAGGGATATTTAATTCAAAACCTTCTGGACCTGTTAGAAGTCATGCTGCTGCAGGACATTATAGAGGGAGGAAAACCAAAAAAGAAGAGTCCCAGATGGCGTCATCATGGAAATTCATGCGTCCATTCAAGCCATCAACAGTTGGACTCTCTATGGATTTGTTATTTAGGAGCAAATAATCAGTTTCTTTTGATTTTGGCATCCTAGAGTGAGGACAGCTCTGATTTAGGCCATGATACACCTTTTTTTGCATATCGCTATGGTTTTCAAATTTTTTTTTCCCGTCGCTCCTATTTTTTGTATTTCATTTGGCATAAAGTTGTTGTATAATCTACATTACAACGGGGATCCTTACATTTGTATATTTAGAGGATTAAATTCTCTGCTGTACAGTTTCTGGGAGAACTTCTTCAGGACCTTTCCAAATGTTTCGGCTTAGC

>C100047_65

TTTTTTTTAATAAACCTTCACCTTCAATCACCTTACTATTTTAAGATATAAAACTGTACATAGTTGTTTGATGCATGATAACGAAATTCAGCTTTCTAAAGCTTTAACTCTTCACCAAGCACATACAGCACTAGTTGGTGGGCAACTTTTCAAATGCACCCACATGGATAGATATACTAACATATTTATGGCAAGAAGCCAACATGTAAATCCTACGCTACATCTTTTTGGCACTTTTGCCGTAATATTGAAATTCTATACTCTATTTTTCTTACTCTTATACACATAAAACCAGACACCACAAGTGAGGACCCTACTTGCTCCACAATCTGCCATTGAGTTTAATTACCGAAAGTGGTTCCAAGCAGGTACACCTCTTACCTGTACCACGATGCAAGAAATGGAAACATCTCTTCAGTGGGGAGGGGAAGAAAATGTTCATAACTTAGTAATCAAGTTTGTGAGATGATCTCATTTTTTGCACATGTTTATGATTCTCCTTCCTTGAATTCATTTTGCTTCCTTTGCTTTCTCATATCTAATAATGATCTGTATGCAAGCCTATGATGGAGGGATAACACTGAAGCTTTTCATCATCACAAGATTGAGGAAATGGTTATTATCATTTATCATTATTATTATTGTTATGATGCATCCTATTTTGCACAGCTTTTACAGCTGCAGCTCTGGCTGCGTTGGCTGCTTTATTAGCTGCTGCCACTACTTTATTCATCCCGTCATCCACCTTTGCCATGTTGTAAGCTTTCTCTGCAGCAGAATGTGCATCCTGTACTGCATTGGAAACCTTAGCATGGTCCACACCACATGGAGATCCAGTAGGACTATTATGTCTTTTAGGATCATCAAGAATCCCATTCTCCCAGTGACCACATTGTGTTTCCCCATTTCTGAAAGTGTACATTCCCAGTCCTTGTCTTCTTCCTTCATGCCATGCCCCTTCATACCGATGACCATTCTGAAATTGATAAACTCCAAAACCATGCATCTTGTCTGCAAAATATTCACCGGCGTATATGTCCCCATTTCTGAAATGGTATTGTCCGTGTCCATGTTTGACACCCCATTTGAACTCACCAACATAGCGGCTCCCATCATTGCAAGTGTGGACTCCAAATCCATGACACTGCCCATTAGACCACTCACCACCGTACACATCACCACTGTAGAACCTATAGATCCCCATTCCATGCCTCAAACCCTCCCTATACTGCCCTCTATACCTGCTCCCCCTTGCCCATGTCTCAACACCATATCCATCATACTTCCCATCAACCCAATCCCCCTCATACCTTCCACTCATGTGATAGTGATAAACCCCACTCCCCCAACACTTCCCCTTCTGGAATTCACCCTCGTACACATCCCCATTGCTGTAAACTTGCACCCAACACCCCCAACTCGTGCTCTTGATTTGCTTAGGGTTCGACCCAATAGACCACACCACAGGGTGAGCAGAGGAGGAGGAGGATGAAGAAAAAGAAGACGATGATGAGAACTTGGTGGGGAAGAATCGAGTGAAGAGCAAGAGTGCCAGATTGAGGGAAATCAACAGGGCTGCTGACAAAGCGATGACAGAGAAGAAATCCAAAGGGCTAGGGTTTGACAAGAGAAAGTAGATGGAAGGAAGGGGGAGGAAGATGAGGAAACGGAAGCGAGGGAGAAGCTTCGAAGTTGGAAACTTTGCGCCAAAGGGTGTCGGGAAGAGGGGGTAAGGGAGGTTCATGACGGGTATTCTTTTGCGGGGCGAGGTTTGTGATGGGTTATTGGAGAAGCGTTCATGGTGAAAGGGAGTGTTTGGTGAGTTTGGTGGCGGTGGATGGGGAGAGGAGGGTGAAACCTGAATTAGGAAGTGGGTTTGCGTTTGTTTTTGGTTTTGGTGAAGAGGAGTTGAAGTGGCATCCTCAGAAGAGAGGTGGGAGTTCTCTTCTCCTATGTGAAGGTCGGTCTTGTGGTGGTGATGCATTAACGCACAAAGGAATTGACACTGCAACACTGCTTTGTTGCTTGTTGTGTGAATATCTAAGTGTGTATGAAGAGTGTTTTGCGATTGTGATTATGGTTTTGTTTTGCTTATGTGTATGTTGGTAGTTGCTGCTGGTGTTTGTGCCTCTGCTTTATTTTGTTGGACTGAGAGGAAAAAGGGAGGTGGAAAAGTGCAGTTCGTGCTTGTTTTGGGGTCCAATGAGATGGCTATGCCAAGGCCAAAGCCAAAGCCAAT

>C100049_65

TGAAAACTTAAAGCATCCAAAATGAAAAGTTTTAACTCCCAAATATGTAACAGTGCTGTTGCTTGAGGTAGTCAAATACAGTTGTGAAATGTGCAATGAAACATACAACACATAGTGGTTTCTTCCAAGATCAGAGTTGCATACACAGCCCACACTCACAAAATTGGCGGGCAAATACACGAGATTTGATACCCTGTCGCTAATTTTGAAGAAAAGAAGTTTCCAAGAGAGAATTGGTACTCTAGAAAATAGTTTTTTTCCTTCATACATGATTGTGCATATAATGGTTCAACCAAGAGGTTCAATCATCTTAAGTATTCATCAAGCAGTAACAGAATTATACGCAAAAGTTTGTTGCGATTTACCATTTATACTTGTATTACTAGATAGGCTAGGATGGTGCCTATGGGGACTTGACCTCGTAATAGAATTGAGGTGCATTGCTACCTGGTGCACTGGACGAAGTGAAACAGAGGTTCTGAGGAATTGAGTTCATTGATTTTGTTGAAAAGATGGCATTTGGTGGAAAGGTAGCTGAAATTGAGAATCCTGATGATGCTGCAGTAGAGAACGGAGGTACTGGGGTGGCAGTAACCTGACCATGTGTTTGCCAAGCCCAGTTGGGGACTCCTTTTGAAGGATCTATGCCGATTCTCTCTGCTGTTTCCAGATTAGGAAAGAAAGTGGAATATGCGGCATTCCATACAGAAGGACGCTCTTCAGTTACAACATGCCTTTTCAAGGAAGGATCACCAATAACCGAATTAACATTAGTCTCCATCCTTGAAATTCTTCCCGGGTGCGTGTTGTAAGGGACTGGCTGAAACTGAAGATGCCCCCTGTTTTCTTTGGGACCGTGTCCTGGGGTTGCTATTCCCAGATTGAGATCAAGATCGTGACTGTCACCTTCATTAATAGCTTCAGGTTTCATTTCCCTCTCATAAGTACTTGGCTCAAAGTTAGTCACTGCCTCTCTTCCATTGCACTTGATAGCTGCCTTATCATAAGCCTTTTTCCCAAGAAATTGACCCATTCGAGCTTCCCACCGGCCACATTTGTGAAGTGTTACTCCCCGGTATTTTGAACTCCCCCTTGAGAAACCAGTACTGTGACGGCGGAGTATGTGCACGAACTCCTCCTTGGAAAGACTCTTCATCTGTTTTAGATCCTCCTCATAATCAACAAGATTAAAATTGATATCAGCATCAAGTCCCCTGAACTTAATTGCAGCTCGATCATAGGCCCTAGCAGCGGCATGAGCGGTGTCAAATCCACCCAAGTAGACTTGTTTCCCGCAATCCCAGATATGCGATTCCCATCTTCCGGTCCTTCTATAGAAGGTGACTCCTCTGTACTGAGAGCTTCGAGACCGTGGACCTCTCCTACTCTTCTTCACCTGAGGCTGTTGCTGAACCTGAACCACCTTCACCTCACAGTTTTGACGATCCAGAGAGAGATCCATCAAACTCTTCCTAGCCTGAAACGACGAAGTCGAACTCTGCCCTTGCCAATTATCTGCGCTCGCCGGAAACAGTTCCTTCGTCGCGGTGACAGCGACGTCGTTCGCTCCTTCGACCTTGAGGATTCCGAAATTGAAGGTGAACACGTCACCGGCACGTGTCGAGCAAGAGTCCTCGTTGCTCGACCCCTCAGCATTCACCACCGAGGAATTCGAAGTCCCCGAAGAAGCTTCGGGAAACTTGTCCAACAGCATCACCGAGTCGTCGTTCTGAGTCGACTCGGCATTCAGATTAAGATCCAACATGACGGCTTAATCTTCTTCTACTAAGTATTTTTCAACTCTGATTTTCTTTCTATTCTTTTATTTCTAATACTTTCGCGCTTTATTATTATTAATTCGAACTACAACATAGCAAACTAATATTGCTTACAGGAGATTCTCTATATCTCTTCCAAATGATGGTTATAACAAGTAAAATAATAGAAAGAAACAACAACCAGTTATACACGTAAAATGTAACCTAATCGGTTGAAGACGCAATATTCGGAAGGTGTAGTTAGGGAGTATAAAGCGCTTCTTCGATGGACAAGGAATCTGCAGTGGAAGAGAGGAAAAAGAGAATTATAAGGTTTTCGGGTCACTGTGAGTTGGACCAAGAATTTCTCTCCGGTGAGCAAAAAGAGCGAAGGATGAGAGAGAAAAGGAAACTCACAGAAGGCGTGAGAAGGAAGCACTGGGAGGTGTGGTAGTTACATTTGTCATTTCATGGCGATGATT

>C100051_65

TCACACTCCACAACACACATGATTGGTGTTGTAAACAAATTGCCGCCCTTCCCCTTCCAGCAAAATTCCGCCACCCACAACCTTAAACGTTCCGATCACCACCTTTTACATTTTCAAAACCCCATTCATTCATTCATTCGTTCCTTCATCTTCTTTCTCTCAACCTTCAACTTCATCTCTTACCCATAACCCCCAAAAACCACTTTCCAAAAAACTACACACTCTTCTTTGCTTATCTCCCATTAATGCATTTTTTTATTTTCCTTTATGGACCCACCTTCCTCTCTTCCTCATCCTCACTGTTGAACGCAGCCCCACCTCTTTTAAACCTTCACCACCCATGAACGAACGAATGAATGAATGAATGTATGTATGTGTGTGTGCTTTGTTCCCACCCTAAGAGATGAGCAAGAGCGTTTCTCCAAAGGACTCGGGCGACAACCCCGTGACCCACTTTGATTTTCTCAAGAAATTTAAACGCTTTTCCCCTCTCGACTCTTCTCTTGGAGTCCTTGCTTTCTTCTTCGTCGCTTCTCTCTTCATCGCTTGCTTCTTCTACTTGGATAAAGGCTTCCGCTCCCGAGCCACCACTATCCTTGACTTCGATTTTTCCTCGTCTTCAACTTCTTCTGTTACTTCCTCTTCTGTTACTTCTTCTTCTTCTGCTGCTTCTGACCTGGTTCAGTTTCTGAGCCCTGAGGGCGATGCCTGTGATGTCTTTGATGGGAATTGGGTTTGGGATGAGACTTACCCTCTTTATAATTCCGCAAACTGTTCCTTCTTGGATCAAGGTTTTCGCTGCTCCGAGAATGGCAGGCCTGACACCTTCTACACTAAATGGCGATGGCAACCCAAAGATTGTAACTTGCCCAGATTCGATGCAGGGAAGATGTTGGAAAAGCTACAGAACAAGCGACTAGTTTTTGTTGGTGATTCAATTGGAAGGAACCAATGGGAATCACTACTTTGTATGCTCTCTTCGGCAATTCCAAATAAGGCTAGGGTCTACGAGGTCAATGGGAGCCCAATTACAAAGCACACAGGCTTCTTGGCATTCAAGTTTGAAGATTTCAACTGCACTATTGAATACTATAGATCACCGTTTCTTGTAGTGCAGGGACGACCTCCTCATGGTGCACCTGATGGGGTAAGAATGACTTTGAGAGTCGATCATATGGACTGGATTTCACATAAGTGGAGAGATGCAGATGTGCTGGTTCTCAATGCTGGACATTGGTGGAATTATGAGAAGACTGTCAAAATAGGCTGTTACTTCCAAATAGGGGAGAAAGTGAAGATGAATATGACTACCGAAGATGCATTCAGAAAATCAATAGAGACAGTAGTTGATTGGATTGCCAATGAAGTAAACATCAACAGAACTTACGTCCTTTTCCGAACTTATGCTCCTGTCCATTTCAGGGGTGGTGACTGGAATACTGGTGGTGGTTGTCACTTGGAAACATTACCAGATTTAGGTTTCTTGCCAGCAGTATCCGACATCCATTACAGAACTGTCGTTGATGCCTTGTCAGAACGCACAAATAAATCAGAGTTCTTAAATCTGGATTTGCTTAATGTCACACAGATGTCAATACGCCGGAGAGATGGCCATGCATCTATTTACTATCTCGGGCCAGACGGTACAGCCCCAATGCAGCGGCAAGACTGTAGCCATTGGTGCTTACCTGGTGTTCCCGATTCATGGAATGAGCTTCTTTATGCACTTCTTCTCAAGAGGGAGGATTTTTGGGAACAGAGAAAGAATACCACAAATGTTTCTCAAGTTCCAGTATAATTGATATATACAGTTTGCAGAGAATGTTATAAGTTATACAAAACCATGACAAGCCTTTTTGAGTGAGGTGAAGCTGCAAGTTGAATTTGGCATCTTTCTGGTCAGTCTCACTAGTTGGTGCACCTAACATTTTGTTAGTTTGGTCGATGTTACAGAATTTTAGTCTTAGCAACCAAAGAAATTTATATGTTTTAATTTCCTACAAAATGATTGTTTGACCCTCTTTGTAGTGGTCTGCCATTGTAGGTGAACATATCTAGATGGGATGGGAACTTAATTTTGGATGGTGAGTTTCAGGGATATCTTGTACAGAAAAAAGAAAAAATAACAGCTGATATTCAAACTTGATTGGTCAGAAGGCATACAGAATTTTATTTTTCTTTTTGTAAATTTCATATCATCCATGATGTTGCTGGTTCTACATTGTATATAACATTTTTT

>C100053_65

GTGTAATAATATAATAGAATATAGGGTAACATTTCATCACCATGTGGATATGATAGATTATGAGTTGACGTGCGCGTGCCTTGAAAAGACTGAAGATGCTTCAATTTTGGTTTCACCTCAACTCACCAAGATATTCCTCAACGTAATCCAATCTATAGCTACCAAGCTACTTGGTCATAAGAAAGCGAAGCACACAAAAGAAAAACACGTTGTTAGCCACAGAAAATTGATGCTTCCTATTTAGGCCTCAGTGTCAGTCCATTAAAACTTTTGTTTCATAGGAATTTTGCTCGGGGAGTAGTTACTGTTGTATTGTGGTCGATAGCCTAAACGTGGAAGTTTCAGTCTTTGGTTGGAACTAGGAACTTCTTGTTGGCTTGTTTAGTCTAGTCACAAAAATGACAATGGTTTCTGTTCCAAAAGTGGTGCTTGGTTTAATTGCATTTGCAATCTTCTGGGTGTTAGCAGTTTTCCCAGCTGTGCCTTTTCTACCTATTGGGAGAACTGCAGGGTCCCTCCTTGGAGCCATGCTTATGGTCCTATTCCAAGTTATTACTCCAGATGAAGCTTATGCCACCATTGATCTTCCAATTCTCGGTCTTCTTTTTGGGACAATGGTTGTTAGTGTCTATCTTGAAAGAGCAGACATGTTTAAGTATATAGGGAAGTTGCTGGCTTGGAAGAGTAGGGGTGCAAAGGACTTACTTTGTAGGATATGTGTTATTTCTGCTGTCTCAAGTGCTCTCTTCACAAATGACACCTCTTGTGTTGTTTTAACTGAATTCATATTGAAAATTGCTAGGCAGCATAACCTTCCACCAACACCCTTCCTACTTGCCCTTGCTTCAAGTGCTAATATTGGGTCCTCAGCAACTCCAATCGGTAACCCCCAAAACCTGGTTATAGCTGTTCAGAGTAAAATATCATTTGGGAAGTTTCTGATTGGTATTCTTCCAGCTATGGTGGCGGGAGTGGTAACAAATGCTATAATTCTTCTGATCATGTATTGGAAGTTATTGTCTGTTCACAAGGACGAAGAAGATGCAGGGGCAGAAGCTGTGGTAGAGGAGTATGATTCCCATCGGTTTTCTCCGGCCACTATGTCTCATTTTTCATCCTTAAATTCTCAAGAATGGAATTCCCATCTTGAAGCTATTAGTGTTCAGAACTCTCCTCAACAGATTCTGAGAAACCGATCGATTGCAAATGCAAGTGAAATTAATGGGGTTTCTAGCAACACTTTTGATTCTGCAAGAATTTCAAGTTTACCGAGGGATGGAATAAATGGTGTGCCATCTCTGACAAAGGAGGAAACTAGCCCTACAAGTTCCTCAGCAACCGTGGATACACTTATTCACCCTTCGGAAAGAAAAAACGATTTTATCATAAGATGGAAGAGGGTATTGTGGAAATCTTGTGTTTACATCATCACAGTTGGAATGTTGGTTGCATTACTTTTAGGTTTAAATATGTCGTGGACTGCAATTACTGCGGCACTTGCTCTGATAGTTCTTGATTTCAAAGATGCTAGACCAAGTTTAGAGAAGGTTTCATATTCACTTTTGATATTCTTTTGTGGAATGTTTATCACGGTAGATGGCCTCAATAAAACTGGGATTCCAAGTGCTTTGTGGGACATAATGGAGCCCTATTCTCGTGTCGATCGTGCTAGTGGAGTTGCAATTCTTGCAATTGTCATACTTGTCCTATCAAATTTGGCTTCAAATGTACCAACAGTTCTCTTGCTTGGAGGACGTGTAGCAGCCTCAGCTGCTGCAATTTCCAAAGCAGATGAGAAGAAGGCATGGCTGATATTGGCTTGGGCCAGCACCATATCAGGGAATCTCTCACTGTTGGGATCGGCTGCTAACTTGATAGTGTGTGAACAGGCTATTCGTGCTCCAAACCTGCCATACACGTTAACCTTCTGGAGTCATTTCAAATTCGGCATTCCTTCCACAATCATAGTCACAGCTATAGGTTTGACATTCATAAGATGATAATGGCATTTTATGGAAAAAGGGTCTTGTAAGTTGAAGATATTTGAACAAAATGAAAAAGGATGGAAATCAGGGTTCATGTCAATATGGTTTTAACTTATGGATTCATTATCAAGAAAATGGATGGTGATGTTTTTTGCTGTCCGTTGTTTATGTAAAATAAAATATGTGCCGTTAGTTGCCATGGGATGCTTTGGAATTTAGTAAGAAAAGGAAAAACAACTTCAAGTGAATTTTAC

>C100055_65

CACCGAGTGAGATAAAATTGGGAACGATGAGATGTTACACATATGTGTATATATCAAAAGGTATGAAATTACTGAAATATGTACATAAAAGACAATATCTTTGCAACTCTCAAAATAAACATACATCCTCATTGAAGATTGATTCTCACAAACAACCAATAACATTAGCAGGTAGATGGAACGTTAGCTAATCTATCAAGTTTTAAGTGCCCATTTTGGTGGTCAAGCCCTTCCTTTCCGACCCCAGATATGTACACCTGCATTTCACATTGTTCAACGAGTTCAAAAATGCATGTGTCTCCAAAATTGATGTCATTGTCACGAACAAAGGCCATCCACCCCCCACAAAAAGTGTGAACTGTTCGGCCTTTTGCATCTGGGACAGAGTTCACTGTCCAACATTCACCTCTTGAATTACGAAGTATAACTTCAGTTTTGTAAGTTGGAAGATGTGCAGCCGAGAACTGGTACGGGATTTTCAAAGTGTATGACCCACTGACATTGAACTTTTTCATGATTTTAACAAAACTAGGAAAAGGGGAACTAAAAGCTTGAGCTACTCTTTGTTCATCGAGTGCAAACAACATTCTCAAACCAGCTTTCGCCTGTGAGCTCAGTTCATCTTCAACACGATGTCTCCTCGGAGTAGCTGTCACCAACTTTTTATGGACAGATTTGGACTGAGAGCAAAGACCCAACTTCTTAGAAGCATTTGAAGCTCTACCAGTTTTCTTTAGGTCAATGGATAAAACAGTTTCTTGACCAATCTTTGGCCATTTTTTATCTGATAGATCAACTTTTCTTATGCACTTTGAGTTGACTTTTGCATTCATTGGGATATATCTGGGAGTTCCAGCATGTCCTGCACTAGGCTTACTAATGGCCACTCTTCCAACCTGGCAGTCTAGCCCATCTTTACCAACCCCAGCAATGTGAACACGTAATTCACACTCGTGGACAAGCTCAAAAATACAAATATCTCCAACCTTAATGTTATTACCACGAACAAAAGCCATCCATCCTCCACAAAGTGTGTGACTAGTATGCACTCTAGTTGTTGGCACAGAATTGACAGTCCAATGTTCTCCTTTCAAATTATGAAGAATAATCTTTATCTTACAATTGGGAAGGTGTGCCATTGAAAACTGATATGGGATATTCAGAGTATATGAACCACTGACATTGAAGCTTTTCATGATTCTAACAAAATACGGAAAATTGGAAGCAAATGACTGAGCAACCTTTTTCTCATCTTGCGCTGATACATTTGATAATGAAACTCCACCAAAATAACAACGATCATCTTCCTTTCCACTTCTATACACGATGGCAGTCTCTGCATTTCTATTGTCCATATGAAAAGGCACAGGGACATCATTAGCCAAACACGCTTTAGAGGTTTCTTCAGTTTCTCTGAACATTCTAATCGGTCTTACTACCTCATATCGCAATAGTTCTTTGCCAACAACACCCAGTTGAAGTTCCTGGTTCTCATTTATACAACCTCTCGTTTTCTTTGAAACACCATCAAATGCAATATCCAAAGAAGAACTCTCCTTTATATCTCTTTTCTGTCCTATAATATTATTGAAGTCACTCGAACTTTGACTGCATTTAGAAAGAAATGCTGCCTCTTTCTCACATGCATCCTTATCAAATACTTGCACGGTAAAATGCAAGTGACCTTCGTATCTGAAAACCAAAAGCTCACCACATTCTAGACGATGATCCACCACAAATGTCGACCATCCATGATGGAAGAATAAATCATTGTCCTGCTTAAGCAATTGCACATTCCATACGTTTCCACTAGGACCAGTCAATGAAAATGACCCGTATGTCCTTCCCTCCATGCGACAAACAAATCCATCTGGAATTTTCAGTCTGTGAGAACTAAAGGCAGAGGAATAAACCTCATAAAAATGAGGCTTTCTCACATTTAACTGCCCCCCACAGTCACACCCCATCAATCAAACCCTATTCCTCTCTTTCCCTCTCTTGGTTTTGATTCTAGCAACGAATTTTGAGTAATGTGTTGCCACTTCTCAACATCAACAACCCCAGAGCATCTGATATCACTTTTATTTCGTTTTATGGAAAATCACTTCGCCCTGATCATAGCCAATCGAATATCTTGGGTAGTTTCGTGTGAAGGAACTTGCAGAGTAGAAGTTGTGGCAATGCTAATTTCTCTCTCTTTCGTTTTCGTTTTCATTTTCATTTGGGTTTCATCCTGCTTC

>C100057_65

GTTTTCCAACTCACTTAAGCCAATTTTCATGTTTAAATACATACAAAAACATTGTGTAGTATACAAATTTTCTCTCCCATCTCCATCCACAAATACTGTGCCTAAACTACATGTGAGAACTCCATCCACAAATACTGAGTAGCATATGATGCTTAAATTATGGAAATACAAGCAAAACTGAATGGATTTAAATTGATCCACTGTCAAGCCATTTATTTTTACACTACTCTAATCATGCATTATCACATTGTAAAATGTAGCTTGACTATGATAAAAACTACTCGAAAACTGTGTTAAGAACATGATTTGACTGTAGTATAAAAACGCCATGTACTGACAAAGGATCAAAATTGAACTCAACAAAAAATCCTGGTCTTAGAGGGCACGTGGTGATGCTTCATTCACCTAAGTGAGGGCCATATTCCGAACTGGTGCAGCAAACCCTTGTCTGTAGTGACTTCAAAAAACAAAAGAAGAAGCACAGCCAGCATTGCTACTCTTCCATTCCAAGTTTCAGCACTTTTTGTCCAACCCCATTCCCACACTATAACTGGTGGTGGCAACTCCTTGCGTTGTGTATCATATGCTGCCAATAACTCTTCTACACTGCCCAGTGGAACAAGGGACTGTCGAGCTTCAAGGTCTGAGGCCGCCAGGGCACCAACATATGGGAGACTCTCAATGACAGCATCTGCCAAATCAGAAATGAAGGTAGGTTCACATCCTAGAGCAGGAACACGGCCCCAATTTTCTACACCAGATTCTAGAGCCAACTCTTTGTATTCAACATCGATTTCTTCTAGAGTTTCAATATGCTCACTGACAAAGCTAATTGGAACAGCCAGCAGACTTTTCACTCCCTTTTTTCCAAGTTCAATTATTGTCTCGTCTGTATAGGGTCTTAACCATTCTATTGGTCCAACTCTACTCTGATAAGCAAGGGTGTAGGCATTAGTTATCTTCCTTGTCTCGAGTTCTTCCATGATCAAATCCACACATTCCTCCATTTCAACCTTGTATGGATCACCAGCCTCTTCCACATAAGCAAGAGGCACCCCATGTGCACTGAAGAATATCATGACCTCCTGAGGGCAGTCAAAACTCTTCAGCTCTTTCTCGATTAAATTTGCCATGGCCTTTATGTATCCTTGACGTTGATACCATGATGGTATTACTGTGTGCTGCATGTTGACTAGATACTCATCTTCTCTGAAGATATTCTCCAGTAGACGTAGGCTTGAGCCACTGGTCGATATTGAAAATTGAGGATAAAGTGGAAGAACAACAAGCTTTGTAATTCCATCTCTTTTAATCTGCTCAATAGCTTCTTCAGTGAAAGGATGCCAGTAACGCATTCCAACATACACTTTAGCTGGGACATTCTTTTCCCAAAGAGATTTCCTCAACTCTTCAGCCTGTGCATCAGTCATACGTCTGAGAGGAGAGCCACCACCGATTGAAGCATAACCTTCTTTGCTCTTTGGGGCCCTTACAACAGATACAAATTGGGCCAATGGCTTTTGAAGAAAACTAAATATCCTTGGCAATCGTATTATATCCGGGTCGGCAAAAAGGTTAAACAAAAAAGGCTGCACATCATCTAGAGTCTCTGGACCTCCAAGGTTTAACAGTAACACTCCAATCTTATCATCACCAATGCGAGTTGTATCAGAAACATCTTGAGCTGTAGAAGTTGCTAAAGATCCTACTGAGAACAACTGTTTGTTGAGTGATTGGTTAGAAGCCAGGAGTTGAGCTTCAGACCACCATGGAGAACATCTGGCAGATTTGTTATTTTTCAAGGGATTAGTGTTAAAAGAAGCCTCTACATGAGCTCCAAAGCAGCCATACTTCTTCTGAGAGGTGCAGATTGTCTGTGGCAACAACAGAGGACACTTGAGGTTACGAGAAGCATGAGTGAGGCGTGGAGGAGGACGAAGCACACAGGAGGAAGAAGAAGAAGATGCTGTTGATGAAGGGGCATGAATTGGAGAGTTCATAGCTCAATAATACCAGTGATAACTCAGCACAGCCACCAATGTATGGCCTTAGCAACACATAAATGGGAAAGTCCAAGGCTATGAACAATAATTACCCAGTAATCAATATAGCGAAAAGATGATAACTTTACTCAATTCCAATTAATTTGGAGGAAAAGGAGAAAGCACTAGCAAGAGGGTAGTTAGGTGAGAATGAGATTGGAGAGGAAGAGCTACACATTCTTTATTTTTATTTTTTTC

>C100061_65

GAATTTCCGAGATTTGATTGCAAGTGAAACTGATACATTTGATTAAGATTGATCCAAATTACCTATAAGGAATACATCTCGTGTTATAGTTAAACAATGAGTGTAAGTAAATAAAAATCAGAAAACTACAAGTGAGAGAAAAGACATTCACTTGAGGTTCTTATTTGGCTATATTTCAATTAGATCTACCTATTTATATGTATTGTGACACAATTTGATGCCTTACTGGTACATGGGCTGTGCTGGTGTAATTGTCGAGAACTCCATGTACTGTTTACAGTTGAACTTGGAAAAATGATTGTTGGAAGTCTCTTGCTTTCTTCGAGATAAGGAAAATCAACTGTGTAATGCAGTCCTCGGCTCTCGCGCCTAGAAAGGGCACTGCTGATAACCAGCTTTGCACAGCAAAAGAGGTTTCTCATCTCACAGATCTCAGGGCCTGCCATTGTTGGCTTCCATCCATGCTGAAACAAGCACTCTTCCCATTTAGCCTCCAAGTTACCAATTTTTTGCTTGGCGGTCTCTAATCTCATTGTGGACCGAACAATTCCTACATAGTTCCACATGATGGATTTCAATTCTGTCCTTAATTCTTTTGTCATTGATAAAATCTTGTCAGTGACATTACTTCCAAGCGACAAAGGCACAGCAGGTCTAGGCCACAAGTTTGATGCAGTCAGATCAAGGCTAGAGTTCTTCATCCAATCAACTGAGGGCTGCACAGCTCTTCTTGCAAAAACCAGTGCCTCAAGCAATGAGTTGCTAGCAAGTCTGTTTGCTCCATGCAAACCTGTGCATGCTACCTCACCAGCTACATATAGACCTTTCACATTGGTCTCCCCTTGGAGACCAGCTTGAACTCCTCCACACATGTAATGAGCAGCTGGAACAACCGGGATTGGATGACGAGTTATGTCCAAACCATGCTGGAGACACGTAGAAGAAATGTTTGGAAAATGGGAGAGAATTTCCTCCTTCGGCTTGTGACTTATATCAAGGAGCACATACTTCTCATCTCGCTTTTTAAGTTGATCATCTATGCTTCTAGCCACAACATCCCTTGGAGCAAGCTCTGCCCTCTCATCATACATGGGCATGAATCTTTCCATGCCTAAATTATAAAGGATGCCCCCATCACCTCTGACAGCTTCGGATATCAGAAATGCATTTTCCCTAGGCTTGGTTGGTTTAATAGGAAGCCCTTCATCAGCTAAGGCAGTTGGATGGAACTGTACAAACTCCATGTTGGAAATCACAGCTTGAGCTCTGTGTGCCATGGCAATCCCATCTCCAGTGGCTACCAGAGGATTTGTAGTTTTGGGATAAATATGCCCAGCTCCACCAGATGCAAGTAAAGTCACCTTAGAAAGAAACCTTATCACCTCTAGGGTTTTAGTATTCAGAGTGTCAACGCCAAGACAAATTATGTCAGATCCATCCTGACAAGTAAGAAGATCAATAGCAAAATGGTGTTCAAACACAAAAATATTAGGATTGTTGATAACTGCCTTCAGTAGAGCTCGTTCAATCTCTTTTCCAGTCATATCAGCAGCATGAACAATTCTATGATGTGAATGACCTCCCTCCCTCATTAGATGCAAGTTACCATCTTCCCCATGGTCAAATGATGCACCCATAGCAATTAATTCTCTGATTCTTTCAGGTCCTTCAGTACAGACCACTCGGACACTTTCCTCATCACAAAGATAAGCTCCAGCCACAATGGTGTCCTTCATATGACTCTCCACAGAATCTGAAGGGCATAGCACAGCACTAACACCACCTTGAGCATAATTAGTATTGCATTCATGAGACTCAGCCTTGGTTATCACTGCAACAGATCCATATTTCGCAACTTCAAGTGCATAGCGGAGGCCAGCAATCCCACTCCCAATAATGGAAAAGTCAAAACATTTTGTGGGACTATCTATCTTGCAAGAGGAAATGATTTTGAAGAGTTTCTCTTTCTTTTGAAGTGGAGGTGCAGAAAATGCACGTCTGTGTGTCTGCAGGACCTTGGATACTGCTTTAGACCATGAAAGATCTTTTCGTAAGACTGCCACAATTGGCGCATCAGAAACCAAAGCAGTCCTTCTACAGCTGTTTTCCTTGCAGTTTGTCACTCTAAATTGCAGCGTGCCACTTCCAGCAGGTATACAAGTCGCCATTGCAATACAAGTATGCGACTTGTTGTTGTTATTATTGTCAAAAGAAAACAAAGAAAGTACAATCTGAGTCGCG

>C100063_65

AACACAGTAACTCTCTCTCTCTCTCTCTCTCTCTCTCAGTCACACGCCAACCTCTCTTTTCACATCGCAAATTCAGAAGACATGGAAAATTCCCAAGATCAACCACTCCCTCCTGGTGTTCCTTCGTGGCCAAACTATGCTCATGCCGCTCCTTCACCTCAATACCCCTTTCCTCCCAATGCGCAAGGAACCCTTTCATACTATGGCTTCGCAACCTCAGAGGCCATTGCCAATGTTAAACCAACCCAGCAATATCAAGAAAATGAGTCTTTCAATTATAACCTTGCTCAGAACACTAGAGTGGATGGTGCCACAGTGAGTGCTTCCAATAACACCGCTTCCAACTACTATGGAGCACAGTTCAGTGGCTCGCGCGACATTGATATTGCTGCTCAGGATGCAGTGCTGCGTGAACAAGAAATTGCTATGCAAAATGTCATAAGGAGTCAAAGAGAGGGAAGAACTGCAGTTGCACCTAACAAGGATAATTTGGATGTATTCTCAGAGCGGCGTGACCCCAATGCTTTGAAGGAACATCTGTTGAAAATGGCCACAGAGCATAGGGCTGAAATGGCTGGGAAGCGTGGGAAAGCTGCACGTGCAGAAGAAGGTAACACGGAAATAGGCAATGGATATGGTGTACCTGGTGGAGGTGCCTATTATGATGCTCCAAAACTTAATACTACTGCAGGTAGCAACACTTTCAGTCAGTCTGATCCTATACTTGATGGATGTTTAGAACCAAAACCTGCAGCTAAAGAATTGCCTGAATACCTCAAGCAGAGGTTGAGAGCTAGGGGCATTCTCGAAGATGATTCATGTGCTACAGATCTTAAAGCCACTACAGCTGAAGTTAAGGAAAATCAAAAGTTGCCACCGGGATGGGTTGAGGCAAAAGATCCTAGAAGTGGGGCATGTTATTATTATAATGAAAGTACAAGGAAGACTCAATGGGAAAAACCTCAAGAATCATACTTAACTAAGCAATCACCGTCATCTTCACATCTTCCAGAATATTGGGTGGAGTCATTGGATGAAACAACAGGTCATAAGTATTACTACAATACAAAAACTCATGTATCTCAATGGGAGCGTCCTAAAATAGCTCAGGAAGTTGTTTCAGATCACTCAGACTTGTCTGTTGGTGGTGGGTATGATCAATCATCTAATTTACCACGGTGCATGGGATGTGGTGGTTGGGGTGTTGGCCTCGTTCAAGCATGGGGTTACTGCAATCACTGCACAAGAGTTCTGAATCTTCCTCAATGTCAATATTTGTCCTCAAGTTTGAATAATCAGCAAAGCAGTACTGCACATCCCGGTGAAATTTCAGATAATAATGCTGGCAAGCAGAGGACCAGTTCCAAACCTCCATTTCACAAGGGAAGTAGAAAAGGGGGAAAGAAACGTGCTTATGCGGAGGATGATGAGTTGGATCCCATGGACCCTAGCTCCTATTCTGATGCTCCTCGTGGTGGCTGGGTTGTGGGTCTGAAAGGTGTGCAGCCACGAGCAGCTGACACAACAGCAACAGGTCCTCTCTTCCAACAACGACCATATCCATCACCTGGAGCTGTTTTGCGGAAGAATGCTGAAATTGCTTCTCAGAAGAAGAAACCAAATTCACATTTTACTCCCATATCCAAAAGGGGTGATGGAAGTGATGGGCTTGGTGATGCAGACTAGGACCATCTTATCACAATAATGAGCTCACTTCTCTTGCTCTTTTTGCCAAGTGGATGAAATTTATCAGATACATTTTACTCTATTATCCAGGAGTTAGATAAGGATGCATTGCATTGGCCAAACCATCTGTGCTGTTGTGCAATAATATTGACTTAATGCAAATACTGGACAATTTGCATCACTTCAATGCTACAACCAAACGACTGAACGAAAGCCATATTTGAAGCATTCTGACAGGTGATGATGAATTTGCATTCAGCTTGGGAGACACACATTCATGAGCAGAAACGTCAGAACCATATGATTTTTCACAATTTGAGAATTCTAGAACCTCAAGGTTGAGTTAGAAAGGAAATTCTTACCAGTAATGTCTTGCTTTCATTTTGTTCATCTTAGTTGGTCATGTTATGCTAGAAATATAAATGTTTGTTTTTATGTTACGGTGGGATTTTACGATGGTAGATATATGTAGTTCAGATCTTTTTCACTTTGTTGAAATAGCTGTATACCTTAATTTCTCATACGAACTGGGAGCATTTGATTTAGATTCAGAGA

>C100071_65

CTTTCTCTTTCACCTTTGCTTCTCCTCAACGTTCGGTAGCCTAAAAGTTAGCCCTAACAAAAAAAAATTGAAAAAGGAAAAGGAAATAAAAGAAAGAGAGAGAGAGAGTGTTTACATTTACAAGAATAGGTAGAGGTAGATTACAGTTAAGAGATGAAAGTGGGTTTAATGGGTTTTTCTAGAGAGAGAGAGAAGGGTGTGATGGTCACCAATCCAATGAACAAAGCATTACCCTCTTATTCTTATTCTTGCTGTTGAGTTGTGTTGTGTAGAGAGAGAGAGAGAAAGAGAGAGAGAGAGAGGGTGTGGTGGTGATTCCAGATGCTTTGGAAATGGGAAAAAATGAGCAACAGCATGTGCAGGAAGAAGGGCAGAGTGATGGGAGTGAGGTTGAGGCTCCACTCTGTTGGGTCCTTCTTCGACCATTCAGTTTCAAGTGTCTATTCCTTCTCCTCCTCAGTCTCTCGGCTTTGATTTCTGCAGTCTTTTGGGTTCTTCCTAGACACAACGTTACTCGTAGTTTTGATGCAAAAGATGTAATTAAGCAAAGTGCTACTGTTCAGACGTCCTTCAGACTGGAAAAACCAGTTTCACAGCTTATTCCATATATTGAAACATTAGAATATGATATATATGGTGAAATAGCTCTACCACATACAAAGGTGGCTATCCTGTCCATGCACCAAAGTATTACACCTAATTGTACTGACGTTCTGTTTGGTGTTCTCTCTGTTCCGATGAATGGTTCAATAAATCCAGTTTCCTTGAGTGTGCTGAAGTCATCTTTGATTGAACTATTTCTCAAGCAATCCAACCTGACTTTGACTGCACCAGTATTTGGGAATGCATCAATATTTGAAATCTTGAAGTTTCCTGGAGGATTAACTGTAATACCACCGCAGTCTGCTTATCCTTGGCACATGCCTGAGGCTCTGTTTAATTTTACTATTAACAGTTCAATATCTGAAGTGTTGGAAAATATTGATTACATGAAGAAAAATTTGAAGGATGGATTGAATCTAAACTCTGACGAGAATGTGTACGTGCAAATAGTAAGTGATAATGGCTCAACAATAACTTCTCCAGTAGTAGTCCAGGCTTCAGTCCTGCCGTCCTATGGGAGCCTCATGCCCCAAAGATATAAACAAATAGCTCAGACAATAAATGGCTCTGCTGGCAAAAATCTTGGCCTTGATAACTCGGTTTTTGGCAGGGTTAAAGAAGTCAGACTATCTTCTTTCATGAAGAAAAAACTACATGATTCCTCACCTTCTCCTTCTCCTTCTCCTTCTCCTGCTCCATCCCCACAGTTAATTGATCACACTGAGCCATCAACTTCACCACATCCTGCTAGTCCGTACTCTCCAATATCACCAGCAACTGCCGAGCAACCTCCCTGTTTTAACTGTGAAGTAACTTCACCTGCACCTTCTATTGTGTCTGGGCATCCTCCAGATCCTTGTCCATATAGTGGCTTCATACATCATCCCATCTCTTCACCAATATCTTATTCTAAACCATCCTTTTCCCCTGATTATTCACCTTCTGGTGCCCCTACCTCAAAACCTGCCAGCCACACTACTGAGGAAGCTTCTGATCTCTCACATGTAGCTGATGTGTCCCATGGATCCAAGCTACGGCAGGGTGAAGAAACATCAAAAAAATTGGTGTCTCAGTTACTTGCTCCATCCTCTATATCTTCTGCGGGTGGTGATTTCCAAAGGGAAATCTTGCTAATGGGATTTTGTATGCTATTAATTTCTTTTGTTTTTCTCAATGATACCACATTTTGATGATATGGAGATGTTGCCTGATGAAGGATCCTGGGGACATGCAAGGAATACAGCCTAATGATGCTTTGCAAGTTTTGTTCAGACAATAGAAGGAAGAAGCTTCCGTTCAACTTTAATTTGCCTTAATTTTACTTCAAGCATGCAACCACCGTGTAAAGCTCCGGTGGGTAAAGGGTCAAAGCCAGAAAGTTGGCAGGCCTCTCATCTAGTACTGCATATTCTATGAAGCATGAAATTGATCCCAAGGGATCAACTAACAAACAGCGTGATCAAGAGCAAAATTGGCTTTCAGATGTCAGAAGAACCATATCTTGTATTGGATCAAGTGTATGATATGCAAAGAAATGTGGGTTCTTTGAGCATCTGAATCTGTAAAACATCTACAGTAATATCTAGCAAAGAGATAAATTAGTCACACTTGTATAGCTCTAAGCTTCCCCCTTCTCCCA

>C100073_65

GTTTCTTCAAAACCCTAATTCTGTCAAATTCAGTTTGAGCCTCGAAAAACCTATCTCTCTTTCTCTGTCTTCATCCTCTCTCTAACATTTCGCCATTCAACAGCGATCCCTACATCGTCGTCGTCATCATCAAACGTCGAACCTCTTATATCCGAAACCCTAGGGCTATTGTCTTGTAAGGATGTCTTCTGCATCCCGATCCAAGCCTAAAGACAAAAAAGCTTGCAAGGAAGCCCAGAAGGCTTCTACAAAGCCTACGGGATCTGGTAATACAGTAGCTGGTGTTCCAGCCAGTGCATATAACCCGTTATTAGGAACATTCCACACACTGGACATTTCATCAACACCTACTTCACCTATACATTCTAATGGTCGTTTTCGGAACATAGATGAGACAGATGAACATCCTGTCAACTCATTGGTTGCTGGTGTTGAGTATGATTCTGTTTCTAACAATGGTAGTTGGTCTGGTGATTCCGAAGAACATAAAGAGAAAAGTTCTAATGCCCCTGTTCGATCAGAATCAGTACCAGGAGCTGATAATGACAAGCGAGAGAAAATCCGCCAGAAAAATGAGAGGAAGCATCAAAGACAGAAAGAAAGGCGAGCACAGGAATTGCATGAGCGGTGTACTGGTTACCTTATGTCAAGGAAACTTGAGGCACTTTCTCAGCAGCTTGTGGCAATGGGATTTTCTCATGAAAGGGCAACTATGGCACTGATATTGAATGAAGGTAGGGTTGAGGAATCAGTAGCGTGGCTGTTTGAAGGTGGTGAAGAAGCAGATGGTAACAAGGATACAAACATATGTAGGGGAAATTTGAAAATTGACATATCAGATGAGCTAGCTCGAATTGCAGACATGGTAATCAGGTATGATTGCTCAAAACAGGAGGTTGAAAGAGCAGTTGTTCACTGTGAGGGTGATCTTGATAAAGCTGCTGAAACTTTGAGAGAGTTGAAACTTGATCCACCATCTGCTCCACCAAAACTAGAGGAAACCGGTGATCCTCCCATGAATAACAATGTTAAGCAGTCAGTAGTTGCAACTCAGAGCTCAAGGCCACAAACAAAACCTGTTCCTTTACCAAATCAACCCAAAAAGGATGACAAGGAATTTAACTATACAAAGGCTGCAATGACGATTGGAGTACCTTCAGATTCCAGTAATAGAAATATTCAACCTTTAAAGAGAATCCAACCTAAGTCTGAATGGACAAAGCCCCAACAGGCTATTGTATCAGCTGATAAAAAGTGGCCTAATTCAGGATCTAATCCTTCTGTTTCTTATTCCCTGGCATCACCATTGCAAATGTCACCCCCACCTGCTAAGTCTGAAGCACGATATATGCCTTCTGGAGGTGATTTTAAGAACCTTCAACCTGTAGCAGCCAGGGAACCATTAATCATGATGCAGCGGCCCCAAACTGTAAATGCAAAGCAAGTTCCAGCCACAAGTATGAGTTCATCGCCTCCTGGAATAGCTGCTAGTTGGTATCCAACTAACGGTGTAGATGCTGTGAGACCTAATGGCTTTTTATCTCATGCTCCGAACTACCTCGGTTCTAATCAAAGGTACCACCAACTTCAGTATAAACCTCAACATCAGTTTGTTGGCGGCGGCAGCAGCAATGCAGTAGATCACCAAGCAACCTGCCAAGGGAATAGCAATTGGAATAGAGCAGGTGCAACTCCAACTCTTGCTGCTGCTACTTCTCTAGGACTCTTTTCCGGACTAGGATCAGCAGGCCAGTCAGGGGCAACTTCTCCTGTAGACTGGAGCACTGGTGGGTCGATGCAGTTTGACTATACCAACATAGACTGGTCCTTGGATAGAAGTTTAGCTTCACCAAGGTCAAGCGGTTTATGGCTAGGATTTTCACCATTTTCAAAGAGTAGTGCTCAGATGTATGACTCAAATGCTTCAGGAGTCGTTCAATCATCAACGAGACCGGTTACCCCAAATGGGAGCATAGTTCCCTTGCCTGGACTGCAAGATGGTGGTGTAGCTTCTGCTGAGACATCTGCTGGTTCCCGGGACTGGAGTTCTCCATTTGAAGGGAAAGATCTTTTTAGTTTACCGAGACAGTTTGTTTCTTCTCCTACTCTGTGAAATGAGGGGAGAAAGTTGAATGAATAAAAATGAAAGAAAAACCCAAAAATGTGTGATTTTGATGTTGGTTCTTATGATTCTACAGTTGATGTTTGTTGGTGCTAAGTTTCGGTGGCTGAACCATACAAGAA

>C100075_65

AAAATCAACCTTGGCTTGTATAAACACAGTAGAATATTAACAGGAGACAAATTTTTGGCTTAGATTATTCGTGATAGATACAAAGCTCAGCATAATAGTATAGTACAGAACACCTCTATTTGTATAATGAACAAATTTTAGACATAGAGGCAAAGGACTATAAAGAAAACCTTGTAAACACCCGACCTCAAAGTTCAAACAAGAGTTAGGTCAAGTAGACACTGAGACACGACTAATCAATTGAAGTTCTTGGGAACTTTTACTGTTCCAACATCATGCAACACAGATTGCATGATTCTTAATCCAAAATATGGTAAATAAATGAGTGTTTGAATGGAAAATTGGTACAAGATCTCACACACTATGGCTTTTGCTCCACCTTGGCTCGAATCTTCTGCTCCAAAACTGATATTTCAGTCTCAAGGTTGAACATTCTATTCAAAGCATGCATGTTCTCAAGGGTCTCACTTAAAGTCCTGCTATCACTCCCATCACACCTCAAGTGTTGCATTGGCCCATGAAGCATCTTATTCACTATACCCCTGCTAAGATCATCCACTGCTCTTTGTGTCTTCTTGGTGATATCATCACCCATCTTACCTAGGCACTTTTCAAGCTCAGCCACTCTTATTCTCTCAGCATAGGCCCTCAACTTTTTAATAGTAGGAACAGTTTCCAATGAGTCCCTCCAAGCCTCAAATTGCTTAGATTCTTCACTAATGATTGCTTGAGCTTCCATGGCTTTTCTTAGCCTATCTTCTTTATTAGCTGCCACAACCTCCTTAAGGTCATCAACATTGAACACTCTCACAGACTCAAGATCTGAGACACATGAGCCAACATTCCTAGGAACTGATATATCAATAAAAAAGCGACGGCCTCCAACTTCTTGAGTGGCAGGAGGAAGGTCCTTAACATCATCCTTGAAGAACAATGGACTCTCTGATGCTGTGCTGGTGAAAACTACATCTGCCTCACCAATACATGTGAGCATTTCTGAGAGTGGTTTGTAGATTATCTCAACATCCTTCAGTTCTTCCCGGATTGCCGCAACTCTCTCCTCACTTCTATTGACAACCACCATCTTTGTGCAGCCTTTTGCCACCAAATGCTTGATCACAAGCTTTCCCATTTTCCCTGCTCCAATGATCAACATCCTTGCATTTGCATGAGAGGCCTCAGGTAGCTTCATCAAGGCCAGTTCAACAGCTGCTGAGCTGACAGAAACTGCACCAGCAGCAATGTTTGTCTCAGTTCTAACCCTCTTCCCTACAGTGATTGCATGCTTGAATAGCCCACTAATGTTCCTCCCAAAGCCATTAACTCCTTGTCCAACCTTGACAACTTGCTTCACCTGGGCAAGAATTTGACCTTCTCCCAACACGAGAGAATCAAGACCTGCAGATACTTCAAAAATGTGCTGTGTGGCATCTTTGTTGTAAAGCAGAAACTGATGCTGGCAAAGATCTGCAACTGGGATCCCACTTGTTTTGGACATCCATTCGGTGACTTCTTTGACACCACGGTGCTTGGACAGAGCAACAACATATATCTCCATTCGATTGCAGGTGCTCAGAACAGCTGCTTCTTCAATATGATTCAGACTACAAAGCTCTGCTATGGCTCTAGGCCATTCTGCTTCTGGTATAGCAAGCTTTTCACGCATTTCCACTGGTGTTGTATGCACACTCAGTCCAATAACCATAATGCTGCTCTTTTCCTTTGTATACCTATCAGCTGCAGAGGTCTTAAGCTGCTCAAGAGCAGAGACAGTGGATGGTTTCTTAGTGGGATCAACCGAAACATCAGAAGCAGAAACCTCACACCGAATAGCCCCTCTATGAGTCTGAACAATTGTCTTTCTGTTTTTGCCCAGAAGTGAAAAATGGGTGGTGGTGGTGGTGGCATTGGAGGAAGCACATTTGAGCAACAAAGCCTCCAACTTTGCACCTGAGAAACTAGTTGAAACAGCCATTGAAAATTGAAGGGGAATTGTGAGTGAGTAGCAAAATGAAGTCCCAGATACAAGTTAGGTAGAGTTAGTTGAGGCTGAAGCTAAAACTTCTTCTTTTTTGTGTTATGGTTATGGTTTTGATTTTGGGTTTGGGTTTGGCCTATGTGGAAATGATGCATGAGATGTGGAGGAAGGAAAAGGATTTGGAAGGATTTGAAAGAGTGATTCTCTGAGTTCTGATTGGCTGATACACTACACGTTTTATCCACACTCGTAATCTCACCCTCTCA

>C100079_65

CCATGTTATTCACTTATACTTTGTTGTGTGTACTGCTAAAGTCAAGCAGGTATAGTACAGGGAGACAGAGGGAGAAGAAAGAGCTGGAAACTTGTGAATTCTCTCGTCCTTTGCCCAGGGATAGTGTTAACAAGTAATTCTACGTTACATGTTAACAATCTAGTAAGACTCTTGCTGACTAATTCTCATAGAAATTCATTTCATCATGATATAGAGATAAATAGCCATTCGGGTTTGCGGTCACGGCAAAATTGATGGAATTTACATTCCTAAATTGTTTTAGTATGAAGCTCACATTGAACAGAGAGCAAACATTCTCCTAAACATAACCATTAGACTTTGTTCCTCTTGCATTTCTTTTCCTAATGTCAGCCTCATAATGTACCAGGGCAGCATCCAGAGCCTGCTGCATAGGATGGACAACAAGGGAAACCTGGTGCCGCAAGTCTGACTGTGCAGATACCTTGATTTCCTCGAGTTTAGAATATATTTGTGATATGGAATCTTTCAGTCTTCTTGTCTTATTAACAAGGTTTTGTGTAGTAATTGGATACGAAGACCAAAAATGCCTCAGCAATTCCTGACTGCACACCCAATGATCTAGTAGTTTTTCCTTGGTTGCATTGGGCAATATATCAAGGACACTCTCTTGAGAAGTTTTCCCATGATGATATTTGGTGCTTGAGATACTTTTAGTCAATCCGTTAAGGACCTTAAGAGCAACCTCAGGACTAAAGAGAGGATCCCTTAATCCAGTAGCTTGGATTTTGGATATCGATGCTCTCAAAGAGCCATAGGCCTCCTCAGAGCCCAAACTGCACATCATTTGCTCCATTCCAGGTTGGGAATCATCCAGTGTTTTTACTGCATTAGCTTGTTGGGAATCAAAATAGTCACGGGGATCCTTGATACAGAGTGGTGCATAAGGATGATCATCTTGTGCCTGAAGATCTTCTATAGGGGTCATCTTAGAAATTCTGTTTCGTCTCCCATCGTCATCTACAACCCCATCAGATTCACCCTCTTTTCTCCGAGCAAGAATTTCAGCGACTGTTCTTGGGTGTTCCATCTCCATATCTAAAGTTTTCCCTTCAAGAACAACTGCACCTTGTCTATTAAGATCTTGCAACAATGTTCTCTTATACAGAGAATTTTGGGCTTCAGATATTTCCTTGCAGCCATCTCGGAAGATTCCATGATCAGGAAGATGTGTGTAATCATCTCCTAGGTCTGCTTCCATGTCTAAAGTTGGATCAACTCGTCGAACCTTCTTTCGAGCTTCAATTTCTAATATCTCATCATCTTTCAAAAAGACAGCAAGATCTTCATCTTCAGCAGCCTCAGCAGCTGCTGCAACAGCATTTTTGGTGCTATGAAGATACTCAGCTTTGAAGTATTTATTCCAGAAATCTACCTCATTCATTTTACTAGGTACAAAATTGAGGAATGCCTGGTGGACAGCAGGTTTGAGGGCAAAAATCTGATATTTAATCTCTGGTGTCAACTGAAATTTAACCTGGTTTATCCGTCCATCACTCATAGGCTTTGTGTCAAAGATCAAAGAATTTTTAAAACCAATCCTTTGTTTTAACTTTCTGCTTTCATCTTGATCCAGCAATTTCTTCTTTGTAGCCCAAAATTCTGATTCTGTAAGCTTACCACTAGCTACAAGTTCCTTATGAAGTCTCTGCAACTTGCTATCTTCCTGCAATAGCTTTATCCTGAGTGTCATTTCAGCAGCGCTCAGCTGTTCCTCACAAATAACTTTTGTAGCTTCTCCTTGCACATGTTTGCTAAGGGCAATACCCACCAATTCCCGACAAACATGAAGATCTGCAAAACTTTCTAACTCGAAGATGTAACTTCCCTGAGCATGAGTAAGGTTAAGCCATGGCGGTTTGCCTGATCCCTCTTTAGTATGTTTATGACTCTTTATAAATCTGAACTCCACATCGAGCTTGTTGTTGGAGGTAGGATCATGGGGCTTGAAAACGAACTTATCTTGAGTCAATTTGAGGACGCCGGGGGTGCCAGGGTCTTTGACGGTGGATTTGTATTTGACACGCTTAACCACTTGCCCCGACGACATCGTTTTGGAACTCCGATTGCGCGAACAGTTCAGAGGATTAATTCCGCATTGGGTTGATTGAGGAAGAGGATCAACCACTTCCAATTTCACGAGAATCGGCGATGAAGAGATGAGAGAGAAGGACGGCGCAGTGTAACAAGAAGACGATGAATTATAA

>C100081_65

GTTTCTCTGTATCTGTGTTCTCTGTCATCATCTCCTTCTTAATTACCCTCTCTCTCTCTGTATTTGTCTCTCTCTGCCTCCGATCTATCTCCCCAACGACACCGATCTTCTTCCTCTGCATCGTAACAAAAAGCGATGGATGAGACTTCAGCTCTCATCGAAGCCATTCTCAGGGAGCAAGAGGAAGAAGAAGAAGCTCACAGAAGAAGAAACCACACCACTCAAAACACCGCCGCTAAAAATAACACTAACGAATGGCAAACGGTGTCGTATCAGAAGCGCAACCGCAACAACAAGAAGTCCTCCTCCAAGCAGCCCCTCGCCGACGATAATTTCGCCGCCGATCATTCCTCCGATGTCTTCAGCTCCGTCGAGCGGCATTCCGAAGAGCGCCGCCGCCGCCTCCTTGAGTCTCAGATAGCCGCCGCTTCCGCGGCGACGGACGACGTTCCGAGCAGATCGAAGCGACATTCCGATGACGAAGACGACGGCGATGCGGAGGCGGAAGCCGGCGCCGACCAGAACGGTTCTTCTGAAGTCAAGAAGGCAAAGCAAAAGAAGCCGAAGAAGCCGAAGGTGACTGTGGCCGAAGCTGCTTCCAGAATCAGCGCCGATGAACTGGACTCGTTTCTTGCCGAAATTACCGCTTCGTATGAATCGCAGCAAGATATTATGATGATGCGATTTGCTGATTACTTCGGTCGCGCGTTCTCGTCGGTGAGTGGAGCTCAATTCCCGTGGCTGAAGATGTTCAAAGAGTCCACTGTTGCGAAGATTGTTGACATCCCTCTTTTGCATATATCTGAGGATATTTACAAAATATCAACTGACTGGATCAGTCATCGATCATATGAAGCCCTTGGTTCCTTTGTCATATGGTCATTAGACAGCATTCTTGCTGACCTTACAAGTCATCAGGGAACTGTTAAGGGATCTAAGAAGGTGGTACAACAATCATCGTCAAAATCTCAGGTTGCCATATTTGTGGTTTTAGCAATGGTGCTTCGACGGAAACCTGATGTCATGATTAGTTTATTGCCTATAATGAAGGAGAGTCAAAAATATCAAGGACAGGATAAACTCCCTGTGATTGTTTGGGTGATAACTCAGGCTTCTCAAGGAGATCTAGTTATGGGGTTGTACTTGTGGGTATCTCTTCTCTTACCCATGCTGAATGTGAAGTCTGGCTGCAATCCACTGTCAAGGGACTGGATACTACAGTTGGTTGAGAGAATTATCACATTTCCTAAAGCTCGTTCTATCTTGCTAAATGGGGCTGTCAGAAAGGGGGAGCGTGTCGTGCCCCCATGGGCACTTGATTCGCTATTGAGAGTTACTTTCCCTCTTCCTTCAGCCCGGGTCAAGGCCACTGAAAGATTTGAAGCTGTTTACCCAACATTAAAAGAAGTTGCCCTTGCTGGTTCCCCTGGAAGTAAAGCAATAAAGCATCTTGCACAGCAGATACTCAGTTTAGCAATTAAAGCTTCGGGAGAAGCCAACCCTGACCTGTCAAAGGAGGCAAGTGACATTTTTATTTGGTGTTTGACTCAGAACCCTGAATGTTACAAGCAATGGGATTTGCTTTATATGGATAACCTTGAAGCAAGTATTGTTATATTGAGAAGACTTTCTAGTGAATGGAAGGAATACTTTGTCAAACATACGACTCTAGATCCTTTGAGGGAAACTCTTAAGAGCTTTAGTCAAAAGAATGAAAAGGCGTTGGCTAATGTGGATGATGGTTCTCGCCATGCATTATTAAAGGATGCAGACAAGTACTGCAAGGTCATTTTGGGACGATTGTCGCAAGGCCATGGATGCATGAAGAGCATGGCAGTTTTTTCTGTTGTAATTGCTGTTGGTGCCGTTTACATGTCCCAGAACATGCATCTATGGGATTATAATAAACTGACAGAAATGTTGAACCTCTCCTAGGTTCCAGAGTTATCCTGTGGCTTAGTCGGTGCTTATGCCCAGCGTTAATCACTTCAGCCAATATTGAGGTGCGAGGAATATTAAATTTATTTTAGTCTTCAACTTAGTGAAATCAAACAAAAATAGTAACAAAGCCATGGAAGCTGGTGCTGGCCATTTATATAGGTGTTGTGTGTTCTCAAATTTTAGGTCAATTTGAATTTTTTTTTATTATGAAATTTTATAGTGGCTTGTTATATATTGACTTTCAGATAAAGTGCCAAATGGCTATAGAGAAATGCAAATGAAGGTGAAATTAAGTTGTGCTTGAA

>C100083_65

CGAGAAGAGTATAGAAACTCAATCCGATCTTTGCGACGCGACGAGACGCCGACGATGGGAGCGACCACTTCATCAGAGCCGAGGGTTTCGGCGGAGCAGAAAGAGGCGGAAGCCGTCGCAGCCTCCACCGGAGCTCTTCCAATGCTTCAGAAAGCTTTCTCCAAGCTTGCCAATCCTCAAACAAACGCCGTTCCGTTAGAAAATCTACAGAAACTCTTTAGTTTCGCTGGCGAAGGACGAAGTTGTTCAGGAAGCAACGTGTCAGATTCGTTAACGGTGATGTTGGATCACCTAGGCTCAACCCTAGTGGATCAGTTTTTCGTGCCCGATAAAGGGGGAGTAATAAACTGGGTTGAATTTGTTAAGGGTTACAACAAGTGTTGTGCGAGGGTGTCCGCGTCGGTTTTGCTAAACATGCTTTCGAGAGTGTTCGTTGCTATCTCAGAATGTGCTAATGTACCTATGCATTTGGAGTTTGAATCAGGAGATGCTGATTGTAAGATTAACGGCCACCTTCTTCCTAGTGACGTGTTTTTGCTTCTTGCCCTGTGTTGGGTTATGACGTGGGGTTCTAGAAACTTGAAGGGGGGAGGGAATCTAAGCCTACCGGATCTGAATCATTTGGTGTTGTCTGTGATCACTTCGTGTGGGCAAGTTGAGGGTGGTTTGGATGTGTGGGACTGTGATATCTCGAGCTTGGAAGTTCAGCTTCCTGCAGGGAAGTTCGTAACGTGGGTTATGAGTACCGTGCCGTGCCTCCCCGATTGCTTGAGACTGTATTTTCATGCTAGACTGCAAATGGCAGCAACTGATGGGGTTGAATTGGCTTACTCTAATTCCTCCTCTTCTGGAGATATTTCATCAATGGCTGCACATGATTATCTTTTGACCCAAGGAAGGGCATGGGCAATTTCTATCACCCAGAAAAGTACAGTAACTGAAGAGATTTCTAGAGCATTTATAAGCAATGGAGGTGGAATAGATGATAACCTTGTTTATAGGTCATCCGCTCATGGAAGAGGCCTTGGCAGATTTTGGTCCCGTGTTGAAGGTTACAAAGGTCCTTTGCTAGTTTTGATATCTGCAAGTTCAGGAGAACCTCATGAAGGAAATTCTGTTGATAGGAAATGGGTTATAGGTGCCCTAACAGATCAGGCTTTTGAAAGTAAAGATATCTTCTATGGAAACTTTGGATGTTTATATTCTATTGGCCCAGTGTTTCATGTGTTTCCACCTATTGGTAAGGAAAAGAATTTTGTTTATAGCCACTTGCATCCAACTGGTAAAGTTTACCAACCGCATCCAAAGCCAGTTGGTGTTGCATTTGGAGGAACTCTGGGAAATGAGAGAATATTTATAGATGAAGATTTTGCAAAAGTGATTGTTCGCCATCATGCTATTGACAAAACTTACCGGAGTGGATCCCTCCTTCCGGATCAGGGTTTCCTACCCGTTGAAGGTCTGATTTCAGAAGTTGAAGTTTGGGGACTTGGTGGGAAAGTAGCGAAGGAAGTGCAGAACTCTTACAAAAAGAGAGAAGAACTTTTCACTGAGCAAAGAAGAAAAGTTGATTTGAAGACCTTCGCAAGTTGGGAGGATTCACCAGAGAAAATGATGATGGACATGATATCGGATCCAAATGCTGTTCGAAGGGAAGACCGCTGAGTTGTATCGTTTAGTGTTGCATCACTTTGCTTAACGCAATACTTGACAGGCACAAGCGTGGAATGAATTTCATGCTTTATGATGTGATTGGTCATTATTCTTCAAGCTTGTGATCCCAAGTGGTTGTACAGAACACGAAAAAAGGTGTATGTTAACAAATCTATGGTTTAGTGCACCATATAGGGTTTATATATGTTTACTGTATCGTCCTCAAATGGGATGGAGGTATCGCATTTGGAGAGTGACATAACCCTCTTTCTCATCGTTCCACACCATTATGTGCATAACAAGTCAAACGAGAAACATGGCTGATAGTTTCACGACCAGATTCCAGAAATCGTCGAGCGAGATAGCAGCCTTGTCGTCCGGTGTACATTAATAAATGGAAATCTTAGGTTTACGGTACCTTATTGGTATTTACCATTTGTGCAGGTGTATCTACAAGTGTGTTATTGCTTCATTGTTTTGAATTTTTATTTGATGTTTGATATCAATGGTTTGCCCATTTAAAGTGAACGAAATGTCATCGCGTAGCCATACCTGGAGATTGTTTGGATCATGAGAATTTATATATTAATAAT

>C100087_65

GTTTACAGCTTCCTGACTTAGTGACAGGCATGCTACTTCTCTTTATGCTGTCTAAAAACATGTCTTTTTTCTCCCCCTTGGTCTGACACAAGATTTTTCGGTGAATGAGATTCTAGCATGGAAAGAAGGAAGTGAGTTTCTTTTCTTATTTTGAACACCACAGTGATGAAAGAACATCCATTGGAGAACTGTTAAACGTTCCATATTTACATCTTTGTTGGTTGAAGTTAGAAGCCTCAACCTCAAGTGAAGTGTACTTGCATCTTGTTCCTTGGAAATTCCAATTGAATGGGGACGCCAAGAGGAATAACTCTTCTCTGTTTCACATTGTTCTTGTTTTGGAGCTCCGCAGATGCTTTGCTTTCTCCCAAGGGAGTGAACTTTGAAGTGCAAGCCTTAATGAGCATAAAAGATTCTCTAGCGGATCCACATGGTGTTCTAGATAATTGGGATAGGGATGCAGTTGATCCATGTAGCTGGACTATGGTCACTTGTTCTTCAGAAAACTTGGTCGTTGGACTGGGCACTCCAAGTCAAAGTCTATCTGGTACTCTATCTCCAAGCATAGGCAACTTAACCAACCTTCAGATTGTGCTGCTACAGAGTAACAACATAAGTGGACCAATCCCATCAGAGTTGGGCAAACTTCCTAAGCTTCAAACCCTTGATCTCTCAAACAACTTCTTCAGAGGGGAGATTCCTCCCTCTCTGGGACACCTGAGAAACCTCCAATACCTGAGGCTCAATAACAACAGTCTTGTGGGTGAATGTCCAGAGTCTCTGGCTAACATGACCCAGCTTAATTTTCTTGATTTGTCATACAACAATCTTAGTGGCCCGGTTCCCAGAATTTTAGCCAAATCATTCAGCATTGTTGGAAACCCCCTTGTTTGTGCCACGGGAAAAGAACCAAACTGCCATGGGATGACATTGATGCCTATGTCCATGAACTTAAACAATACCGAACATGCTTTACAATCAGGCAGACCCAAAACTCATAAAATGGCAATTGCCTTTGGCTTGAGTTTGGGATGTCTCTGCCTCATAGTTATTGGTTTTGGAGGTGTTATTTGGTGGAGGCACAAACACAATCAACAAGCATTCTTTGATGTTAAAGACCGGCATCATGAAGAAGTCTACCTTGGAAACTTGAAGAGGTTCCAATTCAGAGAACTCCAGATTTCTACTAACAACTTCAGCAGCAAAAACATATTGGGAAAAGGTGGTTTTGGAAATGTATACAAAGGAGTTCTCCCTGATGGTACTCTTGTCGCTGTCAAGAGGCTTAAAGATGGCAATGCCATTGGAGGAGAGATTCAATTTCAGACCGAAGTTGAAATGATCAGCCTGGCAGTGCACCGAAACCTCCTCAGACTCTATGGATTTTGCATGACGCCAACAGAAAGGCTTTTGGTTTATCCATACATGTCCAATGGCAGTGTTGCTTCCCGTCTCAAGGGTAAACCAGTGCTGGACTGGGGCACCAGGAAGCACATTGCTTTAGGAGCAGCAAGAGGACTACTGTACCTTCATGAGCAATGTGATCCAAAGATAATCCATAGGGATGTGAAAGCTGCCAACATATTGCTTGATGACTACTGTGAAGCTGTAGTTGGAGATTTTGGGTTGGCAAAGCTTTTGGATCACCAAGACTCACATGTTACAACTGCTGTGAGGGGCACTGTGGGACATATTGCCCCAGAGTACCTATCCACAGGACAATCCTCTGAGAAGACTGATGTCTTTGGATTTGGCATTCTACTCCTTGAATTGATCACAGGCCAGAGGGCTCTAGAGTTTGGAAAATCAGCCAACAACAAAGGAGCCATGCTTGATTGGGTAAAGAAAATTCATCAGGAGAAAAAGCTTGAAATGCTAGTGGACAAGGATCTCAAGACCAACTATGACAGAATTGAGCTTGAGGAAATGGTTCAAGTGGCCCTCTTGTGTACTCAGTATCTTCCAGGGCATAGGCCTAAAATGTCTGAAGTGGTACGCATGCTTGAAGGTGATGGACTAGCAGAAAGATGGGAAGCTTCACAAAGAGTTGACACAGCCAAGTGCAAACCCCAAGAGTCCTCTTCATCAGATAGGTATTCTGATCTCACTGATGACTCTTTGTTGTTAGTCCAAGCCATGGAGCTCTCAGGACCCAGGTGAATCAATTGTCTAGTAGTACAATTTTACTTTTATTTTAAAGGTTTTTAAAAGAAAGCATATGAAATTCAAGAACATATGATAGTTGAA

>C100089_65

GCAACCTCACTCACTGATTCTACATCATTTGTCACATTCATTAACCATTCATTACTATGATCAATAGCAAGTGATACACAATATAGTCTCTGCTGTCCCTCTGACCTCCCTCACCTATCTCTCTACATACAATTACCACCTCTTCACTAGAGGCTGTTCAAAGGAGTCAAATCATGAGCCACACACAACCGTTTTGCTTGGTTTTGGAACCAAACGAAGGGTTGGTTGCTCCTAATAAGAGGCTCTCAAGGGTTAAAGACCAACAACTACTACTACATACATACATACATATCCTCATTCCTCACACTCCCTCAAGAGAAAGAAAAAATAAAAAGTTGCAATTAATGGTCCAAGAAAGTGCATATAAAAACCAAACATCCTAGCAAAGGATGATTTTTATCTTTGCAAGTGCGAACTCTTTGTATAGTAGTGGATTAACCCTTTTAGCCACCATGCCGAATGCCCATGAAGTGATTAAAGTCATGATGCTGGACCCTGAGTTGCTTCAACCATGAATCTGCCACACTCAATAGTGACAATAGCCTTTCTTGGTCCCGACCACAATTTCCAGCGACCCACACGTTTCCTCCTTGCATCTTGTACGTTGCCAAACCAAATGCGGGCAGTGCTATGCCTTCCCCTTCCTTTTTCTTCTCAAGGCCACCCTCAATATCATCGTCAAGATCCATACCTTGAAATGAAGATGAAAGCGTGTGATAAGTGAGGAAGCATGTAGAAAGGTCTTTAACTGTTCTTCCCATGGGGATATGATATATTGGGTACCACGCAACAGCCATCCAACTTGCTGGCGAAAGATCAACACTTCTTAATGACATCAACCCTGGGTATCTTTGAGCTAATCCAGTAATCTTATCCATGAGAGGAACTCTCCCATATGGAGTTGCTCTCTCAAAATACTGGCAATAGAGGTGACCCAGTCTGTCATTGAAATGCCAGAGAGAATCTTGCTCAGACCCTCCCTCTTCTGAGGAAGTTCCATCCCACCTCCATAACTTGTCACACTCACTTTCATCGCTGAAAGAATCACTATAGGAATCCCTCGTCTCACAGTCACCTGACTCAGTCTCTTCCCTGAAACTATTGCTGGTGAAAATCTGAATTGCGGAGAGATAAGGCACATAGTACTGCACAAGTGTCTCCCCGCTGGTCAAGGAGATGGGAACTCCTGCTCCATAAGCACTCCATTCGTCATAGCAATTCCAGAGATCCCCCAACGTGAAATATTCCACGTTTTCTCTCTCCCACGGATGCCACAAACGGTTAAGGCTTGTAATCTCGGACTTAGGAAGAAACTGGGATTGAACCACAGGTGTGGTACACCGTACGAAACAGTCAAGGTTAGAATGCATTGACCCTTTATTAAAAATCATGGTGTTGTAGAGAGATAGATGAGAGAAGAAAAAGAGAGAGAGAAGGGAGGGAAGATGGAGCTTTTAGCTTCTCTCCCAACCCTGAAAAACAGAGGGAACCACCCAGATGTAGAAGAAAACAAAACAAAACAAAACAAAAAAAGAGTGAGAGAGAGATTGTGGGGATTGCAATTTTGCCTTCGAAAGAGAATCGAAAATGCAAATCGCCCCCACAATGGAAGACTTCTATTTTTTGACGTTCAGACACCGAATTTCCCGCAAAATCTCAATACCCTTCACTTTTTTATTTTATCTCTCCGTGTCTCTTTCCTTCTCTCTCTTTTTCTCTTCTCAATCAATCAACCAACAATGTCGCAAATTCAACACCCTCGCACGGGGAGACAGATTCTTATGCAAACAAACAATGCTATTTTTCTCTCTCTGTCGCTGTTAGGCCAAGCCTTCAAGAAAAATCAAGACAAGACTGTTTTGTTCAACGTTTAAGAAACACACTTCACAGGAGATAACAATAACACGCTGACGCTTCAACCTTAAATCTTGAGATTAAATCAAACTACCAAAACAAAGCCGCATAGTCACCTTAAAAATGGGAAAGAAAAGACAAACGAGGATAAAAAAACATCATGGACAAAGTCAGAAAGTACTACGTGTATCGAAAATATTCATCAAACAGAAACAAGAGAAGAACTCTTGGGTTTAATTTCCAGAAGTTGTGATGAATTTTGATGAAATGATGACTGAGTAATAGCAGAGGAGATAGATAGATAGATAGATAGATAGATGAAGATTATGAACTATACAAAGCCATAATTGAAGCATCAGAATCCAAGATTTATAACATTTATGAGCTTCATCC

>C100093_65

TTTTTTTGCAGCAAAAAGATTGTATTCCAAGATTCCAATAAAAAATACGCAAGACAAACTTATACTCATAGTTTGAACCAAAACTGACGGTTTTGAATTTGAACCCACAGTGTATTGTACTTTATGGACCCTCATCTTTAGTCAATAAACTGCATTAACAAACTGTCAACTAAAAAGTTCATAAAAAAACATGAGTTCTATTTAATTCCTCGATTTCTTTCTTGACGACTTGGATGAAGGCTTGGTTTGCTCCGAAACTGCATTCTGCCCAGCTCCTTTGGAAGCTCCTTGGTTTGATTTTGGATTTGAACTGTTAGATGAGGCACCAGTATCATGCTTATCTCTAACTACAGCACCTTGAGACCCTCTCACTTGAGCAGCTCTTTTGTCTTTCCTCTTGGGCCTATATGTTGATCTCTCCCGTTTGGGAAGCCACCTTTCTGGATCTGGGGGTGGACCTGGATTTGCAGGGTCAAACCCTTTAGGATACCTTGGCTTTCTCTTTCTCTTTTTCTTAGTTTTTGTCTTGTTCTTCCCTTCTTCATATGTTTCAGAGACACCAACACGAGGGGCGTCAACTTGTTTCACTCCAGATGTACGCTCCAAGCTGTCCACATCAATTCCCTTTAAACCAGGTAGGGCCTTCAGTTGCTTTTCATAAAGCTCTGCCTTGTCAACATCCATTCGGGCAACTGTTGTTACTAGCCCAACTAGTGCTTCTATGCTTCCCTGGCTTTTGACGAGTTCCTCATAGAGTTGAGCAGCTTCCTCTTCTTTCCCATGCCTGAGCTTGAATGACGCAGCTTCTTGCATTATAATATTAAGCTTATTGTCCTCTGTCATAGCATTAGACCACCATTTAGTTGCAGCATCAAGCACAGCAGCTGCACCATCAATGTCACCAGCTCGCTCTTTTAAAGACACAAGGGTGGCAACAGTAGCAGGCATATGTTGGATATCAGATATCTTAACCAGAGAATCGGCTGCAATATGTGGGTGGCCAGCAGCAGCAGCCACCTGAGCCCGGGCTAAGTGAACAACTTTGGACTTCTCAGGGAATTTACTAGCAAACTGTGCTAGTATTTCCTCAGCCCTTCCAGCTTTGTTCTCTCTAACCAAGAGGGCAGCATGTAGCAATACTGGTGTCACACTTTCTGGGAACATATCTGGCAATGCAGAAACAAGTTCTCGTGCCTGCTCAATCTTATTAGCATGAAGAAGTAAAAGAATCCTATTTGCATATATTGCTTCCTTTTCTTTTGCTGAAAGTTTCAAGTCCAGTCCCCGAGCTAGCCTATAGCTTTGACTATCTTTGTCTTTCAGTCGATCAAGTTTCCTTAAGCTATCAGAAACATCTTTTGGACCTTTCAGTGAAACAAGATTGTTCACTGCCACTGCAAGTGATGATTCATCAGCCATATCTCGTTTAATTGTGTCTGTGTAAGCTTCAATAGCATCTTGCTTATGCCCAAGAAGCTGCTGGACATAGGCTAATTGTACAGTTATAGGAGACAATTCAATTTCTATCTCATCATCAGCCAAGTTATCTTCCATCAGGACCTCTTGACCAATTCTTCGGCCTGATAATAAGAGTTGTTCTGCATCTGTGTACTTGTTCCTTGCAATTAAAGAACAAGCAGTATTGTATGCCAATTCAAAGCTGCTAGTTGCTTTAACCCGAAGTGAATCCAGCATTCCTTGAACTTCAGATGACCTGCCTGCCATAACTAGGGCTGCAACAGAATTGATTTCCATGTTATCAATCTTGGAATTTTGGAGCTTCTGGTAGATATCTAAGCAAGCATCCATTTTCCCAAGACGATACAATATCTGACACTCCAAAAGCATAGTTTCATCATTTCTCTCTTGTCTTTTCAAAGACTCCAAAGCTTCATCCAACTTGTTTTGTCTGTATAAGCAGTATGCCTTGAAGAAATGAAAATCATCAAGTTGCTTCCGAGAAGACTTAATGGCGGAAAGCGCATCTTCTAGGCGGTCATTTTTTATCAGTGCCACGACCTTGCATCGAAGCGCGTCCTCATCGTCGGGTGCAATGGCGAGAATCTGATCTGTGAGTTTGACGGCATTGTCGAATGCGGAAGCCTGGATGTGCCTGTTGAGAGTGGTGAAGAGATCTTCGAGGGAAGGGGGAGGGGTGGATTGCGATGGCGCCGCTTTAGGCTTCGGAGCCATTAGGTTTCAGGCGAACCCCAATGTCAATTGTGCAGCTTTACTCACGAAAGAGTACA

>C100095_65

CTCCCCCAATTCAATTCCATTTTCACACTGACTGGTCAAGGTCACTCATCTCTTCACCTTCTCCTTCTCTTCCACCCTCAATTCTTCTCTCTCTTTCCATTTTTCCTTCCCAATTTTCCTTCTCTCATCTTGGTCCTACCCAAAATAAGATTGTCTATCTTGATCATGGGTTTCAATTCTGTCTATAGAAGCTTGCAGGAGTTATTTCCGCAGGTTGATGCCAGGTTACTGAGAGCTGTGGCTATTGAACACCCAAAGGATGCTGATCTCGCTGCTGGAATTGTTCTTGCAGAAGTTATCCCGTTCATGTCCAAAAAGTTACCTCTTGCAACTCCTCTGCAGGAGAATGGCCTTGGAGCCCCACTGAATCTTGAAGGACCTGTTCAATGTGTAGCTGAAAGTGAGGAGGAAGGCAGTAGTTTGAGGCACCGTCAACTTGTTCAAGACATAAATGTGGGACCTTCTTCTGCTCCACATTCTATACATATAGAGACTATCAACACAGCTGACTATTCTTTAGTACCAGATTTGAATGAAGCGCTGGACAAGTCTACTTTGTCAAATTTCACTAATTCAAATGATGGTACTGAAAAGTTTCTTGGAATGGGTGATATGAAAGAGCTAAATGTTTTTCAGAATGCTGAGGATAACTCCATTGAAGAGACCTCAAACAAGATTGCTTTGGAGACGTCAAATGGCTTTAATCAGGAAGATAATGAAAATTTTGATCAGAGACGACTTCATATTGATGTTGAGTCTGAAAACTTGATTTCTTCTGGTATATGTCAAGAGATGGAACCTGAGCGTAATTACCTTAGCAAGGAGGCTACTTCCACTAATAACAATGGAAATAGAACGGGAAACCATTTAAATGAAGAGTGGGTAAATTTTGTAGCTCCTTCAGCTGATGATTATGATGCCACTACAAGTCGCGTACTGGAGGATTGTGAAACTTACTTGATTGAGGTAGAAAGTTCTGGGGCTCAAGCAGTTTCTCATATTCAAGGGAAGAATGAAGATAGTCTTCAATCAGAATTGGATGCTAGGTCGTCTAGTGCTGGTGACAAAACTGGTGATGTTGAAGTTGAGGTTGGTGGGAAGAATGAAGTTAGCCAATATAGCCAAGTATGCAGAATTGATCTGCTTGAGGAAATCATTGATGAGGCTAAAAGTAACAAGAAAACCTTGTTTTCATCTATGGAATCTCTTATCAACTTGATGAGGGATGTGGAACTTCAGGAGAAAGCTGCTGAGCAGGCCAATATAGAAGCTGCTACTGGAGGATCTAATATTCTGGCCATGGTAGAAGGATATAAAACTGTTCTGGAACAAGCTAAAGAAGCAAATGACATGCATGCAGGAGAAGTTTATGGAGAAAAAGCAATCCTGGCAACTGAACTGAAGGAACTTCAATTACGCTTACTCAGCTTGTCTGATGAAAGGGATAAGTCTCTCGCGATTCTAGATGAGATGCGCCAAATTCTTGAAGCACGACTAGCTGCAGCTGAAGAATTGAGGAAAGCAGCTGAGAATGAAAAACTGGAAAAGGAGGAATCTGCTCGAAAGGATCTTGTTGAACAGGAAAAACTGGTGGAGAAGGTACTACATGAATCAGGAAGACTACAGGAGGAGGCAGAAGAGAATTCGAAGTTACGGGAGTTCCTGATGGATCGAGGGCAGGTTGTTGATATGTTGCAGGGAGAAATTTCTGTTATTTGTCAGGATATCAGGCTTCTAAAAGAAAAATTTGATGCAAATCTTCCATTGAATCAATCCTTCACCTCAAGCCAGACCAGTTGCAAGTTAGCTTCGTCAGGTACGTCCCACAAGACGGCGACATCTGATGTAGGTTCTGTGCATAGTGATTCATCTGAAAGAAGTAAGACAAGCCGGGTAACCTCAATTGAAAGCCTGCCTTCCAAAAGTGTGCACGATGAGGAAAAATCCAAAGCTGATCACAATGCTCTTCTTGATGATGGGTGGCATATTTTTGAGAAGGATGCAGAGCTGGATTCTGGTGGATTTTACTGACTGTTTGTTCTACCGACGGAGAAAACAACATAGGATTTCGTGCTATTGTTTATACAACGCAATAAACCTTAGAATCCTTGGATGTCACTGATTAGTTGTGATCTGGATATTTTTGTCTTTTACACTAGTACTCGAGGTTGGCTTCTCTTTGTTTTGCAAACTTTGTATATAAAGTCATTCAAAATTACTTCATTTCTTCTTTGTCACCTGTAATGCTGG

>C100097_65

CTCAGCTTTTTCATTCTGCTCACTTTCTTTGACTTCATAAGGCCTTCTTCGTGGTCCAAGCACAACCGCACATTATACCCATTTTTAGCTTCACTCTGAGTTGCATGAGCTTCTAACTCATTCCTGTGTAAAGTTCCTTCCTTTCGGTTTATTACATTCTCATGATTTGAACCCTCTTCTGTTTCAAAGTTTATTGCAAAAGCAATTCTATTCAACAATTGAATTCTGTACACAAGATGGGGTGTTTCACTGTCCTGAAATACAAGAAGCAAAAGATCAATAAAATCCGATACACCAAATGTGTCAGTGTCAGCCATAGCGAACATGTTCCTACAGTACTGCCTGAACCTGAAACTCGTACTCGTTCCCTGCAGTCTGCACCTCCTAGTTTTAGGAACAGAATGAAATCTACTCACCCCATTAATAAAGTCTCCAATTACAGAACACGGGCATTATCTGCTCCATCAGCTCTTGATGTGGCCAAACAATATACTCTTACCACGGTCAAGGTTGAGGAACAAGAGGAATCAAGATACAATGTGGGATCGCTGAAGGGGCAGCATTCATCTAGTCCACAACCTTTACCTCTTCCATCACACCAGGGTGGTGGTGAATTGAAAGCTACTAGCAGCTTTAAGTCAGGAATAGCTAGTGGTTCTCTATATGCTCCTGATGCAGCAGAACAGGATCCTTTGGCCTCAGTTGAGTATGAGGAACAAGAAGAGGCAAAACACCGAGTTGGATTGGTGAAGGATCATTGTTCATCTAATTCACTACCTTTGCCTCTTCCATCTCCTCAGGGTGGTGGTGTTGCATTGAAGGCTAATGTCAGCTTTAAGTCGGGGATGGCTAGTGCTCCTCTATATGCTTACGGACCTTTGCCTCTTCCACCTACTGGATTGCTTAGAAACTTTCCCTTTGAGGAGATTGCTGCTGCTTGTCACAACTTCTCTTCAGATCGATGCATGTCTGAATGTCTTTCTTCTACCATATATAGTGCTTCATTTGGTGATGATGCTTCAAGTGCAAAGAAGTTTGAAGCCACTGTCACCCGCCTTCATTCGTCAACTCAGGGATTGAAGGAATTCATAAATGATTTAAATACCCTTGCATCTTTGCAACATCCAAACCTCTGTAAATTGCTAGGATTTCATGCACGTGATGGTTCTGAACCAAGGATGTTGGTTTATGAGAGGCTATGCCATGGAAGCTTGGACCACCAGTTGTTTGGGAGATCTGATGGTCCTTCAATTGATTGGAACTCACGAATGAAAGTCGCCATATGTGCTGCACAAGGTCTTACTTTCTTGCATGAAGAAGGGCCTTTCCAGGCAATGTATAATGAATTCTCAGCAGCCAACATACAGATTGACAAAGATTTCAGTGCAAAGCTGTCTGGGTATGGTTGTGTTGGACGAATTCCTGAGGAAGAGATTTCAAGTAGTTCATGTGCTGTTGGAAACCTGTCAGTGGAGACATTGGAAAGAGGAATTCTTACTCCAAAGAGCTATGTTTGGGGTTTTGGAATTATTCTTTTGGAACTACTTACAGGCAGAAAGAATGTTGATAGCCATCACCCCAAGGAAGAGAGGAACTTGGTCAAGTGGAGCCGGCCTTTCCTGGCCGATGATTGTCGATTGTCGCTGATCATGGATCCTCAACTTAAAGGTCGTTTTCCTCCCAAAGCAGCAAGAGCAGTAGCTGACATAGCACAAAGATGCCTCCAAAAGGAGCCATCAGAAAGACCCACCATGAGAACCATTGTTGAGCATCTCAAAGTGGTTCAAGATATGAAGTACTCATGTTGGTTCCCACTGCAAGAGCCAGCAAAACAGATGTCAAGATCACCAAGTCTTAATGGTATCAAATGTCTCACACCGACGTTGAGTTTCTCTCCCTCAGCAGCACCAGTAGCCACACCATCTGTTTCATCTCCAAGATGGTCTGCTGTGCCCACATTGCATCTTCCTCTTTACGCTTGTTCCTCCACTTTCTCTATGGACGAGCTAGGTAGGCCGGAAACCCGGAAGTCATCATCCTCAATGTGTAGGAGAGCTAGTGTTGAAGGATATTGATTGTCAATAATGTTCATTTTTATTTGATTTTGCTTGTTATTATCATTTTTGGTGATTCAACACAATTGTATAGGAGATAAAGGAATGGTTCTTCCATCTTGCTATTTCTCTCCATGAGATGTTATTTTGTAGATAGTGCAAAGGTTGGTTATAATGGCTCCAATTAGTTACCGCG

>C100099_65

AATTAAGCCGCTTGCTTTCATAATTTGCAATGTAAAACGTCGTTTTCTCTCTTACAAACCAACCACAATGGCGAAAGAGCTTCTGCAGCTTCCTCTGCTTCTGCTCCAACAACGGTGTCGTTTTGCACTTCCTTTGCCAATGTTCAACTTCGCATTCTCAGATTCGTCTCTTCCCTCTACCTTTTGAATCGCTTCTATTTCCTTCCTCAACTTCTTCTTACCCGGTCGCAGGAGAGGGAAATCTCTTGCTAATTGATGACATGGAGCAGTATGAAATACTTGAGCAAATTGGAAAAGGGGCTTTTGGTTCTGCTCTTCTTGTGAAACATAAGCATGAGAAGAAAAAATATGTGCTGAAGAAGATTCGCCTTGCCCGCCAAACTGAGCGCTCTCGTAGGTCTGCTCACCAGGAGATGGAACTCCTCTCCAAATTAAGAAACCCATTTATTGTGGAGTATAAAGATTCGTGGGTAGAAAAGGGTTGCTATGTATGCATTATTATAGGTTATTGTGAGGGTGGAGACATGGCGGAAGCTATAAAGAAAGCTAGTGGGGTTATGTTTCCCGAGGAGAAACTATGTAAGTGGCTTGTTCAGCTTCTTATGGCACTTGATTACTTGCATGTGAACCATATTCTTCATCGTGATGTCAAGTGTTCAAATATATTCTTGACAAAAGATCATGACATACGTCTTGGTGATTTTGGACTTGCCAAAATGTTGACTTCCGATGATTTAACTTCCTCTGTTGTGGGAACTCCTAGCTATATGTGCCCTGAGCTCCTTGCTGATATACCTTATGGCTCTAAATCAGATATTTGGTCCTTGGGGTGTTGTATATATGAAATGACCTCGCTTAAGCCTGCATTCAAAGCATTTGATATACAAGCCCTGATTAACAAAATTAACAAGTCAATAGTGGCTCCATTGCCAACAAAGTATTCTGGTGCATTTCGTGGTCTTGTTAAAAGCATGCTGCGGAAAAATCCAGAGCTTAGACCTAGTGCTGCAGAGCTACTCGGGCACCAACATCTTCAACCTTATGTTCTTAAGGTCCATCTCAAAATGAATAGCCCAAGGCGAAATACTTTGCCAGGTCATTGGCCAGAATCCAACTACATGAAGAAAACTAGATTTTTGAAGTCAGAAGATGATCCTGTTTCCGTCTCTGGGTATAAGTGGCATTCTTTTAGCAATGATCGGACTTTAAATCCCAGTGTATCAGGAGCAGAGCAAGATTCTCTATGCTCGACCCTAGAGATAGACTGTACTCCTGATAATTTAAATCAAAGATTTGCTGAACTTTCTATTGGAGACAAACACGACATGAAGTCACTCCATAAGTCAGTTGTTTCCAAAACTTCTAGTAATGCCAAGACTCCTAGGCTAACTGCATCAAAAGCTTCTGCCACCCCCAAGAAATCAATGGTATCCTCAAATAATCATAAGACGTTTCCTGTTTCACACAATACGAAACAACCTGTCCATACAACTCGCAGAGCATCGTTGCCGTTCCCACGAAGTTGTGCCATTCAGCAGCCTCCTCGCAGGCCCAGTATTGGTTTACTTGACCTTGTAAATTCACCCAATATTTCAGTTAACTCTCCCCGGATTGATAGGATAGCAGAATTCCCATTAGCATCATATGAAGACCAATTGTTTCCCATCAATAGATCGTCACCAAACTCGGTACAGGGTTCCTCAGGCTCCCCTCTTTGTGGAAATGATTCAACTTTGATAGACAAATGCACAATCAAGGTATGTGACACATCCTATGTTAGGCCAGGCTGTACGGATGCTTGGCAGGGAATCAAGCACAGCATGTTCAAGAAAAACGATGAGGATAAAAGTGGCTCATCTGATCAAAATGCAACAGCTGGAGCATCAAGCCACAACTCTTCAGACTTGCACCACCGACAGTTTGATACTTCGTCATTCCAACAGAGGGCAGAGGCATTAGAAGGTTTGCTTGAATTTAGTGCAAGATTACTACAGCAGGAAAGGTATGGAGAACTTGGAGTTTTATTAAAACCGTTTGGGCCAGGGAAAGCTTCCCCAAGGGAAACTGCTATTTGGTTGAGCAAGAGTTTCAAAGAGAACACTTTCAACCGAGAACAATCCACCTAAACTGCTGTAACATCTCATATATCATGTTTGTAGAGTTCGGTGTTTCTTTTCGCACATTTAGACAGCTAATTTTTTATAGGTAACAACGTTTTTTCATTTACCCACTTCTTTTCACTTACTGAAC

>C100101_65

CAGCTTTTCAAAATATCTATTCACGGTTCATAGCCACAACAACACTCACTGTTTTTTTTTCTTCTTCTTCTTCTCACTCTAGGGCAACAAAGATTGAAGCAATCTTATATATTATGTTGGTGGTGGTGGGTGCTTTTTGAGATGGATCTCAGGCATTTTAGAAGTGAATTCTTGCCCACAACACTCAACCCATCTTCAACTTACCTTCAATTTTCTCCTCTTAGACCTTTCCACAAAGCCCCAAATCTAAAATTCCAATCACACAAACACTTTGCAGTTGTGTCACTCTCCCAAGGCCCTAAACCAATTAGTGAATCCTTAATATTGGGTCGGAATTCACCTGGCAGAAGAAGGGACCTCAGGTCTTATGCTGGTCGTAGCAAGAAGTCTGGTGGTGCTGGCTCCTCTGGTGGTCGAGTGGAAGGCAGTGCCGAGTTCCGGAATCGGTTGAGGCGCAATGCTAGGGCAAGAACCAAGAAGTATGCTGAGTCTCTATTCTACCGGCTGAAAAATCCTCATGGTGGTGGGAATTATCCTGATAACTTCACTGAGGATGAGCTCCAACAAATTGGCCTTGGCTATGATCGAATGGTCCGGTTCATGGAGAAGGATGACCCAAATTTGCGTCATCCTTACGATTGGTATAAGTATGGTCAATATGGACCCTACTCTTGGCGAGGAGTTGTTGTGGGCGATCCAGTTCGGGGAGGGATCACGGATGAGTGTGTAACATTAATCAGTGAAGTCAAGGACCATGAGGAGTGGGAGAAGATTGAACAAGCTGAAATGGCTGAAGATTATGGGGAAAAATTGAAGCAGCTGGATCAAAGTAAATTAAGGTATTTTTGGGTATTTGTAAGGCACCCAAGGTGGAGACTTACAGAGCTTCCCTGGCAGCAATGGACGTTAGTTTGTGAAGTAGTACTTGAAGCAGGTAAACAGAGATTAGATAAGTGGAATTTGATGGGTAGACTTGGGAACAAGGCTAGGTCATCGATAGGGCAATGTGCTGCATGGATGAGACCTGATATTGTATATGTGAAGAAGCCCGTGTTCCAGTGTAGATTTGAGCCACAGGATGACTTTTTCAAGCCAATAATTCCGTTTCTTAACCCTCAAACTGAACAGGATTTTCTTTTTGAGTTAGAGAATGATGATGGTAGTGTTGAGTTGTGTACTTACTATGGTGGATTGTGTAAGATTGTGAAGGTGAATCAAAAGGCATTTGTCGATGATGTGGTGAATGCATATCAGAAACTGAGTGATGAGAAAAAGTCAAAATGTTTGGAGTTTCTGTTGAAAAACCATCCAGTGCTGTTGTTACATCCATATACGAAAGAGTGGAAGGCTAAGTTGGAGGAGATGGAGCTGGGTTGTGATGCTCCTGATGAAGAAGATGATGCTGTTGTTGATGATCCTGAGGATACTGAGATTCTAGATTGGATTGAGGATGAAGGTAGTGATGAGGATGCTAATGCTACCGATGATGAAGAGGAGGATGAGGAGGAGCTGGTTGATGGTGATAATGATTTTGATCGCATTGAAAACCAAGATCTAGTGATGGACATGGAAGAAGGTGAAGACGGCAAATATGAGGCCATGGAAGAGGATGACACAGACTTTAATTGGAATGAGGAGTTTGAAAAGGCATTGAGTAGTGGAGAAGCAATGGAAAATCTGGCAAAGAAGGCTCTTGATAACAGTAGGAAGTTTGATAAGAAGAAATCAATGGTGATGAACAAAGTAGAGGAAGAAACAGAACAGTCAGTAGATGGAGATGAAACTGCCTTGAGAGGCAAGCGAGCAAAGGTGAGTCCAGAAGAGTGGAAGTATATTGGTGTTGGAAAGTGGAGAAAAAAGATTAAGAGGAGCAGAATTCCTCCAGAGCTATTTTTGCGAGCGGTTGTTAGACCTTTCACTTACAGAAATCTTGTGAAAGAGATTGTTTTGACCAGGCATGCGATTTTGGATGGTGAAATTGGTGCACAAGGTTAAGACATTTTGCCTTTTACGTGAAGGGCCATGTTGTTGTGTACTTGTGTAGGAGTGATTTTGACAAGTCTTTAGATGGTTCAGCATTATTCAATTCCCATCATGTTGTTGAAGTTGAGGAAATAGAGTAGAAAAATATTTAGTGACTCTCTTCTTTATGCTTATGTAGATGCTGAAAAGAATGAAGTATACGTCTTACAGTATACAATATCAGTTTTGGACTACTTGAGAAACTACTTCTATTTATTTATACTGGTG

>C100103_65

TTTTTTTTTTGCAGAGAAAATATAAACTCAATAATATATAGTTCAAGCTTCCTTACAAATCACTACTAATCCACCTTGTACGAAGTTAGACATACGTTGTTTGTGATCAATTTCATGAAAGGATAGCAACACATCAGAGTAATAACTATTATGAGAGGGATCTCCTGAATTTGAAGGAGTGAAGTTCACGAAGAAAGTAGTAGAGCATGGAATAGACCAACTAATATTATACTCATTATGTAAAAGGTATTACACTTATATACAGGACCAATATAACTTGTATGCTATTGGTCTACTCTAGCTATATAGAGAGCAAATTTGACACAAGCAGCAGAAACCAGTATTGTGAATGCAATTCAGAGGATTACATCATCAACCAGGCTTTTTGAAGGCTTTGTGTGTACAGCCCAAAATCATGCACTATATCCTGTGTTTGATGGATGTAATTGGCCAGCGTGATAGCCACCACAGGCTGCCACACAAGCCTCCTCCATGGAGGCCTCAGAAACGCCACTATTGCAAATGTTGGCGAATGCTCGCATGTGTTTCATTCCATACTGAGTGAGTGATCCACAGTGAGTTTCAAACACCCGAACCATTGATTTTAAGCATGTCCAGTCATCAACAAGGGGAAGACCAGGAGTCCTCACGGAGTGTAGGACAGAAGAACCTTTTCCTGGACCATACAATAAAACTCCAATCAATTCCACGCTACTATCTAAGTGTTTCCTATGCTTCACTGTCTCCGCAATCTGTTTGAGGATGTCTGTCTTCTTTTCTGGCTGATGGTTCGATCTCTGATACATTTGCCACATGAAGAAAAGTTCTGCATCTCTCTGGTTAACAACTTCCATTTTAGCTTCTAGTATGTCATTATGTGGAGGGAAGTTCACAGTGGAAGGATCAAAACCTTGATATAAATAAAGCTTTTCAGCTGTGATGTTGGTATCACCATATTGCATCACATGAGAACCAATGGCATAGTCGTTAAGATTTGAAGTACGTTGCTTTACCGATTTGTATTGTTCTTTTACGGATTCTCTTTTCAGATTGTGAGTCTCACTGTCCTCCATCCAAGCAACGCTGTACAAATCCCCGAGACAAGTGATGTATTCTGGAGGTAGAGAAGGATCCATTCCAGGACAATAAGTTCCCCAACTATTCTCTTGTGCATTCGATGCAGTTGTGACATAAATATTCAGATCCTTAGGCATTAGACCCTCAAACATGCTCCCACTTTCACATGCTTCCACGTATATGACCATCTCCTTGTAGCCTCCAGATTCATGTTTCTTCTTCAAGACATCAATAAGATCCAAAGCATAGATGTATGGCATGTTTGGCATCCCAAGAACTCCTGGACCTCCATGATCAGAGTAGTATATAAATATTCTGTCCTCAGGTTTGCTATCAATCACCTTGCCACTTCCTCCCTTCACTTTACTCTTGTCCCCAAGAATAACGGCGAAGAAGTTCTGGGCCGTCACATTCTCACCAGTGTAATCCTTGGGAACACCAGCATAGACATCTGGTCCCTGAGGGTGGTTGATGATGATTCCAGGTCTGGGATTCAAGTCATGGGTAGCTATGTCATCGTACATAAACACCACAATGTTCTCTTCTTTCAGACCACCTTTTATCAGCAACTGGTACGCATGGCACACATCTGCTTGATGCCTGTAGTTTCCGAAGCCATTTGAACCAGCCACGAGAACCGCCCATCGCGTTCCCACTTCATCAGAGTCAGCATCCACCGCTTCTGCCGGTAGCTTTAAGACCGAGTCCCGCTCCTTCCGGTTCGGCCTCGCGCCTGCACCCTGCGCTCTCATCAGCATCACCAACATCCACGACCAAAATACGACGCTACACCACGCCGTTTTGCTTCGAAGAGAGCGATCAACGGCCATGTCAGTGAAACGAAACCCGTAACTTGTCACTCACAGTTGACACAAGAAAAGAGGGAAGGAGCAGAGAGGTTGGTAAAGCAGAGGTGTGAGGTGGAGAGGGTATATAGGGGATTGAGAAGGTTAAAGACAATATGGCATGCAAATTCAGAAGGTGACACGTTAGACACGGTTTAGGGTGCGTGTCTTGTTCGACTGCCTCTGCAAAAATTGGTTTTTTTCTTTTTCTAATGTAGGAGGCTTATTTATGTCTCTCTCTCTGCTCTCTCTTTTTCACAGTAACTTGTCACTCACATTCTTCTTCCTATACCAGCTATGTTTTATTTGCTTTCACAGTTGAATGTTTT

>C100109_65

GAAGAAGTGCGTGGCAAAGGGAAGGGAGGGGGGGTAAGAGAAACGCAGCATCTTACCATAATAATAGCAGCAACGCACGAACGAACAGCACAAACAATGCTCTCTTCCGTCTTCCTTCACAATCCACCACTCTAGCCTTCGTTTCGCTTTGACTATAAAAATGGACAGGTCAACTGGCCGTAGTGGTGGTGGAAGTGTGGACATGTTTCTACCAAATTATAAGTTGGGAAAAACACTTGGCATTGGGTCCTTTGGCAAGGTGAAAATTGCAGAGCATGTACTGACTGGTCATAAAGTTGCTATAAAGATCCTAAACCGTCACAAGATAAGAAACATGGAAATGGAAGAAAAAGTTAGAAGGGAAATCAAAATTTTAAGATTGTTTATGCATCATCACATTATACGACTTTATGAGGTCATAGAAACCCCAACAGACATATATGTTGTTATGGAGTATGTAAAATCTGGAGAGCTCTTTGATTACATAGTAGAGAAGGGTAGGTTGCAAGAGGATGAAGCCCGCCATTTTTTTCAGCAGATAATTTCTGGTGTGGAGTACTGTCACAGGAATATGGTGGTTCATAGAGACCTGAAGCCTGAGAATTTACTTTTGGACTCCAAATTTAATATCAAGATTGCTGATTTTGGCTTGAGCAACATCATGCGTGATGGTCACTTTCTTAAGACAAGTTGCGGAAGCCCTAATTATGCAGCTCCAGAGGTTATCTCTGGGAAGTTGTATGCTGGACCTGAAGTAGATGTCTGGAGCTGTGGTGTAATTTTATATGCTCTTCTCTGTGGCACTCTTCCTTTTGATGATGAAAACATTCCTAATCTCTTCAAAAAAATAAAGGGTGGGATATACACTCTTCCTAGTCATCTATCACCTGGTGCTAGAGATTTGATACCGAGGATGCTTGTGGTTGATCCCATGAAGAGAATGACTATACTTGAGATACGTCAACACCCATGGTTCCAAGTTCGTCTGCCTCGTTATTTAGCAGTGCCACCACCAGATACACTTCAACAAGCCAAAAAGATTGACGAGGAGATTCTTCAAGAAGTGGTTAATATGGGATTTGACAGGAATCAATTGGTTGAATCTCTTGGGAACAGGATACAAAATGAGGGTACTGTAACATACTATTTGTTATTGGACAATCGGTTTCGTGTGTCCAGTGGTTATCTTGGAGCTGAATTTCAAGAGACGATGGATTCTGGTTTCAACCGTCTCCATTCCAGTGAAGTTGCTTCTCCAGTTGTTGGACACCACAGCACAGGGTATATGGATTATCAGGGAGTAGGAATGCGACAACAGTTCCCTGCTGAGAGAAAATGGGCCCTTGGGCTTCAGTCTCGAGCCCAACCACGTGAAATAATGACTGAGGTCCTTAAAGCTCTGCAAGAATTAAATGTTTGTTGGAAGAAGATTGGACACTATAACATGAAGTGCAGATGGGTTGCTAGCATTCCTGGTCAACATGAAGGAATGATTAACAATTCTGTTCATAGTAATCATTACTTTGGAAATGATTCCAGCATTATTGAAGATGAGGCTGTTTCTAAGTCAAATGTGGTCAAGTTTGAAGTGCAGCTTTACAAAACTCGTGATGAGAAGTATCTACTTGATCTTCAAAGGGTCCAGGGCCCGCAGTTTCTTTTCTTGGATCTTTGTGCTGCTTTCCTTTCACAGCTTCGTGTTCTCTAGTGTAAAGCTCGCAAGTCACAAAGGAGACCTTGAGGTGTCTTGTGCTGCATGTGAATAAGCTTACATTGTACATACCAGTGCCCTTTTAGTATGGGTATATCTATCTGTTGGTACTTTTTAGCATACCCCCAGTTTTTATGGGTTATCATGATTGAGGCAACCTACATATGGCATTTGCTTCCTAAATCATGGTGTCTGACTAGGTCTGTCTTTTACTAGTGAATTTAATTTTCTCTATCCCGTGAAATCCCTGAGATTTTTATGGAAGCATTCGCCTCCAACAGAGACATCTGTGTATGGTATTGAACTTAGTGTTGTAATTGACTGCTTATGTCTGTTGCTCGGAAGCCGAGGGGTTGTTTGCCTAGGCTGCATCCAACATGAGCTTGCAGATAAGCATTTTCTCCCCGCTTCCCTACTCAATTACTGCTCCTTTTATGGATGCATGTTATTTGACAAATCATATGTATTTTTGAATCTATGTAAACCATATTTTTCGGGCAGTCTTAATTAGTTATGTTTAATCACGCTGCTTGATCCTTG

>C100111_65

TGCCACGACCACAGTGGTTTGGAATTTACAAAGCTAAATAGTAACAAAGAAAAAAAAAAAAAAACAGCTATGAGAGGGAGAGAGATTGATAATATAAACTGAGAATGCTGATGAATATCAGCTATCTCTATCTAGATATTGTGATGAACTTGATTAGGGACCTCAGGTTGATTATCCAAAGGCATGTATTGTGCCATAATTGCCCGTATCTCTGAATCCATATATCTCTGTATTCTGTACTTGTAAAATGCATATCCTCCAGAAGCAGCAACAACCACGATGAGGATAATCATCCAGATGCTCACCCCTGAAGCAGAATATGCACCTAAACACGTGTCATTTTCTCGTGAGTAGAACAAACCACTACCACATTTGCACTCATAACTTCCCCAGGTGTTTTTGCATTTGCAACTTGGGCATTGACAGGCCGTTTTCTCGTGACACTCATCGATATCTTCACATGACTGGACTCCATCACCTCTGAACCCAGGTGGACAAGTACAGCCTTTTCTGTAGTCATCCAGACAAGCCGAGTAAGCTCTGCCATCTCGAGCTTCCTTCCAACAACCACCATTGTTGAAGTCACAGCTCAAGGTTCCTGATGCTTCACAGTGGGTATATCCATCTCCAACAAATTTCACATTTTGTACAACAGGGCATTCACATACTCTCCCACGGAATGTGTCCCTGCAAGCAGTAATGTTAGAGGAGTCTTCCTTCCAACAACCACCATTGTTTACCAAACACTCATTTGTTTCCATTTCTGGAGTTAGGCAAACTGATGGTTCAGTGGTCTCTAGGAAACCTGCACAGATTGCCTTGAGAACAGCAGGTCTTGACAACTTACCTCTATACTGTCTGTTGTTTATAACAAGAGTAGGCAATATAGTAACATCACCACGACTGCCTTTGCCAATCTGTGCATCCTGCTCAGCCTTAAGAACAGGATTTTCAACATTTGCATGGGGATCACCAACGCAGTCCTTAATCTTCTTTAGATCAGCACCAAGAGATTTAATAACTTGATCTGAGCATTCTTCTGTGTATTTGTTCTCTTTCATGGGGCAACGAATTGAAAAGTCGGTGACATAGTCCCACCATTGCCAAGGCTTTCCACTTTCATTTGCCACTTTATAGAAGCAAGCTTGGCGTAAGTTTTGAACAACAACATCTTTGCCATCATACCCTCTACTGAAATCTTGCTCGGGATCTGGAGCACAGTACCTTCCATTGTTTATGCATTGAGACTTGCACTGTTTGCTCAAGATAAATGCTTCAGGACAAAACCAAGTTATATAGCGAGGTATAAACTGGGTATACCCTTTCTGCTCAAGCAGCTGAGCTACCCCTTTAAAGTCCTTCAGAAAATTAATTAGACTGTCACACTTTGGCCCACACTCGTCGTTGCTATTCGTCCATAACTCATACTCAACTCTATCATCAGGATGTGGAAGAGACTCTCTCCAATCAAGATTCATGTTAACTATCTCCCCATCAGAGAGAGCCTTCTTGATGCTATCCCCCAAGGATTTGCTGATAAGAGCAGAAGGAATACTAATATTGTCCACATAATCATCATTTGCACCTTGCCCTTCTTCAGGAGTGTCCATAGTTATCAATGTTTCTGCCTTGTCATCAGCTACAAGAATAGCTGCTGCTCCACCATTCTGTGCATTCCATGCCTTCAAAGTGAAGTAGCAATCTCCACGATCCACAAGAAGAAAAGTGGGAAGCACTCCTGGCTTGGACTGAAAATTGACATCAGTGAAGTTGAAGCAAGCCTTCTGATTTACCTTTGGATACACTACAGAGCCAACCAATGTTCCTCCATACTTGGGGACCCCAAAATTGCCAATTGCACATTCATATGTCCCCTTCAACGATTTGGGAGTAGTCACTCTCAAACTGTTCTTCTCCACCACAAACCTACCCACAGAACACCCGAACAGCAAAAGCCAAACACACAACAAAAGACTAAGTTTTGTGCCCATCTTCTCCTCCTTCTTCTTCTTCTTCCCCGCACAAGCAAAGGAAGAAGATCAAACTGAAATTCAAAGTGCAATTAATGCAAAGCGTGATCAAAGGGCGAAGACCCAGATGAGGAAAATGAGAAGTGAGGAACAAAAAGGAAGCTTTTGAGTTTGATGATGTTTATGGCGTTCTGGGTCAGCAGAGTGATGCGTGAGAAGGGAAAAGCATCGAAATCTTAAAGGTGTCTAAATAATGGGGAAGACGTGTCAGTTAAGTTCAAC

>C100113_65

CACATATTCAACACCAAAAACCTTAACTTGCAGACCCCCTTTTGAACTGGTCCTGTCTTCATTCACTCCATCCCACATGTGCAGCCATGTTTTTCTCGTTCTTGGAAAGCATTATATTATAAGTACCATTTTTTTCATGGGATATTTGCTTTCTTTCTTTGTACTTTAACATGCCACTGTCCCTCTTTTTCTTAAAAACCGTGCTACTCCCTAAGCACAAGCACATCTTGCTACAAAAGAAGGGGGAGTTTCATCAGTTTCTAGCTAGCATCTATATATAGCTTCCATTAAAAAGGTTACATAAACCAGCTTTTGCCTTAACCAAAAGAAAGAAAAAAACAAGTGTTTTTATCTTAACTCAGTTGACAACTAGTGTGGTGAACCATGAACAACACTATTCCAGGTTGGGATTTTGAGAGTGATACATGTCTCACCAACCAGAGGAAGCACACAGGGCTGGACCAAGAACTTGTAGAGCTCCTATGGCAAAATGGGCAAGTAGTTATGCATAGTCAAACACATAGGAAGCTACTTGGGAATTCATCTAACTTGAGACAGATGCAGAAAAATGATCAATCAACATTAAGGGTTAGTGGGCCATATGGGAACTCAAGCAACTTGGATCAAGAAGAGGCCACCCCGTGGATCCAATTTCCTCTTGAGAACCCTTTGGAACAAGAATTTTGTTCAAACATTTTATCTGAGCTACCACCACCTTGTGATGCTGAATCTTATTACAAGCCAATCAGGCAATTCGAAGAGGAAAAGTTTGACAATATTTTTTCCCCTAGTGACCCCCATCATCCTACTTCATCTTCACAACCACTACCATCTAACATGAAACCCTCCTGTGTTCAGGGACTCCAAGGGAATCCTATGTCTGCTCCAGGATTTCACGTTCCTGATTCATCTCAGAAAATAAATGACTTTGGTACATCACGGAAGGTGCTAAATTTTCCTCATTTTTCAACTCCCCGTAATGTTTCTTCACCATCTCAAAAAACTACTGATAACCTCTCACAAAGTGAGGCTAGAGAGCATTCAGTGATCACGGTTGGTTCAAGTCACTGTGGCAGCAATCACATCCCTCAGGACCGAGATGTAAGCAGGGTTTCAAGAAGTGGTGTTTGGGCCACCACTAATAGTACTACTTTATTTGCTGAGCCTGAAGCTGTAGATTGTGTCCAGAGACCAATTCCTAGGAGTGAGAAAAGAAAATCAGAGATGATTGAAGCAACTGTGACATCATCTTCGGGTGGCTCAGGCAGTACTGGTATGGGAAGAACTTGTTCCCTATCAACAAGAGGCCATGGCCAAAAGAGAAAAGGAACAGAAGAAGAAGCATCAGAGGAACAAAGCGAGGCCACAGAACTTAAATCAGCTGATACAAACAAGTTATCTCAGCGAACGGGGTCTTCCAGAAGGAACCGTGCAGCTGAAGTGCATAATCTATCAGAAAGGAGAAGAAGAGATAGGATTAACGAGAAGATGAGAGCATTGCAGCAACTGATACCTAACAGTAACAAGACAGACAAAGCATCAATGTTAGAAGAGGCAATCGAATACTTGAAATCACTTCAGTTTCAGCTTCAGGTAATGTGGATGGGGGCTGGCATGACACCAGTGATGTTTCCAGGAATTCAGCACTATATGTCACAGATGGGAATTGGAATTGGTGGACCTTCTTTGCCTTCCACTCAGAACCCAATGCAATTACCAAAGGTGCCTCATGATCAAGCCATGTCTGTGCCTCAGATGCCAAACCAGAATTTAATGTGCCAAAATCCAGTTTTGGGTGCTTTCAATTACCAAAACCAGATGCAAAACCCATGCCTTTCAGAACAATATGCACGTTACATGGGTTACCAACTCATGCAAAGTGCCTCTCAGCCAATGAATGCGTGGAGATATGGTTCCCAAGCAATGCAACACAGTGAAACAATGATTGCAGAAGGCAATAATAGCAGCGGACCCATGAGTGGAACAGCTAATATTGATGATGCTGTTAGTGGCAAAACGGGATCTTCCACCTTTAACTGAAGAATGAACAACAATACCTCAAAATTACTCAATTGGGGGGATTATTCATTTGAGTACATCAATCGCAGAAGCACCAAAATGTGCTCCAATTTTATTGAAGCACATGCAGCAATTTTAAGTTATGATGTTTAACTTTAGAGAGATTATATCAGCAGATAGTTTCTAGGGTTTGAAATCAGCTCCTAGGGTTTAGGACCCATCAGCTTCAGGAA

>C100115_65

TTTATTAGTATCTGCTGCCCTTTCATTCCTCCAAGAAAGATACATAAAAGCTTCTTCTTCTCTACTCTCCACTGCAGCCACCTTATCTCCCTTTATCCCTCTCTTTGGTTCTTTCTTTAGTTCTCAGAGGGAACTGAGAGAGAGATAGTCATGGACGTTGTTCTTCAAACCAGAGGCCTTCTCTCTCTCCCCACAAGCCCCATTAACAAGGTCTCACTCTTACACCCCTCCGAAGGTTTAAGGCACAGGCTTTTCTCACCAAAGCCAAAAATCCTTGCTGCCTCTTCTCTTTCATCCAATGGTTTCCAGAAACTCTCACCAAAACCCAATGTATTTGCCCAAAGGGACAAAAATCAAAATTTGTTCCTCTGCAGGGCTGAGGCTGCAGCTGCAGCCTCCTTTGGGGAGTCGGAAACTGAGAAACCAAAGTTTTTGGGTGTTGAAGTTGCCACCTTGAAGAAGATTGTGCCTCTGGGCATGATGTTCTTTTGCATCCTCTTTAACTACACCATCTTGAGAGACACAAAGGATGTGTTGGTTGTGACGGCCAAAGGAAGCAGTGCTGAGATTATACCGTTTCTCAAAACTTGGGTCAACCTTCCCATGGCCATTGGCTTCATGTTGATATACACGAAGTTGGCTAATGTGTTGTCCAAACAAGCTCTATTTTACTCTGTTATCCTTCCCTTCATTGCCTTCTTTGGGGCTTTTGGTTTTGTTCTTTACCCTCTCAGCAATTATATCCACCCGGAGGCGTTTGCCGATAAGCTTCTTAACATCCTTGGACCTCGGTTTCTTGGTCCTCTTGCAATTATGAGGATATGGAGCTTTTGTTTGTTTTATGTCATGGCTGAATTGTGGGGGAGTGTGGTCATTTCTGTGCTATTTTGGGGATTTGCCAATCAGATAACAACTGTTGATGAAGCAAAACGATTCTATCCTTTATTTGGACTTGGGGCCAATGTAGCCCTTGTGTTCTCCGGTAGGACAGTGAAATACTTCTCTAATCTGAGGAAGAATTTAGGTCCTGGAGTTGATGGCTGGGCCATCTCTCTAAAAGCAATGATGAGCATAGTTGTGGGCATGGGACTTGCAATCTGTTTCATATATTGGTGGGTGAATAATTATGTTCCACTTCCTACACGTAGCAAGAAGAAGAAGGAGAAGCCCAAAATGGGAACAATGGAGAGCATAAAGTTCTTGGTGTCTTCAAAATATATAAGGGATCTTGCCACTTTAGTGGTTGCATATGGAATTAGTATTAATCTTGTTGAGGTGACGTGGAAATCAAAGCTCAAAGCTCAGTTTCCTAGCCCCAACGAGTACTCATCTTTCATGGGAGACTTTTCAACTGCGACTGGAATTGCTACATTCACAATGATGCTTCTAAGCCAATATATATTTGATAAGTATGGATGGGGAGTTGCTGCCAAGATTACCCCCACTGTCCTGCTTTTGACAGGAGTTGGTTTCTTCTCTCTCATATTATTTGGAGGCCCAGTTGCTCCTGTTATTGCAAAGTTTGGAATGACTCCACTGCTAGCTGCTGTATACGTGGGTGCCTTGCAAAACATATTCAGCAAGAGTGCGAAATACAGTCTATTCGATCCGTGCAAAGAAATGGCCTACATACCCTTGGATGAGGATACCAAGGTGAAGGGGAAGGCAGCTATTGATGTTGTGTGCAATCCATTGGGGAAGTCTGGAGGAGCTCTAATTCAGCAGTTCATGATCTTAACTTTTGGTTCACTGGCTAATTCAACTCCATACCTGGGAGGGGTGCTTCTGGTGATTGTTCTTGCCTGGTTATCAGCGGCCAAGTCGCTTGATTCCCAGTTTACTGCCTTGCGTAGAGAAGAAGAGCTTGAAAAAGAGATGGAAAGAGCAGCTGCTGTTAAGATACCAGTGGTGGCCAACGATGAGGATGGAAACGGTTCTCTTGCAAGCGTTCCACTGTTGAATCCAACAGTTGGTGGTGACTCCACAATTGCATTGAATTGAAGTAAATTCTTCCCAATAGTTGAAACTTGAACTTCATTAAATTGCAAGGCAGCAGGACCAGCCCAACTCATGAGATGTGTTAGCTCAAACTATTAGTTGTAGGTTATTGTCTAATGAGTTTGATTCTCACTTTTTTAGTTTCATTATTGTTACTTTTGTCCCACAGATAAATGATTTTTCTCTCGAACCTTTCTTATTTGTCAGCACCTAAAAAAAGAAGAAATGAAAGAATGAACACATTTGTAAAAAAA

>C100117_65

CCCACCGAAACACGAGCCACTCACATTTTCTGGTTCCTACGTACAACTTCAACAAAGCCAAAACAAAACTAGTTGGCGATGGTGTTTTGTCCGAACGCACGCACTTTAAAAAGCTTTTGCGAAAGCGCGTTCAGGGACTCGGATGCGACTTTGGTTTTTCAACAGTAACAGCTGAATCATGAACGCCGCAGCGTCAGCAACAGCAGCAGCTAGTAGCAGTTTTTACTCACAGACACACTGCAGACTTTCCTCGGATCTTCGAGGTTCCGTTCCTTTCTCAAGAGTGAAACGTTCCCTGTTCTTCAAGGATGTTCTTCCACGAAATTCTCGGTTTTTCCTAGCCCCGAGGGCTGCTGATCCCAATACAGACAAGGACAGCAATGTGGGCGACGATGAGGTGCCAAAGCAGCAGCGTGTGATGCAACAACCGCCCGTGTCGCTTAGCCTGGACGACGTGAACCCTGTGGGACTGGGGCGAAGATCACGGCAGTTGTTTGATGAGGTTTGGAGAAAATTCTCGGGATTGGGTCAGATTTCTAGGACTATCCGCTCCGATGATCAGGAGGCTCTCGATGCCCTTCTTGTTCGTGAAGGTCCGATGTGCGAGTTTGCAATCCCTGGCGCTCAGAATACTACTGTACTCGTTGTCGGTGCCACTAGCCGCATCGGTCGCATTGTTGTTCGAAAGCTCATGCTCAGGGGTTACGCCGTCAAGGCTTTGGTAAGGAAGGCAGATCAGGAGGTGGTAGACCTGCTTCCTAGGTCAGTAGAGATAGTGATTGGGGATGTTGGTGATCCCGCTACTGTCCAGGCTGCCGTAGAAGGTTGCAACAAAATAATATATTGTGCCACTGCTCGCTCTACTATCACTGGGGATCTCTTCAGGGTTGATCATCGGGGAGTTTATAATCTAACTAAAGCATTTCAGGATCTCAATAATAAATTGGCACAATCACGGGCTGGGAAAAGCAGTAAAAGTAAGCTCTTGATTGCCAAATTCAAATCTGAATCTTCATTGCAAGGATGGAACGTTCGTCAAGGAACTTACTTCCAAGATGTACTTGCCACAAAATACGATGGAGGGATGGATGCTAAATTTGAGTTCACCGAGAACGGTGATGCTGTTTTCTCAGGGTATGTCTTCAATAGAGGAGGCTATGTGGAACTATCAAAAAAGCTTTCCCTACCATTAGGTTATACTCTTGATAGGTATGAGGGTTTGGTTCTCTCTGTTGGTGGCAATGGAAGATCTTATGTATTAATTCTAGAAGCTGGTCCTTCGGCAGATCCAAGTCAGAGTAGATTGTATTTTGCAAGAATTAGTACAAAAGTGGGATTTTGCCGGGTTAGAGTACCATTTTCATCGTTCCGTCCAGTGAAACCAGATGATCCAGTGTTAGATCCTTTTCTTGTACATACTTTGACAATACGCTTTGAGCCAAGAAGACAGAGGCCTACTGAAGTAAATACAACAACGCAGCAAGATTTAAGAAGCTTTAAGCTTATATTGGAATATATTAAAGCCTTGCCTACTGGGCAAGAAACCGATTTTGTCTTAGTTTCATGTTCTGGACTAGGAATTGAGCCTTCCAGACGGGAACAAGTTCTTAAAGCCAAGAGGGCTGGTGAAGACTCCCTGAGAAGATCTGGCCTCGGATACACAATAGTTCGTCCTGGTCCATTGCAGGAAGAACCTGGAGGGCAGCGTGCGCTAATATTTGATCAAGGAAACAGAATATCTCAGGGCATCAGCTGTGCTGATGTAGCTGATATATGTGTGAAGGCACTGCACGATACAACTGCAAGAAACAAAAGCTTTGATGTATGTTATGAGTATATTGCTGAAGATGGAAGGGAGCTTTATGAACTGGTTGCACACTTGCCCGACAAAGCAAATAACTACTTGACACCAGCACTGTCGGTCTTAGAGAAGAATACCTGATCATCCCTTGTTGAGACAAATTACTTTGCAGAAGTAAGATTTCATAAATTTCCTACCCACCTTCTGCTTCTACATGACTGCCTAAAGTGATCCATGTAACTATTTTTCCTGTATATATTCTTGTGTAACCCAAAACTACAAGTTGAGAAATTAAATATGTGTATAAATTCCTTCTTTGAAAATTGAAATATTTCAAAATATAGATTTCAATAGTTAGAACGAATCATGCCAATCAATGATATGGATATGAACATCTTGCCTTGTACAATGAAACAATAGCTGAAAATATATAATTTTGGTTGCTATAAAT

>C100125_65

CAAAAACATATTATATTTTCTTAATCCATAATTTCCCATTTATAATTACAAAAAGATGGCTTCATTTGGTGACAGACCTTACTGGTGAAGCGAAATTTTGTTTCCAAGTCGTTGGTCTTTCACTGTCTCTCTCTCTTATCTGAATCAACCTATCACTGGTGTGACTCGTTGTTGTGACCCGGCGAGCGATTCGTTCCTTTGACTCCTTTTGAAGCAACCCTTTGTCTTTCTTAAGCAATTTCCTGTGATGGGTTTTGGATTTTTCATTTTGATTCCATGTGATCAGCTGCCACGACTGGGAACTGCGTTGTTCAAATTGTAGTTTCTGTTTGGTATGGTGTGTTTGGGGTTGGACATGGTTGAGGTTGACTCTCAAAGATTGGAAGTTGAGCGGAGGGAAGTGGGGATTTTGTTGAGAATTTCTTGTGGGTGGTTATTGCTTTTGTTGTTTCTGAGTCCTGTGGCAGGTTTGAGACCTTTAAGGGATAGAACCAATTCTTGGGGTGATGAGTGGGTTTTTACAAGAAAAGACGAAAGTGACTTAGGTCCATTTTCACAATGGAACATAACTGGAACTTACAGAGGGTCTTGGAAGTTTTTAGATACTACAAATGGCTCTACCAGATTTCCAGACATCAGGAAAATAAATGGAAATTCTGTAATTGAATTAGTTAGTGTGCCCACAAAGATAACCGGTGTACATTATGTGCAGGGGGTAGTCATATTCCATGATGTATTTGACAATGAATACAATGTTGGCGGTGCTCAAATCAGAGTAGAAGGTGTATATATATGGCCTTATCGGCAGCTTCGAATGGTAGCCAACAGTGGAAAAGACATGGGACTAAATCCGGATGGAGACTATATTTTATCCAATCCATATCATCTGCTTGGAGTTTTCTCATCTCAAGTATTCCAAGAATCCTCACGACAAAAGATGTGGAGAAGAAAGCATTCTCCATTGCATGGCATGGAGAAACATTGCAATGTAGAAATCGCTGCACAGGTTTCACGCCTGTCATCATCAAAACATGAAGGGGAGCGTGATTCTTTTCAGCTAGTAGGGTTAATGGAGAGTCCATCTGTGGACGATGATGGGGACTGCTTCTCGCCTTTACAGTTAAATGCAACATCTATTAACATTGAAGTCTACTATAATAAAGCAGTGAACTATACCTTGATGGTTACTTTTATCTCATTTTTGCAAGTTCTTCTATTAATTCGTCAAATGGAGCATAGTAACACTCAATCTGGGGCAGCTAAGGTTTCAATATTAATGATTGGTCAGCAAGCTATAGTAGATGCTTATCTTTGCCTTATACATTTGACTGCTGGTATACTAGTTGAATCCTTGTTCAATGCTTTTGCGACTGCTGCATTTTTCAAGTTTGTGGTATTCTCAATATTTGAGATGAGATATCTCCTTGCCATCTGGAAGGCAAGTAGGCCTTTGAGCAATGGTGAAGGTTGGGAGGCAATGAGGCGTGAACTGTCAGTTTTATACAGCCGATTCTATGGGATTCTATTGGGAGGCATTCTGCTCATGTATGAATTCCACAATCATTTGAGACTTATTCTTCTTCTTGTATACTCCTTTTGGATACCTCAGATAATCACCAATGTTGTTCGTGACTCACGCAAACCATTGCATCCTCATTATATATTAGGCATAACTGTAACTCGGCTAGCAATCCCATTATATATTTTTGGTTGTCCTAACAACTTCTTGCGCATAGAACCAGATCAGAGCTGGTGCGTTTGTTTGGCTATATTTGTTGGACTTCAAGCTGCAATTCTCTTGCTTCAGCACTATTTTGGGTCTTTGTGGTTCATTCCTCGTCAGTTTCTACCTGAGAAATACTGCTATTATAGGAGGTTTGCTCAAGATACAAATCACGCCACAGACTGTGTTATATGCATGACAGCCATTGATCTTTCTCCGCGATCCAATGATTGCATGGTGACCCCTTGTGATCATTTCTTCCACTCTGGTTGTTTGCAAAGATGGATGGATATAAAGATGGAGTGCCCAACATGTCGGCGCCCTCTTCCACCTGCATGAAGAGATTTCTAGTGTTCATATCAATAGGATCAACTCACACAGCCCAACTACATAATTTTGGTTGGGACAGAATCTGTATTGCATACGTATAATTGGAACTTCAGTACAAGACTGAAATACGATACTTCTCACAATTGTATCATGCATCATTGGTATAATTTTAGATTTTTTTCTTTCTTTCAATGTTTATG

>C100127_65

TTTTTTTATTATAATCCTCTTCAGGTGATGTATGCATTGATTGAGTGCTAATATAAAAATTTTACAGTGACAGTGGATTGGAGAGAAGATATAAAGAATCATGTCTTTGGAATACGGGGCTAATCTTGATGCATAAATAACCTTTCCATTTCCTCTAACTTCCCTTGCCTAGATAATCTTCCTCTTTAATCTTCTCATCCCAGTCCATCTCCTATTTCAGAAAAGATCTATTCTTACAGTAAACAGATTTGCACGGGGTCCTCCAACATCTAACAGTTGGGGGGAGGGGGGCATCTTGAGTCTCAGGTGCTGATTTTAAGTACAAGCTATAATATCAATTTGGCTGAAATTTGGTTATGACAAAGTTACAGATGTTACAGTCTTCTAAGATTTGCTATAAGTCTCCTGTAAACTAATACCTAGGATGCTATTCATGCTGGGGTTTACTGGTTTGTTGATTGATCTTCTTGGATGTGAGGGCAAGGAATTTTTGGTTCCCCTCTTGCCCACAAAACCTTGAGTTCACCTCTCTTCTTTCCTAATGACAAAAAGGTTCCAAGTGTAAACGGGACTATATAAAGCAAAGCTGGTTGACCATGTCCATCCATCAAATTCAAAGCCACGTATGTGATAAGGAGACCTAAACCATAAGCAGTCATTGCCCACACGAAGTATCCATCTCGAAGCTTCTTCTTTGCCAACCAATCATACCTTAGTGAAAATGCAACTAGAAGCCCTGGTAAGATGATGTCCCCAAAACCAATGATGCTGTAGCCACCCCAAGGATCAAACATACGTGGTATCTTGAGTAGCATGGGGATACCATCTTCTCCACTCCTATCACCTCGAGCTACCACTATCATCACACTCTCATGGAACCACCATTTAGAGACAAACACCCAGAAGATGTCATATAGGAAGGCACAACTGAGAAGAACAGTTCCAACCTTGAGATTTGGTATCCGCACAATCTGAAGAACTGTAATTATCAATGTGATACCAAGAATATCTTGACCAATCCAAGCAAAAGATACACGGCGATAAACTCCCCAAAGCACCGCAAACACTATGCAGAAGGGAGTAACAGCAACTGTCAGATATGATACAGCACCAAAGAAGGGTACTTTCACAAATGTTTGTGCAGCATGTTGGAACCATCTGAAACAAGATAACAGAGCCACCAAGCAAGTTTGTAGTCCCTCAACCCCACCAATGCAAAATAGAACCACCAGAACTTCAACAAACCATGATGCCATTAATTTGTAAAGCATAACCAGGAAACAAGAAGCAATCACAACAAATGATATCGCCGCTGCAGTACTGATTTCCACATAAGCACTAGAACCAACATTCTCTGCACTTAAATATTCATCTGAAGCATCCTTTAATAGTTTCTCATGCTCAATGGCAGACTCTCTCGCAGTCCAGGCAGACCAGTAAGAAGCACAGAGAATAGTACCAACAGCCATAAGCCATAAAAACACTTCTGCAACATCAACCAATGGACGGTATGGAGAGTACAACTGCACAGACACTACTGAGTTGTTTAGTATGAGATTTTCCAAGTTTTCACCAGCATCTTGTGGAAGCATGACAACAGGTATTCCAATATCAACATCAGTTTCATTTGCTTCACAAACCATCTTGAAAAGTTCTGTACGATAATTTATAATGAGGATGGCCGAAGCACCAGCTTTTTCAGCTATATTTGCCTTGGTTGTGAAACTACATTGTCCTCGGTGCACCAAAATGATCTCGCCAGTGAGCTTATTCTTAGGTTGGCTACAACAATCAGGAGGGTCCGCAATCGCAACTCTAGTATGGTTAGCATGTTTTTCTTTTGATTCCAATGTAGGGCCAAACCTTGCACCAACGCCAACGTACTCACAGCTTTCCACACCATCAATCCAAGTTGGAACTTTAACCAAGACAAAGTTGTTTTCACAGCCAGGCCTCTTGGGAGCAATATTATCATGGTGCACTATGTCCCCAGCAAAAGTGAAAGTGGCAAGCATCATAAATAAAGAACAACACACAGCACAGGAAGCTCCAAGTGAAACCATGTTGATGAGAAGTACTGAGAACCCAGATTCCAGATGCAAATTTGAGGATGGAAACCAAAAGGGGTTTGGGCGTGAAGGGTAAAGATTTTATCTTTGGAAAGAAAGAAAAGAGGAAGAAGGTACAATGGGAATTGGGGTGGAGGGAGAGAGAGAGAAAGAAGAGACACAGAGGTTTTAACTTTTATTTATT

>C100129_65

AGACCAAATAGCAAAGTGATGCTGAATGATCTGATAAGATTTAAAGCTAAGGAATCTGAAACTAGTTAGGAAAAAGCTCATGCAATTAACTCTGCCACTCAACTGCGTTCATTTACAACTTGATTTGCTACAAACATGCCATCTCTTCTCTTGGGAAAACCCGGAGGTTCCATGGTGGTGGCCCTATATTTTAGCTCAGAGGCTCCACGAAAATCTGGATCAACTGATGCTGGCCTAGCATTGTGATCTGCAGAAGCACCGAGTCTATAGGAACTAGCAGCAGCACCACCAGGATACCTTGAAGGAGAGCCGTACCTTCTCGGTGGAGCTCTGAGAGGTCCACCTCTAAGATCCATAACTTCAGTGCTTCTTGAAGTAGAGTCTCCAAAAGATCTGTCTGCAGAGCTTGATGCTAATGGCGCACTGGTGGTAGCTGAAGTGGAAGTGAAAGGATCGTCTGGTCCCCATGATGTAAGTGCTTCCTTCTTCTTGGCAAAAAAGTGCCAAGCACCAAACAAGAAAAGTACAAAGAACAACACAGGACTAGTCCATAGCATGGTGTTGAATTCCCCTTTGAAGATAGGTAGATTAGAATGATACATTCCAACATACCCACCTCCAAGACCAACTACAACAAGTTTTTCACGGTCAGTGCCTATCAAAGGTATTACTCTTGTTTCGGCATCCAAACCTGGGATTGGATTATTCAAGAATGATGATCTAAGCTCATCAAGACTCGAGGAGAAAACAAGCCGAGGCACCCCAACTCTCACATAATGTGGAGAAGAAACATTGTATACGAAAACCTTCTCGGGGTTAACAATCAACAAATACCCCTTGATTGCCTGTAGAGCAAGAGGCTCTTCCATATCAAAATTTTTCTTGTATCTAACCCTGCATTTGAAATTCATCACATCTCCATGCAACAAGACGTAAATTATGTCCCCATCTGAGGTAAAACCATAAGCTTTGGATCGTTCCACGACATCAAAAACATAAGTTCTTGCAACAGAATGGTTCAACCCTTCACACTCAGATTCCCTGACTTTCATGCTCCTTAAATCCAATGAACCTGCCCCAGTTTCTGTCAAGAACATAAGTCTTTGCTTTAAGAAAACCAATGGCCTACTAGAAGCCAGGGCAGAACCATACAAGGAACCATTTTCCATAAAAACCCTGATCTTCCCACTTGTATCAGCTGACATAATGTACTTCGTCCTACCTACATAATGAACTTCCAAGAGAGTCACTGGCAATCCATTTTCATGGTTTTCAGGTGACACAAACTTACCTACATTTTCCATGAAAAGAGAACTATAATCCTCTCCACTAGACCCTCCCTCCCAAACTTTGTGCAACAAAATTTCCCCATTTTGATGACCAGTCACTACAAAGCTCTCATTTTTATAAACCGAAGTATAAGAAACCATTGCAGTAATAGAAGACCCCACCAAGGTATCGAACTCAACTAACACATCTCCATTCCTCAAGAACACATATATTCTTCCCCTCTCATCACTAACTGCAACATACTTACTCAACCCCTCTTGATCCCTAAAAGGCAAAACATTTATACAAGTGACCTCAGAATCCAACTTCACAGCAGATGCAAATTGGAACCTCTCAGACCAAAAAGGTGAGTACTTTGCTACAGACATTCCCTTAGCTTTCTCCCCATCTTGAACTTTTCCCTCAAATTCCCTATCTTCAACTCCTTTACTACTTGATATTGCATCATCACCGTAATCACCACCCTTTCTTTGAGTAAACCTACCCTTTTCTTTTGGAGGGTCAGGTAGCTTTGATTCTAATCTTGCAACTAAATCACTGAGATTTCTCACTAATTCCTCAAGTTTATCCAACAAAACTTGTTGATTCAAATGATTAGTTGTTTGGGGAGGTTCAGGAACAGGTTCCTGCCATGAATCGCGGTCCAGCTTGGGTTCGACTGAACATGATTCGGGGTCTAAAAGGGTGCTAGAAACATGAAGAGAATAGAAAAAGAGCATAAAACAGATAAAAAACTTGCCTTTTTCCCAGGACGCCATTGAATTTGAAGAAGAAAAATGGAGGGTGTGTTGAAAGTTGATGGATCTGGCTATAGGAAATTTAGCAGAAATCTATGTAAGATTTGGGACAGTGTATTGACTCGGGTTGAATTAGGGCTACCATGTGTATTAAGTAACAGAAAACAAGACACAAAACCGAATTGATATCGGTTCAAATGTACTGTAAATAAGGACATATAATCTCC

>C100131_65

TCTTCCGTATTCCCTTTTCCTTCTCTTCAACCAAACCCCTTCTTCCTTAACTCATTTTTTTTTTTTCTTACTTTCTCTCAAACAAATTCACAAACACATAAAAGTGATTGCTTTTTCTTCCAATCCTTGTGTTGCTGATGAAATGGGGGACAAGCACCACCTAGATGACAGCAGCGATTTCACCTCCGAGGACGAAGGCACCGAGGATTACCGCCGCGGCGGCTACCACGCTATCCGAATCGGCGACACCTTCAACGCCGGTCGCTATGTCGTTCAGTCTAAACTCGGTTGGGGCCATTTCTCCACTGTCTGGCTCGCTTGGGACATCAAACATTCTAGATATGTGGCTTTGAAAGTTCAGAAGAGTGCTCAGCACTACACCGAGGCGGCCATGGATGAGATAACGATTTTGCAACAGATTGCGGAGGGAGACCCTGATGATAAGAAATGTGTGGTGAAGCTTTTGGACCATTTCAAGCATTCTGGTCCCAATGGGCAGCATGTCTGCATGGTGTTTGAGTACCTTGGGGACAATCTTTTGACACTTATTAAGTACTCCGATTACCGCGGGTTGCCTATTGCGATGGTTAAGGAGATTTGCTTTCATATTCTGGTTGGATTGGATTACTTGCACAAGCAGCTTTCCATCATACATACTGACTTGAAGCCTGAAAACATCCTGCTCTTGTCGATGATTGATCCATCTAAGGATCCTAGGAAGTCTGGGGCGCAGCTTATTCTTCCTAATAGTAAAGATAAGATGGTGCTGGAGTCCACAGGTGTGAAAGATACGAAGATGTTGAATGGGGATTTGGTTAAGAATCACAAGAAGAAGATTAAGAGAAAAGCTAAGCAAGCAGCCCATGGTTGTGTTGAGAAGGAAGCTTCAGAAGGAGTTGAGGGTAATCCTGAAACTTCGGGGGCTGTGGAGTCGTCTCCAAATGCGAGTTCTTCAAGAGAGCAGGCTTCCAGTTCTGCAGGAACTAGTAGGTTATCAGATGCTGATGGAACAAAGTTGAAAGAGCAGGGTAACAAAAGAGGAAACCGATCTATGAGACAGAAGCTGCTGGGATCAGTTGATCTCAAGTGCAAGTTAGTTGATTTTGGTAATGCCTGCTGGACATATAAACAATTTACTAGTGATATCCAGACAAGACAGTATCGGTGCCCAGAGGTGATCCTTGGGTCAAAATATTCTACATCTGCAGATCTTTGGTCCTTTGCTTGCATTTGTTTTGAGCTTGCGACTGGAGATGTGTTATTTGATCCTCACAGTGGTGACAACTTTGATAGGGATGAGGACCACTTAGCATTAATGATGGAACTTCTTGGAATGATGCCACGTAAGATAGCACTAGGGGGGCGTTATTCCCGTGATTTCTTTAATAGGTATGGTGACCTAAGGCACATCCGGCGGTTGCGATTCTGGCCCTTGAATAAAGTCCTCATGGAGAAGTATGATTTCAGTGAGAAAGATGCAAATGATATGACGGAATTTTTGGTTCCTATCCTTGATTTTGTTCCTGAGAAGCGGCCTACTGCTGGTCAGTGCCTTCTGCATCCATGGATCAATGTAGGTCCACGTCTTTTGGAGCCATCTGTGCCTTCTAATCACAACCCAGCTTCTGAAATTGCTGTTTTGGATCAGAAGAAAAGGGAAAAAGATGAGAGGGAGGCCATGGAGGCAGGAATGGGAAATATTGTTATCAATTCAGATTGTAAATCACTGATGCAATCACCATCAAAGAAAGCCTACCAAGGCACCCAGAAGTAGTTCATCTGGCAAGAGCTCTTTCCTGTGTGACTGTTTCTATGTTCTAAGCTTGCCCCTGCATAATTTCTCTAAATTATTCCATGGGATTTGTTACGGGGCATGTTTGTTTGTTGGGTTGGTTTCCAGAAGTGCTGGCTTGGATGGGGTTTGAGTTGTTTGAAGCCTGGTGTCAGCTAAATCCGTTCGAGTCTTTCACATTGCATCGTCAAAAAGAAGTTGAATACTCCAAGATACCGTTATTATACACCCCCTGCCTTGTTTCTCATGCTTATGATAGGTTATAGATGAGCCTTTGCCAATTGGTTCCATTGACTTGTTTGTGAACATTGTTCATTATCATGTACTCTTGTGCTTAATGATATTGAAAACAATATAGACACTTTCTGGAATTTCAATAATTAATATTTAATTTTTATAGTAACAGGAATGTTGAAAGCAAAGGCGAAGTAAACAATTTCGTTAGAAACAAGGGAAAGATTT

>C100133_65

CCACCATTTTAAACATCTTTATCCATCCGGCCAATAAATCACAATATCCACACCGATAACTTGGGGAATCAATAAAACAATGACCATGTAAATAAACAGTGCTGTACATATGTTCAAGTACAATCATAAACGATTAAACAAATGAACAATAATTATCGCGCAATACACTAAATATACAAAATACCAGAAACAGGACATTGGACTAAGAGTTCCAATCAGCTACTTCAGTCGTTATCCTAGAATGAAATTATAGCCCATGGATGCACAATTTAAATCCAGTAAACAGAATGGATAGAAATCCAGTTCTCAAAATTTCTCAGGTACAATCCAAGTTTAGAACTAGAACACTGTCACGATACAATGTCACACAAAAACGAAAAAATAAAGCCAAAATTTCCTTTACTCACAAACAATAAGGGTAATATATACCACAAATCACGAGACGTATCAAAGTATTTCAGCACTCTTCCTGTTGCAAAGCCAAACAAGGAAACCATTGCAAAGTTCTTCCCCTTATATATAGTCATTTTGTGTCCAGAACAAGGGGCGAGACATGTCATTCCAACAGCTGAATCATAAAAAATTGCAGTTCTTTTAGTCAACTTTCTCATCCACACCTGCCCTTTCAGATACTTCCCCAATCTCATCAGAGCTTGAAGCACTTCCAGGACTACTGCCTGTGATTATACTCTCGGCTGCAGAAGCCCTAATATTCTTAACTGCATCAATCGCACTATTGTCTGTTTTTGGAGAAGTAAGACCAGAGATCCGCTCATCCGATTGCTTCGGTTTGTTTAGGGCATGACAGTGTGGGCAGTAGTATGTGATAAATGGGAAATCCTCCTTCCGAGCTAGACCATTATGCATGTAGCAGTTACCACATATGAGAGCATACGACTGTGTTGGATCCTCACCCACAAGCAAAGCTGCAATTCGTGCAATCCATCCACCATCTTGTGTGCTTGAACTTTGAGGTTGGTGATGCTCAACAACAACAGGCTGATTGTCCTCAAAAGTATGAGTATGATCACTTCCCCCAGGACCAACTTGTTGATCACCAGGAATTGATGTGGCTGCCCCAGGACTAGTGGATCTAGATTGGACTTGTCTCCTATTTCGAAGTCCAGAGGACTGTACAAGTTCAACATCATAGCTCTTCCCTATTGGAGCACTAGGACTGGATTCATCTTCCAGATACATCCTCAAACCAGAATCTGCACTCAACTTAGAAGCTAAAACAGTTGCAGCAGCTGCTTTTGCAGCTGGGTCTGGATCATATCTCTGAATGAGCTGTTGTGTAGTGTAATAATTTGTCTTTTCCTTGAGCTCATCAATTTTTTCTTGCCTCTCAACTCGAAGCCTTTCAAGAATTTTCTGGTCCCTGCGATCACACATCCTTGTGAAGCTGATAAATGCCGAATAGGCAGCAGATGCCAGTGCAGGCAAAAGAAACATTGGCAAAACCCGGATTGCCCTCATTTTCCAATTCATATCAATTGATCTTGTTGTCATAATAGCATAAACTACGGCAACAACCTCAAATATGACAGAAAACATAATAAGATGGCGGGAAATTCTCCTCAAGGATCGCGATCTCCTGTTCATCCTAGTCATAACCTGAGCCTCCTCTTTAGAAATGTATTGGAGTCTCTTCTCAAAATCATCACCGTGTAACCTAAATATCCCGTTCCAAATACGCGAGAGAAAACCTTTGCGCCTCTTCTTTCCACTCTCGGAGGAACTCGAACCACCGGTATCTTTCTTCTCACCTTCATCAATCACCTTATCATCCGCCATCCCCCGCTTCTCTTGATGATCTTAATCACGTTATTGTTCGGATCTTGCAAGTGTGTATTACGATTGGCGGAAAATATGAGGAGTTACGTTTGCTACTTTGCGCACACGACGTGAACACGGATTACGATACGATACGAACACGAAGGTTGCAATCTATTGGAACGGAAACGAATACTTGGATGCTACTCAATCATTCAATTATCGTTGCGATTTTTTGAATTCCCAAGGTATAAGAATTGGGGAACAAACAATATGAAAGGTAAGGTTTCTAAATTGGTACAAATGAAAAGAGAGAAAACGAAAGCTTAAGGATTGGGGTAACGTGAAAATGGTGTTAAGGAAAAGAAAAGAAAAAAAAGAAGATATAGCGTAGTTCTACGGTACGCGTATAGCAATTTTACAAATACGGTGTTGGTGGTTGCTGAACGCCGACCAAGGACGCAAACTTTGATTCTAA

>C100139_65

GCGGCCGGCAACGTCCCCGGCCGGCGGAGCTTCCGACAGACTGTGTGCCGGCACTGGCTGCGCAGCCTGTGCATGAAGGGCGACGCGTGCGGGTTCCTGCACCAGTACGACAAAGCGCGGATGCCTGTGTGCCGGTTCTTCAGGCTCTATGGAGAGTGTCGAGAACAGGATTGCGTGTATAAACACACCAATGAAGACATCAAGGAGTGTAACATGTACAAGTTGGGATTTTGTCCTAATGGGCCTGATTGCCGATATAGGCATGCAAAATCTCCTGGACCTCCACCTCCTGTTGAAGAGGTCCTTCAGAAGATCCAACATTTATATTCCTACAATTATAATTCATCTAACAAATTTTTCCAACAAAGGGGTTCCAGCTATACCCAACAAGCTGAAAAATCCCAGCTTCCTGTAGGAACTAATTCTTCGAATCAAGGGGTGACTGGAAAACCGTTACCAGCAGAGTCCGGAAATGCCCAGCCACAGCAACAAGTTCAACAGTCTCAGCAGCAGCAGGTCAACCAGAGCCAAATACAAAATGTTGCTAATGGCCAGCCTAATCAAGCCAGTAGATCTGCAACCCCTTTACCTCAAGGAATATCTAGGTATTTTATTGTTAAGAGTTGCAATCGTGAAAATTTGGAGTTATCTGTACAACAAGGAGTATGGGCAACTCAAAGGAGCAACGAATCCAAACTAAATGAAGCATTTGACTCTGTGGAAAATGTCATATTGATTTTCTCAGTCAATCGTACTAGACATTTCCAGGGTTGTGCAAAGATGACATCCAGAATTGGTGGTTCTGTTGCTGGGGGTAACTGGAAATATGCTCATGGAACTGCACATTATGGGCGAAACTTCTCTGTTAAATGGTTGAAGTTATGTGAACTGTCATTCCACAAAACTCGACATTTAAGGAATCCATACAATGAAAACTTACCAGTGAAGATAAGTAGAGATTGTCAGGAGCTGGAGCCCTCTATTGGAGAGCAACTGGCATCGTTGCTTTATCTTGAGCCGGATGGTGAACTTATGGCCGTTTCAGTTGCAGCCGAGTCTAAACGGGAAGAAGAGAAAGCAAAGGGAGTGAATCCAGATAATGGAGGAGAAAATCCAGATATTGTTCCATTTGAAGACAATGAAGAAGAGGAAGAGGAAGAAAGTGATGAAGAGGAGGAGAGCTTTGGTCATGCTGTAGGGCCAGCTGGTCAAGGCAGGGGTAGGGGCAGAGGTATGATGTGGCCGCCTCATATGCCTCTTGGGCGTGGAGCCCGACCCATGCCTGGAATGCAGGGCTTTAACCCTGTAATGATGGGTGATGGGTTATCCTATGGACCTGTTGCACCTGATGGTTTTGGTATGCCAGATCTTTTTGGTGTGGGACCTCGTGCTTTTGCTCCATATGGTCCCAGGTTCTCTGGTGATTTTGGAGGCCCCCCAGCAGCCATGATGTTTCGTGGGAGACCCTCTCAACCTGGGATGTTTCCAGGTGGTGGCTTTGGGATGATGATGAATCCTGGACGTGGTCCCTTCATGGGGGGGATGGGGGTTGCAGGTGCAAATCCACCAAGAGGTAGTAGGCCAGTTAATATGCCTCCAATGTTTCCACCACCTCCACCCCTACCTCAGAACTCAAACCGTGTAGCCAAAAGAGATCAAAGAACTACTGATAGAAATGATAGGTATGGTTCAGGATCAGAGCAGGGTAAGAGTCAAGACATGCTCAGCCAAAGTGGTGCACCTGATGATGAGATGCAGTATCAGCAAGGATACAAAGCGAACCAGGATGATCATCCTGCTGTGAACAACTTCCGGAATGATGACAGTGAAAGTGAGGATGAAGCACCTAGGAGGTCAAGACATGGAGAGGGGAAAAAGAAGAGGCGAGGCCCAGAAGAAGTCAACACAAACTATAATCATTAGTTAGCTTATGCTTTCACCAAGCACTACATATTGATGATCCACTAACCAAGGATGCCAGTTTCCAATGCTTTATGTTTGATAGATTGCTTTGTATGAGTTCTTTTCCAACCCTCTTACGACACCTGGAGACTGGGTTGTTTCAGAAAATATTTAAAATTCTGCTCACTGATCATATAATTTTTACATATGGTTCATTGGATATTTATTGTTAAATGTATCGGGATCAAGATGGTAATTTAGTATGCTCGGTGAATGTAAACCTATATGTGTTTGTAAAGGGATCGTATCATTTGAGTTCTCAATGAAAAAGGTGGGATGATTTTATACCAAAA

>C100143_65

GCAAATCGCGACACTGTTGGGTCCGGTCGCACTGTAACACCCTCACCGTTCTGGGCTGTCACATCATACATCACACATAACATTCACATGGCATAGCGACTCACATTCACATGGCGTATCCATTCATTCATTAACGTTTTCTTCATTACTCCCTTCTCTCTTCCACATCCCTCTCTCCCAAGCGCAATGGACATCCGCCGCCGCGCGCCACCCACCGCAAACGACGGCGACGTCCACCACCCTAACCGCGCGAAGAGGGATTCCGCTTCCACACCATCAGCTTCCTCACCTAAGGCCTCCGACGCGCTCCCTCTGCCACTGTACCTCACCAACGCCATCTTCTTCACTCTCTTCTTCTCCGTCGCCTACTTCCTCCTCCACCGTTGGCGCGACAAGATCCGTAGCCACACGCCTCTCCATGTCGTCACACTCTCCGAGATCGCCGCCATTTTCTCCCTCGTAGCATCCTTCATCTACCTCCTCGGCTTCTTCGGCATCGACTTCGTCCAGTCCTTCATCTCACGCGCCTCGCAGGACGTCTGGGACCTCGACGACGCCGTGTCGGAACCCTCGCCCTCGCCCTCGCCCGCTCCGTCAATTACCAAATTGCCGCCTACGGAGTGCTCGACTATGTCTGATGATGATAAGGACATTGTTTGCTCCGTCGTGGACGGCCTGACGCCGTCCTACGCGCTCGAGTCCCGCCTCGGTGACTGCCGCCGTGCGGCCGCGATTCGGCGCGCGGCGCTGGAGAGGCTGACAGGCCGGTCGCTGGAGGGGCTGCCGCTGGAGGGGTTCGATTACGACTCGATTTTGGGGCAGTGCTGCGAGATGCCGGTGGGGTATTTGCAGATTCCTGTGGGGGTGGCGGGGCCGTTGCTGCTGGACGGGTTCGAGTACACGGTGCCGATGGCCACCACGGAGGGGTGCCTTGTTGCCAGCACCAATCGAGGGTGCAAAGCTATCTATGCTTCTGGTGGGGCCAGCAGTGTCGTTTTGAGGGATTGCATGTCGAGGGCACCGGTTGTTAGGTTCTCCACTGCTAAGAGGGCTGCACAGTTGAAGTTCTTCTTGGAGGATCCTCTCAATTTTGAAACCCTTGCCCTTGTTTTCAACAGGTCGAGTAGATTTGCCAGGCTGCAAGGTATTCAGTGTGCTATGGCTGGGAAGAATGTTTATTTGAGATTCAGTTGTAGCACGGGTGATGCAATGGGAATGAACATGGTTTCCAAAGGGGTGCAAAACGTTCTTGATTTTCTTCAGAATGATTTCCCTGACATGGATGTTATTGGCATTTCTGGAAATTATTGTTCGGACAAGAAGCCTGCTGCGGTAAATTGGATCGAGGGACGTGGGAAATCAGTTGTTTGTGAAGCAATTATCAAAGAAGAAGTGGTGAAGAAGGTTTTGAAAACCAACGTGTCTGCCCTGGTGGAGCTTAACGTGCTCAAAAACCTTACTGGTTCCGCTATTGCTGGTGCTCTTGGTGGATTTAATGCCCATGCTAGTAACATTGTTTCTGCAGTCTTTATAGCCACCGGTCAAGATCCAGCACAAAATATTGAAAGTTCCCATTGCATAACCATGATGGAAGCCGTCAACGATGGGAGGGACCTTCACATCTCAGTGACCATGCCTTCCATTGAGGTGGGTACTGTTGGGGGTGGGACGCAACTTGCTTCCCAGTCTGCTTGCTTAAATTTACTTGGTGTGAAGGGTGCGAGCAAGGAGTCACCAGGGTCAAACTCAAGACTTTTGGCTACCATTGTTGCTGGATCTGTTTTAGCAGGAGAGTTGTCTCTGATGTCTGCCATTGCAGCGGGGCAGCTAGTCAACAGCCACATGAAATACAACAGATCAAGCAAAGATGTAACCAAAATATCATCTTGAGGTCGTGGACGTGGTTGTTTGTAGTCATTAATTGTGGATTCTAGAATAAAAGAGGTTTTTTCAGCACAAAATAGAAGTTTGGGAAGGAAAATGGAGAAAAATTAGATTAGGTTGTAGCCTTTAGCTTTGCATTGCTGTCTGTAGATCTTTTATTATTTGGAGAGAGGACAGTAGAGTTCTTCTCTACCTGTTCCCTGTCCCTTTCTGGGTCCTTTCATTCCTGTACACTTTTCGTTGCTTATTCTGCCTATCCATTGAATGGGTGCTAACACTGTTTTTAGCATACCAAAATATTTGTCGTGTTGTATTGTAATTCTTTTCCCCCATATATAATATGCTTAGCTATCTTTCACTGTCAAAAAAAAAA

>C100149_65

TTTTTTTTCCCAAATCATTTTTTCTATTTTTTTCATTAGACTAAGTAGGAACCATACATATATACTTTTATCTGAGTAACAGTATCATCACATTACAATATGTGGCTGCCAATGAATATTAATTGGCCTCTATCATTTGGTAGACGGATAATATTTATGCCTAACACGCAAGATTGACCAAAATTTTGAACCTTACAAATGATGCCCAGCTACATCTACATCGCAGCACTGACAGATCAAAAGCAAATAGCAGCACTGACAGTCAAAAGCAAATAGCAGCACAACTGGTATGGAATATAACAAGGAAGCGCTCAGTTTTATTCATTGTTCATATTCACTACATTTAGTGGCTTGAAGATGTCTGAGGCAACCTGAAAGAGCCTCCAGCATAAAGCATTTTGTAAACAGGCTTAATTCTCAAAGTCAGGATGACGCTCAAGATTATGCCAGCACCACACACTCCAGAAAGAATGAGAAAGGTAAGCTTAAAGCAATTTGGACCGACGCAGGAAACATCAGAATCAAGAAGGCTGACACCATGCTGCTTTGCTGCCTCACTATCATATACGTTCCCTGCTAGCAGAACTGAAAAGAGGAATGCACCAATTGGATTCCCCAACGCCAGGATGCTGCTCAGTACACCGAAGTGCTTTAGACCGAAAAGCTCAGAAACAGTGGGGATCATCACGGAAATTTGAACGCCAAAGCAGACACCAAGAAATGCAACTGCAGGGTAAAGGGTTCCATTGATAGCATAGGCAAATAGAAGGTATACAATGAGCATAATTGTCTGCGTGCACGTCATCCAGATAGTCCTTGGGAGTGTTTTTGTCCTAACAAAATGTTCTGAGACAACTCCTCCACCAAGCCGCCCCAAAAAATTGCAAAAACTGAAAATTGACAGCAAGGTAGTGGTGTCTTCCTCCCCTTGGGCAATACCTATTTGGGCCAGATTATTGAGAACAGTAACCCCAGTGCCAACGCCAACAAAGAAGACTAAAAACAGCAGCCAGAAATCAGCCTTGACTATAGCCTCAGTTAGCTTAAAATCTTCTCCACGTTTAGGCCTTCTCTTCTTCTGCTTCACAGCCCCCTCACCCAAAGCAAGAAGCTCAGCAACCTCGGACGAATCGTCCTGATCATTAAAACTCCCAAGTGCCCCTGCCGACGAAGACGCTAGCAGCGGCTCTGCACTCTCATCTTTCCCTTGAGTTAGAGACTCAGAAGATCCAACTGGTTGCTCTGGTGACTCTGATTTAGAACCATTCCTTGGAAACAATGTCATCTTTAAAGGGATAACAAGCGGAGCCATGACCAGCAGAACCATTACGGCCACCAAAACATATGAAACAGCATCACTAAAGGGAGAAACATTACCAAGTATGGTACTAACAAGAAGGTAAACACCCATGATCACACTGGAAACTTGGACAAACAGAAAATGGTAAGGCTCCGCAGGGTCATCACCAGTGGCCGGAGTACAGGGCCTAACGAGAAACATCATGCTGAAGCAGATAACAGGAACACCAACGGCAAGAAAGGACAGGAACTTAGTAGAATTGTTACGAAGCAGAACGCTGTAAATCTCAGTCCAAACCGCACCACTAAGCCCCCCATAACCTTTCAGAATCCCAGCGACGGAGCCTCTGCTAGCAGGGAAGTTTCTCATGTTGGTCACCACAACCGCAGTGGTTAACCACGCAGTACTGTTAGCAGCAACAAGTAGAGCAAGCCATAGCAGTAAGAAATGCAGGGAGGGGAGGATCTGGGAGACGGCGAGGTAGATGACGCCGTAACCGAGGAAGGCGGAGAGGGAACCGACGATGAGGAGGAGCCAAGGGGGGAACTTGTTGCAGGCGATGCCGGGGAGAAGGCCGAGATTCTCGCCGATGTCGTTGGCCACACCGAGGAGCGTGATGTGGCGCTGGTCGAAGCCCAGCACTGATTTCAAGGAGTGGGAGTAAAGAGGGAAGCTTGCAGTGTTCCCGGCCGCGATCTGAACCCACACGGCAGCGCCCAGCCCCACCCACGGGGGCCGGCTCCCTCCCTTCACCTTCAACATCGGGAAAGTAACCGAACAAACGAAACAATTGAATTCACGCTTTTAACTGCTTTGCTGCATAGTTGTAAGAACACACACGAGGTTGGCGCTTTGGAGCGTGGTGTGTGTGGTGTCCTTGTTATAGAGAGAGAAAGGTTTGAAGTGTGAATGATTGGAATGGGTTTTGGAGAGAGAGAGAGTGGTGACAGAGGTGAAC

>C100153_65

TTTTTCGAACACAAAAAGAAACATAAATTTTATACACAAGTTCCAGACAACAAATACTACAGCAATTTACACAACATTAACCATACAAAGTTTACATGAAAGGATGATACACAAAGTCTTGGGTACTAAAGCTTGAGAAAAACAACATCTAGCTAGAGAGTATAAGTTACAACAAACATAGATAGATATACATACAGAGTCAGAACATAAAGACACACGACAAAGGTATAAAAGCAAATCAGATAACCATTTCATCATTATTTTCTGCATCGCCCATCTCTTCATAGTCACTATCAATATCTGCTTCAAGAAGCCACTTCTCAAATTCATCCACATCATCATAATCATGTGTATATAACCCATCTGAAGTGTCATAGGGATCAGCCCCAAGCTCCTCACCATGGCATGCCACATGTAAGAAAGGTCTGCTTCTAGCTAAAGCATCTACCATGTCTCTATTAACATTTCCGGTCACTCCCAACGATTTGAGCCTTGGAAAATATGGTTTCTTCAACCAGCGAAATGCAAGAGCTGTGATGCCACCACAGTTATAAAGATCCAACAAGCGCAAACTGCTTCCATGTTTGGCATCGTCAATCAGCATTGAAGCTAATGCCATCACTGCAGTATCACCAATTAGAGGGCACTGTCGCATACGGAGTTCAAAGAAGGGAATCCGACTTTTTGCCAGCAGCAGAACTCCATTATCTGAGAGGTTAGGGAGATTAGATAGATCCAGTTCTCTCAATTCCAGCACACAGCATCCATTAAATAGCGCTGTGATGCATTTATCTGTTAGTCTCTTGCAGCCTCGAAGAGACAATGCATACAGTGAACTAATAACAGATGGTCTTAAGTACAAAAGTCCTGCATCAGTTATATCAGAGCCATCTAGTAGTAAAATTTTCAGTCTTGGCAGAGTGCCAATTGCCTGGAGAGCTTCATCCCCGAGGCTCCTGCAATCTCTCAAGTCAAGAATTTTGAGTTCCTTGTTCGATGCCAAACTCAAAACAGCTTGGTTAGTTAACAGATTGCACCGTCTTAAGCTAACATGTGTTAAAGTAAGAGATGTTGCGGAAATATCATGAAAAACTAGATCAGTTAAATGAGTCCCGTGAGTGACCTTAAGCTTGTACAAACGAGTGCAAGAATGCAGGATAGTTTTGAAACCAGTGTCTGTGACACGACAAAAGCCACCGAGGCATATGCTTTCCATGTTTGCGCACTTGTCTGCCATTAGAAGTAATCCCAGATCATTCACTCTTCGAAAGTAGGTAATTAGATACTCTTGGCTTCGAACCAATGAAAGATGTTTCAATCTCCCAAGTTGATTAATTTGTTGAAGACCAGCATTGGTTAGGTCAAACGTAATTCTTGGTTCAATCAATGGTGCGTCTTGAAGATCCAAATGGGTCAAAAAGATTAGACCTTTGGATATTGTGCCAATCATAGCATCAGTTATATAGTCTACATTAAGACATAGTTTCTGAATACTTGGAAGTACGGATGGTTGAACAGGATTTGAGGGCAGATGGGATCCCAAATTTGGGCTCAGCAGTTCAGTGACGGTAACTGAAGAAATGTAGCCAATCTCAAGAGAAGTAAGTTTCTCAGAGGCAGAAGCCCACACTCGAGCAAAGTTGTGACGTAGAAAGAGAGAGACATCAAACATCAGAATCAGTGCTTCCAAGTGGGAACAACCGCTGAGCAACTCCTCCAGATCGGAAATATGAATGGACCTCCCTTTGTTCTCTGCAACGGAACCCAGGTAAAGAGACCTTAGATCCTTGCAGCGGGTGCCGATCTCAGATAGGAGTCTTCCACTGAAATCGGCGCAATTGTGGAGAGAGAGGTCGTGGAGGGAGGGTTTGAGAAGGAAGGAAATGGCGGAATCATCGAGGTGGGCACAATCGACCTTGAGAGTGGTGAGGTGGGGATTGGGCGGCAACAAGGGTCTGAGCAAGTCCCTGGAGGGAGCAATGTCGAGGAGGTGGAAAGAGGGGAGGAAGGAGAGGATGTGACGGGCACAGGAGCGGAAGGTGGAGGAGGTGGAGGCGAGGGAGCATACGGAGGCGATGTCCAGCTTTGTCATGACGGTGGCCACCAGGGCGGCAGGGAGCCGATCCAGGCTCGCCTCCTCCACGCCGCCCGTTTCTTCCTTCGATCCCATCCCACACAGTTTTACGCACTTTCCTCAGATCCTCTCTCTCTTGTTCTTACACATCCTTTCATAATTCATTAATAAAACACGACCCTCA

>C100155_65

GAAGTTGGTGGTTTTAGTATCCATATTTCAGAATGATTGTTTCCCACCCTTCTTTTGCTTTTTGATTACTGAATAGCAATTACTGAAGAGCTGGATCCATGACCCTTTCCATGTTTTGTGAGTTGTTAATCTGAAGATTGAGGTTGAGGAAAAGTAGTGTGTGTTAAGAGTGATGGATAACTCCGAGATCAAGTCACCACAAGAAGGCTCCCTTGAGGGGACACCAGGCAATTCAATGAACAGAAAGAAGCTTGCTATTTACTTCATTGAGTCTGAGGATAGAAGAGTGGCCTTTGGTCGTGGATACACAGCAGGATCTACACCAGTTGATATCCATGGCAAATCCATTGTTGATCTTTCAAAGACTGGGGGTTGGATTGCAGCCTTCTTTATATTTGGAAATGAAATGGCAGAAAGAATGGCTTATTTTGGGCTTTCAGTTAACATGGTGGCCTTTATGTTTTATGTGATGCATAGACCATTCACGAGTTCATCCAACGCAGTAAACAATTTCTTAGGCATATCACAAGCTTCCTCTGTGCTGGGTGGTTTTCTAGCAGATGCATATCTTGGTCGCTACTGGACAATAGCTATCTTCACTACAATCTATCTTGCGGGTTTGACAGGAATAACACTGTGTGCGACAATTGGCAAGTTTGTACCAAACCAAGAAGACTGTGACCAATTTTCTCTGCTTTTGGGAAACTGCGAGCCAGCAGAATCGTGGCAGATGACATACCTCTACACGGTTCTATACATCACAGCGTTTGGAGCAGCAGGGATAAGGCCCTGTGTGTCATCTTTTGGAGCAGACCAATTTGATGAGAGAAGCAAAAACTACAAGGAACACCTTGACAGATTCTTCAACTTCTTCTATCTGTCAGTGACCATAGGGGCAATTGTGGCCTTCACAGCAGTGGTGTACGTGCAGATGCAATATGGATGGGGATCTGCATTTGGGTCACTGGCAATTGCGATGGGAATATCAAACATGGTGTTCTTCATTGGCACTCCCTTGTACAGGCACAGGTTGCCAGGAGGGAGTCCTCTCACTCGGGTTGCTCAAGTTTTGGTTGCTGCATTTCGAAAGAGAAATGCCCCTTTTGGAAGCAGTGAGTTCGTAGGCTTGTACGAGGTTCCTGGCAGACACTCTGCTATCAAAGGAAGTCGAAAAATTCCTCACACTGATGATTTCAGGTTTCTCGACAAGGCAGCTCTGCAATTGAAAGAAGATGGTCCCAATCCAAGTCCTTGGAGGCTCTGTACTGTGACTCAAGTAGAAGAAGTCAAGATCCTTTTGAAACTTATCCCCATACCAGCTTGCACAATCATGCTCAACGTAGTCCTAACAGAGTTTCTCACTCTCTCAGTCCAGCAGGCATACACTTTGAACACCCACTTGGGTCGTTTGAAACTCCCAGTTACATGCATGCCTGTGTTCCCAGGCCTCAGTGTTTTTCTCATATTATCTCTCTACTACACCCTTTTTGTCCCCATCTTTAGACGCATCACGGGTCATCCACACGGTGCATCTCAACTTCAGAGGGTAGGAATTGGGTTGGCAGTTTCTATTCTATCAGTTGCATGGGCTGCAATATTTGAGAGGTACAGAAGAAACTACGCAATAAAACATGAATATGAAGCTAGTTTCCTCACAGCAATGCCTAACCTAAGTGCATATTGGTTGTTGATCCAATACTGCCTCATTGGTGTGGCTGAAGTGTTCTGCATTGTGGGGCTATTGGAGTTCCTATATGAGGAAGCCCCTGATGCCATGAAGAGCATAGGATCTGCTTATGCTGCACTAGCTGGTGGCTTGGGTTGTTTTGTGGCAACTATTATAAACAACATCATTAAATCTGCCACTGGAAACTTAGATAAAGGCCAACCTTCTTGGTTGTCTCAAAACATCAACACCGGGAGTTTTGATTACTTTTACTGGCTTCTCACAGCTTTGAGCATGGTCAACTTTTGCATCTTCATGTATTCGGCACATAGATACAAGTACAGGGCACAGCAAGTTCACGAGATGGAGAAACATGAAATGTCATACGTTGGAAGCTCCTAATTACCAACACAAAGTGCTGTCTTTGTTCCATTGAATAACTTATAGTCTTCTCCTTGTATGGTTAAAAATTACCATTTCATAGAGATTGAAAACGCAAACTAGCTAGGGAGTATTCTGCATAAGATAATGGATTTCGTTTAGTTTAATTAAACACTTACATGCAAGCAAAGATGATAAAAAGATAATTGCA

>C100159_65

ACACATGTTCCCAAATTTCATACCCCAAACGACGTCGATTCCTCTCACTGCGAGATAACCCTCCTGAGAAGAAAACCAGACTTCATCTTCGCAATTATCGCACCGCGCATGGCGGCACCCACGAAGACGGAGCCGTCGCTTTTGTCGCCGACACAGAGGTACGCCGCCGGCGCATTGTTCAGCCTTGCTCTGCACGAGGCTCATCTCAACCAAACCAACCCGTTACCGTTACCCGTTTCGGAGGACTCCCACTCCGAGGAACGAACCAGTAGTAGCTCCAGCAGCGACTCGGTTTCCGATGACCCGGATCTTTGGGTCCACTCCAATTCGGGTTTGCTCCGTCCCGTTTTTAGGTTTCTTGAAATTGATTCCGCTGCGTGGTCTGGACTAGAGGAAACCGCTGGTTCTTCTTCCGCTAAGAATCACGTGGGATCGTTCTTGAGGTTACTTTCACAAGAATCGGACGATGGTTCTTCTCAAACGTTAGATGAAGAACTTGGTTTGTCAAGGGCAGTTAATTGTATCGCACGTGACATGCTGCAAAAATTGGAGTCTTCCGAGTCTAAAAGGGAGAAGCTTCGCGAATATGAGCATCAATGTCGTGAAAAGTTCTCAATCGATGAAGTTCAGCCTCATGGTGAAAAGGTAGATGAGCATTTGGAAGTACAGCAGGTAATGGAAATTCTTCCTTTAGTTGACTGTGAAGAGCCGCAGCAGGGGTCTGTTAGTTGGAATATTGATGAGAGACCAATGGAGCCAATTGAGGAAGTAATGATGTTGAGTGATCAGAGGAAGGTGACAGTTCTATACGAACTCCTGTCTGGTTGTTTGTCTAATTTAGGTGAAGATATCAAGCAGAGAAGGAAAGGCTATGATGCTCGACATCGTGTGGCTCTGAGGTTGCTTGCAACATGGCTTGATGTCAAGTGGACAAAAATGGAGGCCATTGAGACCATGGTTGCTTGTACTGCAATGGCTTTTATTAAAGAGCAAGAATCAAAGAAAGAAGAAAATCATTCAAAAGAAACCAAATGGGCTAAATTGAAGCGGGGTGGTATAATTGGTGCTGCTGCCGTAACTGGTGGAACTCTGTTGGCTATTACTGGTGGGTTAGCTGCACCGGCCATTGCTGCAGGTCTAGGTGCTTTGGCTCCAACATTGGGTACTCTGATCCCTGTAATTGGAACTGGATTTGCTGCAGCTGCTAGCGCTGCAGGAACTGTTGCTGGTTCTGTTGCTGTTGCTGCATCATTTGGAGCTGCTGGAGCTGGGCTTACTGGAAGCAAAATGGCTAGGAGAGTCGGGGGAGTTGATGAGTTTGAATTCAAGGCTATTGGAGAAAACCATAACCAAGGCAGGCTAGGGGTTGAGATCTTAATCTCTGGATTTGTCTTTGAGAAGGAAGATTTTATAAGGCCATGGGAAGGATGGAATGACAACTTGGAAAGGTATGCACTGCAGTGGGAGTCCAAGAATCTGATTGCTGTGAGCACTGCAATTCAAGATTGGCTTACTTCAAGACTTGCGATGGAGCTGATGAAACGAGGGGCAATGATGACTGTTTTGAGCTCACTCTTAACAGCTTTGGCTTGGCCAGCTGCATTACTTGCAGCAACCGATTTTATAGACAGTAAATGGACAATTGCTATCAACAGATCAAACAAAGCTGGTAAGTTGCTTGCTGATGTATTGTTAAGAGGATTGCAAGGAAATAGGCCCGTGACACTTATAGGTTACTCACTTGGGGCAAGAGTTATTTTCAACTGCTTACAGTGTTTGGCTAAAACTGAAAACGGTGCTGAACTAGTAGAAAGAGTTGTACTTCTTGGAGCACCCATCCCAATCAAGGACGAGAACTGGGAAGCAGCTAGAAAGATGGTAGCAGGAAGGTTTGTAAATGCTTATTCGAGGAATGACTGGATGCTTGGAGTTGCCTTCCGTGCCAGTCTATTTACGAAAGGATTAGCTGGAATCCAACCTGTAGATATTCCTGGGATCCAAAATGTCGATGTGACAGATCATATTGAAGGCCATTCGTCTTATCTGTGGGCTACTCCAAAGATCTTAGACCAACTTGAATTTGATGGATACTATCCTGTATACAACAGCATTTTTTGCATATAGTGACCTTTTCATGGGGGTGCTCTTCTTGCATACAGTTGAAGTGATTGCCGGACCTAATCTGCTTAAAGTTTGACACAATTCAGAAATTATGTATGAATTTATTTGTTTATTAGTATTACTATAAGGATGTACTAC

>C100161_65

TGATGATATAAAAATTTGCATGTTCATAAACCCATAATTGTCCAAGTATGAAAAGCACAAACTATTTCATATTGTTGCAATTATTTTACATATGCATGCGTGATAACCCCTCCAAATAAAAGACACTTACATAACCATTGCATGAAAATGAAAAACCGACCGATCAAACCTCAAACTTTGATCATCAATACCGAATGTTGTTCGGTATATACTATGTTTGAAATTGTGACAGAACACATCCAATTCAGCAGCATCAGACACTTTCAAATGATTCGTATATTGAAGTGGAAAAATCTGTGGTTGATCTTGGAGGAGAAGAGAGGAAAGGTTTCGGTGGCATTTCCAAGGAATCCACACTACCTAACAACATGTCAATCACTCTACTTATGGTAGGTCTGTGAGAAGGAATTGTTTGAATGCACCACAAACCCACCATAGTCATTCTCTTTGCTATCTCATTTTCCTCACCAGACAATATGCCATCAAGTCCCAAATCATTGCCCTGCTCAAGCTTCTTGTAAATAACCAATTGTGGGAAATATAGTTCGCTCGAGCGACTAGCTTCAACGTTGATGTTCTTCTGTCCTCCGACCATTTCTAGCAGCATCATTCCATAGCTGTATACATCAGATTTGTGTGAAACACCTCCAAAACTTTTACTGAACACTTCGGGAGCCACATACCCTACTGTCCCTCTAGCATTTGACATTGATATAATGCTTTCATCTCTTGTGCTGAGCTTAGCAAGTCCAAAATCTGATATCTTTGGGCGATAAGTTTCATCCAGGAGGATGTTATGTGGCTTAATGTCAAAATGCAGAATCCGAGTGTTGCATCCTTTATGCAAGTACTCTAGTCCTCGAGCTATGCCTATTGCGATCTGGTGCAATCTATCCCAACTCAAGGATGGAGTAGTGGTTTTATTTTTTTCTGGTGCTTTCTTGTGAATGTACTTCTCAAGTGAACCATTGGACATAAACTCATAGATGAGGGCTTTTCTGCTTCCTTCTAGGCAAAATCCAAGGAGAGAGACAATGTTAACATGGGATGTTTTGCTTATGCTAGCAACCTCATTGACAAACTCTTCGCCATTATCTTTTGACTCGTTCAGTATCTTCACTGCCACCGAGCAACCATTGAGTAATTTTCCTTTGTATACAGAACCATAACCACCTTCCCCCAGTTTAGTTTTGAAGGAGTTGGTCATTTTCTTGACATCAGAGAAACTGTATCTCTTAAGACCCATAGAACCTTGGCTTTCAAGGAAGGCTTCTATAACTCGATCGTTATTTTTTATCTTGCCAAATTGTATTAATATAAATTTCCAAATTCTAGCTTTGTTGCGGCATATGATGACAGCGATTAGAGGTAGTGCAAATCCAGTAGCAACGAAACCTAAAACCAGCTTCAACACTCTGCTCTTCCTTCCACTTTTGTGAGCAGAGCACACTGAACCACGAGTTCCACCCGAGCAATGACAAGAAAACTGAGACGAATCATTCTTATTCGTTCCACATGCTCCTCCAGAATCCCTGCATGCTGTACACTGAGAACTCCACTCTGCATCAAACTTCACATCAAACCCTTGACCCAACGCTTTCTTAAGCGCCTCAATTCCATTCTCTGAATCCCAAACAACACCCTCTGAAACTGGCACCAGAACGCTAACCCCACACTTCTGAAGGCCTGGGAACTGCTTCAGCTGAGTTCCATTGACCACATAGAAAGCCTGTTTATTACTATCATTCCGGCACGTAAAGGTGTTTCCCCCAACAGAATTGTCAGAGGGGCAGTCGTAGAAAACAGTGACATTCTGAGCAGTGGGTAGAAAACTAAAGGGACTGGCACTCAGAGAAGTGTTGGTGAAATTGGAATAGCAGTTATCATAGACAAGGTCTGTTCGCACCATTCTCATGGTGGAAGCATTTTGGTCAATGTCTAGTACATGGAAGTTCTGGGAACCAACTTGAACCGAAGTGTTTTGGTTATGCATGCACGTGAGCTTGAACCCATTACGGCCACAAAACTGGGGTCTGTCCCCTCCCCAGAAAGGATAGGATATGTTTCTGAAAGTTCCACAACTGAAAGACTGGCTGCAAATGGAGTGAGTGTTGTTCTCTTGAGAGTAGGATTGTGGCAAAGTTGCTAAGAAAGCAAGGGCGAGTATGGATGCAAGGAAGAAGGAGGAAGGGGTGGTCATAGTTACATGGATGGACAGAAAAGTTGGAGTGATCTGAATGGTGAAGGCGGGCATGCTTT

>C100167_65

CCTGGCTTTTCATTTTTATTTAGAAGCATCATGTACAGAAAAATATAAGTTTGCTAGAATTCCAGAAAAAGTAATAGGATAATGTTTTTAGAATTTAAATCAACTAATCCTGCATGGTTGATTCCACCCTAAGCAGTATTCCGTGAACCTGATTGAAGCATAGATGCATCATGAGGGAAATTGCAGCTATAAAAACACTGCAGGTTACTCATTCTTTCTTGTAAACTAATGTAGGGTACTCTGGATTCCACATGTTATTCTTCACATATTCTGCGATTTCATCCTCACTAAGTTTCTTTAGCTCCCGAGCATCCATTCCATGATATCCTTCAGCTAGGTCCTCCTCCAAAGCTTCTTTTATAACAGCGGCGGCTACCTTCTCTGTAATATCTCGAATTCTGGATGTTGAAGGGAAAATAATCCCTTTGAGGACCTCCTCTTCACTCATGTATGTAGCAAGACGCCCAGCTGCAGCCTGTAGCATGCCATCAGAGATTATCCTAGCACCTGACAGAAGAGTTCCAAGACCAATGCCTGGAAAGAGGTACATGTTGTTTCCCTGGTTGCAGTGGCCAATATGACCATTTCCAAGATCCACATCAGTGAATGGACTTCCACTTGCAAATATAATGTTGTCACCCAAAATGGAGAATGCTTCTTCAGGGGTGCATTCAGCATTCTTTGTAGGATTTGACATGGCAAATATGGCTGGTCTCGTTGATGTTGAATCCTTGAGGGCCTCTAATACCTCCTTTGAGAACAATCCTCCAACAGCAGATAATCCTAGGAGTACATCGGGCTTCACTTGCTTGACCACTTCAGCAAGGCTTGCTCCTTCTCTCAGTCCTTGGCGATCCATTTCTTTCAAATTTCTTGCAAAAGATAGGGCATCTGGATCTATATTTTCACGTTCTTCTGTGATTAATCCCTTTGCATCAACAACCCAAAACTGACTTTTTGCACTCTCAAAAGCTACTTCATTATTACCCAACATTCTTGCCATTGTTTTTCTAGCTGCATTAAGAACCCCAATTCCAGCACTTCCAGCACCAGCAACAACAATCTTCTGCTTTGGGAAGTCAATCATTGGTCTTCCTTGTGCCCTTACAGCTCCTAGAAGTCCAGCAATTGCAACACCTGCTGTTCCCTGCACATCATCATTGAACATTCTGTAGGTATTTCTATACCTCTGTAATAACTTAAATGCCCATTTGCTTTGGAAATCTTCAAACTGTACAATCACATTTGGCCAGCGAGTAAAGACAGCCTCCATAAATTCATCAACAACAGCCAGATAGTCATCACCATCAAGACGATGTTGCTGCAATCCCAAATATAAGGGATCTTCTATTAACTTCTCATTATTAGTACCAACGTCAATCATCACAGGAAGTACCCTTTGAGGATTTATTCCAGCGGCAGCCACATATAGATCAAGCTTCCCAATGGCAATGCCAATTCCTTGAACTCCAAGATCTCCAAGTCCCAGGATTCTGCTCCCATCAGTAACTACAATCATATCAACCTGCTCAGCTGGCCAGTTATATACCATAGACATCATTTCACCACGATCCTCCGCACTGAAATACATTCCTCTTGGTCTTCTAAACAGTCCACTATAGTTCTGACATACAAGACCCACTGTTGGAGTGTACACTATTGGTGCATATTCCTCTATTTTTGCTATCAAAACCTTATAATACATAGTCTCATTCCTGTCATGCAAGCGATTAAGTATCCGCCACTTGGCCAATGCATTAGGATCAGAAGGTCCATCTCTTGCTTGAACTTCAAGCCTCTTCAGATCAAACATGAAGCGTTCAATCTGTAAGTCAGGAGACATAACATTTGGAGGAAGCAGTCCTCGTAGATCAAGGCGATCTCGTTCTGTCATGGAAAAAGCTGTTCCTTTATTGAACCAAGGATCGTGGAGGATGTCGAGGCTGCGTTTGTGAACGATGGAGGGACGGTGGCCCTCGGTCGTGGTGAACGGCCGCGACAGCAGGTGGGCGCGGGTCACGTGTCGCTTCAGCAGCGACGACGACTCCCTCACGTACTTCAACAACATCGCCATTTCGCTCTGCGATAACGAGATCTAATGAATAGTGCTATGAATCCAGTGACTCGCGCACTCGCACTTATAAGTGTTGAATCGAAATAGCGTTGCGAAATGTGACAAAGCACCAGTGACGCTTGTTCAGAATTGAATTGGGGACGTTGGGGTGGGGACGTGGTGTTTTTTTCTACTGCATCATCATTG

>C100173_65

ATCAGAGATTGCATTGCAAAGTCATAAAAGTCAAGGATGGTTGGTTTATTAGCAATACCTTGAAAGAATAATGGTTACACCAACATAAATATACAGAATAATTTATTTTGGAAACAGTCAAAACACATTGAATACATCTATACCATATCAAGAAATTTCTTCCAATTTTATTTTTTCTTTTTCCATATCCATAGGTTTTCTCGACGCACGCCATTTTATTTGCCTTATTACTGTAGCTCCCCAACTGGTGCCTCATTGAGCCACAGGAAATGAATGAAGTCGTATAATTTTGCACTAACACTGACCTTATATGGTGTTGGGTTAAGTCTCCTCATGTGGTCAGGTCGCATTTGCTCGAGTTGCTTGATGCATCCCTCTCTAATGTCTCTTAGAATTGGTAAGTTGTCTTTTTCTTTATCTGAACTACCAGGCCAGTAACACCTCAGAAGCTCCTCAACTCGCTGTGGCACCACATATGCTCTCTTGGATTCTTGGAAGGGATGACGGCACAGGATTCTTTCACCCACCTTTGGAGGGGGCTCATTTTCTCCTGTCATTATGTCAACAAGGGGATAACCTTCTTTCCCATACAACCTATAGCATCGCTTCTTACATGGAATTGAGACCTTTGATACATCTTCAGAAAGTTTGATCCGAGGTTGATTGTTTATTTCAACCAGCTTGAAAACAACACCTAAAGCAGCTTGAGCGTAACATGTAACCAGATAGGTACCAATTCCAAAGGCATCAACCTCATGACCCTGTTTGTTTAAAGCATCAAGTGTTTCCTCATTGAGGTCATTACTGGCAGTGATTGCCAGCTTCCCAAATCCAGGCACTCCAAATTCCTTCTCAATAGACTGAAAGAATTTCCTGGCCTCACAAGACAGATACGCAAGGTCACCAGAGTCCAATCTAATGCCAACTGCTTTGTACCCTAAATCACCGAGAGCTAATGCAACAGCACAGAAGTTAGGGATTCCACTTCTCATCACGTCATATGTATCAACAAGGGCAAGAAAATCATCAGGAAACGCCAATGCATATGATGTGAATGCTGCCAACTCGCTTTGATTGGTCTCACCAAAAACACCATTTGATAACTGAATTTTGCTCAGCCATGTTTGAACCACACTAACAAAATCATCACATGTACTAGAACCATCTTTTCTGCGAAGTGACTTGTCTGTAATCTCGTCAAGGCCCATGTATGAACTAACGAAGGCATGGGAATGAGTACCACGAAGGGGTATCCCGAATAACTTTCCTGCTGCAACATTGCTTGTTGCATCAAATCCACCAATATAGCAGTACTTTGATGCTCCTACTCCACCATCAGGGCCCTGAGCCCTTCGTAATCCAAACTCCAGAAGAGTTTTGGATTTTCCAGCAACATTACGATGCCTTGCAGCATTAGTAGAAACTAATGATGCATAATTAATTAGATTCACAAAAGGTGTTTCCAGCAATTGAACAACAGCAATTGGGCCTTCCACTCTCATCAGGGGAACCTTGGGAAAAACGACTGTTCCCTCAGGAATAGCATACACCTCAACATCAGAGCAGTCAACTCCTCTAAGATAGTCAAAGAATCCATCCTCACAAGAGGAAGATAAACTTTGCCTGACAAAATCAATCTCCTCCTCAGAGAGCTTGAAATTAGCTATAAACCTTATGCACTCTTCCAAACCAGCGAACACAGTATACTCCCCACCGAACGGATTCTTCCGAAAATACAAATCGAACACAGCACGTTCTTGATGCTTGCCAGCTTTCCAGTACGCGTAAGCCATGGTAAATTGATAGAGATCCGTGAGCAGAGGCGTAACCATCGGGTTGGTAGGTCCGTGTATGGCGCCATTGGAAGGCTTCTTGCTCGGTCCGTTCTCCTTCGCCTCCATTAGGGTTCCCTCTGGCGCACAGACTCTCTGCTGCGAGTGTCAGTCCTTATTTGGTCTCGGTTTTTTCTTTTTACAGTTTTGAGTTGGCGTTGCGTTGTTCTATTACATCGCGCGCGCTCTATTTATACAGTTGATTCTGATGCAAGCGTGAGCGTCACGCACGAAAAGGGAACCTAAGGCGAAGAGAGGAGAGAGAAAGTGGCATATATTTTCCTAGAACAATACTCCTTACTAAGTCCTAAGCTAAGACCGTTTGAACTTCGAACAACGATCAACGAATCAAGAAGAGATAGAGAGAGAAACAATAAGACAAACTGAACCAGGTTGGTTGGTTGTTATGTATTCTACTTCGTTTCGAG

>C100177_65

GTGAAGAAGAAACTATAGTAAAATAATGAGCGAGAAGAATCAAACCAAAACATTGAATTGAAAAAAACAGAGGATGATATAGGTGTAATAAAGCAGAATGAAATTGTTCTTGTTGCTTCTTTTTTAGGCTTTAAAAAAAGCAGAACCGAAAGAGGCAGAGACAGAGAAAGAGAAACAGAGTCGGAAGAAGCTAGGAATGGCGGCGGTTGACCCTCACTCGTTCACGGACTCATCACACCCACCGACGACACACATATCTCTGAATCTCTACCTGGACTTCTCCACCGCCACCGTCCACGCCTCGGGACTCTTCACTCTCTCAAGGCCACACTCCGGCGGGTTCTCCCTGGACACGCGCGGACTCACCATCCACGGCGTGATCGACCCGAGCAGCAGAGCCCCAATCCCCTTCACTCTCTCCTCGCCCGATCGCATCAGAGGCACCCACCTCTCCCTCACTCTCTCCAACCAATCCTCCTTCCTCATTACCTACTCCACCTCCCCCTCCTCCTCAGCACTCCAATGGCTTCTCCCACCCCAAACCTTCACCAAGTCTCACCCTTTCGTCTACACCCAATGCCAGGCCATCCATGCGCGCGCCGTTTTCCCCTGCCAGGACACCCCCGCCGTCCGGATACGCTACTCGGCGCGCCTCAACATCCCTTCTCAGCTCTCCGCTGTCATGGCGGCGCGCCATGTCGAGCGCCGTTCGCCCAGAAACGACGACGCTTTGGACGTGGTTCTGCCCTCGTCGTGGTGCGCTGAAGGGAGGGTGGTGGAGGAGTTCACGATGGAGCAGTCCGTGCCGCCTTACCTCTTCGCGTTCGCAGTTGGGGCGCTTGGAAATAGGGAGGTAGGGCCCAGGACGAGGGTGTACGCTGAGGATGTGGCCACCATTTTGGACTCTGCGGCGGCGGAGTTCGCCGGGACGGAGGAGATGATAAGGGAAGGGGAGAGGCTGTTCGGGCCGTATGAATGGGAGAGGTTTGATTTGCTGGTTTTGCCTCCAAGTTTTCCTTATGGGGGGATGGAGAACCCAAGAATGGTGTTCTTGACTCCCACTGTTGTTAAGGGGGATGCGACCGGGGCGCAGGTTGTGGCTCATGAGCTTGCTCATAGTTGGACTGGGAATTTGATCACCAACACGACCAATGAACATTTCTGGTTGAACGAGGGCTTCACTACTTATGCGGAGAGGAGGATTGTGGAGGTTGTACAAGGAGAACAAAGAGCTGCCTTGAATATTGGAATTGGTTGGAGGGGTTTGAATGAGGATGTTGAGAGGTTCAAAGACAACTTGGAGTTTACAAAGCTAAAAAATAATCAAGAAGGAATTGACCCAGATGATGTGTATTCTCAGGTTCCGTATGAGAAAGGTTTCCAATTCTTATGGCGCATTGAACGCCAGGTTGGGAGACCTGCATTTGATGAGTTTCTGAAGAAATATATTGCGACCTTCAAGTTCAAATCAATTGATACTGAAACATTCATTGACTTCCTTAAAGCTAACATACCTGGCATAGAGAACCAGATTGACTTAGTGTTGTGGACGGAAGGTACTGGCATTCCTCCTGATGCTTATGAACCTGATTCGGCTGTTTATAAACTGATTGTTTCACTGGCAAATGAGTTCATAAATGGGAGGATGCCAAGGGAGGATGAAGTTGCTGATTGGCAAGGTCAGGAGTGGGAGCTCTACTTGGACAACTTGCCGAAATCGATTGAAACTTCTCAGATCATAGACTTGGATTCACGCTACAAGTTATCTGAGTCAAAAGATTACGAGGTAAAGGTGTCATTTCTCCGAAGGGCTATTTCATGTGGATGCAAAACTTACTACAATGAAGTGGAGAAAACCTTAAAAGGAGTTGGGAGGATGAAGTATCTTCGTCCACTTTATACTGCTCTTGTGAAAGACAGTGCGAATGAAGATGGTAAAGTATTTGCCAAAACACTATTTTCAGAGGCTCGTGAGTGTTACCATCCAATTGCTCAAGGGGTTGTTGAGGCCATATTTGCCAAGCATCTGTAGAAGGAATAATTCTTAGAATTTTTCATGTATTTGGACATTTGCAAGTTGTCTTTAGGGTACTCTTATACAATAACTATGGTTTAATTTTCTTCTGACACCTGAACAGCTGCTGCAATTAGAAGTTCTTTCATAATTCTTCTTGTTGGAGCATTTCAGTCATTGGTTTGTAGGGTTCTAAGGAGAGGAAAAAGAGTGGATAAGAACCAGTGATTGATTTCTCCTGTGAGA

>C100181_65

TTTGGCTAATGAAATTCTGACTGTCTAGTATCTTGCATAGTATAAATATCCACCCAATGGTAATGGATCGGACAATTTTAAGCCGAGGCAGAGACCATGTCGTATTTGAGTTCAATAAAGGAAAAATGTGCAATGGGATTAGCATACATATTAGAATGTGCCAACTCGGACAGCAATAAGGATTACCAACCCCAAGTCAAAGACGTCCCTAGCCTCTCCCTAACCCCACCCAATATCAAATTTGGGTAAGCTCTAGCATACAAAACTTAACTTCTTCAATTACACGATGATCATCATACTACCAGTTAAAATCCATCCCGATTGTTCACAACTCATCATGGTTTGCTGGTTGTTCTGAATTCAATAACTCTAGCTTGTGCTGTAACTCCTTCAGCTTTTCCTCCTGACAAAGGGCTGGGGTAGATAATAAAGCTACATATTCGCAACGGCTTGGTTCATCTACATCAGTAATCTCATTTTTCAACCCACATCTTAGCTTGACCTTCAAACTCCTATCAGGACCATTCCAGCATTTATCACCATTAGAAAACACCATTACTTTGTATGAATCCTCAAATTTGTCCCAGCGCCCCAAACGGGTGGTGGAATAGCTCTCCTCTTGAGAAGCCTCTTTATAGGGGCAGACTTTGTAAGTGTACTTGTTCTCCTTGCCCTCAAAGCAATGATCATAGAATGTATAAAACTCCTTTGCTGGACCAAAATCATGTTTTAGCTTTTGCGTCAGACTTGATATTCTAGACTGTATTTTAGACAGCTTGGCACTTGATTCATCATATTCCTTGCGTACACGAGCAGCATCTGACTGATTCACAGGAGCCTGGAATAAATTGACTGCCTGAAAAATATTCCGGACAGTTTTCTGTATTTTCTCTAACCACGAAGGATTATCTGAAAAGTCCGGTTCAGTATCAGAATCAGATTTGTAGGAGGACCTAAGGTCATCATGCTCGTCCTCTTGATATTCCTCATCAACGTCATCTTCATCATCATATTTGTTACTCTCATCATCAGTTTCAGAAGCATAGCCTTCATTTTCGTTATTATTTGTCCCCCTTGGAAGATCTTTGTCATCTTCATTGTCTACAATAGTATCAGTTTCAGCACTTTGCGCATCAGTATGTTCTCCTGTCCAACGTGAAGCAACAAGCCGGCCCAACTCTTCCCTGGATAATCCCTCGGTATTTTCAGATTCGCCATTCCCCTTATTGATGATTTCATCTGAGGACTCCTTGTTTCCAACCTTAACATCGGCTTCAGACTTAACAATAGGTTCTTCCTTGGCTTCGTTTGCATTCTCTTCCACTTTATTATGCAAAGACTCTTCATTTGCAGGACTATCGCTGGCTCCATCATCATTATCAAGTACATCTGCATCCTGATCTGCAGGAGAACTGTCAAGATTTCCAATTTTATCATGGTTATTTTCCACAGCAGTGTCTTCTACATCCGAATGCTTTCCAGCTTCATTTCTATTACCCACATCTTCATCAGCTTTATCTTTTGCTTCATTAGCCTTCTCCTCAGACTCCTTTTTCTGCATCTCTTCCTTTTCTTTCTGTAAACGTTCTTTCTCCTCTGCTTTCTCTATTTGTTCTTTATGATCCTTTAGCTGTTTAACAATCCCTTTAAGTATACTTTCTTCCTTTTTTAATTTTGAAAGTTCTGCCTCATCCTTCTCGATGGCTACTTTAGCTTGTTCAATTTCTTGCTTTCGCAATTTGACACCCTCTTGATAAGTGGCAATCTTTTTTTCCAGTCTATCTCGAGCCACTTTCCCAGCCTCCCAGCAGGTATTTGGACACTTCACTTGACCATCATATTCGTCAGTTCCATCACAGCAATCGCAAATTCCATCATTCACTCTGGATGAAAACAAGTAAACAGGAGAATGTCCTGCATTCCGACAAAAAAATCTTCCACCCGGACATGCAGATGTACCAGGTTCATCGGTGCCATCAGCACAATCGCAAAAATCATCATTGAGCTGCGCCTTGTTGAATTTTCCGGATCCATCTTTACATCTTATAACATCGGAAGCCCTGTAATACTTGTCATCTTCGGGAGCGATTCCGAAGAACGGGTCATTGGGCTTCGAAGAGGAGGAAAATGGGACGCACAACAGCAGAGCAATGAGAAGGATTCGCAGCCGCAGCTTCATGTTCTTGTGGGTTCCGACTAGCGACTGCTAGTGAGGCGTGATAAGTGAAATAAGATTCGAGAAACTACAATTTTTTCAAGAGG

>C100183_65

CGAAAATAAAAAGAACAAAAGGAGAGTCCAGAACAGCATCTAACCAAGTTGTTCACCGTTGGATACTAAAGATTCCGCTCAGAAGCTTACACACGTTGTACACAGTAGAGTGTTACGTTTGTGCATTAACCACTTATTTATCTGTTTCCCACTTGTTTTGTAAAAAACTACACTTTCTGTAGTCTATGGCTTCCCACGTTGCTCTTCTTCGTGCTCCTCCTCTTTCCATCCCCACTCAACGCTTCCATGCCAAACAGATTTGCATAAACGGACTCAAATTCGCCACCACTTCTTCTACTTCTTCTTGCTTTCCGTGCTCAATAACAACACAAAGAGGGTCTTTTTCCTCGGTGATTGCGTGTTCCTCTTCAAATGGAAGAGACCCGGATTCGGTGGGTGATGAAGAGGTACGGAAAGTGGAGCGCATTCTCGAAGAGAAACGACGAGCTGCATTGTCAGCAAGGATTGCGTCAGGGGAATTCACCGTGAAGCAGAAATCTATTTTGCTTTCTCTAATGGAGGGATTGAAAAAGGTGGGGTTACCCAATGAAGTTCTGGAATTTTTGTTTGGTTGGGTTGAAGGGGGTGGTGCGTACCCCAAAATTCCTGAGGCAAAAGGGTCAATCAAAGCTGTTCGAAGTGTTGCCTTCTTCATTCCCTTGTATGAACTTTACCTCACATATGGTGGGATTTTTAGGTTGACGTTTGGACCAAAGTCCTTTCTGATAGTATCTGATCCGACTATTGCAAAACACATATTGAGAGACAATTCAAAGGCTTATTCAAAGGGCATCTTGGCTGAGATCCTAGACTTTGTAATGGGGAAAGGTCTCATCCCAGCTGATGGTGAAATATGGCGAGTTAGACGCCGAGCTATAGTCCCATCATTCCACCAGAAGTATGTAGCGGCTATGATCAGCCTTTTTGGACAAGCTGCAGATAGGCTCTGCCAGAAGCTAGATGCTGCTGCATCGGATGGAGAAGATGCTGAGATGGAATCACTGTTCTCTCGATTGACCTTGGACATCATTGGAAAGGCAGTATTCAATTATGATTTTGATAGTTTATCGAATGACACTGGTATAGTTGAGGCTGTTTATACTGTACTGCGAGAAGCAGAAGATCGAAGTGTTGCTCCAATTCCAGTGTGGGATATCCCAATATGGAAAGATGTATCACCACGTCTAAGGAAGGTTAATGCAGCTCTCAAACTGATCAATGATACGCTTGATGAGCTGATAGCAATATGTAAGAGAATAGTGGATGAAGAAGAATTGCAGTTTCATGAGGAGTACATGAATGAACAAGATCCAAGTATTCTACACTTCTTGTTAGCGTCAGGAGATGATGTGTCAAGTAAGCAACTTCGTGATGACTTAATGACCATGCTCATTGCTGGACATGAAACATCAGCTGCAGTTTTAACTTGGACCTTTTACCTTCTATCCAAGGAGCCTAGAGTCATGTCCAAGCTCCAAGAAGAGGTTGACTCTGTACTTGGGGATAGATGTCCAACTATTGAAGACATGAAGAAACTCAAATATACAACCCGAGTGATCAATGAGTCATTGAGGCTTTACCCACAACCACCTGTGTTAATTCGTCGCTCTCTTGAGGATGATGTTCTTGGAGAGTACCCGATAAAGAGAGGTGAAGATATCTTTATATCTCTTTGGAACCTGCATCGCAGTCCAAAACTGTGGGATGATGCTGACAAGTTTGAACCTGAAAGATGGTCATTAGATGGACCAAACCCTAACGAGACGAATCAAAGCTTCAAATATCTTCCGTTTGGTGGAGGACCACGGAAATGTGTAGGTGATTTGTTTGCTACGTACGAGACTGTAGTAGCACTTGCAATGCTTGTTAGACGGTTCAACTTTCAAATGGCAGTTGGAGCTCCACCTGTTGAGATGACTACTGGGGCTACAATTCATACGACACAAGGGCTGAAGATGACTGTAACACACAGAATAAAACCTCCTATTGTGCCCTCATTACAGATGTCAACTTTGGAAGTGGATCCATCCATAAGCCTTTCAGACCAGGATGAAATAAGTCCGAAAGGCCAAGTTTATCAGGCTCAGGTTCAGGCTCAGCCCTAGTTGCTGCTGTGTAACAAATCATAAACCATACAATACAATATATAAAATGAGTGGAAAGGGGGAAAAGTAGAATTCTTTTCATTCTTTTAGCTTTGAGTGAATTATGTAGTAAGGTGAAGCTTTATGTCCCTAAAAACCCAACAATCCCCCACCCTTG

>C100187_65

GATACCCATATTCCAATTGTGATTAAGGAATTGTAACTGAGGTCACAAGCCAAGCAACCAAGATTCAGCAAATTAAATACATGAAAGATGTAATAATCATGTTAAACAGTATTCTGCCCGGTGAACTTCAAAGAAATTAAATAGACCTAAGATGTAATAATTAATCCTATTCAACAATATTCTGTCTTGTAGATCAACCAGTGAGCCTACATCTCCTAATAATTTACAATACCCCAATATGCCACAAAGTTCTTCATTGTGTTGTCACACTCTTGGCAAGATTTCGAGGCTGGTCCACATTAAATCCCCTAAGAACAGTGAGATGATATGCCAATAACTGTAATGGAACTACATTAATTACAGGTTGAAGACAGTCCTCAACTTGAGGAACTTCAATTACCCTACAAGATTCATTAGGGCATACAGAGATAGCATCCCCTTTTGAACACATAACAACCAGACGACCTCTGCGGGCATGAAGTTGCTGAATAACAGACTGCTGTTTGCTGAAGCAAGCATCACGGGTAGCTACAACAACAATTGGAAGATTTTCGTCCACTAATGCCAAAGGACCATGCTTCATTTCACCAGCAAGTATCCCTTCGCTATGCATAAGTGCCACTTCCTTTACTTTCAATGCTCCCTCAAGAGCCGTTGCATAGTTGTATCCTCTTCCAAAGACGAGAAGAGACTGCTCAGCAATCAACAGCTTTGCTAGATCCTTCATCTCATGGTCAAGCTTCAGCACCTCTCTGACTTCATTTGGTAGGTTAAAAAGACCATCTATGATAGCTTCTCTTCTGTCTTTATTTGAAATTGTATCACCTCCTATTGCAAGAGCTAACATGACCATCACCACTATTTGGCTCGTGTATGCCTTGGTACTTGCCACACCAATCTCAGCACCAGCATTTATATGAACACCACAATGAGTATTCCTTGCTATTGCACTACCAACAGTGTTTGTTATCCCAACACACAATGCTCCATTGTCTAAAGCATATTGTAGTGCAAGTAAAGTGTCAGCAGTTTCACCAGACTGACTAACAAAAACAGCAGTATCTTCTCGGTATATTGGTCCCTCCCGGTCCAATAGATCGCTTGCAATTTCCATAGTAACAGGAACACCAGAAAGTTCTTCCAAAATGGGCCTAGCTGCCAAAGCAGCATTATAACTTGTACCACAACCAATGAAAACTATCCTTCTACTTCGCCTAATTGTTTTGAGGTGATCCTTCAGCCCACCCAACAGAACAGACTTGGATTTGTTGGATCCAAGACGTATAAGCCTTCCCCTCATTGTGGTTGTTAGAGATTCCGGTTGCTCATGAATTTCCTTTTGCATATAATGCTCATAATGACCTTTATTTATTTGCTCAACCTCCATCTCAAGAACAGATAATGCACGTCGCACTGAACAAGTTCTAGAAAGAAAAGACCCATTTTCTCCGATGTTATTGTCAAACTTTAAAATAGAAGCCCCACCGTCCTTAAGATGAACTACTTCACCATCCTCAATAACTAGCACCTTCTTAGTATGCTCAACCACAGCATGGGCATCACTGGACAAAAACAATTCTTTGGGTTTGCCATCTTTTGATAGGAATTTGTTATCCTCAAATGCTGAACCATTGCCTTTATTCTCTGTTACTTCTTTAACACCTAGGAGCAGTGGGCTACCACGCTTGCAAGCAATCAATTCATTAGGATAATGTGAACTTTTGAAAATAAGGGCGTAAGCTCCTTCAAGATGCCTCATAACTTCCAGAACAACCTGACTGAAGGTAACAACTGGACCATCGGCAGCTTCGTTGGCCTTATCGTAAACAAACTTCGCAAGCTTGGGAATAACCTCAGTGTCAGTCTCCGATTCAAAGGTGAAGCCATGACGCAGCAGCGTCTCCTTCAGAACCTCATAGTTAGTGATAACTCCATTGTGAACAACCAAGAACTCGTTTTCAGGACCAGAGGTCTGAGGATGACTGTTCCGCGGAGCAGGCTCTCCGTGAGTGGCCCACCGAGTGTGCGCGATTCCGGCGTGCGTGCTGAAGGGCTCCTCCAAATTCAATTTGATTTCTCCCACTTCTTGGTACACAGATTTGACGAGCGATTCGATGTTTCCTTCTTGCCGGAAAACGAGAGGAGAAGGAGAGAGAGTGAATTTGGAATCGAAACCGCATTGAGGAGATGAATCGATGGCGATTCCCGCGGAATCGTATCCACGATACTCCAATCTTCGCAAGCCATTGAAGAGAACTTGCA

>C100191_65

GGAAGGACAAAGTCAGAGAGTTTGTTGGTTTCACCAACTTGAAGAAAGCTTTGAATAACGAAACGTTAGAGCATTTATGAAGCATCATCCAGATGACAGTGACATGGCCGGACTCGCAGAACCATAGATGCTTCCAACTATAAACTTAGGAAGTTGAAAGAAGAAGATCGTTAGTGTTTTGAGATATCTGAAGATGGGGAAAAAACGTGTAATGTTGCCAGCTGAGGACGTTGACTTGTCCTCAATTAAGTACGAACGTGAAGTGGTTCAAGCTCCTCATTTGACTGGATTTGTGTTTAGATTTTTTGTGAGGATACTTGAAGCTCCGCTGATCGGCCCTATAATAATGAATGTTTTGAAGAAGGAAAATAAAATTGATGAGATATTGCAGCACACTGTGATACCCGAGGAACCCATGTTTAAGCCTGAATATCCACCTCAAGAAATGGAGCCTGGTGTTGTTGTCCTTGAGGAAGATGGGAGACCTGAAGATCGAGTTGAGTCTGCCTTGAAGTGTCTTCCACATTATGATGTTGCTGAATTATGGGAAAATTCATCTGCATCATTTCGCTACTGGAAAATACGCGACTATGCTCATGCTTATCGATCTAGAAAAGTAACCCCATCCATGGTTGCTGAGCGCATCATCTCCATTATAGAGGAGAATGGAAGAAATAAACCTCCTATACCACTATTGATATCCTTTGATGCTGCAGAAGTCCGGGAGCAGGCGGCAGCATCTACTCAAAGGTTTGAATCAGGAAATCCATTATCGATATTGGATGGAATTTTTATGGCCATCAAAGATGACATAGATTGTTATCCTCATCCATCTAAGGGTGCATCAACATGGATGCATGAGGTACGCGCTGTAAAGAAGGATGCAGTCTGTGTTTCAAGACTGCGTAGTTGCGGCGTCATATTTGTAGGGAAGGCAAATATGCACGAGTTAGGAATGGGTACAACAGGAAATAATCCTAATTATGGAACTGCAAGAAATCCTCATGCACTTGATAGGTATACTGGTGGATCTTCTTCAGGTCCAGCTGCAATTGTTGCTTCAGGAATATGTTCTGCTGCATTGGGAACTGATGGTGGAGGTTCAGTCCGTATTCCTTCTTCCCTTTGTGGTGTGGTGGGATTGAAGACAACATATGGGCGGACAAGCATGGAGGGGTCATTATGTGATTCTGGGACTGTGGAAATAATTGGACCGATTGCTTCATCAGTGGAGGATGTCATGCTAGTGTATGCGGCAATGTTGGGGGCATCACCTGCAAATAGAATCAGTTTAAGACCATCACCACCTTGTTTGCCAACTCTTTCATCTAATGATGATTCAAAAGCCTTGGAATCTTTAAGAATAGGAAAGTACACCCCGTGGTTTAATGACGTGCATTCAACTGAAATCTCTGATAAATGTGAGGATGCACTTAATCTGCTGTCGAAGGAGCATGGTTGTGAAATGATAGAAATTGTTATACCAGAGCTTCTTGAGATGCGAACTGCCCATGTCGTTTCCATTGGCTCTGAATGCTTATGTTCACTGAATCCTGATTGTGAAGACGGGAAAGGTGTAAAATTGACATATGATACTCGTACAAGTTTGGCACTTTTTCGGTCATTTACAGCAGCAGATTATGTTGCAGCCCAATGTATTAGACGAAGGAATATGTATTACCACATGGAGATTTTCAAGAAAGTGGATATCATAGTAACTCCGACCACTGGCATGACAGCACCCAGAATACCTCCTAGTGCTCTTAAAAGTGGTGAAACAGATATGCAGACCACAGCTAACCTTATGCAGTTCGTTGTTCCAGCCAATCTTCTGGGACTCCCTGCCGTTTCTGTCCCGGTTGGTTACGATAAAGAAGGACTTCCAATAGGTTTGCAAATAATGGGTCGACCATGGGCGGAAGCTACTATTCTGCGAGTAGCCTCTGCAGTGGAGAAACTCTGCCGTGAGTCAAAGAAAAAGCCCGCCTCATACTACGATATTCTGAAGGCTAACTGAAGCAAAGATATTCTTGCTTCTTGCTGCTTACGGTTTATTATTGGCTGGATTCATTATTTGGGAATCAGCATCCAAATGCTTTGTGGTTTCATCATCCCATTTCAACAAACGGTTTATGATTTGATAAGTTCCTAAAAATAATTTCTGCAGAGAAGAATGGTAATATTTTAAAGACACAATCAAACATTTAGAAAATCATTGGCGAAGTTAATGTGCACATTGTATTTCAGATAAATGTACTGAGTT

>C100193_65

ATAGAATATAAAACATGATACATAATGATTATTAAACAGCCTACGTAGGAGATTTCTTCTAAGGATACAAAGTCGAAAAATTAGAAGACTCGTGCATCTTTTTCATCTTCCTATGCTTCTAAGCCTTCTGCGCCATGATTCAGAAATTCTCATGCCACATATTGTAGTGCTAACATAACTGAGACGAAAATTAATCAGCTTTGTGAGCTCTTGAGAATATTATTTGGCTTATGCTCACGGTCAAACATTGTTCCAACCCTCTGATTCATCTTTCTTGATATTCGTTTTGCTTGCAAGGAGATTGAAGTTGCATGCTCCAACGGCCTCTTCAACAGTTAGATAGATCCATTTCTGCCCTAAATGATTCTGGAACTTCGATTTGTTCAGTTTCTTCATCACCTCGCTTCCAGGATTGACCAAAATAAGTTGAAGCTCTCTTCTCTCCGTAATCTTCTTGACCTCTTCAAGCATACTTATTCCACTTGTATCTATGTTTCCAACACCACTCATATCCATTATAATATACTGCAAACTTGTCTCTCCAGTAGCGTGGATTCTGTCTACCTCTTCATCAATCCACCTTGTGATCCTTTCTCTTAAATAGCTTGCATTGGCAAAGTAAATTGGTGCATCAATCTCTAGGATTAGGATTCCAGGAACATGCTTTGCATTTGGATATTGCTCAACATTTCTGTATATCCCAGAATTTGGAATGTTGCCCAAAACTAATGTCCTTGGCCTTGCAATGAATAGAAGTAGTCGAAGTACAGATATTGCAACAGCTATGACTAAGCCAATTTCAACACTGCCAAAGACCACCCCAAGGTATGCACTCATGCACACAACAAAGTCAAATTTGTCAATCTTCCACAGATGGATGGCTGCTTCATAATCTATGAGGCCAAGCATTGCAGATACAATAATTGCTGAGAGCACCACCAGGGGAGTGTAATGGAACAAGGGTGTTAGAAATAACAATGTCAACATGACTGCAATCGACATTATAATGTTGGATGCTGCTGTCTTGCATCCAGCGTTATAGTTCACGGCTGAGCGTGAAAATGGCCCTGTTGTGAGGTAGCAAGAGGTAAAAGAACCAACTATGTTCATGGTCCCAATAGCTATCATCTCTTTGTTGCCATCAGTATTGTAATTTTTATACATTGCAAAGCTTCTTCCCACTGCTATTCCTTCCGCAAGCGCTATAATGCCAACCACAATCCCAGTTTTCACAGCTGTGGCCATATAAGGTGACACAAATACCAGCTTCGTCACGGATGGTGGATTTAAACCCTTCTTCAATTCTCCGATCACTTCAACTCCGTGCTTCTCGGCGTGAGTGAAATAAACCAATAAACTTCCCAGTATAACGGACGTTAATGGCGCCATCGCCGACACCCAGAAAAACCTTGGTTGTTTTTTGCTGAAGTATCTAGTGATAAGTAGAAAGAAAATGAAGACAAATCCTAACACAGAGCTTTCCCACCTCCACTCATGAGTTTGGGTGAAAACAGAGCGCATCACTGATATGATATCAGCTCCATGGGTGAAATGAACAAGGCCAAGAATGGATTTTAGTTGTTGCAGACAAACCACTGTAGCTGCTCCTCCCATGAACCCTACTATCGTTGCATGTGACAGAAAATCCACGATGAACCCCAACCTAAACAAACCCAAAGCAGCTTGAAAAATTCCAGCAAATAATGTAGCTGTGAAGGCAAGGTGTAAGTAAAGGTTTGGGTCTTGATTGGGATTAACCTCATCACTCAACATGGAACCCATCAGAAGCGATCCAACCGCCACAGTCCCCACCGCCAAATCCCTCGAGCTTCCCATCATCGCATAAATCAACGGTGGTATAAAGCTAGAATAGAGTCCAAGGATTGGAGGGAGGTTGGCGAGCTTGGCATAGCTGATGCCTTGGGGGATGGCCAAGCTAGCTATGGTGATGCCAGCTATGAGGTCGGCTTTGAAGAAGTGGAAGGTGTATTTGGGTGCCCATTCGAAGATGGGGAAGAAATACTGAAGTCCAAGCATCAACTTTTTGGAGGCTGCTTTGTTTTTGAACTGCCTCAGAGGGTCATCTGGGAAGAAAGTTTCCTTCAAGGAGTACTTCAGAGACTTCAAAAACGGTTGAGGCGGTGGAACTTCCACTTTCTGCTGAACACTCTCAACACCCTTCATCTCCAAAGGGTACTCATAATCTACACTACCCATACTATATGCACTAATTTATGTGTTTGTGTGTTAAGGAGGGAAAAAG

>C100197_65

GAAAAGGAAAAGAAAGAATAAGGAGAAACATAGCAACGCAGAAACGCCAATCATCTTCAGAGCCTCTGTTGTGTGGTTCATAGGCAAGTATAGCAACCAATAGAATGTCTCCATCAGTGAAATCGAAGTCCAAGTCCCAGGAGAAGGCCTCTGCAAGGGCTGGTAAGGAACAACAGAAGAGTTCATCAAAGACTTCCGTGTCTCCTACTCATGGAAGTGGCATTCCTGCTAGTCCACACAATCCCATCTTGGGATCATTTCAAACTCCAGAAACAATATTGGTTGCTTCTTCTACTCAAGTTCATGACAACAGTCATTCTCCGAAAGTAGATGATACAGATGAGCATTCTAGTAGTCCTCAAGGAACAGTGTCCGAGTGTGATTCTGTTTCTATCAATGGCAGCTGCTCTGGTGAGTCAGAAGAGTCTAAAGAGAAGGTAGCGGTGACAAACTCTTCTACACGGCCGGATAGCTTACCCGGTTGTGATAATGACAGAAGGGAGAAAATCCGTTTGAAGAATGAGAGGAAGCACCAGAGGCAGAGGGAAAGGAGAGCTCAAGAATTGCATGACCGTTGTCGTGGCTACCTGTTGTCAAGAAAGCTAGAATCGCTTGCACAGCAGTTAGTGGCAATGGGGTTCTCTTCTGAACGAGCAACACTGGCCTTAATGTTAAATGATGGAAAATTAGAGCAATCAGTATCCTGGTTATTTGAAGCAAATGAAGAACAATCACAAACTAAGGATGCTACCAGCCTTGTAAGTGAGGGTAATTTGAAAATTGACATAAGGGATGAGCTTGCTCAGATATCTGCCTTGGAGGTGAAGTACAATTGTTCAAAACAAGAGGTTGAAAGAGCAATTGTGGCTTGTGAAGGTGATCTTCAAAAAGCTGAAAATAACTTAAAAACACAGAAGCATGAATCTCCTGGGACCCCATCAAAGTCCGAAGATTCTTCTCAGAACAATAATAGTCTGGTGAGATCACAAGGATTGCCAGAAGTGTTGGTTTCAATGCAGCAGAGAGGAAGTGAAAGTGACTTTAATCATTACAACAAGGAAGGTGGTTCTGATTCTATGTTCCCTGATCTTGAAATCGGGAATGTACAGTCTCTACCACTGAATCATCCAAATGTAATGACTGAGAAAAGATGGAGTGGTGTTCCAGGATCAAGCCCTTCTTCTGCTATGTTGGCCATGACACCATCAATGCAAGCAGTGTCCCCATTGGTTAAAATGGAGGGTGGCAGGCCTATTGGCATTACAAACGAAGGAAGAATGATCAATCAAGGTCTAGGAAGGGAGCCGGTAATGATGCAACATCTTCAATTCACAAATGCCAAACCAAACTCCATGGTTTCTTTAAATTCCTTTCCTTCTGGAGCAGCAGGGTGGTATGTAAATGGTATTCCAGGTGTTGAAAATGTGAGGTCAAATGGGAAGTTACTACAAACTCAAAGTGTAAGAAGTGTTAGTCCAAATCATTTGGAGCAATTTTGTCAAGCTCCTTACAAAGAATATTCTCATTTCTTTGGACCAGTAGATTACTCATCTCCTGGGGTGGGTGGCTTCTGCAAGCCAATGGGTGCATCATCACCACCTCCACCTTCAATTCCTCCTCGTCACCAAGGTTCATGGAACACCGGCACGCCTTCACCGCCGCTGGCGGTGCCAACTTCCCTTGGACTATTTTCTGGTCATCAGAATGCAGCCAGAACATTCAGCTCACATTCACACATGGATTGGAACACTGGGGGCTTGATGCCAGAATTTGACTACACCAGCATAGATTGGAGTTTGAATGTGCCATCTTCTTCAGGTGGACCGTGTCTAGGGATCTCTTCATTGTTGAGAAACAGTTATGGCAATAGGAGAAGCAGTTCCTGCATGAGTGGATTGCAAAATGTTGGCATGGCTAGGGAAACTTCATCATCAGCTGGATTGAGGGAGTGGACCACTCCTTTTGCTGGGAAGGACTTATTCAGTGTTCCTAGGCAGTTTGTTACCTCTCCTCCTATGTAGTGTTCTTGGGAACTATGGGGTTAGTACCTTGGAAAAAAATGGCTGTCTTGATGGTTGTTTTCTGGTTTTCCAGTGAAATTAATTCATCTTCTTATTTGTGGTCTATGATTTTTTCCAATGGATGCATTTGCATAAAAAGCATAATTCAGTTCTTAATCAATGACCCTTCTTATAAGGATCTATGTCTTCAATTTGATTTGTAACTTGTTGGAAGAGACTAAATAAGCAAAATTTCACTGAGAA

>C100199_65

TCTGGTATGAAATATGAATAGTGAACTAATTACATTCACTGGTATCATCACTAAAACAATGGGGCACCAATGAAAAAGAATAACAAATGCGTACAACCAGCCATACACCTACGAAGCTAACAATGGGGTCCTCAATATGAGGGTAAAGAAAATACAAAATTGAAAACAGGAACAAAGTGAAGAAGTGGGAAAAAAATCTAAAACGGGAGAATAAAATAGTATGGCGGGGCACCCTGAATTTAGTTAAATGTACAGAGAGCATGCTACAGCCTCGACACATGCATATCGTACTCCAGGTAATGTACAGACAGGGCTAAAGGAACACGAGTTTGTCCTCTGGCTTAACAACTTGTTCAGTTCAGCAGGTGCTCATTCCTATATGAAACAGCTCTGTCAACGTTCCGAAAGAGATTCCTGCAAAGGAACAGTTGCCATTCTCTCTTCTCAATTGGCATCATGTACAAAAAAGATGCCACTCCCTTGAAATTCAATCCAAATATATAATCAGATCAAAGTGACCTTCAACAAAATCCTTTGACCGTCGAAAAAGGAGTGTGCCCTCCCACCAGATGGTAAGGGGAAAAAATGAAACAATCACAATATCAGAAATACAGTCAGCATCAAGGCGTGCTCACAAAATTGTAGGTTGCGGAAACACCCATCAAATTCTGAGTAGATCAAGTTGCCGATGAAGATCTTGAATCATCAGTTCTATTAGCACGGCACATACAATCTGGGCATGGAAAGCTGTATATAGAATCATTCGGTCTTTTTAGCTCAACACCCTTGGAATCACTATGTGACCTCATGCTCAGCAGTTGACGCTTAAGACTTTTCCATCCTATATTGGGATTATCAAACAATAATGCCAACTTCCGCTTATCAGGTTTAATTGTCTTAGCATGTCCACCATACAAGTATCTTCTGTGACCCAGCAGAAAATGAGGGAAGTTGCCCCCCTGAGTAGTGACAAACACCTCACTGTGAAGACAAACAGTGTAATCTATAGCAGCCATCCTGGAAGAATAATTCTTAAATGGAGCAAGCTCCTCTTCAGAAGCCAGGGTCTCCTTAGTATGCAAATTAGGAAACATTTGGAGTAGTGGAGCCATAGTTTTTTCAGAATTATATATTTTCCCAGATGCCAGAAAGATAGATGTGTTCTTCGTGAAACCCATTCCCCTCAGCATAAGCCCGACCTCTAATGGGGTAAGGGGACACTTCCCATTGGTCCTTATTGCTCCTGGACGTATAACTCGTCCAGGTTTTGTAAATTTTCCTTTCCAACCTCTTTCTCTAGCTGCAATCATGTCTTCTTTCTCCTGTTTTCCACCGTCGAAAACACAACAAGAGAAAGCAACCATGTCCTCTTCAAAACGAAGATGTACGGAGACATATTTACCACCATTATTGGCACTGTGTTTTCTCATTCTTTCAACCAAAGATTCACCTATGCTCAATATAGGACTTGAAAACCGCAAAGCATCGTAATTTGCTAAGCATCTAAGCCGTTGGACAACTGGAGGAGCATCAAATGACAATCTATTTGCAAATGGTGAGATCCTTATAACCTTTTCTTCAAGTAACTTAGGGAGTACCACATCTCTGTAATACTGAATAGATGACCATGCCTTGATCCTGAAATTGTGAACGTTTGTCATGTTGCTGCCAAATCTTTCCATTAGGTACTCAGGAATCTTATCAACCACCCGGACATCATTTCTTAAGGTATTGACGAAAAATTCTTCATCATAAATGTCCCTGAATTTGCTAGGGTCTTTCCATATGCTATGATAATGAAAGTTGGGGATCACAAGCGTAGCATTGAGATAGCCAGCAACAGCAACTGCATTGCATACGGATGTCCTCTGCTGATTTAAGCCACCATTAGCCTCCACATATATGTATCCATTTGATTCAGGTAGGCCTTCATAAGATCTGTTCACACATGGTTTCCATTCACCACCTTTATAGGGAACCTTCCATATTGTTGATATCGCATCGGCGGAGGAATTGTCGGAATCCATGTCGAGGCGAAGCTTGGCGTAGAGCTGAGGGCTACGGTAGACGGAGCCAGGCGCGGGACGGTGCTTGATGACCGGAACGACGTCGAACGAGGCGGTCCCCATGTAGAGCAGCATCCCGGAGATGTATATGAGAGGAGCGAAGAGGAAAACGCCTTGTCGTCGGAGCAGGACCGATAGGAACATCCAGCTAAGCCGCTGCGCGGCGGTCCGACCGCCCCCCCGGTTCGGAGAGAACCGC

>C100201_65

TTGCTCACAAACTTCAGAACATCTTATTCACCCAAGGGAAATGGTAATGGTATAAACCCAATGTGAAAGTGAACCTATTGAGGTACTCGTTATTTTGCCAACTATCCTCACAATTTGCAATTTTTCACACAGCATAAATACCGGCTTTAACAATTTGCAATTTGCAATTTTTCACACAGCAAAAGTATATATTGAAATACAAAGCACACTAATAATCATGATGCACGACTTTAACAAAAAAAAATATGAATCACTTTTAAACTAATTTGTTCACAACACAAACCAATACCGGCTTCTTTGTGATATACATCCAAGGAATGCTAAAAAATGTGAAGCTTGCAGTTTGAATGTTGCTGTTCTGCATGACAACCTTCCAAAGTTTACCGACTATAAATTGCAAGGAATGCTCATCATGGCACTTCCTCCTCCTTACACTCCAATACAAGAAAACTTCACACACGGACTAGTAGCATGCTTGTGTTGAAACGCAGGCAAATGCGATCTTCATCATCTTGCATAATGTTTTAATCTTCTTAGCACAACTTGTTTATAAAACCTTTGGGTCCGGAAGTATAATACCAAACCAACCAAAAATGTAAGAAAAGCCACACCGGCCAGGATAAAAAATGACGGCATGAAACAGTCGACGCCTAAGCACAAATTATCTTCATCGGCTTGCTTGTCATAAATATATCCAACAACTTTCACAGAAAGTATATAAGATCCAAGTGGGCTTGCTGCAGCAATAGCATTGAAAATGGTACCCATATGCTTTACACCAAAGATCTCCGAAGTGATTGTGGGCATCAAGGACCAATGTGCGCCATAGCAGATGCCCACGAGGACAGGACCCATATACAAATTTCCACGGAAACCACATGCTATAGTTAAATGGCCAAGAACCATTAACCCCAGTGTTGCCGTCATCAACAAGGGCCTTGGCCAACCTTTTCTGTGCATGATATAATCAGATAGATGGCCACCTCCAAAACGACCGAGGAAGTTCCACATACTCCACAGTGACACCAAATTATTAATCTCAATGGTACTATAGCCAAGGGATTGTCCTATTTGGCTCATATTATTTATAGTTGCAAGCCCAGAGCCCAATCCTGATATCATGATCACAAATAACATCCAAAACTCAAGCGTGCACATAGCTTGCAATATGTTCTTTTCTTCTTCATGGGGCAATTCGTCATCTGAAGTCACTTGTACCTGCCCCTCATCACTAGGCAACTCATGGTACTCCACTTGTTCAGCTGATGCAGATGTAGTACCCTTAATTGTGGAAGAACCTCTCTCAATGGTATCGGACTGCGTAAACTTCCGCGTCTCCTCCCAATGGGCCTTGATAGCAATTCCAAAAGGCGATGCTAGCAGAATCATAAGAATCACAAACGCAGAAATCCGTCCCCAATATGTCAAAGTAACCAAGTTCTGTAAAATTATAATGAACATGAGATAGGCAACAATAATCACAGTAACCACAGAGAAAGTATCCAAATGCTTCTTGTAATCACTGCCATGCCCTTCATAAATCCTCAGCAAGAACATAAACAATATGCAGCTGAATGAAGGCAACAAAGCAAGCATCAAAATGTACGTTGCAGGTTGCCCATCAAAAAACGTATGGTAGATTTGGATCAAGATAGCTCCACTCAGTCCAATAAAGCCCTTCATGATACCAATGATGGTGCCGCTATACTCAGGGAAATTGCGCAATCCAGTGACGACATTGGTAGTGTTCAGAAAGGTTTGACCGTTAGCCCCGAGCCACGCGAAGAAGCACATGACGGGCACGGGCGGCGGGCTTATGAGGCCCACGACGGAGGCCCAGATGAACATGAAGCCCACAAAGAACTGGACCGCCCCGGCAGCGAGGACGATCCAGGGCCCAACGAAAGAGTTCCATTTGGACTTAGAGGGCGAGCCGGGGAGGGCAGAGTTGTGGGTGTAGGGGACGACGGCGGAGTAGAGGAGGCCGGAGAGGATGCCGAAGTTGGCGCCAATGTCCTTGAAGACGGAGACGGTGTCGAGGGTGGATTGGTCGTAGCCCTGCGTGGACTTGAGCACGGAGGAGTAGATGCTGAAGGTGTACGATGCGCCGCAGCTCGACTGGATCCAGATGGCGGCGGCGATGCCCGTCCACGGATTCGCCACCCACCCCATCACCATCGATCGGTCCGTGGCAGAATGGTTTCGGTGGGTTTTGTTTTGTTGAGGTGAACTCCTTCCCTGTAAACTTGGAAGAGCAACAAAAAC

>C100205_65

GGAAAAGAATGAAACCTGCATCACAGCTCACACACACACACACTTTCAGTTTGATAAACACAAGATAGAAGAGACATTTGATCTCCCAAATTATAGTTGAGAGTGGTGATAAGTTCTTATCCTACGCCCATTCAATACAAATTTCACCCTCCCAACACCCAAACATTCAACAATCACAATTCTCTCTCTCTTTTTCTCACTCTCTCTTTCTCTCTCTTCTTCTCTCTCACACACACCATGCCGGGTCTTGTTTCTGTAAAAACCCCACCCGAAGCTCAACCGCTCCGAATTTCCGTACCGAACACTCCGACGCAGAGATCGGAAGCGAACAACAGTAACATAAAAACACCTTCTCCACTCCCAAAAAAACCACCGTCTCCATCACCTTCGCGTTCATCCAAGAAAAAAAACCCCGAAACACCTCCAAACCTGATTCTCTCCGATGCATCTCTCGACAACCCCGATCTCGGGCCCTTCCTACTTAAACTCGCCCGCGACACCATCGCCTCCGGCGATGGGCCTGCCAAGGCGCTTGACTTCGCCGTCCGGGCCTCCAAGTCCTTCGAGCGCTGCGCTGTCGAGGGCGAGCCCAGCCTCGACCTCGCCATGAGCCTCCACGTCCTCGCTGCCATCTACTGCAGCCTCGCCCGCTTCGAGGAAGCCGTCCCTGTCCTCGAACGCGCCATCCAGGTCCCCGACGTCGCGCGCGGCGCCGATCACGCCCTCGCCGCCTTCTCCGGCTATATGCAGCTCGGCGACACCTTCTCCATGCTTGGCCAGGTCGACCAATCCATCTCCTGCTACGACCAGGGTCTCCAGATCCAGATCCAAGCCCTGGGCGACACCGATCCCCGCGTCGGTGAAACTTGCCGATACTTGGCGGAGGCGAATGTGCAAGCGATGCAGTTTGATAAAGCGGAGGAGCTGTGTAAGAAAACTCTGGAGATTCATCGTGTGCATAGTGAACCGGCTTCACTTGAGGAAGCTGCCGATAGGAGGCTTATGGCACTGATTTGTGAGGCAAAGGGAGATTACGAATCGGCATTGGAGCATCTTGTGCTTGCTAGCATGGCTATGATTGCAAATGGTCAGGATAATGAGGTTGCGTCGATTGATGTTAGCATTGGGAATATTTACATGTCACTTTGTCGTTTTGATGAGGCTATTTTCTCGTATCAGAAGGCACTTACTGTGTTTAAGTCTGCCAAGGGTGAGAACCACCCTTCTGTTGCGTCTGTCTTTGTGCGGCTGGCTGATTTGTACCACAGGACTGGAAAGCTTCGTGAGTCGAAATCGTACTGTGAGAATGCACTCAGGATTTACTCAAAGCCGGTGCCGGGAACCACTGCGGAGGAGATTGCTGGTGGCTTGACAGAGGTTTCTGCGGTTTTTGAATCTGTGGATGAGCCTGAGGAGGCGCTGAAGCTGTTAAGCAGGGCAATGAAGTTGTTGGAGGATAAGCCGGGGCAGCAGAGCACTATTGCGGGTATTGAGGCACGGATGGGAGTGATGTATTACATGATTGGGAGGTACGAGGACTCGAGGAACTCGTTTGAGAGTGCTGTTGCGAAACTCAGGGCTAGTGGAGAGAGGAAGAGTGCCTTCTTTGGAGTTGTTCTGAACCAGATGGGGTTGGCGTGTGTTCAACTGTTCAAGATAGATGAGGCTGCTGAATTGTTTGAAGAAGCAAGGGGAATTCTTGAACAGGAGTGTGGCCCGTGTCATCAAGATACTCTTGGGGTTTACAGCAATCTTGCAGCTACATATGATGCTATGGGAAGAGTGGGAGATGCGATTGAAATCTTAGAATATGTCCTTAAGTTGAGAGAGGAAAAGCTTGGAATAGCAAATCCTGATTTTGAAGATGAGAAGCGTCGTCTGGCTGAGCTTCTGAAAGAAGCAGGCAAGACACGGGACAGAAAAGCAAAATCTCTGGAAAATCTTATTGATCCTAGTTCGAAGAGGACAAAGAAGGAGGGAACAAAGAGGTGGCCTGGTTTGGGGTTTAGAATTTAACAATATTTTATCAAATATATATATTGAATGCACATTCTGTAACATAGGATATCTTGTATAGGGTTGAATATGTAATTTGGGAGGGAGCTTCCCTTTTTCTTGATATTTGTTCATGCATTTGTTTCTCTTGGTTAATTGTTTTATCATTTGTGTTGTGATCTTTTTAGTTGTACCACAAAATTCCATCCGTTTCCGGAAAATGTTGAAAGCAAATTTTTATTCGAGTGAATAAATTTTGATGAGATTGTG

>C100207_65

TGATGGATTTGGACCATTGGTGACGTAAGCCAAAAACCCTTAAACATTAAACCCTAGCATTTGGGTTCCAAAATCCACCCTTCGAAGGCACCTCAATGGAGGAGCGGAATCATTCTCAAGACAATAGAGAACTCTACGCACTCCTCAACTTGTCTCCGGAAGCTTCCGATGAAGAAATTCGCAGAGCGTATCGTCAATGGGCGCAAGCTTATCACCCCGATAAGTACCAAGCTCCTCACATGAAGGATATTGCTACGGAGAACTTCCAACGCATATGCGAAGCGTATGAGATTCTGTCAGATCCACATAAGAGGCAAATATATGATATCTATGGCATGGAAGGGTTGACTTCTGGTTTGGAACTTGGTCCAAAGCTCGATGGAGCTGAAGAGATTAAGGCAGAGCTCGAGAGATTGAAGAAGATGAAGGAACGTGAAAAAATGGCGGCGCATTTTCTACCTTCTGGTACAATTCTGGCCAATATGTCTTTGCCAAGCTATTTAGACGGTGGTGGCCTCCTCCAAGGAATGGCTATGACAAGTGAAATCCAGTCTCACTTATCCAAGCGCAATGCTTTTACAATTGCTGGTAATTTAGCAGTGAATGGAGGTGAAGGTGGTGGAGCTGCGAATGCTCTATTTAGGCATAAACTTTCAGAAGTTTCGTCAGTAGAGTTTGTGGCTTCAGTTGGTTTGCGTGCACTAATTGGCGTGCAAACAACACGTAATTTATCATCACATTCAGCTGCAACAATGGGCGTAGCAATGTCTTTGAGGGATGGTTCATTGAATCTTTCAAATTTATGGACCCGCCAACTTTCAGAAACAGCAAGTGGACATATAGAGCTTAATTTGGGACCACAATCATCTATAGCTGTTGGGTGGCAAAAGAAAGATCAGAGAAGGACTGCCTCTGGAGAATTGAAGTTTGGCACAGGTTTGTTTGAAGCAGCAGTTCATTGTACTCATCGTTTTTCTCCTAAATCTCTTGGTTGCATTGTGGGAAGAGTTGGGAGTTCTTCCCTTGAGATTGAAGTTGGTGGTGGTAGGAAGTTATCCAAATTCAGCTCAGTACGGTGGTTGTATATAATAGGAATTCAGGGTATTTCCTGGAAATTTGAACTCTATCGTGGGGGTCAGAAGATAATTCTTCCGATTTTGCTGACAAGGCATCTTAACCCTGTGTTTGCTACTGGAGCATTTGTCATTCCTGCATCTCTTTACTTTGTTCTAAAGAAACTTCTTATTAAACCTTATTACCTTAGAAGGAATAAGCAGAAGGCTCTGGAGGAGGAGAAGAAAAGTTCTGCTCAGGTTAAGGAAGCAAGGGCTGCAGCAGAGAAAGCTCAGAAATTACAACAAAATGTGGCTAATAGGAAAAGAAACAGGCAATTAGAAACAGGTGGACTGGTTATTATGAGAGCACTATATGGAAATCAGATAGTTTTAAGCAACTTAAAGTCATCAAGTGAAACAAGTTTAGAATCAACTTCTGATGTCATTGATGTTACGATACCTCTGAATTTTCTTGTTAATGATTCTGGCCAACTCAAGCTTCATGAAGGTGTGAAAAAATCAGGCATTATGGGTTTCTGTGATCCATGTCCAGGAAGACCTAAAGAGTTGTATGTAGAGTACGTTTATTCTGGCAATCAATACCAGGTTTGGGTTGGTGATTATGAAGCATTAGAGATCCCTAAGAGCAGCCACAGGATATAAAGGATTATCATTAACACATCAATATTCCATTGTAACACCTCTTGCAGTTTTGCCTGGTTTACATGCTTGAGGTGTAACATTATCCTGTCAGATGGGATTAAAAGAGGTACTTTTTACAATTTTTGGGCCAGTGGTACCGTTCTTATTTTTTAAATTTTTTATTTTTCTCATAATTACTTCACCCCCAAAATGTATGCAATAAGGACAACTTAGGAGTTGCGTTGCGCTACACTAATAACTGCTGCTGGATCCCAATTTTGATTTGTTAGTCATAATCCTTTAATTGTTGATTCGTTTGCCCATTCACATAAAGCTTCTATCAGGGCAACAGCACTAGGAGTTGATATGATGATAATACAATCAAGTTTTCATGTGGGTTTGTAATTCGTAGATAGTAATACACTTTATAGGACAACAATAACATCTGGGAATAAAACTGAGCGCCGAAACATTCTAAGGTGCTGATTATTTGTGTAGCTGTACCCAATTTAAAGCCCAGATGGCTCTCATGTAACTTCCAATCGAGTATGACTGGCAATTAAATAAGG

>C100211_65

AGCACAGATATGTACAACAATAAACTGCTACAGCTTCCATCATTCTTCTGTTCTGTCTCACGTCTCACGTCCTTAATTTCACCATTTCACACAAACAGCTCTATAAGAACTCAGAAGTGTGAGAGGTGAGAGTGTATTGTCACTCAGAACACCAGAAAGCAAAGGTGAATTGTTGAAGGGGTGGTGGATTTTTGAAGGGAGATAGAGCTTGAAGGAAAATGAATAACTCTGTTTCAGAGAACAGTTTTATCATCGAGAGTGATGAAGAGGATGTGGAGAAGGATTTTAACAAAGAGGGAGATGATGGTAACAATTCTGATTCTTCCAGTTACTCAAACGAAGACCCTCCACAGCGAAAGCCCAGTTCTTACAACATCTCGTGGCCTCAGAGTTACAGGCAGTCTATTGATCTATACAGTAGTGTGCCATCCCCAAACATTGGATTTTTGGGCACACCCTCATTGTCAAGACTAAGCAGTTCTTATCTTTCTACATCACTAACAAGGAGGCACACTCCTGAAACACTTCCTTCAGTGACAAAGCCTCTTTTACAAGACACAGAAGATGAGCAGCATCAGAGACGTAGCTCTCATAACTTGCTTCCTCCTCTTTCTTCCAGAAGATCATCTTTGATTAAGAAGGATTCTAAGGTTTCTCATGAAGTTCCCTCTCGCCACTGCTCTTTTGGACAAGCTGTGCTTAATGGCATAAATGTTCTTTGTGGGGTTGGAATCCTTTCTACCCCTTATGCTGCCAAAGAAGGTGGATGGCTTGGGCTCTCAATCTTGTTCCTTTTCGCAATTATTTCATTTTATACTGGGCTGCTCTTGCGTTCCTGTTTGGACAGTGAACCTGAGCTTGAAACATACCCTGATATTGGCCAAGCTGCTTTTGGTACTACTGGACGTATTGCCATTTCAATAGTATTGTACGTGGAATTATATGCTTGTTGCATTGAATATATAATTTTGGAGAGCGATAACTTGTCTTCCTTGTTTCCAAGTGCACACTTGAATTTGGGTGGAATTGAACTGAATTCACATACACTGTTTGCTTTGATTACTGCCTTGGCTGTTCTTCCTACTGTTTGGCTCCGAGATCTAAGCATTCTCAGTTACATATCCGCTGGTGGAGTTGTTGCATCAGTTTTAGTGGTTCTCTGCTTGTTATGGGTTGGCGTAGACGACGTAGGCTTTCATAGCAAAGGGACCACACTCAATCTTGCAACTTTTCCTGTCGCTGTGGGTCTTTATGGTTACTGCTACTCCGGACATGCTGTGTTCCCAAACATCTATACATCCATGGCAAATCAAAACCAATTCCCTGGAGTGCTCTTAGCATGTTTTGGTATTTGTACCTTATTGTATGCTGGAGCTGCTGTTATGGGATACACAATGTTTGGTGAAGCTATACTCTCTCAGTTTACTCTCAACCTGCCTCAAGAATTGGTCGCAACCAAGATTGCTGTCTGGACTACGGTGGTTAATCCATTTACCAAATATGCGTTGACTATGTCTCCAGTGGCAATGAGTCTTGAGGAGTTGATACCATCAAACCATGCCAAGTCTTATCTATATTCCATCTTCATCAGAACAGGGCTGGTACTTTCCACCCTGGTTATTGGTCTCTCGGTTCCCTTTTTTGGTCTCGTGATGTCATTAATTGGATCATTACTCACAATGCTTGTAACTTTGATACTACCCTGTGTTTGTTTCCTAAAAATCCTGCGGGCCAAAGTGACACGAGTGCAGGCAGCACTTTGCATTACAATCATCACAGTAGGTGTTGTGTGTTCTTCTTTTGGAACATACTCTGCCATCTCTGAGATTGTTAAGAGTTTGAGGGGATGAATTTTTGAGGCTTAAATCCTGAAAGATGTCATATGCCAGCTGTCATCTAGTCCGTCCAGTTATTCTATTTGTTTGGATTCACATCCACAATTTTGAATTATTTTCTCTTGCCACACTTTTCATTCTGTTTTTTATTTTTCTTTTGTGGTTTTTCTGATTCATAGTTACTTGTCATAAAAAGTTTTGCTCGTGTTTGAGGTATTGGTAATCATAGATTGTGGCAGAGAAACGCTTTGCAGTTTATAAGGAGCGATTAAGTGAACTTGTTGAAGAGACATTTTTGTTCTACTTTGAATGTTTTTTTTATCAGCAGTTCTACTTTGAAAGTGTCGTGTACAATCTTTTGGTGACGATAAAGGATCTCTATAACCATTTAATGCAGAAATATTGAGTGACCAAAAGGGCTTCAACATAAA

>C100215_65

TTTCCCAGAAAATCTTGGACATTCTTATTGGGGAAACATTTGAAATTACATAAAGTCTGAGTCTGACCCGAGCAGGGTCTTTCTCTCTGAGGGGGTGACATGAACTTTTCCAGTTAGTAAACTTAAATACAAATGGGTGATTAATGTGAGTGAAGTTTTGCAGAGGGAAGTGAAGCAGAGAAAGATTACATTTAGATTGATTCCATCTCCACTATCCTTGATTAGACCCATCAACAAATTGCTAAGATAGGAAAGTTCCAAATCAGTGTCACCCTCCTTTGCATTTTACCACAATCACTTATCCCCACTAATTAAAACATTCCAAACCTTCCTATTACAGAAAAAAATCATCCATAATTTCGGACATCCATTTCATCTCACCAATGTCAACTCCTTCATCAAAACTACATCACCGTGGGTCCCACTGCTAAAACCAAACCGCCCACAAAATAATACTTCTTCCTTCTTCTCATCCAAGGGCGGCTGACCTAATCTCTGTTTTCGACAGCCAATACCTTCCACTTTCCTTCCCTCCAACCGTAATCCGACACGTGGACATCATATGACCAAAACATTGACCAGCCCCTCATAAACCGGCTCATGTGGGTCCCTTAAGTTAAAATCTCAAAATTTACTTGAAAGAATACTAATTATATAAAATAGTTATAATTTTATGATTTGAATGATTAAGGTGCAGTGAATGTAAACAGAAGATTTAATAAAAATCTATTACTATTATATCAGCATCCAGCATTCCATATATAGACAGTCCACCTATTTGGAAAGAGTACAATGTCACTGTCTTCTTCACCATGGCACTATTTACACCCAACCCCAAAACTTATGTATAATTGCTCTAGAAACCAAACCTATAATGTTCCTCACCTTCTTCTACTTTGGTTGAAAGGAACTTTGGTTGAAAGGAACTTGCTTATTTTGCTACACCATTCTTTTTCTCAAACTATTTCACAGGTGACTTATTTTCTACTTTCTCAACATTTTCCACTTCCTATTGGATTTTTCCTATATGGACAAACACTATTAACAAGTAAGGAACAGAGAAGAACAGAGAAGAGAAGGAAACACTTCCAGCACCGGCCTTTTTCAGCAAAAAAGCTCGAGTGTTGAAACTGCAGGGAGAAAGTTGCATTTTGAGGTAAATATATGAGGGCCATTTTAGCAATAATCTAAAGTTACTATCTGTCTGTCTAGTATTCTATGACTAGTCCTCCCATTGGAGCTCGGTGTGGTATTCCCTTTCTTCTCTTGGCTGTTCTGTAATTAAGAGTTGGTTGTGGTGTGGACGGTGTGAAACTCTGCACCTTGTTGATGTTGTTCTTCCCGTGGCTGCTGTTGCTGCTGTATGAATCCTCTGAATCTGATGCCATTGTTGCTGACTTTTTCTTCTTCTCGAAGTTCCTTGAAGTCCCCACAATCTTGCAACCAAGAGAACAGAATCGGTATGAATCCAGAAGGCTACGCTCACAAACTTCACAAGTGTTGGTGACTCCTTTACCAGGCCTCGGCTGAGGCCTCTCATTCAAGAAAACAACCCTTGCGCTGTTGATAATGTAGGTTTGGACCCCTGCAATGTCCAACACCTTCTGAATCTCGTTCACCCTAATCACATCATGGTATGAGGACCTCCTTATCTGAATAGCACGGTGATCTTTGTGGTAAGCGAGGCAGAGGGAGCAGAGAGCGCCGTTCATACAATCCAAACAGTACATGTTGCACTCGCTCTTGTGCGAATCAGCGTGCAACTTGCATTGAACAAAGAAGCTCTCTTTAAGCAGAGGCTTCAGCCACGGTGGCCACCGGTTGTCCTCGTCATCAGGGCCTCCAGAACCCATGATTCTGCGGTTCTTGGGCTTGATTTCTCGGACAGTGCTGCCTTGGTTTTGGAACGCCAGCTTGAAGGGAGTGTTTGGAAAGAGGTGATGTGTGTTGTGGTGAATTTTCGGAAGTGGAAGAGAGTGTGTTGTTTTGAAGAAGAGAAGGAAGAAAGAGGTTGGTGTTTGAAGAGAGTGAGATTTTAGGGCGGTGTGGGAGGGAATGGGTTCTTGTAGAACTTGGAGGCGGTAGAAGATGTTTGCTAGAGAGAGAAAGAGAGGGAGAGAGAGAAGAGAGAGAGAGAGAGAGAGGGAAAGAGAGAGTATGTATGAGAGAGAAGAGAGGTCGTTCTTATCCACACAACAAAGTCGTCTCACACGACCAAACCTAGCTCCCAACCCAAACCCGACAGATGCGTTCTTGGTAAGGAATCC

>C100217_65

CAACAACATCGCTCAGCTTCCATGTCCATGTGTACTGAAACCACCAATGGAACATTAATTTTGTAGTCCCACGAGTCATGATCGCCTCCACCTCCAACATTGGAACTCACCCTCTCTGCTTTCTCTCCAAACCACTCACACACACGACAAAAGGGTTTCTGTCTCTCACACTCCACTCTCCAGGTATTGCTGTTGCACTTTCTTCCTCGTCAACTAGGACTACCCAGAAGCTCACCTGCCACTTCAACGGCAAAGATGGAAGCTTTGAGCAGCAAGAGGAAGAGGGAGAGCGTGAGGTGCTTTGTGAGGTCCAAGTGGTATCGTGGAGGGAACGCAGAGTTCACGCCCAAATCACAGTCGATGCTGACACTGAATCCGTTTGGAATGCTCTCACTGATTATGAGCGTCTCGCTGATTTCATCCCAAATCTTGTATGGAGTGGGAGAATTCCCTGTCCTTACCCAGGAAGGATATGGTTAGAGCAAAGGGGATTCCAAAGGGCTATGTATTGGCATATAGAAGCTCGTGTTGTCTTGGATCTTCAAGAAGTCATCAATTCAGCATGGGATCGAGAACTTCACTTTTCCATGGTTGATGGGGACTTTAAGAAGTTTGATGGCAAATGGTCCGTAAAATCTGGAACAAGGTCGTCAACTGCTATTTTATCATATGAAGTTAATGTAATACCAAGACTCAATTTTCCTGCTATTTTTTTGGAAAGGATTATCAGATCTGATCTTCCTGTGAACCTTCGAGCTTTGGCATACAGATCTGAAAAGAATTTTATGGGATATCAGAAACTTCCAGTGTCGGAAAATCAGTTGCATAAAACTTATGTGGCCATTAACGGGTCATCTGTTAAAAAGATAAATGGTGCTTTGTGTGAAAGTGTTAAAAAGATAAATGGCGCTTTGTGTGAAAATGATAAGTTGCCTCCTGCAGAGAACAATAAAGAAATTGCCTCCTCTGTTCCTGGTTCTATGCTCCCATCTTCCAGTGAGGTGAGAAGCAACTGGGGTATATTTGGAAAAGTTTGCAGCCTTGACAGACCTCGGATGGTAGATGAAGTTCATCTCCGCAGATTTGATGGACTTCTGGAAAATGGAGGTGTTCATCGCGGTGTTGTTGCAAGCATAACAGTTAAAGCTCCTGTTCGTGAAGTATGGAATATTCTAACTGCCTATGAGACTCTTCCTGAGATAGTTCCAAATTTAGCAATCAGTAAGGTTGTATCACGAGATAACAATAAAGTCCGTGTTCTTCAGGAAGGGTGTAAGGGCCTGCTTTATATGGTGCTTCATGCTCGTGTTGTATTGGAATTGTGTGAATATTTAGAACAGGAGATCAGTTTTGAACAAGTTGAAGGGGACTTTGATTCTTTCCGGGGAAAATGGATTTTTGAGCAACTAGGAAATCATCACACATTGCTAAAGTACTCTGTGGAGTCAAAAATGCGCAAAGATACTTTCCTTTCTGAAGCCATCATGGAGGAGGTCATTTATGAAGATCTCCCATCAAACTTATCTGCAATAAGAGACTATATTGAGAACAGGAATGCCTCAAAATCTTCAGAATTTTGTGAGCAGAATACAAATTCAGGACAACAAATTGTTTCATCAGGCTTTCAAAACGATGACAACACTGGTTCAGCTGAGGAAGTTCCTAATTCCGATGCTCGATGTTCATCTCAGCAAAGACCTAAAGTGCCAGGCTTACAGAGGAATATTGAAGTACTAAAATCTGAACTCCTGCAATTCATTGCAGAACATGGACAGGAAGGATTCATGCCAATGAGGAAGCAACTTCGTTTGCATGGAAGAGTGGATATTGAGAAGGCTATAACTCGCATGGGTGGATTCAGAAAGATTGCAACTGTATTAAACCTTTCTTTGGCTTACAAACACCGCAAACCAAAGGGTTACTGGGACAATCTTGAAAATTTACAGGAAGAGATAAGTAGGTTTCAAAGGAGCTGGGGAATAGACCCTTCATTTATGCCTAGTAGAAAGTCATTTGAACGTGCAGGAAGGTATGATATTGCGCGTGCACTGGAAAAGTGGGGTGGACTACACGAGGTGTCTCGGCTTCTGTCCCTGAAGGTAAGACAAAGGAGCAGACATGATAACATTGGTAAGGATAAGAGGAGTGATCATACAGATAGTGAGATGAAGACACCATGTATTTCTCAAGACACACAAAAGTGGCTTACAAAACTAAAACAGTTGGACATAAATTGGTTTGAATAAAAAAAGAAGATTATTGGTTTA

>C100219_65

GCCGTCTTGTGTTGTTGGTTTGTTACGGTTCTCTCACTTTCTGACTCCATTTTTCTGTTTCTCCGAGACTCTATCGCAGATTCAAGAATGTCTAAAGAAGTTATTGATATTCCTTCAGCGGCTCCTTTTGAGTTTGAGATCTTGGATAGTGATTCTGACATATTTAGGTCTGTTAGAGCCTCATCCTCATCCAATCGTGCAAATCCATGGATTGAACCGGAAAGGTTGAAACTTAGACACAGAATTGGTCGAGGTCTCTTTGGTGATGTTTGGTTAGCAACTCATCATCAGTCAACTGAAGATTATGATGAGTACCATGAAGTTGCTGCTAAGATGTTACATGAAATAAAAGAGGATCATATGAAGATTGTAATAGAGAAGTTCAATGAATTATATTTTAAGTGCCAAGGAGTTGCTAGCATGTCCTGGCTGCACGGAATTTCAATCTTAAACGGAAGGATATGCATCATTATGAATTTGTATGAGGGTTCAATTGGTGACAAGATGGCGGGACTCAAAGAAGGAAGGATTTCATTGCATGATGTTTTAAGGTATGGAATCAATCTGGCTCAAGGTGTTCTAGAACTTCACTCTCTAGGGATCCTCATTCTTAACCTTAAACCATTCAATGTTATTCTCAATGAGAACGATCAAGCAATTTTGGGGGATGTTGGAATTCCCAGTCTTTTGCTCGACTCCTCATTCCTAAGCGCAGATTGGGATAAGAGGCTTGGAACACCAAATTACATGGCTCCAGAGCAATGGGAGCCAGAGGTCAGAGGTCCTATATCGTTTGAAACAGATTCTTGGGGATTTGGTTGCACAATTGTCGAGATGTTGACTGGTAATCAACCTTGGTATGGATGTCCTGTTGGCAGAATATACCAGTCAGTTGTTGAAAAGCACGAAAAACCCAATATTCCTAGTGGCCTTCCTTCTTCAGTTGAGAATATCCTCAGTGCATGCTTTGAGTATGATTTGAGGAATCGCCCTTTAATGGTAGATATTCTGAGTGTCTTTCAAAGTGCTCTGAATGAGTTGGGCAATGATGGAGAATGGAGATATCTAGGAAATGTGAAAGTTAATCCAAAATCAGGTAGCACTTGCTACACTGAGTGGTTCCTCTCAAAGGATCATCTTCAGGTGGGTGACATGGTCCGAACAAGAAAGCTTCCCAATTCCTGCAGACCTAAACACATGGACATCCCTGAAGGGACTGTTGTTAGTTTAGAGGGTAACACAGATCACAGGTATGCCCTCGTAAGGGTCCATGGTAATGACGACCCTATTAGAATTCCTATATCATCCCTTGAACGTGTCACATCTGGCTTGGTAGCTGGAGATTGGGTACAATTGAGGGACGCAAACGAGAAGCACTCACCCGTGGGCATTCTACACGCCATCCATCGTGATCATAGAGTATCAGTTGCATTTCTTGGGCTGCAAACTCCCTGGGATGGAAACACTTCTGAACTCGAAAAGGTCAAACCTTACTGTGTTGGCCAGTTTGTCAGGCTCAAAGACAACATTTCAAGTCCTCGATTCAACTGGCGTCGCAAACGGGGAGGTGCCTGGGCTGCTGGTAGGATCTCATGGATTCTTCCAAATGGAGGTTTGGTTGTCAAGTTCCCAGGGATGCTTCCTTTTGGCAATGAAGCAAACACCTACTTGGCTGATCCAGCTGAGGTTGAAGTTGTTGAGTTCAAGACTTGTCCTGGGATCATAGAGAAGTATCAACATGTAGAGGATCATCACTGGGCCATTAGACCAGTTCTAATTGCATTTGGTCTATTTACTGCTTTCAAATTTGGCATACTAGTTGGAAAGAAAGTGAGGAGGAACAAGGTCAGTGCAGTAGAAATTGGAAGTCAATATCTTGCTGGTCAAAATCAAAATGCCTCCTCTGCTACAACTACTGCTGCTACCACTATGAGATCTTCCAGTGCTACAAAACAAGGCACCACAACTTGGGTTCCTGCAGTTGCTAATATGATTTTGAAGGAAGTTGGTAACTCATCCAATAGCCGGTAGTTTTTCTATTTTTCTATGTTTAGTGTTTCTAAATCAAAACAGTTACTTCATAATGATATAAAATGGTAGGTACTAATTGTACAGGAATTCCATCAGGGCTGACTCTGAATGTTGACGTTGTACATATCCTGCAGAGGATTTGCTCTGTACATAGTCTCCTGCTATGCAAAATGAACACAAAAATTTCTTTACGTGGTTCCTGAATCAATGAAATAAGCAATGAATCAAGATACATG

>C100221_65

ATTTAAATGTGTTTCTTCATATATGTATTTTGTAATAAACTTAAGTCCCAAGTCAAGCATTTTACTTGTCACAACATCACCTTGTATCTAAAATCATAAAAGATAGCAATGTGAAGCCTCATTGTAGTCTCCTGATTACACTCTTCACGTCAAACTTGGATAAGTTGGGAACAGTGGCAAACGTGACCTTCCAAGCAAGGATTTAGACATTATTCCCAAATGCAAAAAAGGGGTTCATGTGAAGAGAGATTACACATGGTCAATGAAACTCCAGGCAGAAATTTTCTCATAAAAAAAGATTGAAGCATAAATAAGCACAACAAACTACTAAAATTGGAGACGTGGATGGACATGTGCATAATGGAGTTGCAGATTCTTTTGAAGTTATTCAAAAGCAAGGTGCGTGAGTGTATGGGTTGCTAAAATTCTCAAGGTTGGCCATGGTTTATTCAAGATAAAAGGGATGGCCACAAAAAGATTATATGAACACAGAGACAATTGATGCAATGTGTGCTAGACCTGAATGTGGAACTGGGATAATCGAAAAATGAAACCTATTTGAGAAACTCCTTGAGTAATGATGGCCTAATGAAAAATTGAGATAAAAAGGAGTAATAATGAGGAATTCATTCACATTGCTTAGCTTGAGGTGTGAGAGATGGTGCTACTTGTGGTAGCACTGGTAGTTGCTGAAGAACTGGAACTCTGAGCAGATGTTTGGGTACTTGGATGAGAAATGGGTCCAACACTTGACAAGGTCAAACCAACTGATCCGGTTGATGTGCTCATTGAAGACATTCTTGATGAAACAGATTCCTTACTGACTCCTGCACCAAGTTCAGGCCGTGACTCTGATGATGAAGAAGAAGGAGCAAGAGTACCAATTGAGGAACCCACAGGATAGGGTGCAACTGGCATGTCGGCCAAGGAAGAGGCAGATGGACTATAACTAAGTGTTCCCATGGGATGATCGAATTTACATGCAGCACCAAACTTGCAAACTCCACTCTGGGTATAATGAGTGCAAGGCTGTGTCCCCGGACGTAAAGGAAGACCCACAGGACTAAGGATTACATTTGCTTTAATTGCACCCTTGTCTGGTGGATGATGATATCTGCAAGATGGACCGAATTTGCATTCCCCTGTTTTCATGTAATAATGACATTCTGGTTGATCAGGTCTTTCTGGAAATTGATGCTCCTTCTGATTAGCACCTGAAGGACCAATTGAGGAACCAGAAGGTTGATAAAGTCCAGTATAAGTAGCTGCTGACGAAGGTAACTGGGTTATCCCATAAAGCTGTGTTGAACCAACATTAGACATAGCACTAGATGGAATTACAGGGTTTGTTGCAGGAGCCTGATAAGGACCCCAGCCTGAAAAAGGGACCATGGTAGGGGACACTACCATAGGCCCGTAGGGACCCTGAACTACTGAGCCAGGTAGCATAGGTGGCCTTGCAACAATTACACCATATTGTTGTTGCGAAGGACCAGACTGAGGCTGTACAGTTGGATATATTGGTGAAGGTACAGGTACAGGCAGAGGAGAAACTGAGGGAACAGCTGATGGTGTTATAACTTGGACACCTGCAGGCTGGGGATGATGGAATTTACATGTTGCACCAAATTTGCACTGTCCAGTTTTCACATAATAGGAACACTCTTTCTCACCCACTCGCAATGGATATCCATAATAATTTAGTGGCACAGGAGTAGCAGTGCCTGCCACCTGCCTAGGATGGTGGTACTTGCAGGATGCACCAAATTTGCATGATCCCGTCCTCATATAGTACTGGCACACAGGCTGTCCCACACGTTCCGGAAACTCCCCTCCAGTCCTTGCCGCAGCTCCAATAACAAGGCCACGGTCACGAGGATGGTTGAATCGACATCGTGTGCCATAACCGCAAAATCCAGTCCTTAAATAGTAGATACAATCGGCCTCATCCGGACGCAGAGGGTACGATTCGGCAGTTGTTAATCCGAGTTGCCACGAGGACTCTTCGATGCCGGCGTCGGTACCGGCAACTGTCCATTCCGGCGACGGATCAGACTGTGACCCTTCGCTTGCCCGGCCGTACCGCTCCATTGTACGGACCAAATGGAAGGAAAAAGGAACGCAATCTGAAGACAGGAATGAGAGTTTCTGATGAAAAGAGTGAGAAACTGCTTCGAATTTTCTCTCTCTCACTCACTCACTAACATACACTACCTCTATGTTTTCTCTTACACTATTAGTTTGGTCTCTTTCTCTCTCTACTTTCTC

>C100227_65

CATAACAAAATAGAAACAAATCTATTTGCCCCAAAACTATCACGAGACGACTCATATATATGTGCACATTTGTCATGAATTATGAATCAATAATATATCCCATCTAGAGAGCCCGTTATCCTACATCATGAACCTATTGATAACACCATTTCCCGTTTATTGATATTTGATCAAAATCAGTGATCATGGTCATGCTTTGGCTTGGCACTTACATTTTATCAATGGTCACATTAGGTATCAAAGCTAGCAGCTCGTGTGACTCTGTACATGGAGCATTCTCCACAGCGTTTCTTTGCTCATGTGTTGCGTTTGTCATGCTACTATTGCACATTCTTTGGAATCTACATTTGGTCAATGTGACTGTACAGGGGACATTTGTCCAAGAATTTCCTTTTATTCAATCCATGAACAGTTATCAGGCTACCTATAATCCTTACAAGAGCACTCTCCATTTCTGAAATGATGGAAACGTATTCTGTTTCACTATGAATCTCTCTTCTCGGACTACCAATAATCCTTACAAGAGCACACTCCATTTCTGAAATGATGGAAGCGTATTCTGTCTCTCACTACGATATCTCTTTGGTATATCTTAGAGATTAGTTTCATAAACTCATGACAGTCCTCACAAGACCTTAGGTTCTTCACAATCCGGATTGTAGACGCTTGACCTATCCTAATTAGTCCATAAGCAAGAGCAAGCTTCTCACTATGGTGAAGCAACTGAACGGCCTTTTCCACATCATCCATCTCAAGCAACACTTCCGAAACTCTGGGATCATACCCTTCTTGTCTTAGCTTATCCACCACCTCATCCAATTTTACAAATATTTTCTCCGCCTCGGGGTGATTGTAATCTCCCATCACAAACTCATGCACACTACCATCAACCTCAATCCTACTAGACCCAGGAGGCTTCACCAGTCCCTTCTTGTTCATCAACTCCCTCACTTCTGCTTTGTTACTCCACTTCCCCGCTGACGCATAAACATTGCTAGCAAGAATGTAACTTCCACTGTCGTCGGCCCTCATGTCTTGTATCTCAAGGCACTTCATCAACCTCTCAGCCCTGTCAGCATCTCCATGAATTTTACAGGCCCATATCAGCGTCCTCCATAGCACCGCATCTGGTTCAATAGGCATTGCATTAACAAAATCTTCAGCTTCCTTCAAACGCCCCGCTCTAGCAAGGAGGTCCACTAAGCAACCAAAATGCTGAATCGTAGGTTTCATCCCGTAACGTTTTTGCACATCATTGAAAAACACGTAACCTTCATGAAACAAGCCTGCATTCCTACACGCGGTTAAAACCGCGGTCACCGTCTTCTCATCAGGCTCAACTCCAGAACTTTCCATGTCAACAAACATGTCAACAGCTTCCTTGGATAACCCGTGGCAGGCAAGGCCAGAAATCATTGCAGTCCAAACGAAAACGTCTTTGTCCACAACATCATCAAACACCTTCCGTGCACTCTCAATGCACCCACTCTTGGCATACATATCAACAAGAGCAGTGGCAACATTGGAAACAGAGTAAACTCCAATTCCCCATTCCTTCAACTTCCCATGCACTTTCCTCCCCAAACTCAAGGCTCCAGAATCCCCACAAGCTCTCAAAACAGACACAACAGTGGCCTCGTTGACCTCTACTCCACATTCCAACATTCGCCGAAACAACGTGATAGCCTCAACGGGTCGGTCATCGTTGACAAGACCATCAATCATGGAGGTCCAGGAAACGACGTCTCTGTGAAGCATTGTATCAAACAGGGCCCGTGCGAGGCCCACATCGCGAAACTCGGAGTACATGTAGATGAGGGCGTTCTGGATATAAAGGTCCGCGGCGAAGCCGAGCTTGATGATGAGGGCGTGGAGCTGTTTGCCCTGAGGAGCGAGTTTGGAACGCGCGCAGCATTTAAGGAGGAAGGGGAAGGTGAAGTTATCGGGAGGGGGGAGGAGGGGGGAGTGGGGATGGAGCATGGAGAGGAAGAGGGAAAGGGCGTGGAGGTGATGGGTGGGGTTAGGGCTGTGGGAGAAGGCGCGAATGAGGGTGTTGTAGTAGTAGGAGTTCAGAGCAGGGTTGGTGGCGAGAAGAAGACGAGCGTAGTTGAGGTCGCCGACGGGGGAGAGAGCGAGGAAGGTGAAGAGGTTGGAGAAGTTGCGCGAGGCGTGGCTGTGCCGAATTCCTAGTTTCTCCACTTGCCCGTGGAGTTGCAGTGCCTCCCTCATGCTTCTCACCTCCATTGTTCTTCCACACTTCAACCCTAAC

>C100229_65

TTTTCCTTCAGAATATATTTTTCCACACATCACACTGGCAAATATCATTTATCTCTGACAGCAACACAACATGGAGGTACAAAAACCTGACTCAAAGGTCCATAAGAAATAAATCCAATTCTTTTCAGTTCTCATGTTTTCACTGACTAAATTGGAAAATATAAACATCCTAAGAATGATGCAAATGGAAAACGGCAAATTGAACAGACTATATAATATGAATCTAGAGACTTGGATTTACAAAAAACTCCAAATCTACCAAATATACAGAGAAGGAAAATGACCTCCAGCAAAAAAAAAAGCCCGGTACCCCTTTTCCTCCACATATGAAATATATTTATATATAACCCCCCTCTTTCGCCCCTCAAATGCTGTCGAAGGAAATGAAAGATAAATAATGAAAAGAATATTGGCAAAAAACTACCGAAAAATCATCAAAATAATGATTCTTTCTTTGCAGCACAATTTTCCCAAACTTCTCAATAAAACCAGCAAGGCTTGGGAGGGACATGGTACAAAACTTGTGCTATTGCTGCTGTTTTTCAGCTTCCATTTTCATCAAGATAACTGCTCAGATTTGTCAACAGATGCCTCGGGTACCAACTTGCCTTTGGTATCATTTGACTTGAAATCTGTATCTGAATCAAGGAAGAGGCAAACCGCGGCACAATCATCAACCTTACAAAAAGGAAATTTTGTCCTCCATCCCTGAACAGCTGACTCAACCAGTGCTCTAGCAGCAGTAGACCGAGGAGCAGATGCTACAATGTCCACGACTTCTTCATTTGATAAAACATCCCATATCCCATCTGTTGCTAATACAACAAATTCATCCTTCTCGGTAATGCGGTGATATGAGATATCTGGAACAGAAATCAATCCAAAGTCCTTCAGGCAAAAATCTCCAAATGCCCGAGCCATAGCAAGACCAGGGAAATCAGAGTTAGGCAGCCAAACTCGAGCCACCTCAGGTTCATTTTGAAGGGAAAAAACCCTTCCTCTACGAAGCCTGATTCTCTCTTCTTCCCTTGGAAGATTTGGCTTAAGGTCAATGGTCAACTGAACAGCAATAAGAGAATCATCGTGATCTCTGGTACCCAATATAGCTCTCGAGTCTCCAATATTTCCAATAACAAGGTCCAGCCCTTGCTTGACCAGGGTAACTGCTGTAGTTCCACTACAAAAGCAGTCAATATCAGCATGCATTTTCAGTTCTTTGTCCATAAGCTTACAAGCTTTCAGAAAGGACTCTCTTAATGTCAAAATAGTATCTGTTGCATCAAGTTCATGATCAGTGGGACTAGTTTTCTCGTCCACCAGTCTAAATCCATTCCCTTCAGATTTATAACTTCCTGTGGCACTGCTATGATCACTGACTCCATCTCTATTCTTATGATTCAAATCCCACTGAGCATTAAGCTTTAAAGGAAAAGAATCCCTAACTTTCTTTGCAACTTTGTGGCCATAAGGTCCATGACCATCAAAAACACCACAAAAAATGGCGTCTTCCTTTGAGCAAAAGTTCTCCCAAACAAGCATAGCATCTTGGTTGATCCCTTTTCTTCCTTGCTTACAATACATGGACGCAACCTCGCTTGACCCATTCAAGAACATCCTCCCAGGCACTTTGAAATCAAAGGACGAGGAAGCCCTCAACCTCCTCCTCTTCCCACCGTCCGTGTTGGATTCAGGCGAATAAGAAAGAAGAGGAGGGGAATTACCACCAGCCCCAACCTCCGAGATGCACGATCCCATTCCCAGAGCCCTCACAATGTTCAGAAAACACTCGAAACCCGCCAAACACTCAGAATCGGAACCCCTAGGCTCCCCCACCATATCCGTCCCATATCGATTCAGCACCTCCACCGCCTGAGTCATGATTGCAATGAAATCCGATTCGACCGAATCAACGCAAATCCAACAGCGTGTCAACCTATGCGACAGTAACCTTGTCGAAAAGCACGTGAATCAGAACTGAAAACGCGTATGCGAAATAAAGTAAAATGAAACCGTGAAATCAGAAAAACTGAATCGAGATTCTCGATCCAGCACTCTCTCCCTTTCTCCTCCTCCGATTCCCTGAAAATCCCTCGCGAAAACCGAAAAACGAATCGATGAATGAAACAGGAATCAGGGTTGAAAAAGAAATGAAGGAGGAGTTTATGTTGGTGTTGTTGAGGAAATCGTTGAGGAAATCGTTGAGGGCAGCGCTACCGTCACGAGCGGGTGCGCAACAGACAGATTTTGTTTCTCCGCCACGAAAAATAAG

>C100233_65

TGGCATTTGTCTTATTTGTTGCGATTCTACCAACCTGAACTCCTAATCCACCAACTTTCTTATTTAGAAAACTGTCAAACGGTGTTGTTGCTATTTGGCTTGGCTATATCTCTATGGCACTAGTCAAAGACCTATGCTAACCCCTCTCTCTGTCTAAACAAAATTCTGAGATTCATACTCATCTGTCTGTGTAAAAAATGGCAGGGTAGGAGGACTTCTATGGTCTGTGAGCTTCTTCTTAGTTTCTAAATCCACAGGGCACAGTCATCCTAGAATAGAAGGTTCAACAAAATTACATCACTGAGCTTCCTCAAGGCGAATCATGTCATTAGTTAGGTCTGCTGATCTAGCTCCTACATCGTATAAAAATGCCAAGCTCTTCTCACTAAAAGGAACTGATGTTAGACCAGGCTTGTCCTCTCAAATTTTTGGTCCTAATAAGCATAGGAGCATGTACATGACAGATTCTTACTCCTGCGAGAGTTATGAGAAGTACTTCCCTGACTCACAAACAGAAGAACTGATAGAACCATCTAGTTCCAGCATCTCTGGAAGTTCAATTCCCCCTGATGTAGCCTCTTCTTACCAGCTCACAGCCAGCTCAGAAGCTTCTGTGGTTGCCAATAACCCAATTGATTGTTCTTTCATGTCTACAATGCATCATGGTTATCAGTCAAATTTTGGATCAGATTTGATGGAGAATGGAAGCCTCGATTCCCGTGACAATGATGGTCTAATGAGTTTAAAACTACAAGCATTAGAGAGGGCATTGCTTGATGATAGTGAGGCTGAGGAGGAGGAGCTGGAAGAGGAAGACATATTTGAGGCTGTTCAAAGCATGGAAATTGATCCAGACATTGCTGAGTGGGCTGATTCCATGCAGAATATGCTCCTCCATGATTCACCAAAGGAGCACTCTTCTTCAGATTCCAGCATCAGCAGCACCCAAGAAATATCACAAACTTCTCAGAACCCCAAGCAGCTGCTTTATGAATGTGCTATAGCACTTTCAGAAGGAAACGAAGAGGAAGGATCATCCATGATAAACAGTCTCAGGCAGATGGTGTCAATTCAAGGAGAGCCTTCTCAAAGAATTGCAGCCTATATGGTTGAGGGACTTGCAGCTCGCTTGGCCGAATCCGGCAAAAGTATTTACAAAGCTCTGAGATGCAAGGAACCTCCTTCTTCAGATCGTCTAGCAGCCATGCAGATACTCTTTGAGGTTTGCCCATGTTTCAAATTTGGATTTATTGCTGCTAACAATGCAATTACAGAGGCAGTGAAAGATGACATGAACATTCATATCATAGACTTTGACATCAATCAAGGAAGTCAGTACATTAATCTAATCCAAGCACTTGCTTCAAGGTCAAGTAAGCCACCCCATGTTAGATTGACAGGGGTTGATGATCCTGAGTCTGTGCAGCGATCTGTTGGAGGCCTAAAGAACATAGGACAAAGACTAGAAAAACTGGCAGAGGCACTTGGTTTACCATTTGAGTTCCGAGCAGTGGCATCAAGGACTTCCATTGTCACCCCATCAATGCTCAACTGTAGTCCCGGGGAAGCACTTGTGGTGAACTTTGCATTCCAGCTTCATCACATGCCTGATGAGAGTGTTTCAACAGTAAACGAACGGGACCAACTTCTTCGGTTGGTGAAGAGCTTGAATCCAAAGCTTGTGACAGTTGTGGAGCAAGATGTGAACACAAACACCACCCCTTTCTTGCCTAGATTTGTTGAAGCCTACACTTACTACTCTGCTGTGTTTGAGTCACTTGACGTTACTCTTCCTAGAGAAAGCCAAGACAGGATGAATGTTGAAAGGCAATGCTTAGCAAGGGACATTGTCAATGTTGTTGCATGTGAGGGTGAAGATAGGATAGAACGGTATGAAGTAGCAGGAAAATGGAGAGCAAGGATGACAATGGCTGGCTTCACTTCATCTCCAATGAGCACAAATGTGACTGATGAAATCCGAAAACTCATTAAGGTGATGTATTGTGACAGGTACAAGATAAAGGAGGAAATGGGTGCACTTCATTTTGGTTGGGAAGACAAAAACTTGATCGTTGCTTCAGCATGGAAGTTACCAAGATAATTAATTATGTTAGCAGACAGAGTTATTGTAGTATTGGTAATGCATAGTTTGCTTGCTTGTTTAGTCTGTAGAATACTTCTGATATTTCTATTGTAGTGTTTTGCTTGCTTCTACACATGTATACTAACTTACAATCTTAAGCTAATAAGAATTTGCATTTCATGTAAGG

>C100239_65

GATAGACACAACATATATCATTCTTGAATGTGTGAAGAAATGGAGGGGAGTGATAAGAGCCTTACTCTGAATACCCTCCATGTCCAAAACCTGCAACTTATCGTTAATAGGTTACACATGCTACTCCACTCCACAGCTTTAGCCTTCTTGTTCTATTACAGGGTCAGTTTTCTTTTCCAACCCTCAGAAGCAAGAGAAAGTCAAAACTTGTTACCATGGCTACTTGTTTTTGCTTCAGAAACCATTCTCTCCTTCATTTGGATCCTTGACCAAGCTTCTCGCTGGCGTCCCGTTTCACGTTCTGTCTTCCCAGAAAGATTGCCGGAGGATCACAAGCTTCCTTCTATTGATGTGTTCATATGCACTGCAGATCCAACTAAGGAACCAACTTTGGATGTGATGAACACTGTTTTGTCAAGCATGGCACTGGATTACCCTCAACACAAACTGCATGTGTATCTTTCAGATGATGGGGGGTCTCCTTTGACTCTGCATGGGATGAGGGAGGCTTGGAAATTTGCTAGGTGGTGGCTTCCCTTTTGCAGAAGGTACAAAATAAAGAACAGGTGCCCCAAGGCTTACTTTTCTGCTTTGGAGGATGATGATGATGGTGATGGTGATTTTGCAAGGAGTAGTTTATACACAGCAGATAAGCACAAGATCAAGGAAAAGTATGAGGCTTTTAAAGAAGAGATAAAGACATTCAGAAAAAATAGGGCATTCTCCAGAGATCACCCATCTGTCATTGAGGTGATTCAAGAGGGTATCACAGATGATGTAGATAATGTGAAAATGCCTCTCCTTGTCTATGTTTCTCGTGAGAAAAAGGCTTCTCATCCCCACCATTTCAAGGCTGGAGCCCTCAATGTCCTTCTTCGTGTCTCTTCGGTGATGAGTAATTCTCCATACATTCTAGTTCTAGACTGTGACATGTTCTGTAATGATCCAACTTCAGCTCGATACGCTATGTGTTTTCACCTTGATCCAAAGATATCATCCTCCTTGTCTTTTGTTCAATTTCCTCAGAAATTCCGCAACATTAGCAAGAATGACATCTATGATGGTCAGCTGAGATCACTATTTACTCTACAATGGCAAGGTATGGATGGGCTTAAGGGACCTGTTATGTCAGGTACAGGTTTTTATATCAAAAGGGTATCACTGTATGGAAGCTGCACTAGGAAAGGAACTGATCTGCTGCAACAAAAAGAACACTTTGGGTCATCTAATGAATTCATTAGCTCACTTGATCAAAACTACACTAGTGATTTGGTATCTGATCAGAAATATGCATTGCTAGATGAACCTCACTTTTTGGCTTCTTGTAGCTATGAAATTGGCACTAAATGGGGAGAAGAGGTTGGTTTCTTATATTATAGTGTGGTAGAAGATTACCTTACTGGATTTATCTTGAATAGTAATGGATGGACTTCAGTTTTCTGTAAACCATCCAGGCCACAGTTCCTAGGTGCAGCTACAACAAATTTGAATGATGTACTAATACAAGGCACTAGATGGTCCAGTGGTTTGTTTGAAAATGGTATAAATAGATTTTGCCCCCTTATGTATTGGCCTACAAGGATGCCACTTCTCCAATGTCTATGTTTTGCATGGCTCACATATTTCCCTCTTTATTGCTTACCTCTTTGGTGTCTTGCTACTATCCCTCAGCTCAGTCTACTAAATGGAATACCCTTGTACCCAAAGGTTTCAGACCCTTTCTGCATTATCTTTATATTCATCTTTCTATCAGCTCTTCTAAAACATTTACAAGAAGTCTGTGTAAGTGGTGGTACAATCCATAAGTGGATCAATGAGCAGAGGATCTGGATGATGAAATCAGTCACTTGCCATCTGTATGGATGTTTGGATGCACTGCTAAAGAAAGTTGGTATAAGAGAAGCTAGTTTCTTGCCCACTAATAAAATAGATAATGAAGAACTAACTATGCTATATCGAATGGATAAATATGATTTCCAAGCATCAAATATCTTTATAGCACCAATGCTTTTTCTCATCACTATCAACATATATTGCTTAGTGGGAGGAGTCTACAGAGTGTTATTGGTGGGTGACACTGACAAAATGTTTATACAGATTTTCCTTGCAGTTTTTATAATTACTATCAACTACCCCTTGATTGAAGGGCTCTTGATGAGGAAGGACAAGGGACGAATTTCAAAGTCAGTTGCTATACCTGTTATCTTGGCTACTCTTGTTCTTTTGTCATTTTTGAAACTACTAGGAAATGCATAACTCTTGTTCTAATTT

>C100245_65

ATTTTACTCATTTTCTCAAGAACAAAAGAAAGTTAAAGGCTAAAAATACAATCATCAGAAGGAGAAAAAAAGTTGTACAATTTTTGGAACATTGTTTTGCTATTTAACAACAGGTAACATTTTTTCCAAAGAAAAATGATAACCTTGGCTTCTTATTTGAACCAAAGTTAAAGTTTAAATCTCCACCTTCATTCTGAACAGCAGCAGTATTGCTTGTAGAGGGGAAGGAAAAAACTACCGCCGGACTAGACTTTTTCAAACCAAATCTATAAGACAGTTCAGTAGATCCTTCTTTTAACTGGTGCTGATCCCCAGTAGAAAAAATAGGCATGATGGATGGAGTTGGGGGCTCAGAGAATACAGAAGAGGATGCTGAAACAGGAAAAGTAAATCCCGAGCTATTATCGGATGCTACTGTCCATCTTGATTCAGGCTTCTTGATAGAAATAGGAGGTACAACTGGTTTAATGGTTGCAGGGTTAATGGGAGGCTTCTTTTCCTGATTTACCCCAAAAGGATGACCTCCACTTGGGAGAGGCATTTTCGTAGCATCATCATTGTCAAGTGTGGGGTTCCTGCTATTATAAGAATTCACATCAGGCGCCAAAGTTTCTTCATTTCTATTGGTAGTTTTATCAGTACTGTCCTTCAATTGCCTATCTACACGGTAGTTTCCCTTGCCTTCAATCACTAGAGTAGATTTCTTTCCTTCAAGCCAATCATATTTATGAGGGCTAACATCATTTAACTTATGCAAGTCATTATCTTCATTTGACAAGATAGTACTGAAATCAGATTCAGAGTTCTTCCATTTTGCTGCCAGCTTGAGCTCAGCAGATTTCTCTTTAGGTGTGGGAATGTTTCTATCGATATGATCTAATATTTTCTTAGCCATCAAACTGGTATGCATGTGTACTGTTGGAACACCCACCGGAAAGCCCAGTGGCTTAAGAGTGGACCCTGTCCCAATAGTTTCCGTGTTCTTTGCATCTGGAATGAAACCTTCAACAACACCAGAACTTGCTCTCTCTGAAGGACCATTCAGTGATGAATAAGCTGGTCTTCTTGAAGAAGATTGTGCACCAAACTTATGCCTAGTCCGACGAATAGGTCCAACAGATCCATATCCACTTTCTGATGCTCCAGTTTTAGATTTATCCTGTAGATACATGGCAGTCTTTGATTTAAGGATGGGAGTTGATGAAATGGGGTTGTTATTTCCTTGCAAGTTAATCAACTTGGACTTCGACTTTGTTAAAAGAGTTCTAGAATAAGGAGTACGGGGGAAATTATGAAGTCCATATCTGCTCCTCTGACTTTGTGGTGTTGTGTAAGCATTCTGCACCACAGCACCCGGCCAACATGTTGATGGCTTCTTTGATGGTGATGGATCATATGGTTTTATTGCAGCTTCAACACCATGCAACACCGTGCTTTCAACAGTATGAATCATGTTGGGACCTGCTTCTAATGCACGAGAATCCATATATGCTCTTGCAATCTCTATTGGTGAAGCACCAATTTCATCCCGAACTTTTGACAGACCAAGAGGTGTTGACGTTCCCCATATAGCACCAACTGACTCTTCATGCCTTTGTCCATTTGAAATTTTTGGAAGCCCCTGTATCAAATTTGTACTTTCTTTTCTTTGTTCAACATTAGAAAGGTCCATCACCCTCGAATTTAACACTGCCACTAAACGATCAAATTCATCTCTAGAAAAATTCTTCCCTTTGACCAGCTGCTCAATATCTGAAAGCCTATTTTTATCAGATTTCTCTGCTTTTTCATCTTGTCTAGACAGACCCAATTCTGAACTGCTTTTCAGTTTACCACTAATTTCATCAGCATTAGCCATTTCAGATGATTTGGGTGCCAAAAGATGTATATCACTTCTGTGTGAACCATCTTCATGACTGTCGTCAGTTTCCCATATATCTTGATCTTCAGCAACAGAGGTAGGGCAAGGGAGAGGAGAGGGAGGAGTCGCAGAGGAGAAGAGGGAAGGGAGAATACGAGTGGCTCCCCCGGCGATGAGGCGGTAGGCAGGATCGACAAGCTTCGATATCCAACCCTGGCGCCTGGTATCCGGCGGACGTGCGTAAGGGCTAGGAGGTGGCTTTCTCGGCAGTGGCTTTCGGAGCTTGCCTCCGGCGCCTCTCTCCTCAGGCATCGGTGCCGTGATTTGTTCAGAAGCCATGAAAGTGGTACAATTTGGAACTACGGAATAGCGAATAAAGAAAAGCCAAGGTAAAGAACAAGGCAAATCAGC

>C100249_65

CTAGAAACAAGGTCCAAAGTTGTTTAGGAAATAAACAATACCAAGATTCATCATATTTCTTCAACTATATATTTATACATAAATCAAGTTACCGAAGGCAACACTTATTTATCTAAGGCTATCGATTCCTATATCTTCACTCTCCTATGATAAAGTCATGAGTCATGACTCTTGTATGAGCTTATGTACAAGAAAATACATTCGCAAGAATGCCTCCTACTATGGCGCCAATTGTTTAAATTCACACCCATTAAACTGTAAAAAGCTTGGAGGTGTGCTGCATGTATATGGAGGGAGGTCTAAAGGTCCACACACAAGTAGGAAATGACTGAACCTGCTCGGCGCTTTGTACGTTGGCTAACCTTCCACTTCTCAACAAGGACCTTCCTTATGTTGGGGCAGCTAGAAACCACGGTGGCCACACCAACTGAAGTTATACCAGAACAATAAACCATGTGGCATGATTCAAGTATTGTGCAACTTTTTACAAGATGGGCTAACCCAACATCTGTTATTTGCCGGCAGTGTGAGAGTACAATTTCTTTCAGCAGTGGACAGTGTTCTCCCACCTCAGCCATAGCCATGTCACCCAAATTCTGTAATACACTCACATCCAAATAACAGAGTTGAGGGCACCCCCTTGCAATTGCTACCACTCCAGCATCTCCAATTTGATGGCAACCACTAACATTCAGATAATGAAGGGAACAGCCCTCAGCTATAGCAACAAGGGCCCCATCGCCCACCCTATCACAGAATCGAATGCTAAGATCCGTTAGCAACTTACAATTCTCTCCCACAGCAATGATCCCCTTGTTCCCAATCTCATAACAGCGACGGATATGAAGCTTCTTTAGATTCTTGCAGCCACTAGCTATGCCACACATGGCTTCATCACCGATACTTGAGCAATCTACCAAGTGAAGAGCTTGCAAGTATTTGCATCCCTGTCCAATCTGAAGAAGGCCAGCATCACCAATTTTTTGGCAGTAAAGCAATGCTAACTCAGAGAGATGCTGGCAAGATTTTCCAACAGATTCTAGCCCCAAAGTTCCAATATTGTGGCATCCATTCACTTCAAGATGAGTAAGTTCCTTGCAGCCGGTAGCAATTGCTTCCAAACCCTTGTCACTTAGGAAATAGCAATCACTTAAAGTCAAGTTCTTTAACTTCTTACATCCATTCCCAATAGCATGCAAACCCTTATCAGTAAATCTCTGAAAACTGTAAAGAGCCAATATCTCCAAAGACAAACAATTAGCTCCCACAACTTTCAAAGCATCATCTGTGAGATTAATACATTGTAGCTTTAGCACTTTCAAATGTGGACATCCTTTAATCACCGCAAGCAGCCCTTTGTTATGGATGAACTCAGAGTCCAATGACAAGGTTTCTAGTGATCTGCAGTGCGACCCCACAGCTTCCATTGAAATGTCAGTTATTTTGGCACAAGCTGCTACTCCAATTGATTTTAAGGAATTTCCCACACCCAATGCTAATTCAACCAAACCCTTGTCAGTCAAGCCTTCACAGAAACGCAGATTCAAATCTTCAAGTTGCTTGCAACACTGTCCAATAGCAGCCAAACCTTGATCTCCAACATAGCAACCCTGCAAATCTAAGGACTTCAAAGAAATGCATTTCCTCGCTAGCGATGACAACCCGTCGCTGGTGACGTTAGAACACCAGATTAACCTAAGCTTCTCGAGTTTGGGAAAACCTTCAGCCAGAGCAGCCAATCCAGAATCAGACAAACAGAGCGAATCGAAATCGTTCTGATCGGAAGAAGAACCGTGTTTATCAGCCACATAGTGCAGCTTCACCCCGGAATTCTCGCTCGCGCGTCTTCTCCCTGTGAAGTTAGTGGGGATAGAAACGGAGAGACGCTCGTCGATGTGGACGGTGGTGATGTTGGAGAAACGCGCAGCGAGGAGGTGAACAAAGAGGTCCGGGGAGCCAGAGGCGCCGATGCGGATGGCGGAGCGTGTGAGGCGTTCGAGACGGAGCCAGCGCGTGCAGACGAGGGAGCAAGCATCCCGGCTCGACTTGGAGTCCAGGCCGCGGAAAATCTCGATGAGCAACTCGTCTGGGAGCAAGGTGTTGATCCAATCGTGGCCTCGCATCGGACCGAACCAATTCACACCGCTTCCAATGACTCAGATTGTTGAGGTTGCAGAAGCTGAATAGCAGATCCAGTTGCTTATCGTACGGTTTTGATATGGTTACCGCCTTTCTTTTTCTGTGGGTGACATTTTAACCCTTCTCTTTCCTTTT

>C100251_65

GCTGGAAGAAGAACGCTTGTTGCTTCTTCTCAAAACGTGATCCCAGGACTACTCATTCATTCATTCACTCTCACCTAACACCCTCTCCTTATATTACCACACTCACATTCTCTCCATCATCACTTCTTCACCAACCATACACTATTTTCAATTCCAATACTAATCACCATGTTAACCGTTGCTCCTTCTCTCGATGCCAACACCAACACCAACGACATCTCTGCAACAAAAACAGACCAAACTCAAACCTCCCATGTCTTCAAATCTAAATTACCCGACATACCTATCTCTAACCACCTCCCTCTTCATGATTACTGCTTCCAAAATATCTCCCAATTTTCCCACCGTCCTTGCCTCATTGTCGGCGCCGCTAACAAAACCTACACCTACGCCGACACTCACCTCCTTTCTCGTAAGATCGCCGCTGGATTATCCAACCTCGGAATCCGCAAAGGTGATGTTGTCATGATCCTCCTCCAGAACTCTGCTGAGTTTGTCCTCTCCTTCCTCGCCTCCTCCATGATCGGCGCTGTTGCCACCACTGCTAACCCTTTCTACACCGCCGCTGAGATCTTCAAACAGTTCACCGCCTCCAAGGCCAAGCTCATCATAACCCAGGCAATGTACGTCGACAAGCTCCGCCACCACGACTCCGGCGTGAACCTCGGCGAGGATTTCAAGGTTGTCACCGTCGACGACCCTCCCGAGAATTGTCTCCACTTCTCTGTCCTCTCCGAGGCGAACGAGAATGACATGCCGGAGGTGGAGATCCACCCCGACGACGCTGTTGCGCTGCCGTTCTCCTCCGGCACCACGGGTTTGCCCAAGGGAGTCATCCTCACGCACAAGAGTTTGACCACGAGCGTCGCGCAGCAGGTCGACGGAGAGAATCCTAACCTCTACCTAACCACCGAGGACGTGCTCCTCTGCGTGCTTCCGCTTTTTCACATATTCTCGCTCAACAGTGTGCTGTTGTGCGCGCTGAGAGCCGGGAGCGGCGTTTTGCTGATGCAGAAGTTCGAGATTGGGACGCTGCTGGAGCTCATACAGCGCCACCGCGTGTCGGTCGCGATGGTGGTGCCGCCGCTGGTGCTGGCGCTGGCGAAGAATCCGATGGTGGCGGATTTTGACCTCAGTTCGATACGGTTGGTTCTCTCCGGTGCTGCTCCGTTGGGGAAGGAACTCGAAGAGGCTCTCCACAATAGGGTGCCTCATGCCGTTTTGGGACAGGGTTACGGCATGACGGAAGCTGGTCCAGTGCTGTCCATGTGCTTGGGCTTTGCAAAGCAACCATTCCCAACGAAATCGGGATCTTGTGGAACTGTTGTGAGAAATGCAGAACTCAAGGTTGTCGACCCTGAAACTGGTCGTTCTCTTGGCTACAACCAACCCGGTGAAATTTGCATCCGAGGGCTCCAGATCATGAAAGGATATCTGGAGGATGAGAAAGCGACAGCATCGACCATCGATGCAGAGGGTTGGCTTCACACCGGTGATGTTGGCTATGTAGACGACGATGATGAAATTTTCATTGTTGACAGGGTGAAAGAACTCATTAAATTTAAAGGCTTCCAGGTTCCCCCTGCAGAACTTGAAGGGCTTCTTGTAAGCCATCCCTCCATTGCAGATGCAGCAGTTGTCCCACAAAAGGATGTTGTTGCCGGTGAACTTCCCGTTGCTTTCGTTGTGAGATCAAATGGCTTTGATCTAACAGAAGAGGCTGTAAAAGAGTTTATAGCCAAACAGGTAGTGTTTTACAAAAGACTGCACAAGGTTTATTTTGTTCATGCTATTCCCAAGTCTCCATCAGGAAAGATATTAAGAAAAGACCTGAGAGCAAAGCTCGAAGCCACCGCAACTCCGACGCCTTAAGGCCAGAACCTACCCTTTTTTTGGCAATATTTTCCCGATTTTTTATTTATATTATTGGTTTCACAAGTGTAATTTGTGTTTCAAAATGCTTCAAGCATAAAGTGAATCCGAGAAGCACCTGTACTTGCATATAATCATTGTATTTTTATGTCCATTGCCTGAGCTTCTCCAGGGTCCATTACAGCTGTATTGTGACTTCAGATCACTAATTTTCGGGACCTTATATTATTGGTGTTATCAGATCCTGCTATTCTTCTCTACCCTTAGCTTTTGTTTTTTGGATGATTTGAGTGGTAGAGAGATTTGGGAGGAATAGATTAAGAGGGAGATTAAAAATGTCCTTTTGTTTCTGTGATACCAAGTTAGGAAAGAAATAAAATTATTTGAACCCTACCACATTCC

>C100255_65

CAGGAGAATGAGGTTGACGTCACTAAGTGCACTTTTCCACATAAAATTGACATATGTAAAAAAAAGGGCAAGCAACTGAGCAGCTAATGACAATACCATGATCCATCCCCAAGCATAGTCTTTGTTTTAATAGTTTAATTTTTGCTATCTTCCAATAAATGTACACATCACATCGAATAATTTTCAGGATGCTTTAATGGTTCCTCAGCAATTTGTCCATAACTAACATTTAACCAGCAACTGCCACTAGAGAAGGCAAGTCTTCCACAGCCAACCTGGGCAGCCAGGACAGGAGCACTCGGTCGCACAACTCTCAGTTCCTAAGTTGAAAATTCAAATACACACACGGTGTTGCCCTACAAGCTTTGACAATGCTGTTCAACTACTTGCTGTACACATTGTTCTTGCAAATGGCATAGGAATTAAGGTTCCATAATACATGTTCTTGGCCAATATTTAAAGCCAAAGCAAGTACACAAAAACTCAACACGGCGGACTTCAATTTGCACAAAGTTATGGTAACATGGGATCTCCAGAAGACGCAAGCATTTGTAAACTTGTTTCTTCAGCCGCCCGTTTAGAAGCATAATCTTGCATGGAATCACCAAAAGATTGACGAAGCCCTTCCTTTACAGCATCGAAGTTCTTGTAAGTGTAACCAACGAAGCTTAGATCTTTGGTAGTTAGCATCTTTCGTGAGGATCCAGACCCACCTCTTGCCGCTGTTGGTGGATCCACCTCATCAAACTTCATGAAGTTTTGGGTATCCAGCTCCCCGTTGACTTGAGGTTTAAATGCTGCCTCCATTTCATACAGTTTATCCCACTCCACACCATTGAACCAAGGATGAGCCTTTATTTCATTTGCCCCTCGAGTACCTAACCTATGATCAACATCACACAGCAACCTATAAATTAGATCCTTGGCCTCAAGTGTTAACTGGGTATCTTCTGGAAATCTTAAATGATTTTTCCAGTGAACAATCTTTCTGCATGTGGTTATTGGGTCATCGGAGTAAAATGGAGGATAACCAACAAGCATTTCATACATTATTGCACCCAGTGACCACCAATCACATTCCATCCCGTAGCCTTTTTTCAGAAGTACTTCAGGAGCAATGTAGTCTGGTGTCCCCACTGTTGAAAACGCCAACTTCCTCCTGTTCATCTGCCAATGTTGAAGCTGTTCACGTGGACTTCTCCAGCTGCTCCGATTGTCCGCATCACGCAAACATCCATCAACATCCATTGGCTCTGCCAAAGTTTCATCATCCATGGTCTGATTTTCATGCAGTGTAGACAAGGCAATACAATCGAGAGGCTTACAGAGACCAAAATCAGAGAGCTTCATGTGACCATTTTTGTCCAGAAGTAGGTTATCAGGTTTAATATCTCTGTGAATGTAATTGTGTTTGTGAATAGATTCTATGGCCAGAACACTTTGTGCAATATAAAATCTAGCAACATTTTCACTTAAGGTGTCTTCCCTCATCAGCAGAGTCATAACGTCACCCCCAGGCAGATACTCCATAATCAAATACAAGTACTCAGCATCCTGAAATGAGTAGTAAAGTTTCACAATGCAATGACTGGCAACTTCAGCCAGCAAGTTCCTCTCAGCTCTAACATGTTCCACCTGGCCTCTCCTAAGCATTTCAGATTTCTTCAGCTTTTTCATAGCATAAATATTTCCAGATTTCTTCTCCCGACACAGTCTAACCTCCCCAAAGGCCCCCCTTCCAATGATGGTCAAAAGTTCAAAATCATTAACACAAATCTTGTGCCTTTTCAATCGCATATATTCAGTCTCCTTCCTCTCTAAATCTTTGATGAGGTTGATCCGCTCCTCATTTGGCACATCTGAAGATGCTAATTTTCTTTCTAACACCCAACGCCTCTCCTTCCTCTCTTGGATATTCTTCATCTGAGCCCTATAATGATTCTCAATGAACTTCTTGGCTGCCGCCACCTTCTCCATGGTCAAGCTTGACCCCAATACCTCCTCCGCTACGTTCTCTTCCTCCTCGAGATCCTCCATCTCTCTCTCTGTCTCTCTGTGAGCGTTGTCTAGCACTCTGAGTGTGAGTGAGAGAGAGAATAATGTGTAACTCCAACTCCAAGTGTATCTATTCTTTATTATTATGTATATAAATCTAACCTATCTGATTCTTCTCAAGCTTCTCTCTCTACTCTCAACACACGCACTTTCTCTCTCTACAAAACACCCTGGAGCTCTTATTTATTGCAACAACGAGGAGATGATGGCAAAGATGCAT

>C100259_65

ATTTGATTGTCAAACAGTAATTTCAATGTAAAGTGTACATCAAACTTGCAGGTGCGACAATGTAAGGGTTACACACTTATTAGTAGGTCAGGCCAGAACTTATTTAGAAAATGATTGAAGGAATCTAAGAACATTGATGAAATTTTAGAAAATTTATTTCTTAAGACAACTGTACAAATCTGATACAACTAGCTATATGTATCCACAAGTAAATGGAATTTTTCAATTCTTATTCTACACATACCAGGCCCCCCTACTGTTGCTGAATTATGTTGTTGAGGTGTGATGGAAATGACAGCCAACTATCAGTGTGAACAAGTGCAAGTCATTGTGACCCCTGCACTGAATTTTCTTTATAAAATAGGTAAACTTTCAAACAATTTAGAGAAACAATCAGCCAGCTTCAATACCAAGTCCAATCATAAGCCAATTTTTATGCACTACGTGACAACCAATTTTTTTTCATGCAGACAATCTTCTGTCATCACATTGCTTCTCTTGTCATCATCCTTCTCTTTGTCAAAAGCAAACTCCAGTTGTTTCTCCACTGACTTGACAATCCCTGTTCGCTTGGATCTTAACCTATATGGGGGAGATGCATTGAATGCCTTATTATTGGGAGTATCAATCCCATTGCCATGGCTTGCTGGACTCCTGGAGAAACGCCTAGAACCAGAATCATCATTGGTTCTCTCTGTTCCATTAGCAGAACCTGGATCATTATCTACCTTCAAAACTTTACTAGGACTAGTGAGTGCTTGGACCCCATTTCTCCTCCTCCTCAATATGGAAGGAGTATTTGGAAAAGTATTAGCCGCCCTTTTCAATATTGATTCTGGTGTCTCAGTGTATAATTCACTACCCTTCGCACGAGGTGGAGTGAAGAAACCAATAGGTGACAAAGTTGGACTCGAACAGTACTCATTCTGCTGGCAGATATAATTTAAATAAAGAGAATCTATGGGAACTGAACCATCTAACTGTGGTGGCTCATAACACAGAAAACCACAGGTTGAACTTTCTTGCAAAGATGTTCCAATTAACCTCGTAGTGCTCATGTCACCATTGTTTGTACAATGCTCAAAAGTTGTGTTTCCATTGAGTCCAGAATTCTCAGATCTTGGCCTATAAGTTATTCCCCGATTAGCTTTCGACACTTGTTCTGAGTTGCTACAGCTAAGATCGATAGTGGAAGATCCAGGCTTGCACTCTACACAGTCAGAATCTGCAGATTCATTTGCAGGAACACTTGAAGAATCACCGACTTCTCTAACTGTTCCTGAGGACTCAAACTGATTCCTTCCACTGTCATCTAGCTTACTAATGGCTGTAGTTTCAGATGATGTTTCAACAGCTGCATTTAATTCTTTATTTGAATAAACAAGTATTGATTTACTAGCAGAACGTCTAATTGTATCTTTGACAGTAACTTGTGGGCTATTCTTGGGAATTGGTGGAAGTCTTCCTGTAGCTAAATAAAAATCTAACTTTTTCTTCAAAGAACTATTCCAATGATTCTTAATTGCATTATCAGTCCTTCCATGAAGGACCTTAGCAATTTCAGCCCATTTGTTCCCATGTATACGATGAGCATTCATCAGGGCTAATTCCTCCTCCAAAGTCCAGGCATCCTTCTTTATGTCAGGGTTAAGGTGATTGTGCCACCGCTCTCGACATTGTTTGCCTATTCGACCAGGTAAAGACTGAGCTATCAAAGACCATTTTGTTGGCCCATATTTTGATACCAGTTCAACAATTTTATCATCCTCCTCCTGTGTCCAAGGTCCTTTAACAAGTTCTGGATTAAGAACTTTTTGCCATCTATGCAGACACTGAACTTCTGATCTATCAGGAAAAAACTCAGCTATTTTCTTCCAACTTTTTCCCTTAAAAACAGCCACAGCATTCCTTAATGTTTCATCCTCCTGTGCTGTCCAACCACCCTTAGCGCGCCTGATAGGACCGGTGGTCCTCCGATGGGAAGGTGATGTGGATGCTGGGCTACATATCCCTGGAGACTTGTGAATTGCACTGCCACTGCCTTCAGATACAGAGGAGCAAGAAGCAGCAGTAGATTGCTTATTCTCCTGACAACACTCCTCCGATTTCACCACCTCAGTCATATCCTTGAAGAGCCCAAAATCCTAAGGCTGTGGAGAAGTGGCGGGAATCAAAGTGAAGCTCATAGAAGAGAGACCACGGTGCTTCTCTCTCTTTCTCTCTCCTCTCTGAGCTAAGGTTTCGAACGTTGAAACAATGGAAAATTGAAATTTCG

>C100261_65

TTAACCAAATGAATCCTTTATTGAGCCGAAACTAACTAACGTGAATAAGCCTTGCTGCCTATAACAACCCGGGTAACCTTGTGTTCACAAGGCATGAATTAGGAAGCATTTTTACACAATAAAAACGCGCCAAACGAAAACGGGCAAAAAAAATATCAAAATTTTGGCGTCGTTAATTTCGCGCAGCCAAAAATAAATATCTGACTGTTTTTAACCACTAAAGCAACGAACTTGAGGAAATGAGGAAAAAATCCGCTACAGAACACTTTTCATTAGAAAACACTTTTCATTAGAAACTTCCATCCTAAGATGCTACTTAAAACTTGAGAACTACGTAGGCAAAATCTTGCCAGCCACTCACTCACTATGCAGTTTTCACTTTTCTAGAAGGAGGCCGCCGAAATAGACTGATTAGATCATCAAGACCCCTAGTGGTGATGACCAAGAAACTGAAGAGCACAATTAAATACGATCCCAATACCAGAAGCAACAGCAAGTCAAATGTGACACTGGCAACCTGATAAATGTTCAGCTTTGCCCTGGTTGAATCGTAGAAAGTGAATATTCCATCTAGCACATCTCTATGCAGATTTACCTCATCAGTGTGATCTTCTAATTCCTTTTTCAGTGCCATGACAAAAGGATCATCTTTTGAGAAAAACGGAGCTACTCGAGGTGTTTGGGACAAAACGTCTAACCATGATCGAATATAAGAAGGATTAACAGCCAAGTTAGTGTTGTCTGCAAATATCTGAACATTCTTTCCTCGATGTCCATAAATATGCCTCGCAAGACTCTCAGCAATTAATTTCACACTTCTGACGATAGCAGCTTCATTGACAAAATGCCTGCCATCAACCAAACCACCAGTCTTTTCTAGCAATTCAGGTGCTGTAGAAAGTTCAGAGAGGGTGGCAGCAGTAACTCTCAGCCTTGAAAACTGTTCATGCTCCCAAGCTACTCGAGGATTGGAAATGTTTATTTTCTTATGCTTCAGATTCACTCTAAAGCCCAGTTCTTCAGCAACACTTGAAAAATCTTCCACAATTTTCTTAATGAATGCATTTTCTGGAGGCTTAGACACATGAATCCACAATTCATTTTCCCAAGAGCCAATACTATCTAAGCAAATAGCATAGTCAATGCTCTCACGTAGACGTTGATCAAAGCTGCGAAGCCACTTACGAGTTCCATTGTAGTTGTAAGGCCCACCAGATGTTAGCCCAAACAGCAGATTATATTGTCCTCTAGTCTTTGGATTAGAATACAAGAGAGAAAACAGCCTAGCTACTTCAAGAAGAGCCACAATGCCACTTCCATTACTATCACTTCCCACTGATAGTGCAGGAGCAGCTCCAAAGGTGTCGTATGATGCTACTATTGCAATGGTGGGTAATTGATGAGCATCATCATCTGTCTTCAGTCCTGCCAACCATCCCTGAATATTTGTAATGGGAGGAGATACAACTTTTCTTGGTTCGGCGGCTGAAACAACAAATTTGTATCCGCCAGTTGTTGCAGTTGCAGGCTGACCAGTGACATCATTCTTCTTGATATCAGCTAACACAGCATCAATATTGTCATCCTCAAATGCAAAATAAACAGGATAAGGTAAGTTGGCGTGTATAAGTATTTGTTCAAGTTCAGCCAGTACATTCTTCAGCGGTTCTTTATGCCCATCTTGATTGTTACTTCCCATACCATCTTTGTTTTCGAAACTGAATATCCGGGGAAGCAAAAATATTAAACCCCCAAGAGGCTTGCTTTCAGCAATATATTCTTTGACAAAAGACATGTTAAGTTCGCGAAGAGGTATGAGGAGGACGGTGCGAGAGAGATCAGCGTGGGGAGAGAAGTGAAGCGAGGCGGCGTGGTGGTTGAGGCTAGCGAGTCGAGATCCGAAAGGAACGCCGGACATGTCGTACTGGATGAGGCGGTAGACGTCGACGACGGTGGCGCCGTAACAGAGGTCGACGCAGGCGACGAGGATGAAGACGAGCGCGATGACGGAGTCGAAAAGCATTTCACGCTCGCGAGGTTTTCGAGGCGCCATAGCCATGGAGCGAGGCGAGATCAGATCTGCTAGCTAGGGTTTCCGAATTCGCGATCGACAAGTGGATAGAAGACGACGATGCCGATCTACACTTAAATATGTCTGTTCCTATCAATCAATATAATATTATTAATTATAATTATTTTTCATTTCAGCAGAAGGCAAGTACACTACCAAACGATCCAAGAGAAAGCCACTGCTGATTCAAGAGATGAAGATGGGAG

>C100263_65

CCCCCGCCGCCGCCGGCAACCGAACCAACCCCTCCCTCTCCACAAACCCTACCCCCGCCGTCTCAGGAGGAGATTCTCGCGAAGCGGCGGAACAAAGATGAAATTCGAAGCGTCTACGAAGGTTACAAGCGGATTCGGCGTTGCTTGCTTCAGAAAGACGCTCCATCCTCAATGGCAGACCTTGAACAGAGTTATCTCGCACTCATCACTTCTTCCAGAGGTTGTATGAGTGTACAAAGAATTGTGGCCGATCTTATTCCTCGGTATGCATGTCACTGTCCAACAGCCTTGGAAGCTGCAGCTAAAGTTGTTATTAATATGCACAACTTTAGTTTGGCATTGATCAGTAGAGGAGAGGATTCCAGTGGTATTGCATTTGAAACTGCTAGAGCCTGTATTTGTGGCCTAGCTGATGTCTGCTGCGTTGCTTCATCGGTGGCCCCAACATCAGCTGTAATTAGAGGAATTTGCTTAGCAGTTTTTCAGAATGTGCTCACCTTTTTTATAGCCTTATTTGAAGGAAAGGATGTCTTACAGATGGTTGATAAGAATTTCTTGAATATGCAAGATACCCCTGAGGTCTTTTCTGAGTTGAAACAAAAGGTTTTAGAGGAGGATGAATCTCCATTGACTAAACTGTCCAAGTTCCGAGTATTATGTCTACTTCGGATTTTTTTCTCATGTCCAAAAGATTTGCTTGCTGCTTGTTTGGATCTGTTAGGTTCTACCACAAAAGAGGGAACTAATAATGAAGGACAGCATTTTTTGAGCCAGGTGACTAGCATGTTTGATGATGATAAAACAGTTCACCTTTTAGATAGAGCAATTGGTGGACCTAAATCATGTACAGACTCTACTGGGTCAGGAATCAGAGACAATGAGGCTGGTGAGGAATTTATGACTGAAGACTACCATGTTTCTGGTGGGGATTCATCTGTTGGCAAGAGCTGCTTGCTTATACGGGTTCTTAACAAGGATCCCCCACTACGGAAATGGATGTTGCGTAGATGTAAGAAATTACTTGACTTGCTCCCAGATGCATCACTGGATATTATGTCAGTACTGCAAGGAATTCTTGAAATGTTTGCTCAACAAACAGATTTGGAAGACTGTCAAGCAGATAGTGATGAAGATAAATCCGATTCTTCAATTTACATGAATAGAAAGTACATGGTTCCTCGAATCTCTGAGGAACATGAAAGCATTGGTGAATCATCTGGAAAAGGGAGCAGTTTGAGAGTTCATGTTGGTTCTACCGATGGTTTTACTGATAAGGTTTCTGATAAATATGTGATGGCTCATAGCTCTGCAGTTTCTCTTGACAGTGTTCCAGCATTAAAAGTGGGTTTGCATTATGATAATGGAGTCTCAAAGCCCATGACCATTGGGGTGGGGGAAGAAGGGTATACACCAAATGTTATATGTTCCACACCTAGGGACTCTGTAAGCCATCAAATATTCTCACCTGCAGTTAGAACACCAGGTAATTTTAGGAGTAATTCATTTGAAGCTAGAAGTGATTTCCTGAATGTAGAGAAAAATCAAGTCTCAAGTATGAATTTCAGTTCACCTCCGTTGAGATCCTCTAGTGGATCTGTTAGTAATTCTTTGGCATCTCCTAATCATCATTTTATGTCACCCACTGCTTCAACAAAAAGTCAAATTGTCTGGTGCTGTGATGGGGATCCTGCTGCCATGGATATTGTCTCTGCTTCTAGGCTGCTATGGATAGGTTATGTGGGACCTGATGTGCCTGAAAGTCATATTAGGTTCCACTTAGAAAGATTTGGTCCTATTGAAAAATTTATTTTCTTCCCAGTAAAAGGATTTGCCATGGTCGAGTACAGAAGGATCATTGATGCAATAAAAACTCGACACTGTTTACCTGGATGTTTTCCTTGCCGTGTAAAATTCATGGATATAGGACTTGGAACTAGGGGTGTAGTGAATGGTATTGCAGTTGGCTCTAGTTCACATATTTATGTTGGAAATATTCCCAGTCAATGGGCCAAGGATGAGATCATGCATGAAACAAGAAAAGTGATCCACAAGGGTCCTCTCACATTCATTGATCTTAGCTGTGAGTTTGCATTACTCATGGAATTTGAAACGCCTGAAGAAGCTACAGCTGTCATGTTGCATCTGAGACAACTCCGAAGAGAAAGAAGTAACTACAACCAGCATTTTGGTCCAGGAACAGTTAATGTTGGAATTGGGCATGCTTATATGGATGGTGCAAGACCTATACCTGCCCCTCCCCCACCCCCTCCTCCACCC

>C100265_65

ATCATTCGGGATCATTCGGGATAAGGATTTAGTCATCACAAAATAAAACTGAAGGTACTGTACTTCATCATGTAGATCTGTAAATTTATAAATTGCATCACCCAAACAGTACAAACCCAGATTAAACTAAAATGCCACATCCGTCTCCGTATTGTGGTTCATATTTTTAACTTTAATTTTAATAAACTAAAAACTTCAATATTGTTTTGTAGCAAATTGCAACAAGGCATTAGATTAACTCCAAAAACAAAGCTTCTTGCTTGTTGGTCAGGCCAAGTTCAGCCAGGGTCGCAGCACTTTCTCCATCACTAAAAGCACGCCTAGGATATGGTCTCACCAGTCTGTAACTGCCTGGTTTCACCACCCTACCGATATCTATGAAGTCGAACAGAGACTGTAGCCTATTAGATCTTAGGAATCGGCGTCCACGACGTTTGCCATCTGGCATTTTTACCATCAAGTTAACAGCATTATCATCATCTGAGGATGGTTCTGGTGGTAGTGAGACTTCTTTGGCTGCTAACTGCGTTTCCAATTCCTGCTCTTCCTGTAATTTCCTGCGAGATTCTTCTGCTCTCCGCCTTTCCTCTTCACGAGCAGCCTCAGCTTCTTCCATAGCCTTCAATTCTTTTTCTTTGTCTGCTTGTAATGATGCAAGATATTCATCATCCTGCTGTTCCCTTATCAGGCGCTGTGCTGCCAGTGATGGTGAAGGTGGGCGATATGGAGCCCGAGGATTGGAACCCCTATTTTGCATAAACTCATGTGGTGCATAAGTATAGCGATATCCAGTCCCTTCCGGTATTCCACCAAACATTGCAGCTTCAAGCATTACTGCTTCATCATGCTCCTCCGAAGATATGCCACCCCACTCATCCGAGGGGAAGGAATTTCTACTATGATGAGGATTACTGCTCTCCTGTGGTGCTGTGCTTGGTAATGAGCTAGCCTCAGCAAATTCAACATCTTTGACTGATCCAGTGGATCCTGAAGACATATGTCTAGATATGTTTCTGACAATTGGCTGCTCCTCTACACCTTCAGCTTCATCATGAAAAGACAAGCTTCCTTCCTGCAATCTTCCATTAGATGTCACTTCCTCTAGTCCAGCCTCTGATGACTTAGATGGCCCTGCTGGTGGTGATTCAGAATCTACTCCTTGTACACGCCACGCTTTTTCTTGCTCAGCTGTCTTCAGGGACAGTGAAACTGCATGGGCAAGCTCAGGATCCTCTAAATAAGATTGCCTTGACTGTGATCCTGATTCACTTGAGTCAATTTGTCTGCCAAGTTTGTGATTTCTGTAATTTTCCTCAGCCTCCCGTTTAGAAGCCTCAATGGCTGCACGAAGCATTTCTTCTTCTATCTCATTGCTATAATCTGGTAAATTCTCAGATTCAGGAGCACTAGGTCTAGCACTGGTATTGAGTGAAGTATCAGCCAAGATCTTATGGTTTTGCTCATCTTGATGTGCAATCTGGGCAGGTGGAGCATTGTCATCATCTTCATCATCAATTATGACAGTCCCATGGATATCTGGACCATGGGCATCAACAGTACCAGTGACGTCTTCAATGGATGGAACATGACCAGATTGAGTAGTGGATTGGTTGCCATCCTTAACCTCTATAGGAATCTCCCTTACCTCTCTAGGATGAGTAACAAATGGTGCCTGGACTGTAGAATCAAGAGGAGAACCAAACATACCTCTCCCAATTGTTGGATCCAGAAGAGAAAACGGATTGATATTTGCTGCTGAAGTTGGAAGTGATGGAATTCTGAGAATATCTGCATCATGTTGATCATCTATATCCATGAAATCATCTTGAGGAAATGCAGTAGAGCTGTTGTGAGGGCCAGTCATTAGATTTCTGTCTCCTTCACTAAAATGTGCATTGACTGCTTCATTGAGATTACCACCATGCTCCTCTAATTTTTGCAGCGCGACTGCTTCTGGCAAGCCAGTGATGCTGACGAAAGTCTCCACCGCTTCTTGATTAGGCCTGGCCATTGCAATCGAAATTGTATGGAACAGAAAACTGCAAAATTCAGAACTTTTTTTTCTTTGTTTTCTTTAAATAAAAAAATAAAAAGAAAAAGGAAAAAAGAATTTGGCTGTTGTGGGTCTCTCTAGGGAGGAAGAGAGAAGGGGTGGGTGGGCCTTTGGAGTGATAGTTTCTTCTGGACGGTGTTTGGAATTGCTTGGTTGTTTGGCTATTTGTATCTCAATGCAAACTCTTCTTGTTAGACAAGTATGCTCTCAAAATGCCTCCACGC

>C100267_65

TACCATTAGCTGTGACAAGGTACAACAGAATTTTTTTTATAATACACAGTAGTGCTACAAATCATGACTTCTTCTATCATGAATTAATTATAGAGGTAACTGTGATACACGAGTTATCGTATAAAAATGAATGAAGAAAGAAACTATGTTTAAATAGGCAAATGGCCTCTCCTATAAAAGAGAAAAAGTAACCCTCAAATTTGAATAGGCAATGCCCTTGTCTACAATGTCTATAATGCATGACACTATGTTTCAAGGATGAACCTGAAGATTATCAGACAAACGAATCAGAAAAATCAAATCACAAATAGAATCATGGTTTGGTTTCTATGACTTTTTGCATTCATCTACAGCATAAAAATCTACTTCCTAATTTGCTCTGTCTGTCTGATCCATGCATGTATCTAGTAAGAAAAAGGGCATGTCTATAAGCCAGAAACAGCATTAGAGTCATCAACCTTACTAGCATAACCATTACCATAGTCATGAGTACTCAATTGGCCGTTAAATTCCATGCCATATATGTGACCCATTTTCACACGTTTGGTCTCTAAGTATCTCTTGTTCTCCGAAGTAATGAGTGTTAATAATGGGATCCTACCCGAGACTGACAAACCATAACCTTTGAGCCCAACATATTTTGCTGGATTGTTGGTCATCAGCTTCATAGATCTAACACCCAAGTCCCTTAAGATCTGAGCGCCAATGCCGTACTCCCTGGAGTCAACAGGCAATCCCAACTCCTCATTGGCTTCTACGGTATCTCGTCCATCATCCTGTAGGTTATAAGCACGAAGCTTGTGGCCCAATCCAATACCCCTTCCTTCGTGTCCACGGAGATATACCAGCACACCCCTACCAGCAGCCTCAATCTGTTGCATTGAGAGTGCAAGCTGATTTCCGCAGTCACATCTGGCAGATCCAAATATGTCTCCGGTGAGACACTCAGAGTGTACCCTCACAAGGACATCTTGTCCATCTCCAATCTCACCCTTAACCATTGCAATATGCTCAATTCCATCTAAAATGGACCTGTAACAGTAAGCTGTGAATGGTCCCCACATTGTGGGTATCCGTGCAGCAGAAGCGCGATCCACCAGCTTCTCTCTTTTTCTTCTATATCTTATTAAGTCAGCAATAGATATAATTTTCAAATTTTCACGCTCTGCAAACTGGCGAAGTTTTGGCAGTCTAGCCATGGAACCATCGTCATCTACAATCTCACACAGAACTGCCACTGGAGCCATGCCAGCAAGTACAGCAAGATCAACTGAAGCTTCTGTATGTCCAGCTCTCTTCAAGACACCACCTTCCCTGTATTTTAGTGGGAAAATATGGCCTGGGCGGTTGAAATCACCTGGAGTAGAATCTCTTGATGCAAGGGTCAAGACTGTTGTTGCCCTATCAGAAGCGGACACACCCGTGGTGGTACCATGTTTAGCATCCACTGTCACAGTGAATGCCGTACAAAGCTTCTCATCATTATCCTTACTGTTTACCATCAAAGGAAGTTCCAATCTATCCAAATCTTCCTCTTTCATGCTTACACAAACTATCCCAGTTCCATGCTTTACAATAAAAGCCATTGCCTCGGGAGTTGCCAATTCTGCTGCCATTATCAAGTCTCCTTCATTTTCTCGATCTTCGTCGTCCACAACCACTACCATCTTTCCCTGGCGAATGTCTTCAATGGCCTCAGGGATGGAAGCAAAACCTGCTGTTGGGCTGTCCAAATCAAATTCATCACCATCAACAGAGAAAACATTGTTTGTTATCCCTGTGTCTGCTGCTGGTGTTCCATTAGATTGTACATCAATTCTAACAGATTCATCTCCAACCAGATTTTTACCTATCACAATGTCATAGCTATTTGGATAGGAAAGTAGGTCTCCTCCTCCAGAGATCAACGTAGCTTTAATTCTACTAACAGCATTATCACTTGAGGGGAATTTAGAATTCATTCGGACAAAAGGAACATCATTGAACAATTTGCAGTGTTTGCTTCCCCGGGGACATGAGAGGGCTGGTGAGGAAGAATAAGAGAGATTGAAAGAAGCCATTGTTTGTGAAGAGGAATGAATGATTTTCAATTCAACTAAGAATAAGACGGGGTCTGTAATTGAATGTATATATATTGCGTAGAAGAAGAGAAGAAAGAGAAAGAAATGCAGCGTGGACTTGGAGTTGGAACTGCAGACACCAACGAACCAAAGAACCAACCTCGAAGCTCTCTGAGAAAACCCCCTTATGGCCCTTCCTCCCACCAATCTTTCTCCTA

>C100271_65

TTTTTTTTCAGAAAATAAAAATATTAACAGTATGCGTTGTATGTATCTGAGGCCACCAACCAACAACAGACAGGGGAAGAGCATGTTTTGGTAACATTCTCTCCTTTCGATGTGTTTCATGATAAAATAAAACATCAGGGACTAGTATACATTGCGTTTCCCGTACACAAATGAAATAAATTGTTACAATAGTAATACAAACAAAATGGCACTTCATGAGATGACTAACGAACTGAGTCCTAAAGTAAGCTTCTAGCACTAACAATAGTGCTTCAAACCACCATTACCACCTCTTTCTCTTTGTGGAAAGAATCAAAGCATGGAAGGAAGGAAAGAAGCAACACCAAAATCGTAATTAAGCATGGAGGGCAGCCACTTCATCTGTCACTCCATCCACCGTCTCATCATTTATCTCCCTAATTGCCTCTTGCTCGGGATCTTTATTTTGTTTCACAACTACACACTTGGAAGCACTAATACTGGCACTCTTTAACAAGCTACGAGCACTGTAGTTTGGTTTGGAGGTTCCATTTTGAGCACCTGTCGCATCAGAGCCTGGAGGACTCCGTAGATTTGATCCACTAGGCTTTTCAGAATCGTTGTTTTGTTTACTTTCATCCTTGGGAGCACAAACTCTCTCTCTAGGTAAGGAGGCGTGCTGTCTCTGCAATGGAGTGCTTCGTTCACCTTTCCCATAATGCTCCTCAAGATGTGCAAACTGTCTCTTGAACCGATCAACCCCACTTGGATACATGAAGCTAGTTTGATCTCCACCACGAAGATATTCCTGGAGCATTTGAGGATGATACTCCAAAATCTCTCGATAAATCAACTCTCTAACATCATCTTTGGTCAATTTCCTTCTCTCAAATTCAAACTCAAGTTTTGAAATGGGTTGAGTGGACGGTTCACGGTCCATGTTCGCCAAACCAATGAAGTAAGGATCAGCTAGTGCCTCTTCAGCTGATGGACGGTCTTTAGGATCAAATGCAAGTAGGCGCTCCAGTAGACAAAGAGCCAATGGATCCGCATTTGGAAATTTTTGGGAAAGTGGAACTGGTTGCTTTTTTCTCATGCTATTGAGATACCTTTTAGCCTTTTCATTTCGAATCCTTGCAATGGACTCGGGAGGAGGAGTGCCAAGCAAATCAGTCATGAGATCCAATTGGTGCACAACATTTTTCCCAGGAAACAATGGCTTCCCAGTGAGCATTTCAGCAAATATGCATCCAATGCTCCATATATCAATTGCAGGAGTGTATTTTGAGAAAAAAGAACCACATAATTCTGGTGCACGGTACCACCGAGTTGCAACATAATCAGTCCAGAATATAGCTGATGGAGCATCATTGAATGAAACTCGAGCAAGCCCAAAATCGCATATTTTTAGTTTGCAGTCAGCGTTAGCAAGAATATTTTTTGGCTTCAAATCACGATGAAACACGTTTGCTGTATGGATATATTTCAGACCCCTAAGAAGTTGGTACAAGAAAAATTGGTGGTGCTCAGGAGTAAGATCATCGTTGGCCTTGATTACTTGATGGAGATCAGATTCCATCAACTCAAACACAACATAGATATCCTTGAACTCTCTCCGAGAAGGAGGAAGCATAATATGCTTAATTTCCACAATATCAGGATGTCTAAGCAACCGCAGGAGCTTAATTTCTCTAAGGATCCGTGTGGCATCGGATACATGCTCAAAAACATCATTAATTTTCTTGATTGCAACCCTTTCACCTGTGTGAGCATCAACGGCAGAACCCACAACACCATAACTTCCTTTTCCAACAACCTCTTGGATCTGGTATTGACTTGCCTCTCCATACTCAGTAAAAAACTCTGACTCTGGGGCACCCTTTTTGTGATGATCCATGGAAGAGGGTCTGAAGGGGATTTGTGCGGGAACCTTGATGGGCTTCAAGGTAGAGAAATCGAAATCAGGGTCAACAATGAGGTCGTGGCTAGGAATGGAAGCCAATTGCGAGCGCAAATCTCTCACGTAGACGCGGCTATTGTTGGCATTGTTGTTGTTTTCAGCAGAAGTAGAAGCGCGTCTTTGAAACAAGCGTCGAATCCCTTCCACGAGCGTTCCTCCACCTCCCATGTATATCAATTCATAGATTATTTCCACTTCACTCTTTCTTCGATCGCCTTTGACTCAATCCAACACACACCACACACACACACACACTCTATATGCGTTTCCTTCTTCTCTTTCTCTCTGCTACCTCAACTCATCACAACCAAACCAAACCAAACCCCATTTCATTTATTT

>C100273_65

TTTTTTTTTTTGGCCAATAAATTGTTATCTTTATAAAGAAATTTTTAAAACAGTTTCCATCCAGATGAAGTCCATACACAAAACTATAATAATTTTGGAGAAACCAAAATGTTGGGCCAAAATTCTGAACAAAATAAAATAAAGAAAAATACCAAATAATCCTGAAATATATAGATTCAGGATCATGAAACAATTTAATCGACTTCTTCAATCTTGGGACCAGCACCACCAGATCCAGATGGAGGAGCATCTTCATCCATGTCTCCACCCACGTCAGGAGCACCACCAGCACCCTGATACATCTTGGCAATAATTGGGTTGCAAATGCTCTCCAATTCCTTCATCTTGTCTTCAAACTCATCTGCTTCAGCAAGCTGGTTACTGTCCAACCACTGAATAGCACTCTCAATGGCATCTTCAATCTTCTTCTTATCGTCCCCAGAAAGCTTAGATGCAATCTTCTCATCCTTAATAGTGTTCCTCATGTTGTAGGCATAATTTTCCAAAGCATTCTTTGCATCAACCTTCTTCTTGTGCTCTTCATCCTCTGCCTTGTACTTCTCTGCCTCCTGAACCATCTTCTCAATCTCCTCCTTTGAAAGTCTACCCTTATCATTGGTAATAGTAATCTTGTTCTTCTGCCCAGTGGTCTTGTCCTCAGCTGAGACATTCAAGATACCATTGGCATCTATATCAAAGCAAACAGTGATCTGAGGAACACCCCTAGGTGCTGGGGGAATGCCAGAAAGTTCAAATTTACCCAACAAATTATTGTCCCTGGTTCTAGCTCTCTCTCCCTCATACACCTGAATCAAGACACCAGGTTGGTTGTCTGAGTAAGTAGAGAATACTTGCTCCTTCTTGGTGGGAATAGTAGTATTCCTTGGAATGAGAACAGTCATGACACCACCGGCAGTTTCCAAACCAAGAGAAAGGGGTGTGACATCCAACAGCAACAGATCCTGAACTTTCTCATTACCCTCACCACTCAATATTGCAGCCTGAACAGCAGCTCCATAGGCAACAGCCTCATCTGGATTGATACTCTTACAAAGTTCCTTGCCATTAAAGAAATCTTGCAACAACTGTTGCACTTTGGGAATTCTGGTGGATCCACCAACTAAAACAACATCATGAACGGTGCTCTTGTCCATCTTGGCATCCCTCAAACACTTCTCAACAGGTTCCATACACTTCCTGAACAGATCCATGTTGAGTTCCTCAAACCTGGCACGGGTGATTGTGGTGTAGAAGTCAACACCTTCGTATAGAGAATCTATCTCAATTGTAGTCTGTGCAGTAGATGAGAGGGTCCTCTTCGCCCTCTCACAGGCAGTTCTCAACCTCCTAAGGGCCCTAGGGTTCCCACTGATGTCCTTCTTGTGCTTTCTCTTAAACTCCTGAACAAAATGGTTAACCATTCTGTTGTCAAAATCTTCACCTCCGAGATGGGTATCTCCTGCAGTGGCCTTAACCTCAAAAATACCCTCCTCAATAGTGAGAAGAGAGACATCAAAGGTTCCACCACCCAAATCAAAGATCAAGACGTTCTTCTCACCCACGCTAGTGGCCTTCTTGTCAAGACCATAAGCAATGGCAGCGGCGGTGGGTTCATTAATGATACGCATGACATTAAGGCCAGCAATGACTCCAGCATCCTTTGTAGCCTGACGCTGGGAGTCGTTGAAGTATGCAGGAACTGTGACCACAGCATTCTTCACAGCTGATCCGAGATAAGCCTCAGCAATTTCACGCATCTTCATGAGGACCATGGAGGAAATCTCCTCGGCTGCGAACTGCTTCTCCTCACCCTTGTAGTTTACAACAATCATTGGCTTGTCAGCAGGACCAGCAACAACTTTAAATGGCCACAGCTTCACATCACTCTGGACGGTTGAATCGGAAAACCTCCTTCCAATTAAACGCTTGGCATCGAAGACAGTGTTGATAGGGTTCATGGCGACCTGATTCTTGGCAGCGTCACCGATCAAACGTTCGGAATCGGTGAAACCGACGTAAGACGGCGTCGTCCTGTTACCCTGGTCATTGGCGATGATTTCTACACGGTCGTGCTGCCACACTCCGACGCAGGAGTAGGTGGTGCCGAGATCGATTCCGATGGCAGGACCATCTCCTTTTCCGGCCATTTCTAGATCCGCGGATGAGATAACAGAATATTTAGGGAGGATGCTTTTGGCTTGTAAAGCGCCGAAACAGAGAAAGAAAAAGAGAAGAAATGAAAAGACTCCAAAGAATGGTTACCCCATCTCCTTTTCCG

>C100277_65

AAAAAAGAAAAGGAAAAAACGAAAGAAGAACAAATAGCTTTGAGAGCATTAACATTTCAGATCTGAGACGGAACATCTTTCTCCGGTTTTATAAATCTTATAGCCATTCAAAGTTTTATTTTTCTCCATATTCATCATACACCACGCCAAATCTCAATACCCTTCAGGTAATTCTGCTTTTTTTGTTTTTATGCTGAATTGTTTGTTCCCGTAAACATTTTTCATTGGGGTTGCGACCCACTAAGTTGGAGCTCAACACAAAGTTAGATTGTTTGTGATAATCATCCACCATATGTAATTAGGAACGCCCATGTTATGAATTTCAGAAGTTTGGGGGTTTTAGTGGGATATTTTCTAGGGTTTTTCATGCAAAATTTGGGGTTTTGGAAATTTAGGCTTGGTTGAGATCCCAGTGAAACCTTTTTGTGGAAAAAGTTTTTATCTTGAACCATATGAGATATTAACCGTGGAAAGAGGGAGGAAGAAAGATGACGGGTGAGAAGTTTTGTGTGAACGAAGAAGAGAAGGGTGTATTGGAATCTGTGTTGGGAGCAGAATCTGTTGCGTTTTTCATTTCAGCACTTTCCAATAATGTCTTTTCCAGCGTGGTTACTCCGTCGGCCGGTGCTGACTCGGCCCTCCGCCAGCGCTTGTGCCAGCTTGTTGAGGGTTCCAAGTGGAACTATGCTGTGTTTTGGCAGGTTACTGGGTTGAAATCTGGTGGTTCTGCCTTGATTTGGGGTGATGGGCATTGCAATGATCCCAAAGGGAAGAGGAGTGGAATTGGGAAGGAGGATGAGCAGGAGGTGAGAAAGATGGTGTTGCAGAAGCTTGATGCCTGTTTTGGTGGGTCTGTGTCGAAAGAGGCTAATTATGCGAGATTGGATAGGGTGTCTGATTTGCTCTTGTTTTACTTATCCTCGATGTGTTATATATTCGGTTTTGATTCGCCATGTGGCCCTGGGAGTTCATTCAAATCTGGTAAATTGATTTGGACTTCTGATGCTGCTGGTTGTTTAAATCAATTAGAATCTAGATCATTTCTAGGGAAAGTGGCTGGTCTTCAAACTGTTGTTTTTATTCCTCTGAAGTCTGGGGTGGTGGAGCTTGGTTCGAATGAAGTGGTGTCTGAAGAGCAGGGTTTTGTGGAGATGGTCAGGACAGCATTTGGGGAATCCAGTCCCGGACAGGCCAAGGTGTTTCCGAAGATTTTTGGGCACGAGTTAAGCCTGGGGGATACAAAATCTCAGTCTATAACTATTAGTTTCTCCCCAAAGGTGGAAGATGATCCTGGTTTTACTTCAGACTCCTTTGAAGTTCAGGCACTAGGGGTGAACCATGCTTATGGAAATTCTTCCAATGGGACTTTAGGGGAAAGTAATGAAGGGAAAATGTTCCCTCAATTGAATCAAATGATGGGTGGGAATTTTAATTCTCAAGCAAGGGTTCCTTGTCTTGATCTTGGTAATGAAGACTCGTCTTCAATTCATGCAGATGAGCGGAAACCCAGAAAAAGAGGTAGAAAACCCGCCAATGGGAGAGAGGAACCACTGAATCATGTTGAAGCAGAGAGACAAAGACGTGAGAAGCTTAACCAGAGGTTTTATGCCTTAAGAGCTGTTGTCCCCAATATATCTAAAATGGACAAGGCATCTTTGCTTGGTGATGCCATTACCTTCATCACTGATCTCCAGATGAAGATCAAAGTGTTGGAAGCTGAGAAGAACATGATTAACAACAAGGACCAGAGGTTATCCTTGCCAGATATGGATTTTCAGGAAAGAGAGGATGACACAGTTGTGACAGTGAGATGCCCCTTAGATACTCACCCTGTTTCTGATGTTGTTAAAACTTTCAGGGAGCATCAAATTGTGGCTCAAGACTCAAGTGTTTCAACTACAGATGATAAAATCATTCATACATTCTCCATAAGAACTGAGGGAGGGGAGACTGCTGCCATACAGTTGAAAGAAAAGCTTGAAGCGTCCCTATCCAAAAATTGACCTTGAGATCATATTTATGTAGCATCTAGTGATGCTATAAATCTTCTTAGAATTATTGTCTGTTTGTCTGGTAGGTTGACATATGATTATTCTTAGTGTACTTTGTTACTTGTAATCTGGTTTAAAATATGTTCAGGGCATCATCAATGACCTTAATATGTGCCTAAGTTGATTAGTAGTGGTTGAATTTCATGTAGATGGATAATGTAAGTTGAATTATACTCTACCATCAATCTAAGTAAGAAAAATGTCATGAAGTTGGAGCTTCCTAAAAAA

>C100279_65

TGGGAGTAGAACACAGTGGATTGTAGTAGGCATGGAAGAGGAAGCGGAAGAAACGAAAAACGCAGTGAGAATATTCGTCGGAGGATTGGCGGAAGCTGTGAGCGCCGAAGACCTTCGAAGCTTATTCTCCTCTCTGGGCACCGTCCAAGCCGTCCAAACCATCCGAACCAAAAGTCGCAGCTTCGCCTACCTCGACTTCCTCTCCGATCCTAAATCCCTTTCCAAGCTTTTCAGCAAGTATAATGGGTGTTTGTGGAAGGGTGGAAGGCTGAGACTGGAGAAGGCCAAAGAAGACTATCTCGCGCGGTTGAAGAGAGAGTGGGAGCAAGACGCTCTTGATGATGCTACTCAACCGTCCCCCACTTCTCCTAAGGAAATGTCCAACAACCCACACTCTTCCAAGTCAAATAGCAAACACCTCAACATTTTCTTTCCTAGATTGAGAAAGGTGAAATCCATACCATTCAGTGGAACTGGCAAGCACAAATATAGTTTTCAGAATATTAAAGTTCCTCCACTCCCTGTGCATTTTTGTGATTGTGAGGAGCATTGCAGTCCTTTTGTCACTGAAAGAGAGAAGCTATCTATTGATGGGGGAGCAGCCGAAAGTGGTGGAATGAATGATGAAGAGATTAGCATAATGAATTCTGTGATGAACAAGCTACTTCAAAAAGAAAAGGTTTCTAGTACTGAAAACCTTGGAAAGGAGAAGGATTCTTATGAATCACCTAATACTTTACAATCTGATGAATTTGAAGATAGTGCAACAGATGAAGATGATCTCATTATTAACATGGAGACGAAGAAAAATAAATCAGCTTTAATAGGGAACTGGGAACTTGAAAGAATCCTGGAATATCAGGAATCATGGTTTAATAAAACAAAAATTGGTAAGGAAGAACCCAACAAGAGTATGCCTCAAGTGGAAAAAAGACATAATAGCAGTCCCAACAAGAACAAGAAGAGGAAATCACTTCCCAAATTGGAAACTGAAAGCAACGCGGAAGTATCATCAAACCCTGGAGGCAAGGGTAACATGCAGACCCTTCTAAATAAGGTGGGATCTGGTGCACAGCCTACTGAGCCAGAAGATGGTTTTGAGGAATTGACTAAAGTTTCATGGTCTCAGAAGTCTTCATGGAAAGAACTTGTTGGTGGTGGAGGCAATACTTCTTTCAGTGCTTCTCTCATATTGCCGAAGTTGGACTCTAGTAAGAATCAACAAAAATCTGATGATCTGTGTGCAGATGTATCTACAAACAACAAAACTGAAAACATGGAAAGGGATGGACACTTAGGGGGCAACCCTACCAATACAGAAGTGATAAAAGAGCCCCCTGAAGCTCAACCTACCGATGAACAAGAGATAGATGTTACAGTAAATCAGCATAATGTGGCACCTCATAAGACTGGCAGAGGAGCTTCATGGCTACAAAAACAATCCTGGACACAAATGGTTAGTGAGAATAACAATTCATTTAGCATTTCACATATTTTGCCAGGCATTACTTTCCCAGATCCAAAGGCTAAGGAGCCCATTGTGGAACCTGCCATTTCCAACTATTGTAAGCATAATGGTGTAGCTAAGGATACTGTTAATGAAGTTGTGAGTGATGGATTTAGATCAAGTCTCATACTAGAAAAGAGTCAGCATATCGGTGCCAATGATATTGCTTTTGCTTCAGTAGTTGAGGAAAAAGTTGAAACAAGTCCCAGGGAAAAATTCACTGAAAATATTGAAATAGGCGAAACATGCTCATTTATGAGAAGTGCTGCATCTTTGAAGGAATGGGCAAAAGCTAAGGCTGCTATTAGTGGATCACTCAAAAGAAAACGGGGTGAGAAGTAAGGTCACAAGGTTTCCATAGGGAAGAAATATATGCCGATTGAGCAACATCATCTTTCACGTGGTTTTGTGGTTTATTCACTGCCAGTTATTGTTCTTTTAGTGCAAGCAGATGAAAGTGAAAGGGAACGAAGGATATTGATTTTCACACACCAGGATAAAAGCAAAATGACATTTGCAATTTGATCACCATTTTCGATACTTTTCTTCACTTATATCAAATCTGGCTTAGACCGGTCTGATTGGGAACCAGTGATATAACTATTCCAAATTGGTTGTTGCATCTAACATGTTTTGTAGCCGTTATGATCCCAACATAACCTGGTCAGTTTTCTTAGTGAAACCAGTGATCCAACCATAGCTGAATTAGAATTAGTCTGATCGAATTTGTGTATTGTGAATTTTTGAGATATTTAAGGTATAGTATGGACGT

>C100281_65

ATTACTTATATCAGTTGCATCATTTCATATGAATTAATCTTAAGGATCTTAAATATGTCTCTTGAGGCTTGAATTGAATTAGCACTTCAACTGAGGCAACAATATGAACAGAAAACATATAAATCAATATTATTTCCCTAAACCCTTAATCAATGACTTAACCCCTTCCTCAAAGCTAGATATGCCACAACTCTATATCCAATCAACATCATACACAATACAACAACATCTTTCCACAAACTTCCAAGCCCTAGACACTTTATGGCAGGAAAATCTCTGATCTTGCAATGTAATCCTTGTCCACCACACTCATAGACCTCATTTACTGAGTATTGCACTCCTACAAGAAGCTTATAGCAGTAGTGACTGAAAGAAATGTACTTCAACCAAGATATAAAGAATGGAATCTGCTGAATGTAGTAACCTCCAGCTAAAAGAAACACCAACATAGTCACTGAAGCTAAGGTTGTGGCTTGTTTCACATCCATTAGAATGGCCCCAAGTGCTAGGCCTATTCCTTGAGAAACTAGCACATTCAAAAGCATGATCAAGAGAGTAAGCACAAATGTAACAAACGAAGGCCTGAGACCACCCATCCAATAAGATATTGTAACGAAGATAGTAGGAAGCACAAGCTCCATTGGTAAGTCCCCAACTGTTCTGGCAACATAATAGCAGGATAAATGGTACATGCCGGATGATCTTTCTTTCACCAACATTGGTCGATCCAGAGGAAATGCAAAGATGGCGTTGAAGAGAGGGAAAAATCCCCAGAAGATGGAGAAGAAGAATAACAGGCCTACCTGGTCTTGTATATGTGAAGGATCAGAATGCCACCATAGAAGTCCTGAGAGAATTGAGACAGAGAAGACTTGGAAGATCCTTAGGCGAGAATAAGATTCATGCCTCCTCTCTTTCAAACCCCTCTCAAGCAGAACCATGAATTGTTCCCACCAGCTAGTGATCCATTGGTTCTCAGAACTTCTAAGTGTTCCTGGAGCCAAAAATGCTAGTTCTCTATGATTTTGTTGAAGCTCTTGTTTTAGAAGAGGATATACATTCTTCTTGTATGATGAAACTAGGAATTGTTTGATTGAAGCTTGATCCTCATGGTGATCTAAATGGTCATCTTCTTTGACATCAACAACTATACCATTAGCAAGGTCAAGAAGAAAATCAGCTGGGTTGATGAAATTGAAAGTGGGCACAAACCCAATGGACTCAAGATAATTCATAACCTCACATGCTTGTCCTGTGAAAATTGGGTACCCATCCGACAACAATAACACCTTATCAAACATCCAATACAACCTGCTCGAAGGTTGGTGAATGGTGGTCACCACTGTCCGACCGAGTCGAGCAAGATTCTGAAGCATTGCCACGATGCGTTGGGCCGTGGTGGAATCCAACCCTGATGTTGGCTCATCAAGTAGCAGCAAACTTGGATTCACCAACATTTCTTGTCCTATGTTCACCCGCTTCCGTTCCCCGCCGGAAATCCCCCGAAACAAGGCGGCACCACCACCAACGAGGTTGTTTCGGCATCGGGACAACCCTAGTTCTCCAATGATCATCTCAGCCTGTTCCATTTTCTCCTCCCTTGTCAAACTTTTTGGAAGTTTCAAAATGGCAGCATAAGTTAGAGTCTCCATCACAGTGAGGTGAGGGTACAACACATCATCTTGGGACACAAACCCTATGTTGCGCCTCATGGAGCTACAAAAAGGGTGGTTGTTGTACGTGATGGCGCCTGAGAGTTTTCCGGCGAGACGGCCGGCGAGGGCTGTGAGTAAAGTGGTCTTGCCACTCCCTGAGGGACCCAACATTGCCATCACCTCCCCTGGCCCCACCATACCCGTCACACCATTCAACACTGTCCTTGTGTGCTTTGTCTTTTGAGAAGATACACAACCATTCTTGTCCCTTCCAAAAGTTATGCTGTAGGAAACATCCTCAAATTTGAGGGTAACAGGGCGAAGGGATTGGTGAAGAACAGAAAATCTGGGTTGTGAATTAGTTTGTGGGTGGTGCTGAGAATTATTTTTGTTGTGAATGTGATTGGCGGCGGAGGAAGGTGGTTCGGCATGGACGGAGCTGTTTTCCGGTCGGTTGGGAGTGGCCGGAATGTTAGATGTAACAGCTGTTTCTTGACGAGGGGGCATCATCGGAGATTAATTTGGTGGTGGTTGAAACTTTGATGGAAATTTTGAGTTAGAAAGTGATGTGAGAAGAAAATGAAGGTGAAGAAGAAGGATAGAGTTAGAAAGTGAAGAAGGGAG

>C100283_65

TTTTTTTGGGAAGAAAAATAAAGATGTCCACATACAATTGCTTACTAATATAGGTAATATGTACAAATAGTATGATATCCAATCCTTACAAGTAGTGCTCCCGGGGATTTAGCGTAAGAATTCTTTCAATAACTAAACAAACGGGTTAGACTAGGAGAACATTTTACAGCTTGAAGTTGATATTTTAGAATGCTACAACTGACAAATCATCAGTAATTATATAACACGTGGACCATAATTCTTAAATATGGTATGCTTCAAAAAAATTGAGTAAAAATACAGGCAACCAAACTGAATACTGAAGGCATAATACAACCAACTATTGTTACAACAATGCACCCCAGACCAACTCTAGCAACTTGAAGACTGATCATTTGGAGATATTTTATATCCAATGAACGATGGATCTTCATTATGTTTCATAGTTGGAGGTGACAAGTTTAACCCCTTCGAATATGAGGGGATGCAGAACCAGAGGACTGGGAAAGTAGCGGCCTTTGGACAAAGGTATGATTTAAGAGTTTAGCAGCACTGGGACGTTCATCTGGATTAACTTTTAGACACTGCAGGATAAAATCCTGTGCATCTCTTGAAAGGGAATCTGGCACAGGAGGGGGCTCACCTCTTCCAATTCTAAACAATGCCTGCATACATTCTAAGTGAGAGTATGGAATTTGGCCCGTTAACATCTCTAACACAGTACATCCCAGACTCCATATATCAGCTGGAAGCCCATAACCTTTTTGCTTCCCTTTTACAACCTCTGGGGCCATCCAGAATGCTGTTCCCTTGCATGATTTAACATCATTTAATTTGATTGCCTTTGCCAATCCAAAATCTGAAAGCTTAACAGATCCATTTGCATCCACCAATATATTTGCACATTTAATATCCCTGTGAACAATATTTCGTTCATGGAGATATTTTAAACCATGCAAAATCTGCCTTGTATATGCAGATACTTGAGAATCTCGAAGATTATATCTCTGGTAGAGGTTTCTAAGAGAACCTTTAGTTACAAGCTCAATAAAGATATAGAGATTTGATGCATCCATTTCAGTGCCAAAGTATTGAACTATGTTCTCATGTTCAAATTGACTCAAAAGTGCAATTTCCTGTTCCAGTTGATATACACTTTGCCTTCCCTGATGCCCTTGGTCAAGTAGTGAAACTTGCTTTACAGCAAAAAAGAATCCATCTTCAGAAATCCCTTCATAGACAAAACCAAACGAGCCACGCCCCAAAAGCTCACCCTTCTGCCAGTTACCCGCAGTAATAACACGCTTGATTCTCCCATTGGGAGATATATTATTAGAACTAAGGCACGTGGTAGTGCTAGAAGAATCATCATCGTTCGAAGTAGAAAACCCAGAAAACTCATCCACAATCTCCGCAATCCTCGCAGCGTTTTCCTCCTCTTCTCTCTCCACCCTCACTCCGCCAACTTCCCCTTCTTCCGTTTCTTCTCGTTCGTCATCTTCATCACCATCAGAAGAACTCAACTCCACGTAACTCTCCTTCCCTCCCTCCCCTTTCGGCGCAAAATCCCGCAACAAATCCCACGTGGAAGACGTTTCGTCCACCACTGAAACCCTAACCCCCGGCGGCGGCTTCAACATCGGAGGCCGAAAACCCTTAATTCCACCGCCGCCGCCTCCGGTGCAACAACCACTGCTCTCAGCAGGAACAACACACTCATCCAAAACCCTAGCCCGATCCTCGCTCTCCACCGCACCAATTTCCTCCGTCTCCTTCCCCTCCTCGTCGAACACCTCACCCTCGTGGCTCCGGCGCGACAGAACCTCGGAGGAGGAACGGAGCTTCATAGCCTCCCACGCGGCGGCCGGAATGGAGAAGTCCTCCGGGCCGGAGAGGCCGAGGCTGCGGCAAATCCGGTCGAACTCGCCCTCAACGCCCTCGATTCGGAAGCTCGTGTGGTCATAGAACTCCATGGTGCTCGTGTAGAGCGACGACAAGGACGTCGAGGACGAGGACGAGGAGGAACATCCGGCGTCGTATTCGAAATGCTTGGCGGCGTTGCGGCGCTCGAGCTTGGGCTGCTGCTTCTTGCCTGGCGTCTTGCTCTTCTTCTGGTCCATGTTTGGGGGTTTCTTTCTATAGCCGAATAGTCGAGGTAGGTGATTCATGGCTGCCCAATTCCATCTAAATCGGCGTCGGATTGGGGAAACGCGGTGTTTAGTGTGTTTAGCCTTTCTGAATGGTGGAGAAGAGGTTGAAGAAAAGAAAATGACGAGTTACGTGAAAGTGAGAGAAAGAAAG

>C100287_65

TGGTGAAAAATAAAGGAAGATGACGACCTTCACATACCACGTAGAGACGGTTCAGCCATGGTTGGTCCTAAGCAGGCTTCACATATTCATCCACCTGCTCGCAGTGCTTGCACTCTGTTACTACCGCATATCCCACTTGTTACTGCAACCACCAACACTGCCATGGATCCTCATCACAGTGGCCGAGCTTGTTCTCTGCATGCTATGGTTCTTTAACCAAGCCTTCCGCTGGCACCCCGTTTCGCGTACTTCCTTGACGGAGAAGCTGCCCAAGGAGGATAAGCTTCCGGGGCTGGACATCTTCGTCTGCACGCTCGACCCTGAGAAGGAGCCCACGGTGCAGGTCATGGACACGGTCATTTCCTCCATTGCCATGGACTATCCCTCTGATAAGCTGGCCGTGTATCTCTCGGACGACGGTGGGTGCCCCGTCACGCTGTATGCGATGAGAGAGGCGGGTGAGTTCGCGAGAGAGTGGGTGCCCTTCTGCAGAAAGTATGGGGTGAAGTTGAGGTGTCCCAAGGTCTTCTTCTCTCCAATGGGAGAAGATGAACACCTTCTTCGCGATGAGGATTTCAAGACCCAGTGTCAACTCATCAAGGGAAAATACGAGAAAATGCAGAAGAACATCGAGAAATTCAGCTCAGATCCTAAGAATCTTGGTATCGTGAGCGACAGGGCTCCTCGTATCGAGATCATAAATGACCAACCAGGATTGCCACTTGTTGTTTATGTGTCTCGCGAAAGAAGGCCATCCCTTCCTCACAAATTCAAAGGAGGAGCTCTCAACACATTGCTCAGAGTCTCGGGTTTTCTCAGTAATGCACCTTATTTTCTAGTAGTGGATTGTGATATGTTTTGCAATGACCCAACCTCTGCCAAACAAGCCATGTGCTTCTTCCTCGATCCTCAAACCTCCAAAACTACTGCATTTGTTCAATTCCCTCAAATGTTTCACAACCTTAGCAAAAAGGACATCTATGATAGTCAAACTAGGACTGCTTTTAAGACAATGTGGCAAGGCATGGATGGACTAAGAGGTCCTGGTCTTTCTGGCAGTGGCAATTACTTGAGCAGAAGTGCGTTACTATTTGGAAGTCCTGACCAGAAAAATGACTATCTGCTAGATGCACAAAAGTACTTTGGCAATTCTACTGGATTCATTGAATCATTGAAGGTCATCCGTGGACAGAAAACTACCAAAACGAACATTTCAAGAGAAGAAAAGTTAAGAGAAGCTCTAGCTGTGTCCTCTTGTTCCTACGAAAACAACACAAAATGGGGTACAGAGGTGGGATTCTCATATGCGATCTTACTAGAGAGTAGTGCTACTGGGTATATTCTTCACACCAGAGGATGGAGATCAGTTTATCTTTACCCCAAAACACCATGTTTCTTAGGGTGTGCCCCCACTGACATCAAAGAAGGCATGCTTCAATTGGTTAAGTGGTTGTCTGAACTTCTCTTGCTTGGTTTCTCCAAATACAGCCCTTTCACTCATGGATTTTCAAGCATGTCCATATTGCATTCCTTTACTTATTGCTTCATAACAATGTCATCCCTCTATGCTATTGTCTTCATCCTCTATGGTATGGTACCTCAAATATGCCTCCTCCAAGGAATCTCTGTCTTCCCAAAGGTGACAGACCCTTGGTTTGCAGTGTTTGCATTTGTGTATGTATCCACTCAGGTTCAACATTTGGTTGAGGTTCTTTCTGGGGAAGGGTCTGTGGCAATGTGGTGGGATGAACAAAGAATTTGGATTCTGAAGTCAGTGATGAGCATATTTGCAATCATAGATGCAGTGAAGAAGGGGTTGGGGCTGAACAAGGTGAAATTCGCCTTGTCAAACAAAGCCATTGACAAAGAGAAGGTGAAGAAATATGAGGAAGGTAGGTTCGATTTCCAAGGTGCAGCTTTGTTCATGGCACCATTGGTTGTGTTACTCATAACCAACATTGTGGGTTTCTTTGGTGGGATATGGAGAGTAGTGCATGTGAAAGATTTTGAAGAAATGTTTGGTCAACTTTTCCTAGTCACCTATGTAATGGTTCTCAGTTATCCCATTCTTGAGGCCATAATAAACATGAAAAGTAAGAGTGGATAGTCAAATTTTGTCATAAATAATTAGACTCATTCAAGAGTCTTAGTTGCATTTCTATCGGACAGTCAAACATCTTCTTCCATCAGTTTAGATGAATATATAGTACTTTATTGCTTAGTAACTAATGTAACCATTTTTCTATAATAAATCAAACTCCAAGTTCTGTTCAAAAAAAA

>C100289_65

TGACACAAAGCTAGGCACTATCTTCAATTTCATATGGCTGGCCCAAATCTATGAAGATAAATTTCAAAAAACTACTTTAACCACGCAATTTAAAAGATCAAATACTTTCAACTCCCAAGTTAGAGACTATAACAAAAATGATGAAAAATAGTAACTTAACACTACCAATTTAACATGTCTTTTCGCATATACTGTGAAAATAGGCTCTACAAAATGTTGAAGTTCTATAACCATATACTGTGCTACTTGTTCAACTGAACAGAAGTTATGTTAGATCAAAAATAAAATAAAAATCTGCAACCATCTACTGTACTAGTTCTATATAAATTCTCCATATGTCATGTTGTATTTTCACACGACCAATAGGCTAAGATAAGATCATTGCTGGAAGAAAGAAAGGCGATGGACCTTGGAACCTATGGCTCTCACTATGATGTGCCAGAGGGAGTGGATATTCGGGGAAGATATGATGCAGAGTTTGCAAAAATCCTGACAAAAGATGCTTTGAAATTCGTTGCTGACCTTCAACGTGAGTTTAGGAACCATATCAAGTACGCACTAGAGAGGAGAAGAGAGGCAAAGAGGAGGTACAATGAAGGGGCTCTTCCAGGATTTGATCCTGCCACCAGGTACATAAGAGAGCAGGAGTGGGTGTGTGCACCTGTTCCACCAGTTGTTGCTGATAGGAAAGTAGAAATCACTGGCCCTGTAGAAAGAAAGATGATCATCAATGCTCTCAACTCTGGAGCTAAAGTTTTTATGGCTGATTTTGAAGATTCACTAACACCCAGCTGGGAAAATCTTATGAGAGGCCAAGTGAACTTGAAGGATGCTGTGGATGGCACCATAAGCTTCCATGACAAAGTAAGAAACAGGGTTTACAAGCTCAATGATCAAACAGCAAAGCTTTTTGTGCGACCAAGAGGTTGGCACCTACCCGAGGCACATATACTCATTGATGGTGAACCTGCAACTGGTTGCCTTGTTGATTTTGGCCTCTTTTTCTACCACAATCACTCAGAATTTCGGCGCACTCAAGGTGCTGGCTTTGGCCCCTTCTTTTATCTTCCCAAAATGGAGCATTCAAGGGAAGCTAAGATATGGAACAGCGTGTTTGAGAAGGCTGAGAAGGTGGCAGGCATCGAAAGAGGAAGCATAAGGGCCACAGTTCTGATAGAAACACTTCCTGCAGTGTTTCAGATGAATGAAATTCTGTATGAGCTGAAGGAGCACTCGTTGGGTCTGAACTGTGGGCGTTGGGATTACATTTTCAGCTATGTGAAGACCTTCCAGGCTCACCCAGATCGCCTCCTACCAGACAGGGTGCAGGTTGGCATGAATCAACACTTCATGAAGAGTTATTCTGATCTCCTCATCAGGACATGCCACAGGCGCGGTGTGCATGCTATGGGAGGAATGGCAGCACAGATTCCAATCAAAGAAGATCCAGTGGCTAATGAATTAGCACTGGAACTTGTGAGGAAGGACAAGCTTAGAGAAGTGAAGGCAGGGCACGATGGGACTTGGGCTGCACACCCTGGTCTCATTCAAGCTTGCATGGAGGTTTTCAACAACCACATGGGCAATGCTCCTAACCAGATAGAGATAGAGCAACGTGAAGATGCTGCGAACATAACTGAGGAAGACCTATTGCAGATACCAAGAGGAGCCCGCACCATGGAAGGTCTCAGATTGAACACAAGGGTGGGGATTCAGTACGTGGCAGCATGGCTCACTGGAAGCGGTTCTGTGCCTCTTTACAACCTGATGGAAGATGCTGCGACTGCTGAGATTAGTAGGGTGCAGAACTGGCAGTGGCTGAAGTATGGAGTGGAACTGAATGGAGATGAAGTTGGAGTGAAAGTGAACAGAGAAGTTTTTGGTAGAGTGGTTGAAGAAGAGATGGGAAGGATTGAAAGGGAAGTGGGGAGAGAGAAGTTCAAGAAGGGAATGTACAAGGAAGCTTGCAAGATCTTCACACGCCAATGCACCTCCCCAGTCCTTGATGATTTTCTAACCCTGGATGCCTATAATAACATAGTCTTGCATTACCCCAAGGAATCATCAAAGCTTTGATTTGAACTAAGAAAAAGGTTGGTTCATTATGTTTAGTTATTGGCTCATTTGAATTCTGTCTTGTGATACTTACACACTCCTTGTCTTTGTATCACTTCATCAGTTCAAGGGACGTTCATAATTATGCTTGAAAGAAAATAATAAATTTCAGATTGTAGCTGCTTTGAATTATTGTTGCTACCAACAATGAAAACAATGTTTATA

>C100293_65

TCTGCCTCTCTTATCAACGTCCTGAATACTTAATGTGACGAAAATCCAGCAGAAGTAATGTGCCGACTACTACCAATCTTCAATTCATTACCTGTATAAGGACTCATTCATAGTCCATGATGTATTTATTGAGATATAGTTTTCCATGACTGTATTCATTTTGAGATTTTAGATGATATAATTTCACTGTCACAATGATGGGGTGAACTAGAATTTTCTCTAAGAAAACTTGTAAAAATACATGTGAGGAGGTGAGATCTAAATTAAGAGGTATACATCAACTGCATGGAATTCTATATCTGCCGAGTTGATGTTGTCCAAACTCCAACCTAATGAGACTTCACATTCTCAACCGTACAACTTGGAATAATGTAGGACGTAAATTCGATCCCTGTGTTGTGCCAATTCCTACTAGCTATCTATAAATTGCGTGATGGAAATTCTATACAAAAATGTATTCAGTATGTTGTCAGGAACGAATCATGATGAATAGGGGTTCTGCCTATGAAATCCCGATCTTGTAGAAGTAAAAGGCTCGGCTTCATCATCGTCATCTCCATTTTGACTTGTAAAGAATGGGGTGAGATAGTTTTTATCTAATGCTGTAAAAGATGCAGCACTCTTGTGGAATTCTTTTAGCTTCATCTTGATTTTGTTCCCTGAAGATGACTCTTCGTCGTAAGGAGGAGCAATATAACCATTGTTTCCATCAAAATTTGTGATAGTACCAACTGAAGCCAAAGGGCTATCATTATCTCCACCTACAACATCTAGAGCTTCCAGCATTGTACCTGTTGAACCTCCAATTAGTAATACCGTTAAAACAACTATTGCAGTAGTTGCAGTGAAGATGGTCTGTCCATGTCCTTCTGGAAGATCATGAACTGATTGCAGAGCAAGCGCAAAAGCCATTGCTCCTCGAAGACCACTATACCAAAGAGCCTTCTGATGTTTAGGAGGTATCTTTCGATGACTGGGTCTGACTAGATTGACCAAATAAGCACAAGAGAAGACATTTGCAGCCCTTGCAATTCCAATAAATATAATGGAGAAGAATATAAATCCAACATGTGACCAGCTATGTTGCTCCATAGCAATATCAAAGCCCATGTATATAAATACAAATGTTTCAGCTAAAGATGATATCAACTCGAAAAAGGCAGAGGCAAATCTTTGAGAACTTTGTGACAAATTGGAATACGTATAATGCTTCATGACTATTCCAGTGAACAGTATTGATACAATACCAGACAGACCAAGGCCTTCAGCAAGCATGTACGAGAAATATGGGAAAAGGACAAAAAGACAGCTCTCCAAGTTCTGAAGGTTGTCAATATCCAATCCTGCATACTTAAATAGCAAAGCAGATATAAATCCAACTCCAACACCTGCTGACATTGACCCTACAAAAGTCTCCAAAAATCGAACAACCACCATGAATAAATTTTGTCCGGATGGATGAGCTTTAACAGATGACATTGTCCTGTACAAAGAAATTGCCATTGCATCATTCAAAACAGATTCTCCAAAAACCAAGGCATATAGATTGACATCAGTGCCCAGCTCCTGAAATATGGACAAAACAGTAACAGGGTCAGTAGCTGATATAAGAGCACCAAACATCAGGCACTCAACGAAAGGTAGCCTATACATTAGAAAGAGCAAACCACCAAGATAAACCAGGATACCCGTAACTATTGAAGCTATAAATGTGCCGAATATAGCAAATGTGACAATTGCTCCAAAATTCGAGAAAAAGGGTTTAGGTGCGAGACTGAACCCAGACTGAAATATGATAGGAGGTAGCAGGAACAGGAAGAAAAACTCCTCGTGAAAATTGAACCACGCCCTGATACTAGTCTCGGTGTCTGAAATGTTTGCTAGAATGCCAACAATTAACCCTATGAGAAGAGAGGCACTTGCTTCGGGGATGATGTAAACCTTCTTGCGACGGAGAACATGGCCGAGGACGAAGGACAGAACGAGCATCATGATCTGCAGAAGGATTCCAACGCCAGCGGCTTGCTGGTCCTTGCCGGGAGCCTTGCGAGCGTCCGCCGGAGAAATTTCAATCTCCGAGGCCATTGTCCTTGCCGGAAGCAATTAACCGATTGCTTCCGTTTGCGATTGGATCGCTGAGTTCTTGACGAATCAGAACAGAACGATGTTCTCTAAACAACTAAGTTTTGATTAGATTCTCTCTTTCTTGTTTTTCTCTTTCAATTCTGTTTCTCCACACTTCTTACCTCTTGGACTCCTCTTCAGCACGGAACATTAAAT

>C100295_65

AAGCATATATAATGATGGTGAATATGCCTTTAGGGTAAACAAGAGGATCCACATATATTTTGATAGTTAAGAGATACTACAAACTATCCAACAAAGTACATTTTATTTTGAAAATATGAGGATACAAGAACAAACTTCCTTCAGAAGTAAAAAAGTTGATGACATCAGAAAAGCAAGAGATCCACATCCAGAGAAGGGGAAGGAAAACCAAAGGCTGCAAAGCAACAATAGACCCAAAGTGCTTAAAACACCAAAAAAAGATGTGTCAAGGCCAGAAGCCAACACTAGGGCAATGTTTAAAAAACTGGGAGGAAAAAAACATGAGCTAAGAAAACTCAGGTTAGATTCATCCTCCCAAAAACCACAATTAGCCAGAGGAGAGCTTCCCCATTCATCTTGGAAAAAGGAGTGTTGTGAAACTAAAAGGGAAAGTTCCAGATATTTTTGGTATTAATTACAACACCAACTTTATCTACATAGTATGTTTGTGCTTCTTTTCTTTCACTGGTCCCTTCTCCCGAATAATATCTGTGAACTGGGATGACGCTGAAGAAGATTATTCTTCAAGGTTGTGCTTGAGTTATCATCTGAGCCTGAGCTAAGCTTTATCTCATATTTTTTTAGCAGTGATGCCAACACTGAGGCAACAAGTTGGATGATAAATTTCTGTCCAATGCAAGCACGTGCACCAAACCCAAAAGGAAGGAATGCTGCATTTTCATTTGGATCATTTAGTACAAATGAACTGAATCCTGTAATAGAAAGGTCTTCGGCAGAACCTGTTCCCTTTGTAACAGATGAGAGAAATCGGTAAGGATTAAAATGACTTGCATCACTCCCCCAACTGAAGTCATCCTTCTGTACCAATTCAACAGGTACAACCAATACAGCTCCAGCAGGTATGGTTACTCCAGTTGCAAGAGTCAAGTCATGTTTCAAAGAACACCTCTGCAGCATGGGACCAGTAGGCAGAAGTCGTGCAGATTCATAAATTGTAGCCAATAACAAAGGCATCCTATAAACATCTTCTTGCTCATATTTCGATGGGTTTCTTCCTACCATGCTAATCTCTGAATAAACCTTATCCTGTATTTCCGTATGCATGACAAGCCTAGTCAATACATTGGTGATTAAAGCAGATGTAGTTTGGCATCCATGAAATATCATGCGCATGACATTCCCACAAGGTTCTTCTTTACCATCTTGATGATCCTTAAGATCACGAAAGAAATAGTAATCTTGGAACTCATAATCAGAATAGCACTCTGCACCATCTGCTGACTTCGTTTCTTTGTTGGAACTTTCATTATGTAGATCTTGGTCAGTACAGTCAAATAGTTTGCAGCCTTTTCTGCAATGTTGAAGGATGTCTTCAGTTAAGCATTTCAACTTTGTGCATAAGCATTGGTACCTCCAAAATCCACGTTTCCAGAAAGGAGTAACATTATAGGAGGCCCAAAAGCAAGCATCTTTGGCGATTGTCATGAACAGTTCCTCATACATTGCAGCCTTCGGAGAGGCCAAGAAGCCATCTCCAAAGAATGTAGCTCCCATTATAGTGAAAGCCATATGCTGAGAAACCAACATGGAATCGATACCTCCTTTTGTTCTGATGTTTTCTATTTTATTTGTGATAAAGTCCACAACCTTCACAGGAATCATGTCATCATTCTTCAGCAATCTTTCATTTAATTCTGTTGCCAATAATTGCCTTCTCTTTTGCACCTTTTCAAAGGAAGGGGCAAAGAGGCTTGATTGCCCAAAGGCAAGATGAAACACTTTTCCCGTAAAAGGCAGCTTATCCTCGGCTTTTATAAGCATCTCTTGGATTAAGGCCGGATCCCTTACCGACACCAAAAGCTGAGTAGGACCTAACCACAGCTTAATAATAGGTCCATACTTTTGATGTGATTCTGCCAACACATCAGTTAAGGTGAAATTTGGCCGAGAAAAGAGCTTGCAGTGGCCAGAGAAAGAGGAGCAGGGGGGGCCAGGAATGGTTCTAGCTCTGCGCCACAACCCAAATAGGCTAAAGAGTCTTCTGAGCAAAAGGGTGGTGACAACAACAAGAGATAGCCATAGAAACGCGTTGAGTTCGCGAGTGGCGAAGTCCCTAAGAAGCTCAGAGGAGTGGAGGACGTGAAGAAGACCCAACAGAGAGAAGCTGTTGGTGTTGTGGTCGATCATTTTCTCAATGGAGAAAGAAAGAGAAGAAGAAATTGATTTGCTTGGTTATGTTATGAACTTAGAACGTTGAACGTTGAATGATTGAAGATGATGG

>C100297_65

GAGATTCAATATCGGAGCTTTTGGAAAGTTTTCTGAGTCAGCTTTGTGAAGAGGCCTTGGAAGGTTGGTTTCAAACTACTTAATATTATATAAGACACAATGGTAGCAAAAATCCGGCAAAGTGCTACTGAGTCTCCTAATCCGAAACCTGAGGTTGGAGAGATCGACACCAGCCCACCTTTTCAGTCTGTTAAAGATGCTGTCTCTCTATTTGGTGAAGGTGCTTTCTCTGGTGAAAAACCAATCATTAAGAAGGCAAAACCTTATTCTGCTGAGAGAGTTTTGGCCAAGGAGACAGAACTTCATGTAGCCCAAAAAGAGCTGAATAAGTTAAGGGAACAAGTAAAGAATGCTGAAACTACTAAGGCTCAAGCCCTTGTGGAGCTTGAAAGGGCTAAAAGAACAGTTGAGGATCTGACACAAAAGATTAAAGTTATTAGTGATTCTAGGGAGTTAGCAATGAAGGCTACAGAAGCTGCAAAGAGTCAGGCAAAACAACTTACAGAAGAAAAGTATGGTATCCCTGACGTAACAGATGGTACTTGGAAGGAAGAATTAGATTCTGCAGTTACAAGGTATGCATCTGTCATGACAGAACTTGCTGCTGCAAAGCAAGTACTGAGCAAGACTCGACAAGAGTATGATTCGTCCTTGGATGCAAGAAAGTCTGCTTTCAAGCAAGCAGCAGAAGCTGGGGATGCATTGAAGGAAAACAGAGAAAGAGCATCTGAGCTTTCTAAAGAAATTTCATCTGTAAAGGAGTCAATTGAACAAGCAAAGCTTGCATCTATTGTTGCACAGCAACAGCAAGGAATGATTTTGGCTGAGAAAGATGTTCTAAGACAATCATATAAAGCTACCCTGGAACAATCTGAAAAGAAATTACTTGCCTTAAAGAAGGAATTCAGTCCTGAGCTTGCCAAAAATCTTGAAATGCAGCTAGCTGAGACCATGAGTGCAATTGGGGCTCTGCAAAAGGAAATGGAAAATAAAAGGACATCGGACTTGGACTCTGTGAAAAGTGTCACTTTGGAGCTTGATGATGCTAAGGAGTCACTGCAGAAAGTAGCAAATGAGGAAAGCTCTCTTAGAAGCTTAGTGGAATCTCTAAAGGTGGAACTGGAGAATGTAAAAAAAGAACATTCTGAATTGAAGGAGAAGGAATCTGAAACTGAATCCATTGTTGGGAATCTGCACGTGAAGCTCCGCAAAAGCAAGTCTGAGCTTGAAGCCTGCATGGCAGAAGAATCTAAAGTTAGAGGTGCATCTGAGGAAATGATCTTGACACTTAGCCAGCTGACATCTGAAACTGAAAATGCCAGGAGGGAAGCAGAAGATATGAAGAACAAGACAGCAGAATTAAAGAAGGAGGCTGAAGTTACCATGCTTGCATTAGAAGAAGCAGAGAAAAAGCTTAAAGTGGCGCTGGAAGAAGCAGAAGCAGCGAAAGCTGCCGAGAAAAGTGCTGTTGACCAGATTACAGTGATAACTGAAAGAACTACTGCTGCACGTGCATCAACATCTGAATCTGGTGCTGTGATTACAATCTCGAAAGAGGAGTTTGACTCGCTAAGCCATAAAGTTGAGGAGTCTGACAAATTAGCAGACATGAAAGTGGCTGCGGCCAAGGCACAGGTTGAAGCTGTGAAGGCTAGTGAAAATGAGGCTCTCAAAAGGTTAGAGACAACTCAAAAGGAGATTGAGGATATAAAGGCTGCAACACAAGAAGCTCTAAAAAAGGCTGAAATGGCTGAAGCAGCAAAAAGGGCAGTGGAGAGTGAGCTCCGAAGATGGCGTGAGAGGGAGCAGAAGAAAGCTGCAGAAGCTGCATCTCGAATTTTGGCTGAAACACAGGTGTCAACAGAATCATCTCCACAGCACTATAGGATTCAAAAGCAGAATGCTCCTCATACAACAGTGGAGGTGAGGAAGTTTGAAAAGGAGAAGGTTTCAGTTTCAAAAAAAGCCCTCTTACCTAATATTAGTGGCATCTTCCAAAGGAAGAAAAACCAGGTTGAGGGTGGCTCTCCGTCTTATCTGCCCGGTGAAAATCCTGTATGAAATATGTAAGGTGTAGCAGTTTGTGCAGCTCTTGAATGTGAGAGAAACATTTATTTGTGTGTAGTTGAGGAATTTGATCAGATTTTGTCGTTGTAGTCTGTTTGAAGAAAAGGATTGTTTAGTGTTTATAGCTTGGTGATAAATTTGGAGATGCATAATGTAAATGGAACCAGAAGTATGATTAAAATCTATTGCTTTTTTCTTTTCTGTTAAAAAAAAA

>C100299_65

ATAAAAACCAAACAACAAATGAATAAAATAGGGACAGAGGAGAGGGATAAGATTTAACTTTTAAGGAATAATAAACAACATACATAAATCAACTTGCGCTCCTCTTCCTCGTCAAACATCATTAGTTTTCAGATTCTGAATCCTTGTTTTTACCCAAAACTGAATCCCAATTCAATCCCATGAGTTCCTCAACTACAGTGAGGAAGCTAGAGGTCGTGTCCCCTGTTCCTGCGGACATAGACATTGCTAACTCCGTTGAACCTGTTCACATATCCCAGATTGCCAAAGACCTCAACCTCAGTCCCAATCACTATGATCTTTACGGTAAATATAAGGCCAAGGTTTTGTTGTCAGTTCTTGATGAGCTTCAAGGATCACAAGATGGGTATTATGTTGTAGTTGGAGGCATTACTCCAACTCCTCTTGGGGAAGGCAAATCTACTACCACAGTGGGGCTTTGTCAAGCTTTAGGTGCTTTTCTTGATAAAAAGGTAGTCACCTGCCTTCGTCAACCATCGCAAGGACCTACTTTTGGCATTAAAGGAGGTGCAGCTGGTGGTGGCTATAGCCAAGTTATTCCTATGGATGAATTCAATCTTCATCTAACAGGAGATATTCATGCAATAACTGCAGCAAACAATCTTCTAGCTGCTGCAATTGATACCCGAATTTTCCATGAGTCAACGCAGTCGGATAAGGCCCTTTTTAACCGGTTGTGCCCTCCAAATAAAGAAGGGAAAAGGAGCTTTAGTGATGTCATGTTCAGGCGTCTTAAGAAGTTTGGCATTTCAAAGACAAACCCGGATGATCTTACACCTGAAGAAGTAAATAAATTTGCGAGGCTTGATATTGACCCAAATTCTATTACATGGAGGAGAGTAATGGACATCAATGATCGGTTCTTGAGAAAAATTACTATTGGCCAGGGACCCGATGAGAAAGGAATGGTAAGAGAAACAGCATTTGATATTTCAGTTGCTAGTGAGATAATGGCTGTTTTGGCGCTGACAACATCCTTAGCTGATATGAGAGAGCGGCTTGGGAAAATGGTTATTGGAAATAGCAAGAGTGGTGACCCTGTCACTGCAGATGATCTAGGCGTTGGAGGTGCCTTAACAGTTTTAATGAAGGATGCCATTCACCCTACCCTCATGCAGACCCTGGAAGGAACTCCTGTTCTTGTTCATGCTGGTCCGTTTGCAAATATTGCTCATGGGAATTCTTCTATTGTGGCTGATAAGATTGCGCTGAAGTTGGTTGGACCGGGTGGATTTGTAGTTACTGAAGCTGGTTTTGGTGCTGATATTGGAGCCGAAAAGTTTATGAACATTAAGTGTCGTTATAGTGGTTTGACACCTCAATGTGCAATTATTGTGGCAACAATAAGGGCACTAAAAATGCACGGTGGAGGGCCAGCTGTTGTTGCTGGAAAACCTCTTGATCATGCATATTTGACTGAAAATGTTGCCCTAGTTGAGGCTGGTTGTGTGAATATGTCACGGCATATATCAAATACAAAATCTTACGGTGTAAATGTTGTAGTTGCTATCAACAAGTTTTCAACTGACACTGAAGCTGAGCTAAATGCTGTTCGGAGTGCTGCTTTAGCTGCTGGAGCTTATGATGCTGTAATTTGTACCCATCACGCGCATGGTGGCAAAGGAGCCGTTGACCTGGGCGTTGCCGTTCAAAAAGCTTGTGAGAATGTGGCACAGCCATTGAAGTTCCTGTATCCTCTGGATCTGAGTATAAAAGAGAAAATAGAGGCAATAGCAAAGTCATATGGAGCCAGTGGCGTTGAGTACTCTGAGCAGGCTGAGAAGCAGATTGAGATGTATAGCAAGCAAGGATTTTCAGGTCTTCCAATCTGCATGGCTAAGACTCAGTATTCTTTCTCAGATAATGCTGCAGCAAAGGGAGCTCCAAGTGGGTTTGTCTTACCCATAAGGGATGTAAGAGCTAGTATAGGTGCTGGATTTATTTATCCTTTAGTTGGAACAATGAGTACCATGCCAGGGCTTCCAACAAGGCCATGCTTCTATGACATTGATATTGACACGACAACTGGGAAAGTCATTGGTCTCTCATAAACCTCAAGTGACTCGCCCTACCACTTGTCTAAAGCTTTATGTCATTTACATATCCCTTTTTGATGTTCATGAATGTTAATGTCACATTAACACTCCTCCTTGATGTTTTCTTCTTGTTTGTTTGCATGCTTTATCTGTATGAATGAAATAAATAATTTAAGGAAAGATTATATCTGTTTTCTTAAAAAAAA

>C100301_65

GGAAAGAAAACACTCTCACCCCATTTACGAGAGTTGAAAATCAAAGAAAGGAAAAGAGAGAGATCAAAGCAAAAGCATATTCTTTTGAAGTATTTATTTCATTCGTTCCTTTCTTCTAGAACACCATTCGGTTCGATTTTGGTTTGGTTTTGTTTCGTATTCCAAGGGAAAGCCTAAGCCTCCGTCACTGTTCAATCCTCGAACTGTATTCCTCGCGTTTTGGTTTTCATTAGGTTTTGTTGCAGTTGAAGTAATATTCGGCGATCGATCTTCAAACATGCCTTCCAGTCACTTGCTTCTCGAGGAACCCATTAGGATGGTCTCCATCCTGGAGCCATCCAAGCCTAGTTTTTTCCCTGCAATGACAAAGATTGTTGGTACCCTGGGTCCTAAATCTAGATCCGTTGAAACGATTTCTAATTGTCTCAAGGCTGGCATGTCTGTTGCTCGATTTGACTTTTCGTGGCATGACCCTGAGTATCATCAGGAGACTTTGGAGAATTTGAAGGTTGCTATCAAGAGTACTAAGAAGCTATGTGCTGTTATGCTGGACACGGTGGGTGCAGAGATGCAGGTTGTTAACAAAAGTGAGAAAGCTATCTCGCTTGAGGCAAATGGTCAGGTTGTTCTAACTCCTGATCACGGACAAGAAGCCTCTTCAGAAATATTGCCTATCAATTTTGATGGGCTGGCTAAGGCTGTGAAGAAGGGGGATACTATTTTTATTGGTCAATACTTGTTCACAGGAAGTGAAACTACTTCTGTATGGCTAGAGGTATCTGAAGTCAAAGGGCAAGATGTTGTTTGTATTATAAAAAATTCAGCTACATTGGCTGGGTCACTGTTCACTTTGCATGCCTCTCAAATTCATATTGATTTGCCTACTCTCACTGAGAAAGACAAGGAGGTTATAAGCACCTGGGGAGTTAAAAATAAAATTGATTTTCTGTCATTGTCATATACTCGACATGCTGAAGATGTTCGCCAGGCACGGGAATTCCTTTCAAAGTTGGGTGATATCAGCCAGACTCAAATTTTTGCAAAGATTGAAAATGTTGAGGGATTGACCCATTTTGATGAGATTTTACAAGAAGCTGACGGAATTATCCTTTCCCGTGGAAATTTGGGTATTGATCTTCCACCAGAGAAGGTGTTTTTATTTCAAAAATCTGCCCTTTACAAGTGTAATATGGCTGGGAAGCCTGCTGTGCTTACACGTGTTGTGGATAGCATGACTGACAACTTGAGACCAACTCGTGCTGAAGCCACTGATGTTGCCAATGCTGTTTTAGATGGTAGTGATGCTATACTTCTGGGTGCTGAGACTTTACGTGGGTTGTACCCTGTTGAGACTATTTCTACTGTTGGCAGAATTTGTTCAGAGGCTGAGAAAGTTTTCAATCAAGACCTTTATTTTAAGAAGACTGTCAAATATGTTGGAGAACCCATGTCCCACTTGGAATCTATTGCATCCTCTGCGGTACGAGCTGCTATTAAGGTGAAGGCATCTATTATAATTTGTTTCACTTCATCTGGAAGGGCTGCGAGATTGATTGCTAAATATAGGCCGACAATGCCAGTTTTATCTGTTGTGATACCCCGACTAAAGACAAATCAGCTGAAGTGGAGCTTTAGTGGAGCTTTTGAGGCAAGACAATCACTTATTGTGAGGGGTCTCTTTCCTATGCTCGCAGATCCTCGACATCCTGCTGAATCAACAAGTGCAACAAATGAGTCTATACTCAAGGTTGCCCTTGATCATGGAAAATCATTGGGAGTTATAAAGTCACACGACCGGGTAGTTGTTTGCCAGAAACTTGGTGATGCATCTGTGGTTAAGATTATTGAGCTTGAAGATTAAAGCACATTCAACCAACTGCAGTTTTAGTGTTATTTATTTATTTTACATCTTTGCCTAGTTTTTCAAAGATGCAGGTCATTCTGCCACAAGTAGAGGTTATCTCTACATGATTTTGATGAAACGAATTTTGGGTGCAAATTATTGCTGGCTGACGAACATAAAATCAAGGGAATGTACCCTTGATGCCAATTTTTGGGTTTAGTATTCATACTAGAACACAGCTCGAAAATGTGATTATTGAATCGGATTACCAAACAAATGATATGCTAATAGCTTGTTATTTCAGACACCAATTGTTTATAAGCATATTTTTATTATCTCTACTCTTTATATGAGCTAGAAGTACGTGTTTAATGGTGAACGGAGTGTGTATCAAAATGATTTATTTATGAAGTGCCTAAACTGCCACGATTCATTCAG

>C100303_65

TCTCCATTGCACCTTCCCTTCTTCTTTTCTCTGTCATTTCTGAGATTCCTTTACTGGTTTTTTTTCTCTTCCATTGGTTTGCACAAATTTCTAGCTACAACACCCAGATATCAATTTCTGTAATTCTGTTTGATTTTGTGATTTTTTAAGGACAGTAAAACACAAACACTTCTGGGTCTATTGGCTTCTGCTTCCTTTAGTTTTGCAAACTGATTTCTGGGGTTTGCTGCAGAATAAAGCCTTCAATACCCACTAATACAAGCCTTCAAAACAACTGGTTTATTTAAAAAAAAATCATTTTATTCTTTTTTGTTAAAAAATTATTTTGCCCCTTTTTTCTGCAATTTTTTCTACATATTATTTCCCCCTTTACTTGACGAAAAGATTGAGACATGGGCAAGCAAGGGCCTTGCTATCACTGTGGAGTTACAAGCACTCCACTTTGGCGTAATGGGCCACCTGAGAAGCCTGTACTATGCAATGCTTGTGGATCTCGATGGAGGACTAAGGGAACACTTGCAAATTATACCCCTTTGCATGCTCGGGCAGAAAATGTTGATTATGAGGATCAAAAGGTTTCCAGAGTGAAAAACATATCATTAAATAAGAGCAAAGAAGTTAAATTGGTCAAGCGAAAGCAGAACTATGATAATTCTGCATCTGGAGGGTTTGTTCCAGATTATAATCAAGGACATCGAAAGGTTGTGGATGAAGATACGAGCAATAGATCAAGCTCAGGATCAGCTGTCTCTAACTCGGAGAGCTGTGCTCAATTTGGTGGCACAGATGCTAGTGATTTGACAGGTCCTGCTCAGTCAGTGGTCTGGGATGCCATGGTGCCTTCAAAAAAGAGGACATGTTCAGGTCGTCCGAAGCCTTCATCTGTTGAGAAGCTTACAAGAGACCTGTGTACTATTCTTCATGAACAACAGTCTTATTTTTCTGCATCTTCTGAAGAGGATCTTCTTTTTGAAAGTGATTCACCAATGGTCTCTGTTGAGATAGGACATGGTAGCATTCTCATAAGGCATCCTAGCTCTATAGCTCGAGATGAAGAGTCTGAGGCTAGCTCTCTTTCAGTTGATAATAAACAATGCCTAATGAATGAAGCATATTCATTCTCTAGTACGATTCCGTTGTATAGTGATCACAGTGGCATGAACTTCTCATCTCATGGAGTGGAAAAGATAAAAAACTCAGCTGGCCAAATTATGCAACAAGTGAAACTTGAAAGGGACAAGTCTCAGCTTGAAAAACTACAAGTTCCTGGAAATCACAATTCACCGCTGTGCTCGATAGATTTAAATGATGTAGTGAACTATGAGGAGTTCATGAGAAACTTGACAAATGAAGAGCAGCAGCAATTACTGAAGTATCTCCCTGTGGTTGATACCGCTAAATTTACTGATAGCCTTAAGAACATGTTCAGTAGCTTCCAATTCAAGGAGGACTTGACTTATTTTCAGCAACTTCTTGGGGAAGGTGTCTTTGACATCTCTTTGTCGGGGACAAAACCTGAAGAATGGAAGACTTTGAAAAGGCTTGTGTTATCTAATCTGTCAAAATCAAAATGGGTTGAACACTATAATTTTCTCAAGAAATGTGAAAACAAAGCTGGAAAAACTATTGGTCCGGGATCTACTGCTATGGAATCAAGTAATGTTACAAATGGCAAGAGAATGCGTGAGCATGACAGTCAAAATCAAAGTTTTACAGAATTGAAGACAACAATGAGGAGCCCCAAAAGGGTGATCATAAAGCCTATCTCTGAGGGCAAAGAAATTGTAGAAGAAGGCTCAAGCTTCAGTCCAAAAAGCCTATTTACTTTGCCCCATGGTATTGGTGGCTTGCACGTGCTGGATTCTTTAAATTATGTTGGTGAGAGTTCTGAGGATCTGCTGTTAGAGGTGCCTTCTAACAGTTCTTTTCCACAGGCAGAGCTCCTGCTCCCAACTTTAAGCTATGGTGCTCAGGTCAGCACCACTAGTAGCTCAGTACACTCACCTGTTACTCATCCTTAATATTCAGTCATAATAGGATGCACATGTTTGCATCTGAAACACCAAGAGACTGTACAAGAGATATGATGGTTGTGTTTTTGTTTCTTTGCAACCTTGAGCTTGGATTGCCAAATGATGGAAGTTGAATGGGCTTTTTTTGTATAGGTTGGTTATAGATTGTAACATATAGTATGAAATGCGGAGTGATCTTCTCCGGAGTTTTGTATGCAATTGTAGTTTATGTTTTGTGAATGATTTTCACACCCTTTATTCCCAGTAGG

>C100315_65

AAGAAATCAAGATGTCAAGTTCCCTAATCCCTACCATTCTCTTCTTGAGAAAAATGAAACAAATAGCATTTTTGCATGCCATAAATAGCATTATTTAAACCTGAACGTTGGAATGACAATGCGTATCTCTCTACTAACAAGTACCTTTTTTGTATTTTGCAACAGAATATCTGTACTAATGAAGAGAAACACCACTATAAGGGAACCTTGAAGGGAACCATACATATTGTTGCTGGTGGTGGTGGGGCAAGCCTTTCAACATTTACTTCCCTAAAAACCAAATGGAGCATCTTTAAAGACTACGACTATGGGTTTGTAAAACTAACTGCTTTTGATCATTCAAATTTGTTGTTTGAGTACAAGAAGAGCAGAGATGGGAAGGTTTATGACTCCTTCAAAATATCACGGGATTATAGAGACATATTGGTCTGCACTATGGATAGCTGCCCAACTATGACAATGGCTTCTTGATTGATGAAATGGTGAAAGCTTCAATGTATCCAGCCATGTTGAAGCAGTGAATTATGAGTAAATTATCTTCCACAAACAGATAGATGTTATATTCGGCTTCAAGTATGGCTTTGGTAGAACGCGCACTCAATTTATTGCTGTTGAAGTTTAAGCTGCAAGCACTTTCTTGTATCTAAAATAAAATAGGAGGGCAAAATACAATGCTATACAAGCTATACCAGTAATTACCAAAACCAAATTGAATCCAGATGGATAGTCAAAAACTGTGGTTTCAAAGTTCATTCCAAATACACCTGCTACAGCAGCAAATACTGCAGCTACCAATGTAGCAGCTGTAAGAAGTAACTCGAACTGTATTAGCTGGTTTTGAATATTTCCCAATTTTATGTTGATAAAATCTTCTGTGTCGTCAATGTATTCTTTGAGCGACAATAGTGTGTTAAGAGTATTATCAATGACAATAAAGTATGCTTCAAGCAACATTTCCAGCGGCTCAATCCTTTCCCCATTGTCAGACGAACCCATTAAACTACCATGTTTACTAGAATTTCCAACGCTGCTGAAAGCCCTCTGCAACATCTGGACTCCACTAATTGTTCGTTCGGGTGAAGCAGGAGCTGATTTTGAAACTACTCTACCTGATGCATGAGTTTGAAAACAATCATTCAAAGTGTAAGTATCAGATCTTCCCTTTTTCTCAGTTAGACACATCTCAGCCATGTCACCATCATCATCCATAAGATGTTCTATTTCATCACGAACCTTCTGAACTCGTTGAGTCAAAGCAAGCAGGTGACCTTTAAATCTCCGAACACGTTCCAGATTTAGTGTACTGATAGATGAGGCTAGTTCATCTAGCACAGGATATATTTCCATTTCCAGTTCATTTACCTGAGCATCTAAAGATGAGCATGTCAATTCTAGAGCCAACTCCAGTGCCCTAAATTCAAAAGGTAGATCATCAGCTTTTTCATTCTCAAGCCGATGGCATAATTCTGACCTGTACTGACCAACACTACCATCCAATGAATTCATCAGGATGACCTCATCAGCAGTGATTATACATCGGATTTGCTCAAGGTTGACTACAATAGCCTTCTCCCGTCCCAATATCGTAGAAGGATAAATAAACATTGGATCCAATAGTCGGAGATCTCTAGAAGGCAAAGAACAATGTCTCATTATAGTTGCCTTGTCAAGTGTCACAGTCTGAAAATTCCCATCCTGACCAATTTTAATCCAAGAGCGACTTCCATGACCTCTCTTCTTCAGGCCTGATATACTAGACCCACGATTTATTTGCGGATTGGAATAAGACCTCTGGCTGCCATCATGAAATAAAGAAATCTCAGGCAGACTGGAACTATAATACCGGTCTTGTGTTTCATCCATAACAATTGCATTACAATTTTCTAGCTTGCCATACTGATACAGAATTATTTTAACTGATATAGTCTCTGTCAGTATTCACTATTCAGCATTCATTTCCATCACAACCTTGATATACAGGTGATAACAAGAACCACCTCTACCACCTGAGGTGATCCATACGTTGAGCCTTTCTCAAATCAAAACCTGAAGGTCGAGAATCAGCAGAAAACGTTTGAAACGTGGAAGATGGTTTGAAGTAAGATTACGAATGGGTTTCTATTCTCAGAGAACAGAGACGAATAAGATTACGAATATCACGTTACCAGAAATGATGAATTGTACCAAAGCGCTTTTCATACGATCTGATTTCACTTCGAAGAGTAAAACAGCTTGACAACGTTATTTTAGTTGTATCTGTAACTCTGTGATGCCGAAGATTCA

>C100319_65

TAGAGAACCTCCCACCATGCACCGCAAACCACCCAACCCATCACGGATATTAAAATTAGCCATAATTATGATCGAGATCCATAGTCAAAGCATACTAACTGTTCCATAAATTACTAGTAGACAATAGTCTGCAACAAAATTCCGACCCACGTAAGTATCACTAATCTCTTTGCAATTATCAAACCGCAACAAACTTCTGACCTAAATAAAGTAAAATAAGGTCAAAATGGGAGCATATTATCATCGCGAGCAGCATAGTCACCTATCATATCTCATCAATCAAGTCCATAAGATCATCAATCTCCCGTCTCAGAGCAGTAATTTTCTGCTGATATGAAGCAATCCGATTCTCAATTTCCCTTCCTCCTAGCTTCTTCTCATAGCCGTCTACAACTGTAGTGTGTTTCAATACAAGTTTCTCCTGCAACTCTCTCTCCTTGGTAACAAGTTCCTCCACCTTTGGTAATATTTGAGCAGCTTGTGGAGAAGATTGCAAAAATGCCAATGGTTGCTTCAACTCTTTAGAGAGCTCTTTCAATGAGCTATCTACTGATTTTCGAGTTGCTTTACAGGCTTGAACGTCTCCTGTCCTAGAAAGATCGTGCAATGACGTTTCTAGCTTGTCATGTGCTGTTAAGCAACGGTTAATGATACCATGGACCTGCTGAATAGTTGCTTGCACCTCGTCCCACTGGAGCTTTGCCAAATAAGATGCAGAAGATTTGGAGATTGATATATCTGCATGTGAGTAGACAATGCATGCAAGAAACAGAAGAAAAAACCCAGAAATCAACATCAAAGGCTCCCTGAGCATAGAAAGACTGTTGAACTTATAGTAGACCTGAAAATGCTCATTATGCTCAGGGACTACATTGTTCTTCTCCAGAACAATAACAGGTCTACCAACAATATCCAAGTGGGAAATCTTCGTCTCCTGCCATTCTTTCACAGGAAATGGAACAGATGCTGAAATGTATTTAGAACCCTCTGGCAAAACAACCTTCACAAAAAGAGTTTCAATCACCAAATCATTAATAGGGGCACCAAAAGAGATATTAAGGAAGCGCTTTCCCTCCAACCCAAATAAGAAGTCCTGAAGTGGCACGCCATAGCCAATAGTAAAGGAAGCTTTCCAGCCACCAAACATGGGGTACCTAGGTTCAATCTCCAGTTCTGTCTTTTTTGAGTCAGCCCATAAACTAGAAGTGGAAATGTTGCCAATTTCATCCCTGTAGTACACAGAATGAGCTCTTGGTGGCAGCTTGGCAACAAGACGCCTAAAGGCTGATGCACCCCGTAAATATGGCCTGGTCTGATAGTCAAGCCTAGAAAATTCTCCTTTGCTCTGAGCACCAGCATGAATAATATCGTAATGCTCTGTGATCTGTACATTTCCCCAGTGGGAAATTTGGATCTCTCGCACCAACTCTTTAGCAACAGCAAAGGGTTGGTTATTCTCAAAATGAATAACTATTGGTACGAATGAGAATGGAGGAAGATTCTCATATGGACCATATTTTAATTCAGATCCCTGGAGTTTGGCATTTTCTAGTTTTGAATAGGACTCTATTCTTGCATCAGGCAACTTAACAGTGAGTGATTGGGCCTTGACAGCATATGGAGAGAGATAGTGTGCACTCTCTTGAAACAGTAGAAGTTGGATGTCAGCCTGGCTGATTTTCTCTGGAAATGGTTGCAATATGTGGGTAAAAACAGCCAAGACATCTAACGTCAAGCTATGTCCATTCCCAAGCCCCTTTGGTAAAGATACGGAATAAATTGTCAAGGCAGGAGGTACATCTTTAGGGTGGACAACTTCAACGGGTAAACCAACACCAGAAGATGTTTTTACTTTACCCTTCCCTTCGCTAACTGTTGCTTTCAAGTATGCCAAGTGTTTTGCCTGGTTTTCAGGAAAGGACAGCAAAATCTCAGAGACAACATCAGATCCCGTATTCTCCACCTTTAGTGAAGTAGTGATTCGCACAATTTGTGATGTCAGATCAATACGACGGTCAACCTTGGCGAGGATCAGATCCGAAGCAGAGAACACGGGCGACGACAGAAAGGCAAATGCAAAGAGAATCGGAAACAGAAATGGAAGCAGATTCAACCTCATGATGCCTCTTTTCATTGTTTTCTTCTTCTTCTTCTTCTTCTTCAACTCATTCACTGCACTCGGGACCGAACAGCCACAAGACGCTGATGTTTTTCTTGTTCAACTCAACTGTATGTAACGAGTAATTTACCACATTGTACTGTGTCTCTGCTGTTAATAAAAAT

>C100321_65

TCTGAAGCCGCCACTACAGAGAGTTGTGGAGGAGAAGTTCATTTCCCTGTTACGGTCGTGCGGAGATTGCACCCGGCTGCACCAGATCCAAGCCCAAATAGTCACACATGGACTCCAGGGCAATGATTACGTCACCCCTAGTTTCATCACGGCGTGCGCGCGTCTCCGTAGAATGGGTTACGCCCGAAGAGTGTTCGACACAACGGCCCAACCAAACGGCGCCACGTGGAACGCCATGTTCCGAGGGTTCGCCCAAGAGGAGTGTCCCTTTGACGTCGTCGTTTTGTTCGCGCAAATGCACCGCGCCGGTGCTTCGCCGAACTGCTTCACGTTCCCGATGGTGGTGAAGTCCTGTGCTCAGGTGAACGCCCTGAGAGAAGGAGAACAGGTTCACTGCGTGGTCGCCAAACGCGGTTTTAAGGCGAACGCGTTTGTGGGAACTGCTTTGATTCACATGTACTCCGTCAGAGGGGAAGTGTCCGTTGGGGATGCTTACAAGGTGTTCGGCGAAATGCGCGACAAGAATGTCTTCGCTTGGACCGCGATCATCGCTGCACACGTGGCGTGCTCTGACATGGCTTCCGCGCGCCGTCTTTTTGACCTGGCGCCGCTGCGCGACGTGGTGCTGTGGAACGTCGTCGTTTCGGGGTACATCGAATTGGGGGATATGGTGGCTGCGAGAGCGCTGTTTGATAAAATGCCGAACCGCGATGTGATGTCGTGGAACACCGTGTTGAATGGTTACGCCTGTAACGGCGAGGTGGAAGCGTTCGAGAAGCTGTTTGATGAAATGCCTGTGAGGAATGTTTATTCTTGGAATGGGTTAATTGGAGGGTACGTTCGGAATGGGCTATTTAACGAAGCTTTGGAGTCTTTTAAACGGATGTTGATGTTAGTAAAAGAAGAAGGTGAAGGTGGTGAGGTTGTGATTGTTCCTAATGATTACACGGTTGTGGCAGTGCTGTCTGCGTGTTCCAGATTAGGGGACCTTGAGATGGGTAAGTGGGTGCATGTTTATGCAGAGAGTATTGGGTATAAGGGTAACTTGTTTGTTGGAAATGCTTTGATTGACATGTATGCAAAATGTGGGGTTATAGAGAAAGCACTTGATGTGTTTAATTGCTTGGATGTGAAGGATATAATAACTTGGAACACCATAATTAATGGCCTTGCTATGCATGGTCATGGAGCCGATGCTTTAAGTTTGTTTGAGGGGATGAAGAGCGCGGGAGAGAAGCCGGATGGTGTTACATTTGTTGGAATTTTATCTGCCTGTACGCATATGGGGTTAGTGAGAGATGGGTTTCTGCATTTCCAATCGATGGTTGATGATTACTCGATTGTGCCTAAGATTGAACATTATGGATGTATGGTGGATTTGTTGGGAAGAGCTGGTCTTATAGATCAGGCTGTGGATTTCGTGAGGAAGATGCCAATGAAACCTGATGCGGTTATATGGGCGGCACTGCTTGGGGCGTGTAGGATGTACAAGAACGTGGAAATAGCAGAACTGGCGCTTCAGCAGCTCATTGAACTTGAACCCAACAACCCTGCAAACTTTGTCATGCTTTCCAACATATACAAGGATCTTGGAAGATGGGAGGATGTTGCCCGGTTGAAGACTGCGATGAGAGATACTGGATTCAAGAAGTTACCGGGATGTAGTGTTATTGGGTGCAATGATAGTGTGGTGGAATTCTATTCCTTGGATGAGAGACATCCTGAAACAGATAGTATATATAGGGCATTGGAGGGATTGACGATTCTGTTAAGATTGCATGGATACGTTCCAAATCTCGTGGATGTTGCTCATGGAAACTGAAGGAGAAGGGAGGTATTTTGAGGATAGTCAAATCTTCATGAACTGGCTCAGCATCTGAAAGACCCAATCAATTCTACAGGCCAAAATCAATTCTTCAAATTCCGGGTCTATTTTGAAAATCAATTCTAAGGCCTTTGAAACAGTTAAGAGTCACGTGAGCAAAATCACTTGAGTCTTTCATCCTCCTGCTGTTTGTTGTCAAACTCAGTTATTGTCAATGATCACTTGGCAATTTGGCGACGCTCACTTCTGCACTGCATGCATGCCTAGCTCCATTTGGCTTCGCCTGAACCTGAGACTAGGGAAATGCCCTTTCTATTGTTGGAAAAATGTATTACATGCACGATTTGGCTTGGAAAGGTTATGAAGCTGTGAATTGATAGATTATACCCATTCTTAGCCAAGTGCTCCGCGATTAAACATTCGCTTTTCAGTGAATTATCTTTTAAGAATGGTTCTTTG

>C100323_65

GTTTGAACAGTTTTTTTAAATGTCATCATAAAACAAATCATGTCCAAATAATGCTTACAATTTAATATATAATATATATACTGAAATTAAATATGCTCTATAATTTAGATATAGGACAGGAATTAGAACTTGTATATGAACTTTCTGACCGAGTTGGACAGGTAATGCGAATCAAACTTCATATCATAGCAATTAAGGAAAACTTTCTGTAAATATGCGGAAATGAGTGCAATGAAACAGCAGTGAATATATAACAAACCAACCATACAGCTGCAGGACCTCCTTCCCAAGCTATAGTTTTATGTCGGAATTTGAGTTGCCATTACCATTCTGGTGCTGGTGCTGGTGCTGTTGCTGTTGAAGATGAATGTTTTGCTGGGATGCAGAGGGAGGCTGTTGCTGCTGGAACATCAGAGGGTTGACAGAATGTTGAGGAATCAAACAGCTAGAACCATGCGAATCTCCACTCAATTCAGCAGTAGCAAGCTTTAGTCTTTGAACCTCAGCAGTAAGCGCCTCATTTAGAGCATCTCGGAGCTTTGCTTGTTGTTCCATGGATTGAAGGCGAAACTTCAACTCGCTATTCTGGTTAGTGAGTCCAGCAGAATCTCTCTGTAACAAAGTAAGCTGTGCAGATAATGTAGTGGCTTCCGTCTGTAGAGTCTGAACCTTATGTTCTAGCTCAGAAATGTACCGCATCTTCCGCTCTTTGGATCGTGCAGCCGATTGCCGGTTGGCCAGAATCCTTTTTGCACGCTTTGGATCGGTCAAGGCAATCTCAGCAAGTTTTTCATTGGCCATAATTTTCTTCAATTCAGGCCCACTGAACTCCCCATTTCCAAACTCCAAGCTGAAGGCGGACGAATTCCCATCAACTCCCCCGGCTGGTGAAATCAAACCGGTTCTCGGCCCTGGCGAAGGTGGCAGCTTCGGCGACTCATCACCAAAGTTCAATTTCGAAATGAAACTATCCATGGAGACACTCCTATAGTGCCTGGTGGTGGGTGCAATGTCCCCACCCGCACTCCTCTTCATCCCTTCCCTCTTCTCACTCCCTCCCTGCCTAACCACACCATCCCCACTCTCGTTAACACTACTCTCAGCTTCATTATCACTACTGTCACCACCATTAGTCTTAGTCCCACTCGCTCTACTATCCAAATCATCGCGATTTTCAGTCCCGTTTTTGTCATCGGTTCCAGAAGAGTTCAACGCATCAAAGCTATCCAAATTCATGTAAGCAGAGAAAAGATCATCCACCACTTCCCCTTCAGGGGATTTCTTCTCCCCAGACCCCTCTACATTACTGTGGTCAACACCCCTGTCCCACGGTGTTTCTCTTTTGACCAATTGAGCAGGTTTAGCCAAAACGGGGTTTTCTCTGCCCCGGAGAGGAATAAGCGGTGGCGAAGATTGCAAAACGGTGGAAAACCCAAACGGAATATCACTGTTAGAGCGTCTATGCGCTTTGCGAGGGGGTAAAGGTAAATTACTGTTGGTTGAAGCGTTGAGTGTTCTGGTGAAAGGTGAGGGTGGCAAGAGGGAATGAGAAGTGACGTCACGATCTTCCATAGACACGTCAGCAGCTTCGGAGACGGAGGTAGAGGAGGAGTCGCGGAAGGGGGAAGGGCTGAGGGGAGGGAGGGAATCGAGGGAGAAAAAGGAAGGTTGCGAGAGTGATCGCGTGTGGGTGGGTGTAGGGGAAATGTTGTGCGACCCCATTTGCTGCTGACGCGTCACCGGGATCTGAGAGTAGGGCGAAATGGGTGGGATCTGGTGCGGGTGCGAAGGTGGTATACCCGCGCGCTTGTTACTGTTCCCACCGTCAAACTGATGCTGATGCTGATGTTGATGTCTGACGCGCATTTGCGAGGGGTTGAATTGCGGGATGCTGAGTTGCTCCATGTTCAACGGCTGTTTCAGAAACGAAGAAGAACAGTGAAGCCTCTGCATCATGTCATTCGTCGCTTCTTCATTTTCACCCATTCTCTTCACCTTCTTCAAATTCCACTTCCAATCAAAATCTTGGCAGTGTTCATCTAAATATTGGAATAGGGAATGGTGAAGAGGCGAATTCAATTCAATTGATTAATAATAGCAGCGTCGGTCTTGGAAGTGTTAACTTTGTCTGACGGAAATGCCGAAGGAGCAGGCGCTTATCTCTCCAAGATTCACTCTTTCTCTCTCCAACAAGAGCAGGAACGAGGGAAGAACGAGAAGAAAAAAAAGCTTTGTGTCTCTCTCTCTCTCTTTCTCTTTCTCTTTCTGCTTTCTCTCTCTACATC

>C100327_65

CTTGCTGCTGGCGTTATTATTGTCACTGTCAGCTGTGATTTTGTTCCCAAATTTTTAATTGTTTCTTATTTGGAAGAAATTTTAGATGCATGAGTCTTAAGTGATCCTTTGCCTTTAGCTTCGGAACAGTTTTAAGTCTTGAGTTTGTGAAAATCGATCTTCGGGCTTCCAGATTTGCTGTGTTTTGATCTGTTTATCCAACCAAATTCATCGAGTTTTGCATCCAGAGAGAGAAATTTTCATTGTTATCACAGAAAGATATTTCAAGTGACTATGGAGAAACAAAGTAGTTTTCGGGCATCTACTATGGAAAAACAGAAGAGTTTTCGTGGATTTATGGAAAAACAGAAAAGTTTTCGCATAGCGATGGAGAAGCAGCTCAGCTTTATGGGAAGCGAAAGGAAGAAGAGCAAGGAATCACCTGGTAAACGTGGTGACTTACCAATTCATTTAGCAGCTCGGGCAGGGAACTTGAGCAGAGTGAAAGAGATAATTCAAAACTGTTCTAATTATGAGACAAAAGATTTGTTGGCAACGCAGAACCTAGAGGGGGAGACCCCTCTTTATGTCGCTTCAGAGAATGGGCATGCTTTGGTTGTCAGTGAGATTCTAAAGTACTTGGACCTGCAAACTGCTTCTATTAAAGCCAGAAATGGTTATGATCCATTCCATATTGCTGCAAAGCAAGGTCATCTTGAGGTGCTAAGAGAACTACTGCACTCCTTTCCCAACTTGGCCATGACCACCGATTTATCCAACTCAACTGCTTTACATACAGCTGCGACTCAAGGTCATATTGATGTGGTTAATCTCCTTCTGGAGTCAGATTCTAACCTTGCTAAAATAGCCAGGAATAATGGTAAAACTGTCCTTCACTCTGCGGCTAGGATGGGGCATTTGGAAGTTGTGAAAGCTTTGCTAAACAAGGATCCAAGCACTGGATTTAGGACTGATAAGAAAGGCCAAACTGCGCTACACATGGCTGTGAAAGGGCAAAATGAAGAAATTTTGCTGGAATTGGTAAAACCTGACCCAGCGGTTTTACGCTTGGAAGATAATAAAGGAAATACAGCATTGCATATTGCGACAAAGAAGGCCCGTACTCAGAATGTTCGCTGCTTATTATCAATGGAGGGTATCAACATCAATGCAACAAACAAGGCTGGAGAGACTCCTCTTGATGTTGCAGAAAAATTTGGAAGTCCTGAACTTGTCTCCATATTGAGGGATGCTGGGGCTGCCAATTCGACTGACCAAGGGAAACCTCCAAATGCATCAAAGCAACTAAAGCAGACTGTCAGTGACATAAAGCATGATGTACAATCGCAACTCCAACAGACACGTCAGACTGGCATGAGGGTTCAGAAAATAGCGAAGAAGCTGAAAAAGCTACACATTAGTGGCCTGAACAACGCAATAAACTCTGCTACTGTTGTTGCTGTTCTTATTGCTACAGTTGCTTTTGCAGCCATCTTCACTGTCCCTGGTCAATATGTTGAAAGCAAAACAGATGGATTTTCACTTGGGCAAGCGAATATAGCAAACAATGCAGCTTTCCTAATATTTTTTGTGTTTGACAGCTTGGCACTGTTCATCTCTCTGGCAGTTGTGGTGGTTCAAACTTCTGTAGTTGTGATTGAGCAAAAGGCAAAGAAGCAGCTAGTTTTTGTCATTAACAAGCTCATGTGGATGGCTTGCCTTTTCATTTCCATTGCCTTCATTTCTCTTACATACGTGGTGGTGGGATCACACTCCAGATGGCTTGCAATATATGCTACAGTGATTGGAAGCTTGATAATGCTATCTACAATTGGCTCCATGTGCTATTGTGTAATTTTGCATAGAATGGAGGAGACAAAATTGAGGGCCGAGAGTCGATCGTTCTCCATGTCTCATGTATCAGACCAAGAGATTTTAAACAGTGAATATAAGAGGATGTACGCACTGTAGGATAGTTGATATATCTGAATCGAATGGCTTGTATCCCTCCAGCATATTCTTGAAGTGGTGATTGGTAGTTTCTACTCATGTCAAATTGTATGAAGATAACCTATGATCATTGTTAGACTACAGATACCGTGAGAAAAGAGGGAAAATGTTATCAAATTTAGCATGATCTTGGTGAAATGATTGTGGTGTTGAAGACTTATTTTCTTTACAATAGGTGGATGCAAAATTGTCATTTATATAGTTCACAATCTCACCTAAGATGCTACCTCGAGCTATATCTGTAATACTCACTTTTCTTACTTATAATTCCAACTTATAACACTTGGTTATATAGCA

>C100329_65

ATTTCGGGTAGTGGATTTTTTCCGACAAAGATGAAAAACTCCGTAAAATTACACCTTTTTACTCTTTTTCCTCTTGAAGAAACAAATTTTATGAAATTGAAACACATCCTATCTATGATTTATTTTGCTGCAAAATAAAATTGAAACTCACTGGGGTGGACATTTAGCAGATGGTTTCACCTTTGCATTTAATCATGGTGGTTTCACCAGTCTGGAAGAACCCTATTGAACCTGTAGCATTTCACAGTGCCAAAAACAAATGAAAATTCTTCGGCATTATTGATTATACTATCCCTCGGAATGCTGTTTGCAGCCTTTTGGTCCATAATGCTTGGTGCCGCGTCTGACAATCTCTTTGCATAAGTAGCAGTAATGGCTTTGGCATGCCCAGCAAAACAAATGATTGTTATTTCCAATCTTTGCATTAAATTGACGACAATTAGGACATGTCAAGCCACGCTGAGGAAAAAGCTCAGCTTGTAATTGTCCTACAGCCTGGCGATGATTTATGCGCTCTTCCCAGGACTCAACCATTTCTCGTGGGAACAGCTCGCATGAACCATCCCTGAAATGTCCATACGGATCTGATGGATCAATTGCTTTGTTGCAGCGGTAACAGAAGTATTGTTCACAATTACCACATTTCATTTTGTTACAACCTTCAGTTCGAGAAATTGCCATGTCACAAGAAGGGCATAGCTTGGAATCGCGATGAATTTCTTTCATACTGAGCATTTCATTAATCTTTTCACGTTCCTTACGCTTTTGATCTTCCTTTAATTGAGATGAATTTTGACGGTCCTGCAAAATCTGAAGCTTCATTTCTAGACTCATGCATGCTATGCCAACATGGCGTCGATCCCTGCAAAGGGTACAAAAGCTAAAATAGCATTTTGGACATTGAGCATGCTGGTCTTCATCCTCTATACAGGGGGTTTCACATCTTGGACAATAAACAACATCAGACATTGATGCAAGTGTTTTTTCCAACATCATGGATTCCCAGCGCTCATAATCTGTGTCATCTAGCATGTGTTTTAAAAGGCCAGGTGAAATCATGACTGTACATTTTGCTTCAGGGCATTGCAGATTACTAACAGTTCCTTCCTTTACATGTATCTGGGCAAAGGTCTGCAAGCATTTGAGGCAAAAAAAATGCTTACATGGTAGCCTGATAAACTCAGAACCTGCATATTCACTAAAGCATATGTTACATTCATGTAATTCTTTGAGGAAGTTCTCATTGAGTCTCTGATCATTGTAACTTCTTAAGAAAGGAATATCAATATCGATGCATTCAGCTCCTGAAATAACACGCTCATCCTGGACAAGATTCATGCCATAAGGCCCAAGTGTGATTTCCTCATCAAATCCCAGATGTGAAAGAGAAGAACCATGTAACCATTCTACCCAAGGGTAAATTACTTCCTGCCCTTTTTGTTCAGCCCATATTGAATCCAACTTGGAGCACAGACTCAGAATCTTGACAGGTTCCAACCACTTAACAGAGAGTGTAAAAATAGGTGGTTGGTGACTAGGGTACGACTTAGGTAATAAACAAGTCAAAACAATCGGTGGAAGATAATGAACTTTGAAAGTGTATAAGAACTCATCTGAACTGCTTCTTACAGTCTCAAGTCGATTAAGAGAATTCAACTTAGTAGTGATCGAAATTTCACCCAAAACATCAATATGTATATGTATCTGAAAACATCGCAAGCCTTTCCATCTCTCAAGGCTGAAAACATTTTCTCCATAAATTGACTCCACCACAAGTATCTCATCTTGCTGCAATTGATCATTGATTCTGATCTGCTCCTCGGACAATTCAGGTTCCTCAATCTTACAGAGCAACTTATCAAGCCTAGTCTGAATATCGTCATCACCATCATCATCACCGCCATCATCATCATTGCCGCCTCCTTCATCAACCTTGCTGGAGCTAGCCACACGATCGATGCCATCAACAGTGTTAAGCTCGGAGGCACGGCCACCGTCCTCGCCATCGCGATTTAGGGATGCGACAGTGCCGTCATCGACATCCTCAACGGAGTGAGAATTGGAGGAAGAAACCGATTTGGACTTGTTCACGACACGGTATTTCAAGTTTAGTTGGTGTGGGCCTCGGTTTCTACCTGCAGGCCTGCGGTGGTTTGGTGAGCCCAAATTGTGGTGAGGGGCAGAAGGTTTTGGAGCGAAGAACGAGTCTTCCGCTTCACCTTGCTGTTTCCCTCTCATGGTGGAACCAATGGGAGGAATGTTATTGTGGTGTGAACTATGTCACCA

>C100331_65

AGAGACGGCCCAAACGTGTTACAATATGGATTTACAGGAATCTCTATGGAAAACTTAAATTTTTTGACACAAAATTGAGATGTTGAAGAACGTTCGTTCAAAAGGACCAAAAAGTATTGCAAGTGTTCGGTTAAAGATATGGTAGTACACAGACGTTATCCCCAAAAGCACCCTTTGCCATCTAACAAGTTGCTCTGGAAGCTAGAAACCCACCCAACCCAGCTTCCCTTATAGTACACAAAAGAACTGCAAAATTACTATAATTTTCTACAGAATGCCCTATGAAATGAAGCAATAGCTCTAGTGGACTTGAGAGGACAAAATGCCTACTCACACTTGATGGATCGATAAATGTGGCGGACAAAGAGCAAAGCAGCACGGAATCCAACAGTTCCAAGCATCAAGAAGAAACCATAGCATATACAGGCCATGTATCCGAAGAAAAATGAGGTCTGCATGAATCCAGACATGTCGGAGCGTGCATAGTAGTAATACAGACAGTAGGCATAGATAAATAACCCAGTTGAACCACCACATAGGAATGATCTCCACCACCACTCATGATCCTCAGCAGCAAGCTGGAAGTATGTCAATGCCACAGTAATGAAAGCAGTAACAATCAAGAGAATAATGAAGACAATGAACAGGATGCTGTAGATGGTGTAAATTCTGTGTCCCCAAACACTGGCAAATATATAGTAAAGTTCAATGTATATAGCACTAAAAGGCAAAAATCCTGCCATTGCCATCTGAGGAATTGATCCACGATACCATGGCAGAGGTGGAATCTCCCTTGGATATTTAGTTGTGCGAACAGGAGCTTGAAATTCGGCCTTGCTATTCTTCCCAGCAATCCCACCCAATACAAGCAAAGGAGATGTAACAAGTGTCCATATGAGAACTATAACCACAATTGTCCCAAATGGGAGGGCAGCAGTAGCCTGGTATGCAATTGCAACAGTGTTCAAAAAGCAGAAAGTCAGAAACAGAGGCCCGCAGAAAAGGCATCCGGTCAACAGCAGATTCCTAACCCAGTTTGTTCCTTCCAACTGACAATAGAACGAAGTTGCAGTATACCCAGCGATCCCTGAAGTAAGTGCATAGATGACAACAAGTGCGGTAAATAGTGCTCCTCGATTGTATGGATAAAACACACCAACAAGTGCCAATATGAATATGAACACAGTAAGTGTAAACAACTGAGTGCCAGATCCAAGAGCAGCTGCAAAAAGAGACTTAAACTTGGGAAACCTAAATACATCCCCGTGGATATATTTCCAGCCAGTCTCTTCCTGATCGTCTACAGTCTCCTCATCATGTGCATACTTTACAAAATCGTTCTTGAGCACCCTCATCAAAATGGTGGCAAGGAACCCAGTCAAGAGCAGCACTGTGACACACGAATTGATAATTGAGAACCAGTGGATTTCCAAATGATGCGGCAGAGACGAGGACTGTGAGTACTTATCCATCCTCTTCTCAAAGGGAGTGTTGGTCTCCTTCCACTTCACTGTATACAGAAACTCGGCATCCACCTCAGTATCCTCAGTGAGATCCACAAGGGCATTGGGATCAGTGCGTACATTGATCTCGATGACCCTGTCCTTGTTGTAGAACACATCGAAGTGAATGTGCTTATACAGGAAGTACCTATACTCACTAGGGTCCTTCCCTTCCTTGTCCACCTTCCCTATGAACCCCCAAATCGGAAGGTCATCGTAGTACATCTGGAAGTAGTAGTCCTTACGGACAGCAGATCTGAACCGCGCCACATCCTCTTTGGATAGCGTAGTATTACAAACAGAAATCGACTCCTTGTCACGCTGAAATTCTAGCTGGTATGGGGCACTAACCAAACGGTCCCCATTCAGGACCTCCCCAAGCGCCTCCTTCTTTTCTTTCAAATTACCTGGAGGACAGAAAGGAAGGTCGAAATAACGATAGGTTTCGCTAGGGTTGTGGAAGGGGCCAACTTTGTTGGCGTAGAGAGGGACGATGTCGCCTTCTTTGTAGCGGTGATCTGAGGAGTCAGATCTGACGGCGGAGAAGGCGGCGAAGGCGGTGAGGAGGAGGAGGAAGAAGGAGGAAGTGGAAGAGAAGAGGTTCTTCATTGGGAAGAGGTTTGGATCTCGAAACCCAACCCCACCGACGAAGAAGAGAAGAGTGGAAGGAGAAGAGTGCGGTGTGAGTGTTTTAATATAAACGGAAAACAAATGAATATGAACGAAGGAATGAATGAATGAATGAAGGAATGAAGAGTATGAAGTGACGTAGACTCACCGCCAC

>C100333_65

TTTTTAAGAACTCCATATGCAACTGTCCTTGGATGTGCTCTGATAGAAGCTTAAAAATAATTGCTACAAGGGATTGGTTATCCTCACCTTTTGAAGTCCACTTAGTGCCCAACACGTCCTTAGCAATCTGGAAACAACATTACTATATTGATCCTTAGACAGATGATGCTAGCTGGGTCATTATTACATAAATACAACTGCAAACTTGATTGCATTGGCTACGTGTAAATAAAGTGAAATAGCCAAATAAGTTATCAAAGTGTGTACAGAAAAATGGTAGGAATCTCATTCATCTATACAAAAATTAAGGCCCGATTTCCCTGATTCTCCTGTTGAGAACTTCAATATTAAGGTACAACTTAAACGGTACAAGACTAGCCGTGGGAAGAGGCAGCAGGATTGCCGTTTGCTACTGTCCCCAAAACTCCATTGCCCTCTGCGGCGGCCTGGTCTTCATTTGCATGGCGAACAAACTCCTCAGGGGTCATGTGAGAACCATGGCATGCACAGACAATTCTAACTTGGTTAGTGTTATATCGGTACGTAACACCAGATATTGTCCTGCCATTTGGACCTGAACCTGAGGTGGATACCCAAGGCAGATTTGGATAGGAACCACATCCACCAAATTTAACATCTGCAGCAAGACCGGGCTTAATATTTGAAAAATCAATGATGGAACCTTCTCCTGTTAATTGGTCTGATATATCTTTGGCCCTAAGGTTTGTGCTTCTTCCTTTCACATCTTCAGGTTGTGAAGAAGAGCCTTCTTCAGTGACACGCTGTTTACCATCTCCTTTGGTACGATCTAATGGCCTTCCTTCGTATGGTTTGGCCTCGGATGTGTTGTTTGAGATTACTTGTAGAGCTGGGTTAGCTGGACCCCTGCCAACATAAGAAGGGTGTAATTGTTGAGGATGAGAAACCAAACCCCACGATTTGTCCTTCTCCAACATGGGAAGCTGAACAGGTGGATATCCAATCATCATCGGCAGGCTCCCATTATTCACTGATTGGGCGACTGGGCGTTCACTGGTTGCAGCAGGCATTACATGCATCACTCCATGTATCTGGGGGCCTGGTGCCCCAACAGAGCTAGACTCTTTTACTGGAGAAGGGTAAGATACATTCATCATATTTACAGGCTGAACAGAAAAGGAAGCACCGTAAGTTATGCTTGCATGCTTAAAATCTTTTTCTGATGACCCAGTAAACCTCTTCTGCCCACTAAAGTCAGTAGCATTTGAATCAGCACTTCCACGAACCTCTTTTGCAGCTTCAGAAGAAGAACCAACTCTGATGAATCCTTTGGAACCATCACTGTGGTGTGAGATGGGCCTGGAGGTGGAGTTCTCAGTTTCAGAGTCACCCACATCTTCATTTTCCGCAGTTGAACCTTCTTCTGTTATAGAAATATGAGATGTTCTAGCCCTGTCATGTAAATCACCGTGGTGGACATCACAGTCATGCTTCTTTGGATTACATATATCATCGAAAGACATTTTTCGCTTATTTGCTGCCTCTGACCGTTTATCACCCTCAATTTCAATAGGTTTACAATTATTTGCAAACAGTAATCCTCTGCCATTTAAATTCAAAGAAGCCTTCTCTTCTTTGGCCTTTGAAAGATCACTAAAGAAGTTCTCCTCAGGTTTTGGTGAATCAGTTTTTCGTGTCCCACTAATAGATTCTGGCTTCTGAGGAGCAGTATCAAACATGCTCTTATAGTCGTCTACAATTTTGCCACCTCTGCCAACTTCTTCAGCCCTAGTATCTGAGGAGCTACCATTCTTACCCTTGGGTTTGGAAGATGAGCCACCACAAGATAAACCCAAGCTGAGCTCAAGCCCACTCTCATCCTCCATATTGTAATATCCAAGCCTATCAAAATTCAAACAGATCAAACCGCTTGAGAGTCTTATCTTCCATAATACTCGTTATTCATCAGCCCACGCACACACCTTTTCACCATAAGCAAATATCTTACTCGTGATCCAAATCTTCCTTCCCTCTCTTACTCTCCCGAGAGGGGAGAAATTCTTGCCTCTGACACCAAACCTGGAGAAGCCAAAAGCCTTAGGCCAAATCACCAGCTACAAGAGCATACCTTTTCTCCCCTCAATTTAGAAACTTTGGTGTACCAGAGCCTTGCTTGAAGGATAAGAAAAATGATTAAGGCTCCAATCGGAGATCCAAAGCTTAGTCTGAAATTGGGAAGAGAGATGATCAAAACCCTAGGTCAAGTCTGCATCTCTCTCTATGTACCAGTGGAAGATTGAGACTGTCA

>C10033_67

GTTGAAGGATTCTAAGGTCAATAAAAGAAAATCCAAACCAGATTTTCCAGTTACAAGAAAAAGTCCATTCACAATTAAACAGCTTCCAACCAAATTATCAACTAATTAATCCTCTGACAGTGTAAAACCAATTTTCCACTTTTCTGAGAGAGAGGATATGGGAAAGGACTATGAGCAAGCTATTGAAGAACTCCAGAAATTGTTGAGGGAGA

>C100341_65

GCCAAGTACAACTTGGAAAACCAAACCTGTTTGTGTATACACACAATCATTGATTTTACTCTCACATGAAGGAACACACGTTAAGATTTAAGAATGTACAATATTCTCTAAAACCATTCCCTCCGTCGTTGAAATCATCTCATCTTGGGGAAAAATTGTAAAGAAAACCTCAAAAAGAAACAAAGGACCTTGGTTTCAAGTAGACATCTACTCAATTAATTACACAATGTGGCTGATATTCAGCATCTGGAATGTACTTCCTAACATAAAATTCAAGGTTGATATACATGACTGTGAGAGAACTGAGAGAGGATAGAACCATGAATTAGTATATACTAAGCACAAAATTTAATGGACCATCCGTAGGAAGTAGAGAAGTAGAGAGCATCCCTGTCTTTTACATTCAGGGACTAAGGAGATTCCCTTTGCATTAATGGTGGAGTAGGTGGGTTCATTGTTTCATCTTTACCTCTTTCAGAAAGCTCTTCTTTTACAGTAGATAGTGGCCTCTCTGGACTACGATATTCCTTCAAGAAACTGCTTGAACTTAGAGGACTAGATGGCTTGATAGGGGAAGAGTAGTTACCCAAAGGAGAACTTGTGCCAGATTTGGTTGATGCCACATCTTCACGAGGCAAAGGTGACATTGGTTGTTGGTCACTATAAGAACCTTCTTTCCTGCATAATTTGTCTTCTACTATGTCATAGCTATTCGCCGTCCTGACTTCTTGAGCAAGAGAGCACCAGCAACAGCATAGCCAAAGTGTGCAATCAGAAGCTGAGGGTTTGCCAAAACAAAAATTATAAGAAGGCAAATTGAACCTTTTTCTCATCTGAATCCTCCAAAAGCCACCATAAAGTGCACCAAAAAAACACAGAATGATCCCAGTAGCTACTAGAGCCTGTCTAACAGTGTCATTCTCAATATTAACAGCTGCCAAGGTAAAAATCCAGAAGGGAGCCATACAGAACAGCATAAAAGTAGCAATGTGAACATACACGTTTCCAAAGCCAAGTCTCTCCATGTTCCAACCAAAGACACAAAATGTACAGAAAAGTGATAGGTATGCTTGAGAAATGTCATTCCAAATGTCAAGTATTCCTCCATTCCACTGTGGTCTATTTTCAATAATCCTTTGTTGCTGGCTGGATGCAAATGAATATTTCTTCTGAAATGATTTCACTCTCAGCTGCTCTTGCCTCTGAGCAACAGAATCTTGAACCTGTGCTTCTTCATCCAACTCAGAATCATAATCTTTACCTAGAGGGCTAAGAATGGAATACAGACCAGCGACTGCAGGCGCACCAATAGCAAAAGCTATGCATATTCCAACTCCTATGGCCGGGCGTTCAGATCTTTTATACCCCAAGTTTAGACCACAAAGTGCATATTGAGCAAAACAGTTCAAATTAAGTAGAATGATCACTACCATCATGTGTGCCCACTCATGGGGCTTATAAGTCCCATTCTTGCAATACACCTTTCTAAGTTTTGCGATATCTTTTGGACTCCACCTGCACAGAAGCACAAGGTCGTAGCACCTCTTTGGATGCTGGTACAAACACATCAGTGTAAACAGAGCATTGAGTATTTGATTGTTCACTTCAAACCATGCATTTCTCTGAGACTTACTTGGCAGCACAGCATTCAACATGCCAGTCATGACAAGGAACAGAATTGCCCCGGAAACAGCAACACACAAGATCCACACAAAAAGGGCTATGTTCATCGGGTCCTTAATCCATTCAACACACATCTTTTTGAGTGAACCCCAGTCAAGTTTCTTAGCAAACATGCCACTGAAGCGCTCCCTCAAGCGAAGAGAACCCGGCGAAGGCACAGCCTGAGAAATCTGGTCCTTTTCTGTTGCAAGCCGCTTGAATTTGGCAGATGCAGACCCAAATTTCAAGAAGTTGATCCTATTGGGAATAGAACTCAAGGTGCCCTGGAAGTTTCTCTGAGGATTTTCATTGCCCACCAGTGTTCTTCTAGAAGTTGAAATATCTATAGAAGCAAACTCATTGACTTTACTTCCATTAGATTCCTCAATTTCATCTTTACCATTATCTACGGAAACCATTCTGATGTGAAGCCTGAGCCTGTAAGGTGACAAAAATGGAAGAAAGAGTTGAATCGAAGGAAGAGTTATTCTGCAAAACTCAGAATGAATAAAGAATCTAAACTATGGAAAGTGCAAACTGTACAACGTAAACAAAGAACATATAAACGAACGGAACAGTTGTTTATTTCTCAGCACCTGGTCAAACAAACAGATTGCTGAATGCTGC

>C100343_65

GGGCAATTTATCCTTCATTATTTTATAAGCCTATGCATTAGATTTTTCTTCATTGGAATAGCCTTGTCTTCTACACTCCCCCATGCTCTGAGATTCTAATCCATTGTGCTTTTCCAACCTTGGAATTTCCTCAAATCTTGACCGAAGATCATGTCAAATTTGTGGCTTATTTCCACTCAGAAGAGAGAACCTTAACAATTCAGGACTATATCTGTTTTGTTGCCATGAGGATCAATTAACAGCTGTCTCCAGGCCACTATCGTCCATTGTTCTGCTTCAAGATACAATCTCCTGAAAGAGATCACTTGAATTACTACAACACCGTTGAGACCCAGGCAATTCTATAATGCAGGCATCAGAGCAACATAGAAGTTCAAGCATGTACTATCAACCATTACAACAAATTGAAGCCTACTGCTTGCCGCAGTATCGAACTCTAAATCAACAGCTATATTACCATGATGGAGGCCATGGAACCCACTTTTCAACTCCAAGTTCTTCTGAACTATACTGTACATTGGAGTCATCTTCTGTAGCTGGCAGTTTTACTCTTTATAACTCCCCTTCAACTGTTACTTTCTCACCTAATGGTAGCCCCATATCGCAGCAAGAGTCTCAGTCATATCCAACTGACCAGTATCATTCCCCTGAGAATACCTATGGCTCTCCATTGAGTGGCTCCTGCATAACTGATGATTTAAGCAGCTTCAAGCACAAGCTGAGAGAGTTGGAAAGTGTAATGCTTGGCCCTGACTCTGATAATCTTGATAGTTATGACAGTGCCATAAGCAATGGAAACAACTTTGCTTCACTTGAGATGGACAGTTGGAGACAAACAATGGTGGCAATTTCTAGCAAAAACCTGAAGCACATCCTTATTGCATGCGCCAAAGCCATTGCAGACAATGATTTGCTTATGGCACAATGGCTGATGGATGAATTGAGGCAGATGGTGTCAGTTTCTGGCGATCCAATTCAACGGTTGGGAGCATACATGCTGGAAGGACTTGTTGCTAGATTGGCTGCTTCTGGGAGTTCAATATACAAAGCTTTAAGATGCAAAGAACCAGAAAGTGCTGAGCTTCTCTCCTACATGCACATACTGTATGAGGTTTGCCCCTACTTCAAATTTGGGTACATGTCTGCAAATGGAGCCATTGCAGAAGCTATGAAAGATGAAGATAGGGTGCACATAATTGATTTCCAAATTAGTCAAGGAAGCCAGTGGATCACTTTAATTCAAGCTTTTGCAGCTAGACCTGGAGGGCCACCCCACATCCGGATTACAGGTATTGATGACTCACAATCAGCTTATGCCCGCGGTGGTGGACTGCACATAGTGGGCAGGAGGTTATCAAAGCTTGCTGAGCATTTTAAGGTACCATTTGAATTTCATGCTGCAGATATCTCTGGTTGTGATGTTCAACTACATAACCTTGGAGTTCGACCGGGGGAAGCTCTGGCTGTGAATTTTGCATTCATGCTACATCACATGCCAGATGAAAGTGTGAGTACTCATAATCACAGGGATAGGCTGCTGAGGTTGGTTAGGAGCCTATCGCCAAAGGTGGTGACACTTGTTGAGCAGGAATCTAACACAAACACTGCTGCATTCTTTCCACGTTTCCTTGAAACTCTGGACTATTACACAGCAATGTTTGAGTCAATAGATGTGACTCTTCCCAGAGATCATAAAGAGAGAATCAATGTTGAGCAGCATTGTTTGGCAAGAGACTTGGTCAACATCATAGCTTGTGAAGGGGTTGAGAGGGTGGAACGGCATGAGGTGCTTGGGAAATGGAGGTCAAGATTTGCAATGGCCGGTTTCACTCCTTACCCTTTGAGTTCACTAGTGAATGGTACCATTAAGAAATTGCTTGAGAACTACAGTAATAGATATAGACTTGAAGAGAGAGATGGAGCTCTTTATCTGGGTTGGATGAATAGAGATTTGGTTGCTTCTTGCGCATGGAAATGAGAACTGGGTAATATGTGACTCCAGAAAGTTTGAGGGGCGACAAAGCAGAACCTGTCCCTCTTCATTTTCTGTTCCATTACTATGTAAGAGTTAATGAGAAAGTTCTCAACATTAAATCCAATCTAATTGTCCATTTTCTTTGTTTCAAGCATTTCAATAGAAATTGTAGGTAGAAAATTTCTTTTTCTGCAATATTAATCAGTCATTTCAAAATCCTTTCTTGCCCCTGAATTCTGAGCACCCCAATGGTTAAATATTATACTTTGGCTTGGTGGCGGGGAAAGTGATTTCTAGAATATTAGGAGAGTGGCA

>C100347_65

TAAACTTCTAACTTTCTATGATCTTTGAATAATCGTTGGAACATATACGAGGGTTGAACAAAGGTTGAAGGTTGAAGGTTCCAGAGTGAGAAAGTGAGAAAAATAGGGAACGAAAAAGGTTTGGTTTTGCTTCTAGATCACGCTTTGTGCAGTTAAAAATAAATTAAAAAAATAGAAGAAGATGTTGCAGTTGGGAGTGTGTTTCGTGGCTCTGCATCATCACCGACCGTCACGCGAATAAGTGAGTAGTGAGTGACTTTATTGGGTGGAGAATTTCGAGAGAATGGCGGAGGTGAGAGGCAGGGACACGTGGACGGAGGAGCTGGCCAGTCTCATAGAGGATTCGGGCGTGCGCTACGGCGGAGATCCCAACGAGTCCTCCGCAACGTCGTTTGAGGTGAGGAGGTCGGTGTACGCGCCGCAGAGCGAGTCGGTGGAGTCCGGGGAGAGCTTGAAGGAGCAGGCCTTGGGGTTCGTGATGGCGTGGTGCGAGATACTCTTGGAGCTCGGGAGAGGCTTCAGGGACATTCTGCGGCAGAACTTGATGAACGAGGATTCGTATGTCGTGAGGAAGTTCGGTGGTCCTTGTTCCAAGGTTTCGAAGAGGTTGAGGTTTCTCAACGAGTTTCTACCCGAGGATCGCGATCCCCTTCACGCTTGGTCCGTTGTGTTCTTCGTTTTCATTCTCGCGCTCGCAGCTATTAGTGTAGATCCCAGTCGGGAGGCAGTGGCTCCTATGATGAAGGTGCGTAAACATCCTCCCAGTGCCAGTCGTGTGCAACTTCCTGATGGTAGATACATGGCTTATCATGAGCAAGGTGTTTCGTCAGACAAGGCTAGATTTTCACTTGTCGCTCCTCATTCTTTTCTTTCATCCAGACTAGCAGGTTTACCGGGAGTCAAAACATCGTTGCTTGAAGAGTACGGCATTCGCTTGGTCACATATGATCTTCCTGGTTTTGGGGAGAGTGATCCTCATCCCAGCAGAAATCTCAACTCATCAGCTATGGATGTTCTTCATCTAGCCAATGCTGTCAATGTTAGTGATAAGTTCTGGTTACTGTGCCTCTCCAGTGGATGCATTCATGCCTGGGCTTCTCTCAAATATATTCCTGAGAGAATTGCAGGTGCAGCAATGTTGGCTCCTATGATTAATCCATATGACCCACACATGACTAAGGAGGAGACAAAAAGAACTTGGGAGAAGTGGCTGCCAAGGAGGAAAATGATGTATTCCTTGGCTCGTAGATTTCCAAAACTTCTCACTTTTTTCTATCGGAAAAGCTTCTTGCCTGAAGAGCATGACAAGATTGAGAAGCTGCTATCTTTCTCTCCAGGAAAGAAGGATAAACTTGTGATTGAAGAACCAGAATTTGAGGAATTTTGGCAAAGGGATGTGGAGGAGTCAGTTCGTCAGGGAAACATGCGACCATTTATAGAAGAAGCTGTTCTGCAGGTATCAAATTGGGGTTTTGACCTTAAAGAACTTCATGTGCAAAAGAAGTGTCAAACAAGAGGCATACTTCTTTGGTTGAAATCCATGTACAGTCAGGCGGACTGTGAATTAGCAGGATTTCTTGGCCTTACACACATATGGCAGGGTCTGGATGATAGGGTGGTCCCACCATCAATGATGGAATACATAGAACGGGTTTTGCCAGAAGCTGTTATCCATAAACTTCCAAACGAGGCCCATTTCTCCTATTTCTATTTCTGTGATCAATGCCATAGGCAGATTTTTGCCACTCTCTTTGGAACTCCTCAAGGTCCAGTTGAACGACAAGAGGAAACAGCATTAGAGGAAGTTAAGGAAGAAGAGGTTTTACAAATACCTTCTTAACTGCTTCTAATGTTACACATAACTGCTGATATGCACGTCTTAGGTTGGATGTAAATACCACAGGCAGCTCCACTTCTCTATGGAGCTTGAAAGAAGAGAATGTTCGTTATAAATGGTCGGAATTTTGAACCATAAGAGCTTTTGGAGAGATATAGGTAAACCAAACCACCAATGTCACACATTGTTTCTCCTTCCTTCCTCTAAGCAAATTGAATCCTAAAAATCTGTCTGATTGGTTCTAGACAGTTCCACTTGTAATCATTTTTTGAAAAACTTACATAATTATTACAAGGTTTTTAGTCATATTTTCGAAGCTAAAGCCACTGAATTACTAATCTATATTCCAACCTTTTCAAGCAAATTCGTCATCTATTTCTTACTTTCTTTTAGTTGGCTAAGAAATTAGAAGACTTAATTCAGGGCTTGAATGACAAAATTTGAACGTAACTCC

>C100349_65

CATCCTTTTAACCCTGTTATCCCTTTGGGCATTCAGACTGTATCTTCTCTGGTTCTTCACAATCAACACCGCACAGCACACCAAGGGAAGGGTTGAGTGACTATGGCGTCTTCACTATCAGCTCAATTGGTTCATTGTCAGAACTCACTTCCACGTCTTCAGGGTAATTTGAGCCGGGTTGGGTCTAAGGTTCAAAGGCCACGTGTGGTCTGTTGCGGTACAGGGTCACAGCTTGGTTACGGTAAAGCATTGGCTTCAAGGTCACACTATGCCTTGAGGTTTCCTGTGAGGCAACAATCTGAAGCAAGGTCAATTAGATATAGGAGTATGACATGTGTCAATGCCACGGACAATGCTTTAGAGCTTCAAGCCAAGGTGACAACCAAGTGTTTCTTTGATGTAGAAGTTGGGGGTGAAGCTGTGGGCAGGATTGTTTTGGGCCTATTCGGAGAGGATGTTCCCAAAACAGCTGAAAATTTCCGTGCTTTGTGCACAGGTGAGAAAGGGTATGGTTACAAAGGAAGCTCCTTCCACCGTATAATCAAAGATTTCATGATTCAGGGGGGAGACTTTACGGAAGGAAATGGAACGGGTGGAATTAGTATCTATGGTCCTAGTTTTAAAGATGAGAGTTTTGCCTTGAAGCATACTGGTCCTGGAGTTCTAAGCATGGCAAATGCTGGTCCTAATACCAATGGTAGTCAATTTTTCATTTGCACTGTAAAGACTCCATGGCTAGACAATCGTCACGTTGTATTTGGTCAAGTCATCGAGGGAATGGATGTTGTGAAAACACTTGAATCCCAGGAAACAAGTAGGTTGGATGTCCCTCGGAAGCCTTGCAGAATTGTTAATTGCGGAGAACTGCCAATAGATGGTTGATCTTAACGCGGAATTTACATCCTTTTTCTTATGATTTTCAGTTCATTTCCCAATGTTCTGATTGTCCCTTATTAGTCATGTTAATGTGTTTGGATTGTCATTACTTTCTGCCAAATTACCCATATTTTGTATGTGAGAAGACTCGTGCTTAATCTCTATAATATAACAATAATATTCTCTTTTCCATTTCTTTATCTATGCTTTTGTTATTTGCGTTTACATGCTCCGAACAATATGATTCTAATTTTTAAGCGTTACTGTATGTGGGGAGACAACTGCTATGCACCGTGGTTGGAATTGCTCGGTAGGTAAAAGTTTGTTTCTTGTGCAAAGCAAATTCTATGCAAGGACCATGGTTTCAACTGCAGGGGGTTCGATCATTTGCTGTTGGTCTTTCTCAACCTCCCATATATCGGGTAATGCTTCTTTGATGTTATGAATGCTGTTCAGTGCATGGTCAGCACCAGGCACCAAATCTGAGCGTCCCACAATAACTGTGTTAAGTCCAGCAGCTTTCCCACTTGCAATGTTTCTAGCACTGTCATCAAAGAACAACGTCTTCCTTGGATCTGCATTAGCAATCTGAATGGCGGCTTCAATGGCTTCCACGGAGGGTTTGCAGAGGATTTGTGTCTTGGAGTTGAAACATCCACTCTCTACCTGGTTACAGTTTTTTTTGTCAAACGAGTTCCTCCATGTTAGGACGTGATTATCAGTTGGTACATTTGTATGATTAGGAGGATTAAGCGTTTCAAAGCATATGATGCTGTCAAAACATTCTTTCAAGCCCAATCTATTGAGCACTTGGTGTGCATGTGCTTCATCTCCATTAGTGAATACTATTTTACGCTGAGGCATGGAAAGCAGAAGGTTCCTTAACACTGGATCTGGCTTCAGTTTCCAGTATGGTAGTCTTCCATGCACGTAGGCATGAAATTCATCATTGTCAAACTCATAACCAATGGCCTTTAGTCCTGCCATAGTGGTCCCATATTCCTTGTAAAAATCCAGGCAAACTTTTGGTACTTCACTTTCTTTCATATGCAAGTGTTTTAACATGTACTCTTCTATGTTCTTCCGACATGCCAAATTGAGACCCGAGCTCAATGGGTAGAGAGTATCGTCCATATCGAAAAGCAAGCATTCGTACTTGAATCCAGCAATCCTGCACGAAGTATCCATCTTAAACAAATTCTAATTTGGTGAACCCAATGAAGAAGATAAAGTATACAGGTATATATGATCCAACCTTCCAATGTATATAGCTCAGTATAACGATTCCACGGAAAAATTATTATCAAGGCTATGAACAGGATGAGGACAATATATTAATCAGAGAACAGAAAAAGGAAGTACAATAATTCGCAAAGAAACGAAACGACTCATGTGCTGTGGTGTGTAGAAGTGTA

>C100353_65

TAGCAACTAATACAAGCACAGGACATTTCCAAGGTTACAAAAGTAACACATAGTCATGATATACATATTGCAACCTGAATCTTCCCTTCTGTTCATGTTCAAATATGTTGCCTGTGCTAAACCCCCCTCATTCCTTTCCTTTCTTTTCCATTGTATAAACACAGGCCTTTTTAGGCACTTATACATTTTTCTTTAGAGTATATTAAAGTACACATATGTATGTATATATTATATTCTATGCCATAACAAGCTACCACATTTCAAAATTGACTCTCCAGCACTGCTTATCTTGCTCTGGAAAGCTGTATGGCTTCTTGATCAAGTGTAGATTCATCATTCCAAACAAATTGGTGAGTCATGAGTGAGAATTCTTGATTCCTAGCCTCTTCAAGTTGCTGCCAGTCAGCCCATCTATCTGCCAACCCCACTCCCTGCAGCATTTTTACCACCTCTGACATGGTTGGACGATCCTCGGGGTAGCCTTGGGTGCAAAGCAATGCAACCTGAAGAATGGTCTCAACTTCCTTTGGATCATAACTCTCCAAATTTCTATCTACAATGTCCTCCAACCTTTTCTCTCTCAGCAGCTTTTTGACATAATCAATCAGAAGAACATCCTCGTCCTCTTCCAGCCGAGAGAGGTCGATTGCACGCTCCCCGGTGACTAGTTCAAGAAGTGTTATGCCATATCCAAATACATCAGTCTTCTCTGAAGATTTTCCAGTGGACAAATATTCTGGGGCAATGTGACCCATTGTGCCACGGACTTGAGTGGTGACATGAGTAATCCTTGCATCAACCAGCTTGGCTAGCCCAAAATCACCAAGAACAGCTTCAAATTCATCATCTAGAAGGATATTTGCAGCCTTTAGATCACGATGTATTATTTTCGGATTGCATTGCTCATGTAGATACTCCAAACCATGGGCTGTGCCAAAAGCCACACGTTTTCTTGTTGGCCAGTCCAAGCCTTTCTCACCTGGTTTTAAATCTCTCAGTCGATAAGCTACACTAAGATTTTCCATGAAAGGGTATACAAGGATTCTTTCAGTTGAGGTTGTGCAGAAGCCAATTAGTCTTAGGAGGTTTCTATGAACTGCAACACTTATAAGTTGAACTTCTCTCTCGAATGCAGCCTCTCCACCAGGGTTGTGATAATCAATGAGGCGTTTCACAGCGACTTTGGTGTTATCAGAGAGTACTCCTTTGTACACTTTTCCAAAGCCACCCTGACCAATTACATTGCCTTCACTGAAATTTTTGGTGGCAAGTTGGAGTTCACGCCAAGAAAATCTTCTCAATTGCCCAAAGGAAATTTTGCTCTCGTCTTCACCTGAAACGTCAACAAAGACTTCCTTTTTGTTCCTATGCATTTGATGGTGGCGATATGTGAGGATAGCCCCAAGACACAGAAGTGCAAAGGCACCACAACTTGCATAACGTACAATTTTTGCCAATTTTGATTTGTGGGTTGAATCTGGATTTTCAGATTTAGAAGCGCATGTCTGGTCGAAGCCAGGACCACACTGAAGGTGCGTATCTGAAAAGTTAAACATTGGAACTGAAAAGAGTTGCATTGGGATCCTTCCTGTAAGGCCATTAGATGAAAGATCTAGATACTTTAGATTGGATAGTTCACCCCATTTATCTGGTATAGAACCTTTAAAATTATTGTCAGCGAGATTTAGATATTGCAGGTCTTTCAAGTTGGAAACGTAATCAGGCAAGGGTCCTGAAAGATTGTTCTTCTGCAATTCCACGCTAACCAAATATTTCAATTTGGTAATTGAGGGAGAAAGTGTTCCAGAGAATCCAACCGAAGCCAGGGCTAGTGATATGACATGTCCATTTCTACAAGTGACATGAGACCAACTGAAGCATGGACTAACTAAATGACTGTCCCAGTCTGTTATTTGCTTATTGGAATCATTGAGGAAATGTAAAAGATCAAGCAGAGCTTCACCTTCTACATCAGGATCTTTGATAGCAGAACTGACTTTCAAGATAGTAAGGAATATGAGCCACCTCGTCAATATTTTGAGAGGACCCCTGCTAAAATTTGGGCGAAACATATAAACTTCTTTGATCTTCAAGGAAAACTCACCTGGTCGAGTCCCTAGATTATTAGTCCATGAGTTTTCAGAGCCAATGATTGCAACACATATATGTTTTCACATCACATCTATTTTCATATATGTTTGCCAAATATCTATGTAACAAGAAGCTAGTTAACAAGCCAATATATGTATGAAGTTGGTAAAGGGACAAAATGAAGAGAAAAAGAGCAAAAG

>C100359_65

GTTAATCCATCACTCAGGTGTTTGCATTATGTATCAATTCAATCGGCATATAATCAATTTCCCCTATAATAGCAATATTCCACCAGATCTCTCTTTTAATATATTCTCATAATTACAAAAATCCATTTCATTGGAGACAAACAAAATAGCTGCAGAGATAAAAAGGCCAACCTAAACCCCATGCATAACATAAAATCTTGAGCCTTCAAAGAGAAAAGGAGGGGTGTGTGGAAATAAGGAATACAAAAAACAAAAAAATCTTAATGCTGACATTGAACACTACAATTTTAATGCCTCCCTGAACTTGATACTGAAGCTATTTTCTAGGCCCTTTTTACCAACCGCTGGAAGATTTCCTTTCTGCATCATGGCCACAAATTCATTGTAATCTATGCGTCCATCATTATCTTCATCAATCTCTTTGATTATCTCTTCCAAACGAACATCTTTTATGCCAAACTCATCACAAGCCTGTTGTAGTTCTTCCTGTGTAATATAGCCACTTCCATCTTTATCAAAGTAAGAAAAGGCTGCAAATAGATTATCTTCTCTTTCGATTTTGTTGCGGTGTAATGTTGCAGCAAGGAACTCGCCATAATCAATTGTCCCACTGTTGTCAATATCAGCCGCTTGCATTAAATCATAAATTTCAGACTCCTTAAGATTAGCACCCACTCTTTTCAAACCAGCTTTAAGTTCTTCAAAAGTGATTTGACCACTGTTGTCTGCATCTATCATGTTGAACATCTCTTTTAAGCCAGCAATTTCTTCTTCAGATAAGCTCTCGGCAATAATTATAAGAGCCATTTTCTTCAGCTTGTTCATAGCAGAAAATTGCTTCAAGCGACTTAATACAGCAGAATCAAGTGGCTTGTCAGGAGCTACACCATCAACTTGAATCCAAGGATGACATAATACTTGATGCGCAGTCAATCTCCTTCTAGGGTCACGAACAAGCATTTTTCGTACTAAATCTTTTGCACTTTCAGAAATGGTAGGCCAGGGATCAGATGAAAAATCTAGATCACCACGCAGAACCTGTTCAAATATTCCTTGTTCATTCTCAGCCCAAAATGGAGGAACTCCACTCAAAAGAATGTAAAGGATAACACCAGCACTCCACACATCTGCCTCAGGACCATAACGCTTTCGCAAAACTTCAGGGGCAACATAGTATGGGCTGCCCACCACATCAGAAAAGATATCACCTGGCTTAAAAAAGACAGACAATCCAAAGTCAATAGTTTTTAGAAGTGATTCTTCTTGCTGATTCACGAAGAGAAAATTCTCAGGTTTAAGGTCTCTGTGCATCACACCAAGAGAATGGCAAGCTTCCACAACCCCAACTATAGTCCTGGTTAGCTCAGCTGCCTGTCTTTCAGTATAATGCCCTCGCTGAATGATCCTATCAAAAAGCTCTCCACCTGCACACAATTCCATCACAACATGAACAGCCACAGCATCCTCATATGCACCTTTGATAGATATAACATTAGGATGCCCAGCCAAATGGTGCATTATCTGAATTTCTCTTCTCACATCCTCAACATCATCATCTGTGACAAGCTTCCTCTTTGCAATAGATTTACAGGCATACTCGTGACCTGTTGCCTTCTCCACACATAAGAATGTCATCCCAAACTGACCCTGTCCAAGTTTTCTCCCAAGAGTGTAGAACTCCTTGAAATTATCAGTCTCTCTTTGCAACACGGAATCAACACGAAGCCCTGCACTAGAAACTCTCTTCACCGAACCATGTTTCCTGTGCTTCTTCTTCTCCTGTTCCTGTGCTGGTTCTATTTCTGATTTTGCCTCTTGTTTGGCTTCTGGTTTCGGCATAGTTATCTGCTCAGGAGGTTTGTTTTGGACAGGCAAAGGCGATTCGGGCACACTAGTTGCCTCCTCTTTTACAGATTCTCTATTAGACACTGATTCATCAGGCAATTGAGACCGCCAAATCGCCGCGGAAACTGATTGAACAAAACCATTTTTTGAAATGCTAGGACCAACACATGTGTTCCCCATATTGCTTCACACTGAATAAATTTCTGCTACAGCTAACAAATATCCAACTTTCATCACTCGGATCTAAAAGCCTACATCATCTGCAAACACAAGACACAAGATCTGAATATGAACTTCAAGTCCTTCTTAGTGAGCATCTAAGTCAAACCCCATATATAGGACCAAAATTTTGCTACAATAATCAGCAAAACCAAAGATTCATCATCATCCAACAAAAGGAGACTTAGCTTGGACCGGAAAGCAGAAAAAAAATCTTTTCATTTTTCA

>C100361_65

GATTAATCAAATATTACGTACAAAATATGGAAATTGGTTTACGTTCAAATTCATAGATAGTTCATAGGTTATCCCGAGAAACACGCCAACAAGAAAGACATCGGAAGCGGTGAGTGTTTCATGCAATTGGAAACCTCTGAATTTGTCTCTGTCTGCGTCATTGCTTCATCAATGGAATGATCGGTGAAATGCCCAAACCAGACACTGTGATTATCTGATTACAATAGTTTCTTCAGGGCGATTCCTCTCTTCAAAATCGTTGCTTGCTGTTGGTGCCTTTCTGGGTTGGTTAATTGGTTTCTTCCACAATGGTAGAGAGAGAAGGGGGGAGAGAGAGAGAGAGAGGAGAAAGAGTTTGACAGAAGATAGAGAGACATACTGAGAAGGGGGTCCTACTGCTTAATTTTTGAGGGGCATCAACAGTCCCTGTCAATGATTTTTACTTGGGTTGGGTGGTGCTGCTGTGTTGCCTCTCTCAATCTCAAATGGTCCATCCAATCAGCGTCAGTATCAAACTCCTTTCTTTCTCACCAATTTGATCAATAAACCAAAACTTCTCTTCGTCTTTGCCCCTTTCACCCTCTTTTTCATCTCTGGGAGGATTTCAAATTGGTCATTAAGGATAGCTCAAAAGCTCTGCCTCTGGTCAAAAGGGACAAGTGGATCTCACTCATATGAAGAGACTTTCAACTTTGGTGGATGTGTTTCTGGTTCTTGCATTGTTCAGTTGTAGCCCATGGACAGTGTGGAGCACCTCGTACTGCAATCAGGTAAACTTGAAGGAAGTTCGGCCTCACAGTGTCAGTATTACCGAATTTGGGGCTGTTGGCGACGGGATCACTCTGAATACAAAAGCATTTCAGAATGCCATCTTCTACCTAAATTCATTTGCTGACAAAGGTGGTGCCAAGCTTTTTGTCCCCGCTGGCCGGTGGTTGACTGGAAGCTTTGATCTCATCAGCCATCTAACATTGTGGTTGGACAGCGATGCAGTAATTCTTGGTTCAATGAACTCTGACGACTGGCCACTTGTGGAGCCTCTACCATCATATGGTCGTGGAAGGGAGTTGCCCGGTGGAAGGCATAGAAGCCTCATTTATGGGTGTAATTTGACTGATGTTGTCATAACAGGTAATAATGGAACTATAGATGGTCAAGGGAGTATCTGGTGGGACAAGTTTAGGAACAAAACATTGGATTATACACGTCCTCATTTGGTCGAGTTGATGAATTCAACAGGGATTCTCATCTCAAATATAACTTTCTTGAACTCACCATTTTGGACTGTTCACCCTGTATATTGCAGCCATGTTACAGTTCATAATGTCACAATCATTGCTCCTCTTAGTTCACCAAATACAGATGGAATTAATCCAGATTCTTCAGACAACGTATGCATTGAAGACTGTTATATAAGCACTGGTGATGATCTGATTTCCGTCAAAAGTGGATGGGATGGATATGGCATTTCATTTGGTCGACCCAGTACAAATATTTACATCCGCAGGCTCATTGGGAAAACAACGAGTGCAGGGATTGCTATCGGAAGTGAGATGTCTGGAGGTGTATCAGAAGTTCATGCAGAAGATATTCACATTTTTGATTCACGTAGTGCAATTAGAATAAAAACTTCTCCTGGAAGGGGTGGTTATGTTAGAAATGTCTATATCTCAAACATGATCTTGGCTAATGTAGATATTGCTATTAGATTCACTGGTTTATATGGAGAACATCCAGATGACAATTATGACCCAGATGCTCTACCGGTTATTGAGAGGATTACTATCAAGGACGTGAAAGGGAGAAAAGTCAAACGTGCGGGCCTTATACAGGGTATTCAAGGTGACAACTTTGTCAATATTTGCCTATCAAACATCACTCTTAAAGTGACCTCAAAATTGCCATGGGACTGCTCTTACGTTAAAGGATACTCCGACCTGGTTTCACCGGAAGCTTGTGAACCTCTCAATGAGCGAATATCCCCTGAGCATTGTTCCGACTGTTGCTATTTACCAAACCACTTAAAGAGTTTGGGTAATCAAAACTTGTGGGCTTCGGTTTAGAACATCAAGCTACCAAAGTACAGTTATAGCTGAAATTTGCTTGGGTTTTCCGACTGTTCCGACAAATTTTATCTGACAGAAGTTGAATGTGAAGCGGATCAGTGGTTGTCAGTCTACAGAATGACACCGAGAGATGATTGTTTCTTGTAACTTGTGTGCAGTAACAAATAATTGAGTGATTTATCAGAAATTGAATTTGAATTTGAATGATTTCATGTCTTGCTTTCCTA

>C100365_65

TATATATATATATATATAATTGTCCTTCTAACTTTTAAGCGAAATCTTAGTTGTGACAAGCTTCAAATGTGCCTGATGATTTTCATAACGGTAGTGAAGGGTATGTTGCTGGAATTGGTCACTCCCATGTAATTGAGGGAACTAGATTTCAAGGGAGATTGAGGACTGTTTATTTCTGTCATCGAGCAGGGAGAGTTTCGGGAAGAAGTGACATGATTAGATTCCTCGCTATCATATTTTTTCGAAGTGTCCCCTATTAAATTATGAATTTTGTGAAGAAACCGTGCGCCAATGGAGGCTGGCACTACACCACGGTTATTATTTCTTGAACTGTGACCGGGGCTATGTCTACGTGAATCATCTCCTGATCCGGAGTCTCCTCTGGGACAGCTTTGCCCTTGTGAAGGTAAAGCATCTTGATCGTGCAAATTTTGTACAAAGGCATGTGTAAGAAGCATTGCTGCCGATGGTCGATCTGCCGGATTCCTTCTGAAGCACTGCTGAAGGAAATCCTGTCCGTCTGAAGACAAATTTTCAGGTATAGCTGGGGATTTGTGCAGTACCTTGAACATGGCTTGCGGCCCTTCATATTCACTCCAAGGAGGGTTTCCTGTCAGCATTTCGATGATAGTGCACCCTAAGCTCCATATATCAATGGCCATAGCAATGTTAGGACTGGATTCTTTCTTTATGGCAGCCTTCATGAGCTCTGGAGCCATCCAGTAGGGACTACCCTTCAATGAAAGTTCATATGATTTCTCTGTCAGAATTTTAGACACCCCGAAATCCGCAAGCTTAACCATGCCTGATGCATCGACCAATAAGTTTGCACCTTTGATATCCCTGTGAATAGTCTTGGTACTATGCAAGTATGCCAATCCAGAGAGAATATGCTTTGTGAAGTTGCGAACCACAGATTCTGTCATAGCTCCACAATGTTCACGCATAAACTTATGAAGTGATCCAGGATGAACATACTCCATATATATGTACAGTCGATCACCATCTAATTCACTTCCATAATACTGCACAATGTTGGGGTGGTGTAATTGACGGAGAATTCTGATTTCCTGTTCAAGTTGCTTTATGGAGTCAGCAGATTTAGGATCATCCGGGAACATTTCCACCTCTTTCATTGCACACGAGGCTCCAGTCTCTAAGTTGGTTGCGTGATAAACAGATCCATATGATCCTCGCCCTATAAGTTTTCCTTTCTGCCATTGACCTTTCATTGAAGATGAATTTTCTGTGGTGAGGTGCACGATACTTGGTTGATGCTGCGGAGATGACTGTGCTGGTGGTAGTGAAGCTCTAGGAGGAAGGGGCAGTGGATGTGCATCAACATGATTATTTTCAGGCAACACTCTTGAGCAAATTTTATGGAGCTGACTCGGACATACCTCTGGGTTGAGGTGGCTCCCTGGGCTGCGCAGAGGAGACCGATCTGGACTCTGAGCAGTTTTAGCCGGTGACACTCTCAAAATGTCATTAAAATCTTGAGGGAAAATGATGGAAGGATCATAGAAATCTACATTGCTGGATCTACGTGGACTAGTAACAGGGCTAGAGAATATGCTGTTGGGAGCACTCTTGGCCGAAAAATGCAATCTCAAGTTGGAAACATTTTCAGAGTCATTATCATGAGAGAGTTCTCTGTGATCTTTACATGAACTAGTATTGCCGGCCAACACTCTTGCAGGCGGAATATTAACTCTCAAATCATATTTGGCATTTGGAGCCACCGGATCGAAAGAAGGTCTACCCAAATTGCTGGCAGACCTCAGACTTCTCACTGCATCATGATCCACCGACCTTCTGGGAAAAGCGAAGCCAGGATGTTCGGAAGGGAGAGATCCGTGGTGATGATGGTCGTGTCGGCGAGTCAACGGGGAGTCGGGACGCGGAAGCGGTTGGGGAATCGCAGAGGAAGACCAATGGTCGGAAGCGGAAGAGGACTTAAAGGAGGGAAGAGGAGAAGAGGGTAGAGAAGTGGTGGAATGCAGACCAGCGCCTTTGTCAGAGAGGTATCGGAGCCTCCCCTGTCGGGTGAGCCTACGTTGTTTAATCGTAGGTAGAGGAGAGGAAGAGTTGTTAGAGTTGGCGGTGCTCATTGCTTGAGAAGAAGTGGAAGAAGAAGAAGCAGAAGTAGAAGAAGGTCGTCATGTGAGAGTGGTGCTTTTGCGTTTCGCGTTTTGCTAAGTTGGTGTGCAGAAAAGCAAAGGCAGAAGAAGCGCCCGATGAAGGAAACACCTACGCTGCTTCTTTGCTTGCAATATTTTCCAATTCAAATTGCC

>C100369_65

GATCTTTCTCTCTGTCTCTCTCTGTTTCTTTCTTTCTTTCTTTCAATCATCTTCCTCCAAAATAGACTCAAACAAAATCACCCCTTTACCCTCATGGCTGATGCCACCAAGTACATTCCCATAGGGGGCACCCTTAACTCAGACCTCAAGAGCCTCTTCTCCATTCCCAAAACCAGAAAAACTCTCACTTTTGCCTATGCTTTTACTTTTGCTTTTGTTGCTTTCACTGTTTTCCTTGCCTTTTGCCCCTCTCCAAACACCTCCTCTCCCTTCTTCACCAACATATTCTCTTCCACTGCCTCTTCCACTTCCTCCACCACTGCCTACAGGTCCCAACTCGCTTCCATTTTCTCTTTCTTCTTCTACAACACCACTTCTTCATCATCCAATTCCTCTACTGCTACTACCTTTTACACCACCCCTTCTAGATCTACCAACAACACTTTTCAATCTCCTCCAACAAGTAAAAAGCCTTTTAAAGCCACCCCTTTTAAAAACCAAACTCAAACCACCCCAACTATAAGCAAGATACCCAGCAATCACACAACAAAACCACATGATCATGTTGTGAATAAAGAACCAACAAGTGTCTCCAACCATACCCGAGTTTCTGGAACTTCTGATGAGGATAAAGCTTTCAAGAAGACAAATCAATCTTCAAATGGTGTTGCTCCTGCGCCCAAGGTTTCAGGGGGAAGTCCCAATAAGAATTTGAGTTCTTCGGGGAAAGGAGGGGACACAGAGAAGGGTGTTGTGCATAGTAATTACACGTCTTCCTTGGTGAAGAAAAAGAAGAATGGGAACAATGAAACGGATTCCAGGGTGGAGGATGAAGTCATTGAGGCTTTGATCAAGTGTGATTTTTTTGATGGGGAATGGATTAAGGATAATTCTTATCCTCTTTATGAACCGGGGTCTTGTAATCTGATCGATGAACAGTTCAATTGTATCCAAAATGGAAGACCTGATAAAGACTATCAGAAATATAAGTGGAAGCCAAAGGGATGCAGTCTTCCAAGGTTGGATGGACATCGAATGCTGAACATGTTGAGAGGCAAGAGGCTAATTTTTGTCGGTGATTCCATCAATAGGAATATGTGGGAGTCTCTCATTTGCATCCTGAGAAACTCTGTGAAAGACAAAAGCAAAGTCTATGAAGCAAATGGAAGAGTCCATTTTAAAGGGGAAGCCTCTTATTCATTCGTATTCGAAGATTATAACTTCTCTGCGGAGCTTTTCGTGTCCCCATTCTTAGTTCAGGAATGGGAAGTTCAAGTAAAGAATGGAACAAAGAAGGAAACACTTCGTCTTGATTTAGTTGGTAAATCTTCAGTTCAATATAAAAGTGCAGATATAATCATCTTCAACACTGGTCACTGGTGGACTCATGATAAAACTTCTAAAGGGAAGGACTATTACCAAGAGGGAAGCCATGTCTATAATGAACTGAATGTTTTAGAGGCATTTCGAAGGGCTATAACCACTTGGAGTAGATGGATTGATGCCAACATAGATTCATCCAAGTCTATGATATTCTTCAGAGGCTATTCCGCATCACATTTCAGTGGAGGGCAATGGAATTCTGGTGGACAATGTGACAGTGAAACTGTGCCAATCAAGAATGAGAAGTATTTGAAGGAATACCCTCCTAAGATGAGGGTATTGGAAAAGGTTTTGAAGTATATGAAAACCCATGTTACCTACCTAAATGTCACTAAAATGACAGATTTCAGGAAGGATGGCCATCCCTCTATTTATAGGAAGCAAAACCTATCACCAGAGGAGAGGAAATCACCATTAAGGTTCCAAGACTGCAGTCACTGGTGCCTCCCTGGTGTTCCTGATGCATGGAATGAGATCCTTTATGCTGAGCTTCTTTTGAGACAGTATCAAAATCAGCATCAGAAGAAGAGACCATAGGTAAACTACACTAAAAATCACTTATATTGAGTGAGTTTAGCTGAAAGCAGAAATAGATGCAATATATAATTTACTATTTTCAGCCCTCCCACAAAAGGATAGAGAAAGAAATAAGGCATATAGAACAGGTTTTTCTAATTTTGTAAAGGTTCACAAGTTCATGTGCAGTAGTTTATGTGTAGTAGTGAAAAAGGATACAAGTTCCTTGCAAGCTGAAACTAAATGAACTTGTTCATAAGAATTATACAAGTTAGCTAGGACATGGGTGGTTAATTTACCATTGTAAACCACCCTCTAAATTTTGTTTCTATAGGTTCAGTTCTACTGTATTTTTCAATTTATGTCACAAATTTTTCCAGATGAATTAGATTTTCA

>C100371_65

TATGACTCTACAAAGTTACTATTCTAGTTGTTATGCTTGATTGCATTATCGGACACAAGCGAAGATACACTAGTATTTGCAACCTCCTTCAAGCATCTTACCATAAAATTAATATAAAAAAATTAATGTTAGGGTTTCTGATGATAAAACAATGAATGTGGAGGTGTGAAAATATTACTTGGCGACTAAGCTAGAGAAGCATAGGCAGCAGCTGCAAACGTACAATATTCCATGATTCCCTCATCTTATCTCCTTCAATTCCCACTCCCATTCCTCTCCCCAACTTTTTTCCCTCTGGAGAACCAGTTTACAATGGGAAAATTAAAAACATTATAGGAGTGATTCTTCTAGTAACATTCAAAGATCAACTTCCATTTTGCCTACTAATATACGCTTCCAATTTCCCTGTTGCGGCCAATATGAGACCTAAAAACAAGGCAGATGGTCTCACTGGCCGTGATTTGAATTCTGGATGGAATTGCACACCTATATAGAATGGATGACTTGGAAGCTCTAAGATCTCCATTCGTTTTCCACTTTCATCCTTCCCAACAAAATGTAGTCCAGCTTCTTCTAAAGTTCCAATGACATCTGGATTTACCTCATATCTGTGCCGATGTCGTTCGTCCACATACTCTGAATTACCGTACAGCTTGGAAGTGATACAATCAGATGTCTGTAAGAGTGTTCTTCGAGATCCAAGTCTCATGGTACTTCCCATATGCGTTCGTGAACCCTCTGGCATGAAAATCACAACAGGATTTGGTGTTTGGGCATCAAACTCTACACTGTTTGCTCTTTCCCAACCCAAAACTGATCTAGCAAACTCAATTACAGAAATTTGCATTCCCAGGCAAATCCCCAGGTAAGGAACATTGTTCTCTCTCGCATATTTGGCAGCAAGCATCATACCTCTCACACCACGATCTCCAAATCCCCCGGGAACCAAGACACAAGCTGCACTCTTCAAAGTCTTCCAGGCTGCAGCATGTGCTTCTGGTGTTGATTTTGCACTCTCATCTTCTAGGTCAGATGCTGCAATCCAGTCAATTGATGGCTTTAAAGAGCGTGCAACACATGCATGCAGAAGGGCCTTTACAACCGATAAATATGAATCTGTTAGACCAACATACTTTCCCACCATTGCAATCCTCACAGATTCAGTAAGACTATCATAGGTCTCAGCCATCTCTGTCCACTGCTGTAAATCAGGAGGTGTAGCTTGGCTGAGTAAGTTAAGCTGCTGCAGAATTGAATGGTGAGCATTTTGGTTTCTCAATAACAGGGGAATATGCCAAATGTTTGGTACATCATGGATGTTCAGAATATTCTCAATTGGAACATGGCAAAATTTCGAGAGTTTTTCCTTGGTACTTTCTAGAAGGGGCTCAGCTGAGCGACTTGTTAAAAGATGAGGTGTTAATCCCAATGCTCGCAGCTCCCGGACACTATGTTGTGTAGGCTTTGTTTTTTGCTCTCCCACTACACCCAACACTGGTATCAGGCTAACATGGATGAGACAGAAGTTATCAGGCCCTACTTGAAAGGACAATTGGCGTAGAGCCTCAATAAATGGCATAGATTCAATATCACCCACAGTGCCTCCCAGCTCAATCACACAAACATCTGCCGGGCCCTCTTTTCCGTCCACGGGAATCACAGCAACCGACTCAATCCAATCTTTAATAGCATCAGTGATGTGCGGAACCACCTGAACAGTTTTCCCGAGATAATCTCCTTTACGTTCCTTCTCAAGAACAGACTGGTATATTTTTCCAGTGGTGATGTTGTTATCTTTGGTAAGCGTGACATCGAGGAAGCGCTCGTAATTTCCCAAATCCAAATCAACCTCTCCACCGTCGTCGAGAACAAAAACCTCTCCGTGCTCGAAGGGAGACATGGTGCCAGCGTCGATGTTCAAGTACGGATCGATTTTGATGGAAGTGACGCGAAGGCCACAGGCCTTGAGGACGACGCCGATGCTGCTGGCGGTGACGCCTTTTCCGAGTCCACTGACGACTCCGCCGCTTATGAGGACGTATTTCATTTGATTTGGGGAAATGAGAGAAGTTAGTAGGCCAAGTGTTTGGGTTTATACCCGAGCGGGAAGGAAGGTTGAGACGGTTATGAAAAAGAATGGTTAGTGCAGTAGGAGAATATGGTGGTGAAGCTCACGGTCCCTTTCGAAATGGTATTTAAAAAACCTAAAAAGGCGTCTCAACTCAAGTCTCGTCTCCACCTCTTCGATCGATCTTGGACTTCTAGCCGCCTACCGCCCACAACTGTCCAACTTGC

>C100375_65

CCCCTAACCGATTGAAATCAATCCAAGTCTATAATTATATTTAATTTTTCATCACACTTTCCAACATAGTTCGTGCCAAAGGATCATAATCATACAATCTACATGGTATCACATAGATGAAGCACAAACTTGGAAAGACTAATCCTGTATTTTCAAACCCCTGAATCTAAGGAAGAACCTTTAGTCTGCACACCTGGAACTATCAACAGCTTCAGGAAATGGAGTGACGTCTATTCTTATTTGGTTTAGTGCGACCTTTGGCAAAAGGTGCAATACCATCGGCACGCACAAAAGGAGAAAGCCGTCCAAGTCTTTTGGACATTGAACTTATGAATGATCTTCGAGGCAAAGGAGGTTTATCAGCAGAAGGTGAAGCACTTACCGCAGGACTGTTAGCTGCAGATTTAAGTGTGGAGGTTTCAATCTCTTTCAGTCTCAATTTCAGTTTCACTAATTCTAATTTCAGGTCTTCATTTTCTCTTCGTAGAGTAGAGAGCTCATTTGAAACTGGATGAAGATCAGAGGAGTATACGTTCAGTTTGGTATCAACCGAAGATTCCCCGCTTCCGTTGCCATTCATGGCATCGCGAAGCCGTTGCTGTTCGTAATAGAGAACTTGAACCACGGTTTGGACAGGTAGCCGGTCATTTTGTGCAGCATGTGCACATGCCTCTCGAGATAACTTCTGACAGTCCATCACGCTGCAGACTTTCTTTCTCTCCATGTCACTTAGAGCAGGATGAGCCTTAAGATAGATATCTATGGCTCTATACATCCCATCTTCTGCTGGTCTGGACTGTTCAGGAATTAATTCTGCCAAACAGGTGAACTTTGGAACTGGTAAATTTCGGTCAGTGGCTATTTCAGCAAGATAGTTCTCCATTAACTTTCCAACCCGTTCCATATCACTCTGCGGAGGAGAAAAGTATTCATCATCTGCATTGTAGATAAAGTGGTTTCCCGTTTGAGATTCTAGGTAATTAGTCATGATCCTCTGCACTGTATCCACGTCAAACAATGTGTCCCCAGTGAAAGAATAGGAAGGAATGAGAAGATCATCTATAACAACTTGCCCCAACTGCATGGCCATCCTCTTCTCCAAATCAAGCCTGCAAGCAACTGTAGTCTCAAGATATATTGCTGCCCGAAGCAGCATAGAAAGAAAGCTTACTGACATTGCATTCTTCTCCCTTGGCAAAAGGCTCACTATTGTCTCTAAAACAACCCTCTTTTCATGCTCTTGTCGCGGCTCAATCTTTTTCCTTGCCTTTCCAAATACATCCAAACCTCGTAGAGATTTCTGAGCATAGAGCATTAGAATGGGACCAATGGCGTACTGTTTAAACCCTCTTGCCATCATTGCGATTATAATTCTTTGGAAAATGTCGATTCTGAGCACAGTCATATCTTCAGCCCACCAATCAACTACTGGTTTCTGGTTTGATGCCACCACCACTCCCCCAGTGCCACTCTCACTTCTTGCAGATGAACAGAATTGACTTTCCTTACATGCTATATATGCAATGGCATCTATGCATCGGCTCACCAACTTTGTTCTCTCTGCTATTGGAAGGAGATTCTCTGACTCTGACATATGCAAAACAGACACAGCCCCTGCAATGGTCTTCAGCGCCACTTCGTTCAAATAAGCATCAGTCCTTCCCACCAAGTTTCCAACTGAATAGTCCTCCGTCATCTCAAGATACTCAGCCACGCAGCGTAGCGTGGCAATGTTTTCAACGTTTATTTCGAAGTTTATTCCGTAGCAGAACTTTGCTGCCAGTTCAAATGCTTCTGCTCCACCAGGAACATCAGAGAGTTCAATGAATGAAACGTCAGCATCGTTAGATTCTGAGACGAGTTTCCTAATGTACCCACACTTGGAGACTAATGGAAACTTATGTAACGAAAAGGAATCTTCCCCAACTTGAACGTTGACATCACTTGGAATCTCCTGCGAGAAAATCCATTCACTTGTTCTCTTCATGGCACTAGAAAGAAGCTCCTTCTTCTTCAGAGACAAGTCACTACCAGCAGGTACAGTTTTCTCTTGCCCACGATCCATCATGGCAGGTACTGATTTTCTAGCTCGATACTTTTTCTATGTTAGAGTTGGTGGTAAAGCAGAAAAAGAGAGGAAACAGATTGAATCCGTAATTGTTTTGGTTCAAGGTAATCTTTTATAACTACAGATTCCTCGTACATTTTCTTCGGGACTAGGAAGACTGAAGGGTAGCTAGATAGAGAAGAGGTATTTTTTTATCCTTTTGGAACTTTAATTTTTTTCTTTTCCAAA

>C100377_65

GAAAAAGGTAAAGTTGGGGTAGGTATAAGATGAGCATTTCCAATGGAGCAAGAAATTGGTGATTCATTTCATTTCTTTCCCAATTGCATTGCAATGATCTGGACTCATTGGTTGGCTTTGCCAGAAGGGGAGCTTGCGTGCTACTCTAAACGCAAGTTTGCAAGATAGCGATGGGTGTTGTATATTTTTTGGGGAAGAATTAGTGAGCATAATGTTTTCTGGAAGATTGGGGGCAGACGAATCTTATTGTCAGGATTTACAGAATTTGAGTGTTTCAAAGCGACTTGTGAGGAGTGTCAGTCAGAGGTTGAGAAAGAAGAATAACAGGAATGCAGCGGAAGAAGATGATGATGTGAATGGAGTTTCTTTAAGATGCTTAAATCTGTATGGACGAGGTGGGGGTTGCAAGGTAGGTGCCGACACCAGTGAGGATTTTGGTGATTCAAGCAGTAGAAGGAGATCTAGTGCCAGTGACGAAGGAAAGGGATATAAGCCGATTTGTGGCCCTGAAGAAACTGCTGTGGATTGCTTTTCATATGGGGTGAAGGACAGGTTTTGGAGGAGACACAACCGAAAGAATTCAGAGCTTGAAGAATTTTTGACAAACAATAGAATGCATATCTTTCTTCCTGATGATATTCTTGAAATGTGTTTGGTTAGGCTCCCATTGACTAGTCTCATGAATGCCCGACTTGTGTGCAAAAAATGGAGGTCCTTGACCACCACTCCTAGATTCCTCCAAATGAGAAGGGAGGGTTCCTATCAAAGTCCATGGTTGTTTCTGTTTGGTGCTGTTAAAGATGGCTTTTGTTCTGGTGAGATACATGCACTGGATGTGTCCATGAATCAATGGCACAGGATAGATGCTCATTTTCTCAGAGGAAGGTTCCTGTTCTCTGTTGCTGGTATACATGATGATATCTTCATTGTTGGAGGATGTTCTAGCTTGACTAACTTTGGGAAAGTGGATAGAAGCTCGTACAGGACACACAAAGGGGTTCTTGTATTTAGCCCATTGACAAAATCTTGGCGTAAAATGCCGTACATGAAATATGCTAGATCAAATCCTATATTAGGAGTCTCTGAGGTCAGTTTGGATTTTCCAACTTGCCAAAGTCATCAAAGTCGGCAGGATAGACGTTTTCCAAGATCAAGGATTGGTGGTGTTTGTGATGTTTATGAGGATCCTCACAAGCTTTCATTGAGACGTCATTGCAGATCTTCTTTTAATGAGACTGAAGCTTCATCTTTGCCCAGTCGAAAGGCATACAAATTCCTTAGACAAAAAAGTGATCATTCAAGCACAAAGAGCAGTAAAAGATTCTTGTTGATAGCTGTAGGAGGTCTGGGATGTTGGGATGAGCCTCTGGACTCTGGAGAAATCTATGATTCTGTGTCAAATAAATGGACCGAAATCCCAAGATTACCTTTTGATTTTGGGGTTGCTCGATCTGGAATTGTTTGTGGCAGGATGTTTTATGTTTATTCTGAAACAGACAAGCTTGCAGCATATGACATAGAACGAGGTATCTGGATTGCAATTCAAGCCACTCCAATCCCTCCTCGTGTACATGAATACTACCCCATACTTGTATCTTCTAATGGCCGCCTTTTCATGCTCTCTGTGTCATGGTGTGAAGGGGATGGTCAAATTGGGCAACGAAACAAGGCTGTTAGAAAACTATGGGAGTTGGATCTCACGTATCTTACCTGGACTGAAGCTTCAGTGCATCCCGATGCCCCAATGGACTGGAGTGCTGTTTTTCTGTCGGATAAAAACCTCATTTTTGGGGTAGAGATGTTCAAAATATTTGGTCGGGTGTTGGGTTTTTTCACTGTTTGTAATGTGTCTGATATCGCAAACTGGAAGCATATTTCAGGGAACCATGTTACTCATGAGCTCGATGGTTCTTCCTGCATGACCAAATCTGTGGCAGTGTTACACCTTTGAAATCCTTTGTGCATGTATGAAGCTAAACAACAGATTCTTAGATCTTCCCTCGCTGTTTTGAGATGCAGCATGACCGGTAAGACCTGCTCTATATTTGCTGCATATAACTGTATATGGGCAAATCTTCTGAACTCCGTGATTCTCAGTTTCTGTGCTTCTCATGTACCTTTTATTTAATTCATTTCTCACCAATCATAAAAGTTGAATTCCACCTGACTTTGCACTTGTATTATTTCTCCTATCCATCGAGTTTCTGGAATTATGGTTTGTGTTATAACTAGTTGCACTAATCAGCCTCCTTGTGACTTTGGCATATGTCTATACTTTATGCAATCCAGGTTACAT

>C100381_65

TTTTGATATACTTGACATTTTATAAAAGAATCAATATACTAGGTCACTATTACATGAGATGAAACTCTATGGCCAATTGTTAAGGAACAACAAAGTCCTACATAACAATGATCAAAGATACATCGCACTGGCAGTGAACATAAACCTTCAGATTTACCACCATCAAGTACCATGCAGTTTTCATCATCATGATATTTCTATTACACTACTACACAGTATTTCCTATCTATGTTTCAGCAAAGCCAGGCTGAAGCTGATATCAAATTTCTGTCCTTCCACCCCAACAACATGGCTCCATCCTTCTCCACTAGAGTATAATGTTCTGAATAGCACCTCAGAAGGCTTCTTATGACAGAATTCACATAAGAGCTCAAAGGACACTGACTGAAGCCAGCCATTGTTAACCTGGATTTCCACTTGCCTAATAGTTCATGTCTCTCAACCCTTTCCTTGCCTTCACAAGCAATAATATTCACTATATCCCTGGCTAGACAATGTTGCTCCACATTGATCCGCTCCTTGCTCTCTCTTGGGAGGGAGACATCAATAGACTCAAAGATTGCCAAGTAGTAATCCAAGGTTTCAATAAACCTGTTGAAGAAAGGGGTTGTGTTGGTGTTTGATTCCTGCTCCACAAGTGTGGTCACCTTGGGAGCAAGTGACTTTACCAATCTCAATAGTCCATCCCTTGGATTACTCACATGAACACTCTCGTCGGCTGTGTGATGGAGCTGCAAGGGGAAGTTCACTGCCAAGGCCTCTCCAGGCCTGATATCAAGCATTTCCCTTGTCACTTCTGGAGCAAAAACGGGCACTCCATGAAACTCAACTGGGATACCAAATTTCTCAGACATCAAGGCCAACCTTTTCCCAACCATCTCCAATCCGTCGCCACGAGCGTATTTAGAAACAGGATCATCAATACCCGTGATCCGAACATGGGGAGCACCACCAGGTCTTGCTGCAAGGGCTTGAAGAAGAGTCATCCATTGAGATCCCTGAGCAATTTGGAAGTCTATGATGTGTATTCGATCCTCGTTTCTGCAGGCTTCAGCAATGGCTCCATTGGCAGCCATGTAACCAAATTTTAAGTAGGGGCAGATCTCAAACAGCAGTTGCATGTAAGAGAGTAATTCTTCACCTTCAGGCTCTCTGCACCTAAGGGCATGATAGATGCTCTTCCCTGATGCTTCCTTCCTTGCAACAAGCCCTTCTACCATGTAAGCACCAAGCCGCTGGATTGGCTCCCCATTGATGGACACGACATCTCTAGCCTTTCCTATCAATTGATCAAAATCTTTCATGTTGTTTTCAGACAGGGCTTTGGCACATGCAATCAGCAATTGCTTCAAATTGTTTGGTGGGAAAGCTTGTGGTGATGGTTCCTCCATCAACTTTTGCCGTTTCTCTACATGCACAACTTCACTTGATAGCCTACTGCTAGGAAGATATGATGGCTGAGTTTGAGTATATTGTGAGCCATTGTTCTCATGGCTCCAGGACTTGTATCTCTGGCTAGATGCCATTGGCCTGCTGCTCTCAGCCAATGTATTTGGTGTGGTAACTTGATCATCATCAGGGGCCATCAGAGCAGTTTCTAGCTCCAGCAAAGCGTGTTGGATTTTCTGATTACAATTCACATTCTGTAGAAGAGAGTTTCTAGAGGAATATAGTGGGAAATTGTCTATGAGATTGTCCACGGATGATATTGATCCATACATATAATGGTTGGTTTCCAGTAAAGAGTTACAAGAGGAGCTGACACCAGAGAGGATTTCTCCTGGACTGTATTGCTCCTTGCTGTCACTCAATGCAGAAAGGGTGTCAGAATCAAAATAAGTGGAAAAGGGGGAATTAGGTGAATTTCCTGTTTCATATTTCAAGGATCCAAGGAGCCTGTTGGGTAGGGATGGAACAGTGGGAAAAGATGACATGTAAGGTAATCCAGCACTAGCAACACCAAAACTGAAAAGCTGTTGTGAGTCCATCCTATGATGATAGCATCGGCTGATTTATGTGACGGGGAGATTTAATAATTTGACTAGCAAATCAGAAAAACACCACCCTCTGCAAACCTCTTAATACTTTGCTTCAGACTTAAAGTCCAACAGTTTCAATAAAAGAATATAGCCTTAAAAGGGTAAACTAAATTGAGCTTTGATGAGTGTTGGGAATTTGTGCAGGTTCGATGATGATGATGATGATGATGTTGTAGCTGTTGTTGTTTTATTAAAGATGGTGTTCTTGTGTATGTGTTTTGGTTTGGATGAAGTTGGGCAGGGGTGTGTGAATGTTAT

>C100387_65

GGCGGTTGTGGTGAAAGGAGAGAGAGATTTGATTTGGGTTCGGAAAGAATCGAACAGGAATGAGTAGTTCGGCGAAGAGGAGTGTCGGGAGCAGCAGAGAGGAAGATCAGCTGATAGTGACTCCATTAGGCGCTGGAAATGAAGTGGGTCGTTCATGCGTTTACATGTCCTACAAAGGCAAAACCGTTTTGTTCGATTGCGGAATCCACCCTGCTTATTCAGGCATGGCTGCATTGCCCTATTTCGACGAGATCGATCCTTCCACCGTCGACGTTCTCCTCATCACTCACTTTCACTTGGACCATGCCGCTTCCTTGCCCTACTTTCTCGAGAAAACTACCTTCAGAGGCCGCGTTTTCATGACCTACGCTACCAAAGCCATTTACAAGCTCCTCTTGTCTGATTTTGTCAAAGTCAGTAAGGTCTCCGTCGAAGATATGCTTTATGACGAACAGGACATCAACCGCTCCATGGATAAAATTGAGGTAATAGATTTCCATCAAACAGTAGAAGTGAATGGTATTCGATTCTGGTGTTATACTGCAGGCCATGTCCTTGGTGCTGCTATGTTCATGGTTGACATAGCTGGTGTTAGAGTACTATATACCGGAGACTATTCACGTGAGGAAGATAGGCATCTCCGAGCTGCTGAGACCCCACAGTTCTCCCCGGATGTTTGCATTATAGAATCCACCTATGGAGTACAGCACCATCAGCCTCGGCACACACGGGAGAAGCGCTTCACTGATGTTATTCACTCAACCATTTCTCAAGGGGGTCGTGTGTTGATTCCAGTATTTGCCCTTGGTCGAGCACAGGAGCTCCTCCTGATCCTTGATGAATATTGGGAAAATCATCCAGAACTCCAGAATATACCCATCTATTATGCATCTCCACTTGCAAAAAAATGTTTGACTGTGTATGAAACATACACCCTTTCAATGAATGACCGGATCAAGAATGCAAAGTCAAATCCCTTTGCCTTCAAGCACGTATCAGCTTTAAGTAGCATTGAAGTTTTCAAAGATGTAGGACCATCTGTTGTGATGGCCAGTCCAGGTGGACTTCAGAGTGGGTTGTCACGGCAATTATTTGATATGTGGTGCTCTGATAAGAAAAATGCTTGTGTTTTACCAGGGTATGTTGTGGAAGGGACATTGGCAAAGACCATCACCAATGAACCCAAGGAAGTTACTCTCATGAATGGACTCACTGCACCACTTAACATGCAGGTGCACTACATTTCCTTTTCTGCTCATGCCGACTCTGCTCAAACAAGTGCATTTTTAGAAGAGCTCAATCCTCCTAACATAATTCTTGTTCATGGGGAAGCTAATGAGATGGGCAGGCTCAAACAGAAGCTCATGAGTCAATTTGCTGATCGCAACACTAAAATTCTTACTCCAAAAAATTGTCAATCTGTTGAGATGTATTTCAATTCCCAGAAAATGGCAAAAACCATAGGGAAGCTAGCTGAAAAAACACCAGAAATTGGTGAAACTGTCAGTGGTTTGCTGGTAAAAAAAGGCTTCACATACCAGATAATGGCACCTGATGATCTCCATGTCTTTTCACAACTATCAACTGCCAACATCACTCAGAGAATTACCATTCCTTATACTTGTGCCTTTAACGTCATTCGGCATAGATTAAAACAGATATATGAGAGTGTTGAGCAGTCAGTGGATGAAGAATCTGGAGTTCCAACACTGCAAGTCCATGACTGCGTTACTGTTAAGCATGAATCTGAAAAACATGTCTCCTTGCATTGGACAGCAGACCCCATTAGTGACATGGTATCAGATTCAATTGTCGCTCTGATTTTAAATATCAGCCGTGATGTTCCAAAAATAATGGATGAATCCGATGCCATTAAAATTGAGGAAGAGAATGAGAAGAAGGCAGAGAAGGTTATGCATGCCCTCCTCGTTTCACTCTTTGGAGATGTGAAGATAGGTGAAAATGGTAAATTGATAATTAACATTGATGGGAATGTAGCAGAGCTCAACAAAGAAAGTGGGGAAGTTGAGAGTGAAAATGAAGGTCTCAAGGAAAGAGTAAAGACAGCATTCCGCCGTATTCAAAGTTCTGTGAAGCCAATTCCTCTTTCTGGACCGTAGTTTATTCCTGTTCTGACTGTCTTGGGAAGGTGAAATCCCACCGTTAAGGAACACTAGCTCACATGAGACAATTATTATAACATGTAGTTACTCTGCCTTTAATATGACGAGCTTGAACACAACAGTCTTTGCGCCATTTTGTTATTCATAAAATGTTTTATTACTATCTTTACATTCTC

>C100389_65

TTTTCTTTTCTCTCACTTGTTCTAATCCAACCACCACCACCTGGTGTGTCCCCTTTCTGGACTAGCCTTTTGCTCTTTCCTTCTTCTCACAAAACCACAATTCACAAATCATCACACCAAAACCCTTCTTTCGTCTTTCTTTCTCTTTCTCAGAATTTCGGGAACGTGGTAGAATGCCGGCGTGGTGGAGTAGAAAGTGGACCAAGAGCAAGGAGGACCACGAGGAGGCCGAGGAGGAGGAAGAGGAAGAGCCACGTGCTGGCTTTCCTTTGAGTTTCATGAAATCTCCTGTCGCTGTTCGGAGGGGTGAGAAGAGTAAGAGTAAGAGGAACAAGAAGAAGCCCAAGAGCTTCGACGAGAAGCTCAAAGGGGTTCCGTTGCCTCGTCCCACGCACAGTCACAGCGATCAAATTCAAACCTTTGGATCTCTCTCGGTGTCCGGTTCCAGCGTTAGCTCCTCCGCCTCCTTCGACGATCACCCAATTTCGCCTCACTTTAATGCCAACAGAGGACAAGAAGAGGTGAGGTTCAATGCGAGGTCAAAAAGTCCTGGTCCTGGATCAAGAGGACCTACTAGTCCTACCTCACCGCTTCATCCAAGGTTGCATGCTTTGAGTCTTGACTCTCCTACGGGGAAGCAAGACGATGGAAGGGGTAAGTGTCATCCGTTGCCTCTTCCACCTGGATCTCCAACTAGTCCTTCCTCTGTGCTTTCCAATTCCATCACACGATCAAATGGACTGTTGGAAAACGCTACCGGCAATGTTTCCAAGTGGAGAAAAGGAAAGCTTCTAGGACGGGGAACATTTGGGCATGTGTATCTGGGATTCAATAGTGAAAATGGACAGATGTGTGCAATAAAAGAAGTCAAGGTTGTTTCTGATGATCAAACATCTAGAGAGTGCCTCAAACAACTTAACCAGGAGATAAATTTGCTAAATCAGCTTTCACACCCTAATATTGTTCAATACTATGGGAGTGAACTGGCAGAAGAATCTCTTTCCGTATATTTGGAATATGTCTCTGGTGGTTCTATCCATAAATTACTTCAAGAGTATGGTCCATTTAAGGAGCTTGTTATTCAAAATTATACCAGACAGATTGTCTCTGGACTTGCCTATCTGCATGCAAGAAATACGGTACACAGGGATATTAAAGGTGCAAACATACTAGTTGATCCTAATGGTGAAATCAAACTGGCTGACTTTGGAATGGCTAAACACATAAACTCTTCTGCCTCAATGCTTTCATTCAAAGGAAGTCCATACTGGATGGCACCTGAGGTTGTGATGAATACAAATGGCTATAGTCTTCCAGTTGATATATGGAGCTTGGGATGCACAATTATTGAAATGGCAACATCGAAACCTCCATGGAATCAGTATGAAGGGGTAGCTGCCATATTTAAAATTGGGAACAGCAAAGATATGCCTGAAATCCCCGAGCATCTCTCGAATGAAGCAAAGAATTTCATCAAGCTATGCTTACAAAGAGAGCCATTAGCTCGCCCAACAGCCCAACAGTTACTAGACCACCCCTTCATTCAAGATCAATCAGCAACAAAAGCTGCAAATGTCAACATAACTAGAGATGCTTTCCCTTACATGTTTGATGGAAGCCGGACACCGCCTGTATTAGAGTCCCATTCCAACCGAACAAGTATAACTTCGCTTGATGGAGATTATGCATCAAAGCCAGCTCTTGCAGCTCCACGTGCATTAAGATGTCCTAGAGATAACACAAGAATGATCACATCTTTACCAGTATCTCCCTCTTCAAGTCCATTGCGACGGTATGGGCCAACGCATCAGAGCGGTTTCTTTTCTCCTCCTCATCCAGCTTACACAATAATGGGACAAAATAGTTACACTTTGAATGACATATGCTCGTATCCCATGAGATCAAATGCCACCTTAACTCTTGATCCTTGGTATGATACATCTCGATACAAAGCCAATACACCACCTGGTGGATCTCCAAGAATGAGACTCATTTGAGTTGAGATTGTACATAGCTATAGAACTTAAATATTTCTGTCTCCCCATATGTCTGTGGATGAACTTCAATGTGGACTCTGAAGCACAAACTATCCAAAAGATTCTGGTCCAATCTTTCTTTATACGTGAAAACCAGATGACAGAAAAAATGATTCTGGCTGTAAGCTCTCGGTGCGTTGGATTAGGGTGGGGAGATGTTGCTGCTTGTAACAATTTCTTGATTATTGTAAATGCGATGTGCTTACTGTGTTTGTAGTAACTTTATGTTGTATAATCCTAGTTCATGGCAGAATAAAAAAAAA

>C100391_65

TAAAAGTTAATGGTTCTTGCAGATAATTGATTGAAAAGAATAACAAAAGCTTTGTTGTTCAAGCCATGGATTGTATTTGTAACACAAACATTGTGGGATTATACATGGTACACAATCTTCCTACTTAAAAGTAGAATGCCAATAGTGGTAACAAGAGTAAAGATTTATGTCATGGCAAATCTGTCTCATTACGTTTCTCATCCTAACACACCAACTATATTTAACTCAAGCATCAAGATAAGTTAGTAAGAACAATTCAGCTTGATCCATCATTGATTTCCTCCAGGATATTTCATGTCACTAGCCTTCAGCCACAGAGATAGAGAGATATATATATATATATATATATATATAGAGAGAGAGAGAGAGAGAGAGAGAGAGCTGCAGCAACTTCCACAGATGCAAAGGCTAATTAATTGCATAACACAACATCAATCAAAGCAAAGCTCACTCTCAGAACACCAAACAATTTCTTCTTAAAAGCAGAATGCCTAAAAAACACTAACAAAACACTTGCAATGCCTACAACCCCATATTGCACATAATTTTGCTGTAGACTTACCACATGAACAATGTAACCTTTTTTTATCATAACCGTAAAATCAATTCTTCCAAGTCCTTTGGGCCATGCTATTTGGCTACACACGACGATTCTCATGTGTCAAACTACTACCACTGTCTCCACTGACACGGTTTGGTTCCCCCACCCTGGCTGCCAAAGCCAAAACTTCAGGGGACATTTCCCAGCTTCCACTTTTTCTTCCCCAGCTAGATCGCCTTGGATGCATTCCAGCACGGTCTGTGTCATCTACTGTCGTCCTTATTTCAGGAGACAATTCACCATCCTTTACAGCCACACGATCGTTGTTTAATTGAGCTTCAGAATCATCAATTCCTGCACCCATGTCCCATACTGTTTTCCTTCCCATCTCTATGTCCTCCCCAACTTTCCCTATGTTTTGGTTCATATAACCTCCACCCACTGATCGAGTAGGTAATGCTGGTTCTTTTGGCACTGTTGCCCTGAAGTTGTTCTTTGACGGGGGAATGCTGATGCAGAATATCTCTTTGAAATTATTCAACACTCCTTTGTTATATGGATTGGCTCGCCTATCATATCGGTATCTGAAGTTTTCATAGGTAGTCTGATTTGTACTTATCAAATACAAATGGAAGGCAGTGAGGCCACCTACAAACCACATCGATATAAAGGTGTAAATTATTAATACTATGGACGCTGGAGTTTTTATCATTGCCTTCCAAATTGTTGTCTCTTCTGCTGTCATAATTCTTACAATATAAACCCAGCAAAACGCAAAAACATATATACACAGTAGCGTAGTTGAGAAGACAAACATGAAGAAGAACCGATAATTACGCAGCCCAATACACTGTCCAACCCAAGGACAGTGATGATCAAACCGTTCCACACAATTGTTACAGATCGAGCAGTGTGAGCATCGAGGAGGCCTGTAGAGCATGCAAGTATCACAATACTTAACCTTAATTGGGATGCCATTGACCTCAACCTCCTTAAAGCGAGGCAATCGCATTTGCGGAGTCTGACCAGCACCAACATCTAGATTACTATCCAAACCTTCTGGCTCCGGAGGATGTGCATTACGCGGAATTATGCCTGGATCTCGGCCAGATGTTAACAAAAGAAGAACTAAGACATAAACTGTGAACGCAACCGCAACAGCCATTATGGATATCCCCCAGTGGTCAGAAAAAGCATCCATCAGTTTTCTGGCAACAAAGACACAGAAAACTGTGACAGGAGCAACAATAAGAAAAATTGTCAGCGCCAATGATCTCACATCTGGTCCAAATATGAACCTTCCCTGAAGAAAAAATTTATTGCTACCCTTCCAAGCTTGGTAGACGCGCAAATCGCCGGATCCTGACCCCGGATCGGAGCGCTGCGGCGGAGGCACCACGTACATGTCGTCGAGGCAGGAGTTCTCAGAAAGGCTCTCTCAGTTGCAGCGATTTCAGAAATTGAGCACCCAAAACGGGAAAGCGATGAAAAAAGGCGAAAACTTGAAGGGTCTCTAGGGTCGGAAATGAAGGGCCATGGGCGAATTCGGAGACCCTTTTGGATAGAGTTTCCGGCGAGTTCAACGGGTCGGGTCGGGTCGGCGACAAGGCATGAGGGGTTGGTTGGGAGGGAAAAGAAGAAGGTGCTGCAGTGCAATGCAATGCAAGTAAGGGTTTTTCTTTTTTCTGTTTTATTTTTTCCTCTTTTTCTCTCACTGTTGTGATGATTGTTGTGTGTTGTGAATTGTGATGTGTGTGTG

>C100395_65

CATTATTTTTCTGTTTATGTTGGTTTCCCTGTTCGAATTTCCCATCGCTTTGGATGCACCATTCTGCGTCCACCGAAGCGCGTTTCGTCGATCTAAAACCATAAACCTCTCTCTCTAAGATCACCTTCCGTTCGTTCCTTTGATCTCTCTTCCGATCCGATCCGATCTATTTTCCACTTCCAAAATGGCGACGATGACGAGCTTGATCGGTCTCATCAATAAGATCCAACGAGCCTGCACCGTTTTGGGTGATTACGGCGGCGAGGGTTTATCGCTTTGGGAGGCTCTTCCCTCCGTCGCCGTCGTCGGTGGCCAGAGTTCGGGAAAATCGTCGGTGCTTGAAAGCGTGGTCGGAAGAGATTTTCTGCCTCGTGGATCTGGAATTGTTACACGGAGACCACTAGTGCTGCAACTTCACAAGACAGACAATGGGACACAGGAGTATGCTGAGTTTCTTCACTTACCGAGAAAGAGATTCACTGACTTTGCTGCTGTGAGGAAGGAAATAGCAGATGAAACAGATCGTATAACTGGGAAGACAAAACAAATTTCTAATATTCCAATTCAGCTCAGCATTTATTCTCCAAATGTTGTAAACTTAACCCTCATAGATCTTCCTGGGCTGACAAAGGTTGCAGTAGAGGGACAACAGGAGAGTATTGTTCAAGATATTGAAAACATGGTCCGCTCTTATGTTGAAAAGCCAAACTGCATTATCTTAGCTATCTCTCCCGCAAATCAAGATATTGCCACTTCAGATGCCATAAAAATCGCAAGAGAAGTTGACCCTTCAGGTGAAAGAACATTTGGTGTTGTTACCAAACTTGATCTTATGGATAAAGGAACTAATGCCGTTGATGTTCTCGAGGGAAGGCAATACAGGCTGCAGCATCCATGGGTTGGAATTGTGAACCGTTCACAAGCTGATATTAATAGAAATGTTGACATGATCGCAGCTCGTAGGAAGGAGCGTGAATATTTTGAGACAAGTCCTGAATATGGACATTTGGCACATAAAATGGGATCAGAGTATCTTGCTAAGCTTTTATCTCAGCATTTGGAGCAAGTTATCAGGCAGAAGATACCTAGTATCATTTCCTTAATAAATAAGACTATTGACGAGCTTAATGCTGAGTTGGACCGTATTGGCAGGCCAATCGCGGTGGATTCAGGGGCCCAATTATACACTATTTTAGAGATGTGTCGGGCATTTGACAAAGTATTTAAAGAACACCTGGATGGAGGACGTCCTGGTGGGGATCGAATATACGGAGTTTTTGATCACCAATTACCAGCTGCTTTAAAAAAGCTTCCTTTTGATCGCCATCTTTCATTGAAAAACGTCCAGAGAATTGTCACTGAAGCTGATGGATATCAGCCACATCTAATTGCTCCAGAGCAGGGTTATAGAAGACTCATTGAAGGATCTATTGGCTATTTTAAAGGCCCAGCTGAAGCCTCTGTGGATGCTGTTCATTTTGTTTTAAAGGAACTTGTACGGAAATCAATTGCAGAAACTGAGGAACTAAAAAGATTTCCAACACTTTCAAGTGATATAGCAACAGCTGCAAATGAATCTCTGGAAAATTTCCGAGAAGAAAGCAGGAAGACAGTTACACGGTTGGTTGATATGGAGTCGAGCTATCTAACAGTGGAGTTTTTTAGGAAGATTCATTTTGAACCAGAAAAAAATCCAAATGGACCACCAAATCCAAATCGAAATGGTCCCCCAAATATGGACAGTTATACTGACAATCACCTGCGCAAAATTGGATCAAATGTAAATTCCTATATCAATATGGTTTGCGATACACTTAAGAATACCATACCCAAGGCCGTGGTTCATTGTCAAGTTAGAGAGGCTAAGCGATCTTTGCTAAGCCACTTTTACGTTCAAGTTGGGAGGAAGGAGAAGGAGAAATTGGGTGCAATGTTGGATGAAGATCCAGCCCTTATGGAAAGGAGAAATCAAATAGCTAAGAGGCTTGAATTATACAAGCAAGCTAGGGATGATATTGACTCCGTGGCTTGGAAATAATTAGATCCCAGAGTTTGCAACAGATGTTGTGAGGAAGATTTGTTATAGTTTTTTATAGTTTTTACTATTTTTTAATCAAAACACGAACAGAGAAGTCCACGGTTATGGCAAGAAGTGGAATTCGTACTTGCGTATAATTCTTTTGACCCACAGACCACAGTGTATAAAGCATCCAAGTTCTATGTTGTGATTTTATGTAACTTACGAGTTGTTAAGATGTTTGATTTTTTCGACCATATTGATTTTGTTAGAAGAACTTATCC

>C100397_65

CTTTGTCAAATAGTGACAAAGTAAATCAAATCCAACCAAAGAGAGAGACTCTAACATGAACCAAAGCTCCTTCTTCCATGTTCATATCACAATCTCAATTCTTCAACCTTTATCAACTTTTTTTCTCATCAATGGGACTGCATGGTTCCAAAATCCGAGGTAATTCTCTCTCTTCACCCCCACCACTGCTCTCTCTGCTTTAGAAAAACAATAATTTTTCGTTTTGCGATTTACATGATCATCTGGGGTTTTCAAATCTTTACCGTTTTGGATCTTGATCTAGGGTATTGGAATTTTTAGCTTTGGGTTATCGGTGATTGAAAAAATTAGGTGTCTGTTCTTTGGTTGGTGTGAGGGTGTGGAATAGATGTTCGAATCTGAAATTTTGCTTGATTTGGGGGTTTGAAGGAATTAGATAGAAGACATGGATTGTGTAGGCCATTCTTCCAAATCCGGGTCTGAAGTTTGTGTTGCTCAATCAGTAGAAAATGGAAGTTTTGGTGGAGATGGGAGGTGTTGCAAGCAAGCTTCCACTCCAAGGAGTGGTGGGTCTAGGAATACTAGTCCAATGGGGCGGGGCGGGGCGAGGAACACGAGCCCTTCGCGGCAGAAGGTTGTGAAGACCAAGCCAAGGGGTTTGGATGAGGAAACTCACGCCACATTCGGTAAGGTAGTGCATGCTGATGTTCAAATGGAGGATAATATATGGGCAATGTTGCCTGAGGACTTGTTGCATGAGATCTTAGCTAGGGTCCCCCCGTTTCTGATTTTCCGGCTCCGATTGGTTTGTAAACGGTGGAATTCGCTGCTTCAAGATAGTAGTTTTCTCAAATTCCATTCCAGTGTGCCATCACATGGGCCGTGTCTCCTTACATTTTGGAAGAACACGCAGATCCCGCAGTGTTCAGTTTTCAGCTTGCCATTGAAAACGTGGTATAGAATACCTTTCACCTTTTTGCCACCATGGGCATTCTGGTTAGTTGGCTCTTCTGGTGGTCTTGTATGCTTTTCTGGACATGATGGGCTAATGTTCAAGACCCTGGTGTGTAACCCCTTAACTCAAACTTGGAGGGCACTGCCTAGTATGCATTACAATCAACAAAGGCAACTGATATTGGTTGTTGATCGGGTGGATCGGTCATTTAAAGTAATAGCCACAAGCGATATTTATGGTGACAAGTCATTGCCAACTGAAGTTTACGACTCAAAGATAGACAGTTGGACAATTCATCAGATTATGCCAGCAGTTAATCTCTGCTCTTCAAAAATGGCATATTGTGACTCCAGATTGTACTTGGAAACCCTTTCACCGCTTGGTCTGATGATGTATAGACTGGACACAGGTCACTGGGAACATATTCCGGCTAAGTTCCCTCGATCTTTGTTGGATGGTTATTTGGTTGCTGGTACCCAGAAGCGTCTTTTTCTTGTTGGAAGGATTGGCCTTTATAGTACCCTACAGAGTATGAGAATTTGGGAATTGGATCATACCAAATTTACGTGGGTGGAGATCAGTAGGATGCCCCCGAAGTATTTTCGTGCTTTATTGAGATTATCAGCTGAGAGATTTGAATGCTTTGGGCAAGACAATTTAATCTGCTTCACTTCTTGGAACCAAGGGAAAGGCCTTCTATATGATGTGGATAAAAAGATCTGGTCATGGATTGGTGGGTGTGCTCTTCAGTCATACAACAATCAAGTTTGTTTCTATGAGCCAAAGTTTGATTCTTCTATCTACTAAGCTAGAGTCTTATGACATCATGTCAGTTGTACATCAACATTTACAAAATTAAGGTTTACCAGTGATTTTCTGGACTTATGTTCTTTGCAATGACATTCTCTCTGACCATTCATAATGTTATTCCTCACAATGGGCTGGTTTTCTTCATCAGTAAAATATCTTACTAGCTGCAATGAATAGCATGCTTGACTCTGAATACTGGTTCAGCTCATAGACAGATTTTGTTGAATGGAATTCAAGATGAAACATAATGCAAGGTAGTGTGACAGCGTGACTGGGATGTGGAGTTGATGTACGCTGCTGAAGGAAAGATTATAAGTTTCAGCATAAGGGTTTGGATGTTTAAAAAGGGGTATGCAGCTGTTGATGTGTGGGGAACATTTTTTTGAAGACCTTTTCTTACCTTACCAATTGGAGAGGCTTTACAGCATTGAAACTGTGCATCGAAAACTTTAACTGCTTTTCTGCTCGACGCCTTACCAGTGTAATGTAACTGTATTAAAGCTTGTCTTGAAACACTGACCATATGTGACTTTCTCGTGTAAATGGATTCCCTCCCG

>C100399_65

GAATTACATATATTCGAGATAGAGGGAGAACAAGCGTACTGTATTACGTATTTTAAATGCCATTATACAACTTAAGAAATTTTAAATACTACATTTGGCTGATCGTTACTGATTGATTACATGTAACGTGACCATGCGACATATCTTATGTGAACAAAATTCTTGGCACAATCTTCTGAGATAATGACATCAAATTAAATCAAAATTGCATATTACATTCCTGATAGCACCCAAATCTGGCGTAATAAATTATCAATTTCTAGTTTTCTTCTTGCCTCGTTTCTTTGCACTAGTTTCTGGTCCTTCAGCCTCCTCAGCTTTCTGTTCAGCTTCTAATAGAGCCAGCTCGTCAAGAATAGGTTTTAGCTCCCTGTACCGTGCTTCGGCAAGATCAAAACCATCCTTACGGAGTTCTTTTCCAGTGTGTTGAGTATGTTCATGGGCAGCCATTTGCCAATACATATTTGTACGCTTGTAGAAATCCCTCAAGGATTCTCCTGGCATTGGTGTTCTCTGTGAATCTGACAAGCCAAGACTAGCTCTAATCTGCTCTATCCGTGACCTCCTTTCTTTCCTCCGCAGATTCTTTCCCACACCTTTGATTAGAGCAACAGCATCTCCCACCTGAACTGAGGCATTCTGAGCCTCCTCTTCCTCCTCTTCCTCATCATCATCCTCATCAGTTGAATTATCACCATCCGATTCATCTTCATCACCATCTTCATCTTCTTCATCATCTTCTGAAGCTTCCACCCATTCAGATTCTGAAGCCGGTATAATGCACTTCCATTCATCAAGTTTGCTAAGGTTCAAAGAATACAAATCATCAAGAGTGATTTCTTGATCCTTAATCTCCATCATGCCACCATATATATATAATGTGTCTCTTCCAACAGCCATGCAAGCATTAATACGTCCACAGGGCTTCACTACCTCAGGCAAAGAACTTTGAATGTCCAATTTATCACCAGATTCCTTGGGCTTTCCATCAGAGTTGGTTAGTTTTTCACCATCAACAATAGACATATTTGATGCTATATCTTTGGATATATCATCAATGTTGCTTTCTTCGTCCTCTGACTCCACAGTTTCTTCTCTTGCTGTACATGCTGGATTTGTCTTCTTGTTGACATCATCAGGACAATTTTGTTCAATTTTTTTTAACTTATCTTTTGTGGACTTCTCCTTTCTCAACTCCAGAGGATACCAGCGATTCGTGTCCAACTGAAACCCGTAAAGCTCATTCAAAAACAAGCTCATCATTACATCACCTTCAACTTCTATATCCACAACGCCACCAAATAGCAGAGCCCTTCTCTTATGAACACACATCGAAAACCCAGCACGGGGTCCAGGAGGCATTCCACTTTTCTTCACCTTGTTCCATTCCCAAGTCTTAGGATCAAGGGACCACATGTCTGAATGAACAATTCCTTTCTCCGAGTTGTTCTTATCAGATGAAACTTCTTTTGAATAACCACCATATAAAAATATGTCATCTTGGTAAACAAATAATTGAAAACCACTACGTGCAGTTGGCCACATTGCTCCGGGCTTAGGCTTTATTTCTTGCCACTTAAATTGATCAAGGTCAAATACAAATAGATCATTGTAGTACCTCACTTCTCTAAGAGTGTCATAGAAGCCACCAAAAAGAATAATCTTGTGCTTGTACAATATCATTCGATGTCCTGAACGAGGACTAGGGCACCCTTTTAAATTAAGCTGCTCCCACTGATTTGTTTTCAGGTCCATCATCCAAAAATCCTTGTAGTGATGAAACCGCTCTTGGTTTGGAGATGTAAACTCACCACCAAAAACATAAATATAATTCTTCCACGCAACCGCTTGGTGAGCACTGCGCGGAGGAGGACTATTAGGACTCGAAACCAATTTCCATTCCAGCTTCTCCACGTCATACCGGAAGAGATCACCGTACACGAAGGTCTTGTTTCCGTTGTAGAATTCGCCGCCGTAGAGAATCAACTCGGTCTCCTTCAACGGATTGACGGTGAGCGAGCAGTTGGAGCGAGGAGAAGGCGCGGGAACGTTGTCGTCGACGTGAACATCCTTCTTCTTCGCTTCCTCCTTTTGAATGCTCAACAAAATGGCGTCGATGTCATCCTCCGGAGAAAGCTTCTTGGTTTCCCTGCGAGCTTTCTTCTCTTCAGCTTTTGCGGTTTTTCTCTCGGTTTTCTCTTTGCCTTTGCCGGGTTTCTTCGTCTTCTTCCCCATCTTACTCACAAAGCACACTAAAGTCTAAACCCCGCGGCGCAACAATGTGCCCTTCCTTAGACCCTGC

>C100401_65

TTTTTTTTTGAGTAAAACTGTGAAGGCATTAGATGAAACTGTCAAACAATTACAAGCTGTTCAGTTTTTTATGTTTAAAGAATACAAGATTTGATTTAGATACATATATACTATGTGTATCACAAAAATTTAAAGAGACACTCTCAAGTACCACTTCATCAATTCTTAATGCTAATCTTGGATCGATAATTGGCCCCAGGCAGCTATATTCACTAGGCCAAGCTCCATTACTTCCATTTCTCCAAGCTTTTGTCTGCAAAATTACCTTGATCTTCCAGTTGATACAAAACGAAGGTTACATGTCACTCAAATCATCAGGGTACTTGCTAGAAAGAAATAACCAATGGCATGGTGCACTTATGGTTTCCATGGCGCTGGAGGTGGTGGTGGTGTGGGAAACATTGGGGGAACAGCAGAGAATATAGTATTTTTGTCAATTGAAGTGCTTTTGTCACCTGACAATGCTACTAGTGCCGCAACTAAACCAGCATTTCCAGCAAGAGTTGGCTCTGTGTAATTGTAGTTTGTACGAACATCGTGGAAGCCATCATGCTTGTCAGGACCAGCAACCATGGCACCAACAATTGTATTTGGGTTAGGCTTGGAAGAATCCCTCCATTTCCATCCACCTTTACAGTTATACTTGACCTTGTTCTTTGGAATAGATGCACCTCTATGGTGGACATGTTTTGGATAGTGATTACCAAAACCTACAATATAGCTCATTTTCCTCGGATTATTCCCAAGAATATAATCAATCTGGGTCTTGGCGAAGTTACGGAGCACATCAGTTGAAAAGAAATTAGGTCCACAATACCATCCAGGTGTATCAGCAGCATCAAGATAATCACTATATAGGGCAGCCAAAAAGGCGGCATTGACAACATATTGCAGAGGCTGAGGCCTACCATGGTTTAATTGAATCAAGCCACCTTTGGTTCTATTAAAGCTAGTGAAAACTGGTAGATAGGAGCACATGATTATGCTTGTCTGATTGTGAAAGGTCCTCAAAATTTCTTCATATGGATAGCCAGGACTCAAGAATAACCTCAAACGACTCAGAAGAACCTGAGCACCAGCAAGTTTGTTATCCCAGCTGAGTACCCCATAATCAGGGCCTCCCCAGAAGGCACCAGCATGCTTGGCCAGGCCAGGAGCAGTAGCAAGCTTAAGGTATGAGGAATTTCCAGTTGCAAAGTACATCCAAGCACCTCCCCAAACAAACTCATCCCAGTAACTGGTGGAGTTGTAAAATATAGCAGCCTCCGAACTCCCAGCACTGTACCTGCCTCTCTGATCCCTCGAAAACTTGAAGAGCGTGGTTGCACCATGAACCAGTTTCTTCGAATAGGCCTTGTTGTCCTTGAAAACAATGGAAGCAGAAGCCAAGGCAGCAGCCATCTCAGCGGCAAGATCTGAACAACTGTGGCATTCAGTCACAGGGCGTTCATAGTCAATGTCCTCCGGACGCATCCAGCAATAGTGATCGTTAGGAGTTGTACTCCCACCAGAAGTATCTCCAACCCCAACCTGAGCAGCAAGGGTGGTGATGGTGTCAGCAGTACTATTGAAACTCTTGAGAAAATAATCAGCACCCCACTTAATGATCTCCTTAACATGATCAAGCTCCCCAGCAGCCTCATACTTTGCACTATACTCAATCACACTCCAACTGAGCATACTAATGGAAAACGCAGCAGGAAAGTTAAACTTGATGGCATCCCCGGCATCATAGTACCCACCCACCAGATCCTTAATCGCAGAAGACGTAGCAGAAGACTTCCCGTCTTGCATACCCGAGTTCCCCCTCCAAGAGACATTGTTATGCTTAGGCAACTTCCCCGATTTTTGGGCGTTGAAGAACATCAGAGCCTTGTGGAGGGCGAGGGTGTAGTTGTCGGGAGGGGGGTGTTTGTGGTGGTGGCGAGGGACGGTGTTGACGATGAGCGCGATGAATCCGGCGAGGAATGCGGCGAAGAGAACGGTGCCGACAGTCCAGACGAAGATCTTGCGGCTGACGATGATGCAGCCGAGATCCACGTACTTCTTCTTCTTCTGCTCGCCGGGGCCGAGGAGCCAACTCTGCTGCGTCTCGTCCAAGGGACGCGAGAGCGCCGCCCTGTCCAGGTCCTGTAGGTTCCGGCTCCGCTCCTCCTCCGTGGCGGAGTCGGTCGCATTGATCTCCAACGGACCACCCCACGGATCTCTACCGTACATACTGTTGTTTTTGTTTTCTCACTCCAACAACGCCACCGACACAACACACAACACACAACAACACAAGACACTGTTTGTTTGAATC

>C100403_65

CTAATGAAATCAATCTGAAATCATCATTAAATTTACAAATAATAACTAAAAACTTCGGCAGTAAAATCAACAATTCTACACTTTATTTTGAGTCTGAGTCGAGTCTTTTTTACTTGAACTTGGTCATGCAATATGCTCTGATTCACATCGCAAACATTGAGTAAACATGGCTGTAGAGTTTTGATGTATAATCAAAGTGATCAGTAGGCTGTAGCTTTGCCATTCTGGCGCTGTGACTGAGTCTGTGTCTGTTCCTGGGAATGACTATTGTGCATTTGAACATGTCTAAGCTGGTTAGGGTGCTGCTGGTGCTGTAACGGGAACATTTGTTGGCTAATAGCAAGCTGTTGAGCCATGCGACTCGAGAGGAGGGACTCTCCGCCAAGTTCTGCAACTGTGCGTCTTAAACGCCGGACTTCAGCATCCAAGGTCTCATTCAGAGCATCTTTCAGTTGGGACTGCTGTTCCATGGCTTGAATCCTCAACTTGTACTCATTATTCTCACTTTTAAGCTCTGAATTATCCATCTGTAATTTGGTAAACTGAGTAGACAATGTGGTTGTCTCTGTCTGAAGTGTTTGGACCTTCAGCTCCAATTCAGAAATGTACCGCATCTTCCGCTCTTTTGACCGAGCAGCAGATAGACGGTTAGCCAAAATCCTTTTTGCACGTTTAGGATCAGCCATGGCTATTTCAGCAAGTTTATCATTCTCCTTTATTTTCCTTAGCTCCTCCGAGCTGAATTCACCATTTCCAAACTCCATGTTTGATTCAGATGTCTTACCATCTACTGAATTGCTAGGGGAGTGTTGACCAACTCGGTTTTGCAAAGGAGGAAGCTTAGGTGATTCATCTCCAATATTAAAATTTCCAATGGAGCTGTCCAACGAGAAACTTCTCCGGTGTCGAGAACCAGGTGCAATGTCTCCATTAGAACTCCTCTTCACTCCTTCCTTCCTCTCCTCCGAACAGCTTGAACTAGCCCCTTGAGCACCAGTTGTTTTCCCATTTGCATGACTCTCAACTTCGTTATCACTGCTTTCAACAGTCTTGGAACCACTGGTTCTGCTATCCAAATCCTTATCTTCCACACCAGAGAAATTCAGACCATCAATGTTGTCCAAATTCATATAAGCAGTAAACAAGTCATCCAGTGCATCATCCTCTTTCCTCCCAACCATAGGTTCCCCACCAAAACCATCAGCACGGTCCATATCCTTAATCGGCTCCTTTAACACTAACTGAATCGGTTTCTCATAACCTGAATTCCCTCCCCTAGAAGCCGAGCCATCTCGATCACTCCAAGTCCTGGAGGGAACCAACTGAGGGGCAGACTGAATAAAATCGGAGATTCCCAAAGGGGAATCACTGCTGGATCGCCTGTGTCCTTTCCGAGGTGGAAGAGAATGGCCAAGGTGAAGTGCAGGACCACGATTAGGCAAAGGGGCATGTGGGTTAGCCAAGCTTTCCTCAGCAGACACATCTGTCGAAATTGTGTCAGAAAGGCAGGGCTCTCTATAAGGGGAAGGACTCAATGGGGGCAAGGAATCCAGGGAGAAAAAAGAGGGCTGAGACAAAGACCTAGAATGGGATGAAGCAGGACTCAAATTGGGAGAACCCAAACGCTGGGAAACCGAATTGGAATGGGATTGGGAGGCATAAAACTGAGGATAGGGTGAAGAAGGTGAAATATTTGTATTAGGGTGAGAGGGTGGTATACCATAGGGTTTCTGATTCTCAGCAACACCCATTTGACTAATAGGCACAGCATTATTCAATCGATTTGGGGGTCTGAGATTTGCTCCTGATGATGAACCAAATACACCACCATTTCGATTCATATTACTTTGATCACCACCTTCCATTGACTCAACAACAACCACTACCCCTTTTACTAAAAAATCACAACCTTCAACAATATTCAACAATCAACACATAAAAACGAACCAAACTGCAAAATCCTAACACACAGTTCACCTAGGCTAGTCTACTGAAACAGTTGCTAATAATTGAAGAAAGTTCTAACCAACACAGTACTTTGCGTACCTGGTCATAGCTAGTACTAATTAAAAAGTGCAAACCCTATCTTGTAACCAGCACGGTGACAAGGACAAGAGTGAATTATATTACAAGCAGTGTGTGTGTGTGTGTGTGTGTTTTGTGTTACAGAAGCAGGAAAATTCCACGGTGGTGCGGAAGAGGTTTCTAGAGGAAGAACATGAGAGGCTGAAGTGTCAGAGGACGAAGATGACGATGTGGTTGTGTTGTGTATGTTAGAAGGTAAATTTGTAATTGTCCCTTTT

>C100405_65

TCTGGTGATGATTCTTTGTAGCAGTTAAAGAACTAGACAGTCATATAAGGCATAAGAGCAGTTATTTATATTGCCTTACAAGTCAACCTACATAAACGGTGATAGGGTACATGGCTTATAAAAATTAGCATTACAGAGCACACTGAGGCATAGAGAGAGGGAAAGCAGTGAAATTGGGGAAATTGTTGAGCCAATGGCAATCATCGAAGGAGCAATAGGGTCCACCCTTTGCTCTCTCAACATTGAGTGGGCGTATATACTTGGGAGACCATGCCGGAGTTCCATCCTCGTATGAATTATGAGTCAGTCTCTTCAAACCCGACCCATCCAATCTCACGCTGAATATTTCACCGTAGGGTTGGTAGTGATGCGGGTTCGAGATTGGCTCAGCGGATATACCAGCATAGTCCGAAGTAAACGTTAAGCTCTTCCCGTCCGGGCTAAAATACGGGTGGTTCGTCCTCCCGCCCGACCCGCTCTGGATCAACTTCCGCAATCCGGTTCCGTCCGGGTGGATCAGGTACAACTCGAAACTCCCGGTTCCTGGGGCATGCCGGTCCGACGCGAAACCAATCCATTTCCCATCCGGTGACCAGTTGCACATGGTGTCAGTCCACGGACCCTCCGTCAATCTTCGAATCCCTTTTCTCTCTCCATCAACGGCGTCCATTATGTAAAGGTTCTTATAACCAGACCGACCCGACCGAAACACGATCCATTTCCCGTCCGGCGAGGATGATGGAAAGGCGTTGTTCTTTCCATCCAAGGTCAACCTCTTCACAATGTTTCCGTGATCAATGTCAATGGACACAATATCAACCTGCGAGCTCTCCGAGGCAAATGTTTCCCCAATCGCAGTGTATATGACACCGCTTCGAACCCTGTCCCAGGCCGTGGAAAACGCCATCGCGTTAGAAACCTTACGCAGATTCGAACCGTCCCGGTTCACTACATAAACCCCCGGCATCTCAACATACGCGATCCGGTCTCCGGAGGGTGAGAAAACCGGGAAAGAACCAACGAACCGGAATAGTGATAGACCGGGGACGGGGCTCCGAACGTTTTCGAGTAAAAACTGGGGGCTACTGTGAGCGTTAGGTTCTCCTCTGCACTTGTGGTACCCCACTCGCGATGAATCTGGCGAGATGAAGGGGTTGAGGTGGTGAGAACGAGGGGAGACCAACCGAGTTAGCTCCGTGAATTCGTTATCAACCAGGTTGAATAATTCGATATGGCGAAAGCTTGAGCCTGGTCTTCTTGTTGCGACGGCGATGAAGTTGTTGTTGCCTGGGGAGGTAGCGGGTGTGAACGCGTGGAGACCCGGTGGAGTGACTCGTTGTATGATAACAGAGTCACTGGAAACGGGACCGTTGGAGGGGAGAATCGCTTTGTAAATGCTCCACCACTGGTCTTCGCCTCTCCGGTGGAAGTAAAGAGTGCGATCGTCCACCCAGGAGGGCCAGCCACCGTGGTCGACGATTTTGATTCGGTGAGTTCCGTCGCGAGTGAGGAAGACGTAGATATCTGTGGTGAGATCCTCTACTTCGCCGGCCCAACCATCTGGGCCGTAGGAAGCGACAGCGGTCCAGAGACCAGAAGGGGAGAGGGCTGGGCTGAAATCAGCGATACCGTAGGGGGTGAGTCTTTGGGTGAGGCCCGATTGGAGGTGGGTGGAGTAAACCGCGGCCCAACTGGCCCGTGGTACAGCAGGGTCCTCGTGTGTGGAAACGTATATTAAAAATTGTCCGTCGGGGGTAAGACTGGGCTTGTCCTTGATGGAAAGTTGAGAAGTGTCGCTGGGCTTGTTCGGTAATAAAGGGATTTGGATTCTCTCTGCGGTAACAGATTCTTGAGGTGCTCTTCTTGTGGTTTTGGAAGTGTAAACTGCGTCGTAGTAAATGGTGGGGGAGCCGTTTCTCTCCGTTACGTATACGAGTTGGAGAGGAGGAGAGTGTGTGGGGAGTTGGATTGAGGAAGCATTGGCAATGAAGTGGCCGTTGAAGTTAACAGAGTGGCCATCGGTGAGTTGAAGTTCTTGGTTAGGATTGAGAGGTTGTTGGCGAATTGGAAGAGAATAGATGTCGAAGGCGTAGTAGGCTCTGCCGAGTGTGGTGAAGATGATGCTGGTGGAACCGTCCTCCTCCGCCTCCGCCTCCGCCCCACACCTTAGCGTTAACAGTAGCACTACAACAAACGGAAAAGGTATGTGCCGTCTCTTCATCGTTTGAGTGATGAGTGTGAGTGTGAGTCTGAGTGATTGTTTGTCTGGAACCAACAGGAAAGTGAGGAAGAAGGCATCTGTG

>C100407_65

TTGAGGTAGATTGATCAGATTCAAATATCTAACAGTTATTTACTAAACGGTACAGAAATAATTTATGAAACTCAATCCGAATGATCTGGCCTGATTTCCCTGAAATCCCAACTTTCATTTGTTCTTTAACTTATTATTCACCCAATAATATGTAACCACGTTATTTCGTGATTACTGTTTAGCAATACTCTACATAGTGTAACAAAAATATTATTAACATATGCAGAAATTGGCATAAGCTTGCTAAAGTTGAGAAGACCAAGAATTTGAATCAACCCAATTATATGCAATAGGGCTCAAAAAAGCATCAGGAATACCATAGGAACTGACCAAGGCAAGTGCATGTGGTCGAAGTTCAGCGCAGAGTTTTGGCACTTCTTTCCTCACCGTAGAAGCATTCTCTGTTGACAAGTGTCCATATCGAAGAAAGGTAGCACCTTCATCCACACTTATCACAGCATACAACGATCTCAATAGACTCAACACATTCTTCAATGAACCAGCTGGTAAAGTTGACTCGGCATCTATGAACGTTTTCAGTATTGCTCGTTCTGAGAAAGCTCTGCCCAAGTCTTCTGCTAGCTGATAACTTAGAATGAAGGCAGACTCTTTGCTTTCTCCACGAGATTGATATTCTGAGACCTCTTCAGCAAAACGCCTCAATAGGTCTCGTTCTCTTAGGTGGAACAGATCAATCTGAAATTCACTGCTCCTAACGGTTGAACTTGTTAGTTGAGATGGGATAACAGGCAAAGGTTTGTTCATATGCTCTAATCCCAAACCACTGAACGGTTTGTTTTTCTTCTGACATGCTATGTATTCTGCAAAGAGTGCCTTGCTTATCTGCTGCATCAGAACATTGTTGTCCCCCTCAAATGTCGATTGCACATCAAATTCACCCATGAATTGACCAACACGATTTTCAGATTTAACTCCTTGGCCTCCGCAGGCTTCACGACATTCCTGGAGAGTACGCATATTATTCCAAGTAAAAGTAGCCTTGTAAGCACTAGAAATAATATGAATTGCTTTGTTTGACTCGGGTGTTCTTTTGACATACATCATTTTGAGATCATTTGCGGCAAAACTCATTGCATATACCTTTGCAAGTAAAGGTAACAGACGCCGTTGATGACTAGGGTAATCAAGCAGTAAAACTTCAGGCCCGTTTGGTGTAATGGAGAATGCCCGCCTTGTCAGTGCATATCTTATAGCAATGGCCAAGCTAATCTTGGACATGTAAACAGCACTAACGGCAATAGTAACACGACCAGAGGTCAAAGGGGCTAAGAAAGCAGCAAACCTCTGATCAACATTTTTTATTGCACTCAAATATTCACCACTTGCTGAAACATCAGCCACAGAATTCAACAAATTCTCCTGGGGTATCCTCACATTATCAAACCAGATACGGCCATTATCAACCCCATTTAAACCAATTTTGTGACCACAATCAGCTATTCGGATGTTTGGACATATGTTTCCATCTGAATCCCTGATTTGGGCAATGAATGCATGCACCCCTTGATTGCTGCCATTTATATTGAGCTGTGAAAAGACTATAGTGTGCGTTGCATGATTTGCGGCGCCACCAATCCAATACTTCTGACCCGATTCACATGGAGTATTGATGACAAATTCTCCTGTGCTTGAATCATAAGTAGTGACTGTTTCAATTCCCCGCACATTACTTCCATGGCCCAACTCAGACATGGCAAAACAACCCTTAATGTCATAGTTTTCAGTAGCACTCAACCACTTGTCATGATGGCGCTTTGTTCCCAAAAACTTTACAGCCCCACCCCAGAGGAAGTAGTGAACACCAAGCTTAACAGCAAGCGAGTGATCGTACATCCCAATAACTTCATGGAGCGCCAGCTTCCTGAGCTCCTCTTCCGGGCCCTCGCCGGTGAGCCACCCTCGGAAAACCCCTCTCTCGACAAGATACTCGATACGCTTCATGGTGGCTTCGCGCTGCTGCTCCATGGACTGGTTGTAGTCGGGGCTGACGAAGATTCTGCCGGCACGCTCGCGGCGGTTGAAGAGGGCGCTCTGCAGGATGATGGAAAATAGCCAGTCGCGGTCCTCCAAATTGTGCCCGTCCATCAGCCTCCGCATCTCCCACTGGTCGAACGCGAATTCGTGGGAAAGCTCCGGGGGGGAGTAGCTCAGGCAGCGGTGAGGGTGGAGAAAGGAGGTAGGCGGTGGTGGTGCGCGGCGGAGGAGGTGATTGGTGAGGATCTCGGTACGGCGGGAGACGCGGTGATCCATGGCTGGAGAGAGTGGAAAGTGGCGAAATCGTG

>C100413_65

CGCCCAACTGTATTATGTATATTTATACAGGTTTGGTTGCGAAATATGCAAAAACTTTACGATTTATATGCACAACTCAATGTATCCGTGGGGTGCAATCTCCTAAGTAAAAGCTGTGTGTAGTTTCCCAGTCAATTCACTGTTTCCGGGTCTGTCCATTTCACTGACAATGAAGTAGGCCAAGAGGGGTTTTTGCCACACCATGTCACCATATGGCTTCAACTAGTAGCGGTTTATCTTGGCGTCATTCACTTCGTTGGATGCATTGCAGAGCGGGATGCACTACTCAAACACCAATGGCCACGAATGAACCAGCCTTGATCTTCAAGACGTGTTCAGTCCAACTAGCTTCCCCATGCAGCTATGCAAGTGGTCATGAAAACCACCAGTCTTTGGCCAGTCAGCTACTCACTGAGTTTGGAGATGTTGTGTCGCCAATGCGAATCCTATTTGGCAATGGTCTTGCTATAAGGCCGTCTGTGACCCAGCCTACCAATTCTCCATCATTTTTGACATGGTTGAATTCCCTTTCCACATAATCCAGAGATATATGCAGCCTCCCACCAGGTTTGGAATCATTCAGGGTTCCTTTTCCTCCAATTCCTGCGTTCTTTTCTAACCAATAAAGGTAATTGACCTGGAACAGTGCCTTGAGCATGTCTTGCTGTGAACAGTTTTCTTTGAGCACAACACAGAATTTTCCCATGTGCTCAGACAAGATGTACCCTTCGTTTTTATAAAGCCCAAAGAGGGCAATCACATCTTCCTTGCTATTGACAATTTCACTGAGCTTGGATCCAAGCAGCAGACGTCGTTCAATTTCAGCAGCTGCGTCTTTTGCTTCAGATGATAAAACAATTGATTGTGCTTTGTTTGCGAAAGTTGCATTTAATATGGGTACAGCTGGAAATAGTGGCTCTTCATCATTGACATCTTTAACTGGAGGTGCCTGGCCACTGAGTAGATATTCACTAAAAACCAAACTTGCACGATAAGGATTTAATGTCCGTAATTGAATAGATTGGTAAGACTTCAGATTGCAGTACATGTGGATCCAAGTCAAGACACTAAATGAAGCAAGAACAAGGGGTGTGGAGGAGCCTATGCAGTTACCCAATCCTATACCGAGTCCAATACCGATAAATCTGCTTGCCATTCCTTGCACTTCCCCTTTAGCAATCACCTCAGCAAAATTCCTTTGAGCAGCAAAACCAGCAAAGAAACAGCTCCTGGTAGAAGCCTGAATCAGAGAAGCTGCGGAGCGAGATGCCCCAGCCAAAGCACCAATGAGCACAAAAAACTGAGGGAAAGCAGGAGTACACATCTCCAAACCAAAGGCAGCATTTTCAAGAAGATCAGCAAACAATCTCCACCCTTTGGGGTTAACATCAAAATGGCGACCAAAGTTTGACAACATGATCTTACTGAGGTACCCAATTCCATCCTTGAGCACCCAATTGATGGCAGCAGCAGTTGGAATAGCCCCTTTTCCCAATCCAACAGCATAAAGCAACGATTGCGTTGCCAGCACCCCACTAACTTGACAAGCTACACCCTGAACTGCTCTCCACAGAGAATACTCCAAGTAATCACTCGTCACACTCTCGGGAAAGCCCTCTGGAAGCATCAAACGCGTGAAGATGTCCCTACACTTGAGCCACACGAAGGTTCCAAATTGTGAAGGCTTGAGGGATTGTAACTCCGACAACAACCCCGGGTGTGCGGACACAAAGACATCGTTGGTGGGGTCAGGGACAAGCCTGGTCCACTTGCCACCTTTGATTTCCCAAACGGATTCGGACAAAAGTTCGTTGTCGGTGGAGGAAGACCAGGTTTTGGCGATTGCAAGTTCAACCGGAAAGAGATGGCAGAAGAAATATGCCGCGGAAGAGCATAGCAGTGAAAGGAAAAGGATGTGATGAGAATTTGAATTTGAATTTGAATCTGAATCGTTAGAATCAAAGGGGTTACCCCAACCACCACCGTTGTTGTTATTGTTATTGTTGTTGTTTCCGCTGCCTTGGGATAACAGAGGAAGGGCCGAAAGGCGAAAGGCGTGAGAAGGGGTTTGTTGTTTGGGACGAAAGGGAACGGCGGCAGCGAAATGTGGAGGCGACGGTGGAAGGCGGTAGCGAAGCGGAAGAGAAGTGAAGAGAGAGGCGGAGAAGGGCGTGCAGATGCAGCCCATTTTGGTGGTTCCGCCGGCACGGGAATGTAAGAGGAAGAAAGGTCAAAACTTTGCCATCATCGCATTCAGGGTAATATCCAATCAACGTGACCGATTATGCTCATTGTTCATGCTGCT

>C100415_65

GTTATTTTCTATTTGTAAATGTGATGCCATCTAAAGACATCCCCAAATTATTGGATTTCCATTACAAATCATATTACAAATAGACAGAAGGACTAAACTTTGTCCTCAGTTGGAAGAAAACATTGACTTCAACTTAGTGCTACTATCTTCAGACAAAACAATCACCTAGAAAAACCCAAAAGGACACGTGCACAAAAAGGAAAAGGAAATGCCAATCATAAGGCTCGATCAGACATATCCTTTAGATAAGACAGCTAGCAGGCGAACCTTTCTTGAGCTGTTAATTTAGCACAAAGCAACAGTTCCTCAGCCCTTTAGCCGAATTCGTTGATTGAAGAACAAAAAAGGACCTCGAAATTGCACAATGATCAATGAATAGAAAACTTCTTTATGTACAGTTTTTACATGTCTAGAGCCTCGGTTGGATATTTCTGCTCCGCACGTAAGTTAAAAATTGAGTCGGTAATTCTGCTAGAAGTGCTTGAACAACTGAAAATCCACTTTGAACCTCTCGAAATGGAACAAATTGGACTATATCACGTGAAGCAACGCGTCCATATGAACTCTCAAGCCTCTCTCCCTTGTCTGCATCTAAAACCTCCATTTCTTTGAAATCAGCACCTCCTACCCCAACAATAAGGATTGATAATGGCAGGTCAGATGCTTTAACAATGGCATCTTTTGTTTCTTGAAGATCTGTCACTACTCCATCTGTGATTATTAACAAAACAAAGTACTTTCTTCCACCATTTGCTACAGATTGGTTGGCAATCAGTGCAGCTGTGCTTATGACAGGTCCAAAAAGGGTAGGTCCTGCAAGAGATACATTATGCAGGGCACTTGTGTAAGCCATCATAATTCCTTGGATCCCTTCCACCTCACAATAATGACTACTTCCATTCAAGTTGAAGCAATGGGAAACAGGACCATCAATTGGTCGTGCCCCAAATCCCCAAGTAGGAAATCGTTTGTCTGAATCATAAAACTGTAACACTTCCCCAACCTCAACAATTGCCCTCTGGTATGAATTTGGCCGTCCTGTAGGATCAATATAATGCAAAGAATCTGGAAGGCGTGGATTTCCATTCGAAGCTGTAAAGTCAACAGCCACCATGAAGTTCAATTCAAATCCCCCCGCCAAGTAATCCAAGAAAGTATATTGGACACTTTCAATAAATTTGTCCACAAATAGTTGGCTCTTTAACATCTTGTTATCATGGTTTTGTCCACCAGAAGATGGCAAACATAAATTTTCTCCTTGGCCATTATTATGAATGTTCTCCAACCCAGCCAAAGATCTCTCCACTTTTCCAATTAAATCATGTTTTCCATTGCTATTAAAGTTATAGCACTCTATTATCAATGTACTGTCCTTGCTTCCTACTTGTTGAATATTCACAAACACTGACTTCCATATTGGGTTGAGATCATTCCTTATAACTTCTGTTTTGCAAATTGGAATTTGGATACCACCTTCTGCAACTTTTGATATCAATAAAAAGGGGTCATTTCTTGAGAAGAGATCCTTGTATTCCAAATCTGAACACCTGAATGCCATCTCTATTGCAGTCTTTGAGCTAATACATTCCTCAGCATGCACCACGAGTTTCCCGCAGTTTTTGGGAAGGGTAGATCTCAAAGAATCTTCTGTGTGTAGATCTATAGTCAATGATTGATCACCTTCTGTGATTATCTGAGACAGTACACAGGTAGCCTCACCAAGAAATTGCTGCTCATCTAGCTCAAGCATCTTTATATCTACATTGTGAAACTGAGTATCAACATCATACACACGAAACACTAAAATCTGAACAACCTCAAAATGATAAATGAGGGTGTGTTTAGTAATCCATGTTGGACTTGATGAATTGAGAACTACTTCTGTTCGACGAAGTTCCTCAAGAGCTCCATTTTTTCCTTTTGCATAAAGAACCATCATCGGATTACTCTTGAAAAGCACATCACGATCACGCAAGCCAGAAGCGGAAAACGACAACTCGATCTGAGAGAAGAGGCCTTGGTGACCGCGAGACCTGAGGAAGTTCTCGACGGCGTCGTTTGGAGCGCCGTCAAGATTGCGGAAATCGCCGGCGGTGCCACCGATGGCAGCGCGGCCTGCGGCGACTTCGGAGAAGCAGTTTCCCATCGAAAAGTGCGCGCCCAGAGTAGTGTGTGTAGCTTGACTTCAGTCTTTCCGTCAAACTCAGCAGTGACGGGAATGAAATAAACAGAAAATTGAAAGGAACAAAAGCAGGCACACAAAAACAGAAGAATATCCAATTATATAATCTAATTTGCAATTTGC

>C100417_65

ATGGCCAAAAAGTTCATGCTACAAGCAGCCATTGAGATTCTCAGAACATGAAAATGTGCATGTTATATCAATGTCAAACGTTTGACTAATTGATCATATATTACTTTCAAGTTAGGCCTCTAAGGCCCTAGCCTCGCATAAATTTTGTTGATCATACTCAGTGGTGATTGCAGCAAAAGAAGAATGATCTATCAATGGTTATTCATAATCAAATTGGTGTACCTTTTTTGCTTTGTTTCTGGTCAGAACAGTAGCAACACACAACCACTTGATTATAGATATGCTTGTTTGGACCAATCTTCTGTTCCTCCCTCTACCACTTACCAAACCAACCTCAACAATTTGATGACTTCCCTCTCATCTGATGCTGTAACCAGCAATGGTTTTGGGAATGCAACTTCAGGTATTGATCAAGACAAGGTTTATGCACTCTACCTTTGCCGTGGTGATGTGAATTCCAGCCTTTGCCACTCATGTGTCCAAAACTCTAGCAACTTGCTCATGCAGCACTGCCCCAATAATGCCTCTGCTGTTTTCTGGTTCCCCTTTTGTCTTCTGAGGTACTCAAACCATAATTTCTTTGGCAACCTCACTCTTACGCCAAGGATTCCGATGTTTGACGCTACGCAGAATTTTACCAGTGCTGGAGAATTTGACAGTGATGCTAAGATTTTGATGAATGGTTTGATACAATTGGGGTCAGAGGCAGCCTTGATGTTTGGGACACATATGTTCAACATAAATGGCACTCAAAGAAGATATGGTTGGGTGCAGTGCAGCAGAGATATTACACCTGAGGAATGCAGAACATGTTTGAGCTATATGCTTGAGGATGTTGACAATTGCTGTCAGGAGAAGAAGGTATGGCGTGTCTTTTCTCCAAGTTGTGTTGTGATGTATGAAACTCAGCCTTTCTTTTTGAATGGTACTAGTCCTGCGCCAGCACCTCAGCCAGCCACAGATGGGGATAACAGAAGGTGGTTGCTTATAGTAATCATTGCTGTAACTGGAACTGTAGTGCTAGTATTGGCTATCATCACATACTACTTCTGCTGCTTGAAAAGGAAAAGAGACAAACAAGCTATACAAGAGGAGGGACAAAATTCAGTGTTTTCTCAAGATCAAACAGATAAGGAGGAGAGTATGAACAATGACCTTCCTATGATGCCTCTGAGTACTATTCTGAAAAGCACAAATAACTTTTCGGATGAACAGAAACTGGGGAAAGGTGGATTTGGACCTGTCTATAAGGGTGTATTACCAGACGGGAGACAAATTGCTGTTAAAAGGCTTTCAAAAACTTCAGCTCAAGGTGCGGATGAATTCAAGAATGAAATAATATTGATTGCGAAATTGCAGCATCGCAACCTTGTGAGACTGTTGGCTGGCTGCGTAGAGCAAAATGAAAAGCTGCTAGTCTATGAATACATGCCAAATTCAAGCCTTGATTTCCACCTATTTGGTACGGAGAAGGGTGAACATCTGGACTGGAAAAATCGATTAAACATTATTAATGGGATTGCTAAAGGGCTTCTATATCTACATGAAGACTCTAGACTAAGGGTTATTCACAGAGATCTGAAAGCAAGCAACATTCTATTAGATCAAGAAATGAATCCTAAAATTTCAGACTTTGGATTAGCAAGAACATTTGGAGGAGATCAGAACCATGCAAACACAATTAGAGTCGTGGGAACTTATGGATACATGGCTCCAGAGTATGCTATGGAAGGTTTGTTTTCAGTGAAGTCAGATGTTTTCAGTTTTGGTGTTCTTATGCTAGAGATCATCAGTGGAAAAAGGAGCAATAAATTCTATCTGACAGACCAAGGCCAAAGCCTTCTCATATATGCATGGAACCTCTGGTGTGAAAGCAAAGGCTTGGAATTGATGGATCCATCAATAGAAAAATCAAGTGTACATGGTGAAGTTCTAAAGTGTATGCACATTGGACTATTGTGTGTGCAAGAAGATGCAGCAGATAGACCAACAATGTCAAGTGTTGTTCATATGCTTGTGAGTGACACAATGAGTCTTCCCAGCCCCACCAGGCCTGCATTTTCTGTTGGAAGAGCTGTCACTGAAAGAGAGTCTTCATCAAACACTTCCATGCATTACTCTGTCAATGAAATAACAGTCTCTGAAGTGATTCCAAGGTGATAAATGGATAGGATGCAAGAAATGTGTATAATTCAGCTATTGAAACACAGAGAAAAGTAATGTATTTAGGAAGATGACAATTTTTTGGTGGGTGTATAAAAATGGTTGAGTTTTCAAGAGTATAACCAACTTTATCAAAATGGAT

>C100421_65

CTTGAGCTTTTAATTTGCTTGCGTGACAAAAAGGGGTTGGTCCTCTTGTTGAATAGCTACTCCTACCCTTTAATTTCGGGAGGTTATTCTTTTATTCTTTCATTTTCGGTTTGAGTTTCAGATCCAAAGGCCAAATCTATCGGTCACGACTTTGGTGCATCCCGGATATGTCATATGATAGTCCAACCAAACACAGCACCCCTCTAAAGCTAGCAACTTGAGACTGTTTTCAAGTTTGAACATCAAAGAACACACACGGGTCCAATTGGAATCCGAAGCAAACAGATGGGGTTCCAAGAAAATGGGAATGGCGAAATGGGTTTGTCTGGAATCCCCTTGGGAGCAAAGAACAAGTACAAGAGGATGAACTCTGAGCTTCCTGAGGACAACGACGATGTTTTGCTGCAACAACAACAAGAAGAAAGGAGGAGCAGTACTAGGAAATATGTTCTTGCCTGTGCCATCTTTGCTTCTCTCAACAATGTGCTTCTTGGCTATGATGTAGGTGTCATGAGTGGAGCTGTTATATTCATCAAAGAAGATCTGAAGATATCTGAGTTTCAAGTAGAGTTTTTGGTTGGTATTCTTAGCATTATTTCTCTATTTGGTAGCCTTGGTGGTGGAAGAACATCAGATATTATTGGTAGAAAATGGACAATGGCCTTAGCTGCAGTTGTCTTCCAAATGGGTGGACTCACAATGACTCTTGCTCCTTCGTATGCAATACTAATGATTGGAAGATTTCTAGCTGGGATTGGAATAGGATTTGGAGTTATGATCTCCCCCATATACATTGCTGAGATATCACCAAACCTGAATAGAGGCTCTCTTACTACCTTCCCGGAGATTTTTATAAATGTAGGAATTATGCTAGGTTATGTGTCAAATTACGCGTTTTCTGGCCTGTCGGTGCACACAAGCTGGAGAGTAATGCTTGCAGTGGGAATTTTGCCCTCAGTGTTCATTGGCTTTGCGCTTTTCATTATTCCTGAGTCACCAAGGTGGTTAGTAATGCAGAACCGAGTTGAAGAAGCAAGATCAGTGCTGTTGAAAACAAATGGAGATGAGAAAGAAGTTGAGGAGAGGCTTGCAGAAATACAACAAGCTGCTGGATTTGCCAATTCTGAAAAGTATGAGGACAAACCTGTGTGGCGTGAATTACTATTTCCTCCTCCTCCTCTTCGCCGAATGCTGATCACTGGACTCGGAATTCAGTGTTTCCAACAGATCTCAGGAATTGATGCAACTGTGTATTACAGTCCTGAAATTTTCCAAGCAGCTGGGATTGAGGACAATTCAAAACTTCTAGCTGCTACTGTTGCTGTAGGCATAACAAAGACTATTTTCATTTTAGTAGCAATCATTCTCATTGACAAACTAGGAAGGAAGCCCCTTCTCATGATAAGCACAATTGGGATGACAGTTTGTTTGTTCTGCATGGGGGCTACACTTGCATTTTTTGGAAAAGGATCATTTGTGATTGCTTTGGCTATTCTGTTTGTTTGTGGGAATGTGGCATTCTTTTCTGTTGGACTAGGCCCTGTGTGCTGGGTTTTGACATCTGAAATCTTCCCTTTGCGCGTGCGTGCTCAAGCTTCTGCACTTGGAGCTGTGGCTAATAGAGTCTGCAGTGGCCTTGTGGCCATGTCCTTTCTTTCCGTGTCTGAGGCAATCAGTGTTGCTGGAACATTCTTTCTGTTTTCTGCGATTTCTGCTCTTGCCATTGCCTTTGTCGTCACACTAGTTCCTGAAACTAAAGGTAAGTCGTTGGAGCAGATAGAGATGATGTTTCAGAATGAGTATGAGAGTCAAGGGAAAGAAATGGAGCTAGGAGATGTTGAACAACTTGTGCAGAACAAAACTGATTTAACAAATTAATTGATTCATTAGCCCCTGCCTTTATGCGACTCTTGTGAATTTTTATTCCATGGTTACACATTATTCACTTGTTATGTCTAAGGCTCTATGCATGGCCTGCTACTTGCGATTCTTGGGAGGACATAACTGATATGAATATGATCTTCTGCTACCAGAAAAATTGAGATATGAGTAGGGAACATAAGGAACATGAAGTGATGAAGATAAAATGCATTCTAAACTTTTCCTAGTTGTACAATTATTCTTATTTTTTATTTACATTAAAGTCAATTATAGCATTGTATATTGAAGGAGATTTTTGGTGGAGATGATCATGTCTCCATATGTATGTTCTGAGACAAAATATAGTGCCTATTACAATGTATCGATGAGGGATATGTAATTCAGATGAAGATTCTTTCACAGATAGTATTCTATTCAACTCTGG

>C100423_65

TGTCCATCATATGGTAATAAAAGTATAATCTCAACTAGTTACAAGAGATGCGGATCATATCTACCATAATCATGTTCTCCGTTAGTACAAAACTTGATTACTACACACAACTTGCATTAAAACCTACAGTGTCAACGGCCTTGTAGATTGTAACCTATACTAACCCTTGCGGTATTTAAGTCAGGGTTCCATCCCTCCACACCTCATCCCCACCTCCAGATCCCAAAAACAAGGAAAAGATTCTGACTATTCATTCAAAGATTGGCATCAACATTCAGATAAGTGACGGCTGAAAATAGTAAGACAAGCACTGATAACATGAACAAGTGCTTAAAATTAAGAACTCCTGCATCTATTCTCCTCACAGCACCAGCATCTGGCACAGTATCTACAAACCTTACTTCATTACTATTTGTGAATGAATTTTTCCAAGTCCTGTCCACCTGGTGATCAGTTGTCAAAGTCACACTAACTCCTTGAGAAGGATGCTTATTTGTCTGCTTTAGCTTCACAACCAATATTTTGTTCTCACTTGCTAAGGAAGATAACTTCTTATTAAGACGCTCTCTCTCAACAGCCAGCTGCAACACCAAATTTTTGAAAACTTTCGTCTGCTTTCCGATATCCTTTGCAGATGCCACTTTTGCTTCCTCCACTTGGTGTAGTGATCCCTCCAAGCAGTCCAATTTGCTCCTCAAGAAGCTTAGTTCCTCTTTTAAATCAGAATTAGATTCAGATAATATGATACATTTCTCCTCTGCACTGTCAGCCCGGCTCTCCGCTTTAGAGACCTTTGACTTAAGATCCTGTATCACAAGTTTCATATCTTTAATTGTGGAATACAACATACTTTGCTTCTCTAGACTAGCTTCAGCAGATGCCACGGCATGCTGCAGCCGTAAATCAGATTCCCTTAGCTGCCTCTCAAGTGACTCTACCCTCTCCGATTTGCCACCACAATCTTTCAAAAGAACTATCTGTCCGTTGAGTTCACCATTAGCCTTCGACAATAACTCACAACGAGTTTCTGCAGTATTAGCCCGACTTTCTGCATTAGATAAATTTTCTTTCAATTCAACAATAATGCCTTCCATGTCCCTCACTTCAGAACACAAAACAGTATACTGTTTCTGATATTCATCAGCAGAAGTCTTCACATTCATTAGCTGGCTTTCAGATTCTTTTAGCTGATTCTCAAGTGAACACACCTTGTCCCTTAAAGTGACTACCTCAGAATTGGCATGAGTTAGTTTATCATTCAATTGTTCAATCTTGTCCGAAGTAGCATCCCTCTCTTTCAATTGTTCTAGAGAAGTTTCAAGCTTAGCTCTGAGTGCAGATTCACGCTGACTCAAACCATTCAAATTGAACTGAGAAATCTGGATTCTACCCAACAGCTCTTTTGAAATTCCTGTGAGTGTCTCACGTGCATTATCTGCCTGGAGCCATCTCTCCCAAGCATCAGTTGCTTCTTCCTCCATCAGAATTAGCTCTTGTTCTAAAGAAGCCATCCTTTGTTTTAGCTTTTCTTCAATTTGCCTTGAAGCAATGAAATTCTTCTCAAGATCCATTTCATTTGCTAAAGATTTCTCTAGCATCCTCAGAACATATCTTTGTTGCTCAGTTGTGTGCATGGTTATTACTGGGCTTACATTTAAAGACTCGTCATCCTCTAAGATGATCTCATCCTCTTCAACGTTACCTACGTTTCCTCCCCTCTTATAAGAAGACAAAGTCCTCTCAAAACTAGCAGACAGTTTCTTAATCTCTGAAAACTGCTCCTCTGAATGCTTCAAGCTTTGTTCAGAAGCAAAATTGTCTTGCCAGGGCTTGCAAGAGGCCGCCCTCTCTCCAGCATCAGCAATCTCAGCATAAAGAATGTACAAGAACCTATCCAATTCCATCACCTCCGAACTCAACACCCCACATAACAGGTCAAACTCCAGAAACTTCCCAACACAACCCACGTCCACATCATAGTCTTCTCTGTCCAAAACAAAGCCCTCCAATTCAACCCCAAGAGTTTCCAACTGCATCACAAAATTGCTCAAGTTTGCAACTTTGTCAGAGAAACATGCCAGATTCAGTTCCAGCCCAGTGAGCGCTGTCACCATGTCATCACCTAGATCCTGAATTGCTTCTCCACTGGATGAAACCCCTCCAAGATCAATATCAACTGTGTCTTTAGCACTTTGAGTATCCATTCTTCTAATCTAAAACTTCAGCAGTTGTTGCTCTGAGCCTGAATTGTTGATTTCCGATAACAGAGACAGTTGCGCCCTAAGCACAACACCATACACTTGCCC

>C100425_65

CTCCTATAAAGTAATAAATTTAATTTTTTTTTCTTGAGGACTTGGAAGAAGTTTATAATGCTCGAGCAATTTCTGATGCCATTTCCACCACAAAATGCTAATGCAATTTCTAACATAGTCAAAAGACTCAACTGCAGAGATAAAAGAAGACACAAATAGTAAAATAAGCAACTAACTAAGCAAGCTGAGCAAGTATCTATGCATGCTTAACTATATTATTCCGCGACAACTATCAACATGACAGTTTGCACTTAACCAAGAACCTAAAGCACAGACAATATAAAAACCAATTTAGGGAAATAATGTTCACTAAGCAGTTGGTTGTGGCACAATCTCTGATATGCCAGGATAAGAAGGTTGTCCCTCTTTCTGCACTACTCCATGTTGAAGTAGCTGTGGCTGCTCTCCTTGCCAGTTCCAAACAATGTCCTTAAGTGCAGGCCTTACTTGTTTCAAAAGATTTTGGTACTCCAATGTGTCTCCACTAGACTTTTTTAACTCCACCAGATGGAAAACTGGGGTGATCTCAAAAATCTCAGCATCAATACCCAAAGGCCCTTTTCTGCCTTCCTTGGAACCTTCCAACTTGAACAGACCTCCATCTTTCTTCTTCAGTTTCAGCCGAAGGCGCTTGCAAATTTCTTCCAACTTTGAGATTATAATAGAGGCCGGCTTGTCAGATGTAAATCGCGCTTCTTTCTTTCTATCAGTCTCCTCAAACAAACCGGACAAGTCAAAGCCTGAGGAATAGGAGATGATGTCAAAAGCATTCAGATTACAAGGTTTTGAGTCTTTTGCTGGTTCTATAGGATCTCCATTCTCACAAGCACCAAATACTCCATCAGCATCCAGAGGAGCTAGTTCTTCGTCTTTGTTCTGAGTAATGGTGGGCTTCTGTAACCCCTTTCTGAACCAAGAACTTTCCATAATTTTGGCCATTGATATCCTGGTATTTGGGTTTGGATCCAAGATTTTCGACAACAATCTGCGCACATCTGGTGCAAACCAGTTAGGAAATTTGAAATCTCCCTTGCCAATCTTCTTGTACATCTCCATCAGATTTGATTCTCTAAATGGAAGAAAACCAGCTAATAGAACATACAAGATCACCCCACATGACCATATGTCAGCTTTGGCTCCATCATAACCCCTTCTGTTTATCACTTCTGGAGCAACATAGGCAGGGGTACCACATGTGGTATGGAGCAATCCATCTTGGTGCTTAGTTTCAGCAAGTGCACTTAACCCGAAATCTGAGACCTTCAAATTCCCATCTTCATCCAGTAGAAGATTTTCTGGTTTCAGATCCCGATGGCACACTCCTCTGCTATGGCAGTAGTCAACAGCACTAATCAATTGCTGAAAATATCTCCTAGCATCATCCTGCTTGAGCTTTCCTTTGGATACCTTGTCAAAGAGCTCACCACCTTTTACATATTCCATGACAAAGTAAATTTTGGTTTTGCTGGCCATTACCTCATAAAGCTCAACCACATGTGGATGCCTGATTAGTCTCATCACTGAAATTTCACGCTTAATCTGATCAATCATCCCAACTTTCAGAATCTTCTCTTTATCAATAATTTTTATGGCCACACTCATGCCAGTTATGAGGTTTCTAGCATGGTAAACCTTTGCAAAGGTTCCTTGGCCTAATAATCTCCCTAGTTCATATCGTTGCATAAGCACACCTCCTTTTTGCTCCATCTCTAGAAACACTTTTAGTGCAAACCAATGCTAATATCACTAGCACTATGTACAGGCAATCACGTTGTCACAAGCAGGAAAGTGCTACTCAAGAAATTCAATGTTCAACCGTCTTCACAAACATCGTGTTTTGAAATTGTAGAATGGCTGAGATTTCTTGGCACCTCAGTTGCTTGAGCGCAAAATAGATCCAAGCAATGGACTAAACTTCCAATGTCATCAAGATTGAATGAGGGTAGCTCAGATGGAGGCACGAATTCATTAGCCTCAGTGAAGGAATTTCTAACAGTCAAGCACATCTTTCAGCATTCCCTGATTTTGGTGATGAACCAGAGAAGACTGAAGAATATGGTCCTATTAGAAAGCTTGAAGAATAATTAGGAATGAAGAAGCACCTGGTTTAGCCTTCCAATCACCCAGCAGCAGAACATATAATGAAAGACACACCTGCAAGATGATTTGAGATGATTTTTTTTCTCTCTCTTTCTCTCTCTCTTTTTCCTGTGTTTTTTTGTTTTTGAGTGCAAGGAGAGAAATGATGATGATGGAGGCGTTATAACAAAGAAGGAGAAAGAAAAGACACAAAAGTTGCTGTCAAG

>C100427_65

CACGCATTTCGCATTTTCACTTCAACCCGACGCATCTCTCTCTTCCCTCTTCACAAGTATGTGAAGATTCTTCGCCTCTATTTCGCTCTGCAATTCATTGCCTTAGATTCTCTACTCTTTGAAGTTTTCCAACAAACACACTTTCAAAGGTTGGCTTCAAAGCCTATTATTATTGTGAATATTATTTTAATTTAAATAAAAGGCACTATGGTGGCCAAAATCAGGCAAAGTTCTGCTAACTCTCCAAATCCTAAAAAACCAGTGGTTGGAGAGATTGACACCAGTCCACCTTTTCAATCTGTTAAAGACGCTGTTTCTCTATTTGGTGAAGGTGCTTTATCTGGAGAAAAACCTGCCATTAAGAAGGCAAAACCTTATTTTGCTCAGAGTGTTTGGGCAAAGGAGTCACAACTTCACATAGCCCAGAAAGAGCTGAAAAAAATAAAGGAACAACTAAAGGATGCTGAAACTACCAAGGCTCAAGTCCTAGTGGAGCTTGAAAAGGCTAAAAGAGTGGTTGAGGATCTCTCTCAAAAGCTTGAAGTTCTCAATGAATCCAGAGAATCAGCAATACAAGCAACAGATGTTTCAAAAAGTCGAGCAAAACAGCTTAAAGAAGAAAAGTGTGGTGATCCTGATGGAACCAACGGTGCTTGGAAAGAAGAGTTAGAAACTGCCGTTAAAAGCTATGCCTCTGTCATTACAGAACTTGATGTTGCAAAGCAAGAACTGAGTAAAATTCGGCAGGGGTATGATTTATCCTTAGAAGCAAGAGTTTCTGCTTTCAAGCAAGCAGCAGAAGCTGAAGATGCAATGAAAGCGAACACCAAAAGAGCATCCGAACTATCTAAAGAAATTTTGGCTGTACTGGAATCAGTTGATCAAATTAATGCTGAATCTGTCCAACTACATTTGCTGCAAGAAGAGACATTAACCGAGCAAAATGTTCTCAGACAATCATATGAAGCCGACCTTGAAGAATCAAAAAGGAATTTGCTTGATTTAAAGAAGGAATTTAATCAAGAGCTTACCAAAAGTCTAGAAGTGCAGCTAAGTGAGACAATGAATGAAATAGGTGTTCTGCAAGAACAAATGGAAAATAGAAAGAAATCAGACCTGGACTCTTTGAGAAGTGCCAGTCTGGATCTTGATGAAGCTAGGGAATCACTGCAGAAAGTAGCAGGACAGGAAGAATCCCTTAGAGCCATGGTGGAAGCTCTTAGAATGGAGCTGGAGAATCTAAAAAGAGAACACACTGCATTGAAAGAGAAAGAAATCAAAACCGAATCCGTTGTTGAGAATCTGCGGGCAGAGCTCCAGAAAAGTGAGTCTGAACTTGAAGTCTACTTGGCAGAAGAATCTAATGCTAGAGGTGCATCAGAGGAAATGATCTTGACATTGAACCAGTTGTTGACTGAAACTGAAAATGCAAGGAGGGAAACAGAAGATATGAAGAATGAGGCAGCAGAATTGAAGATGGAAACTGTAGTTACTAAACTTGTGTTAGAAGATGCTGAAATAAAGCTTAAAGTAGCATTGGAAGAAGCTGAAGTAGCAAAAGCAGCTGAGGCAAGTGCTCTTGATCAGATCAAGGTCTTATCTGTGGGGACCAGTTCTTCTCATTCATCAACCTCTGAATCTGGTGCAAGAATTGCAATCTCAATGGAGGAGTTTGAGTCCTTAGTTCATAAGGTTGAGGAGTCTGATAAATTAGTGGATATCAAAGTGGATGCTGCCAAAGCACAGGTTGAAGCTACAAAGGCTAGTGAAAATGAAGTTCTAAAAAGGTTAGAGGAAACTCAAAAGGAAATTGAGGATATGAAGACTGAAACACAGGCAGTCTTGAAAAGAGCTGAGATGGCTGAAGCAGCAAAGAGGGCAGTGGAGAATGAGCTCAGAAAGTGGCGAGAAAGAGAGCAGAAGAAAGTAGCAGAAGCTGCATCTAGAATATTGGCTGAAACACAGATGAGACCAGAACTATCTCCTCTGCACTATAGGATTCAAAAGCAGAATTCACTACCAAAGGTGGAGATGAGGAAGTTAGAAAAAGGGAAGGTGTCTGTTTCAAAGAAAGTTCTCTTGCCTAACATCAGTGGCATCTTTCACAGGAAAAAGAACCAGGTTGAGGGAGGACCTTCTTCTTAACTTCTTGTTTGTGTGAAAGTTGAGACCAATTTGATCAGATTTTTGTCAACTTGGTATGCGGGAAAAGGATTTTGAAATGTCATTAGTTTGGTCATAAGTATTTTATTAATGTAACTTGCCTGGTATATTATTGTTATTGTATATTGCTACTTCATTTCTGA

>C100429_65

CGTGGGGGTATCAGAGCTTGTAACATCATTTGATCAAATGAGTCAGCGTGTGAGTGACGAGGCTGTGACAAAGGCCATGGAGGAAATAAGAAGTGGAGCTTCTTCACGTAGGCATGGAGATACTTTGCCACACATTCACAAAGTGGGATCTCCTCCTAAGCAGACACTTATCCAAGAGATCAAATATTCTGTGGTTGAGACTTTCTTTCCAGACAGACCCTTCGAGAAATTTAAAGACCAAAGCTGGTCTAGAATGTTTCTATTAGGCCTGCAATCTCTCTTCCCCATTTTTGAATGGGGTAGACATTACAATCTTAAAAAATTCAGAGGTGATTTCATTGCCGGACTCACCATTGCAAGCCTCTGTATTCCTCAGGACATTGCATACGCAAAACTTGCGAATTTGGATCCCCAATATGCACTTTACACGAGTTTTGTGGCTCCTCTTGTGTATGCTTTCATGGGAAGCTCAAGAGATATTGCCATTGGACCCGTAGCTGTGGTTTCCCTCTTGCTTGGGACTACGCTAAACGATGAGATCAGTAACTTCAAAAGTCATGAATACCTGCGTCTTGCATTTACTGCCACCTTCTTCGCAGGAGTCACTCAATTTGCACTTGGTGCTCTCAGGCTTGGTTTCTTGATTGACTTTTTGTCTCATGCTGCCATTGTGGGATTCATGGCTGGAGCTGCCATTACTATTGCACTGCAACAGCTTAAGGGTCTACTTGGCATAAAAGACTTCACTAAGAAAACTGATATTGTTTCTGTCATGCGCTCGGTTTTCAAATCAGCACACCACGGGTGGAATTGGGAGACAATAGTCATTGGATTAGCATTCTTGGTTTTCCTTCTTGTAACCAAGTATATTGCTAAAAAGAATAAGAAATTGTTCTGGGTGGCTGCAATTTCTCCTATGATATCGGTTATATTGTCTACTTTTTTTGTCTATATTACCAGGGCAGACAAGAAAGGAGTGGCAATTGTTAGACATGTTAAGAAGGGTGTGAATCCATCATCTGCTAGCGAGATATTTTTCAGTGGAAAATACCTAGGCCCCGGTATTCGGATTGGTGTGATAGCTGGTATGGTAGCTCTCACGGAAGCTGTAGCAATTGGGAGAACATTTGCTGCCATGAAAGACTATCCACTGGATGGTAACAAAGAAATGATGGCAATGGGAGCAATGAACATTGTTGGTTCTCTAACCTCTTGTTATGTGGCTACAGGCTCATTTTCTCGCTCAGCAGTGAACTACATGGCTGGTTGTAAAACTGCTGTATCAAACGTCGTTATGTCCATTGTTTTGTTATTGACCCTGCTACTCATTACGCCACTATTTAAGTACACACCAAATGCGGTGCTTGCTTCTATTATAATAGCTGCCGTGCTTGGCCTAGTGAACATTGAAGCTATGATTCTTTTATGGAAGATTGACAAGTTCGATTTTGTTGCTTGCATGGGAGCCTTGTTTGGTGTGATCTTCAAAAGTGTGGAGATTGGCCTTCTAATTGCGGTGGCTATATCTTTTGCCAAAATCCTTTTACAAGTGACAAGGCCAAGGACTGCAGTACTAGGGAAGCTTCCAGGAACTACTGTTTACAGGAACGTCCAGCAATACCCCAAAGCAACCCAAATTGATAGCATGCTTATTATCAGGGTTGACTCTGCAATCTACTTCTCAAACTCCAACTATATTAAGGAGAGAATTTTGAGATGGCTGACTGATGAAGAAGCACAAAGGACAACAAGTGGATTGTCCAGAATAGAGTATCTCACTGTTGAGATGTCACCTGTTACTGATATTGACACAAGTGGCATCCATGCCTTTGAAGAGTTATACAAGACCCTTCAGAAAAGAAAAGTCAAGCTTATCTTGGCAAACCCAGGGCCAATTGTGATAGAGAAGCTCCATGCATCAAAGTTAACAGACGTCATTGGAGAAAATAACATATTTCTGACAGTGGCTGATGCTGTTTCAACTTTTGCTCCAAAGGGTGAAAGAGTAGTGTGAAGAAATCAAATAACTACTTCAATTCTACCGTTGAGGTCCAAAGGGTGGGAAATAACTGTTGAAGGAGCTCATTGGAAAGTTTGTTTATGAGCAGAGTGTTATGCAGATTTGTCATCGATAGTTCTGTTTGTTTTTCTTTAATTTATTGCTCTAGATGATGTATAGAACAGAAGGGAAAGAAAAGTGTGTCATGATTGTATCATACCTTGTGTTTATATTATACTTTGTGGAAATAATTATTTTCTTTATGAGATGCAATACAACACATATGCTTTGTTGCAGACGAAAAAAA

>C100431_65

TTTTTTTTTAGAAATTTGAAGTGTGATTTATTTACGGATTTGGGGAACAAGTGGGCACAAGGTTGAGGTGAAGTAAACTTCTGATTCATATAACAATTAAATCACTGCTGGCAGTGGCAATTAATACATATGAAGGAATTATTCATTATTCTGGCACATCCAAACAAAGTACAATGCAGCGGTCTACGTAGCAGCAGCATCCTAATGAAAAGGAGTAGTTCTACTTGGGGCAACCGGGCCATTCAACGTGCCTGGGAGAAAGGGTTTTGAGAACAGGATTCCTATCGAAGAAATTGGTTGGTCTGAGCTCGAATGCAGTGCTCAACAATGGCATTATGGGGAAGTCTTCCTGAGCAGGAACATGATGAATTCCCACAACGTGCCACAACACGATGTCCTTGTTCTTTATGTCTCTGTTCTTTTGAGTCCAAACGGCTAAAGTATCTTCTCCATGGCTGTGATCAACGTAGAGACCACCAGCCCACTTCTCAGTTCTGTTGTATGGAGTGACCCAAACATTAAAGTTTGTAAAAGCTCCACGTATTTGTGGATAATCGTCAGGCGTTAGAAGAGGATGAGCTGGGATGGCTGGAATCAAACGGTATCCGACATCGTTTCCAACTTCTGTCTTCTTGTTAGGGTTCACCACCGAAAGCTCACCCGGTGCAGTCCCAATTGTTATCTTTGCATCGTTCTCGCTCTTGGCAGTTTCAACCTCGGTTGTCCAATAGCTTTTCCTCTTCGAGCTTCCATCTGTCACTCTCACCGTCTTCAAACTTGTCTTCTCAAAAGAGTTGTGTACGCCATCGATGTCAAGGTCAAGATAGTATATGTAGAAGTGGTCGTGGTAGACACCAATGCTGTTTTCTGACACCAGGATTCCATGTTGGTCACTCTTGATCTCATCCTTGTGCTTGATATTCACTCCCTTGATTTCCAATATACCCGACAGAGCAATAGCGGGTTTGATTGAGCCACTTGTCTTAAACTCCCAGTCAATGATGTTGTCGTAGTTACCAACAGTAACAACTGTTCTTACAATCAAGTTCACTTCTGTTCTGGTTTCTACGAACGAATTATTGGGAATTCCAGTTTCGGTGTGACGCCACATGATGTTGCCATATTGTTCAAACACACAGATTGCGTTGGGAACGGGCACTGGATTGCCAGCACCATCGTGAACATATGTGTCAAGGAATTGAGCATTGGATGGGCAATCAAGGTTGGGCACCAATGAGACAGTGGAGAGACCGAACCCAAACTCTCCAGCATCAAAGAATGTCTTAGAGTAAAAGTCCTCTGTTGGGTCTTGGTATGGCACGAACAGCTCAGAAATGTACCCTTTGTATAACACTCTGCGAGACTTGTGCTTGTCCAAATCGTAAATGGAGGCCAGTGATATCACAAGTCCGGCTCTTGAATCGAATCCAATGTGGAACTTCCAGTTGGCCCAGCTAACACTGTGGCCACTGATTTGGAAGCCAGGACCCTGTGGTTGATGGGTTGCTAGACTGTGTTGTTTTGGACCGAATGGTGGTTTCTGGTGTGATAAGCGGTATTCAGTGTTATCAGCGGTTGGTACTGTTACAACACCATCGTCGTGATACTCAACGATCTTCATGAGCTCAAGATCAACGATTATTGTGAGTCCACTGATAGGCCTCACCCAGATATTGGGGCTGCTGGGTTCATTCAAGAAGCATTCCACCTTCACTGTCCTTCTGTTCTCGGTTTCTCCAAACCACCCCTTGGTGAAAGTGGAGCACACCACCTCTGAGAGGTTCAACCCTCTCTTTTTCACTGACTCTTTGAATGGTCCGTATTCCAGTGGCAGCTCTATGGCAAGGGTCTGTTCATCCACGGATAGGGTTGGGAACCCATTTCCTTTGTGAATGTTGTCTGAGAGAATGCGCTTTGCCTTGAGATCAATTAGGATCTCGTGGACTTGGCTGTTGATGATGGTAATGGCCAAGGCCCTACGAGGGACAGTTGTGATGGTTGGTTTGATGGATTCCCATCTGAGGATGTCTTCCTTCTCAGGGTCATCAAGGCCAATGTAGTGGAAGCTCAACCTGTTGCTTGAGGTTGGGTACTTCTTAAGGACTATGGTTTGGACAAGAGTTATCTCTTCTTTGGTCAAAGGGTCTAGTGGATGTTGAAAATGCAGAGGGGTGTCGGACACCACTGCTTGGAAGGAGAAGAGCATGAGAGCAGAGAAAAGCAAAAGTTTCAGGGTGGAAGCCATGATTAGATGAAGATAGCTACCTCAAGCCCTAGCTTGATGTGTGGAATGGAAGCTCTTCCCT

>C100433_65

CAAATTCGTTATGGCAAGAAGCATGCTATGCAACTCGGTGCGGCCAGTTGATAATTTATTATACCAAATACTCCACACTTTTAAACAAGGAATCCGAAAAATATTTTCCATACTGTTTCTCAATACAACTAGTTCGAATTCAACAGTGTAAAAACTGATAACTGCATGATTGTTATCATACGTTCTCCGCAGCGTATTCCACCAACATCGAGCTAATTATGAACATTTTTAACATGTTTACCCCAGTAGCTGTGGTAAGCCAAACAATTAAACATCGTAAGTGTGTAAAACTTACTTTAAAACAGTATTGTACACTTTCACTACCATCTCTCCCACACCAGCATGATCATCAGATATAGTGCCAAAATTGGGCTACATAACAAAGCAAAACCTTAGTATTTCATCCATTTTCTTCTAAATCACAATATCTGTAAATATTTATCTCTAGAAGTTAAAGGGATCTCAACCATTATTGAACCGAAAAGGCTTGTCCAGTCTGGCTTAATCCCACATATCCTTCCTGTGAACTAGTTTTCCATACTGTAGTTTTTCTCTCATTTCACTAGGGTCATCCACTTATCTGATAGTTTTTCCTTCGTATAAAACATTTTTTCTTTTTCTTTTTCTTTTCCACTTTAGCAATCTCTATGAGTTCATTGCTAGATATCGGCTTCACCTTTTGTGCTGCTTAGGCATATCACAATAATCCACAGTTAGGTAGTCATCACGCCATCACAATCACTACAGATGGATAGACTTATATCAAGTTTGCACCAAGGAATGAACAGAATCTAAGGCTCAGTTTTAGCCAAAAGCAGGAATGATATGTCGTCTAATTCACAGCTCATCTATGGTATGTGCCATGTGATTTAAAGAACTGAAAATCCGGGTGATTAACCTGAACTAGCCTTAACCACTTATCGGCAGCCTGCAAGAGGGAACTCACTTGTTGAGTCTCACCAACTCCTTTCTTCATCCAAATAGACCCCTTTAACTTATAGGAAGCCATTGCAAAAGTAGGAATAGAAATCTTTGGAACACCATCCATCTCACTGGGATAAACAACTGTTGGAGCATGAGTATGACCACTTCCTGTCAACGGTGTATGGAGAGTATGGTATGTTAGAAAGCACCCATCTAAATCTTTTAAAGTTGGACCCGTGGGTATTCGATATATAGGATACCATGCCACAGACATCCAACTGACTGGTAATAAATCACAGCTTCTCAATGACTTGAGGGCAGGATAGTGGCGTGCTAGATCTTGTATCTTGTCAGCCAAAGGTTCACGACTATAAGGAGGATCTTGTTCCAAATACTCAAAAAGCAGATCTTGAGGATTCCCAGTTTCGCTATCATCACTAGAAAATCCTTCTTGCATTGTACTGCGTTTATCATTTATAGATAGTGTGTTCATTTGCAGTGAGACACCACCTTTTAGATATTGACTTCTTTGTGTCGAAAACTCGTTCCTTTTTCCATATTCATAATCACTACTTCCTTCACTACATGAATCTTTGTAGTAATCGCCATCACTATCTTCACTGGTGTACCTAGGCTTTGTACTTGGCTTCTCAGCAGATTGAGCATAGAGTTGAATAGCTGACAAATACGGAACATAATACTGAACAACAGATTCACTTTGATCAAGGACCAAGGGCACTCCAGCTCCATACGCACTCCACTCCTTAAAAGATTCCCAGAGATCACTCAGGGCAAAGTAAGACTGATACTCCACATCGCAAAACTTCCATCCTCTCATCGTTGTCTTGGAGAAATACTGAGCCGGAACTAACGGCGTCGTGGAGTCCAAGAACCTATCGATATTACTAACAGACTCTACAGACGGAGAAAGAGTTGGTTTCAATGATTGGTTGAAATTGCTATTGCTATCCTCAGAGACATCGAGCTTGCTCTTCGAAGTTAAATCGGCGTTTTCCGGTTCGCCATTCTTGGCTCTGTGGCCTTGTTTGCGCTGATTCTGGTTCTTCCTCGCCTTCACCGGAATGTAAAAGCGATCCTCGCCGCGAACGCCGCCAAATTGCAGAGCAGTTCCCAGCATTTTCCAATCGTTTTCAGAACTGTGTTGTCCTCCGTTTGCACGCGGAGAAAACGGAGGAAAGTATCGATGAGAAAATTCGTTATCGCAGAGGATCAACAAAGAAAAGGGGAAGAAAGAGTACCGAATAGGAATCAAAGACAGACGAGGAAGAAAAGAAAGAGGAGGAAAGGAAGGAAAGGTTGAAGGCAAAGAAAGAAGACGATAGAGAGAAGAAAAGCTTCAACACTCTCTTTACAAAGAAAAC

>C100435_65

TTTTTTTTATCCATCAAGGAGAAAATATCGATGTAAATATTGATGTACAACTAACACTGTCCAAGCAACCACAAGAAGATACTATCACTGATACAATTCAAAAACTGATTAAACTGTTCCTTCACACAGCTATTACAACTTTTAGACCTATAATAATTATTGTAATTACATTTTATTCATACAACATTCGTAGCATAATATTACAGGATAATGAAAAACTAAGCAAACAGCACCTGAGAGCTTGAAGGAGTCAGATCAGACAAACATTCTGGCGCATCAATTACATGCCATCGAACACTGAAGTTCCATTTTTCCTTACAACTTTCTTTTGGTTTCAGTCTAGAGATATGGCCAACAGCAACCAGAAGGTTTCGACCCACAACATAAATATGACAATCACAAGCATTCACAGCAAAAGGCTTGCATATTTGCTCAGGCAGAGGAGGCCCTTCTATGGCTTCCCAGTAATCGATTTCGGGGTCATAAACCTTTAGCTTCATTCTTTCAAGCTCAGAAACAACAAACAAGTGCCCGTAAACAACGACACTCGAGCCGGTCCACCCTTCTCTGAGTCCAACAGCCATATTTTCCCAGTTATTAGTTCTAGGATCATAGACCTGACCTCTTGGAGAGACATAAAAAGGCCACAACCACCCTTCAGTGACAAGGAGTTTTCCATTGAGAACAGCAGCGTCATAAGATGCCATATTTGTTCCCATGTTGGCAATAGGGCGCCAACTACCACCGAGAGGATCCAACACCTCAGCAGAATCTAGCTCATAAAGATCAGTACTGTTTCCCCCAGCTACATAAATCATCCCATCAATCACTCCACTAGCAAAAAATGACCTAGCAGTGATCATCCTATTCATCACAGTCCACCTATTCTTCTGCATCTCGTATTTCAACACCAAGTCAAGAGGGCAATCCACATCAGAGACCATGCCACCACAAACAAACAGTGTGCCATCACGGGGGATCGAAACACACCTAAAACCATGAGGGCAAACCCTGTCCTTACAAGGCATTGCAGGGATAGTGTGCCAAGAAAAGTGTGTGAGGTCAAGAACCTGCCATTGGATCTTCCCAGTGCACTTATGGTAGGCAAATACAAAAAGCCAAGGGTCTTTCAAACCAAATTGCTTCCTGTTGGTGAAAAACCTCTCTTTGTTACCAAGTAGCATATGCCACCTCTTGCAGACAGCTCTACAAGATGAGTGACTCTGAACCGGAATCCGGAGAAGACAATTGAGAGCAACATCATCAGGAAGGCCAGGAATTAAGGGCTCCTCTCTTAGAGAAAGCTCTAACTCAGGTGATGGATTTGCCAAAGAAGATTGGATCGCCGCTAACCTAAACTTAGGAGATAATGTCATTTGAGGATCTCCTAGTTTGTGTACCGGTGTTTGTTGAGATGAAAATCTAACACGCTGCATCTTTAAATCTCAAATATACAGCTAAACCCCCTGATAAATACTAAAACTTTTTAACTTCTCTAATTTGCAAACTTCTAGTTCATGCTTTTGTATTCTCAAATGGGTTGGAACAATATCCCAACAAATAGTGACACCAAATTTTCAATCCGGATGTTAAGAATCTATTGCAAAGTTACTTGAACTGATTCCCTCATAGATTTTTGTGACAGAAAAAAGGAATCCCCCAAATCAAATCCACAATAAAGATAAGAAGCACCAATTCCGCCACCACAATGATCTAAAAGCTGATATAGGATGCAACTAAAACAAAATTCTCGTCCTTAAAATCTCCAACTTTATAAAAGTAATTTCATACATCAGAAATATGGGGAAATGCCCTCTTCATCAAAAGCCAACACCTTTACTAACTGAGATTCATCAAAATTTTAACTTAGGGAACCCCAAAATCCTTAATTTGCACCAACTGCAAATTTCCCTATTCAAGGCACCCCCAAAATCAACACCTCAGCAAATAAAAATTTCAACGTTATCTGAAATTCCAGTGCAGAAGACCAGAAAAACAAAAGCTAGTGGCTACCCTTCAAATATTTGATTAAGATAAAGACAACCCAGAATTTACGAAGGTGGAAACACTGGAGCCAAACAAACAAGGACAACACAACAAAATCAAGGGAAAAATGAAGTGGACAAAAAACAAAAACTTACAAGAGAGAATCAAGGGAGTGGTAAAGAGGTTGGGGAGAAAGAGAAGATGAGATTGAGGGGCAGACTTTGCTTTTTAGCACAAGAATATAGGGATAAAGAGAAGAAGAGGGTGACCCGTTTTTGATGATTGTTGTGATCAAAGAAAGATTTGATTATTGTGGCAAAC

>C100439_65

GTATTATCGTGCATGGACCGGTTAGGAGGTCGCAAAGTGCTGAGTGCGCAGATGTGATGATTATTATGGGAGTGTCTGCCAACAGAGGACCTACAGCACATGCTGCCCATTGTCCAACTGATTAGTTTACATATTCTTGGGGAAGAAAAGGAATATGCGACTACCATTTCGAATCATAGTGCTGTTGAAAGAAAGAGTAAATCCTAACTTTAACCCATCAGCTTCTGCACTAAATGCAAATTGAGGGTTTAATAACAAATGAAGGAAAGTGAGATATACATATGGGAAATGTACGCCCAAAATTCCAAAATTCTTGCATGGGTAAACTAGGACTTTACAAACTCATGCTGTGACCTAAACAGACTTTGCGTGGGGGAAAAAGAATATAACGCAAAACTAGTATACGCTACACTAGGCTTGAAATTTTCATATGAAACGTGCTCATCTAAGCTACTCAACTTGGCCTTCTTTTTTCTGGAAGTTGGGTCCCCATATACGAATTGTATAATCATCACTTGCAGATGCCAACATTTGTGGTCTTTTGGGGTTCCAGCTCACACAGTTCACCGTCATGGAGTGCCCAGATAGAACCTCTATAGGTGTCCGGTTCCGGTTCCGGCAGTTCCAAATAAATACCTGTGAATTTTCACTACCACTGGCGATAAAAGTGTTATTCAATCCACCAAAGCATGATCTTATCACATACCTCCGTTGCTTGTGGCCCATGAACCTCAATGGCTTATCCCATTTCCCAGCCACATCCCACATATGAATCTCCTGACTGTTTAGATTGACAATGAAGAACTTACTATCTCCTGAAACTGAAAGGGATGTGATTGGGTACTCCTCTGAAATGACTCGCTCAGCATTTGTTCCCATATGCAGAATACGAATTTCTTTATCCATAAAGATGCTGATAAGATACTCACCATCTGGAGTAACAGCAAGATCTACAACCTTGGGCATCCTCATTCCTCTCCATGATTTGATCACGTTTCCATCACAATCCCACATGCAAACGCCCTTTTCAGGGTCAGAACTGCCACATGCAAACTGTTTTGAGTTGGGAAACCAGGCACAAGAGCTGACAACAAAGCCTTGATTCCCAAATGTGTGCTTGCATGTACCTGTTTCAACATCCCACAGCTTCAAAACTTCTGTGTTCCCACATGTAAGTAACTTGGTGTCATCAGGACTCCATGCTACAAACGATACAGCATGCTGGTGACCATTAAGCGTGTGCTTCAGTGTCAATTTCCCATCCTCCAGCACCTTCCATATGATGGCTGTGCAATCATTGGATGAAGAGGCTAGGTACTCCCCATTATTAGAAAATTGAACAAACCAAACTTCATTTTTATGTCCAGTCAATATCTGAGTCGTTCTAGTAGGAATCTGATCCCTGCTGCAGCAATGGTCCTCATAAAGCGATGGCCGATTAGACGAACTATGATACATACACGAATCAACCCAGCCCGTAACAGTAGATTCTACCAAGTGCTCCAACCTTGCTTCAGGCAATGAAATCGGAGGAGGCAACAACTTTTCTAAATCCGCTAACAAATTTCTCCTCAAATCACGTACAACAACACCATCATCATCACCAATTGCACCTAACTCCCTATCCTTGAATGAAAGCAAGCACATAGCCAGACTGTGAACTTTGCAATTGTCCACATCCAAGGCAGAAACCTGTTTCCTTAATACCCCTAAAGCTAAGGCATCTTCACCGCAATTCAAATACTCCATAACACACTGTCTGAAAACAAGAAACATGGCAGATTCCCTTGTTTCCCCCAACACATCCTTTATGGAATTGAGATAATCAATGCTATCGTCCCAATTCCCATTCAATATAAGTGATTCAAGCAACTTAAGCTCATTGGACTTGAACGAAATGCCAGATTCTAATTCCAAAGAAGACGCAGAGTTACTGTAACCTAAGGAATATAGACACTGGATTATGATCCTCACAAATTCGTGCCTTCGAATCAACCCTTTTGAACCTAGTACCTCGCAAGGATTCTCCATGCACGAATAAGATGCATGAATAGAACGGGAACAAATCGAAACGCGAACAATTCGGTGACAGAAATTCGGAGAATTTAGAAAGAGAAAAAACAAAAAGTAAACCCTAGATGCTAAAAGAGTGAAATGATTTTGGAAGGGATTACAAACAACAGTTCGAAACTAGAAGCAACGGAGCAGAGTATGTAACAAGAACTGTCTAGAAGGTAGAAAAGAATAAAGATCGAAGCTAAGGATAGGATCAAGCGTAGAACTCAGAACCGAAATTGAAAATCAAAA

>C100441_65

CAAGAAACCAAGAAAAAATTTCACTACAGGACAAAATAACTGAATATATAAATAACATTTAAAGGACAACTATGTAGATGCAACCCAGGCACCAACATTGTAGCAAATAAAAGCGAATACTCACGAACGAAAATACGATAAAATGCAGGATCTTGAAGCACTACCTTGCATGGGGGGTTGAATTATCCAATATCTCTATCTACAAGGGAATACTTTCTCTGTACAAAGTTGTAATAACATATCTTACACCCAAAGTAAACGACAGGTCTATGCGTATCACAACGATTTAACAGCACCTTCTATGTAAGCAATGGCTTCCGCCTCCGAAAGTTGTTCTCCCTCTACCAACCCTTTAATTAAGGAGTTGGCATACGCTTTATTAGGGGCACGTAATGGTAACTCCCCCGATTTGAAACGTTCAATATCAAGAAGCGAACACGTCATTGTAATTACTGGAACATCCTGCTCCTTTCCTAAGTAGACAACATTACCATACCAAACACCCTTGACAACCTCCACAGAGTTGAACGCCTGGTCAGAGATAGCATTCAAGGTAGTTATATCAAATAAAGGAGATCCCGCATCAAGGCTTAAAGCATTTTCCTGAAATAAAATATCATTGAACTGCTCTAGCGAAATTTTGTACATGCGCATGTAGGTCTTGTGTTCAAAATTTTTCTCAGGATTAAGAAATGCAACGCCACCCAATCCCCATGAATACGATGAATCACGACCAAAGAATATATGACAAGGGAAAGTCTTCCACAGGATCTCATTTGGCAGAGTTCTGTTCACTGAACCAGAACACTGCTTCGCCATACCATCCACCTGTCCACCAGCTAGGTAGCAATTAAATCTTGCTTTCCATATATTTGATCCATATGAGGCATACCATATATAAACATTTGACAATTCAGATCGCCAAGTTCTAGAAACATGAATCTCTTTATCAGCATCAACTTTCAGTTCGGAAAGTGGTTTCATGACATCCTCAAGAAGTTCTTCAAGATCTTTAGAACTCGGTGCACCCTTATTATCCTCAGCCTCGGAATCAGAACTTGAAATTAGAGTATGCGGAGGTGGTGCCGTTTGAGAGATTAACTCTTTTGGTAGTTCTGATGCAAGTGTAGGCCAAAAAGGACTTAACTGCTTGCGAACCGCTTTAATCACACTCCATGTTGACTGAAATGGATAGGCCTTTAAGGATCCAATAACAGGCTTGTCTTTTAGCAAAACTTTCAAGCAAGCATGCACTGATTTTGCAATGGAATCAAGATTATATCCTCCTTCTAAAATCAGTACAATCCTACCTTCAGCAAAATTCATCAACTTCTCCAACAAAACAGAATAACCAAATGGTGTGACACAACATCCTCCCAAAGGGTCACCAACAGCTGCATCAAATCCAGCAGAAACTATAATTATGTCTGGATTAAATTCTTTGACAACAGGAAGCAAGATGTGGTCCCACACAGCAAAGTAATCTGCATCACCACATCTACCATTCTCCCAGGGGACATTTATATTATATCCAGCACCTGATCCTTCTCCAACCTTGGTATAATACCCATCTTCATTAGCAGGATAAAAAGTCCCATACTCATGCCTGTGAACGGAGAAGAATAAAACTCGAGAGTCATTGAAGAACATTTTTTGAGTACCATTTCCATGATGGACATCCCAATCAACAATTAAAATTTTCTTCACACCTAACTCTGGTCTTCCATCTAAGAGATATCTTGCAGCAATTGCCACATTGTTGAAGAGACAAAATCCCATAGCTTCATTATGTTCTGCATGATGACCTGGAGGCCTAACAATGGCAACAGCAGAATCCAATTCCCCACTTGCCACTCTTTCAACAACCTCCACTGCAGAGCCAGCAGCAAGGTACGCAGCTTCAGATGAACCTTCGTTAAAATATATGGAATTCAAACTCGAGGCAATTTGTTGCCTCCGCGAATTGTTCTTGGAGCTAATGTTCTTAATCAGTTTTACATGATTTTCAGAATGCACTAAACGTATATGTTTATCTTCAGCTTTCTTTGCCTCCAGAACGACGCATCGTTGAGGAATGCCGGCGCTTCGGAGTCGGTTCCAAATAGCCCTAAGGCGATTAGGAGTCTCGACGTGGTCCTCGTCGTTCGGCGTGTGGTGCTTACACATCCTCTCGTCGTATAGTAAACCCACACGACGTTGACCGTTGACACTGCTTTCGCTACTCTTCTTCCCACGCTCGCGCTCCATGCCTGCACCTCACTTTTCAATTTTTGTCACTGCAACTGAAGTTCAAGCTTCTCTAACTCGG

>C100443_65

CTTATTTATACCATCACCAACCTGCTTCATAAAACATTGGCCTTCAACTGTGTAACAACCAGGCATAAAACACAAACATATGCTAAAGGTAAGAAACATAAAAACAAAAAAGTTCTTATTGTAATACAAGTGAGGAGCCAAAGTCCAAGACTTCACGGGATCAAACGTTCCTTACTTTCAAAACTCTCCTTCCTTCGAAGCTTCTCCTGTGGCTATGCTCTTCTGCCTGTCTTTCAATTTGATCATCATCACCTTCTTGCTCTCTTCTTTTCTCCCTCTCTTCTTGCTGTCTTTTGGCTTGTTCTTGCTCCCCTTCTGCTACCTCTTCTTCTTCTGGTCTTTTCCCCTTCTCTCTCCTCACTCTCTCCCACCAAGTCGGATCATCTTCTTCTCCTTCTTCCATTTCTCCCTCCCCCTGTCGCTTCTTTTCTCTCTTTTCTTGCTCTCTTCTAGCTTGTTCTTGCTCTTTCTGTGCAGGTGCTGCTTCTGCTTCTCTTTTGGCTTGTTTTTGCTCCTTTTCTGCCTCCTCTTGTCTCCTCATTTCCCTCTTTTCTTGCTCTCTCTTAGCTTCTGCTTCTTCTCCTTTTCTTCTCTTTTCTTGCTCACTTCCAGCTGCTGTTTCTTCTGCTTCCTCTTCTTTTTCCTCTTCTGCTTCCTCCTCTGTTTCCTTCCTTTCTCCCTTTTCTTTCTCTATTTCCCTTTCTTTCTCTCTTTTCTTTTCCCTCTTCTCCTGTTGCCTTTTAGCTTCTTCTTCCTCCCTCACCCTCTCTTTCTCTCTCCCAGCTTCTTCTTGCTCCCCCTCTCTCTCTTCTTCTCTTTCCCTTTCTTTCTCTCCCCCAGCTTCTTCTTGCTCCCTCTCTTCCTCTTCCTCGCGCTTCTTTTCCCTCTCTTCTTGTTGTCTTTTAGCTTCCTGTTCCTCTATCTCCCTCTCTTTCTCTCGTTTCCTGTCTTCCTCCTCTTCCCCTCTTTTTCTCTCCTCCTCCTCTTTCTTTTTCTCCCTTTCTTCCTTCTCTTTTTCCTCCTTCATTATCTTCTCCTCTTCCTCCGCGCAGGAACTGCATTCGAAAATGATGGAATCATCCGCAGAACGTAAAACCTCGTCAACGGTTCTGTCACTCACACCCAAAGAAGTAGCCAGTATCTGCTTGTCCAGAATATGAAGCACCGATCCTTTTCCCGCCAAAAATTTAGGATGGTTGTTCTTTGCTGAAGTGCTGAAGCCAAGAAAAACAAATGGTTCATCGTTGAATGACATCTGAGCCATTGGACGAAACCTAGGCACCACAAAAGTATCACCCGCATTAACCTTAAAGCTCGTGTTTTGACATTCTGTCTCATCTTCATTGCCGCTTCCACATACCATTCGGACCATGCCTTTCCCTTCTACTGTCACTGCCACCTCTGTTGCTTTCTGGTTCCAATGAGGCCCCATTATCGATCCCATGGCCAAATTCACCATCAGAAATCCAATGTTTGTCCGCTTCAATGACTTTAGTTGCTTTTTGGTCACTGCTACGCTCCAACCATTACGATTTCTGAAGTCAGGGTCATTATCAAAGATGTTGTATTTTTCGAGTTTGTTAGAGTTGGACCCAACGCCTAGAAAGTTTTTAAGAAACGAAGTCTGCAGTTCCTGGACTATATTCCTCTTATTTGAAACCGCATGCACTATAGCTGGAGTGTTTCTCTTGCTCATGATTTCTTCAATCAAATCATCTGGCGCCTTTAAAGCTGCTTGCATGATTTTCTTATCAAAGCCCTTAACCAGTTCATTGATTCTTGAGTAAGCACCGACTGACGGATTAAAAGTATTGCCTTCTGTATTGGTAAATATTGCATAAATCCTAAGTTTCCTTCTCTCAGCCTCTAAGTCGCTCTGTATGTAGAAAATAGATTCTTCACTGAGACTGCAGAGATCTCCTTCGCGTAAAGGGATCGTGCCAGAGTCGTCATCATTTGACCAAGTCAACTTTCCACTCCCTGTATGAACATAAAAGACCATATTTGAATGTAGAAGCACAGGAAGAAATAACGAGTTTGGCTCCAATGTGATGAACTGAAGATGGTAGGGTAGTTTCTCTGGGTCTTCCTTGATGTTCACGGCAGTTATCTGTCCGAATTCAGTGACAAGTAATGTCCTCCTCTGATCCTTTTCCACCACAGGTCCCTCTTGAGACTTGTCATGGTGCTCTTCACTTGATGATGCCTTCACTCCACACAAGGAAATAGCAAGGAAGAAGATCAAGACCCAAGATAACGGCAAAGCTTTTTTTTGAGGCTCCATTGCTAACATAGTGAGGGGACTTGGA

>C100445_65

CGGCCCCAAATAGTTACACAATCACATAAAAAAAAGGTCTAATACAAACCAACAAGCACGAAAACTGAGAACGATCATGTTAATGAAGTAATTAACAGATCCCAAACAACAAAACACCAAGAAAACACGCCTATTTCATATACCACTCGCTAGTATTTGTTATAGACAAATATAAACTGCAAAAAGTTAAATATGCAATCCCTTCAAAAATCTGCAATCATTGTGTCTTTTAACCCTTAGATCAGATCAGACATCCTTCTTCTATGACGACCCACAGAAACTCTCAAGTTTTGGTCTGAAGATGTACCCATCCTGCTTCGAGGGGTAGTGACTTTTGATTCCAAATCATTGCATTTAGGCCTTTCTGTGAGAACAGGGTTTGAAATTGGAATACTTTCTGTTTCAGCATGCCGAGATTTTCCATTTGAACTTCGCAGTAGTGCTTTTATGCGGATATCTGAATAACTTAATGATTGGGAATATGAAAAATGGATGGAACTTCTAGAGACCATTGATCTATAACTTTTCAATTTCACAGTGCCAATGTCTCGCTCTGCAGCACTTTTCCTTTGGCGACTTGCAACTGTTGAAGTCATGAATCGAGGCATTGAGTTGGAGAAATTTCTTTTAATGGACTTCTTAGATGCTTTAGGGGTTTCAATCACCTCTTTCACATCATCTTTGGGGCTAATTGAGGCTGTCATGTCATCACTTTCCACAACACTGTATGTGGTGAATAAGAGCTTTTTACTATCATTTAATTTCAATTCAATCTTCTGTATTTCTTCCCTTAAATTCTGCCGTTGTTTTTCGGCTTCCTTGATGTCTTCCTCTAACTCCATAATCTTCTTCTCCCTTTGCTTCTTCACTTCCACAGGCACTTCTTTGTTGGACTCTATTGCTCTCGCTCTCTTTGCAAAGTTCAAGGAACAAATTGACTCACAGACATCTTCGTCTGAAGGGCTTATATGCACGAGCATCAAGACCTTTGAACCATACCCAAGAGAATCTTTCAGTAATTGAGTCAGCTTGCTATTCCTGTAAGGCACATGACACTTCTTCCTCTTCAAAGCTGCAACAACATCAGCTAAAGCTGAAAGAGAAAGATTAATGGCTCTGCCCTCATCCAGTGTTAGTCCTTTGGCTCCAGTTTTAAGCAACCGTTCGCTTCCTCCAAGATCAACCATCCACAATTTACTTACTTCACTTTTGGCTTCCAAAGCATCCCCACGTCGAAATATGCTTATCCTCGTTAAGCAGTGTGACCTGCTTGATGCTTCATTCACATTAGTCCACGAGGTGGATCTGAACCTTCTCCCCTTGTTGTACCACCATTTGGCTTTACCATAATCACTTATTTGCACTTCAGTGAGACCCTCAATTTCAATCAACCCCTTTGGATCTGTTTGGATGTTGAGATTGCACTTCGTCATAGGTTGTTCATGTGGTCTACCAGACGGTCTTGGAGCTAGCAAATCCCTGAGATTGCCCATATAAACTTCCAACATGCTCATTGAAAAGGTAAAAGAAGATGAGTTATCCAAAGAGGCTTGACGAAAGAGCTCTTCAAGAGCACGAGGAATAATTCCAGGTTGCTCGTTTGTACCATCCATGGTGAATGTCTTGCCTGTGCCTGTTTGACCGTAAGCAAACACACACACATTGTGCCCGTCCATTGCAGATCTTAGAATTGGCTCAACCTCAACAAAAACACTTTCTTGGCTTGTTTCTTTAGTAAAAACTTTATCAAACTCAAAATCTTTCCTTGTTCCCCCCAAATTAACCCGAATTTGCTCTGGTCCAGCTGATACCGGTTCAGAATGTCTTCTCTTCTCCGTAACCAGATTTGGTCGAATTCTACAAAATACTCGAATGCTCCCTTTGATGTCTAATATCTTGCTCAATGCTTCTCTCCGCTTTTTATCCAATTTCTTCTGCTTGACCCTCAGCTCCTCAATTTCTCTTTCTAGATTGGATATAAAGTGCTCAAGCTCGTTCTTTTGGTGTTCTGGCACGACACCCACATCAGTGTAAACGGTGGAAACAGGTGGGGACCCATCAACTGAATCTGAATCAGGGTTCAAATCCAACGAGGGTGACTTTAAAACCTCACTTGCATTTTCTGAAGCCAACAGAACCTGCTCTTCTCCGTTAGGAACAGGCATTTTCACTACCCAAGAACATGAAATGAAACACAGCCACCTCGAAGTAAAAGGTGTAGAGAAGAAGTTGGTTATTTTCAATGATTGAACACTTTGGAGGATCCTGATTACTGATTATTAGGAGTGAAGAAAAGGAGTTTTTTTTTG

>C100449_65

TCGGGCGATAAAAAAGAGCATGGAGCTAATTTCACAAATCTAAAACCTGACAAAAACCAATGTAACATGTTACAGTGTGTAGTATTTACACAAAAGAAAAGTAATAAAAAAAAGGCTTACGAGTATTCACAGAGGGGTAAGAAGTTGGTTCCGTTCCAGTTTGCAACTCATGAATGATTATAATAATCACCAAACGTGAAGCGATGGTTCCTGCGGAGGAGATGAGGAACCCTTGAGTCCAGAAGTTCTAGTCTCGTGGAAGTCTTGTATAACTTTGATCAGGAACCGCGCTTTCTTTGCGCCGTGACAACTCCCACCCGTCAACAGCGAATAAAGAGAAGGCGTCACCGAAGGGTCCTTCGCCAGAACCGCCACCACCTCCGCCCCAATATTGACGCAAAGCGACAACAAAATCGAAGCAGAGTGCTCTTTCCCCGCGCGTGACGTCGCAGTTCGCAGCATCCCCGTAATCAATGCCAACGCCGATCCTTCGAATATCGCACGCGCCCCATCAACATTCTCAGCCAGCGCTGCAAGAACCGCCAATGATTCAGCCAGAAGCTCCTCCTTATCCGAAGAAACCAAAATGTCAAGCAGCGCAGGAACCGCACCGGCAGCGACCACCCTTTGGTGATTCCTAGGAAGCAAAAGCAACCCGAAAATCGCAACCACAGCGTTCTTTCTCCCGCAGGTTGTTCCTTCCTTAACTAACTCCACCAACGCCGGAATGACCTCGGGGTTCTCACCTATTATTTTCCGGTACTCTTTCACGGAAGAGAGATAAAACATGATAGCAGCAGCTACTTGACGAGCTTCTAGGCTAACTCCATTTTTCAACACGGAGAGAATTACCGTTAGGCCCCCACTGGTTATGATGTTTTTGGGTCCATTCGGATGTTTAGAGAGCTTCAATAGAGCGGAGATTGCGTTCTCTTGAGTGGATTTGTCGTTAGACGCAGAGGCTAAAAGTTCTAGTAGAGGAGGCACTGTTCCCACTTCATTCAAACACGCCCTATTGAAAATGCTGGTTCTCGCAAGCACGCGAATCTCGTACGCAGCCTTGTGTTTCTGTTCTTGTGTTCCGAACACCAGCCTCCGCGTCAAGAACCACGCTAGGAATTGCACGGCGTGCGCCGCCCCGGGACTAACTGCGACGTTGTTGCGGCTACGGTTACATGGGTTGTTGAGTGAAATGCCGTTGTCGGCGCAGAACTGTTGGATGAGCCTCTTGAGAGTTGTGTTGGGAACCAACTCTGTGTTTGTGAGCTTCTCCCCTGTTTTGGGACATGTGGTGTTCCCAGCTTTGAGCCATCTCTGAATGGAGGCTCGGTCGTAGGTTTGACCCGTCGACACAGTCACCGGATCCGTCATCAACTCGAGGGAAATCGGGCAGCGGAAATCCTCCGGATTAACGGAAGTTAGTAACGTTAACGATAACGGTGCCAGCTCCGTGCTGCATCTCCCTTCGGATTGATTCGTGTCTTCCATAGTTTCGAAAATCACACCTCTGCAGTAACTCAAGAACCCTACCAAGCTACTGAGCAACGGAACCTCTCGCTCGTCACGATCGCGACACTCTAAGTTGATTTCTTCTTCTAGGAAATTAATCTCTTTATTGCAATCCGTCCAGGTTCTAATGTCGAGGTAGTCCATGATTCCCTGCATGGAACTGAGGTGTGGTTCCGTTCCCCTCGAGAAGAGGCGCAGAACAGTGTGGAGGGTTTTGCACGCACTGGCGTCGCTGGGGTCCACTTCGAACTTTGCTTTCCTCGCTTGCCGCGTTACTAACTCCGCCAATTCCCTCACTTCGGTGCACACGTCGAACTCGTGAAGAGGCAGAACGTCGAGGGAGGTGGCGACGGCGCGAATGAGTGCACGGAACTGGGATGCCACGTGATGAGATTTGGCAAGCATTAAAAGTCGCGCGCCTTCGAGTGTGCAATCTTGCATCAAGAAGTGGATCTTTTGTAAGGCGAAGTGGAGTTCTGCGAGGCAGAGGATGGTGGAGTTGGGGATTGATAAACCGTAGTCTCGAATTTCTCGAAGGAGAATTAAGAGTATGCCGATTTGGCGTATCGTTTCTCGTGCGTTGCGGCGTTGAGTGGGGAATAACTGGGGTTGAAAATTGCATATGGATTGGGAGAGATTGATGAGAGAGGCGAGAAGTGTGGGCGGAGATATGTCATCGCAGGGGTGAACCGCCGGGAATGATAGGAGGCGGCGATCCCTTGGAGTCATTGAAGTGAATGAGAGAAGAGGAGAGAAGAGAAGAGAAGAGAAGAGAAGTAAGTTTAGTTGTTTGAAGTTGGA

>C100451_65

CTATTAGCTATCTCTGATTTATATTCTATAGAATGAATATGAACAAGCCATACAACACAAATGACAGCTCCTTCACTTGTCCTCAAGAAAACAAAACGAAGTTTTTAGTGTACAACCTTATTTCTTAAAAGAAATACTCAAAATCCGTGGAAACGCGCTCCTTAGAACAACATTCATATCGCCCAATACATTTAGTCTCATTCTCCCTCACACCCTTGTATTTTCAGCCAAGGACAAAATAAACAAAAATCATCATCCAACATAAGCAACCTTTACAATTACACAGCTACATACAGACTTTTACTATAGCCTTTTGATGACTGTACAAAATATGGAGTAGAAGGATGCACAAAATCAAATGAATTCAAAGCGCATTAGAATCTTCACATTGTTATACTTTTCTCCTTTGCGGGAATATAGTGGAACTGCTCGAATTCCACTTCTTAGTTCCCACACAGGTAAGCACGTTTGGCCACCAAAGTCATCCTTCTCGGACATGTCATACTCATGAACTTCTACACGAAGCAGAGCCAGTTCTGGAACAGAAAGTGGGAACTCAAATACCTCACTCCATGAAGGAGACCAATTATCCTCTATAGTCTTAGTTTTTTTCATAACAGTATCATAAGCGACTCCGGCAATCCCCACTCTGGCATAGAAGTCAGGGGGTGAGAATTGATCAAAGTGCGTATGCTTGAAATCATGAAACCATCCTTCCCCCATATATATGGTCACCTTCAAAGTTTTCTTCACAGGCAAATGAGCTTTAGGATCAAAGACCTCATTATTAAGACCAACTTCTAATAGAAAATCTGGTTTCTTAACATAACCACATCCTCCATTGGCTTTGAACATTCCCTGCATCAACCAAAGAGATCTACCATATCCCTGCATGTTAAATGCAACCATCTGAGCTCCGTGCATCCACCCGATCAGTGGGTTATAATTTGTTGAGGTAATACGAGTGCCTTTTGGATATACTCTCAGTATATTCCGCTGAGTAAACCTGACAATTTCTTTTCCATGAGTTTCAGCAGCCTTTTCAAGTTGTACCTCACTTAAACTTATACGTCTCACTGTATCAGGATCCACTTTCAGACATTCCTTTAACCCCCCTTTAGGCTTCCCAGCATGAATGGCAATTAAACGTCTGTATTTGTCCGATGCATTGTGACTTGACTTTTCTGATTCATCAGTATCGTTCTCATTATAAAGATCATCTTCATCCTCAATGTTCTTGTAATCAGAGATAGTGCCACCTCTCAAACTAGGGACTTCCTTCCCCCATGCTTCTTCATCATCTACAGGCTTTTCCTTTTGTGATTCCACCCCCTTTTCCTGAACGTCTTTTGCTTCAATGTACTCCTTAGGTGGTTTGGTTGATATGATAATCCTTCTTTTGAGTGATTTAGGAGAAGGAAATTCCTTCAAGATTTCAGAGGTAGGAGAAAATAGTATGTCTCCAAATGTTTCTGTAATCATCTCAGCCACTTTAGCCTGAAGATCGGGAGTAAGGTGATCTTCTAAGGTTATTACAACTGGATATTCTGAGGCAACAAAGGCATACTGCTTAATAGACCTCAAACATTTTATGAGTGCCACAGGAGATGTCAAAGTCCTTCCATGAAGAACATCCACATCATCCTTTGATGCATTAGGCCATATATCTAATTCAATCACCCTTACACCCTTCTGCAGTGCATTGATGATAGGGAGATCACTGCAGTCACTGCTAAGTTGGTTCCCAATTAGATAGGAATTGTGACCAGTATAAATGAAGTAATGAGACAACGGTGAAGACATATCTTGGTGCACCCCGAGAGAAGGTGAGAGAGGTGGATTATTATTGTGACTGAAGAGGTAGTTGAAGAAACTCTCAAGATTGAGGCCCCTTCTGTGAAAGATACTGAGATGCTTGTGGCCATCGATGATGGCCTGTGTTTCTTCCTCAGTGGCACTCTCCTCCTTCTGCACCTCAACCAGGAAACTGTGGAGGTGAGAGGCTGTCATGACACCATGCTCATCGGAGTAACGATCGAAAAGGGTCCTTATCTCCGGCGGGGCCTCCGACACGGGGAGCCTGAAGCGGCGGCGGAAGCAAAAACAAAAGCTGTAAGTCTGTTTGGACATGCCTTAATCAATGTTTTGTTTTTCTTCACTCTCTATGTCTGTCTCTCCCAACCGAACTCCTCGAAGAAGATTACTAACGGGGTGAACGCGTAAGGTGCGTGTACTTTGTAGATACTAACATGGGTACGTGTGCGTGTGAGTTGTGTGTAGAAGATAACCTTTTTTGGTCTGAGAATGAAT

>C100453_65

AACACCCTCCTGATGTTTAGTGCATTCATGATTCGTGACGTAACGGGAGTCCACACACTCATTGGAGACAACTGTATATTTTGCTTTTTCTCTGTACGACTGTACCCAAACAAGTGATACACTTACATAGTGCATAGAAATATGTTTTCTCATGTATATATAGTCTCTGCATGATGAGTATTGTGCATACTTGAAGAAGCAGTAAAGATACTCTAGTTTTCACTTTTAAATTCTTAACTCCTATTCCATCCAGGTGGAAAGTAAATAGATTACACTGCTATCCCCTATCTCCAACTAACATTTTACAATGGAGAACAATAATCACAGCAATAGAATCACATCATGCACTCCCCAAAAAGAAAAAAATAAATAATGCAGATCAAGCGCAACCCACTTTCTCCATCATAGTGGAACTGACAACATTGTTTACAAAGGCGTAAAAGCATTGCCTGCAACAATTAGGCATTAAAGTAGATTTCTGAAACTGAGGTGTATTGTCGGAATGGTTGCATACTGTGCTAGAGGAGGATGTACTACACTTTTGCTGCTCAGAATTGCAAGCAGCCAAGATGGTATGGGTGGCCTGCCTTGCAACTACCTTGAAGGTGTCAAAACCTAATTTTTTATCTTTAGTTAAGAGCTTAAGATTTAATTTGACGAGAGACTGGATCTCAGTTTTTGCATCGTCAGTTTTTCTTGTGCTTCCATCCTCACTACTTCTAGCTAATCTCGTTTCTTCATTGACAAGATCCACATCCCTACTACTAGAGGAGAGTGCACTAACAAACTTGCCATGCGAGGCTGAAGTTACCTGGCAAGCATTTCTCTCTTCTTCACGACAGGGAACCCCACCCTGGATATTAGTGCGGACCTTTCTGGACAACGGACATTCACAATATCTTTCTGAATTAGTATGATCCCTTACACGGTGTATCATCTCCTCATATCTAACCCTACTTTGCTTTTTCTCTTCCAACTTTAGAGTTGAGCAGTTTTCCAAATTCTGGACGAGCCTAGAATAGTCACACTGCTTCTCTTTCTTAGTGTTCCTGAAATCTTTGGAATGTTGATCTTTCTGTTCTCGGAAATTACAGTGTGCAATTGATATTTTTCTAGCTCCTCCACTGCATGACGGTTTATCTACTCCCTGTGGAATGCTGCTAGTTTTTTGATGAGTTAGTTGCATCCTCTTTGCCCCGTCCACCATTTTCCGAGCCTTGTCAGCATCTTTAAAGATTCTCTCATTCAACATTTTGTTAGTAGGACCACCTTGGACTGTGGATTGTTGCAGGCTTGTGGAAGCCCTAGAATGCGAACCATCTAATTTGCCATGTGTTAAGGATCTAGAATCTTGTTCTTGACAAACTGTGCTGCTAGCTTGATCCGCATTACAACGGAACTGTAATGAACCACCCCTCAAAGCATTCCAGTTTTCACGGAATGCCTGTATATTACACTGCATATCATGGACGCCTACGGCAGGACTTTTTCCTTTAAATCTACTTAACCTATCTGATGAAGGAATAGCAGAAGGAGATGACAGGTTCTGTTGTAAAGGAGATGCCCTTGGTCTTCTAACCACCCGACTGCCTGTTTCCCTCACAATATCAAAGATATCAATGGTTTCACTTGGCTCAGCCTCTGGCACAACATTTTGTTGGTCTGATTCTTCATTGGCATTGGTCTCCCGGGAAATAGCACAATCATGACAAAACCAATCACCTTCAGGAACAGTATAGCCCAGGCCAACACAGTATGTGTGTGAAGCAGTATCACAAAGATCACAAAGTAGTAGAAGATGTTCATCCGCCCCAGCATGACAAACACCACATTTGGTTTCAGCATAAGAATTGACAGGACCAATTGCCACATTTCCATGAGGATGATAAGCCTGGTCACGATGAGGGATCTTAACGTCCCGAGAGGAGGAAAAGACGCCATGCATAGGGAGCCTGCGAACGTTGGAGAATCTCTGGCGACAAATTGGGCATCGAGACTCGTGCTTGGCCCATTCCATGATGCAGACGAAGCAAAAGTAGTGGCTGCAGCAATCGATTTCCCCTGCGATCGAAACCCCTCGCTCCGCGTAACAAACCCCACAACAAACGCTCTCTTCCTCGTCCTCATCCTCCTCTACTTGGGATCTCAGCCTCTTCTTTGGAGAACCCAACGACGCCGACGACGACCCCATCCTCGCCGGATCTCTCAACTCCGAAGCCCGCAACGCTTCTCCTTCCGATTCCGAATATCACACTGTTCAGAACCACACGGTGAAGATGACAAGCTCCACCACACCGAAACTTTGTTGCCA

>C100455_65

CAAAGTAGCACATCCTCTGCGTTTCTCCTCCAACTGAACAAAAGCATCGTTTGGTAGACTAAACGCTGCGTTGAGAGCGTCAGTGGTGACACCCAAATTTCTGAACTGAACCAAAGGCTGTCTTCATTCGCTTCCAAAGGTTCCGTTCAATTGGAAAGCTCTCTGGAAGGATGAAGGAAGCAAGGACAAAGAAACAGCCTGAGGCTGATTCAGAGATCTCAGCATGGGTTAATAAAAGCCGTAAGCTAGAAAAGAAAAGGGCATTGCAGCTCTCAAAGATTTTCGAGGAGCAGGACAAAATTGCTGTAGAGGGAAGTGATGATGAGGATACAGCCCAACACACTGAGAACCTGGCGGGGCTGAAAGTTCTACATGGCCTTGATAAAGTTATGGAAGGCGGTAGTGTAGTTCTAACTATTAAAGATCAGCCCATACTTGCTGATGGTGATGTTAATGAAGACGTTGATATGCTTGAGAACATCGAAATTGGAGAACAGAAACGACGAGACGACGCTTATAAAGCTGCGAAAAAGAAAACTGGTGTATATGATGATAAGTTCAATGATGATCCCTCCTCGGAGAAGAAAATGCTTCCACAGTATGATGATCCAGCTGCAGAAGAGGGTGTAACTTTGGATGAAAAGGGACGATTCTCAGGTGAAGCTGAGAAGAAACTTGAAGAGCTGCGAAGAAGGTTAACAGGTGTTTCCTCAAGTACCTTTGAAGATCTTACTTCGACTGGAAAGGTATCATCAGATTACTATACTCATGAAGAAATGCTAAAATTTAAGAAGCCAAAGAAGAAAAAATCATTGCGGAAGAAAGATAAGCTGGACATCAATGCCCTTGAAGCTGAAGCTGTCTCTTCAGGATTGGGTGCTGGTGACCTTGGTTCTCGGAACAATGTGAGGAGGCAAGCCATCAAAGATGAGCAAGAAAAATTGGAGGCTAAGATGAGAAGTAATGCTTACCAGTCTGCTTATGCTAAAGCAGATGAAGCCTCCAAATTGCTGCGCCTGGGACAAACTCTCAATGTTAAAACAGAGGAAGATGAAAGTCCAGCTTTTGTAGATGATGATGAGGATCTTCGAAAATCCTTAGAGAAAGCAAGAAGATTAGCTCTTAAAAAGAAGGAGGAAGAAGGGGCATCTGGTCCCCAAGCTATTGCGTTGCTTGCCACCTCAAATCATAATAATGAGACTGATGGTCAAAACCCTACAGCTGGAGACTCACGAGAAAACAAGGTGGTCTTCACAGAGATGGAAGAATTTGTCTGGGGTCTCCATATCGATGAAGAAGCACGTAAACCAGAAAGTGAAGATGTTTTCATGCATGATGATGACGAGGCTATTGTTCCTGATGAAGAAAAGACTAATGAGGCTGGTGGGTGGACCGAAGTTCAAGAAACTAATGAAGATGAACAACCCAACACAGAAGACAAAGAGGAAATAGTTCCTGATGAAACTATCCATGAAGTTGCTGTAGGGAAAGGATTGTCAGGTGCTTTGAAACTGCTTAAAGAGCGAGGGACACTTAAGGAAAGCATTGAATGGGGTGGCAGAAACATGGACAAGAAGAAAAGTAAATTAGTTGGAATTGTAGATGATGACGAGAAGGAAGCTCAGAAGAAAAGGGAGATTCGCATTGAAAGGACTGATGAATTTGGGAGAATTTTGACTCCTAAGGAAGCCTTTCGGATGATTTCTCATAAGTTCCACGGTAAAGGACCTGGAAAAATGAAGCAAGAAAAGCGTATGAAGCAATATCAAGAAGAATTAAAGATGAAGCAAATGAAGAGTTCAGATACACCATCACTGTCTGTGGAGAGAATGAGGGAAGCTCAAGCCCGTTTGCAGACCCCCTATCTTGTTCTTAGTGGTCATGTTAAACCAGGACAAACTAGTGACCCAAAAAGTGGTTTTGCAACTGTTGAGAAGGATCTTCCTGGTGGCTTGACACCTATGCTTGGTGATCGAAAGGTAGAGCATTTTCTGGGAATTAAGAGGAAAGCTGAGCCATCTAGCTCGGATAACCCAAAAAAGCCCAAAACATAATTCCTTGGTCTCGTAGCATTCTAAGCTAATATCCCTGTAAGCTTTTAGAAGTTTGACTCATAGTGTTCATCTATTCTCTTGCTGCCGCAGATAATTAGCAGCAGAATAGAGTTTGAATTTCGTTGGTAAAACTTGATTTTCTTGGAAGATGTATGTTGGTAGATATTTTAAGGACTGGCTTACCACATCAGTTGTTATATGTTTCTGACCATTCCAAGTGGCCCAGAATTCGCTATTCCTTTTGTACAAATAAGAGA

>C100459_65

CTTTGAGCATATCCGAAAAAATTATTAATCAAGATAATTTGACCTCTCTGCTCTCAGAAACCTAGCTTCTTGCTCTGTAGCATGAAGTGGAAAGTGTATGCTGGAACGCTGCTTCCAAGTCAATAAAGCCTCCTACACTCCTCTAATAATTATAAAGCCGGAAGAGTCCTGTAAATTTAACTCAGAAATGCATATCTAGGGGAAATACTTTTAACGAATCTGCTTTATCTGCCGCACAAATACTGGCACACATAACATCTTGAACAAAAATCTCACAGCAGGTCAATGGGGATTCAATTATATTTGGCCGGGCTGGCCGCTTTCAGCTGCTAGTTTTAATCGTTGATGATGTTCCACCACCTTGTAATCTACAATCTCTTTAAACAACTGGGAATCCAAGATGCAGTCTGGACGCCCATAGACAGCAGCTTGGACGCTGGCCATGAGTTTGGCGATCTCACGGCCAGAAAATCCCTCTGTTTTCTTGGCAGCCTCTCTGAAAACATCTTCGGATAAATCTTTTATAGTAATCTTCTGAGGCTGCTTCTTCAGAAAGAAGCCTCTCTTGGAGTCATCATTATTGTCATCACATAAATATTTGTTTAAATAGAGCTTCAATAACTTTAGACGTTCTTCCTCTCCTGGAAGCGGAAATTCAATCACTTCATCAATGCGATCAGTAACTGCACTGTCAAGATCACCAGGTTTGTTTGTCGCAAGGACAAGTACTATGTCTCGAGACTGGTCACCAGTTCTGAATAGCAATGCGTTTAGAGCACTTCGCTGAGCTTCACTCATGTGTGAGCTGTTACGCTCGCAAAGGAAAGCATCTGCCTCATCAATAAAAAGCAATAGGCCTTTTCTAGATTTCTTGGCCCAATCAAATATATCATGAATTTTGGTAACAGCCTGAGCGCCTAGGGGTGCAACATCTCCTCCAGTCATCATAGCATAATCCAAACCCGATCTTCTTGCTATTTCCCTTGCAACCATCGTTTTACCAGTACCCGGAGGCCCATAAAAAAGCATGTTACGAAATGGAGCCTGATGGGCTTTGGTATTTGATGTTGCACGCGCAAGATGCTCTATTCTTCTCTGCAACGATGGATGGAGGATTACGTTTCCAAGACCATTTTTACTCCCAATAGGCTTTTCTGCCCCAGCCAAAGTACTACTATTTAAAAGTTTATTTTTGGCTTGAGAAAGTATCTTTGATCCTGGGAATTTTGCCATAGATGATTCCCGGATCAGTGATGGCTGCCCTAAAATTCGATTAATATAGCCCCAGGTGACTTTAGCACCTTCTCTAGTTGTATATACTCCTGCAGCTAATGCAGTAGCTCCTCCAACAGTCATAAGTAATTTGTCCCTATCAGTCAATAAGGCCCTCAGACCACCTTCAATATGACTGAAAGTTGTGTTGATTGCAGCAAGCCATTTATCTCTTTCACCTTGCAGTCGTTCTACAAGCATTCTCCTGTTATGATCCTCGGTTAACTTTGCTTCATGGGCTCGGCCTTCAGCCTCAGCCATTGCCTTAACTCTAATAGTTTCTCTTTCTATTTCAGCTCTCTCTCTCTCAGTCTGACGCTGCTGAGATTGAATCTGTTCTTCAGTAGCCTGTCTTGCCCGCTCCTTTCTTACAGAAGACTCCTCTTGCATCTTGACCAATTCAACGTTATGTTGCCTTTGAGCTTCATGATCTGTCTGCATTCTTTTCCTGGCCAATTCATCTTCATATCGCAGCACCTGGGCCTGTCTCTGGGCTTGCTCTTGTATTAAATTACGTTGTTCTTCAGCCATTTTCCGCTGCCTTTCAATATCTCCTTGAGACTGAATAAGTTCGTAGTGAACTTTCTCAGCGTCTAATTCAGCGAGGCGAGTTTGCTCCTGCTTTCGCATTAAATCAAACACCTGCTTTGAGTAGGTAGAACTGTTGATTTCACGGAGAGCTTTGGCTCCTCTTTCTAACGATTCCGGGTCGAAACCCGACTTGTTGGGCTCCTCGGGCGCCGGAGACTCCGAATTGGTGTCGGAAGCCTGGTTTGACGGCGGCGGAGTATTGGAAGGTGAAGAAGAGAAGAAAGGGAATCGGAAGGAAGAGTCGGCGTAAGCACGATCGGAGGAGAACGTAGACATGCAAACCGCCGCAGCGGATGTTACCGCCACGCATGAGGTTAACCTTGCAGCAGCCATGGCTTAGAACGCACCTTCCAGATGGATCATGAAGAAGCTTTTCTGTAGCGCGAGCTTAAACCCTTCGTTAGGGTTTAAACTGAAATGGAAAATTTTGAGAAGGGCGTTGCGTGGAG

>C100461_65

CTTTAAAGAAACAAGTCTGCGCCACTGCATTTTACAACAGTCAATCCCTAGTCTCTAAAGCACGCGCCGTCCAACCACCCACCCACCTCCGTCCGCCGGCGAGCCACAGCAATACGTTCACTGCAATGTGAAGCAGAGGGGGCCAAAACCAAATAGAGAGAGGAAAGGGTCTGCATCAAATCTGAGGAAGAATGAGCGGTGGTCGACTTTGCACATTGCTGGGAGAATTGGGGTACGAAGGGTGGGAAGCATTGGATCCTGATAGCTTCGAATGGCCGTTTCAGTATGAAGACACTCGCCCTCTTCTCCACTGGATCTGCTCCAATCTTCGTCCCTCCAATGTCCTCTCCCTCTCTGAACTTTCCCAGTACGAGCAGTTCAAGCAAGAAGGGAAGCTGTTAGAGGGTGAGGATTTGGACTTTGCTTATGACAGCATTTCGGCCTTCTCTGATAGGAGAGACAATCAGGAAGCAGTTTTCGGCGCTGAGGAGGGATTGAAAGATATCAAGGAGGCAACTTTGGCATATAGAGAGGAGGCCTTGGCCTTGCAGAGGCAGCTGAGGCATCTGCAATCTCAGTTTGACATGCTTTCCGGACAGGGCTCAGCATTGACACAAGGAAGACGGCCACGCCTTGCTGCAACCTCTATTGTTAATGGACACCTTGCCAATATCGATGATAGCCTTTCTGTCAGAAATTTACAGATGAATGCCGTTCTTGAAAGAATTGCTTCCACAGCGCATGAGTTGGCACATTATCATTCGGGAGATGAAGATGGTATATATTTGGCATACTCAGACTTCAACCAATTCTTGCTTGGAGACTCATCTTGTCTAAAAGAGCTAAATCAGTGGTTTGCTAAGCAATTGGACACAGGACCATTTCGACTTGTTGCTGAGGAGGGGAAATCTAAATGCTCTTGGGTGAATCTTGATGAGATCTCCAATACCTATGTTAGAGATTTGGAAAAATCACATCATCAACGTGTATCTGAGTTATGCCGGCTCCGCTCAATATTTGGAGTAAGTGAAAAACAATGGGTTGAAGCGCAGGTCGAGAATGCCAAACAGCAGGCTATTTTAATGACGCTTAAATCACAAGTATCATCTGATGAAGCTCACATTCATCTTGATCTTCATTCTCTTAGGAGAAAGCATTCTGAATTGAAAGGAGAACTTTCAAATCTTTATAACCATGAAGAAAAGCTGCTATCAGAGACTATTTCAGATTTGTGCTGGGAGCTGGCTCAACTCCAAGATACATACATTTTACAAGGTGATTATGATTTGAAGGTCATGCGACAAGAGTACTACATTAACCGGCAGAAAGCTTTCATAAACCATCTCATCAATCTGCTTGCCAGGCATCAGTTCTTAAAGATAGCCTGCCAATTGGAAAAGAAGCACATGCTTGGAGCATTTTCATTACTCAAGGTTATTGAGTCTGAGCTGCAAGCATACTTGTCTGCAACTGAGGGACGGGTGGGTCGTTGCCTGGCCCTGATCCAAGCTGCATCTGATGTTCAAGAACAAGGAGGAGTACATGATAGCGATCATTTTTTGCATGCTATCAGAGACCTGCTAAAAATATATTCAAATACACAAGCTTCACTGTCGACATATGTATCAGCTCCTGGTATTGTCCAGCAGATATCAGCTCTTCATTCGGATTTGATGAGCCTTCAGTCGGACCTTGAGAATTCTCTTCCGGAAGACAGAAATAGGTGTATTAATGAACTGTGCAACCTTATTCAAAGTCTGCAGCAACTGCTCTTTGCATCTTCAACAACAGCACAACCAATACTGACCCCTCGGCCATTAATGAAAGAGCTTGATGAAATGGAAAAAATTAACGCAAAGCTGTCTGCTGCGGTTGAAGAGGTGACCCTCGAGCATGTCAAGAAGAATGAGATTGTGAAACATCATTCACAAGAAATTGGACTTCAAAGACGAGTCTTTGTTGATTTCTTCTGCAATCCTGAGCGATTAAGGAGTCAAGTTAGGGAACTGACTGCTCGGGTCAGAGCCTTGCAAATTTCATAGCAATCCTAAAGTGTTTAACTTTCTATGCCTGCGAACCTGGGGTCGAACCCTATAAGGAGCGTTGACTGAGATTCAGACCAGGCATTCGTAGAACAGAATTAGCAATTTTCTTCCCTTTTTCCTTTCTTACTTGATTTGAAAAGGATGCAATCAAATACTTCTCTTTTATTCTGGTAGTGTAGATATTTGTAAGTGTATGTTGTATCATCTCTCCCTATGAAGTTGGAATTTTTGTGTTTTACATGTGCAAATTAACAAGTAAATTACATA

>C100463_65

GGACACTTTATTTTCATCTTCAAGACAGAACCGCACTGAGTTCTACTCGTATCACCAACCGTCACTCACTGTTCTCTTCTTGTATTTTAGACACCCAACCAAATGCACCATTAAATTTCTCCCTATTCGTCCTTTCATTCTCGCTAAATTACGAAACAGCACTCCTCTCCACCAGATCCGGTGGTGGATCAGCGTCGATTCGCTCTCATTCTCCGAATGCCGCTTCAGTTCCTCTGATTTCCGCTGCACCGGAATCCGGAACTGTGCTCTGATTCTTCTGCCTCTTTTGCTTCTCCACAATGCTTCTCTTCAGTCCTATTACTCCCGGTCAGGTCTCGTTCTTGCTCGGGATCACTCCGGTCATTGTGGCATGGATATATTCGGAGATATTGGAGTATCGGAAAAACTCTGTTTCGTCCAGAGCTGGTCACTCGGACATCAACTTAGTTGAAATGGGTGGTGATGCTGTTAAAGACGAAGACAAAGCAGTTCTATTGGAGAGAGGAGCTCTACAGTCAGGATCCCCGAGGGCGCGGAGTTTAACTGCCTCACCGTCAATTATCCGATTTCTTTTGATGGATGAGTATTTCCTTCTTGAAAACCGTTTGACACTTAGAGCGATGTCTGAATTTGGTCTCCTTTTGGCTTACTTTTATCTGTGTGACCGCACAGATTTCTTTGCCTCGTCAAAAAAGAGTTACAATAGGGATCTCTTCCTATTTCTCTATTTTCTCCTCATCATAGTTTCGGCACTGACTTCCTTTAAGATACATCAAGACAAGTCACCATTTTCTGGGAAATCCATCCTTTATTTAAATAGACACCAGACTGAAGAATGGAAAGGCTGGATGCAGGTTTTGTTTTTAATGTACCATTACTTTGCTGCATCTGAAATCTACAATGCGATCCGATTGTTCATTGCTGCATATGTTTGGATGACTGGATTTGGGAACTTCTCATATTATTATGTTCGTAAAGATTTTAGTCTAGCCAGATTTGCTCAGATGATGTGGAGGCTTAATTTCTTTGTGGTGTTCTGTTGTATTGTACTTAACAACAGTTACATGTTGTACTACATCTGCCCCATGCACACTCTTTTCACTCTAATGGTTTATGGGGCACTTGGTATTCTCAACAAGTACAATGAGATTGGGTCAGTCATTGCTGTAAAAATCATTGCTTGCTTCTTGGTTGTTATCTTAGTATGGGAGATACCTGGAGTCTTTGAATGGATTTGGAGTCCATTTACTTTCTTTCTCGGTTATACTGATCCGAATCCTGCCAAATCACATCTTTCTCGCTTGCATGAATGGCATTTTCGCTCTGGGCTTGATCGCTACATATGGATAATTGGAATGATTTATGCCTATTATCATCCAACTGTTGAAAGATGGATGGAAAAATTGGAGGAAGCTGAAATCAAGCGCAGAGTATCAATCAAAGCAACTGTTGTGCTGATATGCTCAGTGGTGGGTTATTTATGGTTTGAGCATATATATAAGCTGGACAAGCTTAGTTACAATAAATACCATCCTTATACCTCGTGGATTCCCATAACGGTTTACATATGCTTGCGGAATGTTACACAAAGTTTTCGTAGCTATACACTGACACTTTTTGCATGGCTTGGGAAGATTACACTGGAGACTTACATCTCCCAAATCCATATCTGGTTAAGGTCAGGTGTTCCAGACGGTCAACCCAAACTGTTGCTTTCCCTGATCCCAGACTACCCAATGTTGAACTTCATGCTTACCACTTCCATATATGTTGCAATTTCCTATAGGCTTTTTGACCTGACAAATACACTGAAAGTGGCATTCGTGCCCAGCAAAGACAACAAGCGTCTTGTACACAATTTAATTACAGCAACAACAATCTCAGTTGTTTTATATTCTTTGTCCCTTGGCTTCCTTAGGATACCTCAAATGTTGGTTTGAAAATTTGAACATGTATATACTGTTGCAAGCTTCTATGATAGTTCCTGTGGAATGGAAGAATACCGGGCAGCTGCTACCATTGGTTTGTCAGGCCAACTCTTTTACCGAAAGATGCATTCATCTCTTTTTTGCAAGATACAGAAGTAAAAGTGAACTTGTACCCATTAAAAGCAAAAATGAATCATTCTAATAGGCTAGCTTCAGTTTTAGGGGTTTGGAAGTTGTATAGATAAGAGGGTGGGTGCATGGCATCAATTATTTTTTTATATTATGCAACCTCATATTTTTTTGTTAAGTGTTGTAAAATAATATCGCTTATATTATCACCTACTATATTATACATCATACAGTAAATGAGAGTTTAGAAGAAGA

>C100465_65

GTCACATAATCAACCTCCAATGCAAAAAAACTTCGAGACAGATAAATAGAGTCTGAAAGCCAGACTAAGAAATCTATTATACAAAGAATGGAGTACAAAAACATGTATGATCATAAAAATAAAAAAAAATACCCCTGCTCGTACAATTTCTCTTTGGCAAGGGTTGCGTTACCCTCAGTATTCGTAGGAAATCAGTACTCTTTGCACACACGTAGTAATACGTTTAATTACATAATGAGTGGAATATAACACACAATCAAACAAAGGAATATTACATACAACGGCGACAGAAGCAGCAATTGTCAAAGTTGCAGCACTAGCACCCACCTAACGATTCGACCGCATCAAATTATTCTACTCAGATGATGATCAGCTTGCTCTATAGATTTCAACATGAAACAAAACTAGGCCACATCCAAATATAAAAACCACTCTAAAAGGATATGATGCAAGGAACCTCCGATAGCTCACTCCAGAAAGTGCTTTCCCAGACTATGCCATACCTCCCTCATGTAGATAATTGGAACCTGCCACTAGGTTTTTTTCTTCTCCATCCAAAGGCAAGGTAATACACAAAGGTCAATCCAGCCAAAATAACAAATGCAATTTGGAATACATTGTACTTGTACAAATATTTAATTCCTCGAAGAGACTCCAAAGTGTGGCCAAATATTTCCTGGGCTGCAGTTGCTTGAGCATAGTAGGCAAAGGCTGTGGAGCAGAACTGAATCACACTAATGGAACAAAGAAGAATAAGGCCCACATTAAACAGAAAAGAGTTCATGAGAGTTGCTCCCCACTTCATGGGATGTATAGTAATGAAAACTAGTCTCAGTCCAAGCATCATGGCACCAGCAATCACAGCAAGGAGAAGGTAGAAGCAGAAAAATGCAAATGCAGCAGTGCCGAGAAGACCCCAGACATCATCAAGCTTGATGAAAACTTGATTCAGGAAAGGAGAAAGAGGAGGATCAATTAATAGGTAGATAATAATATGAATGATCCAGACCACGGAAACAATCAACCCTATAATTCCCAAAACAAATTTTGCCAGATAGCCAAGAACTGTTAGTGCCCATGTTGTCTCAGCCTTTTCCCCTTGAGGATACACCTCTTCAAGAAGCTTTACGTCTTCTTCTAATTGAAACAACTCCTTCTCAACTTCCTTCACATTTTTACGAAACTTTCTACCCTTTGAACCACCTTTTTCTTCCTGACGTAGAGACTCAGCTGCTTTCTTCAGTTCTCTTGCTTTTTTACCCAGTTCAGTGGCTTCCTTGATATACTGTGAGCGAGTGATAACAGCCTTTGGACGCCGGATAAATGAAAATATAAGTCCTAATGGAAGACAAGCAATACCAACACCGCCAAATATAGCAAAAAGCACAGATCCAACTATAGTAGCAAGTGCAACAACATATTCTGGGAAGGTAGTACGCATGGTCCATGTTTTCTCTGATGAAGGACTGGCAGAGTAAGCTGAGCACTGATGAACACCACTTCCAATACATTGTTCACCACTATTGAATGTCCATGAGCTAGGAAATGCAGTTGTGGATGAAGAGAGATGCCTAACAGTAAAGTCCACCTTACCAACCAGCCCATATAAAATACCCAGAACGAGGGCGCACACTATAGCCGTGGTGACCATCCACAACAACGCACTCTTAATCCTCTTCCCCACACTCTTGTCGAGGTCGCCCTCGTAGAAGAACATGGCGAACGGGATGACGAAGAAGACGAGGACGGCGTCGAGGATGTAGACGGCGAGCCAGAGGTCCTTCATGGGGAGCGTGAGGCTGCACGCGCCATTGTAGATCGCGTGGCGGCAGGCTTGACGGTTCGCCACGTCGGCCGGGAGCATGAGGATGGAGATGGCGGCAATGGAGAGACCCAAAACGACGACAAATTTGGGGAAGTAGGCCTGGTTGGCGTCATCGGGATGCTGGTAGTTGACCAAGAGGTACACGTTGACGAGGAACACGATCACGCACACCACCACCGCCACGATCACCAGGGCGAGGTTGAAATCGCCCATCTTAGCTCCCGCGAAATCAGAGGAATCGGTAGATTCGAGTAAGGGTTAGGGTTTGTCGGATCTCGTTCTGAGAGAGAGAAAATAATAGAAGAATTTGGTTTCGTTCCGGTGGTTGTTTGCTACGGTTACCGGTGGTGGTTTTTATGCGAGGGTGGAACTACTGTTATGTCCTCGTGACCGTTGGCACAGAGAATGGGGTTATGGGGGAACAGTGTGGGGTATATGAGTAATTTCCATTGGCGTAGTTCGGTGTATAAGCGAAATTCAAACCAGG

>C100471_65

TTTCGCCACATGTAAACTCTTCACACCTAAAGCCTCCGCGTTTAACACGCTTGATTGGTCCTACAATACAAGCACCACCACAACGGTAACCAGGGAGAAAACTTTCTCTTTATATCGCAATCCATTCCCATCACTTCTGCCACCTCTCTCGAGTCTCTGCAAGTTTATGTACTCTACACACGATCCAACTTCTTCACAACTTTTCTTTCTTTCTCTCTTTCTTTCTTTACCTCATGGAACTCGAGATACGTCGTTTTTGTCTCTTCTTGTTTCTCTTTAGTATCGTCGCCGGGTCTCAGGCCTACACGTTCTACGTTGGTGGAAAAGATGGGTGGGTTTTGTACCCTTCTGAGAATTATAATCACTGGGCTGAAAGGATGAGGTTCCAAGTCAGTGACACTCTTGTTTTTAAGTACAAGAAGGGATCCGACACGGTTCTGGTGGTGAACAAGGAGGATTACGAGAAATGTAACAAGAAGAAGCCGATCAAGAAGTTTGAGGATGGTGAGTCGGAGTTTCAGTTTGATCGGTCAGGTCCTTTTTACTTCATAAGTGGAAAAGATGATAATTGTGAGAAGGGTCAGAAACTGATTATTGTTGTTTTGGCTGTGAGAGAACCTCCTCCGTACTCTCCAACACCTCCAAACACTCCCTATCTCCCAACTCCTCCAAAAGTACCTTATAATCCCCCTTATGTTCCAACACCTCCTCATGTTCCCACTCCTCCAGTAACTCCTTATCCACCCCAAACACCTTCCCCTGTTTATACTCCTCCAAATGTTCCAAACCCTCCAAAAACACCTTCACCTATTCATTATCCTCCCATTGTTCCTCCTACTCCTCCAAAAGCACCTTCACCTACTTACCATCCACCCTATGTTCCAGCACCTCCCAGTGTTCCAACACCTCCCAAAACACCCTCTCCTGTTTACACTCCACCAAATGTTCCTCTTCCTCCAAAAGCTCCTTCCCCAAGTAACCATCCACATCCACCCTATGTTCCAATACCTCCCAAAACACCTTCTCCTACTTACACTCCCCCTAATCTTCCCTATCCTCCAAAAGCTCCTTCCCCTAATAACCATCCACCCTATCTTCCTCAACCTCCACAAACTCCTTCTCCAAATGTTCCCACTCCTCCAAAAACTCCTTCCCCTGCTCACCAACCTTACGTCCCAGTTCCTCCAAACACTCCTTCTCCTATCTCCCAACCTCCATATACTCCAAACCCTCCAAAAACCCCTTCCCCTGGTCACCAACCTTTCGTCCCAATTCCTCCAAACACTCCTTCTCCTATCTCCCAACCTCCATACACTCCAAACCCTCCAAAAACCCCTTCCCCTACCCATCAACCACCTTATGTGCCAACACCACCCAAAACTCCTTCTCCTACTTCCCAACCTCCTTACATCCCATCACCTCCAAACACTGCCTCTCCAATATCCCAACCACCTTACATTCCAACACCACCAAAAACTCCTTCTCCGACTTCCCAACCTCCTTACCTTCCAACACCTCCAAACACTCCTTCCCCAATATCTCAGCCACCTTACATTTCATCTCCACCCACCACCACTCCTTCTCCCATTCCCCCACCTCCAAATACTCATTCCCCAACATCTCAACCACCCTACCCTTATGCCCCAACTCCAAAACCCAATTCCCCTATCCCCCAACCTCCATCATCATCCCCAACTTACCCATCACCACTCTCACCAGCATCACCCTATCCATCCACCACCCCTCCTCCATCATATCCACCTTCACTTTCACCTTCAACCACTTCTTACCCACCTTCCTCCTTAGCTCCGGTTGCTACTCCGGCTGCTACTCCGCTTCCCTCCACTACGTCGCCACCAGCGCCGTCTCCGGGAACCGCCGCTTCTCCTCGGGGAACCGCTGCTTCTCCTACGGGGTCCCTCCCGGCAGCAACAACTCCATCGCCGTCGGAATCATCGTCGAACGAAACGACGCCGGCCCGGCCAAACAGCGCAGCGTCTGTAACGGCGTCGCCTTTAGGAGTGTACTCACTAACCATTCTGGTCGGTGCTGCTCTTAGTACCATTCTTGGTTAGAAAATCAAACTTGTTGTTATTATTAATTTTTCTTCTTCGAACTTGTGTTATTATAGTATATAATCTGCACATTTTATTTTATTAATATTGTTATTTGGACATATGAGATCACATCTCATAATTTAATTTTATTGAGTAATAAGAATCTCAAATCTGTGTATCTATGGTTACGTGCCTTGCCTTCAAATTCCTCAGCTGCTACTTCGGCAACTTTTGTTCTTGTTCCACTATGTTCCCAA

>C100475_65

CTCTGTTACCACTCAGCCCCAAAACAGAGCAAAACAGAGGATCCACTCCTAATGGATCCTCCACCTCTTTCTGCCGCCACCATGCTCGCTACCACCACTCATAAAAATCATCCGGATTCGATAGAGTCTTCATCTCCACGTTCCCGCAATGCAGAAACGTGGAATGATGAGACTTTACCGGTCGTTCCTGGCGCCAAGCTGCGCCTCATGTGCAGCTATGGTGGCCACATCATGCCTCGCCCTCATGACAAGACTCTCTGCTATGTCGGCGGTGACACGAGGATTGTGGTGGTGGACCGCCACTCTTCGTTGAAAGACCTGTGTGTAAGGCTTTCCCGAACCATTCTCCATGGAAGACCCTTCACACTCAAGTACCAGCTTCCCAATGAAGACCTCGATAGCCTCATCACTGTCACCACTGATGAAGATCTTGACAACATGGTGGAAGAGTATGATCGAATTATGGCGAAAGGTTCGGCTTCTTCAAGGCTCAGGTTGTTCCTGTTCTTCAACAAACCGGAGGCCACAGTTTCGATGGGGTCTTTACTTGATGATGTGAAGTCAGAGACATGGTTTGTTGACGCACTCAACAACTCCGGAATGTTAAACAGGGTAGTTTCAGATACTGCTGCAGGGGATTCTTTTGTGAACCTTGATGGTGTTGGTGGAGGTGTTAGTGCTAGTGGTTCAAGCAACAACTTGGAGGCTTTGCCTGATACTAACAATAACAAGGCTAAGAACTTGCCTGATGTGGTGCAATCAAGTCCTGGTTCACCTATGATGGAGAACAGTTCTTCTTCTTCTCCTTCTTTCTCTCCTTCTCTGGCTAATCTGCCTCCGATTCGGGTTCGTGTTGATGACAATGGTAGTAGGCTTCAACAAGAGAACAAGGTTGGGATGATGGTGGAGGAGCAGTTTGCTCGGATGACAACTGCAAGTGGGGTGAAGCCAGATGATGGGTTGGCAAATGTGATTTCTTCTGGAGTGGTTGTGCCTGTAATTCCTGCAGCTGTGACTATGCCATCTGTTGGGGTGGTTACTACTAGTGATAATGTGATGAATAGAGTTGTTTCAGAGGATGAGAGACCAGATCCCGGGTTTCGGAAGCCCCCTTTACCATTGCAGCTTGTGCAGCCAAGGACTAGTGGTGGTTTGGGTTTGCCTTCTCCAGATTCAGTTGCAAGTGATAGTAGTATTGCATCTGCAAATTCTTTCTCCAAGACTGTTTACTATCAAGATCAAGTCCAAGCTGCAGCTGCTTTCCCAAATGCCAAGAGTGAAATATCTGATCAGGTGATTCACGTCCATGGACAAGTTCAGGATTCTGGCTACGCGTTACCCCCACAATTGGATCAAAATAGACAACTTCAGCAGCAGAAGCCCTTGGTCCATGCCAACACTCACTACATCCACCACCCTGCAGCCACAGGTCCAGTGCCAGTTTCATCATACTACCCAGTTTATGCCGCACCATCACCACAACAACTTCACCCTCCAATTGGTCAGCAACAGTACCCAGTTTATATGATGCCTGTTGGACCAACACAAGTAACACAACCATACAACATGGCTGACCCAAATGTGTTAGCCTCAGGGAGGACCCTTATATCACAAAGTGCTGTTACCTCTGCAGCTTACAAAGATGGTACTCCACCAATCTACCCCACCAAGTCAGTTAGCCCTACTATACCTGAGGTGACTCCAACTGTTTACAACACTCCTCCTGTGGCATCAAATCCTGCGTATGCTCCAATACCTCCCAATCAATTTCAGCCACAGTATGTGGGTTTGCCTCAGTTCCATCATCCACCACAGTCAATTGCTATGGCTCCCTCTAGTACTACTACCAATTATGGTTATGAATATGGTGGCCATATGCAAGACCGAGCATACTACACCCAACAACAAACTACTAGTGCTCCGTTGCTTCCTCAGTACCAATCAATGACCCCAGCTGCAGCTGCTGCAGCACTATCAGATGCTTCAAATCAGTTTCCTGCAGACCAAATTCAGCAGCAAAATAGAGCATCTCAGCCAGTATGAATCAAAAGTGGAGAAAAATGAATGAGTTCGGAGGAGGCATATTGGGGAGGGGTGGAGTGAGGGGTAATTGGACTTTACTTTCATTTTTTAACAATGAACATAAGAACCCTACTTTCTACAGAAAAATAAGGACTAAATCATAGGTTATTTCTTTTTATCTGTATGGAAATGAAAGACATGTAAGACCATATTTTGCAAACCTAGCTTTTCTGATAGTCACAGTTACTCAGTTGAATAAAGCAAGTTGCTGCCATTAGCTTTATTTAGGGACA

>C100477_65

TTCTTTATCCTCCTTTTTATTGTGAATTATAATAATTTTATGATCAATCCCATGCAGCTGTCAATTTTGCGGGGAGTGCGAAGCATAGGACAGTTGTGGAGCCCAAGCCAAAAATCATGGCACAACAACAAGATTCCCTATCTAAATAATAGCACAATACAACACCAAATTGAGGGACCCAACTTGACTTTGACATAACCTTAATCTAGTAGGTTAACTACTTGGCACCAATTCTGTTACCAACTTCACTGCTCCTGTTATTGTCAAAATTGAAATTGAGACAGTGACTAAATTACATCTTTAACAACTTTTGACTAAACCAAAGGAATCTCAATGTGCAAAAGAGGTAGAAGGTGTCTCAATTGCATGAGGGGTTGGAGTGGAACCTTCTGATCTAGATTCAGTGGATGATCTATTTTCAGTATTTACTCTACGAATTTCCTCCACCATTCTCACCACCTCACCAATTGTTGGCCTCTGATCTGGTGTTCTCACAACACATGCCATCCCTATTTGTAGCATCTCAACCATTTCCTCCTCAATATTTGGATACCTCAGCAACTCCACATCAAACACTTCTGCAGTCCACTCCTCTCTAACCACAGAATTCACCCATCTAACCAAGTGGACAACTTCCTCACCTCCTTTGGCATGTAAAGGAGACCTCCCAGTTAGAAGCTCAAGCAGCAGCACACCAAAACTGTAGACATCAGACGCCGGCATTGATTTTCGTGTGTCGGTTGCTTCAGGTGCACGGTATCCTGTTGTCCTCAATGCTGGATTCATCAGTGTTGCAAGACCAATGTCAGATAGACAACCATATCCTTGGGAATTGAGGAAGATGTTAGAGGCCTTTATGTTTCCATGGAGGAGTTTCCCTCCTTGTTGAGCATGGATGTGAGCAATGCCTCTTGCTACACCAATTGCTATTTTCAAACGGCTATCCCAGTCTAAAGAAATTCTGCCTCCTCTTTTGCCATGCAACATTGAAGACACACTGCCCTGTTCGTAGTAATCATATACCATAAGTTTCTCCTCCTTTGAATAGTAATATGCCCTTAATGCAGTCACATTATCATGCCTAATCCTGCCCACCAACTCCATCTGTTGTTCAAACTCGCGTTTTCCAACCGTGACATCCTTCAACCTTTTCACTGCCACTGTTGTTGCATCTTCTAGAGCAGCCTTGTAAACTGTCCCAAACGTGCCCTTTCCAAGCACCTCAGCAGATGCTCTCAACAAGTCCTCCAAGTCAAATGCAAGATTGCAACCCTCAAAGAAAACAATTTTGTTCTTGTCTCGACTTTCAGAACCTTCTTTTTTCCTGGATACTTCTCTTTTCTGAGACTTCACTTGTTGTCTATTTGTACCACCTTTCTCATAGCAGCACACAATCACAAAAGCTGCTATCACTGCAATTCCCAGAACACAAGCACCAATAATGATACCCAACAATGCTGGTTCCCTAAGTCCTTTGGATTTTTTCTTTGTTGGATGAGGATTTGGGGGTTGAACAAGAAAAGAAGGGGAAAGTGCATGTGAAGAAGTAAGATTGTTACCAGAAAATGCCCCACTTGGAAATCTCTCAAGGGATTCAGGCACAACTCCACTAAGGTTATTGTTGACAAAATTCACCTCTTGCAGGGTAGGGATATTGAGATCAGGAATATCACCTGAAAGGGAGTTGTTGGCGAGTACTAAAGAAGAGAGATGAGTCAAATTTGAAAGTGAAACTGGGATGCTGCCATTGAAAGAATTGTTGGATAAATTGACAATACTAAGATTCTTCCAAACTGAAAAGTCTGAAGGCAATGGACCAGAAAACTTGTTGAATTGGAGATACAGATAGATCAAATTCTTTAGCTGAGAGAAACCAGAAGGGAAAGAACCAGTTATGCTATTTGATGCAAGACTCACAGTTTGGAGTGCTGAGAGACGGCTAAGAGTGTTGGGTGGGATTGGACCACTCAATCCAGTTCTGGTCAGTTGGAGGGCTATAACTTGTGATTGCTCAGAGTTGCAGGTCACTCCTATCCACCTTTCGCACACAGAAGAACTGTTATCCCAGTTGATCAGATGATGAGAGTGGTTAATGTTGTGAAGGAAATCAAGCAATGCTTGTTTATCTTCCACTGGCTCAGCTCCTACTCCAAACAACATTGCTCCAATCATGAGGAAGAGGCCCAGCATCTTGGCCATTTTTTCTAGCTCCAATGGATGCAAAACAAGGACTCCCCCAGGACACCAAACTGTTGATTGTAACTGATCCTAAGGTGGTGCTGCTTCTGCTTCCCCTTGTTTATACTTTCTAATCT

>C100479_65

AGAAGGAAAAGGAGGATGAATAACTGAAACATAATTCATCCTCTTTTTCTCTCTGTCTGAATATTTTTACAAATTCTACTGAATTCATTCTCAGTTTCGTGTCAGCCGTACCTGATCTCAATCACCGAGACGGAAGTGTCCCTAACAGACAAACCTTTTTACTTTTATTTTTCCTTTTGGGGTAAAACACATAAGCTTTTGCATTTGCAAGCTCATTGTAACAGCTACCAAACCAACCATCATAATCCTTGAATTTCTCATTTGTCCCAAATAAAATTGAATTTTGAGGAACTCTTTATTGACACTCCGTATTCCCAGCTGCAGATTCTCCTCGTCAATTTCAGCATAGTGATTGTGAAAGCTTAACAGAGGGATTGGTGCTGTTGACAGTAGTCCCTGAAATGTATATGTCTGTGGCTACCTCTGCCACACCGCGGGGTGGATTGCCTACTGACAGTGGGGACTCTGTTGTAGCATTAGATCAAGTTCCGCGATGGAATGATGCTGATCAGTCCCTACATTTTCCTGATCCTCTAGCGTTGCCCTTGGGAGCGGACAGTGGTGATGGCAGTGAATCGGTGTCAAAATTTCCTGTTGACCCTGAAGTCAATTCAAAGATATATTTGTGGAGGGGGAATCCCTGGAATCTTGAAGTAGATGCCGTGGTAAATTCAACAAATGAGAATATGGATGAAGCACACAGCAGCCCTGGATTGCATGATGCAGCCGGACCTGGTCTAGCAGAAGAATGTGCGACTCTGGGAGGATGCCGAACAGGGATGGCTAAAGTCACCAAGGCATATGACCTTCCTGCCAGGAAAATTATCCACACCGTTGGTCCAAAGTATGCTATTAAGTACCATAATGCTGCAGAGAATGCTTTAAGTCATTGCTATCGTTCTTGCCTGGAGCTTCTGGTTGAAAATGGGCTTCAAAGCATTGCTATGGGTTGTATATATACGGAGGCAAAGAACTATCCCCGAGAACCAGCTGCACACGTAGCTATAAGAACTGTGCGGCGATTTCTTGAGAAGCAGAAAAACAACGTAACAGCTGTTGTCTTTTGCACTGTGAGCACAACTGACACTGAAATATATAAAAGGTTGCTTCCGCTGTACTTTCCTCGGGATAAACATGAGGAGCAGGTTGCTTTAACTAAACTTCCTGCTGATGTTGGGGATGAAAATGGTGAGACAATCATGGCTGAGCGTAAAATCAGAATAAAGCCTTTGCCAAAAAAGAGTGTTTCAAGACCACCTGAATTTGAGGTTGATCTTCCAATTAGTGATATTGGCTTGGTTAGCAGGAATTCGTCTTATTTAGATTCATTTCTGGATCCTGCCTTCATGTCCATGATTAAAGACCCTGATCTAAGGCGTATGGAGCAGTGGGAGAAAACTGCTGAAGCACAAAGGGGCTGGAATTGTGCTAAATTGCTAGGATATGGCAACCTTGGTGGACCCACATTGTCTGCTGCCGAAGAATATTCTCTACACTCACGATACCTATCTAAAGCAAATTCTTTAAATCTTTCTGAAATTGCAGAAATGAAAATTGTCTATCGTGGAGGGGTAGACAGTGAGGGTCATCCTGTCATGGTTGTTGTAGGGGCTCACTTTTTACTCAGGTGTCTTGATCTGGAACGATTTGTGCTCCATGTGGTAAAGGAGTTTGAGCCTTTAATACAGAAGCCTTTTTCTATTGTCTACTTCCACTCTGCTGCTTCTTTGCAGATACAGCCAGACCTGGGTTGGATGAGAAGATTGCAACAAATACTAGGTCGGAAGCACCAGCGCAACCTGCATGCAATATATGTCCTTCATCCAACTTTTGGACTAAAAGCAGCTGTATTTGGGCTTCAGTTATTTGTGGACAATGTGGTTTGGAAGAAAGTGGTATATGTTGATCGACTTCTGCAGCTCTTCAGATACGTACCTCGTGAACAGTTAACCATCCCAGACTTTGTGTTTCAGCATGATTTGGAGGTAAATGGAGGTACCGGTCTCATTGTGGATCCCAGAACCAAATATGTGTATAACAGACCGTGAAACTCATGTACTTTTATAATTAGTGGATAAAGAAGGGCCATTCACTGGAGTTTCTTCATTAATGTCTTGCCCTTTTGTCGTCAACAGGAAGAGGGCTTGTATTAGTTTGAAATGTGGATGGAAGCATTCCTTTTTTATCTTTTTGACTCTTTGAAAGAGTACTAGTTCCCTTGTATGTATATGTAATTGATTATGGAAGCTAATTGGTTTGATTTCTTGGATTGTTTGTACATTTCAAGGGTGATGAATTGAATAAAATTAGGCTTT

>C100481_65

CACACATTTCCCAAATGGAGGAATTGCAGTCATGAACGCCATTCTCTTACAATCACCACACTCACCCTTTCAACGCGCCGCCGCCATCACCACCGCCGAACCCCGCGACCAGAAGCTTCTCTCGCTCTTGCGTGACCGAAAAACCGAAGAAGCCTGGCAAGCCTACACCCACTCCACGCACCTCCCCAATCCCACCTGTCTCAGTCGCTTAGTCTCTCAGCTGTCTTACCAGAACACTCTCTCCTCCCTCACGCGCGCCCAGTCCATTGTCACGCGCCTCCGCCAAGAGCGCCAGCTCCATCGCCTCGATGCCAACTGCCTCGGCCTCCTCGCGGTCGCTGCCACCAAGGCGGGCCACACGCTCTACGCCGCCTCCGTCCTCCGCTCCATGCTCCGCTCTGGCTACCTCCCCCACGTCAAGGCCTGGACCGCTGTCGTCGCCCGCCTCGCCTCCGCCCCCGACGACGGCCCCGCCGAGGCCCTCAAGCTCTTCCGCTCCGTCACGCGCCGCCTCCGGAAGCTCCCCGACAAAGACATGGCCGCCGCGTCGCGCCCCGACACGACGGCGATCAACGCCGTTCTCAACGCCTGCGCGAACCTCGGCGACGCGAAGGCTTTCTTGCAGGTGTTCGATGAAATGCCACTGTTCGATGTTCAACCTGATGCGCTGAGTTATAATACGATGATGAAGCTGTGTTGTAAGATTGGTAGAAAGGACTTGCTTTCGTTTGTTTTGGAGAGGGTTCTTCAGTTGGGGATTCCTTTTTGTGTGACCACTTTGCAGTCCCTTGTTTCTGCTTATGTTGAGTTTGACGATTTGGAAACTGCCGAGAAGTTGGTTCAGGCAATGAGGGAACAAAGGAGGGATATTTGTAGGGTGCTCAGGGAGTGTAGTAATTTAGAGTATTTGAGTGGAAATGAAAGTGATTATGATAGTAATAATGATAATGATGAGGATGAGGATGAGGAAGAGGATTCTGTTTTTGAAAAATTACTTCCTAATCTGGTGGATCAGAGTGGCAGTGAGGCTGAGCTGCCCTTGTTGCCTAAAGGGTATGCTCCCAACACTAGGACTTACACCACTCTAATGAAGGGTTATATGAATGCAGGGCGTGTGAGTGATACGGTGAGGATGCTTGAGGCTATGCGGCGTCAGGATGATAAGGGTAGTCACCCTGACCATGTTTCCTACACCACTGTGGTTTCAGCTCTTGTGAAGGTGGGTTCCATGGACCGTGCTCGTCAAGTTCTTGCTGAGATGACAAGGATAGGTGTGCCGGCGAACTTGATAACTTACAATATTCTCCTCAAGGGTTATTGCAAACAGCTGCAGGTAGATGAGGCAAAGGAATTGCTTAAGGAGATGGTTGATGATGCAGGGATTCAGCCTGATGTGGTGTCCTATAATATACTCATCGATGGGTGTATATTGGTCGATGACAGTTCTGGGGCTCTTTCCTTCTTTAATGAGATGAGGGCAAGAGGAATAGCTCCCACCAAGATTAGTTACACTACTTTGATGAAGGCTTTTGCGTACTCGGGTCAGCCGAAGCTTGCTCACAAGGTGTTTAATGAGATGGACAATGACCCTCGGGTGAAGGTGGATTTGATCGCGTGGAACATGTTAGTGGAAGGGTATTGCAGGTTGGGGTTGGTGGAAGAGGCAAAGAAAGTGATTCAGAAGATGAAGGAGAGTGGGCTTCACCCTGATGCGGGCACCTATGGCAGTTTAGCCAATGGCATTGCTTTGGCAAGGAAACCTGGAGAGGCCCTTGTGCTTTGGAATGAAGTGAAGGAGAGGTGTGACATGAAAAAGGAAGGGGGGAAATCTGATTCTTCTGTTCCTCCATTCAAGCCAGATGGAGGGCTTTTGGATACTCTGGCTGATATCTGTGTGAGGGCTGCTTTCTTTAGGAAGGCTTTGGAAATCGTGGCTTGCATGGAGGAGAATGGAATAGCTCCCAACAAGACCAAGTTTACTAGAATCTATGTGGAAATGCATTCAAGGATGTTTACTAGTAAGCATGCTTCAAGGGCTAGACAAGACAGAAGAATCGAGAGGAAAAGAGCTGCTGAGGCGTTTAAGTTTTGGCTGGGTTTGCCTAATTCCTATTATGATGGAAGTGAGTGGCGGTTAGAACCTATGGAAGGGTATGGCCCTACCTCTGATTCGGTTTAGTTGCTCCCCTTTCATACATCTCAAAACATGCTGAAGAATATCTGCAACAGTACAGAGGAGAAAGAAATGTAACTTATAAAAGGTGGCTATTGTACTCTACAGTGTAATTTTTCATTAAAACACGGTTTTCCCAGAA

>C100485_65

GGAGAATATTAAACATTTAACGTCACAATCGCACGTATGTCATAAAGACTTGGCTGATACCAATTCAACTGTATACTTACTGGTGTGGCCAACCTCTGTTATGAAGTCAAAAAAACAAAAAATTCAGAAGAAAAGGCTATGGTTCTAAATACTTGTGATTTTAGAAAAGAAGGGTCCATTAAATTGTGTCTTTTCTATCCTATAACATGACAATATCATGTTAGTAAGCAATATGAGAGGATTCAATCCCAAAAAATAATAATATATCATCAGAGCTGTAACGCCAAATTGGGTTGCAACTGAGAACCAGCCTCCACGTTGGAACTACTAGGTGAATTTGTAGTTCCAATCCTTCCATTCAAGTCATTAGCATGTGCAAAAGAATTCTGTTTAGTGGGAACTTCCAACCCCTGCCAAGATGATTTTCCAGCAATTCCTTGAGACCAACTTCCTGGCTCAAACCCAGATTCCCTGGTCAATAAATTTCCGGATTCTAATTGACTTGATTTGCTCACGTATTGTCCCATAACAGAAGGATTAATATTTGTATGCGCAATGCGGCTGCTTATGGGATCCATGTCAAACACTTGAGGAACAACTCCAGCACTCGGAGACCCAGTTAGGTCAGCAAGTCTTACCATCTTCATCTGTGGTGAAAAATTAGAACCTAACCAATCATGTGAACCAAAATCATCAGAGTGCGGCATGGATTCCTGCTGAACCCTCACAGGAGTTACGGAGTCAGACCCACCACCAACGTTGATTGCTGGATTCAACTCTTTTTGGTAATTATTTATAACCGTGTCTCCACTTTGCAAGCATGATCTATTTGCAAGGGGGAATGATCCAGATGGAGAAGAAAATCTACTTCGACAATCCTCTGCTACAGGAGGGTCTGAAGTTCTACCCTCATCACAAACTGGGAAGTGTTGCCTCTGTGACCTGTCATCTTCACTTACCCATCCAGGACCAAATTCATGTCCAGCAGGCAAAACACTTCTGATTTTACTTGCAGCAATCTTCCAAACAACAGGACCAAGATCTGCAGCAAAATGGGCTAGACTTCTTGCATAACTATGCTTCATGTGTATACCTACTGCAAGAAGTTGCTTGACTTCATCTTCAACTGTGGTCAACACAGGTCCTTCATTTCCTGAAGTCGCTGGTATTTTGTAAGAATCGCGCCTGGTCTCATCAACTGCATACTGCTTCTTTCCATATCTTAGCACAGCTTTTACAACAGAAGCTGGAAATTCATTCTCCCATTCAGACCAGCCAGTATTATACCCTCCACTGTTAAAAGTGCTGTTGTAGGCCCTGGCAGATGAATCTGTGGGCTGAAATTTGCTTGGTCCTTTCCTTAGATTATAACCGTTGGAGCCACTAGCAATATCCCCGCCAGAAGCAAGAGTTGCATCAGAAGAAGATTCAGGACCAACACGCTCAGCAGGAGACACACCCAGTGACTTTCTTGAGAGCTTGCCTGGTGGCCTTCCTCTCTGCACAACTTTAGGCTGTGGTTCACTATCATCACTATCTTGTCTCAGATTCTCAAAGTCCTTCCTTGCGATCTCTTGCATGGCTCGAGCCTGCCGGTGGTAAATGGTATCTGATGAATTATATTGCATTGCATTGGAGCAAATCAAGAACACATCTTTCTCAAAATGTTCCAAGTCGGTATATAGTCCTCCATCCAATTTCTTTCTCACAGTTCCAAAATCCATCGGGTGTTTAATAATGTCATGATAATCAGGAAGCTCTTCCGGATCCACAGGCTCAGAGAACACCCCATGTGTGTCCTTTTTTTGAAGCCTGTCAAGGATAAACAGCAAGAGCTTTTTGTCTGGCAAAGGTGTAGTGGGACCGGACTCCCCCTGCGAACCATGTTTACTGTCAGTCGCTTTCGGAACCTTCCCATCCGTTTGGTGGTGTTGGAGAGGGTCAATCTTACGCCTCTTGGGATCGGAATTGAATGGCTGGGAATTGGGGAATAATGTTGGGTGGTGGAGATGGGAATTGAGTCCGACGAGGAGCTTGTGCTTCTTCTCCTTGCGCTCGTCGTCGTCATCGTCGTGGGGGACAACGTTGGTGTTTTTGGAGTGGGAATTGGTGGAATTGGGGTTGTTTTGTCTGTGGTTGTGGTGGTTGTTCTGTTGCTGTTCCTTCTTTAGAGATCGCTTTTGAAGATCCAAAAGAGAGGGGCGTCCCTTCTTCTTCTTCTTCGTCATCGTTTCAGATACCTCGCCCATTGTTTCCTCCTCTTTTCTCGCTCTCCCCAAACAACACACACAAACAAACACGTCCAACACCTTCTCT

>C100487_65

GAAGGTTATAGATTAGATTAGAGAATTGCAAAGAAAGAAAAAGAGAAAATAGAAAAGAAAAAAAAGAGAAATTCAGTGGAATAATCGATGAAAAATTTCTCTATATAATAAACACATATCCACACACCATAATCTCTTTCTTCTGCTGGGGTAACTGCATAGAGAAAACGAGAAGAAACGGTGCGTAGCGCGGAACGCGGAAACACGAGGAAGGAGGAATCGGTAGTGCTCCACTGGCGATCGCTTATCGGCGCCGGCGAACTTCAGATCGCTGCTCTTGCTTCTCCGGAAATTTTGCCATGAGATTTTTTCAGAGGCATTTTTTGTTATAGTCCTGCAGTGCTGTGTTTTGGCTCACTGGTGGAAAATTGTTATATATGGGCTGAGGTGTGCTTTTCATGGGAGGTGTGGAGGATGAAGAACCAGCTTTGAAACGAATGAAAGTATCCTCTAAAGGATTAGTTGGTCTCTCAAACGGTTCGTCGTCAGTGGAGCCTGTTGGAGGCTCTTCAAGTGACTTGATGGCGCGGCCCCTGTCTTCTGAAGGGGACAGCGAAGTTATTGGTTCCAAAGGGGTTATAAAAAGAGAGGAATTTGTTAGGATAATCACAAAGGCATTGTATTCTCTTGGTTACAAGAAGAGTGGGGCACGCCTAGAGGAGGAGTCTGGCATACCTTTGCACTCGTCTGTGGTGAATCTGTTTATGCAGCAAATACTTGATGGCCATTGGGATGAGAGCATAGTCACATTGAATAAAATTGGTCTAGCAGATGAAAATGTTCTCCGCGCAGCTTCATTTTTAATATTGGAGCAGAAGTTTTTTGAACTTCTTGATGGGGAAAAGGTCATGGATGCATTGAAGACATTGAGGACTGAGATTACTCCTCTTTGCTCTGATAGTAGTAGAATTCGTCAACTTTCTTCCTGCATGCTTTCTCCATGTGGCCAGGTTGGTTCTTCAAGGCGAGATATTGTTCAGGTGAGGTCTCGGGCAAAACTTTTGGAGGAATTGCAAAATCTGCTTCCCCCAACAGTACTGATACCTGAAAAGAGATTGCAACATCTAGTTGAACAAGCTCTTATCTTGCAACGAGAGGCTTGCCTGTTTCACAACTCATTGGATAAAGAGATGTCATTGTATTCAGATCATCATTGTGGAAAAACTCAGATACCTTCTAGGACATTACAGATATTAGAAGCACATGATGATGAAGTTTGGTATGTCCAATTTTCCAATAATGGGAAATATTTAGCTTCAGCATCAAATGATCGATCTGCAATCATTTGGGAGGTTGACATGAATGGTGGACTGTCTATGAAACATAAATTGTCTGGTCACCTGAAAGCTGTTTCTTCAGTCTCATGGAGTCCTAATGACCAAGAGCTCCTCACATGTGGTGTGGAGGAGGCTGTTAGGCGTTGGGATGTCTCTACTGGTAAGTGCCTGCAAGTTTATGAAAAAAATGGTCCTGGTCTCGTCTCGTGTGCATGGTTTCCAAGTGGGAAATATATACTTTCTGGCCTCAGTGACAAGAGCATTTGCATGTGGGATTTAGACGGGAAAGAAGTGGAGTCTTGGAAAGGACAGAGAACCCTTAAGATTTCTGACTTGGAAATAACAGGTGATGGGGAACACATTCTGAGTATTTGTAAAGACAATGCAATACTGTTCTTCAACAAAGAAACGAAAGAAGAGAGATTTATTGAAGAGGACCAGACAATAACTTCCTTCTCTTTGTCAAAGGATAGCAGATTCTTGTTGGCTAATCTTTTGAACCAAGAAATACACCTCTGGAATATAGAAGGTGATCCTAAACTCATTGGCAAGTACAGAAGTCATAAACGTACCCGCTTTATTGTCAGATCTTGCTTTGGTGGTCTTAAGCAATCTTTTATTGCTAGTGGTAGTGAGGATTCTCAGGTTTACATATGGCACAGAAACTCGGGGGATCTAGTAGAGGCATTGCCTGGTCATTCTGGAGCTGTTAACTGTGTGAGCTGGAACCCAGCAAACCCTCACATGTTAGCCTCGGCCAGTGATGACAGGACAATTAGGATATGGGGCCTTAAGCGCCTCAATGTAAAGTACCCCCCCAATCTACGGACCAATGGCGTCCATTACTGCAATGGTGGAGGCGAACTTAGATGAACCAACCAAAATGCAAGCTATTTTAATTCCATTTCTTTTTTAACATTGCAATTGTGTAAATACCTTCACTATCCACAAGTAGTAGACTGGACACACAGACAGACATTCATACCAAACATGCACCTCCTGACTTCTCAAAATAAATTCTTGTTTCATTTAAAAAAAAA

>C100493_65

GCACACTAAAATGCACCAATAAGTTATTGAACTTGACATCTTTAAGCAAGGTCTTATGATCAAACCTTGTGGATGCAGTTGAGAAAGATATAAAATTCTGTACACACCCCAGTATTATGGGTTCTCTTATCTTAGAAATAGCATCCACCTTCATACACAAACAATATCTATCCTACAAACAAACAAAGGGACACCAAGAACTGACCAATGCATTATGCATGGCAAAACAAATATTAAAATTAAAAATATCTCCAGCGGTACCAGCCCCTCTGCAATGCCCATCATAGGTATCCAAAAATCCAAGAATCTAAAAAGCTATGGAAGAAAATAAAAAAGAAGCTACCACTTTCACTTTATACATCCCTCCAAATTCTGAATAAGAATCCATCTTAAGGTCATTTGTGTTGTCACCCTCTTCTCTCTTCTCTGGAATCCTGAATCTCACTCCTGTCTGTGTCAGAATATTCCTTCTGGGAGTCCATTTCTCCACTACCCCCTTGATTTCTTCTTCGGTGTCTTCGGTCCTCTCTTGGTAAAGGGTATTCCTCTGATTCCAGCATACGAACGACTTGGCCCATCTTGGGCCTTTTCTCAGAATCTGGATCTACACATCTTAAAGCAGTTAAGAGAGTCCGTTTTAAAGCTCTAGTGGATGGCTTCACCTCAATGTTGGGATCCACCACTTCTTCCGATCTCCTATTTCCAACCATCATCTTCAGCCAATCAACCAGGTTCACTTCATTTGATGGTCGACCATAGTTGACTGGATCTCTTCCAGTAATTCCTTCCAATAGCAAAACACCAAAGCTATAAACATCACTCTTCTCATTTAGAAGGCCAGTATTTGCATATTCAGGAGCCACGTATCCAAAAGTTCCCATAACTCTTGTGGTGACATAACTCTTCCCAGCTCCCAATAACTTTGCCAGGCCAAAATCAGAAACCTTGGCATTGAAATCATCATCAATTAATATGTTGCTTGACTTGACATCACGGTGTACCACCTTTGGCTCAATTGCTTCATGCAAATATGCTAGCGCTTTGGCTGTGCCAAGCAAAATCTTGATACGTGCTTCCCAGGTAAGGTATCCGTGGTGGCGCATAGCTCCATGAAGCCATTGCTCTAAGTTTCCATTATTGACATACTCATAAACTAACATCCTGTGAGTTCCCTCAATGCAGTATCCCAAAAGGCGAACCAAGTTTTTGTGTCGTACATGGCCAATAGCTTCAACTTCCACTCTAAATTCTTTCTCTGCTTGACCAATGTTATTGAGTATCTTTTTAACTGCCACTGGAGTTCCATTGACCAACTGCCCCCTATAAACCACTCCATAACCACCTTCACCAAGGATATTTTCTTTGCAAAAACGGTTTGTGGCAAGTTCAAGATCCCTCAAGGTAAACCAGTGACCCCAACCCAAGTGAGAGAATTCCGGAAGGCCAGATAGAGGTGAGGGAGCTGTTATAGGGTGCGAAGAAGATTGCTTGTACACTGTAAACGTGCCAGAGCTACCTTCTTCTCCTGACTGTGAGTGTGAACCACTTCCATCTTTCTGTATGCAACGAAATGAATCTGAGTGAGTGCTGCTATTCCCATTCTTTATCTTCCCCACACCTAAATGAACCATGACATGGTTTGATTCCTTGTCACTGGACTTGTCCTGAATTGTAAGAAGTATTCCATCACGAGGAGAAAATGCATTAGTTGACACTTGTTCAACCCGCACTTCCTTGATTTCCTTTGAGACAGTTGGTATTTGACTAAGGGGAATCCTGTCTTTCTCTTTTCTAGATTTCTTCTTTGACGTGAGGCAAAGTGAGACCACAGAAAGAATAACTATTATAGACAAACCAACTACAATTCCAATTATTTCCCAAACTTTGAGACCAAAAATGGGAGTTTTCTTCGATAATTCAGCATTAAGACCAGACCCCATTTCTCAGAAATCCTGGATTCTCTTATCAGTACTCATTCAGAAGAACAAGTGAGAAGCCTCAAATCCCAGATCTTTGAAACAGCCCCGTCACAAACTAAGAGGTTCAAACAAGAGAACAAACTCAAAAGGCGAGATCTTTGAGGCCTCCGAATATTGGGGTGTCAGCAAAAGCAAGTATGTCTGTATAAGCAAGAATCCTATTCCCATATATATGTGGAAAATGTGAGAGTGAATCTAAGTTGGAGTGGAGCTATCTGAAGAACAGAGAGAAAGGTGGTCGGGTAAGAAGGAGATGAAGGGAAATGTGAGTGATGATAAAGAGGAAGGGGTGGAGTTATCAGTGGTAGGTGATGAGTTTTGGAATAGATGCCTTCCAA

>C100495_65

GTTTTTTCTCACCCTCTTTCTCATTCTCTTTTTAGTGTAGCATCTTCAAAATTCATTGTTTTGTTTGGTTGCTCAGAAAACTAAAAAAGAAAAATCATTTGACAATTTTTTCCTCATTCTTTCAGTCCTCACTCGTTGGAATCCCAGATCTCCAAACCTTTGCTCTACCACCCTTTTATATGGTTCTAAATGGCGAAAATAAGTTCAGCTGATGGAAGGACTAGAAGCTCTGTACAGATCATCATAGTAGTTGGTCTGTGCTGTTTCTTCTATATATTAGGTGCGTGGCAGAGAAGTGGTTTTGGAAAGGGAGATAGCATAGCTTTGGAGATTACTAAGACGACTAATGAAGAATGTAATATAGTTCCAAATTTAAGTTTTGACTCCCACCATGGTGGAGAAGTTAGTGAAATTGATGAAGCCGAATCAAAGCCTAAGGTATTTAAACCATGTGCTGCTCGTTATACTGATTACACTCCCTGTCAAGATCAAAAGCGTGCCATGACCTTTCCTAGAGAAAACATGATCTATAGAGAGAGACACTGCCCCCCAGAGGAACAGAAGTTACAGTGTATGATCCCTGCACCCAAGGGGTATGTAACACCATTTCCATGGCCGAAGAGCCGTGACTATGTTCCGTTTGCTAATGCTCCCTACAAGAGCCTTACAGTTGAGAAGGCTATTCAGAATTGGATCCAATATGAGGGAAATGTGTTTAAGTTCCCTGGTGGTGGAACACAATTTCCTCAAGGTGCTGATAAATATATCAATCAAATTGCGTCTGTGATACCTATAACTAACGGGACAGTGAGGACGGCACTAGACACCGGTTGTGGGGTTGCCAGTTGGGGTGCATATCTTTGGAGCAGAAATGTTATTGCCATGTCGTTTGCACCAAGGGACAATCATGAAGCACAAGTTCAGTTTGCTCTTGAAAGGGGTGTCCCTGCTGTCATTGGCGTTTTGGGGAGCATAAAGTTGCCTTATCCATCTAGAGCCTTTGACATGGCTCATTGTTCTCGCTGTTTGATACCTTGGGGAGAAAATAATGGAATGTATATGATGGAAGTTGATCGAGTTCTAAGGCCTGGTGGTTATTGGGTGCTCTCTGGTCCTCCAATCAATTGGAAGGTTAACTACAAAGCCTGGCAGAGACCTAAAGAAGACCTTCAGGAAGAACAAAGAAAGATTGAAGAGATTGCTAGGAAACTTTGCTGGGTGAAGAGATCAGAGAAGGCTGAAATGGCCATTTGGCAAAAGGTTGTAGACTCTGAATCATGTCAGAGGAGACAAGATGATTCTAGTGTAGAATTTTGCAAGTCCTCAGATGCCGATGATGTCTGGTATAAGAAAATGGAGGCCTGCATTACTCCCAGTCCTAAAGTTTCTGGTGGGAATCTTAAACCATTTCCAAGCAGGCTTCATGCTATTCCTCCCAGAATTGCTAGTGGCTCAATTCCTGGAGTTTCCTCTGAGACATACCAGGATGATAACAAAAAGTGGAAAAAGCATGTAAATGCTTACAAGAAAATTAACAGACTGTTGGACTCAGGAAGGTACCGTAACATTATGGATATGAATGCTGGATTGGGTAGTTTTGCTGCAGCTATCCATTCTTCAAAGTTGTGGGTCATGAATGTTGTGCCTACTATAGCTGAGACAAACACACTGGGTGTGATATATGAGCGAGGACTAATTGGCATCTATCATGATTGGTGTGAAGCCTTTTCCACATATCCACGAACATATGACCTCATTCATGCTCATGGTCTCTTTAGTCTGTACAGGGATAAATGCAATGCAGAAGATATTCTTCTGGAGATGGACCGGATTTTGCGACCAGAAGGTGCTGTCATATTCCGTGACGAAGTTGATGTGTTAATTAAGGTAAAGAAAATAGTTGGAGGAATGAGATGGGATACTAAAATGGTTGACCATGAGGACGGTCCTCTTGTTCCTGAGAAGGTGCTGATTGCTGTTAAGCAGTATTGGGTTGTTGGTGGAAATTCCACATCAACACAATAATCACTAAACCAAAATTGAAATACTGGCTTGGCCTATGGCTGCCAATACCTTTCTGTATAAGATCTTCACAGAATTCATATGGTATTCTGGCTCAATTGGTCATTGCAACTGTGGCAGAGTGGGCAGTTAAAGAAGTTTTGCACAATTGTCACTTAGCTATGTTTTGATATGTTGTTAATTTATTGATCAAACCTGTGCTTAAGGTCTATACCACTTTGGCAAATTTTGGAGTAAGTTAGTTATATTGTACAATGTTACATTCAGAAGAGAACACCTTCTCATATGTTGCTTAAT

>C100503_65

AGCTTCAGCTTCTCTCTCCACTTCAGCATTCGAAGCTTCAGCTTCTCTCTCCACTTCAGCATTCGTTGCTTCAGCTTCTCTCTCCACTTCACCATTCGAAGCTTCAGCTTCTCTCTCCACTTCCCCATTTGAAGCTTCAGCTTCCCTCTCAACTGCACTATCTGAAGGTTCAGCTTTCCTCTCAATTGCACCATTCAAAGGTTCAGCTTTAGTCTCTAGTGCACCATTTGAAGATTCAGCTTCACTCTCCACTGAACTATGTGATGGTTCAGCTTCACTCTCCATTGCACCATTTGAAGGTTCAGCTTTACTATCCACAGCACCATTTGAAGGTTCAACTTTACTCTCTACAACACCATTTGAAGGCTCGGTTTCACTCTCAACTGCACCACTTGAAGGTTCAACTTCGCCCTCAACTTCACCATTTGAAGCTTCAACACCATGTGATGCCTCAGCTTCATTCACAGCTGAAGGTTCATCTTTACCCTCAACTGCACCATTCGAAGGCCCAGTTACACTTTCAACTGAAACATTTGATAGCTCAGCTTCATTCACAACTTCACCATTTGAAGGTTCAGCTTCACCCTCAGCTGCACCATTCGAAGGTTCAGCTACAATCTCAACTGCACCATTTGATGGCTCGACTTCACTCACAACTGCACCGTTTGAAGATTCAGCTTCACCCTCAACTGCACCATTTGAAGATTCAACTACACTCTCAAGTGCACCATTTGATGGCTCAGTTTCACTCTCAACTGCCCCATTTGAAGGTTCAGTTCCACTCTCCACCGCCCCATTTGAAGGTTCAACTTCCTTCTCCATTGCACCATTTGAGGGTTTAGCTTCACCCTCCACTGCAAAGTTTGAAGGTTCAGCCTCAATCTCCTCTGTACTGTTTTCCATCTTAGACACAGCAGAAGGTTTACTTTCACTCTGCACTGCACCGTTTGAAGGTCCAGCTTCACTATCTACAGCACCCTTTTGTATCTCATGTACATCAACATTAAGTTCACCATCACTATTAATAGCATTGCTTTTCATCTCACAAACATCAACAGAAGGTTCAGCTGTGCTCTCCACTGCAATGTCAGATACAACGTCACCATCTCCATTCACATCTGTGCTATTTAAGGCTGTTGAGTTTTCTCCTTTATGTAGTTCACTTTCTGCTTCCACCTCAGACCTCTCCAACTTAGTGCCTGATTCACCTTCAACAACAACCTCATTCTCCTCAGTGCATTCTCTGGGCTCCTGCGCTGACTGTGCCAAACCAGAGGCAGACTCAACCTGCCCCATCTCTGAACTATTTTGAGCCTCTTCAGGTTCACATTCCTTCACATCTGTAATACAGGAGACGCCACCAACATCATCTGGAACCTCATTCCTCTCAGTGACTTCTTCCACATCATGACTTTCATTAGTTGCAATGTCCTTCTCATCGGGGACATTTGCACTAACAACATTTTCGCTTTCCCCACTTTTATCCACATCTTTATCAGATATTGACTCACTTCTTCTACTCTGCACGTCATCTCCACCGCTCTCATCAGCAGTAGCAGTCAATTCTTCCTCAGCTCCGTCCACCACCGCCTCATCATCGTGAACTTCAACACATTCGAAGTCACGATCACTTTGATTCACGTCAGCATCTCCCTCCACAACATTCACAACGACACTGTTTTCATCTCCGGAAACCTCCGCACGATCCAAAAGCTCGCATTCCTCAACTCCATTGACGTTCGCAAGCGCATCACCGCCAATCTCGCCCTCCGCACCGACACCGTTTTGAACATCCGAAGCACCGACAACAACATATTCCTCGTGATCCGCAACTAACTCCTCCGCAGCAGCATCACGAATCTCACTCTCCACGACGCCGTTCCGAACGGATCCCGCGCCGTCTTCACTATACACACATTTCAATTCACCGTCCGTTTCAGAACCTTCCTCTGCCGTACCGTTAGCCACTGCATCCTTGCAATTTTTCACCGCCACTTCCTCCGACACAGCACCGGCGGCGGCGAGATCCAAATCGCCACCGACAGCGTGCGCAAGTCCATTGGCTTTGACGCCGCTGTTATCGTTGCCAGAATTGCCTTCTTCGCCTCGCACATTCACCACCTGCTCCTCCGTCATTGTGCTCCGGAGATGCAGCGACGGATCAGGCGTGAGCGAACAGATCGAAAGTCCAGATCAGAGAATCAAAGAGAGTAACGGAACGAAACTTGCGCTTTAGGTGGGAATAGTAACAGATTCAGATGCAGATACAAATGCAGATATATAGCCGACACAGAAGAGAGAAAAAAAAGTGAATAGAAT

>C100505_65

CTTTGGTCTGCAAAATGGAACTTGAATTATTTTCACTGACTGAACAGCAATCAGCCAAATCTTTCGTAACTTACAAATAATGTGAAACATGAATAAAAACACGACAAGTATTTTACAATGAAACATGCTTATTAATTGAGGACAGAACAAAATACGAGAACACCCCATTCTCTAGAAATTTTATGCTCTCCCTCTCAAACATAGTTGGCAGGTAAAGAGAAAAAGATTCATCATGGTGTGTGCACATTAGACTCAGACTCAGAAGATGGCCTGTTTTTCAACTCAGGATGCTTAATTTCCTCTATCATTCTAACAACCTCATCCATTCTAGGCCTATTTTCTGGCCCCTTAGCTACACATGCTAATGCAATCTGAAGCATCTGCACCATTTCCTCTTCAACATACTGTCCTCTCAAAAGCTCCTCATCAAACACTTCTGCAGTCCATTCCTCTCTAACAACAGACCTCACCCACCTTGGAAGATCAACCACATCCTCATACCCTGGATACCTCAGTGGGGTCTTCCCTGTCAGCATTTCAAGCAGCAACACACCAAAGCTGTATACATCAGACTTCTGGGTGATCTTCCTTGAGTCAGTGACTTCTGGAGCTCTATAGCCATTGGCTCTAGACATGGTTGCTGGGGTGTTCATTAGAGGTGGCAATCCAACATCAGAGATGCAGCTGTCTAGTTCTTGGGTTATGAGTACATTGTTTGACTTGATGTTGCCATGTGTGAATTTTGGACCCCCCTCAGTGTGGATGAAAGCGATGCCCTTGGCAGCTCCAAGCAAAATCTTCACTCTAGAATCCCAGTCTAGTGGGGTTCTTCCTGCACCCCTGTTTCCGTGCAACAAGAAAAACAAGCTGCCACCTGGCATGTAGTTGTAAACCAGGAGTTTCTCATCTTTGGAATAGTAATAAGCCCTTAGGGGCATCACATTGGGGTGGCTTCCAAATCTCCCAACAATCTCCAACTGCTGCTCAAACTCCTTCTTTCCTACCACAACTTCTTTCAACCTTTTGACTACCACTGTTGTTCCCTCCTCCAAAACAGCCTTGTATGCTGTTCCATAACTTCCTTTTCCAAGAACTTCAGCTGAAGCCTTCAGCAAATCCTCAAGGTCAAAGCTATGAGAGGAACCTTCAAAGAAAAATAGCTTGTTCTTCTCAGCCCCTTGCACCCCACTCCCGAAACTCTTTGAAACCTCGGTCTTTCCAGCACAAGAAGCCTTTCCTTTCAGTATGCCACTGCTTTTACTGTTTTTCTTCCTCTTCAAACAGAACACACAGATCACTACAACTAGTAGAGAGAGGAAGGCAAAGACTCCAATGACTAGAGCAAGGATGGTAGCCAGGCCAAAGCTTTTCTTATGGTTAGTACCTTTTTGGTTTTGGGTGGTTGGTGGAGTGAGTGGCTGGTAATCATCAGTGGAAGGAGATGGAGAAGGGGAGATTGTAGAGCATCGATTTAGTGGTGGTCCACACAAGAGAGAGTTCCCAACAAAAGAAGAATAAGGGAATGTCTTTATGGAATTTGGAATTGAGCCATTCAAGTTATTATAGCTCAAGTTCAAATGTTTGAGACTTGGAAGGTTGAAGTCAGGAATAGCCCCAGATATGGAATTGTTTTGGAGATACAACCAAGTGAGTCTTCTCAGGTTCTGAAATGCAGGTGGGATGCTGTCAGAAAAGGAGTTAAAGGAAATATCCAATGCAAAGAGTTTAGGGGACACACGAGAAGGAATTGGGCCTGAGAAGCTATTGTGCTGAAGGTATACAAATTGGAGTGAAGGTATGGAGAGGATGTTAGAAGGAAGATTTCCTATAAGGCCATTGGAATGAAGACTCAGGACCCTAAGAGCATCTAGATTTCCTATGCTGTTCTCTGGAATGGTCCCAGTTAATCCCATTCCTGGAAGGTGAAGGGCTATGACTCGTGTGCCATTCGAATTGCATGTCACACCAACCCATGAGATGCAAATTGAAGCAGAGGCATTCTTCCAGTTAAGCCTTGGGGCATGTGGAACACTTGAAGCAAACTCCAAAAGAGCTTGTTGGTCTGACTTCAAGTCAGCTACAATCAGGCCATAGGGACACAGCGTGGAGCCTAATAGAACAAGTGCAACAATAGAGAACTGCAACTTCATGGTTATTTTATGATACAAAATCAAGGCAAGTGATGGTTCCAGCTTCTGAGCTCAACTGTTCTTCATTCTCTTAAGGTCTTCCTTTGGAAGTTGCAGCAAATTGTTAGCTGATCCTGTAGCTAAGAAGGTACAATATATTCAAAGTTCAAATTAACTAGTTTTGTTCAGAC

>C100511_65

ATTCGAATGCCAATTCTGTGTGTGACCACCGCATTGCTTCCCTCTATCGTCTTCTTCCCCTTTCTTCAATGGTCTTAGGGTTTCCGGCGTCCATGCATAGCACCTCGCAGTAATGCGTTTTTAATATTTGTTTTCAACTGTATCTAACGATTTACTAGAAGAAGGTCACAATTTCTCCATCTTCGCGTTTCGGTAGCTCGTGAAGTCGAAAGATATTGACACTTTCACGCATCACGAAATATTGCGAACTAAGCAAAATGTCTATGGCTGACGAACCACTATATCCGATAGCAGTGCTTATAGATGAGTTGAAAAATGATGACATTCAACTGCGGTTAAACTCGATTCGCAGGCTATCAACAATTGCGCGAGCACTTGGGGAGGAGCGCACCCGTCGAGAATTAATTCCATTTTTGAGTGAAAATAATGATGATGACGATGAGGTACTTCTTGCAATGGCAGAAGAGTTGGGAGTTTTCATTCCTTATGTTGGGGGAGTGGAACATGCCAGTGTGTTGCTGCTACCGCTGGAGACACTTTGCACTGTTGAGGAGACCTGTGTAAGAGATAAAGCTGTGGAATCACTTTGTAGAATTGGATCTCAAATGAGGGAGAGTGATTTGGTTGAATATTTTATACCATTGGTGAAGAGATTGGCAGGAGGTGAATGGTTTACTGCTCGAGTCTCTGCATGTGGGCTGTTTCATATTGCTTACCCTAGCGCACCAGAAACATCAAAGACCGAATTGAGATCAATATACAGTCAGTTATGTCAGGATGACATGCCTATGGTTAGAAGGTCGGCTGCATCAAATCTTGGTAAATTTGCTGCAACAGTTGAATATGCTCATTTGAAGGCTGATGTCATGTCAATTTTTGAGGATCTTACTCAAGATGATCAAGATTCTGTCAGATTGTTGGCTGTGGAGGGCTGTGCTGCTCTTGGCAAGCTGTTGGAGCCACAAGATTGTGTTGCACATATACTACCAGTTATTGTTAACTTCTCCCAGGATAAGTCTTGGCGTGTGCGTTACATGGTTGCAAATCAATTGTATGAACTTTGTGAAGCTGTAGGACCTGAACCTACCAGAACGGAATTGGTACCTGCTTATGTGCGATTGCTTCGAGACAATGAGGCTGAAGTACGCATAGCAGCTGCTGGAAAAGTGACCAAATTTTGCCGGATTTTAAATCCAGAACTTTCAGTTCAGCATATTCTTCCCTGTGTGAAGGAATTGTCATCAGATTCTTCACAGCATGTCCGCTCTGCTTTGGCTTCAGTAATAATGGGAATGGCTCCAGTGTTAGGAAAGGAAGCAACAATTGAGCAGCTTCTTCCTATTTTCCTTTCACTTTTGAAGGATGAATTTCCTGATGTGCGCCTAAACATTATCAGCAAGCTTGATCAAGTGAATCAGGTTATTGGAATCGATCTGTTATCTCAATCTTTGTTACCAGCCATTGTTGAGCTTGCTGAGGATAGGCATTGGAGGGTCCGGCTAGCAATTATAGAATATATACCCTTATTGGCAAGTCAATTGGGGGTTGGGTTTTTTGACGACAAGCTTGGTGCTCTTTGCATGCAATGGTTGCAGGATAAGGTTCATTCAATTCGTGAAGCAGCTGCTAACAACTTGAAACGCCTTGCTGAAGAATTTGGTCCTGAGTGGGCTATGCAGCACATAATACCTCAGGTTTTGGAGATGAGCAGCAATCCACACTATTTGTATCGGATGACTATTCTTCGCGCTATCTCTCTGCTTGCTCCTGTAATGGGGCCTGAAATCACATGTTCAAACTTGTTACCGGTGGTTGTTGCTGCTTCAAAAGACAGAGTGCCCAACATAAAGTTCAATGTGGCAAAGGTATTAGAGTCCATCTTCCCCATCGTCGATCAGTCTGTGGTCGAGAAAACTATCCGTCCATGCCTAGTTGAGCTCAGTGAGGATCCTGATGTTGATGTGCGATTTTTCTCCAACCAAGCACTACAGGCAATTGATCATGTCATGATGTCTAGCTAGGCAGGAATGGTTCAACACGCATTTAGGTTGAAAACGTCAAGAAAGGATGTAATATTTCCAACAATCATAAGTAACACTGCATAGTTATGTTTAGTGCTTTTCAGTCATAGCTGAATGGTTATTTATCTGTGAGTAACTAGTGATTGACAATCGATGTGATTTAGTTTCCATTATTCCATATATTTTTGTGTTTTTTCTTGATTATATTCTTCATAGATTCTTTATTGGTGTTCCTTGTTGTAATTCTAGGCAATGAATTGCATATTTCAAATACGGTCCAAGCACACCGTAACTGCCACA

>C100517_65

TATCTGAATCTAATACAAAAAATTCCTCAAAGAACAGAAAAATGCATAAAACTGCATGGCATATCGTTCCCTGAAACAACATTAACAATGGAGAAAAGCTGTTGTTGGAATACCTAAACCCCATTGCCTAATAATGTTAATTATGCAGCAACCAATTCCCTAGACTCCAGCCATGACTTGGCTGCTACCTGTGTAGTCAAAAACCAGCCAGCCTTATCTGGTGCCTCGTGGAATGGAGCATCGAGTTCGTCCAAATGTGATTCAACTACACTTGCCAAACCTTTGTCCGAATACCTGTGTTTCCCATGACCAGTATTAATTCCAAGAACTGGTGGAAGATCCTCTCCTGATTCCAAAACCTTAGACAAATCATTTATCCAAGCATGTAATGCAGTGAGCGAAGCTCCAAGGGAGAGACTCTTTAGATGCAGAGACCACTGAGTTTGAGTCCTGGTCTGTATATCCGTATATATTTCACGCGTCAACCCCAGGTCCAGAAGCTGACATGCCAGGTCCAACAAATTCAGGTTGACACAAAGATCAATCAGAGAATTGCAAAAGGGCTTCTTTACTTCATCAGAAATTGAATCGAAGAGTTCAGATGCTTCTTTCCTAAACTCCCCATCACCCTCCTCCAGCCCTTCCACCAAATATCTTACCACAGACCCAAGCTTTGGATTGGCCTTATCCACACAATCTTTTAGCTTAAAAAGTTCTTCTTTCGGTGTTTGGGTCATAACATTCAGAAGACAACCACAGAAACGGTCGTCTGGACGGATGCCCAAATCCAAAAGTTGATCAAACGTCTTAACAACATCATCAGTACGCCCAGCCTTCCCATAGCACTGTACTAGCGATGTCAGAACAAAAATAGTAGGCTCAAAGCCAGATTCAATCATTTCATTCAACATTCTTTCAGCCTCTGAAACGTTTCCACTGCAAGAATATATCGTAATCAAGGACGAGAACGTCCAACTGTCACAAGGGCAAGTTGCAGAACTTTTCATGTCTTCAAAAATCTCAAATGCTTCATCAGCAAGACCAAGATCAGCACACATAGCTAAAAGGGTATTGTAAAGATGAGTGTTCAACTCCATTCCCTTTCCCCTCATTTCCTTATAAACAAAGAGAGCATCTTCGCTGTAACGACCTCTACCATAGGCTCGCAAGAGAGACGCATAAGTCACCCAATTCGGTGTCAATCCATTATTCATCATCTCCGTATATATACTCTTGGCCTGCCAAGGCCTCTTAGCTCTCCCCATGGCATCCAAGAGAGTATTATAAATAACCATGTTAGGTTTAACACCAAGAACCTTCATCTCTTGATAGACATTTAAGCATCCATCGTAATTCCCTGCCAAACCATACATCTTAATCAAGGTGGAGAAGGTAACGGTATCAAGGCGCCATTTCTCGGTCCTTGCACGATCATACAAGCTCAAAGCCATGTCAATGTTACCAGCCTTGCCATAAGCATCAATCATAACCGAGTAGGTAACTTCGTCAGGCTCACACCCAAAAGATGCCATCTTTTCAAACCACTCCACGGCCTTATGGGGCAAAGAACAAATCCTAGCGCAGCTAATTATCGTCGAGAAGGTGACATTATCAAGCCTGACCCCTCTCTGAAGCATTTCATCAACCAGCTTCTCGATGGCATCGAAATCTCTGCTCTTCCGAAATACCTTGAGGGTGACGTTGTAGAGAATGGCCTCTCTGGTGGGTCTAATCATACGCTGAAAGTAGCTGAGAACAAAGGGTACAACAAGGGAATTGGACATGTTGTTGATGACAGTTACAGCGTCTTGCTCTAAAACCCTATCCCCCAAATTCTTTAAAACCAAAGACACGTCCTCTTGAGAAGGATTGCAAGAGTCCAAGGAATTGGCGACATTTACAAGAAAAGTGTACCTGGCATCGTAGGATTTCCTCTCAAGTTGTTTCGCCCTGGGGCTTCGGGGATTCACCCAGATACGACTACTCTTATAGGAAGACGAGGAACTTTGCTCGGGTGGTATCTGTTCGGAAGTGGGTTCGTGCAAGGACACCTGGGGGGCATGGAGAAGGGTTTTGGATTGGGAAGTGAGAGAATGACAAGGAGACGGTTGAAAGGAAGAGGAAGACAAGTTTCGAAACTTGAATTTGCGAGAAGAAGAAGAAGAAGACGAAGATGAAGATGAAGATGAAGAGATGGAGGGAAGATCGTGAAACAGAGAAGACGGAGAAGAGCAAAGATTGTAAGCCATTTTTGCAGCCTATGCTCTGTTCGGTCTCACCTTATCGCGCTCCTCCAACTAATATCTAAAGAAGACAGAAAAT

>C100519_65

ATTCACTAAACCCTAAACAATTTTCTATTTATTGTTGTTGCTGTTATATTCTCTTCAATTTTCTCTTCTTTTTAGAAGAATTGTAATAACAATCAATAATTGAATTGTAAATCTGGAAACTGAAGGTAATTTGAATCGCACTGTGAATTAGAAAATTGAATTGTTGATTGGAAAATGGCGTTGAGCATGACTCACCAGATCGGGAGTTTGGCCGGTGCGGTGGTGACAGATTCCATCTCGAATTCCGGCGATTCAACGACGGCGGCCTCGGCGGTGTGGAAGTCGCCGGCGGCGAATATGAAGTGCAAGGCGGTGAGGGCGGACGGCGTGGAAGGACTGTCGCCGCCGATGACTCCGTGCAGGTCTCCGCCGATGACTCCGTGTAGGTCGCCGGTGGCGAGATCGGTGCGGGCGGATCTGACGGCGGCGTGTCAGGCGTTTGCGACGGAGGCGGCGGAGGAAAAGGAGTATGGCGTTGGAGGGAAGGAGAAGGGAGAGGGAAATGGCGTGCCGGTGTTCGTGATGATGCCGCTGGACAGCGTTACGGCGGGGAACACGGTGAATCGGAGGAAGGCGATGAACGCGGCGATGGCGGCGCTGAAGAGCGCGGGAGTGGAGGGGGTGATGATGGACGTGTGGTGGGGTTTGGTGGAGAGAGAGAAACCTGGAGAGTATAATTGGGGAGGGTATGTGGAGCTGATGGAAATGGCCAAGAAACATGGCCTCAAGGTGCAGGCCGTTATGTCCTTTCATCAATGTGGCGGTAACGTCGGAGACACTTGCAACATTCCCTTGCCCAAATGGGTTGTGGAGGAAATTAACAACGATCCCGATCTTGCATATACTGATCAATGGAGACGAAGAAACTATGAATATCTATCACTTGGATGTGATACTTTGCCTGTGCTCAAGGGTCGAAGCCCAGTTCAATGTTACGCTGATTTCATGCGTTCTTTCCGAGACACTTTCAAGCACCTCCTTGGTGACACTATAGTGGAAATCCAAGTTGGGATGGGACCCGCCGGTGAGCTGCGTTATCCTTCTTACCCGGAGCAAAACGGAACATGGAAATTCCCAGGAATTGGTGCTTTTCAATGCTTTGACAAGTATATGTTGAGTAGCTTAAAAGCTGCTGCTGAAGCTGGGGGTAAGCCAGAATGGGGAAGCACGGGACCTACTGATGCTGGTAACTACAACAACTGGCCAGAAGACACTCAATTTTTCCGCAAAGAAGGTGGAGGCTGGAATAGCGAATATGGTGAATTTTTCCTCTCATGGTATTCTCAGATGCTGCTGGACCATGGTGATAGGATTCTCACATCAGCCACGTCAATCTTTGACGACGCTGGAGTTAAAATCTCAGTAAAGGTTGCTGGTATTCACTGGCACTACGGTTCAAGGTCTCATGCTCCAGAACTCACAGCAGGGTATTACAACACCCGTTTCCGCGATGGCTACCTGCCCATTGCTCAAATGTTGGCTCGCCATGGAGCCATTTTCAACTTCACCTGTATCGAGATGCGAGATCACGAGCAGCCACAGGATGCCCTTTGTGCTCCTGAGAAGCTGGTGAAGCAAGTGGTTCTAGCAACACAGAAGGCGCAGGTCGCACTTGCCGGCGAAAATGCACTGCCACGTTATGATGAATCTGCTCACGAGCAGATCATACGGGCATCGCAGTTGGATGTTGATGGTAACTCAAGTGGCAGAGGAATGTGTGCATTCACATACCTCAGGATGAACCCTCATCTTTTTGAACCAGAGAACTGGAGGAAGTTTGTGGCGTTTGTGAAGAAGATGAAAGAAGGGAAAAGTACAAACAAGTGTTGGGAAGAGGTGGAGAGGGAAGCAGAGCATTTTGTGCATGTTACACAACCTCTTGTGCAAGAGGCTTCAGTGCTGATGCACTGAAAATTGTTGGAACATCCTTGTGTTGATAGGGCTTAGGAAACAGTCACAAGTAGGCTGTGTGAAAGTTTTAGTGAACCAGCAGCCCTGGTTTGTGGCTATGAAGATGTAAAATTTTGAATTACATCCTTGTTTTCTATTATATGCGCCTAGAACTTCTATTTGTTATCCTTCATAATGTATGTAATCTTATAGACATTGGTGTAGTTATTATCTTAAGGTTATTCCAATTTGTACAAAGTAGTTACTTATTTCTATATCAGTAAACTGAATTAGTTTGAAAAAAATGTAATTCCTCAATCAAGTACATGGGGAAGCAACAGATGCTATTTTCTTCTTTGGGAGAATTCATAGTTTCGCAGCTACCTTAACCATAACTTTTTAATGTTCAATTAATTGTTTCAGATAAAAAAA

>C100525_65

AAAGGACATACAATTTACTGTGTCCCAAATGTGGAGTAGGTTAGAAGCAAAGAGTGAGTTGAGAAAAAAGGGGAAGAAAAATGGAGAGGGTGTGGAATGGAATCTGGAAATTGCAATTGGGAATCCTGAAATGAAGGTGAGAGAAGAAGTAGTGAAGTGGAATCTCCCAACTGGAAAGTAATTATAATAAGGTAATGTCGTTGTTTGTTTCTTGTGTAGTTTAGTTAATTTATAATAAAGAATAATGGTGTAGTGTAAACGGGTGCGAGCAATTTAGGGTTTGTGGTGGCATTGGATTTGGATGGATAAAGTTTGTCTAAACAGTGAGGCGGTAGGAGATGAAGAATGCGAGAGTAATGTCCATGTCCATGTCCCTGTTGCTCCTGCTGTTGGAATGGAATTTGAGAGTTACGATGATGTCTATTACTTCTATAATTGGTACGCCAAGGAACAGGGATTTGGGGTTAGGGTCACAAATACCTGGTACCGCAAGACCAAAGAACGTTACAGAGCAAAACTTAGCTGCAGCAGTGCAGGCTTCAAGAAAAGAACTGAAGCTAATCGCCCCAGACCCGAAACCAGAACTGGTTGTCCTGCTATGATCAAGTTCAGGTTGATGGACTCCTCAAGATGGAGAATTATTGAAGTTGAGCTTGACCACAATCACTTGATTAACCCCACCAGTGGCAAGTTTTACAAGTCCCATAAGCAAATTGCTGTTGGCACCAAAAGAACCTTCCACTTAGATGCTACTGCTGCTCAACAGGTTCGCAAAATTAGGATGTTTCGAACTGTCATAATCGATGCTGAGGATGATGCTATTGTACATCCTGATGAAGGGAAATCCCCAAAAGGCCTCTACTCCAACAATCAGTTGAAGCTGAAGAAAGGTGATGCTGAAGCAATTCTGAATTTTTTCAGTCACCAGCAATTGGCGGATCCAGACTTCTTCTATGTGGTGGATGTCAATGAAGGGGGATGTTTCAGGAATTTGTTCTGGGCTGATGCAAAGTCCAGAGTGGCATACAGTTATTTTAGTGATGTTGTGGCAATTGATACTGCGTGTTTGAACGGTGAATATCAAGTTCCCCTGGTTGTGTTCCTTGGGATGAACCACCATAAACAATATATCTTGTTTGGCTGCGGATTACTTGCTTGTGATACGGTTGAATCATACACATGGCTGCTCAGGGCGTGGCTTACGTGTATCTTAGGACGCCCCCCACAAGTTATCATTACCCATCAATGTGGGATCTTGCAAACTGTGGTTGCTGATGTCTTTCCAAGATCTACACACTGCCTTTGCCTGTTTAATATCATGCAAAAAGTTCCTGAAAAACTGGGAGCCTGCATACAGTATGAAGAAATCAAAGCAGCTTTGAGGAGAGCAGTTTATTCCTCTCTGAGGGCTGAGGAATTTGAAGCAAAATGGGAGGACATGATGAAAAGCCACGAAATTAGAGGCAATAAATGGCTTCAGTCTTTATATGAGGAACGGAAACGGTGGGCTCCAGTTTATCTGAAAGAAATCTTCTTGGCAGGAATATTTTCAATTCAACCAAGTGATGTTGTATCATTTTTCTTTGACGGGTACCTTGATGAACAAACTTCTCTGAAAGAATTTTTAGAAAAATATGATCACACTTTACAAACAAAGCGACAGCTTGAAGCTTTGGCAGATTTGGACTCAAAATCTTGTAGCTTTGTACCGAAATCTAGGTCTTACTTTGAGTTACAGGTTTCAAAGTTATATACCAATGAAATGTTAAGAATGTTTGAAAGGGAAGTGGTAGGAATGTTTTCTTGCTTTAACTTCAGACAGATAAATGTTGATGGACCAGTCATCACCTACATTGTGGAGGAACAAGTTGAAGTTGAGGGAAATCAGAGAGATGCAAGAGATTATGAGGTTTGTTATAATGAAGCTGAAATGGAGGTCCTTTGCATCTGTGGTTTGTTCAATTTCAGAGGTTGCTTGTGCAGGCATGCTTTATTTATCTTAAGCCAAAATGGGATAAAAGAGATTCCAGCACAGTACATTGTTTCACGATGGAGAAAAGATATGAAGCGTAGCAATGTTTATGATCACAACTGTAGTGGCATTGATATAAGTAATCCAGTTCATAGGTATGATCATTTATACAGACAGATTGTGAAGGTTGTAGAGGAAGGTAAGAAATCTCATGATCATTACAGGACTGCACTACAAGCAATGGAGAATATATTGAGCAAGCTTCACATTGTAAACGTTGAGGATCCGGACAAAATAATGGACAAGTATTGATATCCGAATAACTAGGGCATCGCCATTGTATATTTGCG

>C100527_65

TCGACTTCCACAGACTTCAACCAGTTCGCTCTCCCAAACTCTGTGTGTGCTTGCTACATTGTCTGGTACCAAAGATGGCAACTTTGACTGCTTCAAGTAACTTGGTCTCTGGAAGTTCTCGTGTTCACCATGGACCAACTTCATCATATGAATCTAAAGCAGTGGCAATGGGACTTAGATATCTAAAGCAGACCAATACTCATAATGGACTGAGAATTTTGAACCCTGTAGATGAACTACTTAACAGGACCCCAATTAAAACCAATGCGGTGCAAGCTATGAGGAAAGGACTTCAAGGCAAGAATGTCAAGTCTACAGGTATAATCATATGTGGCATGAATTTGATTTTTGTTGGAACCGAGGTGGGTCCGTGGAGCAAAACTGGTGGGTTGGGAGATGTTCTTGGGGGTCTTCCGCCAGCATTAGCAGGTAATGGACATCGAGTTATGACTATCGCGCCGCGTTATGACCAATACAAAGATGCCTGGGATACAAGTGTTGTGCTTGAGCTGAAAGTAGGGGACAGAACTGAAAAGGTTCGCTACTTCCATAGCTATAAGAGGGGAGTTGATCGTGTCTTTGTGGATCACCCTTCGTTTCTTGAAAAGGTCTGGGGAAAAACCGGAACCAAACTTTACGGACCAACTACAGGAGATGATTACGAAGACAACCAACTACGTTTTAGCCTATTTTGCCAGGCAGCTTTGGAAGCACCTAGGGTTCTGAGTCTCAATTCCAGTAAATATTTCTCTGGACCATATGGTGAAGATGTAATTTTCATCGCCAACGATTGGCACACAGCCCTTATCCCCTGCTATTTGAAATCTATGTACCAGTCAGTAGGCATATATAAGAATGCTCGAGTTGTTTTTTGTATCCACAACATTGCTTACCAAGGAAGATTTGCATTCACAGACTATGCACTTCTAAATCTCCCTGACCAATTTAAAAGCTCCTTTGACTTTATTGACGGGCATGTTAAACCAGTGATTGGAAGGAAACTCAATTGGATGAAAGCTGGAATTATAGAATCATGGTTTGTCATAACTGTTAGTCCAAACTATGCTAAAGAATTGGTCTCAGGTCCAGACAAAGGAGTGGAACTGGACAACATCCTTCGCAAAATTGAAGAACGATTAATTGGAATTGTGAATGGCATGGATGTTCAGGAGTGGAATCCATCCACTGACAAATATATAGCTGTCAAATACGATGTTTCAACAGTTTTGGAGGCAAAGGCTCTTCTGAAAGAAGCCCTCCAAGCAGAAGTTGGATTGCCGGTGGACAGAAATATTCCTCTCATTGGTTTCATTGGGAGGCTTGAAGAGCAAAAAGGTTCTGATATTCTTGCAGAAGCCATACCGCAATTTATCAAGGAGAATGTTCAGTTGGTAGCCCTAGGAACGGGAAAAAAACAAATGGAAAAACAGCTTGAGGAACTTGAAATAGCATACCCTGATAAGGCCAGAGGAGTGGCAAAATTCAATGTTCCCCTAGCCCACATGATAATCGCTGGAGCTGATTTTATATTGGTTCCTAGCAGATTTGAGCCTTGTGGTCTCATTCAGTTACAAGCTATGCGCTATGGATCAGTACCTATTGTTTCCTCAACCGGTGGGTTAGTTGACACTGTCAAAGAAGGCTTCACTGGATTTCAGATGGGTGCCTTCAATGTTGAATGTGAAGCTGTGGATGCTGCTGATGTGGATGCTATAGCAAAGACTGTCAAAAGGGCCCTTGCAGTCTATGGAACTCCGGCTTTTACAGAAATAATCAAGAATTGCATGGCTCAAGATCTTTCATGGAAGGGACCTGCTAAGAAGTGGGAGGAAGTGCTGCTAAGCTTGGGAGTTCCTGGCAGTGAACCTGGAGTTGATGGAGAAGAAATTGCTCCACAGGCAAAGGAAAATGTGGCAACTCCATAATAAGAACAAATACATGAAGGAAGCCTCTTCTAGATGAGTCTCATGAAGTTCCCTTGAACCGCTGCTTCTTATTATGCTTCTATCCTACTTCCATTTTTTGTTTTCTTCAAAATATATTATAGAAATTAATGTACATGGACACGGTGATTACACTGTTAGAATCAGAGTAATTCACCCCCAATGAGCGTTTTCACACTATTTATAAGAGTATGTCTTCAAGATTAGCATGCCTAAATTAAAGCGTTTCTTAGTAGTCTAGAATGACACAGCCTAACTTGTAATGCTTATTATTTTATCTGCATCTTTGGCTCCAACTATGTTTATTTGGTCCACACTCTTTGCAATCTGTCTCAATAATAACATCTGCAAGATTGAGTTGCATTGTATAAGCCACAAAA

>C100529_65

AATAAATGCTTTATAAAATATTTTTCTGAGATGAACCTAAGGCACCTAGCTCAATTAATTTTTCATGTACACTAATGTAATATAAAGGCATGTTTAGACATTTACAGAACTACAGGCAGAGCCATCTATTTGTCCATACAATGAATATCACTGGTTAGGCGAGAAGAAAACCTCAAGCTCCCATGTATGAAGTGGATAATCACCCACCGTGCTATATTTTACTTCTTGTCCACATCATACAGCCTTTACATTAAGGCAGTTAATGGAACAAGTATATATCAAAACTGAGGATCATAAGAAAAAAGGTTTTTTTGTTCATCTGTTGGATTCAACAGCCATCTACAGAACATCCATGCTGCACTAAGTACTGATAGAAGAGACAACAGTGCCAAGCATCCAAAATGAGAAGGATCATTTGCAATTATTGCAACACCCAGCATTATGGACTCTGCCAAACTAGCAACAGCAATTTCTGTCGTCCCAATGAGATTTGCTTTGGATGATGGGATTCCTGTCTGGAGAATCTGTGCCCCTACAACATCGTAAGACATGTGTCCTAACCGCGATAAAACAATAAAAAATAAGAAAGTAAGAAGTGGGCTCTGATGAGAAATTGATCCACTCCAGTATACGGCAACAGCCATGCTGAGAAGTAAAGCTTGAAATACCAGCCCAACTGCTCCAGCCTTTAAAATGCCAAATTGCTCAACCAGAGTTGAAGACACAAATGTTGCAGCAACACCCATAAAAGCACACATTCCACTGAATCCTCCTATGATAGATGGATGTAGGCCACGCTGTGTTAAAAATGCTGTCAACAAACTGCCTGGCGCGAGAACAATATTAAAGTAGAGGAGCACCCATGCAAGACTAGCAGGAAGAACAGGTTGACCCAGATACTCCTTCCAGCCGAGCTTGATGGCTTCCAGACCTTTAAAAACTATACTCTTAACGTCTGTTGCAGAATCTTCATTCACTGTTCTGCAACAGGTCTGTGAAGGTTTGGGTCTATCAAGAACACCAGTGGAAAGCTTGTTGGTTAAATAAGTGAAAATAATTGTAACAGGCAGAAGCCCCATCATTAAGCCAGAAGCAGCTTTTAAGCAGATTACAGGATGAAATTTAGAAAGAAGGAATCCGAACAGCACTGCCCCAGCTATCTCACATAGGAGATCAATTCGATTTAGAACAGCATTTGCCTGTGCAAGTGCAATCGGCCTATTCACTCCTGCAAGCAACACAACCCAGTCACGCTCATTTGCAACTCCCAAAGCTACACCACATAGTCTCTCAATGGCCCCAGCTGAAACCAATATGACAAACCACGGACGCAGAAGCAGACTAGACAATGAAGTATGTCGCACAGTATGGGCATGAATAATCATTGCCGCAGATAGTAACTGAGCTGCTGCCTGAACGACTGTTAAGCAATTATATGCATACATTCTTGGAAAATGGTCCATAACTTTGCCAACCAAGGGACCACCAACAATTATAGTCACTTTGGTGAAGAAACCCATTACAGCAACCGGCAAAAGGCTTGGATGAATCAAGGCAATGGCAGAAGGCCAAGCAAAATTCCAAAGCTGCTCCACTACATTTGCAGCTATGCAACTAGCATAGAAAGCGTGTAATCCAGCCGGGTGGGCTGGAGTAGCTGAAAGGGCATGTTGCTCCTCCTCGGACAAAACAGGCAATGCTGTCAAGAGAGTATCTACAAATGTGGCTTCAGCGAGCAAGTTCAAAGACTCAGTCTCAAGAATATCAGTTTTGAGTTTAACAAATGGCACCGGACAGTGAGGCTCAACCACCCCTCTTCCCTGACCCTCATCCTCATCGGTGGTCACATGATCCAAATGCACATCAGTATCTGTCAGTGAACACTTGGAACCAAAACACGAAAATCTGTGAGAGGCACGAGGGTAATAGAGAGTCAACCCTCGGCCAGAAGGGAAACGATTGCGTATTGAAGATGAATGAGTCTGACGAAGGGGGAATTTGCCAAAATGGAAAGAGAGGGAGGCCGTAGCAATAGCCATAGCGGCAGCAGCTTGTAGAACTTCAAGCTACCAATCTTCCTCGGATTTATCCAACTCATGCAGTTGCAGTCCCAAAATCACTACGCTGAAGAAACCCCTTTTGCCTCAACGTTCATCCCCAAACAACACTCTTTTCTTTGGTCGAATCGTGAATGCGATTAATCTGGAGATATTTCGCAAACACCTTATCTGTAAGAGGTGTTTGGAAATGTGGGTGGGTGCGTGCGTGCGTGCAGCCTTAGCCTGAATTTTCGTCTAGCGGAGAGTACAACTGGTGTTGTTTG

>C100531_65

CTGGAAATTCACGGTTGGTCATTTGACACGTCTCATATCACATCGTGGTTCTGGCAATCTGCTTCATTTCCCCCCTTTTAACTTTATACGATGTTTACCGTTTTTCTTCACTGATTTCAACTTAGTGGGGTGTTTTGTTTGTTCTACACTAGAGAGATGAAGATTTGTGCTGTGGTACTTGAGCATAATGTACGTTGTGTCACTGATTGAGTGGTTGATGTCAGTGGAAAAATGGATTTGGCATCTGTTCTTTTGCTATTAACTAGTGTGTGCTCTTCTGCGATGGCTGGGCTTGATTCACAAGAAGATGCTCTGTATGCGTTGAAGTTATCATTGAATGCTTCTCCTAACCAGCTTACAAACTGGAATAAAAATCTAGTTGACCCTTGTACTTGGTCCAATGTTGAATGTGACCAGAATAACAACGTTATTCGAGTTTCACTAGAATTTATGGGATTCACAGGATCGTTGACACCAAGAATAGCATCTCTAAAAAGTCTTGCAACTCTTTCTTTGCAAGGGAATAACATCACTGGTGACATACCAAAAGAATTTGGAAATCTTACAAGTTTGATCAGATTAGATTTGGAAAACAACAAATTAACTGGTGAAATACCATATTCCCTTGGTAATCTTAGAAAGCTTCAATTCTTGACATTGAGTCAAAACAATCTCAATGGGACTATTCCAGAATCACTTGCCAGTCTCCCAAGCTTGATCAATGTTATGCTAGATTCAAATGATCTTAGTGGGAAGATTCCGGAGCAGTTATTCAGCATTCCTACGTACAATTTCACTGGAAATAATTTGAATTGTGGTGTGAGTTATCATCATCTTTGCACATCTGATGATGCAGATCAAGGTTCGTCTCATAAAACAAAAATAGGCCTCATAATTGGAACAGTTACAGGTTTAGTTGTTATTCTTTTTCTTGGTTGTCTGCTGTTCTTCTGGTACAAGGGGTTCAAGCGAGAAATTTATGTAGATGTTCCAGGTGAGGTGGATCGCCGAATTACATTTGGTCAAATAAAAAGATTTTCCTGGAAAGAACTACAGATAGCTACAGACAACTTCAGTGAGAAAAATATTTTAGGGCAGGGAGGCTTTGGAAAGGTTTATAAAGGTATTCTAGCGGATGGTACAAAAATTGCTGTCAAAAGGTTAACTGATTACGAAAGTCCTGCGGGTGATGCAGCTTTCCAGAGTGAAGTTGAGTTGATAAGCGTCGCTGTCCATCGGAACCTGTTACGGCTCATTGGTTTTTGTACTACTCCAACTGAACGCCTCTTAGTTTATCCCTTCATGCAAAACTTAAGTGTTGCCTATCGTCTACGAGAACTCAGACCTGGGGAACCCGTTTTAGATTGGCCTACTAGAAAACGAGTTGCTCTAGGAACAGCCCGTGGCCTAGAATATCTTCACGAGCAGTGCAATCCAAGGATTATTCATAGGGATGTCAAGGCAGCTAATATATTACTAGATGGAGATTTTGAAGCAGTTGTTGGTGACTTTGGTTTGGCAAAATTAGTTGATGTTCGAAATACTACTGTGACAACTCAAGTTCGTGGGACAATGGGTCATATAGCTCCAGAATACTTGTCCACTGGAAAATCTTCAGAAAGGACTGATATTTTTGGTTATGGTATTATGCTTTTGGAACTTGTTACAGGTCAACGTGCAATTGACTTTTCACGTTTGGAAGAGGAGGATGACGTGTTATTGCTTGACCATGTTAAGAAACTGCAACGAGAGAAAAGACTAGATGTTATTGTAGACTGCAACCTTAAGAAAAATTACAACATAGGAGAGGTTGAAATGATTGTACAAATTGCATTACTCTGCACACAAGCATCGCCGGAGGATCGTCCAGCAATGTCAGAAGTTGTAAGAATGTTAGAGGGAGAAGGGCTGGCCCAAAGATGGGAAGAATGGCAGCATGTGGAGGTTGCTACGAGGCAAGATTACGAGAGACTACAAAGAAGAATGAACTGGGGGGAAGATTCAATTAATAACCAAGATGCCATTGAGTTGTCTGGTGGAAGATGAGAAATTTAGACCATTACATTTGATGTTGTGTCACACTATCCCCCTTCACAAGAGGTTAATTCGATTAAAAACAGACATAGGTGTTATTGTTTTCCAAGTGTAAAAATATAGGTAATAAACTTCACATTCGGAAAATTATGACTTTTTTATTTAAATAAGGTTTTAACCTTTTTCTTTTTCCTTAGCTAGTGATAAAAGGGAAGGGAATAGAGTTCCCCTTTGGCCTTAGGAATTGGTTGCCAATTTATTGCTGATTCTCAGTTACAGGAATTTGACC

>C100533_65

CTTGTCAACTAAGTTGTTTATCCAAACACACTAAATAACTCTCCCAGGGTATGAAATGAATGTCTTCTCATTGTCTTGAGAGTAAAATTTCAGTGGAACTACTGCATAAAGTCACTGACATAGGGATACATTCAAACATACAGGCAAAAAAAATGTAACGGGCATGACTACACAAGCGTTTCCCAAAACTACAACAAGGAATTCCCCAAAAGTATCTGCAGTCCAGGTATACACAAATAATGGCAAATGTACAAAGGAAAAGAATCAGACACTATTATATACACACGTCCATCTCATTGAACTTTACCTGAGGAGATCATCTTCCACAATCCCTCATTTGATGATTTTTTTTCAAAAACTTCCCACCTCTTCATCAGGTATCACGGTTAAAAGAGAATATTTTACTTTAAGTGATATCTTACCCTTCTCCACAAAAAGCCTTGCCCCAGACACAACCCAATAGCCAGGAAAGTCTTGTGGCCCTCTTGTCATTTCAGTTGTGTCAACAAACCTTAGAAGTTTGGGTGACTGAGTAGGCACAGGAGGCCCCCCAGGGTATAAAGCAGAGTTGATGTTTACATCAGACGGTCGAGGTGGCGGTGGCTTTTGTGGACCTGAGAAGTGAGTGCTGATCAATGTTGAGATTATTCCAGATTTCTGGGTCAGTCCAGGAGAACCTTCCCACTGAGGTTCTTTTACCCTGGTTGCACGGACAACTTTACAGAAATGAAGTCTTAGGAATAGAACTTTCTTAAGACCGGTATCTCCAACTTCGAAGTGAGCACCGGTGACAACAGCATGATCATCATCTGATTGAACAGGAGCAGTGCAAACATGAGAGAAACTCTTCCACTGGACCTTCTCATAGTACTTGCGTTCTGAAGAGTCATTGGAAGCATTCCCATTATGATCATCCTGGAGTTGGAAGGTTTTGGGCAGGGAGGAGAGGTGCTGTAAATGGATTGCCAGGCAGTTACTTCTTTTTCCTTCCAAATACAATCGAAGGCCTGTCACAGGCTTCTTTCCCACATCAACCGGAGTCGTGTTTACATAGAGCTTAGGACCCAGGAAACTGAACTGTAAAGATGCAGTATTTTGAGTTTTCCTTTCAGGACCAAGGGCAAGCTCACCAAATACAGGGGCCCATTGCCTTGGAAGCTGGAACTCCAAAAATTGATGCAGTTCTTCAATTGGTGGTTTATATCGTAAATAGAGGTTCATCGCATGAGTTAAATATCCACTTCCATTTATTCCACCAAGTAGAGATGTAATTGGAACAAATGACATTGATATCACATCAGGTTGAGATAGAACAGTTTGGCACCAGTCACTATGGGACAAGGATTTTTTCCCATTTCCACCTTTTCTCTTGCACATGAACTTGATATCCTGCACCTCTGATTCAAAATAGGAACCAGCTTGAATCTCCATGAAACCTAGTGCATTGTCCTTTTCCTTTTCATTAACCCTCCCACCAATGAAGAATTCATCTGCCATGTCCTTCAATTTTTTCTGTACAACAGCAGGTTGGACAGGAGATGAGTATTGCTGCTTCGCATAGATTATATCAGTTCCTCCTATTTTCACCCCAACTATAACATGAGTTCCATACTTCTCAATGAATCTTGTCAAGGCAGCCGGATCCCAGGACGAAGGAACAGCTTGTTTGACATGATCATGAAGAACTAGCTGGGTTCTCTCAAATGCAATATTGTAGAGTGTGATAGATACACCATCAAAAGCAAGAGTCTTGGTATCGGCAGCATCTTTTTGCCAAACTCCAGTAAACCCAAATGCAATGTTGAAATGGCCCGTTGGAATCTTACCAGACAGTGACAACTCCTGGTTGAACTGCTCTGACATCTGCTGAAAAGAGAGGACATCGGAGCACAATCTCATGCGGTCACCTTTATCGCACTTGATGGATTTGGGGACATTAGGGATAGAAAGCCGAGGAGGGAGTTCAACAGTTCGGAGGTTGTCACCATCGATAGCGATCAATCTGGAGTGGTTCTTGCAGAATTTGAGCCTGAGATCGTTGGTGAGATCGTAACCCAACCCAATGACCCTTATGGCGTCTTCCGCCGCCACCCCCTTCTTTTTCCCGGACATCATCACTTCACGCGCTCATCACCCAAATCGCAGTGAATCAAATAGTGAGGAAGCAGTGTTGGTTGTGTTTGGAGGAATGGAATTTGTGGAGAAGAGAAAGGGAAAAGCAATTGATGGAGTTCACGTGAATTGGAATTGCGAAGAGGGAGAGGAATTGGAAGGTGGCTGTTTGATAATGTCTATAGTCAAAGAAGACGTAGTATTATTGTTA

>C100537_65

TTGGAATTCCTCTTCTACTCCTTCTACCATTTTTCTTTCCCTTTCTCCACCTTTCCATCACTTTCTCTTGTTTCCCCCCATTCACTTTCTCTTGTTTCCCCTTCGCATTTACCCACCGATAGAGACACACAGAAAATCACGTTTCCAACCACGGGAATGGAGGAAAAGCACGCAGCAGCCTTCGAATCCGCCCACGCCGAATCCAACGGTATCTCTCACGGCGCCGACCACGGTTGGCAGAAGGTAACCTACGCTAAGAAACAGAGGAAGAAGACCAACAACAGCGCTAACGGCTCCGATTCTCGACCCAATTCCCACAAGCTCATTCCAAACGGGACCTTGTCCGGCTCCGATGGCGTTTTCCGGTCGCTGGAGCTGCAATCGGAGGACCGGCGCCGGAAAATTCTGGAGGCGCGGAAGGCGGCCGATGCCGCATACGACGAAGATGACGCGCTTGTCAGATCGAAGCAGCGACCTCGCATCGACGACGACGACGACGAGAATGTGGAGCGTTCCGCGGAGAATGGGAAGGTGGAGGAGAAGAAGGTGAAGCAGAAGAAGCTGAAGAAGCCTAAGGTGACGGTAGCGGAGGCGGCGGTGAAGATCGATGCCGCTGATCTGGGCGCTTTCCTCGTTGATATATCGGCATCGTTCGAGAAACAACAGGACATATTGATGATGCGGTTCGCTGATTATTTTGGACGTGCTTTTTCTGCCGTGACCGCATCTCAGTTTCCCTGGGTGAAATTGTTCAGGGAGTCAACCGTGGCTAAGATCACTGATACGCCACTTTCTCACATATCAGATGCTGTATATAAAACATCAACCGACTGGATTAACCAACGTTCTCCTGAGGCACTTAGTTCCTTTCTGCTTTGGTCTTTAGATAGCATTCTTGCTGACTTGAGTAGCCAGCAATCTTCGGCCAAGGGTTCCAAAAAGGCTGTGCAGCAAGTTGCTTCAAAATCTCAGGTTGCCATGTTTGTAGTTTTAGCAATGGTACTGCGCCGGAAGCCTGATGCTCTTATAACTGTGTTACCCGTGTTGAGGGAGAATACAAAGTATCAAGGGCAAGATAAGCTTCCAGTGATTGTATGGATGATTGCTCAGGCCTCTGTAGGAGATCTCTCGGTAGGTTTGTATGCTTGGGCAAGGAATCTCCTACCCATAGTGATTAGCAAAAGTGGTAACCCCCAATCCAGAGATTTGGTTTTGCAGCTGGTGGAAAAAATATTGTCTACTCCCAAAGCCCGGCCTGTTTTAGTAAATAGTGCTGTGAGAAAAGGGGAACGATTAATTCCTCCTTCTGCATTTGAAATTTTGCTCCGGGTTACTTTCCCTCCGTCTTCAACTAGAGTAAAGGCTACTGAAAGATTTGAGGCCATATATCCCACTCTGAAAGAGGTGGCTCTTGGAGGTTCTGCTGGAAGTAAGGCAATGAAACAAGTTTCACTGCAGATATTCAATTTTGCTATCAAAGCAGCTGGAGAAAACAATCCCGAGTTATCAAAAGAAGCAGCTGGTATTTTAATTTGGTCTTTGAGTCAAAACCCCGAATGCTACAAACAATGGGAAAAAGTTTATCATGACAACATTGAAGCAAGTGTTTCAGCTCTGAAAAAGCTTTCTGATGATTGGAAGGAGCAATCAACAAAATTGTCTCCTCATGAGCCACTGAGGGATACCTTAAAGAATTTTAAGCAAAAGAATGAGAAAGTATTGGCTACCGAGACTGATGCTGCTCATCATGCACACTTCAAGGATGCCGATAAATATTGTAAGATAATCTTAGGAAGAGTTTCGCGAAGCCACGGATGCACGTCTTGTTTAACCATTACAGTTCTTGCTTTGGCTGTGGGTGCTGCCGTTTTGTTGTCCCCCGGAATGGAATCTTTGGATTTTAAGAAGCTCTCCGAAGTTTTCAACGTTCAGCACTGAGGTACTTGAGAATATTCCGCGTTGCTAGCAGTTGGTTGCTTCAAACAATTTTCTTTTCAGCCCAACTCATTCGCAATGGGCAAGTAAACCAATAGTCTCGTAATAAAGTGCTTAATGCGGAGATTGTACACTTAGTTCAAATCCTGTTCGGATGACACAGAATAGTTGCGGTCGACAATGGTTTGAGGTTTACTCTAGTTAGACTATTTTATAATTTATTTGTTCTTGTTTCTGTTGAGTCTTAATTTTAGTTATCTTATGCCTTTACATTAACTATGCTGGCAAAATGCCACTGAGGTAACAACAGCTGTATCCTGAAGTTAATTTAGTTGAATCAGAGGAATTTTTGTGGTACATTGCGAAATTTTTTTCTACCTTTTGCTCTG

>C100539_65

GCCTAATGCGAACTTGTGAAGCATTTACATTGACATGACATAGGATAAATATCTGGCTTACGGTTTCCAGATAATAAGCTCAATTTTCATAAATGGAGGATAAACCTCGGCTTGTTTCAAAAAAATGAGCATTACCGCTACAACAGATAACCCATCATCTACTTAAAATCAGAGTACCTCCCATTATATGAGGTTACATCACTTCCAAGAAGCTTTACATTAGTACAAGCGTGGTTAGCTGAACTCCAGGATAAGAACACTGGTGCTATTTAAATAGGCAGAATTCCCCTAAGTGCGAAACGTTTACCTTCCCCGAACACTGGCAGGGGATTGACCACATCTCTCACTGCCAATATCATCATCTACCTCACTGCTATCTAAACAAATTCCATTTTGGTTAACGCTAAGTTCAGAACTAGCATTACTGCTTGTGGCATGTGGAGAGCCTGAAACACTGATTGTTCGGCCATGATGGGATCCAGACCTCACGCTATACAAAGAAGATGCTGGAATATTTGTCATCAATGGCCGTAGATTGCCTGGAATGGTTCTCCTTATATCCATATGCCTAATAGCCATATCCAATGACTTCTTTGAAAGTGTTCTTCCAAAACCTGAGCTATCTGGAGATGAAGTTGACTTGCCGGACAGATTACTACGTGGAGAGTTTTTGTCATCAATCATTGGTGGTGCTAGTTTTCGCATATTGATTACCCTCTCGACCATTTTGGTGCCCATTAAAACAGGACTAACATTGTCATTTACTTTGGAGCGCCCACGACTAACTGCCGGCATAGAGTTTCCACTGATATGAACAGCACCGTTGGAAGCCCGTCCTCTTGAAGGAGAGCACGATTGTCGCCTGGGTCTTCCACTAGAAGAAGGCTCAACAGATGAAGACCGAGAAGTGGGTGCTCCAGGCCTGCCCCTAGTTGCTGACAGTGGCCTATCAGGTAGTGTTGTTCTCAAATTAGGTGGGGCATCAAGTGAAAAACCTGGCATGTCAGACGGCTTCCATGGTCTTGATCTCACTGTTGGTGAGGTGCCACGTGATGGAACTGCTGGCTGCCTTGAGTTAATAGGAGTTGGCTTTCTTGTTGTCCCAGGAGTTGGCTGCCTTGATGTCACAGTAGCTGGCTTGGGGCTCAAAGAAGCTTTAACTGAAGAAGCAGATATACTGGGTTCACTTGAAGATAAAGAGGGTCGCCGGGTTGGGGTAGATGCTCTTGATGTGGACCTGGATGGAGGTAAAGTAGGCCTGCTAGTTGGTGTAGAAGATCTTGCAACAGATCTTGACAAAGGTGTAGAGGATCTTGAAGGAATTGTGGATTTAGCTGCAGGGACTGGATTCTTAGTGGCAGAAGTCAAAGGCTTTGTGGTAGAAACTACAGGCTTGATTGTTGAAATTGTGTTCTTCGATGTAGCTGCTAAGGACCTAGTTGAAGGTAAGGTAGACCGTGTAGGTGTCGAAGGTCTTGATGATTTCGAAGATGCAGTTAGTGTAGGGCGCCCAGTAGGAGTTGCAGGTCGTGATCCTGGACCCCCTGATGATGAAGGCCTCCTAGTTCCACTACTTGAAGAGCTCAACCCAGGAGAGGAAGAAGGTTGTTTAGATACTAAATTAGTCCTCCCAGCAGGCTCTGATTGATGATTTGCTAACCTGGATTTTAATGCTACAGGACGTGTAGTTGGAGTACCAAGCTGACTCATAACAGTCTTTCGACCTTCCATTTCCAAAGAAGGAAAAAGAGGAGTACCAGGAGGAGTGAGAAGCCAGTCATAGTCATTCTTATCATTCTCCGAATTGAGAAAATCATCAACACCAGTCTTCCTCACAGGAGCCGGCGTAGAAGACGAAAGGTTAAATATAGGATTAGCGCTGGGATTCGACCCGAGAGGTGCATCGAACTCCTCCGAGCCGTTAAGAAGAAGATCATTCCGCTCCTTCTCGCGCTTCTTCATCTCCAGAAACAATGCGAGCTCCTCCTCCTTCTCCTTCATCACCGAAGCCCTAAAACCCCCAAATTGCTGCTCCCTACGCTTCAACGCAGAGTGCATTTGCGACTCCTGAGCCCTGAAACTCCGATTCATTCCCAAAATCCAGACGACACGAACCCTAAATTCCCAACCGAAAAACCTAACCGAGTTTTTCTTTCAGCACGCACAGAGAGAACAAGTGAATGCGGTTTCCTACACAGTGAACTCTCTAAAAGCCATAGAGAGAAGAAAGCGCTGAATCTCTCGCCAAGTTCAAATCTCAGCGAGAAAAACAAAACGCTCGCAGCTCGAACGAAACGACGTTGTCATAAAGAGATTAAAAAAAAA

>C100541_65

AAGTTAAAAGCAACATATTAACTTCTCTCAGTCCTCAATCCTCACCACCCCTCCCCACTCCCTATCACCACTATTTTCACCAAAACACTGAAAATCCCTTACTTCTTTAGCCATGCCACAGAACTGCATAGCACCAAAACCAGAATACTGCAATAGTAACCACTCTGCGGAAGGACCCCCAGAGATGACTGAACCCCGTAACTCAACTGTCCTTTCCCATCCCATGCAAACCAATGAACAACAACAACCTTTTCCAAAACTCATTATGTACCCCATAACTCTGAAGTTTGAGGAATTGGTGTACAAAGTTAAACTTGAGCAGAAAGGAGGGTGCTGGGGCAGTAAATGGACTTGCAAGGAGAAGACCATACTGAATGGAATCACTGGTGTGGTGTGTCCAGGAGAAATACTAGCTATGTTGGGGCCGTCAGGTAGTGGCAAAACCACACTTCTCACAGCACTTGGAGGCCGACTCAGCGGCAAACTATCTGGTAAGATCACGTACAACGGCCAGCCTTTCTCTGGTGCGATGAAGAGAAGAACAGGTTTTGTGGCTCAGGATGATGTGCTATACCCTCACCTAACTGTCACTGAAACTCTAGTGTTCACTGCACTTCTAAGGCTTCCCAACAGCTTGACCAGAGATGAAAAAGTTCAACATGTAGAGAGAGTGATCACTGAGCTAGGACTGACTCGTTGCAGGAGCAGCATGATAGGTGGCCCACTTTTTAGAGGAATATCAGGTGGAGAAAAAAAGAGAGTGAGCATAGGCCAAGAAATGCTCATCAACCCAAGCCTCCTGTTGCTGGATGAGCCTACTTCAGGCTTGGATTCCACCACAGCACAAAGGATCCTCAACACAATCAAACGCCTAGCTAGTGGGGGTAGAACTGTTGTCACCACTATTCACCAACCCTCTAGTAGGCTCTACTATATGTTTGATAAGGTGGTCTTGCTCTCTGAAGGCTGCCCCATCTATTATGGTCCTGCTTCAACTGCCTTGGACTACTTTTCATCTGTTGGCTTTTCCACATGCGTGACTGTCAATCCAGCTGATCTCTTGCTTGATCTTGCCAACGGGATTGCCCCTGACTCCAAGCATGCAACTGAACAAAGTGAGGGACTGGAACAGGAAAGGAAGCAGGTGAGAGAATCTCTCATCTCTGCTTATGAGAAGAATATAGCTACGAGGCTGAAAGATGAGGTTTGCAACCTGGAAGTGAACAATCACAATATCACAAAAGATGCTTGTGCAAGAAATTCGATAAAGCCAGAACAATGGAGCACAAGCTGGTGGCATCAGTTCAAAGTACTATTACAAAGGGGAGTGAAGGAGCGGAGGTATGAAGCCTTCAACAGGTTGAGAATATTCCAAGTTGTAAGTGTCGCTTTTCTTGGTGGACTATTGTGGTGGCATACTCCAGAGTCACACATTGAAGATCGGGTTGCCTTGCTCTTTTTCTTTTCTGTTTTCTGGGGATTCTACCCTCTCTACAATGCTGTTTTCACATTCCCTCAAGAGAGAAGAATGCTGATCAAGGAACGGTCGTCAGGAATGTACCGCCTTTCTTCCTACTTTCTTGCCAGAACAATAGGAGACTTGCCACTGGAGCTTGCACTTCCAACTGCATTTGTTTTCATAATCTATTGGATGGGAGGGCTTAAACCTGATCCTATGACTTTTATCCTTTCTCTTCTTGTGGTTCTTTACAGTGTTGTTGTCTCTCAAAGCCTTGGCTTAGCATTTGGTGCTATTTTGATGGAGATTAAACAGGCAACTACTCTAGCCTCTGTGACAACCCTTGTGTTTCTCATTGCTGGAGGGTACTATATACAACAGATTCCTCCATTCATTGTGTGGCTCAAGTACTTGAGCTACAGCTACTATTGTTACAAGCTGCTTTTAGGTGTCCAGTACAATGAAAATGACTATTATGAATGCTCAAAGGGGGAGCTGTGCAAAGTTGCAGATTTTCCTCCTATCAAATCAATGGGCTTGAATCATTTGTGGGTTGATGTGTGCATTATGGCCCTCATGTTGGTGGGTTATCGACTAGTTGCTTATTTCGCACTGCATAGAGTTAGGTAGATCAAAATTATGACGGAGCTCCCATACACACAGCACGAGAACTTGAAGAGTTTTTGTACAAGTCAACTGAGGAATAATGATAGAATACTAGACTTTCGATGGAACTCATGTAATGAAACTTTATCAACTCTCCCTGACTGAGTTGGATGTACAAGACTTATTTTTAATAGATTAGAGAGACTACTAACTCTATGTTTCTTCTTCATCATAATACATTAACGCAAGGGATATGTGTT

>C100543_65

CTAACAGAGTGCTTGAAGTGATTCAAAAATCACAGGAAATTAAATTACAAAAGGGGGATGGTAACAAGAAATCAGTATTTCTTAAGAACAATACAAAAATGAAGGTTCATACCACAAGACAAACATATTCTGTTAGCAAGCAAATAAATGTTCTAGAGTTACAAAGTTATCTAATCATACAAGTATTTACTATTACTAATATCATCATGAGAAATCCAAATAGGCATCTCAAATATTTCCAGATGCTTTCCAGAGTGCTTCTTTGATCATCCTCATTGGTGCTGTTGCTGCTCCTCGAAACTTAGATTTAAGTGTCAATGAGAGAACACCATCACTTGTTGATGATTCAACTGTGTGAGCATCTAAATGTAGGTTGTTAATGGTATCCATGATATCATAGAGAATGTATTCCCTGTAAGGACATTTCATCTCAATCAGAACCTCCTGCTCCTTCACATTGACTTTCACATCCAAAGCCTTTGCTTCTTCAGAAACTATTCTTTCTAGCTCTGTGTCTATCTCATCAATAACACTAGCCTTTCTCTTGTTCATCCAAGGTTTCATTCCCATGCAAATCTTTCTTGTTCCATAGTTATCTGACATCTGTTCTAGAACATCAGGGCATTTTCTCCTGGTTCTTGCTTCAGGATCTGCAACTTCCATGTAAGATTCTAGCTCTTCCACTCTAGTCTCAAGCTTTTTCAAGTACTGAATTGTGTCCCCAAGAATTGAAATCTTCTCCTCCTCACTAATAAAAGACGGAGCCAAAGATTTGACAGCCTGAAAGTTTTTAGTTTCCCTTTTGTTTTCAAAAAATGTTTTCCCCATGAAACCATCATCAGCATTTGCCAATTTACTAGTCCATTCCATTCGGTCATTCTCTTTCTGCTGTGACTTGAGAGAAAAGTAACTTCTGTGCATAAAGGGAACCTTAAACAAAGTCTTCTTTAACATGCTTTGGTGAAACTTCGACCTCTTCCTTTCAGAAACTCTCCCTTTCCTCCATTTTGCAAAACTAGATTTGCAATTTGATTTACCGGCATAAGGGTTTGGTTTAAATGTTGATGAGTTTCCCAAAATAGCACAAAGTGTTTTAATGTAGCATAAATCTTCATCAGCTCCAGCATCCAAGGAGCTTCTCTTTGGTTTATCGCAATCTTGAAGTTCCACAAGTTGAGTTTGGCCTTCATTCTTGGAATCCTTGCCTTGGTTATCAGAAGCTTCAGACATACAATCACAGGAACTCAGGGAATCAGGAGCACCAATGACTAAGGCATCATTCACAAAATGAACTTGAGAGGGCACACCATTGATACCCTCAATCATGGATTCCATAGGGTAATGGTGCTCACAACCATCAGGAGAATCCATATTAGAATCTTCATGGTTATCATCTTGTAATTCATTGATGGGATCGTGATCAAATTTGATTTTTTCTTCAAGGGAACATGGATTCTCCAAAGCCATTGTATCTAACACCCTGTGGTCACCCTTGGTACATGTTGGATATTTGTCATCATGAGGCTTATCAAGCAGAGAGGAAGATTTATCAGAGCATGTAGGCTTTGAGATTTCTAAGAAGCAAGCCTTGACATGTTGAATGAGATTAGGGTCCTCAGTAACCAGTTCAGTTGTGCCTATCTCAATAACGCCTTTCTGGTAGGGAAAACACACCACGGTCTGAATTGATGCACTCTTTGCTAGCAAAGAACGAGAGAAAATTTTACTATCTGCATGTTGAGCATCGCATAACCAGATTGTGTCACCAGTTTCCAGTGCTCTTCCTGGCAAACTTTGATTGTGGTTGAAAACAAAGGACATGCAAACCAAGTAATACCACTCCAAATCTGAGAGGTCCTCTGGAGCTAATGCAGCAGAAGGCCTTTTGGTTTGTGGATCAGCTTCACCTGCAAGAAGAAACTTGTACAGTTCCTTCAGTTGCTCACTCCTCTGCAGGCCTATTTTATCAGCCTTCATTTCCAATTCCATGGCCTGAACTGTCTTTCTTGTCTTAATGTCTCCATTGTAGTACCCTTCCCTCCATTCCAGTACCCTTTCTTCAGTGGTTGAAGGTGCCCAGAAAATCCCATAGCTCCACTGAATGCTTCTCACTGCAACAGCTAGCTGTGTGCATAGGTTTTTCTGTATCTTCTCATGCTTTGGAATCCCCTTGTTGACCATTCTACTTCCTCTGAAATGTCATCAAAATGCAGGCCTATCACGGTTTGGTTAGATAATGGAAAGAGGAAGAGGTTGTTGTAAGTGTGAGAGGTGAAAAAGAAGGGGATGAGAACGAACTTTCAAGGCTGATCCATGAAGAAAAAT

>C100545_65

GGAATTGATCAAGAAGAGAGTGGCAAACGTTTCCTGAATCAAAACTTGAACCAAGCTGTACTAAAAAAAAAACTTTTATTCACTCTTCATGAAACCTTACATCACTAAGCTTACATTTTCAAACCAAAATAAGAACAGAATTGACAGAGTACACAATAACAAAGGACCTGGGCCTTGATTCACACTGCAACAAAAGTTGAAGTTAGTGATACACATCTATAATCTCAGTCAACTTCTTCAATCTTAGGTCCAGCACCAGAAGCAGATCCAGCACCAGGCACGTCATCACCCATAGGCACATCTCCTCCAGCACCACCCTGATACATCTTAGCAATGATAGGATTGCAGATCCCTTCCAACTCCTTCTGTTTGTCCTCAAACTCATCCACTTCAGCCAACTGGTTCCCCTCCAGCCACTGTATCGCATCCTCCACAGCCTTCTCAATCTTCTGCTTATCATCTGCCCCCAGCTTCCCCCCAATCTTCTCATCCTTTATCGTGTTCCTCATGTTGTACGCGTAATTCTCAAGCGAGTTTTTCGCGTCCACCTTCTTCTTCACCTCTTCATCCTCTGCCTTATACCTCTCCGCATCCTTCACCATCTTCTCTATCTCCTCTTTGCTCAACCTACCCTTGTCGTTGGTTATAGTAATCTTGTTCTTCACACCCGCGGTTTTATCCTCCGCAGAGACATTCAAAATCCCATTAGCGTCAATGTCGAAGCAGACATTAATCTGAGGAACCCCTCTTGGTGCAGGAGGGATCCCAGTAAGCTCAAACTTCCCGAGAAGATTGTTGTCCTTTGTTCTAGCACGCTCTCCTTCAAACACTTGGATCAAAACCCCGGGTTGGTTATCAGAATAAGTTGAGAAAATCTGCTCCTTCTTGGTAGGAATCGTTGTGTTCCTCGGAATCAAGACTGTCATAACACCACCAGCAGTTTCAAGCCCAAGACTAAGTGGAGTCACATCCAACAGCAACAAATCCTGAACCTTCTCGTCCCCTTCACCGCTCAAAATCGCAGCCTGAACAGCTGCACCATAAGCAACAGCTTCATCGGGGTTAATACTCTTGCAAAGCTCTTTCCCGTTGAAGAAATCCTGCAAGAGTTGCTGAACCTTCGGGATCCTAGTGGACCCTCCAACAAGAACAACTTCATGAACTTGGCTCTTGTCTATCTTCGCATCACGCAGACACTTCTCCACCGGCTCCATGCACTTCCTGAACAAATCCATGTTCATCTCCTCAAACCTGGCTCTCGTAATTGTTGCATAGAAATCAATCCCTTCGTATAAAGAATCGATTTCAATAGTTGTCTGCGCGGTGGAAGATAACGTCCTCTTGGCTCTCTCACACGCTGTCCTCAACCTCCTCAACGCTCTTGCATTCCCACTAATATCCTTCTTGTGCTTCCTTTTGAACTCTGAAACAAAGTGATTCACCATCCTGTTATCAAAATCTTCACCTCCGAGATGAGTATCACCGGCTGTAGCCTTCACTTCGAAAATCCCCTCCTCGATGGTCAATATCGAAACATCAAAAGTTCCACCACCCAAGTCGAAAATAAGCACGTTCTGTTCACCTTTTCTTGAAGCCTTCTTGTCCAAACCGTACGCAATAGCAGCAGCAGTGGGTTCGTTGATGATTCTCAACACATTCAACCCCGAAATCGCCCCTGCGTCCTTCGTGGCTTGCCTCTGCGAGTCGTTGAAATAAGCAGGGACAGTGATAACAGCGTTCTTGACGGTATGACCAAGAAACGCCTCTGCCACTTCCCTCATCTTGATCAACACCATGGAAGATATCTCTTCGGCGGAGAACTTCTTCTCCTCGCCCTTGTACGTGACCACGATCATGGGCTTGTCGCCAGGACCCGGCACCACCTTAAACGGCCACAGCTTCATGTCGCTTTGAACGGACGAGTCGGAGAATCTGCGGCCGATTAAACGCTTGGCGTCGAAAACGGTGTTTTGCGGATTCATAGCGACTTGATTCTTGGCAGCGTCTCCGATGAGCCTCTCGGTATCGGTGAAAGCCACATAAGAGGGTGTTGTTCGGTTACCTTGGTCATTGGGGATGATCTCAACGCGGTCGTTTTGCCACACACCTACACAGCTGTAGGTCGTACCCAAGTCTATGCCTATGGCTTTGCCTTCCTTCGCAGCCATTGATTACAAATTTTGTAACTTGAGAAAACAAATTCTGAAAATTGGGTGTTTTGTTGTGTGTAGAGTTGTTTCGACTGTGCAGAAACTACGAGTGAGGAGAGAGAGAAGCGAGAGCGCTTTATATAGCAACCGTTAGAGAGTAGAGGGTGAGTTCGAG

>C100547_65

CTCCTCTACTAACAATTAGAAAAAAAAAAGAAAAAATTCGTATAGAAGTTCAAACAAAGTGGGAAATGAAAATGTAAACATGATGTACAAATTTCTGATTTACAAATTAAGAGGGTTCCCAGCTTGTAAGAAGAGATTCAAGGGACTCAACAAACTATTGTCAATATTTGGGGAAGCACACTGGGGAACAGCAATCAGAAGATCGAACTACCCAGAACAATGGGATATTATCGTGAAAGCAAGATAGGCCCATTCTCCGCAGCAGTGTGAGCATTGCCATAATTATTATTATTATTATGCCTTGCCATCTGGATGTCACGTGAGAATTGGGTCCTTGCTTTTGCATATGGAGCTGCCCTTGCTGTTATTTGTTGAACCCGGAGGATATTCTGCCGCCTTTTCCACCAGCAAACTGAACCGGTAATAACAAGTAGAAAAGTAAAAGAAGCACCTAACCCAATACCAACTTTAGCACCAGCAGAAAGGTGAGGTCCACAAGTGGGTAATCCAGGTATGCCACATAGACCTGCATTATCAGTAAAATTAAAGCTTGCCCCATGTAGAAGTCTTCCTCCTAGAGTCGCTGGGACCCTTCCAGACAGGAAGTTGCCATTTAGGTTCAGTCTTTGTAATGATGTCAATTGTCCAAGGCTTTCAGGAATCGATCCATTGAAGAAGTTGTAGGATAGGTCAAGCACTTGCAAGCTAGTTACTGTTCCAAGTGGGTATGGAATAGCCCCACGAATGCTGTTTCCACTCAAGTTTAGGATTTGTAGATTATGAAGTCTGGAGATGTCATTTGGCAAAAATCCCTTCAGACCTTGATTGTCAAGACCAAGTCCATCAATGACCCATTTGTTGCTACTTTTGTCTAATCTGCAATCTGCTCCAGTCCATGGATGTTGTTGGGGAACACAAGGATCACCATTCCACCCAAACCTGGGAGGAAGCCCCAAAGCCTTCTTCAACGTTTGTAAAGCCATAACTTCATCTGATAATGTTTTTGACTCAGCCGTTATAACCTCCAATATCTCAATGGCATTGATTATGGCAAAACCACCTTTTTTTGGTCTCAAAGTTATGGTCAAAGTTCTCCCATTAACAGTAACAGTTTTATTAAGCACAAGGGCAGTAAAACGGTCCCCACTCAATTTCACAATGTCAACATCTTTAAATTGAACATCGCCATTTATCATAATGTTAAAGACTCTTTGTCCAGCGGCAGTCACTGAGTTATCAATCTCAGCAAAATGTAACCAAACAGAATAGTTTTTGTTGGGATCCACATCTAATGTATATGTTAAATCAGGCTGGCTACTAGAACTAACAAGTGCTGATCGATAAAGAGTTTCGGGATAAAAGTTTGGTGGATGTGAAGCCTGTTTGATTCTAGTTTCAACAGATCTCGGTCGATCAGAATCCTCACCAAAAGTTTTAATATGCTGCCAATATCTGTCCCCTCCCCGGGGATCTGCACCATAATCCACACCAAATTTTGACTGCCCAAACCCGCAACTCAATCTTTTTACTGTTCTAAGTATTATCCCTTGACTCCACCCCGGTCCAAAATTATAAGCTTCATCATCAATTTGAAGAATCTCGATTGAAAGAATTGCTGGATCTCCATGACCCGTGCCATGGAAACAGATTGAGACTGAACCATCCATAAGAAATACTTGGGCTTCAGTAAATGCTTGATCATCGTGAGTAGTCCAACCTGATTTCAAAGAATATATTTGAGTTCCTTGAATTGAGATGTCAAATAGAGGTTCATCGGTAACTCTGGCATGTGCAACAAGTCCAAAAAAGATCCTGATTGAGTAGTGTCCTTTTGGCACTCTATTAATGTTGTAGCAATTTGAAGGACCTTCAGAGAAGGGGAAATAGCGTAGAGTCTTCAGAGGAGGGGCAATGAAACTTGTCGTAGATGCATTTGTGGGAATGCCTCCTGTGTATCCAAAGTCTTTATACCAAAGGGTAGTGGTTGGTTTTGTTTGAACATCATTACGAGCCCCACAGCTTATGTGCATGCCATATGGCCCTCGTTGAGCTGCACGCGCAGTAAAGGCAAAGCAGAGGAAGAGCGTCCACAGCAGAAGGAATCGAAACGTTGCTATGTTCGCCATCTTCGCTTTTCTTCTTCTTTTCTCCCTCTCTCTCACATCCTAACTTTTCTAGAGAGAGAGAGCTCGAACGTGTCTGCACCTTGCAGCAACACTGCACAAACAGTTGGAAATGGAAATTCACAAATTCACATTCACTCCCTACTACTTCGTTTAGTTGACTCGTGAACGAATGAAGGAACAGCGGTTACAGTGGTGCTGAG

>C100549_65

TAGGTCTCAATAAATTTAAGATTAACTCAAAAATATACAAATTACTGACGAATTTAGTAGCACTAAAATTTAACTGAATTTCTCAACTCTTACGAAGATGGCGTACATATAGGACCCAGAGGGAGTACAAAACTATGATGCGCGTTGCTTTTTAGGCGAGAAATTCAGCTTCAACCACTTTGATCTGATATATCTGTTAGCATACTCAAATATATTATTAGCCACTTTTTGCAAGAGCCCCAGCCGCAAAAAGAAGATTCCTTCGAGAAAACCGCAATGAGTAAACTGGAGTAGATTTGGTTGGCAGAGTTTTCAGCGATCGAAGTCTGTTAGCATTCCCACTTTTTTCTTCAGCCCTTGAAACCTTGGTGCTCGTATTTACATCCCACAATTTTACAGAACAATCAGCAGATCCAGATGCTAGAATAGAACCTTCAGAGCTGAAAGCGAGGGACCAGACACAGGATGTGTGCCCAATCAAAGGTGTGAGACAGCGCCCACTAGAGAGGTCCCACATCATGATCGTGCCATCTTCATCACCAGATGCCATATAACGACCATCAGGAGACATTGCCAAAGACAAAATCATACCCCTGTGACCAACAAAAACCCGAACGCACTCACCACTCTGCACATCCCATAGTCGAACTGTTTTGTCACTGGAACCAGTTGCAATGTAGTTGCAGTTGGCATGCCATTGCACACAATCAACATCAGACAAGTGCCCTGCCATTATTCTTAAGGGCTGTATCCTATCCATGGACCAAATCCTAGCAGTTCTGTCATGTGAAGAGCTGGCAAAATAATGCCCTACAGGACTAAACTGAACATCCCAGACTGGGTAATTGTGACCCTTATAACAAACAAGATTGGCATTAAGTTTGGTGCTCCATAATCGAATTGTTGAGTCTGCTGAGGACGAAAGGATAAAATCCCCGACAGGACTAAAAGATGCTGCATAAACAGGCCCTGAATGACCTTGAAACAACGTATACTGTCTTTTCCCACCACCTTGCCCTAACGTTTGATCATTTTGTGATGTGTCATTCTCACCCTGCGAAAGAGAACTGGCTAGCGGTTGTCCAAGCTTTGCCATATCCCAAACCTTCAGTGATGAGTCAGAAAATCCTCCAGCAATCAATGATCCATCATGGGATATTGATGAACAGCTTAAACCATTATGAGTATTTATAAATGTGTAAAAGCTAACTGATGGCAATGCAACACTGCTGAGCTGTACACGGTTCCTTAAATCCTCAAGGATGGAGTGTTCCACCTCAGTTGGGATTACGGGTAAGGGGAGTTCAGGCTTAACTCGAGGAGCTGCAGGTGCAGTGTTAGCTTCAGGTTTTGCATTTTTTCCACTTGAACTACCACCCTTGTCCTTTTTTACTTTTTTAACCGAACCACCACCTTGCTTTCCTCCTTCAATGGATCTTTTCTTAGCCTCGTCATTCTCCCCCTCTTTTGCTTCCCCTTCACCCTTTTCAGAGTCTGAAAGCAAGGCCCCTGCCTTCTCCAGCCGTTCTTCAAGAGAATCTTCAAGTAACCCCCAATGAATTTCTTTTTGATTTATCCGGTTTGCTGCTTCCTGGCTGCTTCCAGTAAGGGTAACAGCTTCGGGATCATCAGAAATTAAGCTAGGTTGTCCAGGGGAGACTTGAAAGTTAATATGCTCATTGATAATACCAAGAATAGTAGTCGATTGCGTACTGTGTAGATGTTGCAACAAAAGCTCATAGGAATACTCACATATCTTTATGTTGAATTTGCTCTGTCTAAGCGAGTGAGCGAATTCCATTTCCTGCAGATGCGTAGGAGAAAGAACTCCTTCCAACTTCTGAAGGTCACGCAAGTGCATCATTTCGTGGTCTTCACGGAAAGTATTGAAGAAATTCCGAGCTTCCTGAACGTGTCCTTTCGCGACAAGGTCCATAAAGCAGTGGATAAACACGGGATAAAGCACGCGAAGCAACTCGTGCTTGTACAAATCCAGTGAAGAATAAGTCCATGTTCGCAGTTTGCTGTAGCCGTCGTGGAACCTAGCCGGACCACTCTCCAATTGGGAGAAGGCGAGGAGGTGGTTGGCAGAGTCGGGTTCGAGAAGGGAATTATTGGAAGAGGAATTGGAGGTTTTGTTGTGCTGAAACTCTTCCTGGAAAATCTTCTCGGTTTGCGTGAAACCTTTCTTCTTCAGGTACGCTGTAACGTATCCAACTATTTTATCATCCTCCATTCTTTGATCTCAGACGCCAACCCCTTCTTCGTCTTCTTCTTCTTCTCCTTCTTCTTCTTCTTGCGCGCTATGCACCTTCTACAAATTTCA

>C100551_65

GGCCGAACTAGCAGTTATTTGATTGAACAGTTTCAACTTCTGAAGCAACAACAAAGCAAATAGAGGTGAAGAATGGCCAGTTTTCACGGAGATGTTTACGGGTTGAGGTACATGATAGGTGTTCTATAGTATAAACGTAAACTTGGTATATTTTGTTCCATATTCCTTACCGATTTTGAAACTCGGTCGCACAAATAATCTAAAAAAGAAAAAAACATTCTTTTAATTTCAGAAACATTCGACAAGAATAGTTGTGCAAGAGCTGCAAGTTTAACTTATTCTTTTCTACTTTGAAAGTGGTAGAACATAATCTGTGAGGTCATTTGATATGTGATGAGTCACAATTTGGCCACTTGAGGAAGGAAGTTTAGATCCACGGAGACGACAACCTTCCCAACACTTTTGCCTGAATGGAGATACTCAACGGCGTCAGCAACAGAATGCAGGCCTATAAATCTCTTTGGATCAATAGCAATCTTTAGCTTTCCCGTAGAGTGAAGATTAAATAGGCTGTCAAGATGTTCTTGCCACAAGTGACCATATTGCACCAGGAAAAAGCCAGCCACAGTTTGGCTCTTTGCTAAAAGCTTCTCACATAGGCCAGGGTATTTCGATGGCGTCCAACCCTTTTCTCCTTGATACTGAGAAATCATGCCAATAATAATGAGTCGTCCATGAACTGCCAAAGCATTCAAGCACAAGTTTAACATGTCACCTCCAACAGACTCATAGATGATGTCAACACCTTTTGGGTACTCTTCCCTCAGAACAGTTTTTATATCTTCTTTATGATAGTCTATGACTCTGTCAACTCCCAAATCCTTCAGAAGCTTGGCCTTTGCCCCACCTCCACAAGTTGCAACCACCCTATTTCCTGCTAATTTTGCTATCTGAACAGCAAATTGCCCAGTTCCTCCAGCCGCGGCAGTAACAAGGACTACTTTTCCAGATTCCATCTGTCCTGCCTTTTCTAGTGCAATTGATGCTGTTAATCCTGATGTAAGCATGGCAACGACTTCAGGATCTGGTCTAGGCACTGGAAGGGCATGTTTTGAAGGAATCATTATGAATTCAGCATAGCCTCCAAAAGTCATGAATGCACAAGGCATACCAATTTTCAAGTCAGTGACCGAATCCCCAACTGCTGCAATTATGCCTACAGCCTCAAATCCAGCATCAAATGGAAGACGAGAAGCAATATCATTGTTGTTTCCTCCAAAATAGCGGCCTGAGCTAAAATTTACATCACTAGCATTCACACCAGCATAAATTATCTTAACAAGAACATGCTTTGGCTTGATAGGTAATCTCAGTGGTGTTCGCACTATGCTTGTTGCATTCCTAAAGTTATGAGTCAAGGTGTGAACAACTATTTTCTCAAAACTCTCAGGAAGTTTAATTGATGGAGGTTTGAAATCGGATCTTCTCTTGAAACGAGAGGAACGAACCATGTACTTTGCTTCTTCAGATGGAGTGGGCCAATACTCAAAACCCCGACGATTTGTAATCCATAGGCAATGACCAGCTTTACTTTCATCCGTAATGAGTTCAAAAGCACCTTTGGCCACCATTTCCATAGGTACAAAGCCCCCCATCAAGTTGATGAAATTGGAATCTACCTTTAATCCCATCTCAGTTTCAACAAACTCAGGGCAAAGCACATTGACTCGAATTCCTTGACGTTTGTATAGACGAAGTGCTCTAGAAAACATAACAACACCACCTTTAGAGCCGCTGTAGATTGGGTCAGCCAACATTGGATAAAGACCAGAGGCAGAACCCAAGTTGATAATCACACCAGGTCTTTTGGCAGCTTCCATGGCTTTAATCGCAAGGCGAGTGGAATCAATAAGAGCAATGAAATTCACATTAACAGTGTGTCTCCATGTTCGGGTGCCATCTGTTTGATCCTTTGGGAATGGTATAGAACTACTGATCCCCGCACTGTTAATACAAATGTCTAGACCTCCATAAGTCAAAAAGTGCTTTTCAAATGCAGCAGCTAAATCCCTTGCATTAGTCACATCACATTTCACAAACATGGCAGAGGGAAATTCCAACTTCGAATGAAACTTGGTGTTAATTTTCTCAACGAGGTTTGCAACTTGTCTTCCATTTGCTTCAGACAAATCCACAATAGTGATAAATACACCCTTCTCTGCAAGAGCTAAGGCAAGACCCTTGCCGATTCCAGAGGCACCACCAGTGACTAGTGCTGACAAACCAGGTCTGATCTCCATTGCTATGGTTACTCCACTGCAGGTGCGGTCGGTTATCACGAAAAAATATGAGATTAGTGATACAAACGATATACGATACTATACAT

>C100553_65

AAAAAGAAAAAAAGAAAAAGAAAAAGCGGAAGAACGAAGAAAAGGAAAAGAGAAAGGAGAGCGTTGGTGCCAGATCCTGTAGCGAGTCCGATGAGTCCGATGAGTCCTCCTCCACACTCAGATCTGCGAGTTTCGCTTTCTGTTGGTGTTCTCTGTGTTGCTACAAATTGAATGTTCAATCCCCAATTTCCTTTTTCGAGTTCTCAGAATTTCAAATTTCCTAACTACGACCCCAATATCTAATGTCTTGTCTAGCGCTTTCCTTGCAACCCGCAAATGGATCCGACATCCTTCTCCAGACTCGCGAATGGTTCCCTCCCTCACGCGCCCTCGGCGCCCTCTCTGCGTTTCGCCAGACGCGCCGCGCCCTCGCTGCCAACAAGCACTCCACGCCGGACGACGCCTACGCCGCCGAATCCATCGGTGACGATCCCCTCGCGGCGTCCAGTGGCCAGGTCATCGTCGGCGTGGAGAGTCGCTACCGCGTGGTGTACCGTCTCGTGAACAGCATCTACGTTCTCGGTATAACCGTCGCGGACCACGACAACTCTGTCAACGTGTTCGAGTGCATCCACATCGTCAACCAGGCCGTCAGCGTCGTCGTCACCGCGTGCCGCGGCGTCGATGTCACGCCGGAGAAGCTCAGCCGCAAGTACGCTGAGATCTACATGGCCCTCGACATCGTCCTCCGCGGTGTCAGCAACATCCGCCTCGCTGCCATGCTCGCCACCTTGCACGGCGAGAGCATTGCCAGGATGGTCCACTCCGCCATCGACACCGAGAACAAGATCCGCGGTGCTGATACATGGTCCACGGCGGAGGTGCATTCACTCGAACACCAGGCCTGCATCGACGCCTTGTCCACAGCCTCATTCGAGTTGCCACCGGAGACGCTTGAGGCCGGTGAAGAGATCGCAGCCAGCCTTGCCCCCGCCCCACCGGAGGTGCAGGAGGAGCCACAGCAGAAGCAGGAAGAGTCTCAGGTAGAGGACCCTTTTGCAGCTAGTGATGCAATTAACAAGCCGCAGGAACTTGTTGAAGGGTTCAAGAAAACAAAGGACCCTTCTGCTACTGATTTAACCGCGGCGTTGGAGGGGCTTGATGTCACCACATTGCCTCCTCCTGAGGCTACTCAATCCACGCATATAAACGTGGAGGGTTTTGAAGGGAACTACGGTGGAGTTGAGTTTGGGCACGAGCAAGCTTCTATTGGAGAAGCTTTTGAAGGTTTCAACGATGCTTGGGGTGGTGGACTAGATCCTTCCGAGTTTGTGGGCACAACCAAGCCTCCCAAACCGCAAGGGCTTGGTGGGGTTGAATTGTTGCAGACTGGGCCTGATGCTGCTCCTAAATCCGCTCCTGATAGTGGTGCAGGAACCCCACTCGATAACTTGCTGCTGCTGAATAAAACTGAGATGAAGGGTCCTGAGATGTATATTTCGGAGGTGATTAGCGCAGAGTTCAGGGAATCTTTGCTTGCAAGAGTGGGATTGATGGGTGTTGTTTACCTCAGAACTTTGCCACCCAAAACTGCTGGTGATAAAGAAACTGAATTCTCATTCCGAATTGAGGGAACCAGTGCTGTTAAGAGATTTGTTATTCAGAGTTCGCGCGTTAGTAGCCTTGGTAATGGGTTGTTTCATGTGAGGACTGCTGCCTCTGAGGAACCAATACCAATTATAAAGTATAGTTTGGTTCCTAGGTTGACGCCCTTGCCTTTGAGGGTTCGACTTACGAAACGGCATACTGGGAGTTTGCTTTCTGTGATGATACAATATGCTTCAAATCCTGATTTGTTAGTTCCCTTGCATGATGTTACATTCACTCTAAAACTGCCCGTTGATCCGACTCTGTTGAAGGTTTCTCCAAAAGCTGTGTTAAATAGGACTGAAAGAGAAATTAAATGGCTTGTGCCAGAGATTCCGTTGAAGGGTACTCCTGGTAGGTTGAGAGTTCGGATGCCAGTAGATTCTAGTGAAGATGACGAAGAAATTGAAGTTGTTGGTTATGTCAAATTTTCTGAACAAGTAACTCAGTCATTGTCTGGAGTTTCTGTACGGCCTGCTTCGGAGGGTAAGACAGACTTCTACGAGGTTAGTGACAGACTTGAGAGTGGGATATACATGTGCAATTGATTTTGTAATCATAGATAATTAGCAGGAGCTGTACTTTTTCATTGTGTTCATGAGATTTATGGTTTGGTTGTGTTTATAGCATTCTTGGATATCGATCATGTACTCTGGAATTATCTGAAAGTCTTTGTTTCTCTGTATTGTGACTATGAATTTACTTCAATGGACTGTATTTCCTCTTTGCAATAAAAAAA

>C100555_65

GTGTGTAAAGAAATAGCGGTGAAAGAAAGGTAAACGGGAAAAACAGAATAGAGATAGTGTTCTTCCCCATCTGGTTAGGGTTTACAGACAGGGCTGCATTGCACCCTGAGAAATTGCTAATCAAATAGTCTGTTAATCTGCTACGATGTCTTCGTCGAAAGAGGCGGACCCAACCCTGGGTTACCTCACCCGGAAGGACACGGAGGTGAAGCTTCCGCGTCCAACCAGGGTCAAGAACAAAACGCCCGCTCCAATTCAAATCACCGCCGAGCAAATTCTCCGGGAGGCTCGGGAGCGTCAAGAGGCGGAGATCCGCCCTCCCAAGCAGAAAATCACCGATTCCACCGAGCTCGGCGAATACCGTCTCCGCAAGCGCAAGGAGTTCGAGGACCTTATCCGCCGCGTTCGATGGAACATTGGCGTTTGGATCAAGTACGCTCAGTGGGAGGAATCGCAGAAGGACTTCAAACGCGCGCGTTCCGTTTGGGAGAGAGCGCTGGAGGTGGATTACAAGAATCACACGCTCTGGCTCAAGTACGCGGAGGTCGAGATGAAGAACAAGTTCATAAACCACGCGCGCAACGTTTGGGACCGCGCCGTCACGCTTCTTCCGAGGGTGGATCAGTTGTGGTACAAGTACATTCACATGGAGGAGATGCTTGGCAATGTCGCCGGCGCGAGGCAGGTTTTCGAGAGGTGGATGAAGTGGACGCCGGACCAGCAGGGATGGCTTTCCTACATAAAGTTTGAGCTTAGGTACAATGAAATTGAGCGCGCCAGAGGGATTTTCGAGCGATTTGTTGAGTGCCACCCTAGGGTAGGGGCTTGGATACGCTATGCCAAGTTTGAGATGAAGAATGGCGAGGTTGGGAAGGCTAGGAGCGTGTACGAGAGAGCTGTGGATAAGCTCTCTGATGATGAGGAAGCAGAGCTGTTATTTGTTGCTTTTGCTGAGTTTGAGGAAAGGTGTAAGGAGACTGAGCGTGCAAGAGCTATCTATAAGTTTGCTCTTGATCATATTCCGAAGGGAAGGGCGGAAGATTTGTATAGAAAGTTTGTGGCATTTGAGAAGCAGTATGGGGACAAGGAAGGGATTGAGGATGCCATCGTTGGGAAGAGAAGGTTTCAGTATGAGGATGAAGTGAAGAAGAATCCATTGAATTATGATTCCTGGTTTGATTACATAAGGTTGGAGGAAAGTGTAGGGGATAAGGAAAGGATCAGGGAGGTTTATGAGAGAGCTGTAGCCAATGTTCCCCCGGCCGAGGAGAAGCGCTACTGGCAGCGCTATATCTATTTGTGGATTAATTATGCACTCTATGAAGAGCTAGATGCTGGGGATGCGGAGCGAACAAGGGATGTATACAGGGAGTGCCTCAACCAGATACCTCACAATAAGTTCTCATTTGCAAAAATATGGCTTCTAGCAGCCCAGTTTGAAATACGTCAGCTGAATCTTAAGGCTGCTCGTCAGATATTAGGAAATGCCATTGGAAAGGCTCCTAAAGATAAGATTTTTAAGAAGTATATAGAGATAGAACTGCAGCTTGGTAATATAGACCGGTGCAGAAAACTATATGAAAAATATCTGGAGTGGTCTCCAGAAAATTGTTATGCTTGGAGCAAGTATGCTGAGTTGGAGAGATCATTATCTGAGACTGAACGAGCCCGAGGAATTTTTGAGCTTGCAATCGCTCAACCAGCATTGGACATGCCTGAGCTATTGTGGAAGGCATTCATTGACTTTGAGACTGCTGAGGGTGAATTTGATAGAGCAAGAGCTCTTTATGAAAGGCTTCTTGACCGAACAAAGCACTTGAAGGTATGGATAAGCTATGCGGAATTTGAGGCAACAGCTATGGATATGGACAGTTTAGACATAACGGAACATGAACAAAAGAAGCAATGCATTGAACGTGCTAGAAGGGTGTTTGAGAAAGCTCTCAACTACTTCAGATCATCAGCTCCAGATTTGAAAGAAGAAAGAGCAATGCTATTGGAAAAATGGCTCAACATGGAGACTACATTTGGAGAGCTGGGTGATGTTAGCTTAGTCCAGTCTAAGCTGCCCAAGAAGCTCAAGAAGAGAAGGCATGTTGCCACTGAAGATGGTTCTACCAGAATTGAAGAATTCATCGACTTTTTGTTCCCTGAGGAAAGCCAGACAACTAATCTCAAGATCTTGGAAGCTGCTTACAAATGGAAGAAGCAGAAATTGTCTTCCGATGATTAACAATAATTTGTACTTGTTTTATTTTTAGTGTGTATATATTGGGGAAGATTTAATTCATGTGTAACTAGCAAATACAGGAAGTTCATCTACCTGT

>C100557_65

CAAACACTGACACAACATAGTTGCCCTTCGATTCCTGTGTGGAACAACAAAGCAATCAAGCTCGGACTTAACCCAAATCCCCAACTTGGTATGCACCGAAAGATTCATCTCTCTGCGCTCCTTGAACCTCACCTTCTTGCTCTTGATGTTTGTGTAGGGGGCAGTGTGTGAGGGTTCTGAAGGGTTGCTCCTGAGAATGGCTGACATAGCAGGTCTGGCTGAGGCTGCAGGGGCAAGATTTAGTTCATTGGAGCTCATTGGGCAGGGATCATTCGGCGATGTGTATAAAGGGTTTGACAAGGAGCTAAACAAAGAAGTTGCGATCAAAGTGATTGATTTGGAAGAATCAGAGGATGAAATAGAGGACATTCAGAAGGAAATTTCTGTTCTGTCACAATGTCGATCTGCATACATTACTGAGTATTATGGTTCCTTTCTTAATCAAACTAAACTCTGGATAATAATGGAATACATGGCTGGAGGCTCTGTTGCTGATCTACTGCAATCAGGTCCCCCACTTGATGAAATGTCAATTGCTTGCATTCTACGTGATTTACTTCATGCAATTGATTACCTACATAGTGAAGGAAAAATTCATAGAGACATTAAAGCTGCAAACATTTTATTGACTGAGAATGGTGATGTAAAGGTTGCCGATTTTGGTGTTTCTGCACAGTTAACAAGAACTATATCAAGGAGAAAGACCTTTGTAGGAACACCATTTTGGATGGCACCTGAGGTTATTCAAAATTCAGAAGGATATAATGAAAAGGCAGATATCTGGTCTCTGGGGATTACTGCAATAGAGATGGCAAAAGGGGAGCCTCCACTTGCTGATCTTCATCCAATGAGAGTGCTTTTCATCATTCCCCGAGAGAATCCACCACAGCTAGATGAACACTTTTCTCGCTACATGAAAGAATTTGTTTCATTGTGCTTGAAGAAGGTTCCAGCTGAGAGGCTAAGTGCTAAGGAGCTTCTTAGACATCGTTTCATTCGGAATGCCAGGAAGAGTCCAAAGCTCTTGGAGAGAATAAGGGAGCGTCCAAAGTTCCAAATAAAGGAAGATCAGGCAACACCTAGAAATGTCCCAAGGGGGATGGGGGAGGCATCTGATACTATTAAGGTTGCTAAAGAATTAAGGGTTGATGAGACTAATCAGCCTGGTGGTCAAGGTAAAACCTTGAAAAGTTCTGGGTGGGATTTCAGCATTGGTGGATCACAGGGTACTGGAACCTTCCGAAGTGTGTCAAGGCCACCTCAGTTTAGAGATAAGAAAACTGAAGTTTCAAATCATCAGCTTAACCAAAGAAAAAGTCCAGAGAGTGGCTATCAAGGGGGATCTGGCAATAAAAGTGCACTTAATGAGTCGCTAGAGACTTCTTTTGGAAAAGATTTTGGAGTTCCATACCACGATGAACATCCACATAATCACCTTGAAGATGACGAGTTGTCTGGGAATGGATCAGGAACTGTTGTTGTTCGGTCTCCAAAAGGACCACAGTCATCACTGTTTCGTGACCAAAGTTCCCGCTCTAGCAGCAGCTATGCTTCTTTTGAAGATGTTTCTACTAGCGGAACTGTTGTTTTGCGTAACCAGCACGATGATTATGATTCTCCACAGACACCAAGGTCCCGACTGGGACTTAATAGCAGAAATTTAAATGCCTCATTTGAAGATAGTGCTACAAATCTTGCTGAGGCAAAGGCTGCTATCCAAGGTGGATTGAGGAAAGTGAATTCTAGGGAGAGATTTGCGCTTGGAAAACTTAACAATGATGTACAGGAAAGCAGGAGAGATCAAATGTCAACCAGCTCTGATTCTTCCAGACCATCTCGTGAATACATTGATGCACAAAAGGTTTCAAGATCACATTATGCTATCGATGATGAAGAAAGTGGCAAAATAATCTCATCCTCTGCGCCTTTATCTGTTTTGCTCATTCCTTCATTAAAAGAGGCCATTGCTGAGGATCCAGAAGGGCCAATTGTGCAAATTGTTATCAATGCTCTTATAAACATGGAGAGGACAAAGCCCCAATCCTGTGATGCTCTTGTCAAAAAATTGCTTCAGCGGTTGGCAAGTTCAAAGGAATCTTCATTGAAGGACCTGCAAGAACTGGCTGTCCAAATACTCAGCAAGACTAAGTCAGCAGACGAAACCCGAAATGCAGAGACAGATAACAAGAAGAAACAAAACAAGGAACTTCATTCAAATAGCAATTTAAGCCCCCTCGCAAGATTTCTTCTCTCAAGATGGCAGGGCCAAACCTCGCGGGATCTAAATCCATCATGAACGGAGTCAGAGGTTAATTTTTGTGTGAGGA

>C100559_65

GAATACATATAATTTTCATAGCACAATTTGTTTACACGCCAATGACCTAATCCTGTATAAATATATTCTAGATAAAGAGAATGAACTGAATCTTCATGTATTTCTTGTTCTTTTGCGAATTAATAATGCCACTAAAGTCTGCTCTGAATTGATAGCTCACGCTTCATTTGATCTCTCAAAACTCTCCTTAGTAATTTGTTGGAAGCTGTTCGAGGAAAGTCCGGCACAATTTTAACAAGGCTCACCTTAAACAAAGGATTAAGGTTGCTTTGAATGGCTTTAGCGAATATCCTTTTTAGAGTTTCTGCATTTGAATTGTATCCTTTCCTTAAAACAACAAATATAACCAGTTGTTCTGGGCCTCCATGTGCAGGTGCAACACTGATTGCAGCTGTCTCCAAAATGCATTCATCAGCCCTATCACAGACACGCTCAATTTCTACAGAACTTGTCTTTATTCCACCAAGATTCATTGTGTCATCAGCCCTCCCTTGTACAACGAAATGGCCTTCGGCTGTTCTTTTGATTATATCTCCATGTCGCCTAAGAACCTTTCCTTTGTACATTGGCATTCCCTTAAAGTAAACCTCATCATTATCAGCATTAAGCAATCTATCAGTTGCTCCCATATATATAGGAAACAAGCCCACTTCACCAACACAGGCAACATCCTCCGGATAAGGAACTCCATTTTTGTCAAGGATGACCAAACCAGTTGTCATTGATGCTGTGCTAAATGATCCAAAAGCTTGAGGCTGCAGTGGACTTCCCATGATGTAAGAAGATGCAAGCTCAGTGCCTCCACAACATTCAATGATTGGTTTGTAATAAGACTTTGAAGAAAGCCAGAGGTCATCATCGACATTTGAAGTTTCTCCAGTCGAACAAAATGTTTTTATCTTTGTCCAATCCAAGCCCTCCATGCATTGTGTATTCTTCCATGTTTTTACTAAGCTCGGAACCGTTCCCAAAATAGTTACACCTGCATCCTGAACGAACTTTCCAAAACCATGGCCTAGAGGAGATCCATGGTACAGAGCAAGAGTAGCACCTGATAGAAAGCATGAATATAGAAGAGTTGGGCCCATCACCCATCCTAAGTTTGTAGGCCAGCAATAGACATCTCCTGGTTGAATATCAATGAAAGCCCATCCATCTGCAGCACTTCTTATGGGTGAAAGTTGAGTCCAAGGAATTGCTTTTGGCTCCCCTGTGGTTCCAGATGAAAAAAGTATGTTAGTGACAGAATCAATTGATTGATAGAATGGAGAGTAGTGATGTGACCTGGGATGCTGTCTGCTAGAAGAAAGAAAATCCTTCCATGATAAATCACATTCTCTTAATTGCACACCTAGATCATCACCTATCACAGGAAGCACGATAACTTTACATGCAGCTGCCTCAATCACTCGACTGTACAAAGGGAATTTCCTGCCACCTCTTGATATGAAATCCTGAGTGAAAATACCCTTTGCTTCAGAAACACGCAGGCGAGTTGCAATTTCTTTTGGTACAAAACTATCAGCTATTGATACCACAACACATCCTGCTAGAACAATGGCTAGATATATAATCACTGCATTGACTGTCATTTGCATGTCAATGGCGATTGCATCTCCTTTCGAGAATGTGGCATCTATTGCATTCGCTACCAACATTACTTGTTGCCGGAGTTGTTTAAGCGTGATACTATTAACCTCGGAATCATCAAAACCTTCATCTCTCCAAACAATGGCTAAGCTGTCATCTGGTTTGTAGGGATGTGAACTGGGTTGCACACAGCAATCAGCAATGTTGAGCACAGAACCTGGAAGCCAAGTCCCTCCATGTTTAGAAGGGTCAGAAGTATCTAAAATGCACTTGGGAGGTTCAACAAAGGAGATCGAAAGTTCCTTGAGAACAAGGGGCCAGTACAGCTGAGGGTGTTGAACAGAAAACTTGTGAAAGAGAGGAAAACTGGTAATAGGGTCTTTGTAAGAAGGTCCTAAAAGTTGAGGGGCATGAGTTTCCATGAGACGGCCTAGGTTGGTGCGTTTGGAGTGGTCCAGAGAAGGAAACCAATAGAATGGAAGGGAAGCATCTTGAGCGTGGAAGGCAGAGTGATAAACAGAGTGGTAGAGAAGCTGATGCAAGGAGTGTGGAAAGGATGGTTTGAGCAGTTTGTGAGTGACAAGGTGGCGCCATATGAGTGAGGGGTGGGAGGGGGAGAGACACTGAGACAGAATATCCCTCAAACCGCGGTAAAGTTCATTGGCTTCTGCTACAGACAAGCCTGCATTCACCAAATCTTCCACTTCCAATTCCCTCACGCTCTTCCCCATCTCTTTC

>C100567_65

TCCCTCTTAAAGGCATCACATTACTGGCACAAGTAAATAACAAAAAACACTATCCACTCTTCACTATGTGAATCCTTCTACATATTTCATATACATACAAAGATGAGAAAATTGAAAAGTGCTTCGGCTATAAAAGATCAATAGCCTCATACATGCCATTTCCGGTCCAAGATACTTGGGCTGGCACCACCTTCTGTGCAATCACTAGACTCCACATCTGTTTCACATGTTGCCACTTCTTCATCAACTGTATTTGATTCTGAAACTTGTTTCTCATTTGAACTGTCACAGTCCCTCCCTTTTGGATCAATGCTAAGAAAATCAGTCAAAGATTTCTTCACTCCTGCCCCTCTTTGGGGGAGGTTGTTGTCATTGTTGAGTGGAGACTTCCCTCCCTTGGAAGGACTAATCCCGTGTGATGGAGAAAGGGCAGGCGTGCTGAAAGCCGATTTGTAGGGTGTGCTTGGGACAGGTACTGAGTATGGAACTGGTATACTCCGCTTGTGAGTTACTACACCAACTTTTGAGGGTGAAGAGATTTGATCTGAATGTTTGGTCAAGTCATCTACATAGAGCACATCATCAATCCGTGCTACCATGTTAAATGCCAAACTCTCCAGTACTCGAGAGTAGCTCTCCAAAATGGCTTTTCCAACATCCTTGTTGTATTGGATTTTGCTCATATCCAAGGTTGTTTGAGGCAGACCAGGAAACCGCTGCTTCAAGGAAAGCAAAAGGCTTTCTGCTCTCTCTGCAAACAAATCCCTCTTGTCTGCATCAACCATCAGATCCTTGACCATTTCCCATGATGATCTTGAACTAGACCTGGCACTGATGGCAGGCTTGGAGTTGGTCTTTTTGCGCCAAATATACATGGAAGCCTCAGCTCGGTTCGCAATCTCTATAGCTTGATGCTCAGAAGATAAATCAAGGCAAGCTAGCAAGCACTCAGGAGAAAAATGATCTGATGTGATATAACGATGAATAACATCCCCCAAGCTTACTCTTGCAGTCTTTGGAAGAGATTCCAAGTAGGACTCAGGAATGTCCATTTCTGCTAAAGTGATGCTATTAATGGCCATTGCAGCCTTTAATATTTGGTTTGTGCAATCACGTTTGTGCTGCAAGTGCTTCCTTGAGTTTTCATGGAGGCCACAAGGAGGGATTCGTGGTACAGGAAGCCACCATTTCTCCTCCTGCCGCTGAAGTGCTTGTCGAAAAGAAGATGGACCATCAGCATCAGGAGCTAGAACCCCTTGGTCTACATACCAGAACTCTGTATTGACAAAACTATCTAGAATTTCAAGAAGCAGGTTATCTAATTTGCGTAGAGCAGGAAGATTAACATAAAGATCTGAGCGCGGTCGACAAGTCATGACCTCAAGCTTGCTTCCATCTGGAAATGTCTGCCAATTAGGTGTCAATTCAACTATATGATCACTGACTGAAAGAAACCACTCTATCTCTCTCCGCCACATCGCTTTCTTCTCTGAACGCAGGGGCTCTAATCTCCAAAGTTGCCCAAAGAGGGTAGCACTTAGATTGGTTATGGCATTTGAGATGGCCAAGGCTGTAGGGACCCCATTTCCACAACCTGACATATCTTCTCCAAGTAACAATTTTGCAAACCTCTCCTTCATCATCTCAATCTCTGGTAAGGCTGAAACTTGTTTCTTAAATTCCTTATCCTCCAAAACCAAGTGCTTTTTCTCACCATCTTCAATGTCATGATGACAAGCACAATCTGATGCAGCAATTTCCTGAGCCGTCCACCCCACCGAAGGTGATGAAGATGAGTCCTCAGTGCTACTCCTACTATGCTCCTCATGAGTGGTCTCAGATGACAGAAAATCTGAGCTGAAACTGCTCTCACGACCCTTTTCTTCAATCAAATCAGCAAAAGTTTCTCTCTTACCACCATTTTCACTTCCTTTACTCTCTTCAGCAACCTCACTCTTCTCAGCCAAACTCAAACTCTCCATTGCAGCTGTTTTCCCTTCAACAAAAGCAGATGAAGAAGAAGGAGCATAGAGGACAGAGGTGTTAATCATCATGCCATGGTACTGAATCCTCCTCAAGCAAAACCCTTGTGAGAGTTGAACTCTGAAACACAAACTTCTAAGAGATTTAGATACCCAGAAGACCAGAATGGAAAACGGGTTAGGATAAGTGGACCTTCTGAGCCTCACCAAAGGGGCACAATGCTTCTGATGCTGTTGTTGGATTTGTTTTTGTTCTCTCACTTCTTGTTGGGTGAAAGTACTATCCATAAGTTGTTAACTTCAACCATGTCTCGTGTCTGGTAGTGAAGAGAAGAGAGAGAAAGAGTCTG

>C100569_65

TTTTTTTTATGCACAATTCAAGGTAAAAATTAAAGAAGCTGCAGAGAGATTTAGTACAAGAAATCAGGACACAAACAAGCACATTTGTGTTCATCTCCAGTATGTGCTGTATATGATATTCAATTACAGAAGCCTGTTGCATAAACCCAATACGGAAAGTTTAAGATAAAACATTATATACATTAAATTGTGATGTATTGAATAGGAGTAAAAGGAGGATAACCTTTCTTATGCGACTTAACCCCCTTTCACATTAAAAGAGCCATACAAAAAGGAGCCAGTATATGATATAACAATAAGTGAAGACTCCATTTTAGGTTTCGGTGCATTGGCCTTAGGCAGAAGACATTTGTTTCGAATGGAGGATCCAGGTTTCATCGACGCTGTTGATTGCACATCTGGCATGTGGTAGCAGACTTAACATTTGCATAGGTACAATGTTCACAAGACCAATGATTATCGTCAGTCATTCTGCTGGATATGCTAGTTCGAATAGTTGTTTTTCCTCTTCCTCCTCTCCCCTTGCTGCTCCCTGCTGCATTTTTGGAGCTCGTTGCTTTGCTCGAAGCAGCTCCATTACTATCCACATCAGATAAACCATTCTCCAATGCCCTCACTAAATTCTCAAAGTAGTTTCTAGTCACACTGGTCAATCCAGCTAGTTTGGTGCGGAAGTCATTGAATTCTCTGGATGGGCCATCGGCATTGAGGAATAACTGTAGTTCCTTTTCAGCACACTGATGAAGTCTCTCCAAACCAGATTCTGCCTCACCTTGTAAATACTCAAAGAACTGTTTTTTAGCATGTTCATGCTCCGGTAAATAGTATCCATACGCATATGTCCATTTCAGCACCCTCCTGCATTCAACAATCTGTAACCAGGCCTCCGTTATGAACTTAAGTTGTGATTCAGGCTGGCACTGTGTGTCACTAAGTTTCTCAATATGAACAGTTTGCATCTGGTGTAGATCTGCAAGGGCTTTTTGCCTTGAAGATTGATTGCTGGCCCATCGCTCATAATAATGTGTGTACCTCTCCAACGAATTCTTTGCCATTTCTCTTCTTCTTTCAGTTTCATCATACACACCCTCTTGTTTAGCAGCTTCATAACGATTGCAAGCATAAAAACCACCAGTCCTTTCACCATGGTCTGACCACGCACCAAGGCATAGCCAGCAAAATTCAAATTTACAAGGTGGAGTACAAGTCATATGCATGCACCCTTGGTTTTTTTCAATTGGCCGCTTGCACTTTGGACATGGCTTTGAGTTTGCAAGTATCCAGTTCATGTTTTCGGATTCTGCACTGTTTTTCAAAATCCACTTTGACACAGTGCCACAGTCCACTGGGCGGTGAGCCTCCTCTGTGCAGTTCCAGCAAAAGCCATATGAACAGAGGCAAGATACATCATAATTTCCACTTCCAGCATCAAAAGTAACTGCGTATTCACAACCTGGAGCAGGACACCACTTGGTCTTCTTATTGTCTTCAATATAAGACCTAAGAAGGTAACGGTCATACTTCTCTTTATCTTCATCAGATGCTAAAAGATTAATCATATCTTGACCAACAGCAGCACCACAAGTGGGATCAGGACATCTCAGTACCAAACATCCCGGACCATCATTAATTGATGTGCCAATGTATCCTGCCCAGCACACATAGCAGTAAGGATGACCACAAGCAGCAGATTTAATCCGTGCACGAGGATGTGATTCAAAACAGATGCCACAAGTAAGCTCTCTAGCATTAGAATTCTGGAAAACTGGCTTATCCAACAAACCAACTGTTTTTCGGACTTGATCTTCATCAGCAAACCAAGCATCATGTACCTTACTAACACTCCAATTATAGTGACGAAGTAGGATGCTCGCAGAAACCCGTGGTATTGAAAGAACTGCCGCTACTCTAGTGATGTCATCTTCCTGCCTTTGTCGGATGTCCATTTCCCTGAGTATGGTAAAATTTTGCTCGGGGCGACGAGACTCAATCCGGTCAGAATCATCGGCGTCGTCGAAGTAGTCGTCGGCAACGTCTTCGTAGTCACTGTAGTAATCCATGGGAGCATCCTCGGTGTCGCCACTGTAGAAATCGTCATCGAGCGACTCGATATCGTTAGCATCGTGCATATCGTCCTCGGAATCCATAGGAACCCTAGCGGAAGGGTGGAGGGTTTGGAATTGAAGAACGATCGAAGGGGTTGAGTAGCGGATAGAGAAAATTTTAGGGCACAGCAGTGATGATTAGAGAGGAAGATGAAGGAGCAGGAAAAAGTGTCTTGCTTTATGGTTTCTTCCTTGGATCCTTTCTCCTCCTAACTAACGAGAGGTTT

>C100571_65

TTTTTTACCATAAAAAAAAATAAACATTTCACCATAATATACCATACATAGTTCTGAGGGAGTGCTTGGATATTAAGATGTAAAAAACATTTCTCAAACGAATAGGATGAAAAACCAGATACAGCTCAACAAAATTTGTGAAACTGGTATGTTTGACCCCCGCACATAATCTCAACAGTGGACCGAAAACACCCAATAAAGCATACGCCAGTTAATCTAGTCTTTAAATAAACAAAGTTCACAATTTGCTCTGGCAAACAGTACAATTACCAAAACAACAGCCCAGTGAAGGACCCAAAACTTCTAAGAATAGTGATAAGACTTAAGGAAAGCCAAGAACCTAGCACATCATGAAATCTATCAAAAAGCAATGTCAGTTGAACCTAGATTCTCAGTCAGGAATGATTTGCTCACTGTCAGCATCTGCCTCCGTCTCAATCTTGGGTTTTGAAGGACCTGAAGCTCCAGGGGCCTGCTTCTTCTTGATCCCACTCACTATATCATCAATCCTCAGAAGCATGCAAGCAGCTTCAATGGCAGTCTTAAAAGCTTGCGCCTTCACATTGTAGGCATCCCAGATCTTGCGCTCTTTCATGTCAGTGATACTACCAGTATTTCCATCTATACCAATCCATGCATTTTCTCCATTTGCATGTTTTCCTTGTAGAGCAGTCATAGTCCGGATGACATTTACTCCACAATTTTGTGCCAAAGTTCGTGGTATAGCTTCAAAAGCAATGGCAGCAGCTTCATACGGCCACTTTTCTATGCCCTCGATAGATGAACTCTTCTGTTTCAAGGCAGCTGACACTGTCAACTCTGTTGCACCACCTCCAGGAACAAGTTTTGGATTTTTTATTATGTTCCTTGCAACAGACATGGCATCCTGTAGGTTTCTTTCAACTTCATTTAAGAGATCCTTACTAGCACCCCTTAGTAGTACAGTGCACGCTTTGGGGTCTTTGCAATCAACAATAAATGCGAAGTACTCATCTCCAATTTTCTTAACCTCAAATAACCCAGCACCAGTACCAACATCAGACTCCTGCAGTTCATCTGGTCTGTTCACAATAACAGCACCACATGCCTTTGCAATTCTATTATTATCAGTTTTCCTCAGCCTCCTGATTGCACTAACTCCATGCTTACTCAGATAATGGCAAGCCAAATCACTCAGACCCTTCTCTGTGATTACCAAGTCAGGCTTAAACTTCAATATCTGCATGCACAGCTCCTCAATATATTCTTCTTCCATCTTCAATAAGAGACTCCAGTCTTCTTCTTTGAGCAGTTCAGCATTTGTTTGGTTTTCACCCTTTTTATACTCAAGGGGACAATCAAGAAGAATGATGCGTGGGTTAACAATCTTTCTCCTCATTTTTCCAGGGGCAACCACATCTTTGTTTATCATAACTCCCTTGAGTACTCGTGAATCCTCCAGCTGCCCACCAGGGACCTTCTCGACCTTAATATAATTTTTAATATCTACATCTCTCAAACCTTGGCCAATCTCAACACCTACTGTTGTAGTAGCATCAATAGCTAAATCAGCAATTAAATCCCCAAATTGACCTGTGAATTTGGTACCTATGCAGCTTTTTACTAGCCCCAGCATTATACCTCTATCCTGAGCATCAATAGGCATTGCAATTTTGTCAAGAACAGCAATAGCATCCTCCAAAGCTTTAGTATATGCTCGGCAAATAACTGTAGGGTGAATTTTGTCAATGAATGCATCGGCAACATGAAGCATCTCACCAGCAAGAATGATGACAGATGTTGTTCCATCTCCTACTTCTTCGTCTTGGGTGCGACTTAATTCAATCATAGACTTCGCAGCTGGGTGAGCAAGGTCTAATTCACGTAAGATAGCATTTCCATCATTGGTAACCACAATTCCTCCTTGAGCATCAAGAAGCATTTTTAGCATGGACCTGGGTCCCAGTGTTGTACGGACTACATCAGCAACAGCCTTTGCTGCCTGAATAACACCATATCGCACTTTAGCTCCAGACTCTCGTTTCAAAGAGTCCTTGAGAACTAGAACTGGCGCGTGCATGATTTCTTGGATTCAAAATATTCCGAGAATTTTCGCACGCGAGAACGAAATCCAGCAACAGAGGATGTGCTTCTGAGGAAGCACGTGATAATCTGAACTTGGCTCAAGAAGGTTCGATCGGCACCGGAAGTGGCAAGAGAAACGGTGGTTAGCGGCGGCGGAAGAGGCGCTAGGGCACAGAGAAATTATCGGTTTCGAGAAACTATCGGCGGCAGAGTGTAGTGTTAGTAACTACGCTGAGGTTTTCCGCCAAAATGTGCTAGAAAACCCTTG

>C100573_65

TATGGATTCATATATCTGTAACTAACTTGCTTCATCTTCCTCTTCCTCTTCCTCTTCGTCTTCTTCTCCGTTCTCCTGCTAAATCCAATGTCTCCACCAATTCCTCTCAGTTCCACCTTCTCTTATTCTGCGCATGTTTTTCCTTTCCTCTTTGCTCCCAACAACTTCGCCAGAATCAATCTTCATCCACCTGTCTTTTGCCAATTTCAATCTAAGACAGAGACACCGCTGACAAGAAAGTGTTCCCCCTTTCTAGAAAGTTCATTACTCACTGGTAATGATGTTGTGGCCTCAGAGGAGTGGAAAGCTGTTCCTGATATTTGGAGATCTTCTGCCGAGAAGTATGGTGGCAAAATAGCATTAGTAGATCAGTATCATGATCCTCCTTCAACTATGACATATAAACAGTTGGAGGATGCAATATTGGACTTCGCTGAAGGGTTGAGAGTTATTGGAGTAAGACCCTATGAGAAGCTTGCACTTTTTGCTGATAATTCTTGTCGCTGGCTTGTAGCAGATCAAGGTATGATGGCATGTGGGGCAATCAACGTGGTAAGGGGTTCGAGGTCGTCAATTGAGGAGCTGCTGCAAATATACAACCACTCTGAAAGTGTTGCACTAGCTGTGGACAATCCTGAAATGTTTAATCGGATTGCAAGACTGTTTTATTTGAAGACTTCAATGAAATTTATTATTCTGCTTTGGGGAGAAAAATCAGGCCTAGTCAGTGAAGGAGACAAGGAGGTGCCTGTCTATACTTTCATGGAAGTCATACATTTGGGACAAGAGAGTCGCAGAGTATTGTTTGATCCTCTTGAAGATAGGAAGCACTATACGTATGAAGCAATCAAATCTGATGACATTGCTACTCTTATATACACTAGTGGAACTACTGGAAATCCAAAAGGTGTTATGCTAACCCATCAGAATCTTTTGCATCAGATAAAAAACTTAGGGGATGTTGTACCTGCTGAAGTTGGGGATAGATTTCTAAGCATGTTGCCTTCCTGGCATGCGTATGAAAGAGCTAGTGAGTATTTCATTTTCTCATGCGGTGTTGAACAAGTGTACACAACTGTGAGAAAGTTGAAGGATGATTTGAGGCGTTATCAGCCACATTACTTGATTTCTGTTCCTTTAGTTTATGAAACTTTATACAGTGGGATCCAGAAGCAGATATCTACAAGCTCCCTTGTTAGAAAGCTTGTTGCACTCACATTCATAAGGGTCAGCTTAAGGTACATGGAATGCAAGCGCATTTATGAGGGTAAATGTCTAACAAAGGATCAGAAGCCGCCTTCATATCTCCATTCAATGTTGGATTGGTTATGGGCTAGAGTTATTGCCACAATATTATTTCCAGTTCATTTGCTGGCGAAGAAACTGGTCTACAGTAAAATTCATTCGGCTATTGGAATTTCAAAGGCTGGAATAAGTGGTGGTGGTAGCCTATCTTCTCACGTCGATAGATTTTTTGAGGCTATTGGTGTGAGTGTGCAGAATGGATATGGTTTAACAGAAACTTCACCAGTTGTTGCTGCTCGGCGGCTTAGTTGTAATGTTATTGGGTCTGTTGGGCATCCAATTAAGCATACAGAATTCAAAGTCGTAAATTCTGAGACTGATGAAGTTCTCCCACCTGGTTCAAAGGGAATTTTGAAAGTCCGAGGTCCACAATTGATGAAGGGATACTACAAGAATCCTTCAGCTACAAATCAGGTCTTAGATAGGGATGGCTGGCTGAATACTGGGGATATTGGTTATATTGTTCCCCATCATTCAACTGGGCGGAGTCGTAATTCCAGCGGAGTAATTGTTGTGGATGGTCGTGCTAAAGACACTATCGTACTTTCTACAGGTGAAAATGTTGAACCGGGAGAGCTTGAAGAAGCTGCCATGAGGAGTAGCCTCATACATCAAATTGTTGTTATTGGACAGGATAAGCGACGTCTAGGGGCTGTCATTGTTCCCAACAAAGAAGAAGTTTTAAAGGCAGCAAGGGAGTCATCAATTATAGATTCCAATAGTTCAGATGTCAGTCAGGAAAACGTGAGCAGCCTAATATATAAAGAATTAAAGACCTGGACATCAGAGTCTCCATTTCAAATTGGACCAGTCCTTCTCGTAAATGACCCTTTCACGATTGATAATGGGCTAATGACACCCACCATGAAAATTCGAAGGGATAAAGTTGTGGCTCAATTCGGGGACCAAATAGAGAATTTATACAAGTAAACTCCTAACGTCGAGGTATGGCTTTGACTGTATATTGAAAGTAGGAAAATCATAACAACCTTTTTCCGGGAGACAATTCTTTTACCTCTTACAGAAT

>C100575_65

GTCACTATTAAAGAAGACAAGCAACAGAAAGTGGAAGAGGAGTGCGTATTTGAGTGGTGAGAGGTGAGAGATGGTTAAGCACGTATCCATGGCTTCGCTCGAGCTTCTGCTGCTGTTGATCATTCTCAGCTTCCTCAACTTGAACATTTGTAGAGCACAGGAACAGAGCACCGACCCAGTTTATCTGTATCATAACTGTTCAGGCGGGAACACCACCGCCAACAGTGCCTACCAACTAAGCCTCGGCACCCTCCTCAGTTCCCTATCTTCCAACGCCTCCAGAGAATTCTCCAACAACACAGTCGGAACATCCTCTTCCGACAGGGTGTACGGACTGTTCATGTGCAGGGGAGACGTGCCCTCTACTCTCTGTCAACAGTGCGTGTTGAACGCAACGCGACGACTCCAGTCACAGTGCTCTCTGGCCAAACAGGCCGTGATATGGTACGACGAATGCACGGTTCGCTACTCCAACCGCTCTTTCTTCTCCACGGTGGACACTCGGCCACGTCTCGGACTACTGAACACCGCCAACATATCCAACCAAGAAAGCTTCATGAGCCTGCTTTTCCGAACCATGAACGGAACTGCTGATGAGGCTGCCAACTTTTCCATTGGTGAGAAGAAATACGCGACGAAGCAGGCCAACATTTCTGGGTTTCAGTCGCTATACTGTTTGGCTCAGTGCACACCCGATCTGTCCCCGCAAGACTGCAGAAGGTGTCTGAGTGGTGTGATAGGAGACCTTCCTTGGTGCTGTCAGGGAAAGCAGGGAGGAAGGGTTCTCTATCCTAGCTGCAACGTTAGGTACGAACTCTACCCTTTCTACCGCCAAAACACATCCGCGCCTTCACCCTCACCGCCCCTGCTTCCTCCTCTAACTAATTCCCAAGGAAGCGGTGGGATCTCAGCTACGACCATTGTTGCGATTGTAGTTCCAATTACTGTCGCCGTGCTTCTATTTATAGTGGGAATTTGTTTCTTGAGTAGAAGAGCTAGGAAGAAGCAGGGTTCTGTAAAAGAAGGAAAAACTGTAGATGACATCACCACTGTGGAATCCTTGCAATTCAGTTTCGGCACGATTGAAGCGGCCACAAACAAATTCTCTGCGGACAAGAAGTTGGGCGAGGGTGGATTTGGTGAGGTTTTCAAGGGCATTCTTTCGAGTGGGCAAGAAATAGCTGTCAAGAGACTCTCCAAAAGCTCTGGGCAAGGTGGAGAAGAATTTAAGAATGAAGTGGTGGTGGTTGCCAAGCTTCAACATAGAAATTTAGTGAGGCTTCTCGGTTTTTGCTTGCAGGAAGAAGAGAAGATACTTGTTTATGAATATGTTCCCAACAAAAGTCTCGACTACACTCTCTTCGACCCTGAAAGGCAAAGGGAGTTGGATTGGGGGAGACGTTACAAGATTATTGAAGGGACTGCTCGAGGCATTCAATATCTTCATGAAGATTCTAGGCTCAGAATTATACATCGAGATCTTAAAGCAAGTAACATATTGTTAGATGGAGACATGAATCCAAAAATTTCAGATTTTGGCATGGCAAGAATATTTGGAGTTGATCAAACTCAAGGAAACACCAGCAGAATTGTAGGGACCTATGGTTACATGGCTCCAGAGTATGCAATGCATGGAGAATTCTCTGTTAAGTCTGACGTGTACAGTTTTGGAGTCCTCCTTATGGAGATTATAAGTGGCAAGAAGAATAGCTCTTTCTATCAAACGGATGGTGCTGAGGATCTATTGAGCTATGCTTGGCTTCTTTGGAAGGATGGAACACCTTTGGAACTGATGGACCCCATATTAAGAGAATCCTACAACCAGAATGAAGTTATCAGAAGCATCCATATTGGTCTGCTCTGTGTCCAAGAAGATCCGGAAGACAGACCTACAATGGCAACTATAGTTCTTATGCTAGATAGTAACACTGTTACTCTACCAACTCCTAAGCGGCCTGCATTTTTCATCCACAGTGGAACTGATACAAACATGCCAAAGGTACCGCAATCTGATCAGTCTTTTACAAAATCTTCAGCAGTGTCTATCAATGAGGTCACCGTTAGCGAATTCGATCCTCGATGAGCTTGATTGCTTGAAGACTTTATAATATGTATCATTAACCACTTACTTATTTTGTAGTAGTTTCACATGAGTGCATTTCCAAATCTTGTAACACTTTGTATTGTATTCTATAGTTGCTTATTTTTCAGCACGGAATAACATATGCAGTATTTACACAATGTTAATTAGTATCCACAATATGCGGCTCTGGAAGTTACAGTAGAAACCTATGTGTGCAATAGTAAGGATTTTGCATCTTCTATTGAGAGG

>C100579_65

AATACAACCGCTTATAATTTACATGAAAAAAAAAAAAATGATAGGAACCAAGCTAACTAACTGCCCCTGTAAACAGTTCCCACAATCATGAACTTTGCTAGGAACTTGGACACATGCAGACACACTATTGGATATTTTCCTTACTTCAGATAGAGTAAAAGATGATGGGAAAATGTCACTCCAGAAACTAAGTTTGGAAAAGACACTACTCATTAAGCCCACAAGGTTATCAAAAATAGATGTCTGTATTAATTTTAACTATGATAAGGAAGACGGCTTCATGGAATTTAAATAATCCTCCACAAATGGCAGGATCCGTTAAATGTTTGATATATAATCTGAGCCATGTAGGTGTAACAGCTAATACATTTTAAATGCAATGGCCCATAATGTTCTTCACCTTCTCGTTGGCGCATGCTGCTTTACATACTTAGTGCCATCTAGAATTCTCATGCTAGAGCACTCTCTTTAATAGTTGCTGAATCTCTGACCTTGTTAGTACCCCTCTTCTTTAGCTTGTTGCGGAAGCTTCTTTTCCTGCTAAATGCAGTTTCCACAGGTTGACCACGTTCCTCCGCCGGACGAACTGCATTAACAAGTTCCCTGTTGGATTCAGTGTACATGCTGGTAACAAGTCCAATTTGACCAGCTCTAGCTGTACGACCAACCCTATGCAAAAAGTCTACAGCAGAAGTAGCAAAATCTGCCTGAATAACATGTAAAACATTTGGAATATCAACACCACGGGCAGCAGCATCAGTACATACAAGAACACCACCTTTATCGTGAAAATCAACTAAGGTCTGTGCTCGCTCTTCCAATGTACAATTCTTGTGATAACGTGAGCATTCTATCCCCGATCGGAGTAATATCTTTGCCACAGCTTCAACAGCCTCAACGGTGTTGGCAAAGACCATTGTTCTGTGAATGCCACCAGCGTTAACCAAATCTTCAGATCTGAAACTGTGATTCACAGCCTTTATGAGTTCATCCAATTGAGTATCAACGGTAACTTCTATCCATTTTTGCTCCAATCTTGGATTGTGACGATGAAGATAGTTTCCACAAACCCACTCAGCATCAGGAAACATATATTTCAATATCCCTCCAGCTGTCTTCTTTCCATTCACTGGAAGTGTAGCAGCAACAAAAACATACTGCTTACTACGCTCATAACCTTTCCTCACTCTCCTCCAGTCTCTTTGCTTAACAGATTCATCTTCATTATTAATATCAACAATATCATCCTCTTTATCAGTGGCATCCTCTTCTAAGGTGGCTTCAGTTTGAAGTTCCTCTTCACCTTCAAAAGCATCTTCTAATGACAAGGAAGATTCGTGTTTCATTGATTCATGTTTCAGTGAAAACTCCGCTACTGATTTTTTTGATCGAGACAAGACTTTTTCATCAAAGCGGAGCAAGTTTATCAGGCGGATTACTTTATTCTGGAAGCTCCCACAGAGAAGCATATCAGCTTCATCAAACACCACGTACTTAACACCACGCATAAATTGCATACGGCGGGTTCTATCAAGGTCAACATAATTCAGAAGGGCAGCAGGTGTAGTCACAATGACATCAGGTTCCCGAATTGGCCATCCCTGTCTGCCACAAATGGCTGCCGCACTGACTATCGCCTCACCATCATCTCTACATAGATTGTTAGCCATCCTAACTACCTGCTCACAAAGTTGCACATTCGGACAAAGGATGAGCAGAACTTTCTGCGGCGGGGTAACTTCTTGATCAGACACAGCACGTAGAGAATGTTCCCGGGCATCCCTTAGCTTATCAATTAGAGGAACTAGATAACTGTATGTCTTACCACTCCCAGTCTCCGCTGCAATAATAACATCCTTCCCTGACAGCACAGACGGTACTGAAGAAGCCTGAACGAGAGAGGGTCTATTGAGGCCAATGTCGGAGAGAGCTCGAGAAATGGTATCAGAGAGACCGAGTGAAGTCCACGAAACGTTTTCTTCTGAAAAGAAAGTGTCTCTCCCATTGGAACCTTGTGATGAAGATGATGATGCAACAGCACTTACAAAGGGTCTAGGCTGCGTTTGGCGTGGAGTAGTTAGAGAGAAAGGACGAGGTTTGAATAGAGAAGGGAAGGTGGGTCTGAGGAAGTGGGAGAAGAAGTGGCAGCGAATTGGTGGGTTTTGGAGATGAAGCATCAAAGCAGAACGAGTGACAATCATTGGAAATTTCTTCTTCACTGAAAAATTATCTCTTCGTTTTAACTCTGCTTGGTTCTAAGCAGACAAACAATCACATAACATGATGGATGGGGTTTAGGGTTTTAGGTAGAACGGATGAGATTTTTTTTTTCC

>C100587_65

GCCAAAGTTTCAAAGCCCAAGTTTGGTTCTTGCCTTACCTGAAATCTCAACTCTCTACTCACTTGCAAATGCTCCCAATTTAGGGGTTTCAGCTTCGATTTAGGGTTTCGGTTTCGCTCATCCATGGATTCTATCTACGCGAGCTCGTCGTGTACGGTTCCTCACCTTCTCCGTTGCGACACTCTGCTGTTGCCGCCGGGAACCTTGAATTTAATTGCTCCATCTCCGCTTCGGCGAGCGAAGAGAGCGCGGCGCGGGCGGGTTTTGAGTTCCGCCACGACGAACGGCGCCGTTTCGAGGGCGCTGCAGGGAGTGAGCAGCTCTGCGCTGGAGCAGCTCGACATCGAGCGTGGAGTCTGCATCCCTTTCCGCAAGTACTCGCCCGACATAGTGCGGAATAAGGTATTAGAATCAAGAGGATCCACTTTGTCACTTATGTTGCGGGGAGTGGAGATAGTTTGGAATCTGGGCTTGTACTGGTCTAACTTGGTATATGATTTCCTGGTTGGAAGGGATGAAGAGGTTGTTCCATATCGCGCTCGACAGCTTAGGAACCTCTTGTGTGATCTGGGGCCTTCCTTTATTAAAGCTGGTCAGGTCCTTGCAAACAGGCCAGATATCATTCGAGAAGATTATATGAATGAACTTTGCATCCTTCAAGATGATGTTCCTTCATTTCCCAATCAAATTGCCTTCAGAATCATAGAGGAGGATTTGGGTCAACCTCTTGAAGCTGTTTTCAGCAAAATCTCATCAGAGACAATAGCAGCTGCAAGTTTAGGTCAAGTTTACCGAGCTACTTTACGTGCCACTGGCGAGGATGTTGCTATAAAGGTTCAGAGGCCTGGGATAGAACCCATTATATATCGAGATCTTTTCCTATTCCGCACTTTAGCTTCATTCTTAAATGGCATCAGTATGCAGAAACTGGGATGTAATGCTGAGCTGATTGTTGATGAATTTGGTGAAAAGCTTCTGGAAGAGCTTGATTATACCCTGGAAGCCCGTAATCTTGAAGACTTTCTTGAGAATTTTAAAAATGACCCTACTGTCAAAATCCCCAGGGTTTACAAACAATTTTCTGGTCCACGTGTGCTGGTAATGGAATGGATTGATGGTATTAGGTGCACCAATCCAAAGGCTATCAAAGAAGCTGGTATTGATGTGGATGGATTTTTAACAATTGGAGTGAGTGCTGCCCTACGACAATTGTTGGAATTTGGATTATTTCATGGAGACCCTCACCCTGGAAATATTTTTGCCATGCGTGATGGGCGAATTGCATATGTGGATTTTGGCAATGTTGCTGTTCTCAGTCAGCAAAATAAACAGATTTTAATTGATGCTGTTGTTCACGCTGTGAATGAGGACTATGCTGAGATGGCTAATGATTTCACCAGATTAGGCTTCCTGACACCAGGGACTGATGTCACACCTATAATTCCTGCTTTGGAAGCAATTTGGCAGAACTCTGCTGGAAAAGGACTGTCAGATTTTAATTTTCGTAGTGTCACTGGAAAGTTTAACCAGTTGGTTTACAATTATCCCATTCGAATCCCAGAAAGGTTTTCACTTGTAATTCGATCTTTATTGACCCAAGAAGGCATCTGTTTTACCCTGAAACCTGACTTCAAATTTCTTGAGGTTGCATATCCTTATGTGGCTAAGCGACTACTTACAGACCCAAATCCAGCTCTACGGGAACGCCTCATACAGGTTCTATTCAAAGATGGTCTTTTCCAGTGGAAACGGCTTGAAAACCTTATTGTTCTTGCAAAGGAAAATGTGGCCAAAATGAGTAGCAATCCAGCATTGAAAGTGAAAGACACGCATATACAGAGAGACTGGAAAGTTGAAAGAAAATTGGACCTCACTGACACAATCAAAGATGGAGCTCGCCTTTTCTTTGTTGATGAAGGAATTCGTAGACAGCTTCTTCTTGCCTTGACTGAGGATTCAAAGCTTCATGTTGAAGAGCTTGTCGATGTGTATAGGTTGGTTGAAGATCAAATAGACATACCTTCTGTGGCTGTAGAAGTAGCTAGAGACTTACCAACAGTTGTCAGGGATCTCCTACTTTCATGGAGTGAGTCAATCTTATCTGATAGATAGCAAAATCTTTGGACTACGTGTACATAGCTTATACAATACTACTACTTGTCGGATGAGTATACTTTACACATCTTCTATATTTTCTCTCAATTTTTTTTTCTTTTTTATAGTAAAGGTTTAGTATATAAAATTTTGAATGAATGTGTAGAAAATCAGCAACACATTTGTCTAGGAAACATTATTTTGTTCAATAAATATCAAGTTGTAATTTCACTGGGAAAA

>C100589_65

AAAGGGGTTCTGTGGTGGTTAGTGAAGTTGCTCTGTAAAGTCGATAATAAAAGATACAAAATTTTACAAAAAGTGTATTCATATAGCTTGGGCTCTCATATTTTGGGTTATGTGAAAATCCTACCTTACAAATGAATATCTACAAATACACTTTGGCCTGTAGCTAAAACACAGTTTATATCTAAAAGGGAAGGAGAAGCCGACACAACCTTGCAATTTGTGAAGGATTGCCACGAACTAGCGTGCAGGTACACCAAGCTCGTCGGGGAGATTTGGCTGAGCCACCTCTCCAGCTGCAACACCACCTTGTCTTCGTTGCCTCTCGCGGAGATACAGCACAGTTATCAGGCATGCAAAGAGGGTCAAAATTGTAGCATTTCCTTCCTCCATAAGCACATTCTCCACCCAAGCTTCTAATTTAGGATACAGTTCTGGCAATGAATCAATCACGTGGACCATGAAACTGTCAGCATAGTTCTTACGAATCCACAAGCTTGAAAGCGCTAGAGTGACTGGCAACTTTGCTGCACGATCATGATCTAGAGCTTCATCATAGTAACGCTTGGCTAGATGAAGGTCAAACGGAAGTCCTTGGCCATGCTCATACATGTATCCAAGATTGAACATGGCTTGTGCGTTAGATTGTGATTTGGCGTGCATGTAAGCCTCAGCAGCTCGCTCATAATCCCTGGCCGTACCCCGACCATAGTAATACGCATCTCCAATGAGTAAAGCAGCATGTTCATTACCCTGCTCAGAAGCTTGCCACCATAGAGAATGTGCCCTCTGATGCCTTTCTGCATCAGTGCAAAATCCAGATTCACCCATGCACATGCTACGTTCCCCATATTTATCAAGAATCCAAGCAGCATTACTTTGTGCCACCTCATAGCCCATCTCAGCCATTCTTGAATACAACATAAATGCCTTGCCAAGATCACCTTTTAAGTATGCTTCTAGAGCCCATCTAGACAAAGAACTCCATGGACCCCGCTCTGCAACTAGTTTGTACAGTGCAGTAGCCAAGGGAATATTCTTCTTGAACCCAACACCATTGTGGAAAATCTTAGCCAACTGGTAGAATGCCTTTGGTTGACCATGGTTAGCAGCAACCACGAAGAACTTACAGGCCAGCTTAACATCCCTTTTAACTCCAATCCCCTTGAGATACATGACTCCGAGGTTATAGTGCCCCCCAACCTCCTCATTCTCTGCAGCCTTTTCAAAATACTCTTTTGCTTTAGTGTAATTCTTCTTGTCAACTCCATAGCCTTTGACATACAAATATCCCATCCCATTATAAGCAGAATATAGCTGGTGCCGGGAAGCAAGCGTGAGCCACTCAAACGCTTTGGTGTAGTTTCTCTCAACACCTGCTCCCCTAGCATAGATCTCTCCAAGAAGCTCCATAGATCGTGGCTCTCCCTTCTCCACAGCTTTCGAAAACCATGACAGCGCTTTAGAATGATCACGCCTCAATCCCCTGAGGCCAAAGTAGTAAAAGAGGCCAACTTTGTACATGGCAGCGGCATTCCCCTTCTGGGCTTGATACTCGAGTATCTGAAAGTCCTCGTCCTCTTCCCCCTTCGATTTTCTCAGAGCCTCCTTGTTCTCCTCGGCCCCATTGTGAAGCCTAACCGGTTCAATCACAGGGGAATCTTTCGAAATCAGAAAATTATTCACAGCCACTTCAGCTAATTCAGCATACAGATTAACAGCTTTATCAAACATGTCTTGGCGCGTGTATTTATAAGCCAAAGCCATTTTGGACTGCATGTTGCCACCCTCGGCAGCGAAGTGGTGGTATAAAAACGCCTTCCCCTTGCTTCGCTCTCTCAAAAGCCCCATTTCCCACAAAAACCCTAGAACCGATTGCGCGGCAGGGTGGCCGGTCTCGGCCACGGCGGCGATCTCGGCAGCGCCGTCCTCGATGAGGGCGGGGAGGCCGGACATGAGCTTATCGACGGCGGAGTAGTAGGCGGCGTCGGATTCCGGGATGGGTTGGGGATCTCCGGCAGGTGGCTCGAAGATTGGGCGCCATGAGCCAGGGTCGAGGTCCTCCTCAGATTTGTGGGCGTCTCCGTCGCCGAGCTCATCCCACTCGGCGGAGGAGTCGGGTTCAGCGGGAGGGTCGTCCTTGAAGTCTTCCTGAGAGAGCACGAGCACGAATTGCCGCGCGGAGACGGAGAGAGAGAAAGTGAGGAGAGAGAGAGAGAGCGCGAGAGAGAGCGCGAGAGAGAGGAACAACGACTTCGGGCGAGGCATGGTTCTTGCCGCGGTGCTGTACGGACCACGATTGCTGTCGCCAACAAATTTTGGGCGAGGAAATGCAAT

>C100595_65

TAATACCATCATTAATTCATTAAAATAACTTCAACAATGTGGATATCCAGCATATTCTTATAAACCTTTACTCACTAAATACATGTTCATGTCCTAAACTAGGTCTACAAAACTTTTCTAGTTTAAGATAACAAAAAAAAGAATATTACAACAATTACTTCTCACACTGGATTGGATCGACCATTTATGACATTTTAGCAAACCAGGTAATAAATAATCCCTACATTTCCTCCATGCAAGACATTAATCAACTGGCAACCCTTTTTATAGGGTTAATGTTTTCTGTCAGACACAGTAAGATTTGACAGAATGAATATGTTTACAGCTGTCTGTAGAAAAGAACATATGCCTGATCATGCAGCACCTTGTTTATTCCAATAGAAAAGACAGATGCATCATCAAATCGCAGCCACCGACCATTGGAGTATTGTGCATCAGCTGTGTAGTGTCCCTTTGAAGACTCCCTTCCGTGATGAGTAATTGTAGCAACAAGTTCATACTTTCGGCCCTCAGTAGATGGTGAAACAAGCAAGTCACGACCCAAAACCAACTCAAGAGGAAAGTGCACTGCCTTATGCAACTTGGTGCTTCCCTGGCTTCCGTAACCAAAACGCATCAGGTGCAATATCATTATTTTAGGAAGTGTCACTATTTGTACAGATTTTCTTGCAGTCACAACACCAGCCTTTGCCGTGAGTGATGTTCGGTACCCTTCAAGAGCTTCTGATGCAGAAAACAAGCGAAGAGCATCTTCAATTGTGTGTACAGTGTCAGGATAGATGTCAAGATGGAGTAAGAGATATGGTTGAACAGTGGCCGAATTTCTATTTCCTTTAGCTCTCACCAAACTTCTAAGTTGTCCTCCAAAAATACCACTTAATTCTGATGGGGCAAGGCTTTGAGTCCTTGTTACAGCAGACTTATTCTTCGGCCCAACTGTTTCCCATTCATCATCTTCCACAGAAGAAACAAGAGAAGATTTGCTAACGTCAAGACTTGAAGACTGTCCTTCAAGCTTCAGTAATTCATCATGCATTTGATCCATTACAAAGCTCAGAAATTCTTGAGCATCTTCCTGCCTTGGTCTTCCTGAAATGCTATTTGGCACATCTGGAGTAAAATTTTTTAAAACGCCTTCAAACATAACAGGGCAAAATGGCCTCCCAGACTCGAAAGTATCCGTATCTTGCTTCTTTATGTTAGTTGTCCTTGGCATATCAAATTGCGTTATGAACTCAGCAAATGCCGTTAATGTAGGATATCCAACCTTAGGGAGGTTGCGAGTTCGTAATTGCTGCAAAAGATGAACAAAAGGCGAGCATGACAGTAGGGCCTGCATGGTTGCATTAAGAAAACATAGATTCCCAGAGTTGATAAGGCCTCTAGGCAGTATTTCTTTAACAGGTAAAACAGCATGCCCATTAGGTGCCTTTGGGGGCTCTCGATCAGAGAACTTTAATGAAGAATCATTCCCACTGCCTGCTTTATGTACATTCTTCAAGCTTTCAGTCTCAGAAGCATCTAGGCTCAAATTAGTAAACTTGTTTGAGGGACCATCTTCATTAAGTAATGTAAAAGAATTTGCATTTTTTTCTTCAATTTCATTCACACTACTGGGACTGGTGGGACTACGGGAGAAATTGGTAATGCTGCCATTCTCTTTAATTGTTCCCGACACTTCAGGTGAATTATCACTCACACTTTTCACTCCATTACATTTCTGAGAATCAGAGGGTGGAGCACTAACTGATGCTCTTGATGATTTTTGCAGGTTGTTTGACTCAACAGTAACAGAATTCAGGGAACCAAACTGCAGCTGATTCTTCTCCACGGACTTTTCATTTTTCCCACAAGACTGCTTTGATAGCAGTGATCGGGTTTCATCTTCAGTAAATGACCCAAATAGTAACATCTTAGAATCACTCGTGTTGGTGAGGAGATGGAAGTTGGAAAGCCAAAAGTCGGAAATTGAATATTAAATGGCCAGTATAGGAAAGAGGAAAAGGGTTGGGATGAAAATCAAAAAGTAGGGTTTTTGGAGACCCTGAAGGAGCAGAGGCAGAGGCAAAGGCAGAGGCGGTGATTGAAGACATGAAAGGGAAAAAGGGAGAGAAGAGAGAGAAGAGAGAGAGCATTTAACAAAGGAACCCTCCTCTCGAATTGAGCCTTAGCAGCACCAGCGCGGAACATCGACAGAGAGGAGAGAGAAGATAGAAGATAGAAGAGAGAAGAGAGAAGAGAAAAGCCTCTGAATTTTTGGTTTAGGGTTCGGTTAGGGTTAGTGATGGAATCGGAATTGGAATGGAAAGTAAAAAAGCCACTATCACCCTTCTTC

>C100597_65

TTTTTTTTAGAATCAAGGTAAATGAATAAATTGGAAAGTTCACACCAGTTTATCTACACGTGGGATAGTGCACAAATAGGATCAACAGATATAATTTCTCCTCGCAAAATGAATTGCTGTCGTGTCGACAGTCTTCGACAATTAAGAAATAATATAGTTCAGCACTTGCTAGTTGCTGAATAATACAAATTAGCACTAATATAGATGAGTTTGGGAACAGGAATAACTGGTATAGGGAACCACTAAATAGCCATAATCATGCCTAAAACCTCAGTTTTTTAGTTTACTTCCTGTCAAACTTTGGTATACTTCATCTATTTCCACCTGATAATACTTGAGAAGTTTGTTTCTTTTCTTCTTTAGTGTTGCAGTCACCAAGTCTCTTTCCATGTCAAATGGATGCGGATCTAATATGACCCCCTTGATATGTTCGAAGCCCCTCAGCTTATTTCTCTCAGCTGTCAATTTGAGCTCAGATAGCACGTATTTCTTGAGTTGGTCAAGAGAGCAGAGTTTGGGGAAAGAAGATATGTGACCATTTGTATATGCCCACTTATTGGCAACTTCTTCATTCGGAACTACTACTGCTACTAGCATTGACTTCAAGCTATTCCCATAAACCCAGATATCTTCAACTATTGGAGTGATTCCATAAACATTTTCTAGATGCTCAAGTGCTATATACTCTCCTTGGGTGAGTTTAACAAGATTCTTCTTCCTGTCAATTATCTTAATAACACCATTAGGAAGCATTTCTCCTATGTCCCCTGTGTGAAACCATCCATCTTTAACAGCTTCCCTTGTTAACTCTGGATTTTTGTAGTAACCACTGAAAACAGTTTTCCCTCTTACACATATTTCCCCACATGGAGGAGTTTCAAGAGGGTTGTAACCCATCTCTGGAACCTCCTCCAGCCTTATTTCATTATATATAGATACAGCTCCAACCGTACCAACCATGCACATTTCATCAGGAAAGCCAAGAGTAGTTGGTCCACATGTTTCAGTCAACCCATAGCCTTGGCATACAAAGGCACAAGTAGTGACTCGCAAGAATTCTTCCACCTCAGGGCTCAAGGCTGCACCACCAGAGATTATTAGTCGAACACGACCCCCAAGCCTGGCTTTGACCTTTCTGAAGGCTAATAGATCTGCTAAACGTGATGCTTCTCTTTGCTTATAACCCTTTTTCATCCAACCAAGTTTGTAGTTATAGAGCATGCCGAAAACTGTTCTCCTAACTGGATTGAGTTCTTCCACTGCTTTCTTGATACCTTCATAGACCTTTTCAAAAACCCGTGGGACACCAGCAAACAGTGTTGGCTTTAACTCCATTAAATCATCCCTCAACGCGTTGAGATCCCCATGATAGTAACCAACAGATGCACCCTTACGGAAAAAGTACTCCTCAATTGTGCGATCAAGGATATGAGCCAAGGGTAGGAATGACAAATAGACATCATTCACAGTCATCTTGTCTTCAAATTGTTCCATGAAAAGATCCATTCCTCTTACTAAAGCAATTGTGTTTTCATATGTTAAAACAACTCCTTTAGGGTCTCCACTTGTCCCACTAGTGTACATTATGGTGCAGATGTCATGGCCCTGAGGAGGAAAAGTGCTTTTGGGGTTTGCTTTTCCCAAGTGCAAGAACTCATCCCACGAATATGGCTTAATTCCAATAGCAGAAGCCTTATCTTTCTCTTCCTGTGTTAATGTAGTGAAGCACACCATGGCTTTCAGTCGCTTTGAGGATTTACAATCAGGATTTAAAACCTCTTTAACCTTCTTATCTTGGACGAACACAATATCTACTTCTGCATGATCTATGATAAAATTTACAGCACCAGGTCCAAGGGTGTCATAGAGAGGCACACAAATTAAGCTTTGAGCAGAACAAGCCTCCATTGCCACAATCCATTCAGGGCAATTAGCTCCATAAATTCCAATTCGAGCACCAACTTCGGCACCAGATGCTCGTAATGCAGAACTAATATGCAGAACTTCATCATAAACTTCCTTATATGTCTTCCAAACATATGGTCCAAGCTTTCCATCAACAATTTTACGCCGTCCTAGCATCCGGTTGTTAGGATAATCTTTGACAGACGCACAGAAAAAATCCCAAGTTGTAGAGAAATCAGGATCCAATGGAGGAAATTCATTTTCGGAGAGGAGATTGCGGTACACTGGACCAACAGAAAGGTTACCATGTTTACCTTCACTTCCTTCCTCAACCTTAACTGAAAAAGTCTTCATTTTCCTTTGTTATGAACCTCGCTGCTTCTCTGCAACTCGTGCTGAAACAATGTTTCAGAGTCAGACATGTAGCTAGG

>C100603_65

GTAGAGTTGAACAGCTCCGTGTCTGGCCTTTTAGCGATAAGTCCGGTCATTGTTCAAATGGGGAACAACACCAACATCAACTACAACTCTCCTTCTTCTTCTTCTTCATCACCACTTTGAGTTTAACTCACTTCTCCTTTCTCTCTCTAGAAACCGCGCAATTCTCATATTCCCGGATTTTCTTTTTCATTCTCTTTCCGTTTATCACACACCAACCAACCAGCCCTAGAACATTCTGCTTCTCTTCTACTCACAGCTAAGTCTTTCTTTCGTTAAGAGAGAAAATAAACTGTGCTCTTGATGAGAGTTGAGACGTAAGGAATGACCATTTGGGAAATTCAACGAACTCCTGAAACCAGGCAGCAAAATAAGGAAGAGAGTGTGAGGGGTATCTGAGACCTAGTTCAGTCTTTTGCTTTTGAAAGGCGTTCTGCAACTGCAAAAAGCTAAACAAGGGCCTCCTGATTTGGACCGCGATATATCAGAGCGTGGAAAAGGCCTTTTTATCACTACACAGCCAAAACAGTCTCTGAATTATCATCATCAATGCACAGGACACAATAGCCAACCCCACACCTGCACTCCTCTTTCACTCTTTCTTTCTTTCTCTCTCTCTCTCTCACCCACACACACACTCACATTCTCTCTCTCTTTAACATAAAGAGAAAGGCAAAAGCAAGAGTTTGGTCTCGGTTGATCACCCTCTGATCTGAGCAAGGCATGATTGTGGTATGGAGGAGGATGAGATTCAAGCACTGGTGTGTAAGTTCCCTAGAGTTGCAAACGGAAGGAACGAGGATATTGGAGAGGTGAGAAGAAGAGGTAGTGATGGAGAGAGCACCAACAGGTTCCACAGCTGGCACCACTCTTCGAGGATCATTAGGGTGTCTCGAGCATCTGGTGGGAAAGACAGGCACAGCAAGGTGATGACTTCAAAAGGGTTGAGAGACAGAAGGGTTAGGCTTTCTGTGACCACGGCCATTCAGTTCTATGACCTTCAAGATCGGTTGGGTTATGACCAACCCAGCAAAGCGGTTGAGTGGCTTATCAAATCTGCTGCTGATGCAATTGCTGAACTACCTTCTTTGAACACTTTCCCTGACACCCCCAAGAGCGATGACAAGAGGGCAACCGCTGAAGCTGCGGGGGTACATGACGACAATGTAAATGAGAATGGCAATGGGACCTTTAATCAGCAGAACGACAACAACAATCATAATGATAATAAGAGCCAGAACATGTCTTTGTCCAAGTCTGCTTGTAGTAGCACTTCTGAGACAAGCAAGGGCTCTGGCTTGTCCCTCTCCAGGTCTGACATCCGTGTGAAGGCCAGGGAGAGGGCAAGGGAGAGAACAACCAAGGAGAAAGAAAAGGAAAATGAGAAAGAAAACAATGAGTCTCAGCACAATGTTCCCCCTCCTCATGATCATCGTAACCACCACGATGTGAACCCCATTTCACAAACAGCTTCTTTCACTGAGCTTCTCACCGGTGGGATTGGCAACGCGGTTCCCACAACAGCCAGTCCCAAAGGGTGTGATGAGCCTAACATGATGTTCGACAAAGCAGGTAGACAGCAATGGTGTTCATCAGCAACTATGGACTACTTTCTGGGGCCTTCTTCTTCTAGAACAAACCACCATCATCAATATTCTTCTTCTACTGGGTTCTCATTGGCACAAATACAACTGGGACATTCCCTTCCTGAGGCCTTGAATAATAATGTCTCAGCCGCATTCAACAACGTGAGTGGTGAAAACCACCACCACTCATTAGATCCTCAGCTGCAGCAGCACTTCTCTTTCATTCCGGATCATCACCTAATGTCCGCTGTGGTTACTTCTTCTTCCACTTCTCATCAGCCCAGCGGGAACGACTACAATCTCAACTTCACTATGTCTTCGGGCCTCGCTGGTTACAATAGGGGGACCCTTCAGTCCAATTCTCCGTCTCTGCTGCCTCAGCTGCAGAGGTTTTCGCCGCCTACAGACGGATCCACTCTACCCTTCTTCATTGGAGCTCCTCCACCTGCTCCTGCCATGGAGAATAATAACAACCGTCACCACCATCAATTCTCTTCTGTTTTCGATGGCAGCCGCTTGCAACTTTACTATGCAGATGGAGGCCGCCATTCTGACCAGAAGGGGAAAACTAAGAACTGAATTTTGGATTGCCATCTTCATCTCCAGGTAAGGCAACTAAACAGTGTCATCTTTTTATCTTTTTTCCTCTTTCAAACCCTTTCCTTTTGAATGACAAAACATTATCCAGTTCATTCTGAAACAATTTCTTATTAGACAGTTCCTATGATATCAGAAAATTCTCGGTTTAGTTGCAA

>C100605_65

TAAGCACTTGCTACAAAGAAAAGCATTGTTCCCTCTCAATAGAGCAAAACTGTTGAGTGGATTCTCATAACAAATTTATGGTCTATAGCATTAATAGGTTTATTACAATTTTACTATGCAGCAAATTCGTATCTGCACACACAAATGCTAATATTATACATAAACATATATGTGTATAAGCATCTAATGCTGACTAGTAGTGAAAACATGGAAAATATGAGATTGAATCACTAGATAGCAATGAAGGGTATTTGCTCCATCAACAATCTCCCAATAGGAGCGGCAATTCGACACATATCAACTATTAAACACTTCCTCCTTTGAGCCATTTTCTTTACTAATCTTGAAGTCTTTTTCAAACTCACCCCCATTCCTAAATTCAGTGGATTCTTCTTCCTCCCTTGAGCCATTTTCTTCACCTAACTTACGTACTTCATCAACGGTAGGTCCATAAGCCCAACTGCATATTAGGTAATAGACTATATTAAGAGCACTGAGTCCAGAAATAACCCAGTAGTACTTATCGAAATGACCCTTGTTAATATTATCTGAAATCCAACCCTGTTTTCCCCCACTTGAAGTAACGTTTTGTATCACGCTGAATACAAAACTAGATACCAAGTTTCCTACAGCCATTGCCAATCCAGAAAGGGAAGCAGCAACGCTGGACATACTCTTAGGAAACTCCGTATAATAAAACTCATTTTGGCCTATTGCATTGAATGCTTCAGCAATACCACCCAAGATAAGTTGTGGGAAAAGCCATAGTGCAGACATATCCAACACTGCATGGGCATTATTAAGATATCCCTCCTCGATTGCTCTTTTTCGCCTTGATGTCTCAACAATTGCTGAAACTACCAGGTGGATAAAAGAGAAAACCAAACCAAGTCCCATTCTTCTCTTAGCATTTATCCTAACTTGTTTCCCTCTTATCTTTGATGCTATAGGAAGAATGACTCGGTCATACAGTGCTATCCATACAAATATTGAAAGCACCAAAATTACACTGAAAGAGCCAGGTGGCACTTGAAAGTTCGAGGTGATATGTCTGTTCAGGGATCTAGCTTGCAGTAATCCAAATGAGCCTCCAATGTTAACTGACATCATGATCCCAGTAGACCACAAGGGAATGACTTTAATAATGGCTTTTAGTTCCTCTACTTGATCTACTGTGCAGAGTCTCCATGGATTTGATGCTGAACCATCAGGGGCTATATCTTGTTCCGGATCTTTAATGACACAAGCTCTATTCAGAAACCTTAGTTTATCAGTTGGGACAACAAGATCTGAGTCCTTCTTATGATGGTAAAGTTCAGGAGAGTCCTTAGGTGGTAATGGAAGTTTTCTGTTCTTGTAGGCAACAGCAATCACTTGTGCAAATCCAGTGAACAAGCTGGATTGTACTTTATTCTTTACATAAAGTGGAGAGGCAAGGAGGAAGAAGACGGTAGATAAAAACATAAGTGCTGCTGGAACTCCATAACCCACTTTCCATCCAACATGATCTTGGATGTAAACTATTGCTGTGAGTGCAATGATGACAGAAATAGCAGCAGAAGCATAATACCAGCTGAAGAATGTTTCCAAAACCCTCCGGTTGTTGGGGTTATCTTTTCTGTTGACCTGGTCTGCACCGAATGCTAAGGAACATGCAAGACCACCATTTCCAATAGACATGAGGGCAAGGGCCGAGAGTAAGATTGCCATTTGGCCTCCTGTTGCCGATTTACAGCCTCCAGCTTCATGGCTGCAAGGGGGAGGTCGTGCCTGTGGGATCATAGCTGTTAACCACAGCAGTGTCATTCCCAGAAAACTGATGGCAGAACCTAACCCAACAGCGAGGAATCGACCCAGATAAGAATCAGCAATGAAAGCACCCACCACAGGGGTGAAATTGCTGGTAGCAGAGGACAAGAGAAGTATCTGGGTTGCTTTGCCAAGGTGAAGCTTGTAGCTCCCCATCAAGTACTGTATCATGTTAGGCAAAAGGCCCAAACTTGCCACCCTCGCAAGTGCCTCATTTGCGATGATGAAAGGCATAGTAACAAGACCACCCTTGCGCCTCTCTGGTTGTGAAATGTGTTGGGTAGACACTTCATCTTCAAGGGAACCAAGCTCCACTTCCTTGTCCATTCTCGTTAATCGGTACAAGGCTCGACCAAGGAAAACACACACCACCAAGTGACCAACAACTTAACTAATAATGTTCATGCATAGCACATATATATATGGAGTGATGTCAGTGCTTGCGATCGCTCCGGCAATCAATAATCAATTTGCGTGTTCATACGGCCACTGTCTCCATGCATATCTGGAATCAACCGTCTCTATGTCT

>C100607_65

TTGGGTGTGAAGGAGGCAGGAGATAAGAATGATATTCAGATCTTTCATACAAAATTCAGACGCACAAAAATTCATTTCCGAGACTTCATCCCCTCTTTACATATATTGAATAATCGAAACCATGTTGGGGAATTAGAAACCAGTCCTTCAATGGGTCCCTATTTTATAATGGGATGAAACAGTAACCGCAGGAGTGATATTGTACTAATTATCTAAATTATAAAGACAATCAATTACAGCCGGGGAAAAAATCTCAGGGCTTCCCCAATATAGAAAAGAATACACCCTCATTGTGATTCTACCTGTAAACATTGTTAGGTTCCCAACACATTACCAGAGGCTTCCTCCAAAGATTTATTGTCTTCAGCAATAAACCTATGGAAGTGCTCAGTTCTGACTCAGAATACCTTAATCGCACTTGATATTTCTGTAGATCCTCCTGACAAACAAATTTGAACCAAGAAAACCCACAGCTCCGCATAGAATTCCTAGTCCAAGAGAAAACATCAAAGTGTAACCAAAATAGAAGCTGGTCTGGAAGAAGCCTGACATCTTTGTCTTCACGTAGTAGTAGTATATTGAGTACAAATACACATAAACAGCTGTTGAGGCAGCTGAAAAGAAAGAAGTCCATTGCCAGTGGTAGTTCTCAGCATTTAACAAAAAGTATGTCCCCACAATTGTCACACAAACAGTAACGATGATAAGTATCAGAAAGACTAGCAGCATAAAGCCATATACATAGTACACCTTGTAATTCCAGAAGGAAGTGAATACAAAATACATTTCAATAAATATGCTTCCAAAGGGTAGCAGTCCTCCCATCAGAGAAACCACAGAGGGTGTGAGGTACCACTTCTTTTCAGGAATGGGACGAGGAATAGTCTTTACACGACAGGGATTATTCAGAGCACCACTCCAGTTTCTTCCAACAACTGTGCCAAGAAGTGCTAGAGGGAAAGAAATGAAAGCCCAAATGACAAATACAACCACTATTGTGCCAAATGGAATTGCTGCCAGAGACCCATAGAATATAGCAATGGTGTTTAAGACAAATCCAATTCCAAAGCACATGAATGGGAACAGAGAAGCTGTAAGGATCATGGATTTAATCCAGTTTTTACCGCCATTGCGGGAGTACATTCCCCCACTCACATAACCAGAAATAAAAGATGTAAGAGCATAACACACAATGAAGGTAGTGACAATTGCTCCTCGCCCAACATACAACATCCCAACAATAGCCAACAAGACGACAAGAAGAACCAGCAACGCTAGCTGAGCACCTGTGCCAACAACAGCTGAAAGAATAACTAAGTAGCGAGGAGGCCGAAAAACATCACCATGTACAAGTTTCCAGCCAGACTCTTCACTAACATCCCTCTCCAGACTTTCCAAATCATCATCTTCCCGAGCATATTTTGCGTAGTCATTTCTCAAAGTTCGCATCAATATCATTGACACCAATCCAGTAAGGAAGATAACCATCATGAAAGAGTTAAAAATGGAGAACCAATGAATCTGATGCTCAAAGAAAGGATGGTCCAGGTAGACATCAAAGCGGCGCCCATAAGTGACATTGGTAGAATCCCATTTTACCGAATAAGTCAAGTCTAAATGTTTTCCTACTTCCAAAGGCTTTGGGACATCCTGAGTGAGATTGACGTGAATAATCTGATCATTGTTGTATTTAACAATAATGTTTTTGTGTGTGTAAAGGACATGCTTGCCATCAACATTCTTATCAGAATGTAGCTCACCAACATATCCCCACAGAGGTAGATCATCCATGAAGAATTCAAACCAGTAATTATTCTCTATTGCATCCTTGAACTGTTTCACCTTTGCTTCATCGAGATCTATCCGGCAAAAGACAGTCTTCTCCACATTTCTTTGGAACTTTATCTCAATTTGGCTATCAATGAGTTCATTGCCACCCAGGACCTCACCAAGACCACCCCATTTGTGTCCAGCACTAGCACTACTCAATGGATGACAAAATGGGAGGCTGTAATAGTTGTACGTTTCCTGTGGATTGTTATAAGGACCCACTTTGTTCACCCAAAGGGTAACTGGTTCATCACGTTGATACTTGTGATCTGACTCTGAAGCGAAGGCGGAAGAAAGGAAGAGAGATAAGACGAGCGTCAACAGGAGGGATCGGGATTGGAAGAACATGGCGATGATGGAAGAGAAGAGAGAGAAGATAATGGAAGGAGAAAACTAAACTGAACAGAAAGCCAAACACGGACTATAATGGTAATTTACGTTGTCGAGGAACGAGAAAATAAGGTTACAATTACAATACAAACTACGCTGAATTGAATGAAATGAACGAC

>C100609_65

TTCTCTCTCTCTCTCTCTCTCTCTCACTCACATTGCAGATACTACTCTCGCTTCTAGCTTCTTGTTAATGGCGTCTCAGATCTAACAGTGCGAGCGCTTCGCCTATCTGCTACTTTCTCATCGTGGAAACGACGCCGTTTGGCTGGCTATTGTTAGTTTTTTCCATATGCAGAAAATGGCGTACACGTTTTCGTTTCCGGAGGAGGTGCTGGAGCACGTCTTCTCGTTCATTGAGAGCGAGAGGGACCGGAACGCGATCTCGTTAGTGTGCAAGTCTTGGTACGAGATCGAGCGGTGGTGCAGGAGGAAGGTTTTCGTGGGTAACTGCTACGCGGTGAGCCCGGTCATGGTGATCAAGCGGTTCCCGGAGGTGAGATCCATTGCGCTCAAGGGGAAGCCGCACTTCGCGGATTTCAATTTGGTCCCCGAGGGTTGGGGCGGTTACGTCTGTCCCTGGATCGCCGCCATGGCCTGCGCCTTACCCTGCCTCGAAGAGATCAGACTCAAGAGGATGGTCATCACCGACGAGAGCTTGGAACTCATCGCTAAGTCCTTCAAAAACTTCAAGGTGCTGGTTCTCACTTCGTGTGAGGGTTTCACCACTGATGGACTTGCCGCCATTGCTGCCAACTGCAGGAATTTGAAGGAGTTGGACTTGCAGGAGAGTGAAGTGGATGACCTTAGTAGTGGCCACTGGCTAAGCCATTTTCCTGATTCCTACACTTCTTTAGTTTCTCTTAACATCTCTTGCTTAGGCAGTGAGGTGAGTTTATCTGCACTAGAGCGTTTGCTCGGGAGGTGTCCCAACCTGCAGACCTTGCGCCTCAATCGGGCTGTGCCCCTTGATAAACTACCCAATCTACTTCGTCGTTGTCCTCAGCTGGTTGAGTTAGGCACAGGAGTGTACTCAACTGAGATGCGACCAGAGGTCCTCTCAAACCTGGAAGCAGCATTTTCTGGGTGCAAGCAGTTGAAGAGCTTGTCTGGCTTTTGGGACGTACTCGCATCCTACCTTCCAGCTGTCTACCCCATCTGCTCCAGGCTAACATCGCTAAACTTGAGTTATGCTATTATTCAAAGCTCTGATCTTATCAAGCTCATCAGTCAATGCCCAAATTTGTTGCAGTTATGGGTTCTGGATTACATAGAAGATGCTGGTCTGTATACCCTTGCTGCATCGTGTAAGGGTCTAAGGGAGTTAAGGGTGTTTCCGTCTGACCCATTTGGTTTAGAACCAAATGTTGCATTGACAGAAAAGGGCCTTGTCTCCGTGTCTGAAGGTTGCCCCAAGCTCCAATCAGTTTTATATTTTTGTCGTCAAATGTCTAATGCAGCGCTGCATACAATTGCGCGGAACAGGCCTAATTTGACTCGTTTTCGGCTTTGTATTATTGAGCCTCATACGCCTGACTATCTTACGCATGAATCACTGGATTCCGGTTTTGGAGCTATTGTAGAGCATTGTAAGGATCTGCAACGCCTATCCCTGTCTGGTCTTCTTACTGATCGTGTTTTCGAGTATATTGGGACTTACGGGAAGAAGCTGGAGATGCTATCTGTTGCTTTTGCTGGAGATAGTGATTTGGGACTTCATCATGTGCTCTCTGGTTGTGATAATCTTAGGAAGCTGGAGATCAGGGACTGCCCCTTTGGTGACAATGCCCTTTTGGCAAATGCTGCAAAGCTGGAGACAATGCGATCCCTTTGGATGTCCTCCTGCTCAGTGAGTTATGGAGCATGTAAGCTACTGGGTCAAAAAATGCCCAGACTCAACGTTGAAGTCATTGATGAAAGAGGACCTCCAGATTCAAGGCCAGATAATTGTCATGTTGAGAAGCTTTATATTTACAGGAGTATTGCCGGGCCAAGGTTGGACATGCCTAGTTTTGTGTGGACGATGGAAGATGATTCTTCACTAAGGCTTGAGTGATCACGCTGTGGATTGAGTCGAGGAATCTAATGGCACTTTGATTTGGGAAATTAACAATGGCAGGTACACGCTCAGTAAGCAATTCTGCAGTGAATTTACTGTTGCCTGGCTTATATAGATGCAGTTATGGCTTGTTTTTGAGCTAGGTACAGGAAAGAGAGTACTCCGAAGGCTTGAGCTCGATTGCCATTCATTTTACACTTTGGCTGAGCTTACGGGACGCCCCATTTACCCATTAATAAATAGACTGAAACTCTGTTGTTGTACGTGCGTTCGATACTTATTCATCATACTATAATATTCGCGGACTTCAATTTTTATTTGTATTTTAACTGTCTTTCGTAGTAGGATGTTGGACACAATTTGTCTTTTTCAAACCTTCTCAATTAATAATATTCGTATTCTAACG

>C100613_65

ATCGTAAGTTCGTAACCATGGAAATATGTGAACTTGGACATTAAGTGTCGTTAAATAAATGAGCTTTTCCAGTTCCAGATACAGCTAATTGCGATGGAAATTAGCTTGCAAACAAAATTTTATTCATCGTTCCACACAACTTTATTGACACTTATAAATCAGAATAGCATGATGGATAAATTTCTTAGATGGCAAGAGCATCACGTAGGGAAATTGATTACAGTCTAGCATTGCGTAGTTGTTGCTTAAGCATGGGTTGAATATACTCTTTGAATCCATCAGGAACAACTGAATCATTCAAAGGGTCCTTCCATGCCTCCTCGTACAACTTCGGATACACATTTCCATCGAAACGTCCAAGAATTGAACCAAGGTAGTGATCGGTGTAATTATAAGCATCAACAAGCGCAATTGCATTAGGACGAACCTGTGAATACAAGTTTCTCAGCTGCTCATTGGCAAGTGATCCTTGTTTTGGGTCAATGCAACCAGTGGAAATGAAATCACCCAGATGCTTATGAAGTAGAGACAAAGCATAAACGCTGCAAAGAACTTCTAATATCTGTTTCACTCCCTTTCCAGGTATATCTTGCTGCAACTTCTCAATAAATTTGGAAACAACAATTAGCTGGCAATGAGCAAGAGCCGCCTCAACTAAATCAGCTGCGAGTTCTTGAAAACCCTCTTCAGGATTAGAGAACTTGCTAAGATTTTGAGCACAAGCAACAGACATCCTAGCAGCCCTAGCTTCAAATGCTTCCAACACAACATTCGGCTTCAACCAATCCTCAGCCTTTTTAACATCAGAATGGTATTGCATCAGTTGTTCGAGTCGAGCCATATAAGCTGTAGTACCAACAGGTTTATTTCCAGTGCCCAGCTGAGAAACAGTCTTCATGAGATGCCTTGCCACCTGTAAAAGCAGCACAATATTGTCACCCTCATAGGTGCAGGCAGGAACATAAACAGCAAATAACTCGGGGAGGCCACTGCTACAAAGGTAACCATGACCACCACATAGTTTGCGGCATTCTTCAATTCCATCAGCAGTTGCGGAAGTAGTCAAGGATTTCAACCCAGCAGTGCATGCATGAGCCTCAGGCAATGTTGAAAAATCATTAGCTTGCAATCTTTCTGACACATCCGTATAAAGCCATTTCAGCCACTCACCAGCAAATCTGAAAGCATAAGCAGAAGCCAGCAAAGGGAAGAGCCTAGACTGCTGCGTTTTGTAATCGATCACCTGTGTTTCTAGACCTCCATTATGTGATCCAAACTGTCTTCGAACAGCACTATATCTTGTAGCAATGCAAACTGCTCGAGACAAAGCAACTGAGGCATCAGCTACAATTTTTTGTCTCACATTTACCATAGTACCATAAACTAGTTGTCTTGGAACGTTGGAGTGTACATATTTTCCTTCTCTGGTAACCTGTGAAACCCTCATTAACATTTGATTCCTTGGAATTCGTACATGGTCAAATGTTAGAACTCCATTGTCCATGGTGTTATATGCTGCATTTCCAAATTTCATCCCAATATCACCAATAGTAATGCCTGGAAGAGGTAAGTGATCATCCAAGCTCCGCAGCTGGACAATAAAACCATGCACTCCATGGTCTTGACCATCAGTAATTAGACGGGCATAAACAACAGCATGGGTGGATACTTTACCCAACCCACCTGGCCACCATTTGCTGGAAGTCAATGTGGGGCTATGGATAACAAACTCGTCTGTTGTGGGATCAAATGTTGCAGTTGTTTCTAGTCCTTGAACATTGGATCCATGACCAAGTTCAGTTTGGGCATAGCAACCAATTATTTGCATCCTATAAGCCAGAGGTAACCACTTCTGCTGCTGCTCATCGGTGCCCTGTCCTTGGATAGAAGGCACAAACATTCCCCAATGAAGATCCGTAAATGCAGGTTGATCCACAAAGAATCTGAGCTTATAAGCTTCCTGTTCATTGAGACGGAGCTCGACTATCCTTTTCCAAGCATGAGCTGCTTTTCTCAAAGTGTTTTTAAATAATTCCTTTCTACCAAGATTGACTCTGTCATCCTTTCTGAACACCGGATCGCTAGCAACAAGGCGAGAAATCCGGTCGGAAAGCTCAAAATCTTCACGAGAACCGGCCCAAACGATCTTCATCTCTTCCACATCGAACTGCGCCTTGTTCCTCTCGAAAGCCAAGTGATCAACATCTTCCATTTTCGCGACACCAAACCCAAAGAATGTGTTTCTGAAATCTGATTTCCTACCCACGACCAGCTTTGACTTCAGTGCTCCGTCCTGAAAATGAATGAAGAAGATAATGCAAACGTTGTTTTTGTAACTA

>C100615_65

AAAAAAAACATAAAAGCAATAGAAAAAGCAAAGTTACCTGTAACGACTATGAAACCGGTAGCCACGTTCAGTTCACTTTATGTTCTTCTCTGGTCTCTGCTAGTATCCGCGTTGTTTCCATATTCCACTTTTGTCCTCACTTATTCAACAAACCGCGTCTCCAAAGGGGGCAATTAGGTAATTTAAACATCTCTGCTCCTCTTCTTGGTTCTTGTTCCTGCTGAGTGGAAGTGATGAAGTAGGTTTGGGTGAAAACATGTCCGAAAACGGAGAAGAGAAGTTACTCGCTGTGGCGCGGCACATAGCAAAGACGCTGGGACACAACAACACCATGTCTGATGATATTTTTCAAATATTGTCGAACTTCGATGGAAGGTTCTCAAGGGAGAATCTTTCTGAAAAGGGCGCAGATGCGGATTCCAGAGCCTGCGCCGCACTCGATCATTCCCTTAAATCACTAGACCACCGGATCTCTCAATACGTCTCCTCCGACCGTCCAATCTGGGCTGACGCTGCCGCAGCTGCTGCCTTCCTCGACGCCGTGGACGAGCTCGTTGCTGTCGTTGCCGAGTGGAACCACCTGGCCTCCGACAAGTCTGTCGCCGCGTGCCTCGTACGCGCCGAGGACATGCTCCAGCACGCCATGTTCCGCCTCGAGGACGAGTTCCGCACACTCATGGAGCGTGGCGGCGAGTCGACGGAGAACATGCCGTTCGAATCGGAGGAAGAGGACGAGGATGAAGACGAAGCGAGAAACGGAGGAGTCGAGGAGCAGATTCCGGTGGCGCTGCCCGTCACCGACTTCGACATCGTGATCGATGCGCTGCCGTCGGGGGCGATCAACGACCTCCACGAGATCGCGAAGCGAATGGTGGCCGGCGGGTTTGGGAAGGAGTGCTCGCACGTGTACAGCAGTTGCCGGAGGGAGTTCCTGGAGGAGAGCGTGTCGAGGTTAGGGTTACAGAAGCTCAGCATCGAGGAGGTTCACAAGATGACGTGGCAGGACCTTGAAGATGAGATTGAGAAATGGATTAAAGCCTCCAACGTCGCTCTCAAGATTCTCTTCCCCAGCGAGCGGCGTCTCTGCGACCGCGTCTTCTTCGGATTCGCCTCCGCCGCGGATTTTTCCTTCATGGAGGTTTGCCGCGGATCCGCGATTCATTTGCTGAATTTCGCCGACGCCGTTGCGATCGGGAGCCGATCACCTGAACGGCTGTTCAGAATTCTCGACGTGTTCGAGACGCTGCGTGACCTAATTCCGGAGTTTGAGGCTCTGTTTTCTGATCAGTTCAGCGTGTCGCTCAGGAACGAAGCGATTACGATTTGGAAGAGGCTGGGGGAAGCGATTAGGGGCATTTTTATGGAGCTGGAGAATTTGATTCGCCGGGATCCGGCGAAGATGGCGGTTCCCGGTGGCGGTCTTCACCCAATTACTCGCTACGTGATGAACTACCTCCGTGCCGCGTGCCGGTCACGGCAGAGCTTGGAGCAGGTTTTTGAAGACTACGGGTTGAAAGAGTACCCCAAGCTTGATGACAGAGTGCCCTCGTCTTCTTCTCTATCAGTGCAAATGGATTGGATTATGGAGCTGTTAGAGAGCAATTTGGAAGCGAAGTCGAAGATTTACAAGGACCCTGCTTTGTGCTATGTTTTCTTGATGAATAATGGGAGGTACATTGTTCAGAAAACCAAAGATAGTGAATTGGGAACCCTCTTGGGCGATGATTGGATCAGAAAACACGCTGCAAAAGTTCGACAATTCCATGTGCACTATCAAAGAAGCTCGTGGAGTAGGGTACTGGGGATTCTGAAGCAGGATAGTAACGTCTCACTGCCCCCTAATGGCTTAGCAAAGTCAATGAAGGAGAAGCTGAAGTCGTTTAACGCCATGTTTGAGGATATTTGCAAGGAACAGTCTTCGTGGTATGTTTTTGACGAACAGCTGAGGGAAGAAATAAGAATTTCGCTTGAGAAAATCTTGTTGCCTGCATATGGAAACTTCGTAGGGAGGTTCCAGAATATTCCAGAGATTGGTAAGCATGCTGACAAGTATATAAAATATGGAACAGAGGACATTCAAGCAAGACTTAATGAATTATTTCAGGGATCAAGTGGTAGCCGAAAGTGAAGATGAAACTGCTACTAGTTGTTCGGGTGTATTTGTAGTGTAATTAAAATTAAAATACAATGTTTTGATTATCACAAATGTAGTCTTGTAGTCTTCTTCTATATATACTACTGTATGTTATTCTTCTTAGCATCCATGTGGACGAGGGATTGAAAGGATTTTGAAGCTAAGAGTATACAAAATTAATTTTCTGTGATAATATTTGAGGTGGT

>C100617_65

CTGATCAGCATTGTCTAGGCTCTTCAGCTGTTTCATGGCAATGTATTCTTCTGTTGATCAAGATGCAGAGAAAAAGCCGTGATACTAATGCAAACATTCTATTCATTGCAAAACATTAACGAAGCATTTGAGTAGAAAAACTTAAATCATCTTCGCATTGGTTAAAAAAAATACAAAGGTTTCTTCGCAAAAATACCGATCCCTATATCAAACTTTTTTCCCTTTGTTCAATTCCATTCTTCAACGACCATGCAACTTGCCATGTAACGAAGATGTTGAATCCACGTTAGAGATTCTCTTGTCAAAGGCACAACCTAAGATCCAAAAACATGTTCACACCAAAGGCTGAACAACTTAAATTCTAGGAAACCCACCAGAAAAAGATAAAAGTATTGACAGAATTCATGTTTTTAGTTGCTGTACAAACAAGCTGTATACATTTTTATGCCTCTGAGGTGAAGCTTTTGATTATCTTCATAGAGTCAACTGATTCATGTTGGTTTGTAAATTTTCAGGAACAACATCCTCTAAGATGAAATGCTTAGTTGCACCAAACATGCTGGCTAAATTCAGAATTACACTCTGATATGTGTAACTTGGAGGCAGCACAGGCATCGGAAGAGAAGGGACACATGTCGGAAGGAATTTGTTTTCATTGTAAACTGGTGCATATAGGGTCAACACTGCCTGACAAAACATGAATCTCAAAAGAAGCCTCCTTATGAAGGGATCGCCTAAAATCTGCCCCCAAACAGGATTAAGTGCGTCTGATGTTGCCAATGCTAGTCCCCAGTTATTTAGTGAAGATGTAAGTAACATTTCAGCTCTGTTGAATGTGTCCAAATCAACATCAGTGCCGGAAAGTCCTAACAGAAGACAGAAAGCTTGCAGCGGAGCTGTAAGAAACATGGTGAATAGGCTTCCGTTTGAATTATGTGAGCAATCAGACACAGCAGGAGAACAGCTTGGCGAAAGGAGCATAGCAACTGGCTCTCCTTTTTCTGCATCTGCTATGACCTTAAAAGCTTTGCTGTTCTCATTATCAATGACTAAAAGAAGTGGTCTTCTAGTAAATGGTAGAAAGTCAGAAGGATATATGCAGTTTGATCCTCTATCTCCACGAGGACCAAAGTGTATACAACCAGATTCATTTTGAGGAGCACTACAATATCCTGAAGCTGACAAATACACAAGGAGAATACCATCAGAAGGAAGCTCCTCACATATTGTGGCAAGAACAGCTATAAAATGTGTAAGTGCAGGACGGTATAACACAGCTTTCCGTGGGTTTGCCGGTAATGTAGGGTCAGCAATGTCATGGATGTAACTGATACGACTAGTCCCGGTAGCACCATTCTGACTAAGTTTTGAACTACTAGACTGATAAAATAAGCCACTAGGTTCCCACTCCAGACATTGAAGCATCCTAAAAGTGTCAATCGTGAGCTCTGAATACTTTACCTCATTTTGATGGAAGCTACTTAATACAGCATCTCGTAGTTTCAAATGTCGTTTAGCAATGGCTGCAGCAACATGTGGCAAGGTATCAGGATGAGGGTCTAAAACTAGACTGTATCTTAAGGGTCTGAGATTCATAAAAGCAGTGTCAGCTTTTAAAAATTTGACTGTTTCTTGAACCACCAGCTTCCATTCTTTAAAGTCACTTTCCTGGAACGCTCTCTTGCATTCATCAACTAATACTTTAAGCTGATTAACCAATTGCTGCATCATTTCCCTTCGATTCAAAACCAAACACACCATTATGAATCTAGCGAGAAACCTCAATTGTTTGTTTGCAATATTCACATCCTGAAACAATCCCTCCTTAAAATACTCCCGAGTCAATATGGCCTCATAGAATATATATGACTCTGAGAGATAATTTGCGTCACTTGTTTGCATGTACTGCCCAAAATACAGTTGCCCTATGCGAGATGCAATCTCACCAATTTCCCATCTCCTAAGCCCAGCTTCAACTAGCTTCTGCCGATTCTCCTGCTGAAACTTCCATAATTGTGTGTACACCTTGAACACCTTATAAAAGTATGTATCATGCCTGCTGCGCTGGTAAAAAGGAAGGTCTCTGATTTTGGAGAACTTTTTGTCGGCTTTATCGACGAGAGTCCAGAAAACCTCGCTCACTGGGATGTTCATGCTGCCGCCGCCGCCACTGTTCTGCATTTGCTGCTGCTGCGAACACATCTTCCGCCACCGCCAACGACGTCGTTTGGCTTTGCCAACCGAATCGATGGATTGTGATGAGAACGCAAAAGCAAACACTGTCTGAAAGAAAGAACTACGCGAACTTCTTTGCTCAGTGATGATTGTGCCCACCTTCAG

>C100619_65

TCATTACATGATTATACAAAACTGTTCTTTCATTAGCAATTATCAATAAAACCATTAGATTTTTTGATCAGCAAAAAAGATGACCAAAAGTTACACAAAAAGTTTTCTTTCATGCACCTATACGTTCCGTGTCTAATTTTCCCTCCACGAGCAAAAGAGAGGGGAAGATGGTTTGTCCTTGAAGTTCTCATAGTTATATCTCATATCGCATTCATCCGCAACGAACCAGCTAGTACCTAACCTGTGAAATGATTGATGATGGTCTTCTGCAACAATAATGAATTAAGTCTGATTCCGCACATCAAGCTCCGTCTGCAACATTTTTTAATACATATACATGGGCCGAAGCAGAAATGCCCAGCCTGAAGTTGTAAAAACTATATCTATCTGGCCCTCGAGATTCAAAATGTGGCAAAGATAAACTTCAAACAACAGTGCTGCTAGAGCGAGCTGCCCTTTGAGCTTCTTCCAAGTCCCTTAAAAACCTTTTAGAATTAAGAACAGAAGAGCCACCAAGTATAAAACGAATGCCCGTATTGTTGGCATTTTGTAAAGCAACAGACCGAGACTCCTCATAAGTTGTCCCACCAACAATGAAGATGATCACATCCTGCGGCCTTCCCTGTTGATAGTGATTTCCGACAAACGGGTAGTCCACATCTCTCAACCTCCCTTTGACAATGCTTTCCATGATTTGAAACAGAAGTGGTTGATGCTGGGTGTAAACATTCTCAACACCCTTCAAACCACGAGCCATGTTACGGGCAATATTCATTAGATCTCGATTTCCAAAAAGATCACCTGTTCTCTTGTCAACACCTGCTTGCTTCAGAAGAAACTGGACAAGCCCAGGTTTGTACTTGGCAGATCTGGAAGCCAGTTTGTTGAAGAGCTGCATTAATTGAACAGGGCTATCTTTCTCATATCGTAAAGCATACAACATGACCAAACGTAGTCGGTCCACATCTGATATGCTTTCATTGTTGAGTAGGTTTGTCACTGCCTCAAAAGCAGCTCCTTGTCCTGCGTTGCAAGCCAACTCCTGCTCTGTTTGTGAAACTGACATAAGTTTTCGCTCATCAACTATCTTGCTCAATTCAGTTACTAAAGTCACATGTTTGGTCACATTTCCATGCATTTTTCTGTACTCGGGATAATTATCAACAAATTTGGCCATGTCCTCTATCGTTTGGATGTTCTGGTTACTTTTTGACACTTGCTGAAATTCATCAACCATCCGCTTGATATTCATACCTATATCTCCAAAATTCTCATACATGTTAGCTTTGAAAAAGGAATCTTGTTCTGATGACAACACAACCTCCTCCTGATCCTTTGGAAATTTACCAATGGATTTTAAGTCCACTTTGTTGTCTTCGATTCCTATTAATTCATGAACCATAGCCTGATAGGTCCATTGATTTAGCAACGGGGTTACAGGATCATCCCTCCTATCAATCACTAACAACAGTGGAGAAACTTCCATTCGCCTAAAATCAAAAAGACCACTCTCCTCTTGGTACATCAGTTTAGCTGCTTCCTGCGCTATCCTTTTTGCAATGTCAGATGTCCTTTGATACCTAATTACTGGTCTGCGTTTTAATGCCAAAAAAACAGCTGCAAGACCATCAACAACTCGGTCACAGAAGCGCTGCACTGTTGAAGGATCTACCACAGCTGGAAGCATATATATGTAATGTGACGGCACATGGAAAGTGAAATGATAAGGATCAATTGCAACAAAATCTGCATAGAACTCCTGAACTTGCTGGACAACTTCCTGTTCATCTGAATCAGCAAGTATGTGAATCTGAGTGTCCTTCAATATGTTGGAGAAAAATAGGTGATACTCTCCAAATCTAGGACTAGCCAGCTGACGGCGCAAAAGCTGGATATTCTCTGATGTAGGTCGAAGGAAGTAAACGGCCTTGAGATGCGACATTGATTCGTTTGACTTGGAAATAGAATCCACCAACTCAACCAAAAACACTTCTTTCTGGAGAAGCTCTGACTGCGAATACACAACACTCACAACACCTACCGTTTGAGAATCAAGGATTAGAACCTTCATGCCGGAGATGTCTTGCAAGATGCGGTTGATGTAATCACGCGCGGAGGATGTGACCACCATCTTGTGTGTGTGTGTAGGACTAGAATCTCTCCGCGAAGACGAATTGATTAAACCAGTTCAACTGCAGCGTAGGCGAATCAAGGAGCACGGAAGCGCAGGGGTTCGGAGTTCACAATTCAGAATTCAGATCCACGACCAACGAGAACGATGGGCGCTTCGCTCCAACGCACCGTTTTACAAGTAGAAGTTGGTTCTTGTCTTTATGACAAT

>C100625_65

CTTTGTCCCGCTTTACTCCCTCACTCACTCACACGCTGCCCTGGTTGCGCGTGCCTCTCAACTGCTGCATTTTCTTCGCGCCTTCTTAACACGATCACTGATCATATAGAAATGAGTTCAAAAAGAGGGGGTGGGAGACCCAAAACCATGGCTAACAATGATATGTCCAAAGGGAAAAGCATATCGGAATCTCAGCCAAGTATTGATCAGTTGACTCAGGGTATTGCTGACACCAAGCTAAACTCTGGACAAGATGATGGTGATTGGGAGGTATATGGAAAGAAATCCAAGAACAGAGCTGGAAGCAGTGCCGCAAAACCTTGGGGAGGGCCAGCCCATAATTCTAATCCTAGGGCCTGGGGGAACACAGATATGGCTCAAAAGCCAGGCATCCGTAGTCAGGGTGGAGTTGGAAGATCTTCTGCAAATTCCTGGCAGACACAAAATGCTAACTTTAGGAGACCAGCTGGCAGAGGAAATGGAAGGCCTCAGTTAGCAACAAGTGAAAGCAATTATGTGCCTCCAAATCCTTTAATTCGGCCTCCACTTGAGCATGGCTGGAACTGGCAATCTAGACCTGGTGCAATACATCCCAATGTAAGGGATGAAATTGCACCTGAGGACCTTGAACAGAATAATGATGTAGATGATAATGATGATGGGCAGGAGGAGGACTCTGATGCTTTGGAAGATACTGATGATGATCTGATGAGTGATGACTATGATTCAGATGTTAGTCAAAAGAGCCATGAGACACGTAAGAAGAACAAATGGTTTAGGAAGTTCTTTGTGAATTTGGATGCTTTGACTGTTGAACAAATAAATGAACCAGAAAGACAGTGGCATTGTCCAGCTTGTCAAGGTGGTCCTGGAGCTATTGATTGGTATAGAGGACTGCAGCCCTTGTTGACACATGCCAAAACAAAGGGATCAAAAAGGGTGAAGATTCACAGAGAGCTTGCTATACTTTTGGATGAGGAATTACGCAGAAGGGGTACTTCAGTAATTGCAGCTGGTGAGGCTTTTGGAAAATGGAAGGGTTTGAAAGAAGAGGAAAAAGATCATGAAATAGTTTGGCCTCCAATGGTTATCATTCAGAATACAAAGCTTGAACAGGACGAAAATGATAAGTGGATAGGTATGGGTAACCCAGAACTTCTTGATTATTTTAGCACATATGCTGCTGTGAAAGCACGCCACTCATATGGCCCACAGGGTCATCGAGGGATGAGTGTTTTGATATTTGAAGCCTCAGCCAGTGGTTATCTAGAGGCAGAGCGTCTCCACAAGCATTTTGTAGAGCAAGGAACTGATAAAGATTCTTGGTTCAACCGCCCTATTTTATATCTCCCTGGGGGGAATCGCCAGCTCTATGGATACATGGCCACAAAAGAAGATCTCGACTTTTTCAATAGGCATAGCCAAGGTAAATCTAGGCTCAAATATGACATGAGATCATATCAGGAGATGGTTGTACACCAAATTCGCCAAATGAGTGAAGACAACCAATACCTTATCTATTTTAAGAACAAGGCTGTGAAAAAAGCTAAACACACCAAAGCTCTTGAAGAATCCATTGGTATAATGTCTGAGAAGCTGCGGAAGACAATGGAGGAAAATCGCATTGTAAGGAGGAGAACTAAGATGCAACATGAAGAGACCAAAGAAGAGATGTATATGCAAGAGCAATTCTTCAAGGACCAGATCAAAATCATTCATGATTCAAGGGATGCAAAGGAAGAAGATTTTGAGAGGATGCAACAGGAGGAACGTGAGAAGGTGAAACAGTCCAGTACTGCTGGTCATGTAAATGCTGACGAATATAGACTCAAGGCGGATGAATATCTAAAATTTGTTGAGGTCCAAGATAAAGAAATGGAGAATTTTGTTGCTGAAAAGGAGAAACTGTTGCATGCTCATGAAGTGGGTTATGCTGCAATGAAGCGGAGGCATTGGGAGGAAGAGGTGCAGCATGAGCAGAAGTTTGATGATGAACTAGCCAAGCTCATGGAAAAGTACTCTCCTTCGGAGACAAAGTCCAATGGTATCTGATGTTGGGGTCTTGCGTAGACTCATACAAACATATAAACACATCCAAACAGGTAGAGAAAACAAATAGTGCCCTGACATTTCCAGAGAATTGAGATTTTTACTACATTCAGTATGTCATTTTATTTTCTTTCGTACTTGTCTGACATTTGGGTTCAGCCTGGAGACACAAACCTTGTTTAAATTTTAAATTGCCGACGCCATGCCCTGAATGGATGATGCATGAGATTTTTGGATATTAACGGGGATATTATAATGATTTGGCAAGTTCATTAAAAATCAATTCAACCAAA

>C100627_65

TTTTTTTTAGAAATTCATTCGGCAATTGTAGCACTTAATGATTGGAAATGGGAATGGATTCACATAAGAAATAATCTAGAATGACCCATGAACCCAAAAAAACGACATTTGCAACAAAACAGCTAGCCTTTGTTTGTTCAGCCATCTATCTTCTTTCAAAGTTTAGGAGAGAAGTCAAATCTCACGGCAAGAACCTAAAAAAGAAAGGGAGTGCCCCCAAGGGAAACATTATATTAAGACTATATAAATAGCACTCGGCAGTTGCAAATTATTCAGCACATCAAAATTTAGATAAAGTAATTCAAACTCCACTCAAGTGCCCATAGCAAAGGCTGCAAACAAACAACCAAAGGTACAGGTCGGCTACCCATAGTCCTGGATAGAAACTGGACAAAGAAGAAATAGCATTCTGAGAAGCTTCAGATCTAACCACCTTCAATTGTATTCGTTCATGTAATGACTGTGGGTGCAGAGCGACCGGTGAATTCCTCCATTTTTGAAAGTTTCAATGCAACTTCCACAAGAGGAGCAAGTGCTTCATTTGAAAGTCTCCCAATCTTTGATGCATCCTTCTCATATTGCTCAATGTATAGTCGAATAGTTGCACCTTCTGATCCAGTTCCAGAGAGACGGTAAATCAATCGCGAGCCATCCTCAAATAAGTATCGGATTCCCTGATGTGATGAGATGGAACCATCCACAGGATCTTTGTACTCAAATTCATCAGCGTGGACAACTTTCGAAACATCTGACCTTACCCCCGTAATAATCTCATTGACTTCTGAAAGCGAGGACTGCAGCTTGACCAAATTCGCCATCAGTTCTTTTGCTGCACCTGCATCCACATTTTCGTAGTCATATCGAGTATAATAATGGCGCCCATAAGTAGCCCAATGCTGGCGAACTATGTCTTCAACAGTGACAAGCTTGTCTTCAAGTTTATCTTTGTTTCTATATGCAAGTATAGATAGCCAGGCCAAAACTGCCCAGATTCCATCCTTCTCACGAATATGGTCAGAACCAGTCCCGAAACTTTCTTCACCACAGACTGAACATAATCCAGCATCCATTAAATTACCAAAGAACTTCCAACCTGTGGGGACCTCAAAAAATTTCAAATTCAGATGTTTGGCTACAACATCCAGGGCAGCAGAGGTTGGCATGCTCCTGGCAACACCCTTTAAACCAGCAGAAAAGTATGGTATAGCTTCAACAGCATTTGCAGCAATAATGGCCACGGAATCTGAAGGAGTGACAAAAAACCTTTTACCAAGTATCATGTTGCGATCTGCGTCACCATCAGCAGCAGCACCGAATTCTGGGGGCTCATCTTGTGGTTCTGATTTGCCCAATCCCAGACGAGCAACCAACTCTTTTGCATATGTCAAGTTGGGGTCTGGATGTCCTCCTCCGAAGTCTTCCTTTGGTGTACAGTTCAGTAAAGAGCTTTCTTGTGCCCCAAGCTCATCCACAAAAATACTCTTTGCATATGCTCCAGCAACCCCATGAAGTGCATCATAACAGAATGTGAATTTAGGAGAAGTCAGCAGTTTCCTGATAGATTCAAAATCAAAAATTGACTTCATCAATTTTATGTAATCACTTGCTGAATCAAAAACCTCAACATCAAATGGTCCTTCAGGGCCTGTAAAATTTGTAACACCTGTTGTGGTAATATCCACATCTGGAAGATCTGCAGCAATCAGGTACTCCTTAATCGTTGTTGTGAATTCATATATCTTGTTAGTAATTCCCTCTGGTGCAGGCCCACCGTTTTCCATATTATATTTAATCCCAAAATCCTCATTAGGACCACCGGGATTGTGACTTGCTGTCAGGATAAATGCACCTGTTGCCTTGGCTCCATCAGCTCCAACTCTTTCTCGTATGACAGCAGATACAGCAGGAGTTGAAAGCAATCCATTTTGACCAATCCAAACACGCCTTACACCATTTGCTGCTGACATTTTGGTTATTATCTGAATAGCTTCCTTTGAAAAATAACGACCATCACCAGATACAACTAGTGTTGCACCTCTAACTTTTTCCTCAGTTAATGCATTGAAAGATGCCTGAACAAAGTTATGGAGGTAATGAGGTTGCACAAATACTTTCACCTTCTTGCGGAGACCAGAGGTTCCAGGCTTCTGGCCATCGAAGGGAGTGGTATCCACGCGTGAAACGTTGAAGAGCACCATGGGTGGTTACCTGAAGGAAGGAACAGGAAATCCAGAAGGTTGAGATTAGAGAGAAAGAGTGTTAGTAGAGAAAGTGTGTTAGTGAGAGAAAGGTGCAATGTGTATTTATAGGTGGGAGGAAAAGCGAGGGAGGGGAGAGAAGAG

>C100629_65

GAAAATGATAAGTGAAAAACTTGCAACCAAACAATACACTTATTATCCCTTGTTTGGTTCTACCTAGTGGAGTTACTTCACTTTCCCTATCCACTGGTGGGCCCTCTAGTAGGGCTTCCAAGGCCCACCACTTTAATGGGCTCCTTTAAATACATCCATTAATAAACATAGTCAACGTGACTAAAATACCACTATACTATTACATGAATAATACACACACAATTAAGTATAATTGAGTTAATTTTGAGTATACCCTTTTGTAAAACTTAAATTGTGATAAATTGTTTCTAAAATGGAAGACAAGATATAAGGTTTACATGGTTGATATATGAAAGACAATATTGTGAACAATAAAAGATAACCCCTTTTTTCCTTTATTATAAATTTTGTATGGAAAAAGGTAATCTTTAGGGATTTTTCCATTCAGGGCTACTGAAAGAGTGTTGAAAAAATGTTGAAACAACAAACACCCCTTTGGGTTTTTTTAACCTTTGTCTTTCATAAAAGTTATTGGACCTTCTTTTTTCATACAGAGAGTGTTTGGAATGTGATTATGATTTTAAAAACTAGTCTAAAAGAAGAAACACACAATTACTACATTATTACACAATGCCCAAACATGGTCTTAAGTTTGGACATTGGTCCCCAATTGGACTTGTGAAACTTGGGGATTTCCATTTTCTACACCCTTGTGGGAGTTGGGTTTGGATGTGAAAGTACCAAAAGACCCCTCCATGTCTTCAAGTGTTTTACCCCGTGTCTCAGGAAGAACAAAGTAGAAGAATAGCCACCCAACAGCAGCAATGCCAAAATAGAGGAAGAAAGCTCCACCAATAGTTATGGCTTTAGAGAGGGACAAAAATGTCATTGAAATAACACCACTTGTGGTTCTATTCACTACAACACCCGCAGCTGCACCCTGCGCCCGCAACCTCAATGGAAAGATCTCAGAGCTGTAGACCCACGTGATGGGGCCCGCACCGATCGAAAACGTGGCCACGTAAGCCAACACCATGGATATGCTCAAACCAATGGCCCACATCAGTTTGCTCTTGGAGTGATCGATGATGGTGAGGCTGATCGCGAGTGTGAGAAGCGAGAGCACCATGCCCCCGACACTAGACAATAACAAGGGACGACGACCAACGCGGTCCAACATAAAAGTAGCCACCAAGATGAACATCGTCTTCACGAATCCAACGGCCACGGTTGCAAGAAGCTTCTGGGTGTCGTCGGTGATCCCAGCCTTCTCGAAGATTCTGGGGCTGTACAAAACGACGGCGTCCACGCCGGAGGCTTGTTGGAAGAAGTGAATTCCGAGGGCTGCAATTAAGATGTGACGAATTGCAGGGGTTGGATAGAGGAACAACTCTTTCCACACGCCTTCGCCGGTGCTCTGTTTCGCTACCTCGACAACCTCGTCGTTGCAACTCTCCGGGATTCCTGCGGCTTGTTTGATGTCTGCTAACCTCAGTTGAGCTTCTTCCTTGCTGTCCGAGGTTTTGTTAAGCACTTTTGTTGCCTCTCCCAAACGCCCCCTCATCACCAGCCACCGTGGAGATTCCGGCATCGCCAACACCCCCACTGTTAGCAGCACAGAAGGAACTGCACCGATTCCAAGCATCATTCTCCATCCCAACTTCAAACTCAGCTTTGAAAATGCGTAGTTCGATATGTATCCGAGTAATATCCCTCCATTGATAAAAACCTCTGGGAAGGAAGTGAGGAAGCCACGAGAGGAGGCTGGGGAGACCTCAGCGGTGTAGACAGGGGCGATCATGAGGGCGTAGCCGATGCCGATGCCCGCCACGAAACGGCCGAACATGAGAAAGGAATAGTTGGGGGAGAACCCCATGAGAAGTGCTCCGGCGAAGAAGATGGCGCCGGCGAACACAATGGTGTATCTCCGCCCTATCCAGTCGGAGGTTCTGCCGGCCAGGCACGACCCTATCAGAGAGTAGAGGTTGATGATTCCAAGGAGTATCTCGATTTGCACGTCCGATACCTTTAGATCCTTCTTTATGTACAGGGCTGCACCACTCATCACCCCAATATCATAGCCAAGTAAAATGGATGTCATGGAGGCCAACATGGCGCAACCAAAAGCATACTTGTTTCTTTTGGGCTTCTTGGGAGGATCGAAGTCTCCAAGTGTTTTATGAGCTTCAGCTAGCTTTCCCTCAGTCATGTTGTTGACTTAGTTGAAGTGAATATCAGAGGAATATGAGAGGAAAAGAGGGATATCAGAGAAGAAAAGCTTGTTAAATTCGTAACATGTAGTTGATTTTTGGGTGATATATATGAAGAATGAAGATAAGTGAAGCAGAAGCAGTGCAATGGAG

>C100631_65

CCAGGATCAAACCACATATCAAGTCACTTTTCGCTTACGAATTGAGCTCCTTTCTCGAAAGGATACAATTGAGCTTTCTAAATTTGTCATTTTTCATTAGATAATTTAACGATAGTCCTACAAAATTGGATTAGTGTCATTCATTCCTTTAAAGGAAAAACTCAAATTCTCGTCGTCTCTTTATAAATAGACATTATTGTCGTTTCACTCATGCAGATTTCAACTTGGCTGATGTCAAAGCGAGATGAGAAAGAATTCTGTGCCCCACAAACATCAGGAAAGCTCCCAAAAGACCAAATGCCTTGAGCGTAGTAAACAGGTCCAGCTTCAACCAGAAAAATCCATAAAGTAACAAAACCCCAGCAAGACCAACATGGAAAAGGATCCCAAATGTAGCAGGTACCGTTGAACCAGGGAAATTCTTTACGTTCATCCCCAATCGCAATAACCCAACCAAAAAGCCAAGTAATGGCAGAAGGATTAAACCCAAGAAAATAAGAGAGAGATTCTGGGGAGGAAGCTTTTCAGGAGCCCTGAATATATGTGTAATCTCTTCTTTGGGCCCATATCTTGAGTAAGGGTCAACAGGTGGTGGTGGAGGACGGGCTGCTTTCTCAGGTGCTTCTGGAAGATCTAATTCGAGATGACCAAGTAGCCGTAAGAAAGAGTTCTCCATGACAGCATCACCAACAGTCAACTCAATGTCATATCTTCCTGAGAGATAGAAAAGTTTCTCCACCAAGCCAAGAAAATCAAGAATTATCTCAAACTTGTTGCCGGTGTTTCCAACCACAAAGATGTGTTCAACCTTTGTCTCGTGTCTCAACTTGAAAAATGCCTGATGAGGCTTAAAAGCTTGACCATGAGGTGTTGTCAATTGGAAAGACAACCTCAACTTCTGCAAGTGGTTAGCTGAGAGTGAAACTGCATCATTTCCAGCCAAATCTAGCTTTTTCTGAGTTTCGCCACTTCCAAGATCACTGTCCACAACTGTGATTTCTGCATTGTCAACTTTAATGATCCCAGTGACATATATTGGTACATGAATCTGCCCTCCAGTTGCATAAACTTTTTCACTGCCAGAATCACGAAGCACAATCTCAAAAACAAACACATATGTTCCCACATCCAAATTCTTTGGGAAAGCTTCCAAGACGTGAAATCCAATTTCTTCGTCATACCAAAGCTCCTTGCTGTCAATAATAGCTGAATCTTTTGCACCAGTGCTAAAAGCACGGACAAGCTTAACTGTTAGAGGTGGTGCAGCTGAACCAAGCACTGTATTCACCTTAACCTTAAGCTGGTCTTTATTAGTTAATGAGTAAACTGTTTCGGGGAGTGACAATATCAATGGAACGGAAATCCTGTTGCTCTCTAGGAAAGCTAATGAGTCAACTTGATTGAAGAAGTCCTTGGCATCTCCTGGGATTCCAATGCCCAAGAAAAATTTTGCCAAACCCAATATTGTATCCCCTGGAAGATTTATTTTTCCAGAAGTTACAGCAGCAAATGCAGTAAACCCTCTAACAACTGAAGAAGTTGTGGAAAGTGAACCTTGTTGTTCTTGTCCACCAACAAATTTCTCATCAAAATAAAAGGTTCCATCATCATATTTCTCAATGAGATCAAAAAGCTTTACTATGTCATTTTTCACTATGTTGACCTTAGACTGATCAATTTCAGAAGATGCTAGTGAGATCACTCCAGCAAGAGCTTCAAATGCTAATCCTGCAGCGAAGGTACTGGACTCTGGATTATCAGAACTATAGCGCCATCTTCCATCACTTTGACTAAGAGCCTTGATTGATTGAAAAACCCCATCAACATCAGCAAGATAAGCATCAACATTAGAATCCTGATCCTTTATAAGAACCAAGCCTCCAATTGAGTAGTATATGTCAAGCAAAGTACTCGCATCATGAACAGTGGCTTTAAGTCTTGAAGCAATATCCTCGAAAACCTCCCCATTAACATCACAATTTAAAATGGCGTTAACTTTCAAGGAATAGAACAAATCCTTGAGAGGGGAAGCGGAGCCTAGATTTTCCAACACTTGCTGACAAGTGGCTTCGCTCAGATGAGTCTTCTTCTTTTCGATTCCGAGAATCTCGAACACTCTAAGCGCCTCGTAGGCGTCTTCTAAGCTTGCGGGAGAGGAAGCGAAGCGGTCGATGGCAGCGGATCGGTGAGAAATGGAAGTGGAGAAAGGAAGAAGAAGAAGCAGAAGAAATGCAGATGCTACTAGTGTCACGTGTGTGGCCATGCCTGGAACAGCAATGGATCGGAATAGGAGAGAGTGGCCTCAGTTCGAAGCGGAGGTGAAGACTCTCTCTAATGGATCGC

>C100639_65

GTTCCTTCCTTTTGGATCGGATCTGAGACAAAGAGGGTGTTTCACTCTGTCCAGGGGTGTGTTCAAAGACTCCCCAAAGTTACTTCACTTTGTGTTTCTTCTTCTTCTTCTTCTTCTTCCCTCTCCTCCACAACATGCTTATTCCTCTGTTCCTCCACCGTCCATGAACAAATGAAATTAACACTTTTAGCTCTTCATCCACATTGTTTGATCAGTAGAATCAAAGAAAGCTCAAGGAGGCTTCTAAAGAATCAAACTTTTGAACCTGGGGTGTTTTAGTCTAATGCAGTATTGGTGACTTTGACGAGATACCCTTCATGGAGTCGCCTATTAATGGAGTTGATTCCTTGTCAGAGGTTCAAAATTCAGTTTCTGGGGTACATGATGATCATCTTTTGTCTGGGACTCCCCTACCTTTACGGCCTCCCTTGAGAGCATCTAGAAACCATGAGGGTGGTCATCAAACCAAGACCATCCACCATCAAAACGGCCATGTAATTACTCAAAAACATTCCTGTCAAGAGGGCAATAACTTGAAGGAAGAAGGGTTGCCAACAAAATTGTCCAGCAAGCAGCCACCACTTGATAATTCCAAGGATTGTGAGTCAAATGGTGTGTTAGAATCTGAGACGAAGCGTGCCTTTGCCAATCATGCAAAGAGCTACACTCAACCAGAGGCTACTTGCTGCCCAAGTCCTCAGAACAGTTTCTATTCAACCACTGTCTACTCAGAAGCCAAAGAAAGTTTCACCAACACTGAAGTCAGTGAGTGTGCTAGTGTTGACAAGTCATGTGAAAGTGAAGAAGTGGCGAATTCCAGTGATTTTAATGAGAGCAGGAAGACAAGCATATGTAGAGCAAGTACTGGCAGTGATGCTAGTGATGAGAGTAGCACCAGTAGTTTGAGCAGTGTTTTGTATAAACCTCACAAGGCAAATGATATAAGATGGGAAGCTATTCAAGCTGTACGAATCAGAGATGGGATGTTGGAAATGAGGCATTTCAGGTTGTTGAAGAAATTGGGTTGTGGAGACATAGGGAGTGTGTATCTAGCTGAACTGAGTGGCACAAGAACTTCTTTTGCCATGAAGGTCATGAACAAGACTGAGCTGGCAAACCGCAAGAAGCTCCTGAGGGCTCAGACCGAGAGAGAGATATTGCAGTCCTTAGATCATCCTTTTCTACCCACATTGTATACATACTTTGAGACAGAAACATTCTCCTGCCTGGTAATGGAGTTCTGTCCTGGTGGGGACCTGCATGCACTCAGGCAAAGACAACCTGGGAAGTATTTTTCAGAACATGCTGTCAGGTTTTATGTGGCTGAAGTTCTGCTTGCTTTGGAGTACTTGCACATGCTTGGGATCATCTACAGAGACCTCAAACCTGAAAATGTGTTGGTGAGAGAAGATGGTCACATAATGCTTTCAGATTTTGATCTCTCTCTAAGGTGCTCTGTCAGTCCAACGCTAGTAAAGTCATCAAACAACTTGGAGACAAAAAGCTCAGTATACTGCGTTCAGCCTTCTTGCATTGAGCCAACATGTGTGATGCAGCCAGACTGCATCCAACCGTCATGCTTCACACCCCGCTTTTTATCTGGCAAATCCAAGAAAGGAAAAAAGTCTAAACCAAAGAATGATATACATAACCAGGTGACCCCTCTTCCTGAGCTAATTGCCGAGCCAACAAATGCAAGATCAATGTCCTTTGTCGGAACACACGAGTACTTGGCACCGGAGATCATCAAGGGTGAAGGGCATGGAAGTGCTGTGGACTGGTGGACATTTGGGATATTCTTGTATGAGCTTTTGTTTGGTAGAACACCATTCAAAGGTTCAGCGAATCGTGCAACTCTGTTCAATGTAATTGGTCAGCCTCTAAGGTTTCCAGAATCTCCAACTGTTAGCTTTGCTGCAAGGGACTTGATCAAAGGGTTGCTTGTAAAAGAGCCTCAGCATCGTCTTGCCTATAGACGTGGTGCCACAGAGATAAAACAGCATCCATTCTTTCAGAATGTTAACTGGGCACTGATCCGTTGTGCCAATCCTCCCGAAGTACCAAGGCAGGTTATGAACCTTCCTCAAACGGAAAAGGACCTTGGTGTGAAGCCTTCTGGTAATTATTTAGATATTGATTTCTTTTGATACTTATGTGACTATTTCTTCGTTGGACTAATTTCCAGTTTCCATCTCTCATTTGTGGTGTTTCATGTTTGTCTATCCTTTCCATAAAAGGATGCAATGTTTTCAACTACATTGTTGTTGATCAGCACTTATAATTGTCCATCAAAAACTGTTGTAAGAAATTTCAAAATGCCTTATATTGAGTAACAAGTTTCTG

>C100641_65

CTTTCTGCCTCGCGTGGCCACCATCCACCAAAGTGCGCAATGCTACCATTCAACCAAACCAAACTACCCTAACTCCTCAATCACACTCACTCACCCCAACAATCTTCTGCTTCTGTTTCCTCCAAACCATGATCAACACTAATTCTGTTCCAGATTCTAATTCTCCTAACCAAGATGGGGATTCTGATTCTGACATTGACGCTCTCATCAATGCTCTTCTTGAACCTAACCCTCAATTCCCCAATCTCGATCACCTCCAAGGATCCACCTCTGACGCCAAAGAATCATCCCTCAAAAGAGGATTCGACACCGCCGATGATTCTTCCCACTCTGCAATTACCCTCAAACGCGGTCGTATAGACAATGTCACCTCTCACCATCAAGATCTGCATGCAGCCTTTCAAATTGAAAACCCTATTCCTCCTCCTAACTCCCAGCCTCCTCCGCATGCAACTATAAATGGAGACCCTCAGGTCGAAGGTCAAAGGAAGCGCCCGGTGTTTGACTCAGATTTTCTATCCAGGGCTCTCCGTGAAGCTGAATCGGTCTTTCCGCAGAATTTAGGCGACGAGGTTAACTTTGACATGGAACATGTGAATTTTAATGCACCTTATCTATCACTGAATGATTATAGGAGGATTCGGAACATGGAACGTTTCCGACAGATAGCGAAGGAGAGTGCCACTCACTATGCTAGATTCACCTCCGAGGAGACCTCGGATGAAAGGCCGATGTCTTCTACTGTGCCTCAGGGAATCCAAGATTCCACATCTCCTTTTGCGATCTCGATGAAGGCGATCAAAGACCGAGCGGCGAAAAAGAAGGGGCTTGATTCGTGGGTTGCAAAGAATCAAGAAACGGAGGAGGAGAGGGTGAGGGTTCCCTCTTTGCAAGAACTGTGTCTCAAAATTCTTGCGGACAATGCTGATGCCATGGTTTCTCTTGAGGGTGTTCCGGATGAGTTGAGGCACAATCTTTGCAAACTGCTCTGTGACTCGAGAAAGATGAACAACCACTTTCTACAACTTCTTCTCAGTGGGTCTCCCACTGAGGTTCGCCTGAGAGATTGCTCGTGGTTGACCGAAGAACAGTTCGCCAAATGTTTTCATTTGTGTGACACTGAAAGATTGGAGGTACTGCAACTTGACCAGTGTGGAAGGTGTATACCTGATTACGCATTACTTGGTACACAGACACCAAGGTGGATGCCGAGACTAATTAGTCTGTCCCTCAGTGGTGCATGCCGTCTTTCAGACAAAGGGTTACAAGTGCTAATTTCTTGTGCCCCAGAACTAAGATCAATTAATTTAAGCCAGTGCTCCCTTCTCTCATCTTTCAGTGTCAATATTTTGGCTAACTCCTTGGGATCCCTTCTGAAAGAGTTATATCTTGATGATTGCCTAACCATTGATGCTGCACAAATTGTGCCCGCATTGAAGAAAATTGAACATTTAGAAGTGTTGTCATTGGCTGGCATTCAAACTGTCTCTGATGAATTTATCGGGGATTACATCCCTGCTCGTGGTCGCAACATGAAGGAGCTTGTTTTAAAGGACTGTAGAAAATTAACCAATGCATCAATAAAAGTCATTGCTGAGCACTGTCCGGGGTTGTGCGCACTCGATCTTATGTATTTGGATAACTTGACAGATATATCCATGGGATATCTTACCAATAGCTGTCGAGTTCTTCATACATTGAAGCTCTCCCGTAATCCATTCAGTGATGAAGCAATTGCTGCATTTTTGGAGATTGGTGGAGATTTCTTAAAAGAACTTTCACTTAATAATATTAAGAAGGTTGGGCACCAGACAGCCTTATCACTTGCTACTCATGCAAAAAATTTGCAGACTCTAGATCTCTCTTGGTGCCGAAATTTGACAGACAATGAGTTGGGATTGATTGTAGATAGCTGCTTCTCATTGAGGTTGCTTAAACTTTTTGGATGCTCACTGGTAACAGATGTTTTTCTGAATGGGCACTCAAACCCAGAGATTCAGATCCTTGGACTGAAGTTGTCTCCTTTATTGAAAAATGTCAGAATCCCTAATACTTGTGAAGGTCCTCTGCGTTATTCACCAGTTTAGGTAGATTTAATATATGAATGCGAATTAAAAATGGAACAAATGGTTATTCCTAGTATAAGCAGACATAACTTTCTGCCAACATGGTGCTAACAAGAGGCAAGAAAACAGTGTTGCCGCATTTACCTTTTGTTTTAATGATGCCATTGTTGTTGCAAGTATTCAAAACCAAACAATTCATATTCATTATGTACAGTGGATATGTAAAGTTGATGGAGACAGGTAATAAATTTTTTTTCCAGAAAGAAGAGAACTTGAA

>C100643_65

GCCGGTTAAACATAAAATTAAAATGATTTGGACGTGAGATAGAATTGGTGAGTTGGAAAGCAGAGAGGATAAGAAAAAGGAACAAAAACACGAACACATAGAAGAAATGCTGCTTCGCCAACACTGAACTCAAACAAATTCAATTTCCAACCTCTCTCTTTTTCACTCACAGTGTTGTGAAAATGGCTGTTGCACTATCAAGTACCATTTCCTTATCCTCTGTGCTCTTCTCCACAGTTAAATTCCGTTGCTCATTCAGACAAAGACCCATCATCGCCTCTATCCGTTGCAATCACTCGCACCGACTCTCCTCCTCTTCCTCCAAACCCAATCGGCAAAAGGAAGGTGCAGGCCACAAGAAGCAGCAGTCTAAAGATGAAAGACCTTCAAGAGGGAATGTGGTGGAACCCCAACCAACTTCTTCTAAAGCTTTTGGCATTCTTAGGAAAAATAAGGACCTTATGTTTGATTCCAAGGACCAGCAGGTTGAACCAAGTGATTTACAAGACTCGGCTTTCCTTAATGCTGTTGTAAAGGTTTACTGCACCCATACTGCTCCAGATTATTCACTTCCTTGGCAGAAACAGAGACAATACACAAGTACTGGAAGTGCATTTATGATTGGAGATAGGAAACTACTAACAAATGCACATTGTGTGGAACATGATACCCAGGTCAAAGTAAAGAAAAGAGGGGATGATTCAAAATATGTGGCTAAGGTTTTGGCCAGAGGTATTGACTGTGATATAGCTTTGCTTTCAGTAGAAAGTGATGAGTTTTGGAGAGATGTGGAACCTCTCAGATTCGGACGTTTGCCACATCTTCAGGATTCTGTAACTGTTGTGGGATATCCTCTTGGAGGAGACACAATTTCAGTCACAAAGGGAGTTGTATCTCGTATAGAGGTCACGTCATATGCTCATGGATCGTCTGATTTGCTGGGCATTCAAATTGATGCAGCAATAAATCCAGGTAATAGCGGAGGCCCAGCGTTCAATGACCAAGGGGAGTGCATTGGCGTAGCATTTCAGGTCTTCAGATCTGAAGAGGCTGAGAATATTGGATATGTGATTCCTACAACTGTTGTATCTCATTTTTTGACCGACTATGAAAAGAATGGCAGGTATACTGGTTTCCCTTGCCTTGGAGTACTTATACAGAAGCTAGAGAACCCTGCATTACGAGCATGGTTAAAAGTACAGTCCAATGAGGGTGTATTGGTACGTAGAGTTGAACCTACATCTGATGCAATTAATGTATTAAAGGAGGGTGATGTGATTGTCAGCTTTGATGATGTTCGTGTTGGTAGTGAAGGAACAGTCCCGTTCAGAACAAATGAGCGAATAGCCTTCCACTTCCTTATTAGTCAAAAATTTTCAGGTGATACTGCGGAGCTTGGTATCATCAGAGCAGGGACATTTATGAAAACTAAAATTGTTTTAAACTCACGAGTTCATTTGGTTCCCTATCACATTGATGAAGGTCAACCTTCCTATCTAATAATTGCTGGTTTAGTGTTCACACCTCTCTCAGAGCCATTGATTGAGGAAGAATGTGAAGACTCTATAGGGCTAAAATTATTGGCAAGGGCACGTTATTCATTAGCAAGGTTTAAAGGAGAGCAAATTGTAATTCTTTCACAGGTCTTGGCAAATGAAGTCAACATAGGTTATGAAGATATGGGCAATCAGCAGGTTGTAAAATTTAACGGAACTCGAATTAAAAACATTCATCACCTTGCGCACCTCATTGACTCATGCGAGGACAGGTATCTGCGTTTTGAATTTGAAGATAGCTATGTAGCTGTTTTAGATAGAGAAGCTGTTGCTGGTGCTTCATCCTCCATACTGAGTGATTACGGAATCCCATCTGGAAGATCTTCTGATCTTTCGAAACCTTATGTTGATACCCTAGAAGGTGATCAACCAGCATACCAGGAATTTGGTGATAGCCCAGTTTCAAATTTTGAAATTGGCTCTGATGGACTACTCTGGGCATAAGAAACCTGCACTGAAGATATCACTGAAATTTCACCTGGCAGCGGTATTTGAAGAAAGCAATCTCAATTTTGGGCAGCAGAAAGTGCTGCAACAGTATGAGGCAACCTTGATGCGAACACATTGAATGGTTTTATGCTACACTCGGATATGTTAGAAGCGACCTATTAAATTGATTTTAGAAAGGGGTAGGACTATAGGAAAGAGAGTTCATGATTCTTCCTAATGTAGTTTTATTCTTGTCACTCTTAATTTTATGAACCTGAAAGATCACTTCAGGTTTGATGTCTAAATTTTTCGCACACATTCTATATTTTTAAGAAAATAGTGCACATACGCCTTTGATG

>C100645_65

CAAAGAGACACTGAGCTTACTTTACTTTTACCCCATTCCTATGCAGCTTCATGGGATTGAACTGCAATCACCACTTAGCACCGTTCAAGCACCCTTACAATTCTGCAACTTTTTCTGCAGATTCTCATTCCATCTCGCCTTTTGGGTGAACTAGCCGTGTCAATGAGGCTCTTCCACTTTCCACTCTGCTCCTGAACTGGTCAATCAAAAGATGGAGCGCACCAGGCTAAACATGAGAGGCCGCTGCTCTGGTTCAACTCCTTCAGAAGAGTCTGCTTTGGATCTTGAAAGAAACTGTTGTAGTCATTCTAATCTGCCTTCTCTTAGTCCACCAACGCTTCAACCCTTTGCATCAGCTGGACAGCATTGTGATGGCAGTGCTGCTTACTTCTCATGGCCAAGCCGTGTGCATGATGATGCCGAAGAAAGAGCAAACTACTTTTTAAATCTACAAAAAGGGGTGCTGCCTGAAACCCTTGGTCGGCTGCCAAAGGGGCATCAGGCAACCACATTACTCGAACTTATGACAATCAGGGCATTTCACAGCAAGATACTGCGTTGTTACAGCCTTGGAACAGCAATTGGTTTTCGCATACGGCGAGGTGTATTAACAGATATTCCTGCTATTCTAGTGTTTGTTTCCAGGAAAGTTCACAAGCAATGGCTAAGCCCAATCCAGTGCCTACCTACTGCTCTTGAGGGCCCTGGTGGAGTATGGTGTGATGTGGATGTGGTGGAGTTTTCTTATTTTGGTGCACCTGAGCCAGTTCCAAAAGAACAGCTGTACACAGAGATTGTAGATGACTTGCGTGGGGGTGATCCATGCATCGGTTCAGGATCTCAGGTGGCAAGCCAAGAGACATATGGAACTTTGGGTGCCATTGTGAAAAGCCAAACAGGCAGTCGACAAGTTGGTTTTCTCACGAACCGTCATGTAGCAGTTGATCTAGACTATCCAAATCAAAAGATGTTTCATCCTCTTCCACCCACTTTGGGGCCTGGGGTTTATCTTGGTGCCGTAGAGAGAGCAACATCATTTATAACGGATGAGCTTTGGTATGGCATATTTGCTGGAATAAACCCAGAGACTTTTGTGAGAGCTGATGGTGCATTCATTCCTTTTGCTGATGACTTTGACATGTCCACTGTTACTACTTCAGTGAGGGGTGTTGGAGACATTGGTGATGTGAAAATAATTGACCTACAGGCTCCAATTAGTAGCCTTATTGGAAAACAAGTGGTGAAGGTTGGAAGAAGCTCCGGCTTGACTACAGGAGTTATTTTGGCTTATGCCCTAGAGTACAATGATGAGAAAGGCATATGCTTTCTTACGGATCTTCTTGTTGTCGGTGAGAACCAACAAACTTTTGACCTTGAAGGAGACAGTGGAAGTCTCATCATGTTAAAAGGTGACAATGGTGAGAAACCACGGCCAATTGGGATAATATGGGGAGGAACTGCTAACAGGGGCCGCCTGAAGTTAAAAGTTGGGCAACCTCCTGAGAATTGGACTAGTGGGGTGGATCTAGGACGACTTCTCAATCTGCTTGAACTTGATTTGATAACAACTGATGAAGGACTTCAAGTGGCAGTGCAAGAACAAAGAGCTGTGTCAGCCACAGTAATTGGCTCTACAGTTGGTGATTCTTCTCCTCCTGACGGTGTACTTCCAAAAGATAAAGCTGAAGACAAATATGAGCCACTTGGTCTTCAAATTCAGTCCATTCCCGTAGGAGTTGCACCTAGCAGCCAAGACATGAAACCATCAATCATGGAGACTGAATTTAAATTAGAAGATGGAATCAAGGCTGGTCCAAGTATTGAACACCAGTTCATCCCGAGCTTCATCGGTCGGTCACCATTGCACAAGAACAGCATACATGACAGAACTGCAGCAGAGAACTTATCTTCATTGAGGAATAACTGTGATGAAGATTTGTGTGTTTCACTGCAGCTCGGTGACAATGAGGCAAAGAGAAGGCGTTCTGAAGCCTCAACTAGTACTGAGGAGCCTCAGTGAAACTAGCACAGTATTTGCATAAGCTGGAAAAAAAAAACTGACTTATAAGTTTAATCACCCCAGAATGTGTTGTTTATGACCACTGTGGTACTGTTTTTATGCTTTTAGCAGCTCTTACAATTGTCAATGATGTGACAATGGGCAATGATTTATGGTCTTGAAGGGCCATTTAGTTCCTGAAGATACCTGAATTTCTGAACTGAAGTATGTCACTTTTCAATTAACATGTGCATACTTTGCCAATTAACTGCAAGCTCACATCACATGGCACTGACAATTATTTATATACTGTGATGAACTGATAATGCCTTGAGTTTGATTTTTA

>C100653_65

TCCAGAGAAAGGTACCTGATACAATTTATGCCACATAAAGAATAGTATCTATATGAAAATTTTCTATATAACTTAACAAAATCAGATTATTGAGTTACCAATTTTTCATCTGATAAATATGAACTTGAATGATTCTGGACTTTTATATTATAGAATGAATCAATGTTTTGTTGAGGGTCGCTCTGAATTCGTTCCCTTTCCTGCTTTTGGATTTACGATTCTAGCCACCCTCTCACCACTGCACAGATTTCAAAGTTTGTCATCTTGAACCTTTGTTGGATGAATGCCATAGCTTTTTCTGGCATATCAAAATCTTCTCTTTGTCAACTCCATACTCAGTGTTTTCTTTGCGACATATCCATCGCATGCCCTTGCCAATAGTAGCAATGGCATCCACAAAATAAGTGGACTCTCGAATGATAGCATGCCCACCAGGTCTTAAAATTCGGTCCATGTCCAACAACATATACTTCATTTCACATCTGTGGCTTTCTGCAGTGAAGAGTCCATCAAGATGAAGGAGGTCATAGGTTCGAGGATAGGTTGAAAAAGCCTCACACCAGTCATGGAAAGTTCCAATAAGGCCACGATCATAAATCACAGGCAGTGTGTTGGGACCATAAGATGAGACCACATTCATGACCCACAGGGGATCATTGATCAAGGCTGCAGCAAAAGCCCCATACACTGTATTCATGTCCATCACATTTCTTATTTTGTCACTGCCAAGTTCAGGAAGCAACTTTTTGTAGTGCTGAATCCGTTTTTTCCACTTGCCATCGTCGTGATGGAAAGTACTAATACTACCACTAGTACTAGCACCATGAGCAGTTGTGATCCTTTCAGGGGTAGCATGCAACCTTTCAGGCCATTTAGGCATGTATGGGAGAACAGATTTCTTATATTTTGAATCTGGAACCACAAAACATGCACGAAGTGGAGTGTACCATCCTGAATCTGGTTCAAGACTGTCATCACACTTTGGTGGGTAGTTCTCTTTAGGAAGCTTGTCATAGCAACTGTTATCTTTGGCCTTCTGCCATACAGCAATATCATCCTTTTTATTGTACAATGTAAAGCACATTGAAGTGAGTAAATTCTGCAACTTATCATAATCTGATTTTTGTTCGGCAATAGTTGTGTTCCATCCACGCCACCTGTGTTCGTAGTTGACAGGTGGACCAGACAAAACCCAAAATCCTCCGGGACGGAGAATACGGTGTATTTCCATGAGATAAATACCACCAAATTCAGTCCATGGGATAAGGCATCTGGAGCAATGAGCCATATCAAAGGCGTTTGATGGGAAAGGAAGTCTCTGTGTAGAAATGACACCAAGAATAGCTGGGATACCACGCTCTAGAGCAAATTGAACTTGAGCTTCATGGTTATCTCTTGGGGCAAGAGAAACTGTTAGAATCCCCCTATCCAACAAATCACCACCCCAGCTAGCAACCCCACAACCAGTGTCAATGGCAGTTCTGACTGTCCCATCTTTCATTTCTGGGATTAGATCTTGCATCATATCAACATATTCACCAACACCATTGGGAAACATAGTACCCCCACCGGGAAACAGGAATTTCTCCCCTTCCTTAATCAACCAATGCTGATTAGATTTCTCCTTGTTGATCCAGTCATATGGCACATTCCTGTACCAACACTCGTCTCTACTCTTTGGCCATCTGATTGGAGGCTTGTATCCATCTGGTGGAGGAACCAAGCATTCTTTCCTCTCAAAAATTGGAGGGCAATGGCGTTCTAACAAAGTAAGCCGGTATATGCCATATTTTCTCCATCTCTTTGGATCTGTGCATGGGGTGTAATCTTGATAATCCAAGCCACATTCAGGGAAACTGATGGGTCTCATCAGCAGAGAACCTGAGGTTTGCTTAGGAGTATCAAGGGTCTTTTGAATGGCATTGACCACAACCGCATCCTTCCCAGAACAAAAGATTCCTCCCAAATAGAAAGAAAAACCACATAGAGCAATTAAGGTGACTGCAAAAGTCACAAGCCGGTTCTTGTTTGGATGGTTGATCGGTTTTCCATCTTTATGCTTCATGTTCCAACAAATATTCCACTATCGTTTTACTGTTTTCCTCAGACGCTGGATTCTGGGTGCAGAATGTGAAGGCGCAAAAGGAGTGTAGCCTAAATAAGTGGCATGGAATGTGAAAGAGGGTGAGAGAAAGAGGGTGTGAAGATGGAAGGTTGAAGGGGACCAACCAAAGAGTCAGATTTGGATGTGAGGCTGAACTAAACATAGATCAACCATTTATATAACTATTTCTTCGCCATGTGCTTCTCTTTTCCCTCTGTTTCTTCCACTTTCTTCTTTCCTT

>C100655_65

CGCCCAACTGGTATCTCTTTAATTGAATCAAAATCAATTTAGATAAACAAACAGACGCTGTTTGAAGCCATTATTTCAACCCACACAAGGGCACAGAAGAGGCTTCATGGCAACAGCTGACAAGTATAAAAATGATAACATGATAATAAAGCAGCAGTGCACACAAATGAATTCCAAACTTGAAACAAGTTCCTCCACAAAACTCATGATGATGATGAATCAACTAAGAAGCGTACTTCTTCTCAATCATGACATCGCCACTCTCTGGAATTTCAGCCTTGTTGTGAGTATGACCATGGCAGCACTTGTAACTGACAGAAGCAATCTCCATGGATCTGCCACAACCCGCATCAGGGCAGTTAATGGGGTGGAGAATGTCAGCAGGGTAGTTAGCCAACTGCATGTGGGAACATTCCTGAGGGTAATTCCGGCGCTTCTGCTCATAGTATTCCTTGAAAGCCACGTCAAATCCTGCTTTCTCAAGCACCTTCTCCTTCCATATCTCGAAATCTGCTGCAGTCGCCAGCATTGGTTCCCCACTCCCCAGAAGTTTCACGTTTGACCCTTTGCTCAGCAGAACCCACCCTGGTTGCTGCTGTTTCAGGATCAACAAGCTCTTTATCTCGTCAATGGTGGGGTCCTTGTGTGTCTTCTGAATCATGTTTGCGAACAAGCTCTCTATTCCAATCCAGAAGCGAGGGACGATCCTCGGATCCTCCCTTCCAAATGGGTAGTGCTCTATCACTGCGTCTGCCCTCTTGATGATCTCGTGCCTTCTGATTTTTTCCACTAACTGACTGAACTCTTGCATCCACTTCCTGTCACTACCTCCATAGATGAAGATGAAACTGTCCGCTTTGATAAGGTCTTGAAGTTTGGGATTGACTTTCTTAATTTCGGCCCAGAACCAGTTCCATTTCTGGGTAAGGACGTCATCATCAGAAGGTCTGAAGGGGAAGGCATCGATGCCCCAGACGAAAATCATGTGCATGGCATTGTAGTTCACCACTCTTCCCTGGGGGTTCAGCACAGGGATAATGGGTTTGTTCTTGTAGTTCAAGTCCTCTCTTATGAGTCTGATTCCTGCTAAGGGGTAGAAGTATTCCACCACGTACCACGGCATTTCTAACTTGAGATTATCGTATTCTGCCCTGTGCAGAAGATTCCACTCTTCCACCACGGGCACCCACAAGATCCTGAAGTCCTCTTTCCTGTATCCTTTCACTTCCTTAGGATCATCTTGCAATCCCTCGTAGATTGATTGCAGCAGCCGAATCTCATCTCTGATGCTGTCCAAGCCAGAAATGAACAGCAACACGTGTTTCTTCCTAAACACTTCAAGGCTAACCATTTGTCTAGTGAGGCCATCGTACACATGTGGGTCGTGAACTTCGTTGTGGAAGATAAGAACCTTGAGAACCTCTACAATTTCTGTGGGGGTTTGAAGCAACTTCTTACGCCTCCAGTAGTCTTCTAGGTCACCTATTTTCTTTCTGCTCCTGGTCAGATTCGCCTTCAATTTGCTGATAACGGCGGAAAGCTTGTCATCAAAGTTAGCTATTTCATATCTGTCATCCGATTCACCAAGGAGGAAATCAAAATGATTAGCACAAACAACAGTGGTGACGATTGCCCAGTAGACAGCAACAGGGATCTCTTGCATGGCTTCCGCCAAAGCTGGGACATCCTTCAAGTCATAACCTTTGGTCGAGAGCCTCTCCAACTCAGTGATGCACTCAACAGCGATCAGCAAGTTCTTCACCAAACTGTTGTAGTCAGCAACAGCTTGCCTATTCCTCTCCACGATGTGAACTCGATTCATCTGTGCCAATGATTTTCCAAGATGATCTCCTGTTGGAACCTGGACAAGATGCCAAAAATTTCCATATTCCAAAGCCAAAGCAGCTAGAACTAGTATTGCCTTTCCATCCCACGAGTAGGTCCTCAGCTGCTCAAGTATCGACATTGCTGTCTGGTGTGCGTGATGCTCACCATGAGGTGTGTTCATCATCTGGGAAGCAATATGCTTGAGTTTAAGGAACGGTGGGTCGAAAGTGGACAAAGCAACCTTGTCTTCAATAAAGCCAACGGGAGTGCCAGTCTTAACAGCAACACTATCAGCAACTGCGGTAGCACGCTTGATGATGTTGGAAGTAACGTTGAAAAGCGATTCCACATCGTACCTTTCAGCGGTGTGGACGTGAGTAATGTAGACTTTCTCAAGGATCTGATCATCAGTCCAACCCAATGGGTTCAAAGTGTTGGTTTGAACCAAAGCGGTTGCTGGGGAAAGTGAAGCTGCCAATGCCATTGCTGATGCCTCTCTTTGAAACTCTCAGGTTTTGA

>C100661_65

GGGTAATCGATTTTTCAAAGACAATTAACTAGTACCTATAACATAATTCGTATGGCTATTATTCATTTAACAATGACTGTATGCAAGGTGATTAATAGACACCGGATATTAGAACTGTGAACAACCATCGAAACAATTATTTCTATAATTACTTTTCGATTGAATATACATTCTAAAATGTTGCATGTGAAAAGAACAAACTTGCTTCATGTCAACTTTGACTACCCCATACGGAAATTTCTCATACTAGGCTGAATTCATCTTTAGTATTTGTAAATTTTAGGTCATCTTTGAACTCAGCAGCAAGCTCCTTTCGCAATGCCACTAATCCTTCCCCTGCATTACAGATATAGGCCGAAGCTTGTCTGCCAGACAAAGTTGCACCTTCCATGCTGTCTATGTAGTCCTGTTTTGTGTAAGAACCAGCAAGAAAGAAATTCTTCACTGGTGTCCTTTGATCTGGTCTAAATGGATCTTTGCCAGGTCCCTCGCGGTACAGAGATTGACCAATTTTAACAACAGAGGACCAAGTCACTTCCAAACCTTGAGATGATGGGAACAGCGCCAAAACCTGTTTTGCCACCCTTGCAATGATTTCATCATTTGGTAAGGGCATGTATGGATCTCCTGGCGTCAGAACACATTGGAGCAAAGATCCTTGTCCCTCAATGTAATAATCTTCTGGAGATGAAAGTGCCAGGTCTGCAAAGCAAGAAAAATCAGCATCGGGTGTATAGAGAAGGTTATCCAACCCAACAGCTTTCCTCAGTTGCCTTGACTTTTCTAGATCCTGTAACTCTGTAACCCAACCATTGTATCTGAGCTGCACTGTGACTACAGGAACTCCAACTAGTTCATAGATGTTATTGAAAAACTCCTGTTCCCTCCACTCTGATGGAATTAATCTCTTAATTCCGGGGACATCACAAGCAGCAACGTAAGCGTCAGCTTTCACAATTTGTTTGGCAGTAGCCTTTGACATGGAAAGTCCTGTAACATAAATACTCCCATCTTCAGATTTGTCATAAAGTAGTTCTCTGCATCCCCACCTAAGGTGGAACCTGCCCCCTCTATCTGTGATGTACTTCCTGATGGGGCCACTCAGATAAACGTCTGGTGAACCCTTCAGCATTCGCAAAAGGGAAGCCTCAGTCTTTGTGGCAAACAATGCAAATATGGTGAGCATGCAGCGGGCACTGATATTATCACAGTCAATGAATCCAAGGGCATAGGCAACTGGATCCCACATTTTTTGAATACTCATCCGTGTGCCACCTTTGGATAAAAACCAATCTGAAAAGCTAATACTATCCAAATTCCTTATGTCCCTCAGTGCACCATCTGGATCAACAAGAGCTTTAACAACTGGACTCAGTGCCAGAGCCACAGCATTTCTAGCCTTATCATAGGTATTAAGCTGATTTGTGGTCAAAAATGCCCTTATCCCATGTATTGGTGCTCCCACTGGGAAGCGAAAATCTAGTTCTCCAATTTGACCTCCTTTGTTAACAAAAGTGTGAGTGTGATCCTTCACAAGTAGATTGTTATGCGCACCGACCTTCTTCATCAATCGGAAAAGATTGTTGTAGCAACCGAAGAAAACATGCAATCCCATTTCAATGTGATTCCCACGTTTGTCAACAAAGGAACCAACTTTGCCTCCAATAAAAGACCTTGACTCATATATATCCACCTCGTGGCCCTGATCCAAGAGTTCCACAGCAGTTGACATGCCTGCAAGCCCAGCTCCAATGATAGCCACTTTCAGTTTCGGTCCTCGATAATGTTCAGGTTCCGGCGGAAACAACCCTTTTGGCGCGTTAACGCTCATGTCGGAAACGTTATCATCTAAGGAGCAACGAACTCGACACTTGTGAGTGTTGGTGAAACGGGTAGTGAAGGGGGCAAAGAAGCCAGCAGAGTCTGCTCCTCGAGTTGCGGAGAAGGAAGTGACCGAACATTGAATCATAGAAGCGAGAGCCATAGACGAAACAAAAACACAACAACCTACTCTGTTGCAAAGGAAGGAGTCGGTGTTGTGTTGTATGTGTAAAGCGAATTTGGGGTATCTGGGGAAGCAGATTGGAGGAGATGCCGGGAATGAGAAATTTGTCTTGTAGTAGAGAGGATTGAAGATTGAAAGTGGTGATCACACGTAAATTAGAGGCTGGTGTATGAATTATTAATATAAAAATAAACAATAATAAAAATAATAATAATAATAATTGCAATTGAATGAATATGTTGTTAGCTTTGGCAATTCTTCTGGAATTTGGAAAATGGAAGTGCCACTGCCACTGCCACTTTTGCGATGGATAACGGACCAATGCTCCTCCTCACTCTCCAG

>C100663_65

ATAATACATGTATCTAGCCTATAGATACATGTGTAACTAACATATGACAACTTACTTTCTATTACAGTGTGAATTATTCACAAGCCAACACTAGATAAAACATATGAAGTAACAATAAAGATGAAAGGTGTAGAAACCAATATGCAGAGAAGAAGTTGCTACAACAGGTTGCATGAGATGAAGAAGGGGATGGAGAAGGATGAGTCGTTGTTGCGTTTTCCAGTGAGCAAGTGTAACTGCAGCGGCTTGGACGAACAACACGATAAACACCAGTGCTTGCAAGAATATAAACATCTTTGTTGTTGTCTTCACCAAATGAATAAATGTAGCCCAAAGCTGGAAGAGGGCTTCCAGGGATCGAGTCACATTGGATAGGAGAGTCATGTGCACAACTGAAAGGTATTTTAGTGGTGCTGAAATTTCCACTATTTTGTGGATCTTCGATGGCTGCCCATATTGCACCTGCATATAGATCAGTGTACAAGTACCTTCCATACAGGCAAGGATCAGTTCTAGACCGATAGACATATCCACCAGTGACAGATGCGGATCCTTCTTTCTTGTTTACTTCAGAATGGTTGTATCCAGATATTGGGGGGATTGAATTGATGGAATTGAGGGAGGTGTTGCCACCAGGTGACTCTGTAGGAGTGAAAAGATAGGGTCCCTCATAAACACGCCAACCATAGTTTCCACCCTTTGTGATGAGATCCACCTCCTCATAAAGATCCTGTCCAGCATCTGCACAAAAAAAGTAGGAAGGTCTTTCTGAATCAAAACTGCATCGCCATGGATTTCTTAATCCCAAGGCCCATATTTCAGGTTGCAGATCTTTATCTTCACTGAATGGATTATCCTTAGGAATGGAGTAGCTACCCCAAAGGCCAAGTTTGCTGATTTCTGAAGCACTTGGAATGTTGTTTACATCAAGCCTCATAATCTTTCCAAGCAATGATTTCTTGTTTTGCGCAAAATTGTATGGATCACCTGAACCTCCACCATCTCCCATCATGAAGTATAAATATCCATCAGGTCCAAAGAGAATCTGGCCTCCATGTTGAGATATAAAAGGGAGTCCCATGGTAAATATCCTTCTCACTTCTACTGGTTTAGCACTTGTCGATGATGGCGGAGATGCAGTACCATTTGCAGTATATTCTGCAATAACAGCTTGATATTGACATGGTTGAACACCATTATCATTTCCTAGCTTTGATGGATCACAATCAACATTTGAATTACAAGAACATACTCCATTACATCCAGACCACTTATCTTTATCACAATTAAATGAAGCAAAGAATCGACCATTCTTTGCAAAGTTTGGATGAAATGACATTCCCATCATTCCAAGTTGAGTATCAAAATAAACTTGGTCGGTTAGATCAACAAAGGGACTTGACACATCAAGTTCTAATTGTCCTCCTGATCCCTCATCAGGAATAGTTGCCAGCCAAACCTTACCCGTTTGACTAGAGAAGAATGAGCGGTTAGAGCCATCAGGGTGAGCAACCATAACGAGATAAGATTCATTCCCAACTTTCTCAAAGCACAAACCATGAGGGGGGTTCATAGGAGTTTGAGAGTTGTTTAGTACAATAGGTTCCCCCTCAAAGCAAACCGATTCACTTTTGGAGGCTCCACCAAATGCTTTACAAAAATCAGTTTTGGATTGCCATAATTCAGTTAGTTTGGTGGCATTGGCATTATTACCTGGTGTTCCTCCTGCTTGGCCTTGTAATAAAGGGGAAAATGGTGAGTTTATAATTGACACAGTTTGACATGTATCCCATACTTCAGAGCAGAAGTCCTGCACTGCTGCCTTTGACTGAGAAGAATTTGCTGGGATGGTCGAATTGCAAAGCACGGGAACTGATCTGGGAGTGGATTGAACTGTAAAGAGCTCTGCTGAAAAGGGGTCGCATCTCGCACAGAGTATAGATTTCAAAATAGAGGCACAGCCAGGATCAGATACATTCATCACTTGGAATTGCTTCTGTATCCCTTCATCTTCTGTGGAATTGCAACACGTGCTTCCATAGTAAGGGCAAAATGCGATCGTGGTATTGAGGGTAAATGGTCGCTTTGAGTCAAGGCATAGAGGAAGTGCAGTTGAAGAATCCAAAAGCAGCAAGAACCAACAAAACAATAAGCTGATGAAAGAAACACTCTTCATGGCACAAAAACCAATTGAAAGCGTCTGTACTTCAGTTCACGTGAATCTCAGAACATATTACAAGCATTAGTGGTTGGTTTGGTGAGTTGACAAATGAAGAAAGAGGATAGTATTTAGAAGAAAACAGCTTCATGCGTTTTCAACTTTCTGACTTTCTGCCTCTTATGTCTG

>C100665_65

TTTTTTTTGAATAAAAAACTTTATTTATATAACAGTCGCACTTATAATAATAAGTAGCATTGTAATAAGACTATTACATAAAAACACACATAAAAGGGTTACATTATGGTAACTCCCATGATGTTTTAATCTGTAACACCACCGGATAAAGACATTAGAAAACATAAATATCTGGACCTAAACATTCAATTACATATTAAGAAAAATGTAATAACTGCTTTTGGGTCAAGAGACTTTAGTGTAACAGTCTCACACAATGAATGTCCAAGGAAACACTTGTTTCCTCTTCTCATTTTCACTGGAACCAAATTGAAACCTCTTGCTGTTTGTGTATGTTGTCCATTCCTACACCCTGTATATCCTTAAAATCAAAAGATGATAAATCACACTGGGGACTAAACATCATGTGGTTGGTATCAAAAGGGGCGTTCATGTTCATGTTCATGGCCTTGAACCTGTCAAATTGACCTTCCTCTCGTGCAAAGAAGTTCCCTTCCATCACCAGTCCTTGGCCAGTGAAAAAACTGTCATGACTATCCTGTTGCTGTTGCTGTAAGATGGTCTGAGAAAAATTATGATTTTCTGCAGCTACAAAGTTGTTGGAACTTAAGTTATTGTTTCCGACAACATTTGTATCATAGATTGTCATAAGGTCATTTATCATTTTTTGGCCATCCTCCGGAACTCCAAGTCCAGATACACTGAATGAAGGTTGAACCACACTTGAGGAGGACTGAGATGTAGTGTTGGGTTGAACAAAGGACTGGGGGAATATGACAGGCTTAACCTCAGCAGCATGGAAGTTGGAACCACCACCATACTCTGCAGCACTGTTTCTGTATGCACAATTCAACTGATGATTGTCCCTAGCCGACCTGTCAGGGAAACCAACGCGAGGTTCATTGTAAGGGCATTGGGGATGCTCACAAGTGTATATTTTTTGATCCATCATGAGGTTGAAGTCACCAGGAATCTTCCTCTTCCTGATGAAATCAAGGTTTGTGACAACATCTCCCTTAAATGGATGAGAAATTTGTTGTGGTGGTAGTCTTCCTGTGATTCTCTCCATCCCTAGATTAGATGGATGAAGATGGTCAGGCTTCCGGTCCTCCACATCAAAGTTTGGTTCCTCCTCTGCTCCATCAACATCGTACTCACTGCAATCATTGATGACCATGGACCCATTACCTCCACCAGAGGAAAATGGAGGGCAATAGTCAGGATAAAGCTCTCGAGCCAAGGTTTCCTCTTGGTTGATGATGGCAAGCCATGTTGCACTCTCCTTCGCTGTCATTTTGTCCTGAAGGCATTTAGATTGCCTTACAAGCTTCCGAATCTTGGCTATATCAGGTGACATGTGCTTAATGACAGCAGTAAGAACACCAACCTTCCATGCCTTCTTGAGGTCATGAGGTTTCTTGTAAGGTGGAGGGCCTTGATCTTTGGGAAGACCAATTTGTGGCCACCATTCTTCATTTCCAGTAGGCCACCATGGTGGAGGAACACCCTTCTCTAATGGGAACCTTCTCTGAGGAGGATCACAATGCTGCATAAGTGCTGACAGAAGAGAACCTAATGTTGTGTCCTGTAATTCCTGCAATGTGTGTGGAGTTGGACCAATTGAATTACAACCATCATTCTTCCCTGGAATTGCATTATCAGCCTGATACTTTGCTATGGCAGCAGGACCATTTCGATCAAACCTGACCTTGTCCTTCCACCATTCACGAAGATTGTCGGATGCTCCAGTCACTGGTTTTCCTTTCTCAGGAATTATCCCATAAACAAACCCTTGTGCTTTGCAAACCTCCATCATTTTCAGCATGTACTTAAGTATTCCATCCTGAGCTCTTGACATCTTTTTCCTCCTAGCTTGTTCTTGGGATTGCCTTTGCTTCACTGCATCTGTCCCTTCCTTGGACTTGGTTTGTTCTTTCAATCTTTTGAGGCGCACTTTGTCCCTCCACATCCGCTTCTCGAGCTCATCCACATCAATTTCTTCATCACTGTAATCATCCTCAACAACAGGATCTGGTTCTGTTTGTCTCACAGTAACATCCTCCTCTCCAAGAGGACAACTCAACAAATCCAAATCGCCACAAAATCCAATGTCCTCAAACATCATCATCTTTTCCCAGAAAAAAAACAGCCCAAAGATAGACAAGTGTCCTAGAAAAATCCTTCAATTCAACCTAGCAGATGAAGTGAGTAACAACTGACTTTACACGAGAAGATCCAGAAGTAGGTGTTGTTGTAAGATGAAGAGAGGTCAATATGGAGAGAAAAAAAGAGGGAAGCTTTATAGAGGAAAAGCAGAAAGTGAAGGAAGAAGGAAGAAGAAGAGAGTG

>C100669_65

CTAATTACCCAGACAGGCCGGCGTTCTGAAAGATTCTCTCCTCTTCGGAGGTTCAGCTGTGACTGCTAGAGGAAGCTTGAGTCTGTGCCAAGCCAAGAACTACACTACACTCACTCACTCCCGATTCAAACTGTAGCTATTCAACGACACGGTGCGCGATGCTTCGACACGACGACGTCCGGGTCGTCTCCTCTTCGATCAAAATCCCTCATGCTTCCAAGCGCCCAACCTGTCCTCTGCGAACCGCTCATTCTCACATCGCCAACGCTACCAACTCGATTGCGCAACTCGTTAACTTGTTCGCGTCTCACTCCACTGAGCTGACTCGCTCCGTCCTCCAAAAATCTTCGCTCTTCTGCTCCGTTGCGCTGTCCTTGACCGGCGACAGGAAGAGAGCATGTCCGATTCAGCGTCTGGCGTCGCTGTCTCTGGCGGAGGAGGCGCAGCAGAAGGCTCGACAGAACGAGGAGAGAGTGCTGATTAGCGAAGTGCTGGTTCGAAACAAAGACGGCGAGGAAATGGAGAGAAAGGACCTTGAGGCGGAGGCGGTGCAGGCTCTGAAGGCTTGTCGACCTAACTCGGCTCTCACAGTGCGCGAGGTACAAGAGGATGTGCATCGAATCATCAACAGTGGATATTTTTCGTCGTGCATGCCGGTTGCGGTTGATACGCGAGACGGTATCCGATTAGTATTTCAGGTAGAACCAAATCAAGAATTCCAAGGATTGGTATGTGAAGGAGCTAATGTTCTTCCGGCTAAATTTCTAGAGGACTCAATGCGAGATGGATATGGGAAGATTATTAATTTGAGAAGATTGGATGCAGCAATATCTTCCATTAATAACTGGTACATGGAGCGTGGTCTTTTTGCCATGGTTTCTGCTGTTGAGATTCTCTCTGGAGGTATTCTTCGATTACAAGTTTCAGAGGCTGAGGTCAATAATATTTCCATACGGTTTCTTGACAGGAAGACAGGTGAGATTACTGTGGGGAAGACTAAGCCCGAAACAATACTTAGGCAAATTACTACAAAGAAAGGACAGGTCTATAGCATGCTTGAGGGAAAGAGAGATGTAGAAACTGTATTAACTATGGGAATCATGGAAGATGTTAGCATTATCCCCCAACCAGCAGATACAGGGAAGGTTGATTTGGTGATGAATGTGGTAGAACGTCCCAGTGGAGGATTTTCTGCTGGTGGTGGGATATCAAGTGGGATTACAAATGGTCCACTAAGAGGGCTTATTGGAAGCTTTGCCTATTCTCATCGGAATGTTTTTGGTAAAAACCAGAAGCTCAATATTTCCTTAGAGAGGGGCCAGATTGACTCAGTCTATCGTATAAACTACACAGACCCTTGGATTCAAGGAGACGATAAACGAACATCTAGAACAATAATGATTCAGAATTCAAGAACGCCTGGAACCATTGTTCATGGTAATGTGGATGGTAATGGTAGCCTGACCATTGGCCGCATCACAGGTGGTATAGAGTTTAGTCGCCCAATCAGGCCAAAGTGGAGTGGAACAGCAGGACTTGTTTTCCAGCATGCTGGAGTCCGTGATGAGAAAGGTATTCTTATCATTAAGGATTGCTACAGCAGTCCTCTTACGGCAAGTGGCAATACTCATGATGAGACGTTGCTTGCTAAATTTGAGACTGTTTACACTGGTTCTGGCGACCATGGCTCTTCTATGTTTGTCCTTAACATGGAAAAGGGACTTCCTCTTTTGCCTGAATGGCTGTCCTTCACTAGGGTGAATGCACGTGCTAGGAAGGGTGTTGAGATAGGTCCTGCCCGCCTTCATTTAAGTATCTCTGGTGGTCACGTGGTGGGAAATTTCCCTCCGTATGAAGCTTTTGCCATTGGTGGAACAAACAGTGTGAGAGGCTATGAAGAAGGTAGTGTGGGCTCTGGGCGATCATATGTTGTTGGCTCTGGAGAAATTTCTTTCCCCATGTATGGACCAGTAGAAGGTGTTATATTTTCCGACTATGGAACTGATCTTGGATCAGGCTCAAGTGTACCTGGTGACCCTGCTGGAGCAAGGAAGAAGCCTGGTAGTGGATACGGCTACGGGTTTGGCATCCGTGTGGAATCTCCTTTGGGTCCTTTGCGTCTTGAATATGCCTTTAACGACAAGAAAGACAGGAGGTTTCACTTTGGAGTTGGGCATAGAAGCTAAACGTCCGGAGACGATTCTTGCTAGCTGCTTCTTGGTCTTTGTATATAATCCGTGCCCTCCTTGTAGCTTGCATGGCCTTTTCCTTATTTAGAAAACTTGTGTATCATTTCTCGATAAAAAAAATTACTCAACTGACTCGATCCCCTTAAGTGTTTCTTGTAT

>C100671_65

TTACTATACAACTTTTTATCTCTCATAGTGCTAGTTTCACTAGTTTGTGAACATGGCAACCATTACTCCGCAAAATAGCAGCCAAAGCGAGTTGTGTGTGAGTAACACAGATCCTCTTAAATGGGGTGAGGCAGCAGATTCACTCAAAGGTAGCCACTTAGAGGAAGTAAAATGCATGGTGGCGGATTACCGGAAGGCAGTGATTTCTTTGGGTGGAGGGGAGACACTAACTATATCACAGGTGGCTGCTGTTGCTAACCATTACTTGCAGGCTAAGGTTGGCCTCTCAGAATCTGCAAGGGCAGGCGTTGACGCCAGCTGCGAGTGGATCATGGATAACATTAACAAAGGCACTCCCATTTATGGTGTTACAACCGGCTTCGGTGCTTCCTCCAACAGGCAAACTCAAGAAGGCCGTGCTCTTCAGAAGGAAATGGTTAGGTTTTTAAATTGCGCAATATTTGGCTACGAAACAGAGGTCTCACATACACTGCCAAAATCAGCAACCAGAGCAGCAATGCTTGTGAGGGTTAATACCCTTCTTCAAGGGTATTCGGGAGTTAGATTTGAAATCTTAGAAGCTATCTCCAAACTCCTCAACCACAATATCACCCCTATCTTGCCATTACGTGGTACAATTACTGCGTCTGGTGATCTAATTCCTCTGTCCTACATTGTTGCATTGCTAACCGGTAGGCAAAACAGTAAAGCTGTTGGACCCTCTGGAGAGTCCCTCAATGCTAAGGAAGCTTTCCATTTAGCAGGTCTAGATTGTGGGTTCTTTGAGTTGAGGCCCAAGGAAGGTGTTGCCCTTGTAAATGGCACAGTCGTTGGGTCTGGCGTAGCTTCTATTGTGCTTTTTGAGGCAAACATATTAGCTTTATTAGCGGAAGTTCTATCAGCAGTTTTTGCTGAAGTCATGCAGGGGAAGCCAGAATTTACACATCATCTTATACATAAGCTGAAGCACCATCCTGGTCAAATTGAAGCTGCCGCTATTATGGAACACATTCTACAAGGGAGCTCTTATGTCAAGAATGCTGAACTGCAACAGCCAGATCCGTTGCAGAAGCCTAAAAAAGATCGATATGCTCTTGTAACTTCTCCTCAGTGGCTTGGTCCACAAATTGAAATCATCCGGTTTTCGACCAAATCAATTGAAAGAGAAATAAACTCAGTAAATGACAATCCCTTGATTGATGTTACAAGAAATAAGGTTCTGAATGGTGGTAATTTTCAAGGCACCCCAATTGGAGTTTCAATGGATAATGCACGTTTAGCTGTTGCTTCAATTGGCAAACTCATCTTTGCCCAATTTACTGAACTAGACAATGATTTGTATAATAATGGGCTGCCATCAAATCTCTCTGTTGGTAGAAACCCAAGTCTGGATTATGGTTTTAAGGCATCTGAAGTTGCCATGGCTGCTTATTGTTCTGAACTTCAATATCTAGCAAATCCAGTAACAAGTCATGTGCAAAGTGTGGAGCAGCACAACCAAGATGTTAACTCATTGGGCTTAATTTCGGCTTTGAAAACTGGCGAAGCCATAGAGATATTAAAGCTCATGTCTTCCACTTATTTGGTTGCACTCTGCCAAGCTATTGACTTGAGGCATTTGGAGGAAATTTTCAAGAGCACGGTCAAGAATACTGTAAGCAGAGTTGCACTGAAAACATTAAATACTGAAGATAAAGAAGAAATTAACCTATTTCGACTTTGTGAGAAAGATTTGCTTAAAGTGGTGGATAGAGAATATGTATTTTCATACATTGATGATCCATTAAATGCTAAGTACCCATTGATGCCAAAACTGAAGCAAGTACTTTATGAGCAAGCACACAAAAGTGTCATTAATGACAAGAATGTGAGTTTGTTGATTTTTGAGAAGATTGGAGCTTTTGAGGACGAATTGAAGTCTCTCTTGCCAAAGGAAGTTGAAAGTGCACGGGTGGCTTATGAGAATGGCAATCCAGCAATTCCAAACCGGATTAAGGAGTGCAGGTCATATCCACTCTACAAATTTGTGAGGGAGGAGTTAGAGATAGGGTTGCTCACCGGAGAAAAAACTCTATCACCAGATGAGGAATTTGAAAAAGTATATAAAGCCATGTGTCAAGCAAAAATTATTGACCCAGTTCTGGAATGTCTAGGAGATTGGAACGGGGTTCCCATTCCAATATAGTGTGTAATGTGATAGTGTTCCACAATAACTAGCCAGGATTTTTTTTCGTGATATGCTTTCAGCTTTTATTTTGGGAGAAACTAGTGTCGGTTTCTCGTATTCGCCTTATCTATACGATTTATAAATGAAAAACAAATGTCATCACTTTTAATTATAAAAAAAA

>C100675_65

TTTTTTTTTTTTTTTTTTTGCTATATTTTGAAATGCCTACAGTCAGGGCAGAGTCGAGTTTCGGGGAAGCATGGTGTTTGGAAAATGTTGGATAGTGTCAACTTGCTAACTTTTCAGTCATGTAAGTTAAATTGTTGTGACCATATTAATCTCTGTTCTGCCTTTCATGTTAGCACATGACAATCTGGAATGGTCTCATACATTCTGATTGAGAGGAACTGTCGATTCGGGAACTGGATCTTGGTTACCTTCACTATCAGATGGTTGAGATTCATGTTTTGCGGCAGCCTGCAACACCCGGTTCTGAACATCTAATGATGAAGGTGATGTAGGTAGTCCTAAGCCATTTGATTCTAATTTTCCAGCTGCCCATGAAAATGCTTCAGCTCCAAGTCTAAGAGGAGAAAGCAACAACCTCACATCTTGTTTTAGATCTTCAGAGTTTTCATATGAAGAAACCCGCAAATAGATGGTCAAACCGAGACCAAATGCACCAATTAACTGTAATGTCTTCTCCCACTCTAATAATCCATACAGTCCTGCACCAAGTACCGTACCATAACCTAATAGTTGCCAAGGAGAAGGACTAACATCCTCCAGAATTGCTTCAGCTTCCTCATTGAGTATGGAAAGTGCTGTCTCAGGTTTGAGTTCTTTGATACGGAGACCTTGTTTCATCCAAGACTGAAAGCCGCCTTCAACCAAGTATGTATTCTTAACCCCAATCTTCTTTAAGGATCTTGCGATGCCTTTAGAACGAGTACCATCAGCATCCAACACTATAACCTTCGAACTGTCCTTAACATTCTTCAAATTTCGAATGATAACAGCAATCAAGGAATCATCGAGGTCTCTACCACTCTTCAACAACTTGCGAATGGAACCGTCAACCTCGAGAGGAGTTATACTTGCATATCGAAACCGAGCAGCTCGTCGGAGATCAGGAACACCATCTCTTTCTCGCAATTCCTCAGTGCGGACATCAATAAGTGCAACGTTTGCATCCTCGGACAAAAGCTCGAAAGCAGATTTAGGGGACAAATCTCCTGAATAACCTCCATATGTCCACAACCAATAAAAGGCCCATAATGTGGCTGAAGAGCCTACAAGAACAACAAATGGAATAATTGGGTCATTTGGATCCAAGCCAAAAGTCTTCTCCAGTGAATAGACACCCATGTATATCCTTTGAGTTGCAGAGGCAACAGGCCTCAAGATTTCTGTTGCTTTATCTTCATATACATTGACTGTGTCCTTAATCCCTGCAGGAAGGAGCTCCTTGGCTGACCCATATAGGTAGACAACATAAGAAGCCCCATTTGTTAGGGAACTCTCTACTGCTACAATTGTGCGGCGCAACACATCAATAGCCACAGCAGGTGCTTTATTTGTGACTCCACTCAGCCCACTTGAAAAGCTAGTGAGTTTCTTGTTTGCTATGTCCCCTGATTGATCAACAGCAGAAAATGCCTTACTGAAAGCATTATCTACAGATTTACTAGCAGTTTTAACAACTGAATCTGCAAAGGAAGTAGCTGCATCCAGTGAGCTTCGCAAAGCATTTTCTCCCTGATTGATTGAGGCATTGAATGAGTCGTTGATTCCGGTAAAAAGGTCCCCAACACTTGCTTTTGTGCTGGCTAATGATTCATTGTCCACATTAATTGAACTAGGCCTTGGGGCAAAGTTCTCTGGTATTATATCAGTACTTGATAGTGTTTCTGGTTGTGGTGTTGTCACTAATGTATTTGATCCTTCAGTTGACTTTTGTTCTGCAAAACCCACGAATTCCTCTGGCACAGTAGATACATGATAATTTTCCATGGGTCTGACTTCACCATTACTCAGTGAATGTTGTCCGTCTGTTGTCAGTGGTGAATAACCCCGTGTGCTTGTAATGTATCCTCCGGGTTCACTTGTAGAAGAAAAACTATCAAAAACCGACTTTGTAGCAGACCGCACTGCAAATGAATTTCTGAGTGAATGAGTTCCATATTGCGAAGCCAGATGCACGCCCTCTTCAACCACAAATCTGCTCTCGAGATCCTTTCGAATTGGGTGCAATCCCCCGAACAAGGGAATCTGGGAAGATGTGCAATAACTGGGGGTCGCTGAGCAAACAGGCAACATTCTCATACTGGGAATCGATCACTGAACACATGAATCAAACATTAGTTTATTTAGATTCAGCTAAAGGTTTAGTATTTATTAAAATATATAAAATCTGATGAGAAATTTTTGTGGAATGGTTTGACAGATTATCCGAATGGAAGAGGAGGATATGGTGGAACTCGATCCTATTTGCTTCCTTTTTGTCTCTTATGCTCCCATGTCACCACACAAACTCAC

>C100677_65

ATCAAACTCCCGCCATAAATATCCACCCCAATTGAGCTACAGCTCTTAGGCTACAAATAAATTATTTACACCATGTACATGACTGTCCATGTTACATACATGATACATATACGAAAGAGTGAAGTGAAATATGTTAGAATTGGGAAAACAAATTGAATTATTCAGGCAAAATAGGAAGCCTGGTGATACTGGTGCCATTGCCGCTGCCATCAGTAGACACTGAGGTAGGACTTGTTTTCTTGCCGGCAACAGACGTAGATTTAGAGGGGTTTTTTGAATTTAGCCTCAAGGGAAGAATCTGCAATCTGGGGTTCTGTTCTGTACCTGACTTTTCAGCAGCCAACTGGTAACTTTGCACAAGTTCAAGCTGCAGGGCAATAATTTCAGAACGTCTAGGAAGAAGTTCCACAGCCTCTCCACCAGGAATCACAATGTACTCTATTGCAAGCCGGACCTCCTCCAAGGCATCAATCTCTTCCAGAGATGGTTTTCGTTTCGGCACATCATCCTCAATTTCAATATCCAAGCAGTCATTTAATGGCTTATTTGTTGTACGGCCAAAAGATTCTAGCCCAAGGATCATTCGGACTGCCTTGACCATTTGTGCCATGGTATTGGACTTGATAACAAATACTGGTACATGATGGAATTTCGCAACACCACGGATCCAAGGGTTCTGTCGTATCTCAGAACCAGATGCTAGAATTGCATCAGCAGTTCCAATGTCATCAGTCACATCTATTACATCTTCAAGCCCCATTACCTTTGCTACTTGTAATAGATCAGCTTCAAGAATCTTATAAGTATAAACTTGCATTGGTGAACTCCTCTTTATCACAGGTCCATGAGTACTCCATTTTGAGGACCAAGTTCGTGGATGGTCTTTATCATCCTTACCAGATGCTTTGTCAGAACTTGTGATATTATTGTTACTTAAATCAGATGTTTCTAAAAGATTTTCTTCCGGCACTGGAACATATTTTTCAAAATCGTTGACAGAATCATCCCATTGCCTGACTTCAAATAATGGAGGTTTTCCTGCCAAAATGGCATCCACAGTTGCATCTAATCTATGATGAACACGGCATTCAGTTTTAGAGATCATCTCAATAGCACACGAAAACGTAGGGGGTCCTTTCCTTTCAAGAATTGTCTTCTGCACTTTCCTTTTTCTGGCTTCCTCGTCCCCAAGGGTTACGCTCTCTATGCCCCCAACAAGAATCTGTAAATAAGGATTTTTTATTATGTTTTCAATGGTCATTCCATGTGCGGTTCCAACCAATTGAACTCCTCTTTGAGCAATGGTACTGGCTGCCAATGCTTCAAGCTCTGTTCCAATTTCATCAATTATAATGGTTTCGGGCATGTGATTTTCAACTGCTTCAATCATAACATTATGCTGCATATTCACGTTTGGAACTTGCATCCGCCTGGCTCTGCCTATTCCTGCATGAGGAACATCTCCATCCCCTCCAATTTCATTAGACGTATCCACTATTACAACACGCTTCCTGAACTCATCTGCCAGCATCCTAGCAATCTCTCTAATTAAAGTTGTTTTACCAACTCCAGGAGGTCCGATGACCAAGATAGAACCTCCATTCTCAACCAAATCACGTATGATTTCAGCACTACCACTCACAGCTCGACCAACTCGACAAGTAAGCCCAATGATTTGCATTTTCCGATTCCGAATCGCACTTATCCGATGCAACGACCTATCAATACCCGAGCGGTTGTCATCAGAAAACTCACCAACCTTGGAAATGGCGTGGCGCAAATCTTCGTGCTGGATGGGTTGTTCGGAGATGAACCAGTCGCCGGAGGGGAACCTGGCGAGAGGTTTTCTGCCCAAATCCATGACGACTTCTATGAGGCCTCCAATTTCGCGGTGGCGATAAAGTTCCCTTCGCATCTTCAGAGGGAGAAGTTCGAGGAAGAGGTTGAGGTCCTCCGGGCGCGAGGTGGACGGCGAGGAGCCGTAGAAGCGGTCGGAGGAGGGGCGACGAATCTCCGGCGAGGCCGAACATCGGAGAGCGTTTGGTGGTCGACGGAGGGAGGATATGAAGTGGGAGTTTCTGAGGAAGGCGAGGGTTGAAGTTGGCAGGTGAGGGTGGTAGTGGAGATCTATGAGGAGCACGTGGGAATTTAGCGCCCTCATTTTTCTCTTTATTATTACGTGTTGAGTTGAATGTGCAGTGGTGGTTGTTATGTTATTATGGAGGGAATGATAAATAAAAATAGTGAGAAGAAGGAAGATGAGAAGATTCTATGGTATCTTCTTCTTTTCTTCTTGACTTTCTCTCAGATTTTATTTTACATTATAGAAGGGAGAGGACACAGTTTGTG

>C100679_65

TTAAAGTTCTGTTGGAGAAGATTGTCTAGGTTTCCAAGGTACTACTTGATCTGTCCCTAGTTAACTTCATCACTTTCTATACTATAATTATCTTTTACTGTAATCCATTCCTTTCTTATCATGTATCATCACTATTCCTAGGGTATTGTTCTCACTTTCCTCCTTTTCCTGTTTCTTCTTCTGTATTAAGTTCCCTTCAAGGTTTCATTTAGATTCCTAGTCATATATTGTTAGATTCTTAGAAACAATCTTTGAGATAGTTCAACATGCTTTCCACAAACTCTCTTGTGCAAAATTTTTGGGGTCCTATCTCGGTGTTTTCAAATCAGAACCAAGTAACGGAGGTGATTGAGCTTGATGATTCATCTTCTCCTTCTTCTGGTTCAAGCTCAAATGGAGAACCAACTAAGTACTCCAACCACATCCTCAGATACATTAGCGATATCCTCATGGACGAGGAAGATGACTTGGAGAGCAAACCCTGCATGTTGCAAGAATGTTTGAGACTCCAGGCTGCTGAAAAATCCTTCCTTGATGTCCTTGTCCATCACAGCTACCCTTCCTCTTTCCGTGATGAAAGCCTCGACATCGTAGACTTTGATGAAAATTATGGTCGCACTGCTAGTTTTGAGAGCAATGGCAGCTGCACCACTGATAATTCATATGAGTCTGACTTGTTCAATGGGGTCGGTGAATTTGACTCTTCTTTTCTACGACTGCAAACCCATCTAGTTGATTCCCCAAATGTAGTTCTTGATTCATTTGGGGAGTCACAAGCTGCTAGCTATTTTCATGAAGCTCTTGATCCATTTAGGGTTCGTGATGGATCCTGGAATCTCTTTCCGTCTCATACAAAGCCGCTGACGGTAGAAGAGGGTTCTTCTTCTTCAGAATCCAGAGAAAAGAGAAGCCATCAGATCGATGATGATGCTTTTCACGAACAAGATGGAAGAAGAGGCAGCAAGGTTTCAGCTGTCTTTTCTGACGAGTTAGAGCCACCAGAGATCTTAGATGAGGTGCTGCTTTGTCAAACTGGAAGAAGTGGATCTAGTACTGAACCATCACAGAGTGTTGATTCAGGAGGGTCCAGTGGAAAGGCTACACGCTCACGTTCAAAGAAAGGGTCCACCAAAGCGGGTACTGCTGTAGATTTATGGACCCTTCTAACTCAATGTGCACAGGCAGTGGCAAGCTTTGACCAAAGGAATGCAAATGACTTACTCAGCCAGATTAGGCAACACTCTTCACCTTATGGGGATGGACTCCAGCGTTTGGCTCATTACTTTGCCTATGGCCTTGAGATAAGGTTGGCTGCTGGGACCCCATCATACGTGCCACTAGAAGTGGCAACTGCTGCTGATATGTTGAAAGCTTACAAACTATTTGTTACATCCTCTCCTTTGCAAAGGATGTCAAACTATTTGACAACCAAGACAGTCATTGATCTTGTGAAAAATGAGAGCAGCGTTCATATTATTGACTTTGGCATTTGCTATGGATTCCAGTGGCCCTGCCTTATCAAAAGGCTGTCAGAAAGACATGGTGGTCCTCCAAAGCTTCGTATAACAGGAATTGATCTCCCTCGGCCAGGATTTCGGCCTGCGGAAAGGGTTGAGGAGACAGGGCGTCGTTTGGAAAACTACTGCAAGAAGTTCAATGTCCCTTTTGAATACAATTGCCTTGCACAGAAATGGGAAACTATAAAACTTGGAGATCTCAAGATAGACAGGAATGAAGTAACTGTTGTTAGCTGTTTCTACCGGCTGAAGAACCTGCCTGATGAAACTGTGGCTGTGAACTGTCCAAGAGATGCTGTGTTGAAGTTGATTAGGAAGATCAATCCAAACATCTTCTTCCATGGGGTGGTCAATGGCACTTACAGTGCACCTTTTTTCCTGACACGGTTCAGGGAAGCGCTATACCACTTCTCATCACTGTTTGATATGTTTGAGGCTAATGTGCCTCGTGAAGACTCGCAAAGGGTGATGTTTGAGAAAGGGTCGCTTGGTAGGGATGCCATCAATGTTATAGCGTGTGAGGGGGCTGAGAGGGTTGAGAGACCGGAGACATACAAGCAGTGGCAGGTTAGGAACCTCAGAGCTGGGTTCAAGCAAGTCCATTTTGATCCTCAGTTTGTGAATGATGCAAAACAAATGGTGAAGAGGGAATATCACAAAGATTTTGTAGTGGCTGAGGATGGCAAGTGGGTTTTGCAGGGGTGGAAAGGGCGAATTCTGAATGCCTTATCTGCTTGGACTCCTGCTTAAGGAATGTCCAGAAAGTGTCTTGTAACTAGTAAATCTACTCCTAGGTCGGTTAAAAAAAATGGAATAAACTACTCTTTTGCATT

>C100683_65

TTTTTTTTAAAAAGGAAAAGAATATATTATACAAACGTGTTTAGGTTTATCATTTGGACCGAGCAGAAAGCAGTCCAGTATATGATATGAGGTACGTACATTGACTGTTCCTATATTAACAAGTTCTCCATCCACTTCACCGCTATCTGTCATTGTTATCCTGATTCTCACGGTTTTCATCCCCAGTCCCGGGTGTTTCATTGAAAACTTGTCCTTGCTCTCTCACGTCTCTGAACATGGAAAAAAATGAGTTGAGGCCCTCAGACGAAGCACCTCCTAAGATCCATAATAACAAAGAACTGAACGAATTCATGGATCCAGGGTTAGGACCCCTTTCTGCTTGTCGGATGTCAGCTTGCTCAGGAACCTGTGCCTGGTTTGCAAACCTGTCCCGATTATTTTCTGACCGCGCTTGAGGGTCGCCAGCTCCTGCTCCATTTTGAGGCATTGGTTGATCAAAACCAGGTCTCAATCCTGTTCCAGTAGGCAAATCGGCGTTACTTAATCCCTGGTTTCTTGTGTTCAATCTTGTGATGTGTAACGTTGATGCAAGAATGGTGGAAGAAGTAATGTGGAAATCTGGGTGCTGTTGAAGCTGTGACCGTCTATTTTGCATGTACCTTTGCAATGCTGGTACCTCAAACCGATTCCAAAAGTAAAGGATAAGGTGTTGCATAAAAGCTGCAGTTGTAGAGAGTGCCAAATATGAAAAACCATAAGCATAAGAGAAGAAATATATGTGGAAGACCAGGAAATACAGCAAAAAGAACCGAGGAAAGAACTTCATTGATATTGGTGTTCGGACACTAATGAGAGTAAAAAGTTCGCACAACCAGACAAGAATCAAGACCATGAAGGCAAGAAGCTGATCATCATAAAACTCAAAGAGAAAAAACAGAATCCCAATCATAATAGGAACAAAAACTAGTGATTCAATCACATGTACAAAGATCAGCTGAAATGTTGGAAGGCGATGCCGAGCATGGTGTTGAAGCTGCACTGTGAATTTCAGCATGCGAGTCTGTGTTTCTCTTAATGTAAATGATACTGACATTGTGGTAGTAAAAAATACAAACAAGGACATCATAAGCACGCCACACTTCGTTACCAGGTAGTCTCCAAACCTAGCTGGGCCTTCCGGAGCTTCTTGTGCATAACTAAGGTTATAGAATTCCTTTGTTTGATAGTTATAAATATAACCTTGACCAGGAGAACTCAATAAACTATTGATTAAAATCGTATCATATCCGACAAATCTGTTTATGAGTAGTTGCTGCCACTTGTTTCCAAAGCAGGAGTGCCAAGCTGATATGCTTACATTCACGGTACGGATATTATGATAAGACTTAGCACTTTCAGGTAACAAGAAATATCCCTTTTCCATAGTATACAGATATGTAGGTTCAAATAACTTGCCTTTCTTTTTAAGCCACTGCACTGCATTAGTGTTAAGCTTGTCCAATCGAAGGATATGCATCCATTTGGGAATATCAAGATTGAGGTGTACACCAGCAATATTCCACAGCTTCTGGAAACTTCCAATAATCTGCATTGTATGTCTCCAAATAAATGAGATCCGCCTATACCACTTTTTACCAAAATGTATAATTGCTGCTTTTAATGTCTCTTTGGCAGATAAAGTAAAACTTTTTGGTGGCTCCTCCTTATCAATTTTTATGACAGCATCTTCAACAACAGGTCTGGTGTTTTGATTACTTGTAGAGGTTTCAGCTTGATATTCAAGAAACTCAGTGTCAGTTTTCCAAAACTTGAATACCAATTTACCTCTCCTAGAACCAAAACCAATCCAGTTCCACCAAAATTTGGAAGCAAAAAATGTCAATTTATCTCCACTGGCATCAGAAAACTCCATTTGATCTTCTACAACTTCTGTTTCAGGAGGATCTATTCTGTTTGATTCAGAATCATTATGAGACCACAGGCCGGCACCAGTTATCTTAATCTGAATAAGTTGGGCTTCTGATGTCACGACTTCTGATAGCTCACTTGAACACCCAGGCTGCTGAACGTAATTAGCGTGCATAACAACGAGAATGCAGAATAAGGTTATGGCAAGCAATAGATAGAGATACTCCAGAGCAACTCTGACTTTGGGCGTCAGCATCTGCGAAAACCTCTCCTGCACGCGAATGAAGGTCTGCTCCGGATCCATGGAGCCACTCCACAATAGAAACGAAAATACAGAGAAGAAGATTTCCCCAAAGAATGATGGAAAATCAAAGGTTTTCGATGGAACGATATTTCATGTTGATCATCTTCACGTTTCCCACGGGGGGAATTGGATTTTACAATCACAGATAGGAATAGGAACAGTGAAAATGAATTCAAG

>C100685_65

CTCTAAACCTCATTATTCGTAATCCCAAACACTTCGTTTGGAAGTGAAAAGAAGTTGCTATCAACGGAGGAGCGTCTCCACCGGAAAATAACCCTCTGGCGACAGTTCTAGTTTTGTGCTTCGAACCTCTTCTGTTAGCCGACGGGATCGCAGATTCAATCGATTCCGTTTTCTCATTCTAGTTGATCCAGTTCGTAGGGTTTTATGGTGTCGGTTTATTGGACTTACGGTTGTGATTTGCATTACTCACTGGGTGGTGGCACCGAGAATTGTTGAGTCAGATTATACATGTTTTGAAACATCAAAGAGAGAAATGTCTTATCAGCATAAAAGCTTTTGGATGCCACGGGATGCTGGCTGCATGGCTGAAGAAAACGTGGGATATGAAAATTCTTCCAGAATTGAACCTAAGCGTAGTCATCAGTGGTTTATGGATACAGGTGAACCTGAAATATTTAGCAACAAGAAACAAGCAGTTGAAGATGTTAGTGGTAGACCAATTTCAGGAGTTTCACACGTGAATGTTTCCCAATGGGACACCAATTCAGGATTTCACTCTGTCACAGGTCAATTTTCTGACCGTCTTTTTGGATCTGATCTTGCTAGGACTGTGAATTTGGTTGATAAGAATGTGCCATCTATTGTTAGTGGAAATATGAACATGGGAAGGAAGGATTTTGAGCATCAATATGGTAATGATCCTTCTGTGGGATTGTCAATATCTCATTCCATTGCAGACCCTTCATCATGTCTCAATTTTGGGGGCATCAGAAAAGTTAAAGTTAATCAAGTCAGAGATTCTGACAATTGCATGCCTGCTGCATCCATGGGGCATTCCTATAGCAGGGAGGATAATAGTACAATTTCAGTAGGTGCTGGCTACCATAAGAATGATGGCAATATTTCGTTGGGTCCTACTTATAACAATAGAAATGACAACACCATTGGTATGGGGACCAGAATAAGTAGTAAGACTGATGACAATCTTCTTTCAGTGGCTCACACCTTCAATAAAGGTGATGGGGGTTTTATGTTGATGGGCCACAATTATGGCAAGGGTGACGAAAGTATCTTATCAATGGGTCAGCCCTTTGACAAGGGAGATGGAAATTTCATATCAATGGGTCAATCATATGAGAAAGAAGATGGCAATTTAATATCTCTAGGCACCTCATATTCCAAGGGGCATGAGAGCTTTATATCAATTGGTCCAACCTATGGTAAATCAGGAGAAAATTTCATAACTGTTGCTCCTTATGACAAGGGTACTGATCATTTAATATCAATGGGTCCAACATATGATAAGGTGGATTCAAATATTGCATCAACAGTTCCATCATATGACAGAGGAGATTCTAGTTCTTTACCTGTGGGTCAAAATCACCATAAGGGTCAGAGCAGTACCATATCTTTTGGAGGTTTTCATGACGACTCTGAAGCAAATCCCTCTGGTGGAATCATTAGTGGCTATGATCTGTTGATAGGAAGCCAGAATTCAGCTCAAGGTTTGGATAGTCAGAATGATCTGACTGAATCAAACACTGAATCAATTGTAAATAGCATTCCAAAACCCAATACTAAAAATGATATTGTTGTTAAGAACAAAGAACCTAAGACAACAACCAAGAAGGCTCCTACTAACAACTTCCCTTCAAATGTCAAAAGTTTACTGTCAACTGGTATTTTTGATGGTGTTCAGGTGAAATATGTCTCATGGTCACGGGAGAAAAGTCTTAAAGGAATCATAAAAGGAACTGGGTATTTGTGTTCTTGTGACGACTGCAAGCAGTCGAAGGCTCTTAATGCATATGAGTTTGAGCGTCATGCTGGTGCTAAGACAAAACACCCAAATAATCACATATACTTTGAGAATGGAAAAACCATCTATGCTGTTGTTCAAGAGCTGAAAAACACTCCTCAGGAGATGCTTTTTGATGCAATTCAAAATGTGACTGGCTCTACCATCAACCAAAAGAACTTTCGAATATGGAAAGCATCATACCAAGCTGCTACTCGGGAGCTTCAACGTATCTATGGAAAGGATGAAGTTATAATTCCGTCTTGAGTTCAATTTTCTTCTTGAATGTTCAGAACAGTGGTAGATATGTAATCTGTTCATAGTGAGTGATTGGCAGCTTGAGTTTAAAAGACCATTTAAAATTGAAAAGTTTTAGCTGATACTTCTTGTAGGAACTTGATTGTTTTATTTTTGGTAATGAAGATGTCTTTTTGTGGTTCAGTGCCATTCTTTCCATCAATTTGCTGATGTCTTGTCACCTTTGTAAAATTCCTTGCAAACCATGTTCCTTAGTGAATCTTTCAAGAGATTACATCCAATATTAGGGTTGTC

>C100687_65

TTTTTTTATGCAAAAATTTCGCTATCGTTATTGAAGCAGCCGTGTTCTGACTCCGAAAGTGCTACAAATGAAGAGCCTTCTAGCAAAAAATGGCATTCAACAGTAGATACAAAAGTAATGTTGATTCTTGTAATTATGATGATGATTAATGGGAAGAGACACAAATGCTACAAAACCAGCGCCTACATATCTCACTCACTTCTTGACTTCCTCATACTCAGCCTCAGGAGCTTGGTCACCACCTTGAGAACCCCCAGTTGAGGAACCACCACTAGAACCACCTGACATGTGTTGTCCAATCTTGGAGACAGCTTTGTTCGCTGCATCAAGCTTTGACTTAATTTCATCAGCATTGTCCCCTGCCATTGCCGTTCTCAAATCTGATACTGCATCTTCAATCTCTTTGGCCACTTCGCTGGGAATCTTCTCTCTGTACTCACCTAAACTCTTCTCAATGCTGTAGATGGTTGTATCAGCACTGTTTCTAATGTCAATGAGAGCCTTTCTTTCTTGGTCTCTCTGAGCATGCAACTCTGCTTCTTTGACCATCTTATCGATCTCATCTTCTGAAAGTCCTCCAGATGATCGGATGGTGATTTGCTGTTCTTTCCCAGTGGATTTGTCTTTGGCAGAGACAGTGACAATTCCGTTGGCATCTATGTCAAATGTGACTTCAATCTGAGGGATACCTCTTGGGGCAGGAGGAATGCCAACAAGCTCAAATTCTCCAAGCATTTTGTTGTCTGTAGCCATTTCCCGCTCACCTTGTAGAACCTTGATACCAACCTGAGTCTGATTGTCAGCTGCTGTTGAAAACACCTGGCTTTTTTTAGTAGGAATTGTGGTGTTGCGGTTTATCAACCTGGTAAAAATACCACCCAAAGTCTCAATACCCAGAGAAAGTGGAGTGACATCTAGGAGTAATAGCTCTTTAACATCTCCACGTAAGATACCACCCTGAATAGCTGCTCCCATGGCAACTGCCTCATCAGGATTTACTCCTTTGCTAGGACTTTTCCCAAAGATCTCTGAAACTACCTCCTGGACTTTAGGCACACGGGTCATCCCTCCCACAAGAAGAACCTCATCAACATCCTTGATAGATATGTTAGCATCCTTCAAGCAGCTCTTACAGGGTGACTTTGTCCTTTCAATCAAGTGATTTACCAAAGCCTCAAACTTGGATCTTGTCAATGTGATGTTCAGATGCTTTGCTCCAGATACATCAGCAGTGATGAAAGGAAGATTGATTTCTGTTTGAGAGGTTGAAGATAGTTCTATTTTTGCTTTCTCGGCAGCTTCCCGAAGCCTCTGCAAGGCAAGCCTGTCTTTCGAAAGATCAATACTCTCAGATCTTTTGAATTCATTCACTAGAAAGTCCAGTAAAGCATTATCAAAATCCTCTCCTCCCAAGAAAGTGTCACCATTTGTTGCTTTCACCTCAAAAACACCATTAGAAATCTCTAGGATGGACACATCAAAAGTTCCACCTCCAAGATCAAAAACTGCAATGAGACCCTCCTTGTTGTTCATCCCATATGAAAGTGCAGCAGCAGTGGGCTCATTGATGATTCTCTGGACATCAAGACCAGCAATTCTGCCGGCATCTTTTGTTGCCTGCCTTTGAGCATCATTGAAATAAGCTGGCACGGTAATGACAGCTTTTGAAACTGATTTTCCAAGATATGATTCAGCAGTTTCCTTCATCTTGGTGAGAACAAAAGCACCAATTTGGCTGGGAGAGTACTGCTGCCCATTGGCTTCAACCCAAGCATCTCCATTGGGTGCCCTAACAATCTTGAATGGTACCATCTTCATCTCCTTCTGAGTTTGAGGATCATCAAACCGCCTACCGATCATACGCTTGGTTCCAAAAAGAGTGTTTGTTGGGTTAGTCACAGCCTGACGCTTGGCTGGTGTACCAACAAGAAGCTCCCCTTTCTGGTTGAAGGCAACCACTGATGGTGTTGTTCGAGCTCCTTCAGAATTCTCAATGACTTTAGGATTCTTTCCCTCCATAACAGCAACACATGAATTGGTAGTACCCAGATCAATTCCAATGACATCATTACCAGCAGGCTTTGAACTGAATGGTCGAGCCAAACTAGCCCACTTGTGACCTACATATGATGTATTGCAGCCGCTCGTCAACGATCGAAAGGCGGAGAGCGAAGAAGTGGGAAGGTCACGGCGGCGGAGAGAGCGGAGCAAGGCGGTGGCAGCTGCCATGGCGAATGGAAAGAGTGACAGGAAGGAAGGGTGTGGAATTGGGCGTTAGAGAGGGTTTGGTATGGTATGGTTTGGTTTGCCTTGTCTTTAAAGTAGGGTTTAGGGTTTTGTGCTGACGAAAG

>C100689_65

TTTTTTTTCCAGAAACCTACAAAAAATAACATGTAGTAGGTGTATTTAATAGATAAAAGTGGTGACTTCTCGTTAATTAGAGTCATGACACTACCATCATCAGTAATACATGATAAATATGCTCCAAAACACAGGGAGAAAAAACAAAAACTACTTCGGGTAAATTTCCAAATACAGTCTATTAAAATTATTGCCTAGTATATGAACTTGATATTTTACTTGATGACAACACAGAACTTCTCTGTGACAGGCTACATTTAACATCAGATTCTTAGATTCAAAAGAATATGGTATACCACATTTGGCATGTTATCTCAAGCTTTTATCATGACGACAGTTGTGTGTTGATCTCGACTATAGGCCAAAATTGCACATCTGCACAAACTCCATATCATTGTCCACGTATTTGGTCCATAGATTTTTGTATTGATTCAAAGCAATATCAAGCCAGGGTTTCATGTTTCCGTTGTAATGAATGACTGCAGCATTGTTGATCTCATCCATGCTAATGCTGGGATTGTACCCAAGGCCAAGCACGTGCCAGGATTTGTCCAACGACTTGGTTGTGGAGTAGAAGGTGATCAAACCCGGTGGCAGGGTCCCCGCTTTCCACAAAGTTTGGTCTTCATTCGAGTTCTGCCAATAATGGTAATTATCTGTACACTTTTCCCGCCTCCAAGCATCCAGATTAAATAGATTCATGCCATAGGCCCAAGCACAAGCTTTTGGATTGAACCGCTCCTTGATCAAAGGATGAGAAAAATTTATGTACTGAGCATATCGGTGGAAAGAACCGAAACAGATCTCAACAGCACCATTCACCTTTCCATCCAAATCAATTTTCCACAAACCTGTCAGGTCTTTCTGAACCACAACATCATCATCCAAAAGTAAGATCTTATACAATTTAGGGTACATATCTGGCAAATAAAATCGAAGGTGATCCAACATGGACAGGGACTTAGCATTTTTCATGTTTGCATCATTTGAGGCATTTTCAGGCTGATTCATTTTTGCCGACTCAAGTTGCCTCAAGATAGGGACATAGGATGAATTTAAGAATGTAAATTCTTCTACCGCTTTCACCTCCAAAAATGCACCCCCTTCAATAGGCCTCATCTTAAACCAAACCTTCATTGCCCCAACGTTCATCCTGTTTGCAACAACGTGAAAAACATGCTTCCATGGTTCCACTGCATTCTTCACAACAGATCTCACCACCACAGATACAGCTATTACATTGTCCGAGAATATTGCATAATGATACAAAGTGGGATCTTCAAACTCTGGCTTGGGCTCCTCATCTCTATATTTATCAGGATTTGAAATCTTTTCACCCATCAGCCTCATAGCCAAACAATGCAAGCTCTTGGGAATCGATCGGGCTGAAATCAAGCTTGCCATTGCCCCATTCTTCTTCGCCTTAGCAAGTGACTCATGAACTGCAAATATTGTATCCTTCAACTTCTGAATCTTCAACTGATTATCATAACTCTCTTTCACCTCCACAATGATCATCCGTGCAATCTTCACTCTATCCTTAACCTCTTTCTCAAACTGCCTCAGCACATCCTCATCAATCGGACCATCTGACTCAAACAGCGACTTTTTGTAAACCGGCTTCAACGAAATATCCGAAAAATTGCGCGCCAACTCATCAAAAGTCTTCAATTGCTTAGAAATATCAAGCTTGAGCTTCCTAGCATAAGCACCATAAGCATTTACTAGAGCTATGTGATCTTTAGCTTGCTGGTGTATCAAATCCACTCGGGTCCTAAGAGGGTCAGATTTCAGTGCCAAAAAAGTCCGATGCACATATGCATTACCAGTGGTGGGAAGATCAGATTCATCGTTGGATGTGGAGGGGTTGGTGGTGAAGAGGACCGAAAGGGTGGCGACGAAGAGAAGAGAGAGCATTGCAGAGATGAAGATCCGGAAGGAGAAGAGGCCTCGAAAAGTAGATCCACCTCTGGTTCCTCTGGAAGCAACCGCCATTGGATCTTGAACTTTCAGCAAGTCAGATCCAAGAATTAGAAGAAACCGTGTGTGAAAAGTTGATTAAGTGTGTTCTGATTGTTTTTAATTTTGAATTGAAGGGTGGAAATTGGAAAGTGGAGTTAGCATGGTTGTTTGGGGCACAAAATTAATAGGAGGATGGAGGAATGAGAGAAGAAGGTGATTACGTTGAAGTGAGAGATAGAGATAATGCAATGAATGCAGAGTGTGTAGTTTTGAGATTGTGAAGTGTGTGTTGTGTGTGAATGAGAAGAGGTGGGCTAGTAATGATTCTCAGCCTGTGTGAATCCTGGGACAAATCTGGGCTCCATCTCTCAATGTTTCTTTTATCCTCT

>C100691_65

CCACGAATAAATGAATTATATTTCCCTTTTAGGTGTCAAAGACAACTAATACACAATTTGAAAAACTAGCTCCTAAAAAAATGCTAGATATTTTACTTGCCAATTTCTGTACATCAAAGACTCAGGTCTTGGGACTCATCTCAGTCAAAATTACACAATGACGTACAATGGCACCTATTCAATGAGGAGTGCATTTATGTGTATTTGTTGATGAGGGCCTATCACACACCAACTAGTTGTAAACATGTTATTGCCTCTGCAACACGTCAGTTCAGTTACATCTTTAGTGAAAATCAGGCAAAAATCTTTTCATGCAGATAACTCTGCCTCTGCCCCCACGTATTCCGTATCCTCATTGTCAAGTCGTTTCCTTCTTTGAGCTTCAAGTTTGGTTTGGTTGCCAATAGCATCAGAGATGGCTTTAGGTAACCAAAATCCTGGCTTCTCAAGGAATGGTGCCCGGTCAAGCACCAAAGGCTCTCTCAAAGTTACTTCAACCCCATCAAAGACCCACAATTTCGTGGTTGCTTCACCTTTGAGACCCAACGTGAACCAACCTAAACCCGCTATTGCGATGTCAATGCTGTTGACATCCCAACTAGTCCCAGACACTTTTACTTCTCTTTCTTGCCATGTTCCTAATTCAGCAGCGCGATCATTACCAATGGGAGGCTGTAATCTGATACCGACATGCTTTCTCCAAACCTCATCAGCATTTTCAATTTTCCCCATGTGAAGAGAAACATTTGGTGATGCCCAAACTGTGACATATATTGTTTCAACAGAGGCTTCAACAAGGTCAAGTCTTGTCAAGCCACCAATGTGTATAGCTTGTCCCACCTTAATTCTATATGACCGAGGTCGGAGTTCCTTCCGGATCTCAACCATCTTTTGCTCATCCCGGTTCAATCTCATTGATATTAAATATGGGTGCAAGAGCCCTGGAGTGTCAAACATCTTAGTCTTAGCTGGCAGAACTCCTGCAATTCTCAAGATCCCAAGTGTTGTCCCAGGAATAGGAGCTTCTGTCAGCTTTGTAACTTTAGCTCCTTGCTTCTTTGCAAACGCATTGATCAGTGTAGACTTCCCTGCATTTTGAGATCCAATCACCCACACATTCCCACGAGGACCAGCCAAATCCTTCACGAAGGACAACAAATTCCTTACTCCTAAATCCTTCCTTGAACTGACCAAATAAACCCCATTTAGCTTAGGTGCTCCTCCAGATCTTGCACGGTTTCGAACCCATCTATCTAACCTCGTAGGAGAAACCTGGGAGGGAAGGAGATCAACTTTTGTTGCTACAAGAACAAGCTTCGGCAGCTTCTTGGCTCGCTTTGAGTTATCTTGTATTCTTTCCAATGATTGAAACAAGGATTTCACAGCAATCCTAGGGAATGAACCATCAAAATCAACACAATCCACAACCATGACAACAACAGTAGCACTGCCACTTCCAGAAGGGTTCATCAGCCGCGTCGAAATCAACCTATTGAAATCAAAATCCGGTATCAAATTCTCCGCCGTCTGGTTCTTCACGTGGCCATAGTTTCTCAAGGAATGGCACCGAGCACACACAGTAACCTCTTCTCTCTCCCTGCCAGCCTCCTTAGCCATCCTCTTCTTCTCAGCTTTGGACACCTTCTTCTTCTTTACCTTCTCCAGGTACTCCTCAGTAATATTACCATACCCAACTCCTGCAGGTGCAAACCCATACAATTCCAAATTCTCATCATCTTCACCCAGTAACTTAGCTTCCCAGTCATCAGAATCCCAATCAAACTCATCTGTTAACCCCTCATTCTCTTCTCCAAGCTTAATTTCACTTTCATTCAAAAACCCCACTACGTCATCATCTTCTTCTTCTGCATAATCACCACCACCATCATCATCATCAATCACATCATCATTCTCCTCTGAAAACTCTTCCACTTTCACCTTCCTTGGTTGGTAATACCCTAGAAGGTCAGGGTCCTTATCTTGCATGAAGACTCCACAACCAGGGCATATGGGTCCTCTAGCTTCATCTTCATCCCTCCCCTCACTCAACAACGGCTTTCTACCGTCTCTTCTTATGGCTTTAGCGTGAGAGGAGGCACCCTTCACCGCAAAAGTAATCAAGGACGACACTTTCTCAAAGCGCTTGCTAGTATCTGAGAAATGACGAAAACTTGGAAGTGCACGCTCTTGTGTATTGTTGTTGAAGATGACAAGCTTGGTAGAAGGAAAAACAATTGTAGACAACAACATAGCCATGACTATGAGGGATGAGATAGGGACAATCTTGTTCAGGTTCCCAAGCACGAATGCTTAGAAGAGGGAAGAGGGTAAGGCAAAGCACGGAGTTTTTG

>C100693_65

GCAACTTATTAAATGCATCATATCCAAAATAGTAGTCACCGATACAGTTTGAAGGTTCAGTAGCTTCCCAACATAAAAAAAAAAAGAGAAAATTTAAAATAACCACATCCCTTATCTGAAAACACATGCATCCCAGTTTTTGTTTTTCACATTTAATATCTGCCACCGCCATTCAGATATGGATAGACTTGCAGTAGGAACAGTATACAGACAGACTTTTTGCTGTGGTTAGCTATTTTGCTCGTGCATAGAGAGAATGGGATGTGAAGGGGAGAGAATCTTAACATCAAAACCGTGTTACATTGCCATATCATTGATTGAACATTTCCAGGTCATGCTCGGCCAACATAGCCTTCGGCTGGCTTAGATAAGCATACACAATGATTTTATTGCAAGGGATTCTGAGAAACAAAAAGAAAATTGTTACTATCTTCTTTTTCTTTCATTTTTTTTTTCATCTTACATCACAAAAGAGTAGTAATTTGTGTGATCAAGGCCTGCCACTTGTGGACTTTGACCTGAAAACACATACACCATGTGGATCTCTTTAATCGACTATTTACACGCATGATGATGACAGCTTATATCCACTTAGATGGATGTGTAGAAGCTCGCCTTTTGCGCAGCTCACAAATGTAGTACTCTAGTTTGTCTGGCCGAGGGATACAATTCCAGTACTATGTTATACACGTAACACAAGTACTATAAGCAGTAAAAGTGACCATTTAACAGTTCGAAGACAGCCTGCAGAAGATGATGATAGCCCAGGTTCTGGGGCAAGTTCATTCTTAGGAGGGGGCTCAGCATGAGCAAAAGCTACATTCGGCAAAGGACTCAGGGAAGGTACCGAATGAGAGACATGTGTTGGGGGTTCAATATGCAATTTTGGTGATGCAGCAAGAACAGGGTGGTGTGGATTAATAATCGGGGCAACTGCAGGTGTTGGAGGATGTGCGTGCTTCCCAGAATTATAGGTGGACCTTTTTCTAAAACCAAATCGACAATTAGGAGACTGAGACTGAGAATTTTTTCCTGGTGCAGGAGCACTTTTTGCGGCAGTAGGAGAACCAACCTTGGGTCGAGTCACACCCTTGCTGGGTGTAGGTGCCGGTGTATGTGTATGTGTATGTGCATGTCTAGGTGTAGGTGCAGGTGTATGTGTATGTGCATGTCTAGGTGTAGGTGTTGATGAAGGTGTTAATGAAGATACTGGTGCAGGTACGGGTACAGGAGAAATTGCAGGAGATATATGGGCATCATGGTGATGATGATGATGCTGGTGTTGATGATGGTGGTGATGGTGTGGATGATGTGACAGAGGAGCAGGAGAAGGTGACCATGCAGAACCACTACCATCATTGCCATGAAGGGAGTGTTGCAAGATGGATGAAAGTCGGACCTGCTTAACCCTACCAAATTCAGTGTGATTCAAACCTAGATTATGTGGTCCCATGATGGTTTGTGCCAGTTGCTTTAGCCTCTCTTTCGATGGAGTAATCCCAACTGCAAGTAGAACTGTTGACTGGATAACAGTAGGAGCATCAACTGTTGAACCTTCAGAATTCGATAAGCTGATATACAAGTTCTCAAATGATGTCAGATATAATCCAGACTCTAGCTGACTAGTCAGCTCCTTAAAATTGGATTGTATTTGATAAATGGGGAAATTCAATTTAAAGCTGAATTTTGCTTGCCCTTTCTGTAGAGGAAATGCATTCTGTTGTGGAATTATAGTAATTCCTCCCTTAAATTTCAGCACTTCAAAGGAGTAGGGATCTCCAAACAAGGATGAAGATGTAAGCTGGAGAATTGATTGGTGTATAACCAAGGATGTAAATGATGCTCTTATCAAACTGATGACAGCTGAGGACATTTCTGTGTCTTTATCATCAGAATCAACTGCAAAAATCACTTTTGTCTTGTTTGGCCCAGGTAAGGGTTCTAGAAACAAGACAGCTACTTTGCTAGAGGGAGCATCTATCTCATCAAAAATTTCATCTGCGAGCTGAAATTTGCTCTCTTCTAGCAAAGAAACAGATTTATTGACGATAAAGCTTGCAACAATATCATGATCTTTGAATCTGGAACCCCCATGCAGGTTCCTCCGATCCGCGAGGCTCAGAAAAGGGGGCAACCAAAACGCCGCGGAGAGGAACAGAGCAACCGACACGACAAGTACGACGATGCATCTGGCGCCGACAAGCTTCCGAATCAGAGAACAGCGGCACCGACACTCCGCATTCCGCAGTGGATCCTCGGACGTAGGCAGAGGCAGCTGTTCTTCTTCGCCCTTTCCCATTGCAGAAGAAGAGGAAGAGGGTCACAACCCATTCGCATTGGGTACACCCATT

>C100703_65

TCCAAGTCTGATTGATAAACCTGTCAAACATCCTGACGGCATACCCTGAAGATTAATTCCTGATTCAATATTCTACACTCCGTTGTGTCGTGTTGAAATAAGCAGACATATCATTACTTCAGAAACTACCACAATAATATTTGAAAACAGTGTAACAAATTAACAATCCATCTGCTACTCTACTAATAAACAAAAAGGGTTTTCAAGGGTGGTGGAGTGGGGGAAAAGGTAACTTCCCGGTAGTATTTGCTTCGAGTACTCTCATTGTTAAAGCCTCTAAGGTTCCGGGTTCCCGTTGTAATCATCCACTATATGATGCCTGAAATTAGTCTATCTTCACCGAAGAGTAAATCTTGCGAACAAACCAGAAGCAAGCATAGAAGCCAATAGTTCCCGTCAACACAAAGAAGGCATATGAAACGATTATCATATATCCAAAGTAAAGGATGCCCGAAACCAGCTTTGAAATCTCCAACTTGGTAAAGAAGTAGAAAATTGAGTAGAGGAAAAGGTACAGGGCAGAGGACCCAGCAGTGAGGTAAGATCTCCACCACCAGTTGTAATCCTCACTGCACAATTGGAAGTAGCAAAGCACAATGGTAATCTCGGCACAGGTGATTAATAGGATCACAAACACTATGAAAAGGAAGCCAAAGATGTAATAGAACTGGTTCAGCCATATTGATGTCAGGATAAAGAAGAGCTCGATAAACACTGCACCAAATGGGAGAATGCCTCCAATAAGAATTGAGAAGACAGGTTTCATATACCATGCCTGCTCAGGTACCTGCCTAGGAATTTTATTAGTCTTCACTGGGTCTTCAATTGCTGGTTTTTTAAAACCCAAATAACTGCCCACAAAGACCAAGGGTACTGATATACCAAACCATAAGCAGACCAGAGCAAACATGGTTCCAAAAGGGACTGCTCCGGAGGACTGCTCCCCCCAGATTAAAGCATTAAGCACAAAGAAGACAGCAAAAAGAATGCCCGGAAACATAAAAGCAGTTTTCAAGGTATTCCTTTTCCATTCTGTGCCCTTGAACATCTTGTACAAACGTGCTGAGGAGTAGCCAGCAAATAAGCCCATAAAAACCCACAACAGAACCATCGCAGTCATGAGACCTCCCCGATTAGATGGAGACAAAAACCCTAGCAAAGCAAATATCATTGTCACAACTGTCATTGCAAAGATCTGAACACCAGTACCAACATAAACACAAAGCAAATTTGAATTGACTGGTGGTCTGAAAACATCTCCATGAACAAGTTTCCACCCTGTTTCTTCTTGGGCCTCATCTTGGGTTTCTAGCTGATTATAATTGGCAATATCTCTGTAAAGAGTTCTCATCATGATCATTGCTACCATTCCGGACAAGAAAAGAACAATCATCAGAGAGTTAATGATGGAGAACCAGTGGATCTGATCGTCATTCATTAAAAGATATGTGTCCCACCGTGATGCCCATTTGATATCACTTTCCATGAAGGAAACATCATATGTGAATACGACATCCTTGTCTGTATCAACTTCTTGCGGGACTGTACTACCTTGCATCAAATTCTTGGTATCTTTATTGCACGTTGTTACCTGTGGATTCTTGTCATTCCACTCCTTGTATTCATGATTTATACTGTTTGGGGTAACCTCAAATCCTACAATACGAGCAGAACCAGTTTCCGGATCCTTGTGAAACATGACTCGAAAACTCAAGTGGTTATTGATAAAATATTTCTCCTCCTTGCTCCCTTGATAGTTTCCTTTGAACCCAACACGAAAACCATGTTCATAAGTTGTTGATTGACTTCCATCCCTCCTTTGTCTACGAACAGCAACTGGAAGGTTATCCAAAATCATGTTCACTCGATATTCATCATCAATTTTTTCTTTAAAACTCTTTGCAGATTCAGCATCCAATGTTTTATGACAAACAACAGTGCAAGACTGTTCCTTCCTCATATGAAACGTATATACTGAATTCTCAATGCGATCACCTCTAAGAACCTCTCCCAAATTCTCTGCACTGTTCAATATCGGGGTGGGTTTGCAATAGTTCAAGAAGTAGTAGTCGTATGGAAGTTGAGTCTTTGTAGATGATAGTTTGTTCACTTTGACAGCGAGGGGGTCACCAATTTGGAAATCGCGAGGAGCGACGCCGGGAAGGTAGAAGGAGTGGACGGTGGAGAAGAGGAAGAGAGCGGCGAAGACGAGGGAGATGGCGGTGCTTGCCATCGTCTTCTTCTTCATTGCTTGCTTTGTTTTTCGATGTTGGAGAAAAGAAAAAGAAGGAGGAGATAGAAAGATGCAGTGAAAGTTGGATAAATAGATATTCGCAAAATAAAACCAATTCAG

>C100705_65

GATGGGACTAATCTTAGGCTTAGGCAAGGAAGAACTGCATGAGAGTTAAAAAGAAGATCAAAACGAAGGTGTACATAGTAAGTAGTACATTACACTAATATGGTCGAATGGCACACATGGTTTCTTTTTCTACCCTCAACAAATTGTTAAATTGTATTGAATTTCTCTTCCCGTCTTCTTTGGAATTATCAATCATGATTCATGACCCTGCGGGATCAAAGCAGGACAACGTGCTTCAACAATCAACCCATATACAAAGCAACTATGATGGCTTATATGAGGAGTTCAAAGAACTTCTATAGCAGGAACAGGAACCAATACCATCTTGGTTTGTAAGTCCCTCAAAATCTCAGCCGAAAAAATCAAAAGCTCAACCATTTCGAGACCTGCAGCGGAAACAGTATCCATCTTCTCATCGCCGCCTTCCTGATGCCCCATGTCACTGCCCACGCTGTATCCAATGTAACTAATATCTGCCTCAACCGACCAGCTGGTCGGCAAGGAGCTGCCAAGAGCAGTCCTTAAAAGTTCTGCTTCATGAATAACGTCATTAAGGATTTCTGAAACACCGGAAATTGTACCTTCCGAAGTCACACACTCCTTGGCCTTGTGAAGAGAGAGACGAAGCCTATGAATTGATGAAGTTAAATGTTCCATGTTTTCCATGCTTTCCTTAAGAGTTGCATTTTCAATCTTCATTCTTGCAAAATCCATACTGCTGGATTGATGATTGAATGTTTTCCCTTCATTATTTGAACTACTGTCCACTTCATTTCCACTAACATCCAGCGGGCCAATCCATCGTCTCAAATACGATTTCTTCAGTAAGTTCTTTAAAGTTCCATCTCTTTCATGACTAACAAGCTTTCTGTCTCCAGTGTTGACCACACCTTTCCCTTGTTCAGGTTCATTACCCAATTTTTCCAAAAGCAATTGATGCTCCTGCTTCAATTGAGCCAATTCCATGAGAGCTTTCACCTTTTCATTAGTAACCTGCAACAAGTTCCTCTGAAGTTCTTCAGTTTTTCCTTGGAGGGCCCAATTCACATTTCTCTCCAATAGATGTCTTTCTTCCTGCTGTGATAAAAGCAATAATGCAGAAACCTTCTCTTGCAATGCCATTGCTAAAACATCAGATGCATCACCTTCTAGACCAGCATTCACTGGCAGTGCGCTAGGACTGTTCCTCTTAGACTCATAGATAGGTTTAGCTTCAGGGCTTATATGTATATCCACACCTTCAGACTCTTCTAGTTCTGCATCTCTTTGAGGGGTCACATTTGGTACAGTACTCACATCAAAACTCCGGACTTTTTCATCAATCACATTGATTATGGATTTAATAATGGATTCTCCATCACCAAGTAAATTCATAATTTCATCCCTACACTTAGAATGATAATCTTTCAAGTCTGCTATGCCATTCTCAATCACTTTGTCCATAGAAATTTGTTTCTTTTCCAGTATTTTAACTCGCTTCTTCAAGTGATTTTCAATTTCCAAGCCCACCCTCAGTTTATTTTGAAGATAATCCATGGAATTCCTAAGTCTCTCCAGCTGTTCTTCCAATGCACTAATGTATTTAGAGGTGGAAGCATCATTGAAACTCCACACCTCCTCGGGGTCACCCAAAAGACATGCACATTTCTCATCCCAACTTGTATCTTCAAATTCCTTCACAGAATGTTGCCTGATCTGAAAGAACTTATTAATTACCTTCTTGAAGTCTTCATTTTCCTTCGTATGTACTGCTTGATCAATCTGCAGAGCATCATTAAATCCCTTTAGTTTAAGACAATCTGCAGTGAGCTCCTCTACCCTCTTGTGAACGCCATCTATTTGTTGCAACATCGTCTCCTCCTTTTCTTTGGCCTTTTCAGCCTCAATTATTGCTTGATCCCTTTCAGCGAAAGCAGCAGCCATACAACCTTGGAAAAACTTTACCTGCTTCTCAGCTTCCATATTCTTTGAAACCTCAGCGCTGTGCAGATCTGCCAGTTGACCTTTAATTCTGTATGCCTCAGAAAGCTCCTCTTGTAGATTTAGATTCTCTCTTGAGCAAGCAGCTAACTTCTTTTTCAAACTCTCAATCTCCTGCTCCAATCCAGCTGTCCTTTGAAAATGCATTCGGGTAGCCACAGCAAGATAGCCAGGCCCAGCTTGCTGCATGCATAACTGTTCGATATCTTTGTGTAATTCATCACGCTCCTGTTCCAGTTGTCGTATACGAGCCAACAATGACTCATCATCTGTACTCTCTGTCATTCTGGAGGAAAAGCGAGAAAAAAATTGAAATGAAGGTTCTTCAATTCGCCGATAAAGCTCACCGAATTGGAATGATTTCTGAAATTCT

>C100709_65

CAGGCCTAAGCTCTAAGAGAATTCACTCTCAAACAGACATTCAAAATATACATCCCTTGCATAACTGTCAGTCGTACATATACCACAAAAATACATAATCGTCTGAAAAAGTTAGAACTTACAATTGATGACAATTAACGAGACATATCAATTCCATGCCCATTGTTCACAATGTATCTATACGTAGAATAATCAATCCTTCCGACAAATCTGATGTTCCTATACTTCTTTAAAACACAACTTTGTTCTTGGAAGAACTAGGAATTAGGACGCGTAGAACAGATGGTGATATAAAGACAAGTGGGCAAAGAGAATAATACATTATAAAGACCATCTTTCTAATCACATGAACAAGAGCAGTCTGTCAGGAAAGCGTTAGGCAACCATAAATACCACCACTATTTTACATCAGTCAAGGTCCGTAGTTAAACAATCTTTACATGCAAGAAAAACGTGAACACATCTAGGGGACATTAGTAGTATATCTTTGGCCAGAATCACAAGGTTTTTAGCAGCCACTAGCCAGACATGAATGACATTGCTCGTCTGAAGTCCAAAGGTTTGAGAGCTTCCTGGGGAAAAGGTTTTCCATCTGGTAGCCACAGTCCACCCTCATGAGGTAGTAGCACCTGTGGTTGGGCTTGGCTACTTCTCGCACCTTCAACACCGTCCTTGGATGAAACCTCATTCTTTTTCTTCACAAATTCAAAAAACTCTGCAACTTGGCGTGCATATTGCCTCTTTGCCTCAATATCTTTGCTGAAATCAATATTGGGTTCACAGTCCAGAACCAATGCAGGAACCTTCTGAATGCTTGAATGCATGTGACCACCCTCCAGATAAAAAACACGGTCTCTTATATCAGGGTGTAAAGAGTTGTCAATATGATGGGGTAGTTTATTGACTGATAAAACCCCATGATTACCACTTTGAAAGGGAAATAACCAGCTTTCATGCTTTTCGTGCAGGTCACGGAGATAGTCCAGGGTAACTCCACCTTCTTCTTCTCTCTTCCTTAACATCATTCTCTTATGGCAAGTATCAGGACTTGCCCTAAGATAGATAAAACCATCAGGAATAAGTCCAGGCAAGGAAGACACCACAGGATCAAACCACGAGTCATAAATACTAATCTCCATCTCATTCATCCAATTGGCTTCATGAACAGCTCTGACAAAAACCATTCTGTCACTGAAAACACTCCTCTCCATCAAACGAAGAGGCTTGATTCCACCAGATGACTCTCTTTCCTGCATCACCCTCGTAACAAATACATAGTTCTGAAAGGTGTAGGCGTACCTTTGTGGCTCCGCATAAAAAGCATCCAAAATATTGAAGTGGTCAGGTCCAACATCCTGCCACTTGTTAATGGGTTCAGGAACCACCTCAACAAGGTCACGCAATTCAATAGTTTCATTCGCTATTCTCTGAAGGAAGGTAGTCTTGCCAACGCTGATATTTCCCTCAACACAGAATGTCAATCGCTTCTTCTGCTGAGGCTGCGCAGAGGAATTGTCTTCCAACTCCGCATCAACACTCTTTTTAATGAAAGTAGTTATACTTTCAGCATGGTTCTTGTGGATTATCCCAACAGAACTCTGTAGATACTCAACCATCTTGTCACTGGATTTGCCAAAGAACTTATCCTTGTAGAGTTGCTTGAGTTGAGCAACACCAGCTATACCCTTCTGAACGAGCTTTCTGAAATTTCTAGGACCCACACCGGGGATGGCCAACAAATCAGGATTAGACGGAGCGGGAGATGAAGAAGTAGTAGAATCCTTTTGCCTCCTATTTAGCCTGAAGCTAAGGGGTTTCTTCTCCTTTGCGTGGCTAATCTCCACAGAAGAATCATGAAGAGGGTGAGAGTGGGTAATAAAGGAGCCGTGAGAAGCCGAAAACAAGGAGCGGTTTCGGGTGTGGTTAAGGAGAAGAAGGGTGTTATTGCGGGTGGGGGTGGGGGTTAGACTTAGCCTACAGGCACAGCAAGTATTGGAAGAAAGAGCGAGATAGCGAGAAGAGAGATGGGGTCTGAGAGCTGCGAAGGGCTTCTTCCTGACGGAGAGGGAAGAAGCAGGAGCAGGAGGAGGAGGAGGAGGAGGCGGTGTTGCTGACATTGGTAGAAAGTTTGAAACTTTGGGAAATATGGAGAGGAAGGGGAGCGAAGATGAAGAAGAAGAAGAAGAAGAAGAAGGGATGAGCAGTTTCTGCATTTCATTCACTCACACACACTCACTCACTCTCACTCGGTGGCTGTTCCTGGCGTGTTCTTCATGGTGAGGGCGGGAAGGGTTTTAGGTTTAGCCTCTCTCTCTCTCTCTCTGTGTGTTCGTGTTTGTGTTGTGCTGTCCTCAG

>C100711_65

AAAACACAGTCACAGTCACTGCACTTATTTTGTTCATCTCAGAAGTTGCCTTACTGAATCGAAAGGAGGCATTTTGAGGTCAACCCATTCATTATTTGCGGATCCATGAATTAAATTGTGAATTCCCAATGACCGGCGATTTTCTCCAGACACCCCTCTCACTCAAGATCGCCGCTTTCTTCTTCATCGCCAGCACATTCTTCTACTTCGGCAAGCACTGGTCGGATGGCTACCAACAGCTCGTATTCTTCACCAACCAACGCGACGACCCGGATCCGAACCCGAGCCCGGTCGTTCTCACGTCGCCGAATTACGCAAAATCATTTAACCTCTCCGCCCTAATTGACCAGAACAGCACCCAACCGGCGCCGGAAAATTCCCCGCCGCCTCCGCCGCCGAAGGGGTCGATCGAGCAACTGGGGATCGTGAACGATAATGGCACGAAGTCCGATGAGTTCGAGGTGGGGCATTTCGAATCTGGTATGGTGGATCAGTGGGTGAACGAGACTCAGGTTGGGAAGGAAGGTTCTTCTTCTTCTTCTCATTTTGAATCGGGGATGAAGAAGTTTAGGTTGTGCCCGCCCGAGATGAGAGAGTATATACCTTGTTTGGATAACGAGAACGCGATTCGGAAGCTGCCTTCCACGGAGAAAGGGGAGAGGTTCGAGCGGCATTGCCCTGAGCAAGGTCAAGGGTTGAATTGCTTGGTTCCTGCACCCAATGGGTACCGTATCCCTATTCCATGGCCCAGAAGCCGGGATGAGGTATGGTTCAATAATGTTCCTCATACACGTCTTGTTGAAGATAAAGGAGGCCAAAACTGGATTTCCAGAGACAAGGATAAGTTTAAATTCCCGGGAGGTGGCACTCAGTTTATACATGGGGCAAATGAATACTTGGATCATATTTCAAAGATGATTCCTGATATTACATTTGGTCGGCATATACGTGTTGTTCTTGATGTTGGCTGCGGCGTTGCTAGTTTTGGTGCATACTTACTCTCACGGAATGTTGTCACCATGTCTGTTGCTCCCAAGGATGTTCATGAGAATCAGATTCAGTTTGCTCTTGAGCGTGGTGTACCAGCAATGGCGGCAGCATTTGCAACTAGACGACTGTTATATCCAAGTCAAGCTTTTGACTTGGTACATTGCTCACGCTGTAGAATTAATTGGACTAGGGATGATGGGATACTGCTGCTTGAAGTTAATAGGATGCTAAGAGCAGGAGGGTACTTTGTTTGGGCTGCCCAGCCAGTTTATAAGCATGAAGAAGTTTTAGAACAACAATGGGACGAGATGCTTAATCTTACAACTCGACTCTGTTGGAATTTTTTGAAAAAAGATGGGTACATTGCAGTATGGCAGAAACCGTCTGACAATAGTTGTTATATAAACCGTGAGGCAGGAACTAAACCTCCACTCTGTGATCCAAGTGATGACCCAGACAGTGTTTGGTATGTTGATCTAAAAGCATGCATCTCTGAATTGCCTAAAAACGGCTATGGAGCAAATGTCACTGAATGGCCTGCTCGTTTGCAAACCCCTCCTGATAGGCTTAAAAGCATAAACCTTGATACTTTCTCATCCAGATCAGAGCTATTTAAGGCAGAATCAAAATATTGGAAAGAAATAATAGCAAGCTATGTCCGTGCTTTACACTGGAAACAGATGAGACTAAGAAACATTATGGACATGCGAGCAGGTTTTGGAGGATTTGCAGCAGCACTGATTGAGCAGAACTTTGAAAGCTGGGTTATGAATGTTGTTCCTGTTAGTGGTGCAAACACTCTGCCTGTTATATATGACCGTGGATTGATTGGAGTTGTGCATGACTGGTGTGAGGCATTTGACACCTACCCAAGAACGTATGATTTACTGCATGCTGCCAACCTCCTTTCTGTTGAGAGGAAAAGATGCAATGTCTCATCCATTATGCTTGAGATGGATCGGATACTAAGACCCGGTGGACGTGTATACATCCGTGATACTCTTGAAATCATGGATGAACTTCAAGATATTGCCAAGGCAATAGGCTGGCACGTGATGTTGCGAGAAACGGAGGAGGGGCCAAATGCAAGTTCTAGGGTATTGGTTGGTGAGAAACACTTATTACGCACCTGAAGCCAAATGGGGTAAATTGCATTAATTTTTCTGTGACCTTGCAACTGGAAGTTATGAACAGAACATGGGAAAAACTGATATTTGAGAATTGCTGGAAGATGCAAGTGCAAGTACTACTTGGCATTTGAGAAGTGCAAGAAGATGCAAGTGCATGCATGCACTTCCTTATAAACCTCAGGAGGTGGTGAGCATTTATTTTTGAGAAGTTAGATCAGGTGCCTCATCTAACTTA

>C100713_65

CTAAAATAAGAAAAGGGAACATTGAATAGGAAAATAATGAATAGGAAAACAACGCATCGGGTGCAGACGAATCACCTCAACAAGACACGGAGAGGTGAGGAACTGCAGTGAAAAGGAGAGTTGCTTGAAGAGAGAGTTGCATTTCTGAAGAGGAGAAATGTATAATGAAACTGAAGCCGAATCCGAATCTGAATCTGAGCGCAACAGAATGAGTAGTGGCGGTGATGAAGAACATGGTTTCTCGAAGAGCTTGGCCAAGCAAACCGAGGAGAGTTATCAGATGCAGTTGGCACTCGCCCTTCGTCTTTCTTCTCATTCTTCTTCATCCTCGGATCATCCATCTTCTTCGGTCCAGACGCTCTCACATCATTTTTGGGTGAATGGTTGTTTGCAATACTCTGACAAAATTCTGGATGGCTTTTACCTAATCCATGGGATGGATGCATATACATGGGCTCTAAGTACTGATCTGCAGAATGTTGGTATTATTCCATCATTTGAATCACTGTTGTTAGTTGAACCTGATGATGACTCGTCAATGGTAGTGGTTGTAGTTGATAAATCTAGAGATACTGGTTTGAGAGAACTGCAAAATAGGGTAGCAAGTCTTTCAAATAACTGGATTACCACAAAAGATGCAACTGATCAGCTTGCAAATCTTGTTTGCAATAGGATGGGGGGAGGATCTTTGACTGAGGAAAATTTAGATATACGCTGGAAGGAATGCACCGAACTTCTTAAAAGCTGCATGCACTCTGTCATTCTTCCAATTGGAAGCTTACCCATTGGCCTTTGTGTCCATCGAGCGTTGCTGTTTAAAGTGTTGGCTGACTTAGTCAATCTACCATGCCGTATTGCAAAGGGCTGCAAGTATTGTAGGCAAGATGTGGGAGCTTCATGCATAGTGCAGTTTGGTTCTGACAGGGAGTATATGATTGATTTAATTGGAAGGCCTGGGGCAGCATGCCAACCTGATTCTTCTCTGAATTCTGCATCTTCAATGTTGGTTGCTTCACCATTATGTCATCCAAAGTTCAAGCCAGTTGAAACAGTTGAATCTACAAAGAAACTAGCCAAATTGTATTTCTTGGACAGCCAAGCACTGCATCTCGTATTTGATACTACATCAGAACCTGCTGTTAATCACAGTGCCAGAATGGATCTTCAGTCAACTGAAGCTTTGGGTGCAACTTATGTTGGTGCAAACAGCCATCTGATTTCTTTGAAGGAATATGAGTCTTTCAATGAAGAAGACCAGCCAGTTATGCATTATCCAAGTCATGAAGTTGATCTTGACGAGGAGGACCTGGATATTCCATGGAGTGAACTTATTTTGAAGGAGAACATTGGGACAGGGTCATTTGGAACTGTCCTTCGTGCAGACTGGCGTGGTTCCGATGTTGCTGTGAAGATTCTTAAAGTGCAAGGTTTTGATCCTGGACGATTTGAAGAATTTATAAAGGAGGTCGCACTCATGAAGCGCCTACGGCATCCAAATATTGTACTCTTGATGGGTGCAGTTGTTCAACCCCCAAAGTTATCTATAGTAACAGAATATTTATCTAGAGGCAGCTTGTATGAACTTCTGCACATGGCCAATGTTGGATCATCACTTAGCGAGATGCGTCGTTTAAGTATGGCTTATGATGTGGCAAGTGGAATGAATTATCTTCATCAAATGAAACCTCCCATTGTTCATAGAGATTTGAAGTCTCCAAATCTTTTGGTTGATAATTCGTACACTGTTAAGGTCTGTGATTTTGGTCTTTCACGTACAAAAGCAAACACATTTCTTTCGTCTAAAACCGCAGCCGGGACTCCTGAATGGATGGCACCCGAAGTCATGAGAGGTGAACTGTCAAACGAAAAATGTGATGTATTCAGCTTTGGTGTAATCTTGTGGGAACTAGTGACTCTGCAACAACCGTGGAGAAATTTAAATCCTTCACAGGTTGTTGCAGCTGTTGGTTTTATGGACAAAAGGCTAGATATTCCAATGCATGTGAATCGTGAAGTGGCTGCCTTGATTGAGCTCTCCTGGGCCACGGAACCCTGGAGACGGCCTTCTTTCTCCTATATAATGAAATGTTTGCAGCAAATAATTGCTGATGCTACAAGCGAACGAGTTCATAATTCATACTGATAGTCTTCAGCCTATATACAGACGAGAAACAGCAATATTGAAAATGGCATTGCAATTTATTCAGGACCTTGCTGAAGAACTGAATCACCATTTTATAAAGTAACTCTCCTTGTTTTTGTTGTATATGTGCTTATATGAGCATTATTCACATTGTCATCTATATTGCTATTTGCTTTGTGTACACCTATAATAATAATATAACACTGAGATTC

>C100717_65

GTTGACTATAACGCTGTCCTAAGAACTCTCTCTCGAATCACAGTCGTCGTTGCTGTGGTTTCTCATCTTCTTCCTTCCTTTTTGATCTCTTTTTGTCACTGCCTCAGCCGCCGAAAAACAATGGCCGGAAAAGGAGAGGGTCCTGCTATCGGAATCGATCTTGGAACTACGTACTCCTGTGTCGGGGTCTGGCAACATGACCGTGTTGAAATCATTGCCAACGACCAAGGCAACAGAACGACGCCGTCTTACGTCGGATTCACCGACACCGAGCGTCTCATTGGTGACGCCGCCAAGAACCAAGTCGCCATGAATCCCATCAACACCGTCTTCGATGCCAAGAGGTTGATTGGTCGTAGATTCACTGATGCCCCTGTTCAGAGTGATATCAAATTGTGGCCTTTTAAGGTCATTGCTGGTCCTGCTGATAAGCCAACTATTGTAGTTAACTACAAGGGTGAGGAGAAACAATTTGCTGCTGAAGAAATCTCATCTATGGTGCTCATGAAGATGCGTGAGATTGCTGAGGCTTACTTGGGCTCTACAGTGAAGAATGCTGTTGTTACTGTTCCTGCTTACTTCAATGATTCTCAGCGTCAAGCAACTAAGGATGCTGGTGTTATTGCTGGTCTAAATGTCATGCGTATTATCAATGAGCCTACTGCAGCTGCCATTGCGTATGGTCTTGACAAGAAGGCCACAAGTGTAGGAGAGAAGAATGTGTTGATCTTTGATCTTGGTGGTGGGACATTTGATGTTTCTTTACTTACTATTGAGGAGGGTATCTTTGAGGTGAAAGCCACAGCTGGGGACACCCATCTTGGAGGAGAGGATTTTGATAACAGAATGGTGAACCACTTTGTGCAAGAGTTTAAGAGAAAGAACAAGAAGGACATAAGTGGAAACCCCAGAGCACTTAGAAGGTTGAGGACTGCATGTGAGAGGGCAAAGAGAACCTTATCATCTACTGCCCAGACTACCATTGAAATTGATTCTCTCTACGAGGGAATAGATTTCTACTCTACCATTACTCGGGCCAGATTCGAGGAGCTCAATATGGATCTCTTTAGAAAATGCATGGAACCAGTGGAGAAATGTCTAAGGGATGCTAAGATGGACAAGAGAAGTGTTGATGATGTTGTCCTTGTTGGTGGTTCTACCAGAATTCCCAAGGTTCAACAACTACTCCAGGACTTCTTTAATGGGAAGGAGCTGTGCAAGAGCATTAACCCTGACGAGGCTGTTGCTTATGGTGCGGCTGTTCAGGCTGCAATCTTAAGTGGTGAGGGCAATGAGAAGGTTCAAGATCTTCTCCTTCTTGATGTCACTCCTCTATCTCTTGGGTTGGAGACTGCTGGTGGTGTGATGACTGTCCTGATTCCTAGGAACACTACAATTCCAACAAAGAAGGAACAGGTTTTCTCAACATATTCTGATAACCAGCCAGGTGTGTTGATACAGGTATTTGAGGGTGAGAGAGCAAGGACCAGAGATAACAATTTGTTGGGCAAATTTGAACTTTCTGGCATTCCTCCTGCTCCCAGGGGTGTTCCACAAATTACAGTCTGTTTTGACATTGATGCCAATGGTATCTTGAATGTCTCTGCTGAAGATAAAACCACTGGCCAGAAAAATAAGATTACCATCACCAATGACAAGGGTAGATTGTCAAAGGAAGATATTGAGAAGATGGTTCAGGAGGCTGAGAAGTACAAATCCGAGGATGAAGATCATAAAAAGAAGGTTGAGGCCAAGAACGCCTTGGAAAACTATGCATACAATATGAGGAACACTGTGAAGGATGACAAGATTGGTGGGAAACTGGATCCTGCTGACAAGAAGAAGATTGAGGACGCAATTGAGCAAGCAATCCAATGGCTAGACAGCAACCAGCTTGCAGAGGCAGATGAGTTTGAGGACAAAATGAAGGAATTGGAAAGCATCTGCAATCCTATCATTGCCAAAATGTACCAGGGTGGTGCTGGTCCAGACATGGGTGGTGCTGGTCCTGCAGAAGATGGTTATACTCCTTCTGGTGGAAGTGGTGCCGGCCCCAAGATCGAGGAAGTCGACTAAATAATAGTTGTTGCTTTCCTCCATATCGGTCATTTTTTTTATTGAAGAACTTGTTTGATGTTTTATTTTTGGATTTTAGCTGCATGATTTTTGTTGGATGGGATACGAAATATATTTCCCATCTAAATATGAACCTTGCTTGATTTGAAGCATTATGTTGTTGTCTGCTACTCGATTTATTTAGATGGAGGATACTCAATTTAGATACACTTTGCTTGATTTTTCATCAGATCTTACTTGATATGATATCATTTACGCGAATATGAATACTCTTAATATCA

>C100719_65

TTTTTGCATGAATAATTTTCTGTATTTGTATTTGATATCAATATCATTATTAACAAATGAGACAACATCATCTATTGAGTCACCAAATATGTTAAAGAATGATATCTTGAATTGTGTAGGTGTGGACTTTAATTTTAGTAGCAGCCATCAACCCATCTGATACAAAAGAGGCCAAATAATACAAAATTGAGAAAGCTCTTATTCCATTGAAGCAATTGGGCAATACAATAACGCCAAGTTCCTCATCTATCTGCTATCAGAGATTTGACTAACAGATCTAGACCGTTCAAGTTCATCAGACGGATCCCAATCCAACGTAGAGGATTGAATCTTAGACAAAGTCATGAAAACCTCAGAAATCTGTGGTCTTGAATTGAGGTCACGAGCAACACAGCGTTTGGCAAGTTCAGCCATGGAATAAGCAAGCTCCAAAGGATATTCATTTCTCAAATTTGGATCCATGAAACCTCGAAGTTTTTCTCTTACATTCTCTCCTTCAAGCACCTGATTCACAGTCACCGACAGCATCTGCTCCCCAGATCCATTCTTGTCCCCACCAACAGCTTCTCTCCCCGAAAGAAGCTCCAAAAGCACCACCCCAAACGCAAAAACATCAATCTTTGGAGTAATCAAACCATTCTCAATATACTCAGGTGGCATGTACCCTTGAGTCCCCACCACATGCCTTGTCAGTTGAAACCCCCCATCCTCCCCTTCACCCTCCACCGTTCTCGCCAACCCAAAATTCGAAACCTTTGCCCTGAAATTCCCATCCAAAAGCACGTTCCCACTCTTCAAATTCTTATGCACATGAGGAGGACTAGTATAGTTATGAAGGTAATTAAGCGCGTCAGCAACATCATGAGCAATATGAACCCTCTGCACCCAACTTAGAGACACCGAATTCTCGTACTTCTTATTACCACTATGAAGCCAATCCTCGAGAGAATCGTTCTCAGCAAACTCGTACACAAGATAAGTGTCACCTTTGTGAACACAGAAACCCGACAACCTTATAACATTGAAGTGGTTAATCTTCTTGAGAATGTTGATCTCACCCGACACATCCCCTTTGAGAATCTTAACCGCAGCATAATCACCCTTGAAAGAAGCCCTATAAACAGAACCCTTGATTTTGTTCTCTTCACTGAAGAACCCCGTGGCTTTCTGCAACTCCTCGAACTTGTAAAAACTCAACGACTCAATCGCGTAACGAACCCCTTCCGAAGAAAGAGACCACGACTGCGTCGTCGGAATAGTCACCTCCTTCGTGGTGGAGCCCGAGAAAGCCTTCGCAGCCGGAGTCGGCGACGGCGGTTGCACCCGTCGGCGCCGATAGAAACATAGGAAAAACAGAGCAGCGCAGACAAGAAGCAGCACAACAACCCCAACCACGACTCCGACTATGATCCATTTCTTGGAAGAGTCGGAATCTCCGTCTGCCACCGGAGTCGGAGGCGGCGGGGAGGCCTCTGGCGGAGCTGTTGCTCTCTGTATAGTCGTCGGTGGCTCTGTTTTTAAAGGAACTGAAATCGGCGTGAAGTAGAAAATAAAGGCGGTACTAGAAAGCTCGTTTGCGTCGAGGATGCTCTGTTGGTCAACACCGAAGATTTCAGCAATGGAAGAAACCGTTTCTCCTTGAGAGACCAAGTAAGTTAGCAAGTACTTGAACCCTGCTTCGCTCTGTTTCTGGGTGGGGCAGGCGCACCTGAGAGGCACGTGGATGTTGAGACCTTTAACGATGTCATGTTCGCCGTAGGTGTTTTGGGCCATGAGAGCTTGACACGTGGTGAGGGCCTGGTAGGTGTTGTTGGCGATGGAGAAGTAGGTTTCGCCCTGTATTTTGGTTGTGTAAGAGGCGTTGTGCTGGTAGTAGGGGCCGGAGCAGGAGCAGTTGACGGGGACGGTGATGAGGGTGTCAGCGGGGATGGATTGGACGTCAGTGATGTTGTTGGCGGCGGCGATGTCGGCAGCGGTGGAGTTGAGGAGGTAAGAGATGGTGACAGGGGAGGTGTAATCTGGGACGGAAGATTTGAAGGTGAGGTAGGATTGGCATGATGAAACACTGTTGCATAGGTTGCCCTTTGCGGAAGTGCTGTTGTTCTCGCACCGTAGTTGTGTGTTGTCGACGTACTCTTGTTGGGATTCAGATTTTGGGATGGAGGAACAGAGGAGGAGGAGGAGTAACACTGTTATGGTGGCTGCGGGCCACACCATTTCTGCCATTTTTGTGTGATGAAGAAAAAAATTAGTGCTTTCTTGGTTTGGAGGGTGTGTTGAACTTAAATTCAAAGAACGCGTGAGTCTGTTTTCAAGTTTTATTATTGTTGTGGTGGGAACCAACGACCCCGTCAAACT

>C100721_65

GTTGATATCATGTTAAAATTCCTGTGATAATACATTATCAAACAACAGTAACAAATGTTTGAAGTATATCAAATGAAAAATTCATAACGGAAATGGTATAACATTTGACATAAATATCCCTAAAATTAGACACCACAAAATCATATGGCATTTTTATGCATCTGGAGTTCCCACATGGTTGACAATGATCAACATAATAGCAGAAGACATCAAGCTCCCTAAAAGTCGCTCTGTACAAGAAAGACATCTCATCATTGAGTTCAACACTCCATCCCAAAAAGGCCCGTTGCGTCTAGAATCATTGATTGTCTTGCCTGTGGTCATCATGTTGGTTATCATCAGGTTCAACATCCTCCCTTTCCTCGTAAGTAACCCACGGTAGCATTGACTCAAACAAGACAGCTAATGCATTTCTATTTGCAACACCACGAGGAGCATGGCCATTGCCAGGCAGATTAGCAAATTGGTTTTCATCCTGCATACCCTCCTCCATCATCCTTGGAACGCCCATAAATTGTTGTAGATTTTCTCGCGGAATTGATGAAAGTGAATCCGAGAAATCAGATACCAACAAATGTCCATACTCATTTTTCTCGGACGAAAATGCTTCTTTTCTGACACAAGCCCAATCCTTAACTTCACTTTTATTACTTTCTAGTGTTTCAATCACTAGTTTTGCAGCACCACTAAGAAGTTTTTGCAGATCAGGAAGCCTCCACATAAGATAATTTCTCTCAACATATATATTAATCAAGTGATCTTGAGATGGAATTCCTGTTTGATCTGACCGGAAAAAGGCATGATTGAGAATATCTGTCCATGTGCGATCTTTCAATGGTACTTTTGCAACTAGCTTCTTTATGACTGAAGGATGAAGCATCAATGCTTGTTTCATAAGATCAGACGAAGAAGACTTTTCAGCATTCATACAATCATCTTTGCCGGCCTTGCCCTCAAGGTAAAACCGACAAATGGCAAGGGAATAAGAGAAGTTTGGAAACAACCAAATGGAGTAATCACTTTTATATTCTTCAGAAAACATCTCCAGCCATGCATACTCCTCAGCCCTTAAAGCAAAGTAGTCAATGCAGAAAATAGCCCCCATGGGATCATCAGAATCTAGTGAAAGCAACAATTTGCAGACCTCTAAGGCTGATCTATGACAGCCACGCCTGTTCAAATTTTTCATGTGAGTGAAAAGAGCTGTGAATATAGGTTTGTTTGTATCATGTTTGAACTTCAACTGGCAATTTCCCTGCAGTGGATTGAACATGGGATGCCATGCACATTCAAGGGCATATAGACACCTGGCAATAGTATCTGCAGACATTTGTTGTTCACCCAATACTGTAAAATATTCTGCCATTGTTAGAAGGGAATCTATATGATAAGGACGGTGCTGTAATATGCTCGCTATGGCATTTATGTCATTAATTGCTTTGGCAGCTTCAAACGCTCTCTGAGATTGGGAGTAAGATGGCGAGTGTACATATCTGAAGTAGTTATATCCATTTTTTGTCTCCAGAAACTGCATGGACAAAGAGTCATCACAGCGGAGCCAATTGTCTAATGGAGTGACAAGAACACTCTTTCTAAGATTATAATGTACACGCCCCCTCACCCCTCGCATCTGCCTAGAACTGCTCGCTTGGTTGCTACTCTCAAATGATTTCACAACCTTAGATCCAAATATTCTTTTCAGCTCATTTTCAGCATTCAAATATTTAGGATCCACTTGCAATATGGAGGTTGCCTGCTGTTTAACAGATTTATTTCTGTCTTTGGTCGAAACAGGTTCCTCAGCTGATGAGTTGACATTCAGAGATAAATCTTCTAGAATCGAGTCCAATTCTTTATCATCTCTCATTTTATTTGCAACAGCACTATCCTTGTTTTTCTTCTTTTTCTTTTTCTTCGATTTTGGATTTGATGTGGAAACTTCAGCAGTAGGTTTCAATGAAGATGACTCCTCTTTATCATCATTACTCGCCCATGGTTCATTGGCTGACGCCAACTCATCTCCCTGGTTTTCGGGTTCAGAATCCTGGTCGTTGAGGAGGTCGAAGGGGTTGATGGAGGAACCAGTGATGGGCTCAGAATCGTCCTCTTCTTCTTCTTCTTCGCTAACGTGTTGGGGAAGATGATGTTGCTCATTCAGAACCTTCTTCATCAATCGAGCCGACATCGTTTTTGTTGTTCCAACTGAACCACTTTCACGGCGATGCAGGGGAATTCAGGGGTGCCCAGAAAGACAAAATATCGATTATGGTTCTCAAATTCAATCGCAAGAAACAAAAGAAACGAATTAGAAATGAATGAAAGAAAGAAGAAAACAGCGTTGGTGTTACTCAGTTCATT

>C100723_65

ATAAGTATGAATTTTCAGAATCATATTAGACAACATGCCTTTACCTGCTCACGCTGATATATTATAGGCAAATAGTGGCACCTGCAATACACGAGACCAAAACTCCACCAAATTTTGATGTATAGTCAACTATGGAGGAATAAACATGAAATTCAAATAACCACAAAGCTCATACTGCATGTAACAGTGAACTACGAGAAAATAAAACCCAAAAACAATAAATAGTATACAACTGTTGGATGATGAAAGCTACGTGTGTCTCAGTTTTAAAGATAAATGAACCACAATAATAAGAAACAGAAAGGCAAGCCAGCTTTATAGGTTCTTGGGAGAACAAGTTATCAAGCTTGGGAATTAAGGTAAAATGGACATGGCCAATTTCAATAGTCTCCACATAAGTTTGGTCCTCTAAGTAGTAGCGTCAGGGTGGGCCATAAGTTTGTCAAACCCTGGTTCATCAGGTCTTCTGGTTTAGGCAGTAGGCAGGCTGCGGGCTTCATAGAACTCAGCTGCAGAGACTCATTAAGTTTAGTAAGCATGGTTTCTCCTCCTAGAAACACCACTCTGACTTGGCAACCCAGCAGTACTAGACCGTCCCAAAGAATAACTTCCATAAGCCCCATAAGCACTGCTACTTCCAAGCTGAGAACCACCATAGCTTCCATACATGTCACCACCTGTTAAATCACTCGAGCCACCATACCCACTTGAATACGAATGTCCACGTCCATGTCTTTTACCATCTCTACCATCATAGTTTAGACCATCTGATCCATCTGCTGAAACAGGCAGATATGGCATAACTGGAAGGAATGCAGAGAGAGCACCCTCCCTATCAAAAATATTTGCTCTCAACCGTGTAAGTACATGCACTAGAGCCTCCTTGGCAACATCAAGGTCTCCTGAAATCTGCACCATTTCATCATCTTCAGATGCAATCTTAGGAAGATTTTCCTTTGAGATAATGCGAATGTTAGCTTTTGTAAGCCTCCGCATCTCAGTTATGATAGATCCCCCTTTACCAATAAGGCAACCAATTCGTGAGGTTGATACCAGAAGACGAGTTGTGAAAGAGATGATCCCTGAATCTCTTTCAACCTTCTCACTGCATCGAGGTTGCAATCGCACAGCTGCTTCTATTGTTGGAGAGAAAGTCTCTTCAAAGAACTCTTTAGTAGAAATGGTGATTAAGCATTCCTCTGCTTCTGTTGTTGAACTATCAACTTTGATTGTTGCCCCAGATTCCTGCCTAATTTGATTTATTATCATACCACCTTTTCCTATTACACCTCCAATGTTTCCTGTTGGGCAAACCAATCGAACTGAAAATTCCTTTGAAGATGCCTCATCCCTTGGAGCTGAGTACATTGACCGCGGTGGCCAGTCACCAGTGTCACCTTTATATCCTCCATAAGCACCAACAAGAGGAGCTATCCCCACAATTGGAGCTCCTGGAGTTGGACCCATGAGTGAACCACCAGCAGCATATACGCCAGGAACAGCAGAAGTAAGAAGATGCTGAGTTCGTGAAGGGTTATCATGAAGTCGAGATGCTATTTGATGCAGAGCCTTTTTCACAACTGCAGCATCCCCTGTAATCTGCACAAGCTCATCAGAGCTCAAGGCGCAAAGAGGTAAATGATCATCCTTGAGTATCCGAATCTGGGCCCCAGTCTCACTACGAATGTTCTGAACTATCTGCCCACCTTTTCCAATAACACAACCTATCTGATCAGATGGTACCAGCAGCTTAGCAGTCACTTGGTGACCTCCATCATCATCCTGATCACCATACAAGTCCTCAGCAACCACTCTGTCATGCACCTTAAACAGAGCATCCTGAGCCGGTGACATGTAATTCCCACCACCCTCAACGGAATTGGTCTCATCACTGGGACTATAAATAGTAACAACACGCTCTTCGCAACCCGGCACAGTCTCACCAATCCTAATCTTGGCCTTAGTCTCCATCCTCAGTTGCTTAACAATCTCACCTCCCCTACCAATAACACTGCCAATCTTTCGACCCGGGCACACATATCGAAAAACTGTATCAACCGGATCAATCACAAACTGCTCTCTGTCCTCACCATGATTCCTCCTTTTATTTGGCCCATTATCATAGTCAGATTGAGAGTGAGGGCGCTTACCGTAACCACTCCTTTGGCCACCCATCACCGCAAATTATCGATACCAAACTTCCAAATCAAACTAACAAACACCGTTCACAAGGAGCTAGATATTATGGTGGGATTGGAATTGGGAGGAACGGAGCAGAAGAGTTGTGATTGAGGAGACAAAGGAATCTGTCCTGAATTGCAGTTACGTTAGGGTTGGGTTAATGCTCATTCAATGATGG

>C100725_65

GAACTTGTCCCTCTCAGCATCATCATTATCATCGATTTCTTTTTCTTCTTCTTCTTCTTCTCCCAAATTTCCAATTCGAACCTCATCTCATCAATCAACTCAATGGGAGCCTCTCACAGCCGTGAAGATCCGTTAGAATCATCCGATTCAGAAGAGGAGCAGGAGGAAACCTACCAAGACGCCGTGGAAGGAAGCTCCGAACGGCCACCGAAAACGCTGTCAACTCCGTCATCCCTCGATGACGTAGAGGCCAAGCTAAAAGCCCTCAAACTCAAATATTCCTCCTCCACAACCCCCTCCGTAAAAAACGCCGTCAAACTCTACCTTCACGTCGGCGGCAACACCCCCAAAGCCAAGTGGTTAATCTCCGACAAATTCACCACGTATTCTTTCGTCAAAGCCCACTCTGACGCCGACACAGACGACGAAAACAACGACACTGACGACGACGACGACAGCAACAACGAACAACCATTCTGGGTTATGAAAGTCGGTTCCAAAATCCGGGTCAAGGTGAGCCCTGAGATGGGCCTGAAGAGCTTCCCCGACCAGAAGCGCGTGGACTTCGTGGCGCGTGGCGTCTGGGCGATGAAGTTCTTCAGCGTGCAAGACCAATTCGATTTCATTGATTCCTTCCAGAAGTGTCTCTTCGAGAACACGCACGGCGTGGAGGCGACGGAAGCGAACAAGCTCAAGATCTACGGCAAGGATTTCATGGTGTGGGCGAAACCCGAATCAGCGGACGATTCCATGTGGGAAGACGCAGAGGAAACTTTCTCCAAAAGCCACACTCCGGTTAGGGCAACTCAGGATTTGAAAGAAGAGTTCGAAGAAGCTGCAAACGGTGGCATTCAGAGCATTGCATTGGGTGCTTTAGATAACAGTTTTTTGGTTAGTGATGACGGGATTCAGGTTGTGAAGAACTTCGCACATGGGATTCACGGGAAGGGTGCTTTCGTTAATTTCAGTGATGGGTATCAAAATGGGAGTGGGGTTTCTTCTTCCTATTGTACCCCTAAGAAGACGCTTCTTATGAAGGCTGAGACGAACATGCTTCTGATGAGTCCAATGGGTGGAGGAAAGCTTCACTCGACGGGGCTTCATCAGCTTGATATTGAGACTGGGAAGGTAGTTAGTGAATGGATGTTTGGGAAAGACGGGACAGAGATTACAATGAGGGATATTACCAATGATTGTAAAGGGGCACAGTTGGATCCTTCTGGGTCAACTTTTCTAGGGTTGGATGATAACAGGCTTTGTAGGTGGGATATGAGGGATCGCAATGGGATGGTTCAGAATTTGGCTGATTCGAATGCCCCTGTGTTGAATTGGGCGCAGGGACATCAGTTTTCAAGGGGAACTAATTTTCAGTGCTTTGCTACTACTGGTGATGGGTCCATTGTTGTAGGGTCTTTGGATGGGAAGATAAGACTGTATTCCGTGAACACGATGAGGCAGGCGAAGACTGCGTTTCCTGGCCTTGGATCTCCTATTACTCATGTGGATGTTACCTTTGATGGCAAGTGGATTGTGGGTACTACTGATACCTACTTGATTCTTATCTGTACCCTCTTTACTGACAAAGATGGAAGGACGAAGACCGGTTTTGCCGGACGGATGGGTAACAAGATTGCTGCTCCCAGGTTGTTGAAGCTCACCCCTCTTGATTCCCATTTGGCTGGAGGCAATAACAAGTTCCGGAATGCACAGTTTTCGTGGGTGACAGAGAATGGGAAACAGGAGCGGCATATAGTAGCCACTGTGGGGAAGTTCAGTGTGATATGGAACTTCCAGCAAGTCAAGGATGGTTCTCATGAGTGCTACCGGAACCAGCAGGGTCTTAAGAGCTGCTACTGCTACAAGATAGTCCTTAGAGATGATTCCATTGTTGAGAGCCGTTTTATGCATGACAGGTTTGCAGTCAGTGATTCTCCTGAGGCTCCACTGGTTATAGCTACCCCAATGAAAGTTAGCTCGTTCAGCATATCCGGCAAGCAATGACGCAGCCAGTGAACAATTGTTGCTCTTTTAGCTTAATTAATATCGTTTCATATATTTCATTTAAAGACTTTTCAGTTGTATGTATGATGTTTTCAGGTTTAGCTGATATTAATATCATTACGGATATTAATACAGGAAAATTACTGATGGTTGTCCAATTGTACCAGGATTTTCAGTTAATTCAAGATAATATTAGTTGGTCCCAGAATTGTATCATTTTTATCTGAATTGTTTTTCCTTTTTCTCGTTTACAAGTTCCTTTTGAGTTGTATGTATGATACTTTCAGGTCTTAGCTGATATTATCATCGGATATTCATGCAGGAAAATCAGTAATGGTTGACCCGATTGTACCAGCAA

>C100733_65

TATACAGTACTTGTGGCTGCAGAATAACTCTAAAAATATCCGGGTTTGTTCATCTGATTAGGACGGTGAAAAGTTTAGGGTACGGAAGTGAAATGTGCATGTGACGGGGAATGTACTCTGCATGTGAAATTAGACGCCTGTTTTCCACAGAAAGCTTACCACCAACATCATATCAACTATAGCATCATAAGAACAAACATCAAATCCACACATTTTTTGGTCTTTTTTTTATTCATTTGATTTCTGCCCTCAACTCTACAGAGAGGGAGAAGAAACAAACTGTCTCGGTAAACTAAAAAGGTCTTTCCCTTCAAATGGAGAACTCCATTCCCGAGAACCTGATGTTTCAGCAGAAGATACTCCACCATCTTGCAATCCAGGCAAGGGAACAATGTTACCATTAGAGGGTAATGATCTGATTGCTGGCTGAGCAACCACACCAGAAGCATTTGAACCATATAGAGCACTACTCTTTGTAAAGGGTGAAAGCCCTAGCAATAATGCATTAGACCTTGGTGGAGATAAGCCTCTATCCAAGGACCAGTCTATGTTGGTATAATCAAATTGCATTGACCCACCAGTGCTCCAGTCTACTGGAGAAGTTGACCCTGATGTTGCTGCAGATCCTAGCCCAGAAAAGAGTCCAAGAGAAGAAGCAGCAGCAAGCATTGGAGCCGCTGTGTTTGTTCTATTCCATAGGCTATTCCCTCGGTTGGTTGCTTGCAGATCAACTGAACTGCTGTTACCAGCCACAAACTGTTGAGGAGGCTGACACTGAAGTTGGTGGTACATTGGATTAGAACTGAAATAGTTTGAACTGAGGCTTCTAGAGCTAGAAGGGTGAGACATAAAGCCATTGGACCTCATGACTTCTACACTGTTAGTTGGATACCAACTGGAAGCTATTCCAGGAGGAGAAGAACTCATGCTGGCAGCTGGAATCTGCTTTGCATTTACAGTTTGAGGCCGCTGCATCACAGTCACTGGCTCCCTAGCTGAACCAGGTTGAAGGTTCTTATAATCACCTCCAACAGCCATATAAGCTTCTGTCTTAGCAGATGTTGGTGACACTTGTAATGGTGATGCCAATGAGTAAGAAACTGAAGGATTAGATCCTGCAGTTGGCCACCTCTTGTCAGCTGGTATAGAAGCTTGTTGAGGCTTTGCCCAATCAGACTTGGGTTGGATTCTCTTAAGAGGCTGCACAAGTCTGTTGCTGGATTCCACAGATCCTCCTGGTGTAACTGGTTGCTTGGTATAGTTAAAATCCTTTTCATCCTTTTTTAGTTGATTTGGAGAAAGAATTCGTTTAGTCTGTGGCCTAGCATTCTGACTCACATCTCCTGACTGCTTATAGCCAAAGGAAGGAGGATCACCAATTTCCTCTGCCTTTGGTGGACCAGATGGTCGATCCAGCTTCAATTCTCTCAAAGACTCTGCAGCCTTATCAAGATCACCCTCACAAGCAACAACTGCTCTTTCAACCTCCTGTTTTGAGCAACTGTACTTGATTTCTAGGTCTGCAATTCGAGCAAGTTCTTCTGATATATCAATTTTCAAATTACTCCCACCTATATTTGTATCTTTATGATCATCTGCTTCCTCACCACCTTCAAACAGCCATGCTACTGATTCCTCCACCCTTCCTTCATTCAGCATCAATGCAACTGTTGCGCGCTCGTGAGAAAACCCCATTGCCACAAGCTGTTGAGCAAGTCCCTCTAGTTTCCTTGACATAATATATCCACTGCAACGATCATGCAACTCTTGTGCCCGCCTTTCCTTCTGTCGTTGGTGTTTCCTTTCATTCTTCTGGCGGATTTTCTCTCGTTTGTCATTATCAGCTCCTGGTACTGCCTCCAGTCGAGTAGGATGATTAGAAGATTTCTCTTTATGGTCCTCTGACTCACCAGACCAACTACCATTGTTAGAAACAGAGTCATACTCCACACCAGCTACCACTGAGCCCGCAGGATGTTCATCTGTCTCATCTATATTTCGAAAACGGCCATTCATTTGTACTTGGGAAGTAGATGCCGGTTGCATCTCCAAAGTATGGAATGTTCCTAATAAAGGATTGTATGCACTAGCTGGTACACCAGCTACTGCATTACCAGAGCCTGAAGACTTGGAAACAGACTTTTGAGCTTCCTTGCCTGCCTTTTTGTCTTTAGACTTAGATTTGGAAGGAGACATCTCTTTAGCAGCAAATATGTCCCTGTGGATCGAGCTGTAGAACGGAAAACCTTGGAACTTAGAGCAAGCGAGCGAGAGAGATAGGTGTTAAAATGTTGAAAAGTGAAATTGGGGGAATTAGGGTTTTGATTCAAAGAAACAGCAGAGATCCGAAATTTTGCGAGA

>C100735_65

TTACGTTACTCCATTAAATTTTCACTGCTTTTGGAACACATTGGAACAGATAGGATTTTAATTAAATTCAACGTCATTCTAAATTCCTCTAAATTTTAATATCCCTCTGCATTCTGCATACAACTACTACTTCGTGTCGAGAAAATAGATGTATCATGCTTTCCTAAATACAACACGTCTTTCTCTCTTGTACTCTATTTCATCTCTCTGGTGCATGATTATAAGAGCTCTTCTCACAATAGATTCATTCATCCCCATTCTGCTGAGGTCATCAATCAGCCTCCTTTCTGATATGTGGTTTCCGATCCCAATTCTTCTCTTTATCTGAGTTTCAGCTTGCTTTATCTCATTTGCCATATCAGGTGTAAGATTTATTTGTTGGTTTATCCCGGACTTAGCCGCATCCATTGTGGACACGGTGAAAAGCCTTACTGCTTCTTGCACATTCTCCTCAGTGGCAAGATGAGATAGTTTCATCTTTGCAAGTGCTTCGCTCAACCTGACTATAGCTTCCAGCTGCCTTACAGTAATTGGTATTGCTGCAGCTTCTCCAGTTTCATTCGATTGTTGCCTCATATCCTGTCTGATTTTTACATAATGATTCTGTAATAATGCGGTTGCAGATTCTGATAGACGAGGGTGGCATTCATTTCGGCAATATTGTAAATACCTCTTCAGCCAATTCTCTTCTTTAGAAGTTCTGCTTTCACCCATCGAAGCACCAGGCCGTCCAGCCGATGCATGGACCTTTATGATATGATTAGCAATTATCTTGTCTTGACTGTACATTCTGACATCTTTCACAATAAAAATTAAGTCAAATCGAGAAAGAATGGTTGTCTGCAAGTCAATGTTATCCTGTGCAGTCTTGAGATCATCATAACGTCCAGATGGAGGGTTGGCAGCAGCAAGAACTGAAGTCCGTGAGTTCAGAACAGTTGTTATTCCTGCTTTGGCTATAGATATAGTTTGTTGTTCCATAGCTTCATGAATAGCAACCCTATCTTCTGGTCTCATTTTATCAAACTCATCAATACAGACAACACCTCCATCAGCCAAGACCATGGCTCCTCCTTCTAGATAAAACTCACGCGTACTGCTATCACGTATTACAGAAGCTGTAAGGCCAGCAGCTGATGATCCTTTCCCAGAAGTATAAACAGCAACAGGGGCAGTCTTTTCAACAAACTTCAGAAACTGTGACTTTGCTGTTGATGGATCCCCCAGAAGCAATACATTGATGTCACCTCTTAACCTGACTCCATCAGGCAAATGCTTTCTTGATCCTCCAAACAGAAGACAGGCTACAGCCTTTTTGACATCATCGTGCCCAAATATTGATGGAGCAATCTTAGAGCATATATTTTTATATGCATCAGGTTCAGCAGCAAATTTTTTAAATTCTTCTATCTCATCTTGTGTAAATGCTGCTGGACCCCGAGACTTGGTCTCATTTGTTTCTTCTATGCCTACAACCCTAATATAAGGCTGTCTAACTGCAACAGCTCCTTTGTGGGATGTAGAAGAGTTAGAAGCCTGGTAGACACTATAAATTCCCATGATTGTTAATCTTGAGCCAGGCACTACTGTCTGAACCAGATGACGATCAACAGACAGAAGCAGGTTCCTAGGAAGCTCTCCAGTTGGAACGTCCTCTGGATTTTCCTGCAGCTTCAAAGTTTGTTGGTCAACATATTTGCTCTTGTCGGGGACCACAAGCCAGGGATCAATCGGGCATGGCTCTTCTCCAGGCTGAAGAACATGATCACAAGATCGAGGAACAATGGCTCCCCCAAGTCCTGGCCGACATGGAACCTGCTTCCCCTTCTTACAGTTTTTACATATCAGGGTAACGTAAGTGGCCTTTGCCTTAGTCCTGGAGGCAGCAATAGTAATACCGGCGATTTTAACAAGTTTCGATATGTACTGAGCCCCAAGCGATCTCATGGACACCGGATCCTCCTTGGAGGTGAGCAAAATCTGAACATCACCGGGAGTAGGATCCTCCATAGCTCCAGTATCTCCAGCAACCTTGGTCTTCAAACTCACGAGAACCTGTGCCGCTGCCGTTTCAAACAGCGGCAAAACATCGGCGGGGGAAGAGCGAAGTTTAGCGGGAAGGTCGGAGTCAAAGGTGTCGAGATCTCCCATATCGACGAGAAGGAACTTCCGGTTGTGGAGGAGGCTCTCTCTGTAAGGGAAGACGTTGTTGGAAGTCTCGAAGTTTCGAATAAACTCCTTGAATTTCTGGAGGAGGGAGTGGTTTGAATCGGTGCCGGCCTCGCCGCGGGCGCCGGCTCCATCGTCTCCGGCGAGTGCTTGGTCGCTGTAGTAGACGGCTCCTTCGTCCCACCCTGACAT

>C100737_65

AATCAAACTTCATGTCATACACTTATATATATTCAGTAGTCAAATTCATTTATATTAAAATTGGGAGAGAGAAAAGTACACGTGTAAACAGAATTCCATCATACAAGAATTATATGATGAAAATAGACAAGCCATCTTGAAGACAAAGTACTGTGCACGTAAAATATACAAGCAGGAAAGAAGTAATATATATCTCCATCAATCTGCCAAACAGCTCCGTTCTGTACAGTTATCTCTTCACCAGCAAAACTTCTTTTTCTCGAATTTACTTCTTTCCTGTCTATCTATATATGCCAGAAGTTCTTCTTGTCTGATCAAAGCTTCGTATAAAGCCTTCTTTGTAGCAATCAGTTCCGCTTCCAATGCATCCACACGATAAACAGCAGCATTCAGCAGCTCCTCTTTCTCATGCGGCATCACATTAGGTTTTGACTGTAGCATGTCAACCTTCTCCTCGAGCTCACCAAGGCGTTTCAAAGCAGATGAAATGTGTTCTGTCTTTGTTAATCTAGGAACAGGTGATGGGGGACGAGATTCTTCCGTGTTTATTGAATCAACGGTCATGTTAGGCGTATTTTGGAATGAAGCAGATTTAGTGTCCTTTATTCTCTTAGTCACATGAAGTGCTAGTGAACGAGCAATAGTAAAGATGGCGACAAAGAAGCCCACAATAACAACCCAGATATATGCACAGTTTCCACCAGATTTTCCAGTCCTCAACAGGAAATTTTCTGTTGAGCCATACGAGTTTTGAGTTGTCACTTGTTTCTCCTTCCATCCGAGGTCAACAGCCTTGTCAACCATGGGAACATACTCATCATACTCAGAGAAACCGCCAGAAAGATTAGCCCTACCAATCAATCTTGCTTCTTCATGGACAGGAGTTAACCTAGGGTTTGTATAATTTCCACTTGCTTTGGGAGATGTGATATCTTCGACTTCAGATCCAGATTCTGCTGTTGATGTATCACTACTTCTTATCGGCATTGGAAAGCATGCCTTATCACATTCAATAATGGTGCCCTCATCATTAGAAATTGTAACTATTTGCGTAGAACACTGCACTTCGCCACTAAGAACCATCTTTAAAATGTTTGGATCCTGCCATGGACCTTTATCAGACCTCATACAACCACCTTGATCTATACAAGTGCAACTACCACCTAGGAATTCAGGCAGCTCACTCACATCAATAATTTCAAGTAGTTTGTTCTGGAACTTGTTTCCAAGAACATTTATCTTTGAAGTAGTTTTGGGATCAAGAAATGATTTCACTGTGTTCCAAAGCAGCTTAAAACCAGGACCAGCATTGATTATAAACATTTGACATAATGTTTCTGGATAGTAATCACCATCAATTTTCTGCAGCCGCATGATGAGTTCCCGCGCAGATTTGGTTAGGTTTTTGAAACCCACACCATGAACGTCCAAAATGGTTATACTTGAATCTATATGTCTTTTTGCAGCAATAGAGCATGCGGGAAACTTAACAGCAAACGTTTTCTCAAAACCCTGTACATGGTATCTCAAATACCTTTCTATGGTAGTCACTTGCATAAGCTTACTGGGATCAACTTTGCCTAACCTTTCAATATAAACAGGTCTTCCTTCCTTATCCACACCATGATAACCATGTGGATAGTACTGCAGGACTTCATTCAACTCACTGAATTCAAAATCCTCCATAATCGTATCAGTACCGTAGTCTTTCCTCCATTGGATCATGTTGGCCCACATGTGCTTTGCTTTCTCAATGTCAAACTTCCTTGCTTTCAAGAATCTCAATAACATATGATAGTCATCATGTCTTGATGGCAACAAGTTATCCGACATAAGCGCCTGTCGAAAAGCATCCACTGCTTGGAGCTCCCTAACATCTCGAACATCCTCAATGGAGACCGAATTACTTCGGCTGGCGCTCTTCCTCCTGCTGCTCTTCTTCCTAAGAGAATGCCTGAACTTGCTTGAAGCATTGATTGCCTTCTTTTTCAAACTACCAATCCTCGTTCGACGATCATCCTCTGAATTCTCAAAATCCGATCTCCTCTCCCTCCTTTCGTCATGGCCAGAAAACCCCTCAAAGCAGGGACGTGCGAACCGATCAAGCGGCCCTGACATGTGAAGACTGTTAGATGAATATGAAAGATGTTTTGTGGATGACAGAAAACATCAAATTGAAGGCGCCGACAGATAAAACCAATTCAAGTGGAACAGAATCCTAAGCGAGAACCGAAAACAAAAAAGGATTACAATAATTACGTTTTATTGTAATGGACAGAAGTTGTTTCAAGATAGATAGAAGATGTCGGCAGAGGAGAAAGAGAGGAGAGAAAGAAATTGTTTATCGGTGAAAAACTAATA

>C100743_65

GAAGCCAGTTCTGCCTTGGTACAAAGAAATTTGCTTCAGAATTCTTATTGTTAGTACTTTCCTCACTAAGTAACCATGAAAGATAATTTCAATACAGTTTCAGTAACAATCGACCAAATAACTACGGAGAGATATAATTGACAAAGAATATTTCTAGTACTGATTATCAGCGTAGTAGGGGATATAAATTTAGTAAAAGAAAGGAACTTAGTAAAGAAAAACAAGCATCCTATCTTACATATCCTAGTTGCCAGCATGATCACAAAACTTCAAAAGCTACTAGATTACTATCTTACAAAACCACCATGAAGATAAACTTGCTTAGGAAATCAGTAACTTGTCCTGCCATCACCCTCAGTTATAACGTCCTGGAAGGTTCCATTGCTAGGAATCATGGGCAAGACATGCTCTTGATGGGGCACAATGACATCAAGAAGATACGGACCAGGGGTGTCCAACATTTTCTGAATTGCCGCTCTGAGGTCATCCTTCTTGGCCACACGTGCTGATGGTATCCCACAAGCATCTGCAAACTTCAAGAAATTTGGGAATATGGTGTTCTCATTGGAGGGGTCTCCAAGATATGTGTGTGCTCTATTAGACTTGTAGAACCGATCCTCCCACTGAACAACCATACCCAAGTGCTGATTATTCAACAGCAATATCTTAACTGGGAGCTTCTCCACCTTGATAGTGGCCAGCTCCTGAACATTCATGATAAAACTTCCATCACCATCAATGTCAACCACAACCGCTCCCGGGTTAGCCACCGCCGCACCAATGGCAGCCGGCAATCCAAACCCCATGGCACCAAGACCACCAGATGTTAACCACTGCCTAGGCCTCTTGTACTTGTAAAACTGAGCAGCCCACATCTGGTGCTGCCCAACTCCAGTACTCACAATAGCCTCTCCGTTGGTCAACTCATCAAGCACCTCTATAGCATACTGAGGAGAAATCTCTTCCTCAAAAGTCTTGTAACTCAAAGGAAACTTTCGTTTCTGCTCATTCAGCTCTTCCCTCCAACCTCTAAAATCAAGCTTCCCCCCAGCCACTCTGCTCTCCAACACACTATTAATCCCCTTCAACGCCAACTTCAAATCCGCACATACCGACACATGAGGCTGCTTGTTCTTCCCAATCTCAGCCGAATCAATATCAATGTGAACAATCTTAGCCCGGCTCGCAAAAGCCTCAAGCTTCCCAGTGACACGGTCATCAAACCTCACCCCAAACGCAAGCAAAAGATCACACCTATCCACAGCATAATTTGCATAAACAGTCCCGTGCATCCCAAGCATCTGAAGAGAATTCTCATCAGCAATGGGATAAGCACCAAGGCCCATCAAAGTACTAGCAACAGGAACACCAGTGAGCTGAACAAAACGCCTCAATTCCTCACTAGAGTTCAAACAACCACCACCAACATACAAAACCGGTTTCTTAGCTTCCAAAAGCAATCTCACAATAAGCTCCAAATGGTTCTCATTGGGAGATTTCGGCAACCTAGACATGTAACCAGATAACCTAATCGGTTGATCCCAATTAGGAATGGCAAGCTGTTGCTGAATATCTTTCGGTATATCAATCAACACAGGGCCAGGTCTTCCTGAAGTGGCCAAGAAAAACGCTTCGTTAACGATTCTTGGAATGTCATCAACATCGAGAACGAGGTAATTGTGCTTTGTGATGGAACGTGTTACCTCAACGATGGGGGTTTCTTGGAAGGCGTCGGTGCCAATCATGCGGCGGGGGACCTGGCCGGTAATGGCAACTAGGGGGACGCTGTCGAGGAGGGCGTCGGCGAGGCCGCTGACGAGGTTGGTGGCGCCAGGGCCGGAGGTGGCGATACAGACGCCAGGGAGGCCAGAGGACCGCGCGTAGCCCTCCGCCGCAAACACGCCGCCCTGCTCGTGGCGCGGGAGTACGTTGCGGATAGTGGCGGAGCGCGTGAGCGCCTGGTGGATCTCCATCGACGCGCCACCGGGGTACGCAAAAACGTCCTTCACGCCCTGGCGCTCGAGAGCCTCCACGAGGATGTCAGCACCCTTGCGGGGCTCCTCAAGGCCAAAGCGCGATACGAAAGTTTCAGCAGCGGTGGATTTGACGAGGGCGGCGGCATCAGAGAGAGCGCTGGAGATTTTGAGAGAACGGCGTTGGGAGTGTAAGGGGGCGTTTGGAAGTGGGGAAAATGGGAGGGCGAAACGGAGAAAGGGCTTTGAGGAGGGTGATGAAGGAAAGGCGGTGAACACGGGTTTTGGAGCAGTTGCTGCCTCCATCTTCTGATTGCAAGAAAAAACGTAAAAGCTGTAACAAACAAACAACGCAGACCCTAACTTAAGTACTATAACTCAACCATACAC

>C100745_65

AGTGTCCAAACCAAATTCAAAGAGGACTCAGAAATAGAATCTGAGTGAAAGCAAAAAAAAAAAAAGAAACCAAAAATGAAACAGGGAGTTGACTAACAGTCATGTGAAGCATAGTGACTCAAACATTCTAAACAAAAGCTTTACATGCCTATGAGTAATACAAACTTTTGTAAGAACTTCTCGGTGGTATAAACCTTTTACCTAAGGGGGTCTTGGAAACCAGACCCTCAGAATGGTTAGATGGTGTGATTTAGAATAGCTTGCCTTGTTGGTTTCCACTCTTCATCATTGCAGAGAATTCTTCATAGTTGATTCTACCATCATGATCTGTATCCACTTCAGATATAATTGTATCAACTTCAGATATGATTTCCTTGATTGTGGCATCATCACCCATACCATATTCTTTCATGGCTGATTCCAATTCATCTCTCGTAATATACCCACTGTTGTCTTTATCAAAATACTGGAAGGCCTTGAAAAGTTGGTCATCTCTTTCTAACTTGTGTCTATGCATTGTAGCAGTGATGAATTCTATGTAGTCAATTGAGCCATTTCCATCTACATCAGCCGCATCCATAAGCTGTTTCACTTCAGCCTCTGTAAGCTTTGAGCCAAGTCTTTGCAATCCCGCCTTTAGTTCTTCATAGGTGATTGTACCACTCTTGTCAGTGTCCATATTTGTAAACATTGCCTTCAAACCTTGGATCTCTTCTGGAGACATATTCTCAGCAATGACCTTCAGGGCAAGTTTCTTTAGTTTATTCATTGCTCTGAATTGCTTCATTCTGGAAAGGACTGCACTGTCTATTGGCTTGTCTGAAGCAGTTCCACCTTTAATCCATGGGTGCTCAAGTACTTGAGCAGAGGTAATGCGTTTCTTTGGATCCTGTATAAGCATCCTACGAACGAGATCCTTGGCACTGTTTGAAATGTTAGGCCAAGGTTGACTTTCAAAATCAATGTGACCTTCCAATATGGCATCAAATATTCCCTTCTCAGACTCAGCCCAAAATGGAGGGACTCCACTAAGTAAGATATACAGTATGACTCCTGCACTCCATATATCTATTTCCTTCCCACATCTGCGCCGCAGAACTTCAGGAGCAACATAGTAAGCACTACCAACTATATCCCGATATACCTTTCCTTCTTCAATGAAGACGGACAAACCAAAATCAGTTGCCTTGAGAAGTGCATTTTCATCCCTACTAGATAACAAGAAATTCTCTGGCTTCAGATCCCTATGCATCACACCCATGAAATGACAGATATGAACAACATTTACAATTTGTCTGCATATTGAAGCAGCAGCCCTCTCACTGTAATGTCCCTTGGCAATAATCCTGTCAAAAAGCTCCCCACCTGCACACAGCTCCATCACAACATGAACTGATTGCCTATCCTCATAAGCCCCTTTGAACTCAACAATGTTGGGTTGACCACTCAAATGCTGCATAATCTGAATCTCCCTCTTTATATCCTCCTTATCAGATTTGCTCACAAGTTTTCTCTTAGAAATAGACTTACAGGCATACTGCATCCCAGTTGAAATCTCGGTGCAAAGATATGTCACTCCAAATTGCCCTCTACCCAATTCCTTGCCAAGTGAGTAGAACTGCTTCACATCCTCAAACTGCTTCCCTAGAATTGTGTCTTGCTTTACACTAACCACAGGTTTGGGACTTGGAGCAGCAGGACCAGAAGACTTCCATGGAGTCTGAGGCACTGTCTGAGCAGTGGATGCAGCATGCTTTTCAGCTTGATAAGGTTGATTTGCCGGTACTCTTGATTGGTTGACCAAAGGTTCATGAGTCTTCTGGGTGTGCACACCACCAGTCCCAGCATGTCTGTAACCATTGTGTTCTGGCTCTGAATTTTTGCTTACACAACAACCCATTTTCCCAGATTCTCAAATCCAAGTATTGTGAGGAAAGAAACAAGAAAAGAAAAAAATGAATATTTTTCCCTTTGATCAGACAATCAGCACAAAAATTCAGACTGATTCAGTCAAAACATCAAACAGGTTGAACCCAGATGAGAATTCAAGGAAACTATACCATCTTAAAATTTGGATCAGAAACCTATAAGTAAATCATTTTGAAAATTAAAAAGAAAAAAACTAATAAAAACCTTGTCAGGAACAAGATTAAAAAACCCTCAGATGAGGATTGTGGAAATATATATGATATATGCAGAAGATGTAAAAAGAGAGATAGAGGACAAGTTTGATACTTTTTGATGAAGAAATGTACAAGGAATTATAAAAGTGGAGCCTTGAAAGAGGGTTAAAGAGGGTGAAAGAGAAAGAATGTATAAAAAAAATTGGGATAAATGCTAGTTGTACCTAAGGTGGAGTTCACA

>C100747_65

CAATGGCATATGACAAAACGCTCTGGACTATTCACTTATTAAGATGAGGAAAATTAAATAACACATGATACATTAAACCATCTTAAACGCGTTATTCTCAGGGTGGAAAGAGGAAAGAAGCTTACCAATAAAAACAAACCAAATTGACTCCAATGTTTTGTCTTACAAACTAATGGGATCTACGGGGATAATCAAGTTACTCATCAAACACAAACATTCTTGACAGGCTTTATTTTGATCTCTCATTTAAGGAGCGTCTTCACAATTGACTTGACGTTCAGTTTCTTGAGGTCAGTATAGGACTGTCCACAGCCCACAAACATAACTGGAGCTCCAGACACGTAAACCATTGAAAGTGCAGCTCCCACCTTATCATCAATAGTGTCAAATTTTGTGAGCAAGATCCCATCTATCAATCTGGGTGTGGGAGATGTTGAGAGGTCGGCTAATTTCTGGTTGAATTTTGAAAGCTGATCAACTGCATCATTACCAACAAGTGCTTCTCCAACAAATAAGACAAGGTCAGGATTGTTGAGGTAGATGAGCTTTGACAGGGCTCTCATCAATGGTTCATTATCCTGCATACGGCCAGCTGTATCGACTAGAACCACATCTGAACCATTGCGTGAAGCTTCCTGAATTGCTTCTTTTGCTACAATGGCAGGATCTTTCTCATAGCCCTTCTCAAATATAGGGATCTGAAGTCTACGTGCATGTGTCCGCAGCTGCTCAACAGCTCCCGATCGAAATGTATCACATGCAGCCATCATGACACTAGTGTTGTGTTGCAGTAGCCAGTAAGCAACCTTAGCCAAATTAGTAGATTTTCCAACTCCATTAACTCCAACAAACACAACAACATATGGTTTCCTTTGCTCCTTGGCAGCATGCACATCCCTCAATATGTCAATAGAACGCCTTGGGGTTAAAATACGAACAAGTGCATCTTCCATTGCTGTATGCACTGTTGAAGATATCCTTGTAAATGAAGCTAGCTTTTTGCCTTCAAGACTTGCTGCCACTGACTCGCATAGTTTCTCAGCTATTTCCTCGGCCACATTCTTGGTCATGAGCCTGTCCTTCAGAGCTTTCAAAGCCGGTTCCAAGTCGGACTTCTCCAAATTGGCCTTTCCAGCAATACTCTGCAACATAGACGAAAACCAACCCTTTTTCTTAGCCTCTGGCTTGCTATCCTTTCCAGTATCATCATCATCATCTTCTTCTTCAACCTCACTATCACTGCTAAGAACCTCTTCTTTATCCATCATACTCTCCCCCTGTTCCTTGGCCAAAAAGTCAACCTTTTTATCTCCATCTCCATCCCCATCAAGGTGATCAGTAAAGTCCAGTTTGGTTTGCGGCGGAGAATCATCCCAAACCCTGTTTTTCTTAACCACCTTTTTAGGCTCAGCCTTGGCAACAACAACAGCTTCTTTCTTCTTCCCATTCCCATTCCCATTCCCCCCTTTTCCTTTAAGCTTCTGCAGCCTATTTACATCAAAAGCCCCTATATTAGCACTCTCATTCTCTTTCCCAATATTCACACTACCATTACCACTACTCTTCAAGTTAGCATTGCAATTATTATTAGCTACAACAAAACCACCACTCTCCAATCTACGCCCTTTCTTCCCATCACCACCACCATCACTCTTTTTTTCTGACCCTGCGTTTTGCCCTTGCCCCTGCCACGTGGCGCTTAGCTGTTTCTTTTGAGGAGCCACCGGGGGGAGGTGATTCGTTTTCTTGAAATTCTCAGCGCGAGCCTCGGCCTCAATCCGTAGCTGGCGGAATATCTCGTCAAAATCCGGGTAAGCGGTTTTCTGGGGGTGGTAGAGGCGAGAGAATTCACGCTTGACGGCGGCGAGTAGATCGTCGACGTAGAGTAGGTGGAGGATGCGCTGGTAGACGGCGACGAAGACGAGGCCGAGATCGTTGTGAAAGGTCCATTTGAGGGAGTAGGAGGCTCCAGGTGCGTCGTAGGTGAAGCTCGCCGCACCGGATCTCTCCTCCAGGAGGCACGAGCGGATCAACGTGTCGATCGGAGAGCCCTTGAGGGCGTTGGTGAAGTGGTTGCAGGTCCACAGGATCAAGCCTCCGCGCGTGAATATGAGTAACTGCTCTAACATCTTTGATCACAGAGAGGTTTTTCTGATTCTTGAAATTTGCGAAAACGGAGAAGGAAAAAAAAAATGGAAAAAGTTAGGTTTGATTTGGTTTGCGGTGGATACGGAATACGCAGAAGAAGATGGATTATGGATATCGCTGACACGTGCTGGATTGATTCCAGGACGGTGCAAACCGCGCACGCCGTCGTTGACTGAGATTATCTAAACCAAACGTCGTTTCGAGGCTGATTCTTCTC

>C100749_65

GTTGGAGACACAGAGACCCACCACAAGCCATAACGCCTCTCTCCTTCTCCCCCTGTTTTCTCTTCTTTACTTCTTTCTCTGATAAAACTGTCATCAACCCCTTTCCAATTCTCACACACTCTCTCTCTCTCCCTACTTTTTTATTTTATTTTTTCTTTCATTCTTCTTCTTCTTCTCTTTCCCTTCCAAATATACATAAAATATAGTATTAGTATAATCGATGTGTGTTGAAGGCATATTTGCTTAAAGAACTGAAACATATCATCTTCTTGATCGTTTGAATCTATGGAACAGCTTGTCAACTTCATCATCCGGCCACCAAGAGCTGAATATGACCCCAAAAGTGATTTATTGGATCAGGAGTTCATGCTGAAAGGGAAATGGTATCAGCGGAAGGATGTAGAGATAAAGAACAGTCGGGATGATGTTCTTCAATGCAGCCATTACATGCCTATAGTCAGTCCTGAAGGAAAGCCTCTGCCTTGTGTAATATATTGTCATGGAAACAGTGGATGTAGGGCTGATGCTAGTGAAGCTGCAATAATTTTACTTCCCTCAAATATTACAGTTTTTACTCTGGATTTCTCAGGATCTGGAATTTCTGGAGGGGAGCATGTTACTCTAGGCTGGAATGAAAAGGATGATCTGAAGGCTGTGGTCAACTATCTGCGGGAGGATGGAAATGTCTCTTTGATTGGCTTGTGGGGTCGCTCGATGGGAGCTGTAACTAGCCTTATGTATGGAGCTGAGGATCCTTCGATTGCAGGGATGGTTTTAGACAGTCCCTTCTCTGACTTGGTTGATTTGATGATGGAACTTGTGGATACATATAAAGTTCGTCTGCCAAAATTCACTGTGAAGTTTGCAATCCAGTACATGCGAAGAGCTATTCAAAAGAAGGCAAAATTTGACATAGCGGACCTCAATACCATTAAGGTGGCAAAATCTTGTTTTGTTCCAGCTCTACTTGGGCATGCCATTGAGGATGATTTTATACAGCCCCATCACTCTGATCGGATATTTGAGGCTTACATGGGTGACAAAAACATAATTAAATTTGAGGGAGATCACAATTCTCCACGTCCTCAGTTTTACTTTGATTCCATAAACATCTTTTTTCACAATGTTTTGCAACCTCCGGAAGATGAGGTTGGGGAATCATTTTTTGACCCTGTGAATGATTACTTTGGTAAGGATGTTTGGAGATCTATGCATGAATTAGGCTACAGCAACGAATCATCATCTAAAAACAAAGAACCATCAACAAGCAGTACAATAAATGCCATTGAGCAGGTCCGTTCAAGACGACCAATGAGTAGAATGGAGGTTCCATCTGATATTTCTTCAAAAGATGAACGTCGTGAACATGAGCATGAGAAGTGCGGCGATCATTCTCCATCTCCATCATCAATGATTAGCTTTGAATTATCAAATGGTGATCCTTTTGGACCCCATGTTCCAGCCACATTAGAAGATGATCAGTACGTTGAATATCAACTAGATGACCTTGCAGGTTTTCCATCTACTTCAGAGGAGGAAGAAAGAATGTTCATGGAAGCAGTGATGGAGTCACTGAAGGACCTGGAAGTACGAAATTCAAACGCAGAACAACCAACAAGTAGCGTTAGTACTGTGTCTGTAGCATCTGTAGAGCCGTCAGATAAAGATGCTTCCTCACAAGAGAATTCAAGACCTGTGGAGACAGAATCTTCCTCACTCAAACAAAACACGGATTCAAAATCTAAAACCACTTCCTCTGCATCACAAGAATGTGAGACATTGAAAGGCGAGGCCAATTCCATTTCAGTGAACCATTCTCAAAATGTGGTTTCCAAGCCAAGTCCTGTACCAAGTCTTTCATCAGAAGCACCACAGTTACAGTTGCAGCCTCCACCAGCAGCAGCAACAACAGACACGTCGTCACTAACCGAGTCCAGCAGCACAAGCAGTTCTGCTCGTAGCAGTAGTTCTGCTAGTTTGAAAAGTTCATCAGAAACTGACATATCACACAATACTAAAGCCACAGTAACTGTTGTCAGGAATCCTGCAGGCCATGTCATGGACGGCTTAATGCGTCGTTGGGATTTCAATTTTTTCAAAAACAAATAGTAACAACCCTTACGATCAGATGGGAGATTCTCATTATACGTTGTTTCTGTAACTACAAGTAAAAAAATCATAGTTTGTACCAAAGAAATAGGTTGCTCGTGTGTAAATTTAGTTTGTTTAGATTCTCAACTAACAAATCAAACAATATTCTTTAACTGTAAGTGAGAATTGTCATATTTTGTTTTGTCTTTATGAATTTAAATGGGAAACAATGTCTCCTTTGTATCTACTATCTAGACAAATATTCTTGATTCTA

>C100751_65

GACGAGTATAAAAAGAACAAAGCAGTAAAGTTTTGGTGTAGCTTGTAAGAGGACAAAAATGCCTACTCTGTATGCTCCAACTCATTGGTCCAGTAAACAATTGGTGTGATTCTAGTAGTATCAAGGCAAAAAGACCAGCAAAACTGAAACCATTCCTATTTCGTGTTCTGCTCTCAGCTGTGACTATGCCAAAGCCTTGCCCTAAAAGTTATAGAATGAATAAATAGATGAATGAGTACACAATTATTTCACCAGTATCATGCCCCTCGAAAGAAAAAAATGTCTAAGGAACTATGTTCTTCTGCAACCTTTTAGTTCTCTGAAAATAGAAAAGCTAAAAGATGGCTGGCTGGTGGCTGCTAATTTGACAAACTAAATTGTATAGCACTGCAAATTAAAGTTCGGCAGCGTGATGTATTCACTAAACCAAATCTCAGCAACTTCCACAAACCATTCTTTCGTCCTCAGATATCTGCAGGGTCGTTGATTTCCTTATTCTCAATCGTCACCCCATCAGGAATTTCCAATTTCAAGCCAGGTTTTGCAGCAATAGTCACTTGCCCCTTCAAAGTAATATTAGCTCCAAACCACACATCACCTCTCACCATCAAACTATCCAGCTCAATGATGCTTGGAATGGATTTGAAACGACTTTGGAAGTCACCAACCTTTTCAAATTCAGGTCCCAAGTCTATCACAGGATTTAAAGGGTTAGTTCTAGCTGGATTGCGAGTTAAAACACCTTCTCTACAAGTGTATAGATCCGACTGAAGAAGTAATAGATCTGATGTTGCATCCAATGGAACATAGCGAGATTCAGGCACAGAGACACCAATTACATTATCAAAGAACTTCAAAGCTGAGGGCTTCTTCTGCTTTAGTTCATCAGTATCAACAAACCTCTTGATGGCTCTTAAACTCACCCACATGTTTGTTGTATCAACCAGTTTGAAATTGTCCCTCAAATGTTTATCTTGGTTTCGAGCAATCTCCCGAAGCTTAAATTTCATTGCAGAAAAGATTAAGTTAAACGAATGGCTTGGTGTCACCTCCATGCAGTATTCAATATCATTTATCATCAAATGATTTAGTATGTTTGGATCAACAACTGTTGCCACATTGTCTGACTTCAACACTAGGATATACTCTTTACCCTGTGACAATAATGAATCAAGGGTTCCACCAGTCATGAGTGAACGGAACACATCAACATCATCAAATGGATGCACTTCCTCCTTACTGGAAGGTCCAGCTGATAATTTCAATTCTCCACCTTTACCCTGGTTCAATGTGTGCACTTCAACATTTGATTTTGAATACTTCTCCAAAACCTTTAGAGAACTATCATGAATGTCATTTTTGTTGAAAAGAAGCAATGGAACCCTGCATCCATACTTGGAGTTTAGCGTCTCAATCTGATTAATGATCAAGTCCAGAAATGTCAGTCCATCCCTAATATTAATTGCAGATTCGGGACCCTCAAAATCCATGTTTCTTCCTTGGGCACCACTGAACTTGAAAACAACAAGCTTGTCCAAAAGCCTCTTAATTTCCTCTGTATCTTCAGGTGAGGGAGCTAAACTAGCATAGGGAACTACGGTTATGCCGACCTTATCCAAATCGCTCTTGCTCTTGACCTTGCGTGGAGCTTCTTCTCGAGAATCAATTTCGAGATTGGACTTCTCACGAAGCAGTGTTTTGGTAATCAAATTACAGAACAAATAAACGAATTGAACAGAATCGTTGGCGGGAACAGGATAAGCAATGCGCTTATAGACATCACGGGGCATGGCGGAGTCGACCAATGCAACAACGGGAATTTGAAGCTTGAAAGCCTCATTGATTACGTTTGACTTTCGCTCAGTGTCGATAATGACGATGCAATCGGGAGACTGGGTGGGGCCAAAGATGAGTTTCTTGTTGCGGGAGCGGAACTTCTTGGGACTGTAGCTGTTGGTGAGGAAGCCGCCGGTGCGCCAGAGGGAGTGGGTGGAGGAGGAGTAGGAGCCAACCTTTTTGGTCATGAGGTCGAAGATGTCGTCGAAGAGAGGGTTGGTGTTGATGAACATGAAGCGGGCGTTGTGGCGGGCGAGGGAGGCCATGAAGTTGCAGGCGCTGCGCATGCAAATGAGGGTTTTGTCGGAGTCGATGATGACCATCTTGTTGCGGAAGCCATAGGTGTAATCCTTGAAATGGTGAGTCGCAACTCGTCGACCCACCTGTGCATTCGTGCTCAGCAGCTTCTGAATCACTATCGAATGGATCGTCATCCCTTCCTCCGTCGATTCTCCCTCGCTTCACTTCCTCTTCCACTCCAGTAGGGTTTTTCGACTTGTCTCACTCTTTCCCTTTTTTTACAAAAAAA

>C100753_65

ATTTTTTTGATTCACATCCCCATCGCTGGTGAAGCTCGCAGACGGAAAGAAGCTTCAGTAGCAACTTCTCCGACGGGGAGAACCAATTGGTCTCAAGCCCTATCTCCTTTTGTTTCAATTCACAGTGTATGCTAGTTTTCCCTTGTTCGTGACGGTTGGCAAAACTCTACTGACAAGTTGTTTTTTTTTCTTCTTTATTCAGGAGAGAACATGACATCTAAAAGGCAAATTGAAGTTGGTGATAGAGTAGAGGATGAAGGGCTTTATAATGAACCAAAAAGGCAGAAGCTGGATGAGGATTCTTCCCCCTCTCCTCCTCCAGTCTCCATTGCAAATCCACTTTCTGGGTTGGCCAATAATTATGCTGACATTGATGAGGAGGAAGACTATGTTCGAAGGGAAAAGGGTTCTGTTAATGAGAGGAGGAATGATGGGCATAGATATGGAGAGGCTGGTGATAGTGATGAAGAAGATGGTTCGCATGAACAATTTAGTGGAAGAAACAATCGCCGGGTTGAGGTTCGGAAGGACTGTCCTTATCTTGATACTGTTAATAGACAGGTTTTGGATTTTGACTTTGAAAAATTCTGTTCTGTCTCCCTATCAAATTTAAATGTGTATGCGTGTTTGGTTTGCGGGAAGTATTACCAAGGGAGAGGGAAGAAATCTCATGCGTATACACATAGTCTGGAAGCAGGACACCACGTTTATATCAATCTTCACACTGAAAAAGTCTACTGTCTTCCTGATGGTTATGAGATTAATGATCCTTCATTAGATGACATTCGGCATGTCTTAAATCCAAGATTCACTGCCAAAGAAGTTGAACAACTTGACAAAAATAAGCAGTGGTCTAGGGCACTTGATGGTTCTAGTTACCTTCCTGGAATGGTGGGACTTAATAATATCAAGGAGACTGATTTTGTGAATGTAACAATTCAATCCTTAATGAGAGTTACCCCGTTGAGGAATTTTTTCCTTATCCCTGAAAATTATCAACACTGCAAATCTCCACTTGTTCATCGATTCGGTGAGCTGACTAGGAAAATCTGGCATGCACGCAACTTTAAAGGACAGGTTAGCCCACACGAGTTTCTACAAGCAGTGATGAAAGCGAGTAAGAAAAGGTTTCGGATAGGTGCACAATCTGACCCAGTTGAGTTCATGTCATGGCTTCTTAATACACTACATGCAGATCTTAAGACTTCCAAAAAGAATACCAGCATAATCTATGAGTGTTTTCAGGGTGAACTTGAGGTTGTGAAAGAGATTCCTAACAAAAGGACCATTGATAAGAAAGAAAACATTGAAGACCAGAACAATGTTGAGAAACTTTCAGATGGGGTAAATGAGCGGCATGCATTTGTCAAAGAAACTTCAAATATGCCATTTCTAATGTTAGGATTGGACTTGCCACCACCTCCTCTTTTCAAGGATGTGATGGAAAAAAACATAATACCACAGGTTCCACTCTTTAACATACTGAAGAAGTTTGATGGTGAAACTATCACAGAGGTGGTCCGTCCTGGCATAGCAAGGATGCAGTATCGTGTTACCAGATTGCCTAAGTATATGATTCTTCACATGCGTCGGTTTACTAAGAATAATTTTTTTGTGGAAAAGAATCCTACATTAGTCAACTTTCCTGTGAAGAACCTGGAGTTGAAGGATTACATTCCCTTGCCAACACCAAAAGAAAATGAGAAATTGCGATCTAAATATGATTTAATTGCTAATGTTGTTCATGATGGTAAACCTGGTGAGGGTTTCTATAGGGTATTTGTGCAACGCAAATCAGAAGAACTATGGTATGAGATGCAGGATTTGCACGTGTCTGAAACACTTCCTCATTTGGTTGCACTTTCAGAAGCTTATATGCAGATTTATGAACAGCAGCAGTAAAAAGGCCGAGTTCGAGTCCTTTCTGTGCACCATAACCTGAAGCATATTATGTGTTTAGAATAAGAGAGATTTCATGTGCACATAGCAGTCAACTTGATTAACAGGCTGCAATTGAGTTAGGAAGGTTGGGGTTAACTTAAAATTAATCCCTGTTTCTGAGTAATGAAGCTGTCTAAAACACGTGAGTATTAAACTTGACTGGTTCAACCAATTAATTAATATACAGATGTACTGCACGGCTCCGAGGAACCCACTTGAATTTTTCTAGTACTTAGCTCGTAGTTAGTTCACGATAACACATGGAATGTGATATATCCTTTAAGGGTATTCCAGATTTTGGTTGCGTTGAACATCCTCCAATCCCCTTCAGGGGGGCTGTTTAGAAGATGTTGATGGTACTGTTTGGTCCTTGAACCTTCAAGGTGGCTGATATTATTATAATTTTATATTTTTTGTTGGCAGA

>C100757_65

CTTGGTCACATTTTACTCGGACTAAACATCCTTAGATTACAAACCAGACATTTCCTGACATTTTCTCTGTCCAAACAAACCCCGTTTCATCTGCTTCACTTCACGTGCTTCCTTTCACACACCCTTTTGAGTCTGCTTGCTAAGATATCTCCCTCTCAACATCAGAGCATAACAGCAACGCAGGAAGTGAAGTTCCCTTGAACTGATCATGCACATGCTCCTGCTGAAACTGCTACTCTTCCTCGTATGGGCTTCTTCTCCATCTCTTTCCAGCTCCATGACCCAGCTTGACACTCTCTTGGCCATCAGAGACTCTCTAGACCCAGAGAACCGTGTGTTGCTCTCATGGAACCCCCATTTGGACCCGTGTAGTAGTGCCTCTTTTGAGGGTGTGGCTTGCAATGAGCAAGGCCTTGTTACCAATATTTCACTGCAAGGAAAGGGTCTATATGGAAGGATACCTGATGCTTTAGCTGGTCTCAAGAACTTGACGGGTCTCTACCTTCACTTCAATGCCCTCAATGGGATATTACCAAAGGAAATTACCACTTTGACTCAGCTAAGTGATTTGTATCTCAATGTCAATAACCTCTCTGGTGATATCCCTCGCGAGATTGGCAATATGTCAAATCTTCAAGTTTTGCAGCTTTGTTATAACAAGTTTAGTGGGAGTATACCTACTGAACTGGGAAGGTTGAGTAAACTCAGTGTTGTTGCACTGCAATACAATCAATTAAATAGCGCTATTCCTGCGAGTTTGGGTGAAATGGGGACATTGGCACGATTGGATCTGAGCTTCAATAGCCTTTTTGGTCCAATTCCGGTGACATTAGCTAATGTACCTACGCTGGAGACTCTGGATATTCGAAACAATTCTCTCTCTGGACGTGTCCCTACAGCTCTAAAAAGACTCAAAGGTGGGTTTCAGTACATGAACAATCCTGGTTTATGTGGGACTGGATTTTCCCATTTAGATTATTGCAAAGCAGTAAGCAGTTCAGACCCAGTTAGACCAGAACCATATGAACCTGCTGATATTTCTACCAAAGATCTTCCTGCATCAATTGAACCAAAACCTGAAAGCTGCAGCAGTGGTCAATGTAAAAGGACATCGAAATCTTCAAAAATTGGTTTGGTTTGTACGATCATTGGATTCATTGTTGCTTTAACTCTTGCTGGACTGTTATTGCTCTTATGGCGCCGTCGTCAGAATCAGAAAATTGGAAGTTCTGATAACAGGCTTAGTACGCAAGTGTGCCAGAAAACTGCATCTCCTCTTATAAGTTTGGAGTATTCTAACGGTTGGGACCCATTAGCCAAAGGTCGAAGTGGGTATTCCCAGGAAGTTCTGGAGAGCTTTATTTTTAATCTTGAAGAAGTAGAACGCGCAACTCAAAGCTTCTCAGAAGTAAATTTATTGGAGAAGAATGATTTTTCAGCTGTGTACAGGGGGATTCTGATAGATGGTTCTGTTGTGGCTATAAAGTGCTTTGCCAAGACAAGTTGCAAATCTGATGAAGGTGAGTTCTTGAAAGGTTTGAAGATATTGACCTCGTTAAGGCATGAAAATCTAGTTCGGTTGAGAGGGTTTTGCTGTTCAAAAGGCCGCGGGGAATGTTTTCTGATCTACGACTCTCTCTCAAATGGGAGTTTGTTGCAGTATCTTGATGTTAACAGAAACAGTGGTAGGGTACTTGATTGGTCCACCAGAGTGTTCATCATTCGTGGTATTGCCAAAGGTATCGATTATCTACATAGGAAGAAAGGAAGCAAAGATGGTATAGTTCATCAGAATATATCAGCTGAAAATATACTTCTGGATGCTTGGTATAAGGCTTTACTTGCAGATTCAGGATTGCACAAGGTCCTTGAAGATGATGTTGTTTTTTCTGCCCTCAAAGCTAGTGCTGCCATGGGATATCTGCCACCGGAGTACACGACAACTGGTCGTTTGACTGAAAAGAGTGATGTGTATGCCTTTGGAGTGATTGTTTTCCAGCTTCTCACTGGAAAACGTGATATCAGTCAGTTAAATTGCCAATGGGTAGAAAGTGCTAGTTTGAAAGATATAGTTGATGAGAATCTTGAAGGGATGTTCTCAGAATCAGAAGCCACCAAGCTGAGAAGACTTGCTTTGCTTTGCACCCAGGAATCCCCACATCTTCGACCATCTATGGACACCCTAATGCTAGAACTGGATGATAAATAGTAGCAGTTCCTCACATGTAGCATCCTTGTTTCTCCTTCTTGTTGGAGAAAGAAAAGATGGGACACTGTATAATTAACTTTATGATTTACTAAGAACTGAACTACGTTTAGTTTAATGTGTAATATAGAGCAAAACGTGTAGTGTTAATGGAAATTCC

>C100761_65

TGTGGGAGACAAAGTGGTAGAATACTTAGATACAGAGGGAGAAAATACAAAATGTTCTTAATCACCTTCAACATATATTACAGGAGAAAACAAAAACTCGCCGGTTATACTTATATAACACATGTTCTGAACTCTACAAACACGACCATACCTACACATAGGGTCAAACGAAAATTTAAGCAGCTAGAGCTGCAACTTCCGCAGCCAGATAAAACTCAAAAGGAGAAAAGCTTCTTTCACCTTTTGGACTTCCTTACACTCTGGCCCTCACCCCTGGCTTCCCCTAACCTTGATTTTGGACCATTGCTCTGTGCTGTAGCGGGTCTCTCCTGGAAGTTATTTGAAAATTGTCTAGTGGGACTGGCATAACCTCTTGAAGATCCAAGCCTGGAATCAGCCAATATATGATCCCTTAGACCTTTTACAGATTTATAGCCTTGCATTTCATCCCCATATCCTTTTCCTTGTGCTTTCTTTTCCAGCTCATAAAAGTTTTCCCAATCTATCCCATCTGAATTTTGAAGCTTCTCAGTTTGGACACCCCATTCCATTCCATCTGATATACTCCTTTGCAGGGACGTGCTTCTCCTTGAAGCCCTCCCAGAGGTAGACCTCCTACTACCTTTCACTTCTTCCTCAATTGGATATCGTCTTGTATCAAATCTGCTTTCAGAAGGGTAGGACCACTTATAACTCTTGTTGTTGTTGTCCATATTTAGCTCTATAGAATGAAGATCACTTTCCGCAGATTCCTCCTCACATTCTACTCCATTGTCAACCTCTCCTCCATCATCTTCTTTATCTTCAATGAAATGGGAACTTAATCGGCTTCTGCTCAGATACGCAGCAATTTCTTCATCATTCAAGTGAGTGGAACTCCGACCTTTCTCCTTAACTTGTTTGCTTCCTAGAAAAGCTTCAAGTTGATTCCTGAGCGTATCCACTGCTGCATTCTTTTCCTCAAGCTGATATTTTGCCTCGGAGAGTTTCTTTTGAGCTCTCTCCTCGCGTAACCTATCAGTCAGCTGCATTATTTCCTTTTCTTTCTCTATCTCTTCACAAACTTTTGTGGACACTCTCTTTTGTTGCTCGATCTCAGATTTATCGTCATCAGCATCTCTTGCTAACTCATCACATACTTGCTCAATAATTTCTCTTGCCCTTTTCTCACTTTCAAGTTCTTTCACCACCTTAAGAAGGGATGTTTTGGTGTCAGCCAGTTCTCTCCCAAGCTTCTTGTTCAAGCTTTCAAGACGTCTCCTGAGCTTCCTCTCTACATCAAGTTCTCCAGCAACAGATTCAATTGCAGCCTCAACAATTTCTTGCTCCTTCTTTTTCCAAGAAGCCTTTTCTTCAGCAAAACACTTCATCAAATAATTTATCTCATTCTGATCCGAGCGCTGTTCCTGAATAAGCTGATTGACCTGCAGGCGAGCCCTCTCCAGCTCAGTATGCAAAGCTGAGATTAGGGACATACTAGATGAAGGACGATCTTCATGACCCCACATGCGGTTTATAATTTTAAGTAGCTCTTTGGATGTTGTTAATGCATTACTAACATCTTTTAAACGTGATTTAACTCCAACAGCAGATGAAGCCGGGGTCTGTGCTCGGGATCTGGTTTCAATCTCCATGAGACTGACATTGCTAAGAGAGTCCAAAGGGCCAACATGGTGTTCAGTGATCCTCTGTCTGTGAGAAACAGAAGGAGTTCTTCTATGACGACTCCCACTTCCAGATCGATCCATCCTCTCGGACACAGGACTGTGAGATGGATCGGACAAATGGGGAGGCAAAGAGCCAGAACGCATGGACCTAGCGACCCTTTCTCTAGCTCTGACTTCCTTCTTGGACCTCACACTGGGCGAAGGAATCTCGTTCATCTCCCACAGGGTGGCGGCGAGCTTCCTAGCCGACACCGGAACCTGGCGGGGTTTGGCGGCTGTCTGCGATGGCGGGTACTTGGGAGAGGAGCCCAATGGGCGGAGCGGAGATCTGGAGCTCATGAGCTTCCACGTGGGCACGGGCGTACTGGATCCGCCCCGCTTGCCGACCAGAATAGTCCTCTTGAAGCGGTAGTTGTGCAGCACAGAGGAGGAAGAAGAGGCCGAGGAGGAACAACCTCGTTTTCGGATCTTCCCAGGAATCATGTCCATGCTGCTTGGGTTTTGGTGCCTGAGCATAAGTTAGACTAAGCGGAGTTTTGGGGCGGCGAGATTACAGTGACATAGCAATTGGCGAGTGGAGGAGGAAGAGGAAAGTGTGAAGGAACACGTGTCGGGGTTTGAGTGGTGGGAAGAGGAAGAAGGAAGAAAAGAGAAAGAAAGGGTTAAAGAAAGTAAAGGGTTTGGAGGAGTATTAGCTGAG

>C100763_65

TTTTTTCAAAGAATGTTTTAACAAGTGGATTTTAAGCAGAGATGAGGCAAAAAGGTTTAATGAATCCACTAAATACAGACCAATATTGTCGATGAATCTGTAACCTTTAAATATATAACGAAGAAACTTATAAAAAAAAAAAAGCTTAGTTAGATAAAAGTAAAAGTCTAAAAATGCTGGAGCTGAGAAATGCTCCACTCATTTTCTTTCTTATTAGATTTCCATCCGATATTAAATGCAGATAGCATCACAGGTTTGACATCCGAACATAAAGATTCATCCCTTGCCAACAGAGTTAAGAACCTTCTATCCAAGATGGCGATGGCACACCAATCCTCTTCTCCAATTTGTACCTCAGAAAACAATGCTTTGAAAGATGGTGCTAGTAACTTGAATTGATCACCTATACACGCTTTCATATCTTGGAATGCACCCTCATAAAATATAGGACAAAATCCTAGTCAAGATCCAAGTGTAGAGGACTGAGAGAACAGCACCAACTGGAATAGTGACTACCCATGAAGCCACAATCTCCTTCACAGTTTCTGATCTAACACTGTTAAATCCCCTTGCAAATCCAACACCCATTACTGCACCTACCAGAGTATGGGTTGCAGAGATAGGTAGCCCCAGCTTTGAAGCAAACAGAACCACAGAGGCAGCAGCAAACTCAGCTGCAAATCCTCTGGTTGGTGTAAGTTCTGTAATCTTCTTTCCTATTGTAGCTATCACTCTATAACCCCACATCATTAGCCCTGCAACGATTCCAAATCCTCCCCAAGCGAGAACATCAGTTGGAATAACAATGTTAGCTCCCGTGGCACCACCACCTTGAAGGATAGCCAGTGCACCTGCCAATGGACCTATGGCGTTGGAGACATCATTTCCTCCATGAGCAAATGACATGAAACATGCTGAAAGGACCTGCATGTAACCAAAGACTCCATAAACTATTTCCAATTGAGCACCCTTTGGGCCTGCAACATCATCGAGGAATCCTATATTGTGGTGGACAGTGTCTTCTTTAGGCTCAGGCTTTGGAGTATTTGACTTGACAAGAAGATGCCCGAGCTGTTTTCTGATGATTCTGTCAACCAGAAAAGCACCAACAGTTCCACCGATTAAAGCTTGGGCCAGAGCTAAAGGAAAACTCTTGCTAAGGGGGAAGGCAACAAATGAAATCCCACTAACACCAAGAAATACAGCAATTGGTGCCGCGGCAGCTGCTGCTAGTCCAGGATTAGGTGCACTGTACACAAACCTTCGGATGCACTTATAGACAAGAAATGACACAGCTGCTCCCATCAGTGGTGAGACAACCCACGAAGAGATCACCCTTGCTAGTGAACCCCAGAAAACAGCTCCAGGACCTCCATATGCTAAACCAAACCCCACCATTGCTCCTACTATACAGTGTGTAGTAGATACTGGCCAACCATAATATGATGCAAACTGTAACCAAGTACCAGCAGCAGCGAGAGAAGAGAGCAAGCCAGCGAAGAGCAGAGTATCCTTCCCATTGAACACATTCGCCAACAGAATCCCCTTCTGCATCGTGCTCGTCACGTGCGTTCCCATCAACAGCGCCCCGGAGAATTCCAGCACCGCCGCCGTCAGCACCGCCTGCCGAAGTGTCAGCGCCCCCGATCCCACCGAGGTCCCCATCGCGTTCGCCACGTCGTTGGCGCCAATGTTCCACGCCATGTAGAATCCGAACAGCAGCGTCGCGTAAGATAGCACCTTCGTCTTCAACGCCAGCCCCTGCCCCAGCGACTTCATCATCAGCGGGAAGCTAAGCACCGCCAACGCCATGCATATCGATATGGCCGTCGCGGTTCCCCTGGACACGTTGAAGGCCTTGGCAAGCCCATCCGCCTCATCGCCGTTGGTCTCTCGCGCTCCCTCCTCCCGCCCCTCCGCCTCCCCTTCCGCGAAAGATGACAGCGTGGCGAAGCGCTTGCCGGCGGTCAGGTGGCTGAAGCGGAGAACGTTGATAGAGGAAGGTTTTGGTGGTATGAACGGTGGCGAGGGAAGGAGAGTCTTGAAACGGTTTGAGTGTCGGAGGAAGAAAGGGGAAGTGGTGTGAGAGTTTTGAAGGAAGGGAGTGTGTTTAGTGGAAGCGAAACGACACGAGGGATTCATGTTGGTTAAAAGAAGGAACGAAGAATGATGAGAAAAAGTGTGCGTGTGATTATTCTAAAAGATTGTTTTTGTGGTGGTGGTTGAGGAAGAAGAGGGAGGGGAAGGTGAAAGAGAACATAGCTAGGGAAGTGAGAGTGCGTTTGGAAGAAGCTTGGAGGGAAATGAGTGGTGATCGGTGATTCTCTGTTGCGTTCTCATCAAACACATATCTCTGTAGCTATAAAG

>C100769_65

CTAGAATACACTTTTTCTGAGGAAAAAAGAAAAGAAAAGCTGTGAATAATAACGCAAAGTTCAAAGCAAACAAAAGTAGTTGATCGACCGCTCTTTGATAACTCCGCCGCTTTCTTCTACATTCTCACTGTTCCTAACTTCCAACGGACTCGTCGAGGTTTCATTCTCACTCATATACTCTTCTACTCTTTCCCTCTCTCAGATCTCATTCTCTCTCTCCTTCTCCACTTCACCATTTCCGCTTCTCTCACTCTCCAAAATGCAATCCTCGCTCTCTCTCTCGTTCTCCGCCACCTCCTCCGCCGCCGCCACCGTCTCCAGATCACGCCACTCCTTCCACTTCCCCGCCGCCACGCCTCTCAATCTCCGTTTCTGCGGCTTGCGGCGTGAGGCATTTGATTCAGGTCTCGCTGCTTCTCTCAACCGTCACCACTCCCACCTCCCTCGCCGCCCTCACTCCGCCTCAGTCTCGGCTGCGCTCTCCGCCAATGGCAGTCCTCCCAAATCGTTCGACTACGATTTGCTCATTATCGGCGCTGGGGTTGGCGGCCACGGCGCTGCGCTTCATGCCGTCGAGAAGGGTCTGAAAACCGCTATTGTGGAAGGAGATGTGGTGGGAGGGACATGTGTGAACAGAGGCTGCGTTCCTTCGAAAGCTCTTTTGGCAGTGAGTGGTCGTATGCGAGAACTAAAGAGCGATCATCACTTGAAGTCCTTTGGCTTACAGGTATCTGCGGCTGGGTACGACAGACAAGGAGTCGCTGATCATGCTAATAATCTTGCTTCGAAAATCCGTAATAATTTGACCAACTCAATGAAAGCACTTGGAGTAGACATACTTACTGGTTTTGGAACTATATTGGGTCCTCAAAAGGTGAAAGTTGGTTCTTCAAACAAAATAGTAACTGCAAAAGACATCATCATTGCTACTGGTTCTGTTCCTTTTGTACCGAAGGGTATTGAAGTTGATGGGAAGACTGTGATCACCAGTGACCATGCACTCAAACTGGAGTCTGTTCCTGATTGGATAGCAATTGTAGGAAGTGGATATATTGGCCTTGAATTCAGTGATGTCTACACAGCACTTGGAAGTGAGGTTACTTTTGTAGAGGCTTTAGATCAACTTATGCCTGGATTTGATCCTGAAATTAGCAAGTTGGCTCAGAGGGTCCTTATAAATCCCAGGAATATTGATTATCATACTGGAGTTTTTGCATCCAAGATCACACCAGCAAGAGATGGAAAACCTGTCTTGATTGAACTTATTGATGCAAAAACCAAGGAGCCAAAGGACACTTTGGAGGTGGATGCTGCACTAATAGCAACTGGAAGGGCTCCATTCACACAAGGTCTTGGATTGGAGAATATTGATGTGGTAACACAGCGTGGTTTCGTTCCTGTGGATGAGCGCATGCAAGTAATTGATGCAAATGGCAAGCTGGTACCGCATTTGTTCTGTATTGGTGATGCAAATGGCAAGATGATGCTTGCTCATGCTGCCAGTGCGCAAGGAATTTCAGTGGTTGAACAAGTTACTGGAAGAGATCATGTGCTGAATCATTTAAGCATCCCAGCTGCTTGTTTCACTCATCCTGAAATCAGCATGGTTGGATTGACAGAGCCTCAAGCAAGGGAGAAAGGTGAAAAGGAGGGTTTTGAAGTAAGTGTTGCCAAAACTAGTTTTAAAGCAAACACAAAGGCCCTAGCAGAAAACGAAGGAGAGGGAATTGCCAAGTTGATATACAGACCTGACAATGGAGAGATACTGGGAGTTCATATTTTTGGTTTACATGCAGCAGATCTCATCCATGAAGCATCCAATGCAATAGCATTAGGGACACGTATTCAGGACATAAAATTTGCAGTTCATGCACATCCAACTTTGTCCGAGGTTCTTGATGAACTATTCAAATCAGCAAAGGTTAAAGCAAAAGCTTCTAGCCCAGTAAGTGAACCAGTTGCAGTCTAATCTTCCCAAACAAAGTATAAATTTTGAAGAAATTTACTTGTACTAGGGACTTCCAAAATATAATGCATCTTCAGGAGGAGGACAACGCATTCAGAATCCTGTAAAAGAGGGGAACTGTAGGAGGAGGGAGATCAATAATGGCCTGATAATGGATAAGCGCCCAAGATTACATATACTGGTTTCTTTTCTACTTGGTATCCTTAGTGCCTGTCAAAGATGCTTATCTTCATTATTATTGCATTTTTTAAACTAGTTTACTTCATCTAAATTATAGTCAATGAGTTAAACCTTTTGAGTATGGGATTTTTTTTAGAATGCTTCTAGTTTTGCACCGAATACGAATGTATCATCATTAGTTTTCCGTCAAAGATGTTGATGAAATTATGCTCGTTGACACACA

>C100771_65

AAAAAATAACATCAGACGCCGACGACCAACCAAACCAAGCCTATATTGTCTGTCGACTTTTCTCTCTCTCTCTCTCTGGTCTCAGGCACACAAAGTTACTCTCTCTCTCTCTCTCTCTCTGATTCAGCTTCTTCAGCTTCAGCGAAATCTAGTTCTAACCGTAGATCGTGCTTTTGCTTTTGTGCCGAAGAAGCCACTTCTCTACTAGCGGTTCTTCTTCTTCAATTTTATTCGATTCGCTTGTTTCAAAATTCTTTAAGTGAGGGAGAGAATGTCGGGTGGCACGCAGAAGAGTCTGAGAAAGGCACTCGGGGCACTTAAGGACACGACAACAGTCTCATTGGCTAAGGTCAACAGTGATTACAAGGAATTGGACATTTCTATAGTTAGAGCTACAAATCATGTTGAACGCCCAGCGAAGGAAAAGCATATAAAAGCTATATTTGCAGCTATCTCAGCTACAAGACCTAGGGCAGATGTTGCCTATTGCATCCACGCTCTTGCCAGGCGATTGTCAAAGACACATAACTGGGCAGTTGCGTTGAAAACTTTAATTGTTATTCACCGTGCTCTAAGGGAAGTGGATCCTACATTTCATGAAGAACTCATTAATTATGGGAGAAGTAGAAGTCATATGCTCAATATGGCTCATTTTAAAGATGATTCCAGTCCAAATGCATGGGATTATTCTGCATGGGTGCGCACTTATGCATTATTTTTGGAGGAAAGGCTGGAATGTTTTCGTGTGTTGAAGTATGACATTGAGGCAGACCGCCCTAGGACCAAAGATTTGGAGACTGCAGAACTATTAGAGCAGTTGCCCGCCTTACAACAACTTCTCAATCGGGTTATTGGTTGCCAGCCTCACGGGGCAGCTGTAAATAACTTTGTCATTCAGTTAGCACTCTCAATGGTTGCTTCAGAGAGCATTAAAATTTATCAAGCTATTAGTGATGGCACAGTCAATATGGTTGACAAGTTTTTTGAGATGCAACGTCATGATGCTCTGAAAGCTCTTGATATATACAGGAGGGTTGGGCAGCAGGCTGAGAGATTGTCAGAGTTTTATGAAATATGCCGTAATCTTGATATTGGACGTGGAGAGAAGTTTATTAAAGTTGAGCAGCCCCCTTCATCATTTCTGCAAGCAATGGACGAGTATGTCAAAGACGCTCCCCAGGGACTGATAGTACGCAAGGATCAGGCTATTGAAAACAAAGAAGTTTTAGCTATTGAATACAAAAAGACAACAGAAGTGCAAGAGGAACGCCCACCCTCACCTTCACCCTCACCTCCACCTCCTCCTTCTGAACCTGTGAAAGTGGAAGCACCACCAGTACAACCACCACCTGATCTGTTGAATTTGGAGGATCCAGTTCCAGCTGCTTTAGAGTTGGAAGAGAAGAATGCATTGGCTTTAGCCATTGTTCCCATTGCTGTAGAACAACAACCTTCTGCTGTTTCAAATCCGGCAAATGGAACTACAGGATGGGAATTAGCTCTTGTTACCGCTCCTAGTTCAAACGAGAATGCTACGGCAGCTAGCAAGCTGGCTGGAGGTTTGGACAAGCTTACTCTAGACAGCCTTTACGACGATGCACTGAGGAGAAACAACCAAAATGTCAGCTATAACCCATGGGAGTCAGCATCAGTGGGAAATGCGATGCAACCAACAATGCACGATCCATTTTTTGCTTCTAATGCAGTTGCTGCTCCACATTCTGTTCAGATGGCTGCTATGTCCAACCAACAGCAAGCTTTCATGTATCAGCAGCAGCAGCAAATGATGATGATGGCCCCTCAACAACAATCAATAAATCCTTTCGGAAATACTTATGGAGCCACTGTTCACCCCTATGGCTCAGGTATGCCTGTGCAATCATACAATCCATATACAGGTCTCATCTAGGCATTTCCTGTGGAAAAAAAAAAACAAATCCTTAATAAGCTCAAGGACTCAAGGAAGAAAAGAAATGGATACTCTGATTACTCTCAGCTTTCGGTTCTGAGATTGGGAAAGATGAAGAATAGACTTGTATCATACGTTGTGTGTGTGGCATAGTTAGCTAGAGTGAAAATTCTTTTTGAATAATTAGCAGAGGGAGGGTTTTGATTATATGATTTTTTATGTACAAGGTAAATTGATAATGAGCTTTGATTTAAACCACTTAGTTTCTTTTGTCCCTGATTGATCACGTCAACGCTTTAGCAGAAAATTTGTTCTTGATATATTGGACTACATTGTTCTCATTGCCATTTCCTGTATACGGTGGGAAGTATATTCACTATGTGATTGTTATACAATTTGTTCAATGAAATTATGTCAACTCTAATAAAATGTATCATTTTGGTTTGAGGAACAAAAAAAA

>C100773_65

ATTCATTCGTGCATGTCAACCAGTATCAGCATCACCTTCCTAAACTCAACGACTCTGTTTCGTTCTCTTTCCTCTTTCTCGAAACCCTAATCAGCTTCTTCACTGCCTCTGCACTTCCCTCTGATTCCACTTCTGCTTCAACCAAAAAGATACTGTAGGGCCTGGGCCACCTAATGATAAAATTTCCGATTTCTACATAAGTTGCTGTTGGTATTTTTTTAATCAAATGGATTCAGTGCCTGCTGATGTGTCTAATTCTCATCCTATCAAAGATCCACCCACTTCTTCTCCTGCTAAAGAGGGTGATCGGCAAGCTTCTGCTGCTTTAGTTTCTGAGCAAGATAATTCGGCACACGAGGCACCACCTGTGCTGTCTCCAACTGGCATATCGTCTTGGGCCAGAAACTTGAAAATTTCTCAGTCATTTTCTGGCTCCCAAGATGACTCATCAAGTGGAAATGTTGGGAAATCAACTTTTGCACGCATCACTAGTAATTTGGGATTGAAGTTGTCTCCAAAGTCTTCTGTAGCAGATGACAGTTCCAATGAATCGGCCGTACAATCTAATTTGTTTGGAACCATTACGAAAGGCCTGGTTGACACTTCTAAGAATGCTGTGAAAGCTGTGCAGGTTAAGGCACGCCATGTTGTTTCCCAGAATAAACGCAGATACCAGGAAGGCGGTTTTGATTTAGATATGACATATATCACTGAAAACATCATTGCAATGGGATTCCCTGCTGGTGACATGAGCTCTGGATTTTTTGGCTATGTTGAAGGATTTTACAGAAATCATATGGAAGAAGTGATTAAGTTTTTTGAAACTCACCATAAGGGTAAATACAAAGTTTATAATCTTTGTTCTGAGCGGTTGTATGATGCATCATTGTTTGAGGGGAAGGTGGCTAGCTTCCCTTTTGATGACCACAATTGCCCACCAATTCAACTGATTATATCTTTCTGTCAAAGTGCTTACTCATGGTTAAAACAAGACATTGAGAATGTGGTGGTTGTCCACTGTAAGGCTGGAATGGCAAGGACAGGGTTGATGATTTCTAGTCTTCTATTATTCTTAAAGTTCTTCCCAACTGCTGAGGAATCGATGGATTACTATAATCAGAAAAGATGTGTTGATGGAAAGGGACTTGTTCTTCCTAGTCAGATTAGGTATGTCAAATATTTTGAACGAGTCTTAACTTACTTCAATGGTGAAAACCCTCCTGCCCGCAGGTGCATGCTTAGGGGATTCCGTCTTCACAGATGCCCTTACTGGATTAGGCCCTCTATAACTGTCTCAGATCATAGTGGTGTGTTGTTCTCAACCAAAAAGCATCCGAGAACCAAGGATCTTTTGCCAGAAGATTTTTGGTTCAGTGCACCAAAGAAGGGAGTGATGGTCTTTGCTTTGCCAGGAGAGCCTGGTCTTACAGAGTTGGCTGGGGACATCAAAATTCATTTTCACGATCGCCAAGGAGATTTTTACTGTTGGTTGAACACAACTATGACAGAAAATAGAAAAGTATTGAACACCGGTGATCTTGATGGCTTTGACAAGAGAAAATTGCCTTCTCCGGGATTTATGGTTGAGGTTGTGCTCGTGGATTATAATGGTAATGTTGTAACTTCGCAACCTGAAACTACTACAACGAAATCAGATGAGAGTTCAAGTAACAGTCCTGCTCGTCCTGCCCCAGTGGAAAGGAGCAGCCCTGCACAGAATGCAGACAAAGAATCAGACAGTCATAATAATGATAAAGATGATGTCTTTTCAGATGGCGAGGCAGAGCAATCTGCATCTTCAAAAACCAAGCAAGCAAAAGCACCTTCTGAAGCTGTTGAAACAGTTAAAAATGACAACAGAGGCTCCGACAACAGAATTTCAAATCAAATTGCAAATTTATCGCATGCAACAGAACAAGTTTCCTTGGGAAACAAGAGCTCCACGTCAATTCATCACGCTAGTGAGCCAAGAAGTAATGTTGACGGAAAAACAGTGTCCAGTATCGAGGTGCCCAGCGCAGAAAGTGAGTTTAAGGCCATGGCTGCAGATGCTTCTGTTTTTACCTTTGGAGATGATGAAGACTACGAAAGTGATTGAACTACAACAACAGGGATTACTTTGTTATCTTTATACTTAAATATTTGTACATAAAATTGCTTGCAACTTACAACAATATTTTGGTCATCCATTTGTTCGTTTGTTTGTTGTAATGATAAATCTGCTACTTCATTGGCTTAATTCACAAGTTACAAATAAGATTATGTAGTTGGCCCCCTCCCCCTAGTGCCACAGTCTGTATTGGATACAGCGTCCAATTTAATTTGGAGTCTTATTGCATTTCACTTCTACCTCATTTTTCTTATCTCTCT

>C10077_65

TGCAGGATGGGTATCACCCTGAGTAACCACCAGGTGGGTGGATGCATCATGGGCTGATGAATTCCAATACATTCAGTTCACAGTAGCAGTCTCCATTTGACCAGGCTCACCTGCTTGCAAGAGGTGAAAATCTCCCCAAATCATCAGCATCAACACTTGTTGCCACACCACCAC

>C100777_65

CACACTCAACAAGTGTTACTAGATCTATAAACACATTGTTCTAGAGAAATATTTGTCAGCACAAAACTTCGAAATATTTGTCAGATTCGGGTCCCCCCTCCCTTTTATTTCTTTAGGAGAAGATAATATTTCCAAGATGAAACCAATGTTTTACACAATTATTTCCATTATAAATGTATAAGAACAATAGAGCGATGTTGGCATCGATCACGAGAGCCATGGATGCAACAACAAAGGTCACACTGTTCACCGTCATGCTCTTTTGGAGCTGCGTAGTACAATGCACTCGTCCTCACCCACTTGACCCTCTAACCACTTCTGAGCTCAACCTTGTTCGAACCATCGTCCAAAACGCATACTCAACCTCAAAAACCACCAACCACAACCTCATCTTCAACTACGTAGGTTTGGACGAACCCGACAAGTCCGAAATCCTCTCATGGCTATCCTCAAACCCCAAAACCAAACCATCTTCTCCACCACCTCGTCGAGCCTTCGTCATAGTTCGGTTCCAAAAACAGTCCCACGAAATCAGAGTGGACGTGTCCACACGTTCCATCGTCTCAAAGAATGTCTACGAAGGAAATGGGTACCCTATGCTGACCTTCGGCGAACAAACCGTGGCGTCACAGCTTCTATTCAGCTACGAACCGTTTAAGGAGTCTTTGAGGAAGAGGAGTCTGAACACGTCTCAGGTTGTTTGTGCTTCATTCACTATTGGTTGGTTTGGAGAAGCCACGACCAAAAGGAACGTGAAGGTTAAGTGTTACTACACGAAGGGCACTGCAAATTTGTACGCAAGGCCTTTGGAAGGGGTGGCTGCGGTGGTTGACTTGGATGACATGAAAATTGTTGGTTACAATGATAGATACGTGATTCCAGTGCCAAAAGCAGAGGGTACTGAGTACCGAGCTTCTAAACTGAAGCCACCCTTCGGTCCAAAGCTTAAGGGAATAGCTGTCACGCAACAGGGTGGACCTGGATTCACCATTGAAGGTCATTCCGTAAGTTGGGCCAATTGGGATTTTCACGTAGGATTTGACATTAAGACTGGTCCAATAATTTCTCTAGCTTCAATTTATGATCTCCAAAAGCAGAAACGTCGTCGAGTACTATACAGAGGATTCATATCAGAGTTGTTTGTGCCATACCAAGATCCAACTGAGGAATGGTATTACACAACATACTTTGACTGTGGTGAATATGGCTTTGGACAGTCTATGTCTTCACTACAACCTCTTACTGATTGTCCTCCCAATGCTGCATTCATAGATGCATACTATGCTAGTTCAGATGGAACCCCTGTAAAGATACCTAATGCCTTCTGCATATTTGAAAAATATGCTGGTGATATCATGTGGCGTCACACTGAAATTTCAATCCCAAATGAAGTGATAACTGAAGTCAGGTCAGACGTAAGCCTAGTGGTGAGAATAGTTTCAACTGTGGGCAACTACGACTATGTTATAGATTGGGAATTTAAGCCAAGTGGAAGCATCAAAATTGGGGTTGGACTTACTGGGATATTAGGGCTTAAGGCAGGGACTTACACCCACACAGATCAAATAAAAGAGGACATCTACGGCACACTGCTAGCAGATAATACCATTGGTATCTACCATGATCACTTCTTCGCCTACTATCTTGACCTTGACATTGATGGTGAAGTCAACTCCTTTGTGAAGTCTAACTTAGAGATGGTGAAAGTGAAAGATCAGAACATACCAAGGAAAAGTCGTTGGACAGTTGTAAGTGAAACAGCAAAAACAGAAGCTGATGCGAGAATCAACCTGGGTTTAAAGCCTTCAGAGCTACTTGTGGTAAATCCTAATAGAAGGACAAAACAAGGAAATAGAATAGGGTACCGTTTGCTTCCAGGCCCTGTGGCGCACCCACTTTTGCTTCATGATGATTATCCACAAATCAGAGGTGCCTTCACCAATTACAATGTATGGGTCACACCATACAACAAATCTGAGAAATGGGTAGCAGGGTCATATGTTGATCGTAGCAGAGGTGATGACACTATTGCTGTTTGGAGTTCCAGGGATAGGGAGATTGAGAAGAAGGACATAGTATTGTGGTACATGATGGGATTTCATCATGTTCCTTCCCAAGAGGATTTTCCAGTTATGCCAACATTGAGTGGAGGGTTTGAACTAAGGCCAACCAACTTCTTCGAGAGCAACCCCGTCCTTAAAACAAAATCACCCAAACCTCTGCATTTCCCTAACTCCACTTCTCAACATTAATTGCTTCCTATGCATTTCATTTCACTGAAACAGTCCAACCAAATTTCAAACATTCATGTGTTGTCGTATATTATATGTAGTTTCTTCGTTTTCTACGATTCAGCTAATAAAGAAAAAAT

>C100779_65

CGAAAAATGAAAAGGTTCAGATAATTTTTGTGTTCCCCGCCGGCAGCAGCACGTATTCAGGCCACTTGCCATAACCGCTCAATCCAAACCTTCTCACATTTTTACAAACATCTCAACCCCAATGCTAATAGCTTTGTATTTATCATACAAATTTCCTTAGATTCGTCTCTGTTGCTTCTCCCATTTCTTTGCCATGATGAACTCAGTTGCCCTTATTCGCAATCGCGCCATGAAATCCGTTTCCAGGATTCTCATCGGTTCTCGAAAATCATCGGCTTATGGGTCCACACCTGCAAATTCTGATCTCACTCCTTCCATTGCTAACAATTCGTTGAAACCTCGTTTCAATCATGACCACGCCAATCACCACCCTTGGCAAATGCATCGCATAAAGGGTATTGCTCAGAAAGTTTTTGGATTGCCCTCTTTGAATTTCGCCCCTTTATCAATGCCCTTTAGTTCAATCACGTCGAACTGGGACATCTCCACCGTCAAGGTTCGAAATTGCTCAACCTCTGTGGAGACCCGTGGCCATGACAACAATTTTGAGAGGATTTACGTACAGGGTGGCATGAATAACGTGAAGCCATTGGTGGTGGAAAGTGTTCATGAAGATATTGCGGGTGATAGAAATTTAGGTGGTGGTGTGAATGTGAAGAATAAACGGGAGGAGTCAGAGGTGGAAAAACAAGCATGGAAGTTGTTACAGGAAGCTGTTGTGACTTATTGTGGCAACCCTGTGGGGACTGTGGCTGCAAATGATCCTGGTGACAAGCTACCCTTGAATTATGATCAGGTCTTTCTTAGGGATTTTATCCCTTCAGCACTTGCTTTCTTGCTTAGAGGAGAAAGTGAGATTGTCAAGAACTTTCTTCTTCACACCTTGCAGTTGCAGAGTTGGGAGAAAACAGTGGACTGCTACAGTCCAGGGCAGGGCTTGATGCCTGCAAGTTTCAAAGTTAGAACTGTAGCTCTTGACGGAGATAATCATGAAGAAGTTTTAGATCCTGATTTTGGGGAATCAGCTATAGGTCGCGTTGCACCTGTAGATTCAGGATTGTGGTGGATCATCCTCTTGAGGGCTTATGGGAAGCTCACTGGAGATTGTGGCTTGCAAGAAAGGGCGGATGTTCAAACAGGCTTAAAAATGATCCTTAACTTGTGTTTAACAGATGGTTTCGATATGTTTCCTTCTCTGCTAGTCACTGATGGTTCTTGCATGATAGACAGGAGGATGGGCATTCATGGTCACCCCCTTGAGATCCAAGCGTTATTTTACTCAGCTCTTCGCTGCTCACGTGAGATGCTTGTTGTAACTGAGGGAACCAATGATCTTATTAGAGCTATTAACAACAGACTCAGTGCTCTGTCATTCCACATTAGAGAATATTATTGGGTGGACATGAAAAAGATGAATGAAATATACAGGTATAAAACAGAAGAATACTCCATGGATGCCATCAATAAATTCAACATCTATCCAGAGCAAATTCCATTGTGGTTAATCGATTGGATTCCAGACGAAGGTGGATATCTGATTGGGAATTTGCAGCCAGCTCACATGGACTTCAGGTTTTTCACACTTGGAAATCTTTGGTCCATTGTCTCATCATTGGGCACCCCAAGACAGAACCAGGCTATTTTAAATCTGATAGAAGCAAAATGGGATGATCTTGTGGGCCATATGCCTCTTAAGATATGTTATCCTGCCTTGGATAATGAAGAATGGCGCATAACTACTGGCTGTGACCCAAAGAATACCCCTTGGTCATATCACAATGGTGGATCTTGGCCAACCCTTCTGTGGCAGTTCACGCTGGCATGCATCAAAATGGGGAGAATTGAACTTGCCCAGAAAGCAATTGCTACGGCTGAGAAAAGACTGCCAGTTGATTCTTGGCCTGAATACTATGATACACGCACTGGAAAATTTATTGGAAAACAAGCCCGGATGTATCAAACATGGACCATAGCTGGTTTCCTTACATCCAAGATGCTTTTGAAAGAGCCTGAGATGGCATCCAGGTTATTCTGGGAGGAGGATTATGAGCTTCTAGACATATGTGTTTGTGGACTTAGCAAGAATGGCAGAAAAAGGTGTTCTCGTGGTGCTGCGAGGTCTCATATTCTAGTATGATGAATACCTAAATTCAGAGTTCAGGGGATAATATATTTATTTTATGCGTTTGGTAGGTTATAGTAACAGTTAAATCGGAAGGTTAACGTCTTCTCCATTGGTGTAATTATGGTAAGAAGGTTTTCCTTCGTTCTAGGGTGGAAATTCTCATTTTTTCTAACAATGAGGGGATAGAAGGTTAGAAATATCCTCTTGTTTGTATACGAGCAAATATTCACATTGTTTTATCTCACA

>C100781_65

TGGTTATATAAATTGAAGAGAAATAATTTAAAAAAGAATAGTAAATTTCAGGATGTGTTTACATTGTAAGAAATGAGGGGAACATCTCTGGCTTCAACACTTTACATCTTGGCACTTCAAGTGATAAGTAGACCTGCAAGTATCATTATATTATGTTTTAGCCATATCGTACAACGTGAATGTCACACTATGTAGCAGTAGCAGCTGCCGTAGAGTTTGATTGCACCCAAGAGACTAAATCCTCTCTAGTTGCAGTAAAACAGCCAACTTTTTCCTCACTCTTCCTAAATGGAACACCTAGCTTCCTCAAATGAGAGGCAAAAGAAATATTGAGTCCTTGGGCAAACTTATGAGCACCAGTACCAGTTTGGGCTAGGAATAACTCTGGCAGTGTCTCTTCCCGCTTAACAATTTTGGTGAGTGTCCACATCCATTCTTCAAGTGCAGTTAGAGCGGCACCAACTGATAATGATCTAACGTCTAAGCACCATTCTTCATCAGTTTTGTTGTGTAAACTAGGATATAATCCAAACAAGGTTCCCAAGTAGAGTAGCTCATGGGCTCTCTCTAGAAGATCTTTGTTTCTACATATGTCAATCAAGCAGTTACAGAAGGGTCTTCTCACTTCAAGCGCTGCGCTGCTCATGATACCCTTGAACTCTTCCTTGACAGTCTCAAAACTAGTTTTTTCATCTTCAATCAATTGAATAAAGGCAACCAATTTTGGGTTAGCTTGCTGCAAACAACCAAGGACTTTTTCCTCGTCCTTGCTACCCTGAGAAAGAGATACAACAGATAGCAAGCAACCACATAGCCTATCATCTGGTTTAATTCCTCGCTCAACCGAAGTATCAAAAACTCGTACCAAACCATCAAATTCCATAGCCCTTCCCAAGCACTGAATCAAACAGGTAAACCCCATCACATTGAGTTCAACATCCGACTTGCACATCTCTTCAAACAATTTCATTGCCTTGTCGACATCACCTTGACTCCCGTATATGTTGAGCATCGCCGTGTAACTCCAACTGTCTGGCTTGCAATGTGAAGACTGCTTCATGTCCCTGAACAAAGTTTCAGCTTCCTCCACCAGTCCAACATCCGCACACATATTCAACAATGTATTATACAAGATAAAATCCATAGGCCAGCCATTTTCCTTCATCCGTTCCCACAACTCCAAAGCATCTCTAGACCACCTGGCCTTGCCATATATCTTAATAACTGCCGTCAGAGTCTTCTCATTCGGCACAATCCCTGACTCAATCATTTCTTCAAACAGGCCCCTTGCGAACCCTGGCTTCCCAGCCTTTCCCATTGCCTCCAACAAGGTGTTATACACAACCAAATTAGGCTGCACCCCAACAGACTCCATCTCCTGAAACACGTACCTAATGCCATCATAGTCCCCAGCCTCCCCAAACATCTTCCCCAACACAGAGAATGTGATGGGATCAGGCTTCCACCCAGTTGCTCTACCTCTCTCATACAAACTTATCACCTCTTCAACCTTGCCTAACCTAGCATAAACATCTAAAATACCAGAGTAAGTTACCTCATCAGGCATCAAACCAGTCTTGTACATCCTCTCAAACCAGTGCACAGCCTTGTCATAGAGGTTACACTTCTTGGCACAGGAAATGATGGTGGAGTAAGTGATGTTGTCCAGCGGAACCCCAGTGTCGATCATCTGGTGCGCAAGTTCTTCTATCAGCGCAAACTGTTTCCCGAACCGCAGAGACTTCATTGTGACATTGTAGAAGATGGTTTCCATGGGAAGCAAATTCTGGGCCTTGATCCAATTCAGGAAAAGACAAGTCTTTTGCCATGGCTTCAAGGTGTTGAGTATCAACAACACGTTTTCCCTAGTAAGGGGGCGAGGAATATCCTCCAAAGAAGGCAAGAATTCATCTTCAGAGTAACCACACTCATTCAGTTTGTGAGCAAAGCGTTTCAAATCTCTCAACTGTGGACTGTATGAGTAAGTAGCGCGTTTTTGTCTCTCAGAGGACAACACAGTGCGCTTTGCTTTGGTGGGGTTGACCCATGTGGATTTCGGCTTAGAAAACGCATAGGATTGGTCTCCTGTGACGGTAGATAGGGTTTTGTTGGCAAGGGGTGCGAGTTGGTCGGATAAGGAAGTGTTCTTCTTCTTGTTCTTGTTGTTGTCTGAAGTTGGCGTGGGAGGAGCTTCTGGAGTGGAATTAGAAGTAGCACAGTGTATGAAACATTTTCTAATGTTTTTTTGCCTACAAGTGGTGGTGATGCTGAATCTTGTTCTTTTGGTGTCTGTGAAGTACACATCCACAACGGTAGACAGTGGAGCAGCCATTGGAGTGCTTTGGCTGGTAACCAGTTCCAGGATATTTTTT

>C100783_65

TTTTCTCTCTTTCTCTCTCTCAGCTCAACGATTCCTCCCAAGCCCAACTCCTTTTTTTTGATCGGGCTATGACCAAGGACTTGTGTTAAAGAGCAATTTTTTTTTTACCATTCTTAGGTAAGCAATGGTTGGTGCATCATGGTTCCGAACTCTATGGAAGACTCAACGGAAGGATGATGCTCATTCTGAGAAGGCGGTGCTTGGAGTATTAGCATTTGAAGTTGCAAGCTTGATGTCAAAGCTTGTTAACTTGTGGCAATCTTTGAGTGATAAACAGGTTGCTAAGTTGAGAGAAGAGATAACAAATTCAGTGGGTATCAGAAAGCTTGTTTCAGAAGATGAGAATTTCATTGTACGTTTAATCAGTCTAGAGATGCTTGAGAATATGGCACATGTGGCTGAATCTGTGGCTAGGCTAGGGAAGAAATGCAGTGATCAAAGTTTGAAAGGTTTTGAGAATGCCTTTGATGAATTTATCACTTATGGTTTTGATCCATATCGGTGGGGATTCACTTCCAAGAAAATGGAAAAAAAGGTAAAAAGGATGGAAAAGTTCATATCAACTAATGCAACTTTGTATCAAGAGATGGAACTGCTTGCTGACCTCGAGCAAACTCTTGGAAGAATGAAGGCTTATACTGAGTCAGACGGACGAAATTTAATTGACTATCAAAAGAAGGTTGCGTGGAAGAGGCTGGAGGTTAAGAACTTAAAGGCTAGTTCTCTGTGGGACAGAACATATGATTACACGGTAAACATTTTAGCAAGATCTTTATTTACAATATTCCGTAGAATCAACAATGTATTTGGCATTCAAGAGATAATAGATGTTGGTAAAACCAAGAATCGAAGTGCTTTGAATTCTGATAATGCTAATGGCAGTCGATCAGTTTCTGAATTATTGCATCCTTCAGTCCAACCCTCCAGCAAAGTTCGTGCCACGTTTGCTTCAGGACCCCTTGGTGCTTTTGCTGCTACATCTCGTCCAAATGCTCGAACTAATAAAGCTAGCATGCTTCCTTCAGATGCTGGAGACTCGTCCACAAAGTCAGGCCTAATTTCAGCAAAGAATAGAACTGTCAAACTTTTCTCAGGTCCTCTTGGAAGAAATTCAAAAAAGTCTGCCCCTGATAATGGAACAAATAAAAACAGCAAGATTTGGAACTTTCATGGCCACTCAACTGCTGCAAGTGGGAAGGAAATTAACGCAAGACATAGTCGACTGACTCAAGTAGAACCTTTCAAAGGATCTATACTAGCTGACAGTTCTTTGGTCATTGATAGCCACTCAAGCCTAAATGATGTTCATTTAGCATCACAGAATCCCAATGACCCTAAGGCAAACCTTGTTACTCCTGGAAAAGAGGTTCATCGCCCTCAATCAACTTTCCGTTACCTATGCAGGTTAAAGCCTCCATCTGAATCCCTTGGTGCTGCTTCTTTAGCCCTGCACTATGCAAATGTTATCATCATGATTGAGAAGCTAGCAACATCTCCATATTTGATTGGTCTTGATGCAAGAGACGATCTGTACAACATGTTACCGAGACGTGTGAGAGCTGCTATTAGCATCAAGCTAAAGCCATATTCCAAGGCCATGGCTTCAGCAGTGTATGACGCAGGTCTAGCAGAAGAATGGACCGAAGCAATGACGGGTATATTAGAATGGTTAGCCCCGCTTGCTCACAACATGCTAAGATGGCAATCTGAGAGAAGTTATGAGCAACATTGCTTTGTTTCCCGGACAAATGTGCTGCTTGTGCAAACCCTATACTTTGCAAGTCAAGAAAAAACGGAAGCAATAATCACTGAGCTTCTTGTGGGTCTGAATTATGTGTGGAGATATGCTAGGGAGCTCAATAAAAAAGCTCTGCTAGACTGTGGTAATAGTGGGGTAGATAATGGATATTCTCAACTGAATGGATGAGCACTTTTCGTCAAGTTTCATGGCAGGTTTCGTTTTTCAACAAAAGTGCAATCCCTGACAATTGATTTGGGGTGAATCTTTTCAGGGATACCAATCTTCTGGCTTCTTAAATCTCAGTGAATGTGAAACAAGTGCAGTGGACCATGAGAGAAATACCCACAATACACTGGTATTTAGTGTCAGAAATGCATTCTTGGTGAGGAATGTTCATTTTATATGTTCAGTATTATTTTTCTTTGAGGAGATCCTTGCTGATTTAACTTGGATCGCATATCTCATCTTGGAAAAGTTGCTCTATGGGTGCATAGTTATGAGCTTTATATGTATATAGCTTTATCAGCCGGGATATTTCTATATTGATATCTGTTGAATGCTTTAAACTCCTATGTTGATATCTTTTGAACGGTGAGACAAAGATTTCAAAATGCTTTGTATTTATGGTACG

>C100789_65

CAACAAAACAAGCAACAAGATTAAAAAAAAATAAAGAAGAGACTCAAAAGAGTTCTTCATCAACTGAATCTTCATCCTCTTTCTTTCCCCTTTCTTCTCTCTCGCTCTCGCTTCTTCTTCCTTTTCCGTTCTTCACGATCCACTTTCTCTCTCTCTCTCTGACTCGCTTCTTCTTCTTCTTTTTTCTGCTTCTTCTTATCGTGTGTTTGTGTGTGTGGTGCTGATCGCGTCTGTTGATGGTGATCGAGCAAGACATTGAGAGCTGCGGGAGCAGAGCGGTGCAGTCGTCGCACGCTCACCACCCTCGCCACCACCGCCAGAAACTGGAGGTGTACAATGAGGTGCTGCGGCGGATTCAGGATTCCGATTGCGAGGAGGCGAACGTTCCCGGCTTCGATGATCAGCTTTGGCTTCACTTCAATCGCCTTCCCGCTAGGTACGCGTTGGATGTGAATGTGGAGAGGGCAGAAGATGTTCTAGCTCATAAGAGATTGCTCAAGTTGGCGGAGGACCCTGCTTCTCGACCTGCATTTCAAGTTCGCTTAGTACAGGTATATCCTTTTGGTGGTGCAAATTATGCTGATTCCATACATTCAGATCCTTCCGAGATAGAAGATGCACAGAGTTCTCTTAACTATTCTTTGAAGCAGGGAATTCATCCACCACCTACATTTGGTTCATCTTCTAATCTTGAAGCTCTTGCACTTCAAACAACCAAAAATAACACTGAAGATGGGGGAAGTGCTATGAGTGTAACACCATATTTTCCCAGGCCCATGCACGAGATTACCTTTTCAACAGTTGACAAGCCTAAACTCCTTAGTCAGTTAACATCAATACTTGGTGAGATGGGACTGAATATTCAAGAAGCTCATGCATTTTCCACCACTGATGGATTTTCTCTGGATGTCTTTGTTGTTGAGGGATGGCCTAATGAGGAAACTGAGGAGCTGAAAGGTGTCTTAGAAAAGGAAATTTACAAGGTTAAGGAGCAAAATATGTCAAATCAGGGCATACATTATGCTGTCAATGGACATTGCCAGGCAAGGATGGAACCGTCCCCTCATTGCATTCAAATACCATCCGATGGAGCTGATGTCTGGGAACTTGATACCAACCAGCTGAAATATGAAAACAAAGTTGGCTCTGGGTCATTTGGTGACTTGTATAGAGGTACATATTGCAGTCAAGATGTTGCTATCAAAGTTCTCAAGCCTGAACGCATAAATACTGATATGCTGAAGGAGTTTGCACAGGAAGTTTATATCATGAGGAAGATTCGACACAAGAATGTTGTTCAGTTCATCGGTGCATGTACTAGGCCCCCAAATCTTTGTATTGTTACTGAGTTTATGTCTAAGGGAAGCCTATATGACTTTCTGCACAAACAAAGAGGTGTATTTAAGCTTCCATCTTTGCTAAAAGTAGCAATTGATGTTTCCAAGGGAATGAACTATTTGCACCAAAATAATATAATTCACAGGGACCTCAAGACTGCCAATCTTCTGATGGATGAAAATGAAGTGGTCAAGGTTGCTGATTTTGGGGTTGCTAGAGTGCAAACTCAGTCTGGAGTGATGACGGCTGAAACTGGAACATACCGTTGGATGGCTCCTGAGGTCATTGAACACAAACCATATGACCAGAAGGCGGATGTTTTCAGTTTTGGAATAGCTCTCTGGGAGCTTTTAACTGGAGAACTACCTTACTCTTGCTTGACTCCATTACAAGCAGCAGTTGGCGTGGTGCAGAAGAGCCTACGGCCTACAATTCCAAAAGCCACACAACCAAGACTTTCTGAACTGCTTCAGCGGTGCTGGCAACAAGATCCGACTCTCAGACCAGATTTCTCTGAAATAATAGAAATTCTTCAGCACATAGCAAAAGAGGTCAATGATAACAAGGATAAATCATCCCATGGCTTTCTCTCAGCTCTTAGACGAGCCCATCATTGATATTGATATTGATATTAGAGATTACACGACATTATAGCTTTTTTGTCCTGTACAGTAGTCAGACTCTGACCACAAATATCTATGGAGTTGGTTGTTTCTACTCTAGAAAGATTCCGTTGTATTGTGCTTGCCTTAAATTTCTTTTACAGTCGTCGTCTTTCTTCCATTTGTCTACTCAACTAGTTCGCCTGTGAATAGGATGGATACAAGATTAGTGAAGGTCGAAAATTCAAGGTATCATTTACAATTAATTTTTTGCTAATTGTATTCCAAGCTTTTTGACTGGGAGTTTTTTCGATGCACTCCCTTGTATCCAAACCCTCGAAGTTGACTAGTAGAGGCGTTATATGTTGATACTTGAGATGTACCAAGCTTAACTGTAATAAAATCGGCATGTTATGCAATTATATTCCAAGGG

>C100791_65

AAAGAGATATGTTAGTGTAGAAAGAGCAGCACTACTGATAGAATAACCATAACATACAATTCAAGACCAGCTGAAGGTCATATTACAGGTTTCAGTTTCAGATTAAACAAAGAGAAAGTGTGGTTTATTCAAACAAGACATTGCAAGAAAAAAATTAATATGAGCTACAAGATGTTGTATTTAGGTTTGAGACTAAGCATTGATGTTGAATGAATATCAATCCCGTGAGCAAAATCTAGCGAGGGTAGGGCTTAGTAATTGAATCAGTTTGTGATCCTTGATCAGATTTGATTGTCGATTCGTTTGATGTTGTTACCCTTGAACTATCCCCCCATGATAGAGTGGGTGGAAGGCTTCTAGTTGCACTGTTCACATAAAATGCAGGTTCAGAAGGAATGGGGAGACTGAGAGAATAGCTATTAAGCATGAGCATAATGGTCGCCATGGTTGGTCTGTCAGTTAAATTTTCTTGAACACAAAGCAAACCAATATGGATGCATCTCATCATTTCATTTCGTGAATTGTTGTTTAATGATGGATCTACAATGTTTACAGCTGTCCCCTCCCTCCAGTTTCTCCATGTAAAGCTCAGTAGATCCTCTACATTTTCCCCACGACGAATGCCACTGTTTTTCTGGCCACTTACAATCTCAAGAAGCAGTACACCAAAACTGAAGATATCAGATTTCACTGAAAATTGTCCTTGCATTATATACTCCGGTGCCATGTATCCATAGGTTCCGACAACTCTACTTGTATTTGCTTGAGTTTGATCCACTAAAACCAGTCTTGCCATGCCAAAATCTGCTATCTTAGGATTCATCTCTTCGTCTAAGAGAATATTGCTCGCTTTGAGATCCCGATGTATTATACGCAAAAGAGAATCTTCGTGAAGGTAGAGAAGGCCTCGTGCTATTCCTTTAATGATTTTGTAGCGCATTTCCCAATCCAATTGTGCTTTCTTAGCAGCATCAAATATGAAATAATCAAGGCTTTTGTTAGGAACAAATTCATAGACGAGTAACCTTTCTCTTCCTTCCAAGCAGAAACCAAGTAGCCTAACTAAATTTCGGTGCTGAAGCTTGGCCACTAAAAGCACTTCATTCTTAAATTCTGTATCTCCTTGGCTGGATTCTCTTGACAACCTTTTGACTGCAATCATCTGTCCACTGAGGAGCTTACCCCTGTAAACAGCTCCAAATCCACCTTGTCCAAGCTTATTAGAATCAGAGAAGTCTTCTGTGGCAACTCGTATTGTGTCAAAGTCGAATTGCAATGACTGAGCAATTTCAATGTCATCATCTTCATTAACTAGAAGACTTTTTCTTGCCTTCCTCCTCCTTAAATATCTGCCGAGACAAATGAGGGAAACGACAGCAACAACAACAGTAGGAACAACTACGGCAATGACAATCGGTATTGTGTTGCTGCTGCTATCTTTAGGGGAAGTGTTACTGGAGGAGGAAGGAGGTTCTGGAGATGGTGGTGGTACATCAGGGTCTAATTTAGGCGTAGGATCGTAGAAGATTGCGCTTTCGAATCTAATATTACAACTTGGTCTAAGAACTCTACCACCCATCTTGTTCTCACAGCACGATGGGATATCTGAGATAGCCCCATCCAAGCACCTAGTGCAGTCTTTCTCAGACAAATCAGGGGTGCACTGCACAAGGCCATATACTGTTTGAAAATTTGAAGCAACAGCACTACCCGTAGCATACTTACGACGAGAGTCACCTGATGCAGCCACGTCTGTGAGGTTCCTCATTAAGTTGGAGAGTGCTTGGTTGAACTTATCCGCTTCCGATACATTGTTTGTGTTGTGCAAGAAGAAGGTAGGAGAAGATTCCATGACGCCGAATATTGTGGTGGGAGAGTAACGCAACAAGCATTTGCTAGTGTTCAGCCACAGAAGAGACTCTTTCTGGTTTGGACAAAGCTCTGTGATAGTGAGCGCAGAATCGTTGAGGCAACCGCGGCACTCATCTGGCATAACATCTCCTCTGCAGAGCCCAATGGCGTTTACTTTGTCACTGTTTTGACCATATGAGAAGTTGTAGAAACCATAGTTAATATCTGTGTGGGAAGAGAGAGTGGATAAAAGGGTTTTGAGGTTGCTGTGATAGGTACTATTGACTGTGTAGTTCCCTTGGCTGTTATCACAGTACGCGGAGTTATACTGGGCGCTGGCTTGAGATATTAGAAAACAAAGAACGAAAAGAAGCATGCAAGAAATGGCAGCCATATGCATAAGATGAATGAGAATCTATTCATCAAAGACTGCTTCTGTCACTTTATATATAACCAAAATTTCCATCCATCATTTCCACGATGATACCCGTTGTTGTACTTGTGATTCAATATTACGTCTTT

>C100793_65

TAACAGTAAGAAAAGAGAACGGAATTTTCTATGTGAATCTTTGGCAAATGAAAACATGAAATACACAGAAAGGGATCGAAATCACAATGGATAAATTACAGCTATGAAGAAAGGAAAAAATTTACTATTCTAGGGTTGTGCGTTGGGAGGTATCCCAGATTGGTTACCAGACTGGTAAGGATTATACCCTTGCATCAAAGGCATTTGACCACCTTGAGGTCTGTTAAATGAAGAACCTTGAGGAAAATTGGCATTTGATGGGGGGCCCTGCATATTAGGCATGTTGCCTGGAAAACCACCAAGAGGGTGCCCTCCACTTGTAGGCAAGGATCCTGAATGCATTTGATTCATGCCCACATAAGGACCTTGTGGCATTGGAGTACCCATCTGATTCATCGCCCCTTGCATCCCCATTGGACCAGGCATTGGCATTGGATGTGATGTCGGCACTGATGAAATCCCTGGTAGAGATCCTGGCATGCCAATAGGCATTTGGGGAGGAGGGCGAGCTAAATGCTGGTGAGGGACCATGGGCGAATGGGAAGGGTGCTGTAGTTGTGGCATATTTGGAGGAATAGGCAAAGGACCCATCTGCTGTGGTAGAGCCTGGTGTTGATGTTGTGGCGGCATCTGCTGAGGATTCTGTTGGTATGGTTGTTGGTTGGCATTAAGAAGAGAGGGATGTGGACCAGGGGGAAGAGGAGGAGCACCCATGGAAACAGGATGTGGCTGCTTCTGTTCCCCTTGAGGCATGTCAAGAGAGGGAATAGATAAGGGCATTGCAACTCCAACACCTGGAATGGTACCCTCATTTCGAGTTAGACCAGGGGCAAATGGTCCTGGAGTTGTTGGGCCTTCAGCCATTGGAAAATTACCACCCACCCGACCAGCAACAGGATTTGGGTCAGCATATCCTTGCATGCCCGTGTTAAATCTATCACGAGCCGTGTCTCCTGGCCTATTTCTGCACCAAAACTTTGTTGTATGATCACTGCTGCCACTGCACAGAAGGTATCCAATAGGATGCCATGCAAGATCCCACACGTTATTATCATGAGCATTGGCAATTTCAATTTGTGGAGTTTCATGCCCAACAAGCCAGTGGAAAATGGAACCATCATAACTCCCGCTCACAAAGTACTCTTCATGGAATGGATGCCATGCCAAAGCAGTAACATCTTTGCGATGTCCACGAAAAGATTCAAGCTCCTTCATTGCTCTAATGTCGTAAAGCTTTATTATTTGATCCTTCGAAGCTGTTAACACCCAGTTGCCATTTTGATTCCATTTGACACATAGCACAGTATTTTTGTGGCCATGAAATGAGCAAAGCTCTCTCCCTGTTTTAGCATCCCAGAGTTTCACAAGATTGTCCTTCCCACCTGATACTAATAGGGATTTTGTCGGATGCCAGTCAACACTCTTGACATCCCAACCATGGCCAGTTAATGAGCACTCTTCTTGACAGCGTGCAAAATCCCAAACCTTAACTGTGGTATCGTCGGAACATGAACAGAACTTCAAATCTGTCCTACAAAAACTTAAGTCACGGACTGATTCTTTATGAGCAGATTTGTTGGCTTTAACGTTGTTCATGTTGTTCTGCCAATACTTTATTGCGCCTCCATCATCTCCAGAGACCATCCAGTTGTCATTGTGACTCCAGACCATGGACCTGATTGCTTGATCATGAGCCTGAAGAATCATTTCAAAATTAAAAGATTGCCCATTCCAAAGAGTGAATTCCCCAGTCTGTGAGCCAGTAATAAGTCGCCGGCCTGTTGGAGTCCAAAGTACGCGATTAATGGGGCAACGATTTTTATTTAGAGATGTATGGACAAATTTGGCAGCAAAGCTAGTGGATGGGTTGTCTGGATATCCAACTGCTGGCAACATATCAATTGCTGCAGCTGGGGTAGGTTGCAGTACTGTCCTATCCCTTGAATCCCGCTGCCACATACGGATCTGCATATAGCGCACAACTGTGCTTGTATAATCAACTGCTTTCCTTTGAGTAAGCTTACGTATTCTTTTTGCACCATGGCTATCACTGTGAACATCGTAATGAGGAGGAGGGGGTCCTCCGGGGCCGGGGTGGTGAAACTCGGGGGCGAGGTTCGTCGAAGAAGCGGACCCTTGTCGCATCACCGGTGGCTGCGACGGCGGCGGCGGTCCGCGGTGGAATTCCTGACCTGGCGGCTGGTGGTGGAAATGCGGCTGTTGTTGCTGCTGCTGCTGGTACTGGTGCTGCTGGTGCTGCGGGTCATTGTACATCATTGTCACTGGATTGTGCAGAGGGAGAAAACAGCTATGGAAACAATGCAAGAAATTGGATTGCCGGGGAGAGAGGAAAACCCTAAACCCCGAATTTTG

>C100795_65

TAAAATCAAAACTCAGCCACAACTAGTCCATAGTTTTTTCTAGTTAACGCCAATTTGCTTTCTCTCTCCTTTCTGCTGCACCTGCATCTGCACTGCGCCTCCCTTCTCTGCCAAACAACACAACTCTGACTCAACCATCATACAGACTGTGATTCCTCAAAACATCTGTGAACGGTTGTTGGCTGACAATTATTGCATCTAGCAACTCATATTAAACTGGTGACTATGAGTAATCTGCAAAATTTTACTGCTGAGCTCTGTGTCTCCGAATGACCATCGAGTGACCCTGTGTGGTTGGGAGGAACCTTCGCAATTACTGAGATTGAGTACTTGTTACTCTTTTAGTTTTTCTTTGTGTAGTTTCAAGTTCTTTCTAGATGCAAACGTCTCAGAAACACCCAAGTTCGGCTGGTGTACACTTCTATCACCACCAGCCTGTGCAAGGTATTTACCAAGTGTTGCAAAACAATCTAAGCCATGATACTCATGATAGCAGTAGCCAGGGAACTAGCGTTTCTTTTGAAATCTGCAAGGAGCAATACTTTACTCTGGAATCATGCCCTGCACCCACTACTGGTTTCATGGATTGTGATGATTCTCCTTCCTATGCCAGCGTGTCATCTAAAAGGACTCCATTTTCTCCACAAGGTTCTCAGTCATGCTATTCAGATCACCACCAGTCCTCTGACAACACCTATGGATCACCAATAAGCGGTTTGTCTAGCGTTGATGATGGGCATGAACTGAAGCACAAACTTAGAGAACTGGAGATTTCATTGTTGGGGCCTGATGAATCTAACATTGTAGACAGTTTTGGCTGCCTCGTCAAGGGTAGAAGCCTCTACGGAGCATCTCAGTTGGCTAAACATAACTGGGATCAGATTGCTGAAAATATTTCACAATTTGACCTGAAAGAAGTCTTCACGGTATGTGCACGGGCCGTGTCTGATGATGATATTCCAACAGCAAGATGGTGGATGGATAATGTATTGGGGAAAATGGTATCAGTCTCTGGAGATCCAATTCAGAGGTTGGGGGCTTACTTGTTGGAGGGGCTTAGAGCAAGGTTGGAATCATCAGGGAATCTCATCTACAAATCCTTGAAGTGTGAACAACCAACAAGCCAAGAACTAATGAGTTACATGCACATCCTGTACCAGATTTGCCCATACTGGAAATTTGCATACATATCTGCAAATGCTGTCATTATGGAAGCAATGGCTAATGAGTCCAGGATTCATATAGTGGATTTCCAAATAGCGCAGGGCACACAGTGGTGTTTACTTATCCAGGCTCTTGCACATAGGCCTGGGGGCCCCCCTTCCATTCGTGTAACCGGTGTTGATGATTCGCAGTCGTTTCATGCTCGAGGTGGAGGACTTCAGATTGTAGGAGAAAGGTTGTCAGATTTTGCCAGGTCTTGTGGAGTTCCATTTGAATTCCACAGTGCTGCAATGTCTGGCTGTGAGGTTGTGAGAGGAAATGTTGAAATTCGACCCGGGGAAGCTCTGGCTGTAAATTTTCCTTTTGTTTTGCACCACATGGCAGATGAGAGTGTGAGCACTGAGAATCACAGAGATAGATTGTTGAGGTTGGTGAAGAGCTTGTCTCCCAAGGTTGTAACCCTTGTCGAGCAAGAATCGAACACCAACACATCTCCTTTCTTTTACAGGTTTCTTGAGACCCTGGATTATTACACTGCCATGTTTGAATCCATAGACGTGGCTTGCCTCAGAGATGATAAGAAGCGGATTAGCGCAGAGCAGCACTGTGTGGCACGAGACATTGTCAACATGATAGCTTGTGAGGGGGTTGAGAGGGTGGAGAGGCATGAACTTTTCGGAAAGTGGAGGTCAAGACTTTCCATGGCTGGATTTAAACAGTGCCAATTAAGTTCTTCGGTGATGGGTGCTACCCAAAATTTCTTAAAGGACTTCAGTCAAAATTATAGGCTTGAACATAGAGACGGTGCCCTTTATCTTGGTTGGATGAACAGACATATGGCCACCTCTTCTGCTTGGAGGTGAGGTGAAACTGACTGATGAGTACTTTGGCCTCCTTTTTGACCTTTAATTGAGTATAATTCACCATATGTCCTTAGTTTTTCCATAGGATTTGAGATTTACCTCTGTTCTATATACTGCATTCTGCAGAATCTCAAAGCATTGTTGTAAATGCCAATACTGAAATGATAGAAAGAAAAAAAATATCGTCTTCAATGACTTTAGTGTAATTTTCCTTGCCTCTTCTGCGGGCTTTAGTGTTATTTAACTAATGTCAGATGAGCTAGAAACTCTAATCTTGTTTACTCGCTTTAATTCCTTGTAATGACATTCAGGTCAAGGTTTTGCTCCACTGACTATTCTATCAACC

>C100797_65

TCCTTTCTCTCAATTTCTTCTCTAACAAGTCCACCATTAAACAACAAATAAGTATGATTAGAAAAACAAGGATGCATCATGTAAATGATCATGCATTCTAATAAAGTGACAAGTCTAGCGGTCTATTACCGCTTTCCGTTAGCAGTCTCAGTTTTCCAGTAGTTCAATCTTTCTAATCCGTCCTAACTTTCAATTCATTCTATCATTGGAAGTCTTACCAAGCGAAGGGACATTGCTAAGGCACCTCTGATCACAGCACGTCGAAATCATGCCATCCAATTCATTATTGAAAGCCTTATCACGTCGAAAGCATGCCAGCAAGCTAGTGGTATTAGCGCCTTACCCTTAAGTGAGTAAGAGCTTCTTCCTTGCTTTTGCTCTTTCTTTTTTATATTGATCTGAATTGGATTGATTGAGGGATAGAGTTTGCAAGGCTCACAACTGGATTTCAAATGCCCTACCAATGAATAGATTAGTCTACTCGACAAAAAAATCCTGTTGGTTGAAGGGCCTGGCCTTTGACGCTGCTTCAGGAGAACTTCTTTCATCCACCCTAACAGGTCACCTCACTGGTAGCATTCCCGACACCTGTTCATTGATCTCATATCTATAAGCAGGGGATCTGCGTTCACTATTACGCCTACGCCCGTCCATTCCAAGCTTGATCCCGCCCTTGTACTCGTTACGCTTGTATGGTGTATTGATGTTCCTCTACTGAATTTTGGAACCAGACTGGAGACCTGCTTGCACTTTGCTTTGGAAGTGGGATTCTAACCTTGTAGCACATTTGTAATAAATGGTTTCTGGAGAATTATAGGTGCGCGCATTCGCAAACATTCTTCTGACATCCGCTACAAACATCTCAAATGTTACATAATATAGTTCTGACTCCACCCTCTTAGACATTGTCTTCAAATCCATTGGATCTTTGATGATGTCATAATAATCAGGAACATCCCGGGCATCAACTGGTTCCTTAAATGGCCAAGCATCGGCATGATCGTGCATTGACTTTAGAAGTGATCGCATAAAACCAGACAAATGTTTTTGATTAGTAGCATTTTCAGTAGAAACATTTAGAGTTCTGAATCTTGAATGACCCCACTGATCAGGAGTCCATCCAGCCTCTCTCAAACCAGGGATGTCATCAATAATTCTTTTAGGAATACCAGCTTCTTTCTTCTGAAAATCAATTCCAGGATAGACTATGTGACAGTTTGACAGCTCTCTGATTTTTTCATCAATAGCCTGTCTTTGGCGACGTATCATAGTTGACAAATCAGTGTAAGGGAGCTTTGGATCTATCTTGCATTCCATGAGAATTCCTCCATCATAATCCTTTATATAACCTTGCCATCGATCTTTCTCCAAGTGAATCTCTTTTGTAAATCCCTGTTTGATAAAATAGCCAACAGCATTGTTGTCAGCATATGTGAGAAAATGTGTCAGCCCATCCACATCCCGTGCATACTGTTTTAAGTGATTCATCAATCTGGTACCATAACCCTTTACCTGCTCGTCAGCTGTAATTGCACAAAAGGCTATCTCACCAAACTTCTGGCTAGCATATGGGCGATATGTAATGCCTCCAACAACGTGGTTACGCCTTATAACCATTACAGACTTATGGCTCCTATCCATAACAAGCCGGACAATGTACTCCTTTGGCATATTGGGAAGTTGTCTAGCAAATATATTCTTCAATCCAATCAACCAAACCATATGCTCATCAACACCATCGTTTGAATGGCATACGAATTTAAGCCTTCCTGCTTCCTCCTCCTTCTTCAGACTCTCTTCCCTAGCTGTGTAAGCCCCACTCGTTTGCAAATTATCAGTAAAAATCTTAGTAGGGTCCTCCTTAACCACAATGCCAGGGACAGATCCAACGGCTTCGGCGGCACCCGCTCCGGCGGTTCCGGCGTCTTTTGCGCCGTCCGAGTTCTCAATTTTGACGGCTGAGTTTTCAGTCTTGAGCTTGGTATTGCGGGGAGCAGAGGGAGGGTTATTGAGTCTGGAGGCGGTAAAGGTGCGCATGGAGGAGTCATTGTCGTAGTCGTCCTCGTCATCGTCGACCACGGCGTCCTCGGAGTCGTCGTCGGAGTCGGAGTCGGCGCCGCGGGCGGATATGCTCTCCAGGTCGTCATTGGAGGTGAGAGCGCCGTCGCGGGTGTCGGCGGAGAAGGAGGAGGGCGGGAAGGGAGGCGCGTGGTCCTCAGACGCTCGCTTGCGCTTGTGGATAGAGGAGGTGGCGGAGGCTGAGGCGGAGTGGGAAGGAGAAGGGGACTGGGAACTTCGGGAACGAAGGGGTCCTCCGCCGTGCGACTCCATGTTAGGGTTTTTTCTTTCGCTCGGAATTCGAAAATTGGAGTTT

>C100799_65

GAAAAAACGGAAAAGCTGTGTAAAGTAACAGACGGAGATGGAAGGAAGGCCCGTGAGGTGAGAGTGAGTGTGGGTGTTTATAATCGGTCCCTCTGCTACCCCTTACCTAGCCCCTCACGTACCTTAACATAAACCTTCACCTTCACATTCCCCTTCACCCTCACCTTCACCTTCATTTCTAATCACAACTCAATTTTCCTCTTCATTCTCTCTTTTGAGCTACCACTATAGGACTCATGCTCCTGTAGCGGAGAACATGGCCTCAATCTCCGCCGCCGAACTTAATTTTCTAGTTTTTCGTTACCTTCAGGAATCAGGTTTCACGCATGCTGCTTTTACTTTTGGTTATGAGGCTGGTATCAACAAATCCTCCATTGATGGAAATTTGGTGCCACAAGGTGCTCTTGTTACATTTGTTCAGAAGGGTCTACAGTACTTTGAGATGGAAGCCAACTTGAGTAATTGTGATGCAGACCTGGAAGAAGATTTTTCATTCTTACAACCTTTGGATATTATCACAAAAGATGTGCATGAACTTAGGCAAATAATAAGTGAAAGAAGGAAAAAACTACAAAAGGAAAGAAAGAAAGAAATTGAAAAGGAGCATGAAGGAGAACGTTTACGAGCAAGAGAAAAGGAAAGACGCGAAAGAGAAAAAGAAGTTGAAAAGGATAGACGTGAAAGAGAAAAAGAAGTTGAAAGGGATAGACGTGAAAGGGAGAAGGAAATTGAAAGGGATAGACGTGAAAAGGAGAAGGAAGTTGAAAGGGACAGACGTGAAAGGGAGAAAGAAGTTGAAAAGGAGAGACGTGAAAGGGAGAAGGAAGTTGAAAAGGAGAGACGTGAAAGGGAGAAAGAAGTTGAAAAGGACAGGGAGAAGGTAGAAACAGATAAAGAACGAGAACAACATGGAGATCAGAATGTCAGAGACATGGTCATAGATCATGAAGATAGGGTTCCTGTAAAGCATGAAGAGAATGGAGCAGTTGGAGGGCCAGAACCGATGGACATCTCTACAACATCAACTTCTCAGCTATGTGAAATTCCCAGTTCATGTGTGACAATTTTGGAAGGTCATACGTCAGAGGTGTGTGCTTGTGCATGGAGCCCTACAGGATCTCTTCTTGCATCAGGATCTGGGGATTCGACAGCTCGTATTTGGACAATAGCAGAAGGAAGATGTAAGCCTGGTTCACATAATGGCCCTTTGAGCGTGTTGGTGTTGAAGCATGTTAGGGGTAAAACAAATGAAAAAAGTAAAGATGTTACTACACTTGACTGGAATGGGGAGGGAACACTTCTTGCAACTGGTTCATATGATGGGCAAGCAAGAATTTGGACCACTAATGGTGAACTAAGGAGTACATTAAGCAAACACAAGGGACCAATATTCTCTCTGAAGTGGAATAAGAAAGGCGATTATCTCCTCACTGGAAGTTGTGATCAAACTGCAATTGTGTGGGATGTGAAGGCAGAGGAATGGAAACAACAATTTGAATTTCATTCAGGTCCAACACTTGATGTTGATTGGCGCAACAATGTGTCTTTTGCAACAAGTTCAACAGACAATATGATATATGTTTGCAAAATTGGAGAAACCCGCCCTATAAAGACTTTTGCTGGACACCAGGGTGAAGTTAATTGTGTCAAATGGGACCCCACAGGTTCATTGCTGGCCTCCTGTTCTGATGATATCACAGCAAAGATATGGAGTATGAAGCAGGATACATATCTTCATGACTTACGGGAGCATTCCAAGGAGATATATACCATCCGATGGAGCCCCACTGGTCCTGGCACAAATAACCCTAATCATAAACTGGTGTTGGCCAGTGCTTCATTTGACTCAACTGTAAAGCTATGGGATGTGGAACTCGGGAAACTCATCTACAGCTTGGATGGACACAGGCATCCCGTATATTCTGTTGCTTTCAGTCCCAATGGTGAGTATTTAGTTAGCGGGTCTCTTGACAGATCTATGCACATATGGTCATTGAAGGAAGGCAAGATTGTCAAAACATACACCGGCAATGGAGGCATTTTTGAGGTCTGTTGGAATAAGGAGGGTGATAAGATTGCTGCATGTTTTGCTAATAATACAGTTTGCGTTTTGGATTTCAGAATGTAATATCTATGAAGGACTAGTACATTAAATTATTTCTTTCCCACCCTTGAGATGAGATCGGTGGAAGTGTATTTTGGGTGGGTGGGTAGGTTACCCATTTTAACCATAGTTGTAGGTATAACTCATTGTTCGTGGATCCTGTTGTAATTTTCTATAACATAAGTAAAAAGTAGTTTGTAGGAGTGTATTTTTAATAAGCATAATGTATAGATTTAGTTGGACCATATCCTGTACAAGTATCGCTTGACT

>C100801_65

CGCCACTTCTTCCTCTCCATTTTCCTCTGTCTGTTCCTCATTTCTCTTTACTAAAAGAAAAAAAATAAAAAATTTGCTTTCTCTGATACCCTTTTGTTTCTCCTTTAATTTTGCATCAAGAGCTTTAGCTCTCAGCATGGTTGCACTTGGAGCTGTACATAGTGTGGTCTATGTGCTCCTGTTTTCAGTCACTTTGGTGTTGGGTGGGGACATAGTTCACCATGATGATGTTGCCCCCAGAAGGCCTGGTTGTGATAACAACTTTGTTTTGGTAAAAGTCCCCACTTGGATTGATGGTGAGGAAAAAAGTGAGTATGTTGGTGTTGGTGCAAGATTTGGCCCTACGTTGGAATCAAAAGAAAAACGTGCCAATCTTACTAGAGTTGTCATGGCTGATCCTCCTGATTGTTGTACAAAGCCTAAGAATAAGCTCACCAGTGAGATAATTTTGGTACACCGAGGAAAATGTAGTTTCACTACCAAGGCAAATATTGCTGATGAAGCTGGTGCTTCAGCCATCCTCATTATAAACTACCGTACAGAACTTTTCAAGATGGTTTGTGAAGAAAATGAAACTGATGTTGATATTGGAATACCTGCTGTCATGCTTCCACAGGATGCTGGATTGACTTTGGAAAGGCATATTAAAAATAACTCCAATGTGTCCATACAGTTATACTCTCCACTGCGTCCATTAGTTGATGTTGCGGAAGTATTTTTGTGGCTTATGGCTGTTGGTACCATTTTATGTGCTTCATATTGGTCTGCCTGGAGTGCCAGAGAAGCAGCTATCGAGCAAGAGAAGCTTTTAAAGGATGCTTCAGATGACTATGTTAATGCAGAAAATGTTGGTTCCAGTGGTTATGTGGAGATTAGTACTATAGCGGCAATTTTATTCGTCGTGATTGCTTCATGTTTCTTGGTCATGCTGTATAAATTAATGTCATTCTGGTTTGTTGAAGTTCTGGTGGTTCTGTTTTGCATTGGTGGGGTAGAGGGACTGCAAACTTGTTTGGTGGCTCTGTTGTCATGTTTCAGATGGTTTCGTCAACCTGCCCAGACATTTGTGAAGATACCCTTCTTTGGAGCTGTCTCATATCTGACTCTTGCCGTTACACCATTCTGCATAGTGTTTGCCGTGGTTTGGGCAGTTAATCGCCGTGCATCATGGGCTTGGATTGGTCAGGATATCCTTGGTATCACATTGATAATTACAGTTCTTCAGATTGTTCGCATACCGAATCTCAAGGTTGGAACTGTACTTCTCAGTTGTGCGTTCCTATATGACATCTTCTGGGTATTTGTCTCTAAATGGTGGTTCCATGAGAGCGTGATGATAGTGGTAGCTCGTGGTGATAAGAGTGGAGAAGATGGCATTCCCATGCTTCTCAAGATACCTCGTCTGTTCGACCCTTGGGGTGGTTACAGTATCATCGGATTTGGGGACATAATCTTACCAGGACTTATAGTAGCGTTTTCACTAAGGTATGATTGGTTGGCAAAGAAGAACCTTCGCGCAGGATACTTCTTGTGGGCAATGACTGCTTATGGGTTAGGTCTCCTCATCACATATGTGGCTTTGAACTTGATGGATGGGCATGGTCAACCAGCTTTGCTTTATATAGTTCCATTTACACTTGGCACCTTTTTGTCATTGGGAAAAAAGAGAGGTGAGCTCAAGATTTTATGGACAAGAGGGGAACCAGAAAGACCTTGTTGTCATATCCAAGAGGATAACCAATCAATTAACAGTCACCATTGAACAGCAGCAGACTTGTATCCTCCTAGGTATGTTAGTTTATAGGAGAGTGGTAGGAACTCCTAGGACATTGTGTAACATCTGTAACTTTGTTGTAACAATTTCAGCCAAATGGTATGATAGCATGCACTTAAATTGGCAGCTGAGAAAGATATCACCCAATTGTTCTGATATTCAAAAGAACCATGAAACTGTGTACTGTATGTAACCTTATTTTTTGAGATAGCAGAGGGAGACTCCGTCAGGGTCTGATCAAAACCATGCCTTTGCCAAGCAAATGACAAAGAAGCAACGAGACATGCATAGAAAACTCCAATCCAAGAGCGAAGGACTGTTGAAAGGCTACTAACACGGAGAAGCTGCTGTTTACTATAAGCATAACTTATGTTGTATTATTATTATTACTATTGTTTATGAATTGTATGAGATTTGAACCTGAAGTTAGTTCAATTTTAATTTCTGTAAGATTTTGCATGTTACCAATTTCCTTAAATCTTTCGAGATCGAGAGGAAGGACATTTATCTGTCTAGTCTGTCGTGTTGTATATGCTAGTTCTTGCTTGAAAAACTGGCTAGACCCCCCATCAAGACCTGTTTGAGTTATGAAAGTTAAAT

>C100803_65

TTTTAATGTAAAAGAATCATAAAATAACTTGCTAAGTTGCATAATGCTATGGCTAAATAGAACCAGCATATCAAAAGGTCAGGAAAATTGAAATATTTCTATGGGGATAGAAGATATATAACTTACATGGGAATATTTCTATAGGGAGAATTGGCATGTACAATTAGAATGCAGAGAAAAAGCAATCTAATTTTCCAGTTCACTCCTTATCCAATCTTGTGAACACACCAAAGCTTGGACAGTAGCAGGATTAAGTGAGCTCCAATCACGATCAAGGACCCTTCCACTTTGATTAAACGCCAACTCAGGCGCAACCTTTGCCATTGGAATTCCTAAAACATTGCGCGCCATCATGGATAAGACAGGATATCTGGGAGTGTGAACTCTCCACCAATTCAGTATGTTGAAATCAACATTGCGGGGAAAGAGGGGCTCTTCCAAGTACTTGTCTAGATCTGATTTTGTACGTTCACCCTGTGAAGTTTCATGGAGAAATTTATCAAACCCCATTAATCGATCCCTGGAGTCCTTTGCAGAACCTTGTAAGAGAAGTGGACCATTCCCAACTTGCCAGGCCAGACCTTGATCATGAGATGCTAATGGGGAGCCTATCGAATGTTCATTGTATAGAGCTTTCACACCATCAAAGACCTCTTCAATGCGACTTGCAGACATGCTGCCATAAATTTGTGGATAATAATAATCTACCAACTTCATCTTGAATCGAGGGTCTAACATCGCAGCAACTGCCAACCCTAAGCTACATTTCTCCCAATACTCATCAAACTTGCTTCTCAATCTTGAAGCCAAAGAACTGATATACTCATCTGAATTCTTGCACCATTCAATCAAATGCAACTTAACATCACAGAGCTCAGGGAAATATATATTTGCAGTTGGATACTTACTCTTAGTGAAAACATTTATAACCTCAACAAAAAGCTTCAAGTAGCTAGTAACCGCTGTGATTCTCTCCCACTCTACATCAGATAAACAGACTTTGTAGGCAGCGTCATTTTCTTGCAAGAGAATTAAAACATCCTTGAATTCTAGGGCAACTTCAAGCATTGAGTACGTAGAATTCCATTGTGATGCATTATCTAGACATAAGCCCTTTTGACTCAGGATCCCAACTTCTTTAGCCATCTCATTGAACTTTGCCAGTATAATTTGTGAACTTTTAATGTAACCAATAGTTTCCCGAATCTTAATGAGTATTTCACTTACAGCTCCCAGGGCATGCTGAACCATGGAATTAATAACATTTGCAGCACAGCGTATATCAAATAATTGACCATTACAATAAAGAAACCTGTTTTGCAGAAGTCTATCACCAATTCTAAGAGCAATGTTATCACAGGTAGAACAACTATCCAGTATCATGGAAAACAATTTACGGTCAATATCCCAATACATTAGGCAAGTCATGATAGCTTCTGAAACCATGTCTTCAGTATGACAAGGATCAATCCTTATAAAATTTAATATCCTCCTTCTTAACTGCCAAGATTCATCAATGTAGTTTGATGTCAAACAAAGATACTCTGCATCACCCACAGCATTCCACACATCAGCACTAAGACTGATTTTCCCAGGCAACTTATCCAACATCTCATTCACCTTTTTCCTCTCTCTCTCATAAATCTCAATACAATCAGCCTCAACTCTATTCAACGACACAAGCTCAAATAACGGCTGTAGATTTTTTACAAAAGCTCTGAAACCAACATGCTCAACCATAGCCAATGGATATCCATGTAGAATAATCATCCGAGCAAGGTCAAATCGACTGCGTCTTTGATCAAAGTTACTTGTTCCAGTGTTGACAGAATCATCTTTCAACTGTGTCTGCTCAAATTTAATATTGACAAGACTAAGGGTATTATCATCTTTGTTTGTATCTTGATCTATATTGAAATTTGCAATTGCTAGAGTTCCTTCCTTTCTCTTTTCTCTAGCTGAAATGTATTGTGCTATGCCATGACTAGACCTTCTCTGACACCGAATTAAATGGTTCCTTAGATGTGATGTTCCACTGGTACTTGAACCACTCAGTTTCTTTTTGCAATGCCTACAAACAGCCACACAGGTGTCACCTTTTTTAATCCTATCAAAATCATTCCAAACCACCGATTTTAACCTACTAGATTTGACAATGACAGCTTCAGACATGTCCATCGCATGAACCGTGTCGAAGAATGTGTCGGATTCGGTGTGGTGCGTGCGAAGAATGTAAAAGGGGTTTGAGAGAAAACCCTAATCGGAAGGTGAAAGAGGGAAGAAAGAAAAAGAAAGAAGAGAGAGCAACGTAGACAGACGCAGTAGCGTCCTCCTATTCATCTGCTATTGTTGAAAGTTAAAACGCACAGTTTCA

>C100805_65

CTCGGCACCACACATTGTTTCACTGTCAGTAGCTGAGGCCAAAACCACTGATTTAAAAAAGAATATAGAACAGAGAGGAAGAGAGCAAAATGGGTTTGGGAATTAGAAGAGAGTAGAAACAACCGAGAGAAACACATAGAAAAAGGAGGCATAGCAACCTATTCAAAAGAAGATGCAAGGCTGTGAGGTTGAAGCCATTGGCATAAACTACACCATCCAGACACACAAATCGGAGCACCCTTTTAAAATCTTCAGCAAATCTGCACAACTTGACAGTGAACAAGATGGTAAAGAGCCAGAAGAGGAAGCAGAAGCTGAAGGAAGTTGCGGTGGAGTAAGGCATGTTCTGAAGAATGTGAGCTTCCAAGCCAAGCCATGGGAGATCCTAGCAATTGTTGGGCCAAGTGGAGCTGGGAAATCATCACTGCTTGAGATTCTTGCTGGAAAACATAGCCCTCAAAGTGGTACTGTGTTGGTGAACCACAAGCCTGTGGACAAAGCCCAATTCAGGAAGCTTTCAGGGTATGTGACACAAAAGGACACTTTGTTTCCTTTACTTACAGTTGAAGAAACCCTGATGTTTAGTGCAAAGCTGAGGTTGACGCTTTCTCAGGAGGAACTTTGCTCCAGGGTCAAGTCATTGATCAAGGAGCTTGGACTTGACCACGTTGCTGGCACTAGAATTGGTGATGATAGGGTCCGAGGCATATCTGGTGGTGAGAGGCGCAGAGTTTCCATTGGTGTTGAAGTCATACATGAACCAAAAGTGCTGATTCTTGATGAACCAACTTCAGGGCTTGACAGTACTTCAGCTTTGCAAATAATTGACATGCTTAAGATCATGGCTGACACAAAAGGGAGAACTATAATCCTTAGTATCCATCAACCAGGGTTCAGAATAGTGAAGCTGTTCAATTCATTGTTGTTGTTGGCCAATGGAAGTGTTTTGCACCATGGAACTGCAGAGTTGCTTGGTGTAAATCTAAGGCTAATCGGTTTGGAGCTTCCTCTTCACATCAATGTAGTTGAATTTGCCATTGAGTCCATTGATACAATTCAGCAACAGCAGAAATGTGTGCCAGTTCAGGTGGAAACTCCAAGGCAATTGCCAGGGACAATGCAACTGAAGAAAGTTGATGATGAAGCTGGTGAGAGTAGGAACGGTAAGTTTACTTTGCAACAGCTGTTTCAGCAATCCAAGGTAATTGATGAAGAAACCTACTATGCTGGAATGGATTTCACTTGTGATTTTGCTAATTCCAGATTGAGAGAAACTATGATTCTCACTCATAGGTTCTCCAAGAACATATTTCGTACAAAAGAGCTATTTGCCTGCAGGACAATACAGATGCTGGTTTCTGGGTTGGTTGTAGGTTCCATCTTTTGTAACCTTAAGGATGACCTAGAGGGAGCTTATGAAAGGGTGGGTCTATTTGCTTTCATATTAACGTTTTTGTTGTCAAGCAGCATAGAAGCTCTACCAATTTTTCTGCAAGAAAGGGAGATTCTAATGAAGGAAACATCTTGTGGAAGCTACCGTGTGTCATCCTATGCTATTGCCAATGGCCTAGTTTACTTGCCATTTCTTCTCATCCTAGCCATTTTGTTCTCAATGCCCTTGTACTGGCTTGTTGGTCTCAACCGGAATTTCTTGGCCTTTTTGCACTTTTTGTTGCTGATATGGCTGATTCTTTACACGGCCAATTCAGTTGTGGTGTGTTTTAGTGCTCTGGTGCCGAATTTTATTGTTGGGAATTCAGTGATTGCTGGTGTTATAGGGTCATTCTTCTTGTTCTCTGGGTACTTCATATCAAAGCAAGAAATTCCAAATTACTGGATTTTCATGCATTATATATCCTTGTTTAAGTATCCCTTTGAAGGGTTCCTGATCAATGAGTTCTCCAACTCGGGAAAGTGCTTGGAGTACATGTTTGGGGCATGTATTAAGAGCGGAGAGGATGTGCTTGAAGAAGAAGGGTACGGGGGAGAGAGTAATAGATGGAAGAATGTTGGGGTCACTGTGTGCTTCATCTTGGTTTACAGGTTCATCTCTTATGTTATTCTTAGATACAGATGTTCCCAGAGGGGATTTGGCAAGGTTATCACCATCTAAAAGTCTATGGTGATTCTCTTTCTTTCTCTCTCTCTCTGGTTCACGTGTCTTGGATTTTCCTTCTGATAGGAGCCAATGAAGATATTCTCAAAAAAAAAAAATAGCATAAAAAATTATAGGTTTGGTCAGTGCCAACAAATTGTACTAACAAGATATTATGTTGGTTTTTGCTTTACATGATTACTTAATTGACAACATTGTGTTTCTCTTGTCATTTAGATTCCTACTCCCCTCATTTTATATAATCTCAGGCTTGGCCAATG

>C100809_65

CAAAATTTGCCCTAGAAAATCTTCAACAGCCGCAACATGCTCCTTCCTACCAAACATTCACTTTCTCTCTCCTCTCTCGCCACTCGCCACCGCCACCACCACCACCAGATTCGCGAAGGGGTTTAGGGTTCCGTCCATGGACGCTGTTGAATTGCCTTTACCACTTGACGTGGCACCCGCGCCCAAATTGATGGGATCCGAGGGTTTCTCGCTAATTCAAAACGATGTTGCAAGTATAAAAACAGCAGCCGAGTTTTCCTCCTGCAATAAATTTGTAGAAACTCAACTCTCTAAGAATTCTAGTCAGCTGCCCCATCTCAAGGGTGAAGAGGCACCCAAAAATTCGTCTGTTGGTAATGGAAAAAATTTTCCTGTAATTAATTCCAGATTGGAAGGTGTAACATTACAACGAAAAATGGCAAAATCAAGCAGGAGTAATAGTTCATGTTCAAAGAGGCCACGGATGTCCCAGCCAGAAGATTCTTTGAGCCCTATTGGAATTGAGGAGTCAAAGGATATTTCTGATAAACTTGGGTCAAATAATTTAAACTGTACTTCTCCAGAGAAAGGTCAATTACCAAAGCAAAAGAGCAATGCTAGCAAACGTGGTGAGAAGAGGAATTTTAAAGTACCTTCTGCGAAGGCTAAATTTGATTATTCCTCTATGAAGATGGGCACATCAATATTCAGCTCTACTAGTGGGGGGAACAACTTTTTTGGGCTATATGGTCTGAAACATGATTTTCATGATGTCACAAAGCTTATGGATGAACCACCGTTAGATGAGCTCCTTAAGGGAACTTTTGACTGCCCCATTTTAAGCAAAGATAAAGGGAAGAAAACATCAAATATGAGTGTAAGTTTTTTGAATTCAGTTAGAAAGGCGTGCTCTATCCTTCAGCTCCCGAAACCTGTTCAGTCCCAAAATATGTCTGAGATGGATTACTCCTCCAACATGAAAATGTCCACTTGCCAATTGAGCTCAGTTTGTGCAGCAGAAAGTGTTGGTAATGGGGATAAAGAGCAGTCATTAGATATGTCCTCATGTCAGAAGGATCATTGTAGTGAAACAGAAAGTTCAACTAGTCCGCTTGACTTCCCATTACATCAACCTAAGGATGTTTTGGAACGACTTGCACTCCATACGTTCCAGGACTTGGAGTCTTTGCTGCTTGATGTGTCCAAGCCTGCTGCTACCACAAAGAATAGTAATGACCAACGTTCAGGCAAGCAGGTGTCTCGACGGCCTATCCTTCCAGCCTTTACATGGTCACATGCTTTTGGTGGCCATTCTAGAACCAATTCTGATGCAGTTAAGTTATCAACAAGTAGAAGCATGTGCCAAGGTAAATGGTCGAGGATAGGTGTCATTGCTGGCTCTACTGATACTGATCGTAGTTCCTTCACAAACCTTGATTCATTCAGTTATGACCAGAGCCTTGTTCCTTCATCTGGTAGTTCAGAGAAAAGGAACTTCTCATTTTTATTTTCTAACCTTCCTTTCCATCAGTTGGATTCTTCATCTTCTGTATCTTGTTCAGAAGACTCTCAGGCTAAAGCAGAATTTGGAGGCCAAGTTGATACTAAAGAAAATGATGAGCGTTGTCCAAGAATATTAACTGCTGCACAAACACTTTGTGAAATTGCAACCCACTCACCAAGGCAGAGCTCGGATGGAATTTTAAGATGGCAAAGGAAAACTTCACAAAAAGCCATGAAAGCTTGCCACTATAAATCAAATAAGAAAATTGAAGAGATGTCTTCAAGATCAGTTTCAATGATTGGATCTGACGTGGCCAGAAGTGTGGACCAGATAATACCCTCAAAGAAGCCAAGGTTTTCCATAGTCGAGAACAAGAACAGTGGTCACTCCAGTAATGCTAAGAAAGGACACTTTGTGTGGCCGATTTCAAAGTCAAGTAGATCATTACCTAGTAAACAAGTTAGAGACTCGTTTGTGGAAACCAAACGTACAAATGCCAGCATCTTGAAGCAACATTGTATGATGCCCCCGCCTGCGAGGGATTTAAACAAGGTTCACGATGGTCAGCAGCAGGTAGGAAAATTAGTAATGATGGATTGGAAAAGGGGAAGAGACACCACAGATTGATCAAACAAATTATTGACTGTAGGCACATCAGTAGCTGGCAGCTGTTGTGTGATTAGCAAGTGTTAGGTTAGTAAAGGCTACTTTGTACATATTGTTATGTTGTGGCTGCAAGCCTGCGATGAATTTGTTGTTAGTGTTCATGTTCGGAGGCAAACAGGATTCACATGTTGTAACATTAAAACAGGTGTATAAAACAGCAGAATTCTTTGCTTACCGCAGAGTACGAAGCATTAGTGAGATTAAGTTCCCTCGTTTCTTTCTGTGTG

>C100811_65

ATTCCTTGGTAATGTGATGTTCATAACTGATGCCATGAGAATATCTAATCCATGGCAAGAGGACTGACTTTACAGATACATCACAATCACAATTCCCTTGCATGTTTCTCTTCTCTTCTCTTTCTTTTACTGCACTGGAAATTTCGAATTGGATGTATCTGTATGTGTAACCCTTCTAACGCATCTATTCTCTCTCACTCATAACTCCACTCTTTCTTCTTATCACCCCTACCCACCACTCCCATTCTCAACAAAACTTAGATTTTCTCTCACATTTCCCCAACCCTCTAAGCCTTTTCAATTTTCATCTCTTTTCACAACTGGGTTGTCAGTGTTCCATCTTTTTTGGGTTCTGTGTAAGTCAAAGATCTGGCCTTTTTCTGTTTGGTGTTCCTTTTTTGAGATGTGGTGATCTGGGGTTTGTGCTGCTTGTCCTGGTGAATCTCTCTACACGTTGATAGGGCTTCGGGGTTCTAAGAGATGGGTGCTGATCTTAAAGCAGAATTGTCTGTGAAAACTCCTGTTTTTGATCTTAAGGTTTGGGAAGTAATTGGAATTGCGGTTGGATTGTTTATTATTGTCATTCTATGTGCGCTCTCATTTTGGCTTACTTCAAGGAAGAAATCTAGAAAAGCCAGGGACAGGGTTCCTGTTAGTCATATACCTACTATCTCAAAGGAAATCACAGAAGTGCGGGTTGAGCAAGTGCCGGCCAATGGATTTGTTCCTCGTGAAGGAATACTTCTGACAATTCATGATAAATCCAGTGACAAAGAATCAGACAAAGTTATGCTTCATTTAGGTGTTGGGAAGAAGAAGCATGGGGATAGTGGCAGTCACTCCGATTCGTTTCATTACTTGGAGAAAGATGGTGGAGGGTCACAATCAGGGGAGGAAATTAGCTCTGGCTCGGTTGGAATGTACATGCCGTCTTCTTCATACCCTATAACAGCTCCTTCACCTCTCTCTGGCCTTCCAGAATTCTCTCACTTGGGTTGGGGTCACTGGTTTACTCTGAGGGATCTTGAACTTGCTACAAACCGTTTTTCAAAAGAAAATGTTATTGGTGAGGGTGGGTATGGAGTTGTTTACCGGGGACAGTTGATCAATGGGACTCCAGTGGCAGTTAAAAAGATACTCAATAACATTGGTCAAGCTGAGAAAGAATTTAGAGTGGAAGTTGAAGCTATTGGCCATGTCCGACATAAAAATTTGGTTCGACTTTTGGGGTACTGCATGGAGGGAACTCACAGGATGTTAGTCTATGAATATGTCAGTAATGGAAACTTAGAGCAATGGCTTCACGGAGCTATGCGACATCATGGATATCTTACCTGGGAAGCACGTATGAAGATTCTCCTTGGCACAGCTAAGGGGCTTGCATATTTGCATGAAGCGATTGAGCCAAAGGTGGTACACAGAGATATCAAGTCAAGCAACATACTAGTCGATGATGACTTTAATGCCAAGGTTTCTGATTTTGGCCTGGCCAAGTTACTGGGTGCTGGGAAGAGTCATGTTGCAACACGAGTTATGGGAACCTTTGGATATGTGGCTCCCGAATATGCAAATACTGGTCTTCTAAATGAAAAGAGTGATGTTTATAGCTTTGGTGTTGTGCTACTGGAAGCAATTACTGGAAGAGATCCAGTTGACTATGGCCGCCCCGCACAGGAAGTTAATATGGTTGATTGGCTTAAGATGATGGTTGGACACAGGCGATCAGAAGAAGTGGTGGACCAAAACATTGAGGTGAAGCCATCTACCAGGGCTATGAAACGGGCGCTCTTAACTGCTTTGAGATGTGTAGATCCAGATTCAGAGAAAAGGCCCAAGATGGGTCAAGTTGTGCGTATGCTTGAGTCAGACGAGTACCCTTTAGCTAGAGAGGACCGTAGGCATCGAAGAAATCATGGGGTTAACTCAGACATTGAATCCCATAAGGATAGTTCTGACACGGACGGGAGTGACATTCAGGGTTCTAGATCAGAAAGTGGAAGGTAACAGCAGGCATGACCTAAGAGAATGAGATTCTTTTTTAGGATCAGTGGGTGTATAAAGTGAGTGATTTATTTTATTTTTGTCTGGTTAGATTCTTGGATTTGCCTACCCAAGATGGGCATGTCTAAGGCTGAGCAGGGGATAATATTATTTTTTGTGTTTTCCTTTCCTTTGTTGTAGAAGAATAGGTATTTGTTCTAGAAGGATATATATTGTTTGTGTATGAAGGTGGGTGTTATTTGGGGTATGTACAGAATCTGATAGCTTTCCTAGATTTATTTGATAAATCTGTTGGCCTTGTCACTGGGGAAAAGTAAAATAAACCAATTCATGGCTGTACCTTCTCGTGATTTTTTACGCCTAGATCATTCCGTT

>C100813_65

CGGCGGCGATGGCACAGGCGGTGGGGCGGGGCCGGGTGGTCGGAGACTACGTGGTGGGGAAGCAAATCGGCGCGGGGTCGTTCTCGGTGGTGTGGCACGGAAGGCACAAGGTGCACGGAACCGAGGTGGCGATAAAGGAGATCGCCACGCTTCGCCTCAACAAGAAGTTGCAGGAGAGCCTCATGTCCGAGATTTTCATCTTGAAGCGGATTAACCACCCTAACATAATCTGTTTGCACGATATCATTCAGGTTCCTGGGAAGATACATCTTGTGTTAGAATACTGTAAAGGGGGAGATCTGTCCATGTACATTCATCGCCATGGAAGGGTACCAGAGGCAACTGCAAAACATTTTATGCAGCAGCTGGCTGCTGGTCTACTAGTGCTTCGGGATAACAACCTTATTCATCGGGATCTGAAACCCCAGAACCTTCTTCTCTCCCAAAATGATGAGAAATCAGTGCTGAAGATTGCTGACTTTGGATTTGCAAGATCTCTGCAACCTAGGGGCCTTGCTGAAACCTTGTGTGGTTCACCACTCTATATGGCTCCAGAAATCATGCAACTACAGAAGTATGATGCTAAGGCAGATCTCTGGAGTGTTGGCGCAATCTTATTTCAGCTGGTTACAGGAAGAACGCCTTTTACTGGAAACAATCAAATACAGTTACTACAGAATATTATGAAATCGACAGAATTGCAGTTTCCATCTGATAGCCCAAGTCTGAGTTTTGAATGCAAAGATTTGTGTCAGAAGTTGCTACGTCGGAATCCAGTGGAACGTCTAACTTTTGAAGAATTTTTTAACCACCCATTTCTTTCTCAGAAGCAAAGAGAACAGGATGATGAGCCATTGAGGAGTAGAAGTTCTTCAAGGCTGGTTGGTGGGTTTTCTTCTACAGTTTCTGACCCTTTGAGAAGAACTGATGAAAATTATCAAGAAGATTGTTTGCCTTTTATCCTAGATGACGATTCTAGTGGCCCTGAGGGGAGTCCATCCTTTTCAAAAAAGTCCTCAATAAAGTCCACCTATGGATTTGATCTTAATTCTAAACTACATAAAGCAGAGCCAGCATCTCCTATGTCTAATAACATAAATTCTAATTCAGGATTTGGTAGTATGACGCAAAGATCTGAAAACACTACTAGAAGATTGGTCAACCATAAAATTTCAAGAAATTTCACTGATCCTCTAGATTCTCCAGAACAGATGTTCGCAAGTCCTTATCCAAAAGTTACGGATTCACTGGAGAATATTGATCAAGAATATGTTCTGGTGCCTGGGCCCCCTATAGATGTTTCATCTTCTTCAGTCGGTGCTTCAAGGCCCAGCCATACCACATATAGGTCTGGTAGTTTACCCCATGAGTCTTCTTGTGCAATCACTAGATCAAGTGCTCCAATGCCCATAGTTGGTGTACCTAACAATAGCATATACCAAATTGGAAGTTCAGGAAGTCAGGACTCTGCTCCTGGGACTTCACTTGGATCAATGGATACAGGTGATGAACAGCCATCAGCTCACTGCATGACAAGGGTAAAATCATTGCAACAATGTGCATCTGCGATCACTGAGTTAGTTAATGAAAAGATGGAGGCAGGAAAGCATCTAGAAGCATTTTCTATTCAGCTTGTAATTCTTGCAATCTGGAAGCAAGCACTCCACATATGCCATACACAAGCTGCCTCAGCCATGGAAGGAAGTCCAAGTCAAGAAACTTCAAGATATAGAAGGAGTGCCAGCAGGAAGCATGGTAGTCCTGATTCGGAAGAATGCCTTGATGGAAACATCCTAGGGCCAAAGGATATATTATCTAAAATTGAAAGTGAATTTTTGAGGGAATTTGCACATGCTGAAGAACTTGCCAAGACCATTGAGCCTGGAAACACTGAGATGCCAGATGCAATGGAGACAATATTTCAATCTGCCCTTGCTTTTGGAAGGCATGGGGGTGTAGAAGAGCTCATGGGTGACATGGACAGTGCAGCTGCATTGTATTCAAAAGCTGTCCGTCTGTTAGTTTTCCTTCTAGTGGAAGGACCATCTCTGATTCTTAATCCTCCGTTTTCGCTGACAAACTCGGATCGTTACAGACTTCGAAGTTACATTTATATTCTTAATAACAGACAAGGTTATTCTAGGTCCCAGCGAATGGCATTACTGAAATGTGATGACAGTCAAGATATCCTAAAGGAAAAATTTTAGCAATGGTTATTTATCTCCCATTCCACACAACAAATTATTTATTTGTACAGTTATTCTTTGAGGGAGGGGAAATTGGGGATGCTGATGGGAGTAAACCAGCACAAGGTAGATTGTAAAATAACACATTTCTGTGATTAGAATGATCTTAAAATATGCATTTGATTATGTAT

>C100829_65

TTTTTTTTAAGCAGATAATTGATATATTCATTGTACTGGTAAATCAAAATTTCATACAAAATATGTTCACATTGGTAAATCATATAATTAGTGGTCGTCAAAACCCATCTCACTTCTGGACAATGTTTTTTCACTACAAAGAACCTGGTAATACGGATAAAATGAAAGCAAAGCAGGATCACCACAATGATGTGATACAAAAGATTGGGCAAGTTGGCGTGACGCCAATTACACAAGAAAATGTAAATCTTATATATAAAAAAAAATAACTAACGACAACATGAAGACTGATCTAGCTAGACATCTTGACTTGATCACTAGATTGTAGTGCTTGAGAGGCGAAAAACCTAACATCCACATCTGGATCCTCACTTAGCTCAACCAAACAGGGACGGATGGTATTCTCAACAACAGACTGATCAACAATAGGGATGAGAGACTGCAACACTTTAGCTACGTTGAATTTGATATTGGGTACCCTATCTTTTGATGCATTAACGACCAGAGGGAGCAGTTTTGAAGAAGTAATCTCCGAGCCCAAAACAGGAGCTAGTAGGGAAATTGCTTGTAGGATCGTCATCCGATACAGATAATGTGGATCAGTAACCATGTCCAAAACCTGAGGAATAATGTGCTGCATTGCCCAATCCGGTCCAAATTCTTCTGCTAAGCGTTTGATGTTATTTGCGGCCGCATCACGGATAGAGAATACCTTGTCCTTTAGCCATTGCATGCAAAGAGCACCGAGCTTATCATCAAAAAAGCCTACACCCAATTGGCTTGCCAGTAAGGGTATATATTCTATGATTGCAAGTCGAACTCTCCAGTGCCGATCCTCAGCAAGCTCAACTATAGCTGGCAACAGAGACTGAGAAAGCAAATCGATACCAATAACCTGATTCACTTGATCAAGCTTGCTGATAATATTTAGCCTGACATCAGGAAATTCGTCTTTCAAAAGAGAAAGGAAAATAGGTAGAAGTTGCTCAATTGTTGCATCCTTCCCCAACACAGGTGCCATTCCCATTATAACGGAAGCCAATGCAGACCGAACATGTTGAGAGGAATCGGTTGATAGCTCCTTCACACAGGGTAGAATATGCTGAATGGCAAGTTCGGGATTTAATATGCGGGAGAACTTAGTTACTTTCCCAGCAGCGGCAATACGTACTTCAGCCTCATTATCACGCAGCAGCCTAACATATGCAGGAACCAACTCTGACCTGGTGGGATCAGGACCAACAGCTTCACATAGCTCATATAGTTGATTTGCAACCATGTATCGAACACGCCATGACTTGTCCTGAGAAAAATTGACTATGACAGGGAGAATATGTGCCACACAATCTTGAGGTTCCAATAATTTTCCTAGAGCTGCACAACCCTCCACAGCAAGTAGCCGAACGGAATCTTGATCATCCTGCGTAAGATCCTCAAACACAGACATTATGTCTGATTTCAAGTGAGAAGCTTCAACAGTAGCAGCAAACTTTCCCAAGTTTGTGGCAGCAGATCTCCTAACCATGGGCATATCATCTTGACAGAGCTGGCCATATAGGGCTCTCAGTTCAGTCTTAACCACCTCTGGTGCGCTAGGGTAAACAATATGGAACAAACCGCATGATGAAACTCGTGCTGTGAACCACTCGCCAACAGCCAGCCTCTTAACTAAAGGAATGAAGTGTTCAACCAAATCTTGCTCCCTCATCTGAGCTCCAATCCTACACAGCGACTCCACCGCTTTGTCTCTGACGCAAGTTTCCTCAACGGTGCAGAGCGTCTCCAAGGGAGGAAGAAGCACGTTGGCATGTTCCACGCCTCCGACATAGGGAATGAACACGCCTAATTCCTCGGCCATTGCAAGAAGCACTTCATCGTCATCGTCGTTGTTCTCGCTGAGAAACGGAATCAGCTCCTTCCGCGTGCGCTCCTCGCCGAGCGCGCGAGCGATCGTGGAGAGCCGTCGGATCGAGTTGAGCCGCAGCTGAATGTCCTCGTTCTTCAGCTCGTCGATCAAAACGGCAATCGGATACAGAGGCTGGTCCACCATCGCCATGTTTTTTCACTGGATCCTCGAAAACTGCAACGATCGGAGATCGACGATTCCCTCGATTTCAATTAAGCAATAACTCAAAACACAAAACAACCAAAGGAAAGATAGAGAAAAAAAAAAACTCAAAATCTACAATACACTTCTTGATCTAAGCAATGGAGCTTAGTTCAGAAAGAAATTGAAAAAAGAAAAAAAAAATGAATCAATGTGTTGTTGTTGTGGCAGAGAGAGAGAGGGAAGAAGGAGCAGTGATGTTATGGGACCTGAAAGGAGATTTGTTGAAGGGCGGAGTAGAATGGGAATGTAGCGCGGATCGGATCTGCCGTTC

>C100831_65

GCCACAGCTTGGTATCTTCATGATTGCTCATTGTTGATGCCCATAAGGAACCTTTATTGCTTTTGTTTTCTTCTCACTTGTTAACTTTCGTTTCCCTATATAAACCCTCCTTTGTTCCCTTCTCTTCCCATCTTCCATTGAGTTGATTGATTCCAAACACAAATATGGCAATGAAGAAGCTCTTGTTAGTTGTTCTGTCCCTTTCTTTGGCTCTTGTTGTGGCCAACAGCTTTGATTTTCATGAGAAAGATTTGGCGTCCGAAGAAAGTTTGTGGGACTTGTACGAGAGATGGAGGAGTCACCATACGGTTTCGCGAAGCCTGGATGAGAAGCACAAACGGTTTAACGTGTTCAAAGCAAATGTGATGCATGTCCATAATACTAACAAGATGGATAAGCCTTATAAGCTGAAACTGAACAAGTTCGCTGACATGACCAACCATGAATTCAGGAGTACATATGCTGGTTCAAAGATTGATCACCACAGAATGTTCAAGGGCACGGCACATGGGAACGGTACCTTCATGTATGAGAAGGTTGGTAGTGTTCCACCTTCTGTGGATTGGAGGAAGAAAGGTGCTGTCACTGATGTAAAAAATCAAGGCCAATGTGGTAGTTGCTGGGCGTTTTCAACTATTGTAGCAGTTGAAGGTATTAACCAAATCAAGACAAACAAGCTGGTCTCATTGTCTGAACAAGAGTTGGTGGATTGCGACAAGGAAGAAAACCAAGGATGCAATGGTGGGTTAATGGAATCTGCTTTCGAGTTCATCAAACAGAAAGGAGGCATAACAACAGAAAGTAATTATCCTTACACAGCACAAGAAGGAAGCTGTGATGCATCAAAGGTGAATGACATAGCTGTGTCAATTGATGGCCATGAGAATGTCCCTGCTAACGATGAAAATGCATTGCTCAAAGCTGTTGCCAACCAACCAGTTTCTGTAGCCATTGATGCTGGTGGATCTAATTTTCAGTTCTACTCTGAGGGAGTATTTACGGGTGATTGTAGTACGGATCTAGATCATGGTGTAGCGATTGTGGGGTATGGAACAACTGTTGATGGCACTAATTACTGGATAGTTAGAAACTCTTGGGGACCAGAATGGGGAGAACAAGGTTACATCAGAATGGAAAGGAACATATCTAAAAAGGAGGGACTTTGTGGCATAGCCATGTTGGCTTCCTATCCAATCAAAAACTCTTCTGATAATCCAGCTCGATCTTTCTCCTCTCTTCTTAAAGATGAACTTTGAAAGCTTCCCACAAAAGTTCATATAAGTCTTGGTTGTTGTTTTTCATTTGTATTCAGATATATCCATCAAGAATATATTACACACAGAAATAAGTATTTTAATTTGATTTGAGTGATAGCAGTTTCTCATGTAACCTTCTATAGCCAAGAGTTGAATGCAAAGTGATAACTTTAAAACATCTATATAAATAGCAATGGATCAAGTAGAGTCCAGTTTGCATATGATATGGTCACTTTGAAGATACATCATGACATTTTATTTTTGGATTATACTGTATTATTGGAACACACACTAGATGGCTGATACTAGCCAGACATGATGAAAATTGTAGCAAGCAAGTGATCATATGATTCATCCACCATCAGCCATCAGCCATAGCACCTGATGGGATCAGTGCAACCTTTTTCTGCTGACTGTTTGTTAGATTCACCTTTTCCTCCACCCTTCTCCAGATATTGTTGATGATAATCCTCTGCTTTGTAAAACCTCTTAGCTGGAAGTATTTCTGTCACAATCTTACCCTTCAACTCCAACTGTTTTGCTTCTTTCGATTCCTGAGCTAAACGAGCCTGGATATCATTGTAGTAGTATATTCCTGATCTATACTGTGCACCCACATCGTTGCCCTGGCGATTAATGGAAGTTGGGTCATGTCGAGACCAAAAGAGAGCAAGCAGAGCCTCATAGGGGCAAACCTTCGGGTCGAACTGAACCCGAACAACTTCAACGTGGTTAGTGGTTCCGGTGCAAACCAGTTTGTAATCTGGATCCGGGGTGTGACCCTGAGTATATCCAACCTCGGTCTTCACCACTCCAACAACGCGCTGAAAGGCCAACTCGGCGCCCCAGAAACAACCGGATCCGAATTCGGCGAACTCGTGATCCGGACTATCCGGAACATCCGAATCCGGGTCAAGAGCAGGATTGTTGTGAGTCTCATCGCCTCCACTCGTAGCCATTGCACCCCACCAGTGGTGTGACAGATTGAAGGGTTTAAGAAGATGGAAAAAGGAACAATTCTGAGTAATTAAATATGAATTAATTGGTTTTGATAAAGATAGAGAAGCTCATGGATGGAATGAAACGCTTCTTGTGGTGACTAGGAATCGGAGAATTGAAAAAAAA

>C100833_65

AGAAACCGACAATTTTAACATGACTAATAATTCCAAGTTTCCAACCATCCCATGTGGCTCCCTCCACCTTTCACTGAATTCTCTGCTTGCCTAATCACTCTGCTCTTTTTCTCTGCCCCCTCTTTTTTCTGGTTGTGACTCTCAGTTGAAGGTACTGTTCCAAGAAAAATCTAGAGCAGTGCATTCTGTGAATCTGATTTTCAGATGTGGGAATCTGAGAGTGAGTCAGCTGCAGGACACAAATATGGGGGTGGACTTCTCACTTCAAGTAACCATGGTGTCAAGACAGAAGGGTTTGTGCAAAGAGACCACTCTTGGTATGTTGCTACTGATATTCCAAGTGATTTTCTAGTTCAAATTGGAGAAGCTAGCTTCCACTTGCACAAGTATCCCTTGGTATCTCGAAGTGGAAAGCTGAGTAGAATCATATATGAAACGCAAGACCCAGATTTGAATAAGATAGTTATGGATGATATCCCTGGTGGGGCAGAAGCTTTTGAGCTTGCGGCCAAGTTCTGCTATGGAGTTGCTGTTGATTTAACAGCAGGCAACATCTCAGGGCTAAGATGTGCTGCAGAGTACTTAGAAATGACTGAGGACCTAGAGGAAGGGAATCTTATATTCAAGGCAGAAGCATTCCTTAGCTACGTTGTTTTGTCTTCATGGAGAGACTCCATAGTAGTGTTGAAAAGCTGTGAGAAGCTGTCACCATGGGCTGAAAACCTTCAAATTGTTCGCAGATGCAGCGAGTCCATTGCTTGGAAAGCTTGCGCTAATCCCAAAGGGATAAGGTGGTCCTACACTGGAAGACCAGCGAAAATTTCTAGCCCAACATGGAATGATATGAAGGAGTCAAGCCCAAGTAGGAACCAGCAGGTTCCTCCAGATTGGTGGTTTGAAGATTCATCAATCCTTAGGATTGATCACTTTGTTAGAGTCATCACTGCCATTAAGGTAAAGGGCATGAGATTTGAATTGGTTGGTGCTGCAATAATGCATTATGCAACTAAGTGGCTACCAGGATTGATTAGTGACACAGCAATCCCAGGTGATGAAGCAAGCAACTGCAGTGTGAGTAACAGCAGTAGTAGTGGGGGCAGTAGCTGGAAAGGTGGACTTCATATGGTTGTGACTGGAACAAAAGATGACACTTCAACTGGTCAAGCTAAAGAGCAAAGGATGATCGTCGAGAGCCTTGTCAGCATTATTCCACCTCAGAAGGACAGTGTCTCATGCAGCTTCCTTCTTAGGCTCCTGAGAATGGCAATCATGTTAAAGGTTGCACCTGCACTGGTCACAGAATTAGAGAAAAGGGTGGGAATGCAGTTTGAACAGGCTACACTGGCTGATCTTCTAATTCCTTCTAATAGTAAAGGAGAAACTATGTATGATGTGGATCTTGTTCAGAGGCTGTTAGAACATTTTATTGTTCAGGAACAGACTGAAAGTTCGAGTCCTAACAGACAGTCATTTTCTGATAAACAACATATGGGAATGGGATGTATCCTAAATGCCAAGGCAAGAGTGGCTAGGCTTGTTGACAGTTATCTTACAGAGGTGTCCAGACATAGGAACCTATCCCTGACCAAGTTTCAGGTCCTTGCAGAAGCTTTGCCTGAGTCAGCTAGGACCTGTGATGATGGACTTTACAGAGCAATTGATTCCTATCTCAAGGCACACCCAACACTTTCTGAGCATGAGAGGAAGCGACTCTGTCGTGTAATGGACTGTCAGAAACTCTCCATTGATGCATGCCTGCATGCTGCCCAAAATGAAAGGCTTCCATTAAGGGTTGTAGTGCAAGTTCTATTCGCGGAACAGGTAAAGATAAGCAATGCACTAGCCACCAGTTCTGTGAAAGATGTAGAATCTGAGAGTCATGCAATGATAACAAACCGGAAAACACTTCTAGAAGGGACACCACAATCATTCCAAGAAGGATGGACAGCTGCTAAAAAAGACATCAACACACTAAAGTTTGAGCTCGAGAGTGTAAGAGCCAAGTACATGGAGCTTCAAAATGATATGACTAGTTTGCAGAAACAATTTGATAAGATGCTAAAGCAGAAGCATACTTCTGCATGGAGCAGTGGGTGGAAGAAACTCAGCAAACTTGGAAGGGCAACAAATGTAGTAGAAAATCAGGATGATTCTCCTAAGATTCCTGATTCAATAGAACAAAACAGAAAGACTGCTAGAAGGTGGAGAAACTCAATATCCTGATTCCTGAAATATGAGAATTCTCAATTTCTTTGAAACTCATTCTTCTTCCCTCTGCATATCGTTTAGCAATTTCTACAATATTCTAGCTAAATTGTAATTTGTCACCAAAGCTAATCATATCTTTCAAGTGTGAATTCTACTGGCACTTCCATCACGAC

>C100835_65

AAAGAATATAAAGAAAAAGAGGAAAGAGGTGGATCTGAATTCACATGGGACTCACCCACAATAAGAAAACGTTGCTTTCTTCTGTGTCGCTCAGAGAAGAAGAACAAGCACTTGTTACTGTTTCTTAGTTTAGTGATAGGAACAGAAAGTAGAGAGAGAAGCTAGAGAGAGAACGAGAAAGAGAGAACTCACTACATTTCGAATCGGAGCATGGCGGAGCTCCGCCACTCCTCTTCCCTCGGCGCCCGAGCCACCAATTCTCCGATGAAGCGTGATGCTGTCGGCGTCGCCGGCGCCGGCGATTCCTCCCCTCTCATTCCCGACAGCCACCTCGCTGACGACGACGAGCACGATCGCTACTCTTCCAAGGACCGCGAACGCCACTTGTGCTCCTTCTTCAACGACGACCCTAGGGTTTCGCTTCACAACTCCAGGATCTCTCTCTTTTTCACCTTCCTTCTTCTCCTCGTTGGTTTAGTCTCCGTATTCACAATCTTCCATAAACTCAATTCCCCTTACTTGTGCAAAAAGGACGGGATTGTTCTTCATTGCCCACATGTTAAGGAATCGCCATCTCTTTGGGAAAATCCTTTCTCATCCACGACATCTTGGAAGCCCTGTGCTGAGCGCCGGGATGGTGTTTTAACAGAGCTTCCTCCTGAAAATGAAACAAATGGCTATATATTCATCCATGCTGAAGGTGGTCTAAATCAGCAAAGAATTGCGATATGCAATGCAGTGGCTGTGGCCAAAATAATGAATGCCACCCTTATTTTGCCTGTTTTGAAGCAGGATCAGATTTGGAAAGACCAAACGAAATTTGAAGACATTTTTGATGTGGATCACTTCATTGACTACCTGAAGTATGATGTTCGTATTGTACGTGATATCCCAAGTTGGTTTACTGATAAATCAGAGCTATTTTCTAGCATAAGGCGGACTGTAAAAAACATTCCAAAATATGCACCAGCACAATTTTACATTGATAATGTTCTGCCCCGAATCAAGGAGAAGAAGATTATGGCTCTGAAACCGTTTGTTGACCGGCTGGGATATGACAACGTTCCTCCCGAAATCAACAAGCTGAGATGTAGAGTTAATTATCATGCACTGAAATTTCTTCCTGATATAGAGCAGATGGCTGATTCACTAGCATCAAGAATGAGAAACCGAACTGGCAGTTCAAATCCTTATATGGCTCTGCATCTTAGATTTGAGAAAGGAATGGTGGGACTATCATTCTGTGATTTTGTGGGGACAAGAGACGAGAAAGTCAAAATGGCAGAGTATAGAAAAAAAGAGTGGCCTCGACGGTACAAGAATGGTTCCCATCTGTGGCAATTAGCTCTGCAGAAGCGGAAGGAAGGTAGATGTCCTCTAGAGCCTGGAGAAGTGGCTGTTATTCTTCGGGCTATGGGCTATCCAAAGGAAACTCAAATTTATGTAGCTTCTGGACAGGTGTATGGAGGACAGAACCGGATGGCCCCACTAAGAAATATGTTCCCTAATCTGGTTACCAAGGAGGAGCTAGCAACCAAAGAGGAGTTAGATGGTTTCAGAAAGCATGTGACAAGCCTGGCAGCTCTTGACTTCTTAGTGTGCTTGAAGTCAGATGTATTCGTGATGACCCATGGAGGCAACTTTGCTAAGCTGATCATTGGGGCTCGTAGATACATGGGTCACCGTCTAAAGTCAATTAAGCCTGACAAAGGGCTAATGTCCAAGTCTTTTGGAGATCCATACATGGGTTGGGCTACTTTTGTAGAAGATGTTGTTGTTACTCACCAGACGCGGACTGGATTGCCAGAAGAGACTTTCCCTAACTATGACCTCTGGGAGAACCCTCTGACTCCATGCATGTGTAGAGCCTGATTACAGCAATTTTTTATCAACGACACTGGGACTATACTCAGAATGCATTTGGGAGAAATATGCTATTTAAGTTATGAATTCTATCTATCTCCAGGGTAGACGTCGGAATGTGCGCCTCCTGTCAATGCAAGCTTTGAGGTAGGAGGGGCAGTAAATAGAAATTTGAAATAGAGATAATGGCTTGAAGGGAATGAGATTGGACTGTTGGTTATCATTTTAATGAACAGATTTCCTATTCTGAGTATTCTCTTTGCCGAGACACCAGGTGGATTTTGTCATTTCATCTAACATGTAGCAATTCCCCACTTTTCTTCTTCCAGGAAAAAAATGATTATTTTTTGTACTAAGAAAAATGTGCAAATCATTAATGAACTACATTGCTAGGTTTATTTTACGATGCATTTGGCAGTAAAATGTGAGGGTGAGGCTGATGAAGGTTTTTCATGATTATGTAGGAGGATTTGGCTCCTTAAAAGTGCAGAGTGTAATTAAAGATGCTTTGGAAATTTT

>C100841_65

CTTGTGCTTGCAGTAGTATGATCTAACCCTCAAAAGAAGCACATCTTACTCGTGAAAGATCGATGGCAATAATCGGTTGTGACAGTAATGGACACATGGATGACACCAAGTTCAGCAAACCGATGCCATGGATTGGCATATACATAGCAGCAGCATCTCTTGCCTGTCTCATAGCAATGGCTGCAGATCTCATACACGGAATTCGGGGTCGCAAACTTTGGTTTCCTTGCAAATACTTCTGCTTAAACGCCACTTCCTTGGCCATAATAGCTGTAGCAGTGAAGTTATCAGTGGATCTCAACACCCCTATGCCACACCGCCATGATCAGCTATCAAAACTCAGCAGCAGCGCCCTGATCTGCACAATAATGGCCAATTCCATGCCGACTCTTGGGATCACCGACAACCAAGACACCATGATGAACCTCATGGCCATGGCTCTCCTTGTGGTTACCATGATTGTGAACATCTGCATCCAGTTTGTCACCGGTGTTATCTATTTGTTCTGGGTGGAACATGCTGTCGTCATGCTCCTCATGATAATTTTGTTGATGACCATGACCTCCTCAGCCATAAGTATTCCAAAGATGAAGCACCATTTCGAGCTGAAACATAAGATGAATGAAGACGCCCTTAAGGAGGGTTCAAAACTGTTTGGGGAGGAGGGGAATCAAATCGTCAACAAAATCAGAGATCAACTGATGAAAATTTGGATGATGACCCAGACAAGCAGCCCCCAATTTGTTCTTGGAAGGTCAGTGACTTGTACTGCTTCTGGTGCCTTTTGTCTTCTAAGTACCATGACATTAGCAGAGGCCATTCTTCGATCTTACTTGATGTCATGGTCGTTTGATTTTTGCTCTGGTGACTGTGACTACAAGTGGTCCACCATCTTAATTCTCATAGTTCAGGTTGCTGCAGTGGTGGTGGGCACTATTGCTCCTGCTTTTAGATGGTTCGTTGCCATAACCTATAAGTGCCCAAGTATGAAGAATAAGACCTGCAAGAGAATGTTCCATGTTGAAGGGTACTGGACTACCAAGCTAATCTTAATTAAAGAAAGCCCTCTTAGTTTTCGAATTCATAATAGGCAGTACAGAAAGCTGGCTCATGATGCAAAACTTCTTCTATTGTGTTTCTGTATCAAACTGCAGGTGGGGATTGTGCTAATGAGTAAAGCAACCCAGTACGTTTCAATAACCCTCATGTGCTGGATCTTGTCATGTTATGATCGTTGCAAGAAGTTGAAATCCAAATTGGTTTCTACTGTCTCGAGCGTTAGCTCGGGAACAGAATCAAAGTCTGCCCCAAAGCTGGATCTTAGACGTTTTGTTTTGCATCTTGAAGGCGAGGAAGAGTTAGTGGGGGTGATGTTGAGACAGAATCGTGATGCCACTATTCACTGGGTTCAAGTAGGAGAAAAAAAGCAGCCAAAACTTCTCACTGAATTATTGGAGAAGAAATGTTCTTTTTTTCAAGGATTCAAGGGAGTGGGAACATTTGACAGTGACCAAGTTCTTTCTTTACATTGTGTAGAAGCTCTATATAGTTGGTCACTTCCTCTGGTGACACTAGCTAGCATTGTAGTGGCACTGCCAAACACCAACAAGGATTCGGTCAAAAAATTGATAAGTACTTTAAATGAAGGACTCCCCTTTGTAAAGTTCATAGAAAATAACCTGGACAAAGAAGGAGAATTGTCTAAACTCAGGTCGGCAGCAGAAATTGTGTGGCTTGGAGTTGATCTATATGATAAGTGGTTCGACGTGGATCTTCATGAGCTCTCACTTCAAGATAAGAGCCCCAAAGAGACACTTGAACAACTAGCCAATGCTGCAAAAGTCAGGTATGAGAAATTTAGAGGCAAATATAACCACATATGCGTAAAGTTAAGCCCTTCATTATGGCCCAACAAAGTATCTGCAGCTCACATTATGTACAGAATATGCAAAACTGCTCTCTTAAATCAAGAACTCTTTAGAGACAATTCGAGTGAGAAGCTGTTTGAAGCACTTACAGCGATGATTGCAGATATATTGGGTGCATGTCTCACCAACTTACCCCTTGTTATATCCAACAAGTGTTTGAATAGCACCATTGAAGAGAGGGAGGATACTGTGAGAAATGCAGTTTACATTTTTGGCAAGACCAAGAAGATTATACAAATGACAGAAGAGAGAGCATTTCCCCGAGTGAATTTCTGCAAAGGGACCTATATTGAAGATTGGCGTTTAATGCATAAGCAGAACAAATTATTTCACTTTGTTCCATCTTCACAGGAGAATGACACTCCTACAGATTCTCCCACTTCTAGTACTCCTACAGATTCTCCCACTTCAAGTGATCTTTACCTTAATATTGACTAAGAGCCAGGGCACTGGTG

>C100843_65

CACTCGCTCACATTCTATAAACAAAACCTTCCTCAATCGGAAGAACAAAACCGAAAACCTCAATCTCTTAACCAATCAGTGTCACCAACATGACGAGTGTGATCCTCAGAAGATCCTCTTCCGTAATCTCGCGCCGCTTCTTCGCCGCTCTAGCCTCCGTCGAACCGCTTCCGCACCACCGCCGGCTCGCCGCGCTCCGTCTCGATGGCTCTAGCGACGGAGTTGGAGTCAGATCTTTGCACGCGAACTCTGGACCGTTGAACTTCCGGTCGTCGCCGGTTTCTCGCGCGGCACAGTTCGCCGTGGAGCGCGACTACTCCAATGACGAGGTAATTTGCGTCGCGAACAGCGACGAAGGACTCGAGATCGCGAAGCTCGGGATCTCGAGCGAGATCGTGGATGCTTTGGCCAAGAAAGGGATCGCAAAGCTGTTTCCTATTCAGAGAGCTGTGCTGGAACCTGCAATGCAAGGACGTGATATGATTGGTCGAGCTAGGACTGGAACTGGGAAAACTCTTGCATTTGGGATACCCATCTTGGACAGGATTATTCAGCTTAATGCGAAGCATGGGCGAGGAAAAGATCCTTTGGCTTTGGTTTTGGCTCCGACTAGAGAACTTGCACGGCAAGTTGAGAAGGAATTCAATGAGGCTGCACCTAACCTGGACACAATCTGTCTTTATGGGGGTATGCCCATCCAGCAACAAATGAGGCAGCTTAATTATGGTGTAGATATTGCTGTAGGCACACCTGGTCGAATTATTGATCTTCTCAATAGGGGGGCACTGAATTTGAAGGATGTCAAGTTTGTTGTTCTCGATGAAGCTGATCAGATGCTTCAAGTAGGATTTCAAGAAGCTGTGGAGAAGATCTTGGAGAGATTGTCTCCAAATCGTCAGACTCTTATGTTCTCAGCAACAATGCCACCTTGGATAAAGAGTATTACCCGCAATTACCTAAACAATCCCCTTACCATTGATCTTGTTGGAGATTCTGATCAGAAGTTGGCAGATGGAATCTCACTGTACTCTATTGCAGCTGATGTGCGCACTAAAGCAGGGATTCTTGCACCACTGATAACAGAACATGCAAATGGAGGAAAGTGTATTATTTTCACTCAAACCAAACGTGATGCTGATCGATTATCATATGTCATGGCAAAAAGCCTTAAATGTGAGGCCTTGCATGGAGATATTTCACAAACTCAGAGGGAAAGGACTCTTGCTGGTTTCAGAAACAACAATTTCAATGTTTTAGTGGCAACAGATGTTGCCTCACGTGGCCTTGATATTCCAAATGTGGATCTCGTAATACATTATGATCTTCCCAATAATTCGGAGATATTTGTTCACCGATCTGGACGAACGGGCCGTGCTGGGAAGAAAGGAGCTGCTATTCTTGTTTACTCAGAAGATCAATTCAGGACTGTGAAGACTATTGAGCGTGATGTTGGATGCAAATTTACAGAGCTAGCAAAGATTAATGTTCAAAGTGGATCAGTAGACATGTTTGGTGGAATGAGTGGTGCTCGATATGGTTCTTCAGGAGGTATGAGGGATCGTCAATCTGGAGGTTTTGGTCGCAGTCCTGGATTTGGTCGTTCTGGCAGTTACGGTGACTCTGGATTTGGCCGATCTAGTTATGGAAATTCCAGTGAGACGGGAAGGTTTAGAGGACCAAATTCTAGCCGTTTTGGGAGCCCTGGTTCAAGTGGGGCGGGAGGGGGTTTCGGAGGATTTGGTAGTTCAGACCGATCTGGTGGTTTCGGAGATTTTGGGTGGGGTCAATCTAGTGCATCTGGTGATAGAAAGTCAAGCCAGAGCAGCAGCTTTGGTGGTTCACGTGGCAGTGGTTTCGGTGCTTTTCGGGGATAACCCAAATAGTGAAAGTTACAATAGAGGAAAGCCGTTTTAATTTCATCAACTTGGTAAGTACAGCCATCTTCATATCTTGATGGTTTAATTTTCGCCTCTTGTATAAAGTTAATCTAAATTTTCGGACATCTTGCCAATTTCCCCGTATCTCGGTAATATGACATAAGAATAATACATTCTATTTTGGGAGGCATTACAGTATTTTCTCTTGGATCTTCAGCTAAATCTTCTCCCATAGCAAAAGCCTATATCATCTGTATGTCAATTTTCTGTCTCCATTTCGTCTGTTCCCTTTTCAAGCGGTTTGGTAGATTGTGATTCTAAACGTGTATCCGTGTATGTAAATGCCTTGGAAATTTTGTAGCAGCTGCCTTACAGAGGGGTGTTCCTATTTGTTTAATACGTTCTTCAAAATTCATCATGGGCAAAGATAGAAATTTTTAGAAACTGGTAGGTGTTTTCTGTTCTTGTTATCACAATGTTGCATATCATGTTACAAGGAAATTCTC

>C100847_65

GGTAGGTGGAAGTGAAGGGAGTTGTACCATTTGTTTAATTTTTGGTGTTCCAAACAGAGAAAATTAGATAGACGCGGTAGAAATGACATTTGGCTGTTGGGCCGATAAAGTGAACGGAAATCAATAAATGTTTGATTCATAATGTCATCATTCACTCTTCCATCCGAACACGAGCTAAGTAGTTGAGTTTTTGGAGCGCTTTAAACAAACACGCCATCACCGTGATTTGAAATCGAATAGAAGAAGGCACGTTAGGGTTTCGCATTGCCAGAGAGAAGAAGAAGATGGAAGACGTTGTTGATCACTTGGCTTTCGAGCGGAACAAGACGCAATTCGATGTCGAAGAGATGAAGGTCGTTTGGGCTGGTTCTCGCCACGCTCTTGAAGTCTCCGATAAAATGGCTCGTCTCGTTGCCAGCGATCCGGCGTTCAGAAAGGATGATAGACCCAGGCTTGGTAGGAAGGAGTTATTTAAGAACACTTTGAGGAAAGCAGTTTATGCGTGGAAAAGGATTATTGAGCTCCGTCTAACTGAAGAGGAAGCTTCTATGCTCAGATCTTTTGTGGACCAACCTGCTTTCACGGATCTACATTGGGGAATGTTTATTCCTGCTATCAAAGGACAAGGAACCGAGGAACAGCAGCAGAAGTGGTTGCCTTTGGCTCAAAAGATGCAAATAATTGGATGCTATGCCCAAACGGAACTGGGCCATGGATCTAATGTTCAAGGGCTAGAAACAACTGCAACCTTTGATCCCAAAACAGATGAGTTTGTAATTCATAGCCCCACATTGACTTCCAGCAAATGGTGGCCTGGTGGATTGGGTAAAGTGTCAACACATGCTGTTGTTTATGCCCGACTAATTACTGATGGTCGAGATCATGGAGTGCATGGTTTCATTGTCCAGCTGCGGAGCTTGGATGATCACTTACCTCTTCCAGGCATAACTGTTGGTGATATTGGAATGAAATTTGGAAATGGAGCATATAACTCCATGGATAATGGGGTTCTAAGGTTTGACCATGTACGGATTCCAAGAAATCAAATGTTAATGAGGGTTTCACAGGTAACTAGAGAAGGAAAATATGTACAATCCAGTGTTCCAAGACAATTAGTTTATGGTACTATGGTATTTGTAAGACAAACAATTGTATCTGATGCATCATCTGCTTTATCACGAGCAGTTTGCATTGCTACAAGATATAGTGCTGTTCGAAGACAGTTTGGGTCGAAAAAAGGAAGTCTTGAGACACAGGTGATTGATTATAAAACACAGCAAGCTAGGCTCTTCCCTTTGTTGGCTTCAGCCTTTGCCTTCAGATTTGTTGGTGAATGGTTGAAATGGCTTTATACGGATGTGATGAAAAGATTGCAAGCCAATGATTTTTCAACATTACCTGAGGCTCATGCATGCACTGCAGGATTGAAGTCCTTAACTACTTCAGCAACTGCTGATGGAATTGAAGAATGTCGCAAACTATGCGGTGGTCATGGTTATCTTTGTAGTAGTGGTCTTCCTGAGTTATTTGCAGTGTATATTCCTACCTGCACATATGAAGGAGACAACGTTGTGCTGCTTTTACAGGTGGCAAGGCATCTCGTAAAGAGTATTTCTCAGTTGGGCTCTGGAAAAAAACCTGTTGGTACAACATCTTATATTGGACGAGTGGAACAGCTAATGCAATATCATTCTGATGTTCAGAAAGCGGAGGATTGGTTCAAGCCTAGTGCTGTGCTGGGAGCATTTGAAGCTAGGGCCGCTAAGAAGTTGGTTGCTTGTGCTCAAAATCTTAGCAAGTTTACCAATCTTGAAGAAGGTTTCCTAGAACTGTCGTTTGATCTAATTGAGACAGCTGTTGCTCATTGCCAGTTGATTGTTGTTTCCAAATTTATTGAGAAGTTGCAGCAAGATATACCTGGAAAGGGAGTGAAACATCAATTAGAACTTCTTTGTAGCATTTATGCTTTGTCTCTTCTTCATAAGCATTTGGGTGATTTTCTTTCAACTGGCTGCATCACTCCCGAACAGGGTTCCCTTGCAAATGATCTGCTGAGGTCCTTGTATTCACAGGTTCGTCCTAATGCAATTGGACTTGTTGATTCCTTTAACTACACTGATCACTTCCTTGGTTCGGTTCTTGGGCGCTATGATGGAGATGTGTATCCAAAGCTATACGAGGAGGCATGGAAGGATCCATTGAATGACTCAGTTGTGCCAGATGGCTTTCAAGAATATATTCGACCAATGCTAAAGCAACAACTTCGGAATGCTAGACTCTGAAACATTTTGTTTTTGTACTCTTGATACTGGACAAAGTTATGTATTTTACGGTAATAAAGTTTATGGAAAAGGTAAAATAAATATACATCGATGAAGTTGTCA

>C100851_65

TATATCTATTACTTTTGTCCCTTCTCACATGCATTGGCCCCCACCTCCCTCACGAGACCCCTCTATGATATCGAATATATTAGTTCACACGTGAAATCTTCACGTTTCACTGTGCATGACCGAGTCTACTCCATCACTTGCTTCATTTTTCCATGGTCTAACTCGGTAATGTTGTTCAGTTTATATGCTCCTACTAATAACAGTAGCTAGCTATATATACCACTATGACACAACTTCATCAGTTTCTTTAGCTCTAAGCTACGTTTCATTAAAATGAAAGATTGAAAGTTGAGGCTCTAGATATAGTGTCCACCGTACTGGCTCTGCAGGTTCATGGCTGAGGCTGACCCCATTGCATTAAATTCAACGAGATCTTGCTGAAAAAAGGGTCTCCTCATTGATTCAATGTTATTATCTCCGACACCCAAAAAATCTCGGGTTAAACCAAGTTGATTTTTGTTGTGTGAGCTTATTGAGTGTAATTGCTTTCCAGTAATTGACGCTGTTGTCTCTTCATCCACCGGCATCACCATGTGCTCCAAATCCTTGGTGCCATTCATAACCACAAACGAGTTGTTTGAGTCATCATTTAATAAATAACCACCACCACCACCTCCAAGGTTGGTGCAGCTCTCCACACTCACCAACTCGTTCAGCTTCTGAACCTCAACGACAACGTTGTTCACAACATTGTTGTTGCTGCTGTTGTTTCTATTGCTGACCATGGAGCAAAACGCGTTGTCGTTCACCCTCGTAGATCCCATTTGAACATTTTTCTGCAACAGTGTAGTGGCTGACATGTAGTGAGGTGGGTTCTGATTACTACTCAAGTACAAGTTAGAGTTTGCGCTATGCGACAAGTCTCCTTTGTTTTCTTCTTGTTCTTGCTTCAGTCCATGTGGCGGCAGCACAGACAGGTTGGCACCTCCAAATGATGCTTCTGGGTAACGGTAATTCACAAACTGTGGCCCAAACATGTCCATCGTTTGCACCACATCAGGCACAGGGTTTGGCCCCGAAGAAGTGAAAGCACCTGGTTTGGTTGGGAAGTTAAGAGGGTGATGATGAAGTACTTGTGAATTGGCCTGGTGGTCTAGCCACAGTGGTAGCCTTAGCTTTTGTTGTTGATGTTGATGATGTTGGTTTGCGTCAGCATAATTCACCAACGGTTCTGATGTCCCTGAACCACCGAATTGGTCTGAGTGAAACCCGGAAAAAATCTGAGGCATCCTCGAAGCTTGAGCATTGGTCAAATGATGGTCGTTTCTCAAGTTGCTAAAAGCTGCTGGAACTGTTGATATCCTCACATTCTCTTCAGCCAAAGCGTCACAAAAAGCTCTATGCGTTATGAAGCTATCCTTCCTGGAGAAAAGTGTGCCACAGTCACATTTGTACTCTCTAGTCCCACAAATCTTGCTATGGGCCTTCCAATCGGATTGAACAGCATATTTCTTGGAGCACTTGTCACACTTCCACTTCTTCTCCCCATGCTTTCGGCTATAGTGCTTCTTTATGCCTGTTAAATCTCCCAAGGCTCTAGAGGGGTCATGGTGCACACAACTCTTCTCAGGGCACACATACACCTTCTTCCTCACTTGATCTTTGTTTGATCTCTGCTTCAGCTTCCATGGAAGGTTGTGGCCACGTCTATGCAGCTGCAAATTCTGGTCTCTCTGAAAGCCCTTGCTGCAAATTTCACATATGAAACGGTTGGTGGCCATGAGGGACTTAGGTGACAGAGCTACCACTTCTGCATCTGGATCTGGTGTTCCGGGTAGACTTCTCCTTTTCTTAGCTGGATTTGAACTAGGGTTAGGATTATTAGGGTTTGAGCTTGGGGGTTGAACATGAACCAAGGAGCCTCGGATGGCGTTGGGAGGAACAGAAAAGGATTCTTCAGACATCATTTGTGAAGTGAAGAAAAACTGTGTGTCCTAAACTAGCTACCTATGAGAAATTCCAAAAGAAATAGTAGTTTGAGGAAGGTACTACCAAAACAAATATAAAACCAAAAGGCAACGTGTTAGCTAGCAGCTACCAGTGCTCATGCTGGGAAAAGATAACATAATAAGTAGGCATGTTGAAAGGAAAACTGCATAGATAGATATAATTGATCTTTTTCATTGTGTTGATTTTGTGGTTTGAGACATAGAAAAAGAAAAAGAATATGTGTGTGGAAGAAATTGAAATTGGTTTTTCATGTGAATCGCGCTGAAAGGAAAGTCATAGTTGGGAATGAATGAGAGTTAAATGAAGAGAGAAGAGAATAATGGTTTATAGAATCTTTCTCACTCGATCTCTTCCTTTCTCTCCTTATCTAACACGGCTGCTCTGTGAGGGTCAGTCAGCCATAGACTCTGCGGACCACTTTCTCTCTCTCTTTT

>C100853_65

GTTCTCACCACACGCCTTTCTCATAGCTCTTCTTTCTCATCTCTCTCTCTTCTCTTCCTTTCGAAAGGGGAAACGAATCTCAGGCTCTTCAACTCTTCCAGCCGCCATGGCCGATCGCTTGACGCGTATTGCCATCGTCAGCAACGACAGGTGCAAGCCCAAAAAGTGCCGCCAAGAGTGCAAGAAGAGTTGCCCCGTCGTCAGAACCGGTAGATTGTGTATTGAGGTTACTTCTGCTTCCAAGATTGCTTACATATCTGAGGAATTGTGCATTGGATGTGGTATTTGTGTGAAGAAATGTCCGTTTGAAGCTATTCAGATTATCAACTTGCCGAAGGACTTGGACAAGGATACTACTCATAGATATGGTCCTAATACTTTTAAGCTGCACAGGTTGCCCGTGCCAAGGCCTGGGCAGGTGCTTGGGTTGGTTGGAACGAATGGAATTGGGAAGTCCACTGCCCTTAAAGTTTTGGCTGGGAAGTTGAAGCCGAACTTGGGTAGATTCACTAACCCCCCTGATTGGCAGGAAATTTTAACCTACTTTCGAGGGTCTGAATTGCAGAATTATTTTACTCGGATTCTAGAGGATGATTTGAAGGCTATCATCAAGCCTCAGTATGTTGACCATATTCCAAAAGCTGTGCAAGGAAATGTGGGGCAGGTGCTAGACCAGAAAGATGAGAGAGATATGAAGAAGGAGCTTTGTGCTGATCTTGAGCTTAATCAAGTTATAGATCGTAATGTTGGTGATCTTTCAGGTGGTGAGCTCCAAAGGTTTGCCATTGCAGTTGTTGCGATCCAGAATGCTGAGATATACATGTTCGATGAACCGTCTAGTTATCTTGATGTAAAGCAGAGGCTGAAAGCTGCTCAAGTTATTCGATCACTGCTTAGGCCTAATAGCTATGTAATTGTTGTAGAGCATGATCTCAGTGTCTTGGACTACTTATCAGACTTTATTTGCTGTCTATATGGCAAACCTGGTGCATATGGAGTGGTGACACTTCCTTTTTCAGTCAGAGAAGGAATTAACATTTTCTTGGCTGGTTTTGTTCCAACAGAAAATCTTAGGTTCCGGGATGACTCACTTACCTTCAAGGTTGCTGAGACTCCACAGGAAAGTGCTGAGGAAGCTCAGACATATGCCCGATATAAATACCCAACCATGACCAAAACTCAAGGCAATTTTAAACTTCGTGTTGTTGAAGGGGAATTTACTGATTCTCAAATTATTGTGATGCTGGGTGAGAACGGGACAGGAAAGACAACATTTATTCGTATGCTGGCTGGTTTATTGAAACCTGACAGCGTGGAAGGTGGATCTGAAGTGGAGATGCCTGAATTTAATGTTTCATACAAGCCTCAGAAGATTAGCCCGAAGTTCCAATCAACTGTTAGGCACTTGCTACATCAAAAAATACGAGATGCGTATACTCATCCCCAGTTTGTTTCAGATGTGATGAAGCCACTTCTTATCGAACAATTAATGGACCAAGAAGTTGTGAATCTTTCTGGAGGAGAGTTGCAGAGAGTTGCTCTGTGTCTCTGTCTTGGAAAGCCTGCAGACATCTATCTTATAGATGAACCAAGTGCATATCTAGATTCTGAACAGAGAATTCATGCTGCCAAAGTCATCAAGAGGTTTATTCTTCATGCAAAGAAAACAGCTTTTGTCGTTGAGCATGATTTCATCATGGCAACTTATCTTGCCGACAGAGTTATTGTTTATGCGGGTCAACCATCAATTGATTGTATTGCTAATTCTCCACAATCATTGTTGACTGGGATGAACCTTTTCTTATCGCATCTTGATATCACATTTAGAAGGGACCCAACTAATTTTCGTCCAAGGATCAACAAACTTGATTCAACTAAGGACAGAGAACAAAAGTCTGCTGGCTCGTATTATTACCTTGATGACTGAGTCCATACTTACAGGCTGTTGTTTTTTCACCAATGCAACATTGCATCAGTTTGAGCTTTTCATGGGATCTCAGATTTTGTTAATTTGATGCCTTGAAATATAAGATTGCCATTTTTACCGTTGAAGGCCATCATTCTCAATGGATAGAAGAGCTTGCATACATGCTTATGTTGGTTTTTGTATATTTACCATATTTATGCAGTTACTCATCACCAGTTTTGCTACGACTTGGGTGGGTGAATCTGAATATTACATTAGTTAATTGGTCATGGTTTTCATTGAATGTCCCAAATCCATGGGAACATTCATGACGTCAGACTGGGTTTATCGCCACTTTGGCACATAATTTGAGTATGTAACGAAGTTTTACATTCATTCCCATGTTACTTGATTTAGATAGATATCTCCTGTTTGGAATATATTGCCGTTGAGAGTTTTATTTATTTGAATTCCTTCC

>C100857_65

CAAAGCACACACACTCACTCACACAGAAACAAGACATAAAAATAAAATTATTTGATGAATAACAAAAAAAAGGAAAGATAACTCCATTATTCCATCGAATGAACACCCAGATTATCATTCATTGTCCAATCTTCACTGCAATATTTTCTTGGCAACGGGAAAGTTTGGAGCTATGTCAATGGGTACCTGAACCTTCTCCAACTTTTGTAAAGCAAGTTTGAGTGGCTCTGAAATCACACCATATTTTTGGAGAAGCAAACCAGCAGCCTCCTTATCACCTTTGGCTTGTATGGTCAGTATCTCCTTACTTAAGCTTTCAACTGCACTTTCAATCTTGGAAAAGTCAACAGAAATTGTGTCTTGAGAGTGCAGGATAAAGGCTCCTTTCTCATATAACCAGTTGAATTGCAATGCTTGTCCTTTTCCATGAGCTTCCTCTAAACCAAAACGTACTGACCTGAAGCATCCAGCCAGAAAAGAAACATACATGGACTTCAATAAGCTTTCAGAAAGCAAACCCTTAGAGATTAAGAACCTCAATGCCCAGAGACCTACTATATCAGCTTTTGCTTCTTCCAAAGCTGAGTGAAATTCTTGCAATTCCAATCTCACAGTAGACTTCTGACCATTTGGAAGTGTTATAGTATGAGGTCCAATCCCATGGACGCATTCATGGCAAATTGTATGAGTAAAATATGATTCAAAATCCACAAATTCTTGTAGCTCCTTTGCCACACAGTAACCAGCTATGGGCAGAAGAATATGCTCGAACTTTGCCTCTGAAACATTCTTAAGCATCACCATTGCTGTTCCTCGATCTTTTACAATACGTTCATCATTTGGTAGATTGAAGGCAACAATTTGTGGTCCCTTGACATCCCCTGCATTATAGATAAGTTGAATAACACGAATAGGTGCAGCATTCACATCTTTGGATTTGTACACATTGTCCATGGGAAGATTTTGCTCCAAAAGCTGCAGATTATCGCCAAAGAGTTTGAGCTGATTTGTAGCTTTATCATCTCTGATTCCAACAAATGCTTCGAAGGAAGCCTTATATCCGAAAAGTTTATCCTCGTACGTTTCATATGGACCAATTGTCACCTCCAACTTAGAGTCCAAATCCATCCAAGCCAAATCGGACTCATAATAATCATTTGAAAGGAAAGCATCAGCCTTGCTATGCAGCAGCCTCTTCAGACTTGGTGATTGGGTAATATCTCCAGCTTTGTGCAATAAATCAGCAGCTTTCGTGAGAAGAGACTTATATTCTACTGAGTAAGGCACTATATATAGATCATTATCATAACTTTCTTTGTTATTGAAGTGGGAGATATGAGAATCCAAACGAAGTTCACTGTGTCTTTTAATAACACTAAAAAAGCTTGTTGCCTCTTTTTGTTGATCCTTTTCTAGATTGTCCTTCCATTTCTCAAACTCCAATTTGTCCATGTCCGAAGGGTAGAAATTAGCACCACGAGGTTTTGACACAGGAAATGCTGCTCTATATTCTAGACCTTTCCATTGTTTAACAGGTTTAGTTGCTGTAGAAAGTAACCTTATTGCAGAATCTGCAGTCGTCAAAAATGCATTATCTTCATCAAGGCTAGACCACGGGCTCTTGTTAATCAGATAATAGGACCATTTCAACTTATTTAACTCTGATTCATCAGCATGCTCCTTCAACCAATCTCTAAGAGATGGATTATTGTACCAAGACTCTGCATTAAGGGAGATAGGAGCGTAACGATTTAGCTGCTTCTGCAAAGTCAAACTGCGCGCTTCAGTATTCTCTTTGTACCTCTTGTCAAGTATATCAAAGAGTTGAGCATATTGTCTAGAAACATCATAAGGAACATAATTTGAGTCTTGCTTGGCTAAAAACCCTTTGTAGTCGTCATAAGAGATATATTTTACAGCAGACACATGTGTTTTCTCAAGAGTAAAGGCCTCCAAAGGTATTGGATCAACAGTTGTTACTAGATAAACGTCCTTAGATTCCTTTTTGGTGTATTTTTCATCAATGGCACACTCCTCTTGGAAGACAAATATCAACTCAAATGCCTCCTTGGGAAGGCTTACGCCTAACTCTCTTTTTAGCTCCCTTCGAGCAGTTAGAAGAGATGAATGAGAAGGAACAATGGGAGCAGAAATGGTGATATCCCACAAGCCAGGTCGATTGGCACGTTGTTGGAGAAGCAATTCTTTTGTGCTCTCAGCAAATATCCATACATGAACACCACCATCAACTTCATTTTTTGCACTGCATCTATGGAAGTTGCTTAATCTTAGATTTTTACTGCACCTTTTAGTCACAACATCAACTTCAATCTCTCTTCCAGCCATTCTACCTCTCAGTAGCTGAAGTGGAAGCAAAGAATGAAG

>C100859_65

TCAACCACAAGCAAGAGCTTTAGACACGTTGTACTCTCTCTCTCTCCTCTCTCTGACAACAAGTCTTTGAGTTGAGTTTGAAGCCTCCCTCCATGATTTTCGTTCCGAAGGGCACCAATTAACATCTCTCGTTTCAGGTAGCGCATAAAGCGTATAATTATGAACACAATTGGAGAAAGAATGACAAAGAGAGAGACTTTTGATGATGACCAGGAGAGTTTATTGAAGAACGATGATAGGGCAACAGATGATGACCCTAAGATTTACAGAGGGTGGAAGGTCATGCCCTTCATTATAGGAAATGAGATTTTTGAGAAGCTAGGAGCTATTGGTACTTTATCCAATCTATTGGTCTATCTCACTACAGTATTCAACTTGGAAAACATCACGGCTACAAATATTATCAACATCTTCAATGGCAGCACCAACTTTGCTACATTAATAGGAGCTTTTCTGTCTGATGCCTTTTTCGGTCGCTACAAGATATTGGCATTCTGCACAGTGGCTTCCTTTGTGGGTTTGTTTGCAATCCAACTGACAGCGGCAGTTGAGAAGCTGCATCCACCTCACTGTGAAGAGTCGACCGTATGTCAAGGGCCAACAGAGGGGCAAATGACATTTCTGAAGACTGGTTTAGGTTTGTTGATGATAGGAGCTGCTGGGATTCGACCATGTAACTTAGCATTTGGTGCTGATCAGTTTAACCCTAACACGGATTCTGGGAAAAAGGGGATCAATAGCTTCTTCAATTGGTACTTTTTCACTTTCACTGTTGCTCAGATGATATCCTTAACAATTATTGTCTATATACAGTCAAATGTAAGTTGGGCAGTGGGTCTGGGAATTCCTGCATCTTTGATGTTTGTATCTTCTATAATCTTCTTCATGGGGTCCAAAATGTATGTGAAAGTTAAACCAAGCGGTAGCCCTATAACTAGTATAGTGCAAGTTATAGTGGTTGCAACAAAGAAAAGGAGGCTGAAGCTCCCGGAATATCTATATCCTTCCCTCTTCAACTATGTGGCTCCCAAGCCAATGAATTCTAAACTTCCCTATACATATCAATTCAGGTTCCTTGACAAAGCGGCAATCATGACTCCCCAAGATCAAATCAACCCCAATGGATCTGCTACTGATCCCTGGAATCTTTGCAGTATGCAGCAAGTGGAAGAGGTTAAATGCTTGTTGAGAGTGTTACCCATATGGGTTTCAGGCATTTTGTACTTTGTTGTAATTGTCCAACAGCACACCATTTTAGTGTTCCAAGCCCTTCTATCTGACAGGCGCATTGGGCAAAGCGAGTTCTTGATCCCAGGAGCATCCTACTACGTCTTTCTGATGATTACTGTGGCAATTTGGTTACCAGTCTACGACCGAAAAGTTGTGCCTTTACTCCAAAGGCTCACAGGAAAAGAGGGTGGCATCACACTCCTTCAGAGAATGGGTATTGGAATATTTTTCTCTATACTCAGCATGCTAGTATCTGCCAGGGTTGAACAGCACCGTAGAACCCTGGCTTTGATTAATCCTCTGGGAATAGCAACAAGAAAAGGTGCAATCTCATCAATGTCAGGTCTGTGGCTGATTCCTCAGTTGATATTAGCTGGATTGGCTGAAGCATTCATGACTGTTGCACAAGTTGAGTTTTACTACAAACAATTTCCTGAGAACATGAGAAGTATTGCAGGTTCTCTTTACTATTGTGGACATGCAGGGTCAAGTTATTTGAGTAGTCTGCTTATTTCAGTTATCCACCAAGTCACTGCCAAATCTGAAACTGGGAATTGGTTACCGGAGGATCTTAACAAGGGCAGATTGGATAAGTTCTATTGCCTCATTGCTGCTATAGAAATTATTAATTTAGGTTACTTTGTGTTATGCGCAAGGTGGTTTAGGTACAAAGGCACGAGCAGCAGTTCTATTGAACTCGAAAAAGTGACAAAACAATCGGAAAAGAGTGCTAATGGTCTTAATGATTCGAGTCTATAGCTACTGCGTAGAAATAGGCTACTGCAATTTTTCATCGGATGGAGCAGCAACTAGCAAAGGTTAAAATCAAAATTGAGTATAGCAACAAAATTACACACTCTCTACTTTTTCTATTAGATAGATTTTTTGTGTAGATGCATCAAGACAGATGGGGTGCATTATCTTAAAATAGGTGCCTACATCCAAAACGTTTGTCTTAAAGCACCTAATGACAGTGAAAAAAAAAATTATAGACTGACATGACGATTATATTTTCCACAATTTGTTTCCTACGGGTGAAGATAATGATTGTAACCACCTGAAAGTGGGTCATAGTCGGTGGTCATCCAGCTCATGTTACACGAAAATATCACTTGAGAAAGAAAATAAAAACATTATTGTAGGAATTGTCATTGCT

>C100861_65

ACATACGTGACACCCACCACTCTCCCTCACACACTCACGCAACTCACGACACTCTTTTTTTTTTTCTTAAAAAAAAAAAATCATCAACAGAATCACACTCGAGGTTCCCATGGCGCCAATTCCGTAGCTAACTAACTCGTTTCCTCGAACAACTCGAACAACCGAAATGAAGTCCGCGCGGGTCAAAGAAGCGATTCTCTTTACTCTCCTAACGTGCGTCTTCGCCATTGCAGGTTCGGATCTAGCTTCGGACAGAGCGGGGCTATTGTTACTCCGAAGCGCGGTGGGGGGACGCACGCTGCTATGGAACGCCACCCAAACAAGCCCTTGCTCGTGGACCGGCGTGGTCTGCAGCAACGAGAGAGTCACCATGCTGCGCCTCCCCGGGATGGGCCTCTCCGGTTCGCTTCCCTCCGGTTTGGGAAACCTCACCGAACTGCAGACGCTCTCCCTCCGCTTCAACGCGCTAACCGGACAAATCCCCCTGGATTTCGCCAACCTCAAAGCGCTGCGGAACCTATACCTCCAGGGAAACTTCTTCTCAGGCGAAGTTCCCGATGCGGTGTTCGCGTTGCAGAACCTTGTGAGGTTGAATTTAGGTAACAACAATTTCAGCGGCGAGATTTCTCCCAAGTTCAACGGTCTCACTCGTTTGGCCACTCTCTTTCTCGAACACAACAATTTCACTGGTTCAATCCCTGACCTTAGTGTTCCGCCTCTCGATCAGTTCAACGTCTCGTATAATTCCCTAACCGGTTCCATTCCCAACCAGTTTTCGCGTCTGGACCAAACCGCTTTTCTCGGAAACTCACTCTGCGGGAAGCCCTTACAACTTTGCCCCGGAACCGAGGAGAAGAAGAGTAAATTATCCGGTGGTGCCATTGCGGGTATTGTCATTGGTTCCGTGATTGGTTTGTTGTTGATTCTGCTGCTTTTGTTCTTTTTGTGTAGAAAAAGAAGTGGGAAAAACGATGAAAGTGTGTCATCTGGGAAACGTGATGTGGTAGGTGAAGTGTCTCGTGACAAGAGTGTTGAGAGTGGCGGGAATTCAGGTTCGGCAGTTGCAGGTTCGGTGGAGAAGAGTGAGGTTCGAAGTAGTGGTGGTGGAGATAAGAGTTTGGTGTTTTTTGGGAATGTGAATAGAGTGTTTAGTTTGGATGAGTTGTTGAGGGCTTCTGCGGAAGTGTTGGGGAAAGGAACGTTTGGAACGACTTATAAGGCGACTTTGGAGATGGGTGCGAGTGTGGCGGTGAAGAGGTTGAAGGATGTTACTGCCACAGAGAGGGAATTCAGGGAGAAGATTGAGCAAGTGGGGAAGATGGTTCATCTCAACTTGGTCCCACTAAGGGGTTATTACTTTAGCAGGGATGAGAAGCTTGTTGTCTATGATTACATGCCTATGGGAAGCTTATCTGCTCTCTTACATGCAAATGGTGGAGTGGGGAGGACTCCACTGAATTGGGAAACAAGGTCTGCCATTGCCCTTGGTGCTGCTCGTGGAATTGCATACATTCATTCGCATGGCCCAACATCCTCTCATGGAAACATCAAGTCATCGAATATCCTTCTCACCAAATCTTTTGAAGCTCGTGTCTCTGACTTTGGCCTTGCTTACCTTGCTCTTCCAACCTCTACGCCAAACCGCGTTTCTGGTTACCGAGCACCAGAGATCACTGATGCGCGCAAAGTGTCCCAAAAAGCTGATGTCTATAGCTTTGGTATCATGCTTTTGGAGCTGCTTACAGGGAAGGCTCCTGCTCATTCTTCACTGAATGAAGAAGGGGTGGACCTCCCAAGATGGGTTCAATCTGTGGTTCAAGATGAGTGGAATACTGATGTATTTGACATGGAGCTTCTAAGGTACCAGAGTGTTGAGGAGGAAATGGTCAAGCTCTTGCAGCTTGCCCTTGAATGCACTGCCCAATATCCTGATAAGAGACCTTCAATGGATGTGGTGGCAAGCAAGATTGAGGAAATTTGCCAACCTAGTTTAGAGAAAGAGGAAGGGAAGAATCATGATTTTAAAGATGCTGATAATGGATTTTCCCAACAATATTACTCTGTCGACTCTGGTGTGTCACAAGCTTGAATACAGAATTAAAGAAAGACCTCTTTTATTTCATCATTGCCACACCCAAGCTTGAATATAGAACTGAAGAAAGACCTCATTGTTTCATATTTGGCCTGTTTTTGGCATCGATATTAAACAAGTGTTGTTTGTTTGTTTGTTTATTCATTGAGGGATTATAGAAACCAGCTGTGTATGTCCTTTGTCCTACACTCTTACTACCTTATTCTCCTTAGTCCCTTTTGTCATTCTGAGTCTTGGTTGGGGGTTTACATTCCCCACTTTGTTTACTACTTCATTATATTCCTGAAAAGACGGG

>C100863_65

TCCCACCACTTAATTACCCCTTCTAAGTTGAAGTGGCAATGGGCCAAAGTCAAGATATAATCTAACAGTGACATTAATGATACGGATTAAATAATCAATTAATCTGTAAACCATTATTGCTGCCATCTTCCATCATTATTTTTGCTTTTTATCAGAACTTGCAGGACTATTATCTGTTGCAGCAGCTGCAGCTGCCAATTCCATCAACCGCTCATAATCTATCTCAGTTGATTTAAACTTAAACTTGACGCCAACCTGAATCCATGACTTGATACAAAGAATAGCTTGTGTGCTATTGTGTGGCATACAGATTCGCTGCTTGTCAATTTCATCACCCTTGGCACAGAAAAGTTCTTCAGGCACAACTGAAGTTGCCTGCACAGCCAGGAAATCTCTAGCCATAACAGATAGTCGAGGGTAGCGTGTACTGTTGACCTTCCACCACTCTAGAACATCTGTTGGTATTGGAGCTGGAGCCTCTGATAGGTACTGTGTAAGCTCATCAGTGGCAGAGCTCATGGTTGTTCTTCGCTTCTTGCGTGCTATTTCCTCAGCAAAGGAAACACTTCCTCCTTCTTCAATTTCTTGAGCATTGTATCCAGAACTCATGGATGAGAAATGGCTGGTAGAATAATTTCTCATAAAATGGGTTCTCGCTTCGTCCAAAAAATTTTCTGAGTTTAAACTATCCGGGATCAGCTCTCCCTTTATTCGAGGATCTAGAATTGCTGTCATATAAGTAAATATGTTGCAAACTTGATGAATATAATTTCTGGCCTTTTTAGCCATTTCTTCTGCAGCATTTTTTAGCCATTCTGGGCTGTGACGTGATTCTCTGCAGGAAGTAATGGTTTCTGAGATGTGATCCATGAAGAAAAGGACAAGCCCAACCGTTGGCACCTTACTTGTGCAGATGTTGTTTGTGGTTTTGTAGAATGGTTCAAGATATTGATGCATGATGTTAACCACACTTTTGTCAGAAGACGCCAGCAGCATCCTACTGCCTAGCATTTCCTCGTATTTTCGAATAACAGCATCCACTGATTTACCTGCCTTGCGAATAAGATCAAGCATTTGGTAGTTTCCACTCCACCTTGCTGAAATATCAAGAGGAAATTTCCAAATACCTTCTTGATAAGCAGTGGATAATTGAATAAAATCCTCAGAGATCACTGGAGAGGCATTTAACTCTATTACAAACTCCCGTATCTTAGAAATTACTTGTTTTGCAGATCTCAATCCATCATCTATTATCACATTCAAAGTTCGAGCAGCACAGGGAATATAACAGAAAGGCCCTATTTTCTGACCATCTAAATCCTCTTTCAAAGTATGGCAGGCATGCATGGCACTTGTACTATTATCATGGGTACAGGACAGAATTCTGCTTTCAATATTGTAAATTTTGAGAACTTTTACGAGGGAACGATATATCTCTGCACCTCCACATGGATAGGGTATTCGACAGATATCAAGAAGCAACTTCTGGAAGCACCAGTTTTCGTTGATCCATTGACATGTTACACTCATATAGTAAATCTGTTCAAAGGAGGTCCAGAAATCAAGTGTAATGGACAACTTTGAAGAAACTTGTTCTAATAATGCTCTTACATCTTCCCTCATGCTTCGGAAGACTTCATCAAGTACAGTTCTATACTTGTCACTTGGCCAGAGTTGTATAGATGGATTCAAAAACTTGTAGGAATTCACAAGCCACTTCTCTTCTAGAGTTGAAGGAGGGAGCGTAGCAAGAACAAGCCACCTAACGAGCAACCAATTTAGATGATCATAATCCACCTGGTTTGCTTTTCCTTGAGGTTGAGATTTCTTGACCACAGTGATGGGCCGTGCTGTTGAATTGCTGACAGCATCCCCCGACTTGTCGTAACCTGGATGGCGATTAGCCAGATGCCTTCCCAAATTGCCTGTGGCTGTTGCAATAGAATAGCTCTGTCCACAAAACTTGCATCTGCGAGTCTTGCCATCAAGAGCTGTCTCAAAGAACTTCAAATACACTGATGTCATTGTTTTCTTTCTAGGTTTCAGTGAAGTGACGGGACCTGGCTTATCCGAACAACCCAGCCCAATATTTACAGGATCCATGTTTGAAATGAGTGCCACATCCATCATGGACTTCTGTTCGTCTTTGTAAGTAGTTCTGTAGTGATTGTTAACGCCAGTCCAGTCCATGTAATTAGCATCACCAACAGTCACCTGTCCGAGAGGGAGAGTTGATGAGACTCATAATTAAATTTTGGCGGAGAATTGAATTGCAATTTTCAATCAGAGAGAGATGATTGAAGCTGATGAAGATCGGCGTGGAGTGAGAGAGAAAGACAGAGCGGCGTGGCTGAGGGTAGATGAGATCTGCAGTTTCACGGCTTCGC

>C100865_65

CATGAACATGCCACAGAAGATATTTGTATATACAAACTAAAGTTCAAAAGAAAAAATTTAGGAGACGTAAATCAGTAATCTGAGTAATGGGAAAATTCATAAATACGGTACAATAAAAAAACTAACAAGAACAATTCGTCCTTATTTGTCATATTGGATGATAAATCCAGCAGACCTTGCTTCCTGAATAAAGGAAACACATTTGTTGGTCACTTGGCAGAGAAAGACTAAAATGTCCCAATTCATCACCAATAAACTATCTCCTCGGTGCTCGGGAGACAGGCTTCAAAAGGCTAATCTTAACCCTTGATGCATCGAAACCCTGTGCCTCCATGAGCCGACATATTCTTTGTGCATCCCGCTGAAACCCACCATCAATAAGGCCGCTTATAACTCTCTCAAACTCAGCTTGTCCTAATTCATCCTTCCTAGCTTCCAGAACGCCTAGAATTTGAAGGGCCTTTTTCTCATTTCCAGCCCTTGCATACATATCACAGAGACTCACTTGAAGCTTAAAAGGAGGAGCCTCACCCTGCAGAGCTATTTTTCCTAAAAGCTGCTCAACTTCGTCAACAAGCTGCAACTTACCCAACCAGTCCACAAGAACAGAATAAGTGTCTACCCCAGGTTCAAACCCATCCGTTTCTAACTTCACAAGAAGAGCCAATGCATCATCCAAGTAGTTTTTCTTCTCATAAGCTGCAATCATGCTAGCAGTGCACCTATCATCAGGCTTATGCCCAAGTTTTATCATGTAATCAAAATGGCTTTTTGCCTGATAAGGATCACCAGTAAGTGCATTTGCCTCAATAAGTAGGGTGCATGCCTCCACACTCTGCTGGAACCCTGCAAACTGCATAGCTAATGAAATTCGGTCAGCTCCTTGAGTATCACCACGTTTAGAAAAAGAACGGAGCAATGCCATGTATATTTCCTCTGTGGGTTTAATATTTCCCGTATCCATCTCCCTCATCAATTTCTCGGCCAACTTAGGCTGGTCATTATTCACAAATGCCATGATCATTGACTTGTAGACTTGGAAGTCCGGTTGGAAACCCATGCCCGTCAAGATTTCAAATGCTTCATTTGCACGGGCAAGATCGCCTCCCTTGCAGTACATGTGGACCAACACACTGGCTGTTGAAGCATCTGGTTGTATACCATTTTCGCTCATCTTCTTTAGCAATCTCTCAACATCATCAAAGCAGTTCTCTTTAGCATAGCTATCTATAAGCCTAAAGTAGTCAGAGACATTTGTTTGGAAAGACTCTTCAGTCAATACCATTTCAGCCACCTTCAAGTACAAACTACGATCCTGCGATTTCAATTTGTCAAGCAAATCCATCCATTCCACCCTACTAGGTTGTACAAGCTCCACCCATTCATTCAACAGGCTTTGCAAACCATCATCATCCTCCTTCGACGACAGAATCTTCGATGTAACAATCTTCGCTTTCCCGGTAATTACCTTCGGCTCCTGCGGCCAAAACACCTGCTCTGCCTCATGGATCCTATCAGCCACTTGCGCCCACTTGGGATCTGAGAGATCAAGCCCATAAATCTTGTACCTTATCTTCCCTCTCCTCTTGGGAAGTCCAACAGCAACCTTCTCCTTCTCCCCCTCCTCACTAACATCACTTTTCTTCTCCTCATTCTGTGCCTCTTTCCTCATTCTCTCCAAACCCTCATAGAATTCATGTTCCTCCATCTTCTCACGCGCCCACTTGAACCTAAGCTCCCTAAGCAAATCGCCGCGAATGCCAACTTCACCGGCGAATCTGCACATTTCCTTAACCTCTTCACACTGCAGAAAGCCCTTCATTTTCTCCTTCTTCCTCTCCTTGTTCATATCCTCCAAAACCAAATTGCTAACCTCCCACACCGTTCTCCACAAATCCTCGCTGAAATCCCCCGACGCCCTTTCAACTCCCCCACCCTTCTCCATCTCCGACGTAACCCTCTCCACGATTGCCACGAGCATGCCGTCCACGGTGGCCTTGTCGGAGTCCGGGTATGCTTCCTTCACCTTCTTCCGCATTATCCACACGAATCGCGATAGAAACTCGTTAGTCGCGTCATCTTCGACAACCGCATCGTTTTGAGGGACGTGAACTTTGGGGTCAATGAGGGCGTTGGTGGGTTGATCGGAGTGAAACGTGGTGGTGGTGGTGAACGGCGCGGCAGAAGAGGCGCGTGAAAGCTGGGGGGAATGGAAGGTGGTTGAATGAGATCGAAAGATGGCATTGAGTCTCTTGAATGTGTGTCTTGAAGTGGAAGCTAGAGTTCTCCAACTCATGGTGTCTGAGATTGAATTTTGAAGAGTGAAAATGGAACTGTATTAGGGTTTTAGGTTTATGGAGGTTCACACTGTAATATTCACATTCTCTC

>C100871_65

CCGCTTGCCAACTTTCACATTTGAAGCCTGCCCGGCCGGCCCGTTGTTGAAAAACTCTCTCTTTCACATGCAGTTTCCCACTGCCTCTCTCCTCCACCACCTACACCCCCTTTAGACTGATTTTCAGAACTGGGTTTGTTTTTGTGTTTGTTTTTTACTCATTTATCTGTGTACTTGTGTTGATCCTTTTGTTCTGATTCACAACAGTGGTGTTCTTCTTTTAGTTCTGTTGATTGTGATTGTGATCATGGTCATGATCATGATTTCGGTTGCAGAGTCTTCAATTATTGGAAGTGAGGTTGCTTGAAGAGGCTTTTATTTCTTCTTTCTTTTTCTTTTTTTCATTTTTTTATCCTAAAGTCTGAAACTTGTGGGGACTTTTTAGCATGAAAAGCTCTTTGTAAGAGTTTGTGGAAGATGGGGTTTGGGAATAATGCAATTCAGGCAGGGAGTTTGGATGTGGGGAAGTCAAAGGTAAAGATGCAAAAGGATGGTGCAGAAAAGGAAATTGGGTGTTGTGTTAAATTCTGTTTCATTGGGAGCTGTATACCTTCAAGATCAAAGGTTGATAACTCCATCAGCGGCACTAGTGCTAACAGTGTGGAAAAAACATCTGCATATGAGAAAAACAAGAAGGAAAGAAATGCTCTTCCAGGGTCCTCTACAACAACCAGTAATGCAGAAAGTGTCCCTTCCACCCCAAAATTCAGTGAGGAGTTGAAGGTTTCTTCTCGTTTGAGAAAATTTACATTCAGTGAGCTTAAGTTGGCTACTAGAAATTTCAGGCCAGAGAGTCTTCTTGGTGAGGGAGGGTTTGGATGTGTTTTCAAGGGTTGGATTGAGGAAAATGGAACCGCACCTGTGAAACCTGGTACTGGGCTTACTGTAGCAGTGAAAACTCTCAACCATGATGGACTTCAGGGTCACAAAGAGTGGCTTGCAGAGTTAGACATTCTTGGTGATCTTGTCCATCCGAATCTGGTCAAATTGGTTGGTTTCTGTATTGAAGATGACCAAAGATTGTTGGTTTATGAATGTATGCCCCGTGGAAGTTTGGAGAACCACCTCTTCAGAAAAGGGTCGTTGCCTCTTCCTTGGTCTATCAGAATGAAAATTGCACTTGGTGCTGCAAAAGGTCTTTCTTTTCTCCATGAAGAAGCTCAGCGGCCTGTAATCTATCGTGATTTTAAGACATCTAATATTCTGTTAGATGCGGAATACAATGCAAAGCTCTCTGATTTTGGACTCGCCAAAGATGGTCCTGAAGGGGAAAAGACACACATATCAACTAGAGTCATGGGAACGTATGGTTATGCAGCTCCAGAGTATGTGATGACCGGACATTTGACATCAAAAAGTGATGTCTATAGTTTTGGAGTAGTGCTACTAGAAATGCTCACTGGCCGGCGATCTATCGATAAGAATAGACCAAATGGGGAACACAATCTTGTGGAGTGGGCAAGACCTGTACTTGGGGACCGAAGGATGTTACTACGAATTATTGATCCTCGCCTTGAAGGTCACTTCTCTGTTAAAGGGTCACAGAAGGCTGCCCAGCTGGCTGCTCAATGCCTTAGTCGTGATCCAAAATCCAGACCTATGATGAGTGATGTTGTTCAAGCTCTGAAGCATTTGCAAAACCTCAAGGATATGGCTATCTCAACTTATCATTTCCAGGTTGCGCGAGTAGATCGAACCATGTCCATGCCAAATTCTAAAAATGGTGTGCAAGCACAGTTAACTACTTTATCAAGGAAGGGTCAACCTGCAAGGTCCTTGTCAAGTCCAAAAGGTCCTCATGGTTCTCCATATCATCATTATATCAAGTCACCAAAACCTAATGGATAAGATCATGGTTGTCTCTTTGATTTCCTTTTCTATTCATTCAGTTCCCTCAATTTAGGCTATCAGCTGAACAGAAACTGTTTGTGTTTTTAAAATTGCTCTAAAGTTTGAGGAATCATTAGAGTTTTCTGACCTTATCTTGTGTCAAATAAGGTGGGGGGGACCATGAGACAGTTTACTGGATTACAAAGTTTAGTGGGGACCGAGTTGACCAAACTTGAAGAGAAAACTATACAGTTTTCACATGGGGAATTTGTTACTGTGAAGTTGCTGTACTTTTTCTACCATTCCACAGGCTTTCTTCTTCCTTTCAGAATGTATTTTTGTAAAGAAAACAGGATGGTAGTGTTAACTCATGTTATTCATAGATATTTTTACATACTAATTCGTCAATATACTTTTAGTTGATACTTATCAATGTAATTAAGTGTCGTAGTAGAAACATGCTAGAGTTTGGAATTTGAACGACTTAAAAAGTTCTGAACATCCTTCATTTGTCATCTGTTGGTTATATTTTGTGTGTTAAATGAGTTGTTCAGAGAAAG

>C100873_65

TTTTCGTCATTCCCTAAGCTCAAATCCATACAACCGATGAACACCCTCTTGATTCTTTAGCTGAATTCCTTTTGAAAACCCGGAAGACTGGCTACAGATACGCAAAGCTGCTGATTTGAATGGTTATTATGACCAATCTTTAATAGGCCAATATTAGGATATCTGTTAGCCCAGTGAATTTGTAACTCGTTTAGAATATTGGGCAGGTAGTTTCCTTTCACGTCCCAGGGATTTGATAGTCTTGCTTCCAACATATGCAAGCTCTAGTCTATTGTAGCACTGGCACAAAATAAAATTCCAAATTCCAGAATTCTCTCGTTTCAAATAAAGTTAATGTGTTAACTACAGAGGTCTCAAATCGAATGCTGATTATCAGTATAGTACCCCATTCCTGGTGGTGGAAGTGGCAGGAAACGAGGAGGTGCTGGGGCTTGCTGTGTTACTGGTATTTGTTGTTGCGGAGTTATTTGCAGGGTTTGATGAGTCAAAGGTACCATTGCCCCCACATGAGCTGAGGGTGGGGGTGGTAATGGTGGGATGCTACCATAACCATATGTAGAGATTATCCCTCCTGCTGACTGCACATATTGCTGGGATGACGGATTAACAAGCATGTGATATTGACCTTGTGATGAAGAGGGAGCATTCTGCATGGGTGGTTGGGCTACCAAAACTGAGGGATATGAATGGTTTGGCGTGGCAATCAACTGTTGTGGGGCCATAAATGCATGATGGTGATCGGAGCTAGGCACTGAAATCTCTTGTTTTGATGTGGACTCAGAAGTTAGACTGGCACTTTTAGCCTCTTCTGCTGCAAATGTTGAGAGAACAGAAGTCATGATCAATTGAGATGACGAAGAAGCTGCAAGCTTATCTGCAACCTCAGCAGCAATTACTGCAGCTGACTTTTTAGTTGCTGCTTCTGACTTTGCATTTAACTCAGTAGCTGAAGTTGTTGCCGTAGATGTCTTCTGAGAAGAATCTTCACCATCAAGTAGCTTCCGCATGTTGCAAGCCTCTTCTACTTGTGCTTGTGCAACCTGCATTTGTGTACGAACATTCTCTAGTTCAGATTCCTGCTCACGCAACACTTCTCTTAACTGAGACACAAGAGCTACTCTACTTGCTTCAACCAATTTAAGTTTCGCTATGCATTGTTTCAAAAGATTTTCCTCCTCCTCTAGTTCCTTTGACAAAATATTTCTCTTAGGATCTTTGGCAACGGTGCACACAGTATCAACATCTTTTCCCATCTTCCTCACACGCAGAACAGCAGACTTGCATTTAATCATCTCTTCATTTTCAACGGATTGTTCACTGCGCACCAAATGAAATGCAGACACTATTTTTTCTGCTGCACCTCCTATGGACAGTTTCGATTGGATAGAATGAGAATCCCTTTTCACAATATTTACAGAACGTGACCGCTTTTTGCCAAATTTCAATGGTGGAGGTGCATCTTCTCCAAGCATCAGATCTTTAAGGTTCTTAGCTCGAGATCCAAACACTTTCCTTTCCTCCCATATATCAATCAATCTAGATACCACTTGCTTTCCATGATCATCACCTTTCTCGATAAAATCTTTAAGTGCTGCAGGAAGAACCTTCCAAAACTCTGTCACAAATTCATCATCCCCTTTATGCTTACTGTTCTGTAGGATGTCATTTGCAAGATTCAGAAGGGGGATTCTTTGAACCATCTCTGATTTGTCAAATTGTTTTTTCCATGTGGCAACAACCAGTTCTGCTTTGCTCCGGTGAAATATACACCAATATGACAAAGTTTCTATGCATTGTTGGGTGTTATTAAGCTTGGAGAGCTTGTCTGCAAGTACTTGCTCTCTGAAAACGCTATTCATGTTCTTCAAATCTGCTCCCTCACCAGAATCAATTCAAGTAAAACCTATGTCATATTGAAAAACTAGGAATAAACCATCTCAATTTAACACTAAAACCCTATAACAGAATTGGAACCAAAACGAGCTTTTCAATATCAATACTAGTGCCCAACTCGCTAAAACTTGTGAAAAAAAGGGGCTGGTTTGAAAATCCGAACAAAAAGCGAAAGCGAAGTACAAAGAAGAAGAATTTAAGCAGCGATAGTAGCGGGAACACTGCTGGAAATCGAGAGTAATTAAAATGACCACGTTTTGGGCAAATCAGAATTCAGAGAGATCTTAGAAAAAGAAGAAGAAGAAGAAGAAGAATTAACTAACAGAGAGAGATAGAGAAGCAGATAGAGAGACGTTTCGCTGGGGAATTAGTGAAGAAGGATGGGTGCGTGGTGTTGTGAATTTCGAAATCCCTATAAAGGTTAACTAAATTTTGCAGATAAAAAAGAGGGAAAAATGAAAAGGGAGGTACGAATTAATTGAAGAAAGAAAGATGTG

>C100875_65

ACAAGTAGCATAGTTCAACCAAACACTTCAAACAACCATGAGTTTAACTCCCAGTATGGGAGTCAGCGAACAGGAGCACTTTCATCTTGAGGATACTAGCCAAACAGAGAGCTCCCTTTGGGTGCTGAATCCTCCCAATCCACCACCTCTATGGAACAAGCTATTTAGTCCACTGAAGAAAACCGTGTCCTTTTTTTCATCCAAGAACAAAACTTGCCTTGGACGCGTTGTTTCATTCTTAGAGAGTTTATTTCCTATCCTTACCTGGTTCAAAAATTACAAAGCCTCCAAGTTTAAAAATGATTTGCTAGCAGGTTTAACTCTTGCCAGCCTCAGCATACCTCAGAGTATAGGATATGCTAATTTGGCAAAACTTGATCCTCAATATGGCCTGTACACCAGTGTTGTTCCTCCACTTATCTATGCTGTAATGGGGAGTTCAAGAGAAATAGCAATTGGCCCTGTAGCTGTAGTATCATTACTTCTATCCTCCCTAGTCCCCAAAGTGGTAGATCCTGCTGTTGATCCTGATGCCTATAGAAGTGTTGTCTTTACGGTGACCTTATTTGCTGGAATCTTTCAAGCTGCATTCGGTATTTTCAGGTTGGGGTTTCTTGTGGATTTTCTTTCACATGCTGCACTTGTTGGGTTCATGGCAGGCGCAGCCATTATTATTGGTCTTCAGCAGCTCAAGGGGTTGCTAGGGGTCACTCACTTTACCAACAAAACTGATGTAATATCCGTGTTGGAGTCTGTATATAAGTCACTCCATCAACAAATTACATCAGGAGAAAAATGGTACCCTCTGAATTTTGTCATCGGGTGTTCATTCCTGATTTTCCTTTTAATTGCTCGATTTGTTGGCAGAAGAAACAAAAAACTTTTCTGGTTGCCAGCTGTTGCTCCTCTTATATCAGTTTTACTATCAACTTTAATTGTGTATTTGTCAAAAGCTGATAAGAATGGGGTTAATATAATCAAGCATATCAAAGGAGGCTTAAATCCAAGTTCAGTTCACAAGTTACAATTCCATGGTCAACATGTTGGACAAACAGCTAAAATTGGATTGATTTCTGCTGTTATTGCTCTCACTGAAGCAATAGCAGTTGGTCGATCTTTTGCTTCCATTAAGGGATACCATCTTGATGGCAACAAAGAAATGTTGGCAATGGGATGTATGAACATTGCGGGATCTCTCTCTTCATGCTATGTGGCAACTGGTTCTTTCTCAAGGACTGCAGTAAATTTCAGTGCAGGATGTCAAACATCAGTATCAAATATTGTGATGGCAGTTACTGTGCTTCTGTGCCTTGAATTGTTTACAAGGCTCCTATACTACACTCCTATGGCCATACTTGCTTCTATCATCCTCTCTGCACTTCCTGGATTAATTGACATAAATGAAGCTTGCTATATTTGGAAGGTTGACAAATATGACTTTCTTGCCTGCATTGGTGCCTTCTTCGGTGTCTTGTTTGTATCAGTAGAAATTGGTCTTCTTGTTGCAGTGTCAATCTCATTTGCAAAGATACTAATACAATCAATTCGACCAGGGCTAGAAATTTTAGGCAGAGTTCCAAGAACAGAAGCATTCTGCGATGTTAGTCAATATCCTATGGCCACAATCACTCCAGGCATCTTGGTGATTCGCATAAGCTCTGGCTCACTCTGCTTTGCAAATGCGAATTTTATTAGAGAAAGAATACTGAAGTGGGTCACTGAGGAAGAAAACGAACTTGCCAAGGGAAGGGTCCATGCAGTAGTCTTAGACATGAGCAACCTCATGAATGTTGATACTTCTGGGATTCTAGTACTGGAGGAACTGCACAAGAGGTTGCTTTCGCGTGGTGTACAATTGGCTATGGTGAACCCGAGGTGGCTAGTGATTTACAAGCTGAAAGTGGCACACTTTGTGGATAAAATTGGGAAGAAATGGGTTTTCCTTACTGTTGCTGAAGCCGTAGATGCATGTCTATCTTCAAAATTCCCTGAGCCATGATTTCACTTGCCTTATCTGCAATGTACTGCATCGTTGCCTATCTAACTCCACAAAGTTGTGCATCACATTGTCTTATGTAAAATGTAATGATCGATGTTTGACATTGTTCCCTCCACTATTAGTGGAACTGTGTCCTGTACGGAAAAAGTCATATATGCGAGGGTATTGTTGTACGGAAAAGCTCCTTGGATGGATAATTTTGCTAGGGCGAATGTCAAACAATTAAATATAAATAAAATAAGTATAAGATTGGTATCACATGAAAAGACTAGAAAAGAAAATATGCTACCAGAGGAAAAGAAATGGGTGAACTATTACTTCTTTTATTTATGGTTTTATGTACCTACATGGCTTCTTAACTATCTTCTTATGTTGTAATAAATTCTTTAAATT

>C100877_65

AAAGATAAAAACAACTTTGACTATGACATAAGGTAGAAATTATGAAACTCTCCCTGATTTGCTAAGAATTTAATTCTTGATAGATACAACTAATGGCTGTAATTCTTCAAATGAAAAATAAGCATACAACTAATGTCATAAAATTTACAAGCGACAACCATTTGAAAAATTGCGATTTACAGCCTCTTCTTCCACTTGCCTTTTGTGGTTGAATGATGCCGTTAGTCTTCGGTCACTAACATATATATAATGGTAAGACTATTCATCAAGTATGGCTCGACCAAGATTCGTAGCGAGAGCCAGCCTAATAGAGCTTAGTGCATCAGGGGGTGAGCTAGGCTCAGTATAGTGGGGGCGCAGCTTCGCCAGAGATAAATCTTCAAGTTTGTTCAACAGAAGATTGGTCTCAGAGACACTTAAAAGGGGGCAATCCTTCCAATTAAGCAGCTTCAACGAGTCATACAGAAGAGGCAGCCAAAAGCGCTGAGGAGTCGATGGATTTTTCATAAGCAGTATGAGGGAGCCTACAGCTTGCCTGGCTGTATCAGTTGATTTTCCACCAGATACCTTCTGAAGGGATTTCTTGAAATCCCTATACTTGTGCAAAAATTCAAGACCCCCAGCAGGCTTAGATTCAGAACCCAGTAGCTCAATCATGCCTTCCCATTGCTTGAAGCTTTCATCAGATATTGACTTTCCAACAGAATCAAATAACTGTTGGGCAATCCTATCAAGACAACTTGCATCTTCGGCTTGTTGAAGCCAGAATACTCCAGCACCCTTATGACCATGCTTCCAGTGATATACTCCAGCAATCTTCATGATGTTTGAACTAATATGATCAAGCTCAAACAAACGGCAGATCTCGATGTTCTTAAGCAACAAATTATTGTGTTGAACAGATTGCCTAGACAATAAATTCTCTAACAAGCCCATTCCTTGCTTCATGCATGATGTCAAATATATTGGAGCTATTTGCCAGGTCAATGCATGTGACGATAGAATTTGAGCATATACAAGCCGATGTAGTTCCACTATGCTGATTCCCCCCAAGTTATAACGCTCCTCATGCAGAAGAATCTCTGCTTGCTCACTCCCTGCAGTCAACAATTCTATGGCATGTGCCACCAACCAAGGGCCAAATTCTCTGGAACACTCAGCTAAAACAACCTCAGTACTTTCTTCAAGAATACCAATCATAAGTCCTGCCAATCTATGGGTATTAGATTGTGGTTTCAACTGGATGCATTTCTGGGCCAAATTATACATGCTTTCTATTCCCATTGTAAATGGCCTGATGTATAGAAAATGAGAAATATATAATTCAATCCAATAACACGTAGCCATGCAGAGACTCTCAGTGTTACCCAGCATAATTTGTAGCAAATTTCTCAGGCCCTCTCGTGTTTGCTGATTATCACACTGAATCCAGATTGGGCTGCAATCCAGTTTTGTGATTTGTGATCTCCATTTTTCCCAGGGCTTGATAAAGTCAGGCTTGGATTTATAGCATTCACCCAATTTTCCAACAGATTCAGGACGCAAGCGGGGCATTTTGGAAATAAGAATAGCAACAGCCTCTACAAGTCCATTTTCTAACTCCCGATTACTGAGCTGATCTAATTGGTAAGATCCATGCAACCGCAACATTTTCACCACAATATCAAGCCAGCCAACAGACAGTGCAGATGACAATAAGTCCCAATATCTGGGATCATCCTCAATTACCTGTATGTTGACTAGTTCTTTCTGAAAATCCACAAGCTTGCCATGAATTGTTTCATGTGTACTTGTAAATAGGCTGTCATAATCAGATAACCAATCAACAAGCCTTTCAGGTAGCCATGCTTGAGATTGCTTGTCCACGTAAAATATTTCAATTAACTCCCATGCAGCTTTCAAAGATGTTGGCTCTTCACCTCTCTTTAAGAATGTGTTTGAATCCTCAATTATAGGACTTGGGAGCAACTTTGACCCCCCAAGAAGAGAACCTATCTCCTTGCTATACTGAAGGACATTCTCCCACCAATCCGAATGATACGGAGAAGGAGTCTTCAAGAGAGCGGAAAGAGAGCTCCTCCGACTCTGAAGCAGCGCGAAGGGCGCAACAGAGCCGTACGCAATTCGGCGCCATTGAGCGTCGGTGATCTCTGGATCTTCAGCGGTGAGCTTAACTTCAACGACCTTGGCGGCGGCGTCGGCGAAAAGGGAGAGGCGGAGGGAGTTACCGCGGGACCAGGAGATGGAGAGGCGACGAATCGGAGGGGCGAGGCCATGGTGGAGAGGGTATACAGCCGGCGGGTCTCCGGAGAAGGGGACCATAGCCCCGTTGCCGACGGTGTCCGACGGCATTTTCAAGAGGGGGAGTGTTTTAGGGTTCAGGGGGTTTTGG

>C100881_65

CAAAAACAAAAGAAAAAAAATGAAAATGGATGATTCTGCAAATTCTACTGCCGGTAAAACCAAACTTCTTAATCCGTGGATGCTCCACTTTCAGAAACTCGCTCTGGAACTCAAGTGCCCTCTCTGTTTGGGTTTGTTCAAGAGGCCGGTGTTACTTCCTTGCAATCACTTGTTCTGCGATTCATGCTTAGCTGATTGTATAACGGTTGGTTCTGAATGTGCTGTTTGTAATGCTAAATATGCTCAAACAGATGTAAGGCACGTGCCTTTTGTTGAAAACGTGGTAGCAATATATAGAAGCTTGGATGCCACTTTTTGTGCTAGTTTGTTTCAGCAACGTTCTTCTGGTGATGAGAGGGTTTTAGAGCCATGCCAGGCGATTCTTAATTCTACTTCTAACAATATAAAAGCTAGCAAGATTCCTCAGAATTTGCCTAATTTAAACAAGGTTGGGGTTGGCAAGAATCTCAAATCCAAGATTGCAATGCATGATAAAGCAGAGGAGCTTGAGTTGTCCCGAGGGAGAGGGAAGCCCAATCCAATGCAATCTTCCCAGATGGAAATGGATGTGAATCAAGTGACGCAATCAGCCCCAGATAGCCCTCCTTTTTGTGATACCAAAGGTTCAGATAATGACTGCAGTGATCAAGACAGTGAGCATCCATTACATCCTGATAGACTGGGAAATTCTTCCCTAAAGAGAGCAAGTACAGGAAATGGAAATTTGAAGGTGAAAATGGGACAGTTAAGGTCGGAAAGTTCAGCCTCTGAAACTGAGGGTCTTGATAGGGATCCCAAAAGACAGAAGATTATTGACCTTAAATCTGGAAAGGACCCTGGTGCACAATTTCCCACAAATGCTCCTAATGACTCTTGTCCAACTGGAAGAATCTGTTCATTCTGCCAATCCTCTAAAACCTCTGAGGCTACTGGTCCAATGCTGCATTATGCTAATGGAAATTTAGTTACAGGAGATGCAGCAATGAAGCCATATGTTATACCTGTGCATAGAAGTTGCATTGACTGGGCACCACAAGTGTACTTTGTTGGTGAAGTGGTTAAGAATTTGAAGGCAGAGGTGGCAAGGGGAGCAAAACTTAAATGCAGTAAATGTAATCTAAAAGGAGCTGCTCTTGGCTGCTACGTCAAATCCTGTAGAAGAACTTATCATGTTCCTTGTGCAATGGACATCTCAACTTGTCGTTGGGATCATGAGGATTTTCTCTTGCTCTGTCCCCTCCATTCAAATGTCAAATTTCCATGTGAGAAAACCAGATCTAAGAAGCAGGCAACTCGGAAACATCCCACACCTACTTTGTCTCATCCGCCATCTCACCACCCAAATACTCTTGAAGCTTCAGAAGATGATAGTAAAAAGATGGTTTTCTGTGGATCTGCCTTATCAAATGAAGAAAAGGTACTTTTGGTCAATTATGCAAGCAAGGTTGGTGCAACTGTGACCAAATTCTGGACATCAAATGTTACGCATGTCATTGCGGCAACTGATGCAAATGGAGCATGCTCCAGGACATTGAAAGTTCTCATGGCTATTCTAAATGGACGATGGGTTCTAAATTTGGATTGGGTAAAAGCATGCATGGAAGAGAAAACCCATGTAGAGGAAGAACCATATGAAATTAATGTTGACAACCAGGGGTGTCAAGGTGGCCCAAGAGCTGGCAGACTTAGGGCATTGGCCAATGAGCCAAAGTTATTCAGCGGCTTGAAGTTTTATTTTTCTGGAGATTATGTGTCAACTTATAAGGAAGATCTTGAAGAATTAATTGAAGTGGGTGGAGGTACTGTTTTGAGAAGCAAGGAAGAATTGGTAGTGCAAAGACAAGAATGCAAAGTGAATTCTTCTAAGTTGCTGGTTGTTTACAACCTTGATCCCCCACAAGGATGCAAGTTGGGGGAGGAAGTATCAATTCTTTGGCAAAGGCTAAACGACGCAGAGGATTTAGCTGCTAATACTTTACAGGTTATTGGTCACACGTGGATTCTGGAATCAATTGCTGCATGTAAATTGCAGCTTTTTGTTAACTAAAATGCATTAGGTAGCTGATAGTTTGTCTCACTTGATATGGCGTTCGTTCTGCAAAAAAATTGATCAATCGTGTATAGGATTTAGTTTGAATTTATTTCTTTTTCGGGTCTCTGGCTGTTAGGCTCTTTTGGTTATGTAACATCAAGTAGTTAGTCACCAACTTATGGTTTTGCTTGTTAATTTGTACAATTAACCGGTTACTCAAATTTGATGGATAATGAGAGGCCTCATGCTGTCAGTAAATGCCAAGGCAAATTCTAGAGTTCTACCATGCAAAAAACTAGATGAAGTTCATCGATGTACTACTGTTTAGCATTTATTCAATAATAAGAGATTAACAACCCCGG

>C100883_65

ATTAACGAAACGGAAAAAGAATAGTGTTTTGGGAAATTATGATTGCTGAAAAGGAGTGAGGAATCAATTAGGGTTAACAATCGGAATCAGTTAATGAGATGGAGGAGAAAAGAGCAATGCTCTCACCTCATTCCATACGCCATTTTGTTTCCATTTCGATGATTCCTATGTTCCCCTCCTTCTCCTGCTTTCTCTCTTCTCACTCTATAAATTCAATTACCATTACAATTTTCAATTTAGGAACTGCAAACTAGAATTCTGTTCATTGATTCATCCATCGATCCATCCATCGATCTCGTCTCGAATTCATGGCTCCCAATCCAATGAAGATGAATACGAAGCGATCTGCGAATTCTAGTACAAGTACCATAACAGCAGCTACTTCGTCTCAGGAGATTCTGGTCCGCGAAACGCTGCGTATCAGTGCCAATTTGGCCTCCACCCCTCTCCTCCAATCTCCCTCCTCCCTCACAATGATTTGCTGCGAGGAAATTGATGGCCGTCGTTGGAAGTACGTCGCCGAGACCGATGCCTCCGGTCATTTCAAGAAGAATTCCTTTCGCCCTCTCTCCTTGCACACTCCTCAGGCCGCCCCTCTCGATGAACTTCTCTCTTTCGTTAGATCCTATGTTGTTCCCGAAGGTTTCCCTGACAGTGTTACTCCTTCCTATGTCCCCTACATGACATGGAGGGCTCTTAAGCACTTTTTTGGTGGAGCAATGGGCGTTTTCACAACTCAAACCCTCTTGAGTTCAGTTGGCGTCTCCAGAAACAGGGCTACGCCTGGGGCTGTCGCCATCAACTGGATTCTAAAGGATGGTGCTGGTCGTGTGGGGAAGATGCTTTTTGCTCGTCAAGGAAAGAAATTTGATTATGACCTCAAACAGCTGCGCTTTGCTGGTGATCTTCTGATGGAATTGGGTGCTGGAGTTGAACTGGCAACTGCTGCAGTGCCACATCTATTTCTCCCTTTGGCTTGTGCTGCTAATGTAGTAAAGAATGTTGCTGCTGTAACATCAACATCAACTCGGACACCAATTTACAAAGCCTTTGCCAAAGGAGAAAACATAGGGGATGTCACTGCTAAAGGAGAATGTGTTGGCAATATTGCGGACCTGTTAGGAACTGGTTTGAGCATACTGATGGCCAAAAGAAATCCGTCGCTTGTCACCACATTTTCCCTCCTTTCATGTGGATATATCCTTAGCTCTTACAGAGAGGTTAAATCTGTTGTTTTGCACACACTAAACTTTGCAAGATTCAGTGTGGCAGTAGAATCCTTCCTCAAGACAGGACGAGTTCCCACTTTGCAGGAGGGCAATATGAATGAGAACATATTCAGTTTTCCTTGGAGAGATAGGCCTGTTGTCCTTGGATCAAGAATCAAGGAAGCATTCCAAGACCCTAGTCAATATATTGCCACTGAGCCCTTGTTTGATAGGGAGAGATATATTGTAACATATAACCCCTCAAAACACAAGGTTTATGCTGTGCTCAAGGATCAGGCGAAGCCAGATGACATCCTAAAAGCAGCATTCCACGCTCATGTGCTCTTAAGTTTGATAAATTCATCGAATGAGAGTAAGGCCTCTACTTTGAAGGGAAGGGGGGATCTTAACTCAAACGTGACACATACAGTTGCTGATATTGAGGCTTGTATGGCTGATACTTGCAAGATTGTGGCAAATTCTTATGGTGTTTTCAAGAATAAAGCTAAGGAGCAGGGTTGGACAATGTCAGAATCACTTCTAAATCCTGGTCGAGCTAGGCTGTATCCACCAGATAATAGATGATATTCTCATATTCTCTCTGCTGCTGGGTTGAATGCCAAAGTTCTTGGGACTGCTCTGAATGTGAAATAGGTCGTCCAATTGAAGCTTTTAAAATAACGAAGACATACAATGCTCGTGGAATCGTAGCATCCTTGATCCTCACAAAAGCCAAACGAACTGTTTGCGGATGTTGTGCGTGGATGGCCTTTAACAGAAATATATTTATAGCAATGGTTAGCATGACTGCATATGTCCACGTAAAATACCAATGCTCGTCTCTTTTTTTAGGTCGAAATATACAAATTTGTGACTCTGATCTTTCTATTTTCTTAGTTTTGTTCTTTATTCTTTTTGCAGCACTATTCTGTAGTATAAGTTTTCAATTTCAATTTTGCAGCAGAGTTCAAGAACTTGTTGCACATTTGTCAGTGCAATGAGTTTGATTAAGCTAGGTCCAAAGTATGTGTCTTGTAAATTTTTCGTCGAAGCAGGAAGCATAGGGAATCATAGTGGGTTTGGACTAGAATTTGTAAGCTTGATTTTAAAGATCAGAACTTTGATGGTTGTAAGAACGAAATTTATATTTATATTACTGTGTTATTCATGCGCTGTATCAAAAATTG

>C100885_65

ACATTAAAGCACATAATAACGATAACGAAACAAGGGAGAAGAATCCTAGAGCGAGAGAGATTGTTATTGTGGTGTGTAGTGTAGCAGCTCCGGCAGCTTGAGATGGACGCCGGTTCGGTGCTGAACAAGTCCAGGAGCGGAGGTTCGGAGCGGAGCGATGACAGCAGCGGAGGAGGAGGGATATTTGAGTACTCTGGCTGGATTTACCACTTGGGAGTCAATTCCATTGGCCATGAGTACTGTCATCTTCGATTCCTCTTAATTAGAGGAAAGTATGTCGCCATGTACAAGCGTGATCCTCACGACAACCCTGGCATCAAACCAATTAGGCAGGGAGTTGTAGGACCGACGCTAATGATGGAGGAGCTGGGTCGTCGAAAGGTCAACAATGGGGATCTTTATGTTTTACGGTTTTACAATCGATTAGATGAGACCAAAAAGGGAGATATTGCTTGCGCTACAGCTGGGGAGGCCAGGGGGTGGATGGAAGCATTCGATCAGGCTAAGCAACAGGCCGAGCATGAGCTATCAAGAGGAACTAGTGCCAGAGACAAACTAAACATGGAGACCGAGATCAATCTCGAAGGACATCGACCTAGAGTGAGGCGATATGCCCATGGTTTGAGGAAGCTTATAAAAATTGGCCAAGGCCCAGAGAAATTGTTGCGGCAATCATCAAAGTTGTCTTCTAGAGCAGATGGGTTTGAAGGTGATGGAGGAGATGCAGTTGAAGCACATCAATGGAAATGTGTTTTGACTTTGTCTGGTATTAGAATTTTTGAGGATGTTTCTGATCACAAGAATAGTAAGACTGTCCTTGCAAAGTCTGTTGGTGTTATTGATGCAACTGCAGATACTGTTTTTGAAGTTATTTTGAGCACTGAACAACAGAAAAGATATGAGTGGGACACATTGATATGCGACTTGGAGCTTGTAGATTCTTATGATGGACACTATGATGTTGTTTATGGGATATATGATTCTAAGTATCTATCCCGGTGGCACTCAAAGCAGGATTTTGTCTTCTCTAGGCAATGGTTTCGTGGACAAGATGGAACATATACTATCTTGCAATGTCCAGCTGTACATAAGAAAAAGCCTCAAATACCTGGATATCGACGTGCAAAAGTTAATCCATCTTCCTGGGAGATTAGAAATTTGAACATATCTACAGCATCAAATAGCCCAAGATGTTTAGTGACCCACACTTTAGAGATAGACTCTGCGTCCTGGTCTCGATGGAAGAACAACCAGAACTCAAAATTTCAGAGGAGCATTCCACATGCATTATTGTTCCAAGTGGCAGGTCTGAAGGAATACATTGCTGCCAATCCTGCACTTCACCATGAAAATGCCACCACAGTTGTTCATTCCAAAATCTCGGATGCTTCTATTTCTAGTGCTGAATATGAGGATGAAATGCAGGATGAGTTTTATGATGCAATTACTGCTGATTCGTCAACATCAGATGAAGAAAGTGATGATGATCAAAAACATGTTCTACAGGAGCCAAGAGTCAAACTAAAGAATATTTCATGGGCAATCACAACTTTAGCTTTAAAGCGAACTGCAGCTCCAGATCTAAGCGAAGAATTGGATCCTCTTGTAACTCCTATCACAATTCCAAGTGACTTGCATGGTTCTTTACGCAAAGGAAAGGATGACAATGACACAAACTGTTGGGCTTCTCCTACTGGAAATGGATTTATGATTAGAGGAAAGAACTATCTTAAAGATAGTTCTAAGGTAGTTGGAGGAGATCCTCTTCTCAGGCTTGTAGGAGTCGATTGGTTAACTGTTGATAAATCTGTAGATAGAATTGCTCTTCACCCTAAATGTTTGGTTCAGTCAGAAGCCGGGAAAAAGCTTCCATTTATCCTTGTGATTAATCTCCAGGTTCCTGCTAAACCAAACTACAGTCTGGTTCTATATTATGCAGCTGACAGACCGATAAATAAAAATTCTCTGTTAGCTAAGTTTGCTGGTGGAAGTGATGCGTTTCGGGACTCAAGATTCAAGCTTATTCCAAGTATAGTCGAGGGATATTGGATGGTCAAGAGAGCTGTAGGAACGAAAGCTTGCCTCTTGGGAAAAGCTGTAACCTGTAAATACTTTAGACAGGATAATTTTTTGGAGATTGATGTGGACATTGGATCATCTTCTGTAGCCAGAAGTGTCATTGGCCTTGTCCTGGGATACGTTACAAGTCTAGTTGTCGACCTTGCGATTTTGATAGAGGCACGAGAAGAGTCGGAGCTGCCAGAGTACATTCTTGGAACTGTCCGATTAAACCGTTTGAAACTTGAATCTGCTGTACCATTGGAAGATTAAGTTGCCAGGAATATTAAATTAGCTGATTTCATTCCATTATTGTAACTCAAATTTACACGGGGAA

>C100887_65

CTTCTACTTTCCCTCCTCTTTCTTCAAACTCTGTTTCTTTTGCCACTCTGCATATTTTCGTTGCAACACACAGATTCTGGTTTGCTTGTGCTCTCTCTTAGCCACCTGCTTCTGCTACACACAGTGCAAAAACAAAAAACCATTTTTCAGTGCTCCGTTTCTCATTGAAGAGAGTAATGAAGTACGTGCTGGTGACTGGTGGAGTTGTTAGTGGCCTTGGGAAAGGTGTTACAGCCAGTAGTATTGGTTTGCTTCTCAAAGTTTGTGGACTTAGAGTCACTGCCATTAAAATTGATCCTTACTTAAACACAGATGCGGGAACAATGTCCCCCTTTGAGCATGGAGAAGTGTTTGTGTTGGATGATGGTGGTGAGGTGGACCTGGATCTTGGAAACTATGAGCGTTTTATGGACATCAAGTTGACCCGTGATAATAACATAACTACTGGGAAGGTTTACCAGTCTGTCATTGATAAAGAGAGAAGAGGAGACTATTTGGGAAAAACTGTTCAGGTTGTCCCTCATATCACTGATGCTATTCAAGATTGGATTGAAAGGGTGGCCCACATACCGGTAGATGGAAAGACCGGTCCAGCTGATGTGTGTGTCATAGAATTGGGTGGAACCATAGGAGATATTGAGTCCATGCCATTTATTCAGGCACTAGGACAATTCTCATACAGAGTAGGTGCTGGAAACTTTTGCTTAGTTCATGTCAGCCTTGTTCCTGTTTTAAATGTTGTTGGTGAACAGAAAACAAAACCAACCCAGCATAGTGTTCGTGCACTCCGAGGCCAAGGGTTGACTCCACATATTTTAGCATGCCGCAGCACAATGGTACTGGATGAAAATGCAAAGGGAAAACTCTCTCAATTTTGCCTAATTCCGGCAGAAAACATTGTTACTCTTTATGACGTTCCCAACATCTGGCACATTCCTTTGCTTTTAAGAGAACAAAAGGCTCATGAAGCAATGTTTACAGTGCTGAACCTTCATGGTGTAGCTAAAGAGCCTAATTTAGAGGAATGGACTTGCAGAGCTGAATCTAGTGATTTGCTTCATGAACCAGTTCGTATAGCCTTAGTGGGGAAATATACATGCCTTTCAGATTCATACCTCTCCATTCAAAAGGCCTTACTGCATGCTTCTGTTCATTGTCAAAAGAAACTTGTGGTAGACTGGATTCCAGCTAGCAACCTTGAAAGTGCAACCGCAAAAGAGAATCCCGATGCATATAAGGCAGCATGGAAGTTATTAAAGGGTGCAGACGGTGTTCTGGTTCCAGGAGGGTTTGGGGATAGAGGAGTGCAAGGAAAAATTATTGCAGCTAGATATGCCCGTGAAAACAGGATTCCATTCCTTGGCATTTGTCTTGGAATGCAAATTGCTGTCATAGAGTTTGCACGATCTGTTCTTGGTGTACAAGATGCTAACAGTACTGAATTTGATCCCCATACCAAAAGCCCATACATCATATTTATGCCTGAGGGATCAAAAACACAAATGGGAGGTACCATGCGCCTTGGATCTAGGAGGACGTATTTCCAGACCAAGGAATGCAAATCTGCAAAATTATATGGCTGCAAAAGCTTCATTGATGAGAGACATCGGCATAGATATGAGGTGAATCCAGATTTGGTAGCTCGCCTCGAAAATGCTGGTCTCTCTTTTACTGGGAAGGACGAAACAGGTCAACGCATGGAGATTGTTGAGCTACCTAACCATCCTTATTTTATCGGTGCTCAATTTCATCCTGAATTTAAATCAAGACCAGGAAAACCTTCTCCTTTATTCTTAGGGTTCATTGGAGCAGCATGTGGTCAATTGGATGCTGTCTTACAGAGCATTTCAATTGTTGATAATAGTCTCTCAAAAGGAGTAGGCAGTGATATCTCTGCAATCAAAACATATAGAAAGGGAACTGCAACAAAGACAGCTTATAATAGGACTGAATTTATATATGGCAATTGCAATGGGTTGCATTTCTAAATTTTAACGCTCTTCCTTCATCTTTTAGAGTTTTCAAGTTAATTAGTTAAATATGTGTTGAGGGCTTTCAGTTGTCTAGAAGAGATTGTTTGTACAGTGAGTCAATTGAACCTTGTTAGGTATAGAGTTTTCAGAGGATACAGAGAAGAGCTTACTCCCGGTCTTTTTAATGGGACCAGCTCATGTAAGTTTATAATGGTTTGAGTGGGGAACTATGAAGCTTGTCTTATGTTTCAGGCAATGGAAACTCTTGGTTTTGCAAAGATATTAAGCAGTCTATAGAGAAATTCTTGTGAGCTTCAACTGTTTTCTTATATTTACTGTAATGTGAATTTTCTTGTCATAATGTTTTAGGAGGTGTTTAGTAAATGAAATCTCGATTATTTCTTCTTGATCATAGTAAAAAAA

>C100889_65

CACACATTCACTTCTCTCATCTCTCCTTTTCATTCAAAAATCCTTCTTCCCTTCCCACAGGGATTCAAGAGTAAAGGGTGTTTCTACTTCTGTTTGTTGAGTCTTGACTAAACAACACTCAAAAGTAGACATGACTATGGAAGAGGGGAGGGTGGTGAACCAGGACTACACACAAGATGGAACTGTCAACATTAAAGGCAAACCAATTCTTAGATCCAAAAGTGGTGGCTGGAAAGCTTGCTCCTTTGTTGTTGTATATGAGGTGTTCGAAAGGATGGCTTATTATGGAATATCATCAAATTTGATCCTGTATCTTACAACAAAGCTTCACCAAGGCACAGTAACCTCTGCAAACAATGTCACCAACTGGGTTGGCACCATTTGGATGACACCAATATTGGGTGCATACGTTGCTGACGCTTTTCTTGGTCGCTATTGGACTTTTGTCATAGCCTCTACCATTTATCTCTCGGGTATGTCTCTTCTTACACTGGCAGTGTCCCTTCCAAGCCTAAAGCCACCTCAATGCCTTGAAAAGGATGTGACAAAGTGCATGAAAGCTTCCACCCTACAGCTAGCAGTGTTCTATGGAGCGCTTTACACTCTAGCAGTGGGAACTGGAGGAACCAAGCCCAACATCTCCACTATAGGAGCTGACCAGTTTGATGATTTTCACCCTAAAGAGAAGTTGCATAAGCTCTCTTTCTTCAATTGGTGGATGTTCAGCATCTTCTTTGGGACCCTCTTTGCAAACAGTGTTCTAGTGTATATCCAAGATAATGTGGGCTGGACTCTTGGGTATGCTCTTCCAACTCTTGGCCTTTTAGTGTCAATCATGATCTTCCTGGCAGGCACACCCTTCTATAGGCACAAAGTACCTGCTGGGAGTACCTTCACTAGAATGGCTAGGGTCATTGTGGCTGCTTTGAGGAAATGGGAAGTGCCTGTCCCTAGTAACTCCAAAGAACTATATGAGCTTGACAAGGAAGAGTATGCAAAGAAAGGGAGCTACAGAATTGATCACACTCCAACACTGAGGTTCCTTGATAAGGCCTGCGTCAAAACAGATTCAAACCCTAGTCCATGGATGTTGTGCACTGTTACACAAGTAGAGGAGACAAAACAAATGCTACGGATGATTCCAATCTTGGTTGCTACATTTGTTCCTAGTACAATGATGGCTCAGATAAATACCCTTTTTGTAAAGCAAGGAACTACTCTTGACAGACACATCGGAAACTTCAAAATCCCCGCAGCAAGTTTGGCTGCATTTGTTACCGTGTCCTTGCTTGTGTGTGTTGTGCTCTATGACCGTTTCTTCGTGAAGATCATGCAAAGGTTTACCAAGAACCCCAGAGGCATTACCCTTCTTCAGAGGATGGGAATTGGCCTTGTAATCCACACACTGATCATGATTATTGCATCTGGGACTGAAAGCTATAGACTCAAGGTTGCAAGAGAACATGGGGTAGTTGAAAGTGGAGGACAGGTTCCTTTGAGCATATTCATTTTGCTTCCTCAGTTTATACTAATGGGAACTGCAGATGCCTTCTTAGAGGTTGCCAAAATTGAGTTTTTCTATGATCAAGCCCCGGAACATATGAAGAGTATTGGAACTTCATATTCCACAACTACCTTAGGCATTGGTAACTTCATCAGTTCCTTTCTTCTCTCAACAGTGTCACATCTCACCAAGAAAAATGGTCACAAGGGGTGGATTTTGAACAATCTGAATGAGTCTCATCTTGACTACTACTACGCATTTTTCGCAGTATTAAACTTCCTCAACCTCATCTTCTTCGCGTTTGTTACTAGGTTCTATGTATATAGGGTTGAAGTTTCAGATTCCATTGACGTGCTTGCAAAAGAACTGAAGGAAAAGACAGTGTCCAACGTTGTGAATCCCAAAGACTAGTTGATTGGGTATCTAATTAAAGGGAGAAGATTAAGACCCAATAGTTTCAAAAGTGCTGTTATGTACAAATTTGCATGGCCAAAGAACTAAGTATGTGTGTGTCTGTGGGGTGTGTGCTTGTTGACTTTCGAATGGTTTTGGTCATTGGAAGCCACTTCAGAAGAGAACTTAATCAAGTTATAGTTATTGGGGGTCGTTTTAGCATGTGATCGTTTTTCCTTATCCTTTGTAACCTTCGAGAAATGTCACATGCATCTCGAAAAAAACAAGATTGGAAAAAAAGAAATGTAATGTGTTTGATGTTTATCTTTTTAGATCTTTTCTGGCCACTGAAGCTTCTCTTTAGTTAAGAAATTGTAAAATAAAGGGTTGAAGACAAAATCTATCAGGTTAGATTTTTATTTTCATTCCTCCAATAAACTTGCTTTTTTAATATTAAAGCAACTTCTTTGTCGCAGCTAAAGATTACAGGAAACTGGTA

>C100893_65

TACTTTTATCCCGTCTTTCATACCACTATGTTATAGTAACACAACATTAGACTTCTAGCCACAACCTAAGTCATAAGGACCGAAACCCTAAACAAATGCACTACGTACGATTCAGATGAAAAAAAGAAATGTCCTGCAATGGCATCAGCACCCTGCAATGACACCATCTAGTATCATGATAAAAGTTTAGCAAGTTCATCAACTCTTAATGTTCCTTGTGGTACAACATTCACTAATTGGTCATCAAAATTTGTTTCTCTAGGAATTTTAATACTCTTTGATTTTTTCCGTTCCATAGAAACAGTGGAAACAGTGGGAACAGTGGTAGTTTCTTTATTAGTCTCAGAAGGGAAAACTTCTTTCTTGTCACTCTTTTGAGCCACAACCTTTCTGTTTTTCCCAAAAACTGGTCCAGGTGGAGCTGATTTGGCAAAGGCAACTGCATTTTGATAAATATTGTCCGCATCCAAAGAATCTTCATCTAAAACAAGTGCACTACCTTTCTCAACAGCTTGTTCGAACAGTAATAAAATTTCTTTCATACAAGGAGCAGTTGACCTTGGTGACTGACTGACAGCTGCTTCTCCTATAGCCTCTAGCAGCTTATCAGAATGAGGATCTACCATGCCATTGATATAATTGTCACCACTTCCAACACTTGCATCAGATCCTAAAATTGAATCAGAAAGACCATCACAGCAGGTTTTGCTATGCATTATATCCTCGGCTACATGGTTTTCACTCATAGTTCTCGTCGAGCATTCAGAACTGCTATTATAGCCTGATAAGGTCATACTTGGACTGGGACTCGTGCAAGGGTCTGGCTCAGCACAGGAGTCCGTGGCAATATTTGCGGTTTCAACTTCATAGGTTTTTGTAAGTGAAGCAACTTCAGTTACCCCAGAAATAGATAGTTTGGTATTTTCATTGGGATATGGCACCTCAACCTTATCCAAAGTCACATCACTTGAACTGGAGAAAATGCTAGTTTCATGTGATAAATTGCTTGCATGCTCTACTGAGTTCTTCTGTAGACCTGGTGCTGAGAATTCTGCCTGTAATGGCTGAGGTTCAAAGTTTTTATCGTCAACAATTTTACTGTGTTCCTTGAGGTCATCTCCCAGATCTGGTATAGAGGACTTCCAATTTGGTCCTCTCCACATAAGTATGTGCTCATATTGAAAGGAAAGCAATGTGCACGGTACAAGATCCCTTAGTTTTGCTCCAATCTTTCGATAGTCACTTTTATTTAGTCCCTGACAGTTTATACGCACTAGGTCACACTCTTCAAATGCCTCCCTGACGGTGTTTACTAGGTTAACGTAAACACCATTTTTCCCTAGCCGGCATATGGGAATCAATGTGCGTCCCTTTTGACGCATTTCCGTTACTTTTTCCAGAGTTAGACCTTCAGGAACTCGCGGAATCAACCTAGGATATACTGGACTCACAGGTTTCCACAACATAAGTGGAAAATATGGGCGTGTTCTGTAATTGTAGTTTCTTCCTCGAAACAGGTACAACTTACCACCCTGTCTGAAAATGACTTTACCCCCTGTCTTTTCCTCTAACTGCTGGCAAACGTTATCCATATCCACAGTACACACTCCCAAACACCTGATCTTGCACACCCTCCGCCGCTTCCAATGAGCATGTATATTATCCAACATGTTATGCGTCAAACCATCTCTACCCATATTGAGTTGGCGTTGGGTTTTCATGCAGGATTTAACCAAGTCACGAATCTCCTCCTTCGTCAATGGTTCCCCTAAAATCTCCTCTCTCGACTTCACATACCTAGGTCGTGTCCCCGGCAAATACGGCCCGGGAGACTGCACCGGCTTAACCCCCTTCTTGTGCGGCGGCGGAAGCTCGAAAGAGTCGAACTCCTTCAGCTCCTTCTTACTCGGCGGCAGCGGCGCGCGCCCCGTCCAGGGCCGCGGCATGGTGGTCGGACCGAAGGGCAGGAACGGCGCCTCGCGAATTTTGATTGGCTTGGCTTTGGGGGTTTCCGTGTAGCTGTACTTGAACTCGAACGGGGCACCCTCGATGACGTAGGAGAGTCCATCGTCGCTGATTTTGATGCCTTCGTCGGCGGAGGTTATCCGTCGCGGTTTCGATTTGGGGATGTTGGAGAACCTGAGCGCCGGATGGGACTGCGGGTCCAAGTTGGGCCTAGGTGGGCTTTTGGACCTTTTGGGCGGGCCGGAGGGCCGGTATGTTCGGCGGGCATTGGGGGATCTGTCGGAGCGGGTTTGCGGGTTGCTCCAGCGAGAGAGTCGGAGGTCCGAGGATGGACGCGGGTTGGGGTTGAGGTCGAGGGTTGGGGCGAAGATGGGAAAAGAGTTAGTGTGTGCGAGCTTCAGAGCCATCAAGAAGAAAAAGAACAAGATAAAC

>C100897_65

ATAAGTTTCTTTTGAACGCCAAGTTCTTCAACTTCATAGCTCTTCAATTCACAGGTAAGTCTCATGAGAGTGTGGTGAATCAGTTTTCACGAGACAAAGTTCTCCCAAACACTTCAACATGGCTTCTTCAGCGATGGAAACCTGCAAATCAGAGGATATGCACATGCAGGTAGACATTGAGAAGAACGCACAGGATGTAAGGTCTCAGTGGGTTCTTAACGCTCCTGAGCCCCCAAGTCCATGGCGTGTGGTCGCAGATTCTGTGAAAAAAACCATCTCCCACTATAAACAAAAGTTGTCCTCTCTCACAGATCAGCCCTGCACCACACTCCTCATGTCAGTTCTGCAGGTAGTTTTTCCTATTCTTGCTTGGGGCAGAAACTACACTGCTACTAAGTTCAGGAAAGATCTTCTTGCAGGCCTCACTATTGCAAGCCTCTGTATTCCCCAGAGCATTGGATATGCAACCTTAGCACATCTTGATCCTCAATATGGCCTTTATACAAGTGTGGTACCACCACTTATCTATGCTGTGATGGGAACTTCTAGAGAGATAGCAATTGGTCCTGTGGCAGTGGTTTCTTTGCTGTTATCCTCAATGATGGAGAAACTAGTAGACCCTGCTTCTGATCCAGTGGGTTATACAAGGCTAATTCTGCTTGCCACTCTCTTTTCTGGTATCTTTCAGACTTCCTTCGGACTCTTCAGGCTGGGTTTCCTTGTGGATTTCCTATCCCATGCTGCAATAGTTGGATTTGTGGCAGGAGCAGCCATTGTGATAGGGCTTCAACAGTTGAAGGGACTCTTAGGAATCAGCCATTTTACGACCAAAACCGACATAGTCTCTGTCATTAAAGCTGTCTGGGAAGCAGTTCATAATCCATGGAATCCTCGAAATTTTATCCTCGGATGTTCATTCTTGGTCTTCATCCTAACAACGAGATTTCTGGGTAAAAAGAAGAAGAAACTGTTCTGGTTGGCATCAATCGCTCCTCTTGTATCTGTTATTTTGTCAACTCTACTTGTGTTTTTGACAAGAGCTGATAAAAATGGAGTGAAAATTGTGAAACATGTCAAAGGAGGATTAAATCCAATCTCCATCCATCAATTAGACTTCAACAGCCCCTACATTGGAGAAGTGGCCAAAATTGCACTAGTGGTTGCTGTCGTTGCCCTTACTGAATCTATTGCTGTTGGGCGATCTTTTGCTTCCATCAAAGGATACCAACTTGATGGAAACAAGGAGATGATGTCAATAGGTTTGACAAACATCATAGGATCTTTAACCTCCTGCTACGTCGCAACTGGTTCGTTCTCCCGCACAGCTGTTAATTATGCAGCTGGGTGTGAAACTCTGGTGTCAAACATTGTGATGGCGATCACGGTCTTGATATCACTACAGTTTTTGACAAAGCTCTTGTATTACACTCCTACAGCTATTCTTGCTTCTGTGATCCTATCTGCTCTCCCAGGACTTATTGACGTCAGTGAAGCTTACAAAATCTGGAAGGTTGATAAGATAGACTTTCTTGCTTGTGCTGGTGCCTTCTTCGGAGTACTGTTTGCTTCTGTGGAGATTGGTCTGCTAGTGGCGGTGGTTATATCTTTTTCAAAGATCATTCTGATCTCAATTCGACCAGGCACAGAAACTCTAGGAAAACTTCCGGGAACTGATTTGTTCTGCGATGTCTATCAATATCCCATGGCAGTTAAAATTCCTGGAGTTATGATAATACGAGTCAAGTCTGCGTTGCTTTGCTTTGCAAATGCCAGTTTCGTTAGAGAAAGGATCATCAAATGGGTCTCTGAGGAAGAATCAGAAGATGACAAGGCAAATTCAAGAAGCACTATTCAACTCGTAATTCTTGACACCTCCAATTTGGTGAACATTGACACATCTGGAATTGCTTCTCTAGAGGAACTGCATAAGAGTTTGTCTTCACATGGAAAACAGTTAGCCATTGCAAACCCTAGGTGGCAAGTGATACACAAGCTAAAGGTGTCAAACTTTGTGGGCAAAATCGGTGGCAGAGTATTTCTGACTGTTGAAGAAGCTGTTGGGTGCAAGAGCCGGTGCTAAGTTAGATGATCGCAGCTAGCTAATTCAACTGTTAATGCCTGAGTCTGATGTGTGGCAAAATAAAAAGTTAAATAGCAGTATCTAATATCCATTTCTCTTTGTGGAAAATCGTATCAGTGTTTATGAAGTTTGCTTGTATTTCCAGTCCCAAGAAGGCAAGTACTGATGTACAAACGGGACATTAGAATAAGTCATCTTGTTGTGATCTTGTTGGAGATGCTGTGAGTTTCTGCTCATGTGTGTGCTTAATAAAATTGCAGTTTGAAGTGGTGTGTGTAAAAGTGCTGGAAAATACTTTTGGTCAGTGTTGGTGGAAG

>C100901_65

ATTTACATTAATACTATGGAATAAAAACAAAATTTCAGGAACAGGCTAGAAGCTACATCTTATAAATAGCTGAACTCAAAACACTTATACAAGGGTTTCTGTTGAAGCAACTTTGGTATAAGCTTCATATTTGTACAGAAGAATCTGTATATAATCATACAAACAGAAATTAGATTTAAGAGTCAACCTGAAGGGGCACAGCTACTACGAATTCTACTAAAGAGCAGCTCTCCACATATAAGGATATACATAAACGAACTATCACCATTTCCAAAGAGAAAATGAGGAGCTATGCCTTTAAAACTTTACCCGCCAAAAAGTAAAACCAAAATGGAACTCAGAATTTCTTTTTATTTGTGTGTGGAAACAATTTTAAGGACCCATTAGTATTCTTCCCATGTTCTGGGGGCAGGGATAAGCAGAACGGTTTAAAGATGTACTATCACTTGGATTTTCTCTAGAGCCATATGGGTTAGAGTTGTTATTTAGAACACCAAAGTACACTGGCGCCGTCGAAGAATGGACAAACTTGAAATTGGAATTGCCGGTGTGAGCTTGGCTGTCCCCAAATTCGGAAAGCATCATTCTCCCTTGACTGCTTGGTGTGTCTTCCGCAGCAGGGCTAATTCCCATACCAAGGTCAAGGCTGATGGTATCACTTTCTTCCGGCCTAATTCTTGTCTGTCCCCCAGCAGCACTTACTGGTCCTGCAATGTCATGGCAACTATTATTCCTTGCAGTTGGTACATCATGATTGTGTTTCCCCTCATATGTGGTTATCACAGCTTTTGGATCATGAGATGCCCTTTCCACATGTTTTCTAACGGGGCACCCCGTGTTTGTGCATTTGTAATAACTCCTAGGGTTAGGATTGCCTCTCACCACCTTCTGCCCATACTTGCGCCAGCGGTAACCATCATCCAATATATCAACCTCACTCAGAGTTTGTACAACAACCCGTGGCTCCCTGATGGGCTTAACTACAGGAGTAATGTCAGCATTTCCAAGCTCCATTTTTCTTCGCTTTGAGAAGGGATCATCATCATCAACCTCATCACTATTCCGGTTTGACAAAAATCCTGTACCCTCTTGATCACCATCATTTGTAGCTGCAGGTGATAGCTCTGGAGTACTGTCAGGCTCAGATGGATGAGACACCTGGCCACATATATTGGATCCTCTGTCATCTCGGCTAGTCAAAGAAGCTTTATCAGATCTCTCTTCTTGCATAGTAATGATAGTTCCAGTTGAGTAACGGCGATTTGGTTGAGGTTTTGGGTGATCATGTGTTCCCTTGTAAATTATCTCAGTGATTTGCCCATCATGAGAGCGTTCAAATAGTTTCTTGACTTCGCAGTTAGGATGTGTACATTTGTAGTAACTGCGTGGAAATTCACTTCCTTTAACATGTTTCTGCCCATACTTTCTCCAGTTGTATCCATCATCAGATACTCTCTCAGCTGCAACGGAAAGTCCACTACCTCTATTGTCAACTTGTGATGCTGGAAGCCCAGTTGTTATGTTGCTCCTAGGGTTCAGTTCATCCAAATCACCTTCAACACGGGCATTAACTCCTGAGTTAACCATTTGAACAGGTGATGATAGACTTAATTCATTAGAAGGGACTGCCATCTCATTTTTTACTAATGGTGACGAGTCAAAAGGTTGAGCTTTTCCTTGACCATCTATTTGAGTAGATTTTTCACTTGCATGGTTGTCAGGGTCTGCAGGAGCCATATTTGATGTACAGTGTGGTTTAAACTCAAAGAAGCTGGGTTTTCGGTCATCAACCGTATTGGTATTGAAGCATACAGTGGTAGGAAATGAAGCAGATGCAGCAGAAGTCACAGAACCATGAGCTGTTTGATGAAGCATAGAAAGTGATCCTGTAGTTGGTGAAGGTTCCACCTTCATGTTGGAGAGCAGCACCGGCGACTCGAGAAATGACGTCGGACTTAGGCCCGGCGGAATCGTGACGCACGGCGAGCGCGAGATCGGGAGCTTCGCGGGTGACATGAGTTTGTACCTTGCGCCACCGCCGGCGGCAGGAGCAGCGTCGGCAGAGGAATTGGGCCTGGAGGGAGGTCGGTTGGGATCGTCGGAGATGGGTTCGGCGGCGTCCATTGGGGCGTAGAAGGAAAAAGTGTGTATTAAGGATAGGGAAAGAGAAAAGTGAAGTCAAAGGAGAAGGAAATGGAAGGGCAAAAGAGTAATTTGAGGTGAAGGTATGTATGTCGCAGAGAGAGTGAGACGAAATTGACTCTGTGTAAGAATGGATCTCAAATTGTGAATCTGTGTTGTATTAGGTCTTTTGCTTGTCATTTTAAAACTTCTTTTTTTTCTCTTCTTTCATTCTCTCTTACACTGCTACTGTTCTAACTTCCTCAACTCACCTCCA

>C100903_65

GTGCCCTATAACTAAACTTTAGTTCAACCTAAAGCTACGACCCTTTGATTCACTGCTATAATGCAATTATCACAAACAGAATTGCGTTTGCAGATGATACAACTTCTAAAGAATTCTTTATATACATCTACGAGGGTTTCACTATATCATGATAGACAGCTCGATTTCTTAATTTTTAGAAATCAAAATCTTGATCGGTTGTATGACTATATTATTGTGTAAATTATTCATGCATTGACGGACTCGAATATGCCAGCCACATTAATCAAGCAAACCATTAAGCAATAGTGGTACAAACGAAGCTCATAATGGCTGAAGTTCCTGCTCTCGAACAGCTACATCAACTGGCTCGTATGAGTGCTCAGCTTGCAGTCTGTTTTTCCAAATTAAGAATATTCCTAAGGCTGATATTCCTATAAGCAATGTAAGCAAAACAGCTAAAGCCACAGCCAAAAAATGTTGCTGCCACCAAGTCCGCTTCAATGGAGGCTTGGCATTCCAATCAAGTAACTTATAACTTCCCAATGAGTCAGGAAGTTGCATACGATGTTCAGAAAGCCGGCCAATCATACTCATTGCGGTAGCATTTGAAAAGAAATCAGCATATGGCCCCGGTGTTATGAGCCATTTAGAAAGAGAACCATTTCCAAGAGAAGAAACATTCATTAAGTGAACCTGTGAAGTATTAACATCTAACTCATTAGCAATGAGTCCAGTCAATTCTGTAATGTAAGGCTTCAAATCCACGTAGCTGATGTTAAATGAAATTACAATTATTATTTGAGCAATTTGGAGTTCACCTAGCTGTAAATTATACTGTGATGGGGAGGGGGACGGAGCCACAGAGGGCTCAAATGCTTTGGTCAAATTTGTTCCTTCAGAATTTGGAGACAAGGGTGGTGGGGCATCTAAGACATGTAGTCTTTCCCATAGCTCAGAACAATTAGTCTTCCAGAAACCAATTTTTGTATGCTCACGATCATACATCACAAGAGTGTTACGAACAATAATACCTCCTAAAAGAGTAGTTGGATCATTGTTATTTGAAAAAACCCCTAGACAATATGCACCTTGCACTTTTAAATGCCGAAACAGGTAATTTTCAGGTGACAGTGACAACTTGTGACCATTTCCAAATACCATATCAACCACGGGAAAGCTTTTAGAGAGTTGAGAGACATCACTTCCTGCACCAGAGAAACATATATCATTATAACGGGGATCTGGACCACTTATTCGCTTCAATGAGTGAGTTTCCTTCATAATAGCATGTTTAAATGCAAGAAATGCAGATTCTGGTAGGTATGCATAAGTCGTGCCACTATCCAAGACAGTTCCATGTTTCCCATCAAAAACCTTTGGGTTTAAATTCAGTCGTTTCCCCGCAACATGTACCTCCTTCAAATTTATATTGTAATATGGACTGTCGCCATCCCAAAACTTCGACCAGGATCTGAATGTGTAAAAACCATATCAGCTGGGGGGGATATGCCACCAAGAACCATAGCACCCCCACCAACACCCATACCACCATAGCACAGTGAGAATGAATCACTTATCACCTTTTTCTCAACCAGTTGGTCCATGATACTCAGATCTCCACGGCCCAAACCCATGATGCCATCAGCATGCTGAGTATAAAGATCACCAGTCTCATCATTTTCACAGCCAAAAGTAGCACGTTGAGGGGAAAGCGCACTCTGATTTCCAAAGGACACAACATCCTCTCCAAGGACCCCACTGCTAGTACTCATTTCTGCATACCGTCTCTCATACGTGCACTGCTTCCTGTCTTTGTCACAGTTGCACTGCCATGTGCATTTTATAGGTTGATAGGTTTCTGAATCTTCTGGCCGGAAATTTGGATCCTGGTGTCTACCACAATGTTGGCAAGTAGAGCATGGGACATAGGTCACAGTGCTCCCCGTATCAACAATAAGAGAAAACCGCTGCGGAGGAGTACCGATCCAAAGCTGGGTCGTATAGTACCCGTTTCGAAGGAGATCGTCGTGGAGTCTCATGCGAGCGTTAGGGTGGCCTTGGGACTGCGATCTCTGAAGTTGACGACGCGGATTAGAATCCGAGACGGAATATTCGGGAACAGAGTGGTGTAGAGGGAGAATCATAGCAGGACGAGAACCCTCGTTTGGTAAGAGTAAGACGCTGCCGGAGTCAACGGAGGCAACAAGCCACCGCACAAGGGATAGAATTAGGGACACGACGGAGATGAGTTGAGTCCGTTGCAACGCCATGAGTTAAACTGATTCGATCTTGCAGATCCAAACGAGACACAAAGTGAAGACTGAAGACTCGATGGAGAAAAACGAGTCTCAATCTACACAAAGGAGAGAGGACGAAAGTCAAACTATCCTCTGCTCTGCTGTGTCTGAAACCAAAA

>C100905_65

TTTTTTTTCATTAAAATCACACGGGAAAATATTTATTTAATTAATGAATATAAAATTCAGGAGTTAACAAATGCTAATTGTGCTTAAAAGATTGTACTAAACACCGAAAGGAAGGGAAATGAGAATGATGTTTTCTTTTGGTTCTCTCTTTCGGCGTAATGCAAAAACACAACAACAGAAGGGACCAGAGATAGAGAGGACAAAGAAACAAAGAAACAAACTAGCCTAAGGAAAGAAAACACATTGTCTGCGAAGATGTTGATTAAGGAAAATGTTCAGAAGTTTACCTACAAGTAGGACACACCCACATTAATTGTGGGAATCACAATGTTCTTACGTGTATTGTGTCTACGACGGACGAAGGTACATACATCTTAATTGCCCACGCCTAAAAAGGTAAACAAAAGCTATTCGAATCCACGATAAGAGGAACTGGTGTTACTTGCCATCCTAAGAACAGTTATGCAGATTCATAGTCTAAAACCAACTGAGCAAAAACAAAATCACATCCTTGAGATCTGGAGAGGTATAAGAGCTTCAAGCATTTCATGACTAACGGAAGCTAGGCGCAGCTTTTCCACGGGGCCTTGTTTCTCTAAAAGATATTTCTCACAATTTTCAGCAGCCATAACCCCAGCGGAGTAAGCTCCATGCACAGATCCCTGGTTGTCCAAGCTTACAGCTTCTCCCCCAAAGAATAGATTGCCCAAGGGTGCACGAAGCTTGTCATACACATCGTCTGGTTTTCCTACTAAATCATATGAGTAACATCCAAGAGAGTTTGGATCTGTTCCCCATCGAGACACTAGATACTGAACTGGCTCAGAAGCTTCTGGAAACATCTTCTTGAGCTGTTGCATTACGAATTTTGCAGCTGCCTCATCAGATAGCTTCTCGATGTCATAAGCATACCTTCCAGCTACCATATAGACAAGAACAGGATGGCCTGTTGCTTTGTGAAGATTGAGAAAATAGCCGCAGGCATAAGAGGTTGGAGCAACAGTGCCCAAGAGTTCTACATTAGGCCAAAATACTTTGTCAAACCTTAGGGCAATCTTATTTTCGTTGCCCACACCAAGATCGGAAATTGCTGAAACCTTCCAATCAGGAAGTTTTGGTTCAAATTCAATTAAATTGGCTTTTAGGATTCCAATAGGAACAGTTATGATAGCAGCATCAGCAACAAAGTTCCTGCCATCCTCGACCGTAACCATTACCTTGTTATAACCACTGGATATCTTTTTCACCCTGTGGTTTAAACGTATGTCAATATCTTTTGCAAGAGCTTTTATAACAGGATCATATCCTTGCACCATGAGTCCATGACCACCAGAGAGGACATGTTCCTGATCCCAGGTATTCAGGGAAATCATATCTGCATCAGCAGCAAACCAAGCTTCCATTCTGCATATAAACCATTGTAGCACTTCATGGGCAAGTCCTTTTAGCCTTAGCTCTGGATGCCTATCTAGCACAACTGATATAGCTCGGGAAACTGAAATGTCCTCGGGATGCTCATCCCTCACTTTCCCTGTCTCTTGCAGAATTTTCTTAAAGGTGTCTCCAACTTCGATGACCATTTGCTGTGGAACTTGTTTACCGTCGATGTTAAAAAGCATATAACTTTCCAAATCATGGTCATATAGGACAGAGTTGTCACCACTGGTACGATATAAAGAAAGCCCCAGGCCACGTATCAATGGAGCCAATGGATTTTCATTACAAACTCCATGCAGCCATGAGGCTCCCATGTCAACTGGACAACCAAATGAGAAGTCGGTATGAATGCGACCACCAAGCCTATCTCGTGACTCCAGTAAAGTTACCTTAAAAGATGCATCGTGGAGACTTCGTGCTGCAGCAATTCCAGATATTCCGGCACCAATCACAATAACAGATGGGGGAGGCCTGCTCTGCCCCTCAACGCGTGAGGCAATAGTACCATCGAGGAAATGCGTGGAGAAGAGCTGATTAGGGTCCATGCGTCTGGATGAATTGAAAGCGAGAGAGAGGGAGTTATAGAATGAGATCAAGTTGGATCGGATCGGAAAAGAGTGGTGGTGTGGTTTAGTTTTGTTGCGAAATCAAGGGTGTATAGGAATGGGGAAATTAGAGACTAACTGGAAGGAATTGCAGAAGATTAACAGGAGGATGCCGGCAATGAAGCGGCTAAGGTGAGGAGAGAAACGCAGCTTCTCAAAATATATCATAATCTTACAATTCTTCCGATCAAAATCCAACCGGAGAACCTTCCTTGCATAAGAAATTCGATCGATGCGCAACCACAATCCCGATTTCCGATTCGATCGCTCGTGAACGAGCGAACCCTCTCTCTCTCTCTCTCCCTCCCTCTTTCTCTCTCTGAACGAATGAATGGAATAGCCGACAATGATTTCTC

>C100909_65

TGAAGGCAGCAACTGTATGCCATTGTACGCAACTACTTTACTTTCCCTCTCTCTCACCTCTTAACTCTCATCTTCTCTACTTTCTTCTTCCACAAAAAAAATAGGGACATAAACACAACCCTTTCAATCTCCAATGGCTGATGCCACCAAGTACGTTCCCATCGGTGGTACCAACCTTTCCTCAGACCTCAAGACCCTCTCATCCGTTCTCAAAACCAGAAAAACAGTGGCTTTTGCATACGCTTGCATACTTGCCTTCGTGGCTTTCACTGTTTTCTTAGCCTTTTCCCCTTCTCCAAACGCTTCCTCTCCCTGGTTCACCAACATATTCTCTTCCACATCCACCCCAGATGGTTCCTACAAGCCTCAAATCCCTTCCATTTTCTCACTTCTTTTCCCAAACACTTCTTCACCCTCCAATTCCACTGATCCACTGCATAACAACACTAACACTTCTGGATCTACAGATACCAATGCCGCTTCTCTTTCTACTCCTCAAAACGCCACCTCAGAAACTCAAACAGTCAAAGCAACCTCAAACCGAACACAATCCTCGGTTGTTTCATCCCTAGATGCAGTTCCTTTTCCATCCACAAACCAGACCCAGAATTCTCACGCGCCAACTTTGGAGGTGAAGTCGCCACCACCCCTTGTCGCTAAGGAACCATCTGGGCTTGTCAAAAATCACACTTTTAACTCTGGTGAAGCTAAAGCCATAACCACAAATCAAACTACAAATGCTACTGCATCTGCAACATCTGCAACCAATGTTTCAGTGAGTTTCCCTGTCCAGAATGTTCCACATCAGAACTTGAGTTCTAATTCTTCTGTGAAGGCCGATTCGGTAAAGGGTGTTGCCTTGAATGATAATTACACTGTTCCGCATCAGAAGTCGAGTTCTAATTCTTCTGTGAAGGCCGATTCGGTAAAGGGTGTTGCCTCGAATGATAATTATACGGCTTCTCTGGCGAGGAAACAGAATAATGGAAAGAATCACGCTAAAGGGAACTATCAATCGATGGAGTCTCTCATGAAGTGTGATTTTTTTGATGGAAAGTGGGTTAAGGATGATTCCTATCCTCTCTATAAGCCAAATTCTTGCTCTTTGATAGATGAACAGTTCAATTGTATTAGGAATGGAAGGCCCGATAAGGAATATCAGACATATAAGTGGAAGCCCAAGGGTTGCAGCCTCCCCAGGTTGGATGCGCATCGAATGCTGGAATTGTTGAGAGGGAAGAGACTGGTTTTTGTTGGCGATTCGCTCAATCGGAACATGTGGGAGTCTCTGATTTGCATATTGAAGAGTGCCGTGAAAGATAAAAAAAATGTTTATGAAGCAAATGGAAGAGTTCATTTCAGAGGCGAAGCCTCTTATTCATTTATATTCAAAGATTATCACTTCTCTGTGGAGCTTTTTGTGTCTCCATTTTTGGTTCAAGAAGGGGAAATGACTGATAAGAATGGGACAAAGAAGGAAACACTTCGTCTAGATTTAGTTGGTCAATCTTCGTCTGAATATAAAGATGCAGATATTCTCATCTTCAACACTGGCCACTGGTGGACTCATGATAAAACTTCTAAAGGGCAGGACTATTACCAAGAAGGTAGCCATGTGTATTCTGAGCTAAACGTTCTTGAGGCATTCCGAAGAGCTTTAACAACTTGGAGCAGGTGGGTTGATGCCAACATTAATCCATCCAAGACCATGGTTTTCTTCAGAGGCTATTCTGCTTCTCATTTCAGTGGTGGACAATGGAATTCAGGTGGACAATGTGATAGTGAAACTGATCCAATCGAGAACGAGAAGTACCTAACCGAGTATCCAGATAAGATGATAGTTTTGGAGAAGGTGCTAAAGAATATGAAGACCAGTGTGACCTACCAAAACGTGACCAGAATGACAGATTTCCGAAAGGATGGTCATCCCTCGATATACAGGAAGCAAAATCTAACTCCTGAGGAAAGAAAATCACCATTGAGATACCAAGACTGCAGCCATTGGTGCCTTCCGGGTGTTCCTGATATTTGGAATGAGATTCTCTATGCAGAACTTCTGATGAGAGAGTATCGGAATCAGCATGCCCAGAAGAGAACATAGGTAACAAATAAACTATGAAACTTAATGAGTTAAGTAACATGCGATATACAAAAATTTTTGTTCCCACGCATACAAAACAAACCTTATACAGAAAATGCTTCTATATAAAGGTTCATACAATTAAGTGAACCTATATATATATACACGATTAAGAGGTGTGTGCAATAACTTTGTTCATATGGATGGATACGTAAATTGGTGAGGAGAAGAGAATACAGGGGTAGTTTGTTTGTCATTGTAAATTTTCCTTTCTATTGCATCTACTTTTCCTTTTTAGCCTCTGATTCTTATTGTAAAT

>C100911_65

CATGGAAACAAAAAACTCGAGAAAAAAAAAATCTCCATAATTTTTTTACAGAAATTTAATTTGATAGATAATTCTAAGAAATATGTATATGAACATGAACAAGAACAAGAAATCTGCACGTGCTGGTACCCTGTTCAAGGCACTTGCTATTCTTAGTTTCTTAGCAAAACAGATATGAGGGGAACTTCTCTCTTGAGCACAGAACACTATTGGTAGACATTTACCGTCATATTCAGGGGACAGAATCCACAAGCATCCATATCCATCCATCATAGCCTTTGATTCACCTAATTAATCTCAGAAAGGGGCCTCGAGCGATTCTTCCCAGACGTCTCCACTGGCATTTGACATATCTTCACCATCTTCTTCCATGGAAAGCCTAATATACTCTCTATGTGCAAAACGATCCCACTCAGTCAAATTTGGATCCTCACGTTCCTGACGTATGAGAAATGGATCACGAGTCTCGAAAGCAATAAACTTGTTAAGATTGAAAAGAATATTGAAAACATTCCCAGAAAGTTTGCTTCCTTTAACGTCTCTTAATGTGATATATCCCTCATTCTCTGGTCCAATCATATCTACAATCTGACACAATATATCTTCAAACAGCACAGGCTCTTGGGCCATGCATTCCATCCGATGCAACTGCTCCTCATAAAAGAACTGCATTTCATTTGGCGTCAGCACTCCATTTCCATCCAAATCAATGCATTTGAACCAGTATTCTAGACTAGGTTCGGATGACTTATCTTCCTCAGCAAGTATAAAATAAACAAAATCTTCATAGTTCATCTTCCCTTCAACTCTACTGGTAAATTTCCTTGGAACCTGGGAAAAAATTCTATCAACAATCCGATAGGTAAGAGAATGGTTCCCATATTTAATAAGGTTCTCTCTATCAATGAAAAAGTCATGATCTGTGTCGAGCTCCCAAAATTTGCAGTAAATGACATAAAAATGCTCATATGAGAAGTACCTCAGTACTTTGTTAATGTCCTCTTCCTCGTCTACATGTAGCATTGCATCAATTAGGTTCCCACGTTTCAATTCTCTCAGTGTAAGTCGGCCATTTCCTGATCTATTCATGTAGTAAAATATTCTATGTATGACAGTCTCAGCATATCTTTCCTGAAACTCAGGCGTGCTTTGCAAAAACTCCAATCCAGGGTGAGTGGACAATAGTTCCCTCAGAACAGGTTTGAAATCATCCTGTGCAAGGTATGCTCGACATGGTTGCTTCAGTATGGTATATATTTGTGTGGTTAAATCCATAGCTAACATATTGCCTCGAATCCAATAGTCTATAAATTGATCCCTTGTTATGGTTCCAGTAAAATTGACATCAATCTTTCTGAAAAGAGCAGTGGAGAAAAATGAGGGAAGTTTGCAGAGCTCTTTTGTGACAGACTTAAATTCATGTAATTGCAGACCATCCAAATGACCATAAAATCGACTTTCGATTTGATACAAGAAATGCTCTTTCATTTCATTTGGTGGTGGACGACCATTTTTGAAGTAGAACTGTGGAATGACCTCCCTTACAGGTTCACTGACTAATTTTAGTGGAGATCCTAATGAAGGACTATTTCTCTGTTTTGTGGTACGTGGGGAACCAGATGAACTGCGTGGTGAAAGTGGTGGTGTACTCCCGGCAGGAAACAAGGAAGGCAGAGAGTTGCTTGACAAAGCATTTGAGTTTGTTGAGTTCTCAAGTGCACTCGAAGGAGTTCCTGCCTTTGCATCAGCAATCAATGACTTAACCAGGCGACAAGTATCTGGAAGCGAAAGCCACTGCGAGAATAGCTCCTCCACAACATAAGGACTCGATTTCAGTGCAAAAGAAGACACCTCCTGAAGCTGCAACAAATCTGTGTCCAAAGACACCAACTCCTCAGTTTCCTCCACATCCATCACAACACAATTCTACCTCTCATGCACTCAACAACCCTAATTCAATCACAAATTCTGCACCCCTGCCAAAAACCCTAAACCCCACTTTTTCAACAATCACTCCGATTCCTCCCCCTACCTTTCTTCAACCACTTGGGATAGCAAAACCCAGTAACCAAGTTAACAACTTTCAGCCAAAAACAATCATGGGTACCGGAGAAGAACATAGAGGGTCCTGCAGCTTGGCTTCATGCAGACACGCCCCAGAATTGCAAATTCCAGGAAATTTGAATAGATGAGAGGGAAAAACAGTTTTAGAGAGAATCAGAGAAGGAAAAAATTAGAAGGGATAGAGGAAAAAAATGGAAAATTAAAAAAGAAAAAGAAAAAAAGAAAAGAAAAGAAAAGAAAACCCTTGTTTGGTTTCGGGGTTTGAAAAAGGAATAAGGAAGAGGCAACGAAAACAAAACGAAACGGTAGTGATGAATGAGAAAACAAAAGAAAAC

>C100913_65

GTTTATTTGAAAGCAAGGTTCCTAAACGCAGAGCAAGAACGAAGAAGCAAGATTGAAAAATTGAATCGGAGTGGTGACGTGTAGGACCGAGATCGATTCTCCTCGCTTCTACGTTCTTTCAATTCAACTCAGCACTGCCCCTTCCCTCGCTGAACAATTCTTCGTTAACCATGGGCGCATCATAGCACCGATCAATCCATCACATTAAAATCAATGGCGGAAACCGCATCACCCTCGTCCAAACCCTCACCAATGGACCCTCAAAACCCCGCACAATCCTCCAACCCTACAATCCCTTCTCCTTCCCCTTCGCACAACAACATGGCTTCACCCTCGCTCCCTCCGCTTCCCCAAGACCAACAACAACAACAGCAGCAGCAGCAGCAGCAGCAAAATCTTCATCAACAGTTGAGTCCCCCTCAACAACAACAACCGCTCGTGTCGTCGCAAGCGATGAACACCATCAACGGCATGAACCCTATCTCCAATTTTCAACTCCAGCAGACGCTGCAGCGATCGCCTTCCATCTCGCGCCTCAATCAAATTCAGCCTCAGCAGCAGGCGCAGCAGCAGCAGCAGTTCGGCGTCATGCGCCAGCAGGCCGGATTGTACGGCGGACAGATGAGCTTCGCCACCGCGGGCGGCGGAGCGGGCCAGCAGCAGCAGTTGGGCGGGTCCAATTTGTCGCGCTCCCCGCTGATAGGGCAGAGCGGACACTTCCCCATGTTGTCCGGTGCTGGCACGCAGTTTAACTTGCTCTCCTCGCCAAGACAAAAAGGTGGGCTTGTTCAGCCATCTCAATTCTCCTCAGGTAATTCTGCTGGACAATCACTGCAAGGAATGCAAGCAATGGGCATGCTTGGATCACCAAATCTCACCTCACAACTACGAGCTAATGGAGCCCTGGCTTATGCACAGCAGCTTCGAATGAGTCAGGGCCAAATCAGGCAGCAAATATCACAGCAAAACTCACTTAACACTGGACAGGTTCAAGGTTTACCAAGGTCATCATCCCTTGCTTTTATGAATTCTCAGTTGTCTGGGTTGTCTCAGAATGGGCAACAAGCAATGGTTCATAACTCCTTAACGCAGCAACAATGGCTTAAGCAAATGCCCGCAATTTCTGGCCCTGCCTCACCATTGCGTATTCAACAGCAGAGGCAGCAGCTGGCTTCTTCCGCTCAATTGCAACAAAACTCTATGGGCCTGAATCAACAACAAATTTCCCAGCTGATGCAGCAGCAGAAATCCATAGGGCAGCCTCAGCTGCATCAGCAGCAACAACAACAGCAGCAACAGCAGCCTCAACAGCTGCAACAGCAGCTTATACAACAGCAGTCTCAACAACAGTCTCAGCAACAGGCTTCTGTCCATCAACAGCAACAGTCTCCGAGGATGCCAGGACCTGCGGGCCAAAAGTCATTTAGTCTAACAGGATCACAGCCAGATGCTACTGCATCTGGTGCAACTACACCAGGTGGTAGTTCTAGCCAAGGAACTGAAGCAACAAACCAAGTCCTTGGGAAGAGAAAGATACAGGATTTAGTTGCACAGGTGGATCCACAAGGTACACTAGATCCCGAAGTTATAGATCTTCTTTTAGAGCTTGCCGATGACTTCATTGATTCTACAACTACACACAGTTGCATTTTGGCTAAACATAGAAAATCATCAACTTTGGAGTCCAAGGATTTATTGCTACACCTAGAGAAGAATTGGGATTTAACAATTCCTGGATATTCGAGTGAAGAGAAGAAGTATCAAAGCAAACCTCAATTAAATGACCTCCACAAGAGGCGCCTAGATATGATTCGCACGATGATGGAATCCTCTGCCTCTGAGTCAAATATTAACAGTTCTAAAGAGTTGAGTAGACAGGGCATTTCTAATCCTACCCCTGTGGCAGCTCACCATCTAGTAAGACCCTTGAGTTCAGAGCAGTTAGTTTCTCATTCAGCTGGTTCTCAAATGCTACAGCAAATGACAAGGTTTTAATGAACCTTTGATTTTGATCTTCATGCTGCTAGGTTTGACTGCCTGTATGCCTGGTCTGGGAAGAAATTGCTTGCTGAAACTGATTAGTATACAAGGGAACTGTAATTACTTAGTATTTAGACATTAAAAATAGTTGTTAAAGGATCCCGGTAACTCTTGTTATTGCCCCAACCCTCAATAGTCATTTTGTTTCACTGTTTGATCTTAATTATGGATTTTTAATGTCCATTGGTGCCTGTTTCCGTGTTCATTTTGTAGTTTAAATAATTGGATACATGCTCTGTTTATTATTAACTAGGGAAAACTGGTGTAAAGATTTTCAATTTTGGATCGAGTGTAAAGCAATCATCAAATCGGGGCTTTGAATTTTAATTGTAAAACGGAAACAAAAGAGTATATGCGGCA

>C100915_65

CAGAGTTTGAAGTGTTGAGTTGAAGTTGAAGTTGAAGTTGAAGCAAAAGAAAATCAAAGGATGCATTGGTTGGGTCCCGATGTGCCGGTGTTCCACCCCAATTTCAGTTACAATCACAATCCCTACTCCTACCATGATGAATTTTTTCGAAATGATGATTCTCTGCCCAATCTTAACGATGCCGTTTTGGCCCATCACCACGATGGAACCTCCCCTCTCCCCTTGGGCATGGATTGGAGCCTTCCGCCGCGCGAGTGGGCGGGTCGCAACACTGTTTGGCCACGTCATCCTCCCACCGGTTGGAGCTTCTGCGTCACGCTTCCTTCCTGGGTTACCGTTCCCCAATCGCCACCCTCTGACCCCGTCGTGTTTTTTAGGGTTCAAGTCGGTGTTCAATCTCCCGAAGCAATCACCACTACTCGATTAATATTACGTAGATTTAGTGACTTCGTCAGCCTCTTTTCTGAACTGAAAAAAGAATTTTCTATGAAATATCTGCCTCCACCTCCACCCAAAAAGATGCTCAGAATTAAAAGCCAGACTCTTTTGGAAGAGCGACGGTGCTCACTAGCAGATTGGATCCAGAAGTTGTTATCAGACATTGATGTATCAAGAAGTGCCCCGGTGGCAATATTTCTCGAGCTAGAAGCTGCTGCTAGATCATCCTTCCATGATGTCAACGAGCATCCGGATGAAACCTCTGCTAATAGTACTACACCTGCACAAGTGTTTCAGGATAACTCTCCTGGTTCTGTAATAGCTCATGGTTCATCTGCTGCGACTGTTTCGGGTAATGATGACATTTCTGAGCTGGGAGCACCACAGCTTGGAAAGGCTGAGCATTCTGATCAGATCATAGATGATTCATCATCAGAGCATGGTAAAATTAATCAAAGAGAAACAGCTATTGATCATGCCACATCTGGCGAAGTCTTTGTTAATAAGGATAATAGTACAGACAAAGATGCTATAGCTCTCAACCTAGATGGAACTGAGTTTACACCTGCTGATGTTGCACATGACCATACTGGGAGCCATGAAGCCTCCAGAAACTCGGATTTATTGTTGACTTTTCCATTGGATGAGCGGCACAAGTTAAACAAATTTCTTAATACCCAGAAAGAGAGACTGGTCACTGCAAAAGCAGATGTTGAAGATCTTATAGCAAGACTGAATCAAGAAATGGCTGCAAGACAATATCTTGTAACAAAGGTCAAAGATTTGGAAGTTGAACTTGAAACAACTCGACTGAACTGCCGAGAAAACATGCAGCAGGCTGTTTTAGCTGAAAAGGAAAGGTTTACACAAATGCAGTGGGATATAGAGGAACTTCGGAGGAAATGCCTTGAGACAGAAATGAAATTGAAGTTAGAAGAGGATGAAAGGCTGCTAGCAGAATCTACAAAAGCATCTGTCATTCAGGAGAAGCAAATGTTGCAGCAAGAACTAGATGTTGCTAGAGAACAACTTAAAAACTTGCAGAAACATCATGATGAGTTTGAGGTGAAATCAAAGACAGATTTGAAGGTTCTAATCAAGGAGGTAAAATCCCTTAGAAGTTCTGAGTTAGAACTGAAGCAGCAGCTAGGTGAATTGATCAAGGAGAAGTTAGATCTGGAGAGAATTCTCCAGAAGGAAAAGCAAAGAATGGAAAATTTACATAATGCTAATACAAAGTTGCTGCATGAGTGTGCAATCCTTCAGACGAGGCTTTGCGAGTGCAGTGTAAATTTCCTTGCCGAAGAGGAAGATAAGCTAAATATCGACACTTCGCCATCTGATGCTCTGGATTTATTAGCAACATCAGATAATCGGATTGGTCTCTTGCTTGCAGAGGCACAGCTCTTAGCACAAGATGTAGAGGATGCAGTTGTTGCTGTGGAAGAGACCCGTGATACAACTACCGACAGTACTGGGAAAACTTATGATGAATTGAGGAAGATGCTGGCAAATATGTTTGTAGACAATGCCAGTTTAAGGAAACAAATCAATTCCGTTATTCGTTGTGCTCTAAATGCAAATGTCAATTCTGAAGAGGAAGGGGAAGAAATCCATTTGCAAAAAACTGTTCTAAGCAAGTTTTTAGAAAGATAGCATGTTCAATCCTACACCGTGTCTATTACTGGCTTACATCTGTTAATTATGGCCTATAATTTTTGCACGTGTACGGTTTCGAATTGCGTTCTTTCCGATCCGATCGTCAGTTAGTGAACAAAAAACAAGTCTGCTTGTTTACTTAAAGCTGATTTCAAGCGTTTATTGTTCCGCCAAAAGGGATATGAATAGTTGAAGTCAATGCTATCATTGCTATCGAATCCATCAATTAATTGTAATCTACAAATCTAAAACTCGAATTTAAACCTTCTCCCCACTCCTCCATCAATAAATATTAAAAACCAA

>C100917_65

CGAAGAATGAACACAGAGAAGATGCAGAAAAAAATGGCTTCAGACCTACAAAAGGAGGAAGAACAAACAAGAAGTTGAGAGATTATTCGGATTGAAGAAGAGGAGCCAGAGAAATCAAAAGTTGAGATTAGTGAAAGGAAATTTCCAGAGAAATCTACTGAGAAGGGTAGTCTAGTTTAGTGGAACTGCATTTCAGTTGCTGTCAAATGGCTTCTCTTCTTCCACACAAATCACCTGCTCCTGGTGATTATGAAAGTAAAACTAACTCATCAAGAGTTGAAGCCACCACACTGGAGATAGAAGAAGCATGCACTGCTCCAAAAGGGGCCAAAAGAGGAGAGAGTTCAGGTTGGAGTAATGAGAGAGAAGAGGTGGGTATGTGCTTGACATGGAAGGATGTTTGGGTGACTGCTTCTGTGGGAAAGAATGGAAGCAAATCAATTCTGCAGGGTCTCACTGGTTATGCCAAGCCAGGGCAGCTCTTGGCCATAATGGGTCCTTCTGGTTGTGGAAAGTCCACACTTCTTGATGCCTTAGCTGGGAGATTAGGTTCCAACACAAGCCAAACTGGGGAGATTCTTATCAACGGGAAGAAACAAGCACTAGCATATGGAACCTCAGCATATGTGACTCAAGATGATACCTTATTGACAACCTTAACGGTTGGAGAAGCAGTGCACTATTCTGCTCAACTCCAATTACCAGACAGCATGTCGAAAGAAGAGAAGAAAGAGAGAGCAGAGTTTACAATCAGAGAAATGGGTCTTCAAGATGCCATTAATACAAGAATTGGAGGATGGGGTGTTAAAGGAATAAGTGGGGGTCAGAAAAGGAGAGTGAGCATTTGCATTGAGATTCTAACACGACCAGGACTTCTCTTCCTTGATGAACCAACTAGTGGACTTGATAGTGCAGCTTCCTATTATGTCATGAAAAGAATCGCTACCCTTGATAAAAGAGATGACATGCACAGAACTGTTATTGCCTCAATCCATCAACCTAGTACTGAAGTTTTTCAACTTTTCCACAACCTTTGTCTTCTATCTTCAGGTAGAACTGTTTACTTTGGACCTGCTTCTGCTGCAAGTGAGTTTTTCGCTTCAAATGGTTTTCCTTGTCCTCCTCTCATGAATCCATCAGATCATTTACTGAAAACCATAAACACTGATTTCGATCAGGACATTGACTTGATTTTAGGAGGGAATGAAACCATACCCACCAAAGAAGTGATTCGGATCCTTGTAAATGCGTACAAGTCATCTGAAATGAACCAAGAAGTTCAGAAAGAAGTAGCAGAATTAACTGAAAAGGACACAGGTTCAACAAACAACAAGAGAAAGCATGCTGGCTTCCTTAATCAGTGCTTTGCTCTTACCAGAAGATCCTCCATTAACATGTATCGTGATCTAGGCTATTATTGGTTACGCCTTGGCATATATATTGCATTGGCTATAAGTTTAGCCACTATTTTCTATGATCTGGGTACAAGTCATCGCTCAATTCAGGATAGAGGTTCATTTCTTATGTTTGTATCTTCATTCATAACTTTCATGACAATTGGCGGATTTCCTTCCTTTGTGGAGGATATGAAGGTGTTTGAACGTGAGAGACTAAATGGACATTACAGTGTCACTGCATTTGTGTTTGGGCACACCTTTTCATCAGTCCCATTCTTGTTCTTGGTTTCGATCATTCCTGGATCTATAGCCTATTACCTTCCTGGTCTTCAGAAAGACTTTGGGCACTTTGTATACTTCATCTGTGCTCTCTTTTCTTGCCTAATGTTGGTAGAGAGCCTTATGATGATAGTTGCAAGCATTGTCCCCAACTTTCTAATGGGCATCATAACTGGTGCAGGAATTCAAGGAATGATGATTTTGAGTGGTGGGTTCTTCAGATTACCAAACGATCTTCCAAGGCCATTTTGGAAATACCCAATGTTCTATGTTGCCTTCCATAGATATGCCTACCAAGGATTGTTTAAAAACGAGTTTGAAGGACTGAGATTCGATGCAAATAATGTTGAAGATGGTTCCTACATTACTGGTGAAGAGATTCTAAGAAATACATGGCAAGTAAACATGAGTTACTCAAAGTGGGTTGATCTTGCAATCTTGCTGGGTATGATTATTCTGTATCGAGTTCTCTTCTTGGTTAACATCAAAACTACTGAGAAGCTGAAGCCAATCATTGTATCATTCATGTCATCAAGGCCCTCTCCCGAGCGAACAATACAGATCATGCAGAATCCCAATGGCACTCCTTTAGAAGAAGTTCAAGTTATCGAAAGTTAATGAATCAGGTTGAAGTGTAGGACAAAAAATCATTTTCATATTCGCATAGATATGTGAAAGATCAAGTGATATCTAACTAGTGAATGTATCTTGCTCCAACCAAAGTA

>C100919_65

AGAACTTCAATCTCTCACTCAATTATTACCAGTAGAATACTTTCAATATTACATTATCTATCAAATGAGATCAGCCATTTTTGGCTGTTTACTGCTATTATATATGAAAAACCTGCAAATGCTTATGCTTTTGCTTCTACTTATTTATTAACGCATTAACCTTGGTCGTCATGCTGTGGTAAACACCACAATGGGGCTCCTCACTTGGAATTTACCATCACTCCAAGTCAAAGAGGCTGATACGATGGGTTCTTTTATTGTTCCCTTAACTGTGAGCACAAATGTGCGCTTTTGGTTAAGAGAAGTGAATGATAAAACATTGGGATGTGCTTCAATAATTAGTCCTTCAGGAGCAATCACGTTAGCTTGATATGTGGACGTTGCTGACCCAACATTTGTAACAGTCCTGTTAAAACTGCCACTTACTCCATCGGAGTTGGAGGGTGGGGCAAAAAGTGCAAATGAAGCATAGTTCAGATCCCTTGCTGAACTTGTTTCAGAGCAGCTACTGTTATCTCCTGTGATGAGTTGTAAATTCCTTGAACTATAGCCTTGTCCACATAAAAACCTTACATAGTCTATTTCGCCAGCATCATATACTAAACCTGGGTACAAAGCCTTGGAAGGATCAATTTGGCCTGCGCCATATGCGAATTCTGCGTGAAGGTTAGTCTTGGGATCAAGTTGTTTAGCTGTTGTCATTAGAGCTGAACGAATAGCAGCAGGAGACCATGTTGGGTGGAATGACTTAACATATGCTGCTGCCCCAGAAACATGTGGACAAGACATTGAAGTTCCGGAGATAATATTGAAATTTAATGTTCTATTGTCACCTTCAACATCAGAAGGAGGGGAAACAGGGGACCAACTAGCTAGAATGGCAACTCCAGGAGCCACTAAATCCGGCTTGAGAACTTCAGGTGTAACAAGGTTTGGACCCCTTGAAGAGAAAGAGGCGACAATAGGAGCTGCTGCATCTTTTGTCTCATCAGTCTTAAATATGGTGGCAGTTGGAGTCCTTGTAGAGTTAATGTAGTCAAGTACAGAGGCACCATCGTGCAAATCAAGGTAAGATCCAGGTAATGGAAGAGAAGGAGGAATATCTCGAGTACCTTGACCTTGTATCAGTGCCCCGACAGCACCAGCATCGAAAGGACCTATGGCTTTGTTTCTTATCTCACAGAGAACAATTTTACGGTCAACTAATTTTTTGTCCAACGAACCGCTGTAGCAATACCTTGATGATGATGAGTCGAAACCTTTGCTTGGTGCATCTCCGCCATAGATTATAGGATATAACTCTCCCTTCAGGTCAAATGTATTTATCGAAGTACCCTCATAGGTAATGTTGTTACCTAATTCAACCTTGGTGATAAACTTCCTGTCTATGGTACTAGCAGCCACTGAGATTGACCAAGGCGAAAAATTTCCCAGGGAGGAATATTTTGGACCACTGTTCCCTGCTGAAGTTACTGTCAATACTCCATTTTTATCAGCATGAAATGCTCCAATGGAAATTACATCTCTAAAGTAGTTTTCATCACTGAAACCTCCAAGGGAAACTGATAATATGTCAACCCCATCTGCAATTGCATCATCAAATGCAGCAAGTATGTCTGCATCAGAACAACCTTCATCTAACCAACACACTTTATACACAGCAATGCGGGCTTTTATCGCGCCACCTCTTGCCGTTCCCCGAGCAAGGTCTGACATGCTAGCTGAACTAACTGGATTCCCAGCTGCTGTTGATGCTGTATGAGTGCCATGACCAATTACATCTAATGGAGATTTTGGATCGTCAGGCGTAAAGAATCCATCAGCTTTGTAAATCTTAGCCCCAATGATTTTATTATTGCATGTGAAATTTTTAGAACTTTGGCAGGTGCCCTTCCATTTACTTGGTGGTGGACCGTATCCTTTGTCACTGAAGCTCTCAGATTCTGGCCAGATTCCAGAGTCAAGCAGTCCAATGATGACATCACTCTCAGCTGTTGCTCTCTCTACATCTATTGGAAAGCCAATAAAATCCCATGACCTTGTTGTATGGAGTTGCTTCTTTTCATTAGGAAAGACAGCTACCACTCTATCATGTCTAGCCAATCTGTTAGCTTCCTCCTCCGTAAGCTTAGCAACAAAGCCACCGAAACTTCGTTTGTAGTACTGCTGCACTGACTTGGGCTCAGTATTGCTGAAACTGTCTGCAACTTCTTGTAGCATGCTTGAATAAAGAGCCAACGCAGAACCCTCATCCTTCATGATATTGCCAGTGTAGACAATGTAGCTCTTCAGTTGTGATGATGAGTGACATAGTTGAAGAAATGAAGCAAAACAAAGAAGTATGAGCAAATGGGGAGAGGATCTGGAAGCAGTATCCATTGTCAGGTTAGTAAGAACTCAG

>C100921_65

CTTTTCTCATAACTTGATATCTTACAATAATATTTCATGTTTCAATTATTCAATGCTCACAATGGAAGCATAAAATGAGAAGAAGATGAAGTACCAAAATACATAAAAAAACAACCAAATGACCCGATCATCCTGAATAAGCTTTGCAAAAGTACACAAAAAGGGAAGCCAACACCATGGACTTGGATATGGTGGATAAGGGAATTCCATATTTTCCTTTCCCAAATAACCCTTTCAAAAATGGCCAGTAGCACACAACCAAATATGAACAACATAACACTTCCCCTAGACCAGATCCATGACCATTTCTAACTGGTGCTTGCCATCCCAACCACTTGGTAACCAGTGCTGTGAGTTGAATAAGCAAAATAGTTGTGCCAGGCACAAAAATTGGAGACTTGTTGAAAGTGAATCTACCATTGTTTTCATCAGCACTCTCATCGGATGATGGTTGGTCTTTCTTTGTTATTTCAAAGACAGTGTCACATATCCTTAATTTCTTAAGCACGATGTCCAGAAATCCAAAAAACCAAGCATTCGTGGTGGTTATCCTTGTCATTATTTGGTTGTTCCACCATGTTCGAAATGACAACCCTGTTCTCAAGTACTCTACAAGAGTAGTTATGTTGTAAATCACAAACAAAGTGGTAGGAATCCACTGTCCAAGTTCCTGGGGCCAGAAAGTAGAGTTGTTGATGATGCAATAGGCAGGGAGAAGACTGTAGCATATTTCAGGAACAGATCTTAAGGCCCAAGTGGTGACCCAAAGATAGCCCAAGCACTGTCTGAATTGGAGCTTACCAAATAGGGTTCCCAAAATTGGACAGTGGGTGCCTAGAAGAATATCTAGCAAGCCTGAGGCCCATCTCTTTTGTTGAACCATTACAGTTATGCCATCTCTAGGTGAGCAACCCATAAAGGCTATATGTTCAGGTGCACACACTTCAGATCTCCAACCTTGTGTGTGGATTTTCAGCCCAGTAAGTGCATCTTCTGATGTTGATCCATACATCCAACCCACCTGTTTACCCCAAGCAGTGCCGTATTCATATTCACAACTACCAACTTCTTTCGCAGCCTCAACATATTTCCAGATATTATTATTGGGAGAAAATGTCTTTCCTTCCAAAGCATTAGTAGCTGATTCCACAAACCCCTCTGAACTTCCAAATATCCTTTTCTTTTCTGAGAATTTCCCATTGGTAATGTCATGATTCTTCTTTCCACTTTGAATGTGATCATCAGGAGAAAGGCCATAAATTACTTTTCTTCTGTGCAAGCAATTTGTTCCTACGTAGAATATCCCTTGGAGCCCTGATAATCCACCCCCTACGTACAAAAACTTTGCCACAAGCTGATTTCCAAAAGGATCATCCTTCAATCCATCGTAGAATTGCTGGATACATTGCGAAAATGCAACTTCTTTTTCTCCCTTTGAATCTAATAAAATGCAGAGGGCGTGTAGAACAATCTTTGGATTATGCGCATACATGTCACAGTCTACGTTCAGAATATAGGGAGCATTTGTCATCACTCCAGAAACTCTTGTCAACACATTCATGGCACCAGCTTTATAATGATGTGGATGTTCCTGCTTCTTTTCTCTAGATACGTATACCAAGTGTGGCAACTCGTCTGAAACACCTTTGTTTTCCCATATTACCTTAATAATGGTTGGATGATTACTAAGCTGTGTATTTGAGAAGACAGCAAATTCTCCTCTAAGTGGAATCGAATTTTGTGCTGCATTCTGAATTTTACTACAAAAATTCTCATACTCCTTCTTCAACCTTAGCCAGTCTTGAATGAATTCTGGGGAATCTTGAGTGTTGTTAGGGTTGGTGTTGTCAGAGAAGTATCTGAAGGGCACTCTAAGTTGTATGTTGTAGTTCTTACAGAAAGGTACCCAAAGCTTGGCAAACTTAGAGGCTTCGAGAAGGGCATAGAAGTTAAGAGGGGAGCATCCATCATCAGAAACATAGCAAGCTAGCTTGTTGGCAGGATAGTCAAGTGCCAACAGAGACAAGACAGTGTTGATTGTGATGATAGGTGGCTCAAGCACCGGGTCTGCAGTTGTGACAAATAAGTCCACTGGTGGAAGCTCAGGCTCAGGTACCCTAAGCAAGAGACGCTCTGTGTAGGTTTTGGTGAGTGCAGGAGACCATTTAGTGGAGATGATGATAATCCAAGTGAAAGTGAACCATGACTCGCATATGAAAGCAAGGAAGCAAGGGAAGGTGTAACTGTTATGAGAGAAAATACGGTAGCCAAGAAGCAAGAGGAGGAGGAGCAAAGTGAAGGAATCCATTACTCTTGAAAAAGTGTACTTCACCCATATTTTATCATAAAGAGGGAGAGTGTTTTCGTTGCCCATGTTCAAGAAATGGAAGGATAACTCAAC

>C100923_65

AAAAAAAATATCTCAGTAGACTGTTGTTGCTTCGTCCTTCTTCTGGTCTCTGTATATTTTCTCCTCCCAAACCCGCCCTTTCTCTTCACATCTCAAATTTCCATTCTCTCTTTTTCCATCTGTTAGCTGCTATGGTTCTGTTCGAAGCCTGAGAAGAGGAACGTGTTACATTTCAGTTCTTGAAAAGTCAAGCATATAGCTTAAACATCTTTGGTTTCCCCAATGGCACAAGATAATGTTTTTGGTGCTGACTCACCTCGACGACGTCTATTGCGAGATCAGGTTCAAGTGGTTAAAAGAAAAGATTCTGATCGCTATGAAATTGTTCCTATCCAAGATTCCCTGTCATTTGAGAAAGGATTTTTCATATTCATTCGTGCATGCCAATTGTTGGCTCAAAAGAATGATGGAATCATATTAGTAGGAGTTGCAGGTCCATCTGGAGCAGGGAAAACTGTTTTCACCGAGAAGATCCTCAATTTTATGCCAAGCATAGCTGTTATAACAATGGACAATTATAATGATTCTAGTAGAATTATTGATGGAAACTTTGATGACCCTCGGTTGACGGATTATGATACCTTGCTTGAAAATATACAGGGTCTTAAAGCAGGGAAGCCTGTTCAAGTTCCAATATATGATTTCAAGTCTAGTTCTCGCATAGGTTACAGGACTGTTGATGTCCCTAGCTCTCGTATTGTAATCATAGAAGGCATCTATGCCTTAAGTGAAAAATTAAGACCTTTGCTTGATCTTCGTGTTTCTGTCACGGGTGGAGTTCATTTTGACCTTGTTAAGCGTGTATTACGGGACATTCACCGTGCTGGTCAAGAACCTGAAGAAATAATTCATCAAATCTCTGAAACGGTATATCCTATGTACAAAGCTTATATCGAGCCAGATCTCCAAACAGCACATATAAAAATCATTAACAAGTTTAATCCATTCTCTGGATTTCAGAATCCCACATACATATTGAAGTCAGCAAGAGCTGTGACAGTTGATCAAATCAAGGAAATTATTGACGCACAGCATACTGAAACCAAAGAGGAAACGTATGACATTTTTCTTCTACCGCCTGGGGAAGACCCTGAAGCTTGTCAATCATATTTGAGAATGAGAAACCGTGATGGAAAGTACAATCTCATGTTTGAGGAATGGGTGACAGATAGTCCATTCATAATATCACCTAGAATTACTTTTGAGGTCAGTGTGCGCCTTCTTGGAGGGCTGATGGCCTTAGGATACACAATTGCATCTATCCTGAAAAGAAGCAGCCATATCTTTCGTGATGATAAAGTGACCATAAAAACTGATTGGTTAGAACAACTTAATCGCACATATGTTCAGGTGCAAGGGAAAGACAGGAACTATTGCAAATTTGTAGCAGAGAAACTGGGGTTGGATGGTTCCTATGTTCCTCGCACTTATATTGAGCAAATTCAGCTAGAAAAGCTTGTAAATGATGTGATGGCATTGCCAGACGATCTTAAGACAAAACTCAGCCTAGACGATGACTTGGTTTCAAGCCCCAAAGAAGCCCTTTCCAGAGCTTCTGCAGATAGGAGAATGAAATATCTTAACCGTGGTATTTCACACTCATATTCAACTCAAAGGGACAAGATTCTACCTAAGCTGACTAAACTTGCTATAAATAGCCGAAGATTCGATGGAAGAGCTCTAGAATCACCTGCACCGATTGCTAACCATGGGGTCATCACTCAACTTTCTGATCAAATTTCCACATTAAATGAAAGAATGGATGAATTCACCTCCCGTATTGAGGAATTGAATTCAAAGTTTGCTAGCACAAAAGGTTCAGCCAGTCAACAAAACTTGGCTTCACAGACTGAGGCATGTAATGGCTCAGGCCCCAGTTCCTTCTTTGTAACTGGATTAGGAAATGGTTCATTGACTGGATCATTGCTGCCTAATTCTTCATCGTCTTCTCAATTGGCGAAAGAGTCTCCCCTGATGGATGAGGTATTAGTTATTGCACGAGGACAACGTCAAATCTTGCATCAACTAGACACTCTGAGCAATCTTTTGCATGAATACTTCGGAGAAAGGTCACGCCTTGGAAGACCAAATCAGACAGGTAGAATGCGTGAAGCTGAATCTGTTGCCATCCCCTTGGTTCTGACTCTGGCCATTGGTGTTGTTGGAGTGTTTTTGTTCAGGGGTGTGACATCCCAAAAATAGGGTATGATAATGTATATGTATTTTTTCTCTTCTCGATTGTCATTACTATTTATGAGAGAAACCTCGTAAGTATAGCTTAAAGGATTTCTGAAAACAATTACATCTGCCAGGGACTGCTTCACATCATCAAGCAGTAGTAGTACGACCTAAATGGGCCAATGTTATATATACATATACATATATACATATACATATATAT

>C100925_65

TTTGCATTAAAACATCACATTCCATCTCTACGTACTTACAGTACTACATTTGCCTTTTGACAAACGCATGCATGGTATGCAGACAGAACTCACTCATTTCTGAAAACTCTCATTCTCCCAAAGAAAATTGAACATCTTTCATGTAATTTTTTTCTGCATTGTTTGTGTAAGTTTGAGATCAAAGTTAAATCCGCTGATTCCTCAGCAGATTGCAGAAAACATATGAATGCATCTTCACTTGTTCTCCAACTTGGAGCTATATAAGTTGAAGCATCCCATCCTTTCAAAAAATTGAGATAATAATTAGAACATAAAATAGCATGTTACAAATGCACCAGGAAGAACAACCTCTTTAGATATGATGTTATAAGAGACCAAAAGGTTCAGGCAGGTGCTCAAATCCGTTATCAAATATGGGGCCTTGATCACACTTATCATCAGCACCATGCAGCCAGTTTGTGTTCTTCAAATCTTCTTTGAACAACAGTGCATCATAACGAGAACTCTCATAGATGTCCACTTCACTAACTTTTGCAGAATATTGTCTTTCATCTACCTCTCTTCCTTTCATCATACTCCTATTACTGATACCATTTCCCATATCAAAATTGGATCCATTGTTACTTCTGCTAAGCAGGTTCCCATTCATGTCTACAGATGCTGGAGCACTTGAAACTCGATGAGACTGGCTCTTGGGAGTTGAAGTTCTAATACTCTGAGGGAAGAGTGTGGTGCTTGATAGTGAACGAATATTTCCTGAGCCGTTCCTTATATCCATGTGCTTAATAGCCATATCCAAGGATTTCTTTGATATAGTTCTCCCAAATCCAGTGTTGTCAGTGGCAGTGGTTGAGGCCTTAACAGATTTCCTTGAGGCAAGCTCAGGGGCATGTGAGGCTTTCCTAGGCTCAGGAGCATCAGCATGATGGCCATTAGCATAGCCACGGCTTTTCACCAAAGGTTCTGTCACTCTCCCCCTACTAACAACAGGAGATGAATGTCTCCTTGGCATAGTAACAGAGGAGGCTTGAGTTTCAGAACCATTTGCCTTCAAAGTGGTACCACTGGGACGTGACCTGCCTGCAGAAACAGGCCTATCAGACAATGTTGTTCTAAGGTTTGGTGGTGTATCAAGGGGGAAATCAGGAGGAACAATTGGCTGTGGTGGAGGCCTAACTCGTGGGCTTGGTGAGCTTGGCCTTGATGAAGGTGCTGAACTGCGTCCATTCAACGAAGCACGGCTAGACGAAGACAAAGAACCTGTTGTAGGGGACGGTGAAGGAGATAAAGATGGCATTGAGCTCCTTCGAGTGGGAGTAGAAGGTCGAGACAGTGACCGAGTTGATGGAGCAGAAGGAGAATGCAAGTTTGCAGGAATGTGAGGCCTAGAACTAGGTGTGGATGGTCTTGAGCCTTGTGATGATGGTCTATGCCTTTCAGCAGTGGAGTTGGTGGAAACTGGACGTGGTTTGGAGGGTGTTGAGGACCGTGATACTGTGGGCCGAGAGGAGGGAGTGGAAGGCCTTGATGCAGATGAAGAGCGAGTTATGGGGGAAGATGGCCTGATGTAAGATGAAACTGAAGCTGAACTTGTGTTGAGGATTGAAGAGGAGTTTCTATTTGAAGAATATGAACTGTATTGTGAGTGCGAAGTGGAGATGGAAGAGCGAGTAACAGAACTGCTTCTTGCTGGTCTAGCAGAATTGCTGTTCTCTGATTGTGAGACTGCAAGCCTTGAGGCCTTAGACGTAGATGTCGATCTAGTCAAACTTCTCCTTGGAGGAACTAGGGTTGATTGGGATTCTCCTTCTGATGGAAAAACAGGAGTCCCCGGGGGAGTGAGAAGCCAGTCATAATCATGCTTCCCTCCTTCTGTAGATGATAACAGATCATCAATCCCACTTCTAACCGGTTTTGCTGTTCCAACTGAGAGCCTCCCCAATTTCACCGAAACATCTGAAGATTCATCAGAAGAAGCAAGAGAGAGGGACCGACGGTTGTTGGAGAAAAGATCGAGGTTGTCATCGTGGTTGTTGTTGGCCACCCCATTAAAGCTATGCCCTCTACGGTGATTGTTGAGAGATCCCAGAAGAGACTCTCTCACATTCCTCCCACTCATCATGTTCAACTCAGCAAGATCTAAAACTGCATCACTCAACTCTTACTCACTCACTCGCTCATGTTCCATAGTCAGATCTACCTATCTATCTTCACTAGTGCTTTTGTTCATTTTGTTCATGTTCCATGTCTCAACAAACACAACATTTGTGTAAATGTGTAAATGTTCTCATTGCGTGTTGAGTTAAGATGACGAGATGGGGCATGTCAGAGAGAACTAAAAAATAAGAAAGACACCCACATGGCTTCTTCCTTCTTTCTCCATAATAATATTTCATTTCAT

>C100927_65

TGAAATAATACAACTTAACTTAAAACTAGCATTGAAATAATACAACTTAACTTAAAACTAGCATTGAAATAATATAACTTAACTATTCATGATTTGTTTTGTCTTTGGAAAGTGATTCTGCAATCTTACTGGAAATACAGTATGCTGTAGACTGAATGGTTATCATGGGGTTGACACCAACTGCACTGGGCAAAACACTTCCATCACACACATACAAACCTTTTGCTTCCCAACTCTCACCATTTTCATCAAGTGCACCTTCTTCTTCAGTGGCACCCATTTTACAACTTGACATCTGATGTGCAGTTGTAAACACTGTCCAGAGTTCGTTCCTTGACATTGGACCCCCAACTATTGTGACAGTGTGAAGAAACTCCTCTAAATCTTCCTCCTTGATCCCCCTACATTTGATTCTTTGACCATCACTCCTGTATGTGCCTACTTCCACAGCACCTGCCGCCACCAAAATCCTCAAGGCCTTCCTTAGTCCAATTCTAAGGTTTTCTTTGTCCAATTGGTCAAGTCTGTAAGTTACTCTCCCTTCAGCCTTCACTTCTCCTGACCCATGGTCTCTTATCAATGCAAAAACGTTAGCAGTTCTGGCATACTTTGCCATCCTGTCTTTCATGTCACGCCCTGAAAGCCAGGGAACCAATGCTGAAAATGATCCTGGTCCTAGAGCAGGTGTTTCTATAATGAAGCTTGGGGTGGAATCTTCTGCAAATACCTTGTGGATCGAAGTTATGATCCCACCTTCATAGTTAGTTCCACTGAAGTTTGTCATGTCTTCTGGGAAGTATCCCCATGCAAATTGAACAGGGTGGAGATGGAGATTCCTTCCAATGTTTGGATTTTTCAGTCCACTGGAAATCATTAGAGGAGGAGTGGAGAGAGAGCCACATGCTGAAATAGTTACCTTGGATTCGATTAAGAGTTTCTTTGTTACCTTACTCCTCCAAGTTGCTGCAGCAATCACTCCTGAGCATTTCTTTTTTTTCATTCCACCATTTCCATCTTCAAGTATGAATCTCTCAGCTTTACATCCAGAGAGGATCACTGCACCATTTCTTACAGCATCAACCAACCATGTAGTGTCAGTGCCCTTCTTATCTCCTGTCCTACAACCATAACAGCATGAACCACAATAATGATCTGCTGAGGAATTTATAGGCACTGACTCTACTTTCAAGCCCAATTTCTCACATCCTTGTCTGAGAATTTGATTCTGAAAGCCTTCTTTCTTACACTTCTCTGTCACACCAATCCTTCTGCATACCGAGTCCATGGCAGATTGATAACCAGAGCTTGCAAAAAATGGAATTTTATATTTTTTAGACCATTCCCTCAGAACAGAATCAGGTGTTCTGATGCTTGCAGACCAGTTTATGGCAGTGCCTCCACCAACTGTTGAGCCAGCTAGGATCATCATCTTCCCATCAAGACTTGGCATAATGCCACCTGATTCGTAAAGCTCATCAATGGATGGACCTTCCAGGGAAGAATAATCATGGGAAACAAAATACTCTCCTTTCTCTAGGACGATTACTTTCTGACCAGAGTTTGCAAGAACTGCTGCTGCAACTCCACCACCACAACCAGACCCCACAATGACAACATCACATTTGACCTTGTATAGGTTCTTCTGTTTATCTTCTGTGACTTCAAGGCCTTTTTCAGTGAGAGATTGCATTAAAGTAGAATCAGTTTCGTACATAGTCTCTATTAATCCCTTTTGTAGAGGCCTCTCCTTTCGTGCCAACTTTTCCCTGGTGTCCACCTCATATCCAATTGCTTTCCATACCTGATTATCTCCATTTGCTTCAGCCCTTGAGAAGAAATTGTAGAAAAAAACAAGCTTGATGACCACAAACACTAATCGCAGGGGCACCCAACGCGATTCCCTGGACCAGTTCCTGAGAATCTCTTCTCTCTTCTCCAGGGAAATCTCAGAGAACTTGTGAATGAAAGGCCACTTCCAGTCCAAGCTAAGTCTTCCACAGATCAACAGTGTCCCCAATCTGAAAGACATCATCCAAAGAACCCAATTCACCAGCGACAATGCTTCTGGCAAGGTCCTCTTGTAAAGCAGCTCTGCAGCCTCATCAGGAAAAGGGACCTGAGAAGCAGAAGTGTTGTAGAAGGCTGAGAGAGGTTGTTGGTCAAGTGAAGTTTTCTTATTGAGTGAGTCCAGTGGAAGGGAAGGAAAGAGGGTGTCACATATGGAAGCCATGACATGCATCTGAGAAGGGGATAGCCCATGGTTGTAACCTTTCTCTCTTCTACCTCCTCTCAGCAATGGGTGGGAATTCTCTCTCCCTTCCATTCTCTCTTTTTTTTATTCTTTCTTTGTAAGTCTTTTTAATATGTGAGTTGAAGTAGTTACAACTATGCCTTTTTTATAG

>C100931_65

TATAACTTAACTTTAACCCTATATATATAATATATATATTCTTTTGTCTTCTTTCATAAATTTCATCTCGTCAAGCAAATCTGTGATCTCTCTCCTTTTTTTTCTTTTCTTTTTTTCACTTTGTATGGAAAACCCCACATGACAAAGGGTGTGGTGTGGCTTTTAAATAATTTTTCCCACTTGAAAAGATGAAAGGGCTAGTCCCATCCCATTATCATCATCAATCTACTCACTGAGCTTTCGAGTCATGTCTATAGTGAATGTACTTGTTTTAACTGTTGCAACCTTCTGTTCCTCAGCTTTCTGTCCCATCCACCAATATTCCAAAAGGAATGTGGGACTCGTTGAGTTTGCTTCCAATCAACCAGCACTCTCTTTTTTTCACTCTACTAATTTATCTCCCTGACTGTTCTGGTAAATAACAAGAAAAAGAAAAGGTAGTAAGCAGAAAAAGTGGAAATCTCTCACACCCTCCCCCACATCACAGATTTCAAACCAAATAAGAAAGAGCATAACATGACCTTGAATGATATTTGCACCACCAAATAAAACTTAAAAGGATGATATTAAGGTGTTTTGTGAGGGAGGTGCTGCATGCCTTTTTCTAGGTCTTTTTATAACCTTCTCTTTGTATTAGGTTCTTCAAATGAACTGGTGTTTGTGTTTGTGTTGTTGTCTCCTGACCAGTCAAACAGATGGAATGGATCTAAGGCCTTGGATTCCCTTTGATCATCAGCTGCACCAAAACCTTGAGTAAACAAAAGGTCCAGTGGGTCAGCTTCTATTTCTCCCAACTCAGCAAAGAAGTCCTCAGATTGGTTATTACCCTGATCCATCAAACAAGGCTTGTAAGGGAGTCCATCACTGAACTCTCCCTCATCCATCTCAAGCTGCTTCTCTATGTCCTCCATATTCTCTTCCTTCACAGATGCACTGTTTGCCGATGAATTCCCAGCAACAACTGGGGACACATTGCCCTCACTGTTGTTGCTCTCCTGCTGCTGCTCACCCTTTGCTTTTGGTGTTGTGCTCTTTTGGGGATTTGAGCCATCTGAGCTCTTTGAATTTCCGGCATTGTTCTTGGATGGCTGTGACCTTGTTGAACCAGCCAGAGCATTTCTTTGAGTTGGCCATGGATGGTTGTGCTCTGATGTGTATGTAATAACCAACATGTTTGGGTCTGTTCTGCTCCTCTCAACTTGCTTCCTCGCAGAGCAACCCTTTGAGCTACTGCATCTGTAGTAACCCCTGGGATAAGGTGAACCTTTAATGGGTTTCTGACCATATTTTCTCCAAGCCCACAAATCTGACGGAACTACCTCTCCAGTTTGTCTGCTGTTTGCAGCTGCTGGTGCAGGAATACAAACCACCTTTTTGGCCTGGTTCTTCCTTCGTTTAAGACCCGGATTCCGTGGAGATGAGATCTGCACTCCTGTGGTGTCCACAAGGCAATCCTTGGAGGTGTTTGCATTAACCATGTTGGGTGAAACCACAGCAGGAGGTTTAATTGCCCTTGGAGAAGGTGCCATGGCTGTGGTGGAATCGTAGGGTGAGACCGGTAACTTTGCATTGGGAGAGATCTGAATCATGTTGGAGAATATGTTTTTACGAGGCCTTCTCATGTCATGTTCCTCAAGAATCTTGTGTGCAGAAAAAACAGCGGTACTAGTATTAGCATTAGTATTAACAGCGGCAGCACCAAAACTTGCAGCTGCTTCTTCCAAGCCCCCAGAGAAGTAGGCAGAAGCTGAAGGCATGTCGAGCTCCTGAAGGAAGGGATCTCTCATGGTGGAGAAGGGATCACCAAAGTTGGTGCCTCCTCTGGGGTCTTGAAGAACAGAAGAGAAAGTGATGGGATCAGAAGAAGAAGAGGAAGAAGAGAAGTGATGGTGCCAGTGATCATTATGAGAGAGGGTGTGAGTAATAGTAAGGTGATGAGATGTGGTAGGTAATTCAGAAGAAGAAGTGCCTGTGCTGCAGCTGCTGCTGCTGCTGCTACCGTATGCTCCACCACTGGCTCGGACTATGTCAGTTAAATCACCTTGGTAATTGTCCATGATTATTGGTCCAAAGATGCTGCTGCACATGCTTTTTCTCTGGCTATTAGTGGAACAAGAGCTCGGTGTTGAAGTATAATAAGGAGGAGGAGGAGGAGGAGGAAGGGTGTGTGTGTGACATGTTTGGGAGCATAGATGATGGAGATATGTTGGTGCTTGAGAGAATCAGAGAAAGACATGCAATTGTGGGAATTTTGTTTGGTGTGTGAGAGGAAGAGGAGGGTAGGTTGGGAGGATTAAGAAGGAGGTGTGAGAAGAAAGGCTTTCTCCTGGGAAGATGGGTTTTTGGCTGTTAAGAAGCAAGAAAAAGCAAATTAAGGAACAGGTTTTTGGAGTTGAAACTGAT

>C100933_65

CCACCTCTATATATATAATGGGCATTCAGGATTGATGATATTAAATTGATGTTTCAATATTGATAGACCACAAAGGGATCTAGACCATAAAACCTATATATAACACTAACATTTGGTTTAGGACCACAAGGAAATACTTACATATTAAAATTGATCACATACCCTAGTTATATCTCACATACATACCTTTTCAATCATGTCCATCTCGAGCTACAATAAGTTCTTCTAAGTTATCAGCATCACCAATGTCCACGGGCAGCATTATCTCATCCTCAGTCGTGAAATTATAATCCATGCCAATGGCTCTTAGAAGCTGGTATACTTCAAACATGGTAGGCCTCTCCTTTGGCATTTCTGTAACACAGTTGCATGCAACCTTCAGAAATTGGAAAAGCTCCTGGTCTACTCCATTCCCAACTAACGATTCATCAATGAGATCATGGAGTTTTGCATTGCTAGATTGCTGTGAAATCCATTCTACCAAATTTCCTTTAAAAGTTTCTGGAGCTTTAGCCACATGAGTAGGTCTCTCACCAGTCACCAACTCGAGAAGCACGGTCCCGAAGCTATAAACATCCCCTTTAGGAGTAGCCACCAAAGTTTTTGTATATTCAGGAGCAACATAACCTAAATCCCCAAACTCTCCATTCACAAAAGTACTCAAATGTGTATCAATTGGGTTCATCAATCTGGCAAGGCCAAAATCAGAAATGTTGGGCTCAAAATCATTGTCCAACAAGATGCATTTAGAGCTTATGTTTCGATGGATAATACGGGGATTGCAGCTATGATGAAGCCATGCTAACCCTTTGGCTGCCCCAATTGCAATTTTGAGCCTTAGAGGCCAAGCCATGGTGCACACACCTTCATCAGGATGCAGTTGATCATGGAGGTTACCATTTGGCATATTTTTATAGACCAAAAGCCTCTCCTTTTTAGCCAGGCAAAAACCTAAAAGAGGAACCAGGTTACGATGTTTGACAGTCCCTAGAGTAGCCATCTCAGACATAAATTCTTTCTCTGAGTGTTGAGATTCCTGCAATCTCTTAACCATGAGTGATGTGCCGTCAGGAAGGACAGCTTTGTAAACAGTTCCTGATCTTCCAGACCCGATAATATTGCTTTTACTGAAGTTATTAGTTGCCTTCATGAGATCACTCAAGTTCATTTTTGAAATAGCCTTCTCAAACATAGAAACCTTTATTGTTTTGGTTCCCTTTAGACTTCTTGCCCACTTGTTTCCTTCAGGGTCCTCTTCCTTCTTCCTATAAGAAATACGGCGCACGTAGAAGAACATTCCAATGCCCAAACCTAAAGCTGCAACAGTCACACCACCAACAGCTGCTCCAGCTATAACAGCAGTGTTACTCTTAGCAGAATTGTCCTGGCAAGCAGGCAAAGGAGGACCACACAGGCCTGAATTATTTGCATAAACTGTCGCAGGAGCCACACTTTCCTTAAAGGGTGGAACCGGCCCTGACAAAAAATTATTGGTAACACTAAACGTCTTAAGCCGCGGGAGCAGGCTCAAGTTTGCAGGAATTTGACCAGTGAGTTGGTTTTGGTCAAGTTTAAGACTATTAAGATAGCTACAGTTTGAGAGGGAAGCAGGTATTTCCCCAGTAAACTCATTTGAAGTTAGATCAAGAGATGTCACAAATGTAAGAAGTGTGGATATATCAGTTGGAATGGTTTTAGAGAGTCTGTTATATGAAAAATCTAATCCTGTCATGCTTGAGCAATTTTTAATGCCGCGAGGAAACTCACCCTTGAGTCCCATGTTGGAAAGTTTGAGATTCAAGACCCTGTTCTCATCAGGGTGCCAACACTCAACCCCAATGAACTTGCATATATACCCTTCTGTTTTATTGTCGAAATTCCAGGATTGCAAATAATTGTTGGGGTCCTGAAGTTCAAACTTTACACTTTTCAAGCAGGATATATCACTATCAGTCCCACAAACCATGCCACAGAGAATTAGCAGAAAGAAACTAACAATAACATGAGCTCTGAAAATTTGGCTACCCACAACCATCTTTACCCAAAAGCCAAGAACAAGCAACTATTGAGATAATTCACCAAAATTTGTATCTAATGAAGCTGTAGCATAAAATTTATGGACTCCATAGCACTGCAACCCTCACAAGAGCACATACACACCCTGGGATATTGAGCAAGTCAACTCAGAAATAACATGGAAACATCACTTTGCCCAAAGGAAAGGGCAAGGCAGGGGGAACAGAAGAAAAGGAAGACTAGACTAAGAATTGTGTAGAGAGAGAGAATCATTATATAGTGATGGTGGAAAAGAAGAGAATGTGTTTTTCATGAATTTTGATGAGAACACAAAAGAGAAGATATTCACGAGTCAAACAAATTAAGTTTTTTTTTTTTTTAAGGACA

>C100939_65

GGGAGTTTAAGGTCAAATACTCAATCAATGCAATACAAAACCCCAAAATCAGAATATCCAATTTAGGATTTTTGTTCTTTGATTCTTTCTTCTCTCTGTACTTAGGTCACACAGATACATTTTCAAGGAACTTCCAACTTTTCTCAGGTGGAGAAATAAGAATAATTATACGCCTTGCCTTCGAGGAACTGTAATGGCTCACAGCAGCTCTGATGAGGATACTGATATCAGTGAATCTGAAATTAGTGAGTATGAAGATAAATCCTATGAAGAACTGAAAACTGGAAGTCAGAATTTAAAAACATCAGATGAGACATTCACTTGTCCATACTGTCCTAAAAAGAGAAAACGGGATTATTTGTACAAGGAGCTCCTTCAACATGCTTCTGGGGTGGGTCAGAGTAGTTCACAAAAGAGGAAAGCAAGAGACAAAGCTAATCATTTGGCTTTAGTGAAATATTTAGAAAAGGATCTCATGAATGTAGATGTTCCATCAAAAGACTCAAAATCGGTAGATGAAAGTGATTCTTCTGTTAACTCTAATGATCAGTTTGTGTGGCCTTGGATTGGAATTGTGGTTAATATTCCAACTCGACGGACAGAAGATGGACGCAGTGTTGGGGAGAGTGGTTCCAGGCTGAGGGATGAGTACAGAAGCAGGGGTTTCAATCCAGTTCGAGTCAATCCTTTGTGGAATTTCCGGGGTCACTCTGGAACTGCTCTTGTGGAATTCAATAAAAGCTGGCCAGGCTTAGATAATGCAATGGCATTTGAAAGAGCATATGAATTAGATCATCATGGGAAAAAAGATTGGTTTGCTAATTCTGGGCAGAAGTTTGGTCTTTATGCATGGGTTGCCCGAGCAGATGACTACAAAATGAACAACATCTATGGAGAACATCTGCGGAAGATGGGTGATGTCAAAACCATATCTGAGCTTATGGAAGAAGAAGCTCGAAGGCAAGATAAACTTGTCTCCAATTTGAATAACATTATTCAGGTCAAGAACAAGCACATAAAAGAGATTGAAGTGAGATGCGATGAGACTAGGGACAAAATGAAACTTGCTATGAGGGAAAAAGAAAAACTCATTCATGCTTATAATGAAGAGATACAGAAAATACAGTCAAGTGCCAGGGATCACTTCCAAAGAATTTTTACTGATCACGAAAAGCTTAAAATGCAACTGGAATCTCAGAAAAACGAGCTTGAGTTACGGAAAATTGAATTGGAAAAACGTGAGGCACATAATGAAAGTGAAAGAAAAAAGTTGGCAGAAGAAATCGAGGAGAATGTAACAAAAAATAGTTCTCTTCAAATGGCTGCTATTGAGCAAAAGAAAGCAGATGAAAATGTTATGAAACTAGCTGAAGATCAAAAGAGGCAAAAAGAGCAACTCCATGCTAAAATAATTCAGCTTCAGAAACAACTTGATATGAAACAAGAGCTGGAGTTGGAGATTCAGCAATTAAAAGGATCATTAAGTGTGTTGAAGCACATGGAAGATGACGAAGATGTTGAAATTCTGAAGAAGGTAGATATGCTACAAAAGGATTTAAGAGACAAGGAACAATCACTTCAAGACATAGATGCACTGAACCAAACACTAATAATTAAAGAGCGTGAGAGTAATGATGAGCTGCAGGAAGCTCGACAGGCATTGGTTGATTGCGTAAAAGAGTTACCATCTCATGGAAATATTCGTTTGAAGAGAATGGGGGAACTTGACACTAGACCATTCCTTGAAGCAATGAAGAAAAGATACAATGAAGAGGATGCTGAAGAGAGAGCTTCAGAACTGTGCTCATTGTGGGAAGAGTATCTAAAGGACCCAGATTGGCATCCATTCAAAGTTATCATGGTTGAAGGGAAAGAAAAGGAAATTATTAGAGATGATGATGAAAAGCTGAATGGGTTGAAAAATGATTTGGGTGAAGGTGCATATAAAGCAGTGGTGGAGGCGTTGTTAGAAATAAATGAACATAATCCTAGTGGGCGATACTTAACCTCAGTATTATGGAACTATAGACAAGGAAGGAGAGCAACTCTGAAAGAAGGAGTACAAGTTCTGTTGAATTATTGGAAAGTTCTTAAACGCAAGAGGGGAATATGACGTGAAGGTTGATGTTGGCTTCGAAGAGAATTATGCATCGCAGCATGGTAGCATGCGTCCATAATGGGAGAGAAGCAAACCTAGCGGTTGTGTATAGTAGGTTAAGTAATTTGTGATAATGCCAAACTATTTAATTTCATTTGGCACTTGGTTTCTGTGGTCATGTTGCCTCCAAGCTAACCGCATGCATAGTGGCTGTGTGGGAAAAGACATTGAAGTTAAAGTTATATGACTATCATTTGGCTTTTAATCTTAACTTTATGAGTAAATGATGTTAAACTTGTATCACAT

>C100941_65

CTCAATTTTTTTTTTCATCAAACAATTTCCGTTTTTTATTCCCATCATTTCACTCATATCATTCGCAACACTTTCTCCTTCTTCCGTCGTCACTACTATGTTAAGTTTGGTAGCTATTTCTGCCTTCGATTTAGATTCGCGTAATGTTGTTGCTTACATGTGCCTTTCGTCTCCTATGTCTTAATCATTGCCCTCTATTGCCTACGCTTATTCTTTTCACCTTCTTAATATCCATCGCCTTCAACCCATTCACTTTCTCCCTCTTCTTTTTTTCTCTGTCTCCAAAGTTCGTTTCTTTACATTACAGGGTGTCTAACCAAAGCTACCCTCAAATCTCAATGCTTTCTGTGAAGCCCAACACAACAAGTGCAATGATTCTGGAGGCTTGCAGGAGGATAGAAAGTTTCTGATATTTTGAGATTTTTTTTGGGGGTATTGATTTGGATGATCCTCTCCTTCGAGTTGCTTCAAGGAGAGAGAATTGTTTGGGATGAAAGATGCTGGTGTTGCGGGAAGGAAGGAAAGAAGGTTCTGAATTCTGATATAGAGTAGTTAGAGTCAAAACTGAGAGATTAATTAGGGTGGTGTGTGAATCCAATTCCAATGGAGTCACCAACTGTACAGCCGCAAGTTGCATCAACAAGCACGCCAAGGAAAAAAATGACAAAACAATTGACAGGTAAAAGGGATGATACTCCTTTGCACTCTGCAGCAAGAGCAGGGAATATGAGTGTTCTGAAAGACACAGTTAGTGGCACTGAAGGGGGTGAACTGCGTGCTTTGTTGACAAAGCAGAACCATGCTGGAGAAACGGTGCTTTATGTTGCTGCTGAATATGGTTATGTTGATATGGTTAGGGAGATGATTCAATATTATGATCTTGCTGGTGCTGGAATTAAGGCAAGGAATGGTTTTGATGCACTCCACATTGCTGCCAAACAAGGCGATTTAGATATTGTGAAGATCCTAATGGAGGCTCATTCTGAATTGTCAATGACAGTGGATCCATCCAACACCACAGCCCTTCACACTGCTGCATTACAAGGGCACATTGAGATAGTGAAATTTCTTTTGGAAGCAGGTAGTAGTCTGGCAACCATTGCTAGAAGTAATGGGAAAACGGCTTTGCATTCTGCAGCAAGAAATGGACATTTAGAGGTTGTGAAAGCACTTCTGGAGAAGGAGCCTGCAGTTGCAACGCGGACTGATAAGAAGGGACAGACAGCACTTCACATGGCAGTGAAGGGACAGAATCTTGAGGTGGTGGAGGAGTTGATAAAAGCTGATCCCTCAACAATAAACATGGTTGATAATAAGGGTAACACGGCATTGCATATAACGACCAGGAAGGGCAGGGCTCAGATTGTTAAGTTGCTTCTTGGACAGACAGAAACAGATTCCTTGGCAGTTAATAGGTCTGGCGAAACTGCCCTGGACACTGCTGAAAAAACTGGGAACTCTGAAGTTAAAGACATTCTGCTGGAACATGGTGTTCGAAGCGCCAAAGCCTTAAAGGCTCAGCCAGCAACAGCTACTGCCCGTGAGTTGAAACAAACTGTGAGTGACATAAAACATGAAGTCCACTACCAGTTGGAACACACCCGCCAAACTAGAAGAGGTGTTCAAGGCATAGCCAAACGTATCAACAAAATGCATGCTGAAGGACTCAACAATGCAATAAACTCCACCACTGTGGTTGCAGTCCTCATTGCCACAGTTGCCTTTGCAGCTATTTTCACTGTCCCTGGCCAATTTGCTGATGATCCAAAAGACATTCCTCCAGGTTCAGGAATGACCCTTGGTGAAGCAAACATTGCCCCACAACCTGCATTTCTCATTTTCTTTGTCTTTGATTCCATTGCCCTCTTCATATCTTTAGCTGTGGTGGTGGTGCAAACCTCGGTTGTGATCATAGAAAGCAAGGCAAAGAAGCAGATGATGGCCATCATTAACAAGCTGATGTGGCTGGCTTGTGTGCTTATTTCTGTGGCCTTCTTGGCACTGACATTTGTAGTGGTTGGGAAGGATCAGAGGTGGCTAGCAATTGGAGTCGCTATAATAGGAACAACTATAATGGCCACAACTTTGGGCACCATGTGTTATTGGGTCATTAGGCATCGCATTGAAGCCTCAAATTTAAGGAGCATTCGAAAATCTTCAATGGGAAGCAGGTCAAGGTCTTTTTCAGTATCTGTTATGTCAGATTCTGAGCTACTAAACAATGAGCGTAAGATTCTGTATGCAATATAGAATCACTAATGAAGCCTTGGTTGCACTTTTCATTTACACTCGTTTTTCCAGGGCCTGATGAATTTATATCTATATAACTACACTGAGTTTCATCAGACTTCCCTGATGTTTAATCACATACTTAATACTCTCCTTACAAAATATAGTCTCTTTC

>C100947_65

GCCAAATTGTAATTTTCTATTGCCCGATGTCAACAGCCACTCATTTTGTATACTGTACTGTACAAAGCCTGTAGAAAGAATATATACAAGTGATGTGTAGTTTCAAAACCATTTCAAGCACACACACATGACTACCACTATGAGTGAACTGCTGCCAGGAATCACCAATAATCTAGCAATGCAAACTTCACCTGTAATCCAATCTTACCACCATCAGTCACAAGCTTAGCAGCAGTTACCAACCAATGACCAGGAGTATCATGTGGGCCTCGCACAACCTCAGCTGTCTCCACGTATTTAAGTAGTTTACCAGAGCGAACTGGCACAGGTGGACCGTCTGGATAAACTCCAGAGTTGAGTGCAGTTGGAGCCTGCTTTGGAGGACCAGTGTTGCCATGCTGTGTGAATGAAAATGTTGTGCTCAAATTTGTGAGAAAAGATGTTTTTCTTGAAGCCTCTGGTGCAGCAGTCCACTCTGACTTCCGGATACTGCAATTGGGTATATGAGTGAAGAGAAGACGCAGGTGAAGTACATTCCTTGGCCAACTCCCTTTGCTAAGCAGTTGTGCACCTGTAACAATATAAACACCTCCAGGACTATTCAACCAGTTAGGGTCATGTTTAACCACTGCTGTGCACACATTTGAGAATCTCTTCCATCTTATTCGTTCCAGAAATTGATCACTGGAATCGTTATCATCAGATCCTCGCCACATAGCTGGTATGCTTGAGGTTCCAGAAGAGAGGATCATTTTATTTGGGAGGCTTGAAAGATGATGTATATGCAAAGCAAGTCTATCACATTTCCTTCCCTCCAAGTACAAGCGCATGCCAACCACGGGTTTTTGTTCACTTACCACCTGTGTAGAGATGATATGAAGCTTCGGACCCATGAAACTAAATTGCAGGGAAGGGGAAGCAGTCTTTCTCCGCTGATGCCTGAGTGGCAGCTCGCAAAACATGGGTGCCCATTGCCTTGGAATTTGAAACTCCAAAAAGTATTGTAAATCATCGGGAGAAGGCTTATAGCGTAGATACAGATTGATTGCATGACTAAGATATCCACTTCCCGGAATTCCTGTCAGAAGTGAGGAAATAGGAACAAACTTGAAGAGGATTGCTTCAGGGTTAGAAGGCACTGTCTGAAGCCAATTTGGGTGACTGTGTTTGAACATATCTCCTCCTCTTTTTGAACAAATAATAGTCAGGCCATCCTTGCTTGATGTTTCTGAAATACTTGTGAACTGCATTGTGTTTGATTGCATCACACGGTTAAAGACCTCAGGGACCTTCTGTTTTCCATCTGCCATCTTTCCCTGTAGTAAAGAAGGGCTTCTCACATCTGAGAATAGAAAATCTCCAAGATCCTCTAAATGTCTCCTGAGATCGCCAGGAGGAATCTTTGAAGAATGTTTTTGTTTGACACAGATTACATCTTGACCTCCTACAGCCATACCCACTATTATGTGTGTACCATATGTCTGTATGAACCTAGACAATGAGGCTGGATCCCACTGAGCCGGAACAGACTTCTTTACCTCTTCTTGCAGTACGAGTGGAGAAGCAGTGAGGTGCAAATAGTAGAGTGAAATGAAATACCCATCAAAAGCAAGGTATTTAGTGTCAGCAGCATCACGAAACCAATCACCACTCAAATCAAAAAGCGCATTAAAATACCCAGAAGGAACTTTCCCTTGCACTGCCGATTTCTGATTAAGCAACTCAGACATCTGGTTAAATTCAAGTACATCAGACTTGAAGCGAATTCTATCCCCTTTATCGCACCGAATATTCTCAGAAACTTCTTTAATGGTAACTCCTCCAGTTCCAGGAATCAAGATATCCCTCTTGTTCTGTTCATCAAGCACCACCAACCTCCCTCCACGAATCCCCTTTGCAAACCTCAACCGAAAATCACTGGCCATATCGAACCCCTTCCCCAGACACTCCAATGCCACCATCTCAACACCCTTCTCCACCGCCATCTTCCCAACCCAAAATCAGTTCACTGATTCCAAGAGAGAAAGAGAAAGGTGTTGGCTTTGTTTTAAGGCTACGGAGCCAACCAACCCCAATTCAAACGGGTTAGCTATGCCATGTGGGGCGTGGGCCAAAACAGAAAAGGTGTTGTCTTTGGTGGTTATTCGGAAATGAGTATTGCTAGATGCGGATGGAAGGTTCGGGGAAGACGATGGGATCAGTGAACGTTACGCAGCTTTTAATGAGGGAGATAAAAAAAAGAAGTACTTAGGTTTGATTAGGGTGAAGAGGGATGGAAGAAGTGTAGAGAGGAAGTAGCTAATTAGCTAGCTGCTATGCAATGGAAGTGCGAAGAAAGGGTTGAATTGAATGTAATTGTTGGAGTTTGACTGTGTCAGTTGTATTATTGTGAAAATAGCAACGAG

>C100953_65

TTTTTTTTTTAGATTAAACTCCTTTCTTCATAGTCTTATTTATTTATAAGAAGGCATAATTATTGTTGACAACCAATTTTCACTCTTTTAAATAAAAACAACAGAATTAAAAAGTCCATCAAGGCTTACTAAGCCTAAGACTACTAGCTACGTCCAGAGATTGATCATCAACATTTTTACACCGGTTACAACAGTATGAGTAAAATCACCAGCTAAAGCTAATGGACTCAAAATGCAGATTCTCAGCTAAAGCAGATGAGCAAATGGCTGCAACCTCATGCCTCATTGTCTTGGGTCCTGAAGCAAGAACTCCCACACTTAATCCTTTGTGTTCAACTAGGATTCTTCTTAGGTCAGGTCTTACACCATAATGCACATTGGTAGCGTGAACAAGTGATTGATATGGAAGGCTTTCCAGTTCTCTCTCTGCATTGTAAACAATGGAGTTTGGAGACACTGTTGGTGTAGACCCTTCTATGTTCTGAATCTGTTTTGCTTCTTTAGCATTATGTTTCTTGTTCCAAAGAAAAGCTGCACTAGCAACACTTGCAATGGAAACACATATTACTAGCATGTTAAGGAATGCATTCAAAGGGTACGAGAATATTTGGTTTGAGTTATGATCAATAGGGAAAATGTAGTAACGGGTAATGATCCCTATTAAGATAAGGAAAATGACGAAAGAGGATGATATTATAGCGCCAAGCCAGAGCCAACTGTTTGGACCTAATATAGCATGTACTGGTTCATCAGATGGATTTGGCTTGAACCATATGGTTTGAGGCTGAAGTGCGCCATCTGCTTTAAGCTCCTTGACCCTTGTGATGTAGGCCTCAATTTGTAGTTCCATATTAGAAATGTCAGAAGGGGTGCCAGATATTGGGAGGATCAAATCTAGCATTGACAGAGATGAAGAGTTCTTGAAGGCACAAATTAGGACTACTTTTGGGGTTTTGCATTTGAATGCAGTGTTTAGATATATTAGCTCCCGAATGATGGATATAAAAGGTGTGATGCCACTTCCTCCGCTCACCATCACAAGAGTATCATACCGTAGATAATTGGTTGAAGCAGGTCCATAAGGACCTTCAACAGATACAGAAAGGCGATCTATTGTGGAAGGAGTTGAAAGCAACTGGTATAGCTTCTTGGTCCAAGTTCCTTCACCTTTTATAACAACACTTAGCTTGTCTTGTTCCAAATTACTATTAGAACTAATAGTAAATGGATGCCACTGCAGCTTGGATATGCTTGGTACATTTATGAACATCACACTTGTGGGGTTATAAGTTAATCCATGGCCCTTAGAGAAGTTGAGTTCTACAGCTTCACATGGCAAAACCCGAGCAGAAACCAAACGAACTTGGCGCCTTGATTGAAGAAACCTTAAGTAACGATCAACCATGAAGAGGTAGAAACCAGGGAGCATCATGCAGGCATAGGAAATGCCAACATGAAAGATGAAGAAGACTACGAAGGGGATATAGAGGTGGTGAATGTAGTAGAAGAGCTCAAAAAATTTACGCCTGATGCGGGGAATGGTAACAATCCATAGCAACAAACCAGAAAGCAAAGCTATCTCTCCAGCCACAATTGATATATCATTTTTCTTCCATTCCAGCATCGCAGAAAGTTGATCAATAACTGCCCAATGGATGATGTAGAGCGAGCCGTGAGTAGTGAAGAGCACCATGACAATGTGTCCAAGCCAAATATGGTACTTGATGCAACTCTCAGAGGTGAGGCCAAACAGTGGCAACACGCATGAACCACGAGCCACCGGAAAAAACATGAATACCAAACATATGTTCCCAACGAGACCAAACATTAGTGCTGCACTATCTAGCTTCTCTTCCCATATTTTCTCGCCATCCTCTTTTGCTGATTCGGGGGTGATCTGTGCAAAGCTGTTGCGTAAAGAAACTGCAAAGTACCAGACTAGGAGTGCAATGAACATAATCGAGAATGCTAGCTCTGTGCCAGAAACAATGCCAAGAGGCCCTTTTATAAGCACTGGACGCTTCCATATGGTCACCTCAGCTTTCTTTGAATTGTAGCTTTCTATGTAGGAATCATTTGCTTTTTTGCTTATGTGGAGGTAGAAACACCCCAAAGTAGCTATTAGCAAGACTGGGAAAGAGTACATCAGAAGATTAGAACCTTTTGAACCAAAATAAGTTGTATTGTTAGTATTTGCTCTGATTCTAAGAAGCCACCCTTTTCTATAAGTAGTTGTGGGTGACATTATCCAAATAAAAATCCATCCCAAGAACAAAACCATCACCAACAGCCTTATTGCAAATCGAACCCTGTGAAGTTTTTCCTCAGAGGGTGACCTTTTCATCACAGTTTCTTGAGCCATCTTGATTTCAGGTGACAAATTTGTGTAGTAAAAGACTGCAGTGA

>C100957_65

TATCCATTTAATTTTTCTTAATCAGTATTTGAATGAATAAGGAATGAGACAGTCTGTGCAATTACAACTTCCTCAAGCAACTTTACCAAACTAAATCACATGAAACAAAATAATACCAAACACTAGTTGAATGAGTAAACCACTTACGGGGGAGAAGGGAAGAAAGGGGACAAACGGTACTTCGCATAGCAACATAAAATTAAAATGAAACTACAAAAAGAATTAAGCAAAAGTTATACCAATTAAGAAAAATCCCTCTTCCAGCATGCCTTTTGCTCAGTTGGTGAGAGATGGCAAAACAAACTTCAATCTATCTTTACTGATGAGAACAAGTAGTGCACAAAGTAAAATGACATAAGGAAGCCAATGGTTCCAGTTGATAACATGATTGCAATTGCCATGAGCAGTGAATAGCCAAGATAAAGTATAGCCGAGACAGGTCCACTCAAACTCTGCAGGTCGAAAACCAAGTAGTTAATGGAGTACATGAAGACATAAAGAGCAACAGAGCCTGAAGCAAAGAATGACTTCCACCACCACCGCCAATCTTCCACACAAAGGTGCATATATGTGAGGACCACGGACACTTCAGCACAAACAATAACAAGCAGTAGAAGAACAACCAACAGGAAACCAAATACATAATAGAACCTCCCAAGCCAGATACTGGAAAGGATAAAGAAAAGCTCAATGAAAAGGGTTCCAAATGGAAGAGTTCCTGCACCAAGAACAAGAAGCCATGATGGATATTTACGTGCAGGAATCTCCCTAGGAATCTGGTTAGTTCGCACAGGATATTCAATTTGCTGAGCTCTTGTGCCCATATATCCTCCAATGAGAGTAAGTGGCACTGAAATGCAGAACCAGAGGAAGAACAACTCAAAATAGAGAGAAATGGGAATAGCACCAGTACTTTTGCTACCCCAGAGAAGAAAATTCAGGGCTGTTAGAATAATAAAAGCAATCCCAGGGAAAAAACATGCAGATAACCAAGAAATTGATCTCCACCCTTCTGAAGTTCCCTTAATGGTTCTCCACAGACGTACACTGACATATCCAGAAGCAATTCCCAAGAAAAGATATAAAAGTATCATCCCTGTTAGCAGCATTCCCCTGGAAGCTGGTGACATGAAACCCAGGGCTGCAAAAACAATTGTCTCAGCAGCCATTCCGAGAATCTGAACCCCATCCCCAACCATCACGCAGAGAAGCTTTGGACAATCAGGCTCTCTAAATACATCACCCACAACAAGCTTCCATCCAGAAAGCTCCTCATTCATCTGTGCTTGTGCCTCTTTGTCCAACTCTTCATACCGTGTCAAGTCCCTTCTCACAGTTCTCAAAAAAATGACAAAGACAATACCAGCTAGGAAAAAAATCACCATGAGTGAATTTAGTATAGAGAACCAATGCACCCGGGAACCCTCCATCTTTAAATAAGCATCCCAACGAGATGGCCATCTTATATCACTTTTCACAAACTCAACTTCATAGGTAAATGATATCCTCTCGTGCTCTCTAATCGGTTGGTACTTGTCTAGCTCAGATGGGCAGTTGGTCGAAGTGATATTGTCATACATGTGGAGTTTTGTCATGACTTCCGGGTCCCTCTTAACACTACAAGGGATAACCTGGAAGCCAACAATTTCATATTCAGATACCTTCTTCTTGTCAGCTTCTGAAATAACACCCATACCTTCCTCCCCAGTCCCAATGATTTCAACACCATTCCCTTCATATTCATGAACCAAGACTTTGAATTTGAGATGATTGATGATGTAATCTGCACTACCATCCGGTGGTGTGTACCCAACAGGGAACCCCGTCCACTGGATTTTAATCCCATTTTGATTGGCAAACCTCATAACAGGCAAATTGTCAAGGATCATATTTACTTGATACAGATCACGAGTTCTTTGCTTCAGTAGATTTACCTGAAACTCATTCAAGGGGGGAGTAGTACAAAGGTAGATTGACTGATTAACATTCATGCGGAAAAGGTAGGGAGAGTTATCGATCTGATCTCCCCTGAGGAGCTCTCCAAGATTCTCGGCGCTTTTCTTTACGCCACCAAGCGGCTTGCAGTAAGGGAGACTATAGTAGTCATAGGGAAGCTCAGTCTCAATAGAGGTCAATGAATTAACCTTGGCATATATCTGATCTCCATTAGAGTAAGTGTGCATGTAGCTTCCCGGAAGATAAAATGCATCGCTAATTTGAAGAAACAAACTGACAATGAAGAAAAGGCAAACACAGTAGATCACTGGTTTACTCCTGGTCGTAACCTCCATTATCCACCAAATGTCGAAAAATCAACCTAACTCTGACGATGCATTCAAAACGAAATCATGGATTTTGGCGATACAGATCTCGATGCTCAAACAGAATGTAGCATATGCAGATGCAGA

>C100959_65

TTTTTTTTAGTTGGGAAATAATATACTTCCATTGTCAGTTTAACACAATGTTCTCCGACATAATCAACAAAGGCTTCTTAGTTCGCCCAATACATCATGTCATGCGTGTGAAACTTTACATCCGTGAAATCAAGAACCTGAAAGGTCAAAATCCTTCTACTTAAACTATAGACAGAATGCCCCTACATGAAAGCACATAAAAAAGACCAACTTGAAAGAGCTGTAACATCCAACACTGAGGTTTACAAAGAGAAACAAATTGCACTCATACAGCACATAAATGTAACACTTGATGAGTGTTCATGCGAGAACACCCACAATCCTGATTGTTCATGTATTGGACCGGTCAGCAGAATATGAATTTTTTTTTTGTAAAGATTTATCCATAATTTTTCTCTTTCTGGCTTCGCTGCATACAACCTGTACATAATGAGCACAGTCCGGAGCTATTGAGCACTTGGCGCATCCCTCATGCTTAAATTCAGACTGTTGCGCATAGTCCTCCTACCAATTCCAGCATTGCCTTTTTGCATCATGGCAGCAAATTCACTATAATCAATCCTTCCATCATTATCTTGATCAACTTCTCTAATAATATCTTCAAGAAAAGCATCAGTCATGTTATGTTCTGCACAAGCTTGTTGAAGTTCATCAACGGTAATATAACCACTACCATCCTTATCAAAATATTGGAATGCTGCAATAAGATGTTCTTCACGCTCTAGTTTGTTGAGATGAACTGTTGCAGCAATAAACTCCCCATAATCTATCGTTCCACTTTTGTCCACATCAGCCGCTTCCATCAGATCACGTATTTCTATATCTTTAAGGGTAGACCCATATCTTCTTAGACCAGCTTTGAGTTCATCGAAAGTGATTGCACCACTGTTATCGGTATCCATAGCCTGAAACATTTCTCTCAAACCAGCAATCTCCTCTTCGGATAGACTTTCAGCAATCACCCGCAACGCCATCTTCTTTAGCTTATTCATTGCAGAAAACTGTTTGAGACGAGAAAGGACAGCAGGGTCTATTGCCCTGTCAGGTGCAACTCCATTTTCGCATATCCAAGGATGACATAACACTTGATGAGCAGTCAACCGTTCTGAAGGGCGAGAACACAGCATCTTTCTAATCAGATCTTTTCCACTGTCAGATATTAGAGGCCAAGGATCTGAGTCAAAATCTATATGTCCCTTCAATACGGCATCAAATATACCCTGCTGGGTCTCTGCCCAAAATGGTGGCACACCACTAAGCAATATGTACAGTATGACACCCGCTGTCCACACATCTGCTTCAGGCCCATAATGCTTGAGGAGAACCTCGGGAGCAACATAGTACGGGCTGCCCACTACATCAGTGAAAACTTGACCGGGTTTGAAGAAAACGGAGAGGCCAAAGTCAATTGCTTTAAGAGAGAAATCATCATCTTTATTGACCAACAAAAAGTTTTCAGGCTTGAGATCTCTATGCATGACCCCAAGGGAATGGCAAGCCTCAACAACCCCAACGATAATTCTGGTCAACTCGGCAGCCTTCCTCTCGGTATAGTGGCCCCTCTGGATGATGCGATCAAACAACTCACCCCCAGAACAAAGCTCCATGACAATATGAACATAGAGAGGATCCTCGTAAGCACCCTTGATGGTGACAATGTTCCTATGACCAGCTAAATGATGCATTATCTGAATTTCCCTCCTGACGTCCTCAACGTCCTCCTTGGAAATCAACTTCCTTTTGGAGATGGATTTGCAGGCATATTCAATGGAGGTAGAATTCTCGGTACATAAATAAGTGGTGCCAAATTGTCCCTGTCCCAATTTGCGGCCGAGAGTGTATAGATCACGAATGTTGGGAGTCTTATGGCCCAAGACATAATAAGCTTGGTTGTCGAGGCCGCGGCGCATGATGGCGTCTTTCTTGGAGTTGAAGGGTAGATGGTTGATGGTGGTGGGGGTGGTGTTGTTGTAGTTGTCTGCAGAATTGATATTTTGTTTGGCTGAGGAGGGCTGTTCCGAGTCGGGGCGATCAGAAAGGGTGGTGCGCTTGGAGTGGTCTTCGGGCTGGCTGAAGCCCTGAATATATTTCCCTTTCAAAGATCCGCGGCATGTGTTGCCCATCAAACAAACCAATTATTATATATTCATCCAACGATCTCTCATAAGCTTCCCCTAACCGCCCTGTCCTCTCCTCTCCAGTGCTATGCAGAACGAAACAGAAGGTAAACGAATGGAAAGAGGATAATCGCGTTCCTTTCACTTCTTTGTTTCTGCTCCGACAGATCTCCACAAAATTAGTTTGGGAAAATATTTCTATTTTTTTTCTTGTTAATTTTAGGATGTGATGGTATTGGTTCCTGCCGAGAAACATCGGAGGTCAGTGCTGACCAGCTACGCCCACTTCTC

>C100961_65

TCCTAGTTAAGAAAAAGGATGAAAGAGCATAAGAAAGCATATTGCTTATCCATGTCTGAGTTCAGCTCGTATAAAATTGGCGATGGATGGTAGACACAAGTCAAAATTCAGAATTTTGAACGAGCTGCCATTAAACCTGTCTCTTGATAACCCTAGAGGCTATGCTAGATGTCATTCAGGAGCTTCATAGCAAAATGTCAACCTGTCTTCATAGAGAAGAAAAGAAGCATTGTCAATTTCTAAAATTCAGAATCATCATCATTGAAATCACTGTATTTATCGCCATTGAAATCACCACCATTGTCATCTCTATCAGAGTATTCATCCTCACTAAGTTCTTTTTTCATTTTTTCTATTTTTTCCCCCAATTCAGTTACATGCTGACTCAGAGCCTCGTGCCGTTGCAGGGCTATTGAACGCTGCAATGCCTTTGCCTTTGTTAGAAGGTTTCTAGGCCTTAGAGTCATAGGTCGCCTATAATTCTTTCCACGATAGTAAATAAGAGAAAATCCTTTAGGAACTTTATCTATTGCTACTAGTATTCCACCACTCTCATATTCCAGTAACCTAGCTGTGTCTTCAACAAAAGCAAGGGTCTTTTGTTTTGTTATTAATTTAACAAGTTCTCGATATTTCCAATGCAAATGCATATTCTCAATGACACCATCAAAAACACCGCGTATACCAAGTGGCAAGTATGCCTTCATTCTTAAACCAACTGAACGGAACATAACGCGTTCTTCATCTGTTATTGCTTCCCTATCATAATCAGGACCAACTGGAACCAAAGATGCTTCTATTTTCGCTAATAGCTTTTCTGCTCTAAGCCTTTTGGCCTGGGCAACAGCTAGTTTACTTTCAATTTTTTTGACAAGCTTAGCATTCTTGGCTTTGGCAACCTCTTGCATCATCCTCTCACGTTCTTCAGTAGATATACCCCTTCCCCAGCTTGCTTGAGCCTCATAGAACTCAGCCAATGATCCTGCTTGTGCTGTTGAATTGTCTTCCCTTGATGGAGCTACATCATAAACTTTGCACCTCACCTTCTCCTCAACATCCTGCACATGTTTTGTCATTTCCTGTCTTTCAGCAATAACAGCAGCCACACTGGTTGGAACGAAGTCCTTCCCACGATATATTACAATGTAAAATTTGTTCCGCAGAAGTAAGGTACCTCCTGTTAATTTCTTCAGCTCCTGAGCCATCAGTTCATTGTTTGTATTCACAATACCACGCTTGATTCCAATCTTAGCAACTAAACTTTTCTCCCAAAGCCTGAGAATAGCAGATGCCAGACCTTGAAGATTTTTATTTCTCCCCAAGGCAAAGTGACAAGGAAGAGATTTAGCAAGTTTCCGCATCTTCGTGAGTTCATCATCAGTCAGTCGTGGACGCATTCCAGCAGGAAGAAGCCTAAGAGGCGTCTTGTAACCAGGAACTGTGGGGGGAAGCAAATCAGCATCAATTGGAAGTATTCCAGTGCCCCACCATTCAACAAAACGAGGACCAAAGCCATCAAGCATCCTGTTAAACTCCACTTCCTCTGGCGTCATGTTCTCCTTCCAGAAAACTGCCTCACTCTTCCCCAGCAACAGCACTGCATCACCATCCGGCACAGGAATTTGATCATCTCCTTCCTTTTCATTTCGTTGCATCTGCCCTTGGTAATTCCTACCACGGTACACCAACATATATCTCCCCGACCTCCATATAACTAACCCTCTAGTTCTACACTCAACAATTTGATGAGCCAACTTCATGTTCTGAGCAAGATGCTCGTGAAACTTGAGCCTCACCAACTCGCAGTTGCTCCACTGACGGTGAATCTTGTCCAGCACGGCTCGCGTGAGACCAGCTTTGGGGATATTAACCTTCTCCTTAAAACTCATTCCCAGCGTTCGCAACCGCCGCAGCTCCTTCTCCTCCAGCGATTGCTCCGCTAGTGTCGCGGCATTAACGCTCTTCTTCTTTAGCTTCTCCTTCTGCTGTTCCTCGTGCTCCTCCTCCTCTTCCTCTTGCTTGTCCCACGGCAAAAGCGCCTTGTCGGGACGAAGCCATTCTCGGCGCAGGAAATCGTCGTCGTCGGCGTGGTCGACGGAGGCTAACCCTAAGTTGCGCAAGCGCAGGGCAATTCTCTCGATGGCGTTGGTGGCTTTGTCGACGTGGCGGGAAGTGGGAAGAGGGGCGAACAAATCGTCATGGTCGTCGTGAGGAGTGGTTGGGAGAGAGGAGAAGGTATTGAGAGCGCGAACGCGAATGCAAATTCTGAATGGGAATGTAAATTTGAATTTGGAAGAGGCGAAGAGAGCCGAGGAAGAGGGCGAAGCGAGTGGGAGTGATGATAGTAAAGCATGTGTGATGTTCAATCGCATTTTCAGTAACAACAATCACATTTTCATTTCCACTTTT

>C100963_65

GCCATCATAAGACCATCATTATTTTGTACACTTAAAAAAACCAACAATGCCAACCTTGATAAAATATTTGTGTTAAGGTACACAGTACAGTAGTAACCTTGATAAAAGATTAAGTACATGTTCAGTCTTTTGCAGAGGTAAATATAGAGACTTAAAACTATCCTCCAACACAGCCCTACTCAGATCTTCACAATGTCTCTGATGCTGCTACAGCATTTTTTAAGCAAATATGCTCAGAGGGCCTGTTTTATTTGATGCATCAAGTCCATGTTCACCATTCCGAGTTCCTGTAATAAAGGCCCTTTTAACATTTTTACTGCCTGAATCCGTCAGAATTGTCAATCTTTTCGCTGTCTTCATGAAGTCACTGAAGGGTTCTTCTCCAGTTTGCTTAACAGCACCAGTTGCATCATAGTAGAAGACATGATTCAGAATCTCAGATCTTTCAACCCCAAACATGTTGGAAAGTCTTCTATACAGCTCTTCATAAGAACCAAGCCGTGATAGGTCTAGGGTCCTTCCTACATCTTCTGACTCCAAGAAAACTTTACAGTGACCAGTATCCAAGCCAAGTTGCCAAGAGAACTCAGCAGCAGAAGCTTTCCCTGGAGAAAACTGCTGTGAGAGTGCAGATTGGGAGTCATCCAAAACGCACTTCTGTTTGTCCTTGACATCATCCGGTGATTTTCTCCCACGAGACAACACATCTCTTGAAATTTGTTGCTCAGTGAGTATTGGTTGACCAAAAAGCAAAAACTGATGTTTCTTAACATCGTCAGATTTCTCCAAAGTCTTGCCAGATTTCCCCATGGTCAGCAAGTGTGATAAGCTTTCTTTGCTTTTATCATGGTTGGTCGTGTTACCATTAGAAATCCCACCATACACTCCAAGTTGATGGATATTAGATGGAAGCAACCCCAACTGCAGTTTCTTGTTAAGGTGGAGATCTGATAGTGATGTTCCAATTTGAGCATGCCTGGCTCCCTGTATGCCTGCAGGAGCGTTATCTGAAAAACCACACAAGGGGTTGTTAGGCCCGAGCTGGTTGCCTGAAAACGTTGGTATTGGAAATTGAACATCGAGGGGGAACTCCGGGTGCTGCGGAAACCGCAGCTTCTTTCTTGGTGGAGAGAAGGGTGTGAAATTGATGACTGGCAAGTTTGATACCAATTCAACCAACCACGGGCTAACACGCTTCACATTTTGTAGTAAATCTGGCTCATCCCAAGTTACCTGAAGAAGCCTCCATGGGGAATTAGGCCAGCGGATGGGGTCAAGAACCTGAACAGAAGCAATGGTTCCCATGAACCAACTAATCCTGGAAGCATCCTCAGTCTCAAAGGCCATCTTGAATCTCATCCCAGAACACCACTGAATCCTCATAGCAGCCCTCACAGCAGAGGCCTTAATGCAAAACTCAGGAGTACTAGCTCTTGGGTAATAAACAACCTCAAAAGGTTTATTAGCAGCAGCCAGTGTCAAAGCTTCAACAACAGCCTCAGGCCTCACTTTAGCCCTTCCACTCAAATTCCCACCACCACCACCATTCCTTAAACTCTTATTCTCCTCTCTCATGAAAAAAGAGAAAGGCCCATAAGGTCCAATCCCACAATTCCCATTTCCCCCTCCACTTCCACACCCAGAACCAGAACCAGAAGCAGAGTTCCAAACCTCACACCCAATTCCCTTCTTTGCCCTCCGAATCCCAACACACAAGTCTCCATTCTCAGACCTCAGAAAAACCACAGAATCCCCAGCCACCAGTTTCTTCTGGTTCACGAAACTGCTCCACCCTGTCGTCAGCAAGTGCCGACGTGGCGTCCCCCGGTATATGTGCCGGAACCTCCACGTCTCCCCGTGCACATCCTTCGCCACCACCGTCTGCACCGGCGGCTCCGCCGAGTAGTCCAGCCGCGGAAAAATCGTCTCCGCGCAGTACCGCGGCACAGAAAACCCGCCACCATTGTTGGCGTCGGACTGCGTCAGAGTCTTCGCGAACGACGCGGGTTTCTCCGAAGCCTCGGCCACGTCAGCACCCCCCTCCGAGTCGGGACAGTGTTCGGAGTTTCTGAGAGGAACCAGACTCAGTTTGGCGAAGACCTCGTCGGTTTCTGGGTCGGCCATGAACTTCACCGCTTCAACGTTGCAGAGAATGAGTGGCGGAACCCTGAGATCAATGTTGGTGTGAGCGTGCTCGGCGTGACCCTGTGGGAAGTAGAAGACTTTTGAGTTCACTTGGGGCATCTGAACCATGCCCCCTGCACACGCGTGCCATAGCTGCGGATCCAATACCTTCTCACCCTCCTTCATCGAAAGACAGAAACAGAAGAATAGTCTCTTGAAAGAGACGTTGACGCTCAAACAGAATTATTGAGAGAAGAATTAGTAACGAGCTTCATCACAGAATTA

>C100965_65

AAAACCCTAAACCCCGAATTGCACTCTGTTAGGGTTTTCCTTTTCCATTTTCCATTTTCCATTTTCTTACCACACAAGACCACACCATGCTAACCGCAATCCTCAGAAGAACTTGTTCCACTCTCTCAAGGCGCGCTTTCCCCGCCGCCTTAATCTCCGCCTCCACCGCCGCCAGCAACGACTTCCGGCCACTCTCCGCCGCCTTCATTTCCAGAACCTTTCACTCCAAACCCAGTCCCTTGAATTTCCGCTCCTCTTTATATCATCGTGCAGAATATGCTGTTGACGATATCCCTTACGAAGAAGGGTCGAAAGGGAACGCCGAGGAAGGCCTCGAGATCGCGAAGCTCGGAATCTCTCAGGATATTGTCTCTGCTTTGGCCAAGAAAGGCATCATCAAGCTCTTCCCCATTCAGAGGGCTGTGCTAGAACCTGCTATGCAAGGTCGTGATATGATTGGTCGAGCTAGGACCGGAACTGGGAAGACTCTTGCTTTTGGGATACCCATCATGGATAAGATTATTCAGTTTAATGCTAAGCATGGGCGGGGGAGGGACCCTTTGGCTTTGGTTCTGGCTCCAACTAGAGAGCTTGCTAAACAGGTTGAGAAGGAGTTCTATGAATCAGCGCCTAACTTGGATACAATTTGTGTTTATGGGGGTACGCCCATCTCTCGGCAGATGAGGCAGCTCGATTACGGGGTTGATATTGCTGTGGGAACACCGGGTCGGATTATTGACCTTCTCAACAGAGGTGCTTTAAATCTGAAGGATGTTCAATTTGTTGTTCTTGATGAAGCTGATCAGATGCTTCAAGTGGGCTTTCAGGAAGAAGTGGAGAAGATCTTGGAGAGGTTGCCCCCCAAACGTCAGACTCTTATGTTCTCTGCAACAATGCCATCTTGGATAAAGCAGATTTCACGCAATTACCTGAATGATCCCCTGACCATTGATCTTGTTGGAGATTCTGATCAGAAATTAGCAGATGGAATTTCACTGTACTCCATTGCAACTGATTTGTATGTTAAAGCGGGAATTCTTGCACAGCTAATAACAGAACATGCAAAAGGAGGAAAGTGTATTGTTTTCACTCAAACAAAACGTGACGCAGACCGATTATCATATACAATGGCAAAAAGTGTTCAATGTGAGGCTTTGCATGGAGACATATCACAAGCTCAGAGGGAAAAAACTCTTGCTGGCTTCAGAAATGGACATTTTAATGTTTTGGTGGCAACTGACGTTGCCTCACGTGGGCTTGATATACCTAATGTTGATCTTGTAATACATTATGATCTTCCCAATTCTTCAGAGATATTTGTTCATCGATCTGGACGAACAGGTCGTGCTGGTAAGAAAGGAGCTGCTATTCTTGTGTATACAGAAGATCAGTCAAGGGCTGTTAGGCTTATTGAGCGTGATGTGGGCTGTAGATTTACCGAGCTACCACGAATAGCTGTTGATAGTGCATCAATGGACACAGTTGGCTTGGGTGGTGGGCGATTTGGCTCTTTTGGAGGTTCAAGGGATCGTCGATATGGTGATACAGGTTTTGGCCGTGGTCCTGGGTCTGGCCGTTCTGGTGGCTACAATAGCAGTGGATTTGGCCGCTCTAGCTTTGGAGATTCTGGTGAAAGGTTTGATGGACAGAATTATAATCGTTTTCGTGGCTCTGGTTTAAGTCAATCTGGAGGGGGATTTTCTGGTAATTCATCTGGTGGACCAAGCTCTGGTCGGTTTGGCTCATCTGGTGGCTTTGGCTCAGGTCAATCAGGAAGTAGATCCGGTCGATCATCCAGTGGGTCTAGGTTTAGTCGTCCAGATGATTTTGGGGGATTTGGTGGTTCAGATCGTTCAGGCGGTTTTGGAGATTTTGGCTCAGGCCAACCTAGTGGTTCTCGTGGTAGTAATCAGAACAATAGAAGACCTTTTTAACCTGAAAATATAAAAAATGCACCACTCTTATTTCTTGATGGTTTAGCTCTTGTAGAGTTTGCTTCACTTTCTCTAAAACTTACAGGCGGCTTCTTGGCTAGGCCTTGTATTACTAAGGATTTGAGAAGGTCAAATAACATAAGAATCAGAAGATCTACGGCATATGATTTTGCTTTTCTGGGACTTCTCAGCTGTATTGCTTTTTTAAACATCTATAAAAAGAAGTGAAAGAAAGGAAGGACTGTATTCTGCGGGTTCCACCATCTGCCCTGAATTTTGTATGGCTCATTTACCATCTATGGACATTGAATCTCATAATGATCCTGAAAATGTGTACAAACGTATATGCATGCATGAGCAGTTTTGAAGAGGCAGCTGTACGAAGTACTGATTCAATTTATTTAGTGTATATTTATGTTGCTTAAAGTAGTTAATGGCAGGTAAAGTGTCTTGAATAATTTTATGGAGATATGCA

>C100971_65

GAGTTGCATACGGCACACTCGTTCCGCTCTGTATATAAACCGATGAGAGTTACTTAGTTGTACTCAAAACTATAATCGGTGAGACTGAGAAAGCAAAATCAAGAGAAAATAAAAAATGGAAGACACGCTCTCTGCGTTGAGGTCTTTGATGGTTTCTCACTCTCCTCCACTCGATGCCTTAGTTGTGCCTTCTGAAGATTATCACCAGAGCGAATACGTATCTGCACGAGACAAGCGCCGCGAATTCGTTTCCGGCTTCACTGGAAGCGCTGGCTTAGCGCTTATAACAAAGAATGAAGCGCTGCTGTGGACGGATGGACGATATTTTTTGCAGGCTGAGCAAGAACTTAGTGCTCAGTGGAAGCTAATGCGTATTGGTGAAGATCCTGCTGTAGATATCTGGATAGCTGATAACTTGCGAAAAGAGGCATTTATTGGGGTTGACCCTTGGTGCATTTCAATTGATACTGCTCAAAGATGGGAGCGTGCTTTTGCTGAAAAACAGCAGAAGCTGGTTCAAACCACAAAAAATTTGGTAGATGAAGTTTGGATAAATCGACCACAGGCAGAGATTAATGCTGTTATTGTACATCCACTGAAATTCGCAGGTCGCTCTGTTGCAGATAAGCTGAAAGATTTGAGAAAAAAGCTTCTACAGGAGCAAGCCCGTGGTATAATTTTCACAGCACTTGATGAAGTTGCGTGGTTGTATAATATTCGTGGAAGTGATGTGGCCTACTGTCCTGTTGTTCATGCATTTGCTATTGTGACAGCCAACACTGCTTTCGTTTATGTGGACAAGCGAAAGGTTTCTGTTGAGGTTGAAGCTCATTTAGTGGAGAATGAAATTGAAATTCGAGAGTATACAGCTGTAAGCTCTGATGTGACATTGCTGGCAACCGATGAGCTTGATACTGTATCTACTGTCAAGAGAGCAAATGGTGGACATCAAGCTGAAGAAAATAGCAACAACCTCATATGGGCTGATCCAGGTTCATGCTGTTATGCACTGTATGCAAAGTTGAACCCTGAAACAGTTCTTTTGCAGCAATCACCTTTGGCCCTTGCGAAAGCTTTAAAGAACTCAGTGGAATTGGATGGGTTAAAAAAGGCTCATATTCGGGATGGTGCAGCGGTTGTGCAGTATCTTGTCTGGCTGGATAAGAAGATGCAGGATGTTTATGGAGCTTCTGGATACTTTTTGGAGAAGGATTCTGTGAAAAGGGAGAAAAATTTGCAGTCCTTGAAATTGACTGAGGTGACTGTAAGTGACCAGCTTGAAAGTTTCCGAGCATCCAAAGAGCATTTCAAAGGATTAAGTTTCCCCACTATTTCGTCAGTTGGTCCAAATGCAGCTATCATTCATTATTCTCCAAAGGCAGAAGCATGTGCTGAACTTGATCCTGACAAAATTTATCTCTTTGATTCTGGAGCCCAGTATCTAGATGGAACAACAGATATTACACGAACAGTTCATTTTGGGAAACCTTCAACTCATGAAAAAGCGTGTTATACGGCAGTCCTAAAGGGTCACATTGCACTTGGAAATGCTAAATTTCCCAATGGGACAAATGGGCATGCTCTTGATATTCTTGCCCGAATTCCTTTGTGGAAAGATGGTCTTGATTATCGACATGGTACTGGTCATGGAATTGGGTCCTATCTCAATGTTCATGAAGGACCACACCTAATCAGTTTTAGACCACAGGCTCGAAATGTACCATTGCAATCTTTCATGACTGTTACAGATGAACCTGGTTACTATGAGGATGGAGAGTTTGGGATAAGATTGGAGAATGTGCTTATTGTCAAGGAGGCTGATACAAAATTCAATTTTGGTGATAAAGGTTACTTAGCTTTTGAGCACATTACCTGGGCACCATATCAAACGAAATTGATTGACCTGAACCTTCTAAGTCCTAACGAGATAAATTGGCTGAACTCCTATCACTCTAGATGTAGAGATATTCTAGCGCCATATTTGGATGAAGTTGAAAATGCTTGGCTAAAGAAAGCTACTGAACCTGTAGCTGCATGAGCTGCTTGCGATACTTCAGCAGCATAATTTATGGGCATTGTCTTCCGTATCCTTATTATACTGTAATCTAGATTTTAGATGTCATTGAGATGGCTATTCCTTCAAATTAGTATGCAGCATATATCAATGTTGCATATTTGAGGGTGTACAATGTTCAATTGTCTATCCGCTCCCTCAGGCATCTCTGCATCAGCTCTTTTCTTTTCCCGTATATTGTCTTGTACCTAAAAATGATGAACCACTAGTACAGATGATCATTCTTAGATACTGGTTATTTGAGGATTCAGAAATTGGATTTAACCGTTTTTAGGAGGAATCGTAAAGATAAATTACACTTTCGACTCAGATCAAATATTACAAGGTTAAACAAAA

>C100977_65

AGATCAGAGAACAGTTTCATGTCATTACAGTAAATATTTGCACCTCAGGTACAGCTCCACAGGAACAATTAGTAATTTTGTCAAGTTAAAGGAGCGAGCAGGTTAGAATATTACATCAATTCCATAATTTGTGTACTGCCTGAAATGATGCAATACGTGCGAGTTATACATTTTTCTCAAGGATGTCACCTCTCATGAATAGGAATGTGTTTGATCAGCTGGCCATGTTCATTTATCATATAGAGTGATCCATGACCTGCCCATCTCCTATTACTAATTACTAATAGAAAGTAAACAAAGAAGTGTTTACTTAATTTTATTTGCGAATTAACCAAAAGGGGTACAAAATACATAATTGTAGACCTCTTCTAACATTAGAACCAGAAATAAATTCCAGCACTATATGGATTGATGCCGAGGAAAAATAACCTCTTTTTTTGGGGGAGCCTGCGGGATGAAGCAGCAATAAATTGACAATACTGCAAATATGGAAACAAAGGCCAACTTTAAAATTTCAACCAGCCTCCAATTGTCCTCCAGAATGAAAATTCTACAACGGAGAACATCAATTAGAGAGAAGGACAAGATGAAAGTTGAAAAATCCAGGATACATCGTTGACATCCATCCTGCGGTATCGTTCTTCCCCTACAGCTCCCCAAGTTCAGCAGATTGTTGTCCTTCTCAAATGATGCCTCACAGTGACGATTCCCTAGAGGGACAAAAAGTCAGTCTCATCTGAGCTTTCTTCATATATCACATAGTTGGCATCTTGACCATCCTCATTGCCACTTCCCTGATCACTATTGTAGCTGCTTTCAGATGTACTCATAAAAATAGATCCATCTGAGGTGTGGCTGCTCAATTGTGAGGGCAGACTTATCTCACTATTCATATCAGCATCAAATGTCTCAATATAACTTCCTTCTGCCGATGGATATTCAGCCACCAGACACATATCAGGCCTTGCTTCCAAAACCTGTTTTATAGGCATAATACTAGAGTTGTAGCACCTGTGATACCAGTCAGCCTCGGATGCTAAACCTTCCCTATTGGATATGTCAAGATGTACAAGAAATTTGAAATCTCCAGCAAGAATTGCAGTAAACAGCCTAGTGACTTCCATCTCTTTGAGATGCATGCAATCCCTTAAAATTAAGACCTCAAGTAAATTCCCCCCATTGCAGCTTCCAAGGTTTCTTAAGAAGGTACCAGTGATTGAATAGGATGTTGAAAAGTCAACATACTTTAGTTTCCTAAATCCCTTAACAAGTACACGAACTTTGCAATCATGTAGCTTAAACCCTCTAAGGGATAAAGAAGTTAGATGAGGACAATTTTTTACTAGCTCAATACAGGTTTGTGCTTTCAAGCTGTCATCTCTACCTGTTTTAGAAGACTCAAAAAACAAACGCTCTATCAAGTGGCAGTTTGCACTTACTGACATTAATGTTTGCCTGAGCTCAACATGGAGCCCAACAATTTCCAAATCGAGGAGAGATGGGCATGCTGACAATGCTGCACCAAGGGATGCATTATCCATTCTCTCAACATTGTACAAGTGCAACTTTCTCAAAAGAGCCCCTGGAGCACCACCATCGCCACTTAAAGGGCTTAAACAGCATCTTGTGAGAATGGATGACTCCCTCCCTTGTCGAGATCTCTTATCATTCCATGAAAAGTTAGATACTTCTACAATAGCATTTCTGATGGTACAAACAAATGGTGGACAAGATCCAAGTGATGTAGTAGCGCCAGGGACAACACCAAGCTTGAGAGACCTTAGTGCCTTTCCAGCTCGCTGAATCATCGTTGCAACTACTGCATTGTTAACCTTCGGAACAAGTGTAGTCAAATCAAGATTTGCATAGCACAAAGGATCTTTAGCACATTTGCTAAACAGTGAACAAGTAGCTGATGCATAGCACAGGCTCTGGGTGTCAAGCATTGAGAACACCTTGATGGTGAGGTCGGGAGGGAGGGGCCAGGCGAGGGAATTGTGGTTGGAAGCGCGTTTCCGGGAGGAGTGTTTGGCGGCGTCGAGGAGGAGAGAACCCATTTTGAGGGCGCGATCGATGAGATCGGAGTCGGAGGGGACAGATTGGAGAAGCCTTTCGAAGGAGAGATCGAGTGAGAGAGAAGGGAGGTCGGAGAGGGAGAGAAACGCGTCCAGAAGACGCTCCACATCGCTCAGGTTCTCGACTCTAGGGTTTTGCGACGGACACGGACGCTTCGCCATTGACGAGGACGAGGACGCTGATGACGCACTGAGACAGTGGAGTACAACGGAATCCATGGCCAGGGCAAAGAGTGAGTGAGTGAGTAAGGGAGTGAGTGGTAAGTGGTTTGGTGTGGTTTCTTCACATTCACACGACCCTCACCCTCTCTGATTCCGTGCCTATGGCTATGTACCGC

>C100981_65

ACCAGTATCAGAAAAATAATCACCAGCTACTTTTTCAAACTTGGATTTTACCACTCTAAACTAGTTTAATAACACAGTACTTTGTACAGAGTAAAATAGGAAAGGGAATATGCTTGAGCTGGTTGTAAAATTTGTTTGACAGTAAATCTCAGATAATAGTTCCAAAAGTTGATTAAACAAAAGTGTGATACTTTCAACCACTTGGACATGACATGACCTACAGTAGGTACACCTTCAGGAAGATGGATGGATCATTCATTTGAGAAAGTGAAATTTATGAGACCCCAGCAAGAGTTTGTTTGAACTCCAAATGATGAGAGGGTGCAGAGGGTGGAATTGGATCAGCATCCCTCACCCTGACACATCGACAGCACGAGCTCATCCACTAATGCTGTCACATGAATTTTCCATCAAAATCAGGAACAGTTTCTAGTCCTCAGCAGGACAATTTTACTGCAGAAAGGAATTCATTGGTCCTTCATCCTTTGGTCAAAGGAGCAATTTGAAATTCTCAAGCATCTTCAGGAGAGGAATCTCTTGCTACATGGTTGTCTTCCTCCTCTCCATCCTCTTCATCATAATCAGTGGAAGCAGGTTGTCCATTAAGATCCACATTAACACCATTCAACTTTCTGACAAATTGTCCCCTCACACGAGGCCGCCTTTCTGCGAGCCGTTTTCTGTTGACATACCTAATTTTCTTATCAAAACAGCGCTCTTTCCTTTTCTGTCTAAACTTCATCAATGCTGCTTCTCTCCTATCAACCTTATTTAATTTTACTTCACATGAACTTGAACTTCCTAACGATGGCCAAGAATGAGTTGTAGAAATCTGACCAGATTGTAAGCAAATGCTCATTGGATAATATGGGAAGGATGTCATCCCACTTGCATGAGGAGGACACTGGGGAAGATGATTGTACCGTGCAATCATAGAAGAACCAGAATGACTTTGCAGGTCCTGGATATTCTTTTGATACATTTGTGCTGATGATGGCATCATAACATGATTAACAACTCCTGAAATATAATATGGATAAGCATGTTGAGCAGGCTCGGAACCATGACTTCCATTTCTTGGATGCACTACTCCCTGAGAATGTTCTTCCCTGTAATGCTTTTGCTGCGAAACTTCCATAGAAGCAGGTGGAGTACAAGATCTCTCAATAGAGAAAGAATCAGGGATACTGTTGCTGCTGGGTAAGTCATCTGGTGAGTGGTTTTCAAACATTTCTCCATTTTCACGTGTTTTAAGGTCATTGCCCTCCTGTTCACCACATGCTTGATTTACATCCTCCACCCTAACTTCTGTAGTAGTAGTAGTAGTATTGTCAACATGAGCACTCTCTTCAATGTTGCTATTCAGTGCTGTTGCTTTGACATAGGTGAAAAAGGCTGATGACTCTCCAATCTTCAGTTCACTCTTCTTAGGACCAGATGAAAAATGTGCTGTTCGACGATCACTTATTCCTGGCACATCCGGCTGATATTCTGATACAAGAGCATTGGGAAGCTCCACCACAACAGCAGCACCGGTGGCAATACTAGCCTCTTGTTCTTGTTGGATTGATACTACGGTCTCTGGATTAGTGCTTCTTTTGGACTTGTCATCTGTGTCATCAGAGAACAAGGTTGTACTGTTTGTATTGGCATCACTTGGATCTGATGCTACCAGGTCACACTCATAATTCAATATGTTGTTCTCCACAAGTCCAAGCATGCGTCTCCTTCTCCACATGTGTGTCCACAAATTTAATAGTTCATTTGTGCGTAAAGGTTTTACTAGGTAATCTGCTGCTCCAAGCCTCAAGCATTTTACAACAATAGATACCTCATCCTGTGCAGACATCATTATAACAGGGATTCGGCATAACTCTTTATCCCTTGCTATGTACTTCAACAACTTCATGCCTTTCTTTACTGGAAGGTCGACTTCAGCTAGTATCAGGTCTATATATTGCCCCTCCGCATTCAGTGCATCAATTACTTGCCTAGCTGACCTTACTGAAGTAACCTGGTAAGAACACCTCAAAAGAAGAGTAAAAACCTCTTCAGAACTCTTGGAATCGTTATCACACAACAAAATCCTCACTTTGCTTCTGTCAATAAAGCCATCGCCACTTTTACTATTATTCCCACCACAGTTACTTGCTTTATTGTTCAAATTAATAAGTTCCTCAGTCTTAGTTTCAGCATCCATCGTAACCACTCCTCCCTCTACTAGAAAAAAAAAAAGACCCAGTACTAAACCTCTACTCAAAATCAAATCTTGCAACCACAACCACAATCAACACATATGATATTCAAAACCCATGTGTTCATATCTGCTGATCGTTGTAGCACCAGCAAAATCTAAAGTCTGGGAGAGGCGACGCACGTTAGAAGCTCAGACCAAAAAAAAAGGTCCTTTTTTTT

>C100985_65

TTTCCTTGAACAATAATTAACAACTATAAAGCATTGATACAACAAGAATAAACATTTAGCAGTGAGAACTACAGAGGAACAAAAAACTCCCAATAACCTCGAGAGATTTACAGGAGTAACGAGAGTAATGAGAGTCCTAAAACCGCTTGTTAATCTGCCCTACGTAAACTGAAACTTGATAGATTAATGGTTAATCTACTCTACTTGAACTGAAACTTGACACACTAACAGTTCTTTCCTTCGCCTAAAACACAGTTAATAGGCAAATCAGCAAAGAAGATGATTAGATTGCTGTAGCTGGTAGAAGTGAGGAGCAGCCTCCAGTAGGCCCGAGGGATCTATGGTTATGACATTACGCATATATTGTCGATCAGTTGATACAAGAGAATTGTAAATAACCCACTTTGGATTTACTCTGAATAGCACTGACGATGGGTGAATATAAACCTCCTGTGATCCTCTTAACGTCTTATACATCCCATTATGACTGTATGCCTCTAGATGGCAAGCGTTTGCAAAAAATCCAGCAATGACTGCTTTTCTGACTACCTGCATATCACTTTCACAAGATTTTAAGACTAACCCTATCCTCCTTGCAATTCTTTTGAGTTGCTCTCTAACTTCAAGAACCTTTCTCATGGCATGATAGTTTACATAGTTCTTGTGACACCACTGTGAAGATTTACCAGATTGATGAAACCCCTTATATACATTAAGGAACGTTACATGGTCACCCTCGGCAGCAGCAAATCTCAATTTTGCTTCATCCGATTCCTTTTGTATCCCTCTCCCTGAGATCCAGATGGATTGGACGGAAAGAACAGCAGCAATGGTAATTATCTCCTTTGAACACCCAAGTTGACTGGAAGCTATTATCATTTTTGAGATCATTGGATCAAGTGGAATCTCAGCAACTTGAAATCCAGTTGGTGAAGTCAGCTTAGCATCGTCATCCAAGACCCCAAGTGAGTAGAGAACTTCTAGTGCTCGGATCATTGCTTCAGAAGATGGAGATACTGGCCAGTCAAAGCCCAAGATATTATCAATCCCCAAGGCCTTTAACTGGATTACGCAGGAGACCATATTTGATCTCTGTATCTCTGGAATTCCCTCATTAGGCATGTGATTAAGAAAATATTCTTCTGTATATAGCCTGTAACACTTTCCTGGTCGAACTCGTCCAGCTCTACCAGTTCTTTGTCTAGCAGAAGCCCTAGATATTGGTGCCACAACCAGATTTTCAATATCAGAAATCGGATTGTAGAACCTCTGTTTAGAAAACCCACTGTCAACAACATAGACAATTCCCTCTAATGTGAGTGATGTTTCTGCTATATTAGTAGAAATTACTACTTTCCTCTTTCCACGAGGAGCTGGAGAAAACACTAACTCCTGGTCAGCACGTGGAAGTCCTGAATAGAGAGGCAAAATAACCAATCCTGAAGAGTGTTTTCCAGTAGTTTGAACTTCGTCAGTGAGTAACTGAACAGCTGCATCAATATCATCTTGGCCAGTAAGGAACACCAGAACGTCTCCTGCAGGCTCCCGCTCATGGATCAAGAGTGTTGTTGAAACGGCAGCCTGAACATAGTCTTGGACAGCTTCCTCAGCATAATTAATTTGTACATTGAAGCCTCTACCCTCAACGGATAAGATTGCAGGCTCCACTCGTGGTCCATGATCCCTGTTTTCAGGTTCTCGGCGCTTTTTTCTCATTTGGAAGAAGTCAGACATAGACTTTGCTTCAATAGTAGCAGATGAGATAATCAACCGCAACTCTGGTCGACGACGTTGAATCTTCTTCAATAGTCCAAGCAAAATGTCAGTTGATATTGATCTTTCATGAGCCTCATCCACCATTATAACACTATATTTGGTCAAAAGAGGATCGTCCATCATCTCCCTTAGGAGCACTCCATCAGTTAAAAACTTTAGCACCGTTACGTCCGGTTTGGTTACATCTTCAAACCTAATTGTGTAGCCAACTTCTTCACCAAGTTTTACTCCCATCTCTTCGGCAACTCTGGAAGCAACAGCCTGAACGGCAAGTCGTCTAGGTTGAGTACAGGCGATGAGTCTGCCACCGGCGGCCCAACCTGCTTCTTTCAGGTACTGAGGAATTTGAGTGGTTTTTCCGCTTCCGGTTTCGCCGACGATGATCGTGGTGGCGTGAGTCTCAACTAGGTAGAGAATGGCGGTGCGGTACTTGAACACGGGCAACCGTTCGCGTTGCTTCTCGATGCTGGCGTAGCCATGTCTAGAGGAAGAGGAATGGTGAGAACCGGACAAGAAGAGAACACCTCCTTCTTCATCGTCGACTACGCGACCCTGTGGCCTCTCCGTCCCTGGCTTCCAGAACTGCGACATCTTCAATTGTTCACCACCCCAACACACCACCACTCCCCGGATCCTTCAA

>C100989_65

GCACTTCTTTTCTTTTATCTCATTCGATAGATAGCATCCAATCAACTCAAATTCACAATGTTCCAAAGAGTTTTCTTTATACAAATAGTCATTTATCACTAGTTATTTTAGACATCAACTCAGCATTTGATTGAAAATATTACCATTATTTGCAGCAGAATTTAGTACAACCACAGCACCATAAAAGCAGTCTTCAAATCCTTGCAGCTTCTAGTTGATGCCCACTAGGTTGTAAAAAATAGGCAGTGTTAAGAAACCAGAAAAAAAACACATTAGAGTTGACTTTGATTCAATCCTCTCTACTAAAATTGGATCAGTAATAAAATTATTTTGATAAATTTGCTGTAAGGGTTTTCACTAGGAACCTAATTCCATCATCAATACCCCTCCCCACTTCATTAACACATACAAAAATAGAGGTTGTGGGCGAGCTATTTTTGCATTATCCTGACCCGGCAACCAGAACAGCATCCACTAATATACAGAGAGATTCCCTTGGCTATGAATGCACTAAAAGATATGCCAAGATAATTTCAATAATTCATCGGGACATCAAATACAACGCACCATTCAAGCATAACCCACACTGTAATCAAGATTAAACAGAATGTCCCGCCGGCCTTCTGAACCCAGCCTGAAATTCAAGGAATTAGCCAGTGATTGTACTCGGTCATCACTTTCGTAGTCCATTTCTCTCATGCATCGCCGCCTGACCTTTTTTAGGATCTCTTTTGTCGGTTCAAATCCAGCAGTACTCATATCATCAATCACTGCAAGGGAAGATTTGACATCCCGGTTAATCAAGTGGGCATCAATGAGCAGAGAATATGATTTTGCATTAGGCTTGAGACCCAGACTCAATAGGTGCTCAAACACTTTTGAAGCTTCATGTGTCTTCTTGAGCTTCCCAAAGGCGTACATCAGCCCATTGTAAGAATGAATATCAGGGCTCAACCCAAAAGTAGATCCAATTGACTCAAAAGTTTGGTAGGCTCGATCAAGGTCCCATATGTTTGCACATCCTAAAATGACGCAGTTAAGTGCAACAACAGATTTGTAGGGATGCTCTGCACGGTTTAAACTCTCCAGTTGAAAATAAACATTATCTAGAGTCTCAAAACCCTTCTTGGAGCATGCCATAACCAATGGACGTAATGAAGTAAATGGGCAGAACAATTCTTCAGCTTCTTGACCAGAGTCTCCATAAGCCAACTCATACTCATTCAGAGTACTAAAAGCCTTTTGTAGATTTCCCAATGATGCAAGGGCATATATCTTCGCAAGATATGACTCTGGATTAGGAGCTTTCTTCTTACGCAATGAGCGGTCCAAAACAGCCCATGATGCCCCTAGCAACTCAGAATTATAGGTTCTACCAGCAGTCAAAAGAGTTGACAGAACTAAACCTTCATCTACAGACAGAAATAGAGGCGGTCTTTGTCGTTCACCCTTTACAATCCACTTAGCCATATACTCCAGGCCATAAAAAGCTAATTTACCATTATCCTCTCGAGTTGCAGTTTCAACAATAAAATTGCACAAGTCCCAGTTGGGGCAAAGAGCTTTGTTTTGATCTGATGCCCTGCACCTTTCAATTATTGTCACCAATGTATCAAGCCTTCCATTTCTGACACATGTCCACACACAGTTCATAAATACCTTCATTGACAAGACGTTACCAGATTTCAAAATTTGATCTATATATTTAAATGCTGTATCAATCTGGTCTGTTTGAAAGAGCATTCCAATGACTAAGTCATATGATTCGTCATCAGGGAGAGATTCGTTTCCTGTCTGCAGCATCCGTTGGAGCAATTTATCCGCAGCGACGGTTTCGTTGGCCTGGCACATGGCTTTGAGGACGAGGTTAAACGACGCCGTGTTGGGCTTGACACGGAACTCATCCATCTGCGCGACGAGGTCGAGGAGGTCGGCGGCGGAGGCGCCAAGCATGAGGTTGGCGCGGAGGTAGTGGTTGAAGAGATTGACGTCGGGGCGGTTGGGCTTACCGGTCTTGTCTAGGGCCCTGACCCAGGTCTCGAAGAGCTGTTTTACTTCGGTCCACTCGTGCGAGGCCATGCAGTCTCCGAAGAGGTCGAAGAGCGCGTGAGGGTCGAGCGAGTCGGCGGAGGCGCGGTTGTAGATTCCGCCGAGAGTGAGCGCGTTGGCGGAGGGTGCGGAGGGGGGGTGCGGGGAGCGCCAGTTTTGGTGGTAGAGGGGGCTGCCGGAGGCGGGGTTGGGAGGGAGAGGGGCGGGTTCCTCCAGCTGAGGCTCCTGCGAGAGGAAGGGGAATGTGGATATGGTTTTGACGAAGGAAGAGGGTTTCAGAAGGAGGAGGGAGCGCGTGCGGAAGAGGATCGCCATTTGCGACGCCATGAGAGAGTGACTGTGTTTTCAGGTTTAGGGTTTTGTTTTG

>C100993_65

CTGCAAACAAATGTCACAAAACTCTCAACAACCAATGGCGGGGAATAATGAAGAAGAGGGAGATAGCCTTCACCAGACGCCGTTACGAATCCCGGGATTGCTTTATGTTTCGGTGAAGATGGAGAATCCCAATCCCACTCTCTCTGGGGACCTTCTCCCTCACGTATCTGGCTCTTTCCCGTTAAGCCCTTCTTGGGATCCTTCCAAAGCTCTTTCTATGGAACGGGAATCCGCGTCTGTATGGGAGTTGAGCTTTGTTGTTCCACCTAATCATGAAGCTTTAGAGTTTAAGTTCCTTTTGAAGCCCAAGTATATCGATAACCCTTGCTTTGTCGAGGAGGGTCCAAGCCGAGTACTTATTGGGGGAGCATGGCAAGATGGTGACAGGCTGGCTTTGTTTAGACTTGATAATGATCAAGTTCTTGAGTATCGAGTATTTGTGGAAGCAAAAAGGGTTTCTCCCTTTGATCTTGCAGCTAGTTGGAGGGCTTATCAGGAGAATTTCCGCCTTTCAGCTGTACGTGGGATACCTGATGTCAGCATAAATTCAGAACATCAGACATGTAGTGAGAACATCTCATCTGCAAATTTGGAACTTGATCTTGAGCATTATATTGTCCCATCTCCTTCAACTTCTCCAAGTTCAGCACTTGTATATGCCGCCAACATCACAGAGAATCCCAGATCACTAGGTAGTGGATCTGCCAGCATTTCATCTCCCACTGGAGATAGCGGTGTTCCTATGATCAATCAACTAGCAACAGTGGTAGAGGTTCATGCCCCTGATCCATCTAAGGGGTACCAAAGTCCTGGAATGGTTAAATCGCAGTCTGTGGGAACAATGTCACCTCTGCAAAAGGAAGATGATTCGAGGGGACTTGTTGACAGGGGTGTTGGATTTCCCCGACTTATAAAATCTTCTAGTTCAAATACTTTTGCTACAAATTTTAACTTGGAGACTTATACTAAGAATTCAATTCCTGCTGCTGCTGGAGCTGTTGCAGCTGCTGCGATTGCTGATCAGATGCTAGGCCCCAAGGAACATAGGCATTTGGCGATTGTCATGGTGAGTTTGCCAGCTCGAGGCAAGACTTACACTGCTGCTAAACTTACAAGATATCTTCGATGGTTAGGTCATAATACAAAACACTTCAATGTTGGTAAGTATCGTCGCCTTAAGCATGGCTCTAGTCAGTCTGCAGATTTCTTTAGAGCTGACAATCCTGAAGGTGTGGAGGCACGTAATGAGGTAGCAAAGCTGGCATTTGAAGATATGATATTTTGGATGCAAGAAGGTGGCCAGGTTGGGATATTTGATGCCACAAACAGTAGCAAACAGCGAAGAAACATGCTGATGAAACTGGCTGAAGGTAGATGCAAGATCATTTTTTTGGAAACAATCTGCAATGATGTAGACATAATTGAGAGGAATATACGCTTTAAAATTCAGCAAAGCCCTGACTATGCAGAAGTACCAGATTTTGAGGCTGGATTGCGAGACTTCAAAGAACGTGTAGCCAATTATGAGAAGGTTTATGAGACAGTAGAAGAAGGATCTTACATAAAAATGATTGACATGGCCAGTGGACATGGAGGGCAAATACAAGTGAAAAATATCAGTGGCTACCTACCTGGTCGGATAGTCTTTTTCTTGGTTAATACACATCTTACACCACGCCCAATATTACTTACCCGACATGGAGAAAGTGAGTTTAATGTGAGAGGCAGAATTGGAGGAGACTCTGCATTAAGTGAGGCTGGAGAACTTTATAAGAAGAAGCTTGCTAAATTTGTTGAAAAGCGTCTCAAATCAGAAAGAGCTGCTTGTATATGGACTAGTACACTGCAGCGAACAATTTTGACGGCTGGACCAATTGTTGGATTTCCAAAGATACAATGGCGTGCACTTGATGAGATAAATGCTGGTGTGTGTGATGGCATGACATATGAAGAGATTAAGAAGAACATGCCAGAGGAGTACGAATCACGCTGTAAGGACAAGCTTAGGTATCGTTATCCTCGTGGAGAGTCTTACTTAGATGTTATTCAAAGGTTAGAACCTGTAATTATTGAGCTTGAGCGACAACGAGCACCTGTTGTTTTGATTTCTCACCAGGCAGTTCTGAGGGCATTATATGCTTATTTTACTGACAGACCTCTGAAAGAAATTCCAGATATTGAGGTGCCCCTCCATACGATAATAGAGATACACTTGGGAGTTACAGGTGTGGAAGAGAGAAGATACAAACTAATGGACTGAAAGAATAACTGGAGGGAAAGAAGAGATTTAAGTGAATATTTTTGGTTCTTATGAGAAAAAATAACGATTATCATGTATATTGTAAACTTCTGAAACCCCTTTTAATAATTTTTATGAGCATTATTTAGTTTCCATGGAGAATTTTGAACATCAAAAC

>C100995_65

TGCAATGATAGTGGTCAAATATATTTGTACAGAAAAGGGTGGTGATCCATTAGTCTGTACAGATGGAAAGACGAAAAGCTCAGTCATATCTGGGAAACAAGTCAAATGACGGACATTTAAAAGCGCAAGATAAAATGTTTAGAGATTTAAAAAAATAAAAATAAAAGAGGATAAGAAATAGTTGGCGTAACAAATTAATGGTCAAAGAGTGGGGCAATCTAGGAGAACTTGTTTTGCTGGGAATAACCAGGAACAAACACAGGAAGCTGGCCACCTCTATGTGATCTACCTTGCTGAAAATCCCTTAATGTAGAATCTCTATCTGGAATACCAGGTACAGAACTGCTAATTCTTGTTCCCGTATTTGGTTGTTCATTCACAACGGGACTGCCAAAAAATATAGACCGACCAGTGTACAATAACTCTGTTCCAGATATCTGGGAACTGCTAGGACCTCTTGATGAGGACGAAACAAAGCGTCCGGCAGCTTGATCCCAAAACACCGACGATCTCCTATTATCTGAAACAGCTAAGTTGTTGCTTCTCATTGGCACACGTACCACATTTTCTTGGACTGGATTCATGTTCCCCTCACTTTCTTTAGTTGACGAAGGAGACTGATTTCCTGATGGTTGTTGATACATGGGGTTGAAATGGTCTCTGTTCAAGCTTGGCTTCTGCATTGGTGAAGGAGTAAGGTTTGAGACTTGTGGACTGCTGAAGTTGCTCATACTGTGATGACATGATGCATCTATATCATCCTTGCTTGCTTGACTAGGAGCGTATGAGCTCTTGGAAGGAGATAACCTTGATGTCCCCGCTGTGTCATATTTGTTATGAAATCCTTGATTGCTAATTGGACTGCTTCTTCCACTCACATTGCTACTGGATAAATGATCGACGTCGTATGCATTAGGTCGAGAACTAACAGGACGAAGCACAGATGATGATGCTCTGGCTTTAGTAGCTGCCCTTATTGCCTCATTAGAATCCAATTTTGCAAGTTTCCATGCACTTATCCTCACAGGACGCTGGTTCAACTTTTTCCCTTTGTCAGGTGGTTGTACTGCATCTGGATCAACAGTGGATGGTAGGCGCCCAGGCTCCAAATGTGGTATAATTTCATCCGGGTGATCCATAAAGATCCTTGGAGGTGTACACCATGCACCTTTGTACTGCAAACTCATTCCAACAGAGCTTCTTCCACTCATTGCAGTGACGGCTGAACTAGTAGGAGATGATGGTAGACTCTGTTGTTCACCTCCATCAACTGAAGGACCAGGTGGTTCACTTAGAGTTCTCATTGCAACAACATACTCGTAAGTTGTGATACCCTTCCGTATCAGGATCATATGGAAAAAGAATAATTCTCCCAAAGGCACAGTTGCAAGGAATGAAACTGCTGTGCATATGGCCACTATAGTAGCAAATGGGACCCGAGAGAATCCAGCTCCAAGTTTGTCAGCTATCTGATTCTCTGTACCTTTCTTATCAACGAAGCATCTGACAAGCACTGCAATCCCAACTCCACATTCAACAACAAGCCAAACTAGGCTCACTGCCATAAGACACACAAACGTGATGTAATTCTTCTTTCCCACACAATTGTTCAACCACCGACAATGATGATCAAATCCATCAACACATTTGTCACAACTTCGACAATGCTTACTGAACTTCCGCACCTCAGCATTGCACAAAGTGCAGAACAAAGCATCCTCTTCTCCAGATTGCTGATGCGGAATATTATCTTCATTACTGCAGCAATCTTCTCTAACAAGAAAACCGCAGAAGAAACATCCAACTTTGGAGCACCAACTTGAATTATGCCGATCAGACATTCCTTCTCCCTTTAGTCCTATCTTGCTGGGTTCTGCCAACTCTTCATCATGTTTTGATATATTCTTTGACGACTTGTCACAGTCAAGCTTTACACCTTGATCAGCAGGATCAATGGCTGTGCATCGAACATAAAGAAAGAACACAGAGAGAGCCAATAAAGAGTAAACACCAATAGCCACATATTCATAGATGTCATTTCCAAGAAAAGGAGCAAAGAAGGCATAATATGCAATAGATAGCAAGAAAAATACTGTTATAGCCACGACCTGAAAAGTGTGAAAAGGAAGCTCCCATCCATGACGCCTAGCCATGACCGAGTGAAAAGCCTCTCTAAAGAACTACGAAATCGGAAGCATTTTCAGAGAAAAGGAGCATCTGCTCCACCCTCCAAACTTGGTCCCTCACGGTGTAAAGTGGAAACCGATTTGGAATAACAGAAGAAGGAGTTGTATGAAAAATTCTTAGGCTAGGGTTTGCCAAGTCAAATTCAAAGAAAAAGAAAAGAAAAGAAGAGAAGAAAGAGAGAGAGAAGAAGTAAGAGGTTGTTTGGTGATGTGAGAGTGGGAAGAGAAAAG

>C100999_65

TTTTTTTTGTGAAAGGGAATTCGATTCGTAGGATTTTACAGTAACTATGGAGCTCAGAATTACATGAAACTCAAATGACAATCAATCCTTGATGGAATTATGAAACGATGGAACACATAAAAGGAGTATCAACCTTTTCTCCATCAGTAATTACAAATGTAAATGTTGCAATGTTGAGAAAGTGAACATTAACTTCCCATAATAAGCCTAAATGGGTAGTACGCAATCTCATCACTTTCAGGCTTCATGACAGAGGGCAGTTTTGAATCGGGCATAACAAAGATTTTCCTGTAAGTGAAAGAAACGTACATAACTGTTGAACATCATTATTCCACATACAGAACAATCACGACATTGACATTGAGAAATGGCGCCCTTCCAGTCCAAATAAATGCTTGAAAAATCTTAAGCAGACCTCTTCTGCTTGTACCTTTTGGCAGCAACAACGTACACCAACATATTTAAGAAGCTAAGTCCAGCTAAAAGCCAGAAAAAGTAATCGAGATGACCTTTGTTCAAGTTATCTGGAATCCATCCAGGATTTGCACCTCGTGTTGTCAAGTAAGTCACTATAGTAAGAATAAAAGAGCTCAAGTAATTCCCCAGAGAAGTAGTCAGCAGAGACAAAGCACTACATAAACTCCTCATGGCATCTGGAGATTGGTCATAGAAAAACTCAAGTTGCCCCACAAATGTGAATACTTCTGCTGCACCCAATAAGAAATACTGAGGGACTTGCCAAAATATATTAAGGGGTACAGGAACAGGATTATCAACAAGATCAAGCTCTTCTGCAAGCTGCAGACGTACAATCTCCACTATAGCAGCTGCTGACATGCACAGGACTGAAATAAAAAGGCCAATTCCCATTCTTTGCAACTCTGAAAAGCCCCTCTCTTTGCCTGTAAATTTCCTCGCAATGGGAACAATAACCCTGTCATAGACGGGAACCCAGAAAATAACACTTATCACATCAAAACTAGAGAGGGAAGCAGGAGGAATTTTGAAGGAACCAAAATTTGTTTGCATCATCGTCCCTTGTTCCACAAACAATGTTGACATCTGGGCATAAACAGCAGCAAAAACAATCCCAGTAGCCCAAATTGGAAACATGCGAATCAAGATTTTCAATTCCTCCACCTGTGTCACAGTGCAAAGTCTCCACTTGTTAGAATAGTCACCGGTTTTGCCTTCAGAATCAGTCACTACAGCCGCTCTATCAAGACACTTTAGTTCTTCACTATGCTCCAGTTTCCGACTTCCTTTAATTGCAGAGCTCTTATCTGGTGTCTCATATAGGACACTACTATCCTCAGGTACAACCACATTTCGCTTCCGGAAAGATGCTACCACAACCTGGCACATTCTTGTAATTGGGCTCCCCCCTGGTTTTTGAAACCTGTAAAGGGGCGTGCCTAAAAAGAAGCTTCCAATGGCTAATGCCATAAACAAAGTAGGAATGCCAAATCCAAGACCCCAGCCAGCATTTTCTTGAATCCAAACAATAAAAGTGCTGGATACAAGGGCACCTATGTTGATAGAAAAGTAAAACCAGTTGAAAAAGGATCCCTTCTTAATCCTTTCCTGGGGATCAGTATCATCAAACTGATCTGCCCCAAAAGATGACACGCATGGTTTTATACCGCCAGTCCCAAGTGCAATCATGTAGAGACCAAAGAAGAACACAGCGTATTGGGCAGGAGTAGCTGGAGGGCATGAAGTACCCAAACACTCTGCAGGCTTCAATGCAGGAACCGATGCAGAAAGAGTCAATGTCCCCATTCCTATGACATAAATCGTGGAGAAAACAGCAATGGTCCAGTATCGTCCCCAGTAAGCATCAGCCAAAACGGCTCCAATGAGAGGTGTAAGATAGCAAGTGCCTTGCCAGGTAGTGACATTTCTTGCAGCAGAGACATTTCCTTCATGAAGCTTCTGGGTAAGATAGGTAACAAGATTTGTAGCAATGCCATAGTATGCCAATCGTTCGCAGCACTCATTGCCTAAGATAAATGGGCAAGCTTTCCAATTGCCAGTGTTCCGCTTAAGAACAGGCCTCCCTTTAAAGTCAACTGATCCATCTCCAGTGTATTGTTTTCTCTCATCATCCTGAAGAAGAGCCTCTTCAGCGAGCAATATATCATTCTCCGTGGTACCCATAACTCGTTCCTATCGTAGGAAGAGAAGCAAGAGAAGAACGTGGAATTTATTGACAATTAAAAAAAACTATATCTCGGAATGCGCAAGAAACGCCACAATTGTTTTCTTGTAATACTAACAAGTTGCAAATCGATGGAGTCGCTGTCAGAGAAGAGGGAGAGAGAGACGCAAGTTGAAGAAAGACGAAGAGAGTGAAAGGTGTTGTCTTCCCCAATGGAACCCAACGCGGTGAGAAAGCCGAGAGAGGAAGCAACCAAC

>C101003_65

TTTTTTTGTACCACAAAGCATTTTCATTTGTTGTATACCCTTACGGTGGGGTTAAGGATATAATAGGCCATTACTACTCTTTGCAGAAATAGATAATGAACTAATGGAAAGTACAGTTTTATACATGCTAAGATTTAATCACACTTAGCCTAGACTGAGTCCTTAACATTCGAAAATCTTTTGGCAGTCACAGGACCTGCAATTCGATGTCTACGAGGGCAAGAAACAAAAAGAGAACCTAGGTAAAGATACAATCATCATAATTATACAAGGGAACAAAATCATATCAATCGTTATTGACTAAGATGTCGCTCTCAAAACCACTTAACTATCGCTGAGAAGAACCATGTAGATTGCACAGCCTTCAATCTAACGCGTGTTAAAGATTGCAGCCTAGCTAGACATCATGACATGATCAATAGCCTGAAGTGCTTGATTGGAAAAAAATCTTACATCAACATCCGGATCCTCACTGAGCTCAACCAGACTAGGTCGGATGGTCTTTTCCACAACAGATTGATCCACTATGGGGAAGATTGACTCCAAGACCTTTGCCACATTGAACTTAATGTTGGGCACTCTGTCTTTAGATGCAGTAATAACAACAGGCAATAATTTTGAACAAGTGATTTCAGGGCCCATCACTGGAGCAAGCAGAGAGATGGAATGAAGTATGGTCATTCGATACAAATAATGTGGATTGCTAATCATCTCCAGTACCTGTGGAATTATGTGTTGCATAGCCCACTCAGGACCAAATTCTTCAGCTAGGCGCTTCAAGTTGTTAGCTGCTGCCTCACGGATGGAATGAACCTTGTCCTGTAACCACTGCATACAAAGAGCACCAAGCTTGTCATCGAAAAATCCAACACCCAATTGACTTGCCAATAAGGGTATATATTCTATTATTGCAAGTCGAACCCTCCAATGCCTATCCTCAGCAAGCTCAACAATGGCTGGTAACAAAGATTGAGATAACAAATCGATTCCAATGACCTGATTCACTTGATCGAGCTTGCTTATAATATTGAGGCGCACATCAGGAAATTCATCCTTCAAAAGGGAGAGGAAAATAGGAAGAAGCTGTTCAATTGTTGCATCCTTTCCTAACACTGGAGCCATTCCCATTATCACTGATGCCAAAGCAGAGCGAACATGCTGTGAAGAATCCGTAGACAGTTCCTTCACACAAGGAAGAATATGCTGAATTGAAAGATCAGGATTTAAAATCCTGCAAAACTTGGTTACTTTCCCAGCAGCTGCAATCCGTACTTCAGCCTCATTGTCTCGAAGCAACCGCACATAGGCAGGGACCAATTCCGTTCTGGTAGGTTCAGGGCCTACAGCTTCACAAAGCTCATACAATTGATTTGCAACCATGTATCGCACACGCCACGACTTGTCCTGTGAGAAGTTGACAATAACAGGTAGTATATGTGTGACACAATCTTGTGGCTCCAGCAGCTTCCCAAGTGCAGCACAACCCTCCACAGCCAACAATCTAACAGAGTCTTGATCATCCTTAGTGAGATCCTCAAAAATTGACATAGTGTCAGCCTTCAAATGAGTGTATTCAACAGTAGCAGCAAACTTTCCAAGATTTGATGCAGCAGATCTTCTAACCATTGGCATGTCATCTTGACACAACAGATTGTATATCGATCTCAATTCTGTCTTTGACATTTCTGGTGCACTAGGGTAAGCAATATGAAATAGCCCACATGAAGACACTCGAGCAGTGAACCATTCCCCTGCTGCCAACCTCTTCACCAAAGGGATGAAATACTCAACCAAATCACTTTCCCTCATTTGTGATCCAATCCTACAAAGTGATTCCACAGCTTTATCTCTCACACAAGTTTCCTCAACAGTGCAAAGCGTCTCCAGAGGCGGGAGCAACACATGAGCATGTTCCACTCCCCCGACAAAAGGAACAAACACCCCCAACTCTTCGGCCATAGCAAGAAGTACCTCATCATCATCATCATTATTCTCACCCAAAAACGGTATCAACTCCCTACGCGTTCGCTCCTCTCCAAGAGCCCGGGCAATCGTAGATAGCTTCCGAATTGAGTTCAACCGCAGCTGAATGTCATCATTTTTCAGTTCATCTATAAGCACTGCTATGGGATATAGCGGCTCATCAACCATGGACATCTTCTTTTCCTCTCAAAATTTCGTGTTGCGCGGTAATGTGGATATCTTTAGAATTCGAGGTTCACCGAAACAGAACTGAGAAGAAATTGATCGCGGTACTCTGTGGAATCGTTGAACAGAGTCAAATAGCGATTTCTAAAACTTGTCGTTTTGTTCTTGGGGGGTCAATTATGGCCAATGACGAATGAGTGTTGAGAAAGAAAAAATGAAACTCTAAAAACCCTAACCTTCGGAGGAAGCCAAGGGTGAAGGGAGTTTTCTC

>C101007_65

ATGAAGGTGGTGTCACAACAATGCATCTTCCAGAATAGGCTTCTATCACCTCTCTGCTACCCCTTTCATGCAATCAACAAAATAAATTGACCCCATGTCAAAAGGAGTGGTGTTGGTTGTATTTAACTGACACGATTGTACATTAAGTACAGCTGACCAAAGTTCACCCAAACACTTCAAACAACCATGCATTGAAGCTTCCAAGTTCCAATATGAGAGAACAAGGGGTCTTTCATCTTGAGGAACATAGTCAGACAGAGAGGTCCCAGTGGGTGCTTGATTCTCCCAACCCACCCCCTCTTTGGAAGAAGCTATTTAGTTCTATCAAGGAAACCATTCTGCCTCATGGGAACAAGTTTTGCTTTTCATCCAAGAGGAAAACTTCCCATGGACGTGCAGCCTCATGCTTGCAGAATCTCTTCCCAATCATTAGTTGGTTAAGAGATTACAAAGCCTCCAAGTTCAAAGATGACCTCTTGGCTGGCTTAACTCTTGCCAGCCTTAGCATACCTCAGAGCATAGGATATGCCACTTTAGCAAAAGTTGCTCCTGAATATGGCCTGTATACCAGTGTCATTCCGCCTCTTATTTATGCTATGATGGGGAGCTCAAGAGAAATTGCAATAGGACCTGTGGCTGTAGTGTCAATGCTGTTATCTTCACTGGTTCCAAAAGTGGAAGATCCTGTTGCCAATCCTCAGGCCTACAGAAATTTTGTCTTTACCGTGACTTTCTTCACTGGAATTTTCCAAACTGCATTTGGTGTTTTCAGGTTGGGTTTTCTAGTGGATTTTCTTTCACATGCTGCCCTAGTTGGATTCATGGCAGGTGCTGCCGTCATTATAGGTCTTCAGCAGCTCAAAGGTTTGCTGGGGATTAGTCACTTCACCTCCAAAACAGATGCAGTATCAGTGTTGGCATCTGTTTATAAGTCATTACATCATCAAATTTCCTCTGGAGAAAAATGGAATCCTCTGAATTTTGTCCTCGGGTGTTCATTCTTGATTTTCATTCTAACTACCCGTTTTATTGGCAGAAGAAACAGAAAGTTTTTCTGGTTACCTGCTATTTCTCCCCTTCTATCAGTTATACTTTCAACTTTGATTGTATATTTGTCAAGAGCTGATAAACATGGTGTTAATATAATAAAGCATGTCAAAGGAGGTCTGAATCCAAGTTCACTGCACCAGTTGCAACTCCATGGTCCACATGTTGGACAAGCAGCTAAAATTGGTTTGATTTGTGCAGTTATCGCACTCACTGAAGCTATAGCAGTTGGTCGATCTTTTGCTTCCATTAAGGGATACCATCTTGATGGAAACAAAGAAATGTTATCAATGGGATTCATGAACATTGCAGGATCTTTGACTTCATGCTATGTAGCAACGGGTTCTTTCTCAAGGACTGCAGTGAATTTTAGTGCTGGATGTCAAACAGCAGTGTCAAATATTGTGATGGCTGTTACAGTGTTTGTGTCCCTGGAATTGTTTACTAGGCTCTTATACTACACCCCTGTGGCTATCCTTGCCTCTATCATCCTCTCTGCTCTTCCTGGATTAATTGACCTTAGTGAGGCTTGCTATATTTGGAAGGTTGACAAGTTCGACTTTCTTGCCTGTATTGGTGCTTTTCTTGGGGTCTTGTTTGCAACAGTAGAGATTGGCCTTCTTGTTGCAGTGATAATTTCATTTGCAAAGATACTAATTCAATCAATTCGACCTGGAATAGAAGTTCTAGGTAGAGTTCCAAGAACAGAAGCCTTCTGTGATGTTACTCAATATCCCATGGCCATAAGCACTCCAGGCATCATAGTGATTCGCATAAGTTCTGGCTCACTCTGCTTTGCAAATGCTAATTTTGTCAGAGAAAGAATACTCAAGTGGGTCTCGCAAGATGAAGATGACCTTAAAGAAACCACCAAGGGTCGGGTCCAAGCAGTAATTCTGGACATGACAAACCTGATGAATGTTGATACTTCTGGAATTCTAGCACTTGAGGAACTGCACAAGAGGTTGCTTTCCCGTGGAGTAGAATTAGCTATGGTGAACCCAAGGTGGCTAGTAATCCACAAGCTTAAACTTGCACACTTTGTAGACAAGATTGGAAAAGAATGGGTTTTCCTTACTGTTGGAGAAGCTGTAGACGCATGCCTCTCTGCCAAGATTGCAAAAGCTTGATTGATATCAGGTGTCTGTGCACCAATTCTGAACTTCATCTATTGCACAGGGTTTGGGTTTTGTTTGAAATATTAGTGCATGATTGGATATCTTAGCACATAATTTCGGTCATATATTTTGTATTACACAAGCTGAACATTTATATCCAAAAAGGGATCATGCCATGCTATACTGCAATGCAACCAGGCTGGGGGTTCTAAACCACCTTGTTATAATATCTCAATTTTGGACAGGAAGTGATGT

>C101009_65

CTCGAACCAAAGCATATTTCCCTCCCACTGAGTCTTCTGCAATTTGTCATTTGATATCTCAAGCCATTTCAGCAACATGAGTAGCTACACTCGCCAGTTACCAGCTACTCCTAAGATGCAGCATAGGAAGGAGCGACGCATGTTCTCCACTTCTGATGACAGTGCCATGACCAAGCAAGTTCAGGCCACACATGCCCCTGATGGCCGTGAAATTGATGTCAAACCCATTCTACAACTCGTGGATGAAATTCTGGTTCGGTTCATTGCTCGCACTATTGAGGGTCATGAAGTGAAAAGAGACCAAGATGCACTCGAGATGACTGCTGCTCTGGCTGAATTTGACATGCTTGATTCACTGGCTTACATCATAAACAAAATTTCATGCGAGCTATCATGCAAGTGCTCGGGAGGTGGAGATGCACATTCATCAACGATGGTGCTACTGAATTACATGTCTAGCTATGCATGGCATGCGAAAGTTGTGCTAACCTTAGCAGCCTTTGCTGTTATTTTTGGAGAGTTTTGGCTTGTTGCTCAGTTGAGTGCTGAAAATACTCTTGCCAAGTCTGTGGCCATCCTTAAGCAACTGCCTGATGTTGCTGAGAATTTCATGTCCTTGAAACCCCATTTTGAAGCACTCGTTAGGCTCGTGAAGGCTGCAATGGATGTAACCACTTGCATTGTTAAATTCAAGGAGCTTCCACCTGAATACATTTCAGAGGACACACCTCCTATGTCCGTTGCCAGCACTCACATTCCTATTGCTTCCTATTGGGTCATCAGAAGTATTGTGGCTTGTGCGTCACAAATTGCTAGTCTTGTAGGCACGAGAAATGAGTCCATTTCATCAACCACTGAGGCATGGGAATTATCTAGTCTGGCTCACAAAGTTAGTAGTATTTACGAGCATCTCATGAACCAACTGGGTCTTTGCTACCAATATATAGATGACAAGAGGCATATAGAAGCCTTCCATAATCTGATACATCTTTTCGAGACAGTCCATGTAGACAACATGAAAATTCTCCGAGCACTGATATATGCGAAGGATGATTTCCTTCCACTTGTGGATGGAACTACAAAATCAAGGGTTAGTCTCGAGGTGCTAAGGAGGAAACATGTGTTGCTGCTCATATCAGATCTTGATCTTTCACAAGAAGAGATCCTGGTACTTGATAACTTGTACAAAGATGCACGAGCGAGGGGTGACACATACTATGACATGGTATGGATCCCAGTAGTGGACAAAGCGAACTGGAATGATTTGAACAAACAGAAGTTTGATTATTTGCAGTCACTGATGCCATGGTACAGTGTCTGTGATCCTTTCATCATTGAACCATCATCAATTAAGTACATCAAAGAAGTGTGGAATTTCTCAAAGACTGCTATTCTAGTGGCCCTTGATCCACAAGGCAGATTGTCCTCCCTTAATGCTGTCCATATGATATGGATTTGGGGCAATTTGGCCTTCCCCTTCACAAGTGAGAAAGAAGAATCCTTGTGGAAGCAAGAGATTTGGAGCCTCGAGCTGCTTGTTGATGGCATTGATCCTACAGTTTTAGAATGGATGGCAGACGGAAAACTCATATGTCTGTATGGAGGTGAAGACCTGGAGTGGATTGAGAAATTCACAGCAACAGCATTGAGTGTGGCAAAAGCTGGGAAGTTCGAGCTAGAGATGGCGTACGTGGGAAAGAGCAACGCTAAGGAGAGGATGCAGAAGATGATAAAAACCTTCGCCACAAGAAAGTTCAGTTACTTCTGGCCCAACGTGACGTCCATTTGGTTCTTCTGGACCCGTTTGGAGAGCATGCTGTACTCTAAGTTGCAGCATGGAAGAACGGTGGAAAACGACGACATCATGAGCGAAGTGATGACTGTGCTGAGCTTCGACGGCAGTGATCGAGGGTGGGCGATTTTCTGCAGGGGCGGCACCGAAATGGCCAGAGCCAAGGGAGACGGTGCTCTGAAATGCTTGCAGGACTTCGACAAGTGGGGTCCTAAGATTGAAGAAGTTGGTGTGGTCCAAGCAATGAACGATTATCTCAACCAAAACAAAGCTCCACATCACTGCAATCGCTTGATCCTTCCTGGGAGCACTGGTGGCATACCCCAGAAGGTTGTGTGTGCTGAATGTGGACGCCAAATGGAGAAATACTTCATGTATCGTTGTTGTGTTGAGTAAGACTAATAAGAGTAAGTTGTTTAATGTTGTTTTTAGTGTACTACTGTTACACACATTGTTATTGCTTGACTATGTTTAGTGTTGGTTTGAGAGTTTCATGTTTTTAGTGTCATAACATAAGTTGTTTCTTTGTGAGATATGTAGTGACATGTGACATATGACATGGTTGGTGATAAGATAATGTTTATGTGTACTAGCTGATCCTGAGGTCTCAGAAGCACCATCTCTTTGG

>C101013_65

AACTCATCCCCATGATATTATTTGAAATGGTGAAGAGTGCGTATAGAAGTATTTAATAAGTTTATAGCGTTGGTCAGATTCTGATGATAGTAGAAGGGGTTTGACCCACTGTCTTCTGATATCGGAATTGAATGAATGTCCAAGGCTCAAAGAATAAGAAAGGCCATCCTCAAACTCAAATAAGTCCAACTGAGGGGCTTGGTTGGACCAAAATCCACAAAAGAATGGAGCTTAATCTTTGCCAGTCATTGTATTGGCAGTTTCATGAACCCATTAAGCTATTGAAAAATGCTACAAAAATCGATTTGTAAACTCACTAGACAAGTTTCATTCTTGACCTCCAAACAATGTTGTCAATTTCCAAAATTCAAAGGCTACAAGCTACAAGCAGTTCTTGAAAGCGTTTTTAGGGAAGATGCTAGGCTAGAATTGGTAATTGGGTATATCACACAATGTACAAACAGACAACATCTCAATCTGTCCACCGATAATAAGCATAAAAATTGAAGATGCCCACTCCATATAAACACTGTTGAGCAAGCTCCTCTTTAATTTAATGAACTGAAGCTGTGAATGAGACTTTACAGGCACTCCTACACTACACATACAGCAACATAACATTGCCATGACCCTATAGATTATTCATTCTTATACATCGAGCGAACATTCCGGTGCTCTAGTAGACCTTGATTCTTCAATCTTTGTGGTCACCTCTGCCACTACTAGCCTGGTCAAAAGCAATCCTGCAACTAAAGCTGCTGCCATCAACATTGTGAAAACAGCGCTCCAACTCTTTGCAGATATGTAACCTGTTAACAAGGGTCCAACTGCAGCCCCTATAGAACCGGTTCCATCAATGATTGCTGTGACAGTTGCAAGAGCTCGTGAATTACCCTTCAATGACTTGTGTGTTCCCAGGTCAGCCGAAACTGCAGTGGTTATAAGAGCATAAGGCCCGTTCACAAACATGCCAGTGATGAACATCAACGCTCCATTCACAACCAACGAAACGTGCCCGTAACTTCTATAGAAGAAGAGGGCAGGAATTGCACAGTACATGAAACTTGCTGCTGTAATGGCTCTAGCTTCTAAGCGATCAGAAATGTGGCCGGCTAGAATTCCTCCCAGCACTCCTCCAACGTCAAATAAAGTGGATAGTGTTCCTGATGTCCCACTAGATAGATATTTACCATCAATTGCTGTGTGGCTAACATAGAATGGGAGCCAATATAGAAAGGTATATGCAACCAATTTGGCAAAGAACAGACAGAGAGCAAATGGAGCAACCCCAGGGATTCTCCATGCCTCTAAGAACCCTACTGCTTCTTTCTCCTCAACTGGAGTCTCTTGCCTTAACAAAGGCTCAGCAGCTTCTTCTCCACCTTTTATAGGATAACTGATTTCATCTTCATCTCTATCAGCTCCTACAGACTCAGGTGTAACCGGCAATATAAGGAAAACCACCAAACCAATGAAAGCAGTTATTAGTCCTGGCACAACAAAGGACCAACCCCATCCATAGGTCAACATCGCAGAAGCAATCAAAGAACCAACAATGTTCCCAATTGAAGTGTGAGCATTCCAAATTCCCATGATCAACCCTCTCTTGCTCTTTCCAAACCAGTTACCCACAACAGCAACTACAGACGGCCATCCAGTGGATTGAAACAATCCAGCAAGCATTTGAACCACTAAGAAATAGTAGAAGTTGTGGATGTTTCCCCAGTACCCTACTCCAAACAGTGAAGTAAACAAGCCAGTTCCTACCATCCCCACTGTTAAAAAAATCCTCAAATTACACCGATCACCCAAATGTCCAGAAAAATACATCCCAAAAGCATACACCGCAAGAAAAGCAACATCCAATTGACCAAGAAGGGATGTCCCATTAGATCCATTAAAGGGTGCCCAACCATTTCCATGTTTGGAAGACACACTCCTTAATCCATTTGACTCGGTGACATTGGTTGGCCATAACCGAAAGAAGGTCAGGCCTGTATCTGCTGATTGGGGATCAAGGACACTCTTTACAATACTTGTGGTTTTTCTTGTAGCATGATAACTAGTATAAGCTAAAAATGTTACAATCAGAACGATGGCTTGGTAGGTTTTGAAGGAGAGAGGGCCTCCATTTAAGTACTGCAGAACCCGACTTCCAATGAGCTTGCCACCACTTCTCCGAGCCAACACTTCTGGTGCAGAACCCATGTATTATAATAATAACAGTTGCAACAAAGAGGAAGATGTAGAGCTTCCCCTAGTAGAATATAACCTTGAAGCAAAGAGAAGTTGAAGTGATTCAAGTTCACCACCGTAAAAAGAGATTGCGTCTGCTCGTGTGCTTGGTGACGGTTGCTGAAACTTCGCCAAGTAAAAGGAAAACGCCCTTCTTCTCTTCTCTCTTCAGACTCAGACAGAAAC

>C101015_65

GCTTAATAGAATAGTAGATAATCAAATCAGTGTGTTTCCTAAGAACCTTAAAATGTTCCCGTAATCTTTTAAAGAAGACGCAAATACAAAGTCAGATATACAGGGAGGAGACGGTGCATAAGAAAAACACCGTTAAAGTAGCAATCACTCAAATGGGACAACAATGGACTATGATATGAATATATATATCTGGAAGCTACAATGATTACAAATTAGCAAGAAAGAATAAATATAATAAGCTGCAAGAAGGAGCCTCATGTAAATATTGATCGTTAAAGTGATCAAGATGTAGAATTAAGGCTTCTGCGGGTGATTCCTGCTGCAGAAGCTGGACCTTCACATAAAAAATTCAGTTGTTCAAACTGATCTGTATAGAAGTTATCATCGGGGATTGTGTAGGCGAGGTCTGTTGCAGAAAACACGTCCGTTGGCCCTCACAAATGCTGCTATACCGACACTAATGCCCGCCCACATTAGCGAAAAGAAGAAGTGCCGAAAATCATATTGCACTTGCCTCAAGGACATGGTTGCAAAGAGGAATCCAGTCAAAACCAGAGCCAAAGCCAGAGGAAATGATGCTATAGCTTCAAGGCCTCCTTTCTGAGAAGTGGCAGCTGTATCTCGGCTGACAGAAACTATATAATGTCCTTGTAGGAGATCAATTGCATCCTGCTTTGTTCCATCACGAAAGTTGTTGTAATAATATCGTAGAAGAGCATTACAGCCATCTTGTACTATCCCTTGAACTGTGCGGTGCCCAAACCTGACAAAGTCTCCTTTAAGAGCAGGAGTTCCTGAGTATTGAATACTTATATCATCCCCATGATTAGCCCACAAGATCTTAAACCTTTCATCCAGATTAGGATGTGAACTAATAGTTTCTTCAGCACCAAAGACACCAAGCCTTCTAAGCTGATATTCCAACATATTTCGACCTATCATGCTCTGAGTTACATTTGTACGGTCTAGACAATCAATACAATTGGTCCTAACAACTCCAAGTTGTTCTTTCATTTTCTCTCCCTTTTCATTGAGTAGAAGATATCCGTTTCTCTCCATAAAATCTGAAATTTGTTCATAAAGGATTGAGAGACGTTCAAAATGAACGTGCCCGCAGACATGGTGAAAGTCAAAGTGCACATATCTTACATCATTGCTAGCCACATTCTCCACTGTACTACCAAATTTTTCACACAGTCGCCCTTCTCCTCCATGCTTGTTAACAAGATCAATGGCCAATACTGCCCCGTATTTTTTCCTTAAATCTAAGAAATGTCTCTCTAGGACTCGTGGAGCTTCCTCAAGTTTCAACAACTCAAACTTTGGTTTATATGTTAAGTCAACAATTTGCTGCCAGAGGAGTGGAATTGAACCACGGACCTGAACAAACGAAGCAGTATACCCATTAAACTGCATAATCTGTTCTGATTCCACAAAATTGGCAACATAACCATCAGGATCAGCTCCTCTCCTCCACATCCGAGTTCCATTTCTTCTTGTGCATCTCCTAGCAATCAAAGTGACATCAATAATATCTTTCCCAATGGCTGCTTGATAGTGATGAAAGCTTCCTTGGACTACAGGGAGCAAGTACGGTTCAAGCTTGTTATCTATCAGCACTTCCAAGATGTAATTGTTCCATAGGAATCGAGGCTCTGCCTGTCTCCAAAGTGGAAGCAATCTAGACTCATCACCCAAGTCATTTAACCGCTGAGCACTCAGAGTTAAGTTAGTTTCATAAGAGAAGAACAGACCAGAGCTTCTCTCAGCAACATTAAGCAGTGAAGAAAATTCCATCTCTATTTTCTTCTGTTCAGCAGGGGTGTTTCTAACAGAATTATCACATGGAAAAACCTTCAACTTAGAAATTTTGAAAATTGGATGCCCCAAGTAAGATCCAACAGATTCACGCTCAGTTATAACCAGCAAATATGATCCAGCCAATAGCCTTAACATCCCTACAACACCAAAAATTGTATATATCTTAGGAACTCGAAGCGAGCTGCATTCCGGAACCTCATCGATGAGCTTCATGGAGCCATCGACTCGATTTACAGCCAAAGAAGAACCAGAAGATCCATCCGTAGGCTCAATCACATACTGATCCGGAAATTCCCAGAGCCGCATGCGCGTATACAGCTTCTGCACAGAATCTGCCTTCTCCATCATTATTGAAACTCGAACTCACTGGTTCGCAAGATCGAAGAAACGAAGTGACGATTAAGATCCAAATTCGAATTCGCGTTGGGCTCTTAATACGTGAAGGGAGGGACAGCAACGAAAAAGGAGTGATGCTCCAAACGTGCTATATTCCCTTTTTTCTTTTTAAAGCTAACGGCGTGTTTGGAAGCTTCAGAAAGGGGTAGGATTGCGATGGGCCAAATCCTTGTTCAGATTGGTGGTGTCCAAATGCGGCTTTAG

>C101017_65

CCTGGTAAAGGTTTTCATTTGATTTCTCTTAACTTTACAAAACTTTGCACTACAAACTCATCTTCAACCAAGAAAGTATATATACATTTGAAAAAGCAAAGCAAAAAAAGCCCTTTTCAGCTTTGAAGGCTTCTTCTATCATACATACAATATAAATGACTCAATTGTCACATGTTCTTCAATTTCATATACTTTCTTTAGGAAGCTAGAAAACCAAATGAGAAAGAAAAACAAAAGTGAAAGTAAAAGAGCCAAAAATGACTGAAAAATTCACCTGGGGCCTGATAGTTCCATTGCTTGGACCAACAAAGAAGAGTCATCTGTGAGGTCAGAATACCTATCTGATGAAGAGAGTTCATGTGGTTTGTTGCATTTGGCGTTGTCAGCACTTTGAGAAGCTTCCCATTTCTCTGCAAGGCCATCACCTTCTAGCATGCGTACAACTTCAGACATTCTGGGTCTGTGGCCAGGAAGATACTGTGTGCACAAAAGTGCCACTTGAACAATTTCCTCAAGCTCAATCCTGTCATAGTTGTTCATGAGATCCTTGTCCACAAGCAACTCAAGCTTCTTCTCTTGATGGATTTTCCTTACCCAATCAAGCATGGCTCCTTTTTGGTTGGCTGCTTTTCCAAATTCCAGGGCCCTCTGGCCTGTGATCAATTCAAGAAGCAGAATGCCAAAACCAAAGACATCAGTCTTCTCAGAAGATTGCCCTGTGGAAAGATACTCTGGTGCAATATGCCCCACTGTACCCCTCACTGCAGTTGTGACATGTGAATCTTGGTGATCTAAAAGCTTTGCCAACCCAAAATCTCCTACCACTGCCTCACAATAGTCATCAAGCAATATATTTGCAGCCTTCACATCTCTGTGGATTATCTTTGGATCACACTGCTCATGAAGGTATAGCAGTCCCCTTGCTGCTCCTATGGCAATTTGCTTCCTTGTGCCCCAATCCAACACTGGCTTGCCCTTGAGCCTAGAAGCAACACTGCCATTAGACATGTAAGGATAAACCAAAAGCCTTTCTGTTGGTGTCATGCAAAATCCATACAGTTTGAGGAGGTTTCGGTGCACTGCCAAGCTGATCATTTCAACTTCAGTCTGAAATTGTATATCTCCTCCAATGGCATTGCCATCTTTAAGCCTCTTGACTGCCACAAGAGTGCCATCTGGGAGAATTCCCTTGTAGACATGTCCAAAACCACCCTTTCCCAGAATGTTTTTGTTGCTGAAGTTGTTAGTAGCAATCTGGAGTTCCCTTAATTGGAATCTCTTCAAGTTTCCAAGGTAGACTTCTTCATGATGCTGGTCTTTAACATCAAAGAATGCCTGTTGTTTACGCTTGTGCCTCCTCCATAGAACAAGTCCAAGACCAAAAACTATTAAGCAGAGGCATCCAAGACTCAGACCAAAGGCAATGGCCATTTTGTGAGCCTTTATTCTGCCAGATGGTAAAGCATCCTCAGTACTGTTCAAGTTCATTGACATAGGCATAAGTGTCGTCCCATGGCAATTTTTTTCTTTTTCTGTAGCGCAGACTAGGGGGTTTCCTACAATACTGAATGATTTGGCCAAAATTCTAGGTATAGGACCACTCAGATTATTGTAGGACAAGTCAAGAAAAGCAAGCTGCGCCATGTTAGCTAGTGACACTGGGCATTCTCCATCAAAACTGTTATTATTAAGCCTCAAGTACTGGAGGCTTCTCAGGTGACCCAGAGAGGGAGGAATTTCCCCATTGAAGAAGTTGTCAGAGAGATCAAGAGTTTGAAGCTTGGAAAGTCTTCCTATCTCTGAAGGGATTGGTCCAGTTATGTTGTTATTTTGTAGCACCACAGTCTGAAGATTTGTTAGGTTTCCTATGCTTGGAGATAGAATACCAGATAAATTTTGACTGGGAATGCCTAGGCTAATAACAAGATTCTCAGGGGAACATGTGACCATATTCCAGCTGCATGGATCAACTGCATCCGCATCCCAATTATCTAGAATACCATGAGGATCCACAAGAGAAGCTTTTATGCCCATTAAAGCTTGCACTTCAAAGTTTACTCCCTTAGGAGAAAGCAAAGCATTGGAAGAGCTCCAAAACCAGAAGAAAAACACAAAACAGAGAGAAGCTTCTCCTCCTCTTGGCTTGCCCATTCAATTGAAATATCCCAAGAATCAAATAGAAGTACTGGAAACACCTGATGGTAGTAGTACTAAAGGATAAAGATAATGAGGCTATAACAACAGTTCTCAAATGAGTGAGTGTTCTTTCTTATGGAGTGATTTTCAAAATATGTGCAGAATCCCCATTCACCAAAAATCTTGTGTCAGACCCAAGAAAATAAGACAGGCTTTTGATAATATAAAGGGAACTAGCATAGCATGCCTGTCATAGAGCCAGGTTTTGTGAACAAGATAATGTGT

>C101019_65

CTTTTTATAACCGCGTTTGGTATCAGACATATTTGAAAACCGAAAACCTCCTTTGGTGTATGCAACATCAAACACACCCTTAGTAGTTGTTTTCTGCACTCTCACTCTTCTCCGTGGGCGTCAGTCATGTCCGACGGTCATCTCTTCAACAACATCACCCTTGGTGGCCGCGGAGGCACCAATCCAGGTCAGATAAGGATTTATTCTGGAGGAATTATATGGAAGAGACAGGGTGGTGGTAAATTAATTGAAGTTGATAAATCTGACATAACGGGGGTGACATGGATGAAGGTTCCAAGGACTAACCAACTTGGGGTTCAAATAAAAGATGGATTGTACTACAAATTCACAGGATTTCGTGACCAGGATGTTGCAAGTTTGACCAATTTTTTCCAAAACACCTGTGGAATAACAGTAGAGGAGAAACAACTTTCTGTTAGTGGGCGTAATTGGGGAGAAGTTGATCTAAATGGAAATATGCTGGCTTTTATGGTTGGTTCAAAGCAAGCTTTTGAAGTGTCTTTAGCAGATGTTTCTCAAACACAGCTTCAAGGGAAGAATGATGTGATCTTGGAGTTTCATGTTGATGACACAACTGGAGCTAATGAGAAAGATTCACTGATGGAGATGAGTTTCCATATACCAAATTCCAACACCCAGTTTGTTGGTGATGAAAATCGTCCTCCTGCTCAGGTTTTCCGTGACAAAATAATGTCTATGGCTGATGTTGGTGCTGGAGGTGAAGATGCTGTTGTTACATTTGAGGGTATTGCAATCCTTACACCAAGGGGACGATACAGTGTTGAGTTACACATGTCATTCTTGCGGCTTCAGGGACAAGCTAATGATTTCAAAATTCAGTATAGCAGTGTGGTTCGCTTATTTTTACTTCCTAAGTCTAATCAACCACATACCTTTGTTATTATTAGCCTTGATCCCCCTATTCGGAAGGGACAAACTTTGTACCCTCATATTGTGATGCAGTTTGAAACTGATTATGTAGTTCAAAGTGAATTGGCAATAAATGAAGATCTTTATAACACCAAGTATAAGGATAAGTTGGAGCTGTCTTATAAGGGGCTTATTCATGATGTTTTCACCACAATATTACGCGGCTTATCAGGTGCCAAAGTGACTAAGCCTGGTAAATTTAGGAGTTGTCAAGATGGTTATGCAGTGAAATCATCTTTGAAAGCTGAAGATGGAATTCTGTATCCCCTTGAGAAGAGCTTCTTCTTTCTACCTAAACCTCCAACTCTTATTCTTCATGAAGAGATTGACTATGTGGAATTTGAGAGGCATGCTGCTGGTGGCTCCAATATGCATTATTTTGACCTTCTTATCAGACTAAAATCTGAACAGGAGCATCTCTTCCGTAATATTCAGAGAAATGAGTACCACAATTTGTATGAGTTTATCAGTTCGAAGGGCTTGAAAATTATGAATTTAGGAGATGCCCAACCCACTGTTGGTATAAAGAAGGTTCTTGAGAATGATGATGATGATGCTGTTGATCCACATCTTGAGCGTATAAAAAATGAAGCTGGTGGAGATGAAAGTGACGAGGAGGATTCCGATTTTGTTGCTGACAAAGATGATGAAGGTTCTCCTACTGATGATTCTGGGGCAGATGATTCCGATGCTAGTGATGGTGGTGATGAGAAAGAGAAGCCTGCCAAAAAGGAAGCAAAGAAGGACCTGCCTTCTAAGGCGTCTTCTTCAAAAAAGAAATTAAAAGATGATGAGGATGGAAAGAAAAGAAAACAGAAGAAGAAAAAGGACCCAAATGCACCCAAGAGGGCAATGTCCGGTTTCATGTTCTTTTCTAAATTGGAAAGAGAGAATCTAAAGAAAACTAATCCTGGAATTTCATTTACGGATGTTGGAAGAGTACTTGGAGAGAAATGGAAAAAGATGTCAGCGGAAGAGAAGGAACCATACGAGGCAAAAGCACGTGAAGATAAAAAACGCTACAAGGATGAGATCAGTGGCTACAAGAATCCCCAACCCATGAATATTGATTCTGGAAATGAATCTGACAGTGCCTAAGGCTGTTTCAACACTGAAAAAATGCCGTCAGTTAATACATTTCTATAGGTACGGGTTGTCATGAGATGAAAGCCCATGTAAGCCACACTCCTTATATTTCTCGCTGTTGATGGCATCTACTCATGCTCCGTAAATTGACACAAGTTTTTGCTCTTTGAAATGGCTTGATAACTTGTGAATGAGCTCGAGTTTTGTGCTCTGTTGGTTTGCATGGCTTTACCGAAGACAGAGGAAGGATGAGCTTACTGTATTGATTAGGTTCTATGCTTAATTAAATTAGTGGTCTGTAATTGTGATCCAATTTCTTAATATATTGGCTATAAAATGACAGTACCTCTTGGATTTTTGATCCGCAGATGTCGGATTTGTAAC

>C101023_65

GTTCCTTCTTCCTTTCTCATCCACTTCAACTTAATAACCGTTCCTCTCCTTTCTTTCTTCCCTTCCCCTGACTCAACTCAATCATCACAGAGTGATGGTGGCCTTGTCGCTGTTTGCTAAATCCGAATCCAAAGGTGGCAGTAGATGACGAGAAAAGTTGAAGGCAGAGAAGCATCCCAGACGCAAACAAACTCGGACAATGGTTAGAAACATGACACGCAAGTACTGAAAAGTTGAACTATAAGAAGGAGAGAAGAAGAACACAAGCTGTGCTCTCTACCCCCTTTTATCAATTTTTTTTGGACCTTACCGCTACTTTTCTTGTCCTTTATCGCTTCCTCCCTCTCTCCCCTGTCGTATCCACACACTATATATGCTACTTTCTCCTTCCTTTCTTCTTCCTCTCTATCTTTCTCCGCTCTCTCCCGCCGCATGGTGCTCGATTTGAATACCCAACACCGCCGCATACCCCTCTCTTCTTTATTCCCTCGCCCATGTCGCTCAAGCGCCGCCCCGATGATGGCAAGACTCCAGACGACAAGCGGAGGAAGCCGCCTCCTTTCTCCAGTGTGGTGCGGGACGTGATGAGATTGCAATCACTCGGGCATTTGCTGGAGCCAATTCTAGAGCCATTGGTTCGTAAAGTGGTTAAGGAAGAAGTTGAAGCAGCCCTAAAGAGGCATTTAACCAGCATGAAACAGACCTGTGGAAAGGAGTTGCATACTACTGAATTAAGAAATTTGCAGCTGCAGTTTGAAAACAGCATTTCTCTGCCTGTCTTTACTGGAGGTCGGATTGAAGGAGAGGATGGCTCCAACCTAAGAATAAGTTTAGTTGATGGCCTAACTGGGGAGGTTGTCTGTACAGGACCTGAATCGTTGGCTAAGGTGGAAATTGTAGTCCTTGAGGGTGATTTTGAAGAAGAAAGTGATGTTTGGATGCCCGAAGAGTTCAAGAGCAATATTGTGCGAGAGAGGGAAGGAAAAAAGCCCCTTCTTACAGGAGATGTAATTTTATATCTTAAAGATGGTGTTGGTATGGTGGGTGAAATTTCATATACAGATAATTCAAGCTGGACGAGGAGCCGCAGGTTCAGGCTAGGGGCAAGAGTTGTGGACAGCTTTGATGGTATTAGAATAAGAGAAGCAAAGACGGAGTCATTTATTGTCAGAGATCACAGGGGAGAATTGTATAAGAAACACCATCCTCCTAGTCTGTCTGATGAAGTTTGGAGATTGGAAAAGATAGGGAAGGATGGAGCTTTCCACAAGCGTTTGAGTCGGGAAAAGATCCTTACTGTTGGAGAGTTTCTCACACTTCTCAATCTGGATCCAGCAAAGCTGCGCAATATACTGGGCACTGGGATGTCTGCAAAGATGTGGGAAGTAACTGTGGAGCATGCTCGAACTTGTGTACTTGATACAACTAGGCACGTGTACTTCCCTTCTAATTCTCAACAACATGGAGTAGTCTTCAATGCTGTGGGAGAAGTGACTGGACTCCTTTCTGAATGCGAGTATGTCACAGTAGATAAGCTGTCTGAAACCGAAAAGGTTGAGGCTCAGAACACAGTTACTACTGCATTAAGACAAGGGGAGAAATATTCAACTTTCGAAGATGAAGATTCTCTCATGGATGGGTCTTCACATTTAACTAATGTACTTTACTCCCCAAGCTCTCCCAAGACTGAGGGATCAAGTGCCAACAAGCTTTTGGGTCCCCAAAAGCCTGGAGGATTCAATTACCCACAAGCAAGTGCCTCTTCTCCGGATATCATGTCATCTATTTATTCTGTTGGTGGAACTAGTAGCTTGGATGATTATTGCTTGCCTAATTTCGACGGCATGGGCCTTAGATATGATCAGACCCTCAATTTTCCAGTCCAAGTCTCGAACTCTTTGATCTGTGATACTGATTCAATGGCTCATGCTTTTAGTGATGAAGATCATCTACAATTTTTTGATACAGATCTTCAATCTCACGTTCAAGCTGATTTACAGAGTGCTGTTGATAGCTTCATGCTGGCACGTTCTACAGCCAATGGTGGTGCGCAGAGGAGATGGAGAAAGGTATGCAACGTCCTAAAATGGTTCATGGTTAGGAAACGGGGAAACCAAATAAAGGTAAGGTTGTGAAGAACATGAAGGTTCAGTTACTATCACTAATGTACATTGAATGCGCCAAAAAAATTATTCTGCTCCTTTTATAGAGAAAAAGTTATGGTTTCTCTTAGAGATGTAAATACATCTGTGCATTTGTTCTTATATCTTGCCAGATGTCATAGAATTGCTCTATCTTTTTGTTATTAATTTGTAAATACTTGTATTTTACGGTCAGGAGAGGATTTCTGGTTGTTGGCAGATGCATAGTAATTTCAAGATCCAGTTACGTTTATTCTGGATCATATTTTTGTAAGCATGA

>C101025_65

CAATAATTGAATAGGTTATCAGTTTGCAAAACTGTTGATCATCACTAATGTCATGTGTGAGAAAGTTAATTTATGAGGTCAATGATCCTCTCTGTCAGGCGTCAGCGAGAATAATACATGTTGCAGCTACTGAGAAAGCACAAGGGAGAAAAACAGTATCTATGTCAAGCAAAGGACTAATAGCAACATTCCTAACAACTACTATGGCCAAAAGAATCGCCAAACCTCCCTATCGACACAAAATTCGGATGCAATAATTCATCTGATTTTAGTTCATGAATTGACTATGACAATTTTCTCTTTTTAAAGCTGTCTAAATCCCCATCAAGCACGACAAGATCATCTTCGTCATCAATTACTTCCAACTGCTTTTGATGCTTTCCTTCGGCAGCGGCACTTGAAATGTCAGTTTCATCCGGAAGCTTTCGCTTCTTCAAGTTTGAAACTAAACTTATTTCATCATCCTTCTCAGATTCCACAGCTGTTACTAAAGCATCTGAGGTGCTTGCACCATTGCCAACAGATTTGTCTTTATCTTCTGCTGCTGACACTGGTTGAGTCCATCCTGAGAGAACCATCCCATCAGGTTCTTTCTCTTCATCAAACTCCTCTCTGTGCTTGATGTTAATATTGCAAACAAATTCCTGTTGCATATCCTCTACTGTAAGCATTGTTCCACCAGTTACTGGAGAAGGAAGCTCAGCCAGGACCTTTTCAAGGTTGGCCTCATAAATGGCAATCATGTCGTCCTCAACATCACCAGCTTCATAAAGGAGGTTCGAAGCACACATAATAAGAGGAAGGTTCATCCCCAGTTTTGCCTTGACAATTTTTTCGACAAAGTCTTTCAACTTTGAACGGTTGGTGTTTATTTCAAGTGATAAAGGTGTCTCGGAACAAACATAACAAGACTTGTTAGGTTCAAATGGCTCCACCGGCATAAGCAGCATGTTTCTTGATGGATGTTCCAGACAATATGTCATTCTATAATTTTTAATGTCATTTTGCAGCACTTTGATTGCTTCAATGACAATCAACCCAGCAATAACAGCATTTGTAGTCGCAACAGCATGCACAATATTACCAGCAATACCTTTAGCTTCAAAAAGGCTATGCAGAGGGATGCCAAAGGAAGCAGCCCGTATGTTTGCAGCTGCAGTAACAAATTCAACAGCCAACTGATCATCTTTATCAAATCCTAGATTGCCAATCTCCTTTTCCCTTCTCGTAAAAAATAGTCGCAATGCTTCGAGAAATATTCTAGAATTTTCTCTGAGGCTCCAAATATCCTGTGGGTTCTTCATGCCCAAAGATGTCATGGCAGATACTGATAATTCATCATCACATGCATAATTTCTCTCCAAATTTCCATTTTGCTGAGCCAGTTCATCAGACAGAACATCCCTGCTGTATATAGGCTTTGGTCTATTACGATTTTTCCATGTTTCTTCATTAGACAAAGCTAATTCAATGTTATACCCAAATACATGATCGAAAATTTTCCTTCCATATTGATCAATGTCTTCATCTTTCCTACGTTCAAATACATCCTCTACATTTTCAGAGGAGCTAGCACCATCACTTGATCGAACATTCAAGTCATTTTCCTGATTTTTATCCCCAAATAACTTAGCAAAAAGTAGGTCCTTGGCCCACACAACACAGTGAACAAACTTTGATGGGGTACTTGTGATAGTACAGACAGGATATGTCTTGGGGGCTGGTTTAGGCTGACACTCATAGCACTCTGTTCTGCCCTTGACATGTACGGTTACCTGTCCAAGGAACCCAGTAGTTCCACTTTCAACCAAAGGAACATTAGCTGCCAGGCACAAGCGATTCACATGCCGGCGTGCATCTAGATTGTCAAGTCCATTTAGAACAACGTTAAATTGCTTGAAGAAGTCCACATTGAATTCAGAATCTTTGACATTTGCATGGTATGGTGTAATGTTTATGTGGGGCCTAAATTTTAACACAGCATCCCTAGCAACTTTAGCTTTGGATTGCCCAACATGAGATTGTCGAAATAAAAATTGTCTGTTCAGGTTACTGACTTCTATGGTGTCCATGTCAATGATGTGAATGTCAGGGAAGCCGGAGAGAGCCAGAGTCTTGAGAAGCTCGCAGCCAATTCCACCAGCACCAACCATAAGCACTTTTGCATCCTTGACAACGCAGGAAGAAAAAGAGGGAGAGGCCATTGGAAGTGAGAGTGTGGGGAACGAAGAGAAAAAGAAAAAGAAGAAGAAACGTTGAAATTTCACATGAGAGAGGAGATGAAGAGAAAAAAATCCGACGGGGTTTTTAAAATTGAAAAGGGGAAAGTGGTGCGGGGAATCGAACCGTTGACGTGAAGGAAGTGGCAGAATCTAAAAGGGTCGTCAGGGAAAAAGCGCCAAACCCCAAAATGAAGTCCAA

>C101027_65

TGAACAGATACATTTTTTTTACCGCCTGAAAAATGATGTCAGAAGGCCCTACATATGATGCAGCAACAGAAGCAGAATTTGTAAGGTGAAAGAGAAAGCAGCATACAAAAGCCCTATGTAATTCTATTAATACATATCGTACCTCAGAGTCCATTCATTGCATAGATTCCATGTCCTCATGACTTGAATGCTAGCTAGGTAGGTTGAGGAGGAGAGAATTAAACACCGGTCACTCAGAAACTAAAACTTCACGCAATGCAAATGCAATTTCCTAAGCTACAGCACATGATGATGTAGATGGAGATATGTGTGTAGTGTATATATATATATATATTGGTGAGTAGCTACTAGACGGGTTTATTTGTAGGTAAAGAGAAGCGAAGTGAGACAACAAAGGCAAGAGAGAAGTAGATGATGATGATGATTAGAGAGATGGTATAGAGTAAGTGATGAAGAAAGTCAACAAACACTTGCGGCTAGGCTAATGATTATACGTAGAGGAGAGGGGATGGAGTTTATAATACTAGTAATAATAATAAAGCACAAAACACAAAGCTAGATAAAAGGATGATGGATCGATCGATAGATAATTATAATGATAATAATAAATTATTGTACTTTAAGCTTCTGCAGTAGGGGGATTCTTTTTGGCGAGATTGTGCTTGTTGTTGTGCATCCAAACCTTGAGCACTCTTCTCTTAACCCCAATCTCTTGGCAGAACTGTTGCACCACAGACTCCTCTTGCTTCTGGATTTTCCACCCCACCTTCTCAGCAAAGTTGAGCATTTTCTCCTTCTGCTCCTGGGTGAACTTTGTCCTGAACCTTTTCTTCACTAGCTGGGAGGGTCTAGGCTGCACCATTCCTCCACCGTCTTCTTGTTCATCAGATTCTGAAGGGAGGTGACCAATGCTGTAGGGCATTATCATCTGGTGTGGTGGGACACTTCTAGAAGGAAGGATGTTACCTGTAGGGTAGCCAAGAGCCTCGGGAGGGAGGAGGTTCTTGTGAGGGCCTAGAATGAGTCTTCTTCTGTTGAAAGGGTGGTAGTCACATGAAGAGGGCTCACCCTCCACTTCCTTTCTGTGGAAGTTTCTGTGGCAATGGCAGGCTGAGCAGTTGAGTGCCTCTATGGTGCCTTCTTCTCCACTTGGCATGAACTCTCCACACCCATCAGTGGCATTCCCTCCCATGGCCGCTGCATGGTTCTTCAGGCATTCTCTGTACCTAACCATTTTCTTGTACCCATGATCCTCCTGCTGCATAGTAGGTGTGCCGTTTGAAGGTGCCGTGGAGGAGATAATGTGGTTGTGGCTGTGGTGTGGAGGGTCATGGTGAATCATGAGACCATGACCGTGTCCATTACCATGTCCCCCACCATACGTAGTGCTGTTTATTGGGATTGGGATTTCTCCTTCTTGACTCGAAAGTTCCATTCCTTGCTAGCTAGCTGTTTGCTTCTTTGGAGACCAAAGAAAAAAAAAAAAAGAGAGAAATATAGGGATGTGTATAGGGTGTAGTGTTAAAATGATTGCAATTCTAAGTTTCACCGCTCCTAGGAGTTGGCCTCAAACCTAATTATCAACTGGCGTAATAATCTCTCAAAAGCTAACTAGGAACAAATGAAAATGTTTTGAATCAAAAGAGGGTGTGGTTATGATGGGGTAGCAACAAAATCTAAGTTGAAGAGAAGTAAAGGGAGTGAGATGGAGGTGCGGTTAAGAGTGAAAAGTAGCTAGAGAGGGAGAAAATTAGTGAGTGGTGTTGAAAGCAATTGAGTTGAGTAGCAGCACTATGGCACTACCACTGCCGACCTCATTATCTTCAGGTGAATTAAGAAGAGAAAGTAAGAAAGCACAGACTCAGAAGTAGGGCACACAGGATATGGTCATGAGAAGACAACTTTTTAAGCAAACAAGACACTTTCCCACTCTGGGTATTTTCTAATTTCTATCTCTTGAAAAGGAGACACTCACAGAGACAGAGAGAGAGAGAGAGAAAGAGAGAGAAAGGGCGACAGACAAACTAACCGTCTCTATGTCTCGGTGAGAAGATAATCAATGAAGACAGCTACAAACAAACCCTGTAGCTTTCTTCTTCTTTTTTTAATTTGAGCCAGCCACAAAGTCAATTGGGCGAAGAAGAAGAAGAAGAGGAAACTGACATGGATCAAACACCTTGTGTTGCAGACATGGTTGGTTAGAAGGAAAGAAAAGGATGATTTAGTTTAGAGGGGAATCACAGTAAAAGAGAGTAGAATTAGGGTAATAGAAGGAAGCTGAAGGTGAAGGTGAAGGAGAGAGAGAGAGAGAGAGAAGGAAAGAGTGGAGAGTTTTGAAGGTGGGTTTTGGTAGTGAAGCACTAAACAGGAGGTGCTTATAGAGGTGTGTGGGAGGACAAAAAAGGAGAGGTCCAAAAAAAG

>C101035_65

CATCACCATACATGGCATGAATTTAAAGGAACTAAAAATAACATGCACGACCATCCCTTCCTCAGTGAAGATTTGAATGTGTAACACTCTAACATAGAAAGCTGTAGTCAGTAAAATAGAGATTTTGGATAGACATTGCACGAAGACTGAGAAAACTTGCAGCAATATTTTATGTACAAGATTGTTGTCCAAAGATGTACCTCGCACTGAATGAACTCCAACAAAACCATACGAATGAGAATGCTCGTATTCACATCATATATCCAAGTTTCACCAATCAACCAACAAATTACACTCAATCTCACGTAAAAGCAATAAAACAATACTGCCCAATACACCAATTTTCTCGATTTATGTGGGAAAAAACAAAATCTCATCGTAGTAGTAGTAGTAGTGTTACTGAAATTAAGTTGATGCTCCCACATTTGACAAGAATTGAAAGGTTAATCATCCCCCACTTGTGTACTTGAAATGCATCTCCATGGCCTTGCTCGTGTTCCCTTGTCCAGGAGCATCAGCAAGCCAACACTTTCAGGTGGTAGACTAAACATATACTTGCTAGGATGAGCTCTCCTTCTCCAGAAAACAATTGAGGAGACCCACTTAAAAATTGAAAATAATGAAGAGTTGCCAGCAAAACTGGGTTTCCTTGCCATTTTTCTTTGCGGGGACATATCTTTGCACAGATGTTGAGCAATTTCCTTCAGTAAAACCAGTTGTGCTTTATCCGTGCGCATTGAAATGATTGCCTGCCTCATCGATTCCCTATCAGCCTCAAGTGCCTGAAGCCTCATGTAAAGCTTCTTTACGTAGGGATCCTCAAAATCAGTGGCATGATTCCCTGACTCCCTTGGAGTGGTTACATAGTCTTCGAAATCAACAGCTCCAGCTTTGGAATCTGTGAAACCATTATATGGCGCCCCACAATGAACAGAGTCAATTGTATAAATTCTGTCACTCATATCATCAGCTTCTGATACATCATCTACCTTGGAAGGATCCTCTGACTTTGAAAAATTATCTCTTTTATGGTTACTGATGAGCTTGGGAGAATCCATTATACACTCGGGCCCTGCTTTAACCATCCCACCAAAAGATGTTGAGTCACAGGAAAGTCTCCTAGAATGCTTATTCAGTCTTGGAGACTGTCCAACAATCACTTTCTCTAGAACATTTTTTCCTGTAAAATCCCCTTCCATCTGGCTATAAGTGGGAGTTTTCTCCATTTGAGAAATCCTATTTTCCAAGTTCCTCAATCGGTCACTCGGAGTTTCACCAAAAGCATATTTCTCAATATCCGTGTCATCATTATCAGCATCCGTAGCACCGTGCATCACACTACATTTCAAAGGAGGATATTCATAGGTTGGAAGTTCATATTGATCACCCTCAACCTCGTCCTCAGGAAGACCAAAACTCATCAATCTGTGTTTGTAAGCCTGAACTTCACAAGTAAGTGACTGAATGATCTGTTCCCTCTTATACAATAAATCTTCCAACGACAGAAGTTCCTCCTGATCATGTGTCATCTTCTCCTCCGCAAAACGCTTGAATTGGCGTGCTTCCATTTGAACCTCTGCCTTTTCCCTCTGCAGCCTCAATATCATGGACATGGCCTCATTCGCCGCGGTTGACGAAGAATTTCTCTCCTCCTCAAGCTCAGCATTCAAATCTTGAATGGTTTTCTGCTGCATACTGACCATCTCACGCAAGGCCACACATTCATTCTCAATTTCGACTCGTGCAAAGGTGGGCATGGCCAGTTGGCCATCCATTCTGAACTCATCATGCTTCCGTTTAACAGATCGAATCCATGTCCCCGAAGATTGAGCTAATATAGAACAATCACATCCACAATTGCAACATTTCAACAAATCCCTGGAAGATGGGACTAATTCCGAAGCCATAGAACCAAGTGCATAAAAACCCTTGAACCACTTAAAATCACCAACAACGTTTCACCAAATTAAAAAACAAATCAGCTAACAAAAATGCACACTAATTGAAAAAATTTGGTATTTACTATGACATTCCCTCTGTCTTCCCCTCCTTCTTGCCAATCACCTTCTCCATCGGATCAACAAATAAGGCAAAAAAACCTTGAAAGAGTTGCCGTTGACGAATCAACACCAGCAGCGCCGAAGCCACAAGAAGGAGAAAACGAAGCAAAATTTCCCGGAAAATTCACCTTCTCCGGATAAAATTAGCAACCAAAAAAATCGAAAAAAATTAGAGGAAAAAGAAGTGCACCCAACAACCAATCCGGGAACAGCTGAATCCTGGTTTTCTTGGAATGAAATAATGGGGGAGCGGGAACGGTGTTTGGTGGGAAGGAAAAGTCGAAAGACAATAAAACAAAAAACGAAAAACAAAGGGAATTTATTATTCTTCAGAAACCCTAGATATTTTAATTGGTTTTTTT

>C101039_65

ATTAGATTTGATTGGTTGTGCATATCAATTATCAAGTACCTTCTTGTTAATTTATCTTTTGTTTATCTGATTTGTTGCCATTCCACCAATCTGACCTTCCTAGGGAAACAATTTTTTTTTTAATATATTAAGGAGTCATCAAGAGCTGCTCTTGCTTGCCCTTTTGGGTTGCCTATAATCAATCTCTCTGCTCCTTGGTCAAAAGCCTTAGTTGAGTTTCTTTCTCTGTCTTCTCCAAGAGCATCATCCTCCTCATCTGCCTGAAGTTGCACCAGCAAAGACATCCTAGAAGGTTCAACAAGATTTCATCAGCGAGCACTCGTTTTACTATTTCTCAAGACTGGAATTTTGTATGAGCTGAGTGCATAAAAGGAGAACTATGTCGTTGGTTATATCTACAGAGTTGGCTGATACCTCATATAGGAATGCCAAGCTCTACACGCTAAAGGGAACTGATGTTACACCTGATATGTCCTCCCACAATTTTCTTCCGGATAAGCACAGAAACATGTATATCACAGATTCTTACTCTAGTGAGAGTTATGAAAAGTACTTCCGTGATTCATCTTCAGAAGAACTGATAGAACCTTCTAGTTCCAGCATATCAGGAAATTCAGTTCACCCTGATGGTGCTTCTTCTTACCTGCTCAGGGATAGTTCAGGAGCTTCCGTGATTGTTAATAACCCATTTGACACTTCTGTCCTGTCTACAAGGCGTCGTGATTCCTATCAGTCCAATTTTGGATCAGATTTCGTGGAAAATAGGAGCCCTGATGGCATTGACTTTGATGGTGAAATGAGATTAAAACTACAAGAATTGGAGAGGGCTTTGTTTTCTGATGAAGAAGAGGAGGAGGAAGACATATTTGAGACTGTTCAAAGCATGGAGATTGATCCTGACATGGCTGAGTGGGCCAATCCGCTTCAAGATATGCTGCTCCATGACTCACCGAAGGAGTCCTCATCCTCAGATTCTTCCAATATTAGCAGCATAAGTAGCACCACCAAAGAAACTTCACAAAACTCGCCCCAGACCCCCAAACAATTACTGTATGATTGTGCTAGAGCACTTTCAGAGGGAAATGAAGAGGAAGCCACATCTATGATAAACAATCTCAGGAAAATGGTCTCAATTCAGGGTGATCCTTCTCAAAGAATTGCAGCCTACATGGTAGAAGGGCTTGCAGCTCGCGTTGCTTCATCCGGAAAATGCATCTATCAAGCATTGAGATGCAAGGAACCTCCATCTTCAGATCGCTTGGCAGCTATGCAGATACTATTTGAGGTCTGCCCATGTTTTAAATTCGGATTCATCGCTGCAAATGGTGCGATAGCTGAGGCTGTTAGGGATGAAAAGAAGGTTCACATAATAGACTTTGACATCAGCCAAGGAACTCAGTACATAACTCTAATACAAACACTTGCTTCAATGCCAGGTAGGCCACCTCATGTGAGATTAACCGGGGTGGATGATCCTGAGACTGTGCAGCGCTCCATAGGTGGCATGAACATCATAGGACAAAGGCTTGAAAAGCTGGCAGAAGAACTTGGGTTGCCATTCGAGTTTCGAGCAGTGGCTTCAAAGACCTCAATTGTCTCTCCATCAATGCTCAATTGTCGTCCGGGAGAAGCACTTGTGGTGAACTTTGCATTCCAGCTTCACCACATGCGTGATGAAACTGTATCCACAGTGAACGAGAGAGACCAACTTCTTCGCTTGGTTAAGAGCTTGAATCCAAAACTTGTGACAATTGTGGAGCAGGACATGAACACCAACACCTCCCCTTTTCTCCAAAGATTTGTTGAAGCATACAATTACTACTCTGCTGTGTTTGACACACTTGATGCAACTCTACCAAGGGAGAGCCAGGACAGGATGAATGTTGAAAGGCAATGCTTGGCAAAGGACATTGTCAACATTGTGGCTTGTGAGGGCGAGGATAGAATAGAACGGTATGAAGTCGCCGGAAAATGGAGGGCAAGGTTGACAATGGCTGGCTTCACTCCATCCCCAATGAGCACAAATGTGAGAGAAGCAATTAGGAAACTCATTATTAAGCAGTATTGTGACAGGTTCAAGATAAAGGAGGAAATGGGAGCACTTCACTTTGGGTGGGAAGACAAAAACTTAATTGTTGCTTCAGCATGGAAGTAACCAACACCAATTCTTTTGTTTTGTGAGACTCATTGTAGTGTTGATGTAGTATTTGTTTGCTTGTTTAGTCTGTATCTATATGTTTATGCGTTGTTCAAACTCATGTATACTACTAACGTGCAACTTATGCTAATACAAACTTTCATGTCTAACAAGGATGGATGGATGGATGAAGGTCAATGGAGTATATGACACACATTAAACATTATCAATGCTTAGGCTTGTATTCACATCATTTCTTGTTGGCATAATTTTGGTAACTGAACAAG

>C101041_65

ATTCCAGAGAGTAACTGAGCTCCTGATCACTGGAAAATGTCCTGCTTAAAATATTTGCACAGTCAAGGAATTAGAAATCCAAGAACTTACTTTTGCTTCAACAACTACTTCAAAAATAAATGTACAATGCAGCTGTTGACACAGAATGTGCTACGATCCCATAAATACAAAGTGCTCACTAAAGAATTACAGAATGATAACGATCCCTGTATAAATATGACAGATCCTCATCCAAACTCCCGGCATCCATCCGGTCCTTGCGGAAATTTTACTACCTTGATGGATTTTCAGGAAGCACGAGCATTTGAGTAATTAAGTAATCCCAGATGAAAACCCTCTTCATTTTTCCAACACAAACAGGAAAACACTCAGTTCTTCCATGATAAAATGTCTGATCCTATGGTGTAGTTGATACCAATTTTCGATCATTTGATGTAAGATTCTCTTGGGTCCTTGCTACAGCTTTGCCTTCATCTTTTCCCATTTTTCTTCTGCTGCGCCATAGAAGTCGTATGGGTGTTCCAGGAAAACCTGCGTTTGAACGCAGCTGCTTCTCCATATAGCGCCTGTAAGTCTCAGGAAAAAGTTTCGCATCATTGACGAAGAACACAAATGTAGGTGGCCTTATAGCAGCCTGAGTGCAATAGTAAACACGCCCTCTTTTTCCACCGCGCGTCCTAGGAGGAGGCTTAAAAGCTACTGCTTCCAGAACTATTTGATTCAATATAGAAGTGCCAAGTCTTCTTGATCTCTCCTTTTCAACTTCACTAGCTGCAACAATAATCTTGTCAACACTATGACCAGCTATAGCAGTAGAATAAACAATTGGAGCCCAGTCAAGTGAACGAAGCTTCTCCCTGACATCTTGCTCATAGTGTGATGCAGTCTGCTGCTTTTTATTTGGTATTGTATCCCACTTGTTTACAACTATCACACAACCTTTTCCTTCTTTTTCTATCCTTTCAGCTATCTTGTAGTCCTGTTCTGTGATACAAGCCATGGCCTCAATGACTAGGGCAACAACATCAGAACGACGAATAGCACGAAATGCTCGATTCACTGACAAAGCCTCTGTTGTACTCCCAGCTGAAACTATGGCTGTTCTTTTCCTGATTCCAGCAGTATCAATAAGCTGGAACTTCTGTCCATCTGGTCCAGTAAATTCAGTATCAATAGCATCACGAGTAGTGCCACTGATGGGGCTAACTATTGTTCTGTCCTCGCCAACCAGTGCATTCAAAATACTACTTTTGCCAACATTTGGTCTACCAACAATTGAAATTGCCGGAACATAATCTTCTTCTTCATCAAGAATATTTGATTCCTCAACTTTCTGTAAGCCAGAACAAACAAGGTCAAGAAGCTCTCCAGTTCCAGTCCCAGAGATAGCTGATATTGGAAGTGGTTCAAATCCGAGAGACCAAAATTCAGATGCCTGCATAACTCTTTTGCGGGGAGATTCACACTTGTTAACTGCAAGAATGACATATTTATCTGAGTAGTTTTTACGTAGCCAATCAGCAATCTCCTCGTCAGCTGCTGTTAGACCTGCCTGACCATCAACCAGGAAAATAATAACAGATGATTCTTCTACAGCTGCAGTTGCTTGTCTCTCGATCATTGAGGGCATCCTGGCAACAGCTGCTTCTCTAACAGCAAGTGGAATGCCATCCATACCAATGGTAGTAGTAATTGCCAGCTCTTCCATAACAGTAGCTTGTGACTTTGAAACAGTGATAACCCCACCTGTGTCCACTACCATGAATTCATGCTCTCCCCAATACGATCGACCATATAAACGATCCCTTGTAACCCCAGGTTCATCCACCACAATAGCCCTGTTTCCCCCAACAAGACGATTAAATAACGCTGACTTGCCAACATTTGGCCTTCCAACGATTGCAACCCTTGGGAGAAGATTGTCCGGGATAGATTTTGTTCTACGTGCACTCCTCGTGCGGGATTGAGCCGTTTCCTTGCGATCATTTTTCGCCTCGTCTTCAATGCTCAGAACTTGAGACAGAGAGCTTGAATACGCTTTGGCAGCGTCCTTCGCTTCTTGCTCGAGAGCGATAACATCGAGCTCGTCCAATTCCTCCGATTCTTCGTCGTCGTCGACAGTGCCGGAGAATTCGCGGGCGACAGAGCGGAGGACGGAGCGGGATAGGGTCCGGGAAGTGGGCTTTGGGCGAGAAAGGATTGAAGCGGAGAAGGGAAGAGGTTTGTGGAATGGGGAAATGGGGTTTAGGATTTTAGCGAGAGTGTAAGAAATGGGTGTTAGTGTTAGAGTGTGAGCCATGGCATGGATTGGGAAGTAACTATATCTCTCAATTCTCAATTCTCAATTCTCAATTCTCAGGATTCAACATTTGGCGGGGCGGGGTTTATCTCTATTCTCCTTCTCTCCTCCTCATCGCATTTATT

>C101043_65

GTGGGTGTGGGTTTTCAATTTCAAATAGATTTGTGAGAAGGGAAGTGAAAGAAAGGAGGGAAGAAGAAGAAGAATGATTCTGAGAACTCCACCGCCACCGAAGAGGCCACGCGGCGGCGATGTGGACGGTGACGGCCATGGACAACTGGTCATATACGAGGATCCGCCGGAGTCATCTCAGGAACCCTCGGCTTCGGAGCACATGCTATGCACCTATCAGTGTCGCCAGATGGTCAAATCAGAATTTATTGACGCCTTAAGCAAAGCTGAAAATCAAGCTCGTGATTATCAATCAAAATATGAGACATTGGAGGATAATTTCCAAAAAGTTGAATCCGAGAGGAAGAAATTTCTGGACCAATTCTTATATGCAGAACAGGAACTTGCAGCTGCAAAAGGGCGTGAAGAGGCACTCCAAGAACAACTTTTAAAGGAGGTCACCGAGTCTCAAGAACGATTAAGAAAACAAATCCAATTAAACAGCGAACTCCAGGTTAAGCTCCAGAATGAGATAAACCTTCGCAATAAAGCTGAGTCTCATGCTGCTTCAGCAGAGGAGAAAGCAACATCTGTAGAAGGAAAGCTGGGCCATCTCTCAGAAAGTATAGAGAGGGAAAAAAAGCGACTTCATGACGAGCATTCACAACTGAAAAGTGACTCAAAGTTATCTATTTCTAGAATATCTGCAAATCTTGAACAAATGGAATGCAGAGCTAATAATGCTGAGAGAGAAGCAGAGCTGCTAAAAGAACAACTGAAACATCTTAAGGATCAACTTGATGAGTGTTTGCACCAGAAGATTGAAGTTGAAAAAAAAGTATCTAGTTTAATGTTTCAAGAAGTTTCTTCTACAGAGAGTAATGTTTTAGTCAAACATTTGCAACAGGAGCTTCAGAGCTATGAATCTGAAGTAAGGGAAGCACGGAAACTAAGATCAAGTCATGAGAACATTGACCTTTTGAAGGAGAAATTATTGGAGGAAAAGAGTCACCGGGAAAGGGCAGAATCAGAGTTATCCAAGTTACAGGATATCCAGTTAAACATGAAAAAGTTAGAGGATCAAATATCATCATGGAGAACGATTATTACAGACATACCCGGTGTCTCATGTTTTGAGGACTTGCCTGTTAAATTTGCAGCTTTACAGAAAGAAGTCATTTATAGCACACAGAAGGAGGGTGACATAACTGCTCGCTTGAAACAAATGGAGGTGGCTCTGGATGCTGCTGAAATTGGTAAACAAAATGCTGAAGCTGATGCTGCGTTGGCTAAAGAGAAAACAGAAGTATTGATATCAGAAATTAAACGAATTGAATTAACGCTTGCTGTTGTTACTGAGGAAAGGAATAAATTAAGAAATGTTGCCAATTGGAAGAATGTTGAAACTCTAGATGCATCTAAAATTGAACCTGAATCATTTCTTACGAAGAAAGATGATTGCATTAAAGAATTAGAAAGTACCTTGCATGACCAGAGATTGATTAATAATCGTCAATTAGAGGAAATAAAGTTACTTAACGAAAAGTTGCACGGTGAAGCAAGAAGAGTGAAGTCATTGGAGAGGGAGAGTGACCGACTCCGGTCAGAGATTTCATTACTAGAGGCAAAGTTGGGTCATGGTGATTTTTCTGCTGCTAATACAAAAGTTCTGCGAATGGTGAACACACTTACTGTTGATAATGAGGCCAAACAAACCATAGAGGCACTGCAAACCGAGCTGCAAAAAACAAAAGAGAAGTTAAAAGCTGTGGAAGAATTGAAAAGTCAATCAGGAGAAGCTGGAAAGCTGGTAGACAGCTACATATCGGATAAGATGATGAAATTAAAAGAGCAAATTGCAACACTTGAGAAGCGAGAAGAAAGATACAAGACTGTGTTTGCAGACAGAATTTCAGTTTTCCGGAGGGCATGCTGTGAGCTTTTTGGTTACAAGATTGTGATGGATGAACACCAGCGATCGAATGGAATACCGGTGACACGATTTACCTTGCAATCCATTTATGCACAAAGTGATGATGAGAAGCTTGAATTTGAATATGAATCAGGGAATACTAACATTTTGGCAAATCATTATACATCTCTGCCTGAGGTTTCTCATCAGGTGGAGATATTCATTCGGAAGATGAATTCAATTCCTGCTTTCACTGCCAACATAACGGTGGAGTCTTTCAATAAAAGAACCCTGTCTTAAATTCAAAGACCAACTATACTACTGAATTTTTTTTAGCGGGCAACTTACAGTTATGCTGTATATATCTTGCATCTTCAACTTCGGGAATTTGGTCAGCTAGTAAGGATGTTACGACTGGACATATTTTGTATCCTGACATTGGTGATTGATGGCTTGGATTTGTAAAATCTCACATTATTTTTTTAAATAATTGAATAGTGAGGCGTAATTTTTGGGGTGTAGACGTTCTGAGG

>C101045_65

TGCTAAGACGGGATTCCTTTTTCTCTATACTTTTTACATGCTGCAGCGATACTGTACAAACACACGTAATGCAGAATAAAATGCCATACCCTCTCGGGTTTCCTCTGCCATTAATCTAGGGCGGGTAATTTATGAAGGAGAAGCAGTTTGTAGGAACTATTTTAAGTGCAATGATCTCAGCTGCTATATAATTCAACTGATCTTTCTAACAACTCGTGGTTGTTGCATGAAATTTCTTTAACCCTTGATCCGTTTTTGTATATTTTGAATGCTGGGATGGAGCTCACACCTTCTGACTTTGCTAAGTAAGGGTGGTCTTCGATCTCCACCTTGAGGAAATGAACTGATGGGAATCTTTTGCTTGTTTGCTCCAACATAAGTAACACTTTCTTGTGGGTTGCCTTGTTGCTAAAGAGCACCACAGCCATCCCAGGTGAGGTTACATAATGTCTGAACCGATCATTACTCGAGATGAAAACCAAGTTTGAACCAAATTTCAAGTCCTTAATATCTTCACCTCGCAGCATCTTGAGTTGGAGCTGAGCTTCAAACAAAGCCCTTGCCACTTCCTCGTCCCCTGGCCTTTCTCTCAATAGCATTTCATAGTCTTGAATAGCAGCTTCCCACCGTTCCAACTTGGCATTGCAATCTGCCCTCCTTAACCTTGCTTTGCTATAACCTGGCTGAAACATTAGTGCTGCGTTACAATCCTCAATTGCTTTCTCATATTGGCCAAGCTTAGAACGACAAGCAGCTCTGTTGCATAACAGAACTGAGTTGTGAGGATCATGTTCTAGTCCTTCGTTGTACACGGCATATGCTTCGGTGAATTTTGAGGCCTTGAAGAGCAAGTTCCCACTCATTCTAGCTGATGTTGCTGCTCTGGCCCTCCTGACCACTGCATTCACCTCTCTATTGCCTGGATCAACTTTAGCTGCTTGCTGAGCTGCTGTCACAGCTTCCTCAAACCTGCCAGCGGCCAAGTAAACCAATGCCCCTGTCATCAACAGGTAAGCGCTGCGAGCGGGGCCAAATACCTTGGTACACCAATCAAGTGGAAATTTTGGCATATTTTCATAGATGGCATATGCCTCTTGATATCTTAAGAGCTTCAGCAAGGCTTCAGTTTGCAAAGCATAGACCTGCGGAGCTGAATCAGCACCTAAGGATATTGCAGACTGTGTTTCCCTTAGTATAACACTCCATGCATTGACCTTCCGTGCTTCTATGCATTTGTTAAGGTGATTTTTAAGGGCCTGTGTTTGGAAAGCTAGTATTGAATCAGCACATCCAGTTGATTGATTGCAATCCAGTGCTTTTTCTGCTTCTCCCAACCTGAAATATATTGTGGCCAAACGGTTGTGGGCTCTGACATATGAAGGGTCCAGTTTGATAGATTCCTCACACTCAAAAATGGCTTGTTGAAGCCTTCCTAAACCAATTAAAGCTGCACTTTTGTTGCAATGGTACGTTGCCTTATTTGAATCAAGTGCAATTGCTCGGTCATACAAAACCAAAGCCTCTTCAAATCTTCCTTGCTTGTATGCTTCATTTCCCATGGATTTCAACACCTCAGGGTCCACTTTTTTCTGCCTTGGACTCAAAAACTGTTCAACCTCATTGCCAGTGCTGTTCCTCCTCATGATATTCCCCATTAAGCCTTTGCTTGTATTGGAGTAGGAACTAGGCGAAGGGTTCAACTCTGCATTTTTAGGCATGTACTCCACACTCTTTCTCCTCGGGGTGTTATTTACAAGCAAATTCCCAGTGAGCTTCAATTGAGACTTATTGTTCTCATGGCTAGTCCTTATCCTAGCAAGTGCAAGAGAATTTCCAGTGGGGTCTCTATGTGGCTTGTCCTCAAGATTCTTGTCCTTGTTTTGTTGAATCTTAGGGTGAGTTGAAATTGAAGTTCTCGGGGATGATGATGATGTTGAGCTTCTTGCAACAACATCAGAATGCCTATGATGTTTATTGTTTCGATATGTACAAGGTTGGTGTTGGTGAACAGAGGCACTCTTTGTGGCATGTTTCTGTTCTACACCACTCAGTCTGTGTGGCTCAATGTCAGTGCAGCTTTTTTTTGTAACCTTTAATTCTTCATTTGGGGTCTTCTTTGGTTCTTCACTTTTGCCATCATCATCACTCTGTGGATTCTTCAAGGGTAGGGAGTGAACAGATGCTTTTCTCAGCTTGTGGCTTTTGAGGTGGAGAATTCTCCCCATGAGGCCACACCCCAATTCACTCCCCACCTTGTTGTTGTTCATTGCCTTCGTTGCCATGACTTGTTGAAGAGGTTTTTGAAGTGTTACAGAAACAACTATGTGTTGTTTAGATGGAGGGAAAAACAAAACAAAATCAACCAAAGAATAAAGCACAGCAGCTGAAAGATATCTGAGAGATTATGGATGGGAAGTCTTAAAG

>C101051_65

CTAAGATATTTCACTCAGACAGAGCCATGAGGAGGACAAGGTTACTGTAGCATTGCTCTTGTTGTGTACTTGTTAAAAATAATAACAAAGACTGAACAAGAGCTCCATTAGTGTTGTGTGGTCTTGCTTAGCTCCTGCTTGTTTTTCCTTCTGTCTATTTCCCTCAGACAAGATTAAAGGAGTTTGAGTTGAGGCTCAACCTCTCTTCTGTCTTTCACTCCTCATCACAATATTTTCTGGCTCTTTCTGAAGGCTGCTTTTAGCTGTTCAAGCTGTAAATGCCGCTGAGATAGATTTACATGGGGAAGAAGGGAAGTGGTAGTTCGTGGTTAACTGCTGTCAAAAGGGCATTCAGATCTCCAACCAAAGACAGTGATAAAAGGAGTGGCAGAAGAAGAGAAGACTGTGATCAAGAAGAAGACGAAGAAAAGAAAAGGGAAAAGAAAAGATGGATTTTCAGAAAGACTCACATGAGTCATGAAGGAGTGAACAGCAACAACACTCAACAAAAATTGAAGCATGATGTGGCTGCAGCTAGTGGGGGTTCAAGGACAGACCAGGAACAGAAGCATGCACTTGCAGTGGCGGTGGCTACGGCAGAAGCTGCCATGGCTACCGCCCAGGCAGCCGTGGAGGTGGCAAGATTAACCAAACCTGCTAGCCATGCAAGGGAGCATTATGCTGCTGTTGTGATCCAAACTGCTTTCCGAGGGTATCTGGCAAGAAGAGCACTTCGAGCACTAAAGGGGTTGGTGAAGTTGCAAGCTTTGGTTAGGGGTCACAATGTTAGGAAACAGGCAAAGATGACCCTTAGATGCATGCAGGCTTTGGTTAGAGTACAGGCAAGGGTCCTTGATCAGCGCATAAGGTCTTCACTTGACGGTAGCAGAAAATCCACTTTCAGTGACACGGCCAGCGTTTGGGACTCACGGTATCTTCAGGATATTTCAGATAGGAAATCAATATCAATAGAGGGGAGCAGCATTGCAGATGACTGGGATGAACGTCCTCACACAGTTGAAGAAGTGAAGGCAATGTTACAGCAGAGGAAGGAAGCTGCAATGAAGAGGGACAAGACCTTATCCCAAGCCTTTTCTCATCAGATTTGGAGGAATGGAAGGACATCATCAATTGGAAATGAAGATGAGTTGGAAGAGAGACCAAAATGGCTAGATCGGTGGATGGCCACAAAGCCATGGGAGAATAGAGGAAGGGCTTCAACTGATCAAAGAGACCCCATTAAGACTGTGGAGATTGACACATCTCAGCCTTATTCATATCTAGGAACCAATTACAGAAGATCACATCCAAATTATCAATATAACCCAAACCACCATCAACCACAAAGGCATTCCATTGCTTCCCCGCTCCATAGATCTCACCATAATGGATCCCTTCACCAATCCCCTGCAACCCCTTCTCCTGCCAAATCAAGGCCCATCCAAGTCCGGTCAGCGAGTCCTCGATGTGTTAGAGAAGACAGAAATTACCCCACCTCCCAAACACCAAGCTTGAGGTCCAATTATCACTATACAGGAAACTTGTACCAAAATGGAAGGACTGTTGGAACTGGAACTAGTAGTGGTGGTGCTCCATTGCCTAATTACATGGCAGCAACAGAGTCTGCAAAGGCTCGAATCAGGTCACAAAGCGCCCCGAGGCAGAGGCCATCCACACCAGAGAGGGACAGGGTTGGATCAGCAAAGAAAAGGCTTTCTTTTCCTGCTCCTGACCCTTATGGCGTGGGAGTGAGTTATGGAAATTATGGGCATAGTTTAAGGAGTCCAAGCTTCAAGAGTGTGAGTGGATCACATTTTGGGTTGGAGCAGCAGTCAAACTACTCCTCTTGCTGCACTGAGAGTCTTGGTGGTGGTGAGGTTTCTCCTTCTTCAACTGGTGACCTTAGGAGGTGGTTGAGATGATTTGTTGGATACTTTAATGAGAAGAAACATGACTGGACCCATTAGGTCATGGCCATTCACTTCTTTGATTTTATTTATTTGGGCTTTTGTGATGTTAGAGTTAATTTTAGTTTAACCCTTTCTCACCTCAAAAGAAGGTTATTTACCCCGGGTGACCTGAATCTGATGTTAGGAGCAGAGAAGCAAGTACTTTTTCTTGCTAGAAAACTCAGAACAATTTAATGTGTTCGTTTATCATTCGTTTTTCATACTTTTGTTAAGATGCCAAGATAAAAAGTGAGCCGTATGTGGTGGCCTACCTCACTTGGGATGTTGTGTTGATGTTGAAAGAATTGCCTAGACCATCTGAATAGCTTTTAATGGACTGTCCTGTCACAATAGCATGTAATATTTCGTATTTTAGCATTGCCTTCAGTTAGCAAAATGTACAACCCCCATTTCCTTCGTTGTTTTCTTCTAATCATATGGATAAATTAAACATTGCTTGCAAGAGCAAAGCATATAA

>C101053_65

ATAAAAAGGCAACAATACCCCCCACTCTGCCTTGTGCTTTGGGGACTGCAAGTGCAACCTTGCTTGCAGCTCTCAAAGCTGAAAAAATATTTGCTGTATTCTGCTGCATATTACCACCCACTCACTCACTGCCCCAAAAACACATGCTCTTCCACATCCCTATATATCTTTTCAATTTTCATCATCATCATCATCACCAACTCCAACTCCAACTCTAGCTCTCTCCCAAACCTCCCCACCTCAACTTTCTTATATATTCCTCCACTCTCTTTCCTCACTTCTGCTACCTTTCTCAGACTTCACAAGCCACAAAATGATCACCTTAATAGACTTCTACCATGTCATGACTGCAATGGTGCCACTCTATGTGGCTATGATACTAGCCTATGGTTCAGTTAAGTGGTGGAAGATTTTCTCACCTGACCAATGTTCTGGAATCAACCGTTTTGTGGCACTCTTTGCAGTGCCTCTTCTTTCCTTCCACTTCATAGCATCCAACAACCCTTACAAGATGAACCTTAGGTTCCTAGCTGCTGACACCCTTCAGAAGATCATAATACTAGTCCTTCTTGCAGTTTGGAGCAACTTCACCAAAAGGGGTTGCTTGGAGTGGACCATAACCTTGTTTTCCCTCTCCACCCTCCCAAACACTTTGGTCATGGGCATCCCTTTGCTCAAAGGGATGTATGGTGAGTTCTCAGGGAGCCTAATGGTGCAAATTGTGGTCCTTCAGTGCATCATTTGGTACACCTTGATGTTGTTCATGTTCGAGTTCAGGGGTGCCAGAATGTTAATCTCTGAGCAGTTCCCTGACACTGCCGGCTCCATTGTCTCCATCCATGTGGACTCTGATGTCATGTCACTGGATGGAAGGCAACCACTTGAGACTGAAGCTGAGATCAAAGAAGATGGGAAACTCCATGTTACTGTGAGGAAATCCAATGCCTCAAGATCAGACATCTTCTCTAGAAGGTCGCAGGGTCTGTCTTCCACTACTCCTCGCCCTTCTAACCTCACAAATGCAGAGATATACTCTTTGCAATCTTCTAGGAACCCAACACCGAGAGGGTCTAGCTTCAACCATACTGACTTCTACTCAATGATGGCTGCTGGTGGCAGGAACTCCAACTTTGGAGCCTCTGATGTTTATGGCCTTTCAGCTTCAAGAGGGCCAACTCCGAGGCCTTCTAACTACGATGAAGATAGTGGGAAGCCAAAGTTTCATTACCATGCTGGTGGAACTGGACACTACCCTGCACCAAACCCTGGCATGTTCTCTCCCTCGAATGGGTCCAAAAGTGTTGCTGCTGCAAATGCTAATGCTAATGCTAATGCTAATGCCAAGAAGCCTAATGGGCAGGCTCAGCTGAAGCCTGAGGACGGGAATAGGGACCTTCATATGTTTGTTTGGAGTTCAAGCGCTTCACCAGTTTCTGATGTGTTTGGTGGCCATGAGTATGGAGGTCATGATCAGAAAGAAGTCAAATTGACTGTATCTCCAGGGAAAGTGGAGAATCATAGAGACGCCCAGGAGGACTACTTAGAGAAAGATGAGTTTAGCTTTGGGAATAGAGGAATGGATAGGGAGATGAATCAGCATGAGGGTGAGAAAGTTGGAGATGGGAAGCCAAAAACCATGCCTCCAGCTAGTGTGATGACAAGGCTTATATTGATTATGGTGTGGAGAAAACTCATCAGAAACCCCAACACCTACTCCAGTCTAATTGGCCTCACTTGGTCTCTTGTTTCATTCAGGTGGAATGTTGAGATGCCTGCCATAATAGCAAAGTCTATCTCCATATTGTCAGATGCAGGGCTTGGCATGGCTATGTTCAGTCTTGGTCTTTTCATGGCTTTGCAACCGAGGATCATAGCATGTGGAAATTCCATAGCAGCTTTTGCCATGGCTGTGAGATTCCTTACAGGTCCAGCTGTCATGGCAGCTGCTTCCATTGCTGTTGGACTCAAAGGTGTTCTCCTACACGTTGCCATTGTTCAGGCAGCTCTTCCCCAAGGAATTGTCCCATTTGTCTTTGCTAAGGAATATAATGTACATCCTGATATTCTCAGCACAGCTGTCATTTTTGGGATGTTGATTGCGTTGCCCATAACTCTAGTGTACTACATTTTGATGGGGTTGTGAATGAAAGAAATGATGGATGATACAGAAGATTCACGTGTGCCATCATCACCATCATCATCAACATCATCATCCATGCAATGCAAGATCAGTTTCTTGAGGCTTCTTCAGCATCAGAAAACAAAAGGGTCACAAAAAAGCATTAGAAAAACATCACGAGAATTGGATAGGAAGAACCCCAGGATCAGTTTTTTTTTTCTTTTTTCTTTCTTGATTTTGTATCTGTTTTCTTTTTGAATGAACTGTCCTTAGTGAAAATTAATGTAAAATCATGATGTAGCTAATTT

>C101061_65

GAAATCACAACCTTCATGACAACATACAAGCTCAATAATTTACAGGGGTAGAGAGCTCCAGGCTTGTAAAAGATTTAATACCTGTCGAACAAACAAATACAGCGTGACAAACTCCAAAGTACTCCAGTGTTATCATACCCAATTCCCTGTAGAATCTGTTCCTACCTAACACTTTATATTTATCATTACCCACAACCCTTTTTTTTGTGAGGAAAAATCTCAAATTCAAACTATCAAACTATGCATGATCTAGAGTTTCTGCATATAAGTGGTCTGCACATGTGGAGTGCAAAAATAATGTCACATCTACAATTATCTGTTTCGGTATCGTACCACTTAAATTTTCTCTCCTTGTCTCCTAAGATACCAGAATCTGGTACACGTCCAATAAGCATACCCTATATTCAGTGGCAGAATCTAAATGGCATACTACTCAGCAATTTATTGACCAATCGAAGTTCTGGTAAGAGATATGAACTGGTCCATTATCTCCCAAGAGATGTGTCACAAGACCGGCTTTATTTGGATCCAAGTAATTAAGTATCCACTTCAATATTTCTTTCTCTTGTCCAAAATCTCCACTGAACCATTTGAATATCCGAGTTAGATAAACAATTCTCTTCTCCAGGTCCACTTCAATTCCATCATTCTCAAAAAATTCTCTTGCAGCACCTCTTAACTCGTCTACAACTCTATGTGGTGAGAAGAACCTAACTTTTGGGCTGGATTTTGTACCATTACAGAGTCCAAAGTGCAATAACGGATTCAACTTGACCAGAGCAACCTCTAACCGCTTGTCTCCCGTGCTGAAGGGTTTTACTAAGGAATATGGTGACCTCCGATTGCATCTGAGTATACCATTTTTGATACTATTAAGAGAATAAGGATTGCCTCCAACCAGATACTGGAAATCACTGAAGAAGGATCTCCTATCAATTACACCTTCTGGACACCCTAAGCTGATAACAGCATGGATGACCATTGCATTATATAAATTTAAGAAGAATGCAAGTTTCTCGTTTTCTGATAATTCCAAGAGATTCACTCGCTGCAGATCCTGAGTGATATTTACATACCTACGAAATTCTTCGCTTTTGCTAATGGCCTCATAATCAACATGTTGCCTATCATCAGAAGCATAAGACTCAAGAATGGCAGACATAATCTTGGTAAGCCTAGTGCATATTGCAGCTACAGTAGTTGGTTCACTATCATTTGTGGTACCACGAAAATTAAAACATCTCGGTATAAAAGGTTCATGCTCAAGGAAACGATATAAGTGATTTCCTTCTTCAAAATCATTTTCCCCGAAAACATGATGAATAAAGTGCTTCCTGGATAATTGCTGTCCAATTTCAACGGCCTCGTTACGCGCGCAGTGGAAGTGTTGAATCAACGCCTCCACCAACTCGTTTCCTTCAAAGCAGTTCTTAATCATCTTCAATCTCCTCAACCGGTCTTGAATCGGCAATCTCAGTCGCAGAACTCTAACCACTCCAACCATCTCGTCCGCGTGATCCTCCTCCTCGCAATCGAAACCGTACGACGGCGGCCACGGCGCGTTTCCACCAGCCGCCTTTCCGCCGAGCATCTCCGCCACTCTCCGGTCGAACTCTCCGCTGTTCCGCAGCGAGTTCAGCGCCACCAACCCTCCGATCAGCTTCTCGTTGAAGAAAATCTGAGGCACGGAACCGCTTCCGGTCCTCTCGCGCAGTTCCTTCTCCCTCTCGCCGAAAACGTCCACGTTTATTTCCACGAACCTCAGTCCTTTCTCTCTGAAGAACTTCCGCACCGCGGTGCAATCTCTGCAATCGGACCGCGAGAAGAAGCTGATGCGTCCTCTCAACGGTGCCTCTTCCTCCTCCTCCGGCTTCGCCGTCACCACGACCTTCACGCCGGAGAGATTGAACTCCGTCACTTCGCGCTTCTCGCCGTCGCCGTCGTTGTCCTCTTCCATGGACTGTCGAAGCGAGGATACGCGCTTTACGATCGCAGCGGAGAGGTCACTGCTCCGCTGGAGGAGGAAGTTTCCGACGGAGAAGCTCGGCAGAGCAACATCGTTTTGATTTGATTGGAAATTCTGCGGCGGGACTGGCATGGGAAGGAGGGAGTGTGGGTGGATTAACGTTACTCGGCTGTCACTGTGGTCGTCGTCACCGCCATAACTGTCCGTGATTTCAATTTTGTTGTTATGGTTGGTGACTGATTCGTAACCGTTAGCATGATTTTGGGTTGAAGCGAGATTAGGATTTTGTTGGTTATGAGGTTGTTGAATAAGCGTTGGATTCTCGATTTCTGAGCCCATTATGGAGAGAGAATCATAGATGAAGGGTTATGGAGAGGGTGATTTTTGGAGTTAAGGATTTGAAAATAACAATAAATTAATAGGAGATTGATTTGAACAACGAGTTTCGAATACGAGTTTCCCG

>C101063_65

CTTTGCAAAACATTCAGATCCGTTTATCTGATCCCACATTACCGCCATAAGAGATTTCCTGTTAAGCGAATAAAAACCCGAAACCGAGACATAAAAAAGAGAAGAAGAAAATTCAATACAATTTCTTCAACCAATTCAACGCACTGCACCACAAAATGGAGGCTCCGTTAGGTGGATCCGGATCCGGACCCGAGAGGGACAAATCCACCACACCTCCCTCCTCTTCTCCGATCTCCGTCGTCTCCGCCTTCTGGAAAGATTTTGAGTTGGAGAAGGAGAAATCTGTATTGGATGAACAAGGGCTTCGAATTGCGGAGAATCAGGAGAACAGTCAGAAAAATCGACGCAAGCTTGCAGAGAACACTAGAGATTTTAAAAAGGCTTCTCCTGAGGATAAGTTAAGTTTGTTCAATTCCTTGCTTAAGGGCTACCAGGAAGAGGTTGATAATCTTACCAAGAGAGCCAAGTTTGGAGAAAATGCTTTTCTCAACATCTATCAAAAGCTCTATGAAGCTCCCGATCCTTATCCTGCTCTTGCATCGATTGCTGAGCAAGATCTCAAGTTATCTGAGCTTGAATCTGAGAACCGGAAAATGAAAGTTGAGCTTGAGGAGTTCAGAACAGAGGCAACTCACTTGAAAAATCAGCAGGCTACAATAAGAAGACTTGAGGAGAGAAATAGGCAATTAGAGCTACAGATGGAAGAGAAAGTGAAAGAAATTGTGGAGATTAAACAACGCAGTCTGGCAGAAGAGAACCAGAAAACATTGGAAGTTTTAAAAGAAAGGGAACAAGCACTACAAGACCAATTGCAGCATGCCAAAGACAGCGTTTCTAATATGCAGAAATTGCATGAAATTGCACAAAACCAGTTATTTGAACTCCGTGCCCAATCTGAGGAAGAGAGGGCTGCTAAGCAATCAGAGGTTAGTCTTCTGATAAATGAAGTGGAGCGTGCACAAACAATGCTTCTTAGTTTGGAAAGAGAGAAGGGAGTTCTCCGATCTCAGTTACAAATAGCAAATGAAGATACTGACACTAAGAATAGTGATAGCTTTGACTCAACAAGTGCACTTGAGAATTCTTTAAATGCCAAAGAAAAGCAAATCTCTGAATTGAATATGGAACTACACAATATTGAAACAACTTTATCAAATGAAAGAGATGAACACATTAATGATGTCAAGAAACTTACTGCAATGCTCAATGAAAAGGAGGCTGCCCTTGAGGAGATGAAGAAAGAACTTCAAGCAAGGCCAACAGAAAAAACGGTTGATGATTTGCGAAAGAAAGTGAAAATTTTGCAGGCAGTGGGTTATAATTCAATTGAGGCTGAAGACTGGGAAATGGCTACAAGTGGGGAAGAGATGAGCAAGATGGAGTTTCTTCTTCTTGATAAGAACCGTAAAATGGAACATGAACTAACACAGTTGAAGGTCACAATTTCTGAGAAAACATCTTTACTGGAAACAGCTGAACAGAAGATAGCAGAACTTACAACAAAGGTTAATGAACAACAAAAATTGATACAGAAGTTGGAAGATGATATATTGAAGGGATATAGTTCTAATTCAAAAGATCGTAAAGGGAACTTCCTTGATGATTGGGATCTTTCAGAAGCTGGAGGGGGTGACGCTTCTGAGCATACAGACCAAAGGCAAGTATCCTTGGACCAAGATCAAAGCTCAATGCTTAAAGTGATTTGTAATCAAAGAGATCGATTTAGGAACCGCTTGCGGGAGACAGAAGAGGAAATAAGGCAGTTGAAGGAGAAGATTGGGGTATTAACAGTGGAACTGGAAAAGAGTAAAGCTGATAATGTCAAACTATATGGGAAGATTCGTTATGTACAGGATTATAATATTGAGAAAGTGGTTTCGAGGGGTTCTAAAAAGTATGCTGAAGATCTTGAAAGTGGTTTCACGTCAGACGTCGAATCAAAATACAAGAAGATATATGAGGATGATATAAATCCTTTTGCTGCATTCTCGAAAAAGGAACGGGATCAAAGATACAAGGAGTTGGGATTCAGGGACAGAATTACACTCAGCAGTGGACGTTTTCTCCTTGGTAACAAGTATGCCCGAACTTTCGCATTCTTTTATACCATTGGATTGCATATCTTGGTCTTCACCTGTCTCTACCGAATGTCTGCTCTCAGTTATCTTAGCAATGGCCCAGAGGAATTCCTTGTTGGCGAGAAAAACGTTGATCTTCCACGTGGACTGTAGTGGATATGCAACTCAGTTTTCTTTTCCAGTTATCGATTTCTCAATTGAATGGTGCGTGTGGTCGGAATGCAACCTCTTTTCAGTGGTCTTTCTTTGCAGACAGAACGATAACACATTGTCAATATTCAAATATCAGATGTATTGTACATATGACTGTTGTTTTACTCTATACGCTGTTGCAATGAAATATAGAGGAAT

>C101067_65

TATTAAGACAACTGCAACACTGAATATTCCACGCTATTTTGATTTTGATTTGAAGAGAGAACGAGGGAAGTTTTTGAGTCTGATAATGGCAAATGGTAACGCGCAGCAACCATTGAGCTATTGAAAAGTGTTTCTCCGCTCTGCTGTGTTCGAAGCTCAGTTTTCAGATTTCACCACGCAAACCCTAAGGCTCCCAACTTGCCATTGCTTCGCTCGAAAAGATTTTTGACCAAAACAAGCTGTATAATACGTTGTTGGAGTTTGATTAATAGAGTTACGATGGATTTTGACGAGTATGAGTACTTAGAGAAAACAGTTGAAAATGCTGAAGTTAAGAAGGAGAAGGACAGGGCTAATGGTAGGGACAGAAGTGTGAAGCCCGAAGAGAGAGGGCATAGTCGTAGTTCCAAGCGTAAGAGTGGTGAGAAGGATGATGATGATGTTGATCACCATTCCAAGCATTCGAAATCTGGAGATGATTCCCGCCATTACGAGAGGAAGAGAGAAAGAGGCTCCTCTCGTCATGGATTGCAGTCCAGAGATGGTGAGAAGGAGAAGGAGAAGGAGAAGGATCAGCACAGAAGCTCTGGGGGCAGTAGGGATAGGAAGAGGGATAGGGACAGAGACCATGAGGACAAGGATAGGAAGAGAGGTAGGGAGAGAGACAGAGATCGCGATCGTGACCAAGAAAGGGATAGAAGGGACCGAGGAAGTGAGAGTGAACAGGAACACCGCAGCAGAAGTAGGTCCGAAAGGCATAGGAGTGATCTGGATGACAGAGACAAAGAGACAATTCTTGATAAAGACAGAGAAATAAGCCGAGATAAGGATTACAGAGACAGAGAAAGGGTGAGGGAAACAAGGGAACGGGTTAGGGAAAACAGGCGATCCAAAGAGAAAAAGGAAGAAGCAACAGAACCTGAGGCTGATCCTGAAAGAGATCAACGGACTGTGTTTGCCTATCAGATTTCTCTGAAAGCCGATGAAAGAGATGTATATGAATTCTTCTCAAGGGCTGGCAAGGTGCGAGATGTTCGCTTGATTATGGACCGCAATTCAAGGCGTTCTAAGGGTGTTGGGTATATTGAGTTTTATGATGTCATGTCTGTCCCCATGGCAATAGCCCTTTCGGGCCAACCTCTTCTAGGTCAACCAGTGATGGTGAAGCCATCAGAAGCAGAAAAGAATCTGGTTCAGTCCACGACATCTGTTGCTAGTGGACCTACTGGACTTATAGGTCCTTATTCAGGGGGAGCAAGAAGGCTCTATGTTGGAAATCTACATGTCAGTATGAGTGAAGCTGATATTCGTAAGGTGTTTGAAGCATTTGGTCAGGTTGAGATAGTCCAGCTTCCTCTTGATGAAAGTGGGCAATGCAAAGGTTTTGGATTTGTACAGTTTGCACGGCTTGAAGATGCAAGAAATGCTCAGAGCTTGAATGGTCAATTAGAGATCGGTGGTAGAACAATCAAGGTATCCGCTGTTACTGATCAATCTGGAATGCAAGAATTTGGAGGAAATACTGGTGATTTTGATGATGATGAAGGAGGTGGCTTGTCTCTGAATGCATGTTCTCGAGCAATGCTCATGCAGAAATTGGATCGTAGTGGCGCTGCATCAAGCATGGTTGGTTCCCTGGGTAATTCTGTTGTCAACAACACAGGTCTTAATCTACCAGCCGCAGGAAACATACTGGCAGCGGTGCCCATGAGTTCTCCCCTTTCTTCTGTTCCTGCAGTTGTTGGATTGGCAGGAGGAGGTCTACAAATTCCCACGGCTACAATTCCTTCTATTGATACAATTGGTGTTCCTAGTGAATGCTTAATGTTAAAAAACATGTTTGACCCCAAAGACGAGACTGAACCAGATTTTGATTTGGATATTAAAGAAGATGTTGAAGCAGAATGCTCCAAGTTTGGGACGCTAAAACACATATATGTTGATAAGAAGAGTGCTGGTTTTGTCTACTTACGTTTTGATGATACCCAATCTGCAATAAGTGCTCAGCGGTCCTTGCACGGAAGATGGTTTGCTGGGAAGATGATCACTGTGAACTTCATGGTCCCTCAGTCATACGAGGATAAATTCCCGGACAGCAGATAAATTCATGAGTTGTTGCATGATGAGGAATCTCGAGGCCGAAATAATCATCTCAGAATTTTATAACGGCTTTTACTTGACTTGGATTGGCTAAAAGATGGTGTTTGTAAAAGCTGCAGTTATTAGTTGCATAATTTTTTGGGGGTGTTGGTTTTTTATTCAAGAGTTCTATTTTAGTATTTTACTGATGATGTGGCTTGGCAGTGTTATTGATTGGAACAAGGGTTTTGTGATGCTACAAACATTGATGTAATTCAACTTGGCTGATGCCAATCACAGTTTTGATGTATCGGCACTTTCTTTGTGGCCCATTAGCATAAAGGAACCCCCGA

>C101069_65

AAAAAAAGGAAAGATACTGCGAGGTAACACCGCAAGGAAGCAACGGAAGTGTGCATGACAGCCTTTGTGTGATTGTGACCCCGTTCGTTCGTTCCTCTTTCCCTCCGTCACCATCCCCAAACCCCATACGGTGCCGTTTCCCTGTCCTCTTTCCTTCCTCTCGCTTCTTCCCCCTCCGATGCGCTTCTCCATCTCATTCTGCCTTGTCGTTTTCCTCGCGCTTTGCTTTTCCCTGCGATTCTTCAGTCAACGCTACCGTTTCGCCCTTCTAATTGTCGTTTTCTCGATTCATCCGCACAAAGCACCCTTCTCTCACGGTTTCTTTCGTCACTCTCTCGCTCAGTTACTCCCTTTCATTCCGAGTTCTTCCCAGGAATTCAGCCAAACCGCGCGCGCCTGGTGACGCGAAATCGCACACTGTTGTTTCGTTGAAGAAAACACGTTTCAATCGGTGTTTTTTTATGCATGATCAAGTCACTTGCGGGAATTAGGTTACGTGATGTTCCTTTCTCGCTTTGCAAGCAAAGCAACTATTACCGATGGAAGTAGCTGACTTTTCAGCATCCTGATTTTGCTGAAGGAGGGAGTTTGTTTTCAGTCAAATTGTGTTTAAAGGTCCGAGCGACACTCTGCCATTGTTGATGGAGCGGCCTGGGAGCTTAAACGGCAGTCAGCATGTTATAGAAATAGCTGGGAGTGCCGATGCTTCAACATCATCTGCAACACATCATGATAGGCATTTTAATAGCACAGATGCGACACCGCATGAAGAAAGAATTAGCGGTGCAAGAACACCTCTTTCTCAGCCTTCAGTTTCAGTGACTAGTTTATCTAATGGGTCTAATAGTAGGAATTCGTCATTCATGAGACGTGGGGATACTCGTCGGAATAGGAGTCCTGTGCATTCAGGGTTATGGATATCAATTGAGCTGGTTCTCTTAGTGAGCCAGATTGTTGCATCTGTCGTTGTATTGTCTTTGTCAAGGCATGAACATCCACGTACTCCATTGTTTCAATGGATCATTGGTTATGCTTCTGGATGTGTAGCTACCCTCCCTCTGCTTTATTGGCGGTATTATCATCATAACCAAATGAGAGAGCAAGATTCATCTCAATCAAGGCAAACATCTCCTCGGATTAGTGATCCTTCAGGGACACTCCTTTTCAGTTCCAGAACTAATGGTGGAGAAGATGGTCAGGCTGTTTCATCCTCCAGAAGCAATCAAGCTTCTGTGTTGATGAATAGAAGAATGAAGACTCTAGTGGAATACTTCAAAATATCTTTAGATTGCTTTTTTGCTGTATGGTTTGTTGTTGGAAATGTATGGATCTTTGGGGGACATTCGTCTGCTGATGAAGCTCCTAACTTGTACAGGTTATGTATAGTGTTTCTTGCATTTAGCTGTATCGGTTATGCTATGCCCTTCATTCTCTGTTCAACAATTTGCTGTTGTCTCCCTTGTATAATTTCCATCCTTGGGGTTAGAGAGGACATGTCTCAAAACAGAGGGGCAACTTCCGAATCTATTAATGCTTTGCCAACTTACAAGTTTAAGATGAAGAAAAATAAAAGAAATGGTGAAGGCAACTCTGCTGAAGGTGGAGTTGTTGCTGCAGGAACTGAAAAAGAGCGAGTTATATCTGGAGAAGACGCAGTTTGCTGCATCTGCCTGGCAAAGTATGAAAATAATGATGAGCTGAGAGAATTGCCTTGTTCTCATCTTTTCCACAAAGACTGCGTGGATAAATGGTTGAAGATAAATGCATTATGCCCTCTTTGCAAGAATGATGTTGGTGAGAACATAACAGGATTGGTCTCAGGAGAAGATGCCAGCCAACATCGGAGTGAAAGCAGGATTGATAATGGCCTCCCCAATACCTCAGTTTAGTAGAGTTATATACAAATACACAATTGGATCCACCCTCTCATTTTCCTTGAGAACTGGATAAACTCCTCTGGCAAGTTACTAGAGAGAAAGATCTAATCACCTTTGGCAATGGTGGGTAACTAATCACCTATTTATGAAATTGGTGGACCTTGCACAGGCAATGGTGGGTTCCTACACAATAAACTGCATTTCTCTCATAACAAGATGGCTTTCTCAATGAAAAGGAAATAATCTGAAGCCCCTTTTATACATATGCACACTTTTATACTTTTGTAAAGTTTTTCTATGCACTCCACTCTTTGGAAGGATGCTTGATTGCGGTCATCCATTCCATATCATTTTTGTTGATGTACTTTCCTTTTCATGTTGAAACTCTACGTTGGCCGATAACAACGCATCCCCGTCTGTAGTCTTGTGGTGCAACTAATTGTTCAAGGGACGCTCATCATGGCCATAAGCTGTGTGTGGTATTGACGATGGGAATTAAAGTTAATGATTTCATGTAAATGAGTCACAGGAATATATAACGTGAAGTTAGTGCCA

>C101071_65

ATTTGTGACAAAGAAACGTGTTGGATAATATTTCTGAATCTTCGCATGATGTTCCGTCAGCTATGAAAAATTCCTCTGACATTCCTCTCCGATCTCCATAGCTTGTCTTTCCACACTCTGGAAGTAGTGGAAGGTGAGGTCATTGTTGTGACGGAATAAGGGAGGTGGTGCTAGTTGCTGCTTTTGCTTATAATATTGTAGATGGACCGGAGGAGTTGGCTTTGGCGCAGGAAGTCATCTGAGAAAAGTCCAGGTGAAACGGAGAGTTCTGGTTCAATCTCATCTCTTTCAGAAAGGTTCTCTGACGATCAGGTATATACAACACAAGCTGCTTTATCTCCTGAAGTCACGTCTAAGTCTACACCAAATGACGACGTTAGTACCCCCAAAAAGAGTAAAGAAGAAGTTACGGATGTAAAGATTCTTACAGACAAATTAGCTGCTGCTCTGCTAAATATTAGTGCCAAAGAAGACTTGGTAAAGCAGCATGCTAAAGTTGCAGAAGAAGCTGTATCGGGCTGGGAGAAGGCTGAAAATGAAGTGTCATCTTTAAAACAACAGCTTGATGCTACAAGGCAGAAGAACTCAATTCTGGAAGACCGAGTTGGTCATCTTGATGGGGCACTCAAAGAGTGTATGAGGCAACTTCGACAAGCTAGAGAAGTGCAAGAGCAAAAGATTGTTGAGGCTGTTGTGAATAGTTGTCGTGAATGGGAATCAAATAAATCTGAACTTGAGGGGAAAGTTGCTGATCTTGTAGCTCAACTTCAAACAGCCAAAGCAGATGCTGCTTCATCGATTCGTTTTGATCTTCAGCAGAGGCTAGAGGCTGTTCAGAAAGAGAATTCGAATCTTAAACATGAGCTGCAATCTCGACTAGAGGAACTAGAATTGAGGATAGTTGAGAGGGATTTGAGTTCTCAAGCAGCAGAGACAGCTAGCAAGCAACATTTAGAAAGTGTCAAGAAGGTTGCTAAGCTTGAGGCTGAGTGCCGTAGACTGAAAGCCATGACTCGTAAAACATTTTCTGTCAATGATCACAGGTCTGTGACTGCATCTTCAGTTTATGTCGAGTCTTTCACGGATAGCATGTCGGATAGTGGAGAGAGGTTACTAGCAGTTCAAAGTGATATGCGTAAATTAGGTGGATTGGAGATGAATGAGTGTGAGTTAAGTCGTTTTGATTCATGCTCGTCTTCTTTAGTTATGGAAATTGATCAATTTAAGAACGAAAAGACTGATGGAAAAAATCACATGGTCCCTTCAACTGAGATCAATCTCATGGATGATTTCCTTGAGATGGAAAGGCTTGCTGCATTTCCAGAGAATGAAAGTAGAAGCAATTTTGTAAGGGAAGGAGTTGCATCAGATCAATCTAATGTTGGTCAGGCTACAATGGAAGCTGAAGTAGAAGCTTGGATTCAAAAGAATGTTGAATTGGAGAAGAAGCTGAGGAAAATGGAATCTGAAATGGAAGCTATGATTCAAAAGAATTCTGAATTGGAGCAGAAGCTGGAGAAAATGGAAGCAGGGAAAGTTGAGGTAGAGATGGTTTTAACCGAGTACCATACGCAGCTTGAGACTTCAGACAGTCAGATAAGGGAAGCAGAGTTGAAATTAGCAGAGTTTCAAACTCAGTTAGCTCTCGCAAAAAAATCAAATCAAGAAGCATGTGAAGAACTAAAAGCAACAAAAACAAAGAAGGAGATAGTAGAGTCTACACTCAAACATACTCAAACTGAGGTCGAAGAACTGATTTCGAAAATCCATTCTTTAGAGGAAGAGGTTCAGAAGGAACGAGCTTTGTCTGCTCAGAATTCAATCAAATGTGGAAAGTTGAAGGATGAGCTTTTGAAAATAAAACAAGAAGCTCATGTTCAGCAAGACACCGAGATAAAGCATAAGGAAGTTGTTAATCACGATTTGAAGTTAAAACAGGAAAAGGAACTTGCATTGGCTGCTAGTAGATTTGCTGAGTGCCAGAAAACCATTGCATCTCTTGGACAGCAGTTGAAGTCCCTTGCAACTTTGGAAGACTTTCTACTTGATTCAGACAACCCTATGGAGTCAACTTGTGAAGTCACAAAAGGTCCCCAAAATGGTGAGCTGATGAAACTACAATGTAGTGATTTGAACATACCCAAAAGAGATTCTGAGCAGCCCATTTCTTTAAACTCATCACTTACCAACGAGAAAAGCCGTAGTGGCTTTGGTAAGTTCATCCCCAGAAGCAAGAGTGTAAGCAAAAGAGGAAATATCTAAGGAAAGTGGAGGGAAACTAGAAATGATAAGGATAAATTTGAATTGTAAATTTGAGTTATTCATTTTTATCTCATGGATTCAACTATGAATCCAAAGTTACTTGTCTACATAATAGTAATGGTTGAAGTAAAAATGCTGAAAGCAGAAATAGTAATACATATAAAGTCCAA

>C101073_65

CGCGGAGAGATTCAGAAACAGAACGACTCGGTTTTTCTCAGACCGAGTCCGAGTCGGAGGGCGAGCAAGGCAAACTCAGTAACCTTTTCTTCCTTTGCAATTTACGGTTACACCATTTAACCATTTTTCCCGCACAAACAACACTGACACTGACACCCCCTCTTCTCTCACTACACGCGCGAGATAGTTAGAGAGAGAAAGAAAAAGAGAGAGAGACTTCAGTGTCGAGTTGCCCAGACGCAACGTGCATTTCGCATTGGCACAATACGACGGACACCATCACGCCGTTTTCGTGTCACTCTCGTGTCCGGATCCGTCACGGACATCGTTTTCCTCTTTTCCAATCAGTTGGTTGAAAATCCCAAGACACAGAGCTGGAGCCGCTACAAGATCCCAAGCAATTGAAGGCTCCGCCCGCCGCCGCCTCCTCTAACGGTCAGATCCGGTACCGGACGCCGTCCTCCGCCGAGCTCTTTGACGGCCAGGCTAGCAGCAACTCCCCGCCATCGAACGCCGCCGATCAGCACTCGTCGGAGTTCGAGTCCAGCAAAATGCTGAAGCGCATCGGCCGCCAGGAGTCACTCAGCGACAAGATCTACCGGTTCCGAGGGACGCTGCTCGTCGTCGCGATCCCGCTCCTTCTAATTGTGTTCGTCCTCTACGTCATGCCGTCCAGCTCCTCCAACGAGTCCGCCGGAGACTACGCCCTCGTCAACCGCAAAATGTCCCCTGACAAGAAATCCGGAGGCTCCTACGCCGTCATCTTCGACGCCGGCAGCTCAGGCAGCCGCGTCCACGTCTTCCATTTCGATCAAAATCTAGATCTCGTTCACATTGGCAAAGATCTCGAGCTTTTCGACCAGCTTAAACCAGGTTTGAGTGCGTACGCTCAGGATCCGCAAAAGGCAGCGGAATCTTTGGTTCCGCTTTTGGATAAAGCGGAGAGTGTTGTTCCTCGTGAATTCCGATCCAAGACACCGGTTCGTGTTGGGGCAACTGCGGGTTTGAGGACTTTGGAAGGAGATGCATCAGATAGGATCTTGCAAGCGGTAAGGGATTTGCTTAAGGACAAGAGTAGCCTGAGATCTGAGACTGATGCAGTTACAGTGCTGGATGGAACACAAGAAGGTGCTTATCAATGGGTTACGATCAACTATCTATTGGGAAATTTGGGAAAAGATTATTCGAAGACTGTTGGTGTAGTTGATCTTGGAGGTGGATCTGTTCAAATGGCTTATGCCATCTCAGAGACAGACGCTGCTATGGCACCAAAAGTAGCAGATGGAGGGGATCCATATGTCAAGGAGATGTTCCTGAGGGGAAGGAAATACTACCTCTATGTTCACAGTTACCTGCACTATGGTTTACTAGCTGCTCGTGCAGAGATCTTAAAGGTTTCTGATGATGGTGAAAACGCTTGTATCTTATCTGGCTATGATGGATCCTACAAATATGGGGGAAAGTCGTTTAAGGCTTCATCTTCCTCATCAGGAGCAAGCATAAATGAATGCAAAAGTATTGCTCTTAAGGCTCTCAAAGTTAATGAGTCATCATGTAGTCATATGAAGTGTACTTTTGGTGGGATATGGAATGGTGGAGGGGGGGATGGACAGAAGAATCTTTTTGTAGCGTCTTTTTTCTTTGATCGGGCTGCTGAGGCTGGTTTTGCTGATCCAAACTCGCCAGTTGCGATAGTTCGTCCTGCAGATTTTGAGGATGCAGCTAAGCGGGCATGTCAAACAAAACTTGAGAATGCCAAATCGACTTATCCACAAGTTGATGAGGGGAACCTTCCCTATCTTTGCATGGATCTTATATACCAGTATACGCTGCTTGTTGATGGGTTTGGCATATATCCATGGCAAGAAATTACATTGGTGAAGAAAGTTAAATACGATGATGCCCTCGTCGAGGCAGCATGGCCCCTAGGAAGCGCCATTGAAGCTGTATCATCTACGTAATGTGACTGGCTTCATCACTTAGATTACTCATCAGACATCTAGGGAAACATGAGTTTCATCACTGGGATTTTGTGGTGAAATACTGTCATTCATGATGGTCAGAGATTAACTTTCAAGCTGAATTGAGATTGGATATGGATCTGTGATTCAGAAATAATGGGAGATTATAGCTTCATTATTTTTCGTATGTAATTAATTGCCAACATATCTGATTTAAGATTTTGGAAGATTCATTCGATTCATTAAGTGCATCTAAATATGTTGTCTTAAGAGACGAACAAAATGGTGATTCTATAACACCATTTCATTTTTGTTTCCATAAATGGTATCGTTCGTGATCGTATAGTTAAATCCTTTATTTTTTTTCTCATCTTGTTGAGGACCCAGACCTGGCATGTCGCTTGCGGTAGTGTGGGTAATTAACACTACATTGGTATCATGTTACACGTTAAACTAATTCGATTTTGACCA

>C101077_65

CTTCCATTTCCCTTTTGTTCTCTCTCTATTTTTCTCTTCTTCGTGTTCACTGTAGTGCTCAGGAGCACTTCACCATCACATGCCCACCATAGCAAAACCGGATATTTCTCCCAAATCTAAATCTTTTCACTTCTGTTATTCCAAATCCGGAGAATCCAGATCTTGGGTATAGCTGCTGATCTGGAAATAATTGTTTTTTTTTTTGTTTTCCCTTAACATTATTTTCCCGGAAAACGGTTTCCGGGGAAAGAAGGGTGCAATTGGGGTTTGGAGAAGTTGGAAGATGCGGCGAAGAGCGACGGATTTTCGCAGGCCAGTGCGTAGGAAGGTTCCAGATGTTTTGTGGTGGGCATTGTGCTGTGCAGTGATTCTTCTCTTTGTCTATATTCTGAGCAAAGGTAACAAAATCGAGTCCAGACCTGCTTTGTCCAAGAGAACTTACAAGCATGATAAGATTATGGAAGGCCTTAACATTACTGAAGAGATGTTAAACTCTAACTCAGTCACGAGACAACTCAATGATCAGATATCTCTGGCAAAAGCTTTTGTTGTAATTGCAAAAGAAAGTAACAATCTCCAATTTGCTTGGGAATTAAGTGCCCAGATCCACAATTCACAGATGCTCCTCTCAAATGCTGCCACTAGGCGTGTTCCTTTGACAACCAAGGAAACAGAAAGAGCTATCCATGATATGGCATTATTGTTATACCAAGCCCAGCAGCTCCATTATGATAGTGCAACCATGATCATGAGATTCAAAGCAAAAATTCAAGCCCTGGAGGAACAGATGAATTCTGTTACTGAAAAGAGTTCGAAGTATGGACAAATAGCTGCTGAAGAAGTCCCAAAAAGTCTCTACTGTCTTGGTGTTCGGTTGACAACAGAATGGTTCAGAAACTTTAATTTGCAAAAGAAATTAAAGGACAAAAAGCAAATGGAGATGAAGCTCAAGGATAACAATCTTTACCACTTTTGTGTTTTCTCCGACAACATTCTTGCAACTTCTGTTGTGGTCAATTCAACTGCAATGAATTCTAAAAATCCCAATATGATCGTTTTCCACCTTGTCACAGATGAAATAAACTATGCTGCAATGAAGGCATGGTTTGCCATGAACGATTTCCATGGGGTGACTGTAGAAGTTCAGAAGTTTGAAGACTTTACTTGGTTAAATGCTTCATATGTTCCTGTGCTCAAGCAACTTCAAGACTCGGAAATACAGAGCTACTACTTTTCTGGCAACAGTGGTGATGGAAGAACACCTATCAAGTTTCGTAACCCTAAATATCTATCCATGCTTAATCACCTGAGGTTCTACATACCTGAAGTTTTTCCTGCACTGAAGAAGGTGGTGTTCCTGGATGATGATGTTGTGGTTCAGAAGGATCTTTCTCTTCTTTTTTCCATTGATTTGAATGGTAATGTAAATGGAGCTGTTGAAACATGCATGGAGACATTTCACAGATACCATAAATATTTGAATTACTCTCATCCTCTTATTCACTCGCATTTTGACCCTGATGCTTGCGGATGGGCATTTGGGATGAATGTATTTGATTTGGTTGAATGGAGGGAAAAGAACGTAACTGGCATCTACCACTACTGGCAGGAAAAGAACGTAGACCGGACATTGTGGAAACTTGGTACCTTGCCGCCTGGACTGTTGACATTTTATGGATTAACTGAACCCCTGGATCCATCATGGCACGTTTTGGGTTTTGGCTACACCAATGTTGATCCTCAGTTGATAGAAAGGGGAGCTGTGCTACACTTCAATGGGAATTCCAAACCATGGTTGAAGATTGGGATTGAGAAGTACAAGCCCCTATGGGAAAAATATGTTGAATACTCTCATCCTTTATTGCAACAGTGTAATTTCCACTGATTTTGAATTTTGTTTAAATTGATACGTCAGGCCTTGGTGGAAGATTCAGATTCAGAATTACTTGCCTCAGTTGGTGGGGTAGAAGACTATATGATTGGCTGTTTGTGGAACTGGTAGGCTTGGTGATCCTTACTTGATGACCAGATTCATGTCTTAAGTTGTTGTCAGAAATGCTTGTACAATTTTGAAAGGTCAAACATATCTGGTGGCTTTAACTTGGTGTCTTCATTGAATCTGGATCTTCTGTTGTAGCATATACACGTTCTTCTGTGAAATATTAGACTATATCAGTTCAATTTCTAATTGCAGTCAAGGATGAGATCAGTTGCAAACTTTTTATTGTTCACTACCGTTCCTTACAAGACCAGATGTTCTTTCATGTGGCATTCATTTGCTGAAACTTTGTTCTGTTATTCTTGATGCCACTTTTTCAAAAGAAATTACTGTGATTTAGAGTTATCAACTTCTCATATGTACCTAAAAGAAAGAAACTTCTCATGTGTAACAAAACTATCTAGTTTATTTATTTCAGAAGCAGATATTCCAGCA

>C101079_65

ATAAAGTATTCCTATGAGTGCAGAAGTGAGACATTTCCGTACCCTCAATTTTTTCGGAACTCCTATGTTAACAAACGAGGAACAACACACATCATTCCACTTCTGCGTTCCAAACACAACCATAATCTAAATCCTTTCCTCTTTTTTTCAATTTTTTTTGGTTCTCTTCACCGCAGATCCTCTTCTTTCAGGAATTTCCATTGATGAAGATGCATATCTTGATTCCCTAGGGCCCACCATCACCCAATATTTTCATTATTTCATTTTTGCTTTCTTTATCGAACCAAGTTTATAATATAAAGATTCATCATGGTTACAAAAGGGAACCCTGGGGACAATAGAAATAATAGGAGTCCAATATCTATTTTCATCATAGTTGGCTTGTGTGGTTTCTTCTATATATTGGGTTTGTGGCAACGGAGTGGTTTTGGAAAGGGAGATAGCATAGCTGTTGAGATTACTAAGCAAACAGATTGCAGTGTTCTCTCTGATCTAAACTATGAGACGCACCATGATGTTGATGATGGTTCTGACGCACCACTCAAGGAGTTCAAGCCATGTGATGACCGCTACATTGATTACACGCCTTGTCACGATCAAATGCGGGCAATGACATTTCCCCGAGAAAATATGGCTTATAGAGAAAGGCACTGTTTTCCTGATGAAGAAAGATTGCATTGTCTCATTCCTGCCCCCAGGGGATATGCAACGCCATTTCCATGGCCCATGAGTCGTGATTATGTACCATATGCAAATGCGCCTTACAAGAGCTTGACAGTTGAGAAGGCTGTGCAGAATTGGATACAATATGAAGGAAATGTTTTCAGGTTCCCTGGTGGTGGAACACAGTTTCCCAAGGGAGCTGATGCTTACATCGATGAACTTGCATCTGTTATTCCATTGGACAATGGGATGGTTAGAACTGCATTGGATACTGGTTGTGGGGTTGCTAGTTTTGGCGCATACCTGTTTAAGAAAAATGTTGTTACTATGTCAATTGCACCAAGGGACTCTCATGAAGCACAGGTTCAATTTGCTTTGGAAAGAGGTGTTCCAGCAATCATTGGTGTTCTTGGAACAATAATGCTTCCCTTCCCTTCTGGAGCTTTCGATATGGCACATTGTTCTCGCTGCTTGATTCAATGGGGTTCAAATGAAGGAATGTACATGAAGGAAGTCGACCGGGTTCTTAGACCTGGTGGTTATTGGATATTGGCTGGTCCTCCTATCAACTGGAAAAATAACTATGGTGCATGGCAACGCCCTGAATATGAGCTTGAGGAGGAGCAACGGAAGATTGAGGATTATGCTAGACTTCTATGCTGGGAAAAGAAATATGAGAAGGGTGAAATTGCGATCTGGAGAAAAAAATTACAAAGTGGTGTTTGCACTGAACAAGATACTCAACCTACAATGTGTGAAGCCACCAATTCTGATGATGTCTGGTACAAGAAAATGGAAAATTGTGTAACTCCTAGTAAACCTACTGGTTCATGGAAGCCATTCCAAGAGAGACTCAATGCTGTTCCTTCCAGAATTACTAGTGGGTCTGTTCCTGGTGTATCTGTGGAAACATTTGAAGAGGACAACCGATTATGGAAGAAACATGTGAATGCTTACAAACGGATCAACAAAATCATTAGCTCGGGGAGGTATCGCAACATAATGGACATGAATGCTGGTCTGGGAAGTTTTGCTGCTGCACTGGAATCTCCCAAATTATGGGTTATGAATGTTGTGCCCACAACTGCTGAGAAAGCTAACCTGGGTGTTATATTTGAGCGTGGATTGATTGGCATTTATCACGACTGGTGTGAAGCCTTCTCCACATATCCTAGGACCTATGACCTTATCCATGCAAATGGTGTTTTCAGCCTGTACAAGAATGATGGATGTAGTGCAGAAGACATTCTGTTGGAGATGGACCGTATATTACGGCCTGAAGGAGCAGTCATATTCCGAGATCAAGCAGATGTGCTGATGAAGGTGAAAAGAATTGTTAAAGGAATGAGATGGAATACCAAAATGGTGGATCATGAGGATGGTCCACTGGTTTCAGAGAAGGTGTTGTTTGCTGTCAAGCAGTACTGGGTTGCAGGTGACAACACCACGAACTTGGAGTGATGGCCTAAGCAGTATTGAAATGGAAGAACAGTTCCAAAATGACAGTGGATAAAGTGATTCTAGTTGTGATTTGGGTTCAGGATATTTCCAGCTGAGTAAGATGCTTGGGGCCGGCATGAAATTATCTGTGTCTTAACATCGGTGGTATAGATTTTTCTTGTTTCTTTTGTAAAATTGTAGAGTTAATAGTTGTCCTATTTGTTATTGTTCATAAATCATAGTCATCAGATTTGTTTATTTGGCTTCTCCTGAGTCACATTTTTTACTTCTGAAACAATCAAACAACAAAAGATTCTTAAGATTATCAAGC

>C101081_65

AAAGTAAAAAGAACAAAAGTAATGGAAGGACGTAGCCATGCCCCATTTGTTTGTTTGTAGCTTAAACGGTTAAGAAACATTTTCTCTATACATATGCTAATTATAACATATATCAAATACAATTTTGTATGTTAATTAAGTAAAGTAATTTGAAAATCCAGTTGCAACACTTTTATTTGTGGGTGATGGATTTAATGGAGTGGAGAATTAAGACCATCTTGAGAAGATAGAGAATGGAGGGGTTGATGCCTCTGGGAAGGGAAAGATGGCATCTTCCTGAAAGAGGGTCTTTTTTCAAGCTGTTGGATGGTTCGCTTGGCATAAAGTGTCTGAAATAGTGGTGATGCTCTCTCCTTAAACTTGAGAACAGTGAAGAACAGAAGTCTGTAAACTATGAGAATCACAAATAGAGCTGCTAAGTCCCACCACTTTGAATGGTTTAATTCAATGCCTAACATGTGTGTGATCACATATTTCCCAGTCAATTTTGGGTCACCAGGTATCAGAGGATCAAACTCAAGCCCAAGCAAGTCATTCTTGTAGGATCCCTGTATTGCCCATGAACCATAACTGATATATGAAATTGGGTAGCGCCAAACCGGCTTTGGAAGATCAGACAGCAACCTGAAGAATCCGGAGGTCATCATCATGATTCCTATTATTCCAGCCCCTGTAATTATTCCCATGAGGAAGTTTGGAACAAGTGAAGCCACAACCATCATGAGGCTCTCTATTACGGAAATGCAGCTGTAGATATTGATAGTGAAAAACACAAAGTGACTGATCCCTGGCCTGAATTTCACCATGTTATAGGTGATGGTGCTACTAGTAAGAGCAATAGCAACCAAGAAAGGGAAAGAAGAGAGGAAGTTGGCTAAAATATATGCTGCAACTCCATAGTATCCATTAAGTCTTTCTCGATAAAATACCTTCATTTCTTCAATGAATGATGGAAAGCCCCCAATTGACATGAATGTCATAAATCCTGATATGAATGCACCGCAGGCACCACGGGCCAGGATGGAAGTGTAGCTATAACCAATATCAAAATAAACTGTTCCCACACATATGGATACAATGATGTAAATAATGATCCTCAACCAGTAGTACCCCACATCTCGACACATGTTCACAAATGATCTCTGTGTCAAAGTTGAGAGTTGCTTCCACCAAGTAGCTTGACTTCCTTGTTGAGTCGGAGGTTGAAGCCCTTCATCTGTAGATAGTTCTTGAATTCTGTTCTTTGCCCTTCTGGCATATGTTGAACGCCTATATTTCTCAACTAGCATTGCCTTAATCTCTGCTGTAGCCAAGTTCATGAAAGGATCTGCTGAATTTGGAACATCATGAATTCTCTGAGATCCCTTCAGTGTAGCTGTTACAATGTCGAAATCAGAATTGATGCATCGTAGGAAGTGATCAGAAGGATTTCTCTTACGTGGACAAGGGAAACCAGCTTCAGCAAAAAACTCAATTGCCGATTTTGCTTCTCCAAAGTAAACTGTTTCACCGCCAGATAGCAAGAAAAGGTCATCAAAGAGTGCAAAGACTTCACTGCTGGGCTGGTGGATGGAAGAAATAACAGTTCTTCCATCACGAGTAACATTTCTGAGAGTTTGAACAACAAAAAATGCTGAGGCACTGTCAAGGCCACTGGTGGGTTCATCCAGGAACAGTAAGCGAGGCATTGTAAGGATCTCAAGTGCAATGCTAAGCCTCTTCTTCTCCCCTCCACTTATACCTCTCAAGTGCCAGTTACCAATCAACCTATCAGCGCAATCTTGAAGACCCATTTCTATGATTGTTCCATCCACGATGCTGTTAACTTCTTCTTTGCTCATTGATGTTGGAAGACGTAGATGGGCTGAGTAGGATATGGTTTCCTTCACAGTAAGGGTTCCCAGTAGCACATCCTCTTGAGTTACGTAAGCAACACCGCCGTAACCTAGACCTTTTTTCTTCCCATTTAGAAGAACATTTCCTGTCATAACCACATTTTTTGAGAGTCTGCCTGCAAGTGCATCAAGAAGGGTAGATTTTCCAGAGCCAGAAGGACCCATAATAGCCATGATTCTGCCAGGCTCAGCATAGCCATTGAGGCCATTAAGCAACCTCTTGGTTGGTCCCTTGCCAAAGTTTGGTAGCACGACTCTGAGATCTTGCCAAGCCAAGAAGCTTCCTCTGTCAAAGCCAGTGTATGGAAGATCCCTCTCAGTCTCAGTAGCAGCACCCCAGTTGTTGAAGGCCTTGTTGCTATCGGTGGCTGCTGCCTCAATCTCCATGTTGATATGCAGCTAACTAAGGAAGTTTGACCTAAAGATGATGGAACTTAACATAAAAATAAAGTTAAGGAGATAGGTGGTGTAGGAAGGAAAAGAAAAAGAAAACATTGATAGAGTGATCCGCAACAAATACCAAACAACTAACTATTAAT

>C101085_65

ATCCCTTTACTATAATTTATCAAGGGAGCTGGTAAAAAATACATATCTGATATCTCTTTATGCTTGACAAACAGTATTGGCTGATAACTTTACCCAGTCAAAGTAGCTTCGACGTCTCCTCCGTCTAGATGATTTTTGAGGATGTTCCTAAGATACTCTACTTGCTCAAATGATCCTTGAAGTTCTTCCCACTCATGATGGGATACTGATGCGACTTTCCAACCAGCAGCAGTGATGTATCGGCGTTTTAACATGGTATGTCCTAATGGAACACCAGTATTTCTTGAGAAATGAGTTGGACCATCAATCTCCAGAGCAAGCTTCTTATCAACAAGCACAGCATCCAAAGTGTAACCATCCACAACATATTCTTTGACCCATTCAAGGCCAGTGCTTATGAGTAGGCGTCCAACCTCTTTTTGAAACGATGAAGTTATTTTCTGATTAAACCTTTTAGTTTTTCCAGCAAGAGCAACTTTATCCTCAAACTCACCACATAATGACAACTGAAGATATGGGAATTCAAGCTTCAAGCACTGGTTTACAAGATGAACTTGAGAAGCAAACATGATATCCTCCCTATAAAACTCAGAAACCCTTTCCTCCCCATAATGGCTAAGAGTTTTCCAAACATGAGAAAAGAAACTCCTATCCATTTGTCTAAAGACGGCATAGGACCAAGCTATAGTCCCAAGCTGGTCCCTAGTTAAAGTTAGTACAGGAGAGCCAAAAGATCCATTTGAGGCACCACTTCTATCAACACTAATTTGTTCGTGATTATTTGACGTCTTTCCACCAGTGCAACTTCTCAATTGGCAACGATCTTTGAAGACTGAATCTAGAGAATCAAATACAGGGTCAGCAGGCTCATATAGAGAAGCAAATGCCCACAAAAGCTGAGCTAGCTCTTGTTCTTGAAAAGTGTGAATTATGTCTGAAGCCCTTTTTGACAACTCTGAGAAAAGATCCGGAGCAGAATGCTGCATTGCAGCAAAGGCACCTGCTATATTAGCTATGTTTTGGGAGTTGAATTCCCCAACCTTGGTCAGTGCAACCTCTGCAATTCTATCCATTTCTGAAAGATAAAGCAGTTCACCTCCAATTTTGGACAGTGCCCAAGATATGTTAGAGATCCCTTGAGCAGAGCATTCGGGTAAGGCTGTCATCGCTACACTCACAAGCATGGACATCTCTTTTTGTCGAGCAAAAGCCAGTCTTCGAGTTCTCATCATAGAAATCTTCTCCATATTCTTAGCAATGCGATGAAGAGCAGTAGCAATGTTCAAGGGGGACAGGGGAGATGGGCTTAGGCCTTTTGCAACTGCAACAATTGTTTCATAAGTTACCTCTAACACATCATCTGCAGTTTGTGCTTCTGTGATTGCTTTGTTCAAGTTCACTTCTTTTCTATGATCGGATGGCCCAGAGAATTGAGAAAGCCTATTGACAAATTCTTCCATCGTTTTTTCCTTCTTTTCTATAAACACTCCGTTGGAAAACATACTCACTCTCCTTTTCAAATCATCAACATCATTCAAAGGTTGCATAGGCTGTGCAAAGTCTTCCTCCAAACTAAAGTCAAGATCTTCTATTTCCTCAATTTTCTTAACTGTTTTCTTCTTGCTTTCCAACTTCTTCTTGTCCTTCTTCTTTTTCTTCTTAACAGTAACCAAATTTTCCATCAGATGAGCCATGCCCCTGGCTTCAATTGATTTGAGCGCATTCTTTCTCGCCCTCACACACCAATCCATTTCATCAGTTTCTTCAAGCAATTTTGACCTCTGCTCTTTTTCCCTTTTCTTGGGAGCCCGGTAACCGAATGGATCAAGCTCCCCAAGGAACTCCGATTCCCAGTCCACATTAGATTCCTCTCCTATATCATCATTGTCCACTGCATCAACACTAGCCCCTTGTGAGCTTTTGCTAGTGTCTCTACCTAATTGTGTGCAGTTACTTCTCAGGATGCAGGATTCAGGTCTTCGATTTAAATTCTCAGTTCTCGTCGTGACCACTGGGAAAGTATAACCAAGTCTTGGAGTGAAACCAAAGGGTTTCAAGCAACTTTGGTTAAACAAAGAATGCAACAACCCTTCCATACCTTACCTGAAGAAAACAAATTCAACCTAATAGGTTTGACCTCAATAAAAAAGAAAAAGATCTCTCTTAGTACCCTACTTATTAATGCAATCTCACAGAAGGGATTGTTCCTGCAAGAAGCTTGGTTTTTGAGGCATAAAACAGAACAGCGTTGCGGGAGAGAAGAGAAGGAGTAGAAACTTCAAGTTGGACTCTCTTTGTCAATTGACGGCTGGAGTGTGAAGGATATCGAATCCCAGGCGGTTCTGGTTTGGAAGAAGCAGATTCTGTTCGAAGAAGTGTGTTGGGTTCTCAGATAGAATTGCTGTTTGGATGGAAGTGATGAATGGAAAACTGAAG

>C101091_65

TTTTTTTTTTTGGAGAACATCAACATCATATTTTTCATATTTGTAAGAATCATTACACATGATATGATAAAATTACAATAAACTTCTGATAAAATTGGGCAAAAATGAAGGAATGGGCATTGAACAAGAACCCATAACTTAAATCTGGGTTTAAGGGAAGGTCATTACTGATAAGCACAGGTTGGCAACCCAAGATGTTTTCCAATCCTTTGTGGCTATAAGTCTTTTATCCCCAATTTGAATTTTTAACTCTTCTGATCTGGGTGGAATGGATATGGGCGTATAAATGCAGAATTCATTTGCCAAACTTTTAGTGAGGCTGTCACAGTGGCCTCAGTAGCATTGTTGAACAAGAACAATCTAGCAGCTCCATAGATTGCCTTTGTTGGATAAACCCGAGATGTCACACACGTTCTTCCACCTTGAGCAAAGCTTTCAACAATAGAATGGTCCACCAGCATCCTTAAAGAAAACTTTTCGTCTTTAAGTACTGGAACAGCGCTCCCAAAGATTTGCTTGCGAACATCATTTGCCTGAGAAGACCTTGATTGATCAGAGCAGAAGAAAGTCTTAAGATTTCCATTGCTTCCTTTAACTACATAGAAATACACAGGAGTATACTCAGAAAGCCCCTCATCTGCCAAAACCAGAAGACCAAAAGGTCCTAAGGCACCACGTTGTGCAGCTCCACCACTGGTGCTGCAAGTGAACTCCTCGTTGGATGGAGCTGTTTTCTCTAGGGATTCAGTGTCTATCTCAAACTCCGCAACAACGTCCAACTGTGTGGCTGTTTCAATATCTAGTGACACCACTGACCCTGGTTTAGCCTCCAAATTTTTTAATTCATCACTTCTCAATCTCAAACTTTCCACCTCTGCAACAGGCATCTGAAGCAAGTTGCTGCCAGTCTTCTTATCAAGTCTCACAGTTCTTGGAATACTCTGAACTGAAGCCCAGCCTTTAGCCACATCTGCATATTCACTGTCAGACTCTCCAATCCAACCCCACAAAATTCTTCTACCCTTATTCTGATCATAAAATGTCTTGGATGCGTAGAATATTCCATAGTCATACCTCAATCCAATACCAACATCATTATTAACATCATCCGGTGTGAACAAAACTTTGTTCTCGTCATAAGTCCCTATGGTATAGTAATCATGTCTGTCATCATCCAGGCTCACCTTCATGACATGCTTCACCTCAGCCCCATTAATAGAGGTATCCAATCCATTTTCAGTTTTCGTGGACACAGGGAAGAAGTCCACACACTCCCACATACCAGTTCCAGGGACAGCACGAAGCAACCCCTTCTTAAGCTCATAGGTCTTGAAGTCCTCGGTGTCATAAACCAAGGCAATGCCAGTTTTGTTAAGCTTGGACCCTATGGTGATGCGCCACTTTCCTTCAGAGGTGAGCCATGCAGTTGTAGGGTCACGAAAGTCCTTAGCACCAATGCCGGGTGGTGGGACCAAAACTGGGTTCCCAGGGTGTTTGATCCAATCCACAAGGAGAGGGTCAGAAGGGTCAGCAGGGTATGCAAGGTTTTGAACTTGCACTGACTCGTTGGTGGAACCTGTGTATAACATGATGATTTCACCATTAGGTAAGATGGTGGCTGAACCTGTCCACACACCTTTCTTGTCATACCATTGATCAGCCACCATTGCCAATGGAAGGTGAAGCCAATGGATCATGTCCCTTGACACCGCGTGTCCCCAAACTATGTCACCCCAAATTGCACCGTTCGGATTGTACTGGTAGAAAAAGTGATACCATCCCTTGTAGTACAAAGGACCATTAGGATCGTTCATCCAGTTTTTCTCTGGCTGGAAATGAAAAGAAGTTCTCTGCCAGGACAACATGCTATTGTCCCAAGGAAATGCTTCTGCAGATCCTCCTTCACCAGCGAATAACATGCCCGATGACTTCTCCGACACCCCGGAAGGAACACCCCTTGAAACAGGGTACCATTTGGGAGAAGGCACCGATGTGGGTCTGTGTTGTTCTTGTTCATGGTTGGATGTTGGCGTTGAGACTTGAGCATGAGGCACGTTGGAGGCTCTGTACCCTCCAAAGGCGACAAGGGAAGAGAGCAAGAGCAAGCCGCAAACGACCAGAAGAAGATCTTTGCGAGTGTTTATAGCCGCGTGAGAGGAAGTGGGTAACAACGGTTTGTGATGATCCATGGTGAGAAAGAGATCAAGAAAAGAAAGAAAGAAAGAAAAGAAAGAAAGAGAGAATAGGAAATTGGGTATGTTATGTTATGAGTTATTGGCATATACAGCATGCAAGGATTGGAAGTGTGCAGAAGCTAGCAGGTGACCTGTGAAGTGAATAAGGAGAGTTTGTGGGGAAGATGAGAGGATTTTGCCCACATTGGCACACTTTATAGGACTCGGATCCAGAATGTGAAGAAAACCAATAAAGACAGATT

>C101093_65

CACAGACTCCTTATCCTCCTCACTCTATCTTTCTTTCTCTTTCTCTCTCTCACTCTCTCTTTCTCTCTCTCTCTCTCTCTCTGTGTCTCTGGCAACTTCAACAACTACACAACACAACCTCGCTCTTCCAATGGCGTCTCAGATCTAAACGCTGCTCTGCTCTTAGTATTCGTATTCGTGATATTTTTATTGTGAATCCTCTTCGCTTGGAAAACGACAGCGTTTTGGGTTCTATTCCATTGGACATGCGGCCCAGAGTAGCGTACTCGTTCCCTGAGGAGGTGCTGGAGCACGTGTTCTCCTTCATCGAGTGTGACAAGGACCGTGGCTCGATCTCCCTCGTCTGCAAGTCATGGTACGAGATCGAGCGCTGGTGCCGCCGGAGAGTCTTCGTCGGAAACTGCTACGCCGTCTCTCCGGCCACCGTCGTCAAGCGCTTCCCCAAAGTGAGATCCATCACCATCAAGGGCAAGCCGCACTTCGCGGACTTCAATTTGGTCCCCGAGGGTTGGGGCGCTTACGTCGGCCCTTGGATCAAGGCCATGGCCGCTGCCTACCCCTGGCTCCAGGAGATCAGGCTCAAGAGGATGGTCGTCACCGACGAATGCTTGGAGCTTATCGCGAAATCGTTCAAGAACTTCGGAGTCCTGGTGCTCACCTCTTGCGAGGGTTTCACCACTGATGGACTTGCCGCCATTGCCGCCCATTGCAGGAATTTGAGGGAGTTGGAGTTGCGGGAGAGTGAAGTGGATGACATTTGTGGGCACTGGCTTAGCCATTTCCCCGATTCATACACATCCTTGGTTTCCCTTAACATTTCTTGCTTAGGTAATGAGGTGAATTTGTCTGCCCTAGAGCGTCTGGTTAGTAGGTGTCCCAATCTGCAGACTCTCCGCCTCAACCGTGCTGTGCCCTTAGACAGGCTGGCCAACCTTCTTCGTGGAGCTCCCCAGTTGGTTGAGTTAGGCACAGGAGCCTACACGACTGACATGCGGCCGGAGGTCTTCTCGAACCTGACCGAAGCATTTTCTGGCTGCAGGCAATTGAAAAGCTTGTCTGGATTTTGGGACGTGCTTCCCTCCTACCTTCCTGCTGTTTATCCTATCTGCTCCAACCTGACATCACTAAACTTGAGTTATGCAACTATTCAAAGCAGTGAGCTTATCAAGCTTGTTAGTCAGTGTGAGAATTTACAACGGTTATGGGTGCTGGATTACATAGAAGATGCTGGCCTTGAAGTGATTGCTGCATCGTGTAAGGATCTGAGGGAGTTGAGGGTGTTTCCGTCTGACCCATTTGGGTTAGAACCAAATGTAGCGTTGACAGAGCAGGGCCTTGTTTCTGTGTCTGCAGGCTGCTCCAAACTTCAGTCAGTTCTATATTTTTGTCGTCAAATGACTAATGCTGCCTTGCATACAATTGCCAGGAACAGGCCTAACATGACTCGTTTTAGACTCTGTATTATTGAGCCTCAAATTCCAGACTATCTTACACATGATCCTCTGGATTCTGGTTTTGGAGCTATTGTAGAGCATTGCAAGGATCTTCGGCGCCTTTCCCTTTCTGGGCTTCTTACTGACCGTGTATTTGAGTATATTGGGACTTATGGTAAGAAGCTTGAGATGCTTTCTGTGGCTTTTGCTGGAGAGAGCGATTTGGGACTCCATCATGTGCTGTCTGGTTGTGACAACCTTAGGAAGCTGGAGATCAGGGACTGCCCCTTTGGTGACAAAGCCCTTTTAGCAAATGCTGAAAAGCTGGAGACAATGCGATCCCTTTGGATGTCCGCTTGCTTTGTGAGTTATGGAGCTTGTAAACTGCTGGGCCAGAAATTGCCGATGCTTAATGTTGAAGTCATTGATGAAAGAGGACCTCCAGATTCAAGGCCGGATAGTACTCCTGTGGAGAAGCTATACATATACAGGACTGTTTCAGGGCCAAGATTGGACATGCCAGGCTATGTATGGAGAATGGAAGATGATTCTGCATTAAGAATTTCTTGAGTGATTATGCTGTGGAACGAGCCATGGCATATAATGACAATTTGATATAAGAGTTGTTGATGCAGGTACATGCCTAGAATGCTACTCAGTAATGAATTAAAACTTTTTACCGGCCTATGCTGTTGCTGTTGTGGTTGGTGTTTGAGCTAGGTACAGGAAGGAGGGTATATAAAAAGGCTTGAGCTCGATAGCCATTCATTTTACTCTTTGGCTGAGCAACGGGATACCAATTCTGATAAAGAGATCGGAAACCTTGTTGTTATATGTGGCATAGGATAGTTTTATTATTTTTCCTATAATTATAATATTTTATGGACCTGTGCATAATTTTATATGCATGTTACCCCGCACTTTCTTAGTTAAATATCGGTGGACTTGTTATCATCTATAAAATTATGCGGTGGGAAGGAATAAAATTCTTAATTATATTCTAA

>C101095_65

CAGAAAAGCATGAAATTGCACAAAAGCTTGTAATGATATGCATTATTTATGCAAACACTCCGAATCACCACACCCTCTTTTTCTTTCCCTTCTCTTCCCATACCATGCTATGACAGGTGTTTGCTTTGTTCCTGATTTAGGCATCATAGCCACAAAGAGAGAGAGAAAGAAGGCATGAGAAAATGAATGATCTCCATCATCATTCCTTCCCCATGTGATGTGCCACCCCCCTCTCTAGGGTTTTCAATTTCGATTTCAATCAACCAACCCCAAACCTCAAAGCTCTCACCTTTCCCATCCATTTGCACAATTCCCTGTGCCCTTTTCCTTTTCTCCCAAAAGGGTCTCCGGGGCTGCGCCTCTGTTTGACCGTTCCGGGCTCGCCTCCCCAGGCCTCTAGAGAGAGAGAGAGAAATAACTGCTTTTCTAGAGAGAGAAAGTGACCATGCCAATGTAGAGTGGAGCTTCCCTTGGTTTTTTGTTCTTGTTTTTGCTGTTGGTGGTGACCGAGCAATGTTGGAAATGGAGAAGGATTTTGATTCCAAGCTGAAGGTTCAAGGGAATTCCTCATCTTCCAATGGTGGAGGCAATGTCCAGAGATCCAAGAGCTTCGCTTTTAGGGCTCCTCAAGAGAATTACACTATTCAGGATTTTGAATTGGGCAAGATCTATGGTGTTGGCTCTTATTCTAAGGTTGTGAGGGCAAAGAAGAAGGACACAGGAACTGTGTATGCATTGAAGATCATGGATAAAAAATTTATTACGAAAGAGAACAAAACAGCTTATGTGAAATTGGAACGCATTGTGCTAGATCAGTTGGATCATCCAGGCATTGTGCGGTTATATTTTACATTTCAAGACTCATTTTCTCTATACATGGCACTTGAATCCTGTGAAGGTGGAGAACTTTTTGATCAAATAACCAGGAAAGGTCGTCTAACTGAGGACGAGGCAAGGTTCTATGCAGCTGAAGTTGTTGATGCTCTTGAATACATACATAATTTGGGAGTGATACACCGTGATATTAAGCCAGAGAACTTATTACTTACAGCTGAAGGACATATTAAAATAGCCGATTTTGGGAGTGTGAAGCCTATGCAGGATAGTCAAATCACTGTCCTTCCAAATGCAACGTCAGATGACAAAGCCTGCACTTTTGTGGGAACAGCTGCTTATGTTCCTCCAGAGGTTCTTAATTCCTCTCCGGCAACTTTTGGAAATGACCTATGGGCACTTGGCTGCACATTATACCAAATGCTTTCAGGAACTTCCCCTTTTAAAGACGCAAGTGAATGGCTTATTTTTCAAAGAATTATAGCAAGAGAACTTAGATTTCCGGATTACTTTTCTGATGAAGCAAGGGACCTTATTGATCGTCTGTTGGATTTGGACCCCACCAGGAGACCAGGTGCTGGACCTGATGGTTATGCTATTTTGAAAATACACCCATTTTTCAAGGGGGTTGACTGGGATAACTTGAGGGCTCAAATTCCTCCAAAACTTGCTCCAGAACCTGGGACTCAGTCACCTGCATCTGATGATGTTCATGATTCATCGTGGAGTCCTTCTCACATTGGGGATGGTTCTTCGTCTTTCGTTAGACAACCTGATGGTGCTACATCATCTTCTGATGGGACTAGTCACATAACTAGACTAGCTTCAATTGATTCCTTTGATTCAAAATGGCAACAATTTTTGGAACCTGGGGAATCTGTTCTTATGATCTCTATGGTGAAGAAATTACAAAAACTAACAAGCAAGAAGGTGCAGCTCATCCTCACCAATAAACCAAAGTTGATCTATGTGGACCCGTCAAAGCTAATTGTGAAGGGAAATTTGATATGGTCTGATAATCCAAATGATTTAAGCATCCAAGTGACTACTCCATCACATTTCAAGATTTGCACACCTAAGAAGGTAATGTCGTTTGAAGATGCAAAGCAAAGGGCATGGCAGTGGAAAAAGGCAATTGAAGGACTTCAAAACCGGTGAATTTATTCTGGTTTTGAGAACATATTCTTTTAGGTATCCTCATATTGTTCAGCAAGAAGGGATAAAACAGTTGACTGCCCAACAATCAATTATTCATTAAAAGAAAACAAATTTGCAGATTTTGGAAGCTGGTTCTAGCTATAATTCCTCAACACAGCTTAATTTTGTGATGAGTTCTTCCATCCTCTTTCATGCTTCGAAGGGATGATAAATGTGGAAGTTCTAATTGTAGCTATGTCAGAGGTTGGAGCCAATACTTTTGTAAGAAAAGATTCGTTTTTTATTTTTTATTTTAAATATTATATTAATATTATCAGTTGGAAGATGATATCATTTTGGTTGTCATAATGTATGAATCATTGAATTGATCAATTTATTGTTTCTTCAGCCCTTGGTTAGAAAGTGAAGTCATCTTGAAATTTGAAATGGATTATTTGGCCCTTGG

>C101101_65

TCTCCAGCCAGTCATCATCACATAAATGAGTATTGCAAGAAGAAAATTCAAATTGACCCGGGTAAGCCCGTTTGCAAGACTCTAAAAACAATTAATACTTACACTTCAAATGTCAGATTTTATTAAATGTGTAGTTCTAAGTAGAGGACTGAAACTATAGAATATGGGTTGAAAAGTCAAAGTCAATGATGAATAATTCCCCCAATGAAACAAATACATAAGAATGCTGACTAATGTCTCTTTAGCCTTTTCAAGCTAACACCGGGCACAAGATGTCTGTTCATGAGTCCGCAGTTGAGAAGTTCTCATCAGAATTAATTAGCCACAGAATTAACCATTTTCAGCTCTATCGTACGCATTTGATTTATGCTTTGGTGCAAATGTAAAGCCATCAGGAACCTTGCCAAGTAGCATCTCCGATATTCGTATCCAATCAGCAGCAGAATCTGAGGCCATTAGTGTCTCAAGCTCATCACGCCCATAGTAAGAAGGTGGACGCCAGTTTAATCGTTGCGGAACCACATCCAAAAGACCAACAGGTATATACCTGAAAGTGTAGCTAAGCCATTCCAACAAGAACCGCCTTGTTGTCTCAACCCCTTTTGTGTCAGAACCCCAGTGTTCAAGACCAAAATTCACAAAATCCTTGAGAATATTTAGACGCTCGCCAGAACTGATGTCCCAATGTCTTTGTTCCTTGATCTCCGTAAATATCCAAGGTTTAATCAGTGCACCCCGAGCAATCATGTCCGTAGCCAGTTCAGGGCATTCAGTTCTGTGGTTGTTCCAGTCTACGAATGAAAAAACATCTCCATTACCCACTACTGGCAATGTAGTAGGGGCTTTCCTGGCACACTGGTATATATAGTCCCAGTCAGCAAGCTTGCTATAGCGTTGTTGACGTGACCTACCATGTATTGTTACCGCACTGGCTCCCCAAGAGCCAATGTCTGCTATCAGAGAATCAATGCGATTCTTCCCTTCAAAATAAGCAGTCCGTACCTTGATAGTTATTGGTTTATCCACTGTACTCGAAGCTACTTCTACGATACTTTTCATCCGCATTGGTTTTGTTAAAAGAGCTGATCCCGCACCCTTATTCACAACAATGTCAATAGGGCAACCCATATTTATATCAATAAAATCTATTGTACATTCTTGCTCTATTAGTTCAACTGTTCGTGCCACAGTATCTGGATATGACCCACAGATTTGGACACCAAATAAATCTTCAGACGAATGTCGTCTTAGCAATGCCCATTCTGACGCTTGACCCTGCAAAAGATTTGTGCACATTGCCATTTCACCGCAAGTTACATCAGCACCTAATACTTTACAAACTCTCCGGAAGGGGAGATTGCCAACAGTAGTCAAGGGTGCAAGATACAACTTATCCCTGAAATCAATAAGCTTCTTCTCACGTGAGTGAAGCTTCAAGCTTAGATCAGTTTCTGGGGCTATAGCATCAGCTTCTGATTTTGTGCAACTTCTTTCAACAATTTGCTCAGCAACACTAACACCTTCATTTTCTGCCTCCTTAGGACCACAATTCTCTTCATTTTCTGCCTCTTTAGGACCGCAATTTTCTTCAGTTTTTCTTCGTTTCAGGGGACAAAGTTCATCATCAGATTCAGATTTTCCATATACATCATTTTCAACATCTTTCATAACTTCTGCAGGGCATTCCAACTTAGAACCCGAGTCAGACGCAACAGCACAAGAGCCAGTGTCATCGGAATGATCACACTTGTTAGAACCAGTGTCAACTTCATCTTTATTTTCCAAGTCATTCCTTTTTGACTTAGATGGTCCCAGGAGGCCAAGACTTTTGAGTTTGGCATCTGCCTTGGAGAAGCTCATCTTATTTTTCCACAAAAGCTTTTGAACATCCTTGTTCAATCCATTCACCTCGGAACTATTCTTCAGGCCATTCCGATCGCCAGAAGGCACACCACCTTCATGCGTACTCGAAAATCTACACGCTAAGCCATAAGGGCAAGAGCCTCCAGATTTAATGAAAGGACATTGTCCATCTATATCAGACGGTTTCTGCGCCTTGAAGCCTTCCAAATCATGGCTGAAACGACACTTGTCTTTATAAGGACATGAACTAACATCCCCCGTCTTCGCAATTTCGGGACACAGATTTCGCGCTGACTTTTGTTCCTGACGACGCTCGCGTTTGAGTTGGCGCTTAGACTTCTTCTCCTTGGCTAGAACGGAGGTCTTGTTTTCGTTGGGAATAGAAGCGCCGTCGTTTGGAGCGGAAGACCGAACCGGAGGGGGGCGGAGGAAATCCTTTTTGACGGGAGCTATAGCCCTGGCGACGAGTTGCTGTGGGGTTTGGTCGGCGGCAACAACCCCCGGGACAGAACCTCCTTCCTCACGTTCCTCCTCGTCCATGTTGTGGTAACTGCTACGGTTGTTGGTAGTGTTTG

>C101109_65

TCCACGAATTATTTTAACCTTTTATTGGACAGTTTGCACATTATTACTATTTACATATATATGGGGAAGCATTTACAATTACTTTTCCCCGGCCAGCATCAAAGTTAAAAATATCCAGTGATGACTTGGTTCCATTTGATCATCAGAGTGAGTATCAGTATATTAGGCAGCTGTAATCACCTCCTGTTGGGTTCCTTATTGCAGCATAACACAAAAACTGCAGTTCCTATGAGACTTGTTCCGAGATCAATGGGACAAAAAACACCCAATGACACGCAATATGGTACAAACATAAATCTAATTGAAACTGTCGTTAACTATAACCCTACCTCTTGTGTATCATATCCCTCATTAGATTCTTCTTCAAGTTCTTTTATTAGCCTTTCAATCTGCCTTCTATAAACTGGCATGAAGTGGTCACTGATCATTCTCTGTGAAAGTCTGAGTTTACTATATAGAGATCTGGAAGTTAGAAAAATTCCAATCTTATTCTCATCTAGCTCGCTACTGTCTGAACTTGCAGAGGTTCGGCTATCATCATTTTCATGCTCCGTGTCAGAATAGAGTGTAACAATGTGCATTATCTTTCCTGGAGGGAAAAATCTGTGGATTTCTTTCATTTCTGGTGCAGAGCTCTGGGCTTGCCCTACTTCATCCGCTATAGCTGCTTCCTCTTCCCTTATTTCCTTTACAACATCAGTCTCCTCAACTTCAGGACCGTCATACAGCTCATGCTCCAGTTGATGCCACAACTCCACTTCACTCATCTGATCATCATATCTGTCATGGTCAATCACGTTCTGACCATCACCACGGTCAACATCCACCTCAGTATCAGAACTACTTTCGTTGCCACATTCAATCTCAGAAGACCACTCTTCTATAGATGAAGAAACAGGAAGGTTCATATTCTTTGCATTAATCGCCTTCTTGGGGGACCCAAGAAGCGCTTCGGAACTGTCAATGTTGCTAGAGGAAGATGCAGGGGAACTTCCATCGTCTTTAGAGTTTGAATAAGAACTCAAGGCTCTGCGACGGGGTCCCATGCATGACCAAGAGGACAAGTTAAGGTTTGGGCGTGCCCATGCTGCCTGAGCCATACTCTTGGCTCTCTTCATCACAACCTGGGTACCATTAGAAACTGGCTGCAAAATGGCCCCAGCCCCAGCAACTTTTGCTCTAGCAGTGGCAATCGATGGGAGGCGAGAACCTAATGCTGAAGCAGAACGATAGACAGTACTGAGAATTCTTGTTTGCTCAATTTGATTCCTTAAGTCATTTATCCATGCTGATGCTGTGACCTCAGAACGCAAGTCATCTACAGAAGCAGCTGAGAATGTAGGCACCAAATCAGCTCCATTTATAATAGAAGTAATAAAACTATCACCCGACTCTGCCAACTCCCATGTCATACAAGCAGCTGGAGCAAATGTAACACATGTGGCAACAGACAGTTCCTTTATCTCTCTCAACACATAAGTTAGAATTGCTGCAGTGCCTCCGCCCAAAGAGTGCCCAACAATCTTGACGTTATAGTCAGGGTAGTGGCCAAGTGCTTCAAGGAGACAAGGAGTTGCAAGCTTTGCAATCCATCGAGCAGCAGCAACCATTCCACAATGGGCATAACCTAAAACCAAATTGCTGACACCCCCCTGATGAACAACAGTGTGATGGAATGGCACCACATTTCCTGTAACAGCTGTTAGAGTATCCTTGATACTATGTGTGCCACGAATCAATAGCAAAAGGCATCTCATCTCATGATCAGCTATTATTGTAAAAGCTGGTTTCAAAATTCCTGCTTTGGCTTCCCGAAGGAGCACATTTTCTTCACTGTAGCCAGTTTCTTCTAAAAACAAGGGAAATGGTTTCTTTGAAAAATGCCAACATAGTGTCAACAATTTCAAGAGATACCTAAGTTCAGCAGTAATCTCAGATCCTTTCAGCTGCACACTATCCTTACCACAAAATTCACTGTCAACATGATAGTTCCCCTGCCTCTTGAGCAGAAAGCTGATACCAAACGCCAAATCCCCTATTGGCCATTTCCCAAGTGTCTCCGAATACGTGAACCTGAGAGTTTCTGACAATGTTGAAATTGTCTCCAACCATGTACCAGGGGCTTGGATTAACCGACTAGAAACACGAGTAATGCTTAATGGCGTATCAGTGGGCGTTTCACTACCATTTTCTTCATCATCTTCATCGATTGCATCCTGAGTCTGCAATTTCCGATTCAATGTGTAATACAAAAGGGCAGCTGCACCTGCTGCAGTAGCCATGGTTGCAGTTGCCATGATTTTACTATCCCTGAATAACGTGATTTTGGAGAAATTAGAAGGAGGAAAATAGATTGGTTACGACGGAACCATGGATTGTACCGAAGCCTCTTAATTCTTACGATGTGTGTTTTTATTCTGTCAGAAAATAATAACAATAATTA

>C101111_65

AATATAACACGTGTAACAGTTGTTACATGTGTTTTATTTTCATTGATAAACATGTAACACTTATTAAGTTTTTATTGGAGAACAACGACAATATCTATGGAAAAGTACCTCAACTTTCTCCTTTTGACTGTTACATTCAAGTATAATGAGAATAGTTATGGAACAGTGCCTAAATCTTTCTCCTTTTGAATATTACATCAAATGATAGATTCACGGGGCACTCAGAAAGTTTAATGAGATCCATGCCTTGACAGACCACACAATTTTGGGCAGCCACACATGGACTACTACAGAGTGAAGTTGAGAGTGTGGAAAGTGATGCAGGATTCTGTTCTCTTCGCACTTATCTTAAAACACTCGGATTCCTGCTGGCATGCGAACGCCACCGATTCCTTCATGCTCTCAGGTCCACAAACCAATACTCCAATATTAGACCCACCGCTTTCATTTCGGAGCTTGCCAAATATATCTTTAAAGTTAGGCCTTCCCCCAAAATGCACTTCATGTGCTTCAAGTGCATTACTAGCTTCAGCTGAACTTAGATCAAGGGGTTCAATTTCTCTAGGCGATATATGGGGAATACCTTTCCTCAGTCTTCTCCATCTCAAAATAGCTGCCACCGAGGCGCTGCATGCTAAAGCTAGGACAAAAGCAGCTATAAGAAGTAGATCTACAACCCAAGAGGGAGTCTTTTCTTTGTCCATTTTGGAGCGCTTTCCAGATGGAACTATAATATGGTTGAAACAGATTAGGAAAATAAGGAAAGTAGTGGAGCAAAATCCTGTAATGGCAGCCATCCAAGATGGACTTTCAGGCCCGTATGCCGCATAATTTGAACAAAGGCTGTTCACTTGCAGGGTTCTTACTTTATAGAACTCGTTCAGTAGCTCCCTAATTCCTACCCCTGCTTGTGTTTCTTGGGTTATAAACAATTTTAGGTTCAAATGAAAACTTTCAGTTGATTGGTTGAGTAACATATGTGAGATTGAATGTAACAGACAGAAGTCTTGTGCTTTCTTGATGACATACACAAGCTGTATTCTCGATGGAAATCTACTTTTGCTGGTAGCTGAATAAGCTTCTGTCAAAATGCTCAGAAATGGGGTTATTCCACTTCCTCCAGCAACCAAAAGAAGGGTGTCATATCTCAAAAAGTCTAAAGAAGCAGGTCCGTAAGGTCCTTCGATTGCAACCGGTATTCCCTTCCTTTTATCTTCAGTTTTGTCTAGTTCAGCATGAATCAGGTCATAAAGAGAATTAGTCCACCACCCTTCACATTTTATTATCACTGACAAAATGTGGTCATCGGCCCTGGAACTAGATATTATACTGAAAGAGTGCCATTGAAGACGAGATATGGTTGGTATCTTCAATAATATAACACTTGTAGGGTTATATTTCATCCCTGGATCTTTGGGCAGAATTAGCTCCACAGCTTTTCCAGGAAAAATTCTAGCTGAAACCACGCACGTTTTTGGACTTGATTGTATGATTTGTATCAATTTATCAAGGCTAAAAAGGAATATTCCTGGGAAAACCGTATAGAAGTGCCTATCCCCACCGTGAAACAAGAAAAATACTAGAAAGACTACATATAAATGATGGGTGTAGTAGAAGATTTCAAACTTTCTCCTTCTAATTTGGGGAAGTGAAGTGACCCAAATGACTAGCCCCACAACAAGTGCTATCTCCCCGGCGAGGTATATGCGTCCAGTCTTTTGCCACTTCCAAATCTCATCCTGAATATGGTTGCTGACCCCCCATACCAACAAAGTGCTTGCACCATGTATAGTACCAAAAAGTATCATTGCAGTTCCGACCCAAGTGTGATATCTAACTGAAGCTTCAAATTGAAGCCCAAGCATGCGAAACACAGCCAACCCTCTTAAAATAGGAAGGAGCAACAAAGCCAGGCAAGCTTCTGATAGCAAGCCAAACCGGGTTGCTACTCTGTGATACTTGAGTTGCCATGTATTCAACTTCAGTGATTTGTATGGCATCAGCTTTTTGAAGTCTGCAAAAATACGAGAGTAGTAGGTCCATGCTAAGAAGACAATAAAAAGGAAAGCAACCAGTATTTCAATACTGGATAAGATTCCCAGTGTGCTGTTCACCACCAGTGGATTTGAGAAGAAGTTTGATTTTGATGGACTCCTTGCGCTTCTGCTTTGATATCCAGCTTTCAAATCCAGTAAAAGAAGTCCAATTATTGCAATAGCAATTATAGGAAACGTGTACACAGCGAAGCTAAGGCCATAGTATCCAAAAATTGTATTATTGGCGCTATCTTCTAATTGTTTCCATTTTTTTGTCCATGTTTGGGTGGGCTTAAGAAGCCAAAGAGAAACCCAACCCGCGAATATGAAAATGATCAGAATTTTAAGAATAGCAGCAAGTAAAGTGATGTTGCTTGCCATGTTTGACTGCTTTTCTTTATTTTCAC

>C101115_65

AGGGGTGCAAGGGTATATTACAATCACTGATAAGAACACCAGAAAACAAAAGTTTTGGGAGAATGTCCTGATTTTTAACTCAGAACAACTGTGGTTTATAAAGCCTTTACAGAAAACTGAAAGATACACGTTCTGCCCAAGTTTTACAAACTAACCATGTGGGAAATAGTTTACCAAAATAAATCCTAAAAGAAAATCCCCACAACCAGTTAATATCTAGCTTTTGGGAAGAAAGAACGAAAGAAGCTCCATTCCTCAACCTCTTCAGAAGGGAAAACAAATTTGGGTCCAGACTAGATTTCCCAAAAGGATCAAATTCCTAAAGAAAAATAAAATAAAGTGGTCAAAATTTAACTGGGATACCCATTATCGTCATTTAGAAATTATCACATATCTGGAGAAACAACAAATGCACTAGCATCTTTGAGTACTCTCCTTCCACGCCAATAAATAGCAGTAGCCTTTTCTGATGTCCCCATCTTGGTCTCCATGCGATAATAATCTTGCCATAAGCTGACATTTTGGTCATAAGTTGCGAGTGCAGATTCATACAACTTCCTGGTATTTATGAGACCATCTTTATCTCCTATTGATGCAAGGTTTGTTTCCAACTCAATGCAATGTCTATGTAAAGCAAGACCAGGATGCGGTAAAGCTAGAAATCGTTTATAGATATCTCTGGTCCGCTGAATTCCATCTTTTTGAAGTACAAAACTTACAATGGCAGAAGAAAGGGAAAACCCATTTTCACTGCCACCATCTCTGACCAAAGTAACAACAGAAATCTCCACCAGCTTATCAAAATAATGTCTTTGATTTGCATAGAATTTAAGGGCCTTTAGCCACAAGTTATCTGATTTTGAAACAGAAACTTTCATCAAAATTTGTTGAAGGAGTTCAAAAAGGGTTTGCAAATCAGTATCACTAGGTGTAGAAGAAGTTCTTGTAATGCATCTAATTTCTATAGTAATCCTCAACCCCCATAACTCAACTGATTCTGCAAGTTTTCCACTGCAAAGCTTTCCTGCCAGTTTCCGAGCCTCATCCAATTGTCTCAATTGTAAAAGTAAGGATACGTGCTTGCAAGCAAGATCTTCAGTAATACATCCCATGCTTTCAGCCCTTTCGTATATTGACAGGAGCTGCGATATATAGTTTACAGCATGACCTGATGACCCGATTATATTGGTTTCTCCTTCTTTAGGAGCCACAATATCCGTTAGAAAATTTGCATACAAACTAAACATAGTTCCTGATGGCATATTTTTCAAAGCCTCCTCATAAACCTGAACTGCCTTTTCTACCCGAGGAATTATGACCTCTTCACTTATCTCCTGACCATTTTCAATATTACATTCACGCCTTGCAAGCCAGTCCCAAAATTCCGGTTGTGATAAAAAATCCCTCTTCATGTCATTCAATATCTCCTTACTCATATCTTCATAGTGTGACAAATTTGTACCCTCCAATATATCAAAAAATCGCTTCCTAAGACTGAGACTTGAAGGGACAGCTTCAAGCGCTCCGCCATAGACAGTCTTAAAAATATTCATACCATGTTCTGCAAACAATTCCTGCTTCGTTTCCGATTCATCACTTCCAACATTTGCTCCATCATTATCATCACTCTCCTCTTTTTCATCCAGTGACATGAACAACTCTTTGTTTACATCCCTCCACTGTTTTTCATCAGAACTCCTAGGATCTCGGGTGAGAGTTCCCTCATCCTCACCCAAAGCGACCTTCCGCGCCTTCAACTTATTAAGGTAAGTAAGTTCCATCCGAAGATACTCCACCCAAAGGTCTTCCGAAGTTGGACAAACTCTGAGACCTTCCTGCATCAAAGCACGAGCAGCCGCAACATTCAAGTTATGATCAAATTCCCAAGCCGCAGCATAAATCCAAACTCCCGGAACCTTTGGATGAAACCTAATAGCTTTCGCCAATGCAGTCTTCATCCTGCCATTTTTTCTGATCCTGCAAAACTCGAGATAGCGAAACCAGAGATCGATGTCACCCTTGTAGCGCTTCAAAGCAAACTCGTAAATCTCCATAATCCTACTCAAGCCTGCGAAATCGGACTTGGACTTCTTCAGCTTCTTGTTGCCTTGCTTCATCAATTCACGCGCCACGGACTTCTTGCGGAGCCTGCGGAGAGCATCGAGTTGACTCTCGTACTCGACGTAGGCCAAGAAATCCTGCTTCAAAGGACACGGACGCTTCAGCCGGTACTCGAATTTTCGCCGCTGCTTCACAATCTCGGCAATCTCGCGGCGGTTGAAGAGACCACGCTGCTCGAGGTCTTCGAGCTCGTCCACCATGCGCTCCAATCGGTACTGCACCACGTCCGCCATGTTGCTTGATTACGAACACCGTGAAACGGAGGAGGATGAGCGCCGAAGGAACGATGCTGCGTTTCGTTCGAACCTGAACCTATCTATCTG

>C101117_65

AGATAATCTATACAATCAAACACGACACAAGCGCGCACTGTTACGCTTACCAAAACGCCACTTGTCCATTGTCGTTTCGATCCCATGGATCACACGCCTGCTGCTCAGCGTCGGCTCCGAGCTATTCACGGCCACCTCAACGCCACCGCCGACGACTCTCCCTCCCAACTCCGATCCAATTCTACAGCCGGCGAATTTTTCTCCGAACAAGGATATAGTGTTGTACTCCCAGAGAAATTACAAACAGGAAAGTGGAATGTTTACAGATCTGTACGATCTCCTCTGCAGCTGGTGAGCAAGTTTCCTAATCATCCTGAAATTGGTACCCTGCACGAAAATTTTGTGCGCTCAGTTGATACCTTTCGAGACTACAAATACCTGGGAACACGTGTCCGTGTTGATGGAACTGTTGGGGAGTACAAATGGATGACTTATGGAGAAGCAGGTACAGCCCGGTCAGCAATAGGTTCTGGTTTGCTTTATTATGGAGTCCCAAAGGGCTCCAGTATCGGATTGTATTTTATTAACAGACCAGAGTGGCTCATTGTTGATCATGCTTGCTCGGCATACTCATTTGTATCAGTGCCTCTGTATGATACTCTTGGTCCTGACGCTGTCAAGTATATTGTTAGTCATGCTGTCGTGCAAGTTATATTTTGTGTACCTCAAACATTAAATGTATTGCTGAGCTACTTGTCTGATATTCCAACCGTTCGTCTAATTGTGGTAGTTGGAGGTATGGATGATCAAATTTCATCAGTTCCATCCTCAACCGGAGTTCAGGTTGTTACGTATTCAAAGTTACTTAATCAGGGACGCAGCAATCTCCAGCCCTTTTGTCCACCCAAACCTGATGACATTGCAACCATTTGCTATACAAGTGGAACAACTGGAACCCCAAAAGGTGCTGTCTTGACCCATGGAAACTTTATTGCAAGTGTTGCTGGGAGTACTATAGATGAGACATTTGGCCCTTCTGATGTTTATATATCCTATCTTCCTTTGGCTCACATTTATGAACGAGCAAACCAAGTCATGGCAGTACATTTTGGCATTGCAGTGGGATTCTACCAGGGGGATAGTATGAAACTAATGGATGACGTAGCAGCTCTAAGACCTACTGTCTTCTGTAGTGTTCCTCGACTATATAACAGAATATATGCTGGTATCATTAATGCTGTTAAAACATCTGGTGGTCTGAAGGAGAGACTATTTAATGCTGCCTACAATGCTAAAAGGCAGGCTTTATTGCATGGGAAGACCCCATCTCCAATGTGGGACAGATTGGTTTTCAACAAGATAAAAGAAAAACTTGGAGGACGAGTTCGGTTTATGGCATCAGGTGCCTCACCTTTATCACCTGATATCATGGAATTTTTGAAGATTTGCTTTGGTGGTCGGGTGACTGAAGGATATGGAATGACAGAGACTACTTGTGTTATAAGCTGCATAGATGAGGGTGACGTACTCGGTGGTCATGTTGGCTCTCCTAATCCAGCCTGTGAGATAAAACTTGTGGATGTTCCAGAAATGAACTATACATCTGATGATCTGCCCAATCCCCGTGGTGAAATTTGTGTCAGGGGACCCATTGTTTTTCAAGGTTATCACAAAGATGAAGCTCAGACGAGAGAAGTCATTGATGAAGACGGATGGCTGCATACTGGAGATATTGGAACGTGGTTACCTGGTGGTAGACTAAAAATTATTGATAGGAAGAAGAATATCTTTAAGTTGGCACAAGGCGAGTACATTGCTCCCGAGAAGATTGAAAATGTATATGCCAAATGCAAGTTTGTGGCGCAGTGCTTTGTATATGGTGATAGTCTAAATGCTTCTTTGGTAGCTGTTGTCTCAGTGGATCATGATATCTTGAAAGCATGGGCTGCTTCAGAAGGCATAATGTATAATGATTTAGCACAACTTTGCAATGATCCAAGAGCAAAGGCTGCTGTCTTGGCTGATATGGATGCTGCTGGACGAGATGCGCAGCTTAGAGGTTTTGAATTCGTAAAAGCTGTCACTTTGGTGACAGAACCCTTTACGCTGGAAAATGGTCTGCTTACTCCAACATTCAAGATTAAGAGGCCTCAAGCGAAGGAATATTTCGCAAAAGCAATTTCTGAAATGTACAGTGAGCTCTCAAGAACTGATATTTCTCAGAAAAAGTAGTGAAATTGAGGTTCTCCCTTCACTCCCCTCAACTAATAAATAAAGACACGTGATTCATAGCATCGTAAGGCAACATAGCAGAAGCATCCAATGGGTAACGATCTTGCAACTTTGCTTTGTGATTATAAGAGAGAGTTGTGCTAGAACCCGAGCAAGAGTCGTAGCTTTATTTAGTTCTAGTTGCCTCACGCGATTTTTATGATTTTTTCTTTCTGAATAAACATCTCTTGAATTTTTAATATATTGATTATTATTATTATTATTGGGAAAAA

>C101119_65

ATGGAATATGCCATTTAAATGCAAAAACGATGCTTTTCACGTCCCTAACCATCGTCAAACAAAAAATCGAAAAAGAGTAATTCATAAATCCAATACCAAAATCCAAATCCAAATCCAAATTCAGATCCTTCCAACCACCATGATTTCCGATCATGGCACACTCTCCTTCCTCCTCTCCTTCTTCATTTTCCTCCACTTCCACTTCCCCTGCGCCCACTCCAAACCCTCCCTCTCCGTCACCCCCTCCACGCTCTCAAAATCCGGGGACTCCGTCCACATCCAATGGTCCGGCATCGAAACCCCCTCGGACTTCGATTTCGTCGCCATCTACTCCCCTCCCACCTCCGCACACGACTACTACCTCGGCTACCGATTCCTCTCCGAATCCCCCTCCTGGGAATCCGGCTCCGGCAACATCTCCCTCCCTCTAGTCGACCTCCGCCATAACTACTCCTTCCGGATCTTCCGCTGGACCCGAGCTGAAATCAACCCCAAGCGCCAGGACCACGACCACAACCCCCTCCCCTCCACGCGCCGCCTCCTCGCGTTCTCCGGCGAGGTCGCCTTCGCCCCGGACCGGGGACCCGGACAGATCCACCTGGCCTTCGCGGACCAAGACGACGCCATGCGCGTCATGTACGTCACGCGCAACCCCAAGGAGACCTACGTGCGCTACGGGGAGAAGGAGAACACGCTTGACGCTGTCGCACTCGCACGTGTGGAGCGCTATGAGAGGGAGCACATGTGCGACGCACCAGCCAACTCCAGCGTTGGGTGGAGAGACCCTGGTTATATCCACAACGCGCTTCTTACGAATTTGAAGAAGGGACGCAGGTATTATTATAAGGTTGGAAATGATAATGGAGGTTGGTCTGCAACTCATAGTTTTGTGTCGAGGAACAGTGATTCAGATGAAACAATAGCCTTCCTGTTTGGTGACATGGGAACCACTGTACCATATAATACGTTTCTGCGAACACAGGATGAAAGCATATCAACCATGAAGTGGATCCTCCGTGATGTTGAAGCTCTAGGTGACAAGCCAGCCTTTGTGTCGCACATTGGAGACATTAGTTATGCAAGAGGTTATTCCTGGTTGTGGGACCATTTTTTCAGCCAGGTTGAGCCTGTAGCATCCAAAGTGGCATACCATGTTTGCATTGGCAATCACGAGTATAACTGGCCTTTGCAGCCGTGGAAACCTGATTGGGCCAGTTATGGAAAAGATGGGGGTGGTGAGTGTGGTGTGCCTTACAGTTTAAGGTTCAACATGCCTGGAAACTCTTCAGAACCAACTGGAACTGCGGCCCCACCAACTAGGAACCTTTATTACTCATTTGATATGGGATCAGTACACTTTGTGTATATTTCCACAGAGACCAATTTCCTTCCCGGGAGCAACCAGTACAATTTCTTGAAGCATGATCTGGAATCAGTTGACAGGAACAAAACTCCTTTTGTGGTAGTGCAAGGGCACAGACCCATGTACACTACCAGTTATGAAACTAGGGATGCTGCTTTAAGAGGAAAGATGCTCGAGCACTTGGAACCTCTCTTGGTGAATAACAATGTTTCACTTGCCCTTTGGGGTCATGTTCATAGATATGAGAGATTTTGTGCCCTGAATAACTTCACATGTGGTGATAATGTGGGTGAGAGCGCAGGGGACAACAAAGCATATACTGTTCATATTGTGATTGGAATGGCAGGGCAAGACTGGCAACCCATCTGGCAACCAAGACCAGACCATCCTGATGACCCAATCTTTCCGCAGCCGAAATGGTCCCTGTACCGCGGCGGCGAGTTTGGGTACACAAGACTGGTGGCTTCAAAACAGAAGCTTGTGCTTTCTTATGTGGGAAACCATGATGGTGTGGTGCATGACACGGTGGAAATTCTGGCATCTGGGGAAGTTGTCAGTGGTAACGGAGAATGTAGTTTTTATGCTAATACTAAAGCTAAAAATGAGATTGTGGAATCCACATTGTCATGGTATGTGAAGGGAGCAAGTGTGCTGTTGCTTGGGGCATTCATGGGTTACGTTCTTGGTTTCGTTACAAGTGCCAAGAAGAAGTCTGAAGAGGCCAGGAGCAATTGGACACCAGTGAAGACCGAGGAGACTTGAACTGTATATTAATGTTTACCTCCTTTTTAAGTAGGTCATGACGGTTCACTGAAAAGAAAAACAAAATGAGATTTCCACTCTTTTTTGTTTAAGACAACAAGTTGTCACCTCATACTTGGGACTTGCTTTGTATATATGCATTTCTTTCTTTCTTACTTATGCCATCTAAGGCGTTCCTTATAGGACCTGTGATGTTGTACAGTATTCATGGTAATGTATGATTTGTGGGATGAGACCATCCTATTGTAACAGAAAAAAAAGTTTTAGATGTGAGTGTACTAGTGATACAAAAACTTTTATGTCGGAAAAGGAATTGCCCA

>C101121_65

ACGCTTGTTGTTCTGCATCTCCATGCTCTTCTTTTACCCCTTCTCATTCTGTCAGAGGTCTGATCAATGGCGCTTTGTGGAACTGCACCAAACTCAATTTCGGGGAGTAACAGCGTCGCCTATTCAGCTCAAAATTTGTCGACAAAACTTATTTTACCTGTTCCCCTGCGTTTTACTGGACAGTTCTCTAATCTAGTCAGGAAAAAGCGCAGGGGAGTATGCTGCTCTTCGTTAAGGAATGCTGCAACTGCATCTTCTATGGAGTCACAGGAAGATACTCCAAGTACTTTTTCAGTTTGCTTGGAGGAGGAAGCAGATCATGTTATACGGTTCAAAATGTCAGACTTTAGGATTTTAGATTCAGTTAGCACTGGTCTGGGTGGGCGGTCGGACGAGATAGTTTTTGAAGGCATGGTGAAGGATAGTGGTAGTTCTTTGTATAACACAAGAGTTGTGCTTCGACAGCTTTCTAGTGCCCAAGCTCAGCGTCGAGGGAAACGGGCAATAGAGGTTTTGGGGAAGCTGGTTCGGCGTAAACTTCTGTATCATTCTTACTCAATGCAAGTTCATGGTTATATCTCTTCGCCTGCAAGTGATGATAGTGGTTCATTTATCCTGGTACATGGGTATCATGGCAGTTTTTCTTTGAGGCATTGGCTTGAGCGGGCAGATTGGCTTCCAACTTTAGAGGCCACTCTTGCACTGGATGAAGAGTCTGTTAGAAAGGTAGGAGAAGATACAACTGGAGGTCCGGCAGTTTCTAGGCAGTTGCGACTTATTCGAATATTGATGAGGGATCTTCTTATTGGGGTAAATTACCTGCACAGCCACGGTCTTGCCCATACCGAGTTGAGACTGGAAAATGTTCATATAAGTCCAGTAGACAAACATATCAAAGTAGGCATTTTGGGTAATGCTGCTGACTTTTACAAGGATGGTTCCAATGATAGCTCTTTGGACAATTTGGACAGGCGACAGATGATGATTGCATTTGACATGAGATGTGTGGGCTTCATAATGGCTAAAATGGTGATGCGGGAACTTATGGATCCTTTGGTTTTTGCAAAGTTCAAATCGTTCCTCACAAAGGGGTATGATCCTTCATGTCTACGAGAGCTTATGTTGAAAGTCCTTGGCAGAAGTTCCCCATGTGGAAATGCTGGATTACAAATACTTGATAGGAACTGGGGAGCTGGTTGGCACCTTTTATCTTTATTGCTTGCTACTAAACCTTCTCAAAGGATAAGTTGTTTGGATGCTCTTAGACACCCATTTCTATGTGGGCCAAGATGGCGAGTAGTACCATCAATGGATATTCTCAGATGGGGTCTGGGTTCTACTGCAATGCGTATTACTGAGGAGTACATTTATCGCAAGCCGCAGCGCGGCAGGCTTGCCCATTTCATCGACTTAATGGAGATGTTGAATCCCCATCCAAAGCCAAAGAACTGGCTGGACCTGCTTCCAGGTAAATGGCGTCTATTATACTGCACCGGGAAGCATGTAGGCCTGACTCTTCGCCAACCACCTCTCCGTGTACTTGTTGGCGATGTGCATCTAACAGTGACAAGAGAATCAAAATTGAAAGCTAATCTTTCATTTGACTCTGACATTGGATTCTCTGTGATGATTGGTCAGGACTGGCCTCTTGACAAAGCTGGTAAGAGTGGAAGAGTGCAGGTGAATTCTTCATTTACACTAAGAGCTGGGAGACAGTTATATCTGAATCAAGACAAGACCATAGAGAAGTTCTTCTTTGGCCCTTCCAGCAATGTGGATGCTTTAGCTCAGAAATTTAAGGGCAAGAAATGGAGAAAAGTAATCCCTTTCAAGGAGTTTCCAGCAAGTCTTCCGGCAGCCAAGCTTGCATCAGGTGATATTGATGTGACAATGAATCTCGACGATCCATTAAATCGAAACATTGATACTGCAAAAAGTGTTCTGCAAGAACTAAGAGCACAAATTCCCCCAGAAATGTTTGATTTGACTAAGCTTGTGTGTGGAACTTATGTGGACTCAAGGTTGCTTGTCCTACGAGGGGTTAATGGATCCGCGCTCCTATTTACAAGGTCTTTTGTAGACAGAAACGGTTCTTGTTAGTTGTTGCAGTAGCAGATTCAAAATTATTGTTTGACTGTGTATATTTTTCCTTGAATTGTATAGTTCGGTCCATTGTGATCTCAAATGAACATTTCCTCCCGCCCCAAGAAGTAGTTATCCTGTTAAATGTAAAATTTGCAATTTTTTCAAATATTAGTGTTCTTCGTAAATACTATTAGTTAAAAGCACCTAGGAGGGGGAAGGGAGAATTTAAAGCACTTTATTAGTAAATTAATGCTTTGGAATGTACAATGCTTGAACATTTTGATGATCCTTTTTCAACCTGATATGAAATATTGATCATTGAGGATAAGAATTACATTTAGAGGCAGTCAAACACTTCAA

>C101123_65

GGAAAAAACCGCCCACATCTGACGACAGTAGTGATAGGTGTCTTCCACTACCATCAATATACCAACACTGCTTTCTTTCTGCTTCCTCACTGTGCAGTGAGTGTCATTTCCATTATTTCTATCACCCTATTTTCCCTTGGATTCAAAGTTACCAACTTTTCTAGATATCATCTCATAAAGTCACCATTTTTTACTACCGTTTTGAGGTGACTGGAAAGCTGAACATGTCTAGGGTATCGGTTCATCTGTTGGGTGTTTTGTGGGTATGTTGGTGTTTGGCATCAATTGGAGATGCTCAAATAGAGTACCTGAGATACAAAGACCCAAAGCAACCGGTTGCAACTCGGATTAAGGACTTGATGAGTCGAATGACTTTGGAGGAGAAGATTGGTCAAATGATTCAGATTGATAGGAGTGTTGCCAATGCTAAGGTTATGAAAACCAGTTTCATTGGGAGTGTATTGAGTGGTGGTGGCAGTGAGCCCCTTCCAAGGGCTACTGCCGAAGATTGGGTTAATATGATAAATGAATTTCAGAAAGGAGCTTTGGAGAGTAGATTGGGTATACCAATGATCTATGGTATTGATGCTGTTCATGGACACAATAATGTCTACAATGCTACCATATTTCCCCATAATGTTGGGCTTGGATGTACCAGGGATCCAGATCTGGCACAAAGGATCGGTGCTGCTACTGCTCTTGAAGTTAGAGCAACGGGGATTCCTTATGTTTTTGCTCCATGCATAGCGGTTTGTAGAGATCCAAGATGGGGTCGGTGTTATGAAAGCTATAGTGAGGATGCTAAAATTGTGCAAGAAATGACAGAGATCATACCTGGTCTACAAGGAAGTATCCCTGCCAATTCCAAGAAGGGATTTCCATATGTTGGTGGCAAGACAAAGGTAGCAGCTTGTGCTAAACACTTTGTTGGAGATGGTGGTACAGCAAAGGGAGTAAATGAGAACAACACATTGATTGATTGGCATGGATTGTTGAGCATTCACATGCCTGCCTATTCTGATTCCATTATTAAGGGGGTCTCTACTGTCATGGTTTCTTACTCAAGTTGGAATGGAGTAAAGATGCATGCAAATCGTGATCTAGTCACTGGGTTCCTCAAGAATACCCTTAAGTTTAAGGGATTTGTCATCTCAGATTGGCAAGGTATCGATAGAATCACATCACCACCTGATTCAAATTACACATACTCTGTGCAGGCTTCCATTGAAGCTGGTGTTGATATGGTCATGGTCCCATACAAATATGATGAGTTCATTCAGGATCTTACACTCTTGGTCAAGAGCAATGTCATTCCAATGGAGCGTATTGATGATGCTGTGGAGAGAATTTTGCTTGTAAAGTTCACCATGGGTCTTTTTGAGAACCCTCTAGCTGATACCAGCTTGGTCAATGAGCTTGGAAGCCAGGAACACAGGGACCTAGCAAGGGAAGCTGTGAGAAAATCTCTCGTGCTGCTTAAGAATGGGAAAAATGAAAGTACTCCACTTCTGCCTCTTCCAAAGAAAGTCCCAAAAATCCTAGTTGCTGGTAGCCATGCCGATAATTTGGGCTACCAATGTGGAGGGTGGACAATCAAATGGCAAGGATTCAGTGGCAACAGTGACACTAGAGGAACAACTATTCTCAGTGCCATAAAATCAGCAGTTGATCCAAGCACAGAAGTCGTCTTTCGTGACAACCCTGACAATGAATTCGTTAAATCCAATAATTTTGAATATGCCATTGTTGTAGTTGGTGAGCCTCCTTATGCCGAGACTGCTGGGGACAGCACGACACTTACAATGATCGAATCTGGCCCAAATGTAATCAACAATGTCTGTGGGACTGTTAAGTGTGTGGTCGTCACCATCTCTGGCAGACCGTTTGTGATTGAACCATATGTTTCTAACATAGATGCATTGGTGGCCGCATGGTTACCAGGTACTGAAGGCCAAGGTGTGACAGACGTCCTTTTTGGTGACTATGGTTTCACGGGTAAGCTTGCGAGGACATGGTTCAAATCTGTTGATCAACTCCCGATGAATGTTGGGGATCCTCACTATGATCCTCTTTTTCCTTTTGGTTTTGGACTAACAACCGAATCTGTGAAGGACCTGGTAGCAAGGTCAACCTCAGCCGCTATTGGTGTAAGGGCTTGCATATTTACCATCATGGTGACACTATTCATCAGCTTATGTTTAACTGGCTAAGCACAATTCGTGGGAGCTTATTGGAGCAGGCGGCTATAGAACTAACTTGGGACACTTTCCGGCTTAGTCTTATTCTAGTTTAATTCATTGTATTGAGACATTATTATTGTTCTCGAACTAGTAATTTGAGCACATTCTTTTTCATAATTAAAACTTCGTTCCAAGGATATTATCAGACAATTAGGTATTAAATCTACTAGGTGTTAAGTAATGAAGTTACTTTTTAGAGTACTAAG

>C101125_65

CACCAAACCTCACCCTTTCCTCTTCTTCCTTCATACTCTTCATTCTTATCCCCTCAGCAACAACTTTTCTAGTGTCAACATATATAACATATCTAGTCCTAGTTAGCTAAATCATTTCAAGTTAAGTTACTCTATTTTAACCAGAATCAAATATTTGGTGTTGTGTTAGAGATGGGTAACGCAGACTATGCATACCCGAACGTGGAGAGCGTGCACCGAGTGGCTATTCCACCCCCTCAACCATTCTTCAAGTCCCTCAAGTACTCCTTGAAAGAGACCTTTTTCCCAGATGACCCGTTTAGGAAGTTCAAGAACCAAACACCCTCTATGAAGTTCGTCCTTGGACTTCAGTACTTCTTCCCCATCTTTGAGTGGGCACCCAAATACACCCTCCACTTCTTGAAATCAGACCTCATCTCTGGCATCACCATTGCCAGTTTGGCCATTCCTCAAGGCATCAGCTATGCTAAGCTCGCCAACCTCCCTCCAATTCTTGGACTTTATTCGAGCTTTATACCGCCGTTGATTTATGCTATGATGGGAAGCTCAAGGGATTTGGCAGTGGGAACTGTGGCGGTTGGATCGCTTCTGATGGCTTCCATGTTAGGTCGAGTGGTGAACTTCAATGAGAACCCAAACCTTTTTCTCCACCTGGCTTTCACTGCTACATTCTTTGCTGGAGTTTTGCAGGCATCACTGGGCTTCTTCAGGTTAGGGTTTATTGTAGATTTTCTGTCACATGCAACCATAGTAGGGTTCATGGGGGGAGCAGCCACAGTGGTTTGTCTGCAACAGCTAAAATCAATTCTTGGCCTTGAACATTTCACTCACGAGGCCGATCTCGTCTCAGTGATGCGCTCTGTTTTCACCCAAACTCATGAGTGGAGGTGGGAAAGTGCTGTGTTGGGTTGTTGCTTTATTTTCTTCCTCCTTGTCACAAGATACTTCAGCAAACGACAACCAAAGTTCTTCTGGGTGTCAGCAATGGCACCATTGACGTCCGTTATACTGGGAAGTCTTCTGGTTTATCTAACACACGCAGAGAAGCATGGAGTTCAAGTGATAGGAAAATTGAAGAAGGGGCTAAATCCACCTTCCGTGACTGATTTGGTGTTTGTGTCACCTTATATGGGCACTGCCATTAAAACTGGACTTATCACTGGCATTATAGCACTTGCGGAAGGAATAGCAGTGGGTAGAAGCTTTGCAATGTTTAAAAATTATCATATTGATGGCAACAAAGAGATGATAGCTATTGGAACCATGAACATTTTTGGTTCGTTCACCTCTTGCTATCTGACCACAGGACCTTTTTCACGTTCAGCTGTGAACTACAATGCTGGTTGCAAGACAGCTGCATCAAACATTGTGATGGCAATAGCAGTGATGTTAACATTGTTATTCTTAACACCCTTGTTCCATTTCACTCCCCTAGTGGTGCTATCGGCCATAATTGTGTCAGCAATGCTCGGGCTCATTGATTATCAAGCAGCCATTCATTTGTGGAAAATCGACAAATTTGATTTCTTAGTGTGCTTCAGTGCTTATGTTGGCGTAGTCTTTGGCAGTGTTGAAATTGGCCTAGTCATAGCAGTTGCAGTATCTCTACTTCGAGTACTTCTATTTATTGCAAGGCCAAGGACATTTCTCTTAGGAAACCTTCCAAATTCTGCAGTATACAGAAATGTGGAACAATATCCAAATGCAAATCATATTCCTGGAATTCTAATTCTTGAAATTGATGCTCCCATTTACTTTGCCAATGCAAGCTATTTAAGAGAAAGGATCACAAGGTGGATTGATGAAGAGGAAGATAGAATTAAAGTTTCAGGGCAAACCAGTTTGCAGTATGTTATAATGGATATGACTGCTGTTGGAAACATTGATACAAGCGGAATAAGCATGCTTGAAGAGTGCAAGAAAACTGTTGACAGAAGAGGGTTACAACTTGTTTTGGTGAATCCTGGAAGCGAGGTGATGAAGAAGCTGAACAAATCCAAGTTCATTGACGAACTAGGGCATAAATGGATCTATCTGACAGTTGAAGAGGCTGTTGGTGCATGCAATTTCATGGTCAACACGTACAAACCGAACCCCATGAAAGATGATTCTGAAGGCTGGAACAATGCCTAATAAAAGCATTGTTTAGATCTCGTAAAGCTTACGAGGTTTTATCTTAGTTAACCTTACAGAATGTGGCATGAGAATCATGCTGCATAAGATTTAGAGTGAGTCATAGTTGCATCAGTATTTAAATTTCGCAAGATAATTAGTTGGAAGAGAGCTCCAACACAGGCTGCTGAATCTTTTATATGTATATCATGTTAACTAATAATCTATTCTTATCTCTATCTTTTCATATTTATTTTTATTAGTGTGTGTTCTGGTACAAATTTGACAAACTTTGTACGGATGGTAAATAATGAATATGAAATTTTGAAGGAGA

>C101127_65

AGCGCCAGCCATGAAACGGCACGGAAAGTCCAAAACAGACAAAGTGCGGAAAACTGGTGACAGCACTCTTCAAAGGTGACGTCTTTTCCACTTCAACTTTCAACTGTTTCGTGTTCGAAGCAAAAGCAAGAAAAACCAAATCAAAATTCAGAAGAAGTAGAAGAAGAACAACAACAAAGAAGAAGAAAGAGATGGTAATGGCTGTGGCCAGTGGTGACTTGCTGACTGAGAAAGCTTCGGCGATGAGAGCGTCTCTGGAGAGAAGCCAGACTATCACGGACGACGTCGTTTCGATTCTGGGCTCCTTCGATCACCGCCTCTCTGCCCTCGAAACCGCCATGCGCCCTACCCAGATTAGAACACATTCTATTCGGAAAGCTCATGAGAACATTGATAGGACTTTGAAGACTGCTGAGGCCATATTGGCTCACTTTGATCAATATTACCAGGCAGAAGCAAAAATAATTAAAGGACCACATGAAGATGTGAAGAGCTATCTTGAAGCAATTGATCAGCTGAGACGAAACATTAGATTTTTTGGCAATAAAAAGGGTTTCAAGAATGATGATGGTATTGCCATCCGTGCTAATAATTTGATAGGCGAAGCTATTTCTAAGCTAGAAGAAGAGTTTAGGCGGCTATTATCATCTTATAGCAAACCTGTGGAACCTGAACGCCTCTTTGGTTCTCTTCCAAACACGCTGCGACCTTCATCAGCATCACCAGGTCGTGATGATGATTCCAGTAGCAAGAATCATTCTTATATCCATTCTGAGCCACATAAAAATATTGCTGATGCTGTTGTATACACACCTCCTGTTCTTATACCTCCAAGAATATTGCCGTTGCTCAATAATTTAACCGAGCAAATGGTTCAAGCTGGTCACCAACAACAGTTGCTCAAAATATACAGAGATACCCGTTCTAAAGTATTGGAAGAGAGCCTTCAAAAGCTTGGAGTTGAGAAACTCAGCAAAGATGATGTTCAAAAGTTACAATGGGAGGTTTTGGAAGCCAAAATTGGAAACTGGATTCATTTCATGCGTATTGCTGTCAAATTGTTGTTTGCTGCTGAACGGAAAGTCTGTGATCAGATATTTGAAGGATTTGACTCGCTTAGTGATCAGTGTTTTGCTGAAGTAACTACAAACAGTATTTCCATGCTACTTAGTTTTGGAGAAGCTATTGCCAAAAGCAAGAGATCACCTGAAAAATTATTTGTACTTTTGGACATGTATGAAATATTGCAAGAAATCCATTCAGAGATTGAGATACTCTTTCAAGGCAGAGCTTGCACCAAAATAAGAGAAGCTGTAATGGGTTTGACAAAACAACTTGCACAAACGGCCCAGGAGACCTTTGGAGATTTTGAAGAAGCGGTTGAAAAAGATGCTACAAAGACTGCAGTAACTGATGGAACTGTCCATCCTTTGACAAGCTATGTGATTAACTATGTGAAATTTTTATTTGACTATCGAGCTACCTTAAACCAACTTTTCCAAGGAATTGAAGGTGAAGGTGATTCTTCACAGCTAGCTTCTGTGACAATGCGAATATTGCAAGCTCTTCAAACCAATTTAGATGGAAAATCTAAGCAATATAGAGACCCTGCTTTGACACACCTATTTCTCATGAACAATATTCATTATATTGTCAGATCAGTTCGAAGGTCTGAAGCGAAGGATTTGATGGGGGATGATTGGATACAACGGCATAGGAAGATTGTGCAACAGCATGCCAATCAATACAAAAGAAATGCTTGGGCAAAGATTCTTCAGTCCCTCTCTGTTCAGGGCCTCATCTCATCAAGTGGTGGGGGTAGTACTGCTGGTGGTGATGGGGGCAGCAGTGGTGCTTCAAGAGCAGTTGTTAAAGACAGGTTTAAGACGTTCAATACTATGTTTGAGGAACTTCATCAGAAACAATCCCAGTGGACAGTACCAGACACAGAGTTGCGAGAGTCTCTCATTCTCGCAGTTGCTGAAGTCTTGTTGCCTGCCTATAGATCATTTGTGAAACGTTTCGGGCCTCTAGTGGAGAATGTAAAGAGTAGTCAGAGGTATATCAAATACACTGCTGAAGATCTAGAGCGAATCCTAGGTGAATTTTTTGAAGGAAAAAACATGAATGATTCCAAGCGGTGATAGGATCAAGATTTTACATTAGCATATATAGACTTTTAATGTCTTCGTTTATCTCATTTTGAGATGCATATATGTAATAAGGGATAAATGCCCCCATCAAAAATTCAAGATGGAGTTTGTTCCATTTATCATTGTTTTGATGACACATGCACGTGGTACCAACTTCGTGAGGTAAAAGGTCATAAAGTTCAAAGTAGAGAGGGGGTTCTGCCGAAGAAAAGAGTTACTGTATTAGATAGAATAAAAATATATGTACAACAGATACAAGCTATTTATGTATAGAAGAGATGAGCATGGAAGCG

>C101131_65

TATGATATCCATATTTGAGTTTTGGAGGCTCATAATTTTGAAGTGTGATCGTGCAAGTATGTTTTGAAACGGTGCATCCAAACACAGAATATTTTAACACACTGATATATACACATAATGTTTCATATAGGAAATCTTAAACACCACTTGCTAGTAGTAACTTATGCTTGCATAATTGCTAAGGACATGCATGTTGATTATGGACAATTCTCAACTTCAGCTTTATAAAAAATTATAATCTCTACTGAAAATTTTCTTAAAAAACTAGTTTTCCTGAGCAGTGAAATCTGGCTCAGAAGGCTTCTGAGGCATCAAGGGTAGCAATCTCTGTGGCTGTGGCACTCTGGTGCGTTTACCACTTCGCAAGTGGCGCAATGCAGTTGATGCAAACCTTGATGCATATACAGTGGCAGCAAAGCTAGGTGAGGACCCTTCCTCATTTTCCAAATTTTGTATCCTTTCTTCTGCTTCACGTAACTTCCTCTCTGCCTTCTTTTTTCTGTATCTACGCCATGCTGCTTGTATGAAACATGCACCCCATGTCCTCCATTGCAAGGAATAGAACCTGAAAGTGTGTTGGAGTTGTTTGCTGTTTATAAGACGTCGAAATTGGGAAGCAACAGATTTCAAGTCATCAGGCATGAGAGCAAAGGCTTCAACTTCTGATATAGTTTGCACAGTTCTAGTTGAAATGGGTAAATTTGAGGAAGAGTTGGGGTCCAAGGCCCATGTTAGAAGCTCTTCCCCACAGAAGTCACCAGCCTTGATCTCAAAGGAGTTGAAGAAACCAGTTCTTCCACCATTTGTTGTGGCAGTTGCAAGTTTTCCACGCATGATGAAGAGCATTTCATCAACTGGATCCCCTTCACGAACTATGTGACTTTTCTCTGTGTAGAGGACTGGCTTCAGTTTATCACACAGTGCATCCAACAACTGATTATCCATGTTCTCAAACATTGGCACTTTTTTAAGAAGATCTAAGCAAAGATGGCGCCTTATGTCCCTTCTGAGATCTTTAGGGAGGTTCCGAATTAATGTCTCCTCCTCGACACCCCTATTTTCTTGCCATTTGTATTGTTCATACCGTCTAATTCTTTGCTTCAGGAAATCAGGTAGCATACGGTGGGACATCCACAGTTCTGCATCCCTCCTTTTGATTCTCATCTCTTCAACTCTGACAGTGGTAGATTGTAGATATTTCTGCATGTTTCCAATAAGTGATGCAAATAGAACCAATCCAAAGACAGCTATGAAGATAGCAAAGATTATCTCCCCAACATAAGTACTTGTCTGGAGACCTTGCCCAACAGAACTTACACTGCGCAAACCCCACCAAAAGCAGTAGAAGAACTTGTGATGAAAATCAGTAGTTGATTCTACCACCCGATACTTTAGAGCTTCTACGAACATACCAAAATTGAAAACTTCCAGGTCTACAATGTCTTCTGGATCAGTGATAGGGCAAGAATGACTGAGAAGTGATTGAACAGTAGAATTTTCAGGTCCACAGCTAAGGTAGGATTCATGAAAGAAGGTAGTTTTCTTCAACCGCCTGCGCCAGCACCTCATCCTTGATTCTACTGAAAACAGATACCAGAAAGCTCCAACAACATGACTTGCTAGCATGTAGAGAAAAAGATTGTAAGCTGCTCCAGCCCATGCAGTTTCAGTCAATATGCCAGAAGTGCTTGTTACTTCTTTGAATAAAGGGTAGATCCGCAGAAGTCTTGGTACATACTGGGCTATTATTGTATACTTCAACAAATCCTTTCCCACATAAGGAGCTGAGCATGTTGGGAATACTATCATAGCTAAAATGACCATCTGAGGAAGTGGAATAATTGATAGAATGTCAATGATGAAGTAAGAACTTAAGTATCTCATCACTATGGCTGAACGGTCATCGACAAGCTCACCCCTTCCAAACACACGAGAGGAAGGTGCAATAAACCCGGTTCGAAACTGAAAGATGATGTGAAGAATGTAGAAAAGATCAAAGAATGTGCGGAGAACACTGGCAGTAATCTTCAGTGTTCCATCCAAATCAAGGCATTTTTTCTTGTCATCAATCACTGGGATGTAAAAGAAAAGCGGGTCCACAGAGATTGCCATCACACTTGTGAGTACAAAAATTTTGTTCCACTTCTGAAGTATTGACCCCTGTGGATGAAGAACATGACTCCTACAAGACAAATTCCTTATTGATTCATCACTGACAGAACTTAGACTAGATTGAACTTTTCTTTTGTGAAATCCGTCACTAGTGGAATCATTGTGCTCAATACTTGAAGATGAAAGTGACTTCCAATCCTTAAACCTCACAAATCTCTTTCCCTGAGTCATGGTCAAGCTACTGCTGCATGCAGCGAGAATAATTATGCTTTATAGGGGGAAAACACTTCCTTTGTACCATAGAAGATTCAGGCCCCAGGAACAAGCAGGAAGC

>C101133_65

AAGTAATTCAGCATATAGCATCAGTGTTGATACGAGAAACATTTTCCAGTTCACAAAAAGGGCTATTACAAATGCAGCAACAAGCTGTTACAGTAATCAAACTTGTTCTTGAAAACCTATAAAAATAAATGACAAACAAAATATACTACACAAACAAATCTGAGACATGCCAAAACAGAAAGGTATTCTTTCTTCTTTCTCTTCTTTTGAAAGAACATCTTGGTTTCCTATGATAAAAATGAAATCCAATTGAATTGCAATAACAACCAGCTCATACTAATTTTACTAAAGCTTTCTAAATATTATAACTGGGTACTAAAATGGTGGTAGGATGCTGATATGCAAGATTATCTATCACTCTCACATATAGATCCACTGGGTTTCACCAGATGAGGCTGGTTAATAAGATATACTATAGACTCCTTTATTGATCTAAGATTGTATGAAGTATCCACAAAATAAGAATGGCCATGCACCAACCTATTGGGTCAAGCATTTATTTCTGAATTGCATTGATGCTCCCTGCTGCAACTTCTTCTACCCCAAAACATGCCTGAGCATGTTGTTTTGTCCAGTCTTGGATCTTCCATTTTATCCTGATCCATCGTTGATGCTTGTGACTGGCGAGGCGATTCATCTCGTGTTTCTGCTTCCCGGGGTTCAAATGATGGACAAGGACCATCATTACAAGTACACAGAGGAATTGCTACTGATGGAAATGGAGTACAAGTGACAGGGCAAAGCATCTCTGCCGGAACTGAAGCTACACGGTTCCTTCTAATCTCAGGGGTATGAGAAAGAGACGAGCGATCTAGGTCACGTGGATGTAGCATGCATGGCGTACACAGGCTGAGAGACACATGCATTTTGTTTTCTTTTGCATGGGAACCTGGTAACTTGACAAAAGAGACAATTCCATGTCTGCAAAGGGGGCATGGGATAGAGCCAGGAGGTCCACTCGTTTCAGAAGAAACATTGCTTGTTGAGCAAAGATAGAGTGCACATCTTACACAAAGCTCATGTCTACATCCTTCAGCAGCCACTGAACATGACCTCTCCAGGCAGACAGCACAAACATCAACTTCATTAGAGGAGGTTGTATTTGATTGCAATCCACACTCTCTGGCAATGTTGAGCACACTCATGAGAGGCAAGGACAAATAATTTGAAGAAGGGAATGTTGATATTGTGACTTCAGAAGCGGGAGCCAGCAATGGTTCAAGCCAATGACGTCCCCACATCTTAGCAACATCAAGTGGAAGCCATCCATTGCAATTCAAAGCCATCCGGCTGGCACCTCTTGCAAGGAGGATCTGACAGCATTTTAAATTGCCTCCACAAGCAGCATAATGCAATGGAGTGCTTCCAGCCCCAATTAAATCCATTGATGTTCCATAATGAAATGTTACAGCTGACAAATTTGCATCAAGATCAAGTAGCAGTTGGACACAATCAAAGTACCCATTTAATGCAGCCATATGAAGGGCAGTAATACCAGCATCAGCTGTTTTGTTTACAAACTTGGACAGCACACTCTGTTCATGTTTGTTTTTCACATTTGACCCATCACCTTCTGCATCCATGCGAGCATGTGAAGCTTCAAAAGGAGCACTAGGAACAAAATCTGCCACAACAAGTCTGATGCATCTCGCATGACCGTTGATGGCTGCAAAGTGTAGAGCTGTCCTCCCACTAAGATAATCTGCTTTTGTAACATTACATCTGAAGAGCAGAAGAGTTTGTACAACTTCCCAATGTCCATATCTACAAGCTTGCATCAAGGCCGTCTGCCCACAATAATTTCTTGAGTTAACATCAGCTCCATTTTCAAGCAGCAATGCCACAATCTCATTATGGCCTTTAGAAGCTGCAAAATGAAGAGGGGAATTGAGACCCCCAAAAGTTGAGTACTTTGCAAGACAAGGGTTGCATTCCAGAAGCATCTTTGCCTCAACCAAATCGCCGTCCCTCGCCGCCGACACTAACCTTTCGCCGGACGCCGAGCACCCAAATGAATTCCCCATCTCTCTCTCTCTCTCTCTGAGATCAAATCTTCACAAGGGAGGCTCTGAGCAAAGAAAGAAACAAAATCCAAAAAGGGGAGCAGAATCTAAACGCAATGGGTGTGAATGTATGTGTATTTATGATGTGAAGGAAAATTGGAAACTTTGCTTTTGGATTTGGTGGAGAAACTGGGATGGTGACGTGGCAACCTTTTTCTCTGTTTATCTAATAACTGTGTGAAACAGAGAAGGGTAAAACAAAAAGGAAGGAAAAAGCAAACGTTGAGGGTGGTAGCTAGGAATGTGCTTGAGGTCGGTGTTAATGGTGTTGTTGTGTGGAGCGAAACAAAAGGGAAAGGAGTGTGATAGAAAATGAAGGTTCTTTCTCTCTCTCTCTCTCTCTCTTTCTCTTATTCGTGTTATGTTCTTTCCTTCTGTGTGCG

>C101135_65

CTTCGAATGGTGTAGTGGAGAGTGAAGCTGAAGCTTCGAATGGTGTAGTGGAGAGTGAAGCTGAACCTTCGAATGGTGTAGTGGAGAGTGAAGCTGAACCTTCGAATGGTGTAGTTGAGAGTGAAGCTAAACCTTCGAATGGTGTAGTGGAGAGTGAAGCTAAACCTTCCAATGGTGTAGTGGAGAGTGAAGCTAAACCTTCCAATGGTGTAGTTGATAGTGAAAGTGAATCTTCAGTTGATTTGCGTGACATGAAAAACAATGCAGTGAATAGTGAAGCTGAACCTTCAACTGGTGCATTGAAGAGTGAAACTGAATCTTCTGTTGTATCTGAGATTAAAAAACACCCATCGGAGAGTGAAGATGAACATTCAAAGGAGGCAGTGGATAGTGAAGCTCAACCTGCAGTTGAAGGTGAAGGTAGCAATCAAGGAGATGAAGACACAAGACCTGCCTTGGATGCTTTGGACGTACAGAGTGTGGGTGCAGAGATAGTGAAAAAGCCATTCTACTATTTGATCAGGGTTCCCAGATATGACGATGATGAAAACATTAAAGAGCAGATAGCAAAAACTCTTCACCAAGTAGAGGAGAAGACTAAAATTCGAGATGCTATTCGAGCTGAAAGCCAGACTATAAAGGCCCGTTGTAAGGATTGCGACCAAGAGTTTAAGGCTGCAGTAGCAGCACATAGAGAGGCTCGGGACTTGCTCAAATCCAAACGCCAGGAAATGGATTCGGTTCAATCCACAATGAACAGATTAAACAATGCAATTTCTGTTGGGGATATAGATGGCAAGATAAGAAACATGGAACACATGATCCAGCATGAAACTTTGCCTTTAAATGAAGAAAAGCAATTGATACGCCAGATCAAACAATTGAAGCAGAATCGAGAGGAACTGTCTTCTAATATAAGAAAGCAGGATCAATCACAACAATCTCTAGACCACAAGGATGGCAATATTGAAGAGCACTCTAAGCGTTTACAGCTTTTGAAGAAGGAAATGGAAGTGCTTAGAAATAATGTTGTGAAGTCAGATGTAGCAACTAAAGACGCAAAGAAGAAATATAATGATGAATACGCTAAACTGAATGAAGTGATAGCTCAGTTTAAGGCAGCTGATGAAGTTCGTCAAGAGGCATATGCTAAGTCAGTGACTTTGAAAAAACAATTGCATGAGAAGGGCAAACATTTCTGGGATTACAGAAAGGCTGCAAGTAAAGCGCAGGAACTAGCAGCAGGAGGGAAAAAAGAGGAACTACAATGCTTCTGCGTTGATCAGGTTGAGAGAGTTATGGAGCTATGGAACAAAAATGACGATTTCAGAAGAGACTATGTCCGGTGCAACACTAGGAGTACACTTAGGAGACTGCAAACCCTAGATGGCCGATCACTTGGTCCCGATGAAGAGCCTCCTGTGATTCCTAATGCCATAACTGTAAGAGCTTCCAAAAATATTTCCATGGTCTCTCAGTCAACTCTGGAACAAGAAAAGAAATCCACTTCAGCAGAATCTGTTAATATAAAGGATGAACCTGTTTCGAAGGTTGTGGTACAAAGAACTGAAACTAGTCAGACAACTAAAACTGAAAAGCCTACCAAACCTGTTCCCTTAGAGAAGCCTGTGGCTCGTTGGGGTGACGAGTCTGATGAAGAAGAAGTTAAAAAAGAAGAACCTGTGAGAACGAAGGAGGAAGAGGAACTGTTACTGAAGGCTGAAAAGGCAAGGAAGGAGGAAGAGGAGGCAAAGTTGAAGGAAAAGCGGAGGCTAGAGGAGATTGAGAAAGCTAAGGAGGCCCAGCAGAGGAAAAAGCGCAATGCAGAGAAGGCTCAACAAAGGGCTGCCTTAAAGGCACAAAAAGAAGCTGAACTGAAAGAGAAGGAAAGGGAGAAGAGAGCAAGGAAGAAGGAAAGAAGAAAGGCAGCTTCTGCTGTCACTGCAGAGAATACAGAACAAGAATCTTCTCCTACCTCAGAAACTCTAACAAGAAGCGTGGAAGTATATGATCAGAGTGAGAAACCAGCTGAGGTAACTAAAAAGCCACAGAAACCATCTCAATTCACGAAGCAGACTAAGGCAAAATCTGTACCCCTGGCTCTTCGCAACCGGGGAAAGAGACGAATTCAACCATGGATGTGGGTTCTAATTGCAATTATGGTTGCTGTTGCCTTGTTTTATGTGGGCAATAGCAGCTCTCTGAGATCTTCGCTTCAAGGCTTTGGTTTCTGAGGACACTCCAAAGCAGTCACTAGGTGTGCGTCGGGGAAGTTACCCTTACATATTTATAACATACATACATATAGATGCAATTCTTTCTTGCTCTTGCAATTTTTTCCTGTTAGTTTTTTTGGTCTATAGTCATTATTTTCTTCATATTGAGATTTTCATTTTTTACTTTGGGATGTGATGTGATTGCTGAATGCTTCTGCCACCAATTATGAG

>C101141_65

ATTAAAGTGGTACAACTACCTTTATCCCTCCAAAGACAGTCACATTTCTACAAAAGTCACACCCTATTGACTCATGAGTGCAGAAGCACACCACAGAACTCAACAGTAAAAAAAATAAACAAATCTCATGATGCACTTCCACCTTCATATTCTCTCCTAATGATTTCAAACACAAGTTCATTCCAGGTATCAACAGGGCCTGGCATGCACCAATGCAAGCAATCCTGCGGTGGTGGCCTTCCATCAGGGCCACGCTTTGTAATCTTATTCGGGTCAGGACTCCTGTAAGGGCCAGGGTGACCATCATGCCTGAACTGAAATGCTTCAGTAATATCCATTAGCCTCAACTTCGATCCATTTGTTGCCCTTTCCAAAGCACGGTTAAAACCTGTCACCTGTTGCTCATGCATTATATTAGTGTGCACATTTTCCACTCCTTCACCAGGTGCAAGAGGCCTAACCTTCCCGGTGCATGATCCACCAGTGTTCCATGCCCCACCCTCATAATGGTCAGGTGAGTATGAACGCACAATTGTAAGCCCTTTGTAATTTGGGAGTGTAGCAATAGCAGTTAGAATTGTTTCAACAGATATACCATATGCTTTAATACTGTCAACCTTCATCTCCCGAGACGTGTCTGGCCACCACAACTGTCCTCCCACTATTTCATTGTTCAAAATATACACAGACTTCTTGGCAAACCAATGACCAGAAGAAAGAACCACCACATCAAAGTTTGGGATGTGTTCCATCAACTTCTCATCTGGGGCATCAAGATGGAGCTTGTCTACACCGGCTGGAGCATAATCAAATGGTTCGGATATTAGTTTGACAAGCCACGAGGACCATATCCTGACAATCATAAAAGAAGTGGACCTAAAATAATATCTTTGCATATTATGATTTCCCCGATTCTTTGGCGTCTCTACCTGCCACAGAATACACAACATTGATTCCATCTGGTTTCGGGCAACTGAATCTCCAATGAAAGCCAAGGTCTTCCCTCTCATCAGCTCCAAAAATTTTTTGGGATCAAATCGTGGGAGGTCACATTGAGAGGGCTTCCATCGCCAATTTTCGTAATCCTTGTCAGGCCTCCCATTACCCTGGCAATTCTGCATCTGTGTCAATACAGGGCATGAATTGTTTGTGTATAATGGTCCCGATGGATCATGTATCCACTTTCCATGGTACAGATCACAACCAGCAGAGGATGTGCTTTCTGGCTTCTCTATGGATGCTGGAACAACATTTGATGTGGAATCATCAGAAGACGGAGAGGTCTCATTTGATGCTGTACTTTCAACGGTGGACTGATTGATCAAAGACACATGAGCGGGTTCTTCCATTTTAATATTACCTGTCTCATTGGAAGAATCAGAACTTGCTAATGGCATAGTCAAATCAATTTGTGAATTTTCTTGAGCAGGCAACCCACTATTGGCTACATCTGCCCTTTCTTGAGCAGGCAAATTACTATTGGCTACATCTGCACTTTCTTGAGCAGGTAAGCTACTATCGGATACATCTGCACTGTCGCCAGCCGTAGTCACTGGTTCTCCTGAACTAGAAACTGCAGTATCTTTGTTCACTTCCTTGGTCATTGGAAGGTTCACTGAAGTAGACTCACTCAAGTCTTCTTGCGAGTTTGTTGTAGTATTAGTTTGTTCTATCTTACTATCATTAGACACCTCATTGGAAATTGTGGAAGATTGCAGATCCTCAGAGGGTGGTTTATTATCAACACGACCTAAACTCGTATTCATGTTATCATCATCAATAGAAGATTCATTCACTGAAGACACAGGGAAATCTAACTTCTCTGAACTACCTACACTATAAAAATAACCCTGAACCGTGGCGCTAATTGGAGATGATACAAGAACCCAGGAAGCACAAAATAAGAAAACAGCCAGTGCTCCCACCACAACTACAATCCAAGAAAGAGGCCTTGGATGGGAAGACGCTCTGGGAGAACCTTTGAGGGAAGTCCAAGCCATGTCATTCATTCAATACAATAAAGCTCTACACTAAACCCCGAAGCACGAAATCTTTCAAAACCATACAAAAATCTAAGCTTTCAGTGATGAAAAACACATAAATTAGGTAATTAGTGCCTCAGATTAGATCAATCCCCGAGAGAAACCCCAGAAAGAAAATGTACCAAAAAATCAGATTTCAAACACAGAATGAACGAAAAGCAGATTCCGGGTGTAGAGTGAAGAAAATGGTGAAAATGTTGGATCACCGGAAGAATGAAGAACTTGGGTGGAATGGCAACAAGTTTTGCCTGTGTTTCTGATGCTTGGCCCAATTTGCAAGGAATGGAACGTTATTAAAGAGAAAGGGAGAGAAAGAGTCTCAGATGTAGTTTATCAACAGGAACCTTACCTAAAATTCATGCCCTCTTCTTCTAAGTTCTAGGTTACATACATTTGCTTTTTTTTTC

>C101145_65

ATTCAGTTGGGAGCTATCCCTTCAAATTAATGGAAATGAGTAAATAAATTAGTAAATAAATAAATGAATATAAAAGTGTATGGGATCACATCATGAGTGTCAGTAGTCGCACAGTACACGAATCGTACCTTAATCACCACCATTAATTCATTTTACGGAACAATGATCCCCGTCCCTGACGCTCTCCAAACAGTCCTTAACGCCTCTAAACGTCTTCCTCCACTCACTCTGCCTCTACACGAAGCCCTAGGCAAGGTCTTGGCGCAAGACGTTCGCGCTCCCGATCCTCTCCCGCCCTACGCAGCCTCCATCAAGGACGGTTATGCGGTCCTCGCCGCTGATGGTCCCGGCGAATATCCCGTCGTTGCCGAATCCAGAGCAGGAGATGACGCTCTTGGCGTCATTCTCTCCCCTGGAACTGTCGCTTACGTAACTACCGGAGGACCGATACCTGAAGGTGCTGATGCGGTTGTTCAGGTTGAGAATACTGAAATAGTGAATGATGCCTCCGACGGGACTAAGCGGGTCAGAATATTGGTACAAACCACCAAAGGCAATGATATACGCCCAGTGGGAGTTGATATTGAGAAAGATGCATTAGTGTTGACATGTGGAGAAAGGCTAGGTGCTTCGGAAATTGGCTTGCTTGCTACGGTTGGTGTGACAATGGTTAAGGTGTATCCTACTCCAACAGTTGCTGTTCTTTCTACTGGAGATGAGCTTGTAGACCCAACAACTGGGCATCTGAGTCGTGGTCAGATTAGGGACTCTAATCGTGCCATGCTACTGGCAGCTGCAGTACAACATCAATGCCAAGTTCTGGACTTTGGTATTGCTAAGGATGACGAAGAATGTCAAGGAAGGATCTTGGATAAAGCTTTTGCCTCTGGAATTAATATTCTTATAACTTCAGGAGGTGTTTCAATGGGAGACAAAGATTTTATCAAGCCTTTGCTTGAAAAGCGAGGAAAAGTACATTTTGACAAGGTGTTTATTAAACCAGGCAAGCCATTTACATTTGCAGAGATTGAGTTTCAATCAACAGAAAGCAAAATTCTAGCTTTTGCGCTACCAGGAAATCCTGCCAGTTCTTTAGTTTGCTTCCATCTCTTTGTGGTCCCTGCAATACGCCTACTTGCAGGGTGGACAAATCCTCATCATTTGAGGGTACAAGCTCGACTTCGTCAGCCCATAAAGACAGATCCATTTAGACCAGAATATCATCGTGCTTCTGTTATATGGACTGACAATGATGGAACCGGCAATCCAGGTTTTGTTGCTGTGAGCACTGGTCATCAGGTAAGCAGTCGACTACTTAGTATGAAGTCAGCTAATGCTTTGTTGGAGTTTCCAGCAAAAGGGGGAGTAGTTTCTGCAGGGACCACTGTGTCAGCTATCATAATTTCTGACCTAAGATCCATGGCTTGTGATGAGAATCATACGTCATCAGATGCAGCATTTTCTTTGCCAGGAATTAAATCACACAAAATAACTACTGATTCTTCAGGGGATGCTGAAGTCAGAGTGGCTATTCTTACAGTTAGTGATACAGTTGCTACGGGAGCTGGCCCAGATAGAAGTGGTCCCAGGGCAGTTTCTGTTATTAATTCTTCTTCAGAAAGGCTAGGAGGAGCGAGAGTTGTAGCAACTGCTGTTGTTCCAGATAATGTGGCAAAGATTCAGGACATTGTAAGAAGATGGAGTGATATTGAACAAATGGATCTTATACTTACTCTTGGGGGGACTGGCTTTACCCCACGAGATTTAACGCCAGAAGCTACAAAACCATTGATTGAGAAAGAAACACCTGGTCTTCTGTATGTAATGATGCAAGAGAGTTTAAAGGTGACCCCGTTTGCAATGCTTTCTCGCTCTGCAGCTGGAATCAGAGGATCAACTTTGATCATTAACATGCCTGGAAACCCTAATGCTGTTGCTGAGTGTATGGAAGCTTTATTGCCAGCGCTTAAGCATGGTCTGAAGCAGATCAGGGGAGACAAGAAAGAAAAACATCCTCGTCACGTTCCTCATGCCGAAGCAGTTCCTGCAGATGTGTGGGAACAGAGTTATAGACTAGCCACTGGCGCTGACTCTGATGTCTCTTGTTCCTGTTGCAAGTAAAGTTAATTTATATATTTTCTCCTCTGCTTCAACATTGCGCCCCCATGTTTTTCAAGAGAGGAAAACGAATGGTTCAGTTACCAATCAATGCTCGCCGTTATAAATATAGAATGAAATTGATTGCTTTACAAAAGTAAATGGTGTACCCCTTTATCGAGCTTTAATGATTTTGAAATTTTGTAACTATATCTATACCTTATAAAAGTCATGTCGGACTGTAGGATAGCCATATTGTTGCCTAGTACGCTATTATGATGATTTGCATGCAGTCTTCATGTTGTTCCGCGTATTATGCTATTCGACGGCTTTTCATGAGATGAAGTATCCCG

>C101147_65

TTTTTTTTTTTAATACTAACTTGCTAACTTTCTTCATTTTCCTCTTCAATTGTGTTTAGCAAAAGGCAACAAGTACATAAATTCAAAATAAAAATACAACACCTAACTACATTTGTTGGCAACTAAATGAAACATTTTCTCCGTCCAAACTGTTCCAATAGAACACTCAAATAGCAACAATAACTAAAAAAATCAATGATCTAAGAGCTTTATTACTCTACTTTGAAACTAGATGCTTTATTGCTTTACATGAATTCACGTGTTCCTGGTATCTGTCTTCCACCAGAGAAGCAACCATGCTGCTGAAGAAAAAATTGCAAGCCAAGAGAAACCACAAAATAGCACGCATAATAGCTAACATCTGAGGCTGATCACAAGTGAACAAAATGTGAGGGTTAGTGGTCTACGTCTGAACCAAGGTCACCCCATCGTACGTTGTATCTGGCTGCAAGATAGTGTGCACCAACAGGACCCCTACTACCATAAGGATAGTACTCTGGAATTATCTTCTTCTCTTCTAGTTCCTTCAGTACAGGTGTAAAGAGTGACCAAGCCGCATCCAGTTCATCGCTACGTATAAAGAGTCTTCTTTCTCCTTCAATGGCATCCAACAGTAGCCTCTCATAAGCATCTGGAATCTCCTTTGAATATCTTGCTGCATAGTGAAGATTTAGATTACTGCGGTCCAACTTCATTCCCAGACCTGGAACTTTGTTGTTTATCTTCAAATAAATAGCCTCATCCGGCTGAACTCTTATAACAAGTTCATTGGTAGCCCGATCAAGATCAGTACCAAAATTCCGATTGTACAGATTACCAGGCACATGCCTGAACTGTACCCGTATCTCAGCTCCTTTCTTGTGTAATGCTTTCCCCGCCTTCATCAGAAAAGGTACCCCATCCCATCTTGCATTATCTATAAAGAGGGCAGCTGCAGCAAATGTTGGAGTTAAGCTGCCTGCTGGTACAGTCTTGTCATCAACGTACGCTGGATATGTAACACCTCCTCTAGTGTGGCTCTTATACTGGCCTATAATCACATCCTCAAGTTGCAGGGGTCTCATTGAACGAAGAACCTTGACCTTTTCATTTCTAATATCCTCTGCATCCAAACTAACAGGGGTTTCCATCGCAAAGAGTGCTAGTATTTGAAGTAAATGATTCTGCATAATGTCTCTTATGATACCATAATGGTCAAAGTACCCGCCACGTCCTTCAGTGCCAAAATCTTCCGAGAATATCAACTGGACATTTCTTATATACTGCCTCGACCATAAGGGCTCAAAGATGAGATTTGAGAATCGGAGAACAGAAAGATTTTCCACAAGCTCTTTTCCAAGATAGTGGTCAATCCTAAAAATTTGATCCTCGGTCAGATACTGTTTGAGTGATTTTGTTAATGCAGCTGAAGATTCAGAATCGCGACCAAAAGGCTTTTCAACAATGACCCTGGTCCAACCATTACCGGAAGAAGCTGACGAGCTTGCACATTTTACAGCATCTATAAATATATTAGGAGGAATTGAAAGATAAAACAGGCGATTAGAAGTTCTCCCACCCTCATGTTCCTTCAGCTTCTTGTCTAGCGCTGCAAAGTTTTCCTGAGAATCATATTGACCAGAATGGTAGAAACACCTTTTTAGAAATTGGTCCATCTTCTCATCACAGTTCTCTCTCTTATCTATTCTACAAGTGAGGGTCCTGCTAACCATATTTCTCAATTCTGCATCAGTCATCTTACTCCGTGCATAACCATAGATGGTGAAGTGCTTAGGGAGACAATCCTCATAGTAAAGTGCAAAGAGAGCTGGAAATATCTTCTTCTTAGCAAGGTCCCCGGAGGCTCCAACAACAGTAATACTAACAGTGGACTCATTTTCATCCTTTTCAAAACCGTCCTCAACTCGAGACTCCTGTGGGGACTTAACAGACAACAATTCAACATTCAACTGCTTGTGAGAAGTGCCATTTTCAACAGGAGTCACAGAAGCTGCTACTGTACCTTCCTGTTGCTGGGTGTGGACGGGAACCTGATACGATTGAGGTTGGGATTTGAGAGAGACCTTAGAGGCGAGAAAGCGGTGTGAGAAGGTGACGCGTTGAAGGTATGCGAGGTTAGACGAGAAGGGAGAGGGTTGGAAGGAGGAGGAGGAGGAGGAGGAAGAGGAGGAAGTATCGAGTGAACGACAATGCGTTGAATAGAGCGTGGTGGCCATGAGAGAATGAAGAATTGGTGAAGAGAATAGAAAAATGGGACAAATAAGAAGAGAAAGGGTGTGAGTTCGGAGAGAACGAGGGAAAGGCCCTTGAGATCTCCGAAGGGATGCTGCCTTTCCTTTGCTTTCCTTGTCCCTTTCCCTTCCTCGATGGCAGAAGACAACACACAATGCTCCCACTTTTTGGTCTCTCGGGTTTATTAGGTTTCCCTTTTTTTTCTTTTTCTTTTCTTT

>C101149_65

AAACAACACCAAAACAACACCAAACATTTTTTTTTTCTTTTTCCCACAGCAAAATTCCACGTTCAATTAACCATCTTCCACCCATTTAACTCCCTCACACTTTCACAATTTATCATTCTCTGCACTTTTTATCTCTGCATTTCCTCACACCCCCTCAAGTCAATGGCTCCATCCCAACAGCGTTAGACCCAAACCCCACCTTAAAAATCAAACCTTTCCCCCACCCCCCAGCCTCATTTCCTCTCCATGGATCGCAGCAACACGTTTTCCTCTTTGTTCCTTCTGTTAACCCTGCTGGGGTTTCACGTGTTCCTCGCTTCGTCGCAGACCGAGGACGATGTTAGATGTCTCCGTGGTATCAAAGAAGCCGTTTCGGATCCAGAGGACCACCTCTCTACGTGGCGGTTCGACAACACCACCGTGGGTTTCATCTGCGACTTCGTGGGTGTGTCTTGCTGGAACCAAAGGGAGAACCGGGTCTTGGGTTTAGACCTCCGAGAGTTAAAACTTTCGGGTCGGATCCCCGAGGCTTTGAAGTACTGTGGGAAGAGCCTTCAAAGGTTGGATCTTGCTTCAAATTCTTTCTCCTCCGAGATTCCGCACGAGATCTGTAGTTGGATGCAGTTTTTGGTTTCTATAGATTTATCTGGTAATCAACTCTCCGGGCCTATTCCTCCCACACTTGCGAATTGTTCATATTTGAATGAGTTGGTGTTGTCAGAGAATCAGCTTTCTGGATCAATTCCCTTTGAGTTTGGGAGTTTGAGTAGGCTCAAGAAGTTTTCTGTGGCCAATAATAGGCTTAGTGGGACAATTCCTGAGTTTTTCCATGGGTTTGATAGGGAGGGTTTTGTGGGGAATAGTGGGTTATGTGGGGGTCCTCTTGGATCCAAGTGTGGTGGGATGAGCAAGAAGAATCTTGCTATTATCATTGCTGCTGGGGTGTTTGGTGCTGCTGCTTCGTTGTTGCTGGCTTTTGGCCTGTGGTGGTGGTACCATTTGAGTGGGAAGAGGAAGAAGAAGGGGTATGGGGTTGGTACTAGTGTGGGTGCTGCGGGTGGTGGTGGTGATTGGGCTTTGAAGTTAAAAGGGTATAGGCTTGTGCAAGTTACTCTCTTTCAGAAGCCCATTGTGAAGGTGAAGTTGGGGGATTTGATGTCTGCTACTAATAATTTCAGTGGGGAGAATGTTCTTTTTGCAACAAGGACCGGCACAACGTATAAGGCTGATCTCCCGGATGGTTCCACGCTCGCGGTGAAGCGGTTGAGTGCTTGTAGGATTGGGGAGAAGCAATTTGGAATGGAGATGAACCGGTTGGGGCAGGTTAGGCACCCGAATTTGGCTCCTCTGTTGGGTTACTGTGTTGTGGAGGACGAGAAGTTGTTGGTTTACAAGCACATGTCTAATGGGACGTTGTATTCCTTGTTGCACAAGAATGGTGGGGTTTTGGATTGGTTGATGAGGTTTAGGATTGGGTTGGGCGCGGCGAGGGGGCTTGCGTGGCTGCACCATGGCTGCCACCCTCCAATTATACAGCAGAACATTTGCTCCAATGTGATTCTTGTTGATGAGGAGTTTGATGCTCGGTTGATGGACTTTGGACTAGCAAGGCTCATGGCCTCGGACTCAAACGGTAGTTTTGTGAATGGAGATTTGGGGGAGCTCGGGTACATCGCACCTGAGTATCCAAGTACTTTGGTTGCTTCACTGAAAGGGGATGTGTATGGGTTTGGTGTTTTGCTTTTGGAGTTGGTGACCGGTCGAAAACCGCTTGAAGTGAGCAATGGGGAAGAAGAGTTCAAGGGTAGCTTGGTGGATTGGGTGAACATGCATTCTAATTTGGGAAGGATCAAGGATTGCATTGATAAGGCCATATGTGGAAGGGGGCATGATGAGGAGATTCTGCAGTTTCTGAAAATCGCGTTGAATTGTGTGGTTTCTCGTCCCAAGGATAGGTGGTCCATGTACCAGGTTTATCATTCCTTAAAGAACATATCTAAAGACCAGAGTTTCTTTGAACATGATGATGACTTTCCATTGATCTTTGGTAAGCCAGAACATGAGGCAGCGTAGTAGTTCTTGAGGAAGCATGACAGTATGAGAAGATGATGAGGATGAGATTGTTCCCACTGAATTTCTATTTCATAGTGGAGAGTTCTCTGATGGTGCTAATGTTTATGGTTTGAGCATTTCTTCCATGGGTGACCCTAATCAGGTATAGTTATATTGTATGATATAATAAACTTTCTGGTAGGTTAGATTTTATGAGATCTATTTGTTTCTCTGTTTGTTGTATCCAAATGCAATCTTTCTAATTATGTCCAAGATGATTGGAATATACTTTGGATTTGAATCCTCAAATTATAAATATATGTCCTCAACACCAAAAAAATTGGACATCTACTGATGCACTGTAAAGACATCTCATCATGATGTTATGTTGGTCTAGA

>C101155_65

TTTTTGGGTAAGGTAACTGTAAGACATTAGATCAACGTGTCACTTACATGATGCTCGTTTTCACGTTTAAAGAATTCAAGATTTGTTGATGATACATACATATACATACTACGTATATCGCAAAAAAAAGCAAACAAAAGACAGTCTCAAGTACCATTATCAATTTTTAATGCTAATCTTGAAACGAAGATTGTTCCTAGACAAACTAGATTCACTACGCCTAGTTCCACTACTTCCATTGTTCCATGTGCGACCTTGGCTTCAATCTACCTCCATCTTCAAAATCATCAATACACTGAAGTCACTTGTGGTACTTGGCGGAAAGAGGACAATAAATGACAGAACAACTCATGGTTTCCATGGAGCTGGAGGAGGTGGCGGTGTCGGAAACATTGGGGGAACAGCAGAGAAAAGGGTGTTTTTGTCTATTCCTGAGCTTTTATCACCGGACAAGGCCACAAGCGCAGCAACTAAACCTGCATTGCCTGCAAGAGTAGGCTCCGTGTAGTTGTAGTTGGTTCGGACATCATGAAAACCATCGTGTTTGTCAGGACCAGCAACCATAGCTCCAACAATTGTATGCGGGTTTGGCTTCGATGTGTCCCTCCATTTCCATCCTCCTTTACAGTTGTACTTAATCTTGTTCTTTGGTATAGAAGCACCTCTATGGTGAACATGTTTTGGATAATGATTACCAAAACCTACGATATAGCTCATTTTCCTAGGGTTCTTCCCAAGGATGTAATCAATCTGGGTCTTCGCAAAATCTCGCAGAACATCAGTTGAAAAGAAATTGGGTCCGCAATACCATCCAGGAGTATCAGCAGCATCGAGATAATCACTGTAAAGGGCAGCCAAAAATGCTGCATTGACAACATATTGGAGAGGCTGAGGCCTACCATGGTTTAATTGAATCAAGCCGCCCCTGGTTCTATTAAAGCTGGTGAACATTGGCAGGTAGGAGCACATGATTATGCCAGTCTGGTTGTGAAATGTCCTTAAAATTTCTTCATACGGATAACCAGGACTCAGGAACAACCTCAATCGACTCAGAAGAACCTGAGCACCAGTAAGCTTGTTGTCCCAGCTGAGGACACCATAGTCCGGCCCTCCCCAGAAGGCGCCAGCATGTTTCGCAATGCCGGGAGTGGTAGCCAGCTTCAGGTAAGAAGAATTCCCAGTGGCGAAGTACATCCAAGCCCCTCCCCAAACAAACTCATCCCAGTAACTAGTCGAATTGTAGAACACAGAAGCCTCGGAGCCACCCGCACTGTACCTCCCTCTCTGCATCCTCGAAAACTTGAACAGCGTGGTGGCCCCGTGAACCAGCTTCTGCGAATACGCCCTGTTGTCCTTGAAGACAATCGAGGCCGCCGCCAAGGCCGCCGCCATCTCCGCAGCGAGATCGGAGCAGCTGCTGCACTTCTGGACGGGGCGGTCGTAATCGATGTCCTCGGGTCGCATCCAGCAGTAGTGGTCGTTTGGAGTGGTGCTCCCACCGGAGGTGTCGCCGGAGCCAACCTGCGCGGCGATGAAACCAATGGTGTCGGCGGTGTGGTTGAAGGTCTTGAGAAAGTAATCGGTTCCCCACTTGATGAGTTCCTTGACGTGAGCCAACTCTCCGGCAGCCTCATACTTGGCGCTGTACTCGATGACGCTCCAGCTGAGCATGGTCATGGCGAAGGCCTTGGGGAAGTGAAACTTGATAGCGTCACCGGCGTCATAGTAACCGCCCACCAGATCCTTGATCGCGCTCGACGCACCTTCACCCTTCCCATCTTGCATGCACGAGTTCCCTCTCCATGAAACGTTGTTATGCCTGGGAAGCTTTCCCGATTTTTGGGCGTTGAAGAAGAGGAGGGCTTTGTGGAGGGCGAGAGTGTAGTTATCGGGAGGCGCTGGCTTGTGATGGTGGCGGGGGACAGTTTTGACTATTAGAGTGATTAAACCAGCGAGAACGGCGGAGACGGCGAGAGTGCCAAGCGTCCAGATAAAGATTTTGCGGCTGACGATGATGCATCCCAGATCTACGTATTTCTTCTTCTTCTTCTTCTGTTCTCCGGCACCCAGGAGCCAGCTCTGTTGAGTCTCGTCTAAGGCGGCGCGGTCCAACTCCTGCAGATTGCGGCTGCGCTCGTCGTCGGTGGCGGAGTCCGTGGCGTGGATCTCCAGCGGCCCTCCCCAGGGATCCCTACCGTACATACTCATTTTGGATATCAATCCCTAATCCCTAATCTAACAACGTAATGCAATGTAATGAATGAAGAATTAGAATTGTAGTAACTCTTTTCTCTCTCTCTCCGTGTAGAGAGTGTCGGTTGATGAGACAGAAGGAAGAGAAGATTAGTGAGACATGTGATTGTGACGCTCCTTGTTGTTCTTCTTCAAATCCTCTTTCCTTCTTCTTCTTCCCTTCATTCCTTTTATCTCTTTTTTTCTTCACAAATTAAAT

>C101157_65

TTCCTCAAATTAGTAGAGTTCCATATATGTGAGTGAGCAAAACATATAAAAGGGAATCGTGTTACAAATAACTTACCCAAATCAATGCTACATTAGGATTAAGATATAAAAATGATAAAAGGTTACCAGTTTATTGAAAGGAAGGGTCCCTCCCAGTGCTAAATGGCAAACAACTCAAAAAGCTAATATATTACAAAAAAAGTGTCAATCACGTCCTCAATTTGGACATATCAGATGAGATGCCATTTTGGCATTTGCTTTCCAGATAACTAATAGTATGTCCCATCCTCTCTCCATTGTCTTTGGCTGAGAATACATATCATCTCATCACAAAAGAAGATTATGCTTGAGTTTGAATGCCTAGGGAATCTCATAATTTTTGCCCCTTACTCTAGCCACCAATGAAGCCTAACCGGTGAAGAATGACAGCCAAAATGAGCATGCATATTGCAAAAATCATGCCAGGTTTCCCTAGCAGCCAGTTCTTTGTCTTTCTTGCAGATTCAACTCCTCTTTGAATCCTGTCTGCCCAAAGCATCATTTTATCCAAGCTCTCTGGTGACATGGATGACACAGCTCGCTGAGCTTTTGCTGCCTCTTCTGGTGAAAGCTTAACACCAAATTGTTCACCCATGTTTGCCATCATTTCTGGGCTCATATTTTTAATCATGGATGTGAACATCTTCCGCATAGCTGGATCTTTCATCTGGTTTCTCATCTGTTCTTGTAGATCAGTACTTGACGAAGGAAAGTTTGACTGAGAAGCACTTCTCATATTTGAAAAGACATTATGAGAAGAACTTGATTCCCCAAAATTAGTAGTTTCATTAGTACTTGATGATGGGAAGTTTGACCGAGAAACATTTCTTTCAATCTCATCTACTGCTGCTGATGGAATAGATCCTTTCCCTTTCATTGAGGATGCCATTTCAAACATCTTCTTAAGGTCATCAGCTGGCATTTTACTTATCATATCACTTGCTGCTTTAAACATGTCAGGTGTCACATTGGGGGGCATTGATCCAGGGTTGAAAGAAGAATCAGGGGGACCTCCTCTAAAATATGGGTTGTCCCCTTGAAATGAAGAAGCCATGTCAAGCATTTTTTGCAGTTCATCAGGCGACATCTTACTAATCATATCAGAGGTAGTTTTGATCATATCTGGGGGCACATCTTTGAATTGTCCAGCATTCAAAGAAGCCAGCGTGGAAGGATTGGTGTTTGAAATGAAGTTCTGGAATGACCTGACAGCTTCAGGATCTTTTCTCAATGCATCTAAACTGTCTGCATTTGATTTTGGATTTCCATTGATAGCTGTACTGCTACTCTTAGAGGAGTCACCAGATTTTTTTGGTTCAACCACTGTTTGTTCCAAAGAGCTTCCAGAAGGCACATCCTCAACATCTTCAGTTATTTCTTCAATTACTAGTCTTCTAGATGCTTGCTCACCACCTTCCATTGTCAATTTTTCCTTGGCATCCCGTAAAAGCTCTGCAACTGTATCATCATCAGGGGACACTTCAAGTGCCTTGCTCAGATCAGAGACAGCATCATTTAGTAGACCTAGTTCCTTATATGCTTGACCTCTTCGATAAAGAGCCTTAAGGTTCTTCGCATCATATGCCAGAACCTCAGAACCTTCTTTTATGCACTCATTATACTGCTTTGTCTTCAAGTAACAGGACATCAAGTTGAGAGAACATGCCAAAAGAAGCTTTCTACTTTGGAAAGATGGAATCTCTTTAATGTTTTCTTTAGCAAGCATATACTTCTTCAAAGCTTCACTGAACTTTCCTTGACTGTGAAGTTCATTGCCCTGCTTCTTCAACATCTCAGCTGCACTCAACTGATATTTGATCTGGGCATCAACCCGGGATCGCATGGCTGCCACTTCATCAGGGGACGCATTAGCCATCTTCTCACCAATCTCAGCCATTTCCTCCGGACGGGTATGCTTTAACTGCTCGGCAGCAAGTTTAAAGTCTTCAGAACTCATGTTCTTCATGCTCTCCGAGGCCATCCTCATCAGCTCTGGATTAGACATCATCTGTTGTTGGATCCGAGCCAATTCAGCAGGTGACATGCGACTCATCTGCTCCCGAGCGATTCTCATCAAATCGGGATCCATCATACCGTTGAACATTTTCGATGCCCAATTCAAATCTAAGCAACCCCAGAACGTCAAAAACGAAGGTTGAGACGATTCTCGATGAGGGGAGGGAAAGGGGGTACGTGGCTTCTGCTCTTTCGGATGGGACAAACCTGCAAAGGTTAAGAAACGTTTCCTAGTTATGTGTGTTTTGGGAAACTTGAGAAGAAGGTGTTGTGTCGGGTAAAATTGAGGGGAATAGGGAATTGGAAAAAAGGGTCCGATTTGGAGGTGCCGCCTACCAAACCTGAAAAGAGGAAAACCAAAATAAAACAAAATAAAAGAGGAATGAAGGAGGAAAGAGA

>C101159_65

CGTCATGCAAAATCATGGTCTCCACTTGACCCCAAAACCTGTCCATCATCATCTCCTTAAAAAACAACCAAGTCCACACACTCCAACCAACATCATTCACTTACTGTTGAATACATACTTTGTCAAAATGCATGAATAGAAAGAAACAAATTTAAGTCAAGCAAACCAAAAACCTACCTACACATCCAAATTATGATGAGGGCAAGGTATTAAGAAACAAGAACACATCTTTCCAAATTCTAAGATTACCATATGTATTTATATATTATAATATATATAAACTTCACTAATGATTAAAGTCAAAAGAACGAAGTTCTTTTCTTCTTCTTCCTTCCATCATGTCTTCATAAAGTTGATGTCTGATATGCAATGGACTTCACAAGGCATTGCACAAGGTGGTGGCACTGGCACCTTCTTTGTTGTTGTTCTCTGCCCTTGGACTGCTCCCAGCACTGCTATTGGAAAGTGATCCAAATGCTGTTTTTTGCAACACCCCATTTGGAGATGACCCTAGTGGAGGACTATTGTCCCACCCATCTTTCATGAGGTTGAGAGCAGAAGTGTTCTTGCCACAGTGATCACTTCCAATGCTGTTGTTATTGCTACTACTTAGGTTCAAAACCTCTCCAAGAGGACCACCCATAGAACTCTCCCAAGTGATTGGAATCCAATTGGCTTGCCTAGTGTTTGACTCATTTGGAGCACCACTTCCCACTCCCAGTCCCATTTGAATGGGGTCTAACTCCCTGGAAAGCCTTAGTGGTGACAATGTGAGTTTCTCATTGGAGGGTGAGGAGGTAGTGAATGACATGAAGTCTGAGGAAGACATTGGTATGGAGATTGATAATTGAGTCCTATCTGACTGCATGTTGTCAAGTTCAGGCCAAAGAGAAGAACGATGGTGATGATGAGAGTGTGGTTTGGGAGAGTCATCAATGAAGTGTCTGAGGGAATGTTGTTGTTGGGACTCAGAATCATCCTTTTGAGAAGATGAACCAAAGCTTCTCTTCTGTGAAGGGTTGAGCAAGGAATCTGAAGTGACAAGCCCAAACTCATTGTTGTTCCTTGAGGATGATTCATCATATGGGAGTTGGTGCTTATGAATCATGAAAGGGTCCTTTGGTTTCAGCTCAAGAGTTGGAGGCACCATGGGAAGTGCAGGGCCATCCTGCATCCTCTCACTAGAGTTGTTATTGTTCTCTTTGTTACTCATGAACATCCTATTGATGGTGTTGGCTGAAGAGGAATTAGGTGGAAGAGGGTGGTGCACATTGTTGTGTGCAAAAGTGTTGTTGTTGTTGTTGCCGGGCACCACAATGGAGTTTGAGGAAGCATTAGGAGTAGTAGTGGTTGTTGTTGTCGTGGTGGTAGTGGTGGTGAGGGCATGGCCTGATTGGCCTTCCACAGGCTTTCTTGAACGATGGCGTCCTCTGTTCATGTGCCGCTCGCAATATTTCTGATCAACTACGGCATCTCTTGAGCACCGCCATTTCTTCCCATCTGTTCTCCTACACCTCCCTGGCTCAGGGTCTGTGTTGTTCGAGAATCCCAGATGGAAACCTCCCCATCCCAATGTGTTGGGTCTGAGGAGTCCTGTTGAAAAGTTGCAGAAGGCAGCAGAATCGAGTGCTTTTCTGATGGGTATAAGAAGATGAGATGGTACAGGCACGTTTGCTGTTATGTACTTGTAGATCAAAGCTTGGTGTTCAAGTTCCATCCACTGTGATGGAGTGAATGGCCCTCTCACACCAGCCAAAGCCCCATGCATGCTTATGCTTCCCGAACTGTAACCTGTATTTCTGCTGTAACTAGACAATTGGTGGTAAGAAAAAGGCAATGTGGCATTTGAGGAGGCCTTGTCCACCAACAAAGGCTCTGATTTTGGTGAAGAGAAGCTCAGCATTTGTGGTTGGTTGTGACCCTCAGAGAAAAGTGTGGCATTAGATCTCAGCAGAGAGTTTCTCTGGTGAAAGAGCATTGCTTTAGATGCAGCAGACATGTTGTCATCAGTTTTAGGCACTTTTGAAGTCCTCCACTCATCTTCACTTGCTGTGCCAGATCTCTCTTGCTTGAGCACCCCAGATCCGTACCACTTGTGTTTTGTCTCAGGATCTGAAGCAAGAGAGGAACCAAAGGCACCACTATTATCTGAACCCACCAACCCCTCCAAACCGACCACCCCAAGATCCATCAAGACAAAGAAAGAACCAAAAACAAATAGAAAATAAGAACACACAAATAACTAGAACTAGACACTGATGATAGCATTAACCAGACTAGGCTTCAAGGAGTAAGGGAAGTGTTGGTGATGGGGCATCTCTTCCTGAGAGGTGGTAGACAACAACAAGAAGAAGAGTACACAAGTTGTTGCTGCTCCCTTTGGAAAAGGAAGACACATCTCATGTGGGTGGAGAGAGAAAATGAAAGAGAGAGAAAGAGAGAGGTGAGTGTGGA

>C101167_65

GGTTGTTGGATTTGTTGCAAGTGGAAGTGGAATTAAGCGGTGAGGGAGAGGGAGCGTTGGTGTTGATCAGAAGTAAGTAGTAGTCGGAGTGGAATTGAAAGCAGATGTCATATTGTTGGCACAGTTTGGCTCTTGTGCCAATTTCACAATCACAACCCCTCTTTTCCTGTTCACAAACACATGGTATCTTCTCGAGGCAGAGGGTTCCTTGTTCACGGCTTCAGGAAATGTCCGTGGTGGAGCCATCCTCACAATACCAGTATCAACAACAGAAGTACTACACAAACACAAACACAAACACAAACACAAATACGAATCCCAGTTTCGGAGGGAATGATGATTACGACGAAGACGAAGAAGAGGGAATTTCTAAAATACGAGTCCCCAGACAGAAACACATCCCTGTTTCCAAGTCCCAACTCGTGGATGCCATCCTCTCTGTCATGTTTAACCAACCCCAAGATGATGCCCATCACTTTCGCCTTCTCACTTCATGCTTAGACTCTATACTTCACGCTGAGCACAAAAGCATCTTAGAGGAGATGCGTTCCGATTATCAACTTACCAATTCACTCGAAACACACAATTCACATACACAACTCGTGAATGTACCCAATGGGAATACCAATGATTCAGACATGCTTACCAGTGGCAATGACATCATTGCAATTCAGGAGAACGCCAACCTCTATGACTCTCAGCAGGATCCACAGGAAAAGCCTGTGCTCCCTTACCCTGCGTTGGACCTCACCGCTCTTTTACGATCTCTCGACATAGCAGCTACCGAGAAAGATTATGATAGTGGCTCCAGAGTTACCATTGCTACTCGGTTCCAAAATGCTTTCATGCAGCTTCTTTCAGATGCACAATTTGAAGAACTATCAGCTAGGGACCTGATGTTAACATCTGCATTGAACACAGACTATCTCCTTACCTTGCCAGTATATGTTGATTGGAAAAGAGCATATGAGTCTAATGCCATAATATTTAGGCGAGGTTATGCAACTGAGAAGCAGAAGGGCTTATTAATTGTTGAAAAACTGGACTACTTACAGTCTAAACTTCTGCGAAGAACCTTCTTTGTTATTTCAAAACCACTAACAAAACTTGGCGCCTGGTTTAGTGAGCTTTACGAAAATGCCAGTCAGAGACATAAAATACAAAACTGGACAGAAAGATTTAGACTTTGGCTAAAGGAATTGTCAATATTTCAGAAATCACTACTTTATAATGGCCCTGCTTCAAATGAGCAAATAGGTGTAAATCAAGTATCAAATGTAGAACTTCCTATTTGGCTGGCAGCTCAGAGGGCAGTAGCTCGATATGAAGGGATTTTATCACCAGTTGGTCCTCGTGGAAGGCTCTTGAGGAGAATGCTTTCTTGGATAGGACTTATCCCCCCTTTGCCAGAAACACCTTTTGAGGTTCACAATGATAATAACACTCCTGAACCTTACCTTAGGCCTACTTTTTTATCAAGGATATCACTTAGTGATATATGGAGACCCGCAACAAGGAAATACTGTGGTAATGATCCTTGGAAAATGTTGAAAGCTTCCATTTCCATTCTTTTCTCACAGTCAGTCCTGCAGGAGCCGGCATTTGAAGAATTAATTCTACTATATACCAAGGAAGTTGGTGAAACAAATGCTAAAGATAAAGCTAAGGTTCCATCATTGCAATTAAAGATTTATGAGAGAATTCCTATTCCAGATTTACCGGTAATTTTCCCTCACAAAAAGCTATCATTCCGTATCATAGACACGGTGCGCTTGGATGTTGCCACAATTTTGGGACTTTTGGCATACTTCATAAACTACAAATTTGAGAATGTCTTATCTTCCCCATCAGCACTATTACTTGATGTAGTTGCCGTAAGTGCTCTAATAATATATGGGAGTCGTGTGGTTCTTGGCTACAAACAGACATGGGATAGGTATCAGCTGTTAGTCAACAAGACACTCTATGAAAAAACATTGGCAAGTGGCTTTGGCTCAGTGCATTTTCTTCTAGATGCTTCTGAACAGCAGCAATACAAGGAAGCTGTTTTGGCTTATGCTATCCTGCTCAAAGCAGAGAAGGGTCAGGTTATGTCTCAGCAAAGTGTTGGAGAAAAATGTGAGAGATTCATGTATGAAGTGTTTAAAGTAAAGGTTGAAATGCCTATTGATAAGGCACTCAGTACATTATTAAGGCTGGGCCTAGCAACTGAAACTTGTATTGATGGAAGGCGAGGATTGCTGGCTATACCCTGTCCTAAGGCTTACGAGGTCCTTAAGGAACGATGGAATAGTTTGCTTACTTAAAACGTGCACGTCCCGTAGAGTTCTGGAAACGACCTTCACAGTTTACCAATAAAGGCATTGTATACGGTTAGTATATAATACTCTCATTTTATTCGGTAATTATTCTCTGTAGACAGATAGATGAGACTAAGCATATTCATGCTGCACTGT

>C101169_65

TTGTTCCCTCGTTTGTTCCTACGTTCCTCTCTCTCTCTCTCCTCTCCCTCTCTCTCTCTATCTCTATATTCGTGTCTCTGTCTGCGGCTCCGTACGGATTTCGATACTCCGAGGAAGAGGGGGGCGCCATTTTTCGATCTCGCAGGGAGAAAGCGATATTCGTTTCGCTCTTTTTCACCTATATATTATTGTTGTTGTCATTTATCGTCGTGTCTGAAACGACGCCGTTGCGATTCGGTTGCAGAGGCAATCTAGGGGCAAATTACCTGTGTCTTGAATAGAATGCCAGATTTAGGAGAAGTATTAGCTCAAGAAGATGAGAGGGGTAGAAGTTTGGAGCCCGAAACCTCTGAGGATGAGTGGCGGAAGTCTCGAGCTAGATCTCTTAGGAGAAAAGCAATGACTGCTTCAACAAGACTCACTTATAGTCTCAGGAAGCGTAACACACGAGTAGCAGACAGTGAATTTGCTTCAATTTTCATAGAAGATGTCCGCGATGCTAATGAGGAAAAAGCTGTGAATTCATTTCGTCAGGTGCTACATACAAGAGATCTGCTTCCAGATTCACATGATGATTATCATACAATGCTGAGGTTTCTGAAGGCCAGAAAGTTTGACATTGATAAAACAGTCCAGATGTGGGCTGATATGCTCCGTTGGAGGAAGGAGAATGGAGTAGATTCTATTTTAAAGGACTTTGTATATAAGGAGTATGAAGAGGTTCAGCGTTATTATCCTCATGGTTACCATGGTGTGGACAAGGAGGGCCGGCCGGTTTATATTGAAAGACTTGGTCAAGTTGAACCCAGCAAATTGATGAGTGTCACGACGGTAGATCGATTTTTAAAATATCATGTTCAGGGATTTGAGAAAATGTTTAAACAGAAATTCCCAGCATGTTCTATTGCAGCAAAGAGGCATATAGATAGAACCACAACAATACTTGATGTGCAAGGCTTGAATTGGGTGAGCTTTGGCAAAGTTGCACATGATCTTGTTATGCGCATGCAAAAAGTTGATGGTGATAACTACCCTGAGACATTAAACCAGATGTTCATCGTTAATGCTGGTAGTGGGTTCAAGCTACTATGGAATACTGCAAAAGGTTTTCTTGATCCAAGGACCACAGCCAAAATACATGTCTTAGGCAACAAGTTTCAGAGCAGATTATTAGAGATTATAGACTCAAGCCAACTTCCCGAATTCCTTGGAGGCAGTTGTTCATGTCCAAATGATGGAGGGTGTATTAGATCTAACAAAGGACCTTGGAATGATCCTGACATTTGGAAACTATTACATTCTAGAGAAGCAATGAAGCTGACAAAGTTTGGAAGTTCTTCTGTTGCTAATGAGGTAGATGTGAAGTCGTATGCCAACAAGGTTACAAGCTCTGAAATATCTGAAACTGTATCAGCATCAGCAGCAAGATTGAGTCCTTCAGCTTTCGTACAATCTGTTACTTCTAGTGATAAAAAAACAATCAGTAACTGTGCACCTAGCGAAAACCTAATCGAGCCAGTTAATGCTGTAAGAGAAGTTGGGGATGTTGATTCAACAGGTGATTCATGCAACAGTTTTCTGAGAAGACTACCAGAGAACCCTATTCCCTATATTACAAGTATTTTGGCTCAAATCACAGTCAAATTGCTAACTTGTATTTATGTGGTATTTGCGGCATTGGGGAGATTTTTTATGGTTCGCTCTGTAGACAACCAACCAAGAAACCATGGGAGAACCCAATCAGCACAATCTAATTTACGGGAACATTTAATTACTCCGGCAATAAAAGAGCCACTTTGGCAGAGGTTGCAGAATTTAGAGGCAGTGGTTAGTGAGATGGCCAATAAACCTAAAACAATTCCTCCTGAAAAGGAGGATATTCTTCAAGAATCATTAAGTCGTATAAAATGTATAGAATATGACTTGCAAAAAACAAAGAAGGCCCTGCTAGCAACTGCTTCAAAGCAAGTTGAGCTTGCTGAGTCATTGGAAAGCTTAAAGGAGGGTAGATTTGATGGTACAAACTCATGTTGGCCTAAAAATAGAAGTTATGGCCCAGGAAGATAAGTATCCCTCTTCTCAGCATCAGTTTGCAGAGGTTGCTTGTCCATTGTTTTTTATACTCTACAGCTGAATTACTACCAGACCTAAACTTCCATTGGCTTATATAAAAGAACTGCTCTCATTCTCATAGGTTTGAGGAGAATGGGCTATGGGGAGTAGATAGCCCAATGTATAGAAGGCAACAAGAATTTTTATGAGGATTCTGGACTCCAAACTATTCCTTTCCAATGTATAACTTAGTGTGTTAACCACATTTATTTTCATATTGGCTTTTCTGTACAGAGGCCATAGATACAAAAATATGCTACTCAAAATTCGGATGTATTTTAATACCAGTTGCATATATGCTAAAGAAACATTTTCTGTAATACCTTGTAAACATTAGCAAGAGC

>C101171_65

CGTAATTATTTTTTTTTTCTTTTTGTCAATATGAAATTAATTTGGAATGCTATCAATCAATAATCCAGACATGTTCGTGATACACACAAGTTGGATCCAACATCTACAGGATAACCAGGGCAATGCATTTAAATGCTAAGTAGTATCTGTGTGCCTCCCAAGTTGACCAGTGACCATCTTCCGAGTTTCTTCCATGATTCAATGCAAATGATAGTCACAACTCAAAATCCAATCTCCCTGCATACCTTCCCTTTATTTTAGTTCGTTATTGATTCCCTTCTAAATATCAAATCGGTAGAGGATGTGCAATGGCATCACAGGAAGGTGAGCACATGTGTTCTCCTCTGAGGTGTTTCTGCATGGTTATACCAACATGAAACTTGAACTAACTGTGATTTTTACGTTCTGAACAAATTCCTTTTGCTGTCAAGATAATGCCACCTCCTCAAGCTGGTAAGTTGGCTTCCTTGAGCAGTCTAAAATGTAGTGACCAGTAAATGTACAAGAGTAATGCAAAATGCACATGATGTTGGAACATGGAAATGTACCACTAGGGAGAAACTGCAACGAGACTTGGTGAAGCTTTAGTAAAGCCTCTCTTCAATTATCATTCAGTTGGTCTTGGACATGCTGGAGACTGAACCTCTATGGCTGGGCTCATCTCAGTAGTTCCTGTCCTGGAATTCATTACAGCACCAACATTGATCTCATCTGATCCAACTCCAGATGTACCTGAATTGGAGAGACCTTTGATCTGAACAATGATAATTGTTATATCATCTGTTCTGTTCTCAAATTCCAACCATAGATTATATGACTTTTCTGCAATAGCCGCACATGCATCACGAGGATCCATATAGCTTGCTGCCATATCAACAACAGTTTGGCTTGTAAGGAATTCAAATACGCCATCACTTGCCACAACAAAGAAAAGATGATCAGGTGTAAGATGAACAGTTTTCACCTCAGGAATAGCAGTGACACCAATAGACTCTGCCAAACTATCCCCTATACTTCTTGTAAATGCAGTTCCAGGATACATCCCATTAGGAACCCACAATCTGGGAGGATCACCACCCCGACTCTCTTCATCACCCCAATTCTGGATATCTGGATCCTTGAGCCCTTCCACCTGATCAACACTCAACACCCTTGCCCCACAAAGCTTCACTCTCTCATATTCATCTCTGCGGAAGGGCGTCTGATCAGAGGACAAGTCCTCCGCAACAATGCGGTTCCCATCCTTAACAGCCAGCACCGCTCTCGAATCACCAACATTAGCAACATAAAGGGTGTCACCAATAACAAGCACCGTTATCGCGGTGGTGCCACTCATAGAATCGTCAATCTCACTACAACTACGTAATTCTTGGTTTGTTGCCAAGAATGCAGAACTGTAAGCTTGAACAGGGTTCTCCAACAATGCAGGGTCATTTGCCAACTTCATTACCAACCGATCCTTAACGAAATTTGAACACTGGCTACCAAATTGACCATGCCCATCATAGACACCAAAGAAATGGACATTGGGGTTACCTTGGAGTTGTGTACTAATGCATAAACTATCTTGGTTTTCTTTATCAGGTGAGTCAGGGTAGTACCCCCGCTGAGTGAGAAAAGTGTATTCCAAAATGAAGTTGTGGGAAGGAACGGAAGTTTGCTTAAGTGATCTCTGAGCGAGTATGTTCTTCCTGTGACCACGAATGAAGCCAATGTCTCGGTAATCCCTAGAACCATCCTCTGATGAAGACCGGTGACAACAACAACAACAACACTTGCCATAGACACAACCCATGTTTCAAAGAAAGGCTATGCTATCTTTGAAACAACAACACCTGAAATAGAAAGCACTCACTGAAGCTGTTCACAAATAGTAATGTTTAGGCAAGAAGTACAGTGCTAGGTGAATCTCTAACTCCTCTACATTGTGATAAACAGGTTTAGACACTGATCCACAAACCCTAGAGAGATAACTTAAGATTATGGGCAGGTAATCGCGTTGTGCGATTGGGGGTCAATAAAAGGGGTTTTGTGGATGGATTTGAAGTGAAAAGGGTGAGAAAAGGAGAACCCTAGAGCAACCCCAGGAAGAAGGAAAAGACAGAGAAGATGTATTTTGGCGTTTTGGAGGCAGAATGGTTGATGGGGTTGCCGAAAAAGAATTGTGCTTTTCGTTGGGTTGGGTGCAGAGAAGAGGGAAACCCTGACGCAAAAGGAAGAGTCCAAAGAGAAGTTCACAAGAAGAGATTTGGAATGAGAGGGACAGAAGGGAAGGGAAGTGGAGGAAGAGTAATTGAATTGAGATTCTATTCTAATCCTAATTCTAATCTAAATCTAAATCAAATTCTCAAACTCCCATCACCATCTATGTATGTGATGGATGGTTGTGTTGGTTTGGATATTTGCATGAGTATCCCGTCCTCCTCCATTATCCATTTCTCCCATAAACACAAAAC

>C101173_65

GACTTATAGTGTTCAAGGTTTTCTACTTTTCTAGGACCATCTTAGAATGAATGGATTGCGATAAAAACTAAGTCAGGCATTGAGAATTGTCGTGATTTGATTTCTATCCGATATTTCAATCAACTAGAATAGCAAATATAGTCCACAGTCCACAAACCTTTAAATCGTTGGAAGGAAGGAAAATGGAGGAGACTCTTCCTCTCAGTCTCAGCCATGTCAACAACTCTTTGGTTTTCACCAATAGACTATACATGCTCCTCCATTTTACTGCTATTTGTTTCTTGGTTTATTATAGGCTGCGTTTCTTCTTTCAGGATCCACAAACCCGAGTAACCCCTTTTTTCCCTTGGCTTCTTGTTTTTGCCTCTGAAATCATTCTCTCCTTCATTTGGATTCTTCATCAAGCTTTTCGTTGGCACCCCATTTCCAGAACTGTGTTTCCTGAAAGATTACCTCAAGATGACAAGCTCCCTCCCCTAGATGTGTTCATATGCACGGCAGACCCCACTAAGGAACCTACTTTGGACGTGATGAACACTTTGTTATCCGCCATGGCACTGGATTACCCACCAGAGAAGCTGCATCTGTATGTTTCTGATGATGGAGGTTCACCCGTCACCTTGAATGCCATGAAGGAGGCTTGGAAATTTGCAAGGTGGTGGCTTCCTTTCTGCACGAGATACAGAATAGAAAGTAGGTGCCCCAAGGCTTACTTTTCTGCTTCAGAGAATGATGGTGGTGATTTTGATGCGAGCTTTGAGTTTATTGCAGATAAGAAAATGATTAAGAAAAAATATGAGGCTTTTGAAGAAGAAATAAAGAGAGTAAAAGAAAATCAGAACAACTCTGGAGATACTACTGGCAAAATAAGTCAAAATCATCCACCTATTATAGAGGTGATACAAGAGAATAGCAGCAGCGAAATAGAGCAAGTGGAATTGCCTTTCCTTGTATATGTTTCCCGTGAGAAAAAGCCTTCCCATCCCCATAATTTCAAAGCCGGGGCCCTCAATGCCCTTTACCGAGTCTCTGCTGTTATAAGCAACGCTCCATACATCCTAGTGCTGGATTGTGACATGTTCTGCAATAGTCCAGCTTCAGCACGCCAAGCAATGTGTTTCCACCTTAATCCCAAGATATCTCCTTCGCTTGCTTTTGTTCAATTTCCTCAGAAATATCACAATATAAGCAAGAACGACATATATGACAGTCAGCACAGATCAGCATATAAAGTTCTATGGCAAGGCATGGATGGGCTAATGGGACCAGTATTGTCTGGCACAGGCTTCTATATGAAAAGGGAATCACTATATGGAAATTACAAAATTAAAGACACTGAGCTTGAACTCCAACAATATTTTGGCACATCCAATGAGTTCATCAAATCCTTAAACCAAAACTGCACTACAGATTCTGTCACCCTTGGAAGTACATTGTCTGAAGAAGAGACTTTGCTTTTGGCTTCTTGTAATTACGAAATTGGGACTAAATGGGGTAAAGAGGTTGGATTCCTATATGGTACTGTATGTGAAGATGTTCACACCGGATTCATATTGAATTGCAATGGTTGGAATTCAGTATTGTGCGATCCACCACAGCCACAATTTCTTGGAAATAGTACCACTAATTTGAATGATTTACTAGTTCAAGGCACCAGATGGTACTGTGGACTGCTTGATATTGGTTTAAGCAGGCTTTGCCCTCTTATATGTGGGCCTTTAAGGATGTCTCTACTTCAGAGTCTTTGTTATGCACAACTAACATATTTCCCTCTGTATTGCTTACCCCTTTGGTGTTTAGCAATTGTCCCTCAGCTTTGTCTGCTAAATGGAATTCCCCTGTACCCAAAGGTCTCAGACCCATTTTTCTTCATTTTTCTGTTTATCCCTCTATCAGCTCTCACCAAGCACTTAGTTGAAGTGCTCTCAACTGGAGGAACATTTAGAAAATGGATAATTGAACAAAGGATATGGATGATTGGATCAATCACATCCCATCTATATGGATGTTTGGATGCTCTACTGAAGAAATTTGGTTTAAAAGAGGCTAGTTTCTTGCTAACCAATAAAGTGGAGGATGACCAACAGACTAGGCTTTACCAAATGGACAAATATGATTTTAGAACATCAAACATGTTTCTGGTCCCAATGGTTGCACTCCTCACCATCAACATATCTTGCTTCATAGGGGGCATTTACAGAGTGTTATTATCAGAGGGAAATTGGGACCAGATGCTCATACAGCTTTTCCTTATAGCTTATATCATTGTTGTCAACTATCCAATAATTGAAGGGCTTCTGATAAGGAAAGATGTAGGACGCATTTATCCTTCACTACCCTTACTTGTAACATCAAATATCTTGGCCACAACCATCACTTGTACACTTTATTCTCTACTAAGGAAACTATAAGGTCCCTTCTATGTGCCCAATAACATTCATCCACCTTTGCTTTAAT

>C101177_65

CTTTCTTGCCCAGAGAGAACCCAAAATCAATTAAATCTCCATTATTCCAATTTCAGACACCATCAAAACACACTCAGATTTTAGATTCTCCCTACAATGGAGTCTACACCGGTTAATTGGGAAGCCCTAGACGCTCTCCTCATCGATTTCGCAAAATCGGAAAACCTAATCGAAGAAGACTCCTCCGCCCCCTCCCCCTCTTCCTCCTCCTACCACTCTCGCCTCCTCATCCGCCACATCCGACGCTCCCTCCACACCGGCGCCATTGACGCCGCCGTCCATCTCCTCCGCCTCCACGCCCCTTCCATCCTCACCGACCACAAGATTCTCTTCCGCCTCCACAAACAGAAATTTATTGAGCTTCTTCGGAAAGGGACTGCGGAGGATCGAGACTCTGCTATCGAGTGTCTGAGGACGTCTCTCGCTCCTTGTGCGCTCGATGCTTATCCGGAAGCATACGAGGAATTCAAGCACGTTCTGCTCGCCTTTATATATGACAAGGACGATAAGACTTCTCCCGTGGCAAATGAGTGGTCAGAACGGAGGAGGCTTGACTTAGCTGGATATATGTCCTCTATGTTAAGGGCTCATTTAAATGCCTATGACCCAATTTTTTCTATGGCTCTGAGATATTTAATAAGCATACACAGGGTATATTGTTTACGTCAAGGTATTACATCACCCATCTCTGATCTGACTGAAAGATTGCTCCTTGAAGAGCGTGACCCTCCTGCAACCCCGCAAGACATTTTATATGAAGTTCCACCATTTGATGAGGTTGACATCCAGGCTCTTGCACATGCTGTAGAACTTACCAGACAAGGGGCAATTGATAGCTTGAGATTCAGTAAGGGTGATCTGTTCGTGGCATTTCAGAATGAATTATGTCGGATGAGGTTGGATGTTCCACTTCTTGATCAGCTTGTTAGAGAGTATTGTGTGTACAGAGGCATTGTTGACTCTGCATCTGGGAAGCAACCTATTCCAGAACCTGTGAAATTCAACCAACAAGACCCTGGGTATTGCTCATCAAGGGATTGTTCTGTCGAGATAGATTGTAATGCCAGCAAGCACTCTGATGGTGAAACTTCTGTTACCAATGCTCAGATGGACGGTTCCCCTGAAAATAATGCTGATGTGACGAGCATGAGGCGGATTGATTTTGAAGTACGATATGCTTCTGAACTCACAAGCATCCATGAAGACTGTAGCACCAGTGGCTCGCAGCAACATGAAGAAGCAAGTGCTTTGCAACGAAGTAGGTTGCCTGGAAATGGAGAAAGGAGCAAGCGCAAGCGGTGGAGAGGAAGATATGATGACAATAGTTACATGCCCAATGCTTCTTTGGAAGAGCACCGCAAGCAAGAGCATAGCATAAGTACTGTTATTTCAACTATATCAAAGGAGAAACAGGGCTCTGAAAAGCTTTCTGCACATGATGTAAGTAATGTGGAAGATAGATACGAGATTCTGTTAGGAATGAAGGAATTAGCTAGCAGAGGAATGGCTGCTGAAGCAGTCGAGGAGGTTAATGCTATTGATCCCAATTTCTTTGCCCAGAACTCTGTTTTGCTCTTCCAACTCAAGCAGGTTGAATTCCTTAAGTTGGTCAGCTCTGGTGATTATAATGCTGCTTTAAGGGTTGCTTGCACCCATTTGGGTCCTTTAGCTGCTAGTGATCCTGCCTTACTAAAGCCGTTGAAGGAAACTCTTTTGGCTTTACTACGGCCTAATGAAGATGCTCTTGGAAATGCATTACCCTTACATGCTCTAGCTGCTTCTCTCCAGGTTGCAGTTGGTCGAAGGCTTGGTGTTGAAGAACCTCAACTTATGAAAATAATGCGAGCCACTCTTTATACTCATAATGAGTGGTTTAAACTTCAAATGTGCAAAGATCGTTTTGAAGGTCTTCTGAGGATTGATTCCTTGAAGGAAGCCAACACTCCCTTCCTTGCACCAGTCTCTACATCTAAGTCCTATGCCGATAGCTGCACAAATGGATCTTCTCAGGCTACAGTATCATCAGGTACCAGGATGTCAGAAGATGGTTGCAGTCCAACTCAAGTATCTTCAAGGGACGTCATTTGTGACGAAGGTGCAATACTTAAAGTAATGGAGTTTTTAGCTTTGCCTAGGGCTGATGCCATACATCTGCTTGCACAATACAATGGAAATGCAGAGACAGTCATTCAGCAGATATTTGCGTAGCTGTGCAGGAAATTCGGATCTATTATTTGAAGAAAGGTCAAATTATTTTTTTCAATATTATTTTATTCAAATTTGTGAATTTTTTATACCCCATGTACATATAGCTGCAAAGAGGAAGAAGTCATTGTAATGTTTGAAATCCGAATTGATGGAAACTACTCTCAGTCTGTACAGAATCTATTGATTATTACATCTCAGTTTTAACATGAGAAAGTTGTAATTAATAAAAAAATTATTGGCGTGACTCAAGG

>C101179_65

TTTCAATTCCAAATATGGCTCCTTCAGAAGAATCAGTAGCAGGTGACCCTCTATTTGGTTTTATTATAACAATTACGAAAACTCATCTCGGTTTTCTTCATCTTTTTCTTTCGCCTTCTTCCCCAAACCCTCGCCACACGGGCCAAATACCCTAATAGCTCTGATACTCCCATTCCACGAAATCCCAAATTCCTCAACTTCACACGCCAAACACCGCCTAACGGAACACAACCCCGTTCTCCAATATTACCTTCTTACCCTCTCATCCCACTCCTATGCCTAAGGGTTTGCGCTAACCACAGAGTCGCAAATAATTCGCTCCAACATTGAGTTGCGGAGGGAGCGGTTTTGGTTTGTGAGGGTTTTCCCTCGGGAGTAGTAGGAAGTGCAACAGGAGGAACCTCTGCTGTTGTGTAATAAAAAAGTTTTAATGGAAGATTTGGGAGGTTCCCGGTTTGATGGCCTCAGCAATGCTGTTAGGAGGAAGAGGAGTCAGACTTCACGCAGGCCGCGACCTGATTCCCAGCATGTTTCTGAAGGTCATGAACTGTCTCCCTTGTCCTCAACGCCTTCAGAGGATGCTGGCAAGATTTCTAGTGATGAGAATGTTGGTTACGATGCAAATTCCAAGAGAAAGGAGTTCAATCTTAATAACTGTGTCTCTCAAACCTCGTCGGCTACTGGAGCTGAAGATGATAAATCTCATAAAAAGAGTAAAAAGGATGGTGGGTTTCACGCATTTTATAATAATGAACCTGGACGGAGTGGTTTGCATAATAAACGCTGTAGTGAAGGTGTTCTTGCTCCAGCAAATTGGAAAGGTTCTAGCAAAGCAAAGGATAACATGGATTTAGAGTCGAGAAATGCAGATTTGTATGGTGGAAGGAATCCCGAGAGTACAAGCTTGGCACAGTTTGGTGGCTCTCAGGATGGATTGGGGAATGAGAACCGGGTTAAAAAAGTTAAACTCAAGGTTGGCGGGGTGACACGGACTATTCAAGCCAATTCAGCAACCAATGGTGCTTCGGGCAGTGGATCAACTATGAAGAGCTCTCGCTCATCGGATGCTTCTAGACCACGTCATAAGCAACAGAGTAATTCAGATGATAATAATTCTCCTTCTGATAAGAGGAGTGGTCTACAAGGTGTTCCGTGGAAGGATTTTTCACGGGGTGGTTTTGGTCTTGGGAAGGAGGAATCGTTGATGGGGAAGATTTCTGGAAAGAACACATCTAGTAAGTTGGGAGACAAGTCTGAACCAGTTCGTAAGAGTAAGCGAGTACCAAAGAGACGCGTACTTGATGGGGAATTTGGTGACGATGATGATGAGGATGATGAGATTCGGTACTTGGAAAAGCTGAAAACATCAAAAGTTTCTGCTGTCTATAGAGATGAAGAAGAATTAAGCAAGAAACATCGAAAGTTATCTAGTGTCTCTAATATGGAAAATGCTGCCTCAACAAGGTCTGGCAAAGACAGTAAGAAAAAGTCTAGATCTGATAGAGTGTATGAGGACACAGATTATGAGGATGAAGAGGAATCAGGGTCTGATGGTGAGCTTGAGGATAAGAAAAAGAAAAAACAGAGGAAGGAGTCTGTTGATGTATTGATGGACAATAAGAGGGAAATGACTCTCACGACTCGTCAACGGGCCCTTCAATCAAGCAAAGATGCTTCTGCGTCGAGTGCTAGTTTAATTGAGTTTCCCAATGGATTACCACCTGCCCCGCCTAGAAAGCAAAAGGAGAAGCTTTCAGAGGTAGACCAGCACTTGAAGAAAGCTGAGGCTGCACAGAGGCGTAGAATGCAAGTTGAGAAGGCTGCTAGGGAATCTGAGGCTGAAGCTATCAGAAAAATACTAGGTCAAGACTCTAGTAGAAAAAAGCGAGAAGAGAAAATAAAGAAGCGCCAGGAAGAACTGGCACAGGAAAAGGCAGCAAATGCACGAATGCATGCGTCAAACACCATCAGATGGACAATGAGCTCGATTGGAACGGTCGTGACTTTTCCTGAGGAAATGGGGTTTCCTAGCTTCTTCAACTCCAATTCGATCAGTTACCCTCCACCTCGCGAGAAATGTGCTGGTCCATCTTGTTCAAATCCTTACAAGTATCGGGATTCAAAATCAAAGCTTCCTCTTTGCAGTCTTCAATGCTACAAGGCTGTCCAGGAGAATGTGGTAACTGAAACTACCTGTTGATGTTGTCGATGACCTTATCACTGGCTTTTGCCAGTTATATGGCACCATAGATGTTTTTTAATCTTTCACTGAGGCCTTTAGCAAAGCAACTGTGGGTTGAGAGCGTTTGGTAGGGAGAGCTCATATTGCGGATTATGCATTTGCTTTATTATACTTTATTTTTAATGCTGGTATAAGAGGTTTTGCGAAATGGTATCCAGTTAATTGGGAATGGATACATGTATAGCATGTACCGACCTTTCCTGTTTATTACCCTGGC

>C101181_65

TAATACTCGAATAACAAATAAACAATATTACACATTCTTTTTTTGGAATGGGAATGGGGTGTATAGAACCACCGCACTCATTTCTATCACTAAAGCACATTAGAATGTAGATTAAGTAAATCCTTAAATCAAAGTGGCCTAATCTGTAAAAATGTTTGTTAGGATGTTTCATGAAGCTGTTTTGTCCAGTCAAAATGATTCCACTCTTTGGAAAAGCTTGCAACTGCTTGCTTTTGTACCTCCACCGCCTCGTAGCGTCGTCGCCGGTACATGTCCTCTTCTGCCAAATGCAGCATGCCTCGTATCACATTCAACCCAAGGCTGCTATTAAACTGCTTTTCATCATCAATAACATGGACAAAACCCTTGTTTAAACCAAATTCAACGTGGAAATATGGAAAGTGCTTGGGAATTGAATTACGCAATCCCTTTTGACTTGTATCAATAAGTTTCTTTGCATTGTGCTGACTCCACTCATCTTCAGCTTCATCAATCGCCTTTTTAAAGTACAAAGGAGCCTCTTTGGCAATATCTTGGGGTAAAGGAATGCACTCAACCATACAATGCCGTCGTTGCTGCGCCAATCCCATCACAGTCTCAAGAAACACTACCTCTTTCTCTTGCTTAGCAAACATCATTATTAGGCACTTCTTGAAGTTTCGAATTTCTGTCCATACATTATCGTCCACAGTTCTTGTAGCTGATTCATGCTGAATTGGTAAAATGCAGCAATGACCAGGAACCATGGGCTGCCACTGTGGCAACATAAGATATGTGAAATTTGCAATTGAAACTACAAGATGCATAGGTCGATTTGGGTTTTCTAAACAAAAGAGGCAGCGCTCTTGCTGAGTCAAGAACCGATTAGTCTTTTTTTGGATCTTTTGATCTTCTCCTCCTTGCTTCTTTCTACCCTTTCTGCTTGGACCATCCTCAAAATCATATTCATCATCAGCCTGCGTGGAAACCCTAAACTGCTTGTTGTGCATTATCTTGCGAGCAAGATGCATATCAGTATCATCCTCTCCTTTCTTCTGCTCAGCAGATATCTTTTGCTTAGCATACCTGCTAGAACTACCATTATTTCTTGATCTAATTGAATGATCTTGATTTTCCTGCGTTGTGTTCATAACTTTTATTTCTTGCATCAGTTTCTCAGCTTCTTCATGCTTTCCCTTCAAACGAAGTTGCATAGCCTTAGCTGCCAACTGGTTTGCACTCATCTCATTCTTGATTACTGCACTTCTTTCTCCAGGACTGTTTGCTTCCAATTCAACACTTCCCAAAACAGAGCCATCAGAATTGTTACTCATCTTGCTACCAAAATCATGCATAAAGCTTCCATCATTAGCAAACTTATTTAGACTAGATACTGCAGCAGAGATGACCTCAGCACCCTCGGCTACCACATGTTGACTCTTCCGCTTTCCCCAAGACAAAGAATCTTGAACTTTAGGTGTTCTCATTTCATGGTGCCGAACAGAAACATCCTTTAAATAGTCCCTTATAGAAGCCCTTCGACCATGCTTATCAGAATCTGGCGTATTTTCTTCAGTTACCCCCTTTTGTCTATTTTTTATAGCACGTAGATGAGCACGAGCTGGAGCAGCTGCATGAGATGCAACAGATGCAGTCAACTCACCAAGAGAACTCCACCTTTCTTGCACAACCTCGTCAAAGTTCCGTCCTTCTCGAGCTGCTTGTTCTTGTGCACGTTTTAAGGCTTTAAGTCTCCAACTTGCACCTCCATCCCCAACAAGGGAAGAAGATAGAAGTTGGTCAGCACCAACTTTAGATCCACTTTCTTCTGGGTAACCACTTCCACTATCCTTCAAATATGGATTCAGTTCCTTAGGATTTCCCTTCTTTGACTCCTCAACAGGAACCTCCTCTGACAAATTCTCCTCTGTTTCCGTAACTGCAGGCTTCTTACTTTCAGCCCTGAGCATCCAATCTAGTCCCATTTCTTTTCTGGCAATTTCCATCTCACTCGGAGAATCATCCCTTTTCTTTTTTGCTTTCCTCCTCCTCTTCTTTTCATCTTTGTCACTCTCACTTTCACTTGTGTAAAGAGAAGAATCTTCATCGGACGAATACCACTTCTTTTTCCTAGATCGTTTCTTGATCTTATCAAGCCCGTCATCATCAGAACTGTTCCTAGAGCTCCTCCCTTTCTTCTTATTCTTTTCCCTCCTCCTATCTGATTTTTTCCTTTCTTTCGAGGCAGAATCCAAGTCCTCGTCATGCACCTGGTCCCGGGGAATGAATTTCACACCTGAAAACATTTTCTAGATTCCTAGACTGTACGAATGCCCTAACAAAACAATTAAAGGTACGGCCCCGGCGGTGTTCTCAGGCAACGTGAGATGAGAAGAGAGCCAACGGACCAGCGACGGTGCCTTTTTGTTTAGACGCCGCCGTTTTGAGTTTTCACTCCTGATTGTGGGTGATGCTATTAAC

>C101183_65

GGCAAATGAATCTCCTTGCTGTATAAAAACAAACCCAAAAACTTATCTCCACCCACTCGCTACTCGCCGCAGCAACTCCTCTCCTGAAAAACCAAACCAAACATGGTGGTGGCGGGTGTCTCCCAAGCCATCTCCATCTCAATCTCCACCGCCACCAGAACCCCCTCCTCCTACGATTTCCGCTCCAAAAAACCACTCTCTTCCTTTTTGGGTCTGGCCCACTCTCCTCAGCCCAATCTCTCCATCTCCGCCAGAAGGAACCGCTTTAACACCACCCACTCTTGCCGCTGCACCCTCAATTCCGACGAGGCCTGCCTCAATTGGGATTGGAACCGATGGTGTCGTTATTTCTCCGAAATCGAACAGGCCGAGAACTTTGCCTCTGTCCTCAAGTTTCAACTTGATGATGCTATTGAGAAGGAGGACTTTGAAGAGGCCATTAAGTTGAAGAGGGCTCTATCGGAGGCAACGTCCAAGGACACTGTTGCTGAAATTATGGATCAGTTGAAGAGTGCTATAGATGATGAACGATATCATGATGCTTCAAGATTGTGTAAATGCACTGGAAGTGGACTGGTGGGTTGGTGGGTAGGGTACTCAAAAACCTCAGATGACCCCTTTGGTAGAATTATACATATAAGTCCTGGTATGGGTAGATTCGTTGGCAAGAGTTACAGTCCAAGACAGTTGATGACAGCATCTACTGGAACTCCAATCTTTGAAATTTATGTAGTAAAAAATGCTGATGACACGTATCATATGCAGGTAGTGTATTTGCGACAAGCCAAAGGAAAGTCAAGGAAAAACACTCAACCTATATCTGCTAAAGGCCCATCCAAACCTGAGATGGATAATGTATCTTCAGTTGAGGTGCAGAAAGATGAAGAGAAGGTTGAGAGAAATGATGAGAAAAACAGCAATATTGAGGCAGTAACTGAAGAGGGCATTAAAAGTGTCATAAATTTTCTTAAAGAAAAAATTCCAGGATTGAAAGTCAAAGTTATGAATGTTAATGTTGAAGAGGAAGCGGCAGAGAACAATGATTCTATTCAGCAATTGATGGAGCAAGATAGTAATAAAACTGGCTCCAGTGAGAATCACGAAGACGAAGTTGAAAATCTCGATGAACCTGATGAGGTCACTCTAGAAAGAGATGGAGATTCCACAGATGAAGAAAAGGATTTGGAAATGAAGCTTTTTATTGGTGGGGTTGTACACAACAATGAGGATACTCCTGTCAAGGATGAATTTGTACGTCTTCCAGCTGTAATAAAAGATATGGAAAAGGATGCTTTTGTCTTCCATTTTGCTAGCAAAAATCTAGACTACGGTATCAGAGAAGATAAAATTCCAAATATCAAAGTGGCTGCTTTAGCAGCGCAAGGAGTCTCAGAGCTTATGCCTCCTGATGTCGCTAATGCATTTTGGAGTTCAGAGAAAGTCTCTTCCAAGATCTCAAAAAACATGCGTGATATTGTCAGAGTTGCTATGAGTCAAGCACAGAAAAGAACTAGATTATCTGGGGATACATATTTTAACCGGATTACCTGTTCAAGAGGGGATTCTGATCCTTTTGATGGACTCTATGTTGGCGCGTTTGGCCCTTACGGCATGGAAATAGTTCATTTGAAGCGAAAATTTGGACATTGGAATGAAGTTGACAGCGAAAATAACACTTCCGACCTGGGATTCTTCGAATATGTAGAGGCTGTGAAGTTAACTGGGGATCTTAATGTTCCCGCTGGCCAGGTGACATTCCGTGCAAAAATTGGCAGAGGCAATCGCAACACCAATCGTGGGATGTATCCTGATGATTTAGGAGTGGTTGCTAGTTATAAAGGCCAGGGGAGAATAGCTGACTATGGTTTTCGAAATCCAAAATGGGTCGATGGAGAACTGCTGCAACTTAATGGCAAGGGCATGGGACCATACATGAAAGGTGCAGACCTTGGTTTCCTTTATGTCGTGCCCGAGCAAAGTTTTCTTGTGTTGTTCCACCGGTTGAAACTACCCGAGTGAGCCATGGTTGATGGTTACCCTACTCCTGTATAATTTGTTATCTGATGCGGGGTGGAACATGAAGGATTCTGACACTAGCATGAGGTTACATGTTACACTTTTTTGAGTGGCAGTTTTACCCGACTAGCAGCCACCTTTTTTCTTTTCTAGCCCTTCTGAGTTCATTTTTTATTGTTCCTTTTACCAATCTGTTTGCTGTGTATCATCTTGTGTATATAAATTTTGCTGACTAATATCATAGTATGGTAGGAACTCTGGAAGTACTGTAAGGGATCTTTTATGTGCATAATATGGTTTATGTCTATGTGTGCCTCCTTTCTGGTTTGGACTTCTCATTTGTGGAAGTCCAACATAAGTCTGCATGTATGAATGGGAACAAATAATAAATATATTTGATATTAAAGAACTTCTTCCTGTGGTAATTTATCCACAGATTTGTTA

>C101187_65

CGTGTGACAACTATAAACTCTTGCGTCGCTGCGATTTCTTCTTCCGAAAACGCGAAAATTATCAACTTACGATGCAGCGAGCTCTCATTTCATCTTCTCTTTCACTCTCAACTCGCTCTTTCTTCTCTCCCCTCCCTCTCTTGTTCTCACGACCAAACCCAATTTTCTACAAACCCAAATCCAATAAACCTTCCTTAACCATTCGGAATTGCAGCAGCAGTTCCTTCAAAGTGAAACCCTCTTCGGAAATCAGAAAACCCCTCGCTGAATCCCAACCTGACCCCAAGCTCACTGCGCTCCGCCACCTCTTCTCCAAACCTGATGTCAACATTGACGCCTACGTCATTCCTTCTCAAGACGCTCATCAGAGCGAGTTCATTGCCAAGTGTTACACAAGGAGAGCCTACATATCAGGTTTTACTGGCAGTGCTGGAACTGCTGTGGTTACCAAGGACAAAGCTGCTCTCTGGACGGATGGCAGATATTTTCTCCAGGCGGAGAAGCAGCTAAGCTCCAATTGGATTCTCATGCGAGCTGGAAATCCAGGAGTCCCTACTGCGAGTGAATGGCTCAATGAGGTTTTGGCTCCAGGTTGCAGAGTTGGTATTGATCCCTTTCTTTTTACTTCGGATGCCGCAGAGGAACTTAAGGGGGTTATCTCCAAGAAAGACCATGAGCTTGTTTACTTGTACAATTCGAATCTTGTGGATGAAATATGGAAAGAATCTAGGCCAAAACCTCCAAATAATCCAGTTAGAGTGCATGACTTAAAGTATGCTGGTTTAGATGTTGCATCAAAATTGTCATCTTTGAGGTCAGAACTTGTCAATGCTGGCGCTTCTGCAATTGTCATCTCCATGCTTGATGAAATTGCATGGTTGTTGAACTTGAGAGGCACTGATATTCCACATTCACCGGTTGTGTATGCATACTTGATTGTTAAGATTGATGGGGCAAAATTATTTGTGGATGATTCCAAAGTTACTGAAGAGGTTAGGGATCACTTGAAGAAAGCTGATATACAGATCAGGCCATACAATTCCATTATATCTGAAATTGAAAGGTTGGCAGCACAAGGTGCTGCCCTTTGGTTGGATACTTCATCTGTTAATGCTGCTATTGTAAATGCCTATGGAGCAGCCTGCGACAGATATTATCGGAACCAGGAGAGTAAACACAAAACCAGGAAAAATGGTTTTGATGGATACAATGAGGGCTTGGATATACCCTATGCTATCCATAAAGTCTCTCCTGTTTCTCTTTCAAAGGCCATTAAAAATGAATCAGAGTTAGAAGGGATGCAGAATTGTCATTTAAGGGATGCTGCTGCCCTTGCTCAGTTCTGGGATTGGTTAGAAACAGAAATTACCAAGGATAGAACATTGACAGAAGTAGAGGTTTCAGATAAACTTCTTGACTTTCGCTCAAAACAAGATGGTTTCCTGGATACTAGCTTTGACACAATAAGTGGTTCTGGTCCAAATGGTGCTATCATACACTACAAACCAGAACCAGAAAGCTGTAGTTTTGTGGATGCCAATAAATTGTTCTTATTGGATAGTGGTGCTCAATATGTTGATGGCACAACTGACATAACTCGGACAGTTCATTTTGGCAAGCCTACTGCAAGAGAGAAAGAATGCTTTACCCGAGTCTTGCAGGGCCACATAGCTCTTGATCAGTCAGTCTTCCCAGAAAATACCCCTGGTTTCGTGCTGGATGCTTTTGCCCGTTCCTTTCTTTGGAAAGTTGGACTTGACTACAGGCATGGGACTGGGCATGGTGTAGGAGCTGCACTAAATGTGCATGAAGGCCCTCAAAGTATTAGTCACCGTTTTGGAAATTTGACCCCTCTAGTGAAGGGCATGATAGTTAGCAATGAGCCTGGCTATTATGAAGACCATGCCTTTGGTATTCGGATTGAGAATCTCCTGTATGTAAGAAATGTTGAGACACCAAATCGTTTTGGGGGAATCGAATACCTAGGATTTGAAAAACTTACATATGTACCCATTCAGATTAAATTGGTTGATTTGTCTCTGCTATCGGCTGCTGAAATTGATTGGCTTAACAACTATCACTCACTAGTCTGGGAAAAGGTTTCGCCATTGTTGGATGGCTCTGCTCGCCAATGGCTTTGGAACAACACTCGGTCCGCCCATATCATCCATGAGAGAATTTAATTCATGTTAATGTGCCATGGAACACCTGGTTCACATTCTGGCGTGCTGCCCCTTACCTGTTTTATTTGTCTATATATCGACATAATTCGTACAGGAGACGCTGTGAATAAAATAATGAATAGGGATAACTTGAATTTGATCACAGGATCTTTGCAGGACAAGTGAGCATTTAGCCATTTGCAGCCCTTGATTATGGTTATTCTGTGAGATTCAATGTAATTTTTTTGGCTGTTGTGAAAGCTAACACTCTTAACTCTGAATTATAGAAATGAGAAAAA

>C101189_65

TAAAATGTTAGTCCACTACTCTTCCAGTTTCCATTCAACTCTCTCTCTCTCTCTCTCTCTCCCCTCCGCTTTCATTTCTCTCTCCCCATCTAATCCACCATCCCAAAATCCAATTTTTACTTTAGGGTTTTGAAATCCCAGCTAAAAACCACGCACTGCTTAGCCTGATCGCTGATTAGCGATTGGCGGCGGCCTCCATTTTCAGGCTAGAAAGAAACTTGAAGAGAAATGGTATGCTGAGATATGGCACTTAAAGTTCCTGCTCCTATAGCTGCTGAGATTGCAATTGGGTCCATCGGTCGTGGTTATGATATATCGACTGATATAAGGCTTAAATATTGTAAAGGAGATTCCATAAATTCACGTTTAATTGAAATAGATGAAAATGATATAAGAGAGGTGGTACTACCTGGTGGAGTTTCAATACCAAATGTTTCCAAATCAATTAAATGTGATAAAGGGGAACGCACTCGGTTCAGGTCTGATGTTCTGTCTTTTCAACAGATGTCAGAGCAATTTAATCAGGAGTTATCTTTGACTGGAAAAATTCCATCAGGGCTTTTCAATACAATGTTTGAATTTTCTGGGAGTTGGCAGAGAGATGCTGCCCTTACAAAGTCCCTTGCTTTTGATGGGGTGTTAATTACACTATATACTGTTGCATTAGAGAAGTCTCAAATGGTCCTCTGTGACCACGTTAAAAAAGCTGTACCATCTTCATGGGACCCACCTGCTTTAGCAAGGTTTATTGATACCTTTGGCACCCATATAATTGTTGGTATGAAAATGGGGGGAAAAGATGTAATATATTTGAAGCAGCAACACTCATCAACTCTTCAACCTGCTGATGTTCAGAAAAAATTGAAAGAGATGGCAGATAGGAGGTTTCTAGATGCCAATGGCCACTATACCATTGCTTCTGATCAAGTTTTCCCTAATGACAAGTTTGGAATCAGGGAGCAGAGGCTAACATTTGCTAATATTAGTCCATCAAGTTCTTATTCACATAAGGAGGATATTGTAAGCATTTGCAAGAGGAGGGGTGGGAGTGATGACAGAAATCTATCCCACAATGAGTGGTTACAAACTGTTCAGTATGAGCCTGATGTGATCTCAATGTCATTCATTCCAATTACATCTCTATTGAATGGAGTGCCAGGGAGTGGATTCTTGAGCCATGCCATAAACCTTTATTTACGATATAAACCACCGATTCAAGAGCTCCACCAATTCTTGGAATTCCAGCTGCCAAGGCAGTGGGCACCTGTATTCAGTGATCTTCCACTTGGTCCACAGCGGAAGCAACGGAGTTCTGCATCTTTACAGTTTAGCTTCATGGGCCCAAAGCTTTACGTTAACACCACACAGGTTGATGTAGGTAAGAGGCCAGTGACTGGTCTCCGTCTGTATCTTGAAGGTAAGAAAAGCAATCGGTTAGCCATCCATTTGCAACACCTTTCATCACTTCCAAAAATCTTCCAGCTTGAAGATGATCCAAATGATAACTTTCAGCGGAAGTCTTACGATAGGAGATTCTACGAGAAGGTTCAATGGAAGAATTTCTCTCATGTGTGCACTGCTCCTGTGGAATCTGAGGAGGATCTTTCGATAGTAACTGGAGCACAATTACAAGTTGAGAATTATGGCATAAAAAATATACTCTTCTTAAGACTTCGATTCTCAACTGTGTTGGGAGCTAAAGCAGTGAAGCATCCTGAGTGGGAGGGATCACCCAAGTTGGGAGCCAAGTCAGGACTGATATCCACTCTAATTAGTCAACACTTCACAGCAACATTTCAGAAGCCTCCTCCGCGTCCAGCAGATGTTAATATAAATTCTGCTGTCTATCCGGGCGGCCCTCCTGTTCCTGTCCAAGCTCCCAAGCTTCTCAAATTTGTTGACACAACTGAGATGACCAGAGGACCACAAGAGTCTCCTGGCTATTGGGTTGTCTCTGGAGCAAAGCTTGTCGTTGATAAGGGAAGAATTTCTCTCCGAGTTAAGTATTCTTTGTTGACCATGGTATTACCAGATGAAGACATGTTGGATGACCTGTAGCTCATTCCTTTTTTAGGCCTTGCCTTCAGCAAACTCTCTTCTGCAAGTGTTCATTTTTAAGTTGTGGTAGGTTGGGAAATAAAAGGTAGAGGGTGCTACTAGTTATGACCAAGAAAAATTGCTTTGGTTTTGCTATGCTAATCAGGGAGGCTTTCTTGCAGAGATAAAGAAGGGCAAGTGTAAGTTACTAAAGGACTGCATTGGCTTTCCCCACCTATTGGAGTTCCACTTGTAATGTAGACTTTTACAGTTCAACACAATGACTTGTTGAAAATTACAATTAGAAAATCAATTCCACGCTGGCCAATAGCTTAATCGTGTTTGGATAGGATCACTCTTTGAAATTATGGTGTAAACAGTATTATATATCAATTTAATACGCTGAGAAATTATGTCAAAAAAA

>C101191_65

ATTTCGAAGGTCCACTCTGCTCTCCTTCGTATCAGGCTCAGCCATGGAGCGCGCGGTGGATCATTCTCGAAGCTTCATGAAGGACGTGAAGCGCGTAATCATCAAGGTTGGTACCGCGGTGGTCACTCGCGAAGAAGGAAGGTTAGCGGTTGGAAGATTAGGAGCTCTGTGCGAGCAGATTAAGCAGCTCAACTCTCTCGGATACGACATTATCCTCGTCTCCTCCGGGGCCGTCGGTATTGGCCGCCAAAGACTTCGTTACCGAAAATTAATCAACAGCAGCTTTGCCGACCTTCAGAAACCCCAACTCGAACTCGACGGCAAGGCCTGCGCCGCCGTTGGACAGAACAGTCTCATGGCTCTCTATGATACTCTGTTCACTCAGCTCGACGTGACATCGGCTCAGCTTCTTGTGACGGATAACGATTTTCGAGATAAGGATTTCAGGAAACAACTTACTGAGACTGTGAAATCATTGTTGACGCTGAAGGTTATTCCGGTGTTTAATGAGAATGATGCTGTCAGTACTAGGAAGGCTCCATATGAGGATTCTTCTGGTATATTTTGGGATAATGATAGTTTATCTGCATTATTAGCCTTGGAGTTAAAAGCCGATCTCCTTGTTTTGTTGAGTGATGTAGAAGGTCTTTATAGTGGCCCTCCAAGTGACCCGCATTCAAAGCTTATTCATACATATAATAAGGAAAAACATCAGAATGAAATTACTTTTGGCGACAAGTCTAGAGTGGGAAGAGGTGGAATGACTGCCAAAGTAAAAGCTGCGGTTCATGCAGCTGAAGCTGGCATTCCGGTCGTTATTACCAGTGGTTTTGCAGCTGAGAATATCATTAATGTTCTCCAAGGACAACGTATAGGAACTCTCTTCCATAAAGATGCACATGAGTGGGCCCAAGTAAAAGAGGTTGATGCACGTGAGATGGCTGTTTCAGCCAGGGAATGTTCCAGAAGGCTCCAAGCCTTATCTTCTGAGGAAAGGAAACAGATTTTATTGAAAATAGCTGATGTCTTGGAAGCAAATGAAAAAATAATCAGGACTGAAAATGAAGCTGATGTTACTACTGCACAAGAAGCAGGATATGAAAAATCTTTGGTGGCTAGGCTAGCTTTAAAACCTGGGAAGATTGCAAGTCTTGCAAACAACATGCGAATTATTGCCAATATGGAAGATCCAATTGGTCGAGTATTAAAACGAACTGAGCTTTCAGATGGGCTAATTTTGGAAAAGACATCATCTCCTTTGGGAGTGCTCCTTATTGTTTTTGAGTCACGCCCTGATGCTCTTGTACAGATAGCTTCACTGGCAATCCGAAGCGGGAATGGGCTTCTCTTGAAAGGTGGCAAAGAAGCTAAGCGGTCAAATGCAATTTTGCACAAAGTAATTATTGAGGCCATACCAGACACTGTTGGTGGAAAACTTATTGGACTTGTGACCTCAAGGGAAGAAATCCCTGAGCTACTTAAGCTGGATGACGTAATTGATCTGGTCATTCCAAGAGGCAGCAACAAACTTGTTTCTCAGATCAAGAGTTCAACTAAAATTCCTGTGTTAGGTCATGCTGATGGAATTTGCCATGTCTATGTTGATAAGTCTGCTGATTTGGAGATGGCAAGACGGATTGTACTGGATGCAAAAATAGATTATCCAGCAGCCTGCAATGCCATGGAAACACTTCTTATCCACAAGGATTTGATAGAGAAAGGGCTCAATGATATTGTTCTTGACCTACAAACTGAAGGTGTTATATTATATGGAGGACCAATTGCAAGCTCTCTGTTGAATATTCCACAAGCACACTCATTTCATCATGAGTACAGTTCGCTGGCTTGCACTGTTGAAATTGTGGATGACGTGTATGCAGCTATTGATCATATAAATCTTCACGGAAGTGCACATACTGATTCCATCATTGCTGAAGATAACGAAGTAGCTAATGTGTTCCTACGCCAAGTTGACAGTGCTGCTGTTTTTCACAATGCAAGCACCAGATTCAGTGATGGGGCACGATTTGGACTAGGCGCAGAGGTTGGAATTAGTACGAGCAGAATTCATGCTCGAGGTCCAGTAGGAGTTGAGGGATTGTTAACAACAAGATGGATACTAAAAGGAAGGGGACAAGTAGTAGATGGCGATAGAGGGGTTGTCTACACCCACAAAGGCCTTGCAACTTAATATGAATGGTTTGACTGACTGCTTTGATTCCTTTTGTAGCCTTCTCGTTTACAGTAGAGAACGGTATTAAATTATACGATTGAATAAAACCGGGTAATTTTTATCATCTGCTGGTTCCTTTGCATTAAGATTTATTACTGTCAACAATTATGAAGCCCCCACCTTTACTTGTGCTAGGAAAATACAGAGAATATGAAGTCTCAGCAGCAATGGAATCCATTTATTGCTTTGTTAGCGACAATTGAATGATTACAATTTCAATTTATGAGTT

>C101193_65

TGGGTGCACAATAACAGGATAAAAGTGGTTCAATTCACCAGATGCTCATGAATGCGAACTACGTTGTTGGGATATCATTTTTATTACACCGCTAATAATAAAAAATCATCTGACAAGGAGCCAACTTGCAATTCTCCGAAAGTAACAGAAAACAATCAAGTTAGACCGCAACGTACACCTTAAAATCTACACTTCGACAACTCAGGCAAGTAAAAGATATTGAAACAAAAGAATAACGGTGTTGTCACAATTCAAAATTGTACAGAATAGGTTACATCTGATTCTGAACATGACAGCAGTACCTGAATTTGAAAATCCTTACACAAACGGTCTTCGTCCTTTAAGAAGTATAGTAGATTAATCCAACCCTGATTGCTATAACTAATCAATCTTGACCGAGGAATAGATGAGCCTTGTGAACCAAAAGCATGCGTAAAATCCGATGGATCCAGTTACAACAAAGAAGGCATAAGACGCTATAAGCATGTAACCGAAGTATAATAACCCAGATACCAACTTTGTGATTTCAAGCTTGGTGAAGAAGTAGAATGTTGCATAAAGAAAGAGGTAAAGAGCAGAAGAGCCAGATGTGAGGTAAGACCGCCACCACCACAAGTAATCCTCACTGCACAACTGGAAGTAGCAAAGCACAATGGTTATTTCAGCACAAGTGACAATGAGAATGACGAAGACCAAGAAGAGGAAGCCGAAGATGTAGTAGAACTGATTCAACCAGATTGAAGTGAGGATAAAGAAAAGCTCAATGAAAACAGCTCCAAATGGAAGTATTCCTCCGATCAGAACTGAGAAGATTGGATTCATGTACCATGCCTGCTCAGGGATCTGCCTTGGGATTTTGTTTGTCTTCACAGGATTCTCAATTGCGGGCTTCTTAAATCCAACGTAGGCACCAACAAAAACAAGTGGAACTGAGATCCCAAACCATAAAAAGATCAGGGCAAACATTGTTCCAAATGGCACAGCTCCAGACGATTTCTGTCCCCATATGAGAGCATTTAATACAAAGAAAATGCCCGAAACGATTGCAGGGAACATAATAGCAGTTTTGAGGGCAATTTTCTTCCACTCTGCTCCTTTGAACATTTTATATAAGCGGGCTGAGGCATAACCAGCAAAAATCCCCATGAAAACAAAAAGCAAGAGCATGGCAGTCATTAGCCCACCTCGGTTTGAAGGAGAAAGGAACCCCAGGACGGCAAACATCATTGTTACTAGTATCATTCCAAAAAACTGAACACCGGTTCCAACATAAACACACAGCAAATCTGGGTTATTTGGTGGCCTAAACACATCACCATGGACAAGCTTCCAACCAGTCTCTTCCTGTGCTTCTTCTTGGGTTTCTAATTCATTGTACTTTGAAATATCACGGTAAAGTGTCCGCAGCATTATCATTGCTACCATGCCCGAGAGAAAGAGAACGATCATCAATGAGTTCACAATTGAGAACCAGTGAATTTGGTCATCACTCATCAGCAAATAGGCATCCCATCTTGAAGCCCACTTCACATCACTCTCCTGGAATTCAACATCATAAGTGAAGATAATTTCCTTGTTCTCTTCGACCTCTTGAGGAGTGTTGGAATTGACAACTGTGTGTTTAGCATGAGGATCACAGGTTGTCAAACGGGTGGCCTTTTCATCCCACTTCCCTTCAAATTCATGTTTAACACTGAATGGCTTAACCTCAAAGCCCACAATTCTAGCAGATTCAGTTAGTGTATCTCTATGATACTTGACCGTAAAAGCCAAGTGGTTGTGAATAAAATACTTTTCCTCCTTGCTCCCACTATACTGTCCTTTGAGTCCAACATGGAAACCAAGTTGATAAACAGTAGAGTCAGGTTCCACCCGTTTTATGGGAACAACCAGAGGAAGGTTATCTAGGATCATATTCACTCGATACTCATCATCAATCTTCTCTTTGAACTCTTTTGCAGTTTTGGCATCGATTTTAAGCTTACACACAATATTGCACATTTGTGGTTCACGCATTTTAAACACATATCGAGAATTTTCAATGCGATCACCACGGAGCACTTCTCCAAGATTTTCAGCACTATCCACTATTTTCTCTGGTGGACAATAAGGAAGAGAATAATATGTATACGGAAGCTGGGTCTTCGTTGAAGTTAATTTGTTTACTTTTACTTGCAATGGATCTCCCTTCTGGAAGTCCTGAGGAGCGACGCCGGGGAGATAGAAGCAGAGAGCGCCATGGATGAAGAGCGGAAGAAGGATGGCAGAGAACGCGAGGGATCTCCAAAACGCCATGGCGTTTGAGATCGAAACGACAGAGAGAAAGAGAGAACGATAGATCTGTGTGAGGTCTTTCGAATTCGTATTGTTACGTTCGTTTAGAGCAGAGAGTGATATAATAAGGGGTGAAAGAACGAGGTGAAGCTTTCTCTCCGGTTACTCATGAGTACGGTTATTACTTG

>C101195_65

GATTACTTCAGTACATTTTCCCGCTCCCCTCTCTTTCTCTTGCTCTCTCACTCTGTAGCAATCTTCGCCGCCATGACCACCAAGGACCTTGATTTCGATGCAGACATAGCTCTGGCGAAGGAGTTCCTTTCCAATTTCGCAGATGCCAATGGCGAAGCTAAATACCTGGACATACTGCAAGATGTCGCAAACCACAAAATTAAAGCAGTTCAGATAGACCTTGAGGACTTAATTAACTACAAGGATCTGGACGAAGAATTTCTGAGGCGTGTTACCGAGAATACTCGAAGGTATATTGGGATTTTCTCCAATGCAATTGATGAACTCATGCCAGAGTCCACTGAGGCCTTCACGGATGATGATCATGACATATTGATGACTCAGCGATCTGATGAAGGAGCGGAAGGTGCTGACGGTTCCGATCCACTTCAGAAGATGCCTCCTGAAATCAAGCGCTACTATGAGCTTTATATTAAAGCATCTTCAAAAGGACGACCATTTACAATTAGGGAGGTGAAAGCTTCCAACATTGGTCAGCTTGTAAGAATCTCTGGTATTGTGACACGTTGTTCGGATGTTAAACCATTGATGAAAGTGGCTGTATACACGTGCGAGGATTGTGGATTTGAAATTTACCAGGAAGTAACGGCTCGGGTCTTCATGCCCTTGTTTGAATGTCCATCAAAGCGCTGTGATACAAACAGAAGAAAGGGGAATGTTATCCTTCAACTGAGAGCATCAAAGTTTTTGAGATTTCAAGAGGCCAAAATTCAAGAATTAGCTGAACATGTTCCCAAAGGCCATATTCCACGAACAATGACTGTTCATTTAAGAGGAGAACTCACCAGAAAGGTGGCTCCGGGTGATGTGGTTGAATTATCTGGGATTTTTCTTCCTATACCTTACACTGGTTTTCGAGCAATGAGAGCGGGCTTGGTTGCTGACACTTACTTAGAGGCCATGTCTGTAACTCATTTCAAGAGAAAATATGAAGAATATGAGCTTAGAGGAGACGAGGAAGAGCAGATTGCACGATTAGCAGAGGATGGTGACATCTACAATAAATTGTCACGATCACTTGCTCCTGAAATTTTTGGACATGACGACATTAAAAAGGCACTCCTTCTTCTCCTTGTTGGTGCTCCCCATCGGACCTTGAAAGATGGAATGAAGATTAGAGGAGATTTACATATATGTTTGATGGGTGATCCTGGTGTTGCCAAGAGTCAACTTCTTAAACACATAATCAATGTAGCACCCAGAGGGGTGTACACCACTGGCAGAGGAAGTAGTGGAGTTGGTCTAACTGCTGCTGTTCAAAAAGATCCAGTGACAAATGAGATGGTTCTTGAAGGTGGAGCATTGGTGTTAGCAGATATGGGCATATGTGCCATTGATGAATTTGACAAGATGGACGAATCGGATCGTACAGCTATACACGAAGTTATGGAACAACAGACTGTTAGCATTGCCAAGGCTGGAATCACTACATCTCTGAATGCAAGGACTGCTGTCCTTGCTGCTGCTAATCCAGCATGGGGAAGATATGATCTAAGAAGAACTCCAGCTGAAAATATTAATCTTCCTCATGCCCTCCTATCAAGGTTTGATCTTCTATGGTTAATCCTTGATCGAGCTGATATGGATAATGATCTTGAAATGGCTAGGCATGTTGTATATGTTCACCAAAATAAGGAGTCTCCTGCTCTAGGATTCACTCCTCTTGAACCATCTGTTCTACGTGCATACATATCAGCTGCAAGAAGATTGTCTCCTAGTGTCCCCAGGGAACTGGAAGAGTATATTGCTACTGCATACTCCAGCATTCGTCAAGAAGAAGCAAGGTCCAATGCTCCACATTCCTACACTACTGTCAGAACATTGCTCAGTATTCTCCGCATATCAGCTGCACTAGCAAGACTCCGTTTTTCTGATACTGTTGCTCAGAGTGATGTGGACGAAGCACTGAGGTTAATGCAGATGTCAAAATTCTCTTTGTACTCCGATGACCGTCAAAAATCTGGTCTGGATGCCATATCTGATATTTACTCCATACTGCGAGATGAAGCGGCTAGAGGTAACCGAATGGATGTCAGCTACGCCCATGCACTGAACTGGATATCTAGGAAGGGTTACAGCGAAGCTCAGTTAAAAGAGTGCTTGGAAGAGTATGCAGCACTAAATGTATGGCAGATACATCCTCACACCTTTGACATCAGATTTATTGATGCTTGACGCACTTCTTTTCCCCATTAACATGATTTGAGGCAATCTGGTTTCATGAAGCTGGATAAAAGTGGTTCGTGGACTTGGGGAAATCAAATTCTGTACTTTGGTTTTTATTAGTTTAATTTAGGTTTTTGTATTGTTACAGTTTAGATTTGCATCTTTTACTTAAGTACAGTAATTGCAACATTAAATTTCTTATGCTTAAGTTAGTACCTACTTGAAGCACGGATTAG

>C101199_65

TCTCTCTCTCTCTCTTTCTGTTTTCCTTTTCTATTTCACCTGTGAAACCCTAATACACCTTCTCTCTCTTTCTTTCTTTCTTTCTCAATCTCAAGCAATCCAAAGTCACAACCCAAACAGAGAAACCAACTCACTGATTCTCTCTGCAAACTGGAAACTGGAAGTAGTATTAAATACACAGCCTTGTTTACGTGTGTCCTCCATTACTTGTTATTGGTGAAAGACAAATGAAGTAAAAGTAAGGGAAGAAGGGAGCACACCCACACACTGTACGGGTTTTGCAGATCAGAGGCTCAAAGACGTGGGTGTTGTTGGTACACTCTATCTATATATCTTTTTCTCTCTCACATAACCACAAACACCCTCTTTAATGTTTCTCTCTTCTCCCATCATACCCATACCTCATTCTCCCACTCAGTCATAACCAATCAACCAAATCTCTCTGCCCTCTCCGTTACGTCTTTCCATTTTCGGCTCTCATGGATTCGCCCAACAGTGGCAGTATGCAATCCTCCAGCACCGCCGGTGATGAAGAGTACGATTCACGCGCCGACCCTTCTTCATCCTCATCAATCTCTGCCTTCCTCAACAACAACAACCCCGTTCCTCCGCCTCAACCAACCACGTTAACAACCCACGCTGCTCCCTTCCAAACCCACGTGTTCGACCCTTTATCCAATTACTTGGATCCGACACAAAGATCGCAGTCACACCCCAACACAAACCAAATCCTTAACCTCGACATGGTGTGGACCACGGTTGCCAGATCCGAACCCGATCTCGCCGGCTTGATACCTTGTTCTTCTTCACCCTCACCACAAAACAGCAACAACCAAGGTTTTCTCTTAACTCAACTTGGAGCAGGTCAAAGTCAAACACGCGGAGGAGGAGTTGCTAACGTAGCTGTTTCTGCTTTTCCCACCACTCTCCCTCCGGAAAGTGCTTCTCCACGAGGAGGATTTGAACAAAACAGTACCAACACCAACACCAACCCCAACGTGGTTCGAAACCCGAAGAAGAGGTCCAGAGCTTCTAGGCGTGCACCTACCACTGTGCTCACTACGGACACCACAAATTTCCGAGCCATGGTTCAGGAATTCACTGGCATCCCAGCACCTCCTTTCACTTCCTCACCCTTCCCGAGAACCAGGTTGGATCTCTTTGCTTCTTCTAATGCATCTTCCTCAGCGTTATTAAGATCCGCTTCCTCTCATTTAGAACAACCTCCACCGCAAACACAAACACCTTCTTATCTTCTTCGACCCTTTGCACACAAAGTCCAAGCTCAACCCTCTTCAATCCCACACAACTCTTTTTCTCCCATGTTGAACACCTTGCCTTCTAACAACAATTCAGGTTCCAATTCGACCTCCATTCACTACCAGCAGCACCCTCTAAACATGCACAACCCAATTCTCAGCCTCCAATCCATTATCGGGAACAACAATTCTTCTATTCTAGTTGGTTCTAAAACACAACAACAACAACAACCCTCCTTGGAAATCACGCCCAGTGCTGTGGACTCGCACCTCAAGATGGGTGGTCTGGAGGAACTGGGATTGAGCCATGCGCACGTTGGTGGTCATCATCATCATCATCATCATCAAAACATGAACTTGGTTCCCTCTTCATCGGATGGAGCATTGTCGAGGGTCAACAACAACATAAACATAAACAACAACATGCGTGGTCCTTCTTCGGCAGACTGGGCCCAGGCCCAAAGAATTGGCGGCACCAATGATGGAGGGGTTTTGAGGTCTCTGAGTGGAGGCACTGGCACTGGCACTGGCCCCTTAAACTACAGAAGCAGTGTTTCGGATTTTCATGGAGAGAAGGGAGCACCGGAATGTGCTGTGGCTGCAAGAAGCGAAGGTATGGTTGAATCGTGGATTAATTGTTCTTCAGATTAGAGCAGGAACCAACCATCAAATGGGAAAAGAATATGAACAAAGTAAAAAGGGATGAGATAGATTCATAAATATAATTTCATACGCTTCAATGCATGAGAGATGAGAGACAGAATATTGGTAATTGGTATCATCACCTTAACCATGACCATCAAATTAATTAAACACTTTTTTTTTTGGGTCCATTAGAAGAATTATGCGTAAATAAGTAGCGCTACCTTGTCTTTCTCTACCTTTTTTCTTCTTTTTTATTTGTTATCCTCAAAAAATCTCAATTGTGGTTTTGATTAATATATATACACAGCCATATATATATATATATATACACGAAAGAATCCTGGAGAGGTTTCGATGGTTTGGACGTTTGTTTCATAATTTGTGTCTCTATTTGGTGAACTAGAAATGGCAGCATCAGTTTGTGTTCATGTCACTGTATATGGATTTTGGTTTTGACTTGTGTGAAATTATCGTTCAGAGAATGTACAATGTGCAGCATAGATTGAACAACACAGACAAAACTGTATGTATCCTCTCTTTTCCTGTTTTTGTCGCACCA

>C101201_65

GGGAGAAGGGTTACATCTAGAAAGAAATAATATTAATATTAGAATTAATATTAGTATATGGGCATATAGCAGAACCGACCTTGAAATGATGGGTTCTCTTCTGGGTTGTGGTTGCGGTTGTTTGTGAGGTTGCTGAACTCACTGTAACTGTTCGTTCAGGGAGACTCGAAAACAGGATTTGTTCAATGGGGAAAGACTCTTCCGATTCTGATTCCCATCACAAGCGTCTTGGTCTTTTAAAAGATCAAGTACACTTGGTTAGAAGGAAAGACTGTGATCGCCATGAGATTGCTCCAATCCAAGACCAACTATCATTTGAGAAGGGTTTTTTCATAGTAATTCGTGCATGCCAATTGTTGGCCCAAAAGAATGATGGAATAGTATTGGTAGGAGTGGCAGGTCCTTCCGGGGCAGGCAAGACTGTATTTACTGAAAAGATACTCAACTTCATGCCCAGTATTGCTGTCATTTCAATGGATAATTACAATGATTCCAGTCGAATTGTTGATGGAAACTTTGATGATCCACGCCTAACAGACTACGACACATTACTTCAGAATTTACATGACTTAAAAGAAGGAAAGCCTGTTCAAGTTCCCATATATGATTTCAAGTCCAGTACACGAACAGGATACAGGACTGTAGAAGCTCCAACTTCTCGTATCGTTATTATAGAGGGCATCTATGCCTTGAGTGAAAAGTTGCGACCTCTACTGGACCTTCGAGTCTCTGTTACAGGAGGAGTTCACCTTGACCTTGTAAAACGTGTTATACGTGATATTCAACGTGCTGGTCAAGAACCTGAAGAAATTATTCATCAAATATCTGAGACGGTGTATCCAATGTACAAGGCCTTTATTGAGCCTGATCTCCAGACGGCACATATAAAAATCATCAACAAATTCAATCCATTTACTGGATTCCAGAGCCCAACTTACATATTAAAGTCAGCAAGGAATCTAGCAGCGGATCAAATTAAAGCTGTTCTCTCTGAGGATTATAAGGAAACAACAGAGCAAACTTATGACATATATCTTCTACCACCCGGTGAAGATCCAGAAACTTGCCAATCATATTTGCGGATGCGAAATAAGGATGGGAAATACAGTCTCATGTTTGAGGAATGGGTGACAGATAATCCATTTGTGATATCACCAAGAATTACTTTTGAGGTCAGTGTGCGCCTTCTTGGTGGACTAATGGCCTTGGGATACACAATAGCCACTATCCTTAAGAGAAACAGTCATGTTTTTTCTGATGATAGGGTTTGTGTGAAGCTTGATTGGCTTGAGCAACTTAATAGACATTATGTTCAGGTACAAGGAAGAGATCGCTTAGTTTTAAAATGCATAGGACAACAACTGGGTCTAGATGGTTCCTACATTCCTCGTACCTACATAGAGCAAATTCAAATTGAAAAACTTGTGGATGAGGTTATGGCCCTACCAGATGATTTGAAGACAAAGCTCAGCCTAGATGAGGATTTGGTGTCAAGCCCCAAAGAAGCACTTTCTAGAGCCTCTGCTGACAGAGTTGCCATGAGAAATAAACATCTGATGAGTGGAATATCACGGTCATATACCAATCAAAGGGACAAAAACCTTGCTAAAGGTACTGGATTTGATGTCAACGGTAGAAGGTTTGGTGAAAGAAATTCGGACTCCGGTGCAATGGCAGTTAATCAGGGAGCCATCAATCAGCTGTCAGACCAAATTTCTGCCCTCAATGATCGAATGGATGAGTTTACAAATCGCATCGAAGAGCTGAACTCCAAGCTAAACACCAAGAGAGATTTTCCAAGCCAACAAAATATGTCACTTCAAGCTGAGACATGCAATGGATCTGCTCCCACCTCTTACTTCATCACTAGTTTGGGCAATGGTTCCCTGACTGGATCTAAAATGGCAAATTCCTCTTCATCTTCACAGTTAACTAAGGATTCTCCTGTAATGGATGAGATATCAAGCATTGCACGCAGTCAGCGTCAGATCATGCATCAATTGGACAATCTCAATAATCTTCTCAGGGGGAGCTCAGGGGAGAAGTCTCACCAAACAAGAACCAACAAGAGAAGCATGGATACTGCATCAGATTCCTTGGGAACTCGTGTTATGGCAGTGGTGGCAGTTGGTTGTTTGGGGCTCTTCTTGATGAAGGGTTTGTTGAACCGAAATTGATTCCTCTAGGTATAGTCAGTCAGTGATTTAGATTCAAGATGTTTTTCTTCACTGTGACAACCCTTCTTGATAAGAAAGGTGTTGATGCAATTGACAGTTGAAGAGAGATTCAGGAGGCAGTCTTTTAGGATCATTTGAGGGTCAGTGAGAGAATGATGCATGCCCACTTGAAATTTTGTGACTTCGTGGGCTTACTTTTCATCACAATTTTATGCCATATTATACAAAACAAAGTAGAGTCCATGTTTTAGAATCTTTATCAGTTGGTATACCATATATCTTTCTT

>C101203_65

ATTTTTTACCATATGGCAATGTGAGTCCATGTCACTTGCCTATCACCTACCACTGATAATTCCATGCCTTGTTATTAACCCAACCACACCATTTCCCTCTTCTCACAATTTCATGTGCCTCTTGTGTTCTTCAATTCCCTCCAACAACTGCACAACACAGAAGGGTCGGTCCTTTACCTTTACCTTCTCTTTTTGTTTGTGTGAGGAGGGGGGAATGCTGCTGCTCTGAGAAGGTCCATTTTTGAGATTGGAAACACAGACCCATTGGCACAGTTGAAGAATCCCACAAACCAAAATCATATTTTGACAATGGGGTTTAGCTGGCATGTGCCACTGGGTTCACATCCAAGAGGATTTTGACTGCATAAGAGATCTACTGTTTAAGTGGATTCGGGGTCAGAAAAGAGGCTCCAGAAAAATCTGTGAAGGATTTTGGTTGTGAACAGTGTTTTCTCTTTGAATCTCTGTTAAGTTGCTGCAAGCTGCGAATGGCATCTGATCTAAGTTCGGGGCTGTCCAAGGAAACGTCTGTTTTTGGGTTGAAAGCATGGGAATTAATGGGGATACTAGTTGGGTTGTTCATTATAATCATTCTTGTGGTGGTATCAATATGCCTTACTTCACGAAAGAAGTCTAGAAGAGTCAATGGCATGCTTCCCCTCAGCCACATGTTATCCGTTTCAGATGAGATCAAGGAAATTAGAGTTGATCAAGTTTCATCCAATAGTCATCCTCAGAATGGAGCTTTTATGAGTCTGTATGACAGGTTTAGTGATAGGGACTCTGAAAAGGTTTTGATCCAACCTAAGAATGGGGAAAATAGCAGTCAATCGGGCTCATTTGTTCATCTTAAGAAAGATGATGGTGGCTCTCAATCAGGTGAAGAAAGTGGTGCCAAGTCTGTTTCTACTTACAGGTCTTCTTCGCATCCTATAACTGCACCATCTCCCTTGTCTGGTCTACCTGAATTCTCTCACCTTGGATGGGGACACTGGTTCACATTAAGGGACCTAGAACTTGCAACAAACAGGTTTTCGAAAGACAATGTTATTGGTGAAGGTGGATATGGAGTTGTTTATCAGGGCCAGTTAATCAATGGAAGTCCTGTGGCTGTTAAGAAGCTACTAAATAATCTAGGACAAGCTGAAAAGGAATTTAGAGTGGAAGTTGAGGCTATTGGTCATGTGCGACACAAGAATTTGGTTAGACTTTTGGGTTATTGCATTGAAGGGACTCACAGGTTGTTGGTTTATGAGTATGTTAACAATGGCAATTTAGAGCAATGGCTTCATGGAGCCATGAGGCAGTATGGTTTTCTTACTTGGGATGCTCGGATTAAAATTCTTCTTGGAACAGCTAAAGCGCTATCTTACTTGCACGAGGCAATTGAGCCAAAAGTTGTACATCGAGATATTAAATCAAGCAATATTCTAATTGATGATGACTTCAATGCCAAAATATCTGACTTTGGTCTGGCAAAGTTACTTGGTGCTGGGAAAAGCCATATCACAACTCGAGTCATGGGTACTTTTGGATATGTGGCTCCAGAATATGCCAATTCTGGCTTACTAAATGAGAAGAGTGATGTTTATAGCTTTGGGGTATTGCTCCTTGAAGCAATCACCGGAAGGGACCCAGTGGATTATAGCCGACCAGCAACTGAGGTAAATTTGGTTGACTGGCTCAAGATGATGGTTGGGAATAGGCGTGCAGAAGAGGTCGTAGACCCTAACATTGAGACCAGGCCATCAACAAGTGCCCTTAAACGTGCCCTTTTGACTGCTTTGAGATGTGTTGATCCTGATTCTGAGAAAAGACCAAAGATGAGTCAAGTTGTCCGCATGCTTGAATCAGAGGAATATCCCATACCCCGAGAGGATCGAAGACGCCGAAAGAGTCAACCAGGAAATATGGAGGTGGATGCCCAGAAGGAGGTTTCTGATACAGAGAAGAGTGACAATCCAGATTCAACGTCCAATGGCAGAAGGAACCAAAAGAAGTAGAGCCATGAATCTTAAAGCTGAAGTGCCTACCTATTCCAGGATAAGGTACACTCTTGAAGGGCAATTTTTTACATTCTTTTGAGCTTGAGTTTGGATAACTGCCACGTTTGACATCATCAAAGACATGGAAAAGGAATGAGTTACTTTGGGATGGTTTGTATACCTATTTGTTGTTATAGCATAGGTGTAGATGATAGAATGTAGATTATTGTGTATTAATATGGGATTAAAAAGATCCCTCACACTAGTGTGTACAGCTCATTGCTATTCATACATTTCCCTTTTGTCTTTTTATTTGTTTCACCACTCATTAGGATCATAAGGAATTGATGTTTCCTTGTGTAAAAGATGATCAGAAAATTTTACCAAAGCGTGAAAGACAAAAGATGTGGTTATTGTCTATAATTACAGTGAGGATTTTTCTTGATTATTACGGTAAGCATGGTTATGGTAAGCTCTC

>C101205_65

CATCTATTATTTCATTAATCTGTTTCACTTTTTTTTCAAATGATGATCTCCCCCAATGTGTGCATGAATCTTGATGTCACCCATTTGGAGAAACAAAATGGATCAAAATTGAACTTACAATCCTTACAAAGCATTGCATCTATAACTTTACACTGATAGCAGGATTCAGAACTACCGTTTCACATGTCTTTTCCTATACTTCCATAACTCGTCCTTTTTTCTCTTAGGTTTCACAACACCCATCTTACTTCTTACCACACCAATCAAGGATTTCCTATCCGTTCTGTTTTTAATAGCAGCTTTAACATCCAGAATTGCCCGAAGCTTTTGGTAAGAATTGGCATCCATTACCTGACCATTCTTGACCATCTCCTGGTGATAAAAGAATGCCTGCGCAAAATCTCGAACACGGAGAAATGCATATATGATAGTTGAATAAGTTACAGAATCTGGCTTTATGTTACGATCTGCCATCTCTTCCAACAACTCCGGCAGCTTTGAGTGCCGCCCTCCTCGTGCATATGCATTCATCAGCATATTGTACGTCAACAATGTCGGGTGCAATCCAATTTTCCCAAATTGAGATATTACATCTCTTGCTTCTTTGTAGTGACCATGTTTAGAAAAACCATCAACAAGAGTATTGAATGTCACACGTGTTCCTTCAACTTTCTCCCTCCTCATCAACTTCCATATTTTCATTAATGTTTCAGTATCACCAGCTCGTCTAAATGCATCTAGTAAAGCAGTGTAGGTTTCTATGGATGGTTTAACACCTTCCCTTTGCATGTTTTCAAATGCTGCATAAGCTTTCTCATGCCAGCCATTAATTGAATAAGCATGGATCAGAGCTGTATAAGAATGTGAAGTGGGTTTAATACCATCTTTCCTCATCTTCAAGAATGCATCTGCAGCCATGTCACTCATCTTTTTCTGCTTCCCGTATGCACTGACTAGACAAGTATATGATTTGGCATTTGGCTTCAACCCAGCATCCTGCATTTCAGCAATCAGCTTCTTCACAATCTCCGGCTGCATTTTCCTGCTGTATCCATGCATTAGAATGTTGAACGTAACTTCAGTGGGCTTAATCCCTTTAGCTTTCATCTCAACAAGAAGGCCTTCAGCTTCTTCTACACGGTTGGATTTACAATATGCATCCATCAGGGTGTTGTACACAATTGCATTTGGAGAAACCCCTTTCTTCTCCATTTCAGATAAGATGATGAGAGCTTCCCTCATCAAACCCTCAACACAAAATGACTTTATTAGTGCACCTAGGACTTCATCACCCCATTTAACTCCTTTTCCATTCATTTTTTCAAAAAATTGCCATGCATCTTTTGCACTATGGCCAAGTTTTCTCATAACAATGACCATAATAGAACATGTCACATGATCCGGAAGAACATTATCTGCTTCCATTGACTCATACACCTTCCAAGCATCTTCATACCTGCCACTAGAAAGAAGGCCTGAAATTGCGGCGTTGTAAACATGAGCATCTCTGAACTCCTTGGTGGATGGTAAGTTTGAGAATAAAACCATCAACTTATCAGCCATCCTTGCCTTCCCCAACAAAGGAAACAACACAGTGCAAGCCCGAGGAGTAACTAGTGAAGGTTCCTGCGACCTCATCCACTGAAAGAAATACAAACAAGACACCAGAAGGTGCTCCTCCCCAAGTGATTTCAAAACCTCCCAACACTCTTTCTCACTAACTCTTCCTTCATATTCAACCAAAGCCTCTTCCAGAGTAAGATTCTGTGGCAAGTTCCTTGCAAGCTGAACAATCTCCCCCACAATCCCCTCAGCTAAAGGCAAACCTTTTTTCTCACACACCATTTCTTTCTTTTTTTCAACCAACAAGGAACCATCCTCCAAACCCGTTTCAGAGTGTTCTTCAACAAGGTCACCGGTGTCAGAAGCAAGGTGCCATGATATGCGACGGTTCTTCTGAAGCGACAAACTTCCTTCTTTTCCGGGGTCTTGGAAGGAAACCCTAGTGCGGGTTTTGAAGAATTTGTAGATTGGATCATCTGGGTCATGAGCTTGTTCTTTTTCTTCTTCTTCTTCTATTGTTTCTATGACCTCTTGTTCTTCCGGTTCTGGTTCTGGTTCTTGTTGGAGATATGGGAGGAAAATAGGGGTTGGTGAAGAGAGGGGGGTGTTGGATGGTGATGCACAAAGACATAGGGTTTTGGAGAGTGGGGAGATTGAGATTTTGGAGTGTAAAAGAAAGAAAGGTTTTGGAAGAGTATGAAATTGAAAATGGTGTGAAATGGAAGGGTGAAGGTATGAAGTGGAAGAAGAAGGAAGGAGTGTGGAGTGTGTGAGGTTGGGAGGCATGGCTCTCTGTGAATGCATGCGAAGGTTATCTTCTCCCAAACAATTGCTCATTCCTCCACATCATTCCCTTTCCCAGCTTCACTCCTCTCTGTCTCAAACATCACAACGCATCAAA

>C101207_65

TTTTGGAAAGCGAAGATATTATTGGAAACATGCAGGCTATACTTGAGGTTGCTATAATCCAATTGCCAAACAACAATCCCTATATAAGCTAAACATAGTTCATGATTACGATTGACCAAAATGATTCAAAAGCGAAGAAATATAACAATGTTGCCTTGCTACACAAGGGAACATCAGCATTAACAATATTTATACGGATATTAAACCAAGAGATAGGGAAAATACACAAGGGGGAAGATGCTGATGTGTCCCTGTATGTATCATGCAAAAGAACATATATAGTTTTCAGTGATGCAACGTTTGGTTGGAAGCTGCGGTTTTATGCGAGGGGCACTGATAGCACTTGGCAAAGAGTCCAAATGTGTCAACCTGTTTGATGAAGTTTGCCATGCTAGCCCACCCTCCAAATCCAAATCCCACCACTAGAACCCACACCACCACAAATGCATTGATCACATACATTAACGTCCAGCTTGGGATGAAGAAGGGCAATTTCTCAGCAGCATTCTGTCGTGCAGAAGCAGACCTGTAAGTGAGCATATGGGCAGAAGCAGGGATGACATAAACGGTGAAGGTAACCAAAAGAGCCCCCACAGCAGAGTTAATAGGGCCAAAGAAAGGGAAAATAATAGCCAAAAACCATATTGGTATCACCACAGGCAACCTTGCAAGTGCCCTCAAACATATGCTCTTTGTGTCATGCATTCCTATCACCTTCTCCCACACAAAATACAATGGTGTGCAAGCAAATCCAAAAGTGATAAACTGGTGAATCAGCATTAAGATGACACCAGCATCACGCCAACCAGAGCGGGGGAGAAGAGAGAAGGCATTGGAGTGGTCCAGGAGTTGGTCACCAAAGGCCCAGTAAACCGCAATGGCAGATGGTAGGGTGAGTGTGAATACAAAAAGAGTGGCATAAAGATAGATATATTTGAACTTTTGAGGTTTCCACATTGCATGCATGATTTCCACTGTGACGGCGTGCCCGCCGAAAGTGTAGAGAATGTTGGTGGCTCCCGTGAAATACAAAACCAATTTTTTGGGGCCCGTGTGAGTCACATTTTCAACCTGGCCATGAACGATGGCTGCAATGGTCAAGTACCAAGCTGTGTACGTGGTCATGCCAAGACCAAGGAAAGACCAAATCCTGTAGTTGTGAAATGAAGGTATGAACACTGTTGTGGCACAGCATGCTCCGAATATGTAAGTCCAGGTCCTCTTATCCAAGTGGTCATTTATGTAGTATATATTACTTGCACAAGCTATAAGCTGGATGACAGATCCAAAGAGGAGAAAAGTACAATTGAAGGCCAAGCCAGCGGCTTTCCAGTATGGACCTAGTAAGCCTTCCAGCACTTCAAACCACTGAATGACATGGTTTTTGAAGCTGACATTCTCTTTCTCTTTTCTGCTCCTGTACTCGATGTACAGAATGCTAATCAAATAAGCAGTCCAGCTTCCCATCATTCCATAGAAGACTTGGAATATGATGCCAGAAAGCATTCCCAGCTGAGCAAAAGAGCATGGCAGTGTTAGCAGAACTTGCGCAACCTGATTTGAGGCACAGCTGAACCATGCATCATAGACAGACCCACCATGCCACAGAATGCTTCGCAAACTGGAATGTGAACCACCCACCTTCTCTTCCTCTCTCTCTTCACGCTCAATAGTTTCATTGAGGCTTGACATCATGGTTTCTTCACCTTGCTTCTGGGGTAACATTGTTCCAAGATTATTATAATATAAAGAAAGTCCTAATATTGGTCAATGTTGAAAGATGAAAGTGTTCTCTTATAACACGGAAACAGAGGAAACAGAGAGGTTTTATGGTAATATAGATGAGTGTGTTTGTATGTGTGTGGCAGGGAAGTTTAACTTTTTAAGGCAAAAAGAAAAGATGGTAACAGCACTTGATTGGGTTGGTGTTGCGGTTTTGCTGATAATTTCAGAGGATTTTGATTTTTGAACTGTTTAAACTCAACACAAAAAGCTCCAATTGCAAGAATGGCAGGCCTTAAATATGGCTTTATGCACTACCTGGTGCCGTTAAGATGGTGACTCAGTCAACCTCTTAAATGCGGGTTCTGAACAAGGAATTGGCAGAAGGTGGCAGTTTTCAATGCAGAAGATACTATTACTAACCCAGCAAGCAAGAAATTGAGACAACAAGGGAAAAAGGTTGGTGTTGGGTGGTAACAAACAAGTGAGCAAGAATGAGACCTTGGTGGTGTAAGGGTTGAGTTTGGAGGATCGAGATCTGACAAAAAGGAAGTACACCTGAGAGAGTAAACTTGGAAGTTGGAGGTGTGCTTGCTTGGAACTTGATTAAAAGAAACAATAAAAAATTGAAAAGAGAGCAATGGGAAAGGAAAGGAAAGGAAAGGAAAGGAAGAACTGCTATGATAACTCTTTGGTTTATTCTTGACAGTCACATACATAGATGTTGATTTGGCTTGGCTTG

>C101211_65

GTTGTTGAAGACAAAAACTTCATGGAAAACAAAGCTAAGAGATGCTAATTCGAAAATCCAAGCAGAGAGTTGTTTGGTTAGTTTAATCTTTCACACTTTGATCCTCGTTTTCCTCAAGCAGAGAAAAATAGCAAAAGTTAAACAAATCAGCGTCAACCAAAATTAACAAAAACATCTCTTCTCTTCATGTTTCCCCTCCAATGCAACATGGGTGGTAACTCCGCCGCCATCGCCGTCGTCACCACCATCCTCCTCCTGCTCCCTGCGCCGACTTCCCAAGCGCAGATTCTCAAAACACAACCTTACGTGTTCGGTCCCTTCAACCAATCCTACTTCAACACGTTCGCGGTGTTGCCCACCGCCGCCATCAATCTGGAGGCTCTCCAAGTAACACCAGACAGCACCGGCAACGTGTCCCTCGCTAACCACTCCGGCAGAATCTTTTTCAAGACCCCTTTCACTCTCTGGGACGACGACAATAGCCTCGACGGAAAACTCGTCTCTTTCAACACTTCCTTTCTGATAAACGTGTACCGTCCCAAAAACGAAACTCCCGGCGAGGGCATCACCTTCCTCATCGCGCCTTCTCCCTCCATCCCTCCTCTCAACAGCTACGGCGAGTTTCTTGGCCTCACCAACGCCTCCACCGATGGCAACCGCACCAACCAAATGGTCGCCGTGGAACTTGACACCGTGAAGCAGGACTTCGACCCCGACGACAACCACATCGGGCTGGACATTAACGGCGTGAGGTCCAACGTCACTGTGCCTCTCACTCCCTTGGGGTTCGAGATCGCGCCCAACGTCACGCGCTTCCACGTGCTGTGGGTGGAGTATCACGGTGACCGGAAGGAAATCGACGTGTACATGGCGGTGCAGCCTGATAAGGACGCGCCCATAGTGGCGAAACCAGCTAAGCCCGTTTTGAGTTCATCGCTCGATCTGAAACAAGTGGTGAACAGGGTTTCTTATTTTGGGTTCTCTGCTTCGACCGGTGACAACGTTGAACTAAACTGCGTGCTGAGGTGGAACATTTCTATTGAAGTTTTCCCCGAAAAAAAAGGTATTGGAAATGCTTTGAAGATTGGGTTGGCCGTTGGAGTGCCTCTGGTGGTTCTTCTCGTGGCGGGTGTTGTAGGGTGGGTTTATTGGCTTAGGAGAAAGAATAGAGAAAGTGAGACTCAGATTTTGGGAACTCTCAAGAGTTTACCAGGAACTCCCAGAGAGTTTAGGTTTCAAGAATTGAAGAAAGCGACTAATAATTTCGATCAGAGGCAGAAGCTTGGACAAGGTGGATACGGCGTCGTCTATAGAGGAACACTTCCGAAGGAGAAGTTGGAAGTAGCTGTTAAAATGTTCTCCAGAGATAAGATGAAAAGCGCTGATGATTTCTTGGCTGAACTCACCATTATCAATCGCCTTCGTCACAAAAACCTTGTTCGCTTACTGGGGTGGTGTCACAGAAACGGGGTGTTGCTGTTGGTGTACGATTACATGCCTAATGGCAGTTTGGACAACCACATTTTCTGTGAGGAAGGGAGCAGCACTACCCCACTAAGTTGGCCTCTAAGGTACAAGATCATAACAGGGGTGGCTTCTGCATTGAATTACCTACACAACGAGTATGATCAGAAGGTGGTCCACAGAGATCTTAAAGCCAGTAACATCATGCTGGACTCAGACTTCAATGCTCGTTTAGGTGATTTCGGGTTGGCTCGTGCATTGGAGAATGATAAAACATCATATGCAGAATTGGAAGGAGTGCATGGCACAATGGGATACATTGCACCCGAGTGCTTCCACACGGGGAGGGCCACAAGGGAATCGGATGTTTATGGGTTTGGAGCAGTACTTCTTGAGGTGGTGTGTGGCCAAAGGCCATGGACCAAGAATGAGGGGTATGAGTGTTTGGTGGATTGGGTATGGCTCCTGCATCGTGAGCAAAGAATACTTGATGCAGTGGATCCAAGACTTGAGAATGACTGTGTGGTTGAAGAGGCAGAGAGGATTCTGAAATTAGGATTAGCATGTTCTCACCCCATTGCTAGTGAGCGACCAAAGATGCAAACAATTGTTCAGATCATATCTGGTTCAGTTCCTGTGCCTCATGTTCCACCTTTCAAGCCAGCTTTTGTGTGGCCAGCTATGGATCTATCAAGCCTTGCAAGTGACTTCACACAAACCACAACAACAACTGAGTACACTCCCATGACCAGTAACACACATTCTATGCATGTTGATTTCTCAGATACTGGCTCTCCCGTCTAAAACACCATATATGCATGCATTCCAACTCCATCCCCTCTTTGAAGACAACCTTTTCTCTTTGTTTTTTCGTGATCTCTATGTTGGTCTGCCCTTCCACTTAGTTAACTTTTCTTCCTCTGAAAAAAAAAGGAAGAAAATTTCTTCAGACTTTCCTTTTCTTTAATCTTAATTTTATTTTATTTTTAAGTGGTCAAGTG

>C101213_65

TTTGTGTTAGCATAGACGCCTCTAATTTGTCTTAGCAGGGTTTTACCTGCTTCAACTCATTGCTTCGCATTTTTGTACATGTATCTCAATTTTCCCATTTTACAGATTGTTGCAAAAATGGCACAAAAGCATGCCAGGTATAAAGAAAGGGGAAAAAAAGAGATGAAGAACAAACAAACTTATTCTCTACTAGAATTTTCCTGATCAACTAAATATTCACTAGAAATGTCATCCTGTGGTCTCTTTGCAAACGTATCTCCGTCATATTCTGTATCCTCCTCCGAGTTCTCACCACTGCTCCATTTTCTTTTCATGATTTTGGGGACAATGCGAGTAACTTTAGTCTCCAACATCTCATCATTATTATTTACTCGTTCAAACCGAACAGCGCTCACCTTTCTGGCATTTCCAGCTAGTATCTCAAGTTTGAAAATCTGGTTCTTCTCCTTCTGATCCTGCTGGTCAGCACTGGACTCATTGTTATCATCTTCATCTTCCCTTTCAAGAACTACTTTGATGCACTCACCAGTCCTTGGAATGTATCCAAATGCCTCACACACAAAGCCTGATACTGTCTCATATTGATGACCCTCCGGCATCTTGATGTTCAAATCTTCCGAGAGCTGATCAATAGATGTGTTTGCATCCACATCAAAAACACCCTCAGCTCGCATTACTATGTAACCAGTTTTTTTCTGTATCTCCTCCTTTGAGTCATTTTCATCAAAGATTTCACCAACAATTTCCTCAACAACGTCTTCGAGAGTTACAATCCCCACAGTTCCACCATACTCATTAAGAACAACCGCCATGTGAACCTTCCTGATTCTGAACTCTCTAAGAAGATTCCAAACAGACATTGAATCCGGTACAAAATAAGCAGGTTTGTGAGCCATATCTCCAACTGTAGTACTTTCAAGCAACTCCCCCTTTTGAACATAATCCAGCAGATCCATTGCATATGCTATACCCATTATATTATCAACACGCTGCTCAAAAACAGGTACCCTAGAATATTGATGTGTGACCCACAAGTGATGAAAGTCTACCAGGCTTGAACTTGCATCAATTGCAACAACATCAACCAGAGGTGTCATAACCTCTCTGACATGTGTGTCTTTTATCTCTAGAACATTTTCAATCATATCCTGTTCTTCCTCCTCTATTGCCCCACTTAATTCTGCACCTCTTAGCATCAATTTTAGCTCATCTTCTGTTACATATGGCTCACTTCTTCCTTTTAGGCCAAGCAATTTCAGCATCCCCATTGAAAGATAAGTAACAATTCTTCCTACAGGATACAACACCAGGGAAAGCCATGCCACTGGCCTGACCACAAAACGAGCCACCTCTGTGGCATTATGAACAGCTATGCTTTTTGGAGTGATTTCAGTGAGAAGCAAAATGGCAACAGTCATCACTCCTGTTGCTGCGCTAACACCTGCTTCACCGAACATAGCAGTTGCTGCTTCAGTGACCAGAGCAGTTGCCCCGATATTGACCACCGTTGTGCCAATAAGTATTGTTGTAAGGAATCGAGTAACATCACTTCGGAGTAGTCTGAAAACGCCATTTTCAGACTCCTTTTCAGCCAATTCACGAACCTTCCAAGGCCAAAGTGTGGTGATTGACGTCTCTGCCATAGAGAAAAAGGCTGAAAGGCCCAAAAGCACTGCCAGAACCAAACCTTGCTCCTTAAATATACGAAGAACTTGCAACACTTTAGGCCAAGTATTCCTAAGCAAAAGTATGCTCTGTCCAATAACGCCATATCCTGCATTCACAACACCTTCAACAGCGAAAACTCGTGGACATCCAAACACCAAAATCCCACAAACCGTTGCAGCTAGGATGATACCCGTCTTCAACAATTCATGTATAAAGTTCAAATTGGTTTCGGAAACAGCATTTCTCTCACTCAAACAATTACTGCCACCACCTAAACACCTCAACCGAGTTCTATCTAGTAACTTGGCATCTGCTCCAAAACTTGAAACCTGAAGGTTTGAAAAATTGGGTGGACCAACACAATTGGAAACAAAGGGAAATGGATATCTAGTGCTTCTACCTAAAACTTTGATTGGTATGCTCTTTTGTCGATTTTCATGAGCAGGGTAAAACAATCTGGAACTGGGGTTGAAGACTTGGTGACTCAAAACCCAAGATTTGAGTGCCATCAGTCATAACAGAACAAGAACACCCACCAATTCAAAATCAAAACTTCATGCACAGAACTATAAACAAACCCAATTATGCCAAGAAAGTACTTGTATTTTGCTCAGTAATGAAATCGTGAAAAGGTAAAAGAATTGGATGTGTTATGCAACACAGTAGGGGAACAGCAGCAAAGAATAAACGAATATACGCCAAATAATTTATATTTTTGGGTGTCTTGGAATAAAAAAAAAAAAGGAAATATGAAGGGAGAATCGGAGTAAAATTAGGGTTTGAAAGGGTGTAGTGAA

>C101215_65

CAAAATTATCCATGCCTTTCTCATCGTAAATTCATGCGTTACTAACCTAATCGCATTCCTCTCCTTCCTAATTTGGCTTCAATTTCAATTTCTTAAAATTCAAATCAAAACTCAAAATCAAAAAGAGTTGGTGTGGCATTTCATCCATTTATTCCTCCGACCCACCAAACATTCAAACCAATTTGCTATTTGTAACGAGGGATCGAAACACTGTCAAACTCGACACAAAAAACGCTAGAAAGGTAAATACATAACTGCATATATAAATTAGAAAAGGAGTAGAAGAAAGAAAAGAGAGTGGTGGCGAGAAAAAACACAGCGACAAGAGAGAACGAACCAAAGTGTGCTTCGAAATGTGGCGGTGCGTGGCTCGTGCGAATCACCGATCACTGAGATCTCACGTGTCAAGATTTTTCTCTAGTGGTGTGAACTCCTCGTACAACGTGGTGGATCATAAGTACGATGCGGTTGTCGTGGGTGCTGGTGGTGCAGGCTTGAGAGCCGCCATTGGACTTTCCGAACATGGTTTCAAGACTGCTTGCATCACGAAGCTCTTCCCAACTCGTTCGCACACTGTTGCAGCTCAGGGTGGTATAAATGCTGCATTGGGAAATATGACCGAAGATGATTGGAGGTGGCACATGTACGACACTGTCAAGGGAAGTGATTGGCTTGGGGACCAAGATGCCATCCAATATATGTGTAGGGAGGCACCAAAAGCAGTAATTGAACTTGAGAATTATGGACTGCCATTTTCACGAACTGAAGATGGAAAAATATACCAGCGTGCGTTTGGTGGTCAGAGCTTGAACTTCGGAAAAGGTGGTCAGGCATATCGTTGTGCATGTGCTGCTGATCGAACTGGGCATGCTTTATTGCACACTCTCTATGGCCAAGCTATGAGACATAATACCCAGTTTTTTGTGGAATATTTTGCATTAGATCTATTAATGAACAGTGACGGCAGTTGCCAAGGAGTGATTGCCTTGAATACAGAGGATGGAACATTACATCGTTTCCAGGCAGCTTCAACAATTTTGGCCACAGGGGGTTATGGTAGAGCATATTTTTCTGCCACCTCAGCACATACATGCACTGGAGATGGCAATGCCATGGTTGCACGTGCTGGGATCCCACTTGAGGATTTAGAGTTTGTGCAATTTCACCCTACAGGCATATATGGAGCTGGCTGCCTTATCACGGAAGGTTCTCGTGGTGAAGGTGGAATTCTTAGGAACAGTGAGGGTGAGAGGTTTATGGAACGTTATGCTCCTACCGCAAAGGATCTTGCATCAAGAGATGTTGTTTCTAGGTCAATGACTATGGAAATCCGGGAAGGCCGTGGTGTAGGACCCCTGAAAGATCACATCTATCTCCACTTGAATCACTTACCCCCCGATGTTCTCAAGGAGAGACTACCTGGTATCTCTGAAACAGCTGCTATTTTTGCTGGTGTAGATGTTACTAAGGAGCCTATTCCTGTTTTGCCAACAGTGCATTATAACATGGGTGGTATTCCCACGAACCATCATGGAGAGGTGGTTACTATTAAAGGTGATGATCCAGATGCAGTAGTTCCTGGATTAATGGCTGCTGGGGAAACAGCCTGTGCTTCAGTTCATGGTGCCAATAGGCTTGGTGCAAATTCACTTCTTGACATTGTTGTTTTCGGTAGAGCTTGTGCAAATAGAGTTGCAGAAATCTGTAGGCCAGGAGATAAACAGAAGCCTTTAGAGAAAGATGCCGGTATGAAAACAATTGCAAGGCTGGACAAATTGAGAAATTCAAATGGGTCATTGCCAACTTCCAAAATTCGGTTGAATATGCAACGAGTAATGCAAAACAATGCTGCTGTGTTCCGTACCCAAGAAACATTAGAAGAAGGTTGTCAATTAATTGATAAAACATGGGAGATGTTCCATGATGTCAAGTTGGAGGATCGAGGTTTGATATGGAACACTGACTTGACTGAGGCCATTGAGTTGGAAAATCTTCTGATAAATGCCTGCATCACCATGCACTCTGCTGAAGCAAGAAAAGAGAGTAGAGGTGCTCATGCCCGCGAGGACTTCACGAAAAGAGACGATGAAAACTGGATGAAACACACTTTGGGATATTGGGAGAATGAGAAAGTCCGGTTAGATTACAGGCCAGTGCACATGAACACGTTGGATGATGAGGTGGAATCATTCCCTCCTAAAGCTCGTGTTTATTAATATATTTCCTAAATTCTTACTATGGGTGGTATATACGGATTGTTTCAAGAAGTATGAAATAGCAAGTGCAAGTGAAAAAGGATTCAATAAAGTGTAGTAGATGGATGACTTTGATGATAATCGTTTTATTCATCATCAGTGTTAAACTGGTCTGTTCAGGGGCATCCTCAAATAATACACGATGTCTGTATTGAAAGCTGAGAATATTACCCATAAATCCCATTAAGAGTTGATTTTCTGCACAAAAAA

>C101217_65

TTATTGATACACTTTCAGCTAGTAATAGGAATATAAGAAAATGTGATGTTAATGAGAAAAATTAATGCTTTAGTTCAGATGCAGACTTATTACCGCCTCACTACTAGGCAAAACTTCGATTACTACACATTGAAAAGGCCTCGAGGTAAGGAATGAACTCCAACCTTTAGTACAAAAATAAGTGGTCATATATGACATGCTATATGAAAAAAGATGATTGAGATCCCTAACATCTAGACAATTAAATCTGAATAAAGTGGATACACACCCTTCCTCGCCTCACTTCTTGACCTCCTCATATTCTGCCTCCGGAGCCTGGTCCCCACCCTGAGAACCACCGGCTGAGGAACCGCCACTAGAACCACCTGACATGTGCTCCCCAATCTTGGATACAGCTTTGTTTGCGGCTTCAAGCTTTGACTTAATTTCATCAACATTGTCCCCTGACATCGCGTTTCTCAAATCCGAAACTGCATCCTCAATTTCTTTGGCCACTTCACTGGGAATCTTCTCTCTGTATTCACTTAAACTCTTCTCAATGCTATATATAGTAGTGTCAGCACTATTTCTAATGTCAATGAGAGCCTTTCTCTCTTGATCTTTCTGGGCATGCAACTCTGCTTCTTTGACCATCTTTTCAATCTCATCTTCAGAGAGTCCGCCAGATGACCGGATGGTGATTTGTTGCTCTTTCCCAGTAGACTTGTCTTTGGCAGAGACGGTAACAATTCCATTGGCATCAATGTCAAACGTGACCTCAATCTGAGGTAAACCCCTGGGAGCAGGAGGAATACCAACCAGGTCAAATTCTCCTAGTATTTTGTTGTCTGCAGCCATTTCCCGCTCTCCTTGTAACACCTTGATACCTACCTGAGTTTGATTGTCAGCTGCCGTTGAAAACACCTGACTTTTCTTGGTGGGAATAGTAGTGTTGCGGTTGATCAACCTAGTAAAGATACCACCCAAGGTCTCAATACCCAGAGAAAGTGGTGTCACATCCAGGAGTAGTAACTCTTTAACATCTCCACGTAGGATACCACCTTGGATTGCCGCCCCCATGGCAACTGCCTCATCAGGGTTTACTCCTTTACTGGGAGACTTTCCAAAAATCTCTGAAACCACCTCTTGGACTTTAGGCACCCTAGTCATTCCTCCAACAAGAAGAACCTCGTCAACATCCTTGACAGAGATGTTAGCATCCTTCAAGCAGCTTTTGCATGGTCCCTTGGTCCTTTCGATCAAGTGATTTACCAAAGCCTCAAACTTAGATCTAGTTAATGTTATGTTCAGATGCTTAGCACCAGAAGCATCAGCAGTGATGAAAGGCAGGTTGATCTCAGTTTGAGATGTTGAAGACAGCTCTATCTTAGCTTTCTCAGCAGCTTCACGAAGTCTCTGCAGTGCAAGCCTGTCCTTTGAAAGGTCAATACTCTCGGTTCTTTTGAATTCGTTCACCAGAAAATCCAACAAGGCATTGTCAAAATCCTCTCCTCCCAAGAAAGTGTCACCATTTGTCGCTTTCACCTCAAAAACACCATTCGAAATTTCTAAGATGGATACATCAAATGTCCCACCTCCAAGGTCAAACACGGCAATGAGACCCTCCTTGTTGTTCATCCCATATGAAAGTGCAGCTGCAGTGGGCTCATTGATAATTCTCTGCACATCAAGACCTGAAATTCTACCAGCATCTTTTGTTGCCTGCCTCTGAGCGTCATTGAAGTAAGCCGGTACAGTAATTACAGCCTTAGAAACTGACTTTCCAAGATAAGCTTCTGCAGTTTCCTTCATCTTGGTGAGAACAAACGCACCAATTTGGCTAGGAGAATACTGCTGTCCATTAGCCTCAACCCACGCATCACCATTTGGGGCCTTAACAATCTTGAATGGAACCATTTTCATCTCCTTTTGTGTTTGAGGATCATCAAAGCGTCTACCAATCAAACGCTTGGTACCAAAGAGAGTGTTTGTCGGGTTAGTTACAGCTTGACGCTTGGCTGGGGTACCAACAAGAAGCTCCCCTTTCTGGTTGAAAGCAACAACAGATGGGGTTGTTCGAGCACCCTCAGAATTCTCAATAACTTTGGGATTCTTTCCTTCCATGACGGAAACACAAGAATTGGTGGTACCCAAATCAATACCAATAACATCATTTCCAGCAGGCCTTGAACTGAACGGTCTAGACAAACTAGACCACTTGTGAGCTACATATGCTGGCTTATTGCTTCCCGTCAACGAGCGATAGGCAGAGAAGGTGGCGGAGGCGACATCGCGGCGGCGGAGAGAGCGGAGAACGGCAGCCATAGCCGGAAGGGGAGATTGATAAGAAGGGAAATGGAGATAAGTGAAGCAGGTGAGATGGATTTTTATATGTCTGAGAAGAAGGGTTTAGGGTTTTGGTCCTTGCGTTGGCAAAAGAAAAGAAAACAGTGTCGGCTCTTAAGGATGGTGGCAGATTATTCTAG

>C101219_65

TTTTTTTTAACGGATCATCTCTCAACTTTGTTTCATTGTATTGTATTCTAGTTAATAAGACTTGAGTTCGAACGAGAACTGTTTGTTAGAAATATGCCCGACATAGTAATCCTAATCTACAGCTGCAAACTGTAAATTATACAATTTAGCACATGCAAAATGAAAGTAGGTTTACTCCATCTTGTCGACTGCGATTGGAAGGTATGCAATTTTGGTGTCTTGGATATTAGAAACTTCTTTGATTGCGCCGAGTAGAAAAACCAGTGTCGTCTTCATCTTTGATGAAGGGTTTAAATCTGGCGAGGCCACCACTTCCACTATATGCTTGGCTTCTGTTCGTAGCTTGATGATCTGATGGATCTGGATTGCCACAAATCCCAAGTTTGACGATAAATGTAGCTGTGCTTCCTTTGCCAGGTCCCTCGCTCTCAATCCATATGTGACCTCCCATGAGGTTTACAAATCTTTTACAAATAGCAAGCCCAAGACCTGCACCACTGCTAGGTCGAGCTGGTCCACTCCGAGACTGAGCAAACTTGGTAAAGAGATGAGGAATATCTTGTGGGGGAATCCCACATCCAGAGTCCTTAACCTGGACTCGTAAGTAGAAATGACCATCACTTGATGTTGGATAAAACTCTGGAGGGCGCCAATCTTGTAAAGATTCTGGTTTTGCAATAGTTGCTCTTATAGAAACATAACCCTCCTTGGTGAATTTGACAGCATTACCCACCACATTCAAAAGTGTTTGTGTTAGCCGCTTTTCATCTCCAATGGCATGAGTTGGCAGATCAGGAGCCAGAATTAAAGTAATAGGTAATTTTTTCACAGATGCTATTGGCTTAATCAGTTCAACAATCTCTCCCAAAACACCATGAAGGTTGAATTTTCCCATTTCTAATTCGAGGCTACCATCTTCAAGTCGAGAAAGATCTAGAACATCATTAATGAGTGTCGCCAAAACGTTACTGCTCTTCAACACTGTCTCTATCATAACCCTCTGCTCCGGCGTCAGTTCAGTCTCCAAGAGAAGTGATGACAATGCTATGATTGCATGCATTGGCGTCCTCATTTCATGATTCATAACAGCAAGAAAATCATTGCGAGCATGAATTGCCATCTCTGCCTCTCGCCGAGCCAAATCTAAAGCAACATTCTGCTCCATGAGTTGATCACGGGCTCGCATAGACTCCTCCAAAATAGCAGCATGAGAAAGGGCAACTGCTACCTGATCTGCAACCACATCAACAAGTTCCAACTCATGGTCTCGCCATTTTCTAACACTATCAGTAGGGAGGATGAGAACCATGATTGCATAGCTTTTGGCTGAAATATCCGGCCAGTCATTAATTTGAAAATTGGACAAATTTAAAAGTGGCACCCGAACAGCAACAACTTCAGGCGGCACATATCTTCCCACAAGAGGTCTGATCCGGGCGAGTGGACAGGTGGGTGGAATACGTATAGCTCGAGGGCTGTTGAAAACTTCATTGACAATAGGATTGTTTGTTTGCACTGTAGACCCAACTTGTACATGGTAGGTTAAAGTATGGGAAAGTTGCAGATTCAGACCATTTCTGGAAGGCATCCATAATGCACATTCCTCCAAGCCTAAAGTCCTCCCCAGCTCCACAAGAGTAGTCTTTAAAATTGTATGCCTGTCAAGTGTGCTCCTAATTTCATGAGTCAACATTCTAACATGCCTTCCTGTCTCTTCCTGAGTAAGAATAAGTCCCATCTCCCTGTCAAGCTCTTCAGCCTTGTTCTTGAGGAACGATTCACGCGTCTTGACACTCAACAGATCAGGAATAATGTGCACAAGCATCAGAGCCGTCGCGCAAGACACAATAGCACACGACACTTTGGCAATGGTCATGACAACAGCAACAGCTTTGGAATGCGGAGAAAATGTCCACAGGTTTATGAAATGAGTTGCTCCACAGAGGACGATAAAAGCACCAAACTGCATAAGCACCCATCTATACGGAAAGAAAGCAGACTTCTGAACAAAATAGATGAGCTCCACCGGAATCGAAAAATAAGCGAGAGCAATTAGAACATCCGAGATATACTGATACTTGACAAGAAGTTCATCTGGCGGATATTGTGTATCTATACAATCACAGGACTCCATCATCTAATCCCGCTTCAACGGATCAAAGGAAGAGGAAAGGAGAGGTGGATCAGGTGGTGGAGGAGGCGCCCATTGGATTAGGCGTGACGAAGAAAGAAAGAAAGAAGAAGAAAGATGAAAGAAAGAAAGAAAGAGTGTGTTGTGGTGTGTGTGAGAGTAAGTTAAAAGCATGACATGAAATGAGGCATTGATTAACCTTAACCCCCTCTCTCACGGACAAGCATGGCAAACTCCTACTCCGACTTGGGCGTGCCGTGCCCTTTTTCCCATGCTTCTCTCATTTACTTTTTCCGCTTTACACACGTGCCACATCATTTTCTCATTGGCTGTC

>C101221_65

ATCAGAGTTAAGACTTTTCCTTGTTTCATGAAGACTCATTCGTGTTCTGCTGTTTCCTGCTTCCTTCCTTTTTCCTCATCTTCGTACACTTCTCATTTTCACTACTGTTTTTAGGTTTAGAGACTTTGCGTTGCTTTGCATGTTGTGGTGCTGACAGTATCCACTTCATCTCTATGTCAGAAACGTTCTTTGTAAAGGTTAATTCATTCCACCATCCAAAAGTTGTTGAGGGCACCTTCTCTTTCTTGGCTTTGCTTGTCCTACACTAGCATCACAATGGCCCCCCAACGTTTATGGCCTTATGAGAATGAGGTGTAATCTCTTTGACTGCTGCTACACTAGCACTTCTGACACATTTCCAAAGCAGAGTAAATGGAAAATCTTGCCCTACAAGAAACTTGCAAAAGTCACAGATAACTTCGATCAATCTCATTGTCTTGGAAAAAGAGGCTTTGCCACTGAATATTACGGAAAACTTGAGGATGGCCGCGAGATCACAATCCAGTGCTTCAACGAAGACAAGCACCACATATTGCAACAGTTTATCAATGAAACTGCTATTCTAAATTACTTGCCTCACAAAAATATTGTGTCAATTTATGGGTGTTCTTATCATAACGAGGAATCCCTGCTAGTCCATGAATACATATCCAATGGCAATCTTGCAGCTCATCTTAAAAGTGAAGATGGAAAGAACACACTACCTTGGGAGACACGATTAGATGTAGCCATTGATATTGCAAATGCATTGGACTATCTTCACTATTATGGCATCATCCACCGCAATGTGAAGTCCAGTAATATTCTCCTAGATGTGAACTTCTCTGCCAAACTTGCTAACCTGCATTTATCATGCAAACTTCCAGATGGAGTTCCTGTCTATGCCACCCATGTTACTAATGATATAATTGGAACATGTGGTTATATCGACCCAGAGTATTTGACAAAAGGCCAGCTTAGCATAAAAAACGACGTTTATAGCTTTGGGGTGGTGTTGTTTGAGCTTCTATCATCAAAGCTTGCAAAATACTGGGCTCTAAATGAGGAGGAAAATCTTGCTGCCCTTGTAAGCAAAGCAATTGAAAACCAAGCTTTGATGGAGCTCTTGGATCCAAGACTTGGGTTCCAATCAAACCTTAAGATCAAGCGGAAAATGACTGCTACAGCTGAGCTTGCACTTTTGTGCATGCAATGTCCACAAGAATTAAGGCCAAGCATGGAACAGGTGCTAGAGATTCTCAAAGGCATAAAGCAAGGGAGACAAGAAACAAACACAACAAAAGCTTTGAAAATCTTCCACCATGCTGAACTTGAAGAAGCTACTAACAACTTTGATACTTGCCTTGGAAAGGGAGGCTATGGCACCGTGTACTATGGAAAACTTAAGGATGGGCGAGAGGTTGCAATAAAATGTTTCCATGACGAGAATGAGACAGAAAATACCATTAACCAATTCATGAAAGAAACTGAGATCTTGGTTCTGTTGCACCATCAAAATCTGGTTTTACTTTACGGCCGCACTTCTCGCCATAGCAACAAACACATGCTAGTGTACGAGTACATCTCCAATGGCACTCTTTCTAAACATCTTCATGAATCTTCCTGTGCCAAACTACCATGGCACACTAGATTGAACATTGCCATCGAAACTGCAACTGCCTTGTTATATCTTCATGACTCAGGTATCATCCACCGAGATGTGAAAGGGAGTAATATCTTGCTGGATGAAAATTTTACTGTTAAAGTTGCAGATTTTGGATTCTCACGGTCTCTTCCAGATTATGTCACACACGTTTCCACTATTCCAGTAGGAACTCGTGCTTACATAGATCCTGACTACTATGAATCAGGAAGGGTCAGTGACAAAAGTGATGTCTATAGTTTCGGGGTGGTCTTGTTTGAACTTATATCTTCCAACCCCCCAAGTTTAATGGAAGGCACAGATTATGTTACTCTTGCACAGTTTGCAAAGAGAAAAATCTTAAACAAGGAATTAAACGCGGTTGTGGATCCAAGCTTTTGGTTTGGTGTTGACAGAAACATGATGGAAATGATAACAGCAGTGGCAGAGTTAGCATTTCAGTGTGTTCAGTGTCCCAAGGAACTCAGACCATCCATGAAACAGGTGCTAGAGACCCTCGAGGGCATTAGGAAAGGAACATGGGGATTCAACCAGATAACATAGTCTTTGTTCTTTCTACCAACAAGCCTGATTCTTCCAAAGCATTCATTTGGTTTTCAGTCTAGAAATTGGATTTTGGTTGAATAGATATGGAGTTTTGCTATAGTATAGTAAATTGAGGTTAGCCATGCATCATTGCAAATGATGTGCACGTAAAGGAGTAGGTTGAAAAGGAGGTTTTGATTTAGGGTGTGTATGCTTTAGCTGGATTACTCCTTTTGAGCCACATTGAACGTGTATCCTTGTCTCAGAATTCATGTTCAATGTATTGCTGTAGTTTCATC

>C101223_65

TATAGGTTTTAGATACAACACAACACCAGAACCCCAGAACCTCAGTCTTCAGCAAGCTTAAGACCACTTTAGTTGCAGTTCATATCAAGAGGAACACTGATGTCCAAGCTTTATCAGGGTTAAATTATTCACCTGGCAGTATCAAAGGGTAAAACAATTTATACCAGATTTTAAAACAGTATTAATAGGGCAGGGATATCGTACATCAGGTATTGTATCCGAAAAAATTACACGTGATGTGATATTGCCAAACTGCATTCCTTTGACATGCAAAGCATAACACATAAGACAGCATCGGCAAGCTTGAACCATTAGTCATCAGATAAAGGAAGTCGGAACCTGTGACAAAGTGTAAGCATGAAAAACAACGTCCATCCTGTTAAGAATATTGCCTCAGTTTATAGGAAACATATGAAGCTAAAGGAAGAAGCTTGAAGTCATCCTGTTAAAACTTCTTCAGCCATGTATCACCAGTATAAATGACTGACTCCAGCCAAACAAACCAAGGACCTTCAAGACTTCGGTACAGTGACATTTAAAATTTTAACATCTTGATATGGCTTGTCTGTCTTGTCTGTCTTTACTTTTTCTATACCCTGTACAACATCCATTCCCTTTGCAACTCTACCAAATACAGTGTGCTTGTTGTCCAACCATGGGGTAGCAACTGTGGTGATAAAGAACTGAGAACCATTTGTATTTGGGCCCGCATTTGCCATTGATACAGTGAAGGGCCTATCATGCCTCAAGCTTTTATGAAATTCATCCTCAAATTCTCTCCCCCAAATAGATTGCCCACCAGTGCCATCTCCTAAAGGATCTCCTGTTTGTATCATGAAGCCTTTGATGACCCTATGAAAAATAAGATTGTCATAATAGCCATTTCGGCAGTGAGTCGTGAAATTCTCCACAGTTTTTGGACATTCCTCTGGGTATAATTTCATGTGAATATCACCCATTGTGGTGTGTAGAATCACATTATCAGGAAGTGATGTTGTAACTGACTTTCCAATATCCGAAACTGCCAGAAGCTCATCAGCAGGAGGTTTTTCATTGAACACATCTCTTCCTTTAGTTGCATCTTCAGGCTCCTCTGGCTCTCTTCGACTGAATAAATATATTCTGTGCTTTTTGAAGGCACAACATAGGAGAGTGGGATCTGTCAAAGGCTCCTTGCTCTCATTAGCATTTGCAGCTGCCGAAGGGATCTTTCTTACTCTTTTGCTACTCCTATCACCTTGATATAAAGCTATTCTTAAGAATCTATCATTATTCTCCACCTTTCCAAGTATTCGGGCAACTTTATTCGTGTGCAGATTAACTATTTTTATCCCAAGCAAGGTAGCATATATAAGAAAATTGGAACTTTCATCAAAGACAGCATTTGGAAGTGGTGCACTTTCTGTCTTTTCTATTTCCTTCTCAACAGCCATTCTTCGACCAAAATCAATAGCTTCCAACCGGTATAATGGAGCATCACTTCTTTGGAGATCTTGAGCAACCTCCAGTGATTCATCATAAACTCGCCTTAATTTACCCGTTCTGAACCAAAACACCCGTATCCTTCTATCAGGTGATGTAATGGAAAATTGTTTACCATCTGGACTAACCTCAATAGCTGAAACAGAAGTCTTGCATTTTGCAATTTCAAAGAGGTTTGTATCACTTTTCAGTTTAAAATTGACCTCATCCTCAGGGAATTGAAGTGTTATAGGACTCCAGTATTCAATTATCCCCTTTGCATCTGCGGAAAGTACAGAATCACATACAGGATTGTACTTCATAACTTTGATAGGACCCATGTGTATCTCTTTGGAGATGATGGGATCATTTGAACCAGCTCGTGCATCATATATGTGCACAAAAGATGAGTTCCTATCACTAATGGCAAGCGTAGCTTTGACATCCCCTTGTTTGTAAACCCATTCAACAGCACCAGGAGTATATGGCAAGCGAATCATGACCATCATGTCAAAGTTGACTACGTCATATATTTTTACAGAGCGATCATTTGATATTGTGCAGCAAAGCAGACCATCAATACTAACAGCGAGACCTTCAATTGGACCAAGATGAGATCTGAAGTGTTTGGCAAACTCAATACCAATAGGCTTTTTCTTCCAGAACTTCAAATGCCCATCGGAACTTCCAGTGATGAAAAAATCCGCAGCTGAAACAGCGACATGCGTAACCACATCACGGTGCATATAACTTTTCTCGTACATGTTGGCGGAGGGTAAAGCGTCGAGGTAGGCGTGCTCGAACTGAAGGGGTCGCTTGGGCCGAGCCCGAGGGGCCGGGCCCGGGCCTATCAACTGTTCTTCTTCGTTTGCGTTGGCGCCATTCTCGTGTTCCTCCATCTTCAAACGCAGCACTTCAGTGTAATCGAATGAGGAAGAACCACTAAACTAAGTAACTTTCTCCAAATGCAGAAATTTCAGAGTGATGTGGCGAGGTTATGAAAAAT

>C101225_65

TTTTTTTACACCAATGACTTTTAAACTTTTATAACATCTGTAACATCATGTGATGCGGTAGGAATAAACAAAACATCTCTTCATTAAAATCTGAGGATGATTAGAATATAATACATGTTAAAATTATACATTTTCCACGTCAAGCGCACCTAGCCCATTTGCTTCGATTTCTTCTTACGCCGCTTTCATAATCTTCTTGCATTACATCTCTGAATATCACACAAACAAACAAATAACTATGGAGTAGGAAGGTGATCTTCCACCCTTTCCTCCAAACAGATGCTTCCTTCAACACTTTAAAAAGAACAAAAGGGACAAAATGAACGGAGTATAACAAGACATCTTAGGAATCATCGGGGTTAGTAATCAAAGGTGGTTGGGTATCCTCGTTCTGAGATTGAGTTTCAGTTTGGGTTGGAACTTCCTGCCGTTGCTCAAGTAACCTGCTCATACGCTCTAGCAATTGTGCAGCCTTTCTTTTGCCTCTGTCTGTACCATTTTGAGCCAACTCCAACAATGGACCCATCACCCCCAGCTCTTGGGCCTGTGCTAGATATTGTTGATCTCCAGAACAGAGGTGAACTAAAACAGCAGCTGCATTCTCTTTGTTCCTTGGGGAACCATTCCCAATAAAGTCTACTAAAACTGGAACTGCCTCTGCAGCTCTGATGGTAGCCTTCCCTTCAGGATGGCTAGCCAATATTGCCAAAATAGCTAATGCTTCATCCACCATTCCCCCACTATGTTCAGTCAGCAGTCGCATCAGTGTGGGAATAACGCCAGCCCTCACTGCCTTTCCCTTGTTGCCCTGGTAAATGCACAAATTGAAGAGTGCAGTTGCAGCATCTTTCTTACCCCTTTGGGTACCCTCACTCAGTAGTGTCACCAGTGGTGGTATGGCTCCTGAAGATCCAATTGTAACCTTGTTTTCATCTATAACAGAAAGGCTGAAAAGTGTTGCTGCAGCATTTTCCCGCGCCTCCATGCTTCCCTTCTTCAGCACATGAACTATACCTGGAACTGCTCCTGAAGAAACAATGCTTCCTTTGTTATTTTCATATATAGAAAGATTCAGAAGCGCTGTCACAGCATGTTCTTGAGTGCGGGAGTCAGGCACAGACAAAAGACCAACGAGGAGAGGTATTGCGCCAGCTTCTGCAATGGCAACACGGTTATCTGCATTACGCTTCGCAAGAAGGCGGATCTCACCAGCAGCTGATCTCTGGTCTTCAGGACTGCCAGATGTGAGCTTTTGGAGAAGACAGTCTATCTTACTTCGCTCAGCAGGTGAGTAGGCTGATGTGGATTTACTAGGTTGTGAACTGCTGGGTCGTTTTGGTGGCTCTATGCCATTGGCTTCACACCATTGTGCTATGAGGCTTCGTAAGACATAGTTGGGTGTGAGAACGGTGCTAGTGAGAGTCTGCTGTGTTTTGGGACATGTCCCGTGCCCTGCTTGCAACCATTTCTCGATACATGTACGCTCATAAGTCTGTCCGGTTGAAACAATGACAGGATCCCTCATTAATTCCAGGGAAATTGGACATCGAAAATCATCAGGGATAACAGGAGCCTGATGACTCTTCTCATTTGTTCCAACTCCATAGACTTTTGCAAAAATGCCTTTTCCTCCTAAATTATCATCCTTAACTAGATTTTCTGTTTGCACGTAATCCTTTATCTTCTTCAGTAACATCGATATTTTCTCAATGCGTGCCCCTGGATCCCCACCACTTGATGCCACCATCTCATGCAAAGCAAGTGATTCTTGCGTAAGATCAGCTATTCCCATCAGTTGCAATTTTTCAGCTAATCGACTTAGAACAGATGGATCTGTAGCTGCATCACTGCTGTTATTGTAGAGGGACAACATATCCTCATACAACCTAACATCTGGTTCATCAACCCTCCCTTTAGCTCTTCTGAACTGGGCAAGAACCAGCTCAACCTGCTCTTTAACTTCATCTGATATATCAAGTTTATCATAGGAAATACCACCCAATGACTGTTCCAACCGAGCTGTCACCTCGTAGAATTTATTCATAATTCCATCCCTCTCAAGGACCAAGTAAAGCTTGCTGCCTTCGCTTCCGAATTTGAGAAGTTCCCTGGCGGATTCGAGAGCTTCCTTGAATGCGAGGACAGCTTTGGAAGTGTCCTCGGGGATCGCGTCTTTCATGTCCCTAATCTCCTCGAACATGGGGATCAGCAGCTTCAACCTCCGCGCCAAGTTGCAGTACTGCTTCTTCACGGGAGGCCTGTAATCGGAGATGCTCGCTACCTCGTTCACCAACTCAATCACCTTGCTCGCGTTCTCGCCCTCCATGGTCGCGTGTCTCAGAGCCTCACAGATCCACTATCCAACGCTGATTAGTGGAAAACCCTGGCCGATTGCCGGAGATCGAAGTTGCTGGTCATAGTCGCTCGTCAACGACACGAATTTCGCAGACACTGCAGTTTTAAGAGA

>C101229_65

TTTTCTTCCTCCTCCAACATCACATTAGCGGCTATTCTTTCTATGTTCTCTCATCTCACTTTATTTTCTTTTCTTTTCTTTTTCCGAATCTGAAAATTTTCCTACTAAATCCATTCCCTGTCCTCAAAGACAGATCCGAAACCGGATGCCAAATCTATAACCCCTCTTCTCCCGAGAATATCGTACCCATTAGATTCCTCTTACTTCTTCTATCTACACTACTGCATTTTTTTCATACTATTCGCCGCTATAGAGCACAATTTAGTTTATCTACTCACTGTTACTCCGTATTTTTTCCCACCCTTTTCGCTATTTGATCTCTTTTCGTTGTGAAAGAGGAAAAGGGGTTTCTTTGAACGACCCCAGAATCGCGATTGTGCTTCGATTCAACGAGCAATGGGTGTTTTGTAGATTCTAGTTCCTGATCTTACTTCATTTGATCAAAAGCTTTTTCTTCTGATTTTTGCTGTATTGGATTAGCTTAGCCTGAGTTTTGAGTTATTAAATACACTTTCGGATCTTCGGGTTAGTGATAAATCTTGTTGGATCTGAGATTGTGCTGTTAATAAAGCTGAATCGTTGATGAGACACTCCGAATAGGCAATTTTTTTTATTTTTTATTTTTTATTTGAGGTGCGTAGTGGAGTGTTTTTCATAATTTAAAAAGGATCGGGAGATTGGGTTTGTATCATGGATCATGTTATTGGTGGAAAATTCAAGCTTGGGAGGAAAATTGGGAGTGGATCGTTTGGAGAGCTTTACTTAGGTGTTAATGTACAAAGTGGGGAGGAAGTGGCTGTCAAGCTGGAATCAGTGAAGACCAAGCATCCCCAGCTTCACTATGAGTCAAAATTGTATATGCTTCTCCAAGGGGGAACTGGTATACCCCATCTAAAATGGTTTGGAATTGAGGGTGACTACAATGTGATGGCTATTGACCTCCTTGGGCCAAGTCTTGAAGACTTGTTTAACTATTGTAATAGGAAATTAACTCTGAAAACAGTTCTGATGCTGGCAGATCAGCTAATAAACAGAGTTGAGTACATGCACTCCCGGGGTTTTCTGCATCGTGATATAAAGCCTGACAACTTTTTAATGGGTTTAGGCCGTAAGGCAAATCAGGTATACATAATTGATTATGGTCTTGCAAAAAAGTATCGTGATCTTCAAACACATAAGCATATACCATACAGGGAAAACAAAAACCTCACTGGAACTGCTCGTTATGCAAGTGTTAATACTCATCTGGGAGTTGAACAAAGCAGAAGGGATGATCTAGAATCTCTTGGTTATGTGCTCATGTACTTTTTGAGAGGAAGTCTCCCATGGCAAGGCTTGAAAGCCGGCACTAAAAAGCAAAAATATGATAAGATAAGTGAAAAGAAGAGGCTTACTCCAATAGAGGTCTTATGCAAGTCATATCCATTGGAATTCACATCATACTTCCACTACTGCCGATCATTGCGGTTTGAAGATAAGCCTGATTACTCTTATCTTAAGAGACTCTTTCGGGACCTATTCATCCGAGAAGGGTATCAGTTTGACTATGTATTTGATTGGACAATGTTGAAGTATCCACAAATTGGATCCAGCTCTAGAGCTCGACCAAGTGGGAAACCAATCGTAAACCCAGGACAATCAGGAGAACGACCTTCAGGGGCACCAGAGATTCGAGATAGATTCTCAGGTGCAGTTGAGGCATTTGCGAGGAGGAATGGCTCTGGACTTGGTCTGCATGGTGATCATTCTAGGCATAGGTCTTCGGATGATGTGCCATCGTCGAAAGATGTGGCTGATTCTGAGAGACCTCGTAGCTCTTCTCGTAATGGCAGTTCTTCAAAAAAGCCTGTCTTGTCAAGTAGCCGACCAAGCTCTTCTGGCGAGCCTAGTGAAAGCCGTGCTAACCGACTGGTCTCAAGCAGTGGCCGACTATCAACAACTCAGAGACTTCAACCTGGTTCTGAGTTTAAAACATCGCTAACTCGGGCTTCAGGGACAAGAGGTGGCCGTGATGATACCCTCAGGAGCTTTGAACTCTTATCACTTGGTACAGGAAAAAGGAAATGAAGAACTGTATGATCAGCAGATTTGTTGTGTCAAGGGTCTTAATTCAGCGTTGATACTTTCCTTTGAGATGTTGACACGTTGATTACTCTGTATATTTCCAACCAATATTGAGGCTAAAGCTCATTCAAACTTCTTCATGAAGATTTAACAGACTCTTCTTATACCTCTTTGGATGTTCTTCCATGGTTATTTCAGCATGGACCCATTCATGAAGAGATAGTATGTTACTTAAGGAATTTGTTAATATATGACTACTTGTAATAAATACATTCTCTATTTTTGTGTTGAAGGAAAGGAAAAGAATCTACAAAAGCGCATTTGGAAGTTTTGCACCTTTCAACGCATATTTCGTACCTGAGTATTTTGTTCTAATGTACATTTGAGGGAATGGAATTATTGGC

>C101231_65

CAATAACCAAACCTAAAACCCAACGTTCCTCCTCCATCTTTATTTTCGTCTTCATCTTCGAGCTTGAGCAATGAGAAGACCGAAATCGAAGGAGTTTCGGAAGCAACAGCGACTCTCAGAGCAGGAAGAGATAAACCTTTTGAATTCATGGATCCAGTTTCAGCCACCGGACTCCGGTTCCAACCCAATGTCGCTCCCTCCTCTCCCCAAAAACTCTCCCGTTGCCCGTCTCGAGGACAACACCTACTCGCGCTACGCCGGCGCGTCCCGGTTCGATCAATTCCCGCTCTCCAAGAAAACCAAGGACGCTCTGCGGGAAGCCAAGTTCGTCGCCATGACCGACATCCAGAGAGCGTCTCTCCCCCACGCGCTCTGTGGTCGTGATATCCTCGGCGCCGCCAAAACTGGCTCCGGTAAAACCCTCGCTTTCATTATCCCTGTTTTGGAGAAGTTGTATAGAGAGAGATGGGGACCTGAAGATGGCGTTGGCAGCATCATCATATCGCCTACAAGGGAATTAGCTGGTCAGATATTTGATGTGTTGAAGGTTGTTGGAAAACACCATAATTTTAGTGCTGGCCTTCTCATTGGTGGTCGAAAGGATGTTGACATGGAGAAAGAGCGTGTTAATGAGCTCAACATATTGATCTGCACACCGGGTAGACTGCTACAACACATGGATGAAACTCCCAATTTTGATTGTTCACAGATGCAGGTTTTGGTGCTAGATGAGGCAGATCGCATTCTAGACAGTGGGTTCAAGAAGGAACTAAATGCAATCATTTCTCAACTACCAAAGCGTAGACAAACCTTGCTTTTTTCTGCTACCCAAACAAAATCAATTCAAGATCTTGCCAGACTAAGTTTGAAGGATCCAGAGTATCTGAGTGTGCATGAAGAATCTGTCACTGCCACTCCTTCTCTGTTGAAGCAAATTGTGATGATTGTTCCCCTCGATCAAAAGTTAGATATGCTGTGGAGTTTCATAAAGACACATCTACAGTCTAAAATACTTGTCTTTCTTTCAAGCTGTAAACAGGTAAAATTTGTCTTTGAAGCATTTAAGAAACTGCACCCTGGCATACCCTTGAAGTGCCTTCATGGGAGGATGAAACAGGAAAGAAGAATGGCAATATATTCTGAGTTTTGTGAGAAGCGCTCAGTTCTCTTCTCAACTGATGTGGCCGCAAGAGGCCTTGATTTTAATAAGGCAGTTGACTGGGTTGTTCAGGTGGATTGTCCCGAAAATGTAGCTTCTTACATACATAGAGTTGGTCGCACGGCTCGGTATAAATCTGATGGAAAGTCAGTTTTATTTCTGTTGCCTTCAGAAATGCAAATGCTTGAGAAGTTAAAAGCAGCAAAGGTTCCAGTGCATTTTAATAAGCCAAGGAAAGAACTACTGCAACCAGTTTCTAGTCTGTTGGCATCTTTACTGGTCAAATACCCAGACCTGCAGCATCGGGCTCAGAGGGCGTTCATCACATATTTGAGATCCATTCATTTACAAAAAGATAAAGACATATTTGATGTTTTGAAACTGCCTATTGATGAGTATTCAGCATCGTTAGGTTTACCAATGACTCCCAAGATTCGTTTCCTAAACCAGAAAATTAAATCTAAAGCTGTGTCAGAAAAATCAATTTTAGTTGAACCAGAAGATTCAAGCAAGGAAAATGCCTTGGAGGTTTCAAGAAACAAGATAGACACAGATGCTTTCAAGGATGAAAAAATCGAAAACGATCTTTTTCAATTAGCAGATTCTGCAAATGAAGACAACTTGAAGTCATCTGAAATTGAAGAAATAATACCAGCAACTCGTGTATTGAAGAAAAAGAAGCTCAAGATTAATGTGCATAGGCCAGTTGGGACCAGGGTTGTATTTGACGATGAAGGCAACACACTTCCTCCGCTAGCCAGGATAGCTGACACACAAAGTGGCAAAGAAGCTTTGCTGCTTGACCCAGAACAAAAAGCCGAATACTATAGAAGGATGCGTGATGATTTAAAGAAGGCTGACAAGGAAGACAAGCTTGTAGAGCGGCAGCGGCTTAGAGAAAAAAGAATAAAACAGAAGATGAAATGGAAGGCTGGGAATGAGGAAGAAGAGGTCGACCAAGAAGATATTTCTGGGTCGGAAGGAGACGAAACTGTTAATAAACGGCATAAAAAGAGTAAAGTATACTTTGATAGTGACAGTGACGAGGGTGAAAGAAATCAAATCGTAGGGAATGCACGTACAAATACTGGCGCGGTTTCTTTGGAGGAGCAAGAAGCTCTGGCTTTGAAATTGTTAAATTCTATGCACTCATAGGATATGCAGAATTAAGTTATACGGCATTTTTTGACAGCAAAACAGGTGAAAAATAGAGCAATTTTTTGAATTTTATAGGAGAGGTTTTATGAACAATGAATTTTGCGGCCGGTGTGATATCAAATTTATTTAGTTAAATCTCCTAAAAACATGG

>C101233_65

TTGTCTGCAAAGTTGAAAACTGGCAAACTGGACCTAACTCGTGCAATTTGTTTTCCGCGTTTGCCGCACTCCTTCTCCATTCACTATTCTTCTTCTTCTTCCTCTTCTTCTTCTTCTGTTATTCAGCTCTTAATCTCTTTTGAACCGTACTTTAACTGCTTCCACACAGGTGTTCAACTTTTCCACCGTTGAACACGGGGGAGAAAGTTTCTCAATTGCTTCTCTTCCTTTTCACACCTCAACTTCCCACGTTCTTCAGTTTCTTCTCCAACTTCCCTTCTAAAGTTTTCTTATTAATTCTATTTTCCTATTCAGTTTCAACATGTCTTAAGAAGAGGAGCATTAAACCACCCCTCTATCCGCTACCATGTCTTATTCATCAATTACTGCTATCTCAATTGGCACCCGAAGCAAGAAGATGTGAAGTCTGAACTATGAACTCGGAAGTGGTCAGGATGTGTTTCGGAGTCAAGGGGACCTTGAGTGCTTACCCCGTTGTGTACAATTTTGAATTGCTTCGACAATATTGCTTGGTGAATTGATGGTGAGAGATAATCACTAGTTGTAACTTGGGTACGTCTTACGGAAATGTAAGCTTGATATGCTGGAGCTCCATCGAAGTAGTAAAGATACGTTCAAAAGATGATGAAGAAACAATTGACAGGTATACGGGGTGATTCTCCCCTACAATCTGCAATCCGAGCTGGAAATTTAGAGTTGGTTGTGGAAATCACCTCTCAGAGTCCAGAGGAAGAATTAAAAGAGCTGCTTGCAAAGAAAAACAACTCGTCTGAAACTGCCTTATATGTTGCTGCTGAAAATGGTCATCTTGATATATTGAAGGAATTGATTAGATACCATGATATTGGTTTGGCTGGCTTCAAAGCTCGAAATGGATACGATGCATTCCATATCGCTGCTAAAAATGGACACTTGGAGATATTAAAGATCCTCATGGAGTTCTTTCCTGAAATTTCAATGACTGTTGATCTTTCAAATACTACTGCGTTGCATACTGCTGCAGCACAAGGACATATTGAGGTAGTAAATTTTCTTTTGGAAAAAGGTAGCTGCCTGGTAAATATTGCGAAAAGCAATGGCAAAACTGTGTTGCATTCTGCTGCAAGAAATGGCCACGTTGAGATAGTCCAGGCCCTTCTGAATAAGGAACCTGAAATTGCAGTGAGAATTGATAAGAAGGGCCAGACGGCACTCCATATGGTAGTTAAAGGACAGAATCTTGAGTTGGTGGTTGAGCTTGTGAAACTGAATCCATCTTTGGTTAATATGGTGGATGCCAAGGGAAATACAGCACTGCATATAGCAACCCGGAAGGGTCGTCTACAGGTTGTTCAGAAGCTACTAGATTGCAGAGAAATAGACACAGACGTCATTAACAAATCTGGAGAAACTGCTTTAGATACTGCACAGAAAAATGGTCGCTTGGAAGTCTCCAACTTTCTGCAACACCGTGGAGCTCAAAGTGCCAAGTCCATCAAGTCACCTACTGTAAACACAGCCCTTGAGATCAAACGAACAGTGAGTGACATAAAAAGTGGAGTTCATAACCAGCTGGAACACACTTTTAAAACACAAAGACGCATGCAAGGTATAGCGAAGCGAATTAACAAAATGCACACTGAGGGGCTTAACAATGCTATCAACTCCAACACTCTTGTTGCTATCCTAATTGCAACAGTTGCTTTTGCTGCCATATTTAATGTCCCGGGCCAGTATCCTGAGAAACAAGATGAACTGTCTCCAGTAATGTCTCCTGGGGAAGTGTATATTGCTCCTGATATTGGATTCAAGATATTCATAATCTTTGATTCTACTGCCCTCTTCATATCATTGGCTGTTGTGATTGTCCAAACATCGGTGGTTGTTATTGAAAGGAAAGCAAAGAGACAAATGATGGCAGTTATAAATAAGTTGATGTGGGTAGCCTGTGTGCTAATTTCTGTGGCGTTTATTGCAATGTCGTTCATAATTGTTGGAGATCATAAAGAGTTGGCTATAGCAACCACAGCTCTAGGGACATTGATTATGGCAGCTACTTTAGGAACACTCTGTTATTGGGTGATTGCTCACCGCCTTGAGGCCTCAAGATTGCGAAGTCTTAGGACAACAATGAGCAGTAGGCAATCAATTTCTATGTCAATGATGTCAGGATCGGAGAATGAGTACAAGACAGTGTATGCCGTATAACTATCATCTCTACCATTTCAGAGTTCTCCTCCTTTTTTGTCAAATTGCACTAACATTGATGTAGGTGTTTGTTTCTTAGTAAAACAAACATTAACTAATGCTACAAAAGAATGCCTTTGCAGAACACAGGCAAAAAGTGTAATCATAGTCAACTTGATACGTGAATGATCATTCTAAATTGAAAGTATCGGATGAGGCCTATTTTCTCATTAGCTTCATCAGAAAATAAATATAACACAAACTTATTGTTTCTGTAAAAA

>C101235_65

CTTCACCATAAAAAGAATGCCATAAAACAATATTTTCTTTCAGAGATAATCAAAGTCTGAGACTTCAAACACCACCAAGAATAAGCCATATACATCACATGACTAGTAAGTAGGCCATTAATGACTTGAGGATTTATGCATATAACATGAGACAGTGGTGAGAAGAAGTATCCTGTGGTAACTGAATAAAAAGGCAGTATAGGGCTTCATTTCCCGATTGGACCATTCATTGGCTGTTTCTTCCTGCACTCCACAATTTTCTTGTTACACCTCCCATGTATCCTGAAATGGGCTTTACATTCTGAACTAACCTTTCAGTAATAAAAGAGTATAGAGTGATATTGTTGGAGTGCAGGAATAAAAGACCTCAAAAATACATAAAATGCAATGCGCTCTTGCATTTACTGGACTAATCACTGTCCCAAGCCTCATCTGCATCTTTTGGTGTTCTCCTCCTAGGTATATCACTTCTGATCACATTACTATTTTGTTTTGTAGGGTAGTCCCCTTCATCCCCAGTGTCCTCTTCAGCATCTGAATGCCACAGCCAGTTTTCTGAATCGCTAGGTTCATCTATGGATTCAGATTCAACTTTGGTAAACCCATGCTTCCCTCTCAAACTCTTTGGTGCACCAAAACTATTATGATATTGAGCAGAATCACCAACCTTACCCTTATGATCCCTCACACCACTCTGGTTTTTACTCATTGTTTGGTGTGTATACTCATCATCACTGCTGCTACTCTTGTAGTCATTATCACTGAACTTTCTCAAATGTCCCGAGTCATTATTCTCTTCCTCCTCAGACTCCCACATTGCATTTTCAACTTCACTGAACATGCCTTCTGATTCAGAATCTTCTATGCTCATATTCCTCCCAAATTTAGCTGCAGCATGTGTACTTGAATTTGTGTTGTCTCGGTGTTGTCCCATTCCGGTATAATTCTGCTTCTCTCTCTTCCTCCTACTTTCCTTGAACCTACCTCTACTTCCCACACTGTCAAATTCATCATCATCATCATCATCATTGTCAGAATCATCATTCTCTTCATCATCACTATCAATAGCATGACGCTTGGGATGTGTAGTCTGCCAAACCCTTGAGCCTCTAGCCTCAACATCTTGGTCATCATTTCTAGAAGCATCTGATCTTCTGTATCGTCCACCAAATTCTTCTCTGTTCCAGTATCTAATTCTACCAAACTCTTCTTTTTCTTTAGCCTCTTCAGCTTCATATTCTTTATCTGCTGCATCCTCTATCTCAGCAATGAATCGATCTAGCTCTTCCTGATCACTATCAACAGTAGGCTCTGCCTCTTCCAAACCTTCAGCAGGATCTATTATTGACTGCTTACCTTCACGGAAGACATAAGGGCTTCCTTCTTTCTTATCGATGGCATTGAAGAATGTTGAGGCAACTCTCTGGATGCTGGCCATGGCAGCTGGATCTTCAGGATTTACTCCCATCCGTCGAAGCTGCTGTTCTATTTTCTTTATGTTCAGCTTCTGAGATTCAAGAGCTTGCTCAAATCTGGCCTTAAATAGAGCCTTCCTCTTTGTAAGAGTGTTAATAGGTATCAAATTTTTGGGTTGACGATAATTTCTTCCACGGAACATAATTATTGTTTTCACATTATGAACATTTATAACAATCCCCCCACTCAATCTAGCAAGCATTGAAGCCATTTCTTTTATTTTTTCCTTGGGGAAGTTGTCACAACAAACTTGCACAGTCTCATGAAACTTCCAATGGAGATGCATATTTTGCACAACCCCTCCAAAGACTCCACGAACACCCACTGGAACATAGTTCTTATTCCGGAATCCAATTTTTTTAAATGCTTGCAGCTGTTCAGGTGTTAAGAGCTCTGGATCATGCCGAGGAGTAGGCAGCTCTGGTAGCTCATACTTCTTGAGCTTTTGTAGAAGCAATGCCGCTTTCTTCTTGGCTCTTTTGAGATTGTAAATGAGCCTCTCCTCCTCAGTCATGAGTTTCTTCTTCAACTTTTTCGCTCTCCGAATCTCCCTCAAAGTCTTACCAGACTCTTTCTGCATTCGCCTCTCCACCTTGCTGCGCATACTCCTTCCTTTGGAAGTTGACATTGCCCGGGACAACAATGATCCATTCAAAAGATTCAACTGCTTAACATAGCAATACAAACCCACTCTCTGGCATGCCTCTTCAACTTTAACAATGTTGCTGCACTCAGGTGTGTGAGCTAATGGTACAACATTTTTGTAGAAATTGGATTGAAGTTGAAACAGAGAAGAGAGGGGTTTGAAGCAATGTCTTTGAAGATTTCTGGCACCAAACATTGTTTTCTGTCTCTTCCTTCGGTCACCAATCAGCCACAATTCGCGTTTCTCGTTTCTGGGTTTAACATGAGTGCATAGGCCAAAGCACAACAGGGTTTTGGACTTCCCAAACGCGCCCTAGTTTTTCTCTCTTCCTTTTTTTTTAGGTATG

>C101237_65

GAAAACCATAGGAACCCTACTGCCGGTAGTCCTCTTCCTCCAGTGCACCCGCTGCGCCGTTCTTCTTCAGCTGTTTGTCCCACACACTCCCACTTATCCTGTTCAAAGCAATAGCAATCGTTTGATAATTGTAACATATTATAGATAGTGTTCAACTAGAGCATAATGGGATCAAAAAATGAAAATGGGAAGAACAGCAATGAAAAAAGAAAGAAGAAGGTTAAGGATAATATGAGTTCAAATCATTCAGCACCAAGTGATATCAAGGTTATCAGTGATCCTCGATATGCCAAGGCTCAAACCGATCCTCGGTTTAGGGAGGCTCCAAAGCGTGAAACGAAGGTAGCCATTGACTCTCGCTTCAACCGCATGTTCACTCACAAGAGTTTTTTGCCCTCTTCTGCTCCTGTTGACAAGAGAGGCAAGCCCAAGAACAACCCAACCTCTCAACTTGGTTCTATGCGCCATTACTACAAAATTGATGAAAAGGTAGCAGAGCAGAGTAGTGATGAAGAAGAAGAGAATAAAGAGGAATTGGTGAAAGCCAATCGGTTGAAACCAGAGAGTAATAAAGGTTCTGAGTTGGAGGAGACCAGTGAATCTGATGAGTCTTCATCTGAGTCAGAAGGGACCAGTGAGTCAGAGTCTGCTATAGATACTGATACAGATACAGATACAGATGAAGGGGCAGATGAGGAAGTGTATGAGGAAGAAGCATCTGATGTCCAGGAAGACATAGCGGAGATTGACAAAGAAACACACAGGCTTGCTGTTGTTAACATGGACTGGAGGTTTGTTAAGGCTGTTGATTTGTATGTATTGTTAAGCTCATTTCTCCCACCTAATGGACTGATCAAATCAGTAGCTATCTATCCATCTGAGTTTGGCCTTAAACGTATGAAAGAGGAGGAAATTCATGGACCTATTGGTCTATTTGATGATGAAAATGAAAAAAGTGATGAAGATAATAGCAATGATGCTATTGACAATGAGAAACTGCGTGCATATGAGAAGAGTAGGATGCGGTACTATTTTGCTGTGGTGGAATGCAACTCAGTTGCCACAGCAGATTACATTTACAAGGAATGTGATGGTGCTGAGTTCATGCAGACATCTAATGCACTTGATTTAAGGTTTATCCCAGACAACATGGAATTCAAACACCCACCTCAGGATGTTGCTACTGAGGTACCTGCAAATTACGAGTGTAAAAATTTCTATTCTCGAGCACTTCAGCACAGTGAGGTTACTCTTACTTGGGAGGATGATGAACCTCTTCGGCCAAAGATATTGAATCGAAAACTCAACGATGAGCAGCTAGCTCAGATGGAATTGAAGGAACTCTTAAATTCTGATGAGAGTGAAAGTGATGATAGCAAGGATAATAGTGAGACAGATAATCAGCCTGATAAAATGGAAAAAAAACGAGATAAATACCGTGCTCTGCTCCAGTCTGGCAATGATTCAGACGGAGGTGAACATGATAATATCCAGGATATGGAGGTCACCTTCAATACAGGTTTGGAAGATTTAAGCAAACATATCCTGGAAAAGAAGGATAAAAAATCAGAAACAGTTTGGGATGCATATTTGAGGAAAAAACGTGAGAAAAAGAAGGCTAGGAAAAATAAACCAAAGTATTCATCAGATGATGACAGCGATGATACTGATGAAGAGGCAACTGAAGAAGCAGATGACTTCTTCATCGAGGAGCCCTCTGTCAAGAAGAGGAAAAAGGAACAGAATACAGAAGACGAAGAACATAAGCCCCAAGATAAAGTGAGCAAAGAGGAGCTTGAACTGTTGCTTGCTGATGATAAGGGGACAGATACTAGTCTCAAGGGATACAGTCTAAAATTTAAAAAGGGAAAAGGAAAAAGGAGGGAGAATGCTTTTGAAGAGGAAAAAATACCCAGTAATGCATATGATGATCCACGTTTTGCAGCTCTTTTCTCATCAAACTATGCAATTGATCCTACCGATCCACAATTTAAAAGGAGTGCAACATATGCCAGGCAGCTAGCACAGAAGCAGCAGAAGGATAGCGCAGATCCACCTGCGGAAAGGGAGCCTACAAAACTAAAAGGATTGCAATTGTCTTCTGATGATTCTGGCATTATGAACAAGGGTGAAGAAAAAGTATTAGATGCTTCGAAGTCAAAGAAAGATAAATATGAAATATCATCGTTGGTTAAATCGATTAAGATGAAATCAAAGCAAGTTCAGTTACCCTCTGATAACAAGACAAGGAAAGATGGGAAATCACACATTAAAGGTATGAAGAAAATGAGACACTAGTTCTGAATCTTGTAGTTCAATAAACACTTGAATGCAGGAGAATTAGCCTCCTCTGTATCTTACAGAACCCAGCAGGGGAAAAAGGAATTTAGCAGGTAGTTTAACCTTATTTCAAGGCGTTGAAATTCTTGTTCATGTCTAAATCACCCATTTGAAATTTTGAATCCACAA

>C101245_65

GCAACTAGGTTAAGAGAATAACAACTAGTTAACATGAAAGGGAATGCACTTTTGTTGCACTTATTTTACACTCTATTGCTGTTTCTTGGAGCGTCAAGGTTTTCAGCGGGGAATGATAGTAACCAAGATACCAACAGTAAACAAGTTTACATAGTCTATATGGGATCTGCGGATTCCACCAATGCTTCTCTTCGGAATGACCATGCTCACCTTCTCAATGCAGTCCTGAGAAGGAACGACAAGGCTTTGGTAAGGAACTACAAACATGGTTTCTCAGGGTTCGCAGCTCGCTTATCAAAAGAGGAGGCAAACTCAATTGCTCAGAAACCTGGTGTTGTGTCTGTTTTTCCTGACCCAATTCTGACCCTTCACACAACTCATTCCTGGGATTTCCTCAAATACCAAACTCACGTCAAAATCGATGCCAACCCAAACACGCTCTCCAATTCTTCTACCTCCTCAGACGTTATCCTTGGCATCTTAGACACAGGTATATGGCCAGAGGCAGACAGTTTTAGTGACGAGGGAATGGGTCCTGTTCCATCTCGTTGGAAAGGAACATGCATGAAATCACAAGACTTTAATACCTCTAACTGTAACAGGAAGCTAATTGGCGCAAGATTTTACACTGACCCTAATGGGGACGACGATGACGAGGGCGACAACACCCCACGGGACACGATTGGCCACGGGACCCACGTGGCATCAACGGCTGTGGGCGCCGCCGTGACGAACGCATCGTACTACGGTCTGGCGGCGGGGAGCGCCAAGGGTGGGTCCCCAGAATCAAGATTGGCGGTTTACAGAGTGTGTTCTAACTTTGGTTGCCGTGGGTCCGCCATTCTCGGGGCCTTTGACGATGCCATTGCCGACGGAGTCGATGTTCTGTCGCTGTCGCTCGGTGCATCACCGGGGTTCCAACCCGACTTGACGAACGACCCGATTGCAATCGGAGCGTTCCACGCCGTGGAGCGCGGCATCTTGGTGGCCTGCTCCGCTGGGAATGCTGGACCTAGCTCGTACACGGTTGTGAACGATGCGCCTTGGATTTTAACCGTTGCAGCTTCCACCATCGACCGGGATTTTCAATCCAACGTCGTCTTGGGTGGTAACAAAACCATCACGGGTAGAGCTATAAATTTCTCCCCTCTTTCAAATTCGGCTGAGTACCCAATAATATACAGTGAATCTGCTATGGCGAGCAGCGCTAGTTTATCTGAAGCAAGACAATGTCACCCAGATTCATTAGATGCAAATAAAGTCAAAGGGAAGATTGTGGTATGTGATGGCAGAAATGATGTATATTCAACTAGTGAAAAAATTGAGACGGTGAAAGCGGTGGGAGCAATAGGCTTGGTTCATATTACCGACGAAAATGGAGCAATAGCGTCATATTATGGGGACTTCCCTGCAACGGTTATAAGCTCAAAAGATAGTACCACAATACTCCAATATATCAATTCATCCAACAATCCAGTGGCAACAATTGTACCAACAACTACAGTTCTTGATTATAAACCTGCTCCGCTTGTGCCCAACTTTTCATCAAGAGGACCTTCAACGCTATCAAGCAATATTCTAAAGCCTGATATTGCAGCACCGGGAGTTAATATTCTTGCGGCATGGATTGGAAATAGTGCAGAGGAGGTTCCCAAAGGAAGAAAACCCTCACTGTACAACATAATCTCTGGGACTTCCATGGCCTGCCCCCATGTTTCAGGGCTTGCAAGCAGTGTCAAAACCCGCCACCCCACTTGGAGTGCCTCTGCAATCAAATCTGCCATCATGACTTCAGCAATTCAAAGTGACAACCTGAAGAGACCTATAACAACGGATTCAGGGTCAGTAGGCACACCTTATGACTATGGAGCAGGGGAAATGGCAACATCTGAATCATTGCAGCCAGGGCTAGTTTATGAAACTGACAACATTGACTACTTAAACTTCTTGTGTTACATTGGACTTAACATAACCAATGTTAAGATCATCTCGAGAACTGTTCCTGAAAATTTCAGTTGCCCCAAGGATTCAAGTTCTGATCTAATCTCCAACATCAACTACCCTTCCATAGCAGTAAACTTCACTGGGAAGAGTGTGGTTAATGTGAGTAGAACTGTCACAAATGTTGGTGAAGAAGAGGAAACAGTGTACTCCCCCGTTGTTGAGGCTCCCAGTGGAATAAAAGTCACATTAACTCCAAATAAATTAGAGTTTACAAAAAGTAGTAATAAACTAAGTTACCAAGTCATTTTCTCCTCAACTTTGACTTCTCTGAAGGAAGATCTGTTTGGATCTATTACATGGAGTAACGGCAAATATATGGTTCGAAGTCCTTTTGTATTAACTAAATAGATAAACTAAAAGAAAGTAGCAATAATAAATGCAAGCTAAGTTCTTCATGGTGCCTATTTAACGTGAGTTTGTTAGTTAACTCTTGGATATTATGAAAAATCGTATTATGATTGCAAAAA

>C101247_65

TATTTTCTTTACACTTGCATTTCTATGAAATTCTATTCCGGTTCAGGCCTACTTTCGTTGTTTGATCCTCTGCGCGATTTTACACTTAGTCGGTTATACATTCTTTGGCCATTGCCAAGGCTGAAAAACCATGTATTACCCAAGACTAGATGGGTTTCTTTACTATTTTTGCTGGGATGGAGTGTACCTGCAATAGCTGCGATCCAACAAAACACCTTATTTTTGCCAGAAATTTTGATGTTACAAACTGTATGTGATGACACTGACACTCTTCTGCAATGCATATGGATGATGTTTGATCTAGTCCAGAGTGTACGTGTGTTCACCCAAAAAAGAAGGGTACGCTAGCTAATTCCTATAGCATTTAAAGCTTGTTCTGTGGTCGGTCAAACTTTAGGAAGGAACAAATTTCATGCAGATGGGAGGATGGATTCTTGCAAGAGCTAGAATCGCAGCAGGTAGAATCCATAGTCCTTCCCCACAAATTAGTCCAGAAGCAGCTGCTGGAACCATCAACTCTGCCTTTTTGGAGTCCAGCTTGTGCCACAAAAACACAACCACAGTACCTATGAACATGTCGATTGCAAAGTATGCCCCAACCAGAAATGGTACGGCCATGACCATTGGTAATGGCATCCATTTACCAATCTTTTGGGGTGAAAAGTCTCTGGTCATGTTTGCTACTATGGCAAAAGCGAAGAACCCAAAGCAAAGCTGCAAGCAATGCTGGGGCAATGCTGAGAAACCTTGGACACCTATGATTGCCATGTTTCTGTAAATTAAAGCATAAGGAGCTTTGAATTCTCCATGCGGATTTCCCACATCGAATGCCTTGTAGTATAGGAAGAAGCTGAGAGGAGTTGTCACACAGCCCATTGCAATGCCGACTACTTGGCATATGAACATGGCTCTGGGTGAGGTACGTGTGTAATGAGCAGTCTTGAAATCTTGCATTAGAATGCAAGACACCGAGAGCACGGATTTAATGAGACCGCAACCCACAAGTCCAGCCACCACACCATTTTCTTTTCCTGTAATTGCTGCCAGGGTAAAGAGTGCAACTTTCCCATAATTATATGCCATGTTTATGTCGGTGAGACCTGCTCCAAAAGCATTACAAAATGCCAGAGATGGAGCAAATATATAAGCAACCACTATGTAGTACCATTTCAGCTGAGGAAACATGCGTGGGATTATAATTGTGGAAATGATTGTGAAAACAACATATCCAATGATTCCAATCCGCATGGGAATGTTCTCCCTCAGAAATGTCTCAGCTTGTTTAATGTCCTCAGTTGGATTCCCCTTCCGATCAGCAGCCACATTTGTATTGTTCTTGCTCTTTATTCTGTTATGGACGTTGATGACTGTGGAAATTAGAATCTTGGTGAAGTTGTATATGCCATCACCCAAGATTAAAGCGATTGAAAGAAAAACCTTATAACCGTATAAACCCTTCATGTTACTTTCTTCTAAATTGTCCGGGAACCAGTCTCCTCTAAGCCGATCAATGAGCGGATACATGAGCCCATAAGAGAGCACAGCTCCAAGGAGCAAAGACACGTTCACAAGGTGTGAGCAAATCATTCCTGCTCCAACAAAAGTCATACTAAAGTCGAAATAGAATGTTTGCTTCCAAGCTTGCAATCCAAAGGTGGGGAACTGTTCGAATCCACATTCCTCCATCCCTGAGAAGAACCACTTAAATAAACCCCATAAGAAACTTATAGAAAAATACTTGGCGAACCCCCGTACTTGCTTCTTGGCCATTTTGTCTCCCTGAGTATGGAAACCATTGATGAGAACAGCTGTTGCCAAGCCACTGGGATAAGTTAATTTGAGGTCAACTATCATGATCTTTCTGAGTGGAATCAAGACAAAGAGGCCAACAAAGCAAACCACAAAAAGGTAGGAAGTCATCCAACCTAATCCAGGTTCTTTAATAGCCTTTGGGTTGTTACCCTCGGTTCCTACTCCAGACAACTCATAAGTCGTTCTGTTTAATCCCAACAGATAAGAAGCAAATCCTCCTCCAACAGCAATGCTATAGCATGCAACTGCACAAGTCTGTATGATGGTGTTCTCCTGGCGGCTGAAGGGTTTGGCTACAAAACCAGCCTTGTGCAACACTTTGGTCCAGGTTCGGATAAACACAAAGGCAAGGAGTGCAGCGGAAACATTCAGGTTAGGAACCATGCCAGTTGTTAGGTTGAGCTTCATGACTATTATGCTATATATGATTCCAATCAACATGCTCACAAACAATCCTCTCAGTGTTATCTGTTCTGTCCATGGCTGAGACTGGACTTCAGCAGCAGGTAGCTGATCCTCCAAGTCCTGCTCTCTTTCAACTATTTCTAGTTTCTTCTCTTCTTCCATCTCAAGGCCACTTGATGGGGAGATCATCTCTCTAACTGTAGTAGCAGCTGTATGGCCTATGTAACACAAACTTTGAACATCAGAGGAAACAC

>C101249_65

GAATGGTTGTTCACACTAACGCTATTCTGATGTAATATGGTAAAAATAATGCCATAAAGAAGCTGCAAATTGGTTATTTATTCCTTTAAGGCATTACTTGGTTGAATAGTTCAATGGTTGCATCAAAATATGACTTTTAATGGGTACTCTTGGTCCCCCACAGACAACAAATAAGATCCTTCTTCAATCACCATTGAACCAGCCTCGCTTGCTATGCTAAGATGCTCACAAGGGCTTAGCTCAAATCCCACTTGAGCAGTTTCCCCAGCATTTAACTTCACACTTTGAAACCCCACCAATTGTTTCACCGGATTTCCGTTTCTCACTTGTTCCTTCTTCAAAAACAACAATACAGGATGCTTCCCTGCCATGTTACCATGATTTGTGACTCCCAAAGTGATTGATAATGACATGGTTTGGCATGTTTGTTCTCCCAAGTCTGAAACCAGTTTGTAACGGATGGTTTCAGAATTTTCAGTCATCAAATGGGTGGAGGATTGGTTGATGTAGAGGGTGTTGTGTGAGAGAGAGAGGAGTTTGTAGGAATATTTTGTGTAACTTAGGCCGTAGCCAAATTCATAGACCTTTGGACCAGTGTAAAATCTGTAGGTTCTCCCAGGGTAGCCTGAGGCTGAATCAGCTCTCATCCTCATATCTGTCATCGGTACTTTGATGAAATCCTTTGGGTACCAAGTAATTGGTAGTTTACCACCTGGGTTATGATCACCGAAGATAACCTGGGCCAGGGCCACTCCTCCAAGTTCACCTGGGTACCCAGCCCACAAGATTCCTCCAACTTTGTTATCAAACTTGGCTGAAGTGATATCAACAGGGCCTCCACACAAGAGCACCAAGACAACTGGCCTCTTAGAAGCCTTGGCCACACTCTTGATGAGCTCTTCTTGCTTCCCTGGCAACCCTAGGTACTCTCTATCATGTGACTCCCTCTCTTGACTTTGATCCAACCCCATAACAAGCACCACATAATCCACCTTTTTTGCAACCTCCACTGCCTCTTCTATCCGTGCAGAGGTACATTCTGGGCCATCATCACACCCAGGGTGGTAAGTGGTGTCCTTAGCATAGCCCTCAAAACCTTGCAAAAGGGTCACCAATTTGCAAGGACGACCATAGTAGTTTCCTAGGGAAACTAGTGACGAGGCATTAGCATTTGGCCCAATCAAAGCAACACTTGGCCTTGTTTTTGGAAGTGGGAGAAGTGAGCCAGTGTTCTTTAGAAGGACTATGCCATCTCTTGCTGCTTCAAGAGCCAGTTGGAGGTGTTCTTTGGAACACACTTCGTTGGGACCAATGGTGCCATAGGGAAGCTTCGTGGGGTTCCCATCAAATAGGCCTAGCCTCATTCTTATGGAGAATAGGTTCTGAAGTGCACGGTCGATTTGAGATATGGGCAATTTTTTCTGCGACACTGCTGATTTTCCATGCTGAGTTATGTAATCACCACACTCCACATCCATCCCTGCCCTAAAGACATCGGCTATGGCATCCTCTGCTGTTTTGGCATAACCTTGTTTATCGTGTATGATAGAGACTGCTCCACAATCTGACGTAATATATCCATCAAATTTCCATTGTTGTCTTGCAGTTTTGGTTAAGAGATTATAATTTGCACAGTTTGGAACCCCATTGACACGGTTGTAGGCACACATTATGCCACTGGCTCGACCTTGCTCAATGCAACTCTGAAATGGAGGTTGGTATGTGTCTGCCAAATCTTGCAATGACACACGAGCATCGAAGACAAAACGATCGAGGCCTTTCCATTGGTCGAGATCATAGGCTGTGAAGTGCTTGCAACATGCAGAAGCTTGAAGCCTCTCCCCAAGTTTCCCTCCTTCAAAGGAATCCCCTTGAAGCCCTCTCACGTAAGCCACACCATACTTTGCATTCATCAATGGATCTTCCCCTGCCGTCTCTTGCCCCCTCCCCCACCGTGGATCCCTGAAAACGTTAATGTTCGGAGCCCAAAACGTCATCCCTAAAGCTTGCCCCGCATTGTACACTGCCCTCGCTTCCCTCCCAATCGCCTTGCTGATTTGGTACCAGAGGTTTTGATCGAAGGAAGCGGCAGTGAGAACGACTTGGGGGAAGCTGGTGGCGGATTTGATGGTGCCATTGAATCTGATGCCGAGGCCTGCGTCGGCAACGCCGTGCAAGGCTTCGCTCCACCACTGATATTTGGGGATGCCGAGTCGGGGAATTGCGGGAGCTGAGTTGACGAGTTGGGTGAGTTTCTCGTCGATGGTGAGTCGCGAGACCAGGTCTTTTGCTCTCTGGGGAATGGGGAGCTTGGTGTTGCAGAATGCGTAGGAGTGTGAGTTGGAGGAGGAGTCACAGGAGTAAGGAGGTTGAGTTGAGTCGGCGTGGAGGAGAGTGAGGAGAAGGAAAAAGGTGATAACGGAAAGAGATGGAGGGAGAGGCATGGTGCTCTGAGAAAAGTGTAGATTTGTG

>C101255_65

AGCTGGTAGAAAATCTTTGTTTAATTAGCGATATATCTTACAGTTGGTGCCAGAATAATAATAAGAATTTGCACCAGAGAATACAGAATCGTCCTAAGATTTTACACTGAATAATTGTAAGGGGGAAAAAAATTGAAAAAGACCTGCCCCAGAAGATAAGAAACTGAAATCATGGAAAGGGAGAAGAGGGGTAAAAAATTGAGAAAGGAAAAGACAAAAAAGGGTTCTTTTTGAATAGATCTAGTGATAATGGCTTAATAATAATTCATGGGGTTGTTGAAATTAAATCTTTTGTTGCTGTTGTTGTTGTGTACAGCAGTTTTCCTAATCTTCTGGGCTTCAATCGAGGCGTAATGAAAGGTGAATCTTTCTACTGATCAGAGCTCAACTTTCATCTGGGGTACATTATTTGAACCGTTGAAACTTGGGGATTGTATTGGTGTTTTCCAGTTTACAGTTTGGCCAAACCCCATCTGCACAATGGTGTGGAGATCATCCTCACAGAATGTTAATGGAAACTGAGAGCCACCTTCATTAAACCCATTTAGGGGAGGTAATTGCATGGCAAGATTTTGGCACAAAGAAGTATCTAATGGGTCCACTGAGCAGTGGGTCACCGTTCTGTTAGGTATGTTATTATGGATAACTGGGTTTTGCTGAGGCTGGTGCCCATAAAAGGTTTGTGATGAGGAATCTAACGAGAATATTGGATTTGGGTGTGTAGCCAAAGAATTATTAGATTGAAATACATCTTTTGAAACAAGACTCTCAATACTAAGATCCATCCTTGTGTTGACAGAAGCCAACTTCATGGACAGGAACTCAACTTGACGCTGCAATGACTGAACATAATTTATAATTTCGTCTAGCATAAGTGCTTTGCCAGTGACCTTATTGCAACCTGGTACAAGATCTTGGAGCAGCTTCATCCTTTCACTGATTTTCTCCCTCCGTACACGTTCTGCAAGACTATGGCTGTCAGTGGCTTGGCCTCTTCTTGCTCTCACATGAATGTAATCTTTTGGTGGCTCAGGAGGTTTTGAGTTGCTTTTGTTCTGTTTCTCTTCCACTGCTTTGGACTCTTCCTCAATCTTCACGGTCCCATTGTCATTTCCCTCACCCTCATTTGGCTTGGTACGCTTTGAATTTGAATCCGCGCTAAGTTCAGCTCCCATGGTGGTGGGATTAGTAGAGTTCGAAGTTTCTTTGGTTTTTCCTCTGGAAGATGCTTTTCTTTTCCTGGAATTCGTCATATCTTGGCAAATTTTCATCGCAATTTCTCCATTTGGTGTTTGCTCCGATATTGTAGATTCATCTTGAGAATTGGCTACTTCCATTTTCTCCGGCTCCTGAATTGCTGAATTCTTGTTCTCTTGGGCACCCATTTGAGATCCAAGTGTTTTGAGCAATGGACTACTTGAGATTCTAGATAACTTTCCACCATTTTCCACTACTGGTGTAGATCTCTGAGCCAATTCACCGTTGTTCACCCCCAATTGGACACTCCTATCATTAAAACTCCTGCTGCCAAAGCAAGAAAACTTTGCAGCCCTCTCAGCAAAGCCAGGGTCAGCTGAGAATTCAGCCACACTGGAATTCAAAGTCATTGACTTTCCTCCCAAATTTACCAACCTCTCATTCACCAAACTATGGACAGTGTTCACCTTTGGAGGGGAACTCAAAGGGGTGGTATAACATGAAGTGTTGGGGCTGTTACTGCCATTATTCATGTAAGAAGATGACACAGCCAAAGGATGAGGAGAGTGTTGTGGGATCTCATCAGAGCTCCCAATAGCTCCCAATTTCCCCACCAATTCCCTGATGATGAAATTCTCATTGGAGATGTTGTTGTTGGGGTTGGACGATGCTGCAGGAGAAGAGACCATTGAACTCAGTGCTGAGGAATCAAAGTGGTGAAGTCCATGATCTGTTGACTTGTCCCAATTGGAGTTGTAAAAGCAATCCTGGGTCTGGTCAGAGGCACAATTCAGAAGTGTTGGTTGAATTGCCATGGGGGGTGAGAGTGATTGCCAAGAGGGCACTGAAGAAGGTGGTGAAGGTTCAAAGTGAAGGGTGTGCTGTGTTTGTGAGACCCCAGCATTGAGAAAGAACTGGTTTTCCATTTGCAGATCAAGGAGAGGCGCAGAGGAAAGAAGAGAAAGGAAGTGAATTTGGAGGAGTTGGTTAAATTAAGCTTTATTATGTCCCTGGAAGCTGAAGCAAAATGATTGTGATTTTGGAAATTACATTAGGAAACAAGGAGAGGATTTAAAGGAGGAGGAGTAGTAGTATAGCAATGTGGCAAAGTGGGACAACTGGGTCTAATTGGAGTGATTAATGAAGTGGGTTGGGTTGGAGTTGAGAAGAGAGAGAGAGAGAGAGAGAGAGAGAGAGAGTTGGGTGTGAAAAGGGAAATGTGTTTGGTAAATAAAAAGATGAGAAAGATTGAGAGAGAAGGGAAAGGAAGGGGGGG

>C101257_65

CCCTGATGTAGCTTTTCCAAAACTCAACCAAACAAACTTCTTCGCCTCACGCCACGCAGAGAGAAAAAGAAAGTGTTGTCTCCGTTTTACACAAACAAAACAAAACCACAACCTAAACTCAAAACACTTTCTACTCCCTTCACAATCACAAACCATACCTTCAATCTATGGTTGAAGAAGACATGGCCGAGGATCGCGCTTCTGAACCACCCTCCTTTCCTCCACGCGCCTCCATTCCCTCCGGCGACAACTCCGTTTGGGCTGATGTGTCTCCTCTCCTCCACGCCGCGTGCCAAGATCTTCAAGAGGGAGAGCTTATTCATGGAGACAACTTTAATCTTTTTGCCGCCATGTCCGCGTTGGAGATAATGGACCCGAAGATGGATTCCGGTATTGCTTGTAGATACTACTCTCTTGAAGAAGCTATTGAGAATGGCGTAGCTCCTGTTCCTATTAGTGCTGATAAAACTAGTGATGTTCGATGCATGATTGACATCATGGATCATCTACTAGCTTGTGAGGCAACATGGCATAAGGGTCACTCATTGGGGCAAACTGTCTATTCTTGCCTCTATCTTCTACTACCTGAGAGGACATCGCCGCATGCCCTGTTGCACTCTTACTGTAAAGTCATACGTGCAACCTGCAAAACAATCCTTTCAGTTGTCTCTGAAGCTCGAACACATGAAGAAGAGGATCTATTTACCATGGCATATGGCCTTCCTTTGAATGGAGATGGAGATGAAAAGTGTTTATCACTGCTAAATGCTGTTGAAGAAACAATATCTCGCCAATTACGATCTTGTAAGGCTTCATCTTCAAAAAGAAGAGTGTCAAAAGATATTGAGCCACTCCAAAATAATCCTGATCTGGAAGAAGGCTACTGCAAAGCGCTGTTGTGTCGATTACGTTTTCGTAAGCATTTTAATCATCTTTTGATGTCTATGAAACGACCCCAAGGAAGAGGTTTGGAGCTGGCTAGGAAACACATAGCTTCGTGCATTTCAGAGATTGATTACATTCGAAAATCGTCAGAATTCCTTAGAGTTAATGTTCAAGGAATGTCTGAACAAAACTTAGACAGTACAACAGCATCAGGTTGTCAACCAATTGGCTTTGATGCTAGTTTAAACTGTAGATTATTTGCCCCAACACCGCCAAGGTCAATCAAAATTCTTTGTTGGGAAAAGGCCCTTGAATATTTCATGAAACTCCTTCAAGACCTAGATATAATATGTTCCTACGCATTGGGCCCATCGTTGGAAGCTGCTTTACTCTTTGTGGTTAAATTCCAGAAATCAAAACCTGATTTGGTTGCTAGAGCCCATCTTCAGCTTCTGCTGGTCCAGGATGGGAAACTCTTTGGTCGGGATCCTATTTTTTCTATGATCACTAGGGCTGCTGGATTACCTGAAGTTGCAGAGAAGCATGACATTCAGAAAAATGAATTTATGGTGCAACTAGGACAGTTGGTGATAAATTTACTCAAAATTCTTTGTACAAATGCTGCATGGCAAAGGCGTAAGCTAGGGAAAATGTTACAAGATTGGCGTGTCACCTATGTACAGCTGGAGATAGCTTTTAAAGCAGAGATAGAAAAAGCATCAAAATCTTCAAACAATAAGAAAATATGTTTCAAAATATACCAACACATTCTCGCATGGGTAGAAGAGCAAACTTACTGGATAGCTTTTCGATTTCTCAATTTGGGATTTGAATTGGAACTCTACTCTGTTCATGATTATTGCATGGTATACTGGTATATATATGTTGTTTTGATCAAGCTCGCAGAGAAGAAACATCTCCGGCTGTCATTGAGCAGTGACTCTGCTAAGAAAAAGACAAAGAAGAAAAAGGATTCTAAGGATGGGGAAATGGATTATCAGATCTCTGCAGCTGTTTTGTTTCTTCAAAGCCAAATATATCTAGCTGAAGGGCTAAGCATGATGTTCGCTGCTTTGATGAATGAACACAGAATAGTTCAGCTGCAAAGCCCTTTCAATACAGAACATGAGATATTTATTCAGCAATTCGAGCTTTTACAGAAAGCTTGCGTTCCTGATCACATCTCATATGCGACATTCAAGGAGTCAACTATCCATGCTAACTTCTCTACCCTAGCTATCTGTGACTACTTCAAGGAAGCTCAGAGGATAGCAAAAGAGGTGAAAAGTAGTTTTGCCAAAGACCCTGACACAATGGCCGAGCTGCGCAGAATAGAACAAGTAGCTGAACGCAACAGCATTGCCTTGAATGTTTGCAGGGCAGGAGCCCTTGACCCAAAGTTGAAGATTTCTTTTGCATTCTGTCATCATCCTTTCTATGCTACTGCTATTGTGAAGAGGTCCTGATAGTTGATTGATTCAAACAATTTTGAAACATGCTATAGGTTGTTAGGAGCCAATGAGTTGTGGTGTTAGCAGTTCTAACACTAGTATATTTAAACAATTTTGTGGTCACATGTTAGATTGAAA

>C101261_65

CGCGTTTTCAAATATTTTCGTAATGTATTGTATGGGTCCAAACTGGTTCCTTTGGTCTAAGAACATGGTTTAGAGATTCACCCCTTTGCCATATTTGACAATGTTTTAGCAATTTCCCATCTTTCGATACTTTTGAAGACTGATTATTGGGGCTTTTTAACTTCCTTCAATTTCGTTTCTTTGAAGAAAAACCCAATCTGGGTCGTGCTTTTGCCCCAGTTGACGTTTACTCGCAGCAAGAATGAGCAAAAACTGGAAATCCATCTGCTTCAGGAATCCTCTGTTCCTTCTGGCGTTGTGTTTGTTGTTTCAGAACTTTAGTTTGTGTTGCTCTTTGAACGAAGAAGGTAAAGCTCTTCTGAAGTTCAAGCACGGAATAGTAAGAGACCCTTTTGGTGCTTTGTCAAATTGGGTTCATGATGAAGTAGCAGTTAACCATTGTAATTGGTTTGGAGTTGAGTGCTTTGATGGAAGAGTGGTGATCTTGAATTTGAAAGACCTTTGTCTTGAAGGAACTCTGGTACCTGATCTCGCAAACCTTGTTCACATAAAGTCCATAATTTTGCGGAATAATTCTTTTTATGGAATCATCCCTGAAGGAATTGCTCACTTGAATGAATTGGAGGTTTTGGATTTGGGCTTTAACAACTTCAGTGGACCTCTACCTACAGATCTTGGGAATAATATTCCACTAACAATCCTTTTGCTGGACAGCAATGATCATCTCTGTGGTTTCTCTCCTGAAATTAATGAATTGAAGAAGCTTTCTGAGTATCAAGTAGATGAAAGCCAACTAAGTATGGCAAAACAAGGGCCAGCTTGTACAAGAAGATTCATCAAACGGCATATTGGCAAAAATAAAAAAGGTCCTCGGAGGCTACTGCAATTTGGTCCCCGTGATGGTGGAAGTCCTTTTAACCGTGCTGCTACTTTTCCTGATAGTCCTGCTCTTTTTCCTGATAGTCCTGCTCCATCTCCTTCAGCTCCCTCACCTGCCCCAGCAACACCACCAACGGTTGAGAACCCAGCTTCCATTGACCGAAATGATTCTGCTTCTGCTTCTCCTTCTCCCCTGCCTGAAACACGCTCTGCACCACCATCTGAAAGTAGCTCATCAGACACCAATCTCGTTGGTATTTTGGCTGGAGTTATTGGAGGTTCTGTATTTCTTGTCATTTCAATCGTTGGCATATATCTCTGTAAAACAAACAAGGTAGCTACTGTAAAACCTTGGGCCACAGGATTAAGTGGACAGCTTCAGAAGGCATTCGTAACAGGTGTGCCAAAGCTGAAGAGATCAGAGCTTGAAGCAGCATGTGAAGATTTTAGTAATGTAATTGGCACTTCATCCATTGGTACAGTGTACAAAGGGACTTTGTCTAGTGGTGTTGAAATAGCTGTGGCCTCTGTTGCAGTGACATCATCCAAAGATTGGTCTAAGACTTTAGAAGCTCAATTTAGGAAGAAGATAGATACGTTATCAAAAGTAAACCACAAGAATTTTGTAAATCTTCTTGGACATTGTGAAGAAGACGAGCCTTTCACCAGAATGGTGGTTTTTGAGTACGCCCCGAATGGAACACTCTTTGAGCATTTACATATAAAAGAATCTGAGCACTTAGATTGGGAAGCAAGGTTGAGAGTTGCCATGGGCATGGCTTACTGCCTACAACACCTGCACCAATTGGAGCCTCCTTTGGTACTTGGCAATCTGAATTCTTCAGATGTCCAACTCACTGATGATTGTGCTTCCAAAGTCTCTGATTTGAGTTTCTTAAATGAAATAGCTTCAGCTGCGATAAGACCTTCTTCTAGAGAACAACACCCTGACATGACACCAGCAAGTAACATTTACAGTTTTGGTGTAATATTATTTGAAATGGTAACAGGCAGGCTCCCTTATTCAGTGGACAATGATGGGTCACTTGATGACTGGGCTTCACAATATTTATATGGGAATCAGCCCCTCAAAGAAATGGTGGATCCAACTCTAGCGTCCTTCCAAGAAGAACAACTGGAACAAGTTGATGCCCTGATCAAATCTTGTGTCCATCCTGTTCAAAAGCAAAGACCAACTATGAAAGAAGTTTGTGTGAGATTAAGAGAGATAACAAAAATAACACCTGAAGCAGCTGCTCCAAAACTTTCTCCACTGTGGTGGGCAGAGCTTGAGATTGCTTCTGCAAATGGAAACTGAAATCGGTAAATCAGGTTGTAAGTATGGAGTTCTTACCATCCTCATCCTTTTTCAATCGGTGAAGGCACAAAAATGTATTCACCTGTAGATTAATACATCCTGTGTTTCATCATAGAAGAGTGGCAAATCATATGTACATAAGGGAGAGAAAAAAATGTGATTTTTTCTTTCTTTCTTTCTTTGGGTGCTTTGTTTGGTAATGGTTTATGGAATTAAACTAACAAAAAAAGCTTCACATATCAGTTAATTTCATTTTCTCAGTCTGTTTATTTGGGGA

>C101263_65

TTTTTGAACAATCACCTTATAATTCTTATAACACACTTTGGACCTTGAGCTAATGAACACATGGAATGGGAAGGGCTATACATCTATACATCGTAAGAGTTAAGTAGTTTCAAAAAGCATATTCTCTGAACTCTCATGCTAAATGAAATAATACATGAGACTAGACAAACTAAGTGCACTTCACTTGGCAGTGCAGGTGCATTTCCAAATCAGACCTGCTATTATATATGCCTTCGTGGATAGTCTTAATTTCCAGCTTGAAGTTTCATGCGCCGCAAGGAGAGATATGCCAACAAACGGTAACCAAAAACCATTGCTAGCAGAGCAGCAACCTCTGTAAATCCACTGTCAAGTGTAATCCCATTTATGGTAGGTGTAATGTGTTCATATTGAACCTTGAGCAGAAGTTTATACGTATGGTAGTTGAAAGAGAGGTAGTGAATCCAAGATATGAATATGGGGACTTTCTGCACAAAAAATCCTCCAGCCAGCATGAAAGTCATCACAGTTACTGAAGCCAAAGTTGTTGCCCTTTTCAAGTCCATTAGTGTGGCCCCAATTGCAAGTCCAAGTCCCTGAGCTGCTATGATGCAGAGAAAAACTGTAAGAATACTAAGGAAAAAATGCCCTGCACTGAGTCTCAAATTGGCCATGAAATAAACAACAACTAGGAAAAACACTGGTAACACTAGGTCTAGTGGAAGGTCACTTGTAGTTCTAGCCACGAAATATGCACTTAGCCTGTACATATCCGTTGTTCGTTCCTTAGTCAGCATGGCCCTCTCTTGAGGAAATGTGAATATTGCAGTGAAGACAGGAAAAAATCCCCAAAACACGGCAATAAAGAAAAGAAGTCCTGCCTGATCTTGCAAACCGTTTGGGTTTTTAGCATCTGATTGCCACCAAAGTAATCCTAAGATGACTGCAGTGGAAAGAACTTGGGTGATTCTCAACCAGCTAAAATAGTCGTGCCTCCTTTCTTTGAATCCTCTTGAAAATAATATGGAAAATTGCTCGAGCCAACTTGCCCCCCACTCTCTTTTGCAAGAATAAACCTTAGACTTCAGTTCTTCATCAAGGGGGACAGGAATCATTAACTTTGTCTTCTCTATTTCTGCCACTCGAGAATCATATGCCTCCACCAGATATTCTTGTACAACTGAAGCAGAAGGTTTGCCATTGCATGTTTCAAATTCAGCATTTCCCACCTGCACTTTATCCTTTAACTCGGATGGTACAGAAATATCATTTACGTTTCCATTTGCAAGGTCCAATAAAAACTCTGCTGGGTTCATGGCAATAAGAGGTGCACACCCTATAAACTGGAAATAGTCCATTGCTTCAGATGCTTTTCCAAAGTAAAGCAAACTCCCTTTTCCTAGAAGGATCAACTTATCAAATTTATGGAAGAGTCTGCTTGATGGTTGATGGATTGTTATCACTACGGTTTTACCTGCCTCTGCTATGTCTTGTAGCAACTGAACTATCCTTAAAGCTGTTGTAGAATCCAAACCAGAAGTTGGTTCATCAAGAAACAGAAGAGAAGGGTTGATTATGATCTCATTGCCAATACAAACCCTCTTCCTTTCTCCACCAGATACTCCTCGAACATAGGAACCTCCTATCATAGTGTCTTGGCACCTCTCTAATCCCAGCTCTTCAATGACATCTAAAGCTCGCTTTTTCTTTTGCTCTTTCGTTAACGTAGTTGCCAATCTTAAGCGGGCAGCATACGTCAATGTTTCTCTCACAGTAAGGTGAGGAAACAGAACATCATCTTGCGTCACGAATCCAATCCTGCTCTTTAGAAACTTGGAATATGGTTGGTCATTGTAAGTGATAGAGCCACCAATAGTGGACTGAATTAACCTTCCTCCTAGCAGATTTAACAAAGATGTTTTTCCACTTCCTGAAGGACCCATCAATGCCAAAACTTCCCCAGGATTAACAGAACCAGTGATCCCCTTCAAGATATGCTTCTCCTTAGTTGTAGTTATGCCTTTAATGACTACCTTGTAGGTCACATCTGTGAACTTCAGATATATTGGCAGAGTAGGTTCTGTCTGAAACTTAGACTTATGAGTTCCGGCTTCAATATCCTCCGGGATATCATCGTCGCTGAAAGGTTTTGAATCGGCGATTTCATCGGGAGGGAGAGTGAAGCCAGTAAAGGAGAATGAGAGGCCCAAGCTGGCGCTGGAAGCTCGGCTCAGAGCCACGCCGTTGCCAACCTCATCCACCTCCACCTTCAACTGAGCGCTCCTCGCCTTCCTTATGTGTGCGTTTCTTCCTCCGCCGCCACTCGGTGACGCCGCCGACATTCTCCGGCTTGATTTTCTCGACAGAACTCCTCCACCCTCCGCCGACGGCGGCGATTTCTCGCTTGTCGTCTCCGCCACCTGATCCGATTTCGTTCTCGCCAAGCTGGATGTATTTGCATTTTCCATGCTCACCACTCACCACCAGTTTTTTTTT

>C101265_65

CTTATTTTCACTTTTGACTCTTCCAATAACCATAAACAAAATCACAACCAGAGCTAGCATTCTTTTCTCTCCTTCTAAATTCCAATAACAAACCCCCATAATTCCCGTTCCTTTCCTCTTTCCCTCATTCTCCTCGCAATCCACCGCTTCCTTCTACCTTCCTAGGGTTTCCGCCAATGCCGCCGGACTCCTCCGACCAAGACCTCGACGATCCTGACTTCGAATTCGTCGAGGTTGATCCTTCCGGTCGCTATGGTCGGTACAAGGAAGTTTTAGGAAAAGGGGCTTTCAAGAAAGTATATCGAGCTTTTGATGAGTTGGAAGGGATTGAAGTGGCTTGGAATCAGATTAAGCTGGCGGATCTGTTGCGTAACTCTGAAGATTTCGAGCGACTTTATTCGGAGGTCCATTTGCTCAAGACTTTGAAGCATAAGAATATAATTAAGTTTTACAATTCATGGATTGACTCCAAGAGCGAGAACATCAACTTCATTACTGAAATTTTCACCTCGGGAACGTTGCGCCAATACCGGAAGAAACATAAGCATGTTGATTTGAGAGCTTTGAAGAAATGGTCTAAGCAGATTCTGGAAGGCCTTTTTTATCTTCACAGTAATGATCCTCCGGTCATTCATCGTGACCTGAAGTGTGATAACATTTTTGTCAATGGGAATCAAGGGGAGGTGAAAATTGGTGATTTGGGATTGGCAGCTATCCTCCAGCAGGCCAATTCAGCTCACAGTGTCATAGGAACTCCTGAGTTCATGGCCCCGGAACTTTACGAAGAGGAATACAACGAGCTTGTTGACATCTATGCTTTTGGTATGTGCTTGCTAGAGCTGGTAACAGTTGAGTACCCGTATGTCGAATGTGCCAATGCTGCTCAAATTTACAAGAAAGTGACATCTGGAATAAAGCCAGCATCGTTGGCAAAAGTGAAAGATCCTGAAGTTAAAGCATTTATCGAAAAGTGTATTGCTCATGTATCTGAACGGTTGTCTGCAAAGGACCTCTTGATGGATCCCTTCCTTCAGTCAGATTATGATAATGAAAGTGTAGGCCGATCTTCAAGATCTCAAATCCTTCCTTTAGGAAATACTTCTCATAATCAAGCTATTGCTGTGGATACTTCTATTGAGGCAAGTAGGGAATTCACGGTGGAAGGTCAGAGGAGAGATAGTAATACAATATTTTTAAAATTGCGAATTGCAGATTCCTCAGGTCATATTCGGAATATCCACTTTCCTTTTGATATTGAAGCAGACACTTCAACCTCTGTTGCTAGTGAAATGGTTGAGGAGTTGGAACTTACTGATCAAGATGTTACAACTATTGCTAGGATGATTGACTCTGAAATTCGGTTTCACATTCCTAATTGGAATGTGGGTGAAATTCCTGTTGAAAGTAGGCCAGAGGACTCTCCTTTGAAAGTAGATTCCACTCCTACTCCTAGCAGTCTTGCATTAGAAATATTACCTTCAGGTCGAAAGTACTGGTCAGACTCGCCAAGGGGAGTTGGTGGAAACTCTCCATTTCGTACTTTTGCTTCAAAATTTTCTTTTGCAGTAGATACAATCGCTGAGGAAGGCAGCCCGGTTGCAAATGGTGATATTTCTGAAGATGATGATCGTATTGGTGATAGTAATGCTGAGAAGAAATGTGATGAGACTGTTGATTCCCCTTTCAGTGACAGAAGTATCAAATCAGGAGCAACCAGTGGAAAATCTCCTCTGAAGGAAATATCTGAAAGCGTGAAGGATTTGGAAACAGAATACATCAATCACATGGCAACAAAATTGGAGAATTTGTTGGTTAAACAAAGAGAGGAGTTAGATGAACTGAAGAGGAAGCACCAATTAGCTGTCTCGGATCTTTTGAAGGAACTTACTCCAGAAATTAGCCAGAAGGTTCTAAATATATGCAACCTACATGCCTGACGCTGAAATGTAGGACGAGGCTGGCCCCTTCATTCATCTAAATGCCCTTTTGTGAAGTTTATGGTAGTCCCGGGTTCCTCATTTAGAAAGCCAAAGCTCATTTTAGGAGTGAAATTAAGAAATTGAGATTTTGCATGTTACTTGGAACTTGCATACTGTTAATATTTTGGTTTTTCATAATTCATGCTTAAGGGAGGGTAAATTTTTAATGGTTTGGGTGTTCTTGGTATATTGATGTGAAATGCAGCTGGAGCTGGGTGATAACTAGGGAGTGATCTTTGCAGGAGAGGTTTCGTTGAGGGATGGCTTAACGTCCTCTCCAGCTTGGAGGACTCAATGCTATTTACCCAAAAACCATGGGGATAAAGTAAGAAATTAGCTTTGATAGGTCAGGAGACATGATTATATTCCTATTTCTCATTTGTAAATAGTCTGATACAAATAATTAAACTATAGAAAATGTATACTTGTACATATATATCTGGCTCTCGTTACAATTTACATGCAGGTCTGGAGTTCAAGTACTGGAAACTTGGAA

>C101267_65

CTAGAATTAACCTGGATTAAATCAGATCTCCTGCTTGTGAACAGCATTGGTTCTTAGATTCAATGGAACTTCGACGATTATTTTAATTTTTTGGCTAATCATAAATATAACCAAACGTGTATTTCGTTATATGAAACATTATATTAGTCGCTTACACCACAAACTAATGAGATATTATTATCTTCTTATGTAATCTAATTATTTTCGAAGCCAATCCAATAAAATCATCTTCTGCAAAACTAGCTTCCCAAGTAGGGAATGATGATCTTTTTGTATGTAGTAATTGGCAAACATCCAACCCAGTAATTTTGTATGTAGCAATTGGCAAACATCATCCCAGTAATTTTGTATTGTTTGGTACAAATACCTCTTGGCAATTCCTAGGCTGACCTGCTCCACTTCTTGCCTTACTGTTATCTGTAACCAGTGAAGCGAGTGGCACAACACAATTCCAGGATTCCACTAAACAACAAAGAAACGTGCTACTCTACACTAGTTCCTCAGCAAGAGTGACTCCATACACTGCATCCAAAGTCCAATGATTTACTATTTGAGCCCCCGCTGGCATGGCCTTCACAGTTGGCCATCAAGTTAGAAAAGGATAAATATTTTCATCATGATCACAACAGTGCCTCCAGATTTGTAAACCAAGAACCAAGATCTGCTCTACCATCAAGCCTTATCTACCAATGTGCGCGTGATTATATATCGTCCTTTTTCTAGATCATATATTGTCTTAAACTGAGGTTTCCTCCACCTAGATATAGACCATGCCATTAACAAGACTAAAAAAAGTCCAATAATTGAGAGGAGAGTAGGCGATTCATCACTGAAAATGGAGGCAATCCAGTTATGGTTCTGTATATCTGCATCAGCTGTTGTTTGCAGAATAAAAGCTCCCAAAGCCCAATCAAGCGGAACACTTCCCACCTGATTCGCAACCTTGACCCTCTCATCATCCAAAGCAATTCCAAGACTGTCATGAAGCAGAGCAACAATATATGCTGAAGAGAAGCAATACCGAAGTAAATCTTCCTCATCATGGGAAACATATTTTTCCTTCAGCCTTAACCAATCCTCTCCACAGAAATCTTGCCCAGCGGTCATCAACTTGGAAAGATAGGCCCTTGGCCGCAATCCAAAAAACTTTGATGTGTGATAGAAATTTTCTGTGGCCAAAAAATTCCCCTGAAGATTTGGTATGAATGTTGATCCTATGTCACAATGCTGATAGGAACATGATTCTTTCCCCTTTTGTAGCAGAGTAAGTGCTGCAGATCTGCACTCTGAAAAGTTTCCCCTTGTTTGGACAGTGGAGGACAGATAATGCTTTTTTTCACTCTCTGAGCTAGGAGGGAACTTCCATGACTCAACATTATAAGAGTATCCTTTAGGAGTACAAGGATCTATCCGCAACCTTTCTTGAAGAGATTGAGAAGCCAAGTTAGATTCTCCTAATACAAGTGCTTCTTTCCACGACTCATGAGCAGCATTCAGGCCAAAATGAAGAAAGCTGTGGCTGTAGAGGTTGTAAGTAATATTCCCAAATGTAACAGTTTGTGAGAAAGAAGGAAGCACCACTTCTCTTGATACAAAGGTCACCTGTGCAGAAGCACCACCAAGTTCAATAATCCCAGTTGTATCCAAAGGATCACCTCCAAGAGTGCCAAGTGCATAATTAGCAACAACCCAAGCATATACCCCTTCATCAGAACCTGTGATAACAGAAGCCCAATGGTCCATAAACTCGAACCCAGATTTACGCAGCACCTTCCTACACGATTCCAAAATCCTTTCCTGCACCTCCGCTTCCAGCATCCTCAACCCCGCCGTCGCCATCAACCGAATCTCCGTTTCCACCCAGCTATCCCTCGGGATCCGCCCCTTCGCGAATTCCACCAGCTCCGAAACCGACCTACCCGCGCCATCCGGATCCTCCGCGAACGCCGACAATCCCGGGTTCACGCGCATCGAGGCCAAATCCTCCTTCCCGAACTGCAACGCCCTGCCAGACCGGTACTTGAACACGTGCACGCGCGTGCCAGTGCTCCCGCCGTCGACGATGATCCGGTACCGGAAACTACTCTGCGAGGGGGAGAATGCGTAGTAGGAGAGGAGAAAGAGGAGCGAAAACGAGAGAATGACGAGGAGGACGGACGAGAGGGACCGCGATGGAGCGGGCTTAGGGTTGCGAGCGAAGAGGTTGGTGGAGCGTGTTTTGATCGGATCCATTTCGTCCTTGCGATTCGAACCGGCGCGAGTGCGGACAGTCGATCGGCGCATCAAAGGTCTGAGGGACGGAGAGAATCGGCGGTCATGTTCTCGCCGAAGAGGTTGCCGGCGGCGAGGTTCTCTCTCTAGAGAGGTTAGAGAGAGAGAAGAATAAACGTGATTGTGATTTGTGCCAAGTCCAGAAGTTCAGGCGTTGTCAAATGGTTTCCTTACGAATCCTGATATTCGTTTTGGTTAGGTCCGAG

>C101269_65

TTCTGCTAAACGTGAGAGGTGCAGAAGAATTAGTATGTATCTGTATTCTTCAAACAAAACCCTATTTTAGGGAGAGCGCGCATTTGAAGAGAACAAAGAAGAAGCATAAGCATGCTGTGCTTCTACTTTCATCTTTCGGACTGGGAATAGCGAAATCACGGGGTGCAATCGCATGGTATAGGAGGCCTATGTTAGATGGAAGAGACCTCTCTCTGCTGTGAACAATTGCCGGATGGGGAATGCATTGAGGTTCAGAAAGACGAAGATGGCGCGTTGATTGAATTGGATTGTCAGAATGGTTTTCCTGAGGGGAGAAAGGAGTTTGTTGCACCTGCTGTTGGAATGGAGTTTGAGTCATACGATGATGCTTACAATTATTATATTTGCTATGCCAAGGAGGTGGGGTTTCGTGTACGGGTGAAGAATTCGTGGTTCAAGCGGAATAGCAAAGAGAAGTATGGTGCAGTGCTTTGCTGTAGCAGCCAGGGTTTCAAGAGAATAAAGGATGTTAATCATTTGAGGAAGGAGACGAGAACTGGTTGTCCTGCAATGATAAGGATGAGGTTGGTGGAGTCGCAGAGATGGCGGGTGCTTGAGGTTACGCTTGAACATAACCACATGTTGGGTGCTAAGATACACAGGTTGGTTAAGAAGGTTGGAACTGGGATGAAGAGGAAGTCTTTGCCTAGTTCTGATGCTGAGGGGCAGACGATTAAGTTATATCGTGCACTTGTGATTGATGCCGGAGGTGATGGAATTTCAAACTCCTGTGCGAGAGATGACAGAAGTTTGTGTGAGTTTTCTAATAAGTTGAACCTTAGAAAAGGTGACACACAGGCCATTTATAATTTCCTCTGCCGAATGCAATTGACTAATCCAAACTTCTTCTACTTGATGGATTTCAATGACGAAGGGCATTTGAGGAATGCATTCTGGGTTGATGCGAGGTCCAAGGCTGCATGTGGTTATTTTGGCGATGTTATTTATTTTGACAACACTTATTTATCAAACAAATTTGAAATCCAGCTGGTGACTTTTGTTGGAATAAATCACCATGGTCAATCAGTGTTATTAGGATGTGGCCTGCTTGCAAGTGAAACAACAGAGTCTTATGTATGGTTGTTTAGGACATGGGTTAAATGTTTGCCAGGATGTTCTCCCCAAACTATTATTACGGATAGGTGCAAGGCTTTGCAGAGAGCAATTGTAGAAGTTTTTCCTAGATGTCGTCATTGTTTTGGCTTGTCATTAATTATGAAAAAAGTACCAGAAAAATTGGGAGGGTTGCACAACTATGATGCACTGAGGAAAGCACTGATTAAAGCAGTTTATGAAACATTGAAAGTGATTGAGTTTGAAGCAGCATGGGGATTTATGATTCAACATTTTGGAGTTAGTGATCATGAGTGGCTTCGTTCTCTTTACGAAGATCGAGTTCGTTGGGCTCCAGTATTTTTGAAGGACACATTTTTCGCTGGAATGTCTGCAGCACGCCCTGGTGAGAACATTACTCCTTTTTTTGATAAATATGTGCACAAACAGACGCCTCTGAAGGAGTTTCTTGATAAGTATGAATTAGCTCTTCACAAGAAGCACAAGGAAGAGTCTTCTGCAGATATAGAATCAAGGAGCTCCAGCCCCTTGCTGAAAACTAGATGTTCCTTTGAATTGCAGCTCTCCAGAATGTACACTAGAGAAATGTTCTCGAAATTCCAGTTAGAAGTGGAAGAAGTGTATTCTTGTTTTGGTACAACACAGTTACATGTGGATGGTCCCATCATAATTTTCTTGGTCAAGGAGCGTGTTTTGATTGAGGGAAACAGACGGGAGATTAGGGATTTTGAAGTTTTGTACAGTAGGACAGCAGGTGAAGTTCGTTGCATCTGTAGTTGCTTTAATTTTTATGGATATCTATGTCGGCATGCACTGTGTGTGCTCAATTTTAATGGGGTTGAAGAGATACCTCACAGATACATTCTTTCACGGTGGAAGAAAGATTACAAGCGTCTTTATGTTCCTGATCACAGCCCTGGTACTGCTGATGACACTGACCGCATTCAATGGTCTAATCAGTTATTTAGAAGTGCCTTACAAGTTGTGGAGGAGGGGATACTTTCTCTAGATCATTACAATGTAGCGTTACAATCTTTAGAAGAATCACTGAGTAAGGTTCATGATGTAGAACAGAGACAGGAGTAACAGGCTTTAATGTACTCTAAACAAAATTACATGTTACGTTGTATAACTGCTTAGAGCTGTTTAACCGAGATCTTTTTACGATGTCCATAACTTGTATAGCTGTTTAACCGAGATCTTTGAGTTAGACTATAAGTAATTTCTCTCATGTTATTTATTTACATAGTAATGGTACTGCTTAGGAAGTTGAACAATTGACTACTGTTTCCTGAGTATGTCCTTTTTTCTTCCTTTTTGTTGCTACTGAAATGTCTTCAATACAATAATTAGAACTTCCTGGA

>C101271_65

GAGAATGTTCGGGAAAAGTAAAACTCACAGTAGTAGCAGTGGCGCAGAGAGAGAGAGCAGTGTCTGAGTGAAACATCCATTCCTTCCTCCTCCTCCTCTTCCTCCTCGTGATTCCCGAACATTCTCTTCTTATTCCAGCTCCACGCGCGCCATTTCACTCTCACATTACTCGCGCCCATTCTCCTCCTCCTCCTCTTCCTTCTTCCTCTATAATCTTAGGGTTTGCTTAATGCTGAATGTGGCTTCGCTCTCACACTGACTTTTCGCACTACAATGTCCGGACCTCTCGACCGTTTTGCCAGACCCTGTTTCGAAGGTTTCTCCGGGAGCGATGAGAAGAAGGAGCGGAGATCTGATTTCGAGAACTCCGAGGACGAACGGAGGACCAGAATCGGATCTCTCAAGAAGAAGGCACTGAATGCGTCGTCCAAGTTCAAGCACTCGCTGAAGAAGAAGAGCAGCAGGAGGAAGAGCGATGGCCGTGTCAGCTCTGTTTCCATTGAGGATGTTCGGAATTTCGAGGAGCAGCAGGCGGTTGATGCATTTCGCCAATCCTTGATCATCGACGAATTGCTCCCCGAGAAACACGATGATTACCATGTCATGTTAAGGTTCCTGAAAGCGAGGAAGTTTGATATTGAAAGGGCAAAGCATATGTGGGCCGATATGCTACAATGGAGGAAGGAGTTTGGTGCTGACACTATTATGGAGGATTTTGAGTTCTTAGAATTAAATGAAGTCATGAAGTATTATCCGCACGGCCATCACGGTGTCGATAGGGAGGGAAGGCCTGTTTACATTGAGAGACTTGGGAAAGTGGATCCGAACAAACTTATGCAGGTTACGACCATGGATAGATATGTAAAATACCATGTACGGGAGTTTGAGAAAGCTTTTAAGATAAAGTTTCCTGCTTGCACAATTGCCGCCAAAAGGCACATAGATTCAAGCACCACGATATTAGATGTTCAAGGCGTGGGCCTTAAGAACTTTACAAAATCTGCCCGGGATCTCATTATGCGCTTACAAAAGATTGATGGTGATAATTACCCTGAAACTCTTTGCCAAATGTTTATCATAAATGCCGGCCCTGGATTTAGGTTACTGTGGAACACGGTCAAGTCTTTCCTTGATCCCAAAACAACTTCAAAGATCCATGTTCTTGGAAATAAGTACCAAAGCAAATTACTTGAAGTAATCGATGGCAGTGAGTTGCCAGAATTTCTGGGAGGTACCTGTACATGTGCAGATCAGGGTGGTTGCCTCAGATCTGATAAAGGACCCTGGAAAAACCCTGAAATTTTGAAGATGATTCTCAGTGGTGAAGCACGAAGAACAAGGCCGGTGGTAAAGGTTCTGAACAGTGAAGGGAAGGTTATTGCATATGCCAGACCACAATACCCAATGGTAAAAGGTAGTGACACATCCACAGCTGAATCTGGGTCTGAAGCTGAAGATATTGCTTCTCCAAAAGCAATGAAGAGCTACTCTCACCTGAGGTTGACACCTGTTCGAGAGGAAGCTAAGGTAGTTGGAAAGTCAAGCTATGTTGGTGTTGCTAACTTGGCTGGGTACGATGAATATGTTCCAATGGTGGACAAAGCTGTTGATGCTGGCTGGAAGAAGCAAGCATCACTCCAGAGGGCTCAAACTTCAAAAGGAACACCTTCCCTCCCTGACCCTCCAAAAACTCCGGAAGGGATTCGAGCTCGGATTGTGGTAGCTTTAACTGTTTTCTTTATGACGCTGTTTACTCTCTTCCATTCAGTCGCACTCCGTGTGACCAAGAAACTACCTGCCTCATTATCTAATGAGGATCAGTGCACTTCAGAGCCAACCCTTGATTTAACAAATGAAAACTATTGTTCTCCATCACCAAATCCAGAAGACACAGAGGCAAATCTCCTTTCCTCAATGATGAAAAGGTTAGGTGAGCTTGAAGAGAAGGTTGACACACTTCAATCTAAGCCATCTGAGATGCCTTATGAGAAAGTGGAATTGCTAAATGCTGCTGTATGTCGGGTGGATGCTTTGGAAGCAGAGCTAATTGCAACTAAAAAGGCTCTGTATGAAGCACTAATGAGGCAAGAAGAGCTGCTTGCTTACATTGATAGTCAAGAAGACGCTAAGTCGCGGAAGAAGTTTTGCTGGTGAGTTTTGGTTGGCTGATAGAAAGGGACTCTTGGGGGAGAGGGTGTTTTCGAATCATGGTGTTCCTAGTGCTGACCCTGCACGTTGTGTTGAACCCTGAACACAGATTTTCTGTTTAGAGATTAAACTCGAGAATTGTTTCAAACTCCTGTATAGCTAATGGCTTCTTTGCCGCGGATTAAACACAAGCCTTGTTTTTGCTCTGGCCACCTTTGTTTTATTTGGAGTGATTGTCTTTTGTTGGTTTGAAAAGCGTTTGTTCTTCTGATGATCAATATTTGCATTTAATAGTAATCGGTTTTGGGGGATGTCGAGGGGGAAGAAAGC

>C101279_65

GCGAAAAATGCAATTCCATTGGAAATGAAAAAGTGTTTGAGACCGCAAGGACCCTATCCGCCCCGTAGCATCGCCAATTCAATTCAGAACCCAACTGAGACTGAGTGAGAGAGAGAGACATGGCTTCGTCGCTGTCCTCCGTTATCAACCAACCTCACATTGCTCAACGCTTCAAACTTCACCGTCCATCTTCTTTTGCCACTTTCACAATCTTCCACCACAAACGCAAACACAAGCACCAATACAATGTCTTTTGTCCACACCCATATACCTCATCGCCACGTTCTCGCCCTCTCAATCTCATCATAGCTTCCAGAACCTTCTGTCATTCCAACACTCTCCGCCAGCACTTCATCCCTCAAGCCACGCTCACCGCTTCCGAAGCTCAACCCCGCCTTGTGCCTGATACTGAAGTTCCTCCCACCGGTCGAATATATCATGAGACTTATGGATGTCAAATGAATGTTAATGATATGGAGATCGTTCTTTCTATAATGGAAAAAGCCGGTTACAATGAAGTTGTGAGTGTTCCCGAGAGTGCAGAAATAATATTCATCAATACCTGTGCCATTAGAGAAAACGCAGAACAGAAAGTATGGCAGAGGCTTAATTACTTTTGGTTTCTTAAGAGGCATTGGAAGAGCAACGTTGCTACTGGAAGAGCACAGTCTCTGTGCCCTCCCAAAGTCGTGGTTTTGGGATGTATGGCTGAGAGGTTGAAGGAGAAGATACTTGATTCAGACAAAATGGTTGATGTGGTATGCGGGCCTGATGCCTATAGGGATTTGCCGCGCTTACTGGAGGAGGTTGACTACGGTCAGAAAGGGATCAATACTCTCCTTTCACTTGAGGAGACTTATGCTGATATTAATCCAGTTCGAATCTCCAAGAACTCGGTTACTGCATTTGTCTCAGTGATGAGGGGTTGCAATAATATGTGCTCGTTTTGTATTGTTCCTTTCACTAGAGGCAGAGAGCGTTCGCGGCCTGTGGAGTCAATTGTGAGAGAGGTAGCTGAGCTTTGGAAAGAGGGTGTTAAGGAAGTAACGCTTCTTGGTCAGAATGTGAACAGTTACAACGATGCATCTGGTACTGAGAGAGAGGTTGAATCAGGGTCCAATTGGCAACTTAGTGAAGGGTTTTCCAGTATGGCCAAGGTAAAAAAAATGGGCTTAAGGTTTTCTGATCTACTTGATCGATTGTCCTCTGAATTTCCTGAGATGCGATTCAGATTCACATCTCCACACCCTAAGGATTTTCCAGATGAGTTGCTTTATCTAATGCGAGAAAGACATAATATATGCAAATTGATACATTTACCTGCACAAACTGGAAGTAGCAAAGTACTGGAGAGAATGAGAAGAGGATATACCCGCGAGGCTTACTTGGAACTTGTGCAAAAGATCCGGAGTATTATTCCAGATGTGGCTTTGAGCAGTGATTTCATTTGTGGATTTTGTGGAGAAACAGAGGAGGAACATTCTGAGACGTTGACTCTGGTGAAAGCTGTTGCTTATGATATGGCATTCATGTTTGCTTATAGCATGAGGGAGAAAACTCATGCCCACAGAAATTATGTTGATGATGTTCCTGAGGAAATTAAACAGAGGAGGCTGGCTGAACTTATTGAGACTTTCCGCGAGAGCACAGGAAAGTGTTTTGACTCTCAGATTGGAACCACACAGCTTGTATTAGTCGAAGGACCCAATAAAAGAGCTCCAGATACTGAGCTTATGGGCAAGAGTGACAAGGGCCATCGTGTGTTGTTTGTCAATATGCCAATCGTAGATAGGGAAGATGTTAATACAAAGCGAAATCCTGTGGTTGGTGATTATGTTGAAGTCCGCATAACAAGATCTACGAGGGCATCACTCTTTGGTGAAGCACTTGCTATAACTAAACTAACTTTATTTTACGATCATGTGGATAAAGAAGCTGTTACATGCAGCTTGTAGAACCTTGTAGCATAAATAGTTTATCATGCCAATTTTTGGATAAAGAAGTCGTTCATTAATTGCTTTTTCCCGTTTCGCTGACTACACAACTTGGCAACACATTTTATGGGAGAAGACACTACATGTCAGCTGGATCACGATTTTGTATTCTTTGTTCATCCTCTCTTCACATTAAAAGGCTTCGAGTCTGCATTAAAATATACACTATGGATTACTGAGAAATCCAATTGGTAAACCTGAATAGCAGCATCAAAATAAATAGTCTTATGGAGGATTGTTGTGATTTTTGTCTTTTAACAATGGTTTTTTGTCCATCACATTTATAGGTAAATAGAACATCATGTGCATGGCAGTTTTGTTTATTGCTCTCTGGCTACCTGTCCCTATGTTATGTTTTGGAGCAGCATGGGGAAAAGTGATTGTATTTTCATCCCCAATCTATTGATTTTCTTCTGTCACATTAGGGAGCATTAGACACAATGCGAATAATAACAATTCAAGGATAAATCATTCAATCTGTTGGGTTG

>C101285_65

TTTGGGTTTGGGGATGGGAAGAAAAGAAGAAAAGAAGAGGAGAAGCGAAGAGCGGAGAGGAGGTATGGATGGAGTATGAAGAATAGTTGAATAGAAAAAGGAGAGGGGAAAAAGGATGCAATTGCGATTGTGATCTGATCTGATCTGATCGTAGAGTCGTAGAAGTGGGCAGCCCAATTTGCAATTTGTAATTTGCTATGAGAGGAGAGGAATAGAAAGATGTAATAAGTCGCACACAGACTTATTGTTATCACTAGACCACACCAGATTGAAGATTCAATCCCAATTGGCATGCATGGAGACTTCTCCCGGAATATCTCCACCTTCGCTACTACTTTTGTTCCTCTTTTCGCTTCTGTTTTATGCTTTACATTCTTCAGGCGTTTCAACAACCACGTCCCTCTATGATTGCGACCAATGTTATCGCACCAAGGAACACGATATTTCTACAACTAGAAACCTCCACAATCCCGATGAAAAGGACATTGATATAATTGCAACATACACCGATGCTTCTGGCCATGTTCGACTGGCTAGGTTGAAAATGAGGGACTTATCTGATTCCTGGATCTGGGAAAACCCTACTAATGGAAACAATGATTATCAAAAGAGCTCCCAGGAATTGATGGAATCGTTTCAAACTGATTCAAGATTTGAAGACAATCCCAGGCATTCCTTGGATGAACATTATCCTGAAGAAAACAAAGTTGAAATTCCCCGTTCCTCGTCAATGACACCGTTGAAGATCAAGCGCCAAGTAATGCGGCAAGAAAGAAGGAAAACTCGTGCAGCTGAACTTACTCAGAAAAACAGAGAATCGGACAATCACATTGTATCAGCAGCAATTGAACGTACTGAAGGATTTGATACCACTATCAAGGGAAAGTATAGTATTTGGAGGAGAGAATATGAAAACCCAAATTCTGATTCAACTCTGAAACTCATGCGTGATCAAATAATAATGGCCAAAGCTTATGCTAACATTGCAAAGTCTAAGAACAAAACTGTTCTATACGAAGCTCTTCTCAAGCACTCCAGTGATAGTCAACAAGCTATAGGAAAAGCCAGTTCTGATACCGAGCTTCACCTTGGAGCACTTGATCGAGCAAAAGCTATGGGTCATGTTCTTTCCATAGCAAAGGACCAACTTTATGACTGCCTTGTGGTGGCAAGGAAGTTAAGGGTGATGCTTCAATCAACAGAAGATAGAGTGAATATACAGAAGAAAAGAAGTGCATTCTTGATTCAACTGGCTGCAAAAACTGTGCCTAGACCATTGCATTGCCTTTCCCTCCAACTTGCAGCGGATTATTACCAGCAAGGCTATCATAAGAAAGGAAATCTGGACAAAGAAAAGATTGAAGATCCATCTCTATACCATTATGCTATCTTTTCTGATAATGTACTAGCTACATCTGTGGTTGTTAATTCTACTGTGCAAAATGCAAAGGAACCTGAGAAGCATGTATTCCATATAGTGACGGATAAATTGAATTTTGCAGCAATGAGAATGTGGTTTCTCACGAACCCTCCTTCTAAAGTAACCATTGAAGTTCAGAATATTGATGATTTCAAGTGGCTGAATTCCTCATATTGTTCTGTTCTACGTCAACTTGAATCATCAAGAATCAAGGAATATTACTTTAAAGCTAATCATCCTTCCTCCCTGTCTGTTGGTTCTGACAATCTAAAATACAGAAATCCTAAATACTTATCAATGCTAAATCATTTAAGGTTCTACCTTCCTGAAGTTTATCCAAAATTGAACAGAATTCTATTCTTGGATGATGACATTGTAGTGCAGAGAGACTTGACACCTCTTTGGTCAATTGATTTGAGAGGTATGGTGAATGGTGCTGTGGAGACATGCAAGGAGAGCTTCCATAGGTTTGACAAATACCTAAACTTTAGTAATCCACTGATTTCTAATAACTTCAGTCCTGAGGCCTGTGGCTGGGCATTCGGCATGAATATTTTTGACTTGAAGGAGTGGAAGAAGCGAAACATCACGGGTATCTATCACCGGTGGCAAGATATGAATGAGGATAGAACCCTCTGGAAGCTTGGAACCTTACCCCCCGGACTAATCACCTTTTATAATCTGACCTATCCGTTAGATCGAGGCTGGCATGTTTTGGGACTTGGCTATGACCCAGCTCTGAACGTAACAGAGATAGAGAATGCCGCTGTCATCCACTACAATGGAAATTACAAGCCATGGTTAAATCTGGCTGTTTCCAAATATAAATCGTACTGGTCCAGATATGTTATGTTTGATAATCCATACCTTCGAGTTTGCAACCTTAGCGAATAGTTATTTTTTTTGCCCTACAGTAGCTGATTGTACCATTCGATAATGGTACCACTACAACGTAATATAGTTTGCCTCTTTAAAGGAAAAACTGAATGCGGTTCGGTAAACATTTTGATAGTCCTGAAATTGGCTTTCCTTATTAGTGAAATCCATGACAGTATAAAAAAAA

>C101289_65

TTTTTTTAGTGCCAAAAATAAATAAAGATTTTGCATTGGCCACACAATTTACTATGTAATTTTTTTACAAGAAGTTGTAGTTTCAACATAGGACTTATTCTATCTATGTTTATGGTTTTTCACTTCAGTCAACACAATCATGAACCAAAATATACAGAATGCCAAATGGATTTTGCTTCATAACTTCCATAAATCAGAAAAATCCTTGCAGAGTGGCTTGCGTCATGGTTCACACGGTTGCTCCCTTTGCTTCTTTGTATAGTTGATCAATATGATCCTTATAGTACTTGAGCAATTGTGGTCTCTTCAATTTGAAAGTTGGAGTTATTAAATCTCTCTCCATGTCAAAGGGATTTGGTTCCAGGTGAATGGCTTTTAGCAGCTCAAATCCTCTAAGTTGGTGTTTCTGACCAGTGCTGTTGAGCTCATCCAAAATGTACTTTCTTGCTTTCAGATTGTCACATAATGATGTAAAATCATCACTCACATTGTGCTCTAGTGCCCAATCCTCAATGGCCTTTCTTTCAGGGACCACAACAGCCACCAAGAATGATTCAAAGCTGTTTCCATACACCCAAATCGATGCTATAAGGGGACATTGCAAATACTTGTTTTCAATATTCTCCACAGCAATATATTCTCCTTGAGACAATTTAAAGATGTTCTTCTTCCTATCGATAATTTTCATGGCCCCATTTGGTTGCCATTCTCCAATGTCTCCTGTGTGAAACCAACCATCAACCATAACCTCTCTGGTAAGATCTTCACGTTTGTGATAACCAGAGAACAAGCTATTTCCTCTAAGACAAATTTCTCCGCGGGCTACATTGGAGAGGGCATCATATCCCATCTCTGGCACAGATTCAAGCCTGGCTTCAATGGTTGTCATGGGGACTCCAACTGTTCCTGTCATAGAATACACATCACCTATGGCCGTGAAACACCCGGCACAACTTTCAGTAAGACCGTATCCTTGTGTTAATGTAGATCCACTAGTGACCCTCATAAACTCTTCCACATGCCTAGGCAAAGGAGCAGCTCCTGATAACAGGATGCGAACACGTCCACCTAGTGCTTGTTTTGTCTTATCAAACACAAGCCTATCAAACAGAGGAGCTGCTTTGTGTTGTGGAAGACCCTTCTCCAGATATTTTAACTTGTAGTTGTAAGCACACTGAAACAATGTACTCTGGAGTGCTCCTGCTGAAGAAAGTCTGCTTTTGATACCAGCACAAATTCGATCAAAAACTCTAGGAACACCACAAAATATAGTTGGTTTCAGTGTCTGAACATCTTCCAGCAAGAACCTGACATCGCCTTGCCAAAATCCGATTGATGAGCCCTTGGAGATGCAATAGGTCTCCATTATTTGGTCATATACATGAGCAAGAGGAAGAAAGGAGAAGTATACATCATCTTCTGCTGCCACTCTGTCTGTTAACATAATTATGTGGTCAACAGACAACACTTCAGCCATGAAAGCCTCATTCTTAAGAACGACACCTTTGGGATCTCCAGTTGTTCCACTTGTGTACATTATTGTGCATATGTCAGTCTTCTTTTTCGATGGTAGATCCCAATCCAGACATCCCAACTGGAGAAACTCTCCCCATGAGAAGCAGGACGCACCATGCTCCTCGGCTTCCTTCTTTTGTGTGGTTGAAACACTTCCAAAGCTCACAATAGTTTTAAGATTTGAAGAACACTGCGCAAGACATGACAAAATTGATGGAATTTTTTTTTCCTGAACAAAGGCAATCGAAACTTCAGCATGGTTTATGATAAACTCCACCGCGTTAGGACCAAGGGTGTCATATAAAGGAACATATGACACTGCACAGCTGTTGCAAGCCTCCATTGCAATGATCCATTCAGGGCAGTTGGACCCATATATGCCACAACGATCTCCTGGATTGACACCACGGCTCCTCATGGCAGAACCCATTTTCAAAGCAGCATCATAAACCTCTTGATATGTAAGCCATGAATAGGAACCCACCTTAGATTCTGTTTTTTGACGCCTACCCAGCATTTTGTTGTTGGGGTTCCTCTTAACAGAGTTCCTGAAGAAATCCCAAGGGGACTCAAAATCAGAAGGAACCTCCAAGAGACCATCTTTGGCATAAATGCTCCTATAGACAGGACCAGCTGATGGCTTTGCATCAGTTGCAGGCCTTCCCTCTTCAACCTTCACAGTGAAAACCTCTGGCATTGTAAGATGGAAAGGAATGTGCAGGTCTGTCTCAGAACTGTGTCCAGGGGATGATGTTAATGATTAGTGATGTTAGTCACTAGTGGAGTATAGGTAATAAAGACAAATGGGTGTGGATAGACCAACTTCAAAGGAAGAAGAAGTGCATCTGAACAAGAAATGTGGAGGTTGAGCCAGAGTAATAAATTCAGTGTTTACTTTTGCTACTCATTTTCTGAACAATTTTTGTAGCTGCTTTATTGCACCCACTTTCACCTCCTTTATGGCTTTTG

>C101291_65

TCATTATTTATGTCTACTTTGTATTTCTCAATCTACTAAATATCAATTACTGTTGACAAATACAAATAAAAAACAGTAAGATGAAACTGTTCAGCACTTGAATAAAAAGCATAAGACTATTGGTGAATTGTGATGATTAGCATACACATTTTCTAAATTACTTCACTAAAACAGTAAACTTTGTAAAAATCAAGAACATTTAAACTAAACCAGAAAATATTAAACATCTTAACTTAGAGAAATAAAGAGGACACATGCACTTAGAGCCAATGTTGTGGATTTCACTGCAACAGATACTGGTAATCTTCCGCTGTCTTTTCTTAAAAAGAGTCCCTCATACACAGGTACATTGATGAGGACCAAAACCCCACTTAGTAGAACTTGCAATGCCATTGTTTCAAATATTCTGAGACCCCCTTCATTGATGAACACTTGCTTTAGGAGCATGCCAACAAGGCAAAACAAATTGAGCAATGCAAGTGTTGCTAGTAAAGTGAGCATTGGGGATGAGTTTCCAAACTCCATGATTTCTTTCTCGTATCGTTGGGAAACTTTTTCTTCTGCTACCTTTGCAGATACTACAAAGTTTGATTCAGAAAAGCCGAAAAACTTCAAGATTGTATCAACAAAAGCAAAGAGGTAGGAGCTTGTTCTCTTGTATAGCCATAACCGTAGGTCATTCCACCAACCCTTGACTGTGCCTCCTGACCACAAAAACTCCATGAGGCAGTAAGAGGAATCGCCAAGCGTGACATATACAAAAGGTATGAACCAGGGACTTGACATCTGTGGAAACAAGGGAATGCCTTTGAGGAGATAGAGTGAAGGGATGATGCAGTAATATAGAGTTGGCCAAGAATGAAGCACCCAAAGGTTATAGTAACAATATGCCATTTGGAGTCCTGGGCTGATGAATCCATAAGCATACCATGCAGGACTATACTTAGAAAGCAAAATTTGAAAGCCTCCTTCAGACCATCTCTTATGTTGAACTAGTGTTTCTGGCAAGGTGGTTGGAGCTACACCTAAGAAAGCACTCCTTTGAGGATTATAGTACGCTGTTTTCCATCCTCTGCATTTAATCCACAATCCTGTCATCACATCCTCTACTGAGCAACCATATTGCAGTCCCATCTCTTTTCCCCACAAAGTGTTTTTCTCATAGGTACAACTAGCAAGAGCCTTTGATTTTTCTTCCAGTTCATGCAAGCTTGCTTCTTTCATATGATCAATACTCTTGTACTCATTCCAATCTTTCGTGTATTGGTCACTGAACTTTCTTCCACACAATATCTCTCTTCTGTGAAAGCAGCCAGTTCCAATATACATAGGCCCTCCAAGTGCATCCAAACCATGGAATTCCACCTGATAAATTACTCGTAGAGCACCACTATATAGATCATTCTTCGTGATATTCTCAAAACACTGCGGAGTCTGCACAAAGGCAATTTCATGGCCTTTATTTTCATCCATGAAGAAGCATAGTGCATCCCTTAGAGATTGAGAATTGTTTGAGTACATGTCACAATCTACATTAAGGATAATTTCCCCGTTGCTAATCATTGATGACACTCGTATCAATGAATTCATGGCTCCAGCTTTGAAGTTATGTGCAACTTGAGGTCTCTTCTCGCGAGCCAAATATACCAATATGGGCATAACATTCCCATCTACATCTTTGGCACTCGAGTCCTTTCCATCAAGTAATATCTGAAGAATAGTGTCATGATCTCGTCGAGAAGAATATGAATCCCACTGAGAAAAACCTTTATGCTTTGAGCGTATTTCTTCAGGTACTCTACCCATCTTCCCTATGTTTTCTATTCTACTTTCCATATCTTGGTACAATTTCTTGATGAGAACCAATTCTTTGGCATGAGCGTTTGAGTGAGAGGATGAGGCAACAGTTTTGAAATAAGCAGCAGGTGAGGTGGGGTCAACCTTGAATTTTCTGCAGAATGGAAGCCAGTGTTTGGCAAAGGAAGAAGCCTCTAAGAGAGCATAGAAGGTGATATCTGATGCAGCATCATCTGAAAGATATACACTGAGTTTCTCTGGTGGATAGTCATAAGCCATAACTGATAGCACTGTGTTAATCACCATCACTGCTGGCTCAATTACTGGATCTGCTGTGCACACATAGATGTCCACTTTTGGTAACACCTTCTCATATCTTTGAGATAGTCTATCTCTGAAAGGTTCCCTGAAGAGAGGGTTCCATCTGAAAGGGTGTCTGAGGAGCCAATAAAGACCAAACCATAGTTCAACACAGAACATGCCAATCCAACCCCATTTGCCATGTTCACTTTGTGAGGGTATGTGAGTCACTCTGAAAACCCAGATGAAGCAAATCCCCACAAACAATGACAAAGAAAAGAGTATGTAAAAGACCCTTCCTTTTCCTCTCTTTGTCTCAAACAATGGACAATACTCACCCTTCTCTCCCATTTCTTCTATTTCTTTCTACTCTCTTCTCTATCCATT

>C101293_65

AAAAAGTAAGAAACCAAAATAATTCATAATTAAAGTACACAATAAAAATAGAAAGAAAAAAAAAATGCCACAATATAAGAGAGCCTGTTATAAGACAGTGCTGCCTGATATATATAATCTTACAAGTTGTATTGTTTATTACGGCTGTGAGAATGTCTGAGGTTGCAGTTTTTGAGTTTGTCGACACATGCATGCATTAGTTCCACCTTCAACCCACTGTTGAAACCATGAATGCCACATATGTACAACTTTTTCCACCTTTTTCTCAAAGTGTTCTATGCATTGTAATTTGTCACATTCCAAAAGCAATATCAGCTAGCTATTGCCTATGAGCTTGACCTTGAAATGAGTTGAAAATGTCTCACTTTCAAGCATATAACTCAATTCAACTCAGTGAGCTGTGCTCTCCTTTCTGCAGCTTTTTTTCTGATAAAAATGCCCCACCTTAAAGCAGCTTTGAGCTTAAGCCACCCAACAACAGCTTTCCCTGAAGGCTTCCCCTGATCCTCACTGAATGAATGATACATCTGAGGCTCATACTGAAGGCTATAGGTGTAACAACCCTCATTAGAATGACCAAGATTAGCTGACATGTTGACTCCTGCATTAATTGTCTTAAGTAACCTCTGCATATCATCACTCTCCAACATCTCTGAACTCCTAAGACGAAGTTCTTCTGCAACAATGTCTTCCAACCCTTGTCCATTTCTCGGCCGGGACCAATCTCCAGCAAAATAAGTACCACCATTTTCATGGTTTTCACCTGACATGTAGTTCATGCTGCCAGACAAGGCTATTTGAGAGTCAGATAAAGATGTTCCTACCATCTGAGTGTCAGATCTCCCAAATGAATAGTCAGTCAAAGCTGGTCCGGATGAGTAGTTATTTGTTATTTGCAAGTGTTGATTTGGTTCAGAGGAGGCATATGACACCTTGTTCTTTGCAGAGGAATACTGTTGTTGCTCTGGGAAACTGTTATGGTGCATTATCTGAGTTGCTACAGCTCTTGACCCCTTTTTCGAATTAGTAAGCGAATTTAAAACTTTTCCATCATATTCTACAACTTGGTCCCAATTTTCGTATGCTTTCTTCACCAAGGAATCCACAGACATCTTCTGATTTGGAGTAAGTGATTCTAAAGAGAAGAATTGACCATCAGCAATGAGGCCTCTCAGCTCGTATATATTGTTGAACACAATGCCAGCACTATTGGTTTCATCAGTATAGTAAACAAAGAGCTTACCACCCAAGACACATGTCTTGGCATGCTCCACAGTATTCTCCCACATTCTGTTGGACATTCCACTACCAAGTATACTTCTTAACTTCTGTGGCTCGCGGACAAGAAGGCGTAGGAAATCTTCTACAGTTACTATTTTTGCTTTAATTAACTTTTTGTGAAGTGCCCCATCCTTTGCTATCCGATCCAATCTCCAAACTTCATCATGCAAAGCAGGTGGATAATGTTTCTTGTATAATTCTCCTCTGTGATCTTTAACAGCAAAGGCTTCAGTCTTTCCCTCGCGAATACGAATCTCCTCACAATAACCAGGAGCCACCTTAACACCAAGTCGGAATTTTCTGCTTCTTATCCAGCTAGAATTGTCAGTGAAAGTAAGATCACCTAAGGTTCCTACTCCTTCCTTTAGGCTAACCTGCAAATCTCCAGTTAGAAGTGGCCTTTTCCCCTCACGTTCCTTTACTTCGTGGCTTTCAAAGTGCTCTTTTGTCCAATCATCGTCCACTTCCTCATTAAAGTCACCTTCAAGTACCACCACATTCAATTTAGCAACAGATTGTGGTCCAACATGAACGACACTGCCTGTGTTGGGATCCATTAACACAACATGGATTGCCGATCCTTGCTCTCCCTCAACCTTTCCCCCAGTGAATAAATGAGGTGGCATTCTTGTTCTGAAATGAAGCTGCAGATTCTTTGCCTCTGGTCCTTCTAATCTTGGCGGCAGAGATCTTTCAGCCAATTTATCATGGCCCAATTTTGCCAAAGCACGCTCCACTTCTTCACTGACAATTTTTCTGAGAAGAGGTTCCAAAGATGAGCAAAGTCTCTGCAGACTGTCCACTTTTAATGCTTCCACAATCACACTGGCTAAACCGGGCAGTTTTGGTTTCTTTGACTGTGTAAGATGGTGCTGTGCATCGTCACCCTCCTCAACTAGCTCATACCCTCTTTTCTCCACTCTATTGTTCTTGGAACTTTCCATTTCTAGAAACTTATCCAATCCCAATGATATCTCTTGACACTATCTAAATTTAGAAACTTGGTCTTGTCTTAGGGATCCGTAGGACAGGTGCACCCAAGAACCTTCCCAGCAGAAGCACAAGAGGGGAGCGTAAAAATCTTGGAACCAAAACAAAAACTAAAACCTGAATTCAGAAACACATAAGAGGATGCTGATTTTTTTTGTTTGACATCTGAGGATCTAAGATTACGACAAAGAAAATGTGAACAGTGTAGCATAG

>C101295_65

GCAAGTCCACAGCTTCAAACGCTCTCTTACACCACCAGAGAGTGCTTCAAGTTGTGCCGATTTTTCTCTTCTCACCACATAACAAAACATTTCTTCCCACTTTCCACATTTTATCAATCAACCAGCATAACATGGCTTCCTTTGTTCTGGGGACTAGTTTCCTTCCTCTCTTACATTCTCATGATAAGTACTGCTGCTCCTCAAATATTTCAAACATAACCACCAAAATCTCAGCCACCACTCCACAAAGGATGAACCAAGTGATAGCTGTTGCGGGAGATAACGTTTGTGAGGATATGGACAACAACACATTGGTGAAGAAAAGCTACGAACCAAGTCAAAAGTTGCGGAGATCTCTGAATTTCTCAGGAGAGAAGCCATCGACGCCAGTATTAGACACCATTAATTATCCAATCCACATGAAAAATCTAACCATTCAGGAACTTGAGGAGTTAGCCGATGAACTCCGTGAAGAAATTGTGTATACAGTGTCAAAGACAGGAGGGCATTTAAGTTCAAGCTTAGGTGTGGCAGAATTAACTGTAGCACTTCATCATGTATTCAACACTCCTCAAGATAAGATTATTTGGGATGTTGGTCATCAGACCTATGCTCATAAGATTTTAACGGGAAGAAGATCCAAAATGCACACAATACGACAAACTGGTGGACTTGCAGGATTTCCCAAGAGGGATGAGAGTGTCCATGATGCTTTTGGTGTTGGTCACAGTTCTACTAGCATTTCAGCTGGCTTAGGGATGGCAGTGGCTAGAGACATGCTTGGAAAAGATAATCATATTATTTCTGTTATTGGTGACGGAGCCATGACAGGTGGACAAGCTTACGAGGCAATGAATAATGCTGGTTTTCTTGATACCAATCTTATTATAATCTTAAATGAAAATGAACAAGTTTCCCTTCCCACTGCCACCGTTGATGGCCCAGCTCCACCGGTTGGAGCTCTTAGTAAAGCCCTCGCGAGGCTACATACTAATCCTAAGTTCTGTAAACTACTTGAGGTCGCAAAGGACACGAAGCAAATTGGAAGCCAAGCACAGGAATTTGCATCTAAATTAGATACCTACATTAGAGGGATGGTAGGTGGTGCTAGTGCTTGTTTATTCGAAGAGCTAGGGCTCTTCTACATTGGCCCAGTAGATGGCCATGCCATGGAAGACCTTGTGCACATCCTGAAAAGTGTCAAGGACATGCCCACACTCGGACCTGTTCTCATTCATGTAATCACTGAGAAAGGAAAAGGTTATCAACCAGCTGAAATTGCTGCTGACAAGATGCACGGTGTTGTGAAATTTGAACCCAAGTCAGGGAAGCAGATGAAATCCATAACTAGTACAAAGTCTTACACACAGTATTTTGCAGAGTCTTTAATAGCAGAAGCTGAAGTTGATGAGAAAATTGTTGCCATCCATGCTGCAATGGGAGGGGGTACCGGGCTTAACATGTTTCAAAAGCGCTTTCCCGAAAGGTGTTTTGATGTTGGGATAGCAGAACAACATGCTGTAACCTTTGCTGCAGGCCTAGCTGCTGAGGGCTGTAAACCCTTTTGTGCAATTTATTCTTCCTTTTTACAAAGAGGTTATGATCAGGTAGCACATGATGTGGACCTCCAAAAACTCCCTGTTAGATTTGCTTTGGACAGAGCTGGCCTAGTTGGTGCTGATGGTCCAACTCATTGTGGAGCATTTGACACAACATTCATGGCTTGTTTGCCAAATATGGTAGTCATGGCTCCGTCAGATGAGACTGAACTAATGCACATGATAGCCACTGCTGCAGCCATAGATGATAGGCCTAGTTGCTTCAGGTACCCGAGAGGGAATGGTATAGGTTCCACTCTTCCACCCAACAATAAGGGCATACCACTAGAGGTGGGTAAAGGAAGGGTATTGAAGGAGGGAAGTAGGGTGGCTCTTGTTGGTTATGGAACAATGGTCCAAAGTTGTTTGGAAGCGACTAAGGTTCTTGAAGCTCATAGCATCTCAACAACAGTGGTTGATGCACGATTTTGTAAGCCTCTCGATGGAGATTTGATGAGGCAACTCGCTAGAGAGCATGAGATTCTTATCACAGTAGAGGAGGGATCTGTTGGAGGATTTGGTTCTCATGTTTCTCATTTTCTAGGACTAAATGGTCTTCTTGATGGTAATCTTAAGTGGCGAGCCATGACATTGCCTGACATGTATATAAACCATGGATCTCAAAAGGATCAAATTGAAATGGCAGGACTGAGTTCAAACCACATTGCAGCTACTGTTTTGTCATTGACGAATGTGAATTGGGATAATCGTTTGCTTCTTAGTCTACAAATGTGAGTCTTATAGCACAAGTTGTGAGTTAAGTCCCCTAAATACATGTACATTACTAGCAATAATATTATCTGTATGAGAATAAAGTAATTACAATGTTTTGGTGTAAAAAAAGAAGTTCTTTACCTTTTATCATATGTTTCGGTGTAAAAAGAAG

>C101301_65

CTTTATTATAATCCTCCCCTTCCTTTTATATCATGCCTCAAAGTGTGCACAACCAGCGACTGCTGCTGTTCATACATGTTTTTAATTATGTTACTTGGCTAGCAGATTGCACCAAACTGCTGCCCTAATCCCATCACTAACAATGTAGGCGGCAAAAGAACAATTTATTTAATTATTTTTACCCACTGGGTTCTCCATTTGGTTACACGAAGTTCCAGTTCAGAACATCAAAACTCAACTCTTTCAGGAATCTCTCAAACAGAAATATCCACTTCCGTTAATGGAGATTAAACATTGAAACTAAATTATTGCAGGCAACTTCTCTTCTAATGATGACCCGTATTCAAACCCAACTGTAACATTATGAATTTCGTTAGTCTAACTCAAGCATAATCATCAATGTTACATTCCCCAAGAAAAACCTTGATGCTACGAGGTGATCCGCAGCTGCCTTCGGCAATTAGTGCTCTGTCATTGGCGGCCATGTGCACAATAATCTTGTAAATCATTTCAATATCTTCAGGAACATGGCAACGGTCAGCTACTTCTTCAAGTGTCAATGGTTCAATGGGATCTTTGCAGCTTGCCTCATTTAGCACTGCCAACACTCGCTTCTGAAGTGCCAGTACTTCACCGGCTGCTTTTTTACCAGCTTCCACACCAGGTTGATGATAAGCATTTATGTGGACAAGGGAGGCATAAATTCCCACTGCTCGCTCATAGAGCGCAATAAGAGCACCAACTGTTCTTGGTGTCACTTCTTGTACAGAAACTGTGATGGACTCCCTATCATTAGCATACAGAGCTGACCTTGTTCCCTGTAGCATACCAAACAAGTAGTCACCACAAGTGACACCAGGTTCAAGTTCCCAATCATGACCAGGGGGTCTATCACGTAGCACCTCGATGAATGTCACAAAGAAATTGTGTACACCCTCCCTCAATTGTTGGATGTAGGCGTGTTGATCTGTGCTTCCTTTATTTCCATAGACACTAATTCCTTGATTAACCCGATTACCATCCAAGTCAAACTCCTTGCCTAGAGATTCCATGACCAGCTGCTGCAAGTATCTACTGAATAATAACAAACTGTCCTTGTATGGAAGAATAACCATATCCTTGGATCCTACACCATCTGTAGCCCAATACCAACATAAAGCCAGCAAAGCTGCAGGGTTATTCCTTAACACAGTACTTCTATTTGCCTCGTCCATCAATGATGCACCAGCAAGCATTTCTCTGATATCAATGCCCTGAAGGGCCGCTGGAAGCAGACCAACTGCAGACATCTCCGACGTTCTACCTCCCACCCAATCAAACATAGGAAATCTAGCTAACCAGCCTTCAATTCTGGCAGTGTTATCCAACAAAGAATTTTCTTGTGTTATAGCAACACCCTGTTTTGGAAAATCCAAGCCTGCTTCCCGAAAGGCCTTCTGAACTTCCAACAAACCATTTCTAGTCTCCGGGGTACCTCCACTCTTTGAAATCACAATCACAAGTGTTGAAGCTAGCTCCGGCCCAAGCTGTGCAATGTGATGATCAATTCCAGCAGGATCCGTGTTGTCAACAAATCTTATCTTGAGTGGAGGATTATCAGGAGCCAATGCCTCTGCAACAAACTGTGGTCCAAGAGCAGAACCTCCAATTCCCACAGACAGTATTTGAGTAAATCGACCCTCCGGGGAGGAAGGAGGCTTAATCTTACCACTAACGACGTCGTTAGCGAACTTGGAAATAGTTTCGAGAGTTTTCTCAATCTGCGTCTTGAGGAATGAGTTGGGCGCACGCTTAGGATCCCTCAACCAATAGTGTCCCACCATGCGACCCTCATCGGGATTCGCGATCGCACCCTTCTCCAACTCTTCCATCGCCTTAAACGCCGCTTCAAAACGTGGCTCCATTACCGTTACGAACTCGTCCGTGAACCCTACCCGACTCACGTCCAAGTAGACACCTAGTTCTTTATGCTGGTATAGCCAATCCACGTACCTCCGCCACAGCGCGCGGGGATCTTTCTCCAGCCCCTTCGACATCACCACCGCCCCATCGGAAACCTCCCCAGCCACCGCGCACGGCGGCGCGAGGCTGAGGGTGCGATCTACGTGGCGAGCGGGGAAGGAGACGTGATCTTTGCGAAGAGGGTGGGTCAACGGTGGGGATTGGTTTGGGAATTTTTTGAGAGAGGGGGAAGAGGTGTAAATGCCTGAGATTGATGATGCCATAGTTTTGGGATGGTTGTTGAAAGTTGAAAGCAGGGGTTTTTACGTTAAGGTTGTTAGGAGCATGATATTTGGGAGAATGGAACAAAGAAGTTTTGTTGGAGAAGAGAGCGGTTTTTGTCTTTGTTGAGAACTTGAAAGTTGTTTTTGTAGTTCTGTGTGCTTTCTTTGTGATGTTCACCCAACTCACGCGCCGCTTGGTGGGTCTTGCTATGACACTGAAGTGAGTCAAAAACAATGTATGTTTCTTCTCTTCACCTTCTC

>C101303_65

GTGTCCCAATATAAACATGAACATCATTTTTTCATCTGCATTCATAATGATCAAGGAGTCAACTGAAAATACACAGGATCTCCGCTGAATTTTTAAAATTTTGGAACAGCAATAATAGCCTACTAAATTCCAGTACAACACTTCAAACCTGAATTGATGTCCTGATCTAACAAGCATTTGTATTATTGCTGTCTGAAATTTCTCATACCTGACAAGATTTAAGGGGAAAGAACATATCAATAACAATGGGCAAACAGAAATGTCAAAGAAACACTTGAGAAAATTCCCGGAAATCGCAGATACCCTGGTTACAGCATATCCACTAATCAGAGTGACACCATTTCTCTGAATTGCACATCCTGTAAGAGAAATAAGAGACAGCAGAAAATCTGTGGCCGCTTATAAAATTAGCCACCTGCTTGTGTATTTGTATTGCACTATATATGTTTCTTTCTTTTAGCCTAGCATGAATTTGGGTGCGAAAGACACAAAATAAGACTAGCAACCCAAAATAAAAGCATGCACTTCAGCCCAACTAGAGACTGTGTAAAGAATGCCAGATAAACCCTTCCAATTAGAAGTGCTTCCTTCAACGAATTCTCTCTGTTTCTTAACCAAGCCAGAATACAATGGGACAAATGAAATACCAAACTGTGTCCCTACTCTCCTAAGGCTTGAACTTGAACCAATCACTATTCCTACATCTGCTTCAAGTAGGCAAAGTAAATCACCGACTGAATCTCCGATGTAAACAGTTATTTTCTTCTTGTCATCATTGCAATTTTTCATTATGTTCCGAAAAGCTTCAACCTTGTCAATGGGAGACTGCACCTTCTTAATAATTTCACCCGTGGAAACAGATTCCTCGTAAGTGAACTCATTAGCATGAACATCCAACTCATTTAAATCAGCTGAAGAGAAAGCAGATCTGATGAGGTCAACACACCAGCAGTATGATAGAACATGCACGTTGGCATTCAAATTTTCATTCTTCACAATGCTCTGAAAGAAATTAGTGCATCCATCTTGAAGGATCAGACGCTCTCCAGCACGCTTTATATCTTCTAGACTTATACCCTTGAGTACCCCAGACTCAATAACCCTATTATTTGCTGTGTTCTCAAATTTTGAAAGTTGCTCAAGGGCCGTAGACAATTCTGTATAATCAAAATCGTTCAATTTATCGGAAGGCATAATGCTTTCTATACATTGCTCATACTCCTCTGTATACTGTTTAGATAAAAAGCCCCATGTATTCCTAAGGTCAGAAGATGACATCCGAACAATTTGATCTTCAGGCTGATTCTGATCAGATCTTGGTGCCGTTACTATAGCAATTTCAGCCAAGATGGCAGACGAATCAACAACAGTGCATGTTAAATCAAAATCCGAAAAAATTATGAGATGATCTTCCACAGGCTTATGTTCTTTAGTCAAGGGTACTATAGTCGGCTGAAAGAGTGGCTGAGCAGCGAAGAAATCTATTTCAAGCTTCATTGCTTGGGAATAAAGCTTTTCAATGACATCAAGTTCTTCACCAGTCAAAGAGACACTTAGTTTGTCGAGCAAATCTTCAGTTTGCAGAGTAGTAGCCTGGAAACCATCAGAGGAATAGTTGTCGATCCACTTGTTATACGGGTGAGTACCTTCATTGGAATCCAAAAGTTCCTGGAATTTCTTTCCCAAAACGGCATAAAGCCTCATGCAAGGAGTCATGGCACCTAAAGTATAAGCAGAAATTTTTGTTTTCTCAAATGGTGTAGCAAGTTTACCAGGACCTTTTAGTCCTTCGATCTTCCCAGAAGCTGTAGCCAGCAGGAAATCTGTGTACTTAACAGTTGCAGAATTAATACTGTACTCTTTGGTAAGGTCCAAACCCCATTCCTGTACCAGCGTGTTATGCATCTTCAACTCCTCTAGAACTGCCTTCCTCAACTGACAGATTCCAAGTTTCGCATCGTCATCGTCAGCACACTCTTCAGCCATCTCATACGCTTGAGCAAAGGCGCGTAGGAAATGGACGTCTTGGGCGATGTAATGGTGGAAAGAATCGATGCTCAAATTGCCGGAGGCCAAGGCGATGGCGAAGGGAGTGTACATGGCGAAGATGGATTGGCGCGTGAACTTGATCCAGAAGCGTCTGGCAAGTCCAGCTTCGCTGCTGCTGTGGTTGTGGATGGCTGCCATTGGAATTGACGATCTTGGAACGGAGCGAGGAGAAGCGGAACTCGGCCAGTGAGGAACCAAAGCTCTGAAAGAGATTGAACGCCTCGGGCTCAGGTTCAGATTCAGCGGGCTTTTGATTGGGCTTCGGAGGAACCACCGCATGCGCATCCAATATCAATTACACACACGCGCCGATGCCTCTCTCTCTCTCTTTCTTTCTCTCTCTTTCTTTCTTTCTTTCTTTCCCTCTTTCTTTCTCTCTCTCTCTCTCTCTTTCTCTCTTTCTCTCTCTCTCTCTCTCTCTCTCTCTCTCTCTCTCT

>C101305_65

GTTCATTTAGGGGAATATTACAATTTGCCAATAAATTGAAATGAGAAGAATGGAAAATTGGTTTGGAGGGTTTAGTATAGTATAATAGTGTATGTATGGTGTGAGAAAGAGAACTTCTCTTAAATTCATTTTAGTCCATTAGTTTATATCTTTTCTCTTGGACACCCGTGACTCCCATTTGTATCTCAATTATCGTATGAAGAGGAACCTCAATATGCGGAATTTCATCCAAAGGCCTGTCAGCAAAATATGCATATAGTGCCCTCAAAACTGCCTGGTGAGATATCACAACAACAGGTGCTCGTTGTCGCTCAAGCTCTATAATTACAGGTTCTAACCTTTGAATAACGTCCAAGTAAGATTCGCCACGAGGGTAACGATACCTAAGCTTGTCCTTCTTGCGGGACTCATATTCCCCGGGCATGTTTTTCTTGATTTCGGCGTATGTCATACCATCGCACACCCCTGCATTTATCTCGTCAAGTGCACGCCATTGTATCTTGGGAAAACCAACAATTGGACTGGCTGTCAAAATTGTTCGTTGTAGTGTACTAGTCCATATACAAGCAGCCCGTTCTGATTTGAGACGCTTTCCGACAAACTTGGCAAGTTTCTTTGAATAAAGTTCTCCAGCCTCACTTATTGCAGTGTCTCCTCCAATTCTGCCTCTCACATTATCCTGACTTTCCCCATGTCGAGTAAGTAATATCGGGCGTGGTGTAAGATGTGTATTTACCAAGAAAAAGACAATTCGTCCAGGGAGGTAACCACTGATATTGTTCACTTGTATTTGTCCACCATGTCCACTGACCATATCAATCATTTTTATGTAAGATCCTTCATTTACTGCTTCATAAACCTTCTCATAATTGGCTAGACGATTTTTAAAGTCCTGCAATCCAGCTTCAAAATCTGGCTCTTCGGCATAATCAGGACTTTGCTGAATTTTCAGACGAACATTCCTTTCAATTATATTTACATCATTGCATATTGTTTCAAGGAAGATTATCTTGCATCTTCCTTCTGCCAATTTCATCAGCATATTTCTTCGTTCCTTACTACTGTTGGTGGCATCAAATATCCCAACCTGGCCACCTTCTTGCATCCAAGAGATCATATCTTCAAATGCCAGTGCTGCTACCTCATTGCGTGCCTCCATGCCTTCAGGATTATCGGCTCGAAAGAAATCAGCAGACTGATTAGCTCCATGCTTAAGGCGACGATACTTCCCAACATTGAAGTGTTTGGTATTATGACCTAACCAACGAAGATATCTTGTAAGTTTAGCTGCTGTGAAAGTTTTACCTCGGGCTGGCAGACCCACCAGGATAATTGCCAAATGTCTATCTTCCTTGGGACCCATCATCTGATCAGCAACAGCTGCAGCAGCAACAGCTCCGGCAGCAGCTGGCATTGAATTCTTGGTATCATTGAGATCAGTAGAGAAAATATTAGAACTAGAAGATTTTACAAGTCTAGGAGATCCAACACCTCTATCAACAAAATGTCCCCTCTGACTCTCTTGCCTTTGCAGAGGCGAAAATGTTCCAACTGTCTTGGATTCAACCATTCCAGAACTTTGAAATATCTTAGATGGATCAGGCACAATAACCTCCATCTCCTTGATGGTATATGAACTGCTGCCGGACCCACTAACTAGAGACCTTGGATTCTCTGTCAAGTTAGCTGCATATACATGGGCTGTATTTGCAGAAGTTTCTGGAGTTGGGACAACATAATGCTCAAGATCAAGTTCCAAACCGACAAAAGAACCATTCTCACCACCTATCTGAGGTGCTGAATTTATGCTGACATCAGGCATCCATCTCACAGATGACGGACGGAAATTATCCTGATAAGCCCTCCAACTAGCAGCAAGGTCAAAGGGAGAAACCCTGTCTGCTTTAACAAACACTTGATACTCAAGAACCTCACCGGTATCAAGCCTAAACAATGCAAGCCTGGCATCCTCTTGCAACGCTCCCCCAACAAGCAGGCGGCTTGGACCCTCCTCAACAAAACAAGGTGCATTGCTGTCCCTAGGCTTCAACAGGAATTTGAAATCCAAGGTTTCGTGATTAGGAGGGACAACAAAGCTAAGTTCCCACATTGACACCGATTCCCGTTCCATGAAAAGAGCTTTGGAAGGATCCCAAGAGCCGACTAGAGGAAGAGAGCCATAAACGTGAGGAACAAGGTCTCCAGTGAGCTTGTGATTCTCCATCTTGAGAGATACGTAGAGTTGGCCACCAGCTTGGTCGAGCCTGTCACTCTCTTCCTTGCTCCCTTCCTCCGCGTCCTTCGATGCCCCCGTTCCCATCTCTCTGCGCTGATTCTCCTCTGATGATTCTGCAAAAGCAAGAAAATTGTGTGTTTGAAAATTGTAATTGTTGTGTGTGGATGTGAATGGATTAATGGATAGGAAGAAGAAGAGGGATGGGTGTGTTCGGTGTGATATGGATAAAGAGGATTTGGGAATTGGAAAG

>C101313_65

AGAGAGAGTCTCAATCCTTCAAGGGCAAGTCCTCTCGTCGTGTTTTCTCTTCTCTCTCTTTTCTTCTCTTCTCTTCTCTTCTGTTCTGCTCCGAGATCCGCCATGTCCATCTTCTTCAGATTCTCTCTCCTCTTTCTCCTCTCTCTCTTCCTCTCTCCGGCTTTCGCTTCCGAGTCAGATCACAGGTATCAACCAGATGATCAAGTTATCCTTTGGGTGAACAAAGTTGGTCCTTATAACAATCCACAAGAAACGTACAATTATTACAGCCTACCATTTTGCCGTCCACCTGGTAATCCTGCACACAAATGGGGTGGTCTTGGTGAGGTCCTGGGTGGCAATGAACTCATTGATAGCCAACTTGAGATAAAGTTCCTAGGAAATGTGGAAAAGACAACCTTTTGCCGGGTGGATCTTGACGAGGCAAAGGTTAAACAGTTCAAGGATGCAATCGAGAATAATTACTGGTTTGAATTCTTCATGGATGACCTTCCATTGTGGGGGTATGTTGGGGAGCTACATACTGATAAGAACAGTGATAATGGCAAGCATGTAATTTATGCTCACAAGAACATCAATATTCAATACAACAAAGACCAGATCATTCATGTTAATCTAACTCATGACAACCCAAGGCCACTGGAAGTAGGAAAATCTTTGGACATGACATATTCTGTGAAATGGTCTCCTACAAATGTTACTTTCGCACGACGATTTGATGTATACTTGGACTATCCATTCTTTGAGCATCAGATTCACTGGTTCTCCATTTTTAATTCTTTTATGATGGTCATCTTCCTTACTGGATTGGTGTCAATGATATTAATGCGAACTTTGAGAAATGACTATGCGAAGTATGCTCGGGAAGATGATGATTTGGAAACTCTGGAAAGAGATGTTAGTGAAGAATCTGGCTGGAAACTCGTGCATGGTGATGTTTTTAGGCCTCCTCGCAGTTTGGTTATTCTTTCAGCTGTTGTTGGTACAGGTGCTCAGCTTGCATTGCTGGTTCTTCTTGTCATCTTGTTGGCTATTGTTGGGATGTTGTATGTTGGACGAGGAGCCATTGTCACGACCTTCATTGTATGCTATGCTCTCACATCTTTCATTTCTGGTTATGTGAGTGGGGGGATGTACTCACGAAATGGGGGTAAAAGTTGGATTAAATCCATGATCCTGACAGCATCACTGTTCCCATTCATGTGCTTTGGAATTGGTTTTATTTTAAACACAGTTGCTATATTCTATGGATCTCTAGCTGCAATCCCCTTTGGTACTATGGTTGTTGTCTTTGTTATATGGGCTTTTATCTCTTTCCCCCTAGCACTTCTTGGTACAGTTGTTGGAAGAAACTGGAGTGGTGCTCCAAATAATCCTTGTCGCGTGAAGACCATTCCTCGTCCTATTCCTGAGAAAAAGTGGTACCTCACACCGTCTGTAGTTTCTCTGATGGGAGGACTGCTTCCCTTTGGCAGCATATTCATCGAGATGTATTTTGTATTCACTTCCTTCTGGAATTACAAGGTGTACTATGTATATGGCTTCATGCTACTGGTTTTTCTGATTCTTATCATTGTTACTGTTTGTGTGACAATTGTGGGGACATACTTTTTGCTAAATGCTGAGAACTACCACTGGCAATGGACTTCTTTCTTTTCTGCCGCTTCAACAGCTGTTTACGTGTATTTGTACTCAGTTTACTACTACTATGTGAAGACGAAGATGTCAGGCTTCTTCCAAACCAGCTTTTATTTTGGATACACTTTGATGTTTTGTCTTGGACTGGGAATTCTCTGTGGAGCTGTAGGTTATCTTGGTTCAAATTTGTTTGTGAGGAGGATCTACAGAAACATCAAGTGTGACTAAGGCTTTTCTAGAAGTCTGAGACCAGAGCACTTCCAAATGTTTGTTGCTGAAGACAATAAATTTCTTCGAGGAGGTCTCTGGTCATGTGTGGGGACTAAACAGTGTTTACAGGTAGTAAGTATCACAGGGTTGAGGATGGGATGTATTCTTTCTCTATATTAGGGAATGTATTCTTTCTCTATACTAGGGAAGCCCTGAGATTTTTTTCCCTGACTGTGATTGAAAGCCTTGATTAGAATGCAGGGATTGGTCTTTATAATTTAGATAGTTAGCAAAATATCAGACTCGTTGCTCCTCTTTTTATCCTTTTATAAATAGGTGCCCGATTGACGGACTGATGTCTGATTCCCCTACACGGTTTAGCTATTGTACGTAGAGGGGATGATGTCACGGAAATGAATTTTGTGCTTCTAAATTTTGTATCAAATCTGAATATCCTTTCTAACTACTTCAAGCCTGCACATCTCTCCTTGTTTGACTTTTAAATCATTGACGCTTTCCCCCTCTCCAATGTTTTTTAGGCTGCATATATTTCTTCCTTTGTCTGGATCTGTAGACACGATGTTAACCATGAATAAATGAGATGAACAGTTTGTTCAGCTGCATACTCGGTTTCTTAACGGT

>C101315_65

TGCAGCACTCAAGGTACGTAAATAAATATGCGTAACACAACTATGCTCCGAATACAATTCATAGATGTCTCCAAAATTGAACGAAATAAATAAATACCAACAAAGGAAGCTATAATGTTGCAACTTATAGAATGACAAGTTTAAATAACAATAATGACAAAATGAGGCGAAACTGTATAAAGTAAACTTAACTTGGTTTGCCATCAAGGCACCTTAAACTCCAACAAAAATATCTAAACACGAAATAACAAAACTTTTGGCAGCCACGAAAAATGCCAACAGATGTCTCTAAATTGCAGAAAACCGCATCCCCTGTCCTAGCGTCTATCAGTTTTTCCAGAGGAGCGCTTCCCAGAATATAAGTGCTTGGGCTTAAGTGTAGGTATGACTCTATCAGCTTCTCCACGACGGGCATCCTTGTTTCTCTTCTTGACAGATTTCTTTGCAAGTTTAATTGCCTTGACCTTTTGAGCAGAATCCTTGAAGCCTTCACCAGGTACAACTTCATGTGGCGGGCGTGACACTGACCTAGACCGTGACAGTGATCTACTCAGACGCTGTTTTTTGCTAGGTGTGTCACCATCAATATCCATACCATCTCTTCCATCTGCACTTCTTTCAGGTGATCTTCCCCTTTTCCGCCCTCTTGTAGAGGCAGATCTGCTGCGTATTCTCTTCATTGCCAAAGTCGGATCAAGCCCCAAAGACGACAGCTGCCTTCCCATTCTTTCTGATGTGTACTGTTTGTCCTTGTCAAACTTTCTAGGAACAGTTGGCCGATTCTCGGCATTGCTCTTTTTAATCCTGTGCTGCTGGATGACCAAGCTTTTCTTTTTCCTAATGCTAGCTAAAGCTTCTTGCTGTTCAGGCGTCAACTCAGTTCCATCAATCTCGAAATCATCATCTTCAGCTTCAGCCTCTCTCATCCCTTCTTCTCTTTCCAACTCTTCAACCCTTAGCAGAATATTAGGATCGATGAAATCATACACATTGTGTCCGTCCAAGATTTCAGGCAGTACATCCTCTTTCCATTCATCATTGGCTAAAATGTAATTCTTTCTCAAATTCATTGAGTATACACCAGCCCCACCATTCTCCTCCTCCAGATCCTTTTCAGTCTTTCTCTTTTCCTTCTCAGCTGCTCCCTTAGCTTTAGCTTCTAAAACTGCTGCAGGAATACATGGGGGCCTATCCTTCTGGTCACGAGGCTTTGGTACTGCAACATGAAACCTATTCAAGCAGTCATTCATTTTCTTCGATTTCATCTTTATTTCTACCCTCTGATTCAATAACCTATCACAGGCTGCATTTTTTACAGCAATTACCCCTTCTTCCGTTAAAGTGCTCATGGTCAACAATACACTGTCCTCGTTTGTTGCCTCACCTCCCTGACCAATTATAGTCTTCATGGCTTCAGCTTTCATCTCCATGACCAATTTCATATCTTCCTCAGATATCCCATCCAGTGGTTGCAAGTCAGTCTTGTTGCAGACTATAATCAATGGCTTGTTCATAAACAAGGACTTAATGCTGTGAAACAGAGCAGCCTGCTGAGCAATACTGTAACCACAAGACCCAGAAATGTCCAAGAAAAACAATATTGCAGCTCTCAAATGTGCTAGAGCAGTGATACTGCACATCTCAATAATATTACGATCTTCAAAAGGCCTGTCCAAAATCCCTGGTGTATCAATTACTTGGTATCTCAGGTATTTATAATCTGTATGACCTACAAAGAGAGACTTTGTAGTGAAAGCATAGGGCTGCACATCCACATCAGCTCTTGTAATCTTGTTAATGAATGAGCTCTTGCCAACATTAGGATATCCACAGATCAAGATAGTCCTCGTATTTGGATCAATCGACGGGAGCCTAGCCATGTGCTGTCTGACCTGTTCTAGATAAGCCAAACTCGGACCAATCCTCTTTATCACAGTGCACATGCGGCCAAGAGCAGCCACTTTAAGACACTTGCAACGATAAAGCGAGTCACCATACTTCAGTAATTTCACATAGTCTTTCGCAATCTTACTAATAAGATTCCTGGCAGTGTTGATTTGTCCAAGAGCAAGCTTGTAATGGTCTTTGTTGTATAGCACATGGAGGAGATCGCCATAGAACGGATGAATATCATCAAGCCGGGGGAACTCATCAATGATGGTAGAGAGCTTATCATAAAAATTCTGCTGGGTATACTTCACTTTGCGCATGTAGAATTGGCGGAGCCGCGAAATTGCATATCCTTTGTGCACAACAGTCGGTGTCTGACGCTGGGTACGAGAGAGGATGATGTCAACGAAATCCTTCCCATTTGGCACCACAGTAATCTTCTTAAAATTATACTGCACCATCGTGCCCTATCGATCTTCCCTAAAAGACGTTAGCTTCTAGGGTTTGTGTTGTATACGCGAGCGAAGGGTTTTGCTGTTATGCTATCGATCTTATAGACTCTGTTTTTGTTGGAGGCTGCACACCACAGCTCGCTGCACATCG

>C101319_65

ATAGCTATATAGGCAAAGGATAATGAATATGTATTGTTTGTGCCTGTGATAACACATTTGTTAAAAGGCGACCTCACCAATCCCATCTAAGTCCACCATTGTAATTAAGCATATATACAAGGGATGTAAGAACAGAACTACGGTGGCAAAAATATGAACCTTAATAAGAATATCTAAATGTTGTCATATTTAGCAAAATTACACATGGACATGATGTTTCATTATGGTCCTTGTGTGAGTCTGTGCAACTCATTCTGGTCTGTGAGAGTCTCAATGAGAAGGGGTGGTGCATACTGGGCTGTCCCTTGGTGCTCTTCAGAATCCTGGCAGAATGAGAGAACTATGGTATCAATTGACATCTCCACAACTGCAAAGAAAAGGGTTGCAACAATGTATCCAAGAGCCCAGCAAACCACAACAGGTAAGAGTGGAGACGAGGTCTTGTTATGAGCAGAACTGTATTTGTGAGTGTCTAACATGAGGAACGCAAACAAAGCACATGAGAGGCTGACACAAAGTTTTCCAAGGAACAAAATGACATCTCCAATCACGTTAAGACGTCCAATCTTTAGAATGTTGTTCATTATCAGATCTGTTGCGATTGAAGAAGCATTAAAGAAACTTTTGCCTGTTATAGCAATCATGATGTAGGCATTTCGGTTAACTGATTTGATAGTCCATTCAATACACTTGAGAAAACATTGAGAAGATTGATACGCAGCCTTGCCTATGCAGCTGTCATGCCCATCACTTGAGCCTTTTAGTTTGCGACGAATAGCTTCAAGCAGAAAGCGTATTGATTCTACAAATGATACAATCAGAGAACCAAGAGCCACAGACCCAAGACTGTATCGCATAAGCCGCTTCATGGAAGAAAAGACAGAAAGAAAAGGAATTTCTGGAGATGCTTCACCGTGGTTCCAGTAATAAGAGGCGACAGACCCAGCAATCACAGTTGAGGAGCTAGCTATGAAAAATTGTGTAGCCCAATAACACCCAAATAGGTGGAAAAGGATGGCAACTCCAATATGTGGTGTGTAATGAATGCTATAACCACAACAACGATCACAGTTTACCCGTTTTGCAATAAGATCATACGTGCAGCAGTTAGAGTTGCAAATATTCTGAACAACCTGCCCAGAGCTGAACAAATGGAGAGCAGCTGAAATCCAGAACATGTAAAACACTGCAAGGATACCATATGGTATGAGTGGGAAAATAATGAGGGCTTGAACTTCTCCTATCACTTTTGCAGCAACCTTCAGGACAGATGTTGCCATAAGGATGCGTCGAACAATAGCAATTGATGTAAGAAAGGCAACAACCATGATAAAGGTCATAAGTATAGTGACAGCTCGTATATGGGTCAGCTCCCTCCCATATACACTAATGTAAGGGTCATGTTCACCAATGATGGGAGAAATAGCATCATTTCCAAACCATCCAGCTTTTAGGTAGTAGAACATTGTGACAGATATTATTAGAACATTAAAGAGAACAACTGTTATCCAAGGCATGGCAGCAACAAAATGTCGAATCATCATCAGCCAAATCACAGACAGAAAAAGAGGCAAAATCCCTCCACAAACAATCAGCACAGGCCATGCCTTTCCTATGTCAGCCATATATCTCTTTAAGACAGCTGACCGAGAATTAATAGACTTGTGGATGGACTTATCAATAACAATGTCTTCATTGATTTTAACTCCGCCCATCTGCTGCCAATGCTTCAAAGACGTATTTGATGCTCGAGCAATAAATTGGCAACTCCAGTAAACATTTACACTGGGAAATATGACAGGATAACATGGACCCTGAAGTTGAAGAGAGGAATTTCTCATTTCTGGTGTAAGGTACTCGAAATAATCATAGGTCCTACCAGCCCAATCTTTCATAGAAAGACGAATATCTCCTTCTGGATAATCACACACCCAACTAAGGGAATCTTCAGAAGGTATAGGGCATTCAGACAAGCATATACTGCGGGCATCGACCAGTTTGAATTGGCTATCCTTCAATCCACTCTCATAAACCTGATTAGGATTTTGCCAATATCTGAGCTCCAATTCCCTAAGGCCAGGGTGTGCATGCTTGTCCCCACAGACATTCCCTTTGTAGTCCAGACCATAAGTAAGCCTTAGTGGGTTTCCTTGGTTGAAGCCAAAGCTGGAGTTGACAACCATTGCTACCCAAAAAGCTATAAAGATCACAAGAACAGTGATGTCTCTGCATTTCCTGTTGTGTCTTATAATTCCACCCATTTGGGTAACTCCATCACTTGATGGAAACCTCCCTATTACTGCACCCAAAGGACCCCTCATTCTTCAAGTGTGTGTACGCGTCTCAACCAAGTTGCAAGCTTTGCTTCAATTTAGCATTGGGAGAGAGGAGAGTGAGGAAGAAGAAGAAGGGGCGGAGCAGAAAGAAGCTAGGGGAGCGTGAAAGGGTGCGCAAAAGCAAGAGAATTTAGATTTGGGTTTATATAATAATGAAA

>C101321_65

AATAAATATCATTAAATTTTAATGCAATATTACATCTCCATACAAGCCTTTTGCTACTACATATACACATTGATTTATTGGACAAATCCTTAAGTAACTGTATCAAAGTATACACATACAACGGCTGTGCAGTATGATAACATATCCTTACTCCTATTTGCAAGTTAGTGTTTTTGAGAAGTGAAATCTGGCTCGGTTGGCTTTGGAGGTAACAGAGGCAGTAGTCTCTGTTGCATTCTATTGTGTCTGCTATTTTCTCTCAAGTTTCGCAGTGCATTGGCAGCAAACCTTGATGCATATATGGTTGCACCAAGGCTTAGAGTGGAGCCATCCTCCTTTGCCAAAGCATCTTGTAGCTTGTCTTCTGCTTCGTGCAAAGACCTCTCAATCTTCTTTTTCCAGTATCGACGCCAAGCTGCTTGTATGAAACAGGCAGCCCATGTATTCCACTGTGAGGAATAGAACCTGAAAGCGTGTTGGAGCTGTTTGCTGCTAAGACGGCGAAATTGGGAAGCAACATGCTTCAAGTCATCCGCCATGAGAGCAAAGGCTTCAACTTCTGACATGGTTTCTACTGTCCTAGTTGACATGGGTAGATTCGAGGAGGAGTTAGGATCCAAGGCCCATGTTAGAAGTTCCTCACCACAGAAGGCACCAGCTTTGAGAACCAAGGAGTTGAAGAAACCAGTTCTTCCACCATTTGTTGTCACAGTAGACACTTTTCCACGCATAATGAAAAGCATCTCATCAACTGGATCTCCTTCACGAAAAATGTGGCTCTTCTCAGTATAAAGGACTGGCTTCAGTCTATTGCACATTGCATCCAACAACTGCCCATCCATCTTCTCAAACATTGGCACCTTTTTAACTAAAGCCAGGCACAGGTGGCGCTTTATGTCCCTCCTGAGATCTTTAGGAAGTTTACCAATTAATGCCTCCTCCTCAACACCCCTATTTTCTTGCCATTGGTACTGCTCATACCGTCTAATTCTTTCCTTCAAGTTCTCAGGTAGCATACGGTGGGACATCCACTGTTCTGCGTCCTGTCTCTTCACTCTCATCTCCTCCACTCTAACAGTTGTAGATTGCAGATATTTCTGCATGTTTCCAATAAGTAACGAGAATAAAACCAATCCAAAGATGGCAATGAAGATTGCAAAGATTATCTCTGGGACATAAGTACTTGTCTTGAGATTTTGTCCAAGAGAACTTAAACCGCGCAAACCCCACCAAAAGCAATAGAAGAACTTCTGAGGAAAATCTGTTGTTGATTTTACCACACCTGAATGTAAAGCATCAAAAAATATTCCAAAATTGTAGATGTTTGGGCCTATGATATTATCAGGGTCAACCAAAGAACAAGTTTCGTTGAGACGTGAAATGATATCTTGATTGCGGTTTCTACAACTCAAGTCTCCACTCTTCAACTCTCTGCGCCAGCAACGCACCTCTGCCTCTACCGAAAGCAAATACCAGAAAGCTCCAACCACATGACTTGCTAGCATATAAAGAAAAAGATTATAAGCAGCTCCAGCCCCTGCTGTCTCAGTCAATATGCCAGAAGTTCTAGTTACTTCTCTGAATAGTGGATAGATCCGCAAAAGCCTTGGCACATACTGGATTAATACCGTGTACTTCAACAAATCCTTTGCCACAAATGGATCTGTTTTCATGTTTGGATGTATGGCTAAAACAATCACCTGTGGAAGTGGAATAATTGATAGAATATCAATGATGAAGTGTGAAGTTAAGTATCTTTTCGTTATAGCCACAGGATCATCAATGAGCTCACCCCTTCCAAAAACACGAGAAGAAGGGGGAATGAACCCTGTATTAAACTGAAAGATTATGCGAAGAATATAGAATAGATCAAAGAAGGTGCGGAAAACACTGGCAGTGATCTGCAATGATCCACTCAAATCAAGGCATTTTTGACCATCATTAATCACTGGAATGTAAAAGAAGAACGGGTCCACTGAGACTGCCAACACACACGTTATTACAAAGATTTTGTTCCATTTCTGAAGTATTGGACCCTGTGGATCAAGAATTTTCTTCTTATGTGGCAGCTTTTTCTGTCCATCACTTAAGGGATGAACAACTGAAGCTCTTCTCAAGTTCTTCATCTTCTCTGACCCACTCTCAACCCTTCTTCCTATACTCTTCAAAACAGCACTCACACTTGGTCTTCTTTTGTGATTTCCATCGTTAATAGAGTCTTCTTGCTCCATGCTAGAAGATGACTCTGATTTCCAGTCCTCAAACCTCACAAACCTACGTCCTTTTGCATTCATGATTAGACAGACTACTTCAGCATTCAGTGAGAGCCACTAAGCTTTGCAGAAACAGAAGACTCAGGCCTTAGATATGGAAAGTTTCACTACATGCAGAGGAGGGAGAGTTTGGACGCTTCAGAGTTGAGAGAGAGATGGATGGAATGATAGTGTGTTTGTCGTAAGTAGTGAAAGAAAAAGAGAATGAGAAAGAACAAAGAA

>C101323_65

ATTTGATTTAATATCCTATTGCAAGTTTAAACTTGCAAGACTCTATCATCAGAAAAAAAAACTATACAATATAACAACGATGACCTGCTCATATACATTCCCAAACTGACAACTATATAAACAGTGAATTCTATATGCAGTAGAATACAAAAATCATATAAAAAAAATTGACATCTCAGCCTTCGTTTCCAGAACAGCATATTTCTGAACAACATTGGATGCAGCCAAAGCATCCTAGACCAATGCAGAGTTGTGTCAATGCAGTTGCACAATGGTTGTTCAGCCTGGTACAGCACTGGATGCAGCACATGCAACAGCAGAAACTGCACATTGTTTCACAACAATTTGTGGCCGCCTTAAAGATTTCAGGAACTTCAGGCAAGTTCGATTCATGTCCGGGAGGAGTATTTTCAGCAGTTGCAGTTAAGTTGCCAAAGCCGATACCCAGATATGGTAGTAAAATCATTTTCTGGCCTTGAACGGATGCCTCATAATGGCTCTTTTTAGACACTTCTTTCGTTCCGGCTGATTCCTTGACTTTCTTTAGATGATCGGTCTCAAGTAATACCATTCGTGTATACTCGGACATAAAGCCAGTCTGCAGGCTCAGTTTTGCAACCTTCTGTTCTAGCTGTTTATTTTCCAAAAACCATGCTTGAGCTGTAAGATGCTCTATTTGATCTCGAGCAGAAACCCTTTGTACAGGCATGTCCTTAGCATTTTGTATCTTCATATCTACTTCAAAATTACTGAAATCAGCCAAGATACCTTTGACTTTAAGAGTTTTGGGAAAATTTCCCCTGTATCTTCCATATAAAATCAATGGACCGTCTGATGAAAGATCTGGAATATGAGAAGGGTATACCTCAAGTTCATCAAGATCATCCAATGAATCCAATTTGATATTTGCAAGAATGAGAGATGAAGCTTTGTTAAACAGTGTCAGCATTCGAGCCTCAATCCAATCAACATCCATTGCAGCATCATATTGACCCCTACCAATCATTGCAAGTGTCCGCAGGAAATAATGATTGCAGAATAAACCAATGCCAAAAGTGTAAATTCGAGGGCATATTGACTCTCCATTGATCATACGATTCTTTACCATAGCACAAATTTGTCTCTCATCTTCAACAGTTCCATCTGTAACTAGGAAAATTATTGGAACTGAATTTTGAGAATTGGATAGCATCTCTATTGCCATGTTTAATGGACGTGAAATATTTGTACCACCCCCAGCAACAAAGTTCATTTTAATCCACTCAGTGGCCCTTTCAACAGCATCCCCGGAAGCTAATTCCATCGATTTGGAAAATAGATAAGTCTCTCCATTAAAGGCTACAATATTAAATGAATCATCATGATTAAGCTTGGAGAGAGCAGCCAACAGTGCATTTTTTGTGTCTTCAATTAGCTTTCCCCGCATGCTACCACTGATGTCAATAATGAATATTATTTCCTTTTTTAATACCTTCTCACTTTGAATATCTCCTGGAGAAAGATACAGGTAGAACATTTCTCTTTGGTCAAGATCATGCCCGGATGCAGATTCCAAAAGAACACCACCATTAATATGACTTGAAGAAACAGCGTATGAAAAGCTAAAATCAACTTTAGACCAGGCGAGAACATCAGACTCATATAAGAAGCCCATACTACCCGCATGACGACTCACTTGCTTCATTGGGTGGCTTATAGTCTTGCACAGAAGCTCATCTCCAGTAACAGCATCCAAATTAATTCGTATCTTTTCTCTTTTAGGTATCTTCTTTCCCGCAGGATTGACAAAATCAGGAAAGTTAAACGGCACATTCAAAGAGAACTGGCCATTGGAGTACACTATTTTCTGAGACCAACGAACTTTAATCGACAAATTGGAACCTCCATCAATCTGTGGTATAGTTAAAGTAAATATATTAGGGGTGAGAAACCCTCCGTTTTGAGGTGGCGCGATGTTTTGGTTTCCTTTGTCATCTTCCATGACAACCAGTTCAGTAGAATATGATTTTCTAGGCACGCCAACCTCAACACCTAGAATTGAACCCTGGAGGCCCATGGGAATAGCGATACGGCAATCGCAGGAACGGCTTCCCTTGACACAGTGGAGTCTCCAAGTGCCGGAAACGGTGACAAACGCGGTGTCGTGGTGACAATCAGCTTCCATCTGAATTGCGTACATCTGAAGCGGAATCAGCGCCGGAGGATCACACCTTCCGTGCACGTGCGGCTGGTAGCTAGGGATGTCGGGGTTGTCCACGATCCCTGGATCGTAGATCACCGCGTATACCATCGCCGCGGAAGGGAGGAAAGTGGTGGCGGAGCGGACCATCGGCGGCGGAAGTTTCGGCGGCGACACCGCCCGGTCCTTCCCGAAGTATACGCGCTTCGAGAGCTTCAGTCCGTCCTCGACCGCCTTGGAGAAATCCTCCGCCATGACGGAGTAGAAGAATAAGAAGAAGAAGAGAGAGAGAGAGAGAGAGAGAGAGAGAGAGAGA

>C101325_65

GTTTCTTCTGCATTCATTAATTTTCTTAGTTTCTGTGTTTTGTTCACTTCTTCATTTCCTTTGAACCTTAACCCTTCCAAACCAACATATATATAAATCAATTCTCTCTTCTTTTTCTTTCTGCGGTTTGAACTGAAAGAACCTCACTAAACCCTCTTTCTTTCTAGATCTGTTCGAACCCCTAACCATATCCGCTCCAATTTCCATTTCTCCGAACAATACCAAACCCTAACCCTTTCTCTCTGTATGATATCAAATTGAGGCGTAGTTTTCGTGTTAATTTGTAAATTCTTTCGGTAGTTTAGGTTAGATTGAGTTTCTAGGGTTTTGAATCGGAGAGATGAACCACCACCAAAACAACAATAGAGCGAGGTACCCTCCCGGAATGGGGCTTGGGCGAGGAGGTTTCCATCCCAACATGGGCCACAATCCCAACCTGAACCAGAACCCGAGCCTGAACCAGAATCACGCGTTTCAGCCTCGGCCACCGTACCAGCAACAACCGCAGTACGTGCAGAGGCATTTGATGCAACAGCCGCAGCCGCAGCAGCAGCAGCAGTGGCTGAGAAGGGACGCCAATGCCGTCGATGAGGTTGAGAAGACCGTCCAGTCTGAGGCCGTGGATTCGAGCTCACAAGATTGGAAAGCAAGACTCAAGATTCCACCAGCTGATACACGCTATAAGACTGAGGATGTAACGGCAACAAAAGGAAATGAATTTGAGGATTACTTTTTGAAGCGCGAGCTGCTTATGGGAATATATGAGAAGGGTTTTGAAAGGCCTTCTCCCATCCAAGAAGAAAGCATTCCGATCGCTCTTACTGGTAGTGACATTCTTGCTAGGGCTAAAAATGGAACTGGCAAAACAGCTGCATTTTGCATTCCTGCGTTGGAAAAAATTGACCAGGATAATAATGTTATTCAAGTTGTTATACTGGTCCCAACCCGAGAATTGGCTTTGCAGACTTCTCAAGTGTGTAAAGAACTTGGAAAGCACTTGAAAATTCAAGTTATGGTTACAACAGGTGGTACCAGCCTGAAAGATGATATAATGCGCTTGTATCAACCAGTTCATTTACTAGTTGGAACTCCAGGAAGAATATTAGATCTTGCTAAGAAAGGTGTTTGTATTATGAAAGATTGCTCTATGCTTGTTATGGATGAGGCTGATAAGCTTCTGTCCCCAGAGTTCCAACCTTCTATAGAGCAGCTGATTCATTTTCTACCTACAAACCGCCAAATCTTGATGTTTTCAGCAACATTTCCAGTTACTGTAAAGGATTTCAAGGATAGGTATCTTCATAAACCTTATGTCATCAATCTTATGGATGAGCTAACTCTGAAGGGTATTACGCAATATTATGCATTTGTTGAAGAGAGACAGAAAGTCCATTGCCTAAATACTCTATTTTCTAAGCTGCAAATAAACCAGTCTATCATCTTCTGCAACTCAGTGAATCGAGTGGAACTACTTGCCAAGAAGATCACGGAACTTGGATATTCGTGTTTCTACATTCATGCAAAGATGTTGCAAGACCATCGTAACAGAGTGTTTCATGACTTCCGCAATGGTGCATGTCGAAATCTTGTTTGTACTGATCTTTTTACTAGAGGAATAGACATTCAGGCAGTTAATGTTGTTATTAATTTTGATTTTCCCAAGAACTCTGAGACTTATTTGCACAGGGTTGGTCGTTCAGGGAGGTTTGGGCACCTTGGACTAGCAGTGAACTTGATTACATATGAGGATCGCTTTAATTTATATAGGATTGAACAAGAACTTGGAACTGAAATAAAGCAGATTCCACCGCAAATTGATCAGGCAATTTATTGCCGGTGATTGGTTGTAAATACCAGGGTTGTGTTTACAATTGTGATAATAGCAGTGGTTAGTGTGACCGTGGCACAAGCAGGTGCTTAAGAGGATATGCTAAGCTAGTGAATAGTGTACTATTGGACCCTGGCATGAGAGGATGCACTTTCAAAACTGGGTATTATTGGACCCATTATTGGGTGCTTTTCGATTAATTCTGGTTTTGAGAGCCCTGAAACATTATTGGGACCTTTTAGAGTTTAATATTGGCTTTTAACTGCCAGATAGTTACTGGGTCCAGTTTGGGTTTCCGTTAATTACATTTGCAGATTTACTTTGTCATTACATGTTTTTAGTATTTTAGTTTTATAATTGAATGGTTTAGGGATAATATACCTTATTATGAATATCTTCCTTCCCTATGCTATTGGTCTGCCATCATGGGTTTTCTTATAAATTGTAGATTCTTAGATGTAGAAATCTCAAGCTGAGCTTATGGTTGGTTCATGGCGGTGCTGGTAATTGGATGTCGCCAGGATTTTCATGGCCATTGTCGTTGAAGCCTTGAAGGTCTTACCAAGTTGTGGAGCCTTTCATTTGATTGTTTTATTTTGTGAGGGGCTCATTGGGATCCTCATCAATTGTTAATTTCTTGAGCTATGAAGGCACATGGTACTGAAATA

>C101331_65

AACCATAATAAAAATCTTCCAATTCCATGATCACCAATATTCCAAATACAGCTAAAATTTCATATATTTCCCATCAAACACGGGCAACAAGAATATCATACATAAAGATAACAAATGAGTATACAACCGATAATCCTAGCTTGTACGTGTTCATAGTTTAAGGAAACTATAAACACACTCTTTGTAATTCACTCTGTCATTGATTAAAATTTATTAAGAATCACAAAATAATAAGAGAAACTGATCAAATAAAGAGTGAGGCACACACTATTTGTGAGTCGAAACAATTTTCAACCATTGACAGAGTGTATTAGAATGTACATTGCCATCATTCTTCTCAGATTTTTTGTAGTTATGTATATTCTTTTAAGCAACTAAACATCAGAGAACTATAGTTCTTCACGTCACCAAGAATGACATATGTTGCTACTATGTATGGTACACCTCCTTTACAATTGATAGTTGATGACGGTGATGTCTTGCTCCACTTCACCTCTCCCACAGTGACACAAAGAACACATACTATTACCAGGATATAAAGAGTTAACAAAACATGGCTAGTGGTAGAGTGAAACAGTAAAAGATGGTAGCAGGAAGCAGAACTAGCAATATTCATCTCCTAGCCAATTATCCTTCTCCTTTGGACCAGTTGGCCCCTCCTCACCATAAAGCTCTGTTGGAAATAGTTCAAATAAGTTCTCCACAGAAAATCTAACAAATAGTGGAAGCAGATCTATTATCTTGTCTATCTCAGAACGTGAATCATATACAATAGTAGGACCCCACTCTCTCATATATTGCAACCAACATGGTTCTGTAATGACTCCTTCACCAAGATACTCGGCTGCAACAACCTGATATTTAATGCTTGAATCCACAATATATTTGCTTCGAGCTGCGTCATTGCGTACTCCTATTCCTAGTTTGGATGGTCCCTGAAGGTATGTCCCAGGACGAGGGAAACTAGCATGGCCATCTTTTGATGAATAAACAATTGGCTTATTCCCCTTGATAAACTCCAGATCAAATGCATTTACCCATTGACCTCCACTATGCTGAGAGAAGAAAACAGACCATAGTTCTCCGGTGAAGTTGCTTATCCGAAGGGAGAAGTGCTCCCAGTCACCTACATGTTCACCTACTTTGTTCATCTCAATGTTCATAAGGGCCACTTTAAGTGTTGCAGGCCCATTGAAGGGGCAAAAAACCCACATCACGATATCAGTAAAGGCTCCTCCCAATGCTGGTTTTACATGAACATAGAGTTCTGCACTGTCTATGTTTCCCTTCTTTAGATTGTTTCTTGCTTCTTCATCAGTAGGCAGATCAATCCAAAAGGCACCATCATTAATTCCTCCACTAGGTAAATTTGAGCCCTTATAATCTATGGCTTTACCCTTGCTACTGCCTGCTGCATACAAAACTGCACCATTCTTAAAAAACCATTGCACTGATGATGGCAAGTACTTCTCATCAGGATGAAAGTATACAGTGGGACCATAATGCTGAATAAGTGCGTGAATCTGGTTCTGATTTGGCATCGTGTGCAAGGAAGTATCAAGATTCTTCAAGCATGCAATATCCACCACTTGTTCAGAATCAAAATAGTCACTACCACAGAAAAATGTCCCAACAGACACACCTCTAGCTAACATACCTCTGTCACAGGGTTGTGTATTCCAAACCTGAAATGCATTTTTTGCATATTTTGACTTTACAGTCAGTAATAGATCAGAAGTCTCACAAGTTTCGGTGAGATCAGCTCGCACGCATCTAACTTCTTCAACTGCAGGTTCATTAGGACTGATAGTAACTACTATGCCCATGGCTTTGTAACCAGTTGGAGGGTTTGGCAACCAAAAGTAAACACATTCATCATGTGAATCCATGCTCCATATTAGTGAGTAGCCAAGTGGTTTTTCAAGAGCAGGAGATTCTAACACTGAGACATTAGTTTCATGTGCCATAAGAAAATATCCTCTCAATGGCTGGTCATTAGACTGACAGTAGTGACCAAGGCAGAAAAAGCCTTCTGGAATTTCCAAAGGTCTATAAAACGTGAAACCCAACGATTTCCCACGCAAAATTGTGCATCTCCAAACCTTCTCAAACTTGTCTACTTTTGAAACTTCTATTTCTCCCAGGCATATTCTTCCACTTGCAAAACTACCACCTTGTGGCCATTGAGGAAGGGGTGAAGGCAGAGAGAAGGGAAGTGGATCAGGGTGAGTGAATTCAGGGGCACAATCCCAGCACAGTAAACTCTTGCACCCAAACATCTCTAAGAAAACAAGTATTCTTCAGAGACCCTCTTCCCCAAAAAGAGAATGGTTTTCTTTCTTTTCTTGCCTTCAAAGGAGTCCAAGAAGTTCAAGAAGAGAGAGAAGGAGATTGTTCTTAACAATAAAGGAGAAAAGCCTTCAACTTTCTCTCCTCCCTCTCCTCTTTTCTCCTTTTTCTTACAGAGATTATGCTCTGCTTTAACTTTAGATTCCGG

>C101333_65

GTGGGTCCCGCACACCGCACGCAAGAAAATCTTAAACAAGTTAATTCACAACTCACAAGTGTCTCTGTGATGAACACAACACTGTTCTTCTTTCAACCGTGGAAGCAAAGGCAAAAATACCCACCGAGGTATTTGCATAATCCCTTTTACTTCACTCTCACTGATCCAATTTTTCTCTATAAAGAGAAGTAAAACACTCTGATTCAGCCAACCACTCCACACAAATTCACACCTTTCATTGACTCCTTACATCCCACTTTACTCTTTGCTTCAACAAACATATCCATTGTTGAAAGATTGAATGTTGTGAACAATGGGTTCATTCAAGGCTGTTTCTCTGCTCCAATTGTTCATAGTGCTTTGCCTTGTCTTCCAATGCTTTGCTACTGAAAGTCTTACTGACTTGAGAACAACTCTAATAGTAGATGCTTCTGATGCAACTGGAAGGCCAATACCAGAGACACTTTTTGGCATATTCTTTGAGGAGATCAATCATGCTGGAGCTGGGGGATTGTGGGCTGAGCTTGTGAGCAACAGAGGATTTGAAGCTGGGGGCCCTAACACTCCTTCAAATATTGATCCTTGGACAATTATTGGAAATGAATCATCAATAGTTGTTGAAACTGATCGTACCTCTTGCTTTGACCGTAATAAGGTTGCACTTCGATTGGAGGTGTTATGTGATAGTGAAGGTGACAACGTCTGCCCAGCTAATGGTGTTGGTGTTTATAACCCTGGTTTCTGGGGCATGAATATTGAGCAAGGGAAGAAATACAAAGTGATCTTCTATGTTCGTTCAACTGAATTACTTAATCTAACAGTTTCATTGACTGGATCAAACGGTGTAGGAAACCTGGCTTCATCTGTGATCACGGGTTCTGCTGCTGATTTTTCAAACTGGACAAAGGTGGAGACTTTATTGGTTGCCAAGGCATCGGATCATAACTCAAGACTTCAAATAACAACCACCACCAAAGGTGTGATATGGTTGGACCAAGTGTCTGCTATGCCACTGGACACATACAAGGGACATGGTTTCCGAACTGAACTTTTTGAAATGCTGACAGCTTTGAAACCAAGATTTATCAGATTTCCAGGTGGTTGTTTTGTTGAAGGAGAATGGTTAAGAAATGCATTTCGATGGAAGGCAACTGTTGGACCGTGGGAAGAGAGACCTGGGCACTTTGGTGATGTTTGGATGTACTGGACTGATGATGGACTTGGTTATTTTGAGTTTCTCCAACTTGCAGAAGACTTGGGTGCTTTACCAATATGGGTATTTAATAATGGTGTCAGCCATAATGATCAAGTTGATACATCTGCTGTCTTACCACTTGTGCAAGAAGCCCTTGATGGAATTGAGTTCGCAAGAGGTGATCCCACTTCAAAATGGGGTTCCCTTAGAGCTGCTATGGGACATCCTGAGCCATTTGACCTAAGATATGTTGCTGTTGGGAATGAAGATTGTGGAAAAAAGAACTATCGAGGGAACTACCTGAAATTCTATAGTGCAATAAAAAGTGCTTATCCAGATATCCAAATTATCTCAAACTGTGATGGTTCTTCTCGATCTTTAGATCATCCAGCTGATATGTATGATTATCATGTTTATACAAATGCGAATGACATGTTTTCTAGAGCTAACGCATTTGATGGTACATCACGAAACGGACCAAAGGCTTTTGTAAGTGAGTATGCTGTGACTGGAAAAGATGCTGGGACAGGAAGCCTTTTGGCAGCCCTAGCTGAAGCCGGGTTCCTTATTGGACTGGAAAAGAATAGTGATGTTATCCAAATGGTCAGCTATGCTCCACTTTTTGTTAATGCCAATGATAGGAGGTGGAACCCAGATGCAATTGTGTTCAACTCCTTTCAGGCCTATGGAACTCCTAGTTATTGGGTACAACTATTTTTCTCAGAGTCAAGTGGAGCAACACTTCTAAGTACATCACTTCAATCGAATTCCTCCAATTCACTAGTTGCATCAGCAATAACATTTCAAAGTTCTGTAGATAACAAAAATTACATACGAATAAAGGTTGTGAACTTTGGCACCAGCGCAGTGAACCTCAAAATCTCTCTTGATGGACTAGAACCGAACTCATTGCAGTTATCTGGGTCAACAAAGACTGTGCTTACATCTGGAAATGTAATGGATGAGAACTCTTTCTCACAACCAAAGAAGGTGGTGCCAATCCAGAGTGGAATTCAGAATGTTGGGAAGGACCTGAATGTCACAGTCCCACCTCGTTCATTCACATCATTTGATTTACTGAAAGAATCAACTAATCTAAAGATGAAAGGGAGTGATTCTTCAACGAGGTCTTCCATTTAACGTAATGAAAAATTTGACAAAAAGCAGAAAAAAATGGGATTAAATAATGTTGAAATCTTATGTATCATGTAATTAAATTAACATCGTTGCACTGTGAATAAATATTTACATGCTTTCATTTAATTGTCAGTAATGGCCAACAAACTTGCAGACACAAAAAAAA

>C101335_65

TTGTAGAGTTACAGAGAGTGTGCTGTTATGAGTATGATAATCTCTTGACTTTTCTTGCATTCAGTATATATCTGAATTCGTTTCCCATCAAGGCATATAAAAAACAGCATTAGAATCATGAACTTGTTTGTTGTGGCATTTGGTCCAGCTCCCAAAGAGTCACTAAACATAATTGAGAACATCTTTGAGGACAAAGACCAAAAGCAATGCTATTAGGTACAAGAGGTATGCAGGGAAAGATGTCCAAAGAGGAAAAGTGGGACGAAAACTAGCAGTGAGGCCCATTACTGCACAAACTATTGACACAACCAGTACATCAGCTGAGAATTGCCAAGTGATCTCACGCATGTATATGAGAACAGATATCACCACAAAGCCAAGAATGTTGTTCATGAACACTGCTCCATAAATCTCATACATTGTCTGAGAAGTATTACTACTCATCTTATGACTAGCTTCTTTGATTGCTGAGGTTGCTTCTCTAAAATTTGTAGCCAGGGGGACTAGAATGAATGAGATGAAAAATGATGAAATTCCTGCTTCTTCAGAGAATTTTTGAACACTTGCTATTAAGGGCTCTGCAAGAACAGCCAGCATAATAACCCCCAACACCACATATCCTAAAGCCTGCAACCATGCAGAAGCCCCTTTGCTTTGATTCTCTTCCATTACCTTTTCAACATCTTCCCAGGTATGATGAATTCCCGGAGATGAAGATTTTGAGTTAGCAGCTTCGTTGGGATTTGAGTTCATCCATTTTGTAAAACCTTCAATAAACTCGGTCTCACTAATTTCATCGTCTCGGTTGACATCAAGATCTTGAACAAGTTTTGAAACTGCCTCATCAGCATCTACAACCTTGCTAAACTGGATCCCTTTGACTATTTTTTCCAATTCAGACCTTGAAATGTGGTTATCCTCGTTGACATCAATTTCCTCAAACAACCTTCTAATTGCAGTTTTGTCAGGTGTGCCATCATCTTTTCGCAGCCTGCCAACCATATCATTCTGAGCATGCTTTAAGACTTCAGATATAAGATGTCCCTTCAATTCACGTTCCTTTCTCACGTGTTCGATCCATGGCTTGATAAAACTCTGATACATTTTGTTCAGAGATTCTTTTGGAAGGTATTGTCTATCCAAGGCATGCTTTGTTTGGTTAATGTATTCTGTTAAACCAGTGACAAACTCTTCCTTAGTTATAATTTGGTCACCATTTCGGTCAAAAACTTTTAACAAGTCCGCTATCTGTTCCTCTTTGATATTTGTGTCAGTTTTGTTCCCAAAGAAGAGCTCTTTCACTTCAGAAGCTAACAAGTCCTTGGCCCCATGTTGACTAATTTCATGATACAATCCACTTATTGCAGCCACATTGGGAGTTCCATCCTCGGCCAGGATCTTTTGCAGAGTTTGTTTCTCAACACGTTGAAAAATTCTTAATATTAAGTCATCATGCTTAATATACTCCAGTCTAGTTTTCTCTATCTGAGGCTTAAAGACCTGGTAAATGAAGTACGAAATAAGAAAAGTAACAGCTACTATAAGAGCAACCATTAAGGTCACATTGCGTGCTGTAGAAGAGAAGTGGAACAAACTGGGAATCTGCATTATGATAAGTGGTATAACTGAGAAAACCATGATTATTGCCATCTCTCTAGTATCTACATCCATAGTGATACCATAACCAGTTAGTGATTTTTTTATTCCTCCACCAGAAGTAGAGTCATTTTTCAACTTTTGCTTGCCAATGATAACACATGTTCCCCACACTACTGTTAGAAGTAAGATGGATGATCCAGCCAACAAACCGACACCAGTAGAAGCATACTCTTGTGCACTCTCCTTGTCACTGGAAAGTCCAGTAACAACAAGAATTAAGGACTCAGGAAGAGCACCAAGAATATCAAAGGCACTAGCACCAAAAACACCTGGACCAAGAATCTTAAAGATTTGCTCACCCCCAGCAGCCAAATATGACTCTCCATGGAACAGCAAGTACTCATAAACCAAAATGAGGAAAAGATGGCCAAGTATGTTATTGGAACAAGGCAAGAACCCGTACATTTGCTTACAGTAATGCCCATCAGAAGAAGATTCAACCTTTTGGTCTATGAGTTGTAGATAAGACTCATGGGGTTGAAAGGCATCAACCCCATCAGAAACACTAGGGCGAAGAGAACGGCACTCAACATGAAGTGCCACTAAAAAAGCAAACAAAGTGAAGCGTGCAACCTTTGACATGGTTTTCATTTCATTTCACATGTGCTGAATGAACTGAACTCTATAGAACTTGTAGTTGTGGAAATGCCTTAAGAGATTTGTATCCTCTCACTGTGCTCGCAAGGTCAATTTCTGCTAGGCTAGATATGGGTTGGGATTCACGATCGTACAAACATCACGTGATTATTTAATTCCAAAAATGAGGACCGTTGAAATTATTTTTCAAGAAAAATGTGTGTTTTGCATGGTGCTTCATTGATTAATAATCGATATCAAAC

>C101337_65

CCGAAACTGGTTTTGGTTTTGGTTTAAGTCATCGCAGTGAGGAGGGGCTTGAGGTTCGGGTCCGAGAACTCGAGATCTGATTTATAATTTGGCGACCCTTTTGAAAAAAGAGGACTTCCTTTGCTGCTATTCTCTTCGTTCTCCATTTGGGTTCCTTGGCTTTTGTGAGGGTGTGCTGTAGTTGGAGCCATGGCGGTTGAGGTTGTGGGATTTGAGATGGTTCAAGGACCAGTGGAGAATGGTGCTGAAGGAGGAAAACCTGTTTTACATGAGAAGGAAAATGGAAAGCTGGAAAAAGATGTAGGAGCTGTGGAGGCCATCAAATTTGGGTCACATGGAGATGAATCTGCAAAAGGGAAGGGGAATGATGTTTCAGAGGCGAATGTGCCGAAAGATGCTGCTGAAGAGTGGCCTGCACCTAAGCAGATTCATTCTTATTATTTTGTCAGGTGGCGGCCTTATGAGGATCCAACCATAAAGTCTAAAATCGATTTGCTTGACAAAGAGATTAGCAAGAAAAATCAAGCCCGGTTTCAGATCACTGAAGCACTGAAGGCAAAGCGGTCAGAACGAGGCGAGTTGATTTCTCAAATCAAGTCTCTAAGAGGTGACAGTAGGCAATTTCAGAGTATTGTGGATGAGAAAATAAAAGAGATTGAACCTTTGCAGCAAGCATTGGGCAAGCTGCGTAATTCAAATAATGCGGGTCGCGGTGGTTTGTGTTCATCTGAGGAAGAACTTAATAGTGTTATATATAGTTTGCAATACCGCATACAGCATGAGAGCATTCCATTGACTGAGGAAAAACAACTCCTCCGAGAAATCAAACAGCTTGAGGGGACAAGGGAGAAAGTTATTGCGAATGCTACAATGAGGGCCAAGTTACAGGAATCTATGGGGCAGAAAGAAGCCATTCAAGATCAGGTCAAGCTTATAGGTGGGGATTTGGATGGAGTGAAGAAAGAGAGAGAAGCAATTCGGTCCAAAATCAAGCAAATTGATGATGCCCTGAAAGCCATTGACAAGGATATCCAGTCTCTACAGGAGGAACTGACAGCTATTTCTCAGAAAAGGGACAAATCTTTTGAGAGCATGCAGCAGCTGAGAAAACAGCGTGAGGACGGGAATTCCTATTTCTACCAAAGTCGTACCGTTCTGAACAAAGCACGGGATCTGGCTGCAAAGAAAGATATTAATGCTCTTGATGAAGTTTCACAGTCAGAGGTTGAGAAATTTATGGCACTGTGGAACAGTGACAAAGCATTTAGGAATGATTATGAAAAAAGAATTTTGGCCTCATTGGATATGCGGCAGTTGAGTCGGGATGGACGAATGAGGAACCCGGATGAAAAGCCATTGCTGGAAGAGCCAAAACCTGCTGAAACTGAAACGATACCAAAATCTGTTCCAAAACAGCCAAAGGAGGAGCCTAAGCCTTCTCCACAAGAAACTTTGCCTGCTCAGAAAGAATCCAAAAACAAAGGGAGAGATTTGAAATCCAAACCGGATATTAAGGATTCAGCAGTAACTGATGAATATGAATTCGAAAATCCCCAAAAGGTGACCCCTGCGAAGGAGCATGAAATTAACCCCGCAAAGCTGAAAGAGATTAAGCGTGAGGAGGAAATTTTGAAAGCAAAGCAGGCCCTGGAAAGAAAGAAGAAGTTAGCTGAGAAAGCTGCAGCCAAAGCAGCAATCAGAGCACAAAAGGAAGCCGAGAAGAAGCTTAAGGACCGTGAGAAGAAAGCAAAGAAGAAAGCTTCCGGTGCTGGAGTTGTTGCCAACCCTGAGGATGAACCAAAAGATGAAGTTGCTGAGGCTGCCGAACCAGAAAAGGTTGACGATGACAGTCAAGCCCCGGCTCCAGTGAAGGAAAAGGTCCAAAAGGAGAGTGGCGGCATCAGGTCCAGAAACCGAGCAAGAGGCCCGGATTCAGTCCCAAAAGTTGTTCTTAAGCGTAAGAAGTCTAACAATTACTGGGTGTGGGTTGCAGCTGCTGCTTTGTTAGTCCTCCTTTTGGTGGTGCTTGGATACATCTATCTTTTCTGAGATGTTTAGATGTAGAAGGAGCTGTGAAAAACCAATTTTCTAAGTGGGTGGCTAGGTTTGTGAGGTAGCATAAGTTATATTCATGTTCTACATCCTCTTGGGAGAATTAGATCAAAGACACTCTGTTGTGGGCTCCTGCTTTGGGAAGAAGTTGCATGCATACTTTTTAGCTTGACTATGTACTTTTCAAATTTTGTTCAGTTTTCATGGGATGTTACCGGAAGCTTTAATTTGTTTCTATAGTTAATTGGTCACAGGAACGTACTATGATATACTGAACTTGATGATGAGAGATTTTGCATATTGGTTAACAAAAATATCTGTAGACAGGTTGAACATGACTTGAGATGTGATGGTACAGTGTGAATGAATGTCTTTATTACAAAAGGTACCAATATCAAATACTCAATGAGGTAAGTTTAAATGTTTGCGTGAGCAATATTTGCATG

>C101339_65

TTTTTAGAACAAAGGAGATAGATTGTTCTTGAATGAAGATCATCAATTGAATCACAACGTTAATACACAAGAATCCAAGAAAATAAACTTTCCTAGGGAATAAAACAACAGAAGTAATTCCATCTGTGAGAACTGTTTCATGCACTCTTATATACCACTAAATGTTGCTTCCTGAAAAAAACATGTTCAAGAAATCAATTTGCTTGGGAAAGAAGAAAAAGGGCAGAGTTAATTATGGAGTTTGAGCAGTTGATTCCGCCTTGGATCTATTCTCTTCTGAAGATGGCCGGTTTTCTGAATCGGATTGCCGGATTTCTTCAATCATTCTCACCACTTCCTCCATGGTAGGCCTCATATCTGGAACCTTTGCCACACAGGCCATGGCAATTTGCAACATTTGCACCATCTCTTCTTCAATGTTTTGGTACCTCATTAGCTCCACATCAAAAACCTCAGCTGTCCACTCCTCCCTAACAACAGACTGCACCCACCTTGGGAGATCAACCATGTCATCGCGTCCTGGAGACTGTTGTGGTGCTTTTCCAGTCAGCATTTCCAGAAGGAGAACACCAAAACTGTACACATCTGACTTGTGAGTGTGCTTACGCGTTTCAATCACCTCAGGGGCACGATAACCTGCAGCTCTAGAAGGGGTTGCAGGAACATTCATAAGCGGGGTCAGGCCGAAGTCAGAAATGCAGCCATCGTTATCTTGGTTGAGGAGGACATTCGAGGATTTCACATTTCCGTGAGTGAATTTTGGACCACCAATTGAATGTATGTGAGCAATTCCTCTGGCAATTCCAACAGAGATTTTTATTCGGGAGTTCCAATCTAATGGAGTTCTTCCACTTGCCCTGTTACCATGCAAGAGGGTTGATAGATTGCCATTTGGGATGTAGTCATAAACCAAAAGCTTCTCATCCTTCGAATAATAGTAAGCACGAAGTGGCACAACATTTGGATGCTGTCCAACTCTCCTCACAATCTCCATCTGCTGTTCAAATTCCCTTTTGCCTACCACTACTTCCTTCAACCTTTTCACTACAACTGTTGTTGACTCTTCCAATATAGCCTTATATGCAGTACCATAACTACCCTTTCCAAGAACTTCAGCTGAAGCTCTCAACAAATCCTCAAGATCAAAATTATAAGAACTGCCCTCAAAGAAAACCAACTTATTTTTCTCAGGTTCTTGAACCCCACTTCCAAACTCTTCTTTTGGCTTCTCACTTCTCCCACCACTAGGACCCTTGCCTTTAGTAGCTCTGGGGCTTCCATCATCTTTTTTCTTTAAACAGCAAAGAAGAATGATGAGAGCTACTAAAAGTAATACCACACCCCCCCCAACTGCAATTGCAATGATAGCTGCCTTACTCAGTTTACTCTTGGAGCTATGTCTCGGAGGAGTTGGTGGGGCAGGAGTTGCTGATGGAGGAGGTGCAGGGGGGACTACAGAGCATGACTTTAGAGGTAATCCACATAGAGAGTTACCCTCAAAGGAAATATTGGGGAAAATCTGAAGAGCAGCAGGGATAGAGCCATTCAAATGGTTGTAGCTTAAATTCAAGTGCCTAAGCTTAGTGACATTGAGATTTGGTATCTCTCCAGATAGGGAGTTGTTCTGGAGGTTCAACCTAGTTAGTTGAGTTAAGTTTTGTAATGACTTTGGAATAGCACCGGTGAAGGAATTGTATGACAAATCAAGTACATTGAGGTGGGTGGGCAATGAGGTAGGTATGCTGCCAGAAAGGTTATTATGCTGAAGGTAAAGATATTGTAGAGAAGGAAGAGAAGTGATATCAGGAGGAAGACTTCCACTGAGTAGGTTGGCACGGAGGCTGATATTTTTGAGTGAGTCAATCTTGCCAAGTGTGTTGGCAGGAATGGTGCCGACTAGTCCAATTCCAGGAAGCCGGAGAGAAACAACACGGGTGCCATTTGGATTGCAAGTGACGCCTACCCAAGATGTACAGATTGAAGTAGCTGGATTCCACTTAAGGTCACGGTGATGGGGGATAGAAGCAGCAAAATCAAGAAGGGCTTGCTTGTCAGAACTTAAGTCAGCAATAACCACAGGAAAAAGAATAACAATGATGAAAAGGAAAGGATGCACTCGAGGTGAGTAGAACTTCATGGAGAGTTTTTTGGTGATAAGGTATAAGATATTGAAACTTGAATTTGAAGTAATCACAGCCATCCTTCATATTTTCCTGAAAGGAGGCAAAGAGGAAGAAACACAAGGATTCATGGAACAGCTCAGAGCTTAAACCACAGGGCAAGATGCATTAACACTGAAGAAGATGAGCAAGTTGCAGCCTTATACGATGCTTCAGATTCTCAAGTAATTAACAAATAATGGAGAATCTGAATCTGCTTAACTCTAGATTGGAGCATGGAAGCTCAACACCCCAGAGAATTTCCACGAAATTGAAGGGCATTTTAAGGTTGAGGTTTCAATAGCAGCTAGAAGAAGTGAAGACAAATGGTTCAATAGG

>C101343_65

CCAACCACAAATGTTTGCATTCCATTCATTTCACCACCATAGTTGACCAAACCAACTTTCATTGAAACCATGGCTCTCTCCACCCCTTCCTTCTTCTTCCTCACCTTCTTCTTCTTACTCTTCAATCTCCAACCCTCTCTCTCCCAAGAACAACTCTCATCCTTCAACATCTCACACTCCCCATGGCAACCATCTCAGAACAGAACCCTTCTCTCCCCCAACAAAAACTTCACTGCAGGCTTCTTTCCCGTACCAAACTCCAATCTCTTCACCTTCTCCATTTGGTTCTCTAAAGTCCTTCGAGACCCTCTTGTGTGGAGTGACACCATCCAAGTGAACAGTTCTGGTTCCCTTGTCATCACCTCAAAATTTGAACTTCTCCTCAATGGATCACCCTTTCAAGATACTGCCAATACTAATGCTTCCCAGCTTGTTCTCCATAACAGTGGAGAATTGGTTTTTGGGAACTGGAGCAGCTTCAGCAACCCCACCAACACCATTTTGCCGAACCAGAACTTCACAGGGGTTGAGCTTCAGTCCAACAATGGGAAATTCAGGTTCATCAAGTCTCAGTTTCTGGTCCTCAACTCCACCTCTTCTCAATACTACCAAACGCCATCTGCCTTGGTGAACATGGATGATTCAGGGAAAATGACCATGGCAAGTAGTTCTTTCCTCACTTCAGACTATGGAGACCCCAGGATCAGGAAAGTGGTCCTTGATGATGATGGGAATCTCAGGATCTATGGCTTCTACCCTGAGCAGAATGACAAGTGGGTTGAGGTTTGGAAAGGGATTTGGGAGATGTGCAAGATCAAAGGCAAATGTGAGTCTAATGCCATTTGTGTGCCTAGAGATGATTTGAACACTTCTACTGATTGTGTTTGCCCCTCTGGGTTCATCCCAAACCAAGGCGGGCCTGAAGAAGGTTGCAGAAGGAAGATCTCTCTTTCGCAAAACACCCAATTTCTAAGGCTGGATTATGTGAACTTCACCAGTGATGGTAGTTTGACCGAAATTAAAGCTGGGAACTTCACAATCTGTGAGTCCAGTTGCTCAACAGATAAAACATGTCTTGGGTTTGGGTTCAAATATGATGGATCTGGCTACTGTGTGTGGCTGAGTGGGACAAATCTTCGATATGGGTACTGGTCACCAGGCACTGAGGCTGCCTTTTTTCTCAAAGTTGACAAATCTGAGTCAACACAATCCAACTTCATCGGCATGACTGAGGTGATGCAAACCACATGCCCAGTGAACATAAGCCTGCCACTGCCTCCAAAGGACTCAAACACCACTGCCAGAAACATTGCCATAATATGCACCCTTTTTGCTGCAGAACTCATAGCTGGGGTGGCCTTCTTCTGGTCCTTCCTGAAGAGGTACATCAAGTACAGGGACATGGCCACCACACTAGGCCTTGAGCTTCTCCCTGCTGGGGGTCCCAAGAGGTTCACCTTCTCAGAAATCAAGACTGCCACCAATGACTTCTCCAATCTCATAGGCAAAGGAGGCTTTGGAGATGTCTACAAAGGTGAATTGCCAGACCATAGGGTGGTGGCAGTGAAGTGCCTGAAGAATGTCACAGGAGGAGATGCTGAGTTTTGGGCAGAAGTAACAATCATTGCCAGAATGCACCACCTCAACTTGGTGAGGTTGTGGGGCTTCTGTGCTGAGAAGGGCCAAAGGATACTTGTCTATGAACACATCCCTTGTGGCTCATTGGACAAGTACCTCTTCAGGGTCAAAAAGTCTCACAGTAATAATAACAACCATCTCAAAGACCAATCAAGTACAAACACACCACAAGAAAAGCCAGTTTTGGATTGGAGCATGAGGTACAGAATAGCCCTTGGTGTGGCAAGGGCTATTGCCTACTTGCATGAGGAGTGTTTGGAGTGGGTTTTGCACTGTGACATCAAGCCTGAGAACATTTTACTAGGTGATGATTTTTGCCCCAAGATCTCAGATTTTGGGCTGGCCAAGTTGAGGAAAAAGGAGGACATGGTGACCATGTCAAGGAGGAGGGGCACACCAGGGTACATGGCACCAGAATGGATCACTGCTGACCCAATCACTTCCAAAGCTGATGTGTATAGTTTTGGCATGGTGCTATTGGAGCTTGTGAGTGGAATTAGGAACTTTGAGATCCAAGAGTCTGTGGTTAGAAGTGAGGAGTGGTACTTCCCAGGTTGGGCCTTTGACAAGATGTTTAAGGAGATGAGAGTGGAGGACATTTTGGATGGGCAGATTAGGGATGCTTATGATAGTAGGGCTCACTTTGACATGGTTAATAGGATGGTCAAAACTGCCATGTGGTGTCTACAGGATAGGCCTGAGTTAAGGCCAACTATGGGCAAGGTGGCCAAGATGTTGGAAGGGACTGTGGAGATCATTGAGCCCAAGAAGCCCACTGTTTTCTTCCTTGGAGAAGAGTAGATTCATTTCTTTTCTTATACCAGATTGGATCACAGATATTCTTATGGTTGAATGTTGTAG

>C101353_65

GTTTGGAGTTCAACAACGTTTACCAGTCTTGTGTGTGTGTTTGTGCAGTCTTTCTTCACTTTCTCTTCCTTCTTCAATCAAACCCCAATTCCTCTCTGGTCAGCTCCGTTTACTTTCTCCCTCTTCTCATGCTTTTCATTTCACACCTCACAACGATACACACCAACCGAACCAAACTTCAAATCTCTCTCTACTTCTCTTCAATTTCGCCTTTCAGATTTCCAACCGTCTCACCAACTCCTCAAGCCATTCGCGACGTCGTTTTAGATGGATTCAGATGCATCACCTATCTCGGATTTTTGAACACCGCAAGTGTACCGAGGCCATGGATCGCAAGAACCCGCGGAGGAAGCCCAAGCTCGAGCGCCGCAACGCTCTCAAGTACTCCGAATACGACGCCGGATCGTCTACCTCCGATGACACGCTCTACACGCGCTCCATGGAGTTCTACGACCGCACCAGCTTCCGAATCGAGGGCGTCGAAGGCGAGTTCGATCGAATTTGCAGGAGTTTGGGCCTCTCCGGCCCAGAAGACTTCTCTATTCCCGCTGCGGCCTGGGAAGCCATGAAGTTTCGTTCCTCTTCGGATATTCTCCCGAGACTGAAGCTCGACAACCTCGATATTCCCGAGGAAGAGGAAGAGGACGAGGACGAAGAAGAGGAACCGACGGTAAACGAGGAGAGGATAGTAAAACCACTTGAGAAAGGTGAATTGAGTGAAAAACAAGACGATAGGGATAGGGCTAGGGTTAGGGTTAGTGAGGTAGATGAATTGGGCCCTGGTATTAAGGGGGTTCGTCCGCCGATGCTGAAGCCGCCGCCGGGGGTGAGGGTTCAGATGGTGGATGATAGCGCGTGTTCCACGTGGGACCTATTAAGGGATTTGGCTCCGATAGAGGAAGGGTTACCGTTGAAGCTTCCGGAGCGTGAAAGGGAAGTAGAGAATGTTGCTCCTGAAGGGGAAGTAGAGAGGGAAGTAGCGGAGAAGGAAGAAGGGGAAGTGGGTAGAGCGAGTCCGAAGAGGGAAGAGGAAGAGAATGTGGATAATGCAGCGAGGATTGCGGAGATTGTGGCTGGGCTTTCGGAATCGTGTTCGTTTTCCACGTCGAACGAAGATGATTCTTCTAGTTGTACCACGGATCATACGTCCAATAATATTTCTCCGCAAGGGAGAATCAAGCGGATTATTACTGCCGGAAGCTGGCAGAAGGGTGAGTTTCTTGGGGGTGGCTCGTTTGGCTCCGTTTATGAAGGAATTTCTGATGATGGATTCTTTTTTGCTGTAAAAGAAGTTTCACTGCTTGATCCGGGGACTCAGGGAAAGCAAAGTGTATATCAATTGGAGCAGGAAATAGCACTTTTGAGTCAGTTTGAGCATGACAATATTGTTCAATACTATGGCACAGAAATGGACGAATCAAGGCTGTATATCTTTCTTGAGTTAGTAACCAAAGGTTCACTTAGAAGCCTCTATCAGAAGTATACACTTCGAGATTCCCAAGTATCTGCCTATACAAGACAGATTCTACATGGTTTGAAGTATCTTCATGATCGAAATGTGGTTCACAGGGATATCAAATGTGCAAATATATTGGTGGATGCAAGTGGATCTGTCAAGCTTGCAGATTTTGGATTGGCAAAGGCAACCAAATTGAATGACGTTAAATCAATGAAGGGTACAGCATTCTGGATGGCGCCTGAGGTCGTGAAAGGAAAGAACAAGGGTTATGGGCTTCCAGCTGATATGTGGAGTCTGGGATGCACTGTACTGGAGATGTTAACAGGCCAACTTCCGTACTGTGATTTGGAATGCATGCAAGCATTATTTAGAATTGGAAAAGGTGTTCGACCTTCTATTCCAGACTCTCTTTCCAGAGATGCAACCGATTTTATCCTGCAGTGCCTTCAAGTTAATCCAAATGATCGTCCCACTGCTGCTGAACTTTTAAACCATTCATTTGTCCAAAGGCCACTTTCCCAATCCTCTGGTTCTTCATTTCCTCATATTCATGGCCGGAAGGGTTAAACTATGCTATCTTAACTATGAAGCATTATGGAGTTCTATCCTTCACTGCACGTAACATTGCCAATGTTCAGTCTGGAAGTTGCTGGGGTTCATCTAGGGTCCATTAATGAAGAAATCGTTCATTTGCTTGTGTTGTAACATTCAGAGTTTGCCTGATTGTATCAATTTTTCTGCACGGCTTTGAATCTAACACGTTGCAAGGCACACCAGTGAACCAAGTTATCGGCTTGGCATATCCTATTGGAGAATTGTGCTCAATATGTGTTATCTAATTACTCTGATGATTTGACTGTTGTAGCAAAGGTGGAGTATCAACTTCCAGCTGAAAAAGCTATCCTTGCCTTCCCCCTCATATTACCTGACAAGCGAAAAACTATAGGGCTTGCTTAACTCAGGAGAAATAGTATAGCGCGAATTGGAAACTATTTTATTTGTATATATTTCTTATTTTTTATTATTATTATTAGCATGGCG

>C101357_65

CTGCCGAATTTCCCATTATTGATCCAACCACTAGACCAAATCCTGGTACAAAAATTAAAGATGAAAATGCTGAGAAAGAGTCAGCACTGAACTAAGAATACCATTGTGCCAAATCATGCCATCACTTTAGTCGGATGACAAATATGCAGTACATGAAAAGAATATTTATTCATCCAAATGGCTGCCTATACATGGCTCTTCACAAAAATTACACATAAAAGAGCAGCAAAGTGCCTAATGAGCAAAAATTACCAACGTGCTTGAATCTCAATTGTTGGAAACCTAATGTTAGACGGTACCTTGGCGGGTCTTCTCTCAGGGTATTTAGTAAATAGACCCAAGATATTATTATACAAAAACTAATGAGGCATAACAATCGAAAACTGTTTCAGATGAATAGAAAAATGAGAAAAGGACAAAAATGGGGATGGTATGCTAGTTTGATCCATGTTGTGGTGAACCCACATGTGGCCGTAAGGTCAATAAGTAACATGAAAATTTGGTTTGCGTCGTTATAAAAGAAGTCGTCTTATATCACTGAAGTTTGATTGCAACGCAGAGAGAAATGCACCTCCAACCTTGCCATCAAAAACACGATGATCAGCAGACAACGTTAAGCTCAACTTATTGGCAATCGATGGCTTCTCAACTCCATCATCTCCAATTACTGCTTCCACAACTTTGTTGCCTCTCCCTACAGCAAGAATGCAGGCCTGAGGGGGGTTTATAATAGCACAAAATCTATCCACAGGAAACATTCCTAAGTTTGAAATGCTAAAAGTCCCCCCTTGGAATTCATGTGGCTTCAACTTGCCATCACGTGCCTTTGCAGCTAGTTCCTTGACCTCTGAGGAGATAGCAGAGATTGTCTTCTGATCAGCATTTCTTATTATTGGAGTCATCAAGCCCTTATCCGTGGCAACTGCTATGGATATGTCAATAGAATCATTTAAAATGACCTCACCCTTCTCAACATTCCAGTATGCATTCGCTTCTGGTACATTTTTGAGAGCAGCAGCTACAACTTTGATAACAATGTCATTCACAGAAACTTTAACATCATACTGCTCTTTAAGATCCTTTCGAAGGGAAAGAAGAGGATCCAGTATGACATCTGATGATAAATATAGGTGCGGTGTATTTTGTTTTGATTCTAATAATCTCTTGGCGATCACCTTGCGAATTTGACTATTAGGAAAATCTTCATAAGCATCTGACTGCTTTAAGTCAGACTTTGATTCTTGGGAAGCTACAACTTGTTGATGACTTTGAGATGTTGGCGCCTTTTCTTTAGATGAAGCAGGTTTTGGAGACAATTTTCCAGACTTAATTTCAGAAAGAACATCCCCTTTCAGTAGGGTGCCGTAAGGACCCGTTGCATTCAATGTTGATGCATCCAATCCATACTCTGTAATTAGCAACTTCGCAGCTGGACTGATTCGAGTTATCTTGTTTTTTTGAGCTTTGACCTCACTTTTAGTGTCATTTTGGGGGGCCTTCTGTTGGTTTGTTGAACCGCTGCTAACAGAATTCTTTATAGCTTCAATGTCACTTGCATCTTCAACTGTTATTGCAATAGGGTGCCCAACAGCCACTTCTTTCGAACCTTCTGGAGCAAGTATCTTAGCCAGGTAACCCTCTTCAAGAGTTTCAAATTCAAGAGTAGCTTTGTCTGTCTCTATCTCACATAATATATCACCTACTTCAATCTTGTCTCCTTCTTGTTTCCTCCATTTAGCAATGTTCCCTTGGTTCATTGTTGGAGACAAAGCTGGCATTTCAACAAGGACATGAGGAGGAAGTTCTGATGCATTAATCGTAGTAGAAGTTGATTCTGGTTTCCCCTCATCTGTAACATCCTGATGTGCTGGTTTCGTCTCTTCAACCCCTCTCTCACCCCCCACGGAAGCAGGAACATTTTGAATGTCCTTTTCATCCTCAACTGTTATTGCAATTGGTTGTCCAACAGGCACATCCTTTGAACCATCAGGTACCAATATCTTAGCCAGAAATCCCTCTTCAAGACTTTCAAATTCTAGTGTAGCTTTGTCAGTTTCAATTTCACAAAGCACATCCCCCACTTCTATCTTTTCTCCTTCTTTCTTCCTCCACTTTGCAATATTACCTTGAGTCATTGTAGGAGATAGTGCAGGCATCCCAAGGACTTCGTGAGAAGAATCTGAAGATGAAAAGTATTTGACATCGGTCCACTTCGACTTCAAAGATCTATCACGAATTCCAGTGATCCCTGAGCAAGATGTGGGCCTTATATTTGTATCAACTCCCACGGCAAAAATCGTTGAATTAGAAGCTCGAGAGAGGGATCTGGTGGAAGAAGAGAGGATGCGAAGGGAGCGAGAGAAGAGAGGGTGGCGAAGGCGCGAGAGTGCCATATGCGTCGATTCGATCCGATTCAGAAGAGAAGAAAAGAGAAGAGAGATTGATAAAACGAAATGAAACTCTTCTCTGGTTAGTTACTTTGTCTGAGTACAATAAAAATT

>C101361_65

CAGAGAACCAGTCATAGTACCATAACAAAAGAAGTGTAACACTATCTTGCGCTTCATAAAATTCCGTAAGTAAAAGCACCATAAGGAAAAATGTAAAACAGTGGGATTATCTTCTATAAAATGCGCAGATATTTGCCTCAATAGAACTCTGAAAACTGTGTTCCAGAAAAAATAATAATAATAAAAACAAAGGAGAACAAGCTATTAGCTGTGAATTTCAAAAGGGCTATTCATCAAGCAAAGGTTAGGTTCACCACGAACAAAACCATTTTAAGAACTTCAATGAAGAACTATTTCCTATATCAGATACTTTTGTGGCTAGATGAACCCCTTATTACCCCTCCTAGCCTCATAAGCCCCTCCCAATGAGAAAATTCTGACATCTTACAAGCCCCCATAATAGAACAAACTGCTGTTCTACAGTTTTGGTAAAAGGGGTTCAATCAGCAATGGTTAGAGAAGAATGGATATCTAACCTGACCGACCCTCCTTCCTGCCACCTATTCTAGAAAAAGGTTTTTGCTTCTCATTTAAATTCTTATCAAGTCCCTCTTCATGTCTGGTCACAATCCTTAGAGGGTGGGCTTCCGTGTTGAATACCCATTCATCTAATGGGATGACTGAAGAAGGCGAGAAGAGAAGGTAAAAATACTTCAGGGTCTCCGCAAGAAAGAAGCTTTGCATCATGTTGTCTTTGACTCCACTATTAACATCCTTCAGTCCAACATAGCCTGATTCTATGCGGGAGTTCTTCTCAAAAGCTTGAAATATATTCCAACCCCATTCTTGGTATGTTTTGTTGCCAGTTAGACGCCAGAGGTAAAAGAGAGACTCAACTGTCTCTGGCCTCAGAATGTTCCATGATGTGCCCACACTCATGTCCTGCCCAGAATGAAAGAAATAGTTCTCTCCAGCCAATTTTGTTGGAGTTGATTGGTAGAAGTTATAACATGTCCAAGCAAGCTCTTCTGCAAGTGACAGAAACTTTTGTGAATCATCATCAGCATTGTAACCAAATGATCCCAAAGCAATCATTCCAGGAGCAAAGCATGCTAGTTCATCCATTTTATCTGTGAGAGATCCTCCATTCTTCTCACATATATAAGCAAAAGAAGATGGTGTTGATCTCCGAATCAAACTTAACAGACCTCTCATTGATTTCTCCCACATATCTCTATAATGTTTTACGACTGAAGTCTTATTCCCTTGTATCCAAACTTTGAGCAAGTACTCATAAAAACTATCACCCATGGCACCAAATGTGATAGGAGAGTAGCCCGGTGCCCCACTATGAGGATTAATGTAGATGGGAAGTAATCCATCATCAGGAAATGTTTTATTTAGCTGGGTAATTACGTTTTCCACCTTCTGCTGATACTTTGGATCTCCAGTCCTTTGAGATAGAACAATGAATTCCAGTTGTTCAGTGCCAGAATCTGCCAAGATGCTCTCACCCCCTGTCCAACTAGGGTTGTGTGCTCGTCCATGTGACAAATTAATGATGTTGTAAGGAATTCCTGTAGGCGTATTCCATGCAGGCAGTAATCTGTCTGCAATTTCTCTAGCTTTGTCAAGGAAAACCTTGTCACCCGAAAGATCGTAAGCACTAAGAAGACCACCTACTACTCTTATGGTTGTCTCAAAAACACTTGCTTCATAATCCTTGTTAAAATCCAAAGAGTTTGCGACCCATTCTCGTGCTTTCTGAAACTGCTCATTGAGACCCATAATATATAGTGTATCAAGAGAATCTATTAAAGTTGCTCCAAGACCTCCAAAGCTATTGACACCAACCTTTGATTGTGGCTGAAGTTCATCTTGGCCCCATGCATACTTTTCATAAGATCCCCAAGCATGAAGCATTGCTTCTTTTACTTTTTCCCTCCTCTCGTTATCAATGGGATCATCTAGGACCACTCTACTGGACCTGGAAGCTTTACTTCTCAAGTCAATCTTCCCAGAAGTACCACCTTGTTTGTTATTTAACTCACCCAACAAATTTTGCAGATCAGTCACTTCTTTCCGCAGCTCAGAAACTTCAACCTGGTGCTCTCTGACCAGCGTTTGACGGTCCCAGACAACGAAAGAGACGCAAACGAAACCGATGAAGAGCAAAGCAAGACGCTTTGGGCGCTTCAGGTAATAGGAAGGGTGGCAGTACCTCCACTTGCTGCTCGACACTGATCTACTCCCTCTCGCCATTCTCAAACCAAGATCTAAGGTTCACACCAACCTCGTCCCTTCTTTTGTCGCAGACCAACAATTTTTTAAGGAACAAAACGGCCGGTCGTTTGTTCCTTCAAATCTACGTATCTGTTTGTCGGAATGTGAACTCAGACTTTTCAGGGTTTATAAGAAGATTCGTTTTTACTCTTCGCTACGAAGTTGGAGAAGGATTTTATCAATCCAACCAACGAACGCTTCAATTCAATCTGCAACAATGAAGTGCAGATGAAGACGAAGCAGAGACTTCAATCCTCCCTTTGCTCAATTCAAGTCAAT

>C101363_65

CGACAACTCTTAAATCAGAATTTTCAGTTTAAAGAACGAATACCAGCTTTGTTTTTTCTGGAAGATTTTTGGGAAAATTTCGCGCGCGAGAAACCCTAAAGGGAACCCTGATTCATTCGATTCGCTGTTGGATTCGAATTTGGAACTTGGAAGATTAGGGAGCAATGGCTATTGCAGCTCCAGGGCAATTGAACGTGAATGAATCGCCCACTTGTGGATCCAGGAGTGTTGATTGCTTCGAGAAATTGGAGCAAATTGGCGAGGGCACATATGGTCAGGTTTACATGGCTAAAGAGATTAAAACTGGTGAAATTGTTGCTCTAAAGAAAATACGTATGGACAATGAGAGAGAGGGGTTTCCTATAACTGCTATACGAGAAATCAAAATTCTAAAGAAACTACATCATGAAAATGTGATCAAGCTGAAAGAAATTGTGACTTCTCAAGGTCCTGAGAAAGATGAGCAAGGGAGGCCAGATGGTAACAAATATAAAGGTGGCATCTATATGGTCTTTGAATACATGGACCATGATTTAACAGGTCTGGCTGACCGACCTGGGATGAGATTTACAGTTCCCCAAATTAAGTGTTACATGAGACAACTCTTGACGGGGCTTCACTATTGTCATGTAAATCAAGTACTTCATCGTGATATCAAAGGCTCAAATCTTCTTATAGACAATGAAGGAAATCTTAAGCTTGCAGATTTTGGACTGGCACGATCATTTTCCAATGACCAAAATGCTAATCTTACAAATCGTGTCATTACGTTATGGTACAGACCACCTGAGCTGCTGCTAGGGACAACAAAGTATGGGCCAGCTGTAGATATGTGGTCAGTTGGGTGCATTTTTGCTGAGCTTCTTCAGGGGAAGCCTATCTTTCCTGGAAAAGATGAGCCAGAACAATTAAATAAAATATATGAGCTGTGTGGAGCACCAACTGAAATCAATTGGCCTGGGGTTTCTAAGATACCTTATTATAATAAATTTATGCCAACAAGGCCAATGAAAAGACGTTTGAGGGAAGTTTTCCGGCATTTTGATCACCATGCTCTGGAGTTGTTGGAGAAGATGTTGACACTTGATCCAGCTCAGAGAATTACTGCGAAAGATGCACTTGATGCTGAGTATTTCTGGACAGATCCATTACCATGCGACCCCAAGAGTTTACCCAAGTACGAGTCATCACACGAGTTTCAGACAAAGAAAAAGCGCCAAAAACAACGGGAAAATGAAGAAATGGCTAAGCGTCAGAAAATGCAGCACCCACAGCAGCACACTCGTCTTCCCCCCATTCAGCAGCCTGGACAACATGCCCAAATGCGGTCAGGACCAAACCAACAAATTCATGGCTCTCAACCCCCAGTTGCTGCAGGACCTACTCATCATTATGGGAAGCCTCGAGGTCCATCTGGTGGGCCAGGAAGATATCCTCCCAATGGGAATCAAGGTGGAGGATACAATCACCCAAATCGAGGAGGTCAAGGTGGTAGCGGTTATGGCAGTGGACCTTATCCTCCTCAAGGGCGGGGGGCACCTTATGGGTCCAATAGCATGCCTGGTGGTGGTCCACGCGGTGGCAGTGGCTATGGAGTTGGAGCTCCAAATTATCCTCAACAAGGTGGTCCATATGGGGGTTCAGCCGCAGGTCGTGGCTCAAACATGATGGGTGGAAACCGCAATCAACAACAATATGGTTGGCAGCAGTAAATATACTTTCTTACGATGATCAGTGTTAGCACTATGATTTGGCAATACCCTGATAAATTTGAGAAAGATCGGACTATTAATGGATCAAGTTATTTAGAAGATGAGATCTGACCTCTATGGTTTGTATACTATTGAGACATACTATTCTCATTACTTGAAGTACATGGAAACCTTTGTGATACATGCATGCACCTTCGGTGATGTTTGGTGCTCCTAATCATAAGGGCACCATAAAAGAAGAGTGAACCTCCGATTCTGTATTTCTTTTTATTTTTCACTATACTCATTGAATGCTGTGCATTATGTATGAATTATAGCAGCATACTTTCAAATGTGTTACAAGTAGCTGCATTCTTTTCAGGATACTTGTACATTTTTTTTTAATTAGTTTTATACTTTAGCACATTATAGGCACAATATGTAGTAACAAATAGACAGGGTCAATTTGAATGCTTATGGTAGTGATAGGTTAGCGTAGGGAAGTGAAATTAATGAATTACGAGAGATAGTGGAAGAAGAAATAAACAAATTGTGAAAGAAGCAGAAGTGAAAGATGTGGATGTGGTTCAAGGAGAGAGAAAATATCAGAAAGTGGCAGGTGGCTAGGGGAGACAGAGGATTGTATGATGAGTGTGGTAACCGAATGCCATGCAAGAATAAATTGGCAATTGGTTAATCTGAGTGAAGAAGCTGGTTCTTCTTCAAGAAAGGAATTCTCTAATTTCTGTCATTTGTTTTTCTATTGTTCATCTTGTAAGGAGGTAGTTTCTACTTTCTAATTATTACCCTC

>C101365_65

ACACAACCTCAATTTTGCAGAAACTAGAAAGTATGAACTTCACAAAAAAAAAAATCATATGATACATTCCAGATATTAACAACCTGGCCAAACTTTGGAATTAGGCAGAAATGGTAGAGAAGAATGAAATGTCATAAATGCGTGTTACAACGAATTATGAACAGCAGTTACCAGAAACCTCTTCCCATTTCTTCCCTAGCCCTGCGTGGGCCCTGTCCCCATCCCCCACCACTATTTTCTTCCAAGTTGCGTACTGATTTGGGAACCGATACATCTTTATATGATAGCAAGCAATGCATATCAAAGTCTGGGTCACCTTTGGGAGCTATCCCAATCAAACCTGGGTTGTGAATAAACCTCATAATCAATGGGCCTAGTATCAACAACGTTGTCGACCGTTTGAATGCCCTCTAAAAGAGTTGGTTGAATACTATCATTACTTGAAGGCAGTAATTTATACATGACTAATAAGCCAAGAGTGAATATAGCTTGCAGCTTGGTAGAGTCCCCCCGAACTGAGAAGAATAGGAGCATGGCAAGTAGGAATAGATCAACACAGAAACGCATGATGCATATTACAGTATCCATTAACCTACCATCATTGCCTGCAGGGAAGCCACGAACTCGATAGACAAATAGAAAATTGTACTTGTCAACCACATATCTATACCCAAAATATACAGCACCAACAGGGACCACAAGGGGATTAAATGAACAATATACTAGAGTCAGGGCAAAAATTGTCAAATTAAAGGCATAATACTGTGCAAAATCGAATGTCTGTTTGGGAGCAGGTGAGCTTCCAGTGATTGGATAAACAAAAAGATCTTGTCCCTCAACATTATCCCCATTGGCAATGTCATAAGCATTGTCGTGCATTAGTGGTCTCTGAAGACTACTATCTATGCCCTGATGTTCTAAAGGGTATTCTTCACTTTGTTCCGGCACCAATTGAAGCATGTCATTCTTCCGAAATTTCTGAAGGTTTCTTTTAATCCAAGGAATGGGTGCCAACAGATCATAAGAAATTCCCAAGAAAGTGCTTGTAATCAGAAATGCAAGTGAGGAAAGGCATGACTTGGACAGGAACGATGCACTCATATACTGCTCAATCCTCTTGCAGTCTTCACCATCCAAATAGCACCGACCCATCTTCAAGATGGTACTCTCTAACGACGACTCCACCAAACCCCTCAAGAGAATAAGATTCACAAGGAAGAAGCAAACCATCTTCAACAGTGCAGCCCTTTGCTCCCCAGATACTGTCAAATGCCGTTCAAACTTCGAGAGATATGAAAGAACAGAAGGGATCACTATGTACATGCTAACAAATATGATCACATTGGGTAAAAACTGAAATATAATACTTGCAAGCCAGCTAGAGCTTTGCGCCCAAGCCAACCACAGCTGCGCACTATCCATAGCTTCAGCATTGATAATCCTCCCAGCACTCTGAACAGCAGTGATCACTGAAAGGGGTGAGCTGAAGAACAAAAGCATTAACAGCAAACAAGTGTTCACAAACACTCGCCGCAATTTCAGCGACATCCTCGGAGTCCCCATGTTCTTCCAGTAAATGTCAGTGGCCAAGGGAGCCCTCTCAACCTTCCACTGGTTCCGGCGCAACCGCAACTCCATAACCGAAAAAAACTTCCCAATTCTCCTCCTCTTCTCATTCTGAAAATCCTGAACAGCCTTATTAGCAGTATACACATCCCTGAACATAACAAAAGCAACACCAGCACCAGGCGCACGTCCTTCTTTATAATCAGCCAACTCACTCTCCAATTCAGCCCTCAGCTCCTGCAACTTTCGCAACCTCTCTTCATCACTATAACCAAACCTCCCTATAAAATCAATATACAAATCCTTCAACCGTTTCCAACAACAAACCACCGAACCCCACAATCCGGTGGGGGAGCCACCACCGTCACGCTCATCCTCCGGCAAAAGTCGAGAGTCAATCCTGGCCACCAGCCACGAAATCTCATCCCTCACACGCAACAATTCATTGGCAAGATCATCCAAAGCACACAAATCCATTGGGGCAATAACCTTATAAACCTTCCCCGGATACCTATACTGAAAATACTCGTGCAACACTACCCTATCAGCGGCTATAATTTTTGGCAAACCCTGAACCATTATAGTGAATATAGCAGTGGAATTCGCGGCAGGATCACTCAAATTACCGTACCCGTCCCTGAACCTCGTAATCCTCAAACGCTCCTCGGTTGCTGAAATCCCGAAGTGGACCAAAACCGCCACGACAACCGCGAAGAGGAAGTGAATCCAGAGTAGGGGGGAGCCTTTCTCGATGTGAGTAATCGTGGTTCTGGAGAAGCCGTCGTCGAGGACGGCGGTCCCGGCAGAGAGGTTGAGCGGGAGGAGGACAGAGACGGCGAGCACGGCGAGAGAGAGGAGGAGGGCGCAGCTGCCGCCCTCGATGAGGAGGAACTGCGCGGCATCGGCGCCGCAGTGGCGGGCGATTTCGCGGCTGGTGG

>C101367_65

AATCCCCGTACCCCCTCTTCTCTCTCTCCTTCAAACTCCTTCCTTTTCCCAAGGATTCCCTCCTAACTTGGTGGGATTATCCGAAGTTTCAGTTCCTTCCACTCACCCACATTGCCATTTCCTGTTTCACTCCTCTCATGGAAAAGCTTATTTGCATCTTCCCTTTCCTCCTCCTTCTTCTGTCCCCTCCTTTTGCCTTTGCTGGCCATGACTACGGTCAAGCTCTCAGCAAGAGCCTTCTTTTCTTTGAAGCTCAGAGATCTGGCTACCTTCCCCACAATCAGAGAGTCTCATGGAGAGCACATTCTGGTTTGCAAGATGGCAAAGCAAGCGGGGTGGATCTAGTTGGAGGGTACTATGATGCAGGGGACAATGTGAAGTTCGGGTTACCCATGGCGTTTACCGTTACGATGATGTCGTGGAGCATCATTGAATACGGCAAACAAATGGCTGCTAGCGGCGAGCTTGGTCACGCCATGGAAGCAGTTAAGTGGGGTACGGACTACTTCATCAAAGCACACCCTCAAGCCAATGTTCTCTACGGAGAGGTGGGAGACGGAAACACTGACCATTACTGCTGGCAAAGGCCAGAGGACATGACAACGGACCGTCACGCATACAAGATCGATCCGAGCAATCCCGGGTCGGATCTCGCCGGAGAAACCGCCGCCGCTATGGCCGCCGCATCAATTGTTTTCCGACGCTCTAACCCAGCTTATTCCCACGAACTTCTGAGCCATGCCTACCAGTTGTTTGATTTTGCTGACAAATACAGAGGCAAATATGACAGCAGCATTACTGTTGCCCAGAAGTATTACAAATCAATCAGTGGCTACAATGATGAATTGCTCTGGGCTGCTGCTTGGTTGTATCAAGCATCTAACAATCAGTATTACTTGGATTACCTTGGGAGAAATGGTGATTCCATGGGTGGAACTGGTTGGAGCATGACTGAATTTGGTTGGGATGTCAAGTATGCTGGGGTTCAGACACTGGTTGCCAAGTTTTTGTTGCAGGGCAAAGCTGCTCATCATGCACCAGTTTTTGAGAGATATCAGCAGAAGGCTGAATCTTTCATGTGCTCATGCCTTGGAAAGGGTTCTCGTAACGTTCAGAAGACTCCTGGTGGCCTCATCTTCCGCCAGAGATGGAACAACATGCAGTTTGTCACAAGTGCCTCATTTTTAGCCACTGTCTATTCTGACTACCTTGCTTCTTCTGGTAGAAACTTGAGATGTAATTCAGGCAATGTTGCGCCTGCTGAGCTTCTAAACCTTGCAAAGTCTCAGGTGGACTACCTTCTTGGGGACAACCCCAGGGCAACTAGTTACATGGTGGGTTATGGAAGCAACTTCCCACAAAGGGTTCATCACCGAGGTTCATCCATTGTTTCCATCAAGGTTAACCCTTCATTTGTCAGCTGCCGTGGAGGTTATAATACTTGGTATGGCAGCAAAAAGAGTGACCCCAACCTACTCACTGGTGCTCTTGTTGGTGGACCTGATGCATATGATGACTTTGCTGATGAAAGAGATAATTATGAACAGACAGAGCCAGCAACCTATAACAATGCTCCTCTCATTGGTATTTTAGCACGTCTGAATGGAGGTCATGGTGGCTATAACCAGCTTCTTCCAGTTGCTGTTCCAGCTCCTAAGTCTGTTGTTTCCAAGCCTCAACCATCCCCCAATACCAAGACAACTCCATCTCCAGATGCATGGTCTGGCGCATTTTCTATAGAACAGAAAATGACCACCTCATGGGTTGCCAATGGAAAAACTTATTACAGATACTCAACAGTGGTAACCAACAAATCTAACAAGAGTGTGAATTCTCTAAATCTTTCAATCTCCAAGCTTTATGGTCCAATCTGGGGTCTGATAAAGTCTGGTGATTCGTACACATTCCCATCATGGCTTAGCTCACTAGCAGCAGGCAAAAGCCTTGAATTTGTCTACATCCATTCTACTTCTCCAGCAGATGTCTCAGTGGCTAACTACGTGTTGGCCTGAGAGGGTTGAACTTGGTTTGATGTGACATGGGAATGGTTTACTACTTTGTTGTGTAAGACTGTGTAGCCATACAAATGTGCAGTGAGGAGAGGTTCTTTACGTTTTTACTTTTATATTTTGGTGTCAAATCTTCTTCTCTTGTGTATTTTGCATTTCAGTAGGTTAGATCAAAATAGGTAGACTATATAAGATGGAAGGACAGGAGCAGAAAGGAGTGAAGAGACAAAAAGGCAGAGCTCATCCTCTTTCTTCTCCGCTTTACAACTTATATACAGTGAGTGATACAAGACAGACAGCGAAACAGGGTTGTTGAGAAGTTTTAAAAAAATGAACTTCTCAATATTTGAGAGAAGAGTCATCACTTGTATTTTTCAACAGCTTTGGATGTCTTTTGTGTTTGATGTTGTAAGCTTTTTTGCAGCATTTTGTACTCTCTACAGAGAAACAGTTTGTGTTATATATATTATCATGTCCGTGTGCTGTGCAAAAAAA

>C101373_65

GTTTCACAGTGCACTCTTCCGAAGGAAGAAAAGAGACGATAATGGTGGGTGTTGGCGGGAAAAAAGTGCACCAAATGATCGTCGGTTTCGGCGGCAGTGGCTTCGGCCAGGTCGCCATCGCTATCGCCGTCTCCTTCCTCGTCCGTGTCTTCTCCGCTCCCGGCCCGGCTCTCTCGCCGGAAAACAACCCCGACGACACCCCGGAGAACGGTTCCGACGACGCCGAAGCTCCGGCCTCCGGCAATGTCACACCGGTCACAATCAGCTGGACAAACATCAATTGCTCCCTTTCCGATTCATCCTCCAAGTTCGTGAGGTTTCTACTTAGAAATGTGAGTGGAGAAGCGAGACCTGGAAGGTTATTAGCCATAATGGGACCTTCAGGTTCAGGGAAGACAACGCTGCTTAATGTTCTTGCGGGTCAGCTCACAGCATCTCCTCGGTTGCATTTGAGTGGCGTTTTGGAATTCAATGGAAAGCCTGGTTCGAAGAACGCGTACAAGTTTGCATATGTGAGACAGGAGGATCTCTTTTTCTCGCAGCTCACTGTGCGGGAGACTTTGTCTCTTGCTACCGAACTTCAGCTTCCCAACATATCTTCTGCGGAAGAGAGGGATGAATTCGTGAACAATCTCCTTTTCAAAATAGGCTTGGTTAGTTGTGCTGATACCAATGTTGGTGATGCAAAAGTTCGTGGAATTAGTGGTGGTGAAAAGAAACGCTTATCCTTGGCATGCGAACTGCTTGCTAGCCCATCTGTTATATTTGCCGATGAACCCACAACAGGACTTGATGCCTTCCAGGCTGAGAAAGTCATGGAAACTCTCCAGCAACTTGCACAAGATGGTCATACTGTTATTTGTTCTATACATCAGCCAAGAGGTTCGGTGTATAGTAAATTTGACGACATCATCTTGTTAACAGAGGGTTCACTTGTTTATACTGGTCCTGCTGGTGATGAACCACTGGCATACTTCTCAAAATTTGGGTACCAGTGTCCAGATCATATAAATCCTGCTGAATTTTTGGCTGATCTTATATCAATAGACTACAGTTCTGCTGATAGTGTCTATACCTCTCAAAAAAGAATTAGCGGTCTTGTTGAGTCGTTTTCGCAAGGTCTATCAAGAGTTATATATGCAACTCCAATTACTGTAAACGATTTCTCAAACAGTAGAAAGAAAATCAGCCAGAGGGCAGTAGCAAAGAAGAAAGGAGTTTGGTGGAAACAATTTTGGTTGCTCCTTAAAAGAGCATGGATGCAGGCTTCCCGTGATGCACCAACAAACAAAGTTCGGGCAAGGATGTCAATTGCATCGGCAATTATCTTTGGGTCAGTATTTTGGAGGATGGGAAATTCTCAGACTTCAATACAAGACAGAATGGGGTTGCTTCAGGTTACTGCAATAAACACAGCTATGGCTGCTCTCACAAAGACTGTAGGTGTGTTTCCAAAGGAACGGGCAATTGTAGATAGAGAACGTGCTAAAGGCTCCTATTCCTTGGGTCCCTATCTGTTGTCGAAATTGTTGGCTGAGATTCCAATTGGAGCTGCCTTTCCATTGATGTTTGGTACTGTTTTATATCCAATGGCTCGTCTTCATCCTACTTTGCAGAGATTTGGAAAGTTCTGTGGGATTGTCACTATGGAATCTTTTGCTGCATCTGCTATGGGTCTTACTGTTGGTGCTATGGTTCCAAGCACTGAAGCAGCAATGGCAGTAGGTCCCTCTCTCATGACAGTTTTTATTGTTTTTGGAGGCTACTATGTCAATCCTGAAAATACCCCAATCATCTTCCGTTGGATTCCTAATGTTTCTCTGATTAGATGGGCTTTTCAGGGACTTAGTATCAATGAATTTAGTGGTCTTCAATTTGACCATCAACATTCATTTGATATTCAAACTGGCGAACAGGCGCTAGAGCGATTTTCCTTTGGGAAAAGTAGGATTAGGGATACGGTGATAGCACAAAATAGAATACTACTATTTTGGTATTGCACCACCTATCTTCTCCTCGAGAAAAACAAGCCCAAATACCAGCAACTTGAAACCCCTATTGACGACAACAAACCGCTTTTAAAGCTTGAGGAGCTCGATTCTGAACAAGTTGATCAGACACTTGAAGCTCCGCCAGTCGGCCAAGTTGATTCCAACCAGCCCCTTGAGTCACCTGCAGTAGATCTTGTTGGCCCTTTTGTCCTCGAAGGTGCTAAGTAGAAAGATACCTTTGCAAAGGATGACCAGCAAATACTGAGACAGTAAGCCGTTTCCTATAGAACAAGACCACAATCACAATGTGATAATTGGACATCGAAAACATCGGCTAATGCTTACAGGATATTAGTTTTTTGGTCGAATTACAGGCTGTTAGGTGTAGTAATAAACAGTGATGGTGGATAATCCTGGACTGTCTCATGTTGGTTCATTTGATTATAGTGTTTTTTTTTTTTCATTCAATGATCCGAGTTTATGTGGAAATCATCCATGAGATGGGTTTTTCTTG

>C101377_65

GCCAATAACCCACCATCTTCACATTCACCACGTAGTACTGCCCCCACTCTCAGGCAATAAAACTAGGATAAAGTTCAACTCTTTGTCGCTTTTCCGTTTCTCTCTTCTCTCACTTTCTTCTCACCTTCTTCTCACCTTCTCTGCTGAAAGAGTCTCAACACAAAGCTCCTGCTCTTCTTCTTTTCTTATTGACCTGTGGCTACCGCAGAGACTTTTTTCTGGATCTTCAGGTGCAGAGGCCTTTAAAAGTTAAGAACTTGTTAACTTGAATTGGTGTATACTGCTAGAGGGTAAAGCTTGAAACATATCTCACCACTTTCATTGTCGGAGGATTATAGGTTGGATACATATTTTTCCACGGAAGATGATGATGATGTTTGATGAGATGGGGTTTTGCAATGATTTGGATACTATACCCGCTTCCCTTGGAGAAGAAAATATTACCGCCCGGCAAACTGATCCAGAGGCGATAGTTGAGGATGATTTTAGCGATGAAGAAATTGGAGTAGATGAGCTTGAGCACAGGATGTGGAAGGACAAAATGCTTCTGAAGCGATTGAAGGAACAAAGTAAATCTAAAGAAGGAATTGATGCTGTGAAGCAGAGGCAGTCGCAAGAACAGGCCAGGAGGAAAAAGATGTCCAGGGCTCAAGATGGAATCTTGAAGTATATGCTGAAGATGATGGAGGTTTGTAAGGCCCAAGGATTTGTTTATGGGATAATTCCCGAAAAGGGGAAACCGGTGACGGGAGCATCTGATAATCTTCGTGAATGGTGGAAGGATAAAGTCAGGTTTGATCGTAATGGACCTGCGGCTATAACGAAGTATCAAGCTGATAATGCTATCCTGGGGAGGAACGATGGATGCAATTCGATTGGTCCAACTCCACATACCTTGCAAGAGCTGCAGGACACAACTTTGGGTTCTCTCTTGTCAGCACTCATGCAACACTGTGATCCTCCTCAGAGGCGGTTTCCGTTGGAAAAGGGTGTTCCTCCACCATGGTGGCCCACTGGAAATGAAGTGTGGTGGCCAGAAATTGGTTTACCTAAAGATCATACTCCTCCTCCTTACAAAAAGCCCCATGACCTGAAGAAGGCATGGAAAGTTGGTGTTCTGACTGCAGTTATAAAGCACATGTCCCCGGATATTGCCAAAATTCGCAAGCTTGTGAGGCAGTCCAAATGCCTCCAAGATAAGATGACAGCCAAGGAAAGTGCTACCTGGCTGGCAATCATCAATCAAGAGGAGGCCTTGGCAAGAGAGCTTTACCCTGATTATATTCCCCCATTAGCCTCCACTGGAGGTAGTGGATCTTTGGTTGTCAATGATAGCAATGAGTATGATGTTGAAGGGGGTGAGGATGAGCCTAACTTTGATGTGGAAGACAGGAAACAAGAGAATATTCACATGTCCAATCTGGGGATGGAGAGAATGAGGGGAACTCTGCCGGTTCAGCAGCCCTCTTTTTCAATCAAGGGAGAGACAGTTACAAATTTGGATTTCATTAGGAAGAGGAAGGTCTCTAATGACTTTAACATGATGGATTTGAAAATTTACACGTGTGAACAGCCTCAGTGCCCTTACAGTCAAGTTCACCTTGGTTTCCCTGATAGAATTTCTAGGGACAATCATCGGTTAATTTGTGCATATAGATGTTCTTCAGAATTTGGGGGTCCAAATTTTCATGTTAATGAGGTAAAACCAGTCATATACCCCCAGTCATTTGTTCAACCGAAGCCTACTGCTCAATCTGCTAATATGGTTCCACCTGTAATTGATCTTACTGGACTTGAAGTTTCAGAAGATGGCAAGAAAATGATCAGCGACCTTATGACTAACTATGATACAAATGTTCAAGGCAACAAGAACATAAGTTCCTGTAATCGTGTAGCTGGCGAAAATTTGAACCTTCCTCAGCATGGCATGCAGCAGCAACAGGACAACTTCATTCGCGGTCAAGGAATCACTATTGAAGGAAGCATCTTTGATGAAACTAACGTGTCAAATAATCATAATATGTTTGCAAGAGATGAATGCCAATTTGACCGGTTCAAAGCTTTGAATACCCCCTTTGAGACCAATCATAACAACAACAACAACAATAACTTTCATTCCATGTTTGGGTCCTTTTGTGATTTGGCATCATTTGATTTCAAGGAGGATATGCAAGGAGTTGGGATGGATGCTCTTCAGAAACAGCCAGATTTTTCTATATGGTACCAGTGAAAGTGACAAGTGGAATCCAGAAGCTCAAGCTTATTGTTGTGAACTTTTATTTTCATGTGAAGTTCCTTCAGCTTAAAGTCTTACCTCAATTTTCTTCACATGCACTGCTATAGGCCTAGAACTAGATATTTTATACTTATGCTTTACAGGTCTAGTGTTTGTTACAATAAAAAAACTTACCTATGCTTGTGTGTTTTTCATCTGTCTAGCCTTCTATTACTTATTCCATGTAAGACTGTGATATAAATAAGACTTGAAGTTTCTTCCCAAAAAAAAA

>C101379_65

TTCTGATGCGCGCCAGAATGAAGCCCAACGATCGTACAAACCTTTCTCTCTCTCCCGTTCCCCTAACTAACGCGACGAGTCAAATGCAGCAGCCCAATTCCCGCCAAAATGAATCTCCGGGTGAAGGAAATTCGGCGCCTAATGACTCCGGCTACTCCGAGAGAGAGTTTAAACGCGTCCGTGCCTCAATGCACTCTGCTATATCAATGAACAAAACGGAAGTTCTAGATGATGTGCTTAATAACTTCTCGGAGGGTTATTTTAGTCTTTCTCATGAAAACCGTCGCAAGTTGCTCCTTGTCCTTGCCAGAGAGTATGATCTCAATCGATCACAAGTGCGTGACCTGATAAAGCAGTACCTTGGACTTGAATTACCTGCTGACAAAGCTCAAGTGAGTGGCTCTGAAGATGAAGGCTTGTTTTCTTCTTTCTATCGCATTGAGAGGAATTTGAGGCATGCCCTTCAGCCAGTTTACGAAGTTCTCTTTGAGCGTCTCAACACTCATCCTGGAGGATTGAGGATCTTGTCGATTCTTCGAGAAGATATCCTGACTATTCTCGCAGAGGAAAATATTGCATCTCTGAGAGCGTTGGATTCTTATCTGAAGGAAAAATTTATTACATGGCTTAGTCCTGCTGCCTTAGAGCTTCATCAGATTACATGGGACGATCCTGCTTCTTTGCTGGAGAAAATTGTTGCTTATGAAGCTGTGCACCCTATTAGCAATCTTCTAGATCTTAAGAGAAGGCTGGGAATTGGCCGTCGTTGTTTTGGATATTTACATCCAGCTATTCCTGGTGAACCCCTGATTTTCATTGAAGTTGCGCTACTGAAGGATGTTGCTCAGACTATACAGGAAGTTTTGTGGGATAGTCCTCCAATACCTGAAAGTGAGGCAACATGTGCCTTGTTCTACTCTATATCATCAACTCAGCCTGGCTTAGCAGGGATCAACTTAGGAAAATTTCTTATCAAACGAGTTGTAACACAGGTGAAAAGAGAGATGCCACATATTTCTACATTTGCAACTTTGAGTCCCATCCCGGGATTCATGTCATGGCTTTTATCCAAGTTAGCGGCATCTCAGATAATAGCTGAAGGAGAAAATTTGTCTCAACTGCAAGCTGAGGGATCTAGTTCCACTTTCTATGAAAATATACTTAAACCCGAGGAGGAAGAAGCACTTATGAGTTTACCTAAGGATATTGCCGTTGGAAAAAATGGAATGGATTTGATGTTCAACTTGCTCACTTCTACATCCTCTAAGTGGATCCATTCTCCAGAACTTCGGTCAGCATTAAAAACCCCTTTGATGCGTTTATGTGCCAGGTACCTTCTGCAAGAGAAGAAAAGAGGAAAAGCTTTGGACTCCGTAGCAAATTTTCACTTGCAAAATGGAGCGATGGTTGAAAGAATAAACTGGATGGCTGATCGATCAGAAAAAGGTCTTTCTCAAAGTGGAGGGATCATGGTGAACTATGTGTATAGGTTGGATCACATAGAAGAATATGCCCATTCATACTTCAGCAATGGTGAAATTCATGCCTCAAGTGATCTACATCGCTACGTTGATGTAGTGGATACTGCTTAGTGTCCATTCAAATTTGATAGAAGATAAACTTCAGCATATTTTCAACAACATCATATGTGCTTTGGAAACAAAGCTCGTCGGCAGCGGACAACCTTATTAATACTAAGCTTTACCTTTCTCTGTCTCAACCTCTTTGCTGTCATTTGCAGCCAGAGTGATAAGTAATTGTGAAGTCTGCTGATCGGAGCATAATGGAGGTTTTATCCAAACAATAGGACAAATGCGTCGAAGTCTAAAACAGGATGCTTATATATTTAATTGTTGATTATTCCTGCCCGGAGAACAGAAGACAACTGATCGTGTCGCCAATCCAATTCTTTGGCAAAATTGGGTAGGAGACAGCTTACTAAACTTCCCTACTTTATTTGGTCATAAGATGATGGAGGGGCCACTCTTGTTCGAGAGGTGCAACGTAGAAAAGTTATATTGAATAGGTCATGCTTGTTAAACCTCAAGCTATACATCTGATGTGATATTTGTAGTTACTATACTGACAACGAACCCTATATCTTAGTGAAGGATGATGCTGCTTCTGAAATTTAGCTGTGTTGGTTGGAGATCATGAAGTGTAAAATGTAAACTATCAAAGAGATATTATTTCCTTGAACATTGACGATTGAAGGTAATGCTTGTTTCTTGTTTGTTTCATCGTTGGACAGTGTTTTCACTTTCAGATGTAAAGGAGAATTGATTACGCACAAATCCGTCAAAGCTGTCTTCAGAACCAAGACTTGAAAACAGCACCAATATTTGTGTTGAAATATGATATTTTATATCTGCTTGAAATGTTACTTATTTGTAAGGGAGCAGGCAATTCTGGTGCTCCCAGGTATCCTATTAAGTGTGTTTGTATAAACGATTTGCCTTCCATTGAAAGGGATTTCACACATTCCAAGCGTCAAAAGTGACTTCAGAAT

>C101381_65

GCCTTATTATCTCTTTTTTGCCTCAGCTAGAGTTCTTCATCTGCATTTTTTTTTGTGTAGCCCACTATGAATAATATTGGGAAGTTGGGTGCTATGCAACAACTGATAAAAGGTGGGAGAATGATGCCTGCAGCCGCAGTATCTGATGACAGCGTTTTGGTGAAGAAAATAGTGGCAGATCATAACCCTGAGGGGATGATTGAATATGATGTCAGGCCTCTTCTCCATATAGTTGAGGATGTTCTAATACGTTCCACCTTGAGTTCTGAAGGTGTTACAACGGGTGTTCTTGCACGTGTTGATCATGTGGAAGACCGAAGCTACCGTCCTGGCCAAACAAACATGATAGAGGCTCTATCTGCTAAGATTGATCGAATTTCCTGTGAGATTTCCTACAAGACTCTCGGTGGTGTAGATTCACACTCCACAACAGTTGCAATATTTGACATGCTTACAATCTTCAAGTGGGATGTGAAAATAGTGCTGGCCTTAGCCGCTTTTGCTCTAACTTACGGTGAATTTTGGCTGCTGGCCCAAATTCATGATAAAAACCATCTTGCCAAATCAATGGCAATTCTGAAGCAACTACCTAGTATCATGGAGCATTCAAGCTCGCTGAAACCACGGTTTGATACCCTTAATGATCTAGTTAGCATAATAGTTGAGGTGACCAAGTGTGTGATAGAGTTCCATGATCTGCCATCTCAATACATTACACAAGACATATCAGCCTACACCACTGCCGTTAACTATATCCCAGTTGCTTCCTACTGGACCATCAGAAGTATTGTGGCTTGCGCAGCTCAGATTACCAGCCTCACTACCCTTGGCTATGAGATATTCACATCCACTGATGCTTGGGAGCTATCTACGTTGATTTTCAAGCTCAAAAACATAATAAACCATCTCAGGCAGCTGCTGAATAGCTGCCAAGAACATATTGGGAAAAAGATGGATGCTGAAGCTTACCAAATGCTGCGAGATTTATTTTCAAAACCCCACACAGACAACATGAAGGTTCTCAAGGCTCTGATTTATGCCCAGGATGATCTTCTACCTCTATATGATGGTGTTACCAAGAAAAGGGTTAGCCTTGAGTCATTGAGAAGGAAGAATGTGCTGCTTCTATTTTCAGGCATGGAGATCTCCACCGACGAGCTTCTGATTCTTGACCAGATCTACAACGAATCAAAGGCTCATGCACCGAGAATGGAGAGTAGATATGAGTTGGTTTGGATCCCAATTGTGGACCCAAACTCTGAATGGATAGATCCAAAGCAAAAACAGTTTGAGAGCCTGCAAGAAAACATGTCATGGTACTCAGTGTATCATCCGTCCTTGATAGGCAAGCCAGTCATCTGGTTCATCCAGAGCGAATGGAAATACAAGAATAAGCCAATTCTTGTGGTTTTGGACCCACAAGGGAGAGTTTCTTGCCCCAATGCTATCCACATGATGTGGATTTGGGGAAGTGCTGCCTATCCTTTCACAAGTTCTAGGGAAGAAGCTCTATGGAAGGAAGAGACCTGGAGACTTGAATTGCTAGTAGATGGCATTGATCAAGAAATATTGAACTGGATTAAGGATGGAAAATACATTTTCTTGTTCGGAGGGGATGATCCTGAATGGGTAAGAAGATTTGTGAAGGAAGCCCGTAGGGTTGCAATGGCTACACAAATACCCTTAGAGATGGTATATGTTGGTAAAAGCAACAAAAGGGAGCAAGTGCAGAAAGTCATTGACACAATAATTCGTGATAAACTCAACACTCAATACTGGTCAGAGCAGAGCATGATATGGTTCTTCTGGACCCGTCTTCAGAGTATGCTCTTCTCCAAGCTTCAACTGAAGCAAACCGATGAAGACGACCATGTGATGCAGGAAATCAAGAAGCTTCTCAGCTATGACAAACAGGGTGGATGGATTGTTCTGGCAAGAGGGTCTCACATTGTGATCAATGGGCATGCCACAGCCGGGATACGAACCTTGGTGGAGTATGATGCCGTGTGGAAAGATCATGCTGACAGGGATGGGTTTGAACCAGCTTTTAAGAATCACTATGATAAGGTTCATTCGATTGTCAGTCCTTGCTGCAGGTTCGAGTTCTCCCATGCAATGGGGAGAATCCCAGAGAGGCTCACATGCCCTGAGTGTCGCCGCAACATGCACGTGCTCACCACCTTCCAGTGCTGCCACGATCAAAAAGTTGATGAGGACTTTTTTGTCAGCACTGTTACTCCTCCCACCAATTGATCATTCCTTGTTTACTCACTATTGCTGCCTCTGTTTCTCAGCAATTTCAAGCACAACCTTGTGTGTTTACTACATGTGTCAGTTCTATTTATCACAAATTAATGTAATAAAATGTGAATGGTGAGGTTCGGTCTCCCCACTGAGGGATGAGTAAATTATTATGTGACTTCTGTCAGAAGTTTGTTCTTATTGTACTATTGCATTAAGAGGAGAGTATGTATAAATTGGGTAATTTGGATGATCAATAAAATTACAG

>C101383_65

GAGACATTGTGGTACTCGTTTTTCTTCACTTGGTGTTGGTGCCTTCCCTGGTTGGTTCTACACGAGGCTTTTCAGCTATAGCCTAACCCTCTCTCTATTTCTTTGTCTCTGAGGATTACTTGCATGAAACTAGTAAAGCATTCACTTTCCTGCTTCACATGTCCATGCATATATGCTTGTTTCTGACTTTAGGCCACCACATATCTCTGCACTGCCTTTAGTTCAAGAACGGATGCTTCTGCTTCATCTACTTTCTTCATTTTGTACAGTTACTTCGAAACTGCAGCTTTTTCAGTTGTGAATGCTTTTCTCTGGTTCCTATCATTCAAGCAACATCAATGATACATGTTCACGGATGAGGACACCATATCTACAAATTGAAGTTCTTGCAGAAGTATATGAAAATCAATGAGGTCGTGAGGGCATTGTTATGTGAAAGTTGAGAATTTGTTCAGGCTTTCAAGTAGAGGTTTTTCTTGACCATTGGGTATAGATCATAATAAGTAGATATGAAGTTCATGAAACTTGGAACAAGGCCTGATACTTTCTACACTGAACAAGCTACCAGGAGTCTGGTTTCAGATGTACATACTGACCTTGTGATAAAAATCAATGACACGACTTATCTGCTACACAAGTCTTCACTTCTTCCAAAATGTGGCCTTCTACAAAGACTATGCTCAGATTCTAGTGATTCTGAGAATGTTCCTCTAGAGCTTCATGATATGCCCGGAGGTCCAGATGCTTTTGAACTCTGTGCTAAGTTCTGCTATGGAGTTTCAATCAACATTAGCGCTCATAACTTTGTACCTGCACTCTGTGCTGCTAAGCTCCTACAAATGAATGAGTCCACAGAGAAAGGAAACTTTGTAGGCAAACTTGAGGCTTTCTTCAGTTCATGCATTCTTGAAGGATGGAAGGACTCTATTGCAGCACTGCAGGCCACTGACAAGTTACCGGAGTGGTCCGAGAATCTTGGCATAACAAGAAGATGCATTGATTCAATTATTGAGAAAATTCTTACACCTCCACCACAGGTCAAATGGTCCTACACCTACACTAGGCCTGGTTATACTAGAAAACAGCATCATTCAGTCCCAAAGGATTGGTGGACTGAGGATGTGTCTGATCTTAACATAGATCTTTTCAGATGCATAATAATGGCTATTAGATCAACATATGTGCTCCCGCCACAGCTTATTGGTGAAGCTTTGCATGTCTATGCTTGTAAGTGGCTACCTGGCATCACAAAGCTTAAAAATTCATTCGGTTCTGCAGCAATAACCGAAGAATCCAAAGCAAGTAACCGAAAAATTCTGGAGACAATTGTGAGCATGATTCCTGCAGATAGAGGGTCAGTTTCAGCTGGTTTCTTGTTAAGGCTTCTTAGCATTTCAAACCCTCTTGGTGTCTCCCCAGTGACCAAAACAGAACTGGTGAAGAGAGCCAGTATACAGTTTGAGGAGGCAACAGTGAGTGATCTGCTTTATCCTTCAACATCCTCTTTGGACCAAAATTTTTATGACACTGAGTTAGTTCTAGCAGTGTTGGAAAGTTTCTTGAAGTTTTGGAAAAGAATCTACCCTGGTGCTGTGGATAACAGGCACTTGTTAAAATCAATCAGAAATGTTGGTAAGCTCATTGATTCCTATCTTCAAGTGGTGGCAAAGGATGAAAATATGCCAGTGTCAAAGTTTGTCTCCCTGGCAGAAACTGTGCCAGCTATTGGCAGATTAGAGCATGATGATCTCTACCAGGCTATCAACATCTATCTGAAGGTGCATCCTGACCTGAGCAAGGCAGACAAAAAGCGTTTGTGTGGGATTCTGGAGTGCCAAAGACTGACCCCAGAAGTGCGTGCACATGCAGTCAAAAATGAATTTCTGCCATTAAGAACTGTGGTGCAGCTCCTCTACTTTGAACAAGAAAAAGATTCTAAGGAAACCACTAGTTCCAAGCTGCAAAAATCACATGATCTACTTCTAGGAGCAAAAAAGAGACCAGCCACTAGAGATAGCCATGGCAAGCTGTCTTTAATAAACAAAGAAGAATTCAACAGAGAAGAAGTCACAAGAAGAACCTCTCATGCTGAAGGTAGAGAAAAGGGTCTACACAAAGCTAAACAATCAGATGCTAAGATAGCATTGGACTTGGAAAAAAAAATGGTTATAAGAGGAGACACTGAAGAGATAGGGTCAGAAAAGTTAAGGGGGGCCAAAGAAGAGAGAATGTCAAGCAGCAAACTGGACTTGGATTCTAAGAATAACATACAAAGGGCAAGAAGCAAAAAATCAGAACGTGGCCGTGAAAAGGGAAGATAGAGATAATAGTAATTACAATCTTTGAAACTGAAACATTCAGAGCAATCCTCATGTCCCTATATATGAACTTAGTAGCCTCTATTCTTATCATTTCTTTTTCACGTGTCATAAATGTAAGCAAGGTATTGTCCTTTCTACTTTCTATCACTGTTAAAGAGTTGTATTTATCAAAGAGGTCATTGCAACAAAT

>C101385_65

GCGTTTACCAACGCCACAACACAACACAGTTTCCCTCTCTGAAATTTCTTTAGAGAATGGGGAGGAGTAGCGGAAGAGACCGAAGACGAAGAAGATCGGATGAGTCCGAATCCGAGTCACCGTCCGACTCTGACTCGGACCGACGCCACCGTAGCAGTAGCCGCCGGTCGCGGCGTGACTCCGACGGCGATAGGAAGAAGAAGTCCTCGAGGAACATCACAGAAGAGGAAATCGCGCAGTACATGGCCAAAAAGGCCCAAACAAAGGCTATGAAAGTCGCGAAAAAGTTGAAAACCAGTACGGTATCGGGCTATTCCAACGATTCGAATCCGTTTGGTGACTCCAATCTCAACGAGAAGTTTGTATGGCGCAAAAAGATTGAACGCGATGTTTCTCAAGGTGTGTCTATTGACACGTTTTCAGTTAAGGCCGAGAAAAAGAGACAGAGAGAAAGGATGGCAGAAATTGAAAAAGTGAAAAAGAGAAGAGAAGAAAGGGCACTTGAGAAAGCACGACATGAGGAAGAAATGGCACTGTTAGCTAGAGAACGAGCTCGAGCTGAATTTCAGGACTGGGAAAAGAAAGAAGAAGAGTTCCATTTTGATCAAAGCAAAGTTAGGTCAGAAATTAGATTGCGTGAAGGGCGTGCCAGACCAATTGATGTCCTAACCAAGCATCTCAATGGCTCTGATGATTTGGATATAGAAATAAATGAACCGTATATGGTCTTCAAGGGTCTGACTGTAAACGAAATGAACGAGCTACGTGATGACATCAAAATGCATCTGGACCTTGACAGGGCAACACCAACTCATGTAGAATATTGGGAGGCACTCCTTCTGGTGTGTGATTGGGAGCTAGCTGAAGCTCGAAAAAAGGATGCACTTGATCGAGCTAGAGTGCGTGGAGAAGAACCTCCTGCTGAGCTGCTTGCAGAAGAAAGGGGTCTGCATTCTAGTGTTGAGCCAGACGTGAAGAGGCTTTTGCAGGGGAAGACACATGCAGAATTGGAGGCATTACGGGTTCACATTGAATCAGAAATGCGTACTGGTACAGCAAAGGTGGTTGAGTACTGGGAGGCCATTTTAAAACATCTCCACATTTATAAAGCCAAGGCTTGTTTAAAGGAAATTCATGCTAAATTGCTACGTAAGCATTTGCAATCTCTAGAGAAACCATTGGAGGATGAAGATAAATTGGAGGATGCTAATGTTATGATACCTGAGGAGGAGGAAGATACTGAGGATGATATTAAAGTCCGATCTCCAGATGAATCATTTTCACCGGAGCCCATTAGAGAGGAGCAAGAAGCTGAAGATGAGGCTGGATCATTTTCGCCCCAACTGTTGCATGGTGATGAAAGTGAGGAAGCTATTGACCCTGAAGAGGACAGAGCCATGCTGGAGCGAAATCGTAAGACTGTATTAGAAGAGCAGCAAAGACGAGTTCAGGAAGCAATGGCATCAAAGCCAGCTCCATCTGAAGATAATTTTGAGATCAAGGCCTTAAAAGCTATGGGGGATATGGAAGATGGAGATGCTGTGTTTGGATCTGGTGCTGAAGTGAACCTGGATTCCCAGGTTTATTGGTGGCATGACAAATACAGGCCTAGGAAGCCGAAGTATTTCAACCGTGTTCACACTGGATACGAGTGGAACAAATATAATCAGACTCACTATGATCATGACAACCCACCTCCAAAGATTGTGCAAGGATACAAATTTAATATTTTCTATCCTGATCTTGTAGACAAGACAAAAGCCCCAACCTACACCATTGAGAAAGATGGCAGCAATGGGGAGACTTGCATTATAAGATTCCATGCTGGGCCACCATACGAAGACATAGCTTTTCGCATCGTAAACAAAGAATGGGAATATTCACATAAGAAAGGTTTTAAGTGCACATTTGAACGTGGAATTCTGCACGTGTACTTCAATTTCAAACGCCACCGCTACCGCCGCTAATTTCACACAAGGCTATGGTTCGCTCTGTGTAAAGTTTTCCGACTAGAAAGGCTACGTGGCTGCCACTTTTAATAGTAAATTTTATGCCATGGTGCTGGCACTAGAGATAAATGATGACCAGGAAATTTACAGCTTCGATTGAGACGAAGAAGAAATTCTAAGGTACTTTTAGAGTGATATTCGCACCTGTTATTTGATGCAAATCTTGTCATGATATTTTAAATTTGACTTAAGGAGTAGAACGGTTATTCTGTGCTCATCTAGTGATTATATTTTTAAAGGATTGAGTGTAGTTACTAATAAGTTTATTGAGACCAAGGCTCTTTCCAAGCGGTGAGTTACTAATAACATTCTTTACAATCACTCTGAATCCTGTCTGATTTAACTAGGTTTTCTTTTACATTATTGGAGAATATCGCACTGTCCAACTTTTTCTGTAGAAGAAGTTGATTATTGTACCTTGATGATGGTTGTAATCTCATAAGGTGAAAGAATTTGTGGTAGCAGACATATGATCAGTTATAACAACAATATAATTATTATT

>C101389_65

AAAAGACTAGTGGATAGAGAGCACCATCTTTATTTCTTCTCCCCACTACATATTCAGTATCTATCTTGCGAAAAGAAGAGAAGGTGAGAAGAAAGTAGAGAGAGAGGATGAGAGAAGAAGGTGTGAGAGAAGATGAGTGCTTGTTTGAGACGAAGGAAGCAAGATTTCGAGGAGTGTACAAGGTGTTTGCATCAACAATATTTGCAGCAATTTGTTTGATTTGGGTGTACAGAGTTGAAAACATGGGGAGAGTTAGAAGAGGAAGGTGGGTTTGGATGATTGTTATGTTATCAGAGTTTGCTTTTGGATTCTACTGGTTCATCACTCAATCTGTTCGCTGGAGAATCGTATATCAAACACCTTTCAAGCACAGACTCTTCCACAGATATGATGAGGAGAATTTACCAGAAGTAGACATTTTTGTGTGCACGGCTGATCCAAAACTGGAGCCTCCATGCATGGTGATTAACACAGTATTATCAGCAATGGCATACAATTATCCTGCAAATAAATTGAACGTGTATCTCTCTGATGATGGAGGGTCTGAGTTAACATTCTTTGCTCTCTTAAAGGCCTCCATTTTCTCCAAACATTGGTTACCTTTCTGCAGAAGATTCAATGTTGAACCTAGGTCCCCAGAGGTTTTCTTTACTCATTCCCAAAACTATAGCAGTAGCACTAGCACTGAATATGGCAAAGCATGCTTATTTATCAAGAAATTATACGAAGAAATGAAAAGTGAGATCGAATCAGCCGTAGCAAGTGGAAAGCTTCCAGAGAATGTGAGGAATCAGCACAGAGGATTCTTAGAATGGAATCCAAAAACGACAAAGCAGGATCACCAGCCTATAGTGCAGATTATAATCGATGGAAGGGACAGAAATGCTGTAGATGAAGATGGATTTCAACTTCCAACTGTAGTATACATGGCTCGCGAGAAACGACCCAACCATCCTCATCACTTCAAAGCTGGAGCTGTTAATGCACTTATAAGAGTGTCGTCTGAAATAAGTAACTCCCCTTTCATCCTCATCTTGGACTGTGATATGTACTCCAATAATCCGGATATAATTCCAGAAATACTATGTTTTTTCCTCGATGAAGCGAAAGGTCAAGATATAGCTTATGTACAATTTCCACAAAGCTATAATAACATCACCAAGAATGACCTTTATGGAACTTCTTATCTTGTAACCTCTAAGTTTGAGTTAGCTGGCATATGCGGATATGGAGCAGCCCTTTTCAGTGGAACTGGATGCTTACATCGAAGGGAAAGTCTTTCAGGATCTTACTTAAGAGATTACAAAGAAAAACGGGACATGAAGCCTAAGAGAAACAATAACAGAACAATTGATGAAGTGAATGAAGCATCGAAGGCTTTGGCCACTTGCACCTATGAGGAGGGCACCCAATGGGGAAAGGAGATGGGATTAGTATATGGTGTTCCTGTTGAAGACATTGCGACTGGCCTTGTAATCTCGTGTAGGGGTTGGAAATCTATTTATTACAACCCAGAAAGGAAAGCTTTTATGGGCTTAGCTCCAACAACTTTGGATGTAGCCAGTCTTCAGTATACGAGGTGGTCAGAAGGCATGTTCCAAATTTTCTTCTCCAAGTATTGCCCTTTCATTTATGGGCATGGAAAGATACCCTTGGCTCTACAAATGGGATATTGCAATTACCTCTTTTGGGCTCCTATGTCACTGCCTACTCTTTGTTATGCCATTGTTCTTCCTATTTGCTTGCTTCATGGCATTTCGTTGTTCCCACAGCTTTCAAGCACATGGGTCCTTCCATTTGTGTATGCATTTCTTGCTACCTATGGCTATAGCTTGTGTGAGTATTTGAATTGTGGATCCACGACAAAGGGTTGGTGGAATTTGCAGAGAATAAAGTTCATCCACAGAATCACCTCTTATCTCTTTGGTTTCATTGACACTATGGCCAAGCAATTAGGATTGTCACAGACAAACTTTGTCATCACAGATAAAGTGGTTACTGAGGATGGTCGAAAGAGATATGAACAAGAAGTCATTGATTTTGGAGGCTCTTCGATCATGTTGATCATATTAGCAACAGTGGCTTTGCTAAACCTTTTTGGTCTGGTTGGAGGAATCATGAGGGTTGTAATGGATTTGGAATTCAGCTCAAGCCAATTGATGGTGCAGATTGGAGTGAGTTTATTGGTGGTGATGATGAATTTACCGATTTATGAGGCACTTTTCATTCGTAGTGATAAAGGCTGCATATCATCCTCGATTATGCTGAAGTCCATTGTCTTGGCTTCATTAGCATGTTACCTACCATCTTTCATTTCCTAGTCTTGCTTAACTCCAATGCTTGAAGTGTGGTTCAATAATTCCTAGTTTGGCCTCCTTCACTTGATGTTTTCATGTTTTAAGTGCATAAACTACTTATCTCTGTTAAAGTATTTAGCTTTCCATACATGTAGTCACTATTAGTTTATGGAAACTAAACATTGAAGGATTTCCAATATTTGATTTAATAAACA

>C101391_65

TGGACCCATCTCCCCTACAAACCTTTCCTTTTCTTTTCACTGAGGAACAAAAAGAGAGAAACAGAAGCGACGATAAATCCATCCAAACTCTCGTTTCGTTCGTTCTCGTATTTTTCTGCGAAACCGCTGCTTCGGGATTTTGTGCCGTAAAGCTTAAGGCAGATTGTCACGTTACATTGGCCTCTGCCGAATGAAAATTCGTGGTTCTCGAATCCATCTGGTGCGATAGGGTTAGTGAAGCCTGAATCTGAAGCTTCGCGGACGTGGCTATGCTCCGCAAATTGTTCTTCCGCAAGCCTCCAGATGGCCTTCTCGAGATCTGCGAGAGAGTTTACGTTTTTGATTGCTGCTTCACCACGGATGCTTGGGATGAAGAGAATTACAGATTGTACATGGATGGGATAGTTAGTCAACTTAAGGAGAACTTGCCTGATGCTTTGATTCTGATTTTCAATTTTCGTGAGGAAGATATCAAGAGTCAGATGGCAAGTATCATGTCTGAGTATGATATTACTATCATGGACTATCCTCGGCACTACCAAGGTGTTCCCGTGCTCAAAATGGAGCTCATCCACCACTTTTTGAGGTCTGGTGAAAGCTGGCTCTCGCTTGGGCAACAGAATGTGTTGTTGATGCATTGTGAACGTGGTGGTTGGCCTGTTTTGGCTTTCATGTTGGCTGCATTGTTGATTTATAGAAAGGCTTATACTGGGGAGCAGAGGACTCTCGATATGGTTTACAGGCAGGCTCCACACGAGCTTTTGCATTTGTTGTCACCGTTGAATCCTATCCCTTCCCAACTGAGGTATCTGCTGTATGTTTCCAGGAGGAATGTGGCCTTAGATTGGCCTCCCCTGGACAGGGCACTTATGTTGGACTGCATCATTCTCAGACTCTTCCCAAACTTTGATGGTGAAGGTGGTTGTCATCCCATGTTTAGAATTTATGGACAGGATCCCTTTAGTGCTGATAAAAATCCAAAACTGTTGTATTCAACGCCAAAGAGAAGCAAAAATGTCCGGGCCTATAAGCAGGGAGAGTGCGAACTTATTAAAATTGATATCAATTGCCATATTCAAGGTGATGTTGTCATTGAGACTATTAACTTGAATGGTGAGATGGAGCGTGAAAGGATGATGTTCCGAGTCGTGTTCAACACAGCCTTTGTTAGATCTAATATTTTGATGCTTAACCGGGATGAAATTGACATATTATGGGATGCTAAAGATCACTTTCCAAAAGATTTTAGAGCTGAGATCCTTTTCTCAGAGATGGATGCTGCTGCAGCAGTTATTGCCGATGGTACCTCATGCTTTGAGGAGAAGGAAGGACTGCCAATGGAAGCATTTGCCACGGTTCAAGAGATCTTTAACTATGTAGACTGGATGAACCCCAAGACAGATGCTGCACTAAATGCTCTCCAACTGATGAGTGCTTCAACTATGAATGACAGGTTAGATGAGAAAGGTCCTAGAACGCCTCAGCAGAATTTGAATGAAGAGGTACAGTCTTTATTCTCAACCCAGACATCCCCAGGTAATATGAGTAGAAAAGAGGAAAAGACCAACATGGTCGAGGGTGTTCCGCTACAACCTAGTACATCTAACAGTATCTGTCAAGAGACACCCAGGTCTTCTGAAAGGACCTTGGAATCCAATAAATGTTCAGCTGGATCTACAAATCTTGACATAAAACTGCAGGCACCCAATCCTGCCTTATCTTCTATTGATACTTCTTTTTCTCCTCGAACACCTCCTTTGCGGCCTCAAACCACTAGTGCTAAAGAAGCTCATGATTCTCCTCGCCGAACAGAATCTCCCCCTTCTTATGTTTTGCCTTTGCAATCAAAACACCAATCAGAAGACAGAAGTCATTCAACTCAGTCACAGGCCGACACAATTTCCCATCTTTCAGCTTCAACAATTTCTTCAACTCAGTCCTCTCCTTCACGTTCTTCCAAAAATGTGAATGAGATTCCTCCAATTAGAACAAGTCTAGAGTCTTCCCCTTCACGACCTCCAACTCCACCTCCTCCTCCCACTCCTCCACTAAATGATCATAGACTAGTTCGAGCTAAACCTCCTCCACCTCCTCCTTCTCCAAAGAAAGAACTACATATTCAAGTTGGACCCCTTCCTTCTCCTCTATCACATCTTAATGTGGAACCCCAGATTAGAGATGGACCCTCTCCTCCCCCTTCTCTAAAAGAGGAGCAACCTGCTAGGTTTCAGCCTCCTCCACCTCCTTGTCTTTCAGGGAAAGTTGCAGGTTCTACTATAGTACCACGTCCACCACCTCCACCTCCACCTCCACCACCGTTTTCAACTGCATCACTTTACTCAAATGACACTAATTCATCATTGCAAAAATCTCCAGCTGCTCCACCTCCTCCTGCAGCACCTACTCTCCCCCGTTCTCCTGTAGTACCTGCCCCTCCCCCTCCTCCTGCGGCACCTGCCCCTCCTCCTCCTCCTGCAGCACCTGCCCCTCTCCCTCCTCCTGGAGCACCTGCC

>C101399_65

GGTTTAAATTTTCTATATATTGTGGAATTAAATGAGTAATTTACATTGATTGAAACCACAGGCTAACTTTAACCATGAAAAAATGTAATAATGAAAAGGGGGGAAGGAAATAGGATAGTCTCAAACGCTCATTTGGTTGAATGAGAATCCTAAACCCCATTTTCTCTGCTGCAGAAGAACCTGCCCCAGCCAATGCTTGATAACAATACTAATCTTAACAGAATGCTATGTGAAAAATTATATCCCATTAATCCAAGATTTCAGAAGGCCAAATGCTTTTCCAAATGGAAATTTCAGCCTTACTCTGAATTTTTCTGATTCCCCCATAAAAGCCATCTGCTTCATTTCCTTCTTCTCTATGATCTTTTCTACTGATCGTTGTCTTCCTCCAGCGCGAGTCAGGAGTGTATTGATTGAACTCTAGCACCACCATGTCACTGCTATGGCACCGGCAGTAATTTTCGTTCCCATCGCATAACTCCAAGTGCCCTACAACACCATACCACCAACCATAAGGAAATTCTTTGTTTCTTCTCCACTGAATCTCTACGTGATCGCCTGGATGCAAATCATGTAGACAATCAGAGATATGAAGATCGTGTGGAGGAGTATCAACAGGTGGTGCTCTTAGCCTTTCCCATGGAATGCCATGCTCTACTGCAACTGCCCTTCTTCCATGTGGTGGATACCTGGCTTGGAAGGTGTCAGTTTGAGGATCATAGCTAAGCTCAGCATCATAGCATGATAACATAAATCCTACATGGCCATTCTCTCGGTTATAGACCTGAGCAGGAAACCAGAAGTTGCCAGTCTCAAGAGCAAGATACCATGTCATGAATGAATCAGGAGGTAAATAACTTAACTGCTTGTTGGTATTATTTGTATCCACCTTGGTTCTCATCCATGAAAAAGGCCAATGAAGGGACACAAGCCTCATCAAGCTACTTTCCTTGCCATGTCTAAGAGTTCCAACATTCCCTCTGGAAGCAACGTGCCATTTCCACTCTCTATAAGCAGCTTCACCAATAACTCTACCCCATTTCTGTTTCATGTGCCTCTCCCAGAGGTGATCACTGACACACCTCTCCCTCAAGGACCGGCAAACACCAGCCATTCGACAAAGTGGAGCTGGAGGGAGCTTTTCAAGAATGCATTCCAAGACCAGGTCTGGCAAGTCCAGCACTGACATGTTTTGGGAGTCTTCAATACTCTCCACCTTCGTGGACTTCACTAGAGACATTCTTGTGGGAATTTGACAATAAAAACCAAGCCAAAAGCGGTTCCTAAACAGCTTAGAGATGGACTTCACTAACAAGTCTTTGTAAAAGAAAGGGGAGATCAACCTCACCTCACCAGCCCATGATGGGAGTGGTTTGAGAGAGAGAAGTGGCTTTAAAAACAGAAAGAAGGAGATGCAAGTTATAAAATAAAGTAGCATTAGCACGTAAGATTTCTTTCACAGGTCAAGTTCGAGTTTGGCATATATGATAACACAACCTTAATGCCAATTGTGCAACACCAATGGCTAACAGAAGAAAACCAAAATGACCTCACTACTTGACACAAATTAATGACACACCCTCCCTTCCTTGTTGGAAAGAAACACAAGAAGCAATTTGGAACCTTCAAAAGAAAACACCTTTTAAAGATGGGGTTTTATTGGTTCAATGAAAGCCCACCAAAACCCAATGGTGGGCCTCCTAGACCAAAGTTAGATCACAATAAATTAGGTAGACAACCACACGAATGAACAATGGAAATAAACTCACAGTAGCTTAATGCTAACTCTCTCTTATTGGCTTTCAATAAAGAGGAGAGAACAACACAACTATGTAGTCAACCTAAACAGCTGTCATTCAATGAAGAATGAAGAATCTGTATACAGTGGTCCTTATGAGGATGGGAACATCAACACAGCAAGAAATGGAGGTGGGGGCACCAAGATCTGATAGCAGTTTTATAGCACCTAGAAATGGAATTGAAAAATGAAAAAAAGATGGAAGATGAGGCTATTCAATAAGAACCATATATATATATATATCTGCAGAATCTCTAAATCTTATTGGTAAAAGTGAATAAATCTTCTGGGAATATAGTTGGTGTGTAGCTAGAAAAAAATAGATCTAGCTAGTTGGAACTTATGAAGCGAGAGGCCCACACAACAGCATCAAGATCCACAAAAACAATGGAACCAAGGGAAAGGACAATCAATCCTTAGGGTGGTGCAGACTGATAGATATACAGACACAGAAGAGGATTAAAAAGAGACTCTGGTGAAGAGGATAATGAACGTGAAGGGTGGTAGGGCCTCCCTTCACTCCACCAAATTCTTCACCTCACCTCAATTATTATCCTCTTCTCTGCAAATGCAATCACACAATCCTCTCTCTCTCTCTTCTTCTTTTTTTTTCTCGTTGTTGTTGTTCTTGTTGTGTGTGTGTGTGGGGCAATATAGAGAACAATTGGTGGGCAATGAAAGGAAAAGGGAGAGTTATTTACGCAAAAATAATGGAATGG

>C101401_65

TTTTTTAGGTGGAAAAAGACATGCTCTTTGATTGATATATATATTTCAGAATATCAGTGACAAAACAAGACAGGTAGATGCACCAGACAGACACATTTTCACATCACAAAACTAAAAACCTATGAAAAAAAATAAAAACAAAGAAAAACTTAAGCATCCACCACCATTTGACAGACAAAACAGACACGGACACAAACTCGGACATAACTAAACGAATGACCTTTTGCATTTAGTCCACTTCTTCCATCTTGCTCTCCTCAGCACCTTGCTCTTCCAGTGGGGGCATGTCAACATCATCTCCACCATTGTCATCTTCATCAATACTAAGACCCAGCTTCAACATCCTGTGAATCCTTGAAGCAAAGGTGTTGGGATCATCAAGGCTAAAACCTGAAGTCAAAAGGGCTGTCTCATAGAGAAGCAATACAAGGTCCTTCACAGACTTGTCATTCTTGTCAGCTTCAGCTCTCTTCCTTAATTCCTCCATGATACCATTATCAGGGTTAATCTCCATAGTCTTCTTGCTGGACATGTAGCCACTCATGCTGTTGTCTCTCAGAGCCTGAGCCTTCATGATTCTTTCCATGTTAGCAGTCCATCCATACTCACCAGTGACCAAACAGCAAGGGGAATCAACAATTCTGTCAGAGACAACAACCTTCTCCACTTTGTCTCCCAGAATGTCCTTGATGACCTTGCAGAGCTCATCAAATGATTTCTTTTTCTCCTCTTTCTTCTTTTTCTCTTCTTCAGTTTCATCATCCAATTTCAAACCCTCCTTTGTAGCTGAAACCAATTTCTTGCCGTCGTACTCCTTAAGTTGTCCAACAGCATACTCATCAATGGCATCCACCATGTAGAGAACTTCATAACCCTTCTTCTTAAGTCTCTCCAAGAAGGGTGAGTTTTCCACGGCCTTTTTGCTCTCTCCAGTAATGTAATAAATGTCCTTCTGTCCCTCCTTCATCCTAGTAACATAGTCCTTCAAACTTGTCATTTCATCACCACTCTTGGTAGAGTGGTATCTAAGCAAATCGGCCAATTTAGACCTGTTCTGGCTATCCTCGTGGATACCCAATTTCAAATTCTTCGAAAAAGCATCATAGAACTTGTTGTAATCTTCTTTGTTCTCAGCAATTTCATTGAACATCTCAATGCACTTCTTCACAAGATTCTTCCTGATCACCTTCAAGATCTTGTTCTGTTGCAGCATCTCACGAGAGATGTTGAGGGGCAAGTCATCGGAGTCAACAACACCTTTCACAAATCCAAGATACTCAGGAATGAGCTCCTCACAGTTATCCATGATGAACACCCTCCTGACATAAAGCTTGATATTGTTCATCTTCTTCCTGGTGTCAAACAGATCAAAGGGTGCCCTCTTTGGTACAAAGAGAATAGCCTTGAACTCAAGCTGGCCCTCCACAGAGAAATGCTTCACAGCTAAGTGTTCTTCCCAATCGTTGGTCAGGCTCTTGTAAAAAGAAGCGTATTCATCCTTGGTGATCTCTTCTGGCTTCCGCAGCCAAATTGGTTTCTGCTTGTTGATGAGTTGCCACTCATGAGAAACCTCCTTGATCTTTTTCTTTTTCTTGGAATCTTTCTCCTTTTCCTCGTCAACATCCTCAATGTCCCCTTCTTCCTCTTTTTTGGGCTCATCATCTTCATCATCGCTGATTTCTTTCTCCGTTGTCTTCTCAGTCCAAAGGTAAATAGGATAGCTAATGAATTCAGAGTGCTTCTTGACAAGATCCTTGATCCTCCTCTCCTCCAAGTACTCCAGCTGATCTTCCTTGAGGAAGAGGGTAATCTTGGTTCCCCTTCCGAGTTGCTCGCCATCAGTATCCCTAGTAACAGTGAAAGAGCCACCAGCCTGAGATTCCCAAATGTACTGTTCATCATCATTGTGCTTGGTGGTAACAATGACTTTCTCCGCAACAAGGTAAGCAGAGTAGAACCCCACACCGAACTGCCCAATCATGCTCACATCAGCACCAGCCTGCAGCGCCTCCATGAATTCCTTGGTCCCAGACCTTGCAATGGTACCCAAGTTGTTCACCAAATCTGCTTTAGTCATTCCAATGCCGCTGTCAATAATGGAAAGAGTCTTATTGGCCTTGTCAGGCACGAGCCGAATGAAGAGCTCTGGCTGAGCATCGAGTTTGCTCTTGTCGGTTAAACTCTCGAATCGAATCTTATCCAGAGCATCAGAAGCGTTGCTTATGAGTTCACGCAAGAAAATCTCCTTGTTGCTGTAGAAGGTGTTGATAATGAGGCTCAGAAGCTGGTTGATCTCAGCCTGAAACGCGAAGGTTTCAGCGTCCGCCATTGAATCGCAGAAGATAATCGCAGAGAACGAACGAAATTAACTGCAGAGAAAGAATTGCACGAAGGAAAAGTAGAGAATTGCACAGAGCGAATCGCAGAAAATGTTCTTGTTGAAGGAAGAAGTAAAGAAAGAGGTTTATATAGGAATAGAAATGGGTGTGAAACAAACGGGTTTCTGGAGTGTTCTAC

>C101405_65

AGTATAAGCTAAAAAGTTTCGGTTTATTGTTGAGAAATAAAAAGCTAAAAATGATCTATTAAGTTTTGCAGCCTGAAAAGAAGTGACCCTAACAAGTCTAGGTTTTGTTGATATGCAAAGGAGTTACACTAGTTGGCAGCAATATTTTGTGATGAGCCCTCAAACTAATGACAAACAGAATCGCATAGGTGCAAATGCCGAAACAAAAGCAAATACACAGTTCAAGCAAGATAGCGATACAGTTTTCTATCCACTTTATCCCTCTCCAAAAACTCACGCTCAATAAGGGATTCAATTCTTTTCTTAATAACAACAGGATTAGGCAAAAACCGTGACTGCAGCTGCTTTGTGACCTCAGCAACGATATTATTATGATCTAGAGTTCGTCTTGATTTCATTATCCTCACAATTGCTGCCTCAATCTGTGGCTTTCTGTCTTCCTCCACTCTTTGCCGAGTTTCTAGGTTTTCTGGTTCAGACTCCCTCTGTGCAACAACAGTCCCTATCTTCACCTTGAAGAACTTGCTTGTGAATTTGTCATTAAAGAAGAACGCATCATCCTCTGCTATGTCCTTGCTCATTGGCTCTTTTCGAAGAACATTTTTTCCTTTGACACAGGCAAGAGACTGAAGGCACCTCCTCAAATCTGACATGGGAATGGCAGTAGCTTGCTCTATCTCCTTACAAGTCAACCGCTCAACATTGTTGAAAAGCATGAGTACACACATTTGGTACGTCGAAACATTCAACTCATGCTTTTGGCCCTTACCAAATGTTGCCTTCAAATCAGCAGTCCCCATGTTAGTTTGCCATGACAATCTCCTACCATTGTGTGTGCCAAGATAATATGTTCGAAACCTATCACATACACCCAGTATTTCTGTTGGAAGATTACACGGTGGACTAGGTTGAGTAGGCCATGATCCTGTTGTAAGCACCTGGACAGATAGCGTAGGGCCATCCCCCAACTCTGTACCTTGGCCTGCATAGAAACCCTGCATTGTATCATGAGAAGTCTTCATGTCAGTAAACATACCCTCTAACTTAGAAGTGAATTGATATCCACATTCTGTTTTGAGCTTAACAATCAAACTCCTTTCTGCATCATCAGATATAGTCTTCCCTGAAAGAAGCCTTTTTGCCAAGTGTTGCTTGTAATACTTCTCAAACACATCTTTCTCCTGTAGGTAGCGGAAAAGCATCATGACTTTGTCTAGTACAATCTCCACATCCTCCTCGCCAACCCCTTTTAACCCTCGGCGAAGTTTGTCATCCACAAACAAGGAAATGAACTCTGGAGACCGGGCATTTAAATTGATGAAATATTCAAAAGAGGAATTCAAGGCATTTTGAAATGTCTTGTCATTATTAAAAGACATAGTAATAACCCTGTCATATTTATCCTTCAAATCCAACAGACGTTGAACAAAATCCACCGGATCTCTCAACCTTTCAGGATCCATAATTAGTTGCTTACCTGTATCCCGTATAAAAGAGGTCATGACGTCTTTAACAATTGTGAGCCCAGCAGGCACCCTGCGAAACAAGTTATACATTCTTTGCAAGTCTTCGTATTTGTCATCCACAAGCATACTTACTAGGCCTGAGTTCTCCATATGAACTAGAGTGTGCATATGACTTTCAATCATCTCCTTCTCCACCACATTAGTTATCTTTGACTCACTTCTAGGATCCAAGTAATGAGATACTCTCTCCAACTCTTCATTTAAACGTCTCTCAGCTTTCTTCAGATAATCACCACAATCACAAGACTCAATAAATTTCTGAGACTCACAACAGTAAAAATTTGCTGATACATCAAGAAAATGCTTCTCAAAGTCATGCTGGTAAACAGGTAAACCCAAATCCATAAGCATCTTTATTATATTTCTCATCAGTCCTCTGTTTATTACTTCACCATTCCTTTCTCTAAGTACAAGCTCAAGAAGTGTATCTAGAAGCCTAGCCTGAGTTTTGCTGGAATGGATCACAACATCTCTCCAAAGATTCAATCCAAGTTCATGAACAGGAGTTTTATGGTTGCTTGGTATAAATGTTCTATCCATGTACATCAGTATATCTCGGATCATCTGCAATGCCTTGTTATGATCTACCCACTTCCTATTGAGCTCATCCAAGAAAATTTCTCCTTGAGCAGATTCAATCGATAGAGAAATTTCTGTTAGATGAGAAGACATGGTCATCACAAGTCCTGTGTAAAGTTTCTCCCCAAATTTGTGTAACACCATATTATAAGCATTCCTGTAGAGTTCTTCAAAGCTAAGGCCACTGGCGTTGTGGTTGTATATCTCGTGGATAGCATGTTCTAGAACCTTCCACGTCTTCTCGGCGTACTTAGGATCGACCACCACGCGGTGCTTGAACGCCTCTATCTGGAACGTGCGCTTCTTCTGCGTGCTCATCTTCCCTCACGAATCGGAACCCTACAGCGCCTAGCGACCGGGGCGAGGCGGGGAGGATGAAAAGTCTGGGAAAATTGGGGATTAGGGTTTTTCT

>C101407_65

TTCCAGAAAAATAACAGGGCCTATGAGCCATATCTCATACCCAACAAAAGAATATCCTGTCAAGAAAAAAAAACCATAATACCACAGCAAAGTATACGCAATCAGAAATTGAATGATGTTATCAAATGCCAAAAGAATATTAAAAAAAAAAATCAATCATAATCATCCTAATGAATCACATAACTGGTATCTTCTTCCTTGTTATCCATCTCCTTTTAGCATTTTCTATGGCTTCCTCCCTCATCTTCACAAGCTCATGAAAGCATTGTTGATATTGTATTTCTATTGCTTCAAGCTCCAGCTGCAGCTCGTCTGAAGGATCTTTGTCAACTACATTAAGAGAACCTGTGCTTGACATGTCAAAGTTTTTGGATGGGCTGCAACATGAATTGATTAGAGAGCCAGCTGAGTTCTCTGATTGATTCTTTTCCCCATCTTCTTGCCCACTGAACCTGCCATCTGAATTGGAATTGAATACTTCGAACCCTTCCCAGAATTCATCAAAAATAAAAATTTCAGGCTCGACACCTTTAGAATCTGAAGCTATCGTAATCACACACTCAGCTGATATATCTGATGTAACAGATTCTTGTTTCTCCTGGTCACCTCCATCCACAGGCCGTGGGCGAACTAGATCCTCATACATAGTCTTCATATCATAATCACTTGATTGTAGTGGCCAATGGCAGCTCAATTGTTCCTCATTCAGAACTTCAGAAGAAGTCCCGTACAGATGCTCTGTTACAGATGAAAGGTTCTCACATAAGGGCTTACAGTTGGGCTCGAGCTTAACAATCATGTCTTGTATTAGCTCTGCTATAACAGATACATCCTCATTTGTCAGTTCCAGATGTTCCACCATCTCCTCAGCAATGGAAATTGCTGTATCAGAATCGATATAGAATGGAAAATGGATATTCCTTGCTCCACCACGTGCATCTGCAATTCGCAGTGTAAGTGAAATTGTACTTTCAGCATTTTTCTCCCCTCTTAACCTAAATTCGTTATTCTCAGTCATCCTCACAAGATCAAAAGTTGAAACCTGAGATCTTTCTTCAATTCTTCCCACAGAGGAGCCAGGTGAAGTACGCCTGGAGTTAGAATCTATCTCCATGGGATGAGGCTTGGACATTGGTGGATTCACCAATTTGGTTTGAGGATTATTAGGCAGCTGCAGTGTATCATGATGATTGATCTCCTTTGCGTTTTCAGTAGCAAGAAATGGGTCTTTCAGTAGTTCAGATGCCGACAATCTCACAGATGCTGGAACTAGACACTTCTCTATGAATTGCTTCACTTCAAGATCATTTACTTTAGCAAGGGCAGCAGGCTTTATACCAGAGGTAACTTTCTTATATATCTGAGCTGGATTTTTACATTCACTATATGGGTACTCGCAGGTGATCATTTCTAAGATGCACATGCCAAAGGAATATATGTCAACTAGTTCATTGTATTCCTCCTCATAAAGCTCTGGGGCCATGAATTCTGGTGTACCGATGACACTCCGGGCAGTAGGCTGTTGCAGAACTATTGCCAATCCAAGATCTCCAATCTTAACCAGTCCACTATTCCCATTGACAAAAATATTGTCACATTTCAGGTCCCTGTGAATGATAGGAGGAGTTTGACTGTGCAGAAAGCACAAGCCACGGAGAATCTGCCGTGCCCAGTTCTTAATGGCTTTCATATCCACATTCTTATGCTTCTTGCGGTACTGCCTCAAACTCCCGGAGGTGAACAACTCAGTGATCATGTTGACAGTGGCGGTGGTATCATCAACCCAATAATTGTACAACTTGATGACATTGTCGTGCTTCAAGTTCTTGAGCAAGTGAACCTCCGAGTACAACTTCTCTAACTGCTGCGGGGTCTGCACAACGTCCTCCGCGCTGATCCTGTTCCACGCAACTTCGATTCCGTCCACTTCGTCAAATGCTTTGTATACAGTCTTGAAGGCTCCTTTCCCCAACACTTCATCATACCGGGTGTATCTGGAGCTAGGGTCTTTTTCTAGAACGTGGGAAGAGGCCATGGCGCTAAGATAGAGAGAGAAGAGAGATGAATTATCAAAGAGGGGAAGGGTGAGGTTCAGAAAAGGCCTCGCAAGCCACGGAATAGGAATCGAACGCCGATCGGAATCGGCATTCCAGGGAAGACATGCCGTCGAAGGATCCGATAGGCGGAGAAAGAGGATTGTAAAGGTTGTTGCGCTTAGTCATTGATCCACCCTCCCCCATGATTCCTCTATCTGCATATCTTCACAGCAAATCCAACAAACCCTAATTCTAATCCTAAACCTAATCCAAATTTTCTAATCCAAATTCCATTTCAAACCATCCTCGATCTCAAATATTAGATTTGATTTGATTTGTGAATAACACAACTTGGGAGGAAGGCACACGCTAATTACCTGATACTTCTTCCTTTGCTTTGCTCTGCTTTTCCTATACTTTGGGGAATAGAACAGAACATAACAGCACAGAACACAACACGATAAGATGCACTTTTTGAAACTG

>C101409_65

TCATAGTGTTTATCTTGCCTGAATAGTGAATTCTATATATTGATTACGGATGATTTATGCATATAATATTTGGAAGTTGTTAGTAAGACAGAATCCTAATGACAATCTTACACAAAAATACTATAGCAACAACTGCGAAAGAGAAAATGAAGTGTTATATGCCTTTACTATTCACATTGCCGGAGAGGCTATCCACCCAAAGTATATGTCAAGGGATGACATTTACCAACAAGCTGTTTGATGAAGGTGAGCTGTGTCCACTTGACCAATGAATAGGATCATGCACTGATACAGGGGAGCTGTCCTTCACCTCCACAGAATGAAAAAATCTCTCAATCTTCTGTGGAACTATCAATCTCTCACTCCCTTGGCCAGCCCTTTTAATACCACTCAGTGACTTCTCACTTCCATTGCGAGGTGATGAACAAGTGGACCCAATCGATGAAGCTATGGCAGTTTCCTCCGTAGTTGCCCATCCACTACGTTTAATGTGTTCTAGCTCCTCTGCCACTTCCATCATAGTAGGCCTCATGTCACTATGAAAAGCAAGGCATCTAAATGCAAGCTCAGCTACCTTATGAATAGAATATAGTGTCCAAGCATCTCTATGTGGTTCAAGGAAGGGGTCTATGATATCATCTACAGCACCCCTCCTAATCCTATCTACGGCAAGTGCAGCTAAGTTAACCTCACTCCGAGGTCGAGAAAAATCAACCGCTTTCATGGCAGTTATTATCTCTACCAAGACCACTCCAAAACTGTACACATCACTTTTATCAGAAAGATGGAAGTTCTGGTGGTATTGGGGATCGACATAACCCGGAGTCCCTTGTGGGGCGGTTGAGATATGAGATGTTTCTGTCATCCCGAGCCTTGAAAGCCCAAAATCAGCTATCTTGGATTTGAAGTGATAATCCAAGAGTATATTACTAGATTTTATGTCTCTGTGGTAAATTGGGGGATGAATCGCGGAATGGAGATATGCTATAGCATGAGCAGTTTCAGTAGCAATGGTAAGTCTTATTGTCCACGGTAGTCCCTCGCTCCTCTCTCTTTGCAAGTGCTGAGAAAGTGTTCCATTTTGCATATATTCATAAACAAGGATATGCTCTCCCTTCTCTATGCAGCAACCTAAGAGGCGAACTAAATTTGGGTGACTCACTGAAGAAAGGAGCTTGATCTCATTCATGACTTGGTCAACACTGTTGTTGTCTCTTTGCCTTAACTTCTTTATAGCAACCCATTCATCATTGTGCAGTTTTCCGGCATAAACTGTACCAAATGCCCCAGTTCCCAGCCTGTGTTTCTCAGAGAAAGAATTTGTTGCTTTTTCTACTTCTTTGTAGGGATAGAAAGGTATACTAGAGTTGCCTGCAGCTTCACGTAATTGGCGCTTTACTCTCGTATGTTTTCCCAACCAAATAGAACGGCGCCTGGCACAGTAGCATAGAAGAAACAGACCAGCCAACACCAAAGCTCCAGCTATGATCGCTCCAACAGCAACACCGATTTTGGTTGCTTTTCCACATCCCCCGGAAGTTAATGTTGAAGCACTGCAACTTGAAGCAGAAGCTTTCCGGCAACCGGTGCCGCTTCTGAAGCCGTCACCGGTGAAACCCTGGTTACAATGGCACCGAAACCCGTCTTGGTTAGGGAGCCGTGTGCAGGTAGCGTTGTCAGAACATTCATCACACTGTCCATGAAGCCACCATCCCAACTCAACCATCTGAAACTGCAGCGCAATCTCCTTGCTCTGCCCAAAAGAAACCGCAGAGAACAAATACTTACACTTAGTTTCTATCAAATCGTCGTATGTCACAACATCCACCGCATCTTGTTTCGAAAAGCAACTGATATTACCACTTTGCTTATCGCAGCTCTCCACAAAATTCGAAGCAGGAATAACACACCCACCAAACACCTCGCTGCAATCTTGCACCAGAAAGCTGTTGTTAACCGAGGGAGCAAAATTGCTACGGAAAAGTGGCTCAATGGATTGCATGCTGCGGTTGCATTTTGCAGGGAGATTGACGAATATGTTGCTGTTGGTAACGTTTTGCACCAGAAATTCGTCAAGTTGGATTTGGTTGTCGGAACAGTTTAGTCTGATTTTACAACCTTCGGAGAATCCAAAAGGGAACTGAAGAGTCCGCTTCCCGCATTGCCTATTGCAGGTGAGGTTTTGAGCTTTTGTTGTGCACGCTATCAAGATCACGAGGACTATAACGAGATTTGTACGGTGGTGAAGAAACATTTTTAGATAGCAATTGAAGTTGTTGAACTAGCTTGAAAAGGGTAATCACTATCACACTTCATCACCCTTCCCTGGCTTCAGAGGTTCTGCAAAGGTTGAAATTAATGACTGTAGCAGACGAAGAAATAATTCACCACAGAACACTCTTCCAAGATCGGTTTTGAAGTTGAACAGTGCCAATGGAAAACAATAAAATGAAGTTACAGAGAGAGCATAGGTTTTGTGAATTGAAGGGTAGTCTCATGAAAAAGTTGGCCACATTCACA

>C101411_65

CTCTCAAATTCCAAAATACTATTATTGACAGTTTAAGTACAATATATTTATACAAACGTTATTCTTCTAACAAGTTGAAGGAGGCAGAAGGACATTTCCCACAAAATTTTCGCTCCATCACTTAAGAAAAACACTTAACACTCAATCTATAAGTTAATGTCTCCAATCAGATTCTGTACAGTCAGAAACTAGCATCACTCTTTGAGGTGCTACTGTGATGCTGATGTTACACCAAGTTCACTGCAAGTCAGGCACTTGCATTTGAAGAAACTTTAAGTTCCTTTCACTCTTCTTCACCAACTTAAATGTCACACATAGTGACTTTGTAAATCATCTTGGTCCAGAAAGTTCCATAGCTTGTGCAAGTAGTGAAGAGTCATCAGTGAGGTCAGAGTAGCGCTCTGAAGAGGAGAGTTCATTTCCTCTACTTCTTGTTGATTCAGCCCTCTGAGAAGCTTCCCATTTCTCTGCGAGACCATCTCCTTCAAGCATCCTAACCACTTCAGACATCTTAGGTCTGTGACTTGGAAGGTACTGAGTGCATAAAAGAGCCACTTGCACAATTTCATCAAGCTCAATCCTATCATAGTTGTTTTTCAGGTCCTTGTCAACCAACAAGTCCATTTTTTTCTCTTGATGGATTTTCTTCACCCAGTCTAGCATAGCTCCTTTCTGGTTTGCTGCTTTCCCAAACTCTAGAGCCCTTTGGCCAGATATCAGTTCAAGAAGAAGAATTCCAAACCCAAAAACATCTGTTTTCTCAGAGGATTGACCTGTTGAGAGATACTCAGGGGCTATGTGCCCCACAGTGCCTCTGACTGCTGTAGTCACATGTGAATCCCTGTGATCTAACAGCTTCGCCAACCCAAAATCTCCCACCACCGCCTCACAATAATCATCAAGCAAGATATTTGCTGCCTTCACATCCCTGTGAATAATCTTTGGATCACACTGTTCATGCAAATATAGCAAGCCTCTCCCAGCTCCTAAAGCTATTCTCTTCCTCGTAGGCCAGTCCAAAGCTGGCTTGGCTTTCAGACGGGAAGCAACACTTCCATTTGACATGTAAGGATAAACCAAGAGCCTTTCTGTAGCAGTCATGCAGAATCCGTACAGTCGAAGAAGATTCCGATGGACGGCTAAGCTGATCATCTCAACTTCAGTTTGGAATTGGATCTCACCTCCAATGGCATTACCATCTTTGAGCCTTTTTACTGCTATAACTGTACCATCTTGGAGATACCCTTTGTAGACATTTCCAAAACCACCTTTGCCAATCAAGTTCTTGCTGCTGAAGTTGTTTGTAGCAAGCTGAAGTTCTCTGAAGTGGAACTTCTTGAGGTTTCCAAGGCAAACCTCTTCACGGTGTTGTTCATTAACATCAAAGAATATTTGCTTATTATATCTTTGTCTCCACCAGATGAGGAAGCCAAGTCCCAGAACTAGTAAGCATATGCAGCTTAAGCTTGAAGCAAAAGCCAAGGCTACTTTGCGACTCTTTGGTTTTTTGGTGGATTGTGAATCTTGAGAATTATTCGGAGCTGAAGGAATTGACGCTGTTTTGAAGCAGTTCTGCTCGACTCCAGTGGCACATATCTGAGGATTGCCTACAATATTGAATGTTTTTGCATTTATTCTTGGCACAGGTTCACTCAAATTATTATAAGAGATATCCAGAAAAGCAAGTTGAGTCATGTTAGCCAAAGAGGAAGGAATTGGTCCAGTGAGACTGTTATTGTTTAGCCGCAAATAATTGAGACCCTTCATATGGGAAAGAGAATCGGGTAGTTGACCAGTGAAGGCATTGTCAGAAAGATCAAGAGTTTGAAGCTTTTGGAGCCTTCCAATCTCAGTGGGGATTGGTCCTGATATATTATTATCCTGCAGAAGCACAGTCAGGAGGTTTGGTAAGTTGCCTATGCTTGGTGAGAGTTTGCCAGACATATTTTGGCTTGGAATCCCAAGGGCAGTGACAAAATGATCAGAAGAACATGTGACCATAGCCCAGTTGCACGGATCTACAGCATCACTATCCCAGTTACCCAATACAGAATGAGGATCCACCAGAGCGTTCTTAATGCTCATCAGAGCTTGAACTTCAGAGTTGACACCTTTAGGAGAAAGCAAACCAGCCACTGAAGTCCACAAGAAGAACAAAGCTAAACAAAAGAGAGCTACATCCCCTCTTCTCTCCATTGGGTTGAAGGATCACCAGAAACTTATATTAGTTATTACCACAACAAAACTGGATCAAGTTGTATCTTAAGGAGGAGGCAAGTAAGGAGCAGGTTTTGAATGATTCAGCTAACACCACCAAGTCCAACTTGGTTTGTTTTTGTTACATGTCATGTTTTACATCAGAAGAGGAACTTCCACCAAGTCCAACAGTACCATTCCCACCTGCTTTCAAGCCAGAATTGCACTCACTCTCACCCTTGTGTCAGACTTGAATCAGAGCTAATTGTAAAAAGACTTAAAAGAGAGCCAGCATGCCTGCTTGATGGTTCAACAAAGAAAGCAA

>C101413_65

CTCTCTCTCTCTCTCTCTCTGCTTTTTTCTGCTGATCATAGGATTTGGTTTTCCCAAATCCGGTTGCAATTTAACTGAGCATCAAATTCAATCAAACACTGCCACTCCTCATTCATTGAATCCTGTTTTCTTTTATCTGCTGACTGCAAACACTCCTTGTTGATTTCATTCCAACAACGCCAATTTCATATTGCTGCCAAAGTCCTTGCCTTGTGTTATGATCATTCTTTAGTTATAGAGTTGTTATGTTCCCTTTCTTCTGAGCTCTCAGATTACTCAGTAATCATAATCTTTGTTGTTAGGTTTTTGTGATTTGGCTCTGTATCCCATGAATTGATTTAGAGGTTTTCAATTGCAATGCAATGAGAAGGCGCTCTGACCAAATGGAGAAAGGGGCAGGGAAGAATCAGAATTCCCGCCTTTGCTGTTTGGCTTCCCTTTCTGCTTTCTTCTGGTTCTTGCTCCTTTACTTCCATTTTGTGGTACTTGCACGAGATAATAGCAATACCAATAGCAAGAGTAATTACAATAATCATGTTGATCATCACTCGACCCTATCAACAACACCTGTTTCTGTTGATTATCAACCTGCCCCGATCCATACAGCGTATTCCTCTCAAGTGCAAGCCCCTCCTAGGAAGATTGGCTTCCCTCACCCCGAGGTGCGACAGAGTGAGACTTACACTGACACCCCTCGCACTGAGAAGAGTTTCCCTTTCATGAGAGCAATGAAAGCTGCTGAAAACAAGAGTGATCCCTGTGGGGGGAGGTACATTTATGTGCATGACCTTCCCTCCAGGTTCAATGAAGATATGCTGAAGGAGTGCAGGAGTTTGAGTCTTTGGACTAATATGTGCAAGTTCACCACTAATTCTGGCCTTGGTCCGCCCCTTGAGAATGTTGAAGGGGTGTTCTCTAATACTGGCTGGTATGCTACCAATCAGTTTGCTGTTGATGTTATATTCAGCAATCGGATGAAGCAGTATGAATGCTTGACAAAGGATTCCTCTATTGCTGCTGCGGTTTTTGTACCCTTCTATGCTGGATTTGACATTGCGCGTTATCTTTGGGGTTATAATATTTCAGTGAGGGATGCAGCTTCGCTTGATCTGGTTGACTGGCTCATGAAGAGGCCAGAGTGGAGAATAATGAATGGCAGAGACCATTTTCTTGTTGCAGGAAGGATAACGTGGGATTTCAGGAGACTGTCTGAGGAAGAATCAGATTGGGGCAATAAGCTTTTGTTTTTACCAGCAGCAAAGAATATGTCCATGCTTGTGGTCGAGTCCAGCCCTTGGAATGCGAATGATTTTGGGATTCCGTATCCTACATACTTCCACCCTGCCAAGGATGCTGATGTGTTCAATTGGCAGAATAGAATGAGGCAACTAGAGAGGAAGTGGCTTTTCTCCTTTGCTGGTGCTCCCCGTCCTGGCAACCCCAAATCAATCAGGGGTCAGTTAATTGATCAATGCAGAAGGTCAAAGGTTTGTAAGCTGTTGGAGTGTGATTTTGGAGAAAGCAAATGTCATTCTCCCAGCAGCATAATGCAGATGTTTCAAAGCTCGCTTTTCTGCTTGCAACCCCAGGGTGATTCATACACTCGAAGATCTGCTTTTGATTCAATGTTGGCTGGCTGTATACCTGTCTTCTTCCATCCTGGATCAGCATATACACAGTATACTTGGCATCTTCCTAAGAATTACACCATGTATTCTGTCTTCATTCCAGAGGATGATATCCGTAAGAGGAATATCAGTATAGAGGAAAGGCTTAGTGAGATACCTCCTGAACAAGTGAAGATCATGAGAGAAGAGGTCATTAATCTCATTCCAAGACTAGTATATGCTGATCCTCGCTCCAAATTAGAAACTCTTAAAGATGCATTTGACGTTGCAGTACAAGCAGTAATTGACAAGGTTACAAATTTAAGGAAGGATATCATTGAAGGTCGCACAGATGATGGCTTCATTGAGGAGAACAGTTGGAAATATGCTTTGTTGCCTGAGGGAGAGCATGAGGTAGGACCTCATGAATGGGATCCTTTCTTCTCCAAACCAAAGGATGGTAGTGGAGATTCTAATGATTCCTCAGCTAAAACTGCAAAAAATTCTTGGAAGAATGAACAAGTAAATCAATCATAAAGCGAAGGAGTAAAAAAACTGCAGGATGCTGAAGTTAGGTTTAATGCAGACTTAGAAATTATTTTAATTGGGGAGGAATTACAAATTATAATTAGGACTTTCTGTGAACTTATTGCCGAACATGTCTAAAGATATTGAACAAGCTTCCTCAGTGAAACTCCCGATATGGAGACATATTCTTTGCTTTACACATTTGTGTAAGAGATTTTTTTCTGATTTCTGCTTCGTGTATTCTCCTCACTCCTTAGTGGTTCGTAGAATGCTTTGGGAGGTTTAATAATTCCTTTCATGTAATAGTTTCATTCATTTGATTCATATAGGAAATATAAACATTTATTACGATTAATGGTAAACCCGAACAAGTTTCACCATTGAAG

>C101415_65

CGCAAATTAACAAAAACGGAAGGGGTATATGGCTATTTGCGAAACTCGCAGAATCAGAGTGGATCTGCGAAGAAGAGTGCATCCGCAGAAAGAAAAAGTTGATATAAAAGGCAGTACCAGCTTCGTATTGCATCTGCATCCTCAATCTTAAGCCATTTATGCAATTGTGTGGTTGAATTGTGTGGTTGAATATATGTTATAGAAGTTAATTTTGTTCGAAGTGAAGCGAATTGGTGGATCTGAAGCAGCGATGTCGATGGCGATGGCGAGAGCAAGTTCTGGTCTGCAATACCCAGAGCGGTTCTACGCTGCGGCCTCTTACGTGGGATTTGATGGATCCACCTCTCCAACCAAAACCCTCACTTCCAAATTCGCTAACTCCACCTCTCTTCTTCTCTACAGCTTATACCAGCAGGCTAGTGTAGGACCTTGTAACGTCCCAGAACCCAGTACATGGAAACTTGTGGAGCATAGCAAATGGGCCAGTTGGAACCAGCTTGGGTCCATGTCTTCCACTGAAGCCATGCGTCTTTTTGTGAAAATATTGGAGGAAGAAGATCCCGGTTGGTATTCAAGAGCATCTAACTCTGTTGTAGAACCTGTCATAGACGTGCAAATGAATCATAATTCCAAAGTTGAACCAGTAATTGAGAATGGAAACTCTTATCCAGAGACAAAGACTATTTCCACTCAAAATGGAAGTGAAGTTGGAACACAGGATAAAGATATTGTTGTGGAAGGCTTTGGTTCTGTTGCAGTATATGATCAGTGGATTGCACCTCCAGTATCTGGACAACGTCCAAAAGCCCGATATGAGCATGGAGCTGCAGTTGTACAAGATAAATTGTACATATATGGTGGAAATCACAATGGTCGTTACCTAAATGATCTTCATGTTTTGGATTTAAGAAGTTGGACTTGGTCAAAGATTGTGGCCAAGGCTGGAGATGAGTCCCCCACAACTTCAATCCCTTGTGCTGGCCATTCTTTGATTCCATGGGGAAATAAGCTTCTGTCTGTTGCAGGGCATGCAAAAGATCCTTCTGAAAGTATCCAAGTGAAAGTGCTTGATCTTCAAACCGCTACATGGTCAACTCTAAAGACTTTTGGGAAAGCTCCGGTATCACGTGGAGGCCAATCAGTGACCCTTGTAGGGGGAACCTTGGTAATTTTTGGTGGGCAAGATGCAAAGAGGACTCTTTTGAATGATCTGCATATTCTTGATTTGGAAACCATGACATGGGACGAAATTGATGCTGTTGGGGTGCCGCCTTCTCCAAGGTCCGATCATACTGCTGCTGTTCATGTGGAGCGATACTTACTTATCTTTGGTGGGGGTTCACATGCAACATGCTACAATGATTTACATGTTCTTGATTTGCAAACTATGGAATGGTCTCACCCCACACAGCTGGGTGAAATACCAACACCACGGGCTGGACATGCTGGTGTGACAGTAGGAGAGAACTGGTTCATTGTTGGTGGCGGTGACAACAAGAGTGGGGTCTCTGAAACAGTTGTACTGAATATGTCTACACTGGCTTGGTCAGTGGTAACTTCTGTTCAAGGACGAGTTCCTGTTGCTAGTGAGGGATTGAGTTTGGTTGTAAGCTCCTATAATGGTGAAGATGTACTTATATCCTTTGGAGGGTACAATGGACGTTACAACAATGAAGTCTATGTTCTTAAACCAAGCCACAAATCAACCCTGCAATCCAAAATAATTGAAAATTCAGTGCCAGACAGTGTTTCAGCTATCCCAAATGCCACAAATGCTACCCGAGATGTGGAGTCCGAATTTGAAGCAGGCCATGAAGGGAAAAATTGGGAAGTTGTCCTGGACAATGCTGATCCAGCAAAATCCAAAGGTGATATTATATCAGTCCTGAGGGCTGAGAAAGAAGAGTTGGAATCATCACTCAGCAAGGAGAAGCAGCATGCTCTTCAACTAAAGCAAGAGCTGGCAGAAGCTGAGTCTCGGAACACAGACCTTTACAAGGAACTCCAATCAGTTCGTGGTCAGCTTGCTTCTGAGCAGTCGAGGTGTTTCAAACTTGAGGTGGAGGTTGCTGAATTAGGTCAGAAGCTCCAAACGATTGGGACATTACAGAAGGAGCTGGAGCTCCTCCAGCGGCAGAAGGCTGCATCAGAGCAAGCTGCCTTAAATGCAAAACAGAGGCAGAATTCAGGTGGTGTGTGGGGTTGGCTTGCCGGTACTCCCCCTGATGGAAAAGCAGATGACGAGTGAACCTCTTAGTTGAATAGCAGTGCTACATTACTCTTGGTTTCATTAACCAATTTTATTTCCAATACTTATTGTGTATTAATTCAGTTCTTTTTTAGTGATGTAAACAAGATTTAGCTTCTATTTGACCGAAGGATTTTCTACACCTGAGGATGAGGACGATGGTATAAATAGTAAATGTTACTTTTGCCGCCATTTTTGCTGATGATTGTATTCAAAGTGACATACATTATTGAATGAAAAATATAGAAATTCTTTTGATTTGTTAGAAAAAAA

>C101417_65

GTGTGAGAGTGAGTCTGTTGTTCAACGTATGCTAAGACTCCAAAACCTACTGCTACTGCTAAGGTATGTGGTAACAGCCAACAACAACAAAAACATCTTTTTTTTTCTTCTTCTTCTTCTTCCTCAGACTTCACTAAGTTCTATCATTTCATTTCTTGTTTCCTCTCTTCCAACTTTCAAGGTTTCATTTCTGATGGGTTCATGCGAAGCCACAAATGTGGTCCTTTCCAAGATTAAAAATTTTGATCCTGAAAATGCTTCAAAAATCATGGGCTACCTTCTCATGAACCTTGAAGACTCTGAACTCATTCGTTTGGCTTATAGCCCCGACCCTGTGCTCCACACTCTCATTCTCAGGGTTAAGACCCACATGGGTCTGGCACTCTCCACTCCTTCTTCTCCTTCTCTTCCTCCTTCCCCTCTTAACCCCATAGCAAGACTCACTAGTTCCTCCACCAACCCGTTTTCCAGAGCAGGACCCACCAACGGTTTTGACTTCGCTAGAAACCCATCTTCACCTTCTTCTCACTCTCACGCTTGGAACTTTCCAAACAACAACCCCATTAGTCCAAAGTCCACTCCTTTACTCTCTTATGATAACATCCGAGCCCTCTCACCGCGTGTCAACGGTGATTGTGATTTTGTTGACGAGCAGCAGGTGAATGAGTACTTTCCCTTCCTCAATGAGTCCTCCAAGAACGAAGATTTGGTCGATCCACGCCTTGAATTGGGTGTTGGGGCTCAAAATTGGCACTCCGGAGACTCACATTTGCACAGGAGGAGTTACTCGGCTAGTGATGTTGGTTTTGGGTGTGAAGAAGCTGCACCTGGACTTGGATACAAACCGTGCCTTTACTTTGCCAGAGGGTTTTGTAAAAATGGCACCAATTGTAAGTTTCTTCATGGCGCTTTCACTGATTCACTGGATGCCACTGTTGGCTCTCCAAGTAAGCTTGAGGGGATGGAACAGCAGCGTGAAGATTTTGTTAGGTTCAAGGCCCCACAGCTTCAAAGAATGGGGTCTGGACCTTCTGCAGCCGCACGTGAGAAATACTATGAGTTTTTGATGCAGGAGTCCCAAAGGGCTGCAGCAGCAGCATTCATGATGGGGGAAGAATTCTGCAACTTTGGCTGGGATAGACCTGAAAGGAATGACTTTTTAGCCGCAATTTCTGGTGATAAACCCAACTCTGCCTCGCGACAGATTTACTTGACATTTCCTGCCGAAAGTACATTCAAAGATGAAGATGTCTCGGAGTACTTCAGCAAATTTGGACCTGTTCAAGATGTGAGAATTCCTTATCAGCAAAAGCGGATGTTTGGGTTTGTTACCTTTGTCTATCCAGAGACAGTGAGACAAATATTATCCAAAGGGAATCCTCATTTCATCTGTGATTCACGTGTACTTGTCAAGCCCTACAAGGAGAAGGGAAAAGTCCCTGACAAGAGACAACAACATCAACAACAACAATTAGAGAGGGGAGATCTTGCACCATGTTTGAGCCCTTCTGGATTTGGCTCTAAAGAACCTTATGATTTCCATCTTGGGGCAAGGATGTTATATAATCCGCATGATATCTTATTGAGAAGAAAAATTGAAGAGCAGGCTGAATTGCAGCAAGTCCTTGAACTTCAAGAAAGAAGGCTGAAGAACCTACAGCTTCCCGACTTCAAGAATAATCCTATTCACCATCACCAACGCAGCCTTTCTGTTGGTACTCCTTTGGTCTTGCCCCATCAACTCCATGGTCACATTAATGATGCAGGTCTTTCTCCTGATAGCATTAAAGGAGATATTGCAGGCTATGGTGGCAGCTTCACTTCTACCAATTCTTTAGGTATTGCATCTGAGCAGCAGCAGCAGCCGCAGAAACAAGTTGAGCTAGATGATTCTGAGAGCGGGAATATAAAGGATAGTGGAAATAACAAAAACGTGGATCTTAGTAATAGTGTGGAGCAGGCCCTTCCTGATAGTCTCTTTGCTTCACCAACAAAAGCAGCTGGAGATTATCATGCTGACTTCTCTACATTGGCAGAGGTCAACGAGAGTGCTGCTTTTTCATCTAGCTCATCTTCTTATGACAAGCTAGAACCAACAACCTCCTCTAGCGACGTGGCTTCCCGTTAGTTTTCTTTACTATTCAAGATTTCGATTGTAGGTAACAAGAATAGAAGTGGCCAGCTGGGGATAATTTTTATTAGGATTTATAGAAGCTTATAGAAGAATCTTCTAGATGTATAGCCAAATACACCTAGATCAGAAGATTAGATCCTCTAGAGAAGAATCAAATGAGATCACCACCATGATCAATACATACTACTAACAGCAGGCGATACATGTTGCATACTTGGACAGACATATTAGTTTATAAGACTAGTTTTTCTTTTCTTCCTTTCCTTTTCTGTTGTACTCCCATTCCCCTCCACATAATTATCTATAGATGTGTATGTTTCATCAGACTGAGGCAGAACAAAGTACTGTTGTTTTACTTTAGCTTAATGTATAAAACTTGCGCTACCAA

>C101421_65

ATCTGTTATGAGTTCTCAGTAGTTTTACATAGTTCGACAAATTTAGATCATGTGTAAAAAACTGTATATACAAGTAAACACTAAAACATGACACACGGATATGTGTAAAAATATGACCTGACTTTCCCCTTTAGGGAACTTCGACTTCAAATTCCTCTTCCTAGTTTCTTTAGCAAGGGTCTCTTCAAATTAGAGCAACCAAGTTTTTGGGAAATCGAATTACAGATATGTATACAACTGTTACAAGAATAGTAACAATTGATGATGTAGGAAAGGCAGCATAAGACTCTTATCTGACAGCCTCTGGTGCAGAATTGCAGTAAGGTGTATGTAAGCAAACCAACCTAGTGAATGTTAGACATAAAGTAGCAAACGCAATACCCATAATGAGGCCACAGGTGCTCTTCCTCTTATCCAAACTTAGAGCTCTCTTTACTACATTTCCAAAGCTAGGGATTAATCCACCACAGGCAATAACCATTACGTGATCAAGACCACTGTAGTCAATACCAGTAAGAATAGCTCCTATTGCTGATATTGGACCCATAAATCCAATTATGGTAGCTGTTGCAAGGGAACCATGCCAGCTATCAGTGGCTCCAAATATGCAGCTGGCCACAGCTGCACCTCGAGGAAGCCCGTGTAGAGAGACTGGAAGGACCATGTGACGACCAAGTCCATATGCTTTTGGTGCAGCCACTCCCAATGCAAGTCCTTCAGCTAATGCATGAAAGGCAACTGCACCGCATGATATGAATGATTGAAGGGTGTGAACACTGAGTGGGAAACCAGTTAGTGTAGGCAAGTCATTGACTGAGGCCTTTTTGGAAGCTGCCATCTTCAATACACCAGAAGTAGAAACCTGGATGAAAGCAGCTCCCATTGCCAGGAGTAACAAAATGGGAATAAATCCCAATTTTGAAGACAAAATAAGCTGTATTGGTCGCCAGGCACCAAGAACAAAAGCAATACCACATGCAGTGCCCATGAGGAGAGCATGCTGAAGACGCAATGCAAGAGCACATACAACCAGAATAATTCCTCCAAGTAAAGGGCCAAGACCAAAAAGTAGTGAAACAAAAAAACCAGAAGCATCCTCAGAGTTGTAGTCATGGCTGAAATTCTGAAAGAAGGTGCTGAGAGCTTCCATAAATGCTACAGAAAGGGTTGCAGCTGATGCAACCTGTGAAGCTGAGGCTTCCTTAAAAGCATCAGGAAGCACTTCTGCAATAACCATCCAAATCATGCATCCAGCAGCAAACCCCGTACAAAAAGGAAGAAACTTACTGAATGCATCAGCACAAATAAATGAAGGGACAGCTACAATTGGCTGAGGTAAAGATGTAATTATGCTCCATAACATAGCATTCTGTGGAGAGACACCCCTTGATGCAAGAACCATGCTCACAGCTAATCCTTCTGGAATGTTGTGTACCGCAATAGCCAAAGTTACCAACAGGCCTTGAGAAAAACCCTTTGAGCCAGCAAATGAGACTCCAACCCCAGATCCCTCCCCAAAAGAATGGAGAGTCATTATTCCAATAACGAGAACAACTTTGGCAGCGTCAGCACCTTTTAAATCCAACATGCTTACTTCCCCATATTGCTCAAGAAACTTCTTACATAGCCAAATAAAGATTCCACCAGCTAGAATCCCAGTGACAACCCAATTTCCAGCACCATACTCCTGCCCTTCCTGTATTAGATCAAAGCTCGCAGCCAACATGACACCTGCAGCCATTCCATTGCATAGTCCAGCCCACTGCGGATCAAGCTCCACAAAGAAGAAGGGCACAGCACCTAAACCAGTGGCAGCTGCCATCCCCAGGGTAAACAAGGCAACCGTTGAAACTGAAACCTTATTACTTGCACCTTTCCTACTACCCACCGCACTATTGCCATTCTCAAAATTAAAAGCATTCTCGACACCAGTGCCATCAATTACATTAGTTCCTACATCTTTATGAGGAGCAGCTATGACCTTATGTGAAACCCGATGATCCAATTCCGCATATGAAATCAAGAGAAAACTTAACAAAAACAACAACGGAACACATAATCTTAGCCTCAGATTTGGAGCCATGTGAAAAATTATCACCTTTAAGCCACAAGCCCCAAATCAAATTCTTCAGAAGCTACAACTACAACCCCATAAGCTTCTATGCTATCAGCTCAATTCATGAAATACCAATTGATATCAAATTCCAGAAGGAACGTAATTTGAAAAGACACCCCAAATGCAAGAAGTCAGCATTTGAAGGGTTTACCCCTCGCGACCAATCTACATGCTAGGTTGATTGTTCTGGAGGGGAATCACAGAGGCACAGAACGAAGAAAAAAGGGGAAGACTTTTTGAAAACCCAAATTCGATTATTGATTGGAAACTGAGAGGAGCCACGTTAATTGCAAATGGGATTTTGGGGGCACCATAAAACCAGCGACTTTGCGCACGCAGAAACCCACGCTGACGTCACTCGAACATGAAGTGTGAACGGATTTGGCATCCACAGTTTCAAAACTCAAA

>C101423_65

TGCCCTCAGACTTTTTTCATTTATCACACCAAATATACAATAGGACACCTTCCCGCTGAAATTTACAACGCTGGAAGGATAAGTGATTCTCTAATATAAAAGTTTACAAGAAAAATACAACAAAATGAAGGTGAAGAATAAAAAATAAAAAACTACTATAATGCCCACAAGGTCATAACCAAAGGAGTTTTTATTTGTCCCTTTTCACATTTTCTCTCATTGTAAGCAGAACACTCTCCTTTGGTTGCACGTGAACCCAAATTTGAAGACATCAGAAATCATCTACCAGGTTCTGACTACACATTGAAATATTTAATTCTTGAAACGTAGGTTTAAAATTACTATTGACAACCTATAATTTTGAAGCAACTCCATTGCATAACATTTCATCCAACTCGCCTTGGTGATTGATTATTCCTACGAGGATGATGTTGGGTTCTCCAAAAGAATAAAGAATCAATAAACCAACTAAATTTGCAAAACTAATTGACCGCACATCAACTATGTTCACTTTGTGGAATCAGCAATTCTCCAAATCCGTTCCATCACTACAGAAACATCCTTTTCAGTTGATGTAGCAAAACAAAAACGAAACCATCCAGGTTCACTACAGTAACATGAAGAACCCGGGGTAACATTGATCTTAACAACATTCAATAATCTATCCCACAGCTCAAGCTCCCCCTTTTCACTGTAAGACCGAATCAACCCGCTCATGTCAGCCCAGCAGCAGAAACCACCGCTGCTCTTAGTGCACTCAATTCCCAACTGCTTCAACCCTGTCACAAATATATTATACATTTTTCGCAGCCTCAATCTGTTAACCTCAATAAACTTTTGCACAAAACTTGTATCAGAAAGCATGGAGACAAGCAATCTTTGTGTTGGGGCAGAAACAGTGGCGAACCTTGCCAATTTGCTAGAAGCAGCCACGACATTGTCATTATAGGAGTAGATAACACCCGCCTTCAAACCTGGAAGAGAAAGCTCATTGGAAAAACCAAATACTATATGAACTCTGTCCCGGTCATGATCTTCTGCCTCCATGATTTCTACCATGCTTACAAACTCTTCATTGCCATAAGAAGAGCTTGCAAACATTTCATTAGAGATTATGTGAATGTTCTTCTCTCTAGCAAAGTCTAAAATATCCAACAGTGTTTCCCGACTTAACAATTTGCCGGCAGGATCTGAGGGGTTGGTTATTATAATTCCACGAACTTTTTTACCACGCATCTTTGCTTGATTAAAGGTTCTCTCAAGTGAAGTTATACTTAAATTGAAGTCATCAGCGCTGCGACAGGGAACAGGTACTATCTTAACTCCAGTGCGCCATTTTACAACCCCGTCAAAACCGGGACTAAGAGGCGTGGGAACAAGGAATGCGTTGCCATGATCAGCTAGGCAGAAGCTCAGAATCTCAATGGCAGAGGTAGCACCTGCGGTCAGTACCATTCGCGAAGTGTTGAAGAAAATCGGCTTTTCCAAAACTAGAGACATGAAACCAGCCACCGCCACTTTGAGCTCCATCAATCCATCGAGAGGTTGGTAAGGAACAAGCCTAGAAATGGTGATAGGTGGTGTCCCCAATATAGCAGCGGTTCCATTGCAGTGAATCCAATCTTGAATCAAATCAAGAGATAGGGTGTTTTGGTGCAAAGCAAGTTGAATAACACCATCGGGATTATGAGTTTCGTGGTAGGGATCATCAGCGAACTTGTGGAAACCTAGATTGTAAGAAGAGTTAGCAGCGGTTATCGAATTGGCACGAGGAGACACGTAAGCAGAGGCAGAGGCGTGCCTTGGACTGAGAGAGAGAGAGCGGTGGAAGGTGGTGGAATCCTCCTTATGGTTGTGGGAAGGTGGAGGGTGGTGGTGACGTGTCTTGGTTTTGAAGAAGAGTTGAAGAAGGTAGAATAGGGCACAAGGAATGACTGAACCCCAGAAAAGACCTCCTGTGCCTTGAACAACCCCTTGTAGAGGTACTATCACTCTCATCTCTCTCTCCTACTATTCCTCCAAAACCATTCCTCTGCTATGTCACTGCTATAATCAAATCATATGTTACTAGATTGAGGCCAATGCAATACAAAACAAACTCTTGTTCTTGTTACTCTTCAGTAGCGTGTGGACACTGAACTCAACCCAATGAATTGAATATACTCTAATCTCTTTCTTTGGGATATTCTCAACGCCAAAATAGCCTCACAATTTCTCCCTCTTTTTACCCTATCACCTCTCTCTTTTTAAGCTCTCGTCATTTTACTTACTGCACACTCTAACATCCTTCCAATCACTCTCTTTTGTTTCTTTTCTCCTATCTCCTAATAATGTGGCCAATTGGTTTATGCACCGTTCAATTTCTACGTTGATTAGAAAGACAGATAGAGAGAGTTGCGACACATGAGTGATGAGAAGAACAATGAGCGAAATGAACGGCAAATATGAAACACAAATGGAAGTAAAAGAAACAAAAAGAGAGAGTGTCTAGGCCACAAACTGACATCGCTCACGCACAATCAAT

>C101425_65

CTCTCGATTTGCTCTTTTCAAATTATTGTTGTTTCAAATGTTAGTGCCATCTGCTAACATCGTTTGCAAACATAAAGATGCTAACACAACATCTACGTAAATAGCTAATGGATGCTATAGTGTTTCAGGAAAATGAAAGCATATAGATCACAGAAATTACTTTCGGTTTAAAAGCTTGAGTTACACCATTTCAAGTGCTGCAAAACACAATACGTAATCGAGGTAGCAAGTTTTGACCTTAGACTGGAAGGGTAAATAATGACACATTTTCTATTATTTGGTTGAGTAAAATTAAAGGGAAAAGGAAAGAAAAATTGACCCTTAGGGAAGAAAATCAAAATTTTCCTTCTTCCTCTTTTCTCTTCAATTTAATTTGCAATTTGTGTCTCTCTTCCCCTCATCTTTCCTCTGAATGAAACAGAGGGTAATGCCGTAACTTGCATCAGTGGAAGTGAAGGTCTTCCTCGGAACTTGAGCCTCAGTATCATATGCCCCTCCTCTTGCGGGAAGACTAGAGAGGTTTGGTTATTCATAATTTGCTGGTCGAGACTAGATTTCTACTTTTGTTGTTGTTTGTAGTTGTCTCTTGACTAGTGAAGTATAGACACCGTTCTTGCTGAGAAGCTCATCATGGGTGCCACTCTCAACTATTTGACCATCAGATATAACTGCAACGGTATTGGCAGTTTTAACAGTTGATAACCTATGAGCAATGACTAGAACTGTTCTTCCTTTCATGAGAGACTCCATGGCATCCTGCACTAGGTATTCACTTTCAGCATCTAGGGCACTGGTAGCTTCGTCCAAGAGGAGAATTTTTGGGTTCATAAGCAGGGCTCTAGCAATTGCTATTCTCTGTTTCTGCCCTCCTGAAAGCCTTACCCCACGTTCTCCAACAAAGGTCTGGTATTTTTCTGGGAATTTTGATATAAATTCATGCGCATTGGCCATTTTAGAAGCATTTTCTAAATCAACAGCGTCCACTTTGCCATCAAATCCATAAGCTATGTTCTCTTCTATGGAACAGTTGAAGAGTGTAGGCTCCTGACTCACTATACTGATCTTTCTGTGCAAATGCTTGTGTGATATTTCGACCAAAGGAACCCCATTCAGTAAAATATTTCCCTTGGTTGGGTCATAAAATCTTTCAATTAAGTTCGCAATTGTACTCTTTCCACCACCACTTGGACCAACAAGAGCAACCTTTGAGCCTGGATGCAATTTCAACGTAATGCCCTTGAGCACTGGATGACTAGGGCGTGATGGATAGGCAAACCACACATCATCTAGTTCTACTTCCCCGTCTTCATCACCGAGTGGACATTTACTTCCGGACTTGGGCATGGATGAGGTACGATCCAAAAGTTGGAATACCCTTCTGCTAGCACCTGCAGCTTTCATAACTACTGTATACAATCCTGACAGACCGGATATAGAAGAACCCACTGAGAGACTATAAAGAATGAAAGATGTAAGATCACCCGAGGACATGGAACCCTTGATTGTCAAATTAGCTCCATATATAACAACTACAATAACTGAAAGTGTAGATGCAGCATTAAGGCCTCCAGAAAACAGTCCAACAACTTTAGCTTGTTTAAGTCCAAGATTAAGTGTTTCATCAACTTTCTCTGAGTACCGTGTTATTTCAAAATCTTCTTGTGCAAATGATCTTACTGTTCTTATAGCACCAAACGATTCTTCGGCAATTGATGAAGCTACTGCAGCAGCAGCTTGAGTTTTATGAGAAAGTTCACGCAGGTATCGGCCAAACTTGCGAACAGCAACAGAAAGAACAGGTACAACAGCCAAAGCCAACAAGGTTAACTTCCATGATGTTGCAAACATGAAGCTCAGACCAATAAATGCAGTTGAAAAGTTTCTCAGGGCCTCAGAAAGATTGGTAGTTGCAGCATTCTTTATGATCTGTGTATCTTCAGAAAGCCTACTTAAAAGTTCCCCGGTACGAGTAACATCAAAGAAAGCTATCTCCTGGTTTACAAGGTGACTGAACAAATTCTTTCTAAGCCGTGCAACAACCCTTTCACTAGCAGTATAAAAAAGCCAAGATCGGAGTGCAGTGCAAATTGATCCAAAGACAACGATCAGAAATATTTCTAGTATGGTGCTCTTAACAGCATCCAAAGCTTCATCTCTCTCCTCTGGAGTTCGGATATCCCTTGAGACAATGTCAATTATCTGCCCACCAAATTTTTGAACTAAGATACTTGATGTGGCTGCAATCAAAAGAGCCACAGTACCGATCACCAACTTTCCAGCCTCAGGTTTTGCAAGAGAAAACACTCGACAGAATCCCACATTCGCCGCCGGCACTGCGTCGCCATGTTCAAGGTCTGAGACTTGACCTTCGGAGCCACCATCCCTCTTTCCTCTTCCGCCACGCTCAGACTCCAAGAGAGGGGTTCGTTCACTTCTTACTCCGTTCATTCTCAACCTCTCAAACAAACCTTCTCACACTTCCACTCTCTCTATACAGATAGTAAGTAGTACAAGGCAACACGCC

>C101429_65

TTTTTTTTTTTTTTTTTTCCCTTCGTCTTTAGCCGCGTTTTCCGATCTCGATTTCTTTTCCTTCCTCCCTCGATTTCACCCCCAATTTTGTCGCCACCTCTCCAATCGACTCCGCCTTCGCCTTGCTCTTGCTTTCCTGCGCCGAACCATCCCAATTTTCCCCCCATCGGTTCACGGGATCCGCTTTTAGGGTTTCCGTCGCGGAATATTCCCGATCTGTTTTTCCCAATAGGGGATCTGGAAGGGGTAATTTTCTATTCGATTTGGGTTCTGGGTCTTTGTCGCCTCGTTACGTTTTCTGCGATTTTGTAAATTATTTTCGACGCGTGTTTGATTCGGGTTCGATCCTTCTGGAACTTGCTTTCGGTTGTTAGATCTCGCGTTGGGTAATTCGATTCGGTTCGAATCGTAATACTTGTCCATCATTCTCGTTGGATTCAGAAAGATCTAGGGTTAGGGTTTTCTCGATTTGGGGCTTTTGCTGTTTTGTTGAATTTCTTTGGGGCAATTTCAATTCCTTGAGCAATTAGGATCATCCCGTTTGATTTCTTCCAATTAGAGCTCTTAATTGTCCGATTCAGCCTGCTGCATAAAATTTGGAGGAGACTGTTTTAAGGTAATCATGGTGCAGAAGCGTTCTTTTGATGCTGAGGAAATACTTGAGGGGTCTTTCAAACACCCTAAACATGCAGGACCCAGCCATGAGCTCTTTTCATTATCAGAATCCGTCTTTCCTGATGATGATTACCATATCCACATGCCAAAAACTTCGGAGGATGGATGTACGCAAGTTAGTAGTGAAGGTATTGAGAAGCTTGAAAGTGAAAGTTTCGGTGAACATCCTATAGAGGCTGAACATCCTTTAGAGGCTGGGAACTCTGAGAGAAGCTTTCCTGTTATAGATATTCCTGCTTCTTCATGGGCCACAAGTAGCACAACTGAAGATGTCCACGTAGAACCACCTTTACATCTTGCCCTTTTTCCAGAGTATTTTAGTCCAGAGCGACCAATAAGGACATTTACTCGCTATGAGGACATTTATTCCATACTCCTGGAACACTCTCCTCGTAAACCTGTATCTGTGGGAGCTAATCATCAGGCTGATGTTCCAGCATGGGATTTTTTGGGGGCCACAAATAGATCAAATGCCTCTGCTTCTGTTTCATATTCAGACTTTACTGTTGGGGACAGGGATGAAACTGAGAAAAGACTGATGGGGACCTGTGTCATTCCCTTGCCTCAGATGGAATTATCTAGCAATGATGATCAAGTTGGGAAGGGAAGAACTGAGTGTAGCTGTGAAGATCAGGGATCCATGAGATGTGTCAGACAACATATTGCAGAAGAAAGAGATAAACTTATAAAAACCTTTGGGCCTGAGAAATTCACTGAATTGGGCTTGACTAACATGGGAGAACAAGTGGCAGAAAAATGGAGTGCTGAGGATGAACAGCTATTTCATGAGGTTGTTTTCAATAATCCCGCATCTCTTGACAAGAATTTCTGGAACTATCTTTCTATTGCTTTTCCTTCACGAACCAAAAAGGAAATTGTGAGTTACTACTTTAATGTCTTTATGCTTCGAAGGAGGGCAGAACAGAACAGGAATGACCTATTAAACATTGATAGTGATAATGATGAATGGCAAGGTAGTGACAGCAATGATATTGCTACTCGAGAGGAAGATGAGGACTCTGTCGCTGAGTCCCCTGTATGTCATGATGAGACTTGTATGGCTGACTGCCACGAAAATGATCTGCAAGCCTATGATGAGTATGCTGCTGATGAAACGTGTGCTGGTAATGAAAGAGTGGATTTTACTAGTAGGAGCATCGATGATGATGACTCCAAATATGAACCTGTAGAGAGGCATCACTCTAGTGGTTCTCCTCTAATTCAACCCCAGGATCAACCTATTTGGCAAGATTCGTGTGATGAAAAAGTTAAAGATGATTCATGCACATCTTCTGACGCAGGAGTTGCCTCACAGGAGACTAAGTTGAATACTGAGAATGGGGATCATTGGTGTGGTAACTATAATGGTCTAAGCAATGGTTATAACCAAGGATATGTTTTGGAGCCTTGTGATGCAAAAGTGTGGGATTCTGGCTTTGTATCATGCTCTAAGAATAAGATTGACTTCTTACCAACGTGCAACATGATAGAAGAGGTTTTTGGAGATGGACGAAGGCAAGACATGAGAAGAGTTTGAGCATCTGAGGCTTTTATGAGAAGTGAATCTCAGTTAGCATTCTTTGCTAAGTGATGGATGACACTTCGACTTTTTCCTTTGTATATTTGACATAAGATTCAAAAAGTAGCCTTCATTGTAAAATAGTGTTTTGGAAGGTGATTTAATCTTTCTGAGTTATGTCGTCTCATATATTCTTCTCAAGTCCTTTTGGAACTGTAAAATTTCTTATTCCTTAATTTATGATAGTTTCCACTTTGGGGTGGGATTTAAGATTGGAGGGAGGAAATAGTTTTGGTGGAAAAGAAACCATCACTGATGCAAGAGTTCCGGC

>C101431_65

GGCCAGTAAAATGAGAAACCTATTATTCTATTTTATTATTGCCGGGTATAAAGCAAAATGCTTGCATATAGACAAGTAACAATACAGGTTCAAGCAGAAATAAAATGTGGCTCTCACAAAACGAAGCCAAAAATATGCGCCGCCGGTAGATACTTTCCTCCGTCAGAAGCACTCACGAATATACAAACGCTATATCAATGGATAAAAAGAAAAACAATCTAATTATATCTACTGCACAACCTTAAGGATTGAAGAGACTGGATCCCCCAACATTCAGAGCAATTAGTAATGATATGCTAGTAATCACAGCAAGCCAAAATAAAGCTGACCCTCCAGAGCCTTCAGAATTATCAGAACTAGAAGGAATTGGTTCAGCAGTGGGAACCGGAGACTTTGACACTTTGGATCCAGCACTTGACCCATTATCCAAAACTTTGCCAAATCCTGTTTCAGAAGCTACTATCCCCTGAAATTCTTCGCTAACAATTAATGCTGCCAAAACATCCATAAATGACTCTTTATCTGTTTCATACTCAGAATTTCTTTCATCCCTCTCGTCAGTGACATTTGCTTCTGAATCTGACAACTCGTCATCAGAAACATCAGTATAAATGGAGTCCATCTCAATTTTATCATCTTCCTCATCTTCCACATATGCTTTATATGTTAGCCGAAGTAAAATCTCCCCACAAGAGCGCTTTCCTAAAAATCCCCAACCTCCTTGTAAAACCACAATTTTGTCTGTTGGTACAGTGTCTTTAAGAGATACCAAATCAACCTCTCCAGAACCAATAGTCAAATCTGCAAATCCTAGAGCGTCCTTTACTTGGATGAATAACTTCTGTTTTCTAGGGTTGGAAACAAGCATATGAAAATCCTGATTCCAAATTGGCATTCCTGGGGGTCCAATTACAGTAGTTTGACTGTTCTTTTTGCTGCGTATAACTTGATTACCAAGGCTAAGAACGACATAAGGATCTGTCTTGCCATAGAAAATATAAGAAAGCTTTCGAGCATCCACCAGAGTAACAGATAGTTCACCCACAGAATCTTTGTTTCCTTCTTGCATTTCTCCAGATTTAACATCGTTAGCAACTGGACCAACAGCTTTTCCTTTTTGAAAATCTAAAACTATTCTCTTTGGACGTACAAATAGTTTGGGTAAATCTTCAGTGAGAAGTTTAGTCAAAAACATCGAGAGAACTGGAATTGCCATTAAATTGAACAGACGGAATGGAGATAGTTCAAACTTTATCTTTGGAAGTGAAACAAAAGCCCATGAAACAGCACCCACCCAAGGTTCTGTTGGTATTAGTCGTAATTTTACCCAAAGTTCACCATCGATGTCAAAATCTCGAACACCAACAGGTACGACAATTGGAATGATGCCAAATTTCAGAGAAAGCATCAACAGCATGCGAGCACCACCTGTATACCTAAGGCCTATTTGGTACTGCAAATCGTTGACCCTTCGAGAAGTCCTGCGTTCAACATTCCTAACAGACAAAGGTTCATCTCCTAACGAGAACTGCTTAATCTCAACCCTCTGAACATAATCAGGTTTCTTCAAGTTATCAATCACAGGCTGAAGCAACCCTATAATCCAGTTCTCAATTCCACCTCTATACACCTTCCACAACTTCCCCAACACCATGTTCACCCACTCCACCGACTCCTTCCTCTGCAAATCCTTCTCCAGGAACAGCGAGAAGCTCGTCGGCACCTGCGGCCACACGCCGCGCAGCCCATCCTCGCCGCTGCTCTTGCTCCGCCGCCGCGAAGTCCACAGTTTATCGAACACAACGCCGACGAAGAAGAAGAACACGAACAAGCCTACGACGTTGCGGTTAATCGGAGGGGAGGGAATCGGGTGAATCACCCCTAGCTGCGTCCGCAACTTATCCACAATAGGATCCTCGCGGAAACCGGTGAAGTTTGAACCTAATTGAACCGACGGCGGCGGCGGCGACGACGGTGAAGTTGAATCGCGAAACTCGGTATCATCAACATCGTTATTGTTGTTATTATTGTTGTTGTTGTCGGCGCGGAGTTGATTCGAAACTCGTTTGAGGACGAAGGTTGTGGCGGTTCTCCGCGCGGAATTGGCGAATTCGGAATTCCAGGTTGAGTTGGAAGCGTCGGAGGGAAGAGCGCAGAAGCGAACGGTGCGTTTTCTGCGGAATTTGCGGGAACCAGAATTGGCGAATAAGCGTTTCCTGGTTCTTCTGGAGAAGGGAAATGCGGTGTTACAAGGACAAAGAGAAGGGGAGAGTGAAGAGAAGTGAGAAATGGAAGAAGCGTGTTGGAGAATCATCGATAAGGGGCATGGTAGTTCAAAACCATGAACGAATTGAAATCGAACGTTGTGAATCAATGTAACCTTGAAAGCTTGGGATGATGGAGGGCAATGGAAGTGTAAAGTGCCATGTAATGTAATTAATGTTAGAAATAAAAAAATTGAAACAAAAATGAAAGAGTGCGTTTGTTTGTGTTTATGATTGTGGAAATTGGAAGCTAGATTTATG

>C101433_65

TCTGGGAAAAATATGAAGCATTAGAGTAGGCATGGAGCCGAAAAGAAGCAAAAAAGGAGAATAAAAAACTAACGAGGGAGGAGTTTATTTTCATGGGACTGAAGCATCTCTGCAACGTGGCTCATGGATGGTCTTTCGTTCATGTCATCTTGCACGCACTGCAAAGCCACTTTAACCAAAGCCTCTACTTGTGAAGCGTCGTAGTTTCCTTCCAAGCTGGTATCAATTATCTCCTCGATCCAAAACGAAGGTGTGGGTGAATGCTTGATCTTGTCCCTTATCCACGTCACCAGCCGATGCTGTTCTATGCCCCTACTGTTCTCAAGACTGTGGATTTCCATAGGGCTTCTTCCACTCACCATTTCCAACACCACAATCCCGTAGCTATAAACATCCACCTTAGCAGTAATGCGAAGATTGTAAACCCACTCTGGAGCCATGTACCCTCGAGTCCCTCTTATTCGTGAAAAACTAGAATTCCCACGCTCGTCTCTGTTTAAAAGCTTTGACAACCCAAAATCTGCTACCTTGGGTTGGAAATCTGAGTCAAGGAGAATATTTTGAGGCTTCACATCGCAATGCAAAATCCACTCCAAACACTCTTCGTGTAAGTAAGCCAAACCTTTCGCTGTGCCCACAGCGACGTTGAACCTTTTCTTCCAATCAAGTGCATTAGTAAAGAGATTGTCCGCTAAGGATCCATGTTCCATGTACTCGTACACCAAAAGCCTGTGCTTCCCTTCAACGCAATACCCCCACATGTCAATCAAATTCATGTGGTTCAGCATGCCAATTGTGCTCATTTCAGCCAAAAATTCAGCTTCTCCTTGGGTAGCTTCGTTTAAGCGTTTAATTGCTGCAACACGGTTATCACACAGTGTCCCTTTGTAAACAACCCCTCCTGCTCCTCGTCCAACCTCTTCTTTGAATCCTTTTGTTGCGGTTTTTAGCTCTGCGTAGGTGAACTTTTGAAACACTGGCAGAAAGAAGGTGTCGTTGTTGATCCAGCGTGTCAGGGTGCTTGCTGGTCCTGAACAAGAAGAACCACACGAGAAAAATGGTGGAAAACTCGAACACCCCGAACCCGACAGCAAACCAAACCAAGAAACTCAACGTGGAGTTTTTTCCTGGCAATTGATATAACCTCTTCAACGGTTGAGACAAACCAACGGGGCAATTCAAAGGGGAATGCTTAAGAGTCTTCGTAGACGAGCGCAAAATCGCATTCGGCAACTTTAGATAAATCTCTCCATCGAAATTAGGAGTATCCCGTCCGTTGAATGCCATCGTTTTCGGGTAACAATTGTAAGTAGAAACGTCGTTGAACTTGAACTGAACACCTACACATTTATCACAAAGTGCCAGACATAGATTCTGGCATTCCTTTAAGCTCGAAACTTTTGTCACGTTCCAGTCATAGCCATACCATTCCGAGGTTGGTAGATGGAGAAAACCCAAAGACTCACCGCTGTTGCAAGAAAAATCAGAAGGAGGAAACTCAGGCTCACACCCTTGAGTCCAGTCATTAGGATCCTTAATCTTAAACCCTTTCAAGCAATAGCAAGTCCTTCCAATAACAGGATCGTGACTGCACATGCTGTTAGGTCCACAAATGCCATGAACGGTACACGGTTGGGGAATGACTTGCCAAGTCATCCCCCACGACTTACTCTCCTCGTTGAAGCTATACAAACGCAAGTTACCATCAGAATCCATCTTGAGTCTTCTATACACTTTTTTGGGATAATCAGTGGAGCGGAACTGAAACCCATCAGAGGAAGTGAAGATTCCAAAGGAATCTAAAAAAGCGGTCTTGGTTACATTGTAGGTGGATCTACCTATGTCAATTGGAAGCTGCCACGAGGCTGGCCAGTACACGCTGGAAAAAGTGGTACCTTTGTAGAGTAAGCGAAGAACGTTGTCGTTGTCGAAGTAAAGTTTGTAGAATCCCGAGGAATAGTTCGTTGCACTTCTCGAAGAAACGAGGCTCGCTCGCTCCGTCAGTGGTTGCAATGGAAGAAGGGTGTCCGTTGGGGAATCGAAGCTTTGCCAAATGACGGTGCCTTTGGAAGTGAGGAGAACGAGGTTTCCGTTGTTTCTTAGCTTTAGATGAAGTTGGGAAGAGAAGAGAGTGGCGGTTGACCAGATAACGGTTCCTCCGGCATCCGTCAAAACGAGGTTGTCGTTTTTCCAGAGTGAGAGGAAGGATCCTTTGCCGTTTACGGGTTCGTCACGGTTGGCCATCCATAAAACGGTGGGTTGTTTGGAGCGCGTGAAACAAACGGAGAAGCAGAAGGCGTTCTCACCAACTTGGAAGAAGCTAGCAGAAAAATCACCATTGGACGAAACGAGGGTGTCGCTTTGTTTCTCTACTGAAAGTGACGAACCTTCTTGAAGTGTATCCGTCGCTGCAAATGAAGTTGATATGAAGAGAAGAAGGAGAAAGATTCTGATATGCATTGCTCTACACAGTATAAAGTGCAGTGCATGAATGAATTGATGCAGATTCCAAACCTGATTCGTAT

>C101437_65

CTTAATTTCAACTTAAAAAGCTTACGTCTATTTTCAACATCTTCATTATCCTGATTTTTCTAGCCATTAACTTCAAACCACTGATTCTATTGCCCTTGTTTATTCCTCATTAGATTTTGTTAGTGTCATGCATTGTCAAAACTTCGACCTGTAATCATATAAATATAACTTCACTTGATTGATGCAAACTAGTATTTCGGTTTTGAGTCTTGGAGCCACACAACACACACATTCACACACGCACCAACAACCATGGCTTCTACTTTGTGTTACAATTCTCTTAACCCTTCAACAACTGCTTTCTCAAGAACCCACTTTTCAATTCCCTTGAATAAAGAGCTTCCACTGGATGCTTCACCTCTTATTGGCCATAACTGTTCCCTGGGATGCAGAACCAGGAAGCAGAGGAGGAAAGTGATGTATGTGAAAGGTGCAGTGGTAGAGGCTCCACCAAGTGTTTCACCCTCATCACAAGGTGGGAGTGAGGCCCCTTCTAAGAAGAAGCTTCGGGTACTTGTTGCTGGTGGAGGGATTGGAGGGTTGGTTTTCGCTTTGGCTGCAAAGAGAAAGGGGTTTGAGGTGGTAGTGTTTGAGAAGGATCTGAGTGCTATAAGAGGGGAGGGACAGTATAGGGGTCCAATTCAGATTCAGAGCAATGCTTTGGCTGCTTTGGAGGCTATAGATTCAGAGGTTGCAGAGGAAGTTATGAGAGTTGGTTGCATCACGGGGGATAGAATCAACGGACTTGTAGATGGGGTTTCTGGTTCTTGGTACGTCAAGTTTGATACATTCACTCCTGCTGTGGAACGTGGGCTTCCGGTCACAAGAGTTATTAGTAGAATGGTTTTGCAAGAAATCCTTGCTCGCGCAGTTGGGGAAGATGTCATTATGAATGCTAGTAATGTTGTTAATTTTGTAGATGATGGAAACAAGGTAACAGTAGAACTTGAGAATGGTGAGAAATATGAAGGAGATATCTTGGTTGGAGCGGATGGTATATGGTCCAAGGTGAGGAAGCAATTATTTGGGCCCAAAGAAGCTGTTTACTCTGGCTATACTTGTTACACTGGCATTGCAGATTTTGTGCCTGCTGACATTGAAACTGTTGGATACCGAGTATTCTTGGGACACAAACAATACTTTGTATCTTCAGATGTTGGTGCTGGAAAGATGCAATGGTATGCATTTCACAAAGAACCTCCTGGTGGTGTTGATGGCCCCAACGGAAAAAAGGAAAGGCTGCTTAAGATATTTGAGGGTTGGTGTGATAATGCTGTGGATCTGATAGTAGCCACAGAAGAAGATGCCATTCTTAGAAGAGACATATATGACAGGATACCAACATTGACATGGGGAAAGGGTCGTGTTACTTTGCTTGGTGATTCCGTTCATGCCATGCAGCCAAATATGGGCCAAGGAGGGTGCATGGCTATTGAGGACGGTTATCAACTTGCAGTGGAGTTGGACAATGCTTGGGAACAAAGTATTAAATCAGGGAGTCCAATTGACGTCGATTCTTCCCTAAGGAGCTACGAGAGAGAAAGAAGATTACGAGTTGCCATTATTCATGGAATGGCTAGAATGGCCGCTCTCATGGCTTCCACTTACAAGGCATATCTGGGTGTTGGTCTTGGCCCTTTAGAATTTTTGACCAAGTTTCGCATACCACATCCTGGAAGAGTTGGAGGAAGGTTTTTCGTTGACATCTTCATGCCTCTTATGTTGAGTTGGGTCTTAGGTGGCAATAGCTCCAAACTTGAGGGTAGACCACTAAGTTGCAGGCTCTCAGACAAAGCAAATGATCAGTTACGCCGATGGTTTGAAGATGATGAAGCCCTTGAGCGTGCTACGAATGGAGAGTGGATATTATTACCGCATGGAGATGGAACAAGTCTTTCAAAGCCTATAGTTTTAAGTCGAAATGAGATGCAACCCTTCATAATCGGGAGTGCACCAATGCAAGATCATCCTGGCGCTTCAGTTACAATACCTTCACCTCAGGTTTCTCCAAGGCATGCTCGAATTAACTATAAGGATGGTGCCTTCTTCTTGGTTGATTTACGGAGTGAACATGGCACCTGGATCATTGACAACGAAGGAAAGCAGTACCGGGTACCTCCTAATTATCCTGCTCGCATTCGTTCATCTGATGTTATTCAGTTTGGTTCTGAGAAGGTTTCATTTCGTGTTAAGGTGACAAGATCCGTTCCAAAAATCTCAGAGAATGAAAGGCCACTAACGTTGCAAGAAGTATGACTGGTTCTGTTGAGTTGCAATTTGTAAGTAATGGAAAAGTTTTACAGCGCAAATTAAAGTTGTAAATCTGCGTTATTTTAGGGTGGGGTATATACAATGGTCCAGTCATTTAGGATTTGATCATGTTACATGTCTATATGGACTTTTAAGAGGGATTCTGGGGTATAAATTCGTTACCCCACTACAATACTTTTTTTTGTGTAATAACATTTGTTAGATAGATTTATAGCATGACTCATAATGTTGAATGGAGTAAGCAATGCCTAGCTAA

>C101439_65

AGCAGAATGAGTTGACCATTACACATTCACAGAATTTGGAGATGCCATTTTGAATCAAACAAACTACTTTCTTTCTTTCCTCTCTCACAATCCCAACCACAATCCAATCAAATCTTGCTTTTTAATCCATTTCTATTTCACCGGCACCTTCTACTTCTTGTTCGAAACAGGGAAAGATGGCGATGTTTTGTTGGTGATTTGTGAAACATTGATTAGTAAAGATGGCATGGAGAAAAGGGTGTGAGGAAGTGGCAAATTCGAAGCCTCTGTTCCTCACGATCTACACTGTTGTCATCGTTGGTATCGTGGTTTCCTCTTTCTACGTCTTCTCTGCTATATACTCGAGCAACCCACCTGCCGCTCAATCTTCTGCATGGCTCTCTTCCCTTTCGGGTGAGGATACTCGTGTTACGGATCAGACCCTAAATGTTTCTCGGTCAGCAATGGTGCCTAGTGTGCCCACTCCTTCCTCCGGCCCACAAAATGAATGGGCAAGGTCTATATGGGTTGTTCCACCAACCAATAAAAGGATGCCACCTTTGACGGATTTCCGTCTGACGAAGGCACTGGTTCAGCAAAGGGTGAAGGATAATGTGGTTATAGTGACCTTTGGTAACTACGCGTTCATGGATTTTATTCTGACCTGGGTTAAAAGTTTGAGAGATCTGGAAGTTTCTAATTTCCTTGTTGGTGCAATGGATACCAAATTATTGGAGGCACTGTATTGGAAAGGGATACCGGTTTTTGACATGGGCAGCCATATGAGCACAGTAGATGTTGGCTGGGGGTCTCCAACATTTCATAAAATGGGGAGAGAAAAAGTTATTCTGATAGACTCAATACTTCCTTTTGGTTTCGAGCTGTTGATGTGTGATACTGACATGGTTTGGTTGAAGAACCCACTTCCATATCTTGCTCGTTATCCAGAAGCAGACGTTTTAACATCTAGTGATCAAGTTATACCAACAGTTGTTGACGATAGTTTGGAAGTTTGGGAAGAAGTTAGTGGTGCCTACAACATTGGAATTTTCCATTGGAGACCTACAGAGTCTGCAAAAAAATTGGCAAAGCAATGGAAGGAAATGCTTGTAGCTGATGACAAGATATGGGATCAGAATGGGTTTAATGATATTCTTCACAATCAGTTAGGTCCATCTGTTGATGATGAAAGTGGGCTTGTTTTTGCTTTTGATGGAAAATTGAAGATGGGGATTTTGCCTGCAAGTATCTTCTGTAGTGGACATACATATTTTGTTCAGGCTATGTACCAGCAACTAAGGTTGGAGCCATATGCCGTGCACACAACATTTCAATATGGAGGCACTGAAGGAAAGCGCCATCGACTGCGAGAAGCCATGCACTTCCTTGATCCACCAGAATACTATAATCCTCCTGGGGGGTTCTTATCGTTTAAGCCATATATTCCCAAAAGGTTGATGCTAAGTGGGGAGCATAATGTTGAATCACATTTTACTCTTGTTAATTACCAAATGAAACAGATTAGGACAGCACTTGCAATTGCTTCCCTTTTGAATAGAACACTGGTCATGCCTCCACTATGGTGCAGGATTGATAGGCTATGGTATGGCCATCCTGGTATTTTGGAAGGGTCCATGACTAGACAACCTTTTCTCTGTCCTTTGGACCATGTATTTGAGGTCAATGTCATGCTGAAAAAGCTCCCTGAAGAAGAGTTTGGTCCTCAAATAGATTTTAGAGAGTACTCAATACTTGATAATCCCTCTCTGCCATCAGAGATAAAAAAGTCATGGCTTGATGTCCAGCTCTGTAGAGAAGGAACCCAAGATTGTGGTGTGTCAAATGATACCACCATTGTAGGGGGAGCTCTTAAATTTCCAAAGCACAGCAATGAAGAAACGTTTATGAAAGTATTCTCATCATTCAAGGATATAAAAGTGATCAAGTTCTCTTCAGTGCAAGATGCTTTCCAAGGCTTCAGTGACAAGGTAAGGGAAGATAAATTCAGAAATCGTGTTAAGCGGTATGTGGGCATATGGTGCTGTGTGCCAGATCACAATCCTGGTCACATATATTATGATATGTACTGGGATGAGAAACCAGGATGGAAAGCAATTCCTCCTCAAACTGCCGAGGATGATCATCCCCCTTGGTAAGATCAACATATATTTGTATTTCTTATGACATTGCAATGGAGGTGGGAATAAGAGACATAAATCCAAAGGAGATTTAGTTTCCAACTAATTGTCAACAACAAGATTGGATACAAGTGAAGAATCTGTATCCATATTGAGGAGGTAAGATGTTCATCGAGATTAGACAATTGGTTCTTGTAACATTTAAGAATTTTTTGGTTTGTATGTTACTAATATTGAACCAAGAGAAATGGAGGCCCACAATTTCTTAGAGGCTTCACATTTTGGTCTGTGATGTCCTGGTTGGTATTGACTGTAATTAAATTCTTGAGACATTCAATTGTTACACTCTTTTCTTCCTTATTTTACTTTGAACAGAAATAAGCAATGTTTGTTTTTCTGCTGTCCAAAAA

>C101441_65

TGAATTGCGTACACAGCATTGAATGGTTGTATTCTACAACCAAATTAACTCATTTCCTGCAACTTGTATTTAAAAATATTTATATATAGACCTTATATTTTCGCCAACCACTGGTAGCACTTATTTTATTAGTATACGCTATATTAAAGTGAAAATCTTCATGCTTCCAAGGACTGTTTATCATGTTCATCCCTCACTCAAATAATCTTCACTAACTTTTCCACATGAAGAGGACTGAATGTTGGTGTCTGTTCTGGTCTTCTGAGAATTAATCGTTTCACTAAAGTCTGCTCTGAACTGATAGCTCATGCTTCATTTGATCCCTCAGTACTCTCCTCAGGATCTTGTTGGAAGATGTTCGAGGAAACTCTGGCACAATTTTAACTAGGCTTACCTTAAACAAAGGGTTAAGGTTGCTTTGAATAGCTTTAGTGAATTTCATCTTCAGAGCTTCTGCAGTGGAATTGTATCCTTTCTTTAAAACAACAAATATAACCAGTTGTTCTGGGCCTCTATTTGTAGTTGCCACACCTACTGCAGCTGTCTCCAAAATGCATTCATCAGCCCCATCACAGACACGCTCAATTTCTACAGAACTTGTCTTTATTCCACCAAGATTCATGGTGTCATCTGCCCTCCCTTGTACAACAATATATCCATCAACCGTTCTCTTGATTATATCCCCATGTCTCCTAAGAACCTTTCCTTTATAATTGGGCATTCCCTTAAAGTAAACCTTCTCATGATCAGCATTAAGAATTCTGTCAGATGCTCCCAGAAAGAGAGGAAATAAGCCCACTTCCCCAACACAAGCAACATCATCTGGATAAGGAACTCCATTTTCGTCAAAAATGACAAAACCAGTTGTCATTGATGCTGTGCTAAATGCTCCAAAAGCCTGAGGTTGCAGGGGACTTCCTGCAATATAGCTAGATGCAAGCTCAGTGCCTCCACACAATTCAACAATCGGACTGTAATAAGCTTTTGAAGAAAGCCATAGATCATCATCAACATTTGATGTTTCTCCAGTGGAACAAAATGTTCTTATCTTTGTCCAATCCAAGCCCTCCATACATTTTGTACTCTTCCAAGTTTTTACTAAGCTTGGAACTGTTCCCAAAATGGTAACACCTGCATCCTGAACAAATTTTCCAAAATTACGACCTTGAGGAGACCCATGGTACAAAGCCAAAGTTGCACCAAGTAGAAAACATTGATACAATATTGTCGGTCCCATCACCCATCCTAGATTTGTAGGCCAGCAATAGACATCTCCAGCTTGAACATCAATGGCAGCCCATCCATCAGCAGCACTTCGTATTGGTGCAAGTTGAGTCCAAGGAATAGCTTTTGGATCCCCCGTGGTTCCAGAAGAGAATAGTATATTAGTAACAGAATCAACTGATTGATAGCTTGGAGAGTAAAAATCTGACCTGGAGTTCTGAGTCTGATTTGCAGAAGAGATAAAAGCTTTCCATGATAAGTCTTGTTCTCTTAATTGAACTCCTACATCATCACCCAGCACAGGGAGCACAATAACTTTACAAACAGCTGCCTCAATGACCCGACTGTACAAAGGAAATTTCCTGCCACCTCTGGCTATGAAATCCTGTGTGAAGATACCCTTTGCTTTGGATACGCGAAGGCGAGTTGCAATCTCTTTTGGTGCAAAACTATCAGCTATTGAGACTACAGCGCATCCAGCAAGGACAATGGCTAAATAGATGATAACAGCATTAGCTGTCATTTGCATGTCAATTGCGATTGCATCTCCCTTTGAGAATGTGGTATCTATTGCTTTAGCTACCAACATTACTTGTTGTCGGAGTTGTTTAAGCGTGATATGATTAACCTCAGAATCATCAAAACCTTCATCTCTCCAAACAATGGCTAAACTGTCATCTGGTTTGTAGGGATGTGAACTGGGTTGCACACAGCAATCAGCTATGTTGAGCACAGAACCAGGAAGCCAAGTCCCTCCATGTCTAGAAGGGTCAGAAGTATCTAAAATACACTTGGGAGGTTCAACAAAGGAGACCGAAAGTTCCTTCAGAACAAGGGACCAGTACATCTGAGGGTGTTGAACAGAAAACTTGTGAAAGAGAGGAAAACTGGTAATAGGGTCTTTATAGGAAGGTCCTAAAAGTTGAGGGGCATGAGTTTCCATGAAACGGCCTAGGTTGGTGCGTTTGGAATGGTCCAGAGAAGGAAACCAATAAAGTGGAAGGGAAGCATCTTGTGTGTGGAAAGCAGAGTGATAAACAGAGTGGTAGAGAAGCTGATGCAAGGAGTGTGGAAAGGATGGCTTGAGCAGTTTGTGAGTGACAAGGTGGCGCCATATGAGTGAGGGGTGGGTGGGGGAGAGATACAGAGACAGAATATCCCTCAAAATGCGGTATAGTTCATTGGCTTCTGCTACAGACAAGCCTGCATTCACCAAGTCTTCCACTTCCAATTCCCTAAAGCTCTTCCCCATCTTTTTTTTCTCCTCTTTCAAACTTGGAATTTGAACAAAATCTTCAAGAGG

>C101445_65

GTACAGTACAGTTAGCATTCTCTGCAGGTTTTTGCATGTCCAAAAAATACCAATGTAAAATGAGAACACGTATACAGAAAGAATTGTAAAGCTTTAAAAAATAGAAGCTCTACAGAGAGGAATTGTAGACCTAAAAGGTGCACTGCAACTTTGCAAATGCTGAGAAAACTTCAATGATCAGACTAAAAAGACCTATCTATGGACTGATGTTGCATTCTCGCAAATACACATGATTGTAGTCAACATACTTTGTCCAATAACTTCGAAACTTGGGAATACTTATCTCAAGCCATGGTTTCAAGTTCCCATTATAGTGTACAACAGCAGACCGCTCAATATCCTTTTGGTTAACGTTGGGGTTGTAGCCAAGACCTAAGACATGCCAAGACCGGTTCAGTGGGAAAGTGCGTTTCCAGAATGTTATGAGACCAGGGGGCAGTGTTCCTAACTTCCATAGTTGTCTATCATGATTCAAATTCTGCCAGTTGTGGTACACCTGTGTGATGTTTTGCCTTTTCCATTCCACTAAATCAAATATATTCATACCATATGCCCACCCACAAGCATGTGGGTCAAAATTCTTTGCAATAAGAGGATGTGAGAAGTTGAGATATCGATCAAATCGGTGAAAACTTTCTCCACAAGTTTCTACAGCACCATTTACATTTCCTTTCAAATCAATTGACCACAGATCAGTCAGATCCTTCTGCACAACTATGTCATCATCCAAAAACAGTACTTTGTTGAGCTTCGGAAAGATCTCAGGCAGATAGAAACGGAGGTGGTTCAAGATAGATAAATATTTTGGATTTCGAAACTTCAGATTTGAATCAGAGGTTGCTCGGTGAGCCTTGAAGTAATAATCGATCATGGATTGAGAACCCAACTGCTTAAGAACAGGACTGTAACTTGAATTCAACCATGTAAAGTCTTCAATGTTTTCTACTTGAATGGTTGCCTGGCCTGGTGGATTCACCAAAAACCACATCCTCATCGCTGCATAATTGAGTCTATCAGTAACAATGTGGAAAACATGTTTTGAGGCGTCCTTAGCATTGGAAACAGTTGAGTTCACAACAACGGCTGTTGCTAATATGTTGTCAGAAAATATGGCATAATGGTATAACTGGGGGTCTACTAAATTTTCTTGATTAGGGAGCTGTTGCTGGGAAGATTTCATGTTATGATACTCAGTTGTAAGGCGCAAGGGAAGACAATGAAGACCTTTGGGCAGTGTTTTTGCTGTCACTTGTGTTAAGAACAAGGTCTGCTTCTTGAGAATGCGAAGTTGCTCCTCCGTCGTGTGGACCATGGCCCTAAGCTTCCTCACAACAGCAGCACAATCATCTTGAGCTTGCTTTCCTTTCATCAGTGTTTGTTCCATCGCTTTCATTCTCTCATTTGCATTCCTAGGTAATTCAGAATCTTTGCTTGCATCCCCAAGTGATCGTGAAACTTCTTTCACCCGTAATCGAAGCTCCCGTGTGAGATGAGGATTGCTTTTTACAACTGGAAGGGAAAGAAAGACCTTAGCTTGGATAAGCTGGTCTTTTAGCTGCTTTACCCGATAATCAGATGGTGTTTGATCATTCTGCTTATTGGTTTCAGACAATCTATAACCTTTCTGGCTGACTTTGCTGGAAGTTTCTTTTGTTTGTTCCTTAATTTGAGGATCTTGAGTAGAAGCACTAGTTGATTTAGCAAGCTTCACATCATAGCCATCAACATCAACATCATCAGTCACCAATTTAATGGGGTTCTCCCTTTTGGTCTGATCTTCATCAGTTGTAGTAGACAACACCCTGGCAGACAAATGCTCCCTTGATTTCACCAATTGCAAGCCTCGAGGTAAATTCCTTCTGTTTGATGAATCCTCTTCAGTATACACCACCCTAATAGGTTCTTTAACGATCGTTGAAGTTTCCTGCGGCAGCAGATTCAAGTGGCTAGAGTCTGCTGCACTGAAACCAAAAGCAGTAACATCTTCAACAAACTCCTGTTTTTTCGAAGGAGACTCAAAGGTGCCAAGACGATCAGTGTAGAGCACAATCGGAGCCACAACAGTAATGCACAGCAACAACAGCACAATGTTTCTGACTGCCACCATTTTTCTAGAAAGAGAAAAGAAAAAGCGGCGAGACCCTTTGGACAGAGATGAAGAAAACGAAGAGATAGGAGCCAAGAAGGTAACTTTCCTAGAGGAAAAGAAAATCCACTTTCCCTTTTCCTCCGGGTGTGTTGAAAATTGTCTCTAAGCTTTAGGATGAAATCAGTTACATTGAGCGAAGAAAACAAAACGCGTTTTGAAAGACTGCGATTTTTCTTTGAAAGGCTTCTACCCTTCTCTGATTAAACACATGTTGTCAATGCAGAGAGAGCTGGAGAACGAAGAAGAAGAAGAGCGAAGCAATGGAGGAAAAAAGGGGGAAGTGAGAGAGAAGAAAAGAAGAACAATGGGCAAGTCTTTCTCTGTGACTGTACAAGACAACTTTCTTGGCCTAGCGTATCACTATTTGCTAACGGAAAC

>C101447_65

ATCGTGTTCTTCTTTACTCACACACTCTAAACCCTAGACATGCCGCCATTAGCGTCTCTGCAACTTCGGAGGCTATTACTCCGTTCTCGTTCTTTTTCATTCACATCTCCCTCTCCATCTCCCATTCACCCCCCTCCTCCTCTCTTTCCCCGACATTCCTCTACCCATTTCCACTCCAGAACTCTCTTCACTCCTTCGCTCTTCATAACCAGACATTTCTCATCGGAAACCCAAACAGATCCAATTGCGCGTTCTCTCTCCTCGGAGCTCCTTAAAGAACCGGACTCCGACCCTCTCTCAGTCTCGCAGCGCCTCCACCTCAGCTTCTCCCACATCACTCCAACCCCCAATGTGATTCTCCAAACCCTAAACCTCTCCCCCCAAGCTGGCCGCATTGTATTAGGCTTTCACCAATGGCTCTCTTCCAATCCCCAATTCTCACACACCGACGACACACTCTCCTATTTCGTCGACTACTTTGGCCGCCGCAAGGACTTCAAAGCAACCCATGACATCCTCTCCGGCTGCGGCGCCGCCGGACCTAAGACCCTAGAGTCGGCGATCGACCGCCTCGTCCGCGCCGGCCGGCCCAGCCAGGCGGTTCAGTTCTTCGAGAAAATGGAGAGAGACTACGGGTTGAAGCGCGATCGCTCTTCGCTGAAGGTAGTTGTGGAGAAACTGTGTTCCAAGGGTTTCGCGAGTTACGCGGAGAAGATGGTGAAGGATTTGGCTAGGGAGTTTTTCCCCGACGAAGCCACGTGCGATTTATTGATAAGAGGCTGGTGCGTTGATGGGAAGCTCGAGGAAGCTCAGAGGCTCGCCGGTGAGATGTATCGTGGAGGGTTTGAGCTTGGTGCAGGGGCTTACAATGCCATGCTTGATTGCGTGTGTAAACTTTGTCGCGAGAAGGATCCGTTTCGGCTTGATTCAGAGGCGGAGAAGGTGCTGGTGGAGATGGAGTACCGTGGAGTTCCCAGGAATGTGGAGACTTTCAATGTGTTGATAACGAATTTGTGTAAGATTAGAAAGACTGAGGATGCGTTGAGGTTGTTTCATAGTATGGGAGAGTGGGGGTGTTCTGCTAACGAGTCTACTTTTCTTGTTCTGATCAGGAGTTTGTATCAGGCTGCTAGGTTGGAGGAGGGGGATGAAATGATTGATAGGATGAGATCTGCTGGGTATGGTGAGTTTCTTGATAAGAAGGCTTACTATCAGTTCCTCAGGATTTTGTGTGGCATTGAGAGGATTGATCATGCTTTGAGTGTGTTTTCTATGATGAAGGCTGATGGTTGTGAACCCGGGGTTCAAACTTATGACTTGTTGATGGGGAAATTGGGTGCCCACAATCGTGTTGATAGGGCTAATGCTCTGTTCAATGAAGCAAAGAGTAGAGGGCTGCCTGTGATTCTGAAAGATTATGCTGTTGACCCAAGGTACCTGAAGAAGAAGATTGTCAAGGTTGTGAAGAAGAGGGAAACCTTGCCTGAGAAAATGGCTAGGAAGAGAAGCCGTCTGAAACAGATTCGCTTGAGCTTTGTGAAGAAGCCCAAACGAGTGATGGGTCGTTAATGATCATATTTTTGTACTTCAAAGGTTTTTGGAATAGATTTCCCCATTTAATCATCGTTTTCTTTGCCTCCTGGTTAGTGTTGCCTATTCAGAGATAAATTCTGGTATTGAAATGGTTTCTTGGTAGGAGATATGCGACATATTTAGAAATAATTTTTTGTACTTGCTTTACGTTGGTTCAGCTGCCGGGAGATGCTTGCTGAATCAGTATGCCGGGTTTATAATCAGTTATTGATATTGACATCTGATACTGGTACAATTTAAATGATAAATGAATGTAACAATAATGTGACTGAACTTTAGAAAGTTCTAGATTCACAGAAAGCCAACACATGAATTGATTGATAAAATGTTGTCAGTTTAATCTTGTTTTAGTTAATTGTCTCTTGTCGTACTTTGCCCCATTCATAAGCCAATACCCAATGCGTTTGTAGAAGGTTCATTGCCGAATTTTCAGCACTGGAAACTGGAAAGGCAAGCATAAAGTTTGAGCGCCAGTCAGATTGTAACAGCTCACTTTGTAGTTTATGGAACAAGAAAAGAAGCTGCAAGTTCAGACAGATTACAGGATTATACCTAGATTTATTCTGCTCCAAATGTTTAAAGAAGAATCTCGGTGTAGAATTGGTGGCTGAAGCTCATGCCTAGGCATCATTGTTGTTGCCATTCAAATCATTTGCGAAGACTGCCCTTGCAACTAAAAATAACTATCGTATCTGTGCTGCGACAATAGTTTTCAGTTCTGGCAGTGTTGAAGGGGCTGTTAGAATTCCTCCTAGTATCTCAACTAAACTGAACGCCCATCAGGTTTTTAGATTTTTAGGCAGTTGTTCTGGATATAAAATTTTCCTGCTTGGAATTTTCACTGTACTTGGTACTTCACTTAACAATGTGACTCTTTGTCTAGTTTTATGTAGGTATGATTTGTAGTATTTTGTTGGATTTTTAGTCTTCAATTA

>C101449_65

GGCGGTGGTAGTGGCCGTTGTCGTTGCTTAACCTCACCTCACTGGGCAATCGTTGTTTCTGTAAATCACACCAATCGCGTTTCTCAAAGTTATATACTTATGAATGGCGTGGCGCGAGCTTGGAAAGAAGAGAATATATATGAATTTTACTAGAGCTTTGACTGCAACGCCATTTAGGCATGTGACAGGAGCAAGTTTTGCTTCCATCTTTGCTGCACGATCTGTTTATGCATCAGCAAAATGCGTGACAGATATCATCAACCAATCATCTCAGGGAGTGACTGGTTTTGGCAGTGAGACTAAAGAATGTGGAATACGGTGCTTCCATGCAAGTTCGCAAGTCTGGGCAAGAAGTGATGGACCATTAGGTTTGCAGACCCCAAAGAGGGTGAAATATGTGAGTAGGGATAGTAGAAAGCAGCCACCTGTTGAAGCTCCTTATGTCCACCATAATGTTCCAGCTAAAAAATCTAATCCTGATAAAACAATAGAAATATTTGAAGGCATGACCCTTGTTGAGCTGGCAAAGCGCACTGGGAAATCAGTATCTTCTTTGCAGGGTATCCTCACTAATGTTGGTGAAAAGGTTGAATCAGAGTTTGAGCTTCTCACCATGGATATTGCTGAACTTGCCGCAATGGAGGCTGGCATCAATGTTAAGAGGCTACATTCCACAGAAGGTGCTGAAATGTTACCTCGTCCTGCAGTTGTAACAGTGATGGGTCATGTTGATCATGGTAAAACATCTCTTCTTGATGCCTTGCGTCAAACATCTGTGGCAGCCAGGGAGGCCGGTGGCATTACTCAACATCTAGGTGCTTTTGTGGTGGTCATGCCTTCAGGAGCATCAATCACATTTCTTGATACTCCTGGTCATGCTGCATTTAGTGCAATGCGGGCAAGAGGTGCAGCAGTTACAGATATAGTGGTGCTGGTTGTTGCTGCAGATGATGGTGTCATGCCTCAAACACTTGAAGCTATGTCCCATGCAAAAGCAGCTAATGTACCAATCGTAGTTGCAATTAATAAATGTGATAAACCAGGTGCAAATCCTGAAAAAGTTAAACTGCAGCTTGCTTCAGAGGGCTTGCTGCTGGAGGAGATGGGTGGGGATATTCAGGTTGTTGAAGTTTCAGCAACTGAAAAAATTGGACTGGATAACCTTGAGGAAGCCCTACTACTTCAGGCTGATATGATGGATCTTAAAGCACGAATTGATGGGCCTGCCCAAGCATATGTGGTGGAAGCAAGACTTGACAAAGGACGGGGTCCATTGGTAACTACTATAGTAAAGGCAGGGACTCTAGTTTGTGGTCAGCATGTGGTTGTAGGTTCACAATGGGGAAGAATAAGGGCTATTAAAGATATGGCAGGGAGGTTAACTCAACAAGCAACACCTGCTATGCCTGTTGAAATCGAAGGGCTTCGTGGGCTGCCAATGGCTGGTGATGATGTTATTGTTGTTCATTCTGAGGAAAGAGCTAGAATGCTTAGTTCTGGTAGGCAAAAGAAATATGAGGAGAACAGGCTTAGGAATAAGATGATACAGGACAAACCAACTACTTCAGATGATTCCACGGGGGTTCCACGGAGGGTTGAAATGCCAGTAATAGTAAAAGCAGATGTTCAGGGAACTGTTCAAGCAGTCACTGATGCATTGAAAACTTTAAACAGTGCTCAGGTTTTGGTGAATGTTGTCCATGTTGGCGTTGGGCCACTTTCTCAATCTGATGTGGACTTAGCACAAGCTTGTGGTGCTTGCATAGTTGGATTCAATGTCAAAAGTCCTCCTACTGCTCTTAGTCAGGCAGCAACTCGTGCCAGTATAAAGATAATTTTGCACCGTGTAATTTACCACCTCTTGGAGGACATAGGCAATTTGATAATAGAGAAGGCCCCTGGGACTTCTGAAACCCATGTTGCTGGACAGGCAGAAGTGCTTAACATCTTTGAAATGAAAGGTAAGTCTAAGAGTCCTGATGTGAAGATAGCTGGTTGTCGTGTTATTGATGGTTTTGTAGCAAGATCAGCAACCATGAGGCTTCTGAGGAGTGGGGAAGTTGTGTTTGAAGGACACTGTTCATCTCTTAAGCGGGAGAAGCAGGATGTGGATACTGTGAAAAAGGGAAATGAGTGTGGACTAGTAATTAGCGATTGGTATGATTTCCAGATTGGAGATGTTATTCAGTGTTTGGAACAAGTTGTAAGGAAGCCCAAGTTCATTAAGTCAGAGAGTGGTGCTGTTCGAATTGAGTGCTGATCAAGTTAGTTGAGGATTCTTCAACTCATATTTTTGTGATACATTACACTGAGCCAACAAAATGACTCCATATGGAAAGGTGGAGTAATTATTCTTTGTTGTACCAAAAAAGAAAGTGTAATTAATTTTGAGGCACAATTTGAAACGGTTAATATACTAGGTTTTTTGACAAATGTTTTACACGTATGATCCTTGTTTTTGACTATAAGTGATACCCACTGCAGCTCATATAATTCCTTGAATATGAATGCGTCAGCTGTATCATTTA

>C101453_65

ATTAGCTAGTGCTCTTTCATCATCATCATCATCACCATTCTTTCTCTCTCTCTCTCTCTCTCTCTCCCTCTCTCTTTCTCAATCCTTCCTGCTACACTCTACACGACACAACCAAGCTTGGGAATCATTCTCACTATTCTAATGCACCCTATTGAACTTTGCTCTGACCGAGAGATACCCTTCTTCTGCAATCTTAATCTCGTAGATGATTGGCACAGAATTTACATGTACTAGGGCATGAAAACCTTCAAGCTCATATTTTTCGAGTCCATTTCCGTTGCTCCAACATTGCTGATCGGAGAATTGACCGATGAATCAGAACCGATTGGACTTAAGAGCTCATCATTCTGGATCGACACAATCAGAGGAGTCCGCTCTGGACCTGGAAAGGAGCTACTATGGTCATCTTAACCCTTCATCTAGTCCTTCACCCTTGCAACCTTTTGCTGGAGGTGCTCAGCATTCTGAGAGCACCGCTGCCTACTTTTCTTGGCCTACATTGAGTCGCTGGAATGATGCAGCTGAAGATAGAGCCAACTATTTTGGGAACCTTCAAAAGGGTGTGCTGCCTGAAACTTTGGGCCGATTGCCAACAGGACAGCAAGCTACTACCTTGCTTGAATTGATGACTATTAGGGCATTTCATAGCAAGATCTTACGCCGGTTCAGTCTTGGCACTGCAATTGGATTTCGAATTAGAGGAGGTGTTTTGACTGATATTCCAGCTATTCTTGTCTTCGTTGCTCATAAAGTTCACAGGCAATGGCTCAACCATGTTCAGTGCCTACCTGCTGCCCTTGAGGGACCAGGAGGTGTTTGGTGTGATGTGGATGTTGTGGAGTTCTCCTATTATGGGGCACCTGCACCAACCCTTAAAGAACAACTATATACTGAGCTTGCTGATGGCTTAAGAGGAAGTGATTCATGTGTTGGCTCAGGTTCTCAGGTTGCAAGCCAAGAGACATATGGAACCTTGGGTGCTATTGTCAGAAGCCGAACAGGAAATCGAGAAGTTGGCTTTCTAACAAATCGACATGTGGCTGTTGATTTAGACTATCCAAACCAGAAGATGTTTCATCCATTACCACCCAGCCTCGGACCTGGTGTATATCTTGGTGCAGTTGAGAGAGCAACATCATTTATTACTGATGATCTTTGGTACGGAATTTTCGCAGGAACAAACCCAGAAACATTTGTGCGAGCTGATGGGGCCTTCATCCCATTTGCAGAAGATTTCAATATGAACAATGTAATTACAACTGTAAAAGGCGTAGGTGAGATTGGTGATGTAAACAGCATAGACTTGCAGTCTCCAATCAACAGTCTCATTGGAAGACAAGTGATTAAAGTTGGAAGAAGTTCTGGTTTGACTACTGGGACTATAATGGCCTATGCCCTGGAGTACAATGATGAGAAAGGGATTTGTTTTCTCACTGATTTTCTTGTTGTTGGTGAGAACCAGCAGACATTTGACCTTGAAGGTGACAGTGGAAGCCTCATTCTTTTGACTGGACAAAATGGGGAGAAGCCGCGCCCTGTTGGCATTATCTGGGGTGGGACAGCTAATCGGGGTCGTCTGAAACTAAAAGTTGGTCAACCCCCAGAAAATTGGACAAGTGGAGTTGATCTTGGCAGACTTCTTGATCTCCTTGAACTTGATCTCATTACAACAAATGAAGCACTCCAAGCTTCGGTCCTAGAGCAAAGGAATGGTCCTGCTGCTGGAATTGACTCTACAGTTGCAGAATCCTCTCCTACTGTGCCAATAAAAGAAAAGCTCGAAGAGAGCTTTGAGCCTATTTGCTTGAATATCCCACATGCTCAAGTTGAAGATGAACCCTCTCAAAGAATCAACCCATCATTAAGGCCCTGTGAGTTTCATATCAGAAATGAGATTGAAACAGCACCCAATGTAGAACACCAGTTCATTCCAAGTTATGTGGGCAAGTCTCCTGTACGTCACAGCTACCTGAAAGAAGACATGGAACTGAAAAGTCTAGCAGAACTTAGGAATGGCCCAGATGAAGATAATTTTGTTTGTCTGCATTTAGGAGAACCGGAAATGAAGAGGAGAAAGCACTCAAATCCTTCTTTTTGCATCAAAGAATTAAAGTAACTAGTAGGTTTAAAAGTGAAGTAAAATGCAGCATATATACTCAATGAAGCGACTGTCTCATTTGCATGGCTGTTCTCTATGCTACAGAAACGCAAGGCCTTTAGCATGTGGTGCCTATTATGGGAGGAGCTTCAAAAGCAAAGGATAAACAAGGTTCACTTCTGGATGATATTCTTATTCTTTTTTTTCTTTTCTTTTATAATTATAAATCTTGTTTCGGCAAAGTGATGTTAGTCACTTGTGGGTTATACAATATTTTATAGAGGACAATTAGCTTGCACTGTGTTGAATGATTTATACCACTTTTGTAGACTTTGTATTATAAGCAACCCTGATGGAGAATCAGCTATTGTAAGTTAACGGAATGTGAAACACATCCTTGTTAATCAAGCAGCTTCAGTCTATTAAAAAAAA

>C101455_65

TTTTGGAGAAACAACTATTTCTTCATAAAACTAAACCAAACACCTCAATACAGAAACATCAAGATGACATGGAATACATCGGGTTAAGAAAACTGGAGCTTACATCAAGATCAACTTCATTTACAAAACAGCTGAATGAATGCCTTTTGACTTTTTCTCACTTAGGAACATACCAGGGGAAGGGAGAAGGAGAAAAACAAGTGAGGAGCAATTTGTATAGCCCCACTTCTCAATCAGACACACCACTAAGCCACTACCAAAAACCTAAGCAAAACCTTGCACCAGCCAGAAGACAGTTGACAATAACAACAATGCAAATAATAAGAAAAACATATACATTGCGTTAGCCGAACAAAACTTGCACAGATATGCTGACATTTTAGCACAGCATGAAGCAGTCCAATTACCGAGACAAGCCTTCTTTTCCCTTAGCAAACAAGAGATGATCTTAAAGATGTAGGTAGTTGAGTGCATGATGACCAACGGATGACAGATTCAGTCACCACAAGGTCAAGTTGCTCACACTCACCAAGTCCAATTGTTCTGTTTCTCTTGCTGTGGATTCCCAGTACCTTGTGTCCATTCATTCCACTTGACCTTTTAGCCTCTACAGGCTTGCGATTTGTTTCTTTAATATTGTGTTCAATGCAAAGTTGAGAATCTTGTAGTAAGAGTAACTTTCCCAAGTTAGTTTCTCCAGCAATTGCATTTACATCAGACACATTAGCTGAACACTCTAATTTACAACTCTTGTGTTCAACTACGGGAGAGGGACTGAGGAATGAAAAGCTAAGCATTGAGGGAATGGGAGAAGAGAATTCCAAACCAGAGGAATCGCCTATACTCTTGAATGAAAACCCACCAGAGAGTAGACATGGTGTGACTGGTTCTTCCAAAAAAGAAGAAAATGATGATCTGCATGTGAAAGTAGGCGATTGAGCATCACAAGTAGGTGAGCCAGCGCCAAAGCTGATGTCCTGATTCCTGGACATGAAAGAAGCAGAGAATGAGCTGTCACCAACATCCCATTCTTCGCAACTTTGGATTTGATTTTCACTAAGTTGTTCTGGATTTGTTTGGTTGCTTTCCATCCAGCAATGATTTAAAACCTCTAGGGCAGTGAGTCTTCGAGAAGGGTCTGTGCAAAGCATTCCCCTGATCAAATCCTTAGCTGATTCAGAAATGCGATCCCAAGGTTCCGATGGAAACTTCAGGGCAGCAGCTTTAACAGCTTCAAAAATCCGTGATTTAGTCTTCCCCCAAAACGGAGGCGTCCCACTCAACAGAATGTAAAGAATTACACCTGCGCTCCACACATCAGCAGCCTGGTTATATGCACCAGCTAATACCTCAGGAGCTATGTAGAATGGACTCCCAACTAAACCGTGCAAACTTTGACCAGGCTTGATGTAGGTTGCTAGTCCAAAATCTGCCAATTTAATAGGAGATGAGGAGGATCTCGTTGCTAAGAGAATGTTCTCAGGCTTCAAATCTCTATGAACAACCCCGTTTTCATGACAATACAAAACCACCTGCATAAGGTGCCTAAAGAGAACCCTGGCTTCAGATTCTGAAAACCATCCATGCTTCTCTAATCGGTGAAAAAGTTCTCCTCCAGCACAAAGCTCCATCACCAAGTGAACAAAACCTTCCTCTTCATAAACTGCTTTGAGATCCACAACGTTGGGGTGTCCAGATAGCCTAGCCATTATCTCTATCTCAAGTTTGACACTCCTCAAATCATCCGAAGTAACCAACCTATCTTTAGCAATTGATTTGCAGGCCAAAACCTCTCCTGTCAACTTGTCAGAACAGGCCCTTATGACACCAAACTGCCCCCAACCCAATTGCTCGCCGAGAATGTACCGATCTTTTAAATTAGAGACTTGACTTGTTTCTAATATGATCTCAGTCAAGCCCGCTACTTTGTAACAATTGTATGAAGTACAAGGTTCATGGCTGCTGTTGCTCTGGGCAACAGCCATACCAATTTCTTCCTCCCTCTCTCCTCAACAAACCAAGAAATATAAGTAACTATTTAATTTGCTTACTGCAACAAAACTCGTTGAAGAGTTGAAACTTGCTCTTCAGGAGACAGAACCAGCTTCTTGGCAAAATAAAACACAAACGTCAACCTTTGATTTTTCTCACTTTTCTCACTTACAAACCAAAGCTCTCCATTGCAGAGTCAAAATCTTTGAAGAAACTTAACGGGTTTATATTTGACAAAGAAAACACACAACGTATGACACACGATAGCATGAATCTGAACCAGTCAGAATTTGGGACCACTTTTCTCCCTTTTCCAAAATCACTTGATCGGTTGCAAAGAGAAAGGTTAAAAAGTGGAAAAGGGAGGGTGTGAGGAGAGGGAAGGAAGAGAGAGAAAGAGGCCGTTGGAGCAGTGGTTGAACTCGAGGGAAGAGAGAGCAACCATGGGCTCCAAATAGGGACCTCTTGGGGGACTCCACACAAAATAACAGTAACAACTTCGTTGCTGCTTTGTTTGGATGTGTCTTTTTGAAATTTCG

>C101457_65

TTCTCTTGCGCTTTTCTCCTTCCCTTTCCACGTCTCCCTCTTCCCTACGCCACGCCCCGTGCACACTGCGCCCTTCCCTTAACCCAATCCCGCGCACGTTAGCCCCCCCTTTTCTCAATTCCAATTTAGCCGACTCCTCTCACCCAATTATAGCCAATCATCATTTCCTTACTCTTTTCCCCCCTTTTGCTAACGTGCGCCTCTGAAGAGTGCCCTTTGATCTTTTTCGGTTTTATTGGATTGAGGGGTTGAGGGATTTCTTGAGTGAGTGACTGGGCTTCTGATTTGACTATGGTGCTGGCAAGGGCGGTTGTGGAAATGAGAGGTTTTCTGAGAGTTCTTTAGCGTCGTCGTTTTGATGTTAGGGGTAGTGGGTGAGTGAGTGGGTGATGTTATTTCTTTAAAAAAATTGATTTTTTGAGTTGGGTATGGAGTCTGGTGAGGAGATTAATTTGAATATAGAAAGTGGTGGTGGGAATAATGGGAAGAGTAGTGATGGGTTCATTGATAGAAGTAAGGTGCGGATTTTGTTGTGCGATAACGATTCCAAGAGTTCTCAAGAGGTTTTCACGCTTCTTTTGAGGTGTTCTTATCAGGTGACCTCAGTAAAGTCGCCGAGACAGGTAATTGACGCACTGAATGCAGAGGGGCAACATATAGATATCATACTGGCTGAACTTGACCTTCCAACGAAGAAGGGCATGAAGATGTTGAAGTACATAGCACGGGATAAAGAGTTCCGCAGAATTCCTGTTATAATGATGTCCGCACAAGATGAGGTATCTGTTGTTGTTAAGTGCCTGAGACTTGGAGCAGCCGACTATCTAGTAAAGCCTTTACGAACTAATGAACTATTAAATTTGTGGACGCACATGTGGAGACGGAGGCGCATGCTTGGACTTGTGGAGAAGAACATCTTGAATTATGATTTTGATCTGGTAGCATCAGACCCTAGTGATGCCAATACAAACAGTACCACCTTGTTCTCTGATGATACAGATGACAGGTCCAAGAGAAGCACCAATCCAGAGGTGGGATTATCAGTCCAACAAGAGCAAGAGTCGACTATTGCAATTGCTGCTGCTGTTGAGGAACCTCCAGAGCCTCATGCATCAGAACAAGGGCTTGATGTGGATGGATTTAATGATCACCGAACAGCACATTTTTCATCCGGTCCGAAGAAGAGTGAACTAAGGATAGGGGAGTCATCAGCCTTTTTCACGTATGTTAAAGCATCTATACTGAAAAGCAACTTTGAAGGGGTAGACAACAATGGTTCTGCACATGTGAGGATGGAAGTTATGCATCAAGGATGTGCTCAACAAGGGGTTAATGACCTTCAAACACGTGAAAATGGAGAGACTTGTGAAAGTCAATCGCAAGATGACTTACCCAGCAGCAGTAGTATACCTGATTCTCTCTCTATTGAAAGATCTTGTACTCCACCTGCATCAATGGAAGTTTCACCGCAAAAGCACAAGGAAGAAAATTTCCATCAGGGTGTGATGCATCCAAGAAATGGGACTCATTGTTCTGAGCATGATGTATCTGGCATGGCGTCCCAACATGCTTATCCATATTATATTTCAGGAGTTGTTAATCATGTTATGATGCCTTCATCAGCACAAATGTATCACCAAAAGAATATGCAGGACCTACAGAACCATCCTAGTACAGCAGCTATGATTGCACAATACAATCATCTTCCCCAAGGTGGTCCTCATGGAACGGGGATGACATCTTTCCCGTACTACCCAATGAGTATATGCTTACAACCTGGTCAGGTTCCTCAACCCCATTCATGGCCATCATTTGGAAGCTCAAGTTCATCAGAAGCAAAATTAAGTAAAGTTGACAGGAGAGAAGCAGCATTGATGAAGTTCAGACAGAAAAGGAAAGAACGCTGCTTTGATAAGAAAATTAGGTATGTTAACCGGAAACGACTTGCTGAAAGACGGCCTCGTGTGAGGGGACAGTTTGTCAGAAAGTTAAACGGTGCCAATGTGGATCTCAATGGACAGCCTGCATCAATTGATTATGACGAAGATGACGAAGAGGATGAAGATGACCAGGGAGCAAGAGATTCTTCTCCTGAGGATGCTTGAGCATGTCAAATTGCTACTTTTACTTGAGAAAATGAAGGGCCAATCAATTTCTTCCTGCACTAATATTGTCCTGTGAAAAGGATTCCTTACTTAAATGGAAAGTTCTGACCAAAGCATGAAATTATGATCATGAGCATGTGCATGTGCTCTATCATGTATCTGGGGTGCTGATTCAATATGCACCCTCATCTTTTACAGTTGAAACAAGCTCTTGCCTGGTCAATTTCCAATAGCAGCTCATTAAATGATCCATCCTTCTGAGCTATTTCTGAAGGTATTACCTCCTGCTGGTAATGTCTACTGCGGCTGAGAGTATCATGCTTACTTTTTATTGAAACACTTACAAGCAGCTTGTTCCTGCTTATTTTATGGAGAAATGTCATGACATTTTAAAAAAATTGACCTGAGAAATGAGTTATTTGTCCCG

>C101459_65

CAAGTAGTGGTAGCATATCTCGGAGTATTTATTTAAGCCCGTGTCGCTTCCACACGATATCTTCCTCTATCTCCACGTCCTCGCGATACCATGACTTCACTTTCTGCCCTAAACTTCTCTCTGCAATATCCTCCTGCTCGGAGAGACGACACCGTCGTTGAAGATTTCCATGGCGTCAAAATTGCCGATCCTTACCGATGGCTCGAAGATCCTGATGCGGAAGAAGTGAAAGAGTTCGTAGAGAAACAGGTTAAACTGACGGATTCGGTTCTTCAAGAATGCGAAACAAGAGGGAAGCTTAGGGAAACGATAACTAAGCTTTTCGATCATCCTCGCTACGAGGCGCCATTTAAACAAGCCGATAAGTACTTCTACTTTCACAACACTGGGCTTCAGCCACAAAATATCCTATATGTGCAGGAGAGTTTGGAGGGTGAGGCAGAGGTGTTGCTCGATCCGAATGGCTTCAGTGAAGACGGAACTGTGTCGCTGAGCACTCTGTCAGTGAGCGAAGATGGCAAGTACTTGGCTTATGCGATTAGTTCAAGCGGTAGCGATTGGACGACTATAAAAGTGCTGCGGATTGAGGATAGGAATATTGAACCTGATACTTTATCGTGGGTAAAGTTTTCCTCTATTAGTTGGACACATGACAACAAGGGTTTCTTTTATAGCCGCTATCCAGCACCAAAAGATGGAGAACTAGTAGATGCAGGGACTGAAACAAATGCTAACCTTCATCATGAGCTCTATTACCATTTTCTGGGTACAGATCAATCAGAAGATATTTTGTGCTGGAAAGATCCTGAAAATCCTAAATATTTGTTTGGAGGAAGTGTTACTGAAGATGGAAAGTACATCCTTCTCTATATCGACGAAGGGTGCGATCCAGTTAACAAACTTTACTACTGTGACTTGTCCAAAATTCCTAATGCGCTGGAAGGCTTTCGGAATGGAAATTCCCTTCTTCCATTTGTCAAACTTATTGATAACTTTGATGCCAAGTATGAAGTCATTGCCAATGATGACACTGTGTTTACATTTTTAACTAATAAAGATGCTCCAAAATATAAATTAATCCGAGTGGATTTGAAAGAGCCAACTGCTTGGGCTGATGTTCTCCAAGAGTCTGAAAAAGATGTACTTGAGTCAGCGTGTGCTGTAAATGGTAACCAACTGATAGTCAGTTACTTGAGTGATGTAAAGTATCTTCTGCAAGTTAGAGACTTAAAAACAGGTTCCTTGCTGCATCAATTACCAATTGACATCGGCTCAGTTTCTAAAGTTTCTGGGCGGCGTGAAGATAGTGTGGTTTTCTTTAGCTTTACAAGCTTTTTAACACCCAGTATCATCTACCAGTGCAACCTGGGAACAGAGGTTCCTGATATGAAGATATTTCGTGAAATTGTTGTCCCTGGGTTTGATCGGTCTGAATTTGATGTTAAACAGGATTTTGTGAGTAGTAAAGATGGTACCAAGATCCCGGTGTTCATTATCGCCAAAAGGGATATTATTTTGGATGGTTCACACCCATGTTTATTATATGGATATGGTGGTTTTAACATCAGTATTACACCATATTTCGGTGTCAGTCGCATTGTACTCGCAAGACATTTAGGTGTTGTTTCCTGCATAGCAAATATTCGTGGCGGAGGAGAATATGGGGAGGAATGGCATAAAGCAGGCTCCCTTGCAAGAAAGCAGAATTGCTTTGATGACTTTATTTCTGCAGCTGAATACCTTGTATCTACTGGTTATACTCAACCCAAAAGGTTATGCATTGAAGGTGGAAGCAATGGTGGACTTCTTGTTGGTGCATGTATAAATCAGAGACCTGATTTATTTGGTTGCGCTCTAGCTCATGTTGGTGTTATGGACATGCTACGGTTCCACAAATTTACCATAGGTCATGCATGGACCTCAGATTATGGTTGTTCAGACAAGGAGGAAGAGTTTCATTGGCTAATCAAATACTCACCACTACATAATGTTCGGAGACCGTGGGAACAAAATCCTGATCAGTCTATTCAGTATCCATCGACTATGCTGTTGACAGCTGATCATGATGATCGTGTTGTGCCACTGCACACATTGAAGCTATTAGCGACCATGCAGCATGTCCTCTGTACGAGCTTGGAAGAAAGTCCCCAGACAAACCCTATAATTGGTCGTATTGACTGCAAGTCTGGACATGGGGCTGGACGTCCAACACAGAAAATGATCGATGAAGCTGCTGACCGGTACAGTTTTATGGCTAAAATGTTAGAAGTTCATTGGATTGAATAGAACCCACTCAGTCACAGGTTAGAGAGAACAATTACAATGGATTCTTTCTTTCAGATGATACCCTTAAAACCATTTTATTTCTGTTCTTCACTTCTACCAAGAAAATACCTTGAACACGAGATTGTCAGAGAAGTGGTCAATGGGTGTTAGATTTTAAGTTATTAATGCTACGCCCCTATGGATTTGTCAATTATTTAATTGGCATTAGGTTCACTAAAGTCCATTAAAATATTTTTTTTCCTAGAG

>C101463_65

AAAAAAGAAAAAAAGAAAAGAAACGAAACTTTGTAACGTAAAACCTAATTAGGATTCCGAGGCAGAGAATCCTGGATTGTTCGAAGTGTTGAGCTACATTCCCCTCTGAGCTACGTTCCTCACTCTGTCGTTAGGGTTTCGAGTGGTCTTGTTCTCAAACAAGCAGGTGTTGTGTGATATTTTGTAAAATGGGTGAGACTGGAAAACGATATCATTCACAGAGAGACCATGATGGGGACAGGAAAAATCAAAAGAGACGTGTGAATGAGAGAGATGACAGGGGTACTGATGAATTGATTGTTTATAGGATACTGTGTCCTGATGAAGTTATTGGTAGTGTTATTGGGAAGAACGGTAAAGTGATAAATTCAATAAGGCAAGAAACCAGAGCAAAAGTTATGGTTGTGGATCCATTTCCTGGTGCCAAGGACCGGGTTATAATGATCTACTGCTATGTTAAGGAGAAGGAAGAAGTTGAGATTGATGATGAGTCCACTGGTAAAGAACCTTTATGTGCTGCTCAAGATGCTCTTCTCAAGGTTCATGTTGCCATTGTGAATTCCATCACAGCTCTTGGAGATTCTGAGAAGAAAAGGAAGGATAGAGATGAATGTCAAATCCTTGTTCCATCAAGCCAGTCTGCAAATATCATTGGTAAGGCTGGGGCTACCATTAAAAAGCTGAGAAGTAAAACAAGAGCAAATATCATGGTTACTGCCAAGGATGCAGCAGACCCAGCACACTCATGTGCTATGGATTTTGATAATTTTGTCTTGATCACTGGTGAATCAGAAGCTGTTAAAAGAGCACTATTTGCAATTTCCTCTATTATGTACAAGTTTGGTCCAAAGGAGGACATCTCTCTTGACACAACTGTACCAGAAGCCCCACCTAGTATTATTATTCCCTCTGATGTTCCAATATATCCTCCTGGTGGACTATATCCAGCTTCAGATCCTATTGTCACACCTAGAGCAGTTCCTCCAATTATAGGTGCAACTAATGTACAAGATCTGCAAGGTTATGCTGATGCTGGAAATACATGGCCTATATACTCATCTGCCCTTCCAGTCGTTTCTGCCCCTGGTGCTTCTCAGTCAAAGGAGTTAATTGTTAGAATTTTGTGTCCGTCTGACAAGATTGGGCGTGTCATTGGGAAGGGTGGTAGTAGAATTAAAAGTATGAGGCAAGCAAGTGGTGCTCATATTGAGGTTGATGACTCCAAGGCTAATTATGATGAGTGTTTAATCATTATAACTACAACAGAGTCGCCAAGTGATCTAAAGTCCATGGCAGTTGAAGCTGTTCTGCTGATGCAAGGGATGATAAATGATGAAGATGATACCACTGTTTCAATACGGCTTCTAGTTCCATCCAAGGTGATAGGTTGCCTTATTGGTAAAAGTGGCTCAATCATAAAGGAAATTCGGAAGAGAACTAGGGCTGATGTTCGAATCTGTAAAACTGACAAGCCAAAATGTGCAGACGTGAAAGTTGAACTTGTTGAGGTGGGTGGCTCAGTTGATTGTGTAAGAGATGCACTAATCCAGATTATCTTAAGACTCAGAGATGATGTACTGAGAGAAAGAGATACTGGCCATAATCCTTCTATAGGTATAGGTGCTGAATCCTTGTACTCAGGAAGTACTGGTTTTTCATTGCCATCTGTGCTTCCTTCAGTTCCTCCTGTTGCTGCTCCATTGGTTTATGATCAGCGGGCTGAAAGTGGAGCTGGCCTTGGCATGCTTTCTTCAAGCAGCCTTTATGGATATGGATCTTTGTCGATGGGGGAAAATGGCTACGGCTCTATATCCTCGTTTGCCAACAAGCTATATGGAGGTTTGCCTCCCCCATCAACTCTGGATATGTTGATTCCTGCTAATGCTGTTGGTAAAGTGCTGGGTAAAGGAGGGGCAAATATAGCCAATATCAGGAAGATTTCAGGAGCAACAGTTGAGATATCTGACTCCAAATATGGCCGTGGTGATCGTATTGCTCTTATATCTGGTACACCTGAACAAAAGCGCACTGCTGAAAACTTGATTCAGGCATTTATAATGGCAGCCTGAATTATTGAAGTTCTTCTTGGGGTATTGAAGGTGAAAAGTTTAATTCATGAAGTATGTCCTTGTTCCTGTCCTTAGTTCTGTTAGGTTGGTCCTGGAGAGACAATGTTCTCTAGGGTTGTTCAGCTTGACTCCTGCTTCTTCCACAACAGTGGTTAACAAGTTCATGCTTGAGGATCCTTTGCTGATTTTGTATGTAGGAATTTAATGTTCCTTTCCTTTGTTTCTTCTTAATCTACTCTCATCTGGGTTTTATCTTAGAAATCTAGGATCACTCTGTCCTTTCATAATGTTTGTCTTGGACTAGTGTGGAGTCATCACTATCTCCGTACATTATTCAGTGGAACCATAAAGCTGTGTTATTATGTTTCTGATGGCTCACCGTCTTATTCTCCTTTCTTGCTGAAAATATCTTCCCTATTGATGCAAAATAGATGCTGAGTCTCTAGCTATCTGTGCTTTCTCGC

>C101467_65

TCAGAATCGGATCCAAGTCCAATCACGCATCATCACCGGGTTCTCTCTCTATCTCCCTTCTCTCCAAACCCTATCATTTCCTGCCGGACCCTCCGATCGGATTCTAACACCGCAACCACCACCCTCTCTCTCTCTAAAGTGTTGGTAATCTGAAGCCTATGTGATGGAAATATCAAATGATTCAGGTACTAAGAAACCAAAAAGGCTGACATCCGTTGTCTGGAATCACTTTGAAAGGGTTAGAAAGGCTGACATCTGCTATGCTGTTTGTGTGCATTGTAACAAGAGACTTAGTGGATCAAGTAACAGCGGAACTACTCATCTGAGAAATCACTTGATGCGGTGTCTGAAAAGATCAAATTTTGATGTGTCTCAATTACTCGCAGCAAAGAGAAGGAAGAAAGATAATTCCATCAGCCTAGCAAACATTAGTTTTGATGAAGGTCAGAGGAAAGAAGAATATATAAAGCCAACAATCATCAAATTTGAACAGGAGCATAAGAAAGATGATATCATTAACTTCGGTAGTAGTAAATTTGATCAGGAAAGGAGTCAACTCGATCTTGCACGCATGATCATATTACATGGATACCCATTGAGCTTGGTTGAACAAGTAGGATTCAAGGTCTTTGTGAAGAACCTCCAGCCTCTGTTTGAGTTCATGCCGAACAGTGCTGTAGAGATTTCTTGTATAGATATCTACAGGAGGGAGAAAGAGAAAGTGTATGATATGATAAACAGACTACATGGTAGGATTAATCTGTCTATTGAAATGTGGTCTTCAACAGAAAATTCTTCGTATCTGTGCCTATCTGCTCATTATATTGATGAGGAATGGACATTACAGAAGAAAATCTTGAACTTTGTCACACTTGATTCTTCGCATACTGAAGATTTACTTCCAGAAGTGATTATCAAATGTCTCAATGAATGGGATATTGACTGCAAGCTTTTTGCCTTGACACTTGATGATTGTTCCATCGATGATGACATCACCCTTCGAATTAAGGAGCGAGTTTCTGAGAAAAGGCCTTTCTTAAGTACTCGTCAGTTACTTGACATACGTTCCGCAGCACATCTCATAAATTCCATTGTTCAAGATGCCATGGATGCATTGCAGGAGGTGATCCAAAAGATTCGAGAGAGCATCAAATATGTTAGAAGTTCACAAGTAGTGCAAGGAAAATTTAATGAAATTGCTCAACATGCTACAATTAACACTCAAAAGGTCTTATTACTTGATTTTCCAGTTCAGTGGAAGTCTACGTATCTCATGCTTGAAACTGCGCTAGAATACAGGAGTGCTTTTTCTCTCTTCCAAGATCACGATCCCTGCTATTCATCAACTCTAACTGATGAGGAGTGGGAATGGGCCACTTCTGTTACTGGATACTTAAAACTTTTAGTTGAAATTACGAATGTATTTTCAGGCAACAAATTTCCTACGGCTAACATATATTTCCCTGAGATTTGTGATGTCCATATTCAGTTAATTGATTGGTGCAGAAGTTCAGATAATTTTCTTACTCCCATGGCTTTGAAGATGAAAGCCAAATTTGACAAGTACTGGAGCAAGTGCAGTTTGGCTTTAGCTGTAGCTGCAGTTCTAGATCCCCGGTTTAAAATGAAGTTGGTTGAGTACTACTATTCCCTAATTTATGGCAGCACTGCTCTGGAGCGTATCAAGGAAGTTTCTGATGGTATCAAAGAACTTTTTAATGCATATTCTATCTGTTCAACTATGATTGATCAAGGCTCAGCCTTGCCTGGCAGCAGTTTACCTAGTACTAGTTGTAGTTCTAGAGACAGGCTAAAAGGCTTTGACAGATTCCTTCATGAGACATCACAGAGTCAGTCCATGACATCAGACTTGGACAAGTATTTAGAGGAACCAATCTTTCCGCGTAATTCTGATTTTAACATATTGAACTGGTGGAAAGTCCACATGCCAAGGTACCCAATTTTGTCTATGATGGCACGTGATGTTCTTGGGACTCCAATGTCAACTTTGGCACCGGAATTGGCATTTAGCACTGGGGGCAGAGTGCTAGATTCTTCTCGTAGTTCACTAAACCCGGATACAAGAGAGGCTTTGATATGCACCCAAGATTGGCTCCGAAATGAATCAGGAGATCTAAATCCATCCCCAATCCATTCTGCTCTGCCTCTTCTCATTGAATGAAGTTAATTTTACCATGGTCTTATCGAAGAAGCATCTTAGAATTCTCAAGTTTTCCCTTGTTCCCCGCGTCATTTGTTGCTGGGAAGTGATCTTTCTTGCAAATATTCTGTGAAGATGCACTGTATATTCTTAGAGCAACAATGTAGATTTTGCTTTATACTCATAGATTTCTTCCCCGCGAGATGCTTTTATTGGACTAGCATTTGGCTGAATCTGATTTTGTATAGAGGGTTGCATGTCCTTGATATCAAAATCTCTCCTTGCCTGGAAGTTTTCTTTCGCTTCACTGTTCATACAATATGGTGGCGCTAAATGAATGATCTTAACAAATAAAGATCACTTCAGAGTGGCA

>C101469_65

GGGAAAAAGAAAGTAGTAAGAAGAAGAAAAAGACATGGAAAAGAAAAAAGAAAAAGAAAAACTGAGTAGGAACAAGATTGGTAGTGACTTGACAACTGTTATAGTTTCCATTTTTGAAGAACACTCTTACTCTAATTTATTCCAGAATGGGAGTTTATATTTCTGTTTTGGGTTTATAGTTCATTCTCTTGCATTAAATTATTTAATTATCAACCTTTGAAACTACATTCAAGTTCAATTCAAATACGATAGGTGCCTCTGCTCCTGCAGCACCTCCAGAAGCCACAACTGTTTTTCTCTCTCACCTCTCTGAAGATCATCACTTCAATAAATGCACGCCCTTTGAAGTTTGAAGACTTGTTCTTCCTTTTCAAAACCAACCCGGATCAGAAAAAGTCGTAAAGTTATTATCTTTGTCTACTTCAGAAGTACCCAGACAATCATAGTCCTTGTGGTTTTGCAATCTGTTTCTGGGAATCCTTTTTTCATTTCTCCTCTCTCATTCTGTGGTGGTTTGTTTGTGCTGAATGCTAATGTCTGGCTCAAAGTACACAAAACTATCTCTTTGTCTGGTTTCATGGTTGTGGTGTGGTTCTGTGGCTGTGGCTTCAAGTGAGAGTAGTATCGGTTCCAAAGACCTTGATCAGACACCAACATGGGCCGTTGCTTGTGTCTGCACTGTTTTCATCTTGATATCCTTAACACTGGAGAAAAGCCTTCACAAAGTGGGAACGTGGCTACAGGAAAAGCACAAAAAGGCTTTGCTTGAGGCTTTGGAAAAGGTCAAAGCTGAGTTGATGATTCTTGGTTTCCTTTCACTGCTTCTGACTTTCGGGCAGAGTTACATTGTTAAAATATGTATTCCTACTGATATGGCAGACAAACTGTTGCCATGTCCGTATGTTGGTACCGACAAAGAATCAAGTAGTGAAGAGGAACACCATAGGAAACTTCTTTCTTATGAACGCAGATATTTGTCAGAGGATGCTACCCCTTACCAATGCAAGAAGGGACATCAATCGCTTATATCTGTCAATGGATTGCACCAGCTACACATCCTCATATTCTTCTTAGCAGTCCTTCATGTGTTTTACAGTGCTATAACAATGCTTCTTGGGAGACTAAAGATTCGAGGATGGAAGGCATGGGAGGAGGAGACATCATCTCATGGATATGAGTTTGCCAATGATCCTTCAAGATTCCGGCTTACACATGAAACATCATTTGTGAGAGCTCATGCTTCTTTTTGGACAAGATATTCAATCTTCTTCTATATAGGATGCTTTTTTAGGCAATTTTATAGGTCTGTGGGCAAGGCTGACTACCTGGCATTGCGCAATGGATTTATTGCTGTCCACCTGGCTCCGGGAAGTAAATTTAACTTCCAAAAGTATATCAAAAGATCCTTGGAGGATGACTTCAAGGTAGTTGTTGGAGTGAGTCCTGTCCTCTGGGCTTCATTTGTTGTTTTCTTGCTTCTAAATGTTAATGGATGGCATGCTATGTTCTGGGCATCCTTAATTCCTGTTGTGATAATTTTAGCTGTTGGTACGAAACTTCAAGCCGCATTGGCAAAGATGGCTATTGAAATAACAGAAAGACATGCAGTTGTCCAAGGGATTCCTCTTGTTCAAGGCTCAGACAGATATTTTTGGTTTGGCCGGCCTCAGTTAGTTCTTCATCTTATCCATTTTGCTTTGTTTCAGAATGCGTTCCAAATAACATATTTCTTGTGGATATGGTATTCTTTTGGGCTGAGAAATTGTTTCCATGCTGACTACGAGCTTGCAATAGTGAAAGTAGCTTTAGGGCTTGGGGTACTATGCCTCTGCAGCTATATCACCCTTCCGTTATATGCTCTTGTTACTCAGATGGGCTCAAGAATGAAAAAATCAATATTTGATGAACAAACATCAAAGGCATTAAAGAAATGGCGCATGGCAGTGAAAAAGAAGCAGGGAGTGAAACTTGGAAACTCCAAGGTGCGAGCCATGGATGGAAGCACCACTGATTCGACAGTACACTCTTCTGGTCCCACACTACACCGTTTCAAAACTACTGGTCACTCAACTCACACCATGTCAGCCTATGCTGATGACCAAGATGATTACCAATCTGACACTGAGCTTTCTACCATTTCACCAACAGCAAACTTGATAGTAAGAGTGGACCGTGATGAGCAAGAACCAGAAGAAGTTGAACACCACCCAACAGCCAACAATCAAGAGCTATCACGTTTGTCAAGCCTGGAAAGAAGCAAGAAATAGCTTGAGAACTTACTACATTCAACCATTTTTTGTTCTTACAAAATATATCTTGTCCTTGTTTAGAGCTTTCTAGATATTCTTATACTTAATATATAGCCAGTTTTGTACATTCTTGTTCTAAAACACTTACCGGCATTTGAAACTTCAACCTTGCTCAAAAAATCATGCTTGAGCCTTGTGATATTTTTATTTGTGTGCTTGAGCAAGAGCTCATAAGTAGAGGACAGAGGCTCATAAGGAGCAAGTAGAGGACAAAATTGTTGGGTG

>C101471_65

GTTTCAAACTAAAACTTCTGTATCCAAAAAAACTCACGTTCTCAACAATTTACACGTTTAGATGTCCAAAAAAACAAAAACTGCCATTAATTACCTTTTCACTATTTAACGTCAATTACACTCTATAAAAAAAAAAGGCATTCTTGACATCTTTTCTGTCTTGGTTCCCCTTCCAAAACGAAGCTATACAACAATTCATGGTATCTGAAAAAGGGCAGGACAGGAAGCACATAACAAGTTAGGAACACATAAATTGCAGAGCTTCCCGTGTACGGCCTAGCAAACTCAATGCCGTGGCAGCAATGTGATGTCCACTCAACCCCGCTTGGTCAAGCTGTTCATCTGGTGATGCATGCTCAATATATCTGTCAGGTAAGACAATGGGTCGCCACTTAATTCTTCCATCGAGTAGTCCATTAACTGCAATAAACTGAGCAACATGGGAACCAAATCCTCCAATAGATCCTTCCTCAACAGTAATTAGAAAAGAGTGATATTTACAAAGCTGCCTCAGAAGTTTGATGTCAAGGGGCTTGCAGAACCTTGCATCAGCAACAGTCACCTCAACGCCAAGTTTTGTAAGGAGTGAATGAGCCTTTAGGCAGTTCTGAACCATCGATCCATATCCTAGGAGAGCAACATCTTTACCCTCAACAAGAACTCTTCCTCTTCCTATCTTGATGGGGATTCCATCGCCTACAGTGAAGCCTTTCCCAACCAGGGCACCCCTTGGATACCGAAAGCAGACTGGTTGATTGTTGGTATGAGTAGCAGTAGCTACCATGTGCATGAGTTCAACCTCGTCAGATGGTGCCATGACAATCATGTTTGGTAAGCATGACATAAAGGTTATATCAAATGCTCCACATTGAAGGGGACCATCAGAACCTACCAACCCTGCACTTGTAATGACAAAGCGCACTGGAATTCTCTGTTGATCAACATCGTGTACCACCTGGTCATAGGCTCTTTGCAGAAAGGAAGAAGGTATAATGCAAAATGGCTTCAATCCACCACATGCCAAACCAGAAGCAAATGTGACTGCATGTTGCTCAGCCATTCCCACATCAAAAAACCTATCAGGAAATTTTCCCCGAAACAGTTCAAGGGATGACTCCATCGTTATTCCTGCATGAACAACCACAATATCTTTGTCTTTCTCTGCTTCTGCAACCAAAGTCTCCACAAAGCAATCGCCATATGTTTGAGGCCGAACAACATTACCTAACGAATCAGATTTAAAAGGATCATCACCCTGTTGCCTATTTGTTATATCACTTTTCTGACAGCTTTCATTTCCCTGATTTTCATCAGTTATCACATGTACCAAGACGGGTCCTATTGAATCAAAGGAAGCAACTTCTTGCAGAACACATGTTAGATCCTCAATATTGTGTCCATCCACTGGGCCAATGTAGTACAACCCAAGCTCCTCAAAGAGAGTAGAACCTAGGGGACCTATCATACCACGAGCATATTCATCAACTTTAGCCGCCAACTCATGCATACCTCTACCAATTCGTTTTGTAACACCCTTAGCAGCTTCTCTAAATCTTCGGAAAGATTTACTGGACTGGAGCTTGCTCAGTGTACTAGATAGGGCATTTACAGATGTCTTTGGGCCCTCCTCAATTTTTGGAAGTAAAGAGTGACGGCTATCATTCAAAATCACAACCATATTTGAGTCCAAATATCCTGCATTGCTCATTGCCTCGTACGCCTGACCAGCCATAGTTGTCCAATTGCTGATAACTGCAACTACACGTTCTTGCCTCCCCTTAATATCCCGAGCAACTGCCATGCCAAGTCCAGCAGAAATACTGTTGCATCCATGACCTGCACCAAATGCATCATATTCACTCTCAAATCGGGAAGTAAAACCAGAAAGACCGTTCTTTCTTCTCATTGTTGCCATGAGAGATCGCCTTCCTGTAAGAATCTTATGCGCATATGTTTGATCCCCAACATCCCACAATATTCTGTCTACTGGAGCATTGAAAACATGATGTATTGCAACTGTTAGCTCTACCACTTCCATGCTTGCTTTAGGTGATATCTGTGTACCAGACATAATAGAAGATAAATCTGAACGGATTTCAACTGCTAGTTGTTTCAGTTCCTTAAGAGATAAGTTCTTCAAGCAAGAAGGGTTTTCAACCATGTCAAGAATGGGTGTTGGAACTTTCTCCCAGTAGTAATCATCACTATCAAGCTGTGCACATACTCGATCAACTAATCCCTTTGAAGTAGAACGAGGAGACATGGAAAGTGTAGAGAGGTCCACGTGGAGGGGGAACATGGAGGTTGAGCAATCCACTCTGCGACCCAGGGAGTGGAAAGGAACTCCAAAAGGGCATGTAGAAGAAGGAGTAGCAGCCATGGAGAAAGTACCAACCAAAGTGTTGTTACTAAAATCTTCAAAATTCACAGTTCAGGGAGAACCATGATTATTGATTCCAGAGCTATTAGCTATTTCGCATGAACAATGATGAACCCAAAATATCAAAAAGGAAAAAAAAAAAAAAATTTATATGA

>C101473_65

TATCAGTATTATTAATGCTTATTGTTGCATATTCTACATAAACTTTTGAAAGAGGACAAACTTTTTCAAAGCTCCTATTTAAACATTTATGCAACTAAAGGACTAAAGTTCTTAGAGCTGCGATTTGACTTGGTGCAGCTATTTGCATCGTCCAAAGCCTGGCCATGTGCTCCAAAAGAAAAATACCGGTTCATCATTTTCTTATTCAAAGAGCTCTGCTTATAGTTAAACTCTAGTCTACCACCACCATCCATTGGTCGATTGGAGCAAGAGGGTCCCATTAAATCAACACGATTTTGCTTATTATTCGAAGCTTGAGAAGATATAGATAGTTCTTGAAAAAGACTAGAAGTTGAATGAACAGGTAGAGGACCTTGTGGCTTTACCTTAATCACGCCTTGCATTAGGCCTGCATGAAAATATTTTTTGGCTGAAGGACTTGGAAGATAAGTCCTTATAGATAGTGTTCTTTTGTTGTTCGGAAGAGAACACATTTTTGAGTATCTTTGCCTCAATGGGATAGGAAGGTCTTCCAAGTTGTTCCTTGAACTAAAAGAACTCACAGAAACATCATCTTCCAACTCCGAGGCTGCTTCTTTTGTGTCTCTTATTCCATGCCACTGTAGCAGAAATTTTAAGCGCCTTGCACCATAATTGACAGAACTAGAGTTGCTGTTGCATGGGGAATTGGCTGAGTCATAATTGTTTATTTCACTTGACCATGAATTTGATTCTACACTTGCCTGATCATAATTCGTATCACATGAATTCTCAATTCCCGTGTACAAGAATATCTCAGAATCAGATATTCTAAATGATGTTCCAGGACTGGCTCCAATACCATGCGTCCTAAGATGGTTAACAAGAGCATTTCTTGTCATATAGTCCTTACAATTAGAGATCCCTCCTGCAGAAATTAATTGCTTGATTAAAATGTCAGAAGATGCAGATCTTGATCTTTGCTTAAGATGATCCAAAGCGGTCAGGCCATGAGCATCACGAATGTTCAAATCAATTGATGAAACAGAAATCAGTAATTCCACCACCTCACATTTGATGTTATGAATAACAGCAACATGAAGAGCTGTTCTCCCTTCATTGTTCCTGACATTGATGATGTCTTTCATGCTCATAATCTTTTCAGTTGTCAATTGCTTCATGAGCTCAATGTGCTTGTCCAGTCTACAGAAACCAGGGCTTCTAAAACCAACCACTGCCAAGTGAAGAAAAGTATCTCCATAGTTATTGGTTAAAGTTGCTAAAGAGGGAGATGCTCCAATCAGAATCTCTACCACAGGTAAGTAACCTCTGTAAGAAGCCACATGTAATGCTGTATTGCCTTGAGCATTGGTTGAGTTGATGATATCATAGGATTTAATTAGATTTCTCACTACCTCAACCTGGCCTCTTGCAGCTGCTGCATGCAAGACAGTAGATCCTTGGGCATCTCTATAAGACAAAATCTGAGAAACAGAAACACTTCCTAGAAGCTGCTTCAGAATTTCCCAATTCCCTCCTCTGGAAGCAGCATGAATGGCTCGATTCATCACTTCCCTCTTGAAATCCTTGGAACCTTCATCCAACTTCTCTTCCAATTCTGCTTCACTTCCACCAAGGCAATCCTTTCTAGAGAGAGCAGAATGAAGCAGGAGTTTAAAGACCTCACAGTTCTTGCCCCTTGCTGCAGCATACAGCATGTCAGTGACACCATACTCTCCTTCACCAAACACCAACAAAGGGTCTCTCCCCAACAACTGAAGAACAAAGTCCACATCCCCAGCCGAAGCAGCAGTGTAGAGAAGCCATCCACCATAGCCTGAACGGAGGAGAGAGTTGTGCCCCCTTCCTATCTCACACTCAAGCATAAGATTCCTAGCAACCTTGGACCGGCACTTGGCAACATCCTCAAACTTAGACTCGTCGTCCCATACGGCTTCAAGGCGGCGAATACGGCGAAGGGAAGTGAGCTTGATGAGGAGGTTGGTGTCAAGGTGGAGAAGCTCAGTGACCAGATCATAGAGGCCATTTGCAGCAGCATAATCAATGGGAGACGCATACCACCATTGGTCTCCTGTGCTTTCCCATCGAAGAGGAAAGTGAGAGGGAGGCATAGTAAAGGTAAAACTAGTACCTCAAATCAGAGATAACCCTTTTACAACAAAGGACAATCACTACCACTTTGTGCAATTCTAGTTCCAAACAGCAGTGTAAAGAGGCCTTTCAGACCCCTCATAAAAGCAAAAAGTGAAAGCAAGCACACCACTAACAGAAAATATGCTTATTCTCTTGGACTAAATAAAAGTTATTGGAAAGGAAAAATGAAAATCCTTGGAAGTGAGGGTGTGGTAAATTAACCTTGTGAACAGTGAAGTACAAGGAAACAAAAGTTGGATTCCACAATTGGCCAACATGAAATGAATTTAAACGGTGTCCCTTGCTGAAGTTGTAGCAAAAGCAAAACGGTGGAAGGAAGAGAAAGATATGGTGGCAGGATGTTAGATGACAAAAGGAAAATGGCCACTAGATAGGATTTGTG

>C101475_65

TTGAGTGGTAACATTTCCATAAATACAGCAATTTCTCCCAAAAGAAAGATAAAAACAAAGACTTATCCAATTGCTAGTCAATAATGCACTTATAATGGTTTGCTTATAAACATAATACAGTGTGCTGGTAAACATAAAAATAGTTCCACAAAGTTGTTATCCTCATTACAACAAGGCTGAGTGTGAATGTGGGTTATGTTCCAAACATTTTTAAGGTCAAAAATATCACTTCAAAGATGTCTGCTGCCTGCCATCAGGGATTGCATATAGCACAATAGGTTGTGAGGGACGAATGAAGGGTGCATCACACCTGAAATATCCCTTTCTCTCAAGTTGCAATACATCTCCACGCTGAAGATTTCGCATGTTGGAGTCTCCATATGCTAAAGTCTCCTTTTTGGTACATGGGTTAAGCACATCAATGAAATCCTCCCCTTCTTCAAGCTTTTTCTTTGTAATTAGGTAATCAAACTCCACCAATGTCAGGCTAACTAGTTCCTCTATCTCAGGTAGCCAAGTGAGTTTCAATTTGGTTGTCTTCACAGATCCTTCAAGATGCAAAACACCACTTAACCCTGTGATATTTCCATCCTGGTCTTTCTCTATGTCCTTCACGATGGCATTTCCCCAATCCATCAGTGTTACCTCCTCACCTGCTGATATAGACTCGGCATCAACATGGTCAATCCAGATCCTTTTAGTGTAAGTTGTAGCTTTATCTCCGGCAGCTTCATATTTCTTGTGTCGAGGTATGATGCGGACAAATGGTTTCTCAGGACCATCAGAGAGAGTCAACAACACACGTCTGTCTGCAATGACAGCAGTGTGTCTAGGACAGACAGGGTCAATAATCTTCTTATTAATGGTCCAGAGCTTGTCCCATTCCATGAGATTGAGATTTTTTGATGCCCCTTGCTCAACAATAAACTGAATCAGTGCTTCAATTTTCAAACCTCTACGCACAATTCCTTGCACTGTAGGAAACCGTGCATCATCCCATCCATCAACTTTCCCATTTTGTACAAACCACAGAAGCTTTCGCTTGCTGAGGAGAGTGTAGACCATATTCAACCGGCTGAATTCATAGATGAGAACTCTTCTAAGACCCATGTCATCTTGAATCCGGTAATACTGAGCATTGCGATCATGGTATTCACTTGATCGCAGGGCATGCGTGATTCCTTCTCTAGCATCAACATATGGACAAGCAAAATCATAAGTTGGATACACTTTATACTTGGATCCAATTCTATGATGAGGCATTGGATTGCAACGATAATAAACAGGATCTCGAAGTGATTTGTTTGGGTCCTGCATATCCAACTTGCCACGGACACAACACTGCAAGCCCCTCTCTGTTCCTGCAATCATTTCCTTCCACAATTTTAAGTTCTCCTCCACGCTATTATTTCTGCATTTAGATTCTATGCCATCCATTCTCTCTTTTTGCATTTGTTCACGTGGAGTGTCATCAACATATGCTTTACCCTGTAGAATTAATTTTTCAGCCATTTCCATCAACTCAGGGAAGTAATCTGATGTATATGTAATTTCTTCATATTTAATGCCCAATGTTTCAATATCTTTCACCAGGTTGTCCACAAATTCATTGCTTTCTTTAGCAGGATTAGTATCATCAAAACGCACAATAACTTGACCCTGGTATCTCTCAGCAAAATATTTGTTTAACAGAGCTGCTTTTGAGTGTCCAATATGAAGATAGCCACTTGGTTCAGGTGCAAATCGCAAGCGAACTTTTCCAACTTCAGCATCTGGAAGATCTATTTCTACTGAAGGTTTGCTTCCTCCTTTGTTATTCTCAGATACATCCCCATTCGCATTCTTCACTCTATCGGTGATTACTGACTGGTCTTTTAACTTGTTTGCTGTTGGCTCTCCCGATCCTCTTTTGCCAACATATACTGACGTGACTTCATTCAAGGCAGAACCATGTTCTGTCACTATTGAATTGAACCATCGTACAAGATTTGTATATTTCTTTGACTTCCTTAGACTTTCCCATCTCTTTCCAGAACCTGCAAGACCCGCCCAGATTGCTATGTCTGCAATTGACAAAGAATAACCAACCAAAAATGTGCGCTTCTCCAAATACTCATCTATATATTTGCATGCATTCTCAAAAGCAGGGCCTGATGAGAGGATAGGAGCATACTCCAGCCATTCATCAATCTGGCTAGATTCAAATGCATTTTGCCCATAGAAATTGGGAAGACTGGCAACTCGCCCAATATAACGTAGGAGAGCATATGCTCCTTGCAACTTTAGCCCATCGGAGAAGAGAAATGTAGGGGCTGAGTCAGGAGAGAGAGAAGTATCAATCGAGGGAGAGATTCCGGCGAGCTTGGCGGCGGCGATTACCTGTAACGGCGGAGAAGATGCGGCGAAAGCAAAGGTTTTGATGTCCATGCTCCGATAGTTTAACGGTAAAAAGATACGACAGACAAGTATGTTGGTAGCTCCTTAAGAGGTTCGTCTAGGTTAAGGTGTTAAAGCAAACTTGGTTTTTATCTTCTTC

>C101477_65

CCCCATTAAAAAATTCAGAATATGGTGGCTTGGAAGGGATATACAAAAGAGTTTAATTTTGATACCATCAACAATGGCTCAACACAATTTTGTTTCAGTTTACAAACTGACAAGAACACAATTTACAACACAATACATACATTTTCTCTGCTCGTACAATCCAAAGCACCAGACAGTAATAAAAAGGTTATGTGGTGCACAATATCCCTGCACTCTACTCAACTGGCTTCACTGGGGTTATTGATAACCTCAATAGCCTGGGATGAAGCACTTGAAAATACCATCAGTGAATGGAAAAGATGGGGAAGTGTAGTTTGGAGGTGATTTAGGGTCATGGCCCTACTACACTGAACAGCATCCAAATACTTAGCTCTCTCACTCTCCACTTGCTTCTTAATGTCTTCTGTTTCAGTTTTCTTCAGATGTATTGGATGCCTGGGGCTTGTATCAGCGATATCTCCATCTATTCTTTGCTGCATCTCAGCCAAGATGTCCACGCATTTTTGGAATTTCCCTTCAAGTTTCTTTAACCTTTTCAGAATGTTATGTTCCTCGGTCTGTTGGGAAGTTATAGAACGGATGCATGACAAAAGGCTTTTTATTGCCTCAGAGGTCTCCTTCTCTGGTAGGTTGTTAAGCCCTTGCTCCCACTGTTCACAAATGGTAAGAACAGAATAACGATTGCTACATTCTTGACCATCCCCGAGGCTTTCGGTAAGCTTAATCCATTCATAGAGGGTCCTAACATACTCCCGTTGAGATTTTACAAGTTTGCAAAAGCTGTTATACCAATAGGAGGCCTCAGTCTGAAATTGAATTGTAGCCTGGTGATGATAATCAGAACTTAGTATCGTGTTTTGATTATCACTTAGGTTACTCAACTGCTGGGAGATGAGCGTTTGGGCTTCATGTGACTCATGCATTGTTCTCCACATTTGTGCCAACCCTGCAGTTAATGCAACCAGCTGGGGTAAAAGCTCCTCATCTATCATTTCCAAAATTGATGAAGTTGTTTCACTGATGCACTGCCGTAAGTTTATTAAATCAGACTCCAACTTCTCAACACATGATCGAGTTTTGTCAATTTTGACCATGTCAAGGTTCTCATCCTCTTGTTTACGCAATAATGAGGACTTCCTCTCGAATTCCAAAGCAACAATTCCCTCCTCCTTTACTGCCTTGTAAAGTTTCTTCTCTGCCGCATATAATCTTTTAAGTGTGGCACAATGAGCTCCAGGCTTACATGGTTCACTATGACCAGAAAATTCAGCACCATCTTTGGATGGTGGTGATTTTGAATACCTACTCCATGACAGTACACTGAAGACCTTTGCAGAATTGCCTCCCTTCCTATCATGACGCCCAGAATTCTGTCGCAGAAGAGTATCCCCTCTACTGATATCAATAAGAACAGCAATTTCCTTTCTACATCCAGATGCTTTCAGGAAATGGTCATCCAACTCTTTACCTACACCGTCCAATGTTTTCCCACTTCTACAAACCGTCACAGGCATAGCTGTAGCGTCTTTTCTATACAAACTTATTGAAGAGGAATTTTCATCAACTGGTTCTATCAACTTTTGCTTTCCCCTCAATTTTTCAGGAATTACAGCAGCTTCAGCTTCTTCATCCTCAAATTCTGTTTTGGTTTCTTCCCAATTATCCTCCTCCACTAATTCTACTATTTCTTTTCTATCAGGGTATCGATACAGCCGTAATGAGCTCAAACTTGGATCAATTTGCAGTGGAGGAACATTGTTGTAATCTGTCTCTAGTATCTCATCTTCGTTCACCAGATTCTTGTCAGCAAGAAAAGAAGGCGGCGGCGGTGGTAGTAGAGTCAATGGAAGTATATGGGGAGCTTGAGAAGCTGGTTCAACCATGCCATTAGAGGCAGTTTCAAACTCAATTGTATCAGACTCAGTGAATTGCCTCAGGGTGGAACCAGTGTTCCTCAATGCTGTCAAATAAGCAAATAGAGAATCACAAAACTCCCCTCTAATTTTCACCAACTGTTTAATTAACTTCTTCCTTTCCTTGCACCTGCCAACCTTCTCATCCTCATCGATACTTGACCAAACACAGCCCATTTTGGAATATTCAAACCTCACAAGTCACAAGGGTATCCAAAATAACTATATAACAAACATTGTATTGCCAAGAGCTCAGATGGTATGCTTCATCACTAGATTCCACTATCCAATAGTCTTCACAGCGTTTTCAATATTTCAATAACGAAAAATTGCCACCTATATCAGAAGCAGAGCACGTGGTTGAAGTTTTCTGGTGCTGGTGCTGCATACAAGAAGAAGAAGAATAAGAAGACAACCTTGAAGGGTGTGTGCAAAGATGAAGATGTTCCGCATGTGATGATAGAGAGAAAACACTTGAGCAGCTTTGCATTGCCAAGGGAAATAGAGAAATGTATTGGGGTGGTAACTATGAGAGAGAAAGAAAGAGAACACCTTTCTCAAAAGCTTTATTGGACTTCGACATATACCTGTTAGCATGGGACACTGATTTGGTTTTGTTTTCAA

>C101481_65

AAAAAGCTTTGCAAGTATTAAAGAGCAGGCACAATACCAGAGTCTGCCTTCCTTGTTTTGTGTGTCTCTGTCTCCAAGATTCTGCTGAGGTGTTAGGGGAATATATTACAAACTCCATGTGCGGTGTTTCCAAGGGGAAACCATCTGAAATTGGTTTAGCAATGGAGGATGAGTGTGGAAAAGAAGGGATGTATCATAAAATTTCTGCTTTGCTTGAGTTTTCTGCTGCGGATGATCTAATTGGCTTCAGAGAGGCGGTTGAAAAGGAGGCTCATGATGTTGATGAGGTGGGTTTGTGGTATGGAAGGCGTGCTGGGTCAAAGAAAATAGGTTATGAAGAGAGGACTCCTCTCATGGTTGCTTCTATGTTTGGAAGCGTTGATGTATCAACTTATATTATTGGAACAGGTCGTGTTGATGTTAATCGGGCCAGTAGGTCAGATGGGGCTACTGCTCTTCATTGTGCTGTTGCTGGGGGTTCTGCTGCTTCTGTTGAGGTTGTCAAGCTTTTGCTTGATGCATCTGCTGATGTTAGTGCTGTTGATGCCGAGGGTAACCGGCCAATTGACTTGATTGTCTCTGTGGCTAATTCCATCTTCAATCCAAGGGCCAGGGTGCTACGGGCCCTCCTGGAGGGTACCTGTGATGCTGATCAGGCATGTTTGTCACTTCCAGAAGTGATTGACCAAATTGAGGAACAGAGGCAAGATATTAGCACACCTCGTGTTTCAAAAGATTATCCCATCGATCTCTCCCTTCCAGACATAAAGAATGGGATATATGGCACTGATGAGTTTAGGATGTATACATTCAAGGTGAAGCCTTGTTCGAGGGCGTATTCTCATGACTGGACTGAGTGCCCCTTTGTGCACCCTGGGGAGAACGCAAGGCGACGTGATCCGAGGAAGTACCATTACAGCTGTGTCCCTTGTCCTGAGTTTAGGAAGGGATCTTGCAGCAAAGGCGATGGCTGTGAGTATGCTCATGGTATTTTTGAGTGCTGGCTTCACCCTGCCCAGTACAGGACACGGCTCTGCAAGGATGAGAGTGGATGCACAAGGAGAGTTTGCTTCTTTGCTCACAAGCCAGAGGAGCTTCGCCCCTTGTATGCTTCTACTGGTTCTGCTATTCCTTCTCCAAGGTCCTATTCTGCTAGTGCTTCTGCATTGGAGATGAGTTCAGTTAGCCCAATTGCACTTGGTTCTCCATCTGTCTTGATACCACCTGCCTCAACACCACCTTTGACTCCATCTGGAGCAACTTCTCCGATTACTGGTTCCATTTGGTCTCAATCTAATGTTTCAGTCCCTACCCTGCAGCTTCCCAAAAGCAGATTGAAGACTGCGTCAACTGCAAGAGACGTTGATTTAGATTTTGAACTGCTTGGACTTGAAACTCATCGGCGTAGACATCAGCTAATGATGGATGAGATGTCTGGTCTTTCCTCCCCTTCCAACTGGAAAAATTCCATGCCTAATAGTCCATCTTTTCATGTTTCTTTGAGTGATCACATTGGGGAGTTAAATAGGCTTTCGGGGGTGAAGCCTTCTAACCTTGAAGATACTTTTGGATCTCTTGAACCATCAATTTTGTCTAAATTCCATGGAATCTCACTTGATGTTGCAGGAACTCAGTTACAATCTCCATCTGGAATCCAAATGCGTCCGAATATGAACCAGCAACTGGGAGGCTATTCATCCGGCCTTTCCACCTCAAACATGGTTGGATCGCGATCCTTCAGGGTTGATCCTTCTGGTGAAGGAGCCTCAGTGGCTTTGAATCCAAGAGCTGCTGCCTTTGCAAAGCGAAGCCACAGCTTTATCGAGCGTAGCATGGTGAACCATCATTCTGAGCTTCCTTCACCAAAGCCCTCTACCTTCTCTAACTGGGGCTCACCTGATGGAAAACTGGATTGGGCCATAAATGGAGAAGAGCTGAATAAGCTGAGGAAATCTGCTTCCTTTGGATTTCGAAGCAGCAATAGTCCTCTTCCAAAGGACTCAACCAAAATGGCAGCAAATGTTGATGATGAGCCAGACGTGTCTTGGGTTAACTCACTTGTGAAGGATGCTCCACCAGAATCTGGTGTCGAAGATCAGCTAAAACAATGCCATAATGGAACAGAATCAATTCCAGCGTGGTTGGAGCAAATGTACTTGGATCAGGAGCAAATGGTAGCGTGATTGCAATTCCCTTTCCCGCAGAAGCAGCTCCTTCTTGCAACTCAACAGTTTATTTATTTAATGATTTGTTCTTTTGAATTTTATAGAATTTATTTAGATGATGACAATTGCAAAAAAGATGGTGAAATGGTCTACAATGCTAGGATATAGAAATGGGCAGGACGCAAGTTTTCTGCTTCAGTAACTACCCGTTACTGGGTTAATTTATTCCATTTTTTTTCCTGATCTTTTTTTCTTTTATATGTTCTATTGTGTTGTAAGTTGTAACTTTCCATTGACTGAATCACAACAATGTAGTGGGGATATAATATTCAATATAATGAGACTGGAGATGTAATAATTTTATTAATGCTC

>C101483_65

CAGTTTTCATTTTCTTAAAAGGGAGAATATTGACCCGAAGTTTCTTTCAATTACCCGATCTATACAGTATGAGGAGTTGAATGAAGACATCTTGTTAGTAGTGGGCATACTAACCAGATGAAATCTTAAAATGAAACCATCGAGCGAAACTACTGTTTAATGTTACTTTGGTATGCACTAAAGAATCATGTAATTGATTGATGTTTAATAACTACACTATCACTATCGTCACTAACCAAATATATGTTTGGGAAGGGATAAATCATACAAGGCTGGATATTACATGATTAGCCATGTCCTGTCACGTCATGATGAGCCACTAAGAATAAATGCTTGGAGAAGGCTCTGCGCTGCTTTGGTTTCGTCAGGTGAACCAGATATGACAATAATCCTGTCACTTGTTCCAGGACGTGGTTCATGGACAAAAACCTTCGCACCCGATATCTGTCTCAGTCGAGCCAAATTGCTACCATTTTCCCCATATATAGAGTCAATAGTATCATCAGGAACCACAATTTCTACTGTTGTATTAGTCACGATAGCAGATTTACTTCCACTGACAAGTTCTAAGCTTCCTTTTCGAGATGTCAATCCCCAATTAAAATCATTAATTCCTCTTGAATTTATCCCAGCCATTGTCGGTGGTGTCCATAACCCTGGTGATGAAGGACAATCAAAATTTCTAGGCCATAGACCTGGTGAAGAAGGACGGTCTAAATTTCTTGTCCATAAGCCTGGAGAAGAAGGACAATCTAAATTTCTAGAAAGAGCAAGATGATCAATGTCATGAGCAAAAGTATGTCTACTCAGACTATGAGAAGTGGCTGCTGGTAGTTGGCTTCCAAGAGGAACAACACCCTGCAGTCTTCCATAGGGGCTGTTATCGATTCGTATAGAAGAAGCGCTCCTCGTTCCAGCACTATTCCGAGTGCTGGCAAAAATATTATCTCGCAGTCTACCAGTTGTATTATATAATGCATCTTGCACACTTGAAAACTCTCCTGATATCTGCACAACTTGATCATTATCTGACGCACATTTTGGCACCTGATCAGTCCCAATTATTCTTATGTTAGCCCCAGTTACATTCCGCATTTCTGTTATTATTACTCCACCTTTTCCTAACAAACAACCGACTTGGCTTGATGGGACTACAAGACGTGCTGTAACAATTGACCTGTTACTTAACTCCACCTTCTCAAAACCAGATTCAATGGACCTGGAGTAAACAAGCACAGCAGCCTTTTGTGCTGGGGAATATCTTGACTCAGGATTCTCGGAAGCAGTAATAGTAATTATTCTATCCTCGCACCCAGCTATTGAAGGACCAATACTTATTGTAGCCCCGGTCTCACTCTGAAGAGCTCTAACAATATTGCCTCCCTTTCCAATAACTCCTCCAACTCTATCATTTGAGCAAAGTATTCTGAAGGTGACTTCCTGCTGAAGTGCTTTTGGTTCCAGGGAGGAGACTCTATTAACTTCCGCTGGCAATGAATGAACTCTGGTAGCATAACTGTTAGAGCAACTGGATAAAGTAGACAGTGCAGAGCTCCTCAACAAAAGACGATCTACAGTCCCATGTGGTACTGCAGGTAAAGTCTCAGGAGGAAATGCATCTATTGCTTCATAATGAAGCATTTCATAAGGTTTGCTTCCCATTATCTTTGTTCTATCAACAGGAGGACAATCTTGAATACGTTGGGAGACCGCAACAAGTGCCTTCTTTACTGATGATAATCTGCCTTCAATCTCAATGATTTCATCCGAAGCGGAGGCACAAGGCGGCAAATCATCTGTTAAAACCCTAATTTTACATCCAGTGTTTCTCTTGATCCTCTCCACCACTCTTCCGCCCTTGCCAATGACGGAGCCAGCTTGCGCTGTGTCCACCACAAGCCTGCAAGACATCAGCCTGTCCCCGACGTCAAGGCCTTCCGTCTCCGCTGCCACATCGAGGATCTTATCGAACACCATCATCAGCGCTTCCTGCGCCTTCGACACCTCAACGACCTCATCGTTTTCCAGAAGAACCTTGCCGCCAAGGGCAGCGTCGGCAATGACCAGGATTACTCGGTCGGGCGACTCCGGCGACGCGTCCTCGATTCGGATCTTGGCGCCGGTGGATTGCTGGAGTTTTTTTATGACGGAGCCGGACTTGCCAATAACACCACCGATTCTGGACGCGTTGCAGAGGAGGCGGAACACCGTGTGGCCGGGCGGTACAGAGAGCGGCGGCGGCGGAGGCCTGGAGGACCTGGAGCGTTTGGAAGGTCCATTTGGATGAAACGGATTGGGGTCGGGTGCGGAACGCTTAGCTGGCGGAGTACGAAAGGTGCTTCCCATAGTTCTCTTCTTCCTCTCTATGACGAAATTCAAAATTTGAGCAAATTTTCGAAAGCAAGAAGAGTTGCACAAACAGGAAGCAGAAGAGAGAGTTCTGAACTCTGACGCTAAAATGCGCAGAGGGAGAAATTCCGGTGCACACTGAGCCTAGGGTTTCCCAAGGCGCGGAAACGAAGGCTTTGCTTTGCCAC

>C101487_65

ATTACTTTCATTCTTCTGTCTTTACCACCACCTAACTGCATACGCTTCTCTCTTTTCATTGCCTGGTTCTCTCTATTCAACCATGAATGGATCCAATTTGTTTGATTTCTACTAGCGTACAGTGGTGTTATTGGGTGTAAAAGTCAATCTGCTGGTTTCGAACCAAGTTTCAACAATGATTCTTTCTGCCCTTTTAACTTCCGTGGGAATTAATCTTGGCCTCTGTTTGTTATTTTTCACCCTATATTCCATATTGAGAAAGCAGCCTGGTAATATCACTGTCTATGCACCACGCCTAGTTGCTGAAGGAAAAGTTAAAGAGGGTGGTCATTTTAACTTAGAAAGATTGTTACCTACTGCTGGTTGGGTGAAGCAGGCATGGGAGCCTTCCGAGGAAGATTTCTTATCAACGTCAGGCTTAGATGCTTTTGTCTTCATGCGCATATTTATCTTTAGTTTGAAAGTGTTTGGTGTTGGTGGAATTATTGGCATGTTTGTTCTTCTTCCAATTAATTATGCGGGGACTCAGCTTATTGATGATTCAGATTTTCAGCACAAGTCTTTGGACTCGTTCAGTATTTCTAATGTTAATAATGGCTCAAACAGGTTATGGGTCCACTTTTCTGCTGCATATATTTTCACTGGAATTGTTTGCTATCTTCTTTTTTGTGAGTATTTGTACCTTTCGTCCAAAAGAATTGCCTATTTCTATTCATCCAAGCCTCAGCCGCAGCAGTTTACCTTATTAGTTAGTGGTATTCCTGTTCCACCTGGAAGTACATGTCATGATACTGTTGAGCGTTTTTTCCTGGATTATCACCCTTCTACATATCTCTCACATTCAGTGATCCGTCGAAACAACAAACTTCAAAGTCTTATTAATGATGCAGATAAACTGTACAAAAAGCTTACCAATCTGAAACAAAAAAATGATGCTCCTAAAAGGCACACCCGTGATGGGTGTTTGGGAATTTGTGGCCGTAAAGTTGATATTGTGGATCACTATGAAAGGAGATTGGGTGATATAGAAGATAATGTGAGAATTGAACAGTCCTCGCTAGAAGCAAAGGAAGTTCGAGCTGCCTTTGTCTCATTTAAGACACGATTTGGTGCTGCAATAGCTTTACGCATTCAAGAAAGTGTCAATCCCACGGAATGGGTTACTGAGAAAGCTCCTGAGCCTCGTGATGTTTATTGGCCTTTCTTTACCGTTTCATTCCTTAAAAGATGGATCAGCAAGGTGGTAGTTTTTGTTGCGTGTACTTCTATTACAATTCTGTTTTTGATCCCAGTTGCAATAGTACAAGGTCTTACCCATCTTGATCAATTGGAGACGTGGTTCCCTTTTTTGAAAGACATACTAAGACTGTCAGTTGTTAGCCAAGTTATAACAGGATACCTTCCCAGTTTGATTCTTCAGCTGTTTCTGTCATTTGTTCCACCTACTATGATTATGCTTTCATCCATGCAAGGATACATTTCATGGAGTCAAATACAAAAAAGTGCATGCACTAAAGTATTATGGTTTACCATTTGGAACATATTCTTTGCAAATGTACTATCAGGGTCAGCTCTCTATCGAGTGAATATCTTTCTTGAGCCAAAAGAGATTCCCAGAATACTAGCGGAAGCTGTACCATCCCAGGCATCCTTCTTCATAGCATATGTTGTGACTTCTGGATGGACCACCATAGCATCAGAACTCTTTCGATTAACTACACTTATTTCCAATTTTATAAGCAGAACATTTTGTAGAAACAGCAATGATGATTTTGATCCTCCTTCAATACCTTACCATAGTGAAATTCCTAGGATTCGTCTCTTTGGTCTTCTTGGTGTAACATACTTCATCCTTGCTCCTCTGATACTGCCATTTCTCTTGATCTACTTTTGTTTGGGATACATCATTTTCCGCAACCAGATGTTGAAAGTTTATGTGCCAAAGTTTGAGACTGGAGGAGAGTTTTGGCCTACAGTGCACAACTCCACAATTTTTTCCTTGATATTGATGCATATAATAGGCATTGGTATTTTTGGTTTGAAGAAGCTTCCTCTTGCATCCATATTGACTCTTCCTCTTCCAATTCTCACACTTCTATTCAATGAGTATTGCCAGAAACGGTTCTTCCCTATATTCAAGGATTATCCTGCTGAGTGTTTGATTAAGAAGGACAGAGCAGATCAAAACGAGCATAATATGTCTGAATTTTATGATGAGTTGGCCAATGCATACAATGATCCAGCTCTGATGCCAAAGAAGTATTCTGAGAGGTCTGATAGTCACAGATCTCCACTTCTGAGTAGTCCATAACTACATTCCTATGGCCTTGTCTAAAACTGTTTACAGGGTATGAATTTCCCCTTTGACCTGCTAACTGGTTTATTTGATTGTCCTTGTTGTATCATGGATGTCTGTTGTGTTCTTTGTAAATTGTTGATTGATAATTTGTGTTTGTAACGAGTGTAGAAACTTGTAACATCAGTATAAGCAATTGCAAGGCCTTATTTGTGAGAATTAGCATTCATAGTGAAAGAAAA

>C101489_65

TTTTTTTAATAAAATAGCTGAATGATTTAATTTCCATCTAGGTGTCATGAGGTAGTGTTCTCAAATAACATGTATATTGAGCTTGTGTATGCTTAGATGAGGTTTTACATGAGGAAACAGCCCACCGACTTTCTATATAGAAAGCACTTGAAATTCTGGAACAAAGGCAGGGGCAAAAGGGAAAAGCCCCGGTAAAGTGAGTTTATTACTGAGTTTGTCTATAAAGCAAGTAAAAATGTATCTAATATTTGACTTAATTTAAAGTGTATTGTAAGCTTGTCTTACTAAAATATAAGGGTAGATACACCTTTACTTACTCATCTTAGAAACTAACTCACTTTAGAGGAGTGTCGTTGTGGAAAAGGTACAAAACTAATCCAAGATTTCTTGGAGACACTTTTTTATAAAGGCCATGGTTTATGGTGAAAACTTCCATGGTGTATCTTTTTTCTACTTTTTGAGGAGGGGTTTTAATCTTCCAAGACATGTTTAGTAATGTGATTAGTGTTCCATCCGTTTCTATATTTCCATGGCTATAAAGGCTACACTTTCTTTGACATGTAATCTTCAGTCAAGAACTTTCTCCCCGGTTGAAAAGATATTCCACACCAATGTGCCTATTATGTACAATGCAACAGCAACCTTAAAAACATCATTCCAAGAACCTCGCTGAAGTATTAAACCAGTTGCAACTGTACCGAAGACACCAGCAAGAACTCCTGCCGTATTTGATAGTCCAAGCAATACACCAGCATAACGGGGTCCAATGTCTTGGTGATTGGAATAGAGACCGGATTGTGAGAATGCATCAGATCCCTGACTACACGCCATGCACAGTACAGCCATGGCAGGTGTTCTGACATGGCTCAGCTGTGAAAGAAAAAAGGCAGGACCCAGAAACCCTATTGATTGCATGATCTTTCGAACAATTGTTATGGAGAGGCCTTTTCTCACAAGTGTGTCAGCAATCCAACCACCAATATTTGCAAAAGCAGCCATGGTTAACCATGGAAGAACACATAAGAGCCCGGATTCGGTGAGGTTGAACTTCAGAACCTGATTATAATAAGTAGGCATCCAGGTTAAAAGAATAAAAGTCCCCCAATTGTGACAGAAATGGGAGATTATAAGAGCCCAAACAGGTGCTTTTGATAAAATTAATTTCCAAGGAATGGAAGAAACAGGTGCATTTGACACATTGCCTTCTAGTATGAACCTTTTCTCCTCTACCCCAAGATCAGGGTCATCTTTTGGGGAGCTATGTGCCTTTCTTAACCACAAGGCAAACCAGATACTTCCAAGGGATCCAAAAGAGTAAAAAACTGATGGCCACCCAAATTTTTGGATTAGCAATGGCGAGAATGCCAAGCCAACCACAGAACCAAGGTACATGCCACTATATACTAGGGCAAGTGATCTGCTTCTCTCTGAAACTGGAATCCACTTGGAAAGCATATTATTCATAGCAGGCATGGCAACACCCTCACCAATCCCCATAAATGCACGCATAATAAGCAAACAAGGGAGCCCAATTTTTGCAGCAATAGGTGTTAGAATTGTTGCCATTGACCACCAGACAACTCCAAAACCTAGTACTAGCTTGCCACCAAGTTTGTCTGCCCATATGCCACCAAGGATCTGAGTAAGAAGGTAGCCCCAGAAAAAAGATGACTGAATTAGGCCAACTGTTGCACTGTTCCAGTTAAATTCTTGTGACATTGGAAGTATTGCTATGCTCATATTCACGCGGTCCATGTTGCACAGCAGAAAAGCTGCAAAACATAGCAATACAATAACCCAACGCTTAGGAAACTGCTGCCACCAAGAAGATACTTGTGGAACATTCCCTTCCAGGAGAATGGCTTCACCTGTCCCTTCAGTTGATGCTCGAGGATCCACCTCCGATTCTGTAATATCATACTCCTCAGACTTATAATGAACACGAGCCGGATTCGCCTTACCAAGTTTGTTTAAACCTAGTCCATATACTGCTCTTTGTTGAATAAGGCTACTGCTATAGGGATCTGAAGAGAGGTAGCATTGACATCTTCTGCTGGCCTTAAGTTTCATGTTCATGCAGTGAATGGATTTCTGGGACATTGGAGAACAATGCTGCACTTCATCATGAAATGTTGGTATTGGTTTTGAAACTTTCTCATTCAAAGGTTCTCTAGCATGAAAACACATCCCATGCCAGCATCTTGAGAAAGGATCCTTTGCAATCTGGCCACCATAACGTGCAACAAATACACAAGAAATGCCCCCTCTTTGAGGTGATAGATCCTTTCCCAATTGATATACATTTCCTGGTCCAATGAAAGAAGTGAAGTTTCTGTTGGAAACCAAACCACCCAGAGCCATAGGTAATGCAAGTGAGGATCTATGAAAATGGGAATTGAAATGAGAGAAGAAGGGTCTTACACCATGACACAGCACAAAAACATAATTCAAATTCAGCAAAATGATGTCATGTCTTACATGGATAATGGAAATCTCTCCCTGTCTCAGGTATTGGATAAGCAAAACAGGAACCTGTTT

>C101493_65

CTTCTTCACTTTAACTTCCATACTTCTCACTAAGTCCTTCCCAATTCCTGTTGTTGACTCTTTCCTTCTCCGTCTCTTCTATTTTCGCTACCTTCTTCCTCCTCCGCTGACAAAACTAGGAACGATTCTGCCCAGCGCCGTTCTTGCACCGGTTCCCCGCAATTCGGTTGGTTCGGATCCGATGGGTAGCGTCGTGGTGGAAGACTTGGATACTGACACAGTGTATGTGGCGGTGGGAAGGAACGCGGAGAAGACGCAACAACTGTTGCATTGGGCGGTTAAGAATTTATCTGGCAAGAATGTTTGCCTTCTTCATATTCACCAACCTCACTCCCTCAATTCGTTCTCAGACAGAAATCTCTCTGGGTATGAACCAAAAGACCTTCCAATCCAGGCATTCCAGGAGCATGGAACTCAAACAGTGCACGAACTTCTAGACCAATACATTCTCACTCTGGTCCCGGCAGGAGTAAGAGCCTGTAAAATGTTGATTGAGATGGATGATATTGAGAAAGGGATTGCAAAAGCTATTGCTCAACACAATATTAGATGGCTTGTAGTGGGAGCAGCATCAGACGGAGACAACATGGGCAAACTGGCTAAGCAAGATTCGGAGAAAGCTATTTTTGAACGTGAACAAGCATTGCTATCCTGTAATATTTGGTTCATTTGTAAGGGCAACCTCATATGTACTAGGGTATATAGTAAGCATACTTCTAACACAGAGACTGGACCTGCATTGTTGGTGCTCAATTCAAATACAGAAGCCAAACAATCAGAAAAGACTAAATCAGAATCGATTCCTGATGCATTGAAATATTTAGATTCTGATGATATGAAAGAAATCCAAAGTATCAGCTCCCACCGCTCCTTAAATTCAAAATGGTCCTTTAATAATGTCTCACACAGGTCAAAGTTGGCAGATGTGATGTTTCATGAGGATGAAGCATTTGAAAGCTGGTGTGCTAAAGAAATAATAAGAAGAAAAGAAGTTCAAGAACAATTGGCAAGGGCAAAGCAAGAAGTACAGAAAATGAAGAATCAGCAAGATGAAATACTTGAAGAATTGCAAATGGTCAAAGACCAAAATTCAGCACTTAAGAACCAAATTTCTGAGTCCCAGTGTACGGCAACAGAGTTTGAGGAGAAGATCATATCAGCTGTCGACCTCTTGATAAGTTTCAGGGAAAAGAGAGATAGGGTTAGAATAGAGCATGCAAATGCAGTTAGGGAAGTCCAAGTGCTTAGAAAATTTGGTGAAGCAGATACTTCGTTCTCTTATCAGGTAGAATTCCCTGCATTTTCATTCGTGGAGATCAATGAGGCAACTCATGATTTTGATCCATCTTGGAAGATTGGAGAGGGAAGGTATGGAAGTGTTTACAAGGGACTGCTTCGCAATATGCATGTCGCTATAAAAATGTTACCCTCTTATGGTCGTCAGAGTCTATCAGAGTTCCAACATCAGGTAGAGGTCTTGAGTAGGGTAAGACATCCAAACCTGTTAACACTAATTGGAAGCTGTGCAGACTCTAGGTCACTTGTGTATGAGTACCTAAACAATGGCAGCCTTGAAAATCACCTCGCCCGCAAAGAAAAAAATCCACTTCCATGGCAAATTCGAGTATCCATTGCTACAGACATATGCTCTGCTCTAATCTTCCTTCACTCCAGCAAACCTTGTATTATCCATGGGAATTTGAAACCTAGTAAGATTCTCCTTGATGCTAACTATGTTGCCAAACTGAGTGACTTGGGCATCCCTAGTTTAGTCCAGCAAAGTTTGGATCCAGCTGATGCAAGCACAATATATGATAGCCCAGATGAACGTTTGGCGTATGTGGATCCAGAGTATTTCGTCACTGGCAAGTTTAAACCAGAATCAGATGTATATTCATTTGGAGTCATATTGTTACAACTTCTAACTGGAAGATCACTTTTGGGGTTAGTTAGGGATATGAAGTGTGCATTAGAAAAGGAAAACCTTAAAGCAGTCTTGGACTTTTCCGCTGGTGAATGGCCACTTTATCAAACTGAACAGCTGGCATATTTAGCATTGAGGTGTTGTGAAAAGAGTTGGTTGAACCGGCCAGACCTTGTGTCAGAAATCTGGAGTGTTCTTGAACCATTCAAAGCTACTTGCATTGACTCATCCCCATATTTGATTTCTAAGAAGCTTCGTCGTGTTCCTTCCCATTTTGTCTGCCCCATTGTCCAGGAAGTAATGGAAGATCCATACATAGCTGCAGATGGCTTTACGTATGAAGCAGAGGCAATAAGAGGGTGGTTGAATAGTGGCCACGACACTTCCCCCATGACAAACCTCAAGCTTGATCACATGGATTTGGTACCTAACTATGCTCTACACAATGCAATTCTGGAGTGGCAGCAACAGTAAACCTTCACTGCCCATTATACTTGCACATATTACAATGGTGTAAATCACACTTATCACGTAAAAATTAAAATTTAAAGTGAGCAAGTAAGGGTGTATTTACACTTGAATTTTAAAGAGTTAAAATCATAATGCATTTTAAAA

>C101499_65

CGCCTCTTTATTGTAGTAATCTTTAGTACAAAGTATCTGTTCCATTCCAGAATTACAAAACTATAGAATAGAAGTAAACTTATTAAAGTTTATTACAAACATTGCTTTTATTTGCTGGATTTTTCTATGGGAATGGCATTGTCTTTTTTGAGTTGACAACATCTCAAGTTAAACAGAAGTAGGAGGCACTAAAACATTGTTACGATATTCAGTCAAAGCCCTCATTGGAAAAACATTTCTGTAGAGTGGGTAATTCAAGAAGCAATTTCTGTTGTACACCCCCATAGGTTCTTGCTGGGGCCAATCGCCATCTTCTAACTGAGAATTGATGAGTAACTTTGCAGCACGATGAAGGGGAGTTGGATCTCTCTTAGCCTGGCCAGAATGAATCAAAGCCATTAGAGCCCAAGCTGTTTGTGAAACATTTGATCGATTTCCTTCAAGAGGCACGTACATCTTCATTGGACATGAAAGATAACTCTCTCCCCATCCACCATCATCATTTTGTATTTCCAGTAGAAATTTCGCAGCATTACGAATAGCAACACAGTTGGTGTAAGTCTTGCCAGCAGCAGCTAGACCTCCAAGTGCGAAATAGGAAGCATATGTGAAGCAAACTCCCCAGTTTCCATGCCAAGAACCATTTGGTAATTGTTCATCTTCAATGAATTGTGCTGCTTTCACAATGAAATTCTCTATCTCTTCCTTTCTATGCTCTGGATATAGTTTTTTGAACAATACAAAAGTCTGAATTGATGCTGCAGTACACTCAATATACTCGTGCTCAACTACTATGTCAGGAAGAAATTCCGAGGGATTCAGTAGTTCCAACCATTTTTGACCTCTTGCTGGCTCCCACACAGTTAAACCTCCATTTTTACTCTGAAGTGACAATATAAAATTGACTGAGTCATAGAACCTCTCAGGTTCCATTTTTTCTCCCACAATCTCTTGTGGTAAGGTTGATAAAAGTAAACAACACTTCAGACATTCTGCAGTGCCATCAGAAATTTGTAATGCATGGTCTCTGTCAGCTAAGGTCCATGATCCTTTAGAAATGTGATGAAACATGCTCTTAAAATCTCCTGAACGATTTTCTATGACCTGAGATTTCTTGATAAAATCATGTGCTTTTGCAAGTGTGGGACCAAGATCATCAATTAGGTTAGTAGCAAGCAAAGCTTGAGTAATTAAACCAGCATCCCATGACTGTGTACTAAGTGCCTGTATACGCATTCCATCTTCTGAAAGACATAAATAATCAGGGATTCTTGCAAGATGCTTCTTAATAGAATCTCCATTTGGATCCTCTACCCAACAAGCAAGCATACACAAAGCCTTGTTGAAACACCCGCCAGTTATATATCGTGTATTTTCATCTTCACAATGCAGAAGTTTCATAGCTACTTGGAGTGCTTTTTCTCTAACCAACTTATTGAAAGGCCATCGCGTAAGAAGTGGTTCCGTGAATACATAACAGCCATCCCATATCAGATCTTGTATCCAGCTATGGGTATGGTAAAGATCTTCCTTTGCACATTTGTGACGTGCTTTCTTCCAAGTGCTTTCATCATAAGGTTCAGTGAAGAGTTCTTCTCTCAAATTTGTGATTAGTGTAGTGGTTGGACACGCGAATCTTTTTCCATATAAATAAGACATGGGCAAGTATACACTTCGACAATAATACCACATTTTAGCAGGATGTATGGGGAGAAAAGTAGGAAGAATCCAAAACTCAGGGGGCATTGGGTTGCATCCACTCCAATCAACTATGCCAAGTACCGCAAGCCAAAATTTTTCCCAGGAAGGTACATGTGTTACGCTGCCATGATCATGAATCCACTTTCTTGCTTTAGCACAAGCATTGTCTTCACCTCCATTAGGACCTTCTCCAAGAATTCGCATGCATATATAATTGAGTGTGGTACCAAACATGCTGCTAGGACCCTCTATATGTATTCCCCAACCTCCATCCTTGTTCTGGTGATTATATAAGTAACGAAGAATTTCTTTACGATGTTCTTCTGAAATTACAGAATCAAGATGTCCTGTGATGTAGATAGAAATGATCATTGTAGGAGTGAAAAAGTTGGAACCGGAAAGATGAGCAGGCCAATGGCCATCACTAGCTTGTAGTGACGACAGGTAATGAGAGGCCCTCTTTATTGTCATAGTAGCCTTCTCCAATGTCATTTTCTCATCATCTTCTACCTCCACTTTCTTTATTACTTGTTTGAATTTTTTTTCCCTTAAAATCTGAAAGCGCCAAAGTCGATCAGCACAAGCCTTGACCTGAAAACGATTATGATAAAAATGTTGACGAGCTGCTTCCACCTCAGCTCGTTCTTCAGGAGTGCCTGCTTCAGGATCAAACTCCCATACTTGTCTTCCCACGAAATTATTTGTGCTAAATATGTATGGATCCTTCCCTCCATCAGCTATCTTCAACCTCCACATTCTCTTTCAGTTGCTCCTGATGAAGTTGTGAAGGATAAATCTTGAATAATTGAGTCTTAATATTTTGAAAGTATAAGAATTT

>C101501_65

GTGCCACTCAATTTCGGAATTTCCAATAACAAAACCAATTCATTAATAGCCTCATACTCATACTCCTTCTTCCTCATCCCTCTTTTCTTTTCTCTTCTGTGTAAACGAACCCAGAGAGAGAGAGTGAGAGAGTGAGGGAGCAATGGAGAAAATGGCTGCACTTACTACTATCCACTCCTCCCTCTTCCCCACCAATACCACTCTTCCAAAGACAGCATCTTTTCAACCCTTCTTCTCTACCCACCTTCTTGGCCTACCCTTATCTTCTTCTTCTTTTTCTGCCGCCAAAAGATTATCTTCCTCCTCTTGCAGACCCATCAAATGTTCTGTTTCCGAAGCCACAGAACCCAAAACTGAGACGAAGAGGCAATTGCTGAGAAGAGGGGATGTGAGAAACATAGCAATTGTTGCTCATGTTGATCATGGAAAAACTACTCTTGTTGATGCCATGCTCAAGCAAACTAAGGTATTTCGTGATAATCAATTTGTGCAGGAAAGGATAATGGACTCAAATGATTTGGAGCGCGAAAGAGGGATCACAATACTGAGTAAAAATACATCTGTCACCTATAAAGACACAAAGATCAATATAATAGATACTCCAGGTCACTCTGATTTTGGGGGTGAAGTGGAGCGTATCTTAAATATGGTGGAAGGGATCCTTCTAGTGGTAGATTCTGTTGAAGGACCCATGCCACAGACAAGGTTTGTCTTGAAGAAGGCTTTAGAGTTTGGTCATTCAGTTGTTGTGGTTGTGAATAAAATAGATAGACCATCTGCTCGCCCAGATTTTGTTGTCAATTCTACTTTTGAACTCTTCATTGAACTGAATGCCACAGATGAACAGTGTGACTTTCAAGTAATATATGCAAGTGGGATAAAAGGGAAAGCAGGGTTGTCTCCTGAAAATCTGGCAGAAGACCTTGGTCCATTGTTTGAATCCATCATTAGATGCATCCCTGGACCACGCATTGACAAAGATGGTGCACTTCAAATGCTGGTGACAAATATTGAGTATGATGAACATAAAGGGCGTATAGCAATTGGGCGTGTGCAAGCTGGAGTTTTAGAAAAAGGCATGGATGTTCGAGTATGTACTTCAGAAGATTCATGTAGATTTGGAAGAATTAGTGAGCTCTACGTGTATGACAAATTCAGTAGGGTTCCTGCAGATAATGTGGAAGCAGGTGATATATGTGCAGTGTGTGGAATTACTGACATTCAGATTGGGGAGACAATTGCTGATAAAGTATCTGGGAAGCCGCTACCTTCCATCAAGGTGGAAGAACCAACAGTGAAAATGTCTTTCTCCATAAACACTTCACCTTTTGTTGGCCGTGAGGGAAAGTATGTTACTAGTAGGAATTTAAGGGATAGGCTTTATAAGGAACTTGAGCGGAATCTGGCTATGAAAGTTGAAGATGGTGAAACAGCTGATACATTTGTTGTCAGTGGGCGTGGAACTTTGCATATCACTATACTCATAGAAAATATGCGAAGAGAAGGTTATGAATTCATGGTTGGGCCTCCCAAAGTTATCAACAAGAACGTGAATGAACAACTGTTGGAACCATATGAGATTGCAACTGTGGAGGTACCAGAAGAGCACATGGGAGCTGTTGTTGAACTTCTTGGCAAGAGACGAGGACAAATGTTTGATATGCAAGGAGTTGGATCGGAAGGAACTACGCTACTCAAATATAAGATCCCAACCCGAGGCCTTCTTGGATTGAGGAATTCAATTTTAACTGCCTCCCGTGGCACGGCTATTCTCAACACCGTCTTTGATAGCTATGGACCTTGGGCTGGTGACATTAGTACTCGCGATCAAGGCTCATTGGTTGCTTTTGAGGATGGAGCAAGTACTTCTTATGCAATCTCTAGTTCACAGGAGAGGGGACAGATGTTTATTGGTCCTGGTGCGGAAGTTTATAAAGGTCAAATTGTTGGCATCCATCAGCGACCTGGGGACTTGTCCTTGAATGTTTGTAAGAAGAAAGCTGCTACAAACATTCGTTCCAACAAGGAACAAACAGTGATTCTTGACACACCATTGGACTATAGTTTGGATGACTGCATTGAATACATCCAAGAAGATGAACTTGTAGAGGTCACTCCCGCAAGTATCCGAATGTGCAAGAATCCAAAATTAGCAAAGAAAGGAAGGTAGACTTTCACTGTGAAGCAGTAACTTGCAAATGCTTATTGAGTTACCAGTTACCACTGCTGAAGCTGCATAACTCATTAATGCCGTTGCTGCTAAATTGATGAAGAAGTAAATCACTACCATTGATGTAGTGATCACATGTATTCTTCCAAAGCTTCCGGCAGAATAATATAGGAGCTGGCTGAAAACCGTATTCAATATTCATTATAGCTAATTGGTCACACTTGATTTAAAGGGATAAAGTTACACTGAATTTTGTTTACTTGCTTTGTAAAGATATTTTAGCAGTAAGGAACACGAAGATATTAATTCTTCATATTGGTTGGCAAATAGCAATACAAAAAATTATTGTTCTTTGTTTTGAAAAAAAAAA

>C101503_65

TTTACATGGAATAAACATGAAGAAGACCCTGTCGACCAAAAATTCTCAATAAAACATTTAATAGAAGTTGTACACGGGCTTTAAATCAAAATTAAATCCTTACTCTCGTTTACCATACTTGCAATTTTCAGATGATATGACAATGTAATCACGATGATATCAGCTGAACTACAATACTAACAGAAGTCCAAGTATAAACGCAGTCCAACTGATAAACAAAATAAACAATGTAGACTATTACTGCTACAGTTTATATAGTTCTACCATCCAAGAGGGAAAAGGATGAAAATTATGGTTCTATCATGTTAGGAAAAGACTTCTCATTCTTCATCAGAGCCTGAACTTTCTGCACCTTGACCATGTTTATTCACGGTGCCATTGGCAGCTTGATGCTTATTTTTTGTGTTTTGATCATCTTCTTCATCTTCACTGTACTCACTCTCGTAGTCATCATCATACTCTGCATCCTCATTGTCCTCGTCCTCCTCAACACCATCCTCCATGATAGGTCCTTCATCAGCCAAAACAGCACCCCTGGTGCCAGCCCGAGTCCGTTTCAAAATTTTGAGCTTTAAATTTGTCCTTCTGTCACAGCCCAACCAAGAATCACACAAACAGTAGCAAGTCAAACTGTAGTTACCCTCTGAAGGGGCCTGGAACTTGCCCAATACCAATCTAGAGCCCCCTTTCACCTTCTCAGCTGCTTCTGCAACTGCCTTACTAGTCTCCTTCACATTTGCTCCTGACCCCTCCATAGATTCCTCAATTGCCTTAGATGCAGCAGTTAGAGCAGCAGCTTCATCCATGAAACTAACCTTCTGAGAAAACCACACATTATTTGAAACAGAATCCGCAAGCAAAAACCAGTAATTTTCTTCTTTCTGAAATGGGTAGTAGGGAGCATGTGGAAGAGCACCAATCAAGCCATTGCCCCTTTTAACATTTATCCAAGCATGGATTGTCACAATGTCACCCTCTTGTATACCCTCTTCACCCTCAGTCTCACAAGTTACATCAAGTGTCAAGGAAGGCATCATGTCCAGTACCGTCTCAATGTCTTGCACTTCAGCAGAACTTAACCCACCTGTTTGAATAAGCAGGTCAGCTCGCTCCTGAGAGTCCATGTCATGAAGTTCCTGAAATGTTCTCACCTTCTTGCGGGCCACTTTCTTAATTATAGTCTCACTAATATGTGGCAACTGCAGAAATGGTGCAACGCCTTCAGGTGATCCACCTGTAGACTTTCTAGCACTGAGAGGAACAGCCTGGACGATACATTGAGAAAGCTCCACAACACCAATTGCAGGCCTCAGCCATCCATGCCCCTGAGCATTCCGGGGTATAACTGCCATCTTCATTAATTCTTCAAGAAGGCGGGGTGCTGTCTCTAAAATTCTTCTGAAATCACTTTGTAGAGATGGAGACAAGGCTGAAAATTCACGGGTCAACTGAGCTTGAACTAACAGCTCTGTCTTAACCAGTGCAGGATGCTGCTTCCAAAACTTAGCCTGTTCTTGCTTGATGTTCTTGAGGTCAAGATTCAACTCACTTCTTACCAACATAAAAAGCTTCTGAAGAGGTTCATCATCAGTCCTACGAACTGGAATTTCCTTATATTCAGCAGCCTTGATGAAAACATCCATGACTTTGCTGGGGGCCAAAGAAGGTTTCATGAGGTAGTAGTAATTGGAAAGTGTTTGATGCATCACATAGTTGCCTGTATACTTCGACGATCTGGAGAGATAAACAACAGCAATAACCAATGGCAAGAGAATACACACACCAACAATCCAAAGCAGAAGTATTCCACCAGATGCCCCATCAATATTTAGCAGGAATTGTGGCAGAGCTATACCCATTTGAAATCCCTGCCGGCCATCAGGATGGCCATATTTTTCATAATTTTCACGAGCTATTGGATCTGTAAGAGCTTGATAAGCCTTGGCAATGTACTCAACAAAGTATTTGTGTGCCTCTGGATCAGGATTTTTATCCGGATGGTATTGAATTGAAAGTCTCCTATACTTTTTCTTTATTTCAGACTCTGCTGCTCCAGGCTCTAATCCTAGAATATTAAACGGATCGAAAATTTCAATCTCACGACTCATGGTCTTTATGTAATAAACCAGAATAATCATAATCACCCAAAGCAGTAATAATGTCATGTTACTACAGGTTGAGACATTTGAAATCCTCTTGAATATTGATTTATGGTACTTTCCCGACCGCGAGCATTCAGAGCACTGACAGTGTATGCTCTTTGATTTCTTAGAGGCAGCTCGGCACAACTTTGTTATTGTATATGGCACTATAGGAATGGCCATTATTGTCAAAATGAATATTGGGAACAATGCACTATTCTCTTCTGAAGCAGCCATCGTTCCCTTCTACACCCAACAACCTCGATAGTTACCAGACCTGAGTCTTGGGATTCGGAAAGGTGGAATGGAAGGTCAACTTCTATGGGAGAGGGAAATTGGAGGAGATGGAAACTGAGAACGCAGAAGTGAGGGAGATGAGATCGCAGCAGACAGACAACAC

>C101505_65

TGTTCACAAAACAGACTCCAGTCTCTCCCTAATACAAGAACCTAGTCTTTTTACAGATCGATCATCAAAAGAGATATCAATCTCCTTAATCTTAGACAGAAACTCCATCGCTTGTTCATGCTTCCCTGCTTTACAATAACCATCAACGACAATCTTGTAAGTTAGTTCACTAGGCCTGCAATTGTGCTCAATCATAAATCTAATGACTTCAATTGCTTCATCAAAGAATTCCATCCCTGCATAACCTGACAAGAAAGTATTGTATGTAACTATAGTTGGTTGAATCCCTCTAGAGGTCATCTCTGAGAGAACTCTAATAGCATCCTGCATTAGCCCTTTTTTACAAAATCCCTTAATAACAGTATTATAAGACACAACATCTGGCTCTGGACCAGAGTTATGAATTCCCTTGAGTATTTCTTCTGCTTTCGAACACTCACCCTCTCGGACATACAAATCCATCAAGCAGTTATAGGTGAAAAGATTTGGCTGCAGTCCACTTTGATGAATAAAATGTATCATTTCGTGGGCCTTCGAAAACATCTTGTTCCGGGCAAACATTGAAAGCATGGAATTAATGACAACCAAATCAGGTTTGTATCCATACTTTTGCAATTGATCAAACGCCCTTTCCATTCCCCTAAGGTGTCTGCACTTGTGGTTTGATAGGACAAGGGTTCTCAAAAGAATCCAACTAGGAAAGACATGACCATCATAAATTTCTTTCTCAATCTTCTCTATCCCGTTGACATTCCTAGCCTTGGAATAACAATGGAGCAACAGTGAGTATGAAGTTTCATTAGGCTTAAAGCCCTTACAACGCATGTCCACAATGACAGATTCTGCTGCTTTCCAATTGCCTTGCCGAGCAAGAGCATTTAGAAGAGAATTATAAGTTGTTACACATGGTGTAAAGCCTGCTTTAATCATTTCCCCATACATTTTTGCAGAATCAACTTCAGATCCACACCGAGCATATGCACTAATCAGTGTATTGAACGTGTCTTTGTCAGGCTCAAATCCACAGTTTTTCATTTCCCTCAAGACCTTATTAACATAATTGTGCTTACCCTCCTCACCGCATACAGCAAGCATAGTGTTCCACGTAGCACGATTAGGAGCACATCCATTCAATTTCATGTCACAGAGAACCTTAATAACATCTTCTGTTCTTGACTTCTTGCCTAGCATGGCAAGAACAGAGTTGTACGTGTATACATTGGGAGCACAACCCAAGTCCTTCATTTGGCTGAACAAACTTAATGCCTCGTCCTCCCTTCCCGCCTTACCATAGGCATCTATTACAGTTGTATAAGTAATAGCATTTGGCATTACCCCTTTGCTTGCCATTGTATCTATCACAGCCTTCCCTTGGTCGAGAAACCCAGCTCTTACATATGTTGCGGCGAGCTCATTGTAAGTAACAGAATCAGGAGGGCAATTATTATCATCCATTTCTTTCAAGATGCTCAAAGCCTCAGCGTATATTCCAGCCTTTCCAAAAACCTGCAACATAGAATTATACGTAACAGTTCCCGGTTTATAGCCATTCAATTTCAAGTCAGAGAAAAACTTCCTAGCTTCATCTAGCATTCCCTCTCTCCCACAAGCAGAAATCACAGTGCTGCAGGTAAACTCATCAAACTCAAGTCCTTTAATCCTCATCTCATCCAACAACTCCAAGATTCTACTCCAAGAACGACCCATCTTGCCATAAACATCAAGCATAACATTGTAAGTGACCAAAGTTGGATCAAGACCAAACTCATTCATTTTGCCAAACAACTCAATAGCCCGTTTATACTTGCCAGTGCGAGCATACGAATGAAGAACAGTGGTGTAAGCCCGGACATCAAGTGAGTATTGCTCCACAGGTATTAAATCAAACAACTTGGATGCAATTGAATGCTGAGACTCTCTCCCCAATATCCTAACCATCAATTCAACAACCTGATTGTCCAACCTCAAATTTTGTTCACTCCCAAAATGCAACCAACCCCACTCAAACAGCAAAAGAGCCCTCTCCCAGTTCCCAGAACGGTCCAAAGCTTTCAACAGGCCTTGTCGGATAAAAATCCAAATTTTGCATCATCAAGCTGGTCTTGGTCAACTACGGGGGCAGAAGATAAAGAGGGGTGCTTTGAATGATGAGAGTTGTCATCCTGAGAAGCAGGCACAAGTGTGATGGTGTGAGTAGTGACGGGAACTGAAGAGAGATGCTGAAGATGTTGGAGAAGGGAATCCAATTGGAAAGACGGAGGAGGAGGTGGCTGAGACTGTGGAGGTAAAAAAGTTGGTTTGAATTTAAAAGGTTGTTGGGTTGGTCTTGGTGCAGGGGCCGGTAAAACAGGTCTATTGGGGAAGAGGGTACCCTCCATTGGCACCGGGAAGAGCAATAACAAGAAAAGGGTGAGTTTAATGGGTTTAGGTTTTGAAGTTTGGGCATAAGGATGAGATTAAAGGAGAATGGATTGAGGGGTTGGATGATTTTGAGTCTACAAAAAGAGTGGAGTGTGTGTGTGGAGAAAATGAAGATAAAGAG

>C101509_65

AAAAAAAGGGTGAATATTTAGAAGGAAAAGGAAGGGTGAATAAAATTAAGGGGAGGGAAGAAGAAAGAAGAAGAAGGAATGGGGAATATGAAGAGAATCGAGTCTGAATCACACCAAGTGGCGTGACATGGTGTAATCATATCTATCAATAGACTCCTCTTTCTCTCTCTCATCAAATCTCAATTCTTATTATCTCTTCATTCATTTGTATTCCCTCCTGAGAGGGTCAACAGGGACGGCCATGACCCAAACCCTCTCTCGTTCTCTTCCGTTTTTTCTTGCCTTTGTGGAAAAACCCTAGAAATTAACCAGCAAATCCCCTTTTTTCCATTCTCCGATTTTTGTTTTATTTTAATTTTTGGTTCAATCGCCAACCCCTCCTTTTAAGTCAACGGGAACACCCCTTAATTTGGTTTCGTAAATCTCGATCTATGTCATCCGAGATCGAGGTCGTCGAGGAGGTGCAGTCGCGCGATGAACAATCGCCGGCGTCGGGCGTCGCTGATGGCGTGCCGGAGGAGAGCCTCCAGAACGACGTGTACACGGCGGCGGCGTACGGGGATTTGGAGAAGCTGCAGAGATTGGTGGAGCAAGAGGGTTGCCCCGTGACCGAGCCCGATGGGTTGGGTTACTACGCTCTTCAGTGGGCTGCACTCAACAATCGTACTGCCGCTGCTCAGTACATCATTGAGCATGGGGGAGATGTACATGCAACGGACCAAACTGGACAGACCGCACTACATTGGAGTGCGGTTCGGGGTGCTATCCAGGCTGCGGAGCTATTACTACAGGAGGGTGCTCGAGTGAGTGCTGCCGACATGAATGGATATCAGACAACGCATGTTGCTGCACAATTTGGTCAGACAGCTTTCCTTTACCACATTGTTTCAAAATGGAACGCTGACCCTGATGTCCCTGATAATGATGGAAGAAGCCCCTTGCATTGGGCTGCTTACAAAGGTTTTGCCGATTGTATACGTCTTTTGTTATTTCTTGATGCACACAGGGGACGCCAAGATAAAGGGGGTTGCACTCCTCTTCATTGGGCTGCTATTAGGGGCAACTTGGAGGCCTGTACTGTGTTAGTGCAGGCTGGCAAGAAGGAAGATCTGATGCTGGCTGATAATACAGGCCTTACACCAGCACAACTTGCTTCCGATAAGAATCACAGACAAGTTGCTTTTTTCCTTGGAAATGCCCGAAGGCTGCTTGACAAACGCTGTGATGGGAACAGCCGCCTAGGAAGAATTTCCAAATTAGGACTTGCACCTGTCCTTTGGTGCATAATTTTTGTGCTCTTGGTTACTTATATTCATTCGGTCATCTTGGCAACAAAAATGCCAAATTTTACAGCTGTAACTGGTTTTCTTGCATGGTTTGGAGTTTTGCTTGCAACTGTTGGTTTGGTTATGTTTTACAAGTGTAGCAGCAAGGATCCTGGTTATATCAGAATGAGTATGCATGACACCCAGGATACAAAAGATGATGAGCCTCTTTTGAAGATTGAGATAAATAATCCTGCTTTGCTAGCTGGAAACTGGTCACAGCTTTGTGCAACATGCAAGATTGTCAGGCCTCTTCGAGCAAAACACTGCTCTACGTGCGATCGTTGTGTGGAACAATTTGATCATCATTGTCCTTGGGTGTCCAATTGCATTGGCAAGAAGAACAAATGGGATTTCTTTGCTTTTCTTATTCTTGAAGTTTCTGCTATGTTGATTACTGGGGGAGTTTGTCTTACAAGAGTGTTAACTGATCCACTTGCACCTCATTCATTTGGGGCATGGATTCAGTATGTTGGTAAGAATCACACTGGTGCAATATCATTTCTAATAGCAGATTTTTTCCTCTTCTTTGGTGTGTTTGCTTTGACAGTTGTGCAGGCTAGTCAGATATCCCGCAATATTACTGCAAATGAAATGGCAAATGCCATGCGATACAGTTACCTCAGAGGTCCAGGTGGTAGATTTAGAAACCCATATGATCATGGTATCAAGAAAAACTGTTCTGATTTCTTGATCAAGGGTTACAATGAAGATGTGGAGCGCATTGAAGGTCAGTCAGAGGAGGGTATAGGAATGATGAACATTGCAAGAAGTTCGAACATAACAAATGGAGACTCACACTCTCATTCTGAATATGCAAGGGGCAATGGAAATGGGAATTATGTTGTTAATGTGAATTCCAACAGTACCAACTCCAAAATACATAATGGTCATGTTCATTCTTCTCATTGTAGCCATAATAACCATGGCAAAACCAGAAATGAAAATGTTCCCTTGGGCTTAGGTCTTGGCCTTGGACGGAACAGCAGATCTGTTTCACCCTCATCGTGATATTTACATATTTGGTTGAACTTAACCTGTAATGTATCATAGCAAAAATGTGGGTATGCTGCTGGTCTGGGTTGGGTGGTTTTGCATTAATTCAATTTATTGTGGCTCTTGTCTACCACACCCATGTGCAATTTTGTGCTGTTACATTTAGTCATCTTCATTTTTCTACTTCTGTATCTAATTTAAAAGTATTGCCTTGAAAGCTA

>C101511_65

CCTGTCTCAGTTTTCTGAATTCCGCGACACTGACTCACTGAGTTGCGTTTTTCCTGGAAAATTGGGAACCTCAGAAAATTCCTGATTCTCGGAGGAAATTCGCAAAATGGCGCTTAATCTCTGCGATCACCGTCATCTCACATGAATCTCCTCTCCGGTTTCGCTTCACTCTCTGGAAATTGGAGTCCAATTTGCAGGGAATCCCTCCTTCCTTTCTCAGCATCAACGATTTTTTCCAAGCATAGAAGAAAAAATTACAGATATGAAAAAAATCATGTAATCAATTCAAGGAGATATGTGATCAGCTAGTCTTCATTTTTCTAAATTTGTGTTTTCTAAAACTCTTGGTTTCTGGAAAGGAACTTAGGATATAAATATATTAGATATGTAGTTCTTGTTTACTGCTTAGGTTCAGGCATGAGGGAGGGATCAGGAAGAATAATAGCAGTCGAGGCTATCCTTTTTGTGCTTTCCTAGTAAATCTTGGATGGCAAACGGTTGAATTTTGCTTTTGGTTTTGAGGTTCATAACTTGTTTATTTGAGCCAGCAAGATTCTGAACCTGTAAATTGATAATGGCTTGTGTGGCACTTCGTCCTGTGCGTGAAGAATCTTTCAAAACCGGTGGTGAATTCACCAGCTGTTTCAGCCCTCTTTTGAGGGACTCAATTTTGATTTACCTAACTGTTGGTGGATCTGTGGTTCCTATGCGTATTATGGAGACAGACTCAATTGCTTCAGTGAAGCTGAGAATTCAAACCTTCAAAGGATTTTTTGTAAAAAAGCAGAAACTAGTTTTTGAAGGGAAGGAGTTGGCTCGTAACAGATCTTGTATCCGTGATTATGGTGTTGGTGATGGGAATGTTCTGCACCTGGTGCTGAGGCTTTCTAATCTCAAAGCTATTACAGTTAGGACTGTGTGTGGAAAGGAGTTTGGGCTTTATGTTGAGAAGAGTAGGAGCGTGGGTTATGTGAAGCAGCAGATTGCAAAAAAAGGGCAAGGATTAGTGGAGCTCAAGGATCAAGAACTGATATGTGAGGGTGAGGCACTTGAAGACCAGAGACTTATTGAGGATATCTGTAAGGACAATGATGCTGTAATTCATTTTATGGTCAGGATATCAGATGCCAAGGTAAGGACTAAACCAGTTGAGAAGGATTTTGAATTATCAATTGAGGCATCATATGCAAACAATCTGGTGCCTAATTTACCTGCAGACCGGTTAGGGTCAGTCTCTGTAACAAATGAGATCCTGGAGAGGAATCAATTATCGACGGATTATCTTGTGGAACCAATTATTACGAATTCCAATATAAAAATTCCACCAGTGATCGACGAGCTAATTAAGATCACTTCGGAGGGGCTAGAAAAAGGCTGTAAACCAATTCAATCTTCAGAGGGATCAGGAGGAGCTTATCTCATGCAAGATTCATCTGGCTTGAAGTATATTTCTGTTTTCAAACCCATTGATGAAGAGCCAATGGCAATTAATAACCCTCGAGGCCTACCTATTTCCAAGGATGGTGAAGGGCTAAAGAAAGGTACACGGGTAGGGGAAGGTGCTTTGAGAGAAGTTGCAGCTTACATTTTGGACCATCCTAGGAAAGGACCACGCTCATATTATAACAATGAGGAACCAGGATTTGCTGGAGTTCCTCCTACAGTCATGGTCAAATGCCTGCATAAAGGATTTCATAATGTCGAAGGTTATCAGAATGTTGAAGGTAATGCTAAAATAGGATCGCTTCAGATGTTTATGAGGAATATAGGAAGTTGTGAAGACATGGGCCCTAGTGCTTTTCCTGTGGAAGAGGTACACAAGATCTCTGTGCTGGATATAAGGTTGGTAAATGCAGATAGGCACGCAGGCAACATTTTGATTGCCAAGGATGGTGAAGACAGTCCAACTATTCTCATTCCAATTGATCATGGCTATTGCTTGCCTGAGAGCTTTGAAGACTGCACTTTTGACTGGTTATATTGGCCACAAGCTAAGGAGCCTTACTCTGCAGACACCATTGATTACATAAAATCTCTGGATGCTGAAGAAGATATAAAGCTTTTGAAGTTTCATGGATGGGATCTTCCTCCTAAATGTGCCCAGATCCTTCGTATATCAACCATGCTTCTTCAGAAAGGTGCGGAGAGAGGACTCACTCCTTTTGCCATAGGAAGCATCATGTGTAGAGAAACACTGAAGAAAAAATCTGTCATTGAAGAGATTGTGCGGGAAGCTGAGGAAGCTACATTTCCTGGAGCAAGTGAAGCCGCATTTCTTGATCTCGTTTCAGTGATCATGGATAGCCATTTGGATGAGCTATATTCCCGTGACTGAATATACATAATTCCAATACTTACTACTATTTTGATAATACAGTGACTAAGTGATCTTCCTTCTTTCGTCTTCCAAGAGGATCCCTCAATCTCGTTTAGTACACTCTGCTTAAAACTTGCCTGCTCATCACCAATCAAGCATTTCATGCTTGTATTGCAATATCAATTTCTCTTTTATATATATATATATATCTCTGATAACATGGAA

>C101513_65

CAACACACGCCCTACCCTTCAATTGCTTCTTTCTTACTCACTGAACCCAACCCCTCTTACTTTGCATTCCCAGATTCATTTCGCACAATCTTTAGTTCACGTATTACGCTAACACAACAACCTTTTCATTCCATTCATTTACTTTTCCGGATTCTATCTTGCCTTTTGGAAGTTGGAATAGAATTAGAGCTGGGTGGATGGAGGATGAGAAGAGGAGGAAGAAGAACAAGAAGAAGAGGAACAAGCAAAGTAAGAATGCGGAAAATGGGGTTGGAGAAACTGCCAGTAGGGATCAGAATCTGGTGAGTAATGGAAAGGATGAGCATACTGATCTTTCTGAGACTGCCAATGGGAAAAACTCGAATGGGGTTGGAGAGACATCAATTAGGGATATGAATCTGGCAAATAATGGTAAGGATGAGCCTGCTCATCTTTCGGGAGTCACCAATGAGCAAAGTATGAATGAAGATCGGAATGGGGTTGTTGAAGCCCCCAATAGTGATCAGAATCTGGTGAAAAGTGGAAAGGATGAGAATAGTCTATCATTGGAGTATGCACATGGGCAGAGTACTAGTATGGATTTGAATGGTCATCTGCCAAACGGTAAAGAATGTATCATATCAGAAGAGGCAATCTGGAAATTAAAAGAAGAAAATGATTTACACATTCAAAATGAGGCATTTTCAAAAGAGACAATCAGGAAATTAAAAGCAGAAAAAGATATGCACATTCAAAAGGAGTTCATAATGGAAGAGACAATCAGAAAATTAACAGAAAAAAATGACATGCATATGCAGAATAAGGTCATATCAGAAGAGACGATTAGAAATTTGAAAGAAGAAAATGAGATGCACATGCAGAAGGAGGCCCTATCATACGAGACAATAAGAAAATTGAAAGAAGAAAACGAGGTGCACGTTCAGAAAGAGGCCATATCAAAGGAGACAATTAGAAAATTCAAAGAAGAAAATGATAAACTGGTTCAGAAAGAGGTTACCTTGGAAGAGATCATAAGCAATCTAAGAACTGATAATGAATTGCAGACACAAAAACAAACTAGTCTGGAATTGAGAATTGGACAGTTGCAAAGCGAGAATAATTCCTTAGTTCAAAGAGAGTCGGGATTGATGGAAAAAACTAATCAGTTGCTGAATGAAAAGGAGAGTTTAGAGCGAAAAATAAATCTTCTGGAGAATGATTTTAGTTCTTCTAGTGAGAAAGAGGTTGGACTGGAAATAATAATTGCACAGTTGCAGAGTGAGAATAATTCCTTACTTCAGAAAGAGGCTATATTGGAAGAAAAAAACAATCAGTTGTTGAATGAAAAGGCAGTTTTAAGCCTGAAGGGGGAGAGTCTAGAAGAAAAAATATATCTTCTGGAGAGCGAGTTGAGTTCTTTTGTTGAGAAGGAGAACGCAACTAAAGAAACTATTTCAAGCCTGAGTGGAAACATTGCTGTGGTACAACAGCAGGTGGCAGAGTTGGAGGAGTCCAGGAATAATCTTTTGCTAGAGAACCAGCAATTGAGGGAAAAAGTGTCAAGGCTTCAGTCAACAGTCCAGAATCTTGAAAACAGCAAAGCTTCTTCCAATTCATGGGACACATCTCAAAAGAATCTTGCTTCGGAAAATGAGGACTTGAAGTCTCAACTTGATGGTACCTTTACTTTAGTGGAGAAATTGATGGCAGAAAATGCAGAACTTGTTGAGAAGGTCACCAAGTTATGTGTTGAGCGGGAACAACTAAGTGCAGAAATTGAACATTCTGGAGTTACAGGGTCTGATGGGATCAATGAATTCACTATTGGTGTTGCCATTCCTATACCTGAATCTTCTGAGATTGCAACTGTATCAGCTGCAGAGATTAATTCTTTGGAAGATACATTGGTGAAAGATAATGGTAAATCAAGTGGTGCTAAGCATGTTGTTGGGGTAATGTCAAACTCATCGTTGGTATCTGATGATACTGGAGAAATCGTGCAAATTCCGTTGGATGATAGTGAAGCACAAGATCCAGATTTGCATGACGATGACAAGAACGTGGAAAATGATGATGTGCCTATCACAGATGCTCCTCTTATTGGTGCTCCATTTCGCCTGATATCATTTGTTGCTAAGTATGTTAGTGGTGCTGACTTGGTTGAGCAAACCTCTTCAAACACCGCTCGTTGACTTCTTATACTGGATTCTGGATAGCACCCCTTTTTTGGCAGTCTTTGAACATACAATAACCTTACATACATTAGGCAGAATACCAATATAGGTTGGCCTAAATTGGGCCATTTTAGTGTTTGTATGTTTATTCTTTGGACACTAGGAATTATTTTACCGGGAAAAAGTATAGTTTAACTTTACGATGCGTGTTCTTACTTCATATTGCTTTCATAGTTGGAAATGATACCCTTCAAAAGATGGCAGCATGAATATGAATGTAGTAGTTGGTGAATGGTTTCTGCTGTTGTCATAAATTCTTTCATTTTGGTGAGTGATGTATTGCAAAATTTGTGATGATTAATATTTTTGGATAAATTGCAAAATTGAGC

>C101515_65

TCTTCCAACCCAAGTTGTCAAATGTTCCTCTTTTCTCCAATTCTCCAATGGGCAACCACCAAGGTGTCATCATTTCTTCTCTCCTCCTTTAATGGTTTTGCTTTGCTTCCTTTTTTTTAAAAAAAAAAAAAATCAATTTCAATTTGCTCTTTCTCTCTCTCTAAAGTCTTACCCTCCCTTCTTCTAGTTCTCTTGTTGAACACACTCTTATGCTTGGTAGGTTGTGCCGTTGAGGAGATGGGCCCTAAACTCCACTGAAGTATGATGGTAAGAACCAAAGGGGTCTCTTTTCCTCTTTCAGTGCTTCCCCTTTGCCCCCAATTTCGCGCTGTCTCGCATTTAGGTTTTCCCCTCTTTAGACTCACCAGATAAGATTTAGGGCATTTGATTTGACTAGATTCTTGGCAATTTTCGATGTGGAGGTTCTCGTATTCCACATGGATTTCATTGATCACCCTTTGTGATTGATTTGGTTCCTCCACGCTTGCCTCGTTGTTGTGAGAGAACGCAAGTTAAATTGAACTGGGAGGAAGAAAAAGAAGAAGAAGGTGGTTATGGGACTGGATTCTCTCGAATTTCGTGTTGGCTAAGGCGTTTGAGGGATAAAGACTCGACTTTTAGTGGTGCTGGTTGTGGAGTTATCTTTGTCGGAAGGGGAATTTGAAGCCCAGGAAGATGGACAGAGTTATTCAGCCTCCTTTGGTTGATACAACTGCATGTCTATGCAGAGTAGATACAGGCCTAAAAACTGTTGCTGGAGCAAAGAAGTATGTCCCTGGAACAAAGCTCTGTCTTCGGCCTGACATTAAACCATCCATTCACCCAACTAGAAACAAGCCAGCTCGTGGTGATAGAAGCCGCAATCAATCCCCACTACTTCCAGGACTCCCTGATGATCTAGCTATTGCTTGCCTAATTCGAGTCCCACGCGTTGAGCATCGTAAACTCCGGCTAGTCTGCAAAAGATGGTATCGTCTTTTGGTTGGTAACTTCTTTTATTCTCTCCGCAAGAGTCTTGGAATTGCAGAAGAGTGGATATATGTTATTAAGAGGGACCGAGATGGGAAAATCTCATGGCATGCCTTTGATCCTGTGTACCAACTGTGGCAGCCCCTGCCACCTGTTCCAAGGGAATATTCTGGAGCTCTTGGTTTTGGCTGTGCTGTTCTCAATGGCTGTCACCTGTACTTGTTTGGTGGGAAAGACCCACTAAAGGGATCCATGAGACGTGTCATATTTTACAGTGCTAGGACAAATAAATGGCACCGTGCCCCAGATATGCTTCGGAGGCGGCACTTCTTTGGCTCCTGTGTCATAAACAATTGTCTGTATGTGGCTGGTGGGGAGAATGAAGGTGTGCACAGATCCTTGAGATCAGCTGAAGTGTATGATCCCAACAAGAATAGATGGTCATTTATTTCAGATATGAGCACTGCAATGGTGCCTTTCATAGGAGTTGTCTATGATGGGAAGTGGTTCCTTAAGGGACTTGGTCCTCATCGGCAGGTTCTGGGTGAGGTCTACCAGCCAGAGAATGATAGCTGGTACCCGATTTATGATGGAATGGTTTCTGGCTGGAGGAACCCTAGCACTACCCTAAATGGAAAGCTCTATGCCTTGGACTGCAAGGATGGCTGCAAAATTAGGGTTTATGATGAGGTCAGTGATTCATGGAGCAAGCATATTGACAGTAAAATGCATTTGGGGAGCTCGCGAGCTTTGGAGGCTGCTGCTCTTGTCCCCCTGAATGGGAAACTCTGTATCATCCGTAATAATATGAGCATTTCTCTGGTTGATGTTTCAAAACTTGAAGATTTGAAAGGATCTTCTGCTGAACAATTATGGGAAACTATTGCTGGGAAAGGACAGTTCAAGACATTGGTCACAAATCTGTGGTCTAGCCTTGCTGGGAGAAACCGACTCAAAAGTCACATAGTTCACTGTCAGGTTCTTCAAGCTTAGAGGTTGTATAGTTCTAAAAAATCACAATGAGCTTTGGTAACCAGTTTAAGTATCTCATCTCGTGGTATATGTTCGTGAGAAGCTGGGTCAAATCTTGCCAGAACACCATGGTGCAGTCTATTATTTTGTTTCATCTCAAACCCTTGACATCATGCTCATCACAAGAGGTGAAACATAAATGAGATGTTTTACTAATCATATAGTGTTACAGATATTGATACATAGTCGTTACATTTTGAAAAATTCAGTAGGTTGCTTACCAGAGCACATGGTTTATCATTAGCTTGTCTTCTGCATTGCTGCAACCAATTTATGACTGGTGTGTTGGACTGGGGAGATGAATAATGCTTAGAGTCAGTTTGGTATTCTTATGAAACTGAGGGTGAAAAATTCAAGAATTAATTTTAAATTTAATTTCATATATATAAAAACACATCTATGAATTTTTCACCCTCATTTTCTGTAGTGGCACCCAGCTGACTAACAACTCCTAATATTAAATACAACTCCTTGCATTACGTGTTTATATATGAGCACAAGTGGAGTGGAGCTCACGGGTGAAAAATTTTTAGGGTTGCCTC

>C101517_65

CTTAGAGAAGCGTAAGGGCCATAAGAAGCTTGGCCATGCATCACTAACAACAATAAATCTATCTATATTTCGAAGAACGTTGCAAGGACATTTAATTACAAAAATGATAAAAGGGAACTATTTGATAGAGAAGGGGATAAGAAAAAAATTGAAAAGAAAAAACGAGACAGATATTCTGCTTCGTAAACCTCTAATCCTACATGATATCACGTTAGCTTGACTGTTCAAGATCAGGGAGAGATGTTGGCTGAAGATTTGTTTTGGTGGGAAGTTGCCCTGTAGACTGCTGCTTAAGGGACCTAGGCCTTTCTTCTCCCTGCAAGGAAGCTACCTTCTCAGGAACTAAATGGGCTCCTTCCAACTCAAGTTCTGGTTTTCTCTTTACCTTCTTTGTTAAAACAGCTTCTGCAATCCTGACATCATCCAGGGAACTGCTTGAACTTCCCTTAGCTTTTTCTTGTTTTAGACCATTTATTGGACTGGGGACCCGGCCAGTTGTGCTTGTATTAGAAACTTGCTTGTTCCCTGAAGTAAAAGCATGACTGCTAGACTCTGGAGCTGATCTCTCTCGTAGGTTCTGCTGGGAAGTAATTGAATTGGTATCAAATCGAACGTTTTCCTCTTGCTTAGGTACCAGCAACTTTTTCCTCTTAATTTTTTCCTGATCCTTATGCTTGTTGTACAGTGCTCTGCGCCTCTCTTTTGCCCTGCAAATTCCACGTTTGATCCCATGGTTGTCCATGTAACCACTGGGCCATAATTCTGCAAGCTCAGCATACAACTTTCTGATCTGTGGACCTGCATTTTCATCCAACCCATCGACAAAAAGATCATAGAGATCACAAATCTTATCCTCCAATGCAGCATCCATAGCAAACTTTCTTTTAGTTATTGTTTTTCCGTCAGGAGCAAATTCTTGAAAATCACCAGATGCTTCACCCTTCTGCTGCTGCTTGGATTCGAGAGTTGGGGGCTGCATCTTAATCATATCAACAACTTCCTTCTTTATTTGTTGAAACCTATTATCATCCTCCTGCTTTGCTGAGAGACCCATACTGATCATTATTTTTAAGTTTCTCTTTAATGTTCTTAGCTGAATCAGATGCCCAAGAATACTCATCAGACGGTTAATTAACTCTTTTGATACTTTCCCATGGGTTGCCGCTAGTCTAGCAACTTTAGCAAGCTTTAGCTTTATTTCTCTAGGCAACCTCCTTTTAACAGCATCCTGGTTATCCACAGCTGGTGGCCTGGATTCTGCAACCATTTTTTCCAACTCACGAAGAGCCTTCTCAAGCATTGAAGTTTTTGGCCTAACACTAGAGCCCTCTTTCTTCTGCATGTTTTCAGATTTTGTTGCATGGGTAGTAGACTTTCCCTCTGACAAGTTAAGATCTGGCAATTCACAGATACCACTTTTTTCTTTTGTCCGTATAATATTTTCCATATCATCAATACTATTTGGGGGTCTTCCAGGTTGGGATTTGGAATGTGCATAAGCACTTTTATCATTGTATTTCTGATGAGATGCATCAAACGATCCAGTTCCATCTTTACCTTTATCACTAATGTTCTTAGATTGAAAAGCCCCTATCTTCTGATTGTCAGCATCTTTGGCATCTGTTACACCAGGAAAATCATCACTTGATGTTTTCAAAGAGATGGAAGGGTCCAATGTTGGTTTAGTATCAGCAGTTTTCTTTTTTGAAATAATTCCAGAGACATCTGATTGATTCGAAAGTTTCAAGTCTTCATAATGTTCATCAGGTGCAACCAAATTCTCAGACAAATTTAACATATTTTTTGCCTGTAGTGAAGCTGTTTTGCCAGATGCTGGCCTGCCAACTTTTACATTTTTATTTGATCCATGACCATCATTGTTTTCACCAGCATTCTTCAATATATCTTTTCTGCGCCTTTTCTTTGGTTGCTGGTTAGGTAGTGCAGGAGGTTCATTTATGCGCTCCAGTTTCCCCCTATTTACAAAGAACCCATCATGTTTGATTGCAGAATTATCAACCTCAAAATATTCATCCAGTTCAGCATCATCTATAAAAGAGTCTTCGGTATCATACTGATCATCATCAGGAACATCAAGCAGATCCTCATCATCACTACTATCCTTGCCCATGTAAAGGCGTTCAATCTTCTCTATTACAGCACTAAAACGGTTTGGCTGAGAAGGATCGTTTTCTTCAACCTCATCAGGTTGACCCGGAGCGATTGCGACTTGTTGTGGTGCCGAATTGGATCCATTAGGCTTGTTAGAGTCCTTCATCAGCTTCTTCCACGAAACTATGGTGGTCTCACCAGGCCGGAGCTCCACCGTGAACATTTGGCGGTCGCCTTTCTTCACGAACGACGACGACGCCCTCTTCTCCTCCGCCATAGGAGTCCAATTCACCGATGATACAGAGATTCGAGAATTAGGGCGCGAGAAAGAAACATTAGACAGAGAACAAGGGAATTGCGCGGGATTCAAAAATGAATTGCTGGAAATTAAAGAAAATCGAATAAATGGAGTGTGTCCGTTGGGGTTTTAAC

>C101519_65

CTTAGTTTCACTTTCTTCATCTCTTTCCATGGCCAATCAAAATACCCTTCCTCTTTACCAGAAAACATGGCTCAAACACACCTTCTCAAGAGCAGTGGATTCCCTCATCTTCCTCCTCCTTCTTATTCTTCTTGGTTACCGTCTTTTCTCTTATCACCATTATTCTTTCCCATGGTTTGTTGCATTTCTATGCGAATCATGGTTCACCCTCACTTGGCTCACCACCATTAGCACAAAATGGACTCCTGCACAAATCACCACACACCTTGACCGTCTCTTTCTTCGGGTAGGTGAGCTTCTGCAAGTGGACGTGTTTGTGACAACAGCAGATCCTGTGCTTGAGCCACCTATCATCACAGTCAACACTGTGTTGTCTCTTTTGGCCCTTGATTACCCTGCTAACAAGCTAGCTTGCTATGTTTCTGATGATGGTTGTTCTCATCTTACCTACTATGCCCTTGTGGAAGCATCCAAATTTGCTAAATTTTGGGTACCTTTCTGCAAGAAGTACAATGTACAAGTTAGAGCACCCTTTAGATACTTTTCTGAGGAAGCCACAGTTGAGAAGAATGACTTTCCAGAGTTTGAACAAGAATGGTTAAAAATGAAGGAAGAGTATGAACAACTTAGCCGTAAAATTCAGAATGCCTCCGAAGAGTTGAATCCGTGTCCACTCGTTGGAGAATATGAAGTTTTCTCAAAAGCACAACTAAGAAATCACCAAACCATAATTAAGGTCATATGGGAGAACATAGAAGGACTTTCTGATGAAGTACCTCACTTAATCTACATATCTCGAGAAAAAAGGCCACAATATCCTCATCATTACAAAGCTGGTGCCATGAATGTGTTGACAAGAGTCTCAGGGTTGATAACAAATGCTCCCTATATCCTAAACGTAGATTGTGACATGTATGTGAACAATCCCAAGATTGCACTACACGCACTGTGCATTTTGCTTGATTCAAAGGGAGAAAAAGAAGTTGCATTTGTTCAATGTCCCCAAAGATTCTATGATACAGTAAAGGATGATGCTTATGGAAATCAGCTGGTGGCTTTGCCTATGTACATAGGAGGTGGATTTGCAGGACTTCAGGGGATTATATACGCAGGAACAAATTGCTTTCACAGAAGAAAAGTTATTTACGGCCTTTCTCCTGATCATGATATTCAAACTACAAAGAAGGATTTTGGTTTCATTGATGGAACAATATCAGAAAAGAGAAGAGTGGAAATATTTGGAGCTTCAAAAGGGTTTGTTGAATCAGCTAAGGATGCTTTAGAAAGGACGACATTTACTCCAAATGAAAACCTTTTCAAACCTATCGAGCTTGAGGCAGCCAACCAAGTTGCTACTTGTGATTATGAGTACAGCACCGCATGGGGTAAAAAGGTGGGATGGTTATATGGATCAACATCAGAGGATGTACTTACTGGGCTGGTGATGCATACAAAAGGTTGGAGATCTGAACTCTGTTCACCAGATCCAATGGCCTTCATGGGCTGTTCACCTCAAGATAACCTGGGCCAAATGGCCCAACATAAGAGGTGGTCCTCAGGGTTGTTTGACATTTTCCTCAGCAGCCACTGTCCAATTTTTGGCACTCTATTTGGTAAGCTCCAATTCAGAGAGAGCTTGGCTTATATATGGCTCACAAATTGGGCTTTACGGTCTCTCCCTGAAATATCTTATGCCCTTCTCCCTGCCTACTGCATCATTACCAATTCCACTTTCTTGCCTAATAAGGAAGCGGGCATGTGGTTCCCTATCAGTGTATTTGTGATGTACCACGTAGGTACATTATCAGAGCACTTGATATCAGGGTTGTCAGCTCGAACATGGTGGAACAACCAGAGAATGGGAAGAATAACAACCATGACTTCATGCTTCTACGGATTTTTGGACATCCTCCTTAAGCGCTTGAGGATATCTGACACTGTCTTCGAAATAACAAAGAAAGACCAACCTTCTAACAATGAAAACGTGGGAAGGTTCATATTCAACGAGTCTCCCATTTTTGTACCTGGCACAGCCGTTTTGGTCATTCAACTCACAGCGCTAGTTACTACCGTGTGGCGGTGGCAACAAGTTGAAAACGATGGTGGATATGGGCTAGGTGAGGTGTTTTGCAGTGCTTATGTGGTCCTCTGCTACTTGCCTTTGTTGAAAGGACTGTTTGGGAAAGGAAAGTATGGAATTCCCTTATCCACAATATGCAAGGCAACGCTACTAGCTTTCTTCTTCGTTCAGCTCTGCAGGAGTACCATTACCAAGTGATCAGTTTTCACTTGGTCCACTTTGATCTGTTGTAATTCCAAGGTTTCGTCGAATTATGTTCACATTGTAGACGTATGTGGTATTGATTTTTGACAGAACATTGAATATCATCAAGTTCTACCAGTGGGAAGATCTCTAACAAAAGTCACTTACTGATGTTCTATTTTGCAAATCACAAGTCAATGCAAACCTTTGTTTTAAAGTGTTTCGTCCTACTTTCACTTCAGATTTTTTTTTTTAATGGTGTATCCAAAAAAGGAAAGAAA

>C101521_65

CAAACGTCTTGTGCTTTCATAAACATAGCCAAATAATAATAAGGGAGTCCGATGATTCAAGTCACTAGTCTGAAAAGGCAGGCAGCTCATTTGCAATACTTGTCGAACCAGATCGCAATGTTAAGGTAACTTTCAAAGTCGGATTGTGGTCTTTCAATTCCATGAACATCATTTGGAAACACGATGACTTTAGTTGGGACTCCTTTCTCACTTAAAGCCCGAGCATATTGTAGTCCAGTCGAAATTGGAACACGGAGATCTTGAGCACCTAGCAGAAAAAGCGTTGGTGTTTTTGCCTTTGAGAGGTGTGAAATAGGAGATTTGCTATAAAATAGGGTTAGATCCTCTGCTGAAGGTGCTTCAGTAAACTTATCTCTACCCTTGGTTCCAAACGTCTCCACATAGCACCAATCAGGGATATCAGTTGTACCAACCATCAGTGCAAGATTACACACAGGATTTCTAGCAGCTGCTGCAACAAATTTTTCAGGTGCCTGGCCAATCAAGTGGGTTGTCAGAAAGCCACCATGTGAGCCACCAAGCACTGCAATCTTTGAAGGACTGGCAAGTCCCAAGTTAATGACATGATCAATAGCAGTGAGCACATCGCTAACATCCTGAGAGCCGATTTTTCCCGGAAGAGATTGCAAGGCTTCCTCGCCAAATCCTGTTGAGCCTCTGTAATTTACAATTAACAAGCTATATCCAACCGAAGAAAGATAAGCCAATGGCTTTGAAAAGCTAGACAATGAGACAGAGTGTGGTCCCCCATGAAGGACAACAATTAGTGGATCACATGCATCACTTTTCTTAGTTTTTGATGACACAAATATAGCTTCATAAGGTTGACTAGCACCTTTTGTTAAACTTTCAGAAACATCCTTGACAGAGATCTTCATTACACTGAATGTAAGAGAGGAGATCAAGGATCTAACCTTGTCGGAGCACTTAAATATAGGATTTGAGACATCTGACCAACTCCATTCTTTATTAGTTGCATTTTTAACTATAGCTCCATACTTGATTTGAGGAACATCTACTGGACTGCTAGAAACAGCAAGAATATTGTCTCCATCCAATGCAAGGAGACTCCAAGAGAAATTTGAGTTGGAAGGGGTGATCCGCAATATTTCTCCACTCAACACATTGATGGAAAGTAAAACTTGATTGCTTTGCCAGACAGAAGCTACAACAAAACTCTGGCCATCGGAAAGCCATGGATCACTGAGGATATTTGAACAGTAAAGCCCAGGGAAGCTGCCATCCTCCGCAAACAACACAACTGGAACCACTTCATGAATTTCAGAAGATTGATAAAGCTTCGTATCCTTAGGCCAATCAATTCTATGAAGTGAATCTGTAGCAGAATGGGCTCCAGAATCAACTGAACTTCTTGCAGATACAAATACAAGAAACTTTCCATCTGGGCTGAACCGTGGAAAATATGCGCTACTTATGTTTTGAGTTAGGTTAACAGCCTGAACATCTTCAGTTGAGTGAGTCTCAGTTTCATCAGCTTTTGACTCATGGTGTGGTGCTCTAACAGCATATAATGCACACGGCCTGTTATAGCAGTATTTAATACCAAGTTTTCTCGTCTCAAATGACCATCCAACAAAAACCAAATATTGCACAGAGCCTTCAATTGATGGAGCCCACACAACTTGTCCAACACTCAAAGACTTATCAATTCCTTTGACGGCCTGAACCTCTCCACTGGTGATGTTGATGACAAAAAGGGCAGGCTGCCTCTTCCCTGCATATGTCTCTCCCCAGTCCTCCTCCCATTCCCCTTGACCTTTCCAGGTACCACAATCCTTGTCTGAAGAACCACCTTTCTGGTAGCCACCCAGATCATTGAATGTGGGCTTTGCAGGGGCTGGCTCTTCAGCTACATATGCTATACATGTTTCATCCAAATTCCAAGAGATCCCCTCAAACCATCCATCAGTGTATACAGAACCGTGCTTGGATTGAGGGACATGGAACTCCTTTTCCAGTTGAGAGGAGCTCCAAATTTCAAAGCGGCAAGGACCTTCACTCTCGGGATTCCGAACAATTAGCAGTTTAGAGGCAGAAGGAGACGGAACCATTGCGGAGACTCCACACATTTCGATCGGGAAGGGAGCCCAGTGAAACGCGACTGAGCCGTCGTTTTGTCTGAGAAGGGAAGCGGAGACGATGGACTTCTTCCTCTTGTTGGCCAAAAGGTTCGATTGACTAACGCAGAACATTCCCTGCGAAGCCAAACCGGTGTGAGAGTTGAAGAGCCATGCCTTGTCGACGGTGGGGATGGCGGTGAATTGGTGAAGTAGTTCGGAGTGAAGCGTGTATTCTTGCTCCGTGGCTTGGTGTGCCTTGTCCATGGATGAAATTGTTGGGAACGAAAGCCTCCTTCTGCTGCAGATTGAAGCAAATAAAATGAATAATGGTATGATGATAAATGATATCGAAATGGATAATTTTTGTCAACGATTACGCAGTGAGACGAAGAGAGGGTGTTGGAGTTAGCGTGTTGCGAATGGAGGATAAATAAATAGGATT

>C101523_65

CGCGGGGTTAACAGCACCAAAAATAACCCTCAACTCAACTCAACCTCTTCTCTTTTCTTCTCTTCTCTTTCACGCCGCCGAAGCTGCAACTTAACCACCGGTCACGATTAGGTTTTTCATTCAACCAACATGGACGCGGCAAATGATACAGAGAGGAATTTGGATGGAGAGAACAGTGTGAGACGAAGGAGCAGAAAAAAATCCATGCCTGAGAGTGGAGCACGAGAGCATAAGGAGCAATTGCAGAAACTTTCGGAGAAGGACCCAGAATTTTACGAGTTCTTGAAAGAGCATGACCAGGAGTTGCTTCAATTCAGTGATGATGATCTAGATGAAGATGTGGGTACTGACATAGAAGATGGAGATCTACAGCTAGATGAGGAGGCTAGTGAGGATGAGACTCAACAGAAGGAACAAAAATCCTCTAAAGTTATAACTACTTCTATGGTTGATTTGTGGTGCAAATCAATTCAAGAAAATGGGAGCTTAAGTGCAGTGCGTTCCTTGATGAGGGCTTTTAGGACAGCATGCCACTATGGTGATGATGGGGGGAATGAATCTATGACAAAGCTTAGTGTAATGTCTAGCACTGTATTCAACAAAATAATGTTGACTGTACTTACTGAAATGGATGGAATACTAAGAAAATTGTTTAAGCTTCCTGCTTCTGGCGGAAAGAAAGAGAACATAACAGATTTAATGGCCACGAAACATTGGAGTAGTTATGGACATTTAGTGAAGTCTTACCTCGGAAATGCTCTTCATGTTTTGAACCAAATGACTGACACAGAAATGATATCATTTACTTTACGTCGGCTGAAATATTCTTTATTGTTTTTGGCTGCTGTTCCTTCACTCCTGAGGAAATACATTAAGGTGGTCCTTCATTTCTGGGGTACTGGTGGAGGTGCTCTTCCAGTTGTTTCATTTCTATTTATGAGAGATCTATGTATCCGTATTGGATCTGGCTGTGTAGATGAATGTTTCAAAGGAATTTACAAAGCTTATGTTTTGAATTGCCACTTTGTAAATGCTGTGAAACTTAAACATATCCGTTTTCTTGGCAATTGTGTTATTGAGCTTCTTGGTGTTGATCTTCCAACTGCATATCAACATGCCTTCATTTACATTCGGCAACTGGCTATGATATTAAGGGATGCACTTAATACAAAAACTAAGGAAGCTTTTCGCAAGGTTTACGAGTGGAAATTCATCAATTGTCTTGAACTTTGGACTGGTGCTATCTGTGCCTACAGTTCAGAATCTGACTTTAAGCAACTTGCATATCCACTGACCCAAATAATTTCTGGCGTAGCCCGTCTGGTTCCCACAGCCAGATATTTTCCCCTTAGGTTGAGATGTGTAAGAATGCTGAACCAGATTGCAGCTTCTACTCACTCCTTTATACCAGTGTCTATGCTCCTTTTAGACATGCTGGAAATGAAAGAATTGAATAGACCCCCTACCGGAGGTGTTGGCAAAGCTGTTGACTTACGCAGTATACTGAAGGTTAGCAAGCTGACTCTAAAGACACGAGCATTTCAAGAGGCATGTGTTATTTCTGTGGTTGAAGAGCTAGCTGAACACTTGACACAGTGGAGTTATTCCGTTGCATTCATGGAATTGTCTTTTATTCCACTTGTAAGGCTGCGTAGCTTTTGCAAATCGACAAAAGTAGAGAGGTTTCGAAAAGAAATGAGGCAGCTTATACGCCAGATTGAAGCAAGTGCTGACTATGTGAATGGGAAGCGCATGTCAATTTCCTTTTTACCCAATGACCCTGTTGCAGCATCTTTTCTCGAGGATGAAAAAAAGTCAGCTTCTAGTGCTTTGTCAAAGTATGTCCAAACACTCCGCCAGAGAGCAGAGCAAAAAAATAGTTCATTAATGGAATCCAGTGTTCTAGTGGGAGAAGAATCCTCTAAATTTGGAAATGAAATATCAGAAAGTGATGAAGAGGATGCAAAAAAGAATGAGAAGGGTGCTGCCGTCTTTAGTTCATCCTGGTTACCCGGAAATGAATCCAAGATAAAACAACCTAAAGAAACAAAAGGAAAAAGGAGAAAACGGCAGAAAGAGAAAGCCATTGATGACGATGTTGTGGAGGATTTGGTACTTAGTTCTGACGAAGATATTCCTTCAAGCCACAGCCCTTCTGCTGACAAAAATGATGATGCAGATCATTTACCTTCAAAACGAAAACGCAAGCAGAAGAAGCACAGGACAAAAAGGTTGAAAAAAAATTAATTTCACTTGGAGGGCAAATAACTATTCAGGATTCATGCAACCTCTCTCTCTATTCAGGATTTCATGTGAGAGAAAGTTTTGACAGTTTTATCAATCGTCACCATTTTACCTGGAGTAAAGACAGAAATATCTGAATGTAATGGCAGCTGCAATTATATTTTTGTATGTCATTCCACTGTGAATGGAAAATGAGTGGACAAATTGTGACGTGGCACTACGTGTATTTTTCTGGTGCCATTGTTGGAGCAAAATGAACTATTATTCTGAGATGGAATATTGGGAAATACCTTTTGCGCTG

>C101527_65

TTCATTATTGCCAGTTATTAACAGAATATGGAAGGCACCAATCTAAACTGAGCTTCAAATTTGTAACTATCGAAATTATATTACAAATAATAAACGACATACAGCACCCTGTGTCTAAATTGGTTGACCGAAGGTGCAATCAATCTAACTCACAAAGTCGTATAGAGTGAAAGGGTTAAAGAAAGGGAGAAAAATATAAATACATTTCCTTGGAAGCCATTGTAGTAAAACACAAGGGGGCTTAGATCTCCCGTAGGGCTTGCTCATCACTTCCTCGTTGGGATCCTCCATGATCACTACTACTAAATGTTCTCTTGCTCATGTTAGCTCGCTGCACTAATCGCACCAGTGCTTGAACCACTTCTGACATTGGTGGTCGGAATTCAGGCTCCGGCTGCACGCAAAGAGCAATAACATCCGCAAATCGAGAAAGAGACTTAACAGGATATAGCCCTTTCAATGCAGGATCAACCATTTTAGCCAATGCGTCAATATCATGGAGTTGAGGTGTTGCCCATCGAACCAAAGACTGCTCAGATCTTGGCCTTGAGCTATCAAATGGCTTCCGTCCGCTGAGAAGTTCCAACATAACAACCCCAAAGCTGTAGACATCACTTTGCAAAGTATACTGACCAGTCAAGGCAACTTCAGGTGCATCATATCCAGATCCAACATTATGGCTCAATATCTGGTCTGCGTTTGGAATATAACTTGCCAATCCACTATCTGAAAGATGAGGATTGAGCTCTGCATCAAGTAAGATGTTGGCTGACTTAATATTCTTATGAACAATTGATGGCGAACTAACTTCATGTAGGTACTCTAAAGCACGTGCAGTGCCCAAAGCAATCTTGACACGGGAATTCCATATCAATGGTTTACTATATTCGTCTGATAGGTGAAGGAAGTCATGCAGTGATCCATTTTTATGAAATTCGTAGACCAAGAGGTGTTGTCCATACTCTGAGCAATAACCTACAAGCTCTGTCACATTTGGATGATGCAAAAGGGAGATGCTTGAAATTATTTCTATAAAATCATCAGATAAATCATTGGGAAGGACAGATGAATCTATCTTCTTCACCGCAAGAACCTGTCCATCATCAAATTGAGCACAGTAAACACGCCCAAAAGACCCCTCACCAACAAGGTGATCCACACTGAAGCTACCAGTAGCAATCTGCAGTTCAGCAATAGAAAATGATTTCACATTTGCAGGAGCTGTTACTGTCTTCTTCACAATTGGGGGCTTCGTGGAGAATTCTTCATCATCAAATGATTTATGGCGATCAATAGGTGGGGGTTTAAGATTTATTGAGGCAGAAGTATCAAACGTCTTCAAGTCAGATATAGAGGACATTTGCTTGGACTTCTCTTCATGCACTTCATTTGAAGGAAGTGGAGCCAAGGATTGATTGTCCAGCTTTTCTAAATCTGAAGATGTCTTCTTGGATTTTCTCTTCACCAGAAAGAATGCTACTATTGCCCCCACAACTATGACAGAGATTACAATTCCAGCTATGGCACCACCTCCTACACCGGATCTTTTGCTTTCATCAACTGAGCTGCTGCTACTGCCAACATCAGAGGGGGTTTTGCTTCCCCCTCCAGATTTGTGGTGGTTGTTGCTTTTAGGTACTGGAGGCGTCCCAGGAGGAGGTGGCGGTGCAGGCCCAGAGCTCCAGGCATTACCTCCGCTCTGTAGGTTTATGTTCTTCAACTGTTGTGGTATCCATCCAGTAAAATTATTGTTTTCCACATTCAGATTATCCAGAGGAAGATTAGCAAGGACATCAATAGTGCCTGTAAACTGATTGTTTTGTAGATTCATGGTTGTTATGCGTGAAAGTGAGCTCATAGTCTGAGGGAGTTCACCAGTTAGAGAATTGAAAGAGAGATCCAATGTGGAGAGGGTAGAAAGATTAAGAAAGTTAACAGCCAGTCCTTGCTGGAGCTGATTGTGACCAAGATTCAGGTCTGTAAGGGCAGTCAAGTTTGAAATAGAGTACGGGACTGTCCCTGTGATGTTATTATGAGCTAGATTTAATCGCTGCATATATGGAGGGAGTTGGTAGGGTATGAGGCCGCCAAGACTGTTGCTACTCATGTCTAGGTCGGTCAAAGATGTCAGGACTTGTAATCCATACGGCAGCGACCCAGTTAGTCCAAGATTAGATAACTTAATCTCTTTAACACGGTTGCCTGAGCAAGTAATGCCTTTCCAAGATTGTCCACATGGATCATCACTGTTAGCAGGCCAACCAAGCTGGGATGGTGAGTTCATATTTTGAAACAAAGATCTCACAGCAGCAGCATCATTTGGATCTGTGGCGGCAGTGGCACCATTCGGCATCCAACAAAAAATGCAGGTAGTGATGCACAGCAACGGCAACAACCTCCGAAGCTCCACCATCTCACAAAATTCTTCCTACCCAGAAAGCTGAAAGGGATTTTCAGTCACCCCCTTTTTGGCAAGTTCTAATCTTTTCCTCTAATTATTTTATGGGTCTATGCTAACACTCCCCCCATAGCCAAGTAAAGCTGGC

>C101529_65

TTGCACGGAATTTAAAAAAGAAAAATATCTAGGGAAAGGAATCTGTGGATTGAATTACAAGATAGGACTCAATTTCAGTTGTACAGTTTATTGATAACTGAAACCAATGTTTACAAGGTTTGACCAGAGAAAGCTGGTTCTTTCTGGGGAAGCACCAACTTATGTCGTCATCTTACACAAGGCACCACCAGAGACTACTACTTCTCCAATACAAAGAAACTTCTCTTGTACTCCACAAGGATTGCTCCACCTGGCCAAACTATTCACAAGCCAACATTCTTCTAGAACAATCTTGTTATACCATACTGAACCGGTCCAACCTTTCTGTGAGCAGTTGTACTAATTCCACCATATTGATGAGCCTTTGATTGCAGCAACTTTGAGTCAATGGCTATTCGGTCATCCTGCTCTATCTTGTTCACTCCTGCTCGTGTCATGAAAAAAGGGTTGGAATCAATATTTATAGCTATGTCTGGTGCTATTTTGGCATTCACACAGCATTGCTGTGCAAGTTTTGATTGTAAAGACACACTCTTGTCAGCTTCTGTTGCTGACCTTTCTTGTTCCCCAGAGGTTAATCTTTGATAATGATTCGTTGGGGGAGTAGGTTGAGAAGGAAGAACAGAACCTCTCATAAATGTCCTGGGCTCATATGAACCCTTGACAACACTCGCATACTCGTATGGAATAACTGGCCCAACAACTTTTCCTGGTTTTGCTACTGAAAATTTTTGCAGACCCTGAATGGACCTTGACACGGGATTTTCTGTATCCGTGGAATTGTTGTATTCCTCAGTGGTTTGCCTGATTTTGCTGGAAATAATATTTGATTGCTCTTTTGAGGGTACTATATTTGAATGTATTATTGTTGACCTGGGAAGTGATACATGCTTTCTTTCAGGTGGCATAACTGGACAACTTTTTCCACCAGTTTCCTCAAGATGAGCAAACTGCTTTTTGAATTGATCAACAGCACTTGGGTAAAGAAAATTAGTTCTCTCTGTTCCATTCATGTAGTCTTTCAATAGTTGAGGATGGTACTCTAGAATCTCGCGAAAAATTAACTCTCCAATTTCTTCCTTTGTGACTCTTCTCCTTTCGAATTCAAATTCCATTTTTGTAATAGGCTGGCATGATGGTTCCCTCTCAACTTTCGCAAGACCCTTGAAGTAAGGATCAGCTAATGCCTCTTCAGCAGTAGGCCGGTCTTTTGGATCAAAAGCCAGCAACTTTTCTAGTAGTCGTAGTGCTAAAGGATCTGCATTAGGAAATTTTTGTGCAAATGGTATAGGCTGTTTTTTTCTCATACTAGTCAGGTATCTCCGTGCCTTATCATTGCGTACCCGGGATATAGTATCCAGTGAGGGAGTACCAAGCAGATCTGTCATCAGATCCAACTGATGGACAACATTTTTTCCAGGAAAAAGAGGCTTTCCTATTAATACTTCAGCAAAGATGCAACCTATACTCCAAATATCAATTGCTGGAGTATACCTTGAGTAAAAGGATCCACAGAGCTCTGGTGCTCTATACCATCTTGTAGCAACATAATCCGTCCAAAATACAGTTGTTGGTGTGTCACTAAAAGCAACTCTAGCTAACCCAAAGTCACATATTTTAAGTTTACAGTTTGCATTGGCTAATATATTCTTTGGCTTCAAGTCTCGATGATAGACATTTGCAGTGTGAATATACTTCAATGCTCGAAGTAACTGGTAAAGAAAAAATTGATAGTGCTCCTTTGTTAAGTCATCATTGGCTTTAATGACTTGATGCAGATCAGACTCCATTAGCTCAAAAACAACATAAATATCTTTAAAATCCCTCCTCGAGGGAGGCAACATAACATGCTTAATTTCAACAATATCAGGATGTCGAAGAAGTCTAAGCAACTTTATCTCACGGAGAATACGTGCAGCATCAGAGACATGCTCGAAGATATCATGAATCTTTTTAATTGCTACTTTCTCTCCAGTATGAGTGTCAATAGCTGAACAAACAACACCATAACTTCCTTTCCCAATGACCTCTTGAATTTTGTACCTGTTGGCATCGCCATAATCAGAGAAAAATTCCATCTCCATTGAACCCTTTTTTCTGTGATCTTGCTGCATTCTGAGACAACACCCACTACAAATTAAGCCACCAATAGGAAACACATATAAAGGTAAGTTTCAATTTTGACATAAAACAAACCCAAGCACATTTGCCCCACCTACCCCAGCTCCTTCAACAAACACAGAATCCAAATACAAGCCCACCTCCAATCCCAACACCAAACTGCCACATGAAACAACGTAGATCTAACTCAGTGACAAAAACTCACCCATCTCTCTCTCTCACCCCTCACTCAGATCCCAGAGGAGCTTCTTCAACCAAAAGCGCGTTTGAAGAAGCAAAAATGAATGGCCCAGAAGAAGAGTACACCGACACAGGGAGAAAGAGTGAAAATTTTGAAAGGCTTAGTTGTTGTAATGAGTTGTTGGTTGAGTTGTATGAGAGGGTGGCTCTTCTCTCTCTGCTTCGCATTCGCTTGTGAGATACGGA

>C101533_65

AAAAGCTTAAGTTAATAAGTGAGAACAAATAAATGGTCTTATATTTCTGACACGAATGAGAGCTCTCTATACACAGATACATGGCCTTGTCAGAGGCATGTAAAACCAATACAAGTCTACATTCTTTAGTAGAGAGAAAAATGAAGACTCCAAACCCCAGTTTCTTATCCTGAGGAGAATAGGCAAAACAATGATGAATCAACTAGTAAGAGGGCCACGTCATATCCCAGATCTCACATAATCTCCACCTGAATCACAGAGATGCATGATTCCGATCACCGAAATAAAACATTCTAACAAGGGAGTCTGTTTTAAATTATATCAGTAACCGGTAGTAGATGAAACCAAAACATTATGCTCCTTTAAACGTTTCTGAAAATGAGCTAAACGGGTTTGCAACTGCAGGATGCCAGCAGCCTTCTGAGAAAGAATGGCATTTAGTCTTGTTATATAATCATCCAGCTGATTCCCTGGTTGATCTGCTTCAACCAAGAGGTTCATCTCCTCTCTAACAATATTCATAGTCTCCTCTACTTGCGTCCGGTGAGCATTTACAAGGTCCTCTTCTTCCTGTAAGAGGGCATTTAAATCATCCTCAGCGTGTGTGGTTTGAGGCTCAACTTTTGGAAGGTCCTTCCACTTCATCAGACCGCTGGGTTTCTTCAATTTGTCCTCTGTTGTAGCATATGACTCCATCTTTCCATTTTTCTTCAAAGATGGTTTCACCTGCTCGTAATACTCTTCAGGGGGACTAAAATCATCCCCTTCATTTTCATCAGGCCATCCATCTGTTGTGCGATCCTCATAGGATGATCCAGTAATGGAAGATAAGGGAACCGTACTTGATTCTTTAAGGTTGAAATTTGATGATATAGCATCTTTCTTTGTGTTGTTTCCTTTTGACAGGCTTTTCACCCTGTCAGCATATCTTAATGTATTCAGAGTATGTTCGCATGAACCAGTGCTTGGCGATATGCATGAGATCATGACAGTGCGGGAGTTGCCAACAAATGAATCCCTCAAAACTTCAGTCAGTTTACTGCCTCTGAATGGGATGTGTCCTTGGTCATTATCGAGAGCTCTTATGCATTCCTTTAGGGCAAGTAAGCTCTTGTTGATTTCAGCACCTTCTATTCTTGTCTGTTTGTCATTATCTGTGGTATCTGCTCCCCGTTCGCTTCCAGCAAGATCTATGAAGGAGAGTTTGCCAACCAAACGGGGAGGTTTGGATTCATTTCCATCAACTGACCTCTTGATAGCAAGTTGAAGTATTGCATGAGACCGTGATGATTCCTCATTTGCACCTGTGGTTCCTGTACTTCTTGTTGAATTTCCTTTCTCAATCAGTTCTTTGATGTTCTCCACATCTGATACTCGGTACTCTTGCAAACCCACAATGCAAACTTGTTGCTTACCATCCTCCCTCATGCAAAGTTTTTTTCTATCATTAAGAAGATCAAAAAGCTTGCCGCCATATATTTCAAAGAAGCTCACAAACAATTGAAATCCCTGGTTTCTGTAAGTATGATGCATCAATCTAAGGATGTCCCTTGATGCTTTAAGCGGCAGTGGCTTCATAGTGTAAGTTTTTCCACTTCCTGTTTGTCCATATGCAAAGCATGTTGCCTTAGTGCGTTCAAAGATTATTGGAACTATGGGCTCTACAGTTTCACGATACACCTCATCATTCGTAACCTCTTCATTCAAAACAGCATCAAAAACAAATTCATGCTTTTCTACATACTGAGTTAAGTCAACCTTCAATTTAGTCTCGTGCACTGTTAAAGAATTGGAATATGTTTCTATAATGTCTTCCTCATTTTTTGCTAATTCTTTCTTATTCATTGGTCTTTTACGAACCACAACTTTAATCTTTGCTACAGAATTAGATTTCTCTTTTTCTGCAGGAAAACTCTTCAATATGTTTTCAGGAGCACGAGCTCTGCCAGTCTGTTTGCTCATCATGTAAGGTTCAGAGTCATCAAAGCTTCTACCTCGGCCAGGTTGATACAGGGAGTGTGTATCATATGCATTGGAGGCTGGCATCTCAGGAGGAAGAAGCTCGGTGTCAAACGAATGCAGATCTAACAAGCCGGGGCTGAAATCACCAGACAAATCATCGCCATTCTTTTTCTGCATCGAAGCACGGTAACCACCGGGAGGAGTGGAAGGCTCCACATAATACTCATTTCCTCCGTTAAAGCTCCTCTGCGCGTTCCGGTACATTCTTCCTCCCTGCCCTCCGCCGTATAAGTTATAGTCCTGAAGCGGAGGAAGTTGATTGGTGGAAGATTGAAGGTGTTGGAGGCCAGCAGATTGGAGCCACCTGTTGCCGTTGGAGGAACCGTCGAGGAAGTTATCGGAGTACTGGCGCTGGTGGTGCACCCCGGACGCACCAGATCTCAGCCCCTGTCTCCCTGCACCGTTCATCTTCCTTGGCGATGATCAGATCGGAACGGAACGGTGAGAGGGAGGTGATCAGTTACCATACAACGACAGTGGACTGGACTGGAGTGAAACCGAAGATATAGGAAGGAACGAATAGATA

>C101539_65

CGGGCAAGTGTGATTTACACTTTTGACTAGTATACCATCTCATCTCATGTATATATATATATATAAGCAAAAGGGAGACCAATCTGTAGAACGATCCTAGCAACAATTAATTACATTAAGAAAACCCTGCACATTTCAGCAAGCAAATCTTATTATCAAAGGCCTGATCTCCAAAAAAATTCATGCTTCAGGAATGGGTGGAAGTAGTTCCTCAGCAAACTGAAGATGCCCATGATTAACTTTAGAAGATTTAACAATTCTTTCCCACTTAGAATCAGGTAATGCTATCTTGGGAGTTTGAGTTGGTGCATATAAGTGGGTAGCAAGATTAGACCGTAAAAGCATGTTCCTAGAGGATACTGTAGGTAAAGTGCGCTTAAGCCAAGACTCTGAAGGAGCTTTTGGTGAAGGAAGGGCAAGAGGATGATGCTTCATAGAGCTTGCATTTGAAGTCTCTCCATGGCCCAACTTCATATGCAATTGACTTTCTAAGTCAATCTTTGTGTCACCTACCACCTTTGAGCAAGTCAAAGTACTAGTATCCTCACTAGATCCAAGAGTGCGTTCTTGATCAAATACTTTGACAGCTGATAGGCACTTTGAATCAAATTTCATATCACTAGTAAACTTCTTATTTCTCCGTGAAACAGGATTCTGAACGAGACCATTAGAACCTTCCTTCACGCTAAAACATTTGCTTTCAGACTCTATTTTCTTGGATTCAACCATTTCTGGACTTGAAATTGCAACCAAATCATGATCTAAATTACTCCCTTGGTAATTAGGCTTTGTCAATTCTTGCTCTTCTTGGCGTATTTTGTTGGAATGATTTTTCATTTCCATTTTTATATCATCATTTGAATTATCAGAACAAACCAGGACGGATGAATCAAGAGAGGCTGAAAGTAGTACAGCCTTGTCATCCACAATACCCAAGTGTTTGCTATCCTCAATTGAAGAATTTATAGGAAGATTTTTATCAATAATACCAGTGTCTTTTCTTGAAGTATCAAAATCATCTCCTCTGTGATTGGTGTCCGAAGAACATGAACTTGAAGATTTGATCTTATGTACAGAATCTACATATAGAGTTTTCTCAACAACAGGGCTTTCACAGCTAGACTCAAATTGAGAGCTCTCAGAGGCTAACGATTTGCTGCAACCTCTGCGAGGTGGATCAATACCATGCTCAGATTTTTCTGCTGCACCAAGGAAATCTTTCTGTTTTGTAAGGCCGGATTGAGAATTTAGAGACATATTCCCATAATTAGCAGTTCTACCATGCTCTTTTGCAGTTCTTCTGTGAGAAGCAATTGATTTACTCTGCACTCCATGTCCTGTAGAACTTGGAATCCTTCCTTCCATTCTTAATCCTGGAACTGGATTCAAAAGGCAGAACCTAGGAAATAGTCCACAAACTTTGGGTGCATAGTTTTCATATCCATCATTATCATCACTTTCATCTTCACTTTCTTCCCTACCAATATCTTGGGCATAATGTGGCAAAACCTTTTGCCATTTCTCATTAAGGGGGCGGGAATCTGCTCCAATTACTACTTTCCTTACCAGTTTTGGTTGTTCTAGAGTAACAAGTGGCTTCCTAGAGTGGTGTTGAAGTGAAGGTGTTTCAGAAGCCATTGCCTTTGCTGCAGGCAAGAACCTGCCAATCATGAAATCACGCGCCTGTTGATCTGTTGAAAAACTTCCAGATGGCTGGACCTCTTGATCATCCCATTCACTTAAACCACTCACACTACAGCTCATGAAGAATGATTCAGTTCTGGAAAGTGTATCAAGAGCATCTAGATATGTCTCATCGTCATTGTCTGAACCAGAACTTGCCTTCTCCTCAATTATTTCTTTGGGGCTTTCATGTTTTGTCACTTTATCATCCAAAGAAGCAGCATGCTGAGAATCTGAAACACTGCTTCCTGTCCTGGATTGAGTAACTGATGTAACTTTAGGAACTTTATCAGAATCTTGCTGATCAACTTTCAAAACCCTCCCAGGTGGGAGCTTTGGAGCAAGGGAAGGCCGTTCAACAACCTGTTTTTGTAGTCTGCTTTCATTTTTAGGCCTACCAGGAGTCTTCTCCCATACAAAAGGAACAGTTCCAGGGTTAGTCACTGGACCTGATTTTAAATCTGATTTGTAGAAAGGAAGAGGAGGTCTTTTGGGTACTGCCTTATCTGGTTTCCTTTTATTGTCATTTTCAGAGGCCACTGTTGATGAGATTCTCCTCACTGATAAAAGAGGCTGATTAAAATCTAACTGTTTTTCTTCCATTAGAGTCTTTAGTATCATCTAATTGGTTTCTTGGTAAAATGACCAGACCCGGAAATCAAATCACTCTCTTCCTCTGGAAAGTTGGTGAAGGAAACGTTCAAAGAATCAAAGGCAGAGTCAAAAGGGAGGAAGAAGAAGAAGATAGCAACCGAAGAAGTTTCGTAGCTTAAAGAAGAAGAAGAGAGAGAAAGAGAAAGTGAGAAAGTAGTAAGTGTTTGAGAATAAACTCGTTTTGTTGGGTTGTGTTATTGCTTCAGCTTAG

>C101545_65

CAGTATTATTACAACGTTCCTTTACCAACGTTAAAGCTGTAGTCAACAATTGTTGACTAATTGTCAATTGAGGTCTGAGTTTGCGGTTTCAGTGAAAGATCCCACTCTGTTAGACTTCTGATTCTGATTCCATGTTAGGGTTTTAAAGGATCCAATTCTAGCTTCCTCTTTCCCATGGAATTGAAAAAGGGAAAGCTTGTCAGGTTCTACTCGGATGGCAAAAAGCATAAGAAACCACTTTGGGGAGCAACTGAGCCTGCAGCGTATAAACCTAGCAATTTGCTACCATTGGGGAAAAATAGAATTGCTGAAACATTTAGAATTGGAGGATCGAAGGTATTCCCGGAGGACCACGAGCCGTGGCGTAAAAGAATACTTGACCCTGGTAGTGAGATTGTGTTGAAATGGAACCGGGTCTTCATAGTTTCATGCTTGGTGGCTCTTTTTGTTGATCCATTGTACTTTTATTTGCCAAGTGTGGTAGAAAATACCGGGTCTTCATGTGTAAGGACAGACCTAACATTGCGCATTGTTGTGACCTTTCTGCGCACTATTGCGGACCTTTTTTATCTATTGCACCTGATAATTAAGTTCAGAACAGCATATGTGGCACCAAGCTCACGGGTATTTGGCAGGGGAGAGCTTGTCATGGACCCTAAGAAGATTGCAAGGAGATATTTCAGATCTGATTTCTTCATTGATTTTATTGCAACACTCCCTCTACCTCAGATGGTCATCTGGTTTATTATCCCAGCAACAAGAAGCCCTCAAACTGATCACAAGAATAATGCACTTGCATTGATTGTTTTGCTCCAATATGTTCCAAGATTATATTTGATTTTTCCATTAAGTTCTCAAATAATCAAAGCTACAGGAGTGGTTACGAAGACTGCTTGGGCAGGAGCTGCATATAACTTGCTACTTTATATGTTAGCTAGCCATGTTTTAGGGGCTTCTTGGTATCTTCTATCTTTGGACCGATACACTACTTGTTGGAAATCCTTCTGCAAAAAGGAACAAAATCCTAAAAATTGCTTTGATTACCTGGACTGCAGTTCTTTGAATGTTGAGCTGCGCAAGATATGGGCAAATAGTACAAATGTGTTTAGCAGCTGTGATCCCAGTAATCCTGATATTAATTTCAAGTATGGAATATTTGAAAATGCAGTAAAGAAACATGTTGTTTCTTCAAACTTTATTCCAAAATACCTTTATTGTTTATGGTGGGGACTGCAGCAATTAAGTTCTTATGGGCAGAACCTAGAAACCAGCACGTTTATTGGAGAAACATCATTTGCGATTGTCATTGCCATCTTAGGTCTTGTATTATTTTCCCATTTGATTGGGAACATGCAGACATATTTGCAATCTATTACCATAAGGCTTGAGGAGTGGAGGCTCAAGCGAAGAGACACAGAGGAGTGGATGAGGCATCGCCAACTCCCTGAAGATTTGAGAAGTCGTGTTAGACGATTTGTTCAATATAAATGGCTTGCTACTCGTGGAGTTGATGAGGAAACCATTCTACGTGCATTACCTGCTGATCTTCGCCGTGATATTCAACGCCACCTGTGCTTAGACCTTGTTAGAAGAGTTCCCTTCTTCTCACAGATGGATGATCAGCTTCTGGATGCAATATGTGAGCGGCTGGTATCGTCTCTGAGCACTCAAGGCACCTACATTGTTCGTGAGGGTGACCCCGTAACTGAAATGCTTTTTATCATTAGAGGTAGACTGGATAGTTCTACTACAAATGGTGGCCGGACTGGTTTCTTCAACTCAATCATATTGAGACCTGGAGATTTTTGTGGCGAGGAACTACTTTCTTGGGCTTTACTCCCAAAATCAACCATTAATTTGCCTTCTTCAACCAGGACCGTTAAAGCCCTAAGTGAGGTTGAAGCTTTTGCTCTTCGGGCTGAAGACCTCAAATTTGTTGCCAATCAATTTAGACGCCTTCACAGCAAGAAGCTGCAGCACACTTTTCGATTTTATTCACACCATTGGAGGACTTGGGCAGCCTGCTTTATACAGGCTGCTTGGCGCCGATATAAGAAAAGGATATCTATGAAAGACCTCAGTTTGAGGGAACCCATTCCTTTAGACGAGACAGTAGCTAGTGAGAGAGAGCATGAGGACTATTCTACCGGTTCAAATTCAACTCAAGCTAAATTGAACCTTGGGGCTACAATTCTTGCTTCGAGATTTGCTGCTAACACACGAAGAGGAGCCTTGAAGATTAAAGATGACATGCCCAAGTTACCGAAGCCAGAAGAACCTGACTTTTCTACTGAAGCCGATGATGATTAGTTTCACATACTTAGTGGCATTCGTTTTCAGAGTCAGTTGCAATCTACACATTTCAGTATAGATAATGAATCTGTAGGTCACAAATCCGGCGTATCCCCCTCTGCTAAAGACCCTTGCTGGTAGTTGATGTATTTTAATGAGTAAGATACAAAAATGGGATATAAGGTTATGCCCAGCAAATGGCTGTAATGTATATTACTCAGTGGTGTTTAATCTGATGTATAATTTTTACAAAAAAAA

>C101553_65

TTCCAGTAACATAATCTATGTGACATTATAAAATATTACAAAATTACTTGCAATGAGGAAATCATACAAAACTTCAAGCATTAAATCTTGGGGAACCTGCTAACATTCTTAACGCTATCATTCTCTTTAAAATAATTTCTTCTAAGGGGAAAAAAATTGTCTTTAACTGTTTGATGTCTTTACTTTCTTCGGCGAAGCCTCAGAAAGGTCACCATCTCTTCGTCGTTTCTTCACTTTCTTCTCGGTTGCTGCAGTAGCAGCAGTATCAAACTCACTCTCAGCCTTTTCCTTTTTAGACCTCTTCTTCCTTTTAGAGTCATCATCATCTCCACTATAAGCATTTTTCTTACTTTTGTACTTTTCTTTTTTTGGGTTTTTCTTCGAATTTTGGAAGCTCAAAAATGGTGCCCCGGAACCTAGAGATTGGTCGATTTCTTTCCTCATTTCCTTTACAGTTTTTGGATTTGATTTATTATTCGAGGTGTCAGCAAGAAAGGGATCATTTTTCGTAAACTTTGTATCATTGGAACCAAACATAGTATAAAAATCCTTATAAGTAGATTCTTTGGATGCTTGTTTTGACCTCCAGTGATCAGGACTCGCAGCAAATCCGTCAACATCTAGTCTCCGTAATAGGTAAGAGCCCTGCTTTGTCTTGGCAAGATCACTTCGCACAGCCAACACCTCAGACACTATAGCCTCTCTTAGTGACAAATTACTGGCAGTGAAGCACTTTTCAATGGTAAAAGCACCAGATGAGTGGAGTGCGACCTCTCCAAAATGCCCTTGAAGTTTGGTAACCAATCTGCGCTTCTGTTTCCCAGAAGCTCCTGAACATAGAAAAGCCTCAATAACATGTGATCCTCTTGAATCTCCCACTGCTTCAAGCACGTGAGTTGCTTCCATGGAAGTTATACTTGTGATATAAGGTTGTATATATTCGCTTCTAAACCGGAATACTGTCTGTAAGATCAGAGAACCCACGACATTCATTTTAGCACCACTTTGCCAGCTCCAGCTGGACTTATCTTCATATGAGAAACAACTATCAAGAAACATGAGTCGTGGAACAATCCCTTTTGGAGACTCATCGACTGGACATACCGCTTCAGCAAGGACTTGACAGCACTTGTGTTCATTAACATGAAGCCTTTCACTGGCAGCAACAAGTGAAGCAACAACTGCTGACCTTCCCATTTTGAAAAGACCCTCCAAATTTGGCCCAAGTTCTTCCCAAATAAACTCCATCAGATCTTTATTGTTTGCATAAGAAATTAATGCTTGAACAACAAAGTTCCCATGTTGATGAGATGATAACTCAAACAAGGAATTCCTGAATACTTTGGTGAATAATTCATTGAACAAGGCCTCAGGTGATACTTCCAAAACAACCTCCATTAGATGGCTAAATTTAGATTCTTTTAACAAATTCTTGAGATCTGCAACTACTTTAGTGTCTATAAAATTGTCCTCTGCATTATTCTTATCCTTGCAGCCAAGAAGGAAAGGAATCACACGTAACAACTCTTCATCATCACCTGCCAATACCCTCAGCATTGTCTGAAAAACCAAACTACTAAATTGGTCAACTTGTAGTGTCTTAATACATTTCTTAGCATGCTTTAACATTTCTGACACAAGTAATTTCAACAAATTTGGAAATCCAGGCTGAAAATTTGTTGCATCATCTTTCTTTGAAAACTCCTTCGAATTTAACCGGTCTGCTAATACTGTAGTTGATTTTGATAAATAATATCCGCACTTATCTAATGGTACTCCTCTCAAGAGGCACAGAAGAGTCCGCAGCACATGAGAACCATAGCAGTTACACATTACATTAACGGAATTTGCTGCAATCACCTTGCATATCACGGTTAAAGCCTCTTCAACCACAGGGCGAGCCTCATCGTCTTGGAGATGAACGGCTAAAGACTTGATGGCGGTCTCAGCGACATGAGAACCAGATCTATCCATTGCAATGTAGGGGAAGTCGTTAGCAGAAGTGTGAAGGAAATCACATAGGTGATCTACATCACAACCCTGGAGAATGGTCTCTAAAGTGTGGCTAACAATATAATCGGTTGCTATTTCGAATTCTTTCCCCTTGGTTTCTTGGAGAGCATTAGCACATATCAGTGACCGTTCCTCCAATTGTACCCCCTCAGTTTCAAAAAGATTAGCAATTTCTGAAAAGTATTGGGTTGTTTCAGGGTCAAGCTGTTTCCTAACACGGGATTGAGGCGGTGAAGTGAAGTTGTTGGAGTTAGAGTTGGTGTTGGTGCGTTTGGGATTGGAATCGAGTTCATGAGCCGCTCCTCCTTTCTTATGCTTTCTCCTCTTGATTTTGGTTTCTCTGAGGGATTCATAATCGTGGTCGCCCATCGATTTTCTTGACACCAACCCTTTTGAACCAACAGAAACCATAAGCACCCTGTAAGTTGAGGTTGTGGTTGAGGCGTATGCTACTGCTGCTGCTGTTTCTGCTTCCGGCGGTGGCGCGCGACGGAAGGTATGAAATATCACTCACAATTTGAAACCAACGTTGTAAACC

>C101557_65

CTTCATAATAATAATAGACAACTTAAGGTTAAATTCTGAAGTTTTTGTTTTCTTTTCAACAATCTTCTGAAACTAAGTAGTAAGGATTGGTGGCATGTAATCAGATTTGACCCCAAGAACAGGAGCTTTGGTGTCAGGGAAGAACTCAAATGCTTTCAGTTGTGTCACACTTCCTTCGCTGGAAATTGCAATTGGATAGCGAAGAAGGTGCCCAGGAAGGTCTTGTTCAAGAGGCTCAGTAGTGTAGAGATCCCAATACTTGTCACTCATCTGGTTTATCTTCTTAATACATTCTTCATTTTCAGGGTGGAGGAAGGAGTCATCAAGCATGCCAAGGTGCTCATACCACAATGACATGCGGAAACCATGGATCTGACCCCTTGCAGGCTGCATTCTTGCTAGATGATAGGGTTGATATGCACCCATTGCAACCTCAGAGTCTCTACCACCATCCATTGATCTCTGGTTTATGTTGGCAGATCCAATGATTATGTATTCATCATCAACTATCATCAACTTGGCATGAACGTAAATCATGAAGCGTCGAGATTCTTGAGCTCTCATATAATCTGTATCAGGATCTGGTCTTTCTGGAGGCTCGTACTCTCCTTCTTTCTTCACCTCCCGATTACCAAGGCAGAAGAATGTCAAATAGTTCCGAGGATCTTCCTGAATTCCCTTGGCCTTTAGTGCTCCAACAACATCCTTATACATCATATCCATTGTTCTCCTCTGCCAGTCCAATATTGCCTGAACTGTTCCTTTCTCCGGGTAACCTTCTGGCCACATTGGAACCACAATATACACACTGAATCTTTCCCCAGCTTCAATCTTACTAACAATCTTAAGTGAAAGCTCCCTCGGGATGAGATGCAATGCACCTATGTCTTCAGGCTTAATGCCATCAGCACTCCAAGCATAGCAGCTACCAAGGAAGTACTGATTCTCAATGTAGATGAAATTCTTTGCACGTCGAATGGCGTTAATATAAGCATCTTGAATCCCTCTGTCAATTATATTATCCTTCCCACTAATGAGTCCAGATTTAGCAGCTTCTTCAGGAGTCTCGGGGAAGCCAAAAGCAGCCCCACCATCAATGGATCTAAATAACTGCACGTTCCATGTCTCTTGATCATCGGGGTAAGTTACTGCGGAAGGAGGAATGATGACATCTTGGAGGTCTCTCAGAGGAATCAGTAAGTCCTTTCCACCTTGCTTCTTCCATCTTTGCTCAAAGTTGAATAAAACATCCCATGCAATTGGCCCTTCAAGCCGAGAGTGAATGTCATGCCAAGGTTCCCTTGGACCACCTTTTTTCATTGAAGTATGTGGAAAATTAGGCTGATGAAAATCATCATGATGTGCTGTGCCCAAGGTTCTGAAGAGTGAGTGAAATTGAGTGTCATATCTTCCATCACAGAGGTCAATACCCCCCACAAAACTCACAATTCTTCGCTTGTCTGATCTTCCACTTGGCAAAGCACTGTCCACTACCACAATTTTCTGATGGTGTGTAAACATGGAGAAAATTTCTATGTCCTGAACAACGCTTCCACCATCATCAGGGTTGCGAGGGCATAGAACACAGTGCACATCACTGTCGTAGAAATAGTTCCCGGTGTCTTCATCATGAGTAGCCATCAACCCATCTTCTTTTAACAAAGGAACCGAAGTTCTATCATCCCAAACAAGCATCAAAACCCTGACACCGTCACGTGCTTTTTTCTTTAGAAGCTCACCAAGTGTTGCATCTCCTCCAGACCTTGGTCTCCTAGAATCTCTTACCAACGTAATCTCAGTGTAAACAGACCATCCAGTAATGTATATTAAGTGCTGTGCGTTGTAGATTGCATCAAAAACATCTTCCCAACATCTATGAGGTTGGTAAATGTGGCCTCCAGCAAGTGGAATTATGGGAACAAAGTTGTCTGGAACATGAGCATCTTGGTACAAAGAAACCCTACACCCTTGTCTTTGTGAGAAGTAAGTGTAAGGAACCCCGGGGAACTTAGGACTTCTAATGCCTTGGCCCCAGTTGAGGTCTTTTGAAACATCAAAGTACTGCAGTTTCACGTGGATCCTAGAATTTGAATGTATTGGTTTTTTATGTTCATCCAATATTTCAACCCATCTATCTATTTCTTTGCCATCCAAGATCTCCTCAACGGGCACATATGCTCTTCCAATCAAGGTTGCACCAATAGGGTTGTCATCTTTCACTGTGAATATGATATTTGATGCCATATGAGCACAATATATGTGAAAAGACTCGCACCATTTAGGATTTGTGTGCTCCTTTTCTATAATTCTAGTTCTTCCCACGCGTGCCTTCTCTAAATCAATGGTTGCATAAAGTTTAGTAATTCCTTTCCCAATCCCAATACTCTCCTCCATGTTATGTATCAGCTTGGTTAAAATATTACCACCATTACCAGCCTTGAGGTTATCCACCTCATAGATTGTGGCATGGAGGGTCCCATGAAGCAGAATTTGTGCCATCACTTAGTTACCTTTGCAGAGCAGAAAGATTAGAATTAAATAATGAATGGATG

>C101563_65

TTTGCGATAAAAGGGTAAAATATATATACTTGGATTGTTTGGATCACTCAGTACAACACAAAACTGTTAATTATTCCTTTTCCCAATGAAAAATAACGAAGTCAGCCACATTTAAGATTCTAAAACAAATTAATAAGTCACGTGAAACTTTGATACAAACATCTTCCCATGCATGACTGTGTTGTTTCAAACTGTGATGGGATTTGATATTGTGGATCGGAAAATCTCATACATGAGTCTTAGCTGTGATGGTACCAGTACTATAAGTGGTATGCTTCCGAAAAAGGCGCCACAAATAAAGAGTAGTTTATAGGCAGTGTATTTGTGTATGACTAAAGCGAGTGCAGCACAAAAGACAACAATCACGGACACCACAGACACGAAGAGGAAGAAATAGCCGAATAATATCTTCAAGGGCAAACTTTTTAGGAAGTCAATTTCAGTGAAACGTGATGTCTGGACCTCCATGAATATCAAGACTGAAGTGGCAGAAGTGAAAAGAGATATTGCATCTGCTACCACAAATATTATAAATATGGTTTCATTTAAGAGAATGGGAACACCAGTTTCTTGATTGTTTCCTCCTGGGACAGTGAATGCTGCAGCGAACGAGATAGTAGCAATGAGAGTGCCCACAATTGTAAAAGAAGCTGCTGAGTCCTTTGCCCATTTCTCTCCTTCTTTCACCAGCTCCTCGTGCGTTTCAGTAAATAGCTCATGAGGCTTCTTATCGTCCTCATTTTTCTCTTCTTTATACTTGGGATGCACAAGTTCCTCCACCGCCTTAAACCATTGAATTTCCCTTTGCATCTGCAAAGCAGCACCCGGTCTGAGATTAATATCGGAGAATTGGCCTAAATGTCCAGCCAGGTGCAATAGATTATTGCCAAACATGTCTGTGCTACGGTCAATCATTATATTCTTCTTTAGATTTAGAGAATGTATGAGTTGGAACACATTTTTTTTGCGATGAAGAACGGCATACGAAAATACGCTTCTCCCACGACTATCCATGGCCCACAAGAGGTCAGGGTTAGCCTTCGTCATAACATTTATAAACTCAACAATTCCATACCTGGCTGCATCCAACATAGCACGGTGCAGCTCTGAATCATTAGAACCCTCAACTCCAAGTGTGAAGTAGAGTAATATTTGTAGAACTTGATAATGTGTCCGTTTCTTTTCATAAATTTTCCTAATTCCGAATATATTTAGAACTTTGAAAAGTTGAAGGAACAAACAAACAATTTCGGCAAGTACCCAACCCAAAAGCTTTAGGGGCAGAAGCAGAAGATTCATAACCGAGCAGCAGCAGCTACCCAAAAAATTCTTCTTATGGCGTCGCTCATCAGGCAGTATTGCAGTGGAAGCAGACTTGAAGCGGTTTTGATTGTTGTTTTCTACTCTTAGCATGCTGTAAACGAATTGCTCTGGAAATCGAAAGTTGGAACCACTGAAGAAGGCAGAAGGCATTTGAGCCAATTTATGCAAGGGGCCAAAGGATTCATTGTGCCATGGCATGTTTGAATCAAGTATGCTCAAAGCTACGTCGAATATTTCAGCTTTGATACATCGTTCAAGCAGCAAAACTCGAACGTCGGAATCTTGTCCGCTGAGAACGTCGTGCGGAGTTTTGCTGTAGAGGTAGCGAGTCATGTCTTTGTATCCCATTGCAGCTGCCAGAAGCAGAGGGATTTCACCGTTATCATTCCTTGTACCGAGCAGTTCCTTGCTGCCTCTTTCCACCATGCGCTTTGCAATAACCACGTCTCCGGTGTAATGAGCAGCGAGGGCAAGGGCTGTATAACCGTTCCAGTCCTGCATTCCTACTAATCTCTTTCCTCCCAAATCCGTTAACTTCTCCACCATGTCCACATTCCCTGCCACTGTTGCAATGTGAAGAATTGTTCGACCTGAACCCGACACCGAAGACACCGCATTCATATCATTGTCCAGGAAACGCAATACGCTTTTCCAATCACCTTCTTCCGCATACTTGAACAGAGGTCCACTCGCATGAAAACGATCCTCTACCAGACCTTGTTGAATATTTGGTAGGGCTTGACTCTTAGAAAAAGAGTCGTAACGTGCGGACAAGTAATTCCAAGCACTAGCAGCAGTTTCGAAATGTTTAATTTCATCGAAAATCTTTGATCCACAGGTTACCAGAATGATATGCAGAGCCTCTCCATTCATCCTCCGAGCTTCCAGTTCAGTGGAAGCAGAACACCTCTCCACAACAACATCCCACAGATTCTTCCCTATCAGATAGCTTTTCACTAGAGTTGCCCATTCTTCGTAGTTGTTATATCTTGTGAGTACTTCACGAACTATTCCGCTAGCTGCAACATTTCTACCTTCCATCAATCAAATTTGGCTCCTTCAGTTCTTCCACTTTTAATTCAGAAAGAAGAAATATGAGTCTCACCAGAAACTAGGAAGACAAACTTGAAATTTGCAACCCCATTTATTCCTTGCTCTGGGAGATGTATTTAGCACGTATAGACAAACTTGAAACTTCCAAGTAGCTTAATCATTGAAGTCAATTTCAAA

>C101565_65

GAAACTTAGTGGACATTAAATGGAAGAAGCCCTTCCTTTCCCCGGCACTTTCTTTTGGTTTCAGTCTCCATTCTCTCTCTTCTTCTTCTTCTTCTTATTATTATTATTATTATTACTAACAACACTTTACACATTTCACAACACAAACACAAATCTCTCTCCATATTCCATTCTTTTTATTACCTTCTCCCTTTCTCTATTCTCAGATCCATTGCTTTCTCTCACAGTTTACGCCACTGCTTCCAAACACGCCCTTCTCTCATAGTTTCACCAGCTAGCTAGAACTTGCTCTCGCAAATGGCAGCTTCTGGGGCTCATTCTAGCAAGCAAATGAGGAGGTTGGAATCGAGGAAGTCCCATTCTTGGTGGTGGGATAGTCACATCAGTCCCAAGAATTCCAAGTGGCTTTCAGAGAACCTTGAGGAGATGGACCGGAGTGTGAAGCGCATGTTGAAGCTGATAGAAGAGGATGCAGATTCATTTGCCAAAAAGGCTGAAATGTATTATCAGAAGAGGCCAGAATTGGTTGCCCTGGTTGAGGAATTTTACCGTGGTTACCGGGCTTTGGCTGAACGATATGATCATGTGACTGTAGAGTTACGGAAGAATATACCCTCTGATCTCCAATCCCAAGGTTCTGGTATTTCTGATGCTGGTTCTGAACCGTCCTCCGCATGGCCTTCTCCAATTCCAAAGAGGGGAGGTCGTCTTAAATCTAGTAACCGAGCTGCTGGGTTTGATTACTTTCTTGGTTCTAGTGGAAATGGTTCTGATGCACAAAAAGATGGGGATGAATCCTCTACTTTGACTGATTCTGAGGAGGAATCTGATGATTCGTCAGTTAATAATTATTCTGGTTTCTTACAGAATGGTAGTGATCTGGGGATAAACAGAAGAATAATGGAGTTGGAAACTGAGCTCCGTGAGGTAAAAGGAAAGCTATGGATGCAGGAAGAGGAACAGGCAGAGGTTGCATCTAGGGGATCAAGAAATGAGAATAGTGAAGATTTTTACTCCAAAATTAATGCATATGGACAAGAACTGATGACTGTTAATGAGAAATTAAGGCTTTCAGAAGAAGAAATTACTAAACTGAAGATTGAGCTAGAAAAATACAGACCCTTCAATGCAGAAAGTATGGAGGTTGGTTTTGAGTTTTCATCAACTAAAGAGCACGCCAATAATGGAGGGGAAGACCTGGAACAAAAGATGATAGAGGTTGACGGAAGCATTGATGGTGTGGATAAGGAATTGTTTGAGCAAAATGGTGAGATTGAGTCACTTACAAGGGAGCTAAGAATTACCAAAGAAAATCTCAAAGCTTCAGAAAAACAAATTACTTCACTGAAGTTTGAGGCCAATAAATCATCTGAAAGAATTCAGCAGTTGCAGGATCAGCTTGACTTGGCTAGTAAAGAGATTGCTACGTGGAAAACCAAATTCAATAGTGAGAAAAGAGAGAGCACCAAACTCAATGAAAGACTTGTAAGGTTAAGGACTAGTCTATCAGACCGTGACAATGAGATTAGGGATTTGAAAACAGCAGTATCTGATGCTGAGCAGAAAATCTTCCCGGAAAAAGCTCAGTTGAAGTCTGAAATGTCAAAGTTGCTGGAGGAACGGACACATGTAGAAGAGCAGATCAGAGACTGGGAATGCCGAGGCCGATCATTTGAAGAGGAAATTAGAAAGATCCAGTCTGAAAAAATAGAGATGGAGGAGGCACTCAAGGGTGAGATTCAACTGTTAAAGGCTGACATTGAGCAGAGAGAGAACAACATAAAAGATCTCAACACAAGCCTTGATTCTCTAAAATTAGAGAAAGATAATCTCCGTGTTGAAGTCTGTTCGCTCAAGGAAGAGGTGAACTCCAAAGATGGTAGGATTGAACACCTGAACAGCCATTTGAATCAGCTGCACATGGAACATGTACAACTGATTGCTGGAATGGAGGAGGCACACAAACAAGTGGAAGAGCTAAAATCAAAAGCTAAGCAACTTGAAGAGGAGGTTGAGAGGCAAAATACTGTGATCTTAGAAGGAGCAGAAGAGAAACGTGAGGCCATAAGGCAGCTATGTTTTTCGCTCGAGCACTATAGAAATAGCTACAATGTGCTTAGACAGCATGTTATGGGGCACAAACGGGTTCCGATTTTGGCTGCCTAATGAATAAATGTGACGGTGATCTTGTATATTGTTTTGGTTATCTTCATTTCTCTGTCTTGGAATGTTTTTCTTATGCTTATGATTCTAGTGTATATGAGTCAAGGTCATGTGTACTAGTATGGAGAAGTGATGACCATGTTAGTTTTACAATACATATGCTGAGGCCACTGCCAAGTTTCATCGCTTTTTGTAAGAAGCTGTGCCATAGCACCTTTCTTTAAGGGTTCATGAGAGATTGAAATTTGTTTAGTTTGGTCTTAATATTTGTGTAACACCCACCCGTCTGGTCTGATGTGAATGGTTGTGCAACCTTTTTTTATTTATTTTTCAAAGTTGTTGCATGCTGGAATTCTCTTATAGTAAATATAAAATGTTATTGGTAATGTA

>C101567_65

TTTTTTTTCATTTTTACTGTAATAAATATTTTTCATTAAATACATTCAATCAATCACCTTCATAAAGCTAAATTGCTAAACTTATGCAATACTTTTTTCCTTTATCTACCCCTCACACAATATGCTAAACTTGTTGAACTTGATCAATGTCAGAATATAAAGGTTTCTATTTCACTAAATAATTGGTGAATTTTATTTGAGATTTCCACCGTCCTAAAAAACTGGTTTCAGAACATTAACTTTGCCCAGTGAGTAATGCTCAAATGGTGTTCACTGTTCTGTAACCAGCAGTTTCTTCAGCTCCTTTTTATTTACCTTCCCCATGGCATTGCGAGGGAGTTTGTCCCACACGATTAGTTGAGTTGGAATCTTGTAAGGTGCAATTTTGTCTTTGGCCCAGGTGGATAATTCTTCTAGGCTTAGAACAGGCTTTGATTCTTGATCTCGCTTCCGTTTAACATCAGCATGTGGCACAATGATTGCACCAACAATCTCTCCATAGTCTTTGTCTGGTAATCCTAAAACGCAACATTCTGAGACAGCTGGATGCTCTATGATAACTGATTCAATTTCTAATGCAGACAGCTTATAGCCTCCAGCCTTGATTATATCAGCATTAGTACGTCCTAAAATGATGTAATATCCATCTTCGTCTGTAGTAACTGCATCTCCAGTCTTAAAGAATCCATCATCAGTAAATGATTCCTTCGTTACCTCAGGAAGTTTCCAATATTCTTTAAACAATGAAGGGCTTTTAACGCAAAGCTCACCCATTCCAGTATTATCATTCTCATTCTCTTCATCTGCAATAATCTTGACCTGTATACCAGGAAATGGTTTGCCCACAGTTCCTGGCTTGCGCTCACCTTTCAATGGATTCGACAATGCCATAACAAATTCAGTCATGCCATATCGTTCCAATAAGCGATGGCCAGTGATGTCCTCCCATTCTTGCATAACAGGTAGTGGAAGTGCAGAGGAACCACACATCATAAGACGCAAGTTATTTGCAGCAGACGCTGAAACATCTTGTAGCTCAGGATCCATTGCATGATAACCTTGTATCAATCGAGCATAGATAGTAGGAACTCCAGTGAACACAGTTATAGCATCATCAGCCTTAGAACCATCAGTTGGATATGACTCACGCCATCTCTGCCAAACTCCCCTCACACTAAATTTTGGCAAAAACTCAACCGTGGAACCTGCATAGAGAGGGGCCATTAAACCATTGAAAAGCCCATGAACATGATGTAGTGGTAGACAATGCAGAAACTTGTCAGCAGATGTGTACCCCCATGCTTTTGTCAATATTTGGACCTGGGAAAAGATGCTTCTGTGTGTATGAACAACTCCCTTAGGTTTACCTGTAGTCCCACTAGTGTACAAAATCAGTGCGGGATCTTCACTTGATCTTCCAAAATTATCCAACAAAATTCTGTTTGCATGAATTTCCCCATTCTGTGAATGTTCATCTTTGCTTTTCTCTGAAGACTTGTTGCCAACAAGTGGAAGATGAAAAAATTGAGAAGAGCTTTTATCGGCTACCCTTTGCATTAATTCACTATGCTCTTCAGTACTCAGTATAGCAGATATATCCGAATTATTAATCACGTACAAGAGCTCAACTTCTGGATAGCTGGTTGCAAGGGGAACAGCAACACCTCCACTGAGCCAAATCCCAAGTATTCCAGCAACAAATTCAGCAGAGGGTTTGGCTACAATTCCTATTCGAGCTCCACCAAGATTTCCTGTCTGTGCATCACTGGCACACAAGAGATTTGATATCCTCTGTGCTGATGACATGAGTTGCTTGTAACTATAACTCTTCTGATCAGCTCTGATAGCAACATTTTCAAGCGCTGTAGGTTCTTGCTTAGAAATTGCTTTGACCACCTCCATTAATGTAGCAGGTGCAGATGAAGAGAGTGGACGAAGCTGGGAGAAAGAGAACCTGGGGTACGAAACAAGGTTCGGGAAAAGAGGGAAAGAAGAGGAAGTTATGAAGGCATGGGAAGAAGCAGAACGTATGTGTAGAGGGAGAGGGAGGAGAGTGAGGTGTTTGTTGAAAGCTTTCAATGAGCGACTCATATTTAGGTGGCTGGTTGATATTGTGACCAACTAATTGAGCGGTGGAGTTAGGTGGAAACTGCGTGCTTCTAATTCTATTTCCCACGTTCAATCTTTTACCACCTTAATTTGTCTGCTTCTAAGTCAAATGTGGCAATTTCGGAGGCTATAATAAACCTTATTCAATTCCAATTCGAATACTAATACTGTTGCTCTCTTTTTTTTCTGGGTTTCCTCTGTTTTGGGGGACCTGCTGATTATTGTTTCTTAGGGTTCTGCACACTGATTGGCACCATGATATTAACGTGAACGTGATTGTGTTACCACTTGCAAGTTGTTACCACATCTCGCACTTGTGCTGTGCCTTTTGATGATAGACTCCTTTGTGGTTGGAATTAGCTTAATACAGAAACAAAAGATCTCTTTTTCTTCTGTGGTATCGATGGGATTCTCATAGTACTCAAAACAAACAGTGAATTTCTT

>C101569_65

TAATAATATTACTATACACAGAAATCGTGTACTGTTAAAAAATCATGCACCCAATGTTTATTGTCCAAAAATATAAAATAAACGGGACACACAAATGAGGCCAAAATCGAATGATGCTATTTATGAGGGATAAAAAAAATGGTCCCGAAATCATCATTTTGTATGACTATATACAGTTCACTGTTATAACTATCTCAATGACTTCCTATGCAACTTGGATATGCAAAATGCTTGATATCTCAGTTAATCTCAATGGATGCTTTGTTCTGAGCTAACTTGAAAACAGTGCTCCTTAACACCCAGTATTTAATATGATTGTGGTTCAATTCAATGAATGTCTAATATGATTATATTTCTCCATCTTCATCAAGGAAGATCTCTGGATGTACTGCTGCACTAGTGAAATTTAGAGCTCTTTTTAGCTTGCGATGGTCAAATACTTCAACTTCAACCAAGAAGGCTGAACTAACTGGTCTATGATCTGAGAGCTTAATTTCTGCACGTCCATATTGTAGTTGTTTTATTCCTTTTCCTAGCCACAATATACGATCACACCAGGCTGGAGATCTCCTTTTTGCCCCTTCTTTTGGGCTCTGACCGACATATCTATCAGAGTTAATTTCATACTTGTAAGTGGGTGGGAAGTTTATCAACCCCTCTTTCCATCCATCAAACACATGCCCAATACGCAATTCTTTACTTAGCTGGTCATAGTTCATCAACTCATCCCACTTCTTTAGAGCCACCAATTTTCTAACCTCAGCATCCAACATATTGATACGATAATTCAAATCACCGAACCAGAAAATCTGGTCGTGAGATGGGATTGTTTGTGGTTGATCTGCATCAAAGGCAGGCGAGAAGCAAGTGCGTCTCAGAATTTCATGAACATCAGAGTTTCTCCTATTTTCAGCCCCTTCCTTCTGACCAGAACTCAAATGGGAACAAACAAAGCACAGACGTGACTGAAAGAGAGACATACTAATCGAAACTGACCCCTTGTTTCCCATGTAGCCCATAAGACCAACTCCAACCGGGGAAACGTTCAAATTGTTAACGTGCCTTCTCAATCTCCTCTGAACCCAAACAGAGACATATATCCCTACCATTTGCTTGCTCACAATCCGGACATACCTTGGACTTCCACAAGATTTCATGGTACCTAGTTCACTATCATCTTCGGCGTTCGGCATTTCAATAAAAGTATCGTCACCCTCAGCTGATAACACATCAGATACAGAATCAACTACTTCAGGTATCGCTTGCTGTTCTTCCCACAACAAGCCCAAGTTTCCAGAACTGTGGTGTGAACGTTTCAGTCCAACACCATATATCAAAGTACTTTCAGTCCTGTTAAAGCCAATTCTGGCTGAACTGCTTAGTACTCTCCGCAACTTTGAGCTGGAATCAACAATTTGGGGGATTGCATCTAATGAGCGTTCAGGCCAGTCAATATCAATGCCATATATTCTCCTCAACTGTAAGTTATTCTCTATATCAATTATGCTCTTCACTTCCTGCTGTTTCACCACGTTATTGTCAACTGTTCCTACAAATTCCTCAATCATCATATCTATTGGAATTGGATTATCAACATCTATAGTGTCTGCTAGGAGATCAGCAGCAGAAGAAGTTCTTAACACTGGAGAAGGAGGGGCACTGTAGCTTTTGTGTTTACTGTCAGGCTCAGAAGATTTGTTTAGAGATCTCCTAATGATTGCCTCCCATTTTTGGATAGGTGTATTATCTTCTGCTCCTAGCACATTTCCGGCACTCAGTGGAACTACCTCCTGAAAACCAAGAATGTAAATATCAGCTGGGTCTTCAGTACAAAGCCAGTCATCAATTTCAAGATCTTCATAAGGATGTCTTCCAGCAACATTCCACGTGCCAATTGTTACCCTCACTTCCTTTGTGTTTATGTACTGAACGCGCAGAGTTTCTGATTTTCCTCTTCTGTGTCTTATCTTCTGGCCCTTACTAGATGCATCTGATGTTTGACTTGAGAATATGGATGGAATCCCTTGTGTTCTAAGGGGGGGTTTATCTTCACGCATCCGTGAGCCTCTAAGAGAGCAAGCATCATCTTCACTCTCAGTTTCAGTGTCCACTTCATCTTCACTAAAATCATTGGCCTTGGGCTTGATGTTTAACCATTTCTTCATCACAATGGAAGGCCAGAAAGCCTCAGAGCGTTTTCCTCTCTTTGCCTTCATTGTTTGAGGTCAAAAGACCACAACAGAACCCAGAAGCACTATTGCGTATTATGCTGGACTTTGGAAGCACAAGCTCTTATATATGGAATCAGTCACTGACATTTAGTTGGTCCATTAAATGGATGATGCTTAGCTCAAATGGACAACTAAAGTAAACTAAGATTTCATCATGTCTGTCTGGGACAGCTGTAGACCCATTGGTTAGTTGATCCACCTTATATGGTTATTTATATTCTTTCTCTCTCTCTCTCTCAGAAGGTACTGATTTAGAAGCTGACAAACTCTGTAATTAATAAAAAAAGAATGAAGAAGAAGAAGAAATTGTCCCACAAAGTGA

>C101571_65

TTACACTTCCAAAATTTCCCTTGCGATATGACTTTGTCTGTAATAACTTTAATGAAGGAACAAAATAATAAAGATTTCATTGTATGCCAATAAAAATCTTAGTTCGCCAGTGGAGTTTTGCCTCTGATAAGTACCTTCCCTCACAAGCCAATTTGGACAGAAGGACAAGAAAACGAAACCAAGAATCTTGTGTTACTATAAGCACAATATTATCTTTGAACCAATATATACATACATGCATAGTGTTTTTACTCAACCCAATTTATGTCCAACTGTTTTAGTTCTGTAAGCCATTTTTGTGGATCTTGAGAAACAGTGGGTCTAGATGCTGTCTTGATCTCAATATCTACATCAGCTGATTCTACATCATCCTCAACTTTCTTATCCTTGTCAAGGTTGCCCTGTTTGCTTCTTTGTCTCCTCACCTTGAGGGACAGAAGGCGAGAAACCTGATTAAGTCCACCCCACTTTTCCAGTGCACGAGCTATATCAAAGCGCCCTGCACGTTCAAATGACTTTCTGCTGGGCATAAATGAAGGGTCCATTCCCCAGCTCGTTTGAAATCTACTTATCTCGTCATGCAAATTTTCAAGATTGTCCCAGTAACCCTTTGGTTTGCGGTGTTTGTAAGCCAGAGAGAGGTTCATTATCAATGCAATCTTTCTGAACCCACCCATGCGATTTATAGCCTTCTCAATATCAATTCTTCCATGCAAACGAAGTTGCTTCCTCATTGGCATGAATCCTTCTTGTCCATGTTCTGCAATGAATTTCAGGATTTCAGATTTTAGGACTTCAATGTCCCTTTGTAATCCTGGAACTCTTGGTCTTTGTTGAGATAAGCTTTGAGCATTGCAATCAGACAACTCTTCAGTTGAACAATAGCTGTCATCATCTGCAAAGCCAGATGGAGCAGTTTGCTGCCTTGAATTTGTGTTTTGTTTACTTGCTTCAAAAATATTTGATGCTGTCTTGTTCTCGATATAGTCTCTTATTGCACACAGGTTTGACGGGAGATCTTCATAAATGACCTCTTCCATGATAGCTTCAGAAAGGAAAGTGTCTTTGCGCATTTTTGACTCCACAGAGTACTTTAGCAGTGTATGATGATTTCCTAGTTGCTCAAAAGTCCACTTTCCCTGGAATGAGTCAAAGTCCCCTTCAACCTGTTCAAAACTGATCTCTTGTTCAAAATATTCACACAAGTCTAGCACAACACGAGCATGAAGCACCATATAAAGCAGGCCCTTACACCCTTCCTGGAGAATGCGGACTTTATTGTTATCCCGTGATAAAATCTTACTGATTGCTAAATTTGGAACTATTTCAGGAAGAGTCTCATAGCAAGACATGGCGTTCCATACATCACAAACAGGAGCTTTAACTGTTATGCTTGCAAAAACACAGCGATGGACACCCCCATTTTCCAAAAGTCCATCAAATCTACGGAGATGAATTTCATCCACCACACAAGGCCTGTCAAGGCTGCAAGTTTTTCCAAATGCACCCCAATTATTGATGTTCACCTCACTGGAAGACAAGGGTAAGGAACCAGAAATTGAGAGGACTAAATCTTCTTTGTTTTCTCCAGGAGAGGACAGCTCATTATCTTCACAGAAAGAATTATTTGTCTTTTGGGTAGATGACCTATAAACATTACTAGAAGTTTTATCCAAGTTATTTTCTGGCAGGGAAAGTTTCTGGTTTCCTGAAATATTCCTTTCGACTCTCTGTGCCAAGGCGCGGAGGTTCACAGGAAGATCAGATCTGATAATCCTTTCCAAGAAAATAGCTGGGAAATTGAATCTTGGTATAACATTAACTTCATAAGATACATTAGTTGATGAAGATCTAGTTCCAGATTTTACAGACCATTTGCCCTCAAACTTCTTAAAGTCTCCGTCAACCATGGAAAAGTGAAGTTCTCGGTCCCATGCAGAATTGATGAATTCTTGAAGATCCAACACAACTCGAGCTTCTATGTGCCAATACATTGACCTTTGAAATCCTCTTTGCTCTAACCATATCCGTCCAGGATATGGGCAAGGTATTTTTCCACTCCACACAAGGTTTGGAATGAAGTCAGCGAGATGCTCATAATCAGTGAGAGCATTCCAAATGGATTCAATGTCAGCATTGACAGAAATTTCAGCCTTAACTCTCCTTTCCCTCCAAGAAACCACTTGAACCTCACACTGCACCTCACGTTCACCCTCTTCATTTTCTTCCTCTACCCCATTATGGTTACCATTCCCTACTTCTTTCTCACACCGGACTCTGGTTCTGCACTGAGTTGAGGAAACCGCAATGCCCTTGTGAGCAGAGTGGAAGGGGAAGAAGAGGGTGAGAGATAGAAGCGGTGGTTTGGATAAGAAGCGCAGAGAAAGAGGTTGAGCTCCTCCAAGGTTGGTGGTAGAACTTGTGCAAGTGGTGATCATGTCATGAGAGACAGAGAGAGCACAGTGAGTGGTAGAGAAGCTGAGAAGGTTTAGTTGTTGATGACGCTGTCGTTCATGTCATGCCATTGTTGAGTGAGAGCATGCATGGATAATAG

>C101573_65

ATTTACAAACACCATGAAAACGATTCAATTCGGATGGGGATTTAGATGTAGCCTTCATAAATTACAGAACTGTGACCACCTTGTACCTTGCATGAAAAAAGAAAATATATCTTGCAACTAACCATTCGAATTGCAAAGTAGATTTGACAGATGAGAGGTGAGGAAGTTTCCAAATCGCTCTAGTTAAGAAAGATTTATGCATATATACATATATTTATTCTTATGGAGATATAATTTACCATAAAATAGAAAATGGACAACTAAATTAATGTTCATGTTAGTTGACCGACGAGAACTAGCGAGGCATGTTTGATGACTCTCGAGACTCTCCAAACTTCGTGCTGTACACGATGCCATGATTCTGCAGTATGTAGATTTTTGGCCATTTTAACAGCATTGTCGATTCCAGGGAAAGATTGAGAACCATAGTCATTATGTTTTGATGGTCCATAGATCAAATGTTTATACCATGACATTCCAAAGAGGCCATCTCTGTCAGTGAATGCACGCTCAGCCATCATCAATCTATCATTCAGTTCTCTCACTTTCAAGTGGTCCTTTTTCCATGTTATCCAACTTTTACTTACTTCTATTTCCTTTCTCTGGTCATTAATCTTGATTGCTGCTTTCTCAAGCTCCTTGATAGACTTCAATATAGGTGACAAATTTATGTCTTTATTTGAAATCTCATCCTCCAGATTCTCCATGCTAAGCTGGAGCTCCTTAGCATAAGATAGATAATCAAAAGGTAGGAATTCCTCATCTGCTAACCAAAGTGCCACTAGACCCCAAACACTTGCGCCTGCAACATGCCTTCGAAACATAGGATCCCCGAATTTTTCCATCCATACGAAGTCATCATAGAGTGAATGGTATACTGGGTATCCTGCAATATCTCCTCCAAAGGCTATGTCAGCAGCTGGGATTCCTACATGTTGCAAGAAAGAAGCATAATCTGATCCTCCACTTCCTAACCTCCCAAACAAGGGAGAACTACCGGAGCTAATCCAAGATTCATAAATGCTCTGTGATGAGTTATCAGGGTCTTTGACCTTCTGAGTTGCTTTTATTATCAATTCATCTAGCTGTGGAGTAGCACGAACATTGAACCCCGGTCCACCTACTAGACAGTCAGCATTCAAGTAAGCAACTGCTCTTGAAACTAGAATTTCCCTATTCTCCTCTACCCATTCAGTTGATCCTATAAGGCCATATTCCTCAGCATCCCAATTGCATAATAGAATTGTTCTTCTAGGTTTCCACCCTTTTTTCTGAAGCTTCCCTAGCCTCTGGGCCACCTCAAGTAGTGCTGCAGTGCCACTATTGGGATCAACCGCTCCAAATGTCCAGGCATCCCTATGGTTACCTAGTATGACAAATCGATCAGGCTCTTCTACCCCTTCAATTACACCAATAACATTTTGAATTGTGGCTATAACATCCTGCCCCATGTAAGTAAGGTTCAGAATTCCTGGTCCTGGTCCAAGCCTGTAAACAGGGGCATCCTTGCTTCCCTGCCAATCATCCTCTGCAACAGGTCCATCAAGTGACCTCATTATCTTCTCCCCATCAGCAGCTGACACAGGCAATGAAGGTATGAGAGGAACTTCACCTCCCTTCTCCACCTCATCCTTGCTCAACCTCTCAAACTCTCCACCACTACTTGCCCAACCAGGAGTTGTGGGGTCCCCTAACCCACCATACACTGTCCCCACCTGAACCCCACTTGGTGGCATCCACTTCTCATCAGGAAACCACTTTGCCTCACCACCATAGTCCTTCTTATCTGAATATATCACCACCCCAACAGCGCCTGCCTCATAAGCATTCTTCACAATGTCCCCTCTATATATCTTCCCATGCCTTGCCAACACTACCGTGCCAGAAACATTCACCCGATTTCTTTCTTTCAGAGTCAAATAGTCCTCCACTCTTCCATAGTTCACATAACACACAGGAGCAGCCACAGTGCCCGACTTGGCATATGCGTGGAACGTGGGGACAACCTCGTCTGCCACGTCAGCATAAGGATCACCCTCGTAGATTTCTTGGTGGAGAGTGAAGATAGTTGGGGGTTGTGGTGGCGGGTTTGTTAGAACCAACGAACGTGACAAAGGGTATGTGAGGACAACCTCGTAGGACGCTATGTGTGATGGTATGTTTGAGGAGGTGAAAACTGAGAGAACATATGATGCAGCTTCTCCGTTGGCTTCAGAACCTGCGATGTGAGGTCTGCGGGTGAGAGTTTTAAGGTGGTTGGATATTGAAGCATTGTCTGAGAGGGAATCGGATATGAAGAGAGAGTGGTACGTGGATTTTGGTGTTGGTGTGATCAGAAGAAACAAGTAAGAGGTGGCTATGGCTAATAAAGTTGTGGTAGTGCTTGTTTTCATGCTTAACTCTCTCCGGAGTTTGAATCCTTTTTTTCTTTTTTGCTGTTTGTTTGATGTTTGATGCAGTTAATCTTCAACACTTGGCTTCTCTAGTGAGAGAGAGATTCACATGTTGTGATTGGTTTTGTATTGTAAGATTGTGACGTTAACAAAACATGATC

>C101575_65

CGGAAAGTGGCATACTTTAATTATGCTTATTGGAAAATCATGATCTTTTTTGTCTTAAGAGATGTGTTAATTACAGTAATATAATCAATTAATATAGATACGTTACAATGCCTTGAGTTGCAAGAATAAATAAATCTCTTTATTCACTCATGATTCTTCAGCCTGACCTGGGAATCCAAAACAACAGTTCTAGTGTAGTTATTTACAGCTTCCTATAAACCACAATGCCAACAGAACTGAAAGCCATCTGAAAATTAGATGTATCTTTCAGGTCCCAGGTCAAGATCCTTATTCCATTTCCTGTTTCTATTTGAACAAAAGAGCTTCTTTTGGCATTTCAAGAAGAGACAGGACTGTGGCCGCAATGTGCTTTGATGAAAGCCCAGCTTGTTCAACCTGATCTTGGGGTGATCCATGATCAATGTACCTATCAGGAAGCATCATTGCTCTCCACTTTAGAGGTCCATCTAGGATTCCAGATAAGCTCAAGAATTGAGAAACATGTGATCCAAAACCACCAATAGAACCCTCTTCCACTGTGATGAGTATTTCATGCTCTTTAGCCAGTAGCCTAATGAGATCCGTATCCAAAGGTTTGCAAAACCTAGCATCAGCGACTGTAAGATTAACGCCTACTTCTTTAAGCAATTCTGAGGCATGCCTGCATTGTTGGACTACAGAACCATATCCTAAAATAGCAACTCTGCTTCCTTCCGTCAGAATTCTGCCTTTTCCTATCTCAAGGACGGTTCCTTTGTTATTGAGTGGCAAAGTGGTTCCAATTCCATTTCCCCTTGGAAATCTGAAGCAGCTTGGTCTGTCATCTATAGCTGCTGCTGTTGCAACCATGTGCATCAGTTCAGCTTCATCAGAAGGAGCCATGACCACCATGTTGGGCAGACAAGCCATGTAAGCGATATCAAATGCTCCACAATGGGTTGGTCCATCTGCTCCAACCAAACCAGCCCTATCCATAGCAAATCGGACAGGTAGCTTTTGAAGATCCACATCATGGACAACCTGATCATATCCACGTTGAAGGAATGATGAATAAATGGCACAGAAGGGTTTGAGTCCTTCAGCAGCTAAACCAGCAGCAAATGTAACAGCATGTTGTTCAGCTATCCCCACATCGAAACAGCGGTCGGGAAACTTTTTGTGGAAATAATTTAGGCCGGTACCACCACCCATTGCTGCATGAATGGCCACTATCTTGTTGTCTATTTCAGCTTCCTTTATCAAAGACTCAGCAAAGTACTGTGTGTATGAAAGTGTTGGGGATTTTGACTTAAACTGCTGGCCTGTTTTTGGATCAAACTTTACAACCCCGTGCATTCTATCTGCTGCTTTTTCTGCTGGGGGGTATCCCTTCCCTTTTTCTGTCACAATATGAATCAAAACTGGACCTGGAGCAGGCATTGCTTTGACTTTTTCAAAGATGGTGACCAGATCTTCGATGTTATGACCATCCACAGGGCCTATGTAGTATAAACCAAGCTCCTCAAAAAGAGTAGAGCCAGAACCGCTGATCATGCCTCTTGCATACTCATCTACTTTTGCTGCAACCTGGTGTGTTTGTCCTCCTATTTGCTTTGTGATGCTTTTTGCAGCTTCTCTAAGTTTACGGAATTCGGTACTTGCTTGTATTTTGCTCAAAGCACTACTGAGGGCTCCAACAGGAGTTGCAGGGCCATCAAGTGTGGCAGTTGGTAAAGAAACTTGCTTGTTGTCATTAAGTATTACTATCATGTTAGAATCAAGGAACCCTGCATTGTTCATGGCCTCATAAGCTTGGCCTGCGGTCAATGCTCCATCTCCTATTACTGATATGATGCTGTTCTTCTTTCCCAATAGATCGCGTGCAACAGCCATGCCAAGACCAGCAGATATACTTGTAGAACTGTGCCCTACCCCAAAAGCATCATGAACACTCTCATCCCTTTTAGGAAAACCGGCTAGCCCTGAAGTCTTCCTAATGGTGTGCATCTTGGACCTTCTACCTGTGAGAATCTTGTGTGGGTAAGCCTGATGACCAACATCCCATATAATTTTGTCTTCAGGGGTGTCGAAAACATGATGCAAAGCCACTGCTAACTCCACCACTCCCAAGCTTGAGCTAAGATGGCCACCAGTGTTTGACACACTGTGTACAATATCTGCCCTCAGCTCTGCTGCAAGTTGCTCAAGATCCTGAGTTGACAGATTCTTCATGTGAATTGGGTAGTTGACTGTGTCCAACAATGGTGTAGCTGGTTTCTCTCCTGAGTAATTGATCTTCCACCCGTCTTTCTCTTTCCTGATAATAGTCTTCTCTATGTCACCTGAGTCACTTGCTGAAGCTCTCACACAGAACTTGTTTCTGCTACCATAGTATGGACTTGGAGCTTTAGGTTTGTGGCATGGAGAGAAAGAATAGTTTGGCTTAACAAAGGTACCACCACAGAAGGCCATGGCTATTCAAAGCAGTGAGAGAAAGAGATGGGAATTGTGAAAGGTGTTGGAAGTAGCAGAAAGTAAGAGAGCAGATTATGAAGCAGAGGTACCTTCCCGGTGG

>C101577_65

GACAATTGTTGGTGTAGTGTAGTGGAGGGTTCTGAATTGCGTCTTTGATTGGAATCGCTGACTCTGTAATTGGGTCAGTTTGAAAGTTTCAAAATAGGGCAGAGAAAGCGTGGTTTTTTCCATTTTTTATTTTAAGGGTTCAAGGAGGGGAAATTCCCAGCTTTGTTATGGAAGGAGGACAACAAATTGATGCATGAGTATCACTTGGTGACTTGCATGATGGAGTTGTAGGTGGAGGATTGTGATCGTGGTTCTGATTGAAGGATAATAATAATAATAATAATATTGTATAGTTGAATAATAGCTTTGTGGTGCAACATGGGGTGCATATCTTCCAAGAGTGCAGCTGTTGATGACAGCAGGGAGGGTGTGGCAAGGGAATTATCTTCATCTAGCAAAAGGGCCTCGGAGATGAAGATTTCTGCATCGAATTCCAAGAAAAGGGTCGATGGACTTTGGGGGAAAGATAAGGTTTTAGATGGAGCTGATATGAAAGTTTCATTAATTGACAAGGAATCCGGTGGTTCGATGCGGTTGTGTGATTATCAGAATGGGAAGAAGAAAAAGGAGAAACCTGAATCGGCTGTTCTTCATCATCCTGGTCTTGGAAGGGTTCCAAAGGGTTTAGAAGGAGAGCAGGTTGCAGCAGGTTGGCCAACTTGGCTTTCTTCTGTTGCTGGTGAAGCTATCCAAGGGTGGATACCACGGAGTGCAGATACTTTTGAGAGGTTTGGTAAAATTGGCCAAGGAACCTACAGTTCTGTGTATAAGGCACGTGATTTGACTCACCAAAAGATCGTTGCATTGAAGAGGGTACGCTTTGATAATTTTGATGCTGAGAGTGTCAAGTTTATGGCAAGGGAAATCCTTGTTCTGCGTAGGCTTGACCATCCAAATATAATAAAGTTGGAAGGATTGATAACGTCGCAGACATCTCGCAGCTTGTACCTTGTTTTTGAATACATGGAACATGATCTTACAGGACTTGCATCAAGTCCTAGCATTAAGTTTTCTGAACCACAGGTTAAATGCTACATGCAGCAGCTTCTTAGTGGATTGAATCATTGTCATAGTCGAGGTGTCCTCCACCGTGACATAAAGGGTTCAAATCTTCTCATTGACAATAATGGCACCTTAAAAATTGCAGATTTTGGTCTGGCAAATTTTATTGACCCCCATCACAAAGTTCCATTGACCAGCCGTGTAGTAACTCTGTGGTATAGACCGCCAGAACTTTTACTTGGAGCATCTAATTATGGAGTAGCAGTAGATTTGTGGAGCACTGGTTGCATACTGGGGGAATTATATTGTGGCAGGCCTATTTTGCCTGGAAAAACAGAGGTTGAGCAATTGCATAGGATTTTTAAACTTTGTGGCTCACCATCTGAGGACTATTGGCGTAAGCTGCGGTCACCACATTCAACAGTATTCAGGCCTCCCCACCATTATAGGCGTTGTGTTGCTGAAACATTTAAAGAATATCCATCTGCTGCCACAAGGCTAATCGAGACTCTACTTGCTTTAGATCCCACTCTTCGAGGAACAGCGGCAACAGCCCTAAAGAGTGAGTTCTTTTCAAAGGAACCCCTTCCTTGTGATCCATCAAGTTTACCAAAATATCCTCCCAGCAAAGAAATTGACACTAAATTGTGGGAAGAAGCAGCAAGGCAAGGAGCTGATGGGAGAAAGGAGAAGAAAAATGGACCAAGAGTTCGACAAGAGAAAGAACCTCAGACATTTATATTATCCAAAGACAATGTGGACTCTTGCATATCAACGCAGCAAGGGCAACGCCTTCCAAACTCAAGGAGTCAGAACGAATTTTTCAACCCTCACAGAGAACCTGTGTCTGGACATTTGGTTTTTCCACACAAACCGTGTCTTTATGAGAGGCTTATGCATTCAGGTCCATTGGTTCCAGGCAATGGCTGGGAAATGGCTGGAAAGGAAGCTAGTGAGCAGCCTCATGTTTCAAGCAAAGTCAACCTACCAAAACTATCTAGAGTGGCATCTAGGACTTCATTGTCTGGAGATAAAAAAGAAAACTCTGTTTCCTCGAGGTTCCAAGAAGCAATTCAAGCACAAAAATCTTTTGAGTCAACTAATGTTGGATCAGAGTCAAGAAGAAGGCATGATAAAAAACGCCATTCTCAGAGAATTGATCACAGGCAAATCGAAAATGGAAAAGTCTCAACTCAAACATTAATTCGGGAGGGGCATGGACCCATGGGAAACAATATCTGCCACCTCTCTGGTCCATTACTGGTTTCATCAAATAATATGGATCAGATGCTCAAAGAGCGTGATCGAAAGATCCAAGAATATTCGAGACGAGCACGAATGTACAAGTCAGGTGAGATAGCTCGTGCAAACCAAAATTAGGATAATTCTTTGTCAATTACATATAAATTTTTTTTTTTTTTCTAGTTACACAGCTAGATATATTTTCAATGACAGCAAAGTTTATATAAAAATTACAGAGAGAAGGGAAGGAGTGTGTGTTCGCTGTTCAAGCACTCTTCTCTTGTAAATTACAGATATAGTGTAAATTGAGG

>C101585_65

ATTTGCTTTTGTCATTTATAAGGAAATTACACTCTTCACAACTATACAATATTTGTCATCTATGAAAGAAGAAGCTCTGCTATAAGCACAACATTATCAGTTACAGTTTGAGTTCTCCAACCTTTGTGCATCAGCTTTGCATCTGCACTAGGGGAACATCGACAATTCTTTTTCGATGACAATAATGTTCTTCACAAATGGGTCTAAAACAACATCTTCCACTGTCAATATCTTCAAATGAAGAATAAATCTATACGGATAATAAATACAGGAGCTATTCCCCTAACAAACAGGACAATTTGCGAAATTGCCTACATAAGTTTACAGCCAGCTTTACACGAGGGAAAGAAATGGAAAATGTTCAAATATGCAAATGGATTGAGAAACATTTTCCTTCCCTTTCCCTCGTACAAAAAATTTAAAACCACACAACACTTCTATATGTATATATGAATAAAATTAACAGGGATTCTTGTTTCTTGATACTCAAGTAAAACTTTCTGAGTGCTTGGACTTGAGGGAACGCCGTGCCCATTCAATGGAAGCTCTTGGTGTGGATCCAGTGCGGTTGAGGGATTCTGGTTGAGCATCTGAAGGCCGAGGAACAAGCTTACACCCAATTTTCCTGGCAACCAACCGAGGAGAACTTCGGGAACTGTGAGATTTGGTCACAGTTTTCATTTCTTGCTTTATTTTACTGAACTCTTGTTCAAGTTCTCCTACTCTAGAACTCATCCTATCCATATCCACCTTTAAAACCTGGTTTTCACGCACAACAGTCACCCATCCATCTCTTTGTACAATTTCACCGGCCGTATCGCCTATGGCAGTTACAGGAACAGGAGCTGCTGGAGCGATTTCACCATCCAATGCATTGAGACATCCGGCTAATGCTGTTCTTAAATGCAGTTGTTCAAAGAACAGCACTTGAAGAACAACTCTGAGAGGTAATCTGTCATTTTGGGATGCATGAGCATAAGCATGGATTGAAAGTTTCTGGTAGTCAATGATGTTGCATAGTTCTTCCTTCTCTCTATCAGAGAGCCAAGGATGCGCCTTGAAATATATGTCCAGTGCCCTGTAAAGTCCATCATGCAATAATCTTGACGACTCAGGGAGGGCCTCTGCAAGTCTGCCTATCTTTCCCGGTTGTAAATTAACATCAGAAGCAATTTCTGCAATATAGCTATCTATCAACTTTGAAACCTTCCTCAACGACTCAGATGATGGTGAAGCTGATGTTTGCTGGTCACGCGATGAAGGAGAAAAGGCAGTTAAATTTGATTCTGTAGATATAAAATAATGGACTATCTGTTCAATACAGTCGGTGTTATACAAAGCGTCAGAATCTGAATAGGTAGGAATTAGAAGACTATTCAAAGTTGCAAGTTCCAATTGCATCCCTATTCTCCTCTCCAAAGAATCCTTGCAAGTTTGGCTGACATTCAATATCAAAGCCACACGAAGAAGTCCCAGCAGGAAACGACAATAAGATTTTCCCTTCTTGTCAGGAAGAAGTTTCTCAATGCTTTCCAACACAACCCTCTGATCAACTGTGGCAGGCGTCAAGCTGAAACTTGCAACTGTTCTGGTCTTGCCACCTTGACCACCCTCCCACCGACCTAGACCTGGCAAGTGCTTTCTAGAATAGTACATTATGGCACCTGCCAGGTTTTCAGGTCTTATACCTCTTGCTTGCATTGTCTTAATTAGCCTCTCAAACAAACTCACACTGAGATATGAGATGTCTTCAAACCACCAATCAGATTCTGAGCTCCGAATTCTGGCACCAGTATTTATACCATTCCAAAGAATGCTTCCACCAGGGCTCTGAAAACTCCCATACATCATCATGGGCCATCCAAACAAACTTGGGTCCGTACAAACCATCATAGATAGAGCATTCAAACACTTGCCCACTAACTGAAGCTTTTCAGCTCTAGGTAGAACCGGCTCGGAACTTTGGAGAGCCAAAATACAATCTTTCCAGCTGCGCAATATGTTTTTATGGAAAAAATTCTCTGATTTTGACAACAAGTTTCCTTCTCCAAATTCATCTGTCATTTCAAGGTATTCTGCAGCACAGTGCACCAATACTACATTTCTGGCTGTCAGTTCCACACGATACCCGTAACAGAATTTGGCTGCAATTAAGAAAGTGTCAGGACCACCAGGAAATTCTTCCAGCACCATTTTCAGAGTCTTCTTATCGGTATCCTTGGACTCGTCATGCGCTTTAGCAATTTTTCCACTTTTTGATACAAGAGGAAACTTATGAAGATGGAAAGTGACACCCTCGATGGAAACAGTTATATCACTTGGCAATCCCCCATTGCAGAACCAATCACTGCCTTCTCGACGAAACCCGGAAAGCTTCCCAGTAGGAGCCATGGCTGAGGGAAGGAAAGACCCTCTTTTTCCCCAGTTACTCTACCAACACAACATCATTGTTTTTTCCTAAATTGTTGCCTCTTAATTCTAAAAAGAAAAAGGGAAAAAGAGAAAGGCACAGATTAATAGTGTATCTTTTATATGGAAGAAAAAAAAACTAGCCAAAG

>C101587_65

CGAAAATCTTGAAAGAGAGTTTGCAGATTACTGTACGCTAGGTACTCTGCCCCTTTAATCCGTTTCTCTCTCTTTTTTTTCTTCTTTCTCTTCCCTTGTCGTCTTCTCGTTGCCTTCTTCTCTTCTTTAATACTAATTATTTAATTAAAGAAAAAAGAAACACAGTAATTGAAACAGTTGAAGAATCATCCTCATCCCCAATCAAACCACCCATTTCAATCTAGAGATCTCCTTCTGGTCAAAGGAGAACTGTTGGATCGTTCCCAACAATTATTAGTATGATGCAAGAGCCCAGTTCTAGTGGAACTGATACGAATCATCAATCTTCTTTGAACCATGTGCAAAATGCAGTAGATTTCCGATTTTCAGATTATAGGGGATCTTTTGGTGAGACTGCATGTTTATGTGGTACTGGTCATGATGTTCAAAGCTTCAGTGGTTGGAATTCTGGGGAATCTAGTTCTAGACGGAATCTAATCAACCAAAGCAATGGGGAAGATCTAAAATCAGAACATGGTTTATCTTCTTTATATAATGCTACTACTGAGGATGGTGTCAAGTCTGAGGAAGGACAGTTTGAACCAAATAGAGTAATTTTTCCTGTTAGTTCAAATAATAATCTGCATGGCAATCAATCTAGAGTTTACCCTTCCTTTTCGCAAGGTTCCAGTTCTAATCATATTACCCAAAATATTAGTCTAGATATGGGACATGTTGCTAACGCTGCTGATCGTGGGAAGGGCAAAGAAGCTAGTAGTAGTGTTGATGACCACAATACTAGTGGAATAGATAGAGAAAAGACATTGTTTGGTAGTGCTTCAAGTAATCACACAGGAGCTTCATCTGAAAACTCTGGATACATGGCTTGGGGTGATAGTCTTAGTTCAAGTTCATTAGTTAATTGGGGTCCTTCCTGCAAGAGAAAGGCTCTTGAAGGTAGTTCTAGGCAACTGTGCTCAGGGGGAAGCTCAAGCACTCTTGTACAATCCGAAAATGGTTATTGGCCTACTGATCCTATTGATCTTAATGCTTCTAGCAACTTAAGTGATGTTACACTTTTGGAGGATATTCCTGTTACTAGTAGTCCTCCATTATTGCAGAATGTAAGAAATGAAGTGAGACAAAAAGCTTCCAATGCATTCCCTTTGATAAGTATTGCAGAAAATATGGAAAGTCCTGTAAGAAATTTCAATAGAAGAATGAGCCAAATACAGGATCAGGAATCTATACCTCTCAATTTACCATCTATAGGGAGTTACAGGCATCATCATCATTCTTCTCTGAATCAAATACGTGGCTCTCACTCACTTAATGATTCATTGGAATTGAGGTTAACAGCTGGAGTAACAACTGCTAATTCTGGTACTTCTTTAAACCAATCACCAGCCCAGCGCATCCATTCTTTTCCTTGGAATAGAACTGCCAATCGTAGAGGGGCCAGACCTTCATCTTCTTATAACTCTGGAGAAAGGGTTGTTCGAGCAGATTTTAATTTGAGAATGTTTCCCAGAGATAGTACTGAGCACCCCATGAATGTGCCTGCATCTTCAGAATATGAACCTACAAGTTGGCAGACGCCATCTGCTAATGTGAACTATTCTGGAGGTGTGCCTCCTCCATCCTGGATTGGATCTAGTTCCAATGTCCAATCTTCACCTAACTCTGGCTGGAATTTTAATCATGAGGTCACAACTGAAAATATGCAGAGTGTGTTAGAGTTCAGTCCTTGGTCCCTTTTTCCATCAATTAGCTCTGCATCTGGTGTTCATAATGGTCATTCTATCCCATCTTCTTCTGGTCCTCCCTCATTTACCCAGGGTTCTAGCAACAATCAATCCTATCAAAGAACAACATTGTTAATGGAAAGAAGGGGTGGTGATGTTCTCTCTAGACCACGTTCACTGCAAGCATTGACCTTTGACAATGAGGGCAGACGAAGACTACTATCTGAGATTCGCCAGGTCTTGATGGCTATGCGGAGGGGTGAGAACTTACGAGCCGAGGATTATATACTATTGGACCCTTTCCTACATCATGGCATGGCTGAAACACATGACAGACATAGAGAAATGCGCCTTGATGTTGACAACATGTCTTATGAGGAATTGTTGGCATTGGAGGAGCGTATAGGAGATGTTAGCACTGGATTGAGTGAGGATGTCATTATCAAATTAATGAAACAACGACTTTACATGCCAATGATGATAGAGTCTTCTGCAGCTGTGGAACCTTGTTGTATTTGTCAGGAGGAATTTGTTGATGGAGAGAATGTTGGATCACTGGATTGTGGGCATGAGTTTCACAGTGTCTGCATCAAGCAGTGGCTAATGCAGAAGAACATGTGCCCCATTTGCAAAACAACAGCCTTAGCTACATGATAGAGAGAACCTTTATCCTATGTCAGTTTGATCTTTATCTGGCTCTATTTACAACTATTTTTACTTTATGAAATTATTTCTTAAACAGGATCTCTCATCTGTTATGATTAGTACTTGTGTCTTGTTAATGCTACTTCCAGTCTTCCTTAATTTCATCAGTGAAAACAAAATGTTAAAGAG

>C101589_65

GAAAATTTTCAGTTGCAAACTACAAGCAACGCCCAACAGGATAGTTTGACTTTACATTGTACTCTCCACAGGTAAAAATAGAATGTGGACATACATTTTGTATACATAGTTTCAAGTCACAACAGTGTGACCCGTCTAAGCATTTAAGTGTAGAACAAAGACGGTTCTGATCCCTATTTGGCACCCCAAAATCCACAATAATCTTGTCAACATCAACATTTAAACAATTGAGAAACAAACCACGGCAATGAAATCATAGAAGCGATATCTAAGAGCACGAAGAATTCCTCATCCACCACACAAATCATTTGCTCATTAGCACCATCAATTACCAGAAGATACGAAAACATTATACTCATTTAAACGTCTCTGAAACTGCGCCAACTGTGTCTGCAATTGAAAGATTCCTGCAGCCTTTTGTGATAAAATGGTATTCAATTTGGAAATATAATCATCCAATTGATTTCCTGGTTGGTCTGCTTCAACAAGTAAATTCATCTCCTCCCTAACAATGTCTATGGTGTCCTCCACCTGTTTCCGATGAGCTGTTACTAGATCTTCCTCTTCCTTCAGGAGGGCATTTAAATTATCATCCGGATCAGAATGATTAGTCTCAAAATATGATGCATCCACATGTTTAACACGAGATTCTATCTTCCTCTTGTCCTCCACAGCAGATAATTGGTTGGTATCCACCCTCTTACTTGTTTTTCTTGTTCTGTCTTGTTCATATGTTGGTCCTAGGTAATCGAATTCATTTGCAGTTCTATCATTTTGACTACCTCTATGACCATTTTGTGGATCAGAATACACAGATGGCGCCAAATTTCCCCCCATTCTACCACTTGGAACATGGTCCACATTATTTGGAGAGGGTTCTCTTTCCGACTGTTTGGGCCAACCAAATCGATTCCTATCACTGGAAACATACGTTATTTCATCCTCCAAGGTATCATCATGAGCTAAAATTGAAGACCCAGGCAATACAGTGGACTCTCTAAGGTTTGAGGAAGAAAGGGGATCCCTTCTAGAGTTGTTCCCTTTTGACAGGCTCTTCACTCTGTCAGCATATCTTAATGTGTTAAGAGTATGCTCGCATGAACCTGAGCTAGGCGAAATGCATGATATCATTACAGTGCGCGAATTACCAACAAAAGAATCTCGCAGAACTTCCGTCAATTTACTTCCTCTGAAAGGGATATGCCCTTGGTCATTGTCTAGAGCTCTAATGCATTCTTTAAGGGCAAGCAAGCTTTTATTTATTTCTGCCCCTTCAATCCTAGTCTGCTTGTCATTATCTGTAGTATCTGCACCACGTTCACTTCCAGCCAGATCTATAAAAGATAATTTGCCCACAAGTCGAGCAGGCTTTGATTCAGTCCCATCAGCTGATCTCTTAATACAAAGTTGAAGTATGGCATGTGATCGAGAGGATTCCTCATTTGCTCCAGTTGTACCAGTACTTCTTGTGGAATTTCCTTTCTCAATAAATTCCTTGATTGTCTCGACTTTAGATACTCTATATTCTTGCAAGCCAACAATGCAAACCTGTTGTTTCCCATCCTCCCTCATGCAAAGTTTTTTTCGATCATTGAGGAGATCAAAAAGTTTTCCCCCATATATTTCGAAGAAACTAACAAACAATTGGAAACCTTGGTTCCGGTAGGTGTGGTGCATTAATCTTAAAATATCTTGGGAAGCTTTGAGAGGCAATGGTTGCATAGTATATGTCTTCCCACTGCCAGTTTGACCATATGCAAAGCAAGTTGCTTTTGTTCGTTGGAAAATCAGTGGAACAATTGGCTCCACAGTCTCAGCATACACTTCATCATTTGAAACATCTTCATTCAGCACAGCATCAAAAACAAATTCATGTTTCTCCACATATTCTGTTAAGTCAACCTTCAGTTTTCTTTCATGCACTGTGAGAAAATTTGAATCTATGGAAATAATGTCTTCCTCTCTCTTTGCTATTTCCTTCTTATTTAGGGGTCTCTTCCGGACCGCAACTTTGATCTTGGCAACATTGTTTGCTCTCTCTTTATCTGTTGGGATACTTTTAAGGAAACTGTTCTCAGGCAATCCCCGAGACCTTTGTACAAGTTTGTTACCAGAGAGGATTGACTCATAGTCATCAAAACTCTCAGGTAGAAGCTCGGTATCAAAGGAATGCAGATCAAGCAAGCCACTGCTCAAAACACGATCATCGTCAATTTTCCTCAAGCTCGAAGATCGAGTCCCGAACTCCTGCGCGAAAGTCGAAGAGTGTAACCAGCTTCTGTGAGAGTCGAGGATGAAGTTGTCGGAACTTTGACGGTGGTGGTGCACGGCAGAATTGGATCGCGGCATTGCTCTCCCAACCACACTCATTCACTTTGAATTTCGCAATCGATATCTCGTTCTATGACGATATGAGAAATGGAATCAGAGACTCGTTAATCTATAAACGAATATGAACGCTTCAACGCTTATTATCATTGCTCTCTCTCTCTATCTTGTCCCTTTTGAAAAAATCTCGGGAAACAAACAGTGCTGAGAGGAGAGAAAGTGAAAGAAGAG

>C101593_65

CTCTCTCTCTCTCTTACCACTCCAACCATGGCTTCCTCTTCCTCTCTCTCCCTCTCCCAAGCCCTTCTCCACCCCGCCCCTCTCCCCTCCTCTCACCGCCCCACCCTCCGCTTCTCCCCCTCACTCCGCCCCTCCCCTTCCTCCGCCCCTCTCTCCATCCGACGGCGGGCCGCTACCGTGCGCGCCTCCGTCCCCGTCAAGACTGTCGAGAAGGCCACCTCCGATGTCGCGCTGGTCGAGAAGTCCGTCAACACGATCCGCTTTCTCGCCATCGACGCCGTCGAGAAGGCCAATTCCGGCCACCCGGGTCTCCCCATGGGATGCGCCCCCATGGGTCACCTGCTCTATGACGAGGTCATGAAGTACAACCCGAAGAATCCTAAGTGGTTCAACCGCGATCGCTTTGTTTTGTCCGCGGGTCACGGGTGCATGCTCCAGTACGCGCTGCTGCACCTCGCTGGCTTCGATAGCGTTAAGGAAGAGGATTTGAGGGAATTTCGCCAATGGGGGAGCAGAACTCCAGGACACCCAGAGAATTTTGAGACTCCTGGAGTTGAAGTCACAACAGGTCCTCTTGGTCAGGGGATTGCCAATGCTGTTGGTTTGGCCTTGGCGGAGAAGCACTTGGCTGCGAGATTCAACAAGCCTGATAATGAGATTGTTGATCATTACACATATGCTATACTGGGTGATGGTTGTCAAATGGAGGGAATTGCAAATGAAGCGTGCTCACTTGCTGGCCACTGGGGACTGGGGAAGCTGATAGCATTTTATGATGACAATCACATTTCTATTGATGGTAACACAGAGATTGCATTTACGGAGAGTGTTGATAGTCGTTTTGAGGGACTTGGGTGGCACGTTATTTGGGTGAAGAATGGAAATAATGGCTATGATGATATTCGTGCTGCCATTGAGGAAGCAAAATCAGTCAAAGACAAACCCACATTGATCAAGATCACAACAACAATTGGTTATGGTTCTCCCAACAAGGCTAACTCCTACAGTGTGCATGGAAGTGCACTGGGTGCCAAAGAAGTTGATGCCACAAGGAAGAACCTTGGATGGCCATATGAGCCATTCCACGTGCCTGAGGATGTCAAAAAGCATTGGAGTCGCCACACCCCTGAGGGTGCTGCACTTGAAGCTGAGTGGAATGCTAAGTTTGCTGAGTATGAAAAGAAATACAAGGAAGAAGCTGCGGAATTGAAATCTATAATCAATGGTGAATTACCTGCTGGATGGGAGAAAGCACTTCCGACATACACTCCAGAGAGCCCAGCTGATGCCACCAGAAATCTGTCTCAGCAAAACCTTAATGCCCTTGCGAAGGTTCTTCCCGGTCTGCTTGGGGGAAGTGCAGATCTTGCTTCTTCCAACATGACCTTGCTAAAAATGTTTGGGGACTTCCAAAAGAATACTCCAGCAGAACGCAATGTTAGATTTGGTGTTAGAGAACACGGAATGGGAGCAATCTGCAACGGCATTGCTCTTCACAGCCCTGGACTGATTCCTTATTGTGCAACTTTCTTTGTTTTTACTGACTACATGAGAGGTGCCATAAGGCTTTCTGCACTATCCGAGGCTGGGGTTATTTATGTCATGACCCATGATTCAATAGGACTCGGAGAGGATGGTCCAACCCACCAGCCTATAGAGCACCTGGCAAGCTTCCGGGCGATGCCAAATATTTTGATGCTTCGACCTGCTGATGGTAATGAAACAGCTGGGGCATATAAAGTTGCTGTGCTCAACAGGAAGAGACCCTCCATCCTTGCTCTTTCCAGGCAGAAACTGCCCAACCTTCCCGGATCTTCTATCGAAGGAGTTGAAAAGGGTGGTTACACTATTTCAGATAACTCCACTGGCAACAAGCCTGATGTCATTTTGATTGGAACTGGTTCAGAATTAGAAATTGCTGCCAAAGCTGCTGAGGACCTAAGGAAGGAAGGGAAGGCAGTTAGAGTTGTCTCCCTTGTTTCTTGGGAACTTTTTGATGAGCAATCAGAAGCCTACAAGGAGAGTGTTTTGCCTGCTGCTGTGTCAGCCAGAGTTAGCATCGAGGCAGGATCAACATTTGGGTGGGAGAAAATTGTTGGATCCAAGGGAAAAGCCATAGGTATTGATCGTTTTGGAGCTAGTGCTCCAGCAGGAAAAATATACAAAGAATTCGGTATCACTAAGGAAGCCGTTATTGCTGCAGCAAAAGAGCTTATCTAGATGTTTTAGTTTCTTTCTGTTCATGTAGAACTTCTCATTTTTTTTTCTTATGGTTTTGGTTGATTTTTACAAAGAGGGGTTGTTTCAAGAGCATTCCTTGCCACGAATAAGAAAAGGTTATGTATGTATCCTTTGATCGGGATTCTTAGTGAAGAAGCAACATAGATCAAGGAATTTTGTGAACTTTGTAATACCAATTTGACAATCAAGATTTGCACTTTCACGTGGTTAAATTCAATGTTGGTCATGATCATCATAGATGCTAGTGTATACAAATTTGTTTCACTTGCAGATACATTCTTAAGCATTTGTGTTTGGATGGCCGTGTTTTAGGAAGTTG

>C101599_65

CTGTGATCTCTTTATTGTGATCATTATTATGATCCAGTATCAATAATGATGAGGACAATCATATATACAGTTTCTTGGACAAAATTGGAGGATAAGAAACAGAAAAGAGAATGAAACAATTTTCGCAGGAATCAATATCTCACTTCTATTTTCTTGTGTTAGGAACGAAACTCCTATCTTCCCTGTCCACAATAACCTTAACTAGCATTTACAGTGCTGCAAAATCTATTCTGATGCTGTTGATGGAACTCTGAGTATTTGCTGAAGAAAGCGTGCTGCAAGACGTTGAGTGATTCCTCCAACAACACGCCCCCCAAGTCTGCGTGCCTCAGGTTGCTCAAGGACCTGTAGTATTGGCAGTAAAATAGCTGGATCAAAATCATGTGAGGATTGAAGAAGGCCCCATATTCTAAGAACTTGATCTCTAAGCTCTATCATGCTTTGCATTTCAGTTTCTGACATCATAAAGGGTCCATTTGCATTTCCATTGAAGACAAGGGTCCTAACAAAGTCAGGAATATATCGGTATGTTTCCGACATTGTGCCAATGGTAAAGGCTTCAGATACTCGAGCAGCCTCTTTTATAACTAGATTCCTCAGCTCTTCACCATCTGTACTCAATAATACCTTCAGGACAGGTTGTAAAGCCTCTTTAGCAGAGAAGTCTCTATCCTTTCTCCCCTGGGCTAGCAAGTTTTCAAGTCTATTCCACCTGAACTTCCCATCCTTGAAAAGCAATTCAATAAGGGCATCTCTTAGGTATGGATTTGGATCTGTTAGGAGCCTTTTAGCAAAATATGGGTATGATGCAGCAAGTACCTTAAAGTTGGGATCAGCATACAATGCTAAACCTTCCAAAACAGTGAGAGACCTTAATATTAATGCGTAGTATGCTGGAACATTAAATGGATACTGATACAAAACATTTCCCAGACCATCTACTAGTGTCTTGAAGTTAAGCTCACTCACAGTATAATTAAGTGCATCATCAAAAAAGTTTCTAAGTGCTGGCACAATTGGAGATACATCAACATCCCTTGATAAGAAATCAAGATCATAGTAGTCACGAGCCATGGCCTCATAATCTCGGTTAACCAAGTGAACAACATGACCAATTATGGCATATCTTGCTTCTTCCGGAGTCTCACTCATCATGCCAAAATCAAGAAAAGCAAGTTTTCCCTCAGGTGTTGCCAAAAGATTCCCAGGATGAGGATCTGCATGAAAATATCCATACTCGAGTAACTGTCTGAGACTGCACTGTATGCCTGCGTTTACCAGATCCAAGACTTTTAGTCCTTGTCTCTCAATTGCCTCTTGCTCATTTAGTTTTACTCCATCAACCCACTCCATTGTTAGTACTTTAGCACTTGTATAGTCCCAGAAAACATCAGGAACAAAGATATTTTCCTTGTCAGCATACAAATTTTTGAATCTCCTTGCATTTTGTCCCTCCTGCACATAGTTGAGCTCTTGAAAGACTCTACGTGCAAATTCATCAATAAGTGCAACAACATCACTGGTGATAAAGTCCACATATTTATTTATGAGAAACCCTAGACCTCTAATCAGGTAGAAGTCCAATCCTATAGCTTCTTCAATGTCAGGGCGTTGCACCTTAACAGCAACAAGTTTCCCAGAGTACTTCAACTGACCCTTATAAACTTGACCCAAACTGGCTGCTGCTACAGCCCTTGGTGATATAGAAGAGAAAATAGAGTCAATAGATAGTCCTAGTTCCCTCTCAATGCATTCAAAGGCCTGCTCGTCGGGAAACGTTGGCAATCCATCTTGAAGTTCGGAGAGCTCCTCCAGATACTCAGGTGGACATAGATCAGGCCTGGTAGACAACCCCTGACCCAATTTGACAAAAGTTGGTCCCAGCCGAGTGAATGTATCCCTAAGCTCAATTGCACGAATCCTTTTATTCCGATCAAGCATCCCACTTTTCTGGTCAACCAACAGCTTCAACCCAAACAAGCCCACCGCACTCAGAATCTGAAGGGCCCTTCCCACCACCTTGATGGGTTGAGAGCCATATTTGGAGGCCACAAGCTGAGGTGTGTAGACAGAAGCATTGGCAGCCCGACCCATGGCTCTGGCCTCGGCTTGAATGTCATCGGCACGGTTCTCAGGAAGCGCAAGCACCTTGATTGATCCATCTCTTGGAGAAGGAGAAGGAGGTGAACTTGGTGGTGTTGCCTCAACCAAAGCACCACGTGGTTGAGGATTTGGCCATGTCAAGCTTGTTCTAGTTCTGGTTCTAGGTCTTGGTCTTGTGATGGTGATGATTTTGAAAGAGAGTGATGGGGTGGGGTTTGAGAAGGAACAAAAAGGTTGACCCGTGGGGGTAGAAGATAGCATTGATGCAACTGTAGCCATGAGCACTTGCTTCCCTGCTCCACTTTGATGCTGTAAAAAGCCAAATCTGAAACCTTTGTGGATAATGGAATGACCAATTGAAGAAGTTTGGGAAGAAGTAGTGAATTTGGCACAAAGCAAAATGGGGGTCTAGGTAATTTCGGTTTGGATTATGGGAATGGATATGGGAACTGAATCATCT

>C101609_65

AAATTTCGTTCTTCTTTCTTCTATTCAGCCTTAACTGATACAACACCTTCCTCTGCTTCACTTCTCTCACCTGCATTCTATAGCAGGTTGAGAACTTATACAACTTTCTCTTTAACTTCGATCACTTTCACATCACCAATGGCCTGCTTAGAACTCTCCAAAGAGGTAAAGTTCAAAGATGACACAGAAGAGCTAACTCTCGATGGAAGTGTTGATTGGCATGGTCGTCCAGCAATCAGAGCCAAATCTGGGAGATGGGTTGCTGGAACCATAGTACTATTGAACCAAGGTTTAGCAACCTTAGCATTCTTTGGAGTAGGGGTGAACCTAGTGTTGTTCCTGACAAGAGTGATGGGGCAAAATAATGCTGATGCTGCAAACAATGTGAGCAAGTGGACTGGCACAGTTTATATCTTCTCTCTTGTAGGTGCTTTTCTAAGTGATTCTTACTGGGGAAGATACAAAACTTGTGCCATCTTTCAGGTCATCTTTGTAATAGGCCTAGTGTCCTTGTCCCTTTCATCGTACATCTCCTTGATTAGGCCAAAAGGTTGTGGGAATGAAACAATTCCATGTGGGAAACATTCAAGCTTGGAGATGGGGATGTTCTACCTCTCAATCTATCTCATTGCCTTAGGGAATGGTGGGTATCAGCCAAATATTGCCACATTTGGAGCTGACCAGTTTGACGAGGAGCACTCAAAGGAGGGCTACTCAAAGGTTGCCTTCTTTAGCTACTTCTACCTGGCTTTGAACCTTGGTTCACTTTTCTCAAACACAATTCTAGGCTATTTTGAAGATGAAGGACTGTGGGCACTTGGGTTCTGGGTGTCTGCAGGTTCTGCTTTTGCCGCACTTGTCTTATTTCTTCTTGGGACCCCAAGATATAGACACTTCAAACCCAGTGGCAATCCTCTTTCAAGGTTCGGCCAAGTCCTTGTTGCCGCATCAAGGAAATGGGGCGCTCAGATGACATCAAATGAAGAGGATCTATATGTCATGGATGAAAATGAATCTCCCACCAATGGCAACAGAAAGATTCTCCACACTGAGGGATTCAAGTTTCTGGACAGAGCAGCGTTTATATCTTCCAGAGATCTAGAAGACCAAAAAGGAGGCATTCATAACCCCTGGCGTCTCTGCCCTATAACTCAAGTTGAAGAAGTGAAGTGTATACTAAGACTTCTTCCAATTTGGCTCTGCACCATAATATACTCAGTGGTTTTCACACAAATGGCTTCTCTTTTTGTGGAGCAAGGGGCTGCCATGAAAACTACAATTTCCCATTTCAGAATACCACCTGCAAGCATGTCTAGCTTTGACATCCTCAGTGTAGCTGTCTTCATTTTCTTCTACCGTCGAGTGATTGATCCACTTGTCGGAAGACTTAAAAAGACAAGTTCCAAGGGACTTACTGAACTTCAGAGAATGGGAATTGGGCTTGTTATAGCTGTAATGGCAATGGTTTCAGCTGGAATAGTTGAATGCTACAGGCTTAAGTATGCAAAAGCGGGATGCCCCCACTGCAGTGGCACAAGCTCTCTAAGCATCTTCTGGCAAATTCCTCAGTATGCACTTATTGGAGCTTCGGAGGTTTTTATGTATGTAGGCCAGTTAGAGTTCTTCAATGCTCAGACACCAGATGGCTTAAAGAGCTTTGGAAGTGCCCTTTGCATGACGTCCATCTCTCTTGGGAACTACGTAAGTAGCTTACTTGTTAGTATAGTTATGAAGATCTCCACCGAGGATCACATGCCAGGGTGGATCCCTGGAAACTTAAACAGAGGTCACCTAGATAGGTTTTACTTCCTCTTAGCTGCCTTGACATCAGTAGACTTGATCGTTTATATTATATGTGCAAAGTGGTTCAAAAGTATACAACTGGAAGGGAAGTATGAAGAGAATGATATGCCTGATAGCTATAAAGTATAATTTGTGATCGACAACAAACAAAGGCTATGGTAACAGCATATATAAATAAGAGAAAGGAGGGATCAACAACTAATCAAATTAACTTGAGCTATAGTTGCTATTGGAAGAAAAGTGGCTGGTTTCGGTCAGAGAAGCCCTTTTACCAGAGTCTCTTATAACTGTAGTCACTGTTTTTGTTTGAACTAACGGTTTGCTTGGTTTGAGAAAACCTTTTGTTTTCATTTTTTGTTTATGCAAATAATTACTAATATGTTACCTTGTTTCCAATTTGTATCCTTGCTTTCAAAATTTTGTATAGCAAATGGTAAAAACAACAAAATGTTGTTTACAACCAGTTCTTGTACAAAATTTTGAAATCCAAATAAAGTGAAAATAAGGTAGCATTTTTGTAATTATTTACAAAACTGAAAAAAAAACAATAATCTTTTCACAAACCAAACAAAGCCCCTATATTTTCAGTTTATGTTTTTGTATCCAGGTTGTGTAAAACTAGACTTTTTAAAGAGACAATGACAGTTTCAGGGCCACTTTAAATCTCCTTCCTTTTAGTCTTTGTACGTTCCATTGTTAACACTGGAAATGTCATTTTGATTTCCAACTGTGAAATATTATTCTCCTATTACCAACTAAAAA

>C101613_65

AGAAAATATTTGGAGCAAAAAGGAGCAAAACTTTTATACTATGTTTCCTTGACCACCTGATGGGTTTGTGGGTTTTGACTATCATCTATAAGATGCAATATGTACATGATAGCTCTATCCTATGTGAAACCAAAAATTAAACTTGATACATCATCCCAACTTCCAGAGATGATGCATGAGGCACACCAGTGACAAAGGAGGGTGCCCTGCTGTTTCTTCTTAGGAATTCATACACTAAATTAATGGCTATTTTCTTCACCATGCTAGAACCTGGTTTTGCTCTCATGTGTGTCTGTCCTATAATGTAAGCTACCCCAGCCTCCCTTGCTTCCATCACCTCCTCCAACTCTTCCATTGCACCTATGTCAATCTTTGGACTCTCTGGTACCAAAAACCTTACCCTTTTCTTCTGCTGCTGGATCACCTTGGGTGACTTTATCTCCTTCAGTTCTGATGGCCCTGGCTTGTCTAAATTCTCAACATTATCTACATTATCTACATTATCCTCACTCATCAAAAGGGAATGTGTAGAACATGTTCCAACAACTGCCATTCTGTCATCCTTTCCGGGCTCATCATTTGAAGAGTTCAATCCAGTTTTCTCTGTGCGAATGAACTCTGCTATGCTGCACAAAAGATCATTCTCAAACTCAACATCATCTTTGTGGACATCACGGTATCCATATCGCACTATGCACCGGTAGAGTCTGAACTCTTTTGGACCAACGCGACCAACTAGAAACCTTTCATCAGATCTAACATGTGGAACTGGCACATGTTTGATGCAGAGGAAAACTAGGACCTGATGGAAGGCTGGGAGGTTGGTTACAAAGTGGGAGAAGATTACTGGAATTCCAGAAACTAGCTCGGTGTGTATAAGGCCAACCCCACGCACTCGAACTATACCTATGCTGGGACCTAGGCCGAGAAGCCAGTTGATGGAAACCTTGTTTTGAACATCAAATTCATACTTTTTGAGTGTGCCATAGTGCCAAACATACATGAAAGTCAGAAATACAAGTGCCAATGCTATAGGCACCCATGCTCCTTGCAGGAACTTGATAAGAGAAGCAGAGAAGAAAAGAGCCTCAATGGTGCCAAATATGAAAACAAATCCAAGTGCAAGGAGGACATTCTGGCGCCAGCATAGCACAATAACCAGTGACATCAAACAGGTGGTCACCAGCATCACTGTGATAACTGCCAAACCTGAGGCATTACCCAGTCGCTTTGTGTCTCTGAAACAAATAGTTACGGCCAAACACAACATCATCAATAGCCAATTGATCTCAGGGATATATATTTGCCCATGAATTTTTGATGATGTATGAATCACTTTAACTCTTGGGAAACAACTTAATGCGGAGCACTGCTTGATGATTGAGAATGTTCCAGTGATAATGGCCTGACTTCCCACAACAGCAGCAAGTATGGCTATAACCAGAACAGGCCATCTCAATTTCTCTGGTACAGATACGTAAAACCCAAAGTGATAATCTTGCTCAATGTCATGATGTTTAGATAGATAAGCAGCTTGCCCCATATACGCAAGAATTAAAGATGGATAAACCACAGAAGTAAAAGCAATCTTGATTGATAATTGGCTGAAGTGCCCAAGATCAGCAAACATGGCTTCTGATCCTGTGATGCACAGTAAAATTCCACCAAGGGCCATCCAACCTCCTTTCTGAGTTTTCCTTAAGAGATGGAAAGCGTAATATGGAGAGAGCGCTTTGTATACATGTGGATTCCAGTAAAATATATTGTATATTCCAATGGTACTAATGCAGAAAAGCCACACGATAATTACAGGAGCAAACAAGAAGCCAACTCTGTGCGTGCCATAATGTTGAAGAGCAAACAAGCCTATCAATATGATGCATGAAGCTGGAACTTCAACATATGCGTGCGTCTTCTTGGACATTGAAAGCTCCAATCCAGAAACAGCCGAGAAAACAGAAATAGCAGGAGTGAGGATGCCATCCCCAATGACCATGCAAGTCCCAATCAGAGCCAGAACGAGCAAAACCCTCTGCAGAACCTTGCGCTTCTCGAACGTGCTTTTGAGCCGCGACGCGAAGCTCCTCTCGGGAGCCGCGCCGCGCGAATCCTTCTTATACTCCGTCAACTCCTCGTCGGCGACCTGGCAATTCGGCAGCGAACTCACCCGCGCGTGGCGGCACAGTAACGAGTACAACGCGAACGTGCCTCCCTCGCCGTTATCATCAGCTTTGAGCACAACAAACACGTACTTAACCAAAGGAATCAGCGTTATGGTCCAAAACACCAGCGACAAAACGCCGAAAACCTCGTCGTTGCTCTCCGTGTGCTTAATGTCCTCCGCAAAAGTGCTTCTGAAAACGTACAACGGCGAAATGCTCAAATCACCGTACACCACACCCAGACTCTGGTACGCTAGAGTCAGAACCGTTCTCCACGACTCTCTCTTAACAGAGTACCGACGAGTTCCACCTTCTAGATCCATCATCGCATGCGTATTCGAAGTCTCAGAGAAGCATTCTTGAAGAAAAATTGAAGATCACCCAATTGCTGAAACTAGTTCAAC

>C101617_65

GTGCTATTATTATGTCATATGGCAAGGCATTATATTTTTATTTGCATCTATATACAATTATGGGCAGGCATTGTTCTAAGTTCATGAAAACAAGAGGAGATAACAAGAGTCAAACTATTTAACATTAAACAGAAAAGGTGCCATTAACTTAAATAGATGTACTGAGTATAGTTTTGGTGTATGTATATGTATCAATCAAGCTTCAAAATTTGGTTTTATCCAACAGAATCTTCAGTTCAGGTTGCGGCCTCAAGCAACAACACCAGATGGCACAACACCAGATGGCTCGTTCACTGCTACTGTTGTTGCTACTACTGGAGGTAGAAATTTCTCAATGTCCTTTGCTCCAGTTTGAGCAAACTCTTTGACAAGCACCTTCAAAATTCCGGATAAAGAATCCTTGAATCTCTGTGAGCAGCTTCTGTCTCCCTCATCCACGTCCTGAACACCTCCTTTGGCCAAACATCCAACTTGTGCACCTTCCATGTATGCCTTACAAGCCACCAGAATATCACTAGCTCTGCTGCAGAAATGCCCCTTAACAAAATCCTCAAAATTCTGTGGAGGCCTTCGTATCATATACATCATCGTCCTCAATGACAGAATGAATGTGTCCTCGTTATACAGAAAAGACATCTTTTCACCATTTGCTGAACCACTCATGTGTGCATATCCAGGTTCATTAAAGTAAGGCTTTGTATTCAGGATTAGACCTTGTATGGAGACCAGAACCTGTAAAATCGTTGAGAGACCTGGTTGCCACTTCTCATTTTTGTTGCCACTCCAAGTGTTAAGCAGACTAAGGCATACTTTGCCACAATTATACAAATTTGGGTTGAGCCGCAGACCTCCAGAGTGGTAATGGACTTGCGGCGGTACACCGGGATAGGCACTGGGAAAGAAAACATCAAAAAAGAATAGGCCATCATGATATGGAGTCCCTTCAGCTCCAATAATCACAGCCCTTAGAAGATCCATCCTTGACTCATAAACTCTGACAAATATTGAATCTGGCAAATCCTTCTGCAAACTCTTCCACTCGTCTTGTATTTTTTTAGCCCAAATCTTTGGAGGCTGCTTCATTGAAGAATTGTTGTGAACAAAGTAATGATCTGAAGTGTCTTCAACAGTGTCAAATTGTTTAAAACTTTGAAATTTCCTCATAATTTCATCTCTGGCTTCATCCGAGATTGTCACAGGAGGGCCATCCACTGCTGCTATTCCATTAAATCCATCTCCAGCAGAATTGTGAACCCAGTTATCAAATCCATGCTCAGGATTGCCAATAAGAGGATCAAGTGGATAATAAATAATATTAGAAGAACTTTTATAATGAGGAGAAAATGGTTTAGCATTTTGAGAAATCTTCCACCAAGATTTTGCAGGTTCTATTCCCGGAGTATGCAATGATCCTTGGTTTGCAATGAAATTTGAATGAGAGGAAATGCTACTGCCAGCTGCCTTCATAGCAGTTTTAAATGGCCCCCCATATGATGGCTGACCTGCATGTGGCAGCTTCGCAGCCTCTAAATTTTCAACTAAATTGAGGTATGGTTTCTTTTTACTATAAAAAGGCCCCGAAAACCAGTGAGACTTAGATGATTCTACTCCTACTGAAGTATTTGAAGTACGTCCTCTGAGTTTTGAAGTAGCTGATTTCTTCTTAGACGGGGCAGAATGAGAAAAAAATTGAGGAGTAGACAACTCTGTTCCAGAGGAGTGATTAACATTATCCATTTTGATTTGAATGTCAGTACCACCCATTGAAGATGCTTGAATTTTAGAGTTAGTTGGCTCTAATGACACAGAAGGCTGAGAAGAGGATGTCATGGGACTTAATGCAGCATTTGATTGCATATGATGCCAAGGATAAAAGGTACTATTTCCAGTCTTCTTAAAACCTACATCATATCCTGGTGGCAACCAAGTAAAAGGTGCTTCAATTCCAGGAGGTATATCCACATTATCAAAATGTTTCTGTAATAATGCATACTCATCTACATCCATATAGTCCTCTGACAAAAGATCACTGTAGTCATCGTCATCATATGATAGATCAGAACCATGACCATCAACATTGACCAAATTATTTGACACTGAAGGGAGATTGTTAGAAGATGCAATCCCACTAACTGATCCAAAATTTTCCACAGCAGACAGAGAGAATGGATAATCAGACAAATTCACAACTTCGAGATCATCATGAACAGCATCGATTGTCTTCCCTTTGTTACTATTATTAACTTTTTCACCAATTATCACTACATCCTCAGAATCATCATCATTGTCAATGTCGATCACATCATGCAAATTGACCTTGTTGTTATGATAGAAATTTGAAGGTTGGTAAATTGGAGGAGGAATCTCGATGACGTCAGGGTCCATAGTGCTCCCCGAATGTTGAATTGAATGATCCTTTGGCTATTTGTTTGAAGGAAGAGAGAAAGAGTGAGTGAGGGAAGAGTTTAGGATTGTGGATGCGGGTGTTATATATTGATATGGAGAAGAGAGAGAAAGTGTGAGAAAGTGTGTGAAAGTTAAAAATAAAAGAGAGAGAAAGACAAAG

>C101619_65

TTCCTCTTTCAAATTCGGTGGGGAAACATGATTGATTAATTAAATATATTTTTACATCAAAGGTAAAATGAAATTCATCAATCCTGGGTATGGAAATCCGTAATTTACTTATTTATTTTATTAGGGGAGGGATTGACTATTGATTAGATTCCAGCTTTAGGATTTCCTGAACCAGCCGCTGCAACTCCCATCCCACCAACACCTTTTGATGTAGATGTTACTGTGTCACGGACTGCATAATCTTTAACATCTTTAACATCCAATGCACTCACAATTTCATCAATTTTCCTAAATTGATATGGCCATCTTGCATTATAGAGAAACTGGCGAAGATCAAACCTATTATCCTCTGGTGAATAACTCTCAGCATGGTAGGAAGGTGTCAAGACAGTCATTTTGTGTCTATTGATAGAATAATACTTGAAGAAGTGCTTTACTTTTTCAGCCACTTGTGATGGAGTCAATCTTGCACTCCATCTGTAGCATAGATTCTGGAACATGGAAACTGGACCACAACGGAAAATTTTCCGCAGTCTTCCAAAAACTGATAGTTCCTCGTAAGTCATCCCCATATCAACTTCATCAAGCTGACTGTAATTAGAACGTATAGGCTCCAGTTCAGCTGTGGGTGGAGCTGCTTCAATATCTGCTAAAGATGAGTAACCAAGATGGGTGGCTGCCCATCGCAGGAATACCCGAAGGTCCTGCTTACTTATACTACCAATAGGATTTATATCTGCTGAGCTGCAGTCATACTTGGTCAAGTAACCGCGTAACGCTTCATCCACATTAGAGCTCCCCAAGACAAGATAAAATCCAGGTTTGCTATGAACCCAAGGTAAGAGTGATGCTAACATGAAAGCTAGCACCATTCTGATTCTAGCTTGAATGTTCTGCAAGCTTAAATTCTCAATATTAGATCCACCATCAACCTTATACCGTGGTCGTTTTCCTGTAAGTGTTTGGAACAATGACAAAAAGGCAGACACAACTACATCAATGCTAACATCAAGATGCCATGAACCAATTTCATCAGCCAACAACTTGGCTCGAGACCTTGTCATTTGTGAACTATTTTCAGATCCCATAAACACAGTATAGAATATTCTTTTAGCAAATTCTCTGCTGTCTGTAGGATACTCGCCATCCTTATAGTTTCCTATTCGTATTGCATCAGCTTTAACTTGCTCATCCCCATTTGCAATTTCTTTCACAACCAGTTGGCACATGCATCCAACAATTGCAGCAACAGAAGAACTGTCAGCTCCACCAGAAAGGGGAAGCAGAAAACCAGATGCTCCACTTCTTCTTAGGTAGTCCCATAACCAGCAACCAGGTCCAAATGCTATTTCCTCCTGCGGAGTGTGGTACTTTATCTTAAGTGGGAGAGAAAGGCATATTTTAAGGTTAAACGGAAGACAGAGACTGTAAGGTACTTCCACTGAAGGAACCTTCGTTTTACAACTTGCTTGCTCTTGGAAGCTACTTAAAGATCCTCTAAGGCTTGCAACCACATCTAGATCTATCTGAGCAACTACAACCTCAACATCCTTTAAAGAAAATTGTGAACCCTGAGCGACAACATCTCCATTTACCACAACACAGGCACAGCCATCATAGTAAAGGCGACTTCCGTCACAGCCTTGTTGATTACTATACATGTAAACCCCTCCACGAGTATGAGTGGCTCCTATAAAAGCATGAATACGTACGTCAAGCTTTCTAAGTTGGTGGTGACTTCCACTTGCATTCATAATCACTTCAACCCCATTCAATGCAAGCTCAGAATGAGGGGGATCTGGACTAAAAAGCTCTTCACAAACTTCGGCAGCTATAGCTGTGTCGTGAAACTTCATGAACCCATAACCGAATGGAACCGATTTCTGGCCTAAAGCCTGAGAAATTTCAAAGGGCAACTGAAAATCCACAAGCTGATCTCTTTGCTTCCACGCTGTGAACCATCGAAGTTCTCTATAATTGCCATCATTGGCAAGGCACATCTTTGGTCGTATCATAATAATCTTCCGGTTCAGACAGAAAACCTGACAATTGTAGCGCTCCGAACCTTGGATAATCGGCATGCCGAAGCTACACAAAATGCCATCAGTCCAATCTCCAAGCAGCAAATCTTTCAAGAACTCCCATGAATGGTTAACTGTGTCGAGTTCTAAGAAGTGGTCTTCGCAGCCATAACCAGGAATCTCGAGTTCGGGACCGAGCCTAATAGCTGCGCCGGCTTCTTTGGCCTTCGCTATGGACTCCTTGATTTGCTTTACGTTGCAATCGAAGTCCATCGCCCATTGGTTGAGACTGCATGTGGCAACCTTCAAGTGTCTCATCGCAGAACAGAAGAGGTTTTGATTCAGAATTTTGGGTTCCTGAACGTCGCAGATTACAATTTACAATGGTTGTAGTGAAAATTCCTAGGGGCTTTTTAAATTCTACTCTGTGCTTAGAACTTAGCTTCGATTCCACGAAGTTACTACGAAGATTATGGGTAGAGTTTGGTTGGGTTTGATTCGTTGTTCTTTCTCCCTGAGCCTTGGACGGGTTCTTTTTTTTTTTT

>C101621_65

AAACTACCTTCCTTCTAGAACAAAATCAAGTTACATTCAAAACTACCTTCCACTCTGTTTTCACCATCGAAGATCTGGGTACCTTCACCATTCATTCTTCAGCCCATTTACGATTTTCTGCAGACGCTACTTCCCACATGATGAAGCACTGCCTCTCCCATGGCTGTGGCTTGAACCATTGCGGCATTGGTTGATTTTATTTAATTTACACGAAAGTTTGATTCTTTTCATCTTTCCCTCAAAACCCGCCAACTACTGTCCAACAGGTTGCCACGTTGCTTTCTTTGGATTGGTGGGTTTGCCTTCACGTTTTGGGTTTGTTTGTGGGTTATGGGTTTTTTGAGGGGCTTGTTGTAGAGTATGGTGTATTATTGTTTTTGAACATGAAGCGATTGAAAAATGAACCTTCCAATTCGAGGAGGTTTAGGTTGTTTCATTTTTTGGTTGGTGTTGGGGTGCTATACTTGGTTTTTATATCGTGTAATTTTTCACAGTTTATGAAAGTCGTGTCATCGTTGAGTGGGGATGAGAGTTACGATGGAGTTGGATTGGATAGGGTGGCAGCTATTAGGGGCACTGATGATGCAGATTTGAGCAAGCCTTTTGTGAGTTCTGTTTATAAAGATGCTTTTCATTGGAAATTAATGGATGACAGGGACCAGGATGCTCCCTTGAGGCCAAAGGAGGAACCAATGAAGGAGGAAGATCATGGCCTTGAATCTGTGAAGCATATGGCCCAGGGATATGGTAGGCCTACCGGGGACATCTTGCGGCGAAGGAACATGACTGGTGATTTTTCTGTGCTTGAGAGAATGGCAGATGAGGCTTGGACATTGGGGTTGAAGGCTTGGAAGGAATTGGAGCAGGTTGATAACATGGAGATTGGAGAAAGAATTGAAGGAAAAACCGAGGCATGTCCTTCTTGGATATCAATGAGCAGGGAAGATTTGTTGAAGGGGGATGGGTTGATGTTTATTCCTTGTGGTCTTGCTGCTGGTTCTTCAATCACTGTGGTAGGGACACCCCGTCATGCTCATAAGGAGTATGCTTCTGTGCGAGCAAGGTCGAGGTCCACGTGGAAAGGTGATGGATTGGTATTAGTTTCGGTTTCACAGTTTGTTCTTGAATTACAAGGGCTAAAGTCAGTGGAAGGGGAAGATCCTCCTAAGATTCTTCACTTGAATCCCAGGTTAAGAGGGGATTGGAGCAAACGACCAGTTATTGAGCATAATACTTGTTATCGAATGCACTGGGGAACAGCTCAAAGATGTGATGGTTTGCCATCTGAGGATGCTGAAGAAATGCTTGTTGATGGGTATAGACGATGTGAAAAATGGATGCGGAATGACATTTTAGACTCAAAAGAGTCCAAGACAACGTCATGGTTTAAGCGATTTATAGGGCGTGAGCAGAAGCCCGAAGTGACATGGCCATATCCATTTATAGAGGGTAGAATGTTTGTGCTTACATTACGTGCTGGTGTTGATGGATACCACATTAATGTTGGGGGTCGCCATATGACTTCATTTCCATATCGGACAGGTTTTACACTTGAAGATGCTACAGGATTGTTGGTTAAAGGGGACCTTGATGTTCATTCAGTTTATGCCACTTCTCTCCCTACTTCACATCCAAGTTTCTCACCTCAAACAGTACTAGAAATGTCAGAGACCTGGAAAGCCAGTGCTCTACCTAAACATGCTATTAGACTCTTTATTGGAGTTCTTTCTGCATCAAATCACTTTGCAGAACGTATGGCAGTTCGAAAAACATGGATGCAAGCAGCTGCTATCAAGTCTTCGGATGTGGTAGTACGATTCTTTGTGGCATTGAATCCAAGGAAGGAAGTAAATACGGTGCTGAGGAAGGAAGCTGCTTACTTTGGTGACATCGTCATTTTGCCCTTTATGGACCGCTATGAGCTAGTTGTGCTTAAAACCATGGCTATCTGTGAGTTTGGGATTCAGAATGTGACTGCTGCATATATCATGAAAGTTGATGATGACACTTTCGTCAGGGTGGATACTATCTTAAAAGAAATTGAAGCTGTACCCCGGAAGAAGCCCCTTTATATGGGCAATCTCAATCTCTTGCATCGCCCTATGAGAAACGGCAAATGGGCTGTTTCATATGAGGAGTGGCCCGAAGAAGTATATCCTCCTTATGCAAATGGCCCTGCATATATACTTTCCAGAGACATTGTTACTTTCATCATATCTCAACACAAGGAGAGGAAGTTGCGGCTCTTTAAGATGGAGGATGTAAGCATGGGAATGTGGGTTGGGAGATTCAACAGTACAGTGGCCGCGGTTCAGTACTCCCACAACTGGAAGTTTTGTCAGTATGGATGCATGGAAGGGTACTTCACTGCACATTATCAATCACCAAGGCAAATGGTCTGTCTACGGGACAAATTGTTCCAAGGTCGTGCAAGATGCTGCAGCTTCAGATGATTTGCCATTGTATATTTGAAACTCTTGTACCTACTTCACAGATTAGTATTGGAGAAAGTTATTACACATTCAATGTGAAAGAAATATAATTTAGCATTGTATGTACATGGTCTT

>C101623_65

AATAATAATAATAATAATAATAATAATAATAATAATAATATAGTACTTCTATGAATTAAATGACTAGCCAATCAAGACACACCCCTCTTCGAGTGAGTGATGAAGCATTGATCAACCTTGTGGTGCCAGGTGCTACCATCAATCCCAGTTAATCGAAATTGGGGTCCCAACCACGTGCTTCCCATCGGACCATTCTAACCTTCCAAAGGTACTAACTCTCTGTTGGCCCGAACTCGAGGATGAAAAGGTTACCACGAAAGATTTTTTCTCATTTTCCTTCAAACTTAGCGTTTGCGGTTCAACCGAGATTTTCACAGATGCTGAGTCTGACGTCACTGAAGCCTTGTATGTCCCTGCAGGCCCCACGTTAGTGAGGGTCCGCGTGTGCTTAACCACGCCAGATCCACTCCCTGATTCAAACAACACAGCGAACGAAGGGTAGTTAAGGTCGTTCACACTGTACTGCTTTCCCGCGTCGCACTGGAATTTTCTCTTAGCCAGGGTGTTTATTTCAGTGGCAGAGTAGTTTAACGCGCAGAGAAAACCAAGATAATCATCCACTGTTAGATCATAGACCAGGCCTGGATTAAGGGCAGCCACGGGGTCCACGTGTCCCGCTCCGTGATCGAACGGTGTCGACGGTTTTCCTGTTGCACTGTCTTGCAACTTCGCACCGCTTTTGTAAACTGTATAAGCCGTTGTCATTAGAGCCGATCGAACCGCGGCTGGGCTCCACCCGGGGTGAGCCGATTTGATCAAAGCGGCTAAACCGCTCACGTGAGGACAGGACATGGAGGTGCCAGAGATGATGTTGAAATCCACGCGCCTGTTATCAACGGGCAAACCGGTGGGACCCACGGCCTTGGACCACCCCGCTAAGATGTTCACACCTGGCGCGATCAGATCTGGTTTCAAGATCTGCGGCGTGATTGAATTGGGACCTCGTGAACTAAAAGCTGCAACCACCGGTGATGGCTGAATCCCCACCTTCGTTCCCTCGAACAAAATCCTCACCGTCGGTTTCGCCTCGGAAAACAGATACTTCTTGATGGCGTCGCCGGCTTTCTCCCCAACAGCCGAAGCCGGCAATAGATGAGCATCGGCCACCAGCTCCTCACCGTTGGCGGCGGTGTTGCTCAACACCATTCCCAAAGCTCCGGCGGATTTCACCACCGAACCTTTCTGAACCCTAGCAGTTAATCCACGGTCACATAACACAATCTTTCCGGCTACTTTCTCCGGCGACAAGGTTCCCGTCACGCACAAGTTCCCGTTCGTAGCGTTGCTGGCATTCCCTGCGTAAACAAACGGTAACGGCGTATCAGGTAAAGCGTTACCTCGGTAGAGCGACACGCCGGAGAAGTTAAGTCCATTCCCGATAGCAACATAAGCAGGGAAGTCGCGATCCAGTGTTCCAGCACCCACGGTGGTAATCCATGGTGCCACATTGGAGAGACTGTAAGGACTAGGACCAGCATTCCCAGCGGAGCAAGAAACCAAAATCCCTTTCTCCATCGCGGAAAAAGCTCCGACAGCGACACTGTCTCTGTAATAATCAGCCATGCCACCACCGAGAGACAACGACAGCACATCAACGTTGTCAAGTATCGCCCTCTCAATCGCCGCAAGAATATCGGAACTGAAACACCCTCCCTTCCAGCAAACTTTGTACGCAGCGACTCTCGCGCGCGTGGCCATCCCACGCGCCGTCCCGGAAGCGTAACCGAAGAGACTCGCGCCAGTAACCACCGATCCCGCGGCCGTGCTCGCGGTGTGGGTGCCGTGACCATCGTCGTCGCGAGGGGACCTAGACTCCTCAGTCTCGTTAATGGGACCCAACATGGCCTCACAACCCTTGGGAAAGAACCTGGCTCCGATCAACTTTCTGTTGCAGTTGGAGGCGCTGAAGTTTGTTCCCGTTTCGCACTCGCCTTTCCAGCTACTGGGGACAGGTCCAAGCCCCGTGTCGTCAAAGCTCTTGCTCTCGGGCCAAACGCCAGTGTCAAGGACTCCGACGATGACGTCACTCGCTGCGCTCGACTCGGGGAACATGTCGGCGCTCTTATCGAGTCCGAGGAACTGCGGCGTCCTCGTTGTGTGAAGTTCATACCTCATCTCCGGCAACACCGCCAGAATCCCCGTTTGGCTCTGAAGCAAACGCGCTTCCTCTGCGGTTAACCTAGTGGCGTAACCGTGGATCGCGTTATCGTAAGTGTAAATGATTTCCGCTGAGTCAGAGACGGATTTGAGCGACGACTCATACCACAGAGCATGGTGTTCAAAGCTTTGGGGCATTTCTGATTTGGCGACATGTACAATGTAAGTAGCCTTCTCCGGTTCTGCTGCCTGGGCCACAGCGTAAAGACCCAGAAAGAGTAGAAAGGAGAGAAAAGATCTGGGAAATGAACCCATTAGAGAGTATGAGGGAGATGGGTAATGGAAAATGAAAAATAGATGTGGTGGATTTATGGAGTTGTGTTTTTAATGCACACTTTATGGGAGTGTGCCCGTTTAGCAAGGTAAGGAAGCGGTGGGTGGTTGTCTGCTTCTCAAAATAATTGCTTTGAG

>C101625_65

CTTATTTACTCTTCCTTCTGCTCCTTCAGAACAAGCCAGCTTCAATGGAGCACTGGGCGGTGAGGGAGGAGGACGAGGAAGAAGAAGAACAACAACTGCAACATCATACAGCTTTGAAAGGAAGACAACCCTTGCAACAGGGAAAAGATGGTAATGTTACTGCTGAAGCACCTCTCTTTTTTTCTTTAGTGGAAGAACAACCTGTTCCTCACAAGGAGGAAGTGCTTCATACTCACAAAGAAGAACAGAAAGAGCAACCTACTCAGAAGGTTGAAGAAACAAACAATGCTGTTGCTGAAAACGGCAATAACTTCACCGATGATAGTGAAACAGTTGGGCTTGGAATCATAGCTGAGATTGTAGAAAATAGCATAAATGGAGAGGCTATAAGCAGTGCCAGCATAATTGTTGATGCATCTCAGCATACGAATAGTGTTTATTTTGACAAACAACTAGGCAATAATATTGATGGTGCAAAGTCTGCTCTTCCAAAAGGTCTTGGAGAGGGAGACTTTGATATACAGGTCGAAAATTCTGACATTCAGTGTAGTCCATGCAATGGAGTCAACAATCATTTCATGGAAATTGTTAATGAAAAAGTCCCTTCCTCCGGCTCAGAGTTACCACCTCTACCTAATTCATCTGAAATACAGGCTACTGCAACTGTGGACAATTTTGGTGATGAAAGCAGTGAAAAAGCATATCCTAACCTGACAGAAGAAATTGACCAGCAACAGAAAGAATTTGATGTTGAGGCAGTGTTGGCAAAACAAGAGACACATGACTTGTTTTGTCCTAATTGCAAGTCTTGTATTACTAAAAGGGTTATCCTCAGGAAGAGAAAAAGCACTATTCCTATTCCCAACTTAGAAACTAAAGCAAAGCGTGACAAGCCAGCCATTGAGGTGGTTGATAGTTCTGTAGATGAAGGCAACCAAGGTGATCATGCAATTGCAACAACTGATGATGTAGGTACAGTAGAGCCACCTGCTGATAATTATGAGCCTGAAAGAGAACCAGAATTATTCAGATGTTTATCATGCTTAAGCTTCTTTATTCCTATGAGAAATGGATTCAAACTGTTCCCAAGTTTTGGTGGTAAGCCTGAAACTTCACAAAAACTTTCGGTTATACCAGCATCCAATGTGGAAAATCCTTCCATTGATGCAGTTTCCAATGCAAATTGGTTCTTCTCTCTGTTTACTTCAATTAAGGGAAGAAAAGCTAGTGCCCAAGGTGACGCTTCTATTGAAGGTTCTAGAACAGATCCCGCTTCAATTGAAGATTCTAGAATTGATAATCCAGAAAGTCCACCTGCTAATACACCTATTAGCCACGAAGGAGTAAATTCTTCGATTCCCTCAATAGTGGAGTCAATTGAAAAGATTGAATCATGGATAGAAAAAAGCAAGAAATCCAATGTGGCACTGCTGAATGAGCCATTGGTTGAGGAGAATGATTCCAGCACACAGAGTTCCTGTGATGTGGAAGACAAAAATCGTGATTCAGTGGATGTGATAAAGACAGATGTTGTTACAGATATATCCAAGCTTGATAGTGTTGTAGTAGCAACTGTTGACACCACTGAAGTACTTTTTGATGCTGGGAAACCCTCAAAAGATGACATTCTAAAACCATATGAAGGGTCTCCAATCTTGGACAAGGCACAGAAAGACGTAGACAAGACACTGGAAATAGCTCAGGATAGTAATTCCTCTTTGATGAAAGAAGAAGAATTACCAGATCAATCATTTGGCAGTGAAGTTGTTGCAAATGATACTGCGACAGAAGCACATACTGAAATCTATATTGGAGAACAACCAAGGGCCGAAGTTGGTGAGCACCAAGAGTGGGAAATGCTTAAAAGCATTGTATACGGTGGTTTAGTTGAATCAATCACTAGTCTTGGGGTTGTGTCATCTGCTGCTTCTTCTGGTGCTGCTCCATTGAATATTATAGCATTGGGGCTGGCGAATATCATCGGTGGACTTTTTGTTATTGGCCATAATCTCATGGATTTGAAAAATGGGAATCCACCACAAACGAATACACAACAAGATCAGTATCAAGAACTACTTGGCCGTAGAGAAAACTTCATACTTCATGCTTTTCTAGCTGTTTTATCATTCTTAATTTTTGGTTCTGTCCCACTTGTTGTCTACGGCCTCTTAATTTCAAAGCAGTACTATGATGAAGTTAAGATTGCAGCTGTGACAGCCACCTCTTTTGTGTGCATCATACTGCTTGCTATGGGAAAATCTTACACCAGTAGACCACCCAAATCCTATGTCAAAACAGTGTCACATTATGTTTCCTTGGCACTTGCAACCTCGGGAATCTCTTACATAGCAGGTGATCTAGTTAAGGATCTCCTATATAAAATCACTGGCTCAGAATCTGGTAATGTTCTTGCCATGCCCTTGTCAGACACAACAAGAATGGAACCAGCGTGGATGTCTTATTGAAGTTTATCTTTAAATGCTCCAGCTGCTGCTGCTGCTGCTTCTAGCCCAATAAGGGTCTTGAAAATATATATGGTAGCATGTTACAGTTCAAAACAACGTTCTCA

>C101627_65

ATGATAGTAATGGATAGTATTTGCCAAAATTGAAGACAGGGTTAACTTAAGATTCCTTGTTTGTACAACTTGTCACTACCTTCCACTGGTAATTGACCTAATGCTGGATACCGTTTCAGCTGCTCTAGTGCATCAGATTTCATCAGAACCCCAGCATGCCAATGCCAAGGTTGAAGATAAGTTGAATCAATGGATGAGGACCATGAAGATGGATGAAGTATCACAGATCCCCAATCTCTACTTCCAACCCTTGCTTCCTTTAATCTTCTTCTCACCAACAAGCCTCCCAAGTCTTGTTTCTTCATTTCTGATACACTTCTGAGTATTGAAATGCAAAGGAGAAGCATCAGCACTGCATCTTCTTCAGGCGATACTTCAACAACCAATAGCCTCCAATTTAGAAGTGCAGTTGCTTTTCCATCTGGGTTATCTTCTGTGAAACGTACAACTGTTAGAAAACTCTCCTCATCTTCCTCTTCCTCCTTCCCTTTCTCTTCTAACTCTATTTTCATGTCTTCACCTTTCTTTGACTTTGTTTTCTTTACTTGATATTGCATTTGCCTCCCTCTCAATAACTGCACCGATGACTCTGGAGAGGCTTGATTTAGCAAGCTAAAGGTGAGATCTGACACTGATTGTGACGACTCCCATTGTATTGTCAGTTCATCTCCTGTAGAAAAGTTCCATGCAGCTTTACATTGTTCTGTAGGTTCCTTTGGCGTTGCAGTGGCCACCAACTTCTCAGGGGCTCTACCAATGGAACCAGCAACATAAGACCAAGATCCTTCACGTATCTCTATAATCCTATCTTCCCATTTTACAGCTGAAGGAATTTCACTCCCTCTTCTCCAAAATCCTCCTCCTATCCTAATTCTAACAACAAAACACACTCTCCCTGCATGATCAAGAACTGTGCGAGACAACCATCGACCTTCTTGTGGACGATAACTATTCATTTTCAGAATCACATCAGATATCATTGCCCCAGAATCGTCAGTGACTCGATCAGGTACACATTTCAACAAGTATGGCGCTTGAACTGGTGGAGTTATTGATGTAACAACGTTCACCTGTTGACTCACTTCCTTTTCCAAAGTTAAAGAATCTGCTCTCAATAAATCATTCCAGCTGAATGAAACAGTGTCCAGCAGGCTACTTCCTCTTAGACATTTACCACCATGACGACGGTACTCAATCATAACTCCCTTAGTACCAAATTCACAGTAAAAGTGCCAAGCTTTTTTCCATGAATCATTGGTGAAATTGGAGAAGGGTTTTTCTAGCTTTAGTTCACCATAGCACCTTGTTATTCGGAGGCGTAAGAAATCACGGTTTATATCCTTCTGCAATGTTCTTATTCTTTGCTTAAGTCTCACAAACACACAGGCCTCAAGTAAAAATCTTGGAAGCATGGACCTGTATTTTGTATTGACATCTGTGCCTGATTCTTCCCAATAGACAGGGGATTTGATTGAAGTAACACCTTCCAATGTGAAAGGCACCTCTCCACCAGCTTTCTCATAAGGTTGATTGAATGCCCTATCCCACAATTTCTTGGTTTCTTCAAACTCCTTTTCCTTCACGGTTTCAGACAGAGTTACCACGTTCTGCAGATCACCTTCTACAGCTAATGCTTTCAAATCTTCAAAATACACTGTTGGGTAGCTCTGGTGGGTCAGCCACATTAAGAAGATGTCCGAAGTAGGAACGAAACGCGAAGAAAACTCACGCGCGAACCTCTGCAGCATAAACAAGAAAGCCTTGTATCGCTGTCTCGCGGCAATCAAGTAGACAACCTCAGACCTGTACGGCTCTACGAACATGGAGCCCAACAACAACCTCTGCTTCTCAACTTCCTTAAACACACCCTCCTTTAAACTCCCCTGAACAACCTGGGAACCCGAATCCTGCGAATCCGAACTCGCCTCGTTCTCAAACGACTCCAACGGGTATCTACTGCTCCAAATCTCCATGCACCGCATCAACGCGTACTCTCTGTTCTCTTCGTCGAAAATTCCCGCTCTCCCTATCAGCTTCGAGAATCTCCTCTCGCAGTATTCCCTGTAACTCACGGGGTTTAAAGTGTGACAGAACCAAACCCATTGGACGTCAAGGGGAGGAAGAATCACAGGAGGAGCAGAATCCGGAAGCGTGAGATCAGCGATCAACGGCATCCAGAAATCATGGTACCTTCTTATGGCTTCCACCATAATGGGCGTGTGGTGAAGCCACACCGAATCGGCCACGGTTCTCAAGAAGGCGATATTTCGCCTGGCCGCCGAGACGAGATCGATCCCGAGACGTATGGCGGCGATTTCCGGTAACTCGCCGAGGCTTGTGGAGGAGGTGGTGGGGGAGATTTCGAAAGCGTCGAGATTGTCGGACATGGTAATGAAGTGTGAATGAAAAGTTGAAGAGCAGAAGTATGGTAGATTTTTAGTGAGTGAGAAACATGATTGAGTTGAGTAATGAGAGGAAACACTATCTGGTGAAATTGATGAAGACGTTGTTGTTTGTCGGAATTTGGAACAAAGAGAATAAAGAGTAAGTAGAGATGGAGTTGATTAAG

>C101629_65

ATAACAATAATATATAGGATTTGCATTAACGTATTCTTCTTCGGAGGAGGGGGAGAAGAAGACGAAGAAAAGAAGGGTTGCAATTGCAACCGTATTCTGTTAAGCTCCAAAATCCAATTCCCTCTCTAACCAAACCTCCATTCAGGTTTTATGTTTGACGGAAGAGTTCAATCAAATGAGTTTAACCATGATAGATTTGGGAATTTCATTTCTGAGGTTGATCACTAGCCCTGATGCTTCTGGTGCTTCTATTCTTGGATGGTTCATCGCTGGATCATTTGGACTAATGGCAGCCATATATGCTGTTCTCAAGTGGCAGCGAAGGTCCTCGTTGAATTGGATTAAAGCTGCAGCAAGAGAAAAGAAGAAAGTTTGGAAAAAGTTTAAAGTGCCACTATCGGATCATTCTTGGGTTGAAGATTTTACTAAAAGGGAACAGCCATCCACTTGTTGTTTCTGCTTGACTTCCCTTTGGCCTTCTCAAAATTTAGGTACAACAGCCTCACCGCATACCCCTCTCCATCGTTGCTCTGTTTGTGGTGTCGCAGCTCATTTCCTTTGTTCTCAGTTTGCAGCAAAGGATTGCAAATGTGTGGCTCAGGCTGGTTTTGGCCATATTCGACACCACTGGTCAGAAAGATGGGTTAATGTGGATGAAAATCATGAGATGTCTGCCTTCTGTTTTTACTGTGATGAGCCATGTGGTGTTCCGTTTGTTAAAGCTTCTCCAACATGGCATTGCCAGTGGTGTCAGCGCCTCATTCATGTTAAATGTCATAACAAATTGACTAGAGATTCGGGTGACTTTTGTGATTTGGGTCCTTTGAGGCGAATTATCCTTTCTCCTCTTTCTGTCAAAGAAGTTGATGAAGATCAGAAAGGAGGAGGACGATTAAGTTCTATTATAACCTCTTCTGTTAATGGCCAGATTAGAAAGCGGCGTAATCGCAATAAAAATGGAAGTGGCTACAATGCTAATGGTAAGTCACGTGGTTCCTCAGTTACTGATGCAACACTCTTGCAATATGTGTTGAATGGTCTCAACTGGAATAAGCCCAGTAATGAAAAGTGTTTTGATCAATTGAGTCATGGTAGAGTAGTAGGAAACAGGTTAACTTCTAACCATAGCCACATCAAGAAATACACACTGGTTGATTTGCCACAAGATGCAAGCCCACTTTTGGTCTTCATAAATGCCAGGAGTGGTGGTCAGCTTGGGCATTCTCTGCATAGGAAATTGAACATGCTATTAAATCCTGTCCAGATCTTTGAATTGAGTGCTTCTCAGGGCCCTGAAGTGGGTCTGGAATTCTTCAAAAGTGTACGATATTTTAGAGTACTGGTATGTGGTGGAGATGGCACGGTTGCATGGGTGCTTGATGCTATAGAAAGACACACTTACGAGTCACCTCCACCTGTGGCAATTTTTCCCCTTGGCACTGGAAATGATTTGTCTAGGGTACTGAATTGGGGAAGAGGCTTCTCTACCCTTAATGGAGAAGGCGGATTGACCATGCTTTTGCATGACATTAGCAATGCAGCAGTTACTATGCTAGATCGCTGGAATATTAAAATCTCAGAAGAAAGCGCTGAAGGAAAATCAAATAAAGTGAAAACTAAATCCATGATGAACTATCTAGGCATTGGATGTGATGCAAAGGTTGCATATAAATTTCATGTTACACGACAAATAAATCCTGAAAAGTTTTCTAGCCAGTTTTTGAATAAATTGCGATATGCAAAAGAAGGAGCAAGAGATATTATGGACAGAACTTGTGCTGACTTGCCATGGCAAGTATGGCTTGAAGTTGACGGAAGAGACTTTGAGATTCCAAAGGATTCTGAGGGCTTAATTGTGCTCAATATCGGGAGCTATATGGGTGGAGTAGATCTTTGGCAAAATGGTTATGAGCATGATGATGATTTTAGTCTGCAATCCATGCATGATAAAATGCTGGAGGTGGTATGTGTATGTGGAGCATGGCACCTAGGAAAACTTCAGGTTGGACTTTCACAAGCAAGAAGGCTTGCTCAAGGTAAAGTCATCAAGATACACTGTTCAAGTCCTTTCCCAGTTCAAATTGATGGGGAGCCATTTATCCTACAACCAGGATTCTTAGAAATTACTCATCGCGGACAGGCATTCATGATGAGGAAGACTTCAGAAGATGAGCCTAAGGGAAAAGCAGCTGCTATTATGACAGAGGTATTACTTGATGCCGAGTACAAGGGCATTATTAATGCATCTCAGAAGAAAGTTCTTCTCCAGGATATGGCCATCAATCTTTCATAAAGATCCATCTCTGGCTCAGCACGCATTATACATAGTTTTACCCTATACCATATACATGGGTTTTTGACTTTTTGCCTTCTAATCCTTTTTATAGGACCAATTTTGGTAATATATTGGAAATAGAGCAGAAAGATTAGCTCCACGGTGGCAAATTTAGTAAGAGTGTGAAAAATGTGCGGCGGCTTCATCGAGGTTTCAGTTTACTGTTTGCGTTGCGTCTAACACCCCACCCTGTGTATATTCTAACCCGGCAAAATATCTTTTCATCAAATCCTTT

>C101633_65

GGCGCCCTCTTTCTTCCTCTGCTTCTTCAGAAACAAAGAAAGAACTAGTCAATAACCACACGCAAATAAGGAATAATGGCATCCCATATTGTTGGATACCCTCGCATGGGCCCCAAGAGAGAGCTGAAGTTTGCTTTAGAGTCATTCTGGGATGGAAAGAGCAGTGCAGAGGATTTGCAGAAGGTGGCTTCTGATCTCAGAGCATCCATCTGGAAACAGATGACAGATGCTGGGATCAAGTACATACCCAGCAACACTTTCTCTTACTATGATCAGGTGCTCGATACCACCGCCATGCTTGGCGCAGTTCCACCCAGATATGGGTGGACAGGTGGCGAGATCGGGTTTGATACTTTCTTCTCCATGGCTAGAGGAAACTCGTCTGTGCCGGCCATGGAGATGACTAAGTGGTTTGACACTAACTACCACTTTATTGTCCCTGAATTGGGACCTGATATGAATTTCACATATGCTTCCCACAAGGCAGTGAATGAGTACAAGGAGGCCAAGGCGCTTGGAGTAGATACAGTTCCAGTCCTCGTCGGCCCTGTAACATACTTGCTGCTCTCCAAACCTGCCAAGGGTGTTGATAATTCCTTCTGTCTTCTTTCTCTCCTCCCGAAAATCCTTGCTGTCTATAAGGAAGTTGTGGCTGATCTGAAGGCAGCTGGTGCTTCATGGATTCAATTTGATGAACCTATCCTTGTCTTAGACCTTGACTCTCAAAAGTTGCAAGCAATTTCTGCAGCATACGCGGAACTTGAATCTGCTTTATCTGGCTTAAATGTTCTTATTGAGACCTACTTCGCTGACGTTCCTGCTGAGGCATACCAGACCCTCACATCTCTTAATGGAGTCACAGCATTTGGATTTGATTTAGTCCGCGGAACTAAGACTCTTGATCTAATCAAGGGTTCATTTCCTAGTGGAAAATACTTGTTTGCTGGAGTGGTTGATGGAAGGAACATTTGGGCTAATAATCTTGCTGCTTCACTCGATACATTAAATAGTCTTGAGGCCATTGTGGGCAAAGATAAACTTGTTGCATCCACCTCCTGCTCACTTCTTCACACTGCTGTTGATCTCTGTAATGAGACCAAGTTGGATAATGAGATCAAATCATGGCTGGCTTTCGCAGCCCAAAAAATTGTTGAAGTAAATGCATTGGCCAAGGCATTGGCTGGTCAAAAGGATGAGGCCTTTTTCTCTTCTAATGCTTCTGCTCAGGCTTCAAGAAAGTACTCTCCAAGAGTGACTAATGAGGCTGTTCAGAAGGCCGCTGCTGCATTGAAAGGTTCTGATCATCGCCGCGCCACAAATGTCAGCACTAGATTGGATGCTCAACAAAAGAAGCTCAACCTTCCTATCCTCCCAACCACCACTATTGGATCCTTCCCTCAGACTGTTGAACTGAGGAGGGTGCGTCGTGAATACAAGGCTGGCAAGATCTCCGAGGAGGAGTATGTCAATGCAATTAAAGAGGAAATCCGCAAAGTTGTTGAACTCCAAGAGCAGCTTGATATTGATGTCCTGGTACACGGGGAGCCTGAGAGGAATGATATGGTGGAGTACTTTGGTGAGCAGTTATCAGGCTTTGCCTTTACCGTTAATGGCTGGGTGCAATCTTATGGATCTCGTTGTGTGAAGCCACCAATCATCTACGGTGATGTGAGTCGCCCCAAGCCAATGACTGTCTTCTGGTCATCCACTGCTCAGAGCATGACCAAACGCCCGATGAAGGGAATGCTTACTGGCCCCGTCACTATTCTCAACTGGTCCTTTGTTCGAAATGACCAGCCTAGGTCTGAGACCACCTATCAAATTGCTTTGGCTATCAAGGACGAAGTAGAAGATCTTGAAAAGGCTGGTATTACTGTTATCCAAATTGATGAGGCTGCTTTGAGAGAGGGGTTACCATTGAGAAAGTCAGAGCAAGCTAACTACTTGGACTGGGCTGTTCATTCCTTCAGGATCACCAATGTCGGGGTGCAGGATACGACTCAGATCCACACCCACATGTGCTACTCTAACTTCAATGACATCATCCACTCAATCATTGATATGGATGCTGATGTAATCACCATTGAGAACTCTCGCTCTGATGAAAAGCTCCTGTCAGTGTTCCGCGAAGGAGTTAAGTATGGAGCTGGAATTGGCCCTGGAGTGTACGACATTCACTCCCCAAGAATACCACCAACTGAAGAAATTGCTGATAGGATCAACAAGATGCTTGCAGTGCTTGAGCGAAACATTTTGTGGGTCAACCCAGATTGTGGGCTCAAGACTCGTAAGTACCCTGAGGTGAATCCAGCCCTCACAAACATGGTTGCTGCCACAAAACTCATCCGCAACCAGCTTGCCAAGTGAATGGTGTCACTAAAGTAGAGTCTCAGCAATCACAATGGTTCTGCTGGATTCACGGAGGAAGGAAAATTTGCATTTTTTGGTTGTTGCAATAACTGAGTGTTGAATATTTAGCCGCTTAGTACCATCATTACTTTATTTTCTGGCATTGCCTCAATGTTATATTGATATGCATATGCAATAATGTACTTTTTTTTCATGCTTCAA

>C101639_65

CTTCATAATTCCAGTAATGGGAAACCCTTTTTTTCAATTCCAGTGAGTCTCCACACATTTTCTAACATGAAACTGCGACATGATTGTGTAGTAGAATCACAGATGTTTTTGTAATAAAAATACAAAATATAAAGGTTAGACATAACAGAAATTATAAATTTGCAAAATTGTATATTCCCTCCCATGTATGACAGCTGAGACTAACAGCAGAAAAATGTCATCCAGGTGTCTTCCAATTTACCAAAGAGTGGTCTCTGTTCTGTCCACAAAATATGTTTAACTGTAACTCACTTTCCTTTACTTTCCTAGGATGATTGGAGTATCTTCTTATAATCTTGGAACGTGCCATGGAATGGTGACACTCTTCCTTCGGATACAACCCATAGTTCCTCCACGCTTCCAGAGATAAGGTGTTCATCGTGACTAACCATGAGAATGCCTCCTTGGAACAGCACAAGACCTTGGATAAGCGCCTCGACAGCATCCAAATCCAGATGATTGGAAGGCTCATCAAGCAATATTATGTGCGGCTTCTTAAAAGTAATCTTTGCAAAGGCAACTCTGCTTTTCTGACCACCTGACAAAGTATACATTGGCTGCAGTGCGAGATTTCCAGTTACACCAAAAGAACCTAAGTGAGCTCGAAGTTTCTGTTCTGGAACTCCAGGATAGCAACGCATCATATACAAAAGAGGATTTGAAGATAAATCCAGTCCATCAACATGGTGCTGACTGAACACTGCTATACGAACCTTAGCAGAGCGGAAGACAGTCCCAGAACTGGGCTGAAGTTCCCCAGCAATTAACTTTAGTATAGTTGATTTGCCAATACCATTTGGTCCAACCATTGCGATGCGGCTGTCGAGATCAATCCCAAAGTTCAAATTCTTAAAAAGAATGGGGCCCCCAGGATAACCAAATGATGCATCACTGAAACTTATTATCGGTGGACCAGGTCTATCATCTGGAGTGGGGAACTCAAATTTGTAGTCAGGATCATTAACAATTTCATCCACATGGCCCATTCGCTCCAAAGCCTTGATTCTAGATTGAACAAGTGATGCCCTCTTTGCATTATAACGGAACTTGTCAATGAAGGTCTGCATATGAGATCTTGCACGCTCATTTGCCTCCAATGCTTTTTGCTGGTTTTTAATTTGTTCTTCTCGAGTCTTCTCAAAAGTATCATAATTCCCTTTATAAGTAGTCAGCTTTTGGTTTTGTAAGTGAACTATATCAGTAACCACCGTGTTTAAAAATTCTCTAGCATGTGAAACAACAATAAATGTTTTTGGCCATTTAACCAAGTATGCCTCAAGCCATAAGACAGCATGAAGATCAAGATGATTCGTAGGCTCATCAAGAAGCAATATATCAGGCTCTATAAACAGAGCACGAGCAAGGGCTATTCGCATCCGCCATCCTCCGGAAAATGTTTTTGTTGCCCTCTTCTGCATCTCTGGAGAGAAACTCAAACCCGCTAGTATAGATGCTGCTCGTGCCTCTGCAGAATCAGCATCAATGTGTTCAAGCCTCTTATATATTTCCTCAAGCCTTTGTGAGATGTTATCCCTGCCAGCCACTCCATTTGAGTCACCCTTTTCAATTTTGTCCTCAAATTCCCTCTGTTTGGCAACTAACTGAGCTTCTTCATCAATAAGCTGAGTCCTCTCAATGTCAGAGTTGAGAACACACTGCAAGGCTGTGATATTATCACCTGTCACCTCTTGCTCCACGTGCAATATTTGGCAATTCCTTGGAACACCATCAATGGCATGCATAGCCATGTGTCTAAGAAAAGTGGTTTTCCCTGTCCCGTTCCTTCCTACCAAACCATAATGCCTTCCAAACGAAAGCGTGACACAACCATCCACAATTAAATCCCGCCCCCCAACAGAGATATTGAAATTCTCCATGTGAATGTCCTTCACGTTTGGTCCCCCAGTATTCTCATGCCTCACACACACCACTGGCATCCCCGCTCGTGCCGCCTCCATCTCTGCCAAGTGCATTTGGTATTGTGCCTCTCGCTGGCGTTCATCCTTGCGCTTCCTTCTCTCGAGCTTTAGACGGTCGCGTTCCGATAGCAGAGGGCCGTCGACGGGCTCGGCCTTCTTCTTAGGAGCTTCGACGTCGTCTAAACCTTCGTTCATTCGAAACGGTGCCGCCAGGCTTCGCACAGCAGGCTTCGCCTTCACCAAACCGTGCTTCCCGAACTTGTCGCACAGTGTGCTACACACAGAGCGGCACTCGGAGAAATCATCGACGCAGCCGGCGGCGACGAGGAGCTCGCCGAGGGCATCGAAGGCACCTTCGCCGTCGAGGCCGAAGTCGAAGTCATCGTCGGCGAGGACGTTGACAATGTAATCGACGATTGGCTGGTCGACGTCGACGACTCTTTGCCCCAAAACGTCGTGCACCACTGATCTCGCCACCTCCGTCATCTTCCGCCACCACTCAGCTTCTACTTTGTTCTGCTCTGCGTGGTGCTGGTTTTGCCCTTTAGGGGCGAAAATGGAAGTAGAATAAGGTTTTGGAATAATTACACTCCACACCTCCTACTTTTGAAAATT

>C101641_65

TTTTTTTTTTGAATAATTCAAGAAAGCCCTTTTGCATATATTTTTGAAACAGCAATTACAACGCAATATGACAATACAGCTCGAGTCATTTTGTAAAAAGGGTATGACACATGAAGCCATAATATATACAACTTCCTAGGGGTGGAAAACTGCTTCAAAGCAGTGCACTACTGCTGGACTTGGACCATTGATGTTTCAAACAACTAACTAGTTGAACTCACTTCTTACAACTAGTCCTTTCCATCTTGAGCCTTTGGAGATCCATTTCCACCCTGGAATGCAGCCATTTCCTTTGCCTCTCGAGATGTCACATTGTCAATCTCTTCATCAATGGGAAGTGTCCTTTTTGATCCTTCCAATGCAACACCAAGCACTATATTCTCTTCAAGAACAGTCCTAGATGCATGAGGCTGTGGAGCAGGTTTCTTTTCCCCTTGATGTTTTAATGGTATATTGGTATTAAGTCCACCAGTTTTGTCAGCATTGTTAGAAGAGGAGGACACTACATTGTCAGACTCAATATTTGGCTTTATGTTTTTAGTAGTCTGACCCTTGGATTGTTTGGCATCTATGTCCTTGTGGGCTACATTTTCTACTACCGAACCGGATACTTTAGATTTAGAATAAGATTCTGGAACCTCCACGTTTTCCTTGTTTGAATCTGATTTCAGTGGCTTGTTTTCACCCACTTCAGGATCAGGTTTGGGTGTCACAACTGTCTTTCCGTTTGTGTCAGAGTTAGATGTTACATCTGTCTTAGAGTTTCCATTTGCATCAGAGTTAGATGTCTTATTATCTTGATCAACAGCTGGTCGTGCTGATCGGCTTTTCATCTTATCATCGCCATTGATTTTGTAAGACGGCTCAATCACTAATAGTGGACGATTAGGTACTGTGCCAGCACCACGACCAAATGTGGAATCTGCAAACGGTATATTGTCTAAGTCAGCATCACCGTAAATTTTCTGCAGGGTACGGACCGGTGTTGCAAGTCTGGCTCGGTGATGGCCTATAACTCTAAGAAGGTCCAACAGTATTGCTTCCTTAACGCACAGATATTCTTCAAAATGTGAGGTCTTAACAAAACAAGACACCAGAATCAAAAGAGCCTGATTTTCAGGATTTATGTTGTCCAAAAACACTCTCCTGTGCAACCTCTGCTGCTCAACTTGAGGATTTTTGGCTAATACTTTCCGCATGTCAGCAACAATGTTATTAATCTTATTTACGTCCAAATGACTAATGGCTAGATGGGTTTTAATTCGCCAATGCGTTTTTTGACTAAGGTTCCGCACAACATTCACTGTAAATTTGTGGTTTGGAATGTGAACAGCTTCACGATCTTCACCTCTAATAATTGTTGGTGACCACCAGCCAACATGCTCAACAGTACCAGAAACCTCATATCCTTCAATCTTTGTTTGAATCCATTCATTAACCACAAAGGGCCGAGTTGCATGAATCATCACACTTGAAAGGAAGTTTGTAAAAATCTCACGACCAGCAAGGGTCAACAAAACTGTCCCAAGACCTCCTGCAGTAACCCATTTCTGGGTAGAGAAGCCTAACAATTCCATGAACAATGAAAACGCAGCAATCCATACCGCAGAATAAACAGCTTTGCCAGCAAATTGGAATCCCATATTTCTCGTCTCACTTGCATCAGTACCCTCTGCTAAGAATTTCTGTGATTGTTGAATTAAACTTGACAAACAATAGGCAAAGGCCAGTACTGTTGACAATGATCTTACAAAATTCAGAAGCCGTTCCTTAACAACCTGGCCAGGTTCTGAAGGTAGAATTAATGGCTCCAATACTCTGCAAATAAGTATTGCTCCAGTCCATAACAGTAATGGCCGAAGGTAAGAAGTTACGATATGGTGAGTGGTACTTTTCTTCCAACTATTATCACTCCTCTGAAAAAGTATTTTCCTTGTCTGGAACAATAATGGACCAACACCCCATATTGCAAAAATGATAATGCCAATTGCAGGAATCAACTTTACTATGATAGGACTATTTTGTAACACATTACTAGACCTAGCCAACACTGTAGCAACCACCTTAACAGCTGGAAGATCATTTGGTTGGCCTGACACTAAAAAACAATGACACTTAAAAGATCGGGCTTGGCTTGATAATGTATGTGTTGGCTTGTTTATATGACGAAGGTGTTGAAAACCCCTGCACTCCTGTCTCATGAACGAGATGGGATATGAAGGACCTGTATTAAATAAATGTAACTTGCCATGTCCTGTTGCCCTCGAATGTTTATTGCAGCCCAGGTTCCTGCAAAGTCCCAATCCATGGGACAACTGCAGGGAACCAGGAAGAGCCATTACTATATTGAAACAGATATCCAAGGCCTCACTGGTAGCTGTAGAATGTATTAATACGGTATTTCAGACTTTTACACAATTCAACAAGTTCTACACAGACCAGTGCAGCTGATTCACTTAGATAATCTTTCAATTCAGCTACCTGTGACGAAGAAGCTCCGTCTCTCAAAGCTTCCACCTGCAAAGGTTTTTTGTCTACCAGCTTTTACGAGGCGCTGCGCAAAGGAACAGAAC

>C101647_65

TAATTTGTTTCATTATTCTAGAACGCACTCCTCCTGCATATGCACCCTGCTATTTGTGCGGCATAAAATAATGTAATCTTTGGCTTCTAAATGATTACCGAATGAGTTTCTCTTACTTCAATTCAATTGCATGTGGCACTGCTGTTCTCGGAAGCAAATACAGCTCATCTACCATCACTACCAACACACCATCATTGCAATGACCCCAATTTGGATTTCCTTTCTCTTAACTACTCCTGAACACAAGAGGGACATGTATCTGATATGGCCCTGGACCCACTGCTTTTCTAGCTAATCCCCCTAATTCATCACTCTTTCTTTGTTCTTGAAGATTCGTACTATGGTTTTGGAGGACATTGTGCTCTCTGTCTGTCGATCTTTAACTAGCTTGCTCAATTTTAGGATCAAGAAGTGAGTTGTTGTGCTGAACTGTTGATCTTCGTCTCTCTTCAACCAAGAAAATGGACAAGAAGCTGCCCCTTTTCTACATTTTTTCAGCAGCCTTGGTGATGGAAGCAGTGTTGGTTAGTGTTGTGGCTGAACCAGTGGAAGATAAGCAAGCTCTGCTTGATTTCCTTGACAACATGAATCACTCTCCTCATCTCAACTGGGATGAGAACACTTCTGTTTGCCAAAGCTGGAGAGGAGTGACTTGCAACTCTGACAAGTCTCGAGTCATAGCCCTTAGATTGCCAGGAGCTGGTTTGAGTGGTCCCATCCCTCTCAACACTCTCAGTCGGCTCTCAGCACTTGAGATTGTGAGTCTTAGATCAAATGGTATATCAGGCCCTTTCCCTGATGGTTTCTCTGAGCTGAAAAATTTGACCAGCCTCTATCTTCAATCTAACAAGTTTTCTGGGCCACTGCCATTGGATTTTTCAGTTTGGAATAATCTTAGTGTTGTCAATTTATCCAACAATTATTTTAATGGGAGCATCCCCTTTTCAATTTCAAATCTGACTCATCTCGCTTCATTAGACCTTGCCAACAACTCCCTTACGGGTGAGATTCCTGATCTCAATACTCCTAGCCTGCAGGAGCTGAATTTAGCTAACAATAACCTTAGTGGGGTTGTGCCTAAATCTCTTCTTAGATTTCCTAGTTCAGCCTTTGCTGGTAACAATCTTACGTCAGCAACAGCACTTCCTCCAGCCTTTCCTGTACAACCACCAGCTGCTCATCCATCAGAAAAATCCAAAGGACTTAGTGAACCTGCATTGTTGGGTATCATCATCGGTGCTTCTGTGTTGGGGTTTTTGGTGATTGCAGGTTTCATGATTGTGTGCTGCTATCAGAATGCAGACGTGGATGTGCAACCAATGAAATCCCAGAAAAAACAAGCCTCTCTGAAAACAGAATCTTCTGGAAGTCAAGACAAAAACAACAAAATTGTCTTCTTTGAGGGTTGCAATCTTGCATTTGATCTAGAGGACCTGTTGAGAGCGTCTGCTGAGATTCTTGGAAAGGGTACCTTTGGCATGACATATAAGGCTGCTCTAGAGGATGCAACCACTGTGGTGGTCAAGAGGTTGAAGGAGGTCGCAGTTGGAAAACGAGATTTTGAACAGCAGATGGAGGTGGTGGGGAGAGTTAAGCACGACAATGTGGATTCAGTGAGGGCATATTACTACTCAAAGGAGGAGAAACTAATAGTATATGATTACTATCAACAAGGCAGCGTTTCTGCAATGTTACATGGCAAAGGAGGGGAAGGTAGAAGTCCTTTAGACTGGGATAGCCGTTTGAGAATTGCAATTGGTGCAGCAAGAGGCATTGCTCACATCCATGCACAACATGGAGGGAAACTCGTTCACGGAAACATAAAATCCTCAAACATCTTCCTCAACTCACAAGGATACGGATGCATATCTGATATTGGCTTGGCAACGTTGATGAGTCCAATACCCGTCCCAGCAATGAGAGCAACTGGATACCGTGCACAGGAAGTAACAGACACGCGCAAAGCAACCCATGCATCTGATGTCTACAGTTTTGGGGTGCTACTACTTGAGCTTCTGACTGGGAAATCGCCCATAAACAGCACAGAAGGTGAGCAGTTTGTCCACTTGGTTAGATGGGTGAATTCAGTTGTTAGAGAGGAGTGGACTGCAGAAGTGTTTGACGTAGAGCTGTTGAGGTATCCCAACATAGAGGAAGAAATGGTGGGGATGCTACAAATAGGGATGGCTTGTGCTGCAAGAATACCGGATCAGAGACCAAAGATGCCTGATGTGGTGAGAATGATGGAGGAGATTCGGCGTGTAAATACACCAAATCTACCATCCACCGAGTCTAGATCAGAAGTTTCTACACCCACACCTCGTGCAGTAGACATACCTTCTACCTCAGTTCAACAATGAGATTCTTTTGTTCTGCTCAATAGTTGTTAAGATGTAATCAATTAAATCATATTCATAACAGTAGAAAATATCAACCAAAATGGACTTGTGATCCTAGGAAGTTAATAAAACATATTTGTGCTGAGTAGTTAAGCTACCGGATTTCGCCGATGGTATATGAAACTGGGTCTCTTTGCTAGTAATTATTGTATTGTATTATGCAGTCAGGGAATCT

>C101651_65

CTAAGTGCTAAATCAAGCGACGACGACGGTGGAAGTCGTATCGTGTTCTCCTCCATTCACTATTTCTGCTTTGGCAATTTTCCCTCAAAATGCTGTTCATACAAGGAAAGAGAATTTTGTAGCTCAATGAAGCAAAAGAATAGATGGCAGACAAAGTTGAGGCGTATCCATTACTTGCTTTAGATGACAGGAAGGATCAATCCCTAAAATCTGCAATATAAATCCTTCAATTCCTGTTAGTCATTGTAATGGAAGTTGGAGCAGTGGACCCCTATACAACTAATCCAGGTCCAATTGATGGTTTAGTTCTGTATGACCAAGACAAGCATGTCTCCACAGCAGTTTGGGATGGACAGGAGCGTGGTGCCCTCAGATGTCATGAACATACTTCTAAGCTTGATCAGTGGACCCTTACTCATAAACAGGTTGAATTGGTAGAGAAGGCTGGATTTGGATACCTTAGGTCAATCCCAGCTATTAGTCTAGACAATCCACTTATCTCTGCTCTAGTTGAAAGGTGGAGAAGAGAAACAAACACATTTCACTTGAATGTTGGTGAAATGACAGTAACCCTTAAAGATGTTGCACTCTTGCTTGGGCTGGCTATTGATGGAGAACCTGTAATTGGTATTACCTATACAGCATGCAGTTCTGTTTGTGAAAAGTATCTTGGTAGATCACCAGAATCTGGTTACACAAGTGGTGGAATGGTGAAACTAAGTTGGTTGAAAGAGTTCTTCTCTCGCTGTCCTGAAGATGCTCCAATAGAAGTGATTGAGCAACATACTCGTGCATATCTTCTTTATCTTGTAGGTAGCACCATATTCTCCACAACTACAGGGAACAAAGTCCCTGTGATGTATTTGCCTTTATTTGAGAATTTTGCTAGATGTGGGCAATATGCCTGGGGTGCTGCAGCATTGGCTTTCTTGTACAGAGCACTGGGAAATGCCTCTCTAAAAACTCAAAGTACCATTAGTGGCTGTTTAACACTACTTCAGTGTTGGAGTTACTTTCACCTAAATATTGGACGACCAAAGCTCAATCTTGAGCTGATGCATGACCGATTTCCTTTTGTGCTTAGATGGAAAGGAAAACAAAGTGGGCCAACTGCAAATCGAGATGTAGTTTTCTATCGGAAGGCTCTGGATTCCCTAAAACCGTGTGATGTGGAGTGGCTCCCATATCGAAACATGGACAGCATGGTAATTCCAGAACATATCAAAAGCACTTTAATTCTTGGGAGGTCAAAGACAATGCTGATATGCTTTGACAAGGCAGAAAGACATCTTCCAAATCGATGCTTAAGGCAATATGGCATGCTTCAATCAATTCCGGATGATGTGGAGCGGTGGGAGAGGAAGAGTAGAGGAGTTGATGGTGGCGTTGATTTGTCAGGGAAAATGGAATCGGAGCTTAATGAGTGGATGGACCGGCAGGTGCATATTGTTGATGGGGATGAGGGTGTAGATGAAAGTGAGTACATGGAGTGGTATCTGAGAATTACTCGTAAGTTCATTGGAAGGCCTATTTCTCTGTCAACCGAGTTTCAGAGAACGAATGCTGGTCTGAGGGATATTGCACACATAGCGGATACCTTTTCAATGAAGGGGCTAGATCCTCAACAAATTGAATCGATATCAAGGATAAGATGTATTGCACATGAATGTTTGAGAGACCAAATTGGTGGTCCAGTCATGGTGTCAGGGAGTCCCCAAGTTGAACTAGGTAAAAGGGTCAGAGGAAAGGAGAGGGTTAGAAGAAAAGGAGGAGTGGGCAAACGAATGCGAAAGGATGGTGTGATTCATTATAACACCGTTAGTGAGGATGACCAACCTCAGTATTATGGCACTGCTATTGAGGTTGGCCAGTTACATTTAAGTCACATTGAAAGAGAGCTGGATCATGCACAATTATGTACTGTTGACAATGCAGTCAGTGTTGTCCAGTTGATTCGTGCAGATGCTGATGGCGAGAATATGCAGCTCTGTGATACACACATTGATGTTGATGAATCAGAGCTGGGGTATACTGCAGGTGAAGAAAATAATGAGGAGCCAGATGATGTAGCTGCTGAATTTAGCCATGAAGAATTAAAGCACAGAGCTGGTGAAGAAATAAAGCAAGAGCTTAATCATGTGACTGGTGAGGAAAACATCGAGGAGCTGAATGCAGGCCATGAGATTCATATTGTGCAACATTCTGATAATATGGTGATTGATAATTCACAACTTTGTGATGTAAGTCATGATGACATTGATGATAGGACGCTTCCGGATACTGCTGCCCAGTTCGGTCACTCACAACTTGGTGACACAAGTTTGGTCATTCAACAACAAAACAATCCAACTGACTGTCTTGTTAATTCTGAGCTTTCCCATATTAACCCTGAAAGTGAACTTTCATCTACTAAAGCAGTAATGGAAGTGTCTCAACATTCTTCTATTGAAACTCATGAGGATATTTCACAGAATGGTGATTGTAGTGTTGCTGTATAGGGTAAATACCTTACAATATTGTAGAGAAGCAAAATTTTTTAGGCTTACTCAATTTAAAGGTGTGATATCGTGCCTTG

>C101653_65

ACTTGGTCGTTTTCTTTTGTTCCAACTTTTCCCCTCTTTTCACTCTTCACCTTTTTGGTTTGATTTTTGTATCTTCAACACCTTCCCCTGGTCTCAGCTTCGGTTCTTTCCCATGTCTTTTTGTTTTTAAAAGCAGAGACTCATCCCAATATTCCAACACATGCTCATTCTACATTTTGCTTAACCACTCACTCTTTCATAATGGCCAAGGAACCTTCTCTATGCCTCTTCTTCCTCTCTTTTCTCATTCTCTTCTCTTCCGGGTCTGCATCTTCACCCTCTGTTGGTACCTCATTCCTCCTCAAAAGCAAGATCTCCCAATCTGAGATTCGGTTTCCTGTTGCTGATTGCAAGCTTTTGCGCACTCTCACCAACACCAACTTGTCAGTTGATTTCTTCTTAAACGAAAGCCTGGTTGAAAATTTTGTTTCTTCAAAATCATCTCCAGTTTCATGGTTGAAAGTTCATCTTTTGGACATTCTTCCAGAAGTGGACATCAGAAGCATCATTGTTCGATGTGGCAGTGAGTGCTTAGGCCGAGATGAGATGCCTTTACTCATACCTGTTTTGAAATCAATTCATTCTTTTCTCAGCAATCTTCAGCTAGGGAGGGAAGTTAAAGTCTCGGTGGCATTTCCACTTTCATTCTTAGAGAATTTGAATGCATCATATGAAAATGATCTTCGTAGGATTGTTGTGTTTGTAAAGAAAATAGATTCATTTATCATGATAGAAGACACCATTGATGGGGAATTAGGCATTGTTCAGTCTGTTATTGAAAGAGCCACGCTTGCTGCATCTATTCTTCCTTGCAAAGATGTTCCTGTGGTTCTCACAATCAAGAGCCGTGATATTCCCAGTTTAATGGAATTAGCTCAATTTAGTGAGGCAGTATCCAAATATCTTGAAGCAGGATCTCATTTTACAAAAAGAATATTTGCATTGTATGCAGAAGTACAGACAACCAAAGGTTTTGTACTGGGAGATCTCGAAAGAAAAGAAGCAGAAGGGATAATTCCTCTATTCCTAAGAGAAAAGCCAGGCAAAGTCCACATCAGAAGAATTCTCCAAGACACAACCAACTTACCTACCACAGTTTCCCCCACAAATCCAACCCCACCTACACCAGTCATCTCACCTCCAGATACACCAACCATTATCACAGTTCCTTCCACCAATCCTGTCACAGTTTCCCCTACTAATCCAGGGGCAACACCTGTGACAGTTCCTTCTACTACACCAGCTGTTCCCTTGGCCCCAACAAATCCAGCCAATCAAGGAGCTCAACCAGTTACCAACCCTGTGACTTCTTATCCACCCCCATCAGGAAATGGTCCTGTCACAAATGCCCTTCCTCCTCCTCCTAACACAAATGCTCAAGCCGGAGGGGGGCAGAGTTGGTGTGTGGCAAAGACTGGAGTTTCAGAAGCTACTCTTCAATCAGCTCTGGACTATGCTTGTGGAATGCCAAGTGTAGACTGTTCACAGATACAGCAAGGTGGGAGTTGTTACAATCCAAATTCACTACAAAACCATGCTTCTTTTGCCTTCAACAGCTACTACCAGAAGAATCCTGCGTCAACAAGCTGTGACTTTGGAGGCACTGCAACCATAGTTAACAGCAATCCGAGCTCGGGTTCTTGCATTTACCCATCATCTTCAGGGGCAGGCACATCCGGCTCCTCACCTTCAGTTGTAGGGTCCCCTAGCCCTCCTGATTTAAATACCTCGAATTCAGCTGGTCTGAAACCTTTTCTTGACAGTATGGTTTTGGTGGTAATATTCTTAGTCAGCGTAAGACTCCCTATATAAGTTTTCCACAAGAATCAACAGTGAAATTCAATACACTTCGTGCTGGAGTTAGTTGTGTTGTAATATTACATGCAGTGTACAGATCTAAAACTGCAGAATGACAGTAGAATGAATAGAAGAAGTGAGACTACGTGATGATGACAGGTTATGGTCTGGTTCAGGTACAAGAGTAGTGTGTAATTCAGTGTGCATTGGTCAGAGTTTGTGTTGGAAATTCATTTGCATATAAATGAGGGTTTGTTACTATTTTGCAAAATGATTCACGTGGATATGTAGAGTTAGAATCCAGGTTGGTTAACTCTTAGATTGTCATTGGAACAATTTTACACTATGAAAGAGTCTAATATAAGAGCTTGTGATGATGTAGAAGATTACTAAAGAGGTGTCCCGTTTCATGTAATCATTTCTGTGAAGAAACAATTATGATATTGGTAAATTGTGAAGGTTTATTGGGAGACTCCAAAGGGCTCTGAAGATGATCTTAGCAGACTTCTCATCATCATTTGTCGAAGAGCATGCATGCTTTATTGGTTTTGAATCATCATACTCAGCAACATGCTTTTTATTGTCCATATTCTAAAACTCCTATTCTTAAATGAGTTTACTTAGAATGAGTTACTATTTGTTAAGATAATGTTGTCCGTGTAATATCATGACATAATTTGGCCAAATTCCCTTTCCTCTATTTATCTCATAAAATAAACGGGTTAACCTAAATCTGGTGAATTGGTGTAATGTTCAGTCTATTAGAACCATAAAATTG

>C101655_65

GCCACGTCTTGGAGATAGAAACACCATCAAAATCTATGGCATAGATTGAAGCAGGTAGCAGAAGCCAAACCAAGCGCTATAATCTCTATTTCTTACTTGATTTTCCTCAAACTTATATGTACACGATAAACCACACTTACACGAAAGGATCTGTTTAATTTAAGTGTGCAGTGTGCTGTGTGCAGGAGTGGCATAGATAGCATAAGAGGAAGAGAGATCAAACGTATCTGCATCAGGCACTGAATCATTCCGAGTTAAGATGGCATTCGCCACCAGCGCGTTACTATCTTCAAACTTTCTGGGAAGGAAGGTTCGAGTATCGCCCCCAACCCCCAAAACAACGAAACAATCGACTCCATTAGCGTTTGTTTTGAAGAGAAGCATCTTAAATTCTCAGAAACCGAATTCGGAAAAACTGAATTCTGCATCTTCACAAGCTGCTTTGGCAGCCCTTATCTTTTCCTCCTCCACTCTCTCTGCAACCCCACACGCCCTAGCCGCTGATAACGTCACCCCTCCCCCTGTAATCGAAGCGCAACCGAGCCAATTAAACCCCTCCAACTCGAACTCCTCACCTTTCTCTCAGAACCTTCTCACAGCACCCAAACCCCAAGCCTCTTCGGACCTCCCCGAAGGCACCAATTGGCGCTACAGCGAGTTCTTGAACGCTGTTAAGAAGGGTAAAGTCGAGCGAGTCAGGTTCAGCAAAGACGGTTCCGCCCTCCAACTAACCGCCGTTGACGGCCGTCGAGCCTCCGTTATCGTCCCCAATGACCCCGACCTCATCGACATCCTCGCTATGAACGGCGTCGACATTTCCGTCGCCGAAGCCGAATCGCCTAACGGCTTGTTCAACATCATAGGCAACTTGCTCTTCCCCTTTCTAGCATTCGCCGGCTTATTCTTCCTCTTCCGCAGAGCCCAAGGCGGCCCCGGTGGTCCTGGCGGGCTTGGCGGGCCCATGGACTTCGGCCGGAATAAATCCAAATTCCAAGAAGTCCCGGAAACCGGGGTTACCTTCGGTGACGTGGCGGGCGCGGACCAAGCCAAGCTTGAACTGCAAGAGGTTGTTGATTTTTTGAAGAACCCTGATAAGTACACTGCGCTGGGTGCGAAGATTCCGAAGGGGTGTCTTCTGGTGGGGCCACCGGGAACCGGGAAGACGCTTCTAGCTAGGGCTGTGGCGGGGGAGGCGGGAGTGCCGTTTTTCTCGTGCGCAGCTTCGGAATTCGTGGAGCTCTTTGTGGGGGTTGGTGCGTCGAGGGTGAGGGATTTGTTTGAGAAGGCGAAGAATAAGGCACCGTGCATTGTTTTCATTGATGAGATTGACGCCGTGGGTAGGCAGAGAGGGGCGGGGCTTGGAGGTGGCAATGATGAGAGGGAACAGACTATTAACCAGCTTCTGACGGAGATGGATGGGTTTTCTGGTAATTCTGGAGTTATTGTTTTGGCTGCAACTAACAGACCTGATGTTCTTGATTCTGCATTGTTAAGGCCTGGCCGGTTCGATAGGCAAGTTACTGTAGATAGACCTGATGTTGCCGGCAGAGTTAAGATCCTTCAGGTGCATTCAAGAGGAAAGGCGCTTGCAAAGGATGTTGATTTCGAAAAAATTGCAAGGAGAACCCCTGGATTTACTGGGGCAGATCTGCAGAACCTGATGAATGAAGCGGCTATTCTTGCAGCCAGGCGTGACCTCAAGGAAATAAGTAAAGATGAGATATCTGATGCTCTTGAAAGGATCATAGCTGGACCTGAAAAGAAAAACGCTGTGGTCTCAGATGAGAAGAAGAAATTAGTAGCATATCACGAGGCTGGCCATGCTTTAGTAGGTGCTTTAATGCCTGAATATGACCCCGTAGCCAAGATATCAATTATTCCTCGTGGCCAAGCTGGTGGTCTCACATTCTTTGCACCCAGCGAAGAGAGGCTTGAGTCTGGATTGTACAGTAGAAGCTACTTGGAAAATCAGATGGCAGTTGCTCTGGGTGGAAGGGTCGCCGAAGAGGTTATTTTTGGCCGGGAAAATGTCACGACTGGAGCATCAAATGACTTCATGCAAGTTTCACGAGTGGCAAGGCAGATGGTTGAGAGATTTGGGTTCAGCAAAAAAATTGGACAAGTTGCCATTGGTGGACCTGGTGGAAATCCATTCTTGGGTCAGCAGATGTCAACGCAAAAGGATTACTCCATGGCAACTGCAGATGTAGTGGATGCGGAAGTAAGGGAACTGGTAGAGAGAGCATATTCTAGGGCAACAAATATTATCACAACTCACATTGACATCCTTCACAAGCTTGCTCAACTCCTCATAGAAAAAGAAACTGTTGATGGAGAAGAGTTCATGAGTCTGTTCATTGATGGAAAGGCCGAATTATATGTTGCATAATCATACATGTAAGTCATAGAATCATGTTTGTACTATAAGATACGCGAGACATGTACACTGTAAGAATCAATTAAATACTTTATCAAATTCAAGCACAAACAAAATAGCACAACAGCTGACGAAAAAAAAAAAAAAGAAATATGGTTCAGCTCCTGTTTTACCCCCTTTCTAACTAATGAAGTCTATT

>C101657_65

TGTGCACAATAGCATTTTACTAATAACTTGACCATGTCCCGAGTTAATGACGCCATAGTCTCCAATTGATTTAATAAATACCACATTCTATGTTTCTAGATATCATAGGTACACAAAATCAAATTTGTAATCTCCCAAAATTGCAGTCACCGGGTCAAATGTTAACAGGTCCTTGCTATAACTTTCAACTTCTTAGGAGCAAGCGAGTATCAGGTATGCTATTCAATTTATGTCAAGAACAGCAACCTAAACGAATCTTGAAAATGGTCATTCCTATCTCAGTCATCAGTCTGGAAGATAACCTTGAATATTCAGTAGTGTAAGCTGGTATGGAATCTTTACCACATCTATAGCTGATTTATACCAAAACTGTCGAGTATCACTTGTGTAAGAGTCGATATTAGATCTTCCTAGTACTTGACAACTCCAACATTTGGAGCCTTGCCCAGGACAGACTCAACATCTCCATGAAATAAGCCGAATTTTTTTGCCACGGCAAAACCAGCATGCAGTCCATCTACTGCTGCACTGACGATCCCACCAGCATAGCCTGCTCCTTCACCAACTGGATACAGTCCTTTTACTGAAGTGCTCTCATAAGAATCAATATTGCGCGGAATTTGGATTGGGGAACTAGTCCTAGTCTCTACACCATGAAGGAGAGCCTCATTGCAGATAAATCCTGGTAACTCCTTGTCAAATGTTACAAGTGAATGTTTCAAAGCTTCGGTTATATGAATGGGAAATAGCTGATGGAGGTTTGCTGCCTTCACACCCAACCGATAACTTGAGGGGGGTACAGATGTTACTGGCAACTTGTTCTCAAGAAAGTCTGTAACTGTCTGGACAGGCACTATAAAATTTCCTCCTCCCATCATAGCAGCTCTTTGTTCAAACTCTCTCTGAAATTCGACCCCAGCAAGAGGACCATAATAATTCAAAGCTTCAAAGTCCTTTGTTGTGACGGTAACCACAAGTGCAGCATTTGCCCACTTTGATGCTCTCCGAGAAAATGACATGCCATTAATGCATATTTCTGATGGACTTGTACTAGTGAGAACAACCTGCCCACCTGGACACATGCAGAAAGAGTAGCAACTACGACTTGCCACCCCTGAATTAGATACTTCGTTGAAATCCTCCTTGTCAATATAATTGGCAACTTTGTAATCTGCCACTGGAATTTTACCACGTCCATGGCATACCTCGGAAGCCAATTCAGCATACTGTATGCTATTTATTAGCTCTTGAGGATGCTCAATCCGTAGCCCTACGGCAAAATCTTTTGGTATTAATTCCACATTGTGAGCATGAAGCACTTCATATATATCACGGGCAGAATGACCAACAGCCAGAATAACTGCATCATATTCCATCTTCTTACTCGTTAAACGTAGTTTATCAGCTGATTCAGATACCATAACCCCCAGAACATGTCCATCTTTTATAACTAAATCATCTACTCTTGTCCCAAACTTTATGGTGACACCCAAATCTTGCAGATGTTCCCTAAAGTTGCGAAGCAATGGAACCAACCTATCTGTTCCTAAGTGAGGCTTTCCATCAATCAATATCTGTTTTGGAGCTCCAAACTGCACTAGAGTTTTCATAACTGCAAGAACACTGCCACTGTTGCGTCCAATCCTGGTTACCAACTTCCCATCACTCCAGGTACCTGCACCACCCTCCCCAAAGCAGAAGTTACTTTCCAATTCTAAAACTCGACGAACTACAAGAGCACCAATATCTCGCCCTCTTTTCTCTACTGGTTGACCTCTTTCTATCAGTGTAACATCTGCACCTAGTTCAGCAAGAACAAGAGCTGAAAATAGGCCAGAAGGCCCACTACCCACTACAGCAATTTTTGGCTTTTTAGTAGCCTGGTTCTTGTACAACTCCATTGAGAAATTGCTTCGTCCATTTCCTCCAGTCAAAACAGCTTCTTTATTTTCTTTACAGTCACGAATGACACTTTCCAGATCAGCAAAATCTCTTTCATCATGCACACGTTCCACAAGGCCAACTTTAGGCTCCAGCCGTGAAATAAAGTCCCAACAACGAGGCTCCAGACTGATGAGTTTTTGGACATCCATATCCACAGTATGTACAAATTTGGGCTCTTTTAATTTCTTCCTAGCATCAAAGGACTTCCTAACGACGGTGAATGCTTGTGGCGGCAAGAGAGAAGCAACCGGGAACTTGATAACTTTGGCAATTTCTTGGAGAAGGGCATCGGAAACATGGAGAGAGTCCTTGCCAGGGTCTTGATCGAGTGGAACGGCGAGTTTGAAGAGCCTCCATGTACCCTCAAACTTGCTCTTCTCCTTCGACTCAGATAAAAGTTCTTTGCGCTTCGTCCTCAGCTTCTTCTTCTCCGACGGGTACCTCTGTTTACCGGTTCGCTTCGCACACAAGATGCGCCATGTTTGACTGCCGGTGTAAGGAACACGTCCATGAGTGGGACCGCGAAACCAGACAGCATTGTGGTGGTGCTTTATCAAGCAGGTGCAGGAGGAAGAGGAGGGGGAGGGAACAAGGAACATCTTCCGATGTTGGTCTTGGTGATTTCAAGTGAC

>C101663_65

GAAAATTTTCTGAATCCCTAATTTTTCTCCTCCTGCCACGGGCGCACCGACGACCATGCTATCCTCCAAACCCCCGCGCCGGAAAATCGTTCCGGCGAACGGAGATGAGTCTGCCGATAAACTGGACCAGCTTCTCTTATCCTCCGCCATCTGCAACAACGAGGACCTAGGTCCGTTCATCCGCAAGGCTTTTGCCTCCGGCAAGCCAGAGACGCTCCATCACCACCTCCGTCACTTCGCACGCTCGAAGGAGTCCGAGATCGAGGAAGTGTGCAAGGCTCACTACCAGGACTTCATCCTCGCCGTCGACGATCTACGATCTCTCCTATCCGACGTCGATTCACTGAAGTCCTCCCTCTCCGACTCCAACTCGCGACTCCAGTCCGTCGCGCGCCCACTCCTCTCGTCGCTCGACGCCTTTGTCGAGACGCGCAACGTCTCGAAGAACGTGAACCTCGCCATCGACTCCGTCCGCATGTGTGTCAAGCTGATGGAGGTTTGCGCGCGGGCCAACCGCCACCTTGCAGACGACAACTTCTACATGGCGCTGAAGTGCGTGGACGCGATCGAGCGGGAATACTTGGACCAAACGGCGTCGTCCACACTGAAGCGGATGCTGGAGAAGAAGATTCCGGAGATTAGGTCGTACATCGAGAGGAAGGTGAACAAGGAATTCGGGGACTGGTTGGTGGAGATCCGAGTCGTGAGTCGCAATTTAGGTCAATTGGCGATAGGTCAAGCCTCGTCAGCGAGGCAGAGAGAAGAGGATCTAAGAATTAAGCAGCGGCAAGCGGAGGAACAGAGTAGACTCAGTGTTAGGGACTGTATTTACGCTTTAGAGGAAGAGGATGAAGACGGAATCATCGCCGGCGGGATCGGCGAAGACGGTGGTGGGGTCGCCGGGTTTGATTTGACTTCGTTGTACAGGGCATACCATATTCATCAGACTTTAGGGTTAGAGGATCGGTTTAAGCAGTATTATTTTGAGAACCGGAAGCTTCAGTTGACTTCGGACTTTCAGGTCTCGTCGATGACTCCTTTTCTGGAGTCGCATCAAACATTCTTTGCACAGATTGCTGGGTTCTTTGTGGTGGAGGATCGCGTGTTGAGGACCGGAGGTGGGTTGATATCAAAATTGGAGGTTGAGAATCTGTGGGAGATTGCTGTTAGTAAGATGTGTTCTGTGTTGGAGGATCAGTTCTCCAGAATGCAAACAGCTAATCATCTTTTGCTTATTAAGGATTATGTGAGTCTGTTAGGAGTAACGCTGCGGAGATATGGGTACCCCATTGATGCGTTGCTTGATGTTTTGAGCAAACACAGAGATAAGTACCACGAGCTACTTTTGTCTGATTGTAGGAAGCAGATTGCGGAGGCGGTTGCGGCTGATAAATTTGAGCAGATGTTGATGAAGAAAGAGTATGAGTATTCCATGAATGTACTTTCTTTCCAGATACAGACGACGGACATCATACCGGCTTTTCCCTATGTTGCTCCGTTTTCAACCACTGTGCCGGATTGTTGTAGGATTGTGCGGTCATTCATTGAGGATTCTGTGAGTTTCATGTCTTATGGCGGGCAGCTTGAGTTCTATGAAGTTGTTAAGAAGTATTTAGACAGGCTTTTGATTGAGGTTCTGGATGAAGCTTTAGTGAAGCTTATCAATACATCGATTAATGGTGTCTCCCAGGCGATGCAAATGGCAGCGAATATGGCTGTCTTGGAGCGTGCTTGTGATTTTTTCTTTCGCCATGCTGCACAGCTTTCGGGTGTTCCCTTGAGATTGGTGGAGAGAAGCAGAAGGCAGTTTCCCCTAAGAAAAGCTCGTGATGCTGCAGAAGAGATGCTCTCTGGGCTACTCAAAGCTAAAGTTGATGGATTTATGACCTTGATCGAGAATGTAAATTGGATGTGTGACGAGGTACCTCAGAGTGGAAATGAATATGTAAATGAGGTTATAATTTATTTGGAAATTTTGGTTTCAACTGCTCAACAGATATTGCCACCTCAAGTCCTTAAGAGAGTATTGCAGGAAGTTTTTGCTCACATATCCGACAAGATAGTTGGGACCTTAGTTAGTGATTCTGTCAAGAGGTTTAATGTCAATGCTATTAATGGAATTGAAGTAGACATTCGTCTCTTAGAATCATTTGCTGATACTCAGGCTTCTCTTTTCTCTGATGGGGATGTTGATGTTTTGAAAGCATCACTTGCCAGTTCAAAGCAACTGATTAATTTGCTATTGAGTAATCACCCAGAGAACTTCTTGAATCCAGTTATAAGGGAAAGGAGTTACAATACTTTGGACCACAAGAAAGTGGTGATTGTTTCAGAAAAGTTGAGGGATCCTTCGGATCGGCTATTTGGAACCTTTGGTAGTCGTGGAACCAGGCAGAATCCGAAAAGGAAATCATTGGATACATTGATAAAAAGACTTAGGGATGTTAGCTGATTAAGCTTATAGCTGTATGTAAGGTACTTTCTCTTGTTTTATTTTCTTGTTTTGTCTCTAGTCCATTTTGACTATGAATTATTTAATAGTGAGTTGTTGATAAATTTATTTGGTTTAAGGTCAGGC

>C101667_65

TTTCCTGATTGTTTTTTTTTGGGTGTTTCTGCTTCCTTCAGCTCTCTCTCTATGCACCTCCCTTCAAAATCTCAAAAACCACTCTTTCTCTCTCTTTCTCTCTCTCACGATCACACCGAGAGAACCAAACCAAACCGAACTCCTTCACCGCCTCTCTCGCCGTCGCCTTCTCCGGCCACCCCGTCCTCCTACCGGAGCAGCTCCTCAATCGTCGCAATCAATCCCTAACCGACAAAACCTTCCCTCTTTAGCTCCTCTTTTCAACCTCTGCCGGAATCCGCCAATTAGGTTGTGATCAACCTCTCACAGATTAGAGGGGCAGGGGCTGTAAGTGAGGATGAATTTGCAACAAGCTGGGCAGCCCAAATCCTCCAATGGATATGGCCGTCGAAAATCTGAAAGAGAAGGGGCAATTAAGTCTGAGAATAAAATCCTGTCTGGAAAATTAAATGCCAACAGATTGGCAAATACAGGTGCAGTGACTGGCAGTAAAGGTGGCAGTTACGAGAGTCCTTCACATGATCGATTAGTATATGTAACAACATGTCTTATTGGGCACCAGGTGGAAGTCCAGGTGAAAAATGGATCTACATACTCTGGAGTCTTTCATGCAACAAACACAGACAAAGATTTTGGAATCATTTTGAAAATGGCTCGCTTAACAAAAGATGGTTCTTCACGAGGACAGAAGTCTAGTGTGGAATTTGTCAGCAAGCCCCCGTCAAAGATTTTAATTATACCTGCTAAAGACCTTGTCCAAGTTACAGCACAGGATGTTGCTATTGCCAAAGATGGTTTACCTCATGAATCTCACCATGATATGCATCAGGAAATCATGGTTGATTCATTGATATCTCAATCTCGTCACATTGAGCTAGGGAGAGAATTAAAACCCTGGGTACCTGATGAAGATGATCCACAATGCCCAGAACTGGATAACATATTTGATGGCCATTGGAATAGGGGATGGGATCAGTTTGAAACGAATGAGGCATTGTTTGGTGTAAAAAGCACGTTCAATGAGGAACTCTATACAACAAAGCTTGAAAAAGGGCCTCAGACACGAGAACTGGAAAAGCAAGCTTTAAGAATAGCAAGAGAAATTGAGGGTGAGGAAACCCAAGATCTTCATCTTGCGGAGGAAAGAGGCTTTCACTTGCATGAAGACTTTGATATTGATGAAGAAACTAGATTCTCTTCAGTCTATAGGGGTAAACGTGCTGATGACAGTGGATTTGATGAGGATGTACTGTTTGATAACCACAATAATGATACTTTTGGTGGTGTGTTTGGTTCGGTTGTTAAGAGGCCTAGTGAATTAAGTGGTGGAAAAGGAAACGATGGAGCTCGAACATTGGCAAATTCCTCATCCATGGATCACACACAATCATCTCAGTCAAGTACCAGTGTGGATTTAAGCCGTTCTGGTTCTTATGATCATGCTAAGCAGTTAGCATCTGAACTCCCTGTCATAAACTATTCATCAGCGGATGGGGAAAGCAGGATTCTGGAGAATTCAATTATTAATCAACATGGAGACAATGACACCAAGGAAGAAAATCTGATTCAAGCCGAGGATGTTCAACTGTCAAAATCTGAGGATTTACAGGGACCACACTACTTGAAGAAGGATGCCTCTGATAAAGGCGTATTATCTCCTAATGCCAGTTCCTATGCCCCTTCATCTCATACTTCATCAAAAACCCATGAGAAGATGGGATCACATGGAGATTTGATTGAGGGTTCTGCATCTGGCAAGGTTAATGGGGAAACAAAATCTGTAAATTCACGCGGGAGCCCGTATGGGTCAGATTCAGTTGGAGGAACGGCAGCATCTTCTGGTCCTGGTTTATCTCCAAGTTCATCCGTTGGTTCTCTGTCTTCTGATAAATCAACTCTGAATCCCAATGCAAAGGAATTCAAGCTTAACCCTAATGCAAAGAGTTTTATTCCATCGCCTGCACGGCCTCCCACTCCAGTGTCCGATGGTTCCTTCTATTTTCCAACTACTGTACCAAGTATGCCAGGCATGCCCATGGGTATTGGAATTGGACCTACTTTTGCTGGTCCACAACAAATGATGTATAATCCTCAGGTAGCGCAAATGCCCTCTCAAGCATATTATCATCCGAATGCACCACAGTATGGACAGCTGCTTGGTCATCCTAGGCAAGCTTTATACATGCCGAGTTATCTGCCTGAAATGCCATACAAGGGACGGGACTATTGACGGCTTGGCCATTCGCATTCTTGTTGGCAAGTCAAATCCTTGCTTGAAAAGAATAAACAAAGTGGTTGTCCTGATAAGTTTTAAACGAGGATATCTGCAGCTGATGCGAAAATCTCTGAAGGGCTTATATTATGTTATTTGCCAAGTTAGCTAATTCTGGGATATAATTTTATGACCTTGTTTTAACCCATGCATATACATCTTTTGGGGTTTCTCAGCTTTGGTCGTGTAATAGATATAATCTGATTGACCTTTCCCATCCCTCTTTATTTTTTTTTCCCCTCTTCAATGCTATACGAAGTTTACTTCTTTTAACCTCAACTTATTCATCTGACACTCGGACATATCTGC

>C101671_65

GCCCCGTTCAATAGGTTTCTGGATCATTTTTCAATGCTCACACAAATAACATGACAACAATATGACATATGTTACAATAGATTTACAGAGTGAACAGAACAGGAACCAAGTTACTTTTCAACGGCCCAAACCATAACAAATTCTACGAGATAACAAACTGTAAACAGATTTTATATATATCCTCTCTGCTTCCACAAAAATGTTTATGAAGAAATTGAATTTGGCTCCTCGGTTTTAGCCAACTTTGCTCGTATAAGTTTGTCCAATATTGGAGGTTCCAATGGAACCGGATCTAGACACCGTTCTGCAGTAGCAACAATCACCTCATCTAGCAAGAAGTCGATTGCAGCTTCATCTTCAACTATATCCCTTAAAGGGATCCTTAGGAACTGGATATCCACTTGAATTTGCTGGAATCCACTTCGGTTAAAAGTCTGGAGTCGGACAAATTCTTGAAAACTTTTAAGGCACAATTTAACTAAAGTTGTAACAACTGACTCCTGTGTATATTCTACTTTTGTGAAAATTTCAACTTTCTGCTTAAACAATTTTGCTAGATGCGTTTCCAAAAGCTGGCTCCTTGCCCTTTGAGTATTCGACCTACCCAGTTTCTCCTCACGTAATGGGTTGCTCCTTGATGAAGCACTACTTCCATTACTATCAGTCCTGTGGTGCTTACGTCTACCTTGAGGCAAAATCTGCTTCACTTCCTTTATTATAACTTCAAGCTCTTGAAGAAACAAATCAACAAACATATGAACTTCTCTTGGCTCCTTATGCTTCACCCAATTAGGGGTGGTAAACCTCTTCTTCAGGAGAAATGATACCCTCTGAGTTCTCATATTTACGTACAGGTGCAAGAACTTTTCACCAGCTGACCGAAATTTTCGACATATTTCTCCAGGAACAAAGGCAGGTCCAGATTCATGGCCTCTAATGCTACCACCAGAGAAGGAAGCAGCTATCTCCTCGGTTACTTTTGGGATGACAGTTTGTTCAATGAAAGCAGACAATTGGGCCAGCACTAGGACAAGGCCAGCAAAAGCCTTATCCCCTTGAGGTCCCTCTGCCAAACCGTGTGCTTGAATAGATGGGTTACTTCTACCGGAAAACCGAAGGAACTGATCCTCTAGTTGCCTAAAAAAGTCTTGAAGTCCTTCTTGAACCCAATCAATGATTAACTCTCTTAATCTAATTAGGATCCCAGAGTCATCATCTAAAATTTTGCGGAAATCCAACAAGACATTTATGCTTCCTTGGAGAACTGATTTTGTACTTGCATCAAGAACAACATCAAGAGAGCACTGTTCTGCTCCGTCCTTTTTCAGGATTTGTAAGAGGGAGCCTGAGATGTCTTGCAGAAGATGAGAAAATGCACTTCTAACAAATGATGTGACAACAACCTTTGCAGACTCAAGAGAATGGTTAGAAAGAGCTGCTTCCTCCAAGACTTCATCTATCAGGAGCACATCATTCCAGATAACTCGAAGGACACCAAGTAGATCTTCAGGACAAATCCGTGTCTTTACATATTCCTCAGCAATTACAAAGTTCTTGGTAACCAAGTCTTGAGCAAGTTTAACCAACTGCTCATCGGAATCAGGAAATATGACTCGAAATGCCCGAACTGCCTCCACAAATTCATGAATAGCTGCCTCGTGAGCAGAATTGTCCTCAGAAGCATTGTTTATTTCTACAGGGTTCAGTCGAATGTCTGTAATAGACTGTTCTAACTTTTCAAATAACTTCGTCTTCAAATTGTTCACCGGAAAATCCAACTGCTTAAGAAGCACTGCTGCATCAGCTCTCACTTGTATTGATTCAGAATCTGAAAATAGCTTCCCCTGTAAGTTTTTTACTATGATAGCTATTGCTTCTTCAGAAGCATGCTTACAATCCTGGAAAGATGAATCTCCATATGCCTTAAGAATTGGCATTGCTCCAGTATAAAACCTGACTGCATCTGCATAGGCTTCTGACTTGATGCACTTGCCTAGCCTATCGGGTAGATCATATATGAACTGAACTTTACGAAGAAGATTACAAGTCCGATGTAGCTTCTCAATATGTTCCCTTTTGTCAAATAGAGAAGTGTTGACACTGTCACTTCTTGACTGCACAGACATGATCTTCTCAAGAAGCTGTTCCATATTCGTCTCCATCCCAGAAATGTTACTCTTCATCCTCTTTATAGTGTCAGTGGCACTGATGAACTTGTTGTAATTCTCGTAGACTAGCATTTGCAAGTCGGTGTCGAGATTCTTGATTTCAGCTGCCATTTCAACGTGGCGTTGGAGCAGCCCTTCCAAATTGGACTTGTACGCCAAGATATTCATGTACTGATCGGGATCGAAGGAGGTGGAATTGATATCGTCGAGGGAAGCGTGTTTGGAGTTGGTGTTGGAGTTGGAAGGGTCTGGGGAGTAGAAACTCGATAGCAGATCTCTCATCCTCTTCGCTTTGTCGTCCATTGGTACTTCATCAGCCCCCATTTTTTCCTTTTCTCTTCTCGTCTTCCTCTGCTGAATCGGTTGAACCAAGTCGCTTCTCCCTGATCTGTGAAATGTAATGAAACGGGTATG

>C101679_65

CCCGTGATAAGGATGATTGATGATGAAACTAAAACGAAGAATTATATGGTGTTAATCATGCTACAATGTCTTCATTAGGCAACAGCCCTTTTACCTTTTCTCCACCACAAAGACAAAAAAAATAGTTTCTTGGCGGCAAATGATACGGGGCAATAAATAAAAACAAGATAAAAACATGGTGATTGTAAATTGGTATTGTCATAACTAGTGCTATGTAACTGGCAATTGTTTTAATTTTCTCACTCACTCGTGTTTGTTGTGAATGGAGAGAAAATGGTGAAAATTACAGCCATTCCATCAAGTGCTCTGCAATTCCACTTTGCAGGAATCCAATACTGTCTGTCTCCTAGGAATTGGTCTTTTAGTGAGTCCCAATTATCGACCAAAGATTTAATAGCCATTGATCTGAGGCCTTGATGTAGGACCAACAAGCCGTGAAAAAGGCACTTGTGTCCTGCTGCTGCCAGTGTTCAGCCCCCTTTGACCTGAAAACCTATCATTTAGTTTTGGATCTGATACAGATCTCGAGAATGCTTGTGCAAGTTCAACGGCCGAAGCTGCTCTGCCATACCGTATGCCAGCAGCACCCGGTGATGCTTTTGGCTGCTGCTGCTCCACAGGCTTGCGCCATGTCTCCGGTGAAGGTTGCCTCTCTGAAGCCTGTGGCCTATCAGTCTCCCTTACTGTTCTCCGGGTATCACCTCGCCAATTTCTCCTCTGAGCATGAAGAACCCTTTCACCATCTACCCTTTGCTCCTTCCTTTCAGGTTTTCTGCCTGTCCTTTGATCGGGATGAGCATCCTCGGCCTTTTCACTGTATCGAACCTGGCTTGAATGTTCAGGATGATGTTTCTCGGCCCTCACAATATTGTTTTCAACCTTATTTGGATGCTCGACCACATCATAATTGTTGATAGCCACATCATCAATCCCTCGCTCCTTCAACACTAGTTCTCGTGGGCGAGCTCCACCAAATAAAGCATTCCTGTCTCTTTCACTGTTACCGTCCAACTGTTCAATGGGATGAGACCGAGGCTTCAAATTCAGCTTCGGGCGTTGTCCTGCCTCCTTACCACTGTCAGACCCAGCTGATACAGGCTTCACCAAATTTGCATGGCCATGTGCCTGATAAACGGTTTCAACATGACCGGAATCATTAACCTGACGATTACCCTGTGCATATTCTATAACAGCATGTTCTGAACTATCCAATGGCTTTGCCCTTGGTAGCAACTTCAGTTTTGGTCGCTCAGTTGGTTCAGAAGGCACAGGGTGCAGCAAATCAGATCCAATAACTTTGCCATCATTTCGAGACAGCTGAACCCCATCAGAGTGGTAAGGTGCACTAGTAGGTCGTACATCTGAATACCTGGAAACCCCAGACCTTTCATAATTTAGTAACTCATTCCTACCACCTTGCATTTGATTATCAATGCCTAACCCCCTTTCAGCATGTACTGCTAGCATAGCATCATAATACTCCTTGTCACCAACTGCATCCATCCTATTAAATGCACCAACACCATTAACACGTGGTCTATTCTCCACTTCAGGAGATCTAACCATCTCAGCATCTGTCTGATAATGTACAGCCTTTGACTGCCATCTACCAGAAGACACCTTCTCAAGAGCACTCGCATGAGCCAACTTGGAGGCAGCACTAGCACTAGACCAAGCCGGTTGCTCCGGCTCAACAGCACTTGCCATTTCTTTCCTCATTGACCACGCATTTGGATAACCACCCCCACCACCAACTGCTGCAGTTCCCTGCTCCTTACTACCTCCTAAACTCTGAGGATTCATCCCTACGTGCACAGCATCCGTAAGCCTCGCCGAGTATGAATTACCCATCCCAACAGGAGTTTGAGACACTGGAGCCACTTGCCTGCCCAATGAACTCCCTCCCAGTCCATACTCCGGCCTCATCTCAACACGAGAAGGCGGACCACCACGAATGCTCTCATCACTGATAGTCCTCCGTGGCACCGAAACTCCATCAAGGGGCTTCCTCTCATCCTCATCAAAATTCCTACCAATTGGGGCAGTGTGTGACAGAAACGGGCTTTTGTCATCGTAATGCCTAGTCGCCGGCGACGTGTGCGGCCGAGACCAATGATCCGACCGCCCCATCGGTGTCCCCCACGCCGAAGGCCGATCAAAACCAGAACGATCCGAAGCCCTTCCAGTCACGCCGGGAGCAGAAGGTAGAGCGAGATCAGAGGGTATAGAACCGCCATGAAAGTCCTTGAGCGTCATGGTGTTGCCGCTAACTTTCTTCTTCGACATTTTCCCTTTCTCCCTCACAAAAATTTAAAAAGAAAAAAAAAATTCAAAAAATTAGTGAAGAGAATCTCAATCCTAAATCCTAAAATTCAGGCTCGAGGTTGCTTTCGAAAGCGAAGGGAGAGAGAGAGGGAAGAGTAAGGAGGGTAGCCCCACGAGTGGGAATGGAGAAACAGAGAAGAACAAAACAGGAACGCGATTGGTATTGGGTTTGGTTTGGGGTGCGAAACCAAAAGTGAAGAAGATGGGCGACGAGTGTTGTGAGAGAATGATTGTGAATGATATTCGACACTGTGCCATTT

>C101681_65

CAAAAGGACAGCATAACTCCGAAACTCTGACAATCATTACATATACTACACACTAAGTGGGACAATAAGGTTTTGCCATGGACCCTAACCTGATTATGAACACATACAAGCCTGTGTAAAGAGAACAAGATTATTCTTGCCTATCTGCTATCACCTTCTATGAGGATTTTGCAACCACTTTTGAGATCCTTCAAAGAAATCCAAAAGGTAGTTTTCCAAATCATCAGCAGTGTACTTAATGATTTTGTCACTTATGTGATTGTTGTGCCTTCCAACAAACGTTCTATAAGCTTGGATTACTTTGAGAGAAATAGAAATCCGCAAATCTTCTCGAAGCTGAATATCTGGGATAAGCCAAGCTGTCTGGATCCTGTAAACATCCTCAAAGCCAAGGTAGAAGCTTCGTAGCCTCTCTTTAAGAAGACTTTTTGAGACCGAATTTGTACCCGGAACATGAATTCCTTCATCCTTAAGCAAGTAAAGAATGGAACTCCAACTAGCCCGCTCATATTTCATTGCATGCTGCTGAAACTTCCAGTTGCGCTTTCGAATCCATTCATCTCCATATACGAGCCTGAGTTCAGACCCCTTAACCTTGTCAGCCATATAATGTAGGTTGTTCATCAAGAACAAGTGCTGCAAGGAGACTTCCTTGTATAACTTGGACTTTTCCTCAAGGTTGCTTTCCAAGATCGATGCGATTGATCGGAAGTGAAGGGACATTGAGGAAACTCTACTTGGAGATCCCTGGCTTTTGCTATCTTCTTCTGTACCAGGGCTCATGTCAGGTGAAAGTGAAATGGATTCCTCTTCTTCTTGGTCCTTCAGAAGTAGATTGAGTATGTCACTGTAGTCTGTAAGGGTTCTCATATAATTCATAACATATTTGGTTAAAGGATGTATCCCACCACCTACAAAAGGGGTTGATGATGCATTTGTTGCAATGGCATTTTCAAATTCAAAAAATGTTGCCCTCACACAGTCGCCTAATCTCTTCAGAACTTCATGACATTCAATTTTAACCGAGGAACCAACCTCATCTGAATACAAAGCATCAATATCTGGCATAAGGTCTTGAAGAACCTCATACATGTCAAGAACACGGAACAGTTTCTCAGGTTGGTGAGGGCCAATGGACATGGCCTCACCGAAATTCAGAAGCTGCAATATTGAAGCCTTAGATGCATCAACAAAACAAGCTAGACTAACAAGCTCGCCCTCCCCAAAAATCTGGTCACTAAGCCACCTCTCACTTGCAAGATACACCCTAACAAAGATTTTCACAGCCCAAATCCATCTTTTGATTTTGGAATTCAAAGTACCCCACTCCATTTTTAAGACGTCCTCGATACTGAGCTTTTCCATTTCAAGAATGAAGAGGCACTCATCTAGGGCATCCCTCCGGACAATGATATAAGCATTGGAACATTCCTGGCAATAGTTAGAAGCAAAAAGTAAGTTTGCAATACACCTGAGATCCGGGATCACGGCTGGATAAACCAAATCAATGATATGCTCCTCAGAGGCTCTGCTGACACTATCTCTTTGAAGTGATTCTTCAACTGATTCATCACCTATAGAGATTATGGAGTTCTCATCAACAGCATCTTCCTCACTAGACCTAAATGAGACATATTCAGGCTCAAAAGGTTGCCTGTTCTGGATAAGCAAGTTCCTGAATTCTTCTTCAAGGCGTGCCATGGCTGTTTGAAGAACACTATAAGCCTTCTGCATAAACTCGTACTCTTGATCCTCTTTCTTCAAATTCAAACTTTCCAACTTGTCAATCAACCTGCGAGCTTCGTTAGCAGCATTCAGATACTCAGATGCTTCTTCAGGTCCCAAATCCCAAATCATGGATTGATCCTCCTCCCATCTCATAATTTTCTCCTGAATCACATTAAGCCTTTCTTCAATAGCACTAAGACCCTCATCATCATCAGCACCACCACCACCACCACCATCATGATCATCACCACCCTCCCCTCCTTGCCCTTGTTTCCCCTCTTCCTTCTCACTAGGTATGGATATGGAGGACAATCTAGTCCCAAGATCTGCCAGAATTTTCTTGGCATCACTGGTAAGGGTCTTATTTGGTCCCAGTGCCTTCACAATATGCCTTACTGCAGCAATCAAGTTCTCCTCTCTTTCTAGCTCCCCAATCACAGGCTCAGACTCCTCTACAGCCATTGAAAAACCCCACTTCAACAAACAACAATCCCCAAAACCACTTCAAACCTTTTCAACTAACCACCCTCGACTCTTCCTTCTCCAAAATCCTCCAATACACCCCCTTCAACAATTTACTCACCAACCAAATTCAACACCAAAAGTTCAAGTAGCTAAGAAGAGAAATCTCCTTATGCCTTTACCTCCCTCCTGGATATCACGAACACCGGACAAAAATCTGAGTGAAGAAGAAGCCGGAGAATTGAAAGGTGAGCAAGATCTGAAGGAGAGAAGAAGTATCAAAAGGACTGAAGGCTGTATTTTGACTTTTATGAATGCAGACACAGTTGTGTCAATATGGAGAAAGGGAAGGAGAAAGAAGAGCTCTTTTTGGAGCTTTAAAACAGAAAAATAAA

>C101683_65

AATTTATCCATGTTTTTAAAATGGCTAACCTATTATGTCCCTTTAGGCGAAGAAACTATAGTTAGCATCCAGTTGCTTCTAAAAATGAAGTTGACTGCATAACACGTAAAAGATAGACAAGAACCGCTAAGTACAGTATAAAATGAAACGGTTTTCCTCAAGATATACAGATTTTTCCTACTTCCTAGCAAACTCAATTCGATTTTGTGTGCAACTATATGTCGAACGGTTGTCCTCTAAATCGACTTCCATTGCTCGCTTCTGATCACCCTTTTACAGATTCAAGTACAAGTCCAAAACATTCCCAAAATTCAGCACCAAACTGAGGTCATTATGTCCTTAGAAATGCAGAAGATGATCGTATGATTAAATTTTTTATGTCTTTTGACTATAGTCCGGCCAGTATTGAGAAAGAAGCGCCTTTTCTGTTTCCTGTTTTTGGGCTTTATCTGCAGCTGTCAAATTGAGCAGCAACAGCTCAATGGGAACGTGATGAAGGCAAAGAACCATAAGAAGTCAACAGGTACGTCAGCACTAGGCACCTTCACTAATCTGATATAATTTCCTCCAAGTCAGGATCCTCGAAGTTCATAGCATCATAGTCTGAAACTGTGTCATTGAATGTACCCTTATCTGCTAGATCATTCTGATCATCATTATCATCATCATCATCATCCATATCTGGCCATTCAGGCTCATTGTCAACATCCGGTTCATCATTTGATGCATTTCTCTTTGTTGTATTATGCTTTCCATGTTTTCTAAGTCCTGAATTTTTCTCCACTTTGGCTGTGGAATCTTCACAAATGCAGGTCGATGGATTCTCATGAAACCCTCCTCTACCTGGTCTAACCTCCTTTGGCTCCCCCATGAAGCCTCTCTGGAAATTACGGACACTAGAAGCAAAAACTTCCCAAGTCATATTACTTCTATTCAAGAACAATCGATAGAGCTTTTTAGCATTTGGACGAATGGTTGGTTTATGCCCAAAGTATCTCCTTCGTCCAGCTAACATTTTTGCCATATTTCCATTATTATTGGTTACAAATACATCACTTTCATCACAGACCATGAAGTCAAGTGCAGCCATGCGAGAAGAAAAAGAAGAAAATGCTTCTAATTCTTCCTTGGTAGCAATGGTCTCTTTTGAATGGTAATTAGGAAATAATGCTTTGAGAGGTGCCAGTGTTCTTTCCCCTCCATATACTTCCCCTGATGCAACGTAAATATGAACATCAGAACCATATCCCAATGCTCTTAGCATGAGACCAACTTCTTCTGGGGTTAATGGGCATCTTCCCTGCCTCCTTGCCCTATCAGGATTGCTTCTATGCAAAGTCTTCCACCTCCTCCTTATTGCGCCTAGTTCCTTCTTCTCCTTCTCCCCTCCACCATAATCACATCCAGAAAATGCAAGCATGTCAGGTTCAAATCTCAGGTGCAGTGCAATATAATGTTGGCTTCTCATCCTCATCCAGTGGACCAACTTTTCACCCATTGCATGTATTGGGTTGGTAAATCTTAAAGCATGGTAGTTAGCTCTACATCTCAACTTCTGATATTCCGTATCCAACCTGTTTGCAAGCCTGTAGTCAAACTTGCTGAGCTGGACAGCACGCTTTTTCAGGAGTACAGGTAATATACGAGTTATATAACATCTTTCATTGCACTTCCTTGGAACCCGCATATTGTATGCAGATAATGGTTTCCTACCCTTACTGGGAAGTTGCTTGATAATCTTGACATCTTTCGACAGATAAGATATAAACCAATCAACATCAAAGATCTGTGAGAAGTTACTAGAATCTTTCCAGAAAGATCGTTGATCCAATTTTGGAACAACAAGAGTAGCATTCAAAATTCGTGCAGCAACAACAGCATCTGTAATCCCAGTTCTTTGTTGATTCAAGCCTCCACTTGTTGCAATCAACAAATATCGATTCGGTTGTGTAATCACTTGAGCTTTTTGAAACTTGTTGCCGGAACTACTACAGCCGTAGAAGTGCCCTGAATTTCGAGAAGTCCAGATATCGCGTCCCAACTTTCCTCCATCACTCGGAACGTGGAACACAGGATTTTCAATCGCCTCATCCTCTTGAATGTTAATCGAAGTGTGTTGGCGTCTCCGGTGCATACGATCGGTTTCGACGGGAGAAGGAGCTAAAAACGAGAGGAGAGCGAAGAGAAAGAGTAGCGCGGCGGCGACGGCGGAAAGCACCGGGATCACTGGACGGAATCGTTGGTAATAGTGTTGACGCCGCCGCTGATATGCCATATCTGGTTACCTTCCCTCTACGGTCCGCGTGTGTGCTTATTGGAAGCACCAGCTTAATACGATTATTACAATTTCATGTTCGTTATATACAAATAATTAATAATTAATTTTCAACCAGACACGGCTACAGCATGGCATTGCGTGTGGCGTGCTGCGCTGTGATAATAGATCTGTTCGCTGCGTTGCAAGAGAGACGGTTACAGAAGGAGAGTGTGAGTGAGAATGCGAGTAGGAGTAGGAGTGGATGTCTCTTTCTTTCTCTCTTTTTTTCCTTCTTCTCTCACTGCAAGGCTACCTGCTAAGGTCTTTG

>C101687_65

GCAAGGTTGGGTTTCTTTTAGAGGGAACACATACATAACCATTAAGATAAGGAACTGCACTCTATTATTTTAATTGGTTTAGCTTTGCTTGTGAAGTTGGCTTCGTTTTCTGCAATTCCCATTCCACAAAGCCACACCCTGCATCACCCCCCAAAAAACTAACAGAAAAGCAAAAAAGAAAAGAAAGGAAAAAGAAAAACCAAAATTTAAAACAACATATTATTATTAATATCGCTGTAATTATTGTTACAGTTTTCATTTGTCTTGTCCCTTTATTTTTCGATTGGATCTGAGGCTCTTCAGAGTTGCAGAAAGGCACACCAAAGTTGCTTCACTTTCTTGGAGTGTGGTTTCTCTTTGTTCAGAGCAAACTCGCACACAACTTGTGGAATCCACAGCCATGGTAGAGGGTTTCTTGCTCTGCAATTTCATTGCCATTTTTCTGCTTTGATCAGCTCATCAAGGATTTCTTAATGGGGTCACCTGTTAATGGAGTGGATTCTTTACCAGAGTTTCAGAATTCAGTTTCTGTGATAGATCATGATCCTCCTTCAACATCAGGGATTTCCCGGCTTTCTCGGCCGCCGTTAAGCACATCTAGAAACTATGGGGCTTCTTCCAGTCAGCATAACTATGGAATGAAGACCATCCACCATCAAAAGCATTCCTCTCAGGAGGACAACGTGGAGCGCGATGGATTGCCCAATATGGAGAAGCAGTATTATAACACAAATAGCAAATCTGAGTATGTGAGCCTCAACAAAGCTGTTGAAAGTTGTTTAGGAAAGAATATCTCCAGAATGGAAACAAAATTAAGCATCAAGCAACCGTTAGATGCGTCCAAGAATTGTGATTCTAGTGGCGTATTAGAATCTGATAATTGCAAATTAGGTTCGTCAAAAGGTGTTGTTGATCGAACAAAGAGTTACTGTCAATCAGAGATTACCTTTTGTCCAAGTCCTCAGAATAGTTTCTACTCAGAAGCCAAGGAAAGTTTTGCCAATACTGGAGTCAGTGAATGTGTTAGTGTTGATAAGTCAGTTGAAAGTGGAGAAGTTACTAATTCCTGTGAATTTAACGAGAGCAGGAAGACCAGCATCTGTAGAGGCAGCACTGGAAGTGACGTTAGTGATGAGAGCAGCACTAGTAGTTTGAGCAGTGCTTTGTATAAGCCCCACAAGGCAAATGATGTAAGATGGGAAGCAATTCAAGCGATTCGAGCCCGTGATGGGGTGCTGGAAATGAGACATTTCAGGTTATTGAAGAAATTGGGTTGTGGAGACATAGGAAGTGTATATCTAGCTGAATTAAGTGGCACGAGGACTTGTTTTGCAATGAAAGTAATGAACAAAACCGAGCTGGCAAGTCGCAAAAAGCTTGTTAGGTCTCAGACAGAGAGAGAGATACTGCAATCTCTAGATCATCCATTTCTACCCACATTGTATACGCACTTTGAGACAGAGACATTCTCCTGCTTGGTAATGGAATTTTGCCCTGGTGGGGACTTGCATGCACTCAGGCAAAGACAACCGGGGAAGTATTTTTCTGAGATTGCTGCCAGGTTTTATGTGGCAGAAGTTCTCCTTGCTTTGGAGTACTTGCACATGCTTGGGGTTATCTACAGAGACCTCAAGCCTGAGAATGTATTGGTGAGAGAAGATGGTCATATAATGCTTTCAGATTTTGATCTCTCTCTAAGGTGTGCTGTCAGTCCAACTCTGGTCAAGTCTTCAAACTCTACCTTGGAGACAAAAAGCTCAGGATACTGCATTCAACCTGCCTGCATTGAGCCAACTTGTGTAATACAGCCAGACTGCATTCAACCATCTTGTTTCACACCACGATTTCTCTCCAAATCTAAGAAAGAAAAAAAGTTGAAATCAAAGAATGATGTGCAGAATCAGGTGACCCCTCTCCCCGAGCTTATTGCCGAGCCCACAAACGCCCGATCAATGTCTTTTGTGGGGACACACGAGTACCTGGCACCAGAAATCATCAAGGGTGAAGGGCATGGCAGTGCTGTAGACTGGTGGACATTTGGGATATTCTTATATGAGCTCTTGTTTGGTAGAACACCGTTCAAAGGTTCGGCAAACCGTGCCACGCTATTCAATGTTGTTGGGCAGCCCTTGAAATTTCCAGAATCTCCTACTGTGAGCTTTGCTGCCAGGGACTTGATCAGGGGTTTACTTGTGAAAGAGCCTCAGAATCGCCTTGCTTATAGACGTGGAGCAACAGAAATAAAACAACATCCATTTTTTCATAATGTTAACTGGGCACTGATCCGATGTGCAAATCCTCCAGAGGTTCCAAGACAGGCCATGAAAGCACTTGCAGCTGAAAAAGTGCCTGGAGTGAAGCCTTCTGGTAACTATTTAGATATTGATTTCTTTTGATCTTAATGTCTGTTTTCTCATTTGAACTAATTTTCATCTTTTATTTGTGTTGTTTGTCCATGGTTCTTTCCTTTGTATAAAATGATGCAACTCTTTCATCTCCATTGATCAATGCTTGTAATTGTCCATGATAAACTGCTGTAAGAACCTCCAAAATGCATGAAATTGAAGAACAAAGTTTCTGGTCTCT

>C101691_65

GTGGGAAAACCAAACAGAGCAACGAACAGAGTGAGTATGGGTCCCGACGGCATGGTGGCCGCGGCGGAGGCACTGGTGGCGTCCGGTCGGAGCTCGGAGAAGCTGAGTCTTCATTCACTCCAATCGAAAATGAAGTGCGATCCGGAAGGCTACGAATCGGAGCTGCTTCTTATCTATAACCAATTCAATTCCTCCCTCGAACTCTTTCAGAAACAAGCCGCTATGAACTTCACCTCCGTCACCGGCATCGGCAGCGACCCCACCGTTGCCAAGGACCTTGGTGACAGGGCCATGTTCCTCTCCCACGTCACTCCTTTTTACCCCAAACACCTCGCTGATTTCCCTCGCAAACTCGCCGATTTGCTTCGCTGTGCCGCGCGCACTTTACCTTCTGGACTTCGCTGCCACCTCACTCACGCGCTCATTCTCCTCGCTAATAGAAAGATTGTTGATGTAGGAGAAACGCTGTCGTTGTTCATGGAACTTCAAACCTTAGGTGACAGGACGCTGAAGAAGCTGGCGTTTGATCATGTTGTTCATAGTATAAGGCGCATGAACCAAAAGCATAAGAACGAAGCCAAGAACCGTGCGCTTCAGAATATCTTGTTTGAATTGCTTCAGAAAGAGGAGGAAGAGCCGGCCAAGAGGGCACTTGTGACCCTCTGTGAGCTTCACAGGAGAAAAGTGTGGTTTGATGAAAGAACAGCAAATGCAATTTGCACTGCTTCCTTCCATCCATCGTCGAGGATTATGATTGCAGCTTTATCATTTCTGCTTGATTACGAGAAGATTCAAGATGATGATGATAGTGATGATTCGGGCAGTGATGATGAAACAAATGAATCTCCTCAAGTTGTTCTCAGCAGGGAGACCCTTTATAAGGCAAGCCACCAAGGTACAGCAGCTAGCAAAAAGAAAAAGAAAGCAAAACTACAACGCGCCATACGCAGCATGAAAAAGCATCAGCGCATGTCATCTGAAAGAAGCAATAACAGTTATTATTCACCACTTAACCATTTGAAAGATGCTCAGGGTTTTGCTGAGAAGCTGTTCTCACGTCTAAAGAATTGTAATGAACGATTTGAGGTTAAGATGATGATCTTGAAATTGATTGCTCGAACAGTTGGACTTCACCGATTATTTTTGCTAGATTTCTATCCTTTTCTTCAGAAATATATTCAGCCACATCAACGCGACATTACAAATTTGCTTGCAGCAGTTGTTCAGGCTTGCCATGACATGGTTCCTCCTGATGCTGTTGAGCCGTTGTTCAAACAAATAGTAAATCAATTTGTGCATGATCGATCACGTCCAGAGGCTATTACTGTTGGACTAAATGCAGTCAGGGAGATATGCATGCGCATGCCATTGTTAATGAATGAAGATTTATTACAAGATCTTGCACTATATAAGAAGTCACGTGAGAAGGCAGTTTCAGTGGCAGCTCGATCTCTTATTGGGTTATTCAGAGAGGTTTGCCCCTCTCTACTTGTTAAGAAGGACCGTGGACGGCCTATTGACCCCAAGGCAAGACCAAAGGCCTATGGAGAAATCACTGTTGCGACTGATGTTTCTGGTGTTGAGTTATTGCAGACTGTTGACAATGATGAGCAGGAGAGTGATCATTCTGATGATTCTGCATGTAGTGTCTCGGACAATGACCAGGAAAATGATCTGATGAGTATTAATGATGATGATGAGAACCAGTTATCCAGCGATGACACTGAAAGTGATGATGATGAAGCCAAAGGTAACGATGTTATATCAGAAGATGAAGATGAGAGAAGTTCTGATATTGAAGCTGGTGATAGTGATACTGATGAAGATGTTGAGGTTAAGGGGGATGACAGCGATGAAGATGATGATGATGTTTCAGATCATGAAGATGGTGATATGGATTATTTAAGTGATGATAGCAATGTGGAGACAAAACGTAAATTGACAGACTCAGCAAAAAAAAGGAAGTTTACTGATTTTGATGGGGGACTTATTGCTGCTGATACAAGTCTTCGTGCTTTGAAGAAATTGGCAGGGGCAAAAGTGGAGGATGTCCTACCGGAATCTCAAGATGGAATTCTTTCTAATGAAGACTTTAAGCGAATCAAAGAATTAAAGGCAAAAAGAGAAGCAAAAAATGCATTGGCTCAGCAAGGGCTAGCGAAGTCATCAGGAATTAAGATTCCAAGCTCTGATCAACTAAGTCTAAAGCGAGTGGATGGTGCAATGCTGGAAGTTCATGTAAGGAAAAAGATGAGTAAAGATGAAAGGTTGGCATTGGTTAGAGCAGGGAGGGAGGAAAGAGGAAAATATCATGCTCGAACTGCTGTAAAACAGAATAAGACGGGTGGCCTTAGCAACCGACAAAAGGAGCACAAGAAGAAAATGCCCTTGGCTGCAAAGAGAGATAAGGTATCAAGAACTCGAATAGAGAAAAAGAAGAAAAATCAGCGTTCTGGTAAACAGTTCCGCGGAAGGAAAGCGTGGAAGCAATAAATATGCTGTTATACAACCAACTGAAATTTTGTTTTGTAATGAACCCTTTTCACTCTTTCTATTTTTTTTCCAATCTTATTTTATGGTGATTAAAA

>C101695_65

GCTGAATGCTAATTACAGAACATAAACGTCCTAGCTATTATTCCCTTTTCCTATTGAACCCTGATGGATTTGTTTACTAGTTTAAGCTTTATATTAACAGAACAAAAATTTACAAACCTAGACATATTATAAAGTAACCTTTTAAATTAAAATATAAAAGAAAGAAAAAAAAGGTTTAAAGGAAAAATGGGACATTGCAAACAAGTGAACAAGCCTGTTACTGTGGTACGTACCACTAGTTGCCACCATCAACACGCCCTTTCAGAAAACTTTCTTTACTTTATTGGTATCACTAAAGTTTGTTGCAGGCCCTTGTTTCTGTCTAACTCCAACAGGTTCTTGCCATGTTTCATCACATCCTGCTTCATCTTCCCCCCATTCTTCTCTCTCGAAATAATGCTGCTGATAATATCTAAAGGCCACAAATAGCACGAACACTGCCCAAATGCGAGCAACAACTGAAGATAAATTCCAGACAAGGCCAAAAATGCCAAGAGCCACAGCTTTCTTGTGGGTGCATGAATCCCAAGCATCCCGGAATCGACGTATCCAAAAATTTCCATATGCAGTTAACTGTGCAGAATACGAAGAGCATATCTTTGGTACAGTAAAAACTCCAAAAAAGCCCAATTTGGCCATCTTCCAAATGGTTATGTAGCTGCCACATCTGGCTAAAACAAAGAGCAAAACAGCCAACTTTATGGTGGTACCCGGATCTCCAGAAAACAGGGCGTTGAGTTTAGACAGAAACTCGTTCAAGAAAGGGAGAATCAATTTTAGCATCCAAATTGCATCTTCTTCTCCCACTGCATAATTTGAGTCCTCAACTCCTATGACACCCCTGCAAATTAGGGATCTATATAGGAAGATAACAGCCAGATAAACAAGACTTATATAGGACGTCACTGAGATAAGACTCAGATTGATATCCTTTGCATAAGAAGATGAGACTATGATGAATGTCCCAAATCCAAAAACAAATGCTGATCTTGAAGCGTCTCTCCACATGATTAAATCCACTAGACTCTGCAATTTGCTTTGGGTTTGTGGAAAGCTACGGTATTCTTCCGCCTTCGGAATTGAATTAACTTTGGAGAAATTTTGGTGAATTGTGGAATTCCTTCTAATCGTAGGAGATTGCTTCAGACCAAGAGGAACAGAAACTGGCTTTCTTTCCTTTTTGTGATGAAACCGCATGTGGGCACTCACAATCCTCTTAGGTTCTGGTTCATTCGGAATCTTCTTAGCTTCTTGTTGTTTCAGAATCTTCTTTGGTTCTGGTTCATTCACAACCACCTTCTTTTCTGGTTCATTCTCAACAATCTTCTTGTCCTGTTCATTCCCAACAACCTCATTTTTTTCTGTCTCCTTGACAACCTTAGTTTCTGCAATACCAATTTCCTTCACATCAATTCTCTCCTCCATCTCAATCTCAATCTCTTCCCCCACCTCCTCTTCTTCATCTCCTTCATCTGCTTCATCAACTGCAACTTCATCTGAATCACCACCAACCTGAACCGACAATTCTGTTGAACATTGGACCACATTCTCTGAGCTGCTAGATATGACCTTCTCTTGACACACATCATCCAAATCCATGCAATTATCATCGTCAGCGTCATTCTTTCCACTCTCAACCTCAATCTTTTCATCCGTAGGTTCATCAGAAACTCGATCCAACTCGGATTTCGTCTTCCTCAATTGAATTGAACTACCAGGAGAAGCATTCTTCTTTATTGAATCTGACTTGGTCTTCCTCAATTGTGCTGAACTCCTCTCATGCCCCTCATCCGATTCACCGCTTTTCTGTGTCTCAGATCTCATCTTTCTGGTGTGAATTGGGCTTCTCTCAAGTTTATCTGCAAAAGCAGCGATCTTTCTGGTGTGAATTGGGCTCTTCTTGATTCCATCAGAGGATGAAATGCGCAGCTCCTTACACTGCTCCTCGGAACTCTTCGGGGTCTCTGTTTTCCCTCTTGCCACCTGAATTGGGATCTTATTATCCGACCCCTCCGAGGTCTCAGATTTCCAAGTTTTCCTCTTTCCACATCCAATTGGGCTTTTTTTCAATCTAGCACCACCCCCATTTTCTTCAGGATTTTCTTCTGCGTTATACACCTTGATCCCCCCTCTCACTTCATCACTCTTCATCCTGCTCTCCCACACTGACCCTGCAACCACACTCCCTCTAACTCCTACCCTCCTTTTGCCCACTTCCATCCCCAATTTATCTCCTTTTTCTACCTCTCTTTCTACCTGGCAATCAGTACTCCAATGGGTCATAGGTGGTTAGTGGATAAGAGGCTTCTAGCTCAAGCAATGGCGGTTGGTGGGGTGCAACATTGGGATCCCTTTGTAGTGAGAGAGAGAGAGAGAGAGATCAAAAGTAGGGTGAGAGTAGAGACAGTAGTTGGTCACTTTTCTTTTCCTTTTTTATTTTCTCTCTTCTTGGATGATAACTGTGTTATCTCAGGGTAAGGAAAAGCAAAGGTAAATTAGGTGGGGTTGAATGATTAGGGTGTTGAGGAAGAGAAGAGCCCCCTTTTTTTAGCATTGATTTGATTCCCTATAATCTCCCCTCACTCTTAC

>C101697_65

TTCCTTTTTCTTGATGCTGCTGTAAATAATTGCAGGATAAAACATACATAAAACCAAATTGTCATTGTCGATTCATATACGTTGAGCTCGACAAAACCAAGCTGTACTGACTTAAGGACCATACCTCTCAAAAACAGAGGCATCAAAACAATCGACTTCATCTGGCCCCATCACATAAAATCCTAGCCGAAGACAATTAAAAACTATACAGATACATATATACAAAGGCTATACACTCAACGTACACCAAAATTCACAGTTATGTTACATGAAACTGTCGGACAGTCCCTTGCTACTGCCTCATCTCTGCAAGATGTGCTGAATTTACAGGTCTCGTTAGCTTCCTCATCAGATGCAGGTGATTTTTTAGTCTCCTCACGATACAAGTTAACACATCTCATACAAACAAACTTAGAAGGCATGCCATCAGAATTATCAATTCCAGCGCATCTGGTATGCTGCCAAACGCCACAGGTATCACATGCTAGCATTTTCTCTCCATCATCATCTTTAGCCCCACAAGTGCAATCAACCTTCCACACCTCGGTTCCTCTTTCCATTCGGAATCGACTAAGCCCATGTTTGGCTGGGCATTTACCTTGAATTCGGACTGATCCACTTGTTCCAAGCAAGAACTTTACGGTGAGCGAATCGTTGATTGACCCGTATCCTAGGAGTTCTTCAGCTTGAAACCTCTTATACATAGCATACACCTCTTGAAAAGCACTAGTGGCTACGCTCTTGAGCTCACCAACGGTTGCAGTCAAAGGCAACACAATGAGCTCTGGCGGTGGGGGTGGATCGTCCTTAGGCTGATCAGAAAGCTCCGCATGACACCAAAGCCTAATGACAGAAGGCGGTTCATCGGCCATTTCATATGGCTTGTAATCCTTCATAAATTGTTTACAGTCAAGGAGCTTCCTGGCTGAATCAGCAACCCTTTTCCTCATAATCCTAGGCCTGTACGAAACCATTTTGTCAGGATGAATTATTGAATCGAATAAAAATGTCAAATCAGAGATCAGTTGCTCTTCTGTTGGATAGCTTGAATTTGTGCTTAAACCATTACTCCAAGGTCCCAACCTGAATTCGATGGCACTGGAAACGGGATTGCATCGTGAACAAACCACCATCCCATTGGCAGCTAATTTCCCAGGCAAGTGCTTAAAACAGTAGTCAAGAAGCTCTGGAGATGAAACACCCCTGCATACTGCCCCCTTGAGAGCACGCCTTGATACCCATTTGGACTCGGTACAAGCACCCGATGCAAGCAATACCTTGACAAGAGCATGTTGCACCTCGTCAACATCTTTTCCCGTCCAAGTACACAACAAATTACTGGTCTGTTTAAATGTTCTCTTGGCCATTGGCTTACGACATTCACGAATCAATGTCAACAAAAAGGAAAAGAGATCTTGAATGTTTAGAAGTTCAGTTTCTGCCAAAGACTGGTAAAGAGAAATGACACACTCCAGGCGGCTTCGTGGTCCACGACCATGGAATGAAAATGATGACAAGAGCATACTTGACAGGGTATTCACTGCATTCTTGTACGCATCTTGGGTAAGAGCGTAGCTGCCAGTTCCAAATTCATAAGCCCAATTGCCATACCAGGAGTGTCCTTTGGTGATTGCATGAAGCAAGCGGTATTCCAGCCCAAATTTCTTGGACAAATCCATCACGCTAACCTTCCTAACAGAAATTGCAGTACATAGCCTATCCCAGAAATCCATAATATCAGATCCAGAAAGAAGTTTCGAGCCACCTTCTCTTCCACTGAGAGTAAGCAGGTGACCATAACCATTGGCATGGACCACACCATGCAGAAGATGGGTGTTATCTTCAATCTGATGATACACCCAGTCCTCAAGGTCATCAACTGTGGTGGCAAAGTTACACGATCTACACCTTGTTTCAGATAATTGGAGAAGGTTTCCACATCTTGAACAAGGTCTCTGATATGGTTCTACTGCATTATTCGCAGCCCTTATTATGAAATGGTATCGCTTTCGACATACCGGATGCCCACTCCATCCAACGACTCGGCACTGGTCACAATAGACCGAAGTGCGAGAACGAGTAACGTCCTCTTCAACAATGTCAAGAGTAACCACTGCGGGCGAGAGATCGGGACCGTCGACGAGGTCTCCCACACGGAACAAGATCTGCCACGTCATAAGAGACGGAAATAAGGAAGGAGGGAAAGTGATGCGCGCATGGTCTGAGAGGAAGCTTTGGACACATGTCCGGAAGGGTTCGGCGGAGGAGGCGTCGGAGGTGGCCGGAAAAGTGAGGAAATCATAGAGATCAGCCGTGACCCTCCTCTTCAACCTCTTGGATGGTCTCTCGTTGACTACCATGGTCCAGTCTGTGGGTTTTGGAAAAGACGTTAAAGGGAAGGTTGAGAGAAGATTAGAGAAAAGAGATCTGATCTAGAGATAAGCAAAGGATTGATTATGTATATGTATTGAGAGAGTGTATGGAAAGTGAAACAGAAACATAAGAGACGGCGGCGTTTTTGTTGGTGTTGGCGCTGTGGTTTAGAGTTTTGGTTTGGAGGGAGTAGAAGAAGAAGAAGAAGGCGCTGGTT

>C101703_65

TTTTTTTTAATAATAATATCACATTTTCTCTTCATTAGTCAAAGGTGAATTCACAAAACTAGAGTTGAGAAGCAAAACTCGATCAAAAGGAATTATACGATGTAAATGGCTGAACATCGTTCCGTTCCCATCAACTTGTATGGTCTGTTGTATCCCTATTACACTTGAAACATTTACGCAGGGATATTTTTCTCCCAATGACGAAACATAATAAAATGAACACAAAAATACGATAACCAATTGCCATAAGAAACAAAACCAACAAATTTTTCCACTTCGAATTACTGTCAGGGGAGATATTGTACACACTTTGGAGAGCTTGAAACCCAGATATGGTCCTTACCTGCCCCACTGCAAAGGAGGTGCCTAGATACTCGTTCTCCAATAGCCCCTGAATAGAGTATGTATGAAAGGATATATACGACATTGGATACATCCACACAGGTCCAGGCAAAGCATTTCGAATTCTAAAATAGCCTGCTGAAAGCATCATGACCACGTGTATGCACAACAGTGTCAAGACACTCCAAAATACATCTTGCCACAAAGTAGCCACAACTAACATAATTCCTTCATTCAGCAGCAAAGTCATGAAAAAATTGAGCACAAAGTACATTAACAAGCTAAATTGATCTTCCAGCCCGACGAGAAAGTAGAAGACTAGACTTGATGAAATGGAGATGAGAAAAAGAAACGGGATGCTGGACAAGAGCTGAGCAAGTAAAAAAACAATTGTACTGGAGTGCTGGTTTGATTCTTCACAGGAATATATCTTGATTTCTTTCATTAGTGCAGGCACCCTAGCAATGCTTAGAAGTGAACAGAATGATACAAATACAAAAATTGCCGCAACTCTGGTCACAACAGAAGACAGAGAATGCCCAAGACCAGAAAATACAGTGCCAATACACAGTGTGAGAAGCATGTAAAGAATAAGATGGAGCCAGTAATATTTCCATTCCCTTGACACAACTAATAAAGATCTCCATGTCAAAACTGCTATACGAGTAGCATTGCTAGCTTTGCCCTTGCTTTTGAGAACTGGACCTTCCTTCTCAGTGAGTTTCAGAATCATAGTTTCAACAGCAGCTGCATCCGCTGATGACTTATAAGTTGCTTCAAGCGTGCGTATAGCAACAGCAGTGTCCATGTTTACGGCAGAAAAATCTCCATTGTCATCCTGCCAGTTTTTACACATTGCGATTATCCTGTCAAAATCTGTATTAATTGCACGTAGAAAGTGATCAGAAGGACTTTGCATGATTGGGCAAGGAAATCCAGCATTTGAGAAGTGCTGCAAGCAAGCTAATGTTTCTCCAAAGAAGAGTGTATTTCCATTTGAAAGTAGACATATACGGTCAAAAAGGCCAAAGACTTCTGTGCTGCTCTGGTATATGGTCAAGATGAGAGTGTAACCAGTGCTTGCAAGTCTCTTCAATGTAACCATCATTAAAAGTGCCGAGACACTATCCAAATGATAAAGAGGTTCGTCTATAAATAAAATACGTGGCCTCATCACAAGCTCCCTAGCAATGCTAACAAGCCTTCTCTCCCCACTAGGAAGTCCTTTCATATAACAATGTCCACCGATAAGTTTATTTGCATGATCACCTAAAGACATTGCATGTATAGCATCCTCTACCACACTTTTTTTCTGACAAAAGAAACCAGGAAGCTGAAGAAGGGCTGAATAATAGAGAAACTCCCGCACAGTGAGGGATCCAATCAGAGTGGTTTCTCTCTCCACATAACCATAAGACCCATAGGGCATTTGTGATTTTGCTCCATTCACAAACACTTCACCGTACATCCTGGCAGAAGAATGCAATCTTCCTGCAATGGCTCGCAATAAGGTAGATTTCCCTGATTTAGCAGGCCCCATAATTACGGTCATTGTCCCGGGTAATGCATAACCAGTTGAACTCTTAATAACTTTATCAGAGTACTTCCTTTTACCCTTTATTGTAATGGTCAAATCTTTCCAGGCAACAGAAGCGCCAGCAATTTTCCGCGGAATTACTGCACCCTCTGGTAGACGTGGGGATGGAAGGGAACCACTGTTTAGCTTTGAAAGAGAAGGAGAAGCTGGTGTTGTTGCAACATTAATAGAGTCACCTCCTTCCTCAACTCTAACATCAATATCAGTGTCATCCCACTCAGGTGAATCCTCAAAAGATATGGGCTGCCTGAGTGAACCAGGCTTACGCAGGTAGAAGAAGTTACTTGACGGCACCCTACTTGCGGGGCTGCTTGCCGACGATGATGAAGATCTATAATTATCTGATTGAGACTGTATTTCTTCCATAATTTCCAGTTACTTTTACCTCACATCAAATTTCAAGCATCATTCACAAAGCAGCAGAATTCCCCATACTAATGCAACTGTGCATTCAAGCTGTTAGTGATGCACATCTCTCATACAGAACATTTTCTGTACACACAGAACACTATCTACCAACAACCCGGATCTAGGCAAAGCATGTGCTGCGAGAGTGAATGTGAAAGAGTGAAAGAGAGAAAGAGAGTGAAGAAGCGGAATGAACTACAACACTACTCGTAGCTTATGTGAGAGAAAGAATGTGATTTGACCCGC

>C101705_65

GCACGGTCCTCACGAAATCAAAGATTTAACAGCGCCAAATCAAATTCCCTTCTCAACCCCAATTCCTCTGATTCAACCATGTGGGGCACAATCGCCAACTTCAAGGAGAATTTGAACAAAATCGCACTCGACGTTCACTACGACGACGACGACGAACACGACGTCGTCAGCCCCGCCGTCTCCGATCGCCGTAATTCTCACACCTTCGCGCATTCCAATTCGCTTACCATGTCTCCCCCGCCCAACGGAAATTCCGATCACCCCTACGCTTCTGAGATAGAACAATATAAAGCAGAAATCCAGAGACTTCAAGCTTCTGAGGCAGAGATTAAAGCACTATCTGTTAATTATGCAGCTCTATTAAAAGAAAAAGAGGATCATATTGTGAAATTGAATAAAGAAAATGGCTCATTAAAACAGAATTTGAATGCAAGCCCAGTATCTACAAATGGTGCTTACACACTAAAGGGAAATAGTCACCAATCATCTGATCGACAGCATAGGTTCTCAACTCAAATTAAAAATCGTAATGCTAAAATCAATGGAACTACATCTGCTTTGGAATCCGATGCTATTCAAAGTAACATGGTATCTAAACATTCAAACTCACAAGTAAAGGACAAGGAGCTAGCAGATATGGTAGAGGGAAAAAGTAGCCCCACAGCAGCTGTACAACATTTGCATGAGATACAGAAAATGAAGTTGGAACTAGAGCAAGAATACAAAAAATTGGCAAATATTCAGCTTAAATTCCAGGAGGAAGAGAAATTGAACAAGTCTTTCCAGGAGGAGCTGAAATTATTAAAGTTGGAAAGAGACAAAACAAAAAATGAGATGAGCAAATTACACAATGAATTGAATGGGAAAATATCAGAAATAAATCGACTGCAAATGGAGTTGAGTAGACGGGAAGATGAAGAAGCAAGTGGTGCTGTGGATGGCTTCAAGAGACTAATTGAAACTTTAGAGAAGGAAAACGCTACTCTTAAGTTGGAAAAGGATGAACTTGAGGCTGCGCTCAAATCAAGTAGGATGTCTGGTAAAATGTCACCTGATGCTTCTCAGATCCAGGAAAGTGTTCCAAGCTGTAACAATCATTTACCAGATCCTTCCAAAAGTTTTCCAGGGAAAGAAGAGTTGGAAAGATCCTTACATAAGCTAAGTAAAGAATTGGAGGAAACACACAGGGAGAGGGACAAAGCAGTACAACAATTGACCCGACTTAAACAGCATTTGTTAGAAAAGGAATCTGAAGAGTCAGAAAAAATGGACGAGGATAGCAAAATCATTGAAGAACTTCGTGATAGCAATAATTATTTAAGGGCTCAAATATCACATCTTGAGAGAACTCTGAAACAGGCAATTGTCAGTCAGGAGAAACTGAAGATAGCAAATGACAATGAAATTGTCAAATCCAGGGAAATCATTGATGACTTGAACAAGAAGCTTACTGACTGTATGAGCACCATAGATGCTAAGAATACTGAACTTCTAAATCTACAGACAGCTCTTGGACAATATTATGCCGAAATTGAAGCTAAGGAACGTTTAGAAGGAGATCTGGCTCGTGCAAAGGAAGAAAATTCTAAGCTTTCTCAACTATTGAAAGATGCAGATTGCAGAGCAGATATATTGATCAGTGAAAAAGAAGAAAGTTTAGCTAAGCTTTCTCAATCTGAGAAGGTACAATCAGAATGGAGAAGTAGAGTAAGCAAGCTCGAAGATGACAATTCCAGATTGAGGCGAGCTGTTGAACAGAGTATGACACGGCTAAATAGAATGTCAGTGGATTCAGATTTTCTTGTTGACAGGCGCATTGTGATAAAATTACTTGTTACTTATTTCCAGAGAAACCACAGCAAAGAGGTTTTGGATCTAATGGTTCGCATGCTAGGATTCTCTAATGAGGACAAACAAAGAATAGGTGTTGCTCAACAAGGTCCAGGAAAAGGTGTTGTCCGTGGTGTTCTTGGGTTGCCTGGCCGCCTTGTTGGAGGTATCTTGGGAGGAAGTGGTTCTACTGAATCAGCTGCAAATGCAGGAGCAGATAACCAGTCCTTTGCAGATCTGTGGGTTGATTTTCTTCTCAAGGAAACAGAAGAAAGAGAGAAGAGGGAGTCAGCAGGAACTACAAATAAATCCATCGATGATTCACATGATAAAAGTCCTTATACTAATTCTTATCCGCCACATTCCAATCAAAGGTTTAGCACTGGAACAGTACCAGTTAACTTACCCACTAATCAAAACATCAGTTCTCTCCCTCGTGAATACTTTCCGCATTCCGAACAACTTGGCTCAGAGTTTTCAACAGTTCCCCTTACATCATCATCATCTTCATCAGATAGCAAAAGTACTAGTTCAAAAGTCCTTCCCAGATACTAAGTATACATTTTTTTGTAATTAATCAAAAGGTATAGGTCAATGATAAATTGTAGACATGTAATCTTGTTCAATTAATTCTTTCATTTCATTGCCCTATTTGTCCTGTGTGGATATTATTGCATAATTTGAAACGCTGGAACTGTGGAAAACTACCTCATGTATATGTCATGAAATATCACTCCCTTACCTCAACAAATATATATGCACA

>C101709_65

TTGCTAGGAAATCTATATTATATATCATTATAACTATATTTATAAAAATAAAGTGAACTGTGTACCATCCCGATTCATCGTGTTTCTATGTATGCTCTACCGAAACTTATTAGGCTTGAAATGTGTATTCCGTTGCCTCAGGTTTCGTTGGTCTAATCCAACCTAGGTGTGAGCTTGCACAAAGTCTTCTCTTTGGTGAAAATATTTTCTGTGGTGCCAAGGACCACCTTTGTTGCTGAAGGTTTAGATGATTCAACAGAATAGCCTTCTTTGTCGGCACTTTGTTTTCTTCAAGTTCTCCAGCAATTCAGCAATCTCAAGCTGCTTCATCACTGCAGCATGTAGTACCCTCTTGGTATTTTCAAGGTCAAACTCAACAGATTTAATCCTATCCATGGAATCCATAAGCATTCGCTCCTTCTCCTGAGGCATACCATCAGGTTTGTTGTTAAGTTCCTCAAAGATCTTTTCTAGTTTCTGAATACGTTGTATACATGGAAGAATATATTCTCCTTCAGAAGCTGCTTCAACAGCTGCTGAATGGTTATTGATGTTATATTCCCTGGTAATTGATGGGTGAATATTGTTCTGCATTCTCCAAAAATCGAATGTTAAATAGCGGAGGAGTGTAACTAGTTTTTCCGTGATAAATGTCAGTACTCTTGCCACATATAAGACATTTATTTTCTCTACTTTTTCCTTAACAATGCTGAACCAATTGCTGCCAGAAGTACCTCCTGAATCTGTTCTACAAGCAACATTCCCTACATCATTAGTTTGCATTGGCTGCTCCCGATTAAGGTGAAAGTGATCACTTTCAATCACTTTCTCCACTGCTAGAGAACTATCATCACAACTGTAGTAGCCATTGAGATCTGGTGCTTTAACTTCTTCACGAACTGGGGCTAAATAAGGATAAGGACAGCTCCTGTATCTGTAGGGAGAGGAGTATTCATTCATATCAGACCCTGATTTGACTGTAGATGTATCACTGCATCGTTCCTTCAATGGGTGCATCTGAAAGGAGTCAATTTTTTGTTGTCCATCAGGCACTTTGATGATTTGCCTCACAAATGTTGCCTCATTACGTACCAGCTTCATTATGTCAGGATCATTCCATGGACCCTTGTTAGACCGAAGACATCCACCTTCTGAAGCACATGTACAAGAACCACCCAGAAAGTCTGGCAACTGACTAGAGTCAATGACTTCCAGTAATTTATATAAAGACTTAGTTTCAACAATCTGTATCTTCGCAATAGTTTTGGAATCAAGAAACTTCTGTGCAGCAGGCCAAAGCATCTTCTTAAAGCCAGAACCAGCATTGACGATATACATTTGATGTAATGTCTCAGGATAGTAATTGCTATCTATCTTTGTCATAGCAGACAAGAGATTTGCAGCAGTACGTGAGAAATTTTTCATTCCCAGGCCCTGTACATCCAATATTGTTGTTGTTGAAGAAATCCGTCTCTTTGCTGCAATGGAACATGCTGGAAATTTCTCATGCAGAGTCCTCTCAAACTCCTGGACATGATATTTCAAATATCGATCTATTGTGGTGATGTGCATTAGGCGGCTTGGATGGGCTTTCCCAAGTCTCTCAATGTAAACTGGCCTACCTTCCCTATCAACCCCATGGTACCCTTGAGGATAATACTGCAATACTTCTTCCAGCTCTCCAAATTCAAAATCCTCAAGGATAGTATCAGTTCCATATTCCTTTCGCCACATAAGCATTTCTTCCCACATCTGAATTGTCTTCTCAATGTTAAACTCCCTAGCTTTAAGAAATCTTAACAACGTATGGTAGTCATCATGCCTAGAAGGCAAAGATCCCCTTTCAACAAGTTTTTGGCGCAATTCATGGACAGCACTCTCCTCCCCGGCATCTCTAACATCCTCGATGGACACCGACGGAACCCGATAATCAATTTTCTTTTTCCCTCTTTTCTTAAGAGAATGAGTGAATCTGGAAGAAGCATTCATCGCCTTCTTCTTCAAGGTCCCAATTCTCGATCGCCGCCTCTCGTCTTCCGAGTTCTCAACATCAGACCTCCTCTCTCTACTCTCGTCATTACTACATTGCCCTTCAAACCCTGATATTGACATGTTAGTCCCAAAACAAAACCAATCACAATTGATCCAATCGTCGAATCAGCGAATCAAATTCACACCAAACTCCGCCATACCATAAACTACTAATAATATTTCATCGAATTGTAAGTTACAACAACTCTTAAAATTAAAAATTAAACCAAAACGTAAAATGCAGGGTGATCAAATCAAATAGAATCAAACAATCCAGAGATGAACTTAACACGGTCCGAGAATCATCGTTTAAGAACCATTTTCTAGCTGTTTCACTCGTTCATTCCTAAACCAAAACCAAAATCACAAAAACTACTAAACGCAGCAAAGAAACAAAAATCTCAGAATTCGAAAACGACTCGCCGTTTCGCGGGGTGAAAATTATCGGCGATTGCAGAGTAGGAAGGGAATGTTAAGAAGTGAGGAAGAAGAAGAGTTTAATTTATTTTGATTTATATTTAAATTAAGAGTTGCTTTTGAATGAAGTGTGAGATGAGAAGTTGG

>C101713_65

ACTGGCTTTATACAATATATCATACAAATTGTGGGGTAGTAAAATGAACAATAGAACCGGGGAGAACCCAAATTTTTTTGGATGCAGGTGAGGTCAACACCAAGTATATACAAATTAAAATAAAAATGTACCTCTACCTCCCCCTTACCCTCTCCTATCGGGCATAAAAGAAAATAATAAAAAAAAAGAAATAAAGAATACAAGGCTTTCCATTCCATCCTTGGATATTGTATTTTACACCAAACTTCCACCACTATTAAGTAGCTTCTCCTGGTACTCAAATATCAAGTCCGTAACTGCAACTTCCTGAACCTTTCATGCGGTTGCCATAAAAACTAAAAGAGCTAATACCTCAGTCACTGATAGTATTTTTATCTAGAGTACCAAAAAACTCTTCAATCAAATTGGACGTAGGTAAGAAGTCTAAATCTGTACTTGGAGCGGGACATGTTGTGTACTCATGATACCAGTCTTTGGAAACACAGGGATCCATCAAATAAGCATGTTCCATCACATTGCTAGACAAATCAGGTCTTCCCTGCATACGTGGACTCTGATCACATTTAAATCCCCTAGCTTGCAAAGCAGAACTAGCATCTCCAAAACCATGAGAATGGCACGACACCATATTGGCATCACACTCAAAGGACATACAGGAGTCATCCTGGACAACAATATTGTAGTTCAGAATCCCAGGTGCCCCACTGACATGCCCATCAGGATTACTCCAACCCTCCACCTGTGAGTTCACCTTGCAATTTGATGAACTTCGATTAATCTCACCCTCTAACTCAGTGGTGTTTCCCATTTCACATCCAGTTAAACCAAGAGCATTATCTACATTTTCATCGTCACTGTTATCATCATCTTCTTCATCAGAATAATTATCTTGGTTTTCTACATGGACATCTTGGTCACCAAGAGATTCAATGGCAGAATCATCTGAATTTTCAAAACCAAAAATTCCAGGGCTTCTTGTATTACATTCATCATCATCGCTATCAATATCCAAAAATCTAGACCTGTTCTGAGCAGCCCTCCTCTGCAGCATAAAAACATTAAAATAGTAGCTGACTATTTCCTTGTTGGTTCGAGAACAGAAAGTGACTGACAAATGCTTCCAAAAGTTTCTACCCAATGATGCAGGATTGCTGTAGACAACCTCATGAAACATGTCTTCTTCTTCTTCAGTCCATTGTTGTGCGACGTCCTCTCCCATGTCACAAAATCCCAAGTTCACAAACTTCTCCTTACCAAAAGTCTCCATCAAATTTTCACGTGCTTCCCTAACATGCTGTCTTATACATCTGATGGAGCCACGATCCAAGCAGTTACACTCTTTCCTGCCTCGTCCACTCTCATTAGTAGAAGAATAAGAGCTAGATTCATGCATTGAGAGTAGAGAAGTCCCCATGAGTCTCTCTTCATCTTCATCAACAGTTTGAGTTGATAGTAAGCCAGAGGAGGGACTGTCATGATTATATTTGCTTAATTCTGACATCTTATTCACTTTACTCCCCCAAACAGGAATGATAGCTTGGTGATTTGGACCGACAGGAACTGATTTTCTGGGCGAGCAATCAAAGGCCAAATAGTCATCATCCAAAGGAACAAATGCTTTCTGAGGAATATCAAATTCCAAACAATCTGACGAAGCAGATGATAATGACGTAGCCCCAGAGCCAGTATCATCCTCGCCAGAACAACTGCTGAAATGGCCATTGGTTTTAACATCCTTGTCACCAGTATACGGAGATTCCTTAGAAGCATCTGTTCCAATTGGATCATACCATTGAATATTGTAAAAGCCATCCTCACCATCACCTGTAATAACAGTCTTTGCAAAGGCCTTATTGGGTGTAACAATATCATCTAATGAAACCAGTTCATTGTTGCATTCAAAACGTTTTGCCTTGTTCAAAGAAAGATCCTCAAGTTCATTAGCCTCAAGACATCTCTTATACCCCATTCTGGTTCTTGTTAATTCTTCCAAATTCAAATTTAAGACCACCGGCCAACACAGCACTTGGGAGAATAGTTTAGGGTCAACAGAAAATCAAAAGCCTAATTGGATACAAAATCACACAGCACAGAATGTCCCCAGATGCCAAAGAGTGTATCCCAATTGAATTTCCCAATTGCAATAACAACAAATTGTATTTCAAACCTAACCCTAAATTTTTGACTCGTTCTGAAAACTATCAAATTGAAAACAATAGGACCACTACAATTTCGTCAGGTATGAGAAGGAATCAAACCCCTCAAGTAATTTACAGAAAATCCCACCAGAAATTCAAATATCAGAAAACCCTAAAATTGACCGGTCCGAAACCCTAAACTCGTAAACGCGTCCAGAAATTATGTCGAATTAGGAGGAAATATCAAGCGAATAAGAACCAACACCAGGATTTTTGGGGAATACACAAGAAAATACAGCGATCGTGTCACCTTGAAAGACCGAATCGAAACCCTAACCCTAGACCTCGACAAAGATTGAGAAAAGGTGACAATTTTGGAATCAAAAGGAAGAAGGTAGCGGGAATTACACGAAATAGGGACTGGATTGAAGAATTACAAACAATAATTAAAAGAAAAT

>C101715_65

ATAACTAGCTTAGTTCCAGTTCACAGGAAACCAAGATCCGGCAAATGTACACTTACAAGACAGCTAAATAATTTCATGTACCTCTTATTTATCCAGACTAGATACATGATGAATAGGGAACTATACAGCATATTAATCCTTAAAATGCTGACAATAACTTGAATAGGACTTCCCTATGTTGGAAAAAAGACATGCCTAATATATTGTGCTACAACTGTAAGATTATTGTTCCCATATAACAATGTACATGTAACCTATGAATTTCTATCCACACCAGTATTCAGCAAAGCCACATTACTTGGTGTTCTCAATGGAGGCCTATTAATCTTCTCGAGTAATCTACTAAATACTAACTGGACTGGTATGCAAGCTACTGTAGTGGACCCAGAAATATCTGGTTTAGTCAATCTATCTGTTTGATAACTACTGCTATCATGTAATGACTTTCTACTAAAATTATGCTCAGTAGATAATGAGGACAGAGTTCTAGTTTTTTGGCAAGATGACAAGCTCAATTTCTTTGGCACCTTTTTTAAGGACTTTCTGTCAGCTGAAGAACGATGACTTTTAAGGCCACTCCTCTTTTGAAAACCAGCCTGGTGACTGGAACTTCCAGCATTGAAACTTCTATATTCAAAGCTTTGTTCTTGATCTGAAGAGGGAAACTCTGCACCACCTTCAGCACCATTTGATGATGGCACATGGGGGCGCGGGACACGGAAATTTGCAGTTCTCCTTGCAGTCCTCCATGCTGGTGGAAGTACACTACTTCCGTCAATACCAGTCCCATCATTACTTAAGTCATCAATTGTAGGAAATAGAATATCAGATGAACCATCCTTGAGAGCCTCAACCTGGATTGGGTGCCCTACTATTGTCCTCCCATCTAATTTACTCATGAGAGAAATGAAGGGTACACGCTCTTTCCGATAGCTGGCTTGAACCTTCAAATCTACATTAACTAGGATCGGCCTCACCCTCCCACCAAAATGATAACGGTCACCATACGATGGAGAATATGCTTTAACATCCCAATGTCCTTTAGAAGCTAGATGATCATCCCAGGCCAAATCATTCCAAGTCACACCACGATGAATTTTACTTTGACTTCTTGATACGGATCTAGAGGATAGAGGGTAATCATCTTCCAGTCCAAAATTTTGATCATCATCATCAAAGGTATCACCAAAATCAAATTTGTAGCGGTGCAAACTTGGATCTATCCTATTGTGCCTTAAATGACTATTCTTTCCTTCAACATCTGCTCCTGCTCCATACATACATTTTCCATCAGAAGCACCAACAGACCTCTTTACAAGATTCCGATTATTCCTTTTCCCTTTTAGTTGCCATTTAGACACAGCTTCGTTACATGTTACTGAGTCACGAGGATAAAGGTGAGGCATGTCACTGGAATGTGCTAATTCATCAAGCTCCTCACTGCTAGTGCTTTCATGTTCTTCAGCATCATGACCAGTTTCTGAAAATATTGTCATCTCTTCATCCAAGTCAGGTTCTGTCTCAGAAGAATCAGAATCAGAATCCGTACAAGCTGTTTCCGAAGAATCGGATTCAACATCATTTATAAAAGTATATTCAGTCTCTCCAACCATAGAACCATGATAAGGAAACTCCCTTCTCCGTTGGGAAGGTGACATCTCAACACGGGGTAGAGTCTCTCTGTAATCAAGAGAATCACTAGATTCGGTCGGAAAATACACACATCTGCTCCTCTTTGCTCGCGAAGGTCCTATGTGCCCAGCCCCATTGATGTGAAGAGTACTCCCAGCGCGAATACCACCGCTTGCAAGACCCCGAGCACCATCATCAGCCGTACGTTTCTGATAAATGTTAGGATCAACAAAAGATGGAAGCTTTTTTTTTGCAGGAGCAATTCTGAGCCCAAAATCCTGCAAGTCTCTCATCCGAGGTATTGTTTCAGAATAATCCACTTCCCCGCGCAGCTGATTGCCATCCTTGGCCATTTCTGGAGGAAAGAAACCTCCGGCAGACTCATTCTCACAATACATAAACGAATTTGCATGGTTTTCATTGTCATTTCCCAGTGTTTCCGGTGGCGATGCAACTACAACTTTTTTAACAGTATTTGGTGGTCTAGCACCTGATCTCCCTTGCTTTTTGAAAATCTGTTTCTCGAGCTCAAGTGCATGAAGAATGGCATCTTCACGGCGCGCATACTTCTCTCTCTTCTTCAGCGGTCCCCCCTGAGCAGATTCAGCCTTTTCAATACAATCATCAAACTCACCACACCGAAATGCCTTAACACGCTTGGATTTCTCCAAATTGTACCAATCCACGCTGGCGTCTTCTCTGCCGAGGAGCTTGACGGGGGTTCCGGATCGGGGCGAGGTGAGGTGAGTGGCGGAGAGGTCGTCGGGGCCGAGGATTTGACCCGGCCACCAGGACCCGTTCCTCCGACGCACCCACACGATGGATCCAACGCCGCAATCCATCATCCGCTTCCCCGGCCAACCGATGCGCGCAGGATCCGGGAACCGTCGTCGTAACTGGAGGATCCGAATAGAGGAAAATGGGTTAGGGCAAATGTTCCCATTCAGATTTGGGGAAATTTCAT

>C101719_65

TCTCTCTCTCTCTCTCTCTCTCCACTGTCTCTCACCAACAACCTTGTAACTCTCCCCGAGGCTTTGGTCTTCCTTTCCCATTCTTCAACACTTCTTTTCTCACTTTACTGTTTAAGCTTTTCTCTCCCCTCCACACACGGCCTAGCTTGCATTGTCTTTCTCTCTATCTTTCCACCTCTCTTTTATTCTCTTCTCTTCTCTTTCCCATAACTCTAAACCCAGTTACATACCTTGCTCACTACCTTCTTCTTTCCTTTCCATTTTGGATTTGCTCCAACGGTCACTTAGTTGGAGGAACAAGAGATGTTCCCTTTTGTTTTGTTGTTTTACTTTTTCCTTCTTTCCTTCAACTCTCTAGCACCTTTAGTGCATTCTCTGAATGCTGAAGGCTCTGTGCTTTTGACACTAAAGCAGTCCCTCACAGACCCACAAGGTTCCATGAGTAACTGGAATTCCTCTGATGAAAACCCTTGTTCATGGAATGGAATCAGTTGCAAGGACCAAGCTGTTATGGCCATCAGCATCCCAAAGAGGAAACTTTATGGCTCTCTCCCTTCTTCTCTGGGATCTCTCTCCCAGCTCCGCCATGTCAACTTCAGGAACAACATGCTCTTCGGAAACTTGCCTGCACAGCTCTTTCAAGCTCAGGGACTGCAAAGCTTGGTGCTTTATGGCAATTCACTTTCTGGGTCTGTTCCTAGTGAGATTCAGAATCTCAGGTACCTTCAAACTCTAGATTTATCACAAAATTTCTTTAATGGGTTATTGCCTGCTGCAATTGTCCAATGCAAAAGACTCAAGGCTCTTGTTCTCAGTCAGAACAATTTCACTGGCCCCTTGCCAGATGGTTTTGGTACTGGTTTGGTTTCTCTGGAAAAACTAGACCTTTCTTTCAATCACTTCAATGGGTCAATTCCCAGTGACTTGGGAAATCTGTCAAGCCTGCGAGGTACTGTTGATTTGTCTCATAATCATTTTAGTGGTTCAATTCCAGCTAGCCTTGGTAATCTGCCTGAGAAGGTTTACATTGATCTTACTTTCAACAATTTAAATGGTCCGATACCTCAAAATGGTGCTTTGATGAATAGAGGACCAACTGCCTTTATTGGCAATCCTGGTCTATGTGGTCCTCCTTTGAAGAATTCATGTGGTTCTGATACTCCATCTGCTAGTTCACCTTCCTCGTTTCCATTTATGCCAAATAGCTACCCAACTCAGGGTACTGGTAATGGTTCCATGGGAAGTGAGAAAAACAAAGGGCTAAGTAAGGGAGCTGTGGTTAGCATTGTTGTGGGTGATATAATTGGAATTTGCCTCTTAGGTTTACTGTTCTCTTTTTGCTACTCAAGGGTTTGCGGTTTTAACCAGGATCTGGATGAAGATGGAGTTAACAAGGGAAGGAAAGAGAGGAAAGAGTGCTTCTGCTTCAGAAAGGATGAATCTGAGGCCCTGTCTGAGAATAATGTTGAGCAATATGATCTTGTCCCATTAGATTCCCATGTGACTTTTGATTTGGATGAGCTTCTCAAGGCTTCAGCTTTTGTTCTGGGTAAGAGTGGAATAGGGATTATGTACAAAGTGGTGCTTGAAGATGGGCTTGCTTTGGCTGTGAGGAGATTGGGGGAGGGAGGTTCTCAAAGGTTTAAAGAGTTTCAAACAGAGGTAGAAGCAATAGGGAAGTTAAGACATCCAAATATTGCAACTCTGAGAGCCTATTACTGGTCTGTTGACGAGAAGCTTCTCATATATGATTACATATCCAATGGAAGCCTTGATACAGCAATTCATGGGAAGGCTGGACTTGTCACATTTACACCACTTTCTTGGTCCTATCGATTGAAGATCATGAAAGGAACCGCAAAAGGATTGGTCTATCTGCATGAATTCAGCCCCAAAAAATATGTCCATGGAGACCTCAAGCCAAGTAACATACTTCTTGGACACAACATGGAACCCCACATTTCTGACTTTGGAGTGGGGCGCCTTGCAAACATTGCTGGAGGGTCCCCGACCTTGCAATCCAACCGAGTGGCTGCAGAGAAACAACATGGAAGACAAAAGAGCATCTCGACTGAAGTCACTACCAATGTGTTGGGAAACGGTTATATGGCTCCAGAAGCACTGAAAGTGGTGAAGCCATCACAGAAGTGGGATGTTTACTCATATGGTGTGATATTGTTGGAAATGATCACAGGAAAATCATCCATTGTCCAAGTGGGGAACTCAGAAATGGACCTTGTTCAGTGGATTCAGTTCTGCATTGAGGAAAAGAAGCCACTCTTGGAGGTTTTAGACCCTTACTTGGCTGAAGATGCAGATAAGGAGGAGGAGATTATTGGAGTTTTGAAGATTGCAATGGCTTGTGTCCATAACAGCCCTGAAAAGAGACCTACAATGAGGCACGTGCTTGATGCTTTGGATAGATTGACTATTTCCTCTGATTGAGAAGTTTCAGGGAGCTGTGCACTGTGAATGCTGGTCATTTGTGAAGCTTTTGCCTTTAACTTTTCTTGGTCATGTTTAACAATGTCTTCAAGCTGATGAATCCCTTGTAATTGGAGTACTAGAAGAGAAATTTAATTTGTTTGAGCAAAAATC

>C101723_65

TTTTTTTTAGGGAGAAATGTTCCAGCATTATCCATAGAAACATAGTAACTGTCAAAAACATTATTCTATCAACCTGATTCCGGTATCTGATGACACTGACTATTACAGCATTTTACTGATCACAGGTAAGTGATCAGTTTACACAATCACTATATACACTTGAGATTTTACTATTTTTATGGAAAGGTTGACTACTATTGTTCTAATAAAGAGAGTTGTAGCTGTAGCAGGTAGCAATAATGGATGAGACGCAGATGAGGAATGCCGGTAGTAAGGTGGTATTGAGGAGTTTACTTTCACTGAAATACCCAGAGATTGTTATTGTTCCATCTGCAATTACTCGAGCTAGAGTTCCAGCTTCTGTTGACAGCAATCCCCCATTGAAGGTTCCCCTTGAAAGCCTTGATGACATCATTTTTGATAGAAGTGAAAGGTTCACTCCTTCTAGAACTTCAGCAGACACAAATGTGATAAGGGCTGATCCAACATATTGAGTCACTGAATAAGGAATCATTATATGGAAGCTTAGGAGTAGTCCAATGCAAACCATGATTTCTGATGTCAGTAGAACTTGCCTTTCTTCAAACATGTTGCTGATGTAGTTTCCAACAACAATATTTACTGGAAGCACTGTAAGACCAAGGCATGCAAGAAAAATGGATACGTTGCTGGTTGACCAAATAAAATAATATTCCGTGATAAGGCTTGATTCAGCAAGTACGATCTCCATTGCATACTTGAGCATAAAATACACAAATAGTTGAACCTTTACTGATGGTGTGAGTAACTTGTATGCCACCACAATAGAAGTCACCGGTTTCGTAGTTTCTTCAGCATTTTCATTTTCTTCTTCTCCTTCTTCATCCTGCTCTTTTTCTTCAGTGTTCATTAGTAATGGTTGTGTATGCCCATTCTCTACTGCAACATGTACGGCTGGTCCTGTATCTGCTTCATATAGCACAACATTTCCTTGATTTTCATGGGTGGGTTCCTTGAAACAAATCCACAACCACATCAGATAGACAAGCCATGCAAGAGACATTACCCAGCCAGGCAGAGTGTCTTGGTTCATGGTGAACCTGTAAATCCTAAAATCTGTCTGTAACAAACAAGCAAGAGCTGGACCACAAGCCATTCCCAGAGCACTTGCACTAACAAAACCTGCTGAAGCCTGCATCCGAAGTTTCAATGGTACACAGTCACTGATATAACGCCTATTAACAGCCCTTGCAGAGCCTAACCCACAGAAAAGACGTCCCATCAGTAGAACTACTATTGAATTCATATCAAAAGCCAACGCATACAAGGTGTTTCCAATTAGAAGAACAATGCTACTGAATACGAGTGGTCTTAGATATGATCGGTTTGACCATGCACTGAAATAAACTGAAGAAAACATCTGTGCAACAGCCATTGTACCAATGACCACACCACACACACTTGCTGCAGCTCCAAGGTTCAAAGTATAGTTGTCTGCCGTAGGTACAATTATGTAAGTGTTCACCATGTACAAAAATGTATTTGCCAAGTTCAAAAGAAGTGACATAAAATGATATCTTTCATCAGCAATATGATCTTCAGATGGAGATGGTAGCTCCTCTTGCATGATAAAAGCATGTCTTCCCAAAAACTGAAGAAAGTTTGTTGAGTTTGATAATCTGCCAACAGCTTCTTTGATAGAATTTAGAACAGGATCCTGATGAGTATAAGAAGGCTGGTCATATATAGAAATGTAGCTTCCTTGACATTGTTTTAAATCTTGAAGGTCAGCCAGACCATGAGATAAGACTCCAACAACTGCCCCAATGCCCACATGCCTGAAAACCTGCCTTAATTGGGAATAAGGATGATTTGCACGTGTCTTGACATAATAGTCTGCGAATTTATAGCCAAACCGTTTATCAAACTTCTTCAAGATCTTTCTCAAACCAATAGCATTCATTTCCACAAAATTAAGAAGCCTTAAAAGATCTCGTCCCACATCTCTATATGCTTCTTGGAGTTCACAGATGTTTATACTATTTGGCTGCTGGAAAAGAGTATGGTGCTCTTGTCCAATATCTGACAGCCGATGTGCAAGTACTCCTTGTTGTTCAAGCAGAAACAACACAATTTTTTCAATCTGATTGTCTAGGAGCATTGAAAAGTCTCTGAGAACATTATGTCGATTTTGAGCTCCAACCTCCAATTGTTCTACATATCTCTTGACTTTCTTCTTCATTAACTTATAGTTGATATAATAACCTTTCCATTCTTGAATCTGTAATTCTCTCAACTTCTTACCGTAGCCAACCATACTCAAGAAAAAATGTCAGTGAACTGATAACAATAGCAAAAACTCTCTTTCCTCTCAGAGATCAATACTTCAATTGGAAGACCACAAACAGAAGGAACACTTGTAAGTTGTAAGGGTACAGTATCATGGACTTTGCAAGCTCAAAGGCAGAGCTGAGATGAAGATTGAAGATATCATTCCCTCCACTTCAATAATGAGTGTAAGTTTTTTGCTTTAGTACTTTAGGCACTTGAGTCTGAGAGAAGCTGAACTCTATGGAGAAAAATTTACCTAAATGCAAGTATAGAGAGTAAGTTTACGACCGAA

>C101725_65

TTCTTCTCTGTTCCAGTACAACTGAACAAGAAACTTTTCTTCCTATCTCTCTCTGTCTCTGTTCATTATTTCACGCATATATTCCTTCAAATTCATTCTACTTTCTCTCATCCAATCATTCACGCACCCTTTCTCTCTTCCCTTTCGCACATTCTTTGATCCTCTTTTTCTTCACGCCAACTCTCTTCTGGGTTTCTTCATCTTCAGATTCTCATCACCACCTTACTATTGTTATCTTCTATTTCTTTATTGGTGTTTGGTTGCTGAGAAACTCCCAGAAAATAATCAGTCTTCCATTGTATTGATCCTATCTTTACTCACAGCATTTTCTCTTGTTTGTGTAGAGTCTAGAAAGAAACTCAGAGTACCTGACCCACTTCTGAATTTTCTTTCTTTTCTTTGTCCTTCATTGGAAAAACTAAAAAAGACCTTTTTTTTAATCTGGGTCTGGTCTTTTTCTTTTCTTAATCATACCATATTTTCTTTCGTTTTCTTGGGAAAGTTAGTTTGTTGAGAGTAGTGGAAAATGTTGGGGACCGCTTTGCAGTTTGGAGGTTTTTGTGGTGATGATCGATTTTACATTCCAGTGAAGGCAAGAAAGAACCAAAATCAGAATCAGCGTAAACCGGTGAGAGAGAAGGATGGTGAAACTGAGAGTGCTGATTCAGTTTCAAAGAGGGAACTTGTTACTTCTGAGAATGGGAATCTCAATGAGTCTTCATACTTGTTGAGCAAACCATCTTCCTGCCCTTCTGTAGAGCCTGCTAACAACATTGATAGGTTTCTAGAGTCCACAACTCCTTTAGTCCCAGCTCAATATTTAGCTAAGACAACAATGAGAGGTTGGAAGACTTGTGATGTGGAGTATCAATCTTACTTTCTCCTTATTGATCTCTGGGAATCGTTTAAGGAGTGGAGTGTATATGGAGCAGGAGTCCCTCTGATACTTGATCAAAGACAGTCTGTTGTCCAATATTATGTTCCTTATTTGTCGGCTATTCAACTATATGGTCAATCAGATAAGAAATCAAATATTAAGCCAAGGTACATCAGTGAAGACAGCGATGTTGATTACTACAGAGATTCAAGCAGTGATGGAAGCAGTGACAGTGAATTTGGAAAAAGAACTGAACTTTTATTAGCACAAAGAAATGGTCAATATCATAGAGGTGACGCCTCGATTCAAATGAGCCGGCTATCTGTACATGATAAGCACATTAGAATGCAAGAGGGATTTTCTAGCGATGATAGTGAAACTGGGAATCCTCAAGATCTTCTTTTTGAGTATTTTGATCACGATCCTCCTTATAGTCGTGAACCATTGACTGACAAGATACTCGATCTTGCACGGCGCTATCCTAGCCTCAAGTCACTGAGAAGTTGTGATTTATTGCCAGTCAGTTGGATGTCTGTAGCATGGTATCCTATATATCGAATACCCAGTGGTCCAACATTAAAAGATTTAGATGCCTGCTTTCTTACATACCATACACTCCATACACCCTTGACAGGAAATGGAGGTACTCAAGGACCAACTTTGGTTTATCCAAATGACATGGATGGTGTACCAAAGATTTCTTTACCTACTTTTGCAATGGCTTCCTACAAGTTAAAGGGGTCTATATGGACACAGAATGGAGCTAGTGAGAGTCAAGTAGCAAATTCTCTCTTGCAGGCTGCAGATAACTGGTTAAGGCTGCTTCAAGTGAATCACCCAGATTATCAGTTCTTCAAATCACACGGCACATACCACAGGTGAACCAAATGAGATGACATGTCATTACCTTCTGATGGCTAAATTTTAAAGCTGTTAGCGTTAAATTTCTGTGTTCATTGCTCAATGCAAACTTGGTATGGTCCATGCATCAATAGAGACTGATGGAGTTGGCACTTCTAATGTGGATTCCTGTGCCTGTTACACATCCTTAAGAAGCACAAAAGATGAGCTGGTCTAAGGTGAAGAGTGAAAATAGAGATTGCTGAAGTGAAAAAAAAATAAAGAAGAAAAAGAGATGGGATAAAGTGAAAATAAAAAGAAAGAGAAGATGTTTTGTATGGAGAAAAACTGTTACATAAGTGTAAAACAGGGAGATAATGCTCAACATCTCAAAACAGGTTTGCAAGGAAGAATATATGTGGGATTAAGATAGATGATGATCCTAGACTTTTCATCTAAAATAATGATTGAGTTATTGGTCTTAAATTATATACTGGTAGTTATGTTATGTTTGTGATTCAGAAAAAAATTGATGAAATATGTAATTTCTGTATAACAAGGTTCAATTTTGGATCCAAATCAGTGTATCTGAGAACACCTTGTTACTTGGGTCAGAGGACATTGGATGGTGCTGTACAATATTAAAGTTATATTATTTGGAAGAGTAATTAGTGTCAGTGACATGTTTGGTTAGGATTAATTGGGGGAGAAATGGTTCTGTTCCCCCCAAAGGTTTTCTGAAAAACATTATAAGAGAATAAGCTGTCACCCAATGATCACAGTGTGATTTGACTCTTCAAGGGCAAAATTGTTAAACATGGAGTGTGTAAATGGGCACATTGGAGCGTCAATGGTCCCACCCAATGAAATATTGTTCAGTTTTCA

>C101727_65

GCCACGTTAGCCATCGCTGTTCTCACTCTCACTCGCATTTCTTCTTCTTCTTCCAACTCCTTCTCTCGCACCCTCAATCATTTTACCACAAAGTTCGTATCTTTAAGAACCCCACATCAACGACATCGTTTCTTCTGCCGCAACTGCAAAAGGGCAATGACATCTTCACCAGCCACCATCCCTTCCATTTCTGAGAGAGTTACAGCACCCTATGGCTCCTGGAAGTCCCCCATCACCACCGACGTTGTCTCCGGCTCCGCTAAGAGGCTCGGAGGCACCGCCGTCGACGCCCGCGGCCGCCTCGTTTGGCTGGAATCACGTCCGGCTGAATCAGGACGGGGGGTTCTTGTGGTTGAGGCGGAAAATCCGGGAGGTGAGGCTGTGGACATTACACCTAAGGAGTTTGGAGTGAGAACATTGGCTCAGGAATATGGAGGTGGTGCTTTTACTGTTTCAGGGGATGTTGTCTTCTTTGCAAATTACAAAGATCAGAGGTTGTACAAGCAGTCCATCAGTTCTCCGGATGTGCCTCCTGTACCTCTTACTCCGGATTACGGTAGCCCAGTAGTCAGCTATGCTGATGGAATATTTGATTCACGCCTTAACCGATTTGTCAGTGTAAGGGAAGATCGTCGAGAGAGCAGTCAAAATCCGTCTACAACAATCGTATCTATAGCACTTGGCAGCAAGGATGTTCAGGAACCACAGGTACTAGTTGGTGGGAGTGATTTCTATGCTTTCCCACGTCTTGATCCAAAAAGTGAAAGAATAGCTTGGATTCAGTGGAGTCATCCCTACATGCCATGGGATAAATCGGAACTTTGGGTTGGCTATATCTCTGAAAATGGAGAGATCTACAAGAGGCTGTGTGTTGCTGGGAATGATCCTGCACTTTTGGAGTCTCCAACTGAGCCCAAGTGGTCTTCTGATGGGGAGCTGTTTTTCATTACTGATAGGGGAAATGGTTTTTGGAATCTCCATAAATGGATTGAGTCTGAGAACAAGGTCTTGCCTGTTTATTCTTTGGATGCTGAGTTTGCAAGGCCACTGTGGAATTTTGGTATGAATTCTTATGAATTTCTTCAAAGTCATACACAGAAAAACTTAATTGCTTGCAGTTACAGACAGCAGGGGAAGTCATATCTTGGAATTATTGATGATGTGCAGGGCTCAAAACTAACTGTGCTGGATATCCCTTTCACAGATATAGATAACATTACTTCTGGTAATGATTATCTTTATGTGGAGGGAGCATCTGCTGTTCATCCATCATCAGTGGCCAAGGTGACTTTAGATAATGAGAAATCAAAAGCACTTGATTTCATCATTATTTGGTCATCCTCACCGGATAGTTTGAAGTATATTTCTTACTTCAGTAAACCTGAGTTGATTGAATTCCCAACTGAAGTTCCTGGTCAAAATGCTTATGCATACTTTTACCCACCGTCTAATCCTGATTTCCAAGCTAGTGAAGAAGAAAAGCCTCCATTATTATTAAAGAGCCATGGAGGACCAACTGCTGAAGCCCGTGGAATTTTAAACTTGAGTATTCAGTACTGGACTAGTCGGGGTTGGGCTTTCGTTGATGTTAATTATGGTGGAAGCACTGGTTATGGCAGGGAGTTCCGAGAACGGCTTTTGGGACGTTGGGGAATAGTTGATGTCAATGACTGTTGTAGTTGTGCTACATACTTGGTGAACAGTGGAAAGGTAGACGGGGAGCGGCTTTGTATTACAGGGGGCTCTGCTGGTGGGTATACCACCTTAGCTGCTCTTGCTTTTAAAGAAACTTTTAAGGCCGGCGCTTCACTGTATGGCGTAGCTGACGTGAACATGTTGAGAGCAGAAACTCATAAATTTGAATCCCATTATATCGATAGACTGGGTGGAGGTGAGAAGGGGTGCTATGAAAGATCACCAATCAATCATGTTGACAAATTTTCTTGTCCCATTATTTTATTTCAAGGATTGGAGGACAAGGTTGTACCACCAGAACAAGCTCGGAAAATTTACAAGGCTCTAAAGGAAAAAGGTGTGCCAGTTGCTCTTGTTGAGTATGAAGGAGAACAACATGGTTTCCGAAAGGCTGAGAACATTAAGTTCACACTTGAGCAACAAATGGTCTTCTTTGCACGATTGGTTGGACACTTCAATGTTGCAGATGATATTACTCCCATCAAAATTGAAAACTTTGATTGAGAATGGCCTTTTTAGAGGTGCTGCTGATGTTGGCTTTCTTGGCATCCATGTTGAAGAAGGTTTTAAGAAATCTTTGTTCTCAAATCCATTTTGTGCTTTGCTGAACTTCAGGTTTGCCTGAATTTTTTGCCTCACGAACTTCTTCTGCCACAGCACAAACTACCCCATAGCCGTAGTGTTTTTGACTTACAAATTTATTAAGTTCAATGCTCTACTCTGTGTATTGGTTTTATCTGATCCATAGATAATTAGTGTCCAGTAGTACCAGGCACAAATTTGTAATTGTATAAGTTGGAGTTCCTGTGGAAACTATATAAAATCCAATATTAAGGGATGAAAGCTGATTCTGTACCTTGTGACATTGATCAACCTCCTTACCAAATAAATCAGTAGTGGAAAATGG

>C101729_65

GGGAGAGAGAAAGACTAAGGATCCTCAATTCATTCTTCAGTATATAGCACAACAAACAGCCTGATATTTCTGTAGAACATGAGATGCTAACTCACAACAAAAGCAATTCATAATAAATACATTACTTGACAAATATTAACTACATTTTTGGCCTCACAAGAAGCAGAAACATGAAAACATAACAGCAGGGAGATTTACTCTGTGCCAGCCACAAGCAATGCAGTTCCCGCAACCACCGACAGCTAGAAAGTTTGTAGAATATAGCCTCGTTGACAATTATAATCTGGACTCACATCCTAAGCCCACTCATATACTATCATTGTCCATTCCTTGGTGAAGACAAAAAATTGGAAGAGCTGGGGAGGCCAGCAAACACATTTGAGTAGGACTTTGTCAGATATGAAAAATTGGTAACCGGAGGCAGAGAGGATACCCTTTCACCCTTCTTCAAAAGTTCTTGCAAGTTAAGTCTTTGCAAGGTCAAAACAGCTCTCGACTCCTTCCAGATGAAAGCAAGGATGCCATTTTGCAGAACAAAGAGGGAACCAGGCTTTGACCCAGGGTATCTGAACCCATAACCACTTGCAATACCCTCTCCCACAAAAGCATGGCTGACATTAACAGGAACCAAGTCTGCATCAGTGTCCCAGGGAAAGAGTTCGGACGAAGGTTCATTGATATTTGCTGTAAACATTGCTGAACCATTAGGATGCAAAACATAGTGGGTGCCTTCTAAAATTTTTGTTGCAATGAGAAGCTGCTGTCCCTGGAACTCCTCATAAGAGAGCAGAAGAAAGAACAAAGCTTTTCCAGGCTTGTCTTCAGAAGGCTTTCCAGGAGGCCAACCAAAAGTTCCTCCCCATAAACCAGCATATTCTTGGTCATCTCTGGGAACTCGCAATGGCAACTTGTAAAGCGCAAAGCGACTGTCGTGCATATTTGGCCATGCTCGGTAAGAAGATAGCTTCACAGTAGATGCTTTTAACGTTAGTCTTACTGTGTCACTCGATCTAAGGAATTCTTGAAGCCGCGCATGCCTTATCTCACCTCTTGACATAAGCTTTTTGGAAACTGAATGATTGCCATTCATTGAATTTGATCTTCCAAGCATTTGTTTGATGCCATTCCAGAAATATCCACGAAGACCTTTCCTCTTGATGCATTGAGCGTTACCATCCTCCTCAGGCAGAGGATTAGAGATAGACCTTGATCGGTCAAATCTCGCCCTAACATCACCCAATTCAAACTGGGTTGGATCAACTGCAGCTTGAGAACTCGCCTTATACTCATCCATCTGACCACTACCAACCTTATACATTTGAGTAAGGCGTGATCTTCTTTCCCACAAAAGTACCAAATCCTTCTGAAAATTCTCCTCATCATCCCTCAACCTCGGAAACAACGGCCCAATCATATTATCTGGAACCTTTTGTCGAACATGGCTAGTAGTAACCTCAAGCAACTTTCTCCTATCACTAAAAGCTAACTTCCCAAAACCAACCATCCCTTCACCATTTTCAACCACCCTTTGTGAACTAGAAACATCGCTATTCGACCTACAAACCTTCCTCGACGATGACGACTCCACGCAAGAGTGATGGACAAAGCTCTTACTCTGCAACAAAGCACTAACACTGCTACACCACCCAGGTTCAACCTCAAGCAACAGTGCATTACAGGACTTGTCTGCAAGCATGACCGAACCCGGATAAACATAATTCACCCCCTTCTCCCTCCCATGAAGAAAAAACATCATGGAACCATCAAAATCACTAACAATCTCAAACACAGAAGCCCACAGTATGGGACCCTCTTCAATCCCCAAAGGACCAAGCTCCTGTGGAATTATCCTGCACCCAACAACAGAAACAAAACCAGGCATCACATACACCACGTTCCCAAGCTCAGGATTCTGGTGAACCCATATTCCAAGCAACGGTTTCACACCCACCAGAAAACGACAAAGCGCCTTGTAAGAAGCCACGCCCTTTCTCCATTCCACAAGGTCCTTGTGGGGCAGTATGCCCAAATCGTCACATTGTGTGAGCCAGAGCTTCTCAGAGGCCACTAGAGCGTTCAAACTCTTGCAGCAAAGACACAGATTGCACACGTCTCTCGGCGACAGGAACCGAGAAACGACGGCGAAAACATCGTCCGGCAGAAACAAAAGCAAGTTCGAATCAGAATGTTCGGGTTCCATGCCGATTCGGCAAAATAAAAATCAAATCTTGCAAGCTTCAGCGCATGCCCACAAATCGAAACAGCGGAAAAAAACGAAAGGGTTTGGGTGAACGAAGAACAAACCCTGGAAATTAGTCTTTTTTCAGGCGGCACGTGGAAGAACCCTGCACTTGACGGGGAGTACTGTGATTCTTCATATTCCGTGATTGAGAAGGGTTTGGTGGGTTGGCTTGAATTATTGGATGCGTGATAGAGACAGTTTGCCAGAAATGGAGAGAGTGAAAAAGATGGGGTGGAAATGGGGGAGCGAAAAAGTGAGGAGTGTTGTTGGTGTTTTGGATTCTCCAATGAAGATTAAGGTGTAGAGAGAGAAGTGGAAGAGAAGAAGAGTCCAAGAAGAGAAAGCCTTGGTTGCATCGTTG

>C101731_65

AAGATCGCAATGAAGCTGCAACTGGAACAGCAATTTAAAACTTCGGTAATACATAAAAAATCATGGCATGCCTCTGGACATGTTTTTGGGAGCTCCATATAAAACAAAATGCAATCTAAGCATGTAAACATATACTTTCTGATGTACAATACACAATATGTTTTCCTTCTAAGTTGTTTTGTTCAACTATATGGCTGCAAAAAGTTAAAACCACAAACTTCAGTGATGTTTCACACATTTACAAGCCAGTCACTTTTCACAAGATACCAACCAAGTCCAATTCAAACAAGACCTCAATTCCCTGAGAGAAGAGTATCAACCTTCGCATTTGCTGCTCTGGACAAGAATTTCATACAAATTGGTGGCTTGACTCCAGCCAGAGCAAGTATTGAAGCAGGAAGAGTCCATATTCCATCTCCACATATCAAACCAGAAGCTACAGCTGGTGCAAAAGCATCTGCCTTAGCCTTGTTAATCCTCTCCCACACATACAATATCAGACTTCCAACACACATGTCAATGGCAAAGTATGGCCCTATGTAGAAAGGTATTGCCATGGCCATTGGAAGTGGAATGAACCTCCCTTTCTTACCAAGAAAATCCTTAAACAAGTTTATCACAACGGCTGCAGCGAAGAATATGTAACAAAGCAAGAGGCAGTTTTTTGGTAGAGAGCCAAAACCTTGCACCCCCAATATGGCCATATTACGGTATATGATTGCATATGGGGCAGGATATTCACTTGAAGGTTTCCCAAGGTCAGGAAAAGCTTTGTAGAAAATCCAAAATACAGAAGGAGAAACTATACAACCCATTGTTGTGCCAATTATTTGGCTCACAAACATGGAGCGTGGCGAAGCCAGCGTAAGGTAGCCAGTCTTAAAATCCTGCATCAGGTCTGAGGCTGTGGAAACAATGTTCATCATCACTCCACACGCAGCTAGGCCAGCAAGAACTCCACCGTTGGCACCGGCCCATGCTCCAATTGTGAAGATGGCCAGCTTTCCATAAGTGGATGCAAGGGACCAATCAGTTAGTCCACAACCATAAGCATTGCAAAATGCCAAGGTGGGAGCAATTAGATAGATAACAATTATGTAGTACCATTTTAGTTGGGAGAAGATGTGTGGCAGAGTGGCTGTTGAGATGGCAGCAATAGCAACATAACCTGCAACTGCAAACCATGTGGGAATTTGATCTTTAAGGAAAAGTTGGACACGGCGCTGATCATCATATGAAAGCTGAGAATTTGAGGGGGAATCTTGATCTGCAACAGGAAGTACATTCTCCCTTCGTTTTTCTCGGATTTGATGATACAACCCCCATAGGGTATGTGTTATCACCTTTATGAAGTTATATAAACCATCTCCTAGGATCAAGGCAATGGCTATAAATACCCTATAACCTTGGATGCCATGAAGATTGCCTTCGCCAAGACCTTTATCATACCAATGACCCTCTTTGGTTTTTATGAGAGGCCACATTACTCCCCAAGAAAGAATTCCCCCAAGAAGCACTGATATATTTATGATATATGGACAAATCATTCCCACTCCAATGTAGATTGCAGAAAAATCAAAATAAAACCTGTTTTCATATGCTTTAAGCCCCAATGAAGGGAAGGCTTGAAATCCACACTGGTCAGTAGCTGTATAAAACCATTGAAAGAAACCCCATAAAAAGCTCATACTAAAGAATTTTCCCAACATTTTCACTTGCTTCTTTGCAAGTTTGGCTCCTTGAGGAGTGTGGAAGCTGTTGATGAGATGTGCAGTTGCAGTACCACTTGGATATGTCAATTTGAAATCAACAACCATAATCTTTCGGAGAGGCACAACAGAGAAGAGGCCTAGAAAGCTAACAACAAAGAGAAAAGCTATAATCCACCCTATTTTTGGGTCCTTATAATCACTTTTGTCTGTGGATTGCCTAGCTATTTCTTCACTCATTCCAAAGAGGTAACTTCCAAATCCTCCGCTAAAAGCTATGCCAGAGGAAGCCACAACACAGGTTTGGATGACAGTGTTCTCTTGCCTTGTGAAGGGTTGTCTCAACATGCCAGATTTTTCCAAGAACTTGGTCCAAGTCTTCACAAAGAAGAACCCCAGAAGGCCAGCAGAGACATTGAGAGAGGGAATAATACCAGTGGTGAGGTTGAGCTTCATCACGATGAAACTGAACAATATGCTGAGTGCAAAGCTGACCACAAAGGCTCTCACAGTCAATTGGTTCCTCCATGATGGCACCAGAAGGTGCTGGAACACCCTCTCCACCGAAACCTCTTCCTCTTTGAGTTTGGTGCTACCCTTTAGGCTCAGCTTGTTATCATTCTCATCAGCATGATCAAGGGACTCAAAACCATTGTCCACCACTCTATCCCCACTTCCATTTCGAGCCATGATTGTGGATTATCCTTGGAAAATGAAGTTGCTTAAATCCCACCAATTTTTTTATGTCAGTTCGGAATCTCCAAGAATGGTGAAACTTCCTAGATACTACTACCCTCCTCTTGTCTTAGGTTAAAGTTGGTGTTTAACCTTTATTCCAAGGGTTGAGTTGGAATGTGCATGGTTGTTTACTTACTAGTATTTATTGATTCTCTC

>C101733_65

ATAAAAGGGAGGCGAGAGTGAATTCGACATACAAGAATGCCCCATTCTTCAATCTATTCATCTTCCAACAATGGCTGAGCAAAATCTTCCTTTTCAATAACCTTCTCTTCTTTCATCCGCATTGTCGCTGTTCTTGTTCGCCCTTCAAGGTGCCATGGCTGAGGTGGGGAAGATACTGTACATAGTGGTGGTCGATGATGGCCAGAAGAAAGACTCCTTTCCCTACACGCGTCCAGTTCTCCAGAGCACCCTCCAGCTTATTGGATGCAAAGCGCGTCACGCCTTCAAGATTAGCCAAAGAGTTTTTGAACTCACAAGGAAAGCGAATTCTACTGATAATTTGCAGCCGGAAATAGAGCCTTTATCAGCCCAGGCCGAGAAAAAAGATTCGGTAGAAACTAAGAAAATAAACAAATCTATTCATTCTGCTGATGCTGGCTTGGATAGAGCTGACTTACGCAACCAGTTACTTTCTGGGAAAGATTATAAACTTAGGAGTGTATCGTTTGAGTTGTACAAAAGGCGCACATCTGCATTCATCCGAAGAGAAATATTTCTAGATATTGTCTGTGATGCTCTGGCAGAGTACAAGTATGTGGGTCCCAACCAGAGAGCAGACTTGGTATTAGCCTGCAGGATTCGTGAACGGAAAGAGTCTGTGACAGTGCTATTGTGTGGTACAAGTGGCTGTGGCAAATCTACGTTGTCTGCATTGCTGGGAGGTAGGTTGGGAATCACAACAGTGGTATCGACTGATTCTATTAGGCACATGATGAGAAGTTTTGCTAATGAGAAAGAAAACCCCTTGTTGTGGGCTTCTACATACCATGCAGGGGAGTGTTTGGATCCTGTCGCTGTTGCAGAAGCAAAGGCCAGAAGGAAAGCAAAAAAGCTGGCTGGTGTGTCACATTCGCTTCCCAAGGATGAAGCAACTGAGGGTAACAATTCTAGCAAAACTGATAATAGGATCTCGGAGACTAGCTCTGGTCCTACTGAACTGCTAAGTCCAAAACAAATGGCTATTGAAGGATTCAAGGCACAGAGTGAGATGGTGATTGACAGTCTTGATAGGCTAATCACGGCATGGGAAGAACGAAAAGAGTCTGTAGTTGTCGAGGGTGTTCACTTGAGCCTCAACTTTGTTATGGGACTCATGAAGAAACATCCTTCAATCATACCGTTCATGATACACATTACAAATGAGGACAAACACTTGGAGAGATTTGCTGTACGTGCGAAGTATATGACACTGGACCCAGCTAAAAACAAGTATGTGAAGTATATTAGAAACATCAGAACAATCCAGGATTATCTCTGCAAGCGTGCTGAAAAGCATTTAGTTCCCAAAATAAATAACACAAACGTTGACAAGAGTGTAGCAGCCATCCATGCAACGGTATTTAGCTGTCTTAGAAGGCGTGAGATGGGGGAACAGCTATATGATCCTGTTAGAAATACTGTGACAGTTGTTTATGAGGAATATAGAAATCAATGTTCAGCAAATTCTTTGACCGCAAAAGGAATGCTTCAGTTGATCCAAAGACAAGGTTCTTCAAGGAATCTAATGGCCCTTGTTAATACTGATGGGTCTGTAGCAAAGGCTTGGCCTGTTAATTTAGTTGACAGTAATGGGAAGCCTGTATGGTGCCATGGACCAGAGAATGGTATTTGTCATCCAATGTATGGTCCCTTAAGAATTGGCAAGGCAGAACCAGTAAATCTTCAGTTTGGTCTTTACGGAATCAGTGCTTGGCCAAGTGATGGTGGCACTAGTCGTGCTGGAAGTGTAGACGAGTCTAGAGCCGATGGGACTGATACCGGTAGTAGATATGTTTCCTCTTGCTGTAGCTCACCAAGGATGTCAGATGTCGCTGCAAAGGAGCTCAAGGAAGACTTCTCAGTGCATGGTAGTGATGAGGAGATTGACGATCAACCTGAGGTGGGAAGTGATGAGGATTTCAGTGACGAGGCGGACAAACATGCCCATGAAGAGGTAGGATCAGTTGACGAGGAATCAACAAAATCTGATGAAGAGTACGATGATCTTGCAATGCAAGACGTGGTGGAAAATGGGTATTGGTCGGACGATGATGATGAGTTCAGGAATAGGGTTGGTGTTGTTGGCGGGGAGTTAGGAAGCAAAATGCATGGGGGAAACAGGTATCGTCGAAACCTGGATCTTTTCCATAGAACTAGAAGTGAACCAGTGGCAGTTCCAGAGCCACAATGCTCCTATTCTTCTTTGCTCGTTGAAAAGAATGAGAGGAAAGCCAAATTAAGAACACGTTCTCTTAGTATTCCTGCATTCAAACATAGGTCAGCAATGAATGATCCTATCCTTTCTGGTGCTCCTCAGAGGTAGCATCCCATCCCCTCTTCAACCCAACTCCTAATCATTCAGATTTTTTTGTGCTTTTTCTTAACTGTCTCACGATGTCCCATGTGTATATAGTAGAGCGTCCAAAACCTTGGTCGTCTTTTAGTTATTTCGTTGCTTCATGTGGGTGAACTCATGTTTTCACATTTTTCTTGAACATTTTTCTGAGAATGTATATATAATGTGTATTGAATTCGTGAAATCAACCTTGTCTCCTCTAAAAAAA

>C101737_65

GAAAAAAGTTTCATTTATTTCTCTATATTGATATGAAACTATATCATGTAATCTTTATCTAAATAATCAATATTCGAAGTTCCCTTTGCAAAGGGAATTTATCATACAACAAAATCTGGTTGGTTATCTTAATGATGGGTTATATTAATGATTACCGTGCTATATAGAGAAGCAAAGACCATGCAAGGACCTAGCTTTAGGAATAGTTGCAACTTGGAATTGCTCAAAAGACAGCAGACAAATTTCGGTTTTCACATCACATTATTATTTCCCAAGTTTATCAAATTGACCGTCTACTTTCTCGGGGATGAAGAATCTGGTCAACGAGCGTCTCTGGAAGCTTTTTAGTAGCCTGGAAGTTCCTCATGTGCAGAATAGGCTCACATCCAACATCTGCCACTGATTTGCCATTTGGTGTTTGTAACTTAAACCGCTCTGCCTTGTATGCAGAAAGGATATTTTCTTCATCAGGTAGGTGATGGCTAATTTTCAGGAGTGAGCTGAGAGAATTCATCTTCATATATGGACAAGATGCACAGCCACCGTGGATGGAACAACCCTCCCCGCTGGCTATTCCTGGTACAACGGGAAGTATGACATCACCCACCTTGGCAGAATGAACACCTGAGGATAAACTTGAGGATGTTTTTGAGATTGAGTCGGACGAAACTGGAAAGACTATTTCAACAGTAACTTTTTCTTTTTCAGAAGAGGACTTCGCAGGCTCCAACAAACTACGAACTGTAGCCACAATTGAAGTCACCATCCCAGATTCTGTTCCTAAAACAAATTGAAGATGATCATTTATATTTCTGTCCAAAGCTTCCTGAACCCTATCCTTTATGAAATCTAAAATATTCTGAGTGGAGCCTACTACGCCCATTCCCCTTCTCTTTGCTTCCATTGCCAATGAAAACATCTCTCCAGGAACCTCAAGATGAGCAGTGAGGAATGCATCACAATACATTTCTTTTATATTCTCCACAACTTCATGACCAAATAGATGGTGAACAATGCATGTTCCATCCTGAAAATAGTGAAGGCGAGGTAGTAATGATCTAATAGAATCTTGACTGTGCTCAGGATGTATTGCAGCAATCTCTTCATCAGACATCTTTGCCATTTGTTGGAACAATTCTTTAATATTTGCACCCATGTAAGAATCAGGTCCATACCATATGCTCAAATCTGGAACTTGAGCAAATGCCTGTAGGATAGTCTGGACAACATTTGATGAAGTACAAGTTATTGTAGGCACAAGCTCATGAGCAAATGCCTTTGTCTCTAACTTAGTGTTAATGTATATGACATGCAAAGAAGTAGACCCAGAAGCAGCCGAAAGATATTCCATGTAAGTAGGAGTAGCTGCAGCATCAGCCAGAGAACAGCCAATTTGCTCATCTGACATCCTATACACATCAACCTCACTGAAACCAGCTTGATCAAGGATGGCACGCACATTTTCTGACATAAAATCTACACCTAACACTGTTATGAATTGGCATCCAGCTTTTGCCATTTTAACTGCAGTATCTGCCATGACCAGTGAATCAGATATATGGATATGTGGCCACTGCTTCTGAGCAGCAGTTAAAATGCCTTGGACCTCGGGATCCATGTAAAAGTGTGCAACAACACCAATTTTCTTCTCCTTCAGCACATTGACTAGTTCATTAACTTTTAACTCATTAGGGAACATATATTTTGCCTGGGCTTCAGCGTAGCTACCCTTGGGAAAAACCCCATCCGAGGTAACAACCAATGAAGGAAAAGTGTCAAAGGGAACCTTCCCTTCCCTCTGAGCCACCAGCTGCTTGGTCCTCTTCTGCATGCTGACCAGCACGTTGTGCCAAGTGTTCACCCTCGAACGCCCACTCCCTCCCGGCAACCCCACATTCAGACCAACCAAATCATCGGTGCTCACTTTCATAACCTCGTCCGGCTCCGAACCATCCAGCAACCACACCAGACACCCGCAGAACCCCCTCGTAATCTCCGAATCACTGTCCGCCGCCACCCGCACCTTCCCCTCCTTGTCGATCCTCACCTCCACCCACACGCGCGCGGTGCACCCCATCACCCGGTTCGCGTCGACCCGACCCGACTCCGCCATCGGCGCCATCGCCCCCGCGTAGTGAAGCAGCCGCTTCACCCGCTCCACCGGCTCCCGCAGCGACCGGAACTCCGCCGCGAGGTGCTCTAGCTTCGACGGAATGACGAGCTCCGAGGCGGTGAGCGAGGAGCAGGAAATCACAGGGCTAGGTTTGATCGACCTTGAACGGCGTTGGAGGCATTTGAAGGGTTTAAGTAACGGTAGGTGGCTCTTGAGAGATTGGAGGATGAAGGGTTTGGGGTTGCGTGATGTTGAAAAGAAAAAGGAAGCGGAGACAGTGGTGTTCGGAGTGACAACGGCCATGGCTAGTTAGTAGGAATGCGTGGAAGGGGGGTTGAATTGGAGGGAGAGGGCGAAAGGGTTAAAATAACTGCAAAAGTGGGAACGGTTGAGAAGAAACAAGTTGAAGAATCAGTGAAGGGCCCAAAGGGGATGATGATTTGTTGAAGTGCACATAGGTCCCCATTTGTTATAGCATCTTGTCAGATATT

>C101739_65

CAGCACATTTGGTGAATGGTAAAACTGTAAGGGTATAAACTAACGACTAATATAAATGAATTTTACAAAACTTGTCTATTGACAGAATAACATTTGGGATTTATGCACAAGGAAGAAATAATTTAACAGGCAAAACAAGCTCTATAAATAACTCAATGGAATCGTACTTCAAGTGAGTTTACGCCATATCCCTCCCTTGGATACTGTTAAGCACTTCACTCGTCGGAAAACGAAACCAACCTTTGGAGGTTTTCCGTCAACAAGTGAATTATGATAGATCTCTTTGCACTCTTGAATGACCTCTTTGGCGTCCTTCCGAGGAATGCCTTCTCGTAAACCAACAAGTTTCTCGACAACTTCAGGCGGGCAGTCAGGCTGATGTTCAAGTATATTTGTGTAGATGAGTGTGAAGGTATCCAGGCTCTCGGCTGAAGCAAGTTCCCTCAAGTCGCTGAGCACACTCACTCTGTTTTCAACTTTAGAAACACTGATATGTTCCCTAAAGAAATCCATAATAACCTCTTCATCTAGCCTCATCCGTTCAATAGTTTCTTCCTTAATGTAATTTTTCTGTGTTAAAAGATGATCAACATAAACAATCACAGTTTCTTCCAGGCAGGCTTCAACAAATCTCCTAAAAGATCGTTCTTCAATATACATTTTTACATCACCAAAATAATCACCAAATGTTGCAACCAAGTACTCAGTAACCTGCCCCTCACTCCATTCTTTCTGGTATAGCTTCACCAGCAACTCCTGGACACCAGGATCTTCAAATATCACACTAACAGTTTGATGCACAGCTTCCTTAGCTACCTCAAGAAAACCCTTACAGGTATCTTCAAAATTAACCTGCTCAGCATAGTTCTGTGGAAGGGCTTCTATGGTGCTATTACTTAGCTCCATTGCAAGGTCATAACAGCGCAAATTGTTGTTAATCATAGCGCATAAAGGTTCTAGACCAATTTCAGAAGCTGGTTCTTCAAGTCTCTTCTTTTCAGCTGCTTGAAAATCAATCATCACCTGAATGGTTGCCAAGGCAATTCTGTATAACATGAGATCAGTGCTATTATCCCGAACAATTTGTACTTGCTCACCAAGTATCCTGAAGAGATCAACAGCAGCAGGGGTATACAACTTCCCATCTTCAGTTTTCTTTGGGGGCTGTGTTCTATCTGCCTCCAAAATATTCAAGTACCATTTCCTAGTTGTAGCTTGCATTCTCTCAACATAGGAGTTCATTAAAGGATCCATTGAACCACTCTCAGAGCACACTTGGGCTAGAGAGTCGTCAACTCCAAGCCCAATCAGATTCTCTTGGTATTCAACAACCCAGCCAGTTACCTTCAATATCTCAATGTTTGTTAATTCATTCGCCCTGTCGCTAAGCAATCTTAGCATCTGAATAAACCTCTCAGTGTAAAGATTTACCATTAGTTGGAATATTTCATATCTTGGAGGAAAGCAGGGGGCTACATAGTCATAGACATCTCCAAGCTCATCCCCAATCGCTCGTGCTTCCTCTAAAGCTGCTTTCAAGTCCTCAAACACAAGCTCATTGAGAAGCTTGTTAAACCGTTCTTCTACCGTCTTTCTTATTTGCTCATAGCATTTATCCTTATAGCCTTTCCCTTGAACCTTCAGTTTCTGCTGTGTTAAATTTTTTGAAGAAGCCATTGTACTTGTAGATTTTACGCCGTGAGGATTTACAACTGGTGCCATTGCACCATCCCCTTCAGCCTCTGCTGCTTCTTCTGCAATCTGTTGATCAAGTATCTCTTGCATTTCAACAACCCTTATAGCACGAACTAGAGTCTGTGGGCTTTCTTTAGAGAGTTTATAAAAATTGGAAATGTGTTCCCATAGAGTTTTCTCAAAGGTCTCCCAGGTTCGATCTACATCTTCAAAATATTCTCTTAGCCTTCCAACCTCTTCCTTGTGAGACGCCGCAGCTGCTAACGCAAACCTTCGCTTTCCATCCAAAGCTGTTAACCTCTCATAAGTGTTGACAATTTCTTTGTCATCACTCAAAGAATCCCGAGCCTCTGCTGCCTCAACAGAGATAGACATCATACCTTCAACATCCTTCAAGGTTGTGTTTAAGTTATTTCTTGCATTGCTAAGTAGCTTTATCTGATCATGGTTGTCAATTAACGTTTGGCATTCCTGACATAACTTCTCAATAGAGACAAAATTTTCACGAAGCTGATTTATCGTCTTTTCAGAGAAAGATAGTGATTTTAGTCCTGCCTGTGACTGCTCAACCTGTTCAGCAACCATTGTACTAAGTTGTGCATCATTGGCCTGTTGGCGCGAAATGTAATCAGCCTTGATGGATGAGATCGAATGAAGAAGCTCCGGAAGAGGCAACAGCTTTGCGACCTCGCGAACCGCTGCCTCCTTCGCTTCCACGCCGAGATCCTCGGCCATCATCGTGCCTGATTTTGGGAAAATTGAGCACGGATCACCTCCGAGTGCAGGAGATTTACGGTGTTGACGAGGTGCGAGTTCAAGAAAGAGAAAGTTCAAGAAGAAAAATGAACTTTCACAGAAAATAACCAAACCAGAATTGGAAATGGGTGGTAACAACCGAGTGAAGAAGCACG

>C101747_65

GTGAAGTAGAAGATAAGAATAGAAAAGGTAGACTCATTGCCATTGCTGAACTGCAATGCAATGAACACAAAAAAATTTAATGCCTTTTAACTTTTTCAGGCTTGGCTTCTTCTTGTGCTTATTAAAGATTCTTTGTTAGTAACTACTGGCATCCTTCTCTAGTGTTCTTGTGCTAAATACAGCATAGGCAACATGATCTTCAGGCCACTCCTACTATTCCTGGCCTTTATGGTGACAAATTCAGTAGCTGTCATGGAGAAGCAGACATATATAATACACATGGACAAGAGCAAGATCAAAGCCTCAGTCCATTCCCAAGACAAAACAAAACCTTGGTTCAAATCAGTCATTGATTTCATCTCTGAAGCTTCATTTGAAGAAGAAGAGATAGCCCCTCAACTCCTTTATGTTTATGAAACCAGCCTGTTTGGTTTTGCTGCTCAACTTTCAAATAATCAGCTTGAGTACTTGAACAAAGTGGATGGCTTCCTAGCAGCCATACCTGATGAACTATTACCCCTTCACACAACATACAGCCCCCATTTTCTAGGCCTACAGGAGGGAAAAGGACTTTGGAGTGCTTCTAACTTGGCCTCAGATGTGATCATAGGAGTCCTTGATACAGGAATATGGCCTGAACACATCAGTTTCCAAGACACAGGTTTGTCCAAAGTACCCTCTCGCTGGAAAGGAGCTTGTGAAGCAGGCACCAACTTCTCTGCCTCAAGTTGTAACAAGAAGCTTGTTGGTGCAAGAGTCTTTCTACATGGGTATGAAAAATTTGCAGGAAGAATCAATGAAACATTGGACTATCGTTCTCCTAGAGATGCTCAAGGACATGGATCACACACAGCCTCAACTGCAGCTGGAAACATGGTAAGTAATGCCAGCTTTGCCGGCTTGGCCGGTGGTTCAGCAACTGGAACGAGGTATACCTCAAGAATAGCTGCTTATAAAGTATGCTGGCGTTTAGGCTGTGTTAACTCTGACATATTAGCAGCTATTGATAAAGCTGTTGCTGATGGTGTAGATGTATTGTCACTCTCATTAGGCGGCATTGCCAGGCCTTATTACAATGATAGCATTGCCATAGCCTCATTTGGAGCAACACAAAGAGGAGTTTTTGTTTCTTGCTCTGCAGGCAATTCAGGCCCTTCTAGTTCCACTGTTGGAAATGTTGCCCCATGGATCATGACTGTTGCTGCTAGCTACACTGACAGGAGCTTTCCAACTCAAGTGAAGCTTGGAAATGGAAAGGTTTTCAAAGGGTCATCATTGTACAAGGGCAAGCAAACAAACCAATTGCCTCTTGTATATGGAAATTCCTCCAAAGCACTAAAGACAGCACAGTATTGCACCCCAGGTTCACTTGATCCAAAGTTTGTCAAAGGAAAAATAGTTGCCTGCGAACGTGGAATAAACAGTAGAACTGGAAAGGGAGAAGAAGTTAAGATGGCAGGGGGAGCAGGAATGATACTACTCAACTCAGAAAACCAAGGAGAGGAACTTTTTGCTGATCCTCATGTTTTGCCAGGCACATCTTTAGGGGCCTCAGCAAGTACAACAATTAGAAGTTACATTCACTCAGCAAAAGCACCAACAGCTTCAATTTCCTTCCTGGGGACAACATATGGAGACCCTGCACCAGTTATGGCAGCATTTTCTTCTAGAGGACCCAGTGCAGTGGGAGGAGATGTGATCAAGCCAGATGTTACTGCACCTGGTGTGAACATCTTGGCTGCTTGGCCTCCCACAAGTAGCCCTAGCCTGCTCCAGAGTGATAAAAGAAGGGTACTATTTAACATAGTCTCAGGTACCTCAATGTCATGTCCTCATGTTAGTGGCATAGCTGCACTAATCAAATCTGTGCATAAGGATTGGTCACCTGCAGCTATCAAATCTGCTCTGATGACTACTGCTTCCACGTTAAACAACAAAGGTGCTGCAATTGCTGACTATGGTTCAAGCAATTCAGCATTTGCTGACCCCTTTGCATTTGGTTCAGGCCATGTTAACCCTGAAAGAGCTTCTGATCCAGGGTTAGTCTATGACATCACCACCAAAGATTACTTAAATTACTTGTGCAGCCTGAAATACACATCTTCCCAGATTGCTTTATTGTCAAAACGTAATTTTAAATGTGCCAAAAAATCAGCTCTTCAGGCTGGTGACTTGAACTACCCCTCATTTGCTGTGCTATTTGACACTAGTGCTCTAAATGCTAGTGTGACATACAAGAGGGTAGTTACAAATGTAGGGAACCCCAGAAATTCTTACGAAGTGAAAGTGGAAGAACCAAAGGGAGTATCTGTCACGGTTGAACCTAGGAATATTAGTTTCAGAAAAACTGGTGAGAAATTGAGTTATAAGGTGAGTTTTGTTTCAAATGGGAAAACAGCTGTTACTGGAAGTTCATCATTTGGATCACTTACTTGGGTATCAGGTAAATATGCTGTTAGAAGTCCTATAGCAGTCACATGGCAATGATAGGAAAGGTCTTCGTAATGGTGTATTTGAGAGAAACAGAAAAATACATGTTAAGCTCATAGCTAGCAGGGGAACTTTATTTGATGAATTATGATGCATAATACTATTTCTGTAACTTTGT

>C101749_65

AAAAAAACGTAAACACAACAACACGATAGACCAGGATCGGAGAGAAGAAAAAGAAGCAACCGGCAGAGAACACCGAAGAAGGAGAGGATAAATAGAGAGAGAGAAGAGAGAGAGAGAGAGCGAGAGAGAGAAAGAGAGAGAGTGTGAGAGAAGGTGATGACGATGGACAGAGAGAAGGAGAGAGAGATAGAGCTGGAGAGTGCAATGTACACGAATTGCTTGTTGTTGGGACTGGATCCGGCGATTATCGGAGTCGGAGCATCCAACGCGACTCCTCGGGTCGGACATTTCCGTCACTCGAACCCTAAATTGGGGGAACAGCTTCTCTACTTCATCCTTTCCTCTCTCAGAGGTCCAATTCAATCCGCCAAAGATTTCGATAAGGTCTGGCCGATTTTCGATTCCGCGCAGTCGCGCGATTTTCGCAAGGTTGTGCAAGGGATTATTAGCGAGCTTGAGTCGCAGGGAGCGTTACCGAGGAGTAATTCGAGGGTTTCTTCACTTGCTACCTGCTGTGGACCTAGGTTTGTCGAGCTTCTATGGCAACTTTCTCTGCATGCTTTACGAGAGGTTCATCGAAGGACATTTACTGCTGACATATCTTCTAACCCGTTGCCTGCACCATTGACTGATGTAGCCTTCTCACATGCAGCTACTCTACTTCCAGTTACAAAGGCAAGAATAGCCCTTGAAAGAAGAAAGTTTCTTAAAAATGCAGAAATGGCTGTACAACGGCAGGCTATGTGGTCAAATTTGGCTCATGAAATGACAGCTGAATTTCGTGGGTTATGTGCTGAAGAGGCTTATTTGCAACAAGAATTGGAAAAACTGCATGACTTGAGGAATAAAGTGAAGTTGGAAGGAGAGCTCTGGGACGATCTCGTCTCTAGTTCAAGTCAGAATTCCCATTTAGTTTCAAAGGCCACTCGACTATGGGAATCTTTATTAGCCCGGAAAAGTCAACATGAAGTTCTTGCATCTGGTCCCATCGAGGACTTGATAGCTCATCGAGAGCATCGGTATCGAATTTCTGGATCATCTTTGCTTGCAGCAATGGACCAGAGTTCTCAAGCTCCTTATTCAGATGTATTATCTGGTCAGCCTGGGGATTTGCCTGTGATTGATAACAAAGAAGAGAATGATGGATCACATTTTAGCAATGAGACATTAACAAGAGGGGATGATAGAACTGGAAGAGTCCACCAAACTGTTGATGTGGCAGAGGTTATACGGCGATGGACCCATGCCTTGCAGCGGATCCACAAACAATCACTTCATCTGGCAAAAGCAAATGATGGAGAGGGTCCAGATATATTGCGCAGTGGACAGGAAGAGGGTTCAAGTGGCCATGCTGAGTCTTTAGCAGCAACCCTTGCAGAGCATCAGCAACATTTAGCTAGCTTCCAGGTGCTTATTAACCAACTCAAGGACGTTGCTCCAACCATACAGAAGTCCATCTCAGAGTGTACAGAAAAGGTTAACTGTATAGCCTCAAATCTACCTCCAATGAGCAGACACAATGGTCGGTCAACATCACCTATCCAGTCACAGAGTAGTGGGAGGATGGACAATAGCACTGATGATGTTAGTGAGGTGACTTCAAGATTATCTAATATTCAGATTGACAAGGTTTCTGTCAGTCCTCCCACTTTAAAACTTCCTCAATTGTTTAGTTTGACCCCATCTTCTGGAAAAGCTGGAAATGTCCAAAGGCGGCAGAGTAATGCTCCTCAAACTAGTCAAACAGAGAACCTCTCTGACAACAAATCGTTGGATCCTCCTTCAAATAATGAAGTACCGAGTTCAGCTGAAGATAGTGACAGTTCCTATGTCCAGAATTTGAGGAGGTCGGTAAGAGAAGCTGCACTGTCATTGCGGTCATGTAATTCAGATTCATCACGGGATAGTCAATCTGATGGAAGTTCTGAGCACTTCTTTGTCCCTCTTTCAGAAACCGGCTTTTCTCATTTAGATTCAGAGAAAAGGGGACCTTCATTGAGGAGCAAAAGATTATTTGTATCACAAATGGATGACTCCTTGCTAGAGAGTCATGCATCCGTTGGGCATGGGGTGAGCAAGTTTGATGAACTTCCAGATATGTTGAATGATTTGGAAAGGCTTTCTGATTATGACAATGTAAATGGGTTTCTCTCGTATACTGGTTCAAATGCAACATCTGATGCGCAGCGGTCAATTTTTGACTTTGATGGTGCACAGGATCAAGTCTTCTCACCGCCTCTGTTAATGGATTCATCACTTTTGACAGATTCATTTGAAGACTTGCTGGCCCCCTTCTCGGAGACTGAAACAGCCTTGATAGACCATTGAAAACTTTCTGGACAACCTTCCAAACTTAATTGAAGAAACGGCTTAATTGATGCGGTGGATTGATATTCTTTTGTTGGGATATATACTCCATTCACCTTGTATAACACGCTACTTTTATGTATTATTTTTGTTTGGTATATTTTTTAACATCTATTTACTCTGTTGATGCCCTACTTCCGAGAGAGGCACCATAAGTGACCCGTGTTCTTTATTGGTGAATGATGATTTACTTTGGGATGAAGATGCCAAAATCCAAAATGCAAGCTGGGTTGAAGTGTTT

>C101751_65

CGCCAGAATTGTTTAAAGTCACATCCTACAATTTAACATAATATTCCAAGTTTGGCTGCCCTTACTAGTTATTTAAAGCTGAGTTTGGGCATCATTTTCTACATATATATGTATATATATAAAATAAAGCATGCATGTACTTAGACTAAACCATATGAGGGAATCTCAAGCTCATTTATGAGCTCTGCAAACAGGTAAATCTTCATCGAAATTGGGAGGCACTCCAAGGATTGGATTTCTCTCAAAGAAATTGACAGGCTTCAAATCAAAACTTGATGATACAAGAGGCATGATAGGATAGTCCTCTTGGCATGGTATATGATGAAATCCTATTGTGTACCACACCACTATGTCCTTATTCTCAATTGGACGATCCCTGTCTGACCACACTTGAAGAGTATCATCTCCGTTGCTCTGGTAAACGAATAAGCCACCAGCCCATTGCTCACTTTTGTTGTAAGGAGTGACCCATATTTGATTGTTGGTAAAAGCTGCTCTTTTCTGTGGTGGGTCTTCTGGATCCAGCAGGCTAGCTGCTGTGGCACCTGGAACCAATTTGTACCCAACAGGGTTCCCAACCCTTGTTTTCTTCGATGGATTCACCACATGAAATTCAGATGGGTCATAGAGTTGAAGTCTAATTTGAGCATCTTTCTCTGTCTTTGCCACTTTTTTAACAGCTTTTAAATAACTCTTTCTAGGTGATTCTCCTGGACTGGTATCTTGCTTCTTTATGTTTACCTTAACAAATGAATTATCAGATCCATCAACGTCCATGTCTAGGTGATATGTGATAAAATGATCATGGATTACACCAATAATGTTTTCACTCAAAAGGGTTCCATAAAGATATTCTTTGTCAGGGACTTGGTCCATATTCTCATAGGTAGTTCCTTTAACCATTAATATACCACTCAGTCCAACCTTTGATCTGATTAGCCCATCGGTTTGAAATTCCCAGTCCACAATATAGTCATAGTTTGCCACTGCAGCTGCCATCCTAACAACTAGTGTCACCTTTGGCCTCACTTCAGTAACCTTGAGGTTAGTGATGGGGCACTCAGCATGTCGCCAAGCAATGTCACCAGCATAACTCTCAAAAATGCAAATCATGTTGGGTTGGACATAAGGTGTTCCATCAGAAGAGGCAAACACACCGTCCATGTAGTAAGCATTCCGTGGGCAATCATTCAAGGGGTCTAGTGGCATTGCTTGCAACCCAAATCCATACTCACCAGCATCCATGTAAGTCTTGAAGTACCACCCTTCTGAGGGATCCATGTAAGGCACAAATAACTCAGATGTGAACCCTTTGTACATGACATTTCTCAGCTTTGATGTGTCAGGGTCCTTCACCTTGACTTGTGAGATTATGGTTCCAGCTCTAGGGTCAGGTCTCAGATGGAATTCCCAGTTTGCCCATTTCACCAAGTGTCCATCAACAGTGAAGCTTGGACCTTTTGGTTGCTCCAAGGATATTGGATTAAGCAGCTTCAACTCCCCATTCAGTTTCTGAATGGAGTAACGGTAATCAGTGTTAACACCCTTAGCCACAGGGATGTTTTGCCCATCATCTGAAATAGCAACCAACTCTTTTCTATCCATGTCAACCAAGGCAGTGACACCCTCAATTGGTTTCATGTAGAAGTTCACAGTGCCTTCTTTGGAATAGCACTGCACCTTAATCAACTTTCTATTCTCCTCCACTGGTGTCCCATACCACCCTGAAGAGAGTGGCAAGCATTCAAGGTCTGCTAAATTGACACCTCGTTTAGTGATTGAACGGTTGAACTCAGTGCTCTTGAGGGGAACCTCTAGCACCCCCACCATTTCTTCCACAGTCATGGTGGGGTACCCCGAAGATGAGCCGGTTTCGAGGCTGGTCACCTGGCTGGTTTCGAGGTCTACAATGAGCACATAGGAGACCCCCTTCACAATTGCAACCACGGAAGCCTTCCTAGGCAAAGATGAATCACCCTTTTTCCATTTGAGGACTAGTTTTTTGTCTGGTTCTTCAAGGACAACGGAGTGGAGAGTGTAGCTGGATGAGGACTTGAAGAGAGGGTGGGAAGAGAGGATGTTCCTGACCCTGTTGAACTCCTGAATAGTTAGAGGGTCAAGAGGGTGTTGAGGAACTTCTGATTGGTGATCATAAGTACGGGATGATGGTTTCTTGATCGGATTAGGCCGTGCTGAAGAGTAGAAACGGTTCTTTGAGCTGCACCATCCAGAGTAAAGATTGCAGTTTAGAACTTCCTTGCTGGGAAATGATGCATGTTGAAGCCAAGTAACAAGTAAAACTATCGCCACACCGGTTGAAAGAACCAGGAAGAGCCGCAAGTTTTTGGTTTCCATGGATAGATATCACGAGTGAGAGAGAGTTTGTGTGAGCAGTATTATTGTATATCTGATTTTGAAGTAGACTTTTTCTTGAGAAAGATTCACTTGAGTACAAATATGTATAGTCAGAGACTATTTCTCCTCATGAAAGATAAAGCAGTGAAGTGACAAACGTTATAAACATGCATTGGCTTATTTCTCTGTTATTGCAGAGGACATTTTGTTTCAGTAAGAGACCAAGAATCATTTATAAACATTTGTGTCTGAA

>C101753_65

ATTTCAGTAGATTTATATGCTTCAAATTTGAACATGTTCACTCTTACACATTTGTGTAGAGAGATGGAAGGTGTACTAAACCTGCAATAGTGTGAAAATATTTACATATAGCTCAAGATTCTTATAGCAGAATGTTTATGAATGTGATTGCTAATAATCAATCCTCCCACGACATTATGCCCATAATCATCGCAGGTACTGCCATGTTTGATTGCAACTATTTCTTCCTATGTATATATTTGAATGTTATTATGAACTTCTAACTGGCACTGGCTGACAGGAAGCTCATGCAGATTGGGGGACGAATTTTTAACAAAGCTAGAATGGATGAAGGGAGAATCCACAATCCATCTCCACAAATCAAACCAGAAGCAACTGCAGGAACCATCAAACCTGCCTCGCTTCTGTTTAGTTTGTGCCACAAAAACACAACCAAACTACCCATGCACATGTCAATGGCAAAGTAACCACCAACTAGGAAAGGAACAGCCATTGCCATTGGAAGTGGAATCCATTTCGCAACCTTTTTTGGGGCAATATCTCTGACCAAGTTGGCTACTATGGCAAATGCAAAAAATCCATAGCAGAGTTGCAGGCAGTGCTTAGGGAGGGAAGAAAAGCCTTCCACTCCAAGAATTGCCATGTTTCTATAAATGATGGCATATGGAGCCTTGTAATCCCCATTTGGGTTCCCCACATCAAAAGCCTTGTAGAAAAGGAAGAATGAGAGAGGAGCAACAACACAACCTATTGCTGTCCCAATTGCTTGGCTTAGCAGCATTGAACGGGGGGAAGTGAAAGTGAGATGGCCAGTCTTGAAATCATGCATCAAATCAGATGAAATGGAGACAATAGATTTTATCAGGCCACACCCCACAAGTCCAGCAACTACACCATCATTTTTTCCAGCCAAGGCTGCAAGCACAAAGAGAGCCACTTTCCCATAGTTATAGGCCATGTTCATGTCTGTTAAACCTGCACCATAAGCATTGCAGAAGCCAAGAGAGGGAGCGAAAAGATACGCAAACACCACGTAATACCACTTCAACTGCGGGAACATCAAAGGGATTACAATGATAGAAATGATGGAGAACAATATGTACCCAGCACATGCCAACCATATAGGAATGCTCTCTCTTGCAAACACTTCATTCCTTCTAAGATCATCCATAGGGAGCGGTTTCTGGTTATCTGAAAATGTATTAAGGTTCTTCCTTCTCACGGAGGCGTGGATGTTCGTGGCAGTGAAATAGAGGACTTTGACAAAATTGTAAAGCCCATCACCAAGAATCAATGCAATGGAAATAAAAACCTTATAGCCATTAAGACTCTTCATGCTACTTTCTGGTATACTTGCCGGAAACCATTCGCCTTTTAGTCCCCGGATTAACGGCCACATAATGCCCCATGAAATCACAGCACCCAGAAGCAACGATAAATTTACAAGATGGGAACAAATCATTCCTGCTCCGACATATGTCATGCTGAAATCAAAATAGAATGAATTTTTCCATGCTTTCAATCCAAAAGTGGGAAACTGAACAAATCCACAACTATCTCCACCCGTATAAAACCATTGAAAGAAAGCCCAGAGGAAACTGGTTGAGAAGAATTTCAAGAAACCATGAACCTGCTTCTTGGCCATCAAATCTCCTTTAGGAGTATGGAACCCGTTGATAAGAACAGCAGTAGCAGTTCCACTTGGATAAGTTAATTTGTAGTCTATTATCATAATCTTTCTAATGGGAACCAACGCCAACAAGCCAACAAAGCTGGTGACAAAGAGAAAGGCTGTCATCCATCCAATTCCAGGCTCCTTAATACTCGCAGGATTATTCCCCTCTGTATCAACCCCTGCTAGCTCATATGTCCTCCTATTCAAACCCAAGAGGTAGGATCCAAAACCACCTCCTACCGCGATGCTATAGCAAGCGACCGCACAAGTCTGGATTACGGTATTCTCCTGTCTAGTGAAAGGTGTTGAAACAATTTTGGCCTTTGCAAGAATCTTAGTCCAAGCTCTAATAAACACAAAACCAAGGAGAGCAGCTGAGACATTGAGATTAGGGATTAACCCAGTTGTGAGGTTCAGCTTCATCACAATCATACTATAAATTATTCCAATCAGAAAGCTAGCTACCAGTCCCCGAATTGTAATCTGTCTTGGCCATGGTGCTATTTTACACACGTCATCAGGCACAACCGGGGCCTCTTCAATGTCCCCTCTTCCGAGATTCTCAATCTCTTTGAGTTCTTCGTTGCTTATTGCATTAGTACCCATTGGATTCTTCAAATCCTCAACCTGAGTTAATGATCAGTCGCTCACTACTAAGCTGTGTTTCAAATTCCAATGATGGAGAATTGAATCTCTCGGTGGTGGCAGGGACCAGTGCTTGAATTGAATTGTGGAGCGGTGAGAATAACTAAGCGCGTGCAAAGGAAGCCATAAAAACAAGGAGGGAGCGGAGAGGTAGAAAAGAGTGAAGCGAGGTAAAGAACGCCAGGGGAAACGCGGAATTTATAATTGTCATTGGAACCAAAGGGTAGCTAACCTTACAAGACAATATTGGTATGTTTCGCTGCATGTAGTACAACGTAGTGGGTGTTGT

>C101755_65

CAAACTGATTAAACACTCTATCTAATATTAGAAGGAACATTTGTACATGTAATAAGTGAATCTATTGAAGATATTTTTGCAGCCATACAACCTATGACAAAGTGTGGCTAAAGAGTACATACAACCTAGACATAATATGTTTGGTCTGCTGGAGAGGTATAAGCACGACTTAGACGCTTCTTTCTGTATCTCCATGCAACCTGAATACGGCTTGCTGCAAGGGATCTCCAATAAGGTGATACATACCTTAGTGCTCCTTGAACATGAGGGTTCCTCAAGAATCTTGTGAAAAGAATTGTGAGTTCCTCAAGGTCTCCTGCGCGAAGTGAAAATGCCTCCACATTTGTTAAGCATCTTACAGTTCTGTTGCTTAGCAACCTCTGCCCTTGAAGCCTTACTTTTTTGCCATCTGTGGTTACAGAAGAATGTTCAAGATACCATGTCAAAAGTTCTTCGCCACAAGCATCCCCTTCAGATAAGGGAACAATAGTTCCATCATCTCCAAAGCTCTCCAATTTCCCGCGTACAACAAAGACCATCTTCTCTACAAGACCGCCTTGACTCAAAATTTTACTTCCTTTGATGTATGTCTTTTGTTTTAGTCTCTCACAAATTGCATCTAAGATAGGCTCATCCATCAGGGCAAAGATTCGAACTTTTTTAACAAACTTGAAGAGATGTCGTCTTATGTCTGTCTGGAGATCTTCTGGCAAATTCTCCAGAAGCATTTCTTCATTCACCCCTCTTGTTGCTGCCCAACTATACCGTTCAGCCTGTCGTACTCTCCTTCTTAGATCTTCTGGCAAGCGACGATGGCTCATCCATTGCTCAACATCACGACCTCTAAGTTGCATTTCTAACCTCCTGCGTCCAAGAGCCTGAAGAAAGTTCTGTATGTTTCCAATGAGAAGCGCAAAAAGTAAGAGTCCCAAACCTATGATGGCCATTGTAAAAAGAACTTCCCACACAAAAGAGCTTGGGGTTTGATTACCAGCCAGAGTACTGATTTGCTGGAAGCCCCAAAACAGTGCAAATACATATTTGTTGACCACCTTAGTTTCTATAGTAAGTGGTACGGCATTTACATAGATCCCATATGAAAAAGCACGGGAAGTGGCATTCAAACAAGCAGAGGCATTCACATTTTCGCTCCACAGATCTGACTTACGGCCAGATGCACGGCCACAATCAATAAATGTTGAGCATCCACGAATATTAGAATCACGGCAGGCATTTCGCAAGCATTGATTAACCCTCTGTAGACCAAAGAGGTACCAGAAAGAACCAACAACATGGCCAGATAGCATAAAAATGAGAAGATTTATAATGAAATTTGCCCAGGCTGACTCAAAAATGAATCCTGTTGGAGACTGGCCAATTAGCAGAGGCAGAAACCTGAATAATCTGGGAAAATACTGCACAAGGATAGCTGCACGCAGAAGATTTTTAGCATAATTTGCTCCAGACGATCCCAGGTAATTTGGTAGGACAGATAATATCATTACCTGAGGAAGAGGAAATACAACAAATAGGTCGATAAAAAGATAACTCTTCAGATAATTAAGAGCAATTTTCTTGGGATGGTCAACTAAATCTCCAGCACCTACTACCGTTGACTCAGGAGAAACATAAGCCAACCTAAACTGGAGAAGAATGTTGAAGAAATATATCAAATCATTAATGCTTCTAAATAAAACAAGTGTTGTTGTCATTGTCCAATTGATAGCAATACATTTATCATCCTCTTTCACATAGATTAAGAAGAAAAATAATGGATCAACAAAAATTGCCACCAAGCAAAAAATGGCCAAAATCTTGTTCCATTGTTGTACAACTTTTGCGTGAGGATTCATAACTCCTGGAACATATGAATAGCAGAAGGAAAAAATTTTTCTTCCAAAACCCTTGGCATCCCCATAAAGAATATTATGGAACTTGGGATCAAATATAGCTGAAGTTCTTCGAGCTTTTGACTGAGCAGCCTTGAAATAAGTGGGGCAAGTTGTACAATAAGGGTCATTACACATTCCAAGTTGCCCTGACCTCATTAAGTGTTCATTCTTTCTATCATAGTCGTTATCCCAACGATTTTCATTTCTCCCGTTGAAACTAGAAAAATTTTCTGTCTTACTCTCCACCACTTTGCTTCCTGTCGCAGCTATATTCTGTCCTGAAAGATTTGCAGTTCCATGGGTAGCATACAGTGGACCACTCATCTGCACAAAGGGGGTTTTTCTTGCACTACGAAGTGGACCAGTATGGCCCACAAGACTAGTTTCTTTGTCATATGATTCCAAGGAAGCCATAGGAATCGAAATTGAAGCACTTCGTGTCCTAGATACTAGCCTCCGGAAATTAGAATCCACGACATCATCAGATAGTTGAGCCCGTGTTTCAGACAGCATAGGCACCTCATCTTTTTCAAAACGAGCCATGTTTGCGTCGTGATTCTCCATTTTTTATGAAACCAGAAAGAGCAAGAGCTGGTTCGAGAGGAACTGAATTCCCAGTGAAAACCCCACCAAGAATTCAAGTTAAAGAATGCATTTTTGGGAGCTTGGTGATCATGGAGGAAAGCAGAAAGGAATTTCTGACAGAGAGTAGTTGAG

>C101767_65

TTCCTTTTAATACTATATAATCAGTTTCCCTATTCACTTGCCATCGTTGAATCCAAAGAAGCTAGAAAATTAAAAATCCAGAAGTACAGTGAGTATCACAACGAGTTCTGAAAGCATATACCTATCTAACATTTTTCTACTGTATTGAAATGAGATGAGACAGTTTGAATTACAACATTCTAAAGAAACTTAAACTAAAATACTTGAAACATAATGATCTCAAACACCAGTTGTAATACTTGAATGAGTACACTACTTTGGGAAACAGGCAGAAGGAGAACAAACAGCAGATCCCACAATGAATGAAACAGCATCTGTTGCCCTGCTCTGCGATGTGATCATGATACAAAAAGGACTCAGCAGAAGTTATATCGATCAGCCTTTTGCATAGTTAATGCACAATGACAGCAAGAACCTCAATCTATCTTCACTGAGGAGAACAAGTAGTGCACAAAGTAGAACGACATAAGGAAGCCGATGGTGCCGGTTGACAACATGATTGCAATGGCCATGAGTAGTGAATAGCCAAGGTAAAGGGTAGCTGAGACTGGTCCACTCAAACTCTGCAGGTCGAAGACCAAGTAGTTAATGGAGTATAAGAAGACGTAAAGAGACACAGAACCTGAAGCAAAGAATGCCTTCCACCACCACTGCCAATCTTCCACACACAGATGCATATAAGTGAGGACAACAGACACTTCAGCACAAACAATAATTAGCAATAGAAGAACAACCAACAGGAAACCAAACACATAATAGAACCTCCCAAGCCAAATACTGGAAAGGATGAAGAAAAGCTCAATGAAGAGGGTTCCAAATGGAAGAGTTCCAGCACCAAGAACAAGAAGCCACGATGGGTATTTACGCGCTGGAATTTCCCTTGGAATTTGGTTGGTGCGCACAGGATATTCAATTGGCTGAGCTTTTGTCCCCATAAATCCTCCAATGAGGGTAAGAGGTACTGAAATGCAGAACCAGAGGAAGAACAGCTCAAAATACAAGGAAATGGGAATGGCACCAGTACTGTTACTGCTCCATAGAATGAAATTCAGTATTGTAAGAATAATGAAAGCAATTCCAGGAAAAAAACATGCAGCCGACCAGGAAACTGATCTCCACCCTTCCACTGTTCCTTTAATTGTCCTCCATACGCGTACACTGACGTAGCCTGCAGCAATGCCTAGAATAAGATACAAAAGGATCATCCCAGTTAGTAGCATTCCTCGGGAAGCCGGCGACATGAAGCCAAGTGCTGCAAAAACAATAGTAACACCAGCCATTCCAAGAATTTGAACCCCATCACCAACCATCACACAGAGAAGCTTTGAGCAATTAGGCTCCCTAAACACATCACCCACAACAAGTTTCCATCCAGAGAGCTCCTCGTTCATCTGAGCTTGAGTCTCTTTGTCCAGTTCCTCATACCTAGTCAAATCCCTTCGGACAGTTCTTAGGAAAATGACAAAAACAATACCAGCAAGGAAAGAAATAACCATAAGTGAATTTAAGATTGAGAACCAATGAACTCGGGCACCCTCCATCTTCAAATAAGCATCCCAACGTGATGGCCATCTTATATCACTTTTCACAAATTCAACGTCATATGTGAATGATATCCTTTCGTGCTCTTTTATCACTTGATACTTGTCAAGCTCAGCCGGGCAGTTTATAGGAGAAAGAGTATCGTACTTGTTGCGCTTAGCCATGACCTCAGGATCATATTTAATGCTACAGGGGACAACCTGAAAACCAACTATTTCATACCCGGATGCCTTTTTCTTGTCAGATTCAGAAATAACACCCAAACCTTCTTCCCCAGTCCCCACAATTTCAACCCCACTTCCTTCGTATTCATGAACCAAGACCGTAAACTTAAGGTGGTTAATGATGTAATCTGCACCACCATCAGGTGGAGTGTACCCAACAGGGAACCCTGTCCACTGGATTTTAACCCCATTTTGGGTGGTAAACCTCATAACAGGCAAGTTGTCAAGAATCATATTCACTTGATACAGATCCCGCGTCCTTTGTTTGAGTAGCTTCACCTCATGCTCATTCAACGGGGAAGTAGTGCAGAGGTAAACCGTCTCGTTAACATTCATCCGGAATCGGTAAGGCGAGTTATCAATCTGATCTCCCATCAGAAGTTCTCCAAGATTCTCGGCACTTTTCTTTATACCGCCATCCGGTGGGCAGTATGGGAGGCTGTAGTAACTGAAGGGAAGCTCAGTTTCAATTGAAGTCAACGAATTAACTTTGGCAAATATGAGATCTTTATTTGAGTAAGTGTGCATATAGCTTCCAGGGAGATAAAAACCATTAACAACTTGAGCAAAGACGACGACAAAAAGGAAAACCCAGCGGCAGATGAAGGGTTTTCGCAACTCCATTTCCTCCAATTTTCAAAGCAAAACAACAACAGCAGATGCAGAAAATCTTCACTCTGAATCTTCCAATAAGCTAACCCCGACACCAATACCAACACCGGTGCAGTCAATACCAAATCGTGGAATTTTGGCGATATATTGAGTTTGAGAGAGGGAGAGAGAGATAGAGAGAGATCTCTCGCAACGACAGATGATTTTTGTGTTGGAATCGTAGTCGAACGACAAAG

>C101769_65

TGATAGTTTATATTTTATAGACATATATATGTTACACTAACTTACAAAATAAAATAAATAAATGAAATGCCATTTTGGGCCTCTATCAAAAAGGTCATTTCATGTTAATTTTTCCTTCACTTTCATCCTTTTTTTATCTTACAGAACCAAGCATCCACAGCCATTAATTTGTCCACAACTATTGTTCTTCAGAAGTAAAATCAGGCTCAGCAGGCTTCTGCAGCAATATGGGTGATATTCTCTCCGGCACCCTGGTCTTTCTTGTGCTATTTCTACGTAAAAGTCTGAGTGCATTAGCTGCAAACCTTGAAGCATATATCGTGGCACCTAGGCTAGGTGAACTACCACCTGCTTTGGCCAATGCATCTTGCAACCTATTCTCTTCTTCCCTGAGAGACTCTTCCAACTTCTTTTTACTATAGCGCCTCCATGCTGCTTGTATGAAACATGCAGCCCATGTACGCCATTGTTGCGAATAAAACCTGAAAGTGTGACGAAGCTGCTTACTATGAAGGCGCCGGAACTGTGATGCAACAAACTTCAAGTCATCGGCTTTTAGAGCAAATGCTTCCACTTCTGAAAGAGTTTGGACAGTCCTAGTTGATATTGGAAGGTTGGATGAGGAGTGAGGATCTAATGCCCATGTAAGAAGTTCCTCTCCACAAAAGTCACCAGCTTTGAGGTATTCAGAGTTAAAGAAGCCTGTTCTTCCACCATTAGTAGTCACAGTCAATAGCTTCCCCCGCATTATGAAGAGCATCTCATCAACAGGATCTCCTTCCCGGGCAATGTAGCTTTCTTCTGTGTATAGCACTGGCTTGAGTCTGTCACACATTGCATCCAGAAGTTGCTCATCCATTTTCTCAAACATTGGCACCCTCATCAGCAAAGCCAAGCAAAGATGCCGCTTAATATCCCTTCTCAAATCCTTTGGAAGATCTCGTATCAAATTATCTTCATCAACACCTCTGGTTTCTTGCCATTTGTACTGCTCATGTCGTCTGATTCGGTCTCTCAGGCTGTCAGGGAGTAATCGATGAGACATCCACTGTTCAGCATCTCTCCTTTTCACCCTCATTTCCTCCAATCTGGTAGTTGTTGACTGCAAATATGTCTGCATATTTCCAATGAGGAACGCGAATAATACCAAACCAGCAATTGAAATGAAAATTGCAAAGCAGATTTCCCAAACATAGGTACTTGTTGCAAGGTTCTGACCAAGAGAACTTAAATTTCGTAGGCCCCACCAAAAACAATAAAAGAACTTCTGTGGGAAATCTCTTGACTCCACTACTCCAGATTGAAGGGCATCAAGAAATATTCCAAAATTAAAGAGTGTTGTATTTTGATCCTGTGTTGGGCAAGAAGCATTCAGGAATACTGAAATTGTGCCCAAAACTTCATGATTATCACAATACATAGCTGCTGTGTTACATGTCCTATTTCTTCGACAAGCATCTTGCCAGCATGTGGTTTCTCTTTCTATAGAAAACAAGTACCAAAAGGCACCAATAACATGACTTGCTAGCATGTAAAGAAAGAGATTGAATGCAGCTCCAGCCCAAGCTGTTTCAGTGAGAATGCCAGAGGTCCTAGTAACTTCTTTATATAATGGAATTAGTCGCAAAAATCGTGGGATATATTGGAAGAAGACAATGAATTTCAGCAAGGTCTTTGTATTTAGTGATTCAGATCCCCTCAACTTTGGAATGATTATTAGAATTGCCATCTGTGGGAGGGGAAGAACGGCAAGAATGTCAATTAAGAAGTATGATGATATATACCGCATTGCTATTGCCCAAGCATCTTCAACCAAAACACCTCTTCCAAATACTCGAGAAGAGGGAGCAATGAATCCAGTACGAAATTGGAAAATTATATGGATTATATAAAAAATATCAGAGAAAGATCTTAAAACAGTTGCTGTAATTTCCATTTTTCTGTCCAGAGAAAGGCATTTATTGTTATCATCAATCACAGGAACATAGAAGAACAAAGGATCGAGTGACACAGCAATAAGACATGATAATACAAATATCTTATTCCACTTTTGAAGGAAAGGGCCCTGCGGATCAAGGATTTTCTTTCTAGAACTAAAATTTCTAGAGAGAACTCTGTTATAGGGAAGAGATTTGAATGATTGTCCAAATCTCTTCATCCCCTCTGAACTAGATTCCAACCCCCTTTGAAACTTCTCGGAAACTGAATTTAGTGTATTCTTGATTCTCCCCGGGCGTTCTACATAAATTGCAGGAGAGCTGCTCTCGGAACCCTTGTCTGAATTCCAATCCTGAAATCTAACAAATTTGTCTTGCTGAAAGTTCATGACTGTAACCTAGTTCAAAAACAAGATAGAATTTGACAAATAATTGTTACGCAAATGTCACAGGACAGTGAATAGCTGCTTCATACACCTCAAAATCTCACAGCCTACCCCCAAAGGAGAGTAACGAAGGGAAGAGCGTGAGAAGTAGTTGCAGTGAACATGTGATTGATTGCAAGGAGAAACAGGAAAGAAGAGGTTGTAAGCGTGAGAGAGAGAAGGAAGATGAAGGTTCGTGGAAAGCGGGAAGAGAGCGATGGTGGTGTAGACAATAAAGAGAGACAGTTGGTG

>C101771_65

GTTTTTACAGTTCAGTTCATCCCTTGGCTAATGATAATTAATGATTTTGATTATGCATATTTAACTTTATTTTTCTCTCTTTTTTTTACATTCTTCACTTGGAATGTCATGGACCCGTTAGCAGCTCATACACTAAACAAATTCAAGTACATGGTTTTCTGGACAACTCTAGCTTTCCATGAAGCCCCCGTCTCTTGCATGTTCCCATCATTCTAAACAGAATGCAAATACCAAGGTTTCCTGGCGCTTCTTCTCTGACACTAACATATTGAAAAGGGAGTTCGGTACAAGTGTACAACAAAGTTTGCTACCTTTTCCCTATTGATGTTGACATGGAGCTAGTTTATAATGTAAATATGTTAACTTTACTCCCAAATTTCTATGAAATTCCCAAATTTTGGCGAGACAATTGGAGGTGACTAAACTAGTGGGATGATAATTCAAACCTCACCAGTCCGATTAAGAAAGTAATAGGACAGTTTTAGTGCAAAGGCAGGCTTGATTCTCTTCTGCTGCTTCTTTCTTTGTTGCTCAATGTAGACTCCACAAGTTTAACTTTCTTCGAATTTTCACTATTTACAAGAGAAAGTTTTTCAAGCGATGATTTGAGTATGGATATCTGATTGTCTCCAGCATGAATTTTCTTCCCTGGTGTTTTCAGTTGTTGATTAGCTTTCGAAGATTGCAAGTGCCTTCTCAGTCTTGCACTTAGCGCATCGACAGAAGAATGCAAAGCATCCAACTCAACTTCCTTGAAGCGGTCAAGCTCAGATTTAAACTGCCGCTCTGCTCTGGACAGTGGTTTTTTGTGAGCAAAAGGTAAGTTCCTTAATTGTTGCAAGCGCTCTTCCAAAGAATTGTGCATTTGAATAGCACGATCTAACCTTTTCTGTAATATAGCCTCTTTCTCCGCAACTTTCAAAAGTTTCTGTTGTGCATCCCCTAAACGAGAATGTTGGTCATTAATGATTTTCTTCAACTGAGGTGCGTGATGCTTGAGCTCCAGGTACACCTTATGTCCATACTCCACATAAGTTTCATGAAACAGCTTGAAATACTGATGTAATGCTGATCGACCTTCGATTGAATCAGCTGCAACAGAGCGCAAACTTGGAGAGGCATGGGGAACAAGTACCTCCTTCGGTCCACTAAGTAGTTCTTTGCTAATTATTGTCTGTATATCTCTCTCTTTTGATTCCCCCTCTGATGAAATGGGTTTCTTCTCCATTTCAATGCTTACTGGAAGCAAGAGGTTCCAACTTTTCATCTCAAGCACAACACACTCGAGAGAAAGTGTAATAGCTACAATCCATGAATATCCAAATGAATCTGATAATGACACAAAGCCACAAAGTGAGGGTTCTGAGGTGTATCCACTCTGGCAAGTATTTAATACAGGGTGCACAGATGGTGTTTTCATTGTATCATCCTTGCCATTTGACTGACTTGTAAATGGAAGAAAATGTAAAACTATTGAGTCAATCCCTCCATCATGAAGGGAATATATTCTTTCTGGCATAAGCATATCAACGAACAACGAAATATTATATCCACTTTCTGCTCTTCGTGGCAGAGCCAAATCAACAATAGCCAGTCTCAGCAAAGGAGGTGGGTTACCCAACCAGGCATTGCGATCAAGCTTCCACAGACTGCTGCTGGTATTTGACTCACAAATCATAGCAAGGCCAAGAATTTGGTCATGGGAATCAACACGAAGGCGAGGTGGACTGCCAACACACCAGACAGGTTGGATCTCATCGGCTAGAGCATCTATCTGCAGTTGCCCACCACTCCAGGCAGTCACCAAAATAGAGTCTTTGCTAACTAAATTGTAGAGAAAACTAACTGCACGACCTTCACATTCTGCACTACAACCAACTGAATCTTCATTTCCATCCTGACCTACTCTTCGCAGAGGTCCCTGCAAGACAAGAGATGTGTCAAATAAAGCATAAGCATGAGCTCTTAGCAAAGACAGGCTGTCTCCTTCAGTTTCTTGGTTCTGTAACTCGGGAAATGCTGCTTCTAACCAGGAGATTGCCAACTTTGAATTACTTGCAGCAACAGAGTTGGCTGACATTATTCCAAATGTGTGAGCATCATTGTATATTTCTACCAAAGACTCACATTTGAAGAGACTTCCAAATGGAACAACTGGGCAAAGTACATAGATAGCCCCATTACTGAACAAAATAAAAACACTAAACCTGTCCCATAAGTGGTCTCCCCCAAAAGAAAAATCAACTGGGCACACTGATGAGGCATTTCTAGATTTACCAGGCTCAACAGGCTGTAAGTAGTATTCTTGCTCTGGTTGCAAAGGATCCACAGACAGGTTGAAAAGTCGGAAAACTGAGTCAGAAGAAAGAATTCCCAAATGAGTATCACTGTACGGGTGCCACAATGCCTGTAACACCCGAATATCATTGCCACCAGTAGAATATGTCTGAGAACCAACAGTAATAGTCCTGCAAATCAAGTTCACGTCCTTCTTAGAGGCGCGTCCGTAGAGGTACATAACAGAGAGCGTTTCGGAACCGAAAAGGAGTATTGCTGTTCCATTCCTGTTGATGGAGATTTTGTGGACGTCGAAATCGAGCACCGCGTCCGCTTG

>C101773_65

TGAAATTAATGGGTTTCAACTAGAGATAATGGTTCAAAGGTACATAGCAAACTTTAGAAAGAAAAATCGAAATTGATAATTTTCAACTAGACATAAAACTAATTCTGCTCTCTACTGTGTGACCATATCAGACACTTCATATAAAAAATCCTGAGGGAAACAACATTTTGATGATTCAGAAGGAACCCAATATTCAGAAGCATAGGACCTCAGGCACATCCAAAGCATACTAGTACACAGGCAAGGTAGGATCCATGATTCTAAATTACAGTTGCTGTCACATCATGATCCTTAACATTGCAAGATGCAGCAGCAGTTGCAGTCACTAGACAAATATTTATGTTGCGGCTGCAATTGAGTGCTGACCATAATATATATGAAAGGTTATCATTGAAGAATGGATGGGAGCAGAGCAATTAGTGGAGAAAAAAATGATATTGCAATACATTTGTATTATAGAATGCACAGTATGGTACAAAGTTAACTAAAACAATGAATAACGGACCATGCTGGAAATACCCATGTATTTTTCAGTAAGAGCCAAACTCAAGTCAACATGAGATCATTCCCCACATTAGGGCGAAGGCAAACACGAACTTCATGCTCTTCTGATCCTGGAAGAAGTTTAGGCACCGTTATCTCAAGCACAGATCCTGGCCCATCATAGTAAGCTTCAATATTGGCATCTTCAGGAATTCGAGTTGGTAGTGGGATTTCACGAACAAACTCTCCAGGAGGAGAGTGCTCTGAGGATGGCTCTGTAAGCTTGAATGTCCTGTCGCTTCTCGTGATAAATGGCTTGCGAGATATGCTCATACAAGAAACCTTTATGATACCGTGAGTGACGGTATTCCTCCAAGTAACTTTCACACTGGGAAGATCAACGAATGGCAAACTGATAATGATCAAGTAACCTTGTTCATCCTCATAAATAGTTTTCGCAGCTGTAGCTGGTCCACTAACATTCTTCATCACCCCACTGAAGTCATTCAGCCAGAGTGATTCACTTGGGTGCATTACCAAATCTGGAATCCGATCAGATGGAGGATTAACTGCCAAGCAACATTCTTCATCATTCCCATAAAAGAAAAAGTCCTTCCTTTTCTTGCTGCTAATAGAAGATAAGTTTATCCCATCACCGTTACTGAGGTTAGACAAATGATTTGATAAATTTAGCACAGATCCATTTAGCAGCTTCTTTGGATGGGGATTTGGAACACTCTTGATTAGGGGAAGTGGTCTCTCAAGCTCGAAGTCTGTGTTGGGAAGATTCCTCCATGAACTGAAGTCGCTAGCTTCTGGTGGGATTGTGAAGTTCAAATCACGGCCAGTGAGTTCAATCCACTTCTTCTGCTCATCCTTATCAAGACTCATAAGGTTGGGTGCTGGAACAACCTCAATGCCATGCACACATTGAGGATTTGAAAGCCCTCTATAATGCTTTCGTTGCATCCGGTGGGATCGAACAAAACCCCGGTCAGCACTAAATGGAAATGGACGCTCTCCTTGGCGGGAATGGCCATTCATGTAACTCCTCAACTGCATCTTACCCAAAGCATTCTCTGGCCTCTCCTTGAAAACCCACATGTACATGTTTTCCATGTCGTGTTGAACCATGAAAACATCAAGCTTGATATCAGACTTATCGAACCCTGAAACACCATTGCTGTCCCTAACAACCTTGGCCTTAGATTTCTCAAGCAATGCTGGCTTGAAGTAAAATCTGAAGAAAAACCAAGCACCCCATATGCTATCAAGCCGCTTGGCGCATTTCCTTCCAACTTTGGGAAGTGGGAGAAAGCTCTCAGTCTCATATCCTTGAGTACCAACAAGACCAACATCCAGCACTGTATCAGAAGGGTCAGGATTCCATGGCTGTGGAGGGGGACTGCGCTCAACCGAGAGGGGCAAATTGATATCAGGTGGACATGAGAGTGTGATTTTGCCATTCATATCCAGATCCAGTTCCTGATGAGAAGAAGGTGCACTTGAATCCATGGACAAGAGTGTGGAGGGGTGATGATTCTCCAATTCCATCGAAAGACAACATGTGAGAAGTGAACCACCCATTGGATTGAACTACCTCATCAGCTTGATGAGTTTTGCTTCTGGTCGGTTCAATGTTGTCATCCCAAAAACAATTGAACAAAACAACCTTATAAACACCGTCATCATTCATATCAATCCCCACAACAATATCAAACCCTCCATTCCTATTCTTTTATTAAGAAAACCTAACTTACGAATAAGATAACAGAATCCCAACACACCCACCAAAGAAAAAACAAATTAAACCATACACTACAAGATAACAATAACGCTATCAATAATAAAAATCCTATCTTTTACCATGGATTCAATTTCAAAATTGAAATTATACATCTTTGTACACAAATACAGTTCTTAATAATTTTCCAAAATATATTTAATTTAAAATTAGTGAGACCCGAAAAGAAAATTGAAGGAGATCTTCAAGGAGTACCTCAGGGAATAAGCATCGAAAAATGAAAAAGTCGAGGAAGCAGCAATGGCTAAGATAGTGGTCTTCTCCATCGTGTCATCCATACCATTGAATTCTACCCTAGACTTTTCTTTTTTTCCTTTTTTTTTTTTT

>C101775_65

TTTTTTTCAGGAAAGTAATTGCTTCATAGAATGTAAAGTACACCCACACAACACAACATATAATAAAGTTAAGACTAAAATCTTAAGACTCACAAACGTATGGTTATACAAAAGCATTCTCATTAATACAGGTGACAGGAAATTATTACCACCCATCGAGACAAAGCTAATCTAATTCTACAATTCATATATTAAATCTTAAACCAACGATCTGTAGACTATTTTACAATTTGAGCTCCTTCAAAAGAATCAGAGTTGCATTTCATTATGAACCTGTACTTTTTTAAATGGGTGTGCTAGCAAGAGCTTCAGATAGCAACAAGACAATAATCACTTCCAGCGCGAACTAGACATAAATCCAAATATGGTCGAGCCACATAAGGCTTCTGCTTTAAATTTTATTGAACCTTCGCAAGGCTTCGACTAGATACACCGTGTAATAATTTGACAAACCCAAGAAAGCTTAATTTCCCATCGGTGTGGCGTATCCAGTCATGAAGAACAACATGAACAGGGATAGAGGGACCAAGTCCAAGTTCTGAAGCAAGCTCTTCAATGACAATTGCCCTGTTTCCATCTTTATCAAAAAGCTCATAGGCACAGCGGGCATGTTGTTCCCAACGATCAAGAGCTTCAAGTTGATGCACACTTAATGCAGCTGCACAGAACTCTTCAAAATCCATCCTTCTATATTGTAAAGAATTCAGCGAGGTAAGAAAGTCAGGGATACGAGACTCTTTCATGGCATCTGTTGCATATTTCATCAAGGCCTTATTAACATTCTCTAAAGATATGCTGCCATTTTTGCTTGGTTCTAGCAATGAAAATTGCTCCCTCAGATAATGGAGTTCATCTGCAGTCAATGTTTTAGACAAGGCCCTTAAGGCAGCCTTGCGTAAGGATGATGATCTCATATATGTCTTCATGAGCTTAAATATTAAAATATCAAGTGGAACTTTTACATTACTGTAATTCCGTATCCAAGGATGACTTAGAGCCTGCGCAGCAGATATCCGCTTGCGTGGGTCCTTATTCAATAATCGTTTGACAAAATCCTTTGCTTCTGAAGACAAAGATGGCCACGGTGTTTCATCAAAGCTAGGATCAGCTTTCAGAACTGCCCTAAAAATACCAGATTCTGTTCGAGCCCAAAATGGGCGACTACCACATAAAAGAATATATGCTATCACGCCTATACTCCACACATCAGCCTCTGTGCTATAAGATCTATGGAGAACTTCAGGCGCCACATAGTATGCACTTCCAACAATATCATTAAGCCTTTCATCTGGTCTGACAAAATCTGATAACCCAAAGTCTATAGCTTTCAACTCAGAACTTTCATCCTTTTTAGTGTACAAAAAATTCTCTGGCTTAAGATCTCGGTGCACCACACCCTGAAGATGACAAAATGCAACAACATTTAGTATCTGCACCATGACAGCCTTTGCATCATCTTCTGAGTATTTCCCTCCTCTTGATAATATCATATCTAAGAGCTCCCCCCCTTCACATAACTCCATTACAATGTAGACATTATCTTGGTCTTCAAAAGCATCATAGAACTGTATCAAATTGCCATGTCCATTCAAAGCTCGCAATATTTTCACCTCCCTCCTGACATCTTCAATAGCAATTGCTGTTGTCATCTTTGCTTTTGGTATGACCTTAACGGCCACTTGTTGACCCTTAAGCTCACCCTTCTTGAACCTAGCAGAACAAGTATACCCAAAATGCCCTCGACCCACTTCTTCACCAACTTCCAACCTACTAGTGAACTCCTTAGAGAACCCAAACCTCTTATCCAAATCCGCTGCACCTCCACTACCATCCTCCCCCTCCTCCGGTATCGCCGCAGCCGCCTTCTTCCCCTGCCGCCGCGCCAGCACCGCCCTTATGTGCTTCGCCGGCGACGGCGGAGGAAACGGCCGCCGGAAGAACCTCCTCGGCGTGGAAGTTGCGCTCCTGCTCCCTCCCGCCGGTGCCGGGGACTTCTTCAGGAAGCGCGCGGGGCTCGGGCTGTAGAACGGGAAGAAGGGGGACTGTTTCCCGGCGGTGACGTCATCTTTCCGGCGGTGGTTCTCGGCGGCGGCGGGACTAACTGGGGTTTTCGGGATTTGCGAAGGGTCGCCTTCGGCCTCGCGAAGCGAGTAAGGGTTCGGCTTCTGCGGCTTCGATGTGCAAACCCCCATTGCTGAAAGATCACAGAAGCTGAGCTACTCAATCATTGGTCACGCCATTGCGAAGAAGCTTCTTCTGGGTCTTGTTGTTTTCTGCTAAAGATTCGAACTTTGCAGAGGGTTTTAAAGAGGGTAGAAGTGGATTTGTGGGGCAGTGGAAGAAGAAGATGATGATGAAGAAGAAGAAGAAGAAGAAGAAGAAGGAGGGTAATTTCACTCGACACGTGGCATGCACGTGAGTATTGGGTTTTGGGTGTTCATTGAATTGGGAAGTAAAGAAAGGTAGCTAAGGAGAGAAGAGAAAGGGTCTGAAATTGAATTAACGAGGAAGAGGATGATAATGAGAACAGTGATGTGCTTAGGTGAGAAGATGGCAAAGGGGGGTTGTATTGTTTCTTGCCACAGGTTTGGCTACATATTGTTCCCCCTTTTGAC

>C101777_65

CGAATATCAGGGTAACTATGGAGTATATGAACTTCCATAAATGATCCAGAAAATGAATTTTTTTTTACACAAACAAAGATTCTTGTTTCTCAATCTCAACCCACGGAAAATAGAACATAATAGAAAAGAAAATTTTAAAAGACAAAAATTTCAAACAAAGCCGTAGATTGAAAATAATTAGCAGGTACCGTCAAAACTTTCAATTTATCATATCCTTAGGTCAGTTGTACTTTGGCATCCTCAATCTGTTTTGCTCCGACAGAGAAGAACTCAATGATCACTTCCAGTGGCATGCCCTTGGTTTCTGGAACTTTCAAAAAGACAAACACCCATGATATGAAGCACACGACAGCATAAATGCCAAAAACACCAGCAAGGCCAACAGAATTGAGCATAACTGGGAGTGTGTAGGTGACAATGATATCACAGATCCAAAAGGTAAGAGCACAAACGGCTATGCAGAGACCACGAACTCGAGTGGGGAAGATCTCTGCACAAAGTATATTAGGAATTGGCCCAAATCCCATGACAAAGAAACAGAAATATACAATAACACTAATGGTTGAGATTGATGCATTTGCAGTGCTGCCCAAATCCACAAGACTCCCCAGGACTAATATGAGAAGAGATACTATTAGGACGGGAATTGTACTAAGCAGCAAAGTCCTTCTGCCAGATATATCCATGAGCCTCATGGCAACGGCTATACAAGGAAGCATCAACAAGGTTGTCACCGCACTAATAAGAAAGGATGAAGAAGTAGAACCAAGGCCTAGGTTTGAAAGAAGATAGCCAACACCTGCCTGCTCAAGAATTTGAGGTGTATAGTAGAGGACGCCATTTATACCAGAGAACTGCTGAAGAATTTGAAGTCCCACCCCCACAATTAATGCATGCTTCACCCCAGGTTCAAAAAGGTCACTCCAACTTGGCCCTTTTGCATTTGTTTCAGATGGATGAATCATAGCTGCTCCAACTGGTCGTTGATGCATAAGCTCCTTGTTATAAAGGGCTGGCTGACTCACCAAAGCAGCAGCCTGTACAACCTCACCATCATTTGGTAAATCACCACCAGGGAGCGAAACCATAGACCCACGTAATCCAGGACCACCATCTTGGTGTAAATATATTCTCTTGAAGCCACTTTCCTTGTTTCCATCTCGGCCCTCTCTTTCAGACCATTTCCATGCCAGCTGCCAACCACCACCAATCCTGGTACTACCAGTGGGTTCTCCGGCATTTCCATGCAAAAGACTACCTTGCCTTGTGCTTGAAAGGTTGCTGTGAGCAGAAGGAGGTATGTCCTTATCCAGACTTGTTGTTTGACGTGACATCAACGGACTGTGCAAATTGTCATCTGAATCACCAGCAGCGGCATCAGAGACATAATCATCACCCTCTCTGGCAAGGCTTTCTTCATCCCAATCTTCATTCCTAGGCTGATTTCCCCCATAACTAAACATACTCCCAAAGTGTGGAAAAAGAGTGCTTCCAGTTTCAGGGAACTTCTCATGAACACTGCCAAAGAGGTTCACTAGAGGGTCCACTAGACCAGTCGGATTAGTCATGCTTCCTTTCCGAGATACAAAGCCAATTGAACTTTGTCCAGTAGCAGGTCTAGCAACCCAGGACTGACCTTGTTCCGGTCCATACAACTTAATCTGATCTTTTTCACTGTAGGGATCCTCTTCATCACCAAGTTCATTGGTTGGACCAATTATATACTCTTCTATGGCTGTATCATCAGCAACTTTTAGACCCTCAACCAGTAAAGCCATTTCACCGGAAACATCTTCCCTTCCACGAAGGCGCTGCAAAACCTTCTTGGCCTCAAGCATTCGGCCTTTACTAACAAGCCATCTCGGAGACTCAGGCAAGAACAACAGTGTCAGTGCAAAGTAAATAAGAGAAGGGATTGAAAGAACACCCAACATGAGTCTCCAGCTTGGCGCCTTTGTCAGTGACATCCCAAACACCATACAATAGGAGAAAAACATTCCCGCGGAACCAGTAAACTGTGGAAGTGTATTGAGTAATCCCCTAATCTCAGGTGGAGCTGTCTCAGATATGTAAAGAGGTACCAAGGTAACTGCCAAACCAATTCCCAAACCATCTAAAAGCCTTGCAAAGAGGAGGATATAAACATTTGGAGACCATAGCATCACAAGAGAACTAACAAAATAAAGGAGAGAGGAAATAATCAAAATAGGACGTCGGCCTAACAAGTCTGATAGGGCTCCAGAGCATGTCGTAACCACAGTGGCTCCAATAAGTGACATAGCCACAATTAGACCTTCAACTGTGGGTTGACTTTCTAATTGAAACTCCCTTTTTATGTACAAGATAGATCCTGCAATAGTCGCATTATCCCATCCTTGCAGTAAGTTACCGATGGCAGCAGCAACGGCAACAAGAACAGCTCCACTCATAGTTTCAATTAGTTTCGTAATCAATCAGCCTAATAATATCAGTTTGGTTTGAAAGGGTGGGGGTTTCAGGTATATGAGATGCAGAAAAGGACATGGGTCACTCACTCACTGCAACATGGATCATAGGGGGAGCTCTCTCTTTCTTTCTTACCTTTTTTTATTCTTAAAATATTAAAAAAAAAAAAAA

>C101779_65

TACATATATACATTTTTCTTATTCTTTGGATTAACACATATGAAAGAGTAGTTACTGCACTTCGACTTAGATTTACAACACCGTAACCTCCTCCTCCTCCTCCATCTTGCACTTTTTGTTTTTTAGTCGTGTCATAATTTATTGAGGGCAACTGAGTGTTTTGACTTCTAATGGCTTATGAGGGTTTTGCTTTAGGAGGCATTTGGGTATTCAAATCTTGACAAAATTTTGGATCTTTGTAGTTTGGCCGGTGGGAACTGTAAGCCCTAATTTTGTTCTTCTGCATCTTTTTATTGTTGTTATCTGTGTTTTCTTTTTCTTTATGGGGACTGGGGATGGAACAAGCGATGCGGATAAGACTGAATGTGATGAACGAATTCAGTGTTTGAAAGATGAGGCCATGAATGGTGAAGTGGCAATTAAAGATGGGAATTGTGTTACTGAAGAGGGACAGCATTTGAAAAGTGAAGCCATGAATGATGGGGTAGCAATTGCTGATGCAAATGGTGTTGCTGGAGAGGTTAGGGTCTTGGAAAGTGAACCCTTTAGTAATGGGGTAACAGTTGGAGATAAAGGGGACTCTGGTCGAGTTGAATGTTTGCGAACTTATAAGAGGCGTAAGAAGCCAAGTTCAAAAGGCAAAGTTCTGGAACAATGCAAAGCAGGCATGGCAACTGCAAGTCGAATAGCAGATCAGGATGTGAAGAAACCGGGTGATCTAGCTTTAGGCGATACTTCGGATGATTGCTCCCAAGGACAATGGGGAAATATTGTACTAAAGCATTTATGTCACTCATTAGGTGATGGCAATGGTGGTGTAGAGCGGTGCATAAGGGAAGCACTTAATCCTAAACATAAACATGCCACTACTGTTATGGAAACATCTATACTTGACAAAGGTGGACAAAAATGCTCTTTGCAATTTGAGCAGCTTTCTCGTAGAACAGGAAAGCAAACTAATCGACATGCAGATGTCATGTGTAATGGATGTTCCAGTGAATCACCTGACCGTGGTGTGACTGAGATGTGTCAGCGTGTGCTCTGTAATATCTTAACCTCAGAAAATTTCAGTTCATTATGTAAGGCTTTACTTGAAAATTTCCAGGGAATGAAGCCTGAAAGTGTATTTGACTTTACCATCATGAACTCAAGAATGAAGGACCAAGCTTATGAGCAATCACCAGCACTCTTCTTGTCAGACATTCAACAGGTTTGGAGAAAGCTTCAAGACACTGGTAACGAGATTGTTGCTCTTGCCAAGAGCCTCTCCAGCATGTCAACAACTTCCTACTCTGAGCTGGTGGGAATTTCAGCACTTAGCACAGTTCAAGATAAGAAGCAACTGTTGTACAGTCGAGAATTTGGCTCTCACATTAAACCAGAGCAGACGCAAGAATGTGCTATGTACAAAATTTGCATTTGCAGCCATTGTGGAGAGAAGGCAGATGGCAAAGACTGCTTGGTTTGTGATTCATGTGAGGAGATATACCATGTGTCCTGTATTGAGCCGGCAGTGAAAAAAATAATACCCCACAAAAGCTGGTATTGTGCGAAGTGTGCTGCTAATGGGATTGGATCTCCACATGAGAATTGTGTGCTATGTGAAAGGTTAAATGATGAACAGACCCCAAATGACGACATACTTGGTGATGAAAGCTTTCCTACAATTGAGGAAACACAAAATGAATTTGAAGAGAATGCAAATTGGCCCTGTGTTGGGATTCAAGTTTCTTTAGTTGAGAAAAAAACACCTAATTGCAAAATTTGTAGAAATGAAGTGGATGGTAGAAAAATAAAAATATGTGGCCATAGGTTTTGCTCCAACAAATACTACCATGTAAGGTGTTTAACAAATAAGCAGTTAAAGTCATATGGTCACTGTTGGTACTGCCCTTCTTGTTTATGCTGTGTTTGCTTAATTGATCGAGATGATGATCAGATTGTTCTCTGTGATGGCTGTGATCATGCATATCACATCTATTGCATGAAGCCTCCACAGACTTCTATTCCAAGAGGGAAATGGTTCTGCAGAAAATGTGATGCTGGAATTCAAGCAATCCACAAGTCCAAAAAGGTGTATGAAAGTAACAAGCCAAGCAGAAATAGGGGAGATGCTGCAAAGCCAAATGCTAATCTTGAAAAGAAACACAGCAATAAACGTGGAAGAGAATTGGAAAGAGGTGGTGCAATGGACATGCTTTTAACCGCAGCCAATACTCTGAATTTTGAGGAGAAAGCGGCGGCAATCCAGTTTGAGTCAAAGAAAACATGAATTTTGGGACGGCTCCTTCCGGTTATTGTTAGTAATGACTTTAACTTCGTATTTGTTGACTGTTAATACACCGTATTTTACATTTGCTACATTTTTTGAATGACTGATATTTTGGTAGGCTGCAAAACTCTCTTAGATTTCCTGACTTTTTGATACATGACATGGAAAGACATGACTACAGAGTCTAAAGAGCCAAAGGCATAATCTGAAACTGCTGTGTTGTAAGTGCCATCTGTGAGAGTAAGTAGGTCAGATACTGTAGTATACATATGGTTTATATGAAATTTATATTTGGCTGCTTTTCTTGTTTCATTCAGAATATTGTATTTGGGGTCGGGATTTTG

>C101791_65

GTTTCCTTTCGGAACCCTCTCCACATTGGAAGATAAGTTTGATCTGCAATTGCATCGCTCATCTCATCGTTTCTTTTTAATTGAAAATTTTTATTCAAAACGCATAAAGCTTTTGTCCTGTTTCTTAAAAAGGTTGTTCTCTTGAGTTGCTTTAAAAGTTCAAAAAAAGTATTTCGCCGGTGCTTGACAAAAGCTGCTCCGTTTGTGGCTTATGGCTATCATATATCTATGATGAAGTTTAAAATCAGCATCTCAATTTTACTCTGGCCCTAGGTACTTTTCCTGCATGAAGTCAACTGCAGGAATCTGTTCCCGCATTTATGTTGATTAACACAATTCGTTATTTGAATTATTATAAACAAGTTTCAACTTGGAACTGTGTGTGGAATAAATTTTAAATTTATGAGCAAGCTTCAATTTTGTTCTAACTAATTGCAGAACGAATTAATGATGACTGGATTCAATTGAATCCTAATTTGTACAATCACGTCCTAGTGCTCTGGATACCTTAATCTTTATGAGCTTGTTCTGTCATTTCAGTGTGTGGTCTCAAAATGTATCCGAGAGAAACCGACAGCAAGCTAGCATTCCACCAAGAGGTTGGAACTCCTATGATTCCTTTTGTTGGACAATATCTGAAGAAGAATTCTTACAGAGTGCTGAAATAGTTTCTCAGCGTCTCCATGATCATGGATATGAGTATGTTGTGGTGGATTACCTCTGGTATAGAAGGAAAGTTGAGGGTGCTTATCATGATTCTCTTGGATTTGATGTGATTGATGAATGGGGGAGGATGATTCCTGATCCAGGAAGGTGGCCTTCTTCTGAAGGTGGGAAAGGATTTGCTGAAGTAGCTAATCAAGTACATAGCATGGGTTTAAAATTTGGGATTCATGTCATGAGAGGGATAAGCACACAAGCTGTCAACGCAAACACCCCTATCCTTGATACAACAACGGGAAGAGCTTACCAAGAATCTGGTCAAGTGTGGTATGCAAAAGACATAGCAATCCCGGAAAGGGCTTGTGCATGGATGCCTCATGGTTTCATGAGTGTGAATACAAAGTTGGGAGCTGGAAAAGCATTTTTGAGATCCCTTTATGAGCAGTATGCTTCATGGGATGTTGATTTTGTGAAACACGACTGTGTATTTGGGGATGACTTTGATTTAAATGAAATAAGCTATGTATCTGAGGTTCTCAAGGAGTTTGATCGCCCCATTGTATATTCTTTGTCTCCTGGAACCAGTGTGACACCAGCCATGGCCAAGGATGTCAGTGGACTGGTCAATATGTATCGTATAACAGGAGATGACTGGGATACATGGTGGGATGTCACAGCACATTTTGATATTGCAAGGGATTTTTCAAATGCTAATTTGATCGGAGCAAAAGGTTTTATGGGGCAATCCTGGCCTGATTTGGACATGCTACCCTTTGGATGGCTAACTGATCCTGGTTCAAATGAAGGTCCACATAGGTTTAGTTCCCTCAATCTGGAAGAAAAGAAGACACAGATGACTCTGTGGTCTATGGCAAAGTCTCCCCTTATGTATGGTGGGGACGTTCGGAAGATTGATCCTAGCACATATGATATTATCACAAATCCTACCCTTCTGGAGATTAATTCTTTTAGCTCAAATAATATGGAGTTTCCTTATATCACAAGTGTGAACAGTGAAGATCAGGATCTTGTTATGAGAAGAAGCAGCCAAGAAATAAAAACAACTTTCACTCATTCATTAGGCCTCACAAGCTGCACCGAGTCAAAGGCAAGCGGTTGGGCTAGTGAAAGTCTTAATCAATATCTTGAAAGAATATGTTGGAAACGGAGTTTAGGAAACAAGCATCTTTCCCCCTTTTGTGTGCACAAAAGAGAACTTTACTACCCATTAGATGAAGTGAGTATGTATCAAGAGTATCATCAAAGGAAATATCATTTAGTTGCAACCAACAGAGTAAAATTTTGCTTGGATGCTTCTCCAAAACATAAGCTCACGTCTAAAGAGTTCAAGAGAGGGACATTTTCTCCCTGCAGGTGGGATTCAAATCAGATGTGGGAATTGAACCCTAATGGGACCCTGGTGAATAGCTATTCTGGCTTGTGTGCAACAGTAGAATCTGTCGCAGATACTATTAATTCTGGTGGCTTGCGCTCTTGGATTGCGGTAGGAAGAAAAGGAGAAGTCTATATTGCTTTCTTCAATCTGAGTGAACAGAAGACGGTAATGTCTGCACAAGCATCTGACCTAGCTAAGTTACTTCCTGGTAGAGACTTCAGTTCTTGTGAGGGCAATGAAGTGTGGAGTGGAGATGCTATAAAAATAACTGAAGGGACGTTGTCAACAGCAGTGGAAATGCATGGAAGTGCGTTAATTGTTCTGAATTGCAACTAGTCCCCAAAAGAAGAAGAATAATGTAAAGAAAAAAATGGCATTTCACTGTCTGGCTTAATTTTTTCCTACTGTATTCCACTCCCTGTGCTTGGATGTTTGTGCCGGGTAAAATATGAATGTAAAATGTGCTCTCATATACAAGTGTTGAACCAGCATTTTGCAAGAGAAAATAAGTTGGTAATAGTTGTGCTGATGACTGAAAGAGGGTGCTGAGGAAAACGCAT

>C101795_65

CTCTTTACCCATAACAACTAGATTGGCTATAAACTAAATAAAATAATTGACATTGCAGGAACAATATTTCTGATGTGTATACACTATAGACAAAACATAAATTGGTTGCCTACATGGTTAATGGGAACTATTATTCTACCGCTCTAGTTCCCACTGCTTTTCCTTTTAGATCCTCCGCGATGTAACAAAAAATTCTGAGGAAGCTTTAGAAATTGGTTTTTCTCTATGTATTAATCCCTATGGACTATACTAAACTGTAGTTCCTTTGTTATCCAACTGATGACAATGGCTGCAATGGATTGGATTCTGGTGGGGAAAAAACAGCAATCAACTTGTCCTTTGTAAGTGGTTCAGATCCTTCGGACTTAATTACAAATGTGTGGAAAACATTATCATTTGCAGCAGCAAGTTTTGATTCTACAACACTGATTTGTGCCTCTTTGAGTGTTTGAATTACTTTGGAAACAGGGTGGGTATCAAGAGGGCAAGTCACCTTTACAATTACCGCATCTTGAGCTGCTTGAATATCCACATCAGGAGGTTCTTTCTCTTGATTTTGATTTTCAGATCTGGAGTTAGCTTCCAAAACTGACCCATCACTGGAAGTGCTTCCAAATCTCTCTCTTTCAGCTTCCATGATCTTTACTTTCGCCTGAAGCTCGTTGATATAGGAAATGGCATCGCCCAACAACGAAGCCTTGTCCATCTTGGAGATATTAGGCACCACAGATCTCAATGCATAGAACCGCTGGTTGAGCTTCTCACGTCTCTGCCTCTCTGCCTCCACATGATTGAGAGGTTCTTCCCTTCCATTAGCAGGCTTCCTACCCCGCTTCCTGGGCTTCCTTTCATCCACAACACTGGGTTGTTCCTCCTTGCAAGAGGCCTCAACATCAGAAAGCTCTGAATCACCAACAACCGGTCTCACAGGAGAAGGCCTAGAAGTAGCTCCGGTAAAGTCAATTTGCATTTGCACCTGCCTTTGAGGTTGATGGCTGTTGCTCAGCCTGAAATCCTGTCTCACACCATTAGCTAGCTCCGGAACAACACTACTCGTTGAAGCCCTAGGAGCAAAAACCTCAACAGGACCAGGCTGCCTCACACCCGAATTCACTGCCCAACCCGCAACATGAAGACCATTCCGAGCATTCTGATAACTAACGTTATTGTTGTCGAGGTTGTTCCCATTGGGGAGGGGATGGCCTGCCCAAGGCCTATTATCCTCCATTTTTCTAACAGCAAGTTTCTCTCTGAAATGCGTCCGATTGCCAATTGCGTTCACAAAAAGCTTGGGAATTGAAACTCCTCCATCTACCTTGTTACCATCCCCAATTGCCAAACCAGTAAAAACCGCATTCCCACCCGCGCTCACGTTCACCGCAGAAGGGTACTGCGAACACTGCGTGGAGAAAACAGATTTAACGGCTTGCACGAGCTCAAAACTCTCGGGTAACACTCTCACAGAACCCAACTCCACCACTCCCAAATCAGTAGGGACCAAGACAATTGTCTGAATTCCAGCAGATTTAACCAAGGACGAACGCACACAGTAGTCAGACACAGATTTCATCCACAAATGCTTCCCAGAGACGAAACACTTACCTGGGCCACCATGCCCGCGTGGGAAAGAGAAGTACATGGAAGCAAGAAAGAACATCTCGGTGTCGGTCACATGGTCGAGGCCAAAAGCGTAATTGTCCTCATCCGAACCTCCAAAAGTCGTGTGCAGCTTCTGCAACACCCTCTTCCTCATCCTCTGCTGCATCTCCTCGTCCACGCGAAGAAGCCTCAGAGTTCCTCTCACCGCACCCTCTTCTTCCTCGTTGGGCTCCCTGCAGCACCCGTCTCCCCAACCCAGAACCCACTCACCGGATTTCGACTGCGATAGCTGCCAGAAAATCGCGTAGTTCCAACTGAAATTGGAGACGTTGGGACGCTCCACCAGATCCGAGAGCTTGTTCTGAAGCCCCTCATCGCTCCCAACCGCCATTAGAACACTCTCATTCGACACCGAGTTCGTGGTTAAAAACTCCAGAGCCCGACCACCCAGAACAGCCCCAACCATGGCCTTCTCCTCCTCATTCCACGCCACGCGCCCTAACCCCGCGTCAACCTTCATCTCTTAAGAAATAAAAAAATAAATAAAATATACCACCCACCAACTAATTATCTCAAATTTTCAACCCCTGAATCCAACACCAGGAACCTCCTCTAATAACCGAAATGCAACTGTTTCTCCTCTTCCATCTCATGAAGGCGCTCTCAGATCTTTTAGATTTGAGGTTGGCGAAGGAAAGCTATAATAAGAACAGACAGAGAGACAAAGATAGAAACAGGAAGTGAGATTTACTTATGAGGTTAAGCTGGGAAAGGGGGGACAAAACTGGACGAGAAAGGAAAGTTTTAGGGGGAATACACATTCGGAGAAAACTGATAGTGGAAGGAAACAAAAGGAGAGACAAGGAGGTGTGTCGCGAAGGAGGGAATGAAAACGAAGGGTTTTTGTTGCTTGGTCGGTAACAGAGTGTGAAAATTGGAGAAAGGGAGAAGGAAGAGAGAAACGAAGGGCAGAGAAACAGAAACAGAAATTCGAGAAGAGAGAGAGAGAAATGAAAGATGC

>C101803_65

AAAACCCTCACTTCCCCTTCTCTGTAACCTGTGTGTGTGCGACGACGCCGTTGCAACCTCCTTTCTTCACTCTCAGAACTCCAAAGCAAGCAATCACAGACAGAGTAAACAACAATGGCGACTCGTGCCGGCGGCATGAAGTCGACGTCAATTAACGGCGTTAAGATGTACACGATAGCGTCGCAGCAACCCTCCCTTGCTTCCTGGCTTCCTTCTAAGAAGCAGAACTCTCATCGCAACGTTAAAAGTTACACGCAGAACCTGCAACTGCTCGAGGACTTGCGCTTCGCCACCGCCGCCACCAAAATCAAGGCCACTCCCGACGGCGAGTACATCATAGCTTCCGGCATCTATCCGCCGCAAGTGAAGGTGTATGAAGTGAGGGAGCTAGGGTTAAAGTTCGAACGCCACCTGGATTCCGAGATCGTTGATTTTCAGGTTCTGACAGATGACTATTCAAAACTTGCATTTTTATGTGCTGATCGCTCTGTTTACCTGCATGCGAAATATGGAAAGCACTATAGTTTGCGGATTCCGAGGATGGGGAGAGATATTACTTATGACTGCTGGTCTTGTGACTTGCTTTGTGCTGCGTCATCCCCAGATTTGTACAGAATAAACTTAGAGCAGGGCCGATTTCTCTCCTCTCTTAGCACACAGTCCCCTGCATTGAATGTAGTTTCTCGAAGCAAGATTCATGGATTAGTTGCCTGTGGTGGTGAAGATGGTGCTGTGGAATGCTTTGACATGCGGGTGAGATCTTCAGTTGGTAGAATCGATGCTGTTGGACCTAGTGGTGATGCTGGCCAGGAGGTCACTGCTTTGGAGTTTGATGAAGATGGTGGGTTCTTAATGGCTGTTGGAAGTAGTGCAGGAAAGGTTCTCATATATGATCTGCGATCATCACATCCTATACGGATACAAGATCATATGTATGGCAGTTCAATATTGGATATTAAGTGGCATCGTACTCTTAACTACGAACAACCAATGTTGATTACCAGTGATAATCATGTTGTTAGGATATGGGATCCTGAAACGGGAGAAGGCTTGACCAGCATTGAACCAACAACAGGAACAATAAATGACGTGTGTACATTTCCTGGCAGTGGTTTGATATTGCTGGCCTTGGACTCTAGTCAAATACCATCTTACTTCATACCATCGCTTGGACCAGCACCTAAATGGTGTTCTTCCCTAGAGAATTTCACTGAGGAGCTAGACATGGGTGGACAAACAACTATTTATGATCATTACAAATTTTTGACCAAGGAGGAGCTTGAGAGATTAAATTTGACCAACCTGATTGGCACCAATCTACTTAGAGCCTACATGCATGGCTTCTTTATTAATCATGCATTATACAAAAAGGCAAAAGCGCTGGTGGATCCTTTTGAATATGAAGCTTATATTGAACAGCAGAAGAGAGAAAAGATAGAAGCTGAGCGTGCCTCACGAATCACGGTCAGGAAAAAGTTACCCAAAGTTAATCGGGCCCTTGCAGCTCGCCTCCTAGAAACTGAAGAAGCTGAAAATGAGAAGAGAGATGCTGATGATGATGAAACTAAGAAGGCATCCAAGAAAAAGAAAGGTGTTAGCATGCAAGATCTCCAAGATGAACGATTTAAAGCGATGTTTATGGATAAGGATTTTGAAATTAACGATTCCTCACAAGAATATCTGGCTTTGCATCCCATGGGTTCTAAGAAGCAAACATCCTTGTTAAAGGAACATTTTGAACCTGTCATGTCAGATGATGATCAAAGTCTAGGTGATTCTGATGCATCAACATCATCACATGATGAACCTGCAAATGGAGTGAAAGACAAATCTCGGGTTCCTAGAATGTATGAAATTAAGAATGAGCAGCATGCAGAGGCATTCTGGAGCCAAAAATCATTTGCAGGGGAGGAGTCACTTCCTATGGGAGATAGAGTGGCAGCTCTCAAATATGACCAACAATCTTCTCGTGTTCCAAATGGTGTTAAGCAGGGTCCAGGAGGATCACGGGAGATCACCTTCACCTCTAGAAGCAAAGCTAAGTACAAGGAAGATGAGGAAGATAAAGAAGTTCCACACAGGAAAAAGAGGGGAGTACAATCCTTGGGGCTTAAGCCCCATAGACCAGTGTTTCGTGGTCAAGGGAGAGGGAAACGAGGGAGCGGCAGAAGAGGACGTCGATAACGACCTTAGACACAGTAATTTAATTCTTCGTAGATCTTGCTTCCTTGATCGTTATGGCTCAGTTTTCCATCCATGTATGATCTTGTGGGTTGAAGGCGCACAGGGTAGTAGGCATGGCTGCAGGGCCAGACGACCTCTGATACATATTATGTAGCTCAATATCTTAAGGTTCTTGTTTACATTTTTTATCTTTTCCCCATTTTTGGATGCTCTATCAAGATAAACTATTTTGTGTGGAACCTTCCTTAATTACTACAATTTTGTTGTTCAAAAGCCGAGGATAGGAGAGGAGGTATGAGTAATTTCAAATGCTAATCATAGGGATTTATCTCCCTTTATGTGAGAATGTAACATGTACTCTCAATTTTGTGTTTTAAGTATTTTCACCCATTGGGCCTGAATTCTCTCCTTTATGTTTAGTTGTGGGTTCGACTGCC

>C101807_65

CAGAAGTCTACATGAATATCTTAGAAATATAAGAGCAAATATAAAAGGTATATATTTTAGAAGCCATGACTTAGCTATTGGTTATGTTATCCACAGATCACTCATTACCATCGTAATAGAAAACAAGTTTACTGGTTAAATAGAAAGATAGAAAGACTAATGCCAATATCCTATTTTGTCAAGACTGAACACTTGCTTTACCATTATCATTTTCCACAGCAATCTTGTTTTCATAATCATCCTTGGATGAGGATTCCAACTCTTCCCTTAGTCTCTCAATCTTTTCCTTCAGTGCTGTTACATCCAATGCAGCGAGAATCCTCTCTTTAATACTTGCCTCCTTCTTTTCCAAATCCTCAGAATTTGAGCCTTGAGCTATCTCTCTCTCCAACTCCTTGATTTGGCCCTTGAGACCCTCTTCATTTACCACTCTATCAATTTCATTCATGATCTCATTCTTCAAATCAGTTATTTTCTGTTTTATGTCTTCTGGTGCACTCACCACACTTCTCTCCATTACTCCAACAATTTGCAAGTTAGCTGATTTCAACACCTCTTCCAACTCTTTCTTGACTTCCACAGCTTTCTCTACCAATTCCTTGTCTGGTGATTCCCCTTTTGATATCTTTGCCTGAGCAAGCTTCAAGCTCTCTATTTTTGCTTTGGTATCATCTGCAAGTTTCTGATTGAGTTCTTGTTTCAATTTTTCTTGTTTGAGTTTCATCTCAATAAGCCTGTTGACAGTGTCTAGTTTCTGAAGCTTCTGTTTCAATTCAAGATAAGCCCCTGGCTGAGACAACTTGGTCTTTATTTCTTGCATTATTTTCTCCACCTTCTCCTGTATAAGGGGTTGGTTTGAGGAGGCTTTAGACAATTCCATCTTCACAGATTGAACTTTTTCTGCTAACCCCATTGAAATGAGTGCTTTAGTTATCTCCTGATCTGCTTCCTTCATAAGCTTTTGTATTGCTTCCTTTGAGAAGGAATCAAATTCAGTTGGTCCCTTTGCTTCTAAAATTTTCTTCTTGAGACTTTGGAGCTCTGACTCAATATCAATAATGTTGGATGACTTGACCTCGGATGGTTTCATGTTGCGTTTCCTCTCTGGTTCCACAGGTATGCCTTCTTGGAAGCGTCCAATTGACCGAAACTTGAGATGTCTATGGTGTAGCAACTCTTCTGTATCCATTTTTGTCAACTCCTCCATGGCCTCAGTGATTGCATGCTTGATTTGTTGAGAAGTCCATGTAGGATCAGCATGTGCCCCACCCAGTGGCTCAGGAATGATACCATCAGCTATGCCGAGTCTGTAATGTTCTTGCGCTGTTATTCTTAGCTTTTCAGCTGCCTTAGGAGCAGCTTGGGAAGATTTCCACAATATTGCAGCACAAGCTTCAGGACTTGCAACATAAAAGGCAGAATTCTCCAGCATAAACAATTTATTAGCACAGGCTATGGCAAGTGCACCACCTGAACCACCTTCCCCAGTTACGACTGTTATTATTGGTACCTTAAGACCAAACATAGTCCTCAAATTCTTGGCTATTGCCTCACCTTGACCAAGTTCCTCAGACTTGAGATCAGCAAAGGCACCAGGAGTGTCAACAAATGTAATGATGGGGAATTGATGGTGATCAGCATATTTCATCATGCGCAATGCTTTTCGGTATCCATGAGGAGTTGGCATTGCAAAGTTCCGAGCAATGTTTTCCTTTGTGTTCCTGCCTTTCTGATGACCAATGAACATGTAACTCTTTCCATTCATACTCCCTATACCAGTCACAATTGCTGGATCATCATAGCCAGCACGGTCTCCATGAAGCTCCATCCACTTGTCTGTGATATTAAGTATATGATCAAGTACTGTTGGCCTATTCGGATGCCGAGCAATCATCAGCCGTTGAATAGGTGTTAAGTGTGTGTATAGATCTTTCAGAGCCTGTTGATACTTACTCTCCAGAGCCCCAATTTGATTACTGAAATCTAAGCCTGTGTCATCTGCCATTCTACGTACCTCTATAACCTTCTTTTCTAGATCAATCAGTGGCTTCTCAAAATCCAGTGTCACTGTTTTTGGCTTCTCTGCCAAAGGCTTAAAGTGAGAAAGGTAAGTTAAATGTCCACTATTGATAATTGGATCCATATTATCAGGCCATGGATAATCATGCTTCTTTCCCTTTCTAATCTTTGCAACAATATGAAACTTTCGGTCTTTTTTATTCCTTGAAACGCTTAAATACTTTGGCCAGATAACTATGGCCCTTTTGGATGAACATCCAAGAAAATTATTATCAAGGTAGTTTTTTCCAATGCATGGATGTATCAAATCAATGGGACGATCTTCAAGTCTTCTTCCACTTATATAATTTTCACATATCAGTGATGAGGTATTCATCTTCATCAGCTGAAATTCTTGTGCGTTGGATGTGTTTAGGCTTCCGTTTTCCTTGGAAAATCCTAAGAATTCTGAGCTGTGAACTTCAAGAACAGAAGCTTTTGAATGCTGATTAATCAGTGATGAATTATATGAAGTGGGAAGACAATAAAGATGGCAAAAATGGACGGTTTTTTGCTCTGTCTATGATTTCGGATGCTGTTGTGCTGTTTCCAACTCACTGGGAG

>C101815_65

TTTTTTTTTGGTATAAAAATATCTTTTTCACATATGAAGTATCAATAATGATACATGGTTTACAAATTACAATTGTCACCTATATCCTTATTTTACATTTGCATTTTACAGGCAAATAATGTCTTTGTTTTTGTTTATTCTTGTTCTTTGATATGTTTTTGCTATTTGCAAAAAGTTGGCTGCTCCTGCCAAATTTGCTTGACAGTTAATTAATTAATGGCAGAATGAATGGTATTCAGTTCTTGATTTGATGCACATGTTGGACAGAAATAGAATGTTTGAGATCACCAATGTGGAGTTCATGTTGACCGGTTGGGATTCTTCGAATCCCGAACTGGTCCACGACGGTCAAGTGTTTGCACACATGAACACCAATCTTCACTCGCTGCTTTGACCCAGCAGGAACATGGGTTTTCTGGAAGTTCACCAATTGTTTAATTTGAGACCATTCTCCTCGTGGTGGTTTCGAGAATACGAGAAGGGTGTGGGTCCCATCCATGGAGCCTTCGTTTTTTACATCCACGTGGAATTCCATCTCCGATGTGTCGTCACAATTGGCGTGACTCACCCTAACTGCTTGGCTTGAAAGGGAGGAGTTAGAGAAGGCTTGGAGGGTGGTTAAGGAGACCGAGACCTGTTTTGGGGCAACGGCTAAGGTCTGGGTGAATCTTGAGTAACTTAATCCGTGTCCGAATGGGAACACCACGGGTCCTTTATAGAATCTGTAGGTTCTTCCTGGGTACCCTGTTGCTGGGTTTGGACGCATGTCCATGATCGTCATCGGCACTTTAGCCAAATACCCTTCTGGATACCATGTCATGGGTAATTTTCCACCTGGGTTAGTTTGACCGAAGATGACGTCAGCTATGGCAGTTCCCCCAGCTTGACCAGGATAACCAACCCACAAAATAGCACTGATCTTTGGGTCATTTACAGCAAAGGAGACATCGACTGGGCCACCAGACATGATGACCAAGATAACCGGGCCTCTGGCGGCCCTAGCAACCCTAGCAACAAGCTCCTGTTGAAGGCCCGGTAAGAGAAGCCCAAGTCTATCTCTTGTTTCGGCTTCTACGGACTGGTCAAGGCCCATTACTAACACAGTGGCATCAACTTGCCTAGCCACGGTCTCCGCTGCTCCAAATAGTTCGTTCCCCCTACAAGCCACACCCCTACACCCCTCCTGATGAGCGGTCTTCACATACCTCCCAATCCCTTGCAAAGGACTTGTATAACCACACGCCACACCAGCATAGTTCCCTATCATTGTAACGGTGGCGTCCGAATTGGGTCCAATGACTCCTACGCTACGGATTCGCGTAGGGGATAGTGGTAGCGTGTTTCCTTTGTTCTGTAACAAGACTATACTCTCCCTAGCTGCTTGAAGGGCCAGTTGTTGATGGGCAGGGGTGCACACGTCTCTTGGGCCCAAGTTTCCGTATGGTTGGGCTGAGGGTTCGCCGTCGAACATGCCCAATCTCATTTGGACAGTGATAAGGTTGGCCAAGGCAAGGTTAAGATCGTTTTCGGAAATGAGTCCCTTCCTAATGGCAGAATCGGTGTGTATTGCCAAGAATGGCCCACAGTCCAAGTCCAGGCCTGCTTTTATGGCCTCCGCTGCTGCCTCCTCCGGTGTTCTTGTGTAGTGTTGGTTGTCATAGAACACTCCAACTGAGTCACAGTCCGAGACTATGTACCCATTGAGGTGCCATTGGCCACGGATGGTGTTGCGAAGGAGATCAGGGTCAGCACACGTGGGCTTCCCATTCACCTGATTGTAAGAACACATCACGCTCGCCACTTGTCCCTCCAACACACAGGCTTTAAATGGCACGTCATAAGTGTCCTCCAGATCCTGTTTGCTCACCTTGGCGTTGAAATGAAACCTGTCTACACCATTCCAGTTATCAAGATCATAGGCAGTGTAGTGTTTACAACAAGCAGCGACCTTCAGGCGGTTTCCGCCACCGTCTCCTTGGAGTCCTCGGACATAGCTGGCCGCGTATTTTGCTGCCAAGGTTGGGTCTTCGCCCGGAGTCTCTTGACCGCGACCCCACCGTGGGTCGCGGAAAATGTTCACATTAGGACTCCAATACGTCAATCCAGCCTGTCCCCCGTTGTACATTGCTCTTGCTTCATCAGACACCACCCGTCCAATTTCGCGCCAGAGAGATTGATTGAAGGAAGCAGCGGTTGTGATGACTTGAGGGAACATGGTGGCGCCAGGGAAGGACCCACCGAACTTGGTGCCGGGGCCCACATTGGAGACGCCGTGAAGAGCCTCGGACCACCACTCGTAGCCTTGGATTCCGAGCCTGGGGATGGCGATGGCGTTGTTGACGACGAGTCTAATCTTTTCAGGCAATGTGAGTCTGGCGATGAGGTCCTGCACCCTCACGTGGAGGGGCACGTGCGTGTTGCAAAACCTGAAACCCCTGGTGAGTCCGTTCCGAGGGTCGCATGCGAAGGGCACCCTCCCTTCGGCGGCCCTTGGGAAGGACAACGCGACGCAGAGCACTAGCGCAATGGCTAGTAATGGGGTTTTGGTGTGGTTTTGAGAAGCCATGGAATGAAGATGAAGAATGAGTGAGTGAGGAGTGATGGTGAGTAGGGTGGGTTATATAT

>C101819_65

CAAAGCTTCTGGTTTATTCAGTTACCACACATTGCAAAGCACATTAAACCTAATAATTCATGAGTGTAGTGTCAGTACAACCTGTTGAGCACTCCACGTACCACATCGATGAAAAGCCTATGATAGACTAAATCCGCACAACTGATAGTAACAAAAATCATCACCAAATTCAACAGGACAGGTGCAAGCCCCATAATATATCATTCATCATTGGAAGACTTGTGTATTTGAACATTTAAAATATGGATCTAAAAAAGATTTCTCCAAAGTCGTCTCAGCCGGCAACTTTTCTTGTCAAACTTCTCTCAGTTGGAATTTGTTGTCCGGACCTGAGTATGAAGTTTCCAGATTCAGAACTCCAATTGACTCCAGCCTTCATTGGAGGCTGTGGCCGGCTTTGTCCCAAAGACTGTCTTCGTGTACCAATGGACATATTGGCCAACTTTTCAGCTGTTTCTGAAACCCCGCGTGCAGTCCTCCCCTTGTTTATAGTAGTCGGGCTGTCTTTTCCGGGTAATCGATATTTTGATTGTTGAGTAGTCTTCAATGACTTCTTGTTCTTAATTCCATCCTCGTTTGCTAAATTCAGCTTCCGTTGCACACCTGAAGCTATAGAAGCTTGTAATTTTGGTGAAGAAAAATTGTTGGTGATTTTTGTATTAGGCAAAGCACCAGAGTATCTCTTGAGCCCCTGCCGATCCAGTGAACCCCTGGTCCCAGCAGATGGAGGAGTTCTGGTCATAGCTCTTGTGCGAAGGGATGGAGGGATGTAAAAGCAACTCTGAAAGAAAGGATGTTGAAGAGCCTCTGCTGCTGTTGGTCTCTTGCAGGGATCCCATGAACAAAGTGATGTAACAAGGCTGATTGCATCATCACTTCTCGATGGGATCAGAGCAGAGAGATGTACACTAGCAAGCTGCGGAAACTGATAGTTGATATCCCTGGCAAGCTTAAGTCCATCTGCCCAAGATTCAGTTGTTGGACTACCTATTACACTGCATATTTTGTAGATCTCATCTGCTTCACTGGTACCGGGGAATAGAGGACGGAGAGTAAACAACTCAGCCATAATAGCACCCATTGCCCACATATCAACTTTAGAACCATACAGATGGGACTGAAGTAGGACTTCAGGAGCACGATACCACCGTGTGGAGACATACTCAGTATAGGGTGGTTGTGAACTGATCTCTCGGACTAGGCCAAAATCAGCAACCTTGATGACATCCTTGGTGACCAGCAAGTTCTCTGGCTTCAGATCACGATGAAAGTATCCACGCTGGTGCATGTAAGCTAAACCTTGGAAAACTTGAAAACACCAATTTCTAACATCATTTTCAGAAAATGGCTTTTCCCTGTTTTTCATAAGTTGGTAAAGGTTGTATTCCATATACTCAAAAACAAGGCATAGAGTGTCACATTCTCGAATAACTTCCTTCAGCTTCACAATATTTGAATGATTCATTTTTCGCAATGACTTGACTTCTCTCAGGTTTACACACTCCTCCCAAGAGTAATATTTCTTCTTCATTTTTTTTATTGCAACAACTTCACCACTTTGCTTATTAATGGCTCTCCAAACACTCCCAAATGTTCCATCACCAACTTCCTTAATTAACTTGTACCTCTCCATTTTCCCACAGAACCCAAAGCAGCTCTTATTCAAAAAGCAATGGCAAACACACTAAAGATAGATGGCATTTATATCTAGCAGACAACGCCAAGAAAAAGGTAGAAAATGTTGACAGATATTAGATGGTCCAGCAAATCTCACTGTCAATTCTCTGTCATGGCAATGAAATAAAAAACATAAACTTTCTGATGCAAGGGCAGCATAGCCTTATTGATTAATAGACCCACAGCTTCGTAGTAGTAGCAGGATGCATGTGGTAGAATCAAATTAGTCCTGGAGAACATACTATCAATTCCCAGGTCCAACCAAAAATGATTGATCAAAGCAAAATTAATCTTCAGCTAAATTCATCAAGATCCAAGATTTGAGGACTTCTATAATAGTCACTTCATCTAATCTACATAGTTTCACATTTGTCAGGTAAATAATATTTAGTCACCACCAATATCCATTAGAAACAACACCGTGATGGTATATAAGTACTGTCTGCACTGTTTCAACGCAATTGTGTATCTAATATACTTGAGAAGCAGGCAGCATCCAAGATTCACACATCTATATACTTCAGCCCCCAGAAGTGGACTTTTCAGATGTTTCAACTGGATATTGGGTCAGCAATGCATTGTATGTGTTCCGTAAAGATGCAGCACTGGTCTAAGAAGAAACAAGCATTGAGTACACTGTGCCGATCATTAACACAGAACAAAAAACTAGGTGCAATTGACAAATGAAACAAGAAGAAAATCTCCAAGGGCGAAGTATCGGCGGTCAAGCACTTCTTGGAATGAAAGCAACCAATACTGGAAGCAACTTGACAAGAAACAAATTCCTTCCATGTCTCAAAAAATGTATCAGGAACCTTAAGATTCAAATCCAGATCACCCTGAAGAGGTCGAAGGAAGAGGACCGTCACGAAGGACGGAAAGAGGTGGAATCCAGAGTTTCCCTCTCTCTCTCTCTCTCTGCCACCAAACCCAACCCAATGCCAATAA

>C101821_65

TAAACTTTGAAGTTTGACCTCCCGAAATTGGGCCATTTTCCTAACCCTAGCCACGCTTCACCGCCATCATCGTCCACCTTCCGGAAAAGAACCAACCCCAATCTTTCTCACTCCTTTCCCCTATCTCCAATTCACAACTCTCTCTCTCTCTCTCAAATTCGAGATCCTTCCCACCCATCTCTCTCCCAACGAAACCCTATGCACCGAAGATAATTTCGAGTTCTTCTACCCACCTGGAGTTCTGTGGATCCTTCTAAAGCCAGATCGAGGTAAACCCTACTCGATTCAGTGGTTTTTCATTTTCATTTTTATTTTATTTTCCTGGCAATGCAAATTCGGAGTTGGTGGTGACTCACGCAAGTTATGAATTTGGACGCTTGAATTTGTTGGTTATGCCCCCAGTTGGTTCACTCGTTAAGATCGAGAGTTAAAGATAGGATCTCTTCATTTTGGTATTGAGGTCTTTGCATTTGTAATTGTTTTGCCCCTTTGTGGTTGACCAGTGAATTAGGTTTGGGTAGTGATGTCTGTGAACAATAATAATCTTTCTAAGGGCATTGGGGCCTCTTCCTCATCCTTTGGCAATGCTGGAATACCCTCCAATTCTATACCTTCAAACCATGGTTTTTCACAGTCCCAAGGACAAGCCCAGATTCCCGTTGGTTTTCAAGGGCAATTTCCAATGTCCCAGGCCCATGCTATTGTTCAAGCTCAGTCCAAGGCCCAGGCTCAGGCCCAGGCCCAGGCCCAGGCACAAGCAGCTGCAGCCCATGCTCAACTCCATGCTCATTTGCAAGCACAGGGGTTGAGTCTAAATCAGAACCAGGGTGGTGGTTTGGGGACGTCGCCTATTTCCACACCGGGTAATGCAAGTGGGAAACGGATCCCTCTGAAACCCCCAATTCGACCAGTTGGATTTTCCCCTATCAACAGCTTCTCGCCTTTGAGACCGATGGAGCTCACACCTGCTGCACGGAGGAAGAAGCAGAAGCTTCCTGAGAAACAGCTGCAAGATAAAGTGGCAGCTATACTGCCCGAGTCTGCACTGTATACACAGCTTCTCGAGTTTGAGTCTCGTGTTGATGCTGCACTTGCTAGAAAGAAGGCTGACATCCAGGAAGCACTCAAAAACCCGCCCTGTATTCAGAAAACTCTTCGCATCTATGTATTTAACACTTTTGCTAATCAAATTCGCACAATTCCAAAGAAGCCTAGTACGGAGCCTCCAACGTGGACGCTGAAGATAGTTGGAAGGATATTGGAGGATGGTGTAGACCCTGATCAGCCAGGAGTGGTTCAGAAGTCGTCTCCTTTGTATCCAAAGTTTTCAGCTTTCTTCAAAAGAGTAACCATTTCTTTAGATCAGAGACTATATCCTGATAATCACATTATTATGTGGGAGAATGCTCGATCACCTGCTCCGCATGAAGGTTTTGAAGTAAAGAGGAAAGGGGATAAAGAATTTACAGTGAATATACGGCTGGAAATGAATTATTTGCCAGAGAAGTTTAAGCTTTCGCCCGCTTTGACAGAAGTTCTTGGTATTGAGGTTGATACCCGCCCAAGAATTGTTGCTGCAATCTGGCACTATGTGAAGGCCAGGAAGTTGCAAAACCCAAATGACCCTTCTTTTTTTCACTGTGATCAGCCTCTTCAGAAAGTATTTGGAGAAGAAAAGATGAAGTTTACAATGGTTTCACAGAAAATATCTTCGCATTTGTTCCCTCCCCAGCCTATTCTTTTGGAACACAAGATCAAGCTCTCGGGAAACACTCCAGCTGGGACTGCTTGTTATGATGTGATGGTTGATGTTCCTTTCCCAATTCAGAGGGAGTTGTCTGCTTTGTTGGCTAATGTGGAAAAGAACAAAGAGATTGAGACATGTGATGAAGCTATATGTGGAATCATTAGAAAAATTCATGAGCACCGTAGGAGACGGTCATTCTTTCTTGGTTTCAGTCAATCGCCGGTAGAATTTATTAATGCATTGATTGAATCTCAAAGCAGGGATCTGAAACTTGTATCCGGTGAACCTAGTCGCAATGCAGAAAAAGAGCGTCGATCAGATTTCTTCAACCAACCATGGGTTGAAGATGCTGTTATTCGATATCTGAATCGCAAACCAGCTGCAGGAAGTGATGCTCCAGGAAGCACATGACACCAGGAGTTCTAGGGCCCCATATTTAGTAATTCATCCCTTTTGGCGATGTATTTGAAGATATATATTATGAGAATTTCTGAAGTAGCTAGTTTTCCAACTTCCATGCTGTATTAGTGACCGGAAGGAATGCTGCTCCATAGTAATATATACAATATGTTGTTGTATGGGTTAGTGTGAGCACAGGTAGTTGCAGTGCTGAAAATTGTTTGTTAGGTGATAAATGGTTCCACAGTTTCACGTCTAGTTGTAGTCAACTGTGGTGGATTCTTTGGTTACCTCAACAGTTCATTTCTCCGCGCAAGATACTTATCATTATGAAAAATTGTATTAGGAAGGATTGATTGGTGTACAGTATCTAAAGTTGTCTTGCGACAACACGAGAAAACAAATGTTGCAACGCATGGCTCGTATTTCATCCATCCATAGGTTCTGTTGACATTGAATTTTGCACTGTTACTTGCCCAA

>C101825_65

GAACCAAAACTGGAATGGCGGCTATGACTGGAAAGAGGATTGATAAGCTGAAGAATAATAATGAGAACTTGCATGAAGTTCGTTCACATTTATGATTATTAATATATACATAATCTAGACAGATAAAAATACCCTATTTTACTTAGCTGCTATCTCCATTCCACCCACCCAGAGATTTGGTGTGAACTGTCCCTAGCTGGTTCTACTACTCCATGAAGCTTCCCATCTTGATTACCTCAAAATTCATACTAAGCTACTACCCAAAAAAGAAAAGAAATTAATATTATTACTATTTTACAGAAACAGCTACCGGGAAGGCTTAAAATATCCCAGAGCAGCACAACTTACAAAGAGCTAGCTAGCTAGCTAACATGACAAACACATATCATATAATCTCATCTCAACTCAACGCCGCAAAGTGCAGACTGGTTATCATCGTACATCCCCAACCTTAGCCGATAGGGCCGAACGAAGCTGCTCCTGCGTGTAAAACCTACTCCCCATCTTCACCGTCGCTTGTTGGATCATCAAATCATTCACCACAGAAACGCTAGCGTGGTGCACGTCCAAATCCAACTCCATCAGCGCCGCCATCAACCTCGCCGCGGGGTGGTTCTTCTTGCTGCATTGGATCCTTATCATTGCGTCCCACCCAATAATCTTCACGTCGATGTCCAAGTCAATCAACGCCTGATTCGAGGACTTAATGTTACTATTACCCGCGTGCTTAGTATGATTAGAAGAAACGTTGTCACTTGATTTCTCGAGTTCCTTTTTCACGCCTTCAAGTTGCTTCTGCAACCCATCTTTATCTGATTCCAGGGTTTGAAGCTTGGACTTCAGCTCCGTGATGTAAGATATGGCGTCGCCCAAAAGCGACGCTTTATCCATCTTGGACACGTTGGGAACCACAGCACGAAGCGCGTAGAACCTCTGATTCAGCTTCTCCCTCCTCTGCCTCTCCGCTTCCACGTGATTCAACGGTTCCTCTCTTCCATTCGCCGGCTTCCGCCCTCTCTTCCGGGGCCGCTTCTCCGGCTCCACCACAGGATCCTTTACCACCGACGCTTCCAGATCCGAGTGGTCCGAATCGCCGCCGCCACCGCCGCCAGACTTCAAATTCGTGGCCGGAAGAATCGTTCGGGAGGTGAACGAAAGCATTCCGTCGTCGTTGCTGCCTCGCGAATTCGGCGACCGTCTCTTCCCGTTGTTGTTGTTATTGTTGTTCTCATCCGCAGCAGCAACAAAAGGTGATTGACCGGAGAAGAAATTGGAATTGGAATTGGAGTTCCCATTGACGCCGCCGCCGTAAGAGGTTCTCTTGCTCTCGCCGAAGCTGAGAATTTCGCAGGATTCGGGCTTCAGGGAATGCTGATTGTGAGTGTTGCCACTCTTGGGATCAAAACCGTACTCAGAGAAGTTCAATTCTCTGGAGAACACGCTCTGATTCTGGGGAATAGCGTGGATAGAGCTTGGGGTTTCAGTTAGGGTACTCGAACCGGGGGTTTGAATAGAACTCTCCAACTGCATCGTCTTTGAAATCCCGTGAGTACTGTTAGGAGGGGGAACCGAAACAGAAACGGAAACAGAAGGGGTTGCAGCGACAGTGTTAATGGAATCCCTAACTTCAGGATCGTTGAGCCAAAGCGAGGAGGGGTCGTTTTCTCCTTGGTCGGCGCTGGTAGCAGGCCAAGAAGAACCCATATCGAAGTTGTTGTTACTAAAATTGAAGAGAACCTTCACCTTGTTCATCAGATCCGAGTTCTGGTAAATTAACTCAGTGGAGCCCAGCTCAACGACGCCGTTCGCCGAGGGTATGCAGACTAGGGTTTGGAGGCCGAAGACTTGTCCTTGGCGGGCTCTCTCGCAGGTGGAGGCGGAGAGGCGGTCAGCGCCGGTGACCCAGACGGGGGTGGAGTTGAAGAAGGCCTGGCCGGGCAAGCCAGCGCCGTTGATGAAGGACTGGGTCATGGAGACGAGGAAGAACCACTCGGTGTCAGTGACCTCCTCGTCTACGTCGTCAGAGGCGGCGGAGGAGGAGCCGGAGATGAGGGAGTTAAGCTCGCGGAGGACCTTCTTCCGGTGTTCCTGCTCGGCGGCGGAGGTGGCTTTGGCTTTGGCCTTGGCCTTGGCCTTGTCGTCGTCGCCTTTGTAGTAACCGTCGCCCCAGCCGAGGAGCGTGGAGCCGGAATAGTCGTAGGAGGACTGCCAAAAGATGGCGTAGGTCCAGCTCTCCCCGGGGCCCTCGATGAGGGCCTGGAGGCGGTGTTGGAGCGTGTCCTGGTTGAAGACAGCGGCAGACTGCGGCGGCGCTGGCGGGGGCCATATGGAGGAGAGATCGGAGGACGACATGAAAGCCTCCATGACTGAGGAATTATCGTCCGTCCAGAGATTCATCCACTCGCGAACGCTGAGTTGGTTGATGTTGGTGATGGATGATTGGGGATTGGGGATTGGGGATTGGGGATTGGTGATTGGGGTTTGTGGAATTGCAACTGGATTGGATTGGGAGTGGTAGAAGTGGAGAGAAAAAAGAGGGTGAAGAGGGGATAAAACAGAAAGCTAGCTAGCCAACAAGAGAGAATGCATTTGGGTTTTGAATTTATTTGGGCTGCATTTAGGGTGAA

>C101833_65

GCTGAGTAGTAGTTTACTTGGAAAGCTACTCCAATAAAGTCCAAAACACCCTCAATAATAATAATAATAATAATAATATAACTCAAACGTATATTTCTTCACGCATTTGTTAGTGCAGGCAACGAGGAATGAGTGAACACTTTGGCCCGAAGGAGGCAGTAAAATTGTCTTTCCCATTAGAGAGATCTGACAGTAAAGTGAATTCCTTTCACATAAAAAAAGAAATAATGAAGGACTACAAAATTGGGTTTTACAAAGACGAAATCGACGTAAGCTTCCCAACTAAACCTCGACAAGAATAAAATGTTCTGGTCCAAGTCAAGCTAAAAAAACTCACTCTTGACCGTATTCAAGGTGAAGGACCTCTTCTTTTACATCAATGACTTCCTCCACTTCAATGTCCTTTGCCTCGTATTCCTCCTCTTCATCGCTAGCTTCCCCGTCCCTAAGCTTTTGCCTCTGTAAGCTCTCTTCTTCGTGCATCTGCTTCCATTCATTGACCCACTTGTCCCAATCTTCTTTCAACATCCTACGTTTCTCACGCTCTTGCTCACTCAGAAGCAGCGAAACATCTTGATCTTCTGCCTCATATTTCTTGCTGTACTTCTTCAAGTTCTTGGCAATCTCTTCCTCTTTCTCGGGGCTCAAGAAAGATGGTGGCCTTGGTCTCCACAAGAACTGAAAGAAGTGATCCTTGAGAATCCGATATAGATGCTTGCCATTGAAAGACCATATATTGAAACCATTTTCCATTTCATGAACTGAAGTCACAGCAGTTGCAACATACCTTCCAGTTGGATCCCATTCAATATCTGTTGCCATAAAATGTTCAGCAGTAGCCATGGTTTCAAGTTCATCAACATTGTAAAATTCCAACTGCCCATTGAACCCTTTCAACCCAGCTAGTACAATGTAGCGTCCCGCAGGTGACCAAAATAGAGCATTTGCTTGCTTGCCTTTCAGAGTAGTGAGTTTTGAAACTCGGCTGTTCTGACCAGTACGCATGGAGTAAATGCTTACATCAGGCTTAGGGTTATCACCATGAATAACGGCAAACCTATGGCCTTTTGGCTCCCAAGCAAACGCAATGATCTTATCATTCTTATTCTCAAGCTCCAATACTTCAATTGGTATATCTCGTTCTTTTATGCGGAAAAGCTCAAAGCCAGTGTAAGTGCTTTTTTTCGTTTTAGTATAGCGTTCTACATTAACAGCAAGGTAGTCTCCATTGCTTTGCCAGTACATCTTGCAGTCACTGACACTAAAAAGATTCTTCTGCCTGAGTTCTTCTTTGCTGGGGACTTGAATCAGACTTACCCTGGCGGGCTGGTTACCACCCCCCATCTCAGGAACAAAGAGAGCTATAATTGGATCAGTTGGAGACCAGCAGAAATCCATTATATTTTCAACCTTCAAGGACTTCTTGTCAACGAGAGAAAAGGTCTCTGTCTCATACACACAGAGCACATTTTTTCCCATCCTTGCAAAGTATTTATCATCTTTTCCACCACTCCATTTGAAAACAGGCCATGAAACTCCTGTGACACCCCCAGCCCCTCCAACTGCAAAATCATCGGCACTACCTTTAAAGTCCCTCATAACTTTGCCAGTTCTCACATCAAATATATTAATCACAACCCTATTGGCATCTCGAGGATTGCTTGGTTCATGGCTACTATAAGTTACCAGATACTTTTCACCAGGTGAAAAATCAATAAGTTTTACCTGAGGATGTGCATATCGCATAAGCCTATTAAAGCTTGTAGAACCTCCCCAAACAGCTGCCCCTTGTCTGTGAACAGTAGCCAAGTATGTCCCCAACGGAGACCATTGGACAAAACTTTCGGTCCAAAATGCACGCTTATAAACAGGATCAGGCTTCAAATGCCTTGCATCATTCCACAAAACCTCAGTATCTGAACCAGCACGAATCACAAACTGGTCCCTGGCCTTAGCATCAGTGAGCCAGTGTTGAAGATTTTCCCCTGGAGTATATGGCTTAGTTTCAGGAGGAGCCCACTCATTCGGAACCTTCATGAACCTATCAAAGTCATCGAACATGCTGACACAAAATATATGTGCACGATCCAATTTGTACCCATGAGTCTTCTCTTTAGCAAGCTCTGCTTCCTGAGGAGTATTGTACTCAATGAAGCAGTAACCTAGGGTTTTTTCAGTTTCAGGATCAACCGGCATCCAGAGGCCATCCTCTTTGATAACACCAATCTGACTATAAATTTTCCGGACAACTCCTTCGAGTTTTTCAAACTTCTCCCTCGGTACGACGGGGAGGTTATCCACGACAATGATGTTGCCAAATCCAGACTCGAACTCGAGATTATCCTCCTGATAAACTTCCTCGTCATCGCTGACGATTCCACAATTTTCGCCGGGAGGAAGTCGAATGGAGTCGAGATCGATAGTGGAGAGATCGACGCCTAGACGCAGCGCCGTATCTTCGATCTCCTTCATTACCATGACGTCCGCCATGATTGCAGCGACGAGGTGTTATCGTTATGGAGGCAGAGTATGCAGAAGAGAGAGTTTGGCTTCTAGGGTTCTCTCCAGTTTCATACAGGTGCGGCGCAGGGTAAGATACGTTTTCCTCTTCCTCTGGATTAAATTGGAA

>C101839_65

AAGAAAGGAAGTAAAGATATAATATATAAAAATGTTTGCCAAGTTTAAAAGGTTGATAAGCAGAAGCCACAGGGTGAAGGGGAGAGTGGTGTTGATGAGAAAGAACGTGCTTGACTTCAACAGCTTAACGAGTGTGAGTGGGATTATTGGGACTGGAGTCAATATTTTTGGCTCTGCCTTTGATGGTATGGCTGCCATTACAGGTCACTCTGTCAGTCTCCAGCTTATCAGTGCTACCCAGACTAATGCTAGTGGATATGGACTAGTTGGAAAGAAAGCATACTTGGAAGGTATTTTCTCCTCGAGACCAACTTTGGGAGCTGGCCAATGTGCATTTGATGTTTATTTCAAATGGGATGCTGACATGGGAATTCCTGGAGCATTTCTCATACAAAATCATATGCAACTTGAGTTCTTCCTAGTGAGTTTGGAGCTTGAAGATATTCCTAATAAAGGAACCATGCACTTTGCTTGCAACTCATGGGTTTACAATTGTAATAGTTATGATAAGGATCGAATTTTCTTTGCCAACGAGGCATACATTACAAATAAAACACCAGCTCCACTAATGACGTACAGAGAAGCAGAACTGCAGAGTTTAAGAGGAAATGGAACTGGAAAACGCAAGACATGGGAGAGAATTTATGATTATGATGTTTACAATGATTTGGGCAATCCAGACAGCAGCAAAAACCTTGCTCGTCCAGTTCTTGGAGGATCAACCACTCATCCCTATCCTCGAAGGGGAAGAACTGGTCGAAAACCAACAAAGAAAGATCCCAAATGCGAGAAACCAGGGAAGGTTTATGTTCCAAGAGATGAAATTTTCGGTCATTTGAAGTCATCAGATTTTCTTGGTTTTGGGATAAAAGCTTTATCTCGAAATGTCTTACCTATCCTCAAATCTTTATTTGATTTAAATTTCCAACCGAATGAGTTTAGTAGCTTCGACGAAGTACGTGAACTCTGTGAAGGTGGAATTAGCCTGCCTACAGACATACTCAGCAAATTTAGTCCCATACCTGTGCTCAAGGAATTCTTCCGCACTGATGGTGAAATTAATATCCTCAAGTTTTCTATTCCTCATTTGATTAAAGTTAATAAATCTGCATGGATAACTGATGAGGAATTTGCCAGAGAGATGCTTGCTGGTGTTAACCCTTGTGTGATTCGTCGTCTACAAGAGTTCCCACCACAGAGCAAGCTGGATCCTTCAGTCTATGGTGATCAAACCAGTAAAATAACAGAAGAACAGTTGGAGATAAACCTTGAAGGGCTAACAGTAGATAAGGCAATTGAAGATCAGAGGCTATTCATATTAGATCACCATGACATATTTATGCAATATTTGGAAAGGCTAAACAATTTGAAGTCAACAAAAGTTTATGCCACGAGGACAATCCTTTTCTTGAAAGATGATGGGACCTTAAAGCCATTAGCCATCGAATTAAGTTTACCACATCCAGGTGGACCGAAACTAGGTTCTCATAGTAGAGTCGTCTTGCCTGCAAAAGAAAAGGTGGAAGGCACAATTTGGCTATTGGCTAAGGCATATGTTATTGTGAATGATTCGTGCTATCATCAACTCATAAGCCACTGGTTGAATACTCACGCTGTGATTGAGCCCTTCATCATAGCAACAAACAGGAATCTCAGTGTTCTTCATCCTGTTTATAAACTTCTACTTCCTCACTATCGTGACACAATGAATATCAATGCACTTGCTCGACAATCACTAATTAATGCTGAGGGCATTATAGAGCAATCCTTTTTGGGTGAGAAGTATGCTATGGAGATCTCTTCAGTAGCATACAAAGACTGGGCGTTCCCTGACCAAGCATTACCCGCTGATCTTATCAAGAGAGGAATGGCTGTTGAAGATCCATCTTCTCCCACTGGCGTTCGTCTTGTGATAGAGGACTACCCTTACGCTGTTGATGGACTTGAAATATGGAATGCTATTAAGACATGGGTCCAAGAGTACATCTCCTTGTTCTATGGCACAGATAATGGAGTTGAAGAAGACCGTGAAATTCAAGCATGGTGGAAAGAAGTTATTCAGAAGGGCCATGCTGACTTAAAAGAGAAGTCATGGTGGCCTGAACTACAGACTCGTCAAGAGTTGATTCAATCTTGTACTACTATTATATGGATTGCTTCGGCTCTTCATGCTGCTGTTAATTTTGGACAATATCCTTATGGTGGTTATATCCTTAATCGTCCAACTCTTAGCAGAAGATTGATCCCGGAAGAAGGAACTCCAGAATATAAGCAGATGGTGAGTGATCATCAAAAGGCTTATTTGAAAACAATTACACCAAAGTATCAGACTCTTATTAACCTTTCGGTTATAGAGATATTGTCAAGACATGCTTCTGATGAGGTCTACCTTGGTCAGAGGGATTACCCAAATTGGATCTCTAATCCAAAAGCAATAGAAGCCTTTAAGAGATTTGGAAAAAGGCTAGAAAATATTGAGAAAAGTATCACAGAAAGGAACCATGATCCAAATCTTAGAAATCGAACTGGGCCAGCTCAATTGCCCTATACTGTGCTTATTCCTACCAGTGAAACAGGGTTGACTTTTAGAGGAATTCCTAATAGTATCTCTATATAGTGAGTGTGTGGCG

>C101843_65

CAGCAGAGTGTCACTGCACGTACTCATCAGCGGTGAAAGTTCATTGAAGATGAACGCATCTTCTTGGATGAAATGAAATTCGTAATTGCGAAGGATCCCGTTAATCTTCTCAACTTTCAGGGTTCGCCTGTTCTCTTTCGCGTCCCTTCCGTTGCGCGCAATTTTCTTCACAGTTCGAGCGCCAAATGGACGCGTGGCTCCCAGCCCCTCAAGTTTTCGTTTTCTTCCTGGAGAGCTTCGTGCGCTTCTTCTTCTTCCTCGAATATGTACGGAGGCTGGGACGATCTCGGCTGTTCAGACGCGCCTGGTGAGTCCCACGCCTTGCGCAATTTTCTCGTTTCTGTTGGAATCGATGATAGGAAGAACGTTTTCGTGTTCCTCTTGGGCCTCGTTTGCGCGATGGCGATTTCTAGGGTTAAGGTTTCTTCTATTGTCGTTCTTCCTGCTTCCGCGATGGTTTTCGCCGTTGGATTCACGCTAGGGTTTTTCCGAAACGGAGCGTTCGGTGAGATGAGGGCGAGTGGGAGTAAGAAGAGGGAGAAGGAGGAGAATTCGAATTTGAATTCGAAGTCATCTTCAGAAAAATTGAGGAGTTTGGTGGAATTTTTTGACGAGCTTGATCTTGTGGTCAATAACTTGAAGAATGATGTACAGAGTGCTATTATGAATAATAAGATTCGAGTGGATGATTTTTATGGTTATATTGAAGTCACAGATAAGATTAAAATTTCGGCTAAGAATGCTAGGAATGTTGTTAGGGCTTTGATTGATAATGAGGAGAACTCTGGTGGTGTTTTGGTCGAGAAAAATAAGAGTGGTAGAAGAAAGAAACAAGTTGGAGAAGGTGGGTACCAGATGCTACAGTCTTTTAGCAGTTTGTTTGGGGAAAATTTGTTTAGCTCCAATTCGGCCAAAGTCAGGGAAAATGTTAAGCAAGAGGCAGTGGATAGAACATTGGATCAAACTAGAGGAAATGGTACATTGCCTCTAGTTGAAGACACGGCTTTGAATTTTGTTGATGAACATAAGGGAAATCGTAAGTTGGATCTGGATCCTTCTCAAGACTCGTCTACTAACTCTGTTATGGATATGAACAAAAATGGAAGAATAAGAACTACTCCCGAGGGAGCAAATTTTGACTTAGGGGACATACGGAGAAGCAAAAATAAATTCTTTGATGATAAAGAGTATAGTTACCGGAACAAAGGATTGAGGTTCACAAATAATCGCTGCTTCTCTTTGAAGATGGATTCCAGCAGCATAACGGACATGTGGGAATCCCATGACAACCTGATTGACTCTGAGAGCTTTAAAGTCAGAACGAAGCACGTGGAAAATGAATCCTCTTTTACGCGTGAGCAGCTTCTCAAACAAGGTCACCAGACTTTCAGGTCATCTCATGACTTGAGGGAGGGTGGTTCTGACAGGTCTCGGTATGAAGATGATACAGTGAATTACGATGATCATCGCCACCTTGCAGATGATTTGTCTGCTCATGAGAATGAATTCAATACTGCTTCATCTACAAAGATTTCGGATGATATGATGTTTGATAGGTATCTTGCTGAAGCAACAGACCTTTTAAAACAAGCAAAAGAGTTTATAAAAGTTAGGCAGGATGAAGAGCAAGCTGAAATCATGTTATACAGGTCTGCAAACATACTATCCAGAGCTGTGGACTTGAAGCCCATGAGTTTGCTGGCTGTAGGCCAGTTAGGAAACACTTACCTTCTTCATGGAGAATTAAAGTTGAAGATTACTCGTGAATTGAGAACTCTTCTTTCTGGTAGCATCCAACAGTCGTCTGGAAAACATAGTAGAATATTGAAGGGACTAAGGAATAAAATCACTAGTAAAGAAGATGTTGCATCATTTCTTATTGATGTTTGTGAAGAGTGTGAAGAGCTACTAGTGCAGGCAGGCAGAAAGTATAGGCTGGCGTTGTCAATTGATGCAAATGATGTGAGAGCCCTGTATAATTGGGGCCTTGCTCTTTCTTTCCGAGGCCAATTAATAGCAGATATTGGTCCGGGTGCTGCATTTGAGGCTGAAAGAGTATTCCTGGCTGCAATTGACAAGTTCGATGCTATGCTGTTAAAAGGCAATGTTTATGCGCCAGATGCTTTGTTCAGATGGGGTGTGACATTGCAGCAGCGATCTCGGTTAAGGCCAGGAAGTAGTAAAGAGAAGGTGAAGTTACTGCAGCAGGCTAAAAGGCTATATGAAGATGCCCTTCATATGGACTCCAATAACATACAAGTGAAAGATGCCTTATCCTCGTGTGTCACTGAGCTTAATTATCGACAGTTTTAGCCAATTCAATCCTTGTTTCCAACTATTTTATATCTGCCTGCCTTGGACCACCACAAGAAGCAGGTATATTTGCATAAGTGTCATTGCACACAAATGTTATGATTCCCAGGGTCGTCGTCGTCTTTGTACTGACTCTCCCTAGAATCAGATAAAGACAGCTCGTTATAATAACACTTATACTAGGGATAAAATGTAATCATTTTATTTATGCACAGTCGTGGACTCACAGGGTTCACTACTTCCCTTGTTGTAATACATACACGTTTTCATTATCATTCTTATAGAAACACGGTGCTAGCCTGATTTGAAACGCTCCAC

>C101851_65

AATTTTTACATGACACTGGTTTTCAGCCATAGAAGACCTAAACAGTAGAGTTTACTACTCAATAGGAGGGCGTATATTGAATGTTGAGGAGAGGGAGAGGGAGAGGAAGAGGAGACATCATCAGCTGAATTCCACACGAAAGCCATGAGGAAGCGGGATTTGGCCATTCTTATGCTCTCCGCTTTCGCTATTTTCTTCACTCTTCAGCAAGATGGCGGTATTTCGTTCAAAGACGCGTGGATGCACCTAACCGATGAGTATCCAATCAAATACGAAGCCGAACGTCTTCCCCCGCCGCTCGTCTCTGATCTCAATGGAGACGGTAAGAAGGAAGTTCTTGTAGCTACTCACGACGCCAAAATTCAGGTCTTGGAGCCCCATAGTAGGCGTGTAGATGAAGGATTCAGTGAGGCGCGTGTGTTGGCCGAGGTGTCTCTGTTGCCTGACAAAGTACGTGTCATGTCTGGGAGACGGCCTGTTGCTATGGCCACCGGCTATATTGACCGGTATAAAATTGGGCAGCCGCAGAAACAGGTGTTGGTTGTAGTAACGTCAGGTTGGTCCATCATGTGTTTTGATTCCAACCTCCAAAAGTTGTGGGAAAATAATTTACAGGAGGATTTTCCACATAATGCTCACCATAGGGAAGTTGCTATCTCTATAAGCAATTATACTCTAAAGCATGGAGATACAGGATTGATAATTGTTGGTGGCAGAATGGAAATGCAGTCACATATTTTTATGGATCCTTTTGAAGAAATGGGAATGGGAGCCAAATTTTCTGAGCAACAACGAAGAAGTACTACTGAAAAGGAGGCTTCCGAAAACTCTGGAACTGTGGATTTACGCCATTTTGCATTTTATGCATATGCTGGTCGATCTGGCGTCGAACGATGGAGCAGAAAAAATGAGAACATTGAAGCACATTCTTCAGACGCATCACAGTTACTTCCACAGCATAACTACAAGCTCGATGTTCATGCTCTGAATACCCGTCAACCTGGAGAGTTTGAATGCAGGGAATTCAGAGAATCAATCCTGGGAGTTATGCCTCATCGTTGGGATAGGAGAGAGGATACTTTGTTGAAGCTTGCCCACTTCAGACGGCACAAGAGAAAAGCATTGAAGAAAACACATGGAAAGTCAATGAGTTACCCATTTCACAAGCCTGAGGAAAACCATCCTCCAGGGAAGGACTCAACCAAAAAAATTTCAAACATAATTGGGAAAGCAGCAAATTATGCTGGTTCAGCAAAATCTAAGAAGCACCTTCCCTATGTTCCCACCATAACCAATCACACTCAGGTTTGGTGGGTTCCTAATGTTGTTGTGGCTCACCAGAAGGAGGGCATAGAAGCTCTTCACCTGGCATCTGGTCGAACAATATGCAAGCTTCATCTTCAGGAAGGTGGTCTACATGCTGATATTAATGGTGATGGAGTTCTAGATCATGTGCAGGCTGTTGGAGGAAATGGTGCTGAGCAGACTGTAGCTAGTGGGTCTATGGAAGTGCTACGTCCTTGTTGGGCCGTTGCAACATCTGGTGTACCAATACGGGAACAACTCTTTAACGTATCTATTTGTCATTATACCCATTTTAACTTATTCCAACATGGAGAACTTTATAGAAGCTACAGTCAAGGTTCAGATGTTGCCTCTCTAGAGGTAGCAACGCCTATTCTAATTCCTCGAAGTTATGGACATAGGCATCGGAAGGGAAGCCATGGCGATGTTATCTTCTTGACAAATCGTGGAGAGATTACATCATACTCCCCTGGCTTGCATGGTCATGATGCTATTTGGCAGTGGCAACAATCAACTGGTGTTACATGGTCAAATCTGCCTTCCCCATCAGGAATGATGGAAGGTGGTCTGGTGATTCCCACACTTAAGCCTCTTTCCTTGCGGTTGCATGATAGTCAGGAAATGATCCTTGCAGCTGGTGAGCAGGAGGCCGTAATAATATCACCGGGAGGAAGTATATTGGCTACAATTGAGCTACCTGCTCCACCAACACACGTTATGATCTGTGAGGACTTTTCAAATGATGGGCTCACTGACCTTATTCTTGTCACCTCTAATGGAGTGTACGGCTTTGTTCAGACAAGGCAACCGGGTGCTCTCTTCTTCAGCATGCTGGTTGGTTGTCTCATAGTCGTGATGGGGGTTATATTTGTTACCCAGCACTTGAATTCCGCAAAGGGGAAGCCTCGTCCCTCAGCGGGTCCTCGGTGAAAAGCTGGAACTGAAATTTATACTACCCCAACTTCACGGTGTTATTGAAGGAACAGATAACAGTGAGGTGTGCTCAACCAGTGGAGAGTCAAGAGTACACAAAATGCGTAGAGCTTGAGGAAGTCTCTATAGGAGGTAGCGAACGGCCCTAGACGGTTGATTGTAAAATTTGGACAGAATGTAAGGAAATTTGATGTTATAATATTATAAGGAAACTTATACTGTCTGCTTCCGGGATTTTTTTACCTTCCGTTACGAGGAAGCTATACTGTACACACTTGTTACCTGTTCAGAATTAGTTGATGCACTAGAGAAACTCACCTAACGGTTTTTCTAACTCTTCCCAAGGATTTCTTGCTTTTAGGGACGTATTTAATGCATCGTACCAAAATTCCTCA

>C101853_65

CTCCAAAGCAAAGAATAGGCCATTATGAAAAGGCAATCAAACTGGTCTGTAGGTGATGTCCAGACACCTTTAATTGATCATCAGGTAGAAAACCAACACCCAATTATAGAAGGACTGATCTTGTTTCATATAACATAACTATAATGTAAGTTTAACTTCAACTGCTATCTCAGTAGGAGTTATCTCAATCTAAACGGGCTCAATATCAAACAAAAATGTAAAAGTGTGCCACTGAAAGGTAAGTATCTCACTCTGATGTCTGTAACCACGATTGCATTTCCAAACCCCCCATATCTGTTTAATTAACATTTAGTTGTCAGGGGACTTGCTACCTTTTGTATCCTCTTTCCTTCATCTTCATTTTCCTTTTTCCATTCTGGGTATATTTCAAATCAAGAGCATAAACACGAAGTACTTATACACTCAATCAAATTAGCCGAATGGCATGAGACCAAATCTCAGGTTTGTGCTAAATCTTCTGATGTTTGCGAACGATGAAGCTGAGATAACATCATAGCACAAAATTGGTGACCTTGCGGAAAACTCTATCAGCACATCCAGACTCTTACTTCCTTTCAGGTTACCTTACCAGCTAATGTGCCTCTCAACAAAATGCCTGACAAGCCAAGATCATCTCTGCAGTGTATTTTGACTCTCCATCCTTTCAGGCTCATTTTTTACCTTTTCGAGCAAGAAATCGTTCCCTAGCAGCTTGGATTCTACTTTCACTATCATCAGCAGTACTAGCAACCTCAACATCCTTCTTGGGTAAATCTGGAAGATTTTTCTCAGGTTCCTCCTCTACATGATGAATACCACCACTTAATTTGTACACATCCAGGGTCTTGAGCTTGTTAATAATGGGTATAACCCATCTCATCTCAACAGCACATACATTTCTCATGTATGGCCGTGGTGTTGCAATTAGTTCATTGTACACAACATAGTCTGGGAATTTTCCCAGGTCATCAAGACTCAATACAGATGACGGATGCACCTGGACTACTTGAGCCTGGAAACCCAGAGTTCGATAACCATTATGATGCATCTTTCTTTCAGCCAGCTGATTTGCATAGCCCACACATAAAGCCTTCCTCAAATTTCGGTAATCTTGCCTGAACTCTTCTCTCTTCCCATTAGCCTCTACATCCAGTGGTCCCTTTGTTATTTTCTGCATTATCTGCGATAACTGCTTGCGGACGTCTCTCACAAACAACATCCCACGCACCTGCAAGCCATTATCTTTGCACCAGCCAATGTCAAAATCAGTTTGATCCCAGCAATGGTATATCTGTAGCAATTGAATGTGATCACCCAATCCAGAACCATCAGGAAGGTTAGATATTGAGTGTTTCCTCTTTTTCTCAGTCTTTCTCTGCCCAGGTAGCAATGTAGTCTCAGCTGATAACATGGCAGCAACAGTCAAAGCCTCGGATAAGCAACCATAATTGTTTGCCTCCATTAAAGTTCTTGATAAGGATGGTTCTAATGGAAGCTCAGCCATTTTCTGTCCAATACTTGTAATTGCACCATTTTCATCAATAGCATCAATCAGGTATAACTGCTTCAAGGCATCTTGTAAGGACTCAGATGATGGAGGATCCAGAAAGTCAAACTTTAGAATGTCAATATCTGGGAGGTCCAATGACTTCAAGTAAAGAACACTGCCAGCAAGGGAAGATCTCTGAATTTCTGGAACTGTAACATCCAAAAATTCCTCATTGTAAACACGGGAGGGATATAGACGGTAGCATTTCCCAGGACGTGTTCGTCCAGCTCGGCCTGCACGCTGATTAGCTTGCACTTTGCTAATTTGAACAACATCAAGGGAATACATGCCACTAGATGGATTGTACTGCCTTTGCTTCACATACCCAGAGTCAATAACATACACTACACCATCAACAGTCAAGGAAGTTTCAGCAATATTTGTGGCAACAATAATTCGTCTACAATTTGGAGGTGGAGGACTAAATACACGCACCTGCAATTCAGGGGGCAAAGAACCATGTAGGGGAAGGATTATAGCATCCATGCAGGAACCTTCCTCAAGAGAACGAACCTTGTCTTCTAACTTAGATACCAACTTCTCTATGTCATCCTGTCCAGTCATGAATATTAAAACATCCCCTTCTGGTTGGCGAATATGTATATCAAGAGCTGTTTTCAGTGAAGACTCGAGATAGCTCGAGGGACGCTCACTGCTATAAAGAACCTCAACTGGGTACAACTTTCCCGGGATGTTCAATACCGGACAATCAGCAAAGAACTTGGATACTTTATCGCCATCAAGAGTCGCAGACGTGATCAAAACTTTGAGATCAGAAGAACGAACTTTAACCAAGCGTTTCATCAGACCCATCAATATATCGGTGTTTAAACTCCGTTCATGCGCCTCATCCAAAATGATAACAGAATACTCATTCAACTCAGGATTTGCTAGACTTTCACGTAGCAGGACTCCATCAGTAAGATACTTTATGCGCGTGTTGTGAGAAGTCCTATCCTCGAATCGAATGGCGTAACCTACTTCCTCGCCGAGCTGAACGCCGAGCTCTTGTGCGACTCGTCTGGCAACGGAGACGGCGGCGACTCTACGGGGCTGAGTGACCGCGATTTTGCCGTAGCCGCGG

>C10185_65

CACATCAAGGAGAACTTTTAGTGCTTCACGGATTGTGTCCTCTGGGATGTTCACAAAAGGCTCCTGGATGCACAAGTTTGATAAGGTTAAAGAAGATTTCAGACTATCAAAAACCTTATGACCCTCGATCCCGAAATGATAAAGCTTGATCCCATTTGACTTGAGAAACTCCATGTTG

>C101865_65

GAAACATACGAAGAGTTCGCAAAAGTTCACGAGCTGCTACTCGCGGCGTCGGGGTTGCCGGAGCGCCTGCACCGGCGCCTCTTCGACAAGCTCTCCAGCGAGTCCTTCGACGGCGGCAACCACTTCCAGATCGAGCCGTGCGAGGACGGCCGTCAGCGGCGCCTCATTCTCACGACGGCGTCGATGGACACGGACTCCGACGTCTTTCTCGTCGATCACGCGTGGACTTTCAGACTCTCCGACGCATACAAACAGCTGCGTGAGGTTCCAGGATTAGCTGGGAGGATGGGTTCTCTGATGTGTGTAGATGTTGATGTCAGTTCGGATGGTGGTGAAGCTGAAGCGAACGGTGAGCATGGTGTTGAGGACACATTGGAGAGTGAAGTTGGTGAGGCAAAGGAGAAAGGAGATGGGACGTTACGGTGGTTGGAGCTTGAAGAACTGAACATTGATGATGAAATGCTTGTGTCTCTAGCCTTGCCAATTAGGTTTCCGGATATAGTGGCACTTAGCCTGCTTGGAAACAAGCTCAATAGTGCCGAAGTGGTAGTTCAGGAAGTTATCAAATTTAAACATCTCAAAGGAATCTGGCTCAATAATAACCCAGTTCTTAAAAATTGTGATGATAAGATCGCAAGGGTGATTCATAAGGAGTTGCCAGAACTGGAAATTTATAATTCAAGTTTCACAAACAATTTTGGAGAGTGGGCCTTGGGCTTTTGTGCAGGGATATATGGAAAGGATAACCCTGTAAATGTTGATCAGGCTGACACTCCACTGCACACTGTGTCTTCTCTTGACCTTTCAAATAGGAACATTCACAATTTGAGGAATAAGGCATTTTCACCCATTTGTTTGCCATCTCTGACGTACTTGAACATTCAAGGAAATCCATTGGAGCAAAACTCAGTTGATGACTTATTAGCTCTGCTGCGGAGATTCCCTTGCTTGCGTTCATTGGAGGTGGACATTCCAGGTCCGCTTGGACGAAGTGCCATTGAAATTCTGGAATATCTTCCAAACATTTCTGAGCTAAATGGTATCAATGCATCCAAAATATTGGAAACTGGAAAGCATGTTATTGATTCGATGCTTCTTCCTCGTCTTCCTGAATGGACTCTTGATGAGCCTCTTGCTGACCGTATTATAAATGCAATGTGGCAATATCTAATGACATATAGACTTGCAGATGAAGAAAAGTTAGATGAAACATCTGTATGGTATTTGATGGATGAGCTAGGCTCAGCTTTGCGGCATAGTGATGAGCCAAATTTCAGAGTGGCACCATTTCTGTTCATGCCAGAGGGCAATCTAGCATCAGCTGTGAGCTTTTCTATTCTATGGCCAACACAGAATGTTCAGAAAGGTGATGAGTGCACGCGTGATTTTCTTCTTGGTATTGGGGAGGATAAACAACGTTCTGCCAGGCTTACTGCCTGGTTCCACACTCCAGAAAACTACTTTATCCATGCATATGAGAAGCACCATAAGAAATTGTTGTCCACTAGTTTATTACCTCCCACCTTTCAATCTTCTGAGACTCGAAGTATTCATCAACGAGGTGGGCGTCCATTACTCGTATACACAGATATACCTCAAGTAGAGGAATACTTGACGCATCCCGAGTTTGCCATCACAAATGAACCAAAAGGTGCAGATATAATATGGACAAGTGTGCAGGTAGATGAGGAGATGAAGAAGGCTACGGGAATTACGGATCAGCAATACGTTAATCAATTTCCTTTTGAAGCATGTCTTGTTATGAAGCATCATTTAGCAGAGACAATCCAAAAGGCACATGGATCTCCTCAATGGTTGCAGCCTACTTATAATCTTGAAACACAGCTATCTCAACTTATTGGTGACTATTGTGTACGCAAAAAGGAAGGACAAGACAATCTTTGGATCTTAAAACCCTGGAACATGGCCCGAACCATTGATACTACTGTAACTGATAATTTGCCTGCAATAATCCGGCTCATGGAAACTGGTCCCAAGATTTGCCAGAAGTATATTGAACAACCTGCTTTGTTCCAAGGGAAAAAGTTTGATCTCCGTTACATAGTACTGGTTCGGAGCATGCATCCTCTGGAGATATTCCTGTCAGATTGTTTCTGGGTTAGGATAGCCAACAACCAATATTCTTTGGCCAGAAGTAGTTTGTTTGAGTATGAAACTCATTTCACTGTTATGAATTATCGTGGAAGAATAAATCATAAGAATGCTAACGAATTTGTGAGAGAATTTGAGGAAGAACATCAAGTTAAATGGTTGGATATACACACAAGAGTAAGGACAATGATTCGATCAGTTTTTGAGGCAGCTGCTGTTGCTCATCCTGAGATGCACAGTCCATCATCAAGGGCAATGTATGGTGTAGATGTCATGTTGGACAACTCTTTCCAACCCAAGTTACTAGAGGTGACTTACTGCCCGGATTGTACAAGAGCTTGCAAATATGACATGGATATTGTGGTTGGAGAGGGAGGCGTTGCCAAAGGTTGTGATTTCTTCAACAATGTGTTCAGTTGTCTCTTTTTGAATAAAACTTCACAAGTTAGTCAATTGTAATTTAGTGGTTTGTGTAATGCCTTTTTCTCGTCGACCAAGTAGTGTTTTTGGTAATTTTCCTCGTG

>C101867_65

TTATGCAAGAGTTTTACGTAGGAAGGGTGTAATTTCATTTTCTAACTAATTACTTGCTTCCGCTGTTGAGTTTACACCTTTAACTTCTCTATGTACAAGACAAAAGCAGTATTTAAACAAAACCATATACAGAACACATTGCACCATGCGTGCATGCATGCATCAATCTATTTTTTCTTCTTTTCCTCTTGTTCTCTTTCAAGTGTAACAGGAGCTGCTGGTACACTGAACCACCATTCCTTTAATCTCCTCGTCCATCTCTCAGTGCTAGCTTTCCTTGGAGGCGAATATCTTGTGAATATCTCCAAGAAGAGTAGCAGAACTATGTATTTAGAAGGCAAGAAAGCCAAAATCAAGGCTGTTGATAGAAGGCCAAAGGCCAACTTCTCTGTGGCCTGAGGAAAAATGGACAGCAACAAGCCGCGAAACTTGAGCAGAATTACGTTGCCATCCTGGATGAGCTGTTCAGCTTGAGAGACAGCATTCTGAACAGCCAAAAGCTGTTCCATTGTATTCATTGGGGGAGGGGCAATTACTTTAACTCCAGGAACTGACCTACCTTGGCTAAAACATCGCGTAATTATCATAAAAACTGCAAGTAACACAAGCACCAGTGCTGCTGCATAGCGAAGCCAACACCTGTAAATGATGTAACTGAAAAACAAACAAAACCCTAAAGACTTCCATGGATCATCCCAATATGCCAAAGATTGAAGAGACCTCCCAAGTTCATTTATAGGAAATAGTAACTCCTTCATGACAGCCAAATTAGTGTCAATACCATCAACTTTAACACCTTCAACTGTCGCTTGTGCAGATATAACCTTTTTATGACTGTTTTTAGATTCTTTTACTGCTATTTCCAATTCAGTCATTTCTCCAACAGAAATTTCACCCACAGCAATCCTGCTCTCATTTGAGTTGTTTGAACTTGCTCCAAATACGAATCCCAAGTTAGAAACCATGTCCAAAGCTGAGATAGAATACATTCCACTACCACCCTTGAGACCATTCTCACGATCTGACTCTCTTAAGTTTGGCAAATTCGCAAGGGTTTCTAATATCAAGTCTCCACCTGGAAGCTGATCACATAAATTAAACATGAGAAGTGCATCATTCTGTATAGGAATCGAGGAACTAACATCCTGAATAGCTTGTAAACGCAAAATTCCTAAAACAGCTTTCCAAAGCGCTTCATCCCGAGCAACCCCTTTGATTCTGTACTTGTTTATGAATTTGTGAACATATAGAATCTCTCGGATGATAGCTAGCCAATAATCACGACGTGCATGACCTTTAAGTTCTGGAAACTCTAGTACAACTGGTTCTGATAGGGACGATGAGCTATAGAAGACTGCCTTGTCAAAAAGTCGAGTACCCCATGGTCCAGTCAACTCAGGTTTAACAACTTGTTTAAGATCTTCTGATAAGTCATATCTTTTTGGTTTGTCATATGATACAACGCGAAGTGCTTCGAAATAAACTGCATGATCTGTCAGGATCAACCTTCCAGGCCAGGTGGATATTCCTACATGCTCAAGGACAGGCTGAGTTGTGACTGTTCCATCAATCTCAAGAATCTTCTCACCTCTAGAAGATCTTACAGCAGCCAGGAGAGACGACTCTGAACTGCTCTTCATTTTTCTTATTGCTCTTTCTAAGCCACTGAGGTACTTGTCATAGACGGGGAACTGAAGGCGACGGCCAGTTGATGAACTCAGCACCTCAAAAAGATTTTCACTAATGATGACATTGGCAATGATAGGAATTGAAGGGGCTATTCGACAGAAAGCTTCTAAACCAACAGATACATCCTCATCAACCTTATTAGTTAAAGGTTGGCTGTCAGCAGCTGGGGCTTCCCAAGCTAACATCATGTCGTATGTCAGACGCCGGAATGTCTTGTCAAAGAGATGACCAGTCATTTGTGTTGTCAGGAAAAGTGCCTTGAAACAGCAATATTCCAATAAGTTCCTCGCATATCGCGATGGATGCTTCATAGCATCAGAAGCCTCCAGATTGAAGCTCTCCTGAAGGTCACTAGAAGAGGTCCTAAGAATTTTTGAGCATCTACGAACAACTATGTTTGCAACAGGAGAGAGCTCTCGTATAAAATTCCTCCCAGCAGAAGGAGAATTTTCCATCTCTTCAAATTCTTCAGCAAAGTAACTTTTCTTGCCAAGCAACCATTTAAATGATCCTTCTTTTACCAAACCTTCAAACATATTTCTGGTTTTGCCGGCTACAGCCATCTGTTTTCACCTCAATCTTTATTTAGATTGGAAAACAAGATACCCGATATCCAATCGATCTTGAAGTTCTTGTCTTTACTCTAAGCGCATAAAAATCTCAACTTTAGCAGATGGGGCAACTTAAAAGTGAGAGATTTTTTACGATCTCACAGAGCGAAGGGATTCTGAACCACCCCATTTCCAAGAAATAAAGAAAACTTGGATTATTAGAAGGATGTAAGGTAATATAAAGAATCGTAGTTGGATTGAGGTTGATGAAAGAAAAGAATGTTCAGTGATGATGGATAAGCACGAATAGAGAGGGAGAGAGAGAGGTTTGCTTTCTGAAAGGGTTAGTATTCGAGTCCATACGCTCTGTAGGGACATAACATAATCAGGTGTTGC

>C101869_65

TTTTCCATCCGTGAAATTATGGCTTGTGAACAAGGGCAAGGGAGAAAGTAAAAGGGTCTTTTTACACTGGTGGTTGAGGAAGGTTCTTTTCTTATTGAGAGATTTGAATGGAACAATGGGAGAGTTGACTAACTAGGAGTTGAAGAAAATGGGTGAATATTGAAGAAAATGATGGAAAAGATAGAAAGTGCTTGTGTGTCTGGTACTTTAAAAATGAAATTTGAGAGAGAGAGGGCAGCATGACACATAAAAAAGTTCAACACCCCAGAGAGCAGTTTTCAAATAAATTCTGTTCTTTTTTCCCTATCAAGTCAACTTGCCTCTAAAGGTCGTGTTTTTGGAACTGGGGTTCTTGGATTTGCTATATATGAATTGAAGTGGAGATGATATTGGTGTAGGGGCTTTCAACTGGTTTGCTGAAATGAAGGTTGTTCTTTCCATCAGCTTCTTGTTTTGTTGCTTGCTGCTGCTTTTGGATTCTTCCATTTCACTCCCTCTATGTGTTGATTCCCGGGCTCCCTTTACCCTGAATACGACACTTGGATTTTGTCCTTACAATGGAAGCACGTGTTGCAACTCCACAGAAGATGCACAGATAGAGAAGCAATTCCAAGTGAACAACGTATCTGATCCTGGCTGTGCCTCAGTTTTGAAATCAATACTCTGTGCGAGGTGCGACCCATTTTCAGCGGAGCTATTTACAGTTCAGTCCTCACCTAGATCAGTTCCTGTGCTTTGCAATTCCACTGTACCAGCAAATTCTTCTCAGTCAAAGACAGAAGTGGAAGATTTTTGTTCTCAAGTATGGAACACATGCAAAGATGTATCCATAGTGAATTCTCCATTTGGCTCTTCATTACAAGGTCAAGCAGGAGCACCAGTTCAAACCAATGCTACCAAACTCACTGATTTATGGCAGTCCAAAACAGACTTTTGCAATGCATTCGGTGGAGAATCTGATAATTCATCAGTGTGCTTTGAAGGGGAACCTGTTTCACTGAATAAAAGTGAGACCTCTATTAGTCCCCCTCATGGCTTGTGCCTCGAGAGAATTGGTAATGGATCTTATCTCAACATGGTTGCTCATCCTGATGGCTCTAACCGTGCATTCTTCTCTAACCAAATGGGTAAGGTTTGGCTGGCAACACTTCCGGAAGAGGGATCAGGAGGAAAAATTGAACTTGATGAATCAAGTCCTTTTGTTGATCTAACTGATCAAGTTTATTTTGACACTCAATTTGGAATGATGGGCATGGCATTTCATCCAGATTTTGCTAAAAATGGACGATTCTTTGCTTCATTTACCTGTGATAAGTCCAAGTGGTCAGGATGCAATGGAAGATGTTCTTGTAATTCTGATGTTAATTGTGATCCATCCAAAATAGGAACTGATCAAGGTGCCCAACCATGTCAATATCAAACTGTTATTGCAGAGTATTCTGCTAATAGCTCTGGATCTCAGCCTTCCTCGGTGAAAAGTGCTAAACCAACTGAGGTGAGAAGAATATTTACCATGGGGCTCCCATTTACATCTCAACATGGAGGTCAAATACTCTTCGGACCTGATGATGGATATTTATACTTAATGATGGGATATGGAGGAGGTGTAGGTGATCCTTACAATTTTGCACAAAACAAGAAATCATTGCTTGGAAAGATTATGAGGCTTGATATAGACAACATCCCAAGTGCAGCAGAAATTAGCAAACTCGGACTTTGGGGTAACTACTCCATTCCAAATGACAATCCATTTAGCGAAGATAAAGATCTGCAACCTGAAATATGGGCCTTAGGAATGAGGAATCCTTGGCGCTGCAGTTTTGATGCAGACAGGCCTTCCTACTTTCTCTGTGCAGATGTTGGACAGGATCTTTACGAGGAGGTGGATCTCATCACAAAGGGTGGAAACTATGGCTGGCGTGTATATGAGGGCCCCTATCTGTTCCACCCTTCACAATCACCTGGAGGAAATACTTCGGTAAATTCCATAAATCCAATTTTCCCAATAATGGGATACAACCATTCTGAATTAAACAAGAATGAAGGATCAGCATCCATCACTGGAGGATATGTCTATCGATCTACCACTGATCCTTGCATGCATGGAAGATACTTGTATGGAGATCTATACGCTGGTGCAATATGGGCAGGCACAGAAGATCCACAAAACAGTGGAAATTTCAGCACCAGCAGACTTCCCTTCAGTTGTGCAAGTGATTCTCCTATAAAGTGTGACTCTGCACCAGGAAACTCCCTTCCAGCTTTAGGCTACATCTTCTCATTTGGGGAAGATAACAACAAAGATGTTTATGTCCTTGCAAGCAGTGGCGTCTACCGAGTTGTTCGGCCAAGTCGTTGCAGTTATGCTTGCTCGCAGGAAAAGGCCACAACCACCACAACACCACCTCCTTCTCCTTCTCCTTCTCATGCTACACCTCGGAGTGTCCTCTCTGGGTATCTGCTTTTGCAATGTTCATTTTTCTTGTTGATTTTGTTGGGTTTTATGTAGAGTTATGTGATCATTGTACATTATGCAGATTTTTTAGAGAATGTTTACCATTTCTTCTGTCTTTTCTACATTTTGGTTCCCTTGCTTTTGCTTTAATAAATGAGTTGAGGTGCTTTTTATTAAAAAAAA

>C101871_65

CCCCAGTAGAAAACAATGAAGTAAATTTTAATTATATACGGAGTAAGGAATACATGATCACATTTAAACAAACTTTACAGCCATAAGAGAGTATCACAATGCAAACAGTCCAGAGAGACATTAATTTTGATGTACAAATGAACCCCCACAGTGTTGTTCCCTCCCCACCAACCTTCTTGGCTTGGCCCGGTTGATACATTTCATTTCAGAAGTTTCCAAGCAGCAAAAGTTTTTACCCAATTCCAAAACCAAATAAACAAAATAGCAAATTACAGTAAGAGACTAGGCAGAGTCAATGAAATCGCCTAATCCCAGACTTGAAACAAAATAATTTATGAAGCAAATGAACAAAATAACCCGGTATGAAAGAATGAAGTCCCCCACCCACCCCCGAATTAAGTTGCACAGTTGAAATGATGAAGAATATATATATATAACAGTAACACCCTTATTATTGTTGTTCACCTTGACATTCTCTCATGAACATGAACATGAATGGTAATTTAGAGACTGTTCCTTGAAGTCACTCTGCGATGGGTTTGGTCTTGGACCTGTAGAGGAGTTTCTTCTTCTTGGATGTTTGGTTATCTATGGTGAGTACAACCTTGCCAGGTTCACCAATTTTGAAACCGTTAGTGATTACGGTCTCATCAGCGGGAGCAATTTTTCTGTTCTTCTGCACAATGACAGTGTATCCATCCTCAGCGCTGGGCACGAATTCAGCTCCGTAGCTCACATCCCAACCCACCACACGTATTTCCCAAACTAGATGGCCTTTCTCAGAAACTGGGAACTCAACAGCATGTTTGGTAGCGGGTTTAATTGTGACCTCCGTAACAGGATCAGCAGTGGTGAATTCCTGTTCACCCTCTCTGCTCAGTCCACCATATTGAACCGGCACCAGCTCCGGAGAAATATATTTGAAAAGGGTTTCAGCAGACTTGGATTGCCCAGCAAACACAAATTTGCTCTTGGTCCTCTGTGTGAAGAACGGACTGATCATCCTAGAAAAGGCAAGGTACCACCATGGAACATTGATAAAAATCTGCTTGGCTACAAACTCAGGGTAGTTATCCTGAAGCAATTGAAGGGCCTGATTGGTGGCCTGCCTGAGTTCCCTCTTGCCGAGTCCCGGAGAGTTCTTAAGGTCGTTGACCTGAACAATGGTGGAGATGGCAGTGGGAGAGAAGTCAAGGCTTCTCACGCTCTTCTCCAGAACCTGAATCCTCCACCTGATGAACTTGTTGCGCTTCTCCTCGTCCGAAAAGGTTTTGTTGTACAACTCCTTATTCTCAAACTCACCAAAGACGTTGTAGTACACCGGGTGCCCTTGTTTGTCGTGTCCATCAGTGAACACCACCTTGTCCCAATCGCTTCCAAGATCTTCCAGCACAAGAGCGTCGATTCCGAATTCCTTCCGCCAACGCACGGTGTTCTTTATCATGTTGAAGGCTTCCTTCACCTTGAAATCCCTCGCTCTCAGAAACTTCAGCAGAATCACATCGCTCCTCTCATCCGCCAGCAGCGGAATTCCCCATATCTCAACTTCCTCCGGACCAACAACCTCTACCGCTGTCGTTTCTTCTCCCTCCTTCTTCTCTTCTGCAACTTCAACCTCCTTCTTCTCTTCAGTCACTTCAACTTCCTTCTTCTCTTCTGTCACTTCCACTTCTTTCTTCTCTTCTGTCACCTCCACTTCCTTCTTCTCTTCTTTAACTTCCACTTCCTTCTTCTCTTCTTTCACTTCCACTTCCTTCTTCTCCTCTATCACTTCGACTTCCTTCTTCTCCTCTGTCACTTCCACTTCCTTCTTCTCTTCTTTCACTTCGACTTCCTTCTTCTCCTCTGTCGTCACTTCCACTTCCTTCTTCTCCTCTATCGCTTCGACTTCCTTCTTCTCCTCTGTCACTTCGACTTCCTTCTTCTCTTCTGTCACTTCGACTTCCTTCTTCTCTTCTGTCACTTCGACTTCCTTCTTCTCTGCCTCTCCTGCTGGCACTTCTGGCTCCTTGACGATGTCTGGTTTCTTCTTCTCCGGTTCTGGTGTGGGGGGCTTGGGAGCAGTTAGGTCATGGTTGTTAAGGGCTTCTTGAACAAGCTTCTTGAGCTCATCAAGAGCTTTCTTCTGCGCCTCGGGGAGGTCGCCAACCACGTTGGTCTCCTCCTTGAACGAAACCGATTCGGAAATCTTGTCTTCAACAGCGGTCTGGGCCTTCTCTGCTTCCCCCACCACTTCTTCGATGCTTGCTTCTGCCTTCTCTACGGTTTCGGTTTCAGCTTCAGGTTGAGGTTCAGGTTCGATGGTGGGTGGTTTCAGTTCAGGTTCAGGTTGAGGTTCAGCAGTGGGAGGGTGGTGGAGAGGAACAGGGTCGACGATGGGCGGTAGTTCTTGGGCGGGAGGTGGGTTTTGGGGTTCTTGGGCCATGGGTGATTGGCTTGGATTGGATGGTGGAAGTAGAAACAAATAAAAGTATGAACAAAAAGAAAGAGTGAGAGGAAGAAGAAAAAGAAGAGAAAGAGAAAGAGATTGGGAAATGGGAAGAGTTGTTGTTGTTGTTGTGTTATGTTAGAGAGGAGTGTGTGATGCCAGCAGATACACACCACTTTCTTTCTCATTTGGTGGGTCCTCTTCACTCCCC

>C101873_65

AACATTTTCTTTGGTGGTTCATATCGTAAAAAATATATTATATATAAATATACTAGTACATAAAACTTTCTCCTGAGAAAGTCAGAGAACATATAAAAATCTAAACTTAAGCTTCCCCGTCAAAAAGAATAAAAAATACTAAAGAGTTACCGATAACAAAACAGTAAAACAGAGTTTGTTAATGGGGGGGACACATTAAATCCATCTTAACTTGAATCCTCCGTGGCCTTACCAACATCAACTATCTCTTCATTTCCACGTGGCAGCCTTGCAGCACTACCAGATAGAGCAAACTCACCATCTCTTTCCACCCCAGTTGCCACGACCACCTCTTCCACCACCATTGGAAAACCTATCATTTGAAAATCTACCGTTCCTGCCTCCTCCACCTCTTCCAAACCTGTGCCCACCACCTCGACCACCACCATCACCAAATCTGCCACCTCTTGATTGATCTCTCTGTTGCAAACGTGGTAATGCTTTCAACACCTCTAGACTTACATTAGCAGCATTTTCCTGACCACTAAGATATGTGTCTAAATCTTCAGCTGGTACATCAAAAACAGCACCATTTCCATCAGCAGTGAGTGAAAGACCTTTCACAGCCTCAACCTTCTCTTCAGGCAAGAATCTTCTCAAGATTCCATAGGCAAAAGAAGGGGTGAAGATTGGTTTCCCAATTTCAAGAAGCAATGTAACATAGTTCTCCATCGAAGTCAGAAGTGATCTTTGCTTTATCTCGGTATAACCAACAGCCTTTGCAAGAGCCTTAGCAAGCAATTCAACAACTGATAAACCAGAAGTCTTCAAAAGCTCTTCAGCAGTAGCTTTGAATGCAGGAATTACACTATCAGAAACTTGGGTAATCATTTCAGCAGCTTCCCCACCAACTGCTTTGGCAATATCATCAGGCCGAGGGGCAGATATGTGTTCAAATTTTACACCGGACTCTCTTTCTATCTTAGATATATTAGATCTTTTTGGATCATAAAGCGTAACAGCAACCCCAGTATTACCTGCTCTTCCTGTTCGTCCAGAACGATGGATATAGGATTCTACCTCCCGTGGGAACTCACACTGGATAATTAACTGAACATCATTGATATCAAGACCTCGAGCTGCCACATTTGTGGCAACTAATGTCATGAATTTCCCAGTCCTAAAACCAGACAGAGTAACCTCACGTTGTGCTTGCTGTATGTCACCATGGAGAGCTCTTGCTCCAGGTAACAACCCTGCAAGCTGAGAAGCAGACTCCTTTGTCTCGGTAAATATAATTGTCCGGCCTCCGCTGCTATAACAGCGAATAATGTCTGGGATAAGTTGGGACCTAGCAGAAGCAGTACAAGGGAGAACAATATGCCTCACATTGGTACTAGCCTTCATTTTTGTATTTCCAACAAGGTCAGCAGTTTTCTTATCTGGCTTCAGAAATTGTGCAGCAATATGCTTAACCCAGTCTGGCAAAGTAGCACTGAAAAGAAGAGTCTGAACTTTATTAACATTTTCAACCTTACCTAAAATCAATTCAACATCTTCAACAAAACCCATCCTCAGCATTTCATCTGCTTCATCAAGGACACGAAATTTCAGTTGGCTTAGGTCTATATTTCCCCTCTCGATATGATCCTTCACACGACCGGGTGTGCCAATTACAATATCAACACCTCTCCTAAGCTTAATTTCTTGAGTGTTATATGGAGCTCCACCATACAAACAACAAGAACTCAGTCCCATCGCCCCACCATAAACTTCAAAATCAGCATGAACCTGGCAAGCCAATTCCCTAGTAGGTAGAAGCACAAGAACGCTAGGAGTCCTCCCGTAGCCAGTCTTTCTTGAAGCTTTGGCCGGACCATTTGTTAAAGACTCTAATATGGGCAACACAAATGCCAGAGTTTTACCCTGACCAGTGCGAGCCCGACCAACCAAATCAGAACCGTCGAGAACTAAATCGAAAGTCATAGCCTGAATCGGGAACAAAGATTCGATCCCCTTCTCCTTTAATTTTTGCCTCAAAGGTTCGGATATCCTGAATTTGGAGACCGCATTAGGATCCTCCTTCTTCTCCTCTTTTTCCGCTGGTTCCATCACCAAAGGCTGTTCCTCGGACTTGGCCTTTTTCTTCTTTTTCTTCTTCTTCTGGTCGTCTTCTCTAGAAGGTTCGGGCTCGACCAGCTCGGAACTAGTGTCGTCGTCGGCATCGTTGGTCGCCGCCATGGTCTCTATATCTGAAGCCTTGCGCTTCTTCGCCTTCTTCTGGAGCTTCCCCGAAACCCCATCATGGTCGGGATCGGGGGCGTTGGGATCGGAAGGGAGTTTGGAGGCACTCTTCTTCTCTGCAATGATGGTTGGGGATTGGTTTTTGGTTTTCTTGGCGCTGAGCCTCTTGTTTTCTTCGGGGGTGGTGTCACTGTCAGAAAGAACGAGTGAAGGCATGGTGATGGCGGCGTAAGGAGAAGGAGAAAGAGACACAGAGAGAGAAAGAAAGAGAGAGATTTTCTCCTGCTTGAAAGGGGGTGTGGCTATGAGGTGCAGAAGCAGGAGAAGAAATAGGAGTAAGCGGCGTCGAGGTTTTCGGGTTTATGGTTTTGGGATGGAAACATTCTCATACACTTCGCAAACCTAATTCTCTTTTTC

>C101875_65

TGTTCGTCTCCGTTCTCCCCGTCCCCACCGGCGGCACAGGCCAACTGCGCCACCATATTTCTAATCACCCTTTTCTCAACTTCACTTCCGAAACCAACCATGGATAAACAAACCGCAGAAAAATCACGTCGGGAACGAAGGAAAGAATCCCGTTTGGCAAAGAATGCATCAAAACACCAATCCTGGCTCCTCCACCAGAAATCTCAAGCGATGAAAAAACATGGCAATAATTCAGAGATGGAAACGAAAAGCAAATCCGACACATCAGTTAATTCTAGTGTCAATGAAACACAAGCTGAGAACTTAGAGTCTTACTCTAGGAAACATGAGATAGATGAAGAATACATAGTGTCAGAACAGGGTGGTTCTGTTGCAAAGAAAATGAAGAAAATGAAGAAGGGTTCTAGCAAAAGTTCCAGGAAGAGCATGCTGGAGATGGGCATGCAGGATGTTTCTCTAGCTGCTGAAAAGGATCTAGGACTGGAAAGGAAGCTTTCCAAGAAGCTCAAGGTGAAGGAAGGAAAATTGAGGGGAATGGATGATGGGCTTAACATATTACTTGAAGGAATGTCGTCTTCTTTTGATTTCATGGGAGAGGGGGCGGTTCCTGGTATTGGAGAATTGCCTGTGAAGAGATTGAAGAAGAGCTCGTCAAGTAAGAAGGACAAGTTGTCAAAGAAAAGAATGAAAGAGGAAGCAATGGATGATGTATCAGGGGTTGTAGAAACTTCTGATGAAGATGTGGAATTGGATGATGTTCCTGACAGTGTGCCTTCAAGGAAGAAGCATAAGAAAAGGAAATTGTCAGGTCAACAGCGGGAAGACTATGCGGAGGATAATGCTGTTGGCATGTCTAAGCCTTTGGAATCATGTGGAATGGAGGGGAAATTGCGGGATGCTCCTGCTGAAGTTCCTGAAAAGAAAGCCAAAGAAAAATATATAGCGCCTCATTTGAGAGCTCGAGCTGGCAGTGAACCTGAAGAGCATACTCAAATTCGAAGACGTGTGCGAGGTCTTCTTAACAGGCTTTCTGAATCTAATGTTGAGTCAATTACTGGGGAATTGTCATTGATCTTTCAGTCTGTTGCTCGTAGTGTTGCTTCACGGATCTTGACAGAGGAAGTTTTGGCATCATGTTCTGGTGGTCCCCGTGGCAATCAACAGTATGCTGCTGTTTTTGCTGCATTTGTTGCTGGGATGGCTTGTTTGGTCGGTGTTGACTTCAGTGCAAAGTTTATGGCTTCCTTTGCTAAATGCTTTGAGGATGAGTACAATAAAGAAGACAATCTCTCCTTGCGGAATCTTACTCTTCTCTTATCCTATTTATGCATATTTGGAGTCTGTTCTAGTGATTTAATATATGACTTTCTGGTTATGCTGAGCAAGCGTTTAGCTGAGACAGATGTCTCTATCATTTTGGCATTGTTACAATGTTGTGGGATGAAAATAAGGGCTGATGATCCTGCTGCCATGAAAGATTTCATTCTTAGCATTCAGAATACATCAAATAAGTTGAAGGTTTCCTCTGAAGATGAAAGTCAAAAGAAAAATAGTAAAAGAATGGACTTCATGCTTGAAATCATATGTGACATCAAGAACAACAAAAGAAAGCCAAATGAGGACTCTGCACATCACACTCGGATTAAGAAGTGGTTACAGAAGTTGAGAGTTGATGACATTATGATTAGAGGGCTTAAATGGAGCAAGTTACTTGATCCTGACAAGAAGGGCCAGTGGTGGTTGTCTGGAGATGCGGCTACTTCAGCAGGTAATGTTGAAGAAGTTGCCAACAGAATAGACAAAGATGTTCTTGAAACCCAAAGAATGCTTCAGCTCGCCGCTGCTCAAAAGATGAACACAGATGCCAGAAGGGCAATCTTCTGTATAATAATGAGTGGAGAGGACTATATTGATGCATTCGAGAAGCTTCTAAGATTGGAATTACCAGGCAAGCAGGACAGAGATATCATGCGTGTTCTTGTGGAGTGTTGCTTGCAAGAGAAAGTTTTTAACAAGTATTATACAGTGCTGGCTTCCAAGTTATGCGAGCATGATAAAAATCACAAGTTCACTCTACAGTTTTGCCTATGGGATCAGCTTAAGGAGCTGGAGTCCATGCCACTTATGAGGTCAATGCACTTGGCAAAATTTGTGGCTGAAATGGTTTCCTCTTTCACTCTCTCCCTCACTTTTTTGAAAGCTGTGGATCTGAATGATATATCCTTGTTGACACCAAGAAGGATAATGCATTTCCGCATTCTGTTTGAGGCCATTTTGGAGCATCCTGAAAATTTGGTATGGAACATATTCACACGGGCAGCAGTGACCCCTGAACTCGAAAGTCTTAGACAAGGCCTTGAGTTTTTTATAAAGGAGTACGTTGTGAAAACTAACAAAGATCTAACACAAAAGTTTAAGTTGGCAAAAAAGGCCCTCAATAATGTGGAAGGAATTTTGATGTAAAAGTAAATTATGAATTCTACCCAATTTTGTAACTTAACTTTTTTTAGAGGAACTGTTAGTTTTAGGGGATGATTTTGAAAATTTTGATTGTCATTGCCTTTATTTGTTACTGTAAAGTATTTTGAATGAGAAGTTCATAGCATGCTAAAGTTTGCCCAGTATACTTTCTTTAGGACA

>C10187_65

TAACCAATTTCTTCTTTCAAAACCCTAATTTCCTCACGGGGAACACCCTCATCCTTAGGATGCAGTGCTCTATTACCAAGAGGAGAAAGAAAAGAGAAATGGAAGATAACAAACGATTTTGCTCTGTGAGTGGCATGAGTAAGAACAAAGCGTTGGTGGAGAGGTTGCAGCGCTATGG

>C101879_65

TGGGAGATACAACAAACTTTGACAGATTTACAGACTCTACAGCTTTCATATTTTACATGCCTCAATTATCAAATTCAGGATCACTCACATTTGTAACCAAAATCTGATTTTCTTTTTCCTAGATAAATAAACACTCAGGTGTATGTGATGCCTTTGTGGGGGAAAGGATTACAGCTAGTAGCTCCTCATTTTCATGTGGATAAATTAAAATTCCTATAAAAAAGGTGGGAATGATGTAGAAATCAGATAGCCTCTATATGGGGATTTATATTATATATAATCTACAAAAGAATCCATCGAGTGCCTCACAAAATTATATATCAACAGCATCAACTGGGAGGGAAACTGTCCTGTGCAGAGAGTGTGGAAGCAATGAGCCAGTGTAAAAACTCTTCGCAGTAACCATACCTTCGCCAGGGGATTCCGTCGCCTCACAATTTTGGGCCCTTTGGGTGTCCGAATTGGGGCTGCTTTCATGGACATGAGGGTGGTTACTGGAATCACAATGCCACTTATTAGTAATGGGTATCAACTGAGTCTCAATGTTGCCTTCTTTTAATGTTTCTGTCAGTGAATGTGACAAGAAGCCATTTTCTGTTTTGTGGTCTTTGTTAGTACTGTGAGATTGCTCAAACTGTCGATCCTTACGATCCCTTAATTCCATTTGGTATTTGGCTCTGAGCCATCTGAGTTCCTTTTGAATCTCTTTCTCATAGTCATCTTCAAGGTCACAATAAGCAGATCGGATAGTAGACAAATCATGACTTCCTGATAAACTTCTTAGAGAATTTGCTGCGTTGAGAGAAACCTTGTTTTCCCAAACATCATGTTCTTTATCCTTAGCTGGAATTGATTTATCTAATTGTTCGTATTGTTCATCAGAATGGCTTTGATCAGACTCTACTGGACTTAGTTCACGACTTTCATGCTGATTCCATAACTCCTGATACTGCAAGCTGTCTAATTGGCTTGATATGTTCAGATCCCCTCTAACATGGTTGTCATACTCTTCAGATTGGAAGGTAATCTCCTCAAACCGCCCATGCATTGAAGCACATCCATGCCTACAACACTCAAGAAGTTGCAAGTTCTTGTCACCTGGATTCTGTGAAAGAAAATCCAGAAAAGAGCCGCTTGACGTATGATTAGACACACAATTGTGACAGAAACCTTGATTTGCAAAACGGGGAGTTTCGTCAATTCCTGGTCCTGGTCTCCATTCAGGTACCAAGGAAGCAATTTCCCCATCAATCATATCAGCTATTCTGGTAACATCCTGATCAGTAATGTCGAGCTCTGCAACCATTTCAGTTGCCACACTTATTGCTGTGTCCATCTCTATGTCAAATGGGAAATAAATATTTCGGATGCGACCTTCTTTGTCAGCAATTCTTAGTCTCAAAAAGATGCCACCATCATCCTTCCTCTTGCCCTTGATGCTTATGTCAACATCTTCAGAAGGTTCATCATGGTACTCAAAAAGTTCAATTCCACTAGGTTCAATCTCAGCCGGATGAGGACACCAGTCTCCTTCATATCCAAAGCCATTTGAGTATTCATTACTGAAGTTACTATAGCTTCGATGAAGATCAAAGAATGGCTGTCTGATGAGAGGACCAAATTCATCCAATTCCCCATTTTCTACAGTTTTCATATCATATTCATAATCATCAATTCGAAGAAATGGATCATCTAGAAGCTCCCTTGCAGAAAGCCTAAGAGACACAGTTGCCAAGCATTTCTCAACAAATTGTCGCACCTCCGGATCCTTCACTCTGTATAAAGCATCTGGCTTTTTCCCAGAAACAACTTTCTTATAGATTTGAGCTGGATGAGTACATTCACTGTAAGGATATTCGAATGTGACCATCTCTAGTATGCACATCCCAAAGGAATAAATGTCAACTAGCTCATTATATGCCTCTTCATATACTTCTGGGGCCATGAACTCGGGCGTCCCTACACAATGAGCTGCATGAGATTTCCGGAGAATAGCGGCCAAACCAAGATCCCCAATCTTCACTTCCCCTTGGTTTCCATTGACAAAAATGTTATCACATTTTAGATCTCTATGGATCACAGGAGGGTCGTGACTATGAAGATAGAGAAGTCCACTCAAGATCTGTCTACACCAATGCTTCACCGCTCTTATGTTAACTCTCTTGTGTTTTAGCCTATACTGTCTAAGTGTACCGGAGGTGAACATTTCAGTCACGAAGTTGATATTCCTATTCGCAGTATCAACCCAAGAAGTGTAGAATTTCATAATGTTCCTGTGCTTCAATGTTTTCAGGAGATGAATTTCACAGTAAAGTCTCTCAAGATCTTCAGGACTTTGCAGAAAATCATACAGCTTGACCTGGTTCCAAGCAACTTCAATCCCTTGATACTCATCAAATGCTCTATATACTGTCTTTGAAGCTCCTTTTCCAAGGATTTCATTGTATCTGCCATATCTGCCAGTTGGATCAACTTCAACAAACTCAGAGTCATCTGGTTCAAGGTGTGTGACCCCATTCATCGTCTGAATTTGCTGCACTTGGTGCTCTCTTTTCCTATGATTAGATTTCTCTCCTATGCCCTTCCTCTTTTGTTATGACACACGAACACAACCCAAAAGGGAGGAAGAGGGAGGAATAAT

>C101881_65

CATCATTCTTCATTCATCTCCCTTAAACCCCAATTCACAAAATCAGCATCTTTTGTCTCACTCATCTCAAACCCCCCAAACAAATCCAGTCCCATAATCATAAATAGAAACAAAAAACATCAAGCTCCGTGTAGCATATCATATCATACCATTTTGGTTGTGTGTGTTGTTTATTGTAATCATCACAACACAATCAATCATCATAGGATGAAGATTGTTGTGATGATGATATTGCTACCCTTGATGTTTGCACTGTTGTCTTTCACGAGGTTGAGTTCAGCAGCACCCTCTCCTTCCGCAACAACCACATGCCCCATGGACTTGAATTATGTGCTGAGAATCCCTTGGAACACTTCTGCTTGTCACAACTTCCAACAAACTTTGGCAGCAAAAAATGGAACAGATGCAAACACTTGTTGCATATCACTGTTGTCCCTCTTTGGCATAGGACTTGCACAGCATCTCAAAGAGACTTCTCAGTTCCAACTCCCCGACCTTGCCACCTCCGATTCATGTATCCAAGACTTCCAAGCCAAGCTCTCTTCCCTCTCTCTCCCCAACAACCTTGCCGACTCCTGCTTTGACCCTCTTCAGTTTGTGATCTCCCCAAACATCTGTGCTGGGATTCAAACCATCCCCGATTGGACCAAAAAGCTTGGTCAGTCCACACCACTCAACACTGCTTGCAAACCGGATCTCACGGATCTCTCCCTCTGTGATTTGTGTCTGGCAGCTGGGTTACAGGTCGAGCAGGAGCTTATTTCCATAGATGGTAATGCTTCACATTCCATAGATTGTTTTTACTTTGCCATTCTCTATGCTGCTGGGATTGTGAATGAGTTTGGACCTGAAAGCAATGGTGCTGTGACTTGTATTTTTAGCATGTCGGTTTATTCACAAGGGGGGTCTGGGGGAAAGAGTCACCAGGCTCTTGTGTTTGGGTTGACAGGAGCTGGAGTTGCTTTGCTGGTGATGTCTTCTTTGTTGGGCCTTTATGTGTGGTATGACAGGAAGCACAGAAGGAAAAAGCTTGAGTCTTTTCAGTTTGATTTCGACCCTGAGGAACAAGGTTCTAGGCGTAGATTGAGGCCCAATACGGGGTCCATTTGGTTCAAAATTGGGGAACTTGAGAAGGCTACTGATAAATTTTCATCTAAGAACTTCATTGGCCGAGGTGGATTTGGGTTGGTTTTCAGGGGAACCTTGCCTGATGGGACTGTGGTGGCGGTTAAAAAAATCTTGGAATCTGATTTTCAAGGGAATGATGAGTTCTGTAATGAGGTGGAGATTATTAGCAACCTGAAGCACCGGAATCTGGTGCCTCTAAGAGGCTGTTGTGTGTCTGAGGAGAGTGAGAATTATGATCAAAGGGAAAGCCAAAGGTATCTTGTGTATGATTACATGCCAAATGGGAACCTGGAGGACCATCTCTTTGTATCATCGGACTCTCAGAAAGCAAAGGGATCGTTGACATGGCCTCAGAGGAAAAGCATAATCTTGGATGTGGCAAAGGGATTGGCCTATTTGCACTATGGAGTTAAACCAGCAATTTACCACAGGGATATCAAGGCCACCAACATACTTCTTGATGAAGAAATGAGAGCAAGAGTTGCTGATTTTGGTCTTGCTAAACAAAGCAGGGAGGGTCAGTCTCATCTCACTACAAGAGTGGCTGGAACTCATGGATATCTAGCCCCGGAATATGCACTCTATGGTCAGCTCACTGAGAAGAGTGATGTCTATAGTTTTGGTGTGGTTGTTTTGGAGATTATGTGTGGGAGAAAGGCTCTTGACTTGTCTTCATCAGGGTCGCCAAGGGCATTCTTGATCACCGATTGGGCTTGGTCATTAGTGAAGGCTGGGAAGTTAGAAGAGGCTCTAGATGCTTCCCTGGTAAAGGATGAGAACTTTCCTAGCTCAAATCCTAAGAGCATAATGGAGAGGTTTTTGCTGGTGGGGATTCTATGCTCCCATGTGATGGTTGCTTTGAGGCCAACAATTGCTGATGCTTTGAAGATGTTGGAAGGTGACATTGAGGTTCCACAAATCCCAGATCGCCCTATGCCTCTTGGCCACCCTTCCTTTTATAATGATGGCAGTACTTTTAGCATATCTCCAGCATTAAGTGGCCCCAAGTTGCAAACTGGAGACATGCTCAGGTAACGAAGAAGTTCCCTTATATTCCTAGAAATGTAGAAAAAAAGTGGAAGCTGTTTCCATTCTAAGGTCTGCCAAAGGTTCTGGTCAAATCATGTTTGGTAATACCACTTCATTTGAGGTTAATAAACTGATGAGTGTCATCAAGGTACAAATAAATTTCCTCCCAACAAACTGCTCTCAAGATCTCAAGATCCAAGCTAGCAATGACTGTAGGATAGGTCTTATGTGCCTCAACAAATCCTATGCAGTGTTATAATGCCAACCATGAAGAAACTTAACAGTTAGCAGACATCATTATAATCAGCAACCGAGGAAGCTAAGGGTCATTTAAATGCCTGAGAATTGGTATATTATAGGAACTTAGGCATAGGTAAAGTGTCTTGTAAAAAGAAGGTTGCATTTTCGTCTCAATTTTCTTGTACAACAAAGTCACACATAAAAACTCTTGTATATATAAAAAAAATGTTAAGTTGGTCGGAGC

>C101883_65

TTCCACAAAGTTTATGTAATGTACAACAATCGTGTTCCTTTTACTATATTGCTCATAACGTACGTACAAAGACAACCTAAATAATCACTCCTGTAAAGAATAAATAACTTATCCACGAAAAGAACTGTTTAGGTTTCCCAAACCAATATACACTGAATATGAAGAAATAAAGACTGCTCAGACATTCAAAGTGTTAAAAAAATCAATTACTAACACAAAGAATTGCTTTTTTCTATACACTTTTCTGCAGGTTGTATGGAAGGTGACCGAATTTATGAAACAGGTATAATAATTTATTCAGCTTGATTTATTCTGGGCTGCTTCTTTTCCATTCTGAAAAATGTAACCTTCTCCATTTCCTGCAGTGTATGTTGTCATTTGAAGAAGTGTGATTATCTTCAACTGCTATATAATTTAACTAATTTTTCTAACAACTCGTGGTTGTTGCCTGGAATTTCTTTAACCCTTGATCCATTCTTGTATATTTTAAAGGCCGGGATGCAGTTCACACCTTCTGATTTTGCCAAGTAGGGGTGGTCTTCAATCTCCACCTTAAGAAAATTAACTGATGGAAATCTCTTGCAGGTTTGCTCCAATACCAACAGCACTTGTTTGTGAGTTGCCTTGTTGCAAAAGAGCACCACAGACATCCCAGGTGATGTTACATAGTGTCTAAACCGATCATTGCTTGAGATGAAAAACAAATTTGAACCAAATTTCAAGTCTTTGATATCTTCACCACGCAGCATCTTGAGTTGAAGCTGAGTCTCAAACAGAGCCCTAGCCACCTCCTCATCCCCTGGCTTTTCTCTTAATAGCATTTCATAGTCTTGAATCGCAGCTTCCCATCGCTCCAACTTGGCATTACAATCTGCCCTCCTCAACCTTGCCTTGCTATAACTAGGCTGAACTATTAGTGCTACGTTGCAATCTTCAATTGCTTTCTCAAATTGACCTAGCTTAGAACGACATGCTGCCCTATTACATAGCAGCACTGAGTTGTATGGATCATGCTCTAGTCCTTCATTGTATACAGCGCATGCTTCCGTGAATTTTGATGCCTTGAAGAGTAAGTTACCACTCATTCTGGCTGATGTCACTGCTCTGGCTCTCCTCACCACTGCATTCACCTCAAAGCTACTTGGATCTAGTTTAGCTGCTTGCTGAGATGCTGTTACAGCATCCTCAAACCTGCCGGCTGCCAAGTAAATTTGCGCGGTTATCAACATCAGGTAAGCACTTCGAGCTGGGCCAAATATTTTTTTGCAACTATCAAGGTCAAATTTTGGCATTTTCTCGTAGGTAGCATATGCCTCTTGATGTCTTAGGAGCTTCAGCAAGGCTTCAGTTTGTAAACAGTAGACCTGTGGAGCGGAATCAGCACCTAATGATACTGCAGCCTGTGTTTCCTTTAATATAGCTTTCCAGTCTTTGACATTCCGAGCTTCAGCGCATTTGCTAAGGTGATTTTGCAGAGCCTGAGCTTGGAAAGCGAGGACGGAATCAACACATGGGCTTGTTTCATTGCAATTCAGTGTCTTTTCTGCTTCTCCGAGTCTGAAATAAATTGTTGCCAAACGGTTGTGGGCTCTGCCATAAGAAGGCTCCAACCTGATAGCTTCCTCACACTCAACAATTGCCTGCAAAAACCTTCCCAAACCTATCAAAGCTGCGCTCTTATTGCAATGATACGTTGCCTTATTGGAGTCAAGAGCAATGGCTCTGTCATACAAAACCAAAGCCTCCTCAAATCTTCCTAGCTTGTAGGCTTCATTTCCCATTGACTTCATCACCTCAGGGTCCGCTCTGCTGTTCCTTGGACTCCGAAATTGCGCAAGCTCATCGCTGTTCTTCCTCATGATATTCCCCATGACGCCTTTGTTGGCGTTACTATTGTAAGCAGAGGAGGCGGACATGGAGTTCAACTCCTTGCTCTTGGTAACACTAGTTCTGGGGCTGCTATTCACAAGCAAATTCCCAGTGAGTGGTAACACAAATTCTTTGGCTGGGGATTTTGTCTCGTTGTTCTGATGATGACCAGCACTTGTACTTATCCTAGCAAGTTCCAGAGAATTACCAGAGTTCTCTTTCTGTTGCTTACTCTCATCATTTGGGTCATGGTTTTGTTGAATTGAGGTTCTTGCAGCGTCTGAGGGTCTCCCATTTGGAGTGTTTCGGTATGCAGAAGGTGCACGCTGACTAGAGCTACTCTTTCTCACAGGATTCTGCTCCCCTTTCAGTGTAGGAATGGTTCCATTGGCGCCGGTATCTCGTGGAACCTTTGGTTCAAGGTTTGGAGGGGGTTTGGATTCGTTTTTGGCCTGGTCCCTCTGTTGTGCAGTGTTGAAAGCCTTCACAGGTAGTGAATGAACAGAGGATTTTCTTGGCCTGTTATTGGTTTTGAGGTGGAGAATTCTCCCCACTAAACCGCAACCCAAATCAAAGTCCATCTTGTTCTTGGTTTTCCCTTCCATGCTTGAAGATCAGTCTCAATAGAGAAAATATCAGTTTCGCAGAAACAACTGCACGTAGGCAAAGAAGCTGAGAGAAATTTGAGAAGGTTATGACAATGAAACAATCAAGTGGTGGCTTGAATACGATGGAGTTTATGTTAGACGTGTCGTTTGTGGGTGGATATATA

>C101887_65

TTGCATACTCAAGACTCAAGGAGTACTGATTACCATAAACTACTGAAATAATACACTTTATCTGAAATTGATAATAGAATTTCATTTATTTCCATAAAGCTCTATTCTAATTTCTCATACATACAATGACAAAAGTTGCTTGTAACATACACTTCATCCTGCAACTTGACAACCCAATAGAAATTACTAATTTCTTCCTTTAATAATCACAACATGATCAATGGGCATGTAATCTGAGCACAGAAGATAATTTCAAAAGGATGAAAACAAGAGAAGAGGTAGAAGGTGTGAAAATCATATGCACAAATCAGAAGTATATCCCTTTCCAACAGAGAGCCATGTCAGGCAGGATTGTATATTCACTTGTCCATAGAATTTTAAAGGGATCATCTAGGACCTGACAGTTCATCTGGTTGGATATGGGAAGTTGAATCTACTATCCAATTTGCATTAGGGTGATGAATGTTGTTGTTGAAGTCTTGCCGGAATGTCTCATCTTTCTGCCATTGCTCCCATTTCTCAGCCAAACCATCACCTTCTAGCATTCTGACCACTTCAGACATCTTTGGCCTTTCCATAGGAGAGCCTTGAGTACATAGTAAGGCCACTTGGATTAACTGCTCTACCTCGTCATCACTATAATTGCCCTGTAAATCTGCATCTACCAGTGTTTCCAATTTCCTGTCTTTGAGAAGTCCTTTAACCCAATCAAGCAACATCACATCATCATCATTGGCAAGTCGAGCTAGATCAAAAGCCCTTTGTCCAGTTATTAGTTCAAGAAGCATCACACCGTATCCAAAAACATCAGTCTTCTCTGAAGACTTTCCAGTTGAAAGGTACTCTGGCGCTATATGTCCAATTGTTCCACGTACAGCAGTAGTAACATGAGTATCTTTATAATCCATAAGTTTTGCTAAACCAAAATCTCCGACAACTGCTTCAAATTCCTCGTCCAACAAAATGTTCGCTGCTTTGACATCACGATGAATAATCTTAGGGTCACAATGATCATGCAAATAAGCAAGCCCCCTTGCAGATCCCAGTGCAATACGCTTCCGTTCTGGCCAGCCAAGTGGTGGTTGGGATTCTTGACGTTCTCGTAAACATGATGCTACACTTCCATTAGCCATGTAAGGATACACAAGCAATCGTTCAGTAGGTGTCATACAAAAACCACGAAGCCGAAGCAAATTCCGATGAACAGCCATGCTGATCATTTCCACTTCAGTTTGAAATTGGAGCTCCCCACCCTGTGTGCGTTCCTCTTTAAGTCTTTTCACTGCTACAAGAGATCCGTCAGCTAAACGTCCTTTATAAACCTTTCCAAATCCACCTCTACCTAGAATGTGTTTGTTACTAAAGTTATCTGTGGCAACTTGCAGCTCACGGAGCGAAAACCTTTTAAGCTGACCAAGGTGAACTTCAGGATCCTCCTCGGCAGGAACATCAAAGAAATGATCCTGAGGTTTCCTTCTTCGCCAATAAGCAAGTGCAATTGCAGGGGCTGCAAATAACAGAGCGGCACCAGCAGCAACTCCTCCAGCAATAGCTCCAGTGTTACTATTTCCTGAAGAAGCTGCTGGCGGTGTTGGTGACATTGGAGATGGAGCGTTTTTTGGTTGTATCAAGTCAGGATTATTTTGATAACTGATGGGGGTAAATAATGAAAATGAACCGTTCACTGGGATCTCCCCTTTTAGAAAGTTGTTTGAGAGATCAAGAACTTGCAAGGAAGAAACATTTGTCAAAGACATGGGTATGCCTCCTGTCAAGCTATTATTGTTGAGACGCAGGAAACGTAGTTTTGCAAGGTTGCCCAACGTAGTAGGTATGGGACCATTTAAAGTATTCAGGTACAGATCCAAGCTCACCAAGTTTGTTAAGTTCCCAAGCTCTTCTGGAATTTTTCCAGTTATATTATTACTATAAAGTTCCAAGTACTGTAAATTTGTGAGCTGACCAAGTTGTGGAACCAGTTGACCAGAAAGTTCTGCATTTCCGAGATCAACACGGGTCACACTGTTATCACTATTGCACGTAACATGAAACCAAGTACAGGGATTGACAAGGGTAGCATCCCAGCTTTGAAGAACATTGTTAGGATCAAGCAAGTTGCTCTTCAATGCATTCAGGGCGTCGCCTTCTTGATTGCCAGAAGCCGTAAGTACCAAATCAAGCACCAAAATTGCCCAAACAAAAAAGGAACTCATAAAAGATGAAGTCACTCTCTCCATGCCTCTTCAACTTCATAAACAATTGACCTTTATGACAACAACCCAATCTTCATAAACAATTGCTTCCCTAATCCACGCGCCCCTCTTGATCTACATGACACCCATCAAGCTCAAAACCATCTTAACCTTGTTGGCCAACCCAAAATCGCTTTGCTGCCCAAAATCGAACTCTGGGCAGCTTCAAGTTCTCTTCTTTCTCCACTTTCTGCTCTTACCCTGGAATCTGAAGCTAAAATTCCAATTGGATGTGTGCAGGTTTAGCCGAAAATGCAGAGAACAGTTTTGAGAGAGAAGGGTCGAGGAAAAGAGAAGGCCCAAGTCAACGAATGTTGCATCCTTTGTTGAGAAATTGCTCCTTCTATTCTAATTATTCTTTTCTTCTTCTTTTTTTAAACTTAAAAAAAT

>C101889_65

GAGAACCTCCCTCTGTTTAGTGTGCCTGGTGGCCATATCTGAGACTGTTTCGAGCTTCTCTTTCATTTTTCTCTCTGTTTTTTCTCTTCTTCTTCTTCAATGGTTATTGTTGTTGTTGAGTCGTCTTTTCTCTCTCTCGATCCAGACATGACATGACCTGACATCTCAGCGATCCAACCACTCAATTCTTGATTCAACTGCCCTGCCTTTCTCAATCGCGTTGATTCTCCCTCCTTCCGTTCACTGTGAGAACGAAGTTACTTGAGCTCCTATTAGGATTGTGTAGTAGAAAGATGACACTTTGAGCAACGTTCCCTCTCTCCATAGGATATCTTAATCTTGTCACTGGAAAAATATATCATGTCTGAAGAGGCTGGATCTATGTTGGTAGTTTATGATGATCCCTCCGACCAGCGCTCACTATCTTTGGATGACGCAAGCAGCACTGAGGAGTCTCCTGATGAAACCAGACTCTCTTTGGAAACAACACATGATGCTATTCCATACATTGGTCAAAGGTTTGCCACTCATGATGCTGCTTATGAATTCTATAGTGACTTTGCCAAAAGGAGTGGCTTCTCAATTAGGCGTCACCGAACAGAAGGAAAAGATGGTGTTGGAAAAGGACTTACTAGGCGCTACTTTGTTTGTCACCGTGCTGGCAACACTCCTGTCAAAACATCAACTGAAAGTAAACCTCAAAGAAATCGTAAGTCTTCTCGTTGTGGGTGTCAGGCTTACATGCGGATTAGCAAAACAACAGAATTTGGAGCTCCAGAATGGCGAGTGACTGGTTTTGCTAACCACCATAATCATGAACTTTTAGAACCAAACCAAGTTCGATTTCTCCCTGCATACAGAACTATATCGGATGCTGACAAGAACCGAATACTAATGTTTGCCAAAACAGGAATATCTGTTCATCAAATGATGAGGCTTATGGAGCTTGAGAAGTGTGTGGAACCTGGATATTTGCCTTTTACTGAAAAAGATGTAAGGAATTTACTCCAGTCATTTAGGAAATTAGATCCAGAAGAAGAAAGCTTAGATTTATTAAGAATGTGCAGAAATATTAAGGAGAAAGATCCTAATTTTAAATTTGATTACACACTTGATGCAAACAACCGTTTGGAAAACATTGCTTGGTCATATGCTTCCTCAATCCAATTGTATGATATATTTGGTGACGCCGTGGTGTTCGATACAACACACCGCCTAACTGCATTTGACATGCCGCTAGGGATATGGGTTGGAATAAATAATTATGGAATGCCTTGCTTTTTTGGCTGCACACTTCTACGGGATGAAACCGTGAGGTCATTTTCCTGGGCACTGAAGGCTTTCTTAGGGTTCATGAATGGGAAGCCTCCACAGACTATATTGACCGACCAAAATATATGTCTCAAAGAAGCACTATCCACAGAAATGCCTACAACAAAACATGCTTTCTGCATATGGATGATAGTAGCTAAGTTTCCATCCTGGTTTAATGCTGTTCTAGGGGAACGGTACAATGAGTGGAAGGCTGAATTTTATAGACTTTATAATCTCGAGTCAGTTGAGGATTTTGAACTAGGCTGGAGGGAAATGGTTTGTTCCTTTGGGCTGCATTCCAATCGGCACATGGTTAATTTATATAATTCACGCTCTCTTTGGGCGTTGCCATTTTTGAGAAGCCATTTTCTTGCTGGAATGACTTCAACTGGTCAGTCAAAGTCAATCAATGCTTTCATTCAACGATTTCTGAGTGCACAAACACGCCTTGCACACTTTGTTGAACAGGTAGCTGTTGCTGTGGATTTTAAAGATCAAACTGGAGAACAACAAACCATGCAGCAGAATCTCCAAAATGTTTGCCTCAAAACAGGGGCCCCCATGGAATCACACGCTGCCACAATCCTCACTCCTTTTGCCTTTTCGAAGCTTCAAGAACAACTTGTGCTGGCTGCACACTATGCATCATTTTCAATTGAAGATGGTTTTCTCGTGAGGCACCACACGAAAGCTGAGGGAGGTCGGAAAGTTTATTGGGTCCCTCAAGAAGGAATTATAAGTTGTAGCTGCCATCAGTTTGAGTTCACTGGTATTCTTTGTCGGCATTCTCTTAGAGTTCTTTCAACAGGAAACTGCTTTCAGATCCCAGATAGATATCTTCCTATTCGATGGCGTCGTATCAGCATGCCCTCTTCTAAGCTTCTTCTAACTGTACCAAACGATCATGCTGAAAGAGTTAAGTTATTACAAAACATGGTTTCATCTCTGATGACAGAATCTTCTAAATCTAAGGAACGGTTAGATATTGCAACCGAACAAGTTTCCATCCTTCTCTCTCGCATAAGAGAGCAACCAATTTCGTTACAAGGTGGTCGAGATATTTCTTCCATTAATAGGAATCTTTGACTCCAGGATACAAGATATGGTTGAGTTGGCAGCTCTGGTTCAATTAATAATTGTTACACCACACTCTTTATGAGAAAGCATAGTTACTGGATTTCTCATGCAAGGTTGAAACTATTTGAAGGGGGTAGAAGGTGTCGATTGACTTCAACTTCTGCAGTGAAATTTCTGTAGATTGGTTAAAGCGATGTTAAGTGAATGGCAATAATTCTATTTTGTACAAATGTATAGTAAATTTGTTAATAC

>C101893_65

GTTTAGCCTAACAAAGGAAATTATAAACACCACTAACATTTCAATATTTACCAACTAAAATCATGAAAAAATAACAATTCCTTCTACGATTTTAACATGTCCATTTGCCTAAAAAAGAGGCCACAAACAAGAACATAATGGAAGAAGTTTCTCTCCTACTCCTCCTCCCTTTTTATTCTTTTCATTGCATGCCTCAATTTTGTTAGCATGTCTACAGAACATCATTTTTTGTACAAGAAAATCAGCTCATCAGTCAGAGTCTGTCTCTCGAAATGGAAGTGGAACTGTCTTCACTGCCCGGTAATGACCAACGTTGTTTAGATGTTCATTTTCCAACCTATAGAAATTCCAATGCCCTCTTCGAATAACCTCAAGTGCAGCTAATAGAAAATCTAGTAGTCTTGATTGAACTTGTCCTACTTTGAAGTGCATGATAGTCTCAACCCATGTCACTCTTAGAACAATATTCAAGACAATAGATATATAGTATATGCTTTTATTCTTTAGAATTAGATCATCTCTGAGCCATGGATTTATGGAATTAGGGTTAAGAAATCCCCAGTCCTTGATGAAATCCCAGTAAAGTTGATACATTGTGGCCACCACAGAAGTGATTAAGACTATAACAAACCATAGATGGTCATGTTGCCTGCTGTATGTAACTCTAGCCCCTGCTGCCACCATTGCCGAAACATACTTACCCATGTTAGCCAAATGGTTCACATCACCATCATCAAACCACCGTCTCACACACTGCAAAGCCCGCCAATAGTATGGTAAAAATGAGATGATATAAGTTATTTCCATATATAGTCTTCCCGAATGACAGGCCTCAGGGTGGTGTGTTTTTAAAACTCTAGAAAAAATGTGACAGCCTGCAGTTTCCAAATGCCTTAGCAATGGAATCTGGCTAGTTAGTTGGTCAGCCATAAAAAAATCTACTAGCAGAACCTTATAAAAGGGAGAGCATACTATGTTGCGAATAACACGGATGAAGCAGTAACGTGTGGGGCGATAAAAGATGTCAAATGGACAAATGAGCAGTGCTATGAAAAACAGAAGAAGAACCCCAGGAATGGCATCAATTTGGGTAGGAGAAAAGTCAGCAGCCCTTAATAGCAGATGTATGACCATTGCTCCAACCACAGTTGTCATTAAGGTAGTGCTCATCAGAAAGGCATCTCTGTGCTTTAGTGCGGTGCTTGGTGAGAATTCAAATATGAAATTGTAATTGATTCTTGTATTCTTCCACATGAACAGGTTGCATCCATACATGAACAAGTGCAGGCTTAACAATGCAAACACACTGAAAACTGGATAGACTGTTTCCATATAAGCTGGCTCGTTACTAGCAGAGAATATGCCGCACAAATGGGCCAAGATTGCGTATACAGAAAACAAGGATACAAAACATCCTGTAGACAACCCGACAAGGAAGGTGACCATATGAGAATCTTTATGTTGTTGGGGTCTTAGAAATTTCATTGCCTTTTTCCTATCGTTGTTGGCAAAGTGCTTGGTGAACACGGATTCCACTTCATCCATTAGTCTAACAACCTTGTCAGAACTAACGAAATGGGATCTCTTCACTTCTTTTAAATAATTTGCAGAAGCTTTTTGACAAGATACCTTGTCAAATTTCTTTAATATTTTTGTAAAAGCCACCAAATTCAGTGAGCTGTAGGTTTTGAGGAGGCCAAGTCCTCTATAGAGCTCCACAAAAGCACTTCTAATCATTTTCTCAGCACATTGGAGCTTTCTCTTGTGAACGAAATCTCCTGTTGGATTGTTGACTAAATCCTCCCACAGCATGCTAGTGATGGCTGTAATGGCCCTAGTTGGGTTGGTCGCTGGAACATCAATTCTCATTGCCATTTTAGGTTTTCCCTTCTTCGTCTTTGTCCTCGTCGCTGAATTAACAAAGCTTATACCATTCTTCTCTAATGTTGTGATTACCTCATCAGTTTGTGAAACTTCTGAACTTGTTTCGTCTGAATCTCCAAAATTTTCTCCACAAACGGAATAATCAGAATCTCGGGTTGGAGAATATTGAGGGGATATTCCGGCGCTGTATGGCTTCGAAGGAGAATTTTTCCGGCGACGGTCACTGATAATTTTCTTGAGGTCAAGCAAAATCTGAAGCTGCTTGTTCAGGGTTTCTCCTCTCTCAAGAAACTCACTCTCTTGTCTCCGGTAAAATTGGTTCACCTTGTTTAGCTCTTCATCCAGCCTCACAAAAAACACACGTACCTCATCCTCTTCCGAAAATAACTGTGCAAGCTCTGTCTCGTAGATTTCTTCTTCGCTGCCCTTTGTGGTTTTCTTCCTCACCTGAATGATGTTGAAATCATGGTGGTCTGAAGCTGAGAGGCTGTGAGTGATGTTCTTGAGAAAGAAGCGAAAGGAATCGAAAATGGAGAGACCAAAGGAAGGATTAACATGGTGGTGGGATTGCTTGGGAAGTTTAGAGAGCTTGATTCTCTTTATCTGCTTCTTGAGCTGCCAGTAGTTGACGAAAGCCTCCTTCCATTCTGGTATGAGTTGAGCTTCTAACTCCTTTGAGAACTTCACCATTTGTGGGTGGATGAAGAGAGTGAGTTTGTGGAGAGAAAAGGTGAAGATAGGTTATATGAAGAAGTGGGT

>C101895_65

TGTTTATTCATTTGTTAAATCTCTCTCTAAAACGCGAAAAACAGATTTAAATCGCAGATATAGAGAGAGAAACAAACAAGACAAAAGAAACATAAGGAGAAAGAAAGCAAGGAAGAACCAAAAGCATCTATTTCTCTCTCTCTCGTTTTCTCACACACTCTCTTTCTCTCTCTCTCGTTTTCCCACACACTCTCTTTCTCTCTCTAAAACCACCATTTTCGAGGTCGAGATTTTCTGGCTCTATATATATGGTGTGTAATCTGTGTTTCATTCTGTGCGCGTGCGATTTGGAATCTGGTGGTGCGAGCAGTTGAATGTGAGTGAAAGAGAGGATTTCGATTTTCGGATTCGCGGTTAGATCGGGGATGGCAGAGGTTTCCGGCGACGGCGCGGCGCTGTCGGTGTCGGGATCGTTCAAGGAGGGAAGAGGCTCGGCGAGGCGCCGCGGCGGAGTGAGGCCGCCGAGCATGGACGCCGACGAGTTCATGAACCTGCTGCACGGTTCGGATCCGGTGAAGGTGGAGCTCAATCGCCTCGAGAATGAAGTTCGAGATAAGGACAGGGAATTGTCGGAAGCGCAGGCTGAGATCAAAGCCTTGAGGCTCTCTGAACGTCTCAGAGAAAAGGCCGTTGAAGAGCTGACAGAAGACCTCTCTAAGGTTGAAGGGAAGCTAAAACTAACAGAATCTCTTCTAGAAAGCAAGAATCTTGAAATAAAAAGAATCAACGATGAAAAGAAAGCATCAATGGCAGCTCAATTTGCAGCTGAAGCCACTCTTCGAAGGGTCCATGCTGCACAGAAAGATGATGACATGCCTCCAATAGAAGCAATTCTTGCTCCTCTGGAGGCGGAACTCAAGCTTGCTCGGCAAGAGATTGCTAAACTACAAGATGATAACAAAGCCTTAGATCGTCTTACAAAATCTAAAGAAGCTGCACTACTTGAAGCTGAGAGGACTGTCCAGTTTGCGTTGGCTAAGGCCTCAATGGTGGATGATCTGCAGAATAAAAATCAAGAGCTAATTAAACAGATTGAAATTTGTCAGGAAGAAAATAAAATTTTGGACAAAATGCATAGACAGAAGGTGGCAGAGGTTGAAAAGCTCACCCAAACTGTGAGAGAACTAGAAGAGGCTGTCCTTGCAGGAGGCGCAGCTGCAAATGCAGTGAGAGATTATCAGCGGAAAGTTCAAGAAATGAATGAAGAAAGAAAAACACTGGACAGGGAGTTGGCTCGGGCCAAGGTAACAGCAAACAGAGTAGCTGTTGTGGTTGCAAATGAATGGAAAGATGCTAATGATAAAGTGATGCCTGTCAAACAATGGCTTGAAGAACGCCGATTCTTGCAGGGAGAAATGCAGCAACTTCGTGACAAGCTTGCTATAGTTGAGCGCGCCGCAAAATCTGAAGCACAGTTGAAAGAAAAATATCATTTGCGACTGAAAGTGCTAGAGGAGAGTTTGAGAGGAAATTCTAACAGCAGTAATCGTGGCACTCCAGAGGGAAGAAGTGTGAGCAATGGTCGTCGTCAATCCCTAGGCGGAGCTGATAACTTCTCAAAACTAACCTCTAATGGGTTTTTACCTAAAAGATCACCATCCTTTCAACTCAGGTCATCCCTATCCTCTAGCACAGTATTGAAGCATGCTAAAGGAACATCTAAATCTTTTGACGGTGGTACAAGATCACTGGAAAGGAGTAAAATGTTGCTGAATGGAACACCTCCAATTTACTCATTCAACCAATCTCTTGAAGAAACCAAAGAGAGAGAGGCAGGTGACAATTGGAAAGGAAATTCAGATGATAAGCCAAATGACTTCCCAGCGGTAGATTCAGTAGACAGTGTTCCCGGTGTTCTATATGACCTGCTGCAAAAGGAAGTCATAACCTTGAGGAAAGCTGGTCATGAGAAAGATCAAAGCCTAAAAGATAAAGACGATGCTATTGAGATGTTAGCAAAGAAGGTAGATACATTGACCAAAGCAATGGAAGTTGAGGCTAAGAAGATGAGAAGGGAAGTAGCTGTTATGGAGAAGGAGGTAGCTGCTATGCGTGTTGAGAAAGAACAAGAGAACAGAGCAAAGCGTTTCAGCAATGTAAAGGGTTCTGTAAACAGTGCTCATCAACAGCTAATTTCTGGAAGAAATGTGACAAGGGGTGGATTAACACGCAGCACCCAATGACAATTAGTGTATTACAGTGGCGCTCGGGAGTAATATTTCATAGGCATCATCATTCTTCTGCTCATCTCTGTTTTTCCCTTGTGTTTTTGTCCAATTGATTGTAAGATTGTAACTGGCATGCCGAGGACCAGTGTTACTGATATTAGCCATGGATTGGGGATAAGAAAAACAATTGCAGGATTCGGTATGCATCTGACCTAGTGTTATGTTCTCTGCTTAGAGAGAGAGCGGCTAAGAAGGCAGGCAATGGAAGGCAATTAACTTGGACTAACCTTTCACTCTTTCAGCATTTCTGAGTGGTGTGGTTGTATAGGTAGCTTTATTCTCTCCCCTTCTACTCCTTTTGACAGTTTATATTTGTAATACGAAGATAGTGTGATTGTCTTGGTTCAGGTTTGTTAATATATACTAGCGGTTGATCCGTGTCATGCACGGAGTATATGTTTGCTTGTGAAATGATA

>C101897_65

TCAATCGAGTGCTTATTCATTACCAATTCCTACAACTTAGATACATGCCCATTGGAAATAACCAACTGAGACGTATGTGTTTTAAATCATCTGAGTCTATCTATCAATATCCTAAAAACAATCCTAGTTCAAATATCATGATAAGAGAATATTAAGACAGCAAAAAATATAAGAAAATCATACAAATGTCCACGAAAACGACTTGTTTTCACAGGAAGTTCCAGCCTCCATCATTGGTGTCAGAAAACTCCGTGATAGCAGAGTATCAAATATACTTCAACCCATCTTGAACTTAAACGAAATAGCAGGGATATCACAATCAGACAAATGAATAGGCCGAATATTTACCAAACATCTCTAAGATAACGTCCATCCATCTGGAGTTTTTTCACAATAGCCTCTGCTTTTGAAGAGTCCACATTTTCCTGCTGCTGAACAATGGTATGCAGAGTCCGATGAACATCTCTTGCCATACCCTTAGCATCACCACAGACATAAAGATAACCTCCCTGAGAAATCAAATTCCACAAATTTGCTGCTTTATCCATCATCTTGTGTTGAACATACTCCTTTTCGGGTCCCTCTCTCGAGAACGCTATTATCAACTCTGATAGAACGCCTTGTTCCACAAAATTCTTTAGCTCATCCTCATAAATAAAATCCATTTGACGATTCCTACATCCAAAGAAGAGTAACGCGGGTCCAAGTTGAATACCATTCTCTTTGAGATCCAATCTTTCCTGTAAAAATCCTCTGAAAGGTGCTAAGCCAGTTCCAGGACCAACCATAATAATAGGAATGGAATGATCAGCTGGTAACTTGAAGTTTGATGTTCTGATAAAAATAGGAGCCCAGCTGCAGTCATGGCTTTTCTCCAAGGGAATAGCACTCTTCATCCAGGTTGAACATACTCCTTTGTGAATTCTTCTAGTAGGAGTTGGACCACACACCAATGCACAAGTTACATGTACCCTTTGCGGGGAAAACCTAGGTGAGGATGAAATAGAATAATAACGAGGTTGTAAGCGAGGAGCTACTGCAGCAAAAAAAACACCAAGGGGTGGCTTTGCTGATGGAAACTCAGCCATAACCTCAAGGAGACTTCTCTGACTTCCAACCACCCATTTGGAGTACTCATCCTTTCCCTGAGGAGATGAGAGGAAATTTAATCTATCTGCTTCACTGGGTTCCGAAGTATGTGCAGCCAAAGCCACTAAAGCAGCCTTGCGTGGGGGGTTCAAGAGATCTGCATAATGTGCTAAAGCAAAGCGCAGTGTGCAAGGGCCAGGAAAAGCAGGTGGTAGGGATCCTCCCAAGGGAGTGCCATCCTCATTATCAGTGTGAAGGGAAAACAACAAATCTATATCCTGACCCAGCAACTCTCCAGCTTCTTTAACAGTTTCATCACAGTTTTCAGCAAAAACACCCACATGATCACCAGATTCATATACTATGCCAGTCCCTGATATGTCAAACTCCAAATGTATGCAAGATCGGTCAGAGTCAGGTTTGTGAAGTTCTCTTTGAACTGCAACATTAACCCTGCAAGGATGGTGAATATCAAACACAGTATTCCCATTTGCCACATTTAGGTGACTATCATTGCAAGATGTGACAGTTGAATCATGAATAACCACTCGATATTCAGGAATAGCAGCCCTATAAGGAGTAGAGACGGTATTTATATCATCCTCATCTCTGAGCAACTGATCCAACTCAGGCCATAGAGATTCCTTCCAAGCAGCAAAATCATCCTCAATGGATTGATCATCATCACCTAGCCCTAGTGCAACAAGACGCTTTGCACCTTGTTCACTAAGTTCTTGATCAACTATTTCACCTATCTTATTAAAATGTTCATATTGTCGGTTACCTAAGCCAAAAACACCATACGTGAGCTGTTGAAGCCAGGTACCCCTCTCATCTTTATCCTCAGTAAACCATTTGTAGAATCTTGCAGCATTGTCAGTTGGTTCTCCATCTCCATATGTTGCCAACATGAAAAATGCAAGTGACTCTTTCTTCAGCTTCTCTTCATATTGATCATCATCCATTGCATAGTCATCCAAGTCAACAACTTTTACAACTGCTTTTTCATATCTAGCCTTGATTTCCTCTGCCAAAGCCTTGGCAAATCCCTCAGCCGTACCGGTTTGAGTTCCAAAGAAAACCGCAACCCTGGTCTTCCCCAACCCAACATCGACTTCATCTTCCTCTTCTTCCCTCACCAGCAACTTCGGCACCGCGAGCGGCTTCTGCTCCTTGCTCCGATCCGAAGATTTCTTCCATAAGAAAACCAGAAGCCCAATCAGCAAGGCGACGGAGGTGGTGAAAATCACCAGTAGCGAATCCGAAACCGAAACGCCCAAAACGGTGCGCACCAGCTCGGAATTCGATGCCATCAGTTAAAGTCAAACAGTCGGAACTAACCGAAGCTCGAAATCAGAATGCGCACAGAGCTCAGCGCACTCCTCTCTCTCTCTCTCTCTCTCGGGAGCCCTAGGAGGTGGTTGAGCGCGAAACACAGAGAGGAAAAGAGAGAGAGCGTATTGGTGCGAAACGAAAGCGGTGCGTTTTGTCTGAGGACCCACTAGGTGGGAAAATAGGGAGGCTTTCTTGCGTGTTTTAAATTGATTGAGGATCC

>C101899_65

ATGTATGTGAGTCAAGTTATTCAATTTGCCACAAAGGATTCAAATGTTCTTAGGTTTACAACCAAGTTAAACTCTTATATACATCTTGATTAGCATGTCATGTCCTTACATTTTGGATGAGTCATGTGTAGTATATTTTATTCGAAGCCGTTCATGATGAACCAACAATCATACTAATCACATCTCATGATAAAGATCCTAATTTATAGCTCCATAATTTATTCAAAGCCCCTTCCTCAAGTACTTTAATGTTGTTCCCAGTATACTTGCATGCATACTGTAAAAGTATTTTGTAGTTTCTTACAACATGATAATCATGCCAAACTGCAATAATCTGAATTGAACCAATCATCTTACAAATCACATCTCATTCCCAGATCCTACTGGAATTCCTTCATCAACTACTTCAAAGTTATTTGTAGCTTTATAATAAAAAATAAGAGGAAAAAAATACAAAGATACTAAATCCATAACCTGGGAATGTCGTGTCTCCAAAGCAATTCAAAGGGATGATAAGGAATTGTCAGTCCATTTCCATGAAACAGCAATTGGACTCCTCACAGAGTGACTTCCATCACTCCATTCAATTGACCCAAAACTGTCAGAAGTATCGGGCGTAACTCTACTAAACGCAATCTCAAATGCCTGTGTCGTGATTCCACTACTGAACACAAGTGTTCTGGGGGACACACTAACTTCAACACCAAGTGGAGCATTCACTTTCGCAGTGTAAACAGCATCCACAGCACTTCCCACATTAGTAACAACCCTTTTATACTTAACCACATCACCTACCCCGCCCAACTGCACAGCGAAAGAGGGGTAATTCAAATCACCCGGACTAGCAAACTTCCCAGTCCTACCCACTTTCCCTTCACAAAAATTTGCAGCAACAGGCTCTCTAGTGAAAACCGCAATCTTCTTAGCATCATACTCAACTGAGCACAAAAACGCCAAATAGTCATCACTATCAGAATCATAAACCAATCCAGGATTAAGAGCCCTATTGGGATCAACATGACCAGCCCCATGAATAAACGGATTTGAATCCTTCCCACTTCCAAGATCCTTGATATTCCCTCCAGAATTATCCACATTATAAGCAGTTGTTATCAAAGCAGACTTAATAGCCGCAGGTGATAACTCAGGATAGGCTTTCCTAAGCAATGCAGCAATGCCACTAGCATGAGGACAAGACATAGAAGTCCCAGAAATTATGTTAAACTCAACCCTCCTAGGATCAATGTCCAAATCTGTAGGCCCCACCCTCCCAGTCCACCCAGCCAAAATGTTAACACCAGGAGCAATAACATCAGGTTTGAGAATCTCCGAAGTGAGAAAGTTGGGTCCACGGCTAGAAAAGGAAGCAACTTGAGGAGCAGAAGGCGAACCCCCAATCACAGTTCCTTTGAACTCAATAGTCGCAGTTGGGTACTGACTCAACCTTATATACTCCTTGATCTTATCCCCTGCAGTTTGACCCACCATAGTAGCTGCCAAAAGATGTGCATCAGCAAGAAGCTCTTCACCATTTTCTGCAGTGTTAGCCAGAATCATTCCCAACCCACCAGCCAGCTTCACTGCACTCCCCTTCTCAACCCGAGCATTCCCACCCCTATCGCAAACAACAATCTTTCCTTGCACTTTAGAAGGTTGCAGAGACCCCAAGTAACAGTACCTGTTCCCAGTATCTTTAGCATACACCAATCGAAGCTTAAAAGCAGGCAAACTTTCACCGTAATACAAAGACACCCCACCGAAAACCCTTCCATCTCCGAGAATAACATTAGCAGGAAACTCTCGATCGACGGTGGAAGCTCCAACGGTTAAAATCCACGGTGCAATGTTTACAGCAGTGAAAGGACCAGGCCCTGAGTTCCCAGCAGAACACGAAACCAGAACATTGTGACGAGCAGCGCCGAAAGCTCCCACAGCGATTGAGTCAAGGTAATACTCCGGCGGGTAACCGGACGTGCCGACGGAGAGTGAAATCACGTGAACTCCGTCGGCGACAGCCTCGTCCATGGCGGCGAGGATGTCGGAATCGAAGCAACCGTATTGCCAGCAGATCTTGTAAGCGGCGATTCTGGCTTTTGTGGCCATTCCTCTGGCTTCGCCTTGCGCGTAGTGAAACAAACTTGCGTTGGAAACGACGGCGCCCGCGGCGGTGGAAGAGGTGTGTGTGCCGTGGCCTTCGGTGTCTCGGGGAGACTTCGATTCGACGGATTCATCGATTGGTCCGTCAAGGAAGGCTTCGTAGCCTTTATAGAACGCTTTGGCGCCGATGATTTTTCTATTGCAGGAGGAAGCAGGGAAGTCGCGTGAGACTTCGCAGGATCCTTTCCACGTGGAGGGAACCGGAGAGAGGTCGAGGTCGGAGAAGCTCCTGAGCTCCGGCCATATTCCGGTGTCGAGGATGCCGACGATGACGTCGTCGGCGTAGTCAGAGTTGGGCCAGAGGCCAAAGGAGTCAGCGAGGCCCAGGAAGCGCGGCGTGTGGGTGGTGTGGGGGTGGCGGACCTGGTCAGGCTGGACGGCGAGGACGGCGGGGTGGTGGCGGAGGTGGGAAGCTTGGGAGGGCGTGAGGCGAACGGAGAAGCCGGCGGCAGCGGCGGAGTAGGTGTAGAGGAGGGTGGGGGTGTGGGG

>C101903_65

CCAAAATCAAAAGCTTTCGCCGCCTTCAAAGTTTCATCCCACCGTGCGTGCGAATGAAAATGCGTGGTGGTGGCGGTGAAGTGGAAGTGAAAAGGTTGTTTATTGGAGCAGGATGCAACAGAATAGTGAACAACGTTTCATGGGGTGCTTCTGGTTTCGTCTCTTTTGGAGCTCACAACGCCGTTGCTATTTTCTGCCCCAAGAGTGCTCAAATTGTGACTACTCTTCCGGGTCACAAGGCAGTTGTGAATTGCACTCACTGGCTCCCAACTTCCAAGTTTCAATTTAAAGCAAAGCAATTGGAGCAGCATTATTTACTTTCTGGGGATGCAGATGGTGCTATTATTTTGTGGGAGATGACCCTTGCTGATGGGAAGTGGAGGCAAGTGTTACAACTGCCACAATTACACAAGAAAGGTGTTACATGCATTAGTGGAATTATGGTTTCCCAAAATGAGGCAATGTTTGCTTCTACTTCTTCAGATTGCACTGTTTGTCTATGGGAACTTGTATTTCCATTGACTGGTAGCGGTGACTGTAAATTGTCATGCCTAGACTCTTTCTCGATTGGATCTAAATCTATGGTAGCCCTATCATTAGCTGAATTGCCTGGAGATGGTGGGCAAATTGTCCTTGCAATGGGAGGATTGGATAACAAGATTCACCTTTATTGTGGTGGAAGGAGAGGAAAGCTTGTACATGCATGTGAGCTAAAAGGTCATACAGATTGGATCCGGAGTTTGGACTTCTCTTTGCCTATCAATGTCAATGGGGAAGTAAACAATATTTTTCTGGTGAGTTCATCACAGGATAAAGGCATACGAATCTGGAAGATGGCGTTACGCAGCTCTATGTTGAATGGAAATGGCATATACAAGAAAGGAGAAATAAGCTTATCATCCTATATAGAAGGTCCTGTACTTTTGGCTGGTTCATCTTCCTTCCAGATATCCTTAGAATCTCTTTTAATTGGACATGAGGATTGGGTATATTCAGTAATGTGGCAACCCCCTTTGGTTTCATCCATAGAAGGGGAAACCTATTATCAACCACAAAGTATCTTATCTGCATCTATGGACAAGACTATGATGATCTGGCAACCTGAAAAGACTTCTGGTGTCTGGATGAATGTGGTCACTGTCGGGGAACTAAGCCACTGTGCTCTGGGGTTCTATGGTGGTCATTGGAGCCCAAATGGAGATTCAATTTTAGCACATGGATATGGCGGATCTTTCCATCTTTGGAAAAATGTTGGTAATGATAATTGGCTGACACAAAAGGTTCCCTCTGGTCATTTTGCACCAGTGACTGATATTGCCTGGGCTAGATCTGGTGATTATATTATATCTGCCAGTCACGACCAGACAACTAGAATTTATGCTCCATGGAAAGTTGAGGCTTCTCTCCAAGATGGAGAATTTTGGCATGAAATATCTCGCCCTCAAGTTCATGGTCATGATATAAATTGCATGGCAATTATTAATGGGAAGGGGAATCATCGTTTTGTCAGTGGAGCTGATGAAAAAGTTGCCAGAGTATTTGAAGCCCCATTATCATTTCTGAAGACATTAAGTAATGCCACTTTGCAGAAGTCTTACTCTTCGGATGATATCTTGGGAAATGTTCAGATTTTGGGTGCAAATATGTCAGCTCTTGGACTATCACAGAAACCTATTTATGTTCATGCTGTGCATGAGACCCCCGAAAGAAGCGGCATCGATGGTATTGACACTCTTGAAACTATTCCTGACGCCATTCCAACTGTGTTCACTGAACCTCCAATTGAAGATCAACTGGCTTGGCATACACTTTGGCCTGAATCACACAAACTCTATGGGCATGGAAATGAACTATTTTCTTTATGCTGCGATCATAAGGGCGAGCTTGTTGCCTCTTCATGTAAGGCCCAGTCTGCAGCTGTTGCAGAGGTATGGCTTTGGCAGGTCGGTTCATGGAAAGCAGTTGGCCGCTTACAATCCCACAGCTTAACAGTAACACAGATGGAATTCTCACATGATGACAACTTTCTTTTGACTGTCTCAAGGGATCGCCAGTTCTCTGTTTTTTCAATCACAAGAACAGGCTCCGGTGAAATTAGTTATAGTCTTCTTGCGAGGCAGGAGGGGCACAAGCGGATTATCTGGTCATGTTCTTGGAATCCACAGGGTTATGAATTCGCAACAGGCTCACGGGACAAGACGGTGAAAATCTGGGCCGTTGAAAAGGACTCATCCATCAGACAGCTTATGACTTTGCCACAATTTATGAGTAGTGTGACAGCATTATCTTGGGTTGGTCTCCATCATCAAAGGAGTAATGGACTTCTAGCTGTTGGAATGGAAAATGGGCAAATAGAATTGTGGAACCTGTCTTATAACAGAGCAGATGATGGAAGCATAGCAGCACCAGGTTTGGCTGCTGCTCTTGCTGTACGTATAGATCCTTTTATCTGTCATGCCTCTACTGTAAACCGTTTAGCGTGGAAGAAAAATGAGGAAGATCACACCAGTATGCAACTTGCTTCTTGCGGAGCTGATAATTGTGTAAGAGTATTTGATTTAACGGTTTAGTAATTATATTATTGTTCCCCCTTCTTCTTTGATATATCTGTAGATTGATTTTTTGATCCCCATAGCTATGGCTAAG

>C101905_65

AGAGTTATTACAAAGCTTTCAGCTTGAGTTGAAGTGATTGAGTTTGGATTTTGGATTTTTTTTTTCTCAGATAAGGAGATTAAAAAGAGAAAAACAAAATGATGGAGGTTGTGATTGTTGCAGTTGCTGCTACACTTGGGAATCTTCTCATGGGATGGGATAGTTCAACCATAGCAGCGGGTATGACCTACATCAAGAAGGATTTCGTCTTGGATGCGACACTTGAAGGACTAATTGTGTCCATGTCGTTTATAACCGGTACTGTTGTGACAATATTCTCTGGGGCAGTGTCTGATATGGTTGGAAGACGCCCTATGCTGATAACATCTTCTGTTATGTTTTTCCTAAGCGGTTTGGTAATGTTGTGGGCTCCTAATGTTATTGTTGTTCTGATAGCGAGGATAGTGGATGGGGTTGCCATTTCTCTGGCTGTTACTCTTAATCCTCTCTACATATCTGAGGTAGCACCGGCCGATATCAGAGGCCAATTGAACACTCTTACTCAGTTTGCTTGTTCTGGTGGAATGTTTTTGGCTTACATTTTGGTGTTCTCGATGTCCTTGACTGCTTCACCTAATTGGAGACTCATGCTTGGAGTTATTTCAATTCCTTCTGTTGCCTACTTTTTACTGGCTGTGTTTTACCTGCCTGAGTCTCCTAGGTGGCTTGTGAGCAAAGGCCGGCTACTTGAGGCTGAAAGAGTTCTGAAAAGGCTTCGCGGCACTGATGATGTTTCAGGGGAATTGGCGTTGCTGGCTGAGGGTCTCAGCCCTGGGGGTGAAGCCACTTCCATAGAAGAGTATGTAGTTGCCCCAGCTAGTGAGATTCTAGTCAACCAGGAAGCAGGGAAAGATTATATCAAATTATATGGTCCTAATGAGGGGGTTACAATGGTTGCTCAACCAGTGAATGGACAGGGTAGCTTGATATCCCGTAGTATGTTGTCTCAGCAAGGAAGCTTTGGAACTCTCACTAGTACTGGTCTTAAGGATCCTATTGTCACTCTCTTTGGAAGTTTGCATGAGAATACCCTCCCTGACAGTGGTGGTTCACGCAGCATGTTAATTCATAATGCCAACAGTATCTTTAGCATGGGAGAGCCAGAGTCCAGTCCCTTTGGTACCAGTGACAACCTGCATGCGCCACTAATGTCATTTCAAGGGGGTGGTGGGGAAAGGGCTTATGGATCCAAGGACATGTTAGGTATGAGGAGCAATAGCAGCCTGAGAAGTAATAGTAGTTTGGCTCATGGGAATGCTATTGAAACTCCTAAAAATACAAACATTGGAGGAGGTTGGCAGTTGGTTTATAAATCAGCTGATGGTGTTGGGGGTGGGAAAAGAGAAGGACTCCAAAGGGTTTATTTGCGTCCAGAACCTGCTGCAGTGTCTCACTCTCAACATTTTTCATCATTTGTTTCAACTTCTGGCTATGACATACCAATAGATGGCGGTGAAGCGTATCAAGCTGCTGCTCTTGTCAGTCAGTCAGTTCTTGGAACTCAAGATTTGTTGCATCTGCCAGAAGTTGCTGCAAAAGGCTCAAAATGGAGTGCTCTGCTAGAACCAGGAGTCAAGCGTGCATTAATTGTTGGCATTGGACTTCAAATTCTTCAACAGGCTGCTGGCATAAATGGATTTCTGTTCTATGCTCCCCAGATTCTTGAGCAGGCAGACGTAGGGGCTCTTTTATCAAATTTGGGCCTTAGTTCTGAATCTGCTTCTTTTCTTGTAAATATCATTACAACATTTTGTATGCTTCCTTGTATAGCTCTTGCAATAAGACTCATGGATATTTCTGGCAGGAGGTCAATCATGCTGTACACAATACCCATTTTGATAGTTAGTCTCCTGGTACTAATAATCAAGCAGTTCTTTGAACTAAATTCTGTCATCAATGCTGCAATCAGTTCTATCAGTGTTGTGGTTTATGAAAGCGTCTTCTGCATGGGCTTCGGCGTTATTCCCAACATCATATGTGCTGAAATCTTCCCAACAAGTGTTCGTGGAATCTGCATTTCCCTGACTTCTCTTACATATTGGGGTTGTACCTTGATTATCACCTTAATATTCCCCAGCCTGCTCCAGCTTCTCGGTCTCACTGGTGTCTTTGGATTATTTGTCGTTGGCTGCATCATTTCATGGATATTTGTCTATTTGAAAGTTCCTGAAACAAAAGGCATGCCTCTAGAAGTTATTATTGAGTTTTTTGCTATTGGTGCAAAGCCTGGTACTGATCCTGCATTAATTGGAATCAAATAGCTGACCCCTTCTTGCATATTGATACGATCATCATAAGAAGCAGCAGCATAAGTTTATAAGTTCAGTTGGTTTTGGCTCCTCCTAAAATGAGTTCAGAAGATTTGGTGGAAAATTGAAACTATTTGGCTTTAGGATATGCTAGCAGCGACTTTGCTTTTTCTTTTTTGTCTGCTTTTGGGAGAAAAAAAATGAGTATGATATAGTAATTTATTTATGCATATTATGATTAACATTTAGAGTATTCCATGTATTCTTTTTATCTTTTCATTCCTTAGCATTTGCTATATGGCTTTTTTCACCTTGCTTTGCAACAGGTGCTGTGATCTAGTGATATTTGAGATCTGCAAAGGTCATCATTATAATAATGGTTGTGTTTTCTACAAAAA

>C101907_65

CTGCTTCTCATTTGGATTTGGAACAAACAACTGTGGTGGGGTTGCGGTGCTCTGTGTGCTGCAATTTCATTTCATACTACTGTTGATTTGAATCAAACCATTTCGAGGGTGTGTTTATTTGTTTTATTCATTCATCAAGGATAGAAAGATGACTGTGGGGGCAGGGATATCTGTTGCCGATGGAAACTTGATGGTGTTGGGGAAGAAAGTGCTGACCCATGTCCATGACAAGGTTGTTGTTACCCCTGCATGTGGGGGTGCTTTGCTCAATGGAGCATTCATTGGAGTTGAATCCCATCAGAAGGGTAGTCGCACAGTCTTCCCAATTGGCAAGCTTGAGGGGTTGCGGTTTATGTGTGCTTTCCGGTTTAAGATGTGGTGGATGACGCAGAGAATGGGCACTTGCGGACAAGAAATCCCCATTGAGACGCAGTTCTTGCTTGTTGAAGCACACAGTGGCTCTGATATTGATGCAGGAGAAGACCATGCTGCTGCTACCTATGCAGTTTTCTTACCCCTTCTGGAAGGAGATTTCAGAGCAGTTCTTCAGGGGAATGATCAAAACGAGATTGAGATCTGTGTGGAAAGTGGTTGTCCTGCTGTGGAGGACTTCGATGGGACTCATTTGGTTTATATTGGGGCTGGATCCGACCCTTATGAAGTCATAACAACTGCTGTCAAAACTGTTGAGAAACACTTACAGACCTTTGTTCATCGCGAGCGAAAGAAGATGCCAGATATGTTGAACTGGTTTGGATGGTGTACATGGGATGCTTTCTACACTAATGTCACTTCAGAGAATGTGAAGCAAGGATTACAGAGCTTCGAGAAAGGTGGAATTCCAGCTAAGTTTGTTATAATTGATGATGGATGGCAATCTGTTGGCATGGACCCCAATGGTACTGAATGGAAATCTGATCATGCAGCCAACTTTGCAAATCGCTTAACCAACATCAAAGAGAATCACAAATTCCAGAAAGATGGCAAGGAAGGTCAGAGCGTAGAGGACCCAGCTTTGGGACTTCGTCATATTACCAATGAAATCAAACAAGAGCATGATATAAAGTACATATATGTGTGGCATGCAATAACTGGATATTGGGGCGGTGTTAAACCTGGGGTTCCCGGCATGGAGCACTACGAGTCACAGATGGCCTTCCCAGTCTCATCTCCTGGAGTTGAATCAAATCAACCAGATGAAGCTTTGACAACCATTGCCATCAATGGGCTTGGCCTTGTGAATCCTGAGAAAGTTTTTCACTTTTATGATGAACTCCACTCATATTTGGCATCTTGTGGTATTGATGGTGTCAAGGTTGATGTTCAGAATATCCTTGAAACTCTTGGAGCAGGACATGGTGGGAGGGTGAAACTTGCAAGGAAATACCACCAGGCATTAGAGGCGTCAATTTCTAGGAACTTCCCTGATAATGGAATCATTTGTTGCATGAGTCACAACACTGATGGACTATACAGCGCAAAGCGTTCAGCTGTTATTAGGGCATCAGATGATTTCTGGCCAAGAGACCCTGCTTCCCACACGATTCACATTGCATCAGTGGCTTACAATACTGTTTTCCTTGGTGAATTTATGCAACCAGACTGGGATATGTTTCACAGCCTACATCCAATGGCTGAATACCATGCAGCAGCACGAGCTGTAGGGGGATGTCCGATTTATGTCAGTGACAAGCCAGGACACCATGACTTTGATCTTTTGAAGAAACTTGCACTACCTGATGGTTCTATATTAAGGGCTAAACTTCCAGGAAGACCAACAAAGGATTGCTTATTTACTGATCCTGCCAGAGATGAAAAGAGTCTTCTGAAGATTTGGAACATGAATGATTTTTCTGGAGTTGTTGCGGTATTCAATTGCCAAGGTGCTGGGTGGTGCAAAGTTGGCAAGAAGAACCTTATCCACGATGAGAATCCAGGCACTGTCACAGGTTTCATTAGAGCTAAAGATGTTGACTATTTATCCAGGATAGCGGATGATAAATGGACAGGAGATGCCATCATATATTCCCATCTTGGTGGAGAAGCGGTTTACCTTCCAAAGGATGTCACCATCCCAGTGACCTTGAAATCCCGAGAATATGAAGTTTTCACAATAGTTCCCGTGAAGGAATTGACCAATGACATCAAATTCGCTCCTATTGGTTTAATAAAGATGTTCAATTCAGGAGGGGCTGTCAAAGAGTTCTTCAGCTTGGGATCTAATGAAAGTACAAATGTAGCTATCAAAGTCCGTGGTTGTGGCAAATTTGGTGCTTATTCATCAGCTCGGCCTAAGTTGATAACAGTTGATTCGGAAGAGGTGGAATTCAAGTATGAGGAAGAATCTGGATTGGTGACTGTTGATTTGAGAGTACCTGAGAAAGAGTTATACCAATGGACTATTTCCATTGATTTTTGAATCTGGGACTTTGTCTCTAGATGAGGGGGTTATATGCTTATAGTGTATCCATAGTTCGTGACAATTTGACGAGTAGATCAGTAGCAGGAAAGTTTGATTTCCTTGTAGCGAATAACCAGAAGTAATAAATAAATATCACAAATTTCTCTATCTATTTCTTATGATATACTCTAGCAGAATGTAAATGCAGTCTCTGCACCGATCTCCATACAGTATTAATTGATTGTTG

>C101909_65

TTTTACTGTAATACTCCATACACAATGATTAACAATAAAAAAAATTACATGATTGGTTTGGTTGGCATTGATTGAATTTGTAAATCACAAATCCAATATATGAGAATCAATCATCATCACGTTTAACTGGTTTGATACGGTAAAGATATGGTGAAGCTAAAAGTCCAAACCAATTTATTGGTTGATAGTTTGGTTTGGATTCTTAAGTTGATGAGATGATTCAAACCAATAAACACTCCGAAGCAATAAACACCCCCTAATGCATCTTCCATGTGTATTATTTTTATACAAACATACTTGAATCACACTAGGTGAATGATGCTACTTTTTTTTACCCTGGGGAGAATGGAAAGTTGCATGCTGAAACCCTTTGCATTTCTTTATGTCATGACCTCTAGTGCCTCTCTTGTTTGTCCAAGTAAATTGAACACTGTTGCTGCAATGTGAGATGGTGTGAGGCCAGCTGCATTCAATTGGTCAGCAGGTGATCCATGGTCAATATAACAATCAGGAAGAACCATTGGCCTCCACTTCAGTTTTCCATCAAGAAGGCCAGCAAGGGCCATGAACTGAGCAACATGAGATCCAAATCCTCCTATTGATCCTTCTTCCACAGTGACCAAAACCTCATGTGATTTTGCAAGGCTGCTTATAAGGGAATGATCCAATGGCTTGCAGAAACGAGCATCCGCCACTGTTACGCGCAAGCCATGATGTTCCAGCAAGGAAGCAGCAGCCAGACAGTTCTGAACAGCTGCTCCATAGCCCAACAATGCCACTCTTTCCCCTTCAATTAGTATCCTACCTTTCCCAATCTCAAGAGGAATGCCTTTATTCCCTGGTGGTAGTTCAACACCCACACCATTTCCCCTCGGGTATCGGAAACAGCTGGGTCGATCATTAATGGCAGCAGCAGTGGCAACCATGTGAAAAAGCTCTGCTTCATCAGAGGGAGCCATCACCACCATGTTAGGGAGGCATGCCATAAAAGTGACATCAAATGCACCACAGTGTGTAGGACCATCAGCCCCAACTAACCCAGCTCGGTCCATTGCAAATCTTACTGGCAGCTTCTGCAAATCTACATCATGCACCACCTGGTCATAAGCTCTCTGCATGAATGATGAGTAAATTGCACAAAAAGGCTTCAGACCTTCACAAGCCAGACCCGCCGCAAATGTAATGGCATGCTGTTCTGCTATCCCCACATCAAAGCATCTTGTTGGGAAACGGCGAAGGAAGATATTCATACCGGTTCCACCACCCATTGCAGCATGGATTCCAACAATGTCTTTGTCAGCTTCCGCCTCTGCAATCAAAGCCTCTGCAAAGTATGTTGTGTATGATTGGGTGGCGGCCGCGGCTTTGAATTGCTTTCCAGTTGCAGGATCAAACTTGGCTACTCCATGGTACTTGTCTGCTGCTCTTTCTGCATATGGGTATCCACGGCCTTTTTCAGTGACAACATGGATCAGCACTGGACCAGTTGTTTTAGTACTTTTAACTTCATTGAGAATGGCAACAAGATCATCTATGCTATGACCATCAACAGGACCTATGTAATAAAGTCCAAGCTCTTCAAATAGTGTTGATCCGGAACCACTGATCATGCCACGCGCATATTCATCAACTTTTGCAGCTAACTCGTGCATTGGGCCACCAATTTGTTTAGTGACTCCCTTAGCAACTTCTCTCAGTTCTCTAAGAGGTCTGTTTGACTGTAACTTACTGAGAGCACTACTCAAAGCACCTACAGGTGGTATGGGACCATCAAGATTAGCAGTTGGTAGGGAGACCTGCTTGTTGTCATTTAGAATAACAATCATGTCAGAATCAAGATATCCAGCGTTATTCATGGCTTCATAAGCTTGACCAGCCGTCATGGCGCCATCACCTATAACAGCAACCACATTGTTCTTTTCTCCCTTCAGATCCCTCCCAATAGCCATTCCCAGTGCTGCTGATATTGTTGTAGAGCTGTGACCAGTGCCAAAACAATCATGTTCACTCTCAGAGCGTTTGGTAAACCCGGCTAGTCCATCTGTCTGCCTCATGGTGTGCATCTTATCCCTTCTACCAGTGAGTATCTTATGTGGATAAGACTGATGACCAACATCCCACAAGATTTTGTCCATAGGAGAATTGAAAACATAGTGAAGGGCAACAGTGAGTTCTACAACACCAAGGCTCGATCCCAAATGACCTCCCGTTCTAGAAACATGGAAAATAACATCAGAACGCAGCTCCTCCGCGAGTTGTTTCAATTCCTTGGTAGAGAGATTCTTCATGTGAATTGGATAGTTTATGGTGTCCAACAGAGGAGTTGGTGGTTTCTGCGAATGATACTCTCCCATCTCTGATAGCGATGCACATATCCCATGTCGCCTTTTCCTCACCTGGTTGTGTCTGCGTGGGGATTGTGTGAGCAGATCTGCACCCCAATGAGAGTGGGGAGAAGGTAAAGCCAAAGGAGTTGATGCTTTGGGATCAGAAGGTGTTGTGTGTCTCAGATGGAGAGGAAATGATAATGCAGAGAGAGCCATGAGAGAAGAGACAGAAAGGGTAGAAGAGAATGAGATTATGGAAATGAATGTGTGTGACTGTGTGAGTAACAAGGTGGAAGAAAGACCTGCACTTCACAGCAGTGA

>C101911_65

CTCACTTCCTACCTCATCTTTTGCCTACCTTCTGTCTGCCTTCTAATGGATTCTCATCTCTTTCATGAAGATAAGAAGAAAACATGGAAGCAAACAATTCTGCTATCATTTCAAATCGTGGGAATAGTTTATGGTCAACTGAGCACTGCACCTTTATATGTATTTGGGACAATGCAAGTGGGAGATCTTAAAGCAGATCCGGAGGTCGTTTATGAACTCTTCTCCTTCATTTTCTGGACACTGACAATAATTTCTTTGATCAAGTATGCCTCTATAGTGTTAAAAGCTGATGATGAAGGAGAGGGTGGTACTGTTGCTTTATACTCACTGCTGTGTAGAAATGCAAAAGTTGGTCTACTCCCATGTGACAGAAGTGCGAACGAGGTTATGCTTTACGAGGAGAAAAGTCATTGTAAGATCAAAGCAGATTCAAGAGCACGAAGGGCCATTGAGAAGCACAAGATCTGTCACTATTTAATATTGTTCTTAGCTCTCTTCGGTTCCTGTATGACCATGGGGGATGCTGTGCTCACTCCAGCCCTTTCAGTGTACTCAGCTTCAACTGGAGTTCAGAGATCCTTGACAGACATATTGCACGACTTATTTACATCGTCACCACATACTAAGAGGGTCGTGTCAAAGGCTCTAAAATTATATGTACCTGTTCCCTTTGCGTGTGCTATATTGGTTGGCCTGTTCATGCTACAGCATTGTGGTACACGTAAAATTGGGATAATGTTTGCACCAATAATTGCTGCTTGGCTGGTGTTTGTTGCTGCTGTGGGAACGTATAACGTTTTTAAGTGGGATGTCAAAATTATCTATAAGATATCCCCATTCTACATATACAAATTCATCAGAAATATTGACGTGCCCAGATGGAGATTGTTAGGGAGCGTCATTTTATGTGTAGCAGGATCGGAGGCAATGTTTGCTGACCTAGGCCATTTCTCCAAGAAATCAATTAAGATTACATTTATATGCTTTATCTATCCGCTTCTTCTTTTATGCTACGCTGGTCAGGCTGCATATATCTCCAAAAATTTACCTTCTCCTGATTTTAATCATCTTAGTCAATCTATGCCAAGTCGTTGCAAACACTTGTACATAGTATTGTCCCTACTTTCTTCAGCTGTGGGAAGCCAAGCAACCATAACAGCGTGTTTCTCCATCATTAACCAGTGCCTCGCATTAAATTGCTTTCCCAGAGTAAAAGTTATTCACACATCGAAAACTATAAATGGGCAGATTTACATCCCCGATGTCAACTGGTTATTGATGATTTTCAGCCTCATCATCACAATTGGTTTCAGAGACATTGTGAAAATTGGCAATGCAACAGCTTTGGCTATTATATGTGGAATGCTTGTGACAACTAGTCTGATGTCACTGATCATTGCTTTGTATTGGGAGAAGAATTTGATGGTATCTGCGTGCTTTCTTATATGTTTTGGCTTCCTTGAGGCTGCATATCTTTCAGCATGTCTGCTACAATTCCACAGGGGAGCGTGGTATCTGGTTGTCCTATTGGCAGTTTCCATGACAGTCATGCTTTCATGGCATTATGGAACTATGAAGAAGTACGAGTTTGATTTGCAAAACAAGGTATCAACTGAATGGCTAATAGATATCAGTCCAGGCCTTGGGATTTCCAGGGTACCCGGAATTGGCTTCATTTACACCGACATAGTAGCAGGAATCCCAGCTTTCTTTTCACATTTCATTACAAATCTTCCTGCATTTCATCAAGTGTTGATCTTGGTGTCATTCAAGTCTATACCAGTGCCTTATGTCTCAGAAAGTGAAAGGTACCTCATAGGTAGGATCGGGCCAAAGGATTACAAAATATACCGCTGCATTGTCAGAAGTGGATACTGTGATCACATAAGGGACACGGGTGATTTTGAGGAGAAGATTATTCGTTCTATAGGGGAATTCATTTCCATTGAACAGAATGACAATGAGTCCATGGCTATCCCAGATGAAAGAATGATCTTCGTTGGACATTCAACAGTTGATGGAAATGCTTTAGTTCCTCTGGATGAAATTGACTCATCATGCATGGGGCAAAGCCAGGTGAACAATGAATCTCAGATAAGTCCGGTAGATGAGGCGTTGGAGAGTAGCAGTCGTTGTAACAAGAGAAAAAAGGTTCGGTTCATGTTGCCGGATCATAGTCCTAAAATGCAGGTATCTGTTAGGAAGGAGCTCTTGGAGCTAATTGATGCCAGGGAGAGTGGCAGTGCCTATTTCTTGGGCCAGTCACATTTAGTGGTGCGGGATGGCGCAAACTTTCTCAAGAGGTTCTTAATCATCGTTTATCGTTTCAGCGAAAAAAATTGTCGTGAACCTCCTGTGGCTCTTAAAATCCCTCATGCTGCTCTTGTAGAAGTTGGCGTGGTATGTACTATATAAGTCCAAGAAATTATGCAGAGTTTAATAGAACCAACACACAAGTCAGAAGTGAGAGTTACAAAAAAAAGTAGGCTAAAAAAACTAAAATATTTCTTACTTGCTCTTAGTTGTAGATCGGAGTCTTGTACCAAAAAAGAATTATAGTCCCTTTTACATGATGTAAACATTATGACGGATAAATGCTGATGTATATCTGGATGATGAGTTATAGGGCGCAAGAGTATAAAAAATTG

>C101913_65

CTGGTCCTCATCCAACACCAGGGTCACCGAATCTTCACTACCAAATCGCAAACTCTCTTACGAATTCATCCACACTTTCTGAAGCGCTCACCTGATTCTTGACGCACACCACATGTTTGAAAAATTGTCTCAATGGCACTATCCTCTCTCCACTGCTACCCTCTTCCTTACACTGTAATAACCCAACGCCACGCAACACCTTTTTCTTTCTCTTTCTCCTCTTTATCTATCTCTTCCACCTTCAGATTATCCTCCTCGCTCTCATCTTCCACTTCCGCCACTCACCACCCTCTCCCCCCGGACTTCTCCCCCTCCCAACTCCTCGACCTCCTCCGCCGCCAGCCCGATGAGTCCTCCGCTCTCCGCCTCTTCCAATGGGCCTCCGCCCAGCCCAATTACTCGGCCCACCCCTCAATCTTCAACGAGCTCCTCCGCCAACTCGCCCGAGTCGGCTCAGTCGACTCAATGCTATCCCTCCTCCACCAGATGCACTCCTCCGCCTGCCCCGTCGACGAGTCCACCTTCCTCATCTTCCTCGAAACCTATGCCAACTTCGAACTCCACGCCGAAATCAACGCCGTCATCAACCTCATGGAACGTGAGTTCGCGCTCAAACCCCACACGCGCTTCTACAACGTCGCCCTCAACCTCCTCGTCAAAGCCAACAAGCTCAAGCTCGTCGAAATCTTGCATTCCAAGATGGTCGCTGACGGCGTCGCGCCCGACGTTTCCACCTTCAACATTTTGATTCGGGCCCTCTGCAAGGCCCACCAGCTGCGGCCCGCCATTCTCATGCTCGAGGACATGCCCAACTACGACCTCAGGCCCGACGAGAAAACGTTCACCACACTCATGCAGGGGTTTATCGAAGAGGGTGATGTGGACGGCGCGTTGAGGATTAAGGAACTCATGGTGGAGTCCGGATGCGGGCTAACGAGTGTTTCCGTGAATGTTTTGGTGAATGGGCTTTGCAGAGAGGGTAGAATTGAAGAAGCTTTGAGATTTATCTATAATGAAGAAGGGTTTTCCCCAGACCAGGTTACGTTCAATGCTTTGGTGAGTGGGTTGTGTAGGACCGGGCATATCAAACAGGGTTTGGAGATGATGGACTTTATGCTTGAGAAAGGGTTTGATTTGGATGTTTATACTTATAACTCTTTAATCGCTGGGTTGTGTAAGTTGGGTGAGATTGATGAGGCTGTTGAAATTCTCAACCACATGGTTTCGAGGGATTGTTCGCCGAATACTGTGACTTTTAACACACTGATTAGTACATTGTGTAAGGAGAATCATGTTGATGCGGCGACGGAGCTTGCCCGTGTTCTTACCAGCAAGGGGTTTTTGCCTGATGTTTGTACATTCAATAGTTTGATACAGGGTTTGTGTTTGACGAGTAACCGGGAGATTGCGATGGAGTTGTTTGAGGAGATGAAGGAGAAAGGGTGTGAGCCTGATGAGTTTACATATAGTATATTGATTGAGAGCCTGTGTTCTGTAAAGAGACTTAAGGAAGCGTTGATGCTGCTGAAAGAGATGGAGAAAAGTGGTTGTGCTAGGAATGTGGTGGTTTATAATACTTTGATTGATGGTTTGTGCAAGAGCAATAGGGTTGAGGAGGCAGAGGAGATTTTTGATCAGATGGAGATGTTGGGGGTGTCGAGAAGTTCAGTGACGTATAACACCCTTATTAATGGCCTGTGTATGTGCAAGAGAGTGGAAGAAGCCGCTCAGCTTATGGATCAGATGATAATGGAAGGGTTAAAGCCTGACAAATTCACTTATACTTCAATGCTTAAGTACTTCTGCCAACAAGGGGATATAAAGAAGGCATCTGATATTGTGCAGAATATGACTTTGAATGGGTGCGAACCGGATATTGTAACCTATGGGACCCTTATTATGGGGCTGTGTAAGGCAGGGAGAGTAGAGGTTGCAAGTAAGCTCCTTAGATCTGTTCAGATGAAAGGTATAGTCTTGACTCCACATGCTTATAACCCTGTGATTCAAGCACTCTTCAAAAGGAAGAGAACAAAAGAAGCCATGAGGCTTTTCAGAGAAATGATGGAAAAAGGTGATCCTCCGGATGCTATATCATACAAGATTGTTTTCCGTGGCCTCTGTAATGGTGGAGGGCCGATAAAAGAGGCTGTTGATTTTACGGTTGAGATGTTGGAGAAAGGAATATTGCCAGAGTTCCCATCATTTGGTTTCTTGGCTGAAGGTCTTTGTTCTTTATCAATGGAGGACACCCTAGTTGAACTCATCAATATGATCATGGAGAAAGGAAGATTCTCACAATCCGAAACATCTATTATCAAAGGCTTCCTCAAGATTCGCAAATTTAATGATGCATTAGCTAATCTTGGTGCTATTTTGGATAGGAAAAGGCCCAGAAGATTTTGAGTACTGAAAGGCTTTGCTCTTGTGATGCTTTATAGAATGTAATGGGAAGGGAAAACACCTTCCTAAACCCAGGACTCCGAGCTATATTACAGACTTGTAGTCAATACATTTCAGCACAAGTGTTGGTTTCATCTGTCTAGCCTCATGATGATACGAGTTCTCCAGTCTTATATTTTCCAGTTTCAAGGGTAAAATGTGGACATGTAACTTGTAGCTAGCATTCATATGAATATTTCTTAATGGGA

>C101917_65

TATACACACAAAGTGTAGAGTCCAAATCTAAATCTGGGAACTACCATTTTCCATCTCTTCTTTGTCTGAAGAATTTCTCTTTCTTGCCTTCGTCTTTTCTCCTTCTTTGCCTCGCGGGTCTTCAATTCAAGTTGGTTGACATGGCTACGCATACACCGAAAAATATCCTCATTACTGGAGCTGCTGGATTCATCGCATCTCATGTTGCCAACCGGCTTATCCGGAATTATCCCCACTATAAAATTGTTGTTCTTGACAAACTTGATTATTGTTCAAATCTGAAGAACCTCATTCCTTCAAAATCATCTCCCAACTTCAAGTTTGTGAAGGGAGATATTGGAAGTGCCGACCTTGTCAACTACCTACTCATTACCGAGTCCATTGACACAATAATGCACTTTGCTGCTCAAACCCATGTTGACAACTCGTTCGGCAACAGCTTTGAGTTCACCAAGAACAACATATATGGTACTCATGTCCTATTAGAAGCCTGCAAGGTAACTGGCCAGATAAAGAGGTTTATCCATGTGAGCACTGATGAGGTCTATGGAGAGACAGATGAGGATGCTGTTGTTGGAAACCATGAGGCTTCTCAGTTACTTCCCACTAATCCATACTCTGCAACAAAAGCTGGGGCAGAAATGCTTGTCATGGCATATGGCAGGTCATATGGGTTACCTGTGATCACAACGCGTGGGAACAATGTTTATGGGCCCAATCAGTTTCCTGAGAAGTTAATTCCAAAGTTCATTCTCCTGGCTATGCAAGGGAAGCCTCTTCCAATCCATGGGGATGGTTCTAATGTAAGGAGTTATTTATATTGTGAAGATGTTGCAGAGGCTTTTGAAGTTATCCTTCACAAGGGAGAGGTTGGGCATGTTTACAATATTGGTACTAAGAAGGAAAGGAGAGTTATTGATGTTGCCAAAGATATATGCAGGCTTTTTAAGATGGACCCGGAGGCTAGTATAAAATTTGTGGAGAACAGACCATTTAATGACCAGAGATACTTTCTTGATGATGAAAAGCTGAAGATCTTGGGTTGGTCTGAGAGGACTACTTGGGAAGAAGGCTTGAAAAAAACCATGGATTGGTACATCAACAATCCAGATTGGTGGGGTGATGTCAGTGGTGCATTGCTTCCTCATCCAAGGATGCTGATGATGCCCGGTGGATTGGAGAGACATTTTGATGGATCTGAAGAAGAAAAACCTGCATCATATGTCTCAACTAATACTCGAATGGTGGTTCCAACATCCAAGAATGTTAACTCTTCTCTGAAACATCCTCTCAAGTTCTTGATCTATGGCAGAACAGGTTGGATTGGGGGTTTGCTGGGGAAATTGTGTGAAAAGCAAGGGATTCCCTATGAATATGGAAGAGGGCGCCTAGAGGATCGATCATCACTTGTGGCTGATATTCAGAATGTGAAGCCTACACACATTTTTAATGCTGCAGGAGTGACTGGCAGACCTAATGTAGATTGGTGTGAATCCCATAAAACAGAAACCATCCGCACCAATGTTGCTGGGACTTTAACATTGGCTGATGTCAGCCGAGAGCATGGACTCTTGATGATAAATTATGCTACTGGATGCATATTTGAGTATGATCAAGCTCATCCAGAGGGTTCTGGCATTGGCTTTAAGGAAGAAGACAAACCCAATTTCATTGGTTCTTTCTATTCCAAAACTAAGGCTATGGTTGAAGAGCTCTTGAAAGAATATGATAATGTGTGCACCCTCAGGGTTCGCATGCCAATTTCATCTGACCTGAGTAATCCACGCAACTTCATCACCAAGATTTCTCGTTATAACAAAGTCGTCAACATTCCAAACAGCATGACCATATTGGATGAACTTTTGCCTATTTCGATTGAGATGGCGAAGCGAAACTTGAGGGGCATCTGGAACTTCACAAATCCTGGGGCAGTGAGCCACAATGAGATTCTTGAGATGTACAGGGATTACATTGATCCAAGCTTCAAGTGGGTTAACTTCAACCTTGAAGAGCAAGCCAAGGTGATCGTAGCTCCACGGAGCAACAATGAGATGGATGGAACCAAACTTAAGAAAGAGTTCCCTGAGGTTCTTCCCATCAAGGAGTCGCTGATCAAGTATGTGTTTGAGCCAAACAAGAAGACTGCTTAAAAGCATTAAGCAATTTCATGTTTTAGACATCACAGGTTATGCATTTTTGTGGCGGGCTACTTAGATTACTCATTAATTCATTGTTATGTCATTGGGTTTTGATAAGAACCATACTGCACAACTAGCCGATGTAGTTCTTGTTCTCGTAGCTATAAGTGGTTCCAGTTTCAGCATAGTTGCATCATGTTACTTTGTTTAGAGGGTGGCAAGTATTATAATTGTGCCAGATATGGTTCCTAAGTTAACATTTCTGCCGCTTTATTCTAGTTTGTAGTGTCCTCCGTTATGAACAGCTTTAGATAATAGGGTTTTGGTACCTAAAAGCTTACTTCATGCAACTTTTGCATCTGGAAGACCTGTATTCTTTTATTGCTCATATATTATATCGCTTTTCTGGGTTATGCAGTTATGCATGTATGGTATTTGCATTGTATATTACTCCGTGAAATGGATGCTACTGAGATATTAATTTAATTTCCTCCTGTTAAGAGTAAAAAAA

>C101921_65

CGCGAATTAAAATAGATGGGACAACTCCCTGATGTGAGCTTAAAACAATGCTCAACAATCGAATGCAATCTTTCAACTGTAAAACCAGAGGTTCTCAAGGGAATTTTTTCAAACTGAAATCTACAAACTATGTAATTACAGACCCATATTAGTATTCAACCAAGGAATCTTATATACATAGGTAACCAAAACCATTCCCACTAAATATTACAACCCTAAATTTGTACGTGGCAATCCAAATATAAAGTCGGAAGAAAACACCTTAAGAATAGCACTGCCCTTTAGCTATCTCCCTACTGGCCTACTCTTCCTTCGCCTAATGCTTCAAGGTAAACAGATTCCACCAACACACACCACCCTACAGCTAATCGTTGTTGACGCAATCAGTGTTACAGTCAGCGGTAATTTAACACACTACAGGTAACCTAAATACAAAACAAATTTCATGAATCACCACCAGCTTCAGCGACTGGATGATCATTGTGTACTTGACGTACAAATTGCCCTTTGACCCGAGGCCGCTGCTCTGCCAATCTTTTCCGGCTTTGATATCGGACCTTTTTCTCGAAGCATCTCTCTTTCCGCTTCAGTCGAAACTTGGTTAGAGCTGCTTCTCTTTGACTGGAGCGATGAGAATCTGTTGCTCTAAATCCATCATAATTATGACAGAGACTATCACTGAAACCTGCGTTGTTCTTGGATACTATAGCTGAAGTAGCATTCCCATCATTTCCACTGTCCATGCTTCCATATGCACTACTATTAACATGATTTGCAGCATCATGGCATAAACTATTACCAGCACTCTGATCAGCAGAAGGAGAATCATGCCTGGCCTGATCCAAATCAGTGTCTTCCTTAACATTATGATTAAGACAAGTAGCATCATTGGAACAGTCATGATATTGATCAGAGTTGTGACTTTCCGGATTGGACTGGGAGGAGGTACTCGTGGGAAACGGAGAACTCTCGTTCTGGCAGACTGGTTTCGAACTCCACGCAGGGTGAGTACCAGATGGTGCGTCGAACACAGAAGTGAAAACATTACCATGTCCCTTGGACTTAAGATCGGAAATGACCCCTGAAACAGGTAAGATTCCACATTGACTGTTTGATAATTTTGGCTTAACTTGTCCATACTGATCAATGACTGTACTGATCATATTCTCTAGGTTCTGATTTGAGCCACCATACTGACAACAATTTCCAGAAGTAGTTCGAGACAATTTATGGGATTCATAAGAATCCCAACTGGAGTTGTTTACTTCAGTGGATGTAATTGATGGTGTTCGAAAAAGAGGGTGCAACAGTTTACTATTACTATACCAAGAAAATGCAGAAGTGTTTGAATGGTTCAATCTTTGAGATTCCTCAGTTGCTTCACTTGCTTGTTTACCTGAGCTTCCAGGAAAATCACTTCTTAAAGAAAGTTCCAATTGCGTAACACCATCAAACTTGGTTGTACCATCGAGACAACAGTTTTCGTTAGGGTGCTTTGGAAGATTTCCAAATGTGGCAATCAAGTCAATAGCTCCTTTAGAAGGTTTCACCCGTTCATCACTGCATCCATGCAACTCGAAACTAATATTAGAATTGCCTATGCCTAAGTCCGATTTTAAGATTTCAATCTCAATTTCAGTATAAGCAGAGCCATGGTCTTTTTCTAGCCTTAAATCCATCGATTCAAAAGTTTTGTCGCACCTTGCAGCTTCTGATACAATCGCAATTGATTTATCTCTTGTTTCATTATTAATCTTAGCTGATTCACTTTCAAATGTGGCAAAGTTTTCATGCTTCAAATCAATATTCTTCTGTTTAGAACTTTTCAGCTGGGGCACATCTTGAGCCTCACTTAATCTCTCACTGCATTCGTTGTTTTTCATAGAGGAAGCCACAGAACCACTAGATTTATTGCTTGCAGAATTGTCTTCGGAGGCAGTTTCAAGGTTTTTCGGTGAAAATGTTGTATTTTGATTAGGAGTGCTATTAGCGTGCCTTCTCCATACATGCTGCCACAAGTTCCCAAGTTCATTCCTCCGAATAGGTTTTATAAGAAAATCAACTGCCCCTTTTAGCATGCATTTCAACACCATGCTTACCGAATCATGAGAAGACATCATTATGACAGGAATGTTTTTACAAATTTCATGCTCCATGATTAAAGAAAGAAGTGCAAATCCAGATATTGCTGGCAGATCCACTTCAGTTATTATGAGATCAAGCTCCGATGCCTTCTTCTTCAACAATTCCCATGCCTTTAATCCGTCAGGAACTGCGATAACTGTGTAACTGCATTTGCGAAGAAGCGCAGAGATGATTTGTCGAGTGGAATGATCAGCTTCTACAAGCAGGACTCTGAGCACCATCCGCGGCAGAAACTTCTCCCACCTAACCACCTCTGCAGAGCGATCGCCATTGTTGTCCGTCCCGTGCTCTTGCATCGTACCGTTCAAGTCAGCCATTCTCTAACAACAGCATGCTTCGTTCCACGGCCGAAGTGAAAAACCTGTTCTTCTTCTTCTTCTTCTTTAGTTGCTTCCGATTCTGATTCACTGATGACCAGCTTTCGCCCACAACCGCGAAAATCGTTAAAAATATCTTCCCCCCAAAATCTCAATCTCTCTCACTGGATCATCAAAAGGTGGATCGCTA

>C101923_65

TCCTCTTCTCATTATTATTCCATTTCGTTATTTTTTCTTAGCCAGCCTTCGGCTTTTTTTTTTTTCCTTCTTAAGTGTGCGCTTTGGGCGTGGGTGAAACGCTGAGGGTTGTTGTCGTTTGGATGGAGAGAGTGGCGTCTTGGGCGGTAACGACGTCGAACGAGAAGAGCGTGTATGTGAGCTGGGAGGAGGTGCTGGTGTCGAGTGACAAAGGGAGGAGGGAGGTTCACTATTTGCTGAAACGACGCGGCGGCGCGGCGGATCTGGCCGTCGTGGGAAAAGAGAAAACCCTAAGACACATGTCTTATCGCTACGCGATTCGAAGCGCGTCGTTGGGGCCTTATTTGAAGCTCAAATCGCGAAGAGAGGTTGTGGATTGGCTGGACTCCGTTGTTTCAGATTCGTCTTCTGGGGATGCGGATATGGAGGGAAAACATGGTTGTGAACCTGAAATTGGAGCCTTGAAGGATAATCAAGAGCAGAAAATGCACAACTGTACAAAGGAATTCTCATGGATAGGGTTTCCATGGACGTGTAGGAAAAGAAGAAAGCACTATCAGGCCTATAAGAGGAATGGTTTTCAGATATCTGTTCATGATTTTGTATTTGTTCTGGCAGAAGAGGACAAGCGTCTAGTTGCCTACTTGGAAGACCTGTATGAGGATTCCAAAGGCAACAAGATGGTTGTGGTGCGCTGGTTTCACAAAATTGATGAGGTTGGTATTGTTTTGCCTCACAGTTTTAGTGACAGAGAGGTTTTCTTTTCTCTTTATCTTCAAGATCTGAGTATTGAATGCATAGATGGATTGGCTTTCGTCCTCAGTCCTCAACATTACGAAAAGTTTCAGAATGAGGCCCGTCGCACCCACTTGGAACCATTCGTGTGCATCCACCAGTTTGATAATGATGATGTCAAACCCTTTGATATAACACAGATTAAAGGTTATTGGAAACAGGAAATACTAAGGTACATGTATACCCAATTGGACTCAAAGTCTAGTGGGAGTTCTGGGCAATCTGACGATGGCCTGGAACTGGATGAAAATCATATGTCTCCTGTTTCGACCAGACCTAAGAAGAGGCTGCGCCTTGCCAAAGATGATGCCAAAGAAGCAATTGACTTAACTGCCCCTAAATTGGAAAATCCGAAAAACATTAAGAATAATACCAAAATTGGTTCTGGAAATAATTCCTTGAAACTGTTTGGGCACACAAACATGAGAACAACCGTCAAAGGGACGAATGAACATTCTTTACAGCATTTGGTTTTAGGTTCCCTAGTTGAGGTCCTCTCTCAAGACAGTGGGATGAGAGGATGCTGGTTTACGGCTTCTGTCGTTAAGAGACACAAATATAAAGTGAAGGTGCAATATCAAGACATTCAAGATGCGATTGATGAAACCAAGAAGCTTGAGGAATGGGTTCTAGCTTCCAGAATTTGTGTGCCTGACAGTCTGGGTCTTCGAATGCATGGGAGGACCATGGTCCGGCCAGCACCACCGTCCAACAAACGGGAATTATCCTGGGTTGGTGGTGTTGGTTCCATTGTTGATGCCTGGTGGCATGATGGATGGTGGGAAGGATTAGTTGTTCAAAAAGACTCTGAAGCTAATTATCATGTTTATTTCCCAGGGGAAAAGGTGGTGTCAGTATTTGGTCCTGGTAACTTAAGACAGGCTCAAGATTGGGTAGGGAATGAGTGGGTGAATGTGAGAGAAAGGCCTGATCTGGTAGCGTCTGTTTTGTCAAGCTTGAAGACACCACAAAACTCGTGCAAATCGAATGACAGCAAATCAACTGCAGCTACAACTAGAGATGGAATACAACCTAAGCTATCAGATACTACTTGCTTGGATTCTGATAGGGATACACCGAGAAAGTCTCTGGTGGTTTCAGACGTTTTGAAGGAAGATTTGTTGCTGCAGTTAAGGTGGAAGACCACCAGAAAGAGGAGACGTAGAGGTACCTCTTATCAGAAGCCACGGTGCACCGAAAGCCATAGGAAAAGGTCGCCAAAGGTTCTGAAATCAAATGCTCCTGACAGTTTCGTGATCCCAGCATCATTGAAAGTTGATCATGATGACTGCAAGTATGTAGGAGGGGATCCTTCCATTTTCACTTCTTCAGTTGTACCTTCTTTAACTAGCATGGTTATGTGTAGGTGACTCCATTGATTCATATCTTAGTTTCCCGGTTTTTTACTTCTCTCACTCCTTTCTTTGAATTGAAAGTACTTGTATTTAAACCCTTAAACCTTAGTGGGCATCCTTTCGATGCCTATCTGGCGCAGATTTTCATCTAGGGTCTCTTCATCCGAGCCCTTGTTGTTAACCTAGAAAATTTACCTTAGTCCTTTTCTTCCCCAGCTTTTGGTTAGTTTGTTTGTGTAGTTTGTGGCATGGCATCGAAGCTTCATTTCAGTTGGGTGTTTGAATCCGAATAGAAGCAGCCCAAGGAGGCGAGGATGATGCATACAAGTTTTGGCATAGGAAAAGTTTAACGTAGTGGAATAACTGAGGTTGCCTTTGACTTTTTAGGATATATCAAATGATCCAATGTAATTTCTGTAGCTTTCTCCTATAAGAATAGAAATTTGCTTGTTCTTTTATTTTTTCAGCTCAATATTGCGTTAATATTTTTAGTTAAATGGAA

>C101927_65

CAAATATCCATTTCTCTCGATCCAACTCATTTCCCTCTCTCTCTCTCTCTCTCTCTCTCTCTCTCCACTCACTCCCGCTCGCGCGGTTCTCTTTCACAGCTTATGGCGGAGGAAGAAGTTGCGGCACCGGCTGCCAGCCCCGTGCCCTCTGATCACAAGCGCAAACTTGAAGATCTGCAACCCGAAACCACCGAATCTAATGCCAACTCCGACACCGAGACCGCTGATGCGGCAGTTCCCGGAGAGACCGAGAACAAGCGTCCCCGCCTCGACGACCAGAGGGATGGCCCCGCTGATACAAATGGGCATCAAGATGAGCAAGTTTCTGAGCAAGCAAAAGAAACTGGGGAAAGATCGGCTGTGGAGGATGTTACTCCCGATCTTGATCAAACTATAACCAAAGATCCTTCAGAGGCAACTGCTGATCAACAAACATCTGGTGAGAATAGCGAGCCAGCTGATGCGAAAGAACTTCCTGTTGAGGACTGTGGACATGAAAGTGCAGAGGAGCCTTCTAAAGAAACTCAAGAAGCGTCTAAAGAATCATCTGAGCAAGATACTTCTGCTGGAGATAAGCAACCCAGTACTGTTGAAACTGCTATTAAGCAAGATGCTTCTTCTGAACAAAAGCAACCCACATCTGAGGCTGAGATAACAACACGAAAAATTGAGGTTCCTAACAATAAGGTTGGTGTACTTATTGGGAAGGCTGGAGATACTATACGATACCTACAGTATAATTCAGGCGCGAAGATTCAGATCACAAGGGATGCTGATGCTGATCCTCACTGTGCAACAAGGTCTGTGGAATTGATAGGAAGTTTGGAAAGTATTGATAAGGCAGAAAAACTAATGAATGCTGTGATTGCAGAGGCTGATGCTGGGGGATCACCTTCACTTGTAGCACGAGGCCTCTCTCCTGCCCAAGCTACTGTTGGATCTGAACAAATTCAGATACAAGTTCCAAATGAAAAGGTTGGATTAATTATAGGGAGAGGTGGGGAAACAATTAAAAGTCTGCAGACCAAATCTGGGGCACGGATCCAGTTGATACCTCAACATCTTCCAGAAGGGGATGATTCTAAAGAAAGAACTGTTCAAGTTACTGGTGATAAGAGGCAAATTGATATTGCACAAGAATTGATAAAGGAAGTCATGAATCAGCCCATCAGACCATCATCCGGTGGCTTTGGCCAGCAGGCCTATCGGCCACCCCGTGGATCTGGTGGCCCACCCCAATGGGGTCAGAGGGGTTCTCATTTTGGCCACCCAACAGCTTATGATTACCAGCATCGTGGGCCATATCCTTCTCATAATCAACCTTATGCTCCTCCATATGGAAACTATCCTCAACATATGGCTCCAAGAAGCAGCTTTGGTTCTGGTTGGGAACAAAGGCCTCAACACAGCTTTCAAGCCCCACCCCCACACAATGGTGGTTATGAGTATTATGGGGGACAAGGGGGTCATCTATCTGATGCTCCACCATCCACACAGCTTCCTAATTCAGCCCCACCACACGGTACTGGTCCTTCACCTCTACCTTCTATGGGTCCAGCCCCAACTCAGGTGAGTTACAACTATGGACAGCCCCAAGGCCAGGATTATGGTCATCAAACAGCTTATTCTCAGGCAGGACTTCCTCAACAAGGTTATGGACAGGGATATGAAGAGTCGAAGTATGAAAATCGTGTTCCTACTCAACATCCCTATGCAGGACATGTAAATTCTCAAACGACCTACCCTCAGACTGGTGCTCAACCTAATTACCCTGCACCGCAGCAGTATGGCAAGCCTCCGCTGTATGGCATGCCATCACAGGGACAGCCACAACAATCATATGGTCACCCTAGACCTACTCAACCTGGCGAAATGCCATATCAAGGTTCTGCTCCTACCCAGTCGTATGGCACGAATGTGCCAACACAACAACCATACCCATATGCAGTATCTGGGCCATCACAAACAGCATATCCTAGTTATGGGTCTGCACCAGCTGCAGATGGCTATAGTCACCCGCAGCCTGCATCTGGTGCACCTTATACCCAGCCACAACCCAGTTATGGTCAGCCAGCTGCCCAGCCTGCTGCCCAGCCTGCAGCCAGTTATGCACAGGTTGGTCCCACTGGTTATGGATCATACCCATCTTCACAGCCAAGTTACCCTGAGCAGCAGGCTCCCAACAATGCAGTGTATGGCTACCAAGCACCCCAAGATCCTGGTTATAGCAGTGGTGCTGCACCAGCATATAGTACAACCCAACCAAGTGGGCAACCAGGTTATGCTCAACCCACTCCAACTCAAACTGGTTATGAACAGTCTAACACACAATCGGCAGGTTATGGGGCTGTGCCAGCAAGTGCTCCCGCTGCTTATGGCAAGACATTGTCACCACAGCCTGCTGCCTATCCCCAATATGACTCCACCCAGGTTTATGGTGCTCCTCGATAATGTCTTTTATGTTAACTAAGTTCTAGTATTAGCATATGTTCTAATTTTATGGCCCCTTTCGGTGTATCCTGCTTTTGATTTTCCTGTAATCTGTACTGCTTTACGTTATTGAACATGGAACATGAAAGTATAATTGTAATAGTCCCGAATGCTCTGATGTTACAATTTCTTCAAAATCAAAGTTTCTCTTTATAAAAAAAA

>C101929_65

CAATGATACTTCAAAATCTCCGCTTAAACTTTCCTCGCGTTTTCTGCATTCCCCTAACGGTTACCGTTCTTCTCTCCTCCGCCCTTCCCTTTCCTCCCGCCGCCGCCACCGCATCATTCTCTCACCACCGCCTTCTCGCCGCCTCTGCATCGCCGATGAATCCCTCGCTCCAGTCTCAGTCTCAGTCTCCACCTCCTCCGGTGGCGAAGAAGGTCGAGCACGCCATGGAGATGTTCGGCGACGTCCGAATCGACAACTACTACTGGCTCCGAGACGATTCCCGCACCGATCCCGAGGTGCTCTCTTACCTCAGCCAGGAGAATGCCTACACCGATTCCGTCATGTCCGGAACTAAGGGATTTGAAGATAAGCTTTTTGCCGAGATAAGAGGAAGGATTAAGGAGGATGATATCTCTGCACCTTCACGCAAAGGACCTTATTACTATTATGAGAGAACTTTGGAAGGGAAGGAGTATGTTCAACATTGTAGGCGCCTTATATCTGATAACCAAAAGGTGCCATCTGTGCATGACACCATGCCCACCGGACCTGAAGCTCCTCAGGAGCATGTTATTTTGGATGAGAATGTCAAGGCACAACATCACCAGTACTACAGTATTGGTGCTTTTGAGGTTAGCCCAAATAACAAGTTGGTAGCATATGCAGAAGACACCAAAGGAGATGAAATATATACTGTTTATGTTATTGATGCTGAGACTCAAGCTACTATTGGAGAGCCTCTTGTTGGTGTAACAGGGTACCTTGAATGGGCTGGTGACAATGCTTTAGTTTACGTCACAATGGATGAAATTCTCAGGCCTGACAAGGCATGGTTTCATGTGCTGGGAACAGAACAATCAAAGGATACTCTTCTTTATGAGGAAAAGGATGATATGTTTTCTCTCGATCTTCAAGCTTCTGAAAGCAAGAAATATTTGTTTGTTGCATCAGAGAGTAAAAATACAAGGTTCAATTTTTATATGGATGTTTCTAAGCCTGAAGAGGGTCTTAAAGTTTTGACACCACGTGTTGAAGGTATTGACACAACTGTCAGCCATCGTGGAGATCATTTCTTTATTAAGAGGAGAAGTGATCAGTTTTTCAATTCGGAGGTTGTAGCTTGTTCTGTCAATAACACTTCCTCAACTACAGTTCTTATTCCTCACAGGGAAAGTGTTAAAATTCAGGAGATACAACTTTTTACTGATCATCTCGTAGCATATGAGAGAGAGAATGGACTGCAAAAAATAACAGTTCATCAACTGCCGCCAGTTGGTGAACCACTAAGGAGCCTTGAAAGCGGCCAAGCAGTCAGTTTTGTTGATCCTGTATATTCAGTGGATTCTTCATATTCAGAGTTTTCATCAAGTATTTTACGGTTTTCTTATAGCTCATTGAAGACTCCTTCCTCTGTATATGATTATGATATGAAGACAGGCATTTCTGTTTTGAAGAAGATTAACCCCGTTCTGGGAGGTTTTGATTCAACACGTTATGTCACTGAAAGGCACTGGGCACCTGCTTCGGATGGAACTTTGATTCCCATATCAATTGTTTACCGTAAAGATATTGTGAAACTTGATGGATCAGATCCATTACTACTGTATGGGTATGGATCCTATGAGATATGCATAGATCCCAGTTTCAAATCAGCAAGGCTTTCATTGTTAGATCGGGGTTTTGTATATGCCATAGCTCATATTCGTGGAGGTGGGGAAATGGGGAGGCAGTGGTATGAGAATGGGAAGCTGTTGAAGAAAAAGAACACTTTTACTGATTTTATTGCCTCTGCTGAATATTTGATTGAAAAAAAATTCTGTTCTAAGGAAAGACTCTGCATTGAAGGAAGAAGTGCTGGTGGTTTGCTAATCGGTGCAGTCCTTAATATGAGACCAGATTTATTCAGGGCTGCTGTTGCTGGAGTACCTTTTGTGGATGTTCTGACGACAATGCTTGATCCAACCATTCCTCTCACTACTTCAGAATGGGAGGAGTGGGGTGATCCTAGAAAGGAGGAATTCTACTTTTACATGAAATCATATTCCCCCGTTGATAACGTGAAGGCACAGAACTATCCACACATTCTTGTTACAGCTGGATTAAATGATCCACGGGTTTTGTATTCTGAACCTGCGAAGTTTGTGGCAAAACTGAGGGATACGAAGACTGATGATAACATACTGTTGTTTAAATGCGAGCTTGGTGCTGGGCATTTTTCTAAGTCGGGAAGATTTGAGAAACTCCAGGAAGATGCCTTCACTTATGCATTCATCATGAAGACTCTAAACATGATTTAGTCTTTAGAGATTCATGTGCGCGGATGCCGTCGAGAGGGTCATTATTACGGGCTTGAGTGAGATCAATTGCGTAAGTAAATACAATCATGGTTCTGAGTGAAAGCCTATAATGTCAGACGTTTCGGTTATCTTAGGAATTAAATTGCAAGCAACTACAGAAGAAAATCCGAAATTACCTTTGCAATTCAGAAAGCATGTCATTGTATTAGTAATTTATATTACAAATGAGGATACGGCAATAAAGGGTGATGCATTTGTGCTTGTTTGGATGTAATATAATAGCTTATCTTTCAAAACTAATGTCTGAATTAGTGTTAACTTGGATTTGGGTGGACAATATCTTATCTACGTCACAG

>C101931_65

CATTTGTCTTCGTCAATCAACATCTTTTGTCCCAAACATTATCTCTTTCAAAAAGAAATAAAAAGGGATGATAATATCAACTAAGTATATGCTATTCACTGGATAGCACCATGCTTCTGTTGATGTTTCCGGTTCTAGCTTCAGCAAATGCCAACAGATTATTATACTACATGAGAAAACTTTCTAAACTGGTCCAAAAATTCTAGACAATGTACAGCACTATACTGTGATCAATTACAATGTATACCGCAGTTGATAATTGCGAAGCCAATCTTCACCTTTTTCATAATAATCCAGTCTACTGAATGTTTGCATATGGAACTGTGAATCTGAGGCAAAGGTAGCAGCCCCACGCCATGCATCCATTACTGGATCCAATGCTCTCACTATGTTTATAGGTGCGCCACATGGTCTAATCATCCGAATTCCAGCTTCCAGGCGTTCAACTATACCAGGGAAAAGAGAACTTCCACCAGTAACAAGTATGGAACTGGTCAGTCTCTCTTCCAATCTTTCATCCTTGTAAGGTAACCTCCTTATTGAGACCCCAGCCATCTCGTCTAATCCTACCTGATCAACCCCAATCCAGTTTGGATTAAACAGTATTTCTGGACATCTGAATCTTTCGACCCCTAGAACAATCTGAAAATCTTCTTTGGTCAGAGGACGAAAGCGTGGCACCTCAGCAGGTTGACTGGTACCGGCATCTAACTTGGGTATAAATGTTGGATCCAAGTCCTGTAGCCTAGAAGAGACGCGGGTCAGCTCTAATTCATCTTCATCAGGTTCTTCATCATCATCGTTACTATAATCTTTGCTCATCAATTTGTAAAGCTGCCAGTCTTCATCTCTAGCACCAAATGTATCTTCACCCTTACCACGGTCAAAAGCTGCAGTTGTCAGGAGACGCATTCTTTCCCTCTGAGCAGCATTCAATCTTTCACCACGGCCAATACCACCGGACAGATTACTTCCGCTTGAATAGCCTCCATTTGTCTTAAGTCGTTTATGTTGGTCAACTCTTTCTGAAAGGTCTTTGTATCTAGCATGCAATTGCTCCAAATAAAGCTCCGGGTTCTCTAGGCGTTTCTCCTCCTCTAGCTGTTGTTTTCTCTCTCGTTCCAATTCCTCTTCATAGCGCTTCTGTTTTAATCGCTGCCTCCCTTCAGCCATAGATTTGAGTGACAACTGTTTCTTCTTTTCATTGAGCTGTTCTTCTGTCAACATGTCATCAGGAATGTTTATAAGAGAATACTTCTCGCTAGCGGCAACATCATCCTTTTCAGTTTCAGCTTGTTCATTCTTTGGTTCACCTTTTGCTTTCCGTAAAGATTGCATAACTTTATTACGTGCAGACTCTATCTCCTGCTTAGTGACATAGCCGGTTTCTGCCAGGAATGGTGGAACCTCACTCTCTTCCACTTGTTCAAGCTGATGTAAAAGAAACTCTAAACCATGCAATTCATTTTCGAGTTCATTTATTTTAGAAGACCTCTTTGCCTCAGCCATTTCTCGAAGCCTTTGGCCTTGTTTCTCTTTAATTGCTGCCTTTCTTGCAATCTCTTCCTCGGAAGGAGGCTCATCAGTTGGGGGTGGAACCCAAGGGAGTTGCCAAAATCTGGTTTTTCTTTCAGCTTCTATGTCTCCGTTCTTAAACAACCGAGCTTCCGAAGCATAATCTTGTGCAATGTAACAATGTTCCATCTTCAGGTCTTCAACCTTTTCCCATGTAAATCTAGTCAAATGATAAGGGTATTTAAGTGAAAGGAGTTGCTTCAAATGATTAGTCACGTGGTATCCACCAATATTGGTACGACAGCATCCTGCATACACAGGCTCCCCGTCCACAAACGGAATCACATGAGTTGTGTTGAATCCAGGACACAAAGCAAGACCATCTTTAGCACAAATTCCTTGCTGTTGATTATATTTATAGCTGAATGCCACATCAACACCAAAAGCAATTGATGGAACTCCATAAGTCTCAAAAAGAAGTTCTGCCATTTTACTACGAGATTGAACTGGGTTGCATACACATTCGGTGATCAGGACAGGATGATCAATCTCAGTAGTTGCACCCATCCGGTCAAATCCAAAATCAAGAATATATTCCATTGTTTCAAACTGGTAGACAACATTACTGTCAAATGCAGAGCGTGGCCCTGAGCGAGTGCAGTCAAAGTATTTCAGTAATGCAGGATCATGGTCACCCACAATAGTGACAGTTTCCCCGGTTACTTTATGGCGCGGCCTTTGAACAATGTTGCGAAAAATAACACGTGGTTCAGCCTCTCCCGCCCAACCTATGCGGAAATAGGAGGCGCCATTGTCTATCACAATGGGAGTGTTGCTATCGAAGAGGTTGAAATCGGATTGACGGTCAACTTTGGAAATAAAAGGCATGGCGCAGTGGTTTATTGAGCTGGATTTCGGTTCTTCTGTGTGCGCGTGACTAGTGAAGAGTGCCGAACCGGTTGGTTAAACCGAACCTGTTTGGTTCTGTGGACGAAGAAGTGAGTCGGTGTGGAGGAAGAGCATGAGAGGATGAACACTAGCACAGCCTTCTTTGACTGCTTTGGCTCTGCGTGTTTTCTCTGTCTCCTCTCGTCTTCTTGCTTTCAACACTTTTCTTTTCTTTGGGGGTTAAGAAAAG

>C101933_65

TCTGCATCTGCATCTCCATCTTCATCTCCTTCTTCATCTCCATCTGAAGAAGCATGTGCACACATAACATAACACAACATTAACATGCGATCTCAACTGTAACGTGACAGAGCTTGAGCCACCATCGTCACACCGACCAACGCAACAATGGCATCGGCCTTCGTCAAGCTCGATGACTCCCCTATGTTCCAGAAACAGTTATTTTCTCTTGAAGAAACAACTGATGAGTTAAAGGATCGGTGCCAGAAACTATATCAAGGGTGTAAGAAGTTTATGACAGCTCTGGGGGAAGCATATAATGGAGAAATATCCTTTGCTGATTCATTAGAAGTATTTGGTGGTGGCCAAGATGACCCTGTTAGTGTATCAATTGGAGGGCCTGTCATAACTAAATTTATCACCACACTGCGTGAGTTAGCTTCTTTTAAAGAGCTTCTTCGTTCACAGGTAGAGCATGTGTTGATTGACCGGCTGATGGAGTTTATGAACATTGATTTGCAAGATGCAAAGGATTCTCGTCGTCGTTTTGACAAAGCTGTGCATTCTTATGATCAGTCACGAGAGAAGTTTGTCTCTTTGAAGAAGAATACACCAGAAGATGTTGTTGCTGAATTAGAAGAGGATCTACAAAATTCAAAATCAACCTTTGAAAAAAGTCGCTTCAATCTAGTAAACTCACTCATGAATATTGAAGCGAAAAAGAAGTATGAGTTCTTGGAGTCCATTAGTGCAATAATGGATGCTCATTTGAGATATTTTAAGCTGGGTCATGACTTACTAAGTCAAATGGAGCCCTATATTCACCAGGTGTTAACATATGCACAGCAATCTAAAGAACTTGCTAATATTGAGCAAGATAAGCTTGCAAAACGAATTCAGGAATATAGAACGCAGGCAGAGCTGGAAAATATACGAGTTTCTAGCAATTTTACAGAAACCATGCCAGGTTCTGAAGGCACCCACGTTGTAGGTTTGAACTCATACAGAAGTTTAGATTCAGGGATGCTGTCTGCAGCCAAAGGGGAGCTTCAAACAGTTAAACAGGGTTATCTGTTAAAACGGTCAGCAAGCTCGCGTGGTGATTGGAAACGGAGGTTCTTTGTACTTGATAGCCAAGGGAATTTATACTATTACAGAGTGAAGGGTGTCAAACCCATGGGCTCTCAGTCATATAATTATACTCGTTCTTCTGAACAAAATAGTGGAATGTTTGGTAGATTCCGTTCAAAACACAACAGAGCTGCATCACTAAATGACGACATTTCAGGTTGTACAGTTGATCTGTGCACATCGACTATAAAGATGGATGCAGAGGATACAGATCTACGACTTTGCTTCCGAATAATTTCTCCCTCAAAAACATACACACTACAGGCCGAAAATGAAGCTGATAGGATGGACTGGGTAAACAAAATTAATAGAGCTATCACATCACTTTTTAGTTCACAGTTTCTTCAACAGCCTCAATATGGTAGAGTAAATTCACAGAATAAAAACTCCACAATTGGTGCTCCTTTGTCAAGTCAATCGGAGGATGGTCAGAAATCATTGAGGGATGACTTATATTCCAAGGAAGCGGGAAGTGTCTCTAAGATTTTAAGGGGAATTCCGGGGAATGATAAATGTGCTGAGTGCAGTGCTCCTAAACCAGAATGGGCATCTCTAAACCTTGGTATATTGCTATGCATTGAATGCTCTGGAGTGCATCGAAATCTTGGTGTTCATGTCTCAAAGGTGAGATCTATAACATTAGATGTTAGGGTTTGGGACAATACGGTTTTGGAGTTATTTGATAACTTAGGTAATGCTTATTGTAATTCTATCTGGGAGGGCTTGCTTCTACTTGATCATGACAGGGTAGGTGAATTACATGTGCCTATTAAACCTTGCTCAACAGATGCCTTCCAAATCAAGGAGAAATACATCCAAGCGAAGTACGTAGAGAGATCACTAATAATCCGAGAAGAGGATATACCCGACAGTCCTTCAGTTTCCATAAGAATCTGGCAAGCAGTTCAGGCTGGAAATGTACGAGAAGTGTATCGCCTCATTGTTACATCAACATCAAATATGATAAACACAAAATATGGTGATGAGGTTCACCATGCTGCTGATGCTGAAGGGCATCAGCATGATCCTGAAGCCTGTCTTAAGGTTGAAGAGACTATTGAGACTGAGAGGTGTTTCCGTGGATGGTCATTATTACATTTGGCATGTCGTACTGACAGTGCACTGATGGTTGAGTTGTTACTTCAATTTGGTGCTGATGTAAACATGTGTGACTATCATGGAAGAACTCCCTTGCACCATTGCATTACAAGTGGGAGAAATAAATTGGCAAAGTTTCTATTGAAAAGGGGTGCAAGGCCGTCAGTTAAAGATGCCGGAGGGCTCACTATACTTGAAAGAGCTATGGAGATGGGAGCAATAACTGACGAAGAGCTATTTATCATGCTTGCTGAAAGCTAGTGAAGACCGATCTGGTGTATATATATATTGAGTGTTGAAGATATGCTGTTGTTCATTAACTAAAGAAAGTTCGATTTCTTATTTAGAATGCCTTATTCATTGAAAGGAATTTCATTAAGTTTTTAATTTATCTGGATCCATATTTGTTGCAGTTACTAATAGAAAACAGTTGACATGTTCTCTG

>C101935_65

CACACTCTCACTCACACGCGCGCACACACCCAACACTCGCACACTCGCACACTCGCACACTCGCACTCGCTCTTTCGAATCACAGAAATCTCAGGCTCACATAACACAACGAACCCAAGAGAAAGAGAGAGAGAGTGAGAGAGGGTTTTATTTTAACGTTTTTGTTTGTTATTCCTTTACAGCTCAACCTGCATTTCTCTCTCACTCAAGCGCATCTCAGATCGAAATCCAGGCGGTGTCTACCTTTGTCTGCGCTATTAATGTTCATGCCAACTAGATAACTACATGGTGGAGCGAGACATTTTTCTCATATGACTTCTATTAGCTATACTATTACTGAGCAGTGTTGCTATGGGCATTTGTCTTTTGAAATGGCAATGGCCGTGGCTTCTTTTTATTCTGCTGATACATGTTGTAATCAATGAGAACGAAGCAATCACTCCCGATGGTGAGGTGCTTCTCAACTTTAGGACAGCAGTTGTTAATTCTGATGGTATCCTATTACAGTGGAGGCCAGAGGATCCTGACCCATGTAAATGGAAAGGAGTGAAATGTGATCCGAAAACCAAAAGAGTAACACATCTGAGTCTATCTCACCATAAGTTAAGTGGGTCTATATCACCTGACCTTGGGAAGCTCGAGAATCTGAGGGTTCTTGCTCTTCATAACAACAACTTCTATGGGACAATTCCGTCAGAATTGGGAAATTGCACTGCGTTGGAGGGAATATTTTTACAGGGTAATTACCTTAGTGGAATCATTCCAAGTGAAATAGGAAACCTTTCCCAGCTCATAAATTTGGATATATCAAGCAATTCCCTCAGTGGGAATATTCCTGCATCACTTGGGAAGTTGTATAATCTCAAAAATTTCAATGTATCAACCAATTTTCTGGTCGGACCTATACCTTCTGATGGTGTCCTTGGCAACTTTAGTGGAAGCTCCTTTGTTGGAAATCGTGGTCTGTGTGGAGTGAAAATTAATTCAACATGTAAAGATGATGGTTCACCGGGGACCAATACTCAATCGACTTCCTCAGACCAAAATCAACTGGGAAAGAAGAAATATTCTGGCCGGCTGCTCATCAGTGCATCAGCAACTGTGGGAGCTCTACTTTTGGTGGCACTTATGTGCTTCTGGGGTTGTTTTCTTTACAAGAAGTTTGGTAAAAACGACAGAATAAGCCTGGCAATGGATGTTGGTGCAGGTGCATCAATTGTGATGTTTCATGGAGACCTGCCATATACTTCCAAAGACATTATTAAGAAACTAGAGACTTTGAATGAAGAGCACATAATAGGAATTGGGGGATTTGGAACTGTTTACAAGCTTGCAATGGATGATGGGAATGTCTTTGCTTTGAAAAGGATTGTAAAGTTGAATGAGGGGTTTGATCGTTTTTTCGAGAGGGAGCTTGAAATTCTTGGTAGCATCAAACATCGATATCTAGTGAACTTACGAGGATATTGCAATTCACCAACATCAAAGTTGTTAATATATGATTACCTACCAGGTGGTAGTCTTGATGAAGCTCTTCACGAAAGAGCTGAACAACTAGATTGGGATTCACGCTTGAATATAATCATGGGAGCTGCAAAGGGGCTTGCCTACTTGCACCATGATTGTTCCCCTAGAATTATACACCGAGACATAAAATCTAGCAACATTTTACTTGATGGAAACTTGGATGCTCGAGTATCTGATTTTGGACTTGCCAAACTGCTGGAGGATGAAGAATCTCACATCACTACAATTGTTGCTGGAACATTTGGTTATCTAGCTCCAGAGTATATGCAAAGTGGCAGAGCAACTGAGAAGTCTGATGTGTATAGCTTTGGTGTGCTGACACTTGAAGTACTGAGTGGAAAGCGGCCAACAGATGCAGCATTCATTGAAAAAGGTCTCAATATTGTTGGTTGGTTGAATTTTCTAATTACAGAAAATAGGCAGAGGGAAATTGTTGACCCACTATGTGAAGGCGTCCAAGTGGAGAGCCTTGATGCCTTGCTCTCTGTGGCTATTCAGTGTGTCTCCTCAAGTCCAGAAGATCGACCCACTATGCACCGAGTAGTGCAATTGTTTGAATCAGAGGTCGTAACTCCATGCCCTAGTGACTTCTATGACTCGAACTCCGATTAGAAAGCTTGCAAAGACAAACTAAAAGTATGTTGTTGTGTTTGCATCCATGTCACTGCAAATTACTTGAAAGTCAGAAGCGACGAAAGAGGCTCAGAGTCCGCAGTGTTACTAGTCATCTCAGTTGCGTTCGTTCTTAACTGGAATTTTTAATGAAAGAGGTCCTTTATTACACCGCTTCATGATAGAAGTTGTGGTTCCCCGTCACATGTCATCATTGGACATGTTCAAACGTTTCTAAGTCTTTGTGAATGGAGTTGCATCTGTTAGTGGATCGGTCGCAGTTCCAGTCTTCCCCCTTGTGTTTTTCTTTTCTCCTTTCTATTTTTCCCCTTTCTCTTTGCTAGCATTGTAATCTTTGTTACTATTTGTTCACTCCAATTTAAGTGTATATGAATGTGTACAAGTTCAGATGGCCTACCCTGGCAAGTGATTATTGTTATTTATTTCTGTAATATATGTCCCATAAAAGAATTCATCTAATGAGTGTGATTCTCACTTTTCATGTTGTTCTAGTG

>C101937_65

GCTCAATTATTTCCAACATAATCAGAAATTCGTCCACCATATAATTATCTGATACAAAAGCGGATACTATTGATGGTTACTTTTTAGCATATGAAAAATTAAAATCTGAAAGAAAAAAATAAAAAAGCCTCAAAAGAGCTAACTGTGTAAACCTAACTATACAGATCATCTTCTTCAGGGGCAGAAGTTGAAAAGGGGTCAGATCCAACGGTCCTAGCACCAGTCTCTGGAAACCTAAAATCACTTCCAAAGCCACGGGATTGTTGTAAGGTCTGAGCAAATGCCTGGTATTTGCGTATATCAGCATCACTAACACTCCTACGCGCAAACTTCATTGACTCTTCAAAATGAGAAGCCTTTATCTCTGCAATATCATCTTCCTCATTGTCTTCATCCATGGCCTCAGGGTTTTCTATTTTCTTCTTCTCACGCTCTATATCCTTTTCTATGTTTTCTCTAATAGCATATTTGCATGCCCGCTGGCATATCTCTGTTATATCAGCGCCACTAAAGCCTTGAGTATACTTAGCTAGCGCTCCCAAATCAACATCTTTTGAGACAGGTGACTTCCTCATGCATGCCTTGAAGATTTGATAACGGGACTCCTCATCAGGGAGAGGAATATAGATGAGTTGATCTAGACGACCAGGCCGAAGAAGTGCTGGGTCAATTATGTCAGGTCTGTTGGTTGCCCCAATTATGAACACCGTCTTTTTGGCAGACATGCCATCCATTTCAGTCAGAAGTTGATTCAGAACTCTATCAGCAGCTCCACCAGCATCCCCAACACTACTTCCTCTCTGGGTAGCAATGGAATCAAGCTCGTCAAAGAAGAGGACACATGGAGCTGATTGTCTGGCCTTGTCAAAAATTTCTCGAACATTGGCCTCACTTTCACCAAACCACATAGTGAGCAATTCTGGGCCTTTCACACTGATAAAGTTAGCTTGACATTCGTTAGCAATTGCTTTGGCCAACAAAGTTTTCCCACATCCTGGAGGACCATAGAACAGAACTCCTTTTGATGGTGACATACCAAACTTCTCAAATTTTTCAGGGTGCTCCACGGGATATTGAACAGTCTCTTGCAGCTCACGCTTGACATTCTCAAGACCTCCAATATCTTCCCAGCTGACATTGGGCACCTCGACAACAGTTTCTCGTAAAGCAGATGGATTGCTAGTTCCTAGAGCAGTATGGAAATGCTCATTTGAAACTGCCATAGAATTCAGTATTTCAGCATCAATACTCTCATCTTCCAAATCAATGACATCCATCTTCTCCCGGATGCATTGCAGAGCAGCTTCAGTGCAAAGGGCAGCAAGGTCAGCACCAACATATCCATGAGTATCTTTTCCAATTCTCTCCAAATCAACATCTTCTGAAAGCTTCATGTTTTTGGTATGTATGCGAAGGACTTCAAGTCTCCCAACTTCATCAGGAACACCAATATCTATTTCCCTATCAAATCTACCAAACCTTCGCAATGCAGGGTCAATGCTATTTGGTCGATTTGTTGCACCCATGACAATAACATGAGCACGAGATTTCAATCCATCCATGAGAGTCAAAAGTTGAGAAACAATCCTCCTTTCAACTTCACCATGTGTTTTCTCCCGCTTGGGTGCAATAGAGTCAATCTCATCAATAAAGATGATGGAGGGGGCATTCTTCTCCGCTTCTTCAAATGCTTTCCTAAGGTTGCTTTCACTCTCGCCAGCCAGTTTGGACATAATCTCTGGGCCATTGATACAAAAGAAGAACGCTCCAGTCTCATTAGCAACAGCTCTTGCTATCAAGGTCTTTCCAGATCCTGGGGGTCCGTATAGCAGGATTCCTTTGGGTGGTTTCACACCAATTGATTTAAAAAGTTGTGGATGTCTAAGTGGAAGCTCTACCAACTCACGAATCTGTGCCATTTGCTTCCTGACACCACCCACATCATCATACCCAACCTCATCCAGCCTTTCTTCATCCTCTCTTCTCACGGGTTCCCCTTCACAGAATATTTCAGTGTCTGGAGCAACCAAACAATACTCCCCAGGATCAGTTTCAATGACCTTAAATTCTACACTTCTCATCCCTCCTCGGACAAGAAATAGATCTCCCTTTCTGACAGGACGATAAGCCTCCAAGAAATATGGTTTCAAATAAGCATCAAAGAGATTGCCAGTGACACCTTCAATGGTATCATCAATGGGAAGAATGTGCACACGCTTCCCATACTGCACATCAGAGCATGTATGCACCGACACAACATCCCCAAGACGAACCCTCAAATTTGACCTCACAACTCTGTTAAGCCTCAACTTCGGCTCCTCACACTTCTCGTCTGCAAGAACAATGCAAACCGTGTCCCTACGTTTCTTCCCCTTGATGAGGATAGTATCGCCGTGAAAAAAATGATTTTTTTCCATGGTCTGAGGGTGCATGGAAACCACGGAGTTTTCATCGGTAACAGCTTCGTCCACGATTAACCGATTCGGAGATTTCTTCCGCTCGAGAATCGCGGTAGAAAAGTCTTTCTTCCCGGATTTCGAGTCGGAGGATTCAACTTGGTGAGACATTGGTGGAATAGAAGATGAAAAGAGAGACAGAGAGAGGAAGAAAAAACAGAGACAGAAAGAAGCAAAAGCAGAGAGAAGGAAGGGAGT

>C101941_65

GCTTTTGATGCCAATCTGTAACAACAAACTAGAATAATACACATATATACAAAATTCATCCACACGTCGAAACAGTGTCTCCAAGGGTAGAAAAATGGTGATTTACCCAGGAGGATGGCAAAAGCGACCACAGAATAATCCTCTCTAACCAGCAACAACACCAAGAAACCTTCAATAACTTGAAAGAAAACACCACCAGTTATGTTCCTCCTTCAGCTATGGGAAAGAAAACTTCTCAAAATCATGGTGTGAAAACCTTTCGGTGCATATTCGTGGTTAGCATGCTTTTCCAAGTACTTTCATCGTCCTTCATGAAGATCTTCAGAAAATACAGCCGTCCTTGAAATGTTGTCATAGTCAAAGGCACTTAGACCAGAGTAAGGACCCGAAGAAAACATTGACGAAGTCCCATCAGACTCATAAGTGGTAGAGGACTGTCTCATGTTGGGCCGTGTATTGGATGAGGCTAATACAGGATCATGACTTTCTGTGATCCGCTGCACCATTTTAAGTGACTGTACCACTTCACCCATTGTAGGTCGCTGGCTTGCTTCAGGAGCAACACAGGCTGCAGCAATAGTGCAAACACGTACAAAATCTTCTTTTGGGTACCTTCCCCCAAGCCTTGGATCAGAAAGTTCTTCCAACCGGTCCTTATCTCTAAGAATAGGCCTTGCCCAAGTAACAAGGTTCTCTTGTCCAGTTGGCTGTGACATATCCACTGGTTTCCTTCCGGTCAACAGTTCGAGTAAGACAACCCCATAACTATAAACGTCACTTTTAACAAGTAGATGTCCAGTCATAGCATACTCAGGAGCTACATACCCAAATGTGCCCATGACTCGAGTAGATAGATAATTAGCCCTGCCTTCAGGTGCCTGTTTAGCTAGACCAAAATCTGCAACTTTAGCATGAAAGTTGTTCTCAAGCAATATGTTGGATGCCTTGAAATCTCTGTGTATAACACAAGGCTGTGAATCTTCGTGCAGGTATGCAAGTCCTCTTGCAGCATCAAGGGCAATCTTCATTCTAGTGTCCCAATCCAAAGGGCAATTTATTCCCAGGGGACCGTGAAGCCAGGCCTCCAAACTTCCATTCGGGACAAGCTCATAGCAAAGTAGATTTTGTGACGAGTCACGATTACTATAGTATCCCACTAGTTTTACAAGGTTACGATGATGCAACCGACTAAGCATCTCAACCTCAACCAAAAATTCTTTGTCACCCTGTTGCCCTCCACTAGTAAGTCTCTTAATTGCAACAGCAGTTCCATCATTCAAGACACCTTTAAAAACTCTACCAAAACCTCCTTCTCCAAGTACACTTGCTAGTTCAAAATTATTTGTAGCTTCTTTAAGTTCTTCATAAGCAATAAAGCGTGTACTGGTTGGATGGGGAAGAGAACCAACAGCTGGAACTGTACTTTCTGTCCTTGGCTTTTCAGTTTCTGCGGGAGGTGTTTTTGTTTTTCGCCGCATCGTGCATAAGCAAAGTATAAGCACACATACAATGGAAATGAAGAGAATGCCAGTCACTATTCCGAGAATAAGAAGCAAGTTTGAATGCCTTCCCCTGTCTGAAGTACTAGATAATGTAGTTGGAGTTGCTTCTCTATGAGGAGCTTTCGTAGGCGATGTAGCGATTGTAGGAGCTTGAGAAGGAGGCGGAGCTTTAAACCAGGTTATATTGAGTACAGTGTAGTTGCCCACAAATCTGCGGTCTAGTCGAACCTTATGCAATGAAAGAGATGAGTTTATTTTAGCGGCTTCATTGGCTGAGAAACTGATCCCTTTATTTGGAGTGATATCCATCGATATATTCAACGTCGATAAGCTCAGTAGGTAAAATTTAATAATTTCAATTTGGGTAGTCTGTAGCTCAAGTTGGGAGGCCAAATTATTGAGAAAAACATTCCAGTCGGGGTTTAGAGAGACATTCAAAAGAAGCAAGTCTAGCTTTATGGGATAAGCACAGTGGCAAACCTCACTCCCTCGTTTCCACACCATGTCTTGTTTGCAACAATCGGAAACATGAGGGGACAGTGGAGGTTGAGCGAGAGCAGAAGGAATTCCATCAGCAATGGGATGCGCAGGGGCAATATTCTTAAACCCAGAACCCGGAGGTTCTAGTTGTGTTCTTCCTTTGGTGAAATTGGCATGTGAAGGAACTGGTGCAGGCAAATGCTTCAACCCAAGAGCATTAGCCAGTAGTGGCAGATCACCATCAACAACAGGTGGTGCAGCAGCTGGAGATAAAGAAGGTGCTTCCTGCAGCAGTGTTCTACCACATGCACCATACTCACCTTCAAACCACACAATGCAAGATAACAACACAAGCAACCATTTCAACCAACCGATTAAAGAGGTGGAAACGGCGAGCAGAGACATCACCGATGCCGGAAACGGTCCAGATCAGATCTACAGGGTTGGGATAAAATCAACGGTGGGAAGTGAGTTGAGGGAGTGAAGTGGGACTCCGAGTGAAGAAGGTGCGCATAACTGAGTTGAATCGGAGTAGTCACTGTTACCGGTTACTTACTGGTTACTTGCCGGTGGCCTGAGGGAGAAGGTTTGGGTTTGGGGAAAAAATGGGTGTTGTGTGGGGTGAGAGACGGTGTTAGTTTTTTGGTGTGGAAGGGGTTAATTGTGTTGTGTTGC

>C101947_65

CTATAATAGTGTTTCATTTATATTTCACGGAAGTGATCTAAATTTCAGTACAGTTATGCATCCCAATAGCCAAAAAGAAAGCCTAAGTGTCATGTGTAAATTGAAGTACATGTGCTCAGCGGGCATAAATTTACAGAGCTTGCACACACTTCATTCTCATATGACTGCTACAATTGAACTACTTATTAATACATGAATACAAAATCAATGGCTGAAGAATTCCAACGGCCTGGAGTCTATTTGCGCAACATTTTTTGTGGCATGGAACTCAAATAAGTTGCATCTGGAAGTTAACTGACGAATCATACACTCTCCTTGGTCCCATCTTCAAGTGCATTACCATCTTCCTTCCAACTCCCCAACTTAACAATGTCCTCAACAAGTATTTTCCCATCTTTATCCTTGGAAAGATTGCTGACAAGTTCTTGGATACCCTCCTTGCCCAATGTATCCTTTAAGTACATTGCAGCAGAGGCTACCTCCTCAGCAGTTACTTTCCCATCATAATCTCTGTCCAGCAGCCTCCAACGGTCACCAATTTTTGCATCTACATCATCTATTTCCTTCTCTAGATTTTGAAGCATAGCATCAACTCTCTCAATGAGTGATGATGACACGTTATCCCCTTCACCAGACTCAGTAGCATGGTCTTGTTCCTCTCGGGCAGCTTTATACGCCTTAAAGGCATCTTTTTCACTATCTGAACCCTCTTTCTTTACCATGCTATTATAGAGTTCTATCTCTTTATTAACCAGCCTAAGAAAATCTTCACGCTCTGTACTTACAGACGACGCAGAAGCTAAAACTGCTAATGCACGACTAATTTCACACAGCTGCTCTTTGTTTTCCAATGCTCTGTCTCTGGCAAGTTGGTGAGCCTCTTTTGCCGTAGAAATATTCATCTCATGCAGGGCCTTATCATCTTGACTAGAACTACTCTCCCTCCTTGCTTGCACCACCTCCTCTCTCTCCTCCTCCTCCTTAATAAGTTCTTCTTGCATTTCAAGGAATTCCAATTTCCTCCTCCTCTCTGAGACAGAATCCTCAGAGGGTAAAGATGTAACCTGTACAGTATCAACAACCTCATCGGGAAGAGAAGAAAGTGTAGCCTGTACAGCTTCTTCTGGTTTCAATTTCCCAGACACAGTAAATGACCTTGAAAGTATCAAAAGGGATGATGGCACAGAGTGATTCAGAGATAAGTCAAGCCAATCTCGAAGCTGTTGGCGCATTTCTTCAACAGAAAATAACCCAAGCATGCCTCGCTCTCTACAATCTTCCCGAAGTTCAGCTTCTGAAAGAGAATCCACACCTTCTGCTTGAATCAATTTATCATCTTCCTTAATCCTTTGTAAATGTTTTCTTAGCATATAGCGCAAGTATGCATCAGTGCCAAAAGGGCTGATTCCCATGTACTTGCACATATTGACTAATCGTGGCCTGCTTATATTATCTAGAGTAAGTTCATCGTTAAACAACTTAGCAAAGCCCAAAATTTCCTCATTAGAGACAGTTGCTCCTCTTCTAATCATGTTTAAAAATTCATCAAGATCTTCAGCTGTTTTCTTTAGTTCCCCACTACGGGAGTTCTGAACTTCTTTTGCCATTTCTTTGACTGTATCCTGAAGGAACTTTGCATACTCTATTCTTGCCTTGAGTCTCCTTTTCAATGCTTCCTGTTCTTTCATCTTGTCTTGAAATGTTGAGGGCAACATGTTGGGGAACAACTTTAGAAACACAGGCAGGAGGAATTCCATAAAGGGAACAATAATGAAAACTGCAAAAGGAACTAGCCTAAAGATATCAGCAGTTGTCCGTGTTAGTTGCTGCCTCTCCCTTCTGGAGAGACTCTTCCCACCTGCAAGCTTCAACAACAGCCTGGAACTAATCCTCACGTCAGCCCAAAGCAGCTTAGAACCCAACCAATAATGCTGCAACGTTGACACAAATTCCCCTTTCCAGTGAACCAGTTTTTTGGCCCAGTCCTCCCTACTCATAGATGCCACAGCTCGCAATGCAGGACCGATTCCCAAAAACGCAGTCCACACTCTCCGCAAAACGCTTTGCACTTCCTTCTGCGACTCTTGCGACCGTTTCGCCATGGCTTTTGCTTTCGCAGTGGTCAAGCCTTCAACGGCCTGATCACACTCCTCCGGAGACGCTTCTTTCCGCTTCTTCGCCACTACCTCATCGTTTTCCTCCTGCTCATGCTTCTTCGCAGCCGCCAACTGCGTCAACCACCGCACACCTATAGGCTCATTGAATCCCGAGAAACTGTCATGCGGTACCGCACTCCCAAATTTTCCTAACCCAAATCGATTGCACAAGTCTCTATACGGCGTCGTTCCGTGCTTACAAGGAATTGGAGGAGTTGTCGTAGCTATAGCACGTTCCAGAGAACGAACACAAGTAGTGGAAGAAGAAAAACCGTTATTCACGTATTGATGGATGAAACTTCTCCTTTTCAGAATCGCTCGCGATGCCATTCTCGAAAGGTTGTTGAGCTAAGTGAAGCACAGAGATGAAGTCTTGTTCGTCCTTCCTACAAAAAGAAAAAGAGAAAACAACAAAAAGGAAAAAGGAGTATTCTCTTTTCTCTCCCTCTCAATCTCTCAATCTCCCACCGCAGAAGCTATGCCAAGAACATGAAATTAAAC

>C101951_65

TTGGGAGGTAGGGTACAAATACTACAAGATATCTATATATATATGTAAATATATGGTCGAACTTTGACACAAAACCACATAGCTCAACTTTTACTCGGCTCGAGAAAATAAGAATACCAATTTCTGCTGTTACAAACACTTTTACAATCATACTAGGAGAAAAAACTCGAACAAACTTTGTGTATAACCATCATTAGTAAAAATTATGACAACACAGCAATTGTCAACTCAGGAACGGCCATGGCTGATTAATAATGTAGCTCTGGCGTAGTCAACACTTAAAATTAAAGCACCACAGTGCGTGCAGTCTTCAACAATACCAACGGTACGTGATACTGATGCACGATAATCATAATCTGCAATCATGGTGGTAGAGGAGTACTTACTCATCATGATTCTATCACTTATGTTCCTCTCAAGGCTGCAATCCTGGCAGCTATATCATCACAGTCAGGTAACTTGGGATGTACATGCCGAGCTTCGGACGATCTCTCAGGCTGAAATGAAGCAGCACGAGCATATACTTTTTTAACTTCCACAGCTTCGGTTTGATCACGGGGAAGTGAAACTGATCTTGGTGGACGAGTAGCCATCTCTGGTGTCTCATCAGGTCCCTCCCTGCTTCCATTCTTATCTATTTGATGTGCATGACGACTTCGAGACTTTCTTCTTGCCTTTTCAGGCACATTCATGGATGGCTTTTTGCTATAATGGATCAGTAATCTATCTATTATCCTTTCCTCTTCATCATTTTTATGCCGCTCATCATCAAACAGGATTTGCAGGCCACGTTTTTGTTCATCTCGTCTCCTGCTCCTTTGTTTTCTCACAACTTCAGCATCTTCACTACTGCCATCATTGTGAATAGATCTTGATTTTGAATGCTTCCGTCTGGTAGATTTTTGTTTTAGTACAACAACTTCTGTAGCATCTTCCTGAACAAAGAGTTCTTTTTCATGACCCTGTTTACTCGGTCTTGCATGTCTAATGGCCTGCAAATCCCGTTCAGAATCATCCAAACCAGATTGGATCCTGTCCACTGTAGGAGCAGTATCAACCTTTTCATGTACCAAAGGGTAAGCAGGATTGCCATCACTACCAATATTTGAAGATACTAAATTGGTTCCATGCGTGCTGTTCTTCAGTTTCGAATTGGGTTTTACGTAAGGAGGTGGGATGGCATTATTGTAGAAGGAATTTACCCTAGGAGTTTCATCTTTTTGAGTTTTTCTCTGAGAGTTATCAACAACGGGTGGGCCATCATCATTAACAGCAAATGGCTCATTCATATTGTTCTTTGAACGAGATCTTGCATGGCCAGGAGATTTCCTAGCAGTGACAGTGTCATGGTCTTCTTTTACACGTCTAGCATTCCCCCAACGATCATGCTGATTGGAACCACCTTCAAACTGTTCCTCTCTTGTATAAGCAGGTGGGATGGCATTATTGTAGAAGGGCTTTACCCTAGGAGTTTCATCCTTTTGACTTTTTCTCTCCGAGTTATCAACATCAAGTATGCCACCGTGATTAACATATGGCTCATTCACATTGTTCTTTGAACAAAATCCTGCATGAACAGGAGATTTCCTAGCAGTGGTAGTATCCTGGCTTTCTTTTACACGCCTAGCATTCCCCCAACTATCGTGCAGATTGGAACCACCTTCAAATTGTTTCTCTTTTGTATCCTTGGAAGAACATCCAATTGGTTTCAGCATACTACCCTCTTTCCAATACCCCCCTCTATCACTCATCTTAGAAGTAAGCTCTTCTCTTCCAGGTAATGGACTGCCATGGCTATCCCTTTTCAAATTGACTTCATCATGACTGCTTCGTGGCTTGGATCCATTTTCAGCATTTGGGAGTTTGGATTTAGAACGAAGATAATCTTCATCTCCTTTTGAGAGAGCCTCGTTTCCGTTCTGAAATTTGTGCCTGCCATTAGGATGGTCATGACTTCTCTCAAACTTGATTCCTTTCAGGGTAGCATCCTTGCCTTGTAAAGGTATATTGCGATCAGTGCTGTGGTTAGACTTAAAATTACCGTGGTCCTGTACCGAAACAGAGGATTTTGACTTCATTAATTCAAAAGCTTTGGAATCCCATTTTATTGAGAACTCTGAAGCAATATTCTGCATCAAGTGAACCTTCTTCTCCAACGTAGCAGACTTTGAATTCAAATTTGTAGCAAACTCTTGATTGACATAACATTCCAGGGAACTTCCATATCTATCCTGAAATATTTGACGAAGGTCACGTAACTCTGGTAGATCAGAAAATCTTGCTGCAGCAAACATCAGAGATGATACAGCCTCCCTGAGCTCCTCCGGGCATCCACTCACTTTCTGCAGCACTGCAAGGTGCTTTAGTACAACGTCACAAGATTGTTCAACAAAATCATAACAAGACGATAAAGTCAGCTCAACAAAGAGTCCTTCAGCCCTTCCATAAGCATTTTCATCCAAACCATTCGCCAGCAGATCAGCGATGTCCTTCGTCAGAAACTTCTCCGTCGCCTTTCGCTTCCGCCTTATCACATCGATCCGATTCTTCGTCAATTTGATCAACGATTTGCATTTCGCGGCGAATCCTCGACCGAGCAGGCCGTCCAACATGTCGGAAGATCGGACTCGGTGACTCGACTCGGTGAACGATTGACTCCG

>C101953_65

GTGCAACTCTGCACTATTACCATCAGCAGTAGAAGTTAAATTACAGCACCATGATTCACAATAGATTTCTCAATATGCATTCATTCATGCCCCAGAACTAATCACAATTCAAAAGACAGTAACAGTGTATCAGCAATTGTTACAACTTGCAGATTTTCTATACCTCTATTCACTTTTGAATATCTACTGTATCAAAAGTAAAACAAGCACTGTGAAAGGGTATATCTATTACAATTTCAGCATTTACCTTTTTCCTTGAAAAACAAAATGAGGTGGCTGATCGATGAATATCTAATGCATGAAAAGATAAATTAACATTTTAAATCCCAAAATGAATTAGGTTATTGAAGACTTGCAGACGGGGCATGTTGTTTTCCTCTGGAGCCAAGGATCGATACAATCTTTGTGAAATTTGTGCAAACAGGGAAGGTGGCGAATAGTTTCTCCCCTGGCTGGGGTCTCAAGACATATAGCACATGGCTCAGTCATGTTGTCATTCTGGATGGTTGACTGCGGAAGGCTATTGATCAGATTGGTAGACGCACCAGTATGTTGGTGATTTCTCTCATCAAGGGCCAGTAACATTTCATAATCATTTTCGTTGAAATCATGTTGTGCATTAAAGATGCCATCAGCCATTTCGATATCTCTGAAATCCCCCATTGAATCCTCTAAAGCTTCCAATATATCAAGTCTCATGTCCAAGTCCATATCCAGAGGAAACTGGGGGCCTCTTCCTCTATATGACATGTTAGGTGTCCGAGTCCTACTAGTAACGCGACTCCTCAACCGTGACATTCTATTAGAGAAAGAAACTTGAGGCATAGCTCTCCTTCTAGAAGAATTTTGATTAGGCCATATTCGAGGCTGTCTATTTGCCCTAGGCAGCCGTGTAGGATGAATTATGTTGTGACTATCAATAGATGTATCCAGAAGATCCTCTGCATGCTGCAACTCCCACGCTAAATGTTCATCAATCCCTCTGTCTTCAATAGAATCATCATGATACAATTGTTCTTGGAGTTCACGAGCCAATATCTCATCTGCTTCCACTTGTCTCGCCCGAGCTTCCAAGCTATTATTGTCATTATCATCTAAATCTTCTGATGATCTGAATCTTGGTGTGGATGACAACTCAACCACTTCACGATCCAAGACATGGCTCCGGGATGAGCTTGCAGAATTCCTTGGTGTCAACTCTACCAATTCCGGAAATGAACTGGATGATTCCCCAGGTGATTCACGTTTTCTTTGCCTTTTAGTCAGAATGTCAGCAGTAGGACGGTTACCAGATGACTGGTCCTCATCTGGAATGGTCACATAACTTGGTTGAGCTGAATGATCTGATTGAGAACCATGTACATGTGTGCTGCTTTGATCACTCCCAATGTTTATTTTGTCCATTCTGATTGTAACCTGTCTATTAACAATTCTTCCAACACCATTATTTCTCGTCAAATTATGCCCATGTGCATCATACAAGTGCTGATTAGCATTCCTCTCATTATGAGTAGTTCTCCATCCACCTTGTCCCCCAGAGTATTGAAGTGAATTTCTTGTATCACTACTGTTACCAGTGACCTCTTCATAACTGATCACAGGGCTGCTGCTAGCGGCGTGAAAAGTTCCAGCATTGCGTCCATGGAATGAAGGAGGAATAAGTACTTCCTTTCCTTTAACTCTACCATTGCTTCTCTCTCTAGCAACTATGTCATCAACACTAATCGGTGACACTGTATTGCTAGAAACTGCATGATCAGCATGCCTCTTTTCAACATCTTCTGTTTGAGGGTTGCTCTGTTCAGCTAATTGTTTTGCCCTTGTTGCAATATTATGAGGTGAAATGCACCCATTTCGCACCAATCTCCTCTGCCTTATACCTCGTGGAGAGGCAGAAACAAATGGAGGCAAAGGCACTTGCTTTTCATTTTGGGGTCGAGAATCATGAGACATAGAAATACCCTTGCCACGGTCCACACATATATTAGGTCCTGGAAGTGTTTTATTATCTAGTTTGCACTTTCCAGTGAAGGAATTTCTAGATGTATCAGAAGAATCCGAAATGGCTGGCAACAAAGAGTTCCCAATAGTTGCTTTCGGTGCTTTCTTGTCTACAGTTGCATGATCTCTAGATCCACGAAGTGAGAAAGCTGGTTTCAGTTGTTGGCACTGTCCATTCTCTTCTGTCAAATCTAAAACAGAAATACCACAGTGAGATGATTTAGAAGAAACCTTGGAGCTTATACTTTTCCCTTTCTCCATCTTCTCAGCTCCATTAGAATTTGCTGTTTGACCTCTCCTAAAGATGGGAGCATTTTGAGAAGGATAAGATTTCTCTGACGGGGACAATGTTTTAAAGTTACTGTTATTCATGTCACCAGAAGAATTAAAAGCCCTGCCCCTCTTTTCAGGATTACCTAAATATTTCTGATCACCACCCCTCACAGTTATTCTATCTGGGGTGTCAGGAACTTCCACAATTTGATCAATTTTCATATCTTCTGCCATATCCTGATTAGTAAAGAGTATTTTTGTGAATTTGAAACATGAAGATGAACAGAGAAGACAAGGAAGAAGGGGCAAGTATTTATCCGTGCGGAAAACGGGAGGGTCGGGTCGATCCGAGTTCAAGATGGATCGCAGAACCCAAACCAAATCCCA

>C101955_65

GTTTTTTCTCCATTGATATTTTCCTTTAACGTTGAATAAGAACATGTCCTTTATATTTGACCCCTCCCTCTGATCCGCAACTCCTCCCACCAAAAAGGTAGAAAATATGCCTCTTTATGTACAATCATAAAAAAAGCAATAAAATTGTTATCCTTCCACCAATCCTCATGTATAAATTAACTTGAAATGCTCCTTAAATTGCAGCCTGAACTCCCCAGAAGAATGTACAATCATAAAAAAGCAAGCAACAAAAAAGGCTTACTGTCACAATCCACAATTTATTCCACTCTGTTTGTACATGTCATAACAGCTGATTTCATCCTTTCTTTATCCAAAGAAGTTTGAACTATCACCTCTTGTATTTTATTTCCAGATCGAGCAAGTAACTTGTACTGATCCTCAAAGATGGATGCCATATAAACCTGAAAAGTTGGGGCTAAACCAGGGCTTCTGAAAAAACGGCCTTTTGAAACAGACTGGACTCCCTCAATCTTTTGCAATTCTTCAACAAGGGATTCAACTTCAGGCCACTCTACATCTGTTTGGGATGCTTCCAATCTCCCATCTAAGGCGTCATGATCCCCAACTATAGCCTCCAAGGAATTCATTAATGGATCAGGCATTATCTCCTTTAGTGATAAAAATATGCGGTCATGAGCAAAGTTTAATTGGTGAACTTTTGCCTCTAGAACCTGACCAATCTTAAAATATGATGCTGGGTTCATAGTGGCATCCCACGATAATTCAGACTGATGAATTAATGCAGAAACTCCTTCAACCTCAACAAAAATTCCAAAGTACGCTATTTTCTGGACACAACATTTCACTATATCCCCAACTTGAAGTCTAGCCATGAGATTTCTCTTCTTCTCAACTAATTCTTCATTCTCTTTCCGTCTTATAGAGAATATGAGCTTTCTTAATTTTCTGTCTGCCACTAACACATTTGCTTTAAGTTTCTGTCCAATAAACGATGATAAGAACCTATTTTTCTCTTGGTCATAAATCCTTAGCAGATCTTCCAATTTCATATCTGCTGAAATTTTATCTTCAACTTTACCACCAATCTCCAGAGATGGAGAAGAATCAGGAGAAAAATTCTTAGTTTCAGCATCAAAACTTGTAATAGTGCCTGAGTTTTGCTTGTATATTGATGGATCCAGGCCCTTCTGTTTTAACCAAGTCTCAAAAGCTAAGAATTTCCACTTGGAAGTAAGATTACGGTATGGCAGAAATCCTACCAAGGAACCAAAAGAAACAATGAAACCTTTGGTATTGCAGCTTACTAATTCCACATCTTCTCTGTCTCCAGCCATAATCAAACCTTCTGCCCTGGTCCAATCATCATCTTCACTTTCCTGGAAATCTGACACACCAACAGCATTCCCTATATCATCGTTGTTTGTGTGACCTCCAAGATTAAGGGTAACGTCCTCTTCATCGAATTTTGAGGGTTGTTTTACATACTGATCTAATCCTTTTGGCTTTGCCTGTAGTGCAGCATCCACAGGCAACTCCAATCTAGAATCAACTGACTTTATATCTGATGATTCACTTAAATCATTTGAGGCCTGATGGAACTCCGGCTGTCTCTGCTCATGTTGCTCCAACCCATCATTTGGTACCATGGCATTCCCTTCTCCAAACTTCTCTTCTTTTTCAAAGCTTGGTCTGTGTGGCCGCTCCAACAATGTAAAGCCACTAATACCATCACCCGGCTCTTCATTCCACATCTTCAACTCACCATCATTGTTCCCTTGGTCCACATTAGATGAAGGCTCTTCAACCGTATCAGTACTCTTGGCAACCGGTGGTTCAGGTTTCCTCAATAGTGTCATGTCGCTAAACTTCTCCTTCAATTGCTCATCCCGCATTTTCAATGACAGATTGGGCCTCATTCTCAACCTTGAGGTCAACGTGCCCTCATCGTCATCATCTTTAAACACGGTCGGCTTCCTCAGAATAACATTGGGAACACTACTCTTCCTAACACTCCCTTCCACATTTTCAGTCCGGACAGGTTTCTTAATCTCCAATGCAGGTTTATTATCATCAGCTTTGAATTTGAATCCCTTAGCAGGAACAGGACGCACCAAACCAAGGTCATCCAGTTTCCTTGATGACTTCCCTCCTTTAGAGCCTTCAAATGGAATTTCCTCCACTTCCACAACTTTACCACCATTCTTATAAAAGGCTTTCTCGATATCAAGATAAGAAGCATCGGGGTTAGCCTTTCTTCCCATTATCTTGGCAAGGGTGAGCTTGGGGTCTTCTCCGATTAACCGACCGAACTTGAGTTCCATAAGGTCGTAACTGTCGAGTTTAGGGGGTTCTTTTTCTTCTTCATTTCGTGATTGTGACGCGAAAACCTTGACTCTCTGATTCAGAGAAATGGTTCGTGTTCTCGTTCTTGTTGTTGTGTGTGATGAAGACCAATGGAGGTTGGTGAAGAAGGGTTTACTACCTGTGAGAGGAAGAGACCCCATTTTGATTTTGCTCTGAATTTCAATTCACAGCGATTGATGTTACTATTGTTATCATCAAAATTGAAAAGGGTATAAGTAATTCTTGCTTCTTGTCTTCTCACTGTCAGAGACCATGGAAAGTGGGTTTGGATAAACGGCTAGCGAGTAGCAAAGAACGGGGAAACACAGAGTTATC

>C101957_65

CAGCACTTCGTGCTTCACACAAACATGAACTTATAATCAACTTGAAACTATTAATGTATAACACAACCAAATAAGCTTAGATCTTGATATCTGATTACAAAGATATTAACAAACCTGAGATGAGAGGGACACAATCACACTATCTGCGTATTACAAATATCAACTGCAAAAAGGAGAAAGGGGGGATATAAAGCTACCTATACAACCACACAGCCACTCAAAGAAAAGCTGAAGGTTAGCTCCAAGTTAATTTTCTTCCTTTGCCTGGCTTCTTAGCCGATCTCTCTCTAAGCAGGAAATACAAGACTCTATGTCAGACGCATACCGATTCCTCAAACTCTCTTCTCACCATCAATCCATGGCCAATAACATAAACAATGGTCGTCGGAATGCCAGTTACAATCTTATAATCAATTGGGGGAAAAAAGACAAAAAATAAAAATAAACAGAGAAGGGTAGAAGAACGATGCTATGAAAGTTCACTTCTTAGTGCCTCTTCAGGAGACATTGTCATTGGGTGCTGCGTGTTAATCCTCCCCGTGCCACATTTCTTCCAGAAACAAGCTGATGCTGAGCACTGTTCACAGGGCCCTTCACATTGCTGAAACGCTTGGCTCTGCTCTCTTGTTCTTTCTCTACACGCATTGCAGCTACCTCCTTCTCCATGACCGCTACTTCTCTTCTCATCTTCTTAGCCTCAACTTCCATGGCTTTGGTCAATGTATCTACTTTCTTCGCTAGCATTTCAATGGCATCATCTTTATCTTTTAGGCTTTGATCTTTCTCGTGACCAGCTTTCCTCAAAGCTAAGACCTCTTTCTGCAGCAAATCATATAAAACCCCAGAAACACTATCGTCCACATCCACAGTTGGGAACTCATTTGGCTTATCATCTGAATTTCCTTTCCAGTTATCATTTTCTTCCCTGTCTTTGGTTCCTTCAGAAGATTGGTTGAATGAGTAACTTGGAGGTTTTCCATTTAGAAGAATTTTACTCCTTTCTAATGACCTTGTGCCACCATCAAATGATTTAGATGTGCCTTTGGCATGCTTCAAAACTGTGCTTGAAGATACAGAAGACCTAAGTTGAATGGATGGTGATCTCTTCAGAAACCCATTAGATGTTAGCTTAGAGATGTTATCAGCTCCACCTAGGGATTGACGTCGAGAAGGCCCATTGCTAATACATCTTCCCTCTGAGGTGCCGCGATTAATACTGTTAGAAGTTTCTCTCAAACTCTCCTGTAGCACTTTAAGCCTTAATTGATATTTTTCTTTTAACTGTGCTTCAGACTTTGCAGTGCGCTCAGTTATAGCAAGCTTGTCCCGAAGTTGCTGCATCTCTCCCTGCAAGAATCGTCGTTCTTCAAGCCATTGTTTTACAGGCATCACTTTGTCATTAGCATCCTTCCATTCATTTGCAACCACTACAGCTACTCTGTTTGCTGTTACCTTGGCACGAGCTAACTCCCGGTCAAGTGTTTTTCTTTCTTCATTCATTTCTTGAACTTTCCGCTGATAATCTCTCACAGCATTGGCAGCTGCACCACCGGCAAGGACAGCCTCTTCCAGTTCTCTCACGGTTTGGGTAAGCTTTTCAACCTCTGCCACCTTCAGTCTATGCATTTTGTCCAAAATTTTATTTTCTTCCTGGCAAATCTCAATCTGTTTAATTAGCTCTTGATTTTTATTTTGGAGGTCATCCACCATGGAGGCCTTAGCCAAGGCAATCTGTACAGTCTTCTCAGCTTCAAGAAGTGCTGCTTCTTTAGATTTGGTAAGACGATCTAAAGCTTTGTTATCATCTTGAAGTTTAGCAATCTCCTGCCGTGCAAGCTTAAGTTCAGCTTCTAGAGGAGCAAGAATGGCCTCTATAGGAGGCATGTCATCATCCTTCTGGGCAGCATGGACCCTCCGAAGAGTGGCTTCAGCAGCAAACTGAGCTGCCATCGATGCTTTCTTTTCATCATTGATTTTCTTTATTTCAAGATTTTTGCTTTCTAGAAGAGATTCTGTTAACTTCAGCTTCCCCTCAACCTTCGACAATTCTTCAGTAAGCTCTTCAACGGCCTTTTCTCTGAGCCGTTCAGAGAGCCTCAAGGCTTTGATCTCTGCTTGCGCTTCCGATAATTCTCTGTCCTTATCTCTAACTTCATTCTCCAACCGATTGAGCTCCACCTTCACCGGATCCGAACCGTGCAACAGGTTCATGAACTCGTCGGCGTCGAGGCTCTGCCTCATCGAAGGCACGCGCCTCCGCGACGAGGTCTTGCCTTCCTTGAACGATCCGGACACCGCCAACGGCGTGGCCACAGTAGCGTCCGCTCCGCCGTCGCCGGAAACCTCCGCCATGGCCGATCTAACCGATGCCGAAAACAGTCACATCCACGTGGTCGCAGATTGGCGGATCCCTAGGCGCGCAGTGAGAGGGAGAGGGAGAGTGGGAGAGAAAATGCAGGTGTGAGAAGGAGACGGTGGTTTTAGAGAGAGAAATCGGGAGATAGATGGTTTCATAGTCTGTGCTTGTTTCTCTCTCTAGATTGTGATTTTAAATCTGGGCTTTCTTTGGACTTTATTTTATTTTTTGTTTATATTTTAATTTATAATTTAAAAATGTTGCTGTGTGTGTGTGTTGCAGTGAGAGTGATAGTGAGATTAAAT

>C101959_65

TGAAACTACATCGATCATAAACCGCAGAGAAAGAAAGTTTTCGTAGCCAGAATTAACGAAGCTTCGATACAAAGAGTGCATCCCAAAATCCAATCCATTGATTCCGCGTAGCTTATGAAGTTTGAACACAGACTTTTATCCCCTCTAACGGGGTCACCGGTTCAGAATGAGAAGGGAAAGTCTCAAATCTTCACGATTGAGGGAAGAAAAGGAACGGAAAAGAAAAGGAATCTTCGTTGTGAAGTCGATTCAAAGCTTTAAATCGCTGTAGTGATCTTTTTGTTTGTTCAGCGGTTTGAAGGTAAAGTTGAGCAATTGTCATGGAGACACAGCAAGAGTTGGAGTGGGCTGAAGCACAGAAAATCGTGCTAAGCGAAAACCTCGTTGCTACGGCTAAACAGCAGCTTCTGTTTCTTGCAGAGGTTGATAGAAATCGTTGCCTTTATGATGATGGCCCACTTTTACACATGGCTATCTACAGGTATAAATATTGTTGGCTTCCTTTACTGGCCAAACATGCTGAGTCATACGTGACAGAAAACCCTTTAGTTGTGCCGCTTGATTGTGAATGGATTTGGCACTGTCATAGGCTTAATCCGGTGCGTTACAAAACTGACTGCACGGAACTTTATGGGAGAATATTAGGCGATTCGAATGTGGTCTCTTCAACTCAGGGAACCAGCAAAGAGGAAAGTGAGAAAGTCTGGAATTCAATGTATCCAAGTGAGCCTTATGAACTTCTTGGTCTGAACAATCTGCAAGGTTTTGCTGAAAATTTCTTGGAAGCCAAACAAAGTACCACCAATTATGATCTGGTTTCAGCAGTTAAAAGACAGACTACTTTCTTCTATCAGGTTTCCAGGCCTTATTGGAATGAGGATACTTTTCTTGAAGGAGCTGTGGCCAGATACAAGGGGTTTCTGCATTTGATCAAGAGGAATAGAGAGAGGCATATAAACCGCTTCACTGTTCCAACTTATGACATTGACCTCATATGGCACTCTCATCAATTGCATCCTGTGTCTTACTGTAACGATCTAGTGGCAATTATGGGCAAAGTATTAGAACATGATGACACAGATTCTGATAGGACCAAGGGCCAAAAGCTAGATGTTGGATTTTCTGAAACCACGACACAGTGGGAAGAGACATTTGGTTCAAGATACTGGAAAGCAGGAGCAATGTACAGGGGTAGTCCCCCATCTCCTCTTACTGTTGATAAGTATAAAATAGTTGGTACTCACTACTCCTGTTCAGCTCCATCCATTGAGACAAACCAAAATCTGATTCAGCTTCCACCCAAACTGCTTGTGCAGGTGATGCTTGAGATTGTTGATGTGAGAAACTTACCATCCGGGCATAAAGGCAAGCTACTTGTGTCCTTCAACAAGAAACAAGAAGATGTGCTTTTCAATACCAAGAAGCAATTAAGCATTTCCTCCAAGTCCCAAGGGAAACAAGTTGCTGTTTTCCAATGTGAAAGAAATGGAGAGTTAGTCCTTGAGCTCATTTCTCGCCCAAGTTTTAAGTTTAGAGGACTAAAGCTAGCTAAAGTATTGGGAAAAACTTCAATCAATCTGGAGGATTTGCAAAGTGTAGCATCTAAACTGCCAATCGAGAAGTGGTTAGATTTAACAAGTGCAGTCAATTTGTCAAAGCCTATTAGCATTCGCATTGGTTTGTCTTTAACTCCTCCAGTTTCAGCACCGTATAAGCTGCACTTTGTTTCTATGCTTCCATTCAAAGCAAGTTATTTTTCCTTCCTGCTTCCGAGAAGGTCCCAGCAAATTAAATGTTGGACCAATGTTGTGAATGAATCTGGCAAAGAGATCATCAGCATCCAGAAAGGGAACCTGTCAACCGAGAAGACAAAGAGTAGTATAAATAAAGAAGTGATTGGCAGGACTGTATCTGGTGAAACCCATCTTCTTGCCGAGTTAAAAGGAACAGTGTGGTCTATAATGAACTCTAACTGGGTGTTACAGCTAAAGAAGACCAGTGATGAAGATGAACGTGTATATGAGCTCTTTGAGCTCACAGGCACTCGAAATGTGGTTATTTTTCCAGGTACAAAATTGGAGTATGGAACTAGAAACTACGGTAATGGAGAAGGAAGTTGCTTTATGACAGCAGTAAAATTCTCTAAAAAATATCCTTATGGAAAAGCAGTTGCTTTGATGGATTTGGCATATGGATTTCTGGAGATCATGGAAGAGTGGCTGGTACTGCCTGCAATATTGTCTGCGTTTGTTCTCTCTAATTTTCCTGAATTCAGTAATGAGAGTGAGAAAGCGAGTGGGACTGATGCATAAAAGGAAAATCAGAAAAGCTGCGAAAAAATACAGGTGGCAAATGTTATGTCCCTTTCCTCTGGAAAAATCCTCAGGATAGGCCACTTGTCCATAAAAGCTTCTACCATTTAATGCAAGTCAGCTTGTCGGCAAAGCACAATTATACAATAAAAAAATTTGTGGAAAGTTTAGACCTGTGTATGTACGGTTAAGATGATTCTTTAAGGGACTGACACCTTAGAGTAATCAGTTTTAGCTTCACAAAATCCATTTGTAATGTACTTTTCATTAGACCAACTCACCTACACTATAATATACCTAGTTTACTCACCTAATTTTGTACCCAAAAAACTAGAAAGTGTTGCCAGAA

>C101961_65

GTTTAGTAATACGCAAATAGATGCATAACCAGTTGATGAATACCCCACAAAAGAAAAAGTTGGATGCTAAAGAATATTCAAGACTTCACTAACAACCTTTTTCCCCTTTCTGCCACCCTCTTGTTTTGTAGTAGAGAGAAAGGGGAAGCAAAGGATTTTGTCATTTGTATTCTCAAAGCTATATGGATATAAACATTACTTTAAAATGGAGCATGGTGGAAACAGGTTCTAAATCCTATGAGATATGGACATCACATCAGCAGTGCAATGGGAGAATCAACATTTGCCACCCTTGCATCCACTAATCATAATCAACTAACAGTAAAAGCTTCCTATTCAACTCGGGTCATCAAACAGCAAAATGTTTTTACATGGACCACAAGGGCAAATTGTGTGCACCTAAGCACTGCTTCTTCTTACACAGGCCACCACTGCATGCACATCCTCAAACATAAACTTGGAAGGTGAATCAGTAATTAAGCAATGAATATTTCACTCTCAAAACAATTTTCCCTTTTTCCACGCCAAGCTTTCCACCAGTGACTAGCCAATGGCCAGGAGGGTCTTGGGGCTCTTTGGTCATCTCAGACAAGTCAACATACTTCACCAACTTGTTCCCTGTGGCATTTTCTCTGAGGGGTGAGTTATCTGAGTTGGAGGTGTTTCCAACATTCACAGTTTTTGAAGGACTGTTTGTGACGTGATCCCATAGCGATCTTCGGATCGTGCAGCCAGGTAACCTTGAATATAAGAGCTTCATGTACAGTACATTCCGTGATCCGAAATCCCACACTCCAAGCTGTGCTCCAGTAACTATGTAGACACCAGAGAAGTCTCCTATGAAGGTTTCAGGGTTCTCAATTGGTGCAGTGCTCACATGAGAAAAGTTCTTCCACTTAACAGGCTCAAACCATCGGCTATCTTGCTCCTCGGGTCCCTGCCACTTAGGAGCACCTATAGCAACATGGCTGTCCCAATATGGATGGAGGATTTTTGGAAGGGATACCAAGTGTTGAACGTGAACGCTCAGCCGATTCTGCTTGCTTCCTTCTAGAAAGAGACGTAATCCAGTTACTGGCCTACGTCCCACTGTAATCTGTTCTTGACTGATGTAAAGTTTCTGGCCCATTATGCTGAATTGCAAAGATGGACACACAGGTTCCTTCCTTTGTTGACCAGGAATCCTGTCTTGTAAAGGAGCCCATACACGAGGAATCTGAAACTCTAGGAAATATCTAAGTTCTTCAATTGGAGGTTTATATTCAAGATAAAGACCAATGGCACGAGTCAAATGCTCCTTGGCAAGTATTTCATCAAGAAGAGCAGTTATAGGACAGAAAGTCATTTCAATGATATCTGGGGAGGACTTCACAGTTGATAGCCATTTACTGTGGTTTTGTTCCAAATCATCTCCTCCTCTTCTTCTAAAAATGACAGTGACATCTTCTTTCCCAGAAGGATATGTTGCAGTTGTAGGTTGGGGGTAAACCCCTTGACTATTGAATGAAAATGGATCAACACCCTTATCCTTGGATTTTGTTTGACCTGAACTTGTTTGGCTATTGATGTCAGAGAACCTTTGATTTCCGATATCCTGAATATAGTTTTTCATCTCCAACTTTGACAAAGGAGAAGTATGGTGTTGTTTAACATAAATAACATCTTTACCACCAATAGTTATAGATGTAATGACATGAGTCCCAAAATTTTCGATAAAACTTGCCAAGGCCGATGGGTCCCAATTGACAGGGACAGCCTTTTTGACATTTCCTTGCAGCGTTAAGTGGGATTTCTTAAGCTGAACCTTTGCAAGAGGAATGTAAAACCCTTCTGTAGACAAGGTTTTTGTGGCTGCAGCATCAACATGTTTTGAACCAGTAAAGCTAAATGCAGCATTAAAACTCCCAAGAGGAAATTTCCCTGATATATTTGCCCTTCCATTAAAGTATTCCACCATCTCCGGAAAAGTGCAGACCCCAGAACTCTGACGCTCCATGGTCTCCTGGGAGCTTCCAATGTCCCTGGAAACGTCAGGAACAGCCACATCATCGTACAACAACAGCTCCCTACGGTGTTCTTCATCAATCTCAACTACCCTAGACCCTGAAACTCCTTTACAATAAAGCAACCTCGTATCAAAGTTCACATCAAAACCCCTCCCTAATGATTGCAAAGCGTTAGTAGCAGTGTGCACCGCTGCTATATGCTCCCCCATGGCTCCGCCGAGCCCCCTCAAAAGACTCTTCTTTAACGCAATAAAAAGAAAACCCAATTCTGAAAGGAACTAGAAAGTGGATTTCTCATCAATCGAAGGGAATTAGCACTACCCACATGAACAAATTCCACAGAACACATAAAAAAGTGCGTGATTTTAACTTTTGCTCTACGTGGGCATGTGACTCATCAAAAGGGTCATGATCAAACCCATCTCTTCAAACCCTCTCTTTCTTCTGTGTGCTCAACCGGTGGTTGACCCAGAGAAAATCATGATGTCCAGCAACAACCCCCACGGATCTAAGAGCTGAAGATTAGATTATTATCCAAAATCTGGTGTCGTTAGGGTGCTAGCTACTTCTTCTTGTGGTTCTTGTCACTGACTTTCTCTCTCTAAAAAGTTTCTCTCTGTAAAATAAAAACCTCTTTTCTCTCTCTACATTGACA

>C101963_65

TTTTTTTTTTCCACCAAAGCCTGGACTCATCTTTATTCACATTATCCTAACAACCCTTTTCCATTATTATTATTATTAGACACCTCAACTAGGTCCCCTCTCAATAGCACCAAGACACCCCAAATCCTGTGATTCAATTTTTACATGGACCCCAAAGCTGGTCACTTACAACAACCCCCTAGACATCATCTATTTTGAGCCACAACATTTTGTGCAAAACTTCAACAAATGGAGCCATGCACTTCATTGAAACTGCTGAGTGCTGAATAGCTTGACCTTTTTTTTAATGTTGTTTTAAGTTGTGTTTTGGCAAGTGGATGAATAGAATCAATGCACTGCCCATTTGTGGTGCATCATGGCGTTGTATGACCAGACCTGATATCATATCATATCAACCATCGACAATCAGCAGCTTAAGGAGAGACATCATGAGTGATCGGCGTAGCGGTGGGGACCACAAGGCAAAGTCAATGGTCCCAACTTCCAGCCAAATCTACCACTGTAGTTGCAACCTGAGAGGGAATTCTGAGAAACAAAATAATTACAAGACTCAACAGGGAGTTGATATATGACTGGCTGGCTGACTGGCCAGCCCCATCATCATCATCATCATCATCATCATCAAAGTCACTACTTTGTGGGAGCCATTGGTGGGGTCCTTGGTACACATCAGGAGGGTTTTGGAGCAGAAGAACCCTTGTTGCCAAGAGGAACGCTGGATATGCCTTGTTTGGAGAGCTCAGCCAACACACTATCCATGGAAGGAGCTTTGAGTCCTAAGGGCAATGGTAGCCATGGCTTGTTAATTGCTTCCTTCACAACCTTGTCTACCACCTCCTCCGCCGGATGATGCTCAAAGGAACTATGTGGCATGCCGAATCTATAATCCACTGCTTTAGGAGTTTGCAATCCAGGTATACTTTGATTGTAGGGAAAACCAGGAGCTTGAGCAGGTGGGAACAATGGGCATCCCCAAGCATCCACATGCACCCCCGGATAAGGCTTCCAGTGCCAGCTTTCTGCAGGATGCCACAAGGGATATGCAGGAGATCCCCAAATCTGTACCCCGGGCGTTTGACTTCCAGGTTGTCCCCACATAGGATATATAGGAGCTGGAGAAGGGGTGTGATTAGAATGATAGGGAGGGTATGCCATGACTGGTCTTTGCAGATAATAGCTCCTTTGCATTGCATCTCTTTGATTATGCCATTTTCGATCTTCTTCCCCAGGTGCACTTTGTCTCTTGTGTATTCTATATTTCTGAAGATGGCTTGCCACATTGTGCCTTGTCAAACCTTCCACTTTCATTATCTCCAATATTCTAGAAGGAATGGCTTGATCAATACCTAGTTGCTCAACAGCCTTTACAAATTTTTTATGCAGTTCAGGCGTCCAGTCTACTTTCATCTTCTTCCGATTAGCTTTAAGCTCACACGAATCATTAAGGACTCCCGTCTTTTTTGGAAATGTATTACCGTCTGCATTGGTGAGAAACTTTTCTTTGTGTTGGTTCGGCGAAACAGCTCTCTTGCCGATAGAACCATTGGCAAAATCCTCTTCCTCCGTAATCTGAGTCTCCTCGGGTTCCATTGGTTGAGGAGAACTTTCAGCATTCAAATTCTCACATGAAGTTTCGACAGATTTGCATTCCCCATCATGTTCCCCACTTTCTTTTTCTGTTGAGCAATTAGTCTGCTCATGGCCATCTCCATCATCCAGTAACCGTGTCCCCTGTTTCAATTGTGGGGTTGACGGAGCCGGATATTTATCACATACAGATTGTTCATTGTCATTATCACTAAACTTGGACACATTCTCTAAATCAATCGACATCGTTCTTTTATGAGTTTTATCTCGTCCATTGTCTGTTTGGAGCTGCAGCACTGATTCTACAGATTCTTTGATAGGTTTTAGTGATTCAGACAGAACATTTGCTCCAGGATTAAAAGCCTTGTGAACAACATGCTGCCAAATATTTTTGAGCTTCTCCTCAGAAAGTGGTTTACTGAGGAACTCAACGGCACCAAGCGCAATGCACTTCATTGTGGTGTTCAGGCACGGGTTCTTTGAAGTCATAATGGTAGGCAAGTCCTTTGCATTCTCAAGAAATTTGAAACCTCCAAGGCTACTGCTTGTATTCACCTCCACTATGGCAACATGGAAGCCTTCAGGACTACTGGAGATTGCTGACAAAGCTTCATTCTCTTCATAGAAGGTAGAAACATTATAGTCCATGGCCTGAAGCTGTTCTCTTATCTCGGCAGCAGAACTTCTATCTCCGTCAAGCAGAAGAACCCTAAGTCCCTTTGGAAAATCTTTCCATGCTTGTAAATCATTGGCAGTGCAAACCATAGCTGGTTTGGCATCGTCACTCAGTCCCCTACTGATGCAACAGATCGATATTGACTAGGAACATCGAATCCCTTCTTGGCTGAAAAGAAAATGTCCCACCAACTCCTCTAGCCAAAGCTGTGTTCTTTCTCCTCCTATTTCATTGAAGACAAAGAAAGCAAATAGGGAGAGGATGAGTTAAGCAGAGGGAAGAGAAGGGGGAGTGAGTAAGGAGGTTTAACTACGGTCGTTGAAGGGTGTCTGTGAACACTGAACAGTTGCCATCGCTCTTTAACAACCACATAATATGCAGAAAAAGAGTCAATCAACT

>C101965_65

CTGACTTTTGACTCCACATCTAAGCAAAGTAGCAAGCGAGAAACAGGAAAAGCGAAACGCAATAGCGGTAGTTGAATTTTTGGATGTGGAATTGTCGCGTTCGAATTGGAGGGAGCGGCGAAGAAAGAGAGAGTGAGAGAAAAGGGTTAGCATAACCGCAGAAGCAGAAGCCGAATCGCGTCCCGATGGAGGCGGTGGCGGAGGGTCTATGGGGACTCGCGGAGTACCACGAGAAGAGAGGAGAAATTGGGAAAGCAGTGAAGTGCCTCGAAGCCATATGTCAAAGTGAAGTGTCGTTTTTCCCAATCGTTGAGGTCAAGACTCGTCTTCGCATTGCCGCACTTCTCCTCCACCATTCGCACAACGTGAACCACGCCAAGTCCCACCTCGAACGTTCCCAATTGCTCTTGAAGTCTATTCCTTCTTGCTTCGAATTGAAGTGCAGAGCGTATAGCTTGCTCAGCCAGTGTTACCATCTCGTGGGAGCCATTCCACCGCAGAAGCAGGTCTTGCACAAGGGACTTGAGCTCACTGCATCTGTTGGATACGAGATTTCAATGAAGTTGTGGTGTTGCAACTTCAATTCACAGCTTGCAAATGCTTTATCTATTGAAGGAGACTACCAGGGTTCAATCTCTGCCTTAGAGTGTGGCTATGTCTGTGCTACTGAAGTATGCTTCCCGGAGTTGCAGATGTTTTTTGCCACTTCAATATTGCATGTCCGACTAATGCAATGGGATGATGATAATTTGGTTGAGCAAGCTGTTAATAAATGCAACGAGATTTGGGAGTTAATTGATCTAGATAAAAGACGACAATGCCCTGGCTTACTATTTTATAATGAATTACTTCACATATTCTACCGATTGCGACTATGTGATTACAAGAATGCTGCACCCCATGTAGACAATCTGGATGCTGCCATGAAGTTTGATATGCAGCAAACACAGCATATACAAGAGCTACTGAAGGAGCTCAATGTTCTAGATCAAAGTCTCTCTCGATCTGACCTTCACTACAGGGATAGGACAGCATTATCTAAAAAGCAAACAATGATTCAGGAGCAACTGAGTAGCATGACTGGCTTGAATTCAATTGGGCAGGAATCACTGCAACCAGTATATTTTGGGAATGTCAGAAGAACCATAGGAGATAAGCTTCAGTTAGCACCTCCTCCTATTGATGGTGAATGGCTTCCTAAAAGTGCTGTCTATGCATTAGTTGATCTAATTGTCGTTGTATTTGGGCGCCCAAAAGGACTTTTTAAGGAGTGTGCAAAACGCATACAGTCTGGGATGCATTTAATACAAGATGAGTTAGTGAAGCTTGGAATTACTGACGGTGTTAGAGAGGTGGATTTGCAACACTCTTCTATCTGGATGGCTGGTGTGTACTTAATGCTACTAATTCAATTTCTTGAAAACAAAGTGGCTATAGAACTCACGCGAGCAGAATTTGTCGAAGCACAAGAGGCTTTGGTACAGATGAAAAATTGGTTCATGCGGTTCCCAACCATATTACAGGCATGTGAATGCATCATTGAGATGCTTAGAGGCCAATATGCTCATTCAGTTGGCTGTTACAACGAAGCTGCTTTTCATTTTATTGAAGCAGTGAAGCTTACAGACAGCAAATCAATGCAAGCTATGTGCCAAGTTTATGCAGCTGTCTCTTACATCTGCATTGGTGATGCTGAATCTTCTTCACAGGCACTTGATTTGATCGGGCCAGTTTATGGAGTAATGGATTCATTTGTAGGAGTTAGAGAAAAGACAGGTGTTCTTTTTGCTTACGGTCTTTTATTGATGAAGCAACAAGATCTACAAGAAGCAAGAAATCGCCTGGCAAGGGGATTGCAGCTGACACATACATATTTAGGAAACCTTCAACTTGTTTCACAATATTTGACAATCCTTGGCAGTTTGGCCCTTGCATTACGTGACACTGTACAAGCCAGGGAGATATTGAGATCTTCTTTGACTTTAGCAAAGAAACTTTATGATATTCCTACTCAGATTTGGGTGCTATCTGTTTTGACAGCTTTATATAAAGAATTAGGTGAAAGGGGAAACGAAATGGAGAATTTTGAGTATCAGACAAAAAAATCAGAAGATTTGCAGAGGAGACTCGCTGATGCTCATGCATCCATTTATCATATTGAAATAATTGACAAAATAAGACTACAAGTTCACCAATTAAACGATTTGGACATCAAGCGTGCAATGGCAGGCCCAACTATGGGAGTCAATCTTGATATCCCAGAATCTATTGGTCTGTCGGCTGCCTTGCCTGCTCCATCATCGTCAAGGCTTGTTGATATAGACACCAGAAGACGAGGAAAACGGAGAATTTAGTGTGTTCCTTGTGTAGGCAAAACCATTATATTTTTGTGAATTAATTGCAAGCTATTGCTCCAAGGTCCAAGTGGGTTGAAGAGTTTAGCAGATAAGGTTTCCAGCTTACCACTTATGCGCATCACTGTAAAAGATTCTGTACAGTTAATTAGGATGTTATTCATAAGTTATAGATGGAATAGATATTAGAAGATACCAATTAACATTAGAATAGGTGTTTATTTCTATTTTATTCTTTTGTATCAAAAGAGAGAAAAAAAAGTAGAATAGGGTTTTTAAGCCAGGTCCTTAGGATTGTGTTTGTTCAGC

>C101967_65

CTTGAGTCCATTGTCATTGTCACCCTAACAATTTTGCTCACTTTTAGTTTTCTCTGTCTCTCATTTCAACCAGCAGCTGGTTGGTTATTCTTTCCACCGGTAGTTGCCAAATCTTGGATTTAAACTCCTCTTTCTTCACCCTCTGTTACCTTTCATTCTTTATTTGCTGCAAAACCAATCTTCTAGTAGCACACAATGCAGACTTTTAGAACCTTTTAAACTTAGTCGCAGCTTATATTCTTTCCCACTTCCTAACTAGTCTTGTCTCTCATGAGTTGTACTCTTTCTTGGAACGTGCACTTTGAATTTTGTTTGATAGAACTGAAGTGGTGAGTGGCTAACAAAACAAGATGAAGCCTTGTACTTCATTGTGGTTGCTCTCTTTGATTTACACGCTCTCTTTTGTGGCTTCTAACACTGTGCCCTCAAATGAAGTTTGGGCCCTTAGGTGCTTTAAAGAAGCTGTTTATGAAGACCCATATCAAGTTTTGTCCAATTGGGATATGGTAGAAGCAGATCCTTGCAACTGGTTTGGCGTCACTTGCACTATGGTTCGAGATCATGTCATCAATCTAAACATATCGGGATCATCATTAAAGGGGTTTCTTGCACCAGAGTTGGGGCAACTCACCTACTTGCAAGAACTAATTTTGCACGACAACAATCTCATTGGAACAATACCCAGAGAGTTGGGCGTGTTGAAATATCTCAGGGTGTTTGATTTGGGAATGAATCAGTTAACAGGACCAATTCCTCCAGAGATTGGAAATCTAACTCAAGTCGTGAAAATAAACTTGCAGTCCAATGGGTTGACTGGTAGGCTGCCTCCAGAGTTTGGAAACTTGAGATTCCTTCAAGAACTTCGGCTGGATAGGAATAGACTTCAAGGAACTGTTCCTGCTGGCAGTAGCTCACATTTTGCTTCAAATATGCATGGGATGTATGCATCAAAGGAGAATGTAACTGGCTTCTGTCGTTCGTCTCAGTTGAAAGTAGCGGACTTCTCATATAATTTTTTAGTTGGTAACATACCCAAATGCTTGGAGTATCTTCCGAGGTCAAGCTTTCAAGGGAATTGCATCCAGAGCCAGGATTTAAAGCAGCGACCTTCTATACAGTGTGCGGGTGCTTCACCTGCCAGTGCCAAGAGCCAGCCAGTGGTGGATCCAAGCCACCAAGCTGCTGCATATGTGTCCAAGCATCACGGAGCATCAAAACCTGTTTGGCTTTTGGCTCTAGAAATAGTAACTGGAACTATGGTGGGCTCTCTCTTTCTGGTTGCTGTTCTTGCTGCTTTTCAGAGATGTAATAAAAAATCATCTATCATTATTCCTTGGAAAAAATCTGGAAGCCAGAAAGACCATAATGCAGTATATATAGACCCAGAGATGCTGAAGGATGTGAGAAGGTATAGCAGACAAGAGCTTGAAGTGGCCTGTGAAGACTTCAGCAACATAATTGGGTCCTCACCAGACAGTGTGGTTTACAAGGGAACAATGAAAGGTGGGCCTGAGATTGCTGTGATTTCCCTCTGCATCAAGGAGGAGCATTGGACAGGATATCTTGAGCTCTATTTTCAGAGAGAGGTCGCAGACTTGGCAAGGTTAAATCATGAGAATATAGGAAAGCTACTTGGCTATTGTAGAGACGACACTCCATTTACAAGGATGTTGATTTTTGATTATGCTTCAAATGGGACACTTCATGATCACCTACATTGTTTTGAAGAAGGATGTCAATTCTCTTGGACACGGCGTATGAAGATTGCCGTAGGCATTGCGCGTGGACTCAAGTATTTACATACTGAAGTGGAGCCTCCATTTACTATCTCAGAGCTGAATTCTAGTGCTGTATACCTTACAGAAGAATTTTCCCCTAAGTTGGTTGATTTTGAAAGTTGGAAAACAATTCTTGAAAGATCAGAAAAGAATTCTGGTTCCATTGGCAGCCAAGGTGCTATCTGTGTTCTTCCAAACTCCCTTGAAGCTCGCCATCTTGATACCAAAGGAAATATATTTGCTTTTGGGGTACTTCTGCTGGAGTTAATCAGCGGGAGACCTCCATACCGTAAGGACAAAGGGTACTTGGTTGACTGGGCCAAGGACTTCCTCGAAAGGCCAGATGAAATGTCAAGCGTGGTGGATCCTGAACTGAAACATTTTAGGCATGAAGACCTCAAAGTGTTATGCGAGGTAATAACTCTCTGTATCAACCCTGATAAAACTGCGCAGCCATCTATGCGAGAATTATGCAGCATGCTGGAGAGCAGAATCGACACATCAGTGAGCTTGGAGCTTAAGTCATCATCTTTGGCTTGGGCCGAACTTGCACTTTTATCTTAAGATATAGTCAAACAAAAAATTAACAGATCTACACTAAAGCAGAGAGACATACACACTAGTAATACTTTTTCTGACTTCTTTCTTCCTTTTCTCCTACTCTTTAATTTCATTTCTATTGTAAATGATTGCTCATAAAGGGTATGTGTTTAGCTTTAACACGAGAAGATCTCGTTGTATAGCACTAGTGGACTAGTACAAGTCCAGAAATTGTTAAATAGCACAAGATTGTATTGGACAAGTGTTTCCTTTGTGTATTATACCGAGCATTGTATTATTTGTCTTGGACCCTTTACCCACGTATTTTTCAGAGATTATTATGAT

>C101971_65

GATCAATAGTGTATTCCAAGGTTTCGAGTATTACACATGACTTAGCCCAATGATGAGTTCATGCACCCAAAAACAGAAATAATTGGAGTAATGGCCTTGGGCTAAAGAACTGGAGGGGGGTAAAACAAAATGCACAGAAGGGGGCATTGAGCTTTGACTCTTACAATATAACCTCTCTTCAGAAAACTAATATTGACTATGCTTTCCCCTTTTACAATTATCAACCATCTTTTCACTTGCTATGTAGCAAGAGGACTCTAAATAGAGGCACAAATGCTGCTACCTCGAGCAGAGAATCTTCTAAAGAAATGCTAAAGCATGCGGAAAATGAATACCCCGGGCTAAATACAACACAATATTTATGTAGCACGTCACAAACACACAAACTTTAAGCACTTGCAGCATTTGGAAAATGAATCCATTGTTGCAGGCCTAAGAATGGACCCTTCAACATTTATTCATACATCTCCCACATAATCATCCAGCGACAACATGGCTCGCTTAAAACGTGCAAGAATATGTATTGCAAAGTCCTGTGTTGGCGCTATTATAGGGGAACAAGGAAACAGGGCGAGTTCTTGGCAGTTCAGGAATGGGCTTATCCATGTTCAGAAGAAACTCGACCACTTGCTCCATTGAGGGTCTTCCTTTGTTCTCATTTAGAGTGCAAAGCAAACCAATTTCGAGAACACGAATTGCCTGCTCCAAGTTGATCAATGATCCCATCCTTCTGTCCACCAGCTTAACTTTCTCATCAATCTGATGCAAGTTCCAAGCATAATCCAATAGGTTCCTCTCTTCTGGATTTCCCTCCTCTTTCTCATCCCTTACCCTTCCAGCTATCACTTCCAACACAAGGACCCCAAACTCAAACACATCTGCACCATGGCACACTGCTGACTCAAACTGTTTTGATTCTGCCCCCAACAGCACAAACCCAAAATCACCCAAAACAGCTCTGAAGTTGACATCTAGAAACACACTGCTGCATTTCAAGTTCTTGTGGGCTAGTTGCTTAGTGTGGAGGAAGCTCAAGCCATCAGCTACATCCTTAATAACTTTGAACCTCCTAGTCCATGGTAGAACACCAGCCCCAAATAACCATTTGTCTAGGCTGCCATTGGGAACAAAGTCATAGACCACCATGATTTCATGGTTGTCTTGACACCAACCTCTGACAGGAAGCAAATTCGGGTGGCGAACATGACCAATGCCTTTGATTTCCTTCAACAACCGCCTCTTGTCTGATCCATGTGTGTGGAGAAACTGAGTTGAGAACCTTTTCACAGCCACCTGGCTTCCATTAGAAAGCTTTCCTCTGTAGTATTCTCCTCTAGAATCACTGCCAAGCAACTCTATCTCACTGAATGATCGAGTTGATGATGAAAGTTGAGAAAATGTGAAGCGGCGTGGTTTGTTTGGAGGCCTTGGCAAGTGAATCTCTGTATCCACTGAAGTGTTTAACTTGGAAGCATTTCTCCTGTGTTTACTAATAAAGTAAAAGCCAAGGAAAAGAGCCAGAGCCAATGCCACAGAAGCCATAAAAATTAAAAAGCTTCTCGGTGGCTCATTCTTAGAAGATTTCTCAGTAGTTGGTGGTGCTGTACTATTCTCAAGTAAGATCTTACCCTGGCAAGTTTCAGTTGAAGGGAAGCGAAGAAAAGCTTGGCTTGTGGAAGTGAAGTTCCAAGAGAGTATGTTGTGGATTTGTGTATGGTTACCTGTGGAAGCTGAGAACCCCACAAACATGTACTCATTTAGATAAGGAGAGAGATCCACAGATTCTGAGAAGATAGGCGTGGAAGGATAATCCTCTTGGTTGGCCCTTCCAAGACGAATGTCCATTCTCTTTTGATGACCATCATAGGTGATCCAAGCGCGGTGAACAAAACCATCTTTAAGGGACACTCCAACATCAGAAACATTGATAACTTTGGTGGATACTATGGTACCTAAGTTGATGCCAACATGGTTGTCATTTGGATCTCCAAACTCTGGGTTCTTTCTTGTGTCAAACTCCACTGCCACTGCCTTGTAGTCATTCTCACAAGCATCATTGAGCATTCCAAGCCATGGCCCTGGCCTTCCCACAGTGAACTCATCAGGAACAATGATGAAAGTGAGGCCACTACCTCCATAAGCAGCTTGCTCATAGGCAGTGGAATTGTTCATTTGAAAAGAGAAAGTTGTTTGAAAGGAAGCTGGTGTCTTGGTGGTTGGATCTAGCAGGCGAATTGGGAAGGAATAGATGCCTCTACCTGCTTGGTGTCTTATGTCTGAGTCTAGTGACTCATCTGGGATTTGAAGAGAACCCTTTTCATTTGAGAACTTGGCACTGCCTAGGAGCTTCACATCATGGACTAGTCTGGGGTTATTGCTGAAGCTGAAATTATAGAAGGAAAAGTGCTTGGTCACATTAATTGGAACATCACCAGCTGAATGAACTGCAACTGGGTATGGCCCTATGGAGGCCAGAACACTAAACAAACAAAGTATGAAGAAGATATTGCATAAGGAAGAAGCCATTGAAAGCAAGGAACAAAGGAATTTCCAATAATTCAGATAGATTATACGGCTTCAGCCTGTGCTAATAATTGTGGTAGTAATGCAACGGATAAAGCTAAGAAGATAGTTGAATTAGGTGTCAAAGGTTAATAAACAAA

>C101973_65

TTTCCCTTAACTTACAGTTGAAAAAAATAACGAACTATGATAAAAGAATTAGAAGTTCTCTTTATGAAAATCGAACTTAGTGCTAGTTTTTCTGGAGAAGTCAAGAGAAAGCCTTTTAAGTTCTCCTACCAATGTGTGCGCCCCACAGTAGAAAACACCAACTCTTTTTCCAGGGTGTTTGAGTGCAGTATGCTTAAAAACACTGCGCCAATTTGGTCTAGCAAAATGTGTCTTGACCCTTGTCCCTGAAACAATATCCACACCACTTTTGGCATGGTGGATAGATTGAAGCATGGTGATCAAAGCTGATCGAGCATCTCCTTCCTCATACACACTTGTGCAATAGTTGTGAAGCTCGATTATTCCATCCTTGTCGTACTCTGCCACATCATCCATCACACCCTTGAACCATTCAAAGGAACCTTGCTCACGAGTAACCCAGTAGAAATAAGCCCTGTTGGTGGCAAAAGATTTCCTCTTGTTGTTCTTAACTCCACTTTCTACCATCCCCTCTTCTAAGTCTTTCTGTTGTTTCATGTTGTTTAGTACGTCTTTGAGGATACTAATCAAAGGGGTGGCTCCAATTCCTAGACCCACTAGAAGGATTACTTCGTAGTTTTTGTAGTCTTGTGCTGGAGCTCCATAAGGACCATCAATCAAAAGCTTTGGCATCCTTGGTATGTTGTTGCCTTGTAACAAATCAGCTCTCAGAAGACCACTCTGGTCACCACTTGCCGGTTGACATGCCTTGGCGAAAACAGCCTTAAGTTGTGATGTCCAATCACCCAAAGTCCGAATGTGAACGCTTACATAGTCATCTCCTGGGGCAGATGTAATAGAGAAAGGATGCCATTGAAATGGAGAAACGTCTGAGCAGTTAACAAAAATATACTGTCCACTAGAGTACTTAAATCCTTGTGGTTTAGACACGTGTAAGGCCAAAACATTTCCTGGGTACACTGCAACCTTCAAAATCTTGACACTTTTGTAGCCAGACCTAAAAGCACGAAGCAATCGCTCGCATGCATATAGTATCACAGGAATTGCAAGATACATCCAAGTCGTTTTCTTGTACCATTCCTTGGAGAGGTACAAGTAGTATCCGTGAACGATGAAAAGGCCATAGACGATGACAAAAAGATGGTGAGAGTACCAAAAGGCGTTGAAGCCAGTGAGCTTTTTGAGAGGCTTGGGGAGTTTCAGCCTGTTTCGGCGGAACCATGGTTGAGCCAAAGTGTAGGCTATCGCCATAAGCACCACAATGGTTATCCCGGTCCAACCCTCTGTCCCTTTCACGAACCACCAATAATCGTTGGGCCTCTCTTCTCCAAAATATGGCCTCATGGGCACATATTCCTCATCTGTTGCATGTAGCAACCTTGGGAAGTCACACGTTAAATGAGCAATTGCATGTATCCCAACCCCAATTGCAATGCCAAAAGCTATCACCTTGTGAAAGTTGATGTTGTCATCAAAGGGAACAGCCATGCCTAGCTTGGTCTTACTTCTGAGCCAAGTGATGGTGTTTCTGCAAACGGGTAAGAGGATTAAGGCCATGTTGAACTTGAGGGTCTCAGCAGCACCTTTGGCTGATGTGACACAATAACCCATGATGTGGAACACGGCACGGTGCTTGTATTGGATGAACTTCCAAGTGAAAAGGGCAGCACAAATTGAAAGCCAAAGAGCAATAATCCAAATCCTTTTCCAATTGTCCTCCACGAAGTAAGCCAGTGCCCGGAAGCCACGCTTTATGGGGTTGTACTCTTTGGTTGGGACCAGTTTCTGGCTCAGCATTTGACTCATCACACGACTATCCGTGGTTATGTGAGTTGATTGTGCTGGGGCTTGCAAAAGAAGCATTTCCAAATTGTGTAGCTCAATGTATCCAAGGTTGTCTGGATCCAACTCCTCCATGATAAGAGCTGCATATTCCTCTGCACGTTCTTGTATTTTTGACAGCTTATTAGCAGAAGCGCTTAAGGCGATAATCTCTTGAACTTCTTCTTGGGTGATTCTTCCGTCGGCATTTTTGTCCACCATATCAAAGAAGGTCTGGAGCCTTGAATCAAAGCTCTGGTCGGAAATTTGTTCCCAAAATTCACGCAACTCATCTTTTGTAATGGAAGCTGATGTTATGCCTCGACGACGAGATAATGCTTCAAACAATTCCCCAGCAAACTCCTTCGACTCGTGCATACCAATGCACTGACCGAAGCGCGTTTTGGGCAGTTTGCCCTCAACGGCCAAGTCATGAAAGCGCTTCTCGACCTGCGACCAACCTTCGGTTCCAACATTTTTGGTCATGAACTTGAAACCTCGGAGAGCGCGAGCGGCACCGGACTTGGACCTATCGACCCTGTCGAACTTCTTGGAGGAGGTCATGCGCTTGAGCTCCTGCGAGACCTGGCGGAGGCGGACGGAGAGCGAGGAGGGGCGCTTCTCGAGGCGGGTGGCGAGGAGGGCGGTCTCGGGGTCGCCACCCCTGATGTTTTGGACGGAAACGGTGTCGTCGCGGACATCGAGCGTGATCTCGACCAACTCCTCGTCGTCCTTGAACCTGGCGCTCTTCTTGCTGCTCTTCTTGTTGGAGACCAATGGGCCGCTCATGGGCCCACTGAAGCCCAGTCGCGTGCTCCTGCTCCCCGTACTCCCCGCGTCGGA

>C101975_65

CACTAAAGCACTTTACCTTAGCATTTTGAGGTGAAAAAGATGGTATAAAATGGTGGGAGGGAAGAATAATTGAGAAATGGGAGTGTAGTGTTGTAGCATCACGTTAGAGGATGGTTCTCCCCTTCACCTTTGCGACCATACTTTTCCTCACACTTCACACTGCGCACTCATCTCAGCCACCATTCGCTTGCGATTGGTCGAACCCTTCCTCCAGATCCTACCCATTCTGCAACCCCAAACTCCCCATTCCCCAACGAACCAAAGACCTTCTCTCCAGACTCACACTCGACGAGAAACTCTCCCAACTCGTCAACACTGCCCCTTCCATCCCCCGCCTCGGCATCCCCGCCTACCAGTGGTGGAGCGAGGCCCTCCACGGCGTTGGAAGTGTGGGCCCCGGCATCCTCTTCAACGGCTCCATCTCCTCCGCCACCAGCTTCCCTCAGGTCATCCTCAGCGCAGCCACCTTCGACTCCCTCCTCTGGTACCGAATCGGACACGCCATTGGAATCGAAGCCCGCGCAATCTACAATGCGGCCCAGGCCCAGGGCCTCACCTTCTGGGCTCCCAATATTAACATCTTCAGAGACCCGCGATGGGGCCGAGGACAAGAGACCCCTGGCGAAGACCCTCTCCTCACTTCTCGCTATGCAGTTTCCTACGTCAGAGGCCTCCAAGGAGATTCCTTCCACGGTGGCAAGCTCAGAGGCCACCTTCAAGCCTCCGCCTGTTGCAAACACTTCACGGCTTACGATTTGGACAATTGGAAAGGCGTCGATCGCTTTCTCTTCGATGCTCGCGTGTCTTTGCAAGATTTAGCCGACACATATCAGCCTCCGTTTCAGAGTTGCGTACAGCAAGGAGGGGCCAGTGGGATAATGTGTGCTTACAACCGCGTGAATGGGGTTCCAAGCTGTGCCGATTTCAACCTGTTAACCAGAACGGCCAGAAAAAAATGGGATTTCCATGGCTATATTACGTCGGATTGTGGAGCAGTGGGGATCATACATGACCAGCAAGGGTTTGCAAAATCAGCGGAAGATGCTGTAGCTGATGTTCTTCGTGCTGGGATGGATGTGGAATGTGGTACTTATTTGACGGATCATGCTAAATCCGCAGTGTTGCAGAAAAAAGTATCCATGTCTCAAATAGACCGTGCCCTTCACAACCTGTTTTCTATTAGAATGAGGCTAGGCCTGTTTGATGGAAATCCAAGCAGGCTCTCATTTGGCATGATTGGTCCCAATCATGTCTGTTCCAAAGAGCACCAATATCTGGCTCTTGAAGCTGCAAGAAATGGTATTGTCCTCTTAAAAAACTCTCCCACACTTCTCCCACTTCCAAAAACAAGCCCCAGCATTTCCTTAGCAGTGATAGGTCCCAATGCCAATGCTTCTCCGCTAACTCTTCTAGGAAACTATGCCGGCCCTCCTTGTAAATTTGTGACAATATTGCAAGGGTTTCAGCACTATGTTAAGAACGCCGTTTATCATCCTGGCTGTAATGGTGGACCAAAGTGTTCTTCGGCCCAAATAGAGCAGGCAGTGGAAGTTGCAAAAAAAGTGGATTATGTCGTGCTGGTAATGGGACTGGATCAAAGTGAAGAGAGGGAAGAACGTGATCGCATTCATCTAGACTTGCCAGGCAAGCAACTTGAACTCATCAACAGCGTTGCTCAAGCTTCTAAGAAACCAGTTATTTTGGTGCTGCTTTGTGGAGGGCCTGTAGACATCAGTTCAGCCAAGTATAACCACAAAATTGGGGGCATCCTCTGGGCTGGTTATCCAGGAGAACTTGGAGGCATTGCACTTGCACAGATTATCTTTGGTGACCACAACCCAGGAGGAAGACTACCGATTACTTGGTACCCAAAAGATTACATCAAAGTACCAATGACAGACATGAGAATGAGAGCTGATCCTTCAACAGGCTATCCTGGGAGAACTTACCGATTTTACAAGGGTCCAAAAGTGTATGAATTTGGCTATGGACTAAGCTACTCAAAGTATTCCTACGAGTTTGTTTCTGTTACACATCGCAATCTTCACTTGAACCAATCATCCACTCATTTGATGGTTGAAAATTCAGAAACCATAAGGTACAAACTAGTTTCGGAGTTGAGTGAACAAACCTGCCAAAGCATATCACTGTCAGTCACAGTGAGAGTTCAAAATCATGGAAACATGGTGGGGAAGCATCCTGTACTGCTGTTTATGAGGCCTAAAAACCAGAAAAGTGGAAGCCCTGTGAAACAGTTGGTGGGTTTCCAGAGTGTGATGCTTGATGCAGGGGAAATGAATCATGTTGGATTTGAGGTAAGTCCTTGTGAACATCTTAGTAGAGCAAATGAGGCTGGTGCAATGATAATAGAAGAAGGGTCTCATCTGTTGCTTGTGGATGATCAAGAGCACCCAATAGATATAATTGTTTGATGCTACAGATTTATTCTTCAACAATGACAAGCTAATTTAGCCAACATGTTTCTGAAACATGCTGCATTTTGGGATTAAGAGACCAAGAAATGTACTATAATTTGATTTGATTCCTGGATCCATGGTATTTTCATCTGAAATTCAGATGAAGGAAATACTATTTCAGTGTATTGAATGTAAAAATGTTGTTCCTCGTTGATTACCTGGTCACTAAATTAAACTGTATGCATCA

>C101977_65

CCACAATTTCATTTTTCGAAGCCCTCACCCCTCCAAACCCTCTCTTCCTTCTTTCTTCTTTTCTTCCTTCTTTCTTCTTCGTTCCCAAATCAAACCTAAATTCCCACCTTCCACACTCTCCACAAATCGACCACTACTATCGCACCCAACACTATTCATTTTTATTTGTTATTTATACATACAGAGGGAGAGGGGGAAATGGATTCGGATCAGGGTAAGTTGTTCATCGGAGGGATTTCGTGGGACACTACGGAAGACAAGCTCAAAGAGCATTTTGGTAACTACGGCGACGTTTTGAGCACTTCTGTTATGCGGGAAAAGAACACTGGCAAGCCCAGAGGTTTTGGTTTCGTCGTCTTCGCGGATCCCACCATTCTTGATAGGGTTCTGGAGGATAAACATATTATAGATGGCAGAACGGTTGATGCCAAAAAGGCTTTCTCAAGAGAGGATCAACAAATTTCTGTCACCTCGAGAGGTGGAAATTCCAATTCTGGTATGAATTCTGGAAATGGAGGAAACATCAGAACTAAAAAGATATTTGTGGGAGGGCTGCCTCCTACCCTGACTGAAGAAAAATTTCGTCAGTACTTTGAGTCTTATGGACATGTAACTGATGTAGTAGTTATGTATGACCAGAATACTGGACGGCCACGAGGATTTGGGTTTATTTCATTTGATACTGAGGAGGCGGTTGATAGGGTTTTGCACAAATCATTTCATGATTTAAATGGTAAACAAGTTGAGGTAAAGCGTGCACTTCCCAAGGATGCCAATCCTGGTGCAAGTGGCCGTATGATGGGTGGTGCTGGAGGTGGTGGTGGGGGAATTGGTGGATATCAAGGATATGGGGCATCTGGGGGTAACCAAAATGCATATGATGGTCGTATGGATTCTAGTAGGTATATGCAGCCTCAAAACGCTGCAGGTGGATTCCCTCCTTATGGTTCTTCTGCCTATAGTGCTCCTGGGTATGGGTATGGTCCGGCTAATAATGGTATTGGATATGGTGCATATGGAAGTTATGGTGGTGCCACTGCTGGGTATGGTGGACCTGCTGCTGCAACATATGGGAATCCTAATGTTCCTAATGCCGCTTATGCTGGTGGTCCACCAGGAGGCCCAAGGAGTTCATGGCCTGCTCAGGCACCTTCTGGTTATGGATCTATGGGCTATGGGAGCACTGCTCCTTGGGGTGCTCCAAGTGCTGGTGCTGGATCTGGTGGCGGTGGTCCTGGTTCAGCAACTGCAGGTCAGTCCCCTGGTGGAGCTGCTGGATATGGGAACCAAGGTTATGGATATGGTGGGTATGGTGGATATGGTGGTAGTGATAGTTCTTATGGGAATTCAAGTGCATATGGAACTGTTGGTGGACGAAGTGGGAGTGCTGCCAACAATAGTGCTAGTGGTCCTGGTGGGGGTGAACTAACAAGTAGTGGTGGCAGTGGTAGCTATATGGGTGGTGGCTATGGGGATGCAAATGGAAATTCAGGTTATGGAAATGCAGCTTGGAGATCTGAACAAACTCATGCTTCTGGAAACTATGGAACCCCACAGGGAAATGGTGGGCAAGTTGGTTATGGTGGTGGCTATGGCGGTGCTCAGTCTCGACAAGCCCAACAGCAGTAATTTTGATAATTGGATATCTTGCTGGATCTTGAACTTCAACTTGAGGATCCCTGACCTTTTAAAAGTCTTAAATCATGAGGTCACAATGAATTCTTCCAGCAGCAAAGGAGTGGCTCAAAGATCGATGCCAATACACAATGGGACTGGTTGGGTTGCTTGAATCCCAGTAGTTTGCAGTATCTGCTTTCGTAGTTTATAATAAGTAGTATGGTTGAAGAGATACTAGATGCTACTTTCTATTTAGTATTTGTTTCTGAGTGTAGCTTTAATTTGCGTGCTTATGATTTTAGTTGATCTGGAATGGTCTGTTCTTGTAGCATTTAAGCTATAGTATGATATTTCACCTTATATGATGGTTTGATGCAGTTTATTTATCTGTGTTGTCTCATTTTTTGGTGGTTGACTTTCATTTACTGTCTGTTCACTTTTTGATTACAGTGTGGGTAGAAAGTCTTATTGTCTAGACTCATTTTAATTGTCATGACAAGTAGAAATATGAAGATGTAGTCAAGCTATTTACTGATAGCAGTGGACACTTGGATTGATCACTTCGTAATGGGTAGCATAATCGTAGTGATAGTAAAAACTTTGAGGATGGCATAATCATTTTCCCCCTGTATTTGCACTTGTGTTGTACAGGAGGAGAGGTGCACGAGTATTGAAGTGGTGGAGCCAAGTGTCCACTCATGCCTAAGGAATGAAATTTGGGCAGTCGTATTGTGTTTTTCTGGACCAACGCATCGCCAAACTGGAGATTCCTGGCTTTTCATGGGATAAAATCCCATAACTTTCAATCTGGTTTGTGGTGGCTGACAGATATTGTGAGAGTTAGCAGCACTGTTGGCCTACTCTATATAATTTTATCCTAATCTTGGGTGTCCAGTTGATTGATACCTTTTGATTATGTGTTTGAAGAAGCTCCGGATATAATTTCATGTCTGAACTATCTGGGAAATTTTCTAATTAGAAGTTTCCACTTTAGCTGCTATCACTTATGATTGTCTTTCAGTTTTATCTTTCTAAGGTGTTTAATTA

>C101983_65

AAAAATTTGGTTTGTCCATCATCTCTTTTCTCTTCTGTGTTGGTTTTTCCTTCTCTCTCCGTCAAAAGTGACGTCTTCCAGAGACAAGAACGGCTTTGTTTCTGGGTCTGAGCGTTCTTAGTGGTTCTGATTCTTTCCCCACTTTAAACTTGTCCGTTTCTCACTCTCCTCAAATCTTATACACAACATCATCATACATACATGCACCATCTTCCTCCCTTCACATTCTCTCTTTACAACCCAACCATTTTCTTTCACTCTTCTACTTTGCAGTCAATTCAACCACCATCTTCGGTTCTGATCCAAAGTTCAGAACTTTACTTTCTGGGTTCGTTTGATGCACTCGCGTGCTGATGTGGGTTACCTTCAAAATACCAGCTTTGAAGCATACATCTCGGGTTGCTAACTAACGCCTTTTGTTGATTTTGGAACAGAAGGTTTTTTTAATAATAAAAAAAGGGTTGTGGCCTTTGTGGGGATTACGCAGATCTGCATGTTATCTATTATTTCACAATAACCAAAACCAGAAAAGAGCAAGAGAGAGAAAAAAAGGAGTTTTGTTTTTTGTTTCTTTGGTTCTCTTCCCATTAAAAAAGAAGGCATCTTTCTTTCTCGTCTTGTCCTGAATTCCTTCCTTTGATTGATGACTGAAGTTGGTTCACTGTCATTTTAGTTATTTCAATCTGAAAAGCGTGCTGGTGTGATTGTTGTGCGCCGGTGAAGAGGTTGCAAAAGTAAAAAAAGAAAAAATTGAAGGTGGAGAAAGTGGTGTTGGAGTATTGTGAAAGGTTTGATTTTTGTTGGTGATTCTCAAAGGGGTTGTGACGGATTCTTCAGGGGAAGACAGTGGGTGTTCGAGTGGTGTGGAGTGCATTCTTGGCTTTCTAAGGGCCCTTAAGATGGGGTCCTGCTTCTCTGCGGAAAGCAGGAGCCCTCATCCCACTTCACCCACTTCATCTTTCAGGAAGCAAAAGAGCAACTCCAAGAGGAGGCTTGGATCAAGAGGTTCTTCCTCTGAGTATTGGAGGAATGAGCCTCTCCATAGGATCCCTGGGAGGATCTTCCTCAATGGCTCCTCCCAAGTTGCATCCTTGTTCACTCAACAAGGGAAGAAGGGCACCAACCAGGATGCTATGGTTGTTTGGGAGAACTTTTGTTCTAGAAAAGATACTATTTTCTGTGGAGTTTTTGATGGCCATGGTCCATTTGGTCATATGGTTGCAAAAAGAGTGAGGGATTCTCTTCCTCTAAAACTGAATTCCCATTGGGAACTTAATGCTTCTGGTGAGGAAGTCCTCAAGGACATTAGCTTCAATACTGCAGCAAGCATGAATTTAGAAGAGGCTGCCTTTGCATCTGCTGATGAGGATTCCCGGGTATCTGTTGATGCTGAAGAAACAGAAAAACACCCAGAAATCTTTCAGACACTTAAGGAATCCTTTTTGAAGGCATTCAAAGTTATGGATAGGGAACTGAAGACCCACCAAAGTATTGATTGTTTTTGTAGCGGAACAACAGCAGTAACTCTGATTAAGCAGGGCCATGATCTTATCGTTGGAAATGTTGGTGACTCTAGAGCTGTGTTGGGTACAAGAGACAAAGATGATTCTCTTGTTGCTGTCCAGTTAACTGTGGATCTGAAACCCAATCTTCCAGCTGAAGAAGAGAGAATCCGCAAATGTAAAGGCCGTGTTTTTGCTCTTCAGGATGAACCCGAAGTTGCTCGCGTCTGGTTGCCAAACAATGACTCACCTGGCCTTGCCATGGCCAGGGCATTTGGTGATTTCTGTCTTAAAGATTTTGGCCTGATATCTGTGCCAGAGATGTCATACAGACGACTCACGGAGAAAGACGAATTCATTGTCTTGGCAACAGATGGGATTTGGGATGTCCTCTCAAACAAAGAAGTGGTGGACATAGTGGCAGCCACATCAAAGCGTACCTCGGCTGCAAGAGCACTGGTAGAGTCAGCTGTTCGTGCATGGAGGTACAAGTATCCAACCTCCAAAGTGGATGACTGTGCTGTGGTGTGCCTCTTTCTGGACTCGGCCTCACACAAAGTGTGTAGTGCTTCCAATGCCAAGTCCAAGGAGCAACCAAGTTCGGCCATCCAAGTCCACAATGGTGACAAGGAAGATGTTTCGGCCCCAACCCTGCTGCCAAGGTCAGGAACATGCAGGGAGAACAATGAAGATAACAACAACAATGAAGACGAGGAAGAGTCTAAGGAAGAGGAAATAGATCCTGAGTCAGAAATAGAGTGGTCTGCACTTGAAGGAGTGTCTCGTGTGAACACATTGCTAAACCTCCCAAGGTTTGAGCCTGACAAAGAAGACAAGGGTACAAGAAAACAAAAATGAAACAAAATGGTTCAACAAGGAAAGGAATGTTAGTTGAGTTGAGATGAGATGGTCTTTTACTTTATATTTCTTTATTTTATTTTGCTTTTGTTTTGTTTTGTCTTCCATCATCATGATATAGATACACACTCTTTTGCACAATTTTCTTTTCTATTTGAATATTGATGTTCCCACCACCCATTGGTGTTCTATTATTCGTTCTGGGTCATATCTTTGGACTAGAGAGGCTGGCCGGGCTGTTTTAGTGTGTCTGGAAATCTGTCCTTCCTCTCATGTGCTTCAAAATTTTGTAAAATCAAACTCTTACCA

>C101987_65

AGATACATTACATGCGGTGCAAAGAGACCCACATACACCAGAACTCTCTGAATGGCGCAAGTTCAAAGATCCCACTTCTGATTTGTCCTTTCCAGCCAATTTAAATTAAAAAAAATCTGTATAATGTTACATGTTCGAAGTCCACTACTACCTTCAAGGTTTAAACTTTCTTGAAGGCAGAAGATTATCCTAGTAGCAGGTATCATCATCAATGACGGTAATAATGGCTTTGCATGGTTATTATTATAAGAATATAAAGTTGGGAAATCAGCGAAGAGCACTACACAATCGCAGTTTTCCTGGCTCAATTTCAGTTCATAAATTGCCAAAGGATGCAAGGTCAAAATCTGAAAAGTCAGAGAATGGTAAATTTCCTAGATTTCTGGTTGAAATGCGACAGACAGAATTGCCTCAATCAAAATATGGTACAAATGGTAGAGTTGTTAAAATGGTTCCAGCAAATGAGGTATTGAAAAGAAAGACAATGTCGGAGAATAAAGTGGAAACGGGGAGCATATTAAACCAAGCTGTTAATGGAGCAAGTTTGGTAAGAAGAGATCCTAGCCCAGCCTTGACAAAGACAATGAAATCTAGAACCTCTAAAGAACTTCCACCAGTGGAAGAGCTAAAAGTTTTGCCCTCAGATGAAGGCTTCAGTTGGGCGAAAGAAAATTACAATTCTGTGCAGAGATCTATTGATGTTTGGTCTTTTGTTATTTCTTTACGTATTCGAATCCTGTTGGACAATGCAAAATGGGCTTATTTGGGTGGCTTTACTGAAGAAAAGCAGAAAAGAAGAAGGCAGAAGACTGCTGCATGGCTAAGGGAGTGTGTATTACAGCTTGGCCCAACTTTCATTAAACTTGGACAGTTATCATCAACAAGGTCGGATTTATTTCCCCAGGAGTTTGTAGATGAGCTTGCAAAATTGCAGGACAGGGTACCTGCTTTTTCTCCAAAGAAAGCAAGTGTCTTTATTGAGAGTGAATTAGGAGCTCCCATTAACGTATTGTTTAAAGAGTTTGAAGATCGGCCCATTGCTGCTGCTAGTCTTGGTCAGGTTCATCGAGCTATTCTACACAATGGAGAAAAAGTAGTAGTCAAAGTTCAAAGGCCTGGCCTAAAGAAGCTATTTGACATTGACTTAAGAAACTTAAAGCTGATTGCAGAATATTTTCAGAGAAGTGAAACTCTAGGTGGTCCAACAAGAGACTGGGTTGGTATATATGAAGAATGTGCAACGATTTTGTATCAAGAAATTGATTATATAAATGAAGGAAAGAATGCTGATAGATTCCGCCGAGATTTTCGAAACATAAAGTGGGTCCGAGTACCTCTGGTTTATTGGGATTATACTGCATCAAAAGTATTGACATTGGAGTATGTACCAGGTATCAAAATAAATGAAGTGGATATTTTAACCTCGCGAGGTTATGATCTACAACGAATCTCATCACGCACAATTGAGGCGTACTTGATTCAGATATTGAGAACAGGTTTCTTTCATGCTGATCCACATCCTGGAAATCTTGCTGTTGATGTTGATGAAGCAATTATTTATTATGACTTTGGCATGATGGGGGAAATAAAATCTTTCACCCGAGAGAGACTGCTTGAACTGTTCTATGCTGTTTATGAGAAGGATGCCAAAAAGGTTATGCAGTGCCTTATAGATCTTGGAGCCCTCCAACCCACCGGTGACCTGTCATCAGTGAGGCGATCTGTACAGTTTTTCTTGGACAATCTTCTAAGCCAGACACCAGATCAGCAACAGACTTTATCTGCAATTGGGGAGGATTTATTTTCAATAGCACAAGATCAGCCATTCCGTTTTCCATCTACCTTCACCTTTGTCATTAGAGCATTTTCTACACTTGAAGGCTTAGGTTACATACTCAATCCAGATTTCTCCTTTGTGAAGATCGCTGCACCTTATGCACAGGAACTTTTAGATATAAGGCAAAAGCGGCGCACAGGGCCACAGCTTGTGGAGGAGATAAGGAAACAAGCAGATGATGCAAGAACCAATTCGATATCAATGCCGTACAGAGTTCAAAGAATAGAAGAATTTGTCAAGCAACTTGAGGCAGGAGATCTGAAACTCCGAGTCCGAGTACTTGAGTCTGAAAGAGCTGCTAGGAAAGCAACAATTTTACAAATGGCTACTATGTATTCGGTATTGGGGGGAACCTTCCTAAATCTTGGCTTCACTTTGAGTAGTCAAGGCAATCAAGCGTTTGCAAATGGATCATTCCTTGGTGCAGGCGTTTTTGCGACACTATTTTTAAGATCTATGCAACGGGTGAAGAAACTTGATAAGTTTGAGAAAATGATTTAATAATTCATTCACGCCAAATTCAATTGCTTCAAGCATATGCATGATAATAGTTTTGATCTTAAATTTATTATTCATCAGCATTAGCGTTCATATTTTACACATTAGCATTATAAGATATACAAGTACAAGTTGTACAGAAACATGATTGGAGGGTGATGAAACTGCAAGAAACTGTTCCTTCATGACAAAGTGAAAATCATAGTTTCATAAGGAGAAAAGAAATGTCAATAACCATTGATAAGATAGTTAAAAAGTTTTCATTCTAATTACGGCTAATTGTATATTGACCTTGCTAGTTTGCATTGTACTATGTCATGTATTCTATATACAAG

>C101989_65

TTTATAAAATCAAATGGCTCACAACGTCTGAACTGCACGGTGCAGTTCCCGGGTTAAAGTCACTGAATCCACTCCCTACTTTCGCCTGTAATTGCAGTCATGCCTTCTTCTCTCACATGCCCAGTATTCATTCTTCCCTCCCAATCTTTTGTTTCTTCCACCACTTTACCAAACAACAAAACCCTAATTTCCTATTTTACTTAATTATCTGTGTTCTTCACCCCAAAGGCAAAACATACTCAAAATTTCTCCTTTTTCATTTTTCCTTGCTATCCTCGTCTCAATTCTCTGATTACCTGATTGCTTCAACTGTTTCTGCCGTTTTCCCTTTTCAAAAACCTCAATCTTTTCACGTGGGTCTGTGGAAAATCGGCGCCTTCCCTCTCTGATCGCGAATGGTTTGCAGTCACAGCAACAATGTCAGATGACATGCTCATTCATTTCTCGTCCAACTCTTCCAACCAATCAGATCAGTCTCTGCCTACAAAGATTGCAAAGTTGGAAGCTCGTATGGTTGGTAAGGGTTCCTCAACCTCTGCTCAGCAACCAGGTTGGTCCTCTGTGTCTGTTCCTTCGGCTGGGAAATTTGGTGGGGCTGCTGAAAGTTCGGCTGAGCCATCCACTTCAAGTGATTCTGATGATGATAATGGTGGGGCATTCTTGATACAAGCCAACACACAGAAGCGCCAAAAACTTCAAGAAGATGGCAACACTAGTGCTTTCGAACGTGTTGAGGTTGTTACTGATGGAAAGCAAACTAGTTTGGATGCTGTGGAGACAAAAATGCATGTTGAAATGAATAGAAAGAAACATGGTCGTGGAAGAGGGGGTTCTGGTTCAGGTAGAGGCCGGGGTACCAGAGTTAATGATCAGACCAAAACACTAATTTCTCCTCCAGCTGTTTTGGCTTCAAACGGTCAGATCGAGAATGTATACAATAAAGAGGAAGTTGCATCTTTACAAGCTAAAGTTGTTGCTTTAGAGGATGAATTGCGAAAATCAAAGCAAGAAGCCTTTGATTATCAGAATCTTACTCAACAGTTAGAAAAGGAGCTGAAGGAGATGACTGATCATGAACAGCTTATGAAGCCTAAGAGAATGAAAATAATATCTGACTTGCTGATATCCGTTTCAAAGGCTGAAAGACAAGAAGCAAGGCTGAAAGTACGACAAGACTCATTGAGACTTGGAAATGTTGGTGTAATAAGAGCTGGAACTGTCATATCTGAGACCTGGGAAGATGGTCAAGCTTTGAAGGATATGAATGCTCAGCTTAAACAATTATTAGAAACGAAGGAGGCAATTGAGCGGCAGCGCAAATTATTTAAGAAAAAGCAATCTGATAAGGGTGACGGACCAGATGCAGAAGGTGGATTACCCGAAGATATTCTAATTCACGATGAAATTTACAAATGTCGATTAGCTAGCCTTAAAAGAGAAGAGGAAATCATTTTGCGAGAGAGGGATCGGTATGAAACAGAGAAGGGGAGGTTAATTCGTGAGATGAAACGTATAAGGGATGAAGATGGTTCACGGTTTAACAACTTTCAGATTCTAAATCATCGTTATGCCCTTCTTAACCTTCTTGGGAAGGGAGGATTCAGTGAGGTGTACAAGGCTTTTGACTTGGTGGACCATAGATATGTTGCCTGCAAGCTTCATGGTTTAAATGCTCAATGGAGTGAAGAGAAGAAGCAGAGTTACATAAGACATGCAATTCGGGAGTATAATATTCACAAGACTCTTGTACATTGTCACATTGTTCGTTTATGGGACATCTTTGAGATTGATCAAAACACATTCTGCACCGTTCTAGAGTATTGTAGTGGGAAGGATCTTGATGCTGTCCTTAAAGCAACACCTATATTACCTGAGAGGGAGGCTAAGGTCATCATAGTTCAGATTTTTCAAGGCCTTATTTACATGAATAAAAGAACACAGAAGATTATCCACTATGATTTGAAGCCTGGAAATGTGTTGTTTGATGAGCTTGGTGTTGCCAAAGTGACTGATTTTGGTCTTAGTAAGATAGTGGAGGATGATGTGGGATCCCAGGGTATGGAACTAACATCCCAGGGAGCTGGAACATATTGGTATTTGCCTCCTGAATGTTTTGAGCTCAGCAAGACACCTCTTATTTCATCAAAGGTTGATGTCTGGTCTGCTGGTATTTTGTATTATCAAATGCTCTTTGGCCGACGCCCTTTCGGGCATGATCAAACCCAAGAGAGAATACTTCGTGAAGACACAATTATTAAGGCACGCAAGGTTGAATTTCCTTCTAGACCTACCATTTCTAACGAGGCAAAGGATTTTATCCGAAGGTGTCTAACATATAATCAGGCTGAGAGACCAGATGTATTAACCATTGCTCAAGACCCGTATCTCACTTTCTCAAAGAAGTAATGGCATTCATTGTCCAGACTCCTCAAAGAAAAATGGAGGATCTCATCTTCTAATCTCAGTTACCGATTCTGTTAAATTGTATAGGCTGTAAAGATTTTGTAATCTTGTCGATGAGGCAGAGAAGGGGTTTTCCCCATTCTGAAAGAAAGTTCAGTTGTTTTGAAGCATCATCTTTTGGTTTAAAATATGGGGGGAGCATATGGCATGTGGGTTTGTTTTGACCTTAAAAAGGGTCAGGCAGTATACAAGCAAGTGAGAAAGCTT

>C101993_65

AAGAATCCCTAACTCATCTGTTCCGTTTTTTTTTTTTTCCGTAATCATAGTTCGTTGCCCCATCAATCTTTCCCCATCTGGGTTCCCCTTGGATTTTGCTTCCAGATTCGATTCAGATTTTCCTTCTCTTTTTCTCTCTCCGTGTGCAAATAAGATAAGAAAAAGAAGAGATTTTGAGATAGGATATGAAGAGAGGGAACGAGGATGAGAAGGTAGTGGGGCCAATGTTCCCGAGGCTGCATGTAAATGATGCAGAGAAAGGAGGGCCAAGAGCACCTCCAAGGAATAAGATGGCCCTCTATGAACAGTTCAGTGTTCCCTCTCAGAGGTTCAATCCACGCCTTCTGCCACTCAACCCAAATTCATCTTCTAACTTAGTTGCTCCACCAACTTCCTCAGCCCAGGGGAATGGGCATGACAGAAGTTCTGCCTACCCAGTCCGTTTGTCTTCACAAACACCTGCTCATCGAGCTGAAAGTTACATTTCTCGCCAATCTGATGGATCAAGATCAAACACTTCAGCGGTGCAGCTAGAAAGGAGAAAGAGAGTTGATGAAGATGATGTTCATGCATACATTTGCTCGAGGATTGGCCAATCTAATGATAAAACGATGAAGAGTTCGAATGGGAAAAAATTCACTCCTTTGGGTGCTAGGAATTTTTCTTCAGTTTCAGTGCAAAATGATGGTGACAAGGATCCAACACGATTTGGCTCCCTTCCTGTTGATATGAGAAAACATGTGAGGAATGGGAATGAAGCTCATCCACATGTAAGCTCAAGTAGACAGCAACCGAAAATGTCTTTCAAAAACAAATCAAGTGGAGAAGTTATTGACAGTCTAGTGATGCAAGCTAAGGTGATTCCAACTCAAGAAGATCAAGATTGTTCTGTGCCAAATATAAGCAGGTTACATCAGGATGATGCTTGTCTACAGCAGGAGTGTGTGGCTGGGTCCCAATCCAATGACATTGAACATGGAAATGGTCTCCTTAACTCTACAAGGCTTATGGATAATGGAAATGCCCTTGTACCAAAAAGTTGTTTTCAACCTGCAGTAAACCAAATCTGTCCAATTGAGGATACCGATGATGCTGAATATCTTGATACTGGGACTGGAGGTCCAATACAGAAGGGAAATTTTGATGAAAGTGGTGATGTTTCCAAGGTCTCCATGGTAACAAATTTATCAAGTTTGATAGTTTCTCCTGATGATGTTGTAGGGATATTAGGTCAAAAACATTTCTGGAAAGCAAGGCGAAAAATTGCCAATCAACAGAGAGTGTTTGCTGTCCAAGTGTTTGAATTGCACAGACTGATTAAGGTCCAGCAGCTGATTGCTGCATCCCCAGATGTTTTGCTTGAAGATGGTGCTTTCTTGGGAAAATTTCCTCTGGAGGGATCTACTCCTAAAACCATTTCATTGGAAGTTGTTGTTGAACCCCGGCAACAAAATCCCAAGCGGAAAAACAATTCAGAGAAGCTGAACCATAAAACAGAAGGTTCTGCTGAGAATGCAGTTGGGAAAAAAACCTCTTTTTCATCCCCCAAAAATGGCAGCCACCTTACAAATCACACCCCTCTTTCAGGAATTCCACAGCAAGCTGATGTAGCTTCTGATAATAGAACAAGTCCCTGGAGTTTCAATCAGTCACCTCAACATCAGTGGCTAATTCCTATCATGTCTCCTTCCGAAGGGCTTGTCTACAAGCCATATCCTGGGCCTGGATTTACTGGAGCAATGCCTGGAGGATGTGGTGGGCCATTCGGACAAGCACCTCTTGGTGCTACTTTCATGAATCCTGCCTATCAATTCCCAGCTTCTCATCAAGTAGTTGGGGTTTCACCATTTGTTTCTCCGGCCAACCACAACTACTTCCCCCCTTATGGCATGCCAGTAGTGAATCAAGCAGCATCAGGATCAGCTGTTGAACAGGTGAACCAGTTTGGTGCACAAGGTTCTCATGGTCAAAATGGTCATTCATCAGTAGAAGGAGCCAATTTTAACACTCACCATAACCAAAGCTCATCTAACTTGCCAGTTCAGAGGAATAGAGCCGTGTCACATGTCAAGAAACTTCACGTGTCTAAGGAGAGACACTTGCAAGGGAGCACGGCAAGTACTCCTAGTGAAACACCACAGGCAATCAGAACAGGGAACATTGCTGAAGGAAGTGATGCTCATTCTCTTTCGCTTCACGCTGTTGAAACTAGACAGCAAACACAAGTCATCAAAGTGGTTCCACATAACGGCAAATCAGCGACGGAATCAGCTGCTAGAATTTTTCAATCCATTCAAGAAGAGAGAAAACAGCATGATTTAGTCTAGTCATGTTTATGCACATGGAACTACTGAGCCTGTGTTTGGTTCTGTTCTGCTTCAGAAATGTGAAAATCAAAGGCTATAACTACTAAATTCGTATGTTGAAAGTGAAAATCAGACATTTGTGTTGTCTAAATGTAGAACAAAACAGGCTTTGAGACGAGTTTGTACAGTGCGTCAAGTCCTGTACTTTTGTTGTTGTTTGACTATATGTATCTTGCTACTTTATTCTACTTTTTCCTGTTCCTTTTGTCCTTGTTACAGCATATTCGTGTGTAGATATTGTTGTAACTATATGAAATGTTAAAAATAATCAATTGAAATATTAGTGGATGTTGCCTCTCTCTACG

>C101995_65

CAATTTTTGGGTAAGATCTGATCAGGGAAAACGGCGAGAAACACGTTAGAGAGGGCGCGGAGAAAGTGATTAGAGAAATTGGGATCCTATCCATAGGACTGATAATCGCTTCAAAGCCACGTTATCGATAACGACCATTAGCGATGCCGTATCGCCAACATCAAAACCCAACCAAACACCCCCTTATGCATCCACACTATATATAATTCCTGGTTCTTGAACAACAACAACAACAACAACACCGACTTTCTTTTCCTTCTCCTATCCAATACCTCCAAACCAAAATGCAAATTCAAAACTCCAAAGCTGACACCCACCCAGCCTCCGAAGCGGAACGGCGAATCCGGACGCTGACCCTACACCTAAGCCCCGTCGCCTGCGTGGCGGCGGAGCAGTTGGATATGGCGGCGTGCGCCGGGCGAGGGAAGCTGACCGTGGACACGCCATCTCTGTCGAGCTACATGCGAGGGAAGAACAGGGACATTCAGGAGAAGGTGTTCGATTACTTCAACGCCAATCCGCACCTTCAAACGCCCGTTGAGATCTCGAAAGACGAGCATCGAGAGCTGTGCATGAAGCAGCTTACGGGACTCGTCAGAGAAGCGGGGATTCGACCCCTCCGTTACGTGGTCAACGACCCCGCCAAGTACTTCGCCATCTTGGAAGCCGTTGGGGGCGTTGACATGTCGCTCGGGATCAAGATGGGGGTCCAGTACAGTCTCTGGGGTGGTTCTGTTCTCAATTTGGGGACAAAGAAGCATAAAGATAAGTACTTTGATGGAATTGATAACTTGGACTACCCCGGTTGTTTTGCTATGACCGAGCTTTACCATGGTTCAAATGTGCAGGGCCTCCAAACTGTTGCCACCTTTGATGTAATCACTGATGAATTTATCATTGATACACCAAATGATGGGGCTATCAAATGGTGGATTGGCAATGCTGCAGTCCATGGAAAGTTTGCCACTGTTTTCGCAAGGTTGAAATTACCCACTTATGACACCAAAGGAGTTACTGATATGGGTGTGCATGCTTTCATAGTTCCCATAAGAGATATGAAGACCCATCAACCACTCCCTGGAATTGAGATACATGATTGTGGTCATAAAGTCGGCCTCAATGGCGTGGATAATGGAGCATTAAGATTCCGCTCGGTAAGAATTCCTCGAGACAATCTTCTAAACCGATTTGGAGATGTCTCTCGTGATGGAAAGTACACAAGTAGTCTTCCTACAGTAAATAAGCGATTTGCTGCAACTCTAGGTGAACTTGTTGGTGGTAGGGTAGGCCTTGCATATTCTTCAGTTAGTGTCTTGAAGGTTTCTGCCACAATTGCCATCAGATATTCTCTACTTCGTCAGCAATTTGGACCTCCAAACCAACCTGAAGTCAGTATTCTTGATTACCAGTCTCAACAGCACAAGCTTATGCCAATGCTTGCTTCAACATATGCTTTTCATTTTGCAACAACAAATTTAGTGGAAAAATACTCTCAAATGAAAAAAACTCATGATGAAGAATTAGTTGCAGATGTCCATGCTCTATCAGCTGGTCTAAAGGCTTATGTAACTTCCTATACTGCAAAGTCACTCAGCATCTGTAGGGAAGCCTGTGGAGGTCATGGTTATGCTGCTGTAAACCGGTTTGGTATTCTGAGGAACGATCATGACATTTTTCAGACATTTGAAGGAGACAACACTGTTCTGCTTCAACAGGTTGCAGCTGATCTCTTGAAACAATACAAGGGAAAGTTCAAAGGAGGAACTTTTGCAGTAACATGGAACTATTTGAGAGAATCCATGAATACTTATCTGTCACAGCCAAATCCGGTTACTGCTCGGTGGGAAGGTGAAGATCATTTACGGGATCCTAAATTTCAATTGGATGCTTTCAGATATCGAACATCTAGATTACTTCAAAGTGTTGCTGTACGACTTCGAAAGCATTCTAAATCCCTTGGGGACTTTGGTGCATGGAATAGATGTTTAAATCACCTTTTGACACTTGCAGAGTCACATATCGAATCAGTCATTCTTGCTAAATTCATTGAAGCCGTGCAGAGTTGTCCTGATCCTAGCTCACAAGCTGCTCTGAAGCTCGTCTGTGATCTTTATGCTCTGGATCGAATATGGAATGACATTGGAACCTACCGAAATGTTGACTATGTGGCTCCTAACAAAGCTAAGGCAATCCACAAACTTACGGAGTACCTTAGTTTCCAAGTGAGAAATATTGCAAGGGAACTTGTTGATGCATTTGATCTTCCGGATCATGTTACACGGGCCCCTATTGCCATGCGGTCAGCAGCTTACTCGCAGTACACACAATATGTTGGATTTTAATAACAAAGGACGCAAAGAATATTCATAGTTTTGGTGGAATTAATTAACCCGATACTTTAATAACAAAGGACGCCCAATATGTGGCCCAGTGTTACAATTGCGGTATACCAGTGGTTGCACCTGACCAAGAAGCCACCACACTATAGCAGAACCATAAATGAAGCGTTGTATAGTTAAGTTTCAGTTGAAGATTAAATTATCCGTGGAAAATAAAAGATTGGTTGGTTTTGTAAATATATTTACCATGTTCTTTCTTTTTATATTTTTGGAAACCAAAACATTGATTGGGAACTATTTACTTAGTTTGTGGATATTACTAATGAAAAAAA

>C101997_65

ATTTTATACAACAGCAATAAAATATTTACAGTCAAAAGAATTGTCAGGGTGCACTTTATTACATCAATCATTTACAAGAAGCAGTCTCCACGCAGGGAAAGATCCAAATGATCAGAGCAAAGATAATTTTACAGGAACTCTAGGCCTGTTTTTTTTTGTCCTTGTCATCGTTACTTTTTACATGGTTCATTAACAGATAGACTTCATACATATTTATTGAAAGCAAAAAGAAAAAGACACTAATAACAAAAGTCAGAAACCAACACACTTCCATCCACATGGCCACTTGCTGCAGACAACCCCTGGCCCTTATGATTAAAGGAGAAGACAGTTTCAGTTTCTGGATGCTGCTTGTGCTGTTGCTTAGTCTGCTGATGGGACCAATATGCGTGGGCCACTAGAACCCTATCTAAAAGTTCTTGGGGAACTGGTTCACCCCTCCTTTGCTGAGGGGATATTCTTGCCGTGTGCACTAGTGTTTTCCATTTATCCTTCAAATCCACATATGTCCGATGCTTTGCATTATCGAAAGCACAAAGCTTAACATCACGCCACCTTCCGGTTCCTAGTTTTTCAACTGCTTGAACCAGTGCTTCTACTTCATCTACAGAAAAAGGCCTACGTATTCGGCGCTGTACCACCTCGGATCGCCTTGGCTTCTCAAGTACAGGTACCATAGCTAGCCCCTCCTTATCCATTTCAAGAACAGTAACCAAATGTTTGGAATCTTCCATACCTTTGTCAACAGAAGTGTTAGCAGGAGAAGGTGCTGAATCATGGTCACTTTCAAAATGGCTACCAAAACTGGTCACTTGATGCTCAGGTAACACGTCAGAATTGCCTTCAATGCTCCGATGAATTACAGCTGGACTGGAAGGGTACCTTATTACAGGCGGAGATATATCAGCGCTAGGACGGGGAGAATGTGCAGCACATACAATCGGTAGGCTTTGTGAAGAATTAGGCTCCAGGGTGAAACCCAAAGCATCAAGCGGGTTATGATGAGAAATTCCGGTTTGAAGTAGAGTTTTACTGTCATCCCTAACTTTCTTTCCCTGGAGAATAACACCAATGTGCAATTCACCTCCGAGTACAGCAGTCACTGCATCCATCACTGTCCTCTTCAACGACCCAATTGTTGCAGTTTCTGAAACCTCTATAAAAAGCTCTGGCACTCTAAATGACTTGATCCTGAGCTTCACATGAGAACCTATAGGTCTTAACTTCTGGTGTGAACAAGCTTCCAAGGATGGTATTCCAGGGTCTCTGTGCATCCCAGGAGGTGAAGCATCCCGACTCATACAATTCTCGGGTGAATAATAAATTCCACCATTCCTGGTAAATCCATTAGAACTTGAAACAGAGTTATAATTAAAAAGCTTCCTCTTTTTGAAAGGAATATTCATCTGAGAACTTTGGCATTTATTGCACTTCCTCTTACTGCGGTAAGTTTGCTTCCAAGCTCCATCACTGTTAGAAAGTGTATCATGCTTCGGTTTTTTAGCAACTTTCTGAAAGTTAGAAGCCAGTCTTTTACTTATACTATTATTACCTATACATGTTTTCGGCCTAAAGGACTTGGTTTTAGTATTAATAGGGTGAGTACACCCGGAAGAGTTTTCGTCATCATCTCTACTAACTACTGGTACATTGTCACAACCTTTGGACAACAAGCTACAGGGAATCCTATCACTATACCCAGACAATTTGGCATTGCCACCTAAACTGATCAATGCAGGAGGACTTTCATCCAGCGGATCCCCAAAACTGTACATCTCAATCCCACTGCCAATTGGCACCTTCTCCAACTCTTGCATCTCATCCTTTCCTTTGCTTGTATCTCCATCTAACTTGAAATGACTATCCTTTTGATAACCAGAAGAATCTAACTTAACTTCGCTAATAACATTTTCCATTTTGATATGGCTATTGCCATCCACCAATTTTTTTGCAATGAACCTCTCTAAGCAACTAGAACTTGTTACTACAGAAGCAATCGCTGTGTGGTCATCAAGTGCAAGATGTGGGAATTCTTTCAAACTTTTTTCGTCAGAAAGCTCAGATTTTGATGGTTTATTTGCATCTTGGAACTCTTTGACAAATCTACACTGATCCTTATCTGATGATGTATCACTAGAAATCGCTGGATTCTTTCTCTCTTGCAATAGTTTACCAGCTACGGTGGCCAATACGTCAAATGCACACTTTTGCTTGTCTTCTGCTTTTTGAAATTTAGCCCTCCTCCTAACTGAGCGAGAAGCTCGGGGCATGACAGGAGCCTGGTAGCCATTAAATCCGTACTCTAACCTCTTCTGCAAGACCATATCTTAGAAACCATGCCAAAACAGACGGATCAAGCACAAATAGCCAGCAATCCAGGACCCACTATCTGATAAATGAGCACTTTACTTCTTGCTTGACTTCACCAGCCACAATTTGAAAGCCTCTTTCCCTTCAAATGACACAATTTTAACCGGACGAAGCGAAATTCCGGCAAATGTAAGATCGGCGAATTGTGAGAGAATCCGAAGTGAACCAACAAAACAGCAACACTAGTTTTTCATCAAGAAACAATATCTCTCATGATTTGGTTTGTTTTTGACTTATTAAACTAAATTGAAAAGAAAAAGAAAACATTTTTTTGCTTTGAATCCCAAAACCCTCTCAAAAAGC

>C101999_65

GTTTAATTTCCATGGAAGTGTTGATCGGGGAAGGGGAGTTGATTAGGGATTAGGGATTAGGGAGGGGGTCTCATCCCGACCCCGTCCCGTTTTCGCTGCCGTTGAAAGATTTCTTTTGACCTCTCCTTCGCCCTTTTCTTCCTTTATATCCCATTTCCCACACCTTTCTCTTTCAAACTACTTTATATTTTAACTTTTTCCGTGCTCCTCGTGTTTTTCATCTTTCAAGCGTTTTCCACCCTCTCCGATCATGGGAATCTGCTTTTCCGCCACCAAAGTCAGCGGCTCCAACGCCACCGCCCTCACCGTCAAGAAAAAAAGATGCAAGCGGGCCGCCAAGCCCAAATCGCAGACGCCCACGCCCACGCCCACGCCCAACTCCGATCAGCACAAGGCAAGTGCGCGCCACGTGCCCTGCGGGAAGCGCACCGATTTCGGTTACGACAAGAATTTCGATACGCGATACACTCTAGGCAAATTGCTGGGACACGGCCAATTCGGTTACACCTACGTCGGAATCGACAAAGCCAATGGCGATCGCGTCGCCGTTAAGAGGCTCGAGAAGAGTAAGATGGATAAGCCTATAGCCGTTGATGATGTTAAGCGAGAGGTCAAGATATTGAAAGCGCTCACTGGCCACGAGAATGTGGTTCAGTTCTATGCTGCTTTTGAAGATGATTCTTACGTGTATATAGTTATGGAGTTATGCGAGGGTGGAGAACTGCTAGATCGAATATTGGCCAAGAAGGATAGCCGTTATACTGAAAAAGATGCAGCGGTGGTTGTGAGGCAGATGCTCAAGGTTGCAGCTGAGTGTCATTTACATGGTATGGTGCACCGGGACATGAAACCAGAGAATTTTCTTTTTAAATCAACCAAAGAAGATTCACCTTTAAAGGCCACAGATTTTGGTTTGTCAGATTTCAGAAAACCTGGAAAGAAGTTTCATGATATTGTTGGCAGTGCTTACTATGTTGCACCAGAAGTGCTAAAACGCAAGTCAGGCCCACAATCAGATGTGTGGAGTATTGGTGTTATTACATACATATTGCTGTGTGGGAGACGCCCATTTTGGGATAAGACTGAGGATGGTATTTTCAAGGAGGTTTTACGGAAGAAGCCTGATTTCCGTCGGAAACCATGGCCAACTATAAGTAATGCTGCAAAAGATTTTGTGAAAAAATTACTGGTAAAGGATCCTGGGGCGAGATTAACTGCTGCTCAAGCTCTATCACATCCATGGGTTAGAGAAGGAGGAGAAGCATTGGAGATTCCTATTGATATATCAGTTCTGAGCAACATGCGGCAGTTTGTGAAATATAGTCGATTGAAACAATTTGCACTAAGGGCATTGGCTAGCACACTTAACGAAGAAGAGTTGGCTGATCTAAAAGATCAATTTGACGCTATAGATGTGGACAAAAATGGTTCTATTAGTTTGGAAGAGATGAGACAGGCTCTTGCGAAAGATCTTCCTTGGAAGTTGAAAGAATCACGTGTACTAGAGATATTGCAAGCGATAGACAGCAACACTGATGGGCTAGTGGATTTTAGTGAGTTTGTAGCTGCTACATTACATGTGCATCAATTGGAGGAAGATTCTGACAAGTGGGAGCAACGGTCACAAGCTGCTTTTGAGAAATTTGACATAGATAAAGATGGCTATATTACTCCAGAGGAACTTAGAATGCATACAGGCTTGAGAGGCTCCATTAATCCTTTGCTCGAGGAAGCTGACATTGACAAAGATGGAAAAATCAGCCTACCCGAGTTTCGAAGACTTCTAAGGACTGCGAGCATGGGTTCTCAAAAGGTTCCAAGCCCAAATGTCCATCAGCGGTGAATCTATAATAGAGGCACTTCGGAGGAAGAGATAAATTTGGAGAAGCGAGTTGAATTCCTCTTAGTATCTTTTGTGTGATTGTGCTGAAGCTCTTAAGATTATCTTCTATTATGCAGATTTTAATCACTCATCTGTGCTGACAATATATGAAACTCAAACTGCAGTTATTAGCAGTGGTATTCCCTGCGCGGAGATTATGTCCACAATGTCACGAGGACTACATCAAGAGGTGTTCTGTAGAGTTTATTTAGACCCTTCGTGATTGGCTTCAGGTATTGGGTTTCAGCAGTATCATTTGTCTCGGGGTTTTATTGGTTGTGCCATGCATGTAATTTGTAACTAGAAATAAAATCTTCTAGCCAAAAATGGAAAAAAAGAAATACTATAAATGAACATGAAATGAAATACGTGGATCAGATTCTTTTCCTTCTCACTGTTTTTTCCATCTCTTCCTTATTTTATTTCCCTGCAGAAAGTGATTGCACCCTGCTGTTCTTAGTAGATAGATACTATTGAAATGATTGTATAAATTTGGTGTACTAGTTTGAGTCTGAAATGTAGTTGTCTATAGTTCTCTTCTCTTAAGAATCAGTCGGGGGCAACAATGTCATTGTGAAATGAAGCCATAAGGGAGTGGTTGATGTCTTTGATGACTTGCGTTGGAGGTCACTCGAGGGCATGTGCTCTTAGTTTCCATTATGGTGTGACAGGTTTGTTTTTTGTAATAGAAATCGAAGCGAGAAGTAGCTGGGAGACTTCTGCAATGTACATGCATCGTTTTTTGTATCATATTCTGTTAGCCTCGTACACAATACATCAAGGTTTTCTTA

>C102001_65

GGAGAAAAGCCACACGCAGACTTTCTCTAACTGAAATATTGTATCGAATTATCTGCCCCTTATTCATTATTTACTAGAGTTTCAATCATAAAAATCTGTCAAAATGGTTAACTTTACAATCGATCAAATGCGTGCCCAAATGGACAAGCCTGCTAACGTCAGAAACATGTCCGTAATTGCCCACGTCGATCACGGCAAATCTACCCTCACCGACTCTTTGGTCGCTGCTGCTGGTATTATTGCTATGGAAGATGCTGGTAATGTCCGTTTGACTGATACCAGACAAGACGAAAAGGACAGAGGTATTACCATCAAGTCCACTGGTATTTCCTTGTACTTTCAATTGCCAGAAGAATGCCCACTTCCTGCCCTCGCTGAAGGTCGTGACTTTTTAATCAACTTGATCGATTCCCCTGGTCACGTCGATTTTTCTTCTGAAGTAACTGCTGCCCTCCGTGTTACTGATGGTGGTTTGGTCGTAGTAGATATGGTCGAAGGTGTTTGCGTACAAACAGAAACTGTCCTTCGTCAAGCTCTTGGTGAACGTATCGTCCCTGTACTCACCATCAACAAATTAGATCGTGGTTTCCTTGAATTACAACTCGAATCTGAAGAAATGTACCAAAACTTCGTCAGAGTAATTGAAAACGCCAACGTTATTATGGCCACATACAAAGACGAACTCGTCGGAGATACCACTTGCTATCCTGAAAAGGGTAACGTAGCCTTTGCTGCTGGTCTCCAAGGATGGGCCTTCACTTTGTCCCAATTCGCTCGTATCTACGCCAAGAAATTCGGTGTTGATGAAACCAAAATGAAGCAAAGATTGTGGGGTGATAACTACTTTGATGCTGAAAACAAGACCTGGATCACCAGAAACGTCTCTTCCTCCGGAAAAGTTTTACCAAGAGCTTTCTGCAAATTTATCTTGGAACCAATCAACCAAATCTTCAAGGCTTGTATGGAACAAGATATGGCTAAACTCGAAAAGATGTTACCTGCCCTCGGTCTCTCTTTGACCTACGAAGATAAACAATTACAACAAAAGAAATTGTTGAAATGTGTTATGCAAAAGTGGTTGCCTGCCGATTATGCTTTATTAGAAATGATGATCGTTCACATGCCATCACCAAAACACGCTCAAAAATACAGAGTTGAAAACTTATACACTGGTCCATTAGATGATCCTACCGCTACTGCCATCAGAAACTGTGACCCTAACGGTCCTTTGGTTATGTACGTATCTAAGATGGTACCTACTCCAGATAAAGGAAGATTTTATGCTTTCGGTCGTGTCTTTGCTGGTACTATCCAATCCGGACAAAAGGTCAGAATTTTGGGTGCCAACTTTGAAGTCGGAAAGAAAGATGATTTATACATCAAGAACATCCAACGTTTAGTAATGATGATGGGTAGAAGAACTGAACCTGTCGAATCCGTACCTGTCGGTAACACTTGTGCTATCGTCGGTATCGATCAATTTTTGGTCAAATCTGGTACTTTGACTTCCGAAGAAGACTCTCACCCAATTTGCACAATGAAATTCTCTGTCTCCCCTGTCGTGCGTGTTGCCGTCGAACCAAAGAACCCTGCTGATTTACCAAAATTGGTAGAAGGTCTCAAGAGATTGTCCAAGTCCGATCCACTTGTCCAATGTACCATCGAAGAATCTGGAGAACACATTGTTGCTGGTGCTGGTGAACTCCACTTAGAAATCTGTATCAAGGATTTACAAGAAGAATTTATGGGCGGTGCTCCAATCACTGTTTCTGAACCTGTTGTATCTTTCAGAGAAACCGTAGGAAACACTTCCGCTGAAGTTTGCTTGTCCAAGTCACCAAACAAGCACAACAGATTATACTGTGTTGCTAGACCAATGGAAGAAGGTTTAGCCGAAAAGATCGACGAAGGAAAAGTATCACCAAAGGATGATCCTAAAATCCGTGGTAGAATTTTGGCTGAAGATTTTGGCTGGGATGTCACTGAAGCCAGAAAAATCTGGTGTTTTGGACCTAACACCAACGGACCTAACGTTGTTGTTGATGTAACTAAGGGAGTTCAATACTTGAACGAAATCAAGGATTCCGTCATTGCCGCTTTCCAATGGGTCACCAAGGAAGGAGTTTTGATGAACGAAAACATGAGAGGAATCAGATTCGATATTATGGATGTCGTTTTACACACTGATGCTATCCACAGAGGTGGTGGACAAATTATCCCAACTGCTCGTCGTGTATTCTTTGCTGCTCAATTGACTGCTCAACCAAGAATCATGGAACCTATCTTCATGTGTGAAATCTCTTGCCCTCAATCTGTCGTCGGTGGTATCTACGCTGTCATGAACAAGAGAAGAGGTCAAATCGTTGAAGAAGTTGCCAGACCTGGTACTCCAATGTGCACTGTCAAATGTTACTTACCTGTCGCTGAATCTTTCGGTTTCACTGCTGATTTAAGAGGTAACACTGGTGGTCAAGCCTTCCCTCAAATGATGTTCGATCACTGGAAGTTGGTACCTGGATCCCCTGACGAAGACTCCAAGACCAAGGACTTCATCAACTTGACAAGAAAGAGAAAGGGTCTCCCTGCTGAAATCCCTGAATTAGAACGTTTCTTAGATAAATTGTAAACAGAAATGTTTTCATGAGTTAAAAAAAATATTAATAAAATAAACCAAA

>C102003_65

CAACCACACACACACACACAGAAGAAAATAAAATAAAATAAAATCTAATCTTGGACTAAAACGAAACTGAAACAAATGAAGATAAAAAGAGCACTTTAGCCTAAATGAATAAAAGAAGCTTCAAGATTTGCAAAGCCAGTCTTGGATGGCAAGTTGAAGTGCGCGGTTGGGAGTAAGAAAAAGGTGATTCAATTTCAAATTTGTCATGGGAGAAGTGTCATGCCCATTTTCCAGCCATTCACGTATGGCATCTCCTTCATAGGTAAATCCGTCTGCCGCAACTTGAGGATCATGCATTATTTCCTGAAGGATTGGACACAAGAAAAAAGATGGCACTGGTCGCTCTTCTGAAGTATGCATCTGCTCCAATTCTCTCACTAGTGTAGGTGTTAAATCTGGTCTGTCTCTACGATTTTGTTGACAGCATTGCAATCCCAGTTCAACCAATCGCGATGCCACGGGCGAGGGCCACTCTCCAGCTGAAGAATCCAGAATTGAAGACAATTTTCCACATGAAACTGCATTGCGTACTAGAATTGCTAATCCAACTGGAGTACTACCAGTGAGGAGTTGTAAAATGATGAGCCCGAAGGAGTAGATGTCAGACTTTGTTGTTAGTATTCCAGTTCTATGAAACTCTGGATCCGTATATGTGAAAGCACCCTTTGGTTCAGTGCTCAAACGGAAACTAGGACGGAGGAGAGACTCCTCAGTGACCAGACTGCAGAACCCAAATCCACAAATTTTGCAACCAAGTGAAGAATCAAGTAGTACAGTTTCTGGCTTCAGATCACCATGGATAATAGTTTCGGGTTTAAAAGAATGTAGGAAGCAGAGTGCACTAGCGATTTCAGCAATTATTCGCGCTCGGTTATTCCATGTCAAAGGGGAATTGTTGCTTTTTCGGAATAGGTAGTCTTGAAGAGTTCCATTGGGGAGGTACTCATATACAATTGACCAGGCTTCAGGGCAAACACCAAGCAAAGTGATTAAATGAGGGTGCTGGAGACTACCAAGAACTTGAACCTCTTGACGAAACTCCAATGGTCCTTGCATGTTATGTTGATGGAACTTTTTTATAGTAACAGTCCTACCCAACATTTCCCCCTTGTATATACAACCAAATCCACCTTGCACAATTTTAAAGCTATTGGAAAAATTACATGTGGCATTTTGAAGATCTGATAATGAAAACTCTGCTAACTCGGGTAATTCTTCAGGAAACCCAATGACCCCATTGCAATGAGCTGCTCCAACTTGCCCTCGACTTCTCCACCGCTCAAGCCAACGTAGGGCTTCCATTTTCTGTCTCCTTATTTGCTGCCTTTCTTGCCATAGGGTTGAGATGGATTCTTGAATTAGTGATAGTTCATCTGCAGCTTCATCGCGCCTACGGTTTGCTTCCTGAGCACGACTATCTAACAGCGCAATATTTCTCATGGTCATTTGTAGCTCGCTAGCAATTTCTTCACTTTCGTTCAAGAGCTTTTGTTGTTCTTGTACTGTAGCTCTCAGTGAATCCTCAGCCTCTTTTCTGAGCTTTAATTCACGCACATGGGCAGATTCAAACAAATTGACCTTCCTTATGGCTTCCATAGCTTCAACTTCTAACCTCTCACACTTTAATAGCTCTGCAAAGGCTTCGTCTGTTACTGCCTCGGCTTCTCTCTTGGTTTCAATAAATTGTTTGTTAATTTTTTCTTCCATAGCCTCTAAATATGTATCTAAATAAGCCCGAGAAGAATTTTGGGGGGAACAATGACTGCTACAACTGGATGCTTTGGATGAAAAAGTGGCTTCGGTTTCAATGATTTCCCCATGAACCCAACTTCTAATTCCAAAACCAGTAGTAGATCTTGCCGAATTTGGTTGAAGATATTCTGAGTCAGATAAATCATTCGCACATTGAAAAGATTTACATCTCAAGCTTTCTGCGGTGGCAATCTCAGGTTGAGCAGACAAGGATGAAGAACATGGTGTTTCAGAAGCCTCTCTTGTCCAAATGTGTTTTCCTTTATAGATAAACCATACTTCACAGAACGGAGGAGCATTTTTGGCAGCAAAATTTGCTTTACCAGAGTTCCTTTTCAATTTCATGCAATTTTCTGGCACTACTCCAATGACAAGCTTCTGAATATTATGCTTAACAACCAAATCAACAATTCCATTTTGGACTTGGTCAGCTTCAGTTACAATAATGCTTGCATTAACCCTAGCTGTGCGGCAAACGGTCAAGTACTTATCTAAAAGCTTCTGGGTCTGCTCTCTCTCCACTCTTCGAAAAGCAGACACCACAGCAGGACTAGCCTGTGATGCAGGCAATTTCCCCAACAGAGTGGGGATCATGAGAGAAGGTTGATAAACATGAACTATAACGATTTCCGCGTTCTGAAAGTGGTTTGAGGTCCATTGAAGCAAGGTAACAGCTTTATCAAGCGATTTGCCAACAGCTACATGAACCTTGGGAAGCATGGGGAGGGGAATCTGAGATTGAGATTGAGATTGAGAAGAAGAGGGGTGGCTGTGGAAGGGAAGGCGCAAAATGGATGCATGGTGTGGGTGCAGAGGCTTCAAAAGTTCCATTGGCGCAGTGCCGGAACTGAATTATTGGATGGTAGGAATAGAAATCGCAACATGGGTTGGACTGGGATGATGCGAAATTGAATTGAAATGAAA

>C102005_65

GAGGGAAGGAAAAAAACCAAACCCAGCAAAAACCCTTCTTCCTCACTCCATTTCTCTCTCTTATCCAATCAACAATGTCTTCCTTCGCTGCCTCCAACAACCTCAGCACCGCCATTCTACCCTCTCGCCACAGCAAACCCTCTCGATCTTCTTCCGTCAAATGCAACCTTTCCCCTTCCAAACCCAACGATTCAAACCCCTCTTCTCCTGCCCCGCCGCTCTTCAAAGCCATCGCCATCTCCTCCGCCGCCTCCATCCTCCTCCACTGCACCCCTCTCACCCCCTTCCTCTCTAATCCTCTTGCTGGCGGAGGAAACAGCGGCGGCGGCGGTGCCGGCGGAGATGGAGGATGGTTCGGTGGAAATGGGGGCGGCGGTGGAGAGGGGTTCTTTTCTAGAATGTTTTCTGCCATTGCTGACGAATCACAGTCTCAGGAGTGGGATTCGCACGGCTTGCCCGCCAACATCGTTGTCCAGTTGAACAAAATGAGTGGGTTCAAGAAGTACAAGGTTTCTGAGATCATGTTCTTCGATCGGAACCGGAGAACGAAAGTGGGCTCCGAGGATTCGTTCTTCGAGATGGTTTCGCTGAGGCCTGGTGGGGTTTACACGAAGGCGCAGCTTCAGAAGGAGCTGGAGACTCTCGCAACTTCTGGTATGTTTGAGAAGGTTGATTTGGAAGGAAAAACCAACCCTGATGGTTCAATTGGGGTTACCATTTCATTCAGCGAGAGTACGTGGCAGTCTGCTGATGGGTTTCGCTGCATCAATGTGGGGTTGATGCAGCAGACAAAGCCTGTTGAGATGGATTCTGATATGACCGATAAGGAGAGGTTGGAGTACTATTTGAGTCAGGAGAGAGAGTACAAGAGGAGAATCGAGAGGGCGAGACCGTGCCTCTTGCCGAGGTATGTGCACGATGAGATTCTTGACATGCTCAAGAGACAAGGCATGGTCAGTGCTAGGTTGCTGCAAAGGATTAGGGACCGAGTGCAAAAATGGTACCATGATGAAGGCTATGCCTGTGCTCAGGTGGTTAATTTTGGGAATCTCAATACCAAGGAAGTTGTCTGTGAGGTTGTTGAAGGGGATATCACCAAATTGGATATTCAGTTCCAGGATAAGCTTGGTAATGTTGTTGAAGGGAACACTCAAGTCCCAGTGATACAAAGAGAGCTTCCTCGGCAGCTACGCCCAGGTTTCACTTTCAACATTGAAGCGGGGAAGCAAGCTTTACGAAATGTGAACTCCCTTGCTTTGTTTTCTAATATTGAGGTGAATCCACGGCCTGATGAGAAAAATGAGGGAGGTATAATTGTTGAAATTAAGCTAAAGGAGTTAGAACAGAAGTCGGCTGAAGTCAGTACTGAGTGGAGTATTGTCCCAGGACGAGGAGGGCATCCCACCCTGGCTTCGCTTCAGCCAGGTGGCACTGTTAGTTTTGAACATAGGAATCTGCAAGGACTGAATAGATCTATTAACGGTTCTATAACGACTAGCAACTTCCTGAATCCTCAGGATGATCTTGCATTTAAGCTAGAGTATGTGCATCCATATTTGGATGGTGTGTACTATTCACGTAACCGGACTCTCCGTGTAAGCTGCTTCAATAGCCGAAAGCTGAGTCCTGTATTCACTGGGGGGCCAGGTGTGGATGAAGTGCCCCCAATTTGGGTTGATCGTACTGGTGTTAAAGCTAATATCACCGAGAATTTCACGCGTCAGAGTAAATTCACATATGGACTTGTAATGGAAGAGATAACAACACGTGATGAAAGTAGTCATATATGTTCAAATGGTCAAAGAGTATTACCTAGTGGAGGAATTAGTGCTGATGGACCTCCAACCACTCTCAGTGGTACTGGCATTGATCGCATGGCATTTTTACAGGCAAATATCACACGCGATAACACACGCTTTGTGAATGGAACTGTTGTTGGAGACAGAAATATGTTCCAGGTAGATCAAGGCCTTGGCATCGGCAGTCAATTCCCGTTCTTTAATCGACACCAGCTAACTGTGACACGATTTGTCCAGTTGATATCCGTGGAGGAAGGGGCTGGTAAACCACCGCCTCCAGTGCTTGTCCTCCATGGCCACTATGGTGGTTGTGTAGGTGATCTCCCCAGCTATGATGCCTTTACTCTTGGGGGTCCCTATTCTGTGAGGGGATACAACATGGGTGAGATTGGAGCGGCCAGAAACATCCTCGAGCTGGCAGCTGAGTTACGGATACCTGTTAAAAGTACGCATGTGTATGCATTTGCAGAACACGGGAACGATCTAGGGAGTTCAAAGGGTGTGAAAGGGAATCCTACAGAAGTTTATAGGCGACTTGGTCATGGGTCGTCTTATGGAGTTGGTGTGAAACTTGGTCTAGTGAGAGCAGAATATGCCGTTGATCACAACTCAGGGACTGGTGCATTATTTTTCCGTTTCGGAGAGAGATTCTGAGGTGCAAATTAGTGACATTAGTATATTGCACAGCCGGCTTTCTTCTCTTGGTTAGTAGGGCTTGGAGGCTATAGATACTCAAAATTTGGTGTTTTTGTTTGCTTTTCCTAAAATTGGTAGAGGGTTCTGTTCTGTGAAATATTTAAATTATTTTTCTCTTATGAATTGGATTTTATTTTGCGGCTAGTCATCAATGCACAGTCCGTGAGAAATAACGGATCCTT

>C102007_65

CTTACAATTACCCATTACACGTTTTTTCCCCAATTCGAAAGGAAACCCTCGAGTCCGACGAATGATTACTGAAAGAGAAAAAGGGAGAAGAAAATTGTAGCAGAAGCTGTATTCATAAGCAAAACAAAATTCGTTTCAGTGTGTTAATTTGCTTTAATCTGCGTTCCTCTGCATCCTATCACTGTCACCACCACCTGCTTGTACATCGTCGCGGGTGTGCTGCGATTTCTTTTTCTGCGACTCTGAGGGTTCAGCTGTGTTGAAGTTCAAGAAACGAAACCCTAGCCTGCGATTCGTGGTTGAAAGGAATGGCCTCATACAGGCCCTTCCCTCCGCAATCGAGTCAAATTCAAAACCCTATTCCAAGTGGGAATCACCAGTATACTAATAATAATCAAAATTGGGGTGCTTATGGAGATCCCTCTAATTCTTCATTCCCCCAAGCCCCTCCAAATTCCAATTATCACCAGCAGCAACAACAACAACAACACCAACATCAAAATCAACATCATGTTCCATATGCACCTCCAAATCCGCATCACCCTCACTATCCATATCCACCACCACCTCCACCGCCACCGGAGGCTTCATACCAGCCTCCACCACCACCGCCTCCTCCCATGTACTATCCCTCTAATAACCAGTATAACAACCAGCCACCTCCCCCACCACCGCCGTTGTCGCCGCCACCACCACCTCCCCCTGCTTCGCCCCCTCCTCCTCCCCCGGCAACTCAGAACAATGAAGAGCGGCGCTTCAAGGATCCTTCCACGTCCGGCCGCCGTGAATATGAGCCTTCTAATCACGGCATTGCTCACAAGCAGCACAAGCACCAGCACCAGCCTCCCGTGCCAGCAAAAAAGGTGAATGGGCCTCCTGGCAGAACTGAGACTGAGGAAGAAAAGAGGTTGAGGAAGAAGAGGGAGTTTGAGAAGCAAAGGCAGGAGGAAAAGCATAGGCAGCAGCTCAAGGAGTCCCAGAACACTGTTCTGCAGAAGACTCACATGTTGTCGTCTGGGAAGGGGCACGGTTTGATTGCAGGGTCTCGAATGGGTGAAAGGAGATCCACTCCCTTGTTGAGTGCCGAGAGGGTTGAAAATAGGTTGAAGAAGCCCACAACATTTTTGTGTAAGCTCAAATTTCGAAATGAACTTCCAGATCCAAGTGCTCAACCTAAGCTTATGGCTTTCAAGAAAGATAAAGATCAATATGCAAAATATACAATCACATCCCTGGAGAAAATGTACAAACCCAAGCTTTTTGTGGAGCCAGATCTAGGGATACCTCTGGACCTGCTTGATCTCAGTGTTTACAATCCTCCCAGTGTCAGACCACCTCTTGCTCCAGAAGATGAAGAATTGTTACGAGATGATGAAGCTGCTGCTCCTATTAAAAAAGATGGCATAAAAAGAAAAGAGAGGCCTACTGATAAAGGTGTAGCATGGCTTGTCAAAACACAATATATATCTCCTTTAAGCATGGAGTCGACAAAACAGTCTTTAACCGAAAAACAAGCTAAGGAACTCAGAGAGATGAAGGGAGGCCGCAACATTTTGGACAATCTCAACAGTAGGGAAAGACAAATCAGGGAAATTGAAGCATCGTTTGAAGCAGCTAAGTCTGATCCTGTTCATGCAACCAACAAAGATTTGTATCCTCTCGAGATTATGCCATTACTGCCCGATTTTGATAGGTATGATGATCAGTTTGTTGTTGCTGCATTTGATAATGCTCCCACAGCTGATTCGGAAATGTATGCTAAGTTGGACAAACCTGTTCGTGATGCCTTTGAATCAAAGGCAGTTATGAAAAGTTATGTTGCAACGAGTTCAGATCCTGCTAATCCTGAGAAGTTTTTGGCATATATGGCCCCGGCACCAGGAGAGCTGTCAAAGGATATATATGATGAAAATGAAGATGTCTCATACTCCTGGATTCGCGAGTATCATTGGGATGTTCGGGGTGATGATGCAGATGATCCTACAACATTCTTGGTTGCTTTTGATGACTCAGAAGCACGTTACTTGCCTCTTCCAACCAAACTTGTTTTAAGAAAAAAGAGGGCCAAAGAGGGAAGATCAGGTGAAGAGGTTGAGCAATGTCCGGTACCTTCAAGAGTGACTGTAAGACGGAGGTCTAGTGTTGCTGCTATTGAGCGGAAGGATACTGGGGTTTATACAAGTTCAAGGGGCAATTCCTCAAAGAGAGCTCGTCTAGAAATGGATGATGGTCTCGAACACCAACATAGAGGTGCTTCACACCAGGACAATTATCAGTCAAGTGGAGCAGAAGATTACTTGTCTGAGTGATTATTTGATGCAAATGCTGCCGCCTTAAAGCAAATTGGTCCAACGAAATGGTAGTTGGATGATTGGCTGCAGATAGCTGAATTTTTAGTGGCGCCTTTGTGCTTCAATGAAGAATGCCAACGACAAATCCCATATTTTGGTATGGCCATTTTTAATTTAATGCACATACATTCAGAGTGTAGGCAGCTGTTAAATTAATATAGATTTTCTTTTCAATACCATTATATTAGAAAAGATTGAAAAATAATGAGTTGTAAAAAGCATGTCAGACCAAATAGAATCCTGTCTTTTGCTCAATCGTGACCTGCTTGCCAATGAAATTATCATTTGGTTTTCCTTGCCCATAATGTTGTGGCATTCTGATTCAGAA

>C102009_65

GTTAAAGGAAACAAAGTAAGGCACATGATGACAAGTTGTTAGGTTACTTGGACTCCCAAGATTAACCGATCAAGTTGCGTGTCCCGGCTGTCACTTGCCCATGATCAATAACAGAACCACCGTTGTTCCGATTCTAAAAATGGTCACAACAATCTAACCCTGTCAAAATCAATCCCCAGATATATTTTCATTCCCCTAACATTAGCCAATCATTGTTTACATCCGAATCAACAACAACAACTCTTTTCATATTTCTTCTTCTCATCATTCTGATTCCACCCACAACCCCCAAGTCTCTCTTTGTTCCTCTCCTGCACGATGGGCACAGAGACCTCATCGGTGGATATTTCGGAGCCCCTTCTTGTGGATCCTCATGGCAACGAGGAGAAGGAGGAGTATTTGCGTGAGGAGGTTCCGGAATGGAAGGAGCAAATAACGATAAGAGGGTTGGTGGTGAGTGCTGTGTTGGGGAGCTTGTTCTGCATCATCACCCACAAGCTCAACCTCACCGTTGGGATCATCCCTTCCCTCAACGTCGCTGCTGGCTTGTTGGGGTTCTTCTTTGTCCGGACCTGGACTGGTTTCTTGACCAAGATGGGATTTTTCACCAAACCCTTTACCAGACAAGAGAACACTGTTATTCAGACGTGTGTGGTTGCTTGCTATGGCCTTGCCTTTAGTGGGGGGTTTGGTTCATCCTTGATTTCCATGGATCAAAGAACATATGAACTCATAGGCCCTGATTACCCCGGGAATAGAGCTGAAGATGTTAAAAACCCAGGATTGGGTTGGATGATGGGTTTCATGTTTGTTGTCAGTTTCCTTGGACTTTTTAGCCTTGTGCCACTTCGTAAGGTTATGGTCTTGGATTATAAGCTTACATATCCCAGTGGGACAGCGACAGCAATGCTAATTAACAGTTTCCATACAAAAACTGGAGCAGAACTTGCAGGGAATCAAGTTCGGCAGCTTGGAAAGTATTTAAGCATAAGTTTCATCTGGAGTTGCTTTAAGTGGTTCTTCAGTGGTATTGGGGATTCGTGTGGATTTGACAACTTCCCTAGTTTTGGTTTGACGCTATTTAGGAACACATTCTACTTTGACTTCAGTCCAACCTATGTTGGATGTGGTCTTATCTGTCCTCACCTAGTAAATTGTTCAGTTCTCCTTGGAGCTATTATATCATGGGGCTTTCTGTGGCCTTTCGTCTCTGAGCATGCTGGGGACTGGTATCCAGCTGACCTTGGTGGCAATGATTTCAAAGGCCTTTACGGATACAAGGTCTTCATATCTATTGCCCTTATTCTAGGGGATGGTATTTACAATCTGGTGAAGATTATACTTATAACTGTCCGGGAAATGTGGAGGGCAAGCTCCAAACGGAACAGCCTTCCTGTTGTGACAGAAGTTCAGGATGATGAGAGTTCTGAACTGCAATCAGAAGAAAAGAGGAGGGATGAAGTATTTTTGAAGGACAGGATACCCACCTGGTTCGCTGCCTCTGGATATGTGGGCTTGGCTGCGATATCCATAGCAACAATACCAATGATTTTCCCTCCTCTCAAATGGTACTTGGTTCTGTGCTCTTATATCCTTGCCCCTGCCCTTGCCTTTTGCAACTCTTACGGCACAGGCCTCACAGACTGGAGCCTAGCATCCACATATGGAAAGATTGGTCTTTTCATCATTGCAGCAGCAGTCGGGCAAAATGGTGGTGTAATAGCAGGCGTAGCAGCAAGTGCTGTTATGATGTCCATCGTGGCAACTGCTGCTGATCTCATGCAAGATTTCAAGACCGGTTACCTCACCCTCTCATCAGCAAAGTCCATGTTTGTAAGCCAATTGATTGGAACAGCGATGGGTTGTGTGATCGCTCCTCTCACTTTCTGGATGTTCTGGACTGCCTTTGATATTGGCTCACCTGATGGCCCTTACAAAGCACCATATGCCGTTATATTCAGGGAAATGGCTATTCTTGGTGTTCAGGGATTCTCAGAGCTTCCAAAGTACTGCCTGGAAATGTGCGGCGCATTCTTTGTGGCAGCCTTGGCTATCAATCTTGCAAGGGATGTGACTCCTAAGAAATTCTCACAATACATACCTATCCCAATGGCAATGGCAGTTCCTTTCTACATTGGTGCTTACTTTGCAGTTGATATGTTTGTTGGAACTGTGATATTATTTGTATGGGAGAGGTTGAATAGAAAGGATGCAGAGGACTATGCTGGAGCAGTTGCTTCTGGATTGATATGTGGTGATGGGATATGGACAATTCCATCTGCAATCCTTTCTATTTTGAGGATTGATCCCCCCATCTGTATGTACTTCGGTCCTTCTTCAAGTAGCTGAAATCGGCAACTCTAAAACAATGTTTATATTGGCCTTGGGTCTGTAAATATCTGGCTGTAACATCAGAGGGAATCTCCACGTTTCATTTCCTCTATGTTTCTCCATGCTTGATGTGTATTCGTAAATATTTAGGGATGTCATTCAGTCCCTGAACTGTCTCTCAGGCAAAAAAGGCACAAAAAGGGAAAGGGAAAGGGGTGTCTTGACTCCCTTTTCCTTTTGATGCATTTATGTATTTCACATTCCTATTCTTAAAGTTGTTTAAGCTCAAAACCAAAGTATGTGTTTAAGAAGTTACGTAGAATGCATCGATAGTGTTAAAATTAAAAAAA

>C102011_65

CCTGAGAACATTCTAAATGAAAAATATATTTATTGCAACTTGGCAAAATTGAATTAGTTCAACTGGTTGGTTTAAAGTAATGTACACCACGAAACAATCCTAAATCTTCACACGTCCATATGCCATTTCAGCAGTGCATGTGCTTAAAATGTAAGCCAAAAGCTTTTCAACTTGTGAACGCCCTATTCTTCAAAATCTTTTCCGCTTCAGTTACACTCAAATGTCTAAACCCATTCTCCTTCAACCACTTCTTCCCCCCTGCCTTACATTCTTGCACTCTATTTTCATCCAACTTCAACAACTTCTGACCTTCACTTGTTTTAGGGAAAAAACCTTCAGGTCTCAAACACCATGCCGCAAATACCCCAAATAAAACATCCAAGTCACTATGAAATCTGTCAACACCACTTCTGTGAAATTTTACATTGCTCACAAGAATACCATTGAAAATCAACCGATCAATTCCAGCTGCCAAATTTCTCCAAACCCCAACGAAATCCCTACCATTCAAACCCACTTCTACCACCGAAGTTTTACTTTGCAGATAATTCAGGGCTTCTACTAGAGCCTTCGACACAGTCCATCCTTCTTCACCCTTTTGCCATTGCTTCTTGTTCTTCACATAATCTCGAGAATGAGCATCAAAACCTCTCAAAATAGCTAGGGATATCTTTTCAACCCACTCTCTTCTGAAGTCTTCCAACTTTTTGATCTCATCATCAAAAATAACCTTTCTGGAACTCTCTGGCAATCCTTCACCATTAATTTCCTTTTCTGCTTCATCATCCTCATTCATCCCCATCTCAAGGAAGAAAACATCCTCAGACCATTCCTTTAAAACAGATTCAAAGTAATGAGCAGCATTGACAGAAATTGTAACCTTTAATACAGCATCATCATCAGTTAAGGCTGTTAAACCTTCAGCTTCTTGGCAGCGGATAAGTATGCAATCAAAAAATTTTCTTACAATAGGAAGACCTGATAACCTGAGAAATTTTGACCTCAAAATTACATTAGGCAATGATCGACATCGATCAATAACAGATGCTAGATGGCGAAGAAAGAAATTAGAAACCAGAGGAGATTTGTGATCATCAGTACAGGATGAAAAAACGGCACTTTCAACTTTCTTTATCCAATTATTCTCATTTTGGATGTCTGGTTTCAACTTATCTAGGGCATCACCTAGCTCTATTTCAGCCCATAGATCAAGCCAGTCTGGACGATCACAAAATACAGACAGAGATGAAATTTTCTGCATGATATCATCATCATCAAAGGATAGCAAGATCCCAGAATTCTCTACCAAAGACTTAATCCTTTTGTCAAAAGCTATCATCAAGTCAATGAGATGTAGCCATGATATTCTAGCAGATGACTGAGTCCCTGTAACACTCTCTTCGTCTAGTTGACTAATATAACTAGGGAAAATTTCTTTCGCCATGTATGTGGTTAAGGAGGTTACCATTGCTGAGATCCATTCTTCTCTACAACTATAACCAAGTAACTTTGCTTCATCAACCAATGGCTGCAACAATTCATCCATAGAGTCAACATAATCCCTTGTGATTTTATATACAAGAGTAAAAATAAATTCCGGTTTATCAATCCATTTTGAGAAATGTCGTTGGGAAGCTAATGATAAGGGATTCACAAGCTCTTCTATTACCCAAAGTGGTTGCCGTAGGGCGACTTCCCTGTCATGGCCCTCAAGTTGCCTAGCTTTCCTTTTCCTTTGCAACTCCTGTAGGTTGCACAAAGCAAGAAAGTTTTCAGAGTACCGGAGTTTAAGATCCACTTGCATGCTAAGCAGTGGATTCGAGACTTGATTTGCAGTCCTTGCATCTGAATTTGAGGAGGTAAAGGCCGAAAGAGGTGGGGGCCATCCAAGCGAAGTAAGAAGTGCTCGATGCTCAGCAATTGCTTGGGGCCTCAAAATAGCAAGAGCTCGGTCCACTCTGTGATCAACAGCTGATACAAGATGCTTCCACTGGGGATGTGCTTTCGTAATTGAAGCTAATATGTCCTCTGTTATTTTCAGTGTTTTAATTGCGAGCATATGCATTTCTTGTGAATTTTGACTAGAAGAATGTTTCCTCATGCTCTTGTTCATGGTAAATGATACAGCATCCTCAATATCACCAACCAAAGTATCAAGTTTCAGTGCTGTCTCTGCATAAACACGAATTGTCTCCAATCTAGCCACTTCCTTCGCCAAAGTCGCAAGTTCCTCTCTGAAACCCTTGCCATCCCCTTCGCCTCTTCCTCCATCTGGCACGACAGTGGAGGAGAGGGCAGTCAGACTATCGGTGACGTGGCCGAAGAGGCCGTGGATTTCGCCGGAGAAGGAGGCGTAAGCGGTGAGACCCGCTCCGAGCCTCCGAGTTAACTCGTCCAGCTCGCGATCCAACTCGGCGCACTGAGTCTGGAGCTCGGAGACGAAGTTGGGAGCATCGGCGAGAGCGCATTGGGTGTGGAATCTGTGGTCAAGGAAGGAGAGTGCTGCGGGTGAGAGATGAGAAGGAGGAGGTAGCGTTTGGAGCGACTCCATCGTGAAGCTCGAATTTCGATTATTGTCGAAGTTTCTTCGCCGCCGTGTGCGGCTGTTCCCCGCTTCCGGTGTCGGATTCCGATGAGCCTCTGAAAGATACGAACCTTCTCACATTGGGCGTGGGTTAGTCG

>C102013_65

CGGCGAGTGGCCGCAGCCGCCCACCACCCCCGGCGGGAGAGGTAATGGTAGCAATGGTGAAAGATTGAAACTTGATTTGTCTTCCATTCAGCATAACAACCCTGATCATAGTAGGAGTAACAATGGTGGGCTTGTGAAGAGGGATAAGATTGCATTTTTTGATAAGGAGTGTTCAAAAGTTGCTGATCATGTGTACCTTGGTGGTGATGCTGTTGCCAAGGATAGGGACATTTTGAAGCACAATGGGATCACTCATGTGTTGAATTGTGTGGGGTTTGTGTGTCCTGAGTATTTCAAGGCTGATTTCGTTTATAGGACTTTGTGGCTGCAGGATAGTCCCTCTGAGGACATTACCAGTATCTTGTATGATGTGTTTGACTACTTTGAGGATGTTAGGGAACAAGGTGGGAGGGTGTTTGTGCACTGTTGTCAAGGGGTGTCTAGGTCTACATCTTTGGTCATTGCTTACCTTATGTGGAGGGAAGGCCAAAGTTTTGATGATGCTTTTCAGTTTGTGAAGGCGGCAAGAGGGATTGCTGATCCGAATATGGGTTTTGCTTGCCAGTTGTTACAGTGCCAGAAAAGGGTCCATGCCTTCCCTCTTAGTCCTAGCTCTTTGCTGAGGATTTACAGGATTGCTCCACACTCACCTTATGATCCTTTGCATCTTGTCCCCAAAATGTTGATGGATCCTTCGTCAGCTGCATTGGACTCCAGAGGTGCGTTTATTGTGCACATTCCTTCGACAATATATGTTTGGATTGGTAAGAATTGTGAGGCCATCATGGAGAGAGACGCCAGAGGGGCTGTGGGTCAGATTGTTCGGTATGAGAAGGTTCAAGGACCAGTTGTGATGCTTAAGGAAGGTGAGGAACCGGCTTACTTCTGGGATGCTTTTTCAAATTTTCTGCCCTTGATGGATAAATCTGGTAATAGAGTAGAAGATGGAAAGCTAACTAGTAAAGTTTTGCCTGGTGAGAGGAGAGTGGAAGCATATGATGTTGATTATGAAGTTTTCAAAAAAGCAATCACGGGAGGATTTGTGCCTCCCTTTGCTTCATCGGAGAATGAACATGAAACCCATCTGCCTGCAAGAGAGAGTAGTTGGAGTGCTTTAAGGCGTAAAGTTGCTTCTGTTTCGGCCCCTAGGTTATCCTTTCCAAGGGTTTATTCAGATTCCATGTTGTGTATTCATACATCAGCAAATTTGTCTCCATCATCATCGCTTTCATCATCGTCATCAACTGTATCATCATCCTCTTCACCGTCTTTTGTTTCTCCAGATTCCATTTCTTCTGATTCTAGCACTCATTCTAAGTTTTTGTCAGAAGTGTCCCCAGATTCTTCCTCCTTGGTTTTGACTCCTATTCCTGTATCTTCATCTTTGTCTAATTTTTCTAATTTGTCAATCTCTTCAAATTCTACTTCTCAAACAGTGTCTAACAGTACAGATATCCTTGGTGTCAAGTTATCACATCCCTGGTCTCAGTCAGCCTCTTTACCATTGAAAAAGCCATCAACTTCCCTTGCTGAACGCAGAGGTAGTTTGTCAAAATCTTTGAAGTTACCACTGAAGAGTGATAAGAAGCAAGTAATTGATAATCCGTCTACCTTTCATAATAGTCAAGAATATGACGTTGTTGTTAATGGCAATGTTAGCTATTTGCAACAATCAGATAGTAAAGATTATTTTTGTGAGTCTAACAACCATGCCAATGACGGAAGTGTGAATTCATCTCAAAAGTGTGAGCTGGCATTGTGTCCAAGCATTGTTGACAGCATGCACCTCAAGGAATCATCACTTGTTCGGGACTCTGTTGAACCTTTGATAGATAGTTCCTCAATAGAAGACTCATTGCTTGGTCAGTCTACAATATTGAAGGGAGCTAATGAGAGTGGCTTACTGCAGTTTAATGTCTCACAGACTTCGGTATATCACTGGCCCAGTTTAGAAAAGATTGAGACATTTGGTACAGGCCATTTGGATTCTGAAGCTGCTTTTGTCATATTCTCTCCCAGTATGCATGTACATGCAGGGAACTTGTTATATATCTGGGTGGGGAGGTCTTTCAATCGTGACGCTTCACAGGTTCACTTGGATATTGATAAACAGTTAGATCGTCTAGGTGCTGTTGACTGGAACCGTATTGGTTGTGATCTCCTTGTTCGGTTTAGTTTGCCAAAGAATACAGTCGTCAAGATTGTTAAAGAGAATGAAGAACCTTCAGAGTTCCTTGCTTTACTCAGTTCATTGTAGCTCAAATCATACTAGGAGAATCCTTGGTTCAAGTAGCTTCTATAACCTTTTCAGCATCTGCAGCTATCTCTATGTATGGCAAATTTTCAGGCTCTAATTGATCTTCCCTGTGAAAAGAAATCCAGTGAGGAGGTTGCCCCATGCCAAGTCAATTGTAGCTCGGCTGATTGTACAGTTCTTTAACACCCTCAACCTAGAAATTTATGTTCAACAACATACTGAAATTTCTTCATATTCTCTCATGTTATTTATAAAAAACAATTCCTTGTTAGCTCTCTTTCTCTCATTGCGGAAAATGATAGGGTGTAGTTTATTTGCTTATATGTGAACAAATACCTGATTGAGAATTTCTAAATTGAAACAATAGCTGAGTGATATAGATGTTCTCAATAAAATAGTATTTACCAGTATCAATCAAATTCTGCA

>C102015_65

TGCATTGTCTTTGTATTTTTTAATTGATTCCATTAAATGGTCGTACAAAACAACCAAGTTGCAATAGCAAGTTACTCAGGCCAGAAGCAGCAACAACATGATAGTGCAGCAGGGAAATAGCTCAAATGAAGTGAGAAAACCTCTAAAGAATCGTGAAGATGTATTTTATGTTAATTAAAATTAAACTGTTGTGCGAACTTAACCTACTTTAACTCAATAGTGAACACACCCTCACTAGATACTTCAGAGACTTTCTCACTTTACAGGAGCCTAATGCTAAACCAATAACCACCATGGCTGCATTTTAAGAGACAGTTTGTTGATGGTATAACTACAATTTTCTAACATGAATTTGAAGAAATCAACATAAATACACAAAAGAAGATAAAAAGAAAGAAAGAAAGACAAGAAAAGTCCCAATTATAAAAGAGGTTCAGAAAGTTCCATTTCATTAGAAGCCTCCTGTTGTGTAGCAGGTCGCTGCTGAAATCCAACAAAGTCCATGCCAAGCCACTGCCATGGCCAACACACATATCTAGGCCGATTTTGGCTCCTTGAATCTTCTCTGTTTTCAAACTCTTCGAGCTGATGAGTAAGCTCCTCAACTCTGTCTCTAAGTCTGGATGACCTCAAGTCTGCTAACTTAAGAGACTTTTCTGCTGCATCTCTGGCTGCCATCGCAGCAGCAGCTGTCTCTTCTTTGAATTTTAGTTTCTTCTCTGATTCTAACATGGATTGCTTAGCCTCTTCAAGTTCCTGCTTAAGTGCTGCCAACTGTGACACAAATTCTTGTTCAACACCTCTGCCTGCATCAGCTTCTTGCTCTGCTGCCACTTTCCATCTGTTAATTTCCTCTTCAGCAGCAGCAATGTCCATCATTAGCCCCTCAATATTTTCATTTGCCACTCTTTCCTTTTCTTCAAGTTCCTCTATCCGATGCATTCTATGATCAAGCTCCTTGGCCTGAGCTTCTATATGCTGCTTAAGTAAGCTCAACTCTGCCCGTAATTCACCCACGGTATGCTTCAGCTCAATGATCTCAAGTTGAGATGCCTTCACAACATCCTCCAGAGCACCAGCCAAAGAGTATATTTCATCCTCCTCTTCTTTTTTCGTTCTATCCAACTTCTCATTTGAAGCTTCAACCTTTCCATCTCCAAGAAGCTTACTGAATTTGAAATCTATCCCAGCCTCCCTCAACCCATTCTCTGCAGCTTGAAACAGCTCGCTCTTTCTAGAAGATGGATCGATTGCCATCCTCTTAGACAAAGCACTCCTAAGCAAAGAACCAATTTGATCCTTCTCCCTAACCAGCCGAGCCACCGTCTCATCTAAACTTTTGATCTCACTATTCTTCTCCTCAACCACATCCTTTGCTTTCTGAACAACAATTTTAGTCAACTCATATATAGATTCCAGCCCAGCCAAAGAGGCACGTATATTCTCTTCAACGTCTGTTTCTTGCGGAACAAACAACGATTCAGATAGCTCCTCAGTGCCACCATCATCAACTATCTTAACAACATTGCAAATCTGGTTATGAATTTTGGAAACAAGACTCAACTGCTCAATCAACAAAGGCCTCTGGGATTCCATCCGAGACTCAAAACTACTCAACTTGTCCTCACACTCACCAACAAACTCCTTCAACTGCAAAGCCTCACTTTGCTCCTTACTCAAATTCTCCGCAACCTCATTCAGCCTCTGGTCCCTAACAGCCAAATCCTTCTCCAAATTCTCAACAATTGAAACCTTCTTCGCAACCTCATCTCTCAAGCCACTGATCGTGGCCTCAAGCTGAGAAACCTCAATGGCAATCTCGTAATTCCGCTGCTCCATCTGCTCCCTGGCCTCGTTCCTAGCTTTCACAGTCGCCTCGTTCTGCTTCAAAAGCTCCTCCACAATCTCGTTGGCACGCTTGACGACGCCGTACGCCACTGCCGCCATGCCGGTGTATTTCTGCGACCGCGGTAGCGGCAGCGCGCTCCCGGCGAAGCTGCTCACCTTCGCCGAGATCTTCTCCATACCGGTGACGAGCATGTGGCTGGTATTCCCAATCTCCGATCGAAGCGTGTCACGTTCCTTCGCCGTTTCTTCTAGTTGCTTCGCTTTTTCTTCTAGTAGCTTCGCCGTCTCCTCCTTCTCCCGAACGGCGTCGTCGCGCTGGCGGCCAAACTCATCGCGCTTCTTTATGGCCTCGTGAGCCAGCGCCTTCAATCGGTTGAATGATACCTGCAATTCCGACTTCGTATTTTCCACCGCAACGCGCGCTTGCCGCTCCCGATCGAGTTCTGCGAGAACCTCCCGGAGTTTCTCGAAGGAGACATCTTCCGAGGAGGGAGCCTTGGTCGCAATAGGAACCGGATCGCCGCCGTCGTCCTCGACGTCGCTAAGCACCGCATCGGCATCAACGTCGCCGGCGCCGGCCATTTAATTTAATAACATAAATATGACACAATTGTATATTTGATAAACCCTAGAAATTGAAGCTAATCAATATTGTAGAAGTGGCAAGTAGAAACTCTAATCTATTGTGAAATTGTCTTCGTGTTATGGTTTATGGTCGCTGATGATTATAGATTGACGAAGAAACACCGCCAAAACGTACCTACCAGTCAGCCTCTGAAGAATTTAGGAATTTCAGATTGAGAATTGAGAGTTTTACTATTTTTTGGAATTTCAAT

>C102017_65

ATAAAGATTACTTTCATAAGATATATCAATCCTTCAATTAGCAAGATTTACAAAAGTGCTGCGGTGATACAATAACCGGTTCTTACAGAACTAATGTGACTACTGTTCAAATATGCAGTAAAAGTAGAAAGCCCTAAGGGAAAGATAAATGATGCAACACAACTGTTATACAGTTACCCGTTGTCACCAAACAATAGAAATAGTTAAAAGATTAATTCTGAAAAGTGGCATCAATCAATCAATTATTGTAATAAATTAGTTCACCTACACTACAGAGTTCAAACAATGACTCCGGTATTGGGATTGTTGACATTAAACAAGAAACTGAGACCTTGATGCAAAACGGGAACACTTCCTTCTACCAGTTTTGCTTAGCATGCAGACACAGTTCTGAAGAAGTTCAAAATCCTCCTCCCAAAACAACAAGGACGCCTTTTCTGGATTCTCTAGAAGCATCTTTGAGGTCAAGAAACCAGCAATGGTCCATGTCTGCATCAAACGTGATTGCTTCCCAATGAACCTTCCATTTCGTGTGTCGTAATATTCGGGCCATCTATCCACTGAAAGCCTTTTCTCTGCTGAATCAACAGCCTTCTGTGCCAAGTCGGGCCTTCCCATCTTCATGCAGGCTAATGTGAACTGCCAGAGAAGCGTTGGCCAAGATCCTCCATTATGATATGACCAAGCGGTATTCTTGGGGTCACAGCCAGTGATGATACGCCATTCCTCACCCTCCAGTGCTGGATAACAAATCTTGAGAGGCATTTGAGCAACAATATCATCCCATTTTGCTTCAATTAAATTCAAAATGCCCTCGTTCTGTCTTGTTGTTCCTAAAGAAGAAACAATGGTCCAAAGGTTTCCAAGTGTGAAAAACCTAAAGTCCATATGAGCAGGTTGCACATTGCCAATGAAGTATCCGCCATCCTCAGATATCCAGTCTACTAGCCATGAAGGAATTTGCTCTGGATAGATGTTAAACTTGTTTACAGCATCAGTAGAATACTCTTCAGTCTTATACCGATAAATTTCATTAATCTTCTTCATATCAACCCAATAATACTCTCTCATGTGAAAGCAGAGTGCACTCAGGCGATTACTAACAGCAGCCACCAGGCTCTTGGTTGCATCATTGACTATCAGCATCTCTCGAGAGCAGCGTAAAGCCGAGTAAAATAACGCCTGGATCTCCAGAGGGTGACCATGAATGCCCATCCTTCTATCAATCATGCAGGAACCATCAGTGACTAATAAAGAAGGAAACATGTCAAATCCATCAGTCAGACACAACTTAAGGACCAGTCTAATGCCTGTTTGGACATCCACTCTATCTTGCAAAGTATAGTCACCCGTTAATTTCCCATAAGCTCTCAACAATATAATCCACCACAATCCTGAATCAACAGGAGCAACACGACCAATAGCTGATTCCCCAAAATCAGGATCCAAAACTTCCTCAAATGCTTCATTGCTTCCATCAAGAGGCACAGTTCTAACCTTGAAGCTTGCTGGCATCAACCCTTGACCAGGGCTGTAGCAGTCAACAGTCTTCTCCCAACTCTGCAATTGCAATGTGTGAAGTAGAAAATTCTTGACAATTTCCCCTTCACCATTGAGCAAAAAGGCAAGCGCCGATGGAACAAAATCACGGATAAAGACTTGATCATAATTCAGAGGTTGTTTGTCAGCGGGATCATTAGCTGCAACAGTGCCAACGGGATTCCCACAGTAAGTGACGACAGCATCCTGAAGCAACTTCCAAGCTTCCTTTTCAACCTCAGAGACTTCCCTCTGAACCTTATTCTCACTCAAATCCTTCAAATTATCTAACTTGACATTTGACTCATCACATCTTTCTCCAGATACTTCCTCCAGTTTACTTTGATCAGTCTCAATTTTTTCAATTACCAATGGCTTCACATTCAAGCCGCTCTGAATGTAAATCTTCTCAAAGCTCGTGTCATGAGCATGAGACTCAACCGATGTAGAGTGATTCCTAAAATCCGACGCTACATGCGGTATCAAGGATACAACCCTAGGCCTAAAACTACCAATATTGACACAAAGACTAGTGCTAAAATCCCTAACAAGACCCAAATTCGAACCGGAAACACTAAAATCTCTCCGATTCGTATTGATCACATTTATGTACCCTACAATCTTAGTATTACAGGTATTATACCTATGGTGATGGGTTGATTTGTGATGACCCGATCTGGACAACATGCCCATGATTGCTGAATCACTAAACTTGGTTGGAGAAAACCCAAAAATCGATGGACTTTTGTAACTACATAAGATTCTACAACAAGGCTTCATGGTAGAGATTCCAATAGAACTACCACTAGTCATTTGGTATGATCAAACCACCACAGCCCAGAAAATGAAACTGCTCTGAACTATTAAAATCAAGACTCTATATACGGTCAAGTCTTCTATGTATATATCCCAGTAACATAAACGTATATGGGGGAAATATGAAAAAGAATGATTATACTTATAAATCTATAAAAGGAAGATTATACTAAGAAAATGGTAATGGAGATTTACAGTGATTTGTAATTTGTAGGTTCACTTACAGTATGAGATGTTGATGAGATCGAGTTGTTTAAATGAGAGACCTAAAAGTGTTTGAGAGTTGAAGAGAAATGAAGAAGAAACTCAGAACGCCATTTTATAAACTCC

>C102019_65

TAGTGCGTGACCTTTCTTTGAAACAAAGCCTCAACACAACACTGTACTCTCTCTCTCTCTCTCTAGAATCTACGTTGCGTTTCTGTCCTCTTCTTCGCGTTCGCGTTCTACGTCGTCGTTTCAGCTGGTTCGTGGATTCTAGGGTTTCGTGGAAGAAGATGACGACCATGGAGAGCTTGATTGGGCTGGTGAACCGAATTCAGAGGGCCTGCACGGTGCTCGGTGACTATGGCGGTGGTGACAACAACACCTTTTCTTCTCTCTGGGAAGCTCTTCCTTCCGTTGCTGTTGTTGGTGGCCAGAGTTCAGGGAAGTCTTCAGTTTTGGAGAGCATTGTTGGTCGTGACTTTTTGCCTAGAGGATCAGGGATTGTGACAAGGCGTCCATTGGTATTGCAGCTTCATAAATTGGAAAGTGGGTCACAAGAGTATGCCGAGTTTCTTCACCTGCCTAGGAGAAAGTTCACTGACTTTTCTTTGGTCCGCCAAGAAATTCAGGATGAAACTGATAGAGTAACAGGGAAGACGAAACAAATATCTCCCATTCCAATTCATCTTAGCATCTATTCACCAAACGTTGTCAACCTAACCTTGATTGATTTACCTGGTTTGACTAAAGTGGCTGTAGAGGGACAACCTGAGAGTATTGTTCAAGAAATTGAAACTATGGTTAGGACATATGTTGAAAAGCCTAATTGCATTATATTAGCAATATCTCCGGCCAATCAAGACATTGCAACTTCTGATGCTATTAAACTTTCTAGGGAAGTTGACCCCACAGGTGAAAGGACATTTGGGGTGTTGACAAAGCTAGATTTGATGGACAAAGGAACTAATGCTTTAGATGTCCTCGAAGGAAGGTCTTACCGTTTGCAACATCCTTGGGTTGGTATAGTAAATCGATCCCAAGCAGACATCAATAGAAATGTAGATATGATTGTTGCTCGACGTAAGGAACGCGAGTATTTTGCATCCAGTTCTGATTATGGGCACTTGGCCAATAAAATGGGTTCAGAATACCTTGCAAAACTTCTCTCTCAGCATTTGGAGTCAGTAATTAGGGCAAGGATACCTAGTATAACATCTTTAATAAATAAAAGCATTGAGGAACTTGAATCGGAGATGGATCATCTTGGGAGGCCTATTGCTCTCGATGCTGGAGCTCAATTATACACCATCCTAGAACTTTGCCGAGCATTTGAACGGATATTCAAGGAACATTTAGATGGAGGGCGGCCTGGAGGAGATAGGATATATAATGTCTTTGACAATCAGCTTCCTGCTGCATTACGGAAGCTTCCATTTGATCGGCATCTTTCTCTCCAGAATGTAAGGAAAGTGGTGTCAGAGGCTGATGGTTATCAACCTCATTTGATTGCCCCGGAGCAAGGTTACAGGCGCTTGATTGAGGGAGCTCTTGGATACTTTAGAGGTCCAGCTGAAGCTTCAGTTGATGCTGTTAACTTTGTTTTGAAAGAACTTGTAAGGAAATCAATAGCTGAAACTCAGGAACTGAAACGCTTCCCAACATTTCAAGCTGAACTTGCTGCAGCAGCAAATGAGGCCTTAGAAAGGTTTCGTGAAGAGAGTAAGAAGACAAATCTTCGGCTTGTAGACATGGAATCTTCTTATCTCACTGTGGATTTCTTCCGGAGACTTCCTCAAGAAGTGGAAAAATCTGGAACTCCTCCTGCCTCAAACATTGATCGTAATGCAGAGGGTCATTTCAGGAGAATTGCATCAAATGTGTCATCATATATAGGGTTGGTGGCAGACACGCTCAGAATAACAATTCCAAAGGCTGTTGTTTATTGTCAGGTCAGACAAGCAAAACAGTCTTTGCTAAACCATTTCTACACACAAATAGGGAAGAAAGAGGGTAAACAGCTGTCGCTAATGTTAGATGAAGACCCTGCATTGATGGAGAGGAGACAACAGTGTGCAAAAAGGCTTGAACTATATAAAGCAGCAAGGGATGAAATTGATTCTGTTTCCTGGGTACGATGAGCTTGGTGCTAACTGAATGTCATCCTCATCTTGTATGATAGCGATCAGTTATGGGTTGGCCGGACCCTGATGTAATTTACTTATTTACTATTCTAGTACTGCTGTTCTTGATTGTAGGATAAGATTGAAGTTAAGTTGGTTGTCTGAATTGTGGTGTACATCCACACAGCATGGTGATATTCAGTGTGATATGTATAATATTTGTGTATTCTTTGTAATCTCATTCTTGGTTGTGTTACATTATCGAGTCTTGCACAAGTGTTTGGTGATCCAATGTTGGTTTCCTTTCCTTGACACTGAAACGGTCAAAATAACAAACTGGCTGAGAAGGCAGTCCCACAGGAACTGATCAAACATCGGCAAAACAGAGATACGCGGATGAGGCACAAATTGAGAGCCAACAATAACTCATCTTATAAAATAGGAGGTATTAAAGCTCATGTTAATGTGGCAAGGTTGAAGACTGCATAAATGATCGTTGAAAAGAGCCAAGGTTGAAGGAATCCATGGGAATACACCTATAAAAAGAATGCCATGTATGACAGGGATGGATTATATAAGAGAATTACATGCCGAAAGGATTCCTAATCAGTTTGTAGATTCCAGTCTTCTGTACAGCGATTTTTATTCTAGCATTGTTTAGACGCTAGGCTGTATTTTCTAGTTGATCTTGGTTAGTG

>C102021_65

GGGAAATAGCCTTAACCCAACCCTAGATCTTCATTCTTCAACAACACCCAACTTCTTCCTTCTTTTCAACTCAACAACATTACATCTCAAACACTTCTTTCTCAGATCTCTTCCAGATTCCCTCACACGCTTCTACTGTAAAAAACACCCCACAATTCTTCCTCTGTAAGTCTTCTTCCTCAACAAATTTCCACGTTTGAGCCTCGAACCCCCTCAATTTTGCATTCCATCTTGTGGGATCCACCTGAATTGCACTGGATTTGAAACCCCACTAAAAAAAAATCATACTTTTTCAACTGGGTATGCTTTGTATGTGACAGTAGTGTGTAGTGCATTGTAGGCATTGAGTTCTCATGGCAATGCCATCGGGAAACGCGGTCATGCCGGAAAAATTGCAGTTTCCGGTTGGCGGTGGTGCCGCTGGTGGCGGTAGTGAGATCCACTATCGCCACCAGCAGTGGTTTGTGGATGAGAGGGATGGTTTAATCGGTTGGCTTCGCAGTGAATTCGCCGCTGCGAATGCCATCATAGACTCCTTGTGCCACCATTTGCGAGTTGTTGGTGAGCCTGGGGAGTATGACATGGTTGTCGGGGCAATTCAGCAGAGGAGGTGTAATTGGACTCAGGTGCTGCTTATGCAGCAGTACTTCTCTGTGTCTGAGGTTGTGTATGCACTGCAGCAGGTAGCCTGGAGGAGGCAGCAGAGGTTTGTGGATCCGGCGAAGGCCGGCTCCAAGGAGTTTAGGAAGTTTGGTTCGGGGTTTACGCAAGGGCAACACAGGAATGAGGCTTCCAAGGAAGGTTATAATTTTAGGAATGAGGCTTCGAAGGAAGGTTATAATTCTAGGAATGAGGCTGCCAAGGAAGGTTATAATTCTAGTGCAGTGGTTGTAGCTGGAGGTGTGGAAAAAGGGACTCGTGTGGTTGATAAGAATGGTGAACTTAACTCTGGTGGCAAGGTTGCGACCGTGGATAACAATAGTATAGCATCTGCTGAAGAGAGGAAAGATACTATAATAAATCATCAATCTGATGGCATTTTGAATGGCTCTGGGAATTCTCAAGGATCTCTGTCTAGCTCAGAATGTGAAGCTGTAAATGAAGAATGTCTATCGAATTCTAAAGGGAACGATTCACATTCTATGCAAAATCAACATCAGAGTCAGATATCTTCTACCATGGGAAAAACTTTTATTGGCAACGAGATGTTTGATGGGAAGATGGTGAATGTGGTTGATGGACTGAAGTTGTATGAAGATTTAGTTGATAGTACTGAAGTTTCAACACTTGTTTCCTTGGTCAATGATCTGAGAGTTGCTGGAAAAAGAGGACAATTTCAAGGAAGTCAGACATTTGTGGTATCAAAAAGGCCCATAAAAGGACGTGGAAGGGAAATGATTCAGTTGGGTGTTCCCATTGCTGATGCACCACCAGATGTGGACAATCTAACAGGAATCTCCAAAGATAAGAAAGTAGAATCTATACCTTCTTTGTTCCAAGATATTATTGACCGTTTGGCTGCCTCGCAAGTGATGACTGTGAAGCCTGATGCTTGCATTGTGGATTTCTTTAATGAGGGTGATCATTCACAGCCCAATAATTGTCCACATTGGTTTGGAAGGCCTGTTTATACACTTTTCCTGACTGAATGTGACGTGACTTTTGGGAGAATAATTGTCTCAGACCATCCTGGGGATTATAGGGGTGCAGTCAAGCTTTCTCTTGCACCTGGGTCCCTTCTGGTGATGCAAGGGAAATCAGCTGATTTGGCTAAACATGCCCTTCCTTCTATTCACAAACAACGCATACTTGTGACCTTCACAAAGTCCCAACCAAAAAGTTCTCTACCAAATGATTCTCAACGACTTAGTCCACCCGTGGCGTCTCATTGGACTCCACCACAGAGCCTATCTCCCAATCACATGCGCCATCAATTAGGCCCGAAGCATTATCCCGCCATTCCAGCAACTGGGGTACTGCCAGCTCCATCCATACGAGCTCCTCCAAATGGTATGCAGCCATTGTTTGTGCCAGCACCTGTTGCATCTCCCATTTCATTTCCTACACCTGTGCCAATCCCACCTGTTTCCACTGGATGGGCCTCAGCTCCTCAGAGGCATCCACCTCCACGAATACCTGTTCCAGGCACCGGAGTCTTCCTCCCCCCACCTGGTTCAGTTACTACTTCATCTCAATACTTACCAGGTACGGTACCTGAAGTGACCCCTAGTGTGGAAACTTCAACCGTGGCGGGAAAGGAAAGTGGGAAATCTAATCATAACACCACAAATTCTTCACCAAAAAGTAAAGTGGATGGAAATATGCTAGGGGGACAAGAATCCAATGGAAATGCAGATGGAACTGAAGTAGTAGGTAAAGAACACGAAAACAATGACACGAGCAATGCAAACCATTGAGCTATGCCTGATAAGAAAGAGAGAAGGAAAGCTTATCTAAAGAAAGGGGTAAGGTACGAACATAACGAGTTTGAAGCAAATGTAAAGAGCGGCAACATTCAACACGAAATGATACAATACTGCAAAAGAGAGGACAAGGGAAGGAGGCAGAACTTTTTGGTTCACTTCTTAATCCCTTGTTTTCTCCATTCCTTTTTGGTTGTCTTATTATATTAACTTTTACAGCAAGGGAAACCCCATTCTTAATTCAGATTTTGATTTTTCCTAAAA

>C102025_65

TCCTGCTGCATTAATCTTGTCTTCTACTACTACTACTGTTTTCACTACAATTGCTGAAACCAAACCTCACTGCAACAAGAATTACAATTACAAAATGGAAGAAAGAGGGAATAGAAAGTTTCCTTTGTGAAGCTATAATTGTCATTCAGCATATAATGGAAAAAGAGACCATTTCCACGGTAAGACACTCGACCTTATAATACAAAAGAAGAAACACTTCATATAGAGAGACACTTAGGTTTAGATTTGGGAGATTCCAGTGAAAACCAAGGCCTGAGTTATTGCTGGTTGGAGTGCACAACAAGGGCACCTTTACATGTTGTATCTTTTTATTTTCACTTATAGGATAGGGACCCCAAAAGCACTATCTAGCACAAATTTAAGGGCAATTTGTAACAACTTTTTTGTAGTGACCATCTCTTGACAAGCTAACTCGGCTGAAAGCCACAGGTCGATCTCTACCATCCTCCACTTCATTTCTTCACCTCATTTCCCTAACGATGGCAAAATCACATTTTCAAATTGTTCCTCAAGGTTCTTCAGCCAGCTTTGGAGAACAGTATGAAACAGCCTCTGCAACAGTGAGGAAGATCTTGTCCTCACCAAGCAAAGCAGCAAAGTTTGATGTGTGGAGTTTGTCTATTACTACTGGCCCAGGATTTGCCAGAACAAGCTGAACATCTCTTTTCTGAAGACTTCTAAATAACTCTTCAAGGGCATGGATGCCGCTTGTATCGATGTCAGTGACCGGTGACATCTCAACTATCAAAAATTGGATTCTTGTTTGGTAGTCTCCTTTCACTTGTTCTTCCTCATCCATCAGCCATCTTAATATCCTTTCTTTAACATAGTTGGAGTTGGAAAAATATATTGCAGAATCAACTCTAATGATCAGTACACCAGGAACCCTTGTGGCTTCTGGGTATTGTTGGATGTTTCTGTAGACAGTAGTCCTAGGAATCTTCCCCAGAATTGCAGTTCGTGGCCTTGTGACTTGTAATAGGATCTTTGCAAAGGATATAGAGACTGCAATTAAAAGTCCTATCTCAACTGAGGCAAAAACAACCCCAAAAAATGCTCCCATGCAAGCAACAAAGTCAAATTTATCAATCTTCCATATCAAAACTGCTGCCTTATAGTCCACAAGGTTGATGACAGCAGAAATGATGATTGTAGCGAGAATGGCATTAGGAGTGTATTCGAAAAGAGGGGTGAGGAATTCCAAGGTTAAGAGCACAACGATAGACATGACAATGTTAGATACTGCCGTTTCGCAGCCAGACATGTAATTTACTGCTGACCGAGAGAAAGAACCCGTTGCCACATAACAGGAAGTCAATGAACCAACAACATTCATAGCACCTAATGCCACCATTTCTTTGTTTCCATCCAGTTGATAGTCCTTCATAGAAGCAAATGTTCTTCCAATTGCTGTGGCTTCAGTCAATGCTATCATGCCAACCACTAAGCCAATTCTGAAACCTTTCCCAAGGTAATCACCAGTAAAATATATTTCCTTCACAGATGACGGATTGATGCCTTTCTCTATATGTTTTACAATTGCAACACCATGCTTGTCTACACGTGTTAAGTATACAAAAAGAGTGGAGAGAACAACAGATATCAACGGGGCAATTGCTGGCACCCAGAAGAATTTCGGGTTCTTCTTTCCAATATACTTGGCAAACAGAAGAAAAGCCAGAAAGGAAGCTCCAATTACTATAGTTTGCCAATTCCATCCATGATGGGCTGATGACAGTACTGAGTGCATCACAGAGATAACATCAGTTTTCTTTGTGAACTTTTGAATCCCAAGGAAGCCCTTAAGCTGTTGAAGGGCAATTGTGATGGCAGCTCCCCCCATAAAACCAACAATTGCAGCATGAGATAGGAAATCGATCAAAAATCCTAACCTTAAAACCCCAAGTGTTGCTTGAGTAATCCCCGCAAAGAAAGTGGCGGTAAAAGCAAGTCGTCGATAATCAACTGGATTCGCAATGGGATCAATCTCACTTTGAAGTAAAGTCCCCAACAAGAGAGAAACCACTGCCACTGGTCCTATTGCAATGTCACGAGAACTACCCATGACAGCATAAATCAGTGGTGGAATAAAACTGGAATACAATCCATACTGTGGATCTAAATTTGCAAGCTTTGCATATCCAATGTCCTGTGGAATGCAGAGACTTGCAATAGTTAGACCAGCAATAAGATCCCCTCTAAGTTTTGTGAGATTATAGGTTCTCCCCCAGCTAAGTATGGGGAAAATGGTTTCAATGCCAAGGATGAGCTTCCGGGACTTAGTTTGATCCTTGAAGGGCCGTAGAGGATCATCTGCGAAAAATGTTTCTTTTACAGTAGATTGGAATTCCTTGAAAAGGTTTTGTCGCGGAGGAATTCCAACCTTGTGTACGTATGGTTCCTGGCCTTGTGAAGATGATAAACTTCTCGCATTCGTCTCTTTTGTTTCAAGATGTTCATCAGCTGAACCACCCATTGAGGACATCCACAATGACTTTCAGCTGAATTAAAGGGAGAAGACGCAGAGAGAATGGTGTAGGTCACTGGTTGAATAGGTAAAGACTTCTTTAGCAGAAAGACTTCTGTTAAGCTAGTTTGAATTATATTACTTCAAGTCAAAAGCTAAAGTCAAGCAGACAAGCCATGAATGTTAATTCAGATTCGCA

>C102027_65

GAATACATTTCCGCATAAATTTCTATAAGCTCTCCAGTTATAATTCTGAATGTACAATAGTTCAGTCTGAACGTATCATACTGATTCAGGAAAGCACATAACTAACCTTACCCTATATCATTAAACCCTTGAGGATAATTGATTTGAGCTAGAAAGCAATAAAGAAGGCAATAAAATGCAAAAGACAAAAAAAATAGATATGTAGATATTTACAACATTGATAAATGAATTAATGGAACTTCAAGATTTGCAAAGCCACCCTTGGATAGCATGTCGAAGTGCATGATTGGGAGTGAGATGCAGGTGACTCAACTTTAAATTAGTCATAGGAGAAGTTTCATGCCCATTTTCCAGCCATTCACTTATGGCCTTCCTTTCATAAGTAAATCCGTCTGCTGCAACTTGAGGATCAAGCATTATTTCCTTAAAGATTGGACACAAGAAAAAAGATGGCACTGGTAGCTCTTCTGAAACAAACAACTGCTCCAATTCTCTCACTAGAGAAGGTGTTAACTCTGGTCTGTCTCTACTGTTCAGTTGGCAGCATTGCAGTCCCAGTTCAGCCAATCGTGTTGCCACGGTGGAATGCCATTCTCCAGCTGAAGAATCCAAAATTGAAGACAATTTTCCACATGAAACTGCTCTGCGTACTTCACCAGCTAATCCAACTGGACTCCTACCAGTGAGGAGTTGCAAAATGATGAGTCCAAAAGAGTATACGTCAGACTTAGGTGTTAGTACTCCAGTTCTCTGAAACTCAGGATCTGTATATGTAAAAGAACCTTTTGGCTCAGTGCTTAAGTGAAAACTAGGAAGATAAAGTGACTCCTCTTTCACCAGCCTGCTGAACCCAAATTCACATATCTTGCAACTAAATCTAACGTCAAGAAGGACAGTTTCAAGCGTCAGCCCACCATGGATAATAGTTTCAGGTTTGGAAGAATGTAGGAAGCAGAGAGCTCTAGCAATTTCAGAAATCCATCTTGCTCTGATACTCCAAGTCAAAGGAAGAAAACTTCCTTTTCGGAATAGGTAATCTTGAAGACTTCCATTGGGATAGTACTCATATACAAATGACCAGGCTTCAGGGCAAACACCAAGCAAAGTAACCAGATGAGGGTGCTGGAGACTACCAAGAACTCGAACCTCTTGATGAAACTCTGTTGATCCTTGCATGTTGTGTGGATGAAGCTTCCTTATAGCAACTGTTCTACCCAACATTTCTCCCTTATAAATAGAACCATATCCACCCTCCATAGCTTTGAAACTCTCAGAAAAATTGCATGTAGCGTTTTGAAGATCAGATAATGAAAATTCTGCCAACTCCGGAAATTCTTCAGCAAATCCAACGATCCCATTGCAGTGATCTGCTCCAACATGTCCACAACCTTTCCACCGCTCAAGCCAGTGCAGGGCTTCCATTTTCTGTCGCCTTATTTGCTGTTTTTCATGCTGCAGGTTTGAGAAGGATGCTTGAATTAGTGATAGTTCATTTGCAGCTTCATCTCGCTTACGGTTGGTTTCATGAGCACAATTGCCTAGAAGGGAAATACTTCTTGTGGTTCTTTCCAGTTCACTAGTAATTTCATCTTTCTCCTCCAAGAACATCTGTTGCTCCTGTATTGTAGCTCTCACCGCATCCTCAACCTCTTTTCTGAGCTTTACTTCATGTGCGTGGGCAGATTCAAACAGGTTGACCTTACTGATGGCCTCAATGGCTTTGAGTTCCAACCTTTTGCATTTCAACAGCTCTACAAGGGCTTTGTCTGTTGCAGCATTAGCTTCTCCTTTACAATCAATAAGTTGACTATCAATTGTTTCTTCCTCAAACTCTGAATGAGTATCTAAATACCAACCAACTGAATGTTGGGGAGAACAATGACTATTACAACCGGATGATTTGGATGAAAAAGTAGCTTCTGTTTCAATAATTTCACTCTGAACTCTACTTCTTGTAGTCCTTGTAGAATTTGATTGAAGACATTCAGAATGGAATAAGTCATTTGTACCATATTGAAAAGATCTACGACTCAAGCTCTCTGCGGTTGTAGTCTCTGGTTGAGCATGGGAAGACACATAACAAGGAGTTTCAGAAGCTTCTCTTGTCCAAATGTGTTTACCTTTATAGACAAACCATATTTCACAGAATGGGGGAGCATTTTTCGCAGCAAAGTTTGCTTTACTGGAATTCCTTTTGACTTTCATGAAATTTTCTGCGGGTATTGCTCCAATAACAAGCTTTCTGATATTATGCACAGTAACCAAATCTACAATCCCTTTCTGGACTTCATCAGCTTCACCAATGACACTACTTGCCTTAACCTTAGCTGTACGGCAAATGCTCAAGTACTTCTCCAGAAGCCTCTTGGTCTCTTCTCTTTCCTCTATTCTATAAGCAGACACAACTTTAGGACTGGCTTGGGATGCTGGCAATTTTCCTAATAGTGTGGGGATCATAGTAGAAGGTTGGTAAACATGAAGCAAATATATCTGTGCTTTGCTGAAATGGGTGAAACACCATTGAAGCAAAGTGGTGGCTTTCTTAAGAGACTTCCCAACTGCCACGTGGACCATCTCATCACATTCAAACTCGTCCATGGCCATGAGGTGGGGTGGTTGAGTTGAGAAGTGTAGAGTGTTAGAAGACTTGCAGCGGAAGATGTGAAGGTTGATAAAGGTCCATAGTTG

>C102029_65

CGGGAAGAATAAGCAATACGGTAGTGACATAAGAAATTCACAGGAAATTTGCAAGAAAGTATTTGAAATGATGGATAAGCATTGATCAAATGGCATAACAATATATATAAAAAGAATTGTGCTGCTTAATCAATGACAAATACTTTGGGTCCACTTGCTAGAGAAAGAAATACATCCACCCCGTAGAGAAATGGGGGAATATATTATAAACTATATTATACAATAAAAAATCCAGCATCCTTTCTTCAACGTTTCTTCCAAGTCTTCTCTCCCCTGATCTAGATAGAAGTTGGAACTCCGTTCAAATCTGTGGATTAACCTGAACTTTGTTCTTTGCAACATGACCTATCCCTAACTATACTGTGTAATGTTGGATGCAGTTGAAATAAATAAAATAATTAAATAAATAAATAAACTTGTCAGCAGAATAACCGAGAAGCATTGTCATGGCAGCCCTTGCTCATGCTACCATCTGCTCCTGCTCTATGTACAGCTGCTCCACCCATGGTGGCAGCATCTCTTTACTGAGATCATATTGTTGCTTCTCAGCACCAAACATCTCAGACCTCTCAGAAGGAACATCCTTAACCAATGAATGAACCCACGAGACATCTGGCTCAACATGTTCTGGCTGAGTCATGGGGGAAGCAGTTACCCCATTGTTTCTGAACCCAAATGAAGCAGATTTCCTCAGCTTGTTCAGCTCATCTCCGTTAACACCCCAATCCAGTTTCCCAGTAGGGGAACTCCAATCTGACAGGGTAGAGGACACCCTGCAGGAAGGGTTGGAAGGTGAAGACAGGCCAAGATGGTGGGTTGCAGCTCCACGATCAATGAAACTTTGGCTTCGCTTCGCGAAGGCAGCAGACCTGGAGTTCATCACTGCAGCTGCCACAGCAGCTGATGAGTCAAACCCAAAAGCTGAGGGCTTCCTAACAGGAGAGGAAGGGATGTTGGATGGATAACTCGCACGAAGGTGGTTCATGTTTTGGCGCATCTGAAGCCCACTTTGTGTGGGTGTTGAAGGCTGCAAAGAAAGTCCATGTAGTTGTGATAACACGGAAGGGTCAGCAGAGGCTAACAGGTCATCAAGGTTGGTTGGATTCAAATCACCAATCCTACTTAATTCCTTGTTGCGGAAAGACGGGGAAGAGATCCTGGCAATCTCTTCTATCAATTGTTGCTGCTGCTGCTGTTGATGGCGAGCAGGACTGTCTAGACCGAGCATTTCCATCTCCATCTCCAGATCCCTGGCACTCAGAGCAGCCTTGAGTCGGCTACCCGGGAGCTGCAGGGATGGTGGAGTGAGGTTTATTTTGTTCTGCCACAAGCTTCCACTCTTTGGAGATGAAGCTGCTGCCAAGGGAGACATAGGTGGGGTTGAAACAGTTGGCATAGGCAAAGATGTGGAACTGAGAGCCAATGGACTCATGGTTGTCATGTCTAGTGCACTAGCTGAATAGGATTTTGGTGAAGGCATAGCTGACCCAGTGGAAGCATACACAGGGCGTAACTCTTCAGGTTTGTGGGCAAAGAAGCAGACTTTTCTAGTGCAGCCAGTCTCATCCTTGCAGAGCCTTGTTCGGTACTGGGCAGGGTGTAGCCAGGACTCAAAAACACCATGAGCATACTCACAGGAATCAGCCTTCTGGCAGGCCCCTTTGCGAAATTCGGGGCAAGGAACGCAGCTATAAGGGTATTTCCTTGGGTCTCTCCTCCTTGCGTTCTCCCCTGGATGAACAAATGGACACTCAGTCCAGTCATGGGAGTAGGCCCTTGAGCAAGGCTTCACCTTGAAACTGTACATCCTAAACTCATCTGTTCCATACACACCGTTGTTGATATCAGGCAAGGATATATCAACAGGATACTCTTTCTTATCACTTCCTTCTTTTCTCTCAGCACCTTCTTTTGATGAGAAAGGAACGGAGAACATCTGCAGCTCCATCTCCTGGCTCATCAGTTCATCTCTTTCACCACCCCTGAGAAACAACTCCATGGCCTTCCTCCTCGATTTGGAGGAAGAATCAAAGGCAGGAGGAATCAGACTAACCGGCTTGTTTCCACTGGCATCAAGGGATTCAGCATCAGCCCCAGCATCTAGCAAGAGCTTCACAATCTCAGGTGCGGATTCAGAGCCCCCAGCAGCAGCACAATGGAGAGCAGTGGCCCTGTCAGAACCACAGGCCCTGTTCACATCAGCACTTCCTGCCTCAAGAATATACTTGAGAACCCTAGTGCTTCCAAATAAAGAAGCAATCATGAGGGGGGTCCTCGTTTCAGATCCCATCTTCTTTGACCCAATCCTTCTACCATACCAAAAGCCAGCCTCGTTGACATCTAAGCCCTTCTCATCCACTTCTCTTTTGAAAGCTTCATAATCATCGGAAGCGGACAGTTCAAGCAAAGCCGAGAAGTTGTAGAATCCAACAAGATTCTGCTTCTGAAGATTATTCTCCATCACTAGGCTTGGGGAAGAAAGCTTACTCTTTGAATCGCTACACATGTCCCAAATCCCTTTAGAGTAGTAGATTGGAGAGGGAGTAGGAGTGGAAGAAGTGTATGTTGGTTGCAAGAAAGCGTTGTTGTTTGTAGAGAAGAAAATCGAGAAGACCTCGATTGAGTGAAGATGAGGGAGAAAGATGGTATGGGAAGGCCCTTTTATTGTCAGCACACCAGCAACAGCTATT

>C102031_65

AAAAGAGAAAATTGGAAGCCAAAACCCACAACAACACACACTATGCTATGCTAAACTCTCTAATCCGTTTCCGCCGTTACGCATCTCCTTACTCTCCTCTTCTTGGTCTTCCTATTAACAGATTCTCCTCCGCCGTCGGCGCCATGGTTGCTCACACTAAGGACGACGCCTACCTTCAGGCGGCGATTCCGAAGCGCATTCATCTGTTCGAGACTATCCAGGCAGAGCAACGCACGCAGCGCCTCTCTCTCTCGCCGGATCCTATCAAGGTCACTCTCCCCGACGGGGGTGTCAGGGAGGCAAAAAAGTGGCTCACAACTCCGCTCGACGTGGCGCGCGAAATCTCCAAGAATTTGGCCAACAACGCGCTCATCGCCAAGGTCAATGGCGTGCTCTGGGACATGACGCGTCCTCTCGAGGACGATTGCCAGCTCCAGATCTTCAAGTTCGACGATGACGAAGGCCGCGACACGTTCTGGCACTCCAGCGCGCACATTCTCGGCCAGTCTCTTGAGACTGAGTATGGATGCAAGCTCTGCATTGGGCCTTGCACTACAAGAGGAGAGGGATTCTATTATGACGCGTTTTACGGTGACTTGGGTCTCAATGACGATCACTTTAAGCAAATTGAAGCTGGCGCATTGAAGGCTGTTGCGGAAAAGCAACCGTTTGAGCGCATTGAAGTTACACGCGATCAAGCACTGGAGATGTTTTCAGATAATAAGTTTAAGATTGAGATTATCAATGATTTGCCTGCCGACAAAACTATCACAGTATACAGATGTGGTCCCCTGGTTGATTTGTGTCGTGGACCCCATATACCTAATACATCTTTTGTCAAAGCAATTGCATGTTTAAAGGCTTCATCAGCATATTGGAGGGGGGACAAAGATCGTGAAAGTTTACAAAGAGTTTATGGCATATCTTATCCTGATCAGAAGAGTTTAAAGGAATACTTGCATCGACTCGAGGAGGCTAAAAAGTATGATCACAGGATTTTGGGTGTGAAACAGGAGCTTATTCTTCATCATGAATGGAGCCCAGGAAGCTGGTTTTTTCTTCCACATGGCACGCGGATCTACAACAAACTCATGGACTTCATTCGAAATCAGTACAAAGACAGGGGCTACCAAGAGGTCATATCTCCAAATGTATTTAACATGGATCTGTGGGTGCAATCTGGTCATGCTGCAAATTATAGAGAGGATATGTTTATCTTAGAGATTGACAAACAAGAGTTTGGGTTGAAACCAATGAATTGCCCAGGGCACTGCCTGATGTTTAAACACAGGGTTCGATCATATAGAGAACTTCCTCTCCGTTTTGCTGATTTTGGGGTTTTGCATCGGAATGAGGCTAGTGGTGCCCTGAGTGGATTAACTCGCGTTAGGAGATTTCAGCAGGATGATGCACATATTTTCTGCAGGGAGTCCCAGATAAAGGATGAAGTGAGGAACGCCTTGAATTTCATCAATTATGTCTATGACATATTTGGTTTCACATATGAGCTGAAGCTTTCAACGAGACCAGAGAAATACCTAGGAGATATTGAAACTTGGGATAAGGCTGAAAGTGCGCTGAAAGAAGCTTTAAATGATTTTGGCAAGCCCTGGCAGTTGAATGAAGGGGATGGGGCATTCTATGGACCCAAGATAGACATCAGTGTATCTGACGCATTAAGTAGGAAATTCCAGTGCGCAACTTTGCAGCTTGACTTCCAGCTTCCTGATCGTTTTAAGTTGGAATTCTCAGCTGAGGATGAAGCCAAAATTGAGAGACCTGTAATGATACATAGAGCAATTCTAGGATCTGTTGAACGCATGTTTGCCATCCTTTTAGAGCACTACAAGGGTAAATGGCCCTTCTGGCTCAGTCCTCGTCAAGCAATTGTATGCCCCGTTTCTGAAAAATCACAAGCTTATGCACTTCAGGTGAGAGATCAGATCCACCAAGCAGGGTATCACGTTGATGCTGATACAACTGATAGGAAGATTCAAAAGAAGGTGCGAGAAGCACAGTTAGCACAATACAACTATATCTTGGTTGTCGGAGAGGAGGAAGCTAATACCGGACAGGTGAGTGTACGAGTTAGAGACAAGGCAGATCATAACGTTATGAGTATTGAGAACCTACTCAAACATTTCCGAGATGAAGCTGCAGCTTTCCATTGATACTTTGCTTATGAAAACTTGAAAGTTTTTAGCCCCAAATATCTTAGTTTTACACATTTGTGTGTGCATTATTTATATTTTTAACCTAACTATCCTGGTTTTATATACATGGTTTGAATGATCACTATTGCATTGCCATCACACTACTAAGTTAATGGGACTTTTGGTTATAGCTGGACCGTAGTCTAGTAGATATTCTTGTATCGTTTTTTTTACGTTCCTTTCGATATAAGCAGTCAAGTTTACATGCCTCTTGCATTTCCTATTTTCTTTTAAGTTGGTGTGTGACATTTGAGGAAAATATACCTTCTGGAGTCGGTCCTCAATGATGATTGTTAGCAACTTGCCTAGGTTTTCATTAGGATCCAGGTTTCTTTTTCTTTGGTGGATTATTGAGCAACATAAGTTCAATATTCATATCCATTTTGAATGCAATGTGTCAGATGCTTGTACTTGGTTCCCTCAACTGAAATGATTTATATATAGCACGAATGATTGTACTCAGTTCCCTCAAATGAA

>C102033_65

ATTTGTTAGATGAAGAATGCCTATAAAAACTCCTTCACTCATATCATTTCATGTTGAAGGAAGATGATGGTAATAATACAAATCATAGCAGCACTTGTTGTTGCAGTTGATGTGTTATACAAGAAAAGAAAAATTGCATATAAGATGGACTTAAGGCCTCCACAGAGTTTTCTGTGCACATAAGATACCCTCCTTATCCTGAGCAAAAGTCATCCTAATCTCCCACTGCATACTTTTCAATATTTCTTCCAATGGATTTAGAATTTCAACTTTGTCACGTATAATGGTCCAACCTCCTGGCCGTAATATACGATCCATCTCAACAACAATGGACACAGGCTGCTTGCACCTGTTTTTGAGCCGTGAGAACAAGTGATCAGCATGCAAAAGGTCATATGTTCTTGGATAAGTTCCAAATGACTCACACCAATCATGGTATATACCAATAAGCCCACGTTCAAAAATTATGGGAAGGGTATCAGGAGCATGAACAGGCACTACATTCATCACCCAGACCTTTTGTTGAGAGAGAGCTACAGCCAATCCTCCGTAGATGGATTTCATGTCCATTACATTCCGGATACTTGTCCAGTTAATGCCCAATCCACTGATATATGACTTATTAACAACAGCATTCCAGTGATTGGTGTCTGCAACCACTTTCTCTTTGTTGTTCACCCAATCAGGATAAGTTTCAAGCCTCTTGGGCCATTCCTCTGGCCACTCTGCCCCGTGTTGTTCAATTCCAGATGGAATGGGGTGCAAACAAGTCTTCATGGGAACATACCAGACAGCATCAGGATTTTCATTTTCTTTGCAAAGTGGTGGAATTTTCTTCCTCCTCAGTTCGTATATATCATTTCCTTCAGGCTTCTGATATATTTTAACTCCAACTTCACCAACATCATCACTTTTGTGAGCCAGAACATTCCAACAAATAGATGCAGTCAATGTTGTCATGGCTTCTTCTTCTTCTATACTGTCATGTTTAGTTGACATAATAAAGTATCCACCAGGTCTTAGAATCCTATTCATCTCCAATAGAAGCTTTCCACCATTGGAATACCAAGGTATGCTGCAACCCGCACAATGTATAGCATCGAAAACTTGACTTGGAAAAGGAAGCCTCCTTCTATTAAAAGGACTAATCATTGCTGGGAAGCCCCGTTCAAGCGCCACCTGAGCTAAGTCCACTAGATCATTCTTCAAGCCTAGTGTTAATGTTAAAACGTCCTTATCAAGGAGAGCAGCTGCAAAGCTCGAATCTGTACATCCAATGTCCAGGACAACACGAATGTTTTTACCCCATTCAATGTCTGGTACCATCTCTTCAATGGACTCAAGATAGTGATGAATCCCTCCCTTGAACTCAGATTGGTTTTGGGGAAAAGTAAGAAACTCTTCAGACTCCACCAACCAACTATGCCTTTTTATGTACGCAGCTAGTTTCGGGTGTGCAACATTCTTATACAATATCTTCAATTTGCTCTCAGGCCAAGGCAATGGAGATCCATACCCTTCATGAGGAAGAGGAACCAGACACATGAAGGGTGTCCTCGGACAACTCCTCTCTGTGTGCCGGTAGCTTGTTACTTTTCCACCACCAACTTCAATATCAATGCAGGGAATGTAATTATGCTTGCTTCTAGAACTACACAATTTCCAGCTATAGCTAGCATTTGGGTCAAACAAGGGTCCTTTCACCTTTCCCTTGCTTTTTTTTCTCAACTCCTCCACATCTTCCACCACCTCAGAATCATCCTTATCCACAGATTCAACCAAATCACTGCCACCATCAGCATCCACATCCACTTGACTCTCTTGATCCACTTTCTCATCTTCACCCTCGACCTCAACCTCCTCTTTCTCTTTCTCTTTCTCTTTCTCTTCTTGGCCATGTTGCGGTTGAGGATCCTCTAACCCTTGATGATTCCCTTTCTCCTTATCCTCCTTATGCACATGTTTCTTCTCTTTCTTGTTTTCCTTTCCCTTGTGTTGTTCAGAGTGAGGACGCGTGGCAGAAGAGTGAGAAGACCCGCTACCGTTACTCTTACTCTTTTTCTCACTCTCACCACCCTTTTTAGATTCATCTCTCAACTTTTGCGGCTTGTGAGAAGAAGAGGACACGGGTTCCGCAATTTCCTCAAAACTCTCCCTTTGGGAAGTGACAGAAGAAGAAGAAGATGAAAACACTGCCCACACAAAGATGAAACAGAGACCCAATACAGTCACAGCTGCCATCTTCACCCACAACCCATGAGGGCGTTTGGCTTGCCGAACCAACCGAGCTATGGCCATATCTCAATCCAAAAAACGAAATTTTGAAATCCAATAATGGATGTAAGCTCTTCGAACTACCACCACCACCCTTTTGAAACGAAGCTGATCTAGGTATATTGGTTCAAAAATGAAAATGTGAAGAGTGAAACAATGGGAATCGATGACAGTTGTTCAAATCTGAGAAGTGAAAAGAACAGGTTTTAGGTGGTGTTGCCTTTATTGAAGAAATGATATGTGTTTCAGAGTGAGGAATGGGAAAGTGGAAAAATTGACAAAGGGGAAGCGTGGGAATGGTTATTGTAGGAAATGAGGAGTTAAAGAGATAGAAGAGGTTCGAAGTGGCATCCTCAGTGGTTGAGGTATCATGGTTAGGCAGAGGACAAGTTCGATCCGTATAAGGTTTGATGCAGA

>C102035_65

GACTAAAGAAACAAAACACAAAACCCACTTTCTCTTTCTCATTCTCTCCAGTGACTGCTACTACCACTACCACCCAATCGCAACTGCTTTATTTGCACTCCACTCAGAACAAAACCCTGATTCCTCTTCTTCTTCTTCTTCATCTTCATCTTCATCTTCCAAATTTCGTTGCTTCCTCCTTCTTCAGCTCATGACGACGTCGTTCGGTGCAGCTGCCACCTCAGCGACGCTCAAGGACCACAAGATTCAGATCCCAAGCTTCCAGGGCTTGAGGTCTGCGTCCGCCGCCTCTGCTCTCACTCGCAATGCCCTCCCGCTTCCTTCTTCATCCACTCGATCTCTCTCCATAACACGCGCTGTTTCCACGCCTGTGCAATCTGAAACTACCACTGTAAAGCGGAGCAAAGTCGAAGTATTTAAAGAACAAAGCAATTTCATAAGATATCCTCTTAATGAGGACATGTTGACGGATGCCCCTAATATAAGTGAAGCTGCCACTCAATTGATCAAGTTTCATGGAAGCTATCAACAGTACAATAGAGAAGAGCGTGGTTCCCGAAGCTACTCTTTCATGATACGGACTAAGAACCCTTGTGGGAAGGTTTCAAATCAACTTTACTTGACCATGGATGATCTTGCTGACCAGTTTGGGATTGGGACGCTTCGGCTGACCACCAGACAAACTTTTCAGCTCCATGGTGTTCTCAAGAAGGATCTCAAAACGGTGATGGGTACCATTATTAGAAATATGGGCTCAACTCTTGGTGCGTGTGGTGACCTGAACAGGAATGTGCTTGCTCCTGCTGCACCCCTCGTCAGAAAGGATTACCTTTTGGCTCAACAAACTGCGGAAAACATTGCTGCGCTTCTGTCTCCTCAGTCTGGTTTCTACTATGATATTTGGGTTGACGGGGAAAAGTTTTTGTCATCAGAACCGCCTGAAGTTGTTCAGGCACGAAATGACAATTCTCATGGTACAAATTTCCCAGATTCACCAGAGCCCATCTACGGTACTCAGTTCCTGCCGAGGAAATTCAAAATTGCTGTTACTGTACCGACTGATAACTCGGTGGATATTCTCACAAATGATATTGGTGTTGTTGTTGTTACCGATGAAGTTGGGGAGCCTCAAGGGTTCAACATATATGTTGGTGGAGGAATGGGAAGAGCTCATAGGATGGAAACCACTTTTCCTCGCTTGGCTGAACCATTAGGTTATGTACCAAAGGAGGATATTTTGTATGCAGTGAAAGCAATTGTTGTCACACAACGAGAAAATGGGAGAAGAGACGACCGCAAGTATAGTAGAATGAAATATTTGATCAGCTCTTGGGGAATTGAAAAGTTTAGAAGTGTAGTTGAGCAATATTACGGAAAGAAATTTGAACCTTTCCGTGTATTGCCAGAATGGGAATTTAAAAGTTATCTTGGCTGGCACGAACAGGGTGATGGCAAATTATTTTATGGGCTTCATGTTGATAATGGTCGTATTGGTGGAAAGATGAAAAAGACATTGAGGGAGGTTATTGAGAAGTATAATTTGAATGTGAGACTCACTCCAAATCAGAACATCATCTTGACTGATGTCCGCGCTGCATGGAAGCGTCCCATTACAACTACTCTTGCTCAGGCTGGTCTGCTGCAACCTAGATTTGTAGATCCTCTCAACATAACAGCAATGGCATGCCCTGCTTTCCCATTATGTCCACTGGCAATTACTGAAGCTGAACGTGGGATACCTAACATTCTTAAGCGGATTCGGGCTGTGTTTGATAAGGTTGGTCTGAGGTATAGTGAGTCTGTAGTTGTAAGGATAACTGGCTGTCCTAATGGTTGCGCTAGACCATACATGGCTGAACTTGGATTAGTTGGTGATGGCCCAAATAGTTACCAGATTTGGCTTGGAGGGAACAAGAAACAAACATCATTAGCTCGAAGTTTTATGGACAAGGTGAAGCTTCACGACCTTGAAAAGGTTTTGGAGCCTTTGTTTTATTATTGGAAGCAAAGGCGTCAATCTAAAGAATCATTTGGTGACTTCACAAACAGAATGGGATTTGATAAGCTTAAGGAACATATTGAGAAATGGGAAGGTCCAGTGGTAGCACCATCACGCCACAACCTAAAGCTTTTTGCTGACAAGGAGACGTATGAAGCAATAGACGAATTAGCAAAGCTTCAAAACAAAAGTGCTCATGAGTTGGCCATGGAAGTTATCCGTAATTATGTTGCTTCCAACCAAAATGGAAAAAGTGAATGATTTCATTTACTTAGCCAAGGAAGATGTTTGTGATGTCATCGAGGTTGACAGGATTGGTGGATGGCCAGTACACAACTCTCTGTTGTTACTGTGTTGTAACTCCGGGATAGACTAAACTAATGTCTGGGTTATTAATTATTTTTTATCTGGAACAGCTTTGCTAGAATCTTTTGGTTCATCATTTTCATCGAGTTTCTGAACACACAATAAGCCTTCCTATCATTTCTGTATCGAATTTGGGCTGTTAGACAGCTTGGTTTTTCTCCGGAATAATGTTTGTTAGGTAAGATCTTTGTAGTTTTTTGTGCTTGCTCCAATAAAGTTCATTAATAGTATTTGTAAATTGCAATCATGTTGTTAGTTTAGTGATTAATTACACAATATGGTGTGGGAACCCTATGTGTAAGTGTTTGTTCACCTGTTCGGTTGGT

>C102037_65

GCAAAATTCCGACGGAGATTATGATTCCATCCATGTAAGACTAATATAATCTTTCTGAAAAAAAACTCATACTAGAGAACTAGATAACCTTTACTGTCTATGTTGAAGCTAAACCATGAAAAACAAAGGAAAACCGACAGCACCGTCAATAAATAGTGGATGTGATGGAAATCTACAACTCGTCCTTGACATCATCAACATCATCATCAACTTCAGGCTTTGGAGTATCTTCTTCTTTTGTCTCACTTTCAGCCTCTGCTTCGTTGTCATCTTCCTCTTCAACTGTTGCTTCAGGACTGATGTCCAAGCTAGACTTCACTGAATCATAAATACGGGAAGCGAAATCCTTTGGATCGTCAAGCAAGAACCCACTTTCGAAGAGTGCAGTCTGGTACATCAGCTGTGCTGTTTGTTTCACATCCACATCCTCGGGGTTCTTTACTACTCTCTCACGGAGCTCCTTGATAATAGGGTGCCTAGGATTGATCTCAAGAACCCTCTTGCCACGCATGTATGCTTGCTTGCTAGCATCTGACAAAGTTTGAGACTGCATGATTCGCTCCATGTTAGCACTCCAACCATATTTGGATGTCACCACTACACAAGGAGTGTTATCTAATCGGTTGGATATCTTCACGTCATCAACATTGTCCTTGGCAAGAGCACTTTTCCACCACTTGGTAAGATCCTTAAAGGATTCCTTCAGTTCCTTGTTTTTGGCATCCTTCCCAAGTTTCAAACCCTCCTTGGACACATTTTGGAACTTCTTATCTTCATAATCCATCAGGTATTGCATCAAGTATTCATCAACTGGATCCGTGAAGAAAATAACCTCGAAATTTTTCTTCTTAAGCCGCTCAAGGAATGGAGATTTTTCCAGTTGTTCTTTGCTGGTTCCGGTAATGTAAAAGATGTCTTTCTGCCCAGCTTTCATTCTAGATATGTACTGATCCAGAGATGTCAATTTACCCTCAGACTTGGTGCTCTCAAATCTGAGCAATTTTGCCAACCGATTTCTGTTGGTGGCATCCTCGATGATACCAAGTTTAATGGACTTACCAAACTCATTCCAGAACTTAACATATTTACCTTTCTTCTCATCGTTGTCCGAGGATGCCTCTTCTTTCTTTTCTTTGTCAGTGGACTCATCAGGATCCTCATCAGCAATCCTACGGATCATATCAAGGGCTTTCCTAATAAGTTTCTTCTTGATTGTCTTTAGACTATTATGTTGTTGAAGCATTTCTCGCGATACATTAAGTGGCAATGTGTCAGAATCAACAAGACCCGTCAAGAAGTTTAGGTACTTGGGCAACAGTTCATCAAATTCATCTGAAATGAACACTCGTCTAACATACAACTTCAGGTTGGATTTATTTGCGTTATAATAGCTCTCGTATAAATCATGAGGTGCCTTAGGTGGTACAAACAGGACTGCCTTGAACTCTACATCACCTTCGGCAGTGAAGTGACTCCATGATAAAGGTTTGTCATCACTGAAATCCTTGGCAAGAGAGTGGTAGAATTTGGTGTATTCTTCCTCTGTAACTTCCTTTGGATTCCTCAGCCATATAGCTTTCACATCATTTAGAAGTTCCCATTCGTAAGTTGTTTCCTTCACTGTCTTTGTCTTAGGCTTTTTGTCTTCATCTTCACCTTTGTCAGCATCTTCATCTTCGGTTTCCTCCTTGGAGCTGGCATCAGCTGAATCCTCTTCATCACTGGAATCATCTTCATCAGCGGGAACCTCCACATCGACCTCTTTGCTTGCCCAAATATAGATAGGGAAGTTAATAAATTCAGAGTACCTCTTCACCAATTCTTTCAGTTTAGATTCTTCCAAGTACTCCCCAGCCTCTTCTTTGAGGTGTAGTCTAATCTCGGTTCCACGTCCTAGAGGTTCGTTCCATGTATCTTCCGAAATTGCAAAAGCTCCATCGGCTTTTGATTCCCATACATATTGCTTATCATCGTTATGCTTGCTAATGACTTCAACGTAGTCAGCCACGAGATACACAGAGTAAAAGCCGACACCGAACTGCCCAATCAGATTGAGATCTCCACTTGTTTGCATTTTCTCAACAAATGCTGAAGTTCCAGATTTGGCAATTGTCCCCAAATTCTTAATCAAATCCTCCTTCGTCATACCAATACCCCTGTCTCGGATCGAGAGAACTTTCTTCTCCTTGTCTAACTTAATCTGAATATCAAGCTTGGTGTTATCACCTTCTCCCAAAACCTCCTTGTCCGTGAGAGAGAGGAACCTAATCTTGTCCAAAGCGTCAGAAGCATTGGAAATCAACTCTCTGAGGAAGATATCCTTGTTACTGTAGAGAGAGTTAATGATAATATCCATGAGTCGTGACACTTCCGCTTGGAACTCAAACTTCTCGGCGTTGCTTCGAAGAGATCTCTTCGAGATCGATTCCGCCTCCCTTTTGACAACATCTGAATCAGTGGAAAGGCCATGAGGAACAGCGCCGATCTTGTCCTCCACCTTCGGTGGATCTACAAGGTCGTCAGAATCGCCTTCGGCATTCGCCTGAAACTTTCGACCTTGATCTGCAAAGAGAAAGAGGAGACAGAGTAGAAGGAGAACGGAAGCAACTGTCCACTTCCTCATCGCAGTGCGCTCACACACAATGCTTCTAGTAGCTTCCACCACCACTGAAGAAGAGAAGAGATCCGTTT

>C102041_65

TTTTTCATTAATAAAAATAATTATTCGTTACCAATTTCTACACATGATACTACATCACCATCCACTGTAACCTGTATGAGTTGACCGATTTTTGAGACTAACTATCAGAGTCCCCCCTTAATTAATCCTAGTCCAGAGGGATCCAACCTATCATAAGAGAAATATAAAGATAACAGAAAATAAAAGGAAAAGGTAATTGTCCAGAAAAGCATTTCTTCTCAAGGAAACCTGTTTAGCCTCCACTGTCGGTGTCCTACATTTCCGTAAAGGCAGCTGCTAATCTCAAAAATATCCTTCAGCCAATCTTGAATTTAGCATATCAAATAGCAAGGATACCACAATCCGAACATATGAACAACCAGAAAAATCACCACACATCCCTAAGGTAACGTCCATCCATCTGGAGTTTTTTCACTATAGCTTCTGCCTTTGTTGAGTCCACGTTTTCCTGCTGCTGGACAATGGAATGAAGAGTTCGATGTACATCTCTGGCCATACCCTTGGCATCACCACAGACGTAAAGATAACCTCCTTGAGAAATCAAACTCCACAGGTGCGCAGCTTGGTCCATCATCTTGTGTTGAACATATTCCTTTTCAGCCCCCTCTCTAGAGAATGCAACTATCAATTCTGAAAGAGAACCTTGTTCCAGAAAACTCTTTAGCTCCTCCTCATAAATGAAATCCATTCGACGATTCCTACATCCAAAGAAGAGTACTGCAGGGCCAAGTTGAATACCATCCTCCACGAGAACATATCTTTCCTGTAAAAATCCCCTGAAAGGTGCGAGACCGGTACCAGGTCCAACCATAATAATAGGGACTGAAGGATCACTTGGTAGCTTGAAATTTGATGGCCTAATAAAAATAGGAGCCCAGCTACAGTCGTGACTTTTTTCTAAGGGAATAGCATTCTTCATCCAAGTTGAACATACACCTCTGTGAATTCTACCAGTGGGAGTTGGACCATACACCAAGGCACATGTTACATGTACCCTTTGAGGAGCAAACCTAGGAGAGGATGATATAGAATAATAACGAGGCTGTAAACGAGGTGCTACGGCAGCAAAAAAGACACCAAGAGGAGGTTTTGCTGACGGAAACTCAGCCATCACCTCAAGAAGACTCCTCTGGTTTCCAACCACCCATTTGGAGTACTCATCCTTCCCCTGAGGAGATGAAAGGAACTTTAATCTTTCTGCTTCACTAGGTTCAGAGGCGTGAGCAGCTAATGCAACTAAAGCAGCCTTTCGTGGAGGGTTCAGTAGATCAGCATAACGTGCTAATGCAGTACGGAGTGCACAAGGGCCAGGGAAAGGAGGTAGGAGAGAACCGCCTAGGGAAGTGCCATCCTCCTTATCTGTGTGAAGAGAAAAAAATAGATCTAAATTCTGACCCAACAACTTCCCAGCTTCTTCAACAGTTTCATCGCAGTTCTCAGCATAAACACCCACATGGTCTCCAGTTTCATATGTTATGCCAGTCCCTGATATATCAAATTCCAGATGTATGCAAGAACGGTCAGACTCAGGTTTGTGAAGCTCCTTTTGTACAGCAACATTCACCCTGCAAGGATGATGAATATCAAAAACTGTATTCCCATTTGGCACAGTTGTGTGATTATCATAGGTTGATGTAATAGTGGGGTCATGAATCACCACTCGATATTCAAGAATAGCAGCTGTATAGGGGGTAGAGACAGTATTTGCATCATCCTCATCTCTGAGCAACTGATCCAACTCTGGCCACAATGTTTCTTTCCAGGCAGAAAAATCATCTTCAATGGATTGATCATCATCACCCAATCCAACTGAAACAAGACGCTTTGCACCTTGTTCAGTAAGTTCTTCATCCACAACTTTACCTATCTTATTAAAATGTTCGTATTGTCTGTTACCTAGGCCAAAAACTCCATATGAGAGCTGTTGAAGCCAGGTTCCCCTCTCGTCTTTCCCCTCAGTAAACCATTTGTAGAATCTTGCGGCATTATCTGTTGGCTCTCCATCTCCATAAGTTGCCAGCATGAAAAATGCTAGAGTCTCTTTCTTCAGCTTCTCCTCATATTGATCATCATCCGCTGCATAGTCATCCAGGTCAACAACTTTCACAGCTGCTTTTTCATACCTTGCCTTGATCTCCTCTGCCAACGCCTTAGCAAAGCCTTCGGCAGTGCCGGTCTGAGTGCCGAAGAAAACAGTGACCTTAGTCTTGCCGGCGGCAACGTCAACTTCGTCGTCCTCGTCCTTCAGAAATCCCCTCGGCACAATCACCGGCTTCACCTCCTTGCTCCGATCCGAAGATTTCTTCCACATAAACAAGAGAAGGCCGATTATGACAGCGGCGGAGGTAGTGACGATGAGAAGCAGCGAGTCCGAAACGGAATCGGCGAGCGAAACGCCGAGGAGCGACTCAACAGCGCGCACCAAATCGGAATTGGAAGCCATCGATTCCACAATTCGGCGGTCAAATTCGGAGTACGGTGCAGGTGGTGAAGGAGACAGTTAAAAGTGCGTGCTCGATTGCCTGAGAGCGAGTGTGAGAGAAACCTAACAGGGAGTCGTAGTAGTTGTAAATGACTCCACGAGAGAGAAAGAAAGAAAAAAAGAGAGAGAGAGTGATGAGTCTATAATAGTGAGAAGAAGAGAGAGGAAAGGACCCAGCCAGAGCTAAACCAGAAACCTTGGGCCACTTTCC

>C102047_65

CGGGCTCCCTCACGTTCTTCTGCTGAGTAAAAAGCAAGTCACACACTCACTCACACAGAAGAGAGAGAGAGAGAGAGAGAGAGAGAGAGAGAGAAAGCAAAAGAGGAAAGAGATATCCCAAAAGAAAGAAGAGGAGAGAGAAATAGAAAGACAGAGAAAAGATTGGTTTGGTGTTGGGTTGTGTTGTTGTATTGTGATATGTTTACAAGTCCAGTCTAGTCTAGTCTTATTCCTAGCACCAACCTTTCTTTCATCTCAACTTCCCTTTCTCATTTCTCTTTTCTCATTCCTCATTTCTCATTTCTCATACACCATGCTCATCTTTCTCATCTTCTTCCTCTCTCTCTCTTCTGCTTCACAGCCTCGCAACCCCGAAGTCGAGGCTTTGATGAACATAAAAGCATCTCTTAATGATCCACACGGCGTGTTGAACAACTGGGATGAATACTCCGTCGACGCTTGCAGTTGGGCCATGATCACCTGCTCTTCTGATTACCTTGTCATAGGCCTAGGAGCGCCTAGCCAATCTCTCTCTGGAACTTTGTCTCCAGCAATCGGAAATCTGTCAAATCTTCGACAAGTATTACTGCAGAACAACAATATCTCAGGCAATATACCACCGGAACTTGGAGCCCTTAACAAGCTTCAGACATTAGACCTTTCCAATAACCGATTCTCAGGGGCGATTCCTGCATCTCTTAGTCAATTGAATAGTCTCCAGTACCTGAGACTCAACAATAATAATTTGTCCGGATCCTTTCCCGAGTCACTGGCAAAAACCCCACAGCTTGCTTTCTTGGACTTGTCATATAACAATCTCAGTGGACCATTGCCCAAGTTCCCAGCCAGGTCATTCAATATTGTGGGGAACCCATTAGTTTGTGGGAGCAGCACTACTGAAGGTTGCTCTGGGTCCGCAACCCTCATACCAATTTCCTTCTCACAAGTGTCATCAGAAGGAAAACACAAGTCCAAAAGACTAGCAATAGCACTTGGGATCAGTCTTAGTTGTGCTTCTCTCATCCTCCTGCTTTTTGGGCTATTTTGGTATAGGAAGAAACGACAGCACGGAGCCATCCTGTATATCAGTGATTATAAGGAAGAGGGAGTTCTTAGCTTGGGGAATCTAAAGAATTTCACTTTCAGAGAGCTTCAACATGCAACGGATAATTTTAGCTCCAAGAACATACTTGGTGCTGGAGGCTTTGGCAATGTCTATAGGGGGAAGCTTGGAGATGGCACAATGGTGGCGGTGAAAAGGCTGAAAGATGTAAATGGCAGTGCTGGCGAATCACAGTTCCAAACAGAGTTGGAGATGATCAGCTTGGCAGTTCATCGCAATTTACTTCGCTTAATTGGATATTGTGCTTCTCCTAATGAGAAACTTTTGGTTTATCCTTATATGTCTAATGGCAGTGTGGCGTCCAGGCTTAGAGGCAAACCAGCTTTAGATTGGAACACTAGAAAAAGGATAGCAATTGGAGCTGCCAGAGGCCTTCTGTATTTGCATGAACAATGTGATCCAAAGATAATACACAGAGATGTCAAGGCCGCCAATGTGCTTCTGGATGACTATTGTGAGGCTGTTGTTGGAGATTTTGGCCTTGCAAAGCTCCTTGACCATGCTGATTCCCATGTGACCACTGCTGTCCGTGGCACTGTTGGACACATTGCACCAGAGTACCTCTCCACCGGCCAATCATCTGAAAAAACAGATGTATTTGGATTTGGCATTCTCTTGTTAGAGCTCATAACCGGAATGACAGCACTCGAGTTTGGGAAAACGGTGAATCAAAAAGGTGCTATGCTAGAGTGGGTTAGGAAAATACTACATGAAAAGAAGGTTGCAGTGTTGGTGGACAAAGAGCTAGGAAACAATTATGATAGAATAGAGGTGGGGGAGATGCTGCAAGTTGCTTTACTGTGTACTCAATATTTGACAGCCCACCGCCCTAAAATGTCCGAAGTGGTTCGAATGCTTGAAGGTGACGGGCTTGCTGAGAAGTGGGCATCATCACATAATTATGGTAATCACGACATGAACCCCAGCCATAGCAACAATGGTAGCGATAGTTCATCCCGCCCTACCTCTGCTTCGAAACATGATGATGATGTTCATGACCGTTCCAGCATGTTTGGCATGACAATGGATGATGATGATGAACAGTCTTTGGAATCCTATGCCATGGAACTCTCTGGTCCTAGATAAAGAGTAAAACACAGGAAGATAGAAAAGGAGTTCACTTTGTCATGAATCTTAAATTAATTAGCTTTGGTTATTCTAGGATAATATAGTCAATCAAATGTATATATATATTCTAAAAAGTTTTGTCATATTGGGTTTGTGTTGTAAATAAACAGTTTACTACTAGCAGTACTAGTGCAGTTGTACCCACTTTCCTGTGCACTTGGTTGCAAAATATATATATAATGGTAGAAAACGCTGTTGGTGGGTGCGCAATACGCACATTAGACTGTGTATAGGGTTGGACAGGGAACATCATCAGATTCTTTGCTCTCGAGTGTGATGCAGCAGAGACAAGAGGGTTTGTTCCGCAGAATGATATTTGACTGAAGATGAAGATGGGCATGCATATCTTGCAGTGTCCTCTCATATGTCTTGTTCATGTATGTTCAAACTTGAAAGCATCAGTGTGTGATGGTCACAGTCTGAGCTTTTGAGCTGTTAAAGGTCA

>C102049_65

TGGCATTATTCCGTTGTCTTCCTTCTCCGTTGCTCACTCTTTTTTTTCTCTTTTCTCTCTCTTTCGCTGGAATTTTAACCTAGATCAAAAGATGACGGTGCTGTGAGAAAAGCGTGAGAGAAAGAGAGAGCGAAAAATTCACAAAGCATCCAAGGAACACGTGAATTCCGACATGAAACGTCTCAGGTTTTAACCCACCACGACAAAGTTGGATCTGTCGTTTACAACCATCGTCGATCTTCGCCGGAAACGACGCTTCGCTGAAACTACCTTACTCTTTCTCCTTACTATTTACAGCTCGCAACTTTCGCGTTCTTTTTGTGCTTCTGTGTTTTGCCTTTTTCCTGTTACTGTTAGAAGACCGTTACGCAACGGAGGTTTTGAAGTGGTTTTCTTGCTTCAGCTGCCTGATGAGGAGCTGATTTGTTGATCTATGAGGTTGTTGTTCTAATTTATCAAGAATGGATAAAGTTTCATCGGATTGTCCATACCCAGGATGCTTTTTTTGTGTCATGAAGGAAGGGAATCCGAGCAAGCGCAGGGCTAGTGTACTGAAATTCTTTAGGGAACTTCCATGTCAAGATGATGATGGTCAGGTTCTCCCTATCAGTGGCCTTTGGAACACGGCTATGGCACATCCAAACGACCCGGAATTCATAGAGCTAGGAATATTTGAATGCATGGCTGCTCTCATATGGAAGGGACTGAAGAACCGGCGTTGGCTTTCTCATGATCAAAATATATATATTCCTTATTATGCAGCTCATATAATTGGTTCCTACACAATGAATATGGAGGAATTTGCAGAAAGTGCCGTGCATGCTGGGGTCATTCCTCCCTTGGTTGAGCTTTTAAGAGGTAGACTAACTTGGGTTGAGCAGAGGGTGGCAGTTAGAGCATTGGGACACTTGGCTACATATGCCAGCACTTTTCCTGCCATAGCAAGCCATGGTGAAATTCTTGAGCTCTCCATTCAATTGGCAATGAGTTCCCTGGAGATAGTTTATTCACATTTTTACCAATATGTTGATAGAAGGCTTAGTTATCATTGTGATCTGCTCACACGTGGTATGGGTGGTGTTGAAATGGAGTCCAGGAAGGCTGAGGAATGGGCTAGTCAGTTGCAGTGTTGGTCCCTTCAGCTAATTAATTGCTTTGCTTTTAAGCATGAATTTCTTCCTACCATATGTAAACCTGAATTTCTGATAAAATTACCTGCCATGTGGGGTGGACTTGTGAATGAGAATTCACCAGCTGGTATCGGCTTATTAAGAACAATTTGTCATCAAAAGCTTGGTAGGGGACCTGTTGCCAGTTGCCCTGGTATTATTGACGCATTGTGTAATATTGCTCGGTCTTCAGATGACTGGCAGTATATGGCTATTGATTGTCTTCTATGGTTGCTTCAAGACCCCAATACATGTCACAAGGTGATCGATAAAGTGGTGCCTGTTTTAGTTGATCTTGCGGAGATTACAACTTTGGGTGATCACAAAAAGCTCGGCGATTCTATTGTTAATGTTCTTCAAGAGTGCATCCAATATCAAGGGTCAGGACGAAGCTCAATTAGTAGTCGTACTAAAGAACAGATTGAGGATATATTAAATTCAAAGCAGAGATTGAAATGGGAAAAGAATATGCCCAAAGAAGATCTGCATATTAAGCAGGCCGCAGCTCTGGTTGTCAAGCTTGAAGGAAATTCTTTGTTCTCGTCTGGAAATATTTCTGGAGCTGCGGCAAAATATTCTGAAGCATTGGCGTTGTGTCCTATGAGATCCAGGAAAGAAAGAGTTGTTCTATATAGTAATCGTGCCCAATGCCATCTTTTGCTGCAACAACCTTTGACGGCCATTAGTGATGCTACCCGTGCACTATGTCTCCATAAACCTGTCAATCGTCATGCCAAAAGCCTCTGGAGACGGGCACAAGCTAATGACATGCTTGGATTAGCGAAAGAGAGTTTATTAGATGCTATTTTATTTATAAACGAGTGCTCTCAGTCAAATGATCCTGATCTTTCACTGAGGCAAAATAAAGTTCCAGATTATGCAGAGCGTTTAGTTAAAAAGCAGATGCGTGCTGCTTGGTTATTTCGAGAGGCAGCAATTAAGCATGGTGGGGTCCATAGTCAGGGTGATGGTGGTGATATGTATGGCCCAGAGACTGATGATTCTGAATGGGAGACAGCTAGTGAAAGTGATATTGGAAATGATGGAAGGGATGATATGGGCGATGATGACGACGGTGATTGGAATAATGATGATGAGAGGAAAGATTATGAAAAACCTTCAATGAAAGATATAAAGCATGGATACAATGTGCAGCTTGCGGAAGAAGAACCTTGACATAGAAATCGTTGCATTTGGTGCAATTGTTCACCGGCATGAATGAAGCAGGGTTGGATGCTTTGCGGGCTTTAAAAGAGAAAATTCTAAGTCGGTCATTGTAGTAAGTGGCGGGTTGAAAGTGATGACTGAGTTTTTGGTCTTACTATTATTTGAGGTTGAGATTTCATATAAAGTTTTCCATGTTTCATATTTGTTCTCCTCATTTTTCTTGTCAAGTGTAAAAATTGAAGATTTCAAGTTTGGAGAACTACATAGCGTTTGGAGGATGTGAAGTTTTTTTTTTTTTTCTTTCGGAGGTTTGTGTATATAAACCCTTTGTGCTTAAGTTTTCTGAGTCCGTTTCCACTA

>C102055_65

TCTCTAATGTAATCATGTCCAATTCAAGACAAATGAAGAGTTTATGCACTGCCATCTGGCTTACTTTGTTCAGCATATGGCCATAACGTTAATACACATGCATGGTTTTACAACTCAATAAAAAGTACATAAAAGATTAATACACGAACTTTGAATGTATCACAACCAATTTAACAAGATAAAATATCTAAATTAGGCGAATAGTTACAATATTTTGGTCTCCACATTAGTGCAACAGCAAACATTCTCTTCTGCTACCTTATGCCAGCATCACTACTAGGGTACATCATGATTCTCAAGTCAATATTGCAGCACACCTTTGGTGCAATAACTTCCCACTGCCTTGCTCCTAAGCGAATGCCCTGGCCGGAAATCGTCTTCAACGTGTCTTGTAAAGGAAGGTCCAGAACAAGTGACTGCAGATGGTTCAGCAGCAGCTACTTCTTTATTTGCAACAGAAACACCCTCAGTTAATGAAAAGCCAAAGACTCTGCATCTACTTTTACACGAGGGAACCTCTTGACTGGCCCGAAGTGCTGAAATGGAATCATGTGAACGTGAACTTTCTAACTGATTATGCAAGTTTGAGACACCAATAGAATTTGTGCCTTCATGAGCTAGTCCACGAAGAGGCTCATAGGGGGTTGATACAAATTTTCCGCCTTTAACTTCGGAAGTGTATGAACGTTGCTGATGGAGTTTACCTTCCATCATCTGACTAATTTTGTTGTTATAATCAGAGTTTGAAACTGGATTGACAGCTTGCTGGAACATTAACACAGATGATGGAGACGAAACTTGCCCAGATGGAACAGGAGGATGCAAATGTGAAGAATTGTTACTCATGTGGGAAGACCATCCATTTCTGGATCTCAGTGCGTGGTAACCATCAAAAGGTCCAAAGTGATCATTTCCACAAGCCTCATCAATAGACAGGACCCTTCCATATGGTTGGCTTGGAAGTATTTCTTGACCTTGCAAGACCTTCTGGAATCTGAAAGATTCATTAAAGCCTATGCCATTGCAGGAAACATTGGAACTCACTTGTGGAATTCCAATGCTATTTCCGGTTGCAGCAATCCTGGGACAGTTTGAGCCAGGATAGCATCTCCTCAGATCAGATAGCTGGTGACTCTGACCATTAATACTATCAAAAGTAGCATTAACACCCAACATTTCTTGACCTTGCAAGACCTTCTGGAACCTTAGTGATTCCCCAAAGTCTGAAGCCCCAATTGCATTGGAAACTGGAAATTCTAGCTTGGTTGAAGCCAATCCAATCTTGGTCCTCTTCAAACCAGCTGATATCAAGTTATTGGCATTAGATGCAGAACCAGATGGCTCAATCTCCCATGGTGAAACCCTATTATGCCTTGTCGCTTCTAAGTCATCCCACCTTACCATTAGGCATCTCCATTTTGATCCAGGCCATCTAACAGGATCCACATCAGTAATTCCAACAATCAATCCTGTAAATCTTCGTTCTGCAGCATCTTCAGTTTCAAAACGCATCCTGAATCTTGTTCCAACTGAATAAGAATAATCAAGGCTCTTCAAGAATTTGTTGATAGGTATAATGAACTCAGATGGGCTGACCCTAGGATTATAGTGAATGCTAAAGGCACATCTTGCTGATAAAGCATTAACCACATCCATCAGACTGCTAAGATTCAATTGCTGGCCAGCAAGAGTTGAAATGGTACTGCCACTTTTCAATTGAGCAGCTCTACGAATTCCCAATCTTAGTTCTCCATCCTCACCCCTAAGAAACAGAACAGCATCTCCAGATACAAGTTTCTTCTTGTTCACAAATGCACTCCACCCAGTGGTAAGCAAGTGTCGTCGCGGTTGCCCCCTGTAAATATGTCGAAACCTCCATTCTTGGCCATGCAGATCCTTGGCCACAAGCTCCTGTGAAGGTCTCTGTTGACTGTAATTCAGGGGTGGAAAGCAATCTTCTGCAGCACGACGAGGCACAGAGAAACCACCATGAGTGCTAGTATCAGAAGCAGTCAAAGTCTTGCAGAACATGTGGGGTGTGGTAGATTTCATCACAGCTTCAACATCCTCGTCTTCACCATCAACATCAATTTCCCCTTCCCGCAACTTCTGATGCGCTTGCTCACTTTCGGGAACCAACACCACCTGACAATACACTTCATCGCTCCCTTCCTCAGCATGGAGTTTGACATCGAGAACGCGACAGAAGAGATGGGAGGGGATGCTAGCAGAAGCAGGGAGCGGAAAATCGTGAAGGTGTTGTTCCAAGTGTCCTTGAGGGAAGTACACGACAAGGGTCCCTTTCTTGGGCAAGGAGATCATAGGACCCGCGCAGGCGTGCCAGAGTTCGAGGCAGACGGAGGAAGGATGAGACGATGGCGTTTCATGGTCCTCGCTGGCGCTGTTGAGATCGATGAGGGCAGGCATGAGGAGGAGGAAAGAGGGAGAAAGAGAGAGAGGAGGTGCAGTAGAGACAGAGGAAGAAGAAGAAGCACAGGAGCAAGTGAGTGGGGGGAGATATTTGAAGGAGTGGTTTCATGCCTCCTGCATTTTCAGAGAGCGTGACCAACTTTGGCATTGCCAAAAGAACCTGACCCAATACTCCAAAAAGAAAGAGACCCTCTTTCTCTGTCTCTGTCTTTCTCTCCCACTGACCTATCACTCTCTCTTCATTTTTCCCACCCTTTTTGGGCTCTTC

>C102057_65

ATGGATGCCATGTGTGAGGGACATTATAATTATTGGACCCTTTATTCACTGGAAGTAGTCATACAAAGTTTTATTAAGACTAGTCAGGGAAACAATCTTCACAAAAAATTAGATAGAGCCATATACATATATATTGGAGTAACATATAAAGCTAAGCTGTATACATTAACTGCCACATTCCCCACCCCAAGGAACTTGTAATAGATACAACAATTCTCTGCCAAGTTATTACCATGATTGGGGCATTAGACAATGTATAATTTTACAGACAACAACATACTATCCAACTTCCTAAATCTCTTTTGCCACCTCCCATTTATTTCAGAATAGACTTTTCAGAATGTAAAAAATACATTACGGAAAATCAGTTCCAAAATAAATAGGAGGCGGCGCAAAAGATTTGTACGGTGCAGGATAAAATACTCCAAAATACTTTGGCCTAAAATACAAATCAGCATACGGTGCGCCCATAGCACAAACAAGCAAGGTATAGGTTTTTTGACCATTTTTCCTTAACATGTGTGCTTGGAAATTGAGATGTACGGAGAGTTTCCTGGGTTTCATCACCTGGTCTGCGTCTAGGAATGCTCAGTATCAGAGCAGATTGATCCCATGTAGCAGCAGTTGCTGTAATCCTATAACCATTATCCCATCTTCTGTGGATTCCCTCACTAGGATAGAGGAAATCAAGTTCAATAACCTGATCAGTGTATCCAGAATTTCGAGACATAACAACACCCCAGCGACTTCCAGCAGTGGCCATTGAGGTCACATGAAAACCCTCTCTCCACTTCTTGTTTATCCATTTGAATGGGAATGAATCACTTACTTTGTATGATTGTTGTGTATAGTGTGTGCCTTTTGACATCACAACAAGAGAGCTCCCATTATTAGCCCCTGCAATAGAAGTTATGTAATAGTTTTTTTCCCACTGCTCCATGATCCATTCCTTATGTAAGAAAAATGGTGACAACTTATAAACTTGACTAGAAAAACCAGTTCCAGCATCCATAATAAGTGCCCAGAGATTGGAACAAGAAGCCACACAACTAATAAGTAGGCCATCTGCAATACCTCTCTCCACATGCTGAGCTAATCTTGCATCCGCTACATTATAATGATACCTTTGCTTCATTGGTTGTCTAGCATTGTAAACTGAAATCCATTGTGTAGCAGGAACCCCCAAACGGACCTTCTTTTTGGGCTGTGAATCATCTTCTTCCTCAATATTCAATCTACCCCTCTTTTGCCCAACCTTTTGAGCACCTTCCGTATTAATTGGCCTCAATGCAGGATTTGGTCCAAGCATACCATCAAACAAAGAAATTAATTTAGAATAGTTAGGTTCTTCATCGAATTTCATGTTCACGACAATCTCAAGGAACTGCCTAAAAGGTGCAGGGCAGAAACAGCATAGCATCTCAGGAGATGTTCCCATCTTTTTTTTGCAAACAAGGAAGGATTTGTTATCACCCTGATAACCTTGCCAAGGTAACCTGCCTTTATGAAGGAAAATGAGTGTATATGCAAGAGATTCAAGATCATCCCTTCTACTAGCAGTTCTTCCCAAATGAGCATGAACACTAGCATATCGAACAGTCCCTCTAAACATATCAGGACGTTGATCATATTCAACATGCTGCCCGCTGGAGGTGTCTCTCCATTTTGTAGCTAATCCAAGGTCTACAAGATACAATTTCTTTTCTTGTGGCGTTGAAGGCTGACCAAGTAAAAAGTTCTCCGGTTTTACATCACCATGCACATAGCCTCTAGAGTGCATCTTCTCCAAGATTGACAAGGACTCCACTGCAATGCAGGAAACCATTTCTGCAGACATCGCCTGACTTGAGGAATTCCATACATCCCACAAGCTGGGACCAAGCATGTCCATAACCATTACGTAATATTCTCCTTGCCTCCCTTTATAGTGAACTTTAGGTATTCCATGACTGCCACCAAGGGTGTTGTATACTTGCCACTCATAAGGGGGGCCATAGTTACAGCCTTTGCTGTTCCTATGCTCAAATTTCAAAGCCACCTCAGTGGCACCAGGACCAGCTGTACGGTCATTACCACCGGTTACTCGACGGCCAACAAAAACCTGTCCAAATCCCCCTTTGCCGAGTTTCCTCTCCACTTTGTAGACAGGCGACCCTCCTACTTGAACCCTCTCCGGGAAAGGAGCTGGGTTAGCGTCATCTTCTTGTGCAACACCCTTATTAGCACTCAGGCCACCACTCTCATCTCCCATCGCGCCGCCTTCAACTTCCTTCTTCGTGTGATCGGATATAACAATGATCGGCTTCTCCTCTTCTAACTGTTTAGCCGCCAAACGAGTCCTCGGCCTCTCCGCCGCCGCCGCCACTTCCCTGGCAACTGCAGCGCGAGTCTTGACGTATCTCCCAGCTGGAGACGGTGGTTCCAAGCTTCTACGACCAACTGGAGCACGACGTCGTCGCGGTCCACTACGAAGCTCCGGCATTCTCCAAACCCATGCAATCGATTCGCAACGCTAAGATCATTATATGGTCATGAAAGCTCCGACTTGCAATTCATAGATGGAATTGTTGGCAAAGCAAAGTAGAGAGAACAGGAGAAATGGATGGAGGATTAGGGTTAGGGTTCGGGTTCGGTGGTGGGGATGAGGAAAGTGCAGTTTAGAAGATTAAGATGAAAAAAGAAAAGGGACACAAAATTCCAA

>C102061_65

CTTTGCTCTCCAAAGACAAATAAACACAATTGCGAGAAACTGTAAACGCGGCGGTGTCGTTGGCCATGGAAACAGCAACAGCAGCAACCGCTTCTCAGAGAAAGCTGAAGCGGAAGCGAACCGTCGCGAAAAGTGATCCTGAACTCGAGCGTATCAACTCGCTTCCATGGAACTCCTCTCTTCCTCGAAATGAGGATGATGACGCTTTCTCCATGTTCATTGGCTCCAACGAACTCGAAGGAGGTTTTCTTTCGCTCGAGGAGATTGATGAGGCTGATTATGGTTTGAATATTCCTGAACCTGAAGATGGCAAGAGGAAGACGAAGAAGAAGAAGTCTATAGAGACTGAAGATGGGAAGAAAGAGCAGCAGAATGGAGTTGATGGTGCTAGTAGTGACGATGTCGAAGCTGAGTTGGACGAGAGTGTGAAGGCTAAAGAGAAGAAAAAGAAGAAGAAGAAGAAGAAGAAGACGAAAAAGAAGGATGCTACGGAAGAAGAAAAAGTTGAGCAGCCAGATGCTGGTTTAGATGCTAATGTGAAAGATGATATCGGTGAAGAGGACAATGATGAAACTGAATTTTATGCATGGAATGAGTTGAGGCTTCATCCTCTCCTGATGAAAGCAATATGCAAGCTTGGTTTCAAGGAACCAACACCGATACAGAAGGCTTGCATTCCAGCTGCTGCTCATCAAGGGAAGGATGTTGTTGGGGCTGCAGAGACAGGATCTGGTAAAACACTTGCGTTTGGATTGCCCATTTTGCAACGCCTTTTGGAGGAGAGAGACAAGGATGCAAACATGGTTGAAGAACCTGAAAAGTCTTCTCGTAAAGGCCTTTTGAGGGCTCTTATTATTGCTCCAACTAGAGAACTTGCACTTCAGGTCACTGATCATCTGAAAGCAGTTGCCAAGTATATTAATGTTAGGGTGACCCCTATAGTTGGGGGGATTTTGGCAGAAAAGCAGGAGAGACTTTTGAAAGCAAAGCCTGAGATTGTTGTTGGGACTCCAGGAAGGTTATGGGAACTTATGTCAGCTGGAGAAAAGCATCTAGTTGAGTTACATTCAGTCTCTTTCTTTGTGCTTGATGAGGCTGATCGGATGGTACAAAGTGGTCATTTTAAGGAGTTGCAGTCTATAATTGATATGCTTCCCATGTCCAATATCTCCACTGAAGATAATTCTCAAAATGTACAAAGTTGTGTTACAGTTTCTAGTTACCAAAAAAAGAAAAGGCAAACACTTGTTTTTTCAGCAACTGTTGCACTGTCTGCTGATTTTCGCAAGAAGCTAAAACGTGGCTCAATTCAACAAAAACAATCATCGACAGATGGATTGAATTCTATCGAAACTCTTTCTGAGCGTGCAGGAATGAGACCCAATGCTGCAATTATTGATCTTACTAATCCGTCTATATTAGCAGCTAAGCTTGAGGAGTCATTTATTGAATGCAGAGAAGAAGATAAAGATGCATATTTGTATTATATTTTGACCGTCCATGGACAAGGTCGCACAATAGTTTTCTGCACGTCAATTGCAGCTTTGCGTCATATTTCTTCCATTCTGCGTATTCTTGGAGTCAATGTTTGGACTCTTCATGCCCAAATGCAGCAGCGTGCACGATTGAAGGCCATTGATCGCTTTCGTGAAAATGGAAATGGCATACTTGTTGCTACAGATGTTGCTGCAAGGGGTCTCGATATTCCTGGTGTTAGAACTGTTGTCCACTATCAGCTTCCACATTCAGCAGAGGTTTATGTTCACAGAAGTGGAAGAACAGCCAGAGCCTCTGCTGAAGGTTGTAGTATTGCTTTAATATCACCGAGGGATACGGCAAAGTTTGCTTCATTGTGCAAGTCATTTTCTAAGGATAACTTCCAGCGGTTTCCTTTAGAGAACTCTTACATGCCAGAGGTCCTAAAACGATTGTCTCTTGCACGGCAAATAGACAAGATAACAAGGAAGGATTCCCAGGTAAAGGCTGAGAAAAGCTGGTTTGATCGGAATGCTAGCTCAGTGGAATTAGTTACTGAAAATTATGACAGTGAAGAGGAACTAGTGAACAAACATAAGCAAATGAAGAAAAGCTCTAAAGAATTGAAGAAATTGCAGGAGGGGCTCAATATGTTGATTTCACGGCCATTACAATCAAAATCATTTTCACATCGGTATTTAGCAGGGGCTGGTGTCACACCCCTCATTCAGGAGCAATTGCAACAATTATCAAGGCAAAAACTAAGTGATCACCAGGGTTCAGGTCTAGGCAAAAAGGGAAAATTGGTTGTGATTGGTCAAGATTGTGTGGATGCACTTCAGGCACTTCGAAGTGCTGGTGAAGAGGTGCGAATGGATATCAAGGATTTGGCTGGAAAACAGAAAAATGTGGAGAATTTAAAAAGAAAGCGAAAAGAGGAGAAAAAACGTTTGCGTGATCAGCGTAGAAAGCAGAAGAAAAAGCTAAAATACGGGGATGAATATAATTAGAATTGTATACATAACTGAGCCATTAGGTAAAGTTTTGCACCAACCAAAAATTGTCATAAGTTGCTCAATTTTTCAAGGCAGCCTCTGTAGTTTGTCCTGCCTAGCTTTTTAGAATTTTGACTCTGTTTGAATATATATATGTATCATACAATATCCGGTATCGTGTAATGGATGTGATTATGTGGTTGAGTTTATATTAATGTCATAGGAAACCC

>C102065_65

GCAATAAATAAATTTGTTATCTTTTTGTACCTATTATCTTCCTCTTCCTCCTCCTTTTCCTCCTTCAACAACACAATCATTTCATTTCTATATTTTGGAACAATTATTTCTAAACACACAACACAGAGACAACAAACGACTGACTGGAACAACATCAACATGGTGAGATAAGAAAGTTGGATCAAATTTCATTAACCAAGAAAATCAAAACAAAAAGAAAAGAGAACCAACAAAACGCAGATAACTGGTTGGGTCGGGTTCGGATTTTTTCGATCCGCTTCTCTGGTTCAACCGTTATTGTTGCGGTGATTGTAGTGGGCATGGAATCTCGAAAATTCATCATTTTTTAGGTTAGGTGTTGCTAGGGTGAGGTCTGAATGGCTGGGATTGATGATAATGTTGCCCTTACTGGTGATTGGGGTCTTGCTAGCCCAAGTCCAAGAACATTTTTCTCTAGAATGTTAGAGGAAGATCATGTAACAAGATCAATCTCAGAACATTCTGGGAGTGGTAGAAATGGGGAATTCTTTTCAGGACCTCATGAACCTAGTGAGACAGGGAGGAGGAACATGAAGGATGGGGCACAAGATGGTGGTGATTCTGGAGCCCAATTGACCAACCCAAGTAGCTTTCAGACTGAGCAGAAGTCGAACACCCGGGGTGGTCTTGTGGAAAGAATGGCTGCTAGAGCTGGGTTTAATGCTCCAAGGTTGAACACTGAAAGCATTAGATCTACTGACCTTTCGCTCAATTCCGACATTCAGTCTCCTTACTTGACCATTCCGCCCGGTCTCAGTCCTACTACACTTTTAGATTCTCCAGTGTTCCTTGCAAATTCACTGGCACAGCCATCTCCAACAACTGGAAAGTTCCTGTTCATGTCAAATGGCAACATGCGGCACTCAGAATCATCGTCTGATGTTCCAGAAAAATGTGAAGATAATGGCTTTGATGATATTTATACGTCATCCTTTGCTTTCAAGCCTACAACAGACTCGGGCTCCTCTTTTTATCATGGCGCTGGAAGAAAAATAAATCCAACTACACTTCCTCAATCCCATCCTAGTATTGAGGTTTCGGTTCAGTCAGAAAATTCTTTTCAATCTCAAAGTGTTGATGCTGTCAAAGTTCAAACTGAGAACAAAAGTGGTCTTCATCTTCAGGCAGACTTTGTTGAGTCGCCTCCTGAAAAAGATATTAAAATTATCCCGGCTGATCAAAGGGCTTTTGATGCTGTTGGTGGCATTGAACATTCTACACCAATTGAAGAGCATGGAGATGAAGAAGGAGATCAAAGAGGCAATGGAGATTCAATGACTGGTGGTGTTGGTGGCGCACTATCTGATGATGGATATAACTGGAGAAAATATGGCCAAAAGCAAGTTAAAGGCAGTGAGTACCCTCGAAGTTACTACAAGTGCACACATCCAAACTGTCAGGTTAAGAAGAAAGTAGAACGATCCCATGAGGGCCATATAACTGAGATCATATACAAGGGAACGCACAACCATCCAAAACCTCCTCCAAATCGACGGTCCGGTATAGGTTCAGTTAACCCTCCTACTGACATGCAAGTGGACAACCCTGAACATGTTGAACCACACAATGTTGGTGATGTCGACATGGGCTGGGGTAATGTACAAAAGGGAAATATAGCTGCAGCTGCTAACTGGAAGCACGAAAACCTTGAAGCAACATCATCAGCATCTGTTGGTCCTGAATACTGCAACCAGTCCACCACTTTGCAGACTCAAAATGGTACTCACATTGACTCAGGAGAGGCAGTTGATGCCTCGTCTACTTTCTCTAACGAAGAGGATGAAGACGATCAAGGAACTCATGGTAGTGTATCATTAGGTTATGATGGGGAAGGAGATGAATCAGAGTCTAAGAGAAGGAAACTGGAATCATATGCAGACCTGAGTGGAGCTACTAGAGCCATCCGTGAGCCTAGAGTTGTTGTACAGACTACCAGTGAAGTAGATATCCTCGATGATGGTTATCGGTGGCGGAAATATGGACAAAAAGTTGTCAAAGGAAATCCCAACCCAAGGAGTTACTACAAATGCACGAATGCAGGGTGCACAGTAAGGAAACATGTGGAGAGAGCATCTCACGACCTTAAATCCGTGATAACTACATACGAGGGAAAGCACAACCATGATGTTCCTGCTGCTCGTGCCAGCAGTCATGTCAATGCCAATGCTTCCAATGCTGGCCAAGCTTCTGGTGTTCTTCAGACACATGTTCATAGACCTGAACCATCTCAAGTACACAATGGCATAGGAAGGCTTGAGAGGCCATCCCTCGGCTCGTTCAACCTACCGGGGAGGCAGCAGCTAGGACCTTCCCATGGCTTCTCCTTCGGCATGAACCAGTCCATGCTATCGAATCTGGCGATGTCAGGGTTAGGCCACGTGCAAGCAAAGCTCCCTGTCATGCCTGTTCATCCATTCTTGGCACAACAACAACAGCAACGCCCTTCAAATGATTTTGGTTTCATGATGCCAAAGGGAGAGCCAAATGTGGAGGCTATTCCTGAACGTGGTGCTGGTCTTAACCTACCAAATGGCTCATCAGTGTACCAAGAAATCATGAGTCGCATGCCCCTTGGACCTCATACGTAAAATTTCATCCCCTGATTCATTCTTCAAAGAAAAAACTAATGTTTACATCTTATAACAAACATATATATATATATA

>C102067_65

ATTTAATTCCTCATTCCTCACAAACATTATACGAGAGGCACGGAAATAAAAGAAAAATAGGTCGGAATAATAATAATCATTATTTTTTCTTTTACTGTCTACAGGTAGAAATAATTGGGACGACAACTAACTAAGTAGAGATTAACGGGCTTGCAATACATGGGTTCGGTATTCTCCCAATGCCCAAATGGGGAATATGTTTCTGTATGCAGAGTATGAGATCATGCAATTCTTGTTGAAGACTCCCATTATTTCCTGTTGCGGAAAGTCACCGTTATCCATTTGGGAATTTATCAAGTATGCAGCTGCGCGGTGCAATGGTGTCGGATCCCTCTTAGCCTGTCCAGCATCAATGAGAGCCAACATCGCCCACCCAGTGTTTACCACATGAGATCTGTTGCCTTCCAGATTTGAATACACCTTATTTTGACAGGACAGATAACTCTCTGCCCAGCCACCAGAAGGCAGCTGCTTAGACAGCAGAAAGTCACAACCTCTCCGGATGCTTGAGCAATTACTGAAACTCCTTCCAGCAGCAATCAATCCTTTTATCCCAAACCAAGCACCATAAGTGAAGCAAACTCCCCAAGAACCATACCATGAACCATCTGAAGCTTGTATTTTTTCAATGAAGTTAGTGGCTTTCTCAATACAACGTTGTATTTCTTCTCGGCGATGCCCGGGATATAATTTCCTAAATGATGCCAAAGCTTGAATTGCTGCTGATGTACATTCCACATAAGGATAATCAATAACGATGTCACCAAAAGTTTCAGCAGGATTGATTATCTCCAACCAATTATAAGATCGTTTAAGCTCGTATGTTGCAAAACCACCATCTTCATTCTGTAGTGAGAGAATGACATTTACAGAATCATACAATCGCTTCACCTCCAATGGCTCACCAACTACATCTGGTGCAATTTTGGACAATAATAGAACAGCTTTCAGTCCTTCAGCTGTGCAATCAGAAATTGGCCATCCGTGATCCGCAGTTGAAAAAGGCCAAGCACCTTTTGAAATGTGACGATACCATTTATTAAGATCACCTGGACAATCTTCTAAAACCTGTGAATTCTTAATATAGGTATGAGCTTTTCTTATAGTTGGACCAAATTCTTCAATGAGGTTACTTGAAATAATTGCTTGGACAGCAAAAGCAGTATCCCATAGTTGACTTCCATTGTAGCCCTGCATTTTCATTCCATCTTCCGCAATCCATAGATAATCATGAATCCTTGGAAGATGCAACTTGAAGGCCTCAGAATTTGGATCTTCCACCCAACAGCAAAGCATATTTAGCACCTTATTTACAGGACCTATGCAAATATATCGTGTATTCTCATCTTCATAATGTATATGCTCCATTGCTGAACTAATAGCCTTTTCCCTCAACTTTTTTCCAGGCCAATGCATCAGAACTGGCTCAAGGAACTTGTGTAGAGATGCCCAAAGAATATCCTGCACGAGTGGGTGAGGATAGTACAAGTCTTCCTTTGCACACAAATTGCGAGCCTGATCCCAATCTATATCATGATATGGTACAGTATAAAGCTCTTTTCTCAAAGATAATATTGTTGGTGTGATTGGACCAACAAACCTCTTTCCATATAAGTAAGACATTGGCAAATAGACCATCCGGCAGTGACACCACATCCTTCCTGGATGAAATGGCAGCGAGTATGGAAGGAGCCATATCTCAGGGGGCAGAGGATTATTTCCAGACCATTCGTACACTCCAAGTACTGAAAGCCACATCTTCCCCCATGACGTTATACAAGTAGCACCACCATGCCCTAGAATCCAGTCACGTGCCTTCTCCATCTCCCCTTCCCCATCATTAGGCCCTTCACCAAGCAATCTCAGAGTAACATAACTCAAGACAGAGCCGAACATGGTGCTTGGACCTTCTATATGCAAACCCCACCCACCATCCTTATTTTGATGGTTATAGAGGTAACGGCACATTTCCTTTCTATGCTCTTCAGTTAAGACCGCATTCAGTGCCCCAGTGATTGACAGAGTAATTACCAAGCCAGGCATCAGAAACATGGGACCTCCGTAATCTCCCGGCCAATGTCCGTCATAACACTGGAGAGTTGAATGGAAAGTTACAGCCCTTCTTAATGTTCTTGTCACAATCTCCTCTGTCACATCCTCAATATCTTTAACTCCGACTTTGGGTAAGACTTCATGTATTGGAGGATTCTCTCTCGCAAACTGGAAGCGCATAAGTAGATCAGAGCTGTGTTTGTTGGTGAAGCGATTGTCGTGAAAATTCTGGCGAGCTTTCTCAATCTCGAGGAGATCTTGCGGCGATCCGAGCTTAGGATCGAACTCCCAGACCTGTCTTCCAACGTGATTGTTGAGTGAGCGAAGCCAAGGACTCCCACCCTCGGCAAACTTGAGCTTCCACATTGTTGTTTCTGTGTTGGCTGGGAAGATGGATCAGAGAAATGAAAATGGGGAAGGTGAAAGTTAATGCAATGAATGGTAATTGACTTAGTATTTGCTTAGAGGACAAGATTATAGCATCTGCTTGCAATGCATTACAGAAGGCAAAGTGGAAATGTTCAAATCCCTGAGTTTAACTTATAACCAACAACTGTTGCCACCAGAGCGTGGCGGAGCTTGCAAAGCACCAAGAAAACTACTGTTACTGCGATGATGGAGGATGGATGATGGTTGATGGATCATTTGTG

>C102069_65

CGTAAACCTGATATTCGGCGAAGATGCGGCAGAGATACCCATCGATTCCAACACACAAGACAATAAATAAAAGGGTGTTTGATGAAGTTTGGTAAAGTAAATAGAAGCTGAACCACCAACGAACCCACCCCAAACCCTCCTTCCAGAACTTTGATGTCCATCTTGCCCTCACAAACCCAAGCGTCATCTTCGTCCTCTTCCTCTGCGCCATCTCCCAACCCTAATCCCCAACATGGCATCGGAATCCCTCTTCCTCAACAACCCTTCCCTTCTCTCTCCCCCCCACTCCACCACGCTCTCGGATCTCTCCAGATCTCCGATAACCCAGGTTCATCGACTCAAGCTGCTGAAGAATCTGGTGGATCTTCCGAAAAGGTGACAGAATTAGGGTCTCCAAGTGGACCGATGTTTTTACCTCAGCAAAACTCTAGAACACGTTCTAGGAGTCACGGGGGAAGACGGGCGGGAATGTTTTCTTCTTATAGAAACCAACAAGCTCCTGGGTCCATGAGTTCTCATGGAAGTACCCCTTTAGCAGGGAGAAAAGCTCAGACTGTGAACGGAAATTACTTGCTAAATTTTCAATATGACCCCATATCGCGTTCCCAATCAGGGGGTCCCCCTCCTCCCCCTGCAACAAGAAGGAATTGGAAGAGGAAGCCATACAATAAAGACTTGTTTTTGCAGGCAAATTACAAGTTCATGGTGCTAGATTCAGGAAATTATGCTCCTGAGTCAATGGATCCAGATAAAATGTTGCAGTGGGAAGATATTATATGTGTGACATATCTGACCCCCTTTTCAATTCAGTGTCCAATTTGTTTGGAGTATCCACTGTGTCCGCAGATAACCTCATGTGGACATATTTTCTGTTTCCCATGTATTCTACAGTACTTAATGATGGGTGAGGATCACAAAGGTGATAGCTGGAAACGGTGTCCTTTGTGCTTTGTGATGATATCTGCCAAGGATTTATATACAGTGCACATAACAAATGTTAAACAATATCAAGTAGGAGAAAATGTTGAGTTCACCTTTTTAACTAGGAAAAAGGATTCATTTACTCTGTCACATAAAAATAAACAAGACACAAATGTCACATCATGTGGTAATGGAGATTTCTGTGATCCCTTTTCTAAGTTTACGCTTACATCAGATGTAGATCTCTCGGTAAGGCATTCAATATCAGATCTAGATGGTTGGCTGGCCAGAGCTGATTCTGGTCTCGTTGATGACCTGGAGAAGCTTCCTTATGTGTGTGCTGCAATGCAACAACTGGAACAGAGGAAGAGGTATTGGAATGAGCAAAAGTCTCATGACAGTGAAAATTCTAAGCTTATTGATCATGGACATCAGATACCATCTACAGCTACAAATTCTATGGATACTGATGATGAAAACTGTTCTAATGGATCAAGAACTTCCTCTACTGATTACCACGACCAAATTAAGGTTACGATGCTGGATAAGTCAACTGCTGGAGCCTGTTCAGACCAAACTTTGGATGTGGAGAAAGAATTAATAGAGCAGGAAATGAATTTATCTTCTTCATATGAGGAGAAAAATGGTATTCAAAGGCCTTCAGATGATGGCGTTATTGAATTGAAGGGAAATGATTCATACAGTTTCTACCAGGCTGCTGATGGTCAGCATCTAATTCTTCATCCTCTTAACATGAAGTGTTTGCTCCACCATTATGGGAGCTATGATATGCTTCCACATAGAATAAATGGAAGGATTCTACAATTGGAGACAGTTACTCAGTCTGAGGCCATGAGGAGGCGATATCGATTCCTAAGTCATTTTCCATTAACCACAACATTTCAGCTTTGTGAAGTTGATTTGAGTGAGTTGCTACCTCCTGAAGCACTGGCTCCATTTATGGATGAAATAAAGAAACGTGCAAATCAGAGGAAGCAACTTGCAAAAAAGGAACGGAAGGAAAAAATAAGGGCTGAAGCTACTGATACCTATTCCCTTCCCATATCATTAAGTAGTCAGTTTACATCTCGAGATGATCCTCCAACATTCTCCATGGATGACTTTGAAGCTCTAGGAAATTCATCTGTATCCTCAAGTCCTCCCATAGCTGGGGAGAGAAAATCTTTCTCGAATGTCACAAGGCTTGGGTTTGCTGCTGCTCATGATTCTCCATCCTTGCAAATTCAAGAAACTAGTGGTCTACATAATAACAAAATGATCATTGATTCTTCTGCCACAACAGGTTCGAGAAACGGGGAGACACAGTCATACTCTAATGTTATATCAAGAGCAGAATCAAATGTAAGCTCGAATGCACCAAAGACTAATGAGTTGGGGAAGAAAGGAAAGAAGCCAAATCGAGTTCTTCTGTCAACAGCTGGAGGTAGACGTTATTGATTACTTGGTAGATGATTTACTCCCGCCTAAGCCAAAATATTTGGCAGATGATTTCAGTGTAGAGTTGTTAACGTTCTAAGGATTACATTGCATGTACTTTATGCGCCTCTAATTACCTTAGTTGTAGATAGGTAGATGTGTGTCGAGAATAAATGTTTTCAGCAATAAACAAGCCATGAAGTTAGAAGTTTAGTTTATTGTTTCTGTGGCTCTGTAGAACTGTGTGCTGTCTTTGCAGATTTTAATTGAATTCAGACTCAATGAGTACTTGTAAGAAAATACTCAAATGTTTAGCATATATGGGCGTTCACTAAAATTGGAAGCG

>C102071_65

TTTCACCTCCCACAATCTGCAGAGCTTAGAGTTGATTCTCTCACTCTCACGAACTTCCACTCGGCGCAAGTTCATTCAGCAACCTCTGAGATGCAGCAGCAAGCAGTTGAGCACCTCGATGTTCTCACCGAAACCGGTGTCAAAACTGGCGTTTCCAAGCCCAGGGGAGACGTGCATCGCGACGGCGACTACCACCGCGCCGTTCACGTGTGGGTATTCGCGGAGCGGACACATGAGTTGCTTCTCCAACGCCGCGCGTCGTGCAAGGACTCATGGCCTGATCTGTGGGACATTTCCAGCGCCGGTCACATTTCCGCCGGCGATTCGTCGCTCACCACTGCTAGGAGGGAGCTTGAAGAAGAGTTGGGTGTAATCCTTCCCAAGGATGCATTTGAATTGATATTTATTTTCCTCCAACACAGTGTCATAAATGATGGAAAATACATAAATAACGAATTTAATGATGTGTATCTGGTAACAACTCTTGATCCTATACCTCTGGAAGCCTTTTCACTCCAGGAATCAGAGGTCTCTGCTGTTAAATATATCTCTTACGAAGAGTATAAAAGGTTACTGGCCAAAGAAGATTCAGATTATGTCCCTTATGATGTAAATGGACAATACGGTCAACTCTTTGATATAATCGAAAAGAGGTACAAGGAAAATACTGTTGCACGCAGTTTGACTCTTCAGAAGCAAATAAGTCGTTATGCTCCTGTTTCCCTTAGTTCAGAGTTGTCTGAGCTTACAGATATGGACAAAGAGGCTCTGGGTTTTGTTGTCAAGGCTGCAGCTGTTATGGATGAAATTTTTCATCAACAGTCATGGTACAGTAATCCAGCTCTTAGAGATTGGTTGAAGGAGCATGCTGATACATCAGAGTTAAATAAGTTGAAATGGTCCTATTATCAGATTAACAAGAGCCCCTGGTCTTGCCTTGATGAAGATGAGGCATTTTTGACAACTGCAGATTCAGCAATAACGTTGGTCTGTAACGCAACTAGGAAAGTAAGAGACTGGAAGGGTGTGGAATACAGAGCAGCATTTCCCATACTGAAACCTGCTGGTGCTAATTTCTACCCACCAGACATGGACAAAATGGAGTTTGAATTATGGAAGGACAGTCTAGAAAAAGATGAAGAGAAAGAGGCAACAGGATTCTTCAGTGTTATTAAAAGGCACAGTGAATTTATTTTAGATTCTCATCAATCTGATAACAAAGCAGGTTCTCATGATCTATATATAATTCCTTACTCTCAAGAGTACCAGCCTCTTCTTGCAAAAGCTGCTGATTTATTGCATAAGGCTGGGGACATTACCAACTCGCCAAGTTTGAAGAGGCTGCTACATAGCAAGGCTGATGCTTTCCTTTCAAATGATTATTATGTCTCAGATATAGCCTGGATAGAATTGGACTCTAAGTTGGATGTCACTATTGGGCCATATGAAACTTATGAAGATAAACTTTTTGGATATAAGGCTACCTTCGAAGCATACATTGGAATCAGGGACGATGAAGCAACAGCTCAATTGAAACTTTTTGGTGATAATCTGCAGCTTCTGGAGGAAAATCTCCCTATGGACAATGCATACAAATCAAAAGATGTGAAGGCAGCACCTATCCGTGTTATCCAACTTCTATATAATGCAGGGGATGTCAAGGGACCACAAACTCTTGCTTTCAATCTACCAAATGACGAGCGTATTGTAAAAGATCGGGGATCATCAATGGTCATGCTTAAGAATGTTTCAGAGGCTAAGTTCAAGCAAATTCTTCTTCCTATAGCTGCTGGCTGTGTTGCAGAGGAGCAACAAGAACATGTGGATTTTGAATCATTCTTTACTTTCACAATTTGCCATGAATGCTGCCATGGAATTGGACCGCATACCATAACACTTCCAAATGGCCAAAAATCTACTGTCAGATTGGAATTACAAGAATTTCACTCAGCTCTGGAAGAAGCAAAAGCGGACATAGTTGGCCTTTGGGCATTGAGGTTTTTAATCTCCCAGGATTTGCTTTCTGAAAGTTTATTGAAGTCAATGTATGTTTCTTTTCTTGCTGGATGCTTCCGCTCAGTACGTTTCGGTTTAGAGGAAGCTCATGGAAAAGGACAAGCATTGCAGTTCAACTGGTTATATGAGAAAGGAGCCTTTGTCTGGGATTCTGAAAGCACAATTTCTGTTGATTTTAGCAAGATTGAGGAAGCAGTTGAAAGCTTGAGCAGGGAGATACTCACCATACAAGCCAAGGGTGATAAAGTGGCTGCTGGTTTACTTCTCCAGAAATACTGCGTGTTGACCAAACCGCTGAAAGTTGCTCTAAAAAAGTTGGAGAATATTCAGGTACCTGTTGATGTAACTCCAACCTTTCCCATTGCCAGCAAAATATTGGAATGAAGGCAAGAGAAGCAATGTGCAAAATTTTCCATGCTACCATCTTTCTGTCAGCCAGAAAAGAAAGGATATTGTCTCTTTGAGCTAGTTCTTACTATGAAATGATGGAATACTTGTTTCTTCCGGGTGTTGCTGTTTTACCTACCGTTCATTGATTCGAAGAAAAACAGTAACATTTTAGACGTTGTTTAATTACAATCTTGTTATTATGCTTATGATGAATGATCCAAGTCATTGAATCGTTTCAATTCCAAAGTTTTGATTCCTTTCTTGTTGATGGAACCTTGGTGGTGGAACATGTTAAGGG

>C102075_65

GTTCTTTTCAATTATTCATTCACATTGTCCTCACAAATTACTATTTATTCCTCACAAAAAAAAAAATCTTTTTTCTAATTGTTTTCCCTGTCTCTTCTGTTTCATAGCTTCTTTCTGGATTTTGGTTTCAGACCCACAAACACAGAGTTGGTGTTTGTGAAGTTTGGAGAAGAAAATGACTGTGGAAAAGAGAATGGGTGATTCCGGTGAAGAGTTTCCCGTCGGAATGCGTGTGCTTGCCGTTGATGATGACCCCATCTGCCTCATGCTTTTGGAAACTCTCCTCCGGAAATGTCAATATCATGTAACCACAACAAATCAAGCAATAACAGCTTTGGCGTTGTTGAGAGAGCACAAAGACAAGTTTGACCTTGTAATCAGTGATGTGCATATGCCAGACATGGATGGATTTAAGCTGCTTGAGCTGGTGGGACTTGAGATGGACCTTCCAGTCATAATGTTGTCTGCAAATGGTGATACGAAGCTGGTGATGAAGGGGATTTCTCATGGAGCCTGTGATTATCTGCTGAAACCCGTGAGAATGGAGGAGCTCAAGAACATTTGGCAGCATGTAATCAGAAGGAAGAAGTTTGACTCAAAGGAGAAGAACAAAACCAGCAACTTGGACAAACCCACTTCTGATAGTGGCAATGGACATGGGTCATCAGGAGCCGTAAATTCAGATCAAAATGAGAAGCTGACTAAGAAACGGAAGGATCAAGATGAAGATGAAGATGAGGAGCAGGAGAATGATCATGACAATGAGGACCCTTCGACCCAGAAGAAGCCTAGGGTGGTTTGGTCTGTGGAGCTGCACCGCAAGTTTGTTGCTGCTGTTAATCAGCTAGGCATTGACAAGGCTGTACCTAAAAAAATTCTTGATCTGATGAATGTTGAAAAGCTTACAAGGGAGAATGTGGCGAGCCATCTCCAGAAGTATAGGCTTTATCTGAAAAGAATTAGTTGCGTGGCTAACCAACAGGCCAATATGGTGGCGGCCTTAGGGGGTGCAGATCCATCCTATTTGAGAATGAGTTCTGTGAGTGGGGTTGGACATCTACAAACATTGAGTAGTTCTGGACCGCTTCATAACAATGGTTTTAGATCCTTTCCACCTGGTGGGATAATTAATAGATTGAACACTCCTGCTGGTTTGAACGTGCATGGATTTCCTTCTGGAGTTCTTCAGTTGAGTCAATCACAAAATTTAAACAACACTAATGATCATCTTAAGTTCCAGTCAGCTATGGTTCCTGTTAATCAGAATGGTGTTCAGGGAATGCCAATGTCTGTTCAAAACAATAAGGGTCTTATTTCTGTTCAAAATTTGACCACTGTTTTTAATGCCAAAACAAATTTTCCCATCTCAAATAAACTCCCAGATCCAAGACCAAAAATAACCACCTCTGGCTCTCGCACTCCAGATGTAAGTATCTCAAACAATCACTTGATGTTGGAACCATGCCCTCAAGGCACACAAGGGAGTGTAAGAATTGAAACTTTATCTTCTTCAGTAGCCTCTCAACATTCAGAATTTTCTTTACCTTTACTGAATCAAGGTAGATATAGTGATAGTTGGATAAATGCTGTGCAGCCATCTGTGATCCAGACAAACCCTTACCCTCCAAGTGAATGTTTTAGACAGACTAATATACCTCCTCCAGATAATATGGCTTCTGTACCCTTGCAAGGTGGTAATCTGAGTGGTCCATCCATTACTTCACTGTCCAGGCAATCCCATGATTCAATGACGGATATGCACTCTGAAGGAGTGACTTTTACCAACAGAGCTGGGCATATAAGCAGTAATGTGCCATTTCAAGGGTGGGATGATCGTAATCAGGATGCTACTCACCATTCAAATAATATATTTGCATCAATAAACTCGTTGACTCCAGTCAATGGTGCTACTGTTCCAGCAGGCCATGCATTACCAAACTCAGCCTCACATAGAAACTTTGACTTCAACTTTTGTGATCCCTTACAAATGAAGCATGATGGATTTGTGGAATTAACTGAGGAAATCTCATCGAAACAACATCAGGGTAATATCCTGAACCAGCAGAAGGCTCAACATAGTCATTTCTCTAGTAATCTTGGTTCATTGGAAGACTTGGTCAGTTCAATGATGAAACAGGAGAAAGACAAGCTGAAATTATTGGATGGAAATCTCATTTGCGATAATTACAATGGTGGGTTATCTATGTGATAGAAGATTTGGCAGCACATACCAACATATGCTTCCACGTTGATGAGTTTTGTAGTTTTTATTATGTTTAACCAAATTTGTCTTGTAGTTTTCTGTAGAGTTTAGCTTCTCCAGATTACTTTTTACTGGTAAAGTTCTTTAGGTTTCTTCCATCAAGGGCTTTCTAATTTAATACTAATGCATTGTTGTCCTAGCCTTTGGGTCCGGCTATGCATTATCAAGTTATTAATCACTTTATTTAATCTCTCCGTAGACATGTTGGCCTGCAAATTGGACCTAAATCTTTACTCACAGTCATGCTTGCACCGAGAAGAGACAGACACAGGGTCAAGGTGGAACTCACCAGACAAATGGGTCCCACTTGAACCCTGGGTTTTTCTCACACCTTTCTGGAGAGAGATTAGGGATTGCAACTTTGTTAAATGTTAGGGATCGATAAACAAGATTATAGTGATTATGATGAGTGACAATAAACACGGATTCCCCCTTTAAAAAAA

>C102077_65

AGATCAAAGTGTCTGAAATATAGCTTTAGATTTGAACGGCTAACTGAGCGTATAGATGTTTTTCATTTTTCTGAACTAGCAAAGACACTGTTAACGGTAATCAGTTTTTCTTTGTAGATAGAACATAATGATGCAACCTAATTTCATTTAATCACCAAAATGCTGCACTTCGATCACAGAAGCTGGAAAATACCAACACCCTTGCCGCAATTATTTTGTCAAAAATCACTGCTTGTCCCCCACGTTTTAGGGTTGAAGTTTTCATTTTACTACTGAGTCACGTTGACAGAATGCATTTTCTTTATCTTTTCCAAAGCACAACTTGTTTCTTTGTCACTTCAACATGAAAAGAAAGCTGCTATAAAGCCACTGGATTCTTCCTCGGCTGTCTCCAGCTCCGGTTTACTTGTTTCTGAAAACAATGTCACAGTTTGATAACAATTCAGATTGAGACCTTGACTCATCTTTCATCTTCTTTTCCTGGGCACAAGAGAAATTTCCCCTGGGTGGTTGTTGGAGTATGGCTGCAGCATATCTTCCGAGTCTGAATCAGAGATTACCGTTATAATCCCAACCGGGGTTAAGGTAGAAACTGAACCTGAAACAGGTTTCTTCTGCGGGTTTGATGTTTCAAATGCTACACTTGGAGTTCCTACAACTAGTTTTGACTTCTTACTATTGTCAAAAACTAAGAGGGATGAAAGATTGTCTTCTTGCGCGATCTCCTCCTTAACCTTTTTATTAGAATCGGGCTTCCATTCTGAAGTTGAATTGCCCAGCAGTGGCGCATCAACTGCACCCGTAGGCAGTTTTAGCACCTCTCCATTGCCCTTCGTTGTACAGAGTGCAGTAACGTCTGGAATAGTGGGACTGGCCCTTGAGAAATTTGAAGTGAGACATTCCTCCTCCAGGTCACAAGAAAACAAACAATTCTGTGAAATATCAACACAAGTGTCATTTTCAATTCCAATGCTTCCTGTTGCTGCACATCCAACCTTTAAGTTTAGCGCCTGAGGTACTTCAGTTCCATCCTTAACGGGTACTACATTAGACAAACTTGCTGCATGTTCTTCTTTCAAACGTAGTGCTGGCTCTTCAAAGGCAGAAGCATTTTGCATAGAATGTCTGACAATGTTGACAATGACATCAAGGCTGCGCTCAACATAAGGGAGCTTTGACCTTCCTACTATTCGCCGTAAATTGCTTATAATGACAGGCATCTGCTTGGCAATTTCAAGCATAATTTTATTTGGCAATACATAGCCAGGCCCCTCATCATCTGCACGAGCATGACCATCACGCCATTCACACAGCCCCGAAACAACGGCAAGCTGCTGAGCATTAAAACCAGCCCCTCGCAACCCATATATATGAAGGTACGAGTTTTCTGTCAGCAGGTCCTTCTCATATAGCTGCATGCAGACATCATAACTGCGCTTGTAAACATCCTGTAGAGGATTATCACCATCTTCAGACTCCTTACACAATGTAAACAATTTGATCCTCATTAAATCATATATATAAAGGAGATAGTGTGTATCCTCTCTGCCATATCTCAGCATAACATCAGGAAGAGGGCGTAATCTCCAATCTGCATTCTGGTATTCCTTGTTTGCAGTAACACCACAGAAGTGTTGCAAAAGATATTGCAGACTGTATCTTTCCAACTTTAACACTCTAGAAGCCTGGCCAGTATCAAACATATTACAAACATAAATGCCAAAATCCCGTTGCAGCCACAAAATATCTCGATCTGCTCCATGCATGACCTTCTTCTTAGTAGGGTCCTTGAAGACTTCCCTCAGGTATGGCCCAACATGATTACGAACCTTCAGAGTGTCTATAACAAAATCTTCAGTCCTGGTTGAGATTTGCATCAAGCAAGTCAAACCTTGGAAAGACCGATAATGATTATGTTCCAAATCCACCGCAAATTCATTAACAGAAGACAGCTTAGCAGCCAACTCCTTCAATCCTTTGACTTCCTCCACAAGCATGAAAGGGGTACATTCTATTGAGGGAGGCTTTACTGGCACAAGATTCTCGATATTTTCTTTATCAACAAAATCCAAAACAGATAGTTTCTCCAGTGGATGAATAAACCTCTGACCATCTTCACTCTTCTCCAACAAAACATGCTCAAAAGGCTGGTTTGCATTATCAACAACAATATTGTACGCATACTGAGGCTTCATAATAGTCGGTATATGAAACGGCACTTTCGTCTTCGCCTCCAACTCGTTTTCACAGTCCTCTCCCTTCTCCTTCTTAATCCTCCGCAACTCGTCCACCAACTCATCAACCAGCTCCAAAACGTCGTCGTTCGCGTTCACAAGCCACTCGTACGCGTCGTCGACGTCAGCGGGAAATGCCGAGCCTTTGCCCCAAATATTCTCCGCGGAACCGATTCTGTCGAGCATCGACTTGGTCCTCCGCGCTATTTCTTCGATCGGGCGTCGGAACTCCGCGAATTTGTAGTAGAAATGAAAGTCCTCGCCGGATGGAATGCATGGCGAGGAAGAGGAAAGCTCCGTTAGGTGTTGATCGAAGTCCATCATAGTTCGTGAAGAAGATAGTCGAAACTTCGATGAAAGGGTTTTATAGTTAGGGTTTCAGGGTTTTCACCATTGTCGATGGAAAGGAAATGAGAAACCAATGTCGCTCTCTCGCTACTCACTGCTTACGTTTGTTTCAGTTTCAGACGAG

>C102079_65

GGGTATTTTCTAACCTTATTGAATAAAAATATAAAATCCGTATCCTGTTTTATTTTCTTTTTTTTGGTGGCAGGATGGGTTTCTGTAAGAACATTATTCATTACAGAAAACCATCTCTCTTTGGTGTTATATTTCAAAGAAAACATACACACAATTTCATATTTAAACTCGGACTAGAACCTGTAACCTGTAACTTAACATCAATAATATACTACGATCTTCCTACTTCATCTGTATCGGCGTCTTCTTTCTTCGTGCCCAATTTGACAATGTCTTCCACCGATATTTTTCCATCGCTATCCTTGGAAAGGTTGCTGATAAGTTCATGAATTCCTTCTTTGCCCAGAGTGTCCTTCAGATACATAGCAGCAGATACAACCTCCTCGGGTGTTACTTTGCCATCATAATCCCTGTCTAGCAACCGCCAGCGGTCTCCAATTTTAGCATCAACATCATCAATTTCTTTTTCAAGTGTCTGGAGCATAGTATCAACCCTGTCAACAAGTGCTGAAGAAACCTTATCACTAATAGCCGCCTCAATAGCACCATCACTTTCCTTCCTTGCAGCTTTATAGGCCTTCATAGCTGCCTGCTCACCTTCTGTACCTTCTTTCCCTACCATACTATCATAGAGGTCCATCTCCTTTCTAACAAGCCTCAAAAACTCTTCACGTTCCCTGCTCACCGAAGATGCTGATGCTAAAACAGCTAATGCGCGGCTGAGCTCCCAGAGGTGCTCCTGTTTATCCAATGTCTTTGTCTTTACCTCTCCTTCTGTTTGTTTGGTTGTTGAAGCCCTCTCCTTCATAGCCACATCCCCTTCACTACTAACAGATTCTACCATTTTGGCCTGTTCTGCCTCCTCTTTCTTTTCCTCCTCCTTGATTAGCTCCTCTTGCATTTCAAGATATTCTAACTTCCTCTTCCTTTCTGAAACAGAATCTTCAGATGGCAAAGTTGTTACCCCAACAGTATCTACAACCTCATCAGGCAACGAAGAGAGTGTAGCTTGAACAGCTTCCTCTGGCCTGACCTTTCCTGAAACAGAAAATGCTCTAGAAAGAATTAAGAGGGAAGATGGCACAGAATGGTTGAGAGACAAATCCAGCCAGTCCCTAAGCTGCTGTCGCATTTCTTCCACTGAAAGCAATCCAAGTAATCCTCGATCTCTACAAGCTTGACGAAGCTCTGCTTCCGAAAGAGATTCAACACCCTCAAGTTGAATCAGTTTATCATCATTCTTGATCTCATGTAATCTTTTGCGGAGCATGTAACGCAAATATGCATCAGTTCCATATGGGCTGATGCCCATATATTTACACATATTTACCAAGCGAGGCCTGCTAATATTATCCAGGGTGAGCTCATCATTAAATAGTTTGGCAAATCCTAAGATTTCATCATTAGATACTCGGGCTCCTGTTCTGACTTTGTTCATAAATTCATCAAGATCTTCAGCTGTCTTCTTCATTTCTCCACTTTGCGAATTCTGTATCTCCTTTGCCATTTCTTTTACAGTATCTTGAAGAAACTTGGCATATTCTATTCTTGCATTTAGCCTTCTTTTCAGTGCCTCCTGTTCTTTCATCTTGTCCTGGAAAGTGGAGGGTAGCATGTTGGGAAACAATTTAAGGAATACTGGCAACAAGAACTCCATGAATGGAACTATGATAAATACAGCGACTGGAACTAGCCTGAAAATATCAGCTGTTGTCCTTGTGAGTTGCTGCCTCTCCCTTCTAGAAAGATTCTTTCCACTGGCAAGTTTCAACATTAATCTGGAGCTTATCCTAATATCAGCCCAAAGTAGTTTCGTACCAAACCAGTAGTGTTGCAGGGTAGATTTAAATTCATCCCACCAATGGCTAAACTTCTTTGCCCAGTCCTCCCTGCTCATTGACATTATAGTTCTGAAAGCTGGACCAATTCCTAAAATCTTAGCCCATAACTTTTTTATAATAGAATCAGCACTCTTCTGTGTTTCTTGCAACTGTTTAGCCTTGGCTTTTGCCTTTATGGTGCTCAAGCCTTCTACAGCTTCATCACATTCCTCTGGCGAAGCTTCCTTCTTTTGTTTAGGAGCTGATCGTTCACTTCCCCTATTACTGCTACCCAGTCCAGATTGATCAGCTGCAGCCGTAGAAATATAACGAGTACATTGGGATATCCATCCCAATCTTGAAAGATAAACTAACTCTATTTTGTCAGATCTAAATCCAGAACTTGACACATTAAAGGAATTATGTGAGATAAATCTTGAAGCTGCACAAGCTGCTAGATCATCTTTCTCGACAGTAAATAATTTTTTTTCCTGTACATGTTCTGTAGTCACAGATGAACAGAAAGGAAACCGGCTCAAACCTTCTAACTCACTAGATTCCAATGGCTGACGATTCCCACCACTCAAGAATCCAAAAACAATGCGACTAGGTTGACTACATAGAGGGTTGAAGAAGAGGCTTCTTTTCTTTTGCAAAATCACTCTTGAAGCCATGACAACAAATTCTAGCTTCTGATGTCAGTACTCCCTAAGCAGAACGCTTAATTTCAATGTCATTGATACAAAGAATGCAGAACTTTATCACCGTCGTTCAGAAAACAGCGCACGGAAGATAGGCGTTTTCACGGCGAAATAATCAGTGTGAAAATGGAGAGAAGCGCAGAAGTGAGCCCAATCGAGAACCGGAGAGAAGTCTTCAATTAAAT

>C102081_65

CGCGTTTTGACTAGGCTTTTTTGTGAAGTCTTCGTGTTCTTGAGAGTGGGGTAGATCCATTGAAGACACAGTTTTTGTTCTTGTTCTTGTTCTGTGAAATGGCATCCTCGTCTTCTTGTGATGTTAGGGAAATCAAATATGATGTTTTTATCAGCTTCAGAGGTACAGATGTTCGCAGAGGTCTGCTCAGCCATTTGAAAAGAGAATTGCGTCGCAGGCAGATTGATGTGTATGTGGATGAGAGACTTGAAAGGGGAGATGAAATATTACCCTCACTTTTCACAGCAATTGAAGAATCACAGATATCATTGATCATATTCTCCAAAGACTATGCTTCATCCAAGTGTTGCTTAGAAGAACTTGCCAAAATTATTGAGTGCAAGCAAAGAAATAAACAAATTGTGCTATCTGTTTTCTTTAACGTAGATCCATCAGAAGTGAGGAACCATGTTGGAGATTATGGAGTTGCTTTAGCTGAACATGAAGAAAAACTGAAGGATAATGCACCCAGCAAGCTACAAACTTGGAGATCTGCTTTGAAAAAGGCTGGTCATTTATCAGGTTTTCACTACCCAACAAATTTTGAGGATGAATCTGATCTCATTGATAAAATCGTGGAAGACATTTCAGAGAAGTTGAGCAAGTTTTGTCCAAGTGAAACAAATGGTCTCATTTCAATTGATCAAAACGTTGTCCAAATTCAATCCTTGTTGATGATGGAATCAAAAGATGTTCTTTTGGTTGGAATTTGGGGCATGGGTGGCATAGGTAAGACGACCATTGCCCGTGCTATATTTGATAAATATTCTCTTCAGTATGATGGTTGTTGCTTCTTGAATGTTAGAGAAGAATTAGAACGACATGGACAGATGAGTCTTTTAAGAGAGAAACTTATATCTGAACTATTGGAGGGAGAAGGTCTTCATAAAACTGGCATAAGCAGAGCAAGATTCTTCAATTCTGCTGGGAGAATGATGGGTCGAAAAAAAGTTCTCGTTGTGCTTGATGATGCGACTACTTCGGAACATATAAAATATCTGGTTGGTGAACCAATTTGCTTTGGTCAAGGGAGTAGAGTTATCACAACAAGTAGAGATAGCCATGTGCTTACAAGTGGAGGAGTTCATCAAATTCATGAGGTCAAGGAAATGAATGCTCGAGATTCTTTCAAACTTTTCTGCTTAAATGCATTCAATAAAAGCCAACCCAAAATGGGATATGAAAAACTAACTGAGGAGGTAGTAAAAATTGCCCAAGGCAATCCGTTGGCTTTAAAAGTTTTAGGTTCAGATTTCCATTCCAGAAGTATAGAGACATGGGAGTGTGCCTTAAGAAAAATCACTAGATATCCCAATGAGAAAATTCAAAGTGTGTTGAGATTCAGTTATGAAGGACTGCATGAAGTAGAGAAAAAGGCATTTCTGGATATTGCATTATTTTTCAAAGGAGAAGACAAAGACTATGTCATAAACCAACTGGATGCTTGGGGATTTCATGGAGCTAGTGGAATAGAGGTTCTCCAAAAAAAAGCTCTGATAACAATTTCAAATGACAATAGAATACAAATGCATGACTTGATAAGACAAATGGGTTGTGATATAGTTCGTCAAGAATCTACTAATTATCCTGGGAGACGCAGTCGATTGTGGAATAGCGAGGAAGTACATAATGTATTGAGATATAATCTGGGAACAAATAAGGTGGAAGCTATGAAAATTGATCTGTCTCAAATTACAGATTTACCTTTGCATGTTGATGCTTTTCAAAAGATGGCTGGTCTAAGATTTCTCAAATTATATTACCCCTTGCATGCTGAGCTCTCTCTGCCGCCTAGCCGTGCTAAAGCAATGTTACAGGACATGGTGGATCCAAAGAGAAGGAAGTTTAATAGATCCAACATGGAGGTGGAACTCCCACAACTCTCCATGGGGACAAATCTATGTGCAGATCTCAAAAGTTGTGATGGAGCACACAATTTTGTGAATTTATGCTGCAGTGACATTAGTAGATCTAAACAGTTAATAGAACTGTCAAACTTTTCAGAGGGAATCAAACTTAAGAGTGAATATCTCTTGGAGTGTGAAAATATGTGCGATGCGCATGCACCTATTTTATCATTGCAAAAACAAATTTCGTCTCTTTTATCTGGGTGCCAAGAACTCATGAACTCAGCAAGCGAGGTTCACATAAAATGTCTTTACTACTTTCTTATTGATTGCTGCTCAGGCCCCAAGGAATTTTCAAGAGCCTCAAAGGAAATGAAGAAGCTATGCATAGCACGTCCTGGAATGGAAATATTGAACTCATCCATGGGGCATCTCATGATCAGCAATGTTGAAGACTCAGATTGGGACTGTGAACAAATGAAGAATCTACCCAAAGACCAACTTTGCTTGATATCTAGCTATTACCTTAAGCTTAAGAGCACGGGACAAAGCAGTGGAAAACCCAAGCTACATATTCTATGCGATGGGTTAAGGTATTATCAACGAATTTCTTTCAGAATGTTATATAAATCACCTTTCTTTGTTCCTTCAAATTGGTATGTTTTATCATGGTGCCGTGACGTAATAATCGAAGGGAGAGCTGGGATTTTGCTGTTAGCTAAAATTCCTCCATGGCCTAGTTACCCCGTTCGGATTCGTATTAAGTAACTACGTGATTATCTCCGGATTAAGGAATAAACAGAAAATACTACCGATATATA

>C102083_65

CTCCACCTATCTTTCCCACATTTTCTTCAATCACTTCACTCTCTTTCCCTTCCTTTTACTCTCCTCAACCAATACCTCTACTCCTCAGTTTGTCTAGTAAATGGTCTTGAGGGTATAACATCTTACTGAAGCTCCAAGCGATTTTGGAATATAAGTCACATAGGAAGAAATCAGCATGCGTTGGTTGGGTCTCTTGGTTTACGTGTATCGTCAAGTTTTACGCTTGAACTTATATTGCAAAGTTATTCTATAGGTCCCCAGTTCCATCAATCTTTTTACTTTGTTACGTTTGGATTTTGGTTCCTTCTTCTTAACTAAGGCTATGGAAGACAACCATTATTTGATAACTTCTTCAGATAAATATAGAATTGGCTGAAAGATAGGGAGTGAAGATAGAGCCTGGGATTTGTGTCTTCAGTATTGTTGTTGTCTCATATTTCTGTAGTTCTAGCTGATTTTGATTTTATAATTAGCTTGGTTCCTTCATTATCACATTCCATTGGGGCTTTTTGGGGTGTGCATTACATTTTACTGTGGTAGGATCCAAAAGAAGTTATGGTGTGCCAAGCAGCGAGCCAAACAAGATTCCGGGCTTTGAAACATGAGAATGGCATTGAAGGTGAACCAACAATTATTGTTAGAGTGATAGCATGCTTTCAACCTTTGCACGATTGTCAGGCTGAGTACTTCCGTCATTTGCTTAAGCCTGTCACGTAGATTGCTTTGCATTTATTTCTCTTTAAGCTGGAATTTGTAGTTTTCTGTTACTGTCATTTGTTCCTACAGAAGCAGCAAATCATTTAAAATCCCATATCAAGACGCCATAACATCTACTGGTCATTTTTTTCATTGAATGGTTAAGGCTCACAGGTCTTGGTCTTACCCACAGCACGTGGCTTGGCCATCACCTTACTTAAATAGCTCCTGCACCTTAGCAGAGCCAAGTTCGTGGGTTCATTCAACGTACATGAACTCTAGCACGTGCATCTTTCCTGCTGTCAGTATATTTCCTGGGTTCACAGCTCCTGCTGTCCCGAATTTGAAGACTGAGCTGACCAATCAAGTGCAAGGATTCCTTCAGCATCCCAGTTCAGAACCATGTTTGAATGATACACGTACTGGAGGGGCTATGCAAAATGAATCTTTGCAGAAGAAGTTACTTATTTTTGACCACTCTGGTAGTAAGACAAGGTTGCTTTATAGTCCCGTCTTCTCTTTTGTCCAGAGTCCAACTGTTACTGCTACCCAACTTACTCAAGTTTATGATGTAAACGAAGAAGCACGAGAAACTAATATTGGCCTAAAGCATTGGCCCATATACACTTCACCAGAAGAGACTGGTAAGGATCATATAGATAATGAAGAAAGCGAAATGCATGAAGACACAGAAGAAATCAATGCATTGCTTTATTCTGATGATGATGATGAGGAGGAGGAGGATGAGGTAACAAGTACAGGTCATTCTCCATTGGTTACTGAAAAGACTTGTGTGATTCAAGAACAGTTTGGGGATATGAAGGAGGTTGCTAACTCTGATTGGACAAATAAAAGGCAGAAGTTAATTGATGGTGGATTCAATATATTACCACCACTTGCTGTCAATGCTAGTTCCGAAAGGCTAAATAAAATGTGTGAGTATGCCAGTGATGCTGAATCAAACTATTCTAGTAGTGGTGAAGTGTGTGCTACAACGCAAACTAAAGAAGGTAAATCAACAGCTGCTGATATTCAATTGAAAAAGGATAAAATCCGAGAATTGTTAAGAGTTCTTGAGAACCTTATTCCTGGAACAAAAGGAAAGAATCCCCTCTTAGTCATCGATGGTACTATTGAGTACTTGGAATCTCTGATGTCCCAAACTGGTACACTGAAATATCACTAAGTGCTCATTGTGGTGGTTACAAAATGGTTTAAGGACACATTGATGGTGAAAAAAGTTGTGTTGAAGTATTAGATTTGTTGCATTATAAGTTTGAAGTTAGCATCCTTGCTTCTTCGATCCTCTTTCTCGAGGTGTATTTATAGTTTAGGAAAAAAGGGGGTGAAAGCAGCAATGGGAAGTACTACACTTTGCCACTACAATATGGGGTTTGGGATTTGAGGTTGATGACCAGTATATTTTATCTTTTAGCTTTTGGAGGATTAAAGGTCAGAGACTGAATGGATGGGTGATGGTAGTGGATGGTTGTGCTGTAAATGAGGACACTTTTTTTCTCCGGAGTGTTGAATTCTCTTGTTGATTGAATGCATGCGCGTTAGGTAACTGGGGTCCCACTGTCCACCCTCCCTTTAATTCCTGATTTTATGCTTTGTCCTCTCTTCCAAGCTTGGCTAGTACACATATCATGCTAGCCGAGACTATAAAGTACCAAGAAAGTTCGACGTTGGGGCGTGCTTGAATGAGGAGTGTGGTGTTTCATCATGACTTGTTGGGGCCCTTTCTTTGGTACACACAGCATGTTGTGGTAAAGGTTTCTTTGTTTTACGTGTTGAGGATGGAATCATAGGTGATTCTATTTTCTACTTTGTAATTGAGAAGTGGCTTGAGTATTATAGGTTTCTTTTTCTGATGATATTATGTATGCATCTGTCGGGTGTGTGATTGTATGAATAATTGGAGCGTCAATGTTTGTATTGGCCTTCATATTTTCTATGTTGTTTGTCATTGTCCTATGTGTTGAATAATTAAATACTACCAATCCCTTCA

>C102089_65

ATACAACAACACAACCAATAACCGCACCTTCCAAAATTCCTGATAAGCTTCGCCTTGTTTCTCGAAAAAATCTGAGACAGAGAGAGAAAAAGAAGACAAAGCTTCAAGATCAGTTTCCGTTCAGCGGTTCAGAGATGCCATAACCAATAACTCACGCCACTTGTTTTATTTTTTTGAAGAAAAGAAAAATTATGAGCTGGCCGTGCTACCCAATGCGATTTTAATTCATGGGGTTTTAATGTCCGTTTAAATCTTCAATCTTTGAGGAAAATAACGCAACCCCGTCTGTTTGTAATTTGTGGGGCAAAAGTAAACGGAATAAGAAAAAAAGAGAAACTGGAAAAGGTGATTTTTTTTTCGAGAAAATTGAGGTTTGAAGCTTGTAACGTGTTGTAGAAGATGTCTTCGCTTCGTCGGAGAAAAGGGCCGGAAATTGAACAGGTTCCGAGTCCGAGTTCGGAGCCGAAGGTTCTAAGCGAAGAGGAGGAAGAAGATGATAAGAATAAGAAGAGTAAGAAGATGAAAGATGAGGTGAAGGAGAAGAAGAAGAAGAAATGGTCGTGCTTGGATAACTGTTGCTGGTGGGTTGGGTGCATTTGCACGTTGTGGTGGCTTCTTCTGTTTCTGTATCAGATGATGCCTTCTTCGATCCCTCAGTACGTGACTGAGGCCTTCACTGGGCCCATGCCTGATCCACCGGGCGTTAAGCTCAAGAAGGAGGGGCTCAGGGCGAAGCACCCTGTTGTTTTTGTACCGGGGATTGTCACTGGGGGGCTTGAACTATGGGAGGGTCATCAGTGTGCAGAAGGGTTATTCAGGAAACGCTTGTGGGGTGGTACCTTTGGAGAAGTATATAAAAGACCTTCATGCTGGGTGGATCACATGTCACTGGACAATGAAACAGGATTGGATCCACCAGGCATAAGAGTCAGGCCTGTCTCTGGACTTGTAGCTGCTGATTACTTTGCTGCAGGGTACTTTGTATGGGCAGTCCTAATTGCTAACTTGGCACGCATTGGTTATGAAGAAAAAACCATGTACATGGCTGCATATGATTGGAGAATAGCATTTCAGAACACTGAGGTTAGGGATCAAACACTTAGTCGGATAAAAAGCAACATAGAACTTATGGTTGCTACTAATGGTGGGAATAAGGCAGTTATTATTCCACATTCAATGGGTGTGTTATACTTTCTTCATTTTATGAAATGGGTTGAAGCACCAGCTCCAATGGGTGGTGGGGGAGGACCAGATTGGTGTTCTAAATATATAAAGGCAGTTATAAACATTGGTGGACCATTTTTAGGCGTTCCCAAGGCTATAGCAGGACTTTTCTCAGCTGAGGCCAGGGATATTGCTGTTGCCAGGACAATAGCTCCAGGTTTTTTAGATAATGATCTGTTTCGGCTTCAAACCTTGCAACATGTAATGAAGATGACCCGTTCTTGGGACTCAACAATGTCAATGATACCAAGAGGAGGTGATACTATATGGGGTGATCTTGATTGGTCACCAGAGGAAGGCTATCACCCTGGCAAGAGAAAGCACAGCAATAACAATAATACTCAGTTAACGAAACAAGAGACAAATGAAACAAATTCTGTTAACTATGGAAGAATGATATCATTCGGAAGAGATGTGGCCGAGGCACCCTCCTCTAAGATTGAGATGACTGACTTCCGGGGTGCCCTCAAGGGTCGCAGTGTTGCTAATACCACTTGTCGTGATGTGTGGACTGAATACCATGAAATGGGAATTGAGGGAGTAAGAGCAGTTGCTGAACATAAGGTTTACACAGCTGGCTCAATCATAGAGCTGCTTAATTTTGTTGCTCCAAAGATGATGGCTCGTGGTAGTGCTCATTTCTCTTACGGAATTGCTGACAATTTGGATGACCCTAAGTATAATCACTACAAGTATTGGTCAAATCCATTGGAAACAAAATTACCAAATGCTCCTGATATGGAAATCTTCTCTATGTATGGAGTTGGCTTACCAACTGAAAGATCTTATGTGTACAAGTTAACTCCCTTTGCTGAGTGTTACATTCCTTTTGAAATTGACACTACACAAGATGGTGGTCATGATGAAGATAGCTGTCTGCGAGGTGGAGTATACACTGTTGATGGGGATGAGACTGTGCCAGTTCTAAGCTCAGGCTTCATGTGTGCTAAGGGTTGGCGTGGAAAAACAAGATTCAACCCTTCTGGCATTCCCACCTACGTTAGAGAATATGATCATTCTCCTCCTGCAAACTTGCTAGAAGGAAGAGGCACTCAAAGTGGTGCTCATGTTGACATCATGGGAAACTTTGCATTGATTGAGGATGTTATAAGAGTGGCAGCAGGGGGCACAGGAGAGGATCTAGGAGGTGATAAGGTTTATTCTGATATCTTTAAATGGTCTGAGAAAATCAAGTTACCATTGTGAAATCAAGCACAATGTCTCTTCTCTTGAAAGAATCACATCAGATTATTATAATTTTTGAAGTATAGTTTCACTGCCACCAGAGTTCTATAAGTTGCACATAGAATTTCTGGTTCTGCTGATCAAATTGTAGTTCCTGGAGGGGTGAACTATGCTAATTGGTTATATTGAAAGTGCTTGTATTGAATATTATGGTTGAGATTTGATTTGTCTTCTATGAGAAACCGAGCAAGGGGCATTATTCTTAATAAGTTCAGCTGGTTGTTTATGATATTGTCCCT

>C102093_65

GCTTCAACCCACTGAGAGAGAAAGAGAGAGAGAGAAAGAGAGAGAGAGAAGCCGCCGTTTCTTCCATTCTCCGCCAAACATTCAACCACCGCTAACCACCACCTCCAACAACCGCAATCTAATTCAATTCAATTGAATTAGGGTTTGAACTTCTTTCCCTCTGCATGGAACCCCAACAAACTCTCCTCCCCATGGACGACGCCGAAGACCCTTCCTCCATTTGGGACTTCTCCTACCTACTCGACTTCAACGTCGACGAAGACCACATCAACAACTCTCTTCCTCTCCCTCCTCCCGTAAACGACGCTCCCGAGATCCCTAACGACCGGGTCCGAAAGCGTGACCCTCGACTCACCTGCTCCAACTTTCTCGCGGGTCGGGTTCCCTGCGCCTGCCCCGAACTCGACGCCAAGCTCGAGGACGAGGAGGGTTTGCCTGGCAAGAAACGCGCCCGCACCGCGCGTGCCTCCGCATCCGCCCGATGCCAGGTCCCTGGCTGCGAGGTCGATATCTCCGAACTCAAAGGCTACCACCGCCGACACAGAGTCTGCCTCCGCTGCGCCAATGCCGCCACCGTCGTCCTCCACGGCGAGCCCAAGAGATACTGCCAGCAGTGCGGAAAGTTTCACGTTTTATCTGATTTCGATGAAGGGAAACGAAGTTGCAGAAGGAAGTTGGAGCGGCATAACACGCGAAGGCGCAGAAAGCCTCCGGTGGACTCCGGAAGTGCTGCTCACGGCGAACTTGAAGCTGTTACTCAGCATGAGGAAAACGATTATGATGTGGAAGCAAGAAAAGATTGTTCAAATATGAGTACTGAGATCAACAACATAGTAGTGTTGCCAGATCATGAAGATGAACCTGTTCCCATTCTGAGATCGACTCCTGATGCCCAGAATGTTAATAGTGATAGTGTTGCGTCTTTGGTTGTTTCTGGCGAAACGCGAGTGAATAGTGGGAACACTTCTAATTCGCCTTCTTACTGTGACAATAAGAGTGCCTATACGTCTATGTGTCAAACAGGTCGGATTTCTTTTAAGCTTTATGATTGGAATCCTGCAGAATTTCCTAGAAGGCTTCGACATCAAATATTCCAATGGTTGGCAAGTATGCCTGTAGAGTTGGAGGGTTATATCCGCCCCGGGTGCACGATCCTGACAATTTTTATTGCAATGCCTAACATTATGTGGATAAATTTACGCAAAGATTCTTTGGAATATGTGAACGATCTTGTTGCTCCCGGAAAGATGCTATCTGGAAGAGGGACTGCACTAGTTCATCTGAATGACATAATCTTCCGTGTTATGAAAGATGAAACTTCTGTGACAAAAGTCGAGGTAAACTTGCAGGCACCGAAGCTTCACTATGTTCACCCCACATGCTTTGAGGCTGGCAAAGCTATGGAGTTTGTTGCTTGTGGAAGTAACTTACTGCAGCCTAAATTTCGGCTTCTTGTATCGTTTTCTGGGAAGTATCTGAAATGTGAGTATTGTGTACCGTCTCCTCATAAATGGACGGAAGACAATGTTTCTTGTGCTTTTGACAATCAGTTATACAAGATATATGTCCCTCATACAGAGGAAAGTCTCTTTGGGCCTGCATTTATTGAGGTGGAGAATGAATCCGGTTTATCCAACTTTATTCCTGTACTCATTGGGAATAAAGAAATTTGTTCTGAAATAAAGACACTGCAACAAAAATTAGATTTATCTCTCCTTTCTAAACAATTCCGTTCTGCATCTGGTGGTTTGATCTGCAGCTCGTGTGAAACATTTGCACTTAGTCACACATCATCTGACTTACTCGTAGATATAGCATGGTTGCTTAAGGACACCACTTCAGAAAATTTTGACAGAGCGATGACAGCTTCACAAATCCAAAGATATTGTCATTTGTTGGATTTTCTGATGTGCAATGACTCAACAATCATTTTGGAAAAAATATTACCAAATTTGATAATCTTGACAGAAAGCATGAAATCCAATTTTGTGATTAGGACGAGTGATGTTGACGCAACGCCACTCCTGAATCACATACACACTGCTAAAAATGCCATATATCAGAAACATCAGAAAAATGGAAGTGTAAGTGTGCACTCTGAAATGGAAGGCTTTAAACTTGCTCAGGGCTGCTCCCAAGATAACAATCTCCCAGTTGCAGTTAACAGTCAGGGTATCCTGTCAAGAGCAGATGCAAAGTGGGGAGTACTGAGAAGTCTAACTTCTAATGACAAAAATGAAAGAATACCTCTTTTGAAGAGAGATATTATAATGAATGTGGAAGAGTTGCCTGAAAGATATGGTCGTCGATGTATAGGCAGGGGGTTCTTGATCTCTCGACCAGCTATTTTTGTGATTGTTTCTATTGCTGTTTGTCTTGGGGTTTGTGTAGCGGTCCTTCATCCAGGAAGGGTTACCGAATTAGCAGTATCCGTTAGAAGGTGTTTGTTTAATTATTAGTGGATGCTACAATATTTTTTGTACTTGTAGTTGGTACATATTTGTTGTAACACTAAATAAACATCAGAATCAGGATGATTAGGTAGTTTATTGCGTTGGAATCTTAATCCAGTTCTCATTTCTGCATTACCCATTTAATCGGATATGCAGAGGGACTTGCAGTTTCTTTGACTGCAACGAGTCTAATCATAGCTAAAATTTGTAATTCATATTATTAAAATTGGGTGTTTATGTTAGAGACGTAAGGTAA

>C102095_65

TTTGAGTAGGATTTAGGAGGGTTTACTAGCATAGAGCGTAGTTAAACAGTGAAGTAAAACGCAGAGTGGGGGAGTGAGAGAAGCAGAGAAGAAGAAAGTGAGAGGATGGGATCAAAGGCGAAGAAGAAAGGCTCAGCAGCAGAAGACATTCTTGAAACCCTTGGAGACTTCACGAGCAAAGAGAACTGGGACAAGTTCTTCACCCTCCGCACCGATTCCTTCGAGTGGTACGCAGAGTGGCCCCAACTCCGCGACCCTCTCCTCTCCCTCCTCCGCACCCTCGACCATCCTCTCCCCCTCCCCCTCCTTGTCCCTGCATGCGGCAACTCCCGCCTCTCCGAGTACCTCTACGACGCCGGCCACACCGCCATCACCAATGTCGACTTCTCCAAGGTCGTCATCTCCGACATGCTCCGTCGCAACGTCCGCGACCGCCCGCTCATGCGCTGGCGCGTCATGGACATCACTGCCATGCAGCTTGAGGACGAGAGCTTCATTGCGGTTATTGATAAAGGTGGATTAGATGCTTTGATGGAGCCCGAGCTTGGCCCCAAATTGGGGAAGCAGTACCTATCTGAGGTGAAGAGAGTTTTAAAGCCTGGGGGCAAGTTTGTGTGCCTCACCTTGGCCGAATCTCATGTACTGAATCTACTTTTCTCTAAGTTCCGACTTGGTTGGAAAATGAGTGTTGATGCCATACCTTTAAAGTCTTCTAGTAAACCTAGCCTTCAAACGTTTATGGTGGTTGTGGAGAAGGAGCTGTCTACTTCAGTGCATCAGATTACGTCATTACTCCATAATTCTTCACTTAATTGTAATTCAAAACAGGTTTCTGGTCTCAATGAAGCTCTTCAAAATGAGAATCAAATTCGTGAGAAGTACTCTAGTGACTCTGAGATATTGTATTCTGTGGAAGATCTGCAAGAGGAGCTGCTAAAGATTTCTCAAGGTCGGCGATTACAGCTAACTTTGGGTGGACAAGGGTACTCCACTTTTTCTTATAGAGCTGTAATTCTTGATGCTGAAGAACAGGCTGACCCTTTTACGTATCACTGTGGGGTTTTCATTGTGCCTAAGATTCGTGCACGTGAATGGCTTTTCTATTCTGAAGAAGGACAGTGGATGGTGGTCAAAAGCTCCAAAGCAGCTCGCCTGATAATGGTTTTCTTGGATGCCAGCCATTCAGATACCAGCATGGAGGGGATTCAGAAGGATTTGTCGCCATTGGTTACGCCTTTGGCACCTGCAGAAAATGAGAATGGAGCTCAAATACCTTTCATGATGGCAAGTGAGGGGCTCAAGGAACGAAACATTATTCACAAGGTCACATCTTCATTAACTGGATCAATTATTGTTGAAGATGTAATTTATGAAAATGTTGATAGTGAAGTCAGTTGTATTTTTCCTTCTAGAGAGTTGGTCTTTCGACGCCTTGTATTCGAGAGAGCTGCAAATTTGGTGCAGTCTGAAGCTCTTCTAAAAGATGAACAGCTGCCTACCAAATTGGTTAGTGAGACAGAGAGGAAGAAAAACAATGCATCTTCCAAATCTAGAAAAAGTGGATCTCAGAGACATAGTGTTGGAGCAAGTAGGCAGTTGACAGTCTATCATGGCTATGTCGCTAGTTCTTATCATACAGGAATTATTTCTGGATTCACGTTAATATCTTCTTACATGGAAAATGTGGCATCGAGTGGGAAAATGGTAAAAGCAGTAATAATAGGTCTTGGAGCAGGTTTACTTCCCATGTTCCTTCACGGGGGTATACCCTCTTTAGAAATTGAGACCGTGGAGCTAGACCCTGTGATTGTTGATATTGCAAAGGACTACTTCAGTTTTGTTGAGGATAAACGCTTAAAGGTGCATATAGCTGATGGAATCCAGTTTGTCCAAGAGATTGACAGTTCTGGAGTAGCTCAGATTCATGGAAAATGTGATGACCCTACTAGCTATACAAAATCTCCTTTGAATGAAAGTTCAACTGTGTCTCATGCTGGTGTAGAAGTGACAAAAGTTGATATTATTATTGTTGATGTTGACTCTTCAGACCCAAGTTCGGGATTGACGTGCCCTGCTCCAGATTTTTTGGATGAGTCTTTTCTTGAGACTGTAAAAGATAAGCTTTCGGAGAATGGTCTCTTCATTGTCAATTTGGTGTCACGGTCCCAAGCCATTAAGGATATGGCTCTATCAAAAATGAAAAAGGTTTTCAGCCACCTCTTCTGCCTCCAGCTTGATGAGGACGTCAATGAGGTTCATTTTGCCCTAAAATCGGAGTCCTGTATTGAGGATAACTGTTTCTCTGAAGCTTCTGTTAAACTTGAAAAATTGTTGGAGTTTAAACACCCGGAGATAGGTCAAAACATTATTAATGCGACAAAAAAGATCAGACGTTTGAAGTGAAACTTGCTCTTCTTCCTTGTTACCGTTTTCAGCATGACACTCTTCATCTGTATGAGATGTGAATGCTTGCTTTCTCAAACGCGGAAGTGATTTCAGCCAAATATTTGTCATTGATCTGCGTACTGTCTGGCACTCATCAAAGTACTGGTTGAGTGATGTAAGTGAATTGTGATAATATTGTAGCGCTGTCACCCTTTATTTCATATGGGTGTCTGCTTCAACTTTATAATAACATTTTCTGGAATAACTCGCTGTGAATACTATTCTATTCTCAAGGCAATATTTTTCGTTTAAATTTGTTTGGCT

>C102097_65

AACTAACCAGGCTTTAGCGTGACAATGCCCGCGCTCGTTCGTCCCTTTGGCACACCGCGTTTTTTCTTCTTTTCAATTCCCTCCCTCCGATTCTCCATTTCATCCCTTTTTCTCACACTCCAACTAGAAAAGCTTCACTCCCTCACTTTTGACTCTCTCTCTTCTTTCTCTCTCTTCACTACTACTAGGGCACACGCACTCACACGCACGCAATGAAGCCCGACTTGTACGAAATCTCCGATGAGGAATGGGAGAATCACTCCTTCAAACCCTCCCGCGTCCTCAAACGACCTCGCACTTCGTCTCCTCCTTCTCCGCCGCCAATCGAATCTTTCGCCTATATCTCTAAGGTCGACGTTTCAAGCGAAAACGACAGCGATTGCGTTGAAATCGCGCCGAACGACGCTGGTTTCCTCGAGGACCTTGAGGACGCCGACGTTGGAGACGCCGACGCCGGTTACGCGGCGGCCTCGCGCGGCCGGAGGTTCATCATCGACGACGACGACGAAGACGCTGAGGAGAACGGCGGAAGAGACGGACGCGTGGCGGAATTGTACGAGGTGGAGTCGAGTGAGGTTGAAGAAGAAGTTGAAGAGTTGAATGAAAACGATGTAGTTGGTAGGGCTTTGCATAAGTGCGCGCGAATCGCTGCGGAGTTGAAGGGCGAACTCTTTGGCTCGTCGGGGACCACCTGCGACAGGTATTCGGAGGTGGAGTCTTCGTCTGTGAGGATCGTGACTCAGGAGGATGTGGATGTTGCGTGCGCGTCTGAGGTTTCGGATTTTCAGCCATTGCTTAAGCCTTATCAGTTGGTTGGTGTCAACTTTTTGCTTCTGTTGTATCGGAAAGGGATTGGAGGAGCCATATTGGCAGATGAAATGGGTCTTGGCAAAACAGTTCAGGCCATCACGTATTTAACTTTGTTGAAACGCTTGCACAATGATTCTGGTCCACATCTTATAGTATGTCCTGCTTCTGTTTTGGAAAACTGGGAAAGGGAACTAAAAAGGTGGTGTCCGTCCTTTTCTATTCTTCAGTATCATGGGGCTGGACGTGCGGCATACTGCAAGGAGTTGAACTCCCTGTCCAAGGCAGGATTGCCTCCTCCATTTAATGTTCTTCTTGTGTGTTATTCACTTTTTGAACGGCACAGTGCACAGCAGAAAGATGATCGTAAAATCCTGAAGCGGTGGAGATGGAGTTGTGTGTTGATGGATGAGGCACATGCTTTGAAGGACAAAAATAGCTTTAGGTGGAAAAATCTGATGGCTGTTGCACGAAATGCAAACCAACGCTTGATGTTGACTGGGACACCACTTCAGAATGACCTTCATGAGTTATGGTCATTGTTGGAGTTTATGATGCCTGATATTTTTGCTTCTGAAGATGTTGACCTAAAAAAGTTACTAAATGCAGAAGATAGAGATTTAATTGGTCGAATGAAGTCCATCTTAGGGCCATTTATTTTGCGCAGACTGAAATCTGATGTGATGCAACAACTTGTTCCAAAAATACAGCAGGTTGAATATGTTATAATGGAAAGGCAACAGGAATCTGCTTACAAGGAAGCCATTGAAGAGTATCGTGCTGTTTCACAAGCTCGGATGGCAAAATGTGCTGAGCTTAATTCAAAATGTGTTGAAGTTCTTCCTCGGCGACAGATAAATAACTATTTTGTTCAGTTTCGCAAGATTGCAAATCATCCCTTGTTAATAAGGCGAATCTACAATGATGAGGATGTCATTCGCTTTGCTAGGAAGCTACATCCGATAGGTGCTTTTGGTTTTGAATGTACTTTGGACAGAGTTATAGAGGAACTTAAAAATTACAATGACTTCTCTATTCACCGGCTTTTACTTCATTATGGTGTCAATGATAGAAAGGGGATCCTTCCGGATAAGCATGTTATGCTTTCTGCAAAATGTCGGGCCTTAGCAGAACTTCTTCCTTCACTAAAGAAGGGTGGTCATCGGGCCTTGATCTTTAGTCAATGGACCTCAATGCTTGACATATTGGAGTGGACTTTGGATGTCATTGGATTGACCTATAGACGGCTTGATGGAAGTACACAGGTAGCGGAGAGGCAAACAATAGTTGATACGTTCAATAACGATACTTCAATATTTGCATGCTTACTTTCCACGAGAGCTGGTGGACAGGGCTTAAACTTAACAGGAGCTGATACAGTTGTAATTCATGACATGGATTTTAACCCACAGATTGATAGGCAAGCTGAAGATCGCTGCCATCGAATCGGTCAAACAAAGCCTGTAACTATATATCGGCTGGTGACCAAGGGAACAGTTGACGAAAATGTGTATGAGATTGCAAAAAGAAAGCTAGTATTAGATGCTGCCGTTCTCGAGTCCATGGAGGAGATTAATGAAGGTGACATGCCCGAGAAGACCATGGGAGAGATATTATCTTCAATTTTATTGAGTTAAAATATTGAGCTAAGAAAAGCAAAGTTTAATTTATCATCATTCCTTCTTTTTGCTCCAATCTTGAATTAATCTGTTGATTTGGGATAGAAGAGAACTTCCCGCCCTACAAATTGGTAAGAATGCGGTAACCATTATCGATGTACATTAGTTTTAATGTTCACGTGTTGGTGACAGTGAATATAGTCTTTAATTAGAACCAATAGGAGAATTTAGTTTAAAAAGTCATGTTAGAGATTAGCAAATTTGTTGAACATTTCTGAAGCCCA

>C102099_65

CGTCACAAAATCGGTAAAAATTGCTAAGTACTAAATTGTATGATAGTAAGTAATATCATGGTTCATTAGCTAGTGAAAAGCATATTCCTGTCGTTCTTTGAAACATGCGTAGGTTCAAGCCGATAGTAAACATGACGGTTAAGCTGACAAAACAGCATTATACCAAAAAAAAAAGAGACCATGATGCCGCAATGCAAATGATAAAAAAAAAAAATTACAATAAGGGTGAAGGCAAAAATGTTCGTGGAGACCTCTCTGTAGAATACCAAAAAAACAATAGATACGTAAAGTAAGATGGATTCTGTGATTCTTCGGGGGAAGCATCTTCATTTCTTAAAACCCCCATGTCAGGGCATCACCAGTGCAGCTCTCTAAGTCTGGAACAAGGTGGTACCAAAGCGAAAGATGTACAAACTAGAAAAAGTCTCGTTTTTTGCGACAATCTGATGCTCGATGCCCAGGTTGTCCACAATTAAAACAGGCCCCTCCGTGCCCGCCTCTGTTTGATGATGAAAACCTGTTTGACCGTCCACCTCCAATTAGCCAGTCATCGCCGCTGTTTTTTCCCATTCGAGACCTGTTGCTGCCTGATCTGAAATTTCTACCGCCTCGTCCAAACTCATCATCAGAGTCTTGGCCTCCTTCCCATCCCCGAGAACTTCTAAAACCTCTTTGATCCCTAGAATTAGAACCTCTACGGTTACTTCTGTCAGAAAACTTCCCATAGAAATCACTAGCAGGTTGATCGTCTTGCAAAGGAGGTAACTTAGTGATTTTGGAAATGGTGTTTCCAGATGGCAGTTCCTTTTTGAGCAACTCTTTAGCAATTTCCTCCGGAAGATCAAAAACAGCTCCTTTAATCCTCTCATCTGCAATTAAATGTATCTTTCCAACTTCATCAGCAGCTGGAGAATAAACATCAGAAAGAAACCCAGTGACAGATCTTGCAGAAAAAAATCTTCCAGAAGTATCTGAATCTCGAGTCAGTTGCAATGTAATCCATCCCTGTTCATGATTGATAAGAGATCGACATGATGGAGGTCGAGAGAATCCACTCATCTGTGCTAGAGCAGCTGCAAGGACACTTGCTCCTTGTTCTTCAACCAGTTTTTGTGCAGTTGCAGTGAAAAATTCAACAGACTCTGGATGGACTCTATGAAGTGCAGCAACAACTTGCTCAGCAGATGACTCCAAAACTTCTTCCATAGCTGGTGGACTAATAAATTCAAATTTGCAGCCAACATCATTCTCAAGGGATCTAACTGTTCTCCTCTGGCTACTGGTGTACATCAAAATGGCAGTACCCTCTTTTCCCGCACGCCCAGTACGACCAGAACGATGTACAAAAGTCTCAGCATCATTGGGAAGTTCATAATGTATAACCAAATCAACATTGGGAATATCAAGACCACGAGCTGCAACATCAGTGGCAACAAGCACTGTGAATTTCCCTTGCCGAAAACCATTCAATGTTCTCTCTCGCTGATGCTGAGATATATCACCATGCAGTGCTTCAGAAGCTATACTACTCGTTAATGCCATCGATACTTCATCAGCATCCTTTTTTGTCTGCGTAAAAACAATAGTCTTTCCGCCCTTTGCATAAACAGTTATAAGATCAGAAAGAACATTCCGTTTTGAAGTGGCAGTGGCTGATAAAGCATAAAGTTTGATCCCTTCAGCAAGTTTCTCTTCTTGTTCTCCAACCAAATCAATTGTCAAAGGATTGTTCAAATACTTTCTGGACAGTTTCTTGACCCAACCAGGCATGGTAGCAGAAAACAGCATGGTCTGGCGCTGCGAGGGGACCTTATCCAAAATGACCTCAACATCCTCCTCAAACCCAACAGCAAGCATCTGATCAGCTTCATCAAGCACCAAATACTGAACCTCACTCAGTTTAAGGCTATCTCCATTCACTAGATCAATTATTCTACCCGGTGTTCCAACCACCACATCAACACCACGCGAAAGCGCACTCTGTTGTGTCACATATGAAACACCCCCATAAACACAAACCGTTTTCAGGTAAGGTGCAGATTCTTGTATCTCCTTCTCAACTTGCTTGGCCAATTCCCTAGTAGGTGCTAGGACCAATGCCTTAGGAAGCCTCCCATGCCTCCTATGAGAACTTTGTTCATCATCATCAGAGAGGCCTTTGAGAATTGGAATTCCAAATGCTAATGTTTTGCCAGTGCCGGTCTTTGCTCGAGCAATGATATCTCTACCTTCTAGTGCAGGCAGCAACACAGCTCTCTGAATGGGAAAGAGGCTGGTGATTCCACGTTTTTGTAGGGCGTGGACGAGAGAGGAAGGTAAAGACAACTTGGAAATGTCAAGTTCGTGGTCGCTGACGTGGACAGGGGTGGTGTCGGGTTGGTGGTCATAGCCAAATTGGTGGCTATTGCTAACGTCGTCAAGAGTTAAACCCTGTAAGGGTTGTTGGGTGAGAATCGAAGAAGTGGGGGTGGCAATGGAAGAAGGAAGGGAAGAGAGTGTGGCGTTACGGGGAAGGGCGTTGAAGGAAACTGAGGTGGTTGGTCTTCTGAGAAGTTGGAGAGAGGGGTTTTTGAATATGGAAATGGAAGAAGAGAGAGGTATAGTGGAAGAAGAAGCCATGTTTGTGTTGGGAAGAAGAAGAAGAAGAAGCGGCGAGGGGATAAGGCCACGCACTTTGTTGCCTCTGAAAGTATGGCCTTTTAAACCTCTC

>C102101_65

GTACATTTAGTAACTCCAAATACATAATTAAACATACAGCAGGTGGAATTATATTCCATGTATATCACATTTATTTAAAAGCAGGGAAGTTTAGAAAGCATAGAAGGCTTAGATAGAGATACTGTTGGGAATTCCTCTGAAAGTCAAACCCTCCTCACTAGAGGGAAACAGCAGAGTGTAAGGCATTTTAGCCGGTCCAGTGCGGTTTCTCAATGTTTCATCATTGTTCTTCTCTACAAGTTTCGCTTCAATCTCTGCAAGCTTCTTTCCGAACCTCTTAAAGGCTTCTAATGGCACCGTATCAGAAGTCCAATAGTCACCACCATCTCTCTCCCCAAGGTAGAATTCATCAGATGCATGCCTTGACAAGATCTCTATAACTGTAAGGTCAATCAGGGTCTCTTTCTTCCCAGTAATAGTTCTCAAAAACTCCTTCTCAGGATTCTTAGCCAATGCATCGTATTCAGCGGACCCTTTCACAGGCATGAATCTTCTGCTAAGAGTTGGCCTGTTAAGGATTAGACCACCATATGGATATTGTCCAAAGTTAACAGCTGCGTGGAGGGCTGAAGCAATCCATATGAGGGTAGTAGAGACTTCAACCAACTCTTCACGTGTTTGCATCTTTTGCCACCATGGCTTATCTTTCAAATCACCATGACCCACCTGAACAAGTTCTTTCCACCAAGCTTGGAGTTCAGGGTCTTTTTGAAGTACCTCATCTGACTTGTAGTAGAATGAGACATATTCTTGCACCCAGGACTTGATGGCATCCCATATCTCTAACCCATCCGAAGCATAAGGATAATCCTCAATCAAAAGCTTAACACCGTGGGGAGCAGATGGATCTTTAACTGCAACTCCTCTCTTGACAAGATCATTGGGTAATGCTTGATCAGGGAAAGCCCAGTCCTTGTAATTCAAAGCGGACATTTCCAAAGAGTATCTACTCCACAAGAATGTTTTCTCTATAATACCGTCTGCATTGACCAAGGACTTGCGGGCGAGTGAATTAATGTTCATGGTGTCACGAAAGTGAGGAAACAGAAGTTTGTAAACAGGGTGAACCACACTCAGTTGCCTGTTTGTTGCTATGACGAAAGGCTCAACAACCGCATGAGTGTTTAGCCAATGGCTAACGATTTGATGGTAGCATGAGTCGTTCACAACAACATAAGCCTTTGCCAATAACCAAATGTAAGCTTCAACTCCGTCGTAGGCAGGCACATAGACTTCGCTAAGAGGGCCATATTCGTCACCCTGACCATGCGGTTTACTTAACTCAATGGCCAACGGAGTCAAAGTTCCATCATCTTTCAAGAAAAATATGGTCCTTGTAGCATAAGTCTTTGTGTTAGATGAATTTATTCTCCTCAAATATGGAATGAGATAGTCATGGTGATCTAAGATGAACAACTTCTTGTTCTCGATAGCCTGCTCAACAGTGAGTCCGCCCAAGTTAGGCTCCAAATGTTCTTTTGTTATTATAGAGGTATGATCACCATAGGTTTGAATGTCTAGCTTGCTCCTTGGTGGGAACTCCTCGAGAATCTTAATGACATTAGGATTGACACCAGCAATAGTTTCTCTTGCAAATTCTTCATCAGTCATCCATGCAGACTTATCCACTTGCATGACTTTAGGTGGTGGATACTTGAGGAACTGTTCGCCATCAGTTCGAAGAATTTCCTTGAGAACTGGTATAGGAGCATATTTGCTAAGAAAGTTTGTAGGCAATGTAACTCCACCTTCATAAAGCTTGCGCACTTCCTCGAAATTATCAAACTCAAGAGTCAAAAGACTTCCATCGAATGCATCAGTCAACACAGGCAACACATCTTGGGATACAGATTTCAAAGCATCAGCGAGAAAATCGGATGACTTCAAGTGACCAAATGCTTCATCTCTTGGAAGGTAAACAAAGGCGCTCGGTTTCTCACTGTTAGGATCTTTTCTAGTTTTTCCTCTTCCGGTTCTTCCTCTACGAGGGTAAGGTAAGACAGAACCTCCAAGAACGGGGCGAGCCAAATCAGCACTTTTATCTGGATCACCCAAATCGTTGTAGACATCATAATCATAGATCCTATCCCATTCCTGACGCTCTCCAGTTCCATCCCCTCTTACAGTCTTCAATTCTTCTTCTCTGTACTTCACAAGTGGGGCTGGTGTGTCGCTAGGAAGATATGTGTTGTTGGCAAAGAAAATGCGATCAGTCTTGTATTTAGTGGAATTGTAAATCCAGGAGTTGCATACGAAGTGAATGGTTCCTTGGTTGGGAATGTCTTCGATGGTGACAGACTTGAGGTAGAACTCATTTTGCATGAAATTCTTAATGTAAAATGCACCAGGAATTCCGAAGTCAGTGTTCCAGTCAAAATTAACATCATATGCTTCTTCCTTAGCTCCCAAGGTTGGCAAAGATATTTGCCCTCTTAACTTTGTAGCACTTCCTACTTTTCCTTTCCCTTGCCCATCAGCCTTGGTAGCACTGATCAACTGGATGGAGATGGAAGTGGCAAAGGCAGTGACAGTGTCGATAACAGAGCCAAAGATGTCGAAGACGGAGTCAACGACGGTGGCAGGATTGGTGATGCTGTTGATATCCAACACATTCTTTCGCATAAGCACCACAGTTCCCTTCACCTTCTGAGTCTTTCCCCCGAATGGAAACATCTTCTTCTCTCTCAATCTTAATCTTCTTCACTTCTCT

>C102103_65

ACATAGTGATAACTGTAGTGTAGTCTTTCTCTGATATTTGAGACAAAGAAAGTATAACCATGGAGGACAAGACTATAGGAAGAAATGGAAGCTCTAGAAAGGGTAGTTTCAGGTTGGGAATAGGAGTGGATGGAAAGAAGGATCACACTGTGGTTGAGCTTGGAAGTATTGACACACACAGACTTCTTCTCAGCGGTGGAGAGAGAGAGAGAAAGCAACAACCTCTCACCAACTCTCTCTCCTCCAGGCAGATGAAAGCCCTCTCTGCTCTCTGTGACACTATCTTACCTTCTGTCGACCACTTTGTTAACACTTCAGATGAATCTGTCAACAACTTCTATCGAATTTCAGCTTCCATGGCAGGCACACCAGAACGATTCGGGGGAGTGATAACTGAGAAACTGCAACATCCAATGACATGGTTTCTGAAGTTTGCGTTATGGTTGTTATCAACATGGTTTGGAACATTAATTTTGTGTGGAATACAATGTCTCTCAACCCAGTTCCCCTTCATCCATAGCTATCCTGAGTTGTCTATACAAACAAGGCAACAGATTATGCGATCATGGTCTCTCAGTTACCTTCGTCACTTCAGAATGTTCTTCAGAACCATCAAACTTCTCACTCTTCTTATCTTCTTTACTCAGATAGATGAATCAGAAGACAACCTTTCATGGAAAGCAATTGGATACTGTGGACCTGATCCAGAATTCAAAGCTCAGTTGAAGAACCACTTTTTAGATGGAAAATCTAAGGGCGGAGAAGAGGATAAGGAAGATGAAGATGCTGAAGAGGTGATAGGACCCCTTTACAAAGGTCTTGTCCATTTGAATTACCCACGAGACATCACTGCAGATGCTCTAAGACGATTTGGATTCCCTGTCTCTGTCATTCGTAGAAAGCATAAAGCTGCAGCAAACTTGTCTTCTCCTTCTTTGGTGATACAATGTGATGCTGTGGTGGTTGGTTCTGGCTCAGGGGGCGGAGTGATAGCTGGTGTTCTAGCAAAAGCTGGTTACAAAGTGTTGGTGTTGGAGAAAGGAGGCTATTCTGCTAGGAACAATCTTTCCCTTCTTGAAGGACCAACCATGGATCAAATGTACCTCAATGGTGGACTGGTTGCAAGTGAAGACATGGGTATGTTTATACTATCAGGATCGACTGTTGGTGGAGGGTCAGCGGTTAACTGGTCTGCATGCATTAAAACCCCTCAGCATGTGTGCAAGGAATGGTGTGAGAAACATGGGCTAGAGTTGTTTGAGAGTGAAATGTATAGAGAAGCCGTGGATGCTGTCTGCGAGAAGATGGGAGTTCAATCTGAGATTGAAGATGAAGGATTCAACAATGCAGTCCTGAGAAAAGGGTGTCAAGAAATGGGGTATCCTGTTAATACAATTCCTAGGAATTCTTCATCAGATCACTACTGTGGCTGGTGTTGCATGGGTTGTAAGGATGGAAGGAAGAAGGGTACCTCAGAAACATGGTTGGTGGACTTGGTGAAATCTGGCAATGGAGCAATTCTTCCAGGTTGTGAGGCAATAGAAGTCTTGCACAAGAAAAAGGAAGGAAGGGATAGAAAGGTTGCACGTGGAGTGGCTTTTGAATTTGAATATAAAGGGATTAAAGACATTTGTGTGGTGGAGTCCAAGGTCACGATTGTAGCATGTGGAGCACTTTGTACTCCAGCATTGCTTAAAAGAAGTGGCTTAAGGAATCAGAACATAGGGAGGCACTTGCATCTTCATCCAGTGACAATGGCTTGGGGCTACTTCCCGGATGAACCTGCTTCTGAGGTGTGGCCAGAGGCATATAAGAAGAGCTATGAAGGAGGGATAATGACAGCAATGTCCTCGGTTGTTGCAGAGTTTGAACAATCTGGATATGGGGCAGTGATCCAAACACCTTCATTGCATCCTGGTATGTTCTCAATTGTCACACCATGGACTTCTGGAATCGATATAAGAGATCGAATGCGAAAATTTTCTAGAACAGCTCACATATTTGCACTGGCAAGGGATAAGGGATCAGGGACTGTGGAATCACCAAAGCGTATCAGTTATACATTGGCAGGTGTAGACGAAAAGAACTTACAGAAAGGAATTGAAAAGGTGCTGAGAATTCTAGCAGCAGCTGGAGCTGAAGAAATTGGGACACATCATAATAAGGGAAGGACCTTAAATGTAAAGCAAGTGAGCTATCATGAATTTGAGAAATTTGTTAAGGAGGAAAGTTCAAGGCCACTGGCAGACCTTTCAACACCATTGTGCTCAGCACATCAGATGGGGAGTTGCCGGATGGGTTCTAATCCAAAGCAATCAGTGGTGAATCAGACAGGAGAAACATGGGAAATGGAAGGCCTTTATGTGGCTGATACAAGTGTCTTTCCCACTGCTTTGGGAGTGAATCCAATGGTCACTGTTCAAGCTATTGCTTATTGCACAGCACAATCTGTTGTAGAAGCTCTCAGAAGGAAAAGAAGTAAATGAGGGGAAAAAAACTTTTGCTGCGTCTGTAGCTCCAATGGTACTGAATATTCATAGCATTGGGCAGAAAAAAACCTTGTTCAAATAACTTGGTGAGAAAAGCCAAGGTTGAAATTCTAACAACTTCATTGATAATTTTATACTTTGTGCATATACTTTATTATAATCTTAACTGCTGATTTTAAATCAAAGGTTATTACTGTATTTTACCCTGTAAAGTGTTCCCA

>C102107_65

TTTGAGAGTTGATGAGAGTGGTGTAGTAATTTGGATCCAAAAGCAGTGTTTGGAATGTCCACTATCCAAATCCCTACTCTGCATTTCCATTCCCTCACCTTCATCACAACAAACCGTTGACTCTGACTCTCCTTCATTCAATCACCAAATCAGATTCAGATCCACTCCCACTCCCTCACTCCCTCACTCACTCACATTCCATTTCCAAATCCTCCATGAAACCCTAATTCCATCAATGGTTGTCACCTCCAAACCCTCTCCAATGGCGCAAGATCTCAAACCCTCCATCAACTCCTTCTCGCTCCCCAAAACCCTAAAGCTCAAGACCAAGCAGCAGGAGCTCCTCATCCGCGTCGCCGCGCTCTCCCTCATCTACGTCCTCGCCTTCATCACTCGCCTCTTCAGCGTCCTCCGCTATGAGTCCATGATCCACGAGTTTGATCCTTACTTCAACTACCGCACCACGCTCTTCCTCACGCGCAATGGCTTCGCCGAGTTCTGGAACTGGTTCGACTCCGAGTCCTGGTATCCTCTCGGTCGCATCATCGGCGGCACGCTCTACCCAGGCCTCATGGTCACCGCCGCCGCCATCCACTCCCTTCTCCATTTTCTCCGCTTCGCCGTACACATCCGCGAGGTCTGCGTTCTCACCGCCCCTTTCTTCGCCTCCAACACCACCCTCGTCGCGTACTTCTTCGCCAAAGAGGTCTGGGACTCCGGCGCTGGCATCGTCGCTGCCGCGCTCATCGCGATCTGCCCCGGCTACATCTCGCGCTCCGTCGCCGGCTCATACGATAACGAGGGCGTGGCGATCTTCGCGCTGTTGCTGACATTTTACCTCTTTGTGAAGGCCGTGAACACTGGGTCGCTGGCGTGGGCTTTGGGTTCCGCGTTTGGGTACTTCTACATGGTGTCGGCGTGGGGAGGTTACGTTTTTATAATCAATCTGGTGCCGCTTTATGTGCTGGTGCTGCTTGTGACGGGGAGGTACTCGTTGAGGTTGTATGTGGCGTATAACTGCATGTATGTGTTGGGGATGCTGCTGGCAATGCAGATTAGGTTTGTGGGGTTCCAGCATGTGCAGTCCGGGGAGCACATGGCGGCAATGGGCGTATTTTTCTTGCTGCAGGTGTTTTTCTTCTTGGATTGGGTGAGGCATTTGCTTAGTGACACCAAGTTGTTTCAGGCCTTTTTGAAGATCACTGTGACTAGTGCCGTTGCTGTTGGTGCTGTTGCTTTGGGAGTGGGCATGGCGTCTGGGTATATTTCTCCGTGGACTGGGAGGTTTTACTCCTTGCTTGACCCAACGTATGCTAAGGATCACATCCCAATTATTGCATCTGTCTCTGAGCATCAGCCAACTGCTTGGTCGTCCTTCATGTTTGATTTCCATATTTTGCTGTTCCTTTTCCCTGCTGGTCTCTATTTTTGCTTCAAGCGTTTGTCTGATGCCACAATCTTTTTAGTTATGTATGGTCTTACAAGCATGTATTTTGCTGGGGTTATGGTTCGGTTGATTCTTGTTGCTACCCCTGCTATGTGCCTCATTAGTGCTATTGCGGTTTCTGCCACTGTGAAGAATTTGACACGGGTAGTGAGGGCCAAGAGCCCAGCTGTTCAAAGTGGATCTACTAAAGGAACTAGCACTGCAAAAGGTTCTTCCAAGGGTGTAGTTGACAACTCCCAGCCTTTCCAAAGGAATGGTGCTATTGTGTTACTCCTTGGTGCTTTCTATTTGCTCAGTAGATATGCTATTCACTGCACTTGGGTCACATCAGAGGCTTACTCATCTCCCTCAATTGTCTTGGCTGCGAGGGGTGCTCATGGCCAAAGGGTCATCTTTGATGATTATCGTGAGGCATACTTTTGGCTTCGCCAGAACACCCCACCAGATGCCAAGGTGATGTCATGGTGGGATTATGGTTATCAAATCACTGCCATGGGAAACAGAACTGTAATTGTTGACAATAACACCTGGAACAACACTCACATAGCTACCGTTGGCCGAGCAATGTCATCATATGAGGATGAAGCTTATGAGATAATCAAATCACTTGATGTTGACTATGTATTGGTCGTGTTTGGTGGTGTTACTGGTTATTCTTCTGATGACATTAATAAATTTCTTTGGATGGTGAGAATTGGTGGTGGAGTTTTTCCTGTGATAAAGGAACCTGATTACCTTGTCAATGGAGAATATCGTGTGGATAAAGGAGCAGCTCCAAAGATGTTGAACTGTCTTATGTACAAGCTATCTTATTATCGGTTTGGGGAGCTGACAACAGAATATGGCAAACCACCTGGATTTGATCGTGCAAGAGGTGTTGAGATTGGAAATAAGGACATAAAGCTTGAGTACTTGGAAGAGGCATTCACTACACAGAATTGGATAGTTCGTATTTATAAAGTGAAACCTCCTAAAAACAGGTGGTAACTCTAATCCCCCTTCCCAAAGCCCAAAAATGCAAAAACAAAGACTGTTTTCTCTGCGGGAAATGATAGCTTTCTAGTGAACTTGAGCACTGAAAATCTGTTTACAGATTTTGGACCATTTTTGAAGTATGTAGAATTAGTTGAGGTCATTTTATGGATTTTTTTTCTTTACTACAGTTTATGACTTTTAAGTCTACTGCCAACTTAACTTATTGGCTTGGTGCCACATGTTTGCCACAAATTATACAGCATTGTCATAATACAACTTTTAGAACCGAT

>C102109_65

GAAAAAGAAAAATAGAAAGACAGAACAATCAACCGTGTAGTTCCCGTTTTCTTTCCCTTTCAAGGAAAACAGGGATTCTCGGTTACCGAGCATCATCTCCTTGTGTTATTTTTTCCCTATCTTCTCTTTCCATTTGTTGGTCAACCTTTTGTTCATTGGTTCACTTTTTCTTTCCCTTCCAAGTCTTCGCTTCTTTGGTTCAAGGGACTGTGAGACCCTTGAGCATACTTCTTGTCAGACAGACCATCTTCTTTGGTTTTTGTTTTTTTCTTCTTTGCAGCGTTTTGAAGCCTCTTCCAAGACTCTCTTACTCAGGGTTTTGATTCTGGTTATTCTCTCAAGCTTCTGCTGAGTTCATCTTTGCTCTTAACTCAAAGGGTCACCCTTTGTGTAATAGTTATTATTCTTTGCCTCTTGATATCCTTTTCCGTGCAAGTTAACTGCTAAAAGTACAAACTTTTTCGTTCTCGTGCCTCAATTTATTTGATGGGTCGTCCCCAGATGAAAAATCACCTCAATTTATGCTTTCCGGGAATCTGAGTTTTGTTGCTTGACTTGCAAGTGACTTTGGAATTGTGGTTGGGGAAACAGTGCCCTTGTGGAATTTTGCTTATTTGAACTTGGTTTGTTTAGCTACAGGATATGTTGGCTAGCTTTTCAATTGAGATGGGTACTTGAAAGGGTTTAATTTCTTCTTTTGCTTCTTAATTAAGTCAACGAGTTCCTTGACTTAATTGGAACTTATGGTATTTGGGGTTATGGATAACTCAAATGATTCATCATCATCCTTGAGTTTTGTATCATCTCACCTCTCACATGGTTCTAGTAATCACAACGTGTCTTCCTCCACCAGCAATGAGCATGGGGCAAATATTGAAATTTTGAGCCTGGGTAAGCTCAGTGGAAGCCTTGAGAAGCTACTGATTGATGCTGAGTATGATTATTGTGATGCTGAGATTCTCGTTGAAGACATCAGTGTGGGTATTCATCGCTGTATATTGGCCTCGCGTAGTCCTTTTTTTCATGAGCTTTTCAAGAAAGGAACTGATAAATCAGGAAAGGAAGGAAAGCCAAGGTATCTCATGTCAGAATTGGTGCCTTATGGCCCTGTTGGATATGAAGCTTTCCAAGTTTTCTTGTACTATATGTATACTGGAAGGCTAAAAGCTTCACCAACAGAAGTGACAACATGTGTTGATGAAACATGTGTCCATGATGCATGTCGACCTGCTATCGATTATGCCTTGGAGCTGATGTATGCTTCTGCCACTTTTCAGATGAAAGAACTGGTTTTGCTTTTTCAGCGGCATCTCCTAAACTTTGTTGAGAAGGCTCTTGTGGAAGATGTCATTCCTATTCTTATGGCTGCTTTCAACTGCCAACTAGACCAGCTTCTCTCTCGGTGCATTCACAGAGTAGCAAGGTCTGATTTTGACGACATATCTTTGGAAAAAGAGCTGCCTCATGAAGTTGTGACTGAAATCAAATCTTGCCGACTCTCGTCTCAGCAAGAAGCCTCAGCTGATGCAATGGAAGTGGAGCCTTTGAGTGAAAAGAGTATCAGGAGAATTCATAAAGCATTGGACTCTGATGATGTTGAGCTACTGAAGCTTCTTCTAAATGAGTCCAGAGTCACCCTAGATGATGCATATGCCCTGCACTATGCCTGTGCCTACAGTGATTCTAAGGTTATTCAAGAAGTTCTTGGTCTAGGCATGGCTGATATTCTTCGTAGAAATTCCCGAGGATACACGGTTCTGCATGTGGCTGCAAGGCGAAAGGATCCATCCATTTTGGTGGCACTGCTGAATAAAGGGGCATCTGCAACAGATACCACTCCAGATGGACAAACTGCTCTTTCAATTTGTCAGAGGTTGACAAGACGCAAAGATTATCATGAGAAAACAATGCGGTGTAAGGAATCTAACAAGGACAGGCTCTGTGTTGATGTCCTGGAGAGGGAGATGAGAAGAAGTTCCATGACTGTGAGTATGTCTGTTTCATCACAGTTAACAGCTGATGATTTGCACATGAGACTGGATTACCTTGAAGATAGAGTTACATTTGCTCGGTTGTTGTTTCCTGCTGAGGCCAGGGTAGCCATTGAGAATGCTGAGGCAGATTCGTCATCAATGTTCGCAAACTCATCACCTTCGAAGGACACAAATGGCAATCTAAAAGAAGTAGATCTAAATGAATCTCCCTCTGCTCGAACCAGAAAACTTCAGTTAAGATTGCATGCCCTTATGAAAACAGTTGAAAATGGTAGGCGCTTCTTTCCCCATTGTTCTGAAGTGCTTGATAAGTTTCTGGAAGATGATATGCCAGATGTTTTCTTCCTAGAGAAGGGCAGTGAAGACGAGAAGAGAATAAAGAAAGCACGCTTCATGGAACTAAAAGATGATGTGCAGAAAGCATTTCACAAAGATATGGCTGAAAATAACCATACAGCTTTTTCATCTTCAGTCTCCTCCTCATCATCTTCAACTCGGAGGGAAGGTCTTAACCACAGGTTGAGGAAAAAATGAACTCCATCATCTTCTGCTAGAGTCAGTGTTGCAACTTCCAACTATAAGAAGCTTAGTAAATAAGATGTTGTGTTAAATTAGAGGGTTTTGCTAATGAACATGCACATTTTTACTAGTTTGATTTTATTTCTTATAGTTTTTGGAACAGATGAAGAAAGAAGAGAAAAAGGAAGAACTATTGGCA

>C102111_65

CCAGGTTTGAGTATCTTTGTTCTCTTCCCACTCTTTTCTGTTCTCAACTGTTGGTCCTTCTCACTTCGTAGTTCTTCTAGTCAACTCAGCAAAACTTGGCACATAATATCAGCTATTCGTTCCCCGACATACCCGTCAAAAAGTGTTCAACTTTTGGTGTTGAGTGAGTGCACCAATCATATTGTGATTAAAGTCTCATACTTTGGGAGTTTCTGAATCTGGGTTTTTCATTTCACGGCTTCTCAGCATCTATTTCAAGATAGCTTATTTACCAACTTCTTGATTTCTGAAATTTCCTTGGAAATATGATTGTGTCTGCTCTATTGACATCTGTTGGCATCAACACCGCTTTGTGTGTGTTGTTCTTCACACTGTATTCTATATTGAGGAAACAACCAAGCAATTATGAAGTTTATGTGCCGCGATTGCTGGCTGAAGGAATATCGAAGAGGAGGAGTCGTTTCAACTTCGAAAGACTAATTCCTTCTGCAGGTTGGGTTGCAAAAGCATGGAGGCTTTCTGAGGAGGAACTGTTGTCATTATCAGGGTTAGATGGTGTGGTGTTTATGCGCATGATAACCTTCAGTTTGAAAATGTTTACCTTTGCTGGGATGATTGGGATGCTTGTGATTCTCCCTGTTAATTGTTGGGGAAATCAGCTTAAAGATATTGATATTGTGGACTTTGTCAACAACTCCTTGGATGTGTTCACTATATCAAATGTAAACAGTGGCTCTCAGTGGTTATGGGTTCACTTCAGTGCTGTATATATTGTCTCTGGATTCACATGCATGCTACTTTTTCTTGAATATAAATACATATCATCTAGAAGAATTTCTTATTTTTATTCTTCCGAACCTCAGCCTCATCATTTCACCATTTTAGTTCGCAGCATTCCCTCCTCCTCCTCTAGCAGCATCAGTTACAGTGTTGAGAGATTCTTTAGTGAGCTTTACCCTTCTACATATCTGTCACATGTGGTTGTTCGCCAAACAAATAAAATCCATAGCCTCCTAAGTGAAGCTAAAAAATTGTATAAAAGAGTTACTCAATTACGATCAGATTGCACTCAGCAGAAGCGTATGAATCGTGGTATTTCTGGACTTTTTGGCCGAAAAACCAATGTTATAGAGGAATATCAAAAGAAATTAGAGGACATTGAGGAGAGTGTGAGATTGAAGCAATCAGAGGCTTCATTGGCAGGAGAAGAGGCTCGAGCTGCCTTTGTGTTCTTCAGGTCTCGTTTGGGTGCTGCAACTGCTTTTCATTTAAAGCAATCAGTCAATCCCACCCACTGGATTACTGAGCTTGCTCCAGAACCTCGTGATGTGTACTGGCCTTTCTTCTCTGAATCATTCATGAGAAGATGGATCTCTAAGTTGGTGGTTGTAGTTGTGTCTACACTTTTCACTATTTCATTCCTTTTACCTGTAGTATTCGTGCAGGGGCTTACCAACCTTAATCAATTGGAAGTTTTGTTTCCCTTCTTGACAAGTATTCTAACCATAAAATTCTTCAATCAAATAGTGACAGGATACCTTCCCAGTCTTATTCTTCAGTTGTTTTTGAAAGTGGTGCCTCCGGCCATGGAATTCCTTTCCTCCATTCAAGGATACATCTCACATAGTGATATAGAAATGAGTGCATCTAGAAAAGTGCTGTGGTTCACAATATGGAATGTGTTTTTTGCATCTGTATTTTCTGGCTCAATTCTATCTATGCTGAAAGCCGTCCTTGATCCGAAGAATATTCCTGGGAAGCTGGCAGTTGCAGTTCCAGTACAGGCATCATTTTTTATTACTTATGTTGTCACGCAAGGATGGACAAGCGTGTCTTCAGAACTCTTCCGAGTAATTCCTTTTATTTTTTGTTGGATTACAAGGCCTTTCACAAGTCCAGATGATGATGATGAATTTGAAGTTCCATCTATTCCATATCACAGGGATATTCCGAGGGTCCTTTTTTTTGGACTTCTTGGTATTACATATTTCTTCCTAGCTCCATTAATTTTGCCATTCCTCTTGGCCTACTTTTGTCTTGCTTACATCATATATCGAAACCAGTTTATAAATGTATATGAGCCTAAATACGATACAGCTGGGAAATTTTGGCCTATTATACACAATTCAATGATATTTTCTCTGGTACTGATGCAAATTATTGCTGTGGGAATCTTTGCATTGAAAAATCTTTCTATGGCATCAACTTTGACGATGCCTCTACCAGTCCTCACACTTCTCTTTAACGAGTACTGCCGAAAACGATTCCTGCCCATATTTGTTGCATACTCTGCAGAGAGTTTGATAAAGAAGGATAGGCAAGACCTAAATGATGGTACAATGACACGGTTTTATGAGAATTTGGTAAATGCTTACAAAGACCCTGCATTGCTTCCACTCCAATACTCACTAAACAATGATAATCTTCGCACTCCCCTTATATCTCAGGCTTAATGCTAAGGCACAATGGTTCAATATTACACCTTTACTGGTGATTCTTTCCTGTATATGATGAGAAATATTCACATTTCAAGAGCAAAGAAGTGTAAAAGGGGCTTGTATATTCCAAACTGAACTTATTTTTTGCCTTGGCTCTTAAAACACTTTTCTTGTTTGTGTATATTTTGTTCTAAACATAATATCTCAGAGCCAAAGAGATGCTTATTGTTGAATTGCCACATTTTGGATAAGGGTTTTTGTTAGTATGTACCTTT

>C102113_65

TCATGGAAATATAACTTCTAAGAAAATTTAAGAACAATGTACAAAGTTGATGGAGATACCCTGAACACTACACCTCCATAAAATGTGGAATGAAGATTAGTACATTGTGCTTTATGAATCCCTCTTCAACCTATAGTTAGTCAAGCTACAGCTCCCAAGCTAAGATACCTCAGTCATTTGAGTTCTAGACAAGTAGCCCAATGCTCAGTCTCTGCTACTCGGCAGAAAGTTAGTGGGGAAGATATTTTATAGCTTAAATGGAAACCCTCCAGTGATTGATGATATATTCCATTGAAGCACGATTTTAGCATTCAACGAAAGCTAGAACGCTGAGAATGTGAAGAAAGAATTTACAAGTCTCGGATATTATGAACAAAACCCTATTGTTGAAGCCTAAGCCGATTGAAGAAGACCAAAAAATGATACTCAGGGGCCCAATACACAAATCCAACAACTGGGCCAGCTTTTAGGTGCTTTCCATCGAGAATCACAAGTTCACCATCAACCCATCTGGGATTCTGAAACCCTGGTTCAGCCAACCTCCCTTCACCTTTGTAGCGAGCAATCACGCCAAACTCTTCAGGTATTATCCCTTTATGAGGTAGTTGATACCTCTTTCCAATTTTTGCACGAAATGCCACCTGGCCAGCTGGTACATAAGGATCCCCTGTTAGCTTCAAAGCTTCTACATACTCATAAAACTCAAGATCTGAAGGCTCCTTAGCTCCATTGTCCTCTTGCCACTGTCCAATTTTACGTCTTAGATGAATAACTTCAGAGGAGTAAAGCCCATGTGCACCAATGTATAGACCATTTAAAGGATCAAAAGAAGTTGGTATTTCGATGCGATTGAAATTGGTTGACCCAGATAATTGGTGATGGTTCTGGGCCTGGCTTAGAGTGAGATTTATCAATTCTCCCACCTCTTTAAGCACCTTTGAGGGTATCTTTCCCCTACCAATAAATTTAGCAAGGTCAAAAATAACATGATCAACTCTGCGACGACCTTGAAACTTAGTTGATTTATTAGATGAAGATTTTCCTTTGTCAAGACCAATCTGCTGATTGACTTCTTTTTCAACAGTAAACGAAAATGAACCACACCCCTTCATATCCAGCTTAGCAGGAACTCGTAGCAAATCTCTAGTGGATAGATTACTGGAAAGTTTCTGAACAAGACCACCAATAGCAATTTTGACAGCAAATTCATTCTGTTCTTCACGTTCAAAAGTTCCCAGACCAGCATTTACTTCAATCTCATCATCTCCCTCCTGGTCAGTCTCAGACTTAATGTCTTCCAGTTCTAGACTTACTGTATCTTTTTCCTTCTCTTCGACTTCATCTTCCTCATCATCTTCATCTTCATCTTCATCTTCATTTTCATCTTCATCTTCATCTTCATTTTCGTCTTCATCTTCATCTTCATCTTCATCTTCATCCTCAATTATCTGCTCAATCACATCAGATAGATCCGTGTCTACTTTGTCTGGAGTAATCACCTTGAAAACCTTCACCTTTACCCCAGGAATCATATCTTTCAAGACATTCTGAAATCCAGGCATTCCCTCAGCTGGATCAGAGCCATCATTTCGATCATCACCATTTTCTGGATCTTCAGTACTAACAACAAACAACTCACTTTTGTCTTCAGTAGATTCCACTGAACTTAATCTCCCAGTAGTATCCAATGTTTTAGAGGACATTGTTGGGGGTCCATTGAAGGCCCCTCTTCGCTTTAAATAGACAGCCTGTGACTTAAATTCACCTTTTTTATCCATTGTAAGAAAAATTTCAAATAGAGGAATCCCAGCAGCAGATGTTGCAAGTTGCCTAGGACTATAACTCCTTGCCACATATCTTCCATGCTCAGGAGTTATGCGAATAATTAGACCATGTGGATCATTAACATCTTCAGAAATACCAGCCCACCAACCCACAAGTCCAGCGCCAGCTTTATCTCTTAAGAAAGCTGCATCACCATACCGCTCCTCTTTTATGGCTCTGTTGAGATAAGACATCACTCTTCCAACACTATCATTGTTAGCTGCAGCTGCAAACGCCACCTTAAGCCTGGCAGCATCTTTGTAATCCTCCAAATACACAGCCCGACTTAACTGAGACTTGAGAATCGAGAGGAGACGCTCTTGCTCGTCGATTTCCTCGAAATGCCGGCGCCACCGATCCCAATCCCAATCCTCGTCGTGGCCAGCGGCGCGGCTACGATCTTCGCCGGCGTTCCCGAAGGACTTCCAGTAGGAATCGACGCGCTTGGCGACCTCGGAGAAGTGGTGCAGCACGGAGTCCCAACCGCTTTTGGCGCCGTTGCGGTCGTCGGAGGAGAGACAGCGGCAGGTTGATAGGGAAGGAAGGGGGAGAAGCGAGGGCGTTCGCGCGGGAAACGGAACGGCAAGCTTCTGGTTAGGGAAGGTGAGGGTTGGAGCGCTCATGGAAGCCATGTGTGTGAGAAGAGAGAGAGAAAGAGAGAGAGAGAGAGAGATAAAGAGAGTGAAATGAAAGAACGAACGAATGGATATCCGTGTGTGTGAGTGTGTGTTTGAAGGAGATTGGAAGGAATATGCTGTGTTGATGTTGTTGCTAAATGGTGAGGTGATAGATTATTGTTGTTGAGTTGTGAATAATGTGAATGTGAAGTGAATGAGAACGAACATGAGGCTGGGAAGTTGGCTGTGAAAATTTGAGGGACGGATCTTCA

>C102115_65

TTTTAGATCAGTAATATAATTTATTAAAACAATCATTCCTCCATTAGTTTTAAATACTTTTTACTCGATTTTAGTAGAGTGGTTTGCAGAGCACATTTCATAAAGATACATTTTGGCTACATTTTAACCCTTTCTGCTCTAGTAAGCATTGGAAGAATACTTTTCCCAGAGAATCTCCTACACATTTCATAGCTTATTGGAATCGTTTTAACCTACAAATTCCACCCATCATACCAGTATTACTACTCAAGACAAAAAAATTCCAGAGTACAAAGCGCGCATCAATGAGCACCTACAAAATCCGTGTCTAACTTTTTAGAGGAAACACGCACCCACTCTCCATTGACAAAAATCATTAAGAATTTGGAACAGAAAAAACATAATTTCTATTAGTAACTAGCTCATTTAGAGTCGGTGAAATCTGCATCAATGACATCACCGTCAGGTCCCTTGTCTGAAGATTCTGAGGGGCCAGAGTCAGCACCAGGTGGTGGAGTAGGGCCTCCTGCTGCTCCAGCAGCTCCTGGCTGGTTGTAAAGGGATTGACCAAGCTGCATAACTTCCTGATTGAGGGCAGCCATGGCATCTTTAATAACTTGGGTGGAACCCCCAGAAATTGCATCTTTAAGTTCCCCTAGTTTTGCTTCAACCTTCTCTTTCACAGGGCCGGGAACCTTGTCCCCAAGCTCTTTCAACTGCTTCTCTGTCTGGTACACTACAGAATCTGCCTGGTTCTTAGTATCAATAGCATCCCTCTTCTCTTTGTCTTCCTTTGAAAATTTCTCAGCTTCCCTTACCATTCTCTCCACCTCATCTGAAGGTAAGGTGCTAGCACCAGTAATGGTAATATCTTGCTTCTTCCCCGTGCCCTTGTCAATAGCAGTGACGGAGAGAATGCCATTGGCATCAATGTCAAATTTCACCTCAATCTGGGGAACTCCACGAGGTGCAGGAGGGATACCATCCAGGCGGAAGCTACCAAGTGATTTATTGTCCCTAACAAATTCTCTCTCACCCTGAAGGACATTGATCTCTACACTAGTCTGTCCATCAGCAGCTGTGGAGAAAACCTCGGACTTTGAGGTGGGAAGAGTAGTGTTTCTAGGGATAATTTTGGTCATCACACCACCTAGAGTTTCCAGACCCAAAGATAATGGAGTGACATCCAACAGCACAATGTCGCTGACATCTCCAGCCAAGACACCGGCCTGAACTGCAGCTCCAAGGGCAACCACTTCATCTGGATTGACAGTGACATTGGGATCCTTGCTAGTCAACTTCTTTACAAGTTCCTGAACTGCTGGGATACGTGTTGATCCACCCACAAGGATAACCTCATCAAGATCCTTAAATGAGAGCTTTGCATCCCTCAATGAATTTTCAACTGGTGTCCTCAACCTGTCCAGAAGATCAGAACAGAGTTCCTCAAATTTGGCCCTTGTTATGGTTGTCTCAATATGTTTCGGACCATCTGCGGTGGCAGTAATAAATGGTAAACTGATGTTAGTTTGAGTCAATGATGAGAGCTCCATTTTGGCTTTCTCGGCTGTCTCAGTGAGACGCTGAAGAGCTTGTTTGTCTTTCAAAAGGTCTATTCCTTCATCTCTCTTGAAGTTGGAAGCCAGCCAGTCAACAATTCTCTTGTCAAAGTCATCACCACCCAAGTGTGTATCCCCAGAGGTAGACAGCACCTCAAACACTCCATCACCAACCTCAAGCACAGAGACATCAAACGTACCACCTCCAAGGTCAAAGACCAAGATAGTTTCATTGTTTTTCCTTTCAAAACCATAGGCCAAGGATGCGGCAGTTGGTTCGTTGATAATCCTAAGAACCTCTAGACCAGCAATCCTACCGGCATCTTTGGTAGCAGTCCTCTGTGAATCATTGAAATAAGCGGGCACAGTAACTACAGCCTTGGTCACTTTATCGTTCAAAAACTTGGAAGCATCATCCACAAGCTTCCTCAACACCTGGGCAGAAATTTCTTCAGCAGCGAACTGTTTACCAATGGCGGGGCAGTCAAGTTTGACGTTGCCGTTGTCATCTCTGATGACTCTGTAAGAAACCTGCTTGGATTCTTCGTCGACCTCGGACATCTTCCTGCCTATGAACCTCTTTACGGAGAAGAAGGTGTTCTCCGGGTTGACGACGGCTTGGCGCTTGGCGATTTGGCCGACGAGCCTGTCGCCGTTCTTGGTGTAGGCAACGACGGAGGGGGTGGTTCTCTGGCCCTCGGCGTTGGTGATGATGGTGGGCTTGCCGCCTTCCATGGCGGCGACGGCGGAGTTGGTGGTTCCCAAGTCAATACCGACGACTTTTTCATTTACGACACGGAGAGGAAGGACCCTCCGGGGTTTGCTCTTAACTTTGATAAAGGCCTTGGTGTTTAACCTCTGACCTAAGAATAGGGTTCGGGAACCGGCGAAGGAGGGGGTTCCGAGGCCGTGGATTTGGGCGCTTGAGCAAGCCATGTTTACTGAGGGTGAAAGGGGGAAAGAGGATATAACCCTAATTCTGGTGGAGTAAGCGGAGAGAGAGAGTAGAGAGAGAAAGAAAGAAAAGGGGTAAGGGTTCGGTTAGGGAGTAGAGAGGGGGGGTTGAAGAAGAATAAGAAGGAGGGTTTTGGGAGAGGATGAGGGTTATGCTACGTCCGATGTGCTGCCACGTGTGTTTATTCTAGAACCATCTCTTCCCTTTCATTTCACCCCGAA

>C102117_65

TTTGAGATGATGAGATGAAGGGAAAATGAAGTGCTTGGAGCACCACTCCTTACAACAACCACTGCACTCATGTCATGGCCTCCAGATTCTCCCTTTCCCTGCAGGCCTTCCATTTCAAGGCCTCCCCCCTTCAAGCCAACTTACCTTCCTCCGTTCAAACCCAAGCTCCTCGTTCAACTCCCGGAGGCCAACCTCAATTACAAGCCTCAGCTTCGGGGTCCGAAGCGGGCCCCGAAGCATGGTCGAGACCACCCCAAACCCACTTACTTACCCAAGAACACAGACAAGCTCTTTTCACGCGATGCTGGCGTGGACTTCTCTTCCGAGCTGAGCACCGCGCAGTGCAACGCAATCCTGAAACGGCTGGAAGAGAGCGCCGACGACGCCAAAACGCTGGCGTTTTTCGAGAGGATGAGGGCAACCGGAAAGCTGGAACGCAACGCCGGCGCTTACAACGTAATGCTTAGGTTGGTGAGCAGAAGGGGAGATTGGGAAGGCGCGGAGAAGCTAATTTCTGAAATGAAGGCCAGTTTCTGTTCCGAATTAAGCTTCCAAGTCTTCAACACCCTTATTTATGCATGCTGCAAACGGAACCTCGTGAAATTGGGGACAAAGTGGTTCCGAATGATGTTGGATTGTGGCGTCGCGCCTAATGTGGCGACCATTGGGATGCTCATGGGCCTTTACCAGAAGGGATGGAACCTTGAGGAGGCGGAATTTGCGATTTCCCAAATGAGAGGGTTTGGAATTGTGTGTGGATCTGCTTATTCTTCAATGATTACAATTTACACACGTTTCAGGTTGTATGAGAAGGCCCTAGGCGTGATCGAGTTGATGAGGAAGGATAAGGTGGTGCCCAATTTGGAGAATTGGTTGGTGATGTTGAATGCTTATAGCCAGCAGGGAAAATTGGAGGATGCTGAGAGAGTGTTGGAGGCAATGCAAGAGGCAGGGTTTAGTGCTAATGTTGTTGCTTACAATACTATGATAACTGGTTATGGGAAGGCTAGTAAAATGGATGGTGCAGAGAGGTTGTTTATTAGGATGCGGCAAAGTTTACAATTGGATCCGGATGAAACTACTTACCGATCCATGATTGAGGGTTGGGGTAGAGCTGATAATTATGTATACGCCACGAGATATTATAAGGAGCTCAAACGGTTGGGGTACAAGCCCAATGCCTCCAACTTGTTTACATTGATTAAGCTTGAAGCTAAATATGGAGATGATGAGGCTGTTTTTGGGATCCTTGATGATATGGTGGATTGTGGATGTCAGTATTCTTCTATAATTGGCACTCTGCTGCATGTGTATGAGAGGGCTGGAAAGGTTCATAAGGTGCCGCATTTGCTCAAAGGTGTGTTTTATCAGCATGCGCTGGTCAACCAGAGTTCTTGCTCCACTCTGGTCATGGCTTATGTAAAGCACCGGCTAGTGGAGGATGGCTTGAAAGTGTTGAATGACAAAAAGTGGCAAGATCCTAGATATGAGGATAACCTGTATCATCTTTTGATTTGCTCGTGCAAAGAGGCAGGTTCGCTGGAGGATGCTGTGAAGATATATAGTCGAATGCCCAAATGTGATAACAACCCAAACATGCACATTGCCTGCACCATGATTGATATTTACAGTGTCATGGGCCTTTTCGAGGATGCAAAAGTGTTATATCTGAAGTTGAAGTCTTCCGGAATTGCCTTGGATATGATTGCTTTCAGCATCGTTGTAAGGATGTATGTCAAAGCTGGATCTTTGAAAGATGCTTGCGCAGTTTTGGATGCTGTTCACGAGCGATCAGATATTGTTCCTGACAAGTTTTTGTTATGTGATATGCTGCGAATTTATCAAAGATGTAACATGGTGGGTAAGTTGACTGATTTATACTACAAAATCTCAAAAAATCGAGAGGATTTTGACCAGGAACTATATAATTGTGTCCTAAACTGCTGTGCTCAGGCTCTGCCAATCGATGAGCTTTCCAGGCTTTTTGATGAGATGGTACAGCGTGGATTTGCACCTAGCACAATTACCTTTAATGTCATGCTTGATGTCTTCGGGAAAGCCAAGCTTTTCAAGAAGGTTTGGAGGCTGTACTGCATGGCTAAGAAACAAGGCCTGGTTGATGTGATCACTTACAATACAATCATAGCTGCATATGGGAAAAATAAAGACTTCAACAACATGTCATTGACAGTCCGGAAAATGGAATTTGACGGGTTTTCAGTTTCCCTCGAAGTATACAATTCAATGCTAGATGCTTATGGGAAAGATGGCCAAATGGAGACATTTAGATCTGTATTGCAAAAGATGAAGGACTCAAATTGTTCCTCTGACCACTACACATACAACACTATAATCAATATCTATGGAGAACAAGGATGGATTAATGAAGTAGCTAACGTGCTTACAGAATTGAAAGAGTGTGGACTCAGACCTGATTTGTGCAGCTATAACACATTGATCAAGGCATATGGGATCGCAGGAATGGTTGAAGAAGCTGTAGGTTTGATCAAGGAAATGAGAAAAAATGGAATAGAACCTGACAAGAAAACCTACACTAATCTCATCACTGCATTGCGACGAAATGATAAATTTTTAGAAGCTGTTAAATGGTCATTATGGATGAAGCAGATGAAATTATGAATTTCATAAACTATGGCATTATCTATAAAGTAGTTTTGGACCTTCTAACCCTCGTTATTTGAAGAATAGTTAGA

>C102119_65

GCTAGATGGCACTTTGTTCCAATCCAAAACATATTTTTCCATAAGGGTGTCAACTCCAGACATTGATCACTTTCTCTGCCTCAACTATTTTGTGTTTGTTGTTCCAATGAGTGAGGAATCTAATCTGCTTAAAGAAAGCACATCAATGAATGACACCATGGTGGAAGAGGTTGAAGAAAGAGATCCAGAAAGCAATCCACTGAATGAGCCACTACTTCTCAAGAGGAACAGGACTCTGTCTTCCAATCCACTTGCTCTGGTTGGAGCAAAAGTTTCCTACATAGAAAGCTTGGACTACGAGATCAATGAGAATGACCTGTTCAAACAAGATTGGAGGAGCAGATCAAGAGCTCAAGTGTTGCAATACATATTCTGGAAATGGACTCTTGCATTCCTTGTTGGACTTCTCACAGGTGTCATAGCCACTCTGATCAATCTTGCAATTGAGAACATTGCAGGGTACAAGCTTCTTGCTGTTGTAAACTTTATCCAAAAAGAAAGGTACTTGACGGGTTTCTTTTATTTCACGGGAATAAATTTCATTTTGACATTTGTTGCTGCTATTCTGTGTGTGTGTTTTGCACCCACAGCTGCTGGACCTGGAATACCAGAAATCAAAGCTTATCTTAATGGGGTTGATACTCCCAACATGTACGGTGCCACAACATTGATTGTTAAGATAATTGGAAGCATTGGAGCTGTATCTGCAGGGCTAGATTTAGGAAAAGAAGGGCCCTTGGTACACATTGGTAGCTGCATTGCTTCCTTACTTGGCCAAGGTGGCCCTGACAATTACAGGATCAAGTGGCGCTGGGTGCGGTATTTCAACAATGATCGTGATCGTCGCGATCTCATCACCTGTGGTGCTTCTTCAGGGGTCTGTGCTGCTTTCAGGGCACCTGTGGGTGGAGTTCTCTTTGCTCTTGAAGAGGTGGCAACTTGGTGGAGAAGTGCTCTCCTCTGGAGAACATTTTTCAGCACAGCTGTGGTTGTGGTAGTGCTAAGGGCCACCATTGAACTTTGCCACAAAGGAAAATGTGGTCTTTTTGGTGAAGGGGGCCTCATCATGTTTGATGTGAGTGATGTCACTGTGACATACAGTATCATGGATATCATCCCTGTTGTTATTATTGGAGTTCTTGGTGGGGTATTGGGAAGCCTTTACAACTATCTTCTCCATAAAGTCCTCCGTCTCTACAATCTCATCAATCAGAAAGGAAAAATGTACAAGCTCCTCCTCAGTCTTTCAGTTGCACTCTTCACATCAATATGCCAATATGGTCTCCCATTCCTAGCAAAATGCACCCCTTGTGATCCTTCCGTTTCAGAAGTGTGCCCCACCAACGGGCGTTCTGGAAACTTTAAGCAATTCAATTGCCCAAAAGGCTACTACAATGATCTTGCTACTCTGCTGCTGACCACGAATGATGATGCTGTTAGAAACATATTCTCAACCAACACTCCTTTTGAATACCAACCTTCCTCCATCCTAATTTTCTTTGCACTCTATTGCATACTGGGACTAATCACCTTTGGAATTGCTGTGCCATCAGGGCTCTTCCTCCCAATCATTCTCATGGGTTCAGGCTATGGCCGGCTTCTTGGAATCCTCATGAGACCTCATACAAACATTGATCAAGGGCTGTTTGCAGTCCTTGGTGCAGCCTCACTCATGGCTGGATCTATGAGAATGACTGTGTCCCTTTGTGTGATCTTCCTTGAACTAACAAACAACCTTCTCCTACTACCAATAACCATGATTGTTCTCCTCATAGCTAAAACTGTTGGAGACAGTTTCAACCCAAGCATCTATGAGATCATACTGCACTTGAAAGGCCTCCCTTTCATTGATTCAAACCCTGAGCCATGGATGAGAAACCTTACTGTGGGAGAGCTTGTTGATGTGAAGCCAGCAGTGGTTAGCTTCAAAGGTGTAGAAAAAGTAGCCAATATAGTTAATGCCTTGAAAAATACTACACATAATGGTTTTCCTGTCATGGATTGTGGAGTGGTGCCAACAATAGGAGTGGCCAATGAGGCTACAGAACTGCATGGAATCATTCTAAGAGCACACCTTATTCAAGTGCTAAAGAAGAAGTGGTTCTTGAAGGAGAAAAGGAGAACTGAGGACTGGGAAGTGAGAGAGAAATTTACATGGGTAGAGCTGGCTGAGAGAGAAGGGAACATTGAAGATGTGGCTGTGACTAAAGAAGAAATGGAGATGTTTGTTGATCTACATCCTCTCACCAATACAACTCCCTTTACAGTTCTTGAGAGTATGTCAGTAGCAAAAGCAATGATTCTCTTCAGACAAGTAGGACTTCGCCATATGCTTGTTGTGCCAAAGTATCAAGCATCTGGGGTGTCTCCTGTGATTGGGATCTTGACCAGGCAGGATCTACTGGCATACAACATTCTGACAGTGTTTCCTCACTTGGCAAAATCAAAGAGGAAGTGATGTTTTCTTATCAAATATCAATGAATGGACAAGCAGCTTTATTGGGACAATTCTCTCTATTCAGCAAGAAGCTTTTCCTCAGTTTTGCATTATTTGGGCCAAGCATGGAGAAGGATAAGATACAACATTGATGTTAAACCATATTCAATTTTTCCTATTTTTGTCTCACAAGTTTTATCTTATTAATGAAATCTATTACTCTTCCTTTACAATAATGGCAATGCTAGTTTGTCTTTTTCCACCTCAGAAACGGGAA

>C102121_65

ATCCTGTGAATTCAAGGAACCCCTGTATTATGTGACATCATGTTAGAACCAAATGGGAGTGACAAGAAAATGTTATTCTGATAACCTCCCCATTCATGGAAGGCAAAAAAATAATGGGAAGAAATTCTGTACAACGGACAAACTATAAATCAACAGGCAAAAAACATATACAGCATGAGACAGTGACACTTTGTGTAGCTTATAGACAAACCAAAATTGTGATTGCAAGCTTTGCTATATGCAGAAAATAAGTTGAAGAGATCATGACTGATGTTCATAACTCCAATGGAATTGCAGACTGTCCAACAGAGAAAGGGAAAGGACTAGTAAACATAGGAAAGCCATGGATGCTTCAGTGCCTGTCTTGCGGTAGGCCTTCTTTTGGGGTTGATGCAGAGTAAGTATCTGATAAAGTTGATAAACATAGTATCAGTAACCTGTAGGTGCTGCTCCAACGACGACTCTTCTGGAATTAAGTACTCCAGTTGATCAGTCTCCTCATTTACATAATAAATATCATATTCTTTGGTGAAGTACTTGTGTGTTTCTTGTCCTTTCACCAACATTTCCATATCAATAGATCCGAACATTCCAATCATGCGTGCCAGGATCATCACGACTGCATCATTTGGAAACAGCACTTCACCAGAGCATAGCTCTGCCAAGATGCAGCCGAGAGACCATATATCAATCTTTTCATCATATTGAAGGCCTAACATCACTTCAGGAGCTCTATAGGATCTGGACTGTACATATAGGCACAGATTGTCTGTTTGGAAGCAACTACTTCCTAGATCAATAACCTTTATCTCACATCTTTTGTAACTTTTGATTAGAATGTTTTCTGGCTTCAGGTCACAGTGAACAATTCCCAAGCTATGCAAGTATTGCAATGCTTCCAAACACTGACGAGTAATGAGCTGCAATCTCTTTAATGTAAAATATGCTTCCCCTCCAGATTCTTGGTTGAACTTCTGAAATTCGTACAAGTTTGCTCGCAGAAGTTCAGTTACAATGAATAAATGCTCCTGATGGTAGAAATAGTCATAAAGGCGCAAAATGTGATGTTCATCAGCTGGATCATGCTTATTGACAAGTTTCAGAAGCTTGATTTCATCTAAGCTTTGATCAAAAAAGTCTTTGTCATTTTTAATAATCTTCAAACAAACATCTATTCCCGTCTGAAGATCATGTGCCTGAACGACCCTACTGAAGGCAGCAGAACCAAGATACTCTGTTATATAATACCTTCCAGCCAAGACTGTATTTAGCACAATGGGAAGTTCCTTGTTTTCTTCAAATCCAGTCCTGTTCTTTCTATGTATAATTCTCAGATCAAATACTTCGTACTCTTCCTCCTGAGTATACTTTAAGAGCTCATCATCAGTGGTATCCTCTCCATCTGCAGGAAGCTCAGGATCTTGGCAGTCTTCAGCTAATTCTCCGCCAACTTGAGCTTTATAGGTGTCAAGTCCATTTGGTAGATTTTTATCAGTAACTTTGATAGTTAAGTCATAGGGACCCTTGTACTCCTTACTGCTGTCGGTTTTAGAGCAGTAGTCAAAACTTTGATTCAGAGTTAGCTCTGAAGTAGAATTAATGTCCCCAACAACCTTCAGATGAAAATCGTTCAGATGGATTTCCTTCAAATTAGGGTAGCCGAAGTTTGTAGGATCCAAACTGCAAGAACCTCCCGAACCAGGACTGCTTTTGCAAAATGGAACTTCACAACTACAGGCAACACTGCGTTTCTCAGCCTTGCCCTCTAATTCCCCTTCAAACTGGTCCTTGTTATCAACATGGCAATACCCTTTTGAATAAGTCACAGTTCCATCCCCTACAGAGGAATGATTATAGAGGCATGCCTTGTCCATGTAACCACCATTACTAGTCCCCTTTAGTTGAAATTCTTCATAGTTGTAACCAAGGTGCAGAGAATTGTCACGTTGGTTTTCCGTTTCTGCGGTCATGACAAACTTATCTTCACTCTGACACGCAAAGAAATCCGGCCCTTCAAAACTAGGAGTCATGAAATAGTCATCATCTTTTTCATTATACCAATACGGTTCATTTTGCATATCAAAATCGTGATAATCACGCGCGGTGCCAAACTGAGATAATCTTTCAGACGAAGACTCGGAATCATTCTGAGTCTGAGATGAGGAACGGATCCCATATGGATTTATGAATTCTGCAGAAGAGACACGAGAAGTGGAAGAACCGATGGTGATGAACTCGTCGGAACTGGATTTGGAGCAGTCGTCGTCCGCGGCGGTGAGCTCAGACGGTCGGGAGAAGGATCGGACTCTGACCGGCGGCGGCGGCGGGACCATGGGGAAGAAGAATTTCTCGTAGTCGAAGTTTCCGAGGTCGGTGTTCTCGATGATGTCTTCTCGAAGCGCGGACTCCGCTTCCGACAAGCCATTCTTCCGCAGAAACTCTAACACTGCTTCGACGCCGTCGGACACAGCCATGGACACGACACAACCGACTCAGAAACCTTATTGTTGTAGTTGATGTTGATTATGATGATGTCTGAGTAGCGCAGTGACATGTGAACTAAGAAACACGGAAGGGATAAAAGCGTAATAAAACGCATGGTTTATGATGTGCATGCTCTGGTAATGGTTGCAAGAAGAGAGAGAAGAATGCGTTAGAAACATGGAAGATTCTCTCTGCGTCTCTCTTTCTCTCTCTATGTTTTCTGTGTAAAAC

>C102123_65

CATAAATGTGAGATCGCATTATGCATTGCATCTACCATATAATTTACTAACAAAACTAAAAGAAAACAGAGAGAATATTGTGTCTTCTATTCTCAAAAATTAAGAATGTTGTTTCATATCTGACCACTCTCATTCTTGGAATGCATAAATAAATTTCATTACAGGATGTTCTAAAATGAGCCTCCTAAATCTCTTGTCGTGATGTGGTGGCAATCTTGCATTATCTTGCTCCAAACCAAGAACACACAGACCATGTAACATTGTGTCTCGAAAAGTTCCTTTCCTTCATGGAGAAACCAAACTTAAATTACTCAGTCAGAATCTGATAGTGGCATCTGGTAAGCATCCGATGGTTCCAAATCTGTCATAGAAGGGGGTGCTTTTTTATTTGACCGACTCCGGAATCGGTCTCTTGGTTTTTCTTTCCTTTCATCCTTGGAACTGGGTTGACTTGATTCAGATGGCTCATTGGAAATAGGTTGATTTGGTTCAGATGGCTGAACTTGTTGGCTCCTACGCAGCCAGATAGTACCCAACGAAATTAATGCACCCAGAAGCAAAAAAGGACCAACTCTTGGATCCTCACCGTATACAGAGAGTCCACTAAGACTACCAAGAATACTCTGCTTCAGATTTCTCATAGGAATATTTTGTGCTGTTTTGGACCAAATAGGTTCTGTGTCATCCGGGACAAGCTTAGGAGTCCGTTGTGCAAAGAATGGTAAGTGTCTAGAGTCACCATCAGAAATAATGTTTGCAAGCCAGTGAGTTATCTGTGAAACATCATCTTCTCCCTTGTACCCAGTGACAAGCTGGCCTGCAAGATCAGAATCATTGATTAGATCATGGACCAGTGATGATTTCCACTTTGTCTTTTCTTCTGTTCTCAAATCAACATCACTATTATTCCTCTGGTAACGAACAATAAATAACCGAGGAATATCAGTTACACTCCGTCGTTGCCCACAAGTGTGATCGCTACCCGCTTGGCCAAGGTAAAATTTGCAGTAATCCTTCTGCTTTTCACCATCAAGCCAAGCAAATGTCAACCGTCTTCGCTTAAATGCATCCACAGCTGGAGCTAAGGATTGATTTTCAGATGATGCATCCACTTGGCTATGCTTAGACAATGTTTCTTGGACCCTGCACATAGTTTCACGCATTTTGTTCAGCTCCAGACTTGGCCTTCCGACCACAATAGCACAATACCATATTACTGTATCATAACCAGCACGAGAATATCCATGGGGATCACAGCCTAACTCCATTGATGTCACACTCCTTAGTTGCGGAAGCTCTTGCTGTTTATTATTTTCCATCAAGTTTAAAAAAATTGAATTGTTGACAGAACCATGGTGAACCACAGGCTTGACACCTGGGTCTTTAAGAAATACAATAGCTGGTGCTGATTCCACCCCAAATGCGCCCCACCAATAGGAAGATTCCTCTTCCCGCCAGAGAATGAATGCAAATGAAGCAAAATCCCAATAATCTTTAGCTGCTTGTCGTATAAATGGAGCAGCACGCTCACCAGATGTTGAGAAAAAGAAGACTTTTACCTTATGATGGCTAGTTTTACCAATAAAATTTGGCACCACAGATTCCCTTGAGTAGTAGTTAATTTGAGGTAAAGCCAATACACTTGTTGCAAACCAATTTGTAACCTTGTCAACAGTAAGCTCACCATTATACCTACTAATACATTTAGCAGATCGACATCCAGCAGGGATGGCAACAAGAGATGGTATACCATTTCTAAAAAATGGTTTTCCAGTTGATCTTCTATCAGCAAGGTATACAGAAAGCTCCTTCTCACCAAGTTCCACAACACCAATGTTTGCAACAACATCCAATAAAGAAGCAACTTTAATCCAGGACTTGGAAAATTGAGCACAACGATCGCTACCTGATGAATACAACTGAATTAGCCATGGCTTTGCATCTGGGAATATATACTGGAAGTTACTAGCTGTAATGACTTTAGAAGAATGATCTACAAATCCAGATTGGGCTTGTAGTAAAGGGAAGTCCAATTCTGAAATATGTTTCCCTGCATAACGCTTGCTGGCACTCTCAACAATATGAAGTTGTTCATCAATACCAAATATGTCATAGTCTCTTTTCCATAAAGGATTTGTCAGCAACTCATATGCATATCGAATCTTTAAAAACTCATGAATACCAAAAACTTCCTCAGCAGAATTCCATTTAGATTCAAGCTTGTTGTAAGCCTCCTTCACTTTGTCAACCGAACTATAGCTTTCAATTTGCAGAACGTCGTAATGAGAAGGAGGGAAGGCATTGGGAATAATGAAGAGCTGATAGAACATGGCTCCGGCGAAGAGAAGAATAGGTAGCGCGTAGGCTCTGACGGTGGAAGCCATAGAGAGTAAGGGTGAGGGAGAAGGATTCACAGAAACCCTAGACATGGTAACAGAATCAGGAGCCGTGATTGTGATTGTGATTGCAGTGAGTGGAATTTGAAAACTGAGTTGGGAGCGATTCGCTCCGTTGGTGATGACTGTATTCGCTCGTTTTCAGTGAGTAGTACTAACGCAAGAAACTTCTTCAGAGTGTGTTTAAGGTTCCTACTAGTAGTACTCTTCTTGCGTCTAGACTACGTCTTCTCCTTCCCTTTTCTTCTATTTCTCTTTGGCAGCTTGAGCTTTAGCAAAAGTTTGAACTTTCAAATTTGAAGAGAACGAATCTAAAAGAG

>C102125_65

CCCCACTCACTCACTCACACTCATTTCATTTTGCAACCAACCATTTCATCTTTCGTGGGACTCAACTCTCTCACCAAGTGATGTTCACTCACTACTTATTTGATGATACTATGCTATCCATTTCTTGAGTTTTATATGGCCAATTCAGATACATAATGCCACATGTCCATGACAAAAACACACACTCCACACACTTTCTTCAATTTGGCTTGCACTGCAAATTCCTGGGATGACCATGTCCCGTGTTTCATGCTATTAAATAGATTTTCTACATTGCATAGAAGGATTTTGTTATCGCATCCTTTTTGAGCTGAAATTAGTAGCGTATGATGTTCCTTGATTGTTGAACTTACTGCATAGAAGAAAGAAAGAGTTCAGTTGATGTTGGTGTGGAACATGATGGATGAAAGCAAGTTGAACATTGAGGCTCCTCTTATGTCAGTCAGACGCTCTTTTGGTACACCTCCATCCTTAACTGAAACAAGTAGTAAGATCTTAGAGAAGCCACAAACACTTTCACACTGTAAATCGGACACCACTTTGGATCAGGTAACAGAACCTGTTGCAGTGCCTTTCAATTGGGAGCATATCCCTGGAAGGCCGAAGGATTATGATGGATCTGAACCTCAACCTCGTATACAAGCGTCAATTACCCCAACCCCGACTCTTCCCCCTGGGAAGTCTACAAATGCTGCCAAACAACCATTGGAAAAGGAGCCTAATGTTACCAATAACTTTAGATCCCGAAGCACACCAAATTCTTCTATCGAGAAAATAGATTGTGGAAAAGAACACAAAGATGAGAAAATAGAGAAGGTGGAAGAGAGCGATGGCTATGATAATGATGATGATGTTTGTTCTGATGCCCTTGAAACACTGTCACCTACAGAACCATTGTCCATGAATTGTAGTGTAAGTGGTGTGAGTGGCCTAGATAATTTGGATTCAAATATTTGTGGAACCTCTTCCACTGATAAACAAGCTAATGATTTCATGATGAGCCGGTTTTTACATGCAGCAAAGGCTATGACTATCCAGCCTCCTCAATATACTTCTTCTAGAAAGCAATCTGTTCTGGCAGAACAACCTAGGGAATTTATCAAATTAGTTCCTGAGCACAAGAAGTCATTTGTTAATAGACATATCACTGACATTGTACCATATAATGGTCAATGTGAAGAGGAGGAAGAAGAGGAAAGTGGTGATGAAACTAATGATTATGACAATATTTCGGCTAAAGGTTGCGGTTTGCTTCCCCGCTTATGTGTTAGAAATTCATTGTGCATATTAAATCCAGTGACTGGGACAAAAATGGGGAATCAGTTGTCCTTGTATTCTGCCTATGAGGTTGAAAAACCTGATAAAAGTTCTCATATTCGCTCTCACAGACCAGCTCCAGTTATAAAGAAGGCTTGGGATGCTATTAATAAGAGGAAAACAAGCTCTAGAGCTTCATCACCAGACAAGCAGGATCTAAGGAAAAAATGGACTAGTGAATCAAGCAGGTATACTTACTCTGGTGAAATGAAGCAGCTAGGTAGGCTCACTCCCTTTCGTCGTTCAAGAGCTGCTGCTGCTGGCATATCTTCTTTTCGAAGTAAACCTCAACCTCTCTTTCCTGGAGCAAAGTCGCTTGGTGATTCTAAACAAGCTGATAATAATCTTTCTGGCAAATTGAAATTCCCTAGTAGAGGATCTGCAAGTATTCAAGAGATGCTATCACAAGGAGCCAAAAGAAGCACAAGTTCTGGGAGCCTTATTATTGAAAAGACATTGTACATAGATAGTTTGAGTGCAGTAAAATCGTCATCCTCTAGTTCACGTTCTTTAGGAAGTGCAACATTGGTAGGAAAAGAAAGAAATTCCTTACTAGATTCTTTTCGAGATATGAAACACCTACAAGCATTGGACGAGAATTTGGATTCTCAAGAGTTGAATTCTGAGGATGCCAATTCTTCAAATTTATCTAGCATGGTATATCTCATGGCCAAAGAAGATAAAACTGAGCGAATGACAGCTGATCAAGAAATTAATCAAGAATCCATGTCCTTGCAACTTGTGCCAAGTTCATTTGACAAAGATGCAGAGATCAACAACAAGGAAATTGTGGTAGTTGATGATCCAGTAAAAGTTGGCACTGAATATGTTCTGCATCCTCTTGCCCCACCGTTGCCAAAGTCACCTTCTGAGTCCTGGCTTTATCGTGCCCTGCCTCTAGTTTCTTCTAAGAATTCATTCCCACATTCGAATCAAGGTACACATTCCAAGGTTAAAAGCCAGGGTTTTAGTAGAGCATCAAGCTATACTAAGTGGGAAACAATAGTGAAATCTTCGAATGTCAATCATGATCAAGTAAGCTGTTCTAAGGAACTAGTCGTGTATAGGTCTCAGCATTCGAAATCTTAGAGATTGAACACTGGCGTTTATATCATGTTATGGTCCAAGCTTTTTATGTCCTTTATTTTTTCATCTGCTCCTCCTATTGGTTGTTGGCATGACAGCATAGTATTTTACATGGAGGAATCAATATAATTTTTGGTGATCAATAAAAGCAAACAAAGCTTGTTTGGGCTAGTGGTACTTTCGGTGGATTCGAAACTTCATAAGCTACCTCCAGAGAAGTGGATGTGCACTACTATCCTGTCTTTTCACTTGTGGTACTTACTGACAGCTTGCTCAGAGCCACGAACCTTTGATTTCATTATCTGCAGCTAATA

>C102127_65

TCGATATACCAACTGAATTCAAAGTGCCCCAACAAACGATCTTGAGGAATCAGCTCTGCATTTGACTTTCACTAGAACGTACTTCAAAGCTCACCATAGTCAATCTGTTCATTAACATATGGTCCAAAAATGCATAGTTTTCCTTTTTGCTGGGATCAACAAGGTGAAAATCAGTGATTTGTCACTGTCAAGTACCACTCAGAATACAAAGCAAATTCATGACCAAGGAAACAGTAATGGACATCTATATCGCTATGGACAACCTTCACATCTTAAGGGGCCTTGTCTCTGCTTTCACCTCAAGTGCTCTAAAAATAGATTCTTGACAATAACATGTATACTTTTGGCATCCTAGAGACATCGGGAATCAACGAGTTTCTACCAATTGTAACAGACAAAGAAGCTTGGAACATATGTGAAATAGTTATTCTGCTTTAGAAGTAACTAACTCCAGTTTCTCAACTTAGAAATTGGACAAAACTTTGAAAAGATTAGCTCCTTAGAGCAGGAAACTGTTACCATTTTTATTGTTTTGTCGCACCAACGGTGCTTCATTTTTGCACATAAACATAAGTACCATATTTTACAAGGGACCTATCGCTCCAAAATTCTTGAACAACCATATGCGACCGAACTCGAGCGAGCTCTGATGTGGATATGCGTGTGACAAACAACCAGCTTATGCACCCAACTTACAGCACATCTTGGTACATATGACAACTTGACCAAGTGATTGTGGAATTGTCTATTATGTGATCACTGTAGGTTGCTCCCTCCCTGTGAAGTCTTTGAGCTTTGACAAGGATATTGCCAAATCTATTAGTGGTTTTAAGGCAATTTGAGCATCCACATCATGTTTAGAGACATCCGGTTCAAACTGTTCGATGTACACTCTAACAGTTGCCCCTGCAGAACCAGTTCCTGATAAACGATATATAATCCTTGAACCATCTGTAAAAACAAACCGAACACCTTGTTTTGTTACCACACTTCCGTCTACAGGATCGGTGTATGTATAATCATCTGCAAACTGGAGGACATAACTTCCATACTTATCACCAGCCTTGCTCTTAGACACAACATCTCGTAGATATTCTATCATCTTATTGGCACCTTCGGATTCACATTCCTCGTAGTCATACCTAGAGAAGAAATTCCTTCCATAAGTTGCCCAGTGCTCCTTCATGACGTCAGATACTGATATCAATTTCTCCCCTGGCTTCTTGTCTTTGTTGCGATGTGCAATAATAGAAAGCCAAGCTAAGACAGCCCAGATTCCGTCTTTCTCACGAATGTGGTCAGAACCTGTTCCAAAACTCTCTTCCCCGCAAACTGACAAATTCCCAGCATCCATAAGATTCCCAAAAAATTTCCAACCAGTGGGTACCTCGAAGAAAGGAAGGTTCAGTTTTTCGGCAACACGATCCAGAGCACCACTTGTTGGCATTGATCGAGCAAGACCCTTAACACCGTTCTTGAAGTATGGAATCGCTCCTACGTTGGCTGCAATAACTGCTACAGAGTCTGAAGGAGTTACAAAGAAACTTCTTCCTAGAATCATATTTCTATCCCCATCCCCATCACTGGCAGCTCCAAAATCAGGTCCATTTTCAGCATACAGAATGTTGACAAGATCCTTTGCATATGTTAGATTAGGATCAGGATGGCCATGTCCAAAATCTTCCAAAGGAATTCCATTTGAAATTGAATCCAGACTAGCACCAAGTTTATCAACGAAGATGGGTTTAGCATAAGCACCAGTAACTGCATGCATGGCATCAAATATAAACCTAAAATCTGGACGAGAAAGAAGACCTTTGATTAGCTGAAAATCAAATACTGTCTCCAGTAGCTCCAGATAGTCAGAGACTGGGTCTATTACTTCCACACTGAAGCTTCCAAACTTCGTAACCCCAACTTTTGATAAGTCAACATCAGGAATGTCAGCTATCCTTATCTCAGAGATCGACAGGGTGTTTCCGTAAATCTTGTCAGTGATGGATTCTGGTGCAGGTTGTCCACTGCTGTAATTAAACTTAATACCCCAATCATATTCAGGCCCACCAGGATTATGGCTTGCACTCATAATAAATCCACCATTTGCCTTTTGCTTTCTTATTACAGCAGAAACAGCTGGTGTTGACAAAATACCTTCCTTTCCAACCAGAATTTTTCCAACACCATTTCCAGCAGCAATTTTGATTATTATCTGTGCAGCTTCCTGATTAAAGTATCGACCATCTCCTCCCAACACCAACAAACCATTCTTGTAATCCTCTGGAGGCAATGAATTAAACAGGGCCTGGATCCAATTTGCAAGGTAATTTTCTTGCATAAACACTTTCACCTTCTTCCGAAGCCCACTAGTACCAGTCTTTTGTCCCTCAATGGGTTTGGTTGGAATCGAATTAATCTTAATGCCTTCAGGTTCAGCAATGGTTGTGGAAGGGGATGAGGATGATGACGTGGCTTTGATGGTTGAACCGTAGCGGATTCTGAAGGGAAGCTTTTGAGGTTTAAAGGAAGAAGGACAAGGGAGGAAGGAGGAAGGTTGGATTTTTGAAAGTGGGGCAGTGGAGTTCTGGGGTTTGTAGGCAGAGAGGATGAACTTGTCAAGTTTAGAAGAGAATGCCATTGACGGGTATGAAGGAAATATTGTTCTTTCAGTCTTTGTGAGAACAGAAGAAACACTGATGCTGTGTTATGCTTGTGAGTGAT

>C102133_65

TTAGGGTTTATCAACTGAAGAAGAAGGAGAGAACAAAGAACAATTGTTTGGGAAGATGGGAAGAAGAAACGGAGGAGGAGGAGGAGGAGGTAGGTCTCTGCGACAAACCCTACGCCGCCGTCTTGACACCTGCAAGTCCAAATACTCCACCGCCGAAGAAATCGTCAACCATCTCCGTTCTACTTACCCGGATTACAACCGAACTAAGCACCAAACCCTGATTCGATTTGTTCAGGAAGCTCTTCATTCCACCGCCAAGCTTAATTGCACTCCCACGCCTAAACATTCTGACGACGATGACTGTGACAATGATGACGACGGAGAATCACGAAGCGCGTCTCGAAAGCGTCGGAAGAAAATCGACGAGGGTGAAGAGAGATTGCAAAAGATGGAAGCGTTACACGTGGGAAGAAGGGTGCAGAGTCGTTCCTCATCGTCCTCATCGTCGTCTGCTCCAGAGAGTGAAGATGAGGAAACTGTGTCAACGTCGGAGGACGCAATTTACGGGGAGAAGGTGGAGCCTGAATTTGATTTGATGAAGGTAATGCTAAGGAACTCGTATATGCCGAAGAAGATGGTAGCAGTGGAAAAGAATGTTGAATTGGAGACCGGGAGTAACAGTAAGGGTATTGCTACCAGTATTCTTGTGAATAAAGAGATGGAAAATAGCAGTAAGGTCACTCTTGTAAACCAGGAGAGAAAGCCAGCTCGTAGTTTGGTTTCAAATGTTGAGTTGAAGAGGAAAGATGGACCGAGCTTTAAGGATTTGGGTGGGATGAAGGAGGTTTTGGAGGAACTGAAGATGGAAGTGATTGTGCCTTTGTTTCATCCCCAATTGCCTAGACAATTGGGAGTGAGGCCAATGGCTGGCATTTTGTTGCATGGACCACCCGGATGTGGGAAGACCAAACTTGCTCATGCTATAGCTAATGAGACTGGACTTCCTTTCTATCAGATTTCAGCTACGGAGGTGGTTTCTGGAGTATCAGGTGCATCTGAAGAAAATATTAGAGAGCTATTTGCTAAAGCTTACAGAACTGCACCCTCGATCATCTTTATTGATGAGATTGATGCAATTGCTTCTAAAAGAGAGAATTTGCAGCGTGAGATGGAAAAACGTATTGTGACACAATTAATGACTTGCATGGATCAATCAAATAGGATTCTTCAACCAGCTTATGATTCAGAGAGTTCTGATGACCGTCATCCCGGCTATGTTCTTGTAATTGGAGCTACAAATAGGCCTGATGCCGTTGAGCCTGCCCTGAGAAGACCAGGTCGGTTTGATCGTGAAATTATCATAAGCAATCCTGATGAATCTGCCAGGGAGGAGATCCTGTCTGTGCTTACTTCCAATCTTAGACTCGAGGGTTTATTTGATCTACAGAAAATAGCCAGGGCTACATCAGGATTTGTTGGTGCTGATTTGGCAGCTTTGGTTGACAAGGCTGGTAATCTGGCAATGAAGAGAATAATTGATGAAAGGAGACGTGAACTATCTCAAGATCTCATGAGTGAGCATGCTGAAGATTGGTGGAGGGAACCTTGGTCGGCCGAAGAAGTAGACAAGCTTGCTATTAAAATGTCTGATTTTGAGGAAGCAGCAAAAAAGGTGCAACCTTCATTAAGAAGAGAAGGATTCTCTACAATTCCTAATGTAAAATGGGAAGACGTTGGGGGGCTTGATCTTTTAAGAAAGGAGTTTGAACGATACATTATAAGGCGTATAAAATATCCTGAAGATTATGAGGGGCTTGGGGTTGATCTCGAGACTGGATTTTTGCTTTATGGACCTCCTGGTTGTGGTAAAACACTGATTGCCAAAGCTGTTGCCAATGAGGCAGGGGCTAGTTTTATCCATATTAAGGGTCCTGAACTTTTAAATAAATATGTTGGAGAAAGTGAGCTTGCAGTTCGATCATTGTTTAGCCGTGCAAGGACATGTGCTCCTTGTATACTTTTTTTCGATGAGGTTGATGCTTTGACAACGAAACGTGGTAAGGAAGGTGGTTGGGTTATTGAACGACTATTGAACCAGTTGCTTATTGAGTTGGATGGCGCAGAGCATCGGCGAGGTGTTTTTGTAATTGGTGCTACAAATAGGCCTGAGGTGATGGATCGTGCTGTCCTACGGCCTGGTAGATTTGGTAAACTTCTTTACGTTCCACTTCCAAGCCCAGACGAGCGAGTTTTGATATTGAAAGCTCTTGCAAGGAGGAAGGCTGTTGATCCCAGTGTGGATTTGAGTGCCATAGCAAGAATGGAAGCTTGCGAAAACCTAAGTGGCGCCGATCTTGCTGCATTGATGAATGAAGCTGCAATGGCTGCTCTTGAAGAGACAACTTCCGATACATTGATGACCATCAATAAAACCATCAAGAGTAATCATTTTGAGGTGGCACTGAGTAAAGTCTCGCCGTCTGTGTCTGACAGGCAAAAGCAGTTCTATCAGCACTTGTCAGAGAGCTTTAAAGCTGCGTAAACAGCATGGAGTTGACTTTTTTGTTAGCAGGTCACATACCCTTATCAATGGGGTGAAAGAAATCATTTATATTAACGTCAATTATGTATTTACTTTTTGTATGAGATGTACATGTTTCCTGCAATCAGGATGTAATTTTTGGCGAATGGTTATAATTTATGGTTCACTTGCGCCATTTTTTTAGACAATGTTACTAAAATATAAATTGGAGGGATGATGTAGGTTGGATGAGTTTGCA

>C102139_65

TTTTTTTTGAAAAGAATAATGTTTCAGAATACGAACCAAATAATCAAGTCAACATTTACAAACCAAAGGGCAAATTGATAGGAGGAAAAAATATTGGCAAACACCTGATTATGGAAAACTTAATCTACAGAAATGCACGGTAACTCCCCCAATAAATTCAGTTTAAGATAAAAATAATGGGGAAACCGATTGCTGCGTAAGGAGAACATCATCAGGAGCAATGACGAAACAGATCTCCATAGAGTACAAAAATCTGCTAGCAATTTCATGATTGACTTAATTTACAAGGATGAAAGTATGCCTCACGAACTATTATGCGATCTGACAGATGAAATTGACTGAGCAGAGACAGATGCAGTAGGGCTGTTAAGCTCCCTCTCCCCATTTATTTCTTTTGGTTTTCCACTAAGTCTCTCCATTGCATCTTGACATATGTCGTTAAACCATTCGTCTTCTGGCAGTTCTCTATACGGATCCATCCCTGTTGCAGAAAATGCTGCCATCGCTTTTGTTATAATCTCATTGACAATTCGTTGCAAATTCCTTGACAAGTACCGACCGTGCGAAGCAAAACATACGACAAACTTCATATCCAAATAGAACTGTTGAAGACCAAGGGGACCTAAAGGCCTGGGCCCTTCCTCAATATCATCCCAAAAGCTCTGGTCTTCTGAAAGCCACAACATTACAGTTTCTGTAAGTCTCATCAAGAGCAGCGTAGCAAATCTTTCCCTCCCCACAAACATATCTGCTGCTATATTGGCCATTCGGTTTAGTTTTACAAAAAGCTCCTGAAAAATCAGTGATGGAAGCCATTCAACTTCATCGGCATTTCCATCCATATTTATGTACATGTCAGCAGTAAGATGACTATCACCTTCCTCAGTAAAAATTAGGTCCAATGCATGTTGACGACAGAATGTATCTTTCAATCTGTCAACTGAACCAACAAGTCTTCTTCTCCATTCTCTTTGTTCAGGATGACGATTTTGTCTTTCTGAGGTTCTTCTTCGATTATCCTCCTTGTAGGCAGCTTGGTTTACGGGGGAAAGCTTCATAGCTGCACGTGGAAGTAGTTCATCAGCTAGTAACGAGGCATTTGCTAGCAATGCAATTTGCTGGGCCTCAGTCTCAGCCATGCGTACAATTTTATTTCCAGAATCTTCAAAGTTTGCTTCTTCCTCCATTGATCCAGGTAATGCTTTTATGAGCATGTTCACGTAGGAGTTAAATACTTGAAACAATCCCTCCAGAGCTTGGCCTCCCAACTGCATGCTAAGCAGTGGTCCTACATCCTCAAAGAAGTCCTGGACCATCAAATTGAAGCGATGGGCACTGCTTGTGAGTTTCTGTTGAAATGCTGTTGCATTACTAATGGATATACTGGAAGGCCTACTACTCTGACGATTAGCTGTAGGAGGGTATGTAAGTACCCAATCATCAGCCGCAGCCAAAGCAGCAGTACTCTCTTGAATTCGTTTCAAATTAGCATCTAGTGCTTGCTCAACACTAGGCCTAAACAGTTTCAACAGCACAGGACAAAGAGCCAGGCCACGAGCTTCCAACAATGAGCAATGACCCAATGCTATTTGAACGCACTCTGCAGCAGCTCTTAAACCTCCTGCAGCTGCTGATGAAGCTAATGCATGCCTTTTCACCAGAAGAGCAAATGCCTCCGTTTGCTTTGTAGCCCACATCACAAGTTCTGAAGAATAAGCTGGCTCCTCACCAAAAATAGCCAAAGAATCACTAGCAGCTTGCGCAACTGCAGAGAATACCAGTTGAGCAAGAGCAGCAGTATATGCTCCACCATATGAGGTGCTTGATGGTCGAAGACTTTGCATGTTATACTGATATCGCTGCTGGTGGGCATTAAGTAACAGGCTGTGGGCATGGGGACCATCTCCAAGTTTTTTAAGGGCGGAAACTGATGCACGGAGCTCAACACCACGTGTGGAGGGCTGACAAGCAGCTTCAGTCAGCTGATCAGCTAACTTTTGTCTACGTTCAGCAATGGAATTCTGTAGAGACAATAGTACCGATGGATTGATAGATTTCATCTCTTTAGCCTCAGAAACTACACGTTCTCCTTCTTCAAGAGCAGCCAAAGCCTCTTCCACTCGCCTTTCAGCCAAGAGAACATCCAAGAGATCAGGAAACTCTACCAACCATTTGTCTAGGTCTGATATCTCCTTATCTTCATAATCTGATGTAGCATTTACAGAAAAACCATCAGAATTTGAAATTGATAGTGAATCGATGTGAACTCCTTCAGCCAAACCATGTATCAAGGCAGCTTGTGTAGAGAGCAGGTTTCTAATAGATGAAAGCTCCCCCTCCAAGTCTGATATCTCCTTAGATGTTCGTATGAAGGCTGCGTAGTTAGCATAGACACTTCGACGCATCTCCTCAGCAGATGCTTTCTTCAAGTCCACCAAATATGTGCACAATTGCTTAATTTCCTTATCGTTGAGAGAGCAATTGGACTGGACATAGGACTCTGCATCGAATTTTTCAGTCTTGAAGGGATTGAGACCTTCTTCAAGCTTTGGTCCATTCTCCTTCGCACTCACCACTGTGCTTCTCGACCGAGATGTCTTCCCCGTCGCCATATCCACAGGTTACAACTGAGACGAGATCACCAACCAGCGAAGTTTAGGGTTCAATTCAAACACTGAAATCCAAAACCTAGATCGCACGAATCAGAGAGGAAGCACCGAG

>C102143_65

CGTTTCCCTTCCTGTAAGAGTTAATATTTGAAGACAACCAAAAAGGAATGGAGCAAAGAAAGGATTTGTATAGAACCAAAAGTTCTACCTTCTTCCCTTCCCTTGTTCTCCCCAGCAGTATGGTGATATCTCGTGTTGCCGCCCTTTACATTTGTTTCAAACTCAAGTTAAGTTCACACCCTATCATGTTCGTGTGATAAGCTTTTCCTCTCTCTTTCTATGTATGGTACAGCTCAATAGCTTGCCGCAGCTTTGTCATTGCTATCCTGCCGTGTCTCTAGAGCTGGTTCATCTTGAGTTCCATCAGTATGGCCATTGCATTCTTGCTTTTGCACTATTCCTTTTGGTGAAGTACTAGTAATATTATGATTAGTTTTCCCATTATCTTTCTCCAGCATGGTAGGAGTTTCGATGCTTGGATTTACTTCAGCCAAAGTACCAGCTGGTAACTGGTGCAGTGCGTTACCTGACCCCGGTGGAGGTAGAAAGACTCCTGTGCCAGGAGCAGGGATTCGAGGAGAGGGGTGCCTTGGAGGAGGAGCAGTTGTCCATCCGACAGAACTGGGTGGGATTGGCACGGGTGAAGGAAACGGCAAGGGAGGTACAACTGGAGCAGTAACAAACAATGGTTGCATGCCAACTTGAGGTGGAATTTGTGGTCGAATGGGTGGAGCTGGCAGCACTCCAGTTGTTGGAAGTGAAGCATAATGCTTGGAGCCTACATGATGACGAACCTGATTGGGAGATCGGCTTGGTGGCGGACCCCAATAAGAAGACGCTGCTGGTGAGCAAAGACGTTGGGCGTCACTTGGTAGCGACTTTTTTGGTTGGGACTTTGTCAAGGTAACAAGTATGCGTTGCTTGCGTATGGATGGAAGAGCATGCTTAGCAAAATCACTGGATTTCCCTTGCATAGCCAGAAGGGACCCAGGGACGAGAGAAAGCTTGAGACTGCCCCTATGATCCCCAGGATGTTCGGAGGCAATTAATCTTCCAAAAGTCATTTCACATTCTGTTAGAAAAAGGATATAAACGGGCCTTCCAAACCATGATGGCCAACTGTGGGGCTGTGAGTGATCACCCTCATTATAGAAATCCACAATGCAACAATCAGGCTTCACAGTCATTACTTGTGATGAAATCATACGCTCAATAATATCTTGAAACAAGGAAGGAATAGGTTCTACATTCATATCTTTTGAGGCTCCTGTCATATTTTCCCCTTCTGCTGGGGCATCAGCAATTGGAACACCCAACTGAATCATTTCCCTTCCATGCCCCTTCATGGGCCTTCTTGAAACTACATATGCCTGACTTCCTTGTAACTGTCCTTTTTTCCCAGAAATCCTTAGATCATTGACCAATGAAACAAGTTTTGAAACTTCAGTGCTATCAAATAAATCTTCATATAACTTCAGTCCATCAGCCACATTCACCATCTTCCCATCAATCATTTCATTGCCAATAAAAGTCTTCGCTATGGTAGAAAAACTTTGGCTCTGATGTTGACTTTCCACAGAATCTGCATCATTCCCTTTCTTCTCTTCACCAGATGTTAAACCCGTATCCTCCATTTTCTCAACCTTGCTACCAGATTTATGTTCTTCACTCTTCTCGACAATTGGAGTCCCTTTCTCCATGTCTCTGGTAAATGTGCCATTGGCATCATGACTATAAGACACTACGCTAGAATTATAACCTTCCTTTGGAGTCTCAAACCTATGCCCATGCCTGTATCCTGGACCAGACTTCCTAACTTCCTTTGCACCCACTTTAACTGGATCCAGAGGCCTCTGTTGCCTCCTCCACGCCACTTGCTGCAGTGAATACGCCACATCAGCCACAGAAAAGTATTGCTGCATAAGCAGGACCTGATTCCAATTACACCGCCTCTGCTGAATGGCCCCAATGACCATATCATACTCCCCAGGATCACCAACCACACGTAAATGGTGACATAGAGAGTCTATGATGGCATTGGCAGCTGCGAATTCGCTTCTCAGCCAACCAATAAGCCCATCCCTCTCATCAACAAGCCATTGCTGGCGGTAATGGTGCTGTTGGATCTCACCACCAGCACCGCCCCCGGCACCACCTCCACCACTTGGAAACTGCATTTTGTCTTGTATTCCCACATTTCCCGATGGCATTGCCATGAGAACTCACTACCAAGAATACACTAAAACCGCATATAAAGCATACCCCAACACAACTAGATCTAGATAAGGAAACAACAAGTCTAAAACATAAAACAGCGCAATTAAGAGGACCCCACTAAATGAAAGGCAGAATTCTGAACAATCAAAGCAAAAACCGGCAAACTAGTCCAGAAACAAGACCACCAAAGAAATGATTCAGGCCAAGAAAGAGTGAAAAAAACTGATAAAATGAAATGGGTTTAGCGTAGAAATCGATAAGAGAGTTAAGGTAAGAATTGTTGAATTGGAGAGATCTGGGTTTGAAAAGGGTGAGTGTTGGATTGAAAGTGAGGAGGTGGTTGAGTTGGAGTGACGCAAAGGGAAGATCTTGAAGAGGAAGATCTAGGGTTAATGAAGAATGTGGAACAAAGAAAAAGGCTTATTGATCACGCATGTGTTATAGTCTTAAATGGTGGCTCATATTCACAAGTCTCGACACAACACACCCTCTCTCTCTCTCTCTCTATTCGTTCCTGTGCATATGAGGACTGGTGGGTGCTGATTTTGGATTTTACAGAGAATAT

>C102153_65

AAAAGTCTTTGGAAATTCAGTCCCTTTGTTCACATCTTTTCTCACTTCGTACGCGGATTCGGCCGAAATTATTAGCATAATCCAATCCTAACTTCATCTCTTCCTTAACTTGCTAATTCCTACTTCTTTAAACATTCAACTTCTTCACTCAACCACCGCGAAACTTTCATAGCATGCCACTACCGTTTCGAAACCCAGAAAACACTTTCCGAGAAAATCTCTCACCCTTTCAAATTTCTCCCCTTTCCTTTGGGGAACTGCTCCTTTCCTGACACCATCATCTGGGTCGCCGCGAATTCAGAAAAAAAATAAAAATCCTCTGATGGCTTCGGCGGCTATATTCTCGTCGCTTCGGCGGCGCCGGTCACCGTCTCTGGAGGCGTTTCTGGCGCCGGTAGACCTTTCTGACGTAGCGCTGCTGGAGACGCTTATCTCCGTCGCTAGCGACGTCGCGTCCTGCTTTTCTGGCCACCGGTTTCCCTTCCAGCGTCGGAACTCGCGTGCTCTAATCGGAAAAGTTGAGATTTTCCGTTCAATGTTAGAGTGTTTGAGAGACTCCGCCGACGGCGGTGTTATTCCCCCTACGGCAGTGCTGTGCCTAAAGGAGCTTTATTTGCTATTATATCGCTCCAAGATTCTCCTTGATTACTGTGCTCGATCGAGCAAGTTGTGGCTCTTGCTTCAGAACCATTGTGTTTCTGGTCACTTCCATGATTTGAGTCAAGAATTTTCGACCCTTTTGGATGTTTTTCCCGTTGGAGAGGTTGGACTCAGTGATGATGTTAGGGAACAGGTTGAGTTGTTGCAGAGACAGTCCAAAAGGGCTAAGTTGTTCATAGATAAGAAGGATGATGCCCTGAGGATAAGGTTCTTTTGGTTTGTTGATGAGTTTGAGAGTGGGAGGGTTCCTGATTCTAATGACTTGAGGTGTTTCTTTGTGGATAAGTTGCAGATGCTTGATGCTAAGAGTTGTAGGGTGGAGGTTGAAGCCTTGGAGGAGCAGATTGTGAACCATGAGGGTGATGTTGAGCCAACAGTTCCTGTGCTTAATGGGATGGTGGCTATCACAAGGTATTGTAGGTTTTTGTTATTTGGGTTTGAGGAAGAATTGGAAATTGAGATTCAGAAGATGGGGAGGAAGAGGTTGATTACTCAGGAAATTGCTGAAACGTTTCTTACTGTTCCTAAGGACTTCTGCTGCCCGATAACTTTGGATTTGATGTGTGATCCTGTAATAATTTCTACTGGTCAAACTTATGATAGGAGGTCTATATGGAGGTGGATGGAGGAGGGGCATTGCACTTGTCCAAAAACTGGCCTGTTACTTTCTCATAACAGGCTTGTTCCTAATCGTGCACTTAGGAATTTGATTATGCAGTGGTGCAGTGCACATGGAGTACCCTATGATCCACCTGAGGGGGTTGATCTATCTGTAGAGATGTTTGCATCAGCTTGTCCCTCCAAAGCTTCCCTTGAAGCTAACCGAGGGACGGCCATGCTTCTCATTCAGCAGCTTGCTGATGGGTCGCAAGCTGCAAAGACTGTGGCTGCTAGGGAGATAAGATTGCTGGCCAAAAATGGAAAGGAAAATCGTGCCTTCATTGCGCAAGCTGGGGCAATTCCTCATCTTAGGAACTTGCTTTCCTCTTCTAATGCTGTTGCACAGGAGAATTCTGTGACTGCACTGCTCAATCTATCCATTTTTGAAAGAAATAAAAGTATGATCATGGAGGAAGGGTGTTTGGGATCAATTGTTGAGGTGTTGCAATTCGGGCACACGACTGAGGCAAGGGAGAATGCTGCTGCAACATTGTTCAGCCTCTCTGCTGTCCATGAGTATAAGAAACGAATTGCGGATATTGTGGGAGCTGTTGAAGCATTGGCATGGTTGTTGCTAGAGGGAACTCAAAGAGGGAAGAAGGATGCTGTCACAGCATTATTTAATCTCTCCACTCACACTGAAAATTGTTTGAGAATGATAGAGGCAGGGGCAGTGAAAGCTATGGTTGAGGCTTTGGGAAATGAAGTAGTTTCTGAGGAAGCAGCTGGTGCCTTGGCCTTGATTGTTCGGCAGCCTGTTGGCGCCATGGCAGTGGTGAGGGAGGAAGCAGCAGTAGCTGGGTTGATAGGAATGATGCGTTGTGGAACGCCAGGAGGTAAAGAGAATGCAGTTGCAGCCTTGCTGGAGTTGTGCAGGAGTGGTGGTGCAGCTGCAACTGAAAGGGTGATTAGGGCGCCCGCCTTGGCCGGATTGCTTCAAACCTTACTTTTTACTGGAACAAAGAGGGCAAGAAGAAAGGCAGCTTCACTTGCTAGAGTGTTTCAGAGATGTGAAAATGCATCTCTACATTATGGTGGATTGGGTGTAGGTTATGCATTTGCTAGCGATTCAGCTTCAAACAGGAATGCTACAAGCTTTGTGAGTGATGTTTCACTTCCAATGTCCATTTCTGTTCCTGTTTTATAACAGTGGTAATACTCATTCCCATGTTTACTGTGTTTCTGATTTTGTTGTAACCCTATTGATTCTTTCATTGACATTTTAAGTCATCAAGTATAGGAATACCAGATGTGTTCTAAAAGAAAGAACGATGTGTTGTTGAGCAGTTAGCTTCAACCCATGTGTAATCAGTTATTGCAAGAAAGAATACAAAACGTTCTAAAGTGGAATTATTAGACCATTTCAGGCGTCCTATTTCTTCTGTGCTTTACTCCTGTTGGCCATCA

>C102165_65

GAAGCAAACACGAAAGAATGGGCAGAGTGAATGTCTTGGGAAACCAAATGGAATATTTACATACTTACAGCAGTCCTTTGATCAATTTGAAAAACATAAATAGGAAAAAGGAGCAGAGGAACAAATATTTCATAAGGGTGTTATTAAAGTTAAAAATGCTCCTACAAGTCTGCAGGTGGATCAAAACCTTGCTTCTGCAGTTCTGCCCTAAATCAATGTCTTCATGTACACTTTAACAATAGAAGCTTGCTAGAACCTTCGCAATCAAAAGCAGCCAAAAACTTGCAAATATGCCTTTTGCATACATGCATTTAATGTTTGTTATTATTCATCTTGATGGATCACGTAAATGATCTTGAGCATCCAGACTAAATCATAATAATATACTACTCCCTGAAGCCTGAATTTTTTACATCAACTAAATTCTATTTTGTTGAAAAATTTAAACTAAGGAACAAACTAACTAATTGCAGAATGATTTGGTTCATACGGAACGCCAACTATTTACATTGGCGACTGCAATTAGAAAGAGATCTGGCAGGATCTGTTCTAAACCGGAATGTTGTCTAAGGAATATCCATCAAAGTCTAACTCATTACTAGATGGTCCAACACCTTCTTGCTGTTTCAGAAATGCAGTGACAAGATCCTCATGACCATACTGATCAGAGAAGAGGTTACTAATCTGGGAGCTTGGATCAGGAAATCTTTCAGCCTTAACCCTTACGGAATTGTCAACAAGGTTACTGATGTGTTGACCAACATTTTGGAGATTACCTTGATGTATGCCTTCCCCTGTGGAGAACATAGCTTTTCCCATTGAAGTAGTATCTCTAGTTTGTACAGACTGTTGCATAGAGGGAAAACCCTGATGGACTAAAACTGATGGTGAGACATGTAAAGGATTTGCATGCTGAGAAGAATCATATGTTATGCCTGTGTTTGTTAAATCCCAATCATGGGACTTCTGATGGTGGAGTTCATTAAACATATCATAACTTGGAACAAATCCACCAGGTCCTTTAATATCAGAGGTACCTTCTTCATGAAACATGCCTTTAGTTGGGATACTAGTTATACCTGGAGTGGTTCTAAGAGGAAAACTACTGACAGACATTTCATTGGTTTGGTTCATGGGAAAACTCAACAATGAAGAGTTTTGTGATACTGTATTATAAGCAGTAGTTATGTTGCTTGTGCCAGTAATCCCACTTCCCAAAACACCATTAGAAATACCATTTGGCACTATAGGCTGCACCAAAGATGATGGAAACCTGGTTACATGAGAATCAGCATTTTCACTTAGCATCTGACCTCTTGGTTGAGACTGAGCCATCTGCATTAACAAGGGGTTGTTCTGCGCAGCTGGAGCATTGACTCGCATGTTCAAGTTACCAAGGGTTTGGGTAGATTGGTGCAAATTGACAAGTTGTTTTGGCTCCATGTTGGTAGGAATTCCATGAAGCAAGTTTATTGGTTTACTGTTGCTTAAATGATGTAGTGATCCTTCTGCAAATCTTAATCTCGGGTTCTCAAAACTGAAAAGGTTCTTTTGATCAGATAGAGGCATAGACACACCAGCTTTACCAGTCGTGCCAAGTCCTGCAGCTTGAAGCTTGGCTAGACTTTGTGCAGGAAGCTGGCCAGCAACTGAAAGAGTTTGAAGATCAATTCCATTGATTGAGGTTGCCCCAAATGTTGCTTCTTGTGAGCTGATGAAGGAGTTGTTCAAGTTACCCTGCTGCTGTGAAACTCCACTCAATCTTCGAAGATACAAACGGTATTTCTGGAGGTGGCTAGCAACATTTTCTCTAGTGAGCCCAGGAACATTCATCAATTCCAAAATCTTTTTAGGAACCGCCTTATCAATTCCAAGTTGATTCACAGCAGCCATAAACTGCTGATGAAGCTCAACAGACCAAACAACTCGCGGCTTCTTCAACGTGGAGGAATCATCCCTCTCATCTCCTTCCTCCTCCTCATCCCTTCTCTTCTTTGAGCTTTTACCTTCATTTACTGAAGATGAGTAATCTCCATCATCACATCCCTTCAGAAGCCGATCTCCTTCTTCCACACTACCCGATTGTTCCACATCTTTCAACCCGTTCTTTCTCTTCCGAATCACATGCTGCCATATGTTCTTCAAAGCCTCAATTCGCACAGGTTTAATCAGGTAATCACAGGCACCATGTTTCACCCCTTTCATCACAACACTCTTTCCATCATCAGCTGACATCATAATAACCGGAAGGTCCATCTCCAACCCAATATGCTCCAGAAGCTTAAATCCATCCATATCTGGCATATGCACATCGCTCAGAACAACATCAAACCCGTTTTTGTTCTCTCTCAGAAGTGACAATGCAACCTCTGCCCGCTTGCATTTCGTCACTTCATAGAGACAAGCACGCAGCATCCTCTCAAGGATCATAAGGCAAGTGGGGTCATCATCCACCACCAGCACCCGGAGACCAGCCGGAAACTGGTCGGAGACGGCGTCACCGGTTTTCAGAGGAGAACTGGAAGTGGATCCCTTGCCGTTGCTGAGATTCATGGTTGAGAAGATCCAAAACAGTAGACAACAACAAAGAGAGACACAAACAGCTTCACATGGTGGAAACTGGCATCATCATGAGAGTGATAAACCAAATACAAATAAAATAAACACGAGAGTGAGTTGTTTAAGGAGTTCAGACGAAGAAACAGAGAAAAGGGTAATGGAATTTCTTTTGGC

>C102167_65

TCCATCTTTACAAACGAGAATTATTTATATTACCATTTGTGCATTATTTTCTAATCACCATAATATTATTATTTCCCAGTACGCAAAAGAGTAATAGTGCTAAATGTGTACCATAATAACTATTGGATATCCAATTTAAAACATTTGAAAAACATAATGCATTATATTTTAGAACTTTGGTTAGTTTCGGTAAGCTGCTGACGCAAAACCCTTCTCATAACCTTGTTGGATGCTGTCCTTGGAAGAGATGACAGTGGTACTACTTGAGAAACCCGAAACAGTGGATTTAGTTTCTTTTGCAGAGCTGAATTAAAAGACATCCTGAATTGGTGTAAATCTTGCTTTGTGACACTTGAATCCTTGAATACCACTGCTATGGTCAACAACTCAGGTCCACCACCAGAAGGTGGTACCCCTATTGCAGCTGTTTCCAGAATACTGCTATCTACTCCATTGCATATGCGTTCAATCTCAACCGAACTCACCTTGATTCCCCCAAGGTTCATTGTGTCATCCGCACGACCATGTGCATGATAGTATCCTCTAGCAGTACGCTCAAATACATCCCCATGCCTCCGTAAAACCTTTCCATTCAATAAAGGCATTCCTTTGAAATAGACACCATAATGGTCAGCATTCAGCAGTGTATTTGATGCCCCAAGCATGAGGGAACCAAGAGCCAGCTCGCCTATTCCTGGAACATTTTGAGGAATAGGATGGCCATTATCATCAAGGATATACAAACTGCAGCACATAGCAGGTGTACTAAAAGCAGATAATGCCTGAGCCTGTAGGAGTGATCCACACACAAATCCACCCCCAATTTCTGTACCACCACAATATTCAATGATAGGCTTGTAGAGAGCTCTCCCCATCAGCCAAAGGTATTCATCTACATTAGATGCTTCACCAGAGGAGCTAAAGCAACGGATAGAAGACCAATCATATCCAGATGTGGAATTTGCACTTTTCCAACTCCGTACAATACTTGGAATAAGACCAAGCATTGTTACTTTAGCATCCTGTACAAACTTGGCAAAGCCAGACCCAAGAGGGGATCCGATATATAAAGCCATTGAAGCTCCATTTAGCAAGGATGCATACACTAGCCATGGCCCCATCATCCATCCAAGATTAGTGGGCCAGCACACCACATCCCCTTTACGAATGTCCATGTGGCACCATGCATCTGCAGCAGCTTTTAGAGGAGTAACATTGGTCCATGGAATTGCCTTTGGATCACCTGTTGTTCCAGATGAAAAGAGAATGTTTGTAATTGTTTCTACTGGTTGTTCTGTAGCTACGAATTCCTTGCCTTTGAGACTATAGACTTTCTCCAAAAAATCATGCCAAGACAGGTCACTGTCGCGCAGTTTCATGCTAAATTCAGATCCTTTGGTAGGAATAACTACTGCCATAGGTGACTGTGCATCTACAACTCTACTGTAAAGGGGAATACTTTTGTCGCCACGTATAATGAGATCCTGTGTGAATATGACTTTTGCTTTTGATATTCTAAGCCTGTTAGATATTTCACTAGCAGCAAAACTATCAGCAATCGATACAACAACGTAACCTGCAAGGATAATAGCTAGGTAGATAACCACAGACTTGACATTCATTGGCATATCAATCGCAATTGCAGATCCTTTCTCCAGATCCAGAGATTTGAGAGCATATGCTACTATCCAAACTTCTTCACGCAGTTCCTGGAGTGTCATCCTTTGTAGAGGGAGGTCATCGTGTTGTTCTTCACGCCATATTATTACTGTATCATTCAAACCTCTTTTGCCATTTACATTTATGCAATTCTGTGCTGGATTAATAGATGCTCCAGGAAGCCATTGACCACTTGGAAATGATAATGGGCCTTCTTCCCCTTGGGGGCTATCACGTAAGATGCATTCAGGTGGTTTGGAAAATGATACATTCATTTCATCCAACACAGATTTCCAATATATCTCAGGGTTTGAGACTGAAAACTTCTGGAAATCATCAAAGCTTGTAATAGGATCTTTATATGCCGAACCCAGAAACTCTTCACCCCTCCTCTCTAGCAACTGCCAAACATTTGTCAAAGCAGCACTTTCCGGGTCGGGTATCCAAGCGGGTGGGTCGGGACCGAAATCCTTGTAGCAACCATAGTACAGCATCTGATGGAAGGAGAAGGGAAGTTCAGGGTTGAGGATGCTGTTGCTGATGTGCTGCCACGTGGCGGGGGTGCCATCTCCGTGAATTTTGATGATTTCTGTGAGACGTTGGTGGAGAGTGGTAGCATCTTCAATTCCAAGGGATTCAATATCCGACACAGTGATGGAACGTAGCGAATTGTAAGCCATGTGTAATAGTAGGATCCAAAAAGTTGCACAATTGCACCACCCACTTCACACTGGACTCTACTTCTTTTAGTTCCCGAAACCCAAAAGCTTCAAACCCGTTTCAATGACAGATTGATACAAAGATAAGTGTTCGAGGTTGCTTCTATGTTCAGAAAACTATGAAAGGTTGAGAATTTATTCACGAAGCATTGGTTAATAGTTAAAATCGTCGTAAAGTTTGTTCTTATCTCGATGACTAGAGTGTATATAAGATTTTGTATTGAAAGGGAAAAGAAAAGGGAATCTGATGTGTGAGAGTAAACTATAGGATCCTTATCCCCACCTGCTTGGACTGGGACAGCCTAGCAACATGGTCACAATCCTGTCAATTCACAATGTTCATGTATGTTCTACG

>C102173_65

CATTCTTCTCCATTTGCATTTCCTCTTTCCCTTTGTCCCCTCTGGCCTCGTGTGCTTCCTCCAACAATTTGTTCTTTGGTTTCAATCCCCAATACCTGTCTCTCTCTCTCTGTCTCTCAAAAACAAACTCAACTCACAGATCCTCAATTTTCACGAATCTTAGAACCTTGCTTTTATTTGCCTTCACATGTTTCATTTCGTTGTGTCTCTTCTCTCCTCTCCTCTTCCTCTGTTGAAGATAGTTAGCCCCTAAGTTTATTACACAGTGAATGCAAATAATGGATATTGCCGAGGTTGAAGAAAATCTTTTTGCAGCAGGCGATGCCAAGTTACATGGAGAAATGTGCAAGTGTCTTTCTGCAATATACTGCAAGATATTGTTATTATTTCCTTCTTTAGAAGCAGCTAGGCCTAGGAGCAAATCTGGAATTCAGGCATTATGTTCATTGCATGTAGCCTTAGAAAAGGCAAAGAATGTTCTTCAGCACTGCTCAGAATCTAGTAAACTTTACTTGGCTATAACCGGTGATTCTGTTCTTCTAAAATTTGAAAAGGCTAAATGTGCTCTTGAGGATAGTCTTAAACGGGTGGAAGATATTGTTCCTCAATCTCTAGGTTGTCAGATTGATGAAATTGTGAATGAACTTGCAAGTATGGTATTTGCACTTGAACCATTAGAGAAGCAAGTTGGGGATGATTTAATTGCATTGCTTCAAAAGGGAAGAAAGCTTAATGACTCTAATGACAGTAATGAACTTGAATGTTTTCATCTAGCTGCTACGACACTTGGAATCACCTCTTCAAGAGCAGCACTTACAGAAAGAAGATCTCTAAAAAAACTCATAGAAAGAGCTCGGGCTGAGGATGACAAGCGGAAAGAATCAATTATTGCTTACCTTTTACACCTTATGAGGAAATATTCCAAATTATTTAGAAGTGAGTTCTCTGATGATAATGATTCTCAGGGTTCTCAACCTTGTTCTCCCACTGTTCAGAGATCTCTTGAGGATGGCTTTCCTGGTGGTCATGGTCATTGTCATGTCTTTGACAAACAACTTTCAAAACTTAGTTCCTTTAATTTTAAGCCAAATAATAGGGAATCACGTCAAATGCCCCTTTTGCCTGAAGAGTTACGATGCCCAATATCTCTGCAACTTATGAGTGATCCTGTTATAATTGCTTCTGGGCAAACATATGAAAGGGTTTGTATAGAGAAATGGTTCAGAGACGGGCACAACACCTGCCCAAAGACTCAACAGAAACTTTCACATCTTTCTTTGACACCTAATTACTGTGTTAAGGGTCTTGTGGCTAGTTGGTGTGAACAGAATGGAGTTCCTATTCCTGAAGGCCCTCCTGAATCTCTTGATTTTAACTACTGGAGATTGGCATTATCAGATAATGAATCGACAAATTCAAAATCCGCAAACAGTGTCGGCTCTTGCAAGTTGAAGGGTGTCAAAGTTGTTCCTGTAGAAGAGAATAGTATCTCAGAGCAAATGGGGGCGAATGCAACTGAAGATTTTTCTGCACAAGAGGAAGATAATGAACGGTATCTTAGTTTTCTTAAAGTCTTGTCAGAAGGGAACAATTGGATGAAGAAGTGCGAAGTTGTTGAACAGTTGAGGCTGTTGCTGCGGGATGATGAGGAAGCCAGGATTTTTATGGGGGCTAATGGATTTGTTGACGCACTTATGCAGTTTCTGCAATCAGCTATGCATGAAGGGAATGTGATGGCTCTAGAAAATGGAGCTATGGCTCTGTTCAACTTGGCTGTGAATAACAATAGAAATAAGGTAATTATGATGTCAGCTGGAATCTTGTCGCTATTGGAGGAAATGATTTCAAAAACTAGTTCCTATGGTTGTGTAGCCGCCCTGTATCTGAATCTTTCTTGCCTTGAAGAAGCCAAGGAAGTGATTGGCATGAGTCAGGCTGTCCAGTTCCTTGTCCAGATTCTTCAAGCCAAGACAGAAGTCCAATGCACGATAGATTCCCTCCATGCACTCTATAACCTTTCTACTTTGTCCTCCAATATTCCGCACCTCCTTTCATGTGGCATCATTGATAGGCTACAATCCCTTCTTGTAGACCAGGGTGACTGCACTTTGACAGAAAAATGCTTAGCTGTTTTGATAAATTTGGCGGTTTCTCCTGAAGGAAGAGAGCAAATGATGTTGGCTCCTGCACTCATAAGTGCCTTGGCTTCCACGCTCGATACCGGTGAGCTCATGGAGCAGGAGCAAGCTGTCTCTTGTCTCCTAATTTTATGTAACAGGAGTGAGGAATGTTGTGAGATGGTCCTGCAAGAAGGGGTTATCCCTGCTCTGGTGTCGTTAACTGTGAATGGAACCTCGAGAGGAAGAGAAAAGGCTCAGAAACTCTTGATGGTCTTCCGAGAGCAGCGACAACGGGACCATTCACCTGCTACAACAAATCAATGTGAACCTGAAAGTACTAATTTGTCTATGCCTCCTCCTGAAACGAAACCGTTATGTAAATCAGTATCTAGAAGAAAGATTGTAGGGAAAGCTTTTAGCTTTCTTTGGAAAAGCAAGAGCTATTCTGTTTACCAGTGTTAAGTCTCATGTAAATTTCTGTTCGATTCTTAACTTTTCTGGGCCTGCTTTTCACCCTCTCACCCGTTTCCACGGGTACTATTTACTTTTTGGTTCCCAAAGAGCTGTACAGGCATGTGTAATTTATGGATTTATTTCCTGTCTCTTCCGTCTTTGTA

>C102175_65

AGATAAGGAACTTTGCTTTGATGATATTTTAAACATGGTAATTATTGTTTCCATTAAAGGGTTCAGGTTACATATGAAGAATAGGGAATGATTTATACAAAGAAAACTCTAGGTCTTTCTAAATTCTTAATCTGACCCTGTGAAGACAAAAAAACTTGATATCATAATAGCCAACTAAAGAAACAATACACGGCTCAACAAATTTCGAAAAAGAAGTAGGATCAAAAGAAGCAGGACACAATTTTCTATCTGAATTATCACTCTGCAGCAGTATCCTTAATGTGATTGCTTTCTTCATTGGATTGTGGCAAAGTTCCCAAATCCACGCGAGATTCTTTATCCTCCTGAGTGATTGAATGATCAGAACTTCCTCGATCATTCATGTTAGAATTGCTATCTCCATCGGAGTGCGCAGATTCCTCCTCTTTTAGTGACTCCACTGTTTCGTTTTGAGTACCATCTTCATTCTCACTAGTCTTGCTCTTAACTGTGTTCTCATTCTCACCCTGCCCTGCATCATTGTTATTTGAAGACTCATCTTTCTTCTCTTCAGGAATGGATGAACCCGAATCATTGTCTTGTGCTTCACCATTAGCATTCCCATTGTCCTTTACATTTTCATTGCTGTTATCTGAGTTTGAGGATTCGTCTTTGTTTTGCTCAGATGTATTAGACTGGCTAGCATCATTGCTGTTATCTGAGTTTGAGGATCCTTCTTTGTTTTGCTCAGATGAAGTTGCCGACTGGCTAGTATCATTGCTGTTATCTGAGTTTGAGGATTCCTCTTTGTTTTGCTCAGATGAAGTATCCGACTGGCTCGCATCACTGCTGTTATCTGAGTTTGAGGATTCCCCTTTGTTTTGCTCAGATGAAGTATCAGACTGGCTAGAATCTTTGTTCTCATTACCACTTCCATCATTGTTTGTGTTGGTTTGATCAGCATTATCATTCTGCGCAGAATTGGTATAACTTTCTACATTTCCGGATTCATCTTTCTTTTGTTCTGAGGAAGTATCAGAAGTGAGTTGAACATCATGGCTAGCATCCTTGTTGTCATTATTGGATGATGCAACATTTTCTTGCTTTCCATCTTGAACAGAATCGTTGTTGGCGTTTTCATTCTGAGCAGAATTACTCGTTGTGTCCTTGTATTCACCATCATTTGCATTGCTGTCACGTGTTTCAGCTTTCTCCCCTGCTGCATCGGAGTTAGAAGAAGAATCTTCTGTTTTTGGTGTTGAATTCTGCTTGCTGGAGTCCTCCTCCACCTTGGAGCTGTTTTCATTTTCTGATGCTTCAGTTGGATGAGTTGATGTGTCAGTCCCATTCTGCAGGGATGAATCTTGTGTTTCTACAGCATCCTTTGTGGAGTCATTTTTCTCTTGCTGCTTCCATTCTGATTCAGAACTTTTCTGTGATTCATTCTCTGACTGTGCCTGATTTTCAGCGTCACTTTCATTTCCTTGGCTGTTTGTTGTGACACTAGAATCAGTCCCTTCTGTAGTTTCAGCATTTTTCTGAGGCTCTAGCTCAAGCTCATTGTTTTCCTTTTTACCAGATTCTTCTTCTGTCTTAGAATTTTCATCTGAGGTGTCTTGCGATTTATGGTCTACAGCACTAGAGGCATTATCTCCAGCATAATTTTCCTCTCTATGCTCCTCCTCATTCTTTCCCGCATCCTGAACCTGATTTTCAGATGCAGTCTCTTCCTGAATTTTGTGTTGCTGTTCCGCACCAGTTTCTCCTTGATTGCTCTCATCTGTCATTTCTTTACTATCTTGCTCATGATTTTCCTCCACGTTGTCCTTCTCTTCAGTTTCATTCTGTTTCACCTCTTCATTCTCCTTATCTTTATTCTCTGTCTCATCATTTTCTTGCTGACTTTTCTCTTCTGTAGTTTCTCCGGTTTCATTGTCCTCCACTTCATTTTTGTTCTCTTCATTCTCCTTCACTGCGTTGTCACTCTCCTGCCCACTCTTTTCTTTATTTTCTCCAGTTTCTTTGTCCTCCACTTCACTTTTGTTGTCTTCATTATCCTTCAATGTACTCTCATCCTCCTTGTTGTCCATATTGTCGTTCTCTTCAACAATTTTTTCATTATGCTGTTCACCTTCCGCTTCTGTCCTAGCATTTTCAGAATTTTCTTCAGTTTCTTGGTCTACCGAATCCTGTCTGTGCTCAGAATTATCTTCGTTGTTACTTTGATCATCTTGCTTTAGGACGTCATCATCCACACCATTAACATCATCCAGTTTGTGTTGCTCTTCGTGCTTGTTTTCTTGTTGTTCATCCTCCTCCTCCTTGTGCCTTGCATCTATCACAGAAGTTTCCTCTACGCGAGGATGAAGGTCCTTCCTCCCAAACTTGATAACCTCACTGCTTGTCTTTGTGCCTTCAACATAGGATGATTTCTTGTCATTAGAGTGTTTGATTTGGTAAACCAGCCAGATACAAACGCCAAGCAGAACACATAACTGTAGAGCATGCTTCACCTTGAAGCCTTTGGATCTTTGACTCCTCCGGGGAGATAACCTGAACATGCTGGAAATTCCTCACCCACCAAATTTTACTTCTTTCAAAGGTACTTTCTATTTGGAGTTCTCTCAGTGGGAATGCTTCTGGATCTGATCCCTTCCCAGCAACACGAATGAAAAGCAGATTTAACAATAATTTTTCACTGACAAAATGGAAATGACAATGCCAGCATATCACTTATTTATAGCAACTCAACAACGGATC

>C102179_65

CATTCATATACGATGGTCAAGGTTTACTCAAGTTACTCTATTGTTTTTCTGTTGAAACATTCTTACTGATTCCTAAAAGAATAACAATCTTAGGTTTTAAAGAAAGGAGCACGAGATACCTCAATATAACACTTCTGGATAATGATGAATCCGGAATAAAGATCATGACAGTTTGAGTGCAACTCAATTCCAAATAAGTTATAGGCCTAAATATAAACACATGACTCTGAAACCCTCGCCTCCGCCTCAAAATTCCTTAAGCACAACCAAGCAGCACTAATTGAAAAAACTCTGAGCAAATAAACAATAACAAGCGATGTAGGAATGAATCATAAAAAAAATGAGACTTCAGCTCAATGAGGATGATATGCACTCTACCCAAGAAAGTGATTAACACAGTAACTTATAATCTAATAACATCTTGGAGGCAATCATAGAAATGAGCAATATTAATCTTCTCTTTGCCTGAATAGATGGCCTCTGCAGATCCACCTTGCTCCCCTTGCGCTGCCATCTCAATCAACATGTATAGCTTCCTGATAGGCATATCATTCAATGCCTCTGCAGCAGAATCAATATCTTCATCAGAAAACACATTCAGCTGTTCGAGGACCTTCCTTGCATCCTTTGTGTTCAAGGTAGGAACGTGGTAAGTAACAGAGAAGGTATCGCAAAATCCAATTGAGTCCAAGAAACCCAGTTCACTTGTTGTGCCAAGAACCATGAGTTTTTTCCCCTTTGGAGGAAGCCGTTTGAGAAGAACAAGCAGTGTCTGAGAAATCAAGTTTGAAAAACGAGGACCAATGGCCACATACTCCAATAATCTCTCAATGTCATCAAGAACTATGACACTCAAGGGTGACCTGTATGCATCCTCAAAAACCTTAATAATCTGTGCACATTTGGTGCTCTCATGTAGACCAATCATTGTTTCAGCTGAAACTATCTTGACATATGGGAAATCACTGTCAATACCAACAGTAGCTGCTAGTGCAGTTTTACCACTGCCACGGGATCCTTCCAGAAGACAAGTGACAAGTGGGCTTCCTTTACTAACTTTAACTTGCTCCACAAGTAGCATTGCTCTTTGATAAATGTGCTTATGTCGATCACCACAGTCCACCATACCATGGAGTCTGCATCTTTCAAGATCATCAGTTGAAGCACCAAATGCGGGAATAACTTCATGGAGTGCATTCAAAAAGTCTTCCATTGTAACCTTGATGTTCTCTTCCTCCACTTGCTTAGTGAGATCATCTAGACTCAATTGTCTATTTAAAGCATATGAAACAGCACTTTTCACAACACCTTCAAGTTCTGCACCACTGTAGTTTTTCGTTCGAGCAGCAAGCTCTTGAAGGTTCACATCAGGAGCTAGAAAAGAATTCTCTTTCATTTTGTTGGTATGAATTTGAAGAATTTGCAATCGACCATTTTCATCAGGAAGGCTTATTTCAACCTGGACTTCCAACCTTCCTGGTCTTAAGAGAGCTTCATCAAGCATATCCTTTCTGTTAGTCATTCCAATAAGCAAAACATTATTTAGTGACTCCACACCATCTATCTTAGTAAGAAGCTGATTTACAATGCTGTCATGAACTCCAGTACCGTCTCGAGTTGAACCTCTTGACTTACAAATAGCATCAATTTCATCAAAGATTATAACATGCAAATCACTTTCATCCCCTCGAGTCCTCTGATCCTGTTCAGCATCAGCAAAAAGGTCTCTCACATTCTTTTCAGTTTCACCAACAAATTTGCTCAAAACTTCAGGGCCATTTACAATCTTTGGTTCCTTCCCATTCAAAATTTTTCCAATTTGACGTGCCATAAGTGTCTTTCCAGTTCCAGGAGGCCCATAAAGAAGCATGCCCTTCACATGCTTGATTCCTAATTTAGATGTCACATGGGGAGGGAAGACACGAGAAGCAAAAGCTCTTCGAAATATATCAGCAAATTCTGCACTCAAGCCACCAATACCCAGTGACTGAAGGTTAAATTCTTTCTGCTTGAAAATGTTACTAGTTGCACCCTCCCGTTGATTGACAATCTTAATTCCACTATCACGTGATGTTTCGAAAACAATGTATGTGTCATCAGAAATCATCCCTCTTTCAAGAGAATTAGACTTTTCTTGGCCTTCAACAGTAGCACCACTGACAGTAAAGCTATAGTTATTTCCATGGTACTCAAATAACACTTTTTGCCCCACAGTCATAACCTGGTTTAAAAATCTCTTCCGAAGTTGCTTAGCCAATAGAACAGCATCAATCTGCTCACTCTTATTCCCCTTTTTAACAAATTCCAAATCAATGGTTAGCAGTGCGAGGTTGAAGTCGTCGGGAGGCACAAATCGAGTTAATTGTACGGAATCACCAGAAGAAACTTTAGCACACCGCCGTTGAATGGAGTTCAACCCAATCTGGTTGCTGCCTATGCTGTCATGAGCAGAGAGAGATAGGACGAAGGAATCGGCGACGGTGGCGAGGTAGAGGTTGGTGTGACCGGGGACGGCGAAGTTGCGAAGATCGGAGGCGGAACAATAGGCGAGGTTAGTGAGGACGAGGTCGCTGGAAGGCGTGTTGGTGACTCTCATCTTGAACGGAGCGGAAGACGATGAGCCGAACAGACTCGCCATTTTTATCTCGACGCGGATCTTGCTCCGCTCTTGTTGCACCTGCAGATCACACAGCAATGGGAAGGGCGCGGGAGAGCGATGCCACAAACAGCTATGTTATG

>C102183_65

GAGTGTTTCTTGTTCTGCTGCTTGTCCCAAAACCCTAACAAAAACCCGTAGTAGCAGTGCGGTGCATCAATGGGGAAAGCGAAGGGGAAGCATCGTTTGGACAAGTACTACCACTTAGCGAAAGAGCATGGTTACAGGTCTCGAGCTTCATGGAAGCTGGTCCAGCTCAACTCCAAGTTCCAATTCCTCGAGTCCGCGCGCGCCGTCCTCGACCTCTGCGCCGCCCCCGGCGGCTGGATGCAGGTGGCGGTCCAGCGCGTCCCCGTAGACCACCTCGTCATCGGTGTGGACCTCGCGCCCATCGCCCCCATCCGTGGCGCCATCGCCATCCAGGAAGACATCACCAAAACTGAGTGCAAGTCCCGCATCAAGAAGCTCATGAACGAGCACGGATGCCGCGCCTTCGACGTCATTTTGCACGACGGCTCTCCTAACGTCGGCGGCGCCTGGGCTCAGGAAGCCATGAGCCAGAACGCCCTCGTCATCGATGCCGTCAAATTAGCCACTCAGTTCTTAGCCCCCAAGGGCATCTTTGTCACCAAAATTTTCAGGTCGCAAGATTACAGCTCTGTGGTGTATTGCTTGAAGCAGTTGTTTGAGAAGGTTGAGGTGGATAAGCCTGCGGCTAGTCGATCTGAATCTGCTGAGATATATGTTTTGGGTCTTAGGTACAAGGCTCCTGCCAAGATTGATCCAAGACTTCTTGATGTGAAGCATCTCTTTCAGGGATCTGTGGAACCGCAGCCTAAGGTGGTGGATGTACTTAGAGATACTAAGCAGAAAAGGCATCGTGATGGCTATGAAGATGGAAACACAACCTTAAGAAAGATTTCTTCAGCTTCAAATTTCATTTGGTCAGATTCTCCTCTGGAGATCCTTGGTTCAGTCACTTCTATAACCTTTACTGATGCAGCTGATTTACCAATCAAGGATCATGAGCTAACAACTGAAGAGGTCAAATCTCTCTGCGATGATTTAAGGGTTTTGGGGAAGCAAGATTTCAAGCATCTTCTAAAGTGGAGGATTCACATTAGAAAGGCTCTTTCACCAACTCAAAAGCCTGACCCTACCACTAAAGAACAGACGGAAAATGAGCAGAAGGTGGATGAAGAAGATAGATTACTCAATGAAATGGAGGAACTGACTAATGTAATGGACCGCAAGAAGAAACGTGCAAAGAAGCTTCTTGCTAAAAGAAGAGCCAAGGACAAGGCACGAAAAGCAACAGGGATGCAAATGGATGCAATAGAGGATGGTTATGTAGATCTGGAGTTGTTTTCTCTTTCCTCTATAAAGGGTAAGAAAGATCTGGTAGCTGTTGACAACACTGAGTATGAAGGTGACGATGGTGAAGTAGAAGATAGTGAGAATGAAGAAATTCATGAAGGGCCTAAGCATTCATCCAGTGACTTGGAAGATTCTGATGAGGAGCGTAAAAGATACAATGAACAAATGGAAGATTTGCTGGATAAAGCTTATGAGCGGTTTGTGATAAGAAAGGAAGGAAGTACAAAGCAGCGGAAGCGACTTAAAAAATCCTATGATGCAGAGGCCCAACTTCTTGAGGGTGGTGAGGATGTTGAGATTGTTCAATCCAAGTATGATTCCGATGAAGATCAGGGTAAGCAAGAAGCAAATCCATTGATGGTGCCACTTAGTGACGGGGCAGAACTCACCCAAGAGGAGATCACGAATAAGTGGTTTAGTCAGGATGTCTTTGCCGAAGCTGCTGAGGAAGGAGACTTTGAGAAGGAGGAGAGTAAAGATGAAATGGATATAGATGATGAACCTAAGGAGAAGACATCCATTGCCAAAAGGGTCAAAGAAAATAAAACAGCAGCTCCAGCAGTGGTAGATCACCCTCAATCTCAAGCATCCAAGAAAGCAGATGATTTTGAAATAGTTCCTGCGCCTGCCACTGATTCAAGTGATGATTCATCCTCTGATGAATCAGACGAAGATATTGAAACCAAAGCTGAGGTATTGGCTTATGCTAAGAAGATGATGAGGAAAAAGCAACGAGAGCAGATACTGGATGATGCATATAACAAGTACATGTTTGATGACGAGGGATTGCCCAAGTGGTTTCTAGATGAAGAGAAGAGACACCGCCAACCAGTAAAGCCTATTTCGAAAGAGGAAATAGCTGCAATGAGGGCGCAATTTAAAGAAATTGATGCCCGGCCTGCAAAGAAGGTGGCAGAGGCCAAAGCACGGAAGAAGCGTGCTGCAATGAAGAAGCTTGAGAAGGTACGGAAGAAGGCGAATGCCATATCAGATCAGACAGAGATATCTGATCGTTCGAAGAGGAAGCAAATTGACCAACTATATAAAAAGGCTGTTCCGAAGAGGCCTAAAAAGGAGTATGTAGTTGCAAAGAAAGGTGTACAAGTAAAGACTGGCAAGGGAAAAGTCCTAGTTGATCGAAGAATGAAGAAGGATGCTAGGAAACGTGGAATGGGTAAGGGAGGAAAAGGAGGTCCCAAGGGCAAGGGCAAGGGCAAGGCTCCAAAGGGCAAAGGACCTTCAAAGGCTTCTTCTAAAAATGGAAAACAGAGGACTAAAGGAAAATAGCCTCATACTCATACATTTTCTAGGCCCAAATTTTGTTTTTCATTTCTTTCCTGCTTGTTATTGTGCAATTAAGTATTATAAATTAAAGAGTAGAATTTTTTTGTCCTTCATAAAGGAAAACGGTAATGTAATGGAATTTTTGTCTTAATTAGCAGCTAGTATAGTTC

>C102187_65

TCTGGCTGTGTTCCTTCCTCTTTCCTTTTTCCCTGTTCTTGTGATGACAGCGACTGAGTCTGAGAGACCGAGATTCCGCCATAAATGCATTCTTCCCGTAACTCCATCTTATTCTTCGATTCCACATCGCTTCTTTCTCTCGCAACAGGTTATGCATATGTGATCAACTATTGAAAGATGTTTGACTGTGGTCACAAGTCAGAATACATGGGTGGCCAGCGGGATAATTTTTTGAGGTTGGATGCCTTGGATTCTACACTATCTTCATCTTCTGATGCTGGAGTTATAAAATCTGGATTTAGTATTGATAAATTAAGCCATGGTGGTCGTGGAAGCAGCACAACTTCTAGGTCTTTTAAAAGGGGAATGAGAAGGGGATCTGAAGGGCTGAAATCTATTGGGCGAACGCTTGGGTTGGGAGTATCTAGGGCAGTGTTCCCGGAAGATCTTAAAGTGTCAGAAAAGAAGATAATTGACCCTCAAAACAAATTCCTGCTCTTATGGAATAAACTTTTTGTTATCTCATGCATTTTGGCTGTGTCTATAGATCCACTGTTTTTTTATCTCCCAGTCATTAATAATTCATTTCATTGTCTTGGCATAGACCGAAAGTTGGCAACAATAGTTACAACGTTAAGGACAGTGATTGATGCTTTCTATCTTATACACATGGCTCTCCAATTTCGTACTGCTTATATTGCTCCATCTTCTCGTGTCTTTGGGCGGGGTGAGCTTGTGATAGATTCGGCACAGATAGCCAAACGATACTTGCAGCGGTATTTCATCATTGATTTCCTATCAGTGTTGCCAATACCACAGATTGTGGTTTGGAGATTTCTTCAGAGGTCAAAAGGTTCGGATGTACTAGCAACAAAGCAGGCCTTGTTATTCATTATCTTGTTTCAATATGTTCCTAGATTTCTACGAATGGTACCATTGACTTCAGAGCTTAAGAGAACAGCTGGTGTCTTCGCCGAAACTGCATGGGCAGGTGCTGCATATTATTTGCTGCTATACATGCTTGCCAGTCATATAGTTGGTGCATTTTGGTATTTGTTAGCTATTGAACGCAATGACTCGTGCTGGCAGAAAGCATGCAGTGAAATTGAAAATGTGTGCAATAAAGATTTCCTTTATTGTGGGAACCAGCACATGGAAGGTTATACTGCTTGGCATGATAGAAGTGAAGGTACTCTAAAGTCAAGGTGCTCTGCAGATGGTGATCCTCCACCTTTTGATTATGGAATTTTTGAGCAAGTCTTGTCTTCTGGCATAATTTCATCTAAGAAATTCATCTCCAAATACTGTTACTGTTTATGGTGGGGGCTCCAGAATTTGAGTACACTTGGCCAGGGGCTTCAAACTAGCACCTATCCTGGCGAGGTTATCTTTTCAATAGCACTAGCTATTTTTGGTCTCATCCTCTTTGCACTTCTGATTGGCAACATGCAGACATATCTTCAGTCTCTCACCATTAGACTCGAGGAAATGAGAGTTAAAAGGCGTGATTCAGAACAGTGGATGCATCATCGCTTGCTCCCACAAGATCTTAGAGAGCGTGTGAGACGGTATGATCAATATAAGTGGCTGGCAACGCGTGGTGTAGATGAAGAAAATTTAGTTCAAAGTCTACCAAAGGATCTTCGAAGAGATATTAAGCGGCATCTCTGTTTGGCCTTAGTGAGGAGGGTTCCACTCTTTGAGAGTATGGATGAGAGATTGCTTGATGCCATCTGTGAGAGACTGAAACCATGTTTATTTACAGAGCATACTTACATTGTTCGGGAAGGAGATCCAGTTGATGAAATGCTTTTCATCATACGTGGTCGTCTTGAGAGTGTTACCACAGATGGCGGGAGGAGTGGATTTTTCAACCGCAGTTTTCTAAAGGAGGCCGACTTCTGCGGGGAGGAGCTTTTAACCTGGGCCCTGGATCCCAAATCTGGTTCTAATCTCCCATCTTCTACTAGAACAGTTAAAGCATTAATGGAGGTTGAAGCTTTTGCCTTAACTGCTGATGAGTTAAAATTTGTTGCCAGTCAATTTAGACGTCTTCACAGTAGACAGGTTCAACACACTTTCCGATTTTACTCTCAGCAATGGAGGACTTGGGCTGCATGCTTTATTCAAGCAGCATGGCGTAGGTATTCTAAAAAGAAAATGATGAAGCTTCGTCAAAAGGAAGAAGATGCTGATGAATCAGAAGGACCTCATGAAAATGTTGGTGGAAGTTCCTACAGCTTTGGTGCTGCCCTGTTAGCCTCAAAGTTTGCTGCACACACTCTTCGTGGTGTACATCGTAATCGACTTGCTAAGACTGCCAGGGAGTTGGTGAAACTACAGAAACCCCCAGAGCCTGACTTCTCTGCAGATGATGCTGACTAAAATTTTCATGTGTAAACTACTTTCTTAAAATGCTTGTTTTCGTTGACTATTGTGATTGAATTTGTGAAGGAAGTTCGAAGGCAGTGTATTATACAATTATCCTGGTTACAGTTGTGAGGGTTTCAAGACTTGTAATGACTCTTCCTCTGTTACCATGTAAATTATAGTAGTACATTAAAGCCAACTGGCTGTCATATTAGACCTGCCACCAGGCATGTAAACTGATGATATATTGTTGTCACGTTTATTGTCGAAGTTACTGGTTGACACTTGACAGAATCAAGATCTGTCTTATTTGTCATCCTTGTGTAATGCTAGTGACATTTAATATACCAGTGTTTTTCCATCGGAAAAAAA

>C102195_65

TCAAAACAAAGTTCTCTTTCTCAGTGCTCTCTCACACACCCTCCTCTCATCCCAACCCGAATACCCTAAATCCAAAATTCTCTAACTCTTTCTCTTTTTCTTCCTTTCTTTTTTTATGAAGCCTCCTTGGCTGTGATTTTTACTTGTTGCTTTTCAGCTCGCTAAAATAGTTGAAATTATTTGGTACATAAGTTAACACGGACAGTGACTTTGAACGATGGATGTTGCTGAGGTTGAAGAAAATCTATTTGCTGCAAGTGATGCTAAGTTACATGGACAAATGTGCAAGAGTCTTTCTATCATATATAGCAAAGTAATGTCTGTATTTCCTTCTTTAGAAGCTGCTAGGCCTAGGAGTAAATCTGGTATTCAGGCATTATGTTCATTGCATGTAGCCTTGGAGAAAGTCAAGAATGTTCTTCAGCACTGCTCAGAGTGTAGTAAACTTTACTTGGCTATAACTGCAGATTCTGTCCTCCTAAAATTTGAGAAGGCTAAATCTGCTCTTGAAGATAGTCTTAGACGGGTGGAAGAAATCGTTCCGCAATCTATTGGGTGTCAGGTTCAGGAGATTGTGAATGAGTTTGTGACAATAGAATTTGCACTGGATCCATCAGAAAAGCAAGTTGGCGATGATTTGATTGCATTGCTTCAACAGGGGAGAAAGTTCAATGATTCTAGTGACAGCAATGAGCTTGAATCTTTTCACCAGGCTGCTACTAGACTCGGAATTACTTCTTCAAGAGCAGCTCTTGCCGAAAGAAGAGCTCTCAAGAAACTCATAGAAAGGGCTCGATCTGAGGAAGACAAGCGGAAGGAATCAATTATTGCATATCTCTTGCATCTTATGAGAAAATATTCCAAATTATTTAGAAGTGACTTCTCAGATGACAATGATTCTCAGGGTTCTGCACCTTGTTCTCCCACTCTTCAGGGATCAATTGAGGATAGTCTTCCGGGTAGTCATTGTCAAGCCTTTGACAGACAGCTTTCAAAATTAAGTTGCTTTAATTTCAAACCAAATAATAGTAGGAAATCAGGGCAGATGCCTCTTCCACCTGAAGAGTTAAGGTGTCCGATATCTCTTCAACTTATGTATGATCCTGTTATCATTGCTTCTGGGCAAACATATGAAAGAGTTTGTATAGAGAAATGGTTTAGTGATGGGCACAACAACTGCCCAAAGACCCAGCAGAAGCTTTCACATCTTTGTTTGACTCCTAATTACTGTGTTAAGGGCCTTGTCACTAGTTGGTGTGAACAAAATGGAGTTCCTATTCCTGAAGGCCCTCCTGAATCTCTTGACCTTAACTACTGGCAATTGTTATTATCAGAGTCCGAATCTACAAATTCAAAATCTGTAGATAGTGTCAGCTATTGCAAGTTGAAGGGTGTTGAATTGGTTCCTTTGGAAGAAAACGGTATCTCAGAGGAATCTGTGGAAAATGGAACTGAAAGTGTGTCTGCACAAGTGGAAGACACTGAACAGTATTTTAGCCTTCTGAAAGTTTTGACTGAGGGGAACAATTGGAGGAGGCAATGCGAAGTGGTAGAACAGTTAAGGTTGTTGCTGAGGGATGATGAGGAAGCCAGGATTTTTATGGGGGCTAATGGGTTTGTTGAAGCACTTTTACACTTTTTGCAATCAGCTGTGCATGAAGGTAGTTTGATGGCTGTGGAAAGAGGAACAATGGCTCTGTTCAACCTGGCTGTGAATAATAATAGAAATAAGGAAATTATGTTATCAGCTGGAGTATTATCATTGTTGGAAGAAATGATTTCTAAAACTAGTTCTTATGGTTGTACAACTGCTCTTTATTTGAATCTCTCTTGCCTTGAAGAAGCCAAGCCAATGATCAGCATGAGTCAGGCTATCCAGTTCCTAATCCAGCTTCTCCAATCTGATTCTGATGTTCAATGCAAGCAAGATGCTCTGCATACTCTCTATAATCTTTCAACTATGCCCTCCAATATTCAATACCTCCTTTCATCTGGCATCATTAGTGGCTTACAATTCCTTCTTGAAGGGGACAGTGATTGCATGTGGACAGAAAGATGCATAGCTGTTTTGATAAATTTAAGTACTTCTGAAGTTGGAAGGGAGGAAATTGTATCAACTCCTGGACTCATTAGTGCATTGGCTTCGATACTGGACACCGGTGAGCTCTTGGAACAGGAGCAAGCTGTCTCTTGTCTCCTAATTTTGTGCAATAGAAGTGAGGAGTGTAGTGACATGGTCCTGCAAGAAGGGGTTATACCAGCATTGGTTTCAATATCAGTGAATGGGACCCCAAGAGGCAGAGAAAAAGCTCAGAAACTCTTGATGCTGTTCCGAGAGCAGCGACGAGACCATTCACCTGTTAAGACACATCAGTGTCCACCTGAAACTAGTGATTTGTCTATGTCGCCAGCTGAAATGAATCCGCTATGCAATTCAATTTCAAGACGAAAGTCTGGGAGAGCTTTTAGCTTTTTCTGGAAAAGCAAGAGCTATTCTGTTTACCAGTGTTAAGCTCCATTGGTTGTTGTAAATCTGTGCTAGCATATTTACACTTTTGTTTTTATATCCTCTTCTACTTCCTGTTCTCATGGGTGCTGCTTAATTTTTGGTTCAAAGCGAAGAAGAGATGTACAGACCTGTGTAATTTATGGATTTTGTTTTCTTCTTTCGCATTCCTCTTTGTAACATCAATTTTTCTAAGTTTTCTTTCTTTGTCTTTTTTTTTGGGTGGGTTCTTGGGCT

>C102197_65

CTTTCTACAAAATAAAGCTTACGTTTCATAACACGGAATAGTTGGGCTCATTTCTGTTGCGGGAACCGTCGAAACAGCAAAGAAACTGTAAACAGCTTTACTTGACGTTTTGTCTATAATACAACAAACAGACTGCCACATTCAATCTCCCTAAAAATAGCTTCCGCTGGATGTCTTCAAATAATAGGTTTCTCATATCTAAAACTATTACATCAAAATTGCATTTCTATTCCAACCGCAAGAGAGGTTTTGTGGATATTAATTCCAGGAATGATACTTCAGCTTAAGATTTTAGAAAGACAAATTGATGTGTCGAAAACAAAAAAAAAAAAAAAACTCCAAAAAACCAGAGCAAGAAACTCCACTACTCGGGTGCTCTTCATTTTGGCTTGCAGGGTTTTCTATTTAAGAAACGAGAAACAAAAATTGGTGATATAGTTGCAGGCCATCCTCCTAGATGCTATCCGCCAATCACACGCCTGTATCCATATTTATATTAATGACAGTAAAGTTTGATCATTTTATCAGCACCAGCTGTAAATAACCATGGTTGTCTAGGGTGAAATTTACAGTCCAGTATCCCTCTCCCATTTGAACTGGTATGACCTCGAAGAATTTCCAAAGGAACAATAAGGGGATTTTGATTCAGATCAGAATAAACCATTCCATGAAAAACATATGCAGTGCAGTCATCTGAACATGAAGCAAATAAGGGATACGTGCGATGAAAGACGACATTGTTGATGTCTTTTGGATGACACTTAAGAATTTTATATGGTTTAGATGAAAGGTCCATGTCAAACCAACACATCTTCCCCTCTTTGCTACCCACAATTAAATTATCACCTCCGGGATGAACTGCAATAGATGATGCTTCACGGAGCCCAGTATCAAGCTTTTTTATGAGTTTAGTCTTTAATAGATCATACACACGGACACTCTTTTTTGTACAAACAAAGAAGATGGAACGGGATGGATGGAAGGTTGATCTAACAGCAAGTCCATGTAACTTGAAAGGAAGTTTTTGTGTAAGCTTCTTGGATAACTGGTGTATCAAAACTGCCCTTGACTCACCTGTTGGAATCACTGTTGAAAAGTAGTCACCTTTCCGGTGCCATTCTACAGCAGTAACAGTCTTGAAGTGCCTTAACCTTAAACCCGTGTGTTTTTCATCTTTAAGCCAGCTTACACTAGGTGCTTTATCGGCAGTGTCATCTGATGCTGTTGGTGAGCCAATCCAGAGAAGCTCCTTAATTCGGTTCTGCACTTCTTCATCCCCCAAATAGGTGTTCAGTAGAAGTACATCTTGTCCCGCAGAAACAGCTAGAATATGCATATCAGGCAGAGGATTCCAGGAAACACAACTAACAGCTTCACCAATCTCCCACCGCCTAAGACATCTGCCAGTTTCAACCTCCCAGACACGGACAGTACCATCACTTGAACCTGAAGCCATCCATTGCCCTGAAGCTTCAACTGAAATTGATGTAACAGCATCTTCATGGCCTTTATACTCAATATAGCATGTTATGGGATAAGGCTTAAGCTCCTTTCGACTTGGAAGCTTAGGCTTTAAAGATTCTGGATCAATATTAAGACGTTTTTTCCGAACTCGGGGGCACAAGTAAAGATCCAAACAACGTTCAAAGGAGTCCTTCATGGCATTCTCATATGCAGGGATGCTTCTCATAGATGTAAACCTTTTTGGAATAAACTTAGGACGGTCTTCCTCAAACATCAATTGATAAGAGTTTATCTCTTCTTGGGTTGGAATGTACTCTAAAGGAGGATTATACGACTCATCATGGCCAGGTAATTTCTGCTTTGGAGCAGGAATGTAAGCCAAATGATTTGCTTTTTCTGTTGAACCAGAATCATCTCCCCACAAGAGATATGGACCATCTTCCTCTTTTGGCTTGTCAAAAGTAATTGTTCCCTTGCGAATTGCTCTAACATACTCTACAACCTTTTTAGCTTCCCACTTTGAAGGAATAAACCTTCTTTTTGGTTCAGGTGCATTAGACAATGGATGCTTCGCATCTTCCCATTTATACCAGTCAACATAATCCGGATATGGATCAAAGTCGGAATGTGGTGCCTGATTCTTGAGCAGTCTACGGACAAGTTTGATTTCATCCTTTGTCAGCTCAACTACCTCATCATTGTACTCATCATAGATCTTCCGCCAATTCTTAGAATCGTCGACATTTGCAAGAAATGAATCCAACTTGTCTTGTTTTTCCTTTTTCTTAATCTTCTTGCCCTTGATGTCGTATCCTATATGAGGTTCATCCTCATACCACTTCAAAGGTACATCTCCGATGGTGTTTCGAGGAGCCACCTCATCCTCGGAGGAGTCACTCTCAGCTCCTTCCTGATGGAGGTCAGAGCTCTCACTTCCACTGTATTCACCATTGACAAAATCCTCATCCAGTGTAGATTCATTTTCTGCAGCTTCTAAATCGCCGGAACCACTATTCTCAGGGGAACCGTCTTCTAATTCTGATTCAGATTGAGAATCCAGCGAGTCAGAAGATTCTTCGTCATCTGCAGAAGAGTCCTCGTAGTCAGAGTCGGAATCGGAAACCGATGGCGAAGGGACCTTCTCGGATGGAAATGGAACTTCGTCGTCGACGTCGTTTTTCGTCATGGTCTTCTTCTTCCCCTTCATCTTCAAAGCTAAGGAGTTAGGGTTTTGTTCAGAGGAGGGAAAGGGTTTTACGGTTAGTTACTCAACCTGCCGC

>C102199_65

CTTAGACCGTGTATGGCTCTTCTTGCCTCCCGCTACTTTCCCTTTGCCGGTTAATCTTGCTCATCACTTTGCTCAGACATCAAACCCTAACCCTAATTCTCATCCCATCTTTCGATATAAATCCCTCATTTCCATTCTTCTGCTCCTCAATTTCGACAAATTTCTTTCTTCTTCGATTTTTCTTTTACATTGCAAGGTTTATATCGCAATTTGAGGCATGAATCGTTTTGTTGGTGTTCCGGTAGAGCATAATACGTAGTGGGTTTACAGAGCTGCGAACGATTGCATGGGTTTAGAGAATGCCGGAGCTTCGTAGCGGAGTGCGCCGTGGACGTGGTCGTGGGCAGAAGGGGTCGGAGCCGACTAGGAAGGAGGTGAAGGCTCGGGCGGCGCCCAGGGGGAGACCAAGGACTAGATTGGCGGCGAAGGAATTGGAGCGCCAGGAGCCGCCGATCCCGATTTCGGAGCCGGCGAAGGAAGATAAAAAGGAAGAGGGCGTGGGCGTGAAAATGGGTGACGAAAGTGGTGGCTTGAGTGGTAATAAGGCTGAGGAAGATGCTGTCACTCCACCTTTTCCAGACAGGGTTCAAGTGGGAGGATCGCCTATGTACAAGGTAGAGAGGAAACTGGGTAAAGGAGGATTCGGTCAGGTATTTGTTGGCCGTCGTGAGCGTGCAACAGGTGCTGGGGCTATGGAGGTGGCTCTGAAATTTGAACATACAAACAGTAAGGGCTGTAACTATGGACCTCCTTATGAGTGGCAAGTGTACAACACCCTTGGGGGTAGTTATGGAATTCCCAAAGTACACTATAAAGGAAGACAAGGAGAATATTATATAATGGTTATGGACATTCTTGGTCCAAGTTTGTGGGATGTCTGGAATTCCTCTAACCAGATGATGATGTCTTCTGAAATGGTCTCATGTATAGCGGTTGAATCCTTATCAATACTCGAGAAGATGCACTCAAAAGGTTATGTACATGGAGATGTAAAACCAGAGAACTTTTTACTTGGTCAGCCGGCTACAGCGCAAGAGAAGAAACTGTTTCTCATTGACCTTGGATTAGCAACAAAATGGAGAGACACCTCCAGTGGACTGCATGTTGACTATGATCAACGCCCTGATATGTTTAGAGGGACTGTTCGGTATGCTAGTGTTCATGCTCATTTGGGGAGAACTGCTAGTAGAAGGGATGATCTTGAATCTCTTGCGTACACTCTTGTCTTCCTTCTTAAGGGTCGATTACCATGGCAAGGTTATCAGGGTGATAGCAAATCCTTCCTTGTTTGCAAAAAGAAGATGGGGACATCTCCTGAGATGCTGTGCTGCATATGCCCTCCCCCTTTTAGACAGTTTCTTGAGATTGTAGTGAATATGAAATTTGATGAAGAACCTAATTATTCCAAGCTCATATCTTTGTTTGATGGTATGATTGGACCCAATCCAGCATTGAGGCCAATTAATACTGAAGGTGCCCTGAAGGTTGGGCAAAAGCGGGGTAGATTGAATATTGAAGAAGATGATTCACAGCCAAAAAAGAAGGTTCGTTTAGGGGTTCCTGCTACACAATGGGTTTCAATCTACAATGCAAGACAACCCATGAAACAAAGGTATCATTACAATGTGTCTGATTCAAGGTTAGAACAGCATGTTGAGAAAGGGATTGCAGATGGCCTTCTTATTAGTTGTGTATCTTCTTGTGGCAATCTTTGGGCCCTTATTATGGATGCTGGAACTGGTTTTACTGCTCAAGTTTACAAGTTGTCAACTTTTTTCTTGCACAAGGAATGGATCATGGAGCAGTGGGATAACAGCTATTACATTACTTCTATTGCAGGGACCATTAATGGCAGCTCTCTTGTGGTGATGTCAACAGGTACGCAGTATACTCAACAATCCTACAAAGTAAGCGAATCTTTCCCCTTCAAATGGATAAACAAGAAGTGGAGAGAAGGTTTTCATGTCACGTCAATGGCTACTGCTGGAAGTCGCTGGGGTGTTGTTATGTCTCGGAATTCTGGATACCTTAAACAGTCGGTTGAACTTGATTTTCTCTATCCAAGTGAGGGCATCCATAAACGTTGGGATAAAGGTTATAGGATTACAGCAACTGCAGCTACATTGGATCAATGTGCTCTTATATTAAGCATTCCGAAGCGGAGGCCCAATGATGAAACTCAGGAAACTCTACGTACAGCTCAATTTCCCAGCACGCATGTCAAGGACAAATGGTCTAAAAACCTCTACCTTTCCTGCTTGTGCTACGGGCGGACAGTATGCTGATTTCTGTGACATGTTAAATCATTTTATTGACAAGCAGATTATGAGTTGTCAATTGCCCAACCACACTAAGAAGAAACTAATTGTGCCAACAGAGAATCATATTTTGTTGTGTTATAAGAAAATATGATTCTGTTCACACAAAGAAGAAACTAATTGTGCCAGCAGAGAATCATATTTGGTTGTGTTATAAGAAAATATGATTCTATTCACTTCCCTTTGGCCGGGGAATGTTGCAAGAGTAATGAATTGTGTTTAGGTATGTAATGTCTCTCCACTATGTATATGCAGATTTTATATTTTTTTGTTTCCGCACGCCGCGTGCTTAATGTAACTAATGGTCAATTAAGTATGTGACGAGATTTTTCAGATTTATCTCCATGACATACTTGTAAAAGGAAACACAATTGCGCTGTATGCCATCTTCTCTTTAAATTTATGAATAAAGAAATTCTTCTCCTA

>C102201_65

ATCAGATTGTGTACAGCTCTGCAAGTTAAAAAATGGCTCCATATACATACATTAAATAGTATGTATATTATAGAAAGTTATATATACAGAAACGAGATTGCATCACCTCAAGTGAATTACAAGCGAACCAAAAAAAAAACAAATGCATGCGTAGTAAGATACTTAATCAATAGACATTTCTTCAAGCTCGCAGCCCAATACAAAACTGAACAAGCAATGGCCTCGAGACTCTGAAAACGGAGCTATGAATGAAATAGAAAACCCACAGCACCCCTAGCCTTATCTGCTGCTTTGGTGCTGTCATGCTGTTTCTGTAGAGATGACACGCTCCTTATAGGCAATGAGGAAGTGTTTGCCAGGTTCATTCTCGTTTCTCCTATAGGCCATGATGAAGTGCTTGCCAGGTTCATACTCGTTTCTCTCACGTTCTATTTCCTACTTTCATGCAAATATCTATTGTATAACAACTGAAATATTGGAACACCTACGACGTTAACAACCGGACCCCACAATAGATGACTGGTCCTCAGGATTGACTACTGCTTCAGAGTCCATCTTGCTGGGTTTATTTTCTTCATTGATTGGAAGTCCTATTTTGGGTGAAAGCTTCTTCACCTCCTCTGCAGTGTAGATGAAAATTTTCCTCACAATGCTACAAAACTCAAGCCATGGATCATCCCCAACCATCATCATATCATCTTCATTATCAGTGTACACGATCTGCCATTTTTTTGTGGATCCACATAGCTCATCCTTGATGTCAAACATTTCTTCCAATTTCCTTAACAGGTCCTCATATCCATCAAACCGCGTTAAATCCACAGCCCTCCCAACAGCCATACCTTGCATGTGAACCTTTGTGCAGCTTCTTATTTGCCTGCTCTGAGACTCCTGGGGAGACCGCAGGCATGATTTTTCAGCATCACAGCTTACCGAAGGTATATCAGATCGATTAATATTTGATGGTTCAGAATGCTGGTCAGACTCAGCATCCAAAGATGGATGAGGGCCATCATCACCCACCCTTCCTGACAAAGCAACCATTGGTAGACCTTCTTCCCCATTGGAATTATCAAGTAACTGAATCCCAAAGAGCCTACAGCCATTTGCAGTCCCTTGTCTTTTCTCTCCAGATTCTTTAAGTGCTACAGGTGAAAAAGATTCTGTAGAGTTTTCCATACTAGACCAATATATTGATTTGTTGGAAGCAGAACAATTCCCACCAAAACCAAGGAAGCCAGTTGCAGTAGAATTGAATTTGGGTGATGGATAGATGTCTCGCCCATGGTCACGATAAGAGAATGGTGCAGAATCAACTGGTGATTTCCATACACCTTGCAGAGAAGAATCAGGCATTGTTGAAGGTAGAATGGGAGGCCGTGATCTCTTGTTTCTTTGGGATGGTTGGGAGTTTGTAGGAGGGGAAGACACAAGTGGCTCCAATTCCCATGGAGAAACTCTATCAGGACGCAAAATAGATGATGGTTCATCCCATTGAACCTTTAATGATCGCCACTCAGAATCAGTCCAGACTGAAGATTTATTATCTCCAACACCCACAATTGTACCACTGAACCTTCTTTCAGGAACTTCATCACCCTCAAATCTCATTTTGAATCTCATCCCAACAGAAAGTTTATGATTTCGAGCTTCAAGATACTTGTTGATACTCACAATAAACTCAGACCGGCTAGTTCTTGGCTTGTAAAATACAGAAAACAAGGTTCCTGTGGCAATGGCATGAGATGCAGTGGCCAGAACACCAAGATGCATGCTGTGACTGGATATAACCGAAGATGGCATGTTGCTTTGCTGTCTCATGAGCCTCCTAACTCCAACTCGCAGTTCTCCATTTTCACCCCTTAAGAATATAAATGCATCACCAGCCACTAATTTTTTTGAACTGACAAAGACACTCCACCCGGTGGTCAGTAAGTGGCGCCTGGGTTGCCCTCGAAAAATATGTCTAAAATGCCATTCATTTCCATGCAGATCAGTTGCAACCAATTCTTGCCATGGTGGCTGCTGAGTCATATCCAGTGGTGGCAGACAATCATCTGCATGTCTTCGAAGAACAGAGAAACCTCCATGAGTGCTAGTGTCAGAAGCAGTGAGTGTCTTGCAAAATGAGTGAACTGTACACCTTGGAGGTTCCAGCAGAGGATCGTCAGGGCTTGTCACCTCGCTTTGATCTGCTTCAGGAAGCAAAGTTATTTGTGCATATACTTCGTCTGTTTCAGGTTCAGCTCGCAGGTGAATATTGACCACTTTACATAATATTTTAGAAGGCAGATTAAAGGAAGGCATTTGCTGCTCCAATCCCTGATTCATAGATGCCTCGAGCTGTTCCATATGACCTTGGGGGAAGTAATAAACTCTCTCCCCTTCACGCGGAAGAGTGACAAGGGGTCCAGCACAGGCATGCCACAATTCCTTGTATAAAGCATCGTTGGTTGAACCTGGCGCAGCGGAAGCCATGTCGAAGACAGGAGAGATGAATTGCTGTTGTGAAATTGGGTGCCCTAACTATTCGATCAGAAGTGAATTGAGACGAAAAAGAACGAAAAAATGGTGCGTGCCGATGGATCCACTCCACCACAATTGCAGAAGCAAGTCGGTAGGGTTTAGGAATTGAAAAGCAAGCACAGATCGAAATTGAATCGCCGATAAGGTCGTGTTCGGTTCAAGCCGACACCACCCCCTCTTTCTATTTCTATACAACGCACCCCACTATAATATTTACT

>C102203_65

TGGCAGGAGAGTACGTACTGCATGTACGTAATGGCTAAGAATTCATTTGGAACACACATTTATCTCAACTCAATTGTTGAGATATTGTGTTACCATTGCTAAACATGGGCAAATATCAAATGAACACAACAACGTGCCCAATATGGAACAATGTAAATAGGTTTGTGCAATGACATTCTCCAATTTTATAGTTTATCAACTTATTTTGCAAAATACTTTTCATAGAGAGCTTGAGAGATAGCTAGAGCATCTCCTTTGGCCTTTACTGGGTACAGCTCATTACCTTCCTGCCATTTGTTTGACTGTGAAATCCATTGTTTTCGCCACTCTATCAGCTGGAACTTCTCATTTTGTCTCAAGCTTTCTAACAAATGGCTAAAATAGGTTGAAGCTCGAGGGAGATAGTAGCTTTCCAAAAGTCCACTCCAAAATTTGTTGGCATAATCATGGAGCTTGCTCTGAGTAGTTTCATTGGTATCAAACCACATTGTCACTTGTGTTCTAGCATTCCATTCATACTGCTTTATCTCACTGGGATTCACTGCCAGTTTCTTAGCACTCTCAATCCAAGTTCCAAGAAGAAAGTTATCATCCGAAGCAAGCAGTACATCAATATCTCTTATCAGTTGAAGGAACTTATTGCTGTGAAATTGCAAAGCTTCAATGTTCTTCTGCTGAAACGAAGTAACTGCCTTGTAGTATAACTGGTTTGCAAATTTTGATAATACTTGCCGTGTCAAGTCAACCAGGTCATACCTATATGTGAGACTTCTGGAAAGATTCTTTCCACCAGCTAGGAATAATTGTAATGCCTTAATTACATCATCAGATGGGTACCACAAATGAGCCTGTGGCTCAGCAGGTGTTTTCTGGAATAAGTACCTTCTATTTCCAGGTGGCAAAAAGTATATCTTCTTCTGGTTATTGGATACGCCAGTTTCAGAATTTGTTGACGGGTCCCAGTCAGGAAACGTTACAATGAAATCATGATTATGGTCAGCAATTCCGTCAGTACAATTGTAAATTGTATGATAAAGAATCTCCCAGGCAGCCTCAACTTGATGAACAGCTTTACCATATCGTCTATTGCAATAACTCTTGATCCATTCCGGAACTTCAACTTTCTTATCTCGAAATGCCATTTCAGACATCAACTCATAAACAATAGGATTCTGCTCTATTCCTTCCATGCACATACCAACACCTACCATTGTTGAGTTTGCACTGACACGAGCATCAATAGGCCCAGAAGAAATTGCATCAAGGGTTCCATACATTTCAATATTGCCACCAAAATTATGAAGCATACACCAGATATATGGAGTACCATAAAACTGAAAGGAAGATTTCCATATTGGTTTTACATCAGCAAATAGATCAAGAACAATCATCTTTCCAAATGGAACAGAATGTAAAATGGCTTCAATTTGAGGAGGCTTCCAAAATGACGAATCAGAGTAGAAGAGCCAGCCTTGCATTAACCAGACAGCATCCTTGTCACCTTTGGAAATCCCTTTATATACTGCAGCTCCCAGAGTTGATATATATTCAGGATCATTTGTAGGCGGGGAGTTTTCGTTGAATGTGTCACAGTTGTAAATGTCAGTTACATCCCCATATTCTTTAATTTGTTTCTTTATAAAAGCCTCCCCTATTTCAACAAATAAAGGATCAGAGGGATCAAGAAGGTAAGTACAGCACCAGCGAGGATCACCGTCAACAGTGTTCCAGTCACCAAGTCTAGTTATTTTTGCCGAAGGGAATATCTTTGTCAAAGCAGCTGGAACATTCCCAGAGAAAGATGGAAGCACTGGTGTCATCCCCAGCTCCAGCATTCGAGAGATTATCTGTTTCTGTAATGCCAATTGTTGATCTAACCAATTTTGAGATAAAGGCCCGCCCCAGCCATGTAAATTTCCCATGCGAGCCCAAGCAAGGAAAGCAGTTCCACCGAAGAAATTATTCAACTCGTCTGAAGTAATGTTAAAATCCTTAAAAACCTTTTGCCAAATCGTTTCCTGCCCAGTAAATGCCAAGGGCAGGTTCACCCCCTGAAGTGCCATCCAGTCTACCTCTTTTTCCCACCTTTCCCAATCCCACCAAACATATGAATAACTAGAGGTAACAACATTTTGGTAATAATTCCATGGAACGGGGCGTTTAATCTTCAGACCCTCATCTTTTAAACGGGGTAGTGACCCTGGCTCAGGGATTGAAGTTGTCTGAACGCCACCTGTTTTATCCCATGAGACATGAGCACCGCACCAATATTTAAGATACCAATGGAGACCCGATGCAATCTCAACAGCAGTGGTTCCGCTAATGATAATCTCAGGCTCATTCAGGCTTGACTTCTTGTGATTGTTTATTAGAAAACAACTGTCTCCGCCACAGACATCCTTGGACACTATTTTGAACTCAAAACTGGACAAATGGGAAGGAAGCAGTCTTTTGAGAAGACCAACGGCTGCGGCTTCTTGCACAGATGGAGGTGCCCTTTTGGAGTCCAAGCGCTGAAGCAAAGGCTCTATGGCTTCATGCTTAGACAGTGCCGCCGGCAGTGAAGGGATGAGAATCAAAAGCAGAAACGCAAATCCCAACCTCAACATTGTTCTCTTCTTCCCACAAACTTCCACAAAGACAAAGGAAAATGTATAGATGGCTGTCGTAGATTTTGTTTGGTGATTTTTGGTTTAGCACCCAAATGGTTGACACGATGAGTGTGGTTTCCTTGGTATTTATCG

>C102205_65

ATAATTTCTTCGCCTTTCGTTTTCCACTTCGGTGAGCACACACACTCACGCACGAGCTTCAAAACCAAACCAACCTAAACTTTGTTCCAACCTCCAAAAGCCCCTTCAAACTTTTTCTGTCGTTCGTGACTCATGCCATCACCGACAATAAAAGGACAACGGTAAAGCACTGAATCATGAAAATGCTGCCACCTTCCACTGCCGGAGAGCCCTGTCACGGTGGCCGGACGGTGGTCGTGGGAGTGAAGATGGACTCCCACAGCACCGAGTTGCTCACTTGGGCTTTGTTCAAAGTGGCTCAGCCCGGTGATGTTGTGCTTGCTCTTCATGTTCTTGGGAATAATGAAATTGTGAATCGAGATGGGAAATCCTCTTTGTTTTCGCTTGTCAAAGCGTTTGACTCTGTGCTTGCCGTTTACGAAGGGTTCTGCAATTTGAAACAGGTGGATCTTAAGTTCAAAATATGTCGGGGTTCATCAGTGAGGAGAATCCTGGTTCGAGAAGCAAATGCTTATTCTGCAACGCACATCATAGTTGGAATTCCTCAGGGTTTTCATAGGATTCGGCCATGTATATCTGTGGCAAGGTACTGTGCTAAGAAGCTACCAAAAGATTGTTGGGTTCTTGCTGTTAATAATGGGAAAATTGTGTTCAACAGAGATGGTTCACCCGCAACTCGTGCTGACACGAAAGGAGTTGATCAGAACGGCAAAACTGGGGTGCTTTGTTCGATTCACAGGACACTCGGCAAGAGTTCAAAAGTGCTAGATGATGGTGGTACCGGAATGCATGAAAAGGGTAGTGGTCAATTTTCAGATCACAGTTTGGCTAAGGCTTTTCTGGATTCTAAAGAATTCGGTGAGAAGAAGGCTTGCTCAATTTGTTCTTCAACCCCAGCACTGTTTGGTTCATGTTGTAACCATTCAGAAGAGTCTTGTGGTGATGCTTGTGATGAGAACCCTTTGGCAATAGTGCCGGTACAGACCAATGATTCTGCTTCTAAACCAGGTTGGCCACTGCTTCATAGGACGATTGTATCTGACAGGAAATGTTCAGAAAGATCGCTGTTTCGCCAGATCTCTGTGGTACAATGGGCAATGCAGTTACCTTCTAGAAATCTTTCATATGCTGCACATCTTGACCATAAAACAAACAATTGTGGCCCAAATAAGGATCAGTTTTTGGGTCTAGATAGCACGAGTGGTGCTCTTGTTCCAGTGGATTCTGAAATAGGGACTGCCTCTTTGCCTGAACACAATTCAAGAAGCATTCCTAAGGAATTGGAGGGCCTTCATGAGAAATACTCATCCAGTTGCAGATTGTTCAAGTACCAAGAGCTTGTATTGGCAACATCAAATTTCTTGCCAGAAAATTTGATTGGAAAAGGAGGAAGTAGTCAGGTATATAGAGGTTGGCTTCCTGATGGCAAGGAACTTGCTGTTAAGATCTTGAAGCCATCTGATGATGTTTTGAAGGAGTTTGTTCTAGAAATAGAAATTATCACTACCTTGCATCACAAAAACATTATTTCCCTCCTTGGCTTCTGCTTTGAGGATGGTAATCTTCTTTTGGTCTATGATTTCTTATCAAGAGGAAGCCTTGAAGAGAACATCCATGGCAATAAGAAAAATCCACTGGTGTTTGGTTGGACTGAGAGATACAAGGTGGCAATGGGTGTTGCAGAGGCTCTGGAGTATCTGCATAATAATGAAGGCCAATCTGTGATCCATCGCGATGTAAAATCATCAAATGTTTTGTTATCAGAGGATTTTGAACCCCAGCTCTCTGACTTTGGACTTGCTAAATGGGCATCAACCTCATCATCACATATAATATGCACAGATGTTGCTGGAACCTTTGGTTACATGGCCCCTGAGTACTTCATGTATGGTAAAGTAAATGACAAGATTGATGTCTACGCATTTGGTGTTGTGCTTCTTGAGCTTCTTTCAGGGAGAAAGCCCATAAGTAGTGATTATCCCAAGGGTCAAGAAAGCCTTGTCATGTGGGCAAGTCCAATTCTAAATAGTGGGAAGGTGTTACAATTGTTAGATCCTAGTTTGGGTGAAAACTATGATCATGAAGAGATGGAAAGGATGGTCTTGGCAGCTACACTTTGTATCAGACGTGCTCCAAGAGCTAGACCTCAAATGAGCCTTATCTCAAAGCTCCTTGGAGGTGATGCTGATGTGATGAAGTGGGCAAGACTAGAAGTTAATGCATTGGAAGCACCAGAAATGCTTGATGATGAAGCTTGTCCAGCTTCAAATCTGCAGTCACACCTTAACCTTGCACTGCGTGATGTGGAGGACGACTCCCTCTCGATGTGCAGCATTGATCAAAACGTCTCATTGGAGGACTACTTGAGAGGAAGGTGGAGCCGTTCATCAAGCTTTGATTAAGCCAACTTCTAGCAGAAGGCTAGAACAAGGATTGTGTTTTTGAATCTAAATCCTTTGTAGATAATTTTGCTGTGTGGCATGTTCCATTGTATCTTTGGCATGTGCTGTGGAAGTTTATAGATGGGTTTAGGATGAGAGGTTGTTTGTGTGTGCCCTCCTCCTTCCCTTTATTGTCTAGGAAACTATGCCTTGTAATTTGTAAGGCTAGTTACCCTTTCTCCTTCTACTTCAATTGGTCCCAGTTTCAACCCTTCACATGATCCAGTGTAAAATATTCTGGTGCCAAAATAAAAGGTGTATCTTCAAACTGCATAATGATTACAGTTCTCGTGTATTAATAAA

>C102207_65

CTATACTCATAATTCATTATTTGATGATTTGAACTCAACAGTGGAACCTGGTGGTTCTGTATCACTTCACAATTTATACAAGACCTGTCCTAAATTCACTTGCTACACATTTTTAACCCACTCCGAATAATAGCAAATACATACAATGCCACCTAGAGAACTGCCAATCCACGAAAAGTCGAAGAAAATTACATTTACTAAAGTACATCAAAGCAAATACAATGACTTCTTCTCACCTGAACTTTGGGAAGGATGATAGAAGGAGAGAGAGAAAGCAATAAATGAAGTGATGTGATGGGAAAAAAATGTAAGAGATGGAGGTGTAAGGTAAGAAGAAAAATAGAATATGGAAAATTGGGATTGGAGAAAATATAACAAAAAGCCCAAAATAATTGAACACCCACTGACCATCTCAGTCATCACCTACTTGTTTTCAGTATTTGTCAAACGTTCACGCAGTTGAACGCATAGATCTGACATTTGGGACAGCAGTTTTAGTTGTTGATCTCGTGAATTCAGTGAGTGAAATTCTTTATCAACTGGATCACTAAGAAGCCGTTGGAGCTGACTACAAAGTTGAGATACTTCTGGGTCATATGTACAAGTTTCAGAAATATGTTGTAGCAATTTACAGCTATTATGATCTTGGGGCAATGATGGATGAGATGGTATAGGAGGAACAAGGAATGGATGTCCGAGTAGCTGAGGTATTCTCCACCTTTGATTGCGATCCCATGTTAGACACCTCATCATAAGATCCAATAGCCATGGATTTGAAACTGGTTCATACGTAATTTCATGATTTGGATCTGTTATCACTTTGAATTTGGCCCAAAATGTCTTGTAATCAGAAAAAGGTGTTCTCCCATATACCATTTGATAAAGGATGCAGCCCAGGGACCAGATATCTGACGACCGGCCACACTTTATGATGTTTCCATTCGCATCAGTCTCATTACACATAAATGCCTCTGGAGACATGTAACTTAGTGTACCAACCTGTGAATCCCGTTGGATGTTGGTTGTGTCATTCATTATTGCTTTGGCTATACCAAAATCAATCAGTTTTAGGGAACCTTTGACAAGAAGGAAGTTAGCTGGCTTCAAGTCTGAGTGCACAATGCGTTCCTCATGAATAGTGTTGACAGCTAGAAGAATTTGCTGCCAATAAAATCGAAGCCAGTTCTCATCGATGGTCTGATTGCTTCCATCTAGTTCCTTCCACTTCTGAGACAACATGTGAGCCAAATCGATTTCCCCATATTCAAGAACCATGTATATACATCCATCATCCTTGACCCGGCCATCTTTATTACTGAAGGAGCCTTTCATAACTTCCTCAAGCAATGCCTTGTCTGTCACCTCATAATCTATAAGCTGTATGATGTTATCCTTTCCCTTTAGCCTATTCAGATATTCAATCTCTTGACAAAACCCAAATGCAGTAGCATAATCACGACCCTTGAGCTTGATCTTTTTAAGGGCATATATCCTACAGTCGGATGAAATCACTTTGTGCACTTCACTGCTTCCACCACTACCTATCTTGCCAAGACGCTGATAAAGCTTGCCATTAACTTTGAAAAACAAATCAGGGTCATAAGTCCTTTTTCGAGCAGCTGGTGCACCTTTACTACTAACATTCTTCTCTCGCTTTTCTACCTTAGAAGACTCCAACTTCACATCTGAAGATGTGGTTGTTGTGGGAAGTGTTACCTGCGGTTGTATAGTGGCAACATCCACAGCCTCTTTCCCTTTACCATCATCCCAAGAAGTATTTTTGGAACTGCCAGATTCATTCAACACCCCCGCTTGTTGGTCCTTAGATCGATTGCTCTCCTTAGACGCCAATTCGGCATCACCAATACAAGAACCAGAAGCTTGGACTTCAGCTTCTGCCGCCCGATCAACCAACACTCTGTTGGTTTCTTTTTGGAAAACATTAACTGATTTCACAACCCCTTCAGTTACAGGACGAGGATTAGCATTAACCTCCCCAAAAGGCTCAGCAGTAACATTCGAGCCACAGTCTAGATACGAGTGAGAATATCGTGTTGTTGAATTTAGCATAGGTGCCGAAGTTGAATGAACTGAGGTGGTTGTAGCACATGAAGGTCCCACCACCGAAGACTGCGCAGCTGGCTGACTCAAAAAACTACTGAATTGCTGCAAATGATCCTGAGTAACCACACTTTTCTTTGGCAACACAGAACTCACTCCACTATCACATCCCAAACTAGCGTCAAAGCCGACCTGTTGAACATTCTCCTCCTGGTTGCATCCAAGCAATGATGCTGCTGCTGCAGATGATGATGATGATGATGCTTCAATTTGGTTGCCTTCAACCCACTCCATTTCTGCGAATCCAAGCGAACCCATATGAGACGATAAATTCTCCAACTCAGTGGTTGCCTTAGCATCCTGGGCAGCAGAATTCGGGTGAACTGAAAATTGGACTTTCTTCTGAGCGTCTAGGTTTAGGGAAGGGACGTTAAGTGAGACCTTGGAAGAAGCTTGTTCTTGAGAATTGGTGGGGTCGGAAGAAGTGTTTGTTAGCTTTCGTTGTGGAAGCAGCGTGCGCCTTGGCCTTATGCTATTGGACTGCATTGAACCGAGTGGACGGTGTCGTTTCAAGGCGGCTTGAACGTGGCGGAGGAAGTCCGGGGAGGAGGAATCCGGGTTAGGCTTCCCATCCATGGAGCAGTCTCCACCGCATTTCTATTCGCTTCTCAATTCGATTCCCCAA

>C102211_65

TCCCATTTCTCACTTGGCAGTGAAAGAATCGCTATCCATATACACGAATTCAACAGTGCTTATATCTTCAACTCAACAATAAATACCTCACATCCCTGAATCAAAACCAACACAACCCCACAATTGAGCAAAACAACCAGAAACAGAGCAATGAAGCCACTGATTCTGAGGTCCCTGCCAACCATTCTTCTTCTGCTGTTGATAACCATCTTTAGCAGCAGCAAAACCATAGCAGAAGAAAAGCAAACACCCCACCACGCCAAAAAGACCTACATAATCCACATGGACAAGTCCACCATGCCCTCAACTTTCTCCGACCACCTCAACTGGTTCGACTCCTCCCTCAAATCAGCATCACCCTCTGCTGAGATCCTCTACACCTACAAACACGTGGCGCACGGCTTCTCCACGAGGCTCACACCCCAAGAAGCAGAAACACTAGCGAAACAACCCGGAATCCTCTCAGTGACCCCAGAACTTCGATACAAACTCCACACAACAAGAACCCCGCAGTTCCTCGGACTCGACAAAAGCACCACGCTTTTACCCGCGTCCCAGCAACAGAGCCAGGTTATCATCGGAGTCCTCGACACCGGCGTCTGGCCTGAGCTCAAGAGCCTCGAGGACACGGGCCTGGGCCCGGTGCCCTCCACGTGGAAAGGCGAGTGTGAAGTTGGCAACAACATGAACTCCTCACACTGCAACAGAAAACTCGTGGGTGCGAGGTTTTTCTCAAAAGGCTACGAATCCGCTCTTGGACCTGTTGACTTAAGTACAGAGTCAAAATCAGCGAGAGATGACGAGGGGCATGGGAGTCACACGCTCACCACAGCAGCAGGTTCGGTGGTTCCCGAAGCTAGCTTCCTCGGTTTGGCGTCAGGAACAGCTCGTGGAATGGCATCGCAAGCGCGCGTGGCGGTCTACAAAGTGTGCTGGCTCGGTGGGTGTTTTACTTCCGACATAGCCGCGGGAATCGACAAGGCCATAGAAGACGGCGTGAACATTTTATCCATGTCCATTGGGGGAAGCTTGATGGAGTATTACAGAGACATAATCGCGATTGGTTCCTTCACTGCCACGTCACACGGGATTTTAGTCTCCACGTCAGCAGGGAACGGTGGGCCTAGTTCATCAACTCTCGCCAACGTGGCACCGTGGCTAACCACAGTGGGAGCGGGAACTATAGACCGCGATTTCCCTGCTTACATAACCCTCGGAAACGGAAAAACATACACCGGAGCGTCGCTTTATAACGGAAAATCCTTGTCGGATTCACCGCTTCCTCTTGTTTACGCTGGGAACGCGAGTAACTCTTCTGTTGGGTACCTTTGCTTGCAAGATTCTCTCATTCCTCAGAAGGTTTCAGGGAAGATTGTGATATGCGAGAGAGGGGGGAATCCGAGAGTGGAAAAGGGTTTGGTGGTTAAGCTTGCGGGAGGTGCTGGAATGATTCTGGCTAACAATGAAGCTTATGGGGAAGAGTTAGTTGCTGATCCTCATCTTCTTCCAGCTGCATCGCTGGGCCAAAAATCAAGCCAGATTTTGAAGAGTTACGTTTCTTCTTCTGCTAATCCAACGGCTAAGATTACTTTCTTAGGGACCCATTTGGAGGTTCAACCTTCTCCAGTTGTAGCGGCTTTTAGCTCCAGAGGACCCAACATGCTCACGCCAAAGATTCTCAAACCGGATTTAATAGCACCAGGAGTGAACATTTTAGCCGGTTGGACCGGCGCGGCTGGACCGACCGGGTTGAGCGCTGACAACCGCCACGTCAGCTTCAACATAATTTCCGGAACCTCCATGTCGTGCCCTCACGTGAGTGGCTTAGCCGCCATCCTCAAAGGCGCCTACCCTCAATGGAGCCCTGCGGCTATTAGATCCGCACTCATGACAACAGCGTACTCGACTTACAAAAACGGCGCAGCAATTCAAGATGTTGCAACTGGACAACCTGCGACGCCGTTTGATTATGGCGCGGGACACGTGGATCCCGTGGCTGCCCTTGATCCTGGTCTTGTTTATGACGCCAACGTGGATGACTACTTGGGATTCTTCTGCGCACTAAACTACACGTCGTTTCAAATCAAGCTTGCTGCGAGGAGGGACTTTACGTGTGACCCGAAGAGGGAGTACAGAGTGGAGGATTTCAATTACCCCTCGTTTGCGGTTCCTTTGGAGACAGCTTCGGGAACTGGGGGTGGTTCGGATGCTCCGAAGACTGTGAAGTACTCTAGGGTTTTGACTAACGTGGGAGCTCCGGGAACATATAAAGCTTCGGTGTTGTCGTCGAGGGATTCGACGGTGAAGATTGTGGTGGAACCGGAGACGCTGAGTTTCGCGAAGGAGTACGAGAAGAAGAGCTATACGGTGTCGTTTACGTACACTTCGATGCCTTCTGGGACGAGTAGCTTTGCGCGTTTGGAATGGACTGATGGAAAGCATAGGGTTGCTAGTCCCATTGCGTTCACCTGGACATGACGTGGTGTGGGCCAGGGAAAATACGTGGCCCAAATAAAAGGGGTCACTTAACTGTCACTGTTTGGGCTTACGGTTTTGGGTCCAAGGAAGCCCGGCCTGGCCCGTCAGCTTGTTATGTGTTTTTATAGTCCAGGAGAATATTTAGTATGGAATAATAATGAAAATAACAACAATAATTGTACATAAAAGAAAAAGAAAAAAAAAGTAATGACACATGCATGTATCATGATGGATTACTGGCTATGAATTATTCATATATCATCTTCCAAC

>C102213_65

AGAGAGAGAGGGAGAGAGAGAAAGAGAAAGAGAAAGAGAGAGAGAGGTAGACATATATCAAATGATGATCATGGCATGAGGGTCATCATCACCCCCCTATCTGATCTTCTATTTCTGAGGAAGTATTTCTCATCCTTGTTCCTAGGTTGGTTTTCATGGTAGCTCTATGTTGGCTTATTAAGAGAACAAATCTGAGATAAACGGAGCTCCTACTTACTCTCAAAACTACCACCGTTGCTGTTCAGAAGTAGCCTCCACCGACAGCCGGAGCCACAACAACCTTACTTCGTTTCCACATACCTAACTAATTATTCCACTCTTCCATTACGGCAAGATCTCTGAGTTATTGAATTATTTTCCAAAGAAAGAAGCAAATGGTTTGTGGATTCTGTTGCGGGATGAAACAACTCAAGAAGCCGAGCATATCCCTTGCAACAATATTCCAACAACGTAAGAAACCTGAAGCAGTATAAGTACTTGGTGAGAAGGCAATTGATGAATTAAGAAGAACATTAGCAACTTGACGAGCTTGAGATAACTAATTTGATTTGATGGCTCGACAACTGTGTGAAGACAAATCATCGTCTGGATTCTGCTACTCTGATGTATCATCGGGAAATCCAACGATGCTAGTGAACCAGATCCAAGGCTTTGTGTCAGACCCGGAGATGTACAACCTCACCACGGGCATGGAAATGATAGGGTTTCCAAAGAGTGACACCAACGCAGTCATGTGGAGAAGTTTCATCCCCAAACCGGGTCCTTCCTCTTCCAAGACCATCGATTCTTCCACCACTTTCTACCACCATGACTACAATAACAACAAGCCTTCAGATTTCACACCAGGGAATATTTCTGAAACAAGTGCCGAGAATCTAATCGTGGGAACTCATGATTCAGCCCCGTGGCAAGACGACAACAACCATAACAGGTTTGATGATTCTTCTTTAAGGTGCGTGTTCCCTTGTGAAGCCAACGAAAGGCCTAGTCAAGGTCTTTCACTCTCTCTCAGTTCAACCAACCCTTCCACTATCGGGTTGCAATCTTTTGAGCTACGACAAACAAGCCATCATCCTGATTTTGTTCCCTCGAGTTCCAGAGAAGGGTTCTTTGGAAAGCCAGTGAGTGTTCAACAACAACAGATGTTACAAGATGGGTATGTGAGTTCTAATTCCAAAGCTGCAAGTGTTTACCAGCAAGGACATTTCCTGGTGAAGAACTCCAAGTATTTGGTGCCTGCACAGGACCTTCTCAACGAGTTTTGTAGCCTAGACGCAAAGCAAAGCGAGGTGGGAAAGCCTAAATCGTTGAAGAAGCAGTGGGAAGAGGATAACAACGGTACGGGGTCCTCGAAGAAGCATTCTCTGACCTCTCTCGAGTTCGTGGAGTTGCAGAAGAGAAAGACAAAGCTGCTTTCGATGCTGGAAGAGGTTGACAGAAGGTACAAGCACTACCGCAATCAGATGAAGAGCGTGGTGTCGTCGTTTGAAGCAGTGGCGGGGAATGGTGCTGCCACAGTGTACTCGGCGTTGGCGCTGAAAGCCATGTCGAGACATTTCAAGTGTCTGAAGGATGGGATTTTGGGGCAGATTCAAGCAACAAGAAAAGCCATGGGAGAGAAAGACCCAGTTGCACCAGGCACAACAAGGGGTGAAACGCCGAGGCTCAAAGTCATTGACCAAGCACTGAGACAACAAAGGGCGTTTCAGCAAATGAGCATGATGGAAACCCATCCTTGGAGACCCCAACGTGGCCTTCCCGAGCGTGCTGTTTCGGTTCTTCGGGCATGGCTTTTTGAACATTTTCTCCATCCGTACCCAAGCGATGTTGATAAACATATTTTAGCACGCCAAACCGGTCTCTCGAGAGGCCAGGTTTCGAATTGGTTCATCAATGCAAGGGTGAGATTGTGGAAGCCAATGGTGGAGGAAATGTACTTGGAGGAAGTGAAGGATCATGAAAACAACATAGCTTCCTCAGAGGGTGCTACTGATCCAGACAATGATATAAACCCAAATGTTCAAAACCCTCCACCTCATTCATCACGATCAGAGGATCAGAAGCCAACACTGGTTCGAATAGACTCCGAGTGTGCATCCTCCATCATCAATAACCACACCCCGGAGAACAAAAACGACCCAAAGGGCCAAGAACAGTGCTTTGGGTCGGTGGAGCTTGATTTTTCATCATACACACACCACTCCTCGGGCATGGTTTCTTTCGGAGGTAATGATCAAAATGGGAACAACCAGAACGGGGTGTCTTTGACGCTAGGATTACAGCAGCATGGAGTGAGCCTAGCGTTCCCGCCAGCAACACAGAGTTCACTGTACTACTCAAACCCAAGGGACCAAATTGAAGATTGCCAACCAGTTCAGTACTCTCTTTTGGATGGCGAAGGACAAAACCTGCCTTACAGAAATCTGATGGGAGCACAACTTCTCCATGATTTGGCAGGATAGATGATGATATGAATTCTTCCTTTGAGGAATTAAGATTAAAATGGCAGCGACTATAATATAATCAAGTAGAAATTAAGATTAGCTTTTTTTCTTCTAAAGTTTTAGCTAGCTAAGTACAAAGTTATATAGTACACAATTAATGCTTAATTCCATACATAAAGCTATATATACTTGGTTAATCAGATAAACCTACGTACAGTTTAATTTGAACATGTGTATTCATGCCTTTAATTTGGAATTGGAATTCGTAAGTCAAGAGATTTACACAGAATTGGTTTACATAATGA

>C102219_65

TAAATTAAAATAGAATACAATCCTCTGTATATACACATGATCATGAATTACATTTCGACCAGGACCATCAGCAAAACTGAAACTTACAATTCTCTCAACCAATAGTCCTAAAAATTAAAGAATCATATATAATTGATATTGATTTGGTATAAAGAATACTATCCTGTGAAGGCAATCCCCAATCAGGTACTGAAGCATTGCCCTGGCTGCTATACTACACTTGAAAACAATGAAGCTTTGCATTGTAGCAAGTTCCACTGTATCCAAGAACCCTCTATTGCTATGTGGGCATAGCAACTAACAGAGTGCCCCAACAAATGAGCCTAAAACCTTGGTTTCAGCCACGAACAGGTAATCCATAATTCTGGTACACTCTTATGTATCCATCCCATCCTGCTGTCACAATTACTTGGCCCCACAAGTGGGAATTAGACATTCCCTTGCAAGCACTTCTCAAGAACTTGTACTCTGATTTACACATTGTAGGAGAAACAACAGTTTGTGTTTGACTTGAGTCCACAAGTGTCTCCTCGGGCCATGTTGCTGAAACCTTTGGGATCAACTCTGACAAAAATCCCCGACTCAAGAAGAAACAATCTGGTGAAGGCAGCGAACACCTTTGATTCACATCCTCTCCTAGCGATGGGGATAAGAGTGTTCCTGGCATTGACTCAATGCCACACCAAGGTACAGCGATTGCTGCTTTGTGGGAGAGAAAACTCTCTGAAGACCAAATTTTCTTTGCTTTAGAAGTACTCCTATCATGGCCAGCATAATTCCAGATACATACATTTGAATCCTCGTTAACCGAGATGATATGTTTCCCGTCAGTGGTGAAAGAAGCATGCATCTGACCTGCACTTCTTAGGCCCTTGAATTTGTAGATTATGTCAACTCCAGAAAGTATACAGACATGTGAATCAGCAGAAGCAACCAATAGTTTGCTTGGGTCACTCGGAGAAAACTGAAAGCCAGTGATCCTTTTCCCTGATGTCTTCTTTTTGCCTCGTAAGCACAACTGAGCATCCAATTGCAGATGATTATCTAGGATATCGTAGAAACGGCAATTGCCTGCCATGGTACCCACAATTGTGCCCTTTCCATCAGGGCGGAAGCACACAGCTGTGACTATCTCTCTGATATCAATGTAATCACTAACCCTACAGTGAACAACTTCCCAGATGCGCACTTTTCCATCAATTGAGCCACTGATGAAAAAATTATCATTGACGAGATTGAAATTGACGCATGTCACATAATTATTGTGGGAGAAAACTCTAAGACATCTGTCAATTCCCACATGCCATAGGCGCACTGTCTTATCAACAGAGGATGACAGTAGAAACCCTCTTTTAGACCAAGCAAGGTCCAAAATGTCACCACTGTGCCCTTGGAACTCGTGCAAAGGTTTTGCCGATATGCGGAAGGTTCTTGGTGGGACAATCACACAGGTTGCTTCAGAGGATCTCCTCAACTTCTCTGTTTTAACTAATTTCTCTTTATCTACACCAAGGGGAGCAACACATGAAAAATTATTTATTTTGAAATATATATTTGACGGATCATTGTCCAGTATGTCCAGTTCACTTGTTCTCTCATCCTCAACCACCTTCCACACACGTACCATGCCATCTTCACCACCACTAGCCAGATATTTTCCATCAAGACTGAACTTCATCGTCAAAATAACACCCTTATGTGCTTTAAACTCCTGTTCAGTGTAAAGGGAAGAAAGCTCCTTAACCCGCTTCCTATACGAATGAACTCGAACTCTTTGGATCCCACTTCTATCCACTGAATTACAGTCTTTGCATTTAGTTTCATCAAGTCCATGATTATGAACAAAACAAGCAATAGAATCCAGTTTCCTTAGCCAACCCCTCTTCATTTTTCTTTTTCCAACTCCTAACAACCTTGTGTTTTCAGCATCTCTCTGCAAATGTCTGCGAACCATCGATGAAGGCCCAATATTTCTCTGAAACTCCTCCAAGCTAATCAATTGGTTCGAACCCAAAACACGCAGTGTACTAAGAGTCCCATCTTGACCCAGCTTATCCACAATATACTGTGTTCCATCATCCAAATTCTTTATCATACACGCCAAATTCTCGCGATTTTCTTGGGAACCGGAAATCTCACCAGACAAGGAATCCAAAACAATCTGGTTTGAGGTTGAGAGAAGACCCTCCTCTACAGCAAGAGAAGTCCTCAACACCGCCCCACTTGTTGCAGTGATTCGGTCAATCCCACAAGGTCGATCTTCCATCTCCTCTCCCTTTATTGAATAATTCAAATCAGACTCCAAACCCATCCATTTCAAGAAATTGAGGCGTCTCTGGTTAACACTTTCAAGGTCTTTTGCCCAAACCTTGTGCCTAGTAACATGTCCGTTATTACCAGAACTAGATTCATCTGATTCTGAACAATCCGAACCCCCATCAGAGACGGAACACAATTCCTCACGAGTATCATAAAATTGATCCTCTCCCTCTTCCATATCCCACCTTTCCCCAATTCCCACCTCTAATCTCAAGCAGATTCCTTTCTTAACGAATAAACAATTCTAAGTTGAACCGATGAGTTTCACTTCAATTGGGGGAAAAGGGTATCTGGGTTATCTTCAAAATTAGAGCTTGATCCAGAAACCGAAACAATTTTCATAGGGATCTTCGCCGTGTTTGTTTGTTCAGTCTGCACTGTGCATCCACAGGAATACCTACCCACACCCACCACTTTGTGGTACGGCAAAG

>C102221_65

GTTATATTACACCCCCTTAATCACTTACAAATGACAAATGACAAATGACAATACTAATCACAAGCCTTCACCTATGAATATATATTTTTGGTGCAAGCTGAACAAATCAAATCATCCAATATACTCTCCTACACCTTAAAGAATGAAACCCCCGCAGCAAATCCCTTCCAAGTATAGAAACAAAATAAATATATATATAACCAGGTTTTCTAGTCTGAAATCCCAACTAGTATAAAACTGGCATCAGAAAAAATAATCCAATCATGGCACCCAAGAATTAGCTGAACAACAGTATGAAGAACAAAAATCCCGGTCTTAGAATGGGATGAAGGCAGCTTCAAGAATATGAAGACAAAAGAAATCAAGCCTCTGAAGAATTTTAAACTAAAGAGAAAACTTAAAACTGTTCCAAGATGGAATGGAGTTTAGGAACACGCCGACGAAACAATGGTTCAAGTCGAAATGATGGATCACAAAACTGCCACCCTTGAGCATCATACATCTCATTCCATGCCTGTACTATCTCATCAATTTTGAACCCTTCAAGTGCTGTTTCATTTGAGCAGTGATTCTTTACAATGAAAGCAATATCTGTTGCAGAAGTTCCAGCTAGTTCAAACTTTTCAACTGCAACTTCTAGACATTCCTCCCATGTTGGAAGGCCAAGCTTATATTCCACAACAGAACGTTCATACAACAAGGTTCCCCATAAAAGGTGTATCTGAGAACTCATCTTTGCAGCTTGATTTGAAGCTTCATCATCAGAAACATCTTTGAAAAGACCATCCAAGCCCATATTCTCTAACTGTTCTTTATATTTATCTGATTTGGATATTCCATTCAAACGCTGCTCCTCAATCTCCTCCCACATCAAGATGCCTTTCTCCATGCTTTCCTCTGCCTTGTTATAAAGTTGTAGAACCTCATCAGAAAAGCCAGCATCTGAGTCCTTGCAGGCCAATGCATAACACCAGCACAGCCTTGCTTGCTCAAACTGCTGATGCCCAAGAGCAAGATATCCTTCATAGAAGTCAGGTTTGATTTTCAAGGCTTCTTCATATCTCATTTCTGCTTTCTTGTACTCGCTTCGTGCCAACTCATATGCAGCTTTTATATGCTCTAAGGAAGTTTCTCTGGCCCCATCCTCCGAAAAGAAACCCTGGTTCCTTGCCCTGGACATTTGAACACTTCCCCAATTAAACAATGCCAAGGCAGCCATCTCTTGAAATTTATCAGCAGCAATTCCAAAAAGTTCTTGAGCATCATTGCTTGATACCGTATCCTCGATCGCTTCTCCATACAGCTTCATTGCAAACTCATGAGTATCTAGATAAGAATCAGACTCAAACCCTACATGGTTCTTGAACAGCCTTGCAAACTGGAGAAGCCAGTCCTCCACAGTAATCATCTTTCTAGCAGCATCTTCATCCCTGCCTTCTTCCATACCCCCATTCTCAACACCATCATTTCGTTTTCCACCGTCGCTTTGCACCTCATCCCCATTGGTTGTTGTTCCATCATAAGCAGGTTCTTGATCAGGACTGACCTCTGTAATATAAAGCCTAAACGATGCCTTCTCTGGAGCAGACTTTTCAGCTATCCTTAGTTCATTTGTGGTAGTAATCGTAACCAAATCACCTTCTTTATCCTTATACTTGACAAGAACCCCCTTCAAACCTGGAAACCGATCCCTAGCTATATCCCTCACCAACTTCGCACTACAATTCACGGGTAACTGAGCCCACCTTATATCTTCTCCAAACACCAACTTCGCGCTCCTAGTGATCGTGACAGGTTTTTCTTCCTCCACAGGCTCAACTTCCACCACCTTTTTTTCTTGCTCAATTGCTTTTGAAACCACCTTTTTTCCTTGCTCAATTGTTTTTGAAACCACCTTTCTTTCTTGCCCAATTGTTTTTGAAACCACCTTTTTATCCTCAACAATAACACCCACTTTTTCCTCCACAACGACCTTGTCCTCAATTTTTTTACCTCTCTTTTTTTTCAGTTTTTGCGAAGAAGGAGAAGGAGAGTGATAAATGGTGGTATCAAAAGCCACCCCTTTATTATCAACAGAGACCCCCTTTTCTTCCGTTGACTTCGTGAGACTCTCGAGAAACTCCAACGCGGTGAGATTATTAGGCTCCATGCTCAAAACAAGATTGACATCCCTGAGCGCCAAATCGAAGCGATTAAGCGCACGATAACACGTGGCTCTTTTCAGAAGAGCTTTGCTGTACCTTGGAGACACTTCCAGAGCCAAATTGCATTCGTGAATGGCTCGAGGGTATTCGCCGAGACCCAATTGCATGTAACACGTGGCCATGTTGGTATGAAGGTGTGCGACATCTATGTGGTTCTTCGGAAGCAGCTTCAGTGCCTTCTCGTACTTGAGCATGGCGCCTTCGTGGTCCTTCTTCTGAAACAACTTGTTACCTTCTTCCCTGAACTCTTGTGACATGTTAATGAACATTGCAGTATCTTCATCGAAAGCTTTGGAAGACTTCGCAGGTTTGGCGTGTGAATTTGCAGCACCCGGATCAGCGATACCCTTCTTCTTCCCTGTGGGCTTTCCCATTAAGAACCAAAAGGAAAGTCGCAATCGAGATTTGATAGAGAGAAACGAATAAAGATAGAAAGGAAGAGGATACAGGTAGATGAGAAAAGAGAACCGAATTGAACGGTTGGTCTTGAGGGGAATGAATATTGTTGTGCAGAAAAATTTTGTGAAGAACAAAACGGCGATTGCG

>C102225_65

CCTCTTTCTTCTACCGTGTAACTGAACACAATTTCAGTCATCACTACAAATATACAATCCAAGAGCATTTCAGTGATCTTCAGAAGAGAGAAAGAGAAACGCATTGCAATTTAGGGAGAGTGAGCGAACATCGAACCATGGATGACGAACTGCTGGAGCTGCAGAGACAATTCGAGTTTGCGCAGCAAGCCAAGTCGAGCATTCGATTATCTGAACGAAACGTCGTCGAATTGGTCCAGAAATTGCAGCACCTCCAAATCATCGATTTCGAGCTTCTCCACACTGTCTCCGGCAAAGAGTACATCACTCTGGATCAACTGAGGAATGAAATGGTGGCGGAGGTGAAGAGATTAGGGCGCGTTTCTTTGATCGATCTCGCTGACGCTACTGGAGTTGACTTGTACTACGTGGAGAAGCAGGCGCAGAGCGTTGTAACGGAACACAAAGAACTCATGTTGACTCAGGGAGAAATCATGTCTGAGTCTTATTGGGACTCAATCGCGGAAGAGGTTAACGAGAGGCTTCAGGAGTGTAGTCAGATTGCGCTGACGGAACTCGCGGCGCAATTGAACGTAGGTTTGGACTTGGTTGCCTCCGTGTTGGAGCCGCGCCTTGGAACAATTGTGAAAGGAAGGCTTGAAGGTGGGCAGTTGTATACTCCTGCCTATGTGAATCGTGTTGGTGCTATGGTTCGAGGCGCTGCCCGGGGAATCACTGTTCCGACGAATTTGACTGTGGTCTGGAGCTCGTTGCAGCAGTTGCTGCAAGAAATTGATGGAACCAGCGGTTTGGCTGTTGAAGGATCTTTCTTCCAGTCATTGTTTAACGGACTTGTGAAGGAAGACGAGGTTTTAGGGTCACTTCGTGCAGGAGTACACTGGACACCAGCTGTATTTGCCATTGCTCAGAGGGAGTTTGTGGAGTCATTCTTTTCTCAGAATTCTTTTATCACCTATGAGGCGCTGCACAGACTTGGAATTCCCCAGCCCATTCAATTCTTGCAGTCCAGATATCCTGAAGGCAAACCTCTAGTTACTATATTTGTTCATCCATCTATGATTGAGATGCTAGATGCTGCTACTGAGGATGCTCTTGAACGTGGTAGCTGGAGTGATTCTCTTTCCTTATTACCATCATCTTTTACACCTCAAGATGCTTCTAAAATGTTGTCCCTCTGTCAATCTGTGCAAAATGCTCTTAAGTCTAACAAAGCACACATTTTTGGGGATTTTTATGTGTTGAGCAGCAGCTTTATAAAGGACATCTGTGATCGTGTTGTGAAAGAGTTGGAGACATTGGGTGTTTTAGGGTCTGCTGGCTCTACTATGCCTGGTGATGTTCAAGTACCCAATGAAGCTAAAGTAGCGCATGAGTCAAGCAGGTTGAATGAGTTCAATGAGATGGCAAGTGATGGTGCTGCCAACAGGCAAGCTGATAAAGGATCAAAGAAGAAAAAGGGGAAAGTCACAGGGAATGTAGCAGTAAATTCATCTGAAAGTGGTGCAGATAACCAGGAACAGACTTTGCCAAAATCTAAGAGAGGCCAGAAAAGGGGCAAGGATACATCTTCACAAACGTCAGATTCAAAGACAGGTTCTAGGAAAGAACAGCTTAAAATGAAAGAGGAAAATCACAGTCCCTCAGAAGAATGGATCATGCAGAAAATTACAGCATTGGTTTCTGATTTTGAAGAACAAGGTATAGATGATCCCGAAATAATTCTTAGGCCTTTGGCTAGCCAATTAAGGCCTACAATAATCAGTTCTTGGATGGAGAAAAAGAAAGCACTGCATACAAACAATGCAGAAAGAATGAAACGTTTGCTTGATAATTTGCAGAAGAAACTTGATGAGTCTTTCTTAAACATGCAGCTGTATGAAAAAGCCTTAGAATTGTTTGAAGATGATCAATCATCTTCTGTTGTTTTGCATAGGCATTTACTAAGGACAGTAGCAGCTCCTATGGTGGATTTGCTTCTTCATAACCTGGAAGAGCACAACAAATTAAAGAATGGACTTGACGTGCAGGGAGCTCCTAATTCTGAATCTGTTTCACTTAGTCCTGCAGACAGAACTGCAATTTCTAAGAGTTTTCCAGGAGCTCTTGCAAAAAAGGCTCTTACTGTTGTTGAATCATTGGAAGGAAAGAGTGTGGAGACATTTATGGCTGCTTTTAGAATTGTTACAGAAGAGAGTGGGCTGCCTTTGAAAAAGCTTGACAAGAAACTAGAAAGAACGCTCTTACATTCACATCGTAAGGAATTGACATCTCAAGTTTCTGCTGAGACTGACCCTGTATCACTTCTGGCAAAAGTTGTGTCTTTACTTTATATCCAGGTTTACCACAAAGCTCTTCAAGCTCCTGGAAGGGCCATTTCCGTGGCTATTTCCCTTTTAAAGGATAAGCTAGACGAGACGGCATTCAAGATTTTAACTGATTATCAAACTGCCACAGTGACTCTTTTGACACTATTAGCTGCTTCACCCGGTGATGAGGAGGACTGTGCATCTGATAGGATTTTGAGTAAAAGGGAGCTTCTAGAGAGCCAAATGGTGGACCTGAAAAGCTTGGTTTTGAGCTCCTCCCAATCTTCATAAATGTTTAGTAAAGTAAGTCCAAAATTTGTTCATAAACCAATTTGTCCAATTACATAAATTGAAGGTATACTGGTCTACAGAAGTTGTGTAGACTAAACCAATTTGCCAAATGACATTTCATCATTTTATTGCTATTAATTACATTAATTTGCC

>C102227_65

CTGATGAAAGATGTGAGTATTTGATTAGAGAGCTAAATGCCACCAAAAAAACAGTTCAAGCTACAAAAATTAATTGTACAAGTATGCACAGCCTGGCAAACATACAAAAGCATTCACCTGTGCCAACAAAGCTTTCTCCCACCCTCCCGCAAAATTTCAAAGGCAAGTTTATACTTTTCAATGGATGTTGCACCAGCATCTATGTATTTAGATGCAGTTTCTCTTAAACTCCATAGCATCAAATTTTTAAGTTCCTGAGTGCTACTCCATGATTCTATGTCAGGAAAGACACCATCCTCGGCATTTCTTGTCCATCGATGTAAGATGTAGCGAGAAGGAACTTCCCTTAACTGTAATATCTGGAAAACCCTCAACACATGTCTACAAAGAACCCCATCATACTCAAACATCTGACAGCTACAACTAATGCTAAGATTTGATGCATTAAACGTAACTACATGTTTCTCCATATCATTTCCACACCTACGCACCATATATCTGCTCAGTCCCCCTTCCTCAAATATTTTAAACCCAAGATAGCTAAAGCACTGCAATAGCTCCTTTTGAAATACTTTGAATACAGCAAGAGTGTAAAGCCTTCTGCATTGTTCTTCTACTGGTTCTTTTGTTTGCAAAATGGGTTGAAAATTAGAAGTATTAAAATCCTCTTTTCTTTCTTCCTCACGGCGTCGCTCAAGACCTCTTTCATACCGAGGAATGAACTCCATGAGAGGTGTCTGGGAATTTAAAAGTGCACCAAAGAAAGAATCAAGGCTTTCATTCATGGGAATGCCAGCAAAAAAGGTGCCCTTTAAATACAATGGAACCCAAGATGCTCGCTTTTCATACATTTCTTTTAGCCAAGCATTGTCCTTCAATCCATATTTGTTGAGTAGCACATTCCATGTAGCGTCAAATTCATCAACTGTCTGACTTTGGTAAACACACTTATCATAGTCTTTCGTAAAGCCATCACCCATCAGACCCATGTTTTCTTGTTCCTTTGCCTTAATTTGCCATAAAGAAAAGCGATGATTGGTTACAGGAAAGACTTTTGCTATTGCCCTCTGGACTGCAATATCTTGATCAGCAATAACTGTCAGAGGCTGCCGGCCAGACATTGCCCTAAGCCAGGTCTGAAACAACCAGGTGAAAGATTCTTCAGATTCATCAGCAACTAAAGCACATCCAAGAAGCACAGGTTGCTTGTGATGATTGACTCCAACAAAAGTAGCAAAAGGTACCAAATAGACAGTTTTTCGATATGATGTATCTAAGACAAGGACATCACCAAACTGACTACATGAATATCTAGATCTGCCATCCGCCCAAAATATGCTCATGCAATTACCATTCTCAACTTCCACAGAATAAAAGAATCCAGTATCTTCTGTTTGTCTTGATTGAAAATACTCTAAAAGAATGTTGTACCACTCACTTCTAATATGATTTTGCCTACCTCCTCTAGGGACAGGGTAGTTATCTATTCTAAACAAGTCACCATTTACAAATCCAGTATCTGCATCGTCATTCAGAATATTAGAAGCAGGAAGACTTTTTGTCCTGCCCTCTTTTTCAGAGTCCAGATCATGGTTATGATCTTTTCGGAGGCGGTCCACTGTCCACTTTCCTGTTGGCTGTCTCTTAATCCTTAAATAAGCCCCACAACCAACTCTTGAAGGGTGCTGGAACCCCTCTTTAGAGCAAACAAATCTGCGAGATGTGATCAATCCATCATTCTTGGAACGAAATAATTGACCAATTCGAACTCCAAAACCCAAATGCGCTGCATATGCATGATAAAATTGGTAGGCTTCGTTAGGTGAACCAAACTCTTGACCCACAAATGGTTCAGTCTTGGACTCTCCTTCAAGTTCTTCCCTTCTCAGACGCTTGAAATCAATAATGCCAAAGGGACAAGAGGATTCATCATTGGCTTCTTCGAGCAATTTCTTCCTTGGCCTGCGAGTAAACTCAGTAAGAGAGTTGACAACTGTACCACCCTTCTGTTGCAATGTTGGAGTGCAGTTTTCACCACTGATTTCAAGATTGTGATTATGGTCCTTATGAAAATGATCAATTACCCACTTTCCAGAACCATTTATTTGCACCCTTATGAATGCTGGACAGCCTGTTCTTGAACTAAGTTGATGTCCCTCCTTCGAGCACACAAATCTTCGAGAAGAAACAGACCCATCAGTTCTTGATCGATATAGCTGACCAATCCGAACTTTAAATCCTGTTCGATTTGCATATGAAGTGTAAAACTTAAGTGCTTCATCTGCTGTGTCAAATTCTAACCCTATGTACGGATCACAAGAAAAATCTATTTCCTCATCCACAATAGCATTATTCATTGCCAGTTCAGTACCAACAGAATGCTCCTTTACAATCATGATCCCAAGTTCTTATCCATTAAATTCTAACTACCAATATGGAACAACCGAGAAAAAAATTCAAGGCAGAAACAGCAGCATTCAAACACTTTCAAGCTTTAAGGAAGTTATATATTAACTTTTTCTTTTCTTCCACCTCCCTCTTTCAAGTCAAGCAGAAGCACATTCCAGTCCCATAAGCTAAGTTGCAACTATTCCGGAAATAACTAGAAGCATTGATAAATGGGTCGGAAGAGATGGAAAACTTTGAAATTGGAAGGACAAATAGAAGAGCGGAATCAGAGAAAGCAAGTAATTTAAAAGAAGATAGAGAAAACCGGGATCGTGTTCCCGTTTCTTCTTCACTTTGAGTTTG

>C102229_65

ATTTTTATTAAAAACAAAAAAAAAAAATATTCTAGTTACATGAAAAAGGTACTATAATTTACTGTTTAATCTTAAAAGTATACATGCATCATCATCCCAGCACCTATCCCCCCTTTGAAACAAAACAAGGTAATGAACTAATTCGTTGACAAGAAATGCACCCGAGCAAGGGTATATTCCTTTCCAGCAATCCAAACACCAGTTTGCGACCACTGTACATCTTCCCAGTCCAGACTTTCCTCTAGAACCTCCACATGTTCAAGAGGCAGAGGGATGCCGATCAACCGCATCACTTTCTGGGGAAGAGGATTCTTACATCGAGACCATCGAAATACATCAACCATGGCATGATCACAATCAGCTTTGTCACCATTTGTTGGATGAAAATGGTGATTTTGAGACTTCTCTGTATCTGGGTCATGCATCTGAGTGGCTGGTATAGCCTTCACCATCTTTTCTCCAATAAAACAGAGCTCGTATCTGCAGGAGTGTGTCTCCAGATACAGAACTTCTCCCTTCTTTAAACGAGCAGAGGTATAGAGTGGCTTTTTTAGACCCTTGTCAGCAACAATGCTTATACTATATTTGATCCAAGGTTCATTGCAGAGGTTGTACTGTATTGTAACTTCACGTACAAATCTTTGTTGCCGCAATGCATTCGAAGCTGCAGCCCTAGTAGTCATTGTCCGTTCAACAGCTACTGGTGCAAGGGTAGGTTCAATGGTTGAGGTATGCGTAAGGCAGGGTTCAAGATCAATAGTTCCACCTCCTTTCTTCACAATGCAGCACCGCTCAACTTTATAACTACAACCAATTGCAGCACCCCAAGCACGGGATCGTACATTGTTTCTCAGGGTGGAAATATAGCAATCTTGAGGAGGTAGCACTCGAATGGTTGTGCGCAACTCTTGTACAGCTACGGGAGGTAGAGAAGCTGTTGGGCGGCAATAACCTGTATGCATGAGAACAGCAACAAGATCTGAGTCATAAGTGTAAACATCAGTGCCCCATAGCTGGCCACCTCTAACTTGACGGTTTGTAGCAGTAACATTCTCAGCTGGTATCCGAATTTCAAGAGTGGGGCCACTATTAGAGCTTTCACTATTTTTTTCCTCTTGAGATTGAGATAATTCATATTCCTTCCATAACTTTATCAGTTCTTGCATGCTTTCACCAACTTTATAAACAACAGAAGATACTTCTGCTTTACCTTGAGACTCTTCATTGTCTGGAGCATGAGCATGGGATCTAAAACGAGCATCACGATTGGCCACCTGAGGGCTTCCCCTTGATCGGTGGATCCTTTTACGATGTTGAACATTATAATTATGGACCTCCCGCTCCTTTTCTCCAGTCCCTTCTCCATGAGCAGTTCCATCTTCTGATTGCTTGTCAATTTTAAAACGCTTCTCAGGCCGATCTCCTTCTAAATCGGCATCCCTTTCTTTCCTCCTCTCTCTAGCTTCTCCATCAACATTTTTCCAGCTATCAACATCTTTCTGTTTCTTCTGTTCAGGTAACACATTTTCTTGCACAGTATTTTTTGCTGATCCATCACCAAGTTCCTTATTATCATTCAAAGGCTCTTTGTCTATGCCATTCCAGGATTCCCTCTTGCTATGATCCTTTTCCCTCTCTTTTGAACTTTCTTTCTCTTTTGGAAGATCTTTCTTCTCTCTCTCCATTTTTTCTACATCTCTATCTTCCTTGGCAGATTCTTTGTTGTCACCACTATTATTGTTAACTGGCGTACTGCTTCTCCGATCATTTCTCTCCTTTTCCCTATCCCCCCAGTCTCGATGCTTTCCATCCTTTCTTTTTCTCTCTTTAAATCTATCTTCACTTTTAGAATCAATTTTGTTTTCCCCAACAGCTTCATGAGTTTCCAAATTTTCCCTTTCATCTGCCATTGAACTGTCTTTTCCAACTTCTGGTGTATTGCCACGTGATGCATGCCAGGAATCCAAACTCCCACCAGGGGCATCAGAAAACCTCTTCCCCCTATACTCCTTTGATTCTTTCCAATTCAAGTGGCTGCTGCTGCTTACCATACCATAGCCATCCTTATCAGTCTTAACGTCAACTTTTGAATCATTATGGCTATCTCGTTCATATCTGATATCCTTGTTGTCGTCTCCTCTGCCATCAACTCGCACATCTTTCTCACTCTTAGCAATCTGAGAATCCCTTCTGGCTTCACCATGCAATTCCTTCTTCTCTGTCTTCATATCACGATTCTCAAACCGGAGATCTCTACTCTCCTTAAAATCCCTAGATTCTATCCTGTTCTCAGTTCCAATGGGATGATCTGTATGAGAATCATTTGAAGATGATGGCATCCGATACACTGTATGAAGAGGAGATCTTCTATCTGCATCACGAGGTTCAGTACGGAGTGTCTTTGCCACCCGAGAATCCTGACCCATATCATATGGAATATGGTGGTCATTTGAAACTGATGGCATCAACTTGGAATATGTACCTGAATCTTGATGTGGAAGCTTAGAAGACTGATGAACAGCATCTTCATGGGATCTCTTAGGTACACCACTCATAGTGAAAACACAGTTGTAAACCTCAAGAAAGTAATCTGACAGCCAAAACAATTCCTATCTCTGCTGTCCCTGCATTCCACAGTAGTCAGTACCTTAATTGTCGCGGAGCCTCTTCTATCGGAAGTGTGTTGTGTATGGCTTTTGAAGGTTTGTGTTGTGTGTTTATGTGTGAGACTGTGTTTGTGACACACTAAATG

>C102233_65

GCCTTTTTTATTTTCCTCCCTCATTTTGAGTTCTTAAAGAAAGTTTCGCCTTTTTAATCACACTCTCACTCTTTTCTTTACTTTGCTCCGACCCTTTTGCACTCCCTTCAGTGATCTCTCGTTTTCCCCATCTGGGTCTCCCCAGTTTTCGCCACAGCACTTGCTCCTCTTGATTCACTTGCATGGCGTCGATGGGTTCTGCACCCTTTCTCGTGGAAGCCAATGCCGACTCCACTTTGATGATGACACTGTTTCACAACAGGAATCTCAAGCTTTCTTCACCTAAATTTGGCTCTGGCTCTGTGAGTGGTTCATCGTTTGAGCATTCAGGGGCAAGAGGGCGAGGGCGTGGAAGAGTAAACCATAGGTGTAGTAGGGGTTTACTGTGTTTGGTGGAGAATAATAAGCGGATAAGAGCTTTGGGAAAGGGTTATGGGACGAGTGACGATAAGGATGAGTCAGATGAAGTGCTTCAGGCCACAATTGAGAAGACTAAGAAGGTTCTTGCTTTGCAAAAGGAGCTGCTTGAACAGATTTCTGAAAGAAAGAAACTGATTTATTCAATAAATAGTGAAAGCACTCCTGAAACCGAAGGAAACAGTATTTCAGATGAGCACAGCAACGAATCCCTGTTGAGTACTGTTGAGAACCAAAATGGTGGCACTGTTTCAAGCAATTACGTCCAATCTAATGAGAAGGAAATACCAGATGTCTCATTTTTAGGCATTGGTCGTGGTTTTGACAAAGGGGAAGAGGAAGACTATAAGTTATCTCTTGCTGAAAATGATTCACCTGCACAGTATCTCGATGAAGAGCTCACTAGCAACAGATATGAGACAAATGCGCCTGTTACCCTACCAATTGAAATCCCAAGTGGCACCAACACCTCCAGTCTTATGATTGAAATACTGGAAGATATGGGTGAATCAAGTTTAGTGGAGGTTGCTAATGAAGCTGACAATGTTGAGAGTGAAGGTGAAAAACCCCCACCGTTGGCTGGGGACAATGTGATGAATGTTATATTGGCTGCAGTAGAATGTGCTCCCTGGTCAAAAACAGGTGGGCTTGGAGATGTTGCTGGATCATTACCTAAGGCTTTGGCTAGGCGTGGACACAGGGTTATGGTTGTAGCACCTCGATATAGTAATTATCCGGAAGCACAAGAGATTGGAGTACGGAAACAGTATAAAGTGGATGGTCAGGACATGGAAGTAACATATTTCCATGCTTTTATTGATGGTGTTGACTTTGTTTTTCTTGACAGTCCAAACTTTCGCAATTTACAGGATAACATATATGGTGGAAACCGAATGGATATTCTAAAACGCATGGTATTGTTTTGCAAGGCAGCTGCTGAGGTTCCATGGCATGCTCCATGTGGTGGTATTTGCTATGGAGATGGAAATTTGGCCTTCATAGCAAATGATTGGCATACTGCATTGCTACCTGTGTATCTGAAAGCATATTATCGTGACAATGGCTTAATGAAGTACACAAGATCTGTTCTTGTGATTCATAACATAGCACACCAGGGTCGGGGACCACTTGATGAGTTCCGCTACACGGATTTACCTGGACACTACATAGACCTTTTCAAATTGTATGATCCTGTTGGAGGTGAGCACTTAAATATCTTTGCAGCCGGTATAAAGGCAGCAGACCGGATTGTCACTGTCAGTCATGGATATTCATGGGAGATCAAAACTTCTGAAGGTGGTTGGGGTTTGCATGGGATCATAAATGAGAATGACTGGAAACTGAGGGGAATTGTCAATGGAATTGACACCAAAGATTGGAACCCCAAGTTTGATGATCACTTGACCTCAGATGGATACACTAACTACACCATTGAGACCCTGCAAAGTGGCAAGCGACAGTGCAAAGCTGCCTTGCAAAAGGAACTTGGTTTGCCTGTTCGGGAGGATGTTCCATTAATCGGATTCATTGGAAGGCTGGATCATCAGAAAGGCATTGATCTTATAGCTGAAGCAATTCCATGGATGATGGGCCAGGATGTTCAACTTGCCATGCTGGGAACCGGAAGGCCCGACTTGGAACAGATGCTTAAGCAAATTGAGTCCCAACACCCTGACAAAATCAGAGCATGGGTTGGCTTTTCTGTGAAGATGGCTCACCGGATAACAGCAGGTGCAGACATATTGCTCATGCCGTCGAGATTTGAGCCATGTGGATTGAATCAACTCTATGCCATGAATTATGGAACAGTTCCGGTTGTACACGCCGTGGGTGGACTGAGGGATACTGTGACGCCTTTTAATCCGTTCGAGGAGTCTGGCCTTGGATGGACATTTGACAGTGCAGAAACTACTAAGCTAATAAATGCATTATCGAACTGCTTGTTGACCTATAGGCAGTATAAGCAGAGCTGGGAAGGGCTTCAATGGCGCGGGATGATGCAGGATCTCAGTTGGGACCATGCTGCTGAGCTGTATGAGGAGGTTCTTGTTGCTGCCAAGTACCAATGGTAAATTCTTGGCTTCTACTCTACCTAAGCAGACTCAGCAATATGAAGTGCATTCATGCTGAGTTATATCATGGTTTGTCCAAGGGAAATGACCTGGTCATTGTGGTCAAGTTATTACAACTTTCTTGATGCAGGTCCTGGAGGAAAATTTTTGCCCTAAACTAAGGATTATGTAATAATCACTTTTGTATGTCGTCTCTGGACATACCAATGTAGCTAAAAAGTCAATTCACCAAAATATGAAATAAGGTTAGAATCAAAAAAAAA

>C102235_65

TTTTTTTTTAATAATAATAATAATTGTCCATAACTGAACATTTTTATTCTAAGTATAACAAGCAAGGCAACAATAAAATTTGTTACTCTAGAACTTGACGAAGGTCCATAGCATAGACATTTGATTTGAAACAAAATGCAGAAAACAATAACTGACTGAACCTGTCCTTGTCCTGCATCCTGTGCTAAACTAAAAACAGGAAAAACTGAAGTAGAAAAATTGAAGACCTCAACGATCACGACGAACGACAACAACAAGCAACACTAACGACAGAAAGGAAATGATAATGCGAAACCCATTTCAAGCAGGCACAACAAGCTTAATAAGACCACTGCACGCCCTCATCTTCGGAAGCGGGAACGGCAGCGGCGTCATCTTCATTAAAGGAAGACAAGGAGACCAGATAAGGCATCCTGTCAAAAGTGCGAGCAAGGCCAGATGCCAGTACCGCAGCACTGTCGTGGCTGACATACTCATGCGCTCGGTGCTTGAGAGTCTCGAACATGAGCTCGGGCTGGTGCTGCATCACTCGAAGAACATCTCCACAGGATGGACCCGGCACGGCCCGCGTAACGGCGAAGCTGTGAGGGCCATCGCTCTGAGACACCCTCACAACGCTGGGACCGCCGAACAAGTTGTGGAATCCACCGAGCGCCTCCTCGTAGGCCCCACCCAAGAACATTCCCAAATAGTAACTACCTCCTTCAACTTCATGCAGCGGCAGGCTGGACTCCCCGTTGATGAACTTGTCAATCTTGCCGTCGCTGTCGCAAGTCAAATCAGAGAGAACACCCCTCACACAAGGCTTCTCGTCCAAGCGATGAATGGGGATAATCGGAAACACCTGTTCAATTCCCCACGCGTCTGGAATGGACGTGAAAACAGACAGGTTCACATGGTATCTCCGAACCGGCTCCCCAGCCCCAACGGCCTTCCTCACCAGTTCACAAAGTCCATCCACCGCTGCCAGCTGTTCCATACTCACTGTCCCCTGTTTGAACTGCTCCACACATCGCTCCTTCAACTCCTCCGTGTACACCAAGCAGCTTTCACAGTCCCCTCGAAACGCCGCCTCTGACACAAAGCGGTAATCCTCCGAAAGATCCTCCGCCATATAATGGCCAGGAAATGCCGGGGAGGCTGCGCTGCTCGTGCCAACCGCCTCGAAAACCAAGACCGAGTGATGAGAAACGATGGCCCTTCCACTCTCGCTGCAGATAACAGGGTGCTTGACGGACCTTCGGTCGCACACACACTGAACTGCGTGAACAACCGCGGCAGCGTACTCCTCGAGACCGTACCCGACCGAAATATCAGAGTCACACGATTTGGAACCGTCGTAATCAATGCCCAGTCCACCTCCAATATCGATGACCCGCATGTTCGCCCCTAGCCGAACCAGTTCGCAATAGATCTGCGCGGCCTCTCCCACACCATCAGCAAGCAACGCTGTCGAAGGAATCTGAGAACCAATATGAAAATGCAGAAGTTGCAAGCAATCCAGCATGCCTGCGAGGTCCAGCTTTTTCACCACCCGCAGAATCTGAGCGGTGGTCAGACCAAACTTGCCCTTCTCCCCTGAAGTCCCACCGAAATGGCCAGAATGCTTGGTTCGGAGCTTGGCACGCAACCCAATCACGGGCTTAATGCAAAGCTTCTTGCTCAATTCAACTATTAAATCGACCTCCTCCTCTTGCTCAAGAACAATCACGGTGTTGAGCGCGAGCTTGTTGGCAACCAGGGCAAGGGAAATGTATTCTGCGTCTTTGAAGCCGTTACAAATCAACAACGCGTCAGGGTTGCCTTTGCACAAACAGCTCATAGCCAAAAGGAGTTCCGGCTTGGATCCTGCCTCCAAACCGAACCGGAAGGGCGAACCGAATTTGACGATGTCCTCTACCACGAAGCGGTCCTGGTTGCATTTAACAGGGTAAACTCCTTGGTAATGAGAACCGTATCCCTCGGACTGGATTGCGTACTCAAACGCCGATTGAAGGGACTCCAAGCGATTCTTGAGAACGTCCGGGAAGCGGACAATGAGGGGAAGCTGAAGGCCGAGGCCGCCGACAGATTTGGAATCGGAGGCCTTCTTAACAATCTTCAGCAAATCGATCTCCTGGTGCGCCAGGGTGGCGGAACCGTGGGAGCGTACGGAGATGTTCCCGGCGGCGTTGACGGAGAAGTAGGGGCCGCCCCAGCCGTCGACGTTGTAGAGAGCGGCGGAGAGAGACGGCGACCAATGGTTGGTGTTGTCGTCAGTCGTTGGCAGAGGTACGCCGGTAAAGGCGACCGGCGCCGGAAAAGAGATGTCACCGGCAAATGCATAGCCGGGAGGTGCTGCAGCATCCACGCAAGCCAAGGCAGGCATCTCTAGTGGCGGCGGCGATCAAAATGAATTAGAAATTAATATTAAAAGGGGAAAAAAGAAAAGAATAGGGTTAGGGTTTTGTTGATAGGGCTGTGTAGGTGGGGGCTTTGAAAGCCGCCGAGGCCGAGGCCCCGGCTTCCCCCGCAAAAGAAGACAGTGAAAGATCTATTGAAAACGGAGAGTCCCCTCTCTTCCTTCCCGAAAAGACTCTGGTATCCCCACGCCTACCCCTCAGAAGCAAGACTCACACAATTGATGAACCCAAACCACACCCTGCACCAGATCTGGTGCGGGAGAGAGATCTAAAACTAGAATGCAGAAAAAAAGGAAACGAATACCGCAAAGAGGGGAACAAGACAGACTAAACGGCTTAAACAATAATTC

>C102237_65

TGAGAACTCCTTCTCCTCTCTCCGGTTATGGCTACTCGTCTCTTCACACTTCCTCTCGCCTCGCAACCCAAGCTCTGCTTCAATTACACTCCCAACCTTGCCGTCCCATTCATTTCTTCCACCAAACGACGTCGCAACCGCCACCGCTACCGCCATGAGATTCTCCCTCCACCATTCGCAATATCCAGTTCCTTCCGGACCGAGATTGGCTCGCCGGAATCACCTATTCTCCAAGGCCAGAGAGAGAGAATAGACTCGCCGGTTCTGCTCGACGTCACCGGCATGATGTGCGGCGCGTGTGTCTCCCGAGTCAAAAACATCCTGTCCGCCGACGAACGAGTCGACTCGGTCGTGGTCAACATGTTGACCGAGACAGCCGCTGTTAAGCTGCTCCGGATCGAGGAGGAACCGGCGAGCGTCGCGGAGAGCCTCGCTCGGAGACTGAGCGACTGCGGATTTCCGACGAAGCGGAGAGCGTCGAGTTCGGGAGTGACGGAAAACGTGAGGAAGTGGAAGGAATTGGTGAAGAAGAAGGAGGAATTGGTCGCTAAGAGTCGAAGCCGCGTGGCGTTCGCTTGGACATTGGTCGCATTGTGCTGTGGAGCGCACGCCTCGCATATCTTTCACTCCTTGGGGATCCACATTGCTCATGGATCGCTTTGGGAGATTCTGCATAGTTCGTATGTAAAAGGTGGTTTAGCTTTGGGGGCCCTAATGGGACCAGGACGAGAGTTACTCTTTGATGGCCTAAATGCATTTAAGAAGGGGTCGCCTAATATGAACTCTCTCGTTGGATTTGGTTCTATAGCTGCTTTCATCATTAGTTCTATCTCGCTACTTAACCCCGGCCTAGGATGGGATGCATCATTCTTTGATGAACCGGTCATGCTTCTTGGTTTCGTGCTTCTGGGACGTTCTCTGGAGGAAAAGGCTAGGATTCAGGCATCTAGCGATATGAATGAACTGCTTTCACTGATATCTACTCAGTCAAGGCTTGTGATTACTTCAACAGAAGGTTCACCTTCTACTGACACTGTGCTTTGCTCTGATGCAATTTTTGTCGAAGTCCCAACTGATGATATTCGAGTAGGTGATTCTGTGTTAGTGTTGCCTGGAGAAACTATTCCTATAGATGGAAAGGTTATTTCGGGAAGAAGTGTTGTAGATGAATCCATGCTTACAGGGGAATCACTTCCTGTATTTAAGGAGAAAGGCCTCACTGTTTCAGCAGGAACTATAAATTGGGATGGTCCTTTAAGGATTGAAGCTTCTTCAACTGGTTCCAACACTATGATATCGAAGATTGTACGCATGGTTGAGGATGCTCAATCACGTGAAGCACCTGTGCAAAGGCTTGCAGATTCAATAGCAGGGCCATTTGTATACAGTGTAATGACTCTGTCAGCAGCAACATTTGCATTTTGGTATTTTGTTGGATCACAGATATTTCCTGATGTTTTGCTCAATGATATTGCGGGTCCAGAAGGAGATCCTTTGCTTTTGAGTTTAAAACTATCTGTAGATGTTTTGGTTGTTTCTTGTCCTTGTGCATTGGGTCTTGCTACGCCCACAGCAATCCTAGTTGGCACCTCACTTGGAGCAAGAAAAGGACTTCTTATTAGGGGAGGGGATGTGCTGGAACGATTGGCCGGAATAAATTATATTGCTCTAGACAAGACAGGAACCCTGACGAAAGGAAAACCAGTTGTATCGGCCGTAGGCTCTATCCATTATGGAGAGTCTGAAATTCTTCGCATTGCTGCTGCAGTGGAGAAGACTGCAGCTCACCCAATAGCAAAGGCTATTATCAATAAAGCTGAGTCATTGGAGTTGATCCTTCCTGTCACAAAGGGACAGTTAGTTGAACCAGGTTTTGGTACTTTAGCAGAAGTAGACGGGCATTTGATTGCAGTTGGGTCTTTGGAATGGGTTCGTGAACGCTTCCAAACAAGAGTGAACCCGTCTGATTTGACAAATTTGGAACATAGTTTGATGAATCATTCATCAAATACATCTTCTAAGTATTCGAATACTGTTGTATACGTTGGACGCGAAGGAGAAGGCATCATTGGTGCTATTGCCATATCTGACACTGTGCGTGAAGATGCTGAATCTACAGTAATGAGGCTCATGCAGAAGGGGATTAAAACGGTCCTCTTGTCAGGAGACAGGGAAGAGGCAGTTGCAACAGTAGCAGATACAGTTGGAATTGAAAATGACTTTGTCAAAGCATCTTTGTCTCCAGAACAGAAATCTAGATTTATTTCCTCTCTAAAAGCTGCCGGACATCATGTAGCGATGGTAGGTGATGGAATAAATGATGCACCCTCTTTGGCCGTAGCTGATGTTGGGATTGCTCTGCAGAATGAAGCTCAAGAGAATGCTGCCTCGGATGCCGCATCTATTATACTTCTAGGGAACAAAATTTCACAAGTTGTCGATGCGCTAGACCTAGCACAGGCCACAATGGCAAAAGTGTACCAAAATTTGTCTTGGGCGGTCGCCTATAATGTTGTTGCCATTCCCATTGCTGCTGGTGCGTTGCTACCCCAATTTGACTTTGCTATGACACCGTCACTTTCAGGAGGACTGATGGCTCTGAGCTCCATCTTCGTGGTTGGCAACTCACTGCTTCTGCAGCTTCACGGATCTCAGATCTCTAGAAAGGCTAAGTCTTGACAGCATATCTTCCCATTTCATCACATATATGTTTTAAAAAAATAAGTATAGAGTATTATAGAGTAACCTGTAATA

>C102239_65

TTTTTCTTCAATTTCATATTTTCTGGTCAGTGGGGAAATACACCACGGAAATGCTACTTTCCATGATTGCTACTTTCAATCATAGTATTTAGACCTTATCCTTCTTAAACCCAACTCGTATTCTCAGTTTCATGTGAAAAAAAAAATCTAAACTTGATTCTGAAAGGAAGAAACTTGGGACTTCTTTCTCTAAGAGATGGCGTTGGCTAAAGTTGATATTGAGGTTGGAGCTGATGGGGTTGCTGTAATCACTATGGGCTACCCACCGGTCAATGCTTTGGCCATTCCAGTTATCAAGGGCTTGAAAGACAGATTTGATGAGGCAGCTAGAAGGAATGATGTGAAAGCTATAGTTTTGACGGGCAAAGGTGGGAGATTTTCTGGTGGCTTTGATATCAGTGTTATGAAGAAGGTTCACGAAACCGGGGATTCCTCGCTTCTCCCTGATGTCTCCGTGGAACTAGTAGTCAATGCAATTGAAGATTCGAAGAAGCCTGTTGTTGCAGCAGTAGAGGGGCTTGCTCTTGGAGGTGGCTTAGAACTGGCCATGGGATGCCATGCACGTGTTGCAGCTCCGAGAGCTCAATTGGGTCTACCAGAGTTGACTCTTGGTGTTATTCCTGGATTTGGAGGAACACAACGTCTTCCAAGGCTTATTGGGCTGTCTAAAGCTGTTGAAATGATGCTGACATCTAGACCTATCTCGTCAGAAGAAGGGAGGAAACTAGGACTTATTGATTCTATTGTGTCTTCTGAGGAGTTACTAAAGGTATCTAGGTTGTGGGCACTTGAAATAGGAGAGAGGCGCAAACCTTGGGTTCGTTCACTTCATAAGACAGACAAAATTGGTTCCCTCTCTGAAGCACGAGAGGTGTTGGGAACTGCCAGGCAACATGTCAAGAAGACTGCTCCACATTTGCCTCAGCAGCTGGCTTGCGTAGATGTGATTGAACATGGAGTAGTTCATGGAGGTTACAGTGGTGTTCTAAAGGAGGCAGAAGTATTCAAGAAATTAGTTCTGTCCGACACTGCAAAAGGTTTAATTAATGTCTTCTTTGCTCAGCGGGCAATAACAAAGGTGCCTGGTGTAACTGATATCGGCCTTAAGCCAAGAAATGTTAAAAAGGCTGCTGTTATTGGAGGAGGTCTAATGGGATCTGGCATTGCTACAGCTCTTCTACTTGGCAATATTCGAGTCTTACTCAAGGAAATCAATTCTGAATTTCTTACAAAAGGAATAAAAACAATAGAAGGTAATGTTACTGACTTAGTAAGAAGAGGAAAGTTGACGAAACAGAAGGCAGATGCTGCATTGTCACTGCTTAAAGGTGTTCTGGATTACACAGAATTTAAAGATGTGGACTTGGTTATTGAGGCTGTAATTGAAAATGTCAGTCTCAAACAAACCATATTTAGTGATCTTGAGAAGATCTGTCCTCCACACTGCATTTTAGCATCAAACACATCTACTATTGACCTTAATATTGTTGGTAAAAAGACCAGCTCTCAAGATCGCATTGCGGGTGCTCATTTTTTCAGTCCTGCCCACATTATGCCTCTTTTGGAGATCATACGAACCGATAAAACTTCTCCACAAGTGATTCTTGATCTGCTAACAGTTGGTAAAATCATAAAGAAGTCTCCCGTTGTGGTGGGTAACTGCACAGGCTTTGCAGTAAATAGAACATTCTTCCCATATTCGCAGGGTGCCAATTTGTTGGTCAACTTGGGTGTGGATCTTTTCAGAATTGACAATTTGATACGCAACTTTGGCTTTCCGATTGGTCCTTTCCAGCTCATGGACGTGGCTGGCTATGGAGTTGCTGTTGCTACATCCAAAGAATTTGCTGTTGCATTTTCTGATCGCATATTTAAATCTCCACTCCTTGATCTTCTGATCAAATCTGGGCGAAATGGCAAAAACAATGGGAAAGGTTGCTACATCTATGAAAAGGGTAGCAAGCCAAAACCTGATCCTTCAATACTACCAATCATCGAGGAGTCTAGAAGACTTACCAATATTATGCCCAATGGAAAGCCTATATCTGTTACTGACCAAGAGGTCGTGGAGATGATACTCTTTCCAGTAGTGAATGAGGCATGCCGTGTTTTAGAAGATGGAGTTGTTATTCGGGCATCGGACCTTGACATTGCATCTGTTCTTGGAATGAGCTTCCCAAATTATCGGGGTGGCATTGTTTTGTGGGGAGATTTGGTTGGGGCTAATCATATCTATACCAGCCTAAAAAAATGGACACAATTATATGGTAACTTCTATAAACCCTCAAGGTATTTGGAAGAAAGAGCGCTCAAAGGCATTCCTTTGAGTGCACCTGCTTCATCTACTCCAAAGTCAGTAGCTCGTCTATGAGAAAGAAGCTCAAGTGTTCAGCGTGTTGGATATTCGTTCTACCATAGAAAATATAACAAATAATTAAAGTTGCTTCCTTCCTACATTAAATAATGTTAAATTTGGTTTTGCAAAAAGAAAAATCTTGCATAAACTAAATCTTATTAATTATGCATATTTAGTAATTGGGATCACCCAAGATTCCCTTTATCGAATGCTGAGTGTAAACCATTTTCCTTAAAGTTTGGTATGCATTTTGATTTTTACTCGTAGCGTGTTTAGATTTGCTACGTATGAGTTATTTTGTTGCTGTTTTTGCGCCCCTTACAGGTTTCAGAAGTAAAAGTGGATTTTAAATGAGAATTCGAACACAATTGTAAGCTTGTAATAAACTTTGATATAAT

>C102241_65

GGCACCCCGAAGACAAGAGTTGAAGATACAGGAGTGATGGAATTCTCCATCTTCAAACTTCTTCTGTCATCATTTCTTCTCTGCACTTTCTTGCAGGAACCCACCAATGCCCTTAGAAAGACCTACATTGTGTACATGGGAGGACATTCTCATGGTCCTGACCCTTCTCTCACTGATCAAGAAGCAGCCACAAATTCCCATCATGATTTACTTGCTTCTGTCTTAGGAAGTCATGAGAAGGTGAAGGAATCAATTATATACTCTTACAACAAGCATATTAATGGCTTCGCTGCCTTACTTGAAGAGGAAGAAGCATCAGGGATTGCAAAGAACCCAGATGTAGTTTCTGTTTTCTTGAGCAAAGAACACAAATTGCATACTACGAGGTCATGGGATTTTCTGGGACTGGAAAAAAATGGACGAGTTCTTGCAAACTCTGCTTGGACTAAAGCAAAATTTGGAGAAAACACAATCATCGCTAATATTGATACAGGAGTTTGGCCGGAACACCCGAGTTTTGGCGACAAAGGATACGGGCCTGTGCCTTCAAAGTGGCGTGGGAATGGTGTTTGTCAGATTCACAGTTTTAACACTACACAGAAAAATTTCTGTAACAGGAAGTTGATTGGAGCAAGAGTTTTCCTGAAGAATCACGAGGCGGAAGTTGGGGTGGTTCAGGGAAGCATACGCAGCGCACGTGACTTCGTAGGGCATGGCACCCACACTCTATCCACAGCAGGTGGTAATTTTGTTCGTGGTGCGAATGTAGAACGTAACGGTAGAGGCACCGCTAAGGGAGGGTCCCCGAGAGCTCGCGTGGCGGCCTACAAAGCATGCTGGAATAAATTTGACGAAGGAGGCTGCCATGAAGCTGACATTCTCCAAGCATTTGATTACGCCATTAATGACGGCGTCGATATCATCTCTGCCTCTGTAGGTTGGTCCACTCCTTACATGGAAGCTTTGTTAACCGATGGTATTTCCATTGGATCATTCCATGCAGTCGCCAAAAACATACCCGTTATTTGCTCTGCCGGCAATGATGGCCCCTCAACTTCATCTGTCACTAATGTCGCACCCTGGTCCTTCACCGTTGCTGCCAGCACCATGGACAGGGATTTCCTCAGCACCATTTCTTTTGGCAAGAATAACCACATTCAGGGTGCCAGTCTTAATCGAGGCTTGCCACGACCCAAGCGAAAGTTTTATCCCATTGTTTTTTCTATCAACGCAAAACTTCGCAATGCCTCCGATGCTGATGCTCGTGTTTGCAAACCAGGAACACTTGATCCTGCCAAAGTAAGGGGAAAAATACTTGTTTGCATTCGAAGTGATACATTACATTCGGTTAGTGAGGGCGAACAGGGTAAACTTGCTGGCGCTGTCGCAGTTTTTGTGCAAAATGATGAGAAGAGTGGAAATTTACGTCTAGCTGAAAATCAAGTTTTACCTGCTGCAAGTATAGGTGGAACGGCTACTGATTATGGAAAAAATACTAGCGGAATAGGTCCTAAGGGCAGCAGCCTGTTTGCTTACTTGGGTGCTGCAAAAACATATCTGGGAGTAAAGCCAGCTCCAATCATGGCTGGATTCTCATCTAGGGGTCCTAGTATCGTGCAACCCTTGATACTAAAGCCTGACATAACTGCTCCGGGTGTGGGTGTAATTGCTGCTTTTTCACAAGCGGCGGGTTCTTCTAATCTTCCATCAGATACACGTAGGACTCTTTTCAATGTGGAACAAGGAACCTCTATGGCTTGTCCTCATATTGCTGGCATTGCGGGTCTTCTCAAAACATATCATCCTACCTGGAGTCCAGCAGCTATCAAATCAGCTATTATGACTACAGCTACAACACTAGATAACACCAATCGACCGATTCGGAACGCATTTGATAAGGTAGCAACTCCATTTGAATACGGTGCAGGACATGTTCGGCCTAACCTTGCAATTGACCCTGGTCTCGTTTATGATCTGGGCACAACCGATTACTTGAACTTCTTATGTGCTTCTGGTTACAACCAAGCTTTACTCAAGTTTTTTGCCAATTTGAAAATCTCTTACAAATGTCCCAAGTCGTACAGAATCGAAGATCTCAACTACCCTTCGATTACTGTGCACCATCCTGCTTCAAAGCCTATAAATGTTACTCGCATTGTTACAAACGTTGGCCCTCCAGGCACATATGTTGTGAACGCACATGGGCCCAAGGGGATTAAGGTTCGTGTTCAACCAACCTCCTTGACTTTTCAGAAAACTGGAGAAAAGAAGAGTTTTCAGGTTATTCTGCAGGCAATTGCAGTACCCCGTGGGTTACCTTTGTTTGGGAATTTGACTTGGACAGATGGCAAACACAATGTAACCAGTCCTATCACAGTGCTGTAACCTTAGAAACTACTCTTACTGTTGTTATGTTGTGATTGTCATTTGTGTGTGTTTGCGATTGCTGTAGGTTTTAAGCGTGTTTCCTACTCTTGTGTTATTGAACTTTGTAACAAAAATAAGGTATGTTTATAATTTTGAAGTATTGAACTTTGTTGCATTTAATGCTCCGACGACCATTTTCTTTCCAAATAAGAAGATAGATGAAGAAAGATATATGAAGATCTTTGAAGAAGCCATCAACTGATATAAACTTCTATGAATACCATAAAAGACATGTTTGTACAAGAAAGATAGAAAAGACCGTACTGTAACAAGTCTAAATGTTGTTGTACAATTTTTTTATGAAGATAGTCATAAACTGATATGAAG

>C102245_65

GTAAAACCCAGAAAAGGAGACTCTAGGCAAAAACCCTCTACCCGCCATGGCTCGCGTGTCCCGGTCCTCCGCACCGCGCCTCCTCTACGCCCTCTGCTCCTCCGCGTCTCGGTCACCGGCAGCGTTTCTCCTGGGAGGCGCCTTCCAGCTCCGGCAGTTCTCGGCCGGTAACGCGGCGCGCGCGAATGCTGAGAAGGATCCGTGGTGGAAGGAGTCGATGGAGCGGCTCCGCAACATCGGGATCTCGGCGCACATCGACTCCGGGAAGACGACGCTGACGGAGCGGGTTCTGTACTACACTGGTCGGATCCACGAGATCCACGAGGTGAGGGGCAGGGACGGTGTTGGCGCCAAGATGGACTCCATGGACTTGGAGAGGGAGAAGGGAATCACCATTCAGTCTGCTGCCACCTACTGCACTTGGAAGGACTATAAGATTAACATTATTGACACACCTGGTCATGTTGATTTTACCATTGAGGTTGAGAGGGCTCTGCGGGTCCTTGATGGTGCCATTCTCGTTCTTTGTAGTGTTGGTGGGGTGCAAAGTCAGTCCATTACTGTTGATCGGCAAATGAGAAGATATGAAGTCCCAAGACTTGCATTTATAAACAAACTTGATCGGATGGGAGCAGATCCATGGAAAGTGTTAAATCAGGCTAGAACTAAGCTTCGACATCATAGTGCTGCAGTGCAAGTTCCAATAGGCTTGGAAGAAGATTTCAAGGGACTTGTTGATCTTGTGCAGTTAAAGGCCTACTACTTTCATGGTGCAAGTGGTGAAAAAATTGTCACTGAAGACGTGCCTGCAGATATGGAAGCATTGGTGGCAGAAAAAAGACGCGAACTTATAGAAACTGTGTCAGAAGTTGACGATAAACTTGCTGAAGCTTTTCTTGGTGACGAGACCATATCAGCTGCTGATCTGGAGGAAGCAGTTCGTAGGGCTACCATAGCGCAGAAATTTATACCAGTATTCATGGGTAGTGCTTTCAAAAATAAGGGGGTACAGCCACTTCTGGATGGTGTACTTAGCTATTTGCCATGTCCAATTGAAGTTAGTAATTATGCCCTTGACCAAACTAAGAATGAGGACAAGGTTGAGTTGCGTGGAAGTCCTGATGGGCCACTTGTGGCATTGGCTTTCAAGTTGGAGGAAGGACGGTTTGGTCAATTAACATATCTTAGAATATATGAGGGTGTAATTCGGAAGGGTGATTTTATTATAAATGTAAACACTGGCAAGAAGATCAAGGTTCCTCGCTTGGTTCGGATGCATTCTGATGAGATGGAGGACATTCAAGAGGCTCATGCTGGGCAAATAGTTGCTGTTTTTGGTGTTGATTGTGCATCAGGAGATACTTTTACTGATGGTAAAGTGAAGTACACGATGACTTCTATGAATGTTCCTGAGCCAGTGATGTCGTTAGCTGTACAGCCTGTCTCTAAAGATTCTGGAGGGCAATTTTCAAAAGCTTTGAATCGCTTCCAAAAGGAAGATCCTACTTTTCGTGTTGGCTTGGACCCAGAGAGTGGGCAGACAATCATTTCTGGAATGGGGGAACTGCATTTAGATATCTATGTTGAACGCATAAGGAGAGAGTATAAGGTTGATGCTTCAGTTGGAAAGCCCCGTGTTAACTTCAGAGAAACTGTTACTCAACGTGCTGATTTTGACTATTTGCATAAGAAGCAAACTGGAGGGCAAGGTCAATATGGACGGGTAATTGGGTATATTGAACCACTTCCTGCAGGGTCATCTACTAAGTTTGAATTTGAAAACATGCTTGTTGGTCAAGCTATTCCATCAAATTTTGTCCCTGCGATTGAGAAGGGTTTCAAAGAAGCTGCCAACTCTGGTGCCTTAATTGGACATCCAGTTGAAAATCTCCGAGTTGTCTTGACAGATGGTGCTGCTCATGCTGTTGATTCCAGTGAACTTGCATTTAAATTGGCTTCTATCTATGCTTTTAGACAGGCTTATACAGCTTCCAGACCGGTTATATTAGAACCTGTTATGCTTGTTGAGCTGAAAGTACCAACAGAATTTCAGGGAGCTGTAGCTGGTGACATCAACAAGAGAAAAGGTGTGATTATTGGAAATGATCAGGAAGGAGATGATTCTGTCATTACGGCTCATGTCCCTCTTAACAATATGTTTGGGTACTCTACTTCTCTTCGCTCAATGACACAGGGCAAAGGTGAATTCACAATGGAATACAAGGAACATTCACCAGTTTCTCATGATGTACAGACGCAATTGATAAACACGTACAAGGGCAACAAGGAGGCTGAATAATCAGCTTTTGTCGATGTGCATGGTCAAAATATTTATGACAATTATTTATCCAAAAAGCTGGACTATAATATATAGATATAGGTTTGTTTATTTATTAGGCTCAACCAATTTTGGAAAGCAAATAAAAAGTTTCATTTTAGATCAGGTGGGTGAATGGTACTTGCTTCAATGTGCAAGTTACACAAGCTCGTATTTGTAGATGAAGATGCTCATATTCAGAGATCTTTGATATGTAATTTTATGTCCATCACCATACATTCTATTCCCCGAATTTTGTACTTAAATTACCAATTTGGTTTTAGGGAAGGGAGGCATGTAGGTTGATTGGTTGAAGGGGGAATGGGACATGGGGTGGGGATCCCAGAAAAACCCTTTTACCAGTGATTGTTTTTTTGGTGCGTGGGTTTTAATTTTATTTTTTAAGGTTTAAGTAGGAAGGTCAAATGTTTTGTTAGA

>C102247_65

GTTGAGACACAAAGCCCAAGCAGTTAACCGCACAAATTGCCAAAACCCTACTAAAAACCGAACGGCGTAACCGCCCTGAAGCAGAAGCAAGCAGCGACGAGAACAATGGGGGGTGGTGGGAGAGGAGGCAGGTCCCGAACTCAAAGAAAGCATTTCCGACAGAACAGAGAGAACGTGTGGAAGCGTTCAAAGTCGGATCCCGATCCTTCTTCAACAGAAAACAATCCTTCGTGGACTCCTTTCGCCACCGAGAACACCGCTTTCGATTTCTACTACAAAGAGCAAGGAATAGTCGACCCCCAACAGTGGGACCAATTCGTTGCTGTTCTCCGAACTCCATTGCCCGCAACCTTCAGAATCAATTCTAGTAGCCAGTTTTCCGACGACATTCGTTCTCAATTGGAGAATGATTTCGTGCATTCTCTTCGTGAAGAGGTTGCTGAAGGGGGTGAGACTGAGGCTATTCGGCCGTTGCTGTGGTACCCTGGGAATTTCGCTTGGCATTCAAATTTTTCTAGGATGCAGCTTAGGAAGAATCAAACGCTTGAGAGGTTCCATGAATTTTTAAAGCTAGAAAACGAGATTGGAAATATCACAAGACAGGAAGCTGTCAGTATGGTTCCTCCCCTCTTCTTGGATGTGCATTCAGATCATTTTGTGCTTGATATGTGTGCTGCACCAGGTTCAAAAACATTTCAATTGCTCGAGATTATACACCAATCAAGTAAAGCGGGATCACTTCCTGATGGAATGGTTGTAGCAAATGATCTCGATGTCCAAAGATGCAATCTTCTCATCCACCAAACAAAGAGAATGTGCACAGCCAATTTAATTGTTACAAATCATGAAGCACAACACTTCCCAGGATGCCGACTAAACAGGAATTATGAAAGGATGGAGTTAGATCACAATATTGGCCAACTATTGTTTGATCGTGTTCTTTGTGATGTTCCATGCAGTGGAGATGGTACCCTTCGCAAGGCACCTGATCTATGGAGAAAATGGAACACAGGGATGGGACATGGCCTTCACAGCCTACAAGTTTTAATTGCTATGCGAGGTATACATTTACTCAAAATTGGTGGAAGAATGGTTTATTCAACCTGCTCAATGAATCCTATTGAGAATGAAGCTGTGGTTGCTGAGGTTTTGCGAAGGTGTGGAGGGTCTGTTAAACTCGTTGATGTCTCTAGTGAGCTTCCACAACTTATTCGTCGACCAGGTCTCAAGAGATGGAAGGTATATGACAAGGGCTCTTGGTTGGTCTCTTATAATGATGTTCCTAAGTTTCGTAGAAGTGTAATTCTTTCCAGTATGTTTCCTTCTGGAAGAAGCCATCATGATGTTGTTGATAGTAGTTGTAATGTTAACGTAGAGGATGTTACAAATGGTATTAATGGGAATGATAGAGATGGTGTTCAACCAGTACAGAATCCTGTGATGTCTAAATCTGTTGAGGAAGTTTCTGATTTCCCTCTAGAGCATTGTATGAGGATAGTGCCTCATGATCAGAACACTGGAGCCTTCTTCATTGCTGTTCTGCAAAAAGTTTCTCCTTTGCCAGTAATTCCAGAAAAAACAAAAATAAAAATTGATGAGCAATATGTAGAACCAGAAAACCATAGTCTTAATGATGCGCAAGTACAACAGATCACTTCACCAGAAAATGCACGTGAAGAAGACTTGAAAGCGGTTTTAGAGGAAAATGTAGATGACAATGAACCCAATACCGAAGACATGGAAGATGATCCTGTTACATGTGAAGAACAGAATACCAAGGAAACTCAAGTGCCTGATAATGTACAAAACACGACGAAGAGGGTTCCAGGCAAAAGAAAGCTACAAATTCAAGGAAAATGGAGGGGTGTTGACCCTGTAGTCTTTTTCAAAGATGAAACAATTATTAATAGTATAAGGAATTTTTATGGAATTGATGAGCGTTTTCCATTCAATGCTCACCTTGTTACAAGAAACAGTGATACAAATCATGTGAAAAGAATTTATTATATCTCCAAGTCAGTCAAGGATGTTCTTGAGTTGAACTTCAAAGTTGGGCAGCAACTTAAAATAACCTCAATTGGCCTGAAGATGTTTGAAAGACAAACGGCACGAGAAGGTAGCTCTGCACCATGTTCTTTCCGGATATCATCCGAAGGATTGCCCCTTATTCTCCCATACATAACCAAACAGATTCTTCATGCATCTCCCGCAGACTTCAAGCATCTTCTGCAGAACAAAGAAGTAAAATTTGAAGATTTTACTGATGCCAAGTTTGGTGAAAAGGCAGCAAACCTATTGCCAGGCTGTTGTGTGGTAATTCTGAGTATAGGAAACACAGTTGCTACAGAGTCTCTCCAAGTGGATGAGTCAACTATAGCCATTGGATGCTGGAAGGGTAGGGCTAGATTGTCTGTGATGGTCACTGCAATGGACTGCCAAGAACTACTTGAAAGGCTTTTAATACGTTTTGACAATGGCAATGGTTCCTCTGGGCATGTCGACAAATCCTCTAATGACGAGGAAGAAATACAACCTGTGCAAGAGTTGAACAGTAACAATGATGACAATGATGGTGTGAAAGCCCTGCTGAGTTAGTCACTACTTAACAGATATTCTTTACTCTGTTGTAACCGATACTTGTTATATATCATTTACAGTAATGGTGATTTTGTTCCTCACCTATGAAACGTTAGTGAAAATTTATTTTGTTTAACGCCGTCATTATTTTCAAAGATAGACTTTGGCTCATATTTGTA

>C102255_65

TGAAGAAACTCAAACTCCTTCTCTCCTCGATGTACATATTTTCAACCATCAAACGTGTCCTATGAAACTCCGTGTTCTAGCCTTCATTTCACCTTGTCACCCTTGTTTTTTGTGTGTTTGGTTTTCCCTTTGTTTTTCTTCTCTCTCTCTCTACAATGGTTCCAAATACAAATACAACTGCACCCTTTTGTTGAAAGATTATAAAAACTCAATTTTTTTTAACTTAGTGTTATCATTACTTGTGGTGGTGGTTGTTTTCTGATTTTCCCTTCCCATGTGCTGACTTGGTTTTCTTCGCCTTGCTTTGTTTTTTCTGTCTATTCGAGTCTATTGGCTCTCCTCTTTCTTTGGTTTTGGGGTTGAAAAGCTCAACTTTTGAGGATTGAAAGAGTTCTCTTCTGCCTTACTGCTGCTGGGTTTGTGTTCATTTCGTTGAGGTGTTCTTGGCCTTCCTGGATCCAACTTCATCTGGTTATCGATTGTATACTATAATCCTTAGTTTTTAGTTGAAGGAAGATGATCGTGAGAAAAGGGTCCGACAATTAATCCTGCATAGTAGAGGTAGAAGATGGAAAATACAGGCCTATGGATTCAGAAAGAGGGCCACAGAAGAAAAGATTCATTGCATGGGTTAGTAAATGATAGAGAAATGTCAGAGGAAAACTCTTCAAAATCACAGTTAAAGAAGTTGATTAAAAGATCAAACTCTCAACGGGCAATAGTTGTAAATGCCATCCAATCACAAATTAGCAAAGTTTTCTTCAAGGATTTATTCTTTCGACGTGTCCATGGATTGGACAATAGGATTCCGAAGCATGTGGTGAGTGTAGATGAGAAATATATCCGTCGCTGCCTGGAATTTATACACAACACTGCATTAAGAGCTACGAACATGGAGACTTTATCAGAAAGCTTCAATACAGCTAAATTTTTTGGTGGAAATGCATATGGTTCAGGACATTTTGTCTTTGAATGTCCTCTTGCCACTGAGAGTGGGAGAGTAGTTATAGGTGCTAATGCTGGGGATCAGTGGACTTTAGGTACAATAATGGGGACCAAGAGTATGATAAATATATTGAATAGTACACTTCTTCAGCAGTTTGGTGCTTCACATAGAAGTGACAATTTGAACAGGATGAACTTTTCTGATCCTGAAGGATTGATATGCTATGATTTTATGGACTCTCCTACTGATTTACCTATCACTTCGTCATATAAATTAGAAAAGGACAAACGATTCGTACGAAGTCATAAGTATGGCTCCATTTCTGTTCATAAAAGGCTTGTTTCCACTTCTAGCATCACCTCTACTTGTTCTGATTGGTTGTCTTCGGCTTCTTCTACTCTATCTCAGGGAATGATCCAATGTACTTGGAAGAATGGGATTCCTCATTTTGTGTTTTCTGCAGATGATCAGAAGGAGGTCTATGTAGCAAAATTTAGGAAGGTAGACTCAACGGATAATAAGGCTTTGGACAATGTGTATGAGTTTCATTTGAACAAGGGTGGCCAAAAGGGTCGCGAGATCCTTGACAGTGATTTACCATTTGTTGGGAAGATGCATGTATCAACAAGTTTTACCCTTTGCCCGAACAATTGTAGAATAAGGGAGACAGAGTTTACATTATTCCTTAACGTTGAAATTAAGGACAGAGAAATGTCCACATCAGAGCATTCTCACAGGAAGAACAAGGGGCTGTCAAAGAAGGTGTCACAAGTTTTTAGAACTAGCCCATCATCAAAACGCAGAACACTCTCAAAGTTTGGTGGATCAAGTTCCATAGCTGAGAGTTGTCCGTGGGAGCCGAATGCTCATGGTGGGACCAATTTATTAGAGACTGACACTCCTCCGAATTGTGAGATGGCTTCCATTGTTGTGAAAAGCCATCTTCCTTGTAAACCAGCAGAGAAAGTAGGAGGGGGGTGGGGTTTAAAATTTCTTAACAAATCTGGTGGAAATCAAGTCACAGTGCCTTCTGAAAGTTGTAATCAAAATAATGGGGACTGTTCAACTAGCATGAGCATTTTAATTCCAGCAGGCTTTCATGGTGGACCAAGAACCAGAAATGGTGGGCCATCCAGTCTGATAGATAGATGGAGATCTGGTGGCCACTGTGACTGTGGTGGTTGGGATGAGGGGTGCCCTCTTACAGTACTTGAGAGAAGATCTAACAAAGCGGATGTTATGACTAAAATAGATACACCATACGAGTGCAAGTCAGTTGATTTGGTTACTCATCAGGGTTCAACTGATTTCGGCCCCACCTTGAGGATGGTAAATGTCCATGATGGTCTTTATTATATTCATTTTCATCCACCTCTGTCTGCACTACAGTCTTTCTCCATTGCTGTGGCAATAATTCATGCACAAAGTCCTACTCTTCAGCTCAATGGTGCGAAGGAGCTGTCATGACTAAAGTTAAATCTGAGGTTATTTAGCAGTCATCATTTTCTTACTGTATTATAAAAAGTGAAGAGAAGATTTAGGATTTTATACTTGAATCAGGTGTTCATTCTATGTTTAGGGTTGTGATTTTAATACAGTCAGATGTTATCTTATTTAGGAGAATTAGTTCTGTTTGTCCTTTCTCACAATTCATAGTTTGTAACCATCTTTCTCCTCGTTCTTTTTGTTGAAGAGGATCGATTGTAAAAGGTTTGTGGAAGAATATTTAGTAAATTATAGCATTGAGATTGAGTTTGTTAATGAGAAGGAAATTGGATTAGCATTTAACACTCATTTATGATTGGAAACTCT

>C102257_65

TATTGTATTAGTGGACGGTTTCATAGCGCGTGACTCACGCGTCTTCTAATAAGGAACAAACCCCAAATATTCCTAAAACCCAAACTTTCCTTCTCCATTAGCAGAAACCCTCACCCATGACCCAGTCAATGCACGCCGTGGAACCGTCGCCGGAGGCGGCGGAGCTGCCGGCGTCCGATCCTTTGTTCGATGAATTCTCCGCCGAGTTCGGATCCCTCCCCGTCCTGTCCATGGACTCGCTCTTCAACACTGACGCGCTTCCCTTCGCCTCAGATCTCGAATTCGGCATGGATTTTGATGACAACAATGGAGAATTCGAAATCACCTTCGACGATCTCGACGACATCTGCATCCCCTCCGACGCCGAGGATTTCCTTCTCGCTGACGCATGTAACCCCGACAACACCTCCGTTTTGCCGCCAATTAAGGAATCTTCCGCGAAGAATTCCGATTCTCCACGTTCTGACGAATCTGTGGTTTCTGGAAATCTGAGCTCCGGCATTTCTAGGTTTTTCAATTCGCAAGCGTCGGAGTCGGTTTCTGAGGGTAATTCGTGCAAAGAGGGTTTGCATGACGCTGTTGATGTTAGGGTTTCGAACATTCCGTCGCAGGATTCTGAATTTTGCGATCGGGAGGAGTCTTCTAATGGACCGGTTTCGTCTCAGGGCTCAGGAAACGGTGGATCGGGTGTATATGAGACCATGAACTCGCCCTCACCTGATTCTGGATCTTTCGAGAGGGACATAACGTCGTCTCGTGCACATGCAGTTATGGATAAAGGTGTGAAATTGGAGGAAATTCCAGGGTGTGATCTGAAGAGGAAGAAAGAGAGATGTGAAGGAAATGCTACCAAGCACAGAAGGTTCTCTTCCTCCTCTGTGGAGAATAAGACCGAGAAACAGTCTCCATCTGATGTGAATGCAATTGATGATGATGATGAGAAAAGGAGGGCAAGGTTGATGAGGAATAGGGAAAGTGCACAGCTTTCTAGGCAGAGGAAGAAGCATTATGTGGAGGAGCTTGAGGAGAAAGTGAGATCAATGAATTCCATCATTGCTGATCTGAGTAGTAAGATTTCATATATGGTGGCTGAGAATGCTACACTTAGACAGCAAGTGGGTGCTGGTGTGATGTGTGCTCCTCCACCACCTGCTCCTGGGATGTACCCTCATCCTCCAATGGCACCCATGCCTTATCCTTGGATGCCTTGTGCTCCCTATGTTGTCAAGCCTCAAGGATCTCAGGTTCCTTTAGTGCCCATTCCTAGGTTGAAGCCTCAGCAACCTGCTTCAGCCCCCAAGGGTAAAAAGTCTGAGAGTAAGAAGAGTGAGGGGAAAACTAAGAAGGTTGCTAGTATTAGTTTCTTGGGTTTGTTTTTCTTTATCATGCTTTTTGGTGGGCTTGTTCCTCTTGTGGATTTTAAGTTTGGTGGTTTAGTGGACAATGTTCCTGGTACCGTTATGTCTAGGTATGTTAGTGATAGGGTGTATTATGGTGGAGGGAAGGTTTGGTCCGTGAATGGTCCTAGGAATGGGTCTGAACGAGATGAGGAAGTAGGGTTTTCTAATGAGAGGTTTAATGTTAAAGACAGAATGAATTATGAGAGAGGCCGGCATTTAGGAGAAGAAAAGGGTGAACGGCAGGGCCCTGATGATTTTGGTCGCCAGGGAAATGCCAGCGAGCCTCTTGTTGCTTCTTTGTATGTTCCTAGGAATGATAAGATGGTAAAGATTGATGGAAACTTGATTATCCATTCCATTATGGCTAGTGAGAAAGCTATGGCATCTCAAACTGCCGAGGCAAAGAAGGAGAAGAAAGAAACAGGTTTGGCCATTCCTAAGGATTGGGATTCTGCATTAGCTATTCCTGAAGTTGCAAGAAAAAGAGGTCAACATCCACATGTGTATAGGGTTCCTTCTGAACAAAGGAAGGCTCTTGGCTCTGGTTCAACCAAAGCTTTGAAGGACCATATGAAGTCCTCTGCAACTGATGGTAAAATGCAACAGTGGTTCCGTGAAGGCCTTGCAGGGCCAATGTTAAGTTCTGGCATGTGCACTGAAGTTTTCCAATTTGATGTTTCTCCGAGTCCTGGAGCTATAGTTCCTGCGACATCAGTAGCCAATGTCTCTACTGGAAAGCGTCAGAATGCTACCTCTGTCAAAAAGACCGGGAATAGAAGAACCCTTCATGGGTTACCAGATTCGCTTACTGGATCTAGTCTGAACATAACTGAAGAACATGTGAAAAACTTGCAGAAAGATCATCTCCATGGTAATGAATCTTCTATGGTGGTTTCAGTCCTTGTTGATCCCAAAGAGGCTGGTGATGGTGATGTTGGTGTTGATGGCATGATGAGACCAAAGTCACTCTCTCGGATTTTCGTGGTTGTGCTAATGGATAGTGTCAAGTATGTCACTTACTCATGCGGTCTGCCTCGTGCTTCTCCCCATTTGGTGACTTCTTATGTATGAGAATCCGTCCTTCCTTCGGTGAAATGTTCTGATATAGATGAATCATCTAACCTGAGTTCTAGTTTTGTAGCAAAGGATATATGTGGGGACTGGGAAGGAAGGAAAGAAACTCAGCTCCAGTTTTGTACATAATAACATATTTAAAGTAGCAGGCTTAAAAATGTAGCTGAGAGAATACAGAGCTCGGCATGAATGGGGTTGTTGACCAACTGTTTGTACGGTTGAGACCTTTCTTAGAAACTTAATAATATATGCCCACCTTTTAATTTATAAGTGGATCCCTTGTCA

>C102259_65

GAAGACCAGAGATGCTTGGTGGTTCTCACACAAAAGCAGAGCAATCAGAAGAACTGCAAAAATATGAATGGATGATCTCCATCATTTTCTCTCTCAATTCTCACCCTCTTCTATTTTAATCAGTTACTTCTCTGTTTCTTGTTGGATGCTTTTCAAAGTTTGAATCTTGAGTTCTAACAAAAGAAAAGATATTATGGGGTCAAAGTTTCCGATGACCCAGTTGGGGACATCACCCAGATACATGATTTCTCCCCACAGGGGTTTTTGTGCATACAAGTATTCGAGAAGAGTATTCTCAGAGCAAAAGTTTCCGTTCAAGTTTGTGCCACAGTCTTTAGGGGATAAATGGAAGCTAAATGACATAAGTACTGGTTCTATTCAAGAAAGATTGAATGTATTGATGTCAAGGACCCAAAATTTTTTGAATGAAGTGACTTTTCCGCTTGCAAAACCCGGTCAAAGTAAGAAGCCTGATCCTAAAACTGATTTTGGATTTCAAGTCATGGAAGACATATTTATGATTGAGCAGACAATTGACCGCAGAACGCCATCTGGGGTTCTTTCTCTAGCTGCTGTAATTTGTATTGAACAATTCAGCAGGATGAATGGATTGACCGGGAAGAAAATGCAGAAAATATTTGGAGCGCTGGTGCCTGAATCTGTATATAATGATGCCCGTTATTTGGTTGAATATTGCTGCTTTAGATTTTTGTCTAGAGATGGTTCTGCTATTCATCCTTCTCTCCAGGACCCTGCGTTCCAGAGGTTGATATTCATAACCATGCTTGCCTGGGGAAATCCTTATACTGATGACCTTTCCAGCAATGCAGAGAAGGCTTCTTTACAGAATAAACTTGTCACTGAGGATGCTTTTGTTCGTATTGCTCCTGCAATATCTGGTGTGGTTGACCGTCCCACAGTGCACAATCTTTTCAAGGCTCTTGCTGGCAACCAAGAGGGCATTTCTATGGGCTCATGGCTGAATTACATTAATGAGTTTGTCAAAGTGCGTCAAAAACAGATATCATATCAGATTCCAGAGTTTCCTCAACTATCTCAGGAGAGAATTTTGTGCATTGGTTTCAACAGTAAGCGGCCAGTTCTGAAATGGGAGAATAATATGGCATGGCCTGGAAAACTTACTCTTACTGATAAAGCAATCTATTTTGAGGCAGTTAGCATACTGGGAGAGAAGAGAGCCATGAGATTGGATCTTACAGATAATGGAATAAAGGTAGAGAAGGCAAAGGTTGGGCCTTTAGGGTCTTCACTTTTTGACTCTGCTGTTTCTATTTCCTCTGGCTCAGGGACAGAGTTGCATAGGTGGGTGCTGGAATTCATTGATTTAGGAGGTGAGATGAGGAGGGACGTTTGGCATGCATTTATCAATGAAGTCATCACTTTACACAGGTTCATACGTGAGTATGGGCCTGATGAGTATGATGAGTCACTGTTTAATGTATATGGAGCTCGCAAAGGAAATGATAGAGCAACAACCTCTGCTATTAATGGTATTGCTAGACTCCAAGCCCTTCAATATTTGCGGAAGTTGCTTGACGATCCCACCAAACTTGTTCAGTTCTCATATTTACAGAATGCACCACTTGGTGACATTGTCCTCCAAACTTTAGCTGTTAATTATTGGGGAGGTCCACTAGTTACAGGATTTGTAAATACCCGCAATCAGTCAGAAAATCGACCTTCTGATGAAATAGCTGATAGCCGCAACCATGTATTTGACATAGATGGAAGTGTCTATTTGCAGAAGTGGATGAAATCTCCATCTTGGGGTTCTAGTCCTTCCACTAATTTTTGGAAGAACGCTTCAGTGAAAGGTTTAATATTGAGTAAGAATCTTGTTGTGGCTGATTTATCACTCATAGAAAGAGCAGCTAAAACATGCAGACTAAAGTACCACGTTGTGGAGAAGACTCAAGCTACCATTGATGCTGCAACTCTACAAGGCATACCCAGCAACATTGATCTTTTCAAGGAGCTAATGTTTCCTTTCGCCTTGATTGTGAAAAACTTTGAAAAGCTTAGGCACTGGGAGGAGCCACACTTAACTGTTGCATTTCTTGGACTCACCTATACAATAATTTATAGAAACCTGCTGTCATATATGTTTCCCATGATGCTGATGATTTTGTCAGTTGGCATGCTGACAATCAGGGCCCTTAAAGAGCAAGGCCGCCTTGGACGAACTTTTGGTGAAGTCACAATACGTGATCAGCCCCCATCCAATACTATACAAAAGATTATAGCAGTAAAAGATGCCATGCGTGATGTTGAGAATTTTATGCAGCAAGTGAATGTCTCCCTCCTCAAAATACGTTCTATTTTACTTTCTGGCCATCCACAGATAACTACTGAGGTTGCACTGATACTGATATCTTCTGCGACCATACTTCTCATTGTCCCTTTCAAGTACATCTTTTCCTTCCTTCTCTTCGATATGTTCACAAGGGAGCTTCAGTTTCGAAGAAAAATGGTTAAGAAGTTCATAAAATTATTGAGAGAACGGTGGCATACAGTGCCTGCTGCCCCCGTATCAATTTTACCATTTGAAAATGAAGAAACCAGACCAGAAACTTACTTGAAGGAAATTGAGGACCAATCAAAATCACAAGGAAATCAGAGCAGTGGAAAGTCCAGGTAGCTAGTGATTCCATTTTCAGTATTAGTTACTAACAAATAATGTAAAGGTTTAATTATATTTTTAGTTCTGAAATTTAGAGTAAATATTATTTTAGTCTCTCA

>C102261_65

TTTCTCTCTCTCTCTCTCTCTCTCTCTCATTGATTACACACAGCCACCGTTTTCTCTTTCTTTTTACTAGCTTTGGTTCCTCTCTTACATACCCTTCAAACCCATGTCCATTTGTAGCATGTTTCCTCTTGGGCACCTCAAAGTTCAAATTTTTCTTACACACTCCTTCCTCTCCAACCTCCACATCCTCTTGGGTCACTTCTAGAAGATCTCTCCCCCAACCCTGTTGGGTGAACAAAAACAAGAAAGAAAAAATGGGAAGCAAGTGGAGAAAATTGAAGCTCGCTCTTGGTTTGGATTCCTGTGTTCACATTCCTAGAGCCTTTGATGATTCCTCCTCGGCCGCTAGGTTTTCCGGCGCCGCCTCACCCACCGTCGTGTCCCCGGCTGGCGACACCTCCGGCTATCGCCCATCAACTCCCACACCGTCGTCCTCCGGCCTCCGGTTATCCAAATCCGGACCCAAATCTCCCAAGGGTACGTGTGCAATATGCCTTTACACAATGAAACCAGGGCAGGGCCATGCCATTTTCACTGCAGAATGTTCTCACTCTTTCCATTTTCATTGCATCACTTCTAATGTGAAACACGGAAACCAGATTTGCCCTGTCTGCCGAGCAAAATGGAAAGAAGTTCCTTTTCAGAGTCCTACTTCTAATGTGCCTCACGACTTGCCTCGAATCAACCATGTAGCTCCTCGAGATGAAGGCTGGTCTACAGTTATACGCAGGGTTCCTTCTCCTCAAGGTGATGCTGCTCGGTCGATTTCATCACTTTATCATGTTACTGAACCAGCTATTTTTGACGACGATGAAGCCCTGGATCAACAAACTTCAATTACCCATCATAAAAATGTAACTGATCATGATATGATAAATACAGTGGAGATCAAAACATATCCTGAGGTTTCAGCTGTTCCAAAATCAGCCTCTCATGATGCCTTCGCCGTGTTAATCCATCTCAAAGCTCCTCATTCAGGGAGAAAACAAAATATTGAATCATCACCTCCAGTTGAGAACACTCGAGCCTCTGTTGACCTTGTTACAGTTCTTGATGTGAGTGGTAGCATGGCAGGTACTAAGCTAGCGCTGCTGAAACGTGCAATGGGTTTTGTCATTCAGAATCTTGGTCCATCAGATCGGCTGTCTGTCATTGCCTTCTCCTCCACAGCACGCCGCATCTTTCCTCTTAGGCTGATGACTGACACTGGACGGCAACAGGCATTGCAGGCTGTCAACTCTTTGGTTTCAAATGGTGGGACAAACATTGCTGAAGGCCTGAGGAAAGGTGCCAAGGTGTTTGCTGACCGGCGGTGGAAGAACTCGGTTAGCAGCATCATATTGCTCTCTGATGGGCAGGATACATATACAGTCAATAGCAGGCCGAATACTGGAACAGATTACCAGTCACTTGTTCCAAACTCCATTCATCGTAATAATGGTGTGGGCATGCAGATACCAGTACATGCATTTGGCTTTGGCTCTGACCATGATGCCACTTCAATGCATTCAATCTCTGAGATATCTGGGGGTACATTTTCTTTTATTGAAGCTGAGGATGTCATTCAGGATGCATTTGCACAGTGTATTGGGGGACTCTTGAGTGTGGTGGTGCAAGAATTACAAGTGGAAGTGGGGTGTGTTCACCCTCATTTGCAACTTAGTTCGGTAAGAGCAGGAAGTTACCAAACAAACTTGATGGCAAATGCAAGACTGGCTACTATCAGTGTAGGAGATTTGTATGCAGAAGAGGAAAGAGACTTTTTGGTGACAGTTAATGTTCCAGTTGATGAGTCAACTGATGAGACGCCGTTGTTGATTGTGAGAGGTCTTTACCGAGAGCCCATAACAAAAGAAATGGTGGCTTTGGAAGAAAACAGTGAAGTTAAAATTCAGAGACCTGACAACGCAGGAGGAGAACTAGTTGTGTCAATAGAGGTAGACAGACAGCGAAACAGGCTTCAAGCTGCTGAGGCAATGGCTGAGGCTAGAGTTGCAGCTGAACGTGGTGATTTGTCCACTGCAGTTTCAGTCCTGGATAACTGTCACAAGGCATTGTTAGAAACTGTTTCTGCTCAAGCTGGGGATAGGTTATGTGTTGCACTTTCTGCAGAGTTGAAGGAGATGCAGGAGAGGATGGCGAATCAACGCGTTTATGAGCAGTCTGGAAGGGCTTATGTTCTGTCAGGATTGAGTTCACATTCATGGCAAAGGGCAACTGCACGCGGTGATTCCACAGACAGCTCTAGCCTTGTGCAAGCATACCAAACTCCTTCAATGGTTGACATGGTGACGCGTTCCCAGACCATGGTTTTTGGGACGCCACAAAATAAACGAGTTCTCAGGCCTGCAAAATCATTTCCTGAGAGGCAGAGAAGACAATAGGAATGAAAAACTGTTTGGAAAATTCTATTTAGCCTATACATATATACTTTCTGAGGAAACCTTCAAGTTTCTTCATATCTTAAGTTACCCTTTTGATTTTAGTTTTGTGAAGGTGGGGTATAATGGAAAGGAAGGAGTTTTAGGTGGATAAAATTCCTGATTTTGCCAATATGAAAAGTGTAAATGCATAGGCTGGAAACGGGGCTCTGCAGAGAATGAATGAATGGCGAAAAAGGATGCACAGGTTGAATATTTCAGTGTGATTCCGAGTGATGTACATAACCTATTGTTTTCAGTTGAATTGTGCGGGTGAATTTCATCACTTGATTATATACATATGATATAATATATGTGCTTCTGATTCTTTTCATTAGTGG

>C102271_65

AACAGATTAAAAAAGTGAAGATATAAAGAAAAAATTATAGAAAAAAATCACGGAAACCCTGTGCATGCCTTTAAAAGCCCTTGTTGTTTAAACAGAGCTTCACATCTCAGGCTTCTTCATCACTCATCTCAGTTTCTCAGTTTCTCATTTCACTCTCAGAATAACACAACAATGGAGCTTCTGTCAAATGGACACAGAACCAAACCCTCCCCCGAGATACCCCTCGTGAATGGCACCGCGTTAACTCACCTCAACTCCCTCTACGAATCTCACTTCTTCGCCAACATCGATCTCTCTAGAGCTTTTGTCTCCAAGGTGAAGCGTCTCATTGTAAAGGTTGGAACAGCTGTTGTTACTCGAAGTGATGGAAGATTAGCACTAGGTAGAATTGGAGCTCTTTGTGAGCAGCTTAAAGAACTTAGCAGTCAAGGATTTGAAGTCATATTGGTAACTTCAGGTGCTGTTGGCCTTGGCAGGCAAAGACTTAGATATCGAAAATTGGCCAATAGCAGTTTTTCTGATCTTCAAAAGCCACAAAGTGAGCTTGATGGAAAAGCATGTGCAGCTGTTGGGCAAAGTAGTCTCATGGCTCTCTATGATACCATGTTCAGCCAGCTTGATGTAACTTCTTCCCAACTTCTTGTGAATGACGGATTTTTTAGGGATGCTGGCTTCAGAAAACAACTTTCAGACACAGTGAACTCATTGTTAGATTTAAGGGTTATCCCCATTTTCAATGAAAATGATGCTGTTAGTACTAGGAAAGCACCATATGAGGATTCTTCTGGTATTTTCTGGGACAATGACAGTTTGGCTGGTCTATTAGCCTTAGAACTTAAAGCTGATCTCCTTGTTTTATTGAGTGATGTTGAGGGCCTTTATAGTGGTCCTCCAAGTGACCCAAATTCAAAGTTGATCCATACATATATAAAAGAGAAACATCAAGGGGAAATTACTTTTGGAGACAAGTCAAGATTGGGCAGAGGGGGTATGACTGCTAAAGTTAATGCTGCTGTTTGTGCTGCTCATGCTGGTATCCCTGTCATTATAACTAGTGGCTATGCTACAAACAACATCATTCGGGTTCTTCAAGGGGAAAAAATAGGTACGGTCTTTCATAAAGATGCTCATTTATGGACCAATATAAAGGAAGTGAGTGCACGTGAAATGGCAGTTTCAGCACGTGAGAGTTCTAGAAGACTTCAGATCCTCAAATACGAAGAAAGGAGTAAAATATTATTGGCAATTGCTGACGCATTGGAGAAAAATGGAAGTATAATAATTCGTGAGAATGAAGCTGATGTTGCTGATGCTGTGACCGCTGGATATGAAAGTTCTCTGATATCACGTTTAACCCTGAAGCATAAGAAGATCTCTAGTCTTGCAAAGTCTGTGCGCATGTTGGCAAATATGCAAGAACCAATTGGTCAAATTTTAAAAAGAACTGAGCTAGCAGATAAACTCATCCTGGAGAAAATATCATGTCCTTTGGGAGTTCTTCTGGTTATATTTGAGTCTCGACCTGATGCCCTTGTTCAGATAGCTGCATTGGCAATTCGAAGTGGGAATGGTTTACTGCTTAAAGGTGGAAAAGAAGCCCGACGATCAAATGCAGTCTTACACAAGGTCATCACTTCGGTGATTCCAAATACTGTTGGTGACAGACTTATTGGCCTTGTGACTTCAAGAGATGAAATTGCAGACCTACTTAAGCTTGATGATGTGATAGATCTTGTGGTCCCTCGAGGCAGCAATAAGCTTGTTTCTCAAATCAAGGAATCAACAAAAATTCCTGTTCTTGGACATGCTGATGGAGTTTGTCATGTATATGTCGACAAATCAGCTAATATAGAAATGGCAAAACAGATTGTTAGAGATGCAAAGACTGATTATCCTGCAGCCTGCAATGCAATGGAAACTCTTCTTGTACACAAGGATCTGTCAAATAATGGTGGACTTAATGAGCTTGTCCTTGAACTCCAACGTGAAGGTGTTAAAGTGTATGGTGGACCAAGAGCTAGTGGCTTACTAAACATTGTTGAAACAAGCAATTTTCATCATGAGTATAGTTCACTTGCTTGCACAGTTGAAGTTGTAGAAGATGTATTTGCTGCCATTAACCACATACATCGACATGGAAGTGCACATACTGAATGCATTGTTACAGAAGATTGTGAAGTTGCTGAGACTTTCTTGAGTCAAGTTGACAGTGCAGCAGTTTTCCACAATGCAAGTACACGATTTTGTGATGGAGCACGCTTTGGGCTTGGTGCAGAGGTTGGAATTAGCACAAGTCGAATTCATGCACGAGGACCTGTAGGAGTAGAGGGCTTGTTGACAAACAGATGGATATTGAGAGGGAGTGGACATGTGGTTGATGGTGATCAAGGGATCAAATACACTTACAAAGAATTGCCACTAAAAGCTTAACTTTGGATTTAGAACTTAGTTCACTTTCAAGCTAATATGCTCCCCTCAACCGAGCAACATCTCTTTTTCTGTTCTGCTATGATATGCAGAAGTTGTTGAGATGTTGGCCATGTATAATGGTAACTATCGCTTGACCCTTTCCAGGGCATTTTTCTTACCTGGAGCAAGTTCAAGGGGGAGGAGATAATATGAATGTTTGTATTCAATTATATTTCACTTTGGTGCTCTTTTATATGATATTCCTAGTCTGTATAACAACAATGCTGTTACAGTTATATTCCTATATATTAGTGAAGATGAATAATTACGCAGAAGTTTTTAATAGAAAAAAAAAA

>C102275_65

GAGGAAGAAGTAGAACAGAAGCAAGCAAGGAAGCATTTTTTTTGTGTGTTGTGAGTTGCGATCATCAAAGCTTTCTCTTTTCTTCAATCACATTCAAATGGACATTGATTTCAAAGACTACCAGTTGCGCTGCGAGCTTCGCGGCCACGAAGACGATGTTCGCGGAATATGCGTGTGCGGCAGCGAGGGCATAGCGACGTCGTCAAGGGACCGGACGGTCAGGTTGTGGTCTCTGGACAGCAACAGCAAGTTCGTGAGTTCCAAGATTCTGTTGGGGCACACTAGCTTTGTGGGACCCTTGGCTTGGATTCCCCCGAATTCGGAGTTTCCACACGGTGGGGTTGTGTCTGGCGGCATGGACACTCTGGTCTTTGTCTGGGACTTGAACACTGGTGAGAAGGTTCACACTCTCAAGGGCCATCAGTTGCAGGTCACTGGCATTGCGTTTGACGAAGGGGATGTTGTGTCCTCTTCTGTTGATTGTACCTTAAGACGGTGGAGGAATGGGCAATCTGTAGAGACGTGGGAGGCTCATAAGGCACCAGTCCAAGCAGTTATTAAGCTGCCATCAGGAGAGCTTGTTACTGGTTCAAGTGACACTACTTTAAAGCTTTGGAGAGGAAAAACTTGTTTACATACCTTCGAAGGACATACAGATACAGTTCGTGGCTTGTCTGTGATGTCTGGTCTTGGAATCCTGTCTGCTTCCCATGATGGTTCTCTCAGACTATGGGCAGTCAGTGGTGAAGTGTTAATGGAGATGGTCGGTCATACTGCTCTTGTTTATTCAGTTGATTCACATGCATCAGGACTTATTGTCAGTGGTAGCGAAGATCGCTTTGCCAAAGTTTGGAAAGATGGAGTTTGTGTTCAGAGCATAGAACATCCTGGTTGCGTTTGGGATGTAAAGTTTATGGAAAATGGGGATATTGTGACAGCATGTTCAGATGGAGTTGTACGCATTTGGACTGTAGATCAGAATAATGTTGCTGACCAGCTAGAGCTTGATTTATACACTTCACAACTTTCTCTATACAAATCTAGTAGAAAGAGGGTTGGAGGATTGAAATTAGAAGAGTTACCAGGCTTGGACGCACTGAAGATCCCAGGAACAACTGATGGCCAGACAAAAGTTGTCAGAGAGGGGGATAATGGGGTGGCATATGGATGGAACATGAGAGAACAGAAGTGGGATAAAATTGGTGAAGTTGTTGATGGACCTGAGGAGTCAAATCGCCAACTTTTTGAGGGTATTCAGTATGATTATGTTTTTGATGTGGATATTGGTGACGGTATGCCAATCCGTAAGCTGCCATACAATCGATCAGATAATCCATATGATGTTGCTGACAAATGGCTACTAAAGGAGAATCTTCCACTTTCTTTTCGTGAGCAAATTGTACAATTCATTCTTCAAAATACTGGACAAAACAATGTTACTTTTGATGCTTCATTTCGTGATCCCTTTACTGGCTCCCATGCTTATGTACCAGGCCAACCTTCTCATTTGTCTGATATTTCTGCGAAACCCACTTTCAAGCATATTCCTAAGAAAGGGATGCTTGTTTTTGATGCAGCTCAGTTTGATGGAATCCTTAAAAAGATTACTGAGTTCAATAATGCATTACAATCTGATCAGGAAAAGCAGAACTTGTCTTTGACTGAGCTCAATGTTTCCAGACTGGGTGCCATTGTTAAAATTCTGAAAGATACTTCACATTATCATTCTAGTAAATTTGGAGACTCTGATATAGACTTGTTACTGAATCTGCTTCGATCATGGCCTATTGCAATGATTTTTCCTGTCATTGATATCGTAAGGATGTTAGTTCTTCATCCAGATGGGGCTATTTTACTTCAAAAGCATCTTGAAGCACAAAACGACATACTAACGGAAGTGATTAAGAAAGTCACTGTAAATCCAACAATTCCTGCAAATTTGTTGACCAGCATTCGAGCTGTAACCAATCTCTTCCGGAATTCATGTTACTACAACTGGTTGCAGAAACATCGTAGTGAGATACTTGATGCTTTCTCAAGTTGTAGTTCGTCTCCAAACAAAAATTTGCAGTTGTCATACTCTACCTTGTTACTGAATTATGCTGTGCTGTTGATTGAATCAAAGGATCAAGAAGGTCAATCTCAAGTTCTGTCAGCAGCTCTTGAGCTTGCTGAAGATGAAAATGTAGAAGTTGATTCTAAATTCCGTGCTTTAGTTGCTGTTGGATCTCTGATGCTTCAGGGTCTTGTCAAAAAAATAGCTCTGGATTTTGATGTTTTGAACATTGCAAAAGCAGCCAAGGGTTCTAAGGATGCCAAGATTGCTGAAGTTGGATCTGACATTGAGCTGTTAGCAAAGCAGAGCTGATTTGGTGTTCCGCCTAATAAAATGAAGGAGCTGTTGCATTTTAGAAGATTCATTGACAGAAGATACATTGAAAGTGACTCAACAGGTGTAGCCTCTCAATTACAGATTTCCACCGATCCTCCCTACTGAGACAATTATAGTTGAAACCCTTGACATGTTTATCAGATAGATTGCGTAAGCCAAATGAGCAATTGAGGTAGCAGTGTAGTCTTTTTCTATCATTTGCTTTAATATTAGGTTTATTCAAATAATCTTATTTAATACTTCATTTGGTGAAGTGAATCAAGTTTATGTGCTACCGGTGTATTTGTTTTTTATTTAAGAACGCACGTTTTGGTCTTATCAATTAAAAAGACGGAGAAGTATATACGAAGGAAAAAAAATCAAGTCATTTAATA

>C102277_65

CGCCCAGTTTGATCACAGGACACTTTAACTTTGCATATTAATGGCAGGACCAAACACGAGCTTAGCACTGATAACTTGTGAAAGGAACATCTGAAATACATGAATATGATTACACGTGCAGTTACAAAATTTGGGATCATATAGGTAGGGGAAAACACTAAATGCACTAGACAAATTTTTAACAATAAAATTAGAACCTCCAACAAGCCTCCCAAATATTTCCCAGCTACTAAAAATGACAGGTCAATTTGTACCCAATAAACAGTTCTTAAGTCACCAAATGGCACCAATGAAAAAACAGGGAGCCTTGCTTCATTTTCAGACCTCTCCACGTTCCAGGGCATATAGAAAAAATTGTGCTTTATATTGTAGTGTATGGCTAATATGGAAACCATTCCATTTCTCCTCATCTAGCGGCACCATCAAATATCTAGCTGACGAAGATCAATCTTGCTTGCAGAATTTCGTATGAGTTCCTTCCTAGTATCCACCTGAGTACCCATAAGAGAGGAGAAAACAATATTTGCTTCTGCTGCATCCTCAACTTTTAATTGCTTCAGTAACCTGCGCTCTGGATCCATGGTCGTTTCCCATAGTTGTAAAGGCATCATCTCTCCCAACCCTTTGAACCTTTGCATGTTGTATGATGCATTAGAAGGAAATGATTTTTGAAGCTTTTTAAGGTCAGCATCATCATAGCAATAGTGCACTTGTTTTCCCCTGACAACCTTGTACAATGGTGGAACACCAACATATATGCAACCTTCATCAAACAAAGCTTTCTGATATCTGAAGAAAAATGTCAGTAGCAAAGTTCGGATGTGAGCTCCATCCACATCAGCATCAGTCAAAATAATAATTTTATGATACCGGAGTGCATCCTTTTTAAAATCCTCTCCCTTCACACCGAGACCAAGACCAAGAATTAAATTTTGGATCTCTTCATTTTTGTACATTGCTGCTTCATCCCTCCTTTCAATATTCAGAATCTTACCCCTGAGAGGAAGAATGGCCTGGAAGCGCCTGTCACGCCCTTGCTTAGCACTTCCTCCAGCTGAATCACCTTCAACTATAAAGATTTCACATTCTTCAGGATTTGAAGATGAACAATCAGCTAGTTTTCCCGGAAGAGATGAAGATCTCAATACACTCTTTTGTCTCACCAATTCTCTTGCTCTTTTTGCTGCCAGAGCTGCCTTAAAAGCATTAAGAGCTTTAGAAAGTATTGAATCAAGCACATCTGGATGCAATTCTAAATACTCAGTAAGATAGTCATGAATAGATTGATCAACCACTTTTCGCACCTCTGGATTTCCTAACCTTGTCTTTGTTTGTCCTTCAAACTCAGGGTTCGGGACCTTGACTGAGACAACACATGTTAGACCTTCTCTCACATGCTCGCCACTTAAAGTAATATCCTTGTCCTTAATAACTTTTGACTTCTTTCCTAGAGCATTCAGCGTTCTTGTTATAGAAGCCTTCATGGCCTCAATATGAGTACCACCATCAATAGTGCGTATACTATTAGCATATCCCAGTATAGTATCTGAATATGCATCTTCACACCATTGAAAAGCTATATCAACTGTGATGCCATCAGTTTCTTTTCTAAAACTCAGAACATCATGAAGTGTTTTCTTATCTGTGTTGAGCCATTTCACATATTCAACCAACCCCCCGGCATAGAAATATCTGTTATATTGAACTTTATGTGGATCATTGTCCTCTTTCTGAAGCGTGATTGTAAGCTTTGGGTTGAGGAAAGCTAACTCCCTAATGCGTCCAGCTATTGTGTTGTAATCAAACTCAATAGCAGTGGTGAACACTTCTTTGTCAGGCCAAAATCTGACACGTGTCCCTCGAAGATCTTTCCTTTCATCCGGAAGCACAAGGCATTTCAGAATTGTCACTGGCTTTCCACGAGAATATGTTTGCGTGTATTCCATTCCATCACGCCAAACTGTTATCTCTAGTGACTCAGACAAGGCATTAACAACAGACAAGCCAACACCGTGTAAACCTCCTGAAACAGAGTACCCACTGTTGGCACCACCAAATTTTCCACCAGCATGCAAGACCGTCAACACTGTCTCCAAGGCAGATTTATTTGTGACTGGATGCAAGTCAGTAGGAATCCCGCGACCATTGTCGGTGATGCTCACAGAATCATCTGCAAGCAAAACAACATCAATCTTGGAAGCAAACCCAGCTTGAGCTTCATCAACAGCATTATCCAAAATTTCATACACCAAATGGTGCAACCCTCGTGGGCCAGTGCTTCCAATGTACATCCCGGGCCTTTTCCTCACAGGGTCCAAGCCTTCAAGCACCTGAATTTGATCAGAACTATAAGCTGTGGAACTTTCACTTTTCTGAAAAGCTTCGGTGGTAGAGAAAGAGGAAATAAACGCCCTTGGAGTAGCCGCAACTGAAACCGAACAGGGAACCCAAAATTTTCTCAGATGAAAACTTGTCCTGGTTTTGGTGAGAGTAGAAGGAGGGAGAGAAGAAAAGCGCGTATGGAAGGAAGAGGAGGTGAAGAAGCGAGAAGCCATGAAGCGAAGCAGAGGAGGGCGATTAAGAAGAACTTGCGCCATCACATTCACATTAGCAAATCAAATACTTTCTTCCCTTTCCCTCCTATGCTACCACTTTGAGTTCACTGACGCTATCTTCTTTCTCTAAACCCTACAACATATTTAAGACAAAACTCAATTCTTAATACTTTTTCCCTCCACAAGATGACTTTGTAAGGGAAAAAAAAAAT

>C102279_65

GTTTGATAGATGAGCCAAGGGATTGTTATATAACATTATGATGAGCCTTTCATAGCAACACTAAGTGTGAAGATTAAGCAGTGGAGGTAAATGACCCCAACACAGGTCGAGTATTTAACACAATATTGGACCCATACAATCACAAAATATGATGAAAAGCTTCTTATGATTTCCTACTTATTACAACAAGATGGCAACTATCAATTTTTACCGAGCTTCAATCTTGCAAAAGGTTAAATTATTGAAATTTATATCACCGGTGAAAGAAATAGCTCAGAAGCATACACCAACAGTTGTCTAGCTTCAATAATCAAAGGTTCAATCCATCTAACTTAGAAAAACTGGCTGAAGGGGGGTTAAAAAGAAATGATGAAAATCTGAAGAATATTATTTTTCTTTTCTGCCGTTCTATCAACACAAGGCTTTTGTTCCCATTTACATGTCTTGTGATTCTTGGTTATCTCCCCCTCGGTTGGATCCTCCTTGATCTGTTCCAATCGTCCTCTTACTCATGTTAGTTCGCTGCACTAAACGAACCAGCGCTTGAACTACTTCTGACATAGGTGGTCGGAATTCCGGCTCCGGCTGAACACAAAGAGCAATAACATCTGCAAATCGAGAAAGAGACTTCACAGGATATAGTCCTTCCAATGCAGGATCAACCATTTTAGCCAGAGCATCAATATCGTGGAGTTGAGGCGTTGCCCATCGAACCAAAGCCTGCTCAGACCTTGGCCTTGAGCTATCAAATGGTTTCCGTCCACTAAGAAGTTCCAACATGATAACTCCAAAGCTAAAGATATCACTCTTAAGAGTATATTGGCCAGACATGCCAACTTCGGGGGCTTCATATCCAGATCCAGAATTATTGTTCAATATCTGATTTGCATTTGGAATATAGCTTGCCAATCCACTGTCGGAAAGATGAGGATTGAGATCTGTATCAAGCAATATGTTGGCTGACTTTATATTCTTATGAACAACAGATGGTGAACAAACTTCATGTAGGTACTCTAAAGCGCGTGCAATACCCAAAGCAATCTTCACACGTGAATTCCAGATCAATGGTTTACTATACTCGTCAGGTAAGTGAAGGAAATCATGCAATGATCCATTTTTATGAAACTCATAGACTAGTAGGTGTTGTCCGTGCTCTGAACAATAACCTACAAGCTCTGTCACATTGGGATGATGCAAATGGGATATGCTTGAAATCAGTTCTACAAAATCTTCAGATAAATCATTGGGAAGGACAGATGAATCTATCTTCTTGACGGCAAGAACCTTTCCATCATCAAATTGAGCACGGTACACACGTCCAAAAGACCCTTCACCAAGAAGTTGATCCACACTGAAGCTTCCAGTAGCAATCTGCAGGTCAGCTATAGAATAAGATTTTACATTTGTAGGAGCTGTAACAGTCTTCCTGACTGTAGTGGGCTTATTGACTGTAGTGGACTTCTTTGAAAACTCATCCTCGTCAAATGATTTATGACGATCAAATGGTGGAGGTTTAAGACTTATTGGGGCCGCAGATGAATCAAACGTCTTCAAGTCAACTATAGATGAAGTTTGAACGGAATTCATTTCATGCACGTCATGTGTATGAGAAGCCAAGGGCTGATTGTCAAGCTTTTCAATGTCTTTAAACGACTTTTTGGATCTTTTCTTCACCAGAAAGAATGCTACTATTGCCCCAACAACCAAGATGGAGATCACTATTCCAGCAATGCCACCACCTCCAATACCAGATTTTTTGCCTCCATTGTCGCTTGAGCCAGCATCTGATTGGGTATGTCCACCAGATTTGTGATTTCGATTTCTTGTTTCTACTGGAGGTGCCCCAGGAGGAGGTGGGGGTGCAGGTCCTGAGCTCCACTCATTACCATCCTTTTGCAGGTTTATGCTATTCAACTGTTCTGGTATCCAGCCTGTAAAATGATTGTTTCCCACATTCAGAATTTCAAGAGGCAGATTAGCAAGGACATTAATGGTGCCTGTAAACTGGTTGTTTTGCAGATACATGGTGTTTATGCTAGAAAGTGAGCTCAAAGTCTGAGGCAGGTCACCAGTCAAAGAATTGAACGAGAGATCCAATGTAGAGAGGGAAGATAGCTTTTGGAAATCAACATTAAGTGCTTGCTGGAGCTGATTGTGACCAAGATTCAGGACTACAAGCGAAGTCTTTTCAGAAAGAGAATAAGGGATTCCCCCATTGAAATTATTATTAGCAAGATTTAAGTACTGCAGCTTAGGAGGAAGTTGGTATGGTATGGTGCCACCAAGATTGTTGTTACTCAAGTCTAGGTTTGTAACCGATGACAAGGCTTCTAGCTGGTAACCCAAAGATCCGGAAAGTGAACGACCAGGTAACTTAATCTCTGTGACTCGATTGCCAGAGCACATGATGCCTTGCCAAGACTGTCCACAAGGATCATCCCCATTCCAATTTAGCTGGGACGGAGAGTTCATACTTTGAAACATAACCTTTAGAGAAGCAACGTCATTTGGATCTGTGTCACCATTGACGGCAAAAATGCAGGTGCTGATGAACATCACCAGCACCAGCCTTGGATCCAACAGCATCTTTCTCGTGAATCAATATTTTCTCGCAAAAATTCTTCCAACCCGAAAGCAAAAAGGATGTGCAATGAACCCCTTTTCGCTAATTTTTAATTTTTCGCAAAAATATTTTATGGGTTGATGCCTATATATGTTGCCACCATTGTCGAGTAAAAGCTGGCAAGCACGAATCGCCTTATCAAAT

>C102281_65

GTCGTTCTCTGATATCGTGGCACACGCTATATTGTAAAATGAAGTCTTAGGTTAATCTCAGCCTAGATATATATGATACAAAGAGAACATGTTTCCATACTGGTCACCAGACATACAACGAATGTTCAATCGAAAGCATGGGGGGAATTTCGTTCATTTCAGCCACCACTATAATAATATTACAAAAAACCTTAGCCCATCAAAGTAGGGAAATATTAAATTTAAACATAGATTGGGTTGAAACTGGAAAGAGCATGCTTGAGAGATAAGTCAGATAACCATGATAGGCTATATCAAAAAGCTCGATTTCTCCCCATGCATAATCTACAACCAGTCTCCCTCCAAAATCATCACCCAAAATGAAGGGGTCAAAAAGTTGTCGACCAGAAACTCCTAATATGTTAGTAAATCACCAATTCCTACCAACATTACACAACCTTTATTGATGTGGTAACTAGACCACATACACAAACCCATATCCCCCACAATGATTAGCACTGTAAAATAGTACAAAATGATGGCTTGAAACACATAGAAGTAAATTGAAGACCATAAAACAAGAGGGGTGAAAATTCATCCTGTAGGTGTGTATCCGCACCTATACCTAAGATGGTATGCAATGCTATGAAAGGAAAGTCAGTCCCAAGCACTAGTGACGCCAGGACCATACCCTCCAGCATACCCACCAGAGCCTCCATATCCACCACTGCTGTTTCCTGCACTGTAATAATCTGAAGAACCTCTACTAAACGAGCCCTCTCTTCGAAAGTCACGACTACCAAACCGACCACCTGAGCGGCGGTTCCTCCCTCCACCAAAAGATCGAGCAGCATACCGTGAGAGCCAAGCAGGTACTTCTTGATTTGCTTCTTGCATCAGATCTGCTAAAGCTCTAGCTAGTGATGAATTGTTATCATTAAAGAATGCAGTTGCAAGGCCTTTCTTTCCAGCTCGCCCTGTTCTTCCAATTCGGTGTACATAATCATCAATATCATTTGGCAGGTCAAAGTTGACCACATGAGCAACATGGGGAATATCAAGACCACGTGCAGCAACATCGGTGGCAACCAAAATGGGGGTGTTACCACTTTTGAATGACCTCAATGCTAATTCTCTTTCCTGCTGCGACCTGTCACCATGAATAGTAGTTGCAGGAAAACCATTGATACACAACCAGTGTTCCAGTGAATCAGCTCCCTTCTTTGTCTCCACAAAAACTAAAGTCAAAGCTTGCTTTCCCTGTACACCATTTGCCCTCTGTGCATGAAGAAGGTCCATTAGGTGACTTCTCTTGTCAGACTCTTGAACATACTCAACTCTTTGGACAATTAGATCAGTACTTGAACCCACTCTTCCAACAGCCAGGAAAATATAATTTGAGAGAAAATCAGAAGCCAATCTCTGTATCTCTTTCGGAAATGTTGCACTGAACAACATAGTCTGTCTAACGCCTGGTGGAGGCATGTCCATTTGTTCTACAATCTTCCTTATTTGTGGCTCAAAACCCATATCCAGCATCCGATCTGCCTCATCCAAGGCCAGATATCGAATCATCTGTAGTGAAACTCTAGCTCTCTCCAGCAAATCTACCAGTCTTCCAGGAGTTGCAACAAGAATGTCCACCCCTCTTTCGAGATCCCGCAGCTGCTGGTGTATTGGTGCTCCACCATAAGCAACAACCACCCTAACACCAGTTTGGTATGAAAACTTCCTAGCCTCTTCATGTATTTGCATTGATAACTCCCTCGTTGGAGAGAGAACAAGCGCTAGCGGATACACAGTTCGCACCCCACGAGGTGGCCTCTGGACAGGTTGGCCCCTCATAATTCCACTGATGATCGGGAAACAGAATGCAGCTGTCTTTCCAGAACCAGTCTGTGCACAAGCCATCAAATCCCGTCCAGCTAGAGATATTGGTATGGCATGCCGCTGAACAGGTGTTGGTTTCACATATTTGCACCTTCTTATATTCTGATTAAGTGCTTCGCCCAAGTCAATCTCTGCAAATGTATTCACAGGTGGGGGCACATTTTCACCGCTGGTCTCCACTGGAATGTCCTCATATGCATCAAAATTTATTCCTGTATTCTCCTGCTCACTAAATGCCTCTTCAGCATTATCCTCTTCTTCAAAGGGATTGACTTCCCGTTCCCTGCCACGATCCCACCCACCACTTTTATTTCCCCATCCAACTGGGCGTCCACCACCACCATACCCAGAGCGATAATCATTTCTGGGTGCAGGCCAACGGGATCCAGAATTACCCGCACCAGCACTAGAGGCAACAGAACCAGAGGAAGGGCCACTGTATGCTGGTGCGGGGGCTTCTGCGGATGGCTGGCGGTTCCTGAGATGGGGAGGAACATATACCGGTCGTGAAGGAGCTAAAGTACTACCAGTTCCCACACTGTTAGCAGAAAAACCGGGACCTGCATTTTCAGCTGCAGAATTTGCAGCCAAATCAGCCCATGAAGTTCGCATAGTCAATCACAAATTCTTGTTGTCAACAGCCGTCTTCAAAATTCAAAGGGAAAACCCTTCCTTTGCAGATTCGTGATAACACCTTTACAAGTAGCCCCAAATAAATAAATAAAAAAGTTGAAAAAGATAAATACACTTCAAAACGAACAAAATTGCTCAGAACCAACCCTGTGTCTGTGTTGGGAAGGTAAAACCCTGAAATGGTGAGAAGAGTGAGAGATGAAGAAGATCGAGAAACCCTAATTTGGTTTTGGTTGCAGAAAGAGAGAGAAGGGAGGGCGCTTTCAG

>C102283_65

CTTTCTCTTCTTGAGAGAAAAAAAGTGTAACTTTGTTGTTGTTATTGGGTTTAAGAATTGCAGCAAGATGTTTTCAGGAGTGACTGGTCTCATCAACAGGGGGCAGAAGCTGAAAGGAACAGTGGTGTTGATGCGTAAGAATGTGTTGGACATTAATGCCCTGACTTCTGTTCAAAGTCCCACCGGCATCATCGGTGGTGCAATCGGTGTTGTGGGTGGTGCCATTGGCAGCACAGTTGACACTCTCACTTCCTTTTTGGGTCGTTCTGTGGCTCTCAGGTTGATCAGTGCCACTGCTGCTGATGCATCTGGAAAGGGAAAGGTGGGAAAGCAAACATTTTTGGAAGGTATTATTACTTCCATAGCAACCTTGGGAGCTGGCCAGTCTGCATTCGATATTCATTTTGAATGGGACAGTGACATGGGAATTCCTGGAGCATTTTACATCGAGAATTTCATGCAAGTTGAATTCTTCCTTGTCAGTTTGACTCTTGAAGACGTTCCTAACCAAGGAACCATCCACTTTGTTTGCAACTCATGGGTTTACAACGACAAAAAGTACAAATCTGACCGCATTTTCTTTGCCAACAAGGCATACCTTCCAAGTGAAACACCGGGTCCACTGGTGAAGTATAGAGAAGAAGAATTGAATACTTTAAGAGGAGATGGAACAGGAGAGCGCAAAGAACATGAAAGAATCTATGACTATGATGTCTACAATGATTTGGGCGATCCTGACTCCAATGACAGGTTGGCTCGTCCAGTTCTTGGAGGATCCACATTGCCTTATCCTCGCAGGGGAAGAACTGGTAGAAAACCAACTAAGAAAGATCCTAAAAGTGAGAGTCGCAGTGACTCTGTTTATCTTCCAAGGGATGAATCATTCGGTCACTTGAAGTCATCAGATTTCCTTGTTTATATACTTAAATCTGCGTCTCAAAATGTGATACCGCAATTACAATCTGCTCTTAGACTACAATTCAATCAACCTGAGTTTACTAGCTTTGATGACGTGCGTGGACTCTATGATGGTGGAATTAAGTTGCCTACTGATGCACTTAGCAAGCTTAGTCCTATACCATTGTTCACTGAACTCTTCCGTACTGATGGGGAGCAGGTCCTTAAGTTTCCAACACCTAAAGTAATTCAAGTGGACCAGTCTGCATGGATGACTGATGAAGAATTTGCAAGAGAGATGATTGCTGGTGTAAATCCTCATATTATTAGAAAACTTCAGGAGTTCCCACCAAAGAGCAAGCTAGATAGCCAACTCTACGGTGATAACACCAGTACAATTACCAGAGAACACTTGGAGCCTAACTTGGGTGGGCTCACTGTAGAACAGGCTATCCAAAACAACAGACTGTTCATACTAGACCACCACGACACACTTATTCCATATTTGAGGCGAATCAACGCAACAGAGACAAAGGCCTATGCTACCAGAACAATCATTTTCTTACAAGATAATGGAACATTAAGGCCATTGGCCATCGAGCTGAGTAAACCACATCCTCAGGGTGATAATTTTGGTCCTATTAGTAATGTTTATCTTCCAGCAAACCAAGGAGTTGAAGCTTCCATTTGGCTACTTGCAAAGGCTTATGTGATCGTAAATGACTCTTGTTATCACCAACTTGTCAGCCATTGGTTAAACACTCATGCAGTTGTTGAACCATTCGTCATAGCAACAAACAGGCATCTTAGTGTGGTTCACCCTATTCACAAACTTCTTCTTCCACATTACCGTGACACAATGAACATCAATGCACTTGCAAGGAATGTCTTGGTCAATGCAGAGGGTATTATAGAATCTACGTTCTTGTGGGGAGGCTATTCTTTAGAAATGTCTGCAGTTGTATACAAGGATTGGGTTTTCCCCGAGCAAGCACTTCCTGCTGATCTTATTAAGAGAGGTCTGGCTGTTGAGGATTCATCTTCTCCACACGGCCTTCGTCTTCTGATAGAGGACTATCCTTATGCTGCTGATGGACTAGAGATATGGGCTACTATCAAGTCATGGGTGCAAGAATACGTCTTTTTCTACTACAAGTCAGATGCTGCTATTACACAAGATACTGAACTCCAAGCCTTTTGGAAAGAACTTGTACAAGTTGGTCATGGTGACAAGAAAAATGAACCATGGTGGGTAAAAATGCAAACTCGTGAAGAGTTGATTGAATCTTGCACCACACTCATATGGACTGCTTCAGCCCTTCATGCAGCTGTCAATTTTGGACAATATCCATATGGTGGCTATATCCTTAACCGACCAACTCTAAGTAGGCAATTCATGCCTGAGATTGGATCCCCGGAGTATGATGAGCTTGCTAAGAACCCTCAAAAGGTGTACTTGAAAACAATTACAGGGAAGAATGAGGCCCTTAAAGACCTTACTATAATAGAGGTCTTGTCAAGGCATGCTTCTGATGAGTTGTACCTTGGACAGAGAGATGGTGGTGAATTTTGGACTTTTGACAAAGAGCCATTAGAGGCCTTCAAGAGGTTTGGAAAGAGGTTGGCAGAAATTGAGCAAAAGCTCATCCAAAGAAATAATGATGAGACACTGAGGAACCGATATGGTCCAGTGAAAATGCCCTACACATTGCTCTATCCTTCCAGTGAAGAAGGATTGACTTTCAGAGGTATTCCTAACAGCATCTCCATCTAAAGAGGTTTATGGTTATGTTGCAATTTAAATAAAAAGAAAAGGAGTCCAATTCCTTTGTTCCCGTTTATGTTTTCTGTGTGTATGTTTGATGTTCTGCAA

>C102289_65

CCATTCTCTGTCACTCTCTAGCTCGTACCAGAGGCTTCGAAAATGGACTTGAGCGAAGAAGTTAGGGCAGCGCACAAGCGAGAGTTCGCCGATTTCTTGGATCAGGATGTGGGAAAGGGCATATACATGGACGAAATCAAAGCCCTAATCAACCACAAGCGCCACCGCCTCATCGTCAACATTTCCGATCTTCACAACTTCCGCGACTTGGGTAACAGGATTATCAGGAATCCAAGTGAGTACATGCAACCGTTCTGCGACGCGGTGACGGACGCTACGCGTGCAATTGATCCCAAGTACTTGAAGGAAGGGGAACAAGTGCTCGTGGGATTTGAAGGTCCCTTTGTTTCTCGCCGAGTCACACCAAGGGAGCTTCTCTCTGAATTCATAGGCTCTATGGTCTGTGTGGAGGGCATTGTCACCAAATGTTCTCTTGTAAGGCCGAAGGTTGTTAAAAGTGTTCATTTCTGCCCCACCACGGGAAACTTCACTTCTCGCGAATATCGTGATATTACATCTAATTTGGGTTTGCCCACAGGATCTGTTTACCCGACAAGGGATGAAAATGGCAACTTACTTGTGACCGAGTATGGATTGTGTAAATACAAAGATCATCAAACTTTGTCCATTCAAGAAGTTCCTGAGAATTCTGCACCTGGTCAACTCCCAAGAACAGTGGATGTTATTGTAGAAGATGACCTTGTTGATTCTTGTAAGCCTGGAGATCGAGTGGCAATTGTGGGAATATATAAGGCTCTTGCCGGGAAAAGTAAAGGCAGTGTTAATGGAGTATTCAGGACTGTGCTCATAGGCAACAATGTTTCTCTTTTAAACAAAGAGGCTAATGCACCATTCTACAGCCCTGAAGATCTCAAAAACATTAAAAAGATAGCTGAAAGAGATGATGCATTTGACCTGCTAAGTAATTCGCTTGCACCTTCTATATATGGACATTCTTGGATAAAGAAAGCAGTGATTTTGTTGATGCTTAGTGGAGTGGAAAAGAACTTAAAGAATGGCACTCACTTGCGAGGTGACATTAACATGATGATGGTTGGTGATCCCTCTGTTGCAAAGTCTCAACTCTTGAGAGCAATTATGAACATTGCTCCCTTGGCCATATCAACTACAGGCCGGGGTTCTTCTGGGGTTGGTTTGACAGCAGCAGTTACTTCAGATCAAGAAACAGGGGAAAGAAGGCTAGAAGCTGGGGCAATGGTTCTTGCCGACAGAGGTGTTGTCTGCATTGATGAATTTGACAAGATGAATGATCAAGATCGTGTTGCTATACATGAAGTTATGGAGCAGCAGACTGTAACTATTGCTAAAGCTGGTATCCATGCATCACTGAATGCCCGATGCAGCGTGGTTGCCGCCGCAAATCCCATATATGGAACTTATGATCGTTCATTGACTCCAACCAAAAATATTGGACTTCCAGACTCCCTGCTTTCTCGATTTGATCTGCTGTTTATTGTGTTAGATCAAATGGACCCTGACATTGATCGTCGAATATCAGAGCATGTACTTCGTATGCATAGATTTCGTTCTGCTGTTGATGGAGGTGAGGCAGCGCTTGATGGGAGCTCAAGATATGGAAGAGAAGACGAAGCTGATGGGGACTCATCTATCTTTGTCAAATATAACAGAATGTTACATGGGAAGAAAACTGACAGAGGTCGCAAGCGTGATACACTTACAATTAAGTTTCTTAAGAAATATATCCACTACGCTAAGCATAGGATTCAACCTGAACTGACTGATGAGGCATCTGAGAACATTGCCACAGCCTATGCTGAACTCAGAAATGCAAGTTCAAATGCAAAGACTGGAGGAACACTTCCCATAACTGCCAGAACTTTAGAAACCATCATACGTCTCGCAACAGCTCATGCCAAATTGAAATTGAGCAGAAAGGTTTCAAAGTCTGATGTTGAAGCTGCTTTGAAGGTTCTAAATTTTGCTATATATCACAAAGAACTTACAGAAATGGAGGAGCGTGAGCAAGAGAGGGAGAAAGAGCTAGACAGGAAGCGCAAGGCTGATCACAATGAAAATGATGGTCCTGATCGTGGTCCTAAAGATAGAAGAGGATCAACATCAGCAGCAGATGCCATTGAAGTAGATGATACCTCAGCAGCTTCAGCTGCTGTTGGACTCACCCCTGCAAGAATTGAAGCATTTAATTCTGTATTTGGTCAGCATATGCGTGCCAACCGCCTGGATCTCATAGCTATTGCAGACTTGGAGGATGTTATCAACAGAGGGGCGGATTCGCCTTACAGCGCCGCAGATATACTACTCCTGTTAGAGAGGTTGCAAGATGACAATAGAGTGATGATTGTTGATGGAATGGTGCATATGATATCATAAAATGTAAGATTGTTGCACAATCTCTCAACGGATATTCAAGAAGGGAGCAGAATTTGCCGCCATTGTAGTACGCAGTTCACAACGAGGACACTTGAGAAGGATTTACAACTTCCAGAGTTGATATTTACTTGCCTTCTGGGAGGTCTTTTCTAACACTTGGACAAGGAAATAGCATAGGCGATTCATTGGCATATTCTTCACAGAACATTTTAAATTATAATGAACTTTGTCATCCCCTGATGGAATTTTTCTTGTTTTGTCCATAATTTGATACTGTCCTTATCTTTATGTATTGCAGTGTTGCTAACTTGCTAAGTATACGTGTAAACAAGGCTTTAGAGAAGACTGGTATCTATAAGTTTATGTTCCGTCATTTGTGATACTTAATTTTATTGACA

>C102291_65

ACCGAGAGCGCCTTCTTCGCTCGCAAAAGAAAATCACAATGGCGTCACCAGCGGCGGCGAGGTCGCCGGTTTCCGCCAGTGCTTCTCCGATCCAACGCCTCTCCACCTTCAAGAATCCCTCCGCCGGCGCTGTGAGTGTCACCACTCCGACATCCTCGGCCCTCGAATCTCTCGCCTCGGATCCCATCTTCTCGGCCTTCCTCTCCCCTTCCTTCTCCTCCACCTCCTTCTCCTCCGCCGCCCTCTCCTCCGGCTCCCCGGCCTCCACCGCCGAGAAACTCCACCATGCGATTCGCCTCCTCGAGAACCAGCTCCGCTCGGAGGTCCTCTCGCGGCACCACGACCTCCTCTCGCAGCTCTCCTCCCTCCACCATGCCGACCACGCCCTCTCCACTCTCCGATCCGCACTCTCCTCGCTCCAGTCCTCCGTCCGCCGCCTCCGCTCCGAGCTCTCCGACCCGCACCGCTCCGTCGCCGCCAAAACCACGCAGCTCTCCAACCTCCACCTTACCACAGAGCTCCTCCAGCACTCCATCCGCGCCCTCCGCCTCTCCAAGAAGCTCCGCGACCTAATGGCCGTCGACCCCGAAAAACTCGATCTCGCCAAGGCCGCGCAGCTGCACTACGAGATCTTAAGCCTCTGCAACGAGTACGACCTGGGCGGCATCGATGCCGTCGACGAAGAGATCAATTGGGTTAGGGAGACCGGAGATTTGCTTCGGAGCGAGGCGATGAAGGTTCTGGAACGCGGAATGGAAGGGTTGAACCAGGCCGAGGTTGGAACAGGATTGCAGGTTTTCTACAATCTGGGAGAATTGAAGGTCACTGTGGAGCAGGTGGTGAACAAGTACAAGGGTTTGGGTGCAAAGAGTGTGAGTGTGGCGTTGGATATGAAGGCTGTTACGGGTGGAAGTGGATACGGTCCCGGTGGCATTAGGGGTTCTGGAACGCCACACATTGGAGGAGGGGCTAAGGCTAGAGAGGCTCTGTGGCATAGGTTGGGGAATTGCATGGATCAGTTGCATTCTATTGCTGTTGCTGTGTGGCATTTGCAAAGGGTGCTCTCCAAGAAACGTGATCCATTTAGCCATGTTCTGTTGCTTGATGAGGTCATTCAGGAAGGTGATCCCATGCTAACAGATCGTGTGTGGGAAGCCATTACTAAGGCTTTTGCAAGCCAAATGAAGTCTGCTTTCACTGCATCAAGTTTTGTTAAAGAGATTTTTACAACGGGATATCCAAAGCTTTACTCCATGATAGAGAATCTACTTGAGAGAATTTCACATGACACAGATGTCAAAGGAGTGTTACCAGCTATCAATTCGTCTGGGAAGGAGCAGATAATTTCAGCTGCTGAAATATTTCAGAATGCATTTTTGGCCCATTGCTTGAGTCGCCTTTCAGATCTAGTTAATTCTGTATTTCCAATGTCTAGTCGTGGTAGTGTTCCCTCCAAGGAACAAATATCAAGGATTATATCACGCATTCAGGAAGAGATAGAAGCTGTTCAGGTGGATGCACGCTTAACTCTTCTTGTTTTGCATGAAATTGGCAAGGTTTTAATTCTTCTTGCAGAACGGGCGGAATACCAGATATCCACTGGTCCTGAATCACGTCAAGTAAGTGGTCCTGCAACACCGGCACAGCTCAAGAACTTTACATTATGTCAGCATCTTCAAGATGTTCATACCCAAATATCATCTATGCTTAAAGGAATGCCTAGTATTGCTGCAGATGTGTTGTCTGCTTCATTAGGTGCAATATACGGTGTTGCCTGTGATTCTGTAACATCTTTATTCCAGGCAATGCTGGATCGTCTTGAGTCTTGCATATTGCAAATACATGATCAGAATTTTGGAGTGCATGGAATGGATGCTGCTATGGACAACAATGCATCACCTTACATGGAGGAGCTCCAGAAGTGTATTCTTCACTTTCGCAGTGAGTTTCTATCTAGGTTGTTGCCTTCCAGAAATTCAGTCGCTCCTGGGGCAGAAAACATATCCACTAGGCTTGTCCAGAGTATGGCTTCACGGGTTCTAGTATTCTTCATCCGACATGCTTCCCTTGTCAGACCACTTTCAGAATCAGGCAAGTTGAGGATGGCTAGGGATATGGCTGAATTGGAGCTAGCTGTGGGCCAAAATTTGTTCCCTGTTGAGCAACTTGGTGCACCATATCGAGCACTTAGAGCATTTCGTCCACTTATTTTCTTGGAAACATCCCATCTTGCATCATCTCCTCTTCTTCAAGATCTGCCACCGAATGTCATACTTCATCATCTGTATACCCGTGCCCCTGAAGAATTGCAGTCACCACTGCAAAGAAACAAACTAACACCATTGCAGTATTCGTTGTGGCTAGATTCTCAATGGGAGGATCAAATCTGGAAGGGCATCAAAGCAACACTTGATGATTATGCTACAAATGTGAGGAGCAGAGGAGATAAGGAGTTTAGCCCTGTTTATCCTCTCATGCTTCAATTAGGTTCGTCTTTGATTGAAAAGGATCAGACTTCCTGAAAGTCTTGATCCAGTAAATAATTTGTAGTCTAGCAAATTAAACCACATCAGCGACTTCCCATTTTTGCTTCTTGAAAGTTGTGGAAAAGGGTTTTGTTGCGGAAACACCATCAAGAGGCTGTTAGTTCTGCTAGCAAGCATGAGGTACAATCGATTAAGTGATTTATAGTTGTTTTTTTTTTCTTGCTTTTCTTTGTTCATTTCTATTCTTCTTCCCGCCTCTTTTTTTTTATGGAATTCCTGTTGTTGG

>C102297_65

AAAAAACCGCATCCCCAGCCGTTACCGTACACCCTCACACACCATCCTCCTTCTCCTCACTCGCTACTCACTCATTCATTCACACTTACACACTCATTTTCACTCTCTCTCTCTCTCTCTCTTACACTTCTTCTTCGTCATCATCTTCTTCTGCTTCTTCATCACCTTGTTTCAATCTCACTTGCTTTTTGCAAATCACATTCTAGGGTTAGGGTTTCCGCATTATGATGTGAAACCCTAGCACGTGTAGTCGACGCTCTCCTCGCAAACCCTCCCCTCCGCCGCGATCTCCGTTCCTCCTCCATGGGAAACTGCTGCAGATCCCCGGCCGCCGTGGCCCGAGAGGACGTCAAATCGAGCTTCTCCAACGCAGACCACCACGGCAAGCGCGGCGCCGGCGCCGACGCCGTCAAGCAGAAGGCGCCGATCACGGTTCTCGCCGGCGTGCCCAAGGAGAACATCGAGGACCGCTACCTCGTCGACCGAGAGCTTGGCCGCGGCGAGTTCGGGGTGACTTACCTCTGCATCGACCGCGACACGCGCGAGCTGCTCGCGTGTAAGAGCATCTCTAAGCGGAAGCTCAGGACCGCCGTCGACGTCGAGGACGTGCGCCGCGAGGTCGCCATCATGCGCCACCTGCCGGAGAGCCCCAGCATCGTGTCGCTCCGCGAGGCCTGCGAGGACGACAATGCCGTCCACCTCGTCATGGAGCTCTGCGAGGGCGGCGAGCTCTTCGACCGGATCGTTGCCCGCGGCCACTACACCGAGCGCGCTGCTGCCGCTGTCACGAGGACGATTGTGGAGGTCGTGCAGCTCTGCCACAAGCATGGGGTCATCCACCGGGACCTGAAACCGGAGAATTTCTTGTTTGCAAACAAGAAGGAGAATTCGCCGCTCAAGGCTATTGACTTCGGGCTTTCCATATTCTTCAAGCCAGGTGAAAGGTTTTCAGAAATTGTTGGAAGTCCATATTATATGGCTCCAGAAGTTCTCAAGCGGAATTATGGACCAGAAATAGATATATGGAGTGCTGGAGTAATTCTCTACATCTTACTGTGTGGTGTTCCCCCATTTTGGGCTGAATCTGAGCAAGGAGTTGCACAGGCCATTCTTCGAGGGCTTATAGATTTCAAAAGGGAACCTTGGCCCAGTATTTCTGAAAGTGCTAAAAGTCTTGTTAAGCAAATGTTAGAACCAGATCCTAAGCTTCGATTAACTGCCAAACAAGTGCTTGAGCATCCCTGGCTCCAAAATGCTAAGAAGGCTCCTAATGTTCCACTTGGAGATGTTGTCAAGTCAAGGCTTAAGCAATTTTCAATGATGAACAGATTCAAAAGAAAAGCCCTTAGGGTCATTGCTGATTTCTTATCCAATGAAGAAGTTGAAGACATTAAAGATATGTTCAAAAAGATGGATAATGATAATGATGGCATTGTTTCCATTGAAGAACTGAAAGCTGGATTTCGAAACTTTGGATCTCAACTTGCTGAGTCTGAAGTTCAGTTGCTTATTGAAGCTGTAGATAGTAATGGTAAGGGAACACTAGACTACGGAGAATTTGTTGCTGTTTCCCTTCATTTAAAAAGGATGGCTAATGACGATCATCTTCACAAGGCCTTCTCCTATTTTGACAAAGATGGGAATGGATATATTGAACCGGATGAGCTACGGAATGCCTTGATGGAAGATGGAGCAGATGATTGTACAGATGTAGCAAATGATATTTTCCAAGAAGTAGACACGGACAAGGATGGACGTATCAGCTACGATGAATTTGTTGCTATGATGAAAACTGGAACGGATTGGAGAAAAGCATCAAGGCATTATTCACGTGGGAGATTCAATAGCTTGAGCTTAAAGTTGATGAAAGACGGTTCTTTAAACTTAGGAAATGAGTAGTTGTCTAAGTGTTTGCCTATTATTCGATCTGGCTTTCCAAATAGTGTAATATTCCTCCTACAATATGGACTTGGCTTCTTTGTGATGATTATTTGGTTCCACTTTTAGAAGGGTTCCTTTCCACTTGGTGCTGTGAGAGTTACGAAGGATTGGGTACGCCAATTATACACGCAATTTGTTACTTGTGTAAAACATGGTGCCTTGGAGCCACTGGTGTGACATGTATGATTCCCCTCGTTTTAACCACTCCTGCCTTCTCTTCTGGTGTGACAATGTTGAATCAAAACTCTTTAGTGCAGTCAGAATATGTTTATCAAAGTGCAAATATCTTGTAGTTTGAATTCAGGACTCTGTTTTGGTCATTCTATCACTCCAACCACTGTGCCCGATAATGGCTAATGGCCAAAAAACGTTTGTTTTCTCCAAGGTGCTTACTCTGGGATGCTGACATTTAATTTGCTTGTCTAGTAGCATGAGCCTGTAAGATATTCTGATTCTGGAACTGTGCAGATATGGATCTTGTGTAAAACAGGGTAACTTTGCCGTTGTTGAATTTGTGGGTACACTGTTTTGGCTACTTACGTGACTGAGTGCTAGGAAGTACAAGTGGCAGGTTTGTTGTATCGTTAGACCTGATCTGTGAATGCAACTGCGTAGGGATAAGAAGACCATTAAAAAAATTAGAAGGAAAATGGTGGAAGAATGTTTTACCGATGCATTTGCAACACTATTGTGTGTCATTCATTGAAGTCTTTCCAGAGTTTAGGGTTTTAGGCCATCCTTGATCAGGAGGTGCATATCTAAAGATTTGAGTCTTGGTCCTATGGATCTTCAGGTATTTCATCCATAGCAACCATAAGCTGTAATGGGCCTAA

>C102299_65

ATCACATAACAATATAACAATAACAATATACATTACAATTACAAATGCTCTCTTTACACTCTCTTCATTCCTTTCTTCTTCTTCCCTTCACCTTCAAACCCCCTTAACTATTACCATTACCATATCTTTTACTTCCACCTTTTTCTTTCCCACACTTAACCCATTTTCCCTTCACTAATCTCTCTCCACTTCTATTCCTCAACTCTGATTTTTTTCCTGTTTTTATTCTGTCATCTTTCATCCAATTGTTAAAGAAGGACCCAAACAAGTTCCTTTAAGCTGGAGCATCAAAATCCCATCACTTTGCTTTCTAGTTTCTGCTCAATCTGGATTTTCCTTTCCTTTTCTCCAACTGAATTTGTACTTGGTTGTACTATTCTCCTTCAAACATAGATCAAGTTGCTTCCTTTCTCTGGATCAAGCACGCTCTTTGCTTCATTTTCGTTGGCCCTTTTGCTCTGAATACGGGTCATTTAAATATTATCAACCAATGATGATTCTGGTGCGAAAAACCCATTGGTTGTTGCTTCTCTTTCTGCTCCACCTTCACTGCAGCACCTGTTACTCCACAGAACCTATGAACTGTACCGACACAAGCCGGGTATGCACTTCTTTTATGGCCTTCAAGCCTGAGCCAAACCAAACGCTTGCTTTGATTCAGAGCATGTTTGATGTGTTGCCTGGTGACATCACAGTTGAAGGCAACGGGTGGGGATACATGTTCATAAGGAAGAATTGTTCTTGTGCTGCGGGTATCAAGAAATACGTGTCGAACACCACTTTCACTGTGAAATCTAATGAGGGGTTGGTGTATGACGTGGTGATGGATGCCTATGATGGCCTTGCTTTCCTTCCCAACACCACCAGGAGGGCCAAGAATGGTGCTGTTGTGTCTCTGAGGTTGTTTTGTGGCTGCTCCAGTGGACTGTGGAACTATATGGTCAGTTATGTGATGAGAGATGGGGATAGTGTTGAGTCCTTGGCAAGCAGGTTTGGGGTTAGCATGGATAGCATTGAGAGTGTGAATGGCATTGGTAATCCGGATAATGTCACTGTTGGTTCACTTTATTATATACCCCTTGATTCCGTTCCTGGTGATCCTTATCCCCTGAATAATGCTGCTCCACCAGTTCCTGTTCCTGCCCCATCCTTTGATAATTTTTCAGTTGATCAAGACAAACATAAGGCTCATTTACCCTACGGATGGATCATTGGGGGCTTAGGAGTTGGTCTTCTTCTAATAATATCAACTGTGATTCTCTGTGTTTGTCTGAGATCATCAAATTGTTTTGCTGATGATGCCAGAACTCACGAGAAAAATGCAGAGGGCAAGATTTCACATAAGTTCCATATTCTAAGGAACCCAAGTTTTCTTTGTGGTTCTGGAAGGTACATATGTGGCAAGCATATTGAACCGAAGCAAACAGATGCTGAATCCAGCAATCACACGATTACCATTCCAAAAGCCTCAACTCTTGGGCCTGACGCATTTGAAATGGATAAGCCTGTTGTTTTTACATATGAAGAGATTTTCTCCTCATCTGATGGTTTCTCTGATTCAAATCTACTTGGGCATGGAACATATGGTTCTGTTTATTATAGCCTCCTTCGTGATCAGGAAGTTGCTATTAAAAGAATGACTGCTACTAAAACAAAAGAATTTATGTCAGAGATGAAAGTTTTGTGCAAGGTTCATCATGCTAATCTGGTAGAATTGATTGGCTATGCAGCTAGTCACGAGGAGCTTTTCCTAGTTTATGAATATGCTCAAAAGGGTCCACTTAGAAGCCATTTGCATGATCCTCAAAGTAAGGGCCATTCCCCACTTTCTTGGATCATGAGGGTGCAGATTGCCCTTGATGCTGCTAGGGGCCTTGAATACATACATGAACACACAAAAACTCATTATGTACACCGTGATATCAAGACCAGTAACATATTACTTGATGCTTCCTTTAGAGCAAAGATTTCAGATTTTGGTTTAGCAAAACTAGTTGGGAAAACAAATGAGGGAGAAATTTCAACTACCAAAGTTGTTGGTACATATGGATATCTTGCCCCAGAATATTTGAGTGAGGGTCTTGCCACAACTAAAAGTGATGTCTATGCATTTGGTGTTGTCCTTTTTGAGATTATATCAGGAAAGGAGGCCATCATTCGATCAGAAGGCACGACGACAAAAAATCCTGAAAGACGTTCACTGGCATCTATAATGCTGGGAGTTCTTAGGAATTCACCTGATTCCATGAGCATGTCAAGCTTGAGAGATTATATTGATCCAAATATGATGGATTTGTATCCCCACGATTGTGTATTTAAGATGGCCACGCTGGCAAAGCAATGTGTGGATGAGGACCCCATCTTACGACCTGATATGAGACAAATTGTGATTTCCCTCTCACAGATTCTCCTTTCTTCTGTTGAGTGGGAAGCAACTCTAGCTGGGAATAGCCAGGTTTTCAGTGGCCTTGTTCAGGGAAGATAGTTTACACAGTCGGATTGATTCTTTTTTTCTTTTTTTCTGTATCCAGCTGCATACCTTTTTGGTTGGTTGATGATAGTGATGGATCCATAGGAACTGAGACATGTGGGTCAATATCTATTTTTGGCCATTTATTTTGCTTAATCTCTTATGAGCCATGCCTTGATTTTTGTTTAGCTGCATTCCTATATGTAGAAAATTTTGATGTATTTTAGAGTGTGATACCAAAGCTAAAGAAGGATCTGATAATTTATTGCTCTGTGATTTGTATTCATATAAGTTGTAATTCGAC

>C102301_65

AAAAAACCTGAGGCAAAAAGAACGAACCAACCCACGATGTCGCCGAGCTTCGTCTTCGACCCTCCCAGCGACGAAGAAATCGAACACTCCGAACGAGAAGAGGAAGAAGAAGACGAAGAACAACCAGAAGAAGCTGAGTCAGGATCAGATTCTGAGTCAGAAGAAGAAGAAGAAGAAGAAGAAGAAGAAGAAGAAGAAGAAGAAGAAGGGCACAAGGAGGCGCGGGTTTCGAAGAAGAAAACACAGTCTCCATGGGACTTCGCTAAATACACGGAATCAGTGGCCGAGGAACACGCTCGCAGGAGCACAACCTCGGTGGACGAGAAAATCTCCAAAGCACTACGACAACGTTCCACGCCTCTCGTTGCTGAACTTGACCACAGCTCCGAGTCTGAACCTGATGAACAAGAAGATTATAGACCGGAGGAAGAAGACGAAGAAGAGGGTAATGGTGGTGATAGCAAGTCCTTTTTTGCTCCTTCTGATGGAGCCTCTTTCCACGCTGATTCCTTCTTGCAGCTTAATTTGTCTCGTCCTTTGCTCCGGGCTTGTGAGGCCCTGGGGTATGCTAAACCAACGCCAATTCAGGCAGCATGTATTCCATTGGCGTTGTCTGGTCGTGATATATGTGGTAGTGCCATTACTGGGTCAGGGAAGACAGCTGCATTTGCACTACCTACGTTAGAGAGGTTGTTGTTCCGTCCAAAACGTATGCGTGCAATAAGGGTCCTCATTCTTACTCCAACCAGAGAGTTGGCAGTCCAAGTTCACAGTATGATAGAGAAGCTTGCTCAGTTTACTGATATAAGGTGTTGCCTGGTTGTTGGGGGTCTGTCAACAAAGGTGCAAGAGGCTGCTTTGAGAACAATGCCAGACATTGTTGTGGCTACTCCAGGACGCATGATTGACCATTTACGCAATGCTATGTCGGTGGATTTGGATGATCTTGCTGTTCTAATCCTTGATGAGGCAGATCGTCTTTTGGAGCTTGGATTTAGTGCTGAAATTCAAGAACTTGTGCGCATGTGTCCCAAAAAAAGGCAGACTATGCTGTTTTCTGCAACAATGACTGAGGAGGTTGATGAACTTATTAAACTTTCCCTGTCAAAGCCCTTGCGTCTTTCTGCCGACCCATCTACAAAACGGCCAGCAACCTTGACTGAGGAAGTTGTTAGAATACGAAGAATGCGTGAAGTAAACCAGGAAGCTGTTCTTCTTGCAATGTGCTCAAAAACTTTTACTTCCAAAGTCATCATCTTCAGTGGAACAAAGCAAGCTGCTCATAGATTAAAGATTATATTAGGATTAGCTGGCTTAAAAGCTGCTGAGCTTCATGGAAATCTTACTCAAGCCCAGCGCCTGGAAGCTTTGGAACAGTTTAGGAAGCAGCAAGTGGATTTTTTGGTTGCAACAGATGTGGCTGCCCGTGGCCTTGACATTATTGGTGTTAAAACGGTTATCAACTTTGCATGTCCACGTGATCTTACTAGCTATGTTCATCGAGTGGGTCGTACTGCAAGAGCTGGAAGAGAAGGATATGCTGTCACTTTTGTGACTGACAATGACCGATCACTTTTAAAAGCCATTGCCAAGAGGGCTGGTTCAAAGTTGAAAAGCCGCATTGTTGCCGAGCAATCTATACTTAAGTGGTCTCATATTATTGAGCAAATGGAGGATCAAATTGATGAAGTACTTCAAGAAGAGAGAGAAGAAAGAGCCCTAAGGAAAGCTGAAATGGAGGCGACGAAGGCCGAAAACATGATTGCACATAGGGAAGAGATCTTTTCCCGTCCCAAAAGAACATGGTTTGTCACAGAGAAGGAGAAAAAGCTTGCTGCAAAAGCATCAAAGGCATCCTCCGCAGAAAAGAACAATAGTTCTGGGAAAGAGGTAATTAGTGCCGAACAAGCTGAAGAGCTCAGAATGAAGGAAAAGAGGAAGCGAGAGCGAGAGAAAAATTTGCCGAGAAAGAAGCGTAGAAAATTGGAAGCAGCTAGAGAGATGTTGGAGGATGAGGAGCAGGATGATGGCAAGCAAGTAGAGGCCAAGGGGACGAATAAAAAAGAAAAGGGTGGAATGACACTTGTTGATCTTGCTTACCGACGGGCAAAAGCAGTGAAGGCTGTCAAGAAGGCATTAGATTCTGGCAAGATTGTGAAGAAGAGTCAAAAGAAATCCAGTAATGCTCCGAGAAAAACTCCTTCAAGGACAGAAGAGATGAGGGATCTATTCCAGACTGACATGAAGGATAAAAAGCCAAAAAGAAGAGGTGTTGGAGGAGGAAAGAAGGCGAAGAGTTCATTCAAAAGCAAATCAAGGTACAAGCGCAAGTAGTCTGTGAAGCTAATAATCCTTGTCATAAACATGTGCGTGGAAGGTCCTTAAACCTGCAATGTGTGTTCTTATTACTGACCAGACATAGTCAAATGCTTATACCATATCTACTCAAGTCATTCTTGGTGATTATATGAGCTCCCACCTGTGAATTTCAACTGATGCATTGGGTGGCCATGGTGATTGGTGATTGGTGATTGGTGTTGTAACCAATGTTGTGGGGTTATGGTAGCAGTAACTACTACTACTACCAATACTATTTTTTTGTCAATTGTATCACGTTAGCTTTTTGTTCTCCATCCCAAATGGTCAGCATAATTTATAGCTGTCCAATTTTGTAAAACTAGCACAGATTTAGTTTTTAATGTTGCCAATAGAAAATTTTGTTTCAGATAACTCTTTAATTTTGTTGCTTCTGCTGTACTTTTATTAAGGCACCAC

>C102303_65

CGAGAATCATTGTTATCTTTATGTCTCGCACAGCTTTTAACAGCACTACATTACAACCTCAAAACATTTCGATTTCCTGAGTGTGGCTACGCGCTCCTTTTCTGTGTGTTGCTGCAATTTCGCTTCTATCACTGGTTTCTCCTCTTTTCAGCATTTTCTGCTGTAAAACACTAAACATGTGACACTCGCGAAGTTGCATTCAACACAACATACGTCTTATTTGTTGTTGTTTTTCTTGTTTTTACCTTAAACTCTCGGAAACATGTAAAACAAGGACTTCGTGCTAGTGTAAAAAGTAACCAACAATTTTTTCCTGGTACTTGTTTCTGTGCGGAAAGTGTGATGTTAACAATAACAAATACGAGAAGAGTGAACATTAGTTGAGTGGAGTGAGTTCAGTTCAATCTCTGTGAATTGTAATGGCCTCGGTCGGGGGTTTTCTAGTCTCACCCGTTGGTGGTGTAAGGTGCGTTACGAAGCAGCGGTTTGGAGTTCGGGCTCAGAGTTTGGAGGTTGGAGAAGGATCGACGGTGTTGTCGACGAAGGGATCTTCGTCTGTGGTGGTGGGGACGCCGAAGAATCAGAATGGTTTGTTGGTTAAAGAGGAAACGAAGATGCGATTGTTGGGAGTTGAAGAGAAGAAGAAGGGTGAAGCAGAAGAAGGTTTGGCACCGCTTTGGGATGATGGGTATGGGAGGAGGAATGTGAAGGATTACTTTGCTGCAGCTAAGGACATTTGCAGATCAGATGGGGGCCCACCTCGGTGGTTTTGCCCAGTTGAATGTGGGCCCCCTTTGAAGGATTCTCCTATTCTTCTGTTTTTGCCTGGGATGGATGGCACGGGATTGGGTCTCACTTTGCACCATCAAGCACTTGGGAAGGTTTTTGAAGTTCGTTGCTTGCATGTCCCAATTCTTGATCGAACACCATTTCAAGGGCTGGTGAAACTTGTTGGAGAAGCTGTTAAGCTTGAACATGCTTTGTCTCCAAATAAACCAATCTATCTGGTAGGTGATTCTTTTGGTGGAAGTCTAGCACTAGCTGTTGCTGCTCACAATCCAACAGTTGACCTGGTACTGATATTATCCAATCCAGCTACTTCCTTTGGCCAATCTCAGTTGCAACCTTTGTTTCCTATCTTAGAGGCTTTACCTGAAGAACTGCATGTTTCTGTTCCCTTTCTTCTTAGTTTAATTATGGGCGATCCAGTGAAGATGGCATCGGTCAATATCGGAAATAGGATTCCTCCTGTCAAAAAAATTGAACAATTATCATACAACCTTACCGCATTGCTGCCATGTCTTCCTGAACTGGCTAATATTATACCAAGGGATACTCTTCTTTGGAAGCTGAAAATTCTCAAATCAGCTGCTGCATATGCTAATTCACGTCTTCATGCTGTTAAAGCTGAAGTACTTGTGCTTGCCAGTGGTAAGGATAACTTGCTTCCCAGCATAAATGAAGCTCAAAGACTCGCAGGATTATTGCGAAACTGCAAAGTTCGTGTCTTTAAGGACAGTGGACACACCCTTCTCCTGGAAGATGGTATTGGTCTATTGACAATCATCAGGGGAACTTGCATGTACCGAAATTCACAAAGGCATGATATGGTCAGGGATTTCATTCCTCCCAGTATGACAGAATTCAGATATGCAATGGATCAAGTGGTTGGGACATTTCGTTCTCTTACCGGATCTGTATTTTTCTCTACCTTGGAGGATGGAAAGATTGTAAAAGGTCTCTCTGGTGTTCCAGATGAAGGTCCTGTCTTATATGTTGGTTATCACATGTTGTTGGGGTTAGAGCTTATCTCACTTACAGATGAATTCTTAAGTGAGAAAGGTATTGCCCTTCGTGGAATAGCCCATCCGGAGCTGTTCATGCCTAAAGTGGAGAGTTTATCAAGTGAGTTTTCTTTGTTTGATTGGGTGAAGATATTTGGAGGGGTGCCTGTTTCTGCAAGCAATCTTTTCAAATTACTTTCCACAAAGTCGCATGTTCTCTTATATCCTGGTGGTGCTCGTGAGGCTCTCCATTATAAGGGGGAAGAATACAAGTTGATTTGGCCTGACCATCCGGAATTTGTGAGAATGGCTGCACGATTTGGTGCCACAATTGTGCCATTTGGAGCTGTGGGGGAAGATGATATAGCCGAAATACTTCTTGACTACAACGACTTAATAAAGATCCCCATACTCAACGATAGCATAAGAAATATGAATCGTGACTCAGTTAAGTTTAGAGATGAGAAAAGTGGTGAGGTAGCAAACTCAAATCTCACTTTTCCGGTGCTTGCACCCAAAATACCAGGTCGCTTTTACTATCTGTTTGGGAAGCCCATAAGAACAAAAGGCATGAAAAATATGCTAAAAGACAGAGAAACTGCCAACCAAATGTACCTGCAGATTAAGTCAGAAGTTGAAAGTAGTTTGAATTACTTGATCAAGAAGAGGGAGGAGGATCCATACAGAAATATCATCGATAGGAAAATATATCAGCTATTTTATCCTCCTGAAACTGATCCAACCCCATCATTTAAGCCCTGAAAAATGTTGATTTATATTGTTGATTCTTATGTAATCATCATTAGTTGTAAACAGATATGTATATGGCCATTATTCTGATAAAATGTGGTCCCAAAAGAAAAAGATATGCAACTTTCTTCAAAGTTCATAATTGATGCCAATGTCAGCAGTCACATATCTCAGTCCTTGCACTGTGTACCAGAAACTGTGCTTCCTATAAGTCTATAACAGAAACACGAGCGAGTTAAAAT

>C102305_65

TCCAACCTCAACAAGACTCTCAGGTTATTATTAAAGCCAATCTCACCATAAATAGTCATCAAAGTAGTTATTCTGCATCCTTTTATAGTGCATCGTACTAAATGGTTCTAACCACAAACTGACACAGTCTTCATATTTTGAATTTTATTGACCGTGATCATATGTAGCATTCCTTAGAGGTGCTTCTTTCCTCTTTGGTTTAGTACCAACTACCCATCACTGAGTTCAGACGCAAGCACATTGTTTATGTTAGATACCAAAATCATTGGAAAGCTTTGAAATGCTTGGTGTATGTCTTCTATTCCTCTCCTTGTTAACCATGTCTTCCACTTTGGAGATGATTACTCCGACCCAACCCCTGAGAGATAGTAAAAATGAGAATGGAACTCTTGTCTCAACAAATGGAACCTTTGAAGCAGGATTCTACAACCATGAAAATTCTAGTAACCGTTACCTCGGAATTTGGTACAAAAACATATCCCCCAGAACAGTTGTTTGGGTAGCCAACAAAGAAACACCACTTCAAGACCACTCTGGAGTCTTAGAAGTAGACAGCGATCAAGGGTTTCTTAGTATCAAAGATGGAACAGGAGCCAAAATATGGTCCTCAAATGCATCACACTCACCAAGTAAACCAGTTGTTGTGGAGTTGCTAGAATCAGGAAACATGGTTGTGAAAGATGGCCATAACAACTTCTTATGGCAAAGCTTTGATTACCCTGGTGACACTTTACTTCCAGGAATGAAAATTGGAAGGAACTTCAAAACTGGTCATCATCGAGCTCTAAGATCTTGGAAATCCTTAGAAGATCCTATTCCAGGTAGTTTTTCATTTGATGTTGATACTCGTGGCCTTCCTCAATTAGTTATCACAGAGGAGGCTAGTAATAAAATAATTTATAGACCAGGGTCATGGAATGGTCTTAGCGTCACAGGACTACCTGGAGAAATAACTGACCCATATACTAAATCCCTTTTTGTTATGAATGAAGATGAAATCTTTTATGAGATTCAACTCTTGAATAGTTCAACTAAACTGAGAAGTAGATTGGTTCCAGAAGGGTATCAATTACGTTTAATATGGTCAGATGAGGGTAAAAAATGGGATGTGCATTACCCTGGTCCATTTGATGTGTGTGCGACATATGGCATGTGTGGTGCAAATACTATTTGCAATGCTAATGGAAATCGACACTGTGAATGCTTGAGTGGATTCCAATCCAAATTTGCAGATTCAACTTGTGATCGAATTACCGGTCCAAATTGTAACAAGGGAGATAAATTTCAGAAGTATGAGAGAATGAAATTGCCTGACACATCTTCATCTTGGTTTGATAAGACAATTAGTCTTCAGGAATGTGAGAAATTGTGCTTGAGCAACTGCAATTGTAGTGCATATGCTCAATTGAATATCAGTGGCGAGGGAAGTGGCTGCTTGCATTGGTTTGATGACATTTTTGACAGCAGGAAACTACCACAGGGTGGACAAGATTTTTATCTACGACTTACTGTTTCAGAGTTTAAAGAGTTTAAAGATCACGGCTTCAAGTGGAAGATAGCAGGAATTTTTGTTGGCTTTACTATGTTCATCATTTTAGCATTTGGATTGATATTTTGTCTTAAGAAGAAACTCAAGCAGTCAGAAACAATTTACTGGAAGGACCAAAATAAGAAGGATGATATTGATTTATCAATATTCGACTTTTTATCCATCTCCAAGGCCACAAATAACTTTTGTGAGAGTAACAAATTAGGACAAGGAGGATTTGGACCAGTGTACAAGGGCATATTATCAGATGGGCAAGAAATTGCAGTTAAGAGACTTTCCAAAACTTCTGGACAAGGTTTGGATGAGTTCAAAAATGAAGTTATGTTGATTGCAAAACTTCAACACCGCAACCTTGTAAAGCTTCTTGGTTGTTGCATTCAGCAAAATGAAAAGATGTTAATTTATGAATTCATGCCAAATAGAAGTTTGGACTACTTTATTTTTGATTCAACCAGACATACATTAATAGGTTGGAATAAGAGATTTGAAATTATCGGTGGAATTGCTCGAGGACTTCTCTATCTTCATCATGACTCTAGACTAAAAATTATTCACAGGGATCTCAAGACTAGTAATGTTCTTCTTGATAGTAATATGAATCCAAAAATATCAGATTTTGGTATGGCCAGAACATTTGGTCTAGATCAAGATGAAGCAAACACTAATAAAGTGATGGGAACATATGGGTATATGCCTCCTGAATACGCTGTACATGGATCTTTTTCAGTCAAATCAGACGTATTCAGCTTTGGTGTGATCGTATTGGAGTTAATAAGAGGGAGGAAAAATAGAGGATTTTCTGACCCACACAATCACCTAAATCTTTTGGGACATGCATGGAGACTATGGATTGAGAAAAGGCCAATGGAACTTATGGATGACTCAATGGACGATCCAGTTAGTCCATCTGAAATATTAAGATATATTCATATTGGTTTGTTATGTGTACAACAAAGACCAGAGGATAGGCCAAACATGTCTTTAGTAGTTTTAATGTTGAATGGTGAAAAGTTATTGCCTGAGCCAAGTCAACCTGGATTTTACACTGGAAGAAATAACTCAACAACGATATGTTCTTCTTCAACAAATTGTGATTCGAATTCCTTCAACGAAATGTCAGAATCGTTGTTTGAGGCAAGATAGATAATTTGCAAAATCATAAATGGTATGAAAGATAGTAAAGTTTCCTAATCACCACTCTCTACAACTACTACTATATGTGTGGAA

>C102309_65

CAATTGGATTTTAATCGACGGCCAATGATCACTCGATGCGCTCGATAACACTGTGCATCCACGGCGCATGATAAGATTTGTTACCCTGTAAATGGTTATCGTTCTCCATGGGATGCGCTTTACTCCTCGACCCTCCCCGCCACTTCTATCTTCATTTCTTTTTCTTTGATTATTTTTTCTGTTCTTAAATTTTGCAGTTAGTTATTGACCGTTGAGCCAACCTCGTACACTCAACGATTCTACCTGGTTTAAACATCTGCAAACCAATTGAGCTGTTTCTACTGGACAAGAATGTCGGAAGGAACCAGTTTGGTGCTGGAATCATCTGAAAATGGCACAGACTTGTCTCAGGATGACATTGACACCATTGACGAAACAGCAGAGGAAATAATTTTGTCAAGACAAACGTCTGTCAACCTTGTTCCTTTTATTGGTCAAAGATTTGTATCTCAAGAAGCAGCTTATGAATTTTATTGCAGTTTTGCTAAACAATGTGGCTTTTCAATTAGACGACACCGAACTCGAGGGAAAGATGGGGTTGGCAGGGGAGTTACGAGGAGGGATTTTACTTGTCATCGTGGTGGTTATCCCCAGATTAAACCTTCTGATGATGGAAAGGTGCAAAGGAACCGCAAATCATCTCGTTGTGGTTGCCAAGCATACATGCGTATTGTGAAAAGGGCAGATTTTGATGTCCCTGAATGGCGTGTTACTGGTTTTAGGAACATACACAATCATGAGTTGTTAAAATCCAATGAGGTGCGCCTTCTTCCAGCATATTGTCCAATATCTCCCGATGACAAGAGTCGTATATGCATGTTTGCTAAAGCTGGAATGTCAGTAAGGCAAATGTTAAGATTGATGGAGCTAGAGAAAGGCGTCAAACTTGGTTGTCTTCCTTTTACAGAGATAGATGTAAGAAACCTGCTGCAGTCTTTCAGAAATGTGGATAGAGATAATGATGCCCTTGATTTGATTGCTATGTGCAAGAGGTTAAAGGATGAAAACCACAACTTCAAGTATGAATTCAAGATAGATAGTAATAACAGGCTTGAACATATGGCATGGTCCTATAGTTCATCCATTCAATCATATGAGGCATTTGGTGATGCTGTAGTTTTTGATACTACCTACCGAGTAGATGCTTATGACATGCTATTGGGAATTTGGCTTGGTGTAGACAATAATGGCATGACTTGTTTCTTTAGTTGTGCGCTTCTACGGGATGAAAACATACAGTCTTTCTCATGGGCTCTAAAGGCTTTCTTGGGGTTCATGAAAGGAAAGGCTCCACAAACAATATTGACAGATCACAACACATGGCTGAAAGAAGCTATTGCAGTTGAATTGCCTGAAACTAAACATGCCTTTTGTATATGGCATATTCTTTCTAAGTTCTCTGACTGGTTTTCTTTACTCCTTGGATCACAATATGATGAGTGGAAAGCCGAGTTCCACCGGCTCTTCAATTTGGAACTGGTGGAAGATTTTGAAGAAGGCTGGAGGCAAATGGTGGATAAATATGGACTCCACACAAATAAGCATATTATTAGCCTATATTCATTAAGGACATTTTGGGCTTTACCATTCTTGAGGCGTTACTTCTTTGCAGGATTGACCAGTACATGTCAATCAGAGTCCATTAATGCTTTCATCCAACGTTTCTTGAGTGCCCAATCTCAGCTAGACCGCTTTGTGGAGCAAGTGGCTGATATCGTTGATTTTAATGACCGAGCTGGAGCGAAGCAAAAGATGCAAAGGAAACTGCAGAAAGTGTGCCTCAAAACTGGTTCACCTATTGAATCCCATGCTGCCACTGTACTTACTCCTTATGGCTTAAGTAAACTTCAGGAAGAGCTAGTTTTGGCGCCACAATATGCATCCTTTCTGGTTGATGAAGGTTGTTTCCAGGTCAGACACCACTCTCAGTCAGATGGAGGGTGCAAAGTATTTTGGGTCCCTTGTCAAGAGCACATAAGCTGCAGCTGCCATCTGTTTGAGTTTTCAGGCATCTTATGTAGACATGTGCTACGTGTCATGTCAACTAATAACTGTTTTCACATCCCTGATCAATATTTACCAGGCCGTTGGCGGGGCATCAATTCGTCTTCTCTTAACCACTTCCGCGGTACAACTAATAGGGATCAGCCTGAAAGGATACAATTTTTGGAATCCATGGTTTCAACATTTTTCGTGGAGTCCATTGAAACTGAGGAACGCCTGGATGTCGCTTGCGAGCAAATTTCCACGGTGCTATCACGCATCAAAACCCTTCCTAGGTCCCCACATGGGGTGAATGACATTGCATATAGCTATCCATCAGATTCGTTAATCCTGCCAGAGGTAGAAGATGCTGATGGAATGATTCACGGCTTTACAATCACAAATCCCCATGAGATCACTACAGGAAAGCTCAAGGAAAGAAGGGCCAGAGATGGAGTCGATCTCACAAGGAAGCGCAGGCAGTTTTCCGCGCCATTGTGTGCTCAATATGGACATGATGCATCTGATTGCTCAATAATGGCGGGTGATAATATGAGTGGAGATGCATTAGGGTACTTGTAGAGTGAGTGGAAGTAGTATTGTTGTACAAAGTAAATGCATTTTTTTGGGGGGTACATAAATGCATTTTTTTTATTTTATTTCAGAAGCATGTAATAGACATTTTTTATATTTTCTTCGCTCCTTTCCTCCGCTCCAGTGATACACATGTTGCCTCCCACTTTTTACATGAGCAAAGGTTTCGGTGCTATTTGCACCACTGTATGAGCATGAGA

>C102311_65

CAGGAACAAGAACAACAAAAACAGAAAAGGAAGTGTTTTAGCAGTGTAGATTAATATGCTGCAGAAGATTCAGAAATGTGAGCAACAAGATGCTGTGCACTTTATGGTGGTGAACATAGATGTAGAGCACAGAAAATGTAAATAAAGCTCGGATCATAATTAATATATCTGCCTTAAGTGAAAGCACAAATCTGAGTTTATTATTAGACCAAAGATCCTATGGTGGCTCTTTCTCCCTCACTACACAAACTATACAGCAATTTGCAAATATTGTACAAGAAAACAAAAGAAACTGCAGATGAACTGCTGTATGAATTGAAATCCCATACACATTGGAGGTCAGCCGGTAGTAAAATGAGTAGGATATTAAAACATGTATGTTCAATGCGGCTTCAACATCCCACTTTATCTCAACATAAACATGATAGTGTATTTTCCCTGCTTCTAGTCTAATTGCTTTTGATATGAATTACCCGGTCTGAGGTAGCTGCCTACAATTCAGCAATTGGTGAGACTGAGGATGCCTTTGCAAGTAAGATGTCCCACTTGTAATACCATTTTCATCTCCTACAAATTGTTCTGAGTAACTGGAAGCTGGGGGAGACCATACTGGAACTACACCTCCATCTCTTTCTCTCCTGGTACCACTCGAAGATTCTATGGCAGAATCTTCAGCTACATTTCTCAGCTCCATTGTAGTATTGTATTGTACTTGTGTATTCCTATGAGCAGTATGCAACATCCGTTCGTTCAATGCAACTCCTGCATCTTGTTGTGCATCTACATTTATTGGCACTTCAGCTCTGCTGCTCTCTTCTGCTGAGTTTCGAAGATCAGAAGTAGTTTCTTCCTCTTCTATTCTCAGCAGTGTGGAAGTGAGGCTTGGAACCATCTGATCCTGGTAGCGTCGAGCAACAGGAAGAGGATTGTTCCTAACAGGTTCATGAGTTTCCTCCATTTGCGTGTCTTGAGGAAAAGCAGCAGGAATTGATTCAGAAGAGTCTCCAAGTCCTTCCTCAGTTCCATTCTCCATTGCAGCTCTTGAAGCTGCCTCTAGTTGCCACATTTCTGATAGTGCCTCTTGTAATCTGGTTTTTGCTGCATCTATTCCCACAATTGGAAGAGGATAGTTCGTTCCTAGCTCAATTCCAGCAGCTTGGAGCACTGATTCTGGTGCATTCCAAGGATGATGTATCCACTCAGTAGGTAATCTTGCAAGTTCTGGAAGCCAGCGTCGCACATATTCTCCATTCGGGTCGAATTTGTAGCCCTCAAACTGTGGATGATCTATTCGGTCAAATTCACGACCATCTGGTAGTGTACCAGATATGTACTGCCAACCAAGAGCATCACTCTCAAGATCTGCATCCAAAAGGGTGTCCCAGAAATATTTCATTCCCCATCTCCAAGGAAGCTGCAGAACCTTCACAAAGAAACTGGAAACTACAACACGTATCCGATCATGCAGCCAACCAGTGGCCCACAACTCCCTCATCCCAGCATCCACCAAAGGGTACCCCGTTCTGCCTTGTCTCCATGCCTTAAAATAACCCTCATTTACAACCCAAGGAAAGAACTTAAGGTGGCCTAGAAGAGGCCTTTCATGACTGTAGGGATGGTTGAAACTAATGTACCTCGAATATTCTCTAAGACCAATAGATTTAAGGAACAAGTTCACACTCTCTTCACTGGCTTTGTTTCCTTCGTTGGCCCACAAAAGTTGTTTGATACGGACGAGATGGAAGACCTTCTTGACACTCAACTCACCAAAGTGCAAATGGGGAGAGAGAAGCGAGGTAGTGGCACTGTCAGCCTTCCTGCAATTTATTGAGTACTCTATCAGTGGACCATTTATGAATGCGGTTAATGCCTTGTTAGCATTGCTCCACCCCGGTGACCATGCTCGAGCAAGAAGTGCATTGCTTGCCTTCTCTAATTCATCTTCAAACACCAATGTATCACAAGGGCATCTCGACACATCACCTGGAATTATTCTTTTAGGTGGAAGAAGAGGTGAGTCGGGGTCATAAGGCATGCTAAGACATCTGTCCCAGAAAGCAGCAAAAGTTGTGAATGGTCGACCATGGAAATCATTCACATCCCACGGTTCATACAGTAAGTCCGCGTTGAAGGAACGCACTGTTATGCCCTGAGCATTCAGAACCTCTTTTGCTCGATGATCCCTTACAAGTGACAGGGGATCATATAAATGATTAAAAAAGAGTTGAGTAGCTCCAGTGGACTTAACAACCTCAAGGAGAGAAGAAACAGTATCAGTGGATCGCTTAGTGATGAGAGGGGTGCCAAGATTCCTGAGAGAAGAATCAAGATGAGCCAGACTTTGTTTGAGCCACCACCTGGAGACTCTGCCAGGGTAGTATTGGCCTTCTTCTTCGGGTGCCCATATGAAAACTGCAACAACAGCACCAGCTCTTACTCCAGCAGCCAGTGCAGGGTTGTCTTCTACCCTCAGATCTCTTCTGAACCAAACTATGCTGCATCCACAACCACCTCTCATCTCTAAAACCTTAAACTACTTTCACACACTCCCCTTCTTCTAACAAGTTTTAACCATCTCATCTCATGTAATAACTTGTCTCTTTACTTTCTGTCTCCTCGGTGTTGAAGAAAACCAAACAAAATATGTTAATCCAGTCTTATCTTTTATTTAGTTCTCTCTCCTCTCTTTCTATCTTGTCCTGGCTTTTGTGTCTAATAACTTGTTTAAGAGGAGATTTGAAGGCATAAATAGGTAAAAGCAACGTGTGATTATTGGG

>C102315_65

CTAATGCTCAGCCAAATACTGATGCACGCTTAATGTCTTATAAACCAAAAGGAATATGGTCAGATCAAATACTTCCCATTTTTTTTTCAAATATATGGCTGGGGGAGAAAAAAGCCAAATAAATAGACATCACATCAGTAAAGGAAGTTGGAATTTTGTCATTTTTTCATGTAGTTGTCCCCATCCTGACATGTACCTCAGAAGAGGATCCAATTGACATCTTTAGAACCTTCAACAAACTGCCATTTAACTCACTCAAAGACAATACTATAGACTTTTTTATAACTACAATATTTTAGAACAATGTCTATTTTCTATATCAATTTTGCATTCATCACTCTTCCAAGCCAGCATTAGAGCATATAGAAATCTAGTTCCCGTGTTGCTTGAGGCCTAGTATTACTGTCATTGCATGTTGTTTGATATTTATGAACAAACACTCCAATATGCTTGAAAGGTAATTTTTCAACTCATCATTGGTAAAGTCCAGCATTTGGCCTATTCGTTGTCATGCCCCACTTTGGGCAGAGATATGAGATACTCGGCCCAGTGGTACTACAATATCCTTCAAAATCTCATATTCTTCCATACCTATTTGAGAACCATTCAAAACCTTGTGAAATAAAGCCCTCCTATCCGTCATGTGTGAGATGAGAGCAGGGATTCTACAAACGGACAATGCATCGACAAACAAAAGAGCAACCACCTGCCATTGTTTTGTTCTGAAAGCTGGAAGTGCATCCACAAACGACATTGTCTCCAGCAAGCGTGCCATCCCTTGCTCCAAGGTAGATATTGGTATTGGCAGTCGAAGACCAGCAACAAGTGCAGGAAAAGCTCGAGAAAGACAACCAGCTAAAGTTTGTTTTATTTCAGATGAACGACCATCGGACAAGACAATTTTACAAGGATATTCTCTCCCATTGACTGATAGATATTCTTCATGAAAACTTTCATCCCTCCCATATATGTAAGCCAAAGAAGATGATGTCATCCACAAAAAGATGGCATTCCACATTGTTGCAAAAGGTGACAAAGTCAAACTAAAACCCTCCGGTGGAGCATCAAACCATGAGTCGTCAGATTCAAAAAAATCAACATCAGAAATTCCAGGCTTTCTTGGCCATTTCAGAGTAACTGAATCATTTTGTAGTATATCATCATCCTCCATAGTACCTTCCTCAACAGCATCATGTGGCTGAGGCAATATAATAATTCCAGCTTCAGAAACAGCATCAGTGACATCTGAGTCTCCAGATGCAACTGCTTCAGATGCTTGGCTCAATGCAATTGCACAAGCTTCTGCTGATGCTTGTCGTAGCATATCCTCTTCATCAGCAACATCTATGTTACCTACTGAGTCAGATTCTTTTCTAATATCTCCAAATTCTTTAACCTCACAAAGATCTTTACTTCCAGCACCATTAATTTTCTCATCCGCCCAAGTAACAGTGCGGCTAGGTTTCTTTTCACCAGCAGATTTAAGGGAAGATTTGAGTTTAGTCAGACATGATCCACCTGCTTTTTCTACTTGGAATTTCTCTTTAACATTGTCAGGATCAAAATTGGAAGTGGAAGCATCACCATTAGTAGTAACTCTACTTGTCTCCCCTTTATGTTTCAGTGATTTCCTTGCAGAATCATTCTTTTCTACATCATATTGTCTTTCTGATATGGAGACACAATGTACGTCTTTCTTTTTAATAGCCAGATTTGGAGAGGATTTGACCACAGCTTCACATGATTTGGATACTTCCTTCTCTTTTTCTGAGGTACTTAAATTTAAAGCACTCTTAAAAGATGAAGACAGATCTTGAATGCTATCCTCATCTTTCCTGACAACTTTATGGTCAACCTTTTCTGGTTGCTTGTCTACAGCTGTTGGTTTCATTTGATGATGTGTTGTGTCTGTTTGACCTGGGGATGCTTTCGAAACACTATACTCATCTTGCATAATTATAGTACTCACGAAGTTCATCTCACTGTTAATTAAATTTTTGTCACTATTTGGCTTGCCATGTCTAGCTTTGGACCCTTTTTTAACATTTTTTGCCGAACCCTTAGATTCACGCTCTCTTGGTTTTGGCACATAACCCTCAATTGCATTTGATGGTCCAACCCACTGCTCCAAAGACACCTCCCCACTGCTTGTTTCAGTTTTCTCCTGGATTTTCAAATTGGACAAACCTAAATCTCCATCCTTTCGCACATTTTCTGTCTGTTCCAGAGCCAAATTCTCAAATAACCTAAGTACATTGTTTAGCTTTTCTGGGTCTAAAGCTGAGCATCTCTCAGCTTGCAAGCTCCCAGCAAAAGCTTTGCTGCTAACAACACAATTTGAAGAACAAAACATGTAAGTCTCTTGTAGGTCATAGACCTTGTGTTCCTTCAGTGAAATCCGGTATCTACCCTTCCTAGGGCGATCAGACGGCAAGCCATTGCAGCAGAGCGGGTAACCACACATATTTGTGATGGACCGTTCGGTTACAACATCTTCATAGTCACTCCTTGACATCAAAGATCCAGCAGCAAACAGCTGATCTTCATTTTGAATGCCCTCAAGGAGTAATAATTGCAATTTTAAAACAGCATCTTTGACAGAAGCAGGTATGTCCTTTGCCATTGAACAAAAGCTTCAGCACCAAATATCTAGAAGAGTAAGAGAATGGAAATGATTTCGGTTAAAAAATGTTTCTCTCGTCTTCCCTCAATTTATTTTGATTTTCTTGCTTCACGACGACGACGTAGGAGAACAACAGTTTCATCGTGCAAATTAGG

>C102317_65

GAAAAAAAAGGATGGCATCGTCATCATCTGATTCCGATCCTGTTCTGAGGATTGACTAACTATTGAGTATGGGAATTGGAAACCAACCCAAAAAAATTGTGGCCGGGCGCTACTTATCCACGCCAGAGCCAGAAATCAGCATAAATTTACAAAAACAAACAACCCTCGCCACCACCACCTTCCCTTCTTTTTCCTCCACGTGTCTCACCACCCATCTTCTCTATTCTCTCTCTCTCCCTCCTTTATTTTACTTCCACTAACATAAAATTACACATCTCGCATTTCCCAACCCAATTTTTCGTTCGTTTCTAGCTCTTGTTGCTATTATTCGTCGAAGAGAAATGAAAATGAAGTATAGATAGATAGATTGTAATAAAATAGGTAGCACTAGGATAGTACTATGGTTAGGAAAGGGGAATTAATTCTTTCCCCAACACCAATTAATTATTACTAAATCTAATCCAAATTCCGGGGTAGGAATAAACATTCTAATTTGGCAAAAGTGGTAAATGCTGGTGAAACTGCATTTCCATTTTCATTTGCAATTCGAATTCGTTGTTGCATTCGCGTTTGCGCCTCAGACGCTAACTCTGCAGTGCCCGCAGCATCTGATTCCCTCCCATTCGCTCCCTTCACCATGTCCACACCTATTTAATCTCTGCACGATGCGTATTCCCAGGAGGCACCTCACCTTTTCTCTCTACAATTTCTCTCCTCAACTCGCACCTCTTTTCTCTCCACGCCTTCGCTTCGCTTCTGGAATCTGCGCCAAACGCCGTCTGCCCTTCGCCAACGCCACGTCACCAGGAGTTAATCCGTTGCATGCTCGATTTTCAATGGACGGAAACCATGAAGTTGGTGTACAGGATTTAGGATTGGTGGATTTGCCTGGCAGTGATGGCGGCAAACAGCCACGTGTATGGTCATCTCCTGAACATGGGTTTAAAACGGAAATAGGCAAGCAGATATTCTGTAATCGGTCACTAAATATGAAGAACATAGTTGCTGTGGGATTTGACATGGATTATACTCTCGCACAGTACAAGCCTGAAACTTTTGAATCTCTTGCTTATCAAGGCACAATTAAAAAGCTGGTTTATGATTTGGGATACCCTCGTGAGTTACTAAATTGGTCTTTTAACTGGAAGTACATGGTCAGGGGCTTGGTTCTTGACAAAAAAAGAGGCAACATACTGAAGATGGATCGCCACAAGTATGTGAAAGTAGCCTATCATGGATTTAGAGAGCTGTCAAAAGAAGATAAAGTTGGAACCTATGGAAATACTTTAATACGTGATTCCTTTGATGAACCCGATTATGCTCTAATTGATACTCTCTTTTCTCTTGCGGAAGCCTACTTGTTTGCTCAGTTGGTTGATTTCAAGGATTGCAATCCTGGCAAGATTCAAGAGGCTGTTGACTATGCTCGCATGTACAAAGATGTTCGAGCTGCAGTTGATTTGTGCCACCGTGACGGAACATTGAAGCAAATGGTTGCTAAAGAACCAAAGAGGTATATTAATGAAGACTCCTCAATAGTGCCGATGCTCGAAATGATCAGAGACTCTGGTCGTGCAACATTTTTAGTGACAAATAGTTTATGGGACTATACAAACATTGTCATGAATTTCCTCTGTGGATCCAGCATGGCAGATGAGAGTAACAGTTTTTTCTGGCTAAAATACTTTGATGTTGTTATCACTGGCAGTGCAAAGCCAGGTTTTTTTCATGAGGAAAATCGTGCCAACCTATTTGAGGTTGTGCCTGAGACCGGAATGCTTCTCAATACAGATAATGGCTCTCCTATGCCCCAGGTGGGTAATACCTCATCAAGGTTATTCGTAGAAGCAAAGGATCATGCTTGTTCAGTTTTCCAGGGAGGCAATGTTGCTCATCTGCATAAGCTGCTTTCCATTGAATCTAGTTCGCAGGTTCTCTACGTGGGGGATCATATTTATGGAGATATATTACGCAGCAAAAAGGTTCTCGGATGGAGAACAATGCTTGTAATCCCAGAGCTTGAGAAGGAAGTTAAACTCCTCTGGGAATCTAGGGATACCCGCAAGGAACTTCAATTCTTGAGGAGTGAGCGTGACCGCATCGAGGATGAAATACATCATTTGAAGTGGTCTCTCAAATTCAAGAATCCAGATGCTGTCTCCAAGCAGAAGTTATCTTCAGAACTTGATACATTGGAGCTTGAAAGAGAGAGAGTGAGATTAAGTCATCAAGAAGCTCAGAGAAAATTACATCAAAGGTTTCACGAGCCATGGGGACAGCTTATGAAAACTGGTTATCAGAATTCTCGTTTTGCTCATCAGGTTGAGAGATTTGCTTGCCTGTATACTAGCCAAGTATCTAATCTAGGCTTGTACTCTCCAGACAAGTATTACAGACCTAGTGAAGATTTCTTGCAGCATGAATTTGGCATTGTGGCTTATGAGACACCAGACATTTAAGTAAGGAATTTGGAGCAGTTTGAGTTTGGAAGGCGAATTCTGCTCTGCCCCGTGTTCAATATCTTGGCTCTCATACCCCTTCTGTTTAAATGGTCAGAGCTCTGGTTAGCATTTCAAAGTGCTAAATAATATTTTGAACGTCTACCACACGGGTTAAAGAAAGAAATATACCACATTTTAGCATTGTATATTGTTTCGTCGGGCACTTAACAAAAGTAATTGGTGCCACTACGCAGTCGGAATTACACATTTACACTAATATAGTTTCATCATTTATTTTTTTTCTGAAATGAGTTTCATCATTTCTTTATGAACTGAG

>C102319_65

TTATCCTCAAAATCTTGTCCAATAAACTGGTTTTATCCTGTTAAAGACCATACACTCTATCCACTTTCCTTGGTCTTTTTCACTCTCACTCTTTACATGATTGTGGCACCTCTTATATGTTTTCCATCATTATTGCACCTCTCAAGCATATATCTTCATCATGTGCACCTTTGTCAAACGCAAACTCTACATCTGATCAGCACTCATGATAGAATAATCTACAATTCAATGTAACATTATCTTAAACCAACTAAAGATCACATATACAGAAGCTCTAGGACTTGAGGGAAGCACAACTACACAAACACAAATTTTCTATAAGCTAGTTCATAAGTTGGCAATCATAAGCTTTTGGAAAGGAAAATGTGTCCTTTGTCAAACAACCAGAAGGCTAACACACAAACCAGTGGGACCAAAAATGCTACTGAAAGAAATGTCAACTTTCTCCAATTTGCAGGCGTATCTGCTGAAATAGATGATGCTACTTCTTCATCCACCTGCCTGAAAGGCCGCATTAAAGTGATCTACTCCTTCTTCATCCGACGATTTAGCACAGCTATCATTGCTTGAGTCATTGTTGGAAGCTTGATCTTCGTTTCTATTATTTAGTCCATTGTTGCAAGCATCTAAAGAGCCATCAATTTTAGTATTCTTTAACTTTTGTACTAAATGTTTATTTTCCTGTTTTAAATAATGTAACTGGTTATTGATGCGCTCTCTTTCAGAGGCTAGTTGCATTACCATATCCATGATAAGCTTGGTTTTAGTATCAATTTCTTTTGCCCTTGAAAATTTTGTATTGCTAGCTTGATCCAAAGATGTTTTCAAGCTTATTGTTCTGGACCTAAGGAGATCCAATTCTTTATTAAGTTCTAAATTTGTTTCAGATAGCACAAAGCATTGCTCAGCAGCACTTTCTTTATTACTTTCAGCTTTTGAAACCTTCGACTTCAACTCTTCAATTAATATTTCCATATCCCATATAGCTGTGTATAACATGTTCTGCTGTTCTTGACTTGCTTCAGAAGACGCCTTTGCATTCTGTAGTTGGATATCTAATTCCCTTAGTTGCTTCTCAAGTGAACCAACCTTTTTCTCTGCCGTGCTAGCACTCCCTTTAAGAAAATTGAGCTCTTCACTAAGCTCCAAGTTGGTCTCAGTTAGCTGTGTAACCTTGGCTTCTGCACTCTCTGCCCGATTTACTGCAATGTCAATGTTTTCTCTCAGAGACTCAACAAAATTTTCCATTTCAATTTGATGTTCATGATATGCTTCATTCTCCGCAATCGCATTATTAATCCGAAGCTCAAAATCCTTCCTCTCTTTCTCAAGAAACTTCACCTTTTCCCTCAAAGCCAACACATCAGTTTTTTCCGTAGTACTCTCAACATTACATGTCTCAAGCTTCTCTAAAGCAGCATCTTTAGCCTTGAGTTGCTCCATCAAATTTTGAACTGTTGATCTCAGCTCATTTTCTCGTTGCATTGAACCATTGAGGTTGAACTCAGTTACTTGAAGGCGTCCCATTAAACCTTTAGAAATCCCCATTAACACCTCTGCGGTAGTGTCTGCCTCTAGAAATCTTCCCCAAACAACTTCTGCTGCTTCTTCCATATAAAATGCTACTTGTTCTGTGTAACGGAGTTTCAACTGTAGCTCCTCATTGTTCCTTGACTCTGCTAACTTCCTTTCAAGGTCTAGCTCTCTCACGAGAGATTTCTCAAGCATTTGCAGAATATATCTTCGTTGTTCAACCATCTGACTATTGGAATTTGCTTTCACATTTGAAAGTTGACCATTTCCTGATAAATTCAAGGGCTTCCCCATTTCGCAATTCTCATTTCGATAAGCCTCAATGGTTTTCTGTAACTGAGATGATTGCATTTTTAACTCCAATAACTGCTGTTGAAACTGCTTCACAGACTCTTCAGAGTCATGCAGTTTTTTGTTCATCATAAGGAAAACCTCAGTCAAATGTCTGCATGAAAATATTTTATGACGAGCATCAACAATTTCTTCTTGGAGAGTGTCCATGAAACTGTCCAGCTCTCTTACCTCAGAATCCAAAATGCCAGACAATAGATCAAATGTCATTGCCTTCTCAAATAAGTGTGCCAAGATGCAGTTATTCTTTGAATCCATTCCTTCAAGATCACTTTCCTCGGCCAATAGATAAATATAAAGCAAATGCAAGTTCACTAGCTTCTCTGAAAAATAGGCTAGGCGATAGTCTATTTCCGTTAAAGCTTGCAGGGATAATTCCTCTTCTTGTACATCATTATCTTTTGTTGAGAACCCCTGGAGTGAGTAGGGTTTTGCAAAAGCAGAATCTGCAGCATAAAAGTTACCTTGAACCTTATTAGCAAATTCATCCATTGCTAGTCTGTATCTTGTAGTTCAGCAACTAGCAGCTAGTCCTTTATCAAAACGCTTCTGGGTCTTCTTATTGCCTCACTCTGACAATGATCTTGAAGAAACTCGACTTAACATAACCCCACATGAAAATTCTGCTGGATAGTCTTTCTGAGCCTCCTAACTACCACTCATTTCCAAATTACCGTTATATAAACATAAGGGTCACTTTTCACATTCCCCAACGCAAAATAAATGAATGAAGTTAACTTATTTTTTATCTTTTCTTCTGGTTCCAACCTGTGTTGATCGATGAGGTGGAAGAGGAAGAACCAAAAGTGCAAACAGCAAAGTGGTCAACCTAGTGCGTGAGAGCGTTGTTTGTTTTGATCTGTGGAAGACAAATCAAAGACTTGAATTAGGATGC

>C102321_65

GCCATAGTTACCAAAAACAGACCCTGAAAATGGATATGAGTGAGTTGAGAAAAGCGGTGGAGGAGGTGGAACTGGTGGATGCTCATGCTCACAACATAGTTTCTCTTGATTCCAACTTTTCCTTTGTCCATGCTTTTTCTGAGGCCGATGGTCACGCACTTACTTTCTCACCACACACTCTTTCTTTCAAGAGAAATTTAAGAGATATTGCTGAACTGTATGGATCCAAGGTATCCTTGCAAGGTGTTGAAGAATACCGTAGAGTATCTGGATTGCAATCCATCTGCTCATCATGCTTCAAAGCTGCAAGGATCACTGCCATACTTATTGACGATGGCTTACAATTGGACAAAAAACATGATATAGAATGGCACAAGAGTTTTACACCTTTTGTTGGTAGAATCTTAAGAATTGAACGGTTGGCCGAGGAAATCCTTGATGAGGACTTGCCTGATGGATCTTCTTGGACTTTGGATTCATTCACTAAAGCATTTGTCTCAAAGTTGAAATCAGTTGCTGGTGAGATCTTTGGATTGAAAAGCATAGCTGCATACCGCACTGGCTTAGAAATCAATACTAATGTTACTGAAAAGGATGCTGAGGAGGGACTGAGGAAAGCATTGATTGCTGGGAAGCCTCTCCGTATTGCAAACAAAAATCTTATTGACTACATCTTCTTACAAAGTTTGGAAGTTGCTCAATCCTATGACCTGCCAATGCAGATACACACAGGGTTTGGGGATAAAGATTTGGATATGCGTCTCTCCAATCCTCTTCATCTACGTGCAGTTCTTGAGGACAAGAGATATTCAAAGTCTCGGATTGTTCTTTTACATGCATCTTATCCATTCTCAAGGGAAGCGTCATATTTAGCCTCTGTTTACTCACAGGTCTACCTTGATTTTGGGCTGGCAATTCCAAAGCTGAGTGTACATGGGATGATTTCTTCACTAAAAGAGCTATTAGAGTTGGCTCCAATAAATAAGGTGATGTTCAGTACTGATGGCTATGCATTTCCTGAAACATTTTACTTAGGTGCAAAGAAGTCTCGCGAAGTTGTTTTCTCTGTTCTACGTGATGCATGCATTGATGGAGATCTCTCAGTTCCTGAGGCTGTGGAAGTTGCAAAGGACATCTTTGCACGAAATGCAATTCACTTCTACAAGATTAGTTCAGCTAACAGTGTTGTTAGTTCACATAGTAATTTGAGCCAAAAGTTGAATGATGGCTTAGACATTGATGTCTCACTTGTTCGTATAATGTGGGCTGATGGTTCAGGACAGCATAGATGTCGAGTGGTTCCTAAAAACCGTTTCAATGATGTCGTAGTTAAGAATGGGGTAGGTTTAGCGTTTGCGGTTATGGGATTCTCTTCTCTTATGGATGGTCCTGCAGACGGGTCTGGTCTAACTGCAGTTGGTGAAACAAGATTAATTCCTGATTTGTCTACTTTAAGGAGAATTCCATGGAATAAAAAAGATGAAATGGTTTTAGCTGATATGTGTGTTAAACCTGGTGAAGCTTGGGAATATTGCCCAAGAGATGCCTTAAGGAGAGCTTCTAAAATTCTGAAAGATGAATTTGACTTGGAAATGAATGCAGGATTTGAGAATGAATTTATTCTCTTAAAGAGGCTAACAAGGGAAGGTAAAGAGGAATGGATACCATTTGACTCGAGTCCATACTGCTCCACATCAGCATTTGATGCTGCTTCCCCCATCTTTCACGAAGTAGTTGATGCTCTTCATTCTTTGGGAATTTCAGTTGAACAGTTACATGGTGAAGCTGCAAAAGGTCAATTTGAGGTGGTTTTGAAGTATACCGTTTGCACTAAAGCTGCAGACAACTTAATTTTCACCCGTGAAGTTATTAGGGCAATCGCCAGAAAGCATGGGTTGCTGGCAACTTTTATTCCGAAGTACGCATTAGATGATTTGGGTTCTGGGTCACATGTTCATCTTAGCTTGTGGCGGAATGGCGAAAATGTTTTTATGGGATCTGATGGATTATCTAAGCATGGAATATCAACTTTGGGAAGGGAATTTATGGCCGGAATTCTACAACATCTTCCTTCAATCTTGGCATTTATAGCACCACTACCTAACAGTTATGATCGATTACAACCCAATACATGGAGTGGTGCGTTCCTTTTCTGGGGAAACGAAAATAAGGAGGCTCCATTGCGTGCTTCATCTCCACCTGGAACTCTCGACGGTCTAGTCAGTAACTTTGAGATGAAATCATTTGATGGTTCTGCCAATCCATATTTGGGCTTAGCTGCCATACTTGCTGCTGGCATTGATGGGCTTCGAAGGCATCTTCCTCTTCCTGAACCTGTTGATACAAATCCAAATCCAGAAATCCTTCAGAGATTGCCAGCATCACTTTCTGAATCTTTGGATGCTCTCCATAAGGATGATGATTTCCTTAAGGAATTTATCAGTGATAAGTTGCTAACTGCCATCAAAGCAATCCGAAAGGCTGAAATTAACCATTACACCAAGCACAAGGATGCATACAAGCAACTCATTCACCGTTACTGAGCTAGTTGTCTTTTGTAGAGTGCATGACTGGTACGAGCACAGTTTAAGATCTGGTGAATTCTTCGCAGTGAACATTTGATGCTAATTGCTTTTACCTCTTGAGCTTTGTGGTTCAGGGTAAAGACAGTAATGACCATATCTGTTTTATCTATTATCTGTATCTTATTGTTACATAATGTATCTTTTAATGTCCTAGTAAGATATATGAACTAATTTATCCTATATCCAGTTCCTCTT

>C102327_65

CAGAGAGAGAGAGAGAGAGAAGTAGCAACCAACCCCACCATCACTCCCTCATCGTTGCGTAACCGTAAGCTCCAATCCCTACCCTCCTCAATTCACATGCAAATTCATCATCTTCTTCCCATATTCTTTCTTATATACTCTTCTTCCATGTGTCTCCCCTGACACACCTCAACCCCACTCCAATTTTCCTCCATCTAACTGCTCCTCCTCCCATGGTTTCTCTCCATCATCAACCATGCCCAACACCTTCTCCTCTCTCCTCTGGTGGATTGGCAAAAAGTTGCGAAGAAGGAACACAGTTAGTAATGGAGACAGTGGCAACGATAACGAGAACGTTAACATTGTCGGTGCCGCTGTTGTTGACGACAATCCATTTGGCAGTACGGTAGAGTGTTACGCTTGCACGCAGGTAGGCGTGCCGGTGTTTCACTCCACCAGCTGCGACAGTTTTAACCAGCCGCAATGGGAGGCTTCGGCTGGGTCCTCTCTTGTTCCGATCCAGAACCGGCCCAATAAGCTTCTCGGGTTTCGAGCTGCGCCTGGGTCGAATATTGGACCGTTTGGGCGGGTTTTGGATCCGCGGAGCAAGCGCGTGCAGAAGTGGAACCGCGCGTTACTGCTGGCGCGTGGGGTGGCGCTGGCGATAGACCCGCTGTTCTTCTACGCGCTGTCGATTGGGAGGCAGGGTTCGCCGTGCTTGTACATGGACGGGGGGTTGGCAGCGATGGTGACGGTGGCGCGGACGTGCGTGGACGCTGTGCACCTCTTGCACGTGTGGCTGCAGTTCAGGCTGGCATACGTGTCGCGAGAGTCGCTGGTGGTGGGGTGCGGGAAACTGGTGTGGGACGCGCGTGAGATCGCGTCGCATTACCTGCGATCGTTGAAGGGGTTCTGGTTCGACGCTTTCGTGATACTCCCAGTTCCCCAGGCTGTGTTTTGGTTGATAGTACCAAAATTGATAAGAGAAGAGCGAATTAAAATCATTATGACCATAATGCTATTGATTTTTTTGTTCCAATTTCTCCCCAAGGTTTACCATAGCATCTGCATGATGAGAAGAATGCAAAAAGTCACTGGCTATATCTTTGGTACCATTTGGTGGGGCTTTGGTCTCAATCTCATAGCTTATTTTATTGCTTCCCACGTTGCTGGAGGGTGCTGGTATGTTCTTGCAATTCAACGAGTTGCGTCATGCCTTCGGCAGCAGTGTGAGAGAACTAATGGATGCAATCTTTCTGTGTCATGCTCAGAGGAGGTATGCTACCAGTCTTTGTCAGGTGCAATAGGAAATTCATGCGGTGGAAACTCAACAGCGATGTTAAGAAAGCCTTTGTGCTTAGATGTTGAAGGACCTTTCAAATATGGGATCTACCAATGGGCACTTCCAGTCATATCAAGCAACTCTTTGGCTGTAAAGATTCTTTATCCCATTTTTTGGGGTTTGATGACCCTCAGCACTTTCGGAAATGATCTTGAACCCACAAGTAACTGGCTAGAAGTGATTTTCAGTATATGCATCGTAGTCAGTGGACTATTGCTTTTCACATTATTGATTGGTAACATTCAGGTATTCTTACATGCTGTCATGGCAAAGAAGAGAAAGATGCAGCTGAGATGTCGAGATATGGAATGGTGGATGAGAAGGAGGCAGTTGCCATCACGATTAAGACAAAGAGTTCGCCATTTTGAACGTCAGAGATGGGCTGCGATGGGAGGAGAAGATGAGATGGAAATGATCAGAGACTTGCCTGAGGGGCTGAGGAGGGACATCAAGCGCCATCTTTGCCTCGACCTCATTAGAAAGGTTCCGTTGTTCCACAACTTGGATGAACTTATTCTTGACAACATCTGTGACAGGGTGAAGCCCCTAGTCTTCTCTAAAGATGAAAAGATAATCAGAGAAGGTGATCCTGTGCCAAGGATGGTGTTCATCGTCCGAGGACGCATAAAACGCAGCCAAAGTCTGAGCAAAGGCATGGTAGCCTCAAGCATCCTTGAGCCAGGAGGGTTTTTGGGTGACGAGCTACTTTCATGGTGCCTTCGCCGGCCGTTTATAGATAGACTTCCGGCCTCCTCAGCCACATTTGTGTGTCTTGAATCAGCAGAAGCCTTTGGCCTTGATGCAGACCACTTGAGGTACATCACTGATCACTTCAGGTATAAGTTTGCCAATGAGAGGCTTAAGAGAACAGCAAGATATTACTCATCCAATTGGAGAACCTGGGCTGCTGTCAACATTCAATTTGCTTGGAGACGTTACAGGCAGAGGACTAGAGGTCCAGTGACCCCTGTAAGGGACAGTGGAGGCACTGAGCGCAGACTCTTGCAATATGCTGCAATGTTTATGTCAATAAGGCCACATGACCATCTTGAATGAAAGCTGCTGTCCATGTTTTCAAATGTTGTTGCTTCCTGACCCTCAATAAAACTGTCCCAAGTCCATTAGACCTTATGATTTTTTAAGTTGTTGTGGAAGTCGTGGAATCATTCATGAAATGCATAAAAAAACATGTATGAGTAATTGCAAAATCATTTAGGCCCCGTTTGTTCATTTTCTTTTAAGTTTGATAGAGTTAATTTTTTTTCATGGGATTTGCACAATCCCCTGTCGAGGCTGCTATGTGATTAATGAATAACTGATTGAGAAGAACTAGTCAGATTTAGTTATATTCTGGTACGAGAATTCACCCTGCTAAACAAAGGGTGACCTGGATTCAATTCTTATGGAATACATGTTTAGAGAGGCAAAGAACTTTAAATGGAACCAGGTCTCATAGTTGAT

>C102329_65

GCAAAATCAATCAGCTTTGTTTTCTTCCGTCACTCCCCAACCCATAAACCCCTCATTCCATTCTTCTCACTCGGCCGCCGGAAAAACCCCTAGTTACGGCGAGTCTCAACCTTCGACTGCTCTCTGCAAGATTTGGCATCCCAACTCGTTGGTGTTCTTTTCATTCTGGATCCGTAGGAATCAGGGCTTTTTATTCTGTATTGTAAGAGATGGGTGTTACAGAGGAATCCTTGGAAACACAATCAGAGGCTAATGAGGACATCATGGCTGCCATTAAAGGTATGAGGCAACAGCTTGAAGCCCGGATAGAGAGCCAACACAAGGCTCACATGGAGATGCTTTCTTCTATACAATCTGTGATACCTAATCTTGTTTCATCCCTCGACCTTTCTCTCAAGGTTGTGTCTTCTTTCAACCATCGTCCTTTTGCTCCTACACCTGCTCTGCCTCTGCCTGATCCTAAATTGAACCCCAAAAAACCTATTGAACTTACTCACCGTTCCAATTGTGAATCCTATGCTGATGGCTCTACTGAAGCTGACTTGACAAATACCAAGAATCAAAAACTCAAAACATCGATTGATTCAAATCCAGCATGTCAAGTAGATTCTGAGAAAGTTAGTCCATTAGCCGTGGTTCGATCACTGGTCGCCGTTTGTCTATTAGGGCGAGTACCCTTCTCACCAATTGATTCTTCCACCGTGTCAAGAAAATTGGAAAATGATCAGACAGTAACACCAGCAGAAAAGGCTGCGCTTCAGGAGCTTGGAGGGGATTCAGGGGCGATACTTGCAGTGGAGATAGCTTTAAGGTCAATGGCAGATGATAATGGTGGTGTTGAAGTGGAGGAATTTGTGGTGAGTGGCAAAGCAAGAATTATGGTTTTGAATATAGACCGAACACGACTTCTGAGAGAATTGCCAGAAAGTGCACAGTATCAGCAACTTGAATCAACTTCTGGAGATGGGAATGTAAATCAGAATCAAGTTCAGCACATCACTAACAGTGGTAGTAATGTGAATGGTAGTTTGCTTGGGATGGGAAGGCCAGTTCTGAGGCCAATGTCTGAAATGTGGATACCCCATGGGGACCCTCATATGTCAGGGTTGCAGCCAATGTTTTCAGGTGGACCAAGGGGAGCACCCAGAGTAATGGGCATGATGGGGACCCATAGAGGTATGAGTATTTCTTCAATGCACAGACTCCCATTGGGGCCAAATGCACAAGGGAGTAGTCCTAATGCAATGTCACAAAAACCAAGAACTTTAGAAGATGATATGAAAGATCTTGAAGCTTTGTTGAATAAGAAATCATTTAGGGAGATGCAAAAATCTAAAACTGGTGAGGAGCTTTTGGACCTAATTCACCGACCAACAGCAAGGGAAACTGCTGTAGCTGCTAAGTTCAAAACAAAAGGTGGTTCCCAGGTCAGGCAATACTGTGACTTACTGACTAAAGAGGATTGCCGACGTCAATCTGGTTCCTTTATAGCATGTGATAAGGTTCATTTTAGACGGATCATTGCTCCTCATACTGACATCAATTTAGGAGACTGTTCTTTTCTTGATACTTGCCGACACATGAAGACATGCAAGTATGTTCATTATGAGTATGACCCTACACCTGATGTGTCTCCAACAATGATGGGTGCCCCTCCTCCTCCCAAACCGCTAAAGCCTCAGCGTGCTGAATATTGCTCTGAAGTTGAACTTGGTGAACCACAATGGATCAACTGTGATATACGTAACTTTAGAATGGACATTTTAGGTCAATTTGGAGTGATAATGGCAGATCCACCATGGGACATTCACATGGAACTGCCTTATGGAACAATGGCTGATGATGAAATGCGCAGTCTTAACGTCCCTGCTTTGCAAACTGATGGGCTTATTTTTCTATGGGTCACTGGACGTGCAATGGAACTTGGACGGGAATGCTTGGAACTTTGGGGATATAAACGAGTTGAGGAAATTATTTGGGTGAAAACAAATCAACTTCAGCGAATAATCAGAACTGGGCGTACGGGCCACTGGCTCAATCACAGTAAGGAACATTGTCTTGTTGGAATAAAGGGTAATCCTGAAGTAAACAGGAACATTGACACAGATGTTATTGTTGCTGAGGTCCGGGAAACAAGTCGCAAGCCAGATGAGATGTATCCTATGCTGGAGAGGATAAGTCCAAGAACAAGAAAGCTAGAACTATTTGCTCGCATGCACAATACTCATGCAGGATGGATGTCTCTTGGTAATCAATTGAATGGTGTAAGGTTGGTTGACGAGGGGTTGCGAGCAAGGTTTAAGGCTGCTTATCCAGATGTGGAGGTGCAGCCACCATCACCTCCCAGGGCTTCTGCTATGGAAGTAGATACTAATGTTGCTGCTCACACGAGGAGTCCATTTGCAGCCACAGAATCAAAGTCCAATTCAACTCAGTTTGCGGAGCCTGCAGCAGCTCCAGAAACTTCCTTTGCTTTGGAGGATAAGTCAATGGCCATTGATGTTGACATTGGTTAAGTTTCCACTTCTGATCATGTTTAGTTGGAGCAATTCCTTGACAAAGCTGAAAGCAGAACAACATTTTGACCTTTTCACCATCGTTAATTTGTACTCTTTCACATTTCTTTTTAGAAAAAGATTAGATTAACAAAGATATTTGGGAAGAAATACTTCATTTAGGCGAGTATGCTTTTGTAAATGAAATAATTATGAGGAGTGCTGTAAAAGTTCCAGTTGTACCTGACTACATTTCTTTAATGTTCAAACATCAATTTTGTTAAAAAA

>C102333_65

TAACTATGGCCGCCTTGGGTTTCTCTTCTACCCTAAACCTCGTTTTCTTCCACTTTCTCTTCTTCTCTCTCTAACGACTCTCCAACTAGCGCTCTGCGCACCGTTTCGGGGGTGTGGCTCCATGGTCGAAACCTATAGGGTCAGGTCTGTCGATTGGAGACCTTCGCCGGTGGTGGCGCTAGCCACCAGCATCGACGGCGCGCGAGTTGCCGCCGCTCGCCAAGATGGCTCGCTCGAAATCTGGCTCGTTTCTCCCGGCGCCATCGGGTGGCACTGTCAGCTGACTATTCACGGAAACCCTAATGGAAGAGTGACGTCGCTTATTTGGTGCCCTGGTGGTGCTGCCGGAAGCCGTTTGTTTTCGTCCAACGTGGATGGCTCGGTTTCTAGGTGGGATCTTTTCCACTTGAAGCAGGAGACGGTGCTGAAATCGGAAGTCTCAATCTGGCAAATGGCGGTGAAGTTTCCTAAAGATGACAATGTAAATGATGAAAGGAATGATGGACGAATGGAGAATGGGAACGGAAATGGTTTCCATGATTTTGACGAACGTGAGAGCAGTGATAGTGACGATGATTCTCATTCACCCGGTCCTCTCGAGCGTTCTGAGAAGGAGCTTCCACGCGTGGCGATTGGTTTTGATGATGGAAGATTGAGAATCTATGAAATCTCTGACACTGATGAATTTATTCTTGTAAAATCTTTGCCCCCGGTCAAAGGACGAGTCTTAAGTGTGACTTGGAGCACAGATGCTAACTACATATATTCAGGAAGCAGTGATGGATTGATACGTTGCTGGAATGCTACATTGGGCAATGAAATCTATAGGATTACAGCTGGACTTGGAGGGTTGGGCAGTGGACAAGAACTTTGCATATGGTCATTACTGTCTTTAAGGAATGGTACACTTGTCAGTGCGGACAGCAGTGGTAGTGTCCAGTTTTGGGACAGCCAACATGGAACTCTCTTGCAAGCTTATTCTTTACACAAGGGACATGTATTTGCATTGGCAGCAGCTCCTAGTCATAATAGGGTGTTTTCTGCCGGTTCTGACGGTCAGGTTATTCTATACAAGCTTTCTGATAGTCAGACTGCATCAGCTGATGATATCAAATATTCTTCTATGACTAAGAGATGGATCTATATTCACCATGTAAGGTCTCATACACATGACGTCAGGGCCCTTTCTGTTGCTGTACCAATAAGTCGTGAAGCTGATATAAACCCAGAAATTAACCCAGAAAAGAGGCTTAGAAGATATCGATGTGAAGAAAAGCCCACCGATTTTAGTTACCATAAGTGGGCCCATTTGGGGGTTCCCATGCTTATCTCAGCAGGAGATGACACAAAACTTTTTGCATACCCAGTGAAAGAATTCACCAAGTTTCCTCCTCATGATATCTGTCCTGCTCCCCAGAGAACACCTATTCAACTAGTGCTCGATTCTGTCTTTAATCAATCCAAAATGCTTTTAGTCCAATCATCAAAACACATAGATGTTCATTTGCTACCACTTAAAAATGTTTGCACCCCTGGGGGCCTTACAAAGACCGAAATAGTTGCTCGAGTCAAGAGTAAGGCATCTCGGAAGATAATTTGCAGCACCATCTCTAATTCAGGGGCGCTATTTGCGTACTCTGATCATAAAAGGCCTAGTCTTTTGGAATTGAAAAGGTGTGAAGTTGGAAAAATCACTTGGGATGTTGATAGAAGGGAGCTTCCTCAGAGATTACCATTTGCCCATTCTATGGTTTTTACTCATGACTCTTCCAAGTTGATAGTAGCAGGTTGCGACCGAAGGATATATGTTGTGGATATTGGGAATTCGGAATTAGTGCATACTTTTACGCCTCGTCGTAAACCTCAAGATGAGGAATTACCTCCGACTGAGCCACCAATAACAAGATTGTTTACTAGTTCTGATGGTCAGTGGTTGGCAGCTGTGAACTGTTTTGGGGATATATATGTATTTAACCTGGAGATACAAAGCCAGCATTGGTTCATTTCAAGATTGGATGGCGCCTCTGTTACAGCTGGTGGATTTCCACCCCAGAATGACAACGTTCTAATAGTTACAACTTCCTCAAATCAAGTGTATGCACTTGACGTAGAGGCCAAGCAGTTAGGAGAATGGTCAAAACGACACACTTTTACTCTTCCTAAGAGATACCAGGAATTTCCTGGTGAAGTTATTGGGCTTTCATTCCCTCCAGCAGAAACTTCATCTTCGTCTTCGGTGGTTGTTTACAGCTCCAGGGCAATGTGCTTCATTGACTTTGGGTTGCCCGTGGAGCAAGATGAAAGTGATATGCGTTACACTCAAGATTCAAGGGCTGGAAATTTACGTTGTTTTAATGTTAAGGGGACTAAACATAAGAGGAAATTTGAGAAGCCTGTTAGGAGGAACTTTCAAGTTTTACCATTAGAGAATCCTGTTTTATTTTTAGCTCATACTTCAAAAAATTCCTTATTTATAGTAGACAAACCCTGGTTGCAAGTGGTCAAGAGTTTAGAAGCCGCACCAGTCCACAGACATATATTTGGGACATGAGCATTAAGCAACCTAGAGAATTGTTCTCAGCTTGGGGGGATATGATGGGGATAAAAGTCCCTTGTTTTTCTTATTTGTCTCGTCATGGTTGGGATCTAGAATCGTGTATTGTGTTTACCATAATCAATTTTGTTCCATTAGTAATAGCTTCTATTAATTTTTTTTTTTTATTTACACAAGCTCTGTACTTTTTATCTTAATTAAATCCCAGATTATTTTTTGCTAAAAAAAA

>C102335_65

TTTTTTTAGGACAAAATATATAATTTTAACCAGCTTACAATTATAAGATGTACTATACAACCATTAAAGTATCATCAACTTCAGGTAACTTGGTTATGAAGAAGAATTCTTAGTTAGTGTCTACTAAAATTACACACTCAGAGAAATTGATTCAATGGGCAGACAAAATTAGGTAATTGAACAAATATCCGTTCAGTTGATTGATAGAATAATCAAATCAATAATATGTGCATGCTAATTAGGGATAACTCCTGACTTAGACTGTCAAAGGCTAAGGACCTAACAAAATACATAACACAGCATTACTCACCATTTAAACTCAGAAATATCTGCCCTTTGGTCTCATTCAATCTGTTTACACCCTCCCCCTCTTCTACAGTACAGACTCAAAAAAGTCATCCAGTCAGAAATTTTTGGGTTGCTTACAGATTTTGTCTTCTCCATATGAAGCATTTCCCCACCTTATTTGCCTAACCAGCAAAACCAAGCCAGTGAATCTTTCCTATGATAAAAGATTTCATATAATAAGGAATGCATGGATTCAGGCAAACCGTGGTTGAGAAGATACAGCTTTTCACCATTAAAGTCTGAAATTTATATACAATCAAAATGAGAATGAATTAGTGATAAGACACTAATCTAATCCCAAGCACTTGCCACACCTTGACCATACCCTCCACCATAATTGCCATATCCCCCACCTGCTGCACCACCAGTTCCACTACCAAAGTAATCTGTTGATTTATTAAATGAGCCCTCTCTCCTAAAATCACGACCACCAAAACGGGATCCCCCTGACCTCCTATTTCTGTGGCCACCACTGTAAATAGCCCTTGCTGCATAACGGGTGAGCCAGGCAGGTACTTCTTGATTTGCCTCTTGCATCAGATCAGCCAATGGTTTAGCCAAGGACAAATTACTTTCATTGAAGAAGGCAGTTGCTAATCCCATTTTACCAGCTCTGCCCGTTCTTCCTATACGGTGCACATAATCATCAATGTCATTGGGAAGATCAAAATTCACCACATGTGCTACCCGAGGGATGTCCAGACCACGTGCTGCAACATCTGTTGCCACTAGAATTGGTGTGTTTCCAGTCTTGAACGACCTCAATGCTAGTTCTCTTTCTTGTTGTGTTCTATCACCATGAATGCTAGCTGCAGGAAATCCATTAACACACAAGCAGTGTTCCAATGCATCAGCTCCTTTCTTTGTTTCCACAAAAACTAATGTCAGACCTTGCTTGCCATATATTCCATTTTCCCTCTGAGCATGAAGAAGATCCATGAGATGACTTCTCTTGTCTGACTCAAGCACATATTCTACTCTTTGAGCAATTAGATCAGTACTTGAACCGACCCTTCCAACAGCCAGAAACACATAACTTGAAAGAAAATCTGATGCAAGTCTCTGTATCTCTTTTGGAAATGTGGCACTAAACAGCAGTGTCTGTCTCATGCCAGGAGGAGGCATGTCCATTTGTTCAACTATTTTTCTTATTTGAGGCTCAAAACCCATGTCCAGCATCCTATCTGCCTCATCAAGAGCTAAATATCTTATCATCTGCAGTGACACCCGAGCCCTCTCAAGCAAATCCACTAATCGTCCTGGAGTAGCCACCAGAATATCCACGCCTCTCTCAAGCTCGCGTAACTGTTGGGTGATTGGAGCTCCTCCATAAGCAACCACCACTTTGACACCTGTTTGATAAGAAAACTTTTTGGCTTCATCATGTATCTGACATGAAAGTTCCCGTGTAGGGGAGAGGATAAGTGCCAGCGGAAAAGCAGTTCTAGCCACGCGTGGCCTTTGAACATATTGCTCCCTCATGATTCCACTTATAATGGGGAAGCAAAAGGCAGCTGTCTTTCCTGATCCAGTCTGAGCACAGGCCATCAAGTCTCGGCCCGAAAGGGAAATTGGAATAGCATATCTCTGAACCGGGGTTGGTTTCACATACTTGCATCTCTGTATATTCTGATTCAACGCCTCACCCAAATCTATTTCGGCAAATGTATTCACAGGAGGGGGCACATTCTCCCCACTAGTCTCCACAGGGATATCATCATAAGCGTCAAAGTTAATCCCAGTGTTCTCTTGCTCATTAAAAGATTGATCCTCAGCTTCATCATTCTCAAAAGGGTTCACCTCACGCCTACCACGGTCCCTCCCCGCCCCTCTACCATTCCAGCCACCCCCAGTCTGATAACCAGAACCATATCCCTGTTGCCTCCCTTGATCAGCTTTAAAGTGGCCACCCTGATGAGGAGCTGGACCACCATGATTCACCCGGTCATTCACCGGCAAAGGAATCACCGGTTGTGACGCCACAGGAACCTCCGACGACCTGTTGCGAAGATGCGGTGGCACATATGCTCCGCGGGGAGGGTAAGCCGAGGAATTGCCATTGCCAGAACCAATGGCTGAGTTGTCAGCAGAATCCGCCCACGAAGTTCTCATAGTTGCAGAAATCGTGACTTTGCTCTTATAACAAGCAAATGCTGAACAGAACAAAAAAAAAAAAACAACAAAACTACGCTTCCCAATCCACGATCCTCTCACGTGCACGCTTTATCACACAACCACGATCAGCTGAACGAATGTTAGAAGAAACGAAGTTGGATTAAGATTTTGTGTGTTTCGAAGGAGTCCTTGGCGAGGATATCGGTGAAATGAAAGGAGATTTTTCTCGGCGTTTTCCGAGTTGAATCAAAATTTTTCCTAATTTTCTCGTCAAGTTTTCTTCGCTGCCTGCCAAACACAAAAGTTTTGTGCGCTGAAAAT

>C102337_65

GCTACACAGCCTTCTCCTAAGCTAAGTTTTAACTTGGTGCGTGTTTGTTTGTTTCTTTCCCTTTCCCTTCATTTCCTCTATAGTTTGGGTGACAACACAAACACAAACAAACACAATGGTGTGTTGATCACTCTCTCTTCTCTCTCCCTCTCTCCATTTGATGTCTCTCCCCACCAAACGGTCGGGCACCGCTGGTTCCTCCCCGTCGCCGCCACCCCCTATGAAAAAGGCCAAGTCGCTCCTCCTTCGTGCCCCCTCCGACGACGCCGCTTTGGACTCTTCCCCCATGCCTCTCGACGACGATCTACCCAATGCCCGCGCCGCCAATCTCTCCCGCAAGAAAGCCACGCCGCCCCAGCCCGCCAAGAAGCTCCTCATCAAGCTCCACAAAGCCAAACCAACATTGCCCACGAATTTTGAGGAGGATACATGGGCGAAACTAAAGTCTGCCATTTGTGCTATATTTTTGAAGCAACCTAACTCCTGTGACTTGGAGAAGCTGTATCAGGCTGTGAATGATCTCTGCCTTTATAAAATGGGTGGAAATCTTTATCAACGGATTGAAAAGGAGTGTGAAGCTCATATATCTGCAGCGCTGCAGTCTTTGGTTGGCCAGAGCCCAGATTTGGTTGTTTTTCTGTCTCTAGTGGAGAGATGTTGGCAGGATCTTTGTGACCAAATGTTGATGATTCGTGGTATAGCCTTGTATCTGGATAGGACATATGTGAAGCAAACGGCAAATGTCCGGTCATTATGGGACATGGGTTTACAACTTTTCCGCAAACATCTTTCATTGTCCCCTGAAGTAGAACACAAAACTGTTACTGGTCTTCTACGAATGATTGAAAGCGAAAGAAAAGGTGAAGCAGTGGATAGAACCCTTCTAAACCATCTTTTGAAGATGTTTACTGCTCTGGGAATTTATGCAGAAAGCTTTGAGAAGCCATTCCTTGAATGCACATCTGAATTTTATGCTGCTGAAGGTGTGAAATACATGCAGCAATCGGATGTCCCAGATTATTTGAAGCACGTGGAGATAAGATTGCAAGAAGAACATGAAAGATGTTTGATCTACTTGGATGCAAGTACTAGGAAGCCATTGATAGCAACGGCAGAAAAGCAACTTCTTGAACGTCATATACCTGCCATTCTTGATAAGGGCTTTGCTATGCTTATGGATGGGAATCGTATTGAAGACCTTCAGAGAATGTATTTACTCTTTTTAAGGGTCAACGCCCTTGAGTCACTGAGGCAGGCAATTAGTTCTTACATTAGGAGAACTGGTCAGGGCATTGTTTTGGACGAGGAGAAAGATAAAGATATGGTTTCATCCCTTTTGGAATTTAAGGCTTCTCTAGACACAACATGGGAAGAAAGCTTTTCCAAAAATGAAGCATTCTGTAACACCATTAAAGATTCATTTGAGCATCTCATTAATCTTCGTCAGAACCGACCTGCAGAGTTGATTGCCAAGTTTTTGGATGAGAAACTCCGTGCAGGTAACAAGGGAACTTCAGAAGAAGAGTTAGAGGGTACACTTGATAAAGTCCTTGTTTTATTCAGGTTCATACAGGGAAAGGATGTGTTTGAGGCATTTTATAAGAAAGATCTTGCAAAAAGACTGCTGTTAGGAAAGAGTGCTTCTATTGATGCCGAGAAATCTATGATTTCCAAGTTGAAGACCGAGTGTGGCAGCCAGTTCACTAACAAGCTAGAAGGGATGTTTAAGGACATTGAATTATCGAAAGAAATAAATGAGTCCTTCAAACAGTCATCCCAGGCTAGGACAAAACTCCCATCGGGGATTGAAATGAGTGTTCACGTCTTGACAACTGGGTACTGGCCAACATATCCACCCATGGATGTTAGACTTCCTCATGAGTTGAACGTTTACCAGGATATTTTCAAAGAGTTCTATCTAAGCAAATACAGTGGAAGGCGTCTGATGTGGCAAAACTCATTAGGTCACTGTGTCTTAAAGGCAGAGTTTCCTAAAGGGAAAAAGGAGTTGGCTGTCTCCCTATTTCAGACTGTTGTTCTGATGTTATTCAATGATGCTGAGAAACTAAGTTTTCAAGATATCAAGGACTCTACTGGTATTGAGGACAAGGAACTGAGAAGGACTCTGCAATCACTTGCCTGTGGAAAAGTTCGTGTCCTACAAAAGTTGCCAAAGGGTAGAGATGTGGAGGATGATGACTCATTTGTTTTTAATGATGGATTTACTGCTCCTCTGTACCGTATAAAGGTGAATGCAATTCAGTTGAAAGAGACAGTGGAGGAGAACACAAGCACAACTGAAAGAGTTTTCCAGGATCGTCAGTATCAGGTTGATGCTGCTATTGTGAGGATAATGAAGACTAGGAAAGTGCTTAGTCATACCCTTCTTATTACCGAACTCTTCCAACAGCTCAAATTTCCAATAAAACCAGCCGATTTGAAGAAAAGGATAGAAAGCCTGATTGATAGGGAGTATCTGGAGAGGGACAAGAACAACCCTCAAATATACAATTACCTGGCATAGTTTATTTTACCTCATTTCACATTTTAAAGTTTCTTTTGCTAAATGACGTGGGTAAAGCTCGATGTTTACTCCCTTGTCATATATGTACAGTAACAGGATTCTCTCCACTTCCTCTATTTTTCTTTTCCCTTCATTTATGGACAACTGTACTTTAATTGTAAAGCATACTTTACCCCTTGAATTTCTTTTCATTTAGATAAAAATTAAAAATTGTTCCCGGAAATGATAGAGAAATTTAAAAGAATTGGTGTTTAACAAGG

>C102339_65

TGATAAGGGAAGAGCTACACATGGCAACTCACCTCTCCAATATGCAATTTCCATTTCCCAGCGAGGCAAATAGACACGTACGCCTCTCCAATATTACTCATTACTCTTATTGATTCATTCATTCATAACGTTCATTCTCACTCTCCATAAATCCTTTCTATGCTTCACCGCATCACCCACTCCTCTTCTCCCTCTCTTCTCCTCCTTCCGCGTCTTCTCTATCACCAACCCTTCCCTTCTTCAAATTTCAAATTCCCTTTCTCACTCTCACCAAAACCTCTCACTCTCAAGGTCCTCTCCATGGCTTCATGTCATTTCGTTCACCACAAGCACACAAACAGACTCGCTTCCGAACAGAGTCCTTACCTTCTCCAACACGCACACAACCCTGTTGATTGGTATCCGTGGGGCGAAGAAGCCTTTGCCGAAGCGCGCAGGCGCGACGCGCCTATCTTCTTGTCAATTGGCTACAGCACTTGTCACTGGTGTCATGTCATGGAGGTCGAGTCTTTCGAGGACGAGGCGGTTGCCAAGTTGTTGAACGATTGGTTTGTTAGCATTAAGGTTGATCGAGAGGAGCGACCAGATGTTGATAAGGTATACATGACGTATGTGCAGGCTTTGTATGGTGGAGGAGGTTGGCCCCTAAGCGTGTTTCTTTCACCCGACTTGAAGCCTTTAATGGGTGGGACTTACTTCCCCCCGGATGATAAGTATGGACGACCTGGATTTAAGACTATACTTAGAAAGGTAAAACAGGCTTGGGATAGTAAGAGGGATATGCTGATTAAGAGTGGAGCATATGCAATTGAACAGCTTTCTGAGGCAATGTCTGCTAGTTCAGATTCTGATAAACTGCCTGATGGGGTTCCGGCTGATGCATTACGTCTGTGCTCTGAGCAGCTCTCTGGGAGCTACGATTCAAAGTTTGGTGGGTTTGGATCTGCACCGAAGTTTCCCAGACCAGTTGAGATAAACTTGATGCTTTATCATTCCAAAAAGTTGGAGGAGACAGGAAAGTTGGATGGAGCTAATGGAAGTCAGAAAATGGTTTTCTTTAGCTTGCAATGCATGGCAAAAGGCGGGATTCATGATCATATTGGAGGAGGATTTCACAGATACAGCGTGGATGAGTGCTGGCACGTTCCACATTTTGAGAAGATGCTATATGATCAAGGGCAGCTTGCCAATGTTTATCTAGATGCGTTTTCAATCACTAAAGATACTTTCTATTCATATATATCACGGGATATCCTTGATTATTTAAGGAGACAAATGATTGGTTTAGAGGGTGAAATCTTTTCAGCAGAGGATGCTGACAGTGCAGAGACAGAAGGAGCTGCAAGAAAAAAGGAAGGAGCCTTTTACATATGGGCAAGCAAAGAGGTTCAAGACATACTTGGAGAGCATGCTGCTCTTTTTGAGGAGCACTATTACATAAAGCAATCCGGTAACTGTGACCTATCTGAAATGAGTGATCCTCACAATGAATTCAAACAAAAGAATGTGCTAATTGAGAGAAAGGAACCTTCGGAACTGGCATCAAAATATGGCATGTCGGTTGAAACATATCAAGAAATTCTTGGAGAGTGTAGGCGTAAGTTGTTTGAAGCTAGGTCAAGGCGTCCAAAGCCACATTTGGATGACAAGGTTATTGTATCTTGGAATGGATTGGCAATCTCATCTTTTGCTAGGGCTTCTAAGATTCTCAAGGGAGAAGCTGAAGGGACCAAATTCTACTTTCCTGTTATTGGCACTGAACCAAAGGAGTACATGGCAATTGCAGAAAAAGCGGCATTTTTTATCAGGATGCAACTTTATGATGTGGAGACTCACAGGCTATACCATAGTTTCAGACGTTCTCCATCTAAAGCTCCTGGTTTCTTGGATGATTATGCTTTTCTGATTTCAGGATTGCTAGATCTCTATGAATTTGGCGGTGGAATTACCTGGCTTCTATGGGCCATTGAATTGCAGGAAACCCAGGATGCTTTGTTTCTTGATAAGGCTGGAGGTGGATATTTCAATAATACAGGAGAAGATCCTTCAGTTCTCCTCCGCGTGAAGGAAGATCATGATGGGGCGGAACCCTCTGGAAATTCAGTTTCTGCTATAAATCTTATAAGATTGGCTTCCATCGTTTCTGGAAATAAGGCTGAGAATTATAAGCGAAATGCAGAACATCTATTGGCAGTTTTTGAGAAAAGATTGAAAGATATGGCTATGGCAGTGCCTCTAATGTGTTGTGCAGCAGACATGTTGCGTGTACCTTCTCGGAAACAAGTGGTCGTGGTTGGTGGAAGAACGTCTGAGGAATTTGAGAACATGCTTGCTGCAGCTCATGCATTATATGATCCGAACAAAACAGTTATTCATATAGATCCCAACAACAAAGAAGAGATGGAATTTTGGGAAGTAAATAACAGCAATGTTGCTCTCATGGCAAAAAATAACTATGCCGTCGACAAGGTTGTAGCTCTTGTTTGCCAAAACTTCACTTGTAGTTCCCCTGTGACTGATCATTCGTCACTTTTGCTCTCCAAAAAACCTTCCTCTTCGTTCACATAAGCTATTGGTTGTGATTTGAGTAGGAAAGTAGTTATCGTCGGTGAAATATCTCCCTTAGATCCACCTTTTCATCTACAGAACCAGTTGGTTCTGAGTTGAGTTGAGGAATTGATGCATAATTGCATACCCCTTTCAGGGGCCACATCTTTTCCTCAAATTTGATACAGAATGATGGAAGTTTGTATACTATGATAATAAAAGGATTTTTGAATAACA

>C102341_65

CATGGAAGCAAATGGCCCCAAAATAGAAAGAAATGAATTGTATACGTACAATATTTACAACTATTTATATACAAAAGCCAGTGAAAGGGAAAGTGGTGTTTCAGCTGCTGATGTCGAAGTATTTTGGATTAGAAGGAAAATGATATTCCACATGCAACTTGCCCTCATCAGAATCTGAATGACGTTGAGGAAACACTACCCTCATATCATCATACAAGTAGAAGCTTCTTTCAGGTTCAACGTCCACAATGTTTCTCACTTCTGCAGAGAGAGTGTCAGATTTGCACTTATGAACAGACCTTGAACGCCTTTTAGGAAAGGGGCACATAAAGCGAAGTTGAAGAGCATAAAGCAAAATGCCAGCCTTTGAACATGTAAGACTATCAAACTCTCCACATTTTGAGGTGAGAAGATCTTTATCTCTGTGACTGGAGGCTGTGATCAACGAGGACTTCGCATCAACTCTGATATCTGAATCTGTTTGGCTTTCCTTACCCATCGTAGACTTTGATTTAGACGCAGTTAAAGTGACCTTTTGCCGTAAGAATGTCTTGGTACCAGCTGGCATGTCACTCAAATCATAATTGCAGAAGAAGGTATGGATAGGTGTCCTCTCTGGATTGCTGAGAACCAGTTGAATACATCCTTTCATAGGTATACGTATACGGGGCTTTTCAGATCGTGATTCCTCCATGCTGAGGGTTCTTTGAAATTTGGCAACTCTAGCCTTGCTTGATAATAGCTTTCCTGAAAGATTTATTGATGAATAATAAAGTAAGTACTTGTCTCCATCCACGCTGGTTACTGCAAATGGTATTTTTTGTGACTGAGGTGAGAAATTGCCGCCAGTTACATTCAGCATGGCAAGAAAGCCTTCAATTTTCTGACTAACTTTCCCAGATAGTAGGCGACCGGATAACAAAGATTCCTCAAATGAACCAACCAGGGACCTTCTAACTGGCAGTCTACTCACAGTTCCACATAACTTGGTATCCTGAGGAGGAAAGCTACCAGGATGAGTCCAATACTCTTTCAAATTTATCATGTTATCAGGAGTAAACAGGTCAGACTTTTGCTGCATATTGATGGAATTGAGCTGTGATTTAGTTGATATCTCCTCTGCAGATAAACTGTCTTGACAAGTTCTATCAAGTGATTGTTCCACATTATTAAAAGTCAAATTTTCATCATCCAAGATCACATCAATATTTCTGCATCCCCCCAAATTTTTATTTTTAGAAGATGTTTTACCCAGAGGAGACAAAGGGAAGGGAGGCGATGACACATTTTTTTGTGGTATAGACAATGCCGCTGTTTGAGACCTTGTTTTAGTTGTTTCTTCAGAAACATTACGTGCTGGAGATGATTTGAAATGCTTATAAGACCAGGGTTCTGTATGTTCACAATGTGAATGGTGATGACTAGATAAAATATGGTTTATACATGATGACTTAGTAAATTCCTTAAAACAAGATGTAGACCAGATCATGGAGGGGATATGATTGTTGTTGTTTTCAGTATGAACCTTCTTATACTCATGTGAAGCATCAGAATTATCATCACCAACCGAACGAATTTGATAAATACCACCGCCAATGTCTAGTGTATCACCTTTAAAATGATCAGTACGCAGCATCCCATTCAAAGGTGACAATAACCGCTTTCTGACTAGAGACTCAGAAGCTTCAGTTGCATCACTGGTGACATTAAACACAGTTGACGAATATCCATTTCCACCATAACCATTAACACGAGGTGTTGATGCACTTATTTGAAAACCTACAGTCCTAGATATAGGTGTCTGCATAGTTTGCCCTGCATTAACAGATAGCCTGCCAATTTGTTCTATGGGATCAATCTTCAACTTAGCATCTTTACACATACTTGAAATATGTGATTCTTTTGAAAGCTCTAGAACATTTTTTCTTTCAGATTTAAGCACATCAATATGCATGCAATTACCCCGGTCTTCTCCAGCCAACAAGTTCACCTCATAGTTGGTTTTGGTGGTTACTCTAGGTGCAGTTTGTACAAATGTACCTAGTGATGCTGCCATTTCCTCAGCAGGGCAGCCTGAAAACACTTGTGGCAACCCCATTTTTATTTTTCAGGCAGGGTTCTTCTAAATTTTCCAAAATGATTAACTTTTTCAATTACTATCTGTCTTTCCTCATCACCGTCTTCTAAATGTATTCATGCATCAGCATTGGTACATTCGCATTCGTGTTTCAACTTTATTCTAAACAAACACACAGCCAGAACCAGTGCAGTTGTAAAAAACTTGATTCCCTCCAACAAAAAGTAAAAATCTTATAGATCAGACTTTACAGCATCATCACACTAGAGGCACTCTCCACAACGTGCGGTTCAAACGACAATCAGAAAATCCATAAGAATGCTCAAAATTCAATCTTCCTTCCCCAATTCACCAACGAAACCAATCAAAGGGACAAACTTTAGCAAACCCCACAACGTTAAAGGACTCGTATGTCTAAGAATGTCCTTCTTCTTCTTCCTCCTCGAAGTGTGTTGTGTTGTTGAAGCTGGGTGGCATTAGCATATCTCACGTAAAACGGGATAGGGTGTTAGGCTGTGGTGGATGACGATACGATATGGATTCGGAGCCAGCGTTGGCGGAGGCAAACAATTTGTGGGGCACGGCTTAGCCAGAATTTTCTGGCTGTCTGTGTTTGTACTTTGAAAACCACAACTTGCAAAAGGGAAACCACAAGTTGGGTTTGCAAGTTCGAGTCAAGTTGCGGTTCTCTTTTTTTTTCTTTCTTTG

>C102347_65

CTTGACTGTTACATTTGACAGATAAAGCTCGACAGAAGATGTTTGGGAATATGGTGAATGCCCTCACCCCCAAGAGAGATAGAATGAGAGGAAGAGTGATATTGATGAAGAAAAATGTGTTGGATTTCAATGACTTGAGTGCCTCTTTGCTTGATCGTCTTCATGAATTTGTGGGAAAACGTGTTTCTCTGCAACTCATAAGTGCTTTTCAAGCTGATCCTGGGAATGGCATGAAAGGTAAACTTGGGAAGCCTGCATATTTGGAAGACTGGATCACCACAATCACACCCTTGACAGCGGGTGAATCGGCATTCACGGTCACGTTTGATTGGGATGACGATGAGGATATAGGAACCCCAGGAGCTTTTCTAATAAGAAATCATCATCACAGTGAGTTCTATCTGAAAAGCATGACACTTGAAAATGTTCCAGGCCATGGTGTCATCCACTTTATCTGCAACTCTTGGGTATACCCTTCTCATAAATATAAAACGGATCGCATTTTCTTCAGCAACAAGACATACCTTCCAAGTGAAACGCCAGAGCCACTTCTAAAGTATAGAGAAGAAGAACTAGAGAGTTTAAGAGGCGATGGAAGAGGGACCCTGCAAGAGTGGGATAGGGTGTATGACTATGCATACTACAATGATTTGGGTGATCCGGATAAGGGTGAACAATATGCTCGTCCAGTTCTAGGAGGGTCCATTGAATATCCCTACCCTCGAAGGGGAAGAACGGGTAGACCACCTACAAAATCAGATGCTAAGACTGAGAGTAGATTGAATTTGGTCATGAGCTTGGACATCTATGTTCCAAGGGATGAAAGATTTGGTCACTTGAAATTGTCAGACTTTCTTGCTTACGCACTGAAGTCCATAGTTCAAGTTATCAAACCTGAGCTGGAATCTCTATTCGACAGCACCCCTGAAGAGTTTGATAGCTTTGAAGATGTGTTTAAACTCTATGAAGGTGGGATCAAGGTGCCTGAAGGTATACTTAAGAATGTTAGGGATAAAATCCCTGCAGAGTTTCTCAAAGAAATTCTCCGAACAGATGGAGAAAGGTTCCTCAAGTTTCCTGTGCCTCAAGTGATTAAAGAAGATAAGTCTGCGTGGAGAACCGATGAAGAATTTGCTAGAGAGATGCTAGCTGGTGTAAACCCTGTTATTATTCGCTGCCTCCAAGAATTCCCACCAGCAAGTAAGCTAGATGCTAAAGTCTATGGCAATCAGACCAGTACAATAAGGAAAGAGAACATTGAGAGAAACATGGATGGGCTCACAGTAGATGAGGCTATCAGACACAAAAAGTTGTTCATATTAGATCACCATGATGCCTTAATACCATACTTGAGGAGGATAAACTCCACTTCTACAAAGACTTATGCCAGCAGGACAATTCTTTTCTTGCAAAATGATGGGACTTTAAAGCCATTGGCCATTGAGTTGAGTTTGCCGCATTCTGAAGGAGACCAATATGGTGTCATTAGTAAGGTTTACACTCCTGCAGATGAAGGTGTTGAAAATTCTATCTGGCAGTTAGCTAAAGCTTATGTACAAGTAAACGACTCAGGCTATCATCAGCTTATTAGCCACTGGTTGAATACTCATGCAGTGATTGAGCCATTCATCATAGCTGCAAATAGACGGCTTAGCGTGCTTCACCCTATTCATAAACTACTGCATCCACACTTTCGTGACACCATGAACATAAACGCACTAGCAAGACAAATCCTCATCAACGGTGGTGGCTTTGTAGAGGCCACAGTTTTTCCATCAAAGTATTCCATGGAGATGTCATCTGTGATTTATAAGAACTGGATTTTCCCTGACCAAGCATTACCCGCAGATCTCATCAAGAGAGGAATGGCTGTGAAAGATTCAGGTTCTCCACACGGCCTTCGACTTGTGATTGAGGATTACCCTTACGCTGTTGACGGGCTAGAGATTTGGTTTGCCATAAAGAACTGGGTTCAGGATTATTGTTCCTTTTACTACAAGGAAGATGACACAGTTAAGAAAGATCCAGAACTACAATCTTGGTGGAAGGAGTTAAGAGAGGAGGGTCACGGTGACAAGAAAAACGAGCCTTGGTGGCCAAAGATGAAGACTCGTGAAGACCTCATTGAAGTGTGCACTATCATTATATGGGTTGCTTCAGCCCTGCATGCATCCACCAACTTTGGCCAATACCCTTATGCAGGTTTCCTCCCAAACCGTCCAACCATAAGCCGAAGATTCATGCCTGAGGAAGGAACTCCAGAATATGATGAACTTGTGAACAATCCTGAGAAGGCTTTTCTGAGAACAATCACAGCACAGTTGCAGACCCTTATAGGCATTTCACTCATAGAAATATTGTCTAGGCACTCTTCTGATGAGGTGTACCTTGGCCAGAGAGACACTCCTAACTGGACTTCTGAAGTGGAACCATTGGAAGCCTTTGAGAGGTTTGGTAAAAAGCTGGCTCAAATTGAGGAAAGGATTGTGACAATGAACAATGATGGGAAACACAAAAATCGAGTTGGACCAGTTAACATGCCTTACACATTACTTTACCCTAGCAGCAAAGCTGGACTCACTGGCATGGGAATTCCCAACAGTGTCGCAATTTGAAAGGGATTGCATGTAGTGTGACTATGATGCATATTTTTTCTTCCATTCTGCTTAAGGATACTTAAGCTTTGTGAATTTCCTATATATTCTTTCTGCATACAGTGTATATCTATTCGTTTACTTTTTATATGTATAATGTAAAAATAAGTTGTTCTGTGGAATGCTAAAAAAA

>C102351_65

GGAAGAGGAGTGGAATGGCATGAAAGAAAGAGGTTTGTTTGGTGTGAGTTGGGTTTTATAGGACAACCGAACAAGCTGACCTCAGAAGGAAGCTGAGGCGGTGGTGTATGATTGCAGCGGCGGAGGATCAATCACAGTTGTTGTAGATGCTGGTATTTATTCGGTGAAGTGGATTTGAATTGAGAAAAGTTGCAGAGAAATAAAGTGAATAGAGAAAGTAAGTGAGAGGAAGAGGAAGAGGATGTGGAGGTTGAAGGTAGGAGACGGAGGAAATGATCCGTACATATTCAGCACGAACAATTTCGTTGGGAGACAGATATGGGAGTATGATCCTGAAGCAGGTACTCCAGAGGAACGAGCTGAGGTTGAAGAAGCTCGTAACAATTTCTACCATAACCGCTTCAAGATCAAGCCCTGTGCTGATCTCCTTTGGCGTTTTCAGGTTTTGAGAGAGAATAACTTCAAACAAACAATACCTAGTGTGAAGATAGAAGATGGAGAAGAAATAACATACGAAAAAGTCACAGCGGCGGTGAGAAGAGGCGCACATCATCTTTCTGGATTGCAGACCAGTGATGGCCATTGGCCTGCACAAATTGCAGGTCCTCTGTTTTTTCTTCCTCCTTTGGTGTTCTGTATGTATATTACAGGACATCTTGAATCAGTATTTCCAGAAGAGTATCGCAAAGAAATTCTCCGCTACATATATTATCACCAGAATGAAGATGGAGGATGGGGACTACACATAGAGGGTCACAGCACCATGTTTTGTACTGCACTAAACTATATATGTATGAGGATTCTTGGAGAAGGACCCAATGGAGGCCATGACAATGCTTGTGCTAGAGCAAGAAAGTGGATTCGAGATCATGGTGGTGTAACACATATACCTTCATGGGGAAAAACTTGGCTTTCGATACTTGGTGTATTTGATTGGTGCGGAAGTAACCCAATGCCCCCAGAGTTTTGGATCCTTCCTTCGTTTCTTCCTATGCATCCAGCTAAGATGTGGTGTTACTGTCGATTGGTGTACATGCCTATGTCTTACTTATATGGAAAGAGGTTTGTTGGACCAATTACACCTCTCATTTTACAATTGAGAGAAGAGCTATTTACTCAACCCTATGAGAAAGTTAATTGGAAGAAAGCCCGTCATCAATGCGCAAAGGAAGATCTTTACTATCCTCATCCTTTGATACAAGATCTGATATGGGATAGTTTATACATATTCACTGAGCCACTACTTACTCGGTGGCCTTTCAACAAGCTGGTTAGAGAAAAAGCTCTTCAAGTAACTATGAAACATATTCATTATGAAGATGAGACTAGTCGATACATTACCATTGGTTGTGTGGAAAAGGTTTTATGCATGCTTGCATGTTGGGTGGAAGATCCGAACGGAGATGCTTTCAAGAAGCATCTTGCAAGGGTGCCAGATTACTTATGGATGTCTGAAGATGGAATGTGCATGCAGAGTTTTGGTAGTCAAGAATGGGATGCTGGTTTTGCTGTTCAAGCTTTGCTTGCTGCTAACCTAGTTGATGAAATTGGTCCCACACTTGCAAAAGGGCATTATTTCATTAAGGAGTCTCAGGTGAAAGATAACCCTTTGGGAGATTTTAAGAGTATGTATCGTCATAATTCTAAAGGATCTTGGACATTCTCTGATCAAGACCATGGATGGCAAGTTTCTGATTGCACCGCGGAAGGCCTGAAGTGTTGTCTACTTCTATCAATGTTGCCACCAGAGATTGTGGGAGAAAAGATGGATACAGAAAGATTATATGACTCAGTCAATATCCTGTTGTCGCTTCAGAGTAAAAAGGGTGGTTTAGCTGCATGGGAGCCTGCAGGAGCTCAAGAATGGTTAGAATTACTCAATCCCACAGAATTTTTTGCGGACATTGTAGTTGAACATGAGTACGTTGAGTGCACTGGATCTGCAATTCAAGCTTTAGTTTTGTTCAAGAAGTTGTATCCAGGACATAGGAAGAAAGAGATAGAGAATTTCATTACCAATGCAGTTCGATTCCTTGAAAATACACAAACAGCTGATGGTTCATGGTACGGAAATTGGGGAGTTTGCTTCACCTATGGTTCTTGGTTTGCACTTGGCGGTCTAGCAGCTGCTGGCAAGACTTACACCAATTGTGCTGCCATTCGTAAAGCAGTTAAATTTCTGCTTACAACACAGAGAGAGGATGGTGGATGGGGGGAGAGTTATCTTTCAAGTCCAAAAAGGATTTATGTACCGTTAGAAGGAAGTCGATCAAATGTTGTACATACAGCATGGGCTCTCATGGGACTGATTCATGCTGGACAGGCAGATAGAGATCCTACTCCTCTTCATCGTGCTGCAAAGTTGCTCATCAATTCTCAGTTGGAAGAGGGTGATTGGCCCCAACAGGAAATCACTGGAGTATTCATGAAAAATTGCATGTTACATTATCCAATGTACAGAGATATTTATCCAATGTGGGCTCTAGCTGAATATCGTAGGCGAGTTCCATTGCCTTCCACTGAAGTTTAAGTTAGAAGGGTATCAGCATAAGAAGACACTCATGATATCTATCAATGAAGATTGGGCCAAATAAAGTTAAACCAGTTGTGCAATCAAGCTTAATCAATTCTTTTCTATTCCTATATGTTTATTTCCTACATATATTGGTACATTTTTTTATTTTATGTTGGTTTGTATATGTATAGATGTTCTTCAATGTTCCTACTAGGATGCAAGAATAATTTTACTTGAATAATATGCATCTACAATTAGAGGCGTCAAAAAAAA

>C102353_65

AAACAAATGAACACAGCTAAGAGCTACCAGAGCATTGATGTTGTGTAGCGAAAACTGGGCGGGAGGGGACGGAATCCTTCATTTCGCCAAGCTCAACTTCAGCCACACACCAACTGTTTTTGTTATTCGCATTCAAAAAGTTGAATACTTTTTCATTTGTTGCATTTGGGATTTTTTTTAACGAGCTGAGCCACAGGTTTATGCTCACCAGAACGTTGTCGTCTCACCAATCACTAACCACCCGTCCGGCACTATCCGCGAGGCCCTAATCTCCGACTGGTGCCCCTTTAGGTGTTACTGACAGGGAAGCCTTCCATTCTTGTTTGCAGATACACCTAAAGCAGTTCGTGAGTTAACAATAAGAAGTAACAATATTCACTCCAATCAGTAGCACTGTCCCCCTTCCCCCACCTCTTCTCTGTTTATATTCCTACAGCCATTGCTTAGCCTTAGCCTTCTTAAAGCGGAACGTTTTTGTGGCAAAAACCAAGGTTTGAATATGCTTCACAGTGCACGCATAGTGTTGTCAGGCAACACCACCATCTCAGCTTTTGCCTTCAGACCCTCTAATACCTCTATTAAGACAACCTCTTCGCTTGGTTTCTACATGGAGCAATTACCTCCTCTAAAGTTAAAGCAGTCTCACCAACCCAAACTCGCCTGTCAAGCAACAACACGTGATGATGATGCTTCTCTAAGGAATGATGAATCACCTGTTCATGGACTATCTGAAGTAGTTGTTGGGGTTCTGGGAGGAGGGCAACTTGGTCGTATGATGTGTCAAGCTGCTTCTCAGATGGCCATTAAAGTCGTGGTTTTGGATCCACAAGAGAATTGCCCTGCTAGTTCCCTCTCTTATCATCATATGGTTGGAAGCTTTGATGACAGCACTACAGTTGAGGAATTTGCCAAGAGATGTGGAGTATTGACTTTTGAAATTGAACATGTTGATGTTGATACATTGGAGAAACTTGAGAAACAGGGAGTTGACTGCCAGCCCAAAGCCTCCACAGTCAGAATTATTCAGGATAAGTATCAGCAAAAGGTTCACTTCTCCAAGCATGACATTCCACTTCCTGAATTTAGGAAGATTGATGATCTTGAAGGTGCTAAGAAAGTAGGGAAACTCTTTGGATATCCTCTTATGATTAAGAGCAGGAGATTAGCTTATGATGGGCGAGGCAATTTTGTTGCTAAAAGTGAAGAGGAACTTTCTTCTGCTGTGGATGCTCTCGGAGGATTTGATCGTGGTTTATATGCTGAAAAATGGGCACCTTTTGTCAAGGAACTAGCTGTCGTTGTGGCAAGAGGAAGAGACAATTCAATTTCATGCTATCCTGTTGTTGAAACTATTCACAGGGACAACATATGTCACATAGTTAAGGCTCCGGCTAATGTGAAATGGAAGACGAAGGAGCTTGCCACTGAAGTTGCTTTCAATGCTGTTAACTCTCTGGAAGGTGCTGGTGTGTTTGCCGTTGAATTGTTCTTGACTAAGGATGGAGAGATTTTATTAAATGAAGTAGCACCTAGACCTCACAATAGTGGGCATCACACAATTGAATCTTGCTATACCTCGCAATTTGAGCAACATTTGCGGGCTGTTGTTGGTCTTCCACTTGGTGATCCATCAATGAAAACTCCAGCTGCTATCATGTACAACATATTAGGCGAAGAAGAGGGGGAGCTTGGTTTTCAATTAGCTCATCAATTGATCAAGAGGGCACTGGCCACCCCTGGTGCTGCTGTTCATTGGTATGATAAGCCAGAAATGAGAAAGCAACGGAAGATGGGTCACATAACCATTGTTGGGCCATCCCTTAACAATATTCAAAGCAATCTTACAAAACTGGTGGAAGGGAAAAGATTAGATGATAAGACTGCTGTTGCTCCACGTGTGGGGATCATAATGGGTTCTGATTCAGATCTGCCTGTTATGAAAAGTGCTGCTGAAATCTTGGACATGTTTGGTGTGCCTTATGAGGTAAGAATAGTTTCAGCGCACAGGACTCCAGAATTGATGTTTTCTTATGCCTCATCTGCTCATGAACGAGGCATACAAGTCATTATTGCTGGTGCTGGTGGTGCTGCTCACTTGCCTGGTATGGTTGCTGCTCTTACTCCCTTGCCTGTTGTTGGCGTTCCTGTGCGTGCTTCTACCTTGGATGGAATTGATTCACTCTTGTCAATTGTCCAGATGCCGCGAGGTGTTCCAGTTGCCACTATTGCAGTTAATAATGCAACTAATGCTGGATTGCTGGCAGTGAGGATATTGGGTGTTGCCGATGATAACCTTCTGTCAAGGATGATTCAATATCAGGAGGACCAAAAAGAAAGCGTCTTGACCAAAGGAGATAAATTACAAAAACATGGTTGGGAATCCTACTTAAACAATAGTTAATTAGGCCCATTAATTTGGTCATTCTCTTTTTTGGCTCGTTTCTCATTTTTGACCAACTACAAAATTTTGGTAGAAAATATTTCATATGGAGGGTGATATATCAATAAGTGGTTTGAGAAACGTTGGAGCTATATGTTCTGTTACCTAAATCAACAGATTTTCTATTTCGTATTGCTAACATGAGGCAAGAAAGGAAAATTATGCAATATGACTGAAAAGTCTTGTATTCACATTGATATTTACTGTTATAAAAATAAGTCTCTGCCGGTGCCTTGACAAATTAGACCAGTTAGAAAGCAAATGACGAGCCAAATTTATAACTTGTATTTTATTTCGTTGGTAGATGAAGTTGATTAAATGCATAATGGGCCACAATTATATATTTGAG

>C102355_65

AATTATTGCAAGATATCTTGGCTAGGCCCGCAGCCTGAACAGTCCTATGAAGAATATATACCAACTTTCCCTGCATTTCTGAAGCAAATTGGAAAGAAGGCTGAGACTAAATTGTGCTGAAACTCGAATGGTGGAGCGAGAGCAAGGTGACAAGATGAATATGCTGATATTCAAGTCCCTTCTGATTTCTTTGTTGCTTGTTTTCTGTAGCAGATACACCATAGCAGAAAAGAAAACCAATCACCAAAGTAAAAGCACATACATAATTCACATGGACAAGTTCAACATGCCTTCAAGCTTCGACGACCATCTCCTCTGGTATGATTCATCACTGAAATCAGTGTCAGAATCTGCAGAGATGCTCTACACTTACCAACATGTAGCTCATGGCTTCTCCACAAGGCTAACTAGCCAAGAAGCAGAGTTACTCTCAAAGCAACCGGGAATTCTATCTGTCATCCCTGAAGTTAGATACGATCTTCACACAACTCGAACACCACAGTTTCTTGGATTGGAGAAAACCATCGCTCTTCCACTGGCCTCTGGAAAACAAAGCGATGTGGTCGTTGGAGTGCTGGACACTGGTGTGTGGCCTGAGCTAAAAAGCTTCGATGACAAGGGTCTTGGGCCAGTTCCAAGTAGCTGGAAAGGTGAGTGTGAGAGAGGTAAGAACTTCAGCCCATCAAACTGTAACAGGAAGCTTGTTGGTGCAAGGTTTTTCGCGAAGGGCTATGAGGCGGCGTTTGGACCAATCGATGAAAGTACAGAATCAAAGTCACCAAGGGATGATGATGGCCATGGAAGTCACACCTCAACAACAGCAGCAGGTTCTGTAGTTGCAGGAGCTAGCCTCTTTGGTTTTGCTAATGGGACAGCAAGAGGCATGGCCACGCAAGCCAGAGTTGCTACATACAAAGTGTGTTGGCTTGGCGGGTGCTTCACATCAGACATGGCGGCTGGCATTGACAAAGCCATTGCAGATGGTGTGAACATCCTTTCCATGTCAATTGGGGGAGGTTTGACTGATTACTACAGAGACACTATAGCAATTGGAACTTTTGCTGCCACTGCCCATGGAATACTGGTTTCCAATTCAGCAGGAAATGGTGGGCCTAGTCAAGCATCTTTGTCTAATGTGGCACCATGGTTAACCACAGTGGGTGCTGGGACTATTGACCGTGACTTCCCTGCCTACGTCACTCTTGGAAATGGGAAGGCATACACTGGGGTGTCCCTTTACAATGGCAAACTCCCACCTAAGTCTCCAATTCCAATTGTTTATGCCGGTAATGTAAGTGTTGATTCAGAAGGAAGTCTGTGCAGCAAAGGTAGTTTGATTGCTGCAAAAGTAGCAGGAAAAATCGTGATATGTGACAGGGGAGGGAATGCTAGGGTGGAAAAGGGTTTGGTGGTGAAGAGTGCTGGAGGGATTGGGATGATACTAGCAAACAGTGAAGAATATGGGGAAGAGTTAGTAGCTGACTCCTTTCTCCTCCCAGCATTAGCTTTGGGCCAGAAATCCAGCAAAGAGGTAAAGAAGTATGTTTTCTCATCTCCCAATCCCACAGCTAAACTTTCATATGGTAGCACCCAGTTAGGGGTTCAACCATCCCCAGTGGTGGCAGCTTTCAGCTCAAGAGGGCCAAATTTGCTAACACCAAAGGTGCTCAAACCAGACCTGATAGCTCCAGGAGTTAACATCCTAGCTGGATGGACGGGTGCAGTTGGACCAACTGGCATGACTGAAGACACCAGGCATGTGGAATTCAACATCATCTCAGGCACATCCATGTCCTGCCCCCATGTCAGTGGGTTAGCTGCCCTTCTTAAGGGCACTCACCCAGAATGGAGCCCTGCAGCTATAAGGTCTGCCCTCATGACCACATCTTACAGAACCTACAAAAATGGGCAAACCATAAAAGATATTGCTACTGGCCTACCGGCTACACCGTTTGATTTTGGGGCTGGACATGTAGATCCAGTGGCTGCTTTTGATCCTGGCCTTGTCTATGATGCCACCGTGGATGACTATTTGAGCTTCTTCTGTGCCTTGAACTACAGTTCCTATCAAATAAAGCTTGTTGCAAGGAGAGATTTTACTTGCAGCAAAAGGAAGAAGTACAGAGTGGAAGATCTCAACTACCCATCTTTTGCTGTTCCTTTTAACACGGCTTTTGGAGTAAAGGGTGGTTCTCAGAAACCTACCACTGTTCAGTACACAAGGACTTTAACAAATGTGGGAGCTCCAGCGACGTATAAAGTTTCATTGACAGAGTCTCCCTCAGTGAAGATTGTGGTTCAACCAGAAACACTAAGTTTCAGTGAACTGAATGAGAAAAAGAGCTACACAGTTACATTCACGTCTTCTTCAAAGCCTTCTGGAACAACCATCTTTGCTTATCTGGAATGGTCTGATGGGAAACACAAGGTTACTAGTCCCATTGCTTTCAGCTGGACTTGACCCTGATTTTTAAAGGCCTTAACTCTGAATTACCTGTGGCAAACGCGGCTGAGTTCAAGAATAATATTTTTCTTTGTTTTGTTCTGCTTTTGGTCTAGCAAAGGGGTATGATGTTATGTGTCTCTTTGCTGTTCTCTGAAAATTAGTAAAGAATGATTGTACAGTAAAGAAACAGGGTAATGGACTAATGGCGTGTCTCAATAAATGAATAATGACACTGAATGGTAATTCGTAGTTACTTAGCTTTGCGCTCGTTGCCGCTTACATGACAGATTTATCTACCAAATATGATTGATCCACCACTGCAGGCCTCTGAATTGCATTAACA

>C102357_65

AAATTTTGACTTTCTAAAGAGATGCTGGGTTAACAGTTTATCTTCAGGTGATCTCGAATGTTGGAACACTGCAGAGAGCAAGGAGTATCATTGACTGCTGCAAAACACCACAAACTCTGGTGAAAGGACTGGGAAGATGGAGAAAGAAGATGATGTGAACCATGGTGATTATGAGCATGACATAGAAATTGAAGGTCTGCCGGATGGGAATGAGGAAAATGGAAGGTACTGGTCAGGGTTATCTGACAGAAGCCTGGCTCATACAAAGCCACTTCTTGAGAAGAGGACCAACACCACCTCACAGATTGCTATAGTTGGTGCCAACCTCAGCCCTATTGAAAGCCTTGACTATGAGGTTTTTGATAATGAAATATTGAAGCATGACTGGAGGTCCAGGAAGAGGTTTCAGATAATCCAATATGTGGTGCTTAAGTGGGGATTTGCTCTTCTTATTGGATTAGGCACAGGATTGGTTGGCTTCTTTAACAGTTTTGCAGTTGAGAACATATCTGGTTTCAAGCTGCTTATTACCACCACTCTCATGTCTAAAAACAGATATCTTGAGGCATTTTTAGCATATGCTGGTACGAATATATGTTTAGCAGCAGCAGCTGCTGCACTTTGTGCTTTCATTGCTCCAGCAGCGGCAGGCTCTGGGATTCCAGAGGTAAAAGCATATCTCAACGGCGTGGATGCTCACTATATATTGGCTCCAAGCACCCTTTTTGTAAAGATATTTGGTTCCATCCTTGGAGTTTCTGCCGGATTTGTGGTGGGCAAAGAGGGACCTATGGTACATACAGGTGCATGCATAGCTTCTTTATTGGGCCAGGGTGGATCTCGCAAGTATCACTTGACTTGCAATTGGCTTAGATATTTCAAAAACGACCGAGACCGACGAGACATGATTACTTGTGGTGCAGCTGCTGGTGTTGCTGCTGCCTTTCGTGCACCAGTAGGTGGTGTTCTCTTTGCTTTAGAAGAGGCAGCTTCATGGTGGCGGAGTGCACTTCTTTGGAGGACATTCTTCACTACAGCAGTAGTTGCTATTGTTTTAAGAGTTGCAATTCAGTTTTGTGCTACAGGAAAATGTGGGCTATTCGGTGAAGGTGGTCTGATATTGTATGATGTTAGTTCAGCCAATATAGCATATAGTGCTAGTGATATGTTTGTAGTGCTACTTTTAGGAGCCGTTGGTGGAATTCTTGGAAGCATATATAATTACCTTGTGGATAAGGTTGTTCGAACATATAGCATCATCAATGAAAAAGGTCCAGCTTTCAAAATCTCTCTTGTTGTCACAGTTGCTCTTTTGACATCATGTTGTTTTTATTTCTTGCCATGGGTTACACACTGCACTCGCTGTCCTTCTGATTCGACAACTGCCTGTCCCTCTATTGATGAGTCTGGAGAATACCAAAACTTTCAATGCCCTCCAGGCTACTATAATGATCTTGCTTCCCTCTTTCTGAACACCAATGATGATGCTATTCGTAATCTATTCAGCCCCAAGATAATCAAAGAGTTTCGTATTTCCAGTTTGTTCATTTACTTTGCTACTATTTACTTTCTTGGGATAATCACTTATGGTATTGCTATTCCATCCGGGCTCTTCATTCCTGTGATACTTGCTGGTGCTGCCTATGGCCGTCTTATTAGCCGGCTCATTGAACCGATCACCAAACTTGACAGAGGACTCTTTGCCCTACTTGGAGCTGCTTCCTTCCTTGGTGGCACTATGAGAATGACAGTTTCTATTTGTGTCATATTGCTTGAGCTCACTAATGATCTCTTGTTACTTCCACTTGTGATGTTGGTCTTATTAGTCTCAAAAACTGTGGCTGACAGCTTCAATAAGGGTGTCTATGATCTAATACTTCAGATCAAAGGACTTCCTTATTTGGAGGCTCATGCAGAACCTTACATGAGGAATTTAGTTACTCGTGATGTTGTTTCAGGTCCATTGATTACCTTTTCTGGCATTGAAAAGGTTGGGAATATATGGCATGTTTTGAATGCTACAGGGCATAATGGGTTTCCTGTCATTGATGAACCACCTTTGACAGATGCACCAGAATTATGTGGGCTTGTACTAAGGTCTCATTTACTAGTGTTGCTAAAGGAAAAGATTTTTTCAAGAGATAGGGATTTCATGGATCAAAGTATTTTCCAAAGAATTTCTGCTTTAGATTTTGGAAAGCCAGGATCAGGAAAGGGGATCAAACTAGAGGATGTTGATATTGATGAGGAAGAGATGGATATGTATGTTGATCTCCATCCCATAACTAATGCATCTCCGTACACTGTGGTTGAGACAATGTCACTTGCCAAAGCTGCTATTCTCTTCCGCCAACATGGACTCAGGCACATGTGTGTTGTTCCAAAGAGCCAAGGGAGACCTCCAATTGTTGGGATTCTAACCCGCCATGATTTTATGCCAGAGCATGTTTTGGGACTTTACCCTCACATCAAGCCTCACAAACAGCATTGAGTGATATCAATGGAAAACATTCTTTCTTCAGTATTGAAGTGCTTCATTCATTAGTTGATCAAAGTTTTCACATACAGTAGTAGAGTGCAATAATTATTCACAAAAACATCTGAAGCAGGAATGCTACTAAGATGTGGCTGCTGCATAGTTTTGATGCGTGATTTAGGAACTGTGTCCATATATTATTTGTTAATCTCTATTCCATAAACTCAGCCTGAAACTTGGATATAGATAGATACTATGAAACATGGAGTTGCAGTATACTGTGGACAGTGATCTCTTCTCTTGTACATGTTGC

>C102359_65

ACATTGCCCCATGTACCAAAATTAAAACTTTCCACACACATGCCTGCACTTAGAGGGAGTCAATGCCTTCTATTTAACCACACTGTTACTCTTTGTACTTTTTCTTTGATGGGTAAGAGTGGTTTTTGTTTTGCCCCTTCTGTCTCCTTTAGTTTGGTTTAAGCCTTTTTCCTTTTGTTTGTTTGTTAGGGGTAGAGAACTGGGTGGTTGACCTAAACTAAGTGGTGATAAGGAATTTAGGTTTCCGGGGATCAGTTTTTATTTTTTGGGAAGTGGGTATTTGTGTTTTTTTTTCTTGTATGTGTGTCCTAAAAATAGTGGAGTGGTTCTAGGGTTTTTGAGGTGGTGGTGTTATTGATTGAATCTGGTGGTGGATTCGGCTCTTTCGGTTTGTTGGGTTGCTGGGAATTTTGATAAAGGTTGTGTCTTTTGAAGAAGGGCTTGGAAAGAAGCTGCATTGTGAATCAATTTCGTGGAGGGTGAATTTGAGGCCAAGTGGTGCTGTGTTGGTACAGTGTTTGTGAAGGGTTCGATTGGGTTTCAAATTCAATTGACCTTAGATCTGTTCTTCACTTGGTTTTATTCATCAATTGTTGTTCTCTTCTGGGGAATCGAGTAAATGGCTTGCGTCAAATTGGGTTCCAAAGCTGATGCTTTCCAGCGTCAAGGACAGGCCTGGTTCTGCACTACTGGGCTTCCGAGTGATATAGTTGTTGAAGTTGGAGAGATGTCCTTCCATCTTCACAAGTTCCCTTTGCTCTCTAGAAGTGGGGTTTTGGAAAGAATGATTGCTGAGGCTTCTGAATCAATGGAAGAATGTGTCATATCCCTCGGTGACATTCCTGGTGGTGCCAAAACATTTGAACTTGTGGCGAAATTTTGCTATGGTGTGAAACTTGAACTTACAGCTTCAAATGTTGTGTACCTCTGGTGTGCTGCAGAGCGTCTAGAAATGACTGAGGATTATGGTGAAGGTAATCTCATTTCACAGGCTGAAGCCTTTTTCAATCAAGTGGTCCTCCGCAGTTGGAAAGACTCTCTGAGGGCACTTCAAAGCTGTGATGATGTTTCAGCCCATGCTGAAGAGCTCCATATTGTGAAAAGGTGCATTGAGTCACTAGCAGCAAAGGCATCTACTGACCCAAATTTATTTGGGTGGCCAGTGCTGGAGCGTGGTGGTCCTTTGCAGAGCCCTGGTGGAAGCGTTTTGTGGAATGGAATAAGTACTGGGGCAAGACCAAAAAATTCAAGTGCAGATTGGTGGTATGAGGATGTATCGATTTTGAGTTTACCTCTTTATAAGAGATTGATAACTGTCATGGAATCTCGTGGCATTAGGCAGGAGATCGTTGCCGGCTCTATTGCTTTCTATGCTAAAACATATTTGCCAGGGTTAAATAGACGTCAGGTTTCTGGTGAGTCCAGCACCAGACTGACGCAGGTAGCTATGGGGTCTCCTCTATCTGAAGATGACCAGAAGATTCTACTGGAAGAGATTGATTCTTTGCTCCCAATGCAGAAGGGCCTGGTCCAAACAAAGTTCTTGTTTGGTCTACTACGAACAGCCATGATTCTACGAGTGAGCCCCTCTTGCATATCTAATTTGGAGAAACGGATCGGCATGCAGCTTGACCAAGCTACTCTGGAAGATCTCTTAATGCCGAATTTCTCTTATTCTATGGAGACACTTTACAATGTTGATTGCGTGCAAAGAATTCTTGACCATTTCCTTGCCATGGATCAGGTTACTGGTGGTGCCTCTCCATGCTCAATTGATGATGGTCAATTGATTGGATCACCTTCACTGACACCAATCACCATGGTAGCCAAGCTGATTGATGGTTACCTCGCAGAGGTTGCCCCGGATATTAATTTAAAACTTCCGAAGTTTCAGGCCCTTGCTGCGGCTGTTCCAGAGTATGCCAGGCCTTTGGATGACGGTTTATATCGGGCTATAGATATTTATTTGAAGTCTCACCCATGGTTGGTGGAGTCTGAGAGAGAGCAGCTATGTAGGCTGATGGATTGCCAGAAGCTCTCATTAGAGGCATGCACCCATGCTGCGCAGAACGAGAGGCTACCGATCAGAATAATATTTCAAGTTCTATTTTTCGAGCAGCTCCAGCTCCGGACTTCAATCGCTGGTTGCTTTCTAGTTTCAGATAATCTGGATGGATCGAGGCAGTTGAGAAGTGGTTTTGTTGGATCTACAGAGGGTGGCTGGGCTTCAGCTGTGAAGGAAAATCAGGTTTTGAAAGTGGGAATGGATAACATGAGAATGAGGGTGTCTGAGCTTGAGAAGGAGTGCTCTAACATGAGGCAGGAGATTGAGAAATTGGGTCGTTCAAAGGGATCAAGCGCTTGGGGAACTGTGTCTAAGAAACTTGGGTTTAAGATGAAGTCTCAGATGTGTAGTGCTCAAGAAGGGTCAGTTAGCAACCAGAACAATGGAAATAGTAAGGTTGAGAAGTTGAAGGAAAGACATGTAAAGCACAAGAAAAGTTCTTCTATTAGTGACAAAGTATCAGTCTCTTCAATTGTTCATTCATAGTGTCTACATCTTAATCTTTCTCACAACCTCTTTCCTCTCATGTTACTCTGTCATATTGACTAATCAATTTCTTGGCCCCTGCTTCTCAATCAAGTGTAACTCAGATGCAAACACAAGTCTCTGCATTGTGTAAACCGTAGATTTAGCTTCCTTTATTTATTTTTCTTTTTTCCTTCCTCTGTCCCACTACATATGCATATAAAGTTTCCATGCACTTTATGTCTCCTACTTCTCCAAAA

>C102363_65

ACTGAATGTACAGAATGTCCTGTTTTACACACACTATTTACCATGAAGCCACCTATGATTTGAGATATGTCAAGCTAGATGCACTCAACTCAGACATTTTTGAATCAGAACTCCCAATCCGAATAACACGATGAAGAAAAATGAAGATTTTATACACTTGCTATACCAGTAAACAAGAATTCAATACCTGTAAGAGTACAATGTGAGCAGTGCCCTATAAGAAATACTTGCGATGAATGGTGAGAATGCAAAATGAATATAGAGTTGGATAACTGAGAAACCTTGTTTTGGTACTTGCAATCGATTATGTGTTTGATAGACCTGTACTGCGAGTTTTTAAGGAAAGAAAGGATTTAACTATACTTCCTTTGCTGGACTGCAACTTGGCTGATAAACTAGTAGAAGGTGTTTTCCATTCATCGTCTCTTTTATGCTGTTTTGAATGAAAACTAGCTCATCTGCTCTCCAATCAAATCAAGCCCCACAAGGAGGAAGGTCACCCCTTGGAAAAGTAGAAAGTAGAAGTGAACTCCTTGTGCAGATAACAGACTCCTGACAGAAGCTGTGAGAAGGAGGAATGCCAAAGTCAGCTCTTTCTTATATATCTTATTAGTTTTCTTAACAGTTGTTGGAGCTGCTTCTTTGTGCTCCATAAACTTTTGTGACTCAATAAGATCACTGTCGGAAGCTCCTCTATGAATCTTTTGTTGTTCTATTGACTTGGTTTCCCTTTCTGCAGCAGCCAGTAAATCAGACTCTGATGACCTGCCAGCTTTCTTTGTGACAACCCATTCATAAGAGCTGCCTAATTGGAACAGACCAGATACCATTGCATTGAATTTGGTGACGGACATGGTGTTTTCGAAAAGGAGGTAGGGAACAATGAAAGGAAAAGATTTTGGAGCAGGAAGAATGTTAAGGAGTGACATAAATACAGGCACATAGCAGATCACCCACAAAGGAAGCTCTGCCTCTGGCACAAACATGGTTAGAGGAAGAATTATGCAAAATAAGGTGAAGGAGTAAAAGGGAAGGATCAATTTCCTTAACAGAAAAAATAGTAGGATTAAGTTTGCTTTCTTCCATGCAGACACCTTAGACTTCACAATTGCTGGAAGACACAACCTAAAAAGTTGCATAGGACCTGAATGCCAGCGGTGTTGCTGCTTCCTATAAGCTTCATATGACTCAGGAACTTCACAGGGCACCTTTACATCATTTAGGAAGATAAATTTCCAACCATTGAGATGAGCTCGGACAGCAATGTCCATATCTTCTACAGTTGTCCTTTCTAGCCAGCCCCCGGACTCTTCTAGGGCCTTGATTCTCCAAACACCAGCAGTTCCATTGAAACCAAAGAAATTGAGAAATACCCCATTCACCTGCTGTTCGACCTCAAAATGGAAGCATAAGTTTATGTTTTGGAGACGAGTCAACAAATTCTCATCCTTGTTCACAAAAGACCATCTAGCTTGGACCAAACCTAGTTCGGGATTGTCCTTGAAATGTGGCACAGTTCGCTTAAGAAAATCAGGATTTGGTTGAAAGTCTGCATCAAAAATTGCAACAAACTCGTAGTCTTTCACATAATCACAGCTCATTGCAGAGTTGAGATTTCCAGCTTTATATCCTGTCCTTACTAAGCGATGCCTATAGATTATATTGATTCCCTTATGGCTCCATTTAGATACCTCTGCCTTAATTAACCACTGTATGCTCTCATCATCAGAATCGTCAAGTACTTGAATCAGTAAACGATCCCTTGGCCAATCAATGCCACAGACTGCAGAAATGGATTGGTCATACACCTCCTTCTCGTTGCACATCGGAATTTGAACCAGAACCATTGGATAATTGCTTGCAGATCCTTCAACATCATCACTCTTGAACGGATCCCCATCAATCTTTGGCTTGACCTTCCTGTATTTGATCCAAAAGCACCCCAAGCAAAGGAGCATGCGATCCACAGACTGGATAAGAAACAAAACAACACAAAATTTGGAAAGTGCCTGTATAGTAGGAGCAATGTACTCGGCACGAAACGTCAACCAAGCAACATAAGCCAAGTGGAGCAATCCTTCCAAATCAGAAGTACGAGGAATGTGCAAAGTGGGGTTCCCAAAATGCCAACCTTGAAGGTGAGCAACAACCTCAAAGGCCAGAACTGCCAAGGAAGTCACTAGGAACACTCTAATGACTTTAAACAGCAACTTGCCTTTTTCCAACTTGTCACTCTCACTCTCCATAGCCACACCTTGTCCATGAATCAACCTCTTCTTAATGGCCCCCAGCAACGCCCAAAGCACAGTGGCAAGCCAAGTGACACAGCCAACAGCTCGGTGAGCCCTGAGAAGCAACACCCAGGTGACTTGCTTAGCATTCTTGCCACGGCTTTTCTCCACTGGCCTGAATGCAGCATCTGCACCATTGATCTCCACAACGGAGAAAGTGGGGTTCTCCATTTTCACCACCACTGGTGTTCCCTTTTGTGTATCCTTGGTCCACCAATTGGAAAAGTCCAACCTCGGAGCCATTACCACACTATGGTGAATTGGACACAGATTCACACTTCACACACTGAACAACACAAGTATGTTACTCTGTGTGTGTGTATAGATATACACTGAGCAAGTACTCAACACACCAACTTCATCAATATTTTCTAGCACACATCATGGTAAAGACTAAAGAGAAAAGACTCAGATCTGGAAACTCATGTAAAAGACTGCCCTAGATTCGTGGTTGCTCTTATTGGATGGAACCCTCTTGAAGGCTCGGCATTTAACAAAAT

>C102367_65

ATTAACTTCTAGCACCAACCACAAGGTAGATAGCTCTTCATTCAACTTGCTTGGAATCAACAAAAACAACCAGGCACTCTTATTACCCGCGCAATGGTGACAAGAGTAAAGATGGCACTCTTCTCCCTTCTCTTAAATATCTAAGGCAGCCAAACACACACACCACAATACAAATAAGCATGAACACTCATCAAATTCTCTTCACTCATCTTTCTCCCTTTGAACTCAATGCCTAAAAACCCCTAGTTTCCTTCTTCCAAGCACGTAACACGCGTCCTATCACTCCATTGCACACAATGAGTCTCATTCTAACAAGTTATCTCCTCCTGTGTTTACTATCATCTGCAAATGCTGAATTAGCCAAGAAGACTTACATCATTCAAATGGATAAGTCAGCCAAGCCTGACTCCTTCTCCAATCACCTTGAATGGTATACTTCAAAAGTGAAATCAATTCTGTCCAACTCAGTAGAAGCTGAGATGGACCAGGAGGAGAGAATCATTTACACTTACCAAACTGCTTTTCATGGAGTGGCTGCCAAGTTGAGCCAAGAAGAAGCTGAGAAGCTACAGGCAGAAGAAGGTGTTGTGGCCATATTCCCTGATACAAAGTACCAACTACACACCACGAGAAGTCCAACTTTCCTTGGGCTTGAACCAACACAAAGCCCTAACGTGTGGTCAGAAAAGCTAGCCAATCATGATGTCGTAGTGGGAGTGCTCGACACTGGGGTTTGGCCTGAGAGTGAGAGCTTCAATGACACAGGCATGAGATCAGTACCTTCTCACTGGAAAGGTGCCTGTGAGACCGGCAGAGGCTTCGAAAAATATCACTGCAACAAGAAGATTGTGGGGGCAAGAATGTTTTACCATGGATATGAAGCAGCAACAGGTAAAATTGATGAGCGAACAGAATACAAATCACCAAGAGATCAAGATGGTCATGGAACTCACACTGCAGCTACAGTTGCTGGCTCTCCAGTGCATGGTGCTAATCTTCTCGGCTATGCTTATGGCACAGCAAGAGGAATGGCACCAGGAGCAAGAATTGCTGCTTACAAAGTATGCTGGACTGGTGGATGTTTCAGCTCAGATATTCTGTCAGCTGTTGATAGAGCCGTGGCTGATGGAGTTGATGTTCTATCCATCTCTTTGGGTGGTGGAGTCTCCTCTTACTACCGTGATAGTTTATCTGTAGCTGCTTTTGGAGCAATGGAGAAAGGTGTTTTTGTTTCATGTTCTGCTGGAAATGCAGGACCTGACCCTGTGAGCCTCACAAATGTGTCACCTTGGATCACCACAGTTGGAGCCAGCACAATGGATAGAGATTTTCCTGCAGATGTTAGGCTTGGAAATGGAAGAAAGATAACCGGAACCTCGCTCTATAAAGGGAGAAGCGTGCTGTCAGTTAAGAAACAATACCCTTTAGTATACATGGGGAATATTAACTCAAGCATCCCTGATCCAAGATCTTTGTGCTTGGAAGGTACTTTGGATCGTAGAATGGTTTCAGGCAAGATTGTTATCTGTGACAGAGGCATTAGTCCAAGAGTGCAGAAGGGTCAAGTGGTGAAAAATGCAGGAGGGGTGGGAATGATTCTGACCAACACTGCAGCTAATGGAGAGGAGCTGGTTGCAGACTGTCACCTCCTTCCAGCGGTTGCAATTGGAGAGAAAGAAGGCAAGGAGCTTAAACATTATGTCTTAACAAGTAAAAAAGCTACTGCAACTCTAGCTTTTCTGGCTACAAGGTTGGGTGTTAGGCCATCTCCTGTAGTGGCAGCATTTTCATCAAGAGGGCCTAATTTTCTTACCCTTGAAATTCTGAAGCCTGATGTGGTGGCTCCTGGGGTGAACATTCTTGCTGCTTGGAGTGGAGCAATTGGTCCTTCAAGTTTGCCAACAGACCACAGGAGGGTGAAGTTCAATATACTCTCAGGAACTTCAATGTCATGTCCTCATGTAAGTGGCATTGCTGCTTTGCTCAAGGGTAGGCATCCAGAATGGAGTCCTGCTGCTATAAAATCTGCTTTGATGACCACAGCTTATGTCCATGACAATACCATCAAGCCTCTCAGAGATGCCTCAAGTCCTGATGCCTCAACTCCTTATGATCATGGTGCAGGACACATCAATCCCGGGAGAGCTCTTGACCCAGGTTTGGTTTATGATATTCAGCCACAGGATTACTTTGAATTCCTGTGCACACAGAAACTAACTCCATCTGAACTTGGAGTTTTTGCTAAGTATTCCAACAGAAGTTGCAGACGCTCTCTTGCAAGTCCAGGGGACTTGAACTACCCAGCCATATCCGTAGTTTTTCCACAGATAAATTCCTCTTCAGTTTTGAGCGTTCACAGAACTGCCACAAATGTTGGCCCTCCTGTTTCCAAGTACCATGTTGTGGTTTCACCTTTCAAAGGCGCTTCTGTTAAAGTTGAACCAGGCATCTTGAGTTTTACCAAAAAATACCAGAAACTATCTTACAAAGTTACATTCACAACACAATCTCGACAAACAGAACCTGAATTTGGAGGTCTGGTATGGAAGGATGGTGTGCACAAGGTAAGAAGTCCAATTGTTATTACATATCTGTCACCAGTATGAAACTTCACTTGGACTCCCTATAAGTTTAGCAGTATATTTTTTTTTTGCAATAAGCAAAATTTTCTGTACTATGAAGTGTACGCATTTTTATTACAGGAAAATGCTGGATATTATCTGCTTTCAATAATGATCTTGCGTGCTAAACTACCAAAAAAAATGATGTTGCATGCTTCTTGCTGCATCATAG

>C102371_65

TTTTTTTGGAAAAATAAAAAATAAGGTTCTTATTATACATCACTGTTAACATAATAAAGTCTAAAAGCTTACAAAACAAAGTAAAGTGAGCATTGAGATATGGTAACCAGGAACTTGCTTACAGTGGTCAACAAGCATTAAGTGCAACCCACTTTCTCAAACATTTCGAAGTCTAAACTAAGAAATCTTAAACACTGAGCAACAAGATGTCTTGACATTAAACATCAAATGAACTTTCTGGTCGTTCATCAATGGCAGAAGATGATTTTGGGGATGGCAGTGGTGAAGTTTCATCAGGTTTCACACTTTGATCAGCAGGCGGTATGCACCTTTTTGAAAAAAATGCAGCAATACTCTTCGGTCCCCCACTGCTTTTTTCAGGTTTAATGGCCAAACCAAGCTTATCCAGAATTTCTTTCTTCACCTGCAAACGGTTATCAACATATTCAGATACTTCGAGTACTTTATTCTTCAGTAAGGACTTGGGCACAGAACGGAATTTCTGTTGCAAAGACACTAAAACTTTATTCATACTTCGAGAACTTTGAATAGTGGTCACAATTATGGGTAGGTCTGAATCAGGAATGACAGCTACACCAGATATTGGACTAGCACCACTTTTACCTACAGAGAGGTAGACCTCTTGTTCCTCGTCTTGCATTTTATCTCTGGATATTTCTATAGTTGAGCTGCCAGGAATTGCGTACATACTCAAGGCCTGCAACCACGTCTGCCCCATCTTAGAAGTACCACTAATACGGTGATCTGATAAAATCTGTTTGTCGCGAGTGATATTTGTTATAATCAAGGGTTGATTTTTCCGGAGAGCATGCTCTGTCAAATTGTTTATATATTTCTGCTGCCGGAGTAAAGCAGAAAACTCCTCATTTACTGCGTCATTCTTACAGCTAGATGAGTTATCAGCACTCTCAATGTCATCATCTATTTCCAATCTGTCCACTTGTACACCCTCATCTTCGGAGAGATAGCCATCTGGTACAAAAAACCCATCTTCACTTTCTTCGTCAGACTTTGAGCATTCCTCTTGACAGTCCTCCTCATCTTTATCACAATCAGAAAGACTTTCACCAGGTTCCTCCTCTTCCCACTCCTCATCACTGCTGACAACATAATCCAGGCTTGGATCCTTCCTTAAAGGATGGCGTGGTCCAACAACATTACTTTTTGTTGGCCAAACACCATAGAAGGCAGGTCTATGAGATTTATCAAACTGAAATAATTGTCTCCTCCGATGGTCCTTCTTGGCATCACGAGGAGAACTATCAGCATTTACTGGACATGAACTGATATTAGAACTATGTTCACCCAATCTTTTCACATGTTTCTCCATGTCCAACTCACCATCATGAGCTTCAGTTTTAATAGCTGTCAGCTTAAGTTCCTTGATAACTTCAATCCTAGGTTGCCGACGTATGCCCCATTTCTGTTTTCTGTTTGAGCGAATTGATTGTCCTAAAGAGCGCCATGAAGAAAAGTGTGACTTGCGAATATCCTCAAGTGTTACATCACTACTTGATGCGAGAGTACAATCCATTGAAAGCGTAGCTGACTCAAACAAACTTTCATTCTTGCCGCTTGACAAATCAGATGCAGTTGATTTCTCTGAAACTTTGTCATTCTCAGATGAGGTATTAGTTTTACATCTTTTCAGAAAACGCTCCATAATTGAGGCTTGCTTTTCTAAACTACGCTTCTGTTTTGATTCAGCTTCAGCTTTCTGTCTACGTCGTTTATCCCTTTCTGTTTCCTCTACCTGCTTCTTTTGCCGTTTCTTCTTTTCAGAAGATTTCACATCATTTCTTGCCTCACCCGGTGACAATTGTAAGTCTGATTCACTCGACAGTGTTTCTCTTTGCAGTTCATTGTGCATTCTTTCTTTCTCTTTCTCTTTCTCAGCTTCTCTTCTATTTCTCTCCAGCTGTTTAATTAGCAATTTATTTTCTTGATCTGCTCTTTTAATGTCCATGTCTTCACTGTTTTTCTGCAACGAACTACCCACATAAGAGCGGATATCTGCCTCAGGAAGAGCTTTACTGAGCTTTGTGGACGCCTTCTTTAAGTCATTACGGTAATTAGGTTCACTCTCTAGCTTTTTCAATGCTGATATCACTTCAGAGACCGCCATAATCCTCTCATGGATTTTTTTCCGGCACATGCGTCGAACACCAAGCTCTCCTCGAACAGATTTCGGAAACAATTTCAAATCCCTAGTCTGCCAACACCAGAGACACGACTCGGTGTAGTCCTCCAATACATCAGCGTCCGCATTAGGCACACCGAAAGTCAATCTCTGTCCCACAGACAACACACTACTCTTCACAGTCGCGTAGGTCACCGGCTCCGCCAGCACAATTGCGCCGCTACCGACCTCGCCGTTCAACCTGTCGTGGATCTCCTCAACTAGCCTCGACAGCGGAAGATCGCTCTCCTCCAACAGAGCCGCAACCACCGCGTTTCGAGAACCGCCGCACTCGCTCAACTTGATGCGTACTTTCTCAGCTGCGGCTTCCTTATAGAAGCGGAACAGCTGATCGAGCTCTTTCTCGAGGGTTTCAATGACCGCTTGTTTCTCTTGGGTACTTTTTAGGTTTTCAAATTCCGATAGGAGCTTCTTCCGCTTCCGTGTGCTCGTCTTGGCACTATTAGATTCCAGATCTTGAGCAGGAGGACGAGGAGGGGTAGAAGCTGTGTCAGCAATGATCTCCATAGAACCCTTCCCAATTTTTCCCAAACCTGTTTAGTTTTGTAATGAATCCGTTTGAATGAAGTTCAC

>C102373_65

CTTGAAGTTACCATCTGAACAATTTATTCCACGTTAGACCGATGCAAATTCATTACAGCTTATTCCCCACAAGTTACGTGGGAGAACGTTCTACTTTTTGCCATACAACTTTACATTAGCTTTCTTCGCATGTTCTGTGAAAATATTAAACTACATCACTTACAAAAAACAAGAAGAACTACAAAATTTAAGGATTGAAAACCTAGCAGGCTAGTACCTAACACAAACAACTGTAATAGAAAGCAGAAACTAAAATTCAAACCACTAATCTATCACCAGCCATCCATAATTTTTTCTACCTACAATCATGAGATGACGGTTTTCACAGTACTGCTTGTCATAGTCTGGTGTGACAAGTTTTCCTTCGTCACATAATCAAACCTGTAAATTCATTCACCAGCCATAGATAGGATTTTCTACCTGCGGCGGCAAGAGATGAAGGTCTTTACAGTGCTGCTTATCATAGTCACGTGTCCCAGGTATTGCTCCTGTGCATAATCTCAGGAATTCATTCCCGGTGAGACAATAGTGAGCAAATGCTAAGCCTCCATCTACTAGTTCTGTTAGATTAGGCATTGAAACTGACTTAGGGATGAGCTTTCCTTTGTCTAGGCTTGACTTTTCACTATTATTTTCCTTATTCAAGATTCTGTCAAGCTCCGAATATTCCATAAATCTTTGAGTAGCAGCCTCCCACGAAAGTTGATATCTTTGCTCAGGTGTTAGAGGATAAGGCTCGTTTTCTAATGCTTCTTTTACTTTTGCCACAAAGTCTTCGGATGTCTTGTACGTCAAACAGTTGGGAAATGACCTGAAGAACTCGTTTGATGGATGATCAGCACAAACTACAAATTTTCCCATGGCAAGTGCCTCAGCTGTAGCTGTGCATAGCACATCGCTAATGCTAGGATTTATAAAAACTTTGTACCCGTGAAGAGAATCGTCTGCATGATCTCTTCCTTTCTGAAAGTTGAGATTCAAATCTAAGTTTCTAGCTGCACGCTGAACTTCATTAGCATCCTCTCCGTTTCCAAACACGTCCAGCTTGAAGCCATCAAGGTCAGCCTTATGCTTTGCTAATAAATCTATCAACTCCTTATATCCCTTTGCCCACACCATCTTTCCCAAGAAATACGCCCCTTTTGTAAAGGATTTCTGCCCTAGCTCCCTCTCTGCAGCAATCTTGTCTCCAATTTTCAAGAATTTTGGATTCACACCATGAACATTGCAAATTACAGACTTGGGTAAATCCTGAGTAGCAGCCGAGAGACGAAGAACCTTGTGGCAGTATGCTCTTGTAACCCAGTTGTTTATGTGTTTCACAAAGAATGCCTGGAGTGCCCCATTTTTCTCCCTCTTGATATATTCTAAGTAATTTGTATGGACAATACCAACAACATGGTTGAATTTATCAGTCCAACGCTTGCCATGATGATACCAATTTAAATGTTCTGGCTCTTCTAAGATTGCAATGTCAGCATCCCTGGATGGTATAAATTGAGAAGTATCTCCAGCAGGTATTATACTCCGTCTTTCTTTTGAGAACTTCCCAGGGTAAAAAGAAATTTTGAAGTCTGCCTTAAAACCAATCCTTTCTTCAAGCCAGTTACGTATATAAACTTCCTGTTCTTCTGGTGAAGTAAAAGTGAGACTGCTGGGATAAACAAGTTCTTGATCTGATTTACAAAGCCATGGAACCAACAGAGTGACTTTTTGTTTTGCAGATTGTGATAAATATGCTGCTCGAAATAGGGGGTTTACAGCTGTTCCAGTCATCCAAGGAAGACTAGCAGTTGTGACTATTGCAACATGTCTTTCACTTTCAGATGGATCATGCTTAGCATGGTCTGTCCAGAATCCACCTTCATAACAGTGTCCTGTGCTCTGAAGCACACTTGCTATACGTAGATTCAATTCATCATTTATGTTTCCATTCCCAACAATGGAAGACTCCTCCTCCCCAGAGAGGGAATGCAATAGAAGATGGTTTTTGCGTTTGCTATACAAAATTTCAACTATCTTGTCACATATGTCTCTTTTCACTCCAAGATGGTCCAAAAATGGCCCAGACTGTTTTACAAAGTAGGCAAGAAGTTCAGGTACATCCAATGGTGGCACTTCCTTTGACTCCTGAGGTTCCTTGCACATTGATTTCAAGCTAGACTTGACTTTCTCCATGAATTCACTGTTCTTGAAAGCCTCAAAAGAAGAACCGCGCTTCTCAAACTCCTTGAGCCTGGTCTTCAGTGCCCGAATCGGTTCCCAATCCTTAGATTCACCCTCGCCGTCATTTTTCCACTCATCCCAGAATAACCTCCTTCCCCTCTTCTCGAAATCCAGAATCCCATCCTCCTCGGCCGACACAATCGCGTTCTTTATCGCGGAAAGGTCGATTCGAATCCGCGTCCTCGGGCGCCACTTCTCAAGAACCTTCTTGCTGAAATCGGGGGAGGAGTAAGCGCGCCGAATCTCCGACAGCTTCGGGCGCAGACTCTTGACGAACTCGATCTCCCTCGGCGGCGGAGAGTGCATGGCCGGCACCGAGAACGGCGGCGTCGCCGAATTGAAGAAGTTCTCGAGCTCGCGGTCGAACGACGTGGCTAGGTCCTTGAACGAGTTCGCTCGGTCACGCATGAGTCGGATATCCGCGTCCGCCGAGTCTCGAACCTCGCGCCAACCCTTGGACAGGAAGGAGAAGGCGCTCGATGAAGTCGGAGTCTGTGGATGCGTCGCCATGGCTCAGTTCCGACACCGGTGCGTTGTTTCGAAGAGTGTATGCAACGCGGAAGAATAAAC

>C102375_65

TTACTTTGAACCATATTATGATATTATATTACACGCATAAACTCCCCCCTAACAGGAGTACATTTATTCCTTCAATGGCATATAAATAGGATATATACAACTAAACAAGATACACAATGGTTTTGGATGACAAAGCCCCTAGTAATGCATATAATGCCGCAGAATGCAACCACGGATTTGCCACACCAAAATAAAAAATTACTACGCAGGATCAAAGCTGTGAAATCCTACGGTTTCTAAAGCATCAGTCATCCTCCCCATCCAGCAATGAGAAGGAGAGAGGGTAGAACTTGTAACCATCACCCTCATAGAATTTGTTTTGGTACTCTACCCAGTGAAGTCGCAAAGCATGCAAGAAGGCACTCAAAGTTTCCATGACAAGCAACACCCCAACGGTAGCAAAAGCAAAGATAATAAAACCCACAATCAGGATGATCACATTGTTGTACCCCCATGCCAAAATGAGAACCTTCTCGTAGAAAACACTTGATAACTCTGAATGAGCAAGACTTAGAGCCCATAGACGAAGGTAAGAAGCTGTATTAGAGACTGCTCCAAGTACAAATTCTATGGTGTGTATGAGTTGATGCACAAAGACTTCACTGAATTCAAATTCCTCGTGACCATGGGAATCGTGATTTGACTCTGCTTGCAAGCTCTCTTCTGTGATTTGAATTGGCGTATAGGATTCACCACGGTGCCTATCTTCGTGTTGCTTCTTCAAAATAAAAGGTTTTGGGATCAGCATCCAGGGTACAGAGACAAGAGCCAGAAGTAAAAACACTTGCTGCAGAATTTTCTGACCAGCAAAGAGTTGATTCTCTAGCAAATCATCTGTAGGACTCAGGAACATGTATATCAATATATGGTATAAATCAGCTTGAGAACCAGTACACCACTTCACAATAATGAGAGCAGATAGGTAACCAAATAGACTGTTGAGAAATATCATCTGGGGAATAAATTGGAACCATACATTCACGCTACTTCCAAAGAATTTGGCATTGCAATAACTCATGATAATTCCAAGGTTCATTTGGGCAACCCCAAGAAGAATTGACATTTTCATTTTCAACGAGTTCAGAAATGGTAATTCACTTCTAGAACCATGCCATACAGGGTCCACTCCAAATGGGTAAGTGCGACGCACTTTTACCAGGCCAACAGTTGTAGCATCCCTGCAGGAAAGGTCACGGCATGCATATGCGGAAGGACCAAAGAGTTCAAATGGAACAGAAAAGAACTCATTATAGATCAAACCAGTATATATTGAGAAGATTGCCATCAACATAATAACATAACGACCACCAAAAGTCATCTCTGTAATGTCTCCAAGTTTCTGGCCAGAAAGTTTCTTCTCCCTGATTATGAAAAAAAGAGCTCCCAGTAGTAAACATATGCCATGTCCCCAGTCACCAAACATGACAGCAAAAAGAAATGGAAAGGTAATTATGGTATATACAGTAGGATTTGCCTCCCGATACTTTGCAACCCCGTATGAATCAATAATTTCTTGAAATGGAGACGTAAATTTATTTGTACGAAAATAGGTAGGTGGCATCTCCCTAGTTTGCAAGACTTGGAAAATGGAACTGACTTGGGAGTTAGACTCAAGTGCTGCCCGTTGCAAAGCATCCTGAATCATCTTTGTTGCGAAAACAGGACTCCAACCTTCAGCCACTAGACATTTTTTTGTCACATCCAGGCTAAGCATGTTCAGGGCATGGTATATGGCTTTCTCCTTCCTCACCAAAAGATTCCATTGCTCAAATTGAACTCCAATAGACTGTAGCAAGTTGCACCGGTGCAGCAATCCGGTATCTATTGCGGTCTTCAATTCTAGAAGTTTTCCTGAAACCTCTGAAATCATTTCAGCTTGTTTTCCTAGTTCCTCAGTAAAAGGGTAACGATTAGCACCAAATGCCTCGCATATTTTCAGAATCTTGGCTTTTGCTTTCTCGCCAGCATAGAAGACCACGAACACATTTTTCTCAACCTGTTCTCCAGACACTGGGTCTGTGACAGGATCCTCAACAATGACCTGCCTAAAATAGACATTGCCTCTGGTAGCACGAAATAAAATTCTCTCAAATGCCATAGACTTTTCCCTAGGGACAAGACCCGCTAAAAACCCCAATCTAACTGGCTTTGATGAATCAGTGGACAATTCTTGATCCTGTAATAATGGTGTTTCAATAGATTCACCACTTTGATGTTGTGATTCATATTCTCTCCGATGCTCTATGGCACGACTTTGAGCCGAGTGCAAAAATTCACCAGCCTTCTGCAGAACAATCTTATACTCCACTAGTTCATTGTAAGAGCGTTGCAACTTTTCACTGTTCACATTGATCTCTGTTAATTCCAACTCAAGTTCAGTAAGTTTTACCTCAAGGTCATCAATGTTTACATCTGGCTGTGTTGTGGAACCAGCAGGTGAAACACCATTCTTCAACATTTGCTCCTTGAAAAATCGCAATTTACGAGCCATTTCTCCGCATCTTTTAATCTGAGTAGCATAAGTTCGTTGAAAAGGACTCTTCTGTGCATTGAGATCTTTGAATTGAAGAAGACGAAGCTCGGCGAGGTAAGAGACGGTGAGGTAAGCGGATTCGACGGGAATGATGAGCTGAAGGAGTTGCATCGGCTCCGAACGGAACAGATCCATCGGCGGACACCAACCACCACGTCCTACTTCTCCCATTCTCACCAAGCTCTCACACCACACACTACACACCTTCTCTCTCTCTCTACAACCTTCCACGTTTTTATTTATAGCTATTACATGACGCTGCA

>C102379_65

ATAGGAGAAGAAACACGAAGACTTAATTCTTCACACAAAAAGTTGCATAAATACCCCATTCTCTGATTCTTGCAAACATCAAGAATCATTCACCTGCTTCCCTTTAATTTCAATAATTCTGATACTCTTTGGTAAAATTAGATCACCGGTGTGCCCAAGGAGCAATCTTTTGAGTGGGTCGCGATGGAACAGAAACACGTGTTGTTCTCAGCATTGAGTGTTGGGGTTGGATTGGGTGTGGGACTTGGATTGAGCACAAGACAGGCTGTTCAAAAATGGGTTGGAGGGAGTTCTGAGTCTGATGAGATTTCTGGGGAGCAAATTATGCTGGAGCTCAGCAACAAAGTCATTGACGGGAAGAACAGCAAAATCACATTTACTGAATTTCCTTATTACTTAAGCGAAAGGGACCGATTTTCGTTAACAAGTGTGGGATATGTTTACTTAAAACAAGACTTTTCCAAGCACATGAAGAACCTTTGTCCAGAGAGCAGAGCTACTTTGCTTTTTGGACCTGCAGAACTTTACCAGCAAAAACTTGCCCAAGCTTTAGCTCATCACTTTGAATCGAAGTTACTGTTGCTAGACATAATTGACTTCTCGTTGGAGATGCAGAGGAAATATGGATGTCCCAGAAAAGAACCATATTTTAAAAGGTCCATTTCTGAGGTGACTCTCGAACGAGTGTCTGGTTTGTTTGGGTCCTTGTCAATCCTTCCTTCAACAGGCAGAACCAGAGGTACACTATTTCGACAGAATAGTGAAATTGAAAATTCCAGCAATCCTCTTAAACTTTGTAGGAACGTCTCTTCTGCATCTGATACGATTAGCTCTTCTCAATATGATCAGTCAGAACCAGCTCCTTTGAAGTGCACAAGCGGCTTCTGTTTTGATGAAAAGATCTTCGTGCAGTCACTTTATAAGGTCTTGGTTTCTATCTCAGAAACCAGTTCCATTATTTTATACATAAAGGATGTTGAGAAGCTCTTTGTTCGATCACCAAGGCTACACAACTTGTTCCAAAATTTAATAAAGAAACTTTCAGGATCAGTGTTAATACTCGGTTCCCAGGTATTTGATTCTGAAGAAGATACAGAAATTGATGACAAGCTCACTATGTTATTCCCTTACAGAATTAAGATCCAACCACCTCAAAATGAGACCAATCTTGCAAACTGGAAAACCAAACTGAAAGAGGACACGAAAACAATTCTATTTCAAGATAGCAGAAACCACATTGCCGAAGTACTTGCAGCAAGTGACATTGATTGTGATGATTTGAATTCAGTCTGCCATGCTGACACTATGCTACTCAGAAATTACATTGAAAAGATTGTGGCATCTGCAGTTTCTCACCACCTTATGGAGACTAAAGACCCAGAATACCGAAATGGAAAATTAGTTATATCAGCCAAGAGTTTGTCCCATGTATTATGTTTGTTCCAGGAAAGTGAAAGTAATTCGGAGACAAAAGATTCAAACAAGGAAGTTCCTCCTGACAACGAGTACGAGAAGCGCATGAGAGCAGACGTTGTGCCTGCAAAAGAGATAGGAGTTAGATTTGAAGATATTGGTGCATTGGATGATATTAAAGAATTACTTCAAGATGAGGTCCTGCTTCCCCTCAGAAGACCAGACTTATTCAAAGGTGGGCTTTTGAAGCCTTATAAAGGTATATTGCTTTTTGGGCCTCCTGGTACAGGAAAAACAATGCTTGCAAAGGCAATTGCAAATGAAGCTGGAGCAAGTTTCATTAATATCTCAATCTCGAACATCACTTCAAAGTGGTTTGGAGAAGATGAAAAGAATGTCCGAGCTCTGTTTTCACTAGCAGCAAAGGTTGCTCCAACTATTATTTTTATTGATGAGGTTGATAGCATGCTTGGACAGAGGACTAAATCTGGAGAGCATGAGGCAATGAGAAAGATTAAAAATGAATTCATGGTCCACTGGGATGGACTATTGTCAAAACCTGGTAAACGAATACTGGTTCTTGCTGCAACTAACAGGCCATATGACCTTGATGAAGCGATTATAAGAAGGTTTGAGCGCAGGATCATGGTTGGTATTCCATCTGCTGAGAACAGGGAAATGATCTTGAAAACTCTCCTAGCTAAGGAAAAATATGAAAATATTGACTTTAAAGAGCTTTCACTCATGACAGAAGGATATACTGGAAGCGATCTTAAGAATTTGTGCACGGCTGCTGCTTATCGACCTGTTAGAGAGCTACTACGACAGGAGAGGTTGAAGGAAAAGGAAAATAAGGAAATGAAGGAAATGAAGGTAGAAATTCAAAACTCAGAAAATGCTTCTGATGCGAAAGAAGCTGAAGAACACCGAGTCATTACTTTGAGGCCTTTAAATATGGAAGACATGAGACAGGCAAAGAGTCAGGTGACTGCTAGTTTTGCAGCAGAAGGATCAATAATGAGGGAGCTGGAGGAGTGGAATGATTTATTTGGAGAAGGAGGTTCAGGGAAGACGAAGCCGGAGCAGCTTACTTATTTCCTTTAGATTGTGAAAAGCTGGTTTTGTTTTGGACATGTCAGTAGTATCTATCACCAAAGGATACCCTTTAACCAGGGAAGGAATCCTCATCAGAGCAGAGAAATTGAACTAAAAGTATGCATATGCAGAAATCAGAAATTAAAGAGATTCATTCAACCCTGAAATGCTATTGGGGTCCAAAGAATGTGTAGATCATCATCATGCATCATGTTTCGGAGAGGGCACTCACTTATGGTTTGCATTGTCCGCAAACCAGTGGCAGCATGTATTTTGGTAATAATCTACAAG

>C102383_65

AGAGAAGCCAGATTTATAGAAATCAAGAGTTTAGGGTTGAGAGTTCTCAGTGAGAAGCTGAGAAAGCAGAACAATCAACAATGGAATCGTATGCCGGCTTCATGGTCGACGAGAAAGCCGTTACCGTTGAGAACGCTTTCTTGGATTTTCTCAAAAGCTTCAAATCTAGCCAACGGAACGAGCTTCAATACGAAGCGGAGATCGAAGTGATGAGAGTCAACGAATCCAACACCATGTTCATCGATTTCGCCCACGTCATCAGATTCAGCGACCTCCTCCAGCAGACTGTCTCCGATGAGTATTTGAGATATGAACCTTATTTGAAGAATGCTTGCAAGAGGTTTGTTATGGAACTCAAGCCATCAGTTGTATCCGACGACGGCCCTAACAAGGATATCAACGTTGCTTTCTATAATATTCCCGTTATTAAGCGGTTAAGGGAATTGGGTACATCAGAAATTGGGAGGCTTGTATCTGTTACAGGAGTTGTGACGAGGACAAGTGAGGTTCGACCTGAACTTCTCCAAGGGACCTTTAAGTGCTTGGAATGCGGTAGTGTTATAAAGAATGTTGAACAGCAATTCAAGTATACTGAGCCAACAATCTGTTCAAATGCTACCTGTAGCAATCGAACAAGGTGGGTATTGCTTCGCCAAGAGAGTAAATTCGCTGATTGGCAGAGGGTCAGGATGCAAGAGACATCCAAAGAGATACCTGCAGGCTCTCTTCCTAGGTCATTGGATGTTATTCTTCGTCATGAGATTGTGGAACAGGCCAGGGCTGGTGACACGGTAATCTTCACAGGTACTGTAGTTGTTATACCCGACATTATGGCATTGGCATCCCCTGGAGAGAGATCAGAGTGTCGTCGTGATGCTTCTCAACGCAAGGGTACCGCTGGAAATGAAGGTGTGAGTGGCCTCAAGGCCCTGGGAGTCAGAGACCTCTCCTATCGTCTAGCTTTTATTGCTAATTCTGTTCAGATTTGTGATGGTAGAAGAGAAATAGACATCAGGAACAGAAAGAAGGATAGTGATGAAGATGATCAACAGTTTACTACACAGGAACTGGATGAAATTCAACGAATGAGGAATACTCCAGATTTTTTCAACAAACTAGTTGAAAGCGTTGCTCCAACTGTTTTTGGTCATCCAGATATAAAGAGAGCTATCCTTCTTATGCTCATGGGTGGTGTTCACAAATTTACTCATGAAGGCATCAACCTCAGAGGAGACATAAATGTTTGCATTGTTGGAGATCCCAGCTGTGCAAAATCTCAATTTCTTAAGTATACTTCAGGCATAGTTCCAAGATCTGTGTATACATCTGGGAAATCATCTTCTGCTGCTGGCTTGACTGCAACTGTTGCCAAGGAACCAGAAACTGGGGAGTTTTGTATTGAGGCTGGTGCTTTGATGCTTGCGGACAACGGCATCTGCTGCATTGACGAATTTGACAAGATGGACATCAGAGATCAGGTTGCAATTCACGAGGCCATGGAACAGCAGACTATTAGTATCACAAAAGCAGGGATACAAGCAACGCTTAATGCTCGTACTTCAATTCTTGCCGCTGCCAATCCTGCCGGGGGACGCTATGATAAATCTAAACCACTTAAGTACAATGTGGCTCTTCCACCAGCTATACTTTCAAGATTTGATCTCGTATATGTTATGATTGATGATCCAGATGATCAAACAGATTATCACATTGCTCATCATATTGTGAGAGTTCATCAAAAGCGAGAAGCTGCTCTTGCTCCAGCATTCACTACAGCAGAACTGAAGCGTTATATAGCATATGCAAAAACTCTTAAACCAAAGCTCACGTCAGATGCGAGAAAACTACTGGTAGATTCGTATGTTGCTCTTCGTAGAGGTGATACAAATCCAGGGAGTAGGGTTGCATATCGTATGACAGTAAGGCAGTTGGAAGCATTAATCAGGCTATCAGAGGCCATTGCACGGTGTCATTTGGAAAATCAGATACAACCTCGCCATGTTCGTCTAGCAGTAAAACTTCTGAAAACTTCCATAATAAGTGTTGAGTCCAGTGAGATCGATCTGTCTGAGTTTCAAGAAGAAAATCAGAATGCGGGAGCAGTTGGTGGTGATGGACATGATAATAATGGAGATGCTAATGACCGAGGTGGTGATGGAAATGATAATAATAGAGATGCTAATGGTGGAAATGATGGAAATCCAGCAGATGGATCTAACTCCCAGGGGATGAAACAAACTAATGGAAATGATGGAAATCCAGCGGATGGATCTAAACCCCAAGGGAAAAAACTCGTTATAAGTGATGAATATTTTCAAAGAGTAACCAGCGCCCTTATCATGCGTCTTAGACAGCATGAAGAGGCTACCATGCAAGGAAATGGATTGGCTGGAATGAGACAAAAAGATTTGATTCAATGGTACGTAGACCAACAAAACGAGAGAAATAACTACAGTTCGATGGAGGAAGTGACAGCTGAAATTTCTAAAATTAAAGCTATTATAGAGAGTTTAATTCGCAGAGAAGGTCATTTGATAGTGGTAGATGATGGGCAAGAGGCGGCAGCTGAAGGAGGAGGCGCACCTAGAAATAACCGAATTTTAGCGGTGGCTCCCAATTATGTCATTGATTGACAGTCTAAGAGTGAAAGAAATTAGAAATCACAGTTGAGCAGCAAATTGGTCCTTATCTCATCAATGTTGTGATGGAAAACTGATGATGATGGTCCTTTATAATACTTTTGTATGTAGAGTATATTTTATTTATTATTCTACCCGTCCTTGTCATCATCTTTTTACTAA

>C102387_65

TATGAAGAGAAGATGAGAGGCAGAGAAGTGGGAAGAAGATAGCCAGAAACATAGGCTAAGATTGGGCGTAACATGTGGACCGCAAGAACAAGGAGTGTATAGATCATCAACTACCCTTTTCCTCTCTAACTCTCAACAACACACCACAACACACTTCTTCTTCTTTACTTCTACTACCACTACACAACCACTCACCTAAGTGGTTGCTTTTGGTTGTTTTGGTTTTGGTTTTGGTTTTGGTTTGGTTCCACTTTTGAACCTCTTAAGCTTAAGTTAAGCCATGGCATCCTCATCCTATCTACACCTGTCTCAGGCCCTTTTGGCCCGTGCTGTGTACCTTCATGGCTCTTCTTCTGATCGTGTCTCACTCTCCTTCCCATCATTCTCTGGCCTCAAGCCACATTCTCCATGCAAAGCAGCCACGTTTTCTGCAGGTTCACGTAGAAGGGGTCCTTCCACCAGTGTTGTTCGAGCCACTGCTGTTGAGACGCTGGACCAGACCACTGAGGTTTCCCTGGTGGAGAAATCCATCAACACCATTCGGTTTCTGGCCATTGATGCAGTTGAGAAGGCCAACTCTGGCCACCCTGGTCTCCCCATGGGGTGTGCTCCAATGGGTCACATTCTCTACGATGAGATCATGAGGTATAATCCTAAGAACCCCACATGGTTCAACCGTGACCGTTTCGTTTTGTCTGCTGGACATGGCTGCATGCTCCAATATGCTCTCCTTCACCTTGCAGGCTATGACAGTGTTCAGGAAGAAGACTTGAAGCAATTCCGACAATGGGAAAGCAGGACTCCCGGACATCCTGAGAACTTTGAGACACTTGGAGTTGAAGTGACCACAGGTCCTCTGGGTCAGGGAATTGCCAATGCAGTTGGATTAGCACTAGCTGAGAAACACTTGGCAGCACGATTTAACAAGCCTGACAATGAGATTGTTGACCATTACACATATGTTATATTGGGTGATGGTTGTCAAATGGAGGGAATTTCGAATGAAGCATGCTCACTTGCCGGTCACTGGGGTCTGGGGAAGCTTATTGCGTTATATGATGACAACCACATTTCCATTGATGGTGACACTGAAATTGCATTCACTGAGAATGTTGATCAACGTTTTGAGGCACTTGGATGGCATGTGATTTGGGTGAAGAATGGAAATACCGGGTATGATGAAATTCGTGCAGCCATTAAGGAAGCAAAGGCTGTCAAAGACAAACCTACTTTGATCAAGGTAACCACTACCATTGGATTTGGTTCTCCAAACAAGGCTAACTCCTACAGTGTTCATGGAAGTGCATTAGGTGCTAAAGAAGTGGATGCTACAAGGAAGAATCTTGGATGGCCATACGAGCCTTTCCATGTGCCAGAAGATGTTAAAAAGCATTGGAGTCGCCATGTCCCTAATGGTTCTAAACTTGAAGCTGAGTGGAATGCCAAGTTTGCTGAATATGAGAAGAAATACAGTGAGGAAGCTACAGAGCTGAAGGGTATTTTTACTGGTGAATTACCAGCTGGTTGGGAGAAAGCACTTCCGACATACACTCCAGAAAGCCCAGGTGATGCTACAAGAAATCTGTCTCAGCAAAATCTAAATGCTCTTGTTAAGGTTCTTCCTGGTCTGCTTGGTGGCAGTGCCGATCTTGCCTCTTCCAACATGACCTTGTTGAAATCGTATGGAGACTTCCAAAAGAATACTCCTGAAGAGCGCAATGTTAGATTCGGTGTCAGAGAACACGGAATGGGAGCAATCTGTAATGGCATTGCTCTTCACAGCCCTGGATTCATTCCATACTGTGCAACTTTCTTTGTCTTCACTGACTACATGAGAGCAGCCATAAGGATTTCTGCACTGAGTGAAGCTGGAGTCATTTATGTGATGACTCATGATTCAATTGGACTTGGAGAGGATGGACCAACTCATCAGCCAATAGAGCACTTGGCAAGCTTCAGGGCAATGCCAAACGTTTTGATGCTTCGTCCAGCTGATGGTAATGAAACTGCTGGATCATACAAAGTTGCCGTGCTTAGCAGGAAGAGACCCTCAATTCTTGCACTTTCTAGGCAAAAGTTGCCCCAACTTCCTGGAACTTCTATCGAGGGAGTTCAAAAGGGTGGCTACATCATTTCAGACAATTCATCAGGTAACAAGCCTGATGTTATCTTGATTGGAACTGGTTCTGAGTTGGAAATTGCTGCTGCTGCTGCTGAGGATCTAAGGAAGGAAGGAAAAGCTGTTAGAGTTGTTTCTTTTGTTAGCTGGGAACTTTTTGATGAGCAGTCAGATGAATACAAGGAGAGTGTTCTCCCTGCTTCTGTATCAGCTAGAGTTAGCATTGAGGCAGGATCAACATTTGGTTGGGGAAAGATTGTTGGAAGCAAAGGAAAGGCCATAGGCATTGATCGATTTGGAGCAAGTGCTCCAGCAGGAAAAATATACAAGGAGTTTGGTATCACCAAGGAAGCTGTTATTGCTGCTGCCAAAGAACTTTCGTAGATCTATTTGTTGAGTTTCTTTTATCTCATTCTAAAACTTGTGGTTTTCACTAGTGGCTTTGGATTACTTTTACATGATTTGTTTTTTGAGATGCCACTTTAGCCACAATAAGGAAGATTAGATTGTTCTTGCATGTGATTGTCAGAGGAGCCACTTACATTAAAGGATGAGTTGAACTAATGAACTTGCATGAATTCGTAACAACTACTTTGGCTTGTGAATTTTGTCTGTCCAGTAGTTCCATTACTGATCCTCACTCTACCACTTAATTTTAAGTCTTCTTGGCTTCCTGGAAAAAAA

>C102391_65

ATTTTCAGCTACAAATGAAAAAAAATATTCTGGTCAATGTTTTATTTTCCCCATTGTTTCTTCTTGACTGAATATAATATTTTGTGACTTTGAAATTCGTCACAAGAAGCAAAGATTTATTGACTGCTTGTTTTGATTAAATTTTCGTCGTTCTATGGTGTTGCCACTCATCGGCTCAGTTTATTTGAATCTCTAATCTCTGAGTTAAGGTACTGTTGCGCTTCAGAAATGGCTTCCGGAACTGTGCCAACTTCGTTTTCCACTCTCAAGACATGGGATTCCAGTCTGGGGTTTGCAAAAAGCATGGATTTTGTGAGAGTGTCCGATTTGAAGAGCATGAAATCTGCGAGGAAAAAGGTATCAATTATCAGGAACTCGAATCCTGGCCAGGATATTGCCGAACTTCAACCCGCATCCCCAGGAAGTCCTCTTCTGGTTCCTAGGCAAAAGTATTGTGAATCAATACACAAAACTGTCAGGAGAAAAACAAGCACAGTGATGGTTGGTAACGTGGCTATTGGTAGCGAGCATCCTATAAGAATTCAGACTATGACTACAACTGATACTAAGGATATTGCTGGAACAGTTGAACAGGTTATGAGAATAGCAGATAAAGGAGCTGATCTTGTGCGGATAACTGTTCAAGGGAAGAAAGAAGCTGATGCATGCTTTGAGATTAAAAACTCCCTTGTGCAGAAAAACTACAACATACCGTTGGTGGCTGATATTCATTTTGCTCCAGCTGTTGCTTTACGGGTAGCTGAATGTTTTGATAAGATTCGTGTAAACCCCGGAAATTTTGCTGATAGACGAGCTCAATTTGAACAATTAGAGTACACAGAAGAAGACTATCAGAAAGAACTTGAGCATATTGAAAAGGTTTTCACACCCTTGGTTGAGAAATGTAAGAAGTATGGAAGAGCAATGCGCATTGGGACAAACCATGGCAGTCTTTCCGATCGTATAATGAGCTACTATGGAGATTCTCCTAGGGGGATGGTAGAATCTGCTTTTGAATTTGCAAGGATATGCCGAAAGTTGGACTTCCACAATTTTGTTTTTTCTATGAAAGCAAGCAACCCGGTTATCATGGTTCAGGCATATCGCTTACTTGTAGCTGAAATGTATGTCCAAGGCTGGGATTATCCATTACACTTGGGAGTTACTGAAGCTGGAGAAGGTGAAGATGGGAGGATGAAGTCTGCAATTGGCATTGGAACTCTTCTTCAGGATGGATTGGGAGATACAATTAGGGTTTCTCTCACAGAACCACCAGAGGAGGAAATAGATCCTTGCAGAAGGTTGGCAAATCTAGGAATGAGAGCTTCTGAACTCCAAAAGGGGGTGGAACCTTTTGAAGAAAAGAACAGACGTTATTTTGACTTCCAGCGCCGATCTGGTCAATTGCCAGTGCAAAAAGAGGGTGAGGAGGTGGATTACAGAGGTGTACTGCACCGTGATGGTTCTGTTCTCATGTCAGTCTCCTTGGATCAGTTAAAGACGCCAGAGCTCCTCTACAAGTCCCTTGCTGCCAAACTCATTGTTGGCATGCCATTTAAGGATCTGGCAACAGTAGATTCAATTTTATTGCGGGAACTTCCTCCAGTGGACGATGCTGAGGCTCGACTAGCACTCAAGAGACTGATAGATATTAGCATGGGGGTTATAACTCCTTTGTCAGAGCAGCTAACAAAGCCATTACCCAATGCCTTGGTTCTAGTAAACCTGAAGGAACTATCTACTGGAGCTTATAAACTTTTGCCACAAGGCACACGCTTGGTTGTGTCAGTAAGAGGTGACGAGTCTTATGAAGAACTGGACATTCTTAAAGGTGTCGATGCTACTATGCTTCTCCACGACCTGCCTTATACAGAGGACAAAATTAGTAGAGTACATGCAGCCAGGCGGTTATTTGAGTACCTATCCGATAATTCTCTAAACTTCCCTGTTATTCATCACATTCAGTTTCCAAATGGGATTCACAGGGATGACTTGGTTATTGGTGCTGGTTCTGATGCCGGAGCCCTTCTGGTTGATGGGCTTGGAGATGGGCTTCTTTTGGAAGCCCCAGACAAGGATTTTGAATTTATTAGAAACACCTCTTTCAATTTGTTGCAAGGCTGCAGAATGAGAAATACAAAGACAGAGTATGTCTCATGTCCATCGTGTGGCAGAACATTGTTTGATCTTCAAGAAGTGAGTGCACAAATCCGGGAGAAGACTTCACATCTCCCTGGGGTTTCGATTGCAATCATGGGATGCATTGTAAATGGACCCGGCGAGATGGCTGATGCAGACTTTGGGTATGTGGGAGGCACTCCTGGGAAGATTGATCTTTATGTTGGAAAGACAGTGGTGAAACGTGGAGTTGTAATGGAGCATGCAACCAATGCCTTGATCGATCTAATAAAAGAGCATGGACGTTGGGTGGACCCTCCTGCAGAGGAGTAAAAGAGAGTTAAATTTTGGGATTGGCATCCAAGGCCAATGTAAGATGATTGATTATTGTGATATCCTATTATTATAGTGTGTAAGTTAAGCAGTGAAAGCTTAAGTTGCAGCATAGTTTGAAATTCCTTCTAGCAAATATCCCAACTATATCTTGGGATGGGGAAATAGACAGTTACATCATGTATACAGACAATACAATGTTGCTTTACCCCCATCAATAATACTAATAAAACCAGTGGGATGATGTATCCGTACCGAATTAGACAAGCAGTATCTCCATTGCTCAGATTTCAAAGGATGATTACCTTTTGGTTGCTATTAACATGGTTTTCATATATCAATGCATGCATGAG

>C102393_65

GCAAAGAAACAAAAACAAGGGAAAAACCTCAGTTGCGGAAAGAAACATAATCTGGAATTCGAGAAGCAAATTAATCGTTTGCGGGTGCTTTGTTCCTCCTCTCGCAGATGGTTAGGGGAGGAAGGTTTAGTGGCAAACGCAATTTCAGAAAAAAGGTTCGGTCAAAGGAGGGAGGTTCCGATGATTCGGATGAGGACTATGTGGTTTCAGATGATGGGAGAGAGGTATCTGACTATTATTGCTCCTCCTTAGATGGATGTGCATCAGAGGAAAGTTTTGTTAGTTTAATGGAAGAGGAGGAGGAGGAATTTCATGAAGTGAGTAATTGCAATAGATCCAAGGCAAAAAAAGGCACTGCTGGTAGGCAGAAAAATGCCAGTAAAAACGTGCACAAGAGGGGAAGGATTAAATATGTAGAAGAAGAAGAAGAGGAGGAGGAGGAGGAGGAGGTGGAAGAAGAAGATGAAGAAGAAGAATACAAAGAGGAGGAGGAGGAAATTCAAGAAGTGAGGAAATTCAACAGGTCAAAGGCCAAAAATGGTGTATGCAGTAAGAAAAGAAGTGCAGGAAAAATCTCACCAAAGAGGGGAAGGAAAATCCATGCAGAACAACTGGAAGAAGAGGAGGAGGAAGAGGTCAAACTGGAGGAGGATGAGGAAGAGAAGGAGAAAGTGGACGGAGGTGGAGATGAGGAAGAAGAGGAGGGAGATAGAGATAGAGAGGATGAAGATTTTGATTATGAGGATGAGGATGATGAATTTTTGCCTGAGGAAGAAGATTATTCAGATGAGGAAGAAGAAATGAGAGGGAGGAAGAAGAACAATGATGTAATGAAAATGGACAAGAAGGTTTTGCAGAAGAAGAGGTCCTCTGTGGTTTCAACCAGAGGTAGAAAAAGGCAGGGTTCTAGACCATCAAAGAAGACCTTGAGAAACAAAAGAAGAAAGAGGGGAAGGTTAGGGAAGAAAGTTAGATGTGAAGATGAAGATGAAGATGATTTTATAGATAATAGCCCAACCATGAGGACAACAAGTAGTAGAAAACGGGGCCAGAAGAGAAAAAGGGTGCTTCTTTCAGACTCAGAATATGCATCTTCTGGATCATCTGATTTTGAGTTTACCATATCTGAAGAAGAGAGGGAACAGGTGAGAGAAGCCAAGAAATTGTGTGGAAATCTGAAAAATAACTTGAGAAGTTCATCCCATCTAATAAACAATGAAGAGGTTGGTGTCCATGTGGAACAAATTCAGCTGCGGAAACCACCAGGTCGGAAGGGTAAGGAAAAGATAGAAGAACCTCAAGGAAGAAAAGGTAAGGAGAAAGTGGAGGATTTAAAAAATGAGATGGGTAAGCAAGTGTGTGGAATTTGTCTATCCGAAGAAGATAAAAGAAGAGTAAGAGGAGTACTAAATTGTTGCACTCACTTCTTTTGTTTTGCTTGCATTATGGAGTGGGCAAAGGTGGAATCTCGTTGCCCATTGTGTAAGCAGAGATTCAAGACAATCAGCAAGCCTGCACGGTCAACTACAGGGATTGATTTAAGAGAAGTGGTGATACAAATTCCTGAACGTGACCAGGTTTATCAGCCCTCTGAAGAAGAGCTAAGGAGCTATATTGATCCATATGAATATGTTATTTGTTCAGAGTGTCATCAAGGTGGAGATGATGGCCTCATGCTACTATGTGATATCTGTGATTCCCCTGCGCACACATATTGTGTTGGTTTGGGGCGGGAAGTTCCTGAAGGTAATTGGTATTGTGATGGCTGTAGACCAGTTGCTCTGGGATCTTCAAGCTCCCAAGCTCAAGAAGGTGCGGCTGATCCAAGGGTGACAATCCAGAACCTTCCCGTTAGACCACCCCCCGCTCTACATGTACGAGAAAGTATAGACCTCAACTTGATTTCGTCACCCCGTGCAGCTTTTAATCAAGGTTTTGGGCATCTTCCATCTTCTAGATTTAGTGGCAGAAATGTTGAAGGATCTTCTCCTGTATCTGGAGGGGCACCAACGTTATCAGAGAGACGCTGGATACACCGTCAAATTCAACAATTACTTTCAATAGATAGGATGACATCTACACCTGGCAGAACCACCAATGGTATTTCAACTACCAGTTCAACTAGTAACTTGTATATCTCTCAAATTGAACAAAGTAGGGTAACCACCACTCTTCATACAAGGACACAAGATGTGGGAACATCATACCACACATTTTTTGATGAGAGATTATGCAACAATACCTCTCCATTAATGCAGAATGGGGCATTATGGCCTGGACTTATGGGGACACCCCCAGTACCAGATTGTGAACAAGTTCATCAGTTCAGCAGATCAAACATTGTCCCTGATGATGGCGTATCCCCTACTGCCAGAGAAGAGAGTAACTTTCACGTTGCGAAGGAGCAGTTGCAGTCGATGGTTAAAAGTCACCTGAAGAGCTTGTCTCAAAATATTGATTTAGGCCACAGTACTTTCAAGGATATTGCAAGGAGTTCTATGCACACCATACTAGCAGCTTGTGATCTTGAGCACAAGAACAGTGAAGTTTGCAATGTACCTCCTCCATCTGCCTGTCCGCACATGGAACTAATGGCTGGTGGGCCAACAAGTCTGATTAAAGGTTGCTGCTCATCGTGCTTTGATTCTTTTGTAGGAGATGTTGTGAAGAGGGTTTTGGATACAAGATTTTCGTCACAGTGGTTACGATTAGGTCTATAGGCATAAAATTATTGCAATGTGTCCAAAGCCACCTTTTTTGTTAGTTCAGAAAGACAAGAACATTTCATTTACTTGTTTATACAA

>C102395_65

TTCTCTTTCTTATATAGGAAGTAATCATCTCCCTTATTCCCAAGTTCAATCTTTTTTTCATCACCCAAGTTTCTTCTTCTGCTTCCCTTTTTGGGTTTAGTTCTCTTTGCTTTGTTGTCCACTAATGGTTACACCAAAGTGTTGCCATAGATTCTTCAAGTTCCGTCCACTGTTTCTTATAACCACTTCCACTTGTGAATTATCCTTGTGAATGGCCTAAAGTGACCCTTACCAAAAAGCCACACCAACCGTATGTGCCTTCAAAATTCTTGACACACGAAGGGCAGTGACAGGAACATGTCAGTTTCATAAGAAATTATTATCCGTTTATTCTCTTCTTCTCACCTGTCTTATCTTGTCAAATCTTGCAGCTCCACTCCCTCCCTCCCTCCCTCTCTCTCTCTCTCTGCACTTCTTTTATTTTCAAAAGAACAAGCAAAATTTAATCAAATGGTTGCTAAGGACTATGATCCCTTGACATGCGCGGTTGGTGTGAAAATGGGTAGGATAATGCAGTGTGTTGGTCACTCAAGGCTTTTCTGGCTGTTTTTTGGGGTCTTCTCCCACGATTACAACGAGGGGAAGCCTCGGATTTAGGATTCTACGACCTTGGAGAATAGCACAGACTGCTGTTTTGTTTGCTTTTGTTCTTTGCTTTTCAACTTTCTCTGCGCCGACGTTGTTGTGCAGACTGTTTAGGTGATCGTTAGTTTAGTTACTTGTTGGAGTGTATAGTTAAGAATTCAACATGTATTCCAATAAAGGGTGTAAAGAGGATCGCAAAGCTGAGCCTCGTTACGTGGAAACGGACCCAAATGGTAGATATGCTCGGGTTGAAGATATTCTAGGAAAAGGAGCAATGAAGACAGTGTATAAAGCAATTGATGAGGTCCTTGGAATAGAGGTTGCATGGAATCAGGTCAAACTCAACGAGGCATTGCGCAAACCAGATGACTTAGAGCGGCTTTATTTAGAGGTTCATCTCCTCAGCACCCTCAAGCACCAATCCATCATGAAATTCTATACCTCTTGGATTGATGTCAACAACAAGACCTTCAACTTCATTACAGAAATGTTTACTTCAGGGTCACTGAGAGAATACAGGAAGAAATATAAGCTCATAGGTCTACAAGCAATAAAGAGTTGGGCTCGCCAAATCTTACAGGGTCTTGTTTATCTGCATGAACATGATCCTCCAGTAATTCATAGGGACCTCAAATGTGATAACATATTTGTTAATGGCCATCTTGGACAAGTAAAAATTGGTGACCTGGGTCTAGCTGCGATTCTCCGTGGTTCCCAACCAGCTCATAGTGTTATAGGCACCCCCGAGTTCATGGCACCAGAATTGTATGACGAAGAATACAATGAACTTGTTGATGTGTACTCATTTGGCATGTGTGTTTTAGAAATGCTTACATCTGATTATCCGTACAGTGAATGTACTAACCCTGCACAGATATACAAAAAAGTGACATCGGGTAAGCTACCAGCAGCCTTTTTCAGGATTGAAGACACAGAAGCACAAAAGTTTATTGGCAAATGTCTAATCGCTGCAGCAAATAGACCGTCAGCAAAAGAATTGTTGAATGATCCATTTCTTTTGTCTGATGATGCATCATCCATGGCAAAGATTGGAATTCAAAATCCCTTTTTAAATTACAGTGAAATGGAAAAACTTCAATTGAGTGATGATTTACCTAGGACTAAAATGTCAATAACTGGGAAGCTGAATTCAGAAGATGACACTATTTTTCTCAAAGTGCAAATATCTGAGAAGGATGGTTCTTGTAGGAATGTATACTTTCCCTTTGACATTTACACAGACACCCCTATTGATGTTGCAATGGAGATGGTGAAAGAGTTGGAAATCACAGATTTGATGCCTTCTGATATTGCAAGTATGATTGAAGGAGAGATATCGGTGCTTCTTCCGGATTGGAGGAATAGTAACTGTCCAGACACCTGCCATACATTCAGTTATAATGATGACAAGGACGATGAAGATCCTCACCATCATTTCCACTCTGTCTCTTCTTGTTCGTCATCCCAAGAATCAATACCAGGATCAGTCAGCAGAGTTGAAAACTTGTTGAATGGATATCATTGGCTCCATGGTGATGTACTTGATGATGCCAGTTCACTCTCTTCACATGGGACATATGCCAACTCAAGCTTTTGTTCAGTGGATGATCAGGAGGAACACAATAAGGCTTCTACAAGGAAAGATAAACATCCCATCATAAAGGGTCATGTGTGTACAAGATTTTCCCCTAATGAAGACCCCATCACTTTGAACTACTGCAAAATTCAAGCAGGACCACAAGCACCTTCTACTAGCAAAAGTAAGAGTATGATAGACAATCGCAGACTAACAAGGAACAAGTCATTAATTGATATGAGAAGCCAGCTACTGCACAGATCACTGGTTGAGGAGATAAACAAAAGAAGATTCAAGACGGTTGGAGCTGTGGAAAATATAGGATTTGTGTCACCTTATGAGGTTACCAACAAAAAGCCAAATCCAACAAGTGGTGCTTTTGATGTGAACTCTTCAAAAAGTAGCAAGGGAAAAAACATGAGAGGAAAAAAAAATTAAAAATGGTTGTCTCTTGAGCTTTGAAATCAGATAGGAAAAAATGGATGAATTCTGTGTCATGGAAAAGAGCAGATGATATGGCTGAGGTGCTATGGAATTGTTAGACTTTAAAGAAAAATGGTATGATTTGAAGAAAAAATCCATCTACAAGGTTGGGCAAAGACATACCCAAAAACAAAGTATAATTAGGATGTTAGGGTTCAAAT

>C102397_65

CAGAGAATTTGTGGGAAAGGAGATAAAGAATGGTCGATAACAAATGATAACCGATGGAATCATCCTTGATTTAATGTAGTATGCTACAATTCAGTTCGAGAATAGAGTAATGACCATTAAACATGTATAATCAATCAGTATCACTCACTCCCTTTACTATCCAACTACAAATGCACTAGAAGATCCTGTAAAGTGACGAATTGATCCAAATAAACCCTTCGCAGCTCCAAAATGTTTGATTCTGTATACACCAGGAGTTACATCCTGTGGGATTCTCCATTCTATAGTGGCTTTACTATGAGAACTGAGTTTAAAAGGTCTGGACCATATGAAGCGTAGGCAGAAATCATCATCATCATAAACAGGAACCCAAGTACTCTTTCCTTGGAGAAATTCCACCAAGGAAAATGTACCTTCAGTCATAAGGTCATTACGAGGGCACCCAGACCAGAAAGTAACAGACACCATATCACCTCGCTTAAAGGTGGAGTTTTTGGGAACATCAGAGCTACAATCCCCAAACTTCACACCAATAGGGGTTGTATCCATCACAACTGGTGTTAATAAGCTTATTTGCTTATCTAGGAGGTCAGGGGGTGGAGGGCCTGGTTCTACAGTCTGACCATTGATGAGAGCACGCGCAAGCCTTGTAAACTCCTGAATGTAAGCACTCAGTGTGTGTGGACCGTATAGCGTGGAAGCACCCTCATATCTCTGCATCTCGTACTCTTCGTACGTAGTCACATACTGTGAATAAGTATTGGTCAACCCCGCTATAACAACATGAATGTTGCTCTCATAGCCTTTGGTGCCACTTAGCACTGACTTGACAGCATCACGAAGACGCCTCCCAGCCATTGTTGTGAATTCTGCAGGTACACTAAGAATAACAAACTGTCCAACTTGGAGGATCTGGATTGGAAGTATTGAAGGTGCCCAATCATATGGTAGCTTCATTTCACCAGTGTCGAGCAAAATGGGCTTTGGCTGATGACAGTCTATTTGTTCCTTGCCTGGTGTTTTAAGTAAGTTGCGGACCAGCATCCAGAAAGGATTTCCCTGATCGTCGCCTTGTTTAAAGTCAAAAGCTCCAGGTCCATCTGTTGTTCCAGCAGCAAATGCAAACCCCATTGCAGCAGGGCATGTCTTTACGACCTTAGAAGCTCCTACTTTAGGAAGATTTACCTCAAGCTGGGAGAAATCTATGAAAGCATGTCGAAAATCAACCTTCCCTTTAATCTGTTCAGATGCTCCACTGAATAGTTCCACTGCTTTTCTAAACTGTCTCTCCCCTATAATACGTGTACTTTCAAATTCATCTGGATAACCAGGTCCTCGGCCATAGCACAATTCATTCTTTCCTCCACATGTACTATGATTGAAGTCACAGGGTAGTCCAGTGTCGATACAAAAAGCTCCAAGCACATTTGGACTAACATCCCCGCAATTTGATTGACAAAATGCAGATACAAATTTAGGCTTGTCAACCTGCCTAAGGACACCTCTCACACGTCTAGCAACACTTGAAATTTTGGCTGCAGGTTTACCAGGAGGAGATTGGAATGAGGCAGCAAGCTCCAGTAATTCATGGTATTTATCATGAATGCCAGGAATTATGTTTGAAATTCTTCGAGGGATTCCATCATTTTCAAATCCAACAGAATCCATTCTTCCAGACCCTTTTCGCTCAAACCAGTCTTCCATAAATCTTGCAGCTGCACCCTTATTATCCCCGCTAATTAACGAGTTTGTTCGGCTCATTGAAGTACCATGAGTAGCAAACCAATTGAAACTTCCCAAAGGACCCCATTCATCATCAACAAACTTCAACAAAGTCATTTCTTTATCAACATCATACTTGTATTTGCTTCTCTCTGCATCAGGATTGTTGAGATAAGCACTAGGACTGCGATTTACACCAGCATCTAAGAGCTCTCCCTTATTGACAAATATTGATCCTGGCTGGAGATTTTCATGGGCTTCGACTATACTCTTCTCAATACCATCGACAATGACATCAAATGACTGCCGAACAAATCCAAGAGATGTCACAATGTACACAACATATTGGAGATAACCCCCAGGACCAGCATGAGTGTGAATTCCACTAATAGCAACATTCTCTTCAGTGTATAAGTTACCATATCTTGCTCGCAGCCTTTCAATTACTTTGATGACAACAAGCTGAGAAGCCATGCAAGCATCGAGGTTCACAAATACTACCCGCTTACCCAAAGGCTGAGCAACAATAAAAGCTCTTGCCCGCAGCCTAAAGTGAACCCCGGATGCAATTTGCTCTGTGTTTGCGTAACCCATCATATTGACATCAGCTGCAGGGCCGGTAATGTCATAGCTTCCAATACCAATCAAGTACTCAGAAATGGAGTGCGCAGTATCACTCCTAAGTAGTAGTAACAGCAACACTAAAAGGGTCCACACTCGCATGGTTACAGATGCCTTCCGAAGATTAATTCTACCACACCCAAATGCAAAGCAAAAACAAGTCCGGGTTTTTAAGCTCGCCCAGTTCTTCTCTTTTCCCACCCAAATGCAAAGCGCGATCAGGAGACCATGATAAAGTAATCTAAGCTAACTGTGGATTGCAGAAAGACTGCCCCAGAAAATGAAAAGGGAAAAAGAAAGAGAGAAGAAGAAGAGTCCAAAGAAGAAAAGGAAATAAAATAAGAGTGGTTTAGTCAAGTGTGGAGGAACTGGCACGGGAAGGCATGATTTGGAGTGAAGGATCATCCATCTACCCTCCTATTTTAAGGTTCACACCTTGGCATTGCATGGATCAACATCAACC

>C102403_65

AGGAAGCACTCGAGAAGAATACAATACAATACAACTTGCTCTATTCGTGGTGCTACCCAATAAGAGCATCTATCCCGAGTTCCCAACCCTGCTTTTGGTACAGCTGGCTTCTTGTCATATTCATACCATTGAATCTCTCACTCGTCCATATGTCCCTTGAGGCCAAACTGCACTATTCATGCTAAATTCTTGTTTACTTTGTCCCCACATTCTTCCGTTTTCCTTCCTCAGTTTGCCTGTGATTGAGTCTTGTCAAGAAATCTAGCCAACACAAATTGAAAACAAGAAAAATCATGTAAATCATACATTGTTAGTTTCTGATTGGCAGTGTTACTCTTAGCCCCAGTGGCAGCCTTTGGAAGTCTATATTGATTTCCTTTGACCCTTTTGGTGGAGAAGAAGATATGGAAGAAACATTTGTACCCTTTGAAGGAATCAAGAATGATCTGAGAGGAAGGTTGATGTGCTACAAGCAAGATTGGACGGGAGGAATCAAAGCAGGTTTAAGGATTTTGGCCCCCACCACATACATATTCTTTGCTTCTGCAATACCAGTCATTTCATTCGGGGAACAACTAGAGCGAGATACTGATGGTGTTCTAACTGCTGTTCAAACATTGGCATCCACTTCAATATGTGGCATTATACACTCAATCCTTGGAGGTCAACCTTTGCTGATTTTAGGAGTGGCAGAACCTACCGTGATCATGTACACATTCATGTTCAATTTTGCGAAAGAGAGACCAGAATTGGGCCGGAATTTGTTTCTGGCATGGAGTGGATGGGTATGTGTCTGGACTGCAGTGTTGCTATTCTTGCTGGCCATCTTAGGGGCTTGCTCTATAATCAACAGGTTTACCCGTATTGCAGGAGAGTTATTTGGCATGCTTATTGCAATGCTCTTCATGCAGCAAGCTATCAAAGGACTTGTGGATGAGTTTCGCATACCAGAAAGAGAAAATCCAAAATCAATAGAGTTTATACCTTCATGGAGGTTTGCTAACGGCATGTTTGCTCTGGTCCTTTCATTTGGCCTTCTCCTCACTGCATTAAGAAGCAGAAAGGCTAGGTCATGGCGTTATGGTACTGGTTGGCTTCGTAGCCTAATAGCAGACTATGGTGTGCCACTTATGGTCGTAATTTGGACAGGTGTGTCTTACCTTCCAGCAGGAAGTGTTCCACATGGTATTCCAAGGCGCCTGTTCAGCCCGAATCCATGGTCACCGGGTGCATATGAGAATTGGACTGTTATTAAGGATATGGTCCATGTTCCTGTTGTCTACATAATCGGAGCATTTATCCCTGCCACCATGATTGCTGTGCTTTATTACTTTGACCATAGTGTAGCATCTCAGCTTTCCCAGCAGAAGGAATTCAATTTGAGAAAGCCATCTTCTTACCATTACGACTTGCTCCTTTTGGGATTTTTGACCTTAATGTGTGGCCTTATTGGAGTTCCTCCAGCAAATGGAGTCATACCACAGTCTCCAATGCATACAAAGAGTCTGGCAACTCTTAAACATCAGTTGCTTCGGAACAAACTGGTGGTAACCGCACGAAAAAGTATGGGAAAGAATGCTAGTTTAGGACAGTTATATGGCAACATGGTAGAAGCCTACCATCAAATGCAGACGCCTCTTATTTACCAGGACCCCTCTGCTCGAGCACAAGGACTAAAAGAACTTAAAGAATCAACCATTCAATCAGCTACTAGTATGGGAAACATGGATGCCCCAGTAGATGAGACTGTGTTTGATGTTGAGAAAGAAATAGATGATTTGCTGCCTGTTGAGGTTAAGGAACAGCGTCTCAGTAACTTGCTTCAAGCAATAATGGTGGGAGGATGCGTTGCGGCCATGCCTGTCCTCAAGAAAATACCAACCTCAGTCCTTTGGGGCTATTTTGCCTTCATGGCCATTGAAAGTTTGCCAGGAAACCAATTCTGGGAAAGAATCTTATTGCTCTTCACTGCACCAAGCAGAAGATACAAAGTTCTTGAGGACTACCATGCTACTTTTGTAGAAACAGTTCCTTTCAAGACAATAGCAACATTTACCATATTCCAAACCATTTACCTTCTTGTATGTTTTGGACTAACATGGATTCCTATTGCTGGGGTCATGTTTCCTATGATGATCATGCTCTTAGTTCCGGTAAGACAGTACTTTCTCCCAAAATTTTTCAAGGGGGCACACCTTCAAGACTTGGATGCAGCAGCGTATGAAGAGCAAACAGCTTTACCATTCAACCTTGCAACACAATCAGAGTTTGGAGGTGGTGCTTCACATGTTGGAGAAGGTGAGATCCTAGATGAGGTTATCACTAGAAGTCGTGGCGAGTTTAGGCACACTAGCAGTCCGAAGATTACAAGCTCCACCCCAACACCAAGAACTGATCCTAAAAGCCATCTAAGCCCAAGTCTCTCATTTAGTTCACGCTTGGGTGAGCTCAAAACTGAGCTGAGTCCTCGATCTGGTGCAAGAGGTCCCCTCAGTCCAAAGGCTGGAGAAGTGAGACTGTCAAATCTGGGAAGAAGTCCTCTTAATCCAGATTCAAAACAACAGGACCACAATTAATAACTAATAATAGAATATTGCAGTCGTATGCCTTAGAAGGACTATTTAGGAGGCTTATTTCTTGCTACTTTCCATTCTAGTTTGTTCTTTAATTTTGCTTGTTTGTAACTTGTAAAGTCCTCTCCCTTGTCCCATATACGACAGATATGAGATATGTTACCAAAGTGTGGCGCTTTCGTTACTATATTTGTGCAAATGTATATCATCATCACAAGTTTGCTTCAATAAATTG

>C102407_65

ATTTTCTTGTCGTAGTGAATGGTTCCTTCCTTACTCTTCCCTATTCACTCTCAATCATACCTTTTTCTCTTCTCACTCTGGTCTACTCTAGACACCCTTTCTTTCCAATCCATCTGGATACTAGTTTCTTCGAGTGCATAATATCAGCCTGAAGCTCTAAGTAATTCTTGATATCGAAAGCCACACAGGAAAAATCATCATGCGTTGGTTGGGTCTTTCTGTTGATGTGTTCCGCCAAGTTATACAGTTGAACTTATATTGTAAAGTTATTCTGTAGGTCACTATTACCAAAAATTCTATAATTTGTTGCTTCTGGTCTCTATGCTTAGCTACTGCCATGGAAACCAATTGTTTGATCTTTTCTTCTGATAAGTATAGAATAGGTTAAAAGATAGGAAGTGAAGGAAGATTCTGTTATTTTTTATTTTCAGTATGTTCTCATCTCTTATTGTTCTATAGTTCTAGCTGATTCTGATTGTGTTCAGATTGGGTATCTTTGTTGTGATATTCTATTGGGCGTTTCTTCTCTGTGTACAATAACATTAGATTGATTGAGTAGTGTTGTGAGAAACAAAAGAAGTTATGGTGTGCCAAGCAGCGAGCCAAACAAGATTCCGAGCATTAAAACATGAGTATGGCATTGAAGGTGAAGCAACAATTATTGTTAGAGTGATAGCATGCTTTCAACCTTTGCATTTTTGTCAGGCTGAGTACTTCCGTCATTTGCTTAAACCTGTCACGTAGATTGAGTCATAGTTCTTTCTCTTTAAACTGGAGTTTGTAGTTTCCCTCTTTTCCCTTTTGTTCCTTAGAAAATCAGCAAGTAAACAAAGATCTCTTACAAGAGCACCATAACATCAACTGGTCATTTTTTGTTAGATGGTTAAGGCTGACAGGTCCTTGCCTTGCTCACACCATGTGGCTTGGCAATCACCTTCTTTGAATAACTTTGGTATGCTGCCTCAGCCCAGCTCACTTGGTCTCCCAACATATCTGAACTCAAGCACTTCTGTCTTACCTCATGGGCTTGCAGCTCCTTCTATCCGAAGTCTGAAGACTGAGCTAACAAATGAAGTGCAAGGATACCTTCAGTATCACAATGCAGACACAAGTTTGAAAGAAACTCATCTTGGAAGAGCTTTACAAAATGAAAATCCTTCACTGCAAAAGAGTTTACTGATCTTCGACCGCACTGATAATAAAACAAGGTTGTTTTATGGTCCCGTCCCTCTTGTTCAGAGTCCAACTGTTACTGCTAAGAAGTTTGCTCAAACTTATAACGTAAATGGGGAAGAACAGGCTGGTAATGAGGTTCAAAAGCATCTTGCCAGTTACAGTTTAATGGAAGGGTCTGTTAAGGATCATGCTGTTACTGAAGAAAGTGAAAAGCATGAAGATACGGAAGAAATAAATGCATTGCTTTATTCCGATGATGACAGCAGTGAATATGATGATGATAATTGTGATGAGGTAACAAGTACAGATCGTTCTCCTTTGGCAACTAAAATGACTTATGCAATACAAGAACAGTTTGAAGACTTGAAAGAGGAGGTTGCTAGCTCTTATTGGCCTAACAAAAGGCTGAAGTTAATTGATGGTGACCACAATGGATCATCCCCACATGCGGACAAATCTAATTTGGTAAAACCAAATGAAACCTGTGAATGTGTTAGTGATGCTGAATCAAAGAACTCTAGTGGCTGGACATATTCTGTTGATAAAACTAAAGCAGATGATTCAGTGGTGTGTGATATCAAATTAAAAAAGGATAAGATCCGCGAATCTTTGAGAGTTCTGGAGAATTTAATTCCTGGTGTGAAAGGAAAGGAGCCAATTTCAGTCATTGATGGAACTATTGAGTACTTGAAAATTTTGATGTCCAAAACTGGTTCTCTTGGGGTGGAATATCACTAAGTGGTACACTCACAGTCAATCAGTCAGTCTGTTACTGCCACTGAGGTTATAGTATGTTGGTTGAAGGACACATTCATGGTGAAATGATGTAGTTCTTTGAATTCTTAACTTTGTTGGTTGATATGTTTATCTTAGCATATTTGCATCTTGTCTTGTTTAATCCTCTCTCTTGAGGTGTACATATATGGTTGGGAAATAAAGGGACTAATGTTGAAATAGATAGTATTGCACTTTGGCACATCAGTTGGAAAAGAATGCAGGATATGGGGTTTCAGTTTGTTGACCAACATGATTATCCTGTAGCTTTTTTTGGAGGATTAAAGGTCACAGGTTGAAGTGATGGAGATGGTAGTGGGGGACTTTGCTGTAAATGAGGACACTTTTTTTCTCTCTAGTGTTACACTCTCTTGGTGATTGAATGCATGCGCATTAGGCAAATGGGGTCCCACTTTCCACCCTCCCTTTTCATTCTTGGGTTTGTGCTTTTGTCCTCTCTTTCAAGGTTGCACGCATATCATGCTGGCATATACTCTAAAGTTACAAGAAAGTTTCCTGATGTTTGGCAGAGTGAGGCTAGTGTTTCATCATGAGTTGCTGGGGTCCTTTGTTTGGATATAAAGCATTTTGTGGTAAAGGTTACTTTGTTTTACATGTTGATGGAGGGAGGAATGATGACAAGCAATTATAGTTTCTGCTTTGTAATTCTCATTGGTGGTCTAGGTCTCTTTTTGATGATGCTATTATGAAAGCTGTCAGATGTGATTGTATGAATAATTGGAATGTGAATGGTTATGGAAGTGGTGTGTGCAAGTTAAAGTGTATCGTGGATGTAGTTAGCTAATGTGGTTTCATGTTTGTACATATAATTTTCTATCAATCTATGATTCCTCATTTCCTG

>C102411_65

TTTTTTTTTCCATAACTGAAAATAACATTAATTTGCAACAGTCAAACTCACCACAGCTACAATGTTCAAACTTTGTCAAGAAACCCGGAAAACATGTATCATAATGCCTATAAAGTGATACAAATTGATAGCATTCTCAACATCAGATTGCTAAAATCTGGAAATCAGTCTTCAGAATAAAACCATCTTTACAGGACCATTACAATAACTGTTCACTCACTATATTTTATTAACAAGCACATAGAATGATTTTAAGTAGTAAGGATGGGTGGCAGGTAGTCAACTTTGTCCCCAAGAACTCGGGCCTTGGTGTCAGGAAAGTATTCAAATCCTGGAAGTTCAGTGACAATTCCTTCGCTGGAAACCCCGATAGGATAGCGGAGTAAGTGGCCAGGAAGGTCATGCTCAAGGGACTCACTTGAATAGAGATCCCAATACTTATCAGCAATCTGGTTTACCTTCCTGATACATTCTGTACTTTCAGGGTACAGGAAGGAGTCATGAAGCATGCCCAGGTGCTCATACCACAATGACATGCGGAAACCATGGATCTGACCCCTTGCAGGCTGCCTCGTGACCAAATGAAAGGGTTGATAAGCACCCATTGCAACCTCAGAGTCTCTTGCACCATCCATTGATCTCTGGTTTATGTTGGCAGATCCAACAATTATATATTCATCATCAACTATCATCATTTTGGCATGAACATATATCATGAAGCGGCGGGCCTCTTGGGCTCTTATATAATCTGTATCAGGATCTGGTGTTTCTGAAGGCTCGTACTCTCCTTCTTTCTTCACCTCCCGGTTACCAAGGCAGAAAAATGTCAAATAGTTCCGAGGATTTTCCTCTATTCCCTTGGCCTGGAGCGCCTGAACAACATCCTTGTACATCATATCCATTGTTCTCCTCTGCCAATCCAATATTGCCTGAACTGATGCACTTTCTGGGACACCCTCTGGCCACATTGGGACAACAACATACACACTGAACCTTTCCCCAGCTTCAATCTTACTAACAATCTTAAGTGAAAGCTCCTTCGGGATTAGATGCAAAGCACCAATGTCTTCAGGCTTAATTCCATCAGGACTCCAATCATAACAGCTTCCAAGGAAATACTGATTCTCAATATAGATGAAATTTTTTGCACGTCTAATGGCATTAATATAAGCATCTTGTATACTACGATCTATTATATTATCCTTCCCACTAACAAGTCCGACTCTAGCAGCTTCTTCAGGTGTATCCGGGAACCCGAAAGCAGCTCCTCCATCAATGGATCTAAATAACTGAACGTTCCAGGTCTCTGGATCATCAAGGTAAGTTACTGGGGATGGAGGAATAATGACATCTTCAAGGTCTCTCAGTTGAACAAGTAAGTCCTTTCCTCCCTGCTTCCTCCACCTTTGCTCAAAGTTGAACAAAACATCCCAAGCAATAGGCCCTTCAAGTCGAGAGTGGATGTCATGCCAAGGTTCCCTGGGACCACCTTTCTTTATTGAAGAACCAGCAAAGTTAGGCTGATGAAAATCATCATGATGTGCTGTGTCCAGGGTTCTGAAAAGAGAGTGGAATTGAGTGTCATATCTTCCATCACAGAGATCAATACCCCCCACAAAACTAACAATCCTTCGCTTATCAGATTCTCCACTCGGCAATTCACTGTCCACGACTACGATTTTCTGGTGATGAGTAAACATGGTGGAAATTTCTAAATCCTGAACAATGCTTCCACCATCATCAGGATTGCGAGGGCATAAAACACAATGCACTTCAGTGCCCCTGAAGTACTCTTCTGTTTCTTGATCATGAGTAGCCATCAGCCCATCTTTTTTTAACAAAGGAACCGAAGTTCTATCATCCCAGACAAGCATTAAAACCCTGACACCTTCTCTTGCTTTTTTCTTTAGAAGCTCACCAAGTGTTTCATCTCCTCCAGGCCTTGGCCTCCTAGAATCCCTTACCAAAGTAATCTCAGTATAGACAGACCAACCGGTAATGTATATCAAGTGCTGTGCTTTAGTGATTGCATCAAAAACATCCTCCCAACATCTATGAGGTTGGTAAGTCTGGCCCCCACTAAGTGGTATTTTAGGGACAAAGTTGTCTGGTACATGAGCATCTTGGTACAGGGAAACCTTACATCCTCTTCTTTGTGAAAAGAAAGTATACGGAACTCCAGGAAATTTAGGACTTCTAATGCCTAGAGCCCAGTTGCGGTCTTTTGAAATATCAAAGTATTGCAGCTTCACGTGGATCTTGGAATTTCCGTGTATTGGATTTTTATGCCCATCCAATATTTCAACCCATCTATCTATTTCTTCACCATGCAAGATCTCCTCAACAGGCACATATGCTCTTCCAATTAATGTTGCCCCAATAGGATTATCATCTTTCACTGTAAATATGATGTTTGATGCCATGTGGGCACAGTAAATGTGAAATGACTCATACCATCTTGGATTACTGTGCTCCTTTTCTATTATTCTGGTCCTTCCCACTCGTGCCTTCTCTAGATCAATAGTTGCATATAGTTTAGTGACACCCTTTCCAAAACCAACAGTCTCCTCGATGTTATGTACGATCTTGGTTAAGAAATTGTCACCACCAAGTTTGAGCTTATCCACTTCATAGATTGTGGCGTGGAGAGTGCCATGGAGCAGAATTTGCGCCATCACTTAGTACAGATGAGAATAGACAGTGTGGAATGGAATCAGAAGATGGTGTTTAAGAAGGAAGAAGAGATGAGAAGGAGTAGGTGAATAGGAGTGGATGAAAGAAGATGAACATTGATTGAAAAAATGAAGAGCAAAA

>C102413_65

CACGAAAGGTGGGCTTTAATCCACCTTGATCTTGTAATAGTAATGTGTGTAATTCTACTCCACCACATATGTTCCAGCTCAATGAGGTACACTCATGCAGTAAACACTCTTCTATTATCCAACCTCGGTTGAAAAATTTATTTGGGTGGCTCTAAATTGCTCCGTACATGGGTTCCACCGAAAAAAATTGTCTTTCATTTCCACTTCAAGCTGGTATTTTTATGTGGCACTGGGCTTCAATTAGCTGCAACAAATTACATTAGTGCGTACTGTGGAATATTCTTTACGAGGTGGAAGAATTTGTGTCCATGGAGAAAAACATTGTTGATTATAGTCCTTTGCTTTCTCCTCGTGGGGATGAAACTGCAAGCAAGACACAATCATCTTCTCTTGTGTCAGCTACTAAATGGACTCTCAAGTCTTTGATCAGCGTGATCTTTGTTTTGTGGACAGCTTTTATTTTTTTTGAACCTGCAAAGCCTGTGAATGACTTGTTTTCAAAATGGAATAATTTCAACCGTGACTCTCCTTTAGGAATCACAGGAGGCATTTTTGTGGTCTTCACTGCCCCATTTCTCATAATTGCATTTCTTGCCATTGCACACCTAAATCTTACTGGGGAGGATCAGCTTCAAGGGAAGAAGAGTTCCAAACATCCAAGGTTCCGCCTATGGACATTTCCTGTGCTTATCAAAGGACCATTTGGGGTTGTTTCTGCCACAGAATTCATTGGGATTGTCCTCTTTTTGCTGTATGTTGTCTGGGCTACCTATGCTTATACTATGCAGACTCTTGGGCGTATCTCTGAATTTGATCAGATATCCTTTAAAGGCAAGAGCATATTCTTTATGGAAATTATGGGACTTCGATCTGGCGCGATAGGATTAATGTGTTTGGCATTCCTATTTGTCCCAATTTCAAGGGGATCTGTTCTTCTCCGCTATATAGATATCCCTTTTGAACATGCCACAAGATATCATGTATGGCTGGGACACCTCACAATGGTGATTTTTACTATCCATGGACTTCTCTATGTTATTGCTTGGGCAATGAATGGACGCCTCGTACAAGAATTGATACAATGGAAAGATATTGGTGTTGCCAATCTCCCAGGAGTTATCAGCCTTCTAGCTGGTTTGCTAATGTGGGTGACCTCTCTTCCTGGAGTACGAACCAGGAACTTTGAGTTGTTCTTCTACACCCACCAGTTATATGTAGTCTTTGTTGTCTTTTTGGCCTTGCATGTTGGTGACTTTATATTCACTATGGCTGCTGGTGGAATATTTCTCTTTGTGCTTGATCGGTTTCTGAGGTTCTGCCAATCACGAAGGACAGTTAATGTAATCTCATCAAGATGCCTTCCATGTGGCACAGTGGAATTGGTTCTGTCAAAACCTCAAAGTTTGAGATACAATGCACTAAGTTTCATTTTCGTCCAAGTCCGGGAACTATCATGGCTGCAATGGCATCCATTCAGTGTTTCTTCTAGCCCTTTGGATGGAAAGCATCACCTTGCTGTTCTCATAAAGGTTCTTGGCAAATGGACAGAGAAGTTGAGGCAAAGGATTGCTGACGTTGATGCACAAAAAGACTCATTAGTCATAACAACTTCAGTGGAGGGACCATATGGGCATGAAGTACCATACCATTTAATGTACGAAAATCTTATATTGGTCGCTGGTGGTATCGGACTTTCACCCTTCCTTGCTATATTGAGTGACATTCTCCACCGTGTTAAGGAGGGGAAACCCTGTCAGCCGAGAAATATTTTACTTGTTTGGGCAGTCAAAAAATCAAACGAGCTTCCACTTCTCTCAACCATTGACATGGAAACAATTTGTCCGTCCTTTTCAGATAAAGTAAACATCGATATTCATATTTATGTCACTAGAGAATCAGATCCTCCAGTGGAAGAGGGGTACACTTACAAACCAATAAAATCTTCACTCTGCCCTATGGCTACTGATTGTGGCATGTCTGTTTTGGTTGGTACAGGAGACAATTTTTGGTCTGGACTTTATGTCATCTCATCTACAGTTGGCTTTGTGATATTGCTTGTTTTGTTGTATGTTTACTACATAACCCCATTTCACATAGAGATATGGTGGTACAAGGGACTACTATATGTAATCTGCATGGTTGCAAGTGTTGTTATCTTTGGTGGTTTAGTGGTTGTATTGTGGCACAATTGGGAAAAGCAAAGTTATTCGAAAGATAAGTCCAATAATGTAAAGGTTGATGATAAGATTCATCAAAATGGCACTTTGGCTCCCAAGGATCAAAGTCAAGTCAGTATTGCAAAGTCAACTGTCATTTGTTATGGTTCCAGGCCAGACTTCAAAGAAATTTATGAATCAATGTCAGAGAAGTGGGGCCTTGTTGATGTGGGTGTCATAGTTTGTGGCCCTTCAACACTTCAGACAAGTGTTGCTGAAGAAATCAGATCACACAGCATGACAAGACAACGCCATCATCCTATCTTCCATTTCCACAGCCATAGCTTTGATCTCTAGCTCCCCAAAAGGAAGATGAAAAACAGCATTTCCTAAAGAAAGCCACATAAATAAACATATATATACGAATATTGTATGTAGCAGGAATCCAATCTACAACCCTTTTCAGGATAAGGTGCTATAATTCTCAGAAGAATTCTAACTTTAAAAGGGGTAATGTGAATATATATTTTATCACTAGACATGTTTATATGCAATTTCAGGGATCATATGATGTTATTCCTCAGTTTTATATGGCTTCTTTCTATCTCATCTCTTAGAAGTGTAAATTACTGAATCAGAACATAGCGTAAATGCGAAA

>C102417_65

TCCTATTTCAAGTTTTGAACTTCTCTCTACAAATCTTTCTGCTCACACTCAAATCCTTCTACGCCATCCCAATTCACTCTTTTTCCGCTTCAACTTTCTTAGACTCCCCTTGCCCTTCCTCTCCTTCTCATAATTTCTCAATTTTTGGGCTCTTTTCATTTCACTGAACAAACTTCTCATATAGGGTTTCGATTATTGAGGGGGAAATTCCTGTTTTGATTGGTTTTTCGGCACCCCATAAACTATTTTTTCCTCCTTATGCCAGTTTTCGGTTTTGGCGATTTTTATTTGTTGAAATTTGTGTTTTTATAAGGTTTATGCTTTGTGGGGGTTTCTGTTCGGAAAAAGTCTAGGTTTTGGTCGAGGTTTATCGGGTGTTGGTCTTTTGGTGTTGATGGCTACCTCGGTTGAAGAAAACAACGGTGAGTATGATAGTGATCCCGAAGAGGCAAAGAGACCGTTAACAATGCGGAGGCGCGAAGCAAGCGATGACGAAGAAGCCGAAGGGGAAGAAGTCACTGATAAGAGGGAAGATCGTAGAGTAGAGGTTCCTTCTGATGACTTAGATGATGAAGGAGGTGTTGAAGATTATGATGATGCTAAAGAAGGAGAAGAAGAATTACAAGAATTGGAAGAAGAAGAAGCAGAAGAAGAAGAAGCAGAAGAAGAAGAGGAAGTTGAAGAAGAAGTGTACGAGGAGAAGGGTGCAAGTGGTGGGGTTCATGTTGAGGGTTCAGTGGTTGTGGTGGCTAAGGAATCTGATGACGATGTACAGCCCCCTTTGGAAGATCCTCTTGAGGACCATTCCGAGGAGAAAAAGCAGACTGAACCATTTGCGGTGCCCACAGCCGGAGCTTTTTACATGCATGATGATCGATTTAGGGACAATGCCGGTGCCCGCAACAGATGGATACAAGGTGGTGGAAGGAGACTGTGGGAGTCCAAAGATGACAAGAAATGGGGACATGATAAATTTGAGGAAATTTTGCACGAAAGGCACTATGAGGAGAGGAGACCTTCTAAGGGTAATTATCGTGGCCGTGGTAATGGTCGAGGCACTGATTTTGGAGGGTATGTTCGAGGAAGCAGGAAAGGATATAATAATGGTAGCAATCAAAATCAGACGCCTAAGAGTGTTGTGAGAGGAAGAGGCCCCCAAAAGTATGAACGAACCAATCAAAGCAATGGTCCATCTCCCCGTGTGCCGAATAAACAGTCTGGGAAATCTCGTGAGAAAAATTCAAATGTTAGTTCGGAGAGGACTTCCACACCTTCTTCCCATGCAGAATCTGACCCTGTGCAAGCTAAAAATCAAGTGCCTGCTTCAAATTTGAATTCAGCATCCCCGCCATATTACCCATCAGGCTCTTCCAACATTGATGTTAATTTGACTCAAAAGAGGGAGGTGCAAATGGGTGGCAGTAACAGGAACATTGGTCCTGTTGTTATGGATGAGAGTTCTCCTGTTCAACAAAGTGATGCTCTGATTCGGGGAAAGAATTTTGTTGATTCCATCAGTTTGGCAAAGTTGTATATTGATGAATCCATCCCTCCATCTGTTGGAAAGCCTTTGAACAATATGCATATGGCACCACCTGGATCTTCTGGAGTCAATGCTTCTCAATCTCCTCAAGTAAGGGCTTCTGGGACTGGCAGGGGTGTATCAATCCCAGTACAGTCAAATTATCAGCGTGCTCCTTCACATAATAAAGTTTCTCCCTCACAATTCCAGGCTATTCAGACAAGTTCTGTTCCAGGGCATAATTCAACTTCCGCACAAGCTACTGCCCCACAGTTGGGCCATAGACCTGGCAGTGGATCTAAGTTGTCATCTCCCCCAAAAACATCCACGGCAACTAATTCACATGATTCTGGAGGAATGGATTCAGTGTCAGAATCAGGTAAAGGTAAAGGTGCTTTAGTTGGGAAGGAGAGGGGAGGTTCCCATGGTGTAGGACGGGGAACTTTTGCTTATGGTGGGGCAAAGGTAATGGGCACGACTGGAAACGTAGGTGTTAGTCATGGAGATCCAAACTTCCCTGCCTTCTTGCCAGTCATGCAATTTGGGGGCCAGCATCCTGGTGGTATTGGAGTTCCTGCTGTTGGCATGGCATTCCCTGGATATGTTGCTCAACCTCAACTAGGTTTGGGAAAGTCTGAAATGACATGGATACCAGTCTTGGCTGGAACTGCAGGAGCTTTAGGGCCTACATACCCATACCTTGCTGTTGATGGTGCAAGCAGTCACCAATCTGGACAGGCCTCTTCAATGGGTACTTCAAGCAAGGAGCATAATGTTAATAAAGCTAATAATGGATTGAAGCCACCTCAGAGATCTGAGCTTGCAAGTGAAGAGTTTGGACAAAGGCAGAATAAATCCCGAAGATATTCAGAGATGAATTTTGGTCAGTGAATATGGTGCACTTTGTTTGAAGTAAGCAGATAAAGGGTGTTCTCCTGGCCCTTTGTCTCTAGCTTTTAATCTGGAACACCTTGGTCTGCAAAGTTTTAGAATTTTTAGTTATATAGCACCTGCTCTTGCTGTTCCCATCGTTGAAGTAGTTGTTAAAGTGTAAGGTTCTTCTACGATTTGGTTTCTTAGTGAAGCATGTGTGAAACACTGGCAGGCTTCTCTAGAATTAAGGATATAGAAAAAGCCATAATTATCATTTTGCAGCAGCCGATTGCATTGTGGGTGAAAAGTTTCTCATGTAGAGGTTTTTGTTATTTATCTGGTTTGACCATGTGCTTGCTCATATCAGCTATAAGTTTTAATGAGTTGTTCCTTTTTTTAAAAATTCAATTGTTTGTTATC

>C102419_65

CTTGAAAGACATCTATTTAAGAGATGAATCAAGACTTCATATCTAATTTATAAACAATAAAAGACCAAGGGAAAGATGAACTTGTCATCAGTGGAAAGCTATCGCCCAGCAAATGCAATTACTCTTTCACATTTTACATCAATTACTTGACTAGTGGCCCCGTCTCCTGAAACTCTGATTCTTTCACTTCTGTACATCCGAATTCTCATTTGTACCGATTTCCTTTCATTCTTGGGCGATCGGTCACCACAAATCAAAGATTAGAAGTATACACTTGTCTTGCTCTAACTGTTGTGACATCAGGAAAGTCCGTAGTGTTACTTTGTACCTACGTTCATCCAACATCTAATAAGGTCGGTTTGTTTTCTTGTTTTGTTGCGCCAGATCAGAATTCACATGGATTTTGGTATTTGGCGATCTACTAGACATTTGTTTCTGCTTTGAGGAAAATTTGAATCTCAAAAGTAATGAGTTTGTCATTACACCAACGGAACTCAGCCCCATGAGAGCCCCTGCAATTGACGGAGTCAGAACTGTTCCATTTACGGGAAATAACGCTCCAGCTGCAATTGGAATTCCTACGATGTTGTATATGAAAGCCCACCAAAGATTCTGTTTTACAGTATTCATTGTCAGCCTGCTAAGCTCCAAGGCATCAACTAGCTGTGAAAGCTGGTTGCGCATTAGTACAATAGAAGACACCTCACTAGCAGCCCCAACACCTCCACCTAATGCAACACCAACATGTGAAGAGGCCAGGGCAGCTGCATCGTTAATTCCATCACCAACCATAGCTACAATGTTCTTATCTTTCTGTAGTTCATTTATGAACTTCTTTTTCTCAGCTGGTTTTACTTCAGATAGCACCTTATCTTTGGGAATTCCAACAAGAGAAGCAACATGCTCAGCAGCATTCCTCTTGTCTCCTGATAGCATGTATACACCTATATTTTGTTTTGACAATCTGTCAACAACGTCTCTAGCATCTTCCCTTATCTCATCTTCAAAATAAATTAGGCCAGCTAAAGTATCATCAATTCCAACATAAACAAAGGATTGGTTATTACAATTCTCCACTTCTTGATGTAAACTGTTAATAACTCCATGCCTGGTGATCCATTCCAATGTACCAACAGAAACTTTTTTGTTATCAATTGTTGCCACTGCACCGGACCCAGGTTCTTCCAGGAATGTTCCATCTATTACCTTAGCATTATGACAATTAACAGCCTGAGCAGCATCCACAATCGCTTTCCCAACTGGATGTACGGAATTCGATTCTACTGCAGCTGCTAGCCTTAGAACTTCAACATCAGACAAAGCATTTTCTTCAGTTTGGCTTGAAATTGCATTTTTAATGCATGTTGGAGTGACAATGTTTGTCACCACAGGTCTACCAACCGTGAGGGTCCCTGTTTTGTCAAACACAACAGTGTTTACCATTGCAAACTTCTCTAGAATGTTTCCACCACGTAATAGTAATCCTCTTTTAGCCCCCAAAGAAGTTCCAACCAGCACTGCAGTAGGTGTAGCTAACCCAAGTGCACATGGACAAGCAACAACCAGAACACTGCAAGCAAGCTGCAAAGCCAGTGAAACTGAACTTCCTTGATATAAAGTAGGAGGCAAAATGTGTGTGCCATACAGACTCCAGAATGCGAATGTGGCAGCAGAGGCTGCCATCACTCCATAAGTGAAGTGTCCAGCCACCTTGTCAGCCAACCGTTGTACAGGAGCTTCCCTACTTTGTGCCTCTTCAACTAGACGAACAATGTCTGCCATAGAAGTTTCACTACCTGGTCTTTGCACTTCCATTGTAAGAGTTCCATTAAGATTTATACTTCCAGCTGCTACCTCACTCCCAGGTACTTTTGTTACAGGCAGTGGTTCACCAGTGAAACTTGATTCATCAATAGTGCTTCTACCAGCTCTCACAATTCCATCAGCCGGAATACGGTCTCCAGGCAACACAATAATTTGGTCTCCAGGAGAAAGACTGTCAGAAGGAACTTCAACAACCGAGCCAGCCTCTGTTTCCCCATTGTTAACTAGAAGGCGAGCTTTTGGTGGCAATAAACTAAGAAGTCCTGTCATATCACTGGTTGCTTTAAGTTTAGCTCTCTGTTCGAGATTCCTTCCTAACAAGACAAATGCAATTAGCATAATTGGCTCCTCAAAGAAAGCCTTCCATCCCAATCTTGGTAGTAAGGCAGCAAATGAACTGACTGTAAATGAAGATAAAGCCCCAAGACCAACTAAGGTGTTCATGTTTGGTGTCCTTTTAAGAAGGCTCTTTAAGCCATCAACGATAAGTTGGCGGCCAGGACCAAGCAATGTAAACAGAGATAAGGATAGATGAAACCCAACAGAATGAAATACGTGGATCCATGGTGCTTTTGCTGCAAAGAAATGAGAGAAATGGCCAACAAGACAGACAGCACATAGAGCCCAAGAAACAGCTAGCTCACGACCACTTTCTCTTAATTGTCTATGTCTTTCCTCCATCTTCCTTTCAAAAATTTGGAGGAAATTGTCCCTTGTAGAATCTCGGAGGCTAGAATTGTAACCACAGCTAGTTAAATGCTCTGCAAGTGCCTCCCCTAATTGCTTTTGCCAATTTGGTGCATTTTTTGCTTCGGATACAGGCCAAATGATTGCTGTCTCTGTAGTTAAATTTACACTAGCAGATGATACCTGTGGTTGATTTTCCAGTATCCTTTTCACAGTCGCTGCACATCCCCCGCACACCATTCCTGAAACATCGAGAATGATAACGTCAGGCGAAAGTGCCGAAAGCTCCTGTGACGCGTTTCCA

>C102427_65

CTTTTTCTCTCCTTCAGCGCCCTTCCCGTTTTCCCTCCATCAGTTGTTATGGCGGTGGAGAAGGAAAAGCTTCAGGAAGAATCCGTCATCCGCCGCTTCTACCAGATTATTCTTAGTTGGGACTACTTTGGTCTCCTCAAGGAATCCAAGAAGCAGAAAAACAACGAGAAGAAAGGGACTGCGAAGTCGACACTCGTGAAGGTGAAGAATCGGTACACAGACATTGATGACTATATTGCCACATACGAACCCCTCATATTTGAAGAAGCCAAATCCCAGATCATTAAGGAGAAAGAGGAGGAAGAGGTGACTGATTGGAAATTGGGGGTGGTAAAAAGTTGGAGTGAGGCAGATGACTTTCACTTCATAGAATTTCCGTGTGAAATCATAGAGGGAGAATCAATATCACAGAATGATCTTTTGCTGCTTTCGAAAGATAAGTTTCTGGATGGCAAAAGATTACCTACTGTTTATGCATTTGCTTTGGTGGAACATGTCCGGAAATTTTTTGAAACAAGACTTGTTCGAGTTAGACTCTATCTTGCTGGGGAGTTCTCCAAATTTAATACAGATAATGTACAATCTTGCCCCAGGCTGTTTAACATGCGATCTCATGTATGTGAGATAGAAAGACAATTATATTTTATGAAGCTGTGCAGCTTATCTACTATTGCTCGTGAATATTTGGCTATACGAACTATAGGCAGTCTCCCGTACAAGGATTTAATATTAAGTGCAGTTGGAGAAAATTTGAGCACAGAAGTCGAGGGATGGAAAATCCCTACACCGTTGAGGGAATACGTTGAAAGTACTTTTAATCAGTACCAACGTGAGGCCATTACAGCTGGTCTATCATCAAAAGCCTTTGTCCTGATACAGGGGCCCCCAGGGACTGGGAAGACTCAGACCATACTTGGGATTCTGAGCACCATTTTGCATGCAACTCCTACAAGAATGCATTCAAAGACCTATGAGCTAAGGCAGGGACCACAATTACCTATAAAGGAGAAACAAAGACATTGGGGATTAGCTTCTCCATGGCTTAGTAATATTAATCCAAGGGATAGTCTTATGCCAGAAGACGGTGATGATGGTTTTTTCCCAACTACTGGAAATGAGCTAAAACCTGAAGCTGTAACTTCGAGTCGTAAGTATCGCGTAAGAGTGTTAGTATGTGCCCCATCAAACTCTGCCCTTGATGAGATTGTGTTGAGGGTTCTCAATGGAGGTGTACACGATGAAAATGACCGTGTTTATTGCCCTAAAATTGTGCGGATTGGTCTCAAAGCACATCATTCTATCAAGGCTGTTTCTCTGGATGAACTTATGAAACAAAAACGTGCTTCTGCCAACAAATCATCCACTAATAAACAGATTAATGGTCCTGCAGGAAGTAATGATGACAGTATCCGGGCTGCACTTTTAGACGAGGCCACTATTGTCTTCTCCACTCTCAGCTTTAGTGGTTCCCATGTTTTCAGTAAACTAAATCGCGGATTTGATGTTGTGATTATAGATGAAGCAGCACAAGCTGTGGAGCCTGCAACTCTTGTTCCGTTAGCCAATCAATGCAAAAAAGTTTTTCTGGTTGGTGATCCAGCTCAACTTCCAGCAACTGTAATTTCAGATATGGCTAAGAATCACGGATATGGCACAAGCTTATTTGAAAGATTGAAGGAGGCTGGTTATCCAGTTAAAATGTTGAAGACTCAATATCGAATGCATCCTGAGATAAGAAGCTTTCCCTCTAGGGAGTTTTATGGAGACTCGTTGGAAGATGGGGATGAGGTGAAATCCCGCACATTACGTGCATGGCATGATTACCGCTGCTTCGGTCCCTTCTGCTTCTTTGATATACATGAGGGAAAAGAGGCTCGACCGTCTGGGAGTGGTTCATGGATAAATGCTGAGGAAGTTGACTTTGTTCTATTCTTGTATCAAAAACTGATTTCACTTTATCCGGCACTGAAATCTGGTAACCAAGTTGCAATTATATCCCCCTATAGTCAACAAGTCAAACTCTTCCAAAAACGTTTTGAAGAGACCTTTGGAATGTCAGCTGAGAAAGTAGTGGATATATGTACTGTTGATGGTTGCCAGGGACGGGAAAAGGACATTGCAATATTTTCATGTGTCAGGGCAAGCAAAGATAAAGGTATAGGGTTTGTCGACGACATCCGACGAATGAATGTTGGGATCACTAGAGCAAAGTCAGCTGTGTTGGTGGTTGGATCTGCCTCAACATTAAGGAGGAGTGAACAATGGAACAAGCTAGTGGAAAGTGCAGAGAAAAGAAACTGTTTATTCAAGGTTTCTCAACCATATTCATCATTCTTCAGTGATGAAAGTCTGACCTCCATGCAAACAAAGGGGGCTGAATTATCTCAGGCAATTGAACCCACTGATGATAACGATGTGCAGCCTAACAATGCTGCAGCATTTGATGATCAAGCTCAAGCTGAGGACAATGACGATGGCGATGTGGACATGAATGATGCGGGTTTTGATGAGGATTAGGACTAGTTGTGTAGTTGAGCAGGTAGGTGTAATTTATGTCGTTTTCCTGTCATGAGGACAATGAAAATGACAGTCATATTTAATCACAAAATGAAATTGTTATTTATCACCAAATTAACAGCTTGTGTCTACTGAACCACGAAGGATGATACAACAAAGACCTTGCGTAATCAATGACTTTCTACTAACGTAGTAAATATTAACTACAACTGAAAATGTACAGCTTTGAACATATATATTAGGTCTGTTTGGTCCCTAGATCTCTGGTGACAAGAAAAAAAGGAACTACAAATCTTCAGG

>C102429_65

TCTTAATACTCTTTGTTTTATTTTATTAGGGTGCAGCTCTAAAGGGCTCTGATCATCGCAGGGTCACGACTATTACTGTGGTAAGAGAGAGAAAGACACAGATATACACAGAGAGAGAAAGAGTGTGTAAAGTGAAGTGAGTGGTATAAATGTACAAACTCTCTATTCAGTGTGTGGCACCGTTTACATTCTCACTCTGCACTCTCTCTTACTCATCCTCTTCTCTGCGCTTCTCTGTTCGTGCCACTTCTTCTCGAGCAATGGCTTCTCATATTGTTGGTTATCCACGCATGGGACCCAAGAGAGAGCTTAAGTTTGCCTTGGAATCCTTTTGGGATGGAAAGAGTAGTGTTGAGGAGCTGCAGAAGGTTGCTGCAGAACTTAGGTCAGTCATCTGGAAGCAGATGGCTGAAGCTGGAACAAAGTATATTCCAAGCAACACCTTCTCGTACTACGATCAAGTACTAGACACAACGGCCATGCTTGGGGCAGTTCCTGCTAGATATAATTGGAATGGTGGGGAGATTGGGTTTGATGTTTACTTCTCAATGGCAAGAGGGAATGCATCCGTACCAGCTATGGAAATGACCAAGTGGTTTGACACCAACTACCATTACATTGTTCCTGAATTGGGTCCTGATGTCAAGTTCTCCTACGCATCCCACAAGGCCGTGGTTGAATACAAAGAGGCCAAAGTTCTGGGAATTAATACTGTACCTGTGCTTGTGGGACCTGTATCCTACTTGTTGCTGTCAAAACCAGCAAAGGGTGTTGAGAAGTCATTTTCCCTTCTTTCCCTAATTGACAAGATCCTTCCTGTCTACAGGGAGGTTGTGGCTGAACTGAAGGCAGCTGGTGCTACTTGGATCCAGTTTGATGAACCTACCCTTGTGAAGGATCTGGATGCCCACCAGTTACAAGTATTTACTCATGCCTATGCAGAGCTAGAATCAAGTTTATCTGGTTTGAATGTTCTGATTGAGACGTACTTTGCTGATGTCCCTGCTGAAGCATACAAAACACTCACCTCTTTGAAGGCTGTTACTGCATACGGGTTTGACATTGTTCGAGGAACAAAGACCCTTGATTTGATCAAGCAAGGATTTCCATCTGGAAAATTTCTTTTTGCTGGTGTTGTTGATGGAAGAAATATTTGGGCCAATAATCTTGCATCTTCTCTGAACACCTTGCAGACACTTGGGGACATTGTTGGGAATGACAAGGTTGTGGTTTCCACATCCTGTTCTCTTCTTCACACTGCAGTGGATCTTGTGAATGAGATCAAGTTGGACCAAGAGATTAAATCTTGGCTTGCATTTGCGGCACAAAAAGTTGTTGAAGTAAATGCCTTGGCCAAGGCATTGTCAGGACAGAAGGATGAGGTTTACTTTTCTGCTAATGCCGCAGCCTTGGCTTCAAGGAAGTCCTCCCCAAGGGTGACAAATGAGGCTGTCCAAAAGGCCGCTGCAGCTCTGAAGGGCTCTGATCATCGCAGGGTCACGAATGTTAGTGCTAGGTTGGATGCTCAACAGAAGAAATTGAATCTTCCTATTCTTCCAACCACTACAATTGGATCTTTCCCTCAGACTGCAGATCTTAGAAGAGTTCGCCGTGAATTCAAGGCTAACAAGATCTCTGAGGAAGAATATATCCGTTTCATTAAGGAGGAAATTAACAATGTTGTGAAGCTCCAGGAAGAACTTGACATTAATGTCTTGGTTCATGGAGAGCCTGAGAGGAATGACATGGTTGAGTACTTTGGAGAGCAATTATCTGGCTTTGCTTTTACTTCCAATGGGTGGGTTCAATCGTATGGATCCCGATGTGTCAAACCTCCCATCATTTATGGTGACGTGAGCCGTCCCAAACCCATGACTGTTTTCTGGTCTTCAACTGCTCAAAGTTTGACCAAACGACCTATGAAAGGAATGCTTACTGGCCCTGTTACTATTCTGAACTGGTCCTTTGTTAGAGATGACCAGCCGAGATTTGAAACATGCTATCAGATTGCTTTGGCTATCAAGGATGAGGTTGAGGATCTTGAGAAAGCTGGTATTACTGTCATCCAGATTGATGAGGCTGCTCTAAGAGAAGGTTTACCTCTGAGGAAGTCTGAGGAGACTTTCTATCTAAACTGGGCAGTTCACTCATTTAGGATTACCAACTGTGGTGTGAAAGACACCACTCAGATTCACACTCATATGTGCTACTCCAACTTCAATGACATCATTCACTCAATCATAGACATGGATGCTGATGTGATCACCATTGAGAACTCCAGATCAGATGAGAAGTTACTTTCAGTCTTCCGCGAGGGAGTGAAATATGGTGCTGGCATTGGTCCTGGCGTTTATGATATTCACTCACCCAGGATTCCACCCACAGATGAAATTGCTGACAGGATCAACAAGATGCTTGCAGTTCTTGAAAGCAACATTCTCTGGGTCAACCCAGATTGTGGCCTCAAGACACGCAAGTACACTGAGGTCAAACCTGCTCTCACCAATATGGTTGCTGCTGCGAAACTTATTCGCGACCAGTTAGCTAGTGCCAAGTGAGTCGAGAACGAGCCAATAAAGTTATCCATGTGAGATGTACTCATGTTGGGAACTGAACCTTCGTATTTCCATAATGTCTTGATACAAGATTTTGTGCTATCTTCTCCCATCTATCTTTTCTCCTACTGTTGTAGGTAATATTTTCAGTCTATAATTCTGCACATGAATAAGCTGATTTATGTTTACCTATTTTATTGGGGAACTGAGGAACCGGAACGAATCCTATACCGATACGCTGACAATGTGTATATCACTCCGAG

>C102431_65

GAGAGAAAGAGACCACAGTTGATTCTCTCATGTTCTGTCTCACCTCTTACCTAAGAACCAGGCAAAGACCTCTTCAAAAGGTCAAACACAAATCCTCCATTAGATAAATTTCTCTCCAATTTCAATTGGAATCCGTAGTTTCATTTCATCCTCCATAATCTTCCAATTCCATTTCTCTTTCCCAATTTCTCTCTCAAGTAACACAAACAATATTCTCTTTCAGCTTATGGTATTGGGTTTCAATTCTCCACTGTGATTCTTCCCTTCTTCGATTATTCCCCGCAATTCCCTCATCTGGGTACCCTCAATCACCTTCCAACTCAGAGGCACCTTTGATCTTGTGTTCAAAGTTTGCAGCTTTGACTTTTTCTCAACATGGATGGGAGCGAGAGCTCGAAGGGCGTGATTTTGAGTCATTTGGTTGAGTGCATCAAGGAGATTTCTGGGTTGCCAGAATGTCAAAACTTTTGCAAGAGGGTGTATGGGAGTTTGGTCCGCAGGGTGAAGCTTTTGAGTCCTCTCTTTGAGGAATTGAAGGATAGTGATGAGTCCCTCAGCGATGAACAACTTGAGAGCTTTGAATCTCTCTTGGTTGCTTTGGATTCAGCTAAAAACCTTTTAAAGGCTGTCAACCAAGGAAGCAAGCTTTATCAGGCCTTGCAGAGGAATGATACAGCTGAAAAATTCCAACAAATAACAGAAAAAATTGAAGCTGTGCTGAGTGAAATTCCTTACAATGAACTTGAGATATCAGAGGAAGTTCGAGAACAGATTGAACTGGTACATTCTCAATTCAAAAGAGCCAAAGCTCAGACAGAATTTGCTGATATACAGCTAGACATGGATATGGCAGTGGCACAAAAAGAAAAAGACCCTGATCCTGCTATACTCAAAAGACTTTCTGAGAAGTTGCATCTGAGGACAATAAATGACCTAAGGAAAGAGTCTACTGAATTACATGAATTATTCATTACAATTGGTGAAGAACCAGGAGACTACTTTGAAATGATCGCATCCCTTTTTAGCAAACTGAGGGCTTGTGTGCTCGCAGAAAATCCTGAAGTCGACACAAGTGAATGTGAAAAATTATCAGTTAAGCACAGGTCTCCTGTGATCCCAGATGATTTTCGATGTCCTATATCTCTTGAACTAATGAAAGATCCTGTTATTGTCTCTACTGGTCAGACATATGAAAGATCTTGCATTCAAAAATGGCTTGATGCTGGTCACAAAACCTGCCCCAAGACACAGCAGACACTTGTGCATACAGCCCTTACTCCTAACTATGTTCTGAAGAGTCTGATTGCTTTGTGGTGTGAAAGCAATGGTATTGAGCTACCAAAGAAGCAAGTGAACTGTAGAACAAAGAAAGGTGGTGGAAGCAGTCATTCTGATTGTGATCGAACTGCTATTACTGCCTTACTGGATAAACTGGCGAGTAACAACATCGAACAGCAAAGGGCAGCTGCTGGTGAGCTCCGGTTGCTGGCTAAGAGGAATGCAGATAACCGGGTATGCATTGCCGAGGCAGGAGCAATACCGCCTCTTGTAGATTTGTTGTCTTCTTCAGACCCTCGAACCCAGGAGCATGCTGTTACAGCGCTTCTCAATCTTTCCATCAATGAGAGTAACAAGGGAACTATAGTAAACGCTGGAGCCATACCGGATATTGTAGATGTACTAAAAAAAGGCAGCATGGAGGCTAGAGAAAATGCAGCTGCAACTCTCTTTAGTTTATCTGTACTAGACGAGAACAAGGTGCAAATAGGTGCAGCTGGAGCTATCCCTGCCCTTATAAAGTTACTTCGTGAAGGTACTCCCAGAGGTAAGAAGGATGCTGCTACTGCAATCTTTAACCTATCTATTTACCAGGGAAACAAAGCAAGAGCTGTAAAAGCTGGTATAGTAGGTCCCCTGATACAATTTCTGAAGGATGCTGGGGGTGGAATGGTAGATGAAGCACTAGCTATATTGGCAATTCTGGCAAGCCACCATGAAGGTAGGGTAGCGATTGGTCAGGCCGAGCCTATCCCTATTCTAGTTGAGGTTATCAGAACCGGCTCTCCACGCAACCGAGAGAATGCTGCCGCTGTCTTGTGGTCACTATGCACCGGAGATCCGCTGCTACTAAAACTAGCAAAAGAACATGGTGCAGAAGCTGCACTGCAGGAGTTATCAGAAAATGGAACTGATAGAGCCAAGAGAAAAGCTGGAAGCATACTAGAACTCTTGCAGCGAATGGAAGGGGATGATAATGTGCGGAATTCTTAGTCTATTCCTTATATATTTTATAGATGCTCCGAGAATTGGAAATGTTACACTTACATCGGGTTTTTTTAGACATTCACACGACACCCATTAATCGTTAAATACATCATATTTGTACTCAGTGTGGAAAAAAGTCCAACCATAAGACCACTTGCTTTAGTTGGCGGATCGATCGAGACTCCACATACTTATATGCCATAGGACAAGAATGTGTTGTTGCGTGTTCCGGTCGAGACCGACCGAGACTCTACATGCTTATATGCCATATGACAAGGATGTATTGTTATATTTAGCAAAAAATCACTTTTTTCATATTTAATTTTGCTTAATTTCACTAGGTGCTCCTTGATATGCTGAATGCGTTTAACAATTAATAGGTGTCATATGGATGTAAAAGAAAGTGAGTGTACGGTATCATTGTTCCAGAGAATTATATGTATGTATAAACCATGGCCACATGATAATAGTGTATATGTATTCATCTTTTGTATTATGTTTGTTTATTTTGAATCCAGTAATGGGCTGGCAAACTTTTGCAGAAACTTTGTATT

>C102433_65

CTATCTCATTAATTAAAGAGGCTTTCTAACAAACAGAATACAAAGTCACACTTTGCTAGTAACGAAGTGCTGTCACAAACTGATAGATAATATGTACAAAAAGAGAAATGAGGAAAGAAAGAAAAGCACCAGCTAACCTAATCAAAGTATAATGTCCTAACTTGACATCCCTTCCATAAGAATGTCAAACAACGCACATATTCTCTCCGTTTTGATGACTGACAGAAATCAGACAACGACTCTAATCAAACTTGGAAGTAAACACGCAGATAAAATTCTAACATGGGAGTGCAAGGCAACAAGAGCAATGACTAGCCTCAAATTTTGCTCTGGGGCACGATGCAAGCTTTAATTGTCTCTGACGGATCACTCAATAGACCTCTCCCAACGCTTGTACCAAGGCCAGAATTCCCCTTCAGCATTGCCATCAGACTTCCACCATGCACCCTGCCTTCGGGAACGGCTACCAACATTGGTGTATGACCATCTTCCACCACTGGGAGATGAGTACTTGCAATTTCATTTAGTTTAGCATCAGAAAAGGTTACCTCACCAGCTGTCTGTGGGTTACTCATTTTGATCATATCCACATCCATATCTTGGTTAATGACTATTTGCTTCAGTTCATCTAGTTTACTACCATGAGTATCTTGAATAACTGGTCCCAAGGATACACCTCCATGAACTCTCTTATCCAGCACTACCTCACTATGAAGTGCACGCGGACCTGTCTTCCCCCTTGCCAATGAATAAGAAGGGCCACCCTGTTGATTGCTATATTCTGACCCAGGATGACTCCAAGAGCATCTCTTCCTTCCTCCACGTGCTTTGCAAAAGTTGGTGTCCCCTTGTGCACTCTTTCCACACCCTTCAAATTTGCACCTCTTGCCTCCCCCATGTTGTACACAAAAATTAGCTCGTCCTCTAGTTCTCTTCATGCAATCAGGTACAGCACACCTCATTCCACCTCCATGTGCCATACAAAAGTCGGTACCTCCATGCACACTTTTTGTACAAATTCCACCACCCTGGTATGCACAGAATTTTCCTCCACCATGGCCTTTGCAAAGTGGCGTGGTCCCCCCAGCACATTTGGCGCACCCTGGTGCTGTACAGCATTTTCCCACAATATGTGCTTTGCAGACCATAATGTTTCCCTGTGTCCCTTTTGTGTAACCAGAAGCTTGACATCGATGCCCTCCACCATGTGAGATGCACAGACCTGCTAAGCCTTCTGCTTTCTTAGTACATTTTTCTGTTTGACATCTCTTGCCACTGCTATGTCGAATACACATTTCAGATTTCCCTCTAGCATCTTGGATACAGCCCTCATGATGACTGCACCTCTGGCCATCAGCATGAGCAGTACAGAAATCTGTATGTCCTTCTGTGCTGTTAGTGCAACCAAGATATTCACATTGCCTGCCTCCTGCATGGGACTTGCAGTACAACGTCCTGCCTTCAGCTCCTTTATTGCAGACGACTTTGTGACACCTCCTACCACCACCATGAGACATACAATGTCCAGAAGCACCTTGGGATCCCTTTCCACATCCCTCAACTTGACATGTCTTAGAATCACTGTTGCAGTGTAGTGGATGTTTCAGCTGTGTTAGTCTGGAAGTACAGGTGATAGAACTTTTTGGTCTGTTTGATGAGAGATCCAGAACGTTCTTCTCAGCCTGGTTTAACATTACACTAGCACCTGGATTAAGTGAAACAAGCCCTGGTTTCCGTCTAGATGAAGTTGATCCCTCATCTGCATTTGGTGTCCCGTTGAATACTAATGCCGTCTCCATGTTCAATTGAAGAGGTGGGACCCGAGAGGGACTTAGGCAAACACTGGTAATATCTGATTTGGAGGGCTGTGTGGAAAGGCTCAACTCAAAATCAATCTTTGGCTGCAACTCAAACATCTTCAAATTTGAACCAACAATGTTTTTTGGACTCTCTACCTTCTCACAGCCAAGATGAAGTGTAAAGTCCAGTTCAATATCCATAGGCATAGACGATTCTTCATCAATCTCTTTGGCTGAAGACATAGCAGTGCAAGAAGTACCTGAACTCTCTTTGCTGTCAGAGGAACTTGTTGAAAGTCCAAGTCCAAGAGGCAAAGAAGAGCAAATGCTTTGACCCATACATCCATCAATTAAATCCCACTTTCTTTTCATTCCCTTTGAAATTGAAGAACTAGGGGAATCAAGTTGTAAGATATCATTAGTACTACAATCAGCTTCCCTTACTTCAACTTGCTTCGACTTACCCAAAATCTTGAAAGCATTCGCAGAAGGGTTAGCTGTGAAACTCAAGTTCTGGAACCTCACATCCATGAAACACTTCCAGTATAATTCTATACCTATCGATACATTTCAACAGAACATTTTCCTGATTTACCCCTACAAAAAGAATTGGATATCTATAGAAGTCAGGTTAGATGCACCTACAGTCATAGGTGTATCTACAAGGGTAGTATGCATCCTCCACTGACAAATCCTGAAGTATCACGTCGATCCATCTAATAGAATAGAAGATTAAACTGATGTTGAGATTATCAGCTGTTGTAGGTCATCTTATTTCAGCAAGATTTGAATCCTTCCAAAACACGGCTTGGAGGTTAATCATCTCGCGGTAGTTAATAATAATTCCTCGAGTTGATTTGATTTAGTTTTGTTGTTTCCGCATAGCTTGGGGAACGATTCTAGGGTTTTCACTTTCGAAAGATTGTGATTATGATTATCTGATAAAAAAAACTCAAGGGAGGAGGGCGACACAAGACATTGATGATCGACAAAGCCACGATCATTTCAGGCATCTCTTCG

>C102437_65

CGGCTGACCAATCAATTTTGTAAGAATTTCTTTGAGCACAATGCATAAACAAGGGCCACTAATACTTCACACATTTTTTTTCTCCCCTACAGTGGAATCGATTTGAATCAGTTGAAGAACTATATCATATCATAATAGATTAGAAAAATTATCAAAATACAAAACGCAAAACAAAAAATCCAAGAAAATTTTTGCTATATTTGTCAGATGGTATCAGTGATGAAAACTTGGGTCTTCAGCTCATGTGGTATAATCATATCAATGTATTCATGAATCATTGAAAGTATCTGGCATGTCTTTGTCTTCTGGAGATGGAGAAGCCTTGGATCCCAATATGCGTTGGCATGTTGAGTTAAACTCCTCTGTCTTGCTCTTAATATCACTGGACCATAATATTGGGTCAGTATTCTTAGGAAAGGATTCTTGTTGCTGGATCTTCTCTTTCAACCACTGTTCTGCTTTATTGCACTCATTGATGATCAGTTCTTTATCCTGGGGTGAAAGGGAATCTGCTGACGTGCGATGCTTTAAAATACACATCGATAAATCCCCTTTAGCTTGCACTCTCTCTTTTTCATCTTTGAACCGATTCTCAATTGGATCTACCAGCTTTTTCAGATCTTCCAGTTTTGAAGAATAAGCATGCTCGGTTTCATCAACACCATCCTCATAAAGCCATTCCTCAGTCTCTTGAAGACTCCTAGATATGTCATCCCTCTCTTGTTCACTCGCAAAGCTTCTATATGTGTGGAAGAGCTTACTCCTCATATCATAGACATAAGACTCCAAGCTATTCTTCTTTTCTTTGGTTTGCTCTATAATTCTGTCTTGTAGGGCCAACTGGAGTTCTTTTTCACGAGCTTCTGAAATTTCTGCCTTTGTCATTCCACCATAGATATTCTCATTTACTGGCACAACAACCCTTCTGTTAGCCTTATCTTTTCTTGCACCATCAGCAGAACTACATGGAGATTCCAACTTCTTATTGGTAAGATCTTCAAACCCATTGGCAACTGTCTCAGGAATGGGATCAATATCCATTGCATCAGAATTTGAATTATAATCACCCGCCATAACCGAATCATCCATTTCATCCTTGATCAATGTAGCAGATTCAATATTGACAATTCCATGCAGATCAAGTGAAACTTTAACTTTAACTCTGCTCTTACTGCCATGGGATCCATGGAAAGGGCCAATCGTGACACAACTAATTTTAGGAGATGTCCCAGGTGGTAGTTCATCTGAATTAACATAGAAAGCTTCCAAATGAAACAAATTACTTCGCTGAAAGGTTATAACTTTAACACTTGGAAAGGGTTGCCCTCTTGGGAAAAGTACACCATTTGATCTCACAGCAATTGGACCTTCATCTGATGAAAGTCCAATTGAAAAGGGAATAACGTCCTGGACCTCATATTCTCTCACACAATAAACAGGACTGAGCATTGCACACTGTAGAGCACAACCACGTGCTACACACTCACTTGCATTCAGCTGCCGACTGGGTTCTCTCTTGAATAAAGAAATTAGTAATGTACTTATAGCTGGAATCCTAGAACCTGAACCAACTAGCTCTACAGAAGAAATCTTCTCCGCCGTCAACTTCGCATCAGTTAATGCTCTGTGGCAAGGAATAGAAACTCTCTCCAGTAATCCTGATGCTAGCTTCTCAAATTCTTCCCTTGAGATAAAGCCCTTGACATCTTTCTCATCCATCAAGCACTCGATATTAAGAGGTGCCTCTAGATTTGCACTCAAAACTTTCTTCAATTTCTCACATGCTGCTCGTAGCCTAAAGCATGCCTTGGTATTAGAATAAACATCAATTTTATACTCTTCCTTGAATTTTGTGGCAAAATGACTAAAAAGAACCTCATCAAAGTCCCTCCCCCCTAAGCTCCTGTCACATGCCTGTGAAAGTATCTTCATCTTCCCAAACTCAAATGATGCAATAGAGACCTGAGTATCACAATGACCAATGTCAATAAACGCAACATAAACTGGACCTGTACTGCTAAAATCTGTTTTGTACATTCCATAACTAAGGGCAGTTGCAGTACAATCATGGATCAATCTCAGAGGTTTCAACCCAGCAATTTTCGCTGCATCAAGATATGCCCGTCTCTGCAAGTCCGTAAAGTATGATGGGATCCCAATAACACAATCTGAAATGGGCATCTCCAAATCTTTTTCGGTCATAGTCTTCAAGTGAGCAAACAGCATGGACAATATTTGAACTGGTGTAAATACATGAATCTCCCCTAAGTACTTCAAGTGAATCAAGATTCCTCCATCTGGACCCTCAGAAGTTTCAAGAGGGAGCATTTTCAGCTCTTTATCCACATCAGGATCTGCAAATTTCCTTCCTATCAGTCTCTTTATTTGAGATATTGTGGACTTGATGTGCATCATAGCAGAAGCAGCACCAGCAGACCCCAAAAGCCGCTGCTTCTCACTGAAGCAGACCACGGCTGGGGTTTCGCGTTTGGATTCAAAATTCAACAAAACATCAATCCCACGTTGTCTGACGACAGCAATAACACAGTTCTCATTACCAATGTCAAACCCCACCACACTCATCTCAACTCACTTGCACTAAACTTGAACCAACTAATCCAATCCAATTCACCCACTCTACTCAACCAACACCCCTCAAATCCAAAACTTCACCAGATTTCACGAAATCAGTCCAGAAACCTAGGCTTCACCACAAATCACTTACTGATGGTAGAATCAAGACGCATTTGGGATTGCACAGTGGGAGTAGTGACGAAGATGGAATACAAAGAGTGAGAAAGAGAGAGAAAAAAAATCACTT

>C102445_65

TTTTTTAGATCAGAGGGGTGTTTATTATTGATTTAGTAGATTTTACTACAAGACAGAGAATTGGAATTGAAGCTAGAATCCTAAACACTCTAAATATTTGCACAACTCAGTCCTAACATAAAATTATTAATTCTAAATCAATTCCAATCCTAAGGAAATTAAAATTCTAAACTACTTTTTTTTGGTGGTCAAATTTTGCACTCTAAAGTATGTCTGTTAAGGCAGAGCTGCATGGAGTTCAGAAAAGCCTAAACAAGGCGTGCGAGCCGTAACAAAGTTAAACAGATTCTGAATCCCGATTAGGTTCCTCCATTTCTGAAGTAAAATTTATTGGCGCCCCATTGTGATATTGTTGCATTACCAAAACGGATGCTGTTCTGGGGTAATCTTGTGATGCCAACAAACCACCAACGGGTCCTAGTTCAGGGCATTCACTGCTTCCCAGAGAACAGGCAACTTCACTGACTGGCCTTGAACCCACAATAAACAGACTGCAGCTATTAAGCTCACCGATAATAGCAAATGTTTCTGCAGCACTTTTAACTATTTTCTCTTCATAGATGATGGAGTCATCATTGGCTATTTTCACCTTAAATTCGTCGAGGAATTCCTCATCCTGTGAGATTAATTTGGTGCTAGAGGAGTAGTTGCCCACATCAACTCTTACAATCTCTCCTTCATTCGTTGGCTCAACTACAAAGCGAATAACCACCAATCTGATGCCAGGATGCTCAGCCATACGGACTCCATAAGCAAGGGCTTCATGATCATCACCACCACCAAAGAAAAGCACCGTAACACGATAAGAAACACTACTTGCAGAGACATGGGATGTACCACCAAGCCCACGATCAACAAAAATTCCAACTGAGCATGGTGCATGCTCAAGTACCCTTTTGTTAACCCATCGAAAATCATTTCTAGTGACATTCAGTGAACCATCCAAACTTTGTTGCTTATGAAATGGAAGAATAATTACTGAGACTCCCTTCCTCTCAGCAGTTGTACAAATGTCTTCATGCATGTTAGCCATAGATGAGATGGCGATCATTGGCCGCATAGACACTTGACTTAGTTGCCTGTAAGCCTCAAACGTTACTATAATATGGTTAGAATCTGCATGAGAACCTTTGTTCCAGAATGGCAACCCATTTTTTCTTGCCTTATGTACCATTAATATAGATGAGGACCTCTCGGAGAATTCTTTAAGGTGCATTGCATACACACAAAGAGCATCACGCTTCTGGATTCCTCTTGAAGCCTCGATCAAATTTATCATTGATGGAATATTTCTTGCACCATTGAAGCAGGCAAGAATCCTCAGTTGGCTGTTTGCATTTTTCCTCCCAATTGTTCTATATTTGTAATCAGATATTCTTCCCTTCCTTGCAGGCTTATAGATAGCAGTCACAATAGGAGTGGTGATGAAGGTAGTGAAGACAGCCATAAGAACCATAATGGCGAAGGTTTGATCATTCAAAACCTTTCTATCTTTGCCAATGTTGAGGACTATTAATTCAACTAGGCCTTTGCAATTCATTAAGAACCCCAACACCAAAGCCTCGTTAAAAGGTACTTTACAGAAAAGGGAAACAACAACAGTCCCAAGAACCTTTCCAAAAGAAGCTGTAAATATAACAAAGGCCAGAAGACCCCATGATTGCAGCCCCTTGATGGTGGTTACATCAGTCTTCAATCCACTTGACACAAAATAGAGCGGGAGAAATAGACCAGACACAAGATCCTCTACTTTCTCCACAAGAGCACTGGCAAACGGTCCATCATTTGGGATCAAAATTCCAACAACAAAAGCACCAAACATGGCATGGATTCCAATAGCATCTGTGACAAACCCAGCAGCCAGAACGGCAGCTAATGTAGAACATATATATACCTCTTCAACTGGTTCACCTTCATGGCATCGTTGGCTTATCCATTTGAAAATTGGAGGGACAATGATTATTGCACATATGACAAAACCACACCCAGCTAAGAAGACCCACAATGAAACAAGAGGAGATCGCTCATGGCCTGACAAGGCAACAGCCAGAGCAAGCAGAATCCAGGCAGCTATATCATTAATTGCTGCAGCTGACATTGCTGTTCTGCCAACATTTGTGGTTAGAAGTTTTAACTCAGCCAAAATACGGGCTAACACAGGAAATGCAGTTATGGACAGAGCAACACCCATAAATACAAGGAATGCGGAATTATCTGAACCTTTGGCTATTGATTGTTTTAGAACATATGATGAACCAATTCCCATTACAAAGGGTAGGCTTATTCCAGCCATAGCAATAGCAAGGACTCGATTTCCAGATTGACGCAGAGATTTAAGATCTAACTCCAGGCCTGCTAAGAATAGAAAGAATATAAGGCCAATGTTTGCTAGAGTATCTAATACTGTAAGACTCCTGGGTGGGAAAACAGCGTGCATATAGCTTTTATTCCGTCCAAGTGCTGATGGACCAAGAAGTATTCCTCCAATAATCTCTGCAATAACTCTAGGTTGCCTTATAGGTTTTAGAAGATATGCCAGTCCCCTCGAGACCACAAGAACAAGGCATATCTGCAAAATAGCAAGGGGAAGTGCAAAATCAAGAGGGTTGTCACCCTGAAACAAGCCATTTGATGTGGCTTTCATAGGCGCCGGGCAAGCATTTCCAGATGTAGCGTTGGAAGCCATTTCGACAGTCCAAAATCAACAGGCGACTGCAGATCCACCTGTTGAACGTGAATGGTTGGTGAAGGAAGAAGATGAATCAGATTCACTTATTGGTACTAAAGGTCCTCGGATTTGATTCCTGCTAGTAGGAAACAGA

>C102455_65

GACATCAGAAAAATGATGCTGTATAAATGTTCTCTATTGTTTGAATTCCTAGAACTGAAAATTCGTTGCTCCATTGTTACTTGAATACCTAAACATGTAATGTATAAATGATAAAAGAAATGTAATGTTCCAAAACGCTTAATTACTGCTGAGAACCAACCAAGAAATGAAGATTGAGCCAACTAGAGGAATGTTTACCATGCCACCAAATATTATCACATCAGCACATGTAGGTGACATGTCAACGCTATTCAAAAACTTACTGCGCCTCTTAATAGTTTAAATTTTCACCTTGTTCAACACCTACCACTGTATATTCATCGATCAAAGCCTCTCGAAGAAATAAAGATAGCCCATTTCCTTTGGAGGATCAATGGAAGCCCCAACCTTGTTGTCGTTGAAAATTACCCATCTGCCATCTTTTAAAACGTGAGCGACATAGTGACCACATAGTGTAGAGGTTCCACTGTGGCTTACAATTCCAATAAGTCGATATTTCCCTCCTCCATCAGGTAGGTTAACATCATTGGAAGTGGATGCAGCATTTGATGGGGTAGCATCCATACTGGAGACTGATGCATTAGGGTTGTTAAATATCCAATCCGTCGCTTTCTCAATGTCACCACCCGATGCCTCCAGGGCCTTTCTAGCAATTTCTTCTTGAAATCCAAATGAAATTAGGGTATCAACTTTTGATTGGTCAATAATTGTCACAGTTTCAGAGCCATGGCCTTTGGAAATAGGAAGGTCAATATCTGGATCATCCATGTGAGATAGCAACCAATTCATTGCCTCTTCTAGTCCAACATTTGATGTATTTATAGCAGCTTTCTGACAATGGAGATGGTTAAAGCCCATGGAAACCAGATGGGCGACAATATCTTCATTGACCAACGTTTTGTTTGAATCTTCGTCAACAGTAACACCATCAGGTAACAGCTCTTCCCCAGGCTGGTGACCTTTGCTGCGCATATGACTGATATCTATGATATCAGGAACATCAATATACACATCAAGCTTTTTGGGTACCCATCCTGCCTCCATAACAAACCTTCGCATATGTAACACCAGGTAATCAGGAAATGAGGTCAGACCTGCGGTCTTCAGTGCTGTAGTTTTTGTCTTCAAAGCAGTACTGAAAAAATCATGTATCTCCTCAGGAGCTGAGAAGTTGGCAAGGCATGTTTCTAAAGGCACCCTTGGACGCACAATCTCATCAGCATTTATTTCCTTTCCTTCTGCAAGTTTCTCTGCTTTCAGTTTGTGAAAGGATTCTAGTTCTTCTTTGTTAGTCGCTTCACCTAATGGGATATTGAGAGAAAGAATGTAGTCATTCCTTCTATTATAAGTAACTTTTCCAGACGAACACAAAATTCTATCTTCAATACCAAATTTGAAACTCCTCGATGGATCCAGTTCAGTTTTCCCAGCATTAGCTCGTTCAACTTGATCAAGAAAATGTAGGAAAAATTCTAAAGCATCCTGTTGTCGCGTAGATGAAAATTCAGGATGGCTGGCAGCTATTACAGATTTAAACATACGTGGACGTATCCCTTCTTGTTTAGCAGTTGCAGTTGAAGTAGCAATGTTTTCTTTTTCATCATTTTCAAATGCTGGAACAGAATATTTTCCGGAAAGTAAACCGTGAGCCAATTTTGTCAGCTGCATATTGAGATCTACAGTTGGATCAGCAGGAGACATCTCAAATGCCTTCTTTAGACTTTGGCTCAAGTAGTACCGTGATATGAAGGATCTTGTTGAAAATACAACTTGCATATTTGCTGCCATGTAACAACTGTTTCCAATATTGACCAATCCTGTATATCCAGGTCCAAAAATTGGATATACTTCTTGTCCACTTTCTTGTATCCGATTCCAATCAAAATTAGTATTTTGATCAAGTTCTCTTTCAGCAGTTGTCATTTCAGTCTTCTGTAGCAATGAAAAGTCAATGCCAAAAAATGCCAGATGCTGCGCTAGTTGGGGATCTAAGACACTTTCATCCTCCGGATAGGAGAAAACATCTGCTGCTTCCAGATCAGCAGTGATAGTCCCAAGCTTTACTGCAAGAGGGTAGCCTGTTTCTCGGTAATGCTCAACAGCATGGTTGTTTCCTCCAGTTCCATCCCAGTTTTTCCTCCCACAAAGTATCATTCCATCAGTCAAGTTTAGCCAAAGATTGTCTGTCTTGTTACATTTAGCACATTTCCACCCGGTTGGTGGAATAACAACACCATTGTCAATCTGTTGTAAATTCATTGCATAGGAACTAACTTGTTTCTTATCAGCAGTCCAAGCAGCAAGTTCCTCCTTTCGTTCAGCACCCTCAGCTAGTAAGATTGCATCAACTGCCAACCTTACCTTCTCTGGCAATTCCACAGAAGGGAATGGAAGAGAAACATACTCAGGCAATATAACTACACTATGAGTTTCCTCATACTCAGGCTCATTATTATCAAATCCGCCATCAATACCTATAGCCAAAAGAGTAGGTTTCTTTGAAGGTCTGTCTTCTGGAACTAACTTCTTAGTCTGCTTTATATGCAAATAGACAGGATTGCCAGTCTTCTCGTAATTCCAATCAACAAAATCCTTCCCAAAAGCAAGAAAAGTACACATGTCGATGAACAAACCACCTTCGGACCTCGGAGTGTCGAAGGAAACGCAACATTCGTTCTTATAGATACGATTGGTCGGTTCGGGGATCCGAACCCGAGCGAGATAGGAGCGGAGCAAATCCAAGGGTTCGATTGTCATCGTGGCGGACGAAAACCCTCACAGGTGCGATTGCGGAAACTAAGAAACCTGAGGAGTTATAAGAAAG

>C102457_65

CTGAAAGTGATTTAGTATTTACAATTGTTAATTTGTCATCCATGCACCCACGCCACAAAGCAGGAAATAAAAAAACTAACGATTATTGTAGAAAAGTTAAAGAGGGAACACGATGATATTCACGAAAATAGCTATACAGACAATGCCTAAATAATCTAGATTCATGAACTGAAAGAGGGACTATTCATATGAAACTTGACTAAGTTACTTGATCACGTTTTCGTTTATGATCCTCAGGCAGTGGAAGATTATCGCCTTCGATGTTCACAGGTATCGAAGTACACAAGTCTTTGTCCACAGGAAAATGGCTGTCGCTAACTGGATTCGCCGGTTCCCCATCTATTGAAATCAAATTAGACTGCTCCTCAATTGAGACATCACCATTGCTTTTCAGTTGTTCCTCAACAGAAAGATCACCATTGCTTTCCTTGACAGATGGATCTCTTGAACTGGTGATTTCTTGAGTATCACCCAGAAGAAGGTCTTCAAGGAAACGTTCTGCGGCAAGACTTGTCTGAGAAATGAATTTCACATCCAAGATTGGGTTTTCAAAATCTACAACAGAGGAACCAACTGAAAATCCAAGAACGTTCTCTATGATGTCCTCATGGAAGGGTTTTTTAGGGACCTCGGTTGCAACAGTATGTTCAGTTCCGACAGAAAGTCCTTCCTCAGTGACGCTATAACAACGTGGGGTCTGATTATCGAGCAATTCACTTTTCTGACCATTGATTTTTCCATTTTCATCTTCTTCAGAATTGTCAATAAAACCTGTCTCTGTATTGAGTTCAGCTAAACTACTAGTTTTCACATACACCTCTTCTTGATCTTCTTTGGCAATGCAAAGTTGGTCATCTTCTTCAGCTTCTTGGGGAGTCTTTTCAAAGTGTTTATAATTAGAATCTGGCTTTGTGTTCTCACCATTTTCCAAAGTATTGGATGAAGCCAACACCTGGATGTGACTAAATTCCTCACTGTATGCGACACAAACAGAGTCATTTTTCACTATCTCGCTATTGCTCTCATGGTCATCATCATCATCTTCATTTAAAAACTCGGGAGCTTTAACAGTTAGACTTTTTGTATACTTTGGAGAGTCTAATGACATAGAAGATAAAAAATTAGCTAATGCAGAAGATAAAGCACCATCAATTGTCTGCTTTCCTTTATCTCTCAAAGAACTCGTTTCTTGTCCCGATGCATTACCTTCTTCATCCTCGCCATCAGGAAACTCAGGAGCTGTAACAACAAGACCTGGGAGCAATTGAGTAGTATTTCCTGAATCAGACATACGAGAGATATATGGTACCTCAGCGTGTAAATGTTTCTCATCTGGTTCACTTTCTCTAGAGACAACATAATCAGGACAATTGTCAACGGTGTTGGTATCTGATTCTATACAAGAAGCATCAGGAGAAGAAATTCTACAATGTGATGGCAATGCAACGTTCCGCAATGTATCCAGTTGCTGCTCAACTCTCTGAAGTCTTGCCTCCATGCTGCTCATGGGCACTACCATCTTCTCTTGAAAGCCCAAACAGATTTCTTCTATCCTGTCCATGCGCACTACCAGCTGTTCCAGGATCCTTTCCACATTGCTACCCGAAGAAAAACCACCATTGCTCTCAGGAATACCAACTTGCGTGGGAACAGCACTACGATTGCACTCCATTTTAGCAAATTGAAAGGGAACATCATTATGGTTGTTCTCCATTTCAGTAATTTTTGAGGGTCCAACTGTGCAAGTGCTGTCCATTTTAGCAGTTTGTGACGGTACTGCAGCAGGGTTACTCTCGATTTTAGCATTTTGTGAGAATTCATCTGGTTGGGATGAACCCACCCAACCTCCTTTTACCTCATTCAACTTCACTTCTTGAGGACCCGTTATGCTAGTGTTTCCTTTCAGCTGAGTTTTAATTACAGAATCAGTAGGAACAGTTGCTTCCAAATCATCCCCCGTAACATGCGGTTTTTCCTTTCTAAGAGCATTTGGATTGCTGAGCCCTGTTGTCTTAGATAATTGCATTAAAGTGGGAATAAACATAGCCATCAGGGAACTTCCGGATGAGTTTTCATTATGGTTTTCTTGGCTTTCTGAATCAGCTGAATCAACAGGATCAGCAAACACATAAATCTCATCAACATAAACACAACCTTTATTCTGAAGCGAGAGCAGCCGAATAGTAACAGAAATGCAAGGATTTGCATCATCGATCTCTGCCGTAGCCTCATAAAGATCCTGAGACTGATATGTTTTGGTGGAATTGAGGTAGGGTTTTGTTTGCGAATCGATGTTGGGAGAATCAGGAAATTTGACCTCAACCCAATCATCTTCAGTTTTAACATTATCGTCACTGAGATTTTGAACAGCGGGGGAACGAAGAACGTGATCGTCTCTAAGAGCAACGCCGCATCGAACTGTGCAAAGATAGTCGTTGTTGGTGCGGGCGTTGGGCGCAAAGTAAATCTCGTAGACTCGAGCAGTGCTTCGAACGTAGATTTGTCTGAGTTCGTGCCTCTCCGCAAAAGTAATCTTGATCTCGCAAGGAGGAGAATCTGGAGAAGGGGAATGAAGAAGGAGAGGAGTGGGATCGGTTTCGCAATCGTCGTTGAGTAGAGAGGAAGAGGATTGGAAAGTGATGCAGTCGCGGATAGAGGCGGAGGATGAAATGGTCCAGTTGCTTTGGGAATTCCAATCTCCCTCCATAACAAAACACAGAGAAGAAGAAGAAGATGATGAAGATGATGATAAATCAATGGCTCCAAGAGTTGTTAGCGGATAAATCAAGCAACCTGCAAAATCCTACGACATGTTTGTTTCTCA

>C102459_65

CACAGAATCAGACAATAGAATCAAACTTATCTACACTTCAAAATCAATCTACCTAAATTAATCAAACAGCATTCTTGGGATGGTACAACTACAAAAAAATATCTGCATCCAAGACACTGCTGCTACATGTCATACCCACTCAACTTTCTAACAATCCTAAATCAGAGAAATATTGGCCTTAATATCAACATGGAGAAAGGCAATGCTGCTGATATCATGATTCTCTGGATTTCCACTCCAAAATTGCTGACAAAAGTGTGTAATTAGGAATTAAATGCTTGTGAGGTAAAGCCATGTTTGTCATTGGTGATTTCTCGTTCTCTTCAAGCCACTTTTCAATTGCTTTGCGATCATATGTATATCCATCAGCAGCAACACAAGGATCATCCATCACATCCTGAAGTATTGGACAGATAAAGTGATTAGGAGGTTTGGATTTAATAGTAACAGTTGAAGCAGAATATTGGGCACTATCAGCAACTTCTTTCAATCTCTCAAGTGTTGGAAGAACTTGATCTATTAAATCAGGACGGTCTCTACGCCGAAGTTCTGCACAGCTCAGTCCTAACAAAGCCAAATCTAGTGTCTCTTGAAACGGCCAAGCTCCTGCCTCTGGATCCAATATATCTGCCAAATTTCCACCATCAATTGCTGTTTCAACCTTATGAGCAAGTGCTATTGCTGGTTTTGCTGTCAATAACTGTAAGATCACCATTCCAAAAGCATAAACATCAGATTTTGGAGATATTAACCCGGTCCTTTGATACTCAGGATCAATGTAGCAGAGTGTCCCAACAGGTGCTGTGTCCTTATACATGGTAGATAAATTATCTGAATGGAGTACTGTAGAAAGGCCTATGTCACCAATCTTGCTGACAAGGTTACAACCAAGCAAGATGTTTGCCGGTTTTAAGTCACGATGAATGATAGGTTCCGGCTTTGAGCTGTGAAGAAAGGCAAGAGCTGATGCAATTTCCCAAGCTATTCGAAACCTCTCAAACCATGGGATTGGGGCAGTGTTGTTTTTTCTCAGCAGTCTGTCCTCCAAGTTACCATTCTCCATATATTCATATACAAGACATCCATGATCAGCACATGCACCAAGAAGAAGAAGCAAATTTGGATGACGAATTCTGCTCAATATCTCTAACTCCTGTTGGAATTGCTTGCTTTTGCAGCTTCCATTAGTGCTGAGAACTTTAACAGCAACAGTTGTGTGATACAAATTGCACTTGTACACTATTCCATATGCTCCCATTCCAATTCTCAGATCTTCAGAGAAAGATGAAGTAGCTGAGACTATTTCATCCCATGTAAACTTCCGGTACTGCAGCATTGTACCAGTTAGAGCATCCTCCAGCTTCTCTTTCTCTTTTGCAGCACGAATGGCTTTCAACTCCGTTTCTTTCCTTTCTGCAGCCTCTCTTTCAACACATTCTTTCAAATATACTGCTTCTCTGGATGCGTCTTGATATTTCTCTCTTTCAAGTTTTGCTAATTCCTTTGCCTTCTCCTCCTTAGCAATAATCTCCTTCATTTTCAAGGTCTCTTCTGACCGTCTCTTGCTTAGGTCATTCAGCTTTCGGGATGCTTCAATTTTCTCACTTTGAGCTACAGCATGCAGTCCTTGGGCATGTCTTAGTTCAATTCTCAACTTTTCCAGCTCAAGGTTAAATTTTGCCTGCCTATTAGGTGATGGAAGTGTAGTTGCTAGTGTCACATCCTTGCTAGAATTTTGATCATACACCCAAGATTCAGTATCTGAAATAAAGCTTTTGCAACTAGAGACTCGACTTAAGGCATGATCAATGTCTGAGTTTCTGGCAGAGCTTGTAAGAGTACTTTCTCTCCCAAGATCTAGAGATTGACCTCTAGAATGGTTCATGTCAGTTAAACTCGAATTTGTGCTTAGAAGGGCACTATTTATACTTGTAAGAGCTTGAAATCGTTGTGTTGCTAAGGAACACGAGTGTAAACCAGCATATGATGAAACTGAGGCAGATTCTGTCTGAGAGGTGGAAGTGTAGTTCGATGAGCTGCTTGAAGACAAACTGGTTTCACTAGCATCATCTATAATGTTTCCAGCAATGGGCATGTCTGATTGTCGTACGGACAATTTTCCCTTTGAGATAGCATAAATCGTGCAAAATCTTGGAGTGCTTACTGAAATTTTTGCAGACATACACTTCTGTTTTCTTTTAAATATGCCATGAGATGAAGCTCCAACAACAAGTTTGGTTATAGCACCCTTAGCCACTTCCTCTGCTATTGCAAAAGCAACATCATCTGATTCAATCATTACAACATCCACATGCACCTTTCTCTGCTCACACATTTTCTTGAAAGGTAGCAGCATTTGGTTTGTTTGCCACTCCACTTCCTTTTTGAATGCAGTTGCTACATCATTACGCACTTGTGAAAGAGGGACCACATTTCCCATTGGCGTTGGAACTCCTTTTATACCAGCATGGACATGTATCAGCTTGAAGATAATCATTCCCTCAGGAACAAACTTATTAAGTGCCCACTGTACCACATGTTTGCTTTTCTTGTTACCCTTGATTGCCAGAGCAACAACAGAAGAAGACGAGGAATGCTGAGATACAACTTTTGCATCTGCCTCCAGAATCATGTCCATCTCTCTTCCTTACAAAGAAGAAAGTAAGTTATCAATCAAACACCAGCTTAGTTGAATGTTGAAACTATGAAGCTTCAAATCCTTGTTGCTAAAGAAGATACTCCCACCACTCTTCTCCAAAATGAAACCATTTCCAAGTTTCAAACAAGAACACCAACTGGGCACCAAAGTTTACCACAGGTTTGTCT

>C102461_65

TAGCACTTGAGATGAGCGAAGAGTATGAGTGAATAAAAAGATCCACATGACAGTGAGGTGCAAAGGGGCGTGACCGTGATTTTGGATCCACATTCACACCCACCACCACCACCATAGCACCCACCCAAAACCAAATCCACGTTCCACATTCAAATCTCTCTCTCTCTTTCCTTTACCCCAAACAACAAGCCACCTATCATCCCCTTCCTCTCTCTCTCTCTCTCTCACACACACACACACACACACACACACTGCTCTTCACAGTGCCTCATATAGTGAATTCCGAATCTCGCCGCCACCGAGCAACAGCGGAATCGTTTGAGCTGAGACGTGCCACGCTACGCTACTTGCTGGCTGGGCGCTTTCGCTCGCTTCCACACTCTCTGCCTCGTCCTCCTCTGATTGCGAATCGCAGATTGATGTCGCAGCTATTTAGTTCAAAGTTGCCTGTTACAGGATTGGTGATGTATACTCACTGAACCAAGATGCCATTCTTAACGAAGATAAAATGGGTTGTTCTTTCCGTTGTCACTTTATCTGTGGCATCTATCATCATTCATTTATCTCTGGCAAAGTTGTGGTCTGTTAACTTAGTGCAGTATAAAGCACTACCTAGCCTTCCTGAAGATTTTGGGTTTGTGTTGGGTAGACAGGTTGTAAAGAATAAGAAGCTATGGGGTTCCATTGAGTCTTTGGAAGCATTACAACCTAATGCCAATGCTAGAATCAATTATTCTGTTCCAAAGGAGCAGAGCAATGGTTTCATATATGCAAAAGTTTTTGGTGGGTTTGCAAAGATAAGATCCTCGATCTCCGATTTGGTTGCTATTTCTAGACTTTTAAATGCTACTCTTGTCATTCCGGAAATTCAAGAAAGTACTCAGTCAAAAGGAATTAGCTCAAAGTTCAGGAGTTTTTCTTATCTTTACAATGAGGAGCAGTTCATAGCGTATCTCAAAAATGATGTAATCATTGCAAAAAGCCTGCCAGAAAGTTTGATGGAAAGGAGAAAAAGAAATGAAATTCCTACATTTAAGGCCACAAGTTCATCTTCTCTAAAGTTTTACATCAAAGAAATTTTACCAAAGCTAAAGAAATCACAGGTTATTGGATTAATTATTGCCAATGGTGGAGCTCTACAGTCAATACTCCCACCTAGTATGGCTGAGATTCAGAGACTAAGATGCAGGGTTGCTTTCCATGCCCTGCAATTTCGTCCAGAGATTCAAAAGCTTGGCCGTCGAATGGTTCACAAGTTAAGATCCCTGGGGCAACCATTCTTGGCATTCCATCCGGGCTTATTGAGAGAAACCTTGGCATACAATGGTTGTGCAGAGCTTTTTCAGGATGTACACACTGAACTCATTCAACATCGAAGGTCCCGTATGATAAAGGAAGGAATTCTTAAGGATGAATTGAATGTGAATTCACACTTGCGAAGAGAAAAAGGTCTATGTCCTCTCATGCCTGAAGAGGTTGGCATTCTCCTTCGAGTAATGGGTTATCCTTCCAAAACAGTCATTTACCTGGCTGGCTCCGAAACATTTGGAGGCCAACGTGTGTTAATTCCTTTGCGGTCTATGTTTATCAATACTCTGGATCGCACTTCCTTGTGCAGTGAGAAAGAGTTGTCTGACTTGGTTGGACCTGAAACACCTCTTCCTCTAAATAGGTTTCAACCACCTCCTTTGAAAAGTGAGAAAGAACTCAAAGAAGCGTGGAAGAAGGCTGGTCCTCGGCCTCGGCCTCTTCCCCCACCCCCAGATAGACCAATTTACCAGCATGAAAAGGAAGGTTGGTATGCATGGATCACTGAGACCCCTACTGAACCTGATCCTTCCCCCATGGACCTGAGGATGAAAGCACACAGGTTACTTTGGGATGCACTTGATTACATTGTTTCTTTGGAAGCAGATGCCTTCTTTCCTGGATTTAACAATGACGGTAGTAGATGGCCAGATTTTTCTAGCCTTGTCATGGGGCATCGACTATATGAGACAGCTTCTTTTAGAACATACCGACCAGACAGGAAAGTTGTTACAGAACTTTTCAACATGACTCGTGAAAATTTGTACCATCCTAAACATAATTGGACAATTTTAGTGCAGCAACATCTAAACAAAAGTTTGACTGAAGAAGGCCTCATAAGGCAATCTCTTTGGTCAAAACCGGCAATGTTCCTTTCTCATCCACTTCCGGAATGCTCCTGTAGACTAGCTTCTACCAAGGCTACTAATCGTGTCAGAGGCGAGGATGGCCAGGTTCTATATGGAGGCGAAGATGTATGCCCCAAATGGATGCAACATGCTAATGATGCAGGTTCATTGGAGAAAGAAGGGGTCAAATCCGAAGATGAAGGGCCGGCAGACTATGAAAGTAATGATTTTGTTGACGAGTCTGAATCTGACAAAAACGGTGGCAAAGCCAATCAGACTCCACTCTGGGATCAAGATGAGGAAATGGATCCAAATGACTAAGAGTGGATGTGCATACATGGTCTATAGTTATACATCATCCAATTTTTCCATAATTTGTGAGAGGCATAGGTTATAACTTCGTGTCACAATCCTATCTTGCTAAAGGGTATATGGGTATTCAAATCTATTCTTTCATTAAATTTGGGTTATTTTTCCAAGGACAAAGGGAACATTTTTCTTTTTCTTTTTCACTTTATACTTACCACTTCCAGCGGAATTTTGTTGTAACATAATACATTACAAAATGAAAGAAAAAAATATCAGCAGTCGGCTTAATTGGAGCACAACAGATGGAGTTTCGAAGAAGAGTTTTTATCTAAAGTTTATGCATGGTATACATGGTTGCTGAC

>C102463_65

GAGCGTGAGTGAAAGGCAGGTTTGTTATGAAGGAGGGTGTTTGAAGAAACCCCAAATTGAATAAAGTGGTTTGATTTGAGGGAAAAGCTTGAATGGAAGGCAGGGTTCCAAAAGTAGGAGGATCAGAAATGGGGTTGGGTTCTGTAACACTGACTAGGGCTTGAATGGGGGTATTTATTATGCTGAAATCAATGTTGTTTGCATAATTGACTGCTTCCATGGACTTCTTTTCAGCTCTTTCTTTGAACAGTCTTTTTTAGCCCCTCTCTCTCTCTCCTGGGGGGGCCTTCTTTATGAATTTTGGTACTTCAAGTTCAGTGTTGGCTTTGGAAATGGTAGCTGCCAAACTCGTGTGAGCCTTCGTACATCTTTTGGGTACTTGGATTTTGTTTTAGCCCTTTAGCAATTTTTCACCATCATTTACTGAAGCTGTTTTATCTCTTCCTGTTTCTAGTCTTCTTCTCTTTTTTACTTCACACACTTGGACAAGTTTCTAGTTCCTTCAGTTCTCAAGTGTGTCGATTCTGGTTAATGCTTGTCATGGAATCATGTTTGTTGAATTTGTGTTGGGGCTTGAAAGGCTCCATCTGTTAGCTTGCTTATTTGAAATTTGACCACTTTTGATCATTTTTCATGCTGGATTGTTTACCATTTGGCCACTGTTGAAGGTACTTCTGTTTAATTTTCTCTGATTATCTGTTAGTTTGGAAAAGATCATAGAAATAATCTGCGAGTAGGGTGGTTTGGTATCACTTGTTAAGGAGGGGTGGATATGAATGACAAAGAAAAGTTTGAGGTGGATAGAAACGAGGATCCCATGGGTTATTCTAGTGGTATGCACTCAGATTGGAGATTCGGGGGTGCAAATCTTGCAAATTCTTCTGCAGGTTTGGTTGCCATGGGAAATTCTATGAATGTCAGCAGAGGGGATTTGATTGGTTCTTCTTCATGTTCCTCTGCTTCGATGGTGGACTCATTTGGTCCAAGCTACTGGGACAACACCGCGTGCTCCCAAAACCTGGGATTTTGTGACATTAATGTCCACAACAATGGGGGTTCTTCGAATACTGCTGGAATAAGAAAAGATGGCTTTGGCTTTGGAAGAGCTGGTCAGGATCATCATGGAACACTTGAAATGGGTTGGAACCCTGCTAATTCTATGTTACCAAATGGCCCAGGGATGTTTCCCCACAGTTTATCTCAGTTTCCAACTGATTCTGGATTCATTGAGAGAGCTGCAAGATTCTCATGCTTCAGTGGAGGGAATTTTATTGATATGGTGAACTCATATGGGATTGCTCAATCAATGGGGCTTTATGGAGCAAGAGATGCTATTGCTGGCCAGGGGTTGAAATCAGTAACTGGAGGGCAATCTCAAGGAGGTGATATGAATGTAGTTGAAGCAACAAAAGATTTGTCTTCATCTGTTGAGCACCTGGCTGCTAAAGGAAGCCCTCTCAAGAGTGATAAGAGAAGTGAAGGTCATGCTATGTCTCAAGATGATGGGAAGCAATCCCTTGTTAGGTCTGCTAATGAGTCTGATAGAGCTGAATCTAGTGATGATGGTGGTGGCCAGGATGATTCTCCAATGTTGGAAGGTACTAGTGGAGAACCTTCTATCAAAGGACTAAACTCAAAGAAAAGGAAAAGAAGTGGGCAGGACGCTGATCATGATAAAGCCAATGGAGCTCCAGAAACTCCTAATGAAGCTGCAAAGGACAGCTCCGATAATCAACAGAAGGTAGACCATCAACCAACTTCAGCAACCAAGGCTTCTGGGAAGAATGCCAAACTGGGGTCTCAAGCTTCTGATCCACCTAAGGAGGAATACATACATGTTAGGGCTCGCCGTGGTCAAGCAACAAATAGTCATAGCCTTGCAGAGAGAGTTAGGAGGGAAAAGATAAGTGAAAGGATGAAGTTTCTTCAAGACCTTGTACCCGGATGCAGCAAGGTCACTGGCAAGGCAGTAATGCTAGATGAAATCATCAACTATGTACAGTCACTTCAAAGACAGGTTGAGTTTTTGTCTATGAAACTTGCAACAGTAAATCCACGGCTGGATTTTAATATTGAAGGACTTCTTGCTAAAGATATTCTTCAACAACGGCCTGGTCCTTCCTCTGCACTAGGGTTTCCTCTAGATATGTCCATGGCTTTTCCTCCATTACATCCATCTCAACCAGGGTTGATTCATCCAATTATTCCCAACATGGCAAACTCATCTGATATTCTTCAAAGGAACATCCATCCACAGTTAGCTCCCTTGAATGGTGGATTCAAGGAGCCAAATCAGCTGTCTGATGTATGGGAGGATGAGCTTCACAATGTCGTACAGATGAGTTTTGCAACAACTGCTCCCCTGACTAGCCAAGATGTTGATGGTACTGTGTCAGCAAGTCAGATGAAAGTTGAACTTTGATGGTTAGTCTACATACATTCATCTTCGCTGTAGCGCGTATTTGACTTTCATTCTTGTCAGATGCAAATAACTAAGCTCAGAGTTTTACACATGGCTCACCAAACTTGAAGGCCATGATCACAATTCCCTGCAATATATTTTAGATGAAATATTACTGAGTAATGTTCTGGTGTCTAGGCCTTGGAGGGCAGTCACATACTCTAGGTTAGAGGATGTAGAGCAGTATTCAAGGATGTTTCCTTTCTTTTGAAGCGTGAATATATGTATCATATGTTTAGAAGTGGAAGTCTATTTTATCAAAATAGGTTTGCTCACTAAATATATTAAAGAATGTAAAGACAGGATCTCGCTGAGTCTGATCAGCCAGGAGAAATGCTAAACCAATTTTTTTTTTTTTTTTTTTTTTT

>C102467_65

CAACAATTAAATTCATAAGCTGTCCTCTATCCTCTATAGACATGAAGAATACCTCAAGCTACAAGAACAAGGAATAAAATGTTATATACAGTTCAGATTCCAACATTTGAGCCACGTTTTCCCCCAAGGGAAGAACACAACACAAGGAGCAATAGATTTGAAAATTGTACTTTTATTTTAACTACATTATTTAGCCTAGAGAACCATGATAAAGTTTATTTACATACTAAAAGAACATACAAACAAACAGGGTAAAAGAAGTACTGCTACATTCTAGTAGCTACCAAAGGTGCTGTCATTAACAAGTTGTCGAGGCACATACATGATTCAGCTGTTTTCATCTTTCTCCAAATTCACACTATGGCTACGTCTTTTCCTCAAGCTCACCTTAGATGACGAAGGGGGCAAGGAAGCCCCACGCACACAGGTTCTCATATGTTTAATCTTTTTGAGATGGTTTGCTATATCCTCCAGAAGCTTCAACCTTCCTTTTAAACTTTCTCCATTCTCTTGGAAGGAAGACAATCTTTGCTTTCCATGTTTGACCCTCCTCAACCTTTCTCCAAGAATTTCAATCTCATTGCCAATCCTTAACTTTTCATTTTCAACTGCACTTGAAGAATCACTTCCATAATCAAAAGACAAAGTTTTGCATACTGAGTTTGAGAGCATGGATAACTGACCGGTGGAGCCATCAACACTATTACTATTTTCTGTGTTATTTATCCTTGGACAATTTCGTAATAATACACTACTGTTCCGTACATTTAGGCTTCCAAATTCAAGATACCTATTTGTGAGCTCACTTGTAACAGTACTCAGTGAACAGCTACTTAACTTTTCATTATCCTCTTCCCTTAGTTCTACGTTGTCTTCAATGACACGGACATCAAGAACATCTGGGTCAGTATCATAAAAGAACCTACAGAGGTTGTCACGCATTTGAGCTCTTTGTTCTTTGTTTTTCTCCTGTGCTTCTCCATCTTCTGTATTGATGTCAGTCTGAATTGAGGAAACATCGGTTATTTCATCTTTCCTAACAGATGATTTGGTGCTTCCATCTTCATATGTTTCAACCGAAATTGGAGTTTTTTGTCCACTTGGATCACGTTGCACCATTGCTTTACCATAAGATTGTTCACTTCCTTTTGAATCCATCTGCCTATAAGCTTCAAGTTCCTTTTCCAAAAAGTGATTCTCTCTTTCCCTTCTTATAAGAATCTCTTGCATAATTTCCATCTCTTCTTCATCATAAGCAACTCTTTCTTCTATCATCCTGTGGTATTGCCTCATTTCCATTTCCATAGATGCCTTCTCCTCCTGCAAGCGCAATATCATAGCCAAGGTTTCATCAGCAGCAGTAGCAGCTGAAGCTCTCTCCTTCTCTAACTCAAGGTAAAGAGAAGCATAGGCTGCTTTCTCTTCTTCAAGTGCATTTTCTAACATCTTGAAACGGTCGTCTTCATTACCGACATTGTGAACATTGTCACACACATTATTCATGTAGTGTTCCAATGACAATAAACATTTGTCCTGTCCTGGACTATCAACCATCGATCCATTAAATTCATAACTGTGACAGGTCTTTTCATCCAAATCACGGCCAGTTTGATCTTCTTCGGCATCAACTACACTAACTCCTTTCCCTGAGGCAGGACTCGTGCTTGCATTGGTTTTACCTCTTATTACACCGACATCAAAAGGCAAGAAAGGAGTGAGGACATGTTCAGATTGCAGATTATCTAAAGGAACAGCTGAAGAGACTTTCCCAGGATCAGAACCACCCCTCCTCCGGCGCCGAATTCCAGACCTACGTTTCATACACATGGCCTTCTTCCCCTTATCATTAAACACGTTTTCCCTATCAATTAAGGGAAGAAAACGCGGACTGGAGCATGAGCTGCCAGATGCCTCGCCTTCCAACTCATCAACCCTATTATCAAGTGTTCTCTCAGCAACCACTTTATCATTGGCACTGCATGAATGATCCTTAACCCGAATCAAATCAAAGGGGAACCTTTTAATCGCCATAACTTGAATAGAACATATCTTCCTTGAAGGCCATTCAAGCAACAGTTTATGCACACAGAAACGGCGATTTCTATACCCAAAACTCCCCTTGCAGGGGCAAGGCAAAAGAAGATCAACAATTCTAAACCATTTGGACGCAAAGAAAGCAAAAGCTGAAGCACACAACAAGAAATAAGCTACAACAAGGTCAATAAAGGCACCAATAAGCCCTCCCAACGTCCAAGAGTGAGTCTCCTGCGAAGCCATCTTCATCTTTAGAGCCCAGAAAGACCATCACATGTGACAAAAGAAACATAACACAAAAACCCACCAAGCAAAATCCAAAATTTTCACGGACCCTTATCCCAAAATCCCAGATTTAATGCATCCATGAGACAATAAAGATGTATTGAACAAAAGGTTCAGATCAAAGCAGGTGGTAGAACAACTTGAAGAAAAAGGCAAAAGCACACAAGAAACTCAAGATCATGGTTTGGAGAAATTAACAATCCAAACCACAATTTTAGTTCAACCCCTTGATTATTTTTCTTTCTACTATGGGAGAAAAATGCTTAAGAGGGTTGGCGCCATGTGGTGACAAAAAAGTAACAAGGATAAAAAAGTGGTACTTTCAGGGATAGAATTTAAAAGGAAAATATACAATAACAATAACCATAATAATAAATGAAAAGAAAACCCAGAAATGAATGAGGGAGAAGGTGACGATGATTGATGGAAGGAATTATGAGATTGGCAGGTCTTAAAGCTTCTTTGTAAAATAGTTTATTATTTCAACCACCAGAGTTGCATCCGATTTCTT

>C102471_65

GAAAATTGCGTAAGAGGGTGATCAGGTGGTGATGCAAATCAGAAACTCAACAACACAGGGAGAAAGAAGAATCGAAGCCGTTTTATTATATTTCTCCGACGATTTGTCTCAGGCGACAGTTCAGAATCGAAGCGACGTCGTTGGTTGGTTCGCTCGTTCGTTCTTCGTTGGGCAATGAAAGTAGCTCAATAAGTGTTGGATCTCTGAACACTACGGGATTCGAATCTTGAGCAACTCGTTCCCGCAATGGATTCGTGGCTCAAAGCCGCTGAAGGTTTATTTGAAGTGGTAGATCGACGAGCGAAGGCTGTTGCCACTGACTTATCAGAGGAGCAGGCGGATTTGAAGTCTCCAGCATCAAATGGGCAAGGATCTCAAGGCAAGAGGACAAAATCAAAACCAACGGCTCAAAGAGGACTATCCAATTCATCTACTATAATTAGTGATACTACCAAGGAGAAAACTGAATCTCCAACAGCACCTGCGATCATAACAACTTCTACAGAAAAAATTGATCCTGAAAATGAAGGGAGCACTTCCACATCTACAAACCAACCAAAGGAGCCACAACCTAGTGAAGCAACATCACCCTCGTTGGACACTTCATCATCAAAAATGCTAGGCAATGATGTTGCTAAACATAACACAGATGATGCAGAACCATTAGTAAATGATGCAAATGTTGGAGTTGCCACTGGTGATACTAATGGTGATCCTACCCAAGAGAATGCTTCAGATATTAGTGAGATGGATCCTCTTGCAGCCTCTAAAGAAATTGAGAGCCCCAGTGGTGAACCCACCAGTACTGGCCAAATTATCAAATCTGGAGATTTAAATGCCAAAAGAAATATGGATCAAGAGAAATCTGAATCTGTGGCTGCTGACATGGCTCCTAATGACACCATACTCAAAGATTCTGATGTAAAAAAAGTTGAATCTGTTGTGGACAGAAAAAATGAAGAGGATCATAAAACTGACATCTCCCCCAAAAAAGTACTGGATCCACTGGATGAGGCTCAAGGATTGCTTAAAACAACAAAATCTACTGGTCAGTCTAAAGAGGCAAGGTTAGCTCGGGTCTGTGCTGGATTGTCATCCCGCCTTCAAGAATACAAATCTGAAAATGCACAGTTGGAGGAACTTCTCACTGCAGAGAGAGAGCTGGGTAAATCATATGAGGCTAGCATAAAACAGCTACAGAAGGATTTGTCTGAAAGTAAAAGGGAAGTTTCAAGAGTTGAAGCAAATATGGCTGAGGCCTTGGCAGCAAAAAATGCTGAAATTGAGGCACTTCTAAGTTCTATGGATGCAGTTAAGAGACAGGCTGCATTGTCGGAAGGAAATCTAGCTTCCATGCAGGCAAGCATGGAATCAATGATGAGAAATCGAGAACTAACAGAAACAAGGATGATGCAGGCTCTAAGAGAGGAGCTAGCATCTGCTGAGCGAAGAGCAGAAGAGGAGCGTGCTGCACATAATGCTACCAAAATGGCTGCTATGGAAAGAGAAGTAGATTTGGAGCATAGAGCAATTGAGTCTTCCACTGCACTTGCAAGGATACAGAGAGTAGCCGATGAGAGGACAGCAAAAGCCACAGAACTTGAGCAGAAGGTGGCACTTCTTGAGGTTGAATGTGCATCTTTAAATCAAGAACTACAAGATATGGAAGCCCGTGTGCGCCGGGAACAGAAAAAGTCTCCAGAAGAGGCCAATCAAGTAATTCAGCTGCAAGCATGGCAAGAAGAATTGGAGCGTGCCCGTCAAGGTCAGAGGGAAGCTGAAAACAAGCTATCTTCCTTGGAGGCTGAAATGCAAAAAATGAGAGTTGAGATGGCTGCCATGAAGAGGGATGCTGAGCATTACTCACGTCAGGAGCATATGGAGCTAGAGAAACGCTACCGGGAACTGACTGACCTTTTGTACTACAAACAAACACAATTAGAAACCATGGTCAGTGAAAAAGCTGCCGCAGAGTTTCAATTGGAGAAGGAAATTAAGCGTCTACAGGAAGCACGGGCAGAGGCTGAACGGAATAGAGTTTCTCGTCGGGCATCGTCATCTTGGGAAGATGAGGCAGAAATAAAATCCCTTGAGCCTCTTCCTATGCATCACCGTCATTTGGCCAGTGCAAGTATTCAGTTGCAAAAAGCAGTGAAACTGTTAGATTCAGGGGCTGTCAGGGCAACAAGATTTCTCTGGCGATATCCTACAGCTCGAGTTTTTCTATTTTTCTATTTGGTTTTTGTACATCTCTTCTTGATGTATCTCTTGCATCGTCTTCAGGCACAAGCTGACACTATGGATGCTAGAGAGGTTGCCGAATCTATGGGACTCTCTAACAAGAATTTACGGTGACTAAAAAATATTCCATCTAGTAGGGCCTACGTCAGGTGTTATTATCTTCCTGACAACAAATATTTTCAAGTTGACATTTCCAACGTGGCTGCTTATGGTGGCTTGATAGTGGTTCAGCAAAAGAATCAAGTATTGCAAAGCAAAAGAGGAACTGTTTGCTTCAGCCGAATGATTATTGAATGGGTGGCAGTTCTCTTTCTTGTGTAGACTGTCCTCAAAATTGAATTGGTTAATTATGTGTTTATTTTTTCTTTGAAAATGTATTTCGAGTGGAACGATGTAGATAACAATATATTGTGATATCCCTTTCCTTCGTTCCAAGGGCAATTGCCAAATCTAAAGAATGCTTGAATGAAGAGAAGTTAGATCTAGATAAATTGTTAACCTTGCCACAGTTACTATTTTTCTCTGTATTATTGGATCATATTTGTAAGACATAAATGTGAATATCATAATGGCAAATATTAGTCTAAAAA

>C102475_65

AGGGAGAGAGAAAGAAGAGGGTTTGTGGAAAATAAGAAGAAGGCAAGATGTTCGGAATCTTAGGAAACAAGGGTCACAAGATTAAGGGAACTTTGGTGATTATGAGAAAGAATGTGTTGGATATCAACAGCCTCACAAGTGTTTCAGGAGTGGTCGGAACCGGAATCAACATCATTGGTGGTGTAGTCGACACTGTCACTGCTCTTGCTTCTCACATCTCTATTCAACTCATTAGTGCTACTAAGACTGATGCACATGGGAAGGGAAAGGTTGGAAAGGCTACAAATTTAAGAGGACAGATATCATTGCCAACGTTGGGAGCTGGGCAAGATGCATATGATGTTAATTTTGAATGGGACAGTGATTTCGGAATTCCTGGTGCATTTTACATTAAGAACTACATGCAAGTTGAGTTCTACCTCAAGTCCCTAGTACTCGAAGACATTCCCAACCACGGAACCATTCACTTCGTCTGCAACTCCTGGGTTTACAATTCAAAAGTCTACAAGACTGATCGCATTTTCTTTGCCAACAACACATATCTTCCAAGCGAGACCCCAGCTGCACTTGTGAAGTACAGAGAAGAAGAGTTGAACACTGTAAGAGGGGATGGAACTGGAGAACGCAAGGAATATGATAGGATTTATGATTACGATGTCTACAATGATTTGGGTGATCCAGATAGTAGTGCTGACTTGGCTCGCCCCGTTCTTGGAGGTTCTGTCTTACCTTACCCTCGTAGAGGAAGAACTGGAAGAGGAAAAACTAAAAAAGATCCCAACAGTGAGAAGCAGGGCTCCTTTGTTTACCTTCCAAGAGACGAAGCATTTGGTCACTTGAAGTCATCTGATTTTCTCACTTATGGATTGAAATCTGTAGCCCAAGATGTGTTGCCTGTGTTGACTGATGCATTCGATGGAAATGTTTTGAGCCTTGAGTTTGATAATTTCGCCGAAGTGCGCAAGCTTTATGAAGGTGGAATTACATTGCCTACTAACTTTCTTAGCAAGATTGCTCCTCTACCTGTGATCAAGGAAATTTTTCGAACTGATGGCGAACAGTTCCTCAAGTATCCACCACCTAAAGTTATGCAAGTGGATAAGTCTGCATGGATGACTGATGAAGAATTTGCAAGAGAAACCATTGCTGGTGTTAATCCTAATGTCATTAAGATTCTTGAGGAGTTTCCACCGCGAAGCAAGCTAGATACTCAAGCCTATGGTGATCATACTTCCATTATAACAAAAGAACATTTAGAGCCTAACTTAGGTGGGCTAACTGTTGAGCAGGCTCTGAGTAGCAAGAAGTTGTTCATTTTGGATCACCATGACTATCTCATTCCATATTTGAGGAAAATAAATCAAACTACCACAAAGACTTATGCCACAAGAACCGTATTGTTCTTGAAAAGTGACGGAACTTTGGCACCATTGGCCATTGAGTTAAGTAAGCCGCATCCTCAGGGTGACGAACATGGTCCTGTTAGCGAAGTTTATGTGCCTTCGAGCGAAGGAGTTGAAGCTTATATTTGGTTACTGGCAAAGGCTTATGTTGTTGTGAATGACTCGTGCTACCATCAACTTGTTAGCCATTGGCTAAACACTCATGCAGTTGTTGAACCATTCATCATTGCAACAAATAGGCAACTGAGTGTGGTTCACCCTATTTACAAACTTCTGTTTCCTCACTATCGTGACACCATGAACATTAACTCACTTGCCCGCAAGTCCTTGGTCAATGCAAACGGTATTATAGAGAAAACATTCTTGTGGGGTAGATACTCTTTGGAATTGTCCGCTGTGATTTATAAGGACTGGTCTCTCTATGATCAGGCATTGCCTAATGATCTTGTCAAGAGAGGAGTTGCAGTTAAGGATCCATCTGCTGCTCATGGTCTTAAGCTTGTGATTGAGGACTATCCTTATGCTACTGACGGGCTAGAGATATGGGAGGCCATCAAGTCTTGGGTGGAAGAATATGTTGCCTTCTACTACAAGACAGACGCGGAACTTCAGCAGGACCCTGAACTCAAAGCTTGGTGGAAAGAACTTGTAGAGGTTGGTCATGGTGATTTGAAAGATAAGCCATGGTGGCCAAAGATGCAAAGTCGTGAAGATTTGGTTCAAGTCTCTACTACCCTCATATGGATTGCTTCAGCTCTTCACGCTGCTGTTAACTTTGGACAGTATCCATATGGTGGTTTGATCCTTAACAGGCCAACTATTAGCAGAAGATTCATGCCTGAGAAAGGGTCTGCTGAGTATGTTGCATTGGGTAAGAATCCTGAGAAGGAGTTTTTGAAAACTATTACTGGGAAGAAAGAGACCCTGATTGACCTTACAGTTATAGAGATCTTGTCAAGGCACACATCTGATGAGTTCTACCTTGGGGAGAGAGATGGTGGTGACTATTGGACTTCTGATGCCGGGCCATTAGAGGCTTTTAAGAGGTTCGGAAAGAAGCTTGCAGAGATTGAAACTAAGCTTGTACAGAAGAACAACGATGAGACATTGAGAAACCGCACTGGACCGGCTAAAATGCCTTACACTGTGCTCTATCCTTCGAGTGAAGAAGGGTTGACTTTCAGAGGAATTCCCAACAGTATCTCCATCTAAGGCTCTATGCTTGCTGCATTACTGTGCCTGTTATCAATAAATGTGTTATAGAAGGAATCTGATTCCTTCATCCACTTATGTATGCTTAAGTTATGTATCTCCAGTGGTTGAATGTTCTCGTGTGTTTGTTATTAATAAAGAATTTGTGTTTGTACTTTGTATAATCCAGAGACTATAATAAACACTGCTACCATTGCAAAATGCATCCAGAAGAAAC

>C102477_65

TCTCTCTCTCTCTCTCTCTCTCTCTCTCTCTCTCTCTCTCTCTTCTCCTTCCTTCAAATATCAACTATAATATCATATATTAGTCAAATCCATTTATCCATCCCCCACAATCTGTTACTGTTAGTGCCGGCTTTCTGTTTCTTTCTCTTCTATAAAGATTCTTCTTTTGCATTCCCTTCTCTTCACTTCTCTTTCTCCTCATCATGGATCTCTACGCTCTCTCTTCATACCCTTCTTTTCTAAACCGGCCAACACCTTCTTCTGATCCTCGTACGTCACTTTCTCAACGGGGCCTCCATTTCCCCGCCCAATCCCCACACCCTCACAGGCTAAACCAGACGAAGAAAAGGTCGAGAAGGGTCTATGCTTCAATGTCAGAGAGTGGGGAGTATTATTCCCAGAGACCGCCAACTCCTCTACTCGACACTGTAAACTTTCCAATTCATATGAAGAATCTCTCTGCCAAGGAGCTGAAGCAACTAGCGGATGAACTGCGTTCTGATGTTATTTTCAGTGTTTCTAGAACTGGGGGGCATTTGGGCTCTAGCCTTGGTGTGGTGGAACTCACTGTGGCCCTCCACTATGTCTTCAATGCCCCTAAGGACAAGATATTGTGGGACGTTGGTCACCAGTCTTACCCACATAAGATACTCACGGGTAGGAGGGATAAGATGCATACCATGAGGCAGACAAATGGGTTATCTGGGTTCACCAAACGTTCTGAGAGTGAATATGATTGTTTTGGCACTGGTCACAGTTCAACCACTATTTCAGCAGGACTTGGAATGGCTATTGGGAGAGATCTGAAGGGAAGAAAGAATAAAGTGGTTGCTGTTATTGGTGATGGAGCCATGACCGCAGGGCAAGCTTATGAAGCCATGAACAATGCTGGATATCTTGATTCTGACATGATTGTCATTCTGAATGACAACAAACAGGTCTCTTTACCCACTGCTACTCTTGATGGACCCATACCACCTGTTGGAGCCTTGAGCAGTGCTCTCAGCAGATTACAGTCAAATAGGCCTCTTAGAGAATTGAGAGAGGTTGCCAAGGGAGTTACTAAACGAATTGGAGGTCCTATGCATGAATTGGCTGCAAAAGTTGACGAGTATGCTCGTGGGATGATCAGTGGTTCTGGATCCACACTCTTTGAAGAGCTTGGACTCTACTATATTGGTCCTGTTGATGGTCATAACATAGACGATCTCGTTGCCATTCTCAATGAAGTGAAAAGTACAAAAATAACTGGTCCTGTACTGATCCATGTTATCACTGAAAAAGGCCGCGGATACCCATATGCAGAAAAGGCAGCAGACAAGTACCATGGGGTTACCAAGTTTGACCCACCAACTGGGAAGCAATTCAAATCCAAGGCTGCCACTCAGTCTTACACCACATACTTTGCAGAGGCTTTGATTGCAGAAGCCGAAGCTGACAAAGACATTGTTGCAATTCATGCTGCTATGGGAGGTGGAACTGGCATGAATATCTTCCATCGCCGTTTCCCAACAAGATGCTTTGATGTGGGGATAGCAGAACAGCATGCTGTTACATTTGCTGCGGGTCTGGCTTGTGAAGGTCTTAAGCCTTTCTGTGTAATTTACTCATCATTCATGCAGAGGGCTTATGACCAGGTGGTGCATGATGTGGACTTACAGAAGCTGCCTGTAAGATTTGCAATGGACAGAGCTGGATTAGTTGGAGCAGATGGTCCCACACATTCTGGTTCTTTTGATGTCACATTTATGGCATGCCTCCCTAACATGGTGGTGATGGCTCCCTCTGATGAAGCCGAACTTTTCCACATGGTTGCCACCTCAGCAGCCATTGATGATCGACCAAGTTGTTTCCGATACCCAAGGGGAAATGGCATTGGTGTGGAACTACCAACAGGAAATAAAGGAACTCCTCTTGAGATTGGGAAAGGAAGGATATTGATCGAAGGGGAAAGAGTGGCTCTTTTGGGCTATGGATCAGCTGTTCAAAACTGTTTGGCTGCGGCTTCCTTAGTAGAACATCATGGCTTGCGCTTAACAGTTGCTGATGCACGCTTCTGTAAGCCACTGGATTGTTCCCTGATTCGCAGTCTGGCAAAATCACATGAGGTTTTGATCACAGTAGAAGAAGGATCAATAGGAGGATTTGCTTCTCATGTTGCTCAGTTCATGGCCCTTGATGGCCTTCTAGATGGCAAATTGAAGTGGAGGCCAATAGTCCTTCCGGATCGTTATATTGACCATGGATCACCTGCTGACCAATTGTTTTCAGCCGGTCTTTCACCATCTCACATAGCAGCAACAGTGTTCAATATACTCGGACAAACAAGAGAAGCACTAGAGCTCACGTCATAAAAGCACAATGGAGGGCTTCAATTTTTCACTTCACACAATGTACAAAGTATAACAACATGAAAAGAAGTGTAATATGTGAATTTTTTAAGTGAATATTGCAGGTTGTCATCAATGATCAATAATATTATAAAATCTGTCCTACCACCTCTTTGTGTTCCGCGTGTCATGATCTGTTGCACTCTCTTTTTTACTTTTCGCTTACATTTTAAAAGTATACTGGTTTGCTTCTCATGCCTCTGGGGCTACAGTGTAGTAATAAGACAACTAGTTATAAAGGAGAAAATTCTTGAACTTTTCTGTGAGTGCAAAAGAAAAGACATTCTGTCCAAACTGAATTTGAAATCTGTCCCGAAGGTGAAGAAAGCAAACATTGCTAATTTGATGAGGAAATTGGTTCATGATAAACAATCAGCCTCAGTTATCATCGATTGTAGAAATGTCTACCAGAGTCAGAGCTAGTCGTCAAAATAGGGACTCT

>C102479_65

CGCCCATATTTAGAAAGGACACATTAAAAGTTTATATATTTATAACCACGAAATGACATACTATGTCTATTTTTATTCCTAGTTGAGGCAAATCGCAAGGTTCATACTCACCAAAGACAGTAAGATTGACAGAGTGAAGAAAGTTTAAGTAGCACTCAAGAGAAGGGACTAATATTTTCTGTATGCCAGTTCCTTCACAGCATCAGCAAGCTTGTTTTCCTTCTGAACACTCTGAACCCAGCTAGCATTGATATTAACCCGCTCAAGGCTCTGCCTTAACGTTCTAGCAATGGAAGGCATAGGATGGTTAGCAAAGAAGTCCTCTACCTCCTTGGCCTTCTCAAAGGAAGCAAACGGTGAGACAGCAGCACTGACAAAGCGAGTTATCAGAAATCCTGAACCATATGTTTTTGTGATGTGTTCCCAATTTTCCTTAAGCCATGCCCAAGCTACATCTCGTCCTTCTCGAGTAACAGCAAGTCCATAGACAGCATCCTGGCTCCGAACCTCAGATGACAACATAAAGTTAAGAGCGTCAAGAATTAGATCTGGATCACGTGAAGATGCCAGAGAGCCAAGAATTCGTGTTTTCTCCTGACTTAGATCAGTTTCTCTATATACTTTCAAAAGGGATTCATAAGCCGTTGGGTTAGATTTGCTTGCCCGTTGCATTACAGCTACATATGTTGCCTTTCTTATATCAGGTGGAAGAAGTGGTGAATTTCTGTTTTCTAAGAATGCCTGAAATCGCTTGCTTGCTTCGTCCAGTGTTAAATCGTGACCAAACACAGCTAGGGCAGACAAAACTTCTCCTCTCAACATTGCATCTAGATGACTTTCTCCTGGTTTAGGCTCCCAACCAAGCCTCTCAGCAGAGTATTGGAAAAGGTTAATAAAGAACTGCTTGAAATAATCTAGTAAGTCAGGTACGGCATCAGCAGCAATGCGTTCAACTTTAAGACTTATAGTTATTAGGTTAGACAGCACGGTATAATCATCTTCCTCCCTGTAAGATCCCATCAAGTTAATCAAAGAGGTCAATGATTCTTGGCGAGCCATACAAAGCGCATATGAATCATCCAAAACACCAAATCGGTCTGAGGAAGATAAAAGTCGTTTCTCTACTGCGTATCTAAGTCTAGCAGCAAGCAGTTCATCATATTTCACCCTGTAAAAACCAGCTTGATCCACATTAAGTTTAATCCAACAATTTACACTATTTTCTACGGTACCAGTGAACTCTTTGATATCATGTGCATCAGACTTTGTTTGCAAGAGGAAACTCTTGCGGACATCATAAGTGCCACAACATAATGTTATTGGAACAATCCATTGGCCTTCTCCTTGAGCACCACTTGACAAGAATTGTGACTGATTAAATTCCAATTTCTGACCATTGACTTTAACAGATACAACAGGATATCCCTTCTGCTTTGTCCACGAGGTCATTAACTTGTTCACAGGTTCACCAGATCCTTCCTCAAGTGCAGCCCATAGATCCTCGGTCTTAGCATTTGAGAAAGCATATTTTTTTATATATGAAGCCAGCGACCTCTGAAAGCATTCGGCACCGAGATAGCTTTGCAGCATCCTAATAACAGACGCACCTTTTCTATAACTTATAGCATCAAATATTTCATCGATCTCACAAGCATGATTTATCTCCACCTCAATTGGGTGGGATTCTTCAAGTCCATCCAACCTAAGACCGTCAGTGGATTCATGAAGAAATTGAGACCATATTTGCCACTCAGGAAACAAGCTATCAGTTGCTAAATAGCTCACCCAAGTTGCAAACCCCTCATTCAGCCATAAATGTGTCCACCACTCCATTGTAACGAGATTGCCAAACCACTGGTGAGCTAGTTCGTGAGTTACAACAGTTGCAACCCTTTGCTTATTTGCAGCAGCAGAATGCTTGTCATCATATAGCAAAGCAGTCTCCCGATAGGTGACCAAACCATAATTCTCCATGGCTCCAGCAGCAAAATCAGGGATGGCAATCATATCCAATTTTGGCAGAGGGTAAGGAGTAGCAAAGTAATTTTTGTATAGTTCCAAAGTATTGACAGCTACATCAAGTGCAAATTTCCCCTGGTTTGCCTTACCAACTTGACAATACACTCGGACTTTGACCCCGTCAGAGGTGTGATCTTCCACATAATCAAACAGACCAACGACAATAGCAACCAAATATGTAGACATGATTGGTGATTCTTGATATGAAACTGTTTTTAGATTTCCATCTGTTATTTCTTCAATAATTGGCATGTTGGAAAGTGCAACAAGCTCTGAAGGCACATCCAATGTGATCTTGAAAGTAGCCTTGCAAGCAGGTTCATCCCAACAAGGAAAGCACCGCCTGGCATCAGCTGGTTCAAACTGTGTAACTGCCATAGTCTTCTTCTCACCATTATATTCATAGGTACTTCTGTAGAACCCTTTCATTTTATCATTCAAGGTTCCTTCAAACTGAATAGTCAATACACCCAACCCAACGGGTATTTTCTCGGGGAACTCCAGTACCAGAATCTCGTCACTTTCAAACAACTCAACTCGCGAAGGCTTAAGAACCGTGGAGGAATTGCCGTTGGTAAATGAAACGGCATCAGCAGAGACGGAGAGTTCAGCGGCATTGAGAACGATGAAAGAGGTGGCGGCGACGATGTCGAGGTTGACGGCAACGGAACCTGCGAAGCGGTGCGCAACGAGGTCGGGCTTGAGCCTAAGGTCGTAGCGTTTGGGAATAGCGAATTTTGGAAGACGAGGTTGTCCTTTGAACTGATCCATCTATTTCTTCTGTTGTAGGTGTCACACAAATTAACACTGAAA

>C102481_65

ATTTAGAATAGCAGAGACGAAGCATCTTCCAATTCAAACCCTGAAATGGACGATAGAGACAAATCCGAAAAGAGAAGCAGAGAGCGTCACTCCGAACGCCATCGCGATTCCGATGACCACCGCCATCATCGATCGGACAGGCATCACAAGCGCGATGCCAAATCCAGGGACAGGGCTTATGACAGAGAAGGAAGCAAGGACAGAGATAGGGATAGGGATAGGGATAGGGCTTACGAAAGAGAGAGTAGGTCGAAGGTGAAGCGCGATGAGGAGAGGGAAGATTCGGTGGAGCCGCGACACTCCTCGCACTCTCACAAGCGCAAAGAGAGGGAGCACAGCGAGGACGGGGACTTCGAGGATAAGAAAATTAGGGTTTCAGTCGAGAGGAGGGAACGGAGGAAGTTTGGGGATAAAGTAAAAAAGGTAGAACAAGAACAAGAAGAAAACGGTGATTATAAAATCGGAGGCATACATGAGGCCAATGTGAAGGACGAAGTCACCAATGGCTCCCATGCCTCTGCTTCTTTCGAAGACACTGCATCTGTCCAAAACGGTGCATTGGGATCACCTGCTGTGGCACCCTTGGGTGTGCCTGAGACATCTTTGGCCCCTTCTTCTTCCTTTCCTATCAAGGTATCTTCGAATTTTACCACAAATGAAAATAAGGGTGTTAGTATTACCAGGTCTCATGAGGTTACTGGAAAATCTAGTACAGATGGGCCGTCTTCAACTGCTGGAAAACCTGGAAGCCTCTCTATTGATGCACTAGCCAAGGCTAAGAAAGCTTTACAAATGCAGAAAGAGTTGGCAGAAAAGCTGAAGAAAATTCCTCAGTTGAACAAGAGTTCTACCCAAAATTTGCAAGGAAGCTCAAGGTTGGGTTCGAATAGTGAATCTACAAAGAGTGAATCTACAGTGCCCTCTTCCATTGCTGGAGTGGCATCAAAAACTTCCACCTCAGCTTCTTTGGGACCTGTTGCAAATATGTCAATTTTCCCGTCAACTGCTTCTGCTACTTTTGCAAATCCTCTAGCTGCTGGCTCTAGCGCTACTGGTATTGCAAATCTACCCAACTTTGAAGCTGTCAGGCGTGCTCAAGAGCTTGCTGCCAGGCTGGGATTTCATCCAGATCCACAATTTGCTCCTCTAATAAATACGTTTCCTGGTCATATGGTAACAGATATTGCTATTCCACAGAAACCCACCAAAGCGCCCGTTCTTCGTCTTGATGCTCAGGGACGTGAAATAGATGAACATGGAAATGTTGTTAATGTGACTAAGCCCAGCAACCTTAGCACATTAAAGGTCAACATTAACAAACAGAAGAAAGACGCATTTGAAATACTAAAACCTGTATTGGATGTTGATCCTGAATCTGATCCTCATTATGATGCAAGGATGGGTATTAATAAAACAAAGCTCCTGCGACCCAAGAGGATGAATTTCCAGTTTGTGGAGGAAGGAAAATGGTCAAAAGATGCTGAATCAATAAAATTGAAGAGTAAATTTGGAGAAGCTCAAGCAAAAGAGCACAGGGCCAAACAAGCACAGTTGGCAAAGGCAAAGGCTGCTCCTGATATAAATCCAAACTTAATAGAAATAACAGAGAGAGTTGTAATTAAAGAAAAAACCAAGGACCAAATTCCAGAAATTGAATGGTGGGACATGCCACTTCTGCATGCTGGAAATTATGGTGATATTGATAATGGGATCATAGGTGAAGATAAACTGAAAGTGGAAAAGATCACATTTTATGTGGAGCATCCTCGACCAATTGAACCGCCTGCTGAGCCTGCACCTCCACCACCTCAACCACTCAAACTTACTAAGCAAGAACAGAAGAAACTCCGGACACAGAGACGTATAGCTAAGGAGAAAGATCGACAAGAGATGATAAGACAGGGCGTCATAGAACCACCAAAACCAAAGGTCAAGATTAGCAATTTAATGAAAGTACTTGGCACTGAAGCAACTCAAGATCCTACTCGGCTTGAAAAGGAAGTTCGAAGTGCAGCTGCTGAGCGTGAACAGGCTCACATAGATAGAAACATTGCACGCAAGCTTACTCCTGCTGAGCTTCGTGAGAAAAAGGAAAGAAAGCTGTTTGATGACCCAAACACACTGGAGACACTTGTCTCCCTTTACAAGATAAATGATCTCTCACATCCAAAGTCCCGCTTTAGAGTTGATGTCAATGCTCAAGAGAACCGCTTGACTGGATGTGCTGTTGTCTGCGATGGTATTAGTGTTGTTGTGGTTGAAGGTGGAAGCAAGTCGATTAAGAGGTATGGGAAACTTATGCTAAGGCGTATTAATTGGAGTGATGTTTCAAAAGAGAAAGAAGAAAATGAAGATTCAGATGATGATAAGCCTGCCAATAAGTGCGTTCTGGTGTGGCAAGGAAGTGTTGCGAAACCCAGCTTCAATAGGTTCAGTGTTCATGATTGCATCACTGAAGCAGCCGGCCGTAAAGTTTTTGTGGATGCTGGTGTTCCTCATTATTGGGACCTAGCTGCGAATTACGTAGAAGATGAAGCTGTTTGACTCGTCTTAGTGTTTTGGTTCTTCGTTATTGGCCTTTCTTGACTAAAGTCTTCGGATGAGTGATCATTTCTATAATTTACATTAAGCAGTGGTTAAAACAAAATAATAAACACGATAACTGCTGGTGCTCCTATGTTTTTCCTTTGGCCAATGAAGTCTATAATCATTAGGAATTTGCTATATAGATCAATTAGTGGTGTAAGCCTTTTGATGTTGGTTTGGATAGGATCCATTCATATATGCTAATGATTGCCTGCCAGCATTTGCATAAATTCTAAGCCTTCGGGAG

>C102483_65

GCCGAATCATTTCTCTTCAGCATCGCGGAGTCACTCGTAGCAAAACTTGCTTCTCATGCTTTTGAAGAAGCTTCTCGAGTGGTGGGATTATACGACAATCTTCGAGACTTTAACAAAACTCTCTCTTTAGTCAAGGCTGTGCTGTTAGATGCTGAACAAAAGCAGGAGCATAACCATGAGCTGCGGGAATGGCTCACTCAGCTCAAAACTGTCTTCTCCGATGCCGAAGATGTATTGGATGAATTTGAGTGCCAAACACTACGAAAGAAAGTGGTCAAAGCTCATGGTAACGCCAGAGACAAGGTAAGTCACTTTTTCTCATGCTCTAATCCACTTGTTTTTCGTTACAAGATGGCTCAGCAAATTAAAGATCTCACTCAGAGACTAGACAAGGTTGCAGCTGATAGACATAAGTTTAGTCTTCAAACAATTGATGTTGATACACGTGTTGTGCATACTAGAGAAATGACACACTCCCGTGTGAGTGATTCGGATGTGATAGGGAGAAAGTACGATAAAGAAAAGATAATAGAACTTTTGATGCAGCAGAATCCTAATGATGAAGATACAAGTCTCTCTGTTATCCCCATTGTCGGGATTGGAGGTTTGGGAAAGACTACACTTGCAAAGTTTGTATTCAATGACAAGAGAATCCAGGAGTGTTTCCCATTAAAGTTGTGGGTGTGTGTTTCTGATAACTTTGACCTTAAGCAATTGATTATCAAAATCATCAATTCTGTGAATGATATAACGTTTGATGATGCTCCTCCTCAGAGACAGAATTTGAACAAGTTGGATCTAGAACAACTGCAACATCAACTTACAAACAAACTTTCTGGTAAAAAAATCTTACTCGTCTTGGACGACGTATGGAACGAAGACCGTGTCAAATGGATTGAATTGCGAAATTTGATTCAAGTAAGTGCAGCAGGAAGTAAAATTCTAGTGACCACACGTAGTCATTCCATTGCTTCCATGATGGGTACTGTTTCATCTCACATTTTAGAAGGCCTTTCTCAGAATGATTCATTGTCTCTTCTCGTCAAATGGGCATTTAAAGAAGGAGAGGAGAATAAGTATCCTAGCCTAGTAAATATTGGGATGGAAATTGTTAAAAAATGTGGAGGGGTTCCTTTGGCTGTGAGAACATTAGGGAGTTTATTGTTCTCCAAGTTTGAGACCACTGAATGGGAATATGTAAAAGATAATGAAATTTGGAATTTGCCACAAAGAAAGGATGACATTTTACCTGCGCTTAAATTAAGTTATGATTTCATGCCATCCTATTTAAGGTCATGTTTTGCATTATTTTCTCTCTATCCAAAGGATTATGAATTCAATAGTTTTGAGATAACTTACCTGTGGAGGGCACTTGGACTCATTGTACTACCAAATACAAACATGATACTAGAAGATGTGGCCAATCAATATTTGGATGAACTAATGTCAAGATCATTTCTACAAGATGTTAGAAGGTTTGGCACCTTTTATAATTTTCAAATACATGATTTGGTGCATGATCTCGCTCTATTTGTTGCAAAAGATGAGTGTCTACTCATTAATTCTTGCAATCAAAATATTTCTGAAAATGTTCGGCATCTGTCTTTTCTTGAAAGCAGTGTGCTTAACAATTTTTTGATGAAAAAATCAGCTGTGAGAACCTTATTATTTCCAAATGGGGCAACAGAAGTCAATGGTGAATCTTTACTTTGTTTGTCAAAGTTTAAATACTTGCGTGTTTTAGATTTAAAATCTTCCACATGCGAGACTTTGCCTCGTAGCATTGCTAAGTTGAAACATTTGACATATTTGGACATTAGTGGCAACCACAAAATTAAGAGACTCCCTCGTTCTGTTTGCAAGCTCCAAAATTTGCTATTGTTCAGACTTGATGGATGCATACATCTCGAAGCATTGCCAGAAGGATTAAAAAAGTTAATAAGTCTCCGACATTTCGAGTTCAGCACAATTCAATCTCATTTACCTGATAACGAAATTGCTAGCCTGGGGCTAAAAAATATGTCTGTTGTATCATGTCATAATTTGGAGTCTATCATCTTCACAGGGGTGAAATTTCCTGTTCTTAAAGCACTGAATATTGAAAACTGTCAGAGTCTAACATCTTTGCCACTCAATGGTAATACTTTTCCCGAATTAGAAACTTTCTTAACTAGTTTCTGTGATAATTTAGACTTGGAAATATCGATGGACCATGAAGAAGAAATCCCCAAGTTGAAGTTGAAAACTCTTATATTAAATGGTTCAGAACTGATGGTCTTGCCTAAATGGCTCCAAGAAGTTGGCAAATCCTTACTATTCTTGGAGATTTCAAACTGGAACAATCTTGAAACATTTCCAGATTGGCTGACATCTCTAACTCATCTGAAAACATTTCATATAATAGATTGTCCAAAACTGGTGTCTCTGCCAGATAACTTTCATCACCTTGCTGCCCTTGAATATTTGAAGATAGAAGGTTGTCCAGAATTGTGTATAAAATGTCAGCCCCATGTTGGAGAATTTTGGCCCAAAATATCACATATTAAAGAGGTTGTCATTGATGAAGGAGAGGAAGAAACAGACGAAGATGAAGACTAAGAAAAGACACAGCTTCAACCGTTATAGTTTTCATAGTTTGTTTTGAACTCTCCTCACAAGTACACTGGTTCACATTAAATTGTGCTTGGATCATGTTTGATGTAGGCCTATTTGATTATATATTTTTACTTTTCAGTTTTTTGTGTGTGAAAATAAATTTCCTTATTGAGGAAGAACTTTCAATTTAATTTGTGTGACCATAAATCTGAGTCTTTATCTCTGTATAATACTGGAA

>C102485_65

TTTTTTAGCAGTTTTGAACTTTTTATTCAGAGAAAACTTTTTATTCTGAGAAACAAACATGTTACATCCTTGAACAGAAACCATTTTTTATTCCTCTCCCCCACTCTTGAGAGTACTCTAAACTACAAGCATTACAAGTTACACCAAAAGCACTTCCCACCCCCATGCCTACTACAACATGGCTAGTCTAGAACTCAGTAATTATGATCAACCCATTTGGTATTCTCACCACCCCCATGACCCTAACAATTCCCTCAATGAGTCAATGCTAATTACAGGAACCCCCTTTTCCTGACAAACCAGGTCTTCAACAAAGGACTCAAACTTGGATCTCTATTGACCATCTGTACATCTCCTCTTGAGGAACTGGGATGGAGAAGGTTGCTAAACCAGTCTCCGAGTCATAGTTGAAGTCAGTTTCAATGCCACCTACCACACACTTCAGTGGGCGTTGAGAGGAGTAAACTCCAAATCTGCCTCTTCCCCTGACTCTAAGAGCTATAGTTGCTGTTGCTGCTCTATTAGGACTGAGAGAAGAGGTTAATTCTGATAACACCTCACCATCAAACAGTTCTGGTTTGTTGTTAGAGGCTCTGTGAATTTCAACCACCTCCACAGCTCCTCCAGTGTTGAACATATCCAGTAGCCCTATTGCTGCAAATGAAATACTTGGTGCTATTTCATGGATTGGACAGAAATGGAAAAGTTCAAACTCAAGAACTTTTAGTGTTACTGGAACTGAAACCCCCTTTGGTAGCCGAATCACCTCACCTGATTTGTAAGCATAAACAATTGTCTCTCCAAGCCATTCAGTACCTGCTACTTGGGTGATGAGGTCAACATCAGAGGCACAAACAGAGCCACTTAGTGTACCTGGAGATGTGTCATGGATGCGGGTTTTCTTCTCTATCTTGCACCACCCAGCACCTTGGCAGTTAAATACACCAACAACTCCAGAGCATTTGTTCATGTTCCAGATTTTGAGCAAGCTAGTCCCATCTCTGGCTGGATCAACAAAGAGAGAATCACGAGTTGGCCTGCCTGGTAACTGTGCTCGGAGAACTGAACCATCAGGAAGAATCAGCTTCTTAAGAAGATCAAAATTGTGGTTGCCTGGCTTGTCACTCACATAAATTGGACATCCACCAATTGCACGAGCTGCAGCATGATATTCTGCTGCAGGGTGTAAACTATGAAACATGTCCCAATCAGGTTGCATGAACTCTCCAAGGAAAAGTGAGTTGTATGCAACAGATGAAATATGGATGGTGTGGGAAGCGGGATCACGAGGGTAGAAATCATCAGAGGCTCTCACAACAGCAGTCTGCTTGGCACTGTAAAGCCCGTCAGTGTTGTGACACATGCATGCAATGCATCCGTTGTCAGTAAAGTTACGAGCAATGGAGGCCTCAAGCGCATGATGATAGCTGCGTGTAAGAGAGACTCTTCCACCATGTCCGGCACCAAGGGTCTCAATGATGTTCTGCACATCAACCTTCACTCCATCCACTCCACATGAAGCCAAGTAAGCATGGAGCTCATTGTAGAAATTGTAAACCTTCTTTGGGTGCACTAGGCCTAGGCCATGCACAGCCAAGCTGTCCATGACAATGTCTGGTTGGTTTCCTAGCACACCTGGTGACTGCACTGGATATGCCAAGGCAGTGTCATAATGCTCCATGCCAGTTGCTGCTGGCTTCACTCCACCCCAATAACCAGCTAGTGCATGCCATACATATACATTTTTCACGTTGTGATGTTGCTTTACTCCATCTACTAGATGCTTCAGACCTGATATCTGCTCATTGCTCTGTGTTTTCTTCTGAAATTTAGTATTCTCTTTGATACCAGTCAACCTGGTAGCAAACTGTGCTCCTTCTTGTACAACACAGTCAACATCCTTTTGTTTACTTTCAATCTGTTGCCAGCCATCATCTATGATGAGGAACCGTGGTGGTGTACCTCCCTCAGATAGACTTTTCAGGCCGTCCTCAACACCCTCAGCTGTGACATCAGTATAGAAAGCATCCCATGTACACCAGCCAAACCAGTCAAGAAAAGATGGCAACCTTTTCTTCTCACGATGAAGAAAAGTCTGCATGTGTTTTTCCACAGCCTTGACTGCTTGATTGATGACTTCAAAGGGGTTGGTCCCAGCATGCATGTAGACCAGGTGAAGGCCTTGATCAGTCTCAACCGCGTTATCCCCACTCTCGAAGCAAATCTCTATCTCGTTCTTGTCATTGCCTTGAAGAACAGCTCGGAATGGACCCTCTAGTAGAGGAAGGAAGACTGTGTAGATGATTGGAGAATTCTCTTCATCTGCTTCACTCTCTTTGCTCTCAATAAGCATGAACTGTGTCTCCAGAGGAACATCCCTCCCACATGTCCCCATTCTCTGAGTCATCCACCATAACTTGAACCGGAAACAACACATGAACCGGAGGTCCTCTAAGACACCCATTGGAAACACATGGAGACTTTTGCTTTGGGAAGCATTAGCACCAACAAAAGCACCAGTCACAAGGCCACTTCCAGATCCAGGAGTCAGCACAACGTTGTCCGGCACTCCAGTTAGTATGGTCTTGCCATGAACAACCAATTTCTTATCGTTAACGGAGATCTTAGGTGTGACAGTCATTTTCGAACAATTCACCACAAGTTAACGTGACGAGAAGGTGAAAATGAAGGAACAATTAACTTAGGAATTATGAGAAGAGGAAAAAGTGTAAAATCCTATTCCAGGAGGAGAAGGTCCTCCAATGGAAGAAGGTGGAGGGAGGGAGAAACACACTCAGCTCAGGGCTGAAGGTTGATGAGTTCTCAGAGTTTG

>C102487_65

ATTGTTTGGAATTTGGGATGAATGCACTCCTTGCAGCGGTTCCTAGAACAGAGTCTAGACTGATAAAGACAACAGTAATAAGCCTCGTTAAGATGAACAAATGCGATTGGCTACAAAATTTTCGATCATTAATTGCTGCTTTTTTGGGTTTTTGGGAACAAGAAGCGACAAAACCCTCCAGAGGATAGCATTCACGTTACTCTCCAAATCTTCTCAAACCCTAAATATATAACGTCGATTCATTTCGCCAAATCCACCACGCCGGTTTCAATCTCAAAACCCGCTTCTCTCTCTCGTTTTCTGTCACTTCAAGCGTTTCGAAGCTGAATCGAGGCTGTGGCAATAGATTGGAGTATAGATTCAATAGGTACAATACAATATGGAAATTTAAAACTCTTTGTAAAATATAAAGATACATATATTTAAATATAGAGAAAGGTGGTTTTGCCGGGTGATCTTTGATTATTGCTACCAGTTTGGCAGTGTATTTGGAGCCTCTGCATATTCTAGTTTAGGAGGTAGGTTTCCATTTTGAGATCCATGATTGATGGATGTGGTTAATGATTTGCAAATGGGCCCTCCAGCAACTGCCAATTTGGCAGGTTCAGCTAGTCCAGAGGATGTAAATTCGTCACAGAAGCAGAAAATGGTGATTACGAAGAAGAAACAAGGTAATGCTGTCGAGTGTGAAGGTCCTGATTTTTCACTGGATAATTCTACACCGAAGATGGATGCTACTATAAATCATCCATTACATGAGTCCCCAGCTTTGCCTAATTTCTTAAGTTGCCAAATAATTGTAGAATTTAATTCAACAAAGCAGAATGAAAAAAGCAAACGGAAGAAGACTACCCATAGTCCTATTGAAGGCAATGATAACAGTTGCGGCACTTCAGAGGATACCTTTTCACCCAAAGCTTCCACTCCTGCATTGGATATGCCGTCAGCACATCCAATTCAATGTTTTTCCAATGATCCAATAACAATTCAATCTTGTGAGGAAGTGGATGTTTGTCAGGGAGATTATGTAGAAAAGGAGCAGAATGTAATCAATACAACTGCAGAAAAGCAGCATGACGTTCCAGAAGTAAGCTGCAATCACATTTCTGTAGCTGCAAGTCCTGAGCCTTTTGTGAAAGAGTATGTGCCACAAGTTGTAAAATTGAACATAGAGCATGACATAAATGATGATATTTCCAACCATGAGGATAAGAATAGTTTTATAAATAATACAAGTCTGATGAAGAAAGATATTGATGACCCAGCAGATTATGACTTGTCTAATAAAAATCTGTGTTATAAAGCGTGTGCAATGGTTAATGCTGATTGTAGGAGAAGCGGTGGTTACCCTAGTCCTGAAATTCCTAACAAGGAACCAACTATTCAGAATCATGACAAGGATGATTCACAACAGTTTTCTGATGGACCATTGAGAGTTGATTTGATGGATGGGGAAACAAAACTTTCAATTGATCAAGTTACCTCACATAGTGGAACTGCGGCTGAGGAAATAAAGTTGAGCACTGACAACATTAATGAGCAAAAATTTATGCTTACATCTTCTGTTGTAGACATTCCTGTGGAGCATCTCGAAGCAGATGGTACACAACTGGATCATACTGAGATTACAAGAAGCAATCCGTGTTGTGCAGCTGAGGAAATAGAGTTGAACACTGACAACATTAATGAGCAAAGATCTGTTACTACGTCATCTGCAGTAGACGTTCATGTGGAGCAACTTGAAACAAATGGTATAGATCATATGGAGATTACAAGAAATGATCTGTGTTGTGAAGGTGATGTTTCTGATTTAAGCTTCGACAAAGTAAGAGATGGTCATTTAAAGATTTCACAATTCTCACTAGACAGAAGTGTTGCTGCCAACCCAAAGAAGAAGCTTCTCATCCTTGATGTGAATGGATTGCTTGCTGATTTCGTCACTGTCAATGTCAATCATACAAGATATAGAAGATATCCAGAGCCAGATTTTGTGCTGAAAGGGAGAAAAGTCTACAAGAGGCCCTTTTGTGACGATTTTCTACAGTTTTGCTTTGACAGATTTCATGTAGGAGTATGGTCATCTAGAGCCAAGAGCAATGTTGATGAAGCAATCAAATTTCTTATGGGGAAATCTGCATCCAAACTGCTCTTTTGTTGGAATCAGTCCCACTGTACTACAACTGAATTCACTACTGTTGAGAATATAAACAAACCCCTTGTATTGAAGGAACTTAGAAAGTTGTGGGAAAAGGAAGAACCCGATCTACCATGGGAGAAGGGCGAGTTTAATGAGTCGAACACATTATTATTGGATGACTCCCCTTACAAGGCACTAATGAATCCTATGCATTCAGCAATATATCCTTATTCTTACCGGTACTACTACACCAGAGACTCGGAATTAGGACCAGGAGGTGATCTTCGGGTTTATCTAGAAGGGCTGGCTATGGCTGAGAATGTGCAAAAGTATGTGTTGGAAAATCCATTTGGCCAACGTCCCATTAGAGAAGCAAATCCATCCTGGGGTTACTACTGTAGAGTTATAGAATCAGTTAAAAAGAAGCGAAAATGCTAGGCCCTCATCAACCGGGTAGGGAGATAACTGCTTGCAGAACAATGCATGACGAAAGCTGTCGAGATGGGTTCTCAATCACATTGATGAGATACAAGCAATGCATCTATTTAATCACTCTGATGAATGAATTGATGTTGAGTTTTGACTTGAAATATATCAGTTTTGTATATGTGCTACTTAAGCTAAACTGACTAGCTGGGCATATATTTCTATTGTCTTCATAATTTTTTGACCTTGGAACGTGTTTGGTTTGCCCTTA

>C102489_65

CTTGTTTAAACCTTGTTTGACAAGAAATAATTTTAAGAAAAGATTATAACAACGTGTGCTTCCGAAGACCTCAATCGCAAAAGTGAGATTGTTGTTTAAAGAGAAGGCGACGTCGTATCCAGTTGTAAGGGAATGTTGTATCATTTTGTTGGATTGGAGAAAGATTAGACCCATTACTCCTCCTCCGTTTACAATATTCTTAGCTTCAGATCTATTCGATTCATATTTTCTTTCTTTCTTAAAATTTGTCTGCTTCGATCAACATATCATTTCTCTCCAGAAGCATGAATCTTTCAAAGATTGCGCGCTCTCTGTCGCGCTCGTCTCGTGCTAGAAATTTATTACACGGTAATGGGAGATTGGGAACCCTTACAGGAATACCACGGACGAATACATGTTCTGAAGAAGCGGAATCCATATTAGGGTTTGTTAGGAGCTACGTTTCTTCTGCCAGAGTTCGCAGTAACGGCATCATTTCCAATTTGCCTGATTTTAAATCTGTTGCTGCAAACCCTAGGATTCGCCGATTGTTCTCCAGTGAAGCCCCGAAGAAAAAGAATTATGAAAAGTTTTATCCCAAAGAAAAGAAGGAAGCTCCGAAGGGAAATGACAAAAAACACGAGTCTAAAGATAACTCGAACACAGATGATCGCGGAAATTTTCAGGAAGCTTTCATGAAGCAAGTTCAAATTTTAATAACCCCATTATTGATTATGGGGCTATTTCTTACATCCTTTTCTTTTGGTCCTCGTGAACAGAAGGAGATTAGCTTTCAGGAGTTTAAAAATAAGCTTTTGGAACCAGGATTAGTAGACCATATTGTTGTCTCAAACAAATCAATTGCCAAAGTATATGTAAGGAACACTCCCCGCAACCAAACAGATAGTGAAGTAGTCCAAGGGACCCTGCCTGCAATGGGATCTGGAGGCCAGTATAAATATTATTTTAATATTGGAAGTGTTGAGTCTTTTGAGGAGAAGCTAGAGGAAGCACAAGAAGCTCTTGGCATTGACTCTCATGATTTTGTGCCTGTAACATATTCTTCTGAAATGGTTTGGTACCAGGAACTGATGAGATTTGCTCCTACACTGTTGCTCTTGGGATCTCTATTGTATATGGGAAGGAGAATGCAAGGAGGAATTGGTGGTGGTGGTGGCGGTAAGGGTGCTCGTGGAATATTCAACATAGGAAAAGCCCATGTTACTAAAGTAGACAAAAATACTAAAAATAAGATTTTTTTTAAAGATGTTGCTGGCTGTGATGAGGCTAAGCTAGAAATTATGGAGTTTGTCCACTTCCTCAAGAATCCTAAGAAATATGAAGAACTTGGTGCCAAAATTCCAAAAGGTGCTCTCTTGGTCGGTCCTCCAGGTACAGGAAAAACACTTTTAGCAAAAGCAACTGCAGGGGAGTCTGGTGTGCCATTTCTTTCGATATCTGGTTCTGATTTCATGGAAATGTTTGTTGGTGTTGGACCATCAAGGGTGAGAAACTTGTTTCTGGAAGCAAGGCAGTGTGCTCCTAGTATAATATTTATTGATGAGATTGATGCAATTGGTCGAGCAAGGGGGCGTGGTGGTTTTTCTGGTGCAAATGATGAGCGTGAAAGTACATTGAATCAATTGTTGGTGGAGATGGATGGTTTTGGAACCACTTCTGGAGTTGTTGTGTTGGCAGGAACAAATAGACCTGATATCTTAGACAAAGCTCTTCTTAGACCTGGGCGATTTGACCGCCAAATAGCAATTGACAAACCTGATATTAAAGGTCGGGACCAGATATTTCAGATCTACTTGAAGAAGATCAAACTTGACCATGAACCTTCGTATTATTCTCAAAGGCTTGCAGCCCTTACTCCTGGATTTGCTGGAGCAGACATAGCTAATGTCTGCAATGAGGCGGCTCTTATTGCTGCAAGAAATGAAGTGACACAAGTCACAATGGACCACTTTGAGTCAGCTATCGATAGGATCATTGGTGGTTTGGAAAAGAAGAACAAGGTTATAAGCAAGGTGGAAAGGCGAACTGTTGCTTACCATGAATCTGGCCATGCTGTTGCAGGTTGGTTCTTGGAACACGCTGAGCCCTTGTTAAAAGTGACTATTGTTCCACGTGGCTCAGCAGCTCTTGGATTTGCACAATATGTTCCAAACGAAAACCTTCTCATGACAAAGGAGCAGCTTTTTGATATGACTTGCATGACACTCGGTGGTCGGGCTGCCGAGCAGGTTCTTATTGGAAGAATATCAACAGGAGCACAAAATGACTTGGAAAAAGTTACCAAGATGACATATGCCCAGGTAGCAGTTTACGGTTTCAGCGACAAAGTGGGTCTCCTCTCGTTCCCTGCAAGGGAAGACTCATTTGAGATGTCCAAACCATACAGTAGTCAAACTGCCGCAATCATTGATAACGAAGTGCGCGATTGGGTGAACAAGGCATACGAACGAACGGTACAGCTAATTGAGGAACACAAGAAACAAGTTTCTGAAATTGCTGAGTTGTTGCTTGAAAAAGAAGTACTTCATCAGGAGGACTTGCTTCGGATTTTGGGTGAGCGACCATTCAAGGCTACTGAACCCACTAATTATGATAGATTTAAGCAAGGCTTTCAAGAAGTGGAGGAAAAGGTAGGAGAAAGCACCATCGTAGATGTGCCCGAGGAAGGTGGCGGATCTTCACCTTTAGAGCCCCAAGTTGTTCCCACATGATGATCAAGTATATGTTTGTTATTGTACATGACTTTGTTGTTTCTTCGTTGACAGGGACAAACAAAACTCATGGTAACTACTAAATAAAAATCTAATTTTTTTTGTTATACGGATATATGCATG

>C102491_65

CTCGAGAGAGAAAAAAAGAAGAGGCATGCTTGTTTATCCAGATGGTACCACCCCAAAGAAAAAGTGTTGCCAAATATAAAGTACCTAAATGTAGTTATATTATATTAGTCCACTTACACACGATCACTACTCTACAATTTTAAATTTGCATAAAATTAAGCAATCCAAACCATCAAGCACCCTAAACCCCACACACTTTCCCCCTCCCAAACCAAAAAACTAAGAAGGTTAAGCTGTCAAATCGCAAGAGATTTGCTGAGTTTCCTCCCTATTCTGTGAATCTTGGCTCAACAAAGTCTCCTCACCCTCCGATGAACAAGCTGAATGCTTGCTTTTCAACTTCTTCACATCTTCCCTTGAACAAATGAAAATCCTCTTCGCCATACTACAGAACTCAGGCCATGGATCATCACCCGCGAGCATCATGTCATTTTCATCATCAGTGAAAGTAATAGCCCATTTCGTTTGCATTCGAAGCTCTCCCTTGATGTCAAAAATTTTCTCAAGCTCATCTATGAGAGCCTCATAATCTTTTAAAACGGTAAGGTCAACTGCACGACCGACAGCAACACCTTGCATTTGCACCTTGGTCCGAGTCCTGGTGGATGGTACTGTGGCCTGCTTGGTATGTCTCTCATTGGGTGATCCATCAGAAATAATTTGCTTCTGCACCTTATTGGACAGTGAATAATTGGGATTCTGACCAGCCTGAGTTTCACATGCAGCAACAGGAATAGAATCTTTGGGACCACTAGAAATTATTGCAGCTGGACATCCCAGTTCTTTATCCGGGAGGGCAACATTAGTAAAATTGTTAGTCAAATTAACTCCAAATAACCAACAATCCATGGGATTCTCACTCCTTTTACCACATTCCACGGGATCATGTGCCAAATCATCATTCGGCCTAGATGAAACAGGGGCATACCCAGAGACAGTTGATTGTGCTGTGACAGCTTTGCTGTTTTTGGGGTCCGGAAAAAGGTTGGAAGAGACACTCAAATGTGGGGAAGAAGGCCACATTCCTTCCACACGGACTCTGGTGGAGCTGCTCTTGTTACCATTCATAGGATTGCCATTAATTTCTTTCGTCCTTGTAGACCATATAACTGGGCTTTCATTGCTTTGGACCTCTGAAGTGCCACCCAATGCTGTGGGGTCATGGGATAAGGATGGTCCATGGTACCACAAAGCTGGAGCAGGGGAATTAGGGCCAATTTCAGAAGATGAAATCTCAGGAGGCCTGGACCTTTTGCTCTTCACCACCTGTTGAGTGACATTCAAAGCAGTGGAAGCAACGAAAGGCTCTATTTCCCACGAAGAAACTCTCTCTGGCCTTGGGATTATTGCTGGTTCATCCCATTGAACCTTCAATGAACGCCACTGAGAGTTCCGCCATCCTGGAGATACATCCCCAACCCCAACAATAGTACCAGAAAACCTTCTCTCAGGTGAGTCCTCCACCTCAAATCTCATCTTGAAACGCATGCCAACTGAAAACTTGCAGTTGACGGCCTCCAGATATTTGTTTAAACCAATAATAAACTGGCTAGTCCTAGGTTTGTAATACACCAGAAACATGGTATTGGTCAAGAAAGCATGAGAGGCAGTGGCAAGGACTCCAAGATGCATACTCTGGCTAGATATCACAGAAGAAGGCATGGGACTTTGCTGTCGGGCTACACGCCTAACACCAACTCTCAGTTCGCCATTCTCTCCCCTTAGAAACACAAAAGCATCTCCAGCAACAAGTCTCTTCGATGTGACAAATGTACTCCAACCAGTAGTGAGCAAGTGCCTCCTTGGTTGACCTCTGAATATATGCTTAAACTTCCACTCAAACCCATGAAGATCCTTTGCTGCCAATTCCTGAGTGGGGGTAGTTTGGGTCATGTCCAATTGAGGCAGGCACTCGGTAGCATGCCTCCGCAGAACCGAAAATCCTCCATGAGTGCTAGTATCAGAAGCAGTTAATATCTTACTAAAAGAGTGAAAACTCTGTTTCTGTGTCTCGGGTGGAATTGGGTCTGGACTCGTTGGCTCATCTTGATTTGACTCCGGGAGCAAAGTGATACGAGCATAAACTTCATCTGTTTCTTGTTCCGCCAGCAACTGAATGTGTACAACACGACAGAGAATCTTGGGTGGGAGATTGAAATGAGGAATTTGCTGGCTCAGTTCCTGGTTCAGTCCCTGATTAGTCGAAGCTTGCAATTGTTCCATGTGACCCTGCGGGAAGTAGAAAACTCTCTCCCCTTCGCAAGGAACATCCACCAAAGGCCCCGCACATAGCTTCCATAGCTGCGTGTACAGATCATCATCACCCACACCTGTTTCTGTCTGAGAACTGGGCACACAACTGAGAGGAGCCTCCAGATGTGCCATTGAAGAACGACGTCGTACAGAGAGAAGAAGAGAAAGAGAAGCAAAAGCTGAAAGCAGGTGCAGAGTGAGAAATAACAGATGAGATTACCCCATGAATACAAAAGAGAGAGAGAAATAGACTGCGAGACAAGACAGTTATACGCGAGTGAGTGAGTAATGATGAAGAGTCAACAAGTTTCCTCCTTTTCGCTTCTCACTTGTGTAACGATCCTGCCACAGAATTTATCTCTTGTCGCTTTTAACTCCCTCGCACTGCTTCAGTTACAGAACTCAAGGCCAGTTTTTCTTTCTCTTTCTTTCGCCCCTCCTTTCTCTACAAAACCCTTTGGAATAACGAAAGCTTTTTTTATAGGTGCCACCGATGATGATTATTTTCCTCCGGAAACAGCTCAATCGACGTTACCAAATTCAAAAGACGTGGTGTGGTACTCGTTTTTTTTTTTTTTTTTT

>C102493_65

TTTTTTTTTGACAGTCAAAACACTCCTTATTATATGCAAACTGAGAGCATCCAAATACAAAGTGCAGACATACATGCAGTAATTACTCACAGACAAGCACATAGCCATGTTATTCGGAAGTACTATGATACATGTGGATAAAGAAAGCACACTCCTTTATATAGAGATACTGTTGGGAATTCCCTTGAAAGTCAACCCTTCCTCACTTGAACGATAGAGTAATGTATATGGAAGTTGAACGGGTCCATATCGGTTTCTCAGACTTGGATCACTGTTCCTTGCAGTGATTTTCCCCTCAATTTCAGTGAGTTTGTTTCCAAACCTTTTGAAGGCTTCTAATGCCTTTGAATCAGTTGTCCAGTTTGGATTATCCCTCTGTCCAAGGTAGACCTCATCAGAAGCATGCCTTGACAATATCTCTATCACTGAGAGGTCAATAAGGGTCTCGAATTTAGGTGTGATTGTTTTCAGATAAGCTTTTTGAAAATCGGTCACCATCTCATCATATTCTTTGGTTCCTGGTTCTGGGATAAATCTTCTGCTGAGAGTTGGACGGTTGAGGATATAGCCTCCATAAGGATATTGTCCAAAATTAACAGCTGCATGGAGAGCAGAAGCAGTCCATATGATAATGGAGGATGATTTAATGAGATCTTCAATAGTCTGCATTTTAGGCCACCAAGGCTTATCTTTTAAGTCAGCATGACCCTTCTCTACAGCTTCCTTCCACCATGCTTGGAGTTCAGTATCTTGTTGAATTGCAGCATCTGTTGGGTAATAAAAGGAAACATACTCTTGCACCCATGACTTAATAGCATCCCATATTTCTAGTCCATCAACAGCATAAGGATAGTCCTCAATCACAAGACGAAGTCCATGGGGAGCAGATGGATCCTCAACTGCCAATCCTCTCTTGATGAGATCTGCTGGCAATGCTTGGTCAGTAAAAACCCAGTTCTTGTAAACGATTGAAGACATCTCCATAGAGTACTTTCCGGGCAAAAATGATTGCTCAATAATGCCCCCTGCATTAATCAAAGATTGCCTAGCAAGTGCATTGATATTTATGGTATCACGGTAGTGAGGTTGGAGAAGTTTATAAATGGGGTGAAGCACACTGAGATGTCTGTTTGTTGCTATGGCAAATGGCTCCATCACTGCATGAGTATTTAACCAATGACTCATGAGCTGATGATAACCAGAGTCATTTACTATTACATGAGCCTTAGCCATTAACCAAATTGTGCTATCAACACCCTCAATTGCAGGCAACACCACTTTGCTCACAGGACCTAAATTATCGCCACCTGGATGAGGCTTGCTTAATTCGATAGCAAGAGGCTTCAAAGTGCCATCATCTTTCAAGAATAGGATTGTTCTGGTGGCATAAGCTCTTGCAATAGGTAGGCTATTTATCCTCGTCAAATATGGAAAGAATGCATCTTGATAATCTAATATGAACAATCTCTTTGCACCAAGTGCCTCATCTACTGTGACCCCACCCATGTTAATCTCCAACTGTTCTTTTGTTATTGTACTGGTTTGATCACCATAGAGAGAGGGATCAAGCGAACTTTTTGGTGGGAATTCTTGAAGAAGACGAACTACATTTGGATTTACACCACCAATTACTTCTCTTGCAAATTCTGCATCTGTCATCCATTCAGACCTTGTAACTTGGATAACATGAGGCAGTGGAAATTGAAGGAAATTTTCACCATCAGTGCGGAAGATTTCCTTGAGGGCAGGTAAGGGGCTAATCTGGCTCAATATATCTGTTGGCAGCTTAATTCCACCCTCATAGAGACTACGCACATCGTCGAAGTTATTAAACTCACTAGATGTGACCTTTAATTCAAAAATTGCAGATTTTAACACAGGTAGCACATCCTGAGATAAGGATTTTATTCCATATGTAAGGAAATCTGATGACTTCAAGTGACCAAAATTTTCATCTCTTGGAACATATATCTCGCCTGGTTTCTCACTGTTGGGATCTGTCCTGGTTCTGTCTCTACCAGATCTAACCCTACGAGGGTAAGGATAGATGGTAGATCCTCCAAGGATTGGACGAGGGTCACCACCATCTGGATTTCCCAGATCATTATACACGTCATAATCATAGATTCTATCATATTCCTGGCGTTTCCCTGTTCCATCTCCTCTAAGAGCCTCCAATTCTTCTTTTCTATACTTGAGTAGTGGAGCTGGTGTTGCACTTGGAAGATATGTATCATTGACAAAGAAAATGCGATTCCTTTTCTTACTGTTGTTGAAGTTATAAATCCATGAGTTACAAACAAAGTGAATGGTTCCATGGTTTGGAATGTCCTCAAGAGTGACACTGACGAGGAAAAACTCATCAGTCCTAAAGTTTTTGATGTAAAATGCTCCTGGAATTCCAAAATTCTCATCCCATTCAAAGGAAACATTGAATGCACCCTGCCTGTCTCCCAACGTTGGTAACGAGGGAATACGACTATCCAAATAGGCTTCCTTTCCAAGTTTCCCATTTCCACTACCGTCAGACTTAGTAGCACTGATCAATTGCAAGGAAACATTACGGCCTAAGAATGAAGTTGCAGCATCAATTGCTCCACCAGTTATGTCGATGATAGTGCCAAAGAAATCTGTGACAGTGTCAGTAACACTCCCACTACCAACAGAAGTTAGGGCGTTGAAGTCCAACACATTCTTGGGCATCAACACCACAGTGCCCTTTATCTTATCCTTGTTATTAAAGATAAACATCTTAATTTGCTACTATATCAACACTGTATAATATTGTTATTCTTTTGGCAAAGAAGGAGGAGAAGCTTGAAGCTTAGTTGTGTG

>C102495_65

TGGGGTTAAACTATAAAGGACAAAATAACCAATATCCAATTTGCTTATGAACCAAAAAAATGCCTCCAACAATATAATCCTGCAACTTCAACAATTTTCTTGATGTACCATATATAGTCCCTAGATTTACTCCAGGCTTGTACAAAACAAAGTTAAGAACAATCTGATACCCACATGTCATCATCCAAGCTGTCAATTTTCTCACCAACAAGAAATGCATGCACCTGATCTCCAATTTGAACCCAACTGCAACCAGGATTTTTCTTAGCTCCCCTTGATCTCATCATCTCCCTAATCTTGTTTGCCTCCTCCCATCTTTCAGCTTCTGCATACATATTTGAAAGGAGAATATAGTATCCACAGTGCTGAGGCTTTAATTTAAACAAATGTTCAGCTGCCAAGTGCCCCAATTCTATGTTCCCATGTATCCTGCATGCTCCAAGAAGTGCACCCCATATATTTGAATCTGGTACAATAGAAAGGCCTCTGATAAGGTCTGCCGCTTCTTCCATTAGACCAGATCTTCCTAGGAGATCAACCATACATGCATAGTGTGTATGTGTTGGTTCAATATTAAGATCACTCATCATCTTAAAGTATTTTCTTCCCTTTTCAATCAGCCCTCCATGGCTACACACAGACAAAACAGCAATAAATGAAACCGAATCATATTCCACACCATCTTCCTTCATTGCTTCAAAAAGGTTCATAGCAGTATCCAACTCACCCCTCATACCATACCCCAAAATCATAGTATTCCAAGAGGCCGCATCCTTGTTTTGAATACTGTCAAAGACCTTTGTAGCGAGATCTATTCGTCCACACCTAGTATACAAATCCAAGAGCGAGTTCGCCACAAAAAGATGAGTATGAAAAAGCTTTCTCACTAGCAGACCATGAATCTCTTTGCCTTGTCTTATGGAAGCTAGATTTGCACAAGCTGCTATGACACCCATGAAAGAAACAATATCAGTAGTCATGCCCAAGAGTCTCATTTCTGAAAACAAACTTAGAGACTCTGAGCAGTCATTTGTCCGAGAATAGCCTATAATTAGTATATTGTAGGAAACTTCATCCCTGTTAGAAATATTGAAGACATTTCGAGCAAGGTTTAAGCATCCACATTTTGAATACATGTCTGTCAGAGCATTAGAGACAAACAAATCCAAGGCAGATCCAACCCGTACAATCCTGGCATGAAGTTCTTTTCCAACATTCAGGAAACCTAATCTTGCACAAGCTGGAAGAACATTTGTGAAGGTTACATTGTTGAGGATTTCTCCCTTAGCTTGCATTTGCCTCACTAATTCTACAGCTTCAAACTCAAGCTTGTTCTGAGCGAAATTGGCAATCATAGCATTCCAAGATACAATGTTTTTTCCTCCCATTTTATTGAATATAGTGGATGCTATTCTTGAAGATCCAGATTTTGCATACATATCTATCAACGAGTTAGCTATAAAAACATCAGACTCAATAGCCATCCTTAAACTAAACCCATGCACTTCCTTTCCCAACTTGAAAAGTCCCAATTCTCCTAATATAGGTAGCATACTAGAAATGGTGACAGAGTTTGGTGTCATGCCCGCACCAATCATCAACCTAAATACATCGAAAGCCTCCATATATTTCCCTCTAAAAGAAAAACTAGTAATCATAGCATTCCAGGAAACCACATTCCTCTCATCAATTTCATCAAAAACTTTCTTGGAAGCCATCTCAGTCCCACACTTCCCGTACACATCAACCAACGCATTTCCAACCTTTACATTTCCCAACAAGCCAACCTTCAATGCATAACAGTGAACAATTCTTGCCATCATCTCATCCTCAGTTTCCGCACACACTGGCAAAACACTCACAATGGTAACCAAATCTGGATGAATCCCCGGCACAGCCACAACCATCTTCCTAAAAAACCTGAGCGCCTCCTTGTAAAACCCATGAAGAGAACACAAGCCAATAACAGTATTCCATGACACCTTATCCCTCTCATGCATTTCATCAAACACCTTCATTGCATCATCAAAAAGCCAACAATTCCCATAAAAGGCCAAAAGGGTATTCCCAACGAAGACATCCCCATCGAACCCAAGCTTAAAGACAATCCCATGAACCTCCCTACCCTTATGAACCTCCACAAAATCTGAACACCCCTTCAGGACAAAAGGGTAGGTGCACTCGTCAGGCTTAACACCAGCACGAACCATGGTGTTATAGGTGATAAATCCATCAAAGACACCAGCAATGGAATTGGCACGGACAATGGTATTCCACAAGAAAGCACTACGGGAGTACGCAACACTGTGTTGGAAGAGAATAAGGGAAGACGTGGGGTGCCCAAAGGAGGCATATTGGAGAATGAGAGAGGCACAGAGGGAGACACTGCGGGGAAGGAAGCCATGGAGGATGGCAAAGGCGTGAACTTGCTTGGTTTGGGAAAGCGTGTAGCAAAGGGTGCACAGCTGGAGAAGGTTTGGGTGATTGAAACTTCGAAGAGAAGGGGAAAAGAGTGAAGAAAAATGGGTGTTTTTGGTAAGTGTTGTTAATGAGGGGAGTTTGATGAGTTTGTGTTTGAATTTGAGAGGGTGTTTGTTGACAACATGGAAATGGAATATCATGGAACAATGTTATGTTTTATCTTGGCCTCAGTGATCAACCAACAAGGTAAAGAACACGGGATAATATCATGCAATAATGTCATGTCTCACCCTATGGTCTGCATCAGCTTCTGCATCATAAAATGTTCGCTCACTTGCTGTTTGAGAACATCCTTATTAGACAATCCCTTTAATTTAAATGTAAATTTGTTCAACAGCCATCAACGTAATAGACA

>C10249_65

GATTTTTGGCCGAAATGTTGCTGAGCTTCCACTGGTTGCTACTAGCAGAATGCATCAAGGAAAAGTAAGCAACTTATTCATGCATGACCGAAAATTGGAAAAAGTCGCTTGATAGGGTTATTTCCAAGTGTTGTTCTCTTGCATAGAGAAGTTGCTGTTTTCTCTAAACGTAGAAAAGCT

>C102501_65
[truncated: 34,634,548 more chars]
