# Supplementary material for: Comprehensive transcriptomic study on horse gram (Macrotyloma uniflorum): De novo assembly, functional characterization and comparative analysis in relation to drought stress
Source: BMC Genomics. 2013 Sep 23;14:647. doi: 10.1186/1471-2164-14-647 (PMC3853109; doi:10.1186/1471-2164-14-647)
Supplement: Supplementary file 19 [file 1471-2164-14-647-S6.odt]

>gi|374671751|gb|JK671299.1|JK671299 SSH-Mu_35 SSH library of moisture-stressed Horsegram cDNA Macrotyloma uniflorum cDNA, mRNA sequence

GCCCCTCCCCTCTTCCGGGGTCTACATGGCACCTGTGAGGAGGGTAAATTCGTGTGGTGGGGGACTGACT

GTGGCGTGTACTCCCCGACATTGAGAGTCTCTCTCTTCCTCTTATAGTCTTGCTGGATCTGGTGGTCCGC

GGGTCTTATGCCTTTAACTGCTTGTGGTCCGTAATTTCCACCGGCCTATTACTTCACCCAGGATCCATCT

ATGACCAAGATTGGGGCATGACTGGT



>gi|374671749|gb|JK671297.1|JK671297 SSH-Mu_31 SSH library of moisture-stressed Horsegram cDNA Macrotyloma uniflorum cDNA, mRNA sequence

ACAACAAAAGTTATGGTGATGAGATCGCAAACGTGAGGAGTCATGCGAGGGGAAAAATAGTCTTGAGAAT

GGCCGGTTTTTTACAACCCTAGGCCCCTCCCCTCTTCAAGGGGTCTGCATGGCAACTGTGAGGAGGGGAG

ATTCAGTGTGGTGGGGGACTGAGTGTGGCAGGGACTCCCCAGCAGTGAGGGTCTCTCTCTTCCTCTTGTG

CTCTTGCTGGGGCTGGTGGTCCAGGGGTCTTACTCCTTGGAGGCCATGTGGGCCATGAGGTCCACCACCC

TGTTGCTGTAGCCAAATTCGTTGTCATACCAGGAAATGAGCTTGACAAAGTGGTCGTTGAGGGCAATGCC

AGCCCCAGCGTCAAAGGTGGAGGAGTGGGTGTCGCTGTTGAAGTCAGAGGAGACCACCTGGTGCTCAGTG

TAGCCCAGGATGCCCTTGAGGGGGCCCTCCGACGCCA



>gi|374671747|gb|JK671295.1|JK671295 SSH-Mu_29 SSH library of moisture-stressed Horsegram cDNA Macrotyloma uniflorum cDNA, mRNA sequence

ACAAAAGTTGGTGATGAAATCGCAAACGTGAGGTGTCATGCGAGGGGAAAAATAGTCATGAGAAGGGGCG

GTTTTTAACAATTCCCTAAGCCCCTCCCCTCTTCAAGGGGTCTACATGGCAACTGTGAGGAGGGGAGATT

CAGTGTGGTGGGGGACTGAGTGTGGCAGGGACTCCCCAGCAGTGAGGGTCTCTCTCTTCCTCTTGTGCTC

TTGCTGGGGCTGGTGGTCCAGGGGTCTTACTCCTTGGAGGCCATGTGGGCCATGAGGTCCACCACCCTGT

TGCTGTAGCCAAATTCGTTGTCATACCAGGAAATGAGCTTGACAAAGTGGTCGTTGAGGGCAATGCCAGC

CCCAGCGTCAAAGGTGGAGGAGTGGGTGTCGCTGTTGAAGTCAGAGGAGACCACCTGGTGCTCAGTGTAA

CCCATGATGC



>gi|374671745|gb|JK671293.1|JK671293 SSH-Mu_27 SSH library of moisture-stressed Horsegram cDNA Macrotyloma uniflorum cDNA, mRNA sequence

AACAAAAGTTTGTGATAAAATCGCAAACGTCAGGAGTTTGCAAGAGGAAAAAGATCGTGAAAATGGGCGT

TTTTTAACAAACCCCTAGGCCCCTCCCCTCTTCAAGGGGTCTACATGGCAACTGTGAGGAGGGGAGATTC

AGTGTGGTGGGGGACTGAGTGTGGCAGGGACTCCCCAACAGTGAGGGTCTCTCTCTTCCTCTTGTGCTCT

TGCTGGGGCTGGTGGTCCAGGGGTCTTACTCCTTGGAGGCCATGTGGGCCATGAGGTCCACCACCCTGTT

GCTGTAACCAAATTCATTTGTCATACCAGGAAATGAGCTTGACAAAGTGGTCGTTGAGGGCAATGCCGGC

CCCAGCGTCAAAGGTGGAGGAGTGGGTGTCGCTGTTGAAGTC



>gi|374671743|gb|JK671291.1|JK671291 SSH-Mu_25 SSH library of moisture-stressed Horsegram cDNA Macrotyloma uniflorum cDNA, mRNA sequence

GGTGATGAAATCGCAAACGTGAGGAGTCATGCGAGAGGAAAAATAGTCATGAGCAGGGGCGGTTTTTTAC

AACTCCCTAGGCCCCTCCCCTCTTCAAGGGGTCTACATGGCAACTGTGAGGAGGGGAGATTCAGTGTGGT

GGGGGACTGAGTGTGGCAGGGACTCCCCAGCAGTGAGGGTCTCTCCCTTCCTCTTGTGCTCTTGCTGGGG

CTGGTGGTCCAGGGGTCTTACTCCTTGGAGGCCATGTGGGCCATGAGGTCCACCACCCTGTTGCTGTAGC

CAAATTCGTTGTCATACCAGGAAATGAGCTTGACAAAGTGTTCGTTGAGGGCAATGCCAGCCCCAGCGTC

AAAGGTGGAGGAGTGGGTGTCGCTGTTGAAGTCTG



>gi|374671741|gb|JK671289.1|JK671289 SSH-Mu_23 SSH library of moisture-stressed Horsegram cDNA Macrotyloma uniflorum cDNA, mRNA sequence

CCGGAAACCGCCATTGCCCTTGCTGTTTCCGGTCCGGGAACCGGCATTGCCCTTGCTGGTTCCGGACCGG

GAACCGGCATTGGCCCTAGCGGTTCCCGGTCCGGAACCCGCCTAGGCCAAACCGGTTCTCCGGATCGGAA

ACGGGAATGGCCCATACCGCTCCCCGACCCGAAAACCGCTTTCACCTTACGGGTTCCGGTCC



>gi|374671739|gb|JK671287.1|JK671287 SSH-Mu_21 SSH library of moisture-stressed Horsegram cDNA Macrotyloma uniflorum cDNA, mRNA sequence

AACAAAAGTTGGTGATGAAATCGCAAACGTGAGGAGTCATGCGAGAGGAAAAATAGTCTTGAGAATGGCC

GGCTTTTTACAACTCCCTAGGCCCCTCCCCTCTTCAAGGGGTCTACATGGCAACTGTGAGGAGGGGAGAT

TCAGTGTGGTGGGGGACTGAGTGTGGCAGGGACTCCCCTGCAGTGAGGGTCTCTCTCTTCCTCTTGTGCT

CTTGCTGGGGCTGGTGGTCCAGGGGTCTTACTCCTTGGAGGCCATGTGGGCCATGAGGTCCACCACCCTG

TTGCTGTATCCTAATTCTTTGTCATACCATTAAATGAGCTTGACTAAGTGGTCTTTGATGGCAATGCCAG

CCCCAGCGTCAAAGGTGGAGGAGTGGGTGTCGCTGTTGAAGTCAGAGGAGACCACCTGGTGCTCAGTGTA

GCCCAGGATGCCCTTGAGGGGGCCCTCCGACGCCA



>gi|374671737|gb|JK671285.1|JK671285 SSH-Mu_19 SSH library of moisture-stressed Horsegram cDNA Macrotyloma uniflorum cDNA, mRNA sequence

GTTCGGGAATAAAATCGGGCCCGAATATTACTTCCCAAAGAAAAATGTTCTGACTTTTTTTCGCCAAGCT

GGGGTAATCGTGATAATAGGTGTTTCGTGTGAAAATGATAACGGCCAACATTTCCCCCCGGGGAAAAAGT

TATACCCTTTTTTTACCCCCAGGGTGGGGGGA



>gi|374671735|gb|JK671283.1|JK671283 SSH-Mu_17 SSH library of moisture-stressed Horsegram cDNA Macrotyloma uniflorum cDNA, mRNA sequence

ACATATGCCATGGAAAACGACGGCCAGGACTGGGCGGCCTTTCATTTCAATGGAAAACGACGGGCAGGAC

TGGGGCGGGGGTTTTACATTGCAAGTAAAAAGACGGCCACCACTGGGGCGGCGGTTTTCATTTCTGAAGG

AAAAAAGACGGCCCCGAGTGGGGGGCGTTTTTATTTTTTAAGAAAAAAAAAGGGCGCCGCGCGGGGGGCG

G



>gi|374671733|gb|JK671281.1|JK671281 SSH-Mu_15 SSH library of moisture-stressed Horsegram cDNA Macrotyloma uniflorum cDNA, mRNA sequence

CGGCCGAGGTGGCGTCGGAGGGCCCCCTCAAGGGCATCCTGGGCTACACTGAGCACCAGGTGGTCTCCTC

TGACTTCAACAGCGACACCCACTCCTCCACCTTTGGCGCTGGGGCTGGCATTGCCCTCAACGACCACTTT

GTCAAGCTCATTTCCTGGTATGACAACGAATTTGGCTACAGCAACAGGGTGGTGGACCTCATGGCCCACA

TGGCCTCCAAGGAGTAAGACCCCTGGACCACCAGCCCCAGCAAGAGCACAAGAGGAAGAGAGAGACCCTC

ACTGCTGGGGAGTCCCTGCCACACTCAGTCCCCCACCACACTGAATCTCCCCTCCTCACAGTTGCCATGT

AGACCCCTTGAAGAGGGGAGGGGCCTAGGGAGCCGCACCTTGTCATGT



>gi|374671731|gb|JK671279.1|JK671279 SSH-Mu_13 SSH library of moisture-stressed Horsegram cDNA Macrotyloma uniflorum cDNA, mRNA sequence

CGGTCCGGAAACCGCCATGGCCCTAGCCGTTTCCGGTCCGGAAACCGCCATGGCCCTTGCCGGTTCCGGG

CCGGGAACCGGCAATGCCCCAGGCGGTTCCGGACCCGGAAACCGCCTTGCACTAGCGGTTCCCGGACCGG

AAAACAGCAAGGCCCATGCCGGTTCCCGATCGGAACAACGCCTTGCCCCACGGGGTTTCCGGTCGGGAAA

CAGCTATGCCCATGGGGTTTCCGGAACCGGAAAACGCTATGCCCCATGGGGGTTT



>gi|374671729|gb|JK671277.1|JK671277 SSH-Mu_11 SSH library of moisture-stressed Horsegram cDNA Macrotyloma uniflorum cDNA, mRNA sequence

GACGCCAAGGTGATGGATAGCTGGGAATTCACCCTTAGCGTGGTCGCGGCCGAGGTGGCGTCGGAGGGCC

CCCTCAAGGGCATCCTGGGCTACACTGAGCACCAGGTGGTCTCCTCTGACTTCAACAGCGACACCCACTC

CTCCACCTTTGACGCTGGGGCTGGCATTGCCCTCAACGACCACTTTGTCAAGCTCATTTCCTGGTATGAC

AACGAATTTGGCTACAGCAACAGGGTGGTGGACCTCATGGCCCACATGGCCTCCAAGGAGTAAGACCCCT

GGACCACCAGCCCCAGCAAGAGCACAAGAGGAAGAGAGAGACCCTCACTGCTGGGGAGTCCCTGCCACAC

TCAGTCCCCCACCACACTGAATCTCCCCTCCTCACAATTGCCATGTAGACCCCTTGAAGAGGGGAGGGGC

CTAGGGAGCCGCACCTTGTCATGT



>gi|374671727|gb|JK671275.1|JK671275 SSH-Mu_8 SSH library of moisture-stressed Horsegram cDNA Macrotyloma uniflorum cDNA, mRNA sequence

GTTGGGACAGGTAAGCGGTAGACCAGGATAAGCTACCACCATGGCAGGTACATGACAAGGTGCGGCTCCC

TATGCCCCTCCCCTCTTCAAGGGGTCTACATGGCAACTGTGAGGAGGGGAGATTCAGTGGGGTGGGGGAC

TGAGTGGGGCAAGGACTCCCCAACATTGAGGGTCTCTCTCTTCCTCTTGTGCTCTTGCTGGGGCTGGTGG

TCCATGGGTCTTACTCCTTGGAGGCCATGTGGGCCATGAGGTCCACCACCCTGTTGCTGTAGCCAAATTC

GTTGTCATACCAGGAAATGATCTTGACAAAGTGGTCGTTGAGGGCAATGCCAGCCCCTGCGTCAAAAGTG

GAAAAGTGTGTGTCACTGTTGAAATCAAAAGATACCACCTGGTGCTCAGTGTATCCCAAGATGCCCTTGA

TGGGGCCCTCCGACGCCACCTCGGACGCGACCTCTC



>gi|374671725|gb|JK671273.1|JK671273 SSH-Mu_6 SSH library of moisture-stressed Horsegram cDNA Macrotyloma uniflorum cDNA, mRNA sequence

TCCCTAGCCCCTCCCCTCTTCAAGGGGTCTACATGGCAACTGTGAGGAGGGGAGATTCAGTGTGGAGGGG

GACTGAGTGTGGCAGGGACTCCCCAGCAGTGAGGGTCTCTCTCTTCCTCTTGTGCTCTTGCTGGGGCTGG

TGGTCCAGGGGTCTTATTCCTTGGAGGCCATGTGGGCCATGAGGTCCACCACCCTGTTGCTGTACCCAAA

TTCGTTGTCTACCCTGAAATGAGCTTTGACAAAGTG



>gi|374671723|gb|JK671271.1|JK671271 SSH-Mu_4 SSH library of moisture-stressed Horsegram cDNA Macrotyloma uniflorum cDNA, mRNA sequence

CTGTCCAGCTGTTTCTGCACGAACAGCTTGCCGATTCGCATTCCTGTCTCGCCAGGCCCAGTTGTTCCTC

CCCCGGCCCCGCCCTGCACCGGCCGGTTCCTGACAGGAAAAGCCGGTCCCCTAGGGGGGATTCCCGGTCG

CGAAAACCGTAGTGGCTTCCCCGGGGTTTCCCGGTCTAAAACACGNTTCGAGCCGCTCGTTCTCGGGACC

GGAACCACCTTGCTCAGCCGTGTTCGCCTGTT



>gi|374671721|gb|JK671269.1|JK671269 SSH-Mu_2 SSH library of moisture-stressed Horsegram cDNA Macrotyloma uniflorum cDNA, mRNA sequence

CATGAAGGCGTTATGGTCATAGCGTGGTGACGATGGGCCACCTAGGGGCGCGAGAGCTAGCTGATATCGG

AGGGTCGCCTCTGACTTCTTGTCGACACAACTCCTCCTGTTAGAAAAGGGGTCTGGCCTTGCCCGCATCG

AGGGCTTGGGGTGGGTCATGTCCTGGAAAGACATATGGGTTTGGATACAGCAACAAGATGGTGGACCTCA

TGGCCAACATGGTCTCGAAAAAAAGAGACCCCTGGACCACAAACCCCTTTGAAACAAAAGATGAAGAGAG

AGACGCTCTCTGCTGGAGAATCCCTTTCTCGCTCGGACACAGGCCAGACTGAATCTCCCCTCCTGGCAAT

TGCGGTGTCGACCCCTTAAATATGTTATGGGGCAAAAAAAGTCTCACCATGACATGTATTATGCCCGGAA

AGGTTGCTCTATAATAAAATATTCTGGTTGTAGACA



>gi|374671752|gb|JK671300.1|JK671300 SSH-Mu_40 SSH library of moisture-stressed Horsegram cDNA Macrotyloma uniflorum cDNA, mRNA sequence

GGCGTCGGAGGGCCCCCTCAAGGGCATCCTGGGCTACACTGAGCACCAGGTGGTCTCCTCTGACTTCAAC

AGCGACACCCACTCCTCCACCTTTGACGCTGGGGCTGGCATTGCCCTCAACGACCACTTTGTCAAGCTCA

TTTCCTGGTATGACAACGAATTTGGCTACAGCAACAGGGTGGTGGACCTCATGGCCCACATGGCCTCCAA

GGAGTAAGACCCCTGGACCACCAGCCCCAGCAAGAGCACAAGAGGAAGAGAGAGACCCTCACTGCTGGGG

AGTCCCTGCCACACTCAGTCCCCCAGCACACTGAATCTCCCCTCCTC



>gi|374671750|gb|JK671298.1|JK671298 SSH-Mu_32 SSH library of moisture-stressed Horsegram cDNA Macrotyloma uniflorum cDNA, mRNA sequence

TGCCCCCGAATTGGTCTCCACGGACACAATTTCACCCGAAACCCCTATGACCTTGACTCCGCCAAGCTTG

GGGTCATCATGGACATCGTTTCTGTGCGAAATTGCTATCGGCTCACAATTTCTCACAGGA



>gi|374671748|gb|JK671296.1|JK671296 SSH-Mu_30 SSH library of moisture-stressed Horsegram cDNA Macrotyloma uniflorum cDNA, mRNA sequence

GGCGTCGGAGGGCCCCCTCAAGGGCATCCTGGGCTACACTGAGCACCAGGTGGTCTCCTCTGACTTCAAC

AGCGACACCCACTCCTCCACCTTTGACGCTGGGGCTGGCATTGCCCTCAACGACCACTTTGTCAAGCTCA

TTTCCTGGTATGACAACGAATTTGGCTACAGCAACAGGGTGGTGGACCTCATGGCCCACATGGCCTCCAA

GGAGTAAGACCCCTGGACCACCAGCCCCAGCAAGAGCACAAGAGGAAGAGAGAGACCCTCACTGCTGGGG

AGTCCCTGCCACACTCAGTCCCCCACCACACTGAATCTCCCCTCCTCACAGTTGCCATGTAAACCCCTTG

AAGAGGGGAGGGGCCTAGGGAGCCGCACCTTGTCATGTA



>gi|374671746|gb|JK671294.1|JK671294 SSH-Mu_28 SSH library of moisture-stressed Horsegram cDNA Macrotyloma uniflorum cDNA, mRNA sequence

ACAACAAAAGTATTGTGATAAAATCGCAAACGTCAGGAGTCATGCGAGGGGAAAAATAGTCATGAGAAGG

GGCGGTTTTTTACAATCCCTAGGCCCCTCCCCTCTTCAAGGGGTCTACATGGCAACTGTGAGGAGGGGAG

ATTCAGTGTGGTGGGGGACTGAGTGTGGCAGGGACTCCCCAGCAGTGAGGGTCTCTCTCTTCCTCTTGTG

CTCTTGCTGGGGCTGGTGGTCCAGGGGTCTTACTCCTTGGAGGCCATGTGGGCCATGAGGTCCACCACCC

TGTTGCTGTAGCCAAATTCCTTGTCTTACCAGGAAATGAGCTTGACAAAGTGGTCGTTGAGGGCAATGCC

AGCCCCAGCGTCAAAGGTGGAGGAGTGGGTGTCGCTGTTGAAGTCAAAGGAGACCACCTGGTGCT



>gi|374671744|gb|JK671292.1|JK671292 SSH-Mu_26 SSH library of moisture-stressed Horsegram cDNA Macrotyloma uniflorum cDNA, mRNA sequence

ACATGACAAGGTGCGGCTCCCTAGGCCCCTCCCCTCTTCAAGGGGTCTACATGGCAACTGTGAGGAGGGG

AGATTCAGTGTGGTGGGGGACTGAGTGTGGCAGGGACTCCCCAGCAGTGAGGGTCTCTCTCTTCCTCTTG

TGCTCTTGCTGGGGCTGGTGGTCCAGGGGTCTTACTCCTTGGAGGCCATGTGGGCCATGAGGTCCACCAC

CCTGTTGCTGTAGCCAAATTCGTTGTCATACCAGGAAATGAGCTTGACAAAGTGGTCGTTGAGGGCAATG

CCAGCCCCAGCGTCAAAGGTGGAGGAGTGGGTGTCGCTGTTGAAGTCAGAGGAGACCACCTGGTGCTCAG

TGTAGCCCAGGATGCCCTTGAGGGGGCCCTCCGACGCC



>gi|374671742|gb|JK671290.1|JK671290 SSH-Mu_24 SSH library of moisture-stressed Horsegram cDNA Macrotyloma uniflorum cDNA, mRNA sequence

GTGGCGTCGGAGGGCCCCCTCAAGGGCATCCTGGGCTACACTGAGCACCAGGTGGTCTCCTCTGACTTCA

ACAGCGACACCCACTCCTCCACCTTTGACGCTGGGGCTGGCATTGCCCTCAACGACCACTTTGTCAAGCT

CATTTCCTGGTATGACAACAAATTTGGCTACAGCAACAGGGTGGTGGACCTCATGGCCCACATGGCCTCC

AAGGAATAAGACCCCTGGACCACCAGCCCCAGCAAGAGCACAAGAGGAAGAGAGAGACCCTCACTGCTGG

GGAGTCCCTGCCACACTCAGTCCCCCACCACACTGAATCTCCCCTCCTCACAATTGCCATGTAGACCCCT

TGAAGAGGGGAGGGGCCTAGGGAGCCGCACCTTGTCATG



>gi|374671740|gb|JK671288.1|JK671288 SSH-Mu_22 SSH library of moisture-stressed Horsegram cDNA Macrotyloma uniflorum cDNA, mRNA sequence

GCTGAGTGGGTCTGCGCAGACCCACTCAGCTGTATAACGACGGCCTGTCCGCCACGAACTGGGTCTGCCT

TTTCCAACTCAGCTGCAGACTGGCCGTTTTTTTGGATCTACGTGGCTCCTTTAAACACACTAGATTCAAT

TTCGTGGGGTACTGATGTAGCTGAGTGACTCCCCAGCAAACCAACTTCTCTCTCATCGTCGTTGTTTTTA

GTTGAGCGGGTCTCCCAAAACTCTTTCTCCTTGAAAAGATGGGCACCTTGCCGTTCAGTTCCTCTTTGCA

GTACCCCTCAGTCGTTGACAGTTCCGCGTTTTTT



>gi|374671738|gb|JK671286.1|JK671286 SSH-Mu_20 SSH library of moisture-stressed Horsegram cDNA Macrotyloma uniflorum cDNA, mRNA sequence

GCACAAGAGGTCGGTCGGTGTAAATGGATATCGTGCAGAAGTCACCCTTAGGCGTGGTCGCGGCCGAGGT

GGCGTCGGAGGGCCCCCTCAAGGGCATCCTGGGCTACACTGAGCACCAGGTGGTCTCCTCTGACTTCAAC

AGCGACACCCACTCCTCCACCTTTGACGCTGGGGCTGGCATTGCCCTCAACGACCACTTTGTCAAGCTCA

TTTCCTGGTATGACAACGAATTTGGCTACAGCAACAGGGTGGTGGACCTCATGGCCCACATGGCCTCCAA

GGAGTAAGACCCCTGGACCACCAGCCCCAGCAAGAGCACAAGAGGAAGAGAGAGACCCTCACTGCTGGGG

AGTCCCTGCCACACTCAGTCCCCCACCACACTGAATCTCCCCTCCTCACAGTTGCCATGTAGACCCCTTG

AAGAGGGGAGGGGCCTAGGGAGCCGCACCTTGTCATGTA



>gi|374671736|gb|JK671284.1|JK671284 SSH-Mu_18 SSH library of moisture-stressed Horsegram cDNA Macrotyloma uniflorum cDNA, mRNA sequence

GGCGTCGGAGGGCCCCCTCAAGGGCATCCTGGGCTACACTGAGCACCAGGTGGTCTCCTCTGACTTCAAC

AGCGACACCCACTCCTCCACCTTTGACGCTGGGGCTGGCATTGCCCTCAACGACCACTTTGTCAAGCTCA

TTTCCTGGTATGACAACGAATTTGGCTACAGCAACAGGGTGGTGGACCTCATGGCCCACATGGCCTCCAA

GGAGTAAGACCCCTGGACCACCAGCCCCAGCAAGAGCACAAGAGGAAGAGAGAGACCCTCACTGCTGGGG

AGTCCCTGCCACACTCAGTCCCCCACCACACTGAATCTCCCCTCCTCACAGTTGCCATGTAGACCCCTTG

AAGAGGGGAGGGGCCTAGGGAGCCGCACCTTGTCATGT



>gi|374671734|gb|JK671282.1|JK671282 SSH-Mu_16 SSH library of moisture-stressed Horsegram cDNA Macrotyloma uniflorum cDNA, mRNA sequence

GAGGGCCCCCTCAAGGGCATCCTGGGCTACACTGAGCACCAGGTGGTCTCCTCTGACTTCAACAGCGACA

CCCACTCCTCCACCTTTGACGCTGGGGCTGGCATTGCCCTCAACGACCACTTTGTCAAGCTCATTTCCTG

GTATGACAACAAATTTGGCTACAGCAACAGGGTGGTGGACCTCATGGCCCACATGGCCTCCAAGGAGTAA

GACCCCTGGACCACCAGCCCCAGCAAGAGCACAAAAGGAAGAGAGAGACCCTCACTGCTGGGGAGTCCCT

GCCACACTCAATCCCCCACCACACTGAATCTCCCCTCCTCACAGTTGCCATGTACACCCCTTGAAGAGGG

GAGGGGCCTGGGGAGCCGCAACTTGTCATGAACATGCCCGGGCGACCGCTCTAAGGGCGAATTCAGCATA

TAT



>gi|374671732|gb|JK671280.1|JK671280 SSH-Mu_14 SSH library of moisture-stressed Horsegram cDNA Macrotyloma uniflorum cDNA, mRNA sequence

CTCACAGGAAAAAACTGTGGACAATCATTACGCCAAGCTCGGTACGAAGCTTGGAGTAATCATGAACTTA

GCCTGTTTCAAAGTGAAATTGATATCTGCTACCAATTTCCCCGAGAAAAAGGGCAATGACTGTTTATTAC

GCCAAAGTTCGTTACCGCAGCTTGTGTTCACACAGGACACAGTTTTTACGATGTTAAAAGTCCAAGCGTG

ACCAAAATCATGCAGAAAAACAGTTTCTGGAATAT



>gi|374671730|gb|JK671278.1|JK671278 SSH-Mu_12 SSH library of moisture-stressed Horsegram cDNA Macrotyloma uniflorum cDNA, mRNA sequence

ACATGACAAGGTGCGGCTCCCTAGGCCCCTCCCCTCTTCAAGGGGTCTACATGGCAACTGTGAGGAGGGG

AGATTCAGTGTGGTGGGGGACTGAGTGTGGCAGGGACTCCCCAGCAGTGAGGGTCTCTCTCTTCCTCTTG

TGCTCTTGCTGGGGCTGGTGGTCCAGGGGTCTTACTCCTTGGAGGCCATGTGGGCCATGAGGTCCACCAC

CCTGTTGCTGTAGCCAAATTCGTTGTCATACCAGGAAATGAGCTTGACAAAGTGGTCGTTGAGGGCAATG

CCAGCCCCAGCGTCAAAGGTGGAGGAGTGGGTGTCGCTGTTGAAGTCAGAGGAGACCACCTGGTGCTCAG

TGTAGCCCAGGATGCCCTTGAGGGGGCCCTCCGACGCC



>gi|374671728|gb|JK671276.1|JK671276 SSH-Mu_9 SSH library of moisture-stressed Horsegram cDNA Macrotyloma uniflorum cDNA, mRNA sequence

ACATGACAAGGTGCGGCTCCCTAGGCCCCTCCCCTCTTCAAGGGGTCTACATGGCAACTGTGAGGAGGGG

AGATTCAGTGTGGTGGGGGACTGAGTGTGGCAGGGACTCCCCAGCAGTGAGGGTCTCTCTCTTCCTCTTG

TGCTCTTGCTGGGGCTGGTGGTCCAGGGGTCTTACTCCTTGGAGGCCATGTGGGCCATGAGGTCCACCAC

CCTGTTGCTGTAGCCAAATTCGTTGTCATACCAGGAAATGAGCTTGACAAAGTGGTCGTTGAGGGCAATG

CCAGCCCCAGCGTCAAAGGTGGAGGAGTGGGTGTCGCTGTTGAAGTCAGAGGAGACCACCTGGTGCTCAG

TGTAGCCCAGGATGCCCTTGAGGGGGCCCTCCGACGCC



>gi|374671726|gb|JK671274.1|JK671274 SSH-Mu_7 SSH library of moisture-stressed Horsegram cDNA Macrotyloma uniflorum cDNA, mRNA sequence

AACAAAAGTATGGTGATGAAATCGCAAACGTGAGGAGTCATGGAAGAGGAAAAAGTGTCGTGAGAATGGG

CGGTTTTAAACAATCAAGGGCATCCTGGGCTACACTGAGCACCAGGTGGTCTCCTCTGACTTCAACAGCG

ACACCCACTCCTCCACCTTTGACGCTGGGGCTGGCATTGCCCTCAACGACCACTTTGTCAAGCTCATTTC

CTGGTATGACAACGAATTTGGCTACAGCAACAGGGTGGTGGACCTCATGGCCCACATGGCCTCCAAGGAG

TAAGACCCCTGGACCACCAGCCCCAGCAAGAGCACAAGAGGAAGAGAGAGACCCTCACTGCTGGGGAGTC

CCTGCCACACTCAGTCCCCCACCACACTGAATCTCCCCTCCTCACAGTTGCCATGTAGACCCCTTGAAGA

GGGGAGGGGCCTAGGGAGCCGCACCTTGTCATGTA



>gi|374671724|gb|JK671272.1|JK671272 SSH-Mu_5 SSH library of moisture-stressed Horsegram cDNA Macrotyloma uniflorum cDNA, mRNA sequence

AGAAATTCTATGGTCATAGCAGCTCCCAGAACATAGATATTCTAGGGACGAAACTGCTTCAGACCAAAAA

ATACCATGGGACAGAAGTGTCTGCCCAAAAAAAATTTCTGGGGCGAGAAAGTTTTTTTGACCCTAAAAAA

TTTTGGGCAAAACGCTTTCTGCACCTATAAAAATTTTATTTGGGAAAAGAGGTTTTTACCATAAAAAATT

CTTTGGGCAGGAACCGTTCCCACCCCCCAAATAATATTTTTGGGGGGGAAAAATGGTTTTGACCCACATA

AAAACTTGTTGGGGGCACGGAGTCTTTCCCACCCATAATATATGTTTTGGGGCGAGGGGTTGTTTTCAAA

ATAAAAATTTTTTGGGGGGGGGGCCGTGTCCTCCCATAATTTTTTTTTTGGGGAAGACGTGTT



>gi|374671722|gb|JK671270.1|JK671270 SSH-Mu_3 SSH library of moisture-stressed Horsegram cDNA Macrotyloma uniflorum cDNA, mRNA sequence

ACTGGCCGTCCTTCCAAAACAAGGACTGACTATTATCTTATCCGGACGCCCCTCCCCTCTTCAGGGGTCT

ACTTGGCACACTGGGGAGGGGAAATTCAGGTGGTGGGTGACTTATTGATAAGGGACCCTCTTCAATAAGG

GTCTCTCTCTTTCTCATGTGCTCTTCCTGGGTCTGAAATCCAGGGGTGTTACTCATTGAAATCCGTGTTG

GCCATGAAGTCCTCCGGCGTGTTTCTTTACCCGGTTCGTTGTCTTAAAAGA



>gi|374671720|gb|JK671268.1|JK671268 SSH-Mu_1 SSH library of moisture-stressed Horsegram cDNA Macrotyloma uniflorum cDNA, mRNA sequence

GAAACAAAAGTATGGTGATAAAATCGCAAACGTGAGGAGTCATGGAAGAGGAAAAAGAGTTGTGAGGGTG

GGTGTTTTAATACAATCAAGGGCATCCTGGGCTACACTGAGCACCAGGTGGTCTCCTCTGACTTCAACAG

CGACACCCACTCCTCCACCTTTGACGCTGGGGCTGGCATTGCCCTCAACGACCACTTTGTCAAGCTCATT

TCCTGGTATGACAACGAATTTGGCTACAGCAACAGGGTGGTGGACCTCATGGCCCACATGGCCTCCAAGG

AGTAAGACCCCTGGACCACCAGCCCCAGCAAGAGCACAAGAGGAAGAGAGAGACCCTCACTGCTGGGGAG

TCCCTGCCACACTCAGTCCCCCACCACACTGAATCTCCCCTCCTCACAGTTGCCATGTAGACCCCTTGAA

GAGGGGAGGGGCCTAGGGAGCCGCACCTTGTCATGTA



>gi|313247888|gb|HS109647.1|HS109647 MuNAC1 Stress responsive NAC TF family genes in horsegram (Macrotylomauniflorum (Lam.)Verdc.) Macrotyloma uniflorum cDNA similar to MAC domain protein (NAC1), mRNA sequence

CTACCTGCCACGTCCAATGGAGAATAGAACAAGCTCTGTACTCCCACCTGGCTTCAGGTTTCACCCCACT

GATGAGGAACTAATTGTGTACTACCTTTGCAACCAAGCCTCATCAAGGCCATGCCCTGCATCAATCATTC

CAGAAGTGGATATCTATAAGTTTGATCCATGGGAATTACCTGATAAAACAGACTTTGGAGAAAAGGAGTG

GTACTTCTTTAGCCCACGAGAGAGGAAGTATCCAAATGGGGTGAGGCCTAATAGAGCAACCGTGTCTGGG

TATTGGAAGGCCACTGGGACAGATAAAGCAATCTATAGTGGGTCTAAGCATGTAGGGGTGAAGAAGGCTT

TGGTTTTTTACAAGGGTAAGCCACCAAAGGGTCTCAAGACAGATTGGATTATGCATGAGTATAGATTAAT

TGGATCAAGAAGACAAGCCAATAGACAAGTTGGATCCATGAGGCTAGATGACTGGGTCTTGTGTAGGATT

TACAAGAAGAAGAATATTGGAAAATCAATGGAGGCAAAAGAGGACTACCCAATAGCCCAAATCAACCTTA

CACCAGCAAACAATAACAGTGAACAAGAATTGGTGAAATTTCCAAGGACTAGTTCCCTTACTCATCTTTT

GGAAATGGATTACTTGGGTCCAATATCACATATATTACCTGATGCCTCATACAATTCAACCTTTGATTTT

CAAATTAACACTGCCAATGGTGGAATAGACCCGTTCGTAAAACCACAGCTGGTTGAAATCCCTTATGCAA

CAGATTCAGGGAAGTACCAGGTGAAACAGAATAGCACCATCAACCCCACCATATTTGTGAACCAAGTGTA

TGATCAAAGAGGATAATCAACGTAAGCGAACTGCCAGCGTTAAGCACCGAAAGTCAATTGACGAA



>gi|313247889|gb|HS109648.1|HS109648 MuNAC4 Stress responsive NAC TF family genes in horsegram (Macrotylomauniflorum (Lam.)Verdc.) Macrotyloma uniflorum cDNA similar to Nac domain protein (NAC4), mRNA sequence

GGTTCTGTGTCGTATATACAACAACGGCTGCAATCTATGGGAGTTCCAGAGGAAGACCCTCTTTCCCAGT

TGAGTCTACCTCCTGGTTATCGCTTCTACCCCACCGACGAGGAGCTTCTCGTTCAGTATCTCTGCCGCAA

GGTCGCCGGCCACCATTTCTCTCTCCCGATCATTGCTGAAATTGATTTGTACAAGCTCGACCCATGGGTT

CTTCCAAGCAAGGCGATTTTCGGGGAGAAAGAGTGGTACTTTTTCAGCCCTCGTGACAGGAAATACCCAA

ACGGGTCTCGACCCAACAGAGTAGCCGGGTCGGGTTATTGGAAAGCCACCGGAACCGACAAGATCATCAC

CACTGAAGGTAGAAAAGTTGGCATGAAAAAAGCCCTCGTTTTTTACGTTGGGAAAGCTCCAAAAGGCACC

AAAACCAATTGGATCATGCACGAGTATCGCCTCCTTGACTCTTCCCGAAAGACAACTGGCACCAAGCTGG

ATGATTGGGTTCTGTGTCGTATATACAAGAAGAACTCGAGTGCACAGAAGGCAGTGCAGAACGACGTGGT

TTCGAGCAGGGAACACACACAATACAGCAACGGTTCGCCGTCGTCGTCATCATCCCATCTGGACGACGTT

CTGGAATCGCTGCCAGCGATCGACGAACGGTGCTTCGCGATGCCACGTGTCAACACGGTGCAGCAACAGC

AGGAGGAGAAGGTGAACGTTCAGAACCTGGGTTCGAGTGGGTTGGTGGATTGGGCCAACCCTGCGGTTCT

GAATTCGGTGGCTGATTTCGTTTCGGAAAATAATCAAGTGGTGCAGGACCATACTCAGGGGATGGTGAAC

TACAACGGCTGCAATGACCTTTATGTCCCCACCTTCTGCCACGTGGACTCCTCGCTTCCGCAAAAGGTTG

AGGAAGAGGTGCAAAGCGGTGTCAGAAACCAAAACAATAACTCGTGGTTTCTTCAGAACGATTTCACACA

GGGGTTTCAAAACCCTGTCGACACGTGTGGGTTTAAGTTCCCGGTTCAGCCGGTCGGGTTCGGGTTCAGG

AATTGAATTACGTTGGGAAAGGGCCAACCCTGCGG



>gi|313247887|gb|HS109646.1|HS109646 MuNAC15 Stress responsive NAC TF family genes in horsegram (Macrotylomauniflorum (Lam.)Verdc.) Macrotyloma uniflorum cDNA similar to NAC domain protein (NAC15), mRNA sequence

AAGCATTGCTTAACCACCGATGGACAAGGTGAATTTTGTGACGAATGGAGTGCTTAGCTTGCCTCCGCGG

TTCCGTTTCTACCCGACTGATGAGGAGCTGGTTCTGCAATACGTGAAGCGCAAGGTCCTCTCCTGCCCTT

TGCCAGCCTCTATCTTTCCTGAGCTTCATGCCTGCAAGTCTGATCCTTGGGATTTGCCAGGTGATTTGGA

GCAAGAGAGATACTTCTTTAGCACCAAAGTGGCCAAATTTCCCAACGGAAATCGCTCCAACAGAGCCACA

AATCCGGGTTATTGGAATGCAACTGGCTTGGAGAAACAAATTGTTACTTTAAAAGCCAACAACCAAGTTG

TCGGAATCAAAAAGACACTTGTTCTCTACAGAGGCAAGCCTCCTAAGGGATCCAGAACTGATTCTATCAT

CCACGAGTATCGCCTCATCCTTAACGCCTCTCAGTCTCAGAGCCATCTTGTTCCCATGGAAAATTCTATT

CTGTGTCGCATATTTTTGAAGAGGAGAATTGGTGCTAAATCTGGGGAGGAGAGCAACTCTAAGTTGGTTT

TCTATGACTTCTCGGCGCAGAACAAGACCGATTCCTCGTCATCGGTCGCTCTTGGAATTACACATGAATC

AGATGAACATGAAGAGAGCAGTAGGCCCAACACCTTCCCTTATGTTATTAGAAGAAATCCTTAAACTGGT

GCTAAAAATGGGGAGCGGTTACGTTAACTTGGAC



>gi|71812245|gb|DR989636.1|DR989636 HDI_L_95.esd Horsegram (VZM-1) drought stressed subtracted cDNA Macrotyloma uniflorum cDNA, mRNA sequence

CAAANCTGAGCTCACGCGGTGGCGGCGCTCTAGAACTAGTGGATCCCCCGGGCTGCAGGAATTCGGCACG

AGGCTGAAATTCCTTTGTTAATTGATCAGAATAAATTATTTGATGTAATACTTAAATTCTGCATAATGGT

CGTTATGTCATGGTGTAGTTTTTCTGCAAAAAAAAAAAAAAAAAAACCCNAGGGGGGGGCCCGGGCCCCA

TTCCGC



>gi|71812243|gb|DR989634.1|DR989634 HDI_L_92.esd Horsegram (VZM-1) drought stressed subtracted cDNA Macrotyloma uniflorum cDNA, mRNA sequence

AGCTGGAGCTCACGCGGNNGCGGCGCTCTAGAACTAGTGGATCCCCCGGGCTGCAGGATCGGCACGAGGC

TGACTTGTGTAAGAATGAGTATCTAAATTAGACTAATAATGTTTGTTTGAAACTTAGTTGAGTTTAAAAA

AAAAAAAAAAAAAAAAAAAAAAAAAAAAAAAAAAAAAAAAAAAAAAAAAAAAAAAAAAAAAAAAAAAAAA

AAAAAAA



>gi|71812241|gb|DR989632.1|DR989632 HDI_L_9.esd Horsegram (VZM-1) drought stressed subtracted cDNA Macrotyloma uniflorum cDNA, mRNA sequence

GGCTGGAGCTCACGCGGTGCNCGGCGTTCTAGAACTAGTGGATCCCCCGGGCTGCAGGATTCGGCACGAG

GTGCCCACTGGTAAATATGTGCCCTCTTGGTTTTTATTTGTGTTTGTACATCGATAAACTACATTTGCCT

TCCAAGGGAAAATGTCTTGTGTAAAAAGAAAATAGCACAGAATGTTGAATAAGTTTACATTACATGCACA

AACTGTTTTCCGCATAAAAAAAAAAAAAAAAAACCCGAGGGGGGGCCC



>gi|71812239|gb|DR989630.1|DR989630 HDI_L_83.esd Horsegram (VZM-1) drought stressed subtracted cDNA Macrotyloma uniflorum cDNA, mRNA sequence

ACAAGCTGGAGCTCACCGCGGTGCGCGGCGTTCTCTAGAACTAGTGGATCCCCCGGGCTGCAGTGAATCG

GCACGAGGGAAATAGTACAGTTTCTCTTGAGTATTTCTGTCTATCATTATGATTTTATTTCCCTTTTATT

TCTGGCTGTTGGGGGTTTCTTTGTTCTTTGCCTTGGTCCATGTTTTTTTATTGCGCAGACTTTAGAGCAT

CTCAAATAGTCCTTGAGTGAATAGTTTGGACTTTAATTCTTGTACATGTAGTTCTAGAGATGGACCCTTC

TACTAAAAAAAAAAAAAAAAAAAAAAAAAAAAAAAAAAAAAAAAAAAAAAAAAAAAAAAAAAAAAAAAAA

AAAAAAAAAAAAAA



>gi|71812237|gb|DR989628.1|DR989628 HDI_L_81.esd Horsegram (VZM-1) drought stressed subtracted cDNA Macrotyloma uniflorum cDNA, mRNA sequence

AAAGCTGGAGCTCACGCGGTGGCGGCGCTCTAGAACTAGTGGATCCCCCGGGCTGCAGGATCGGCACGAG

GCGTATTTGTTATCCTTATGGTCATGTTTGTACAATGTATGTAGATGCAACTCTACTTCAAACTTTATTT

GGTAGGATTATTTTGGTGTAATACTGTGTTGGATTATTAAAAAAAAAAAAAAAAAAAAAAAAAAACCCCG

GGGGGGGGCCCGGGCCCCACCCCCCG



>gi|71812235|gb|DR989626.1|DR989626 HDI_L_8.esd Horsegram (VZM-1) drought stressed subtracted cDNA Macrotyloma uniflorum cDNA, mRNA sequence

ACAGCTGGAGCCACGCGGGGCGGCCGCTCTAGAACTAGTGGATCCCCCGGGCTGCAGGATCGGCACGAGG

CAACATCTAAATGCTCTTCTCCAATCTTAAAATGTACAACACATCTCACTAAAAAAAAAAAAAAAAAAAA

AAAAAAAAAAAAAAAAAAAAAAAAAAAAAAAAAGGGGGGGGGGG



>gi|71812233|gb|DR989624.1|DR989624 HDI_L_72.esd Horsegram (VZM-1) drought stressed subtracted cDNA Macrotyloma uniflorum cDNA, mRNA sequence

CTGGAGCTCACGCGGTGGCGGCGCTCTAGAACTAGTGGATCCCCCGGGCCTGCAGGAATCGGCACGAGGG

AATTGTACTAGGTTGAAAAACAATTAGGCCTCTGCAGAGTTTCAAAATTTGTTTTGTACCCTTCACACAT

GATTTATATATAAGTATAATTCAGAAAGAAAAAAAAAAAAAAAAAAAAAAAAAAAAAAAAAAAAAAAAAA

AAAAAAAAAAAAAAGGGGGGGGGGGGG



>gi|71812231|gb|DR989622.1|DR989622 HDI_L_70.esd Horsegram (VZM-1) drought stressed subtracted cDNA Macrotyloma uniflorum cDNA, mRNA sequence

AAAGCTGGAGCTCACGCGGTGCCGGCGTTCTAGAAACTAGTGGATCCCCCGGGCTGCAGGAATTCGGCAC

GAGGCCGGAAACAGACCGCTGGAGTAACAGTAGTAGCCGGCCGGATGGTGACAGTGTGGCGTGGTGATGA

AAGGAGAAGTGGGTTTGGTGGTTCTGGTGGAGTGTTCAAGGTCATCTTCTACTTGGTCATATTCCAGGAA

TGGGCGTTGATCATTTTGTGCCCTCTGATTTATTGCTGGTGACTTTTTTTGGAATTCTCTCTGTACTCTT

TCCAGTTTACGTCCTAGAATTCTTTTTGATGCTGAGTTGTTAACAATTTATTATCTTAATACTCACAAAC

AAGTCCTCTATTGGGGTTTGAAGGGACACAAAA



>gi|71812229|gb|DR989620.1|DR989620 HDI_L_69.esd Horsegram (VZM-1) drought stressed subtracted cDNA Macrotyloma uniflorum cDNA, mRNA sequence

AGCTGGAGCTCACGCGGTGCCGGCGCTCTAGAACTAGTGGATCCCCCGGGCTGCAGGATCGGCACGAGGC

AGGCAACATCCAAAAAGAAGAAAGAAAATTGAAGGGGAAAAGAGTAGTTAGAGAATAAGAAGAAAAAAGA

AAGTATTACAGAGTGAAAAAACTGTTTCATTATTTGAGTATTACTGTTAGACTTGTTAGAATTAGACATT

AAATAAGATCTTCCATTCTGGTTTCTTTTGAACTATTGCCTGTGATTTTTCTATTGATATTTTAAAGTGC

CCTTTCTTTCTTTGGTTTGTTTTCAAAAAAAAAAAAAAAAACCCCAAGGGGGGGCCCCGGCCCNAATTCG

C



>gi|71812227|gb|DR989618.1|DR989618 HDI_L_67.esd Horsegram (VZM-1) drought stressed subtracted cDNA Macrotyloma uniflorum cDNA, mRNA sequence

TGGAGCTCACGCGGTGGCGGCGCTCTAAACTAGTGGATCCCCCGGGCTGCAGGATCGGCACGAGGGTTGA

ATTGAATGGTAGACTGAACAAATGTGGGGTTATTAGTCCCCGTTTTGATGTGGGTGTCAAAGAGATTGAA

GGTTGGACTGCTAGATTGCTCCCCTCAAGACAGTTTGGGTATATTGTGTTGACTACCTCTGCCGGCATCA

TGGATCATGAAGAAGCTAGGAGAAAGAATGTTGGTGGGAAAGTGCTGGGTTTCTTTTACTAGTCTATTTT

CCTTTGAAAGATTTTGTGCGGATTTCAGTTTTTTTTTTAAGTTATTTTTTTTAACTTACTGCTGTAATGA

TCAATGTTAGACACTTAGTTTATTTAAGTTATAGCATAGCCAGATGTTTTTCCTGATGTTGCTTAAAAAA

AAAAAAAAAAAAAACAAAAAAAA



>gi|71812225|gb|DR989616.1|DR989616 HDI_L_59.esd Horsegram (VZM-1) drought stressed subtracted cDNA Macrotyloma uniflorum cDNA, mRNA sequence

AAGCTGGAGCTCACGCGGTGGCGGCGTTCTAGAACTAGTGGATCCCCCGGGCTGCAGGATTCGATATCAA

GCTTATCGATACCGTCGACTCGAGGGGGGGCCCGGTACCCAATTCGCCCTATAGTGAGTCGTATTACAAT

TCACTGGCCGTCGTTTTACAACG



>gi|71812223|gb|DR989614.1|DR989614 HDI_L_57.esd Horsegram (VZM-1) drought stressed subtracted cDNA Macrotyloma uniflorum cDNA, mRNA sequence

ACNCCCCGGGCTGCAGGATCGGCACGAGGCTTGGATATAATGTTTAGGGATTAAAATTATGTTTTTCAAA

TTTAAAAGATAAAAAATTCTTAAATTTAGAAATTAATATTAGAATTATTTTAAATTTTAAGAAATGAAAA

TATAATTAACTTTTTTTCTTATGGATAATGTGAATTGTAATATTATTAATTTGGGGGTGGTATAATGAAA

GGATTGGTGGGAGAAAAAAAAAAAAAAAAAAAAAAAAAAAAAAAAAACCGGGGGGG



>gi|71812221|gb|DR989612.1|DR989612 HDI_L_55.esd Horsegram (VZM-1) drought stressed subtracted cDNA Macrotyloma uniflorum cDNA, mRNA sequence

AAAGCTGGAGCTCACGCGGTGGCGGCGCTCTAGAACTAGTGGATCCCCCGGGCTGCAGGATCGGCACGAG

GGGGTGCTAGGGTTGTTTAGGGTTAGTGTTGTGAAGTTTATTGTCCTTTCGGTGGGGTGTAGTTTACAGA

TGTTCCTATAACATATGAAATCGATTTTAAGAAAAAAAAAAAAAAAAAACCNCNAGGGGGGGCCCGGTTA

CCCCAATTTG



>gi|71812219|gb|DR989610.1|DR989610 HDI_L_47.esd Horsegram (VZM-1) drought stressed subtracted cDNA Macrotyloma uniflorum cDNA, mRNA sequence

AAGCTGGAGCTCACGCGGTGGCGGCGCTCTAGAACTAGTGGATCCCCCGGGCTGCAGGAATTCGGCACGA

GGATTGCATGTTTAGCTGAAAACGTATGTGCTTTTAAAATTTCCGAGCTCTAGAAAGTTGCTTTTAAAGT

TCCAATGATTCTGAGCTAATGTTTCTTCGTTAATTCCTTTCCTAGTTTCGGAAATGGTTCAATTCTCCCT

GTGTTAATAACCTAAACTAAGTGGGAAATAAAAGTTAGGTTTGATAAAAAAAAAAACAACAACAAAAAAA

AAAAAAACACACCGCGCGGGGGGGGCGCGCCGCGCG



>gi|71812217|gb|DR989608.1|DR989608 HDI_L_45.esd Horsegram (VZM-1) drought stressed subtracted cDNA Macrotyloma uniflorum cDNA, mRNA sequence

ACAAGCTGAGCTCACGCGGTGGCGGCCGNTCTAGAACTAGTGGATCCCCCGGGCTGCAGGATCGGCACGA

GGAAGTGACTCAACATAGCAGGCTATTTACCATCCTTTTTGTCTTTTTTCTAGTTACTTGGAACTTTGCC

TTGGTAATATTTTATTTAATAACTAGACTTTTTTGTGCTTCATTATAATTTCTATCAAATTATCATGGAT

TTGAATTTCATTAATTTATGAATTTTGATGTTTGAATTCAAAAAAAAAAAAAAAAANTTTAAAAAAAAAA

AAAAAACCCCCCGGGGGGGGNCCCGGGACCCCATC



>gi|71812215|gb|DR989606.1|DR989606 HDI_L_43.esd Horsegram (VZM-1) drought stressed subtracted cDNA Macrotyloma uniflorum cDNA, mRNA sequence

GGCTGAGCTCACGCGGTGGCGGCGCTCTAGAACTAGTGGATCCCCCGGGCTGCAGGAATCGGCACGAGGC

TTGGATATAATGTTTAGGGATTAAATTATGTTTTTTCAAATTTTAAAAGATAAAAAATTTCTTAATTAGA

AATATATAGAATATTTATTAGAAT



>gi|71812213|gb|DR989604.1|DR989604 HDI_L_35.esd Horsegram (VZM-1) drought stressed subtracted cDNA Macrotyloma uniflorum cDNA, mRNA sequence

AGCTGGAGCTCACGCGGTGGCGGCGCTCTAGAACTAGTGGATCCCCCGGGCCTGCAGGAATTCGATATCA

AGCTTATCGATACCGTCGACTCGAGGGGGGGCCCGGTACCCAATTCGCCCTATAGTGAGTCGTATTACAA

TTCACTGGCCGTCGTTTTACAAC



>gi|71812211|gb|DR989602.1|DR989602 HDI_L_33.esd Horsegram (VZM-1) drought stressed subtracted cDNA Macrotyloma uniflorum cDNA, mRNA sequence

CAAAGCTGGAAGCTCACCGCGGTTGGCGGCCGCTCTAGAACTAGTTGGATCCCCCGGGCTGCAGGAATTC

GGCACGAGGCCTCGTTGCCGGGGGGGGCCGGTACCCAATTCGCCCTATAGTGAGTCGTATTACAATTCAC

TGGCCGTCGTTTTACAAC



>gi|71812209|gb|DR989600.1|DR989600 HDI_L_31.esd Horsegram (VZM-1) drought stressed subtracted cDNA Macrotyloma uniflorum cDNA, mRNA sequence

AAGCTGGAGCTCACGCGGTGGCGGCGCTCTAGAACTAGTGGATCCCCCGGGCTGCAGGATCGGCACGAGG

CCCATGAGTATCTACACTTCTGAAATCTATATATCAGTCACTACTACTTGTTTTGCTCTAGTCACTTCCA

CTGTTCAATGTATTGTTTATAATTTGTTGTTGTTGCAATCCCAAGGTTGTTCTGGGGTTTAACTTGTACG

TACTTGATGTGTCATTGGATTACATGTACTGTTCAGTTTATATGTTGTTTCAGTCAGTTTTACTACTGCA

TATTATTTATTGTGACCTGAGATATTGTATCAGACTTCTTGTCTTCTAGTTCAATGAACTCTTGAATTTC

TTTGTGGTACAAAAAAAAAACAAAAAAAAA



>gi|71812207|gb|DR989598.1|DR989598 HDI_L_23.esd Horsegram (VZM-1) drought stressed subtracted cDNA Macrotyloma uniflorum cDNA, mRNA sequence

AAGCTGGAGCTCACGCGGTGGCGGCGCTCTAGAACTAGTGGATCCCCCGGGCTGCAGGATTCGGCACGAG

GCATTATTCTCCTTTCTGTTTGCATTTTGATTGATTTGTCAAATCTTTTTAACAAAATGGTGCAATTTTT

TTAAATTTCCGGAGAATAGATAAGTCGTGTCTGCAAAGCACGACATTTAAGTGTAAATCGTGTTTGCAAA

TTGATGTACTAGGTCTTCCTACATTTTTTTGGTTTCTAAAAAAAAAAAAAAAAAAAAAAAAAAAAAAAAA

AAAAAAAAAAAAAAAAAAAA



>gi|71812205|gb|DR989596.1|DR989596 HDI_L_21.esd Horsegram (VZM-1) drought stressed subtracted cDNA Macrotyloma uniflorum cDNA, mRNA sequence

AAGCTGAGCTCACGCGGTGGCGGCGCTTCTAGAACTAGTGGATCCCCCGGGCTGCAGGAATCGGCACGAG

GGCTCTATCCTTCGAGTGAGAAGGGTTGACTTTCAGAGGAATTCCCAACAGTATCTCCATCTAAGGCTCT

ATGCTTGCTGCATTACTGTGCCTGTTATCAATAAATGTGTTATAGAAGGAATCTGATTCCTTCATCCACT

TATGTATGCTTAAGTTATGTATCTCCAGTGGTTGAATGTTCTCGTGTGTTTGTTATTAATAAAGAATTTG

TGTTTGTA



>gi|71812203|gb|DR989594.1|DR989594 HDI_L_19.esd Horsegram (VZM-1) drought stressed subtracted cDNA Macrotyloma uniflorum cDNA, mRNA sequence

GAGCCACGCGGTGGCGGCGCTCTAAACTAGTGGATCCCCCGGGCTGCAGGATCGGCACGAGGCCGGAAAC

AGACCGCTGGAGTAACAGTAGTAGCCGGCCGGATGGTGACAGGTGGCGTGGTGATGAAAGGAGAAGTGGG

TTTGGTGGTTCTGGTGGAGGTTCAAGGTCATCTTCTACTTGGTCATATTCCAGGAATGGGCGTTGATCAT

TTTGTGCCCTCTGATTTATTGCTGGTGACTTTTTTGGAATTCTCTCTGTACTCTTTCCAGTTTACGTCCT

AGAATTCTTTTTGATGCTGAGTTGTTAAATTTATTATCTTAATACTCACAAACAAGTCCTCTATTGGTTT

GAGGAACAAAAAAAAAAAAAAAA



>gi|71812201|gb|DR989592.1|DR989592 HDI_L_11.esd Horsegram (VZM-1) drought stressed subtracted cDNA Macrotyloma uniflorum cDNA, mRNA sequence

AGCTGGAGCTCACGCGGTGCGCGGCGCTCTAGAACTAGTGGATCCCCCGGGCTGCAGGATCGGCACGAGG

AAACATTCTCCCTCCTATTAGGTTTATTCATCACGGATTCAAATCAAAAAAAAAAAAAAAAAAAAAAAAA

AAAAAAAAAAAAAAAAAAAAAAAAAAAAAAAAAAAAAAAAAAAAAAAAAAAAAAAAAAAAAAAAAAAAAA

AAAAAAAAAAAA



>gi|71812199|gb|DR989590.1|DR989590 HDI_L_90.esd Horsegram (VZM-1) drought stressed subtracted cDNA Macrotyloma uniflorum cDNA, mRNA sequence

AAACGCTGGAGCTCACCGTCGGTGGCGGCCGNTCTAGAACTAGTGGATCCCCCGGGCTGCAGGATTCGGG

CCACGCAGGCCTCGTGCCGAATTCGGCCGAGGCCTCGTGCCGTGCATGCATCGTGCATGATGGTAACATC

GGTAAACAATGGTTTGNATTGAACTTGTTGGCAGTGCAAGTGAGTTTAGTTGACCAGAGA



>gi|71812197|gb|DR989588.1|DR989588 HDI_L_88.esd Horsegram (VZM-1) drought stressed subtracted cDNA Macrotyloma uniflorum cDNA, mRNA sequence

AACAAGCCGAGGCTCACCTCGGGGCGTGCGCGCATATAGTCAGTAGGTCCCCTCCGGNACGAGGTCTTGC

AGGCGCCGTCGCATAGCATGCATGGGAAGATCATTTAGTAGGATCTGTGCATGATGTAATGTT



>gi|71812195|gb|DR989586.1|DR989586 HDI_L_86.esd Horsegram (VZM-1) drought stressed subtracted cDNA Macrotyloma uniflorum cDNA, mRNA sequence

AAGCTGGAGCTCACGCGGTGGCGGCGCTCTAGAACTAGTGGATCCCCCGGGCTGCAGGATTCGGCACGAG

GCCTCGTGCCGAATTCGGCACGAGGTAGCGGCCTATTGGCACTATTATTTTGCCTGGCTTGCTGTCACCA

CTGGCAATTGCTTCTCTCTGGAATTAACTATAAGAAATCAGTCCAAGTAAGGCTGCCGCTGTGATGTCTA

GTTTGTGTATAATATTTTGGAGTATGGGGAGAAGTTTAATTCAAGATAACGCCCTATATACTCCGTAGTC

TATATCAGCTTGTAACCTGTCAGTTCTATTTCCCCATATTGTAAAGCAGATGTGCTTTCTTTAAATACAA

GTTTTAAGAAAACCAGCTAAAAAAAAAAAAAAAAAAAAAAAAAAAAAAAAAAAAAAAA



>gi|71812193|gb|DR989584.1|DR989584 HDI_L_78.esd Horsegram (VZM-1) drought stressed subtracted cDNA Macrotyloma uniflorum cDNA, mRNA sequence

AAGCTGGAGCTCACGCGGTGGCGGCCGCTCTAGAACTAGTGGATCCCCCGGGCTGCAGGAATTCGGCACG

AGGCCTCGTGCCGAATTCGGCACGAGGGGGGGGGCCCGGTACCCAATTCGCCCTATAGTGAGTCGTATTA

CAATTCACTGGCCGTCGTTTTACAAC



>gi|71812191|gb|DR989582.1|DR989582 HDI_L_76.esd Horsegram (VZM-1) drought stressed subtracted cDNA Macrotyloma uniflorum cDNA, mRNA sequence

AAAGCTGGAGCTCACGCGGTTGGCGGCGCTCTAGAACTAGTGGATCCCCCGGGCTGCAGGATCGGCACGA

GGCCTCGTGCCGAATTCGGCACGAGGGTTATAGTAAATCGTTGGGTACTTCCTATGTGACAGTTTATAAA

AATAGTGAATTGTAGTGCATTACTGGATTTACAATAAAAAAAAAAAAAAAAAAAAAAAAAAAAAAAAAAA

AAAAAAAAAA



>gi|71812189|gb|DR989580.1|DR989580 HDI_L_74.esd Horsegram (VZM-1) drought stressed subtracted cDNA Macrotyloma uniflorum cDNA, mRNA sequence

AGCTGGAGCTCACGCGGTGGCGGCGCTCTAGAACTAGTGGATCCCCCGGGCTGCAGGATCGGCACGAGGT

TCATATTTTCACTTGTTTAGTGAAAGTCTGTTATTCGAGATATATATATGTAGATAAGTAAAGGTGTGTG

TTGTGTAGCTTGGTGCACAAGCTTGAAGAGAGTTGTGAAGTGTAGGATAGTATTTAGGGCTATGCAGTGT

TCTGTGTCCTAAGTGATCAAATGAACATGAGTTTTTACTGTTTTTAATGGTTGATCTTTCTATTGCCTGT

TTTTTTCANAGTAGTTATGGGACGAATTTCTCTGGAACATGGAGATAATTAATGCTAATTTTTATTTTTA

AAAAACAAAAAACAAAAAAAACCCCANGGGGGGC



>gi|71812187|gb|DR989578.1|DR989578 HDI_L_66.esd Horsegram (VZM-1) drought stressed subtracted cDNA Macrotyloma uniflorum cDNA, mRNA sequence

AAGCTGGAGCTCACGCGGTGGCGGCGCTCTAGAACTAGTGGATCCCCCGGGCTGCAGGATCGGCACGAGG

GTTATCCACCACTAGTTACGTGAGTAAGTAGAAGTTCAAAGATTAGCTCTTATATAATAAATTTTCCTGC

CCCATGTTTGAGGGCACTAGTGTGCCGTGTATGTCTAACATCATTTGACTTGTTAGAAGGCAACTGGTGT

TAGACAAGTAAGTAAATGAAGCTGAATTCTGTTGTGCAAGTAGTATGAGGAAAACATAGGTGAGTTAATG

AAAGAAATTTTACAATGAGTTCTGTACAATCAATGATGGCTATAGTGGATTGTTATATCTCTATAAAAAA

AAAAAAAAAAAA



>gi|71812185|gb|DR989576.1|DR989576 HDI_L_64.esd Horsegram (VZM-1) drought stressed subtracted cDNA Macrotyloma uniflorum cDNA, mRNA sequence

AGCTGGAGCCACCGCGGGGCGGCGCTCTAGAACTAGTTGGATCCCCCGGGCTGCAGGAATCGGCACGAGG

TGTCTACTAAATAAAAATACAAAAAGCCCACCACCAAGTAATGCTGGGTTCATATGGGTTATTTGTATAT

GTTCCTTCTAGTGAGTGGTGTGTGGTTGTGAAGAATCTATTGCTGTTAATTATAATGAATGAGATTTCTG

GTATGCAAAAAAAAAAAAAAAAAAACCCCGGGGGGGNCC



>gi|71812183|gb|DR989574.1|DR989574 HDI_L_62.esd Horsegram (VZM-1) drought stressed subtracted cDNA Macrotyloma uniflorum cDNA, mRNA sequence

AAGCTGGAGCTCACCGCGGTGCGCGGCCGTTCTAGCAAACTAGTGGATCCCCCGGGCTGCAGGATCGGCA

CGAGGGTTGTGTGTATGTGATGATGACAATTCGTCTTTTCCTTTGTAATTTACTATTTATTTATTATTAT

TATTATTATTTCTTTGGTTTGTATGAGATGAGATCAGATTATGACCGTTATTTAATTCCATATTCATATG

CTGCTGCCAAAAAAAAAAAAAAAAAAAAAAAAAAAAAA



>gi|71812181|gb|DR989572.1|DR989572 HDI_L_6.esd Horsegram (VZM-1) drought stressed subtracted cDNA Macrotyloma uniflorum cDNA, mRNA sequence

TAGCTGGAGCTCACGCGGTGCCGGCGTTCTAGAACTAGTGGATCCCCCGGGCTGCAGGATCGGCACGAGG

ATTGTTAAGAGGGTATTACACCAAAGATCAATGAGACCGACCGGTTCTTTAGAATGCTGAAAGCCATAGT

TTTGTAACTTTTGTTAGCAAGTATTTGTTAATATATCAAAGCTTGTGCACACTGTATTGAAGGTTGAAGA

TGGACATGGCCATNAATTCAGTTGTAGAGTTGTATATTGTCTTTTAAATATGATATAATTTTACTTTTGA

AAAAAAAAAAAAAAAAAAAACCCCAGGGGGGGGCCC



>gi|71812179|gb|DR989570.1|DR989570 HDI_L_53.esd Horsegram (VZM-1) drought stressed subtracted cDNA Macrotyloma uniflorum cDNA, mRNA sequence

GCTGGAGCTCACGCGGTGGCGGCGCTCTAGAACTAGTGGATCCCCCGGGCTGCAGGATCGGCACGAGGCC

TCGTGCCGAATTCGGCACGAGGGAAATTATTGCATTTGGATAATGATGAAGCTGTGTTTCTTCAATAAGT

AGACACCACTGGTTTTGAAGTGCAACATTGATGCCAGTTTGAAGATTTTGAGTTTTTTCAAGGTCTTTGT

ATTTAAATTCCATGTTAAACTGGAAGTTGAGGCTATCAATTTGTTTAAATTTTCTTGTCAAGTTTCCAAC

CATTTATTCTTATATTATATTATAACTGAAATTCCACGACCTAAAAAAAAAAAAAAAAAA



>gi|71812177|gb|DR989568.1|DR989568 HDI_L_51.esd Horsegram (VZM-1) drought stressed subtracted cDNA Macrotyloma uniflorum cDNA, mRNA sequence

AAGCTGGAGCTCACGCGGTGCGCGGCGCTCTAAACTAGTGGATCCCCCGGGCTGCAGGATCGGCACGAGG

CCGGAAACAGACCGCTGGAGTAACAGTAGTAGCCGGCCGGATGGTGACAGGTGGCGTGGTGATGAAAGGA

GAAGTGGGTTTGGTGGTTCTGGTGGAGGTTCAAGGTCATCTTCTACTTGGTCATATTCCAGGAATGGGCG

TTGATCATTTTGTGCCCTCTGATTTATTGCTGGTGACTTTTTTGGAATTCTCTCTGTACTCTTTCCAGTT

TACGTCCTAGAATTCTTTTTGATGCTGAGTTGTTAAATTTATTATCTTAATACTCACAAACAAGTCCTCT

ATTGGTTTGAGGAACAAAAAAAAAAAAAAAAACCCCNGGGGGGGCC



>gi|71812175|gb|DR989566.1|DR989566 HDI_L_5.esd Horsegram (VZM-1) drought stressed subtracted cDNA Macrotyloma uniflorum cDNA, mRNA sequence

GCTGAGCTCACGCGGTGCGCGGCGTTCTAGAACTAGTGGATCCCCCGGGCTGCAGGATCGGCACGAGGGT

TGAATTGAATGGTAGACTGAACAAATGTGGGGTTATTAGTCCCCGTTTTGATGTGGGTGTCAAAGAGATT

GAAGGTTGGACTGCTAGATTGCTCCCCTCAAGACAGTTTGGGTATATTGTGTTGACTACCTCTGCCGGCA

TCATGGATCATGAAGAAGCTAGGAGAAAGAATGTTGGTGGGAAAGTGCTGGGTTTCTTTTACTAGTCTAT

TTTCCTTCTGAAAGATTTTCGTGCGGATTTCAGTTTC



>gi|71812173|gb|DR989564.1|DR989564 HDI_L_42.esd Horsegram (VZM-1) drought stressed subtracted cDNA Macrotyloma uniflorum cDNA, mRNA sequence

AAGCTGGAGCTCACGCGGTGGCGGCGCTCTAGAACTAGTGGATCCCCCGGGCTGCAGGAATTCGGCACGA

GGCAGATATATTGATTAATCTCTGAAATTTTTCTTACCTGCAGGCTGCAGTGAGACTCAACAAAATCTCC

AAACTCGCTACTTCCTATCCTTTCAAAAAAAGTAACTGGCTAAGTGATCTGAGCAGTATAGGCAGCTAGA

TCCCTCGGAACAAAACCCAAAAAAAAAAAAAACCNCGGGGGGGGNCC



>gi|71812171|gb|DR989562.1|DR989562 HDI_L_40.esd Horsegram (VZM-1) drought stressed subtracted cDNA Macrotyloma uniflorum cDNA, mRNA sequence

AAAGCTGGAGCTCACCGCGGTTGGCGGCCGCTCTAGAAACTAGTGGATCCCCCGGGCTGCAGGAATTCGG

CACGAGGGTGGCCATGTCTTCCCTNAGCAAGTTCAAGTGTTATGGACTTGTGATGGGTTTATCTAGCCAT

GGTTACTCTTTTTTTTTAAAAAAAAAAAAAAAAAAAAAAAAAAAAAAAAAAAAAAAAAAAAAAAAAAAAA

AAAAAAAAAAGGGGGGGGGGGGGG



>gi|71812169|gb|DR989560.1|DR989560 HDI_L_39.esd Horsegram (VZM-1) drought stressed subtracted cDNA Macrotyloma uniflorum cDNA, mRNA sequence

GCTGGAGCTCACCGCGGGTGGCGGCGCTCTAGAAACTAGTGGATCCCCCGGGCTGCAGGATCGGCACGAG

GCTTAGATGAAGTGCTGAGGACTTATATTCAGATAAATAAAATGAAGGATATATGTTTATCTTCTTCAAA

AAAAAAAAAAAAAAAAAAAAAAAAAAAAAAAAAAAAAAAAAAAAAAAAAAAAAAAAAAAAAAAAAAGGGG

GGGGGGGGG



>gi|71812167|gb|DR989558.1|DR989558 HDI_L_37.esd Horsegram (VZM-1) drought stressed subtracted cDNA Macrotyloma uniflorum cDNA, mRNA sequence

AAGCTGGAGCTCACGCGGTGGCGGCGCTCTAGAACTAGTGGATCCCCCGGGCTGCAGGATTCGGCACGAG

GCCTCGTGCCGAATTCGGCACGAGGCCTTCCCAAGGTGTTAAGTGTTAAATCTGAAGAGGGTTTCTCTGC

CTGATTATGCATGATCTTGATCTCGATCCAAGTGTTCTGGCAATAGACTAGAGAGTGAAAGAAAATATAT

ATATGTGGTATCATCATATATGGTGTGGTTTAACGTCTCTATGTTTTAATGTTAGTAAAAGTACTAGTCC

TAATGGATATGTGATGCTTTGGTTTCATGTCATTCCATTTCAGTTCCTTGGTGTGGTCTCATCTTACGTA

ACGTTAGTTGTTGCAAAAGGCTGTATTTATAATTCAAAGAAGTATCTCATCATGTTTCCTTCTTAAAAAA

AAAAAAAAAAAAAA



>gi|71812165|gb|DR989556.1|DR989556 HDI_L_3.esd Horsegram (VZM-1) drought stressed subtracted cDNA Macrotyloma uniflorum cDNA, mRNA sequence

TATAGCTGGAGCTCACCGCGGTCGCGGCCGTTCTAGAACTAGTGGATCCCCCGNCGCTGCAGTGTAATTC

GGCACGAGGATTTCTCTGCCTAAACTTGACTTGTATGCACGATGCTAAAATCCATTTTGTTTGCTTCACC

ATTCTGTCTATCAACATAGGTTTTCAAATTAAGTCTAATCACCGGATTTCCAGTTATTGCACAGAATATT

TGTATAATCATATTCATTGTTAATGTAAATTGTGATATGACATCAACACCAATGTTTAATTTTCCTGGTA

AAGTTAT



>gi|71812163|gb|DR989554.1|DR989554 HDI_L_28.esd Horsegram (VZM-1) drought stressed subtracted cDNA Macrotyloma uniflorum cDNA, mRNA sequence

AAAGCTGGAGCTCACGCGGTGGCGGCCGCTCTAGAACTAGTTGGATCCCCCGGGCTGCAGGAATTCGATA

TCAAGCTTATCGATACCGTCGACCTCGAGGGGGGGCCCGGTACCCAATTCGCCCTATAGTGAGTCGTATT

ACAATTCACTGGCCGTCGTTTTACAACGA



>gi|71812161|gb|DR989552.1|DR989552 HDI_L_26.esd Horsegram (VZM-1) drought stressed subtracted cDNA Macrotyloma uniflorum cDNA, mRNA sequence

ANAAGCTGAGCCACGCGGGCCGGCCGTTCATAGAACTAGTGGATCCCCGGGCCTGCAGGATCGGCACGAG

GGGGATATTTAGTTCGTCTCAGCATGATGATGTTATAGAAACTTCATATATCTAGTCTGAGGAGGCTAGA

TCTTGGAGGCTATTGCATTTGGATCCTTCCTGTGGGAAACAGAAGATTATATTGACAGTATATATAAGCT

TAAAGATCAGGAGGTAGAANAGTAAAGGAACATGTTGCTCAANGTAAATTTCCTCAATCTTAGGCCAATT

ACTCTATTGTATATACAACGGTCGGTTC



>gi|71812159|gb|DR989550.1|DR989550 HDI_L_2.esd Horsegram (VZM-1) drought stressed subtracted cDNA Macrotyloma uniflorum cDNA, mRNA sequence

AAGCGGAGCCACGCGGTGCCGGCGTTCTAAACTAGTGGATCCCCCGGGCTGCAGGATCGGCACGAGGTTC

TCTTGAGGGGTTCATGGAATCTGGTGGCATCTGAGTTTCAGATAACAAAAAATTATCATATGAAATATAG

GTTTTTTAGTTAATTTTTACGTGACATATGCTCAAAATTTCATTAAATTTATGAAAAATAATGTCATGTT

GAAAATTAATTAGGACTTGGATGGTTTAAAACACAAAAAAAAAAAAAAAAAAAAACACCCCGGGGGGGGG

CCCGCGACCCC



>gi|71812157|gb|DR989548.1|DR989548 HDI_L_16.esd Horsegram (VZM-1) drought stressed subtracted cDNA Macrotyloma uniflorum cDNA, mRNA sequence

GCTGGAGCTCACGCGGTGGCGGCGCTCTAGAACTAGTGGATCCCCCGGGCTGCAGGATTCGGCACGAGGC

CTCGTGCCGAATTCGGCACGAGGGGGGGGGCCCGGTACCCAATTCGCCCTATAGTGAGTCGTATTACAAT

TCACTGGCCGTCGTTTTACAAC



>gi|71812155|gb|DR989546.1|DR989546 HDI_L_14.esd Horsegram (VZM-1) drought stressed subtracted cDNA Macrotyloma uniflorum cDNA, mRNA sequence

AGCTGGAGCTCACGCGGTGCGCGGCGTTCTAGAAACTAGTGGATCCCCCGGGCTGCAGGAATCGGCACGA

GGATAATCTTAGTTATTCCTAATGAGAGTTAGACATGTTGCTTTGAGTTCAGTTATCTGTATTGAATGAA

AATGTCTGTAGACAGATGCACTGGACTTCTGTTGAGCCTATGTGATCTCGTGTTTTCCACTCCATTCTCT

GATATGAATCTCAATTTCTTTAAAAA



>gi|71812153|gb|DR989544.1|DR989544 HDI_L_1.esd Horsegram (VZM-1) drought stressed subtracted cDNA Macrotyloma uniflorum cDNA, mRNA sequence

GGCTGAGCTCACGCGGTGGCGGCGCTCTAGAACTAGTGGATCCCCCGGGGCTGCAGGAATTCGGCACGAG

GCCTCGTGCCGAATTCGGCACGAGGCGAAATTGTTGGTACTTGGGTTCACATGATGTATAAAATAAGCTA

GAAGTGTTGTAAATCGTACAAGAATATTATGAACTGAATTATGTGTATCATATACATAATAATGATCATA

AATTTTTAAAAAAAAAAAAAAAAAACCCCANGGGGGGGCCCCGGTCCCCAATTCCGC



>gi|71812151|gb|DR989542.1|DR989542 HDI_K_93.esd Horsegram (VZM-1) drought stressed subtracted cDNA Macrotyloma uniflorum cDNA, mRNA sequence

GGCTGGAGCTCACGCGGTGGCGGCGCTCTAGAACTAGTGGATCCCCCGGGCTGCAGGAATTCGGCACGAG

GCCTCGTGCCGAATTCGGCACGAGGGACATCCAGCTATTAAACACAAGGGATATACCGCATTTGTGGTTG

TGTGATTTGATACTGGACTAGGGTAGTTTGTATAAATATTTATGTTGTGAATTGTATCGTTTGGTACTAT

TAAAAGTCATCTCCACATAGGATCAGTAAACTTCTAAGATCATGCTGTTTATGTAGTATGTAATNCTGTA

TGCTTGTCAGTCCAGTGCCTTGTTCTTTGTTATGGCAACTTGGAATTGGAAATACTTAAGTATAAAGTTT

TATTACCTTCTATTAACAAAAAAAAAACAAAAAAAAAAAAAAAAAAAAAAAAAAAAAAAAAAAAA



>gi|71812149|gb|DR989540.1|DR989540 HDI_K_91.esd Horsegram (VZM-1) drought stressed subtracted cDNA Macrotyloma uniflorum cDNA, mRNA sequence

AGCTGGAGCTCACGCGGTTGGCGGCGCTCTAGAACTAGTGGATCCCCCGGGCTGCAGGAATTCGGCACGA

GGCCTCGTTGCCGAATTCGGCACGAGGGTAAGGAATAATTCTCTACTATAATAGTATATTTTGTGCTGTC

CACGTCTATAATGAGTCAATGTCTATGTATTGTAATATTTTGTGCATGATTGAATATGTAACCACTTAGG

TCTTTAAAAAAAAAAAAAAAAAAAAAAAAAAAAAAAAAAAAAAA



>gi|71812147|gb|DR989538.1|DR989538 HDI_K_84.esd Horsegram (VZM-1) drought stressed subtracted cDNA Macrotyloma uniflorum cDNA, mRNA sequence

AGCTGGAGCTCACCGCGGTGCGCGGCGTTCTAGAACTAGTGGATCCCCCGGCGCTGCAGGATTCGGCACG

AGGATTATGTTAGCTTTTTAGAATTTCATGTTAGGTTCATTTATCAGTTCAGTCATAGACTCATGTAGTA

TTATGTCTTTTGTTTTCAGTTTTGATGGTTATAGTAAATCGTTGGGTACTTCCTATGTGACAGTTTATAA

AAATAGTGAATTGTAGTGCATTACTGGATTTACAATATATGATAAGGTTTCTGTGATGCTTTTTAAACTA

TACTAAAAAAAAAAAAAAAAAAAAAAA



>gi|71812145|gb|DR989536.1|DR989536 HDI_K_82.esd Horsegram (VZM-1) drought stressed subtracted cDNA Macrotyloma uniflorum cDNA, mRNA sequence

AGCTGGAGCTCACGCGGTGGCGGCCGCTCTAGAAACTAGTGGATCCCCCGGGCTGCAGGATTCGATATCA

AGCTTATCGATACCGTCGACTCGAGGGGGGGCCCGGTACCCAATTCGCCCTATAGTGAGTCGTATTACAA

TTCACTGGCCGTCGTTTTACAACGAGG



>gi|71812143|gb|DR989534.1|DR989534 HDI_K_80.esd Horsegram (VZM-1) drought stressed subtracted cDNA Macrotyloma uniflorum cDNA, mRNA sequence

AAGCTGGAGCTCACGCGGTGGCGGCGCTCTAGAACTAGTGGATCCCCCGGGCTGCAGGATCGGCACGAGG

CTTGAGTACAAGAAGGCTGAGAGGGCTAAGCTGGTGCAGCAGGGATACAACATTGTTGCAGTGGTTGGAG

ACCAATGGTCCGATCTGAGGGGAAAACCCAAGGGAACCAGGAACTTTAAGCTTCCCAACCCCATGTACTC

TATTGAGTAGATCATCTCTGAAGGATCCCAAGCTTGCTCCTATCTACGAAATAAGATACCTTACGTGTTC

TAATGGGAGCATTAACTATGTATCTATGCATGTAGACTTTCTGGTCTTCGGATCTTCAACAAATCACCTA

AGAGACTACACTCCGTTGTTTTCTCTTTTGTGACTTTAATAAATTATGAACCCTTTTTTCCAAAAAAAAA

AAA



>gi|71812141|gb|DR989532.1|DR989532 HDI_K_72.esd Horsegram (VZM-1) drought stressed subtracted cDNA Macrotyloma uniflorum cDNA, mRNA sequence

AAAGCTGGAGCTCACGCGGTGCGCGGCCGNTCTAGAACTAGTGGATCCCCCGGGCTGCAGGATTCGGCAC

GAGGCCTCGTGCCGAATTCGGCACGAGGAGACAATGTTGTAAATTCAATGTCCTATGTATAGAATAGGAT

ACTACGCATGTTGATGCCAGTCTATATAATATAGGACTTCTACCAATACAAGTTTTGTTCTGACTTAGTA

TTTTTTAAAAAAAAAAAAAAAAAAAAAAAAAAAAAAAAAAAAAAAAAAAAAAAANAAAAAAAAAAAAAAA

AAAA



>gi|71812139|gb|DR989530.1|DR989530 HDI_K_7.esd Horsegram (VZM-1) drought stressed subtracted cDNA Macrotyloma uniflorum cDNA, mRNA sequence

AAGCTGGAGCTCACGCGGTGGCGGCGCTCTAGAACTAGTGGATCCCCCGGGCTGCAGGATTCGGCACGAG

GTGAAAGTTTCAGTACACATTTGAAGACTATTCTATAGTCATGGATGGTATAAATAAATGTAGCTTTTGA

TGCTACATAGGTGGTGTACACATTTGAAGTTGAAGACTATTTTATATTTACGTCTGATATAAATAAATGT

ACTTGTTAACGTTACATATGGTGTAGATGGTGTACCCATTCTATTTTATACTTATGAATGATGTAAAAGA

GTGTACTTGTTAACACTACATACGGTGTGAATGGTATACATATTTGGCATTTTTATTTAAAAAAAAAAAA

AAAAACCCCANGGGGGGGCCCGGCCCCCAAAATTTCGCCCCATAATAGTTT



>gi|71812137|gb|DR989528.1|DR989528 HDI_K_67.esd Horsegram (VZM-1) drought stressed subtracted cDNA Macrotyloma uniflorum cDNA, mRNA sequence

AAGCTGGAGCTCACGCGGTGGCGGCGTCTCTAGAAACTAGTGGATCCCCCGGGCTGCAGGAATCGGCACG

AGGCCCGTTTTGATGTGGGTGTCAAAGAGATTGAAGGTTGGACTGCTAGATTGCTCCCCTCAAGACAGTT

TGGGTATATTGTGTTGACTACCTCTGCCGGCATCATGGATCATGAAGAAGCTAGGAGAAAGAATGTTGGT

GGGAAAGTGCTGGGTTTCTTTTACTAGTCTATTTTCCTTTGAAAGATTTTGTGCGGATTTCAGTTTTTTT

TTTAAGTTATTTTTTTTAACTTACTGCTGTAATGATCAATGTTANACACTTAGTTTATTAAGTATAGCAT

AGCAGATGTTTTCTGATG



>gi|71812135|gb|DR989526.1|DR989526 HDI_K_59.esd Horsegram (VZM-1) drought stressed subtracted cDNA Macrotyloma uniflorum cDNA, mRNA sequence

CGCTTGGACGCTCACCGGCGGGTGCGCGGCCGTTTATAGAAACTAGTGGATCCCCCGTGGGCTGCAGTGA

ATTCGGGCACGAGGGGACATATATCACTTTTTATTCCCACTTAGTGGTATCTTA



>gi|71812133|gb|DR989524.1|DR989524 HDI_K_57.esd Horsegram (VZM-1) drought stressed subtracted cDNA Macrotyloma uniflorum cDNA, mRNA sequence

ATAGCTGGAGCCACCGCGGTGCGCGGCCGTTCTAGAACTAGTGGATCCCCCGGGCTGCAGGAATTCGGCA

CGAGGCTGGTTTGGAAGACACGCTCATTCTCACCATGAAGGGTATTGCTGCTGGCCTGCAAAACACTGGT

TAAACTTCACTTTCTGTTTTCTTTTTTGTTCCTCTTCTATTTTCTGTTTTCTTATCAGTAATAATGAGTT

TAGGTGCATGCACCAAAAGGGTGTTTTCCCCCCTCCTTGTATCCATGTTTCCATCACAAGATACTACAAA

GAGATAGCCACTGCACAATGAGTCAATGCATGTATACAACTGATTATGTTCCAATACGAAATTATGTTTT

GATTACTAGTTAAAAAAAAAAAAAAAAACCCAAGGGGGGGCCCGG



>gi|71812131|gb|DR989522.1|DR989522 HDI_K_55.esd Horsegram (VZM-1) drought stressed subtracted cDNA Macrotyloma uniflorum cDNA, mRNA sequence

AAGCTGGAGCTCACGCGGTGCGCGGCCGCTTCTAGAACTAGTGGATCCCCCGGGCTGCAGGAATTCGGCA

CGAGGCTCATGTAGTATTATGTCTTTTGTTTTCAGTTTTGATGGTTATAGTTAAATCGTTGGGTACTTCC

TATGTTGACAGTTTATAAAAATAGTGAATGTTAGTTGCATTACTGGATTTACAATATATGATAAGGTTTC

TGAGAGCTTTTTAAACTAAAAAAAAAAAAAAAAAAAAAAAAAAAAAAAAAAAAAAAAAAAAAAAAAAAAA

AAAAAAAC



>gi|71812129|gb|DR989520.1|DR989520 HDI_K_47.esd Horsegram (VZM-1) drought stressed subtracted cDNA Macrotyloma uniflorum cDNA, mRNA sequence

AAAGCTGGAGCTCACGCGGTGGCGGCGCTCTAGAACTAGTGGATCCCCCGGGCTGCAGGAATTCGGCACG

AGGAGTAAAGTTTGTGAGTTCATTTCATGAGTTAATTCTTGCCCCTTCTTTATAAAAAAAAAAAAAAAAA

AAAAAAAAAAAAAAAAAAAAAAAAAAAAAAAAAAAAAAAAAAAAA



>gi|71812127|gb|DR989518.1|DR989518 HDI_K_45.esd Horsegram (VZM-1) drought stressed subtracted cDNA Macrotyloma uniflorum cDNA, mRNA sequence

AGCTGGAGCTCACGCGGTGGCGGCGCTCTAGAACTAGTGGATCCCCCGGGCTGCAGGATTCGGCACGAGG

GGTTATAACCGCAACGGTGGTGGTGGAGGTTATGGTGGCGGTGGTGGATACGGAGGTGGCGGTGGTGGTG

GTTATGGAGGCGGTAGAGACCGTGGATATGGTGGTGGTGACGGTGGTTCCCGTTACTCGAGGGGGGGCCC

GGTACCCAATTCGCCCTATATGAGTCGTATTACAATTCACTGGCCGTCGTTTTACAA



>gi|71812125|gb|DR989516.1|DR989516 HDI_K_43.esd Horsegram (VZM-1) drought stressed subtracted cDNA Macrotyloma uniflorum cDNA, mRNA sequence

AAAGGCTGGAGCTCCACCGCGGTGGCGGCCGCTCTAGAAACTAGTGGATCCCCCGGGCTTGCAGGAATTC

GGCACGAAGGCGCTCTGATTGCCTGAATTCTGTGCATCTGATGTGTGCTTATTTCGTAGAATGACTTTTG

TTTTGAATACCTGAATTATTTACATGATCTTACGTTATAAATACCTGAGTATNTACGTGGCTTTACTATC

AATAAATAGAGTAAATTAGTAGCTAT



>gi|71812123|gb|DR989514.1|DR989514 HDI_K_35.esd Horsegram (VZM-1) drought stressed subtracted cDNA Macrotyloma uniflorum cDNA, mRNA sequence

AGCTGGAGCTCACGCGGTGCGCGGCGNTCTAGAACTAGTGGATCCCCCGGGCTGCAGGATTCGGCACGAG

GCCTCGTGCCGAATTCGGCACGAGGGTTTGTTGGTTATTGCATTCGTTGTTCCTATGGCACAATACTATG

CTTATGTATCCAAAAAATAAAATAATATAAGTATATCCGTCTTTCAAATTCTCACTCTTTAAATTCAGAT

GTATAAGTGCAGTTGTAACCATGCTCATCTTACAATTGAGTTTGGGAAGGCTGAAGAATAAGATGAGAGT

TTTTTCATGTAAATGCAATTCCATAGTATATGCATTTTGATTATGTTATTAAAAAAAAAACACAAAAAAA

AAAAAAAAAAACCCCANGGGGGGCC



>gi|71812121|gb|DR989512.1|DR989512 HDI_K_33.esd Horsegram (VZM-1) drought stressed subtracted cDNA Macrotyloma uniflorum cDNA, mRNA sequence

AACTGAGCTCACCGCGGTGGCGGCGCTCTAGAACTAGTGGATCCCCCGGGCTGCAGGATTCGGCACGAGG

CCTCGTGCCGAATTCGGCACGAGGCTTTTGATTACTTGGAGAATTCTCTTGGTAAATTTGATGATGGGCC

ATTCTTTCTCGGTCAATTTAGCTGGGTGGATATTGCCTATGTTCCATTTGTTGAAAGATTCCAGATCGTC

TTTTTTGAGGTGTTCAAACATGATATAACTGAAGGAAGACCTAAACTTGCAGCATGGATTGAGGAAGTGA

ACAAGATTAATGCTTATACACAGACAAGAGCTGATCCCAAGGAAATTGTTGATATTTTCAAGAAACGTTT

TTTGGCTCAACAGTGAAAACCGGTTGCAGTGGGCGTCCTGTAAGATGTAGATTATTGACAATAAAGTGTT

ATATGTGTTCATGGGATGCTTCTTTTGTCTGCTAGAGTCGACAATAAAACTTTGATTTGGTTGCTAGAGT

TGCTGCAAATAATTTAATTTCATGGTCGGTGGTGTGTATGTACCAGCTTGTCTCATGTTTGTGCGCTTTT

AATACTGAGTTGCATGGTATTATCATCATGATTCATTATGTTTGTATATGTATCTCCAAACCTTAAGTTA

AATTTAATTTGGTCCTTTTTTTTTATTGGAACCCCATGGGTATTTNGAAATTTGAAAGAAAATTCCTGGT

TGGGGCGGTTTACAAAACAAAAAC



>gi|71812119|gb|DR989510.1|DR989510 HDI_K_22.esd Horsegram (VZM-1) drought stressed subtracted cDNA Macrotyloma uniflorum cDNA, mRNA sequence

AAAGCTGGAGCTCACCGCGGTGCGCGGCGCTCTAGAAACTAGTGGATCCCCCGGGCTGCAGGAATTCGGC

ACGAGGATCCGTAAATTCCTCGACGGCATCTATGTCTCCGAAAAGGGCCACGTTGTTGCTGAGGCTTAAG

GAATCAGCCGAGCCAATAAACAGTGTAAAAAAAAAAAAAAAAAAAAAAAACCCTCAGGGGGGGGCCCGGG

TCCCCAAATTC



>gi|71812117|gb|DR989508.1|DR989508 HDI_K_20.esd Horsegram (VZM-1) drought stressed subtracted cDNA Macrotyloma uniflorum cDNA, mRNA sequence

AAGCTGGAGCTCACGCGGTGGCGGCGCTCTAGAAACTAGTGGATCCCCCGGGCTGCAGGAATTCGGCACG

AGGCCTCGTTGCCGAATTCGGACACGAGGATAAAGTGGACTCATGATCTTCCCATNACCAAGCTTCAAGA

TGCTTATGGCACTTNATGCATGAGGTTTATCTTATACCAATGGCTTACTCTTTTTATTTATATAACGTAA

TNTGTGTTTGTCTCAGTTTGTTAAAG



>gi|71812115|gb|DR989506.1|DR989506 HDI_K_12.esd Horsegram (VZM-1) drought stressed subtracted cDNA Macrotyloma uniflorum cDNA, mRNA sequence

AAGCTGGAGCTCACGCGGTGGCGGCGCTCTAGAACTAGTGGATCCCCCGGGCTGCAGGAATTCGGCACGA

GGGAAGAAAGGAACCCCTCTCCGCCCATGAATTGCACTATGCGAGAAAGAAAGCGGAATCAGAGGATTTG

AAGAAGAGAACGTATTTGCCATACAAACAGGGAATCTTGGGCAGGTTGGCTGGCTTCGGATCGAGAATGA

ATTACCCAGCTATCGAAAGGGAATCCTTTTTTTTTTTTTTTGTGCGTTGAAACAAATTTGATATTCTTTT

GTGTTCTCTTTGTGTTATAATTTTTAATATATACTCTTTTTGTGTGTATTGTGCTTATCAAAAAAAAACA

AAAAAACACCGCAGAGGGGGC



>gi|71812113|gb|DR989504.1|DR989504 HDI_K_10.esd Horsegram (VZM-1) drought stressed subtracted cDNA Macrotyloma uniflorum cDNA, mRNA sequence

AGAAACTAGTGGATCCCCCGGCGCTGCAGGATTCGGCACGAGGATCGTCAATAATTTTATTTTATTATAC

GTATCCCAGTAAAGTGGGAATAATTTCTCTTAAAAGTATAAGTCAAATAATATTATGGGAAAATGTATTG

CTTATTTGCATCTGGTTTATTTCTTTGTACCGATATGATATAAGCAAATAAGAATACAACAGAAGAAAAG

ACAAAGCCGTATGTTGCTGGTCAAAAAAAAAAAAAAAAAAAAAAAAAAAAAAAAAAA



>gi|71812111|gb|DR989502.1|DR989502 HDI_K_89.esd Horsegram (VZM-1) drought stressed subtracted cDNA Macrotyloma uniflorum cDNA, mRNA sequence

AAGCTGGAGCTCACGCGGTGGCGGCGCTCTAGAACTAGTGGATCCCCCGGGCTGCAGGATTCGGCACGAG

GCCTCGTGCCGAATTCGGCACGAGGCTTCTAGCAAATTGGCAAAGAGAGTACACCATGGAGGATATACTG

ACCCAGCTGAAGAAGGAAATGGCGGCTCCTCATAACCGGAAGCTTGTCCAGCCCCCAGAAGGTACCTACT

TTTAGCATTGAAGATGGTATATATGTCAGATGGTATTGCATATGCTGTATGCAATATATAGTGTGTAATG

CTTTGTGGATCCTTCCTTCTGAATTAAAGAACATATAGGGGAATGCCCCAAAAGATATTTCCATCTCCAT

GGCTTCAGAGTTGTTTTGTTGTCTTTGTCACTGTGCTGAAGGGGGACTGTTTGATTAAAATATTGCGTGT

CAAATATTTCGAAAGCAACTCTTTAATTTAAAAACAAAAAAAAAAAAAACCCCGGGGGGG



>gi|71812109|gb|DR989500.1|DR989500 HDI_K_87.esd Horsegram (VZM-1) drought stressed subtracted cDNA Macrotyloma uniflorum cDNA, mRNA sequence

AAAGCTGGAGCTCACGCGGTGGCGGCGCTTCTAGAAACTAGTGGATCCCCCGGGCTGCAGGAATCGGCAC

GAGGATTTCATGTTATTTTGTCTTCTATTAGGCTGTTCTGCATGATGATAATGAAAACACTATATGTATG

TATGTACTTATCCGAGAAGAAAAAACGAGGCTGAGAATTTATTCAGTGTGTTGATAAAAAAAAAAAAAAA

AAAAAAAAAAAAAAAAAAAAAAAAAAAAAAAACCGGGGGGGGGGG



>gi|71812107|gb|DR989498.1|DR989498 HDI_K_85.esd Horsegram (VZM-1) drought stressed subtracted cDNA Macrotyloma uniflorum cDNA, mRNA sequence

AAGCTGAGCTCACGCGGTGGCGGCGCTCTAGAACTAGTGGATCCCCCGGGCTGCAGGAATTCGGCACGAG

GGTTAAGGTTTGAATGGAAATGAACTGATAAGAATATTTCCATTGAATAAAGTTGAAAGCAATTAAAAAC

CTAAAAAAAAAAAAAAAAACCCCCNGGGGGGGCCCCGGTCCCCTTTCGC



>gi|71812105|gb|DR989496.1|DR989496 HDI_K_77.esd Horsegram (VZM-1) drought stressed subtracted cDNA Macrotyloma uniflorum cDNA, mRNA sequence

GCTGGAGCTCCACCGGCGGGTGAGCGGGCCGCTCTTAGAAACTTAGTTGGATCCCCCGGGGCTAGCAGGA

ATTCGGCACGAGGCCTGCCAAGCCTAGGAACAGCCTTTAATGCACAGCACGTAGCTTACTAGCTATAAAC

GTACTATGTTAATAATATAGACTGTAGTATATGAGATATAGGATACAATAATATAAATTTATGTAGCAAG

ATATACGAGATAGTATGTTATAGTATATATATGATAGTACATGTAACATTATTCCATATAAAAACTAAAT

GTTATAAGTATAAA



>gi|71812103|gb|DR989494.1|DR989494 HDI_K_75.esd Horsegram (VZM-1) drought stressed subtracted cDNA Macrotyloma uniflorum cDNA, mRNA sequence

TGGAGCTCACGCGGTGGCGGCGCTCTAGAACTAGTGGATCCCCCGGGCTGCAGGATTCGGCACGAGGATG

TATGCTCAGCAGTCTCACCCCGTACATGGCACCACAAATGTGGCCCCAGCCTCCAGATCAACGTCAACCA

TCCCCAGAACATTAGCTCTGATGTGTCATGGAAATTAAGATAACCAGGCACCAGAATCAGTTGTGAATGT

CAAACTGAATGGTTGGGGAAGTCCATAGCGAGCAGAAGCTGTAGCTGATAGTTTACATGCAATGCAGACT

ATAAACATGTGTAGATAATGTGCTAGGGAAAACTTAACCTTATCTTTGATTTAGCTTGATAAAATGGTAT

TTTTCATGTTTAAATTACAGGTCATCA



>gi|71812101|gb|DR989492.1|DR989492 HDI_K_73.esd Horsegram (VZM-1) drought stressed subtracted cDNA Macrotyloma uniflorum cDNA, mRNA sequence

AAGCTGGAGCTCACGCGGTGGCGGCGCTCTAGAACTAGTGGATCCCCCGGGCTGCAGGATTCGGCACGAG

GGTGATGATCTTGGAGGATCTTTCTGTGAGTACGAGTATCAGAAGAGTGTCTTCTAGAAGCAAACAACAC

TTGTATTGTCATGCCTACTGAATTTTCCATATCTACCCCTATTAATTGTAGTGTTGGTTGTACATAGGTA

TATAGACTATGTGGAGTTTCATTTTCTCTATAAATTTTACAATAATTATACTCTAAATTTGAAAGCTTCC

CGTCTAGACTAGAAATGAAATAAAATAGAAGGAGCATTTCACTTAAAAAAAAAAAAAAAAAAAAAAAAAA

AAAAAAAAAAAAAAAAAAAAAAAAAAAAAA



>gi|71812099|gb|DR989490.1|DR989490 HDI_K_65.esd Horsegram (VZM-1) drought stressed subtracted cDNA Macrotyloma uniflorum cDNA, mRNA sequence

AAAAGTGATCTCACACATATTCTGAAGTATAGCATTGTGCAAATAACCATGTGTGTACATCTCAGCAAGA

ACACAACATTCTAAAACCTCTTCGAAGGTGGGGGCCCGGTATACCCAATTTCGCCCATATAGTCTGGAGT

CTCGTATATTACAATTATCATCGTGAGCGC



>gi|71812097|gb|DR989488.1|DR989488 HDI_K_63.esd Horsegram (VZM-1) drought stressed subtracted cDNA Macrotyloma uniflorum cDNA, mRNA sequence

AGCGGGAGCCATCGGGGCGTCGGCCGCCTATAACATGGTACTCCCGGGCGTAGGAATNGGCACGAGGGAG

GAAATAAAAGTGTCTGTTCAAGGAGTATACAAAGTTTTT



>gi|71812095|gb|DR989486.1|DR989486 HDI_K_61.esd Horsegram (VZM-1) drought stressed subtracted cDNA Macrotyloma uniflorum cDNA, mRNA sequence

AAGCTGGAGCTCACGCGGTGGCGGCGNTCTAGAACTAGTGGATCCCCCGGGCTGCAGGATCGGCACGAGG

AATTGGTAGCTATCATACACAAAGAGCAATAGAAGTAATAGCTACAATATCTGAAATTTTAATCCCAGAG

AGAAGAAAATAGACTCTTATTCAATCATGGGTCATTAATCTCTGCTGTAAGTCAAATCAATGACCTGTAT

ACAGTTATCTAATTTCTTGATTTAAGTGTCAAAAATGAGAAACACGAACTTAATATGGGCATGTCATACA

GATACTCGAGGGGGGGCCCGGTACCCAATTCGCCCTATAGTGAGTCGTATTACAATTCACTGGCCGTCGT

T



>gi|71812093|gb|DR989484.1|DR989484 HDI_K_53.esd Horsegram (VZM-1) drought stressed subtracted cDNA Macrotyloma uniflorum cDNA, mRNA sequence

AAAGCGGTAGCCNTCGCGGGNTCGGCGCCTATAACATGGGTGANCCCGGGCGCAGGAATCGGCACGAGGA

TACATCGCCTTATCATTCATCCAGTCCAACAAATAATATTATAATATTAATAATAATAATACTTAACTGT

TAAAAAAAAAAAAAAAAAAAAAAAAAAAAAAAAAAAAAAAAAAAAAAAAAAAAAAAAAAAAAAAAAAAAA

AAAAAAAAA



>gi|71812091|gb|DR989482.1|DR989482 HDI_K_51.esd Horsegram (VZM-1) drought stressed subtracted cDNA Macrotyloma uniflorum cDNA, mRNA sequence

AAGCTGGAGCTCACCGCGGTGGCGGCGCTCTAGAACTAGTGGATCCCCCGGGCTGCAGGATTCGGCACGA

GGGGCTTCTGAAGCTTCCCAACCGGCTAAATAGCCAAAATGAGTAGTATAAGAGACTTCTTTGTAACTAC

AGATATTTTCCATGAAGTGGAGTTGGCATGCCCTTCGTTTTGTTTTTTGTTGGCTTCTTCTGAACTACAG

CATCTTGAGTTATTGACCGGCAAAATATTTGATATTTTTATTGGATATTAGCCTATCAATATCATTCATA

TATGTAATGCGATGAATAATTTGGACTATCAAAATTATCAATAAATTTCATGTCTATGTTTGTAAAAAAA

AAAAAAAAAA



>gi|71812089|gb|DR989480.1|DR989480 HDI_K_5.esd Horsegram (VZM-1) drought stressed subtracted cDNA Macrotyloma uniflorum cDNA, mRNA sequence

AAGCTGGAGCTCACGCGGTGGCGGCGCTCTAGAACTAGTGGATCCCCCGGGCTGCAGGAATTCGGCACGA

GGCCTCGTGCCGAATTCGGCACGAGGAGAGCATCCTCTGTTGCTCTATTTCATGTATTTTCCCTCAAGTT

TAATGAAGCTTTGTATACATGTATCATGCTGGTTTTAGTGTATTACCTTTCTGCATTTAGGCTTTATATA

ACGAAGTGTTTCTCTATAAAAAAAAAAAAAAAAAAAAAAAACCCCGGGGGGGGG



>gi|71812087|gb|DR989478.1|DR989478 HDI_K_42.esd Horsegram (VZM-1) drought stressed subtracted cDNA Macrotyloma uniflorum cDNA, mRNA sequence

CCGGGCAAAGGATAAGCTTCTTGCCAAGGAAGCTGCCCAGAGGAATGTCTTAAAAGAGTTTCTTATATTT

TGTTGTTTTGCGAATGCTCTTTAGTGTGTTTTTTAATGTTACTCATTTACTTTTTATCTCCATAAAAACA

TTTGTTATTCACATCAGCTAGAATATTTCATTAAAAAAAAAAAAAAAAAAAAAAAAAAAA



>gi|71812085|gb|DR989476.1|DR989476 HDI_K_40.esd Horsegram (VZM-1) drought stressed subtracted cDNA Macrotyloma uniflorum cDNA, mRNA sequence

AAGCTGGAGCCACGCGGGGCGGCGCNCTAGAACTAGTGGATCCCCCGGGCTGCAGGATCGGCACGAGGTT

TTCATTGATCTTTCTATGTTTGTCTCGTGTTCTATACATCTTATTATGAGACCAGTGTGAGTGAATATAT

GTAATCTATGTTCATGTTCATGTTCCTGATTTAATACTAAATTTTGTTTGTGTTCTAAAAAAAAAAAAAA

AAAAAAAAA



>gi|71812083|gb|DR989474.1|DR989474 HDI_K_39.esd Horsegram (VZM-1) drought stressed subtracted cDNA Macrotyloma uniflorum cDNA, mRNA sequence

AAGCTGGAGCTCACGCGGTGGCGGCCGCTCTAGAACTAGTGGATCCCCCGGGCTGCAGGATTCGGCACGA

GGGAGGTTGCAGCTAATGCACTTAAGGAACCTCCAAATATGGAATTGATTTTCAGATCTTTCATTCCACC

AAGCTTGAGTTTTGAGAAGATAGATGATGACCGATTTGAAGATGCATTGTCTTCAATTTCGAGAACAACA

CCTGTTTGATGTAATTATCTGAATTTCAACTGCTTTTTACGTGTTAGTGGTAGTCTAGTAGGATACTTTT

AGATATTTTATTCATTGTTCATAATACGTCTTAGGATGAACCAGGGTGTTGTGTGTTAAATGTTGTGAAT

GTGATTGTGCAAGAAATTGTTGGACGGTCTGCTAATATTTCCATGCAATGTAATGTGATGTTTGTTCGAC

TTGTAATGTGCTATGGTGTTCAGTGTGCAAAAAAAAAAAAAAAAAAAAAA



>gi|71812081|gb|DR989472.1|DR989472 HDI_K_37.esd Horsegram (VZM-1) drought stressed subtracted cDNA Macrotyloma uniflorum cDNA, mRNA sequence

AACCTGAGCTCACGCGGTGGCGGCGCTCTAGAACTAGTTGGATCCCCCGGGCTGCAGGAATTCGGCACGA

GGGCAACTTCCAAAATATCATCCATGAATGAATTTGAGAGCCGTTGATATTGCAATGTAATGTCCCCTTT

TTAATTTCTAAGAGAGGAGTATATGCATACGTTGTAATCGAGAGGTGTGGCATGAGTCATGTTCTGTTGT

AAACACTATAACAACTGTACCTGTGTGCTATAAAGATACGAAGTTAAGTTGTTTTTCTTTTCAGCGTATA

AAAAAAAAAAAAAAAAAACCCAGAGGGGGGGCCCGGTCCCC



>gi|71812079|gb|DR989470.1|DR989470 HDI_K_3.esd Horsegram (VZM-1) drought stressed subtracted cDNA Macrotyloma uniflorum cDNA, mRNA sequence

AAGCTGGAGCTCACGCGGTGGCGGCGCTCTAGAACTAGTGGATCCCCCGGGCTGCAGGATTCGGCACGAG

GGGCTAAAATGCCTTACACTGTGCTCTATCCTTCGAGTGAAGAAGGGTTGACTTTCAGAGGAATTCCCAA

CAGTATCTCCATCTAAGGCTCTATGCTTGCTGCATTACTGTGCCTGTTATCAATAAATGTGTTATAAAGG

AATCTGATTCCTTCATCCACTTATGTATGCTTAAGTTATGTATCTCCAGTGGTTGAATGTTCTCGTGTGT

TTGTTATTAATAAAGAATTTGTGTTTGTACTTTGTATAATCCAGAGACTATAATAAACACTGCTACCATT

GCAAAATGAAAAAAAAAAAAAAAAAAAA



>gi|71812077|gb|DR989468.1|DR989468 HDI_K_26.esd Horsegram (VZM-1) drought stressed subtracted cDNA Macrotyloma uniflorum cDNA, mRNA sequence

AAGCTGGAGCCACGCGGTGGCGGCCGCTCTAGAACTAGTGGATCCCCCGGGCTGCAGGATTCGGCACGAG

GTATATATACTTACTACCTTTCTTGTACTAAAACTAAACCTATGACAGTGTTTATGAAATAAGTAAGAAG

TTTCTGAGTATTTGCTCATTTTCTTTATGTATGATGAAAAATCTCAGGATGATCATAGAGAGTTGAAATG

ATTGAGAAAGATATTTTAACAAGTTTTAATGATGAAGCAGTGAACTCATTTCTGAGTAAAAAAAAAAAAA

AAAAAAAAAAAAAAAAAAAAAAAAAAAAAAAAAAAAAAAAAAAAA



>gi|71812075|gb|DR989466.1|DR989466 HDI_K_2.esd Horsegram (VZM-1) drought stressed subtracted cDNA Macrotyloma uniflorum cDNA, mRNA sequence

AGCTGAGCTCACCGCGGTGGCGGCGCTCTAGAACTAGTGGATCCCCCGGGCTGCAGGATTCGGCACGAGG

AAGGAAATCACTCGTGATCACATTGCGACTGATTGGGACCAACCTCGCGTTCAAGCCCCACCCCGCACCC

GCGCGTTGTTGTGAAAAACTTGCACCTCATTTGAGCCAAGGTTGCATAATGTGGTGCAGAACAATGGAGA

AACACTTCTATATATGTTTCATGAACCTGTTCTCATCATTCCTTTTCCTTTTCCTTTTTGGAGAACTCAT

TTAAGGTTTCTGCTTCCAAAACTCTATGCTCTCTTTGTAAGCATTATATCATGATACTTTGTGGCTACTG

CTGCTGCATTCACTCATTGTATATTCTTCATTTTTAATAAAAAAAAAAAAAAAAAA



>gi|71812073|gb|DR989464.1|DR989464 HDI_K_17.esd Horsegram (VZM-1) drought stressed subtracted cDNA Macrotyloma uniflorum cDNA, mRNA sequence

AAGCTGGAGCTCACGCGGTGGCGGCGCTCTAGAACTAGTGGATCCCCCGGGCTGCAGGATTCGGCACGAG

GTAAAAACCGAGTTGGGCCAGTTCAGTTGCCATACACATTACTCCTTCGTTCAAGTGAGGAAGGGATGAC

TTTCAGAGGAATTCCCAATAGTATCTCTATCTAAAAGGAATGTGTGATCTCCTTATCTATTATGCTTTTG

AATAAGATAGACAATGCAAGCCTGTGTGAAATTATTGTCTATTTGTATGCAGTATGTTGCTCTCATTCAG

ATGGAGTGTTATAAGAGTGTTTTACTACCACTTTGTATTTACTTCATCTTATCTTTATCAGTAATAATTT

TGACAATTTTCAGATATTTCTTTGTACTAAATGAAAATAAATCTTTGCTCAAAAAAAAAAAAAAAAA



>gi|71812071|gb|DR989462.1|DR989462 HDI_K_15.esd Horsegram (VZM-1) drought stressed subtracted cDNA Macrotyloma uniflorum cDNA, mRNA sequence

CAAAGCTGGAGCTCACGCGGTGGCGGCGCTCTAGAACTAGTGGATCCCCCGGGCTGCAGGATTCGGCACG

AGGGTTACAAGAGAAGACTTGACTTTGGAGGCTCTTAAGGAAATTGAGAAGTTGACCAGAGTAAAGAAGA

TGATCGAAGAGGCTGCAAGACCATTTTAACCAACCTGGACATTCTACCTTTACCTGTAATCAAATGGGAC

AATCTAAAAGCATTTTTGAATCTCAAATTTAAAAAATTTTACTCGTTTCTGTAAGAATAAAAAAAAAAAA

AAAAAANAAAAAAAAAAAAAAAAAAAAAAAAAAAAAAAAAAAAATAAAAAAAAAAGGGGGGGGGGNCCA



>gi|71812069|gb|DR989460.1|DR989460 HDI_K_13.esd Horsegram (VZM-1) drought stressed subtracted cDNA Macrotyloma uniflorum cDNA, mRNA sequence

GCTCACGCGGTGGCGGCGCTCTAAACTAGTGGATCCCCCGGGCTGCAGGATTCGGCACGAGGCAGAAGTA

TGCCATGGGCTGATCTGTGTTTAGATATTGTATATTGAATGGCAGGCATCTATGAAAACTTTTGTGGGTT

TGCTTTTCTCTTCCTGTTTTCTGGGGTGAGATTATGTTTCTGTTATGAGGGATAGGAAGGCGGTGTCTTA

ATTTGATAAAAACAAATAACTATGTTCAACCACTGTGATGTTTTATCATGATGCAAACGCATGAACAGGA

TGTTATTTGTGTCATGGCTGTGTTGTAACTTTCTCTATCAAGTTATGTNGAAATAGTATGTAACACACCT

TTCCCTTTATGTTCA



>gi|71812067|gb|DR989458.1|DR989458 HDI_J_95.esd Horsegram (VZM-1) drought stressed subtracted cDNA Macrotyloma uniflorum cDNA, mRNA sequence

AGGCTGGAGCTCACGCGGTGGCGGCCGCTCTAGAACTAGTGGATCCCCCGGGCTGCAGGAATTCGATATC

AAGCTTATCGATACCGTCGACCTCGAGGGGGGGCCCGGTACCCAATTCGCCCTATAGTGAGTCGTATTAC

AATTCACCTGNGGCCCGGTTCGGTTTTTTTACAACGAA



>gi|71812065|gb|DR989456.1|DR989456 HDI_J_93.esd Horsegram (VZM-1) drought stressed subtracted cDNA Macrotyloma uniflorum cDNA, mRNA sequence

AAAGCTGGAGCTCACGCGGTGGCGGCCGCTCTAGAACTAGTGGATCCCCCGGGCTGCAGGATTCGGCACG

AGGAAGGTACTCTCTGTTTGTGTGCCCCTAACCATTGTATCTCTGAATATCCCCACAGTAAAAAGAGAGT

ATTCAAGTGAATTGAAAAAAAAAAAAAAAAAAAAAACCCCNGGGGGGGGCG



>gi|71812063|gb|DR989454.1|DR989454 HDI_J_91.esd Horsegram (VZM-1) drought stressed subtracted cDNA Macrotyloma uniflorum cDNA, mRNA sequence

AAAGCTGGAGCTCACGCGGTGGCGGCCGCTCTAGAACTAGTGGATCCCCCGGGCTGCAGGATTCGGCACG

AGGGAGACTCTGCCTCCAAGAGAGAGAGTTGTGGACTTTTTCAAAACTCGATATGCATCCATCCTTGCTT

CCAAATAGGTTAATTATGAGTCTGATGGATACTCATGAGTCACGATTTAGCCAAAGGTTTTAAATAAGAA

GTTGTTGGCATTCTGATTTCTGTTCTAGATGGCATCATGAAGGTTATTCATGCTGAAATTTCATGACAAA

ACTGTAGTGTTTGTGCTTTATTCCATTTCAAATTTAAGTTCTAGTTATAAAAAAAAAAAAAAAAAAAAAC

CCNAGGGGGGGCGCCCGGTCCCCAATTCCC



>gi|71812061|gb|DR989452.1|DR989452 HDI_J_85.esd Horsegram (VZM-1) drought stressed subtracted cDNA Macrotyloma uniflorum cDNA, mRNA sequence

ACAAGCTGGAGCTCACCGCGGTGGCGGCGCTCTAGAACTAGTGGATCCCCCGGGCTGCAGGATTCGGCAC

GAGGGAAGTGTGTACGAAGGGGCGTTGTTTGTTCTGTTTGAGATGGTTGTTTACAAGTTAGGTGAGGCCT

TGGGTGAGAGCCCTGAAGCCGTTCGATCTCGCCACACTAATCTTGAATGAAATTATATCAGCCGCTAGAT

CTTTCTGAGTTATGTAATGTTAAAGTAATCGTTACATCTTCTTCTTCTGGAAAATAATGCTTGTTACACA

AATTTTGTAATTTTACGGAGAATATAATTTCCGTTCTAAAAAAAAAAAAAAAAA



>gi|71812059|gb|DR989450.1|DR989450 HDI_J_82.esd Horsegram (VZM-1) drought stressed subtracted cDNA Macrotyloma uniflorum cDNA, mRNA sequence

AAAGCTGGAGCTCACGCGGTGGCGGCGCTCTAGAACTAGTGGATCCCCCGGGCTGCAGGAATCGGCACGA

GGATGAACCAGGTGTTGTGTGTTAAATGTTGTGAATGTGATTGTGCAAGAAATTGTTGGACGGTCTGCTA

ATATTTCCATGCAATGTAATGTGATGTTTGTTCGACTTTGTAAAAAAAAAAAAAAAAAACCTCAAGGGGG

GGCCCGGGTACCCAATTTCCGCC



>gi|71812057|gb|DR989448.1|DR989448 HDI_J_80.esd Horsegram (VZM-1) drought stressed subtracted cDNA Macrotyloma uniflorum cDNA, mRNA sequence

AGCTGGAGCTCACCGCGGTGGCGGCCGCTCTAGAAACTAGTGGATCCCCCGGGCTGCAGGATCGGCACGA

GGGAGAAATTTCAGACAGCATAATACAAATGCTTGTTAGATCAGGACATCAATTCAGGTTTGAAGTGTTG

TACTGGAATTTAGTAGGCTATTATTGCTGTTCCAAAATTTTAAAAATTCAGCGGAGATCCTGTGTATTTT

CAGTTGACTCCTTGATCATTATGAATGCAGATGAAAAAATGATGTTCATGTTTATATTGGGACACTTCTT

TTATTTTTGAATAATTTTTGTTAGTTTCAAAAAAAAAAAAAAAACAAAANAAACAAAAAAAAAAAAAAAA

AAAC



>gi|71812055|gb|DR989446.1|DR989446 HDI_J_79.esd Horsegram (VZM-1) drought stressed subtracted cDNA Macrotyloma uniflorum cDNA, mRNA sequence

AAAGCTGGAGCTCACGCGGTGGCGGCGCTCTAGAACTAGTGGATCCCCCGGGCTGCAGGATTCGGCACGA

GGGATTAATAGAGAGGAGGAAGCCTGGGTGGGATGTTTATGCGCTCATTTTGTATAATTTTTTAATGATG

TTAATGTATTGTAATCTAATGCATCTCCACCACAATCTTTCTTTTCTCAAAAAAAAAAAAAAAAAAAAAA

AAAAAAAAAAAAAAAAAAAAAAAAAAAAAAAAAAAAAAAAAAAAAAA



>gi|71812053|gb|DR989444.1|DR989444 HDI_J_70.esd Horsegram (VZM-1) drought stressed subtracted cDNA Macrotyloma uniflorum cDNA, mRNA sequence

AAAGCTGGAGCTCACGCGGTGGCGGCGCTCTAGAACTAGTGGATCCCCCGGGCTGCAGGATCGGCACGAG

GCCTCGTGCCGAATTCGGCACGAGGCTTTTTTTAAGGACTTGGCTGTGGCAGCAAGTTATACAGGTCTGC

CATAACCACAATCTTTTTCCCTTTGGGAAGGATTGTTTTTCTTACCAAGTCCTAGTTTTTTAAGGCTTCA

TTTTTTCGAACCTGGCACTGCATCTACTATATTAGCTAATGGTCTTCATATCTGCCATTTGTACTGTATG

TTATGAAATGTAATAAAATAAAATCAGTCTCATTTCATGAGGCAAAAAAAAAAAAAAAAAAAAAAAAAAA

AAAAA



>gi|71812051|gb|DR989442.1|DR989442 HDI_J_69.esd Horsegram (VZM-1) drought stressed subtracted cDNA Macrotyloma uniflorum cDNA, mRNA sequence

AAAGCTGGAGCTCACGCGGTGGCGGCCGCTCTAGAACTAGTGGATCCCCCGGGCTGCAGGATTCGGCACG

AGGGCCACCATCACCAGAGGACTTAGGGAAATGGCGCGGGAGATGGCACGTAAAAGGAACAGCGGCTGAT

GTTCCATGTGGTCGATTTGGTTGCAGTGCAGTTGTGAGACAGAATCACTGCTTATGTAGTAAATATGTTT

ATTGTTGTAATAATGAATAAGGACTTATAAACTACTGTAGAAATGTTTTTTATTTTTCAAAAGCTTCAAC

AGTTTGGTGCCATGGACTACCTACAACTTCTCTGTCTTACCTTCAATCTAAAAGAAAAATAAATTATATT

AGAGATCCTGTTATAGCTGTTTTTCATAAAAAAAAAAAAAAA



>gi|71812049|gb|DR989440.1|DR989440 HDI_J_61.esd Horsegram (VZM-1) drought stressed subtracted cDNA Macrotyloma uniflorum cDNA, mRNA sequence

AAGCTGGAGCTCACCGCGGTGCGCGGCGTTCTAGAACTAGTGGATCCCCCGGGCTGCAGGATTCGGCACG

AGGCCTCGTGCCGCCTCGTGCCGAATTCGGCACGAGGGTGACTGAAAGGTTCGAGAAGGGAGAAGGAAGT

GTTTAAAACACGTAAGATGTAAGATGTGTGAAGAAGGATGATGTAAAATAAATAAGATATAAGATGTGAA

GATGGATCTATCTTAAGATGAAGTGGCTGAAGGACTTATATTCAGATAAATAAAATGAAGGACTATATGT

TTATCTTCTTCAAAAAAAAAAAAAAAAAAAAAAAAA



>gi|71812047|gb|DR989438.1|DR989438 HDI_J_57.esd Horsegram (VZM-1) drought stressed subtracted cDNA Macrotyloma uniflorum cDNA, mRNA sequence

GTTGCAGACATTCTCAGTAAGCTTCAATACACATTGATGGGTGTGATAAGGATCCTTGTCGACCATAGTC

TTGAATCACTGGAACAATACATCCACCACCACGA



>gi|71812045|gb|DR989436.1|DR989436 HDI_J_49.esd Horsegram (VZM-1) drought stressed subtracted cDNA Macrotyloma uniflorum cDNA, mRNA sequence

GCTGGAGCTCACGCGGTGCGCGGCGTCTCTAGAACTAGTGGATCCCCCGGGCTGCAGGATTCGGCACGAG

GCACAAACCCACTCTCAAAGTCCAACCTTCAAATTAATGCTTCTTTGAAGGAGAAAGCAGTGACAGGACT

CACAGCCGCAACCTTCACAGCCTCCATGGTGATCCCAGAGGTGGCTCATGCTGCTGGATCCGACTTCTCC

CCTTCTCTCAAGAACTTTCTACTCAGCATCTTCGCCGGTGGGGTTGTGCTAGTAGCCATATTTGGAGCAG

TTATTGGAGTTTCCAACTTTGATCCGGTTAAGAGAACTTAAACTATGCTCTACTTCTTTTAATTTCTCTG

CCTTTCTCACTGTTCTCGTTTCCTGTAAGTTTACGCAACTGGTTCTGTTATACGCTTACTATATATCTCA

GTTGCACTTTATTTTGTATCAAAAAAAAAAAAAAAAAAAAAAAAAAAAAAAAAAAAA



>gi|71812043|gb|DR989434.1|DR989434 HDI_J_46.esd Horsegram (VZM-1) drought stressed subtracted cDNA Macrotyloma uniflorum cDNA, mRNA sequence

AGCTGGAGCTCACCGCGGTGGCGGCCGCTCTAGAACTAGTGGATCCCCCGGGCTGCAGGATTCGGCACGA

GGAAGAATTGTAATATATATCATATAAGTTAAGCCACATTTTAGTTTCGGTATTTGCAGCTAAAAAAAAA

AAAAAAAAAAAAAAAAAAAAAAAAAAAAAAAAAAAAAAAAAAAAAAAAAAAAAAAAAAAAAAAAAAAAAA

AAAAAAAAAAAAAAAAAAAA



>gi|71812041|gb|DR989432.1|DR989432 HDI_J_44.esd Horsegram (VZM-1) drought stressed subtracted cDNA Macrotyloma uniflorum cDNA, mRNA sequence

AAGCTGGAGCTCACGCGGTGGCGGCGCTCTAGAACTAGTGGATCCCCCGGGCTGCAGGATTCGGCACGAG

GAGCAAGTTCTTTCAGTGTTGCTTAAAAACTTCAATAGCAGGTTATATATATATATTTGCTATGTGTAAA

TCAACCTTAAGTTTATGCTATCTATGTTTTGAGTTGTCTAGCCTCCCCTTTGTCCACGAGTTTCATAATT

TAAGGGACTTGGTATTCCTGGCAAAAAAAAAAAAAAAAAAGGGGGGGGCC



>gi|71812039|gb|DR989430.1|DR989430 HDI_J_37.esd Horsegram (VZM-1) drought stressed subtracted cDNA Macrotyloma uniflorum cDNA, mRNA sequence

AAGCTGGAGCTCACGCGGTGGCGGCGCTCTAGAACTAGTGGATCCCCCGGGCTGCAGGATTCGGCACGAG

GAAAAAGATAACTCTTTTCACAAATATTCCCACGCATTGATCAGGAAGATATCAATCAATTACAGTCATA

TATAAGTTTCATAATATATCATCTCATATGTTCAAAGCCTTTGTGTCGCACAGCAGAGAAACACCGAATT

ACATTGGCTATTCTGTAAATAAGTGAAGGGCAAGATCAGAAAACCCAAAACAAACTCCTACAAGCTTTTT

GTTAATATATCTTTATATGCCCACCCATGTTTATCCAAGAATGCCTGGACTGCTTGCTTTATGGCCAGGT

TTGGTATTAGGAGTGATGGATCAAGAGGATCTCGAGGGGGGGCCCGGTACCCAATTCGCCCTATAGTGAG

TCGTATTACAATTCACTGGCCGTCGTTTTACAAC



>gi|71812037|gb|DR989428.1|DR989428 HDI_J_34.esd Horsegram (VZM-1) drought stressed subtracted cDNA Macrotyloma uniflorum cDNA, mRNA sequence

AATTAGCTGGAGCTCACCGCGGGCCGGCGTTCTAAACTAGTGGATCCCCGGGCTGCAGGATTCGGCACGA

GGCGGTGGTTCCCGTTACTCGAGAGGAGGTGGTGCTTCGGACGGTGGAAGCTGGAGGAATTAGGGTTGTG

TGATTAGGGTTTAGTTGGTGAAAAAAAAAACACAAAAAAAGGGAAC



>gi|71812035|gb|DR989426.1|DR989426 HDI_J_32.esd Horsegram (VZM-1) drought stressed subtracted cDNA Macrotyloma uniflorum cDNA, mRNA sequence

ATAAGGCTGGAGCCACCGCGGTGCNCGGCGTTCTAGAACTAGTGGATCCCCCGGGCTGCAGGATTCGGCA

CGAGGCCTCGTGCCGAATTCGGCACGAGGCTAATTTGAATAAGGCTGAAGGCAAGAGACAAACAATTGAG

GCAAATCTAGCTCTTAGACGAGCTAGAACACGAGTAGAGGCTATCAATATGATTTCATAACAAGTTGGTG

CGTCCGAAAATCCGCCGAATATCCAAATACAAAAAATAAAAAAGAAACAGTAAGAAAAGATAGAAAAAAA

AAAAAAAAAAATCAAGGGGGGGC



>gi|71812033|gb|DR989424.1|DR989424 HDI_J_25.esd Horsegram (VZM-1) drought stressed subtracted cDNA Macrotyloma uniflorum cDNA, mRNA sequence

ACAAGCTGGAGCTCACCGCGGTGCGCGGCCGCTCTAGAACTAGTGGATCCCCCGGGCTGCAGGATTCGGC

ACGAGGGGGTGGTGCACATGCCCTTATGCATCAGCAAGGACAACATGCTCTTAAACAGGTAGCCATGGGC

ATGGGTGCTCCTGGGGCTGGTTATGATGCGAGGTTTCCGAATGTTGCGACCACCCAGCACCTAAATGTAC

TATCAGGTAAAGAATTGGTTTTAATTGGTTAAACCTTCCCCTGG



>gi|71812031|gb|DR989422.1|DR989422 HDI_J_22.esd Horsegram (VZM-1) drought stressed subtracted cDNA Macrotyloma uniflorum cDNA, mRNA sequence

AGCTGGAGCTCACGCGGTGGCGGCCGCTCTAGAACTAGTGGATCCCCCGGGCTGCAGGATTCGGCACGAG

GCTCCACCTTATTTAGCAAAGATATCTAAGTGTAACTTCTGGATTAACTTTGCTCCATGTCCTGCAGTTA

CTTCAGTACCTTAAATTTTCTGTACCAGTTCTTATTGCTGTATAGGACACTTCCTTATTGTTAAATGATG

AGACCATGTATTGTATTCTCGAAAAAAAAAAAAAAAAAAAAAAAAAAAAAAAAAAAAAAAAAAAAAAAAA

AAA



>gi|71812029|gb|DR989420.1|DR989420 HDI_J_20.esd Horsegram (VZM-1) drought stressed subtracted cDNA Macrotyloma uniflorum cDNA, mRNA sequence

AAGCTGGAGCTCACGCGGTGGCGGCCGCTCTAGAACTAGTGGATCCCCCGGGCTGCAGGATTCGGCACGA

GGCCTCGTGCCGAATTCGGCACGAGGGTCAAGCCATACTGAGCAATGAAAATGGTGGGTTGTTGATGAGC

TATCAGATGTACAAATTTGCGGAGCGTGTACATTTTTTTACATCTTACCATCTTTTTTTGAGAGGAGGAA

TGTTTCGGCAAGATAAGCAGATAAATCTTTCAGCACTCTNGTAATGCATGGTATTAATCTCAGTACTTAT

GTAAAAAAAAAAAAAAAACAAAAATGTGCGCTCCAGGGTATACCGGTATATTTATAAATTANGTTTCTCA

TATAGTTTTCTCTCTCTTNGCTTATAAAAAAAACACAAAAAAAAAAA



>gi|71812027|gb|DR989418.1|DR989418 HDI_J_13.esd Horsegram (VZM-1) drought stressed subtracted cDNA Macrotyloma uniflorum cDNA, mRNA sequence

ANAAGCTGGAGCTCACCGGCGGTCGCGGCCNGTTCTAGAACTAGGTGGATCCCCCGCGGCTGCAGTGAAT

CGGCACGAGGCAAAATAATAAGAATGTATTATTTCGTTTGTAGTAAAAATTTGTGGTGGTGCAACTTATA

GTCTGCAAAAAAAAAAAAAAAAAAAAAAAAAAAAAAAAAAAAAAAAAAAAAAAAAAAAAAAAAAAAAAAA

AAAAAAAAA



>gi|71812025|gb|DR989416.1|DR989416 HDI_J_10.esd Horsegram (VZM-1) drought stressed subtracted cDNA Macrotyloma uniflorum cDNA, mRNA sequence

AAAGCTGGAGCTCACGCGGTGGCGGCCGCTCTAGAACTAGTGGATCCCCCGGGCTGCAGGATTCGGCACG

AGGCCTCGTGCCGAATTCGGCACGAGGGAAAAACAAAAAAATGAGAGAAAAAGTGTGCGTGTATGTGGTT

GGTAATTGGAGTGTTATATGTGGTTTGGTTGGTTGCCCACTACTTTGGTTGTAGATTATCTTAATGAAAT

AAAATTTTAAATTATCAGTATTAGTATTACATGGTAGAGGCTATTGGATATACATGATAATTGGATAGTT

CAAAAACATTTTTTTTTTTTTTTNAAAAAAAAAAAAAAAAAAAAAAAAAAAAAAAA



>gi|71812023|gb|DR989414.1|DR989414 HDI_J_96.esd Horsegram (VZM-1) drought stressed subtracted cDNA Macrotyloma uniflorum cDNA, mRNA sequence

AAGCTGGAGCTCACGCGGTGGCGGCGCTCTAGAACTAGTGGATCCCCCGGGCTGCAGGATTCGGCACGAG

GGAGAAACATGTGGGGAACGGGCTGGCAAACTGATAGTTCCTTTTGTGATATAAATGGCTCTTTTATAGG

CTGGAATTCTTTTCGTTTTAATGATTTCCTAATTGCATTTATAAACTTCTTTTTTTTTATTTTAAGATAT

TTATTGACAACGATCACTCAATTTTAGCATTCATTAATGGATATTTGTTTTTCATGTTGCTAAAAAAAAA

AAAAAAAAAAAAAAAAAAAAAAA



>gi|71812021|gb|DR989412.1|DR989412 HDI_J_89.esd Horsegram (VZM-1) drought stressed subtracted cDNA Macrotyloma uniflorum cDNA, mRNA sequence

AAGCTGGAGCTCACGCGGTGGCGGCGCTCTAGAACTAGTGGATCCCCCGGGCTGCAGGAATCGGCACGAG

GGTCTGTGTGTATGTCGTTTTCGCACTCTGGTGTTAGGTCTAAACACCCTTTAAAATAAAGTGGCCATGT

CTTCCCTTGAGCAAGTTCAAGTGTTATGGACTTGTGATGGGTTTATCTAGCCATGGTTACTCTTTTTTTT

TTAACTATGTGGTTTGTTCAGTGTAAAGTTGGCTAATCAGAATTATCTTTCTCTGCTAATAATATTATTA

TGTTACTTGAAAAAAAAAAA



>gi|71812019|gb|DR989410.1|DR989410 HDI_J_87.esd Horsegram (VZM-1) drought stressed subtracted cDNA Macrotyloma uniflorum cDNA, mRNA sequence

AGCTGGAGCTCACGCGGTGGCGGCGCTCTAAACTAGTGGATCCCCCGGGCTGCAGGATCGGCACGAGGGC

ACAATCAACATACTCTTCTTGGAGGTGTTCATCCAAACCAAGTGCTATATATGCTGATGGTGAACGGGAA

TACCTCGGTATAGCATTCATGAGGATTTTTAAAATATATTGATTAAGGTATGCATAATCATCACAGTTGT

AATTTTTTCACTGTACAAATTAAAATGAGTTTTTTTGGCCTGATTATCAAAAAAAAAAAAAAAAAAAAAA

AAAAAAAAAAAAAAAAAAAAAAAAAAAAAAAAAAAAAAAGGGGGGGGGGGG



>gi|71812017|gb|DR989408.1|DR989408 HDI_J_84.esd Horsegram (VZM-1) drought stressed subtracted cDNA Macrotyloma uniflorum cDNA, mRNA sequence

AGCTGGAGCTCACCGCGGTGGCGGCGCTCTAGAACTAGTGGATCCCCCGGGCTGCAGGATTCGGCACGAG

GCTACCATCAAGTTCTGAAAGATGGATGTGGTATAAGGAAGAAAAAGGCACGAAGGGGAAATTTTCAGTA

GCAGAATATTACTATATATGATGGTAACGTAGAATTAGGTTATGAAAAATAAAGGGTGGTCGACGAGGAC

TCCACTTGTGCGAGTCTCTCTTGGTGTCGGTGCCTCCCTTAGTTGTTTCAGACTTTGGTCCACTTATGTT

ATGTCTTCTGAAATTTGTATCAGTTGGTTGTTGCATGTAATAATGATATATATAGTGCTGTGATTCCTTG

TTATTTTTCCTTCTAAAAAAAAAAAAAAAAAAAAAAAAAAAAACAAAAAA



>gi|71812015|gb|DR989406.1|DR989406 HDI_J_77.esd Horsegram (VZM-1) drought stressed subtracted cDNA Macrotyloma uniflorum cDNA, mRNA sequence

AAGCTGGAGCTCACGCGGTGGCGGCGCTCTAGAACTAGTGGATCCCCCGGGCTGCAGGATCGGCACGAGG

CCAGTTTCTTCCTGGAGGAAGCTGATCCGTATGTATGTTACAAATTTGATGGTTGTCAATTGAAACTGAT

TTGTACTCCGAATTTTACTATAGAAGTAGATGTCTCTATTATTATTGTTTGTGAAAAAAAAAAAAAAAAA

AAAAAAAAAAAAAAAAAAAAAAAAAAAAAAAAAAAAAAAAAAAAAAAAAAAAAAAAA



>gi|71812013|gb|DR989404.1|DR989404 HDI_J_75.esd Horsegram (VZM-1) drought stressed subtracted cDNA Macrotyloma uniflorum cDNA, mRNA sequence

AAAGCTGGAGCTCACCGCGGTGGCGGCGCTCTAGAACTAGTGGATCCCCCGGGCTGCAGGAATTCGGCAC

GAGGCCTCGTGCCGAATTCGGCACGAGGATTGCACTCTATCTATTTAGTACTTCGTCTTTTCCATTCACG

GTAACATATATCCTTCCTTCTAAAATTGGAATTGAATGGTTGAATCC



>gi|71812011|gb|DR989402.1|DR989402 HDI_J_66.esd Horsegram (VZM-1) drought stressed subtracted cDNA Macrotyloma uniflorum cDNA, mRNA sequence

AGCTGGAGCTCACGCGGTGGCGGCGCTCTAGAACTAGTGGATCCCCCGGGCTGCAGGATCGGCACGAGGT

TTTGTAAAAAGCAAGAAACATTAAGTTGGATTGGATACACAAGATGATTCACACTATCTGAAGTACATTG

CAATAACATGTACATCAGCAAACACAAATCTAAAACTCTCGAGGGGGGGCCCGGTACCCAATTCGCCCTA

TAGTGAGTCGTATTACAATTCACTGGCCGTCGTTTTACAAC



>gi|71812009|gb|DR989400.1|DR989400 HDI_J_64.esd Horsegram (VZM-1) drought stressed subtracted cDNA Macrotyloma uniflorum cDNA, mRNA sequence

AAGCTGGAGCCACGCGGTGNCGGCGNTCTAGAACTAGTGGATCCCCCGGGCTGCAGGATCGGCACGAGGT

TGTGTTGTTGGGTTGTTTTGTCTTCTGGGAAAGGAAAGGTGGAAGAGAAGAAGTGATTCCCAAACATGGT

TGCCAGTTTCGATGCACACGTGTCGCTCGAATGGTGAGAGTGAATGAAATCAACT



>gi|71812007|gb|DR989398.1|DR989398 HDI_J_62.esd Horsegram (VZM-1) drought stressed subtracted cDNA Macrotyloma uniflorum cDNA, mRNA sequence

GCTGGAGCTCACGCGGTGGCGGCGCTCTAGAACTAGTGGATCCCCCGGGCTGCAGGAATTCGGCACGAGG

CCTCGTGCCGAATTCGGCACGAGGTTTTAAATAACATCGATATAATGACAAGGGAAATCGAATAAATAAG

ATAGTCAAACATACAAATCCACTTTTTATTAACTTGATATAAAACAGTAGAAGTCAAAAAATTAAAAAAA

ACAACAAATGGATTGTGTAGGACATTCACGAATTGAATAGAGTACTCAAGGAATTGAAAACTTGGCGAAA

AATTCAATAATTTTGTAGGACATTCACGAATTCGAAGTTTGGAAAGGCGCGGGAACATGTTTTCCCCATC

TTCCCTTGATAACGTTATTATGTTCGGCAGCTTCTCGAGGGGGGGCCCGGTACCCAATTCGCCCTATAGT

GAGTCGTATTACAATTCACTGGCCGTCGTTTTACAAACGA



>gi|71812005|gb|DR989396.1|DR989396 HDI_J_6.esd Horsegram (VZM-1) drought stressed subtracted cDNA Macrotyloma uniflorum cDNA, mRNA sequence

AAAGCTGGAGCTCACCGCGGTGGCGGCGCTCTAGAACTAGTGGATCCCCCGGGCTGCAGGATCGGCACGA

GGCCTCGTGCCGAATTCGGCACGAGGGAAACATGTCAAGTGCATGTGTTTTGTTCATTCTTGATGAGATG

AGGAAGAAGTCGAAAGAGGAAGGAAAGGGTAGCACAGGAGAAGGGTTAGAGTGGGGAGTGTTATTCGGGT

TCGGACCGGGTTTAACCGTTGAGACCGTTGTGCTTCACAGCGTTCCCTTGGAGGGGTGATTGAAAAACGG

TAACAGTGATGATGATTGATGGGGTGAGTCTCAGTATGAAAAAAAAAAGTAGTATTTACTGTAATAACTA

ATAAGAAAACCTCCAGAATTTGAGGAAAAGTGTGATATTGTGAATTAGTGTTAGTTTNGATTATGGATTA

TGGTGGCATAATGCAGTATTAGTATGAACAGTTGGCTGATGATGGTTGTGATTGAATTTGTGAAAGTTTT

AGTGCTTAAAAAAAACAAAAAAA



>gi|71812003|gb|DR989394.1|DR989394 HDI_J_53.esd Horsegram (VZM-1) drought stressed subtracted cDNA Macrotyloma uniflorum cDNA, mRNA sequence

CCCTNGTGGACTCCCCCGTGCGCTGCCGGACTTCGGCACGACGGCCTGTGCCGAACTTCGGCCACGGCCT

CGTGCCTGATCCTGCNGCCCCTGCCGGGGCGTTTAT



>gi|71812001|gb|DR989392.1|DR989392 HDI_J_51.esd Horsegram (VZM-1) drought stressed subtracted cDNA Macrotyloma uniflorum cDNA, mRNA sequence

TACGCTGGAGCTCACCGGCGGTGCCGGCGTTCTAGAACTAGTGGATCCCCCGGGCTGCAGGATTCAGGCA

CGAGGCCTCGTGCCGAATTCGGCACGAGGAGAGGATGATGAATGTCATGTACAAAAGAAAGAGTGGATGT

TGAAAGGGCTGAGTGCAAATCCGGGAACAGCAAGCTTCAGTATTTGGACACAAAGTGGAAGACATATAGG

CCTTAAGAAGATAANTGACCAAAGAACCGTAGTTTGCATACAATAAAACTACCATAATATACATCACAGA

ATCAGCAGTATGTATGAATTAGTGTGAAATAAATGCCTGCTTAGACCAAGACCGT



>gi|71811999|gb|DR989390.1|DR989390 HDI_J_5.esd Horsegram (VZM-1) drought stressed subtracted cDNA Macrotyloma uniflorum cDNA, mRNA sequence

AAGCTGGAGCTCACGCGGTGGCGGCGCTCTAGAACTAGTGGATCCCCCGGGCTGCAGGAATTCGGCACGA

GGCCTCGTGCCGAATTCGGCACGAGGATCAGATAGAAATATAGATATGACGTTGTTTACAAGCCTAACAT

ATTGGTGATTTGATTTGGTGTTCTCTTTGCATGCTTCGTTTAGAAACGCCCTGTCAACAATTTAAATGAT

ATATCTCAGACATACAACTATTTATGTCGCCTAACTTGACATGGAAGTGGTAAAAAAAAAGAATAAAAAC

ATTCGTTTCTATAGAGGATCAAGAATGAATGAAGAGTAAATCTTGAGAAGAACCACATGAATTTGGATGT

GAGGGAAACACCAATGCCTTAAAACCAATTATGTCATCACATCGTCCGTATTTTGTAGTTGGTCTGAATG

AAACGTAAAAGTAGTTACTATAACTTATAATCAAGACCAGCTCCGTATTTCCAAGCCTCGAGGGGGGGCC

CGGTACCCAATTCGCCCTATAGTGAGTCGTATTACAATTCACTGGCCGTCGT



>gi|71811997|gb|DR989388.1|DR989388 HDI_J_42.esd Horsegram (VZM-1) drought stressed subtracted cDNA Macrotyloma uniflorum cDNA, mRNA sequence

AAGCTGGAGCTCACGCGGTGCGCGGCGCTTCTAGAACTAGTGGATCCCCCGGGCTGCAGGATCGGCACGA

GGCTTTAACTAGCTCAAATTTAAGGGATTTGTTGAGCGAGTTATAAGCTATGATTGCTTTGTGTTTGTTC

GGTCTTTATATAAATATGTAATTTGAATCATCAATCGATCTCTGCTTATAAATGAAATACTGTTGATGTT

ATATGTGTTAAAAAAAAAAAAAAAAAAAAAAAAAAAAAAAAAAAAAAAAAAACCCCGGGGGGGG



>gi|71811995|gb|DR989386.1|DR989386 HDI_J_40.esd Horsegram (VZM-1) drought stressed subtracted cDNA Macrotyloma uniflorum cDNA, mRNA sequence

GGCTGGAGCTCACCGCGGTGCGCGGCGNTCTAGAACTAGTGGATCCCCCGGGCTGCAGGATTCGGCACGA

GGAACAAATGCCCGAACCTAATATGTTATTGCTATGGGAGCCTGTACAATTACGGGGGGGATGTTTAGTA

CCGATTCTTATAGTACTGTTCGAGGCGTAGATAAGCACAATTC



>gi|71811993|gb|DR989384.1|DR989384 HDI_J_39.esd Horsegram (VZM-1) drought stressed subtracted cDNA Macrotyloma uniflorum cDNA, mRNA sequence

AAGCTGGAGCTCACCGCGGTGCGCGGCCGNTCTAGAACTAGTGGATCCCCCGGGCTGCAGGATCGGCACG

AGGGTTAATCAACATTATTTAGTGCATTGGAGTTGTTACGAACATGGAGACAGCTACATCCTGTTTATGT

AGTCTCTGTTGAACTCACGATTCTTGGAGGGTGGGGTAAANCTTTGGAAGCCCCGTGATATTAANATAAC

ACGTTG



>gi|71811991|gb|DR989382.1|DR989382 HDI_J_36.esd Horsegram (VZM-1) drought stressed subtracted cDNA Macrotyloma uniflorum cDNA, mRNA sequence

AAGCTGGAGCTCACGCGGTGGCGGCGCTCTAGAACTAGTGGATCCCCCGGGCTGCAGGATTCGGCACGAG

GGTCGTTCAAATGCCACTTGCATTTGCCATTCATTTTAGCTGCAGGGTTAGATTGTGATGGAAAGGAGTG

GAGATTGTATGTTGCCGGAACTGGAATTAACGAATGAATGTAATGTCAAGGGCTGAAGAATATTTGGGAA

ATGGGTTCCTCCAACATTCCTCCTTGTAATTC



>gi|71811989|gb|DR989380.1|DR989380 HDI_J_3.esd Horsegram (VZM-1) drought stressed subtracted cDNA Macrotyloma uniflorum cDNA, mRNA sequence

GGGGATGGAGCGAGCGATCTTCTCCTGGTCTGTCATGTGAATCCCTCTGATAGCCCTTCCCGTNGCTGTT

GGGAGTACCGGGGTATCCTTTGTAAGTTGTGACTTTGAGTGAACAAACCGGGAAGTNGCGTTCCTTCCGA

GGCTCTCAT



>gi|71811987|gb|DR989378.1|DR989378 HDI_J_28.esd Horsegram (VZM-1) drought stressed subtracted cDNA Macrotyloma uniflorum cDNA, mRNA sequence

AAGCTGGAGCTCACGCGGTGGCGGCGCTCTAGAACTAGTGGATCCCCCGGGCTGCAGGATCGGCACGAGG

GCAGAGTAGAGCCAGCTGACTTTTGCAATTGATAAATCTCACCTATCCCTCCACCCATGATTATTGACTT

GGGGTTGTACTCATATTTTTGTAGCTGTGAGGAATTTTAATTTGAAGATTGTATGTTCTTGATTGGTAAT

GATACAATTTATGTCCTTATTTGGAAAAATAATTGACATTGGGGTAGATGTTTTATGTGGTTTAGGAGCA

AAAAAAAAAAAAAAAAA



>gi|71811985|gb|DR989376.1|DR989376 HDI_J_26.esd Horsegram (VZM-1) drought stressed subtracted cDNA Macrotyloma uniflorum cDNA, mRNA sequence

GCTCTAGAACTAGTGGATTCCCCCGTGTGGCCTTGGCAGGAATTCCGTGCACGAAAGTGTTTTTTTTTTT

TTTTTTGGCCACAAAACTCGTGGTAAAATTCTCCATATATCTCAGATATAAGAGAGATTGTGCGGACAAA

CCCCCACCAAAAGATATNCAACACAAGATA



>gi|71811983|gb|DR989374.1|DR989374 HDI_J_2.esd Horsegram (VZM-1) drought stressed subtracted cDNA Macrotyloma uniflorum cDNA, mRNA sequence

AAGCTGGAGCTCACGCGGTGGCGGCGCTCTAAACTAGTGGATCCCCCGGGCTGCAGGATCGGCACGAGGA

TCAAGGTTTGTGTGGTGTGTGACTTTTGTGAAGTCCTTTTGTTCATATAATCAGATTTGAGGACCCCTGA

AAAAAGAAAGAAGAAAAAAAAATGAAAAAAAAGAAGGAAAGAAAGACAAAAAAAAAAAAAAAA



>gi|71811981|gb|DR989372.1|DR989372 HDI_J_17.esd Horsegram (VZM-1) drought stressed subtracted cDNA Macrotyloma uniflorum cDNA, mRNA sequence

AAAGCTGGAGCTCACGCGGTGGCGGCGCTCTAGAACTAGTGGATCCCCCGGGCTGCAGGATTCGGCACGA

GGCGTTTTATATCAATTTTTACAATAAATGACTTCTAGTGGAATTTTATTTATGTACTGCTGCCTCTTCA

TGTTTGCACATTCGCCATAAACTTCTATTTGGCCTTTCTCTGAGCTACATGTCGATGTGTACATATATAT

AAATATGTCAATACAGCACAGTCACCATATGATGTGCTGATAATTAGAAATAAGTCCTTTTGTTTTGAAC

AATATATATAGTATTTATACTTTTAAGGCACTTTACTATATTTGGTGTCCAAAAAAAAAAAAAAAA



>gi|71811979|gb|DR989370.1|DR989370 HDI_J_15.esd Horsegram (VZM-1) drought stressed subtracted cDNA Macrotyloma uniflorum cDNA, mRNA sequence

AAGCTGGAGCTCACGCGGTGGCGGCGCTCTAGAACTAGTGGATCCCCCGGGCTGCAGGATCGGCACGAGG

GATTGCCCTCATGTTAGCTGGAAATGCTCGTGAGGACTTCTTTGAGGAAGAAGTCAATGGTGACAAAGGT

GAATATGGCACAGCCTCCTCTGATAATTATTATGATGCTGATTCAGGATTGAAGGGGAGTGCCTGAAGTG

TGCTACTAAGTAGAAATAAATGTTGAAGTGGTACTTCTCTGTTTCCTTTTAATATATATCATTCTGAACG

ATTCCTTGAAATATTGAATGTACATACTTTAAATGGTTGTTCTCGGTGAACATTTACTGTGATTTAGCAC

TACTGAAGAAACACATAGTTGTTTATAAAAAAAAACAAAAAACAAAAAAAAAAAACCACCCGAGGGGGGC

CCCCGAAC



>gi|71811977|gb|DR989368.1|DR989368 HDI_J_12.esd Horsegram (VZM-1) drought stressed subtracted cDNA Macrotyloma uniflorum cDNA, mRNA sequence

AGCTGGAGCTCACGCGGTGGCGGCCGCTCTAGAACTAGTGGATCCCCCGGGCTGCAGGAATTCGGCACGA

GGGAACTACTGAGTAGTCAACCTTCTAACTATAACTCTGATGGCTTCTCATCTGATTGGGAACATGTGGA

AAAAAGCTTGTAGGCCATTTAAAACCGGTGAAAAAGTTTGGGTATATAGTGGTCCATTAAGATTGTTTAT

GCTTTGGAAAATTTACAGATCGTGAAATAAGTGTNTACTCAGGTACGTCCATGTTACAGAATTTAGGTAC

TTATCAGTCTTTGTTACTGTATATGTATATAACGATGTCAGACTTTATGTTTATCTAATGTTTGCCTGCT

TTAAAAAAAACAAAAAAAAAANTTCCA



>gi|71811975|gb|DR989366.1|DR989366 HDI_I_95.esd Horsegram (VZM-1) drought stressed subtracted cDNA Macrotyloma uniflorum cDNA, mRNA sequence

CTGCTGTGTGTGCTGTGCCTGCTCATCAGCCACACTCAGTTGTGCATCNCACACGTGTGCTTGCCAGTGC

AATCCTGTGCCACTGCAGTGCACATCAAGCATNTGTTGCATTGTCAATGCTCTGTCACACCCATCACCTC

ATNNCATNTGC



>gi|71811973|gb|DR989364.1|DR989364 HDI_I_93.esd Horsegram (VZM-1) drought stressed subtracted cDNA Macrotyloma uniflorum cDNA, mRNA sequence

AAGCTGGAGCTCACGCGGTGGCGGCGCTCTAGAACTAGTGGATCCCCCGGGCTGCAGGAATTCGGCACGA

GGCTTTATCATTTTTCTTGGTAGTAGTATCTAATGTCATGATGTATAGGTTCTCTCTAAGATGCCAACAA

GTTCAGCACATTCTAATTTGGTCACTCTTTTCGGCTGCGATTTGGTTTGCTATGATTAATGAAAAATGGG

AATTCTCTCTAAAAAAAAAAAAAAAAAAAAAAAAAAAAAAAAAAAACCCCGGGGGGGGG



>gi|71811971|gb|DR989362.1|DR989362 HDI_I_91.esd Horsegram (VZM-1) drought stressed subtracted cDNA Macrotyloma uniflorum cDNA, mRNA sequence

AAGCTGGAGCTCCACGCGGTGGCGGCCGCTCTAGAACTAGTGGATCCCCCGGGCTGCAGGAATTCGGCAC

GAGGGTTCNACCGACACAGAGAATATGAACAAATAGGTAAATAACTAAAATATCATACTTAAGTGCAGTG

TTTGGGAGTTGAGTATAGAAGGAACTTTAAAGTACTT



>gi|71811969|gb|DR989360.1|DR989360 HDI_I_84.esd Horsegram (VZM-1) drought stressed subtracted cDNA Macrotyloma uniflorum cDNA, mRNA sequence

TGGAGCCACGCGGTGCGCGGCGTCTCTAAACTAGTGGATCCCCCGGGCTGCAGGATTCGGCACGAGGCTG

TGACTCTACTTGGTCTGCTAGCTGGTGGTGTACTTCTTGTCTACAACACAAGTGCTTTGGCACAATAGAC

TTTGCAGATCCATGTTTATGTTACAAGTTACAACTTAGTCCATGTAACTATCTAAAAATCAAGAACTCT



>gi|71811967|gb|DR989358.1|DR989358 HDI_I_82.esd Horsegram (VZM-1) drought stressed subtracted cDNA Macrotyloma uniflorum cDNA, mRNA sequence

GGCTGGAGCTCACGCGGTGGCGGCCGCTCTAGAACTAGTGGATCCCCCGGGCTGCAGGAATCGGCACGAG

GATTGAGTAGATCATCTCTGAAGGATCCCAAGCTTGCTCCTATCTACGAAATAAGATACCTTACGTGTTC

TAATGGGAGCATTAACTATGTATCTATGCATGTTGACTTTCTGGTCTTCGGATCTTCAACAAATCACCTA

AGAGACTACACTCCGTTGTTTTCTCTTTTGTGACTTTAATAAATTATGAACCCTTTTTTCCAATTTAAAA

AAAAAAAAAAAAAAAAAAAAAAAAAAAAAAAAAAAAACGGGGGGGG



>gi|71811965|gb|DR989356.1|DR989356 HDI_I_80.esd Horsegram (VZM-1) drought stressed subtracted cDNA Macrotyloma uniflorum cDNA, mRNA sequence

AAGCTGGAGCTCACGCGGTGGCGGCGCTCTAGAACTAGTGGATCCCCCGGGCTGCAGGAATCGGCACGAG

GCCTCGTGCCGAATTCGGCACGAGGCTGGACTAGGGCTGGTTAGACCTGCAATGAAAACTTGACGCCTTA

TAGATTTGGGTTGTTGGGTGTCAGATACTCCATTCAGGAGAAGAAAATTGTGCTTTAATTTCTCTTAATG

AATTTACCTTGTTACTGTAATAATCATTATCTGTGCTATCTATGATTTTCTACTTTTGTTTATAAAAAAA

AAAAAAAAA



>gi|71811963|gb|DR989354.1|DR989354 HDI_I_79.esd Horsegram (VZM-1) drought stressed subtracted cDNA Macrotyloma uniflorum cDNA, mRNA sequence

AAAGCTGGAGCTCCACGCGGTGCNCGGCGCTCTAGAACTAGTGGATCCCCCGGGCCTGCAGGAATTCGGC

ACGAGGGGAGAAATTTATCAAAAACATATTTAAATTCAAAATTGTGCAAGAGGGACTTCATTGGTGTCAT

TTATTTCTCAAAATCAGTTTAATTCATATTAGCTAAAAGCTAATTAAGTTTGAAAAGTTTATTAAGTGTA

ACAAATATCATATCTTTTGTTCTCTGCTACAATGATGATTGGAATTCCCCAAAAAAAAAAAAAAAAAACC

CAGAGGGGGGGGCGCC



>gi|71811961|gb|DR989352.1|DR989352 HDI_I_71.esd Horsegram (VZM-1) drought stressed subtracted cDNA Macrotyloma uniflorum cDNA, mRNA sequence

AAGCTGGAGCTCACGCGGTGGCGGCGCTCTAGAACTAGTGGATCCCCCGGGCTGCAGGATCGGCACGAGG

ATTTTTTTAATTATTACTATTATTTGTCACCCTTTCCACTAAAGAACCTTTCTTTCTACTTAATGTTCAG

TACATATCTTTCTATTTATCTATTTTTGTCTTTTCGGTTACTCTATCTCCTCCTTTTAACCATCACCGAC

GACATTCGCTTCTTGTAATCCAATGTTAGTTACTCATTTATGTGAATCTTGTTTGTTTCTGCTAAAAAAA

AAAAAAAAAAAAAAAAAAAAAAAAAAAAAAAAAAAAAAAAAAGGGGGGGGGG



>gi|71811959|gb|DR989350.1|DR989350 HDI_I_7.esd Horsegram (VZM-1) drought stressed subtracted cDNA Macrotyloma uniflorum cDNA, mRNA sequence

AAGCTGGAGCTCACGCGGTGGCGGCGCTCTAGAACTAGTGGATCCCCCGGGCTGCAGGATTCGGCACGAG

GCCTCGTGCCGAATTCGGCACGAGGCATTATTATACTACTACTACCAGTGAATAATCATAATCAAATCAA

TGTGATGTTATGTTATGTTATTGCAGTGCACCGTTGTTGTATGTAATGTAATCAATCCACCTCAGTTAGT

TAACTTTTTAAAAAAAAAAAAAAAAAAAAAAAAAAAAAAAAAAAAAAAAAAAA



>gi|71811957|gb|DR989348.1|DR989348 HDI_I_68.esd Horsegram (VZM-1) drought stressed subtracted cDNA Macrotyloma uniflorum cDNA, mRNA sequence

AAAGCTGGAGCTCACGCGGTGGCGGCCGCTCTAGAACTAGTTGGATCCCCCGGGCTGCAGGATCGGCACG

AGGACCTTTTTATGAAAGTTGCCCGTGTATGAGATATCTCGCTCTACAATTGAGTTTGTTGCAGAAACGA

TATCGTTTAGAGTTAATATTTTTATATATCTTGTCCTTTGGGTGCAAAAAAAAAAAAAAAAAAAAAAAAA

AAAAAAAAAAAAAAAAA



>gi|71811955|gb|DR989346.1|DR989346 HDI_I_60.esd Horsegram (VZM-1) drought stressed subtracted cDNA Macrotyloma uniflorum cDNA, mRNA sequence

AAGCTGGAGCTCACGCGGTGGCGGCGCNTCTAGAACTAGTGGATCCCCCGGGCTGCAGGATTCGGCACGA

GGCCTCGTGCCGAATTCGGCACGAGGGAAAAGAAAAAAGATTCAATGAATAAATAATAGTAACATTTGTG

GTTTTTAAACCAGGTTGGGGGGATGGAAGTCGAATTGCCTTTTTCTAGTTTCTCCGGAAAAAATCAATTT

TCCATTTTACAATCCCTTTTTA



>gi|71811953|gb|DR989344.1|DR989344 HDI_I_58.esd Horsegram (VZM-1) drought stressed subtracted cDNA Macrotyloma uniflorum cDNA, mRNA sequence

TTAAGCTGGAGCTCACGCGGTGCCGGCGTTCATAAACTAGTGGATCCCCCGGGCTGCAGGATTCGGCACG

AGGCCTCGTGCCGAATTCGGCACGAGGGGGGAGGTAGTGACATTGATCCACAAGAAGCACAGCAAACTCT

TGAAGTAGCAGAAACTAATTTGAATAAGGCTGAAGGCAAGAGACAAACAATTGAGGCAAATCTAGCTCTT

AGACGAGCTAGAACACGAGATAGAGGCTATCAATATGATTTCCTAACAAGTTGGTGCGTCCGAAAATCCG

CCGAATATCCAAATACAAAAAATAAAAAAGAAACAGTAAGAAAAGATAGAAANAAAAAAAAAAAAAAAAC

ANCACA



>gi|71811951|gb|DR989342.1|DR989342 HDI_I_48.esd Horsegram (VZM-1) drought stressed subtracted cDNA Macrotyloma uniflorum cDNA, mRNA sequence

AAAGCTTGGAGCTCCACGGCGGTTGCGCGGCGTCTCTAGAAACTAGTGGATCCCCCGGGCTGCAGGATTC

GGCACGAGGGTTTGTTTTGCCGGTTATATTTTCAGGTTTCTGCCGAATTCATGTACCCTTAACAGGTTCT

TGATCGGTGGTGGTCATTT



>gi|71811949|gb|DR989340.1|DR989340 HDI_I_46.esd Horsegram (VZM-1) drought stressed subtracted cDNA Macrotyloma uniflorum cDNA, mRNA sequence

ATCTGAGCCACGCGGTCCGGCGTTCATAAACTAGTGGATCCCCCGGGCTGCAGGATTCGGCACGAGGCCT

CGTGCCGAATTCGGCACGAGGGCTTGATGATTTTCTGAATGAACTTGTGCAGAAGCGTGAAGTGTGGTTC

CGAGATGGCAAGTGTAAACCTTACTGTATAGCTTCTGCTGTGGGACAGAAAAATTGCCTCTGTGTTATCC

ATAGGTGATTGTTGTGCTAGGTTGTATTGTGACTATGTTANGTACTAAATGTGTTTCTATCGTGTTATTG

ATGCNGGTGCTGGATTTCAATAGATAATT



>gi|71811947|gb|DR989338.1|DR989338 HDI_I_44.esd Horsegram (VZM-1) drought stressed subtracted cDNA Macrotyloma uniflorum cDNA, mRNA sequence

GCCTAATTAGAAGAAAGCTCCAAGGAAAATTGTATGAGCGAGAGAAAGAACTGACAACTAAAAAGGCCCA

CAGTCAGACTGGAGAAACAGTGCGAGCAGGATAACAGGCTTCACTTGGGGAACATCAATCATCTGCATGG

CATCCCTCCAAATCAGAACAGCTTTCTGAGAGGATTCCNAGTCTGGGTTTTCCCTTTTTACTTC



>gi|71811945|gb|DR989336.1|DR989336 HDI_I_36.esd Horsegram (VZM-1) drought stressed subtracted cDNA Macrotyloma uniflorum cDNA, mRNA sequence

AAGCTGGAGCTCACGCGGTGCGCGGCGNTCTAGAACTAGTGGATCCCCCGGGCTGCAGGATTCGGCACGA

GGTGATCACTGTGTGATGTGGAATGGGTCTATATTGACCCTATTAAAATAAATTTCGTTTGTGTTGGACT

GTATTGTCCCCTTAATAACATGGTACTTTATCTAATATGTATTGTCATGAATATGTAATATATTATTATT

ATTATTAATATCTATGTTTGCCGTATTCTTAATCAAAAAAAAAAAAAAA



>gi|71811943|gb|DR989334.1|DR989334 HDI_I_34.esd Horsegram (VZM-1) drought stressed subtracted cDNA Macrotyloma uniflorum cDNA, mRNA sequence

AAGCTGGAGCTCACGCGGTGGCGGCGCTCTAGAACTAGTGGATCCCCCGGGCTGCAGGATCGGCACGAGG

CTTAGAAGCATAAGCCAGCGCTTGGAAACGCAAGCACATGCACTCCTGTCCTCGCCTGCGGGCAACCTTC

TGAAGATCTCAAAGAGACACTCATCTGGCAAGGATTCAATAGAGGTTTTCTGCTTTTGCTCAAACCATTC

TCCATCAAAAACGAATGGAGCGTTGACGCGCGATCTCTTCCTAGGAGGAAAGTATACATCAACTTGAGGG

CCAAGAGGCAGGAAGAAACTTGCTTCCTTGGGGTTGGCGTATATCGATCCCCCAGGGCAAAAGTCATCAC

CTCCTGAGAACCCAAACGTATGCACAAAGTCAGTAAAGCACAAAATNCACCATTATTCAACATTATTTAA

CACAAGCAGATTCAACCATCTAAACAAAATGATTCCCGTTTTATCCAAACAACACCAGGTTCAGATAAAC

TTTAAACATAAAAACAAACGACAATAAAAATATCAAT



>gi|71811941|gb|DR989332.1|DR989332 HDI_I_32.esd Horsegram (VZM-1) drought stressed subtracted cDNA Macrotyloma uniflorum cDNA, mRNA sequence

AAAGCTGGAGCTCACGCGGTGGCGGCGCTCTAGAACTAGTGGATCCCCCGGGCTGCAGGATTCGGCACGA

GGCCTCGTGCCGAATTCGGCACGAGGGGGGGGGCCCGGTACCCAATTCGCCCTATAGTGAGTCGTATTAC

AATTCACTGGCCGTCGTTTTACAAC



>gi|71811939|gb|DR989330.1|DR989330 HDI_I_24.esd Horsegram (VZM-1) drought stressed subtracted cDNA Macrotyloma uniflorum cDNA, mRNA sequence

GGCTGGAGCTCACGCGGTTGGCGGCGCTCTAGAACTAGTGGATCCCCCGGGCTGCAGGATCGGCACGAGG

CCTCGTTGCCGAATTCGGCACGAGGGTACTTTTAGAATTCTATTTTAAACGTAGTGTTTCCTTTTTGGCG

ATATGGATACTTTGGTAGTGAGTATTTTACTTCATAATTTCGGTGAACTTTTGTTTCAACGGAGATTTAT

CGTTTGAAAACATTTCAAATTTTGTTTGTGATTTTAAAAAAAAAAAAAAAAAAAAAAAAAAAAAAAAAAA

AAAAAAAAAAAAAAAAAAAAAAAAAA



>gi|71811937|gb|DR989328.1|DR989328 HDI_I_22.esd Horsegram (VZM-1) drought stressed subtracted cDNA Macrotyloma uniflorum cDNA, mRNA sequence

AAGCTGGAGCTCACGCGGGTCCGGCGTTCTAGAACTAGTGGATCCCCCGGCGCTGCAGGAATCGGCACGA

GGAAGAATAGTACGTAGTCTTTGGTATAAGTGTTTGGTTTGATTTATGTAAAGGTGTGGTTTAAATTTGC

CTGTTCTGGTAGCTGTAACTGTTGGGCACTCATGTAAGCAAAAACAAGCTTAATTAGCTCATAGCCTCTC

TTAATTTGGCCTATATCACTCTCATATTGTACCACTCTCAGTGTATCCCTCAGTGAAACTCATTTCAATT

CTCTTTTCCCTTGTATACGTAATTAATGTTCAAGTAATGAAAAT



>gi|71811935|gb|DR989326.1|DR989326 HDI_I_20.esd Horsegram (VZM-1) drought stressed subtracted cDNA Macrotyloma uniflorum cDNA, mRNA sequence

AAAGCTGGAGCTCCACGCGGTGGCGGCCGCTCTAGAAACTAGTGGATCCCCCGGGCTGCAGGAATTCGGC

ACGAGGTGATATATCAGTGGAACTTTGCCAAGTTTGTGGCTTCGATAAGAAGGGACAAGTAGTGGGTCGC

GTATTATCCCACGACGTTTGTCTCAGCCGTTGAGCAGAGAAATCCGGTTCAAAACTTCATAGGCGTATTT

CTGATGATAGGCAAACTTCTGGGTCGGAGAGGATATGTGCGAAATGTCATTGATCTTTATGATTCCTTTG

TAGCTAACACATTGGGTCAATTTATGCAACTATGCGACACTTTT



>gi|71811933|gb|DR989324.1|DR989324 HDI_I_12.esd Horsegram (VZM-1) drought stressed subtracted cDNA Macrotyloma uniflorum cDNA, mRNA sequence

TGGAGCTCACGCGGTGGCGGCGCTCTAGAACTAGTGGATCCCCCGGGCTGCAGGATCGGCACGAGGGGCT

TTCGAATCGGTTGGGGGGGAAGGGTGAGAAGGAGATGGGGGATTTGAGACGGAACCTTTTTCCGACGCCG

TTTTCGTTTTTTGTCGGAAGCACGTTCGAGGGTGCGCCGAGGGAGCAGCAGGCGCTGCTTGAGTTGGAGG

ACACGGCGAAGAGGTTGAAGAGGGAGAAGGAGACTCTGAAGAACACTCTTAACTACCTCACTGCTGCTTC

TGCTGTCAAAGATGCTTTTCCTTCTTCTTGATGATGTGAAACTCCATGGGGATATATACTCTA



>gi|71811931|gb|DR989322.1|DR989322 HDI_I_10.esd Horsegram (VZM-1) drought stressed subtracted cDNA Macrotyloma uniflorum cDNA, mRNA sequence

AAGCTGGAGCTCACGCGGTGGCGGCGCTCTAGAACTAGTGGATCCCCCGGGCTGCAGGATCGGCACGAGG

CTGCGGAGGGACTAGTTGAAGGAGGAAAGAAAGAGATTTTCACACCAATGTACTTCTTCTTGGCACGGAA

GCCTGATTCAGACAGTAACTAAACTGGTTGTGTACAAATAGGTTGCTGTTGTTTATTGTGATTCTGGTAG

GATGAATCGATTAATTTTGTCCTTATTGCCGTAGTTTCGTTCTGTTTCGAGTTGCTTGTTTTTGTTTTAG

GTTACCAAAACCTTTGCTACACCTCTAGCGTATTTCTTTTTATCCTCAAAAAAAAAAAAAAAAAAAAAAA

AA



>gi|71811929|gb|DR989320.1|DR989320 HDI_I_89.esd Horsegram (VZM-1) drought stressed subtracted cDNA Macrotyloma uniflorum cDNA, mRNA sequence

AAAGCTGGAGCTCACGCGGTGGCGGCGCTCTAGAACTAGTGGATCCCCCGGGCTGCAGGAATCGGCACGA

GGGACGTCTTGCTTTGTTGGCTTTTGTGGGATTCTGTGTTCAACAATCAGCTTATCCTGGAACTGGACCT

TTGGAGAACTTGGCTACTCACTTGGCTGACCCAT



>gi|71811927|gb|DR989318.1|DR989318 HDI_I_87.esd Horsegram (VZM-1) drought stressed subtracted cDNA Macrotyloma uniflorum cDNA, mRNA sequence

AGCTGGAGCTCACGCGGTGGCGGCGCTCTAGAACTAGTGGATCCCCCGGGCTGCAGGATCGGCACGAGGC

CTCGTGCCGAATTCGGCACGAGGGGGAAGTGCTGTGGATGCTGTTTACACTGCGAAAGTGAATGCTCCAC

TTGGTGTTGAAGTTAGTGTGTCCCCCAGAACACTTGTGTTCAGTAGTGGAATCACGACACAGGCATTTGA

GATTGCGTTTAGTAGAGTTACGCCCGATACTTCTGACAGTTTTGGGTCAATTGAATGGAGTGATGGAAGT

CACTCTGTGAGGAGTCCAATTGCTGTTTCATGGAAATGGACTGACAATTCCTTATCATCCCTTTGAATTG

CTTTGGAGACACGACATTCCCAGGTTATGGATTTAGTATCTTTGTATTTTTTTCCTCTTATTTTTTATTA

TAAAGCTACAAATAACTTTGAAGTAGTTGATGAAGGAATTCCAGTAGGATCTGGGAATGAGATGTGATTT

GTAAGATGATTGGTTCAATTCAGATTATTGCAGTTTGGCAAAAAAAAA



>gi|71811925|gb|DR989316.1|DR989316 HDI_I_85.esd Horsegram (VZM-1) drought stressed subtracted cDNA Macrotyloma uniflorum cDNA, mRNA sequence

AAAGCTGGAGCTCACGCGGTGGCGGCGNTCTAGAACTAGTGGATCCCCCGGGCTGCAGGATTCGGCACGA

GGCCTCGTGCCGAATTCGGCACGAGGATTTTGAGAGTGTTTCTGTGAATTTAGTAACTTTTTGCCACAGT

AGTTCAGCAGCAGGGAATTTTATTCAATTCTAGCAACCATATTAGTAGAATTTTTGTAGTTTGTGTTGTT

TACATCTGATATTAAGAATTCAATATAGAAGAACATGAATAATAATTTACCGAAAAAAAAAAAAAAAAAA

AAAAAAAAAAAAAAAAAAAAAAAAAAAAAAAAAAAAAAAAAAAAAAAAAAAAAA



>gi|71811923|gb|DR989314.1|DR989314 HDI_I_77.esd Horsegram (VZM-1) drought stressed subtracted cDNA Macrotyloma uniflorum cDNA, mRNA sequence

CTGGAGCTCACGCGGTGGCGGCGCTCTAGAACTAGTGGATCCCCCGGGCTGCAGGATCGGCACGAGGGAA

AGGGAGGAGGGGCGCTCTCATCCACGTGTAATGCTGCAGGTGAGATATTCACGATTGCTGCAATCATCAA

TGGCCTTGTTCTTGTCGGGGTTGCTGTTGGGTTTGTGCTCCTGCGAGTTGAAGCATGGGTGGAGGAGTCC

GAGGCTGAGTGAAAGTTTAATCAGATAAGCAAGTAATTCATATCCACAATAGTTGTATGTTCCTACTATA

TTGATGTTGCGCCTGTTCTTCTTTTCATTATGACTTGTATATCTCTATCTATCAAATTTCATCATTTTCT

TGTTTCGTTCGTAAAAAAAAAAAAAAAA



>gi|71811921|gb|DR989312.1|DR989312 HDI_I_75.esd Horsegram (VZM-1) drought stressed subtracted cDNA Macrotyloma uniflorum cDNA, mRNA sequence

AAAAGCTGGAGCTCACGCGGTGGCGGCGCTCTAAACTAGTGGATCCCCCGGGCTGCAGGAATCGGCACGA

GGCCTCGTGCCGAATTCGGCACGAGGCTAAATCTGAAGGAGTGGCTACATGAAGAGATTGATATCTCAGC

TTCATTTCAGATTATGTGCATTCCACTTGGTCTTTGTGGTTGCAAACCAAGTTAATTTGAATAATAGAAA

AGAAACTTGTGATTTGGATTCAAGGTTATTGTGTTTTAAATATGGTACTATTTAGCATTTCATCTTGTAT

CCTTAATGTCTTGGATTCCTTGAATTGTCTTTAATAATAACATATAAAGTTGTTTTTTTAAAAAAAAAAA

AAAAAAAAACNCCAAGGGGGGGCCCGGGC



>gi|71811919|gb|DR989310.1|DR989310 HDI_I_73.esd Horsegram (VZM-1) drought stressed subtracted cDNA Macrotyloma uniflorum cDNA, mRNA sequence

AAAAGCTGGAGCTCACGCGGTGGCGGCGCTCTAAACTAGTGGATCCCCCGGGCTGCAGGATCGGCACGAG

GCCTCGTGCCGAATTCGGCACGAGGTAAACGAGGCTCTAAAGCGTTAAGGCAACAACCATTCCTCTGTTC

ATGTAGTAACATTTCACCTGGTGTGCTTAAAGTTACTCTTAATGTACATGTACTCTTAAGAAAAGGCTCA

TAACAGACCTTAACATAAAAAAAAAAAAAAAAAAAAANNCCCCGGGGGGGGGCCCGGGCCCCATTCC



>gi|71811917|gb|DR989308.1|DR989308 HDI_I_65.esd Horsegram (VZM-1) drought stressed subtracted cDNA Macrotyloma uniflorum cDNA, mRNA sequence

AAGCTGGAGCTCACCGCGGTGGCGGCCGCTCTAGAAACTAGTGGATCCCCCGGGCTGCAGGAATTCGGCA

CGAGGGGTTTCTAGTGTGGAATCTTGCTTAAGACACTTT



>gi|71811915|gb|DR989306.1|DR989306 HDI_I_63.esd Horsegram (VZM-1) drought stressed subtracted cDNA Macrotyloma uniflorum cDNA, mRNA sequence

AACAGCTGGAGCTCACCGCGGTGNCGGCCGTTCTAGAAACTAGTTGGATCCCCCGTGGCTGCAGGAATTC

GGCACGAGGTTTTTTTTTTGTAGTGGATGAAGATACTCATGCGCTTATGTTGTCACAAAACTGGTTAAAA

TTACCATTATCGATAAAGGAATTGCGGCCAAANGCCCACCACAAGGCATACACACGAGAAAT



>gi|71811913|gb|DR989304.1|DR989304 HDI_I_61.esd Horsegram (VZM-1) drought stressed subtracted cDNA Macrotyloma uniflorum cDNA, mRNA sequence

AAGCTGGAGCTCCACGCGGTGGCGGCCGCTCTAGAACTAGTGGATCCCCCGGGCTGCAGGAATTCGGCAC

GAGGCAGCTTTTGCTGAGCGTTTTAGTTTAAAAGGCATATAATACGGGTTTTCTCTGTAATAAGGTATAA

CAACTACTTGCGCAGATTTTTCCAGATCTCCTTGACTCACTCGTAATTTTTCGTTCTGCATTTGGTCAAT

CGATAATTGGTTAGACCTCCGCTTGCTNGTTCAACAAAATACAAAATAAAACAATAACAA



>gi|71811911|gb|DR989302.1|DR989302 HDI_I_54.esd Horsegram (VZM-1) drought stressed subtracted cDNA Macrotyloma uniflorum cDNA, mRNA sequence

TTAGCGGAGCCACGCGGTGCCGGCGTTCATAGAACTAGTGGATCCCCCGGGCTGCAGGATCGGCACGAGG

GAGAGCATGCATGATGATAGTTGCTTGGTCCTTGCTTACTACAAGGACGGTGCCACTGATCCGACATTTC

TGTACTTTGCATATGCCTTGAAGGAGGTTAAGTGCTAAGTGACTCAACATAGCAGGCTATTTACCATCCT

TTTTGCTCTATTATTTCTAGATTACTTGGAACTTTGACCTTGGTAACTATTTATAATTTAATAACTAGAC

TATTTTTATGGTGCATTCACTTAATACAATTATCGTAGTCAAATTATCATGGAATTTGACATTTACATTA

ATTTATATGAATTTATGATGCTNTATGAATTTTCAAACAAAACAACAAAAAACAACACAAACAACCACAA

GAGGGGGGCGCCCGCGGAA



>gi|71811909|gb|DR989300.1|DR989300 HDI_I_52.esd Horsegram (VZM-1) drought stressed subtracted cDNA Macrotyloma uniflorum cDNA, mRNA sequence

AAGCTGGAGCTCACGCGGTGGCGGCGCTCTAGAACTAGTGGATCCCCCGGGCTGCAGGAATTCGGCACGA

GGTTATTCTATTGCATGTCTTGACTAACAAATGATGTAACAGAAGAGAAGAAAAGAAAATAGACACCCAG

AGGTTCAAACAGCTAATCCAAAATGGACATGACTAGTACTGTACAGATGGACCATAAGTATGAACTACTC

TACTGCGTGAGGAACTGTAAGTGGAGTGAAAATGTACAGTTGAGAGTCGTGGACATCAACTCGAGGGGGG

GCCCGGTACCCAATTCGCCCTATAGTGAGTCGTATTACAATTCACTGGCCGTCGTTTTTACAAC



>gi|71811907|gb|DR989298.1|DR989298 HDI_I_50.esd Horsegram (VZM-1) drought stressed subtracted cDNA Macrotyloma uniflorum cDNA, mRNA sequence

AAGCTGGAGCTCACGCGGTGGCGGCGCTCTAGAACTAGTGGATCCCCCGGGCTGCAGGATCGGCACGAGG

GGAAATACCTGGTGAAAGTACTCAATTCGTAGAGCGTAAGCGTGATGTACCCTTTGAGTTATGGCTTGTG

TTATCCGAATTTTAAATTTGAAAGGATAACTTAATCAAAGGTAAAGGGATGTTCAATTGAATTGTATTAC

CGTTGCCACTCCAAAACCCTTTATCTGGATTGTAGAAGTGCGACTCACATTTTTATACTGAATCTTGGCA

CGTGCACAATTTGCTTTATATTTAAAAATATTAAAATTGCTTAATTTTACCAAAAAAAAAAAAAAAAAAA

CCTCAAGGGGGGGCC



>gi|71811905|gb|DR989296.1|DR989296 HDI_I_49.esd Horsegram (VZM-1) drought stressed subtracted cDNA Macrotyloma uniflorum cDNA, mRNA sequence

AGCTGGAGCTCACGCGGTGGCGGCGCTCTAAAACTAGTGGATCCCCCGGGCTGCAGGAATCGGCACGAGG

CCTCGTGCCGAATTCGGCACGAGGGGGAAAAGATGATGAAGGTGAGGACGAAGAAGAAGATGAGAAAAAG

AAGAAGAAGGATAAGAAAGAGAAAGAGAAGGATCACAAAAAGGAAAAGAAGGAGAAAGATGATGGAAAGA

AGGACGGTGAGAAAGAGGATAGCAAGGTTGAAGTTTCTGTAAGAAGATATTGATATAGAAGAAATCACAA

AGGAAGGTGAGAAAAGAAGACAAAGAAAAAAGATGGGAAGGAAGTGAAAGAAGAAAAAAAAAAAAAAAAA

CCCCCAGGGGGGGCCCCGG



>gi|71811903|gb|DR989294.1|DR989294 HDI_I_41.esd Horsegram (VZM-1) drought stressed subtracted cDNA Macrotyloma uniflorum cDNA, mRNA sequence

AAGCTGGAGCTCACGCGGTGGCGGCGCTCTAGAACTAGTGGATCCCCCGGGCTGCAGGAATTCGGCACGA

GGGAAGCTCGGGTGGTCTATGACAGAGAGAGTGGTCGATCACGAGGTTTTGGCTTTGTCACAATGTCCAG

TGAGACTGATGTGAATGATGCAATTGCTGCTCTTGATGGTCAGGACTTGGATGGGAGACCAATCAGAGTG

AATGTTGCAGAGGAAAAGCCTAGACGCAGCTCCTTCTGAGTGGTAACTTGAGAGTAATCTTCCATGTCCG

GCTGATTGAGTTCCCTTTCAATGGTTAAGTATACAGTGTCAATTTTTACCTTCTTTGGCAGCTGGTTTCA

GATTAGATTTGCATATCTTAATGTTATTAATTTGATGTTCCAAATGGAAAATGATTNAAAGTTACACATT

CCAACTCTC



>gi|71811901|gb|DR989292.1|DR989292 HDI_I_4.esd Horsegram (VZM-1) drought stressed subtracted cDNA Macrotyloma uniflorum cDNA, mRNA sequence

AAGCTGGAGCTCACGCGGTGGCGGCGCTCTAGAACTAGTGGATCCCCCGGGCTGCAGGAATTCGGCACGA

GGGTTCAGAGGAGGCCTCAAATGGAGTAGCTGTTAACTCTGATGAAATTGAAGCTGCCACATGAGGCTGC

TGGCGAGTGTTGTGATGTCATAGTAAACAATCTCATTGTTCTGTTCAGTAAAATATCTTTTGTTGGTACA

AAATGTATTTGAATTTCTTGTAATATCTTTCTATTTCTTAATAATAAACTCAGTGGTTTTCTCCTAAAAA

AAAAAAAAAAAAA



>gi|71811899|gb|DR989290.1|DR989290 HDI_I_38.esd Horsegram (VZM-1) drought stressed subtracted cDNA Macrotyloma uniflorum cDNA, mRNA sequence

AAGCTGGAGCTCACGCGGTTGGCGGCGCTCTAGAACTAGTGGATCCCCCGGGCTGCAGGATTCGGCACGA

GGCCTCGTGCCGAATTCGGCACGAGGGTTTAGTGGGGACCGAGTTGACCAAACTTGAAGAGAAAACTATA

CAGTTTTCACATGGGGAATTTGTTACTGTGAAGTTGCTGTACTTTTTCTACCATTCCACAGGCTTTCTTC

TTCCTTTCAGAATGTATTTTTGTAAAGAAAACAGGATGGTAGTGTTAACTCATGTTATTCATAGATATTT

TTACATACTAATTCGTCAATATACTTTTAGTTGATACTTATCAATGTAATTAAGTGTCGTAGTAGAAACA

TGCTAGAGTTTGGAATTTGAACGACTTAAAAAGTTCTGAACATCCTTCATTTGTCAAAAAGGGGGGGCCC

GGTACCCAATTCGCCCTATAGTGAGTCGTATTACAATTCACTGGCCGTCGTTTTACAA



>gi|71811897|gb|DR989288.1|DR989288 HDI_I_30.esd Horsegram (VZM-1) drought stressed subtracted cDNA Macrotyloma uniflorum cDNA, mRNA sequence

AGCTGGAGCTCACGCGGTGGCGGCGCTCTAGAACTAGTGGATCCCCCGGGCTGCAGGATTCGGCACGAGG

CCTCGTTGCCGAATTCGGCACGAGGGCCAGTTTGGAGTTATTGGAAGGCAAAGAGCTTCCGGGAGTCCTT

GCTCTTGATGAGGCCACACCGGTCCCTGTGTAAGACAATTTTCAACTTGGAAAATTTTCCCATCCTCAGT

TTGTTAGTGGTCAATTAAATCCTTGGTTAAAAAAGGAAGCCAATTAATTGTTTGGTAAGGAACCCTTCTC

GGAAGTCTCTGTGTCTCACTGTGTTAAATTTATAATC



>gi|71811895|gb|DR989286.1|DR989286 HDI_I_29.esd Horsegram (VZM-1) drought stressed subtracted cDNA Macrotyloma uniflorum cDNA, mRNA sequence

GCTGGAGCCACGCGGTGGCGGCGCTCTAGAACTAGTGGATCCCCCGGGCTGCAGGATCGGCACGAGGATA

TTGCACATGTAGTGGTGATGCCAAACATCACTATTGAGAAACTGGATGATTTTCTGAACGAGCTTCTGGA

GAAGCGTGCTACCTGGTTTCAAGATGGGAAGGATCAACCTTACTGTATATCGTCAGATGTAGGTGAAAAG

AATTGCCTTTGTGCTTTGCACAAGTAATTGCAGCATCCAATCTAGAGTATGCTCCTGTAGTTGAGTTTTG

GAGTGTTATGCGTGTCTATGTTCTATCCGGATTTTGATGTTTTTAATTACAATTAGAACCTTCTTTGCTA

AAAAAAAAAAAAAAAAAAAAAAAAAAAAAAA



>gi|71811893|gb|DR989284.1|DR989284 HDI_I_27.esd Horsegram (VZM-1) drought stressed subtracted cDNA Macrotyloma uniflorum cDNA, mRNA sequence

AGCTGGAGCTCACGCGGTGGCGGCGCTCTAGAACTAGTGGATCCCCCGGGCTGCAGGAATCGGCACGAGG

CCTCGTGCCGAATTCGGCACGAGGCACAAGGGAACAAATGTTTTCAACTGGGATTGTGGCACTTTAGCGT

TTAGTTTCTGTCTCTGGTGTTGGAAAGTCATATAAAACAAGCAAGAGACTGATATACTTTTAGTTTTGAA

TTCACATCATATAAGACTATAGTACTGTTATATGTGAATGAAAAAATTAAGGCACTTTGGTGTAACCATA

TAGGAAAAATTTTGATCTCGGTAAAATGGTAGGGATTTTGATGTAGTGAATACTAATAAGAGCTTTCTTT

TCTTCTTTTTTTGTATCTTACTGAGAATTATAAATTTGGGATTGCACAAAAAAAAAAAAAAA



>gi|71811891|gb|DR989282.1|DR989282 HDI_I_2.esd Horsegram (VZM-1) drought stressed subtracted cDNA Macrotyloma uniflorum cDNA, mRNA sequence

AAGCTGGAGCTCACGCGGTGGCGGCGCTCTAGAACTAGTGGATCCCCCGGGCTGCAGGATTCGGCACGAG

GCTTGTACTTCCACCATTCATTTCACTATTTAAAGAAGATCTGAAAGGCATTTTGTATCCTACTTTCCTA

ATCTGTAGAGTATGAGATCATGTTTGACAATGATAATAAATCCAGATACTTGTTGATTCTCGAACATAAA

CGCCGTTGGATCAAATTCAGAGCATATTTTTATGCTAAAAAAAAAAAAAAAAAAAAAAAAAA



>gi|71811889|gb|DR989280.1|DR989280 HDI_I_17.esd Horsegram (VZM-1) drought stressed subtracted cDNA Macrotyloma uniflorum cDNA, mRNA sequence

AAGCTGGAGCTCACGCGGGGCGGCGCTCTAGAACTAGTGGATCCCCCGGGCTGCAGGATCGGCACGAGGA

GAAGGTGGTGGCTTGGGTGGTCTTGCTAAATTGGCTGGAGGCTTCTTAAAATAATAGTTGTAGAAGAAAA

TGAAGAAGATGAAGGTCTTTAGTTTTTCTCTTTCTGTTGTTGTTGTTCTTCTTCTTGTATGATGTAAGGT

TGAATAAAGAAAGATATAGTCTGGTATTTGTTGCAGAAGACCTTTTCTCAATTCCACAAACATGTTTGTA

CAAGACTCTTACTATCTTCTCTTTTTCTATGCATTTGTTTCCTTGTTCATGTGTATTAATTATGAATGTT

GAATCCTCTAGGTTTTCTTCTAAAAAAAAAAAAAAAAAAAAAAAAAAACCCAGGGGGG



>gi|71811887|gb|DR989278.1|DR989278 HDI_I_15.esd Horsegram (VZM-1) drought stressed subtracted cDNA Macrotyloma uniflorum cDNA, mRNA sequence

AGCTGGAGCTCACGCGGTGNCGGCGCTCTAGAACTAGTGGATCCCCCGGGCTGCAGGATCGGCACGAGGC

TTATCAGTTTCTCTCTAGTTTTGTTGTATACAACTTTGCATGTATCATCTACTGTTGCCGCCTTTGTACT

TGTACAAAAATTTCTTCTGTTTGTTTTCCCAATCTTCATAGGAAAAACAAGGAAATGAATTGTGTTTAGA

ACGTAACCACACATGAACTGAAAACATGTCAAGTGCCCCAGCTTCGTCCAATGACGAGTTTGAA



>gi|71811885|gb|DR989276.1|DR989276 HDI_I_13.esd Horsegram (VZM-1) drought stressed subtracted cDNA Macrotyloma uniflorum cDNA, mRNA sequence

AGCTGGAGCTCACGCGGTGGCGGCGCTCTAGAACTAGTGGATCCCCCGGGCTGCAGGAATCGGCACGAGG

GACTGGTAAGGAGCTATGCAGCCGGTTGGTTTCTGTGATATGAAACCAATTTGGTTTGAATAAGGCTTGT

TTCGTTTGGGGCTGTGTGCGATTTGTTTTAACTATATAATTTGTGTCTTTCTTCACCTTCTTAATATATC

AAAACAAATTGAGAAAAATGAAAATAAAATATTCTTACATGTTAAAAAAAAAAAAAAAAAAAAAAAAAAA

CCCCGGGGGGGGGCCCCGGCCCCCTCCCCCCT



>gi|71811883|gb|DR989274.1|DR989274 HDI_H_96.esd Horsegram (VZM-1) drought stressed subtracted cDNA Macrotyloma uniflorum cDNA, mRNA sequence

AGCGGAGCCATCGCGGGGGGCGGCCGCCTATAACATGGGTGACCTCCCGGGCCGTCAGGAANTTGGCACG

AGGGGGGGTGGAGAAATAGCAAGATTGTGTGCCCTCTGTAAGCNGTTGTATACGTCCAGAAAT



>gi|71811881|gb|DR989272.1|DR989272 HDI_H_94.esd Horsegram (VZM-1) drought stressed subtracted cDNA Macrotyloma uniflorum cDNA, mRNA sequence

AAGCTGGAGCTCACGCGGTGGCGGCGCTCTAGAACTAGTGGATCCCCCGGGCTGCAGGATTCGATATCAA

GCTTATCGATACCGTCGACTCGAGGGGGGGCCCGGTACCCAATTCGCCCTATAGTGAGTCGTATTACAAT

TCACTGGCCGTCGTTTTACAACGA



>gi|71811879|gb|DR989270.1|DR989270 HDI_H_92.esd Horsegram (VZM-1) drought stressed subtracted cDNA Macrotyloma uniflorum cDNA, mRNA sequence

CCAAAGCTGGAGCTCACGCGGTGGCGGCGCTCTAGAACTAGTGGATCCCCCGGGCTGCAGGATTCGGCAC

GAGGCCTCGTGCCGAATTCGGCACGAGGCTATTGTGAGACTCACCAGGGAGATTGTGCCTGGCATGATTG

TCACTGGCATGGAAGTTGCAGAAATTGATGGTTCGCCAAGAATGGGTCCTACATTTGGGGCGATGATGAT

ATCAGGACAAAAAGCAGCTCATTTGGCATTGAAGGCATTGGGGAGGAACAATGCAATTGATGGAACATGT

GGTGTTGGAAGAGAAGAACCGGCACTTATTTTTGCTTCTGCGGACACTGGAGAGATCGTTGATGCTTAAA

CTTTTTAGTTACATACCTTTTCTTTTCTGTTTTACCTGAAGCTTATCATTTGAATTCTGGAATAAATAAA

GAAAAATGTGAATGATGCAAAAAAAAAAAAAAAAAAAAAAAA



>gi|71811877|gb|DR989268.1|DR989268 HDI_H_9.esd Horsegram (VZM-1) drought stressed subtracted cDNA Macrotyloma uniflorum cDNA, mRNA sequence

AGCTGGAGCTCACGCGGTGGCGGCGCTCTAAACTAGTGGATCCCCCGGGCTGCAGGATTCGGCACGAGGC

CTCGTGCCGAATTCGGCACGAGGGTCGTTTTCGCACTCTGGTGTTAGGTCTAAACACCCTTTAAAATAAA

GTGGCCATGTCTTCCCTTGAGCAAGTTCAAGTGTTATGGACTTGTGATGGGTTTATCTAGCCATGGTTTA

CTCTTTTTTTTTTAACTATGTGGTTTGTTCAGTGTTAAAGTTGGCTAATCAGAATTATCTTTCTCTGCTA

ATAATATTATTATGTTACTTGAAAAAAAAAAAAAAA



>gi|71811875|gb|DR989266.1|DR989266 HDI_H_83.esd Horsegram (VZM-1) drought stressed subtracted cDNA Macrotyloma uniflorum cDNA, mRNA sequence

AAGCTGGAGCTCACGCGGTGGCGGCGCTCTAGAACTAGTGGATCCCCCGGGCTGCAGGATCGGCACGAGG

GTGAATTGAAGAGCCTTTGGAATTATGGCACAGACCCATCCTTGTCCACAGAGCCTTCTGAAGTTTAAAT

GATTACATTCTTAATTCATCTTCAGGATTGAAGAAATTGAACTGCAACCTCAATGATATGGAATGAATGC

TCTGAGGTTAGGAAAGACAGCAGGTGTACAGTACAGGCTACGCAACTATCTTCTCTATTAATGATTGAAT

AATCTCTATATTTGTGGCTGCTTCATAGTTCACTTGTAAAAGGAATGATAAACAAATGGAAAGTAGGCAT

CAATCATTAACCATCAAAAAAAAACAAAAAAAAAAAAAAA



>gi|71811873|gb|DR989264.1|DR989264 HDI_H_81.esd Horsegram (VZM-1) drought stressed subtracted cDNA Macrotyloma uniflorum cDNA, mRNA sequence

AAAGGCTGGAGCTCACGCGGTGGCGGCGCTCTAGAAACTAGTGGATCCCCCGGGCTGCAGGATTCGGCAC

GAGGATTAGTTTCCACTTTAAAGTTGCATTTTGATATTGATGTGTAGATACTATTTGTATGCAGACTATG

CATTTTGCTTTCGTACTCAAACAGTGAATTTATATATGGAAATGATTTGTCAAAAAAAAAAAAAAAAANC

CTCGAGAGGGGGGGCCCCGGCCCCAATTC



>gi|71811871|gb|DR989262.1|DR989262 HDI_H_8.esd Horsegram (VZM-1) drought stressed subtracted cDNA Macrotyloma uniflorum cDNA, mRNA sequence

GCTTGGAGCTTCCACCGCGGTGNGCGGCCGCTCTTAGAAACTTAGTGGATCCCCCGGGCTGCAGGAATTC

GGCACGGAGGCAACAGGTTTTTTTTTTTTTTTTTTTCTCTTCTCTTTCTCCCTCTCTCTCCCTTAAGTGT

GATATCTC



>gi|71811869|gb|DR989260.1|DR989260 HDI_H_72.esd Horsegram (VZM-1) drought stressed subtracted cDNA Macrotyloma uniflorum cDNA, mRNA sequence

AGCTGGAGCTCACGCGGTGGCGGCGCTCTAGAACTAGTGGATCCCCCGGGCTGCAGGATCGGCACGAGGC

CGGTTGTAGCCTAAATTGCATAACAATATGATCCCTGGTTCTACCACCTCCTTGTTTATGTGCCAAAGTT

TAGAGCAGCTGATGCCAGTATTAATGATTGATAAGTGTCGTTGTATAACGAAATTGTTGGTACTTGGGTT

CACATGATGTATAAAATAAGCTAGAAGTGTTGTAAATCGTACAAGAATATTATGAACTGAATTATGTGTA

TCATATACATAATAATGATCATAAATTTTTAGTTACATATTGTAATGAAAAAAAAAAAAAAAAAA



>gi|71811867|gb|DR989258.1|DR989258 HDI_H_70.esd Horsegram (VZM-1) drought stressed subtracted cDNA Macrotyloma uniflorum cDNA, mRNA sequence

AAGCTGGAGCTCACGCGGTGGCGGCGCTCTAAACTAGTGGATCCCCCGGGCTGCAGGATTCGGCACGAGG

CCTCGTGCCGTAATGGTATGAGGACGATGCTAGCGATGACGATGAAGAAACCAAGTCAGGTGGCCCTCGT

ATGCGTTGTATATTGTGTACTACTGCTGCTAGTTGAGTGTGTTTTTGTACGTGTCCACAGTTTGTGTTGC

ATGGTTTAAATCTTGTTAGTTTCGTTAGTTTCCATGCCAAGAAAGAAGCGTGTGGTTTNCATTTGGTGGC

TCAAAAAAAAAAAAAAAAACCNCGAGGGGGGGGCCCGCGGTTCACCCATTTCGC



>gi|71811865|gb|DR989256.1|DR989256 HDI_H_69.esd Horsegram (VZM-1) drought stressed subtracted cDNA Macrotyloma uniflorum cDNA, mRNA sequence

AAAGGCTGGAGCTCACCGGCGGTGGCGGCCGCTCTAGAACTAGTGGATCCCCCGGGCTGCAGGATTCGGC

ACGAGGCCTCGTGCCGAATTCGGCACGAGGGAAAGCCGAAGTTAAGCAACAGTAGAATCCCATGATAGTT

TCGTTACTTTAATTTCTACTCTTGTTAGAGATATTTGAGGATTATGATTGTCGTCTGAGCAAATACTTGG

TTTCTGAAATTTTCTTTTTGGGTTACCTCCCAGCCTAAAATTGGTTTTTTTTGACA



>gi|71811863|gb|DR989254.1|DR989254 HDI_H_60.esd Horsegram (VZM-1) drought stressed subtracted cDNA Macrotyloma uniflorum cDNA, mRNA sequence

AAAGCTGGAGCTCACGCGGTGGCGGCGCTCTAGAACTAGTGGATCCCCCGGGCTGCAGGATCGGCACGAG

GCTTCTCAGCAGCAATCGGCTAGGGTTCCGAGTCGAAGAAGGGTTCGGAGTTTGTTCACCATGAGGGCCA

AGTGGAAGAAGAAGCGTATGAGGAGACTGAAGAGGAAGCGCCGAAAGATGAGACAGAGATCCAAGTAGTG

CGTTAATCTTAGTTTGTTTCTGTTGGATTTTACAATTATTTATGTCCTGAAGTGGGAAACCCAGTAGTTA

GTGTTACTGGTATCTCCTTTTTTTTATGTTTTTGATACATTTTGTTGGCTTTAATTAGTCTATGCTTGGT

TTTGTGTTATTAGTTCGAAACAACGGTGTTACCGCATTTACGAAATCAACCTAATTTCTTATTTGCAAAA

AAAAAAAAAAAAAACCCGAAGGGGGGGCCCGGTTACCCCATTTCGCGCCCCTA



>gi|71811861|gb|DR989252.1|DR989252 HDI_H_57.esd Horsegram (VZM-1) drought stressed subtracted cDNA Macrotyloma uniflorum cDNA, mRNA sequence

GCTGGAGCCACGCGGTGGCGGCCGCTCTAGAACTAGTGGATCCCCCGGGCTGCAGGATTCGGCACGAGGG

CGGAAAGAGCTAGCTGGAGTAGATCACGGCCAACGAAGATAGAGAATGAATTGGATTCCTCAATTTTGGA

AGTGGGATATGGACCAGAACCATGGATGTGCCTGAATCGTGTTTAGTAATTTGTTTGTGCCCTTGTGTTT

ATAATCTGACAAGGTTGATGGTGAAGTCTAATCAAAATTCGATTGGTTTTTTATAAGAATGTGGTTGAAA

TGGTGGGAAAAAGAGATAGAGGAAAGGAAGGGAAAGAACAACCTTTTCTTCTTTTGGGGATGTAATGGAG

TTTACCTACATGTTCTTTCTTCTCTTTATTTTTCTTTTTTATTTTGTCGTCTAGGGCTAGTAGAGCAGTA

AACATCTTTTTGACAAAAAAAAAAAAAAAAA



>gi|71811859|gb|DR989250.1|DR989250 HDI_H_55.esd Horsegram (VZM-1) drought stressed subtracted cDNA Macrotyloma uniflorum cDNA, mRNA sequence

AAAGCTGGAGCTCACGCGGTGGCGGCGCTCTAGAACTAGTGGATCCCCCGGGCTGCAGGAATTCGGCACG

AGGGTTTTAGAAGCTCTCAGCTAAGCACTAAAGAGTGTTTAGACCTTAAATATTTCTTATGGGTATGAAC

AAGCCTCATGTGCTTATGCTGCTCATCATCATGGTTGTTTCAACATCACTTATAGAAGGAAGGACACTTT

CCTTAATATCTGGTCAAGGGTACTCAAAGATTTTTGCAACTCTTGGAGTTGTATGCAAGTGTTGTGATGG

GGTTGGAGGTGCCTGCACCAGTACATGGACAGAATCTTGCAATAATCTGCAGTGTTATCCTTGGAAATCA

CACTAGCTAATTGTATTTTCTGTATCCTTTCTTGTCTTCCATTTACTGTATATCAAGTTTTGAATTATTA

TAAAATTATATACTAGTGAAAAAAAAAAAAAAAAAAAAAAAAAACCCCAGGGGGGG



>gi|71811857|gb|DR989248.1|DR989248 HDI_H_47.esd Horsegram (VZM-1) drought stressed subtracted cDNA Macrotyloma uniflorum cDNA, mRNA sequence

AAAGCTGGAGCTCACGCGGTGGCGGCGCTCTAAACTAGTGGATCCCCCGGGCTGCAGGATTCGGCACGAG

GCTCACTCTGAGTGATAATTTTGTTGATGAATTTTGAGTTAATTGGTGCACCTTGGTCATGGAATATGTT

TCCATAAATGATGATCATGTTTGAGGTAAAACTCTTGCAGTTCAATTAGTGGTTCGATGTTTTAACATGT

GAATGTTTACTTGCTAAATAAAAGTTTATTTAAACAAACATGGATTGGTTAATGCTTATTCCTGTATTCA

AAAAAAAAAAAAAAAAAAAAAAAAAAAAAAAAAA



>gi|71811855|gb|DR989246.1|DR989246 HDI_H_45.esd Horsegram (VZM-1) drought stressed subtracted cDNA Macrotyloma uniflorum cDNA, mRNA sequence

GCTGGATATATGGTAGATTGCTAGTGATTCTTCCACTTTTTCGACTAAGATATCATAGTCATGATGTATA

ACAATTTAGTGTTATGGTCATCATAGAGCACAAATCCAATGAATAAAGAAACTATGAATATGCAGTATAA

AAAAAAAAAAAAAAAAAAAAAAAAAAAAAAAAAAAAAAAAAAAAAAAAGAGAGAGAAAAAAAAAAAAAAA

AAAAAAAAAAAAAAAAAAAAAAAA



>gi|71811853|gb|DR989244.1|DR989244 HDI_H_43.esd Horsegram (VZM-1) drought stressed subtracted cDNA Macrotyloma uniflorum cDNA, mRNA sequence

ATGTTCAGCTTATCTTCAAGCAGTTGCTAACATTTACATTTCGTAATCTACTTGATGGATGAAAAGTGTT

GATACTTCAATAGAATAGAGAAAAAAAAGTAAAAAACATCAAAAAAAAAAAAAAAAAA



>gi|71811851|gb|DR989242.1|DR989242 HDI_H_35.esd Horsegram (VZM-1) drought stressed subtracted cDNA Macrotyloma uniflorum cDNA, mRNA sequence

AAAAGGCTTGGAGCTCACCGCGGTGGCGGCCGCTCTAAACTAGTGGATCCCCCGGGCTGCAGGAATTCGG

CACGAGGGTTCCTTTATATTTTTTTTTTCCTGGTTGTGCAT



>gi|71811849|gb|DR989240.1|DR989240 HDI_H_33.esd Horsegram (VZM-1) drought stressed subtracted cDNA Macrotyloma uniflorum cDNA, mRNA sequence

ATTTGTGCAATAANACCAATGTGATCCATCTCAGCAAACCCAACAACATCTCTAAAACCTCTCTCAGAAG

GGGGGGGGGCGCCCCGGGATGATCCACAACTNTCG



>gi|71811847|gb|DR989238.1|DR989238 HDI_H_31.esd Horsegram (VZM-1) drought stressed subtracted cDNA Macrotyloma uniflorum cDNA, mRNA sequence

TGGAGCTCACGCGGTGGCGGCGCTCTAGAACTAGTGGATCCCCCGGGCTGCAGGATCGGCACGAGGGCTT

GGCAAGGTTAATATATGAATAGGAATAACGGAAGAAAAAAGGATCAGAGACTTGTACTAGTTTGGATAGT

TTGGCACCCAAGCTACAGTCTGTAATAATGTTTGATTGTTTATTTTGCCTTCCTTGCGCAGATGGATTTA

AGTTCTCATGCATGCTGAGGTTCCAACCACACATGCCTACCATATTTTAGGTTGGTGATTTAGATATTTT

TTGTAGGGTTTGGTTTCCATCTATCATGAACTTTATTTTCATAGTGAAAAAAAAAAAAAAAAA



>gi|71811845|gb|DR989236.1|DR989236 HDI_H_23.esd Horsegram (VZM-1) drought stressed subtracted cDNA Macrotyloma uniflorum cDNA, mRNA sequence

TTCGCCCACTGTTATTAAATAATAAACATCTCCCTTTACCCAAAACTTCATCAATATATATCGTGACAAT

AGATAAATAAATAAAAAAACCAACTATATCAATGATATGACACATTCTCCCAAAAAAAAAAAAAAAAAAA

AACACACAAAGAGAGAGAGAGA



>gi|71811843|gb|DR989234.1|DR989234 HDI_H_21.esd Horsegram (VZM-1) drought stressed subtracted cDNA Macrotyloma uniflorum cDNA, mRNA sequence

AAAGCTGGAGCTCACGCGGTGGCGGCGCTCTAGAACTAGTGGATCCCCCGGGCTGCAGGATTCGGCACGA

GGGAACTGAGTAAAGAAGGAGCATGTGAGAAGGCAATATTTCCAGACACAAACAAAGTCATAACCAGATG

TGCTATGATTGGTAACTTGTCAAAAGAAGGGGGTGATCGCTGCGATCTGAATCTTGTTTTAGGATGCACA

GATCCTAGTTCTCACCTGTACAACCCACTAGCCAATGTANATGATGGAACATGTCCAATTGATTTGGATT

CTGAGTCTGAGGAATAGCATTATACTTTGCATATAAATATTCATTTCGATCATACTTTATGTTGGTTCTA

GGTACATAGATAGTGTGTATTGTATACAAATTTCACTCTTTACATTGAATATTATCAATGTCTGGCCCTT

GCTGTAAAAAAAAAAAAAAAAAAAAAAAAAAAAAAAAAAAAAAAAAAAAAAAAAAAAA



>gi|71811841|gb|DR989232.1|DR989232 HDI_H_19.esd Horsegram (VZM-1) drought stressed subtracted cDNA Macrotyloma uniflorum cDNA, mRNA sequence

AAGCTGGAGCTCACGCGGTGGCGGCGCTCTAGAACTAGTGGATCCCCCGGGCTGCAGGAATCGGCACGAG

GGCCACCTGGCAGATCCAATACCCAGACTGAGGACGAACTGGGTTTACCTGCTGTGCCTCGGGCATCCAT

ACGCGGTTAAAGATGCTTGCTTCTTTTTCTTTTTTAATCTTAGTGCAGTCACAAGCAAGCCCTTGTAAAG

ATGTGCAGATCTTGAGATCGTTGATTGATACAGTGATTTAATTGGTTTGATGTTGCCATTGTAGACTTTT

TTCTTTTATTTTTCCTTGTAATAAACCATGTGGAAACGTCCTTGTTATTTGTATGAAACTCATGACGGGA

AAAAAAGGAAATACTTGATATTGTGAAAAAAAAAAAAAAAA



>gi|71811839|gb|DR989230.1|DR989230 HDI_H_11.esd Horsegram (VZM-1) drought stressed subtracted cDNA Macrotyloma uniflorum cDNA, mRNA sequence

AAGGCTGGAGCTCACCGCGGTGGCGGCCGCTCTAGAACTAGTTGGATCCCCCGGGCTGCAGGAATTCGGC

ACGAGGCGAGTTTTTTTTTTTTTTTTTTTTTTTTTTTTTTTTTTTTTTTTTTTTTTTTTTTTTTTTTTTT

TTTTTTTTTTTTTTTTTTTTTTTTTTTTTTTTTTTTTTTTTT



>gi|71811837|gb|DR989228.1|DR989228 HDI_H_90.esd Horsegram (VZM-1) drought stressed subtracted cDNA Macrotyloma uniflorum cDNA, mRNA sequence

AGCTGGAGCTCACGCGGTGGCGGCGCTCTAGAACTAGTGGATCCCCCGGGCTGCAGGATTCGGCACGAGG

CCTCGTGCCGAATTCGGCACGAGGCGGATACACACCTACAGGATGAATTTTCACCCCTCTTGTTTTATGG

TCTTCAATTTACTTCTATGTGTTTCAAGCCATCATTTTGTACTATTTTACAGTGCTAATCATTGTGGGGG

ATATGGGTTTGTGTATGTGGTCTAGTTACCACATCAATAAAGGTTGTGTAATGTTGGTAGGAATTGGTGA

TTTACTAACATATTAGGAGTTTCTGGTCGACAACTTTTTGACCCCTTCATTTTGGGTGATGATTTTGGAG

GGAGACTGGTTGTAGATTATGCATGGGGAGAAATCGAGCTTTTTGATATAGCCTATCATGGTTATCTGAC

TTATCTCTCAAGCATGCTCTTTCCAGTTTCAACCCAATCTATGTTTAAATTTAATATTTCCCTACTTTGA

TGGGCTAAGGTTTTTTGTAATATTATTATAGTGGTGGCTGAAATGAACGAAATTCCCCCCAAAAAAAAAA

A



>gi|71811835|gb|DR989226.1|DR989226 HDI_H_88.esd Horsegram (VZM-1) drought stressed subtracted cDNA Macrotyloma uniflorum cDNA, mRNA sequence

AAGGCTGGAGCTCACGCGGTGGCGGCGCTCTAGAACTAGTGGATCCCCCGGGCTGCAGGATTCGGCACGA

GGCCTCGTGCCGAATTCGGCACGAGGAGAGGATGATGAAGTCATGTACAAAGAAAGAGTGGATGTTGAAA

GGGCTGAGTGCAACTCCGGGAACAGCAAGCTTCGTTTTGGACACAAGTGGAAGACATATAGGCCTTAAGA

AGATAATGACCAAGAACCTAGTTTGCTACAAAAACTACCTAATACATCAAAATCAGCAGTATGTATGATT

GTGTGAAATAAATGCCTGCTTAGCCAGCCGTTATGTTTGTTTAACCAGGGAGTGTTTTATATCTGTAGTA

CATTAGTACTGAACTTTCCCATGTATGAACTATTAATGAAGCATATACATCAGTTATCTAAAAAAAAAAA

AAAA



>gi|71811833|gb|DR989224.1|DR989224 HDI_H_86.esd Horsegram (VZM-1) drought stressed subtracted cDNA Macrotyloma uniflorum cDNA, mRNA sequence

CCAAAGCTGGAGCTCACCGCGGTTGGCGGCGCTCTAGAACTAGTGGATCCCCCGGGCTGCAGGATTCGGC

ACGAGGGTCGTGATGGGAAAGGAAAGGCTAACATCAAAATCGTGGTCGTTGGACATGTCTCTGAGAAATC

AACTGCCACGGAGCTTGGAAGCCCTACTGAGAAGGGTGTGACTGAAAGGTTCGAGAAGGGAGAAGGAAGT

GTTTAAAACACGTAAGATGTAAGATGTGTGAAGAAGGATGATGTAAAATAAATAAGATATAAGATGTGAA

GATGGATCTATCTTAAGAATGAAGTGCTGAAGGACTTATATTCAGAATAAATAAAATGAAGGATATATGT

TTATCTTCTTCAAAAAAAAAAAAAAAAAAAAAAAAAAAAAA



>gi|71811831|gb|DR989222.1|DR989222 HDI_H_78.esd Horsegram (VZM-1) drought stressed subtracted cDNA Macrotyloma uniflorum cDNA, mRNA sequence

AAGCTGGAGCTCACGCGGTGGCGGCGCTCTAGAACTAGTTGGATCCCCCGGGCTGCAGGATTCGGCACGA

GGTTGAGATTTCGTGGATGATTCGAGCCTGCGAGGACATTTGTGGAAATCCAATGGCGAGCTCATGCAAC

TGGAACACTTCCGGTGGGGTTAATTGCTACGCCGACTGCTACAGTTTCCTCGGAATTTTGCATATATGTG

ATGATGTAGGGATATTGTACCAGCTTGTAGAATATGCAAAAGTTACTATTTATGGTGTCTGGGTAGGTCT

GGTTGAAAAAAAAAAAAAAA



>gi|71811829|gb|DR989220.1|DR989220 HDI_H_76.esd Horsegram (VZM-1) drought stressed subtracted cDNA Macrotyloma uniflorum cDNA, mRNA sequence

AGCTGGAGCTCACGCGGTGGCGGCCGCTCTAGAACTAGTGGATCCCCCGGGCTGCAGGATTCGGCACGAG

GCAATTTTGTTGGTTATTATCAAAGTTAACAATCTTATCATACATAGAAATGTGTCTGGATGTTCATGTA

AAACACTTTACATTGTAAGTGCATATAGCATATCCTTTTTTCTCAAAAACTATTTAAGCTCAATGACTGT

AAAGATTTTAAACATAGAACAAGACGGGGGAAGCAGCTAGTTTGTAACTATTGGACTGTAGCAGACTAGC

CACTGGACTTCCTCAACTACCCTATGGAAAGTTCTTTCTTCAGAATCTCTTCATTTTTCTTCTTTCTGAA

TGGTCAATAGTGTTGAACATTAGGGACAAGGAAAAGAAAGCATAAAATATCAATGGCTTCTCACGATGAT

GCTATGCCAGCCAAAAGTTCTCGAGGGGGGGCCCGGTACCCAATTCGCCCTATAGTGAGTCGTATTACAA

TTCACTGGCCGTCGTTT



>gi|71811827|gb|DR989218.1|DR989218 HDI_H_74.esd Horsegram (VZM-1) drought stressed subtracted cDNA Macrotyloma uniflorum cDNA, mRNA sequence

AAAGGCTGGAGCTCACGCGGTGGCGGCGCTCTAGAACTAGTGGATCCCCCGGGCTGCAGGATTCGGCACG

AGGAATAGATATATCTTGTTTATGAATGGATTTGTAAGTTGAGTATGTAATATGTTTCTCCGTTTTAGGT

CCTTGAAGCCAGACTTTTTATGCAATTGGCTGATGGTATTGGAACTCTGTAGGTTCTGCTTGGTTTGATA

TTCCTTTTATTCGTTTTTCATGCTGTGGCCCAGAATTAGGTGCCTTGGTTTAGGAGGACGATAAATTTTA

AGGATATTTTCTCCCAGAGGGATTTATCTGAGTCAAACAAAAAACAAAACAAACCTACAAGGGGTGG



>gi|71811825|gb|DR989216.1|DR989216 HDI_H_66.esd Horsegram (VZM-1) drought stressed subtracted cDNA Macrotyloma uniflorum cDNA, mRNA sequence

AAAGCTGGAGCTCACGCGGTGGCGGCCGCTCTAGAACTAGTGGATCCCCCGGGCTGCAGGATTCGGCACG

AGGCTTCTATTAGACAATATCTTCGTGTGTGATATTGTTTGTGTTCTTTGTAGTGTACCAGAGTTTAGCT

TGTACTATACTGTGTAATGTATTTCCCTCCAGTTGAAAAGAGAATTTGCTTTTTATAGGGCAGATTAAAA

AAAAAAAAAAAAAACCTCCAGAGGGGGGGGCCCGGGTTCACCCATATTCTCGCGCCCTTA



>gi|71811823|gb|DR989214.1|DR989214 HDI_H_64.esd Horsegram (VZM-1) drought stressed subtracted cDNA Macrotyloma uniflorum cDNA, mRNA sequence

AAAGCTGGAGCTCACGCGGTGCGCGGCGNTCTAGAACTAGTGGATCCCCCGGGCTGCAGGAATTCGGCAC

GAGGCCTCGTGCCGAATTCGGCACGAGGAAACATTCGAGCAAAAATGTTCTTTCGAATGAAACTATGCAC

CGCTGTTGTCCAAAATAATAACGCAGGCGATTTGGAGTGTGAGCCAATGCACTTGTTTTCCAAAAGATGA

TTGATCTTTTCTGTTTGTACGACAGACAGTGTTGGGATATTTTCCATTGCCAATGTTCGTCTTCTAAATT

TTTCTAGAGAAATGCATGCAAGCAGTACAATACAAGACCTCTTCAAACTAAAAAAAAAAAAAAAAAAAAC

CCCANGGGGGGNCCCGGTTACCCATTTTC



>gi|71811821|gb|DR989212.1|DR989212 HDI_H_62.esd Horsegram (VZM-1) drought stressed subtracted cDNA Macrotyloma uniflorum cDNA, mRNA sequence

GGTNNGGCCTGGGTGGCCCCTGGGGCGCCTGTTCCTTTATGGGAAAAGCTANGGTTTGTGAATTCCCCCC

TGGGATGGCCATGCCAGTGAAATTTCGGCAACCGAAAGGCGTTTTAGCTTAGGACCAAGTTGTCAAATTT

TATACTTGTACGTATTCGCTACATGTAACGAGGTTAAGAACTGTGTGCACTGCATATTGTCCATTAATCA

TTCTGTGACTC



>gi|71811819|gb|DR989210.1|DR989210 HDI_H_6.esd Horsegram (VZM-1) drought stressed subtracted cDNA Macrotyloma uniflorum cDNA, mRNA sequence

AAAGCTGGAGCTCACGCGGTGGCGGCGCTCTAGAACTAGTGGATCCCCCGGGCTGCAGGATCGGCACGAG

GCTGCGGAGGGACTAGTTGAAGGAGGAAAGAAAGAGATTTTCACACCAATGTACTTCTTCTTGGCACGGA

AGCCTGATTCAGACAGTAACTAAACTGGTTGTGTACAAATAGGTTGCTGTTGTTTATTGTGATTCTGGTA

GGATGAATCGATTAATTTTGTCCTTATTGCCGTAGTTTCGTTCTGTTTCGAGTTGCTTGTTTTTGTTTTA

GGTTACCAAAACCTTTGCTACACCTCTAGCGTATTTCTTTTTATCCTCAAAAAAAAAAAAAAAAAAAAAA

AAAAA



>gi|71811817|gb|DR989208.1|DR989208 HDI_H_53.esd Horsegram (VZM-1) drought stressed subtracted cDNA Macrotyloma uniflorum cDNA, mRNA sequence

CTAANGCTGGAGCTCACCGCGGTGCGCGGCCGNTTCTAGAACTAGTGGATCCCCCGGGCTGCAGGAATTC

GGCACGAGGCTGTGACTCTACTTGGTCTGCTAGCTGGTGGTGTACTTCTTGTCTACAACACAAGTGCTTT

GGCACAATAGACTTTGCAGATCCATGTTTATGTTACAAGTTACAACTTAGTCCATGTAACTATCTAAAAA

TCAAGAACTCTTCATCAAGTACTTTGTGGATATGTGATGTTCCTTCTGTTCTATAAGTGCGTGGATGTGA

AAAAAAAAAAAAAAAAAAAAAAAAAAAAAAAAAAACACAGAGGGGGGG



>gi|71811815|gb|DR989206.1|DR989206 HDI_H_51.esd Horsegram (VZM-1) drought stressed subtracted cDNA Macrotyloma uniflorum cDNA, mRNA sequence

AAAGCTGGAGCTCACGCGGTGGCGGCGCTCTAGAACTAGTGGATCCCCCGGGCTGCAGGATTCGGCACGA

GGCCTCGTGCCGAATTCGGCACGAGGTTGTACTCTTGTGTCATCTCTAGGTAGTTTGTTTTTCGTACTTA

GTTTCAAGTTTCCACCCTCATCTCCCAACTTTTGTTCCCAGAATTGGGTTCTTGATCGACGGTGGCGGCT

CTTTTACTATTATTATGTGTTTTAATGTGTTTTTTTTTGTGTTTGTCAGAACCCCTGATTACATTTTTGT

TAGCGCAGTGAGTTTTATAGCTTTGCTGTGCTGTTTGCTTGCATTTTAAATATGAACTTTTATATTTGTG

TTTTAAAAAAAAAAAAAAAAAAACCCCAAGGGGGGGCC



>gi|71811813|gb|DR989204.1|DR989204 HDI_H_5.esd Horsegram (VZM-1) drought stressed subtracted cDNA Macrotyloma uniflorum cDNA, mRNA sequence

AAGCTGGAGCTCACGCGGTGGCGGCCGCTCTAGAACTAGTGGATCCCCCGGGCTGCAGGATTCGGCACGA

GGGTTTGAGTTGTTTCTGGATATTGAATGTCCATATACTGACGCTGAGTATTTGGGTTAATGGGGTCATG

TTTTATGCAAAAGGGGGGCAGTTATAACCGCAAAGGAAATGTTGTATGCTTTAAACAGGGCTATGGTTGT

AGTTGCCTGAGTACATGTTTTGGCATACTACTGCTTTGGATTTCTGGAGCAAATATGCACCTTTTGCAAC

ATGTAAACATTATAGATGCTTTGATTACATTTTGAGGCTGAGATAAATAGTAAAAAAAAAAAAAAAAAAC

CCAAGGGGGGGCCCGGTATACCCAATTT



>gi|71811811|gb|DR989202.1|DR989202 HDI_H_42.esd Horsegram (VZM-1) drought stressed subtracted cDNA Macrotyloma uniflorum cDNA, mRNA sequence

AAGCTGGAGCTCACGCGGTGGCGGCGCTCTAGAACTAGTGGATCCCCCGGGCTGCAGGAATTCGGCACGA

GGGAGAGTGTTGCCAAGTCTGTTCTTGATCAGCAGAAGGCAAATGAGATGGTGGTGGAGATAAGAAAGAA

CTTAGGCATTTAGTAGGTTCAGGTTCCAGCTTCTGTCACCATATTCAAGTGTTCTCTTCTATTTCTCAGT

TTTAGCCTTCCATCAAATAATATTTGTGTAATAAAAGGCATTTTATGTGCCCAATTCTTGCTTTCAAAAA

AAAAAAAAAAAAAAAAAAAAAAAAAAAAAAAAAAAAAAAAAAAAAAAAAAAAAAAAAAAAAAAAAAAAAA

AAAAAAAAAAAAAAAAAA



>gi|71811809|gb|DR989200.1|DR989200 HDI_H_40.esd Horsegram (VZM-1) drought stressed subtracted cDNA Macrotyloma uniflorum cDNA, mRNA sequence

GACATTAAATATGCATAGTTGATAATTGCTTATGCCAGTTGCATTACCTGTGATATAATATATATTTCGT

TCCTAAAAAAAAAAAAAAAAAAAAAAAAAAAAAAAAAAAAAAAAAAAAAAAAAAAA



>gi|71811807|gb|DR989198.1|DR989198 HDI_H_39.esd Horsegram (VZM-1) drought stressed subtracted cDNA Macrotyloma uniflorum cDNA, mRNA sequence

AAAAGCTGGAGCTCACGCGGTGGCGGCGCTCTAGAACTAGTGGATCCCCCGGGCTGCAGGATTCGGCACG

AGGCCTCGTGCCGAATTCGGCACGAGGGTGGTGGTGACGGTGGTTCCCGTTACTCGAGAGGAGGTGGTGC

TTCGGACGGTGGAAGCTGGAGGAATTAGGGTTGTGTGATTAGGGTTTAGTTGGTGAAAAAAAAAAAAAAA

AAACTCCCAAGGGGGGGCCCCGGTCCCCATTTCCCCCC



>gi|71811805|gb|DR989196.1|DR989196 HDI_H_30.esd Horsegram (VZM-1) drought stressed subtracted cDNA Macrotyloma uniflorum cDNA, mRNA sequence

AGCTGGAGCTCACGCGGTGGCGGCGCTCTAGAACTAGTGGATCCCCCGGGCTGCAGGAATTCGGCACGAG

GCGGAAACATCATTATTCCACAATCGATTTTGCCTTGAAAAATCCAAGATTTGTTTTAATAGCTACCTGT

ATAACTCATGTAACAACTTGTAATGAATCAGAATCTGGCTATGTTGCTAAAAAAAAAAAAAAAAAAAACA



>gi|71811803|gb|DR989194.1|DR989194 HDI_H_29.esd Horsegram (VZM-1) drought stressed subtracted cDNA Macrotyloma uniflorum cDNA, mRNA sequence

AAGCTGGAGCTCACGCGGTGGCGGCGCTCTAGAACTAGTGGATCCCCCGGGCTGCAGGAATTCGGCACGA

GGCTGTGACTCTACTTGGTCTGCTAGCTGGTGGTGTACTTCTTGTCTACAACACAAGTGCTTTGGCACAA

TAGACTTTGCAGATCCATGTTTATGTTACAAGTTACAACTTAGTCCATGTAACTATCTAAAAATCAAGAA

CTCTTCATCAAGTACTTTGTGGATATGTGATGTTCCTTCTGTTCTATAAGTGCGTGGATGTGAAAAAAAA

AAAAAAAAAAAAAAAAAAAAAAAAAAAACCCCGGGGGGG



>gi|71811801|gb|DR989192.1|DR989192 HDI_H_27.esd Horsegram (VZM-1) drought stressed subtracted cDNA Macrotyloma uniflorum cDNA, mRNA sequence

AAAGCTGGAGCTCCACGCGGTTGGCGGCCGCTCTAGAACTAGTGGATCCCCCGGGCTGCAGGAATTCGGC

ACGAGGGGAGAACTAGTCTGTAGTTTTTTTTTTGTGGTGTCTCTTAATTGTGGATTTTGTCTTTTAAGTT

TTAAATTTTATTTCTATATTAATTTTGCAGATTTAACTCTGCCTTTATTTTGTTATGGTGTTTGTTTAGC

TACTGGGAAAGCAGCTAAAAAAAAAAAAAAAAAAAAAAAAAAAAAAAAAAAAAA



>gi|71811799|gb|DR989190.1|DR989190 HDI_H_25.esd Horsegram (VZM-1) drought stressed subtracted cDNA Macrotyloma uniflorum cDNA, mRNA sequence

AAAGCTGGAGCTCACGCGGTGGCGGCGCTCTAGAACTAGTGGATCCCCCGGGCTGCAGGATCGGCACGAG

GCTGATTCCTTCATCCACTTATGTATGCTTAAGTTATGTATCTCCAGTGGTTGAATGTTCTCGTGTGTTT

GTTATTAATAAAGAATTTGTGTTTGTACTTTGTATAATCCAGAGACTATAATAAACACTGCTACCATTGC

AAAAAAAAAAAAAAAAAAAAACCCGANAGGGGGGGCCCCGGTTCCCCATTTCCGCCCCTATA



>gi|71811797|gb|DR989188.1|DR989188 HDI_H_18.esd Horsegram (VZM-1) drought stressed subtracted cDNA Macrotyloma uniflorum cDNA, mRNA sequence

AAGGCTGGAGCTCACGCGGTGCGCGGCCGTCTCTAGAACTAGTGGATCCCCCGGGCTGCAGGAATTCGGC

ACGAGGCAATAATTGAGTTTTGATAAACTTTTGTTATTAATGAAAAAATGTATTCAGAAAAGTATGTTAC

TTATATTTATAATGGAATTGATTGTGGCATGGTTTGTTATTTATAGATGAATTTCAGGGAATCATTATAT

TTGCATGTTAGTAGAATTTGAGGGTGCTTAAAAAATTATGCTGTTGTATTGTGTTGCAGCAATTAAATAA

GATTTTCAAAAAAAAAAAAAAAAAAACCCCGAGGGGGGGCGCCCGGGTTCCCCA



>gi|71811795|gb|DR989186.1|DR989186 HDI_H_16.esd Horsegram (VZM-1) drought stressed subtracted cDNA Macrotyloma uniflorum cDNA, mRNA sequence

AAACACTCTCCCTCTCCCCCTATTGTGACACAAGAAAGACACCCATTCACCCAAAGAGTCTCCAAAGAAG

AAAAGAGCCCTCAGTGCCTCCTCTCCTATAAGCGAGGGGTATAAAAATAATCCNCACTGTGCTTTTTCCA

ACAAAACA



>gi|71811793|gb|DR989184.1|DR989184 HDI_H_14.esd Horsegram (VZM-1) drought stressed subtracted cDNA Macrotyloma uniflorum cDNA, mRNA sequence

AAGCTGGAGCTCACGCGGTTGGCGGCGCTCTAGAACTAGTGGATCCCCCGGGCTGCAGGAATTCGGCACG

AGGATGAGTTGGTGGGAATGAAGAAGTGTAATGAAGTTGCTGTTGGTGTTCCGTTTTGGTGGAGCTACCC

TAACGTTGTTTGAAGATTTGTAATGTTTATTTTTTATTTGTTTATTTTGTTGGTGAGGTGGGTCAACGCC

CAAATTTTTTTATTTGTTGAATATTGGTCAAGGAGGTGTGAAAAGTTAAGTTGAATATTATGACAAAGTT

CCGTATTAAAAAAAAAAAAAAAAAAAAAAAA



>gi|71811791|gb|DR989182.1|DR989182 HDI_H_1.esd Horsegram (VZM-1) drought stressed subtracted cDNA Macrotyloma uniflorum cDNA, mRNA sequence

AAAAAGCTGGAGCTCACGCGGTGGCGGCGCTCTAGAACTAGTGGATCCCCCGGGCTGCAGGATTCGGCAC

GAGGGCGAGTCGAACTCGGTGGAAAATGGAGGGTTTTCTTGTGAGAGGAGAAGAAGAGGGTTTGTCTTGG

TGGTGTTGGTTTTGATGTTTTAGGGCTTCGTCGAGGCAAAGGTTTTGGATGTCTTTGAAGGAAGAGCAAG

AGAATGTGGGAGAGGCTGATGTTTGATTTGTTGATTTTCCCCGCGGTCGCCACATTGAAGGTGGGAAGCT

TCAATCTTCTCTTTTTCTCTGTTTGTTTGATTCTCTTCTTCACTTGGATTTAGGGAACTTTGATTTTGAG

AAAAAAAAAAAAAAAA



>gi|71811789|gb|DR989180.1|DR989180 HDI_G_M8.esd Horsegram (VZM-1) drought stressed subtracted cDNA Macrotyloma uniflorum cDNA, mRNA sequence

AAGCTGGAGCTCACCGCGGCTGGCGGCGCTCTAGAACTAGTGGATCCCCCGGGCTGCAGGAATTCGGCAC

GAGGCCTCGTGCCGAATTCGGCACGAGGGAGCATTGCCTATGTTCAGGGCATTGTAGTGTGGATTGAAGT

GCAAAATTGTGTGTTTGCAGTTTGGTAGAATTGTAGTTGTAATCATACAGCTGCAATAGAAGATTATTTT

ATTTGTCAATTGAAAAAAAAAAAAAAAACAAAAAAAAAAAAAAAAAAAAAAAAAAAAAAACNACAGGGGG

GGGG



>gi|71811787|gb|DR989178.1|DR989178 HDI_G_M10.esd Horsegram (VZM-1) drought stressed subtracted cDNA Macrotyloma uniflorum cDNA, mRNA sequence

AGCTGGAGCTCACGCGGCGGCGGCGTATCTAAACTAGTGGATCCCCCGCGGCTGCAGTGAATCGGCACGA

GGGTTGTGTGTATGTGATGATGACCAATTCGTCTTTTCCTTTGTAATTTACTATTTATTTATTATTATTA

TTATTATTTCTTTGGTTTGTATGANATGAGATCAGATTATGACCGTTATTTAATTTTATATTTATATGCT

GCTGCCAAAAAAAAAAAAAAAAAAAAAAAAAAAAAAAAAAAAAAAAAAAAAAAAAAAAAAAAAAAAA



>gi|71811785|gb|DR989176.1|DR989176 HDI_G_82.esd Horsegram (VZM-1) drought stressed subtracted cDNA Macrotyloma uniflorum cDNA, mRNA sequence

AGCTCACCGCGGCTGGCGGCGCTCTAGAACTAGTGGATCCCCCGGGCTGCAGGATTCGGCACGAGGCTTG

GATATAATGTTTAGGGATTAAAATTATGTTTTTTCAAATTTAAAAGATAAAAAATTTCTTAAATTTAGAA

ATTAATATTAGAATTATTTTAAATTTTAAGAAATGAAAATATAATTAACTTTTTTTTCTTATGGATAATG

TGAATTGTAATATTACTTAATTTGGGGGTGGTATAATGAAAGGATTGGTGGGAGAAAAAAAAAAAAAAAA

AAAAAAAAAAAAAAAA



>gi|71811783|gb|DR989174.1|DR989174 HDI_G_8.esd Horsegram (VZM-1) drought stressed subtracted cDNA Macrotyloma uniflorum cDNA, mRNA sequence

AGAACTGGAGCTCACGCGGCGGCGGTTTAACTAGAACTAGTGGATCCCCCGGGCTGCGNTGGAATTCGGC

ACGAGGAGGAAGTAGAATACCTTCTCAGGAATGTATGGATTCCTATGCTTGGAATTCAGAGTTGGAGCAC

GGTTTCGTGTACCGTGAGCACAACAGGTCACCTGGATACTATGATGGAAGGTACTGGACCATGTGGAAGC

TGCCTATGTTTGGGTGCACTGATGCTTCTCAGGTCTTGAAGGAGCTCGAAGAGGCCAGGACTGCACACCC

CAACGGTTTCGTCCGTATCATTGGATTTGACAATGTTCGCCAAGTCCAGTGCATCAGTTTCATTGCCTAC

AAGCCACCAAACTACTAAGTCTCTGAAATTTCCAAAACATTTGTAGCCACTCCACTTTGTTTGTTTGTAC

TTAAACCAAATTCCCATTTAGTTTTTGCTTATTTAGAGATTNCATCAATCCTGTAATTTTGGTT



>gi|71811781|gb|DR989172.1|DR989172 HDI_G_72.esd Horsegram (VZM-1) drought stressed subtracted cDNA Macrotyloma uniflorum cDNA, mRNA sequence

CTAGAACTAGTGGATCCCCGGGCTGCANGAATTCGGCACGAGGATCAACTAGNCCCGAAAATGGATGGCG

CTAAAGCGCGCTGACCTATACCCAGGCCGTCGGGGCAAGGACACAGTGCCACNGATGAGTAGTGAGGGCG

CGGGTGTCGTG



>gi|71811779|gb|DR989170.1|DR989170 HDI_G_70.esd Horsegram (VZM-1) drought stressed subtracted cDNA Macrotyloma uniflorum cDNA, mRNA sequence

AAGCTGAGCTCACGCGGCTGGCGGCGCTCTAGAACTAGTGGATCCCCCGGGCTGCAGGAATTCGGCACGA

GGCCTCGTGCCGAATTCGGCACGAGGGGGAATTGTGGCGGTGATGGCATGGGAGCATAGAGTAGGTGAAA

TTAATGGTGAAGTTCTGGTAGTTATTATTGTCAGTGATTATTAGTACTCTTAGAAATTAGATAACTCATA

CTAGTATTAATTGTTCAGTACTTTTATTTTATTTAATTGGTGTCTGAAGTAATTGGTAAGCACTTTAATT

GGGTGTAGGTAGGTGTAAATATGAAGTCAATGGTCAATTGTAGGAGAGTGAGTTATTATTGGAGGGAAAG

GAAACCATGATTTGGATAGTTTAATACGTAAATGGTTCGTCAAAACTAAAATCAAAACAAAAATTCACCT

CTCTTTCTTTCTACTAAAAAAAAACCCAAGGGGGGGGGGGGGGGCCCCCGTTTCCCCCT



>gi|71811777|gb|DR989168.1|DR989168 HDI_G_69.esd Horsegram (VZM-1) drought stressed subtracted cDNA Macrotyloma uniflorum cDNA, mRNA sequence

AGCTCACCGCGGCTGGCGGCTGCTCTAGAACTAGTGGATCCCCCGGGCTGCAGGAATCGGCACGAGGCAC

TGTCTCAGAGAACTTCCAGTTTTGGCTACGGATGAGAAGTACAATGAAGCTATCTATAGCACTGCTAAAC

GTCTAGTTGCTGCCATTGATGGCCTTCCAGATCCCGGTGGGCCTACATTCAAAGACAACAAACGTGAGTC

TAACTTCAAAACCAAGCAAGAGACAGAAGAGAAACGTGGACAATTCACTCTTGTAGTGGGAGGTTTGTTG

GTTATTGCATTCGTTGTTCCTATGGCACAATACTATGCTTATGTATCCAAAAAATAAAATAATATAAGTA

TATCCGTCTTTCAAATTCTCACTCTTTAAATTCAGATGTATAAGTGCAGTTGTAACCATGCTCATCTTAC

AATTGAGTTTGGGAAGGCTGAAGAATAAGATGAGAGTTTTTTCATGTAAATGCAATTCCATAGTATATGC

ATTTTGATTATGTTATTATCCAGTTGGGTTTTTAAAGTCAGGTAGAACGAGTTCTAGTTCTCCATTTCTC

TCCCAAAAAAAAAAAAAAAAAAAAA



>gi|71811775|gb|DR989166.1|DR989166 HDI_G_67.esd Horsegram (VZM-1) drought stressed subtracted cDNA Macrotyloma uniflorum cDNA, mRNA sequence

CTGGAGCTCACGCGGCTGGCGGCGCTCTAAACTAGTGGATCCCCCGGGCTGCAGGAATTCGGCACGAGGG

GAAAGCGGGGTAAACGCCACTGCATTGAACGTAGGACCGGGACAGACCTCTGTGCGCCCTGGCCGGTTTT

TGGCGCCAAAAATCAAAAAGAAAGAAAGTGAGGGAGAGAGAGATCTCGTTAGTCCTGGTATTGTAGCCGC

GGGTTGTCGTCGGCCCGACGAACTTGGGGAGGGAAAGGTAAAGCTGGCGGAGACCCCAGCTTAGAGTAAC

TGCCCGAGAAGGTCAGTGAGGTTCCAACTGTAGTGAACTGAAAGATCTTTCAGTTTACTACAGTTGGAAG

AAGACAGGCGGGACTGTGAACCGAACGAGGGCACAGCAAGTTCCAGTGGTGGGCTGTCGGTCTGGTCCCA

TTTTAGACATAGTAAGAAGAGTAGCAATGCTAGATTAATAAAATAAAGAAAGAAGGAGTCAAGCCATAAC

TCTGCCCTAAGCATTGAGCTTACGCGAACCAAGCTGCGAAAAAGACTGACTTTTCATTCAATTCAAAAAG

GGTAGCCTTTCCATGCCAAGCCAGCACATGGACGGATTGGTACTAGAATTCCTCGGACCATGAGCAGTGG

TCCAAGG



>gi|71811773|gb|DR989164.1|DR989164 HDI_G_59.esd Horsegram (VZM-1) drought stressed subtracted cDNA Macrotyloma uniflorum cDNA, mRNA sequence

CTGAGCTCACCGCGGCTGGCGGCGCTCTAGAACTAGTGGATCCCCCGGGCTGCAGGAATTCGGCACGAGG

CCTCGTGCCGAATTCGGCACGAGGGTGCCAAC



>gi|71811771|gb|DR989162.1|DR989162 HDI_G_57.esd Horsegram (VZM-1) drought stressed subtracted cDNA Macrotyloma uniflorum cDNA, mRNA sequence

AACTGAGCTCACCGCGGCTGGCGGCGCTCTAGAACTAGTGGATCCCCCGGGCTGCAGGAATCGGCACGAG

GATTTTTTAAGATAGGTCCTCCCTCCCTCTTTCTTCTCTCCTTTCTCAACCCGCGAAGCTGCAACCGTCG

TGAAACTCGCCGTCCAGGTGTTTTAATTTTTGTGGTACTAGAATTGAACATACTCTGCCCAGTTCTCAGC

TTAAAGCATGGATCCGGAGGCTGCACGAGCTGCACGAGAATCTCTAGACCTGGCATTCCATATGTCCAAT

ATACTTGATACGGGTCTAGACCGACACACACTTTCTGTTCTAATTGCACTTTGTGATCTTGGAGTCAATC

CTGAAGCACTTGCTGCTGTTGTCAAGGAACTAAGAAAAGAGAAACCGTCATTATCATCATCACTTCCTGA

GGCTCCTTCTTTGCCGTAAAATAAATTGATTTTTTTATTAGATGTTCTGTATTTGAAGTTGATCCATCCT

GTTTGTCTGTTCTTGATCTGGCATTGAATTTCTCTGTATGCTTTGCTTGTTCTTTTTGTATGAGCTTCAT

TTGATGTATAGTGGCATTGTTTTAACACAAGATTGATGCGGTTGCAAATAGGAAACTCATATTTGAACGG

TCTATCTTTTGAGATAAATTTTCCTTACTAAAAGATTTAGTNCCCAAAAAAAAAAACAC



>gi|71811769|gb|DR989160.1|DR989160 HDI_G_55.esd Horsegram (VZM-1) drought stressed subtracted cDNA Macrotyloma uniflorum cDNA, mRNA sequence

AAGGGTAAGCGGNCACGTGTGTAAAAGATTCGGTGGCGANATTCGTAATAATAGCTATAGCCTGTTTGAG

AATCGATGGACCGGAATCTTCGAACTATTTGGTCATTGCCGGGCGAATGATGCTCCTACTGAGTCATAAA

ACTGCCTCAGATTGTACAGTGCCAACAGCTCTTGTTCCTAGTAGTAGCTGACTATGTGCTAGCCATTTGG

TGCTTGTGCTCAATATCATGGTATTGGACCAAGTTAATGAAGGGTAGTGGTTGTNCTCTTGGAGTTTGAG

TATCTAGATGCTTTGCGCTCCCTACGACTAGTAGCTCTAGTGTGATGCTTATTGAAGGGCGCATTCGTGG

AT



>gi|71811767|gb|DR989158.1|DR989158 HDI_G_47.esd Horsegram (VZM-1) drought stressed subtracted cDNA Macrotyloma uniflorum cDNA, mRNA sequence

AAGCTGGAGCTCACCGCGGCTGGCGGNTGCTCTAGAACTAGTGGATCCCCCGGGCTGCAGGAATTCGGCA

CGAGGCCTCGTGCCGAATTCGGCACGAGGGAACATTCGTTTCTCCCTAAGTTTAAGTCGTGATGGGAAAG

GAAAAGGCTAACATCAAAATCGTGGTCGTTGGACATGTCTCTGAGAAATCAACTGCCACGGAGCTTGGAA

GCCCTACTGAGAAGGGTGTGACTGAAAGGTTCGAGAAGGGAGAAGGAAGTGTTTAAAACACGTAAGATGT

AAGATGTGTGAAGAAGGATGATGTAAAATAAATAAGATATAAGATGTGAAGATGGATCTATCTTAAGATG

AAGTGCTGAAGGACTTATATTCAGATAAATAAAAT



>gi|71811765|gb|DR989156.1|DR989156 HDI_G_45.esd Horsegram (VZM-1) drought stressed subtracted cDNA Macrotyloma uniflorum cDNA, mRNA sequence

AAGCTGAGCTCACGCGGCTGGCGGCGCTCTAGAACTAGTGGATCCCCCGGGCTGCAGGAATTCGGCACGA

GGCCTCGTGCCGAATTCGGCACGAGGATTTAGATGGATTACTCTCCTGCTGACTCAGGAATTCAATTTTG

CTGACAGCCTTCACATTTGGGACACTCTTTTAAGCGATCCTGATGGTCCACAGGAGACTCTTCTTCGGGT

ATGCTGTGCAATGCTTGTCCTCGTGCGCAAGCGCCTCCTTGCGGGTGATTTTACATCAAATCTGAAGTTA

TTGCAAAATTATCCCACCACAAACATTAGTCATTTGCTCTATGTTGCCAACAAGTTTCGTGTACAGTCAG

TTTAATGTTTATCTCCCATTATCTTTTGCATTTTGCTTTTGTACTTTTAATTTCTCTTTCAAATTCAATA

CTTGTAAATGACTTTTATGTGTGAGCCTCAGAGCAGTGACTGTTTTTTTATTTATTTTTAGTTATATTTT

GTACGTAAAAAAAAAAAAAAAAA



>gi|71811763|gb|DR989154.1|DR989154 HDI_G_43.esd Horsegram (VZM-1) drought stressed subtracted cDNA Macrotyloma uniflorum cDNA, mRNA sequence

CTGGAGCTCACGCGGCTGGCGGCGCTCTAGAACTAGTGGATCCCCCGGGCTGCAGGAATTCGGCACGAGG

CCTCGTGCCGAATTCGGCACGAGGAAAAGGAGCTGAAGGGAATCCTCTCAGTCTGTGATGAGCCTCTTGT

GTCTGTTGACTTCAGGTGCACTGATGTGTCCTCAACCGTTGACTCATCGTTGACCATGGTCATGGGAGAT

GACATGGTGAAGGTGATTGCTTGGTATGACAATGAAGTTGGGGATACTCCCAAAGGGTTGTGAGATTTGG

CGTGCACATTGTTGCCAACAAATGGAAGTGATCAAAGCTCATTGGTAATTAATTATGTACCAAATACCAT

TTGTAATTTTGTATGCTTACCACCACTACTACTACAACAACACTTGTTGCTTCATTTTTCCTTCTAAACA

AGTACTGAGAAACAGAACCATCAATTTTCTCTGCCTTCTCATTGTGCAATAAAATTCTTACCTTGAAATC

TGAAA



>gi|71811761|gb|DR989152.1|DR989152 HDI_G_34.esd Horsegram (VZM-1) drought stressed subtracted cDNA Macrotyloma uniflorum cDNA, mRNA sequence

AAGCTGGAGCTCACGCGGCTGGCGGCGCTCTAGAACTAGTGGATCCCCCGGGCTGCAGGAATCGGCACGA

GGCTCTTACTTAGTTCTGACACCTGCAGCATTAAATTTCTTTGTTAACTACGCTGAAGGTGCTGTTGAAT

CACTATGGTCCATTGATCAATACTTTGAATTTGTTCTTGTGCTTATGTTCAGCACAGGTTTATCTTTCCA

GGTACCTGTCATACAATTTCTATTGGGACAACTTGGACTGGTGTCCTGAGACCAGATGTTATCTGTTTGG

AGGTATGTTGTGGTTGGTGCAGTAGTGGCAGCTGCTATAGTTACGCCATCCACTGATCCTCTCACTCAAG

TTCTTCTGGCAACACCTTTATTGGGTCTTTACCTAGGTGGCGCATGGATGGTCAAGCTTACTGGACGGTG

ACCTTAGGTCCCATCTTAATTGTATGCCAGAATTCAGTCTGAGCTGATGCTAATGTACATTCACATAGCA

ATCTAGTGTATATTTTTACTCGTACCAGAGCATTACTCTTCCTATAATCCAAATTATTTAAACATTTAAT

TCTGCATTAAAA



>gi|71811759|gb|DR989150.1|DR989150 HDI_G_32.esd Horsegram (VZM-1) drought stressed subtracted cDNA Macrotyloma uniflorum cDNA, mRNA sequence

GCTGAGCCACCGCGGCGGCGGCGCTCTAGAACTAGTGGATCCCCCGGGCTGCAGGAATTCGGCACGAGGA

GAATATCTTCCTTTACTTCATTGACTCTCAATTCTGAGGTCTCCAAAGAGAAAACCTTGACCACGGCTGG

GAGATGATGTGATAAGTTTGTAAACAAAACTCATCATCTGACTCCATTGTACAGTGTATAGCTTTGTTTT

GGTGCAATAATATATCTTCATGTAGTATACTTGAGAGAAAGCAGAGGTCATTGAAGATCAAGTCAAATAA

TATGTATTCCTCCATGGCATGTCCTTTTTGATCCATGCCTTGCTAGGATTAGGGAAGTTAGAAATTGTAG

AATTAGTAGTTACCCTTTTTGTGTGAGGAGGAATCATTTCCTATAATTGTAAAATGAATGTCATTTATCA

ATGGATAATTTGTTTCTTTTTCTGAACAAAAAAAAAAAAAAAAAAAAAAAAAAAAAAAAGGGGGGGGG



>gi|71811757|gb|DR989148.1|DR989148 HDI_G_24.esd Horsegram (VZM-1) drought stressed subtracted cDNA Macrotyloma uniflorum cDNA, mRNA sequence

GACGGGGCCATCNTCGTGTAGAAGGTCACTGAACCCACAATACTACCCCGGTGGACAGATTTCGACCCAT

ATGGGACCTCGCTAGACGACCCAGAGAGCTTTACGACTGAGACTCAAGGTGAAGGGAACATCNANGAAAC

GAGTAGGTTGGCCATGATTCTCCAATGTACAGAGGTTCTTCAGTCACAGGCCATTGTCACCAGGAAAGGG

CCCATTGGAGAACCTCGCCGAACACCTTGACAAGACCCAGTCAACAACAACGACACTGGGACCTACGCCA

ACCAACTNCGTCCCCGAGACAGTGAGCTTTGAAAGAGAATGACATTGTGATAACTACA



>gi|71811755|gb|DR989146.1|DR989146 HDI_G_22.esd Horsegram (VZM-1) drought stressed subtracted cDNA Macrotyloma uniflorum cDNA, mRNA sequence

CTAGAACTAGTGGATCCCCCGGGCTGCAGGAATCGGCACGAGGCTGAAATCATTGAGGAGCTACAGAAAC

ACCAGTCAAGCACTGACCCTAATGGTATGCTTATTGACATATGAAATGTCCGGATACTCTTATTGTTGTG

TATATTAAATACTGATTTTTGAAATTACAGTTAATTGCCAGTCTAGTTGTAATATTGTATGTTTCTATTT

CTAGTTGTGGAGTGGGCTACGGAAGCTCTCCAAGGCCTCGGAAAAGGTGCTCCTTTTTCCCTTTCCTTGA

CCAACAAGTACTTCTCTGCAGTTGCTTCTGCAGTCGGGAAGAATGATGGTGAACTATCCACGGTAAGCAC

TCTTTTTAAGGGAGAGGTTGGTTGATTTCCGTGTGTGCAAAAAACTAATTTTTGTGTATTCCATCTGCGT

TTGGGAAATTCCAGCTTAATGGCGTAATGAAAACTGAGTATCGTATTGCCTTGAGGTCTTCTCTACGACA

TGATTTTTTCACGAAGGTGTCCGAGCCAGTGCTGGTTTGACAAGGATCCAGGTTCTCCATTTTTCTTATA

ATT



>gi|71811753|gb|DR989144.1|DR989144 HDI_G_20.esd Horsegram (VZM-1) drought stressed subtracted cDNA Macrotyloma uniflorum cDNA, mRNA sequence

CGAGCTGGAGCTCACCGCGGCGGCGGCCGCTCTAGAACTAGTGGATCCCCCGGGCTGCAGGAATTCGGCA

CGAGGTTTAAAACAAAAACTTCTTAAAAATGGGTTTAGTTAGAAGAAGGAATAAGAAAAAAAATTATATT

AAAGATTTTTATATTAATTTGTTTTAGCTATAGATTACTCCTAGTTCTTAAAAATAATTTAAATTTTATT

AGAAACAATTTAATTATAAATATTTTTTGAAATCACTTTTCTTTTAGTTAAAAATTCCAAGTATTTTTTT

TTATCTGTTTCTCTAAATAATTATTTTAAATATTTTGTACACAATAATCTGTTCAAATTTTCTACATATT

TCTAAAAATTAATTAAAATATGTTATAATTTACTTATTTTTAATATAATTAGAAAACAAAAATAAAGATA

TTAGCTTCATTTTTAATCATTTCACTTATTTATCATTAAATAAAAAGAGACAAAACATACANTAATGTGA

GAGAGTTTGTTTTTCAAAATCAATTTTCTAAAAGTTT



>gi|71811751|gb|DR989142.1|DR989142 HDI_G_12.esd Horsegram (VZM-1) drought stressed subtracted cDNA Macrotyloma uniflorum cDNA, mRNA sequence

CTAAACTAGGTGGATCCCAACGGGTGGTAAATTCGGCACGAGGACATATGAGGACAACTATTTTACCAGC

GGCGGAAGAAATNCCTGCTATTATGGATAGTATCAAAAAGTTCAATGATAGAATTCATGGAGCAATCCGA

AGACAATAGAGGTTATTCAAGGTTGTAAGAAAATCATCTTAAAAAATGTATTATACTCTTAGTGCTTTTG

GAAAGCACTATAATCCATACCACCCCACATCCCTTAAAAAAAACATAACCTCCGT



>gi|71811749|gb|DR989140.1|DR989140 HDI_G_10.esd Horsegram (VZM-1) drought stressed subtracted cDNA Macrotyloma uniflorum cDNA, mRNA sequence

CTAGAACTAGTGGATCCCCCGGGCTGCGTGAATTCGGCACGAGGCCTCGTGCCGAATTCGGCACGAGGAT

TTAGATGGATTACTCTCCTGCTGACTCAGGAATTCAATTTTGCTGACAGCCTTCACATTTGGGACACTCT

TTTAAGCGATCCTGATGGTCCACAGGAGACTCTTCTTCGGGTATGCTGTGCAATGCTTGTCCTCGTGCGC

AAGCGCCTCCTTGCGGGTGATTTTACATCAAATCTGAAGTTATTGCAAAATTATCCCACCACAAACATTA

GTCATTTGCTCTATGTTGCCAACAAGTTTCGTGTACAGTCAGTTTAATGTTTATCTCCCATTATCTTTTG

CATTTTGCTTTTGTACTTTTAATTTCTCTTTCAAATTCAATACTTGTAAATGACTTTTATGTGTGAGCCT

CAGAGCAGTGACTGTTTTTTTATTTATTTTTAGTTATATTTTGTACGTAAAAAAAAAAAAAAAAACCCCC

AGGGGGGGGCCCGG



>gi|71811747|gb|DR989138.1|DR989138 HDI_G_M4.esd Horsegram (VZM-1) drought stressed subtracted cDNA Macrotyloma uniflorum cDNA, mRNA sequence

AGCTGGAGCTCACCGGCGGCTGGCGGTNCTGATCTAGAACTAGTGGATCCCCCGGGGCTGCNTGGAATTC

GGCACGAGGCCTCGTGCCGCCTCGTGCCGAATTCGGCACGAGGGGGGGGGCCCGGTACCCAATTCGCCCT

ATAGTAGAGTCTGTATTACAATTCACTGGCCGTCGTTTTAC



>gi|71811745|gb|DR989136.1|DR989136 HDI_G_M2.esd Horsegram (VZM-1) drought stressed subtracted cDNA Macrotyloma uniflorum cDNA, mRNA sequence

CTGGAGCNCACGCGGCTGGCGGCCGCTCTAGAACTAGTGGATCCCCCGGGCTGCAGGAATTCGGCACGAG

GACAATGTTATGTTGGAGTACTAGCAATAAATAATGTTGAGCTGAAGCTCTAATAAATGTAATGCTTTTC

AGGTGTTGCCTAAGACCCAAAATGTTGTAATAACATGGTCCATGTTTCGTCCTAAAAA



>gi|71811743|gb|DR989134.1|DR989134 HDI_G_78.esd Horsegram (VZM-1) drought stressed subtracted cDNA Macrotyloma uniflorum cDNA, mRNA sequence

GCTGGAGCTCCACCGCGGCTGGCGGCCTGCTCTAGAAACTAGTTGGATCCCCCGTGTGCTGCAGGAATTC

GTGCACGAGTGCCCTCGTGCCTGGTTTGCCGCAGGAACAGAGGCTATAGCTAAGAAACTGGCAGAGCTGA

GTGGCAAGGGAGTAACAACCATCATTGGAGGAGGTGACTCTGTGGCCTTGCTTGTGG



>gi|71811741|gb|DR989132.1|DR989132 HDI_G_74.esd Horsegram (VZM-1) drought stressed subtracted cDNA Macrotyloma uniflorum cDNA, mRNA sequence

GAGCTGGAGCTCACGCGGCGGCGGCGCTCTAGAACTAGTGGATCCCCCGGGCTGCAGGAATCGGCACGAG

GGGACCATAGAAGGCTGCTTGCATCATATGACATGGAACCTGAGTCAGGTAACTATATCAATCCAGATAG

ACCCATGCTTGATGTGCAGTTGAAAAGTATTACTTAGATGGGTGGAGGCAGGAAATGACTTTGTTTTTCA

GCTGTGGAATTTTGCATGATGAGGTAAAAAGCCTTGGCATGGAAACTGGCTGAAAGTAAATGCAACTGCC

TATCATCTCAGCAAAGGAAAACTTTTTTAAATTCCCATAATTCAATTTAGTCATGTATATAATAAAAGGA

AACTTAGAAATGTATAGGGTACATAGTTTTGTGTAGTTATGCACAGTTATCTGTGTTCTAGATTCAGCCA

AACTTGTAATATCTGAATAGACGAAACAATTCCCTATTAAACACTTTCTAAAGTTGTACAGCATAAATTT

TTTTTTACCACCAGGATATGAACATGTGTAATAAGAACTCACTGAGATTTATTCAAAAAAAAAAAA



>gi|71811739|gb|DR989130.1|DR989130 HDI_G_66.esd Horsegram (VZM-1) drought stressed subtracted cDNA Macrotyloma uniflorum cDNA, mRNA sequence

CTGAGCCACCGCGGCTGGCGGCCGCTCTAGAACTAGTGGATCCCCCGGGCTGCAGGAATTCGGCACGAGG

GAAGAATTCTACAATTTTCCGTCCCTTGATGACTGAGGAAGTAAGGGAAAAGAAAGTTGAATCTTGGTGC

AAAGCTGTTAGTAAAACTTTTGACTTAGCTGATCTNGCTCTTTGATGCTC



>gi|71811737|gb|DR989128.1|DR989128 HDI_G_64.esd Horsegram (VZM-1) drought stressed subtracted cDNA Macrotyloma uniflorum cDNA, mRNA sequence

AAGAAAGCTGGAGCTCACCGCGGCTGGCGGCCGTATCTAGAACTAGTGGATCCCCCGGGCTGCATNGGAA

TTCGGCACGAGGCCTCGTGCCGAATTCGGCATCGAG



>gi|71811735|gb|DR989126.1|DR989126 HDI_G_62.esd Horsegram (VZM-1) drought stressed subtracted cDNA Macrotyloma uniflorum cDNA, mRNA sequence

AANAAGCTGGAGCTCACCGCGGCTGGCGGNGCTCTAGAACTAGTGGATCCCCCGGGCTGCAGTGAATTCG

GCACGAGGCCTCGTGCCGAATTCGGCACGAGGATTTAGATGGATTACTCTCCTGCTGACTCAGGAATTCA

ATTTTGCTGACAGCCTTCACATTTGGGACACTCTTTTAAGCGATCCTGATGGTCCACAGGAGACTCTTCT

TCGGGTATGCTGTGCAATGCTTGTCCTCGTGCGCAAGCGCCTCCTTGCGGGTGATTTTACATCAAATCTG

AAGTTATTGCAAAATTATCCCACCACAAACATTAGTCATTTGCTCTATGTTGCCAACAAGTTTCGTGTAC

AGTCAGTTTAATGTTTATCTCCCATTATCTTTTGCATTTTGCTTTTGTACTTTTAATTTCTCTTTCAAAT

TCAATACTTGTAAATGACTTTTATGTGTGAGCCTCAGAGCAGTGACTGT



>gi|71811733|gb|DR989124.1|DR989124 HDI_G_6.esd Horsegram (VZM-1) drought stressed subtracted cDNA Macrotyloma uniflorum cDNA, mRNA sequence

AGCTGGAGCTCACGCGGCGGCGGCTGCTCTAGAACTAGTGGATCCCCCGGGCTGCAGGATTCGGCACGAG

GCCTCGTGCCGAATTCGGCACGAGGATTTAGATGGATTACTCTCCTGCTGACTCAGGAATTCAATTTTGC

TGACAGCCTTCACATTTGGGACACTCTTTTAAGCGATCCTGATGGTCCACAGGAGACTCTTCTTCGGGTA

TGCTGTGCAATGCTTGTCCTCGTGCGCAAGCGCCTCCTTGCGGGTGATTTTACATCAAATCTGAAGTTAT

TGCAAAATTATCCCACCACAAACATTAGTCATTTGCTCTATGTTGCCAACAAGTTTCGTGTACAGTCAGT

TTAATGTTTATCTCCCATTATCTTTTGCATTTTGCTTTTGTACTTTTAATTTCTCTTTCAAATTCAAATA

CTTGTAAATGACTTTTATGTGTGAGCCTCAGAGCACGTGACTG



>gi|71811731|gb|DR989122.1|DR989122 HDI_G_53.esd Horsegram (VZM-1) drought stressed subtracted cDNA Macrotyloma uniflorum cDNA, mRNA sequence

TCTAGAACTAGTGGATCCCCCGGGCTGCAGGATTCGGCACGAGGCCTCGTGCCGAATTCGGCACGAGGCC

CGAAGGCGTGCTTGGCCCACCCCAAACCGGCCACCTCGCCAGGCTCGAGTTTCGAAGACGCCTCGAGAGA

GACGCAGAAGCCCGCGAAGCCTTCGAACGTCAAGTCAATGAAGAGAAAGAACGCCGTCAAACCCTTCGAC

AATCTCGTATTGTTCCCGATACTCCCGAAGAGCTCATTGAATACTTTCTTGACACTGAAGCTCAGGAGAT

TGAGTTTGAGATTGCAAGAATGAGACCGCGATTGGATGAGGGATTCTTTGCGCAACTGAAATTTGAGTTA

GGACAGCTTCGATTTGCCGTTAACAAAACGGAGCACATGGAGGAAAGACAGATTGAACTGGAGGCTCTGG

AGAAAGCCATTCAGGAAGGAATAGAAGCCTATGATAAGATGCAAGGTGAACTCGTAAAAGCCAGGGCAGG

CCTAACTAAAATCTTGACATCAAAGGATGTGAAAGCAACTTTGCTGGACTATGGTTGAAAAGGAATGAAC

TTTAATAGATCTTTGTTGGCACTTCTAGAATGACACACATAGGCAAATTGCACATCAAGGGTANCCAGAA

AACAGGTTGCAGAAATACATGGAGAAAGCTCCGGGGGTTC



>gi|71811729|gb|DR989120.1|DR989120 HDI_G_51.esd Horsegram (VZM-1) drought stressed subtracted cDNA Macrotyloma uniflorum cDNA, mRNA sequence

GCTGGAGCTCACGCGGTGGCGGCCGCTCTAGAACTAGTGGATCCCCCGGGCTGCAGGAATTCGGCACGAG

GCTGAAGCTGAAGCAAAACAAGCCACCACCTATTGGCCCCAAGAGAGGTGCTAAGGTGAAGATTCTTAGG

AAGGAATCTTATTGGTACAAAGGAACTGGTTCAGTGGTTGCAGTTGATCAGGACCCCAAGACTCGCTACC

CTGTTGTGGTTCGATTCAACAAAGTCAACTATGCCAATGTATCAACAAACAACTATGCTTTGGATGAGAT

CGAGGAAGTCGAATGAGTCCAATTTAATTTTAATTTTGATGCTTGTCATGTTATCGCAGTTACTAGTCTA

AAGTTTTTTCTTGTCACCAAAAAAAAAAAAA



>gi|71811727|gb|DR989118.1|DR989118 HDI_G_49.esd Horsegram (VZM-1) drought stressed subtracted cDNA Macrotyloma uniflorum cDNA, mRNA sequence

GCTGGAGCTCACCGCGGTGGCGGCGCTCTAGAACTAGTGGATCCCCCGGGCTGCAGGAATTCGGCACGAG

GGACCAATGGTCCGATCTGAGGGGAAAACCCAAGGGAACCAGGAACTTTAAGCTTCCCAACCCCATGTAC

TCTATTGAGTAGATCATCTCTGAAGGATCCCAAGCTTGCTCCTATCTACGAAATAAGATACCTTACGTGT

TCTAATGGGAGCATTAACTATGTATCTATGCATGTGACTTTCTGGTCTTCGGATCTTCAACAAATCACCT

AAGAGACTACACTCCGTTGTTTTCTCTTTTGTGACTTTAATAAATTATGAACCCTTTTTTCCAAAAAAAA

AAAAAAAA



>gi|71811725|gb|DR989116.1|DR989116 HDI_G_41.esd Horsegram (VZM-1) drought stressed subtracted cDNA Macrotyloma uniflorum cDNA, mRNA sequence

GCTGGAGCTCACGCGGCGGCGGCGCTCTAGAACTAGTGGATCCCCCGGGCTGCAGGATTCGGCACGAGGC

CTCGTTGCCGAATTCGGCACGAGGTTAAAGCAATATGGACTCATCTGCTGAGGAATTTCGAACTGGAGTT

GGTGTCACCTTTTCCTGAGATCGACTGGAATGCCATGGTTGTGGGAGTTAAGGGAAAAGTGATGGTCCGA

TACAAGCGGAAGGCGCTTTCAGTTAACCAATAGGTTTAATGTTTCATTGTCTGGCATGTTTTTATTGTAA

GCAGTTATTGTTAGTCTTTTCTGGATCAGCTTCTGTCCAACTTCACCGTTTATTTGACGGTGGATATCTA

GAATCTTGGATTTGGTTTTTATTTTGTAATTTTGCTAAGTAATTTTAACTTGTTTGTTTTGTGTCTTAGA

TATTAATAGTTACTTGGGTTAAAGTGAAATGGTTAAAGTAAAAAAAAAAAAAAAAAANCCCAGGGGGGGC

CCGGT



>gi|71811723|gb|DR989114.1|DR989114 HDI_G_4.esd Horsegram (VZM-1) drought stressed subtracted cDNA Macrotyloma uniflorum cDNA, mRNA sequence

AAAGAAAGCTGGAGCTCACCGCGGCTGGCGGCNTNTTCTAGAACTAGTGGATCCCCCGGGGCTGCTNGAA

TTCGGCACGAGGGAAAGGCCTTAGATGATACGCTTCGTGCATTCTGTAAATCCTGATCTTTTTTGCTCCA

TGTTTTTGTTGCAGCTTTTTTTCACCCATTTCTTTTGC



>gi|71811721|gb|DR989112.1|DR989112 HDI_G_38.esd Horsegram (VZM-1) drought stressed subtracted cDNA Macrotyloma uniflorum cDNA, mRNA sequence

AGCTGGAGCTCCACGCGGCTGGCGGCCGCTCTAGAACTAGTGGATCCCCCGGGCTGCAGGATTCGGCACG

AGGCTCTCATCCAGATTGAGATGTGAACTCCAAGGAAGAAGTTGCGTGGTACGCTGGGAAGCGAATGGCT

TACATTTACAAAGCCAAGGTAAAGAAGAACGGAAGCCACTATCGCTGCATTTGGGGTAAGGTCACCAGGC

CTCATGGTAACAGCGGTGTCGTTAGAGCTAAGTTCAAATCAAATCTGCCGCCAAGATCGATGGGAGCAAG

GGTTAGAGTGTTTATGTATCCAAGCAGTATATGAGATTCTCAGGACTAGTTGGTCGTGGAGCTTGTTTGT

CCGGGTACTTAAAAGGAATTTGCATTATGCGAGTTTTAATGTTTTTTGTAGCTCATGAGCAGAATAGTAT

CTCGTTTAGGATGGCGAATGAGCCCCGTGTTTTTTTTCTTCATTAAGGATTATGAGTCTCAATTTATAAA

TGGATTTTTT



>gi|71811719|gb|DR989110.1|DR989110 HDI_G_30.esd Horsegram (VZM-1) drought stressed subtracted cDNA Macrotyloma uniflorum cDNA, mRNA sequence

CCTTGCTGACTCTCGGATTCTTTTGCTGACGCCTTCCTTTTGGGACCTCTTTTGCGATCNTGTTGGTTCC

GGGAACTCTTCTCGGGTATGCTGTGCAATGCTTGTCCTCGTGCGCAAGCGCCTCCTTGCGGGTGATTTTA

CATCAAATCTGAAGTTATTGCAAAATTATCCCACCACAAACATTAGTCATTTGCTCTATGTTGCCAACAA

GTTTCGTGTACAGTCAGTTNTAATGTTTATCTCCCATTATCTTTTGCATTTTGCTTTTGTACTTTTAATT

TCTCTTTCAAATTCAATACTTGTAAATGACTTTTATGTGTGAGCCTCAGAGCAGTGACTGTTTTTTTATT

TATTTTTAGTTATATTTTGTACGTAAAAAAAAAAAAAAAAACCCCCGNGGGGGGGGCCCGGT



>gi|71811717|gb|DR989108.1|DR989108 HDI_G_28.esd Horsegram (VZM-1) drought stressed subtracted cDNA Macrotyloma uniflorum cDNA, mRNA sequence

AAGCTGGAGCCACGCGGCTGGCGGCTTGCTCTAGAACTAGTGGATCCCCCGGGCTGCAGGAATTCGGCAC

GAGGCCTCGTGCCGAATTCGGCACGAGGATTTAGATGGATTACTCTCCTGCTGACTCAGGAATTCAATTT

TGCTGACAGCCTTCACATTTGGGACACTCTTTTAAGCGATCCTGATGGTCCACAGGAGAACTCTTTCTTC

CGGGGTATGCTGTGCAATGCTTGTCCTCGTGCGCCAGCGCCTCTCTTGCGGGTGATTTTACATCNCATCT

GCAAGATTATTGCAGACATTATCCCACCA



>gi|71811715|gb|DR989106.1|DR989106 HDI_G_26.esd Horsegram (VZM-1) drought stressed subtracted cDNA Macrotyloma uniflorum cDNA, mRNA sequence

CTAGAACTAGTGGATCCCCCGGGCTGCATGGAATCGGCACGAGGCCTCGTGCCGAATTCGGCACGAGGGA

AAAATCCCAGAAGAAGATCCTCCAATACTGGACACTGAAGATGTTGGACCAACGAGAGTGGTTGGGTATT

TCATGTTGGGATGGTTTGCGTTGTCAATTGTGTTATCTATTGCACTAAATATATAGAAGAAAGACTAATT

CTATAATGTTTTAAGCTTAGTCTGGTGAAGGAATGTTTTTCTTTTTTCTCTTTTTGTATGATATGATATG

AAATGAAAGGTAATCTTTTGTTTAAAAAACAAAAAAAAAAAAAAC



>gi|71811713|gb|DR989104.1|DR989104 HDI_G_2.esd Horsegram (VZM-1) drought stressed subtracted cDNA Macrotyloma uniflorum cDNA, mRNA sequence

AAGCTGGAGCTCACGCGGCTGGCGGNCTGCTCTAGAACTAGTGGATCCCCCGGGCTGCAGGAATTCGGCA

CGAGGCCTCGTTGCCGAATTCGGCACGAGGGTCATTTCATGATTCAACAGCTTGGTTTGCAGGGGACGCC

TCCCCGGTCCACAGACTGGGGTCGTCCAGATTCAAGAAATACCGGTTTTGAGCTGACAGGAAGTGGCGGG

TCATGGGGCGCACCAGTCTCCTGAGAATGGGGGCTATGAGCAAATTCCCGATTGAAAACTCAATATGTCT

TTTGGATTTGATAGCAGTAATCAATGTGAGATAATGTATGATATCTCTAATACANCGCCGTCTTTAGTCT

CGTGATGCCGTTCACAGTATGCACCA



>gi|71811711|gb|DR989102.1|DR989102 HDI_G_17.esd Horsegram (VZM-1) drought stressed subtracted cDNA Macrotyloma uniflorum cDNA, mRNA sequence

ATGAAGCTGAGCTCACGCGGCTGGCGGCTGCATCTAGAACTAGTGGATCCCCCGGGCTGCAGAATTCGGC

ACGAGGCCTCGTGCCGATAAAGCACATGCTGTACTTGAGAAAGATAACAAGTCAGGCTGAGAAACTGAAA

CGATTTGCCAATCGGGCGGTTGCTGAGAGCAAAAGGCAGAAAATCAATGGTAGCCACCCAGGAAGGAGCT

GTGCATTTGACTTTGAAAGTGAACTACCATGGCCTATAGTCATAGAAGATCTGGAATGCTCAGGGGCACA

TGCTCATAGAGATAGATATGCAACGAGCATGGACTTTTCTTGGAGATAGCTCAGGTGATTCGGAAGCTGG

ATGTCACCATCTTGAACGGGATCTTGGAAAACCGTTCTAGCAACAGCTGGGCTTGTTTCATTGTTGAGGT

TCCAAGAGGTTTTCACAGAATGGATGTTCTATGTCCTTTACTGCATCTTCTGCAGCTTAGAAGAAATCCT

ATCTCGTACAAAAGCTGATCATGCCTGTTCTCAGTTTCAAATGGCAACTTCTATTTAAACGAAAGTTACT

ATAATATTATAGAGGCGACTTGTGTACATTTCGAGTTTACATGCCCCTGGTTAGTGTACTTTGCTACATT

TTGGTTGTACAATGATAAGGCTATTACCTAAAACTTGTAAACCNACAGG



>gi|71811709|gb|DR989100.1|DR989100 HDI_G_15.esd Horsegram (VZM-1) drought stressed subtracted cDNA Macrotyloma uniflorum cDNA, mRNA sequence

AAGCTGGAGCCACCGCGGCTGGCGGCTGCTCTAGAACTAGTGGATCCCCCGGGCTGCAGGAATTCGGCAC

GAGGCCTCGTGCCGAATTCGGCACGAGGGTTTTTTTTCAAAAAATAATCTTACCTTTAAGTTAAAAGGCT

TAAATAATATTAAAAGACGAAAAGACCCTATAGAACTTAAATATAAGCGAAGTAACAAAACCCAAAAAAT

TTTTCTGGGGCAGAAGAAAAATTAAAATTTTTTTTTTTAAAAACAATTTAAAGAAAACTATATCCTTAAA

TACAACAAGAAAAAAGAAAAAGTTACCTTAGGGATAACAGAGTTAAAATTTTTGAGAGACCAAATCGAAA

AAAATTATTGCTACCTCGAATGTTGAATTAAGGGAAACATTAACAGAAAAAAGTTAAAAAGTAAGGTCTG

TTCGACCTTAAAAAAACCTTACAGTGCATTTGAGTTAANGACCGGCGAAAAAGCCAGGTTGGGTTCTCCT

ATCTTTAAA



>gi|71811707|gb|DR989098.1|DR989098 HDI_G_13.esd Horsegram (VZM-1) drought stressed subtracted cDNA Macrotyloma uniflorum cDNA, mRNA sequence

CTAGAACTAGTGGATCCCCCGGGCTGCNAGGAATTCGGCACGAGGCCTATGAGGGCTTGAATGATACAAG

TATGCTGTGGGGAGTCAAGTTCTACAATGATTTTCTTTCTACAGCTGGACCTCTTGGTAACGTTCAATCA

GAAATCTTACTTAGAAAAGACAAATCAACCTTCACATTTGATAAGGGGTGGGCATTCCCGAGAAGGATTT

ATTTCAATGGGGATAACTGTGTTATGCCTCCTCCAGATGCTTACCCTTGGCTGCCAAATGCAAGTTCTAA

AATAGTTTTCTCTTTACTAAGTACAGTCCTAGCCACGTTAGCTTCTTTGTTAATCTTATTTAACCAATCA

TAGAGGATATAGAGATACCTTCTACTCTTTTGGAGTATTGTATCTTTCTCTTCTGCACCAATTTGTTAAA

GCTAAAGTCATCCCCATTGTCAAAACATGCATAGAAGCTGCTACCGAAGCAATAAAAAGTGTCAAAAGAA

TGCATAAATGCTTGGCCACCAAGAAATTCTGTCCTTGCATTCACCTTGGGGAATTGAATGTTTACCCTCA

TATNTGTAACACCATCCTTT



>gi|71811705|gb|DR989096.1|DR989096 HDI_F_95.esd Horsegram (VZM-1) drought stressed subtracted cDNA Macrotyloma uniflorum cDNA, mRNA sequence

AGCTGGAGCTCACGCGGTGGCGGCGCTCTAGAACTAGTGGATCCCCCGGGCTGCAGGAATTCGGCACGAG

GCCTCGTGCCGAATTCGGCACGAGGGGCCAACCTCTACAGGCTCTTAACAGATTATGTGTACAATTCATG

GAAGCTATTCACACTTCTTGCCACCTTTGTTCTCTTGGCCATGACGGCTCTTCAAACGTTTTGCTCGGCT

TATGATTGTCCCAGTTATTTTAGACCCAAATGAGGTTTTCATATCATTTCTACTTTTAAGTATAGCAGAT

AAATGACTGGTAACGGTTTCGAGTGTATAATGTTTCTGTAAAATTAAGTGCAGCGTAAGTTGTAATGTAT

GCTTCATGCAAGTTATCCAACTGAAACCATCTAATATATGATTTCTATTTCTCTCAAAAAAAAAAAAA



>gi|71811703|gb|DR989094.1|DR989094 HDI_F_93.esd Horsegram (VZM-1) drought stressed subtracted cDNA Macrotyloma uniflorum cDNA, mRNA sequence

CAAAGCTGGAGCTCACCGCGGTGGCGGCGCTCTAGAACTAGTGGATCCCCCGGGCTGCAGGAATTCGGCA

CGAGGTTCTAATAGAATATCTTTCTCTTACCAGATTATATCTCAATACATTATGTACTTCCTCGCTATTC

CACAATCGACTGCGTCTCCCAGGATAATTAGTAGATTCTTGACGAACTATATCACAACCCATTTGTCTTA

TCAAGTCATGCATTTGTATTCTATTGTCATTTGAAATTGTTATCAGAGCTTTTTTTTGGAGAACCTCTAT

TCCACTAGCTCCATGAAATCCCCAAGCATCCAGTTGGTTTATGACATAGTCTTTGTCTTCTCCTTTGAAA

AATAATGCAATATCCAGAAATGCCTTTTTCTCTACTTCATGCAGTCCTTCATAACTGAATCTCAACACAC

TTTGAATTTTCTCATTGGGATATCTAGTGATTTTTCTTAAGGCACACTCCCATGTCTCTATACTTCTGGA

ATGGAAATCTGAACCTAAAACTTTTAAAGCCAACGGATTGCCTTGGGCAATTTTTTACTACCTCCTCAGT

TAGTTTTTCATATCCCATTTTGGGTTGGCTTNATTGAATGCATTTAAGCAGAAAAGTTGAAAGAATCTCG

AGGGGGGGCCCGGTACCCAATTCGCCCTATAGTGAGTCGTATTACAATC



>gi|71811701|gb|DR989092.1|DR989092 HDI_F_91.esd Horsegram (VZM-1) drought stressed subtracted cDNA Macrotyloma uniflorum cDNA, mRNA sequence

CTGGAGCTCACGCGGTGGCGGCGCTCTAGAACTAGTGGATCCCCCGGGCTGCAGGAATTCGGCACGAGGC

TTCTTGGTGCTATTTCTCTCCCTAGTGCTGGAATGCTTATTCCCTACACCTACTTCTTCGTTCCTCCAGG

TTCAGGTTCTTCATCTGGCGGTACTGTTGCCAAGGATGCTGTTGGAAATGATGTAATCGCAGAGAATTGG

CTCAAGACACATGGACCTGGTGACCGTACACTTACACAAGGATTGAAAGGAGATCCTACCTATCTTGTGG

TGGAGAAAGACAGAACCCTAGCAACATTTGCAATTAACGCGGTGTGCACTCATCTCGGATGCGTCGTACC

ATGGAATACAGCTGAGAAAAAGTTCATTTGCCCATGTCATGGATCTCAGTACAATGACCAAGGCAGAGTT

GTGAGAGGACCTTGTTCCCTTGTCTCTGGCATTAGCACATTGTGATATAGATGATGGGAAGGTGGTGTTT

GTTCCTTGGACTGAAACAGATTTCAGAACAGGTGATGCTCCATGGTGGGCTTAGACTCCTTTAAACGCCG

TTCCCTTTTGNTATGTCACTATGTAATTTTAACATTATGAACATTCCAAAACAATCC



>gi|71811699|gb|DR989090.1|DR989090 HDI_F_84.esd Horsegram (VZM-1) drought stressed subtracted cDNA Macrotyloma uniflorum cDNA, mRNA sequence

CTAGAACTAGTGGATCCCCCGGGCTGCAGGATTCGGCACGAGGCCTCGTGCCGAATTCGGCACGAGGGCC

CTAAAGACATAGGAACATCACCAACATGTCCATAATCTGTAAAAATTATAGGCTTCGTGAGTTTCATTGC

AGATACACCACTTTAGCATCGTTGGCGGTTTAAATGATATCAATCTATTCCTTTATTTTGCTTTTAAGAT

AAGATGTTTGTTAGATTGGAAATTCAGGATGTCTTGTATACACTGTATACTATTTTGCAATACCAGGAAA

GGGAACAAAATAATAGAATTTCTAGAACTCCTCTACTACTTTAAATGGTTGAGAAAATTTAA



>gi|71811697|gb|DR989088.1|DR989088 HDI_F_82.esd Horsegram (VZM-1) drought stressed subtracted cDNA Macrotyloma uniflorum cDNA, mRNA sequence

GAGCTCACCGCGGTGGCGGCGCTCTAGAACTAGTGGATCCCCCGGGCTGCAGGATTCGGCACGAGGTGCC

TTTGACACCCACAAAGAGTGTCACCCATCTCACCAACTTCCCTACCAAATTGAAAGCCCCACGCTCCTCC

CCTCGTTACTCTCCAATAAGGGTTGCAACTCTGGAGCCTGATACTTCAACAGTGGACTACAACTCAGCCT

TCTCAGTGTTTCCAGCAGAAGCCTGTGAAACTGTAGGTGGAGAAGCCTGCATGGCAAACATGTATCCTGA

AGCCAAACTGCAGTCACAAGCTAATAAAACCACACCACGAGTCGCTACAGAGAGCGTTGAGAGAGAGTAT

CTTGAATACAATGATCCTAAAACGGTATTTCCGGGAGAGGCTTGTGATGATCTTGGAGGATCTTTCTGTG

AGTACGAGTATCAGAAGAGTGTCTTCTAGAGCAAACAACACTTGTATTGTCATGCCTACTGAATTTTCCA

TATCTACCCCTATTAATTGTAGTGTTGGTTGTACATAGGTATATAGACTATGTGGAGTTTCATTTTCTCT

ATAAATTTTACAATAATTATACTCTAAATTTGAAAGCTTCCCGTCTAGACTAGAAATGAANATAAATAGA

AGGAGCATTTCACTTAAAAAAAAAAAAAAAAAACCGAGGGGGGC



>gi|71811695|gb|DR989086.1|DR989086 HDI_F_8.esd Horsegram (VZM-1) drought stressed subtracted cDNA Macrotyloma uniflorum cDNA, mRNA sequence

AAGCTGGAGCTCACGCGGTGGCGGCCCGCTCTTTTAACTAGTGGATCCCCCGGGCTGCAGGAATTCTGGC

ACGAGGCCTCGTGCCGAATTCGGCACGAGGAATGGCCTCTTGGAAGAAGACCATCACAACACCCTTCAAA

AAAGCTTGCACTTTTTTCAATCAGCAGCCACCAAGGGATCAAAAGAAGTCTCAAACAGAGCAAGAGACGC

GAGTGATGGATCTGCACGGGGAAGTGATGGCATGTGGGTATGAAGATGTTCAAGTTATGTGGTCAATTTT

GGATAAGTCCAAATCCACAAACTGCAATATAACCTCTTCGACCTGACCCTATCCTTCTGCTACGAATTAA

TCAAAAATGGCACTCCAGCAATTAGCGAATGTTTTTGTATGCTAATGTTAAGGTCATGATGAATGTAATT

CTCATCACAGAAAACGAACACACAACAATAAAA



>gi|71811693|gb|DR989084.1|DR989084 HDI_F_72.esd Horsegram (VZM-1) drought stressed subtracted cDNA Macrotyloma uniflorum cDNA, mRNA sequence

AAAGCTGGAGCCACGCGGTGGCGGCGCTCTAGAACTAGTGGATCCCCCGGGCTGCAGGATTCGGCACGAG

GGGTCCAGTGAGAATGGAAATGATCAAACGGCTTGGATGGTGGGTGCTGGTTACTGGGTGTGGAGGCGAC

CCTCTGATGGATCGTCAGGTGGAGCTGGTTAGGTTGATGGAAAATAAGGGTGTGAGAGTTGTGGGGCATT

TCACAGAAGGGGATTATCATGGGGTTCAAGATAGTGATCCATTGAAGGCAAAGGAACTATACGGGGTAAT

CAAAACTTTTATTTCAGCTTTAAAATTGTAAGCTATTTTAATTGATTTTTGTACTTTAATGTTTCTGTCC

ATTATTGACTGTTCATGTATTAAGTAATGTTGTCTTATATGATATAATTAATCTCAAAAAAAAAAAAAAA

A



>gi|71811691|gb|DR989082.1|DR989082 HDI_F_70.esd Horsegram (VZM-1) drought stressed subtracted cDNA Macrotyloma uniflorum cDNA, mRNA sequence

GGAGCTCACGCGGTGGCGGCGCTTCTAGAACTAGTGGATCCCCCGGGCTGCAGGAATTCGGCACGAGGAT

TCAGAACCTAAGAAGGAGAAAGGAGAATGTGCTTCTTTCNNTGATACTCCTCCCCAAGCTATATACCACC

GTTAACCGTAGCCTGTGCGCCGTGCGATGGTGGCTCACATACACCGGGACTCAAGGTCCATTGAGGTGGC

TTCCCAAGCCAGGAATACCAATCAATGACATTACTTCGAGTTTGCAAAATCAATACTGGTGGAAGAGCAT

CGAATGCATTCATGGTGATGGCCTGACAGTTGAGGAAGAATGAAGTTCGAAACTACTATTCTTACCTGCC

AGACTCTCACTGACGCCCAAGTGTGGTCAAGAGAGAAGTAGAATACCTTCTCAGGGAATGGATGGATTCC

TTGCTTGGAATTCGAGTTGGAGGCACGGTTTCGTGTACCGTGAGCACAACAGGTCACCTGGATACTATGA

TGGAAGGTACTGGACCATGTGGAAGCTGCCTATTGTTTGGGTGCCCTGATTGCTTCTCAGGTCTTGAAGG

AGCTCGAAAG



>gi|71811689|gb|DR989080.1|DR989080 HDI_F_69.esd Horsegram (VZM-1) drought stressed subtracted cDNA Macrotyloma uniflorum cDNA, mRNA sequence

AGCTGGAGCTCACGCGGTGGCGGCCGCTCTAGAACTAGTGGATCCCCCGGGCTGCAGGAATTCGGCACGA

GGGGACGGTATATTCATGTACTTCGAAGATAATGCTGGTGTTATCGTGAATCCCAAGGGTGAAATGAAAG

GTTCTGCAATTACTGGACCAATTGGAAAGGAGTGTGCTGATCTCTGGCCCAGGATTGCAAGTGCTGCTAA

TGCCATTGTTTGAGTTGGGTCTCAGTTTTTGTAATGCTTCTGTTTCTTTGAGATTTTGATATTTATTTAG

GTTGAATTGCAGACAATGTGGCACATATAGTTTATCTTTGTTTGACTTCTCAGGGAAGTGTTTTGATGGA

TATTATTGATAGTTACAAGAGTTTGTTTTTGGGTAAAAAAAAAAAAAAAAAAA



>gi|71811687|gb|DR989078.1|DR989078 HDI_F_67.esd Horsegram (VZM-1) drought stressed subtracted cDNA Macrotyloma uniflorum cDNA, mRNA sequence

AAAGCTGGAGCTCACCGCGGTGGCGGCGNTTCTAGAACTAGTGGATCCCCCGGGCTGCAGGATTCGGCAC

GAGGCTCCAATCTCAGGGGAGATTGTGGAGGTTAACAAAAAGCTCACAGAAACGCCTGGCCTGGTCAACT

CAAGCCCATACGATGATGGATGGATGATCAAAGTAAAGCCAAGCGATCCTTCTGAATTAGAGTCCTTGTT

GGGTCCAAAGGAATACACAAAATTTTGTGAGGAGGAAGATGGTAGTCATTGAAGTTTGAATTGGCTCCTG

CACTCATATGTGGGACTCACTGTTTGATATTCATGTGTTTATTATTATCTTATTGTCTTCCTGAACAGTT

TTTTAATATGCAATAGAACTGTTTC



>gi|71811685|gb|DR989076.1|DR989076 HDI_F_59.esd Horsegram (VZM-1) drought stressed subtracted cDNA Macrotyloma uniflorum cDNA, mRNA sequence

CTGGAGCTCACGCGGTGGCGGCCGNTCTAGAACTAGTGGATCCCCCGGGCTGCAGGATTCGGCACGAGGC

AAAAACGGTTACATTGTCATCAAGAACAGGCCATGCAAGGTTGTGGAGGTTTCACACTTCCAAAACTGGC

AAGCATGGACACGCAAAGTGTCACTTTGTTGGAATTGACATCTTCACTGGCAAAAAGCTTGAGGATATTG

TGCCATCTTCCCATAACTGTGATGTTCCTCATGTCAATCGTACTGACTACCAGCTTATAGATATCTCAGA

GGATGGATTTGTGAGTCTGCTGACTGAGACTGGTGGTACTAAGGATGACCTTAGGCTTCCAACCGATAGA

AAATCTGCTTCCGCAGATCAAGGATGGATTTGGTGAGGGCAAAGATCTGGTTGTGTCTGTTATGTCTTCT

ATGGGAGAGGAGCAGATTTGTGCCCTTAAGGACATTGGTCCCAAGTAATAGTTCCATGTCAGTTTAATGC

TGGAAAACTGAAGTTGTAACTGAGATGATATAGTATTTATTTCTATTTTTCACTTTTATGTTGTAGGTTT

GTATGTTAAATCAGCCAAAGTTTGTACCATTCTAACAAGATATGGTCAAAATTGGATGGCTTTATTCTGG

GAATCTTGACCTAT



>gi|71811683|gb|DR989074.1|DR989074 HDI_F_57.esd Horsegram (VZM-1) drought stressed subtracted cDNA Macrotyloma uniflorum cDNA, mRNA sequence

AAAGCTGAGCTCACCGCGGTTGCGCGGCGTTCATAGAACTAGTGGATCCCCCGGCNCTGCAGNTAATTCG

GCACGAGGAAGACACACTCTATGTCCACTCTCTGCGTCAATTCTACCGCGAATGCGACGTTTACGGCACC

GTGGACTTCATCTTTGGCAATGCAGCAGTGGTGCTCCAGAACTGCAACTTGTACGCACGCAAGCCTGACC

CAAACCAGAAGAATCTGTTCACTGCACAAGGTAGAGAAGACCCTAACCAAAACACTGGCATTTCCATCAT

CAACTGCAAGGTAGCAGCAGCTGCTGATTTGATCCCTGTCAAATCTGAGTTCAAGAATTACTTGGGTCGT

CCCTGGAAAAAGTATTCGAGGACTGTTTATCTCAACTCTTTGAATGGAGGATCTGATAGACCCTGCAGGA

ATTGGTTGGGAATGGGATTGGGCCACGGTTTGCCTTTGGATACTTTGTANTTCATTGGAGAAGTTCCACC

ATTGAAGTCCCGGTGCCAATTACCAGTCCGCGAAGTTACGTTGCCTGGGTTCTNCAG



>gi|71811681|gb|DR989072.1|DR989072 HDI_F_48.esd Horsegram (VZM-1) drought stressed subtracted cDNA Macrotyloma uniflorum cDNA, mRNA sequence

CTGAGCCACGCGGTGCGCGGCGTTCTAGAACTAGTGGATCCCCCGGGCTGCAGGAATTCGGCACGAGGGA

AAGGAGCTGCTATTCTTGTGTATACAGAAGATCAGTCAAGGGCTGTTAGGCTTATTGAGCGTGATGTGGG

CTGTAGATTTACCGAGCTACCACGAATAGCTGTTGATAGTGCATCAATGGACACAGTTGGCTTGGGTGGT

GGGCGATTTGGCTCTTNTTAGGAGGTTCAAGGGATCGTCGATATGGTGATACAGGTTTTGGCCGTGGTCC

TGGGTCTGGCCGTTCTGGTGGCTACAATAGCAGTGGATTTGGCCGCTCTAGCTTTGGAGATTCTGGTGAA

AGGTTTGATGGACAGAATTATAATCGTTTTCGTGGCTCTGGTTTAAGTCAATCTGGAGGGGGATTTTCTG

GTAATTCATCTGGTGGACCAAGCTCTGGTCGGTTTGGCTCATCTGGTGGCTTTGGCTCAGGTCAATCAGG

AAGTAGATCCGGTCGATCATCCAGTGGGTCTAGGTTTAGTCGTCCAGATGATTATAGGGGGATTTCGAGT

CGGTTCAGATACGTCTACAGGCGGTTTAAGGAGATCTTGGCTCCAGGCCANACCTAGTGGTTCCTCGTTG

GTAGTAATCAGACACCAATAGAAGAACTTTTNAAACCTGAAAAATATCAAAANTGNACCACTC



>gi|71811679|gb|DR989070.1|DR989070 HDI_F_44.esd Horsegram (VZM-1) drought stressed subtracted cDNA Macrotyloma uniflorum cDNA, mRNA sequence

GGACTGGAGCTCACGCGGTCGCGGCNTTCATAGAACTAGTGGATCCCCCGGGCTGCAGGAATTCGGCACG

AGGCCTCGTGCCGAATTCGGCACGAGGGTCAGAGTACCCTGAAGGCAAAATTGACAAGTGAGCAGCAGTA

TGCAGTCGTTGGGAGAATCCTACATTGAGTTGGTCAATAAATTAGATGAAGCGCTTACTTGCTTCATTTG

AAGGCCCTCCATTTAGCATTTGCAAAGGCTTTGTGAGATCCTATTGGATGCAAAAAACATTTATCCAAAT

CGTTTCAAAGCTTGCTTTAGCTCTTGAAAAGAACTTATTACGTTACATCTACATTGACAATCTGCACTGA

TCCATACCCGCAAGCTACAGCGAAGGAGCCCGTTGACCAACGAGAAGGCAAATGAAAAGCAGAATCAGCA

ATCCGATACTGCACAGAATGGCATTGAACCTCTGGTGTCTGACAANAGACGAACGTAATGACTGAGGCTG

ATCGTGGNGTGACTGATATGACCATCGATATGGAATCTTTTGANAGGCGATNCATCGTCCANAAACAAAT

TCTCGAGCCTCAATGCTAACTACATGTCATTGAGCTTCAATCGCGTTCATTGTGACATCCTTTACACCCC

CATAAAACTCTGATGAGCCT



>gi|71811677|gb|DR989068.1|DR989068 HDI_F_36.esd Horsegram (VZM-1) drought stressed subtracted cDNA Macrotyloma uniflorum cDNA, mRNA sequence

GGATTTAGGTAGGTGCTCGATTTGATGCGCGCTTTGTACTCTGGAATTTATTTGTCTTGAGTAGTAATAC

TAGGTATGATGGGTGGAATTTGTAGGTTAAAACGATTCCAATTAAGCTATGACAATGTGTAGGAGATTCT

CTGGGACAAGTATTCTTCCAATGCTTACTAGAGCAGACAAGGGTTAAAATGTAACAAAACAAAAAAACAA

AACA



>gi|71811675|gb|DR989066.1|DR989066 HDI_F_34.esd Horsegram (VZM-1) drought stressed subtracted cDNA Macrotyloma uniflorum cDNA, mRNA sequence

GGAGCTCACGCGGTGGCGGCGCTCTAGAACTAGTGGATCCCCCGGGCTGCAGGAATTCGGCACGAGGAGC

AATTGATAGGCTTCCTTTGATTCCTGGGATTCTTGAGATTGTAGGCATTGGCTTCACTGGGTGGTTTGTA

TATAAGAACGTAGTTTTCAAGCCAGACAGGGAAGCTTTGATACAGAAAGTAAAAGAAACATACAATGAAA

TACTCGGGAGCAACTGAAAGAGGTAGGAAATGCTGAGATGATGTAGACATGAAACAATCATATCTTGCAG

CTTCCTGTTGAAGATTTCATAATGCTTCATTTTTTCGCCATAAGCTTCTGACCATACTTTATCCCAAGTA

ATGTGTGTTCGAAAAAAATTGTATTTCATATATCTTAATCCACTTTTAAGACAATCTTATTTCCTTAAAA

AAAAAAAAAAAAAAA



>gi|71811673|gb|DR989064.1|DR989064 HDI_F_32.esd Horsegram (VZM-1) drought stressed subtracted cDNA Macrotyloma uniflorum cDNA, mRNA sequence

AATAGGCTGGGAGCTCACGCGGTGCGCGGCCGTTCTAAACTAGTGGATCCCCCGGGCTGCAGGAATTCGG

CACGAGGCCTCGTGCCGAATTCGGCACGAGGGGCCTAAGGAATAAGGCAAATGTTTTTCCCCACATCCAG

ACCAGCTTTTAGTAGAGCTACAGGTGATGCATTGAGGATTAAGGGAACACTGAGATTGCTCTCTCTCTAC

TAAACATTTGTTATGCCACTTTGGAGGAGTTTCAATAGGTTGTAGTTGTTAATTGATCAAATCCCCTTTG

CTGTCATTGTACCAAAAGAAACGGAACCATCGTCTTAAACCTGATTATGGTGAGCATGTGTAATGTATTG

TTTAAATAATNCCCTTGAGCACACATTTTATAAATCTTGGCTTGTAAAACAAAAGGAAACAGTACCATAT

GTTGAAGGAGAAGGAAATAAAATATACCTAATTA



>gi|71811671|gb|DR989062.1|DR989062 HDI_F_24.esd Horsegram (VZM-1) drought stressed subtracted cDNA Macrotyloma uniflorum cDNA, mRNA sequence

TTCCCCTCTCGTATCGACATTTGTCTNAGTTTGCGCGACCACATCTTTCAATTCATTGACCTTTCCAGAG

AGGTATCCAAGCATCTCTATGTTCGTGCCGTCGCTAGAGGGAAAATACCTCTT



>gi|71811669|gb|DR989060.1|DR989060 HDI_F_22.esd Horsegram (VZM-1) drought stressed subtracted cDNA Macrotyloma uniflorum cDNA, mRNA sequence

AGCTGGAGCTCACGCGGTGCGCGGCGNTCTAGAACTAGTGGATCCCCCGGGCTGCAGGATTCGGCACGAG

GCTTGGTGTGATAAACTTCTTTGGAATGGTATTCACCTTGTTAGTACCAGAATCAAAGGGAAAATCACTG

GAAGAGTTGAGTGGGGAGAATGAAGAAGATAGTCCTGAGGCTATAGAGATGGCAGGGTCTGCTAGGACGG

TTCCAGTTTAATCATAGTCTCTGATTAACAAAGTAGTTCAAAATTCAAGTAATGCCACTATTTTATGACA

ATTTTAGTTCCGGATTTCTACCGCCAGATGTGATCTATATGATGTAGATATATGTTTCTCGGTGTTGTAA

GTTTAAATCAATAACGAAGTGTGTGTATTTTATTTTTGTTTTTGATTAAAAGTGGTGTTTAAATCATATG

TGTATTTCATATCTGTAGCTAAAGTTATTGAAGAACAAGTTGTCTCGTGTTCCTTCAAAAA



>gi|71811667|gb|DR989058.1|DR989058 HDI_F_20.esd Horsegram (VZM-1) drought stressed subtracted cDNA Macrotyloma uniflorum cDNA, mRNA sequence

AGCTGGAGCTCACGCGGTGGCGGCGCTCTAGAACTAGTGGATCCCCCGGGCTGCAGGAATTCGGCACGAG

GCCTCGTGCCGAATTCGGCACGAGGCTTTTCTATAATAGAGAGTACTATTATACAAATCTTTTTCTATTG

TAAAATACAATCAAATTGGATTCGATACTAAACATCAAATCGATATCTATCTATATAGATAGATATCTCA

AAAAAAGGAAAAATATGGAAAAATTACTCTAAGGGGGATTCTTTGTCTTTTCCTAGAGTCCGATTCCTAT

TCCTTCTTGTTTGTGTTGAAATAGAAATATTCTAAAAATATCCATAAAAGAATAGTTTTGAATCCCTTCC

TGAAAAATCCTATATCTTAGTTTCTATTTTTTCCAACTTAGAATCTTTCTGCCAGATAATCTTTTATTGT

ATATAATATGTACGTAAACAAATCAAAAAAGAGAGGGGATTCATAAATTTGATTAAACAAATTTAATTTC

TTTAATTTGAACTTGTAA



>gi|71811665|gb|DR989056.1|DR989056 HDI_F_12.esd Horsegram (VZM-1) drought stressed subtracted cDNA Macrotyloma uniflorum cDNA, mRNA sequence

TGAGCTCACGCGGTGGCGGCGCTCTAGAACTAGTGGATCCCCCGGGCTGCAGGAATTCGGCACGAGGATT

TGCAGCTGTGCCCTTTTTGCGGTCAACCTTTTTCTGTTGTTGGAGATGAGTTTGTGAAGGAATCCGTGAA

GTTTTCCAACCAATCTACAACATTTGGACAAGCATTCAATAATTTCACCCGTTCTAGAAAATGAAAAAGA

TTCTGGTAGAGCAATTGATGTTGAAGCTGAAATTAAAGATGTCGACTGAGTCTTCACAGCTATAACATGT

TGGTAGGTGTGAAGTAATGAGCAAATTTTGAGTTGAACACAGTGACTATGCTGAACACAAATCTTGATCG

GTTAAAGAACAGGCTGTGGCTAGTATTTTCCATTTGTACTGAAGACACAATGAATTACACATTCAAGAGA

GAAGAGTGTGATGAGCAAAGTAACACATTCTAGAGAGGAGTGTGATGAGCAGAGTAACATTGCAATTGTA

ATATAAGTTTTGACAGTGATGTTTCTCAGGTCACACCTCTATAATTTTGGTGTACCAGGATACTGCTTCA

TTTTTCCTTTCATTGTTTTTTACATAAAATGTTATAGGAGAAATCGAGATGCTTCATTTTCAATGTCAGG

ACATGGCAGTCTTGACAAC



>gi|71811663|gb|DR989054.1|DR989054 HDI_F_10.esd Horsegram (VZM-1) drought stressed subtracted cDNA Macrotyloma uniflorum cDNA, mRNA sequence

GAGCCACGCGGTGGCGGCGCTCTAGAACTAGTGGATCCCCGGGCTGCAGGAATCGGCACGAGGGCTCTCA

GCTAAGCACTAAGAGTGTTTAGACCTTAAATATTTCTTATGGGTATGAACAAGCCTCATGTGCTTATGCT

GCTCATCATCATGGTTGTTTCAACATCACTTATAGAAGGAAGGACACTTTCCTTAATATCTGGTCAAGGG

TACTCAAAGATTTTTGCAACTCTTGGAGTTGTATGCAAGTGTTGTGATGGGGTTGGAGGTGCCTGCACCA

GTACATGGACAGAATCTTGCAATAATCTGCAGTGTTATCCTTGGAAATCACACTAGCTAATTGTATTTTC

TGTATCCTTTCTTGTCTTCCATTTACTGTATATCAAGTTTTGAATTATTATAAAATTATATACTAGTGAA

GTCTGTGTTCTGTACTGTATAATGTGAACAGTTTGACTTTCTGAAAAGAACTTTGAGAATTATTCATGGT

TCTTAATTTGTGGAACAAAACAAAAATTCCCACATTAAAACCAAGGGTCCATTTAAAAAATTGGGGGCTT

TGGGTAATTTGGAAAAACCAACCCCAAACCAAAAA



>gi|71811661|gb|DR989052.1|DR989052 HDI_F_89.esd Horsegram (VZM-1) drought stressed subtracted cDNA Macrotyloma uniflorum cDNA, mRNA sequence

AAAGCTGGAGCTCACGCGGTGGCGGCGCTCTAGAACTAGTGGATCCCCCGGGCTGCACGGATTCGGCACG

AGGAGATTACAGTGTGATCGGTTTTGGATTGATCGGATGGATAGCACCTTCCAGCATACCGGCTATCGAC

GGTAAAAGTCTGACTGGCCTCTTCTTTGAGAGCATTGGAACTGAGCTCGCTCACTTCCCCACTCCTCCTG

CTCTTACTTCTTCGTTTTGGTTATGGCTGGTTACGTGGCATCTTGGGTTGTTCCTGGTTCTCACTTTTGG

CCAAATTGGTTTCAAGGGAAGGACTGAAGACTACTTTTGAATTCCAAACCTATGCTCTGTTCTTACTTTT

TTTTTTTGGGTTTCCTACTTTTGTGCCTGCANTTACAATCTATGATGTATAACTCTATGCTACCTTTTCC

CTTAAAAATCTGTGTAANATGGTCTTCATAATGTGGTATATTATTAAAACAAAAACAAACAAAAA



>gi|71811659|gb|DR989050.1|DR989050 HDI_F_87.esd Horsegram (VZM-1) drought stressed subtracted cDNA Macrotyloma uniflorum cDNA, mRNA sequence

CTGGAGCCACCGCGGTGCGCGGCCGTCTCTAGAACTAGTGGATCCCCCGGGCTGCAGGAATTCGGCACGA

GGATTGTGTTCATTGGTGCTTGCCTGGTCCTATAGACACCTGGAGTGATTTTCTTCTGGGAATGTTGAAG

ATGGAGGGTGTGAGATCAGCTGAAGAGAAGCTTCATTTGCGTTCACACTGACAAATGAAATTCATCTCAT

ACATTTATCAGATATTTACGTAATCTTCCTTGTAATTGTAAACTTCATTGCCACATGTATTGTAAATCTT

TTTAAATTATGATCAAATTTGATTAACCCCTTTTAGTCATATTTTTTTATGTTGATTCATATACATTTTT

GGTTTCAAAAAAAAAAAAAAAAAAA



>gi|71811657|gb|DR989048.1|DR989048 HDI_F_85.esd Horsegram (VZM-1) drought stressed subtracted cDNA Macrotyloma uniflorum cDNA, mRNA sequence

AGCTGAGCCACGCGGTGGCGGCGCTTCTAGAACTAGTGGATCCCCCGGGCTGCAGGAATTCGGCACGAGG

GTTGAATTGAATGGTAGACTGAACAAATGTGGGGTTATTAGTCCCCGTTTTGATGTGGGTGTCAAAGAGA

TTGAAGGTTGGACTGCTAGAATTGCTCCCCTCAAGACAGTTTGGGTATATTGTGTTGACTAACCTTCTTG

GCCCGGGCCAT



>gi|71811655|gb|DR989046.1|DR989046 HDI_F_77.esd Horsegram (VZM-1) drought stressed subtracted cDNA Macrotyloma uniflorum cDNA, mRNA sequence

AAGCTGGAGCTCACGCGGTGGCGGCCGCTCTAGAACTAGTGGATCCCCCGGGCTGCAGGATTCGGCACGA

GGAAACACTGGACAATATGCTGGCAACCGTAACAGGTATGACCACTCACAGCATTATGATGAAAAGGTTC

TGAATAACAGTGGCACTTTCAATGGTAATGGCAACGGTGGAACTATCGAGGGTGGCTTTAACGCCAAAAC

AAGGAACTACTACCGTCGTCGTTGAGACTATTAGTTTATCTTGTATTTCGTATATGCTGGGAAACACGAT

ATGCTACAATGTTATGTTGGAGTACTAGCAATAAATAATGTTGAGCTGAAGCTCTAATAAATGTAATGCT

TTTCAGGTGTTGCCTAAGACCCAAAATGTTGTAATAACATGGTCCATGTTTCGTCCTAAAAAAAAAAAAA

AAAAAAAAAAAAAAAAAAAAAAAAAAAAAAAAAAAAAAAAAAA



>gi|71811653|gb|DR989044.1|DR989044 HDI_F_75.esd Horsegram (VZM-1) drought stressed subtracted cDNA Macrotyloma uniflorum cDNA, mRNA sequence

TTGATACATTTATTATATCGCTACATCCTAATAACACGTAAGTCATATCATTTCGCGCGTTCTATTTACC

TATAGTCGCTGTTCTGGGTTTACATTAATTGTCTTGCGTGTCTTTAAAGTTGATATGATACTGTGCCACG

TTGTAGTGAAGAGAATGGATGGTTATGTGTGGAGAATCGTATTTCCCGTCTCTAATTAGTGCTCGTTGTG

GTCCTAACTACGTAATGTAATGGCCACCTTAATTGGTTAACCTTGGGGTTAANCCAAGTAGGAAGGTTAA

CCTTG



>gi|71811651|gb|DR989042.1|DR989042 HDI_F_66.esd Horsegram (VZM-1) drought stressed subtracted cDNA Macrotyloma uniflorum cDNA, mRNA sequence

AAAGCTGGAGCTCACGCGGTGGCGGCGCTCTAGAACTAGTGGATCCCCCGGGCTGCAGGATTCGGCACGA

GGCCTCGTGCCGAATTCGGCACGAGGGAAGAACGGAAGCCACTATCGCTGCATTTGGGGTAAGGTCACCA

GGCCTCATGGTAACAGCGGTGTCGTTAGAGCTAAGTTCAAATCAAATCTGCCGCCAAGATCGATGGGAGC

AAGGGTTAGAGTGTTTATGTATCCAAGCAGTATATGAGATTCTCAGGACTAGTTGGTCGTGGAGCTTGTT

TGTCCGGGTACTTAAAAGGAATTTGCATTATGCGAGTTTTAATGTTTTTTGTAGCTCATGAGCAGAATAG

TATCTCGTTTAGGATGGCGAATGAGCCCCGTGTTTTTTTTCTTCATTAAGGATTATGAGTCTCAATTTAT

AAATGGATTTTTTTTAAAAAAAAAAAAAAAA



>gi|71811649|gb|DR989040.1|DR989040 HDI_F_64.esd Horsegram (VZM-1) drought stressed subtracted cDNA Macrotyloma uniflorum cDNA, mRNA sequence

CCAAAAGCTGGAGCTCACGGCGGTGGCGGCCGCTCTAGAACTAGTGGATCCCCCGGGCTGCAGGAATTCG

GCACGAGGGTTGAATTGAATGGTAGACTGAACAAATGTGGGGTTATTAGTCCCCGTTTTGATGTGGGTGT

CAAAGAGATTGAAGGTTGGACTGCTAGATTGCTCCCCTCAAGACAGTTTGGGTATATTGTGTTGACTACC

TCTGCCGGCATCATGGATCATGAAGAAGCTAGGAGAAAGAATGTTGGTGGGAAAGTGCTGGGTTTCTTTT

ACTAGTCTATTTTCCTTTGAAAGATTTTGTGCGGATTTCAGTTTTTTTTTTAAGTTATTTTTTTTAACTT

ACTGCTGTAATGATCAATGTTAGACACTTAGTTGTATTAAGTATAGCATAGCAGATGTTTTTCTGATGTG

CTGAAAAAAAAAAAAAAAAAAAAAAAAAAAAAAAACAAGGGGGGGGG



>gi|71811647|gb|DR989038.1|DR989038 HDI_F_62.esd Horsegram (VZM-1) drought stressed subtracted cDNA Macrotyloma uniflorum cDNA, mRNA sequence

AAAGCTGGAGCTCACGCGGTGGCGGCGCTCTAGAACTAGTGGATCCCCCGGGCTGCAGGATTCGGCACGA

GGATTGTGTTCATTGGTGCTTGCCTGGTCCTATAGACACCTGGAGTGATTTTCTTCTGGGAATGTTGAAG

ATGGAGGGTGTGAGATCAGCTGAAGAGAAGCTTCATTTGCGTTCACACTGACAAATGAAATTCATCTCAT

ACATTTATCAGATATTTACGTAATCTTCCTTGTAATTGTAAACTTCATTGCCACATGTATTGTAAATCTT

TTTAAATTATGATCAAATTTGATTAACCCCTTTTAGTCATATTTTTTTATGTTGATTCATATACATTTTT

GGTTTCAAAAAAAAAAAAAAAAAAAAC



>gi|71811645|gb|DR989036.1|DR989036 HDI_F_6.esd Horsegram (VZM-1) drought stressed subtracted cDNA Macrotyloma uniflorum cDNA, mRNA sequence

AATAGGCTGGAGCTCACCGCGGTGCGCGGCCGNTCTAGAACTAGTGGATCCCCCGGGCTGCAGGATTCGG

CACGAGGCCTCGTGCCGAATTCGGCACGAGGAGGACATTGGAGGAGATTCAGTGGTCCTTCCGACGATGA

TTTTGTTCGGTGGGCGTCCTGCAAATGCTCGGATTCTGTACATGCAAATACTGGGTGGACCTGTAAATGC

AAATGTGTAGCTGAATGCTACCATAATTTGGAACAGAAGAAAAAAAAACCCTGAAAAGGGGCAAAAAAGA

AGAAAGAAAACTATATATCTTTTACTATTATTCCTTCCCCTCATGCCATGGAAAAAGATATTATGATCAT

TGTATATATAATACCAATAGTTGCATCACCATTTTTAGGGGAAGGTGAAATAAATTAATAGATTTGTATT

TTGTGTTTTAAAACCTATTCATGTATTAAGGC



>gi|71811643|gb|DR989034.1|DR989034 HDI_F_53.esd Horsegram (VZM-1) drought stressed subtracted cDNA Macrotyloma uniflorum cDNA, mRNA sequence

GCTGGAGCTCACGCGGGGCGGCCGCTCTAGAAACTAGTGGATCCCCCGGGCTGCAGGATTCGGCACGAGG

GGACCATAGAAGGCTGCTTGCATCATATGACATGGAACCTGAGTCAGGTAACTATATCAATCCAGATAGA

CCCATGCTTGATGTGCAGTTGAAAAGTATTACTTAGATGGGTGGAGGCAGGAAATGACTTTGTTTTTCAG

CTGTGGAATTTTGCATGATGAGGTAAAAAGCCTTGGCATGGAAACTGGCTGAAAGTAAATGCAACTGCCT

ATCATCTCAGCAAAGGAAAACTTTTTTAAATTCCCATAATTCAATTTAGTCATGTATATAATAAAAGGAA

ACTTAGAAATGTATAGGGTACATAGTTTTGTGTAGTTATGCACAGTTATCTGTGTTCTAGATTCAGCCAA

ACTTGTAATATCTGAATAGAGAAACAATTCCCTATTAAACACTTTCTAAAGTTGTACAGCATAAATTTTT

TTTTACCACCAGGATATGAACATGTGTAATAAGAACTCAACTGAGATTTATTCAAAAAAAAAAAAAAAAA



>gi|71811641|gb|DR989032.1|DR989032 HDI_F_51.esd Horsegram (VZM-1) drought stressed subtracted cDNA Macrotyloma uniflorum cDNA, mRNA sequence

TGGAGCTCACGCGGTGGCGGCGCTCTAGAAACTAGTGGATCCCCCGGGCTGCAGGATTCGGCACGAGGCC

TCGTGCCGAATTCGGCACGAGGGAACATTCGTTTCTCCCTAAGTTTAAGTCGTGATGGGAAAGGAAAAGG

CTAACATCAAAATCGTGGTCGTTGGACATGTCTCTGAGAAATCAACTGCCACGGAGCTTGGAAGCCCTAC

TGAGAAGGGTGTGACTGAAAGGTTCGAGAAGGGAGAAGGAAGTGTTTAAAACACGTAAGATGTAAGATGT

GTGAAGAAGGATGATGTAAAATAAATAAGATATAAGATGTGAAGATGGATCTATCTTAAGATGAAGTGCT

GAAGGACTTATATTCAGATAAATAAAATGAAGGATATATGTT



>gi|71811639|gb|DR989030.1|DR989030 HDI_F_5.esd Horsegram (VZM-1) drought stressed subtracted cDNA Macrotyloma uniflorum cDNA, mRNA sequence

GGCTGAGCTCACGCGGTGCGCGGCGTTCTAGAACTAGTGGATCCCCCGGGCTGCAGGATTCGGCACGAGG

CCTCGTGCCGAATTCGGCACGAGGAGGACATTGGAGGAGATTCAGTGGTCCTTCCGACGATGATTTTGTT

CGGTGGGCGTCCTGCAAATGCTCGGTTCTGTACATGCAAATACTGGGTGGACCTGTAAATGCAAATGTGT

AGCTGAATGCTACCATAATTTGGAAAGAAGAACACAAAAAACCCTGACAAAGGGGCAAAAAAGAAGAAAG

AAAACTATATATCTTTTCTTTTTCCTTCCCCTCATGCCATGGAAAAGATATTATGATCATANGTATTATA

ATACCAATAGTTGCATCACC



>gi|71811637|gb|DR989028.1|DR989028 HDI_F_41.esd Horsegram (VZM-1) drought stressed subtracted cDNA Macrotyloma uniflorum cDNA, mRNA sequence

AGCTGGAGCTCACGCGGTGGCGGCGCTTCTAGAACTAGTGGATCCCCCGGGCTGCAGGAATTCGGCACGA

GGGTAGTGATCTTTTTCGTTTCTCTTTTCATCTTCGGATTCCTATCTAACGATCCAGGACGTAATCCAGG

ACGTGAAGAATAAGAAAAAAGTGGATTCTGTGTTTTTCTTGCTTGTGCGGAATTTTTAAATATTTTTAGG

ATTTTATCTATTTGAACATCTTTAACTAGTAAATAAAAAAACAAGTTAAAAAAAAATATCAAATTATCAA

CGGAAAGAGAGGGATTCGAACCCTCGGTACAAGAATTTGTACAACGGATTAGCAATCCGACGCTTTAGTC

CACTCAGCCATCTCTCCCCAATTGAAAAAAAAAAAAAAAAA



>gi|71811635|gb|DR989026.1|DR989026 HDI_F_4.esd Horsegram (VZM-1) drought stressed subtracted cDNA Macrotyloma uniflorum cDNA, mRNA sequence

AAGCTGGAGCTCACGCGGTGGCGGCCGCTCTAGAACTTAGTGGATCCCCCGGGCTGCAGGATTCGGCACG

AGGGAAATTGTCCCTGCCACTCTGAAGAAACAGGCCAAGCCACCTGGCAGGCCCTTTGGTGGCCCATCCG

GTGATCGCCCTAGGGGTCCACCTCGCTTTGACGGCGAGCGGAGGTTTGGCGGTGATCGTGATGGATATCG

TGGAGGTCCCCGAGGACCTGGTGGAGACTTTGGAGGAGACAAGGGAGGAGCTCCCGCTGATTACCGGCCC

TCTTTTGGGGGACCTGGCGGAAGGCCTGGCTTTGGTCGTGGATCTGGTGGTTTTGGAGCGCCAACCAGCT

CAAATCTTCCTTAAGTATAATGTTTTCTACTTTATTTTTTGTTTAGTTTTCTAGAGAAAATGTATCTGTA

CTTGGTGTCTATGATAGATTTCCCTCTCCTATAGATTGTTTTATGCGCTTGACACATACTTTATATTCAG

TTTTTGGTTCAATGAAGATTTTGAATTGATAAGTTTTTCTTTCAGTGGGGATTTTTAAA



>gi|71811633|gb|DR989024.1|DR989024 HDI_F_38.esd Horsegram (VZM-1) drought stressed subtracted cDNA Macrotyloma uniflorum cDNA, mRNA sequence

CTGGAGCTCACGCGGTGGCGGCCGCTCTAGAACTAGTGGATCCCCCGGGCTGCAGGATTCGGCACGAGGC

CTCGTGCCGAATTCGGCACGAGGCAGACAAGAAGACCCGTCACTATCTGCCTAATGGATTCAAGAAGTTT

GTTGTCCACAATGTCAAGGATCTGGAACTTCTGATGATGCACAACAGGACATATTGTGCTGAGATAGCAC

ACAATGTGTCAACCAGGAAAAGAAAGGAGATTGTAGAACGTGCTGCACAGTTGGATGTTGTTGTTACCAA

CAAATTAGCCAGGTTGCGCAGCCAAGAAGATGAGTGATTGGCTGAAAATCTTTTCAAAGTTATTGTGTTA

TGTTATCTTTGTTTCGGATCTAGAGATTTTCAGTGTTTGATTAGTTGAGTTATAACTTGTTTGACTCCTC

CTTATTAGTAGTCATTTTATGCCAAACAAAACAAAACAAAAAA



>gi|71811631|gb|DR989022.1|DR989022 HDI_F_30.esd Horsegram (VZM-1) drought stressed subtracted cDNA Macrotyloma uniflorum cDNA, mRNA sequence

CTGAGCTCACGCGGTGCGCGGCGTNTCTAGAACTAGTGGATCCCCCGGGCTGCAGGAATTCGGCACGAGG

CTTACGTTAAATGGCGTTATTACTTTATAAAATGGTAGGTTCATTGGGTTTTTTCTGCGATACAAACAAG

AAAATATTTCTTGATGAAAAGGTAAGTAAGTGAATTGAAAGGTAAAGACGAATATATATACGAATATATA

TATAGTATTCTATATATAAGAATAATGTATAACATAGTAGATAAAGAGGTGGTGGCACCAGTAGCAATTA

GCCTATTGTATATAACAAATATAGTATTAGCAATTAGCCGTGATTTGAGCAGCCCCAACATTCAGCAGGT

TTTCCTTTTCTTCTATTTTGTCAGAGTCAGTTGGAAACCTGGATTTCATTCAGCCAGCAAGGAGGGAAGA

AGAATTGGTCAAGCTATTTTTATAAAATATATAACTATCACTATGTATACTAACTACAGGGATTANTCGA

TCAATATTGATTGATTCAGATTGTAACAATCATTGAACGGACTATTAGTGAATTATCATAAA



>gi|71811629|gb|DR989020.1|DR989020 HDI_F_29.esd Horsegram (VZM-1) drought stressed subtracted cDNA Macrotyloma uniflorum cDNA, mRNA sequence

GCTGGAGCTCACGCGGTGCGCGGCCGTTCTAGAAACTAGTGGATCCCCCGGGCTGCAGGAATTCGGCACG

AGGGCCTTGTGCAGGAAGAGAACGAGGAGGAAGCGATGGCGGAACATGGTGGCCGTGGGATCTCAGTTGC

AGTGCGTGCAACTTGGTTGGAATTATTGATTTGATGTTCTGGATAGAGAAGTGAGATATTTTAAGAAGAA

AAAGTGGAGAGAGTAGTTAGATAAAAGATAATGAAATTTTGACTAATTAGGCTTTTATTTTTGGATGGAT

TTTATTGAAATAGGTAAAATTTTAGAAAATAACAAAAAAAAAAAA



>gi|71811627|gb|DR989018.1|DR989018 HDI_F_26.esd Horsegram (VZM-1) drought stressed subtracted cDNA Macrotyloma uniflorum cDNA, mRNA sequence

AAGCTGGAGCTCACGCGGTGCGCGGCCGTNTCTAGAACTAGTGGATCCCCCGGGCTGCAGGAATTCGGCA

CGAGGATGGACGATGATGTTCCTGCTGGTGGAAGTGGTGCTGGTCCAAAGATTGAGGAAGTAGACTAAAT

TTAGTTAATTCCTGCTGGAGTTACTATTTATTTTGTAAGACCCGTTTGACGTGTTTTTTCTTGGATTTAG

GTTTATGGAAGTATTTCGTAGTGTATGTTTGTCAGTATCGGTCACTTCTATTCTACTTTAGTTTTAGTTG

TTGTGACATATACAAGCTTTTAAGCTCGTGTTCTTTTATTTATTTAGTAAGTATCATGTTTTCGTGGTTA

TAAAATACAAAACAACAAAACAAAAAAACATCCCAAGGGGGG



>gi|71811625|gb|DR989016.1|DR989016 HDI_F_2.esd Horsegram (VZM-1) drought stressed subtracted cDNA Macrotyloma uniflorum cDNA, mRNA sequence

AGCTGGAGCTCACGCGGTGGCGGCCGCTCTAGAACTAGTGGATCCCCCGGGCTGCAGGAATTCGGCACGA

GGCCTCGTGCCGAATTCGGCACGAGGACGGGCTTCACGGATCTAACGGTGACCCTGGCTGCATACGATAC

TGCGCCGGGTTCTCGATCCCATCCAACGGCACAGCAAGGGTGAGCTGGTCCAAGGAGAGGCTGGTAGCCG

TTGATGATGCTGAGTTCTGGCTCAAGTATGAGATGGTGGATAGCAACATCGGGTTCAGTTCCTACGAGTC

AACGATGAAGGTCCTGAGCAATGATGATGATGATGAAGATGAATCGAGTGGATGCATGTTGGAATGGTCC

TTTACTGTTGACCCTGTGGAAGGGATGGAACTTGAGGAGCTGGTTCGGAAGTACCATGTGGGCCTTCAGC

TTATGGCCCAGAAAATGGAGGAAGAAATTCGAAGTTTGGTGAGTAGAATTATGTAGTATAGATAATCAAG

ACATTTTCTTTTTGTTTTTTTTTTTACTAAATTTATCCTGATAACNTTATCCTTTTTTATACCACGAACA

ATGTTTCATGTTC



>gi|71811623|gb|DR989014.1|DR989014 HDI_F_16.esd Horsegram (VZM-1) drought stressed subtracted cDNA Macrotyloma uniflorum cDNA, mRNA sequence

GCTGGAGCTCACGCGGTGGCGGCGTTCTAGAAACTAGTGGATCCCCCGGGCTGCAGGAATTCGGCACGAG

GCCTCGTGCCGAATTCGGCACGAGGAGGACATTGGAGGAGATTCAGTGGTCCTTCCGACGATGATTTTGT

TCGGTGGGCGTCCTGCAAATGCTCGTGTTCTGTACATGCAAATACTGGGTGGACCATGTAAATGCAAATG

TGTATGCTGAATGCTACCATAATTTGGAAAGAAGAAACAAAAAACCCTGAAAAGGGGC



>gi|71811621|gb|DR989012.1|DR989012 HDI_F_14.esd Horsegram (VZM-1) drought stressed subtracted cDNA Macrotyloma uniflorum cDNA, mRNA sequence

AAGCTGGAGCTCACGCGGTGGCGGCGCTCTAGAACTAGTGGATCCCCCGGGCTGCAGGAATTCGGCACGA

GGCTCAGACAAGCCTCTCAGGCTCCCCTTGCAGGATGTCTACAAGATTGGTGGTATTGGTACCGTGCCAG

TGGGACGTGTTGAGACCGGTGTTTTGAAGCCCGGTATGGTGGTGACCTTTGCTCCCACAGGGTTTGACAA

CTGAAGTTAAGTCTGTTGAGATGCACCACGAGGCTCTCCAAGAGGCTCTCCCAGGTGACAATGTTGGGTT

CAACGTGAAGAACGTTGCAGTGAAGGATCTCAAGCGTGGTTTCGTTGCATCCAACTCCAAGGATGACCCT

GCCAAGGAAGCTGCCAACTTCACATCCCAAGTCATTATCATGAACCACCCTGGCCAGATTGGGAACGGCT

ATGCACCAGTGCTCGATTGCCACACTTCCCACATTGCTGTCAAGTTCGCCGAACTCGTGACCAAGATCGA

CAGGCGATCTGGTAAGGAGCTCGAGAAGGAGCCCAAATTTTTGAAGAATGGTGATGCAGGTTTCGTTAAG

ATGATTCCAACCAAGCCCATGGTGGCTTGAAACCTTCTCTGAGTATCCTCCCCTTGGTCGATTTTGCCGT

GAGGGACATGCGTCAAACCGTGGCTGTTGGACGTCATCAAAGAGTGTCGAGCAACGACAGGANCCTACTT

GGAGACCAAGGTCACCAGGGCTG



>gi|71811619|gb|DR989010.1|DR989010 HDI_F_1.esd Horsegram (VZM-1) drought stressed subtracted cDNA Macrotyloma uniflorum cDNA, mRNA sequence

AAAGCTGGAGCTCACCGCGGTTGGCGGCCGCTCTAGAACTAGTTGGATCCCCCGGGCTGCAGGAATTCGG

CACGAGGCATGTCCAAAACGCTGGANCAGATGATGCAAGGTAATATTTGGATGTCAACTAAATCTCTGGG

TTTGGCACAAGGAATGACACTTTTTCTCAGGTTTCAGATAGGATCATCTCATGGAAGATTCATTCTCNGC

TCCTAAAGATTTTTCAAACTCCCAGCTTAGAGGGCTTAAGGTTGTGTTAGCTGATGATGATGATGTAAAC

AGGACTGTGACCAAGAAGCTGCTTGAGAAACTGGGGTGTCAAGTAACTGCTGTTTCATCAGGATTTGAAT

GTCTTGGTGCTATAAGTGCCTCTGGTAACTCTTTCAAAATTATTCTGTTGGATCTTCACATGCCTGAAAT

GGATGGTTTTGAAGTTGCAAGAAGGATTCGAAAATTCCAAAGCCGTAATTGGCCCTTGATCATAGCTTTT

ACTGCAAGTGCAGAACAACACGTCAAGGAACAGATGTCTACGGGTGGGAATGAATGGATTGATTCAAAAA

ACCTATCCTCCTTCAGGGAGATGGCAGATGAACTTAGAACAGTCCTGCAACGTGCAGGGTGAAAACATGT

CATGAGAACAATTTTAGTAATCTCAAATTTNTAGGGTTCTCAAAATTTCATTACTTATGCCATTCTGAAA

GGAGAACCTGGAAATTTCCTTANCAAACTAAATATACTAGATAGCNATNCTACGTTCATTTTGATACCCT

TCCTGGTTGACCTTTTGCCGTCACACGGAATATGGCTTCGAAA



>gi|71811617|gb|DR989008.1|DR989008 HDI_E_95.esd Horsegram (VZM-1) drought stressed subtracted cDNA Macrotyloma uniflorum cDNA, mRNA sequence

AAAGCTGGAGCTCACGCGGTTGGCGGCGTCTCTAGAACTAGTGGATCCCCCGGGCTGCAGGATTCGGCAC

GAGGCCTCGTGCCGAATTCGGCACGAGGTTCGTGTTAGAGGTGGCAATGTAAAATGGAGGGCATTGAGGT

TGGATACTGGAAATTACTCATGGGGTAGTGAGGCAGTAACCCGCAAGACTCGTATTCTAGATGTTGTTTA

CAATGCCTCAAACAATGAGCTTGTGAGAACTCAGACTCTGGTTAAGAGTGCTATTGTTCAGGTTGATGCT

GCTCCCTTCAAGCAGTGGTACCTTCAGCACTATGGTGTAGAAATTGGCAGAAAAAAAAAGCCGCAACCAA

GAAGGACTCTGCTGAGGAGGGTGAGGCTGCTGCTGAAGAAACAAGAAACAGTAAACCATGTGCAGAGAAA

ACTTGAGAAGCGACAGAAAGAACGCNNAACTTGATTCCCANCATTGAAAGAGCAAATTTGGTGGTTGGCC

GAATTTGTTGGGCATGTACTTTCTTCTACGGCCCAAGACAAATTGG



>gi|71811615|gb|DR989006.1|DR989006 HDI_E_92.esd Horsegram (VZM-1) drought stressed subtracted cDNA Macrotyloma uniflorum cDNA, mRNA sequence

GCTGGAGCTCACGCGGTGGCGGCCGCTCTAGAACTAGTTGGATCCCCCGGGCTGCAGGAATTCGGCACGA

GGAGAAGATAGCAGTCAAAAAACAAAAACTAAGAGCATTGTTTGTTCTGATTGTGATGGAAATGGTGCAA

TATTATGTACTCAATGTAAAGGTACTGGCGTTAATTCTGTAGATCACTTCAATGGACAATTTAAAGCCGG

TGGATTGTGTTGGCTGTGCAGGGGTAAAAAAGATATCTTGTGTGGATCTTGTAATGGAGCTGGCTTTGTT

GGTGGATTCATGAGCACATTTGATGATTAAAAGTTTGGCTAGATTGAATTAAAGATTCCTAATGACTATT

TGTTCCTTCGTTGGTCTTTTTGTAATGTGTATTCAAATAAGATTTTGTCTTAATTCATAATTATTCTGTT

ATCTATTTAGGGAACATTACTATTTCATTATTATTGGAACTTTTGCCAAATATGAAATTCATTTTGATTT

TAAAACAAAAAAAAA



>gi|71811613|gb|DR989004.1|DR989004 HDI_E_81.esd Horsegram (VZM-1) drought stressed subtracted cDNA Macrotyloma uniflorum cDNA, mRNA sequence

GCTGAGCTCACGCGGTGCGCGGCGTTCTAGAACTAGTGGATCCCCCGGGCTGCAGGAATTCGGCACGAGG

AATACTTATAACTCCATTGTTGATTGGTATTGTAAGCTAAACCGTCGAGATGAAGCCAGCAGTTTTGTTA

AAAGCCTTCGAAATATTGACCCACATGTTTCCAAGGAGGAGGAAACTAGGTTACTTGAGAGAATAGCAAA

AATGTGGCCATAAGTTGTTTCGTGATCTGTGATATGCTGTACTGGAATTTAAAGTGTTTCTTGACCTCAT

GTTTTTAGATGCCCTTAGTCTCTGCTTCCTTCTTGGTTTCCATGTTATATAATATTAGGGAATGTAGCTG

TAAATGACGGTGATCCTTTATGGGCTTTATCTGCAGAAAAAGGAAGCAGTGTGTTTCCGCATT



>gi|71811611|gb|DR989002.1|DR989002 HDI_E_8.esd Horsegram (VZM-1) drought stressed subtracted cDNA Macrotyloma uniflorum cDNA, mRNA sequence

AAGCTTGAGCNNACGCGGTTGCGGCCGTTCTAGAACTAGTGGATCCCCCGGGCTGCAGGAATTCGGCACG

AGGGATTGAGGGAGGACACTCCTTGGACTGGACCATGCA



>gi|71811609|gb|DR989000.1|DR989000 HDI_E_71.esd Horsegram (VZM-1) drought stressed subtracted cDNA Macrotyloma uniflorum cDNA, mRNA sequence

CAAGCTGGAGCTCACCGCGGTGGCGGCCGCTCTAGAACTAGTGGATCCCCCGGGCTGCAGGATTCGGCAC

GAGGCCTCGTGCCGAATTCGGCACGAGGCTCCTCCCGGTGCTTCTCTTGACTGCTACATAGTATAAAAGA

AAAAGAAAAAGATAAAGAGAGAGAAAGTGATGAGAGCGGAGATGAAGGATGAAACTCAAGCTGTGGTGAA

TGATAATGGTTACTCCGGCAAATTCTCACCACTCCTTGCTTCCCAAGATCGTGATTACCTTCTCTCTCCA

ACTGGGGCTCAGGTTCTCTATCTCTCTCATAACTCACTAGTTTTTGTCAGAGTTAATTGTTCAGAGAGTC

CTTCGGGTAATACCTGAAGATAAATAGTATATACTATGTATAAACAAAAAAAGAGTGTAAGTAAGCTCCT

ACGGTTATACTTGTATCTTTTAGTTCCACTTTTGGCTCAGATGGTGGTCACTGGTCAGTTTTCGTCCTTT

ATATTATCTTGATCTTGATATGATTTAACATCTGTGTGCTTT



>gi|71811607|gb|DR988998.1|DR988998 HDI_E_7.esd Horsegram (VZM-1) drought stressed subtracted cDNA Macrotyloma uniflorum cDNA, mRNA sequence

CAGCTGGAGCTCACCGCGGTGCGCGGCCGCTCTAGAACTAGTGGATCCCCCGGGCTGCAGGAATTCGGCA

CGAGGAAAATATGTCTCTGGAGATTAGAGTGTATAGAATAATCTTGCACATTAGTTTGGTACTGCTGTTA

AAGCTTGGCCCTGTCACAGAGATGCTACACAATAGCATAAATCCAAACAGACTTTGTGAAGGACAAATAG

TGCAGGGTGTGTATGACGTAGGAGTGGCCATAACTCAGGAATACCGATGTACCTTAATGTCCTGACGTTT

TGTTTTGTTTTTTTTTTTTTTTTTTTCCCTATCTCTGAAGAGAGGCTTCCCCTAGAC



>gi|71811605|gb|DR988996.1|DR988996 HDI_E_68.esd Horsegram (VZM-1) drought stressed subtracted cDNA Macrotyloma uniflorum cDNA, mRNA sequence

AGCTGGAGCTCACCGCGGTGCGCGGCCGCTTCTAGAACTAGTGGATCCCCCGGGCTGCAGGCTTTCTGGT

CTTCGGATCTTCAACCAAATCACTAAGAGACTACACTCCGTTGTTTTCTCTTTTGTGACTTTAATAAAAA

AAAAAAAAAAAAAAAAAAAAAAAAAAAAAAAAAAAAAAAAAAAAAAAAAAAAAAAAAAAAAAAAAAAAAA

AAAAAAAAAAAA



>gi|71811603|gb|DR988994.1|DR988994 HDI_E_60.esd Horsegram (VZM-1) drought stressed subtracted cDNA Macrotyloma uniflorum cDNA, mRNA sequence

GAAAGCTGGAGCTCACGCGGTGCGCGGCCGCTCTAGAACTAGTGGATCCCCCGGGCTGCAGGATTCGGCA

CGAGGCCTCGTGCCGAATTCGGCACGAGGCGGCACGAGGATTGTCCTAAGCTTCTGGGTCTATCTCCAAA

CTCCCCTGATGCTGCGATCTTCAGGAATGCTTCGTTGAGAATGAATTCGAGTTCAACAGTGGGAAGTGCA

ACACCAGTTTCTCACAGCAGCCACAAGGCCTCCTTTGGAACGATGCTTCGACCTTCTCTGGTGCCACACC

TACCTGCACTGTTCTGCGCCATTCTCATCCTACCTCCTTCCAATTTCTTCTGTCAATATTAATTTATTTG

TGATGGGTGCTTTTAATTAATGGCTCTTTAAATGAAAGTAGAGGTGGTAGCTCTTTTGTTATTACTGTAA

TGCTACTCTGCATGAATTCAATTAATATTCAGGTTGCACCAAAAAAAAAGGGGGGGCCCGGTACCCAATT

CGCCCTATAGTGAGTCGTATTACAAT



>gi|71811601|gb|DR988992.1|DR988992 HDI_E_58.esd Horsegram (VZM-1) drought stressed subtracted cDNA Macrotyloma uniflorum cDNA, mRNA sequence

CTCTACGAGTGCCTTGCTGATAGGAATATGTGCATTCATGGCCTAAATGTACACGGATGAAATGCCACCT

CCTTAATGACCAAAAGGCGCGGTCGAAAATCTGCGTACATATATGACCACACATGACCACTGACCAGCTG

GAAATACAATCCTGANNGGATGGGCGTGACATATCAGAACATGCTATAGACCGATGGATTTAGAGGATAT

CAAGCTGGTCTGACTGATAATTAGACAGAAGATAGACACTTGTTGACAGAGTGNCAACTGAAGTGGCCTC

CATGTCGGGTGCGGGCGTACTGTGACGCCCCCTAATATTATTGGGTCACGGTCCTGTTCCCCACTTATTA

TGGTGTTATNTTCCGTNCGCTTCTTATTTGNGGT



>gi|71811599|gb|DR988990.1|DR988990 HDI_E_56.esd Horsegram (VZM-1) drought stressed subtracted cDNA Macrotyloma uniflorum cDNA, mRNA sequence

AAGCTGGAGCTCACGCGGTGCGCGGCGCTCTAGAACTAGTGGATCCCCCGGGCTGCAGGATTCGGCACGA

GGATCTCATCCTCCTCATGAGAACTTTGAAAAAAATTGGTGTGATGATATGAAAAGAATAAAGTTTGCTT

CCTTTTCGTAATGTATTAATGTATAAGAATAAGGTTTCACTGCCAAAGTTGGTTTAGTTCTCTGCAACCT

GACTTGTGGAATAAAGGGGCTGATACTCATTTTCACCCTGGTGGGGATGAACCAACTTACATATATCAGC

ATGAGGAGCTTTGAAAACAATGGTGTGATAATATGATAAGAATAAGGCTTTATTGCCAAAGTTGAGTTAA

TTCTATGTAACTTGACTTTGTGTGTTGAATGTTTCTTTTCTTTCTTCATGTAATGTAATGCATTTGATTC

AGTAATAAAAGGATCGTTTAG



>gi|71811597|gb|DR988988.1|DR988988 HDI_E_47.esd Horsegram (VZM-1) drought stressed subtracted cDNA Macrotyloma uniflorum cDNA, mRNA sequence

AAGCTTGGAGCTCACCGCGGTTGCGCGGCGCTCTAGAACTAGTGGATCCCCCGGGCTGCAGGAATTCGGC

ACGAGGACAAAATTTTCTTGATTTGGACAGTACTCCCAAGGATGAGCCCCATGAGGACCATGTCTCCTCC

TCCTCAGTAGCTAGGCCTCGGTCAAGTTTCCAGAGAAGGTCTCCTGTTCCTCGTGTAAAATACCAGCCCG

TCTCTAGCAATGAGGATGATATGAATGTCCTACCAGTAGTTATTGAGGATGGAGAGAACAATCACGATCA

GATAGATTTGAAGCATGATGAGGAACCAGAGCCTGACTCTGAATTAGATATTGATTCAAAGGATGAGTTG

TTCTTCCCAAACATAGGGATAGCTGAACTGTCAGAGGATGATGATTCTGAGCAAACCGATAATGATGAAA

GTGTGGAGGAGCAGCCTGCAGCTTCAACACCAAGATTTGAGTGCTCTGAAGCTGTCTGAATTAGAGGGCA

CTTGCNANNGTCTCGAGGCCTGCAACGGGTTCTCCAAACATGAAGAAAGAGTGACCATCTT



>gi|71811595|gb|DR988986.1|DR988986 HDI_E_45.esd Horsegram (VZM-1) drought stressed subtracted cDNA Macrotyloma uniflorum cDNA, mRNA sequence

AAAGCTGGAGCTCACGCGGTGGCGGCCGTCTCTAGAACTAGTGGATCCCCCGGGCTGCAGGAATTCGGCA

CGAGGGCTGCATCCTCCATGGCTCTCTCTTCCCCCTCCCTGGCTGGCAAGGCCGTCAAGCTTGCCCCATC

AGCCCCCGAAGTCGGGAGGGTCAGCATGAGGAAAACCGTCACCAAGCAGGTCTCCTCCGGAAGCCCATGG

TACGGCCCAGACCGTGTCAAGTACTTGGGCCCATTCTCCGGCGAGCCCCCGTCCTACCTCACTGGCGAGT

TCCCAGGTGACTACGGTTGGGACACTGCTGGGCTTTCCGCTGACCCAGAAACCTTCGCCAGGAACCGTGA

GTTGGAAGTCATCCACTCCAGGTGGGCCATGCTCGGAGCCTTGGGCTGCGTCTTCCCCGAGCTCTTGTCC

CGCAACGGCGTCAAATTCGGCGAGGCCGTGTGGTTCAAGGCTGGCTCTCAGATCTTCAGCGAGGGTGGGC

TCGACTACTTGGGCAACCCAAGCCTGATCCACGCTCAGAGCATCCTCGCCATCTGGGCTACCCAAGTTAT

CCTCATGGGCGCCGTTGAGGGTTACCGTATTGCCGGCGGGCCTCTCGGTGAGGTCACTGACCCCATCTAC

CCCGTGTGGCAGCTTCGACCCATTGGGCCTCGCTGACGACCCAGAGGCTTTCGCTGAGCTCAAGGTGAAG

GAACTCAAGAACGGTAGGTTGGCCATGTTCTCCATGTTCGGGTTCTTCGTCCAGGCCATTGTCCCCGGAA



>gi|71811593|gb|DR988984.1|DR988984 HDI_E_43.esd Horsegram (VZM-1) drought stressed subtracted cDNA Macrotyloma uniflorum cDNA, mRNA sequence

AGCTGGAGCTCACCGCGGTGCGCGGCCGCTCTAGAACTAGTGGATCCCCCGGGCTGCAGGAATTCGGCAC

GAGGAAGAAACACAGAGAGAGAGAGAGATAGAGAAAGATGAGTATAGAAGGTGGTTGTGTTGGTTTTTGC

TACACTTTTGGTAGCATCGCATGGCGCTTCTTTCAGAAGCTTCCCTCTGCTACTGAAAACAGATGGTGAT

GATGAGAGTTACCAAGATATAAGATGCGCAAGTTGGAGACTTGCTGCGGAAACACGCAACATCTTAACCT

GGGAAACCATTCCTGAAGAGTGTGTAGAAGCCACAGCCAAATACATTGAAGGAAAACAATACAGATCAGA

CTCCAAAACAGTTAACCAACAAGTTTACTTTTACGCTAGAGAGCGCGAAGTCCATGACAATGACGTAATT

GTGTTCAGCATTGATGGAACTTTACTCTCCAATGTCCCATACTATTCTCAACATGGATATGGAGTGGAGA

AATTCAATTCCACACGTTATGATGAAGAGTTTTGTTCTCAAGGGTGATGCACCAGCTTTGCCTGAGACTC

TTAAGAACTACAGAAAGCTGGTGAGTCTTGGCTACAGATCATCATATTATCAGGAAACGAAAAGAGAAAC

AAGGGGAAAGTAACTGAAGCCAACTTTTAAAGGCAGGCNTGGGTTTTCGGACCCATGGGAGAAAGTGGCA

TCCTTCAAGGGANGCCTTCTAACCAGTGCACCAAATTGCTCTTGGAGTCACAGAAAGG



>gi|71811591|gb|DR988982.1|DR988982 HDI_E_35.esd Horsegram (VZM-1) drought stressed subtracted cDNA Macrotyloma uniflorum cDNA, mRNA sequence

AAAGCTGGAGCTCACGCGGTGCGCGGCCGTTCTAGAACTAGTGGATCCCCCGGGCTGCAGGATTCGGCAC

GAGGGCAAGAAGCCATTGGTTTTAAGATAAAGAATGCAATGCTGTATCCGCGTGAAGTAGAGAAAGGACT

ATGGCCAGAAGATTATTCTCTCTCTGATCATGCTAGACTCACAGCTGTGTTTTCACCAGCAAGGATGAGA

TGTTCTGGCACACAAAACTGAACCATACCTACCTACCTGGCTCCTAAAATTGCATTAATAGTATATAGGC

CTAATTAGAAATTGACCAAAAAAAAACATTGCTGAACTTCCAGACGAAC



>gi|71811589|gb|DR988980.1|DR988980 HDI_E_32.esd Horsegram (VZM-1) drought stressed subtracted cDNA Macrotyloma uniflorum cDNA, mRNA sequence

AAGCTGGAGCTCACCGCGGGGCGGCGCTCTAGAACTAGTGGATCCCCCGGGCTGCAGGAATTCGGCACGA

GGGCAAAATCTTAGAAATAAGGGAATCATTGATAAACCCGGGCACAGTATTTTCTAGAAGTCCCTAAAGG

AAATCCGGAAATAAACCAACTTAACAAAACCAGCATCCTCCATATGTAATGTATTTCAAGAACAAATTCA

AAGGCTACAAATAGAACCTAAGCCTCGGTTGTAGGATAGTCAAACACAACATCTTTGCCTGAAAACTTCC

TGTATACTGCAGCAAAAGTCTCCAACTTGTACTCAGTGTTGTTCCTCTCCTTGGGGTCCAAAAACACCTT

CATAATCTTGGAACCATCAATTCTAAACCTAACACGCTTTCCAACAATCTCAGCAGGTAAGACAATGTCC

TCCAGCATTGCCTCATGCACAGCAGTGAGTGTACGGCTGCGAGGACGCTGTACGGCTGAACCTTTCTTTG

GTGGCCTCAATATCCTCCGGGTGGCAATCAGGACTACATCCTTCCCGCTAAATTTTTTCTCAAGTTCTCT

CACAAGCCTGACATGGATCTTCCTGAATCCTTTCCTTAATCTGTAGGGGACATGGATAACCACAGCCTTG

CGGTTACCAGAAACATCATTTGGACAGCTGAATTTCATGTATAATCTTTCAGATCACTTTTCAGCTCATT

GTGGTATCTCGAGGGGGGCCGGTACCCCAATTCGCCCTATAGTGAGTCGTATACATTCACTGGCCGT



>gi|71811587|gb|DR988978.1|DR988978 HDI_E_23.esd Horsegram (VZM-1) drought stressed subtracted cDNA Macrotyloma uniflorum cDNA, mRNA sequence

AAAGCTGGAGCTCACGCGGTGGCGGCGCTCTAGAACTAGTGGATCCCCCGGGCTGCAGGATTCGGCACGA

GGCCTCGTGCCGCCTCGTGCCGAATTCGGCACGAGGCAAGATTGGGGAGACCTTTCACGTGGGAGGGCAC

AAGAAAGAGGAGGAGCACAAGCCAGAGCACCATGGAGAGGAGCACAAAGAAGGGTTCGTAGAGAAGATCA

AGGACAAGATCCACGGTGAAGGTGCTGAGGGGGAGAAAAAGAAGAAGGAGAAGAAGAAGAAGAAACATGA

AGATGGCCATGGCCATGACAGCAGCAGCAGCAGTGACAGCTGATTAGATCTCACTGCTGCATCACCTATA

TATATTAAGGTGTATGGACCATAGTAGTGATGGCTGTGACTTCTTTTTTCTTTCTTTCCTTCTTGTGATT

TGCATTTATGAGTGAAAAACAAAAAAATGAGAGAAAAAGTGTGCGTGTATGTGGTTGGTAATTGGAGTGT

TATATGTGGTTTGGTTGGTTGCCCACTACTTTGGTTGTAGATTATCTTAATGAAATTAAAATTTTCAAAT

TTAT



>gi|71811585|gb|DR988976.1|DR988976 HDI_E_21.esd Horsegram (VZM-1) drought stressed subtracted cDNA Macrotyloma uniflorum cDNA, mRNA sequence

TGGAGCTCACGCGGTGGCGGCGCTCTAGAACTAGTGGATCCCCCGGGCTGCAGGAATTCGGCACGAGGGA

AGTCCTCAGTTAGAGGACTACCCTCCCTTTCAAGGAACTCCTCTTCATTCAGAGTTGTTGCCAGTGGTGG

CAAGAAGATCAAGACTGACAAGCCTTTTGGAATTAATGGTGGCATGAACTTGAGGGATGGACTTGATGCA

TCTGGCAGGAAAGGAAAGGGAAAGGGTGTATACCAATATGTTGACAAATACGGTGCTAATGTTGATGGAT

ACAGTCCTATCTATGACACCAAGGACTGGTCTCCAACTGGTGATGTCTACGCTGGAGGTACGACTGGCTT

GGCTATCTGGGCTGTGACTCTACTTGGTCTGCTAGCTGGTGGTGTACTTCTTGTCTACAACACAAGTGCT

TTGGCACAATAGACTTTGCAGATCCATGTTTATGTTACAAGTTACAACTTAGTCCATGTAACTATCTAAA

AATCAAGAACTCTTCATCAAGTACTTTGTGGATATGTGATGTT



>gi|71811583|gb|DR988974.1|DR988974 HDI_E_19.esd Horsegram (VZM-1) drought stressed subtracted cDNA Macrotyloma uniflorum cDNA, mRNA sequence

AGCTGGAGCTCACGCGGTGGCGGCCGCTCTAGAAACTAGTGGATCCCCCGGGCTGCAGGAATTCGGCACG

AGGCCTCGTGCCGAATTCGGCACGAGGTAAGGTTGGAGATATTATGACTGAAGAGAACAAACTTATCACA

GTCACCCCTGATACCAGAGTTCTACAGGCTATGCAACTGATGACTGATAACAGAATTAGGCACATTCCTG

TGATTAATGAAAAGGGAATGATTGGCATGGTGTCCATTGGAGATGTGGTTCGTGCTGTGGTGCGTGAGCA

CCGACAAGAGGTTGAGCGTTTGAATGCTTTCATACAAGGGGGTTATTAGATAAACATGAAAATCCCTGAA

GGGCACTCAAGCTAATTTGGATTAAGTAATAAGCCTGAGTGCTTGTAAATAATATGGTATGGCCACAGTG

CCTTCTTGTTTTGCTTCCCTGTAATTTCAGTTTTATGGACATTAACCCCAATGGCCTAGCAGCATGTTAA

TAATAACAACTGGACGCAGTTTGAATTTGTTTTCAAAAAAAAAAAAAAAAAAAAC



>gi|71811581|gb|DR988972.1|DR988972 HDI_E_11.esd Horsegram (VZM-1) drought stressed subtracted cDNA Macrotyloma uniflorum cDNA, mRNA sequence

AAGCTGGAGCTCACGCGGTGGCGGCCGCTCTAGAACTAGTGGATCCCCCGGGCTGCAGGAATTCGGCACG

AGGATTAAACGTTCTGCTCTTTGTGTGGCAGATTCCCACTGGAATTGACATTGTGTTCAAGGTGTTTGAG

TTCACAAAATGGCCTTGAATGTAAGATTCTAAGGTGTAATCCCTGCTATTTTACTTTCAATTTTGCTTTC

TGGCATTATCAAATAACACAAATTTTGACATTTCCCACTTTCCTTTAGCCATGTACCATGATTGAGTGCC

AGACAAGCATTGTATAATTGCATTATACCATTATTCTTTAGTGTAAATTGGCATATAAAGAACTTCTTAC

ATTCGCAAAAAAAAAAAAAAAAAAAAAAAAAAAAAAAAAAAAAAAAAAAAAAA



>gi|71811579|gb|DR988970.1|DR988970 HDI_E_90.esd Horsegram (VZM-1) drought stressed subtracted cDNA Macrotyloma uniflorum cDNA, mRNA sequence

TAGCTGAGCTCACGCGGTGCCGGCCGTTCTAAACTAGTGGATCCCCCGGGCTGCAGGATTCGGCACGAGG

CTGCATATTAGAGAAGCAGCTATGGACACGCGACACTGGAGATAAAAAATAGGACTCATGCAATCTACAA

CTGGTATCGCAATGATGACGGCAAGAGAGTACCAGCTGATTCTTTGATATTGCTTAACCAGTATTGGGGA

AGCAATGAAGGAAAACAGAGTGCGAGTTATTGACGAAGTTGGCCATTCAATAGGAACTTGGAGCACTCGT

AAATTCGGTGTTTATTAAAAAATAAAAATGGATCTTCTGGCTAATATATGTAGTTACAGTAGTTATACGA

GTGTGCGTAACACAGGTTGTCAGAANGGGTTCTATGTACCTGGCTTGTACTTTTCAATGCTTGTATTCTG

CAACACTAATATTAAATAAACAGTGC



>gi|71811577|gb|DR988968.1|DR988968 HDI_E_78.esd Horsegram (VZM-1) drought stressed subtracted cDNA Macrotyloma uniflorum cDNA, mRNA sequence

AAAGCTGGAGCTCACGCGGTGGCGGCCGCTCTAGAAACTAGTGGATCCCCCGGGCTGCAGGATTCGGCAC

GAGGTGGATGCTCGTAGGGGATGTACCGTGGGAAATGTTCACTGACACTTGCAAAAGGCTGAAAATTATG

AAGGGTTCTGATGCCATTGGCTTAGCTCCCAGGGCCATGGAAAAGTCCAAAAGCAGGAGTTAGGCTGTCC

GGGACTGCCTTTTGTCTAATCGAAGCTTTATTGCTGGTGGATTCGGAGTGGGAAAAGCAAAGGAAGATGA

CTGAATTACCTTCACCTGTTGTTTCTATGTTTTCAAGTCCCTTAAGAGTTTGTCTTGGTCTTGTGTTAAT

TTGTTACCACTATAATATCATATGAAGCTACATTGGAAGATTAGTATTTGTGGAGTCTGTAATCATTATA

GGAATCTGTTTGTTTGGATGAATCGAATATCTGTTACAGCCAAAAAAAAAAAAAAAAAAA



>gi|71811575|gb|DR988966.1|DR988966 HDI_E_76.esd Horsegram (VZM-1) drought stressed subtracted cDNA Macrotyloma uniflorum cDNA, mRNA sequence

AAACTGGAGCTCCACGCGGTGCGCGGCGCTCTAGAACTAGTGGATCCCCCGGGCTGCAGGATTCGGCACG

AGGCAAGCGTTTCAAGTCCTGGTAGCTTCAGGGAAAGGAAGGTGCAAAGGATGTGGTCATGGTTGATCCT

GTGGAAGCCAAGCGTTTGGCTGCCAAACAAATGGAAAGAATTAAAGCAAAAGAGAAACTCAAGAGAAGAC

GCCAAATTGAGGCAATTAATGGAGCATGGGCAATGATCGGTCTCACGGCTGGCCTAGTTATTGAAGGGCA

AACTGGAAAAAGTATCCTGACTCAGCTGCAAGACTACTTAGGTACAATTGTTAGTTTTTTTGTGCAGTAG

ACTTCTTCCAAGAGGTCCATGGATACATAGTAAGGACTGCAGAATTCTTTCTCACATTCTTTGTAGTTAT

TTTAACTTTATATCTTTTATCTTAAAAGTATGCCAACGATGCTTTTTCTTCAAAAAAAAAAAAAAAAAAA

AAAAAAAAAAAAAAAA



>gi|71811573|gb|DR988964.1|DR988964 HDI_E_74.esd Horsegram (VZM-1) drought stressed subtracted cDNA Macrotyloma uniflorum cDNA, mRNA sequence

AAAGCTGGAGCTCACGCGGTGGCGGCGCTCTAGAACTAGTGGATCCCCCGGGCTGCAGGATTCGGCACGA

GGCGCCCATTGAGCTTTGATTATTCACAGAATTACTAGATGAGGACGATTAGGGGGCAGTTTTCCATTTG

GCTGCAGAAGTTATTTTAGCTGTTAAAGATTCCAGAGGTAGATACAATTTTTCTGTACTGTTGTTATAGA

CCCATGTTCCTATTTTAGGTTTTCCCTTTTGATGGTGGTAGTGAACTTTGGATATCTGGTACTCTTCTAG

TGACAGTTTTATACTGATTTGATTTGTAGTGAAATACTGTTTTTCAATTATATGTGGACTTAGTCTAAAT

TTCGGTTGAAATGTATGTGGATTTGTTGTAATGTTACTAGG



>gi|71811571|gb|DR988962.1|DR988962 HDI_E_66.esd Horsegram (VZM-1) drought stressed subtracted cDNA Macrotyloma uniflorum cDNA, mRNA sequence

AAAAGCTGGAGCCTCCACCGCGGGTGCGCGGCCGCTCTAGAACTTAGTGGATCCCCCGGGCTGCAGGAAT

TCGGCACGAGGCCCTCAGCTTCCGGGATCTTCATGTACACGTATCGNCACATCTCGCCCCGCTCGCTCAT

CTCCATACGCTNCCTATGCGCTCTCGTGCCTCCCGTCCTCTCAGAACACGCGCTCTGTCCCCTCATCATC

GCCGTCGTCTCGATATNGCGCTCCTCCCACANAACTGCACGTCGCACCGCTTTTCGCCTCACCGGAGACA

GACGACGACATCATCGCGTATAC



>gi|71811569|gb|DR988960.1|DR988960 HDI_E_64.esd Horsegram (VZM-1) drought stressed subtracted cDNA Macrotyloma uniflorum cDNA, mRNA sequence

AAGCTGGAGCTCACGCGGTGGCGGCCGCTCTAGAACTAGTGGATCCCCCGGGCTGCAGGATTCGGCACGA

GGGTTATGACGAAGACAGACAAAACTTGCAAAGGCTCAGTTGGAGATGATCATGTCTAGTAATGGACTAT

CGGAGAATGTGTTTGAGATTGCCTCGAAAAGCTTGGCTGCTTAAATGTTAATGACATGAAGAAAACTGCC

AAGCTGTCTTTTAGACGGTTCTGGTGTATTTGTGGCGAAAACAATTGCTGCAACGATCTCTATCCTGTGC

AAGCTTTGGCGCAATAACGGGTAATAACGGGTGTGGGTCTCTTTTTTTCATGCACGTCTTAACTCGGTGA

TTTCTGTATCGAAGTCCCTAAGATGTGTAATGCTTTATGCAAGAATTAAAGGTAAACTAATTTATCTTGG

TGATTAAAAACGAAAAAAAAAAAAAAA



>gi|71811567|gb|DR988958.1|DR988958 HDI_E_62.esd Horsegram (VZM-1) drought stressed subtracted cDNA Macrotyloma uniflorum cDNA, mRNA sequence

TGGAGCTCACGCGGTGGCGGCGCTCTAGAACTAGTGGATCCCCCGGGCTGCAGGAATTCGGCACGAGGCC

TCGTGCCGAATTCGGCACGAGGGTACATTGCAATAACATGTACATCAGCAAACACAAATCTAAAACTCTC

GAGGGGGGGCCCGGTACCCAATTCGCCCTATAGTGAGTCGTATTACAATTCACTGGCCGTCGTTTTACAA

CGA



>gi|71811565|gb|DR988956.1|DR988956 HDI_E_6.esd Horsegram (VZM-1) drought stressed subtracted cDNA Macrotyloma uniflorum cDNA, mRNA sequence

AGCTGGAGCTCACGCGGTGCGCGGCGNTCTAGAACTAGTGGATCCCCCGGGCTGCAGGATTCGGCACGAG

GCTCTGTTCCTGATACTGTTTAAAGATTTTATCTTTAATCTGTTTTTATGTGGATGTGTGAGAAATCTCA

CAAATGTATAGAATCTCTCTCTATATATATATGTGTATCTAATGCGTTTGACCAACTCTGGTAATGTTTT

GCGGGTTTGCCATGTGAGGAAAGGAATTAAAAATAGTGCACAAAAGTGAGTGCCGGTTATGAAACTGGCT

ATTGAAAATGGCGCTGCGGTCCTCTGACACGTTCTGCATTTTGGGTCCGTTGCTTCTAATGTCAAATACT

CGAGAGTCTTCACAAAAGGGGTTCCCTGCTTCTTTGTCTGTTCCTGTTTTTTTACAATTCTGTACAAACT

TCTTTTTTATTTCAAAAATATTATATTATCTCAAAAAAAAAAAAAAAAAAAACCCGAAGGGGGGNCC



>gi|71811563|gb|DR988954.1|DR988954 HDI_E_53.esd Horsegram (VZM-1) drought stressed subtracted cDNA Macrotyloma uniflorum cDNA, mRNA sequence

AAAGCTGGAGCTCACGCGGTGGCGGCGCTCTAGAACTAGTGGATCCCCCGGGCTGCAGGATTCGGCACGA

GGGGAGTTAGTTCATAGAATCTAGCACCAATCCCTTCTCATTGGGTTTTGTTTAAAGTGGTTAGCTCCTA

ACAGGCTTTACTCCTAGGGTTTTTGGGTTTTGGGTTTTAGGGTTTCTGCCTAAGATGCTTATATCTTTTT

GGTTTTTTGTTCGGGCTTTATCCTTCTCTCTTTGCCTTTTTCTCCTTTCTTTTTCCTTTTACTTGGTCGG

TCGTTTCCTATCTTGTAAATTTAGATCTACTTTGTTGCAGAGCAGCTTTCTTTGTTACTTCCAACAGGGG

TAGAAGCAAGGTGGGAAATACTGAAATATAGTTGGTTTTTAAGTTTTTCGTTTTAATTTAGTATGTTTAT

TTTCTTTCTGATGATTGACATGGGCATACTGGAGAAAGAAATATTTGTGGACAATCTGTTATGTAATAGG

GTGTTTTCTGCTTTCTGGCTTATGAAGGTATATTGTCTTCCTGCTCAAAAAAAAAAAAAAA



>gi|71811561|gb|DR988952.1|DR988952 HDI_E_51.esd Horsegram (VZM-1) drought stressed subtracted cDNA Macrotyloma uniflorum cDNA, mRNA sequence

AAGCTGGAGCTCACGCGGTGGCGGCGCTCTAGAACTAGTGGATCCCCCGGGCTGCAGGATTCGGCACGAG

GCCGGTTATCGGATTCGAACCGATGACCATCGCATTACAAATGCGATGCTCTAACCTCTGAGCTAAACCG

GCTAACGTAACCGAAATATACACATGCAGAAGAATTTAAAAAAAGGGATCTTAGTTATCAATTTTGAGTG

ATTTTTAACTTTCTAACATAATTGAAATAAATACAAACATAGACATAGAAAATATAGACAATCTTTTTAT

TATTGAATATGTTAGTCCATAAAGTGTAAGATAAGGATTAAGAATTAATATTAATATAATCTATTTCGAT

CTAATATTCTATATATATTCTTCAAATTATATATTTCAAAAATATTATTGATTATATTAGTATTGAATAG

TCAATTTGAGTAAATATTAAGGAATTCTAGATTTCTAAATAAATGTCTAATTCTACATTAAAAGAATGGC

CACTAGATTCGTTTTAGTTATATCAATTGTATATGAATAATATGAATAAATGGATTGTTTATTTCATTTC

AGCAAAAACAAAA



>gi|71811559|gb|DR988950.1|DR988950 HDI_E_5.esd Horsegram (VZM-1) drought stressed subtracted cDNA Macrotyloma uniflorum cDNA, mRNA sequence

AAAGCTGGAGCTCACGCGGTGGCGGCGCTCTAGAACTTAGTGGATCCCCCGGGCTGCAGGAATTCGGCAC

GAGGAGCAATTCAGGCATTGAATGGGACAGTAATTGGGAAGCAGACAGTGCGCCTTTCTTGGGGTCGCAG

TCCAGGAAATAAGCATTGGAGAAGTGATCCCAATGGAGGCCATTATGGTGGTGGTCAGGGTTATGGAGGC

CACGGATTTGCTGTGAGACAGAATCAGGATATAGCCATGCAACCTGCTGCCGCTGCTATTCAAGGGGCTT

CCTGACGGGGTTAGCAGAACTGTGTGTTGAAGTAGGATGGATTTTATTGCTCCCCATTAAACTGTAGTAT

TATTTTTAAATTTGTAGCTGGGGGTTATACGGTATGCGAGGTTCTTAATTAAGATTGTATTAAAATGTCT

GAAATTTTGGATTGAACCCGACCATATATATGACTCGGGTATTTTTGTTTTTTTAAATCCTTAAAAACAT

ATTGGTAGCACAGACAGCTAAGATTATTTTAACAAAAAAACAAAAAAAAAAACCCAAGGGGGGNC



>gi|71811557|gb|DR988948.1|DR988948 HDI_E_42.esd Horsegram (VZM-1) drought stressed subtracted cDNA Macrotyloma uniflorum cDNA, mRNA sequence

AAGCTGGAGCTCACGCGGTGCGCGGCCGTTCTAGAACTAGTGGATCCCCCGGGCTGCAGGATTCGGCACG

AGGCATTCACAGAGCTTCCTCACCCAACAGCTATAAGCTTTTGTTCTGCAATTTTGACCGCAGCCTCTGC

GGTAATGTTGGGATTGTTAGGGATGATGCAGGAAACAGGCTTTTGGCCATCACTCAGGGCAAAGAACTGG

AGTTTGTTCTTGAGGAACTTCCATCATCATCTGCTGCATCTGCATGAGCCACTTTCAATGAAAATTCCTG

CACTTTCTACTTCTGAGAGACGTGCTTTGTTATGCTTTGGATGTAATTATCTTATGTTTTCCAACACTAA

ATAAATATCCAAAGCTTATGTTTTCCAAAAAAAAAAAAAAAAAAAAAAAAAAAAAAAAAAAAAAAAAAAA

AAAAAAAAA



>gi|71811555|gb|DR988946.1|DR988946 HDI_E_40.esd Horsegram (VZM-1) drought stressed subtracted cDNA Macrotyloma uniflorum cDNA, mRNA sequence

AAGCTGGAGCTCACGCGGTGCGCGGCGTCTCTAGAAACTAGTGGATCCCCCGGGCTGCAGGATTCGGCAC

GAGGCTTTTTCAGTGGATCATCCATGCTTGTGTTTCATTATGGTGGCCTTATCCTTTCCTTGACTTGTCA

TCCCCATATGCAGCCTTATGGTACATTGGTGTGGGTGTAATGCACATTCCCTGCTACGCAGTCTTTGCTT

TGATAGTGAAGTTGAAACACTTGTGGTTGTCAAAATTGTTTCCTGGGTCCTGTCAGTTTGTAAGGTGATC

ATACATTCATGATTAGTCATCGGAGTAGAGCTACCTTCAACATGGCTAAAGCAGATTTTATCTTAGAAGA

TCAGATTAGACACCCCCACCAACCAACAGTGTTTAGATTTGTGTGACATGTATTTGAATTTGCCAATTCC

TATTGAATACATGAAGCTTCTCTGCTCTAACCATGCTTGTGATATCTTCCATCCGAGATATAAACCGTCC

ACAATATCCTCTTATATTTGATCAAAT



>gi|71811553|gb|DR988944.1|DR988944 HDI_E_39.esd Horsegram (VZM-1) drought stressed subtracted cDNA Macrotyloma uniflorum cDNA, mRNA sequence

AAAGCTGAGCCACGCGGTGGCGGCGCTCTAGAACTAGTGGATCCCCCGGGCTGCAGGATTCGGCACGAGG

CTTTTTTCATATTCGATCATCTCGTTTTGTCAGATGACACCAATGGCTACCGATAAAATTTTCAAGCCAT

CCAAGTGGTTCTCGAACAAAACTCTTAAGCTAAGTCTTCATCGACGAAGACCAAGATCTTCCAACTCATC

ATCATCATCATCACAATCATCGAGTGCTTCTCCACGATCCCCCATGTCAACATGCACAACCCCCAAGAAG

GGAGAGATGAACGGGCTCATGGAAGCTTTTCGTTACTTGGATGGTGATGGAGATGGGAAAATCTCGGCCT

ATGAGCTGAGGTCGTATTTTGGGTCCATTGGGGAGCACATGTCTCATGAGGAGGTTGAAGAGGTGATCCA

TGAGCTCGACTCCGATGGGGATAACATGTTGGACTTGGAGGATTTCACGAAGCTGATGAAGAAGGATGGT

GGTGATGGCGATGAGGGGGATCTGAGAAAGGCTTTTGAGATGTTTGTAGTGGGAGAACAGAAGGGTGTGG

TTGCATCACCCCAAAGGGGTTGCAGAGGATGTTGCATCGTCTTGGAGATGACAAGTCCCATGATGAGTGT

GTTGCCATGATTGGTGCCTTTGATATTGATCACAACGGTGTTTCTTGATTTCGATGAGTTTTTACCCACA

AATGATGGCCTAAATCAATAATCCCTTATTCCTAATGTACTATGCTTCATTTCCATTCCAGACCGATTAA

GGGGTTGATGAATGGATTGTTTCACCTACTTGAACTTGAACTTTATGTAATTTATGTACGTGANGTAATC

CCCAGCCANTCCTGNTATA



>gi|71811551|gb|DR988942.1|DR988942 HDI_E_37.esd Horsegram (VZM-1) drought stressed subtracted cDNA Macrotyloma uniflorum cDNA, mRNA sequence

AAGCTGGAGCTCACGCGGTGGCGGCGCTCTAGAACTAGTGGATCCCCCGGGCTGCAGGAATTCGGCACGA

GGGCCAAGGTCATCGAGTACTGTAAGAAGCACGTTGAGGCGAATAGCGCCGATGAAAAGCCCTCTGAGGA

TGAACTCAAGAACTGGGACGCTGATTTCGTCAAGGTTGATCAGGCTACGCTCTTCGACCTCATCCTGGCT

GCAAACTATCTGAACATCAAGAACCTTTTGGACCTTACCTGCCAGACTGTGGCAGACATGATAAAGGGGA

AGACTCCTGAGGAAATTCGAAAGACATTTAACATTAAGAATGACTTCACCCCTGATGAAGAAGAGGAAGT

CCGTCGGGAAAATCAATGGGCTTTTGAATGAGGTTTTGATTTTTCAATTAAACAATGGGTGGGGATTAAG

CCATAGTTGGTAGTTATGATGTTACTGACTATTTTGTTTATGTTTGATATGTTGCCGTATAAGACTTATT

TTACTAGTTCAACTTTGTGGGGCAATCTTGAAATTTGTAAGTAATAGTTGTATTTCCT



>gi|71811549|gb|DR988940.1|DR988940 HDI_E_3.esd Horsegram (VZM-1) drought stressed subtracted cDNA Macrotyloma uniflorum cDNA, mRNA sequence

AAGCTGGAGCTCACGCGGTGGCGGCGCTCTAGAACTAGTGGATCCCCCGGGCTGCAGGATTCGGCACGAG

GCCTCGTGCCGAATTCGGCACGAGGAAGAGGACCAAGTTAGGCCTCAGAGACTTTATCTCACTGGATGAA

TTTTTTCAGACTATTGATATATCCAAGAGCAAAGCAGTTAACATGATCAAGTTCAGTGCAAGGGGATTGA

GTTTGTTGTCTGGACAGCTTAGTATTGAAGCCTCTTTCAAGATTTCTTCCTCAACAAGGGTTGACATTAA

CTTCGAAAACTCCACTATCACTCCTGATCAGTTGATGAATCTATTCCGGAAAAATTATGATCTTCTGCTG

AGCATCTTCAATCCAGAGGGATGGCTGGAAATCACATATGTTGATGATTCCATGAGAATAGGAAGGGACG

ACAAAAGCAACATCTTTGTCCTGGAAAGATTTGATAGGTAGTGATAACTAGAAACTTCTTCTCTTCCACT

TCAATGTTCAGAACTTAACCAAGTATGCAAGCCAT



>gi|71811547|gb|DR988938.1|DR988938 HDI_E_28.esd Horsegram (VZM-1) drought stressed subtracted cDNA Macrotyloma uniflorum cDNA, mRNA sequence

AAGCTGGAGCTCACGCGGTGGCGGCGCTCTAGAACTAGTGGATCCCCCGGGCTGCAGGATTCGGCACGAG

GTGGAAACGGCTATGCCCCTGTTCTGGACTGCCACACCTCCCACATTGCTGTCAAGTTTGCTGAACTTAT

GACTAAGATTGACAGGCGATCTGGCAAAGAGCTCGAGAAGGAGCCTAAGTTCTTGAAGAATGGTGATGCT

GGTTTTGTGAAGATGATTCCAACCAAACCAATGGTGGTTGAAACATTCTCTGAGTATCCTCCACTTGGAC

GCTTTGCTGTGAGGGACATGCGTCAAACTGTTGCTGTGGGAGTCATCAAGAGCGTAGAGAAGAAGGAGCC

CAGTGGAGCCAAGGTCACCAAGGCTGCACAGAAGAAGAAGTGAATCGTGCGGGGTGGTTCATCAGGGGAT

GGTTACAATAAATGCTGGTTTCTTGCTTGTACTCTTGTGTCATCTCTAGGTAGTTTGTTTTTCGTACTTA

GTTTCAAGTTTCCACCCTCATCTCCCAACTTTTGTTCCCAGAATTGGGTTCTTGATCGACGGTGGCGGCT

CTTTTACTATTAA



>gi|71811545|gb|DR988936.1|DR988936 HDI_E_26.esd Horsegram (VZM-1) drought stressed subtracted cDNA Macrotyloma uniflorum cDNA, mRNA sequence

CAAAGGAGCTCGACAGGAGGCCAGGGCACTGGCAGCACCGCCGAACGGGTTTCGTCCGTATCATTGGGAT

TTGACACATGTTACGCCAAGTCCCAGTGCCAATCAGTTTCATTGCCTACAAGCCACCGAAATACTACTAA

GTCTCTGAAATTTCCCAAAACATTTGTAGCCACTCCACTTTGTTTGTTTGTACTTAAACCAAATTCCCAT

TCTGTTTTTGCTTTTTGAGATTTCATCATCCTGTAATTTTGGTTTCTGGTTTTCGGGAACTTTCATTTGG

GACACATGAAATTGGAATGAAGGAAACCCTAAATTGGACATTTAACAACACAACA



>gi|71811543|gb|DR988934.1|DR988934 HDI_E_2.esd Horsegram (VZM-1) drought stressed subtracted cDNA Macrotyloma uniflorum cDNA, mRNA sequence

ACAGCTGGAGCTCACCGCGGTGCGCGGCCGCTCTAGAACTAGTGGATCCCCCGGGCTGCAGGATTCGGCA

CGAGGTGGGACGAGGAGTGTCAATTACTTCCCTTCAAGGCATGATCCTGTTCGTCATGCCGAAAGGTTCC

CCATACCTCCTCCTATCCTCTCTGGAAGGCGTGAAAAGTGTGGGAATTACAGTGAGAACCCACTTCAAGC

CAGCCCCGGGAGAAGAGATATCCGATACTTTGTGGCCCCTGTACAAGCGCGCAGAATAAGAAT



>gi|71811541|gb|DR988932.1|DR988932 HDI_E_17.esd Horsegram (VZM-1) drought stressed subtracted cDNA Macrotyloma uniflorum cDNA, mRNA sequence

AGCTGGAGCTCACGCGGTGGCGGCGCTCTAGAACTAGTGGATCCCCCGGGCTGCAGGATTCGGCACGAGG

GAAAGATACAACCATGAGATAGTTGCAATAGAATTCAAGAAAATTCTTCAGGGTCTTCAAGGTACGCGAG

GCCAATTGAAAGAGACGAAGGTCCTCGAAGAAGGCCGCAGAATGATTTCTCAAGAAAGCGTGTTTTTTTC

ACAATAGCAATCACCGGTATTGCTGTTGTCCTCGGTTCTATTTTTTTCCAGAGAAAGACAGATCTCAGCA

ATGTGCAATAACAGCGGAGAGAAGAGAATATATGTTAAATTTGCTTTATCTGTATAATTTATTTCCATAC

AACACAAGGTCGAATGAGATATACTTATGGGATATCCTTCACCTGCCGACCAAACACATTGGCATTGTCA

TTGTCATTGAATTACTATTCTGCTCTTCTATTTGATGCTGAGAACTGCCAACTATCAA



>gi|71811539|gb|DR988930.1|DR988930 HDI_E_15.esd Horsegram (VZM-1) drought stressed subtracted cDNA Macrotyloma uniflorum cDNA, mRNA sequence

AACAAGCTGGAGCTCACCGCGGTGNCGGCGCTCTAGAACTAGTGGATCCCCCGGGCTGCAGGAATTCGGC

ACGAGGTGGCTGCGTTGCTTTGTGCGCTTATATGTGCTTTGGGACTCAACTCAATTGTGCGATGTGCGCT

CAGATGTAGCCGNAGGTTTGCTTTTGAGACCCCAGAGGAGACTGCGGCACGTTTGGTGGCAAAAGGCCTA

AAGAAGAGTGCATTGCATCAGATCCCTATAGTTGTTTATGGTTCAGGTAGTGTTGGTATTGCAGCCACAG

ATTGTCCAATTTGCCTTGGGGAGTTTGTGGACTGGGGAGAACAGTAAGAGTGTTGCCAAAATGTAACCAT

GGATTCCATGTAAGGTGCATAGATACATGGCTTCTTTCACAACATCCTCTTGTCCATAAATTTGCAGACA

ATCTATTGCTTGAACAACCAACTATTTCTGGAGCTGTAGCAAGCAGGAACCAGTAACCCATGCCAGGGAN

ATGCTTTAGGAGAGACATGCAACCATGTGTCTTTAGCAGGTGGCTGTGGA



>gi|71811537|gb|DR988928.1|DR988928 HDI_E_13.esd Horsegram (VZM-1) drought stressed subtracted cDNA Macrotyloma uniflorum cDNA, mRNA sequence

AAAGCTGGAGCTCACGCGGTGCCGGCCGTTCTAGAACTAGTGGATCCCCCGGCNCTGCAGGTAATTCGGC

ACGAGGCAAAGAACACTGGTTCTAGGCTTGTTGTAAGGGCTTCAGATGAGGCTGCACCGGCCCCTGCAAC

CGCCACACCCCCAGCTGAAGCTGAAGCAAAACCAAAGCCACCACCTATTGGCCCCAAGAGAGGTGCTAAG

GTGAAGATTCTTAGGAAGGAATCTTATTGGTACAAAGGAACTGGTTCAGTGGTTGCAGTTGATCAGGACC

CCAAGACTCGCTACCCTGTTGTGGTTCGATTCAACAAAGTCAACTATGCCAATGTATCAACAAACAACTA

TGCTTTGGATGAGATCGAGGAAGTCGAATGAGTCCAATTTAATTTTAATTTTGATGCTTGTCATGTTATC

GCAGTTACTAGTCTAAAGTTTTTTCTTGTCACCAAAAAAAAAAAAA



>gi|71811535|gb|DR988926.1|DR988926 HDI_D_95.esd Horsegram (VZM-1) drought stressed subtracted cDNA Macrotyloma uniflorum cDNA, mRNA sequence

AACCCTCGGGTTGATGAAGATAGTCAAACACAACTATCTTTGCCTGAAAACTTCCTGTATACTGCAGCAA

CAAGTCTCCAACTTGTACTCAGTGTTGTTCCTCTCCTTGGGGTCCAAAAACACCTTCATAATCTTGGAAC

CATCAATTCTAAACCTAACACGCTTTCCAACAATCTCAGCAGGTAAGACAATGTCCTCCAGCATTGCCTC

ATGCACAGCAGGTGAGTGTACGGCTGCGGAGGACGCTGTACGGCTTGAACCTTTTCTTTGGGTTGGGCCT

CAATATCCTCCGGGGTGGGCAACTCGGACTACCTTCCTTTCCCGCTTAAATTTTTTCCTTCACAGTTTCT

CTTCACCAAGCCTTGGACCTGGATCTTTCCTTGAAATCCTTTTCCTTTATCCTTGTAGGGGACCTNGATA

CCACCAGGCCTTGCGGGTTACCGNAAACCATTCATTTTGGACACAGCTTGAAATTTTCTTGTTTTTAACT

TCNTTTTTCCGAATTCCGCCTTTTTTTCAAGGCCTCACAT



>gi|71811533|gb|DR988924.1|DR988924 HDI_D_93.esd Horsegram (VZM-1) drought stressed subtracted cDNA Macrotyloma uniflorum cDNA, mRNA sequence

TCACCGCGGTGGCGGCGCTCTAGAACTAGTGGATCCCCCGGGCTGCAGGATTCGGCACGAGGCCGAACTT

CATGTGCCAGGCGGTGACTTCACCGCCGGAAACGGCACCGGCGGCGAGTCGATCTACGGTGCGAAGTTTG

CCGACGAGAACTTTGTGAAGAAGCACACCGGCCCTGGCATACTGTCCATGGCGAACGCCGGTCCTGGAAC

TAACGGATCTCAGTTCTTCATCTGCACGACGAAGACGGATTGGCTCGACGGCAAGCACGTAGTGTTCGGT

CAGGTCGTTGAGGGGCTGAACGTGGTGAAAGATATCGAGAAGGTCGGATCCAGCTCCGGCAAGACCGCAA

GGCCGGTCGTCGTCGCTGACTGCGGTCAACTGTCTTAGATGGACGGCGTTGACCCGAGGCGGTTATCTCT

GTGGTGGTGGCTCTGAATCTGTGTCGTTTTCGGTGTTTTTTTTGCTTTTTCTCGTCTTATAGGGGATTGT

GGCGTTATGGATAATTTCGAACCCTTCCCTCTGAATCCTTTCCTTTCTAATATCTGTATGTGGTCATAGA

GTGAATGAGTCTGTCATGAGAAACATAAAAATGATGAGCTACCTTCAATAAAAACCCTTTAGGGACCTTT

TATTATTCGGCCTAAAAAAAAAAAAA



>gi|71811531|gb|DR988922.1|DR988922 HDI_D_91.esd Horsegram (VZM-1) drought stressed subtracted cDNA Macrotyloma uniflorum cDNA, mRNA sequence

CCGCTGACCTGCTGCCAAAGCTGGAGCTCACGCGGTGCCGCGGCCGNTCTAGAAACTAGTGGATCCCCCG

GGCTGCAGGAATTCGGCACGAGGGAACAATCGTTTCTCTAAGTTTAAGTCGTGATGGGAAAGGAAAAGGC

TAACATCAAAATTCCGTTGTCGTTGGACATGTCTCTGAGAAATCAACTGCCACGGAGCCTTGGAAGCCCC

TACTGAGAAGGGTGTGACCTGAAAGGTTCGAGAAGGGAGAAGGAAGTGTTTAAAACACGTTAGATATAAG

ATGTGTGAAGAAGGATGATGTAAAGATAAATAAGATGTAAGATGTCAAGGATGGATCTTAAGACGAAGTG

ATGAAGGACTTATATTCAGATAAATAAAATGAACTATATATGTTTATCTTCTTCAAAAAAACAAAAAAAA

AAAACCCTCAAGGGGGGGCACCCGGTTAC



>gi|71811529|gb|DR988920.1|DR988920 HDI_D_84.esd Horsegram (VZM-1) drought stressed subtracted cDNA Macrotyloma uniflorum cDNA, mRNA sequence

ATGAAATGGACATGGGTACCATGTATTGAATGATTCAAGCCAAGGTGAGTAAACTTCACCGTGACTCAAT

TTTAATTGAGGGGAATCTCTTGGTTGCCTTCCACTGGAGTGACGTACACTGCTGCATTCACAGTTTAACC

ATAAAACTGTTACGCCTTGTCACTCTTGTACTTATATTACGGATATAATTCAAAGTCGCATTTTAATAAG

TTTATATCAGAAAAAAGGTTATAGGCCATTTGAACACAATCAGGTTTAATTG



>gi|71811527|gb|DR988918.1|DR988918 HDI_D_81.esd Horsegram (VZM-1) drought stressed subtracted cDNA Macrotyloma uniflorum cDNA, mRNA sequence

GGGAGCTCACGCGGGCCGGCCGTTCTAGAACTAGTGGATCCCCCGGCGCTGCAGGATTCGGCACGAGGCC

TCGTGCCGAATTCGGCACGAGGACTCACTGTGAGAGCGAAATCGCTTATCAAGAAGAAGAATGAAGGACG

CCGGAAAGAGCCACAAGGCGAACGGAAAGCTGTCGCCGGAGACGCGGAAGGATCGGAAGTCAGCGACAGG

GATGAGCGGATCGCCGAAGAAGGGAGGCCACGGCGGGAAGTTCACGTGGATCGGCAACGGCTATGAGCAT

GTCGAGATCGCGATGGAGCACGGTGCTATGGACGCCAAGGATCCGAACTTCGAGGATCCGGCGGAGATTG

CCGCGGTGTGACCGTGGTGGTTTCCGGTGACCAGATCTGTACATAGTAATCGGCGAGAGACGTAGCTTTC

GAATTGAATAATGTGAAGCGAATCGGAGGAGAAGCGTTTTCGAGTGCGTTTTCTTTGATTGACTGTTAAT

GATATGTCTGGTACTTGTCTTGTGTTTTTTGTACATAGGCCTGCGAAATGCAGTCATACTCGCAGTCGCG

ATTTCATGTTTCTACGAGCTTCTGTTAACATAACTTGGATTTTAATTTTCAGTTTTA



>gi|71811525|gb|DR988916.1|DR988916 HDI_D_8.esd Horsegram (VZM-1) drought stressed subtracted cDNA Macrotyloma uniflorum cDNA, mRNA sequence

AGCTGGAGCTCACGCGGTGCGCGGCGCTCTAGAACTTAGTGGATCCCCCGGGCTCGAGGATCGGCCAGAG

GCGAAATGTTTTTGGATGAGGAAGTGTTCAGAGTCTAAAGTNCCAAACTTTCAGCCGTTGAAAGGTTAAG

GCCAAGAAAGATAGAGTCAAAAAACAAAAACTAAGAGCATTGTTTGTTCTGATTGTGATGGAAATGGTGC

AATATTATGTATCAATGTAAAGGTACTGGTGTTAATTTTGTAGATTATTTAATGGAAATTTTAAAGCCGG

TGGATTGTGTTGGCTGTGCAGGGGTAAAAAAGATATCTCTGTGTGGATTCTTGTAATGGAGCTGGCTTTG

TTGGTGGATTCATGAGCACATTTGATGATTTAAAAGTTTGGNACATTCGAATTCAAAGATTCCTAATGAC

TATTTGTTCCTTCGTTGGTCCTTTTTGTAATGTGTATTCACAATAAANATTTTGTCCTTTAAATTCATAA

TTATTCCTGTTATCTATTTAGGGAACATTACTATTTCACTTATTATTTGGAACTTTTTGCCCC



>gi|71811523|gb|DR988914.1|DR988914 HDI_D_72.esd Horsegram (VZM-1) drought stressed subtracted cDNA Macrotyloma uniflorum cDNA, mRNA sequence

CAAGCTGGAGCTCACCGCGGTGCGCGGCCGTCTCTAGAACTAGTGGATCCCCCGGGCTGCAGGAATTCGG

CACGAGGATTAAGGGTCTCACTGATAAATTTAAGGAGAAGGACACACGTTATACGGGTTCAGCTACACTT

TCATATGATGCCTTCATGACCATGGTCCTTCCTTTTCTTGTATCTTATGATTGATACTCGGTCGTCAAGA

ATTTGCAAAGGTTCTTTGGGTGCCTACATTCACAATTGAGNTGTGTTTATAGACATCTTGAGTATGTGAA

GAAAGATAGTTGTNATAATTGTTGTAANT



>gi|71811521|gb|DR988912.1|DR988912 HDI_D_70.esd Horsegram (VZM-1) drought stressed subtracted cDNA Macrotyloma uniflorum cDNA, mRNA sequence

AAGCTGGAGCTCACCGCGGTGCGCGGCCGNTCTAGAACTAGTGGATCCCCCGGGCTGCAGGATTCGGCAC

GAGGCCTCGTGCCGAATTCGGCACGAGGGGCGCCGGAACCTTCATGGCCAACCACTTCGACCGCCACTAC

TGCGGCAAGTGCGGCCTCACCTATGTCTACCAGAAGGCCGGTGCCTAATTCGTAATGGATTTCGTCATTT

TATTATTAATTTTAGTATTTAAAGTTTTGGTTTATGTTTTGTTCGTTTAGTGTTGAGACTGTTTGGGTTT

TAATTAAACTTTTATGGATACTCTCTCGTTTTGGATTATCTGTTATTCGCTTTAATATATTGTTAGTTCG

GATGAAAAAAAAAAAAAAAAAAAAAAAAAAAAAAAAAAAAAAAA



>gi|71811519|gb|DR988910.1|DR988910 HDI_D_69.esd Horsegram (VZM-1) drought stressed subtracted cDNA Macrotyloma uniflorum cDNA, mRNA sequence

GAGCTCACGCGGTGCNCGGCCGNCTCTAGAACTAGTGGATCCCCCGGGCTGCAGGATTCGGCACGAGGCC

ATAATCACCAATTGCCATGGCAAGCTGAATAGTTCGGGTGGGGGAGAGCCATATGTGATGGATGAAGCGG

GTTTTGATGAGTATGATGCAGTTGAAGACGAGAGTGACGAGGAGGATGCAGAGAGTAGTGTGGATTTGTT

GATCAGATTCTTGCAGAGCATGTTTAAGAAAGTTTCTAAGCGAGCCAAGAAAGCTTCTCGCTCTGTTTTG

CCCACAGTAATATCGCCCCAGCTCGTTTCTTTTGCTGTTGATGGAACTCTGCTACTTGCTTCACTTTCTG

TTGTCAAAGCACTGCTTGAGGTCATGTGCACTCTTGGAGGCACTGTATTTGCTGCAATTCTGGTCCTGCG

TGTGATTTGGGCAGCAGTTTCTTACTTCCAGTCAAGTGGGAATAGCTTCAATCAAGGGGGAAATTCATTT

GGTGCCGTAGCCTAAAGGCCCTAACCTCTCTAAACTTTTAGCAGAAGATGGCCATTGTAAGTTTTTTTTA

GATGACAATGAGATTTGAACTTATGATCTTGTATAAAATTATTCGACAATTCCTTTGTCACTNAGGCCCT

AATGGCTTATTCCAAGGTTAGTTCGATGGCAAAAAAAA



>gi|71811517|gb|DR988908.1|DR988908 HDI_D_60.esd Horsegram (VZM-1) drought stressed subtracted cDNA Macrotyloma uniflorum cDNA, mRNA sequence

AAAGCTGGAGCTCACGCGGTGGCGGCCGCTCTAGAACTAGTGGATCCCCCGGGCTGCAGGAATTCGGCAC

GAGGCCTTCGTGCCGAAATTCAGGCACTGAGGGTGGCCNACAGGTCTGTGGAAGAAGAAGTTCTGAAACT

CTTTCTGTACCTGCCAGACCGTCACTGACTGCCCAGATTAGGCAAAAGGAAGATACGTAATACCTATCTC

AGGAATGGATGGATTCCTTGCTTGGAATTCGAGTTGGAGTCACGGTTTCGTGTAGCCGTGAGCACAACAG

GTCACCTGGATACTATGGATGGAAGGTACTGGATCCATGTGGAAGCTAGCCTATGTTTAGGGTGCACTGA

TGCTATCTCAGGTCTTGAAGGAGCTCGAAGAGGCCAGGACTGCTACGACCCCAACGGTTTCGTCCGTATC

ATTGGATTTGACAATGTTCGCCAAGTCCAGTGCATCAGTTTCATTGCCTACAAGCCACCAAACTACTAAG

TCTCTGAAATTTCCAAAACATTTGTAGCCACTCCACTTTGTTTGTTTGTACTTAAACCAAATTCCCATTT

GTTTTTGCTTTTTGAGATTTCATCATCCTGTAATTTTGGTTTCTGTTTTCGGACTTCATTGGAAACTGAA

TGGATGAGAACTAATGAATAAAACAAAACAAAAAAACACCTCCAAGGGGGGCCCCGGTNCACCCAATTCG



>gi|71811515|gb|DR988906.1|DR988906 HDI_D_58.esd Horsegram (VZM-1) drought stressed subtracted cDNA Macrotyloma uniflorum cDNA, mRNA sequence

AAAGCTGGAGCTCACGCGGTGGCGGCCGCTCTAGAACTAGTGGATCCCCCGGGCTGCAGGATTCGGCACG

AGGCCTCGTGCCGAATTCGGCACGAGGGATATTCTTCGAGATGCTTTTTCTCAAACAGAGGCTCATATGA

ATCACTATTATGAGGGATGTACAGCAACAGTGCTTCTGGTGTGGACTGATGGTGATGAAAATTTCTTTGC

GCAATGTGCTAATGTTGGGGACTCTGCCTGTATTATGAGTTTGAATGGCAAGCAAATCAAGATGACAGAA

GATCACAAAATAACGAATTATTCTGAAAGACTCAGAATCGAAGAGACAGGTGAACCATTAAAGGATGGGG

AAACACGTCTATATGGTATAAATCTGGCAAGAATGCTTGGGGACAAATTCCTGAAACAGCAGGATACCCG

GTTCAGTTCAGAACCTTACATAAGTGAAGTTGTGCATATTGATCAAGCAAGCAAGGCTTTTGCTATTTTA

GCTAGTGATGGGCTGTGGGATGTCATCAGCGTGAAGAAGGCAATTCAGCTTGTGTTGCAGATGAGGGAGA

GATGCTACTCAGAGAGAGAGAATACAGCAGAAAAGACTGCTAGCTTGTTGTTGAATGACGGCTAAAACAC

TCAGAACAAAGGATAATACCTCTGTAATTTCCTTAGCATTTTTGATACCTTCCACAAAATTTCTCTGTAA

CGTTGAATCCCTAGTTATGGACTTAGGAATAGNTGTTACCAGGGAAATTNTTTACAGTANCATTATTTTT

TTGGTTCCCATGGGCTTCTTCACAAAAAAAAAGCAANAAAACAAGCAAAANAAACA



>gi|71811513|gb|DR988904.1|DR988904 HDI_D_56.esd Horsegram (VZM-1) drought stressed subtracted cDNA Macrotyloma uniflorum cDNA, mRNA sequence

AAAGCTGGAGCTCACCGCGGTGCGCGGCCGNTTCTAGAACTAGTGGATCCCCCGGGCTGCAGGAATTCGG

CACGAGGCCTCGTGCCGAATTCGGCACGAGGGCAGGCGCAGCGGGCTCGCTTGCAGCTGGTGGGCTTGTG

GTGATCCTGAGCATCTGCCTCACAGTCTATGGGATCTCGTCGTTCAACGAAGGAGACCCATCGACGGCCC

CTTCGCTGACCTTAACGGGCCGCAAGAAGCAGCCCGATCAGCTCCAAACTGCTGATGGGTGGGCCAAGTT

CACCGGGGGCTTCTTTTTTGGAGGCATTTCGGGGGGTTATTTGGGCCCTTCCTTTCCTTCCTTCTTATTG

TTCTTGTGGACCTTCTCCTTTATTACTCTCAGTGTGAAGGGCGCTCTTTCTCTTGTGTTCTCTCTTTTTA

TTATTCATTCTCTATGAATGGACAATGAATTGAGGGTTATTTGTGCACTAGAGATGCATGTAAATGATGA

TGTATTTTATGACTCTCAACATCTTACAAATGCTAAAAAACAAAAACAAAAAAACATCTCAAGGGGGGGC

CCGNATACCCAATATT



>gi|71811511|gb|DR988902.1|DR988902 HDI_D_47.esd Horsegram (VZM-1) drought stressed subtracted cDNA Macrotyloma uniflorum cDNA, mRNA sequence

CTCCTCAACTGCTGATGGGTGGGCCAAGTTCACCGGGGGCGTTCTTTTTTGGAGGCATTTCGGGGGTTAT

TTGGGCCTACTTTCTCCTCTATGTCTTGGACCTTCCTTACTACATCAAGTGAAGGGGCTCTTTTCTTTGT

TTCTCTTTTTATTATTCATTCTCTATGAATGGACAAAGAATAAGAGGTTATNAAGTAGCACATAGAGATG

CATGTAAATGACTGATGTATTGAAATGACTCTCAAACCATCTTACAAAATGCTAAAA



>gi|71811509|gb|DR988900.1|DR988900 HDI_D_45.esd Horsegram (VZM-1) drought stressed subtracted cDNA Macrotyloma uniflorum cDNA, mRNA sequence

CGCGGGCCGGCCGTTCTAGAACTAGTGGATCCCCCGGCGCTGCAGTGATTCGGCACGAGGCCTCGTGCCG

AATTCGGCACGAGGAGCAAGATCTCAGGGTGAGTCTTCTAATAAAAGTTCAGTGGATGAAAGCTGGGATG

CATTGATTGAGCGTCATTTTAACAAGGATGAGCATGGCCACACTGTGCTGAAGAAAGAATAATTAAGTCC

TTTAAAGACTTGAGAATATGTTATATTTATAGTGTATAAAGCAAAATTCGTTCATTCTTATTGTCCCTCT

CATGGGGAATGTTAAATTTGTTAGATGTTCATGGTAATCAGATTCATGTGTAGTCAACTTTTAAATAAAA

GGAAGGATTCTAATAGTAAAAAAAAAAAAAAAAAAAAANCCCCNGGGGGGNCCCGGTTCCCCAATTNTC



>gi|71811507|gb|DR988898.1|DR988898 HDI_D_43.esd Horsegram (VZM-1) drought stressed subtracted cDNA Macrotyloma uniflorum cDNA, mRNA sequence

AAGCTGGAGCTCACCGCGGTGCGCGGCCGCTCTAGAACTAGTGGATCCCCCGGGCTGCAGGAATTCGGCA

CGAGGGGTATGGGGAGCACAAGCCAGAGCACCATGGAGAGGAGCACAAAGAAGGGTTCGTAGAGAAGATC

AAGGACAAGATCCACGGTGAAGGTGCTGAGGGGGAGAAAAAGAAGAAGGAGAAGAAGAAGAAGAAACATG

AAGATGGCCATGGCCATGACAGCAGCAGCAGCAGTGACAGTGATTAGATATCACTGCTGCATCACCTATA

TATATTAAGGTGTATGGACCATAGTAGTGATGGCTGTGACTTCTTTTTTCTTTCTTTCCTTCTTGTGATT

TGCATTTATGAGTGAAAAACAAAAAAATGAGAGAAAAAGTGTGCGTGTATGTGGTTGGTAATTGGAGTGT

TATATGTGGTTTGGTTGGTTGCCCACTACTTTGGTTGTAGATTATCTTAATGAACATAAAATTTTAAATT

ATCAGTATTA



>gi|71811505|gb|DR988896.1|DR988896 HDI_D_34.esd Horsegram (VZM-1) drought stressed subtracted cDNA Macrotyloma uniflorum cDNA, mRNA sequence

AAGCTGGAGCTCACGCGGTGCGCGGCGTTCTAGAACTAGTGGATCCCCCGGGCTGCAGGATTCGGCACGA

GGCCTCGTGCCGAATTCGGCACGAGGGTTATTCTCTCTCTGAGCTTCACTCTCTTCACCTCTGCGAAAGG

GAGAAAAATGGGGTTTTCCTCCTTTCTGGGTCGCGTTCTCTTTGCCTCCCTCTTCATCCTCTCCGCATGG

CAGTTGTTTAATGAATTTGATGCCAATGGTGGACCCATTTCAAAGGAGTTGATTCCCAAGCTCACTATTG

TGAAGAAAAATTTGTCCTCCAAATTGGGGGTAGCACTACCCGATATTGATGCCCGGCAATTCATTGCTAC

TATCATATTTCTTAAGGGGGTTGGTGGGATCTTGTTTGTGTTTGGCAGCACATTTGGATCTTTTCTACTG

CTTTTGCATTTGGCGCTTACTACTCCACTTCTGTACGATTTCTACAACTATAGATCTGGCAATCCTAAGT

ATAATCAGCTGCTGAATGAATTCATACTGAACACAGCACTTTTTGGGGCATTGTTATTCTTTATTGGAAT

GAAGAACTCAATTCCCAGGAGTCAGCTCAGGAAGAAGACCCCTAAAGCTAAGACAGTTTAGAATACTANG

AGGAACAAGAAGTATTAGTGTACCAGAATGCTTAGTTTTCGGCATTCNAGTATTTTGATCTTCGGCTTGA

GTTTGGAATAGTTACTTTAGGAAACCCTTTAATTTNAAGGAAA



>gi|71811503|gb|DR988894.1|DR988894 HDI_D_32.esd Horsegram (VZM-1) drought stressed subtracted cDNA Macrotyloma uniflorum cDNA, mRNA sequence

AAGCTGGAGCTCACGCGGTGGCGGCCGCTCTAGAACTAGTGGATCCCCCGGGCTGCAGGATTCGGCACGA

GGGTTCACCCGAAGAGTTCCTCTCTAAGGTGGATTATTTACTAGGAAAACAGGCCTTCTTTGGCGAAACT

CAAGCTGAGGGTGGTTTTGATCCAAATGCTGTGGCCACGGCTAACATCTTAGAGACTGCAACACCTGTGG

TTGATGGGAAGCAGTACTATGTTTTGACTGTGTTGACAAGGACTGCTGATGGAGATGAAGGTGGGAAGCA

CCAACTAATTAAGCAACCGTAAAAGATGGAAAACTGTACATTTGCAAGGCTCAAGCTGGAGACAAGAGGT

GGTTTAAGGGAGCAAGAAAATTTGTGGAGAGCACAGCAAGTTCTTTCAGTGTTGCTTAAAAACTTCAATA

GCAGGTTATATATATATATTTGCTATGTGTAAATCAACCTTAAGTTTATGCTATCTATGTTTTGAGTTGT

CTAGCCTCCCCTTTGTCCACGAGTTTCATAATTTAAGGGACTTGGTATTCCTGGCATTGTAAAGACCAAC

AAA



>gi|71811501|gb|DR988892.1|DR988892 HDI_D_24.esd Horsegram (VZM-1) drought stressed subtracted cDNA Macrotyloma uniflorum cDNA, mRNA sequence

AGCAGACTGTTACATCATGTCAATCCGACTTCAGCAAATTCACTATATATCTTTGGATTGATTGTTGTAC

TCCGGTAAATTCTTTCTTTGTTACTTTTACCGTTTTCATATTTTTATGGTTCTATATCTTTTGTAACTTT

TGTGTAATCCTCATGTCAAATGTTGAAAATCTGGAGAGGAATTTTATTTGTTGACAGTTGTATTTTGATT

CTTAGTTTTTTTTTTTTTCCTTTTCC



>gi|71811499|gb|DR988890.1|DR988890 HDI_D_22.esd Horsegram (VZM-1) drought stressed subtracted cDNA Macrotyloma uniflorum cDNA, mRNA sequence

AAGCTGGAGCTCACGCGGTGGCGGCGCTCTAGAACTAGTGGATCCCCCGGGCTGCAGGATTCGGCACGAG

GTACACAAGTCCAAAGCTGAGAAGGCAAGAGAGAAGACCTTATCTGATCAGTTTGAGGCCAAACGTGCCA

AGAACAAGGCCAGCAGAGAAAGGAAAATTGCTCGGAGAGAGGAGCGATTGGCCCAAGGTCCAGGAGAAAG

GTCAGCAGCTGCACCCAGTGCTCCTGCTGCTACAGTGTCACAGTCTGCCCAAGCACCAAAGAAATCCAAG

AAGTGAGCCACATATTCTGATATTGGCTGGTAGAATTTTTGGTACTGTCATTCTCAGTTACTATTCTAAA

GTAGTTTTGACGAGTATTGAGGAGTTTCTGTGTTGGCGTAGACCTTCTTTAGTTTTTATTCAAGTATATG

AAATTATAACATTCTGCTTTTTA



>gi|71811497|gb|DR988888.1|DR988888 HDI_D_20.esd Horsegram (VZM-1) drought stressed subtracted cDNA Macrotyloma uniflorum cDNA, mRNA sequence

AAGCTGGAGCTCACGCGGTGGCGGCGCTCTAGAACTAGTGGATCCCCCGGGCTGCAGGATTCGGCACGAG

GCCTCGTGCCGAATTCGGCACGAGGGCAGGCGCAGCGGGCTCGCTTGCAGCTGGTGGGCTTGTGGTGATC

CTGAGCATCTGCCTCACAGTCTATGGGATCTCGTCGTTCAACGAAGGAGACCCATCGACGGCCCCTTCGC

TGACCTTAACGGGCCGCAAGAAGCAGCCCGATCAGCTCCAAACTGCTGATGGGTGGGCCAAGTTCACCGG

GGGCTTCTTTTTTGGAGGCATTTCGGGGGTTATTTGGGCCTACTTTCTCCTCTATGTCTTGGACCTTCCT

TACTACATCAAGTGAAGGGGCTCTTTTCTTTGTTTCTCTTTTTATTATTCATTCTCTATGAATGGACAAT

GAATTGAGGGTTATTTGTGCACTAGAGATGCATGTAAATGATGATGTATTTTATGACTCTCAACATCTTA

CAAATGCTAAAAAAAAAAAAAAAAAAACACTCAAGGGGGGGCCCGGTAACCCAATTTCGCCCTATAGTGA

NTCGCTATTACAATTCACTGGCCGTCGTTAAAACACAAG



>gi|71811495|gb|DR988886.1|DR988886 HDI_D_12.esd Horsegram (VZM-1) drought stressed subtracted cDNA Macrotyloma uniflorum cDNA, mRNA sequence

GAGCTCACGCGGTGGCGGCGCTCTAGAACTAGTGGATCCCCCGGGCTGCAGGATCGGCACGAGGCCTCGT

GCCGAATTCGGCACGAGGCAAATAAGGAGGAACGTGAGAAGGGCACAGAGGAATCACTAGAGGCTCTTCA

ATTTCTGGAGAATGAACTGAAACACAAGTTCTTTGGTGGAGAGAGTATTGGAATAGTGGACATAGCCGCA

AGCTTCATAGCTTTCTGGCTCCCTGCGATTGAAGAGGCTGTGGGGTTGAAACTGGTGAGCTCTCAGAAGT

TTCCCAAGCTTTCTAAATGGAGTGAAGAATACACAAACCATCCTGTTGTCAAAGAGACTCTGCCTCCAAG

AGAGAGAGTTGTGGACTTTTTCAAAACTCGATATGCATCCATCCTTGCTTCCAAATAGGTTAATTATGAG

TCTGATGGATACTCATGAGTCACGATTTAGCCAAAGGTTTTAAATAAGAAGTTGTTGGCATTCTGATTTC

TGTTCTAGATGGCATCATGAAGGTTATTCATGCTGAAATTTCATGAAAAAACTGTAGTGTTTGTGCTTTA

TTCCATTTCAAATTTAAGTTCTAGTATAAAAAAAAAAAAAAAA



>gi|71811493|gb|DR988884.1|DR988884 HDI_D_90.esd Horsegram (VZM-1) drought stressed subtracted cDNA Macrotyloma uniflorum cDNA, mRNA sequence

AAGCTGGAGCTCACGCGGTGGCGGCGCTCTAGAACTAGTGGATCCCCCGGGCTGCAGGATTCGGCACGAG

GATTTGCAGCTGTGCCCTTTTTGCGGTCAACCTTTTTCTGTTGTTGGAGATGAGTTTGTGAAGGAATCCG

TGAAGTTTTCCAACCAATCTACAACATTTGGACAAGCATTCAATAATTTCACCCGTTCTAGAAATGAAAA

AGATTCTGGTAGAGCAATTGATGTTGAAGCTGAAATTAAAGATGTCGACTGAGTCTTCACAGCTATAACA

TGTTGGTAGGTGTGAAGTAATGAGCAAATTTTGAGTTGAACACAGTGACTATGCTGAACACAAATCTTGA

TCGGTTAAAGAACAGGCTGTGGCTAGTATTTTCCATTTGTACTGAAGACACAATGAATTACACATTCAAG

AGAGAAGAGTGTGATGAGCAAAGTAACACATTCTAGAGAGGAGTGTGATGAGCAGAGTAACATTGCAATT

GTAATATAAGTTTTGACAGTGATGTTTCTCAGGTCACACCTCTATAATTTTGGTGTACCAGGATACTGCT

TCATTTTTCCTTTCAATTGTTTTTACAATAAAATGTTTAGGAGAAATCGAGATGCTTCATTTCAATTGTC

AGGACATGGCAGTCTTGACAACTCTCTTCTATTTANGTTCAAAACCCTTTACAAGACAAGGAAAATCAAG

TAGTACATAATCTCCATG



>gi|71811491|gb|DR988882.1|DR988882 HDI_D_88.esd Horsegram (VZM-1) drought stressed subtracted cDNA Macrotyloma uniflorum cDNA, mRNA sequence

AGGCTGAGAATAATGATTGTGGAAGACTTCATTCTGCGCTGCTTCTAGCCATTTCTTTCCACCGAAAGTA

AGAGCATCCGTGCAGCAATTGCTGAAAATCTTCCAGAACCAGAACTTAGCAAGTCTTGTACCTGTGTTGG

ACAGATTTGTGGAATTCCATCTATCATTAAACATGATTTCACCGGAGACTCATAAAGCTGTTAGCGAGGT

CATTGAATCGTGTAGAATTCGATGAAAGCTCCAAAGGGAAAACTCTGTACAGCCACAGCTTAATTTAGGG

TTTGTTCGTTATTCCGGAGGAAATGGGACTCCCTTACCCCGAGTCTGACCAACTTTTATTCTCTGCTTCA

CAGACGCTTATACCCTTATTTTATATATCACCATGCTGGTGATGATGCTGAGT



>gi|71811489|gb|DR988880.1|DR988880 HDI_D_86.esd Horsegram (VZM-1) drought stressed subtracted cDNA Macrotyloma uniflorum cDNA, mRNA sequence

TAGGCCTGACCCAATTCCAGAGCTGTGATCAATAGTAGATTCAGTATTTTAGCTCTATTAACGTAGGGAC

GCTGCACGTTGATACCAAAAACAGTATACCTAATAACTTGCAGCCAATAGTCCAAGTTATCAACTAAAGA

ACTGACCGTTCATCGGAAAGACGAAGTCACAGTGATCAAATGCTCNTTTTCTTGAAGTTTA



>gi|71811487|gb|DR988878.1|DR988878 HDI_D_78.esd Horsegram (VZM-1) drought stressed subtracted cDNA Macrotyloma uniflorum cDNA, mRNA sequence

AGGANGTTTGTANTAAAACAACCATCAGTTGGAGGGGCGGCACACCATATACTTCCTGCCTCCCAACATG

ACTTAATGATCTATATCAATTTGTTTGTGTGAGGAGAGAGCATGCATGAATGATAGTTGCTTGGTCCTTG

CTTACTACAAGGACGGTGCCATCTGATCCGACATTTCTGTACTTTGCATATGCCTTGAAGGAGGTTAAGT

GCTAAGTGACTCAACATAGCAGGCTATTTACCATCCTTTTTGTCTTTTTTCTAGTTACTTGGAACTTTGC

CTTGGTAAGTATTTTAATTTTAAGTAACCTAGAACCTTTTTTTGTGCTTCCATTAATAATTTTCCTATCC

AAGTTAATCATGGCACTTTGAATTTTCCATTAACTTTACTGGAATTNTCGGATGTTTGAACTTTCCATTT

TTGCAA



>gi|71811485|gb|DR988876.1|DR988876 HDI_D_76.esd Horsegram (VZM-1) drought stressed subtracted cDNA Macrotyloma uniflorum cDNA, mRNA sequence

AAAGCTGGAGCTCACGCGGTGCGCGGCGNTCTAGAACTAGTGGATCCCCCGGGCTGCAGGATTCGGCACG

AGGGTTGAGTTTTCTGCTGGCAAATCTATACTTGTCATTGGAGGTGCTGGTGGAGTAGGATCCCTTGTTA

TTCAGCTTGCCAAGCACGTTTTTGGGGCCTCAAAGGTTGCAGCCACAGCCAGTACTGCAAAACTGGATTT

GTTAAGGAGCTTGGGAGCTGACTTAGCCATTGATTATACAAAGGAGAATATTGAAGATCTGGAAGAAAAG

TTTGATGTAGTGTATGATGCTGTAGGGCCAAGTGAGATTGAGAAAGCATTGAAGGCTGTTAAAGAAAGTG

GCAAAGATTCTGTCAATAGCAACCTTTGGAAATCCTGCAGTTATCTCGATAACACTCATTTCAGATAGTG

CTGTGTTGGAGAAACTACAACCTTACTTAGAGAGTGGGAAGGTGAAGCCATTACTGGACCCTAAGGGTCC

CTTCTACGTANTTCTCAAACTGTGGAGGCATTTGCCATACTTGAAGACTAATAGAGCCATTGGGAAAGTG

GTCCATACATCCCCCATTCCCATAAGGGGTCAAGCAGTTGGGACAATAA



>gi|71811483|gb|DR988874.1|DR988874 HDI_D_74.esd Horsegram (VZM-1) drought stressed subtracted cDNA Macrotyloma uniflorum cDNA, mRNA sequence

AAAGCTGGAGCTCACCGCGGTGGCGGCCGCTCTAGAACTAGTGGATCCCCCGGGCTGCAGGATTCGGCAC

GAGGCCTCGTGCCGAATTCGGCACGAGGCAGCTATAAGCTTTTGTTCTGCAATTTTGACCGCAGCCTCTG

CGGTAATGTTGGGATTGTTAGGGATGATGCAGGAAACAGGCTTTTGGCCATCACTCAGGGCAAAGAACTG

GAGTTTGTTCTTGAGGAACTTCCATCACTCATCTGCTGCATCTGCATGAGCCACTTTCAATGAAAATTCC

TGCACTTTCTACTTCTGAGAGACGTGCTTTGTTATGCTTTGGATGTAATTATCTTATGTTTTCCAACACT

AAA



>gi|71811481|gb|DR988872.1|DR988872 HDI_D_65.esd Horsegram (VZM-1) drought stressed subtracted cDNA Macrotyloma uniflorum cDNA, mRNA sequence

AAGCTGGAGCTCACGCGGTGGCGGCGCTCTAGAAACTAGTGGATCCCCCGGGCTGCAGGATTCGGCACGA

GGCCTCGTGCCGAATTCGGCACGAGGGTTCGTTGGATCGGAGGAGGTTGCGCCCTGATAGCTGAACTTCA

GGCTTTGAAACATGGTCTTCGTATGGGATGGGATCTTGGTTATAAAAAGATAATTTGTGAGCAACTTTAT

GGTAATGATAATATTTTTCATAACTGGAATATTGGTGGTGATGTGAACGTACATACCAATAAGGCCTTAG

TTTTGGAGATAAAGGAATTGTGTAGGTCGACATTGGCTAATACTGCTGGAATTTACCCAATAGAAGCAAA

TGTGGCTGCTGATTTACTGGCAATGAATTATGATCAAAACTGGCTTAAATCACCAGAAGATCTTTGTATT

GCTCAAGGACACACAGTTTTGGCTTAGCTTCTGGTAGTTGTCTTTTACTTCCTTATGTACCAAAAAGAAA

GTGCCTCATTAACCTTGGCATTCCATTTCTACTTAAGTGTATTTTTAAATTTTCTTACATTGCTAGTTAT

GTTTGTTATCGGTTTGTTTCCGTTTGTAAGTGTTCGTATGCAACACTTATTCTGTAAAACATATTTTCTG

CAATATATGTAGCTGGTCAGTTTCTGTTTTAA



>gi|71811479|gb|DR988870.1|DR988870 HDI_D_63.esd Horsegram (VZM-1) drought stressed subtracted cDNA Macrotyloma uniflorum cDNA, mRNA sequence

AAAGGCTGGAGCTCACCGCGGTGGCGGCCGCTCTAAACTAGTTGGATCCCCCGGGCTGCAGGAATTCGGC

ACGAGGTTTTTTTTTTTTAAAACACACCAAATTATAAAAGTTTCATATTTATAAAAGTGACAAGCCAAAC

AGCCAACAGCCAAAGCCTATTAAAACTCACCGTGGCGCCATAACCAAAAAGTGTAATCTCAGGGGTTTCT

CGTGACAAAACATACAATAAAAAAAACCAACATTTAAAACACATTAATTAATTTAGTATAAAAGAAGCCG

CCACCGTCTCGAACTCAAGAAACCCAATTCTCGTGGGAAAACAAAAGTTGGGAAGACATGCAGGGTTGGA

AAAACTTGAAAAACTAAGTTACAAAAAAAAACAAAGCCTACCTTAAAGAAAGTGACACAAGAAGTTACAA

NGCAGAGAAAAAACCGAGGCATTTATGTGTTAACCTATATCCCCNGTGAAAGTGAAACCACCCGCGCAA



>gi|71811477|gb|DR988868.1|DR988868 HDI_D_6.esd Horsegram (VZM-1) drought stressed subtracted cDNA Macrotyloma uniflorum cDNA, mRNA sequence

ACAAGCTGGAGCTCACCGCGGTGGCGGCCGCTCTAGAAACTAGTGGATCCCCCGGGCTGCAGGAATTCGG

CACGAGGTTTTGAATGAAAAAAAAAAAACACTATATAATTCAAATGTGAACCCAACCATGTGAAGAACAG

TCTCCAGGATTTATTCCACCCATTCACGTGTTTTATTTACTACACCCTTAACAGCCCTGTGGTATAATTT

CGACCAAAAAACTTAATTATTCCTGTGTTAAGCATTTAGCCCGATGTGTTTTCTCGATGTGTCTCCAATT

TATCCACCATTGGGTGAATGTGTGGGTTCTGGTCTCTATAGGAAAACGGGCTATACTGTGCTCTCCAGTA

TTTGTGGCCGTGGTCTCCACTCCCTTTTGTGCCCCAAAGCGTATATCTCTTTCTCTATACTTATAGGCCA

CCCTGTGTATATTATAGGAATCTAAACATCTCCAATTAAATCTAGAAATGTGAAAATATATNTCGTTGAA

TTAAAGAAAACAGCCCAGTTATA



>gi|71811475|gb|DR988866.1|DR988866 HDI_D_53.esd Horsegram (VZM-1) drought stressed subtracted cDNA Macrotyloma uniflorum cDNA, mRNA sequence

GCTCACGCGGTGGCGGCGCTCTAGAACTAGTGGATCCCCCGGGCTGCAGGATTCGGCACGAGGCCTCGTT

GCCGGGACCTCCTTACAGCTGACGAGCTTCTCTCTGACTCCTTCCGCTACACTGAAATTTGAGCAATGGA

AGTGTTGTGGGAAGTTGAGGGAAAGTGGGTTGTTAAAGGAGCAGTTGATGTAGACATTGGAGCAAACCCT

TCTGCCGAAGGTGGAGATGATGAGGGTGTCGATGATCAGGCTGTTAA



>gi|71811473|gb|DR988864.1|DR988864 HDI_D_51.esd Horsegram (VZM-1) drought stressed subtracted cDNA Macrotyloma uniflorum cDNA, mRNA sequence

AAAAGCTNGGAGCCTACGCGGTCCCGGCCGTTCTANAAACTAGTGGATCCCCCGGGCTGCAGGTAATTCG

GCACGAGGCACAGTATTCAGACATACCCACGGAGAATCTGGAATCAAAACACAAAGGAGAATAATTATCA

ATGCAGGGGAAATTC



>gi|71811471|gb|DR988862.1|DR988862 HDI_D_5.esd Horsegram (VZM-1) drought stressed subtracted cDNA Macrotyloma uniflorum cDNA, mRNA sequence

AAGCTGGAGCTCACGCGGTGGCGGCCGCTCTAGAACTAGTGGATCCCCCGGGCTGCAGGATTCGGCACGA

GGCCTCGTGCCGAATTCGGCACGAGGTCAAAGTCCGTGGTTGTGGCAAATTTGGTGCTTATTCATCAGCT

CGGCCTAAGTTGATAACAGTTGATTCGGAAGAGGTGGAATTCAAGTATGAGGAAGAATCTGGATTGGTGA

CTGTTGATTTGAGAGTACCTGAGAAAGAGTTATACCAATGGACTATTTCCATTGATTTTTGAATCTGGGA

CTTTGTCTCTAGATGAGGGGGTTATATGCTTATAGTGTATCCATAGTTCGTGACAATTTGACGAGTAGAT

CAGTAACAGGAAAGTTTGATTTCCT



>gi|71811469|gb|DR988860.1|DR988860 HDI_D_42.esd Horsegram (VZM-1) drought stressed subtracted cDNA Macrotyloma uniflorum cDNA, mRNA sequence

TCGCTCTTTTTAACTTTGTCAACTTTACAATNTTACCAGCTTCCACTTGCACCATCCGCCCTATGCTGGT

TGTATCTGAACGAATCAGTACCGAGANATCCTGNGATTTCAATCTCTG



>gi|71811467|gb|DR988858.1|DR988858 HDI_D_40.esd Horsegram (VZM-1) drought stressed subtracted cDNA Macrotyloma uniflorum cDNA, mRNA sequence

AAACTGGAGCTCCACGCGGTGGCGGCGCTTCTAGAACTAGTGGATCCCCCGGGCTGCAGGATTCGGCACG

AGGCCTCGTGCCGAATTCGGCACGAGGCATAAGCAAACAAACAAGGAATGTGCAAAATAGGTAGGTTTTG

GTGTGCCAGCTGTGTGGTTTTGGCAGATTTTCCGTGAAGCGTAAGCTTGGAAAGTAATTTGTTTGGAAGA

TGGATGGGATCATCTTGGGGGAAGTAAAGGGAGCGTGTTGAGAGTGAGAGACCCTTTTGTCGTGTTGGTG

AATAAATGTTCTGCTATCTTATCCCCTGAAGATGCTTTGTAGCTGTTGCATGACATTTTAATACTTTTGT

ATGATTATGTTAGTACAGACCTGGGTATGCNAATCTCAAGAAGAACAAGCGTCGTGTTGTGACATCGCGT

AAAACA



>gi|71811465|gb|DR988856.1|DR988856 HDI_D_39.esd Horsegram (VZM-1) drought stressed subtracted cDNA Macrotyloma uniflorum cDNA, mRNA sequence

AAGCTGGAGCTCACGCGGTGCGCGGCCGTCTCTAGAACTAGTGGATCCCCCGGGCTGCAGGATTCGGCAC

GAGGCTGCACTGAAGAATTAACATATATTAGCAAGCATGGCAGTCACATTCTACGAGCTTTACCTCTGCC

TCTGGATTGAAGAAACTTGATGAGTACCTTCTCTCACGCAGTTACATCACTGGAGTACCAAGACTTCAAA

GGATGATCTCACTGTCTATGCAGCTTTCCCAACTGATTCCATCAGCTGATTATGTGAATGTATCCAGGTG

GTACAAACACATTGATGCATTGTNTGAGAATCTCTGGTGTTTCTGGTGAGGGATCCGGTGTCACTGTTGA

GGGATCTCTTGTTGCGGAGCCTGTTGCGACTCCCCCAGCTGCTGACACAAAGGCTGCTGCTGCTGAGGAT

GATGACNATGATGATGTGGATTTGTTTGGTGAAGAGACAGAGGAAGAGAAGAAGGCAGCTGAGGAACGCG

CAGCTGCAGTGAAGGCATCTGGAAAAAAGAAAGAGTCTGGGAAATCATCTGTTCTGATGGATGTGAAACC

ATGGGACGATGAACACCGACATGAAGAAGCTTGAGGAAGCAGTGAGATCTGTTAGCCATGGAAAGGCTTG

ACTTTTGGGGTGCATCCAAAACTTGTTCCAAGTAGGATAACGGAATCACAGAAACTAGCAAACTTAATGC

T



>gi|71811463|gb|DR988854.1|DR988854 HDI_D_30.esd Horsegram (VZM-1) drought stressed subtracted cDNA Macrotyloma uniflorum cDNA, mRNA sequence

TTGCTCCCCACANAAAACATGTTGTTGTGTCGCCACCATCGTGCTCATCATCTCTCTTAGAATACTCGTG

TATACATCTCCCAATTTTCCAAATATCTCCTCTTATCAGGGTATGGATGAAAGTTTAAATCCTCAAAAGC

CGCCTGGGGGGCTTCGAATCTGCTATCGAGACTCGTATAGCTCTGCCATTCAAGTCCACACCATCCAAGG

ACTGAATTGCACTGCTGACCTCTTCGGGACTACTGTATGTCACAAATCCAAAGCCTCTTGATCGTCCACT

GTCCCTGTCATAGATTACCCTAGCTTCCAAAACCTTTCCTTTTCCCCCAAACAATGTCTCGAGGGGGGGC

CCGGTACCCAATTCGCCCTATAGTGAGTCGTATTACAATTCACTGGCCGTCGTTAACAAACCGAGAAA



>gi|71811461|gb|DR988852.1|DR988852 HDI_D_29.esd Horsegram (VZM-1) drought stressed subtracted cDNA Macrotyloma uniflorum cDNA, mRNA sequence

AAGCTGGAGCTCACGCGGTGGCGGCCGCTCTAGAACTAGTGGATCCCCCGGGCTGCAGGATTCGGCACGA

GGCCTCGTGCCGAATTCGGCACGAGGGATTGTTGCTGCTCAACTTGAGAGCTACAACTCCTGAAGCATAT

GAGGAGCTGATTGAATTTCTTGGTAGACACTCGTTGAATGATGGTGACAAGTTCTGTGCCAGCATGTTCA

GAGAATCATCAAGACATAAGAACTTAGCCCTACGCATAATGGAGGTGAGATCAGCATACTGCAAAAATGA

TTTTGAATGGGACAACTTGAAGCGCCTAGCTTTCAAGATGGTCTCAGAATCTAACACGAGGCTCATGACG

GAATATGTCTTAGAAACCAGTCATGTTGAAAGCGAGAAGTGAATAGTCTTGCACCAAAGCATTAGCCCCT

TCATTCCATCTGATCGAACAGGAGCTACGGCAATGTACCTCTTTATATGTACAGAAAGAACTCATCTACA

TGGTTACCGATCAATTTTATACTCCAGTGTTAGCAAGGGTTATGATTAAAAAACAGTTAGATAATAAAAT

GGTTAGACAAATTGCTGATACAGATTGATAATCAACAACATATTCTATTGTTAAACCTTCTATAACTGAA

G



>gi|71811459|gb|DR988850.1|DR988850 HDI_D_27.esd Horsegram (VZM-1) drought stressed subtracted cDNA Macrotyloma uniflorum cDNA, mRNA sequence

AAAGCTGGAGCTCACCGCGGTTGGCGGCCGCTCTAGAACTAGTGGATCCCCCGGGCTGCAGGAATCGGCA

CGAGGTTTCAACGCCCGCAACGTAGCTTCACTCCCAGGCAAATGTGGCGTCTCAATCCCCTACAAGATCA

GCACCTCCACCAACTGTGCTACCATCAAGTTCTGAAAGATGGATGTGGTATAAGGAAGAAAAAGGCACGA

AGGGGAAATTTTCAGTAGCAGAATATTACTATATATGATGGTAACGTAGAATTAGGTTATGAAAAATAAA

GGGTGGTCGACGAGGACTCCACTTGTGCGAGTCTCTCTTGGTGTCGGTGCCTCCCTTAGTTGTTTCAGAC

TTTGGTCCACTTATGTTATGTCTTCTGAAATTTGTATCAGTTGGTTGTTGCATGTAATAATGATATATAT

AGTG



>gi|71811457|gb|DR988848.1|DR988848 HDI_D_25.esd Horsegram (VZM-1) drought stressed subtracted cDNA Macrotyloma uniflorum cDNA, mRNA sequence

AAGCTGGAGCTCACGCGGTGGCGGCGTCTCTAGAACTAGTGGATCCCCCGGGCTGCAGGATTCGGCACGA

GGGTACGGAGTTAAGGGACACTTGCTTAATGCTGGCCTCTGCCAACTCTGTAAAGGGGACGTTGTTGCTA

TAGCTAATGCATTAGAGCGATATCAGGAATTAGATCCAACATTTTCAGGAACACGGGAATATAAATTGTT

GGCGGATATTGCTGCTGCTATTGATGAAGAAGATGTTGGGAAGTTTACTGATGTTGTGAAGGAATTTGAT

AGCATGACACCTCTGGATTCTTGGAAGACCACACTTCTTTTGAGGGTGAAGGAAAAGCTGAAAGCCAAAG

AACTTGAGGAGGATGATCTTACTTGAATTATACCTTTTATATTCTGGTGGCTGGACCTATTGTTTGATGA

TTTACATTGTACTATGCGTGTCTGTTCAGTTGTTCATCTTAGCCATCTAAAAAAAAAAAAAAA



>gi|71811455|gb|DR988846.1|DR988846 HDI_D_18.esd Horsegram (VZM-1) drought stressed subtracted cDNA Macrotyloma uniflorum cDNA, mRNA sequence

AAAAAGCTGGAGCTCACGCGGTGCGCGGCGCTCTAGAACTAGTGGATCCCCCGGGCTGCAGGATTCGGCA

CGAGGGTCACCTGGATACTATGATGGAAGGTACTGGACCATGTGGAAGCTGCCTATGTTTGGGTGCACTG

ATGCTTCTCAGGTCTTGAAGGAGCTCGAAGAGGCCAGGACTGCACACCCCAACGGTTTCGTCCGTATCAT

TGGATTTGACAATGTTCGCCAAGTCCAGTGCATCAGTTTCATTGCCTACAAGCCACCAAACTACTAAGTC

TCTGAAATTTCCAAAACATTTGTAGCCACTCCACTTTGTTTGTTTGTACTTAAACCAAATTCCCATTTGT

TTTTGCTTTTTGAGATTTCATCATCCTGTAATTTTGGTTTCTGTTTTCGGACTTCATTGGAAATGAATGG

ATGAGAACTAATGAATAAGCTGTTGTGTTGTGTTGCTTTGTTTCAAAAAAAAAAAAAAAAAAAAAAAAA



>gi|71811453|gb|DR988844.1|DR988844 HDI_D_16.esd Horsegram (VZM-1) drought stressed subtracted cDNA Macrotyloma uniflorum cDNA, mRNA sequence

AAGCTGGAGCTCACGCGGTGCGCGGCCGCTCTAGAACTAGTGGATCCCCCGGGCTGCAGGATTCGGCACG

AGGCACGGCTAGGGTTGTGTCAAGGCTGTGATCAAAGGGTACAAAGATGGGTTCAACCAGATTGAGTCCT

TGGTTGATGTAGAGGTGGCATTGGAGGGTCTCTTTCTGAGATTGTGAGGGCTTACCCTCACATCAAGGCC

ATCAACTTTGACTTGCCTCATGTGGTTGCCACTGCACCTAAGTTTGATGGAATCACCCATGTTGGAGGTG

ACATGTTCGTCTCCGTTCCTAGTGCTGATGCCATTTACATGAAGTGGATTCTGCATGACTGGAGCGACGA

GCACTGCATCAAGATCTTGAAGAATTGCAGGAAGGCAATACCAGAGAAGACAGGAAAGGTGATCATCGTG

GATCATGTACTTCGACCCGAAGGGAACGAACTCTTCACCGACGTTGGCATTGCATTTGACATGATGCTTC

TCGCTCACAACGCTGGTGGCAAAGAGAGGACGGAAGAGAATTGGAAGTGGCTATTCAAAGAAACAGGATT

CGCCCGCTACAACCATCATCAGATCAACGCTCTCCCCTCCATCATTGAGGCGTTTCCAATCTGAAGAAAG

CCACTTCTGCATGTGGCTCTTGTTCATTGGTTCGTTGGTCTGTTNATTACACCAGCGTTGTGATCTTCGG

TGTTACTATCCTGTGGGGTTTCCCTTAGCCCTTATTGTTTGTTCTACT



>gi|71811451|gb|DR988842.1|DR988842 HDI_D_14.esd Horsegram (VZM-1) drought stressed subtracted cDNA Macrotyloma uniflorum cDNA, mRNA sequence

ACAAGCTGGAGCTCACCGCGGTGGCGGCGNTTCTAGAACTAGTGGATCCCCCGGGCTGCAGGAATTCGGC

ACGAGGCACTTCCGTAGTTACTCCTTCCAGCTTAGAAAACAGGAACAAAAAAAAAAGTGGCAACAATAGC

TGGTGGTGTTGAGTCTCTTTCAAGCCCTTAGCAGAAGTATTTGCTAATGCTAAGGGATCAGAGTCAGCGC

AAAAGGCCCAGGCAACGATTAGGTTCCCGCTCTTCAGGCCCATGCAAAAGTTTGCCCGGTTGGTTGGGGT

TGGCGAAGGCCTGGCCCATTCGCGCAACGCCGGAAAAAGAATATCGGAGACAAGATCGAAGAAGAGCATT

AAAGAAGCCGCGGAAGGAAGAACGTTGCTCCGGAAGGCGGGGCTGACGCGGAATTGTGCGGCGGCCGTTG

GGATGAGGTGGAAGGAGCTGAAGCCGCGGCAGCGAAGCCAGCGCGAGAAGATGCGCAGAAAGCAATCCGA

CCCGNTGGAGACCTACTGCAAGGACAACCCGGAAACCGATNAGTGTCGCACCTATGATAATTAAAGTTAC

CTTGTGTTACATGATTCAGACAACCTTGT



>gi|71811449|gb|DR988840.1|DR988840 HDI_D_1.esd Horsegram (VZM-1) drought stressed subtracted cDNA Macrotyloma uniflorum cDNA, mRNA sequence

AGCTGGAGCTCACGCGGTGGCGGCCGCTCTAGAACTAGTGGATCCCCCGGGCTGCAGGATTCGGCACGAG

GGAAGGACTTCAAGATCCGCCTGCAGGGGACTACCGAGGGGCGGAAGGGGCGGCTGGCGGTCACGGCGGA

GGTCTTCGAGGTGGCGCCCGAGGTGGCGGTGGTGGAGTTTTCTAAGTCCGCCGGTGATACCTTGGAATAC

GCGAAGTTTTGCGACGAGGACGTTAGGCCTGCGCTGAAGGACATTGTCTGGACGTGGCAAGGGGATGCCA

CGTGTGCTGTTAATATCCCCGGCTGTCACGGTGAGGAGTGTCAAAAGCAGGTCATTAGCACTGTATAATT

AATTAATCAAAATTATAATTATAATTATAATTAGCGTGACAGACGGATGGTTCTAGAAACATAGCACGTT

CTAACGAATTTTCCTTTC



>gi|71811447|gb|DR988838.1|DR988838 HDI_C_95.esd Horsegram (VZM-1) drought stressed subtracted cDNA Macrotyloma uniflorum cDNA, mRNA sequence

AAGCTGGAGCTCACGCGGTGGCGGCGCTCTAGAACTAGTTGGATCCCCCGGGCTGCAGGAATTCGGCACG

AGGGTCGTGATGGTATCATTTTATTTTCTTATCATGNGAGGGAACAGAAAATTGGAGCAATTTGCTGTGA

TCCATCACGAAGGTTAGTTGCTGATTGCATTCTGATGGATGCTGATACGGCTGTTGTTTCGGATCGGAAA

GGAGGCANCTTGCTATTTTATGTTCAGATTCATTTTGGAAGGACCAATGCCAAGGTAACTGAATGCAACN

TGACACTAAGCTGTGCTTATGNCATGGCTGACATAGCCTTNACGCGTCCGGAACGGGCTCATATTCATAC

AGACTTCCAGCGGATGACGTNNTTGCAAGGCGGCAATTGACCCCAAGACTAAATGTCGATTCAACTAACA

AGAAATACTATTCATTTGCCAGTAACCCTGTTAAGGGAAGGCATNATGATCTTTCANTTCCTTTTATTCA

CACGGGGAAGCAAATCNCTTGAAGCCTAATTTAANAAAGCCCAGTTGCCAAAGAAAAGAACTTGGTTTAT

GGTCCCATTCAAGTTTTGAACAAGNCGNCCTGGTTCCTCANGGNAAACTTGGATTCCTTACATGGAAGTT

T



>gi|71811445|gb|DR988836.1|DR988836 HDI_C_93.esd Horsegram (VZM-1) drought stressed subtracted cDNA Macrotyloma uniflorum cDNA, mRNA sequence

AAGCTGGAGCTCACGCGGTGCGCGGCGNTCTAGAACTAGTGGATCCCCCGGGCTGCAGGATTCGGCACGA

GGGGACCATGGAAGAAGCATCATGGGAGCAGAGGCTGCAAGCCTTAACCCACATCCTAACGAGTCCCACA

ACCACCCCATCCCTCCACTCTCAATTCTTCATTGCCACCCAGATACCCTGCTACCTCAACTGGGACTACC

CTCCCTTCCTCTGCTCCAACCCCAACCTTCTCAGAACATGGCTCCGCTCATTCTTCCTCAAAAGGGTCTT

CGGAACCACTCCTCAAACCTCATGGAGATCCAAGTGCCCCTACCACCAACCCCCTCCCTTGATTCTTGCA

GAGGGAGTCGAGGAACCCCACTGGGGACCCCAAGAAAGAAGAGCTTATGTTAGGAAGAGAATGGCTAGGA

AACTTCGCAAAAATGTTAACCCTGTTTTACACATTGTACATACCAAATCTTTTGTTGTTGTCGGCTATGA

TTTGGAACCCCTTTCAATCTCAAGATTGATTCTTA



>gi|71811443|gb|DR988834.1|DR988834 HDI_C_91.esd Horsegram (VZM-1) drought stressed subtracted cDNA Macrotyloma uniflorum cDNA, mRNA sequence

AGCTGGAGCTCACGCGGTGGCGGCCGCTCTAGAACTAGTGGATCCCCCGGGCTGCAGGAATTCGGCACGA

GGTTCTCCTAAAATTCCAACCCAGATAACTTTTGTCCTTCCTCAGCCACACAATTCTTGCTTTATAGCAC

TCAAGCAGCTTCAATGAAGAAAGTAGAGTTAAAGATGGACTTTACATTGTGATGATAGAATCAAGCAAAA

AGCTATGAAGACAGCATCTGGGCTTNCAGGGGTTTGAATCAGTTTCTGTTGATGCGAAAGACATGAAATT

GATCTTGTTGGGTGACATTGATCCAGTGAAAGCAGTGTCGAAGCTACGAAAGTTGTGTTATACTGAAATA

GTTTCAGTTGGACCAGCAAAAGAGAAGAAGGAGGAGCCACCAAAATTACCTGTTCCTCTGAAACTCAATG

AAGCCTATTATCCCCTTTATTATCATATGACTCCACAGTATAGTCAAAGTTACTATGTTTACGATGAGAA

TCCTAGTGGCTGTGTTCCATCCTGGCTTNAAAATTAAAACCCTTTTTTAACTATT



>gi|71811441|gb|DR988832.1|DR988832 HDI_C_83.esd Horsegram (VZM-1) drought stressed subtracted cDNA Macrotyloma uniflorum cDNA, mRNA sequence

ATGAGCCACGCGGTGGCTGGCGCTCTAGAAACTAGTGGATCCCCCGTGTGCTGCAGGAATTCGGCACGAG

GATTTCATTTTCTCACTTCTTCAAACAAGTTTCACCGGAGTAAGCAACTTGCATGGGAAAGGAATGGCTT

CAACTTCAGCACTTCAATGTCTCTTCCATTAACTCATCGTCANCACCAAAAAAGGGTGTTGCCCCTTACC

GAGTTTGTAGAGGCCGACT



>gi|71811439|gb|DR988830.1|DR988830 HDI_C_81.esd Horsegram (VZM-1) drought stressed subtracted cDNA Macrotyloma uniflorum cDNA, mRNA sequence

AAGCTGGAGCTCACGCGGTGNGCGGCGTCTCTAGAACTAGTGGATCCCCCGGGCTGCAGGATTCGGCACG

AGGCTTGCAAGTGAGGTTGGAGAGCGAAAGCTTCAAGCTGAGATGGACCTTAATTAGCATTAGTGGCAGG

AATAATAATATTAATTAACTATTATATGTAGTAAAAAGGCTTTAAAATTATGTATGTCTGTGTGTATGTC

GTTTTCGCACTCTGGTGTTAGGTCTAAACACCCTTTACAAAGTAAAGTCGGCCATCGTCTTCCCCGT



>gi|71811437|gb|DR988828.1|DR988828 HDI_C_8.esd Horsegram (VZM-1) drought stressed subtracted cDNA Macrotyloma uniflorum cDNA, mRNA sequence

AAGCTGGAGCTCACGCGGTGGCGGCGCTCTAGAACTAGTGGATCCCCCGGGCTGCAGGATTCGGCACGAG

GCTCCAGAAGACTGGGACAGGGGGCTGGTATTAAAGTTGTAGATGAATACTAGTTCCTCTTTTGTGGATA

CGTACTTCTCAATGTCACTAGTTTCAGTAGGATTTGTTTTCATTGTCTACGGGTCCAGAAATGTTCCACA

GTTATTTATGAGCCGAGGTGCCCAGAAGGAAGAAAGTTGATGGACTAAGAATGTCAGGGAAAAAAACAAC

GAAAAAGAAAAAATGATGTTCATCCATGTGGTCATATACAATGTTTGGTTCTGATCATTAGTCAGTCATT

GATTGCCACCCGAAGTTTACATAGGGACTGTGATTGACGTTTATCATTATACAATCAAATAGAGTACATG

CAACATTTGTATACTCTTTTGATCTATATCATTCAATCAAGATGAACTTGAAGAAGATAAATCTTTATAA



>gi|71811435|gb|DR988826.1|DR988826 HDI_C_71.esd Horsegram (VZM-1) drought stressed subtracted cDNA Macrotyloma uniflorum cDNA, mRNA sequence

AANNGGGTATCCGCTTGANCAGAGAACCGGGGCAATTNCGCAGTATATTCCGACGGAGCGTTGCAGCTTT

CGTGATAGCATGTCCATACACGCTCAAATACAATGTCCACCATCACGTGGAGTGATCTCCAACGAGTAGG

CATCTACAAACCTCAAGGGTGTGACCGATCAAGATCTTGCTCCTCTGGCTCAACATCGTCGAGGTCACGC

GTCAGGGTGACGATATCTACTTCTCTGTCGGCATCGCTTCCGCCGACTTTGGCGTCGAGAATTTCCTTTG

AAGAAGCCCCCCCAGTTGCGTGCTTGNTGGCTTCGATTTGCAAACANACTTCCCCTTAGCGGTGATGTTT

CTTTCGATTTGACTTCCCCTTTTCTGTTATTCATTGGATATTCAGCTCTGTTGCACCTGTAGATCTTCTA

TCTATCTATCTTTNTACTTTTACTTTACGTTAATTCGCCGGTTACCGGGTATNTCTCCCCAAATGTTCCC

TGGGT



>gi|71811433|gb|DR988824.1|DR988824 HDI_C_69.esd Horsegram (VZM-1) drought stressed subtracted cDNA Macrotyloma uniflorum cDNA, mRNA sequence

AAAGCTGGAGCTCACGCGGTGGCGGCGCTCTAGAACTAGTGGATCCCCCGTGGCTGCAGGAATTCGGCAC

GAGGCCTCGTGCCGAATTCGGCACGAGGGTTAAACTATCTATTCTTACTCAAGAACATTCCAGAGCC



>gi|71811431|gb|DR988822.1|DR988822 HDI_C_59.esd Horsegram (VZM-1) drought stressed subtracted cDNA Macrotyloma uniflorum cDNA, mRNA sequence

CGACGAATGTATAAGTACACCAGCGCCTACAATGCCTACCAGTTGTACAATGAAAGCCACGCAGTGTATA

AGCCTCCATTACAAGTCCAAAGCCACCAGTGTACAAGCCCAAAGCCAAAGCCACNCATACAAGAAGCCAC

CTTATGGCAAGTACCCACCAGTTGAAGTAAACGATCAGTTCTGAAGCATGCAACGTTGCATGTCAACTAT

GTACTAAAATAAAGAGGTTCAGTTACTGCTATGTTGCTTTAGTAAATTTCATCTTTATTCATGCACTCAA

TTGTTCTTGGCACCTGCACTTCAATCTTTTGTACACTGTTATCTGTGGCCAAGAACAAGAGCCTCCTCTG

TGTGTCACTTGGNTGATATCCAGTCAAGGAGAACATGATG



>gi|71811429|gb|DR988820.1|DR988820 HDI_C_57.esd Horsegram (VZM-1) drought stressed subtracted cDNA Macrotyloma uniflorum cDNA, mRNA sequence

AGACTTATGGATCATGGGAGTTGCACCTGTGAAAGCTCAATTCGAAGGTGCTGAAATGGGTAGTTTCTGC

TGAGAAACTGATGGCTGCAAGTGTGGTCCAAACTGCACATGCAACCCCTGTACTTGCAAGTGAGGTTGGA

GAGCGAAAGCTTCCAAGCTGAGATGGACCTTAATTAGCACTTAGGTGGCCAGGAATAACTATAATAACAT

AACTATTATATGTAGTTAAAAAGCTTAAATTATGTATGTCTGTGTGTATGTCG



>gi|71811427|gb|DR988818.1|DR988818 HDI_C_55.esd Horsegram (VZM-1) drought stressed subtracted cDNA Macrotyloma uniflorum cDNA, mRNA sequence

ACNCCATTTCGACCCTGCTCTTGCGCCGCTGTCATGCATTGCTGAGCCATGGAGGCTCTTATCAGTGAAT

TCTTCCATGGTGCTGAAGAGGGTGAAGGATGAGTACGGTTTTGGAGCAATGGGGTGGTGTGTTTAGGTGG

GTTTAGGAATGTGAGGGCTGTGCATGATGGAAAGGGGTTGAAAATGGAAATTGGATGAGGACCAATATGA

TGGGAACCAGTAATTGGGAATGTGAATGCTGACCCAGAGACCGAATGGTCAGAGTTCTCAAATGGATCGT

TATCTATCTAGTCAATGCTGTTCGTCG



>gi|71811425|gb|DR988816.1|DR988816 HDI_C_45.esd Horsegram (VZM-1) drought stressed subtracted cDNA Macrotyloma uniflorum cDNA, mRNA sequence

AGCTGGAGCTCACGGCGGTGCGCGGCCGTTCTAGAACTAGTGGATCCCCCGGGCTGCAGGATTCGGCACG

AGGCTCCTCCCGCCGGCGTCAAGCCGGCGCCTTGGCTCTCTTTTCCGTCCTCGGAATCCCTCTCGCGATT

ACTTACAGCATACCCTTTGCTCTGGCATCAATATTCTCCACCACCTCNGGGGCAGGACAAGGGTTATCTC

TAGGAGTCCTCAATCTTGCAATTGTCATTACCCACCAGAATG



>gi|71811423|gb|DR988814.1|DR988814 HDI_C_43.esd Horsegram (VZM-1) drought stressed subtracted cDNA Macrotyloma uniflorum cDNA, mRNA sequence

AAAGCTGGAGCTCACGCGGTGGCGGCGCTTCTAGAACTAGTGGATCCCCCGGGCTGCAGGAATTCGGCAC

GAGGCCTCGTGCCGAATTCGGCACGAGGTTTTTTTTTCGCCTTCAAAATTAATTATACACCCAAATAATA

AAAATAATAATGACAACAAACAGAAAATAACGGAATTAAAAAAAATTAAGTTGCCTTCGTTCTTATATAT

ATTTTCACTTTTTTTTAATAAAAAAAACATGATCATGATCATCATCTGACATGTCATCATCCCAATCNCA

ACCACACCATCACCGCTGNNAGCTCCATCATACGAGAGAAGTCCT



>gi|71811421|gb|DR988812.1|DR988812 HDI_C_35.esd Horsegram (VZM-1) drought stressed subtracted cDNA Macrotyloma uniflorum cDNA, mRNA sequence

AAAACTGAGCTCACGCGGTGGCGGCGCTCTAGAAACTAGTGGATCCCCCGGGCTGCAGGAATTCGGCACG

AGGCCTCGTGCCGAATCGGCACGAGGCTATTCCAGAGCAGTCTGT



>gi|71811419|gb|DR988810.1|DR988810 HDI_C_33.esd Horsegram (VZM-1) drought stressed subtracted cDNA Macrotyloma uniflorum cDNA, mRNA sequence

CAGCTGAGCTCACCGCGGTGGCGGCGTCTCTAGAACTAGTTGGATCCCCCGGGCTGCAGGAATTCGGCAC

GAGGCCTCGTGCCGAATTCGATATCAAGCTTATCGATACCGTCGACCTCAGGGGGGGCCCGGTACCCAAT

TCGCCCTATAGTGAGTCGTATTACAATTCACTGGCCGTCTTTTACAAGAA



>gi|71811417|gb|DR988808.1|DR988808 HDI_C_31.esd Horsegram (VZM-1) drought stressed subtracted cDNA Macrotyloma uniflorum cDNA, mRNA sequence

TGAGCTCACGCGGTGGCGGCGCTCTAGAACTAGTGGATCCCCCGGGCTGCAGGAATTCGGCACGAGGCCT

CGTGCCGAATTCGGCACGAGGCTTCTCTATCCCTCTCCTGGTTTTCTACTCTGTATGTTGTTGTTTTGTC

GCACGCAGTCTCGCCTCGCGCATTCTCTTCTCCATTTTAAAACTTCAGCTGCACACAGGTCACATTCATC

TGAAAACGCCGTTTTCATTTCCGTTTTTTCCATTTCTCCATACTTGTTTCCGATTAAAACTCGTTGTTTT

CGGTGCTTGCAGCTTCCGTTACCTGACGGACATGGCGAGCGAGGGCGGGAAGAGCTTCGCCCGTCGGGAC

CGTCTGCGCGAGATCGAAGTGAAGG



>gi|71811415|gb|DR988806.1|DR988806 HDI_C_22.esd Horsegram (VZM-1) drought stressed subtracted cDNA Macrotyloma uniflorum cDNA, mRNA sequence

ACAGCTGGAGCTCACGCGGTGGCGGCGTCTCTAGAACTAGTGGATCCCCCGGGCTGCAGGAATTCGGCAC

GAGGCCTCGTGCCGAATTCGGCACGAGGATAACACCTCGTCATGACAAACCTGTGCTGTAGTTAACGTTT

AACACTTTTCTGCGTGTTTACTTGTTTAAATTTAG



>gi|71811413|gb|DR988804.1|DR988804 HDI_C_20.esd Horsegram (VZM-1) drought stressed subtracted cDNA Macrotyloma uniflorum cDNA, mRNA sequence

GGCACATTAAGGAAAGCCGGAAGTGCTTTGATAGTGAAGCGGGCGACNTCTGGCTCGTGCGCCATTCTGG

TGTGGAAAGTGCCAGCAATTCTGGTCTTTTGGCTCCTATTTGACCTTTTCCCAGTGTTCGNTGGGGCAGC

CGGGAAAAATTCTTGTGGCTTNTTCT



>gi|71811411|gb|DR988802.1|DR988802 HDI_C_12.esd Horsegram (VZM-1) drought stressed subtracted cDNA Macrotyloma uniflorum cDNA, mRNA sequence

AAGCTGGAGCTCACGCGGTGGCGGCGCTCTAGAACTAGTTGGATCCCCCGGGCTGCAGGATTCGGCACGA

GGAGATAATGTCCTCCCCATCATCGAAGACATCATGGCCGTCGATTGGGTTGCCGATGGCCTCGGCACTC

TCGTTCCGCCCCCGGGCTCTGCGCTTCGTCTCAGCTTCTTGAAATTGAAGGAGGGCGCGGCTGGGGACGA

GGTTTTGGGTGTGGTCAGAGGAATTCCCGATATTTTCAAGGAGATTAGTGAGTTCAGTGTCGGGGAGAAT

TTCTCGCCGGGTAGAGCCAAGGGTTTCTCGATTGCTTCGCTCGCGGTTTTCCCGGGACCGAGCGAGCTGG

AGGCCGTGGATTCGAATCAGGAGTTGGTGAATTACCAGAAGGATAAGGTTAGGGATCACATAGAGAGTGT

TGTCGTCGTTGATTACGTGGTGCCCCCACCTCCTCCTGCTCAGACTGCGAGCCTTTGATGAGGTTATTCA

TGTTCTCTGCTCTGTAACTGTCATTTATGTTTCTTTGCCAGAACAATGAATGAATAATGAGTGTTGATGT

GATGGTTATGATTCACAAAAAAAA



>gi|71811409|gb|DR988800.1|DR988800 HDI_C_10.esd Horsegram (VZM-1) drought stressed subtracted cDNA Macrotyloma uniflorum cDNA, mRNA sequence

AAAGCTGGAGCTCCACGGCGGTGGCGGCCGCTCTAGAACTAGTGGATCCCCCGGGCTGCAGGAATTCGGC

ACGAGGCCTCGCTGCCTGAATTCATGCAGCGAGGGCTTCACATTCGCTCTGCTACTTATCTGCTGGCTGA

TGAATTCTGAATCATT



>gi|71811407|gb|DR988798.1|DR988798 HDI_C_88.esd Horsegram (VZM-1) drought stressed subtracted cDNA Macrotyloma uniflorum cDNA, mRNA sequence

CCACGCGGTGNGCGGCGTTCTAGAACTAGTGGATCCCCCGGGCTGCAGGATCGGCACGAGGCTGAAGCCA

ACTTAAAGCAGGCTGGTTTCGACACATGGGAGAAGTTGATCCTCAAGGAGCCATCTAACAGTGCACCAAA

TGCTCTTGAGTACAAGAAGGCTGAGAGGGCTAAGCTGGTGCAGCAGGGATACAACATTGTTGCAGTGGTT

GGAGACCAATGGTCCGATCTGAGGGGAAAACCCAAGGGAACCAGGAACCTTAAGCTTCCCAACCCCATGT

ACTCTATTGAGTAGATCATCTCTGAAGGATCCCAAGCTTGCTCCTATCTACGAAATAAGATACCTTACGT

GTTCTAATGGGAGCATTAACTATGTATCTATGCATGTGACTTTCTGGTCTTCGGATCTTCAACAAATCAC

CTAAGAGACTACACTCCGTTGTTTTCTCTTTTGTGACTTTAATAAATTATG



>gi|71811405|gb|DR988796.1|DR988796 HDI_C_86.esd Horsegram (VZM-1) drought stressed subtracted cDNA Macrotyloma uniflorum cDNA, mRNA sequence

AAGCTGGAGCTCACGCGGTGCGCGGCCGTTCTAGAACTAGTGGATCCCCCGGGCTGCAGTTGATTCGGCA

CGAGGAAAACTGATGCCAAACGTANGAGTCGAGTGAAACACCCAGGAAGCACCGGTCACCTGTGTATGCC

TACCACTCACTGCGACCTGGTTGCCGGTGCATGTCTGATTGGAGGTCCAAC



>gi|71811403|gb|DR988794.1|DR988794 HDI_C_78.esd Horsegram (VZM-1) drought stressed subtracted cDNA Macrotyloma uniflorum cDNA, mRNA sequence

ATTCAGTTGGCGAATCTTGGCGGCCAATGGACACTTTGTTTGGCATTTGGTAAGTTTGAATTAGGTATAA

AGTTTANCTTGGGGGAAAAGGTAATTATTAGAAAGAGGGTTTCTTTCNAA



>gi|71811401|gb|DR988792.1|DR988792 HDI_C_75.esd Horsegram (VZM-1) drought stressed subtracted cDNA Macrotyloma uniflorum cDNA, mRNA sequence

AAAGCTGGAGCTCACGCGGTGGCGGCCGCTTCTAGAACTAGTGGATCCCCCGGGCTGCAGGATTCGGCAC

GAGGGGCGACTGGAACGACCAAAGTGAGTGCCGCAGTTAAAGTCGCAGTTCCCCAGGCCCCACGTCTCAG

CATCAAGGCTTCCCTCAAAGACCTCGGAGCCGCCGTTGTGGCCACCGCCGCCACCGCCGTTCTTGCGAGC

AATGCGATGGCCCTTGAAATCTTGCTGGGTGGCGATGACGGGTCTCTGGCCTTCGTTCCGAACAATTTCT

CCGTGGCGGCCGGTGAAAAGATCGTGTTCAAGAACAACGCAGGGTTCCCCCACAACGTTGTTTTCGACGA

GGACGAGGTTCCAAGTGGGGTGGATGCAGGGAAAATCTCTATGCCGGAGGAAGACTTGCTGAACGCACCT

GGTGAGACTTACAGCGTGACTTTGGATACGAAGGGAACATACAGTTTCTACTGCTCGCCTCACCAAGGAG

CTGGTATGGTCGGAAAAGTTACTGTTAACTAAATCTATCTCTCAACAACAAAATTCTCTTGTGGTTCGTT

CTTGTGATTTNGTTAATCGTAGAAAGAGCTTATTCACATCGTTCTTGTTATCATGGAACTATATATATTA

TGTATGGGATGAATGAACCG



>gi|71811399|gb|DR988790.1|DR988790 HDI_C_73.esd Horsegram (VZM-1) drought stressed subtracted cDNA Macrotyloma uniflorum cDNA, mRNA sequence

AGCTGGAGCTCACGCGGTGCGCGGCGNTCTAGAACTAGTGGATCCCCCGGGCTGCAGGATTCGGCACGAG

GCCTCGTGCCGAATTCGGCACGAGGTGTAATAGAAGAGAATCTATTCTCTTTCTTTGTCGTTTTTTAATT

ATCTTGGAGATTTCATAATGCTTACTCTAAAACTATTTGTTTATACGGTAGTGATCTTTCCGTTTCTCTT

TTCATCTTCGGATTCCTATCTAACGATCCAGGACGTAATCCAGGACGTGAAGAATAAGAAAAAAGTGGAT

TCTGTGTTTTTCTTGCTTGTGCGGAATTTTTAAATATTTTTAGGATTTTATCTATTTGAACATCTTTAAC

TAGTAAATAAAAAAACAAGTTAAAANAAAATATCAAATTATCAACGGAAAGAAGAGGGATTCTGAACCCT

CGGTACAAGAATTTAGTACAACGGATTACGCAATCCGACAGCTTTAGTCCACTCAGCCATCTCTCACCCA

ATTGAAAAAAAACAAAAAAAAA



>gi|71811397|gb|DR988788.1|DR988788 HDI_C_64.esd Horsegram (VZM-1) drought stressed subtracted cDNA Macrotyloma uniflorum cDNA, mRNA sequence

ACAGCTGGAGCTCACCGCGGTCGCGGCGTTCATAGAACTAGTGGATCCCCCGGGCTGCAGTAATTCGGCA

CGAGGCCATCGTNTGCCGGAATTCGGCACGAGGGATCGGCGCGTAAAATTGACAACGTATGGAGGAGCGT

GTCCATCCTGGAAGTAACTCTGCCATAAGACCCATCGGTGCCCAGAATGTTTGACAANACCTCTTCCCAA

ACACTCCACACTGTACTACGATGGGATGTACAGTGGAAGAGACCAGGGAGCAGGCACCGACTGAAGATGT

NTACAGAGGGCGACGTGGGTATGCATGTAGA



>gi|71811395|gb|DR988786.1|DR988786 HDI_C_61.esd Horsegram (VZM-1) drought stressed subtracted cDNA Macrotyloma uniflorum cDNA, mRNA sequence

ACAACGCTGGAGCTCACCGGACGGTCCCGGCCGTTCATAGAAACTAGTGGATCCCCCGGCGCTGCAGGNA

ATTCGGCACGAGGCCTCGTGCCGACGATTCGGCACGAGGAGANCTTGACCCTGAGAAAAGGTTGTACCCA

GGAGGGAAATTCTTCGATCCTCTGGGTTTTGAGCCAACGACCCTGAAGAAAAAGCCAGACTTCAAACGTT

GCAGAGATCAAGCATTCCCGCTTGGACCATGGATGGTCTNCCATCATCTTTAGCCATATCAAGCTGCTGT

AACAGGAAAAGGCCCTATCA



>gi|71811393|gb|DR988784.1|DR988784 HDI_C_54.esd Horsegram (VZM-1) drought stressed subtracted cDNA Macrotyloma uniflorum cDNA, mRNA sequence

AAGGGCTATGCTGATGTAAGTTTTTGGTCTCGAAGGCAAAGACGTGCTACATAGGCTAATAGAAATGTGG

GAATGCTATNAGATTGTGAGATGTTGAATGGAGTTGCATGCCATAGCACAAATGACGNATACGTGCGGAT

AGCGTAGATATAATCGACACCATGCATAGCCACTATAATGTTGCGTAGCTAANTCTCGCATGACGCAATT

TTCCTTTTACACTTTCTCTGTGGTGTATCTAGTATAAAAACGTATTTGTACTTCCCAATTTA



>gi|71811391|gb|DR988782.1|DR988782 HDI_C_52.esd Horsegram (VZM-1) drought stressed subtracted cDNA Macrotyloma uniflorum cDNA, mRNA sequence

CAAAGCTGGAGCTCCACGCGGTGCGCGGCCGTTCTAGAAACTAGTTGGATCCCCCGGGCTGCAGGAATTC

GGCACGAGGCCTCGTGCCGAATTCGGCACGAGGAAAAGTAGGAGTTGCCAATGTCATGAGCCACATATCT

ACTGGTGGTGGTGCCAGTTTGGAGTTATTGGAAGGCAAAGAGCTTCCGGGAGTCCTTGCTCTTGATGAGG

CCACACCGGTCCCTGTGTAAGACAATTTTCAACTTGGAAAATTTTCCCATCCTCAGTTGTTAGTGTCATT

AATCTTGTAAAAGAGCATATGTTGTAGGACCCTTCGGAGTCTGTCACTGTAAATTTAATCATGCTTGAGT

TATTGGCTTCACACATAAATTGCGTGTACCTGTAAACCCTGGCTGAGAGCCATAAATTCAAATAAATCCT

TGAGTTGCTGCCTGAAAAAAAANAAAACAAAAAAAAC



>gi|71811389|gb|DR988780.1|DR988780 HDI_C_50.esd Horsegram (VZM-1) drought stressed subtracted cDNA Macrotyloma uniflorum cDNA, mRNA sequence

AAGTAACAAGCACACAAGGAGATAATTCTTCAAAGTGTTATTTTCTTATTAACCCCGCACGTTTTTGTGA

AACCCACAGGTACTTGGCAATGATCTTCTGATCATATGTTCAAAATATGGAATGTTGATGGTTTTACACT

AGCAAAAAATCTTATAGGTCATCAACGCTGGGTGTGGGACTGTGTCTTCTCTGTGGATGGTGCCCTATCT

TATTACAGTTCCTCTGATACAATGCTAGACTCTGGTCTATGTCAACTGGCGAAAGATATTAAAGTTTACA

AGGACATCATAAAGCTACAATTTGTTGTGCCTACACGATGGGGCCGAACCGCAACATCTGAGATAATGGT

TCATAAAGTTATTTTTCTTCCATTAGATACAACNTTGTTTTTGTATATAATAATCTGTCTCTTGGTTGTG

AAGTTACTATAGTTCCTGAACTGTAATCCTCGGTANAGGGTGAAA



>gi|71811387|gb|DR988778.1|DR988778 HDI_C_41.esd Horsegram (VZM-1) drought stressed subtracted cDNA Macrotyloma uniflorum cDNA, mRNA sequence

AAAGCTGGAGCTCACGCGGTGCGCGGCCGCTCTAGAACTAGTGGATCCCCCGGGCTGCAGGAATTCGGCA

CGAGGGTTGAAAGCTGTACAATTAGCTGTTTTANAGTACATGTTTTACACACTTATCCGAATGTTTAATT

TGTTTGAGCGTTACCACGGCTACGTGTGGGTATAACATGTTAAAGATAGTGTTCAATAATTNTAGCTCGG

ATAGTTTGGTC



>gi|71811385|gb|DR988776.1|DR988776 HDI_C_4.esd Horsegram (VZM-1) drought stressed subtracted cDNA Macrotyloma uniflorum cDNA, mRNA sequence

AAAAGCTGGAGCTCACGCGGTGGCGGCGCTCTAGAACTAGTGGATCCCCCGGGCTGCAGGATTCGGCACG

AGGGTGAATCATCTTGTGTATCACAAAGGAAAAAAGCAAGTTCTTTGCAACAATGGCCGCTGCATCCTCC

ATGGCTCTCTCTTCCCCCTCCCTGGCTGGCAAGGCCGTCAAGCTTGCCCCATCAGCCCCCGAAGTCGGGA

GGGTCAGCATGAGGAAAACCGTCACCAAGCAGGTCTCCTCCGGAAGCCCATGGTACGGCCCAGACCGTGT

CAAGTACTTGGGCCCATTCTCCGGCGAGCCCCCGTCCTACCTCACTGGCGAGTTCCCAGGTGACTACGGT

TGGGACACTGCTGGGCTTTCCGCTGACCCAGAAACCTTCGCCAGGAACCGTGAGTTGGAAGTCATCCACT

CCAGTGTGGGCCATGCTCGGAGCCTTGTGGCTGCGTCTTCCCCGAGCTCTTGTCCCGCAACCGGCGTCAA

ATTCGGCGAGGCCGTGTGGTTCAAGGCTGGCTCTCAGATCTTCAGCGAGGGTGGGCTCGACTACTTGGGC

AACCCAAGCCTGATCCACGGCTCAGGAGCTCCTCGCCATCTTGGGCTAACCCNAGTTATCCTTCATGGGG

CGCCGGTTGACAGGTTTCCGGTATTGCCGCTGGGCCCTCTCGGTGAAGGGTCAACTTGAACCCCGATTTT

ACCCAGGTTGGCGTTCGACCCATTTTGGGTGGCCTTCGCTTGACGAACCCCCAAAGGGT



>gi|71811383|gb|DR988774.1|DR988774 HDI_C_30.esd Horsegram (VZM-1) drought stressed subtracted cDNA Macrotyloma uniflorum cDNA, mRNA sequence

GCGAATTCGGCACGAGGGTCTCTATGAGGGTCTGGATTGGCTTTCTAACAACATTGCCAGCAAGGCATGA

GACATTTGAAAAATTTTGGTCTTGTGTGGGAATTTCATGCGAGTCTGGCGGTTCTTGGAGAAAGATGCTT

ATCCTTTCTAGCTAAGGTTGTAATAGCTGAATGCTTATTAGAAGTATATTCTCTTCTGTGTTAACTTGGT

TTGTATGATTGCTTAAATTAGCTAATACTTTTAGCTATAATTGGACCTCTTGCAAAAAAAAAAAAAAAAA

AAAAAAAAAAAAAAAAAAA



>gi|71811381|gb|DR988772.1|DR988772 HDI_C_29.esd Horsegram (VZM-1) drought stressed subtracted cDNA Macrotyloma uniflorum cDNA, mRNA sequence

AAGCTGGAGCTCACGCGGTGGCGGCCGCTCTAGAACTAGTGGATCCCCCGGGCTGCAGGAATTCGGCACG

AGGATTTAGAGTAAGGAAATCAAAACACTAAAGAGAAACGGTAATTACTGAGTACAATTACAAATTACAG

TCTTTCTCTTTTCATTCTTTTTTTTTTCTCTTTTTTTCCTTCCCTTCTCACTTTATTTATCATCCTTCTG

CCTCGCGTGGCTGAACTCGGTCACTAACTCACTTCACTCCAACAAGTTCAATCAAACCATTTTTAACCGA

ATTTACCATCATCACGAATCATCATCCTATCCAAGGAAACACCAATTAACTCTCACAGTGTGTCTCACCA

GTTGCAGCGTGGTTTTAAACCTCGGGGGACACCCAGCGAAGTTTGAAACCCACTTAGGAACCTCGGGCCG

AAGGGAAAGCTTTCCACGCGGAAGGNTGGCCGAAATTTAAAAGAGCCCTANNGTGTGTGTCCCCCAGACC

CCAAGACATGTGAGAGGCAGACCGGTTATATCTCTCTCCTCTCCAATC



>gi|71811379|gb|DR988770.1|DR988770 HDI_C_26.esd Horsegram (VZM-1) drought stressed subtracted cDNA Macrotyloma uniflorum cDNA, mRNA sequence

CCACGGCTGGNGACGCCTCCACCCGGGCGGAGTGCGCGGCCGCTTCTACTACACCTACGTAGGACTCCCC

CCGAGAGCTAGCACGTGACCTTCGGCACCGACGAGCCTCGTAGCCGACACTTCGAGCCCGACGAGAGCAC

ACGTCTAAGGTAGCCACGCACGCCGCGGTACACTTCCACGCTCCACACTTACGCGGTTACTACTATTTAC

ACGTATGTTGCACGTTTACACACACACACCTCCGACGAGAGAGAGAGAGCCCAGAGTAACCCCACACTAT

ACAGCCCCTAACTAACAGTAAGACAGTACAGTAACTATAAACCCACACCTTATTACACACCTAAAG



>gi|71811377|gb|DR988768.1|DR988768 HDI_C_18.esd Horsegram (VZM-1) drought stressed subtracted cDNA Macrotyloma uniflorum cDNA, mRNA sequence

ATTCATTTGCATGTATTTTGGAAGAACATATTGAGCTTCTGGAGGTTCAGAAATCTTATAAAGCCACGGG

TATATTTCCATTGGGCTTAATACTTTCTGGTTGGGTCNTATNCCCCCTCTTGGGGCGGTTATGGTGGTCC

ATACCCTTTTTTTTTNGGAACCACCACTTTGGGTTTTGGNTTGAAT



>gi|71811375|gb|DR988766.1|DR988766 HDI_C_16.esd Horsegram (VZM-1) drought stressed subtracted cDNA Macrotyloma uniflorum cDNA, mRNA sequence

CGCAAAAGCTGGAGCTCCACGGCGGTGCGCGGCCGTTCTCTAGAACTAGTTGGATCCCCCGGGCTGCAGG

ATTCGGCACGAGGGAGACAGGGCTTAAGCTGGCTTAGACAGATAAACCCGCTGGTTCGGTTCCTCTCAAG

CTAAGATGCCTTCAATTCATACCCGGGTTGACTTTCAGCTCTGGTCTCATCAGTATTATAGACCGAAGGT

TGTTGTCTTAGAAAGGATGCAACTAAGCCGCTTGATAAAGTAGATAGAGAGAAGGGAGTGTTGATCCGGC

AGTTTCAACACAAGTTCCTCTGTTATGGCTCGCTTGAGCCCAACTGGTTCGCATGGACACGCAACTGGAA

ATAGGATATGCTTCTTAACCGGTCTTGTTCAAAGTGAAACTATTTCAGATCTTTTCAACTCAGGTTTATT

TGGATTCGTGTCAGATTCTTTGGTTACAGTTTTCCTGCTTGCTAATTCAAGAAAAACCAAGAGTTGTAAA

TAAGGTGGTCACAAGAAGGTCAGAACTGCCGGGTTCCCTGTAGGCAAAGGGACGTAACCCCACTCTAAAA

GTTGTCGGTTCCCCTCGGGGAGGGTTGAAAAAGCTCTTCACAATTAGGAAACAAAAAGAACGAATTGTCA

AGGTTGTCGGCAATATCTGCACCCCGGGCTCAGCTCAAGGTGCAAATCCAAGCAAGTTGAGAAGAGCCAG

GCGAACAAGCCAAGGTGCGAAGCCACTTTA



>gi|71811373|gb|DR988764.1|DR988764 HDI_C_14.esd Horsegram (VZM-1) drought stressed subtracted cDNA Macrotyloma uniflorum cDNA, mRNA sequence

GCTGGAGCTCACGCGGTGGCGGCGCTCTAGAACTAGTGGATCCCCCGGGCTGCAGGATTCGGCACGAGGG

AATTCAAAAACTCAAGTCATTTCTTCTGCTTCGCTTTCATTCCCAGCCAAACATGGCAGACAAATGCGGC

AACTGTGACTGCAGTGACAAGAGTCAGTGCACGAAGAGAAACATCTATGGTGTTGACATTGTAGAGACTG

AGAAGAGCTACATGGAGACTGTTGTCATGGATGGTGCAGCTGCTGAGCATGATGGGAAGTGCAACTGCGG

CACTAGCTGCAGTTGTGTGGACTGCAAGTGTGGCCATTAAGGAATGAATGAATGAAAATGTGATTGCCAT

GTGTTTGGCAATACAAACGTTCAAATAATGTAACCTTCATATGAATAAATGTGTGCTTTTATCCTAAAAA

AAAAAAAAAAAAAA



>gi|71811371|gb|DR988762.1|DR988762 HDI_C_1.esd Horsegram (VZM-1) drought stressed subtracted cDNA Macrotyloma uniflorum cDNA, mRNA sequence

AAGCTGGAGCTCACGCGGTGCCGGCCGTTCTAGAACTAGTGGATCCCCCGGGCTGCAGTGATTCGGCACG

AGGGCCACCATCACCAGAGGACTTAGGGAAATGGCGCGGGAGATGGCACGTAAAAGGAACAGCGGCTGAT

GTTCCATGTGGTCGATTTGGTTGCAGTGCAGTTGTGAGACAGAATCACTGCTTATGTAGTAAATATGTTT

ATTGTTGTAATAATGAATAAGGACTTATAAACTACTGTAGAAATGTTTTTTATTTTTCAAAAGCTTCAAC

AGTTTGGTGCCATGGACTACCTACAACTTCTCTGTCTTACCTTCAATCTAAAAGAAAAATAAATTATATT

AGAGATCCTGTTATAGCTGTTTTTCATAAAAAAAAAAAAAAAAAACNCAAGGGGGGGCCCCGGAACCCA



>gi|71811369|gb|DR988760.1|DR988760 HDI_B_95.esd Horsegram (VZM-1) drought stressed subtracted cDNA Macrotyloma uniflorum cDNA, mRNA sequence

GAGGATATTGAAGAGGATTAAGGCTAGGCTGCCATTNACGGAAATGTAGGACAACCGACCAGTGGAGGCT

AAACAGGTTTCCTTTGAGGAAACGAAATGCTTTTTCTGGACAAATTACCCTTTTTCCCTTTTTAATTTTC

CTGTTATCTTTATATTTCTCTTATGATAGTTGGATCCTCTGGACTAGAATTATTTAAGGGGGGGACTCTG

ATAGTTTAGTCTCAAAAATCGGTCACCTCATACCGGGTTACAGTGGAATGGGTGATGTTAGTATTCCATG

TTGTAGAAATTGGTTAACCCGGAAAATTTAAACTTTCATTTATTTTAAATTTTTAANTGAANAAAATTGG

TTAATTTTAAATTT



>gi|71811367|gb|DR988758.1|DR988758 HDI_B_93.esd Horsegram (VZM-1) drought stressed subtracted cDNA Macrotyloma uniflorum cDNA, mRNA sequence

AAGCTGGAGCTCACGCGGTGGCGGCCGCTCTAGAACTAGTGGATCCCCCGGGCTGCAGGAATTCGGCACG

AGGATTGAGCTCGTCGAGCCGGAGATTCTCCGCTTCAAGGCCTTCGAGCCTATCCTCCTCCTCGGTCGTC

ACCGCTTCGCTGGCGTCGACATGCGCATCCGTGTCAAGGGCGGTGGTCACACCTCCCAGATCTACGCCAT

CCGTCAGAGCATCGCCAAGGCTCTCGTTGCTTACTACCAGAAGTACGTCGACGAGCAGTCCAAGAAAGAG

ATCAAAGACATCCTCGTCAGGTATGACCGCACACTCCTTGTCGCTGATCCCCGTCGCTGCGAGCCCAAGA

AGTTCGGTGGTCGCGGTGCTCGCGCCAGGTTCCAGAAATCCTACCGTTGATTTCAACCCCTTTCCCTTTC

TCTTTTTATGTTATCGAAATGTCTTGATAGTACTCTCTGTCTTATTTGTGTTGCGCTGAGTTTTGAGTTT

TTTAATCAGATTTTGAATACTCTTATTATATAATGCTTTAGTTGAGTTTAAATTCTGTTGTTAATT



>gi|71811365|gb|DR988756.1|DR988756 HDI_B_91.esd Horsegram (VZM-1) drought stressed subtracted cDNA Macrotyloma uniflorum cDNA, mRNA sequence

AAAGCTGGAGCTCACGCGGTGGCGGCCGCTCTAGAACTAGTGGATCCCCCGGGCTGCAGGAATTCGGCAC

GAGGGTTTATGTCAGCACCAANGGATGATAATGTTGAAGCCCCAGAAAAATTGGTGGGAGATGATAATGC

AAGACTCTATCGACCATTTAAGTTTACCAGAAAACTTTTGGGGGGAATTATAGAAATT



>gi|71811363|gb|DR988754.1|DR988754 HDI_B_84.esd Horsegram (VZM-1) drought stressed subtracted cDNA Macrotyloma uniflorum cDNA, mRNA sequence

AAGCTGGAGCTCACGCGGTGCGCGGCGTTCTAGAACTAGTGGATCCCCCGGGCTGCAGGAATTCGGCACG

AGGCCTCAAGCCACATCCATTTCTCCCTCCACTGAAGCCACTTCCACCTTTTCCTAAAATTCCTCCAAAG

CACTTTTTCCATCACCCCAAATGGCCTCCAATTCCACCGTCTTCTTCTCATCCTTAGGCTACATTGCAGA

GCTAGATTACTCTTGGGTCTACAAAATATGCGTGCATGTTTTCTGTCTTTATATGTGCTTTCTGTATGAT

CAAGGATTGAGGAAGTCTATTATGCTAATAAAGCCTTTGAAGGAGGATAGGGTAGGTGATGTGAAGAAAA

ACATCAGATGAGAATAGAGAAAACAAAAACAGAGCGATATGATTAAGAAGTTTGAAATTATTGGGAGTTT

GAGTGTGATAAG



>gi|71811361|gb|DR988752.1|DR988752 HDI_B_82.esd Horsegram (VZM-1) drought stressed subtracted cDNA Macrotyloma uniflorum cDNA, mRNA sequence

AAAGCTGGAGCTCACCGCGGTGGCGGCCGCTCTAGAAACTAGTGGATCCCCCGGGCTGCAGGATTCGGCA

CGAGGGTGAAGCCCAATATTCGATTACCTGGGGTGTCACGCTGCAACTTCTGGACAGTTTGCGTTTGAGG

CCACTAGGTTGGTTCTGATTCAGATTATTGCTTAATTCCAAGGGAATTTCGTTGAACCCTATTACCTCGC

TTTACTATATTGCTCCCTGCTGTTTGGTAGTTCTTGACCGTGCCCTGGATTGTCATGGAGTACCCTTCGC

TGAGGGACAACTCCAGCTTCCATTTGGATTTCGCGATTTTCGGGACCAATTCCGCATGCGCTTTTGCGTT

GAACCTTGCTGTTTTCTTGCTGGTGGGAAAGACCTCAGCATTGACTATGAATGTTGCTGGGGTGGTCAAC

GGATTGGCTCTTGATTGCCTTCTCTTGGTCTGTGATAAAGGACACTGTGACCCCAATTAACTTGATTGGT

TACGGGTTGGCGTAATTTGGGGGTTGCGTACTATAATCACTGCAAGTTGCAGGCCCTGAAGGCTTCGGAG

GCGCAGAAGAAGGCCCAGCAGGCTGATGAAGAGGCTGGGAGGCTGTTGGAACAGAAGGATGGGGAAGCAC

TGGAAGGAAGAACGACAACCAGAAACTGATTCCACTCATGGTCTCATTTTGGTCACTCCTCTATAATTGT

TGGCACGAGGAGGT



>gi|71811359|gb|DR988750.1|DR988750 HDI_B_80.esd Horsegram (VZM-1) drought stressed subtracted cDNA Macrotyloma uniflorum cDNA, mRNA sequence

AAAGCTGGAGCTCACGCGGTTGGCGGCGCTTCTAGAACTAGTGGATCCCCCGGGCTGCAGGATTCGGCAC

GAGGCCTCGTGCCGAATTCGGCACGAGGCTCATCTTTCTCCGCCGCAGACTCCACCATGGAAGACCCCTC

CAATCACCGCCGCTCCAAGAAGCTACCTA



>gi|71811357|gb|DR988748.1|DR988748 HDI_B_79.esd Horsegram (VZM-1) drought stressed subtracted cDNA Macrotyloma uniflorum cDNA, mRNA sequence

AAGCTGGAGCTCACGCGGTGGCGGCGCTCTAGAACTAGTGGATCCCCCGGGCTGCAGGAATTCGGCACGA

GGAAGAAACTGGATGTTCTTTCCAATGAGGTTTTCATCAAGGCTCTCGTCAGCAGTGGCCGAACTTGCAT

CTTGGTTTCCGAAGAAGACGAAGATGCGATAATCGTGGAGCCTTCTACGCGTGGAAAGTACTGTGTTGTT

TTCGACCCATTGG



>gi|71811355|gb|DR988746.1|DR988746 HDI_B_71.esd Horsegram (VZM-1) drought stressed subtracted cDNA Macrotyloma uniflorum cDNA, mRNA sequence

AAGCTGGAGCTCACGCGGTGGCGGCGTTCTAGAACTAGTGGATCCCCCGGGCTGCAGGATTCGGCACGAG

GTAATAATATTGCTTCCTCACAGAAGAATTCCATCTGCAGGCTGCAAGAATTTCCAATAGCGCGCAGGCA

TTCTCTTCAAAGGTTTCTTGAGAAGCGGCGAGATAGGCTGGGTAGCAAAGCTCCTTATCCTTCCTCATCA

ACTACAACAGTGGCTGATAACATAGAGAACAACTTCTGTGCTGATAATGCACCGGAACTGATTTCCTTGA

ATCTAGCAGAGGAGGAATTTCAGCCAACTGTCTCTGCCTCTTGAGCATAACTAGTGTTTGTTCTATGTTA

TGTGGTGTGAGTGTGGAAGACCATGAGTTTGCATGTAGAACCACAGAAACTAATATAATTGTATTGTTAG

GTTCTCTATAAATGTGTTTTATATGTAACATAGCCTGAAATGGTCTGTAACAGTGTGACTTGAAAGTTGC

TTATATCTCTGCACGTAGGAAATATGCTGTGTGTTCTGATCACCCATATTAAACTGTTTTTCCTATATTT

ATGTGGACA



>gi|71811353|gb|DR988744.1|DR988744 HDI_B_7.esd Horsegram (VZM-1) drought stressed subtracted cDNA Macrotyloma uniflorum cDNA, mRNA sequence

AGCTGGAGCTCACGCGGTGGCGGCGCTCTAGAACTAGTGGATCCCCCGGGCTGCAGGAATTCGGCACGAG

GCCTCGTGCCGAATTCGGCACGAGGGTGATGTTGGTGAATGACGCTGATTTGCAAGAATGTCTAGAAATA

TTGGATGATATTGGCAAGTGTAGCGTCAGGTTTCTTGTTCGTGATTTGCCTTCTGTTTTAAGCAGTTCTG

GGAGTAGCAATAGCTACTTGGGAGGAAGCTTATAGCATATTGGATGTGGTGGGCGACTTCAATGTGAAAA

TTCAAGTTTCGCGTTGACAAGATGTGGTTTTTCAGAATCTTCCAAAGATTCGGCATGTGGAGGAGTTTGA

ATCTTAGCCACAGCCACCTATAACAAGTGCAAAGCTTCATTTCTGAAAATAGCTTTTTGGAAGAGAGAAA

GACGCGGTTCAATGGAAGTACTGCCTGTTAAATACATAGTTCTGTATATATTGAACTCGTAGTAGCAGAA

TAGAAATTTGTAGTGGTAGTAATAGTAGTTTAGGGTATAAACAGTTCTGTGATGCTGTGATAGCTCTTTG

TAATTGTATAGATAGATAGTATCTAGATAGATTGGTTGATTTCCCATGAGATAATATAACT



>gi|71811351|gb|DR988742.1|DR988742 HDI_B_67.esd Horsegram (VZM-1) drought stressed subtracted cDNA Macrotyloma uniflorum cDNA, mRNA sequence

AAAGGCTGGAGCTCACCGCGGTGCGCGGCCGCTCTAGAAACTAGTGGATCCCCCGGGCTGCAGGAATTCG

GCACGAGGCCTCGTGCCGAATTCGGCACGAGGGTCAAATGCAAACCGTAATAAAGAGACAGGAAAGGCAA

AAGAGTCAAAACCAGAGCCACCTAAAGAAAAAGAAGAGTCAAAATTTCAAGCTTTTACTGGGAAGAAGTA

TTCTTTACGGGGTTAATTCATATCAATTAATTGAATGTCCTTTTACTTTTAAAGTAAGTTACTTTGTTGA

TCTCTGCATGGATCCATGTCTATTGCAAAAACTTGTATTTTTTACTGTATTATAAGCAGTAGATAGACCA

GTAGACCCCACAGAAAGTACTTAGATAATCTTGATTACTTGTGAATGAAGCTTATTTCTATCGCAAAAAA

AAAAAAAAAAAAACNCAAGGGGGGGCCCGG



>gi|71811349|gb|DR988740.1|DR988740 HDI_B_59.esd Horsegram (VZM-1) drought stressed subtracted cDNA Macrotyloma uniflorum cDNA, mRNA sequence

AGCTGGGAGCTCACGCGGATGCCGGCCGTTCANAGAAACTAGTGGATCCCCCGGCGCTGCAGGATTCGGC

ACGAGGATGGCCAGCACCTTCTCGAAAGACGAGCTTCAACTTCTTTGCGAATATAGTTAAAGAGATTCTT

ATTCGAGGAGTCAAATGTGCAGCCTGTCAATAGTCCAGTGACGGATTATGTGGTGATATTCATGGTCAGT

TTCATGATCTGATGACACACCATCTATTCCCAAGACTTGGTG



>gi|71811347|gb|DR988738.1|DR988738 HDI_B_57.esd Horsegram (VZM-1) drought stressed subtracted cDNA Macrotyloma uniflorum cDNA, mRNA sequence

GGTCGAACTGAGGAAGTGAATATCAAAAGCAGCAAAGACATTAGGCGAGGATCCTNCTTAACAGGAGACA

AACTTACNCATTAATGATGAGCACGGGGAAACTGATGTACACTCTGGGACAATTATTGTAAAAATT



>gi|71811345|gb|DR988736.1|DR988736 HDI_B_48.esd Horsegram (VZM-1) drought stressed subtracted cDNA Macrotyloma uniflorum cDNA, mRNA sequence

CAAGCTGGAGCTCACCGCGGTTGGCGGCCGCTCTAGAACTAGTTGGATCCCCCGGGCTGCAGGAATTCGA

TATCAAGCTTATCGATACCGTCGACCTCGAGGGGGGGCCCGGTACCCAATTCGCCCTATAGTGAGTCGTA

TTACAATTCACTGGCCGTCGTTTTACAACGAA



>gi|71811343|gb|DR988734.1|DR988734 HDI_B_46.esd Horsegram (VZM-1) drought stressed subtracted cDNA Macrotyloma uniflorum cDNA, mRNA sequence

AAGCTGGAGCTCACGCGGTGCGCGGCGNTTCTAGAACTAGTTGGATCCCCCGGGCTGCAGGAATCGGCAC

GAGGGCTTAGACTCCTTAAAGCGTTCCTTTGTTGTCATATGTATTTAAATATGAAATTCAAACAATCTGT

TTTGTATCAATTCATAAATATATTTTGTAATCAAATCGATATTCTGTGAACTGATGCTTTGATGGTAAGG

AACCTGGAAAAGTCACTTTTTC



>gi|71811341|gb|DR988732.1|DR988732 HDI_B_36.esd Horsegram (VZM-1) drought stressed subtracted cDNA Macrotyloma uniflorum cDNA, mRNA sequence

AAGCTGGAGCTCACGCGGTGGCGGCGCTCTAGAAACTAGTTGGATCCCCCGGGCTGCAGGAATTCGGCAC

GAGGCCTCGTGCCGAATTCGGCACGAGGGATCACTGAGCTTTACATCCCTAGGAAGTGCTCTGCTACAAA

CCGATTGATTACTGCGAAAGACCATGCTTCGGTTCAGATTAACATTGGTCATGTTGATGAGAATGGTATC

TACAATGGCCAATTCACCACCTTTGCCCTCTGTGGCTTCATCCGTGCTCAGGGTGATGCTGACAGTGCCC

TAGATCGCTCGTGGCAGAAGAAGAAAGCCGAAGTTAAGCAACAGTAGAATCCCATGATAGTTTCGTTACT

TTAATTTCTACTCTTGTTAGAGATATTTGAGGATTATGATTGTCGTCTTGAGCAAATACTTGTTCTGAAT

TTTCTTTGGTACCTCCAGCTAAAATTGTTTTTTGACAGTGTGACATTTCGTTGTTAAGTATTTGGTTCTC

TTATATTTCAATTCTTAAAAAAAAAACAAAAAAAAAAAAAAA



>gi|71811339|gb|DR988730.1|DR988730 HDI_B_33.esd Horsegram (VZM-1) drought stressed subtracted cDNA Macrotyloma uniflorum cDNA, mRNA sequence

AGCTGAGCTCACCGCGGTGGCGGCCGCTCTAGAACTAGTGGATCCCCCGGGCTGCAGGAATTCGGCACGA

GGCTTTTTTTTTAGTACGGTAGGTTTTTAAGTAACAGATAAAACAGACAGTGTAAAAAAAAGGAAAGAAC

CCTGGTCTAAAAAAGGGGGAACCCT



>gi|71811337|gb|DR988728.1|DR988728 HDI_B_24.esd Horsegram (VZM-1) drought stressed subtracted cDNA Macrotyloma uniflorum cDNA, mRNA sequence

CTGGTAAGTTAAGAGGGTAGAAAGAGTTTGAAACCCGTGAAATTCTCGTTTGCTTTTATCGATCCATCGA

ATCAGTGTTTCGAAGACCCAGGATTCTATTTTANTCATACGATTCCCAATAAATACATTCACGTTTATTA

TTTNTCCTTTATCCAGGGGAATTAGATTTGGGTTATCAACTTTGGGATNNANTATTAGAATGGTG



>gi|71811335|gb|DR988726.1|DR988726 HDI_B_20.esd Horsegram (VZM-1) drought stressed subtracted cDNA Macrotyloma uniflorum cDNA, mRNA sequence

AAAAGCTTGGTAGCTCACGTCGGTGCGCGGCCGTTCTAGAACTAGTGGATCCCCCGCNGCTGCAGTNAAT

TCGGCACGAGGCCTCGTGCCGTGCAACTCTTGCTTCTTGTCAAACACCCTTTTTCATGGCAGCAAAGACT

TTGAGGATGATGTATTTGTGATGCTTCACAAAACCTCCACCAGGCCTTGAACCTAAAAAGGGAAGTAGAA

AGCTATTCCACACAAGTTTCCCAAACTCGACTGACTGTGCTGAATCGAGTTG



>gi|71811333|gb|DR988724.1|DR988724 HDI_B_12.esd Horsegram (VZM-1) drought stressed subtracted cDNA Macrotyloma uniflorum cDNA, mRNA sequence

AAAGCTGGAGCTCACCGCGGTGCGCGGCGTNTCTAGAACTAGTGGATCCCCCGGGCTGCAGGAATTCGGC

ACGAGGCCTCGTGCCGAATTCGGCACGAGGAACTGCGCTACTATCAAGTTTTGAAGTGGAATCAGAGACT

GAGGGCACCGGAAACTACTCACTATAGTATGAGTATATCTCGTTAATAGTATTATGAATAAGACAGAAGG

TGATGAGGGCTCCACTTATATGCACCTCTTCTCTTCTCTTTGTACTGCACATTTTATAGTATTGAATGCC

CTTTAATTATGTT



>gi|71811331|gb|DR988722.1|DR988722 HDI_B_10.esd Horsegram (VZM-1) drought stressed subtracted cDNA Macrotyloma uniflorum cDNA, mRNA sequence

AGCTGGAGCTCACGCGGTGGCGGCGCTCTAGAACTAGTGGATCCCCCGGGCTGCAGGATTCGGCACGAGG

CCTCGTGCCGAATTCGGCACGAGGGGAATTTTCGAAAGCCTTTACAAAGTCGTTATGCGTCGCAACTCTG

TCTATGTTACCTTTGTCATCGCTGGCGCCTTCCTCGGAGAACGGGCTGTTGATTATGGAGTCCACAAGCT

ATGGGAGGCTAATAATGTTGGGAAACGATATGAAGATATTCCTGTGCTAGGGCAAAGGCAGTCAGAAGAA

TAACCAGTTTGTTAGCCTTTTGCAAGTTTTATTATATTCATGAAATATGAAGTGAACATTAAGAGCTGGA

AGCAGTTTCCATTTTTACAATAAATACCTTATGCTTGTCTCTTTCAGGCTTAACTCTTATGCGTGGTTTC

ATCACCGGCCTTTGAACCATTGCATAATTCTGTATTTTTTTGAATGATTTATCCGGAATCATTGGTTATG

CAAATTGTAGACTTTCTACTTAAAAAAAAAAAAAAAAACNCGAGGGGGGGCCCGG



>gi|71811329|gb|DR988720.1|DR988720 HDI_B_88.esd Horsegram (VZM-1) drought stressed subtracted cDNA Macrotyloma uniflorum cDNA, mRNA sequence

AAAAGCTGGAGCTCACGCGGTCCGGCNTTCATAAACTAGTGGATCCCCCGGGCTGCAGTGATTCGGCACG

AGGAAGAGACAAATGAAACAAATCTGTTAACTATGGAAGAATGATATCATTCGGAAGAGATGTGGCCGAG

GCACCCTCCTCTAAGATTGAAATGACTGACTTCCAGGGGTGCCCTCAAGGGTCGCAGTGTTGCTAATACC

ACTTGTCGATGATGTGTGGACTGAATACCATGAAATGGGAATTGAGGGAGTAAGAGCAGTTGCTGAACAT

AAGGTTTACACAGCTGGCTCAAATCATAGAGCTGCTTAATTATTAGTTAGCTCCAAAGATGATGGCTCGT

GGTAGTGCTCATTTCTCTTACGGAATTGCTGACAATTCGGATGACCCTAAGTATAATCACTACCAAGTAT

TGGTACAACATCCATATGGAAACAACACATTACACAAATGCTCCATGCATATGGAACATCTTCCTCTATG

TATGGCAGTATGGACTTTACCAACCNTGAAAAGATCTCTATAGTGTAACA



>gi|71811327|gb|DR988718.1|DR988718 HDI_B_85.esd Horsegram (VZM-1) drought stressed subtracted cDNA Macrotyloma uniflorum cDNA, mRNA sequence

GCNTTCTAGAACTAGTGGATCCCCCGGGCTGCAGGAATTCGGCACGAGGTGGACTTTTGGAAACATGTTA

TTAGTCTGTGAATATTTTTATGCGGTATTTGTAGGTCAAATTTTCATAACTGGAGTCTGATTTGTTCATA

TTTAAAGAGTTTTGTTCATATTGTGGTAAGCCTTTATTCAGACATCTTTGGTTTAATTTCGGTTTAGACA

TTTTGGCTTAGTCTTAGTTGGAGAATCTTTAGTTCTTTCAACTCAATCTTGTATGACCTTGACATTTTTT

TGTCACTTCGATCGAAGCAGGTTCATCCTCTACTGAGTTGCTGGAAAAAAAAAAAAAA



>gi|71811325|gb|DR988716.1|DR988716 HDI_B_77.esd Horsegram (VZM-1) drought stressed subtracted cDNA Macrotyloma uniflorum cDNA, mRNA sequence

AAAGCTGGAGCTCACGCGGTGCGCGGCGNTCTAGAACTAGTGGATCCCCCGGGCTGCAGGAATTCGGCAC

GAGGATTGGTTTGTATGCGGTTACTTGGGCGTCTTATAGAGAAAGACACGCAGCTGCTGCAGGTATATCT

CATGGTTCTTGGGTCTCTGAATCACTTCTCCCAGACAAGAGTTCTTATAGAGTAAACTTTTTTTCAGGCC

CCCCCAGCATGTCCCCAAAGCCTTCTGATTAACGAATTCAATTGTGGATTTCCTTGTAAAAAGTTGTCTC

ATCATTCATTGTTGTCTTTATATATAGTTGTTTTGTAGAATTCAAGAGAATCGACGCTAAATTTTTTTAT

GAACTTATCTACAAATTTGACTAGGTTTACATCTTAAAAAAACAAAAAAAAAAAAAAAACCCCGGGGGGG

CCCCGGAC



>gi|71811323|gb|DR988714.1|DR988714 HDI_B_75.esd Horsegram (VZM-1) drought stressed subtracted cDNA Macrotyloma uniflorum cDNA, mRNA sequence

AAGCTGGAGCTCACGCGGTGCGCGGCGTNTCTAGAACTAGTGGATCCCCCGGGCTGCAGGATTCGGCACG

AGGCCATTCTTCGGCGGCTTATTTGCGTAAATAAGGAAGGGCATATCCAAATAAATAATAGATAGAAAGG

GACGCCTTTTAAGCTACGAAAAAAGGAAGTGGCAAAGGGTCTTTTTCGTCTTTATGATGTATAGAATTTT

TTTAGGATTCTACTTGGATTCGGTACTTACTTAAGTTCAGCATCGAAGGCGGGACTCTTTCCCTAGATAG

AGAGATGGTCTAATTCAAATCACGACCAAGAGAGGTNTCATCTTCTCCCGATATCTTAATATAACA



>gi|71811321|gb|DR988712.1|DR988712 HDI_B_73.esd Horsegram (VZM-1) drought stressed subtracted cDNA Macrotyloma uniflorum cDNA, mRNA sequence

TGGAATGAATGGAGCCAAATGTGGTTGGCTAACTTTGACATGGTCTTGTGGCTTGCATAACTTTGCAATG

GANGTTGGGTTACACATCAAGGTTTGGCATTGTCTATGTTGGATTTCAAAACCCTCAAGAGATACCCCAG

ATGTCAGCTTACTGGTTCAAGCAGCTAAATGCCAGAAAGAAGCATTAATAGCTTAGCTCNAACATCTATT

TTCTAAGCTTCTGGTTGCTTCAGAAATCATGTTTAGTGGTTTTGGTTGAGTTAAAAGT



>gi|71811319|gb|DR988710.1|DR988710 HDI_B_65.esd Horsegram (VZM-1) drought stressed subtracted cDNA Macrotyloma uniflorum cDNA, mRNA sequence

CAAAAGCTGGAGCTCCACCGCGGTGGCGGCCGTTTCTAGAACTAGTGGATCCCCCGGGCTGCAGGAATTC

GGCACGAGGCACTCGGTTGTCCTGAAGTAACG



>gi|71811317|gb|DR988708.1|DR988708 HDI_B_61.esd Horsegram (VZM-1) drought stressed subtracted cDNA Macrotyloma uniflorum cDNA, mRNA sequence

ACAAGCTGGAGCTCACCGCGGTGCGCGGCCTTCTAGAACTAGTGGATCCCCCGGGCTGCAGTGAATTCGG

CACGAGGTTCAGTTTCAACTCTACAATGGCAACCATCTCAGCTGCTACCACAACCCCTTCGATCACCCGC

GCATGTCTTGTGCAAAAGCGGTCTCTTGGAGTCTCATCTCCCGTTCTTGGATTGCCAACAATGGGTAAGA

TGGGAAGAGTGAGGTGCTCCATGGAGGAAAAGCCTTCTGTGAAGGAAAGCAGCTCAAGCATAGGGATGGG

GGCATCATTTGATGGCAGCCGCCTGTGCTGCAGCCATGTCAGGCCCAGCCAATTGCCTTGGTGGATGAAA

GACTGAGCACTGAAGGAACCGGGCTTCCTTTAGGGCTCAGCAACAACCTTCTTGGTTGGATCCTGTTCGG

TGTCTATTGGTCTTATATGGGGTCTCTACTTTGTCTACACTTCCAGTCTTGAAGAGGATGAAGAGTCCGG

AATGTCCCTTTATAAATCCTGGGATACGAGTGATGATGGACTTGTAGACATTGCGAACGAGTCCACATGA

TCTTCC



>gi|71811315|gb|DR988706.1|DR988706 HDI_B_54.esd Horsegram (VZM-1) drought stressed subtracted cDNA Macrotyloma uniflorum cDNA, mRNA sequence

CGGCCGTTCTAGAACTAGTGGATCCCCCGGGCTGCAGGATTCGGCACGAGGCCTCGTGCCGAATTCGGCA

CGAGGACAAACTTAACTTCCAGCGATTGGAACTAAACAACCAGAAGTTATTCCTTGGAGGACTGAGTATT

GGATTTGGTGACTTGAAAGCAACTGCTGAGAACTTGCCACCAGCTATTAAAGAAGTAAAAGTATCAGAAG

GACTACTGGGGAAGGCTGCTTCCACCTGTTGTAGCCGATTGGCTGATACCTGTTGTTGCATGTGTCTGCT

GCAGTCTTGCAATTTTGTGAATGACCAGTGCACCATTGTTTGCGCTCAGTTTGTGCAGCTCTGGCCTGTT

TTGAGTTAATCAAATGTGGTGTTGAGCTCTGCGAGTTGTGATNNGTTTATAGTGAAATTATATATGCTTA

AGTTATAGAGAAACTGTTTGATATTACAAATTGTTAAAGATGTATATATGAATTATCTAAGCTCTTGTGC

TATGAATA



>gi|71811313|gb|DR988704.1|DR988704 HDI_B_52.esd Horsegram (VZM-1) drought stressed subtracted cDNA Macrotyloma uniflorum cDNA, mRNA sequence

AGCTGGAGCTCACGCGGTGGCGGCCGCTCTAGAACTAGTGGATCCCCCGGGCTGCAGGATTCGGCACGAG

GTCGAAAAGTAAAGATCCTTAAAGCTCCAAAGTTTGATCTTGGGAAACTGATGGAGGTTCACGGCGATTA

CTCAGAAGATGTTGGTACAAAGGTAGACAGGCCTGTTGATGAAACAATTGCAGAGGAACCCACCGAAATT

GTTGGAGCATGATTCAAGAGATACTGCATTTGTAGGTTTTCTGTTTCGGATGTTATCATTGAAAATTACG

TTTGAAACTTCAGTCTAGTTTGTGTAAGCCGAGTAAACCAGTTTTGTTTTGACAATCAGACCAGTTTTTG

GCTCTTAATATTGAGTTATTGACATATTAAAAAAAAAAAAAAAAA



>gi|71811311|gb|DR988702.1|DR988702 HDI_B_50.esd Horsegram (VZM-1) drought stressed subtracted cDNA Macrotyloma uniflorum cDNA, mRNA sequence

AAAGCTGGAGCTCACGCGGTGCGCGGCGTTCTAGAACTAGTGGATCCCCCGGGCTGCAGGAATTCGGCAC

GAGGGGAAAAGGGAGGAAGACCATTTATTGGAACTGTTGAAGTCCATTCAACCTGAATTTGAGCATCTTA

AGGATGTTAATAAGACTTATTTGAATAAATGTCATGTGTATGATGACTTTATATTATGCTGTGTTCTAGT

CATGTTTGCTGCAATATATGTGTTATGTTATAATAATTAAGGTTCAATGCTTTAGGGGTTCTATGTAACT

CCTGCTTGCAAGGGCCATGGCCATGGATCATGTAATGAGGTTAAACTAATGACTTTGAGTTCTATGTAAC

TCCTTCCGAAGGTCATATTGTCCATGGATATAAATTTCAATGTACGAAATTAATAATGGATCTCTTGTTT

ATT



>gi|71811309|gb|DR988700.1|DR988700 HDI_B_42.esd Horsegram (VZM-1) drought stressed subtracted cDNA Macrotyloma uniflorum cDNA, mRNA sequence

AAGGCTGGAGCTCACCGCGGTGGCGGCCGCTCTAGAACTTAGTGGATCCCCCGGGCTGCAGGAATTCGGC

ACGAGGCCANTTTCAATGCCAACCTCAATGGTTGCCATTGTTAGGACTAAAGTGTCTTTTCACCCNAATT

ATCANCTACGCAAATGGTAAGCCATTTCGTCTCGATCATCTATAAGAAGGAAGCACGTGGGATGCCCGTC

GCCTTGTACNATCATCTAAGTGTTGTCNAGCTACTCCAATTCTGCTCCCTTATTGACATGGATCCAAACA

CACCAGAATGGATCCCTTTGGGTGCCTAGAAATTGCTCNATAATAGTAATAAAGACAGACAGGTCCAAGT

ATAGAGACAACGTGCTAGGAGCTGGCACTCAGAAGTATGCAATGGGCTAGCATGTTGGAATGAGGCATTA

TTATAGGATAGGACGTGTTCCTTCCATTTGTTAAAAGACATTAATGTATAATCTATCTTTCCATTCACGT

TGTTGACTTATATCGAGGATAGGTCTCATGTTGCCCACATATCTGTGAAGACAATAGGGAGGATTTTTAG

TCTGTTTGTATGAAACTTCTCCTGTGAGTGCTGGAAAATATCAGTAAGTGATGTTCTTCAGTGTGTTCAC

AGTTATTTATCTTTGCATACTATGATATGATTCCTAGTTCTTAAAGACAATCA



>gi|71811307|gb|DR988698.1|DR988698 HDI_B_40.esd Horsegram (VZM-1) drought stressed subtracted cDNA Macrotyloma uniflorum cDNA, mRNA sequence

AAAGGCTGGAGCTACACGCGGTTGCGCGGCGNTCTAGAACTAGTGGATCCCCCGGGCTGCAGGATTCGGC

ACGAGGCCTCGTGCCGAATTCGGCACGAGGATAACGCTCTCTTCCTCTTCCTCTTCATCTTCCTCTTCCT

CTTCCCACTCCTTCTTCTTTCTTCCTAATAATCTTCATCTGTCACAGTAATTAATATTATCTTACCTTCT

TTCTACAAAAAAAAAAAAAAAAAAAAAAAA



>gi|71811305|gb|DR988696.1|DR988696 HDI_B_39.esd Horsegram (VZM-1) drought stressed subtracted cDNA Macrotyloma uniflorum cDNA, mRNA sequence

AAAGCTGGAGCTCACGCGGTGGCGGCGTCTCTAGAACTAGTGGATCCCCCGGGCTGCAGGATTCGGCACG

AGGTGGATGCCCAGGAGGTGAAGGTGAAGACGAGCATGATGTTGTCTGGTTTTGGCTAGAGAAAGGCAAG

CCTTATGAATGTCCAGTGTGCTCACAGTATTTTGAGCTGAAAGTGGTCGGACCTGGTGGTGACTCCTTAG

TGGACATGGCGATCACCATTGACTTTCAGTCCCGCAAGTTTTCTTTCAGAAATAAAATTTGCAAAGAGAA

CTAGAAGACAAATTCACCTTTCAAAGTTACGAGGCCTTGTTTTGTTCGGATTTAATTTGCCCATACCTTG

CAGTTTTGCGACTTTAGAATCACGAGAGTTTCTGCCATTGTTTACTGGACACATGCTGGTCATTCCATTG

GGCTATCAAATCCTGTAAGGATATTTGTTTTGGCTCTGCCAAATAATTGATGGCAACA



>gi|71811303|gb|DR988694.1|DR988694 HDI_B_37.esd Horsegram (VZM-1) drought stressed subtracted cDNA Macrotyloma uniflorum cDNA, mRNA sequence

GCGGCCGTTCTAAACTAGTGGATCCCCCGGGCTGCAGTGAATTCGGCACGAGGGTCAGTTTCTGCTTTTG

CTCCAGTAGCAAATCCTATAAATTGTCCTTGGGGACATAAGGCATTCACAAATTATCTAGGTGACAATAA

ATCTGATTGGGAGGAGTATGATGCCACTTCTTTGGTGACGAAGTTTCCCAATGTCTCTTCCTCCATTTTG

ATTGATCAGCGGGGAAGATGACAAATTTTTGACTGATCAACAACTGCTGCCTCACAAGTTTGAAGAGGCG

TGACAGAAAAGCCAATGTCCCCCTGTTGTTACGTTTCCAACCTGGTTATGACCACGGTTACTATTTCATA

GCCACATTCATAGATGATCACATCCAACATCATGCTCAAGCTCTTAGGCTTAACTTAAGTTGTGTGACAC

TTTTCATGAGTAAGTCATTCTTATAAACTCTCTGAAACTTACTCAGCTGTTAATTTTTCTCCTTAGTACT

TGTGAGTTG



>gi|71811301|gb|DR988692.1|DR988692 HDI_B_3.esd Horsegram (VZM-1) drought stressed subtracted cDNA Macrotyloma uniflorum cDNA, mRNA sequence

AAAAAGCTGGAGCTCACGCGGTGGCGGCCGCTCTAGAAACTAGTGGATCCCCCGGGCTGCAGGAATTCGG

CACGAGGGTCCTTTCCCACGCTTAGGTCACGAAAGAGAGAGAATCTAAGACACACAACACACAAACAGAG

AGAGGAAATCATCTTCTCATGGCTACCAGCAACATGGCATCAGCAGCATCTGGGTTTGTCTTGTCTCTTA

ATGTTGCAGCTTCCACCTCAAACTCACCTGCTTCTAGGGTTATCTTCCCCACAAAGAACAACGCTGGTTC

TAGGCTTGTTGTAAGGGCTTCAGATGAGGCTGCAGCGTCCGCACCTGCAACCGCCACCCCTCCAGCTGAA

GGTGAAGCAAAGCCAAAGCCACCACCAATTGGTCCCAAGAGAGGTGCTAAGGTGAAGATTCTTAGGAAGG

AATCGTATTGGTACAAAGGAACTGGTTCAGTGGTTGCAGTTGATCAGGACCCCAAGACTCGCTACCCTGT

TGTGGTTCGATTCAACAAAGTCAACTATGCCAACGTATCAACAAACAACTATGCTTTGGATGAGATCGTG

GAAGTCGAATGAGTCTAATTTAATTGTAATTTTGACTCTTGTCATGCTATTGCAGTTTCTAGCAAGGTTT

TTTCATGTCAAAAAAGTCTGTATCATATTACATGTTTGAATGAAGTCTTGTTG



>gi|71811299|gb|DR988690.1|DR988690 HDI_B_28.esd Horsegram (VZM-1) drought stressed subtracted cDNA Macrotyloma uniflorum cDNA, mRNA sequence

AGCTTGGAGCTCACCGCGGTTCGCGGCCGTTTATAGAAACTAGATGGATCCCCCGTGCGCTGCAGTGAAT

TCGGCACGAGGCCTCGTGCCGACATTCGGCACGAGGCTCGGTGTCAAGTTGAAAGCTGGAAAGTTC



>gi|71811297|gb|DR988688.1|DR988688 HDI_B_26.esd Horsegram (VZM-1) drought stressed subtracted cDNA Macrotyloma uniflorum cDNA, mRNA sequence

ACAAGCTGGAGCTCACCGCGGTGCGCGGCGTCTCTAGAACTAGTGGATCCCCCGGGCTGCAGGAATTCGG

CACGAGGGAAGAATAACGTTTGTTATTTCATTTGTATTGAACTTTGAATTAATGTAGAAGAAAAGTAGAA

AAAAAATATCTTCAACAATCACAAAAAGTATATTTATGGATTTGGCATCTAATTGAATTCTAAAGAATAG

AAAATATCCTTTATGAATTATTAGAAAAGCCTTTGATAATATTCCGATTTGAAGTGGATCTTTAGTTTGT

AATCCAAAACTAATAATGTGAAAAGTTAAGAACCTTCTTATACTATTTTACTATAAAACATTGACTATAA

AAAATTTNGACTATGCCCAAACTTTACAAAACTTTCTATCGGATTTGTTGACTACATCGAGACAATCAAT

GAAGAAAGAAGAAAAAAATCTAGTACTAGATTTTGACACTGATTGTGAAATTTCATTGCTCTGTTAGAAG

AAGGATAGCTATACTGATTCGGTATACTCAAAAGACACCGTTGGTACAAAATGGACGATCTAACAAGGAT

TACTTTTCAGTAAATTTCGACTTACTGATCTCGTCATTTTATGGAATCGAATCCCCCCTTTTGACTGTAC

AAGAATATGTGGAGTTCAAGATGTCTGGAAGCACAGGAGAACGTTCTTTGGCTGATATTATTAC



>gi|71811295|gb|DR988686.1|DR988686 HDI_B_2.esd Horsegram (VZM-1) drought stressed subtracted cDNA Macrotyloma uniflorum cDNA, mRNA sequence

CCCTCTCCATGTGTCTCAATCTCNATCATCGTAGCATCATACCCCGAGTCTTATAATAAGAATACTCTCA

AGAGAGATGGATTTTGTNGATGTCTGCTGACTGAGACTGGTGGTACTAAGGATGACCTTAGGCTTCCAAC

CGATGAAAATCTGCTTTCGCAGATCAAGGATGGATTTGGTGAGGGCAAAGATCTGGTTGTGTCTGTTATG

TCTTCTATGGGAGAGGAGCAGATTTGTGCCCTTAAGGACATTGGTCCCAAGTAACTAGTTTCCATGTCAG

TTTTAATGCTGGAACAACTGAAGTTGTAAACTGAGATGATATAGTATTTATTTCTATTTTTTCACTTTTT

ACTGTTAGTAGGTATGGTATGTTACACATCNGCCAAGTTTGTACCATTCTAACAAGATATTGTCAA



>gi|71811293|gb|DR988684.1|DR988684 HDI_B_17.esd Horsegram (VZM-1) drought stressed subtracted cDNA Macrotyloma uniflorum cDNA, mRNA sequence

AAGCTGGAGCTCACGCGGTGGCGGCGCTCTAGAACTAGTGGATCCCCCGGGCTGCAGGAATTCGGCACGA

GGGTCAGATTCACCTTGAATCAAGATCCCAGAGTGGCATTTTGTGGGTACAGCATTCCTCATCCTTCAGA

TAATCGTGTTAATATCAGAATCCAGACAACAGGGGATCCAGCACGCGACGTATTAAAAGATTCATCTCAA

GATCTGATGCTTATGTGTCAGCATGTTAGGAGCACTTTTGATAAGGCAGTCAGTGATTTTAAAATCAGCA

AGGCTGGGAAGGATAAAACGGATTTTGCTTAAAGTTGATTTTGGTTCGCGTAGTTGATCTAGATTGTTGG

GTATAAAAGTATCCGTAGCGAACTTATCTGCCTATCTTATATTATGCCTATGCAGCAGCTACGCTAAAAT

TTGATGAGCAGGAAAAGGCATGTGCCAAACTACAGTCGTTGAATATGTTCTCAGAAAGCAAACTTAATTA

CTGAAACTAAAAAAACAAAAAAAA



>gi|71811291|gb|DR988682.1|DR988682 HDI_B_15.esd Horsegram (VZM-1) drought stressed subtracted cDNA Macrotyloma uniflorum cDNA, mRNA sequence

AAAAGCTTGGAGCTCACGCGGTGCGCGGCGTTCTAGAAACTAGTGGATCCCCCGGGCTGCAGGAATTCGG

CACGAGGCCTCGTGCCGAATTCGGCACGAGGCGGTGCTCCTGTTTACCTGGCTGCAGTTCTTGAATACCT

AGCTGCTGAGGTTCTTGAGTTGGCTGGGAATGCTGCCCGTGACAACAAGAAGAACAGGATTATCCCAAGA

CATGTTCTTTTGGCTGTTAGGAATGATGAGGAATTGGGGAAATTGCTTGCTGGTGTGACCATTGCCCATG

GATGGTGTGCTGCCTAACATCAACCCTGTATTGTTGCCTAAGTAAGACTGAGAAGTCTGCCAAGGAACCC

AAATCTCCATCTAAGGCCACTAAGTCTCCCAAGAAGTCTTAAATCATAA



>gi|71811289|gb|DR988680.1|DR988680 HDI_B_13.esd Horsegram (VZM-1) drought stressed subtracted cDNA Macrotyloma uniflorum cDNA, mRNA sequence

AGCTGGAGCTCCACGCGGTTGGCGGCCGCTCTAGAAACTAGTGGATCCCCCGGGCTGCAGGAATTCGGCA

CGAGGCCTCGTGCCGAATTCGGCACGAGGGCTGTATCCATTTTTGGTTACCTTTTGTGCATATGTATATA

TGAGGTTGAAACCCTGGAATGATGTGAGATAGCTGTGTGTGGTTTAACAGCTCAGTTTAAGTTTGTTTAC

TGTTAGATTAGTAAGCTGTAAATAAGTAATAAACTAGTATAGGGGTGCTTTTGTTACGTGTACTGTGTAA

ATAAGTCTTTTCATTGTTTGTTCAACTA



>gi|71812246|gb|DR989637.1|DR989637 HDI_L_96.esd Horsegram (VZM-1) drought stressed subtracted cDNA Macrotyloma uniflorum cDNA, mRNA sequence

AAAAGCTGGAGCTCACCGCGGTGCGCGGCCGTTCTAGAACTAGTGGATCCCCCGGGCTGCAGGATTCGGC

ACGAGGCCTCGTTGCCGAATTCGGCACGAGGCATGAGGGAATGCCCCGATGATTAGGTATTTGACCAACA

GTCTGCAGTATCATTCACAATACTCTGCAGCTTGTAATGTTGGTGACTGAGGAGGTATATGGTACATGGA

GCGATGTTGTCTCGAGATGGCAAAGTCAACATGTCCAGTATTCGACGTACCACGAACAACCACTTNGAGT

CTGGGACTGGCTTCAAGAGTGAACATATTACTTTACCCCCAATGTCATTCGTATATACTCTGTGCATAGT

CTCAACTTCTAGACAAGAGGAGAATTTTCTTGTTTTCTCACAAATAGATAACAGAGAGATAACTAACAG



>gi|71812244|gb|DR989635.1|DR989635 HDI_L_93.esd Horsegram (VZM-1) drought stressed subtracted cDNA Macrotyloma uniflorum cDNA, mRNA sequence

AAAGCTGGAGCTCACGCGGTGGCGGCGCTCTAGAACTAGTTGGATCCCCCGGGCTGCAGGAATCGGCACG

AGGTTTGTAAAAAGCAAGAAACATTAAGTTGGATTGGATACACAAGATGATTCACACTATCTGAAGTACA

TTGCAATAACATGTACATCAGCAAACACAAATCTAAAACTCTCGAGGGGGGGCCCGGTACCCAATTCGCC

CTATAGTGAGTCGTATTACAATTCACTGGCC



>gi|71812242|gb|DR989633.1|DR989633 HDI_L_91.esd Horsegram (VZM-1) drought stressed subtracted cDNA Macrotyloma uniflorum cDNA, mRNA sequence

AAAAGGCTGGGAGCTCCACCGCGGGGTTGGGCGGGCCGCTCTAGAAACTTAGGTTGGATTCCCCCGTGGC

CTTGCAGTGAATTCGGCACGAGGTTTTTTTTTTTTTTTTTTTTTTTTTTTTTTTTTTTTTTTTGTGTGTG

TGAGAGAGAAAAAGCGCG



>gi|71812240|gb|DR989631.1|DR989631 HDI_L_84.esd Horsegram (VZM-1) drought stressed subtracted cDNA Macrotyloma uniflorum cDNA, mRNA sequence

ACAAGCTGGAGCTCACGCGGTGGCGGCGCTCTAGAACTAGTGGATCCCCCGGGCTGCAGGATCGGCACGA

GGGGATGGATGATCAAAGTAAAGCAAGCGATCCTTCTGAATTAGAGTCCTTGTTGGGTCCAAAGGAATAC

ACAAAATTTTGTGAGGAGGAAGATGGTAGTCATTGAAGTTTGAATTGGCTCCTGCACTCATATGTGGGAC

TCACTGTTTGATATTCATGTGTTTATTATTATCTTATTGTCTTCCTGAACAGTTTTTTAATATGCAATAG

AACTGTTTCTTTCCAAAAAAAAAAAAAAAAAAAAAAAAAAAAAAAAAAAAAACCGGGGGG



>gi|71812238|gb|DR989629.1|DR989629 HDI_L_82.esd Horsegram (VZM-1) drought stressed subtracted cDNA Macrotyloma uniflorum cDNA, mRNA sequence

AAGCTGGAGCTCACGCGGTGGCGGCGCTCTAGAACTAGTGGATCCCCCGGGCTGCAGGAATCGGCACGAG

GCTCTAGCCAAGACTTTCAATTATGTTTCATATGGAATAAATTCACCCTATGATGATTTAAGCTTTGTTT

TGTAGTAGTTTGTGAGCCTTGTAACCAATCTCTATATGTAGAATAAGACTTTTTGTTGTTGTTGTACACT

TGTACTAGTGCTTTGTGAGTATTAATTTGTCAACCTGAAAAAAAAAAAAAAAAAAA



>gi|71812236|gb|DR989627.1|DR989627 HDI_L_80.esd Horsegram (VZM-1) drought stressed subtracted cDNA Macrotyloma uniflorum cDNA, mRNA sequence

AAGCTGGAGCTCACGCGGTGGCGGCGCTCTAGAACTAGTGGATCCCCCGGGCTGCAGGAATCGGCACGAG

GCTTCATTTTCAGTAACGTCATCTCTTGTCGGAAACCTCTTAAACCAGCTCCTTTTCTCCTCCATCGTAG

CACAACCTTGTCCAAGATCCCAACTGCCCATATTACCTTGTAATGCTTCCTAACCTGGTAACCCCTCACA

TGAGCCTGTATTTTCACTACTTTTTGGCGCAATGATAAGAAATCTTTGCGACCTTTCCAACCACGATATT

TCTTCTGAATTGATAAAGCAGCCGAATTATGTTCACGCGAGTTTCGAAAGGCAAGTTTTGACATAGCAGA

AATTTCTGGAATACTACCAATGCCACCTAAACTCGAGGGGGGGCCCGGTACCCAAATTCGCCCTATAGTG

AGTCGTATTACAATTCACTGGACCGTCGTTT



>gi|71812234|gb|DR989625.1|DR989625 HDI_L_79.esd Horsegram (VZM-1) drought stressed subtracted cDNA Macrotyloma uniflorum cDNA, mRNA sequence

GCTGGAGCTCACGCGGTGCGCGGCCGNTTCTAGAACTAGTGGATCCCCCGGGCTGCAGGATTCGGCACGA

GGGTGGTTCTCGTTTTTGACCTCTGAAATGAATCTGTCTACTATTTTTTATAAAAAAAAAAAAAAAAAAA

AAAAAAAAAAAAAAAAAAAAAAAAAAAAAAAAAAAAAAAAAAAAAAAAAAAAAAAAAAAAAAAAAAAAAA



>gi|71812232|gb|DR989623.1|DR989623 HDI_L_71.esd Horsegram (VZM-1) drought stressed subtracted cDNA Macrotyloma uniflorum cDNA, mRNA sequence

AACTAGCTGGAGCCACGCGGCCGCGGCGTTATAGAAACTAGTGGATCCCCCGCCGCTGCAGTTATTCGGC

ACGAGGCCTCGTGCCGAATTCGGCACGAGGTGCACTACTAGTATACATGTTGTATCCAGACAGAGAATTA

TGTATATCTATGTTAAGAGTTATAAATAAAGAAATAATGGTGTTTAGAGTGGCAAAAAAAAAAAAAAAAA

AA



>gi|71812230|gb|DR989621.1|DR989621 HDI_L_7.esd Horsegram (VZM-1) drought stressed subtracted cDNA Macrotyloma uniflorum cDNA, mRNA sequence

TTCATTAATCCAGTTGGTGCGTCCGAAAATCCGCCGAATATCCAAATACAAAAAATAAAAAAGAAACAGT

AAGAACAAGATAAAACACACAACAGAAAAAAAACTCGAGGGCGGGGCCCGGTACCCAATTCGCCCATATA

GATGAGTCGTATTACAACTTCACCTGCGCCGTCGTCTTTACAACAGAA



>gi|71812228|gb|DR989619.1|DR989619 HDI_L_68.esd Horsegram (VZM-1) drought stressed subtracted cDNA Macrotyloma uniflorum cDNA, mRNA sequence

AGCTGGAGCCACGCGGTGGCGGCGCTCTAAACTAGTGGATCCCCCGGGCTGCAGGAATCGGCACGAGGCT

CGGTTGGTACTTGGTTGGATCTTGTGTTGCCAACTGGTCAGAACTTAACATTCCAGCCTTGCAGGATGTC

GAATAGGCCAAGAAACTCAAAGAAAGTTTTGTAGGCTTGATTTGATAGGAAGAGAATTAAGCTAGCCTCA

CTAAATTCAATTGTGTAGAGATTTGGAAAAGGAAATAATGTGCAATGATGTCTCTGTTTGTTCAAAAAAA

AAAAAAAAAAAAAAAAAAAAAAAAAA



>gi|71812226|gb|DR989617.1|DR989617 HDI_L_60.esd Horsegram (VZM-1) drought stressed subtracted cDNA Macrotyloma uniflorum cDNA, mRNA sequence

AAGCTGGAGCTCACGCGGTGCCGGCGCTCTAGAACTAGTGGATCCCCCGGGCTGCAGGAATCGGCACGAG

GCATTATTCTCCTTTCTGTTTTGCATTTTGATTGATTTGTCAAATCTTTTTAACAAAATGGTGCAATTTT

TTTAAATTTCCGGAGATAGATAAGTCGTGTCTGCAAAGCACGACATTTAAGTGTAAATCGTGTTTGCAAA

TTGATGTACTAGGTCTTCCTACATTTTTTTGGTTTCTAAAAAAAAAAAAAAAAAAAAAAAAAAAAAAAAA

AAAA



>gi|71812224|gb|DR989615.1|DR989615 HDI_L_58.esd Horsegram (VZM-1) drought stressed subtracted cDNA Macrotyloma uniflorum cDNA, mRNA sequence

AAGCTGGAGCTCACCGCGGTGGCGGCGNTCTAGAACTAGTGGATCCCCCGGGCTGCAGGAATTCGATATC

AAGCTTATCGATACCGTCGACCTCGAGGGGGGGCCCGGTACCCAATTCGCCCTATAGTGAGTCGTATTAC

AATTCACTGGCCGTCGTTTTACAACG



>gi|71812222|gb|DR989613.1|DR989613 HDI_L_56.esd Horsegram (VZM-1) drought stressed subtracted cDNA Macrotyloma uniflorum cDNA, mRNA sequence

TAGCTGGAGCTCACGCGGTGGCGGCCGCTNTAGAACTAGTGGATCCCCCGGGCTGCAGGAATTCGGCACG

AGGCCTCTGTGCCGAATTCGGCACGAGGCCTG



>gi|71812220|gb|DR989611.1|DR989611 HDI_L_48.esd Horsegram (VZM-1) drought stressed subtracted cDNA Macrotyloma uniflorum cDNA, mRNA sequence

AAGCTGGAGCTCACGCGGTGGCGGCGNTCTAGAACTAGTGGATCCCCCGGGCTGCAGGAATTCGGCACGA

GGCTTGGATATAATGTTTAGGGATTAAAATTATGTTTTTTCAAATTTAAAAGATAAAAAATTTCTTAAAT

TTAGAAATTAATATTAGAATTATTTTAAATTTTAAGAAATGAAAATATAATTAACTTTTTTTTCTTATGG

ATAATGTGAATTGTAATATTACTTAATTTGGGGGTGGTATAATGAAAGGATTGGTGGGAGAAAAAAAAAA

AAAAAAAAAAAAAAAAAAAAAAAAACCGGGGGG



>gi|71812218|gb|DR989609.1|DR989609 HDI_L_46.esd Horsegram (VZM-1) drought stressed subtracted cDNA Macrotyloma uniflorum cDNA, mRNA sequence

AAGCTGGAGCTCACGCGGTGGCGGCGCTCTAGAACTAGTTGGATCCCCCGGGCTGCAGGATCGGCACGAG

GCCTCGTGCCGAATTCGGCACGAGGGTAAGGAATAATTCTCTACTATAATAGTATATTTTGTGCTGTCCA

CGTCTATAATGAGTCAATGTCTATGTATTGTAATATTTTGTGCATGAATGAATATGTAACCACTTAGGTC

TTTAAAAAAAAAAAAAAAAAAAAAA



>gi|71812216|gb|DR989607.1|DR989607 HDI_L_44.esd Horsegram (VZM-1) drought stressed subtracted cDNA Macrotyloma uniflorum cDNA, mRNA sequence

AAGCTGGAGCTCACGCGGTGCNCGGCGTTCTAGAACTAGTGGATCCCCCGGGCTGCAGGATCGGCACGAG

GGGCAGCAGAAAGTAGCAGATGAGGACACAGTAAGTTCTGATCAGTTATGGATCCATAAACTTAATATAT

TTGGGATTGTTTTATGATATCCGAACGCGCACCTTCGCTACCTTCTGTTTAATATAGAGATTAGTTTTCC

CATGGAGTGTATCCATATTATGTTCTTTAAGTTGAGGAATAATGTTGTTGCCAGATATTGAAAATT



>gi|71812214|gb|DR989605.1|DR989605 HDI_L_36.esd Horsegram (VZM-1) drought stressed subtracted cDNA Macrotyloma uniflorum cDNA, mRNA sequence

AAGGCTGGAGCTCACGCGGTGGCGGCGCTCTAGAACTAGTGGATCCCCCGGGCTGCAGGAATCGGCACGA

GGGAAAGGCCTTAGATGATACGCTTCGTGCATTCTGTAAATCCTGATCTTTTTTGCTCCATGTTTTTGTT

GCAGCTTTATCCAACCAATTCCTTTTAGCCTTTTAGTGAGTTTTTTATCTTGGTGATAAACCCTCACTGG

TGGTTAATTTTTAATTGGTG



>gi|71812212|gb|DR989603.1|DR989603 HDI_L_34.esd Horsegram (VZM-1) drought stressed subtracted cDNA Macrotyloma uniflorum cDNA, mRNA sequence

AGCTGGAGCCACGCGGTGGCGGCGCTCTAGAACTAGTGGATCCCCCGGGCTGCAGGATCGGCACGAGGAT

TGTGTTCATTGGTGCTTGCCTGGTCCTATAGACACCTGGAGTGATTTTCTTCTGGGAATGTTGAAGATGG

AGGGTGTGAGATCAGCTGAAGAGAAGCTTCATTTGCGTTCACACTGACAAATGAAATTCATCTCATACAT

TTATCAGATATTTACGTAATCTTCCTTGTAATTGTAAACTTCATTGCCACATGTATTGTAAATCTTTTTA

AATTATGATCAAATTTGATTAACCCCTTTTAGTCATATTTTTTTATGTTGATTCATATACATTTTTGGTT

TCAAAAAAAAAAAAAAAAAAAA



>gi|71812210|gb|DR989601.1|DR989601 HDI_L_32.esd Horsegram (VZM-1) drought stressed subtracted cDNA Macrotyloma uniflorum cDNA, mRNA sequence

AGCTGGAGCTCACGCGGTGGCGGCGNTCTAGAACTAGTGGATCCCCCGGGCTGCAGGATCGGCACGAGGT

GCCAAGTGAATGGTATAAGAAGTAGAATCTCACAAGTTATGGCTCTGCTTCATATACACCATCAAAGGGA

AATTTTCTATATTGGTTGTTTCAATAACTGCGTGTGGAATATTTTTAGGTGTTTTAGCATATGCTCTATG

AGCAATTGATTCCCCCTCAATCCCTCCCCTTTTTTCCCAACTCTGTTTTTCCTAATATCTGTTGTATCTT

TGTGGCTTTG



>gi|71812208|gb|DR989599.1|DR989599 HDI_L_24.esd Horsegram (VZM-1) drought stressed subtracted cDNA Macrotyloma uniflorum cDNA, mRNA sequence

ACAGCTGAGCTCACCGCGGTGGCGGCGCTCTAGAACTAGTGGATCCCCCGGGCTGCAGGATCGGCACGAG

GCCTCGTTGCCGAATTCGGCACGAGGAGAAAAGAACATGCTAGCTATTCTTATGTTTGGCATTAATAACA

TGTAATGTAAAACTTTCAAATGTTCACGTACTTTGTTGTTGTTTGCTTCAAGCATTCATGTCCCAAATGG

AAATAATTCCAAATTGAATATTAAAAAAAAAAAAAAAAAACCCGAAGGGGGGGCCCCGGTACCCAATTTC

GC



>gi|71812206|gb|DR989597.1|DR989597 HDI_L_22.esd Horsegram (VZM-1) drought stressed subtracted cDNA Macrotyloma uniflorum cDNA, mRNA sequence

AGCTGAGCTCACGCGGTGGCGGCGCTCTAGAACTAGTGGATCCCCCGGGCTGCAGGATCGGCACGAGGCC

GGAAACAGACCGCTGGAGTAACAGTAGTAGCCGGCCGGATGGTGACAGGTGGCGTGGTGATGAAAGGAGA

AGTGGGTTTGGTGGTTCTGGTGGAGGTTCAAGGTCATCTTCTACTTGGTCATATTCCAGGAATGGGCGTT

GATCATTTTGTGCCCTCTGATTTATTGCTGGTGACTTTTTTGGAATTCTCTCTGTACTCTTTCCAGTTTA

CGTCCTAGAATTCTTTTTGATGCTGAGTTGTTAAATTTATTATCTTAATACTCACAAACAAGTCCTCTAT

TGGTTTGAGGAACAAAAAAAAAAAAAAAAAACCCANGGGGGGNCCCGGTAC



>gi|71812204|gb|DR989595.1|DR989595 HDI_L_20.esd Horsegram (VZM-1) drought stressed subtracted cDNA Macrotyloma uniflorum cDNA, mRNA sequence

AAAGCTGGAGCTCACCGCGGTTGCNGGGCGTTNTAGAACTAGGTGGATCCCCCGGGCTGCAGGAATTCGG

CACGAGGCCTCGTGCCGAATTCGGCACGAGGTCTTTGTACAGCATTATATCATGATACTTNGTGGCTAAC

TGCTGCTGCNATTCACTCATTGTATATTACTTCATTTCTT



>gi|71812202|gb|DR989593.1|DR989593 HDI_L_12.esd Horsegram (VZM-1) drought stressed subtracted cDNA Macrotyloma uniflorum cDNA, mRNA sequence

AATCANCTGATGATATTTNGTATATACCATACGTTAGAGTTACATTTCATAAAATAAACCAAACATATAT

ATATATATATATAGACTTTNAAAAAAAAAAAAAAAAAAAAAAAAAAAAAAAAAAAAAAAAAAAAAAAAAA

AAAAAACAAAAAA



>gi|71812200|gb|DR989591.1|DR989591 HDI_L_10.esd Horsegram (VZM-1) drought stressed subtracted cDNA Macrotyloma uniflorum cDNA, mRNA sequence

AGCTGGAGCCACGGCGGTGGCGGCCGCTCTAGAAACTAGTTGGATCCCCCGGGCTGCAGGATCGGCACGA

GGCGTATTTGTTATCCTTAAATGGTCATGTTTGTACAATGGTATGTAGATGCAACTCTACTTCAAACTTT

ATTTGGTAGGATTATTTTGGTGTAATACTTGTGTGGATTATTAAAAAAAAAAAAAAAAAAAAAAAAAACC

CCANGGGGGGGCGCCCGGGCCCCACCCCCCG



>gi|71812198|gb|DR989589.1|DR989589 HDI_L_89.esd Horsegram (VZM-1) drought stressed subtracted cDNA Macrotyloma uniflorum cDNA, mRNA sequence

AGCTGGAGCTCACCGCGGTGGCGGCGCTCTAGAACTAGTGGATCCCCCGGGCTGCAGGATTCGGCACGAG

GGTTTGTAAAAAGCAAGAAACATTAAGTTGGATTGGATACACAAGATGATTCACACTATCTGAAGTACAT

TGCAATAACATGTACATCAGCAAACACAAATCTAAAACTCTCCAAGGGGGGGCCCGGT



>gi|71812196|gb|DR989587.1|DR989587 HDI_L_87.esd Horsegram (VZM-1) drought stressed subtracted cDNA Macrotyloma uniflorum cDNA, mRNA sequence

AGCTGGAGCTCACGCGGTGGCGGCGCTCTAGAACTAGTGGATCCCCCGGGCTGCAGGAATTCGGCACGAG

GGCTATCTGTGCGAACTTTACACCAGAGACAATGCTATCCTTTGCTGCTAATGTGGCAGGTGCCAAGTGC

AGGGATCTAGGAGCACGAAGTGGTCTTCCTTATCGTGCAGATCCACGCTTGGAATCCTTTATATTGGAGA

AATTGACTAGTTCATCTTAACATGTAATAATAGACACCTGTATTCTGTTTCCAAAGCTAGGTCTTCGGTT

AGTTATGCAATAGTACTGATTTTCCAGTGGTCGTGCCTTGTGCATACGGAGAAGTCTTCATTGGGATTAT

CTGCTTTTTAGTTTTTATTTTCTTTCAGTTGTAAACTTCCCATTATGGATTACAATGAAATTATAAGATG

GCGTATTTCGGTGCTAAAAAAAAAAAAAAAAAAA



>gi|71812194|gb|DR989585.1|DR989585 HDI_L_85.esd Horsegram (VZM-1) drought stressed subtracted cDNA Macrotyloma uniflorum cDNA, mRNA sequence

AAGCTGGAGCTCACGCGGTGGCGGCGCTCTAGAACTAGTGGATCCCCCGGGCTGCAGGATCGGCACGAGG

CTGAGTAATATCGCTGAATCAAAATGTTTCTCGTGTAATATTTCTTTCATCAATTTTTGGAGCATGTATA

CAAATATTTGGATAGTTAGAATCTGCTGGCTAGATGCTGACTGCTTGTTTAGAAGTGAGTGAATACGAAT

TCTAATTGAAAGCATTAACGTAAAAAAAAAAAAAAAAAAAAAAAAAAAAAAAAAAAAAAAAAAAAAAAAA

AAAAAAAAAAAAAAAAA



>gi|71812192|gb|DR989583.1|DR989583 HDI_L_77.esd Horsegram (VZM-1) drought stressed subtracted cDNA Macrotyloma uniflorum cDNA, mRNA sequence

GCTGGAGCTCACGCGGTGGCGGCCGCTCTAGAACTAGTGGATCCCCCGGGCTGCAGGAATCGGCACGAGG

AACAGCTATAAGTTTTTGTTCTGCAATTTTGACGGCAGCCTCTGTGGTAATGTTGGAATTGCTAACGACG

ATGCAGGAAATAGGGTTTTGGTCATCACTCAGGACAAACCATTCGAGTTTGTTCTTGAGCAACTACCATC

ATCATCTACTGCATCTGCATAACCACTGTCCATGAAAATTTCTGCACTTTCTCTTTGTGAAAGACGTGTC

TTTGTTATGCTTTGGATGTAATTAACTTCACCAAGACTAAATAAAATATCCAAAGCTTATTTATGTCTTC

AAAAAAAAAAAAAAAA



>gi|71812190|gb|DR989581.1|DR989581 HDI_L_75.esd Horsegram (VZM-1) drought stressed subtracted cDNA Macrotyloma uniflorum cDNA, mRNA sequence

AAGCTGGAGCTCACGCGGTGGCGGCGCTCTAGAACTAGTGGATCCCCCGGGCTGCAGGATCGGCACGAGG

CTGCTATACAATCACTACATTCCGAATAGTCAATTTAGATAAATACATAGATAGATCCCTGTCTACCCTC

TACGTTGTGAGTGTTAGGGTGATGGCTTCATCTTGATTTATTGCTGCATTTGTGATGCTCTGGGAAGGGA

TCAAAATGGAAATAAATAACAATAAGGCATGTATTGTGTTTTGATGCAGGCTCAGTTTCATCTTTGATAA

CTATAGATACTATTAGTGTTTATTGTATTGATGTTGCTGACAACATACTATATGGTAATGCAGTAAATTA

GTTTCTGTTTTATACAAAAAAAAAAAAAAAAA



>gi|71812188|gb|DR989579.1|DR989579 HDI_L_73.esd Horsegram (VZM-1) drought stressed subtracted cDNA Macrotyloma uniflorum cDNA, mRNA sequence

AAGCTGGAGCTCACGCGGTGGCGGCGCTCTAGAACTAGTGGATCCCCCGGGCTGCAGGATCGGCACGAGG

GTTGTGTGTATGTGATGATACCAATTCGTCTTTTCCTTTGTAATTTACTATTTATTTATTATTATTATTA

TTATTTCTTTGGTTTGTATGAGATGAGATCAGATTATGACCGTTATTTAATTTTATATTTATATGCTGCT

GCCAAAAAAAAAAAAAAAAAAAAAAAAAAAAAAAA



>gi|71812186|gb|DR989577.1|DR989577 HDI_L_65.esd Horsegram (VZM-1) drought stressed subtracted cDNA Macrotyloma uniflorum cDNA, mRNA sequence

AGCTGGAGCTCACGCGGTGGCGGCGCTCTAGAACTAGTGGATCCCCCGGGCTGCAGGATCGGCACGAGGG

GTTCGGGTTCGGCCCCCAGAGTTGCAAAAGTAAAGCTTAGTGTAGTAATTAACATGTTTAGTGGACCATC

GTTTATTGTTATTATTATTTATTTGTCGTTGGGTTAATGCAGCTGAAAAAAACTATTCATTTTTTTTTAA

AAAAAAAAAAAAAAAAACCCCNNGGGGGGGCCCCGGACCCCAATTCCGC



>gi|71812184|gb|DR989575.1|DR989575 HDI_L_63.esd Horsegram (VZM-1) drought stressed subtracted cDNA Macrotyloma uniflorum cDNA, mRNA sequence

AGCTGGAGCTCACGCGGTGGCGGCGCTCTAGAACTAGTGGATCCCCCGGGCTGCAGGATCGGCACGAGGC

CTCGTTGCCGAATTCGGCACGAGGGTAAGGAATAATTCTCTACTATAATAGTATATTTTGTGCTGTCCAC

GTCTATAATGAGTCAATGTCTATGTATTGTAATATTTTGTGCATGAATGAATATGTAACCACTTAGGTCT

TTAAAAAAAAAAAAAAAAAAAAAAAAAAAAAAAAAAAAAAAAAAAAAAAAAA



>gi|71812182|gb|DR989573.1|DR989573 HDI_L_61.esd Horsegram (VZM-1) drought stressed subtracted cDNA Macrotyloma uniflorum cDNA, mRNA sequence

ATAGTATAGAAAGGTGTTGCTTGCTTGTGTTGCATATTACAACCGCACGAATCAGCAGGATTCTGCTTGT

GTTCCCCTCGAGTTGCGCATAGTTTGCGCTTGCCTCACTTCTCCATAACTTATTAGTACTATTCTACTCT

TGATATTGTGAGCCAGTTGAGTACTACTACTCAATCGCTCGTCTTCTTCTTGTATTTTTAATCGGGNTTG

TGATTGCATCCTCTCTTTCCCGAGAACACTTTCCATCATCCCTAGATCACCCTTCCTAAATCCTCTATTT

TTATATCCGCTCCTCATTATAATATNCCCTTTTTTTTCTGAATGGTAATCGTATGCTTGGCTTATCATTT

CTATTTTTATTCGTTAAANATTATCTTTCCTTCCACAACCAATGTTTCTCAACTTTATTCAGGGACTGTA

TAA



>gi|71812180|gb|DR989571.1|DR989571 HDI_L_54.esd Horsegram (VZM-1) drought stressed subtracted cDNA Macrotyloma uniflorum cDNA, mRNA sequence

AGCTCACGCGGTGGCGGCGTTCTAGAACTAGTGGATCCCCCGGGCTGCAGGATTCGGCACGAGGCCTCGT

GCCGAATTCGGCACGAGGAGAAGAGTGTCTTCTAGAAGCAAACAACACTTGTATTGTCATGCCTACTGAA

TTTTCCATATCTACCCCTATTAATTGTAGTGTTGGTTGTACATAGGTATATAGACTATGTGGAGTTTCAT

TTTCTCTAAAAAAAAAAAAAAAAAAAAAAAAAAAAAAAAAAAAAAAAAAAAAAAAAAAAAAAAAAAA



>gi|71812178|gb|DR989569.1|DR989569 HDI_L_52.esd Horsegram (VZM-1) drought stressed subtracted cDNA Macrotyloma uniflorum cDNA, mRNA sequence

AGCTGAGCCACGCGGGCGCGGCGTTCTAAACTAGTGGATCCCCCGGGCTGCAGGAATCGGCACGAGGGAA

AGGTTCGAGAAGGGAGAAGGAAGTGTTTAAAACACGTAAGATGTAAGATGTGTGAAGAAGGATGATGTAA

AATAAATAAGATATAAGATGTGAAGATGGATCTATCTTAAGATGAAGTGCTGAAGGACTTATATTCAGAT

AAATAAAA



>gi|71812176|gb|DR989567.1|DR989567 HDI_L_50.esd Horsegram (VZM-1) drought stressed subtracted cDNA Macrotyloma uniflorum cDNA, mRNA sequence

CTGGAGCTCACGCGGTGGCGGCGCTCTAGAACTAGTGGATCCCCCGGGCTGCAGGATCGGCACGAGGCGG

CACCTTCCAGAACGAATCACCGATCGCAACGTGATGGGCGTTCCGGCAGTGTTCGTAAACGGGAAAGAGT

TTGGTCAGGGCCGCATGACGTTGACTGAAATCGTTGCCAAAATTGATACTGGCGCGGAAAAACGTGCGGC

AGAAGAGCTGAACAAGCGTGATGCTTATGACGTATTAATCGTCGGTTCCGGCCCGGCGGGTGCAGCGGCA

GCAATTTACTCCGCACGTAAAGGCATCCGTACCGGTCCGAGGGGGGGCCCGGTACCCAATTCGCCCTATA

GTGAGTCGTATTACAATTCACTGGCCGTCGTTT



>gi|71812174|gb|DR989565.1|DR989565 HDI_L_49.esd Horsegram (VZM-1) drought stressed subtracted cDNA Macrotyloma uniflorum cDNA, mRNA sequence

GCTGGAGCTCACCGCGGTGGCGGCCGCTCTAGAACTAGTGGATCCCCCGGGCTGCAGGATTCGGCACGAG

GGTATACCAACACACCTGCTTTTTTCTAGCTAAACGTTAATTAACCCATGATATCATTGTAATCAGAGTA

ATCATGGTGCAAATTGGGTTTCTTTTGTAATCTCTGAAGATGTAACAGCATGATTTGTTAATAGTAGATA

ATCTAATAGCCACTTTTTTTTTGCTTTTTAAAAAAAAAAAAAAAAAAAAAAAAAAAAAAAAAAAAAAAAA

AAAA



>gi|71812172|gb|DR989563.1|DR989563 HDI_L_41.esd Horsegram (VZM-1) drought stressed subtracted cDNA Macrotyloma uniflorum cDNA, mRNA sequence

AAAGGCTGGAGCTCCACCGCGGTGCGCGGCCGCTTCTAGAACTAGTGGATCCCCCGGGCTGCAGGAATTC

GGCACGAGGGTTATACCAAATCATACAGCCTGACTTTTTTACTGAGCTTAAGACGTTAATATAACCCATC

GTATCATCATTGTGAATCAGAGTTAATCATGGTGCAGAGATTAGGGTTTACTTTTGGTAAGTCTACTAGA

AGATGTAGACAGCATGATATATGTGTAATAGTAGATAAATCTAATAGCCACGTTTTTATTTTGCTTTTTA

AAAAAAAAAAAAAAAAAAAAAAAAAAAAAAAAAAAAAAAAAAAAAAAAAAAAAAAAAAAAAA



>gi|71812170|gb|DR989561.1|DR989561 HDI_L_4.esd Horsegram (VZM-1) drought stressed subtracted cDNA Macrotyloma uniflorum cDNA, mRNA sequence

TAGCTGGAGCTCACGCGGTGCGCGGCCGTTCTAGAAACTAGTTGGATCCCCCGGGCTGCAGGAATTCGGC

ACGAGGCTCTCTCTCTCTCTCTCTCTCTCATTCTATATATACACAGCTGTGTATACATTAACACAGATGT

TGCTGAAGTGTATTGTATTTCATTTCACCCTNTTGTAAGTTGATGCCGATGGAAGTTGGAAATAAAGTCC

ATAGGTATGATTTATATAACCATAAAACACAATCTTCGTGCAAAAAAAAAAAAAAAAAAAAATAAAAAAA

AAAAAAAAAAAAACACCCCCGGGGGGGGC



>gi|71812168|gb|DR989559.1|DR989559 HDI_L_38.esd Horsegram (VZM-1) drought stressed subtracted cDNA Macrotyloma uniflorum cDNA, mRNA sequence

AGGCTGGAGCTCACCGCGGTGGCGGCGCTCTAGAAACTAGTGGATCCCCCGGGCTGCAGGAATCGGCACG

AGGCTTGGATATAATGTTTAGGGATTAAAATTATGTTTTTTCAAATTTAAAAGATAAAAAATTCTTAAAT

TTAGAAATTAATATTAGAATTATTTTAAATTTTAAGAAATGAAAATATAATTACTTTTTTTCTTATGGAT

AATGTGAATTGTAATATTCTTAATTTGGGGGCTGGTATAACTGAAAGGATTGGTGGGAGAAAAAAAAAAA

AAAAAAAA



>gi|71812166|gb|DR989557.1|DR989557 HDI_L_30.esd Horsegram (VZM-1) drought stressed subtracted cDNA Macrotyloma uniflorum cDNA, mRNA sequence

TGCAGATGCACAGAATTAATGAGTACAATGAGATTTTTAGACTGTTCCAACATGNTGAGGAGAAATGTAT

TTTTGGCTGGCACGATTGGAGTTTTTCCACAGCATTTGGGCCAAAGGTTTANGGCTTAATTTTATTCCAT

TTGTGGGGTTTGAAATCCTTTTTTAGTTTAAAGTTAATTAAAAATTGGTTTAA



>gi|71812164|gb|DR989555.1|DR989555 HDI_L_29.esd Horsegram (VZM-1) drought stressed subtracted cDNA Macrotyloma uniflorum cDNA, mRNA sequence

TCATTGCCTGCAACTATTCTTACATTTCTAGAAGAAGACCGTGCATCTTTGTCTAATCGCTCTTGAGAAT

GATAAATGTCATCNTGTATAGTTGCTTCCTAGAGCATCGTTAAATTTAAACATTAATCCGATATATGCCT

ATACTTGCTTTTTATCCTAAAAATA



>gi|71812162|gb|DR989553.1|DR989553 HDI_L_27.esd Horsegram (VZM-1) drought stressed subtracted cDNA Macrotyloma uniflorum cDNA, mRNA sequence

AAAGCTGGAGCTCACGCGGTGGCGGCGCTCTAGAACTAGTGGATCCCCCGGGCTGCAGGAATTCGGCACG

AGGTAAAACGTTATTCCCACCAATCGGATTTTCGGAGAGATATAATCAAAGGATCTATCCGTGCTCAAAG

ACGCAAAACGGTTACTTGGGAATTTTTTCAAAAAAGGGTGCATTCGCCTCTTTTTTTTGATAAAATTGAA

AAATCATTATTCTTTTCTTTTCATAGTTTCAAATCAATGAAAATATTTTTTATGTTAAAAATTTGGACTC

GAAAAAAGACAGAATTTAAAATTTTAGGTTATATAGAGGAAAAAGCAAAAAAAAGTCCGAAAAAAGAGGA

GGAAAAAAAAAAAAAAA



>gi|71812160|gb|DR989551.1|DR989551 HDI_L_25.esd Horsegram (VZM-1) drought stressed subtracted cDNA Macrotyloma uniflorum cDNA, mRNA sequence

AAAGCTGGAGCTCACCGCGGTTGGCGGCCGCTCTAGAACTAGTGGATCCCCCGGGCTGCAGGAATTCGGC

ACGAGGCCTCGTGCCGAATTCGGCACGAGGGGCAAGCGGGAAATAGCTCTATGGTTCAAGGAAGGCGAAT

TATGTGAATGGACTCCTGTTCAAGCACCATGGCTGAGAGAATAATAAATCACAGATTCCCAGTTACAATG

ATTCATGCCATTTCTAGTTTCAATAATCAGATCAATTGTATTCAGTGGCAAATGTAGCACTACGTT



>gi|71812158|gb|DR989549.1|DR989549 HDI_L_17.esd Horsegram (VZM-1) drought stressed subtracted cDNA Macrotyloma uniflorum cDNA, mRNA sequence

AAAGCTGGAGCTCACCGCGGTGGCGGCGCTCTAGAACTAGTGGATCCCCCGGGGCTGCAGGATCGGCACG

AGGCCTCGTGCCGCCTCGTGCCGAATTCGGCACGAGGGGGGGGGCCCGGTACCCAATTCGCCCTATAGTG

AGTCGTATTACAATTCACTGGCCGTCGTTTTACAA



>gi|71812156|gb|DR989547.1|DR989547 HDI_L_15.esd Horsegram (VZM-1) drought stressed subtracted cDNA Macrotyloma uniflorum cDNA, mRNA sequence

GCTGGAGCTCACGCGGTGCGCGGCGTTCATAAACTAGTGGATCCCCCGGGCTGCAGGAATCGGCACGAGG

CCTCGATGCCGAATTCGGCACGAGGGTTTGCCAACACCGCTCCAAAAACCCTTCCAAATAAAACCAGTCA

TGTTTCGTTCCTCTTTAATTGTGTTTGACTCTATCTATGTACATGTGTGTGTCTGATGTTGAAGTTGAAG

ATTTGTGATGGTAAAAAAAAAAAAAAAAAACCCTCAAGGGGGGGGCCCGG



>gi|71812154|gb|DR989545.1|DR989545 HDI_L_13.esd Horsegram (VZM-1) drought stressed subtracted cDNA Macrotyloma uniflorum cDNA, mRNA sequence

GAACCAAGTGGAGTATATTTGTGGAACCAACACGTGGAAACGAGACTTTTAGACCTTCCGTATCCCAGTT

ACTACATTAATGAAAACGTACCGAATATTAAGTAAATAGAATAGTTAACAATAAGTGGAAGTGAACGTCA

AAAAAAAAAAAAAAAAAAAANAAAAAAAAAAAAAAAAAAAAAAAAAAAAAAAAAAAAAAAAAAAGGGGGG

GGGGCG



>gi|71812152|gb|DR989543.1|DR989543 HDI_K_94.esd Horsegram (VZM-1) drought stressed subtracted cDNA Macrotyloma uniflorum cDNA, mRNA sequence

TGGAGCTCACGCGGTGGCGGCGCTCTAGAACTAGTGGATCCCCCGGGCTGCAGGATCGGCACGAGGATTG

TGTTCATTGGTGCTTGCCTGGTCCTATAGACACCTGGAGTGATTTTCTTCTGGGAATGTTGAAGATGGAG

GGTGTGAGATCAGCTGAAGAGAAGCTTCATTTGCGTTCACACTGACAAATGAAATTCATCTCATACATTT

ATCAGATATTTACGTAATCTTCCTTGTAATTGTAAACTTCATTGCCACATGTATTGTAAATCTTTTTAAA

TTATGATCAAATTTGATTAACCCCTTTTAGTCATATTTTTTTATGTTGATTCATATACATTTTTGGTTTC

AAAAAAAAAAAAAAAAAA



>gi|71812150|gb|DR989541.1|DR989541 HDI_K_92.esd Horsegram (VZM-1) drought stressed subtracted cDNA Macrotyloma uniflorum cDNA, mRNA sequence

GCTGGAGCTCACGCGGTGCGCGGCGCTCTAGAACTAGTGGATCCCCCGGGCTGCAGGATCGGCACGAGGG

TTGTGTGTATGTGATGATGACCAATTCGTCTTTTCCTTTGTAATTTACTATTTATTTATTATTATTATTA

TTATTTCTTTGGTTTGTATGAGATGAGATCAGATTATGACCGTTATTTAATTTTATATTTATATGCTGCT

GCCAAAAAAAAAAAAAAAAAAAAAAAAAAAAAAAAAAAAAAAAAAAAAAAAAAAAAAAAAAAAAAAGGGG

GGGGGGGGG



>gi|71812148|gb|DR989539.1|DR989539 HDI_K_9.esd Horsegram (VZM-1) drought stressed subtracted cDNA Macrotyloma uniflorum cDNA, mRNA sequence

AGCTGGAGCTCACGCGGTGGCGGCGCTCTAGAACTAGTGGATCCCCCGGGCTGCAGGATTCGGCACGAGG

GTTCTGAAGTGGGAAACCCAGTAGTTAGTGTTACTGGTATCTCCTTTTTTTTATGTTTTTGATACATTTT

GTTGGCTTTAATTAGTCTATGCTGGTTTTGTGTTATNTAGTTCGAAACAACGTGTTACCGCATTTACGAA

CTCAACCTAATTTCTTATTTGCCAAAAAAAAAAACAAAAAAAAAANACCCCGGGGGGGGCCCCGNCCCCC

TCCCCCCCCC



>gi|71812146|gb|DR989537.1|DR989537 HDI_K_83.esd Horsegram (VZM-1) drought stressed subtracted cDNA Macrotyloma uniflorum cDNA, mRNA sequence

GAAAAGCTGGAGCTCACCGCGGTGGCGGCGCTCTAGAACTAGTGGATCCCCCGGGCTGCAGGAATTCGGC

ACGAGGGACCAATGGAGTGATATTTTGGGAACCAACACTGGAGACAGGACTTTTAAGCTTCCTGATCCCA

TGTACTACATTAGTTGAAAATCTGACCAGATATTAATGAAATAAGATATGTAACAATAATGGAATGGAAC

TGCAAAAAAAAAAAAAAAAAAAAAAAAAAAAAAAAAAAAAAAAAAAAAAAAAAAAAAAAAAAAAAAGGGG

GGGG



>gi|71812144|gb|DR989535.1|DR989535 HDI_K_81.esd Horsegram (VZM-1) drought stressed subtracted cDNA Macrotyloma uniflorum cDNA, mRNA sequence

AGCTGGAGCTCACGCGGTGGCGGCGCTCTAAACTAGTGGATCCCCCGGGCTGCAGGATTCGGCACGAGGC

GCCGATTCGTCACCGCCGTGGTGGGATTCGGTAAGAAGCGTGGACCAAACTCGTCAGAGAAGTGAGGGGC

ATCTGGATGATATGGTTTTTTTAGTTTGCAAAATGTATCTTTTATTTTGTTCTTATTTGATTGATGACTG

ATAGTTAAGATTATGCATTAATGAAAGAGTTTTTTTGCTAGTTAATATCCAAAAAAAAAAAAAAAAAACC

CAAGGGGGGCG



>gi|71812142|gb|DR989533.1|DR989533 HDI_K_8.esd Horsegram (VZM-1) drought stressed subtracted cDNA Macrotyloma uniflorum cDNA, mRNA sequence

AAGCTGAGCTCACGCGGTGGCGGCGCTCTAAACTAGTGGATCCCCCGGGCTGCAGGATTCGGCACGAGGC

CTCGTGCCGAATTCGGCACGAGGTATTTGGAAAGCAAATGGTGCTTGGCTAAGCAAATTCTAAGGTGTAA

CCGTGCCTTGGCATTGGCTCTGAATGTTCTTTTCTTGGATAAGTTTTATGTAGAACTTCATCAAGTAAAA

TATAGTGCAAGTTAATTAGTGGAAGTTTATAGGAAAAAAAAAAAAAAAAAAAACCCCGNGGGGGGGCGCC

CGCGGTTCCCCATTCGC



>gi|71812140|gb|DR989531.1|DR989531 HDI_K_71.esd Horsegram (VZM-1) drought stressed subtracted cDNA Macrotyloma uniflorum cDNA, mRNA sequence

AAGCTGGAGCTCACGCGGTGGCGGCGCTCTAGAACTAGTGGATCCCCCGGGCTGCAGGATTCGGCACGAG

GCCTCGTGCCGAATTCGGCACGAGGCTCTTGAGTACAAGAAGGCTGAGAGGGCTAAGCTGGTGCAGCAGG

GATACAACATTGTTGCAGTGGTTGGAGACCAATGGTCCGATCTGAGGGGAAAACCCAAGGGAACCAGGAA

CTTTAAGCTTCCCAACCCCATGTACTCTATTGAGTAGATCATCTCTGAAGGATCCCAAGCTTGCTCCTAT

CTACGAAATAAGATACCTTACGTGTTCTAATGGGAGCATTAACTATGTATCTATGCATGTGACTTTCTGG

TCTTCGGATCTTCAACAAATCACCTAAGAGACTACACTCCGTTGTTTTCTCTTTTGTGACTTTAATAAAT

TATGAACCCTTTTTTCCAAAAAAAAAAAAAAAA



>gi|71812138|gb|DR989529.1|DR989529 HDI_K_69.esd Horsegram (VZM-1) drought stressed subtracted cDNA Macrotyloma uniflorum cDNA, mRNA sequence

AAAGCTGGAGCTCACGCGGTGGCGGCGCTCTAGAACTAGTGGATCCCCCGGGCTGCAGGAATTCGGCACG

AGGGTCTAGTATTTCATTTGCAAGCTTAAAAGCTTTCTAATAATGTCAACCTCCTTTGTGTTGCCACTGA

ACACAAATTCGATCTCTTTCTTTGACTTTGGGTTATGAAGATAATCAAAATTTGATAGTTTAATTCTCGA

TAAAAAAAAAAAAAAAAA



>gi|71812136|gb|DR989527.1|DR989527 HDI_K_60.esd Horsegram (VZM-1) drought stressed subtracted cDNA Macrotyloma uniflorum cDNA, mRNA sequence

AAGCTGGAGCTCACGCGGTGNCGGCGCTCTAGAACTAGTGGATCCCCCGGGGCTGCAGGATTCGGCACGA

GGCGGATCTTCAACAAATCACTAAGAGACTACACTCCGTTGTTTTCTCTTTTGTGACTTTAATAAATTAT

GAACCCTTTTTTCCAAAAAAAAAAAAAAAAAAAAAAAAAAAAAAAAAAAAAAAAAAAAAAAAAAAAAAAA

AAAAAAAAGGGGGGGGGG



>gi|71812134|gb|DR989525.1|DR989525 HDI_K_58.esd Horsegram (VZM-1) drought stressed subtracted cDNA Macrotyloma uniflorum cDNA, mRNA sequence

CGGCCGTTCTAAACTAGTGGATCCCCCGGGCTGCAGGAATCGGCACGAGGGGTTTCTGGAGTTATGCCCA

TGCAGGGTCAGACTAGGAACATGACCCCGGGACATTATGCTGGGGGCTCTGTACAATATCCACCATCCAG

TATCTATGGTATTGGGCAAGTTCCACCTCCAGTTAACGGTATGGTGACGACAATGGGAGCGAGTAAACCT

CAGTCAGGAGCCCCAGTGTCATCTAGCACTACACAGTCAGCGAAGGATTATGATTTTTCCTCTTTAACAC

AGGGGATGTTTGCGAAACACTGATTGGTGTTTTTTGTTTGGCAATTCCGGCTATGTCATATACCATAGAA

GAAAATTAGTAAATTTACAGGAAGTGCGCTTTCTTTTGTCAC



>gi|71812132|gb|DR989523.1|DR989523 HDI_K_56.esd Horsegram (VZM-1) drought stressed subtracted cDNA Macrotyloma uniflorum cDNA, mRNA sequence

AGCTGGAGCTCACCGCGGTGGCGGCGCTCTAGAAACTAGTGGATCCCCCGGGCTGCAGGAATTCGGCACG

AGGGTTTTTTCTTGTCACCAAAAAAAAAAAAAA



>gi|71812130|gb|DR989521.1|DR989521 HDI_K_48.esd Horsegram (VZM-1) drought stressed subtracted cDNA Macrotyloma uniflorum cDNA, mRNA sequence

AGGCTGGAGCTCACGCGGTNGCGGCGCTCTAGAACTAGTGGATCCCCCGGGCTGCAGGAATTCGGCACGA

GGTTAATGCATGTGTCTTGTCTTTGTTCCCTTAGATGGTTTTACACCTTGAGTTTGAGCTTACAGTAGTG

AGTTAATGAATATCTCGCTACCGTTATCGTGTAAAACTTGTGCTTAGTTAAAAGTTTTATGTTTGGTTCC

AAAAAAAAAAAAAAAAAAAAAAAAAAAAAAAAAAAAAA



>gi|71812128|gb|DR989519.1|DR989519 HDI_K_46.esd Horsegram (VZM-1) drought stressed subtracted cDNA Macrotyloma uniflorum cDNA, mRNA sequence

GACAAGGCTTGGAGCCACGCGGTTGCGGCGTTCTAGAACTAGTGGATCCCCCGGGCTGCAGGAATTCGGC

ACGAGGACCATTCTTGCCCCAATCAAGTGATCTCTTTTAGAGCAATGAAACTGTAGAAATGAAATTTTAT

TTACACAGAAAAAAAA



>gi|71812126|gb|DR989517.1|DR989517 HDI_K_44.esd Horsegram (VZM-1) drought stressed subtracted cDNA Macrotyloma uniflorum cDNA, mRNA sequence

ACTGAGCTCACCGCGGTTGGCGGCGCTCTAGAACTAGTGGATCCCCCGGGCTGCAGGAATTCGGCACGAG

GGAGTAGATCATCTCTGAAGGATCCCAAGCTTGCTCCTATCTACGAAATAAGATACCTTACGTGTTCTAA

TGGGAGCATTAACTATGTATCTATGCATGTGACTTTCTGGTCTTCGGATCTTCAACAAATCACCTAAGAG

ACTACACTCCGGTTGTTTTCTCTTTTGTGACTTTAATAAATTATGAACCCTTTTTTCCAAAAAAAAAAAA

AAAAAAAAAAAAAAAAAAAAAAAAAAAAAAAAAAAAAAAAAAAAAAAGGGGGGGG



>gi|71812124|gb|DR989515.1|DR989515 HDI_K_36.esd Horsegram (VZM-1) drought stressed subtracted cDNA Macrotyloma uniflorum cDNA, mRNA sequence

AAAGGCTGGAGCTCACCGGCGGTGCGCGGCCGTCTCTAGAAACTAGTTGGATCCCCCGGGCTGCAGGAAT

TCGGCACGAGGATTTATTTTGTGACTCTTGTTAAATATGTTGCAAAAAGATACCAGCTGTCCGTTTGATT

ATCAAATGAAATGGGGTTTACACACTTGATATTAAAAAAAAAAAAAAAAAAAAAAAAAAAAAAAAAAAAA

AAAAAAAAAAAAAAAAAAAAAAAAAAAAAAAAAAAAAGGGGGGGGG



>gi|71812122|gb|DR989513.1|DR989513 HDI_K_34.esd Horsegram (VZM-1) drought stressed subtracted cDNA Macrotyloma uniflorum cDNA, mRNA sequence

AAGCTGGAGCCACGCGGNGCGGCGCTCTAGAACTAGTGGATCCCCCGGGCTGCAGGAATTCGGCACGAGG

CTAAGTCTCTGAAATTTCCAAAACATTTGTAGCCACTCCACTTTGTTTGTTTGTACTTAAACCAAATTCC

CATTTGTTTTTGCTTTTTGAGAAAAAAAAAAAAAAAAAAAAAAAAAAAAAAAAAAAAAAAAAAAAAAAAA

AAAAAAAAAAAAAA



>gi|71812120|gb|DR989511.1|DR989511 HDI_K_23.esd Horsegram (VZM-1) drought stressed subtracted cDNA Macrotyloma uniflorum cDNA, mRNA sequence

GGAGCTCACGCGGTGGCGGCGCTCTAGAACTAGTGGATCCCCCGGGCTGCAGGATTCGGCACGAGGGACG

GTTCTGCATGCAGCTACGTTGCGGTTGATGTGAACGCTAACAGACAGAGGCGTCTGGTGCTGACTCAGGT

GAGGAGGGTCGCTTGTGGATTCGGTTTGAGAAACTTTCATATTCTTCAACTGCAACTAATGAACCACAAC

TAACCCTGAAAAGTTATGTGTGATTTCTCTTAGTTCACACAACTGTACTAAGTAAGCACTACATAAATGC

TTACTTTGTTGTAATAAATTATGGCATGACTAAATAAAATAAGGTTAATCTTATTTCTCTTTTATTTTTC

AAAAAAAAAAAAAAA



>gi|71812118|gb|DR989509.1|DR989509 HDI_K_21.esd Horsegram (VZM-1) drought stressed subtracted cDNA Macrotyloma uniflorum cDNA, mRNA sequence

GCTGGAGCTCACGCGGTGGCGGCGTCTCTAGAACTAGTGGATCCCCCGGGCTGCAGGAATTCGGCACGAG

GGTCTTACTGGGAAAGCATTCAACATGATCAGTTTTCCTGATTCATGGTAGAGGACGACCACATATGCTT

TAAAAAAAATGTCAAAAGCCTTTATATGTATGAATCCAGAAAATCTTGATCGTTATCTTCTTGAAATGTA

TAGATACATATGTAATAAGGCTTCAAATATCAAATTTCCTAACA



>gi|71812116|gb|DR989507.1|DR989507 HDI_K_19.esd Horsegram (VZM-1) drought stressed subtracted cDNA Macrotyloma uniflorum cDNA, mRNA sequence

AAAGCTGGAGCTCACCGCGGTGGCGGCCGCTCTAGAAACTAGTGGATCCCCCGGGCTGCAGGATTCGGCA

CGAGGTATGCTTGCTGCATTACTGTGCCTGTTATCAATAAATGTGTTTATAGAAGGAATCTGATTCCTTC

ATCCACTTATGTATGCTTAAGTTATGTATCTCCAGTGGTTGAATGTTCTCGTGTTGTTTGTTATTAATAA

AGAATTGTGTTGTACTTGTATAAAAAAAAAAAAAAAAAAAAAAAAAAAAAAAAAAAACCCCGGGGGGGGG

C



>gi|71812114|gb|DR989505.1|DR989505 HDI_K_11.esd Horsegram (VZM-1) drought stressed subtracted cDNA Macrotyloma uniflorum cDNA, mRNA sequence

AAGCTGGAGCTCACGCGGTGGCGGCGCTCTAGAACTAGTGGATCCCCCGGGCTGCAGGATTCGGCACGAG

GCCTCGTGCCGAATTCGGCACGAGGAAAGTTTTCAGTGACAAATTCAAACTGTTTCTTAAGATCTTAATA

GAAAAAGCAAAATGGATCATCAAAAGTATTCTCGAAAGTATTCGAGAAAGTATAGTATCCCTCACGAAAA

AAATAAAAAAATTTTTTAAATTTATCTTTATTCAATTGAAACCCCAAAAAAGAGATGAATTGAGCGAAAA

AAAAAAAGATTCAACAATTTTGAAAAATATTCCAATGATTTCTGAAGCAACCTTTTCAAATCAATCGAAA

AATTCGACAAATTTTTCATTGAAAAAAAAATCTATAAAGGATATGAATTGCTAAAAGAAAAGTAATTTAT

CAAAAAAATTCGAAAAAATTGAAAAAAAAAAAAAAAAC



>gi|71812112|gb|DR989503.1|DR989503 HDI_K_90.esd Horsegram (VZM-1) drought stressed subtracted cDNA Macrotyloma uniflorum cDNA, mRNA sequence

GCTGGAGCTCACCGCGGTGGCGGCGCTCTAGAACTAGTTGGATCCCCCGGGGCTGCAGGATTCGGCACGA

GGCCTCGTGCCGAATTCGGCACGAGGGGGGGGGCCCGGTACCCAATTCGCCCTATAGTGAGTCGTATTAC

AATTCACTGGCCGTCGTTTTACAACGA



>gi|71812110|gb|DR989501.1|DR989501 HDI_K_88.esd Horsegram (VZM-1) drought stressed subtracted cDNA Macrotyloma uniflorum cDNA, mRNA sequence

TGGAGCTCACCGCGGTGGCGGCGCTCTAGAACTAGTGGATCCCCCGGGCTGCAGGATCGGCACGAGGCCT

CGTGCCGAATTCGGCACGAGGGTAAGGAATAATTCTCTACTATAATAGTATATTTTGTGCTGTCCACGTC

TATAATGAGTCAATGTCTATGTATTGTAATATTTTGTGCATGAATGAATATGTAACCACTTAGGTCTTTA

AAAAAAAAAAAAAAAAAAAAAAAAAAAAAAAAAAAAAAAAA



>gi|71812108|gb|DR989499.1|DR989499 HDI_K_86.esd Horsegram (VZM-1) drought stressed subtracted cDNA Macrotyloma uniflorum cDNA, mRNA sequence

AAAGGCTGGAGCCACGCGGTGGCGGCGCTCTAGAACTAGTGGATCCCCCGGGCTGCAGGATCGGCACGAG

GATCATTTCTCGGGGTTGCAATTCAGATTAGATTTACGTATTTGCTATTATGAATGTAATGTCTAGATGA

AAAAGTAAGTTGTAATTCTGACTCCTAAGATCTTGAACAAAGTCTACTTCGTTGTGTTTTTCTTAAAAAA

AAAAAAAAAAAAAAACCCCGGGGGGGGGCCCGGGCCCCTTCCCCC



>gi|71812106|gb|DR989497.1|DR989497 HDI_K_78.esd Horsegram (VZM-1) drought stressed subtracted cDNA Macrotyloma uniflorum cDNA, mRNA sequence

TGGAGCTCACGCGGTGGCGGCCGCTCTAGAACTAGTGGATCCCCCGGGCTGCAGGATTCGGCACGAGGGC

CACCATCACCAGAGGACTTAGGGAAATGGCGCGGGAGATGGCACGTAAAAGGAACAGCGGCTGATGTTCC

ATGTGGTCGATTTGGTTGCAGTGCAGTTGTGAGACAGAATCACTGCTTATGTAGTAAATATGTTTATTGT

TGTAATAATGAATAAGGACTTATAAACTACTGTAGAAATGTTTTTTATTTTTCAAAAGCTTCAACAGTTT

GGTGCCATGGACTACCTACAACTTCTCTGTCTTACCTTCAATCTAAAAGAAAAATAAATTATATTAGAGA

TCCTGTTATAGCTGTTTTTCATAAAAAAAAAAAAAAA



>gi|71812104|gb|DR989495.1|DR989495 HDI_K_76.esd Horsegram (VZM-1) drought stressed subtracted cDNA Macrotyloma uniflorum cDNA, mRNA sequence

AAGGCTGGAGCTCACGCGGTGCGCGGCGCTCTAGAACTAGTGGATCCCCCGGGCTGCAGGATCGGCACGA

GGCCTCGTTGCCGAATTCGGCACGAGGGCATGGAAAGTTCGGCAAGCATGGTGGTGGGTTCAAGAAGTGG

AAGTGAGGATTGTATATGCAAAACTATGACTCTAAAATGCTGCTCTGTGTAGAGCTTTTTGCTTTAGAAA

TAAACCATACTTCGAATGATGTGATCAATCGATCTATGACACTATATATATGGGACCTGGTTTCTTGTTT

TAAAAAAAAAAAAAAAAAACCCAGGGGGGGGCCCGGCCCCATTC



>gi|71812102|gb|DR989493.1|DR989493 HDI_K_74.esd Horsegram (VZM-1) drought stressed subtracted cDNA Macrotyloma uniflorum cDNA, mRNA sequence

AAGCTGGAGTCACGCGGTTGGCGGCGCTCTAGAACTAGTGGATCCCCCGGGCTGCAGGATCGGCACGAGG

CAACGATTAGGTTCCCGCTCTTCAGGCCCATGCAAAAGTTGCCCGGTTTGGTTGGGGTGGCGAAGGCCTG

GCCCATTCGCGCAACGCCGGAAAAGATATCGGAGAAGATCGAAGAGAGCATAAAGAGCGCGGAGGAGACG

TGCTCCG



>gi|71812100|gb|DR989491.1|DR989491 HDI_K_66.esd Horsegram (VZM-1) drought stressed subtracted cDNA Macrotyloma uniflorum cDNA, mRNA sequence

CTGGAGCTCACGCGGTGCGCGGCGTTCTAGAACTAGTGGATCCCCCGGGCTGCAGGATTCGGCACGAGGC

AAATATGTTGTAGTGGGGTTTCATCAATGTTCTGAGTTAAATTTAGATTCATCATCAGTTCAAACCCTAT

AATCATGTCATATTTATTTTTTTAGGGTCCTTTAGTACTAGACATTGTGGAAGAAAAGTATCTTATGTAT

TTTTCTACTTGTTTACTTACATGTACCCCTCAAGAAATCCTATATGCTTATGGATGACTTGTTACTGTGC

TGTATTTACATCATGAATACATATACCCTGTGCATTTGAATGCTTTAGTAAAAAAAAAAAAAAAAAAAAA

AAAAAA



>gi|71812098|gb|DR989489.1|DR989489 HDI_K_64.esd Horsegram (VZM-1) drought stressed subtracted cDNA Macrotyloma uniflorum cDNA, mRNA sequence

AAAGGCTGGAGCTCACGCGGTGCGCGGCCGCTCTAGAACTAGTGGATCCCCCGGGGCTGCAGGATTCGGC

ACGAGGGGAAATTGTCAAATAATCAATTCCTTTACACTTTTAGTTGGCACCCCTAGCTAACATGCTTGGA

AAGTTGTCACTACGAACATGTAATTTAATCATGTTTTGGTTGCAAAAAAAAAAAAAAAAAAAAAAAAAAA

AAAAAAAAAAAAAACGGGGGGGGGGG



>gi|71812096|gb|DR989487.1|DR989487 HDI_K_62.esd Horsegram (VZM-1) drought stressed subtracted cDNA Macrotyloma uniflorum cDNA, mRNA sequence

GGCTGGAGCTCACCGCGGTTGCNCGGCCGTTCTAGAAACTAGTGGATCCCCCGGGGCTGCAGGATTCGGC

ACGAGGAGAGAGAGAGAGAGAGAGAGAGAGAGAGAGAGAGAGAGAGAGAGAGA



>gi|71812094|gb|DR989485.1|DR989485 HDI_K_6.esd Horsegram (VZM-1) drought stressed subtracted cDNA Macrotyloma uniflorum cDNA, mRNA sequence

AAGCTGGAGCTCACGCGGTGGCGGCCGCTCTAGAACTAGTGGATCCCCCGGGCTGCAGGATTCGGCACGA

GGGAATACTTGCCAATAAGACCTATGTTGACTACTTTGACTACCCTTATGATTCTCAGATTTTCAGCGAT

GAGAATCCAAATGCATGCACTGTAATGTAGATCAGTCTTATTATGTACTAATTAACTGGTGTTTTAGTAG

CTGATCAAGTGAAGCAACTTTCTGTTGTTGGACCGTGGAATAATGCGTAGTAGTGATGAATATTTTACTG

CTTTGTATATGATTTCAACGGCATCCCTAAGAGTTGGCATTTCTAAAAAAAAAAAAAAAAAAA



>gi|71812092|gb|DR989483.1|DR989483 HDI_K_52.esd Horsegram (VZM-1) drought stressed subtracted cDNA Macrotyloma uniflorum cDNA, mRNA sequence

AAAGCTGGAGCTCACCGCGGTGGCGGCGCTCTAGAACTAGTGGATCCCCCGGGCTGCAGGATTCGGCACG

AGGATTGGCTTCATCAAGTATCGCTAAAGCGCTGTTGTCACCTCCGAACCAATATTATGTTCTGGCACTA

GGATATTTTGAGGATTTTATGTTTGAAGAAGCTTGAGACTTTTTTTCTTTTTGCATCTAATTAATTATCA

GACGGAATTTGTGGATCTAAAAAAACTTTTATATATATTGTTTTGAATTATTGACTTCTA



>gi|71812090|gb|DR989481.1|DR989481 HDI_K_50.esd Horsegram (VZM-1) drought stressed subtracted cDNA Macrotyloma uniflorum cDNA, mRNA sequence

GTAGTTGTACATGGNCGCAGGATATAAAGAAGAAGAACTAGTAGATAATTAGTTTCGTTAATAAAAAAGA

AGAACAAAAAAATAATATAGTATAGTTAAAAA



>gi|71812088|gb|DR989479.1|DR989479 HDI_K_49.esd Horsegram (VZM-1) drought stressed subtracted cDNA Macrotyloma uniflorum cDNA, mRNA sequence

AAGCTGGAGCTCACGCGGTGGCGGCGCTCTAGAACTAGTGGATCCCCCGGGCTGCAGGATTCGGCACGAG

GCCTCGTGCCGAATTCGGCACGAGGAGCACTCGTAAATTCGGTGTTTATTAAAAAATAAAAATGGATCTT

CTGGCTAATATATGTAGTTCAGTAGTTTACGAGTGTGCGTAACACAGGTTGTCAGAAAGGGTTCTATGTA

CCTGGCTTGTACTTTTCAATGCTTGTATTCTGCAACACTAATATTAAATAAACGTGCTTTTAGAAAGAAA

AAAAAAAAAAAAAAAAAAAAAAAAAAAAAAAAAAAAAAAAAAAAAAA



>gi|71812086|gb|DR989477.1|DR989477 HDI_K_41.esd Horsegram (VZM-1) drought stressed subtracted cDNA Macrotyloma uniflorum cDNA, mRNA sequence

AGGCTGGAGCTCACGCGGTGGCGGCGCTCTAGAACTAGTGGATCCCCCGGGCTGCAGGATTCGGCACGAG

GCCTCGTGCCGAATTCGGCACGAGGTATTCTCGAAAGTATTCGAGAAAGTATAGTATCCCTCACGAAAAA

AATAAAAAAATTTTTTAAATTTATCTTTATTCAATTGAAACCCCAAAAAAGAGATGAATTGAGCGAAAAA

AAAAAAGGATTCAACAATTTTGAAAAATATTCCAAGTGATTTCTGAAGCCAACCTTTTCAAATCAATCGA

AAAATTTCGACAAATTTTTCATTGAAAAAAAAATCTATAAAGGAATATGAAATGCTAAAAGAAAAAGTAA

TTATCAAACAAAATCGAACAAATGAAAAAAAAAAAA



>gi|71812084|gb|DR989475.1|DR989475 HDI_K_4.esd Horsegram (VZM-1) drought stressed subtracted cDNA Macrotyloma uniflorum cDNA, mRNA sequence

AAGCTGGAGCTCACGCGGTGGCGGCGCTCTAGAACTAGTGGATCCCCCGGGCTGCAGGATCGGCACGAGG

GAGCAAACAACACTTGTATTGTCATGCCTACTGAATTTTCCATATCTACCCCTATTAATTGTAGTGTTGG

TTGTACATAGGTATATAGACTATGTGGAGTTTCATTTTCTCTATAAATTTTACAATAATTATACTCTAAA

TTTGAAAGCTTCCCGTCTAGACTAGAAATGAAATAAAATAGAAGGAGCATTTCACTTAAAAAAAAAAAAA

AAAAAAAAAAAAAAAAAAA



>gi|71812082|gb|DR989473.1|DR989473 HDI_K_38.esd Horsegram (VZM-1) drought stressed subtracted cDNA Macrotyloma uniflorum cDNA, mRNA sequence

AAGCTGGAGCTCACCGCGGTGGCGGCCGCTCTAAACTAGTGGATCCCCCGGGCTGCAGGATTCGGCACGA

GGATTCCACAAAATTTTCTTAACGAGTTTAGAGTAGTTTAACTATAATTTTTTTTCAGTTCCAAAGCTTG

ATTTTGATTTTAAAAGTGATGAGTTGTATGCATACAACATAATTATAATAATAATCATAATAATAATATA

TACTAGTGTAAAAAAAAAAAAAAAAAACCCCAAGGGGGGGCCCCGGTTCCCCAATTC



>gi|71812080|gb|DR989471.1|DR989471 HDI_K_30.esd Horsegram (VZM-1) drought stressed subtracted cDNA Macrotyloma uniflorum cDNA, mRNA sequence

AAGCTGGAGCTCACGCGGTGGCGGCGCTCTAGAAACTAGTGGATCCCCCGGGCTGCAGGATCGGCACGAG

GGATTGATTGCACACATTCTAAAGGTGGGCTTTGTGTAGATTCTGTATTTATTATACCTAGTTGATTTGA

GAGAGCGCACCAGAAGCGGCATTATGAAATAACCACACTGAATAGGAATTCTTTTTTTCCAAAAAAAAAA

AAAAAAAAAAAAAAAAAAAAAAAAAAAAAAAAAAAAAAAACGGGGGGGGG



>gi|71812078|gb|DR989469.1|DR989469 HDI_K_28.esd Horsegram (VZM-1) drought stressed subtracted cDNA Macrotyloma uniflorum cDNA, mRNA sequence

AGCTGGAGCTCACGCGGTGGCGGCGCTCTAAACTAGTGGATCCCCCGGGCTGCAGGATCGGCACGAGGGT

GATGAAGATGAGGATGAGGACGAGGACGAGGACTATTTTGACTTCAGTATTCTCAACGGTGCCGATTCTA

ATTTATCAGATCAGCCTCATGTTAATGGGACCGAGTCTTCTACAGTATCAGGTGAGGGCATGTTTGAGGA

TTGATTGATGCAGCATTATTTAAAATGTATAGTTAGGCTGAGAAATGTCCATAAATTTTGTCAGTGTACC

ATGTATTCACAACGGCAATCTAATTTATTTGTACGTTTACAAAAAAAAAAAAAAAAAAAAAAAAAAAAAA

AAAAAAAAAAAAAAAAAAAAAA



>gi|71812076|gb|DR989467.1|DR989467 HDI_K_25.esd Horsegram (VZM-1) drought stressed subtracted cDNA Macrotyloma uniflorum cDNA, mRNA sequence

AAAGCTGGAGCTCACCGCGGTTGGCGGCCGNTCTAGAACTAGTGGATCCCCCGGGCTGCAGGATTCGGCA

CGAGGCCTCGTGCCGAGAAGGGTGACTTTCAGAGGAATTCCCAACAGTATCTCCATCTAAGGCTCTATGC

TTGCTGCATTACTGTGCCTGTTATCAATAAATGTGTTATAAGAAAGGAATCTGATTCCTTCATCCACATT

ATGTATGCTTAAGTTATAGTATCTCCAGTGGTTGAAATGTTCTCGTGTGTTTGTTNTTAATAAAGAA



>gi|71812074|gb|DR989465.1|DR989465 HDI_K_18.esd Horsegram (VZM-1) drought stressed subtracted cDNA Macrotyloma uniflorum cDNA, mRNA sequence

AAAAGGCTTGGAGCTCCACGCGGTGCGCGGCCGCTCTAGAAACTTAGTGGATCCCCCGGGCTGCAGGAAT

TCGGCACGAGGTTTTTTTTTTTTTTTTTTTTTTTTTTTTTTTTTTTTTTTTTTTTTTTTTTTTTTTTTTT

TT



>gi|71812072|gb|DR989463.1|DR989463 HDI_K_16.esd Horsegram (VZM-1) drought stressed subtracted cDNA Macrotyloma uniflorum cDNA, mRNA sequence

AAGCTGGAGCTCACGCGGTGGCGGCGCTCTAGAACTAGTGGATCCCCCGGGCTGCAGGATTCGGCACGAG

GGGTTTCTGTCTTCCACGGTAGAGAGTACGCTTTTGTTTGGTGTACAGTTAAGTTTATTTTTCTTCTATG

ATGGTTAATTAAGGAAGGTGAAAGGGGACTTATCTCCCTTTGTTATTTGTGTACGGGTTATATATACGGA

GAAGCTAGTTCTGTGCGGAATTCTGTAAATTCCGTTGGTGATGACTGTAGCACAAACGGTTCCTATAAGG

CTACAACAAAATTGTAGTCTCAAAAAAAAAAAAAAAAAAAAAAAAAAAAAAAAAAAAAAAAAAAAAAAAA



>gi|71812070|gb|DR989461.1|DR989461 HDI_K_14.esd Horsegram (VZM-1) drought stressed subtracted cDNA Macrotyloma uniflorum cDNA, mRNA sequence

AAGCTGGAGCTCACGCGGTGGCGGCGCTCTAGAACTAGTGGATCCCCCGGGCTGCAGGATTCGGCACGAG

GTTTTTTTTTATCAATTTCACCGAGGTGAGTCCTATACGCTTACTTCACAATATACAATACAAAACAGTA

TCAAGAGAATGATATCAAACTTAACATGAGAAAATGACTTTCATTACTTCGCTACCTGGGGAACATATAA

AAGTAAAAGAACTATATAAATTATCCAGCTGAGGAACCCCAAAATTCCNAACCGTGGGCCCAAAAAAAAA

AAAAAAACCCGGGGGGGGGCCCGCGGCCCCATTTCCGC



>gi|71812068|gb|DR989459.1|DR989459 HDI_K_1.esd Horsegram (VZM-1) drought stressed subtracted cDNA Macrotyloma uniflorum cDNA, mRNA sequence

TCTGCCGTCTTTACTTTTCTTATATTGCATAGAATTCATTAGCAGTTGTTTATTTTTACATCTTAAGTAC

CGATGTGCAGTTTTTTGAATACTTGTTTGAGAATAATAGTCTTGATGTCAAGCTGCTTGTCAATCATATT

GTTGGAGGATGTTATATAAGAATTACTTTCCAAAATATATGTTCTTAAGTCCTATCTTGTAAGTTGTAAA

GTGAATGTGTAAAATTGTAGTTTGCTATATTTACTAAAAAAAAAAAAAAAAACTCGAAGGGGGGGCCCGG

TTCACCCAATTTCGCC



>gi|71812066|gb|DR989457.1|DR989457 HDI_J_94.esd Horsegram (VZM-1) drought stressed subtracted cDNA Macrotyloma uniflorum cDNA, mRNA sequence

AGCTGGAGCTCACGCGGTGGCGGCCGCTCTAGAACTAGTGGATCCCCCGGGCTGCAGGATTCGGCACGAG

GCTGCGGAGGGACTAGTTGAAGGAGGAAAGAAAGAGATTTTCACACCAATGTACTTCTTCTTGGCACGGA

AGCCTGATTCAGACAGTAACTAAACTGGTTGTGTACAAATAGGTTGCTGTTGTTTATTGTGATTCTGGTA

GGATGAATCGATTAATTTTGTCCTTATTGCCGTAGTTTCGTTCTGTTTCGAGTTGCTTGTTTTTGTTTTA

GGTTACCAAAACCTTTGCTACACCTCTAGCGTATTTCTTTTTATCCTCAAAAAAAAAAAAAAAAAAAAAA

AAA



>gi|71812064|gb|DR989455.1|DR989455 HDI_J_92.esd Horsegram (VZM-1) drought stressed subtracted cDNA Macrotyloma uniflorum cDNA, mRNA sequence

CTGGAGCTCACGCGGTGGCGGCGCTCTAGAACTAGTTGGATCCCCCGGGCTGCAGGAATTCGGCACGAGG

CCGGAAACTACTCACTATAGTATGAGTATATCTCGTTTAATAGTATTATGAATAAGACAGAAGGTGATGA

GGGCTCCACTTATATGCACCTCTTCTCTTCTCTTTGTACTGCACATTTTATAGTATTGAATGCCCTTTAA

TTATGTTGCAAAAAAAAAAAAAAAAAAAAAAAAAAAAAAAAAAAAAAAAAAAAAAAAAAAAAAAAAAAAA

AAA



>gi|71812062|gb|DR989453.1|DR989453 HDI_J_9.esd Horsegram (VZM-1) drought stressed subtracted cDNA Macrotyloma uniflorum cDNA, mRNA sequence

AAGGCTGGAGCTCACCGCGGTGGCGGCCGCTTCTAGAACTAGTGGATTCCCCCGGGCTGCAGGAATTCGG

CACGAGGGTTTTTTTTTTTTTTTTTTTTTTTTTTTTTTTTTTTTTTTTTTTTGTGTGTGTTTTATAA



>gi|71812060|gb|DR989451.1|DR989451 HDI_J_83.esd Horsegram (VZM-1) drought stressed subtracted cDNA Macrotyloma uniflorum cDNA, mRNA sequence

CAAAGCTGGAGCTCCACGCGGTGGCGGCGCTCTAGAAACTAGTGGATCCCCCGGGCTGCAGGATTCGGCA

CGAGGTTTCGTTTCTCCCTAAGTTAAGTCGTGATGGGAAAGGAAAAGGCTAACATCAAAATCGTGGTCGT

TGGACATGTCTCTGAGAAATCAACTGCCACGGAGCTTGGAAGCCCTACTGAGAAAGGGTGTGACTGAAAG

GTTCGAGAAGGGAGAAGGAAGTGTTTAAAACACGTAAGATGTAAGATGTGTGAAGAAGGATGATGTAAAA

TAAATAAGATATAAGATGTGAAGATGGATCTATCTTAAGATGAAGTGCTGAAGGACTTATATTCAGATAA

ATAAAATGAAGGATATATGTTTATCTTCTTCAAAAAAAAAAAAAAAAAAAAAAAAAAAAAAAAAAAAA



>gi|71812058|gb|DR989449.1|DR989449 HDI_J_81.esd Horsegram (VZM-1) drought stressed subtracted cDNA Macrotyloma uniflorum cDNA, mRNA sequence

AAGCTGGAGCTCACGCGGTGGCGGCGCTCTAGAACTAGTGGATCCCCCGGGCTGCAGGATTCGGCACGAG

GGAATATATCATTTTTCTTCCCCTTAGTGTATCTTATCCTCCCTGAAATCGAAACAAGTGTACTGCTGAA

CCTTGATAGTACTGAGAAATTTGAAATTTTTCTTCTGCAAAAAAAAAAAAAAAAAAAAAAAAAAAAAAAA

AAAAAAAAAAAAAAAAAAAAAAAAAAAAAAAAAAAAAAAAA



>gi|71812056|gb|DR989447.1|DR989447 HDI_J_8.esd Horsegram (VZM-1) drought stressed subtracted cDNA Macrotyloma uniflorum cDNA, mRNA sequence

AAGCTGGAGCTCACGCGGTGGCGGCCGCTCTAGAACTAGTGGATCCCCCGGGCTGCAGGATTCGGCACGA

GGCCTCGTTGCCGAATTCGGCACGAGGTTGTCACAAATGAGGGCCTTAGGCTGTATGTACCAAAACAACG

ACCGCAATTTAATGTTTTTCATTTCATATGATGATCCTTCGACTCTCTGTACTATTTGTTCATGAAAACA

ATCTACTCTGCCTTTTCTCTTAAAAAAAAAAAAAAAAAAAACCCGAGAGGGGGGGCCCCGGTTACACCCA

TTTCGCCCC



>gi|71812054|gb|DR989445.1|DR989445 HDI_J_73.esd Horsegram (VZM-1) drought stressed subtracted cDNA Macrotyloma uniflorum cDNA, mRNA sequence

AGCTGGAGCTCACGCGGTGGCGGCCGCTCTAGAACTAGTGGATCCCCCGGGCTGCAGGATTCGGCACGAG

GGTCAGTGTCATTGTTAGTTCACTAGAATATTGTAAGTCAATAAAATAAGTTCATGTCTCCTTATGCATG

TGTCCATCTTTTCTTTTTCTTTTGTGTAAGTAGGAGTCATGTTTGTCTGTTGTCACTGCTTGTCCAATGT

GCCATAATATCCTTCAATTGTTGAAGGTTACTGGTTAATGGTTTATCCAGCGACAATTCTATGATATTAA

CTATGGCTTAAAAAAAAAAAAAAAAAACCTCAGAGGGGGGGGCCCGGGTACCCAATTTC



>gi|71812052|gb|DR989443.1|DR989443 HDI_J_7.esd Horsegram (VZM-1) drought stressed subtracted cDNA Macrotyloma uniflorum cDNA, mRNA sequence

AAGGCTGGAGCTCACCGCGGTGGCGGCCGCTCTAGAACTAGTTGGATCCCCCGGGCTGCAGGAATTCGGC

ACGAGGGTGACGCAGATGCTGACATGCCACCTCTGGAGGATGCCGATGCCGATGCCGATGCTGAGGGCAG

CAAGATGGAAGAAGTTGATTAAGTCTAACTTTTGTAATAGTTTGCAGAACAGTCTTTTATTTTTAATGTT

TTTGTTTTTTAAATTTGGAAATTTTGAAACTTTGGCCAAAAAAAAAAAAAAAAAAAAAAAAAAAAAAAAA

AAAAAAAAAAAAAAAAAAAAAAAAAAAAAAAAAAAAAAAAAAA



>gi|71812050|gb|DR989441.1|DR989441 HDI_J_67.esd Horsegram (VZM-1) drought stressed subtracted cDNA Macrotyloma uniflorum cDNA, mRNA sequence

AAGCTGGAGCTCACGCGGTGGCGGCCGCTCTAGAACTAGTGGATCCCCCGGGCTGCAGGATTCGGCACGA

GGGTAGCCCTATGCTGATTTTTTGCGGGGCCTATGATCAATGTTTTTGCCCATGCTTACTATTATCATAA

TTAAGAATATAATTTTGGACAAAAAAGTTACTGTATATGTAGCTTGGTTAGCATAGTTTCGGTATAATAT

TGAAATCATTTCTTCTCAAAAAAAAAAAAAAAAAAAACCCGAAGGGGGGGCCC



>gi|71812048|gb|DR989439.1|DR989439 HDI_J_59.esd Horsegram (VZM-1) drought stressed subtracted cDNA Macrotyloma uniflorum cDNA, mRNA sequence

AAGGCTGGAGCTCACGCGGTGGCGGCCGCTCTAGAACTAGTGGATCCCCCGGGCTGCAGGATTCGGCACG

AGGGTCTCTGAGAAATCAACTGCCACGGAGCTTGGAAGCCCTACTGAGAAGGGTGTGACTGAAAGGTTCG

AGAAGGGAGAAGGAAGTGTTTAAAACACGTTAGAATATAAGAATGTGTGAAGAAGGATGATGTAAGATAA

ATAAGAATGTAAGATGTCAAGGATGGATCTTAAGACGAAGTGATGAAGGACTTATATTCAGATAAATAAA

ATGAACTATATATGTTTATCTTCTTCAAAAAAAAAAAAAAAAAAAAAAAAAAAAAAAAAAAAAAAAAAAA

AAAAAAA



>gi|71812046|gb|DR989437.1|DR989437 HDI_J_55.esd Horsegram (VZM-1) drought stressed subtracted cDNA Macrotyloma uniflorum cDNA, mRNA sequence

AGCTGGAGCTCACGCGGTGGCGGCCGCTCTAGAACTAGTGGATCCCCCGGGCTGCAGGATTCGGCACGAG

GGGAAAAGCCAAGCTTGTAGCTATCCAAGATGTTGAATTGCCACCACTGGAAAATGGATCAACAAACTGA

TTAAGCTCATTGCCTGACAAGTATGCAGTCATTTAGGAGTCTTGTAAGCCATTTGATGCTTCGACAAACT

TCTCCATAACTACTTCTAAGAGAGAAATATATAACAGGAGAAAGAAAGATTTTTTTTTTATAAATTAAAA

TTAATTTATGTTGTACAACATGCTCTAATTCTATTATCTATTAGTAACTTTCTTTCCTATAAATATGTTT

AATGAAAAAGTTAATCTTGTCAAAAAAAAAAAAAAAAAAAAAAAAAAAAAA



>gi|71812044|gb|DR989435.1|DR989435 HDI_J_47.esd Horsegram (VZM-1) drought stressed subtracted cDNA Macrotyloma uniflorum cDNA, mRNA sequence

AAAGCTGGAGCTCACGCGGTGGCGGCCGCTCTAGAACTAGTGGATCCCCCGGGCTGCAGGAATTCGGCAC

GAGGCTAAGAATTATGATTCTGCAGTTTTATTTGACTCTTTAGAACCTAATCACGAGAATTTGGGGAAGG

GGTAGTAAGGGCAAGAGAATTGGTAATAAGGATGGCTAAAGGTGTTTCTAGAC



>gi|71812042|gb|DR989433.1|DR989433 HDI_J_45.esd Horsegram (VZM-1) drought stressed subtracted cDNA Macrotyloma uniflorum cDNA, mRNA sequence

AAGCTGGAGCTCACCGCGGTGGCGGCCGCTCTAGAACTAGTGGATCCCCCGGGCTGCAGGAATTCGGCAC

GAGGCGTTATTTGGATAGATGGAATTAATGAAGGAACAAAGTTTCATCAAAGTTTGTTATTAATGGAAGC

ATTTATCTTTCATGTTTCCCCCTAACCAAACACATCAAAGTTTGCATATCACTCCACACTCCATTTCTCT

CCCATCAATTCAGCCTATCCATATATTGTTATATTTGCCCTAGGCATATATAGATTTGTAATAGCAGATT

CTAGGTTCATATGTATCGTTCCCCCTTAAAATCAATCAAAAAAAAAAAAAAA



>gi|71812040|gb|DR989431.1|DR989431 HDI_J_43.esd Horsegram (VZM-1) drought stressed subtracted cDNA Macrotyloma uniflorum cDNA, mRNA sequence

CTGGAGCTCACGCGGTGGCGGCGCTCTAGAACTAGTGGATCCCCCGGGCTGCAGGATTCGGCACGAGGGT

GGCTTGGGTGGTCTTGCTAAATTGGCTGGAGGCTTCTTAAAATAATAGTTGTAGAAGAAAATGAAGAAGA

TGAAGGTCTTTAGTTTTTCTCTTTCTGTTGTTGTTGTTCTTCTTCTTGTATGATGTAAGGTTGAATAAAC

GAAAGATATAGGTCTGGTTATTTGTTGCAGAAAACAAAAAAAAAAACAAAA



>gi|71812038|gb|DR989429.1|DR989429 HDI_J_35.esd Horsegram (VZM-1) drought stressed subtracted cDNA Macrotyloma uniflorum cDNA, mRNA sequence

AAGCTGGAGCTCACGCGGTGGCGGCGCTCTAGAACTAGTGGATCCCCCGGGCTGCAGGATTCGGCACGAG

GGTTATCAAAAGCTTTCAAAGCTTCTGAGAGAAGGAAACTGGCGAATGAGGGATACAGAGTGGTTGGAAA

CATTGGAGACCAATGGAGTGATCTTGTGGGAGATGTAAGAGGGTTTAGGACCTTTAAGCTTCCAAATCCC

TTGTACTATGATTGAGCTTTCAAGCTCTAACTTTGCTCCCAATTATGAAATAAAATCTAACAAATTGGGA

CGCTGCATGTGACTTTATGTTGTTGGGATGTACTTTGAAACACTAGTTGGAGAACTCAAAAAAAAAAAAA

AAAAAA



>gi|71812036|gb|DR989427.1|DR989427 HDI_J_33.esd Horsegram (VZM-1) drought stressed subtracted cDNA Macrotyloma uniflorum cDNA, mRNA sequence

AAGCTGGAGCTCACCGCGGTGGCGGCGCTCTAGAACTAGTGGATCCCCCGGGCTGCAGGATTCGGCACGA

GGGAGAAAAGGGTGGAAGAACAACCAGCTGAGCCTGCTGCTAAACAGGAAGAGAAACCTGCTGAACCAGC

TGAGAAAGAACCACCAAAGCCTTGAAAATTTGGTTCAGAACAGCATGTAGCTGCTTCTTGAAGATGCAGC

AGATATACGTGTATTCTTAGTTTGCATATACTCAAAATTGTCACATACCATTCCCTTATTCTTTCATTCC

TGTGTAAAATTGGTTTTGTGCTGCTTTAGTTTTGATATTGTTGCAGATTTGGTGATTATAATATGCATGT

GAATGTTTTATGGTGAAAAAAAAAAAAAAAAAAAAAAAAAAAAAAAAAAAAAAAAAAA



>gi|71812034|gb|DR989425.1|DR989425 HDI_J_31.esd Horsegram (VZM-1) drought stressed subtracted cDNA Macrotyloma uniflorum cDNA, mRNA sequence

CAAATATGCTGAGCCACCGCGGGCCGGCCGTTCATAGAACTAGTGGATCCCCCGGGCTGCAGGATTACGG

CACGAGGGGATCATACTCTTTGAAGATGAAGATGTTCGATGGAACACAAACATGAGTTAACTTGCATTAC

GTTA



>gi|71812032|gb|DR989423.1|DR989423 HDI_J_23.esd Horsegram (VZM-1) drought stressed subtracted cDNA Macrotyloma uniflorum cDNA, mRNA sequence

AAGCTGGAGCTCACGCGGTTGGCGGCCGCTCTAGAACTAGTGGATCCCCCGGGCTGCAGGATTCGGCACG

AGGGACAGAAATAGAATTGAATTACATAGTTGGAGGAAGACTAAAACATAAAAACATTGAGAAAAAGGTA

ACCCGGAGTCATTCTCCCTGGCCGAAGCAAGAAAAAAAACTCGAGGGGGGGCCCGGTACCCAATTCGCCC

TATAGTGAGTCCGTATTACAATTCACTGGCCGTCGTTTTACAACAGAAGCTACGCAGAAT



>gi|71812030|gb|DR989421.1|DR989421 HDI_J_21.esd Horsegram (VZM-1) drought stressed subtracted cDNA Macrotyloma uniflorum cDNA, mRNA sequence

AAAGGCTGGAGCTCACGCGGTGGCGGCGCTCTAGAACTAGTGGATCCCCCGGGCTGCAGGATCGGCACGA

GGCCTCGTGCCGAATTCGGCACGAGGGTGTTGAGTAGCCTTAATTATATTTGAATTCATTTGTACATTGT

TACATGATCAAGTTATTTTATTATTCCCTCAAAAAAAAAAAAAAAAAAAAAAAAAAAAAAAAAAAAAAAA

A



>gi|71812028|gb|DR989419.1|DR989419 HDI_J_19.esd Horsegram (VZM-1) drought stressed subtracted cDNA Macrotyloma uniflorum cDNA, mRNA sequence

AAAAGCTGGAGCTCACGCGGTGGCGGCGCTCTAGAAACTAGTGGATCCCCCGGGCTGCAGGATTCGGCAC

GAGGGAGAGGAGCAGGATACAGATCATTGGAAACATTGGAGACCAATGGAGTGATATTTTGGGAACCAAC

ACTGGAGACAGGACTTTTAAGCTTCCTGATCCCATGTACTACATTAGTTGAAAATCTGACCAGATATTAA

TGAAATAAGATATGTAACAATAATGGAATGGAACTGCATTATTAACCTATGGGCCATGCATCTTGTTTTA

TCTGAGACAGATTCCACAAGCACTGATATTTTCAATGAATTATATCTGTTCTTAGTTTAAAAAAAAAAAA

AAAAAAAAAAAAAAAAAAAAAAAAAAAAAAAAAAAAAAAAAAAAAAAAAAAAAAAAAA



>gi|71812026|gb|DR989417.1|DR989417 HDI_J_11.esd Horsegram (VZM-1) drought stressed subtracted cDNA Macrotyloma uniflorum cDNA, mRNA sequence

AAGCTGGAGCTCACGCGGTGGCGGCCGCTCTAGAACTAGTGGATCCCCCGGGCTGCAGGAATTCGGCACG

AGGGATCCAAATGCCTCAAACTTCGACCCAACTGCAAGAAGTGATGATGGAACCTGCTTATACACCGCCT

GAGAACTTAAGCATTCTCCACTGAGGAGAAGCTAGGACTTGAGATATCGAATAAACTTTATCTTGTTCAT

ACTGGATTGTTATCCCTTTTGTAATTTTTGAGTTATATTGTCTACATTTTACAACTTAGAATGTTAAGTC

TTGATGCTTTTCTCAAAAAAAAAAAAAAAAAAATTCCGAGAGGGGGGNCCCGGTACCCCATTTCCC



>gi|71812024|gb|DR989415.1|DR989415 HDI_J_1.esd Horsegram (VZM-1) drought stressed subtracted cDNA Macrotyloma uniflorum cDNA, mRNA sequence

AAAGCTGGAGCTCACGTCGGTGCGCGGCGCTCTAGAACTAGTGGATCCCCCGTGGCTGCAGGAATTCGGC

ACGAGGTTTTTTTTTTTTTTTTTTTTTTTTTTTTTTTTTTTTTTTTTTTTTTTTTT



>gi|71812022|gb|DR989413.1|DR989413 HDI_J_90.esd Horsegram (VZM-1) drought stressed subtracted cDNA Macrotyloma uniflorum cDNA, mRNA sequence

AAGCTGGAGCTCACGCGGTGGCGGCGCTCTAGAACTAGTGGATCCCCCGGGCTGCAGGATCGGCACGAGG

AAAAAGAAGAATTAGAACAAACTTTGAAGAAAAAAAAGAAGAATTAGAACACAAAAGTTCTGAAGAACAA

AAATTAGAAGAACATTTTGAAGAAAAAAAAGATGACTTAAAAGAAGAGTTTGAAGAAAAAAAAGAAGAGT

TAGAACAACACTTTGAAGAAAAAAAAGAAGAATTAGAACAACACTTTGAAAAAAAAAAAAAAAAACCCCG

GGGGGGGCGCCCGGGCCCCATTCCCCC



>gi|71812020|gb|DR989411.1|DR989411 HDI_J_88.esd Horsegram (VZM-1) drought stressed subtracted cDNA Macrotyloma uniflorum cDNA, mRNA sequence

AAGCTGGAGCTCACGCGGTGGCGGCGCTCTAGAACTAGTGGATCCCCCGGGCTGCAGGATCGGCACGAGG

ATTAGGTCCTAATGAAATCAAATCTATCTATCTATCTATGCTGTAAAAATCCATTTCTTTCTCCTTCACC

CTTTTCTTTATTATCTCTTTCTTGTATTTTTCTAGATTCATTTCTCTTTCTTTTTACAAAACAAAAAAAG

AGAGAGTTTCAAAAGTTAAAATTAGAGATTATGGGGTTTTGAACCATATTTAGATGCATTTAGTGGTTTT

TAACAAAAAAAAAAAAAAAAACCCCNGGGGGGGCCCCGGGTCCCCCAATTCCCCCC



>gi|71812018|gb|DR989409.1|DR989409 HDI_J_86.esd Horsegram (VZM-1) drought stressed subtracted cDNA Macrotyloma uniflorum cDNA, mRNA sequence

AAGCTGGAGCTCACGCGGTGGCGGCGCTCTAGAACTAGTGGATCCCCCGGGCTGCAGGATCGGCACGAGG

CGGAAGCTCATTGTCTACATCACAAACTCTTACTTTCATGTGAAGTTCCTTCAGCTAAAAGTCTTACTGC

AATTTTCTTCACATGTAATTCTGTATGTCTAGAACTAGATATTCTATGCTTATCCTTCACAGGTCTAGTA

TTTGTTACAATAAAAAACTTACATGTTCCCCTTAAGTAGCCCCATGCTTGTGTGGTGTT



>gi|71812016|gb|DR989407.1|DR989407 HDI_J_78.esd Horsegram (VZM-1) drought stressed subtracted cDNA Macrotyloma uniflorum cDNA, mRNA sequence

AACTGAGCTCACGCGGTGGCGGCGCTCTAGAACTAGTGGATCCCCCGGGCTGCAGGATTCGGCACGAGGA

GATTGTGTGGGATTTTGTTTCTCTTTTTTGGCTTAATCAGATGTTCATGTGCTCTTATTATAAATCTGCA

AAAATCAGATCTGGATTGGCTTACATTTTCCTTATATTGCATAGTGAATTATTGCTAGTTAAGAGTGATG

ATTAATTTCTAGTTAACGTAGGATTTGTGAAAAGTCTTTCTATAAAGAATGAATGCTATAATTAAGCCGT

AAAAAAAAAAAAAAAAAACTCTCCAAGGGGGGGCCCGGTTACCCCAATTTG



>gi|71812014|gb|DR989405.1|DR989405 HDI_J_76.esd Horsegram (VZM-1) drought stressed subtracted cDNA Macrotyloma uniflorum cDNA, mRNA sequence

AAGCTGGAGCTCACCGCGGTGGCGGCCGCTCTAGAACTAGTGGATCCCCCGGGCTGCAGGATTCGGCACG

AGGGCTACATGAGGTTGGATCAGATCATCGTATTCACCGAACAGATATTGAGAGTGAAGATCTATCACTG

CACCCTGATTACTAAAAAAAAAAAAAAAAAAACTCANGGGGGGGC



>gi|71812012|gb|DR989403.1|DR989403 HDI_J_74.esd Horsegram (VZM-1) drought stressed subtracted cDNA Macrotyloma uniflorum cDNA, mRNA sequence

AAGCTGGAGCTCACGCGGTGGCGGCGCTCTAGAACTAGTGGATCCCCCGGGCTGCAGGATTCGGCACGAG

GCAAGGCTGCTGCACCCTCTGAACCTGCAGACTCCAAAGCTGAAGAAGGTGGTGGCTTGGGTGGTCTTGC

TAAATTGGCTGGAGGCTTCTTAAAATAATAGTTGTAGAAGAAAATGAAGAAGATGAAGGTCTTTAGTTTT

TCTCTTTCTGTTGTTGTTGTTCTTCTTCTTGTATGATGTAAGGTTGAATAAAGAACAGATATAGTCCTGG

TATTTGGTTGCCAANAACCTTTTCCTCTCAATTCCCAACAACACTGTTTTTGTGTACAAAAAAAAAAAAA

AAAAAAAAAANAAAGGGGGGGGCCAACAA



>gi|71812010|gb|DR989401.1|DR989401 HDI_J_65.esd Horsegram (VZM-1) drought stressed subtracted cDNA Macrotyloma uniflorum cDNA, mRNA sequence

AAGCTGGAGCTCACGCGGTGGCGGCGCTCTAGAACTAGTGGATCCCCCGGGCTGCAGGATCGGCACGAGG

GAAGCTTATATACATGTGACTTAGATGAGGAGATATCAAACAGTTGGAAGATCAGTTGAAAGGAAAAAGA

AGAGCGTATGCTTGACAAAATGGGTGGATGGATGGATGGATAGATCGGTCTGATTAGATTAGTTGTGGTA

GTTGATAGCTGGCAACCCTACCTCTCATATTATCGACAGATTCTATCTCTATATAACTTTTTAACTTCAA

AGAAAAAAAATGAGATAACATTACTGAAAAAAAAAAAAAAAAAAAAAAAAAAAAAAAAAAAAAAAAAAAA

AAAAAAAAAAAAA



>gi|71812008|gb|DR989399.1|DR989399 HDI_J_63.esd Horsegram (VZM-1) drought stressed subtracted cDNA Macrotyloma uniflorum cDNA, mRNA sequence

AAAGCTGGAGCTCACGCGGTGGCGGCGCTCTAGAACTAGTGGATCCCCCGGGCTGCAGGATTCGGCACGA

GGCCTCGTGCCGAATTCGGCACGAGGGGAAGACAGAGACATAGGAAAAATCAAGTTCCTTGTAAATAATT

AGATCTGCCAGTCATGAGTGACATCTTAATTCTCAAGGTTTAATTTTTCTCAAGCACATGCATTTCCTTA

AAGTCTGATATTCTGAAATTCCTTTGTTAAATTGATCAGAATAAATTATTT



>gi|71812006|gb|DR989397.1|DR989397 HDI_J_60.esd Horsegram (VZM-1) drought stressed subtracted cDNA Macrotyloma uniflorum cDNA, mRNA sequence

AAAGCTGGAGCTCACGCGGTGCNCGGCGTTCATAGAACTAGTGGATCCCCGGGCTGCAGGATCGGCACGA

GGGTTACTTCTGTGTTTTCTGTATGTGCACAACTCAGTGAGTTTCAAAGTTTCAATAGATATATCAATCT

TCTCAATGTTTTACCATCAAAAAAAAAAAAAAAAAAAACCCNGGGGGGGGCCGGGA



>gi|71812004|gb|DR989395.1|DR989395 HDI_J_54.esd Horsegram (VZM-1) drought stressed subtracted cDNA Macrotyloma uniflorum cDNA, mRNA sequence

AAGCTGGAGCTCACGCGGTGGCGGCCGCTCTAGAACTAGTTGGATCCCCCGGGCTGCAGGATCGGCACGA

GGCCTCGTGCCGAATCGGCACGAGGCAGAAGGCTGGTGCCTAATTCGTAATGGATTTCGTTACTTTTACC

ATTATTATTAGTATATAAAGTTTTGGTATAATGTTTTGTTGGTTTAGTGTTGTGACATGATTGGTTAAAA

CTTCATGAATGCTCTTAGTTCGCAT



>gi|71812002|gb|DR989393.1|DR989393 HDI_J_52.esd Horsegram (VZM-1) drought stressed subtracted cDNA Macrotyloma uniflorum cDNA, mRNA sequence

AAAGCTGGAGCTCACGCGGTGGCGGCCGCTCTAGAAACTAGTGGATCCCCCGGGCTGCAGGAATTCGGCA

CGAGGGAATGGAGTAGATTAGAGTTTATGCTTCAATAAGCATCGTACGTGTCTGCGTTGTCAAGTGAGTA

TTACTTTGATCAGGAGTTTTACTACTGGCTTTGGATTTACGTAGTTAATAGAATTGATGTACTGATATTG

GTTCGGGTTAATGAACATTTTTCTCTTCTTTACCAAAAAAAAAAAAAAAAAAAAAAAAAA



>gi|71812000|gb|DR989391.1|DR989391 HDI_J_50.esd Horsegram (VZM-1) drought stressed subtracted cDNA Macrotyloma uniflorum cDNA, mRNA sequence

GCTGGAGCTCACGCGGTGGCGGCGCTCTAGAACTAGTGGATCCCCCGGGCTGCAGGATTCGGCACGAGGC

CTCAAAGCACTACGGTGAACGTGTTATTAATAACAGTGGTACTTTCAATGGTAATGGCAATGGAGGCTAT

ATCGAGGGTGGCTTTAACTCCTCCACGAGTAACTACTACAATTAGTCTTCTGCATTTCCTATATGCTGCA

CAAAATGGTCTACTATGTTATGCTGTATTACCAATAAATGTAATGGTTTCCCAGTTATGCCTAAACCTTA

TTATGTACCTTGTAATGGTGGTTGTTAATAATAAAGACCTCTGATGTTGCAGTAAAAAAAAAAAAAAAAA

AAAAAAAAAAAAAAAAAAAAAAAAAAAAAAAAAAAAA



>gi|71811998|gb|DR989389.1|DR989389 HDI_J_48.esd Horsegram (VZM-1) drought stressed subtracted cDNA Macrotyloma uniflorum cDNA, mRNA sequence

AAAGCTGGAGCTCACCGCGGTGGCGGCGCTCTAGAACTAGTGGATCCCCCGGGCTGCAGGATCGGCACGA

GGATGATAAGATGCAAGGTGAACTCGTAAAAGCCAGGGCAGGCCTAACTAAAATCTTGACATCAAAGGAT

GTGAAAGCAACTTTGCTGGATATGGTTGAAAAGAATGAACTTAATAGATCTTTGTTGGCACTTCTAGATG

AAAACATAGCAAATGCACATAAGGGTAACCAGAAACAGGTTGCAGAATACATGGAGAAGCTCCGGGGGTC

TGTTATTAGGTACCTTACAGTTTAGTGCATATAGTACGTTTCTTCCCGCTTTTCTATTAGTTTTGGTGGA

AAATTGGTGGTAATTGGATTACTTGGTATCAAATTAAAAGTTCCTAAGA



>gi|71811996|gb|DR989387.1|DR989387 HDI_J_41.esd Horsegram (VZM-1) drought stressed subtracted cDNA Macrotyloma uniflorum cDNA, mRNA sequence

AAGCTGGAGCTCACGCGGTGGCGGCGTTCTAGAAACTAGTGGATCCCCCGGGCTGCAGGATCGGCACGAG

GGTCCACGTCTATAATGAGTCATGTCTATGTATTGTAATATTTTGTGCATGAATGAATATGTAACCACTT

AGGTCTTAAAAAAAAAAAAAAAAAAAAAAAAAAAAAAAAAAAAAAAAAAAAAAAAAAAAAAAAAAAAAAA

AAAAAAAA



>gi|71811994|gb|DR989385.1|DR989385 HDI_J_4.esd Horsegram (VZM-1) drought stressed subtracted cDNA Macrotyloma uniflorum cDNA, mRNA sequence

AACTGGAGCTCACGCGGTGGCGGCGCTCTAGAACTAGTGGATCCCCCGGGCTGCAGGATTCGGCACGAGG

CCTCGTGCCGAATTCGGCACGAGGGCAGGCGCAGCGGGCTCGCTTGCAGCTGGTGGGCTTGTGGTGATCC

TGAGCATCTGCCTCACAGTCTATGGGATCTCGTCGTTCAACGAAGGAGACCCATCGACGGCCCCTTCGCT

GACCTTAACGGGCCGCAAGAAGCAGCCCGATCAGCTCCAAACTGCTGATGGGTGGGCCAAGTTCACCGGG

GGCTTCTTTTTTGGAGGCATTTCGGGGGTTATTTGGGCCTACTTTCTCCTCTATGTCTTGGACCTTCCTT

ACTACATCAAGTGAAGGGCTCTTTTCTTTGTTTCTCTTTTTATTATTCATTCTCTATGAATGGACAATGA

ATTGAGGGTTATTTGTGCACTAGAGATGCATGTAAATGATGATGTATTTTATGACTCTCAACATCTTACA

AATGCTAAAAAAAAAAAAAAAAAACCCCNGGGGGGGNCCCCGGTCCCCATTTCCC



>gi|71811992|gb|DR989383.1|DR989383 HDI_J_38.esd Horsegram (VZM-1) drought stressed subtracted cDNA Macrotyloma uniflorum cDNA, mRNA sequence

AAGCTGGAGCTCACGCGGTGGCGGCGCTCTAGAACTAGTGGATCCCCCGGGCTGCAGGATTCGGCACGAG

GCCTCGTGCCGAATTCGGCACGAGGCGAGAGCGACGGGGATCAGGAGGCTTTCGTTAATATATTTTATCT

TTCAATGAGCTTTGAAATTGAAGAGGTTGCTGTTAATGCTTCAACTTTCTTTTTCATTCACACCTCACAA

TGCTCTGGACTTCTGGGATTGTTGTTTGGATGGATCATTGATACCAGTATTTTGATATGCAATACTCAGC

ACATTTTGCAAACTCTTTATGTTCCTAAATGCTGATCTTTTGGTCTAGTGAAATCTTATGCATACTTGAC

TTGCGGTCAAAAAAAAAAAAAAAAAAAAA



>gi|71811990|gb|DR989381.1|DR989381 HDI_J_30.esd Horsegram (VZM-1) drought stressed subtracted cDNA Macrotyloma uniflorum cDNA, mRNA sequence

AAGCTGAGCTCACCGCGGTGGCGGCCGCTCTAAAACTAGTGGATCCCCCGGGCTGCAGGAATCGGCACGA

GGGTGATTAGATATCACTGCTGCATCACCTATATATATTAAGGTGTATGGACCATAGTAGTGATGGCTGT

GACTTCTTTTTTCTTTCTTTCCTTCTTGTGATTTGCATTTATGAGTGAAAAACAAAAAAATGAGAGAAAA

AGTGTGCGTGTATGTGGTTGGTAATTGGAGTGTTATATGTGGTTTGGTTGGTTGCCCACTACTTTGGTTG

TAGATTATCTTAATGAAATAAAATTTTAAATTATCAGTATTAGTATTAAAAAAAAAAAAAAAAACACCTC

AAGGGGGGGCCCGGGTTNCCCAATTTCCGCC



>gi|71811988|gb|DR989379.1|DR989379 HDI_J_29.esd Horsegram (VZM-1) drought stressed subtracted cDNA Macrotyloma uniflorum cDNA, mRNA sequence

AAAAGCTGGAGCTCACGCGGTGGCGGCGCTCTAGAAACTAGTGGATCCCCGGGCTGCAGGAATTCGGCAC

GAGGCCTCGTGCCGAATTCGGCACGAGGCTTATCCTAAAATCCCATTTCCAATTCTCGTTTCTTCATTTC

CCCAAAACAACGTTTCAAAAAAAAAAAAAAAAAAACTCTCGCAAGGGGGGCGCCCGCGGTATACCCCATA

TTNNGCGCCCCCATATATATGTGTGAGGTTGCGTATATTATACA



>gi|71811986|gb|DR989377.1|DR989377 HDI_J_27.esd Horsegram (VZM-1) drought stressed subtracted cDNA Macrotyloma uniflorum cDNA, mRNA sequence

AAGCTGGAGCTCACGCGGTGGCGGCGCTCTAGAACTAGTTGGATCCCCCGGGCTGCAGGATCGGCACGAG

GAGAACTTGAAGTGGTGTCAGTTCTGGAATAGCTTTGATTTCTGTTAAGTACCCTGTTATATGAGGGTCG

AGTCCATTTCGTACACCAAAACTTGAAGTGTCGAAATGTTAATAGTCAGTGGCTAAAAAAAAAAAAAAAA

AAAAAAAAAAAAAAAAAAAAAAAAAAAAA



>gi|71811984|gb|DR989375.1|DR989375 HDI_J_24.esd Horsegram (VZM-1) drought stressed subtracted cDNA Macrotyloma uniflorum cDNA, mRNA sequence

AAAGCTGGAGCTCACGCGGTGGCGGCCGCTCTAGAAACTAGTGGATCCCCCGGGCTGCAGGATTCGGCAC

GAGGACACTTTCGTTGTAACTTATGTTAAATCATATGTACTGGTTTTTCTGTTTTGTTTTTTTGTGATGT

CTGAACAACATGCTATTACTAGTTAGTAGCTACATGAGGTTGGATCAGATCATCGTATTCACCGAACAGA

TATTGAGAGTGAAGATCTATCACTGCACCCTGATTACTATACCTTTGCGTTTTCTGTTTTCTTATTGTTC

TTTAAACCAAAAATAGCTGATATATATGTTGTCTTCCATAAACATTGGAGCTGTAACTTCTCAGTAGTAA

TTATTGGATCTTCATGATAAGAGTTTTAATTGAAAGTTA



>gi|71811982|gb|DR989373.1|DR989373 HDI_J_18.esd Horsegram (VZM-1) drought stressed subtracted cDNA Macrotyloma uniflorum cDNA, mRNA sequence

AAGCTGGAGCTCACGCGGTGGCGGCGCTCTAGAACTAGTGGATCCCCCGGGCTGCAGGATTCGGCACGAG

GCAATAATTGAAGAGACGGCATAAATTTTCTTTACAGCGTCCCTCTAGTAACTCCTTACTTTAAAAATGT

GACCAAATCTTTACCTACACCATTATCGCCAATGACATGTGCATTTAAATTGATGTGGGGACGTGGATGT

GGGGCGGTGCGGTCAGAAGAAGATGAGGAAGAACATCGTGTGTCTGTAGGGTGTGTGTGTTTGTGTGTGT

GTGTGTCAGCGGTCAGTGAAGAGACTCGCCCATTTGTGTTGTTTTGAATCCTATGTTTCACGATCAAAAT

GATACTACTCGAGGGGGGGCCCGGTACCCAATTCGCCCTATAGTGAGTCGTATTACAATTCACTGGCCGT

CGTTTTACAAC



>gi|71811980|gb|DR989371.1|DR989371 HDI_J_16.esd Horsegram (VZM-1) drought stressed subtracted cDNA Macrotyloma uniflorum cDNA, mRNA sequence

AAAAGCTGGAGCTCACGCGGTTGGCGGCCGCTTCTAGAACTAGTTGGATCCCCCGGGGCTGCAGGATTCG

GCACGAGGTGGAACTGGTTCTATATGTTCTGCTTAGGTTGTCATGTCTAACAGAATTTGTATAGGGAAAT

GATGGACATGGAGGCTACGTTTAGTATGGCTGATTTTACTCTCTTGTGTCTATTTTGCCAGCTTCTTGGT

GGGGAATGCTAAGAAGTTACATGTTTGATACTTGTGATTTTCTTCTAAATGTTTTTGAGGTTTGCCTTGT

AGTGTTACTCACACTGCACAAAAAAAAAAAAAAAAAAAAAAAAAAAAAAAAAAAAAAAAAAAAAAAAAAA

AAAAAA



>gi|71811978|gb|DR989369.1|DR989369 HDI_J_14.esd Horsegram (VZM-1) drought stressed subtracted cDNA Macrotyloma uniflorum cDNA, mRNA sequence

AAGCTGGAGCTCACGCGGTGGCGGCGCTCTAGAACTAGTGGATCCCCCGGGCTGCAGGATTCGGCACGAG

GGTATCATTGGATTTGACATGTTCGCCAAGTCCAGTGCATCAGTTTCATTGCCTACAAGCCACCAAACTA

CTAAGTCTCTGAAATTTCCAAAACATTTGTAGCCACTCCACTTTGTTTGTTTGTACTTAAACCAAATTCC

CATTTGTTTTTGCTTTTTGAGATTTCATCATCCTGTAATTTTGGTTTCTGTTTTCGGACTTCATTGGAAA

TTGATTGGATAAGACCTATTGATTAAAAAAAAAAAAAANTATAAAAAAAAAAAAAAAAAAAAAAAAA



>gi|71811976|gb|DR989367.1|DR989367 HDI_I_96.esd Horsegram (VZM-1) drought stressed subtracted cDNA Macrotyloma uniflorum cDNA, mRNA sequence

AAGCTGGAGCTCACCGCGGTGCGCGGCGNTCTAGAACTAGTGGATCCCCCGGGCTGCAGGAATTCGGCAC

GAGGATCAGGCAACTTCACATATGCCATATGGCTGTGACTACTGTGTGTTGGTTATTGTGTTAAATTTGA

GAAAATGAGAAACATTTAATAATTATTCAGATGCTTGTGTAGATGACGGATAATGAGCAAAGAATCATCA

TC



>gi|71811974|gb|DR989365.1|DR989365 HDI_I_94.esd Horsegram (VZM-1) drought stressed subtracted cDNA Macrotyloma uniflorum cDNA, mRNA sequence

AAGCTGGAGCTCACGCGGTGGCGGCGCTCTAGAACTAGTGGATCCCCCGGGCTGCAGGATTCGGCACGAG

GGTGATGATCTTGGAGGATCTTTCTGTGAGTACGAGTATCAGAAGAGTGTCTTCTAGAGCAAACAACACT

TGTATTGTCATGCCTACTGAATTTTCCATATCTACCCCTATTAATTGTAGTGTTGGTTGTACATAGGTAT

ATAGACTATGTGGA



>gi|71811972|gb|DR989363.1|DR989363 HDI_I_92.esd Horsegram (VZM-1) drought stressed subtracted cDNA Macrotyloma uniflorum cDNA, mRNA sequence

AAGCTGGAGCTCACGCGGTGGCGGCCGCTCTAGAACTAGTGGATCCCCCGGGCTGCAGGATCGGCACGAG

GGCAAGGTGGGAAATACTGAATATAGTTGGTTTTTAAGTTTTTCGTTTTAATTTAGTATGTTTATTTTCT

TTCTGATGATTGACATGGGCATACTGGAAAAGAAAATATTTGTGGACAATCTGTTATGTAATAGGTTGTT

TTCTGCTTTCTGGCTTAAAAAAAAAAAAAAAAAAAAAAAAAAAAAAAAAAAAAAAAAA



>gi|71811970|gb|DR989361.1|DR989361 HDI_I_9.esd Horsegram (VZM-1) drought stressed subtracted cDNA Macrotyloma uniflorum cDNA, mRNA sequence

AAAGGCTGGAGCTCACGCGGTGGCGGCCGNTCTAGAAACTAGTGGATCCCCCGGGCTGCAGGATTCGGCA

CGAGGCCTCGTGCCGAATTCGGCACGAGGCAAGAGTTAACAACTCTGGTTAGTGGGAATCGAATGTCAAT

GTCATGTGGTCCGTGGTAGATTTACGAACCGTGAACAGCTCGAAAAGAAAAGTCGTGCAGTGTACTATTT

AACTATTATTAATCTTTTAAATTTATGTTTAAAAAAAAAAAAAAAAACCCTCAGGGGGGGCCCCGGCCC



>gi|71811968|gb|DR989359.1|DR989359 HDI_I_83.esd Horsegram (VZM-1) drought stressed subtracted cDNA Macrotyloma uniflorum cDNA, mRNA sequence

AAGCTGGAGCTCACGCGGTGGCGGCCGCTCTAGAACTAGTGGATCCCCCGGGCTGCAGGATTCGGCACGA

GGGTTATAGAAGGAATCTGATTCCTTCATCCACTTATGTATGCTTAAGTTATGTATCTCCAGTGGTTGAA

TG



>gi|71811966|gb|DR989357.1|DR989357 HDI_I_81.esd Horsegram (VZM-1) drought stressed subtracted cDNA Macrotyloma uniflorum cDNA, mRNA sequence

AAGCTGGAGCTCACGCGGTGGCGGCGCTCTAGAACTAGTGGATCCCCCGGGCTGCAGGATCGGCACGAGG

GTTTACCAAAAGAATATACAATAGTAATAATAAAATATCATCTTAGTGCATGTTTGGAGTTGAGTTAGAA

GAACTTTAAAGTACTTCTAATGTACTAATGTAGTATATATTCTTCTTTCTTTACTGTGTGTATTGTAATT

CTTACTGTATGGAATTCCTAAAAGAATTTTCCCAACTTTATACAAACATTAAAAAAAAAAAAAAAAAACC

CAGAGGGGGGGCCC



>gi|71811964|gb|DR989355.1|DR989355 HDI_I_8.esd Horsegram (VZM-1) drought stressed subtracted cDNA Macrotyloma uniflorum cDNA, mRNA sequence

GCTGGAGCTCACGCGGTGCGCGGCGCTTCTAGAACTAGTGGATCCCCCGGGCTGCAGGATCGGCACGAGG

CCGACTTCATGGGTCCAGTTGCTGCAGTNGTTGGTAGGTTTCACTGTCTTCTTTGCCTTTGTATTTTCCT

TCTGCATCAAGACACTGAACTTCCAATCAAGATAAAAGAACTACAATGTTGGAAGTGTAAGTTATGCTAT

GGAAAGTTGCAACGGTGTAATGACTTCTGTATGTAAATAACTGAGGCTGGAGTTCATGCTCAAGATTTAT

GGAACTTGTTTCCTAAGCATAATAGATGACCACGATAGTTTTGCTAGCAAGAAGGTTGTTCAGTAAATGT

ACAATCATGTATTATATAGTATGGAGTATGGGTACCTGGAAGAAANTTAGCTTTTTCTCATGGTCGATAC

TTGCAACAATATTATTAGTAATAAATAAAAACTTATTCTTTTTCAGCAAGAAAAAAAAAAAAAAAACCAC



>gi|71811962|gb|DR989353.1|DR989353 HDI_I_72.esd Horsegram (VZM-1) drought stressed subtracted cDNA Macrotyloma uniflorum cDNA, mRNA sequence

AAAGCTGGAGCTCACGCGGTGGCGGCCGCTCTAGAACTAGTGGATCCCCCGGGCTGCAGGAATTCGGCAC

GAGGGAGACTGGAGAGGTTATTGGGGTGTTCGAGAGTGTTCAGCCTTCTGATACTGATTTGGTGGGCTAA

AGCTCCTAAAGATGTCAAAGGAATCCAAGGTTG



>gi|71811960|gb|DR989351.1|DR989351 HDI_I_70.esd Horsegram (VZM-1) drought stressed subtracted cDNA Macrotyloma uniflorum cDNA, mRNA sequence

AAGCTGGAGCTCACGCGGTGCGCGGCGCTCTAGAACTAGTGGATCCCCCGGGCTGCAGGAATTCGGCACG

AGGACAGCAGCAGCAGCAGTGACAGTGATTAGATATCACTGCTGCATCACCTATATATATTAAGGTGTAT

GGACCATAGTAGTGATGGCTGTGACTTCTTTTTTCTTTCTTTCCTTCTTGTGATTTGCATTTATGAGTGA

AAAACAAAAAAATGAGCAGCAAAAAGTGTGCGTGTATGTGGTTGGTAATTGGAGTGTTATATGTGGTTTG

GTTGGTTGCCCACTACTTTGCGTTGTAGNATTATCTTAATGAAATAACAATTTTAAATTT



>gi|71811958|gb|DR989349.1|DR989349 HDI_I_69.esd Horsegram (VZM-1) drought stressed subtracted cDNA Macrotyloma uniflorum cDNA, mRNA sequence

AAAGCTGGAGCTCACGCGGTGGCGGCGNTCTAGAACTAGTGGATCCCCCGGGCTGCAGGAATTCGGCACG

AGGCCTCGTGCCGAATTCGGCACGAGGATCTTTCTATGTTTGTCTCGTGTTTCTATACATCTTATTATGA

GACCAGTGTGAGTGAATATATGTAATCTATGTTTCATGTTTCATGTTTCCTGATTTAATACTAAATTTTG

TTGTGTTTCTATTTTCTGTTTGCCAAATAAAATATCAAGGATCGGTTTAGGAGAAAAAAAAAAAAAAAAA

ANCCCAGGGGGGGGCCCNCGGCCCNATTTCCC



>gi|71811956|gb|DR989347.1|DR989347 HDI_I_67.esd Horsegram (VZM-1) drought stressed subtracted cDNA Macrotyloma uniflorum cDNA, mRNA sequence

AGCGGTACCTACGCGGGCNGCGGCGCTTATAACATGGTGACTNCCCGGGCGTCAGGAATCGGCACGAGGG

GTGCGCNGCAACGANACCATNTTCCCCNTGTCTTTTTCT



>gi|71811954|gb|DR989345.1|DR989345 HDI_I_59.esd Horsegram (VZM-1) drought stressed subtracted cDNA Macrotyloma uniflorum cDNA, mRNA sequence

GCTGGAGCTCACGCGGTGNGCGGCGTTCTAGAACTAGTGGATCCCCCGGGCTGCAGGATCGGCACGAGGA

CAACCCGGAAACCGATGAGTGTCGCACCTATGATAATTAAGTTACCTTGTGTNACATGAATCAGACAACC

TTGTATTAATTAATNCGTATTTATAAGCATAATATTGAAGCTGATACTACTTCGTATGGTTCAAAAAAAA

AAAAAAAAAAAAAAAAAAAAAAAAA



>gi|71811952|gb|DR989343.1|DR989343 HDI_I_56.esd Horsegram (VZM-1) drought stressed subtracted cDNA Macrotyloma uniflorum cDNA, mRNA sequence

TAGCTGAGCTCACGCGGGCCGGCGTTCATAAACTAGTGGATCCCCGGGCCCTGCAGGAATTCGGCACGAG

GCCTCGTGCCGAATTCGGCACGAGGTGGTGGTTATGCTGGGAAAGGATATGATCAAGTTTATCTCTTTGA

ATGATTGTAGATGTTGGGAGTTTCCCTAGAGTGTAATTTTGTGCACAATAAATTATAGAAGGGTGGATGT

ATTTAGAGGAATAACAGTTTATGCTCAGTGATGCTTTTAAGATGCTTAATATCAGGGCAATGCTCTGCTT

ATCAATTAGATATATATATTTTCTTATTGCCCCAAAAAAAAAAAAAAAC



>gi|71811950|gb|DR989341.1|DR989341 HDI_I_47.esd Horsegram (VZM-1) drought stressed subtracted cDNA Macrotyloma uniflorum cDNA, mRNA sequence

AAAGCTGGAGCTCACGCGGTGGCGGCGCTCTAGAACTAGTGGATCCCCCGGGCTGCAGGAATCGGCACGA

GGCTCAGCTGTAGATATAGGTATTTTGAGAATACGTCTTAAAGACCAATGGTTAACAATGGCTCTGATGG

GGGGGGTTGCTAGAATAAACAATAATGAGATCACTGTTTTAGTAAATGATGCGGAAAAGGGTAGTGACAT

TGATCCACAAGAAGCACAGCAAACTCTTGAAATAGCAGAAACTAATTTGAATAAGGCTGAAGGCAAGAGA

CAAACAATTGAGGCAAATCTAGCTCTTAGACGAGCTAGAACACGAGTAGAGGCTATCAATATGATTTCAT

AACAAGTTGGTGCGTCCGAAAATCCGCCGAATATCCAAATACAAAAAAAAAAAAAAAAA



>gi|71811948|gb|DR989339.1|DR989339 HDI_I_45.esd Horsegram (VZM-1) drought stressed subtracted cDNA Macrotyloma uniflorum cDNA, mRNA sequence

GCTGGAGCTCCACGCGGTGGCGGCGTTCTAGAACTAGTGGATCCCCCGGGCTGCAGGAATTCGGCACGAG

GCCTCGTGCCGAATTCGGCACGAGGCCCACGTTGTCCTGGCACATCTACTCCTGAAAGGTTTGGCATGAT

AAGAGCACCTACTCCTCTATTTATGGCACCAGCTGTGAGAATTAGATCAGTGGTTCCAGTCTGCTCAGCT

CCTTCAAGGAGATCCATGACAGAAGAAGCGTCCAAGAGCAAGGAAAAAAAAAAAAAAAAA



>gi|71811946|gb|DR989337.1|DR989337 HDI_I_43.esd Horsegram (VZM-1) drought stressed subtracted cDNA Macrotyloma uniflorum cDNA, mRNA sequence

AAAGCTGGAGCTCCACGCGGTGGCGGCCGCTCTAGAACTAGTGGATCCCCCGGGCTGCAGGATTCGGCAC

GAGGCCTCGTGCCGAATTCGGCACGAGGGCGGTTTTGAGGATTTAAACTTTCATCCATACCTGAAAGAGG

AGATTTTGGAAGTTGGGAGTTATACAGATTCAAGAAGTAGACAGTGGCCAACAGTTTGGGGGAAACAATA

CACCTACATGCATTGAGTACTACTCGTAAACACAATAGTAATATCTGACCAGTTTCTTCCTGGAGGAAAG

CTGATCCGTATGTATGTTACAAATTTGATGGTTGTCAATTGAAACTGATTTGTACTCCGAATTTTACTAT

AGAAGTAGATGTCTCTATTATTATTGTTTGTGAAGTATTGTCACCACCATTTTTGTCTAGG



>gi|71811944|gb|DR989335.1|DR989335 HDI_I_35.esd Horsegram (VZM-1) drought stressed subtracted cDNA Macrotyloma uniflorum cDNA, mRNA sequence

GCTCACGCGGTGGCGGCGCTCTAGAACTAGTGGATCCCCCGGGCTGCAGGACTGGGATGATGAATTTACT

CAATATCCACTGGTGATGGGGTTGATTCTCGGCACCGGTGTTGGCGGCGGGCTGATTTTCAACGGCAAAC

CGATTAAAACGTGCGGTTGATGTACATAATCTTGCAGAGCTTCGTATCGCTGGCTCAACAGGAATTGATG

CCGAAGGCCCGGATAGTGGTAAACATGATGTCGACTTAACCACCATCGTTTATTCACCACCGCTGAAGGA

TAACTGGCGCGGTTTTGCTGGATTCGGTTATGCCGATGGACAATTTAGCGAAGGAAAAGGGATTGTTCGC

GACTGGCTTGCGGGTGTTGAGTGGCGGTCACGTAATATCTGGCTCGAGGGGGGGCCCGGTACCCAATTCG

CCCTATAGTGAGTCGTATTACAATTCACTGGCCGTCGTTTTACAACGA



>gi|71811942|gb|DR989333.1|DR989333 HDI_I_33.esd Horsegram (VZM-1) drought stressed subtracted cDNA Macrotyloma uniflorum cDNA, mRNA sequence

AGCTGGAGCTCACGCGGTGGCGGCGCTCTAAACTAGTGGATCCCCCGGGCTGCAGGATCGGCACGAGGAT

TGCTCCTTATAGGGGATCCTCGTAAAGATATCGAGGAAAATCCAAACTGCTTGTTAATAGTGACACCCAA

AGGGGGCCATCTAGGGTGGGTTGCTGGTGATGAAGCTCCACTGGGAGCCCCTTGGACTGATCCTTTGGTA

ATGGAGTTTATTCAGTATTTAGACAGAGATGCAGTTAAAGCTCCAGAAGCTAATAGTAATCCAGGAAGTA

AAGAGGCCTTGTACCATCTTGAAGTGTAATATTGGTTGATCTTTTCCAGACATCAAATGCATCCTGGAAG

CTCACCAGAAAGGATATCAATATATCTTGCAATATTCTTTAGCCCATTTGTAATCCCTAATGTTTTGGAA

G



>gi|71811940|gb|DR989331.1|DR989331 HDI_I_31.esd Horsegram (VZM-1) drought stressed subtracted cDNA Macrotyloma uniflorum cDNA, mRNA sequence

GCTGGAGCTCACGCGGTGGCGGCGCTCTAGAACTAGTGGATCCCCCGGGCTGCAGGATCGGCACGAGGGA

CACAACAGCCATTGTTACTTTCCATTGATTGAGCCGAAGCTTTGGCTTTTCTCGGATAATTTTCCATTGG

GTACAAGAAAGTCTCAACTTTTCACCGAAAGAGCAAGGATCTGAGATGTGTGGAGGTGCGATTATCTCCG

ACTTCATTCCGGTAGGACCTGCTGGCCGGTCCCGGCGCGTGACCCCGGACATCCTGTGGCCGAATTTGAG

TAAGCGGTTCTCGAAGCAGCTGCTGGACGATGATTTCGAGGCGGGGTTCAGAGAATTCAAGGATGACTCC

GAAATCGAGGAGGATGATGATGAGGATGAAGAAGGGGAGGATTTGATGGTAGTTGGTGTGAAGAAGCCCT

TTGGCTTTTCTCGCCCCAACAAGGCTTCTAAGCCTCTCTCTCATGGATCAGCAACTGTAAAATCTGTGGA

ATCAAAGGGGCA



>gi|71811938|gb|DR989329.1|DR989329 HDI_I_23.esd Horsegram (VZM-1) drought stressed subtracted cDNA Macrotyloma uniflorum cDNA, mRNA sequence

AAGCTGGAGCTCACGCGGTGGCGGCGCTCTAGAACTAGTGGATCCCCCGGGCTGCAGGAATTCGGCACGA

GGCCTCGTGCCGAATTCGGCACGAGGCACTCACTGGCTTCTTTGAGAAGTTGGAGAAGGAGAAGACAAAG

TTTGGAGGTTGATATCATCATCAACAACACGAATGAATGAATCAATAATCCATGGTGATGCTGCTTCTGC

ATGATGGATTATGGAAGCAGCCTAGTTAGGTTCTTTTGGTTTTTCCCACAGACCCATTAGTTAGATAATT

TTTTTTTTCCTTTTAATTTTTCTGTTCTTTGCTGTACTTTTGGAGGTTGGGATCAAGGAAAATGTCTCCC

ATGTTCTTAAAAAAAAAAAAAAAAAACCCACAAGGGGGGGCCCCGGTATACCCAATTTCGCCCCTA



>gi|71811936|gb|DR989327.1|DR989327 HDI_I_21.esd Horsegram (VZM-1) drought stressed subtracted cDNA Macrotyloma uniflorum cDNA, mRNA sequence

TGGAGCTCACGCGGTGGCGGCGCTCTAGAACTAGTGGATCCCCCGGGCTGCAGGAATCGGCACGAGGCCT

CGTGCCGCAGAACCTCAATCCTTATCTCTTTGATCCTGATATGGTGGAAACCACTAGAAAAAGTGGTAAA

TGGCTGTTATATTTTACGTTCCTACATGCTTTTGATGCTAATGACAGCCATATGTTGTAACTTGTAACAT

GCTTTTGATTGCCACTGTATGGTAGTAACTTAACTGCCTCCTTCTGATAAGTGATGAGAAGGACCATGCA

GTGGATCAAACCATTTTCTGTACAAGTCTATTTACAAAAAAAAAAAAAAA



>gi|71811934|gb|DR989325.1|DR989325 HDI_I_19.esd Horsegram (VZM-1) drought stressed subtracted cDNA Macrotyloma uniflorum cDNA, mRNA sequence

AAGGCTGGAGCTCACGCGGTGGCGGCGCTCTAGAACTAGTGGATCCCCCGGGCTGCAGGATCGGCACGAG

GATTTAAATTGGGTGTCATCGAATCCTTCATACTTTTTTATGTTTTTCTTTTCATGATTCCCTCGATGGT

GAGTTGATGCTACTCTTAAGCATAAAGCCAACATATAAAGAATGGGTATAATTTTTTCCTATTAAAAAAA

AAAAAAAAAAAAAAAAAAAAAAAAAAA



>gi|71811932|gb|DR989323.1|DR989323 HDI_I_11.esd Horsegram (VZM-1) drought stressed subtracted cDNA Macrotyloma uniflorum cDNA, mRNA sequence

AAGCTGGAGCTCACGCGGTGCGCGGCGTTCTAGAACTAGTGGATCCCCCGGGCTGCAGGATTCGGCACGA

GGGTTAGCAAGGGTTATGATTAAAAAACAGTTAGATAATAAAATGGTTAGACAAATTGCTGATACAGATT

GATAATCAACAACATATTCTATTGTTAAACCTTCTATAACTGAAAAAAAAAAAAAAAAAAAAAAAAAAA



>gi|71811930|gb|DR989321.1|DR989321 HDI_I_90.esd Horsegram (VZM-1) drought stressed subtracted cDNA Macrotyloma uniflorum cDNA, mRNA sequence

AGCTGGAGCTCACGCGGTGGCGGCGCTCTAAACTAGTGGATCCCCGGGCTGCAGGATCGGCACGAGGTAT

GGATCCCCCCTACGTTATAGAGTGACCAGATGTAATAACATGAGGAAGTTTCAGTTGCTGATGAATACAT

AATAAGCTTGAGAAGCTTCCCTTTGAAGCACTTCTCATGGATGTGCTTCAAGCTTTTGAATTTATGTAAG

AGTGTTTGACGCAGTTTGTGTAACTGATTTGCCTTTGGATGCAAAATAAATGGAAATTGAAGTCCAAAAA

AAAAAAAAAAAACCCCAGGGGGGGNCCCGGGCCCCAATTCCC



>gi|71811928|gb|DR989319.1|DR989319 HDI_I_88.esd Horsegram (VZM-1) drought stressed subtracted cDNA Macrotyloma uniflorum cDNA, mRNA sequence

AAGCTGGAGCTCACGCGGTTGGCGGCGCTCTAAACTAGTGGATCCCCCGGGCTGCAGGATCGGCACGAGG

CCTCGTTGCCGAATTCGGCACGAGGAGAGGATGATGAAGTCATGTACAAAGAAAGAGTGGATGTTGAAAG

GGCTGAGTGCAACTCCGGGAACAGCAAGCTTCGTTTTGGACACAAGTGGAAGACATATAGGCCTTAAGAA

GATAATGACCAAGAACCTAGTTTGCTACAAAAACTACCTAATACATCAAAATCAGCAGTATGTATGATTG

TGTGAAATAAATGCCTGCTTAGCCAGCCGTTATGTTTGTTTAACCAGGGAGTGTTTTATATCTGTAGTAC

ATTAGTACTGAACTTTCCCATGTATGAACTATTAATGAAGCATATACATCAGTTATCTAAAAAAAAAAAA

AAAAAACCCAAGGGGGGGCCCGGTACC



>gi|71811926|gb|DR989317.1|DR989317 HDI_I_86.esd Horsegram (VZM-1) drought stressed subtracted cDNA Macrotyloma uniflorum cDNA, mRNA sequence

AAGCTGGAGCTCACGCGGTGGCGGCGCTCTAGAACTAGTGGATCCCCCGGGCTGCAGGATCGGCACGAGG

GGCTTTTATGATAGAGGAGCCAGTGAAGGAAGTGAAGAATAAAGAAGAAGAAGAAGAAGAACACAAGGAA

GTGAAGAAGGAAGAGCTGAAGGAAGTGAAACAAGTTGGTAGTACTGTTGAAGTCCAAGAGGTTTGATTCT

CCCTCTTGTGGCTGGTATTGGCTAGGTATGANAATGATATGATATGATATGATATGATAATAAGTTTATT

ATTTCACAGGTTTTATGTCAAAGACTTGCACTAAGTTGTACTGCTTTCCATTTCTTTCTTGTTCTATAGC

TAGGCTCTAGCTAGCTGTGTATCAGCCCTAGCTAGCTGCACTTTGAAAGAAAAAACAATGCTCAAGTTAT

AAAATTTATTCCCTTTAAGTTTCTAAAAAAAAAAAAAAAAA



>gi|71811924|gb|DR989315.1|DR989315 HDI_I_78.esd Horsegram (VZM-1) drought stressed subtracted cDNA Macrotyloma uniflorum cDNA, mRNA sequence

AAAGCTTGGAGCTCACCGCGGTTGCGCGGCCGCTTCTAGAAACTAGTTGGATCCCCCGTGTGCTGCAGGA

ATTCGGCACGAGGCCTTCGTGCCGAATTTCGGCACGAGGTTGTTTTTTTTTTTTTTTTTTTTTTTTTTTT

TTTTTTTGT



>gi|71811922|gb|DR989313.1|DR989313 HDI_I_76.esd Horsegram (VZM-1) drought stressed subtracted cDNA Macrotyloma uniflorum cDNA, mRNA sequence

TGGAGCCACGCGGTGGCGGCGCTCTAGAACTAGTGGATCCCCCGGGCTGCAGGATCGGCACGAGGGAAGA

ATAATATCTGGTCTTATCATGGAGGAGGAAGAATGCTCAACCTTTCTTACGTTTTCCGTACTTATGTTTG

CTTGTGTCATGTTCTTTATTTCCAATAAAGGGTCTTGTTGGCCCCATGTGTTGAACTAGTATCTATCTAA

GCTTGTAAGCTTGTTGCGAAGGGTGCATGCTTGTCTGGACTAGTATAGCATCTACGCGTCAGTGTTTGGT

ACTTGAGTATTTTGGTTCTTGATGATTATGTTTTATGCTTTAATAAAACGTGGTGTCTTGATAATATAAA

AAAAAAAAAAAAAAAAAAAAAAAAAAAAAAAAAAAAAAAAAAAAAAAAAA



>gi|71811920|gb|DR989311.1|DR989311 HDI_I_74.esd Horsegram (VZM-1) drought stressed subtracted cDNA Macrotyloma uniflorum cDNA, mRNA sequence

TGGAGCTCACGCGGTGGCGGCGCTCTAGAACTAGTGGATCCCCCGGGCTGCAGGATCGGCACGAGGGTTG

ACAATGCGAAGAAGTATGAGCCCAAGTACAGGCTTATCAGGAATGGACTTGATACTAAGGTAGAGAAATC

AAGGAAGCAAATGAAGGAGAGAAAGAATAGAGCTAAGAAGATTCGTGGTGTAAAGAAGACCAAGGCTTCC

GATGCTGCCAAGGGTGGAAAAAAGAAATGAGTTTTCAAACATTCTGTTCTGATTTTAAGGTTCTATTGCT

ATTTTCTAATGGTTATTTTATTTTATTTTATAATAGTTTCCTAGAATGAGATATTTTGCTGTAAGTGTTG

ATCCTGAATA



>gi|71811918|gb|DR989309.1|DR989309 HDI_I_66.esd Horsegram (VZM-1) drought stressed subtracted cDNA Macrotyloma uniflorum cDNA, mRNA sequence

AGCTGGAGCTCACGCGGTGCGCGGCGCTCTAGAACTAGTGGATCCCCCGGGCTGCAGGATTCGGCACGAG

GGAAATTTAGCACCATGTAGTGGTGGCTTAATAACATTTGAAGGGCTTTCTCCCCCCGAAAACAGGAAAT

CAAAAGATGGGCATTTTTATTTTTTTTAATCTTGTTGTAATTTCGTTTACATGTACATTCCTACCTTCCT

CCTATTTTGCACATTCCATTGTGAATTCTCGTGTAAACATTTGATTTTATGAAAGCATTAACAAGAAGTT

GATTGTCAAAAAAAAAAAAAAAA



>gi|71811916|gb|DR989307.1|DR989307 HDI_I_64.esd Horsegram (VZM-1) drought stressed subtracted cDNA Macrotyloma uniflorum cDNA, mRNA sequence

TAAGCTGGAGCTCACGCGGTGNGCGGCGTTCTAGAACTAGTGGATCCCCCGGGCTGCAGGAATCGGCACG

AGGCTGGAGTTGAATCTGTCCAACCAACTGGCTGGGAATAAGTTATATATGTATTTATTGATGCTATGAT

TTTGCCCCGTGCTGCAATTTTATTTACTCTTTTGTCATTTCTTTGAAGTAAAATATGGATCAGTCTTTAT

TTGTAGCAAAGGAACTTTTTTAGTTTGAGTGTCAGAAATATTATTATTAACATGGTATATTCGGTAAGAT

TTTATTTTATTTTGCATAGAAATCATGGTTCGTGAATGTTTTGGTAAAAACAAAAAAAAAAAAAAAAAAA

AAAAAACAAAAAAAA



>gi|71811914|gb|DR989305.1|DR989305 HDI_I_62.esd Horsegram (VZM-1) drought stressed subtracted cDNA Macrotyloma uniflorum cDNA, mRNA sequence

AAAGCTGGAGCTCACGCGGTGGCGGCGNTCTAGAACTAGTGGATCCCCCGGGCTGCAGGAATCGGCACGA

GGGTTTGTTTTGCCGGTTATATTTTCAGGTTTCTGCCGGAATTCATGTACCCTTAACAGGGTTCTTGATC

GGTGGTGGTCAATTTTAATTACTGTTTGTGTCCGAGAGTACTGTTTTTCATGTAAGAGTCTGACGATTTC

AATTTTCACAAAGCAGCCATAGGCTTTCTAGTCTTTTTTCTTTAGCCTGCCTGAGAGATGTTTTAAAAAC

AACAAAAAAAAAA



>gi|71811912|gb|DR989303.1|DR989303 HDI_I_6.esd Horsegram (VZM-1) drought stressed subtracted cDNA Macrotyloma uniflorum cDNA, mRNA sequence

AAAGCTGGAGCTCCACGCGGTGGCGGCGCTCTAGAAACTAGTGGATCCCCCGGGCTGCAGGAATTCGGCA

CGAGGCCTCGTGCCGAATTCGGCACGAGGCGGCACGAGGGTTTTTTTTTTTTTGTGTANAAAANACAAAA

AACACATTTAAGTTGTGAGATTGTGAGATATCCACACAAGNAATGTGATTCTCACACTATTCTGAAGGTT

AACATTGNGCAATTAACCATGTGATCCATCTACAGCAAAACACAATATT



>gi|71811910|gb|DR989301.1|DR989301 HDI_I_53.esd Horsegram (VZM-1) drought stressed subtracted cDNA Macrotyloma uniflorum cDNA, mRNA sequence

GGAAAACATGTTCTGTTTGTTGACTCTTAATGTGNGTTTTTAGAACACCTGACTTGAACCAAGTGCTATA

GACTGTAAAATAACGAGACAGTAACTATACTTCTGTAAACCAATAAAATCCTTTCCTGTTTTAAAAAAAA

AAAAAAAAAAAACTCTCGAGGGGGGGGCCCGCGGT



>gi|71811908|gb|DR989299.1|DR989299 HDI_I_51.esd Horsegram (VZM-1) drought stressed subtracted cDNA Macrotyloma uniflorum cDNA, mRNA sequence

TGGAGCTCACGCGGTGCGCGGCGTTCTAGAACTAGTGGATCCCCCGGGCTGCAGGAATTCGGCACGAGGG

TTAGAGAGAGAAAAAGCACAACCCATTTTTCTCTCTCATTTTTTTTGGGATTTTCTCTCTTTCTTTCTGC

AACTGCAACAAATCATGGGTTCAACCAATCTAGTACTCTTCTCAGCCTTGTTCTTCATTTTGGTCGGTTT

TGCAACCTCGGATGTTAACCAAGACAAAGCAGAATGTACGGACAAACTCCTTGGTCTTGCTGGTTGTCTC

CCCTACGTCAGTGGTGATGCCAAA



>gi|71811906|gb|DR989297.1|DR989297 HDI_I_5.esd Horsegram (VZM-1) drought stressed subtracted cDNA Macrotyloma uniflorum cDNA, mRNA sequence

AAGCTGGAGCTCACGCGGTGGCGGCGCTCTAGAAACTAGTGGATCCCCCGGGCTGCAGGATTCGGCACGA

GGGTTTGAAATCCCCTATAAAGAGTGGGATTTATGGTTGGTTATGAGACTGCTGCAACAAGTGAATAAAA

CTTCTATAGTGTATAGTATTTCCAAATTAAAAAAAAAAAAAAAAAAAAAAAA



>gi|71811904|gb|DR989295.1|DR989295 HDI_I_42.esd Horsegram (VZM-1) drought stressed subtracted cDNA Macrotyloma uniflorum cDNA, mRNA sequence

TTGAAAGGCATATGGCTTGCGAAGAGACTGACGAACAAGAAAAGCGATTTTGCGTTGCGATGGGACATAC

TTGCATAGCATAACATTTATGCGCTCGCGTACGCGTATTGCTCTCGCATAAATTATGCGCTCAACAACCA

TATTGCATCATACTTATAAAAGTTTCGT



>gi|71811902|gb|DR989293.1|DR989293 HDI_I_40.esd Horsegram (VZM-1) drought stressed subtracted cDNA Macrotyloma uniflorum cDNA, mRNA sequence

AAGCTGGAGCTCACCGCGGTGCCGGCCGTTCTAGAAACTAGTGGATCCCCCGGGCTGCAGGAATTCGGCA

CGAGGGAATAAGGCTAACATCAAAATCGTGGTCGTTGGAGCATGTCTCTGAGTAAATCGANACTGCCACG

GAGTCGTTGGAAGCCCTACTGAGAAGGGTGTGACTGANAGGTTCGAGAAGGGAGAAGGAAGTTGTTTAAA

ACACGTAGAGACTGTAAGATGTGTGAAGAAGGATGATGTAAAATAAATAAGATATAAGATGTGAAGATGG

ATCTATCTTAAGATGAAGATGCTGAAGGACTTATATTT



>gi|71811900|gb|DR989291.1|DR989291 HDI_I_39.esd Horsegram (VZM-1) drought stressed subtracted cDNA Macrotyloma uniflorum cDNA, mRNA sequence

AAGCTGGAGCTCACGCGGTGCGCGGCGTTTCTAAACTAGTGGATCCCCCGGGCTGCAGGAATTCGGCACG

AGGCCTCGTGCCGAATTCGGCACGAGGTTTTTTTTAATCGGCCAAGTGAAAGGAGGCATTTAAGAAAATG

ATGGGGCGGAAGAATGGTAAAATCATGGACATCGTCATCATGAATCATGAAACTCCAGGAAACAAGGAAA

AATTTTGCTGCAGACTCAACCTAATCAAGTTTTCTTAAAACTACAGATGAAGTCGGGGGTTGGGGGAGGG

GGAGATTTCAAAAACAATGAAGTCGACATGATAAACGAGTTAAAGACTAACAAAAAAAGGGGGGGCCCGG

TACCCAATTCGCCCTATAGTGAGTCGTATTACAATTCACTGGCCGTCGTTTTACAA



>gi|71811898|gb|DR989289.1|DR989289 HDI_I_37.esd Horsegram (VZM-1) drought stressed subtracted cDNA Macrotyloma uniflorum cDNA, mRNA sequence

AAAGCTGGAGCTCACGCGGTGGCGGCGCTCTAGAACTAGTGGATCCCCCGGGCTGCAGGAATCGGCACGA

GGCCTCGTGCCGAATTCGGCACGAGGGACCTTTTTTATGATGTCAGTTGGCTTAGAAGATGAACTGTATG

CTGAAGATGACTATTGGATTAATGGCTATGAGAGAAACAAGTGCTTATCGTGATCCTCGGACAATATACC

ATTTGTGAGTAAAGGTGTATATCATATCTCATATATGTATATCATAAACAAAACACAATCATCATCCTTG

TTATCTTCTGTCAAAAGTATCTAAGAATGAATATTCTCTGGTTTCTCATCAAAAAAAAAAAAAAAAAAAA

ACCCCGGGGGGGGC



>gi|71811896|gb|DR989287.1|DR989287 HDI_I_3.esd Horsegram (VZM-1) drought stressed subtracted cDNA Macrotyloma uniflorum cDNA, mRNA sequence

AAGCTGGAGCTCACCGCGGTGGCGGCCGCTCTAGAACTAGTGGATCCCCCGGGCTGCAGGAATTCGGCAC

GAGGCAATCTTGTGCTATGGTCCANGAAAATTGATCACCAAACTGTTTGTTGATCATGTGCGTGATGCTT

CTCAGGTTTCATCTTCTTGGAGAATGTTTCGTTGNATTTGTACTGGTGTAATGTTTATAGAACTATAAAA

AGTTATGGATTTTTATCCATTTAATAAAAAAAAAAAAAAAAAAAACCCAGAGGGGGGGCCCCGGGCCCCT



>gi|71811894|gb|DR989285.1|DR989285 HDI_I_28.esd Horsegram (VZM-1) drought stressed subtracted cDNA Macrotyloma uniflorum cDNA, mRNA sequence

AGCTGGAGCTCACGCGGTGGCGGCGCTCTAGAACTAGTGGATCCCCCGGGCTGCAGGAATCGGCACGAGG

CTTGTTTTAATTTATATAGGAGAAGAGGATGAAAAGGGAGGTCGGCGACAGAGGAAAGTGTTCAATCCCA

GTTTTGTGCCAGTTGAAGAATTTATATGTAAAATTTTTTAGGATTACTGCGACTGGTAAAAAGACAAAAT

AAGTGAAAAGAGGAAGTAAGTATTCTATAGTATATTTTGTGAGATTTATACAAACTTTCCTCACATATTG

GCTATCTCGAAGTATAGAAACTTTGGTGAAAAAAAAAAAAAAAAAAAAAAAAAAAAAAAA



>gi|71811892|gb|DR989283.1|DR989283 HDI_I_26.esd Horsegram (VZM-1) drought stressed subtracted cDNA Macrotyloma uniflorum cDNA, mRNA sequence

AAGCTGGAGCTCACGCGGTGGCGGCGCTCTAGAACTAGTGGATCCCCCGGGCTGCAGGATCGGCACGAGG

GCCACCATCACCAGAGGACTTAGGGAAATGGCGCGGGAGATGGCACGTAAAAGGAACAGCGGCTGATGTT

CCATGTGGTCGATTTGGTTGCAGTGCAGTTGTGAGACAGAATCACTGCTTATGTAGTAAATATGTTTATT

GTTGTAATAATGAATAAGGACTTATAAACTACTGTAGAAATGTTTTTTATTTTTCAAAAGCTTCAACAGT

TTGGTGCCATGGACTACCTACAACTTCTCTGTCTTACCTTCAATCTAAAAGAAAAATAAATTATATTAGA

GATCCTGTTATAGCTGTTTTTCATAAAAAAAAAAAAAAAA



>gi|71811890|gb|DR989281.1|DR989281 HDI_I_18.esd Horsegram (VZM-1) drought stressed subtracted cDNA Macrotyloma uniflorum cDNA, mRNA sequence

AAGCTGGAGCTCACGCGGTGGCGGCGCTCTAGAACTAGTGGATCCCCCGGGCTGCAGGATCGGCACGAGG

TGCCAATAAAGCGTCCTTGCATTCATGGGAATATGGGATGCATCTTTAATCAAATATCGTCTGTGTTTAT

TTGCTAGAGTTGCAAGAGAGTTTGATAATTATGTTCATTTTTCAGCTTGTTTGTTTGATGTCACAATAAT

GTTTATGTACCAGCTTGTGATCACAGTTCATTGTTATATAATTATGCTATTTTGTTAATTAAAAAAAAAA

AAAAAAAAACCCAAGGGGGGGCCCGGGTACCCAA



>gi|71811888|gb|DR989279.1|DR989279 HDI_I_16.esd Horsegram (VZM-1) drought stressed subtracted cDNA Macrotyloma uniflorum cDNA, mRNA sequence

GTTCTAGAACTAGTGGATCCCCCGGGCTGCAGGAATTCGGCACGAGGGTCGTTTTCGCACTCTGGTGTTA

GGTCTAAACACCCTTTAAAATAAAGTGGCCATGTCTTCCCTTGAGCAAGTTCAAGTGTTATGGACTTGTG

ATGGGTTTATCTAGCCATGGTTACTCTTTTTTTTTTAACTATGTGGTTTGTTCAGTGTAAAGTTGGCTAA

TCAGAATTATCTTTCTCTTGCATAAAAAAAAAAAAAAAA



>gi|71811886|gb|DR989277.1|DR989277 HDI_I_14.esd Horsegram (VZM-1) drought stressed subtracted cDNA Macrotyloma uniflorum cDNA, mRNA sequence

AAGCTGGAGCTCACGCGGTGGCGGCGCTCTAGAACTAGTGGATCCCCCGGGCTGCAGGAATCGGCACGAG

GCTCTCATGGTATATAATTTGATTTGGGAGCAGTTACGAGATTAATTTGATTCCTCGTATTTTTTTTCAT

TAATGTTTTAACGTCATTAGTTTAAATGTGTGAAAAGAATATATTAGGTTCCACCAGATACTATAATTCC

TATCTATCTGTATGTAGTTTTGGGTTTTTCACTTTATATAATAAAATCTTGTCTTGGGCTGTAGCCCATT

TTAAAAAAAAAAAAAAAAAAACCNCAAAAGGGGGGGCCCGGGAT



>gi|71811884|gb|DR989275.1|DR989275 HDI_I_1.esd Horsegram (VZM-1) drought stressed subtracted cDNA Macrotyloma uniflorum cDNA, mRNA sequence

AAGCTGGAGCTCACCGCGGTTGGCGGCCGCTCTAGAACTAGTGGATCCCCCGGGCTGCAGGATTCGGCAC

GAGGTTTTGTACCAAAGAGCATCTAATTTTCATATACTTAATATTAAAATAAAAAAACAAACTTAAGATT

CTGCCATGAACCATAAACTAATAGCATAACCATTACCAGATAAAACCTAAAGATAAATTTCTCAGGCAGT

TATAGCTGCATTATGCAAACAAAAGAAAGCATGCAGAAAACATATAACCTCTACCATAAATTGCACTACT

GTTGGGCACATTGCACTCGAGGGGGGGCCCGGTACCCAATTCGCCCTATAGTGAGTCGTATTACAATTCA

CTGGCCGTCGTTTTACAACGA



>gi|71811882|gb|DR989273.1|DR989273 HDI_H_95.esd Horsegram (VZM-1) drought stressed subtracted cDNA Macrotyloma uniflorum cDNA, mRNA sequence

AAGCTGGAGCTCACGCGGTGGCGGCGCTCTAGAACTAGTGGATCCCCCGGGCTGCAGGAATTCGGCACGA

GGCAAGAACCCTCTACTAGTCGGAGCGATCCGAGAGAACCTCTGCGAACATGTTGCTTTACCAGGACCTC

CTTACAGCTGACGAGCTTCTCTCTGACTCCTTCCGCTACACTGAAATTGAGAATGGAATGTTGTGGGAAG

TTGAGGGAAAGTGGGTTGTTAAAGGAGCAGTTGATGTAGACATTGGAGCAAACCCTTCTGCCGAAGGTGG

AGATGATGAGGGTGTCGATGATCAGCTGTTAAAGTTGTTGATATTGTTGACACCTTTAGAACTCCAGAAC

AACCTGCTTATGACAAGAAACAATTTGTTACCTTTATGAAAAGATTTATCAAGAATTTGACTCCCAAACT

TGATGCAGAGAAACAGGAGTTGTTTTAAAAAACACATTGAAGGGGGCAACCAAATTCCTGCTCCCAAAGC

TTAAAGATTTTCCATTTTTTGTTGGGGAGANCATTGCCTGATGATAG



>gi|71811880|gb|DR989271.1|DR989271 HDI_H_93.esd Horsegram (VZM-1) drought stressed subtracted cDNA Macrotyloma uniflorum cDNA, mRNA sequence

AAAGCTGGAGCTCACGCGGTGGCGGCGCTCTAGAACTAGTGGATCCCCCGGGCTGCAGGATCGGCACGAG

GGAAAGACAGAACCCTAGCACATTTGCAATTAACGCGGTGTGCACTCATCTCGGATGCGTCGTACCATGG

AATACAGCTGAGAAAAAGTTCATTTGCCCATGTCATGGATCTCAGTACAATGACCAAGGCAGAGTTGTGA

GAGGACCTGCTCCCTTGTCTCTGGCATTAGCACATTGTGATATAGATGATGGGAAGGTGGTGTTTGTTCC

TTGGACTGAAACAGATTTCAGAACAGGTGATGCTCCATGGTGGGCTTAGACTCCTTAAAGCCGTTCCTTT

GTTGTCATATGTATTTAAATATGAAATTCAAACAATCTGTTTTGTATCAATTCATAAATATATTTTGTAA

TCAAATCGATATTCTGTGAAAAAAAAAAAAAAAAAAACCCCANGGGGGGCCC



>gi|71811878|gb|DR989269.1|DR989269 HDI_H_91.esd Horsegram (VZM-1) drought stressed subtracted cDNA Macrotyloma uniflorum cDNA, mRNA sequence

AAGCTGGAGCTCACGCGGTTGGCGGCGCTCTACAACTAGTGGATCCCCCGGGCTGCAGGAATCGGCACGA

GGGTTTTGGACTTTATTGGAGTAGCTTTCCAAGTAAACTACTACTCAGCTTTGTACTCTAGTTTTTCAAA

ATATTGCTCATTTATTATTTCATTAGAAATTTTGAGGAAGTGTGCAATTGAAAAAAAAAAAAAAAAAAAA

AAAAAAAAAAAAAAAAAAAAAAAAA



>gi|71811876|gb|DR989267.1|DR989267 HDI_H_84.esd Horsegram (VZM-1) drought stressed subtracted cDNA Macrotyloma uniflorum cDNA, mRNA sequence

CCCCGGCGTTCTAGAAACTTAGTGGATCCCCCGGCGCTGCAGGATTCGGCACGAGGGAGTGGTGGGCTCG

ACTACTTGAGGCAACCCAAGCCTGATCCACGCTCAGAGCATCCGTCGACCATCATGGGCTACCCAAANNT

TATCCTCATGGTGCGCCGATTGAGGGTTACCGTAATTGCCG



>gi|71811874|gb|DR989265.1|DR989265 HDI_H_82.esd Horsegram (VZM-1) drought stressed subtracted cDNA Macrotyloma uniflorum cDNA, mRNA sequence

AAAGCTGGAGCTCACCGCGGTGGCGGCCGCTCTAGAACTAGTTGGATCCCCCGGGCTGCAGGAATTCGGC

ACGAGGCTGCAATTGATGAAACCAAGCAAGGGCAATGGGAAATGATAGATTCCAGGCCAATGATGTATGC

TAGGCCTCCCATGGCAGTCAATTACATGTACCCTCCTCCGTATAGCTACCCTCCTCCTCCTCCTCCTGAC

CCATATTCCAACTTTTTCACGTGATGAGAATACTTCCAGCTGCAGTATTATGTGAAGAGTAATGGGCGAT

GGTGTTGACAANAGAGGGTTGTATGGTTAAGGTTGCAAAGTCCTTTCTAATCTGATGATGCAATATTATA

CAATGGANATGAGTNTCAATTTTA



>gi|71811872|gb|DR989263.1|DR989263 HDI_H_80.esd Horsegram (VZM-1) drought stressed subtracted cDNA Macrotyloma uniflorum cDNA, mRNA sequence

TGGAGCTCACGCGGTGGCGGCGCTCTAGAACTAGTGGATCCCCCGGGCTGCAGGATTCGGCACGAGGGCG

GATGCTGCGGCTACCTTTAAGGAGCTGTTGACAAGACATAAATCTACTGTAGCTGAATTCCTTTCCAAGA

ATTATGAATGGTTTTTTGCAGAATATAACACTAAGCTGTTGGAATCTTCCAATTATATTACAAGGCGCCA

AGCTGTCAAGTTATTGGGAGATATATTACTTGAACGTTCAAATTCAGCAGTAATGACACGATATGTGAGC

TCGAGGGGGGGCCCGGTACCCAATTCGCCCTATAGTGAGTCGTATTACAATTCACTGGCCGTCGTTTTAC

AACG



>gi|71811870|gb|DR989261.1|DR989261 HDI_H_79.esd Horsegram (VZM-1) drought stressed subtracted cDNA Macrotyloma uniflorum cDNA, mRNA sequence

TCACTACTTCAGCGTGCTCATCCATACCAAAGAAGAGAGAGGCCACTTCGACAAATGCTACAAGTCTTGG

ATCCCACCACCAACGCCTGTGACTGGGATGTTTGGGAGAGACTGGTTTATTGCCATGAGTGTTTAGGCAA

GCCCACGTAAAATTATGTATGCATACCCCAATTTCTCTATACAGTCTGATGGGAAGTACTACCCCGTAGA

TTGATGAACGGCAAGAGATGCATGGCACACAAATCTTTGAACAGAATCTCTATTTTTCTCTTTTGCTGCT

TCCTTCTTTGCCTTGATNTGTCTTCTAAGTGACGCATTAGGATATATATTATCTCAATATCTATTATTGT

ATACATTGTAAAATGCAAGTTTTAAAAGCTTGATAGATTCAAACAAAAAAAAAAAAAAAACC



>gi|71811868|gb|DR989259.1|DR989259 HDI_H_71.esd Horsegram (VZM-1) drought stressed subtracted cDNA Macrotyloma uniflorum cDNA, mRNA sequence

GCGGAGCTCACGCGGTGGCGGCGCTCTAGAACTAGTGGATCCCCCGGGCTGCAGGATCGGCACGAGGAAA

GCTTCAAGCTGAGATGACCTTAATTAGCATTAGTGGCAGGAATAATAATAATAATTAACTATTATATGTA

GTTAAAAAGCTTAAAATTATGTATGTCTGTGTGTATGTCGTTTTCGCACTCTGGTGTTAGGTCTAAACAC

CCTTTAAAATAAAGTGGCCATGTCTTCCCTTGAGCAAGTTCAAGTGTTATGGACTTGTGATGGGTTTATC

TAGCCATGGTTACTCTTTTTTTTTTAACTATGTGGTTTGTTCAGTGTAAAGTTGGCTAATCAGAATTATC

TTTCTCTGCTAAAAAAAAAAAAAAAAAAAAAAA



>gi|71811866|gb|DR989257.1|DR989257 HDI_H_7.esd Horsegram (VZM-1) drought stressed subtracted cDNA Macrotyloma uniflorum cDNA, mRNA sequence

AAGGCTGGAGCTCCACCGGCGGTGGCGGCCGCTCTAGAACTAGTGGATCCCCCGGGCTGCAGGAATTCGG

CACGAGGCCCGGCTGCTCTTTCCAGTGGAAGGCCCAGAGGTAGGCCTCCTAAGGTGAAGCCTCAGTTGAC

AGAAGTCAGTGTTTGAGTCATAGAATTGTTTTTTTTTTTTTTTTATATTTGGGA



>gi|71811864|gb|DR989255.1|DR989255 HDI_H_68.esd Horsegram (VZM-1) drought stressed subtracted cDNA Macrotyloma uniflorum cDNA, mRNA sequence

AAAGCTGGAGCTCACCGCGGTTGGCGGCCGTCTCTAGAACTAGTGGATCCCCCGGGCTGCAGGATTCGGC

ACGAGGCGAAAACGCTACATCATTATTCCCGGCACGGAGCCATCTCCTCAAAGCTCAAGACCGCTCCCCG

TAGATCCCCAGCAGCGACCTTCTCCTCATACCTCGAGGGGGGGCCCGGTACCCAATTTCGCCCTATAGTG

AGTCGTATTACAATTCACTGGCCGTCGTTTTACAACGAC



>gi|71811862|gb|DR989253.1|DR989253 HDI_H_59.esd Horsegram (VZM-1) drought stressed subtracted cDNA Macrotyloma uniflorum cDNA, mRNA sequence

AAGCTGGAGCTCACGCGGTGCGCGGCGTTCTAGAACTAGTGGATCCCCCGGGCTGCAGGATTCGGCACGA

GGGAAGCATCAAAATGAATGTTGTCATCACCTTCCCTTGAGGAAATTGTTCACTATGACCTGTGGGCACG

AAAATATGCTGCTAGTTTGGAAGACTCTGCCAGCTTCACACCTATTCTTGCATGAGAAGGATTAGACTAG

GGATGTCGCTCATTCCTCCCACCTCCCTGCTTTCACATCTTTGGCCATTGAACAATTTTATGCAATGAAT

GTAGAATGATGCCTTCCCCAGATTTTGAAAAATAAAAAGTTTTTTATAAAAAAAAAAAAAAAAAAAAAAA

AAAAAAAAAAAAAAA



>gi|71811860|gb|DR989251.1|DR989251 HDI_H_56.esd Horsegram (VZM-1) drought stressed subtracted cDNA Macrotyloma uniflorum cDNA, mRNA sequence

AAGCTGGAGCTCACGCGGTTGGCGGCGCTCTAGAACTAGTGGATCCCCCGGGCTGCAGGATCGGCACGAG

GCGTCGTTCAACGAAGGAGACCCATCGACGGCCCCTTCGCTGACCTTAACGGGCCGCAAGAAGCAGCCCG

ATCAGCTCCAAACTGCTGATGGGTGGGCCAAGTTCACCGGGGGCTTCTTTTTTGGAGGCATTTCGGGGGT

TATTTGGGCCTACTTTCTCCTCTATGTCTTGGACCTTCCTTACTACATCAAGTGAAGGGGCTCTTTTCTT

TGTTTCTCTTTTTATTATTCATTCTCTATGAATGGACAATGAATTGAGGGTTATTTGTGCACTAGAGATG

CATGTAAATGATAGATGTATTTTATGACTCTCAACATCTTACAAAAAAAAAAAAAAAAAAAAAAA



>gi|71811858|gb|DR989249.1|DR989249 HDI_H_48.esd Horsegram (VZM-1) drought stressed subtracted cDNA Macrotyloma uniflorum cDNA, mRNA sequence

AAAGCTGGAGCTCACGCGGTGGCGGCGCTCTAGAACTAGTGGATCCCCCGGGCTGCAGGATCGGCACGAG

GGTTTGTTTTGCCGGTTATATTTTCAGGTTTCTGCCGAATTCATGTACCCTTAACAGGGTTCTTGATCGG

TGGTGGTCAATTTTAATTACTGTTTGTGTCCGAGAGTACTGTTTTTCATGTAAGAGTCTGAGATTTCAAT

TTTCAAAAGCAGCCATAGGCTTTTAGTCTTTTTTCTTTAGCCTGCTGAGAGATGTTTTTAAAAAAAAAAA

AAAAAAAAAACCCCNNGGGGGGGCCCCGGTTACCCCATTTCC



>gi|71811856|gb|DR989247.1|DR989247 HDI_H_46.esd Horsegram (VZM-1) drought stressed subtracted cDNA Macrotyloma uniflorum cDNA, mRNA sequence

AAGCTGGAGCTCCACGCGGTGCGCGGCGCTCTAGAACTAGTGGATCCCCCGGCGCTGCAGGATTCGGCAC

GAGGCCTCGTGCCGAATTCGGCACGAGGTCTCTTTGTACTGCACATTTTATAGTATTGAATGCCCTTTAA

TTATGTTGCAAAAAAAAAAAAAAAAAAAAAAAAAAAAAAAAAAAAAAAAAAAAAAAAAAAAAAAAAAAAA

AAAAAAAAAAAAAAAAAAAAAAAAAAAAAAAAAAAAAAAAAAAA



>gi|71811854|gb|DR989245.1|DR989245 HDI_H_44.esd Horsegram (VZM-1) drought stressed subtracted cDNA Macrotyloma uniflorum cDNA, mRNA sequence

AAGCTGGAGCTCACGCGGTGGCGGCGCTCTAGAACTAGTGGATCCCCCGGGCTGCAGGAATCGGCACGAG

GTGAAACTATTTGTTGTAGGTTTATATATAAGTTCTTATAGAATAAGCTTGCTAGCTACTTTCTCAAGTC

ACAATTTTTTTGTCCTCTTAGATCAAATGAATTTCTTTACCTGTTAATCATGGTTTACTGTTTAGTGAAC

CGCATTTGTTTTAGGAGTCGTTGTAGTGTTCTGTACTCTTTCTTATATGCTCTTCATTGGTACGGGCTTG

GCCTTGGCCTTGGTTTTGGGTGTCAAAAAAAAAAAAAAAAAAAAAAAAAAAAAAAAAAAAAAAAAAAAAA

CAACAACCCGGGGGGGGCC



>gi|71811852|gb|DR989243.1|DR989243 HDI_H_36.esd Horsegram (VZM-1) drought stressed subtracted cDNA Macrotyloma uniflorum cDNA, mRNA sequence

AAACTGGAGCTCACGCGGTGGCGGCCGCTCTAGAACTAGTGGATCCCCCGGGCTGACGGAATTCGGCACG

AGGCCTTCGTGCCGAATTCGGACCGAGGGGTGTATGCCTACTAGTATAACTGTTGTATCCGCGAGAATTA

TGTATATTTCANTGTTTAANGAAGTTTATAAATTAAAAGAAATAATGGTTGTTTAGAAGTTGGCAAACAA

AAAAAACAAAAAAACTNCAGGGGGGGGCCCGCGGTGTC



>gi|71811850|gb|DR989241.1|DR989241 HDI_H_34.esd Horsegram (VZM-1) drought stressed subtracted cDNA Macrotyloma uniflorum cDNA, mRNA sequence

AAGCTGGAGCTCACGCGGTGGCGGCGCTCTAGAACTAGTGGATCCCCCGGGCTGCAGGATCGGCACGAGG

CTTGTTATTTAGCATGAACTTGTAGGGAGCATTGCTGAAGATGCAAACCATAGTTTTGCTTTGGATTAAC

GCCTCAACATGTGTTTACTCAAGCAACAATGTAATGTAAAATGATGCAGCAGAGTGATGTAACTTTTTGA

CTCTGCGGTGCCAATAGTCACCAGGAAATGAAATGTGTCTCGTTTCTATTTCATCACTCTGATACTGTAC

ACATATGAAAAGCTTAAAAATTATACTATAATAATTATGAAAAAAAAAAACTATGTAAAAAAAAAAAAAA

AA



>gi|71811848|gb|DR989239.1|DR989239 HDI_H_32.esd Horsegram (VZM-1) drought stressed subtracted cDNA Macrotyloma uniflorum cDNA, mRNA sequence

AAGCTGGAGCTCACGCGGTGGCGGCGCTCTAGAACTAGTGGATCCCCCGGGCTGCAGGATTCGGCACGAG

GCCTCGTGCCGCCTCGTGCCGAATTCGGCACGAGGATGCCTTATAAGGTTACAATACCTGAGCCTCTGTT

CAGCTGAGGCCGGTGTTGGAACTAGCATCACTATCTTGTATAAGTGCAAAAAAATGTAAAAGAAAAAAAA

AAAAAAAAAACTCCAAGGGGGGGNCCCGGGCC



>gi|71811846|gb|DR989237.1|DR989237 HDI_H_24.esd Horsegram (VZM-1) drought stressed subtracted cDNA Macrotyloma uniflorum cDNA, mRNA sequence

AAGCTGGAGCTCACGCGGTGGCGGCGCTCTAGAACTAGTGGATCCCCCGGGCTGCAGGATCGGCACGAGG

AGAAGGGTGTGACTGAAAGGTTCGAGAAGGGAGAAGGAAGTGTTTAAAACACGTAAGATGTAAGATGTGT

GAAGAAGGATGATGTAAAATAAATAAGATATAAGATGTGAAGATGGATCTATCTTAAGATGAAGTGCTGA

AGGACTTATATTCAGAATAAATAAAATGAAAGGATATATGTTTTATCTTCTTTCAAAAAAAAAAAAAAAA

AAAAAAAAAAAAAAAAAAAAAAAACCCCGGGGGGGGCGCGCNCCGCCCCCCCCTCCCCCCTTTTTTTTGG

CT



>gi|71811844|gb|DR989235.1|DR989235 HDI_H_22.esd Horsegram (VZM-1) drought stressed subtracted cDNA Macrotyloma uniflorum cDNA, mRNA sequence

AAGCTGGAGCTCACGCGGTGGCGGCGCTCTAGAACTAGTGGATCCCCCGGGCTGCAGGATTCGGCACGAG

GCCTCGTGCCGAATTCGGCACGAGGAACAATAGTATGTGTGTCTTTTTTGTTTGAACTTAGAATGTCTGC

GTCTTTACTTTTCTTATATTGCATAGATTCATAGCAGTGTTTATTTTTACATCTTAAGTACCGATGTGCA

GTTTTTTGAATACTTGTTTGAGAATAATAGTCTTGATGTCAAGCTGCTTGTCAATCATATTGTTGGAGGA

TGTTATATAAGAATTACTTTCCAAAATATATGTTCTTAAGTCCTAAAAAAAAAAAAAAAAANTCTCCGAA

GGGGGGGCCCGGTNCCCATTTCG



>gi|71811842|gb|DR989233.1|DR989233 HDI_H_20.esd Horsegram (VZM-1) drought stressed subtracted cDNA Macrotyloma uniflorum cDNA, mRNA sequence

GCTGGAGCCACGCGGTGGCGGCCGCTCTAAACTAGTGGATCCCCCGGGCTGCAGGATTCGGCACGAGGGT

TTGTTTTGCCGGTTATATTTTCAGGTTTCTGCCGAATTCATGTACCCTTAACAGGGTTCTTGATCGGTGG

TGGTCAATTTTAATTACTGTTTGTGTCCGAGAGTACTGTTTTTCATGTAAGAGTCTGAGATTTCAATTTT

CAAAAGCAGCCATAGGCTTTTAGTCTTTTTTCTTTAGCTGCTGAGAGATGTTTTAAAAAAAAAAAAAAAA

AAAAACCCNGGGGGGGGCCCGGGNCCCCAAT



>gi|71811840|gb|DR989231.1|DR989231 HDI_H_12.esd Horsegram (VZM-1) drought stressed subtracted cDNA Macrotyloma uniflorum cDNA, mRNA sequence

AAAGCTGGAGCTCACCGCGGTGCGCGGCGCTCTAGAACTAGTGGATCCCCCGGGCTGCAGGATCGGCACG

AGGTTTAAACATCTACTTTCTTTCTTAATGCGAGCTTTACTGTTGACATCAAGTTTTGATTCTGTAGAAC

AAAACTGAAACTATTATTATTATTGCTTTTTTTTTTTTTTNTGGTTTTCTTTTTA



>gi|71811838|gb|DR989229.1|DR989229 HDI_H_10.esd Horsegram (VZM-1) drought stressed subtracted cDNA Macrotyloma uniflorum cDNA, mRNA sequence

AGCTGGAGCTCACGCGGTGGCGGCCGCTCTAGAACTAGTTGGATCCCCCGGGCTGCAGGATCGGCACGAG

GGACTTCTAAACTTAATCCTTTGGATGAAGCACGAGCGCGTGCTGAGAGCTTAAGGAGCTACAAAGCTGA

TCTCCAGAAAATGAAAAAAAAAAAAAAAAANCNCNGGGGGGGGCCNGGG



>gi|71811836|gb|DR989227.1|DR989227 HDI_H_89.esd Horsegram (VZM-1) drought stressed subtracted cDNA Macrotyloma uniflorum cDNA, mRNA sequence

AAGGCTGGAGCTCACCGCGGTGGCGGCCGCTCTAGAACTAGTGGATCCCCCGGGCTGCAGGAATTCGGCA

CGAGGTGATAATAACGAAGTATGTCTTGATGTTTGGCTCAATCAACTTGGCTTGGAATTTAGAATAAAGT

GGCGGGTTTACCTTTTATTCCAANTGTCCGGNACANTTTGTGCAAGAATG



>gi|71811834|gb|DR989225.1|DR989225 HDI_H_87.esd Horsegram (VZM-1) drought stressed subtracted cDNA Macrotyloma uniflorum cDNA, mRNA sequence

AAAAGCTGGAGCTCACGCGGTGGCGGCGCTCTAGAACTAGTTGGATCCCCCGGGCTGCAGGAATTCGGCA

CGAGGGGAAAGCTGATCCGTATGTATGTTACAAATTTGATGGTTGTCAATTGAAACTGATTTGTACTCCG

AATTTTACTATAGAAGTAGATGTCTCTATTATTATTGTTTGTGAAGTATTGTCACCACCATTTTTGTCTA

GGAAAAAAAAAAAAAAAAAAAAAAAAAAAAAAAAAAAAAAAAAAAAAAAAAAAAAAAAAAAAAAAAAAAA

AAAAAAA



>gi|71811832|gb|DR989223.1|DR989223 HDI_H_85.esd Horsegram (VZM-1) drought stressed subtracted cDNA Macrotyloma uniflorum cDNA, mRNA sequence

CTGGAGCTCACGCGGTGGCGGCGCTCTAGAACTAGTGGATCCCCCGGGCTGCAGGATTCGGCACGAGGAA

ATTGCATAACAATATGATCCCTGGTCTACCACCTCCTTGTTATGTGCCAAAGTTTAGAGCAGCTGATGCC

AGTATTAATGATTGATAAGTGTCGTTGTATAACGAAATTGTTGGTACTTGGGTTCACATGATGTATAAAA

TAAGCTAGAAGTGTTGTAAATCGTACAAGAATATTATGAACTGAATTATGTGTATCATATACATAATAAT

GATCATAAATTTTTAGTTAAAAAAAAAAAAAA



>gi|71811830|gb|DR989221.1|DR989221 HDI_H_77.esd Horsegram (VZM-1) drought stressed subtracted cDNA Macrotyloma uniflorum cDNA, mRNA sequence

AAGCTGGAGCTCACGCGGTGGCGGCGCTCTAGAACTAGTGGATCCCCCGGGCTGCAGGATTCGGCACGAG

GAGATAAATAATTCATTATCTATCTATATCTATATATAGATACATAGATAAGCAAATATACTTAAAAAAA

ATGCATCTATTCTTTAATCATCTAGGAAAAGTATATCTACAGATACCTAAATAGATACTCATACAAATGA

ATCTTGTGCCAGGAACCAGATTTGAACTGGTGACACGAGGATTTTCAGTCCTCTGCTCTACCAACTGAGC

TATCCTGACCATTCTTGTCAGACTTTTCTTTTAAAAAATTTTTGAAGTATATGTCAATGAATCTATATCA

ACTAAGTTAAAAAAAAAAAAAAAAA



>gi|71811828|gb|DR989219.1|DR989219 HDI_H_75.esd Horsegram (VZM-1) drought stressed subtracted cDNA Macrotyloma uniflorum cDNA, mRNA sequence

AAGCTGGAGCTCACGCGGTGGCGGCGCTCTAGAACTAGTGGATCCCCCGGGCTGCAGGATCGGCACGAGG

CATTATGTTTTCAGAATTATTGGTCTGTTACTGACTGAAATATTACTAGTGTGTTTGGTTTTTGTGAGCT

AGTGAATGTAAAAATGCTAAATAACTAGCATCCTATGTTTTTGTTCTTAAGTTTTGATAATTAACCAGAT

TTGCCTGGAACTTTGGCATTTGGTTAAAAAAAAAAAAAAAAAAAAAAAAAAAAAAAAAAA



>gi|71811826|gb|DR989217.1|DR989217 HDI_H_73.esd Horsegram (VZM-1) drought stressed subtracted cDNA Macrotyloma uniflorum cDNA, mRNA sequence

ACAAGCTGGAGCTCACCGCGGTTGGCGGCCGCTCTAGAACTAGTGGATCCCCCGGGCTGCAGGATTCGGC

ACGAGGCCTCGTGCCGAATTCGGCACGAGGGGGCGTAGTAAGCGACGAAATGCTTCGGGGAGTTGAAAAT

AAGCGTAGATCCGGAGATTCCCGATATAGGTCAACCTTTCGAACTGCTGCTGAATCCACGGGCAGGCAAG

AGACAACCTGGTGAACTGAAACATCTTAGTAGCCAGAGGAAAAAAAAAAAAAAAACTCTCNAGGGGGGGC

CCGGGTTCACCCAAATTTCGCGCCCTTA



>gi|71811824|gb|DR989215.1|DR989215 HDI_H_65.esd Horsegram (VZM-1) drought stressed subtracted cDNA Macrotyloma uniflorum cDNA, mRNA sequence

AAAGCTGGAGCTCACGCGGTGGCGGCGCTCTAGAACTAGTGGATCCCCCGGGCTGCAGGATTCGGCACGA

GGCTTCAGATAGCTAGGCACTTTCTTTCCGCAATCTCTGACTAATAGATCTGCGCCTGTTATCCTCTGTC

ATTACTTTTCTCCTCGTTCCCTGTTTCGTGGTCAATCCCTGCTTAGAATTAGAATCAGAAAAATGAAAGA

AAAATTAATCCTATGGCTTGCCGTTTTAAAAAAAAAAAAAAAAAACTCTCGAGGGGGGGCCCGGGTTACC

CAATTTCGGCCCTTA



>gi|71811822|gb|DR989213.1|DR989213 HDI_H_63.esd Horsegram (VZM-1) drought stressed subtracted cDNA Macrotyloma uniflorum cDNA, mRNA sequence

AAGGCTGGAGCTCACCGCGGTTGGCGGCCGCTCTAGAACTAGTGGATCCCCCGGGCTGCAGGAATTCGGC

ACGAGGCTAGTCTCGAGGTTTTGTTTTTTTTTTTTTTCTCCTCTTTTTTCTCCTTNTTTGTGGGTCTCCC

TCTTTTTCCTTTTTCCAGAATTTGGTTCTCCTTTCTCTTGAGAAAATTTGGGAATTGGAAGGGTTGTATA

AAAATCTCAGAATTTTGCGGGGAAATATACCCTCGGGAAAAGAAATTTTTGTTCTCCATGAATAGTTGAT

AAATTAGTTACATNTCCTCCAGAAAGAAGGCGTTTACTCGTCTCGTTATAGCTCGGTTAAAAGTGAATTA

GAAGTATATAAATTTTGAAAAA



>gi|71811820|gb|DR989211.1|DR989211 HDI_H_61.esd Horsegram (VZM-1) drought stressed subtracted cDNA Macrotyloma uniflorum cDNA, mRNA sequence

AAGCTGGAGCTCACGCGGTGCGCGGCCGTCTCTAGAACTAGTGGATCCCCCGGGCTGCAGGATTCGGCAC

GAGGCCTCGCTGCCGTAAATTTATACTATATTCACAAACATGAATGATTTGGAAGGATGTTTATTCCCTT

TACACGAAAGTACATAAACAAAGTGAGATGGCTTGATAAGCAAGGAA



>gi|71811818|gb|DR989209.1|DR989209 HDI_H_54.esd Horsegram (VZM-1) drought stressed subtracted cDNA Macrotyloma uniflorum cDNA, mRNA sequence

AAGCTGGAGCTCACGCGGTGGCGGCGCTCTAGAACTAGTGGATCCCCCGGGCTGCAGGATCGGCACGAGG

AGCTAAAGTGCTTGAATGGTTGAGAGAACATGCAGATATTCAGTATATAACTACATAAGAGATGAGCACC

AGCCAAAACAAATCAGGAAAATGAAATTGCAGGTTCGTTTGAAAGAATTTTGTTTCAGCGGATGTAAGTT

ATGAGAAAATAAATTATTCTTGATTGAATTTTGAAGAAAGGTTATTAAGAATCGAACAATGTATTACAAG

TTTGTAACTTGTGACTTTTACTATAAATTAATAGTAGATAGCACTAACAAAAAACAGTATTGAAAAACAT

CAAAAAAAAAAAAAAAAAAAACCCAGAGGGGGGGCCCGGGANCCCAATTCCGCCCAA



>gi|71811816|gb|DR989207.1|DR989207 HDI_H_52.esd Horsegram (VZM-1) drought stressed subtracted cDNA Macrotyloma uniflorum cDNA, mRNA sequence

CTGGAGCTCACGCGGTGGCGGCGCTTCTAGAACTAGTGGATCCCCCGGGCTGCAGGAATTCGGCACGAGG

CCTCGTGCCGAATTCGGCACGAGGAAGAAACACCATCATCTCTTTGGGTGAATGAACATGATAATAATAT

TCAAGCTCAGAACTATCACCAATCATGCGTGTGTCTCTACTTATGTTCCATTTTCCAAGTGATTTTAAAT

AATTTCTGAGCTAGTTTGAAGAGTGTTTCATTACTTCATGTATTTAATGTACATGTGCTTTATGTTGACT

ATGTTAATAATAATAATAATATGATA



>gi|71811814|gb|DR989205.1|DR989205 HDI_H_50.esd Horsegram (VZM-1) drought stressed subtracted cDNA Macrotyloma uniflorum cDNA, mRNA sequence

AAAGCTGGAGCCACGCGGTGGCGGCGCTTCTAGAACTAGTGGATCCCCCGGGCTGCAGGAATTCGGCACG

AGGGTGAGTTCCAAAAATAAATTGCAGTGTGATCACTTTGGTGGTGGTTATGCTTCATGCATGCATGGGG

TGTCACACTAGGTTTAGGGTTTAGGGTTGTTATTATTATATATCATTGGAGATGATGTTATAGAAAGCTT

GACTTTAATAAAAGCCATGTTCCTTTATATATAAAAAAAAAAAAAAAAACTCAAGGGGGGGCCCGGGTAC

CCAATTTCGC



>gi|71811812|gb|DR989203.1|DR989203 HDI_H_49.esd Horsegram (VZM-1) drought stressed subtracted cDNA Macrotyloma uniflorum cDNA, mRNA sequence

AGCTGGAGCTCACGCGGTGGCGGCCGCTCTAGAACTAGTGGATCCCCCGGGCTGCAGGATTCGGCACGAG

GTCTTATTACGCATCTTTTATTTTTCTACACATGGGAACTGACACCGTATATTGATGAGAATCAATCTAG

TGACTATTTGTTCTGGTTGACCTTGCATATATATATATATGTGCGCATATCTTTGAATAATAATCATTAA

TCACAAATTGTAGTTAAAAAAAAAAAAAAAAAAAAAAAAAAAAAAAAAAAAAAAAAAAAAAAAA



>gi|71811810|gb|DR989201.1|DR989201 HDI_H_41.esd Horsegram (VZM-1) drought stressed subtracted cDNA Macrotyloma uniflorum cDNA, mRNA sequence

CTGGAGCTCACGCGGTGGCGGCCGCTCTAGAACTAGTGGATCCCCCGGGCTGCAGGAATTCGGCACGAGG

CCTCGTGCCGAATTCGGCACGAGGGTTCTTTCTGTTAGTAAAGCAGAGAAAGGTTTGCTGGTAAAGAGTG

GTCTGGTGGGTGGTATGAGTATCATACTCTTCTTTCTCTCCACACATATATACATTATATGAGGAGAACC

AATTGAGGGGTTATAGAATAATGTATAAAATTGCAGTAAATATTGGTATTATTTTCTGTTTCCGAGTATG

GTGGTGTCAAGAACATATACATCGGAAGTGAAGAGTCTTCTTCAATTGAACACTTTTTTTTTCTTCGGGA

ATTCAAAATTAATAATGTTAAAAAAAAAGGGGGGGCCCGGTACCCAATTCGCCCTATAGTGAGTCGTATT

ACAATTCACTGGCCGTCGTTTACAA



>gi|71811808|gb|DR989199.1|DR989199 HDI_H_4.esd Horsegram (VZM-1) drought stressed subtracted cDNA Macrotyloma uniflorum cDNA, mRNA sequence

AAGCTGGAGCTCACGCGGGCCGGGCGTTATAGAACTAGTGGATCCCCCGCCGCTGCAGTATTCGGCACGA

GGATGGACGATGATGTTCCTGCTGGTGGAAGCNGGTGCTGGTCCAAAGATTGAGGAAGTAGACTAAATTT

AGTTAATTCCTGCTGGAGTTACTATTTATTTTGTAAGACCCGTTTGACGTGTTTTTTCTTGGATTTAGGT

TTATGGAAGTATTTCGTAGTGTATGTTTGTCAGTATCGGTCACTTCTATTCTACTTTAGTTTTAGTTGTT

GTGACATATACTNNAGCTTTTAAGCTCGTGTTCTTTTTTTATTTAGTAAGTATCATGTTTTGTGGTTATA

AAATACAAAAAAAAAAAAAAAAAAACCCTCAAGGGGGGGCCCGGATCCCCAATTCTCGCCCTA



>gi|71811806|gb|DR989197.1|DR989197 HDI_H_38.esd Horsegram (VZM-1) drought stressed subtracted cDNA Macrotyloma uniflorum cDNA, mRNA sequence

AAGCTGGAGCTCACGCGGTGGCGGCGCTCTAGAACTAGTGGATCCCCCGGGCTGCAGGATTCGGCACGAG

GCCTCGTTGCCGAATTCGGCACGAGGGAGAGAGAGTGAACTAGTCTCGAGGGGGGGCCCGGTACCCAATT

TCGCCCTATAGTGAGTCGTATTACAATTCACTAGGCCGTCGTTTTACAAC



>gi|71811804|gb|DR989195.1|DR989195 HDI_H_3.esd Horsegram (VZM-1) drought stressed subtracted cDNA Macrotyloma uniflorum cDNA, mRNA sequence

AGGCTGGAGCTCACGGCGGTGGCGGCCGCTCTAGAACTAGTGGATCCCCCGGGCTGCAGGAATTCGGCAC

GAGGCCATTGGCTTGAGTTGTGTGTTCGATGAAGCACACGGGAGTGGTGACCGAAGCTCTATGCTTTGTT

TTTTTTCTTCTTTTTTTTCCTCTGTCTTTGGAGTTTACTGATTAATTATTACCAGTAATTTGTTGGTGGT

GGCCATAGTTGATTAGGAGATGGGTGACGAACATGTTTTGTTTGTTTGTTTCTTCTGAGAAGCTATCTAG

AATATTTTTTTTGGAAAAAAAAAAAAAAAAAAAAAAAAAAAAAAAAAAAAAAAC



>gi|71811802|gb|DR989193.1|DR989193 HDI_H_28.esd Horsegram (VZM-1) drought stressed subtracted cDNA Macrotyloma uniflorum cDNA, mRNA sequence

AAGCTGGAGCTCACGCGGTGGCGGCGCTCTAGAACTAGTGGATCCCCCGGGCTGCAGGATTCGGCACGAG

GCCTGATCTCCGTAAGAAGGTGTTGCCCGCGGTCATCGTTCGTCAGCGTAAGCCATGGCGCCGAAAGGAC

GGTGTTTTCATGTACTTCGAAGATAATGCTGGTGTTATCGTGAATCCTAAGGGTGAAATGAAAGGTTCCG

CCATTACTGGACCAATTGGAAAAGAGTGTGCTGATCTATGGCCCAGGATTGCAAGTGCTGCCAATGCTAT

TGTTTAAGCTGGGTTTCTAGTGGTTTTTCTAATGTTTCTTCTAATATTTATTTAGGTTGAATTGCAGACA

ATGCAGCACGCATAGTTATAATAGTTTTATATATGGTTATTCGTGATAAAAAAACAAACAAACAAAAAC



>gi|71811800|gb|DR989191.1|DR989191 HDI_H_26.esd Horsegram (VZM-1) drought stressed subtracted cDNA Macrotyloma uniflorum cDNA, mRNA sequence

AAAAGCTGGAGCTCACCGCGGTGGCGGCGCTCTAGAACTAGTGGATCCCCCGGGCTGCAGGATTCGGCAC

GAGGGTTGTAATTAATCAAATATTTCACTTAGCTGGATTAGTACTTGTAAAACTGGTAACTTGAATCGGA

GAGGCTTAGCAGAATGTTTCTGCTTGATCAAAAAAAAAAAAAAAAAAAAAAAAAAAACCCCGGGGGGGGG

CCCCGGGCCCCCATTTCCC



>gi|71811798|gb|DR989189.1|DR989189 HDI_H_2.esd Horsegram (VZM-1) drought stressed subtracted cDNA Macrotyloma uniflorum cDNA, mRNA sequence

TCAGTTGCTTGACCATAGNTCTAATTGACTAGATAGATGATAATTGCGTCTCATATGGTAACNCACTGNA

CATAGGTCATCATACTCGACGTGAATATTCACTACAACTCTCGGATAGCTTGCAGGCAACNAATTGATAT

GAATGGCATGACGTATAAGAACACGACGA



>gi|71811796|gb|DR989187.1|DR989187 HDI_H_17.esd Horsegram (VZM-1) drought stressed subtracted cDNA Macrotyloma uniflorum cDNA, mRNA sequence

TGCTGGAGCTCCACGCGGTGCGCGGCCGTCTCTAGCAACTAGTGGATCCCCCGGGCTGCAGGAATTCGGC

ACGAGGCGCGTCTGATGCGCTGAGATTTGCTGGCTACACGAGGGTGGATTNCTCGGTGTGTGCGTGGTGA

GCAGTGTGGTGCCTCTGTTTACTCGGNGGAAGTGACGTGTGTGTAGCCTTTTNGTGAGCTGTGTTGGTAA

AAGTCTATGGAGGTGAAAGTTANGGGGTTATAGTTGATGAATTATAGAGAGATTCNAGGG



>gi|71811794|gb|DR989185.1|DR989185 HDI_H_15.esd Horsegram (VZM-1) drought stressed subtracted cDNA Macrotyloma uniflorum cDNA, mRNA sequence

AAAAGCTGGAGCTCACGTCGGTGGCGGCGCTCTAGAACTAGTGGATCCCCCGGGCTGCAGGAATTCGGCA

CGAGGCCTCGTGCCGAATTCGGCACGAGGTTTCATTGTATGAAAGTATATGTACATCCAAAAGCTTGAGT

TTCAGTTATGAAAAATAAATATTTCTTCAAGCTCGTAAAAAAAAAA



>gi|71811792|gb|DR989183.1|DR989183 HDI_H_13.esd Horsegram (VZM-1) drought stressed subtracted cDNA Macrotyloma uniflorum cDNA, mRNA sequence

GCTGAGCTCACGCGGTGCGCGGCGCTTCTAGAACTAGTGGATCCCCCGGGCTGCAGGAATTCGGCACGAG

GGAAAGTTATATGGATATCAAATCGCCAAGCAAGAAGGCATCTGAGGAGCTGGCACGTGAGTCGCTTATT

GCTATCTCTGATTCTTTACCTGATAAAATTCTGGATTCAGATATTGTGCCTGAAAGCAAGGACGCAGATG

GCTTTGCAAGACCAAATGGTGATTGGGATGACAAGTTTAGGTCTGAATTGATTTCAATCTCCTATGTTGA

ATCTCCTGATGTCAAAATCTGATAACATTACAGGGGAGTCTAGACTGTTACATAAGCTACACTTGTCTTG

TTATGTATTTGAATTTTTGCTGTGCAACTATGAATTGTTCTACGGTTATACTATGAACATATTCAGACCA

AAAAAAAAAAACACAAA



>gi|71811790|gb|DR989181.1|DR989181 HDI_G_M9.esd Horsegram (VZM-1) drought stressed subtracted cDNA Macrotyloma uniflorum cDNA, mRNA sequence

TCTAGAACTAGTGGATCCCCCGGGCTGCATGAATTCGGCACGAGGCCTCGTGCCGAATTCGGCACGAGGG

GGAATTGTGGCGGTGATGGCATGGGAGCATAGAGTAGGTGAAATTAATGGTGAAGTTCTGGTAGTTATTA

TTGTCAGTGATTATTAGTACTCTTAGAAATTAGATAACTCATACTAGTATTAATTGTTCAGTACTTTTAT

TTTATTTAATTGGTGTCTGAAGTAATTGGTAAGCACTTTAATTGGGTGTAGGTAGGTGTAAATATGAAGT

CAATGGTCAATTGTAGGAGAGTGAGTTATTATTGGAGGGAAAGGAAACCATGATTTGGATAGTTTAATAC

GTAAATGGTTCGTCAAAACTAAAATCAACAACAAAAATTCACCTCTCTTTCTTTCTAAAAAAACNAAAAA

AAAACCCAAGGGGGGGC



>gi|71811788|gb|DR989179.1|DR989179 HDI_G_M7.esd Horsegram (VZM-1) drought stressed subtracted cDNA Macrotyloma uniflorum cDNA, mRNA sequence

TAAGGAACTAGTGGATCCCCCGGTGCTGCAGGAATTCGGCACGAGGCCTCGTGCCGAATTCGGCACGAGG

GTAAGGAATAATTCTCTACTATAATAGTATATTTTGTGGCTGTCCACGTCTATAATGAGTCAATGGTCCT

ATGTATTGGTATATTTTTGGTGGCC



>gi|71811786|gb|DR989177.1|DR989177 HDI_G_9.esd Horsegram (VZM-1) drought stressed subtracted cDNA Macrotyloma uniflorum cDNA, mRNA sequence

AGAACTGGAGCTCACGCGNCGGCGGTTTATCTAGAACTAGTGGATCCCCCGGGCTGCANGAATTCGGCAC

GAGGCCGGAAACAGACCGCTGGAGTAACAGTAGTAGCCGGCCGGATGGTGACAGGTGGCGTGGTGATGAA

AGGAGAAGTGGGTTTGGTGGTTCTGGTGGAGGTTCAAGGTCATCTTCTACTTGGTCATATTCCAGAATGG

GCGTTGATCATTTTGTGCCCTCTGATTTATTGCTGGTGACTTTTTTGGAATTCTCTCTGTACTCTTTCCA

GTTTACGTCCTAGAATTCTTTTTGATGCTGAGTTGTTAAATTTATTATCTTAATACTCACAAACAAGTCC

TCTATTGGTTTGAGGAACAAAAAAAAAAAAAA



>gi|71811784|gb|DR989175.1|DR989175 HDI_G_80.esd Horsegram (VZM-1) drought stressed subtracted cDNA Macrotyloma uniflorum cDNA, mRNA sequence

GCTGAGCTCACGCGGCTGGCGGCGCTCTAGAACTAGTGGATCCCCCGGGCTGCAGGAATTCGGCACGAGG

TGAGAATTTCTATATGACTGACGCCATAACCAGGGCATCAAAGATAATGGCACAATGCAGTGCTACGCTG

TTGAAGAAGTGAACATGGATTTTTTATTTTTTGGTTTCAATAATGAAGATTCACGTGGCCGTGCTAGACT

TTGCATGCCAAAAACTTATTGGATGTTGGAAGTATCCAATAAATTAAAACTAGGATTTCTCTGTTCCATT

TGTAATATGCGGTGTGAAAGCACCACTATTTAGAACTGTTGCCATGTACAATTTGAGTTATCTGACTGAT

TGTGAGAGTTTACTCGAGTCATCAATTTTCCGTAGTCAAAAAAAAAAAAAAA



>gi|71811782|gb|DR989173.1|DR989173 HDI_G_79.esd Horsegram (VZM-1) drought stressed subtracted cDNA Macrotyloma uniflorum cDNA, mRNA sequence

CTGGAGCTCACCGCGGCTGGCGGCTGCTCTAGAACTAGTGGATCCCCCGGGCTGCAGGAATTCGGCACGA

GGCTCGTTTGTCCCTCTGATCTCTGGTGATGGCAAGCCTGAAGATTGCATGCATGGTTGCGGTGGTGTGC

ATGGTCGTTCTGAGTGCACCCATGGCACATGCGATCACATGCGGGCAAGTGGCCAGTTCCGTGGGTCCAT

GCATTACCTACATCAGGAGCGGTGGAAACCCATCGGCAGGGTGCTGCAACGGAGTGAGGAGCCTGAACGC

CGCCGCCAAGACCACTGCTGACCGCCAGGCCACGTGCAACTGCCTGAAAACCATTGCCACTTAAAATTGG

GGGCTTTCAACGCCCGCAACGTAGCTTCATCTCCCAGGCAAATGTGGCGTCTCAATCCCCTACAAGTATC

AGCACCTCCACCAAACTGTGCTACCATCAAGTTCTGAAAGATGGATGTGGTATAAGGAAGAAAAAGGCAC

GAAGGGGAAATTTTCAGTAGCAGAATATTACTATAT



>gi|71811780|gb|DR989171.1|DR989171 HDI_G_71.esd Horsegram (VZM-1) drought stressed subtracted cDNA Macrotyloma uniflorum cDNA, mRNA sequence

CTGAGCCACCGCGGCTGGCGGCGCTCTAGAACTAGTGGATCCCCCGGGCTGCAGGAATTCGGCACGAGGC

CTCGTGCCGAATTCGGCACGAGGGAACATTCGTTTCTCCCTAAGTTTAAGTCGTGATGGGAAAGGAAAAG

GCTAACATCAAAATCGTGGTCGTTGGACATGTCTCTGAGAAATCAACTGCCACGGAGCTTGGAAGCCCTA

CTGAGAAGGGTGTGACTGAAAGGTTCGAGAAGGGAGAAGGAAGTGTTTAAAACACGTAAGATGTAAGATG

TGTGAAGAAGGATGATGTAAAATAAATAAGATATAAGATGTGAAGATGGATCTATCTTAAGATGAAGTGC

TGAAGGACTTATATTCAGATAAATAAAATGAAGGATATATGTTTATCTTCTTCAAAAAAA



>gi|71811778|gb|DR989169.1|DR989169 HDI_G_7.esd Horsegram (VZM-1) drought stressed subtracted cDNA Macrotyloma uniflorum cDNA, mRNA sequence

AACTGGAGCTCACCGCGGCTGGCGGCGCTCTAGAACTAGTGGATCCCCCGGGCTGCAGGAATTCGGCACG

AGGGAAAGAAACACCACCACCTCTTATGGCATGATACACTAAACCAACAACACATTNTACTTTATAATTA

TACTATGCAACCTAATAAGAGCATATATTTCCCTCTATTTACCTAATATGCTTTTCGCGGTCTTACATAT

CGTTTGCGTGTCTGTGTGAAATTCTCATTATAGTCTTTCCACTTCTATTTTGAGAATATGAGTACTGGCT

CTATCGTTGTAGTGAGAATGATATTCCTTCATATGTTGTTGTAGACGTAATCGTATTCTCGCACTATTAG

TTATCGTTGGGTCAAACACGTTATGAACGGTCACAATTATTA



>gi|71811776|gb|DR989167.1|DR989167 HDI_G_68.esd Horsegram (VZM-1) drought stressed subtracted cDNA Macrotyloma uniflorum cDNA, mRNA sequence

GCTGAGCTCACCGCGGCTGGCGGCTGCTCTAGAACTAGTGGATCCCCCGGGCTGCAGGAATCGGCACGAG

GCACTGTCTCAGAGAACTTCCAGTTTTGGCTACGGATGAGAAGTACAATGAAGCTATCTATAGCACTGCT

AAACGTCTAGTTGCTGCCATTGATGGCCTTCCAGATCCCGGTGGGCCTACATTCAAAGACAACAAACGTG

AGTCTAACTTCAAAACCAAGCAAGAGACAGAAGAGAAACGTGGACAATTCACTCTTGTAGTGGGAGGTTT

GTTGGTTATTGCATTCGTTGTTCCTATGGCACAATACTATGCTTATGTATCCAAAAAATAAAATAATATA

AGTATATCCGTCTTTCAAATTCTCACTCTTTAAATTCAGATGTATAAGTGCAGTTGTAACCATGCTCATC

TTACAATTGAGTTTGGGAAGGCTGAAGAATAAGATGACGAGTTTTTCTCATGTNAATGCAACTTCCATAG

TATATGCATTTTCGATTATGTTCATTATCCAGCTTGGGTTTTTCAAAGTCACGGTAGAACGAGTTCTAGT

TCTCCAATTTCTCTCCCAAAAA



>gi|71811774|gb|DR989165.1|DR989165 HDI_G_60.esd Horsegram (VZM-1) drought stressed subtracted cDNA Macrotyloma uniflorum cDNA, mRNA sequence

AAGCTGGAGCTCACGCGGCTGGCGGCCGCTCTAGAACTAGTGGATCCCCCGGGCTGCAGGAATTCGGCAC

GAGGGCGAAGCTGAGGGAGCAAAAGCCATGCTTTTTTTTGTGGTTAACCCTTA



>gi|71811772|gb|DR989163.1|DR989163 HDI_G_58.esd Horsegram (VZM-1) drought stressed subtracted cDNA Macrotyloma uniflorum cDNA, mRNA sequence

TATCTAGAACTAGTGGATCCCCCGGGCTGCATGGAATCGGCACGAGGGGACCTCGAGGCTGTCTCACGCG

CCTCGGCGCGTCGACGTCGCCCTCATGCTCAGCGAGCTCGATGGCGGCCCCGGCGGCTGCATAACCGTAG

AGGCCCTCATGAACCACATCGGGTCATGTCTAGAGTCCGGGTCGGACCCGGACGAGCTGAAGGAGGCGTT

CGCCGTGTTCGACACGGATCGCGACGGGAGGATCTCGGCGGAGGAGCTGCTTCGGGTGTTCCGGGCGATC

GGCGACGAGCGGTGCACTTTAAGGAGTGCCGGCGCATGATAGAGGTGGTGGACAAGAACGGAGACGGGTT

CGTGTGCTTCGAGGACTTCTCTCGTATGATGGAGCTGCAGCGGTGATGGTGTGGTTGTGATTGTGATGAT

GACATGTCAAGATGATGATCATGATCATGTTTTTTTTTATTAACACAACACAGTGAAAATATATATAAGA

CCGCAAGGCACACTTTAATTTTTTTATAATCCGTTAATTTCTGTATTGTTGTCATTATTATTTTAATTAT

AGGGGTGTATAATTAATTTTTGAAGGGCGAAAAAGTGCGTTCCCACTCAGAAAAACAAAAAAAAAACTCG

ACGGGGGCCCGATCA



>gi|71811770|gb|DR989161.1|DR989161 HDI_G_56.esd Horsegram (VZM-1) drought stressed subtracted cDNA Macrotyloma uniflorum cDNA, mRNA sequence

AGCTCACCGCGGCGGCGGCTGCTCTAGAACTAGTGGATCCCCCGGGCTGCAGGAATTCGGCACGAGGCTC

TTGAGTACAAGAGGCTGAGAGGGCTAAGCTGGTGCAGCAGGGATACAACATTGTTGCAGTGGTTGGAGAC

CAATGGTCCGATCTGAGGGGAAAACCCAAGGGAACCAGGAACTTTAAGCTTCCCAACCCCATGTACTCTA

TTGAGTAGATCATCTCTGAAGGATCCCAAGCTTGCTCCTATCTACGAAATAAGATACCTTACGTGTTCTA

ATGGGAGCATTAACTATGTATCTATGCATGTGACTTTCTGGTCTTCGGATCTTCAACAAATCACCTAAGA

GACTACACTCCGTTGTTTTCTCTTTTGTGACTTTAATAAATTATGAACCCTTTTTTCCAAAAAAAAAAAA

AAAAAAAAAAAAAAA



>gi|71811768|gb|DR989159.1|DR989159 HDI_G_48.esd Horsegram (VZM-1) drought stressed subtracted cDNA Macrotyloma uniflorum cDNA, mRNA sequence

AAGCTGGAGCTCACGCGGTGGCGGCGCTCTAGAACTAGTGGATCCCCCGGGCTGCAGGAATTCGGCACGA

GGGTTCTCTTGTTGCAGCAGATAGATTGTAGTGCTGCATGTGCTGCGAGGTGCCGTTTGGCATCTCGTCA

ACGCATGTGTCACAGAGCGTGTGGAACCTGCTGCAAACGCTGCAACTGCGTGCCACCGGGAACTGCCGGG

AACCAAGAAGTGTGTCCATGCTATGCCAGTCTCACCACCCACGGTGGCAGACGCAAGTGCCCTTAGAGAT

TTCCATGTCAAGTGTCAACCCATGCAATGCAAGTTATCACTATTTATATATGTCCTTGCTTTGGTGTGTT

GCTTGCTTCCAATAAAATTTCAATGTTGTGTGAAAAAAAAAAAAAAAAAAAA



>gi|71811766|gb|DR989157.1|DR989157 HDI_G_46.esd Horsegram (VZM-1) drought stressed subtracted cDNA Macrotyloma uniflorum cDNA, mRNA sequence

CTGGAGCTCACCGCGGCTGGCGGCTGCTCTAGAACTAGTGGATCCCCCGGGCTGCAGGAATTCGGCACGA

GGCTGTTCTTCCCTAAGAAAGTAGGAACACCAAGGAAAAGTGAGATTGTATTCATAGCTCCTACTGGGGA

AGAGATCACTTCCAAAAAGCAATTGGAGCAGTACCTAAAGGCACATCCTGGGAATCCTGCAATCTCGGAG

TTTGACTGGGGAACTGGTGAGACCCCTAGGCGATCACGCAAGGATTAGCGAGAAGGTCAAATCGACTCCA

CCAGCAGAGACTGAGCCTCCAAGGAAACGATCCCGGAAATCATCAGGTTCAAAGAAGGATAACAAAGAAA

ACGAACCCGTTGCACATTGATGATACAAGCAANAATAAAATCTATGCCCACTCTTCCTTTTGCTTTTAAG

TCTGTATATGCTTTACACATTTAGACATTTGATTTTATTTTTCAGAAAAAAAAAAGGGGGGGCCCGGTAC

CCAATTCGCCCTATAAGTGAGTCGTATTACAATTCACTGGCCGCCG



>gi|71811764|gb|DR989155.1|DR989155 HDI_G_44.esd Horsegram (VZM-1) drought stressed subtracted cDNA Macrotyloma uniflorum cDNA, mRNA sequence

CTGCTCTAGAACTAGTGGATCCCCCGGGCTGCAGGAATTCGGCACGAGGGTGGTTCAAGGCTGGCTCTCA

GATCTTCAGCGAGGGTGGGCTCGACTACTTGGGCAACCCAAGCCTGATCCACGCTCAGAGCATCCTCGCC

ATCTGGGCTACCCAAGTTATCCTCATGGGCGCCGTTGAGGGTTACCGTATTGCCGGCGGGCCTCTCGGTG

AGGTCACTGACCCCATCTACCCCGGTGGCAGCTTCGACCCATTGGGCCTCGCTGACGACCCAGAGGCTTT

CGCTGAGCTCACAGGTGAAGGAACTCAAGAACGGTAGTTGGCCATGTTCTCCATGTTCGGGTTCTTCGTC

CAGGCCATTGTCACCGGAACAGGGCCCATTGGAGAACCTCGCCGACCACCTTGCAGACCCAGTCAACAAC

AACGCCTGGGCCTACGACCACCAACTTCGTCCCCGGAAAGTGAGCTTGACAGAGAATGACTTGTGTACTC

GAAGGGGGGCCCGGTACCAATTTCGCCTATAGTGAGTCGTA



>gi|71811762|gb|DR989153.1|DR989153 HDI_G_36.esd Horsegram (VZM-1) drought stressed subtracted cDNA Macrotyloma uniflorum cDNA, mRNA sequence

AAGCTGGAGCTCACGCGGTGGCGGCGCTCTAGAACTAGTGGATCCCCCGGGCTGCAGGAATTCGGCACGA

GGCCTCGTGCCGAATTCGGCACGAGGATTTAGATGGATTACTCTCCTGCTGACTCAGGAATTCAATTTTG

CTGACAGCCTTCACATTTGGGACACTCTTTTAAGCGATCCTGATGGTCCACAGGAGACTCTTCTTCGGGT

ATGCTGTGCAATGCTTGTCCTCGTGCGCAAGCGCCTCCTTGCGGGTGATTTTACATCAAATCTGAAGGTT

TATTGGCAAAAATTTATCCCACCAACAAACATTAAGTTCATTTGCTCTTATGTTTGCCAACAAGTTTTCG

TGTACAGTCAGTTTAATGTTTATCTCCCATTATCTTTGTGCATTTTGCTTTTGTACTTTTAATTTCTCTT

TCAAATTCAATACTTGTAAATGACTTTTATGTGTGAGCCTCAGAGCAGTGACTGTTTTTTTATTTATTTT

TAGTTATATTTTGTACGTAAAAAAAAAAAAAAAAACCCCCGNGGGGGGGCCCGGTCCCCA



>gi|71811760|gb|DR989151.1|DR989151 HDI_G_33.esd Horsegram (VZM-1) drought stressed subtracted cDNA Macrotyloma uniflorum cDNA, mRNA sequence

AAGCTGGAGCTCACGCGGCTGGCGGCGCTCTAGAACTAGTGGATCCCCCGGGCTGCAGGAATCGGCACGA

GGCTGAAGTGTCTCGAACTGTAGCTGTTAAAAAAGTATGGGAGTACATCAAGCAGCAGAATCTTCAGAAT

CCTTCTAATAAAAAGGAGGTCTTTTGCGATGCAAAGCTGAAGACCATTTTTGGTGGAAAAGATAAGGTTG

GGTTCACCGAGATTGCTAGATTGCTGTCTGACCATTTTGTGAAATGAGCCTGATATTCAGCTTTGATGGT

ACATTTAATGATGCTGATAACTTGTTTTAGTACGTTAAATCGGTTATTTGGTCTTCAAGTACATGGTTTG

TGACCAAGCGCTAAGAAACATGGAAATTTGTGGTATCGAACTTGGGGGGTTTTGAACAGTGGTGTTATTT

TGTTTACTGCCTGCTTTCACATTGTTAGGANTTT



>gi|71811758|gb|DR989149.1|DR989149 HDI_G_31.esd Horsegram (VZM-1) drought stressed subtracted cDNA Macrotyloma uniflorum cDNA, mRNA sequence

TGAGCTCACGCGGCTGGCGGCTGCTCTAGAACTAGTGGATCCCCCGGGCTGCAGGAATTCGGCACGAGGC

CTCGTGCCGAATTCGGCACGAGGGAACATTCGTTTCTCCCTAAGTTTAAGTCGTGATGGGAAAGGAAAAG

GCTAACATCAAAATCGTGGTCGTTGGACATGTCTCTGAGAAATCAACTGCCACGGAGCTTGGAAGCCCTA

CTGAGAAGGGTGTGACTGAAAGGTTCGAGAAGGGAGAAGGAAGTGTTTAAAACACGTAAGATGTAAGATG

TGTGAAGAAGGATGATGTAAAATAAATAAGATATAAGATGTGAAGATGGATCTATCTTAAGATGAAGTGC

TGAAGGACTTATATTCAGATAAATAAAATGAAGGATAT



>gi|71811756|gb|DR989147.1|DR989147 HDI_G_23.esd Horsegram (VZM-1) drought stressed subtracted cDNA Macrotyloma uniflorum cDNA, mRNA sequence

CTAGAACTAGTGGATCCCCCGGGCTGCAGGAATCGGCACGAGGGTAAGAACTATCCTGTGACTGTTAACC

TATGGCAACCAGAGAAAAATGCCTTTGAATGGGCCACATCTTTGAAAGCCCCTCACACAGAGAATGCAAT

TCGGGTTACACAGTCCAGTGCAAACTTTTTTGTGAGTGAAGCTAGGAAATCCTCAAACAAACCTGATAAT

CAGAAAGTGCTAGACAATCTCATCTACAATTATAAACCCACTTATGGTGGTTATGCTGGGAAAGGATATG

ATCAAGTTTATCTCTTTGAATGATTGTAGATGTTGGGAGTTTCCCTAGAGTGTAATTTTGTGCACAATAA

ATTATGAAGGGTGGATGTATTTGAGGAATAACAGTTTATGCTCAGTGATGCTTTTAAGATGCTTAATATC

AGGGCAATGCTCTGCTTATCAATTAGATATATATATTTTCTTATTGCCCCAAT



>gi|71811754|gb|DR989145.1|DR989145 HDI_G_21.esd Horsegram (VZM-1) drought stressed subtracted cDNA Macrotyloma uniflorum cDNA, mRNA sequence

CTCACGGGCTGGCGGCTGCTCTAGAACTAGTGGATCCCCCGGGCTGCAGGATTCGGCACGAGGGNCACGA

GGAAGAGAATAACAGCGAAGCATGTTTGTGGCATGAGAAATTGCGAATGGCCTCTCCAACACTCCTTTTG

CCTCTTCTCCTTTGTCTGATATTTTTCGATAACTTTCACCACTCTTCTTCCTTCGTCTCTTTCCGTTGAG

AATCCCAAGGAAGACATAATAGTGTCAGTCGCCCAAAGGAACATTCACCGCAGGCTTTCGCGCCGTCGGA

GAAAACGCTTTCTGTTTCGCAATATGGTACACTCGACCACCTCACACCGTTGTTTGGATGGCCAACCGGG

ACCACCCGGTAAACGGAAAGCGCTCCACGCTTTCCCTTCTCAATACACCGGTAACCTCGTACTAACCGAC

GCCGACCAGTTCCAAGTGTGGTCCACCAACACCGCCAACTTTTCCAAACACGTCCGGTTACATTTGCACG

ACTCCGGCAACCTCGTACTTCGCGATGATTCCTCCAATGTTGATTTGTGGCAGAGCTTCGATTTTCCCAA

CCGACACGCTTCTTCCAATCACCCTCTGAGAGGGAGCACCACCTCGTTTCCTCGAGGGGGGGCCGGTACC

CCATTTCGGCCCTATAGT



>gi|71811752|gb|DR989143.1|DR989143 HDI_G_19.esd Horsegram (VZM-1) drought stressed subtracted cDNA Macrotyloma uniflorum cDNA, mRNA sequence

GAAGCTGAGCCACCGCGGCTGGCGGCGCTCTAGAACTAGTGGATCCCCCGGGCTGCAGGAATTCGGCACG

AGGTGTTTTCTGACACACTGTGGTTGGAACTCCACATTGGAAGCTTTGAGCTTAGGAGTTCCAGTGATTG

CAATGCCACTGTGGACAGACCAGATCACAAATGCAAAACTTATAAAGGATGTCTGGAAAATTGGAATCAA

AGCTGATGCTGATGAGAAAGGTATAGTCAGAAGAGAAACTATCACACATTGCATAAAGGAAATGTTGGAG

ACTGAAAAAGGAACTGAAATAAAGAAAAATTCCATCAAATGGAAGAATCTGGCCAAGAGTAATGTTGATG

AGGGAGGAAATTCTGATAAAATATTGCAGAATTTGTGGATGAATTGGTTCATCGCTGTGCTACATTAACA

TTGATGGGGGAGACAGTCCTGATAGAAGTTCTTTTCCTCTGTCAAAAGTATGCTATGCTAATTCTACTAA

GAATAACTCTATCGTCGGTCTCTTTAATAGGTAACCAGANAACAGGTTGTCATTTAACATTAGGTGTGAA

CAATTTGTTAGCTTCTGAAGTGGAACATTCGTNATNNAGCATATTGTTCTTTGTCACATTGTGTACGCAT

GATTTGCTCTATCGTAATAANATTATTCNCTCTTAAAAAAAAAAAAAACCCCGGGGG



>gi|71811750|gb|DR989141.1|DR989141 HDI_G_11.esd Horsegram (VZM-1) drought stressed subtracted cDNA Macrotyloma uniflorum cDNA, mRNA sequence

TCTAACANTGTCTCTTAACAGAATTATGTGTACAATTCATGGAAAGACTATTCACACATTCTTGCCACCT

TTGATTCTCTTGAGCCCATGACGGACTCTTCAAACGATT



>gi|71811748|gb|DR989139.1|DR989139 HDI_G_M5.esd Horsegram (VZM-1) drought stressed subtracted cDNA Macrotyloma uniflorum cDNA, mRNA sequence

GGGTCCAAGGAATACACAAAACTTTTGTGAGTGAGCGAANATGGTAGTCCATTGCAAGTTTGACNCTTCG

GCTCCTGAACACATCCATACTGTGGGACTACACCTGTTTGACTCATTCCATGCTGCTTTATTATTACTCT

TCATTGTCTTCC



>gi|71811746|gb|DR989137.1|DR989137 HDI_G_M3.esd Horsegram (VZM-1) drought stressed subtracted cDNA Macrotyloma uniflorum cDNA, mRNA sequence

TGGAGCTCACGCGGTGGCGGCGCTCTAGAACTAGTGGATCCCCCGGGCTGCAGGAATTCGGCACGAGGCC

TCGTGCCGAATTCGGCACGAGGGTAAGGAATAATTCTCTACTATAATAGTATATTTTGTGCTGTCCACGT

CTATAATGAGTCAATGTCTATGTATTGTAATATTTTGTGCATGAATGAATATGTAACCACTTAGGTCTTT

AA



>gi|71811744|gb|DR989135.1|DR989135 HDI_G_M1.esd Horsegram (VZM-1) drought stressed subtracted cDNA Macrotyloma uniflorum cDNA, mRNA sequence

AAAGCTGGAGCTCCACGTCGGCTGGCGGGCTTAACTAGAACTAGTGGATCCCCCCGGCGTGGTAAATTCG

GCACGAGGCCTCGTGCCGAATTCGGCACGAGGTTAACACTTTCCCGTTCTTTGGGGTTTGATATCTTGAC

CATTTATATGGATGATGATGTTCTGAAACATGGACCCACAAAAGGAGGGCA



>gi|71811742|gb|DR989133.1|DR989133 HDI_G_76.esd Horsegram (VZM-1) drought stressed subtracted cDNA Macrotyloma uniflorum cDNA, mRNA sequence

AGCTGGAGCTCCACGCGGCTGGCGGCCGCTCTAGAACTAGTGGATCCCCCGGGCTGCAGGAATTCGGCAC

GAGGCCTCGTGCCGAATTCGGCACGAGGGCTCAGGACTGCTCCAAAATGCCTCTGTGCTGTTCTGTTGTC

TCCTCTGGCCAAGCAAGCTAAGATCAACCTTGCCACTGCTATCACCATTCCAAAGAGATGCAACATAAAG

AACCGTCCTGCAGGAAAGAAATGTGGAAAGTACACCCTACCCTGAAATCAAAACAACAGAATAATACACA

ATATGCAGTGTTCAGCAGATTAGAGAGCTAAATAAAGTGCAGTGAAATGGCATCTTCAACTGTGACCTGT

TTCGGTGGTCATACTGTGTTCTTGTGTTGTTTCAATCACATAATTTATGTCTTCTTTGT



>gi|71811740|gb|DR989131.1|DR989131 HDI_G_73.esd Horsegram (VZM-1) drought stressed subtracted cDNA Macrotyloma uniflorum cDNA, mRNA sequence

AAGCTGGAGCTCACCGCGGCGGCGGCGCTCTAGAACTAGTGGATCCCCCGGGCTGCAGGAATTCGGCACG

AGGTTCAACAGAATTTCACCACTTTATTCTCACATCATTTTAATATGATGTGGTAATCAAGTCAATTCTT

GTTGAATGTTTGTCTGAATGTAGATGTTTTATACTCATGGTTTGTAATTGTGTGTATAAATTAAGCTTAT

ACATAAATATTTACACTATGTCACTCTAAGTTTCTGATATTATAGCTGTCTTTGGATATGTTAAAGAGTG

AGATATATAAAAACTCTCTAGAGTTGAATATGGTGTATGCACTACTAGTATACATGTTGTATCCAGCAGA

GAATTATGTATATCTATGTTAAGAGTTATAAATAAAGAAATAATGGTGTTTAGAGTGGCAAAAAAAAAAA

AAAAAAAAAAAAAAAAAA



>gi|71811738|gb|DR989129.1|DR989129 HDI_G_65.esd Horsegram (VZM-1) drought stressed subtracted cDNA Macrotyloma uniflorum cDNA, mRNA sequence

CTCTAGAACTAGTGGATCCCCCGGGCTGCAGGAATTCGGCACGAGGCCTCGTGCCGAATTCGGCACGAGG

GAACATTCGTTTCTCCCTAAGTTTAAGTCGTGATGGGAAAGGAAAAGGCTAACATCAAAATCGTGGTCGT

TGGACATGTCTCTGAGAAATCAACTGCCACGGAGCTTGGAAGCCCTACTGAGAAGGGTGTGACTGAAAGG

TTCGAGAAGGGAGAAGGAAGTGTTTAAAACACGTAAGATGTAAGATGTGTGAAGAAGGATGATGTAAAAT

AAATAAGATATAAGATGTGAAGATGGATCTATCTTAAGATGAAGTGCTGAAGGACTTATATTCAGATAAA

TAAAATGAAGGATATATGTTT



>gi|71811736|gb|DR989127.1|DR989127 HDI_G_63.esd Horsegram (VZM-1) drought stressed subtracted cDNA Macrotyloma uniflorum cDNA, mRNA sequence

CTAGAACTAGTGGATCCCCCGGGCTGCAGGAATTCGGCACGAGGCCAGCACCCAGGGTTTCCTCAAATCA

CAGTGTGCTTTGACATTGATGCCAATGGTATCTTGAATGTCTCTGCCGAAGACAAAACTACTGGCCAAAA

GAACAAGATCACCATTACCAACGATAAGGGTAGATTGTCAAAGGAAGAAATTGAGAAGATGGTTCAAGAG

GCCGAGAAGTACAAGTCTGAAGATGAAGAGCATAAAAAGAAGGTTGAGGCTAAGAATGCATTGGAGAACT

ATGCATACAACATGAGGAATACTGTGAAGGATGAGAAGATTGGAGGAAAACTTGACCCAGCTGACAAGAA

GAAGATTGAGGATGCTGTTGAGCAGACCATCCAGTGGCTAAACAGCAACCACCTTGGAGAGGCAGATGAG

TTTGAGGACAAACATGAAGGAATTGGAAAGCATCTGCAACCCTATCATAGCCAAGATGTACCAGGGTGGT

GCTGGTCCAGACGCAGGAGGGGCAATGGACGATGATGTTCCTGCTGGTGAAGTGGTGCTGGTCCAAAGAT

TGAGGAACGTTCAGAACTT



>gi|71811734|gb|DR989125.1|DR989125 HDI_G_61.esd Horsegram (VZM-1) drought stressed subtracted cDNA Macrotyloma uniflorum cDNA, mRNA sequence

CTGGAGCTCACCGCGGTGGCGGCGCTCTAGAACTAGTGGATCCCCCGGGCTGCAGGAATTCGGCACGAGG

GACACCTCCATCATACCCTGGTGGGCATGGATGAAAAGGTTCCATCTCCCTGAGGCTGAACTACTCAATG

GCCGTGCCGCCATGATAGGATTCTTTATGGCATACCTGGTTGATAGCTTGACGGGAGTAGGCCTAGTTGA

TCAAATGGGTAACTTCTTTTGCAAAACTCTGCTGTTTGTAGCAGTGGTCGGAGTACTTCTGATCCGAAAG

AATGAGGACCTTGAAAACCTTAAGAAGCTATTAGATGAGACTACATTATATGACAAGCAATGGCAAGCAA

CTTGGCAGGATGAGAATTCTAGCACTTCCAAAAATGAGTAGTGGAATTGCTGTTCTGTAGTTCTCTCTCC

TTCTGTTTCTTAATACAGTTTTCTATTGATCTCTTTAGTACAAATCAAGACATGGATATGAAATTAGACC

TTGTATTTCAATTCCTTCTCTTGTCCTAGCTCCAAATGACCTAAATAGTGTGTGTTAGTTCCAATGCAGG

ATTTTCTT



>gi|71811732|gb|DR989123.1|DR989123 HDI_G_54.esd Horsegram (VZM-1) drought stressed subtracted cDNA Macrotyloma uniflorum cDNA, mRNA sequence

CTGGAGCCACGCGGCGGCGGCGCTCTAGAACTAGTGGATCCCCCGGGCTGCAGGAATTCGGCACGAGGGT

TGGCATAAAATACTTCCTAGCTGAACCAACAAAGCTGCAACAAGGGGGTAAAGCTGTGAAGTTTGATTGA

GAGAGAAGTTTCTAGAAGCTGGAAGGGGAACAAAGACTACTGAAGATAGTGCTGTAGGATAAGAAGGCAT

TTTGTTTAAACGAGGGATTGGTTTCAATGCTCTGTATGATTTCGTTGCTTTATGTTGCCATCTTCGAAAG

TGTGGACTTTGCCAGGAAGAGCAGATTCACATAACATATCCTAAAGGGAAGTCTGTTCTTATTTTACCCA

GCCGTTAGATAATATTAACATGTATAGTTGCAGTTTGTTTATTCATTTAAAACTGATTTTTGCTTGCAAT

TTTTATCAAGTGAAATTCAAGATGATTTATTGGGTTGTAATTTTGAATGTGATCAGTTCTTGGAAAGTGT

AGCCGGTTTATATTAATAATATAAATTTTATCAGATAATAATAAA



>gi|71811730|gb|DR989121.1|DR989121 HDI_G_52.esd Horsegram (VZM-1) drought stressed subtracted cDNA Macrotyloma uniflorum cDNA, mRNA sequence

AAGCTGGAGCTCACGCGGCTGGCGGCGCTCTAGAACTAGTGGATCCCCCGGGCTGCAGGAATCGGCACGA

GGCCTCGTGCCGAATTCGGCACGAGGTAGCGGCCTATTGGCACTATTATTTTGCCTGGCTTGCTGTCACC

ACTGGCAATTGCTTCTCTCTGGAATTAACTATAAGAAATCAGTCCAAGTAAGGCTGCCGCTGTGATGTCT

AGTTTGTGTATAATATTTTGGAGTATGGGGAGAAGTTTAAATTCTCAAGATAACGCCCTATATACTCCGT

AGTCTATATCAGCTTGTAACCTGTCAGTTCTATTTCCCCATATTGTAAAGCAGATGTGCTTTCTTTAAAT

ACAAGTTTTAAGAAAACCAGCTAAAAAAAAAAAAAAAAAAAAAAAAAAAAAAAAAAAAAAAA



>gi|71811728|gb|DR989119.1|DR989119 HDI_G_50.esd Horsegram (VZM-1) drought stressed subtracted cDNA Macrotyloma uniflorum cDNA, mRNA sequence

AAAGCTGGAGCTCACGCGGCTGGCGGCTGCTCTAGAACTAGTGGATCCCCCGGGCTGCAGGAATTCGGCA

CGAGGCCTCGTTGCCGAATTCGGCACGAGGGAACATTCGTTTCTCCCTAAGTTTAAGTCGTGATGGGAAA

GGAAAAGGCTAACATCAAAATCGTGGTCGTTGGACATGTCTCTGAGAAATCAACTGCCACGGAGCTTGGA

AGCCCTACTGAGAAGGGTGTGACTGAAAGGTTCGAGAAGGGAGAAGGAAGTGTTTAAAACACGTAAGATG

TAAGATGTGTGAAGAAGGATGATGTAAAATAAATAAGATATAAGATGTGAAGATGGATCTATCTTAAGAT

GAAGTGCTGAAGGACTTATATTCAGATAAATAAAATG



>gi|71811726|gb|DR989117.1|DR989117 HDI_G_42.esd Horsegram (VZM-1) drought stressed subtracted cDNA Macrotyloma uniflorum cDNA, mRNA sequence

ACTGGAGCTCCACGCGGCTGGCGGCCGCTCTAGAACTAGTGGATCCCCCGTGGCTGCAGGAATTCGGCAC

GAGGCACTGTCTCAGAGAACTTCCAGTTTTGGCTACGGATGAGAAGTACAATGAAGCTATCTA



>gi|71811724|gb|DR989115.1|DR989115 HDI_G_40.esd Horsegram (VZM-1) drought stressed subtracted cDNA Macrotyloma uniflorum cDNA, mRNA sequence

GCGGAGCCACGCGGCGGCGGCGCTCTAGAACTAGTGGATCCCCCGGGCTGCAGGAATTCGGCACGAGGCC

TCGATGCCGAATTCGGCACGAGGAGAGACCAGGAGCNGGGCACCGCTGAGATGTTGTACGATGGGCGCGT

GGGTTGCATGTAAATATTGTCGTTTGTTCGTGAATCGCATGCCACGTTTGACATCTACTAGCCATTGATG

TTTGTCAGAATTTATTGGTCTGTTACTGAGCTGAAAGTATTACTAGTGTGTTTGGTTTGTCGTGAGCTAG

TGAATGTAAAAATGCTAGATAACTAGCATCCTATGTTTCCGTACTTAAGTTTTAGATAATTAACCAGATT

TGCCTGGAACTTTGGCATTTGGTTAATCTTTTCACCCTTTTGTTTTAAGTAGTTATTATCGAATATAAAT

AAAAATTGTGTTTTTGTACTTGAAAAAA



>gi|71811722|gb|DR989113.1|DR989113 HDI_G_39.esd Horsegram (VZM-1) drought stressed subtracted cDNA Macrotyloma uniflorum cDNA, mRNA sequence

GCTGGAGCTCACGCGGCTGGCGGCGCTCTAGAACTAGTGGATCCCCCGGGCTGCAGGAATTCGGCACGAG

GCACTGTCTCAGAGAACTTCCAGTTTTGGCTACGGATGAGAAGTACAATGAAGCTATCTATAGCACTGCT

AAACGTCTAGTTGCTGCCATTGATGGCCTTCCAGATCCCGGTGGGCCTACATTCAAAGACAACAAACGTG

AGTCTAACTTCAAAACCAAGCAAGAGACAGAAGAGAAACGTGGACAATTCACTCTTGTAGTGGGAGGTTT

GTTGGTTATTGCATTCGTTGTTCCTATGGCACAATACTATGCTTATGTATCCAAAAAATAAAATAATATA

AGTATATCCGTCTTTCAAATTCTCACTCTTTAAATTCAGATGTATAAGTGCAGTTGTAACCATGCTCATC

TTACAATTGAGTTTGGGAAGGCTGAAGAATAAGATGAGAGTTTTTTCATGTAAATGCAATTCCATAGTAT

ATGCATTTTGATTATGTTATTATCCAGTTGGGTTTTTAAAGTCAGGTAAACGAGTTCTAGTTCTCCATTT

CTCTCCCAAAAAAAAAAAAAAAAAAAAAAACA



>gi|71811720|gb|DR989111.1|DR989111 HDI_G_37.esd Horsegram (VZM-1) drought stressed subtracted cDNA Macrotyloma uniflorum cDNA, mRNA sequence

AAGCTGGAGCTCACGCGGCTGGCGGCGCTCTAGAACTAGTGGATCCCCCGGGCTGCAGGAATTCGGCACG

AGGGTTGGCATAAAATACTTCCTAGCTGAACCAACAAAGCTGCAACAAGGGGGTAAAGCTGTGAAGTTTG

ATTGAGAGAGAAGTTTCTAGAAGCTGGAAGGGGAACAAAGACTACTGAAGATAGTGCTGTAGATAAGAAG

GCATTTTGTTTAAACGAGGGATTGGTTTCAATGCTCTGTATGATTTCGTTGCTTTATGTTGCCATCTTCG

AAAGTGTGGACTTTGCCAGGAAGAGCAGATTCACATAACATATCCTAAAGGGAAGTCTGTTCTTATTTTA

CCCAGCCGTTAGATAATATTAACATGTATAGTTGCAGTTTGTTTATTCATTTAAAACTGATTTTTGCTTG

CAATTTTTATCAAGTGAAATTCAAGATGATTTATTGGGTTGTAATTTTGAATGTGATCAGTTCTTGGAAN

AGTGTAGGCCGGTTTATATTAATAATATAAATTTATCAGATAATAATAA



>gi|71811718|gb|DR989109.1|DR989109 HDI_G_3.esd Horsegram (VZM-1) drought stressed subtracted cDNA Macrotyloma uniflorum cDNA, mRNA sequence

GCTGAGCTCACCGCGGCTGGCGGCGCTCTAGAACTAGTGGATCCCCCGGGCTGCAGGAATTCGGCACGAG

GGGTGGCCAGACATGGTGCAGATAAGTTTTTGCTTCACCTTGTTGAGAGTCTTTATGACAGCATTAAAGA

ATAGAAAGCAGCATAACAGAGAGCACCAGTTACTTTAATTTGACTGCAAGGATATAGATATGCTTTGAGC

ATGCATACTATGCCAAAGCTGGGAGAGAAGCACAACCTGAACTATGGACATAAATCAAAGGAGAGAAATT

GTATAAAGGAATTTCTTGCTTATCCGAATCCAGTTTCTTTCAGATGTTATAAAGTTCAATTATTCGTTAA

GTCATATTTTTCTGCCTATGATTGCTTTGCTATGAAAGCAAATAAAGCCACGTTGAAATTGATTGTACTT

CAATAGTACTATATCTCATGTTAATTGATGCATTTGATATTCAGCAGTGTCAAAAAAAAAAAAA



>gi|71811716|gb|DR989107.1|DR989107 HDI_G_27.esd Horsegram (VZM-1) drought stressed subtracted cDNA Macrotyloma uniflorum cDNA, mRNA sequence

AGCCACGCGGCTGGCGGCGCTCTAAACTAGTGGATCCCCCGGGCTGCAGGAATCGGCACGAGGAGCAATT

GATAGGCTTCTTTGATTCCTGGGATTCTTGAGATTGTAGGCATTGGCTTCACTGGGTGGTTTGTATATAA

GAACGTAGTTTTCAAGCCAGACAGGGAAGCTTTGATACAGAAAGTAAAAGAAACATACAATGAAATACTC

GGGAGCAACTGAAAGAGGTAGGAAATGCTGAGATGATGTAGACATGAAACAATCATATCTTGCAGCTTCC

TGTTGAAGATTTCATAATGCTTCATTTTTTCGCCATAAGCTTCTGACCATACTTTATCCCAAGTAATGTG

TGTTCGAAAAAAATTGTATTTCATATATCTTAATCCACTTTTAAGACAATCTTATTTCCTTAAAAAAAAA

AAAAAAAAAA



>gi|71811714|gb|DR989105.1|DR989105 HDI_G_25.esd Horsegram (VZM-1) drought stressed subtracted cDNA Macrotyloma uniflorum cDNA, mRNA sequence

GCTGGAGCCACGCGGCTGGCGGCTGCTCTAGAACTAGTGGATCCCCCGGGCTGCAGGAATTCGGCACGAG

GGGACCATAGAAGGCTGCTTGCATCATATGACATGGAACCTGAGTCAGGTAACTATATCAATCCAGATAG

ACCCATGCTTGATGTGCAGTTGAAAAGTATTACTTAGATGGGTGGAGGCAGGAAATGACTTTGTTTTTCA

GCTGTGGAATTTTGCATGATGAGGTAAAAACGCCTTGGCATGGAAACTGGCTGAAAGTAAATGCAACTGC

CTATCATCTCAGCAAAGGAAAACTTTTTATAAATTCCCATAATTCAATTTAGTCATGTATATAATAAAAG

GAAACTTAGAAATGTATAGGGTACATAGTTTTGTAGTAGTNATGCACAGTTATCTGTGTACTAGATTCAG

CCAAACTTGTAATATCTGAATAGAGAAACAATACCCTATTAAACACTTTCTAAAGTTGTACAGCAT



>gi|71811712|gb|DR989103.1|DR989103 HDI_G_18.esd Horsegram (VZM-1) drought stressed subtracted cDNA Macrotyloma uniflorum cDNA, mRNA sequence

TTGAAAACTCAATATGTCTTTTGGATTTGATAGCAGTAATCAATGTGAGATAGATGTATGATATCTCTAA

TACAAGCCGTCTTTGTCTCGTGATGCCGTTTACAGTATGCACCATTCATTAAAGATCGTGGCTTCTGAAA

ATTAATACTAATGCTTGTCCTTGACAATATTGTTCTGCCTCATTTTGGTGTTGTATTATAAATCAGGGTT

GCTTACTTCATTTTTGCGGGCATGTTAGTTTATACCGGGATAAATATGATAGATATATGTTTCCTAGATT

AAAAAAAAAAAAAAAAAA



>gi|71811710|gb|DR989101.1|DR989101 HDI_G_16.esd Horsegram (VZM-1) drought stressed subtracted cDNA Macrotyloma uniflorum cDNA, mRNA sequence

AATCGACGCTGAGCTCACCGGCGGCGGCGGTGACTCTAGAACTAGTGGATCCCCCGGGCTGCATGAATTA

CGGCACGAGGCCTCGTGCCAGCAATTCGGCACGAGGGATTCCTCTTAAAGATGTAAATTGAGGCGCTAAA

TACACACTACTTTATGAAGTTTATTACAATTAACACTGCCGAGAAACACAACTAATTCCAACCATTTAAT

GATTCAAAATATATAAGCGGTGTTACAATCTTGTGATNGTGAGGAATCCTATGTTGAAATGTATCCGAGG

CAGACACGTNTTATGTTAGACAAGTCTCATGATCAGAGATTATTACATTCTTCAGGTTCTTCTTCCTCCT

GGAGAAGATTTACTCTATNGTCAATGAGCCCAGAAAACCACGCAAATCTATGAGACAATTGACATGAGCC

TACAAGTTCACAACCACCTGTTATATAGCCAAGAGC



>gi|71811708|gb|DR989099.1|DR989099 HDI_G_14.esd Horsegram (VZM-1) drought stressed subtracted cDNA Macrotyloma uniflorum cDNA, mRNA sequence

AAGCTGGAGCTCACGCGGTGGCGGCGCTCTAGAACTAGTGGATCCCCCGGGCTGCAGGAATTCGGCACGA

GGGTTCCCAAGACGGGCACGCAAGGTCAAGGCTGGTGATTCTACTCCTGAGGAACTTGCAAATGCTACAC

AAGTCCAGGGTTCATACTTACCTATTGCGAGGGAGAAGCCTTCTGTTGAACTTGTGAAGGTCACTGACGA

CATGAAGGCATTCAAAGCTTATTACAAGCTTAGACTTGAACGCACAAACAAACGCCATTATGGTGCCAGA

CTGAAGAAGGCCGCTGAGGCTGAAAAGGAAGACAAAAAGTGAAGACCTTTGCAGTATCTTGATATAATAG

TTGTTGCGAGTCAATTTTTGCTCGAGATTAGTTTTTATTAAGAACATGTTCAGTCGAAAGCATGGATCTC

TTCACCTATTTTTATTTTATGTACTGTTATTCAAAAAAAAAAAAAAA



>gi|71811706|gb|DR989097.1|DR989097 HDI_F_96.esd Horsegram (VZM-1) drought stressed subtracted cDNA Macrotyloma uniflorum cDNA, mRNA sequence

AAAGCTGGAGCTCACGCGGTGCGCGGCGTTCTAGAACTAGTGGATCCCCCGGCGCTGCAGGAATTCGGCA

CGAGGCCTCGTGCCGAATTCGGCACGAGGATTTAGATGGATTACTCTCCTGCTGACTCAGGAATTCAATT

TTGCTGACAGCCTTCACATTTGGGACACTCTTTTAAGCGATCCTGATGGTCCACAGGAGACTCTTCTTCG

GGTATGCTGTGCAATGCTTGTCCTCGTGCGCAAGCGCCTCCTTGCGGGTGATTTTACATCAAATCTGAAG

TTATTGCAAAATTATCCCACCACAAACATTAGTCATTTGCTCTATGTTGCCAACAAGTTTCGTGTACAGT

CAGTTTAATGTTTATCTCCCATTATCTTTTGCATTTTGCTTTTGTACTTTTAATTTCTCTTTCAAATTCA

ATACTTGTAAATGACTTTTATGTGTGAGCCTCAGAGCAGTGACTGTTTTTTTATTTATTTTTAGTTATAT

TTTGTACGTAAAAAAAAAAAAAAAAAACCCTCAAGGGGGGGCCCGGTTNC



>gi|71811704|gb|DR989095.1|DR989095 HDI_F_94.esd Horsegram (VZM-1) drought stressed subtracted cDNA Macrotyloma uniflorum cDNA, mRNA sequence

GCTGAGCTCACCGCGGTGCGCGGCGCTCTAGAACTAGTGGATCCCCCGGGCTGCAGGATTCGGCACGAGG

AGGAACACTACATGCAAGAGAAAATAGAGGAGATTTAAGGACTTGGGATGATGCACTAAGGAAAAGCACT

CAAGAATTCCAACATCGATCTTACAATCTCTTTACCTGCAACTGCCACTCGTTTGTTGCCAACAATTTAA

ACAGGTTGGATTTCTTGTCTGGTGGATGGAATGTGGTGAACCTTGCAATTTTTATTTTATTCAATGGACG

TTGGGTCAGCAAAGCATCTATGCTTAGTTCTATCTTACCATTTGTGGTTGTATTTTTTCTTGGAGTCACA

TTCGGGGGCTTCACTTTCTTAAAGTTTTGGTTCTTTTTCACTTTCATTCTTATTGGTTGGTTCCTTCTTG

GTACTTATTGCTTCAAGAACCTGATTCAATTGTAATGTCTTTTTCTCTGGTTCAATTTTGTATACAACAA

TAATACGGTACTATGTTGGTGAAACATGACTAATTAATCATATACTTTGTGTTCAGATAAAAAAAAAAAA

CAAAAA



>gi|71811702|gb|DR989093.1|DR989093 HDI_F_92.esd Horsegram (VZM-1) drought stressed subtracted cDNA Macrotyloma uniflorum cDNA, mRNA sequence

AAAGGCTGGAGCTCACCGCGGTGGCGGCCGCTCTAGAACTAGTGGATCCCCCGGGCTGCAGGAATTCGGC

ACGAGGCCTCGTGCCGCCCAGATGCAGATCTTAGCCAGGAAATTGTTAAAAAGGTTCTTCCTATTTCTTA

ATATATATGTATGTAATTTTCATTTCAATACCCCCACCCCCCACCAAAGAAGAATTTATAGTTTCTACTT

TTTCTTCTTTGGATGTGCCTCTTGTTTAATCGCAACACTGTTCAATGTAAAATCCGAGATTTACGTATGA

ATTGGACCCCCTT



>gi|71811700|gb|DR989091.1|DR989091 HDI_F_9.esd Horsegram (VZM-1) drought stressed subtracted cDNA Macrotyloma uniflorum cDNA, mRNA sequence

GAGNCACGCGGTGGCGGCGCTCTAGAAACTAGTGGATCCCCCGGGCTGCAGGAATTCGGCACGAGGCATT

GTTGAAGGGCCAAACTTTGAATTTGCTACAGAGACTCATGAGGAGCTTTTATATGACAAAGAGAAGCTGC

TTGAAAACGGGGACCGATGGGAGACTGAAATTGCTGAGAACCTGAGATCTGAAAGCCTTTACCGCTGAAC

TTTACTACGTGATCCTTGCCTGTTGCTTTTTCGGTCTAATTGGGCAATGATAAATAAAATGACCACTGAA

TGGTTTTAAAGAACTGTATGCAATTTACGATTGGTACATCAGTTTTTTCTTGTCAATGTTTTGCATTGCT

GTTAAGTATACAATATTGATGACGTTTATCTCCTTGAAATCTTGAACAAATGTGGAAATAATTGAATTTT

TCAACAAATAGCAACTTTTAGGTTTGTATTGTAGGCGTAATTTATCCTTAGAAAATAGAAGTACTGATGC

TGTATTCAGAATANAAAAAATTGACGGATTCGGTGATTGCGAGCATGTACTTCATTCACTGTGAAATAGT

TAGAAAG



>gi|71811698|gb|DR989089.1|DR989089 HDI_F_83.esd Horsegram (VZM-1) drought stressed subtracted cDNA Macrotyloma uniflorum cDNA, mRNA sequence

CAGCTGGAGCTCACCGCGGGCCGGGCCTGTTATAGAAACTAGTGGATCCCCCGGCGCTGCAGTGTAATTC

GGCACGAGGGCCTCAGATGACGCATATTTGCGAGCGGCAATGGCTGGATGACGCTCTATTAC



>gi|71811696|gb|DR989087.1|DR989087 HDI_F_81.esd Horsegram (VZM-1) drought stressed subtracted cDNA Macrotyloma uniflorum cDNA, mRNA sequence

AGCTGAGCTCACGCGGTGGCGGCGCTCTAGAACTAGTGGATCCCCCGGGCTGCAGGATTCGGCACGAGGG

TTGAATTGAATGGTAGACTGAACAAATGTGGGGTTATTAGTCCCCGTTTTGATGTGGGTGTCAAAGAGAT

TGAAGGTTGGACTGCTAGATTGCTCCCCTCAAGACAGTTTGGGTATATTGTGTTGACTACCTCTGCCGGC

ATCATGGATCATGAAGAAGCTAGGAGAAAGAATGTTGGTGGGAAAGTGCTGGGTTTCTTTTACTAGTCTA

TTTTCCTTTGAAAGATTTTGTGCGGATTTCAGTTTTTTTTTTAAGTTATTTTTTTTAACTTACTGCTGTA

ATGATCAATGTTAGACACTTAGTTTATTAAGTATAGCATAGCAGATGTTTTTCTGATGTGCTAAAAAAAA

AAAAAAAAAAAAAAAAAAAAAA



>gi|71811694|gb|DR989085.1|DR989085 HDI_F_79.esd Horsegram (VZM-1) drought stressed subtracted cDNA Macrotyloma uniflorum cDNA, mRNA sequence

AGCTGGAGCTCACGCGGTGGCGGCGCTTCTAGAACTAGTGGATCCCCCGGGCTGCAGGAATTCGGCACGA

GGGTTCGCCGGTCCTTGTTTTCTCCTAGTCCTGATGCTATTGGAAATCCAAGCAATTCTTATGCTTCCTT

ACGCTCTGGTCTTTCTACATCTGAACCAAGGGCGTTATCATCTGGGACTGGGGCAAATCCGAGATCATCT

TCATGGATGGAGAGGCAAGGAGACAGTGAATTTGGAATTCCTTATTCACTACGTACGTTGGCTGTTGCAA

GTGAAGGAAGTAGTAGACTAGTATCCGAGCTCCGCCATGTGCTTGGGCCTTATGCGTAGAGGCGGGAATG

TGCGGTTCGAGGATGTTGTGATCCTGGATCATCAGTCGTTTCTTTCTGGAATAGCTGATGTTCATGATCG

ACACAGGGATATGCGTCTTGATGTTGATAACATGTCTTACGAGGAGTTGTTGGCTCTGGAAGAGCGCATT

GGTAATGTGAGTACTGGATTGAGTGAGGAAACCGTATTGAAACTCTTGAAACAGAAAAAGCACTCGGTTG

AAAACAGAATCTCAGATTGATGCAGAACCCTGTGTGTNNGTCAGGAGGATTATGGTGATGGACGATGATA

TTGGAACCTTTGATTGC



>gi|71811692|gb|DR989083.1|DR989083 HDI_F_71.esd Horsegram (VZM-1) drought stressed subtracted cDNA Macrotyloma uniflorum cDNA, mRNA sequence

CTGGAGCTCACGCGGTGGCGGCGCTTCTAGAACTAGTGGATCCCCCGGGCTGCAGGATTCGGCACGAGGA

AAAGAGGAAGTGGCTCCGTGTGGTGGACACTAATCTCAAATCACCAGACGACTTTGTTCTTGATGGTGTC

CCTATCCTAGGAAACACGTATAATATAGCTCCATACTCCTCCATTCTTCTCGAAGCCAAGTTTTGATTGC

AGAGGCTGTTGTATAGTGTATGCATTAAAAGGAAAAAGAGAAAAGCACTAGAAATAATATTTTTTTTGTT

TTTTTTGGTACAGCTATATGTAGTGTTCTTACTGAGAGGTTAAGTGTAAGACAGAGTGTTTTANAGAGAC

TTTCACACTTCTACTATGTTATGCTGAAAGCTTGTCGTCCAATGGATGGGAAGTTACCTGTTTATTGTAC

TCCGACAATCAATTCAGTATATATTCCAGTATATATTCCAG



>gi|71811690|gb|DR989081.1|DR989081 HDI_F_7.esd Horsegram (VZM-1) drought stressed subtracted cDNA Macrotyloma uniflorum cDNA, mRNA sequence

AAAGCTGGAGCTCACGCGGTGGCGGCGCTCTAGAACTAGTGGATCCCCCGGGCTGCAGGAATCGGCACGA

GGGTCCACCAAAGGTAGAAAGCAGCTTCAATGCTGAAATCACTGTGGGAAATGGTGTGGCTGGTTGATTG

CGTAGCCACAAGAAAAGGATGCACAAACAAGCAGTTGAAATATCATTGCAAGAATAAGACAGAAGTTCAT

TTGAATTGTCCCTTTGTTTATTTATCATGTGTATCTTCATCTTATCATTTTTATTGGAGGCTATTATTGA

TTTGGAGGTGAAAACTGTTTTACCTACTACATTTGTCTATTTGTATTCCCATTCATGTATCTATCTACTC

ACAATTTGTGCTGAGGGGATGAAAGGTGGTTCATGATAGTACTATGGACAAAAGAACATTCAGCTGTCAG

AATGACTTTCAGAGCTAAAAAAAAAAAAAAAAAAAAA



>gi|71811688|gb|DR989079.1|DR989079 HDI_F_68.esd Horsegram (VZM-1) drought stressed subtracted cDNA Macrotyloma uniflorum cDNA, mRNA sequence

AAAGCTGGAGCTCACCGCGGTGGCGGCGCTCTAGAAACTAGTGGATCCCCCGGGCTGCAGGAATTCGGCA

CGAGGCCTCGTGCCGAATTCGGCACGAGGATTTAGATGGATTACTCTCCTGCTGACTCAGGAATTCAATT

TTGCTGACAGCCTTCACATTTGGGACACTCTTTTAAGCGATCCTGATGGTCCACAGGAGACTCTTCTTCG

GGTATGCTGTGCAATGCTTGTCCTCGTGCGCAAGCGCCTCCTTGCGGGTGATTTTACATCAAATCTGAAG

TTATTGCAAAATTATCCCACCACAAACATTAGTCATTTGCTCTATGTTGCCAACAAGTTTCGTGTACAGT

CAGTTTAATGTTTATCTCCCATTATCTTTTGCATTTTGCTTTTGTACTTTTAATTTCTCTTTCAAATTCA

ATACTTGTAAATGACTTTTATGTGTGAGCCTCAGAGCAGTGACTGTTTTTTTATTTATTTTTAGTTATAT

TCTTGTACGTAAAAAAAACAAACAAAAAAACCCCAAGGGGGG



>gi|71811686|gb|DR989077.1|DR989077 HDI_F_60.esd Horsegram (VZM-1) drought stressed subtracted cDNA Macrotyloma uniflorum cDNA, mRNA sequence

CGTTCTTAGAACTAGTGGATCCCCCGGGCTGCAGGAATTCAGGCACAGAGGCCTCGAAGGCACCTTTTTC

CCAAATGGAGTGGTCACACCTCAGCTCACATCTTCTCACGC



>gi|71811684|gb|DR989075.1|DR989075 HDI_F_58.esd Horsegram (VZM-1) drought stressed subtracted cDNA Macrotyloma uniflorum cDNA, mRNA sequence

AAAAGCTGGAGCTCACGCGGTGGCGGCGCTCTAGAAACTAGTGGATCCCCCGGGCTGCAGGAATTCGGCA

CGAGGCAGAACCATGCAACTGATAGTGAGAAGAAGGTTAACTGTACATTGACAAGAGATGAGAATTTCAA

CTTCAGAAGCAGCATATTGGCCAGATCTTCATGGCCTTCTTTACAATGCACTGCCATCAGATATATTTTT

ATGGGGTCACCTTTTTCTCTCTTTCCATGTTGTAGATGATAAGGATTCTGTCATAGTAAAGGCTAAGGTC

CTAGAAAACTGGCAAGGTTGTAGAGATAGTAGGGATTTGCTTGTTGCAGCAGATGGCTGTCTCTCTTCCA

TCCGCAAGAAATATCTCCCTGATTTTAAACTCAGATATTCAGGCTATTGTGCATGGAGAGGGGTTCTTGA

TTTTTCAAAAATTGAGAATTCAGAAACCATCACTGCTTTCAGAAAGGCATACCCTGATCTAGGAAAATGC

TTGTACTTTGACTTGGCCTCAGGCTCACATACTGTGCTGTATGAGTTAAAAAACAAAAAATTCACTTGGG

TTTGGTACGAGAATCAGGCCAAGAAGCCTGAAAGTCAAGTGCGGACATCACGTGACACATGAACAACGTC

AAATAAGTGACCTTGATTCCAGAAAGAATGCCGCAAGAAAGCAGAACAACGGTTTTGAATTCCCTTGGCA

GTTTTGTCGAACAGTCCTGAAAACGAAACCATGGGGAACATTCCTTGAATTTTCATATTATGAAT



>gi|71811682|gb|DR989073.1|DR989073 HDI_F_56.esd Horsegram (VZM-1) drought stressed subtracted cDNA Macrotyloma uniflorum cDNA, mRNA sequence

AGCTGAGCTCACCGCGGTGCGCGGCCGCTCTAGAACTAGTGGATCCCCCGGGCTGCAGGAATTCGGCACG

AGGGTTGGCATAAAATACTTCCTAGCTGAACCAACAAAGCTGCAACAAGGGGGTAAAGCTGTGAAGTTTG

ATTGAGAGAGAAGTTTCTAGAAGCTGGAAGGGGAACAAAGACTACTGAAGATAGTGCTGTAGGATAAGAA

GGCATTTTGTTTAAACGAGGGATTGGTTTCAATGCTCTGTATGATTTCGTTGCTTTATGTTGCCATCTTC

GAAAGTGTGGACTTTGCCAGGAAGAGCAGATTCACATAACATATCCTAACAGGGAAGTCTGTTCTTATTT

TACCCAGCCGT



>gi|71811680|gb|DR989071.1|DR989071 HDI_F_47.esd Horsegram (VZM-1) drought stressed subtracted cDNA Macrotyloma uniflorum cDNA, mRNA sequence

AAGGCTGGAAGCTCACCGCGGTGCGCGGCCGTTCTAGAACTAGTGGATCCCCCGGGCTGCAGGAATTCGG

CACGAGGGTGGTTCAAGGCTGGCTCTCAGATCTTCAGCGAGGGTGGGCTCGACTACTTGGGCAACCCAAG

CCTGATCCACGCTCAGAGCATCCTCGCCATCTAGGGCTACCCAAGTTATCCTCATGGGCAGCCGTTGAGT

GGTTACCGTATTGCCGGCGGGCCTCTCGGTGAGGTCACTGACCCCATACTACCCCGGTGGCAGCTNTCGA

NCCCATTGGGCCTCGCTGACGACCCANAGGCTTTCTGCTGAGCTCAAGGTGAACGGAACTCAAGAACGGT

AGGTTGGCCATGTTCTACCATGTTCGGGTTCTTCGTCCAGGCCATTGTCACCGGAAAGGGCCCATTGGAG

AACCTCGACCGACACCTTGCAGACCCAGTCAACAACAACGCCTGGGCCTACGCCACCAACTTCGTCCCCG

GAAAGTGAGCTTGAAGAGAATGACTTGTGTACTCGANGGGGGGCCCGGTACCCAATTCGCCCTATAGTGA

GTCGTATTACCAATT



>gi|71811678|gb|DR989069.1|DR989069 HDI_F_43.esd Horsegram (VZM-1) drought stressed subtracted cDNA Macrotyloma uniflorum cDNA, mRNA sequence

AAGCTGGAGCTCACGCGGTGCGCGGCCGNCTCTAGAAACTAGTGGATCCCCCGGGCTGCAGGATTCGGCA

CGAGGGAAGATCAATGCAATTGCCAAAAAATGGGAACCCACTATGGTTTTGCCTCAGGCTGATAGAGTTC

TTATTCGTTTGGAGGAGCTATCAGATAGAACAGCAGGGGGAGTTTTGCTGCCCAAGTCAGCTGTGAAATT

TGAGCGATATCTCGTGGGAGAAATCCTAAATGTTGGTGCTGAGGCTGGTGAACTAAAGGCTGGAACCAAG

GTATTATTCACTGACATGAGTGCTTATGAGGTGGATTTGGGGACTGATGAAAAGCACTGCTTCTGTAAAG

CAAGTGACTTGTTGGCTGTGGTTGAGTAGGGTTTGGCCACTGTGCACTATTTTTTCTAATATCTTTGACA

AATGTATTTTCATTAGAATAAACCATTTTTATTCATTGGTGTTAAAATATTATATCCCTTTTATGCTATA

ATAA



>gi|71811676|gb|DR989067.1|DR989067 HDI_F_35.esd Horsegram (VZM-1) drought stressed subtracted cDNA Macrotyloma uniflorum cDNA, mRNA sequence

AAGCTGGAGCTCACGCGGTGGCGGCGNTCTAGAACTAGTGGATCCCCCGGGCTGCAGGATTCGGCACGAG

GAACTTTCTCAAAGGCAAAGCGCTCGCTTTGCGCGACCGGTGCCAGCGACATCAGGTTTCCGGCGTAGGT

CAGCTTATCGACCACTACCACCGCGTCGCTCGTTTCGTTGATTCGGCACGAGGGTTCCTTCCATTTGTTA

AAAGACATTAATGTATAATCTATCTTTCCATTCACGTTGTTGACTTATATCGAGGATAGGTCTCATGTTG

CCCACATATCTGTGAAGACAATAGGGAGGATTTTTAGTCTGTTTGTATGAAACTTCTCCTGTGAGTGCTG

GAAAATATCAGTAAGTGATGTTCTTCAGTGTGTTCACAGTTATTTATCTTTGCATACTATGATATGA



>gi|71811674|gb|DR989065.1|DR989065 HDI_F_33.esd Horsegram (VZM-1) drought stressed subtracted cDNA Macrotyloma uniflorum cDNA, mRNA sequence

CATNCCTCATCTATCATCCTTGGTGTCGGTTCTGTCTCCGTTGGCCCTANGTGCCGATTGTGGTCTTATA

TGGGCGCATGCGATCTGCTGTTGTGATGCCTTCGCGTGCCTCATTGTTGCCTGGACCGTCATATTCCGTT

TAGATCGTGCTAGATTATCATNTCTTTTGTCCCAATAGGGCAAATATTATCATATCTCG



>gi|71811672|gb|DR989063.1|DR989063 HDI_F_31.esd Horsegram (VZM-1) drought stressed subtracted cDNA Macrotyloma uniflorum cDNA, mRNA sequence

AAGCTGGAGCTCACGCGGTGGCGGCCGCTCTAGAACTAGTGGATCCCCCGGGCTGCAGGAATTCGGCACG

AGGATCAAATGGAGCATCGAATATGAGAGTTCTCATGAGGAAGTTGATCCTCCGTTTGGCTTCCTCGAGT

TCTTGCACAAAGGAAGCAGAGATATTGATGGTCATCTTCTGAAGGCATAGCAGCAAAAGTTATGAACAAA

ATAAGAAATATAAAATAATGGGGTTGTTGTAAGGATGAAGTGATTATGTTTACAATCCGTGTGATGTTTG

TGTGGAATAAACATGGAGATGCATGTTCGTGATGGCTTTGAAGTGTATTAGATTATATGTTAACGTACTC

ATTATGATGTGAATGCATATATAGATATAATTTATACTTTGTTTAAAAAAAAAAAAAAAAAAA



>gi|71811670|gb|DR989061.1|DR989061 HDI_F_23.esd Horsegram (VZM-1) drought stressed subtracted cDNA Macrotyloma uniflorum cDNA, mRNA sequence

AAGCTGGAGCTCACGCGGTGGCGGCGCTCTAGAACTAGTGGATCCCCCGGGCTGCAGGAATTCGGCACGA

GGCCTCGTGCCGAATTCGGCACGAGGGAGATTCCGATCTTGGGCACCTGACAGGCAAGATAGATATATTC

GCCGATGGGTTGATGCTTTATCCGACCCACGTGTCACCCATGAGATCCGCAGTGTTTGGATATCATATTG

GTCTCAGGCTGACCGTTCTCTAGGTCAAAAGATAGCATCTCATCTGAACATGAGGCCAAATATTTAATGT

TGTTGTCAACCCCAGAATGATTGTAAGAGTTGCAGTCTTGTAAAGAGTACGATAAGGATGTGAATTTGGA

ACCTTTGGAAGGATTAATCCTTTTCTTACAATATTATGTTGTATAACACGTAGTACTCCATGCATGCAAC

TCGTTGAGCTATCTCGATAAAATGCCGTTTGTATGTGTTAGTATTTCTGTACCTAATCGAAGTGTTTCCC

TGCTATTGTTGTGGACTGTGTTCCTCCTC



>gi|71811668|gb|DR989059.1|DR989059 HDI_F_21.esd Horsegram (VZM-1) drought stressed subtracted cDNA Macrotyloma uniflorum cDNA, mRNA sequence

GCTGGAGCTCACGCGGTGCGCGGCGTTCTAGAACTAGTGGATCCCCCGGGCTGCAGGAATTCGGCACGAG

GCCTCGTGCCGAATTCGGCACGAGGAGGACATTGGAGGAGATTCAGTGGTCCTTCCGACGATGATTTTGT

TCGGTGGGCGTCCTGCAAATGCTCGTGTTACTGTACATGCAAATACTGGGTGGACCATGTAAATGCAAAT

GTGTAGCTGAATGCTACCATAATTTGGAAAGAAGAAAAAAAAACCCTGAAAAGGGGCAAAAAANAAGAAA

GANACACTATATATCTTTTCTTTATTCCTTTCCCCTTCATGCCATTGGAAAAGATATTATGATCATTGTA

TTATAATACCAATAGTTGCATCACCATTTTNTAGGGGAAGGTGAAATAAATAATGATTNG



>gi|71811666|gb|DR989057.1|DR989057 HDI_F_19.esd Horsegram (VZM-1) drought stressed subtracted cDNA Macrotyloma uniflorum cDNA, mRNA sequence

AAGCTGGAGCTCACGCGGTGGCGGCGCTCTAGAACTAGTGGATCCCCCGGGCTGCAGGAATTCGGCACGA

GGACGAAAACCTTGATGTCCTTTTAGACTTTTCTTTTTCCTTTTTCAGTTTTGTGGTTTAGACTTGGCTT

GAAAATGATGATTGATTATTTATATATTTCGTTCGTAGAACGTAGGACATGTACCATAATTGTCCCTGTT

CGGTACTTTCTTGAGCCTAGACCTTAGCTCGTAAAATTGTTATGACAATCCAATAAATCATAATTCTTGC

CGTCCTGGTGGTTGTTGGGCTCCCAATTGAATCAATCCCCACATTCTACATGTAATGTCTTGTGTAACCC

TATTCACGTTCATTTGAGTATATTACTAACTTGTTTTGGAGCGCCTTT



>gi|71811664|gb|DR989055.1|DR989055 HDI_F_11.esd Horsegram (VZM-1) drought stressed subtracted cDNA Macrotyloma uniflorum cDNA, mRNA sequence

AAATGAGCTCACGCGGTGGCGGCGCTCTAGAACTAGTGGATCCCCCGGGCTGCAGGAATCGGCACGAGGA

TTTGCAGCTGTGCCCTTTTTGCGGTCAACCTTTTTCTGTTGTTGGAGATGAGTTTGTGAAGGAATCCGTG

AAGTTTTCCAACCAATCTACAACATTTGGACAAGCATTCAATAATTTCACCCGTTCTAGAAATGAAAAAG

ATTCTGGTAGAGCAATTGATGTTGAAGCTGAAATTAAAGATGTCGACTGAGTCTTCACAGCTATAACATG

TTGGTAGGTGTGAAGTAATGAGCAAATTTTGAGTTGAACACAGTGACTATGCTGAACACAAATCTTGATC

GGTTAAAGAACAGGCTGTGGCTAGTATTTTCCATTTGTACTGAAGACACAATGAATTACACATTCAAGAG

AGAAGAGTGTGATGAGCAAAGTAACACATTCTAGAGAGGAGTGTGATGAGCAGAGTAACATTGCAATTGT

AATATAAGTTTTGACAGTGATGTTTCTCAGGTCACACCTCTATAATTTTGGTGTACCAGGATACTGCTTC

ATTTTTCCTTTCATTGTTCTTCTACATAACAATGTTTAGGAGCAAATCGAGATGCTTCATTTCAATGTCA

GGACATGGGCAGTCCTTGACAACTCTCTTCTATTAAGTTTCAAAACCCTTTACAGA



>gi|71811662|gb|DR989053.1|DR989053 HDI_F_90.esd Horsegram (VZM-1) drought stressed subtracted cDNA Macrotyloma uniflorum cDNA, mRNA sequence

TTAACTAGTGACCCGGGGCTTCAGGATTCGGCACAGGCCTCGTGCCGGAATTCCGGCACAGGGGCAAGCG

GGAAATACTCTATGTTCAAGGTANNNGCGAATTATGGTGAATGGGACTCCTGGTTCAAGGCACCATGGGC

TGGAGAGAATAATAAATCACAGGAGTTCCCAGGTTACAAGTGGATTCATGGCCAGTTTCTAGGTTTCAAT

AAGTCAGGATCAATTGGTATTCANGGTGGGCAAAGTGTAAGCACTACGTTAATT



>gi|71811660|gb|DR989051.1|DR989051 HDI_F_88.esd Horsegram (VZM-1) drought stressed subtracted cDNA Macrotyloma uniflorum cDNA, mRNA sequence

CTGGAGCTCACGCGGTGGCGGCGCTCTAGAACTAGTGGATCCCCCGGGCTGCAGGATTCGGCACGAGGCC

TCGTGCCGAATTCGGCACGAGGATTTAGATGGATTACTCTCCTGCTGACTCAGGAATTCAATTTTGCTGA

CAGCCTTCACATTTGGGACACTCTTTTAAGCGATCCTGATGGTCCACAGGAGACTCTTCTTCGGGTATGC

TGTGCAATGCTTGTCCTCGTGCGCAAGCGCCTCCTTGCGGGTGATTTTACATCAAATCTGAAGTTATTGC

AAAATTATCCCACCACAAACATTAGTCATTTGCTCTATGTTGCCAACAAGTTTCGTGTACAGTCAGTTTA

ATGTTTATCTCCCATTATCTTTTGCATTTTGCTTTTGTACTTTTAATTTCTCTTTCAAATTCAATACTTG

TAAATGACTTTTATGTGTGAGCCTCAGAGCAGTGACTGTTTTTTTATTTATTTTTAGTTATATTTTGTAC

GTAAAAAAAAAAAAAAAAA



>gi|71811658|gb|DR989049.1|DR989049 HDI_F_86.esd Horsegram (VZM-1) drought stressed subtracted cDNA Macrotyloma uniflorum cDNA, mRNA sequence

CTGGAGCTCACCGCGGTGGCGGCGCTCTAGAACTAGTGGATCCCCCGGGCTGCAGGAATTCGGCACGAGG

ATTGGACACAAATGTCTTTGAGGGAAGGAGAGGAAAGGGTAAAGTTGAAGTAACTATTGCAGGAGGAAGA

GTTGTCTGGGAAAATAATGAACTGAAGGTTGCTCCTGGAACTGGGAGGTATATAAAAATGCCTCCCTTTA

GCTATCTGTTTGATGGAATTGAAAAAAAGGATGATTTTTATCTAAATTCCCTTCAAGCTCCAGTGAAGCG

AGCCAAATCCAGCTCTTAAGAGTTTCTGATATCCTTTTAGTCAGGGTGGGGGACAAAGACATGTATAAGT

GTTTCTGATCTAGTTTTTTGCTAAAATAAAGTAAAATATGTGTATATGTACATCACATGTAAATGATATG

CTACATGTCACCCTTTCAACTCATGTAATGTACATTGCTTACCCTCTAAATCTATTAGAGAATAAGAGAC

AATGGGTTTAAGAATAAAAAAAAAGGGGGGGCCCGGTACCCAATTCGCCCTATAGTGAGTCGTATTACAA



>gi|71811656|gb|DR989047.1|DR989047 HDI_F_78.esd Horsegram (VZM-1) drought stressed subtracted cDNA Macrotyloma uniflorum cDNA, mRNA sequence

ATATGCTGAGCTCACCGGCGGTGCGNCGGCCNTTCTAGAAACTAGTGGATCCCCCGGGCTGCAGTGATTC

GGCACGAGGATCAACTAGCCCCGAAAATGGATGGCGCTAAAGCGCGCGACCTATACCCGGCCGTCGGGGC

AAGGACCAGGCCCCGATAGAGTAGGAGGGCGCGGCCGGTCGCTGTAAAACCCGGGGCGCGAGCCTGGGCG

GAGCGGTCGTCGGTGCAGATCTTGGTGGTAGTAGCAAATATTCAAATGAGAACTTTGAAGGCCGAAGAGG

GGAAAGGTTCCATGTGAACGGCACTTGCACATGGGTTAGTCGATCCTAAGGGACGGGGGAAGCCCGTCTG

ATAGCGCTCTGAGCGCGTACTCCGAAAGGGAATCGGGTTAAAATTCCTGAACCGGGACGTGGCGGCTGAC

GGCAAACAGTTAGGGAGTCCGGAGACGTCGGTGAGGGCCCCGGAAAGAGTTATCTTTTCTTGTTTAACAG

ACCTGCCCACCCTGGAAACGGCTCAGCCGGAGGTAAGGTCCAGCGGCTGGAAGAGCACCGCACCGTCGCG

TGGTGTCCGGTGCGCCCATCGGCGGCCACTTGAAACATCCGGAGTGACCGAGGTGCCTCCCAACGCCTGG

TCGTAACTCATAAACCGCATCAGGTCTCCAAAAGTGAACAGCCTCCTGA



>gi|71811654|gb|DR989045.1|DR989045 HDI_F_76.esd Horsegram (VZM-1) drought stressed subtracted cDNA Macrotyloma uniflorum cDNA, mRNA sequence

AAAGCTGGAGCTCACGCGGTGGCGGCGCTTCTAGAACTAGTGGATCCCCCGGGCTGCAGGATTCGGCACG

AGGGTGGTTCAAGGCTGGCTCTCAGATCTTCAGCGAGGGTGGGCTCGACTACTTGGGCAACCCAAGCCTG

ATCCACGCTCAGAGCATCCTCGCCATCTGGGCTACCCAAGTTATCCTCATGGGCGCCGTTGAGGGTTACC

GTATTGCCGGCGGGCCTCTCGGTGAGGTCACTGACCCCATCTACCCCGGTGGCAGCTTCGACCCATTGGG

CCTCGCTGACGACCCAGAGGCTTTCGCTGAGCTCAAGGTGAAGGAACTCAAGAACGGTAGGTTGGCCATG

TTCTCCATGTTCGGGTTCTTCGTCCAGGCCATTGTCACCGGAAAGGGCCCATTGGAGAACCTCGCCGACC

ACCTTGCAGACCCAGTCAACAACAACGCCTGGGCCTACGCCACCAACTTCGTCCCCGGAAAGTGAGCTTG

AAGAGAATGACTTGTGTACTCGAGGGGGGGCCCGGTACCCAATTCGCCCTATAGTGAGTCGTATTACAAT

T



>gi|71811652|gb|DR989043.1|DR989043 HDI_F_73.esd Horsegram (VZM-1) drought stressed subtracted cDNA Macrotyloma uniflorum cDNA, mRNA sequence

AAGCTGGAGCTCACCGCGGTGGCGGCCGCTCTAGAACTAGTGGATCCCCCGGGCTGCAGGAATTCGGCAC

GAGGCCTCGTGCCGAATTCGGCACGAGGGAACATTCGTTTCTCCCTAAGTTTAAGTCGTGATGGGAAAGG

AAAAGGCTAACATCAAAATCGTGGTCGTTGGACATGTCTCTGAGAAATCAACTGCCACGGAGCTTGGAAG

CCCTACTGAGAAGGGTGTGACTGAAAGGTTCGAGAAGGGAGAAGGAAGTGTTTAAAACACGTAAGATGTA

AGATGTGTGAAGAAGGATGATGTAAAATAAATAAGATATAAGATGTGAAGATGGATCTATCTTAAGATGA

AGTGCTGAAGGACTTATATTCAGATAAATAAAATGAAGGATA



>gi|71811650|gb|DR989041.1|DR989041 HDI_F_65.esd Horsegram (VZM-1) drought stressed subtracted cDNA Macrotyloma uniflorum cDNA, mRNA sequence

AAGCTGGAGCTCACGCGGTGGCGGCGCTCTAGAACTAGTGGATCCCCCGGGCTGCAGGATTCGGCACGAG

GCCTCGTGCCGAATTCGGCACGAGGCTGTCTCAAGTGTCAGTATAGTTGCAGAGGGAACTCTAGATCTAG

ATGAGGTTGACGATTGGCTTGAAAGAGTGATTGAAGAAAAAGGAGAGGACCTATACAGAATGAAAGGTGT

CTTGTCAGTGGATGGTTCTGATCAGAGATATGTATTTCAGGGGGTGCATTCCATGTTGGATGGCTGTCCA

GGAAAAACATGGGAACATAACGAGAAGAGGATAAACAAGCTCGTTTTCATTGGAAGGAATTTGGATGAAA

CTGCCCTTAGAAAAGGCTTTAGAGGTTGTATAGTATAGCATTACAAAATTGTTTGTGTCAGGTTCTGAAG

CAGCAGCAGCCTTGATTTTAGAATTTCCAACCAGTGCATGGTAGCACCATCATAAAATCTGGTTGGTTTG

TGCTTGTATTGGATGCATTGATAGAGTCGCTTATTATGTAGAATGAATGATTATCATATGCATTGGTCGC

TGGATTCAAATTGACTTAAA



>gi|71811648|gb|DR989039.1|DR989039 HDI_F_63.esd Horsegram (VZM-1) drought stressed subtracted cDNA Macrotyloma uniflorum cDNA, mRNA sequence

AAAAGCTGGAGCTCACCGCGGTTGGCGGCCGCTCTAGAACTAGTGGATCCCCCGGGCTGCAGGAATTCGG

CACGAGGCCTCGTGCCGAATTCGGCACGAGGGGCCAACTCGTCATATCGATGTGCATCCTTAACACGATT

ATGTGTATCACATCTCATTGGAAGCTACTCTCATNACTTTCTTTGCCAGCCTTTATGTTACTCTTGGCCA

TGACGGCTCTTCAAACGTTTTGCTCGGCTTATGATTGTCCCAGTTATTTTAGACCCAAATGAGGTTTTCA

TATCATTTCTACTTTTAAGTATAGCAGATAAATGACTGGTAACGGTTTCGAGTGTATAATGTTTCTGTAA

AATTAAGTGCAGCGTAAGTTGTAATGTATGCTTCATGCAAGTTATCCAACTGAAACCATCTAATATATGA

TTTCTATTTCTCTCAAAAAAAAAAAAAAAAAAACNCCAAGGGGGGGCGC



>gi|71811646|gb|DR989037.1|DR989037 HDI_F_61.esd Horsegram (VZM-1) drought stressed subtracted cDNA Macrotyloma uniflorum cDNA, mRNA sequence

AAGCTGGAGCTCACGCGGTGGCGGCGCTCTAGAACTAGTGGATCCCCCGGGCTGCAGGAATTCGGCACGA

GGCCTCGTGCCGAATTCGGCACGAGGGAAGAGAACACACCACCACCTCTTTGGCTTGATACATAATACCA

TGAAACACTTTATTTATAATTATATATCGCAACCAATAACGAGCATATATTTCNCCGTCTATTTACCTAT

AGTGCTTTCTGGGTTTACATATTGTTTGCGTGTCTTTAAAGTTATATAGTACTGTGCCACGTTGTAGTGT

ATTAGAGAATGGATGGTTTGTGTGGAGAATGTTTCCGTCTCATATGTGTGTGGCATACTCTATTATGCAC

TATGTACTGGTAATCAGTTGAGTACATTAGTAGTATCTGAGATGATAAATATATATTGTGAAATGGTTTG

AGATCAAAAA



>gi|71811644|gb|DR989035.1|DR989035 HDI_F_54.esd Horsegram (VZM-1) drought stressed subtracted cDNA Macrotyloma uniflorum cDNA, mRNA sequence

GGCTGGAGCTCACGCGGTGCGCGGCCGTTCTAGAACTAGTGGATCCCCCGGGCTGCAGGATTCGGCACGA

GGCTAGTGGAGAGACAATTACGACGCCTTTGCCCCAGTCTATTAAGGGTCCAATGCCAAAAATTCTTTGG

ATAAGGATGCACTAGTGAGGCAGATGGGTGTGAATCCCTCAAGTCAGAATCCTAACTTGTTCAGCGACTT

GCTTGGCTAATCTGTTTTACTAAGCCAATTCTCGATCATGATCCTTGAACAAATAGATGGCCT



>gi|71811642|gb|DR989033.1|DR989033 HDI_F_52.esd Horsegram (VZM-1) drought stressed subtracted cDNA Macrotyloma uniflorum cDNA, mRNA sequence

TGGAGCTCACGCGGTGGCGGCCGCTCTAGAACTAGTGGATCCCCCGGGCTGCAGGAATTCGGCACGAGGC

AGAATCAACAGAAATATTCTGGTGGAAAGGTGGATAATGGCCATTTTGCAAATTCATCATCTGGTCATAA

GAGTGCCAATGAGCAGCTTCCTGCAAGCATAAGCTTTGTGGATGTCGCAGATATGACTGAGGATGAATGG

ACATTGTTTCTGGACAAGTTTCAAGAGCTGCTGTATCCGGCATTTGCCAAGAGGAAATCCATAACCTTAG

GTCAGAGGCAGAGGCAGAGGCTTGGTACTTCTTGCCAGTTTTGAAGTTGAGCAATACGATGATATGATAG

GGAGGACTATAGATGAAGAAGAATAAGGCTTTGTAGGTTTTAGGGAATGCATGAATGGTTGTTAAAAATA

GTTCTGGTTTATGAGATTTGAATGAGTGCTTTGGTTGGGAGAGAGAGTAGGAAAACCTGCTGTAAAATAT

GCTTGGCATCAGCTAAGTGGTGAGCCTATTAGTTGTTTGTATTTTGATTTCATGATTTCATTTCTTCTTT

TCCCCTTCTTCTCTTTTGTAGGATAAAATTGTGAACATGAACATTTGTGTATATAT



>gi|71811640|gb|DR989031.1|DR989031 HDI_F_50.esd Horsegram (VZM-1) drought stressed subtracted cDNA Macrotyloma uniflorum cDNA, mRNA sequence

AAAGCTGGAGCTCACGCGGTGGCGGCGCTCTAGAACTAGTGGATCCCCCGGGCTGCAGGAATTCGGCACG

AGGACACTCTGAAAACAACCGGTTGTGGTTTGCCCCCCGGTCCAGGTGGCTCAATCGGTGCACTGGAAGG

AGTGAGCTACCTGGTTGTTGTGGGCATAGTGGGTTGGTCTCTCTACACTAAAGCCAAAACTGGTTCTGGA

CTTCCAAATGGTCCTTTTGGTTTGTTGGGTGCCGTTGAAGGCCTTTCCTTTTTGGCGTTGCTTGGCATTG

TGGTTGTGTTCGGCTTGCAGTACCTTGACAAGGGTTTCATTCCTGGTCCTCTTCCTGCAGATCAGTGCTT

TGGTTAGGTTCTCTAGTAACCATTCCATGATGTAATATGAGCCACTACAATGCTATCCTTCTACTTCTTC

TGGATAAGGTCGTAACATCTGTCTTAGAATGTCCTTTTCCACTGCCTCTTTGTTTCATACTCTGTGTCTA

TATTTTCCTTGTAAGAGTTCACGAGCATAAATGGAAATTAATTGTAATGTGTTGTGCTTTAACAAAAAAA

AACAAAAAAAACCCAGAAGGGGGGGCCCGGTAACCCAA



>gi|71811638|gb|DR989029.1|DR989029 HDI_F_49.esd Horsegram (VZM-1) drought stressed subtracted cDNA Macrotyloma uniflorum cDNA, mRNA sequence

GCTGGAGCTCACGCGGTGGCGGCGTCTCTAGAACTAGTGGATCCCCCGGGCTGCAGGATTCGGCACGAGG

AACTTTCTCAAAGGCAAAGCGCTCGCTTTGCGCGACCGGTGCCAGCGACATCAGGTTTCCGGCGTAGGTC

AGCTTATCGACCACTACCACCGCGTCGCTCGTTTCGTTGATTCGGCACGAGGGTTCCTTCCATTTGTTAA

AAGACATTAATGTATAATCTATCTTTCCATTCACGTTGTTGACTTATATCGAGGATAGGTCTCATGTTGC

CCACATATCTGTGAAGACAATAGGGAGGATTTTTAGTCTGTTTGTATGAAACTTCTCCTGTGAGTGCTGG

AAAATATCAGTAAGTGATGTTCTTCAGTGTGTTCACAGTTATTTATCTTTGCATACTATGATATGATTCC

TAGTTCTTAGA



>gi|71811636|gb|DR989027.1|DR989027 HDI_F_40.esd Horsegram (VZM-1) drought stressed subtracted cDNA Macrotyloma uniflorum cDNA, mRNA sequence

AGCTGGAGCTCACGCGGTGCGCGGCCGTTCTAGAAACTAGTGGATCCCCCGGGCTGCAGGAATTCGGCAC

GAGGCCTCGTGCCGAATTCGGCACGAGGGATTAGTCAAACACGTATTGATCGCAAAGTTCAAGGACGATG

TCACTCCAGAGAGGATTGAAGAACTTATCAAGGACTATGCCAACCTAGTGAATCTCATCCCACCCATGAA

ATCATTCCATTGGGGCAAGGATGTAAGTGCTGAAAACCTGCATCAAGGCTTCACTCATATCTTCGAATCA

ACCTTTGAGAGTACAGAAGCCGTTGCAGAGTATGTAGCTCATCCTGCTCATGTTGAATATGCAAATTTGC

TCCTTCCCAAGTTGGAGAAAGTGGTTGTGATTGATTATAAGCCTACCAGATACCTTTGATTGGAAAAGTA

ATGCGTCTTTCACTGTTGGTGTTCTCATACCATTAACACTCTGGGTTTCCCTCTCAATCAGTGTATGCAC

TGCAATGATCCCTCTTTATCTATTCATGGTATTCTGGTGTATTCTGGATCCTTTGACATTTCCTTGGAAA

AATCCTTTGTTTGGATACAGAACTTTCGTTTTCCTGTCCAAAACATACAACAATCGAAAAACTCGCAGNG

GGGGGGCCCC



>gi|71811634|gb|DR989025.1|DR989025 HDI_F_39.esd Horsegram (VZM-1) drought stressed subtracted cDNA Macrotyloma uniflorum cDNA, mRNA sequence

GGCTGGAGCTCCACCGGCGGTGCNCGGCCGTTCTAGAAACTAGTGGATCCCCCGNCGCTGCAGTAATTCG

GCACGAGGCCTCGTGCCGAATTCGGCACGAGGAAGAAAGTATTAACAAATTGATGGGTTCATCCGANAAT

TAAAAAAAAAAATGAATACAGAAAAATGTGTAGAAAAGAAAAAACCAAAATTACTTCGTGTAAATTTACG

TATTATANAGTCCACTAATTTNGTCTAAACCTATGT



>gi|71811632|gb|DR989023.1|DR989023 HDI_F_37.esd Horsegram (VZM-1) drought stressed subtracted cDNA Macrotyloma uniflorum cDNA, mRNA sequence

AAGCTGGAGCTCACGCGGTGGCGGCGCTCTAGAACTAGTGGATCCCCCGGGCTGCAGGATTCGGCACGAG

GCCTCGTGCCGAATTCGGCACGAGGTGACATTCATTCAATTTTCCTTGTCAAGCAGAAAAGCAGTTTTAT

GGTGTTCACTTCAGTGAGGAGTGATCTGTCAGTGTACAAATCGATTTAGTTTTACTCTTTTAGTCCAAAA

TTGTGTACGTTAGCGATGTAAATGTTGATCCAGTGGTATATTTCCCCCCCTCATAAGATAGAGTAGTATT

TAAATTTTACTTGAGAGTGCCTAGAGGCACCTATACTGAGTTTTCATTTAAAGCCCTTATGCTTTAGATA

AACTGAAAGGAAAATGCTTTGTAAATATTGAACGATTGGTTAGAATACATGTTATTCATTTTACTGAGCC

ATATCCGTTTATTTGAAGT



>gi|71811630|gb|DR989021.1|DR989021 HDI_F_3.esd Horsegram (VZM-1) drought stressed subtracted cDNA Macrotyloma uniflorum cDNA, mRNA sequence

AAGCTGGAGCTCACGCGGTGGCGGCCGCTCTAGAACTAGTGGATCCCCCGGGCTGCAGGAATTCGGCACG

AGGCCTCGTGCCGAATTCGGCACGAGGTGCATTTACTTTCTTCTGGATCACAGATTCATGCCCCTTGACA

GTGAAAGCGGTGGTGAATAGGCCACCATTTCAGGTTCTCAGCCTTGCAAGTTCCATATCTGCCAGTCTTC

AAATCTGAAGGGAACATATATAATGTCATTTTCATTTCAAGCTAAAAAAATGGAGAAAAAAAGAAATCCG

GTTGATAATAATGGTCAAAGTTGAGTTATTGGACTTTAAGAAGCGGCTTATAGCAAGAGATGAGATAATA

AGAAGCCTGGGAGTATAAAAGACTTTATTAATGCCTCCCCTCCTCAACTTGATCATTTACTCTTACCAAG

TTTATTGCTCTGCGATCTCTATCTGGGCTGCATTAATTTAGAAACTGCTGCTTAATGGTAATCCATAGCA

TGCTGCTGTGTTAAAAATAATGTACTCTATCTTGTGGATTTTGGTAGTTTCAATCGCGTGTATGATCCTT

GATATATGAAACTTTAAGAACCTTATTCTATCTTTAGA



>gi|71811628|gb|DR989019.1|DR989019 HDI_F_27.esd Horsegram (VZM-1) drought stressed subtracted cDNA Macrotyloma uniflorum cDNA, mRNA sequence

CAAAGCTGGAGCTCACCGCGGTGCGCGGCGTTCTAGAACTAGTTGGATCCCCCGGGCTGCAGGAATTCGG

CACGAGGCCTCGTGCCGAATTCGGCACGAGGCTCACATCTAAAACTTTTTTTTCTGTTACAATAGGATGG

TCTCATCCCACAAATCATACATTACCATGAATACTGTACAACATCACAGGTCCTATAAGGAACGCCTTAG

ATGGCATAAGTAAGAAAGAAAGAAATGCATATATACAAAGCAAGTCCCAAGTATGAGGTGACAACTTGTT

GTCTTAAACAAAAAAGAGTGGAAATCTCATTTTGTTTTTCTTTTCAGTGAACCGTCATGACCTACTTAAA

AAGGAGGTAAACATTANTATACAGTTCAAGTCTCCTCGGTCTTCACTGGTGTCCAATTGCTCCTGGCCTC

TTCAGACTTCTTCTTGGCACTTGTAACGAAACCAAGGAACGTAACCCATGAATGCCCCAAGCAAGCAAGC

ACACTTGCTCCCTTCACATACCATGACAATGTGGAT



>gi|71811626|gb|DR989017.1|DR989017 HDI_F_25.esd Horsegram (VZM-1) drought stressed subtracted cDNA Macrotyloma uniflorum cDNA, mRNA sequence

AAAGCTGGAGCTCACGCGGTGGCGGCGCTCTAGAACTAGTGGATCCCCCGGGCTGCAGGAATTCGGCACG

AGGGGCGACCGAACCAAAAGCTGCCACCGACGACGTCAAGATCGATCTCTTCGAAGACGATGATGAATTT

GAAGAGTTTGAAATCAATGAAGAGTGGGATGACAATGAGGAAGGGAAAGAAGTGACTCAACAGTGGGAGG

ATGATTGGGATGACGATGATGTTAGTGATGACTTTTCTCTTCAGTTCAAGAANGGAGTTGGANGAGCAAC

ACTGATAAGAATTAAACCACCCGACTTAGGTATTTTAGCTCTTGGAAGGAGATGAAAACTATGCCTTGTA

AGGAAGGAAATGTAAGAACATAGGGACAGAATTTTCTATTCTTTAAATNCTCTACCTGGACATCTCTAAC

CAGAAGA



>gi|71811624|gb|DR989015.1|DR989015 HDI_F_18.esd Horsegram (VZM-1) drought stressed subtracted cDNA Macrotyloma uniflorum cDNA, mRNA sequence

GCTGGAGCTCACGCGGTGGCGGCCGCTCTAGAACTAGTGGATCCCCCGGGCTGCAGGAATTCGGCACGAG

GACGGATTGGCTCGACGGCAAGCACGTAGTGTTCGGTCAGGTCGTTGAGGGGCTGAACGTGGTGAAAGAT

ATCGAGAAGGTCGGATCCAGCTCCGGCAAGACCGCAAGGCCGGTCGTCGTCGCTGACTGCGGTCAACTGT

CTTAGATGGACGGCGTTGACCCGAGGCGGTTATCTCTGTGGTGGTGGCTCTGAATCTGTGTCGTTTTCGG

TGTTTTTTTTGCTTTTTCTCGTCTTATAGGGGATTGTGGCGTTATGGATAATTTCGAACCCTTCCCTCTG

AATCCTTTCCTTTCTAATATCTGTATGTGGTCATAGAGTGAATGAGTCTGTCATGAGAAACATAAAAATG

ATGAGCTACCTTCAATAAAACCTTAGGACCTTTATTATTCGCCTAAAAAAAAAAAAAAAAAAAAAAAAAA

AAATAAAAAA



>gi|71811622|gb|DR989013.1|DR989013 HDI_F_15.esd Horsegram (VZM-1) drought stressed subtracted cDNA Macrotyloma uniflorum cDNA, mRNA sequence

AATTCGGCACGAGAGTGAGTGACATTGTGGAGGAAGAATTCAATGTGGTTCCTTCCGACGAGTGATCTCT

TGTTCGTGCTGGGCAGATCCTGCAAATGCTCGGATTCTTGTACATGACAAATTACCTAGGGTTGTGACCA

TGTAATATGCAAAAATAGCTGCTATGCTGAATGTCTACCATAATTTGGCAAAGAAGAAACAAAAAAGACA

CCCTGAAAGAGGGGCAGACATACAGNAAGGAACAGGAAACACTAAT



>gi|71811620|gb|DR989011.1|DR989011 HDI_F_13.esd Horsegram (VZM-1) drought stressed subtracted cDNA Macrotyloma uniflorum cDNA, mRNA sequence

TGGAGCTCACGCGGTGGCGGCGNTCTAGAACTAGTGGATCCCCCGGGCTGCAGGAATTCGGCACGAGGCC

TCGTGCCGAATTCGGCACGAGGCTTTCATGGTCAGTAACTAACGAGCTTTTGACACAAGCCTTTCAAGAA

TATGGAACAGTGGTTGGAGCTAGGGTCTTGTATGATGGAGAAACTGGAAGGTCACGTGGCTATGGCTTTG

TTTGCTTTTCCACAAAAGCAGAGATGGAATCAGCCCTTGAAGCCTTGAATGACGTGGAACTAGAAGGAAG

GGCTATGCGTGTAAGTTTGGCTCAAGGAAAACGTGCACAAGGCTAATAGAAATTTGGGAAGTATTAGATG

TGAGATGTTGAAGGATGATGCCATAGAAATGAGAAGGGGATGCTAGTATATCAACCATGATGCACTTAAT

TGCTGCAATCTGCTGAGAATTTTCCTTTTCATTTCTTGTGGTGTATTGTATAAACTATTTACTCCCATTT

TCAAAAGATCTACTCACTTGAATGCTGTTAAAAAAA



>gi|71811618|gb|DR989009.1|DR989009 HDI_E_96.esd Horsegram (VZM-1) drought stressed subtracted cDNA Macrotyloma uniflorum cDNA, mRNA sequence

AAAAGCTGGAGCTCACCGTCGTGTGCGCGGCCGTTCTAGAACTAGTGGATCCCCCGGGCTGCAGGATTCT

GGCACGAGGTGCAGCTTGGTGGCCAACAAAGGGTTAGAANAGAGAGAGCTCCATAGCGTCTTGCATGCCA

ACAAATGATCNTTTCAGTTCACCGTACTCACCAACACCATTATCCAAAACTGCACATTGCTGAAATGAAG

AAGTTCGGCAA



>gi|71811616|gb|DR989007.1|DR989007 HDI_E_93.esd Horsegram (VZM-1) drought stressed subtracted cDNA Macrotyloma uniflorum cDNA, mRNA sequence

AAGGCTGGAGCTCACGCGGTGGCGGCGNCTCTAGAACTAGTGGATCCCCCGGGCTGCAGGAATTCGGCAC

GAGGCCTCGTGCCGATATTCGGCACGAGGAGGACATTGGANGAGATTCAGTGGTCCTTCCGACGTATGAT

TCTTGTTCGT



>gi|71811614|gb|DR989005.1|DR989005 HDI_E_82.esd Horsegram (VZM-1) drought stressed subtracted cDNA Macrotyloma uniflorum cDNA, mRNA sequence

CTACTGCGGCCGGGCCGTTATAGAACTAGTGGATCCCCCGTGCGCTGCAGGTAATTCGGCACGAGTGCCT

CGATGCCGAATACGGACACTGAGGAGTGACACTTGGAGGAAGATTCAGTGGTCCTTCCGACGTATGATTT

GTGCGGATGGGCGTCCTGCAAATGCTCGTGATNCTGATACATGACAAAAATTACCTGGTGTTGTGACCAT

GGTA



>gi|71811612|gb|DR989003.1|DR989003 HDI_E_80.esd Horsegram (VZM-1) drought stressed subtracted cDNA Macrotyloma uniflorum cDNA, mRNA sequence

ATCAAAAGCTATGTATGGCATGAAGAAAGGCGTATACCATATAGCATGAGATCATGATATATCTATCTAT

TCCATGTCTCGCTGCGCGAGATTGCCTGACACGCAAAGTTTCATCCATGGTCGCGTNCTCTTCTACCAAG

CATCCAATTTATCCAAAATG



>gi|71811610|gb|DR989001.1|DR989001 HDI_E_79.esd Horsegram (VZM-1) drought stressed subtracted cDNA Macrotyloma uniflorum cDNA, mRNA sequence

GGAGCTCACGCGGTGGCGGCGCTCTAGAACTAGTGGATCCCCCGGGCTGCAGGAATTCGGCACGAGGGTG

GAATATATATGTTACTGATGTTGGGCAGTGGCAACACTTTGATATGCTTTTTAAGGGCCTTTAGCGTGCA

GGATGGCT



>gi|71811608|gb|DR988999.1|DR988999 HDI_E_70.esd Horsegram (VZM-1) drought stressed subtracted cDNA Macrotyloma uniflorum cDNA, mRNA sequence

CACGCGGTGGCGGCGCTCTAGAACTAGTGGATCCCCCGGGCTGCAGGATTCGGCACGAGGGCAGAAGCAA

GTCATTGTGGAGAAGCTTGGTGAGCCAGCCCAAGGCTATGAAGATTTTGCTGGTAGCCTTCCTGCTGACG

AGTGCCGCTATGCTGTTTATGATTTTGAGTATATGACTGAAGGGAATGTCCCTAAAAGCAGAATTTTTTT

CATTGCGTGGTCTCCTGATACATCAAGGGTCAGGAGCAAGATGATTTATGCAAGCTCCAAAGACAGATTC

AAGAGGGAGCTGGATGGTATTCAAGTAGAGTTACAAGCAACTGATCCTACTGAGATGGGTCTTGATGTGT

TCAAAAGCCGTGCCAACTAAATGACTATATAAATAGTACGCTT



>gi|71811606|gb|DR988997.1|DR988997 HDI_E_69.esd Horsegram (VZM-1) drought stressed subtracted cDNA Macrotyloma uniflorum cDNA, mRNA sequence

ACTGGGTTTTCGTTAAATCTGATGTTATGATTCGTTTTACAATCTGCAGGTTATCAACGATCGTGAGACA

GGAAGGTCAAGAGGGTATTGGATTTGTGACCTTCGCCCCGGCACCCAGGCCAATGAAACGATGCGATCCA

CGGGATGAACGGCCACGACCTATGACGGCCGCCACATCACTGTNCAACGAAGCTCNAACTCGCCGGCAGN

CCGCGG



>gi|71811604|gb|DR988995.1|DR988995 HDI_E_67.esd Horsegram (VZM-1) drought stressed subtracted cDNA Macrotyloma uniflorum cDNA, mRNA sequence

AGCTGGAGCTCACGCGGTGGCGGCGCTCTAGAACTAGTGGATCCCCCGGGCTGCAGGAATTCGGCACGAG

GCCCAAGCACCAAAGAAATCCAAGAAGTGAGCCACATATTCTGATATTGGCTGGTAGAATTTTTGGTACT

GTCATTCTCAGTTACTATTCTAAAGTAGTTTTGACGAGTATTGAGGAGTTTCTGTGTTGGCGTAGACCTT

CTTTAGTTTTTATTCAAGTATATGAAATTATAACATTCTGCTCTTTCGTGCTGGTGTTTTGGCTTCTTTT

TTTTGTATGGATATCATTATTGTTGCGGAAATACCTTGAGTTAATTTTTTTTCTGGTTAAAAAAAAAAAA

AAAAAAAAAAAAAAAAA



>gi|71811602|gb|DR988993.1|DR988993 HDI_E_59.esd Horsegram (VZM-1) drought stressed subtracted cDNA Macrotyloma uniflorum cDNA, mRNA sequence

AAAGCTGGAGCTCACGCGGTGGCGGCGCTCTAGAACTAGTGGATCCCCCGGGCTGCAGGATTCGGCACGA

GGATTTGCAGCTGTGCCCTTTTTGCGGTCAACCTTTTTCTGTTGTTGGAGATGAGTTTGTGAAGGAATCC

GTGAAGTTTTCCAACCAATCTACAACATTTGGACAAGCATTCAATAATTTCACCCGTTCTAGAAATGAAA

AAGATTCTGGTAGAGCAATTGATGTTGAAGCTGAAATTAAAGATGTCGACTGAGTCTTCACAGCTATAAC

ATGTTGGTAGGTGTGAAGTAATGAGCAAATTTTGAGTTGAACACAGTGACTATGCTGAACACAAATCTTG

ATCGGTTAAAGAACAGGCTGTGGCTAGTATTTTCCATTTGTACTGAAGACACAATGAATTACACATTCAA

GAGAGAAGAGTGTGATGAGCAAAGTAACACATTCTAGAGAGGAGTGTGATGAGCAGAGTAACATTGCAAT

TGTAATATAAGTTTTGACAGTGATGTTTCTCAGGTCACACCTCTATAATTTTGGTGTACCAGGATACTGC

TTCATTTTTCCTTTCATTGTTTTTACATAAAATGTTTAGGAGAAATCGAGATGCTTCATTTCAATGTCAG

GACATGGCAGTCTTGACAACTCTCTTCTATTAGTTCAAACCCTTTACAAGACAAGGAAAATA



>gi|71811600|gb|DR988991.1|DR988991 HDI_E_57.esd Horsegram (VZM-1) drought stressed subtracted cDNA Macrotyloma uniflorum cDNA, mRNA sequence

AAAGCTGGAGCTCACGCGGTGCGCGGCCGCTCTAGAAACTAGTGGATCCCCCGGGCTGCAGGAATTCGGC

ACGAGGCTTCTTTCCAACAAGTGTTTCCACGGGATGAAGTAGCTGAGGCAGCAATGCTTCTGATGGAGTT

ATCTTGTGGTTTTGTCCACTTATAGTAACTACATCTAGCTAGAATATTTTTGCCATGTTTTTGTTTTTCA

CTTTTGTATAGAAGTATCTAGTAATTATTTTTGTTTGCTTGTGCCTTGTGAGTTCTGAAGTCGAGCTGGT

TTATTTGCAGCAGAACCAAAGTATTGTCGGCTACTTATACCTATATTTGTGATGATTGATCAGTGAAAGT

TTGTGTTAGCGATATGAT



>gi|71811598|gb|DR988989.1|DR988989 HDI_E_55.esd Horsegram (VZM-1) drought stressed subtracted cDNA Macrotyloma uniflorum cDNA, mRNA sequence

AAAGCTGAGCTCACGCGGTGCGCGGCGCTTCTAGAACTAGTGGATCCCCCGGGCTGCAGGATTCGGCACG

AGGCTCAAAGAAGGCGGGTGACACTTGCCCAACTGCTATTCATGATTTTGAAGGAGATCAAACTTACAGT

GAGAAGCCAGGACATGCATTTTCTTTAACAGCAACCTTTGACGATGTGAATCCTTCGAGCTATGACGCTC

TTGTCATCCCTGGAGGTCGAGCACCTGAGTATTTGGCTTTGAACGAGTCAGTCATTGCCTTGGTGAAGCA

TTTCTTTGAAAACAAGAAGCCAGTGGCATCTATATGTCACGGCCAACAGATTTTATCTGCAGCTGGTGTT

CTCAAGGGAAGGAAATGCACTGCTTATCCGGCTGTGAAGCTTAATGTGGTTCTGTCAGGGGCCACATGGC

TGGAGCCTGACCCTATAAGCCGATGCTTCACTGATGGAAATCTGGTTACTGGAGCTGCATGGCCAGGGCA

CCCTGAGTTCATTTCTCAGTTGATGGCACTCCTTGGTATTAAAGTATCTTTCTAGCTGCACTTTGTGATG

TAATTAATTACTGGTGAATAAAGCTTCGTGGTTTTCCATAAGGCATTTTGTATCAATATTATAATATGTG

GATGCGTTTACAGTATTGTATTTGAAGGACCTATAATGT



>gi|71811596|gb|DR988987.1|DR988987 HDI_E_46.esd Horsegram (VZM-1) drought stressed subtracted cDNA Macrotyloma uniflorum cDNA, mRNA sequence

CCGCGGTGCGCGGCGTTCTAGAACTAGTGGATCCCCCGGGCTGCAGGATCGGCACGAGGGTCAAAAGAAG

GAGCAGTCGGATTCATCAACAAATGAGGATGACGATGATTGGGATGGGGAAGATCCTGAAGAAGGTGTCA

TTTATGTGGAATAGATGCCTCCCGATGCATCAGTACTGTCACTGTGCCTGTTTGCTATGGACAGAACTTT

CAATCTTGATAGTGCTCAGAAACACTTACTAAAATGCTTGCTGGAAACATGCTGTTCTAGTGTTCTCAAG

TGACAATATCATCTGATCACAAATTGCATTGTCAACCCCGAAGTGATGTAGACCAATTTTTGGGTTTTCT

CCCCATATATGACTTAACCCAGATGTTACTATACAAGAGGACTTGCTGTATAAATTCTTTTTTCTTGCTT

TACTTATTCCTGTAAGTGGAATTTATTATGTTTGCTATTGCTATCCGTATTTAGGTATCTTCAACTAATT

ACACTCAACTAGATGCATGCAAATA



>gi|71811594|gb|DR988985.1|DR988985 HDI_E_44.esd Horsegram (VZM-1) drought stressed subtracted cDNA Macrotyloma uniflorum cDNA, mRNA sequence

AAAGCTGGAGCTCACGCGGTGCGCGGCCGTTCTAGAACTAGTGGATCCCCCGGGCTGCAGGAATTCGGCA

CGAGGCTCGTTCGCCTTCATCCAAACTTCCCATTCTCCAAATTTCATTCCCCAAAGTCCCAACCCTGAAA

CTGAAACTCCCAATTTCAAAGCCCCAGATGTTGTCCCTCCTCGGGGGAATAGCACCACTGGTCTTGGCCC

GGCCCTCCCTCGCAGCAGAAATAGAGAAAGCTGCACTCTTCGACTTCAACCTGACACTGCCCATTATAAT

GGTGGAGTTTCTGCTGCTGATGGTTGCTCTGGACAAGATATGGTTCACCCCACTTGGGAAATTCATGGAC

GAGAGGGACGCGGCGATCAGGGAGAAGCTGAGCAGCGTGAAGGACACATCGGAGGAGGTGAAGCAGTTGG

AGGAGAAGGCCAATGCCGTGATGGTGGCTGCTCGGGCGGAGATAGCGGCGGCGCTGAACGCGATGAAGGC

GGAGACGCAAGCTGAGGTGGAGCAAAGATTGCAGAGGGAAGGAAGAAAGTGGAGGCTGAGCTGCAGGAGG

CCTCTGGCCTAACTTGGAGAGGCACAAGGAAGAAACCGTCAAGGCCCTTGATTCCCAGATTGCAGATCTT

AGCCAGGAAATTGTTAAAAAGGTTCTTCCTATTTCTTAATATATATGTATGTAATTTTCATTTCAATACC

CCCACCCCAACAAAGAGAAT



>gi|71811592|gb|DR988983.1|DR988983 HDI_E_36.esd Horsegram (VZM-1) drought stressed subtracted cDNA Macrotyloma uniflorum cDNA, mRNA sequence

CAAAGCTGGAGCTCCACGCGGTGCGCGGCCGCTCTAGAACTAGTGGATCCCCCGGGCTGCAGGATTCGGC

ACGAGGTTTGTATTTTAAAAGTAGACAAAAAATGAAGTTCGAGATTACAAGGGAAAAATTAAATTGGTCA

GAAAAGGATGCATCAAATGTGACCTATAACAGTATGTTGTATCAAACTAAGATTCCCTAGTCTAAAAGCT

AGAGCCTTTGTTCTTCAATGTACATGGTCGTGTGATTCTGTAATTCTCTAATAATATACAAAGTATGTTG

TTTAAAATGTTTACCCACTGTCAATCTTTTCCATTTACATAAACGAATTAAGAGGACCATTCTTTAGCCC

GAACTAAAATTACCATTCATTAATTGATTTGGGGTTTCTCTGTTGCCAACTTATATCTCTGTTAATAAAG

TCATCAACTGACATGGTTCCAAGCTCTCCTCCAAACCTAGATCTAACTGTGACTGTTTCAGTTTCAACTT

CCTTTGAGCCTACAACAACCATCAATGGAATTTTCTGCTTTTCTGCATTTCTTATGAGTTTGGGTAGGCG

CTCTCCATGGACAACTTCAACTCTGATGCCATACATTTTCAGTTTGTTGGCTACAGCATTGCAATATTCA

AGCTGGGCATCAGTAACAGGTAACACTCGAAGGGGGGGCCCGGTACCAAATCCGCNC



>gi|71811590|gb|DR988981.1|DR988981 HDI_E_33.esd Horsegram (VZM-1) drought stressed subtracted cDNA Macrotyloma uniflorum cDNA, mRNA sequence

TGAGCTCACGCGGTGGCGGCGCTCTAGAACTAGTGGATCCCCCGGGCTGCAGGATTCGGCACGAGGGGAC

ACTGATGTTATGGGAACCATCTTTAGTGTGCCTCCTTTGTCTCCACTCTCTCCATTAATGGTTGTTTGAG

ATCATGGCCTTTTGCTCTTTCAATCAAGTTGAGCTGGTTTAAAAGAAGAAAGAACTACAGAGAGAGATGA

TGCTGCTGATTATCCTCTCTGATTTAGGTTGGTAATTTGCCTATAAACCAAAATGGTAGTGCTAATAGAT

TGAAGATATATCTGGGTTTGTTTAGTTCATGTAAATGTAAATATAGGATAGTATGAAGCAAGATTTTTTT

GTTTGTGACAAAAAAATTTCTCATGCTTATCTTCTTCCCTTTTTCATCATCATGTTCCTTGGTCAAATTA

TGTACTGTACTTGAAGCAATATTGAAATTTGTTTATTCGTTTATTGAAAAAAACAAAAAAAAAAAAAAAA

AAAAAAAAAAAAAAAAAAAAAAAACAAAAAAA



>gi|71811588|gb|DR988979.1|DR988979 HDI_E_31.esd Horsegram (VZM-1) drought stressed subtracted cDNA Macrotyloma uniflorum cDNA, mRNA sequence

AGCTGGAGCTCACGCGGTGGCGGCGCTCTAGAAACTAGTGGATCCCCCGGGCTGCAGGAATTCGGCACGA

GGCTCACATTTTAGGAAAGATGATGAAATTATTGTTGGTTGTCAGCTTGGTAAAAGATCTATGATGGCTG

CAAGTGACTTGCTAGCTGCTGGCTTCACAGGACTCACAGACATGGCTGGTGGTTTTGCAGCTTGGACACA

AAATGGACTTCCAACAGAACAATGAAGTGTTCTGTTGGACCTGCAGCTGTAAATAGAATAAGTTTTGGGA

GGAGTCGCATACATGGAAATGCATAATTCCCCAAGGCAGTGGCAATCTGATACTGAGTAAAACAAAATGA

AATCTGATACGGAAAAGTTCTTGTAAATGTGGAAAAGGGGGGGTTGTGTATTTGTCAATATAAAGAACGA

AGAAAAAGTTAATATTTATAAAACTTTGCAAAANAAAAACAAAAAAACC



>gi|71811586|gb|DR988977.1|DR988977 HDI_E_22.esd Horsegram (VZM-1) drought stressed subtracted cDNA Macrotyloma uniflorum cDNA, mRNA sequence

GGGGCGGCCGCTCTAGAACTAGTGGATCCCCCGGGCTGCAGGATTCGGCACGAGGAGCAATTCAGGCATT

GAATGGGACAGTAATTGGGAAGCAGACAGTGCGCCTTTCTTGGGGTCGCAGTCCAGGAAATAAGCATTGG

AGAAGTGATCCCAATGGAGGCCATTATGGTGGTGGTCAGGGTTATGGAGGCCACGGATTTGCTGTGAGAC

AGAATCAGGATATAGCCATGCAACCTGCTGCCGCTGCTATTCAAGGGGCTTCCTGACGGGGTTAGCAGAA

CTGTGTGTTGAAGTAGGATGGATTTTATTGCTCCCCATTAAACTGTAGTATTATTTTTAAATTTGTAGCT

GGGGGTTATACGGTATGCGAGGTTCTTAATTAAGATTGTATTAAAATGTCTGAAATTTTGGATTGAACCC

GACCATATATATGACTCGGGTATTTTTGTTTTTTTAAATCCTTAAAAACATATTGGTAGCACAGACAGCT

AAGATTATTTTAAAAAAAAAAAAAAAAA



>gi|71811584|gb|DR988975.1|DR988975 HDI_E_20.esd Horsegram (VZM-1) drought stressed subtracted cDNA Macrotyloma uniflorum cDNA, mRNA sequence

CTGAGCTCACGCGGTGGCGGCGCTCTAGAAACTAGTGGATCCCCCGGGCTGCAGGATTCGGCACGAGGAT

TTTTGTGGTTGAGAATCTGAACAATCGTTTCTCTAAGTTTAAGTCGTGATGGGAAAGGAAAAGGCTAACA

TCAAAATCGTTGTCGTTGGACATGTCTCTGAGAAATCAACTGCCACGGAGCTTGGAAGCCCTACTGAGAA

GGGTGTGACTGAAAGGTTCGAGAAGGGAGAAGGAAGTGTTTAAAACACGTTAGATATAAGATGTGTGAAG

AAGGATGATGTAAGATAAATAAGATGTAAGATGTCAAGGATGGATCTTAAGACGAAGTGATGAAGGACTT

ATATTCAGATAAATAAAATGAACTATATATGTTTATCTTCTTCAAAAAAAAAAAAAAAAAAA



>gi|71811582|gb|DR988973.1|DR988973 HDI_E_12.esd Horsegram (VZM-1) drought stressed subtracted cDNA Macrotyloma uniflorum cDNA, mRNA sequence

ACAAGCTTGGAGCTCACCGCGGTGGCGGCCGCTCTAGAACTAGTGGATCCCCCGGGCTGCAGGATTCGGC

ACGAGGCCTCGTGCCGAATTCGGCACGAGGGAGAAGCTCGAGCCCTCCGAGTTCGACGAGTGGATCAGAG

AAGTGGACGTCGGTTCGGATGGCAAGATCCGCTACGAGGATTTCATCGCCAGAATGGTCGCTAACGTAGT

AACACCCAATCCAGAATCTCTTTTGAGTGTTATCTCTTTTCTGTATTACTGTTTATTCAGTTTACTCTGT

TGTGTTTGCTCTTGAGATGGACCAGTTGATTACAGTGATTGTGCTTATTTTATATGGTTTGTAGCTAACC

GAGGGATTGTTTTANGGAAACGGTGGCATA



>gi|71811580|gb|DR988971.1|DR988971 HDI_E_10.esd Horsegram (VZM-1) drought stressed subtracted cDNA Macrotyloma uniflorum cDNA, mRNA sequence

CCACGCGGTGGCGGCCGCTCTAGAACTAGTGGATCCCCCGGGCTGCAGGATTCGGCACGAGGCAGAATCC

CAGAAACTCGTATCCTCGTTTTGTTCGCTTCAGTTTACATCACCATGTCGGATTCTGCCGCCACACCGCT

TGACCAGCACGCCACCGGTTCTTCCCCTGCACCGGCGGTTCACATCATCTACACCGAGAAGCCACAGGAT

GAGGAGCCTGAGGCTTACCACATCCGAACCCTCTCCGCTGTTCTTGGCAGCGAGGAGGCTGCCAAGGAAG

CTTTGTTGTACAGTTACAAAACTGCAGCTAGTGGGTTTTCTGCTAAGCTTACTCCTGAGCAGGTTGAACA

GATTTCAAAGCAACCTGGTGTTCTTCAAGTTGTTCCTAGCAGGACATATCAGCTCCATGGAGCAAATAAC

CTGCAGTAGAGAATGTTCATCTATACATGTATCCCATTCCAAACCGTTTTCTACACCATGTATAAACTAA

ACTAGTGTGGCTCAGCCTTTATACTTGTGCCAATAAACATGGGATCAATATT



>gi|71811578|gb|DR988969.1|DR988969 HDI_E_89.esd Horsegram (VZM-1) drought stressed subtracted cDNA Macrotyloma uniflorum cDNA, mRNA sequence

ATTTAGCTGGGAGCTCACGCGGTGCGCGGCGTTCTAGAAACTAGTGGATCCCCCGGGCTGCAGGATTCGG

CACGAGGGAATTTCTTGCAGTTTGACCTGCTCCTAGGAGAGGAGAACCTGATGTGACTCGTAGAACGCCT

GATTACTTCTTATAATGTAGCTGTTGAATGTCATACCGATATATCATGGTTACCTCTACTGCGCTAATTT

CAGGTAGTTGCGCGCATCCTCATCATGAGATTGCTGTAAGCATTTAGTTCCATGGATATACACGTTCCGT

GCGTNATGGGGTTGTCCAAAGACTGCTCTTGATGTATAATAGGCAGGTAACATTCCACTGATCTGTTACA

CTCGACAACTTGCTGGGACACCTTGTCTGGAGAAGTCAGNCCAAAATGATGACTATGTCAGAATTCATAA

TTGGTTCCAAGCCAAAATGTAGGAATAT



>gi|71811576|gb|DR988967.1|DR988967 HDI_E_77.esd Horsegram (VZM-1) drought stressed subtracted cDNA Macrotyloma uniflorum cDNA, mRNA sequence

AAAGCTGAGCTCACGCGGTGGCGGCCGCTCTAGAACTAGTGGATCCCCCGGGCTGCAGGATTCGGCACGA

GGGAGACTTCCAGAAGGAGAAGAAGCTCGAGGACAACGACTTCACCGGCGCTGTTTTCGAGGGCAAGTAT

TACGGCCCCGATGAGTTTAAGAAGCTTGAGACTTTGCCCACGCGCGCCGAGATTTATGCCACTCTTTTGG

GGGCTCTCAATAGCCCTGCTTCTGCTCTCGTCGGTACCCTTCAGGCTCCTGCTAGAGAACTTCTGATGGT

TCTCAAGGCGCATGTTAAGAACCTTGAAGAACAACAAGGTGTTCCGCAATAGATACTCTTATTTCAGTTT

ATGTGTATGCCTTTTCTTCATTGCGATTATTTGTTTTCTTCTGTTGTTCTATTATTTATGACTGTGTAGA

AAATTTACTTGCTGAAGGAAAAGGAAATTTAGAATGGTGCAAAAAAAAAAAAAAAAAA



>gi|71811574|gb|DR988965.1|DR988965 HDI_E_75.esd Horsegram (VZM-1) drought stressed subtracted cDNA Macrotyloma uniflorum cDNA, mRNA sequence

AAAGCTGGAGCTCCACGCGGTGGCGGCCGCTCTAGAAACTAGTGGATCCCCCGGGCTGCAGGAATTCGGC

ACGAGGCCTCGTTGCCGATATTCGCGCGACGTAGGGGAGGGTCCGAGTAGCGCCTAATTACTATTAGCGG

TTATGGTCATACAAAGACAGTCTAGGAACAAGCATACGCAGAAATGTAAAGGATGAACGGTAGACATTAA

GAAACCTAACCAACATGTCAAATGAAGTTTATAAACGACCCATACCACAAGGCGGGTTCTGTAGAGAGGA

GTGCATCAGTCTGAACACCTTCGA



>gi|71811572|gb|DR988963.1|DR988963 HDI_E_73.esd Horsegram (VZM-1) drought stressed subtracted cDNA Macrotyloma uniflorum cDNA, mRNA sequence

AAAAGCTGGAGCTCACGCGGTGGCGGCGCTCTAGAAACTAGTGGATCCCCCGGGCTGCAGGATTCGGCAC

GAGGATGGGGTACGATATCAAGAATGATTTGCTCCAGTTTTAGGGTATAACTAAGAACAAGATTACTGAA

TTATGTTGTGTATGTGTATTATTGTGTTGTTGTGTATCGGATGTTATTGGAATAGTCCTTTGTTTCAGTN

TCGTTTATAAGGGTTAAAAATCAAGAATGAAAAATTGTATATGACCGGGTGCTGATTGGGAGGTAACCCT

TCCTCCTTTTTCACTCTGTAGGTGTAGAAAGGTATGTCAAGATTTTTTGACTACCTTAGTGTAAATAAGA

TTAAAGTTATATATGCTTAAAAAAAAAAAAAAAACATACATAAAAAAGAACANAAGAGGGGAGG



>gi|71811570|gb|DR988961.1|DR988961 HDI_E_65.esd Horsegram (VZM-1) drought stressed subtracted cDNA Macrotyloma uniflorum cDNA, mRNA sequence

AGCTGGAGCTCACGCGGTGGCGGCGCTCTAGAACTAGTGGATCCCCCGGGCTGCAGGATTCGGCACGAGG

GTCAGAGGATAAGTAAGGAGAGGAAGGTGAGAGAAGAAACACAGCCTCTTAGAAAAGCTTCACCGATCTG

ACAGCAGCTCTAGCTCTTCAAGCTGAGCGAGGAAGTGAGAACGTATGGGGAGTAAAAAGAAGAAGAAGAA

GAAGGAAAAGAAGAAGATCGAGGAGAAAGGTAGAGGGTGGTCATCATCACAAGGAGGACACGATAGTGCT

TCCAGCTTGAGAAAGTTGAGGTTGTCGCAAACAGCACAGTCTGAGGAAAAGAAGGGGTTCCTGGACAAGA

TTAAGGAGAAGCTACCAGGGCACAATGAAGACAGAGGAGGGCACAGCTCCTCCTCCTCCACCACCTGCTG

CTACATCATCATGCAACATGGTGAGGGTGTTCATCATGAAGGAGAGACGAAGTGAGAAGAAAGGTATCTT

AGAGAAGATTAAAGAGAAGGCTTCCTGGTTACCATCCCAAGACAGCAGGAGGAGTAAAGAAAAGGAGAGT

GGTGCTCACTGAGATTGAGACAAGGCTTCTTTGTTGGGTTGGTTGGAGATGATTGTGGTGTGCTTTGTTT

TCATTTCTCNTCGTCAAGCCTTGTGGGGTTTCTTATCTTTTGTGTTTCTTGATGCTGTCTTTGCTTTTAT

TATTGTGTACGTGCTCCTATTTTTTTCTTCTTTCCTTTTCCTATTTTTGTTTAC



>gi|71811568|gb|DR988959.1|DR988959 HDI_E_63.esd Horsegram (VZM-1) drought stressed subtracted cDNA Macrotyloma uniflorum cDNA, mRNA sequence

AAGCTGGAGCTCACGCGGTGGCGGCGCTCTAGAACTAGTTGGATCCCCCGGGCTGCAGGATTCGGCACGA

GGCTGAGATGTGTACGAGGGCGCGTGGGTTGCATGTAAATATTGTCGTTTTTCGTGAATCGCATGCCACG

TTTGCATCTACTACCATTATGTTTTCAGAATTTATTGGTCTGTTACTGACTGAAATATTACTAGTGTGTT

TGGTTTTTGTGAGCTAGTGAATGTAAAAATGCTAAATAACTAGCATCCTATGTTTTTGTTCTTAAGTTTT

GATAATTAACCAGATTTGCCTGGAACTTTGGCATTTGGTTAATCTTTTCACCCTTTTGTTTTAAGTATTA

TTATCGAAATAAATAAAATTGTGTTTTGTAAAAAAAAAAAAAAAAAAAAAA



>gi|71811566|gb|DR988957.1|DR988957 HDI_E_61.esd Horsegram (VZM-1) drought stressed subtracted cDNA Macrotyloma uniflorum cDNA, mRNA sequence

AAAGCTGGAGCTCACCGCGGTGGCGGCGCTCTAGAACTAGTGGATCCCCCGGGCTGCAGGAATTCGGCAC

GAGGATGATATTAATTTTGTTGAGCTTTATTCGGGGAACAGATCAAAGTGAAGGAAAGTCAAAACTGAAC

AGATACATATATGCATATGAACAACACATGGATGGGAGCAATGAAATAATGGAGAATGTGGATCAACTTG

TTTGGCAGAATCTTCTTCCTGATTCCAGCTACCTCAAATTACTGTTCCTGGTTATTAGAGTGGTACATGA

TAGACCTGGATATCCCTTTTGAGAGTAATGAGCAATCCTTCCTGGGATATCTATTAGATAGGTCATGTGT

ATCTTACATGACATGCCAGCAGCTTGTGTATCTTTAGTGTAAATGTAATTTTAATACTGCTGTCTGAATG

ATAAAAATTTATTAATTCTTCATAAAAAAAA



>gi|71811564|gb|DR988955.1|DR988955 HDI_E_54.esd Horsegram (VZM-1) drought stressed subtracted cDNA Macrotyloma uniflorum cDNA, mRNA sequence

AAGCTGGAGCTCACCGCGGTTGCGCGGCCGTTCATAGAAACTAGTTGGATCCCCCGGGCTGCAGGAATTC

GGCACGAGGCCTCGTGCCGAATTCGGCACGAGGCCAAATTTGAGTTGAAAGGGGATGATATGGTTTACGC

GCAGCTAAACATACCCGATGAAGAGAAAACCCCTTTACCCGTAGACGAAGATTTGCCTGGGATGGGACAG

TACTATTGCCCTCACTGCGATCGGTACTTTGCCAATGTAACTGTGAGGGATGAGCACTTTAAGACCAAGC

GACACAAGAAACGTGTAAAACAAATGATGGGTCCAGCACCACATACTCAACTTGATGCCGAATTAGCTGC

AGGGATGGGTATGCCAGACAATGGACTAAATCTTATGTCTATGTAACTTCGTTTATTGGTTGTAGGTAGA

AAGATTGTCATAGGAGCTGGAGTTATAAGATATAGTGGTTGCTATAACATTACAACATAAGTAATATTAT

ATTTTGAATGCT



>gi|71811562|gb|DR988953.1|DR988953 HDI_E_52.esd Horsegram (VZM-1) drought stressed subtracted cDNA Macrotyloma uniflorum cDNA, mRNA sequence

AGCTGGAGCTCACGCGGTGGCGGCGCTCTAGAACTAGTGGATCCCCCGGGCTGCAGGATTCGGCACGAGG

ATTTGCAGCTGTGCCCTTTTTGCGGTCAACCTTTTTCTGTTGTTGGAGATGAGTTTGTGAAGGAATCCGT

GAAGTTTTCCAACCAATCTACAACATTTGGACAAGCATTCAATAATTTCACCCGTTCTAGAAATGAAAAA

GATTCTGGTAGAGCAATTGATGTTGAAGCTGAAATTAAAGATGTCGACTGAGTCTTCACAGCTATAACAT

GTTGGTAGGTGTGAAGTAATGAGCAAATTTTGAGTTGAACACAGTGACTATGCTGAACACAAATCTTGAT

CGGTTAAAGAACAGGCTGTGGCTAGTATTTTCCATTTGTACTGAAGACACAATGAATTACACATTCAAGA

GAGAAGAGTGTGATGAGCAAAGTAACACATTCTAGAGAGGAGTGTGATGAGCAGAGTAACATTGCAATTG

TAATATAAGTTTTGACAGTGATGTTTCTCAGGTCACACCTCTATAATTTTGGTGTACCAGGATACTGCTT

CATTTTTCCTTTCATTGTTTTTACATAAAATGTTTAGGAGAAATCGAGATGCTTCATTTCAATGTCAGGA

CCTGGCAGTCTTGACAACTCTCTTCTATTAGTTCAAAACCCTTTACACAGACCAGGGAAATAAAGTAGTA

CCTAATCTCAATGTTTACTGTTTAACAAACAAA



>gi|71811560|gb|DR988951.1|DR988951 HDI_E_50.esd Horsegram (VZM-1) drought stressed subtracted cDNA Macrotyloma uniflorum cDNA, mRNA sequence

TGGAGCTCACGCGGTGGCGGCGCTCTAGAACTAGTGGATCCCCCGGGCTGCAGGATTCGGCACGAGGCAC

TGCTGGGCTTTCCGCTGACCCAGAAACCTTCGCCAGGAACCGTGAGTTGGAAGTCATCCACTCCAGGTGG

GCCATGCTCGGAGCCTTGGGCTGCGTCTTCCCCGAGCTCTTGTCCCGCAACGGCGTCAAATTCGGCGAGG

CCGTGTGGTTCAAGGCTGGCTCTCAGATCTTCAGCGAGGGTGGGCTCGACTACTTGGGCAACCCAAGCCT

GATCCACGCTCAGAGCATCCTCGCCATCTGGGCTACCCAAGTTATCCTCATGGGCGCCGTTGAGGGTTAC

CGTATTGCCGGCGGGCCTCTCGGTGAGGTCACTGACCCCATCTACCCCGGTGGCAGCTTCGACCCATTGG

GCCTCGCTGACGACCCAGAGGCTTTCGCTGAGCTCAAGGTGAAGGAACTCAAGAACGGTAGGTTGGCCAT

GTTCTCCATGTTCGGGTTCTTCGTCCAGGCCATTGTCACCGGAAAGGGCCCATTGGAGAACCTCGCCGAC

CACCTTGCAGACCCAGTCAACAACAACGCCTGGGCCTACGCCACCAACTTCGTCCCCGGAAAGTGAGCTT

GAACGAGAATGACTTGTGTACTCGAGGGGGGGCCCGGTACCCAATTCGCCCTATAGTGAGTCGTATTACA

ATTCACTGGCCGTCGTTA



>gi|71811558|gb|DR988949.1|DR988949 HDI_E_49.esd Horsegram (VZM-1) drought stressed subtracted cDNA Macrotyloma uniflorum cDNA, mRNA sequence

CTGGAGCTCACGCGGTGGCGGCCGTCTCTAGAACTAGTTGGATCCCCCGGGCTGCAGGAATTCGGCACGA

GGGGAGGATCATCCTGGAGGTGATGAAGTTTTGTTGTCTGCCACAGGAAAAGATGCAACCAATGATTTTG

AAGACGTGGGACACAGTGATTCTGCAAGAGAGATGATGGATAAATACTACATTGGTCAGATTGATGCATC

AACTGTTCCCCAAAAGCGTACTTACGTTCCACCTCAGCAAGCTCAGTACAATCCTGATAAGACTTCAGAA

TTTGTGATCAAGATCTTGCAGTTCCTGGTCCCTCTACTGATATTGGGCTTGGCCTTTGTTGTCCGGCACT

ACACCAAGAAAGAGTAATTCCTTCTATATCACATTTGACCACTGTTAAGGGTAGCTATTTTATAGTGTCA

TTTGAAAAAACAGTTTTTAGTTTCTTTATGGGATCTGAACATTGAATCCTGGATCAGACTAGATCAGACT

ATTGTTGAGTAAGCCAATGTTTTCCAACTTTCTTTGGCAATGCTATTAGTGTGATT



>gi|71811556|gb|DR988947.1|DR988947 HDI_E_41.esd Horsegram (VZM-1) drought stressed subtracted cDNA Macrotyloma uniflorum cDNA, mRNA sequence

AAGCTGGAGCTCACGCGGGGCGGCGCTCTAGAACTAGTGGATCCCCCGGGCTGCAGGATCGGCACGAGGA

TTGCGGCTGAGAATCTGAACATTCGTTTCTCCCTAAGTTTAAGTCGTGATGGGAAAGGAAAAGGCTAACA

TCAAAATCGTGGTCGTTGGACATGTCTCTGAGAAATCAACTGCCACGGAGCTTGGAAGCCCTACTGAGAA

GGGTGTGACTGAAAGGTTCGAGAAGGGAGAAGGAAGTGTTTAAAACACGTAAGATGTAAGATGTGTGAAG

AAGGATGATGTAAAATAAATAAGATATAAGATGTGAAGATGGATCTATCTTAAGATGAAGTGCTGAAGGA

CTTATATTCAGATAAATAAAATGAAGGATATATGTTAAAAAAAAAAAAAAAAAAAAAAAAAAAAAAAAAA

AAAAAAAAAAAA



>gi|71811554|gb|DR988945.1|DR988945 HDI_E_4.esd Horsegram (VZM-1) drought stressed subtracted cDNA Macrotyloma uniflorum cDNA, mRNA sequence

AAGCTGGAGCTCACGCGGTGGCGGCCGCTCTAGAACTAGTGGATCCCCCGGGCTGCAGGATTCGGCACGA

GGAGTTCAAGCAGCTATCATGGATTGTTCACAGAATCCGATGAATATTACTAAATATCAAAATGATAAAG

AGGTTATGGATGTCTTCAATAAAATATCAGAACTCTTTCCTGGAGTAGGTTCACCATGATTCATTTATGC

ATTGCTGTTTGCTGGGTCAAGTTATGCATGCCTCTCCTGATGAACAATGAGAGTACCTCTGCCTAACCAA

ACACATTGAACCATGCAAAACTCTCATACTCACGGTTACAGCTCCATTCTGACATGCGGATAGTAACTGT

GTCTGGTCTGGGCCAAATATTGCCATAGTTGAATTTTTTTGTTTCACTAAGTAGTTGAGATTGTACATTC

ATGTTTTAAGTGTATCTGCAATGTGTTTTGAGGCAGTGTATCTGCAATGTGTTTTGAGGCAGGTCCCGTA

AAAATTGTGGATGCTTCACATAATANTTTTTCACTAAAGCATAAGCTAGGAGTTAA



>gi|71811552|gb|DR988943.1|DR988943 HDI_E_38.esd Horsegram (VZM-1) drought stressed subtracted cDNA Macrotyloma uniflorum cDNA, mRNA sequence

AAGCTGGAGCTCACGCGGTGGCGGCCGCTCTAGAAACTAGTGGATCCCCCGGGCTGCAGGATTCGGCACG

AGGCATTTTTGTGGTTGATAGCAATGATAGAGATCGAGTGGTTGAGGCAAGGGATGAGTTGCACAGGATG

CTGAATGAGGATGAACTTAGAGATGCTGTTTTGCTTGTTTTTGCCAACAAGCAAGATCTTCCTAATGCAA

TGAATGCTGCAGAAATAACCGACAAGCTTGGTCTTCATTCACTCCGGCAACGCCACTGGTATATCCAGAG

CACTTGTGCAACTTCTGGAGAGGGTCTCTATGAGGGTCTGGATTGGCTTTCTAACAACATTGCCAGCAAG

GCATGAGACATTTGAAAAATTTTGGTCTTGTGTGGGAATTTCATGCGAGTCTGGCGGTTCTTGGAGAAAG

ATGCTTATCCTTTCTAGCTAAGGTTGTAATAGCTGAATGCTTATTAGAAGTATATTCTCTTCTGTGTTAA

CTTGGTTTGTATGATTGCTTAAATTAGCTAATACTTTTAGCTATAATTGGACCTCTTGCAATCTTTTTGG

TTAAAAAAAAAAAAAAA



>gi|71811550|gb|DR988941.1|DR988941 HDI_E_30.esd Horsegram (VZM-1) drought stressed subtracted cDNA Macrotyloma uniflorum cDNA, mRNA sequence

AAGCTGGAGCTCACGCGGTGCGCGGCGTCTCTAGAACTAGTGGATCCCCCGGGCTGCAGGATTCGGCACG

AGGCAAGCTTGCTGAAGCTGAGGAAAAAACTGCTGGCGGTTTGTTGCTTACAGAGGCAACCAAGGAGAAA

CCTTCGATTGGAACAGTGATAGCAGTTGGTCCAGGGCATTTGGACGAGGAAGGCAACAGAAAACCGTTGT

CCGTGACTCCGGGGAACACAGTTTTGTACTCGAAATATGCTGGAAATGACTTCAAGGGAAAAGATGGTGA

CTATATTTACCTTTGGANGGGTTCCTTCCAAGGAATTGGTTCCAATTGGCCTTGGTTCCTTCCTTCCCTT

AAGGGAAACCACTTCCNGCTTTGGCCTTGGTTACTCTTCCAATATTTTCCGTGTTCCTATCTCTCGAAGG

AAAGATGTNCAATATTTTTAATATGGTATATAGACCTATTGAAAATATTATACGTTGATTGCATTCTTGT

NCCTTGGACAATTGCGATATCATTAGATTGGGCACCTAATGTTGAATTAAGTCCTGCAATATCTTGAAAA

AACAAAAACAAAAAAAAAAAAA



>gi|71811548|gb|DR988939.1|DR988939 HDI_E_29.esd Horsegram (VZM-1) drought stressed subtracted cDNA Macrotyloma uniflorum cDNA, mRNA sequence

AGCTGGAGCTCACGCGGTGGCGGCCGCTCTAGAAACTAGTGGATCCCCCGGGCTGCAGGATTCGGCACGA

GGAGCAATTCAGGCATTGAATGGGACAGTAATTGGGAAGCAGACAGTGCGCCTTTCTTGGGGTCGCAGTC

CAGGAAATAAGCATTGGAGAAGTGATCCCAATGGAGGCCATTATGGTGGTGGTCAGGTTATGGAGGCCAC

GGATTTGCTGTGAGACAGAATCAGGATATAGCCATGCAACCTGCTGCCGCTGCTATTCAAGGGGCTTCCT

GACGGGGTTAGCAGAACTGTGTGTTGAAGTAGGATGGATTTTATTGCTCCCCATTAAACTGTAGTATTAT

TTTTAAATTTGTAGCTGGGGGTTATACGGTATGCGAGGTTCTTAATTAAGATTGTATTAAAATGTCTGAA

ATTTTGGATTGAACCCGACCATATATATGACTCGGGTATTTTTGTTTTTTTAAATCCTTAAAAACATATT

GGTAGCACAGACAGCTAAGATTATTTTAAAAAAAAAAAAAAAAA



>gi|71811546|gb|DR988937.1|DR988937 HDI_E_27.esd Horsegram (VZM-1) drought stressed subtracted cDNA Macrotyloma uniflorum cDNA, mRNA sequence

AAGCTGGAGCTCACGCGGTGGCGGCCGCTCTAGAACTAGTGGATCCCCCGGGCTGCAGGATTCGGCACGA

GGGCAAAATCTTAGAAATAAGGGAATCATTGATAAACCCGGGCACAGTATTTTCTAGAAGTCCCTAAAGG

AAATCCGGAAATAAACCAACTTAACAAAACCAGCATCCTCCATATGTAATGTATTTCAAGAACAAATTCA

AAGGCTACAAATAGAACCTAAGCCTCGGTTGTAGGATAGTCAAACACAACATCTTTGCCTGAAAACTTCC

TGTATACTGCAGCAAAAGTCTCCAACTTGTACTCAGTGTTGTTCCTCTCCTTGGGGTCCAAAAACACCTT

CATAATCTTGGAACCATCAATTCTAAACCTAACACGCTTTCCAACAATCTCAGCAGGTAAGACAATGTCC

TCCAGCATTGCCTCATGCACAGCAGTGAGTGTACGGCTGCGAGGACGCTGTACGGCTGAACCTTTCTTTG

GTGGCCTCAATATCCTCCGGGTGGCAATCAGGACTACATCCTTCCCGCTAAATTTTTTCTCAAGTTCTCT

CACAAGCCTGACCATGGATCTTCCTGAATCCTTTCCTTAATCTGTAGGGGACATGGGATAACCACAGGCC

TTGCGGTTACCAGGAAACATCATTTTTGGACACGCTGAAATTTATTGTATAAATCTTTCCAGATCCACTT

TTCAGCTCATTGGTGGGTATCTCCGAAGGGGGGGGGGGGCCCCGGGTTAACCCAATTCGGACCCTTAATT

AGTTGAAAGTTCCGNATTTACACAATTTCGCACTTGGGGCCCGGT



>gi|71811544|gb|DR988935.1|DR988935 HDI_E_25.esd Horsegram (VZM-1) drought stressed subtracted cDNA Macrotyloma uniflorum cDNA, mRNA sequence

GCTGGAGCTCACGCGGTGGCGGCGCTCTAGAACTTAGTGGATCCCCCGGGCTGCAGGAATTCGGCACGAG

GCTTCAATCCGATATTCCTGTATTGGTTGAATTCTGGGCTCCATGGTGCGGTCCCTGCCGGATGATCCAC

CCGATAATTGACGAGCTGGCGAAGGATTATGTTGGTAAGCTGAAGTGTTACAAACTCAATACTGATGAGA

GCCCTTCAACTGCTAGTCGTTATGGGATTCGAAGTATCCCAACTGTCATCATTTTCAAGAATGGTGAGAA

GAAAGATACGGTTATTGGAGCTGTGCCCAAGACAACATTGACCTCAAGTATAGAAAAATTCTTGTGAGGT

GCTAAGTAAAGGCTTCAGCTGGAAGTAAAAGCTATTTTTCTCGTTAGCTTCCTCTCATCTAAGCCAAATA

TCTCAGGTTGGGATGATTTGTTTCTTAAATAACCATTTATGGTCCTGTATAGTTATGTATGCAAACCATA

CTTTCTGTTTTCTTTAGCTAATTATAGTTAACAATGTG



>gi|71811542|gb|DR988933.1|DR988933 HDI_E_18.esd Horsegram (VZM-1) drought stressed subtracted cDNA Macrotyloma uniflorum cDNA, mRNA sequence

AAAGCTGGAGCTCACGCGGTGGCGGCCGCTCTAGAAACTAGTGGATCCCCCGGGCTGCAGGATTCGGCAC

GAGGTTTAGTTAATGGTGGTGGTTCAAGGAAACCAAGGAAATTCAGTAGCAGGAGTATTGGGTTCTTCAG

CAATTCTGGGAGCCATAGCTTTGGTTCCAGGAGCATGTATAGGGGTAGGAGTGCACCTCTGACATGTAAA

CTTACTAGTTCACTGGCTGCAGTCTTGGCTCAGATGCTATCCCACAGGGCAACTCATGTTTGGGTGACTG

AGGACGAGAATGACGATGTTTTGGTCGGTGTGGTTGGATATACTGATATTTTAGCTGCTGTAACTAAACC

ACCTACTACCATGATTCCTGTTAATAGATCTACAGAGGGGTTTGGAAATGAAATTCAGTGTTGATTTTTT

TGCTTTCAACTTTTTCAATGAGACTACTCTTATTAGCAAAAAAAAAAAAAAAAAAAAAAAAAAAAAAAAA

AAAAAAAAAAAAAA



>gi|71811540|gb|DR988931.1|DR988931 HDI_E_16.esd Horsegram (VZM-1) drought stressed subtracted cDNA Macrotyloma uniflorum cDNA, mRNA sequence

GCTGGAGCTCACGCGGTGGCGGCCGCTCTAGAACTAGTGGATCCCCCGGGCTGCAGGATCGGCACGAGGA

TTTGCAGCTGTGCCCTTTTTGCGGTCAACCTTTTTCTGTTGTTGGAGATGAGTTTGTGAAGGAATCCGTG

AAGTTTTCCAACCAATCTACAACATTTGGACAAGCATTCAATAATTTCACCCGTTCTAGAAATGAAAAAG

ATTCTGGTAGAGCAATTGATGTTGAAGCTGAAATTAAAGATGTCGACTGAGTCTTCACAGCTATAACATG

TTGGTAGGTGTGAAGTAATGAGCAAATTTTGAGTTGAACACAGTGACTATGCTGAACACAAATCTTGATC

GGTTAAAGAACAGGCTGTGGCTAGTATTTTCCATTTGTACTGAAGACACAATGAATTACACATTCAAGAG

AGAAGAGTGTGATGAGCAAAGTAACACATTCTAGAGAGGAGTGTGATGAGCAGAGTAACATTGCAATTGT

AATATAAGTTTTGACAGTGATGTTTCTCAGGTCACACCTCTATAATTTTGGTGTACCAGGATACTGCTTC

ATTTTTCCTTTCATTGTTTTTACATAAAATGTTTAGGAGAAATCGAGATGCTTCATTTCAATGTCAGGAC

ATGGCAGTCTTGACAACTCTCTTTCTATTAGTTCAAAACCCTTTACAAGAACCAGGGAAATAAGTAGTAC

ATAATCTCAATGTTTACTGTTTAAAAAAAAAAAAAAAA



>gi|71811538|gb|DR988929.1|DR988929 HDI_E_14.esd Horsegram (VZM-1) drought stressed subtracted cDNA Macrotyloma uniflorum cDNA, mRNA sequence

TGGAGCTCACGCGGTGGCGGCCGCTCTAGAAACTAGTGGATCCCCCGGGCTGCAGGATTCGGCACGAGGT

TCAGTTTCAACTCTACAATGGCAACCATCTCAGCTGCTACCACAACCCCTTCGATCACCCGCGCATGTCT

TGTGCAAAAGCGGTCTCTTGGAGTCTCATCTCCCGTTCTTGGATTGCCAACAATGGGTAAGATGGGAAGA

GTGAGGTGCTCCATGGAGGAAAAGCCTTCTGTGAAGGAAAGCAGCTCAAGCATAGGGATGGGGGCATCTT

TGATGGCAGCCGCCTGTGCTGCAGCCATGTCAGGCCCAGCCATTGCCTTGGTGGATGAGAGACTGAGCAC

TGAAGGAACCGGGCTTCCTTTTGGGCTCAGCAACAACCTTCTTGGTTGGATCCTGTTCGGTGTCTTTGGT

CTTATATGGGGTCTCTACTTTGTCTACACTTCCAGTCTTGAAGAGGATGAAGAGTCCGGAATGTCCCTTT

AAATCCTGGGTAGGTGATGATGGATTGTAGAAATTGCGAAGAGTCCACTGATCTTCTGTTGTGTTAAAAC



>gi|71811536|gb|DR988927.1|DR988927 HDI_D_96.esd Horsegram (VZM-1) drought stressed subtracted cDNA Macrotyloma uniflorum cDNA, mRNA sequence

AGCTGGAGCTCACGCGGTGGCGGCGCTCTAGAACTAGTGGATCCCCCGGGCTGCAGGATTCGGCACGAGG

GTTCAAGTCTCTACTACCCTCATATGGATTGCTTCAGCTCTTCACGCTGCTGTTAACTTTGGACAGTATC

CATATGGTGGTTTGATCCTTAACAGGCCAACTATTAGCAGAAGATTCATGCCTGAGAAAGGGTCTGCTGA

GTATGTTGCATTGGGTAAGAATCCTGAGAAGGAGTTTTTGAAAACTATTACTGGGAAGAAAGAGACCCTG

ATTGACCTTACAGTTATAGAGATCTTGTCAAGGGCACACATCTGTACT



>gi|71811534|gb|DR988925.1|DR988925 HDI_D_94.esd Horsegram (VZM-1) drought stressed subtracted cDNA Macrotyloma uniflorum cDNA, mRNA sequence

AAAAGCTGGAGCTCACGCGGTGGCGGCGCTCTAGAACTAGTGGATCCCCCGGGCTGCAGGATTCGGCACG

AGGCCTCGTGCCGAATTCGGCACGAGGCACAGGCATACCTACTTGAAGGGACCCATGGATAAGATTACAT

CTGTGGCCATACCTCTGGCTTTGGCTGCATCATCACTTTATCTGATTGGACGAGGGATCTATAATATGTC

ACATGGAATTGGAAAGAAAGAATGACCTGGGAATTTCAGATTGAGGAGGTGGGCGATCACGCAAAACAAC

CATAGCCTTCTTATTATCAACCAGTGGTTTTCTTTGCATTTATGTTTGTTCTCTTAATAAAATTGCTTAG

TTGCAGATTTTGACTGCCATTGGGGTTTTATAACTTTAATGAAAGTTGTTCTTTGTATGATTGATATAAT

CAAATTGGGTGAATTGG



>gi|71811532|gb|DR988923.1|DR988923 HDI_D_92.esd Horsegram (VZM-1) drought stressed subtracted cDNA Macrotyloma uniflorum cDNA, mRNA sequence

AAGCTGGAGCTCACGCGGTGGCGGCGCTCTAGAACTAGTGGATCCCCCGGGCTGCAGGATTCGGCACGAG

GCCTCGTGCCGAATTCGGCACGAGGATAAAAAATCAATGAATTCATGATAAAACCCTTGTTTTTACAAAT

TTTTTTTGTCAATAAGGGGATCAAATGGTAAAGTTCTTTTGTTGGTAAATTGGAGGATTAGAAACATGAC

TATTGCTTTCCAATTAGCTGTTTTTGCATTAATTGCTATTTCATTGATTTTATTGATTAGTGTACCTGTT

GTATTTGCTTCTCCTGAAGGTTGGTCAAATAAAAAAAATGTTCTATTTTCCGGTACATCATTATGGATTG

GATTAGTGTTTCTAGTGGGTATTCTTAATTCTCTCATTTCTTGAAGTTCTATTTATTCTAGATTAAAAAC

GAAATGACCCCTCCCTCATAATTCTTTCGGATTGTCAGGTATATAAAAAAGGGAATGGATCAGTTCC



>gi|71811530|gb|DR988921.1|DR988921 HDI_D_9.esd Horsegram (VZM-1) drought stressed subtracted cDNA Macrotyloma uniflorum cDNA, mRNA sequence

AGGCTGGAGCTCACGCGGTGCGCGGCCGCTCTAGAACTAGTGGATCCCCCGGGCTGCAGGATTCGGCACG

AGGTAGATGAGGAGGGAAGTACTCTGAGAAAGAATGCAAAGGAGATGGGGAAGGTTTTCAGTTCTGAAGA

ACTCCATAATAAGTACATTGAAGATTTCATTGCTGCCCTTCACAAGTATAGGAATCCTTCCACCATCTAA

GCAGGGTTTATGCCTTCCATGTTCTTAATTTTCTCTTCTGTGTGCTTTGTTTGCATTGGGTCAAGAAAAA

GAAAAACAATAGTTCAAGATGTTTCTTTTCCTCCTCAATGGAAAATTTGGACTGTTGGTTTGTTCAAATT

TTCCTTTAATCTTTATCTTTATATCACTTATTGGATAAACAAATAATGATCTTCTGTTTTTTTATTAGTA

AAAAAAAAAAAAAAAAAAA



>gi|71811528|gb|DR988919.1|DR988919 HDI_D_83.esd Horsegram (VZM-1) drought stressed subtracted cDNA Macrotyloma uniflorum cDNA, mRNA sequence

AAGCTGGAGCTCACGCGGTGCGCGGCCGTCTCTAGAAACTAGTGGATCCCCCGGGCTGCAGGAATTCGGC

ACGAGGGGCATGTCAGCTACTGTGTGTTGGGTTTCAAATTTGATTGTCTCAGAGACATTTCTTTCAATTG

CGGAGGGTATAGGGATTGGCTCTACTTTCCTGATTATCGGTGGTGTAACTGTGCTTGCATTTGTATTTGT

GGTTGTGTTATTGTTCCCCGAAGAACCAAGGG



>gi|71811526|gb|DR988917.1|DR988917 HDI_D_80.esd Horsegram (VZM-1) drought stressed subtracted cDNA Macrotyloma uniflorum cDNA, mRNA sequence

AAAGCTGGAGCTCACGCGGTGGCGGCGCTCTAGAACTAGTGGATCCCCCGGGCTGCAGGATTCGGCACGA

GGGTGAAGCCTCAAAACATGTTTGTCTTCCACCATATGCATACTTGTATAAATTACATAATTCAACTTTG

GAAGAATCAATCGGAAGCACCACAATGGCCACATATTCTAAGATGCTTAGGAGATGCCACCAGAATTGCA

ACTTGCAATGATTGTATTATTATGATCATTATTGTAACATTGCTTCAAAATTCAATCATGAAAATGACTA

TGCTTTTTTATCAAAAAAAAAAAAAAAAAAAAAAAAAAAAAA



>gi|71811524|gb|DR988915.1|DR988915 HDI_D_79.esd Horsegram (VZM-1) drought stressed subtracted cDNA Macrotyloma uniflorum cDNA, mRNA sequence

AAAGCTGGAGCTCACGCGGTGCGCGGCGCTCTAGAACTAGTGGATCCCCCGGGCTGCAGGAATTCGGCAC

GAGGCCAAAAAGTGAGAGACACTAAGTCACACAACACAGCAACATGGCATCAGCAGCATCTGGGTTTGTG

TTGTCTCTTAATGTTAATGTTGCAGCTGCCACCACAAACTCATCCCCTTCAAGGGTCATGTTCCCCTCAA

AGAACACTGGTTCTAGGCTTGTTGTAAGGGCTTCAGATGAGGCTGCACCGGCCCCTGCAACCGCCACACC

CCCAGCTGAAGCTGAAGCAAAACCAAAGCCACCACCTATTGGCCCCAAGAGAGGTGCTAAGGTGAAGATT

CTTAGGAAGGAATCTTATTGGTACAAAGGAACTGGTTCAGTGGTTGCAGTTGATCAGGACCCCAAGACTC

GGCTTACCCCTGGTTGGTGGGTTCCGA



>gi|71811522|gb|DR988913.1|DR988913 HDI_D_71.esd Horsegram (VZM-1) drought stressed subtracted cDNA Macrotyloma uniflorum cDNA, mRNA sequence

CAGAGTTCTTTGCAACAGAGTGGCCGCTGCAGTCCCTCTCAGTGGCTCTCGTCTTGCCCCCTCCCTCTGG

CTGGCTNATCGGCCTGCTCCACGCTTCTGCTCCGCCATTCGAGCCGCGCCGGATAGATCGAGGGAGCGGT

CATCGCAATGACGCGAAAACTCGGTCACCGAGATGCATGGTCGTCCGTCCAAGAGAAAGTCCACATGGTA

CGGTCCTCCAGTACCGATGTCAACGTACTTTGGNGCCCCATNTCTCGCTGGTCGAAGCTCCCTCTGTCGC

TACCTTCATCCTGGCGAANGCTTTCCCACAAAGTGCTGACACTATCCAGTGTTGTGGGACCATTCCCTAG

CCATTGTGGGCCATTTTCCCTGACGTTGATCGC



>gi|71811520|gb|DR988911.1|DR988911 HDI_D_7.esd Horsegram (VZM-1) drought stressed subtracted cDNA Macrotyloma uniflorum cDNA, mRNA sequence

AAGCTGGAGCTCACGCGGTGGCGGCCGCTCTAGAACTAGTTGGATCCCCCGGGCTGCAGGAATTCGGCAC

GAGGCTCTCANCTATCATCTCACTCTGACTACTTNCTGTCTCTTTTAGCTGCATTGTGAAGCTCTCCCCT

GCTCTGCTGCTTCTCTCTCTTCCTGTCACGGCGTTTGTCAGCTACTGCTCAAGTTATGCCACTGAGGCTN

AGCTGCGCTGGACTCCAGTACCCGAGTGTATGGTATCCTGTCTCTTCGCCCGTCGTGTCTCTGGTGCTGG

CTATCTGGGAATCTGATGGGATTTCAACTTGTGCTTCGTAATATCTGCATCAACCCTGAAGTGTCAGTGG

TTGAACAACGTGCAGTGGAAGTAATAGCGCTGTGCAGTCTTGGAGTAACTTTGCTGAGTGGAGTAGAAGT

ATACCGAGCATTTCCTGAGGAAGTATGTGCGCAACAGAACTCCAGAGATTATGCCCTCCCTCAACAACTT

CTTTACTGACCCAAATCGCAACTAAGTTGCTTTGAATAAACAAAGTTTAATGTGAGATGTGAATTCTATT

ATGTTAAGGACAGTTTTTATCGTGCATTAGAAAACAAAATGTGGTCGCCTTCTCTGGCCTTACAGTGTGT

TTGCTGTGCTTGGGAATAAATGATATTGGTTTTTGATGCATTAAAAAAAC



>gi|71811518|gb|DR988909.1|DR988909 HDI_D_68.esd Horsegram (VZM-1) drought stressed subtracted cDNA Macrotyloma uniflorum cDNA, mRNA sequence

AAGCTGGAGCTCACGCGGTGCGCGGCGCTCTAGAACTAGTGGATCCCCCGGGCTGCAGGATTCGGCACGA

GGGTGGAGCCGGCGTGGGAGGAGGATCCGCTTGTGAAGGCGGCGTTTGAGAAGTTCAGAGGAAAATTGGT

TGAGCTTGAAGGGCTGGTTGATCAGAGGAATGCGGATAGGACGAGGAGGAACCGGAACGGCGCGGGGATT

GTGCCCTACGAACTTCTGAAGCCCTTGTCGGAGGCCGGAGTTACGGGAAAGGGTGTTCCTTATAGCATCT

CCATTTGATATTGTTCCTTTCCTATATCTATATGTGTGTGTGTGGCGTGCATGTTTCTCTCTCTCTCTAT

ATGTTCGAAGGAGCACATGAATTATTAGCATGCGTGTGTCTGAATAAAATGACATACACAAAGTTTGATC

TGCAAAAAAAAAAAAAAAAAACCCTCAAGGGGGGGCGCCCGGTACCCAATTTCGCCCATATAGTAANTCG

TATTTACAATTCTCACT



>gi|71811516|gb|DR988907.1|DR988907 HDI_D_59.esd Horsegram (VZM-1) drought stressed subtracted cDNA Macrotyloma uniflorum cDNA, mRNA sequence

GAGCTCACGCGGTGGCGGCGCTCTAGAACTAGTGGATCCCCCGGGCTGCAGGATTCGGCACGAGGCCTCG

TGCCGAATTCGGCACGAGGGTGAATCGTACTGACTACCAGCTAATTGATATCTCTGAGGATGGATTTGTG

AGTCTGCTTACTGAAAATGGGAACACTAAGGATGACCTCAAGCTCCCCACTGATGATAATTTGCTGGCTC

AGATAAAGGATGGATTTGATCAGGGCAAGGACCTTGTTGTGTCTGTCATGTCTGCTATGGGTGAGGAACA

GATTTGTGCCCTGAAGGATATTGGGCCAAAGAACTAGCTTTTGGTGCTGGCTGTTTGTTGTTTCTATTTA

AGCAAAAATATTTTTGTAAACCTTTATATTGCTTTGTTCAAGTCCTGGCTTATAGATTCTAGTCAGACTA

GTCTTAACAATGGTGTTTGTTGTGGATGTGTTGACAGAAACTATATCACATTTTCCTGGTTAAAAAAAAA

AAAAAAAANCCCAGGGGGGGCCCGGTACCCAATTTCGCCCATAATATTAGAGTCGTATTTACAATTTCAC

TGGCCGTCNTTT



>gi|71811514|gb|DR988905.1|DR988905 HDI_D_57.esd Horsegram (VZM-1) drought stressed subtracted cDNA Macrotyloma uniflorum cDNA, mRNA sequence

TTAGTGGAGCTCACCGCGGTGCNCGGCCGTTCTAGAACTAGTGGATCCCCCGGCGCTGCAGGATTCGGCA

CGAGGCCTCGTGCCGAATTCGGCACGAGGGCTAATGCTAAGTGGATCAGAGTCAGCGCATACAAGGCCCA

GGCAACGATTAGGTTCCCGCTTCTTCAGCGCCCATGTCAAAAGTTGCCCGGTTTGGTTGGGGTGGCGAAG

GCCTGGCCCATTTCGCGTCAACGTCCGGATAAAGATATCGGAGCAAGATCGAAGAGAGCATAATAGAGCG

CGGAGGAGACGTGCTCCGGAGAGCGGGGCTGACGCTGGAATGTGCGGCGGCAGTGGGATGAGGTGGCAGG

AGCTGATGCGCGGCAGCGAGCCACGCGAGAGATGCGCAGAAGCAATCCGACCCGCTTGGAGACCTACTGC

GAAGGACAACCCGGAAACCGATGAGTGTCGCATCCATATGCATACATTAACAGTTACCCTTTGT



>gi|71811512|gb|DR988903.1|DR988903 HDI_D_55.esd Horsegram (VZM-1) drought stressed subtracted cDNA Macrotyloma uniflorum cDNA, mRNA sequence

AAAAAAGCTGGAGCTCACGCGGTGCGCCGGCCGTTCTAGAACTAGTGGATCCCCCGGGCTGCAGGATTCG

GCACGAGGCACAAATCAACTCCACCATATGCAACACGTTGCTGATAAAATTCATGAAGCCAAACTCCATG

AAGGTGATGATTGGCATACCTCTGATTCGGTCAAACACTGGTCTTATACAGTAGACGGTGAGGCAATTAC

ATGTCATGAAACTATTGAAGCCATTGATGAGCAGAAGAAAACAATTGTGTTCAAGCTCTATGGTGAACAG

TATTGATGATAAATATACAGCTCTTTAANCTAATCTTTGAAGCGATTGAGAAAGATGGTGGCAATGCTGC

AATTAGATGGAGTGTAGAAATATGAGAAGGTGAGTGAGGATG



>gi|71811510|gb|DR988901.1|DR988901 HDI_D_46.esd Horsegram (VZM-1) drought stressed subtracted cDNA Macrotyloma uniflorum cDNA, mRNA sequence

CTGGAGCTCACCGCGGTGCNCGGCGTTCTAGAACTAGTGGATCCCCCGGGCTGCAGGATTCGGCACGAGG

CCTCGTGCCGAATTCGGCACGAGGGGAGAGCTCTGACACCATTGACAATGTGAAGGCGAAAATTCAGGAC

AAGGAGGGCATCCCACCGGACCAGCAGAGGTTGATCTTTGCAGTGGAAACAGTTGGAGGATGGGAGTGAC

CCTTGCTTGATTACAACATTCAAGAAAGGAGTCCANCCTTCCCAACCTTGGTTCC



>gi|71811508|gb|DR988899.1|DR988899 HDI_D_44.esd Horsegram (VZM-1) drought stressed subtracted cDNA Macrotyloma uniflorum cDNA, mRNA sequence

TGCGATGGGCAATCANTCTTGAGCGGGCAAGCTAANGGGAGCGATGTTGAGCCAGTTGAGCAGTGAGCCC

TTTATGCTCGTGTTGGTGAACTACACATGTTCTGCCTATCTTTATCCCCTGAAAGCATGCCTTATGATAG

ACNTGGTTGCCATTGACACTTTTAAATAACTA



>gi|71811506|gb|DR988897.1|DR988897 HDI_D_36.esd Horsegram (VZM-1) drought stressed subtracted cDNA Macrotyloma uniflorum cDNA, mRNA sequence

CCTCCTGCTCCAAGGAAAAGCTAGATGCACTTATTGATAAATGCAAGGATCGGACCTCTTTGACTTATAT

GGCTGTGAATAAAGACGGGAGCTGGAAATCTAATGTGGGTCAAATTGATGTTAATGCTGTGACATGGGGT

GTCTTTCCAGCTAAGGAGATAATACAACCAACCATTGTAGATCCTGTCAGCTTCAATGTATGGAAGGATG

AGGCCTTTGAAATTTGGTCAAGAGGATGGGCTAGCTTGTACCCTGAGGGTGATGCATCTAGAAAATTGGT

TGAAGAGGTGGCTGGACGCTATTTCTTGGTCAGTTTGGTGGACAACGATTACGTCAATGGTGATCTTTNT

TGCTGCCTTTGTGGATTTCTGACAAGAGTTTCTTATTANATTAGTTCTTTAAGTAATGTCCACTTACTGT

NGGTTGTCAAACTTTCCATAT



>gi|71811504|gb|DR988895.1|DR988895 HDI_D_33.esd Horsegram (VZM-1) drought stressed subtracted cDNA Macrotyloma uniflorum cDNA, mRNA sequence

AAAGCTGGAGCTCACGCGGTGGCGGCGCTCTAGAACTAGTGGATCCCCCGGGCTGCAGGATTCGGCACGA

GGCCTCGTGCCGGACAAGAGTCAAAATTACAATTAAATTAGACTCATTCGACTTCCACGATCTCATCCAA

AGCATAGTTGTTTGTTGATACGTTGGCATAGTTGACTTTGTTGAATCGAACCACAACAGGGTAGCGAGTC

TTGGGGTCCTGATCAACTGCAACCACTGAACCAGTTCCTTTGTACCAATACGATTCCTTCCTAAGAATCT

TCACCTTAGCACCTCTCTTGGGACCAATTGGTGGAGCCCGGGGAGGCGCCGCCTTACGCCGGCGCGTTGG

ATTGTGTTCTGAAGACGGTGCGCGCGGAGGGTCCCATGGCCCTCTACAAGGGTTTCATTCCTACGATCTC

GAGGCAGGGACCCTTCACTGTTGTGCTGTTCGTGACACTGGAACAAGTTCGCAAGTTGCTTAAGGATTTT

TGAACGGTAGATCGATGAAGATGATGACGAAAAATGCTTCTTTCCTTTTTCACTAAAGATCTGGTCCTTT

TCCTTAG



>gi|71811502|gb|DR988893.1|DR988893 HDI_D_31.esd Horsegram (VZM-1) drought stressed subtracted cDNA Macrotyloma uniflorum cDNA, mRNA sequence

AAGGCTGGAGCTCACGCGGTGGCGGCCGCTCTAGAACTAGTGGATCCCCCGGGCTGCAGGATTCGGCACG

AGGCCTCGTGCCGAATTCGGCACGAGGAGAAAATGGTCAAAAAATATGTACTCGGATATTTTGGAGATAG

ATGGAAGTCCCCTTCCAAAGCTCCCTGAAGAAGAAGAAGCTGTTGGAATTATTACAATGGAAGATGTCAT

TGAAGAGCTTCTACAGGAGGAGATTTTTGATGAGACAGATCATCATTTTGAAGACTCATGACAGTGGCTT

TTCCATAAAACCATGTTCATATTATTGTTTGGACCTTATAGATTTTAACTAGATAAAATACGGTTTAGGG

TGAGAGCTGCTGTATAGAGAACTGAGTGGTTATGGGGACGTGTAAGCCATTTTGGTGGACGAAATGTTGG

GACCAGACAGCTGTTAATACAGCGAACCTTTTGCGATGTGCGTGCGTATGTTGGTTGTCTTTATCACAAT

GAAGCATGCCAGCAAATCATGGTGTGTTTTTGTGAGTATGAGTGTTTTCCTCTTTTTACTTGTATGGGTT

GCATATTGTGTTTAACCCGTAAGACGGAAAGTTATTTGGGGCCCATT



>gi|71811500|gb|DR988891.1|DR988891 HDI_D_23.esd Horsegram (VZM-1) drought stressed subtracted cDNA Macrotyloma uniflorum cDNA, mRNA sequence

AGCTGGAGCTCACGCGGTGGCGGCGCTCTAGAACTAGTGGATCCCCCGGGCTGCAGGATTCGGCACGAGG

CGAAAACCCTAATCCTGAAGCACTGCAAGTGTGATATGGCAAGAGCCATTTCCTCTTCCTGCGCTCGCTT

AACCATGCGCCTTCTCTCCACCGCTTCCACCACCACCTTCACCGCCACATTACCCAAACCCTCATCTCTC

TCACTCCCTTCGCGTCGATTCCTCTTCCCTCTCTCCCATGCCATCGTTCTTCCCTCTACCCGCGTCGCCG

GGATCCGCTGCCGCGTCAACCGTGCTGGCGACTCCGCCTACTCGCCGCTCAACTCGGGGTCGTCTTCGTT

CAGCGACCGCCCTCCCACCGAGATGGCCCCTCTCTTCCCCGGTTGCGACTACACCATTGGCTCATCGTCA

TGGATAAACCCGGTGGCGAGGGCGCCACCAAGCAGCAGATGATCGATTGCTACATCCAAACCCTCGCCAA

AGTTCTCGGAAGCGAAGAGGAAGCAAAGAAGAAGATTTATAATGTTTCCTGCGAGAGGTACTTTGGGTTC

GGCTGCGAGATTGACGAGGAAACCTCTAATAAGTTAGAAGGGTTGCCTGGAGTTTTGTTTGTGCTACCGG

ATTCGTATGTGGATCCTGAGAACAGGACTATGGCGCTGAATTATTTGGTCATGGAGAGATCGTCCAGAAG

GTCACCTGAAAAGGCAGAAANAAGGTGGAAACCACAGCCACCAAAAGGGCTTCCAGACCAGACCACAGGA

TACCATGATCGAACCAACGGTACGTCCGCCGCAAAAAAACCACACAGTGAATTG



>gi|71811498|gb|DR988889.1|DR988889 HDI_D_21.esd Horsegram (VZM-1) drought stressed subtracted cDNA Macrotyloma uniflorum cDNA, mRNA sequence

AGCTCACGCGGTGGCGGCCGCTCTAGAACTAGTGGATCCCCCGGGCTGCAGGATTCGGCACGAGGCCTCG

TGCCGAATTCGGCACGAGGGCATGTCCCAGCTGGGCGGCAACCTACTCGCCCTGCTGCTGCAAAGCCAAC

TCCTGAAGAGGATGAATTGGCAGCATTGCAGGCTGAGATGGCGCTTTGAGCAGAATTTTGCAGGCTTTTT

GTGCATGACGACAGTNTGGCTTCAATGGAGAATTGTAACGTGGACACTTCTGTTTCTCTCCTGCTTGCTT

ATCTTTGTAAAATTATCGAACAGACGAGAGAATGTATATAAAAGGTGGGGAAAAAATTGAACATGAAAGT

CATTCATAAAGCANAAACTTACTACTTCGTAAAAAAAAAAGA



>gi|71811496|gb|DR988887.1|DR988887 HDI_D_19.esd Horsegram (VZM-1) drought stressed subtracted cDNA Macrotyloma uniflorum cDNA, mRNA sequence

AGCTGGAGCTCACGCGGTGGCGGCCGCTCTAGAACTAGTGGATCCCCCGGGCTGCAGGATTCGGCACGAG

GCTGCAGTGCATTCAACCTGTTGGTCAACAATGGGGCAGCTGCTGGCCAAGTAATATATCATACACATAT

TCACATAATTCCCCGTAAAGCGTACGATTGTTTATGGGCTTCTGAGAGTTTGCTAAGACGACGCGTTAAT

TTAGATGATGAGAAAGTTTCTCAGCTTGCAGCTCGTATTCAGAAGCATTTATTGCCAGCTGACATGTGCC

AGGAAAACAAGAATGAAAATTTTTGTTCGAGTAAGAGTTAGTTGGATTATTCATCTGTTGTAACTTAAAG

TTTATATTTTCATTTAGAGGAATGATGTATGCCCATAAAAATATTTAATGTAACACTCAGTTAAAATTGG

GGTATGAGATTTTTTAACCGTTCTTTTAAACGGAACTAAAAAATTCTCAACTACTCAACTATTGAGGGTT

ATGTCAGATATTTTTTTGTACAAAAAAACAAAAAAAAACA



>gi|71811494|gb|DR988885.1|DR988885 HDI_D_11.esd Horsegram (VZM-1) drought stressed subtracted cDNA Macrotyloma uniflorum cDNA, mRNA sequence

ACAAGCTGAGCTCACCGCGGTGCGCGGCGCTCTAGAACTAGTGGATCCCCCGGGCTGCAGGATTCGGCAC

GAGGGGATCCTTTGGTGGGTTTGGGAAAAGGGGGTGTTGTTGTGTCGTTTGCGGATGCGAGGGTTTTGCC

ATGGAAGGCTAATAGGTGGTACAAGGAGGAGGCTATATTGGTGGTGGCTGACAGGAGTAAGAGAGAAGTT

GGTGCTGATGATGGGTTTTATCTTGTGAATGGTTATGGTGATGGTGGGGGTTTGAAGGTTGAGAGGGGTT

TCACCTTAAAGGAACAGGGTTTCACTCAGAGTGTAGGAACTGTCTTGTTGGTTGTTAGACCACCTAAAGA

TGAGCATGATGATCAATTGAGTGACGAAGATTGGGAGTGAGTCTTCTCAAAGGAATTAATGGAACAGGAC

ATCATCATATATATGTTTTTCTCAATTCATGATTCTCAACTTAAACTCAGCTTCAATCCCTTGCCTTTGT

TCTTTTTGTCATGCTAAATAAGGTTCTATTCATTTAAAAAAAAAAAAAAAAAACCCAAGGGGGGCC



>gi|71811492|gb|DR988883.1|DR988883 HDI_D_89.esd Horsegram (VZM-1) drought stressed subtracted cDNA Macrotyloma uniflorum cDNA, mRNA sequence

TGGCTATTCCTATCCGNTTCAAAGGAGGACGTATGCCTATGACACATATTTCATTCTCACGTATCTCTAC

GAGGGGTTGCATGCTGAGTCATGTATCCATATGGTGAACCTCCGTTGGTCATAAGAGAACCCTGTCCACG

AGGTAACTTTTCTGATGCCCTCTCTATTCAATGCGAGCGCTTNCACGAGAAGCATTGCGCGTGCCATCTA

CACCTTCGTTCCAAAGGGCAAACACAAAAGAAAATTGACCGGTNCNC



>gi|71811490|gb|DR988881.1|DR988881 HDI_D_87.esd Horsegram (VZM-1) drought stressed subtracted cDNA Macrotyloma uniflorum cDNA, mRNA sequence

AAAAGCTGGAGCTCACCGCGGTGGCGGCCGCTCTAGAACTAGTGGATCCCCCGGGCTGCAGGATTCGGCA

CGAGGACACTTGGAAAATCTAAAGCAACAGCAAATGGTTCTAAAAAGGATACTGTAGAATCTGAGAATCA

AGGCAAAGCTAGTAAAGAATCTGAAAGTGGAACATCTGGTACTTCTGAATTGAGTAAAGAAATCCCTGAA

GTTTCTAGAAACAGTTTAGATAGCCGTCTTGAGAAATCTAAAAAAGCAGAGAAACCAGAAAAATCTCGTA

TGAGCACAGACCATGTTGATAGGTTTAGGAGAAGAAAGGGGTTAGTTTGGTAGTCTCTCACAGGAACTGT

ATCCAAAATAATTTATCTGCTAGTATTCACTTGGCAGGTAATCTAATTCCACTCTTCAGAATAATGATGC

CACTTCTAAATGAATATATTGAGTGAGTTTTCTATTTCCTTTAGCTCCATAATTCAGCTGTATTGAAAGC

AATATGCATTTCTATGGCCTGTAAAATCAACAGCTTGATCTAAATGCAATCTTTTTAGTATTTTCTTTAA

AAAAAAAAAAAAAAAAA



>gi|71811488|gb|DR988879.1|DR988879 HDI_D_85.esd Horsegram (VZM-1) drought stressed subtracted cDNA Macrotyloma uniflorum cDNA, mRNA sequence

AAAAGCTGGAGCTCACGCGGTGGCGGCCGCTCTAGAAACTAGTGGATCCCCCGGGCTGCAGGAATCGGCA

CGAGGCAATCAAAATATGGTTTTTGGGGATGGAATTTGTGGCGTCAACCTATAGGTTTCCTCGTTTTTCT

AATCTCTTCCCTAGCAGAATGTGAAAGATTACCTTTTGATTTACCGGAAGCGGAAGAAGAACTAATAGCG

GGTTATCAAACCGAATATTCTGGTATACAAATTTGGTTAATTTTACATTGCTTCCTATTTAAACCTAATT

AGTTTCTTCCTTATTTGTAACAGTTCTTTATTTAGGTGGTTCAAATATCTCTATTCCGTACATCTTCGTT

TCTAACTTTTTTGAAATAAATAAAGCATACGGCGTTTTTGTAACCATAATAGGTATCTTTATTACACTAG

TTAAAACTTATTTATTCTTATTCATTTCTATCACAACAAGATGGACTTTGCCTCGGCTAAGAATAGATAC

ACATTATTAAATCTCTTGGGTGGAAGTTTCTTTTACCCATTTCTCTCGGTAATCTACTTATTTAACACAC

TTCGNCTCACACTGTTTTCACT



>gi|71811486|gb|DR988877.1|DR988877 HDI_D_77.esd Horsegram (VZM-1) drought stressed subtracted cDNA Macrotyloma uniflorum cDNA, mRNA sequence

CACAGCTGAGCTCACGCGGTTGGCGGCGCTTCTAGAACTAGTGGATCCCCCGGGCTGCAGGATCGGCACG

AGGATGGAACATGGGAGAGATGATTGGGCTAATTCATCAAGTGAATTTGGGACTGGGTGGGTGGACATGG

ATTTGGTG



>gi|71811484|gb|DR988875.1|DR988875 HDI_D_75.esd Horsegram (VZM-1) drought stressed subtracted cDNA Macrotyloma uniflorum cDNA, mRNA sequence

AGCTGGAGCTCACGCGGTGGCGGCGCTCTAGAACTAGTGGATCCCCCGGGCTGCAGGATTCGGCACGAGG

CCTCGTGCCGAATTCGGCACGAGGGCGCGAAGTCCATGACAATGACGTAATTGTGTTCAGCATTGATGGA

ACTTTACTCTCCAATGTCCCATACTATTCTCAACATGGATATGGAGTGGAGAAATTCAATTCCACACGTT

ATGATGAAGAGTTTGTTCTCAAGGGTGATGCACCAGCTTTGCCTGAGACTCTTAAGAACTACAGAAAGCT

GGTGAGTCTTGGCTACAAGATCATCATATTATCAGGAAGAAAAGAGAACAAAAGGGAAGTAACTGAAGCC

AACTTAAAGCAGGCTGGTTTCGACACATGGGAGAAGTTGATCCTCAAGGAGCCATCTAACAGTGCACCAA

ATGCTCTTGAGTACAAGAAGGCTGANNAGGGCTAAGCTGGTGCAGCAGGGATACAACATTGTTGCAGTGG

TTGGAGACCAATGGTCCGATCTGAGGGGAAAACCCAAGGGAACCAGGAACTTTAAGCTTCACCAACCCCA

TGTACTCTATTCGAGTAGAATCATCTCTGAAGGATCCCAAGCTTGCTCCTATCTACGAAATACAGATACC

TTACGTGTTCTAATGGGAGCATTAACTATGTATACTATGCATGTGACTTTC



>gi|71811482|gb|DR988873.1|DR988873 HDI_D_73.esd Horsegram (VZM-1) drought stressed subtracted cDNA Macrotyloma uniflorum cDNA, mRNA sequence

AAAGGCTGGAGCTCACGCGGTGCNCGGCGCTCTAGAACTAGTGGATCCCCCGGCGCTGCAGGAATTCGGC

ACGAGGCTCGGATGCGTCGTACCTATGGAATACAGCTGAGAAAAAGTTCATTTGCCCATGTCATGGATCT

CAGTTAACAAAGTTGAACCCAAAGGCCAAGAAGTTTGTAGAGAAGGAACCTTGTTCCCCTT



>gi|71811480|gb|DR988871.1|DR988871 HDI_D_64.esd Horsegram (VZM-1) drought stressed subtracted cDNA Macrotyloma uniflorum cDNA, mRNA sequence

AAGCTGGAGCTCACGCGGTGCGCGGCCGCTCTAGAACTAGTGGATCCCCCGGGCTGCAGGATTCGGCACG

AGGTTTTTATTACCTCTTTTGAGTTTTAACAAATGGCAACCACTGTGATGACCACTCTCCCCCAATTCAG

TGGTCTTAGACCCCAATTCTCAGCTGCCCCTTTGCAGAACTTGGTTGCTGTCCAACCCATGAGGCGCAAG

GGAAAGGGTGCTTTGGGAGCAAGATGTGACTACATTGGTTCATCCACTAATCTGATTATGGTGGCTTCTA

CAAGCTTGATGCTGTTTGCTGGAAGGTTTGGATTGGCTCCATCGGCAAACAGGAAAGCCACTGCAGGGCT

AAAGCTGGAGGTGAGGGACTCAGGCTTGCAGACTGGTGACCCTGCTGGCTTCACCCTTGCTGACACCTTG

GCCTGTGGAGCTGTTGGCCACATCATTGGGGTTGGTGTTGTTCTTGGTCTCAAGAACATTGGTGCCCTTT

AGACCAACTGTAAAGTTAGTGCCT



>gi|71811478|gb|DR988869.1|DR988869 HDI_D_62.esd Horsegram (VZM-1) drought stressed subtracted cDNA Macrotyloma uniflorum cDNA, mRNA sequence

AGCTGGAGCTCACGCGGTGGCGGCCGCTCTAGAAACTAGTGGATCCCCCGGGCTGCAGGAATCGGCACGA

GGGCGGGTCATTTGATTGGCATGAAATTTGGAATGGCTCTACTTGACGATCTAAACCATTTGAAAAATAA

ACGTATTCGTTCCGTAGCAGATCTATTACAAGATCAATTTGGATTGGCCCTGGTTCGTTTAGAAAATATG

GTTAGAGGAACTATATGTGGAGCAATTAGACATAAATTGATACCGACTCCTCAGAATTTGGTGACCTCAA

CTCCATTAACAACAACTTATGAATCTTTTTTTGGATTACATCCATTATCTCAAGTTTTGGATCAAACTAA

TCCATTGACCAAAATAGTTCATGGGAGAAAATTGAGTTATTTGGGCCCTGGAGGATTGACGGGGCGAACT

GCGAGTTTTCGGATACGAGATATCCACCCTAGTCACTATGGACGTATTTGTCCAATTGACACGTCTGAAG

GAATCAATGTTGGACTTATTGGATCTCTAGCAATTCATGCGAGGATTGGTATTACGGGATCCATAGAAAG

TC



>gi|71811476|gb|DR988867.1|DR988867 HDI_D_54.esd Horsegram (VZM-1) drought stressed subtracted cDNA Macrotyloma uniflorum cDNA, mRNA sequence

ATCGTCAGCGTGCTACCACATAGCTCCCTTCGATCACCGCGGACNGCATGTCTTGTGCAAAAGCAGGTCT

CATTGAGAGTCATCAGTCTCCCGTTCTTGAGATTGCCAACAATGGGTAACGATGGGAAGAGTGAGGTGCG

TACCATGGATGGAAAAGCCTATCTGTGAAAGGAACAGCAGCTC



>gi|71811474|gb|DR988865.1|DR988865 HDI_D_52.esd Horsegram (VZM-1) drought stressed subtracted cDNA Macrotyloma uniflorum cDNA, mRNA sequence

GAAACTAGTGGATCCCCCGGGCTGCAGGATTCGGCACGAGGGGCTTGTGATGATCTTGGAGGATCTTTCT

GTGAGTACGAGTATCAGAAGAGTGTCTTCTAGAGCAAACAACACTTGTATTGTCATGCCTACTGAATTTT

CCATATCTACCCCTATTAATTGTAGTGTTGGTTGTACATAGGTATATAGACTATGTGGAGTTTCATTTTC

TCTATAAATTTTACAATAATTATACTCTAAATTTGAAAGCTTCCCGTCTAGACTAGAAATGAAATAAAAT

AAAGGAGCATTTCAC



>gi|71811472|gb|DR988863.1|DR988863 HDI_D_50.esd Horsegram (VZM-1) drought stressed subtracted cDNA Macrotyloma uniflorum cDNA, mRNA sequence

AACAAAGCTGGAGCTCACCGGCGGTGCNCGGCGTTCTAGAACTAGTGGATCCCCCGGGCTGCAGGATTCG

GCACGAGGCCTCGTGCCGAATTCGGCACGAGGGCATTATTGGTAAAAATTTAGGTGGTATTTCTGATTTA

GAAACATAAAATTCATATAGTTAGATGTTGAAGATGATTTTTTTGAAATAAAAAGTTTCAATAAAAAAAA

GGTTTCAGCGGAAGAAAATAATTTTTGGGATGCATTTGATGATGAAAAACTAACTTTCAAAAAAAATAAA

GCCTACAAGTTTTAAGCATCAAATTGAGTATTTCAGCAGCCCTATTTGATAAGCCATATTAAGCTCAAAT

CGCTTCAAAGAATAAATCCTAATAATATTCAATCGATTCAAATCGACAGAACAGCAACAAATATTATAAT

AAACCTAATGTAAACAAACAAGCATAGCACTATAAAT



>gi|71811470|gb|DR988861.1|DR988861 HDI_D_49.esd Horsegram (VZM-1) drought stressed subtracted cDNA Macrotyloma uniflorum cDNA, mRNA sequence

CNCCGGGCCGGTTTTCATAAGAAACTAGGTGTGATCCCCCGTGCCGCTGCAGGTAATATCGGGGCACCGG

AAGTGTAGCACAATTGAGGACCCTTGGCCAAAACAGAGCGATGTCCCTGTGA



>gi|71811468|gb|DR988859.1|DR988859 HDI_D_41.esd Horsegram (VZM-1) drought stressed subtracted cDNA Macrotyloma uniflorum cDNA, mRNA sequence

AAGGCTGGAGCTCACGCGGTGGCGGCCGCTCTAGAACTAGTGGATCCCCCGGGCTGCAGGATTCGGCACG

AGGCCTCGTGCCGAATTCGGCACGAGGTGAGGATGAATTAAAACATGCGGTTGCATTTGTTCGACCAGTT

AGTGTGGCCTTTCAGGTGGTTAATGGGTTCCGATTCTATGAGAAAGGAGTTTACACCAGTGAAATTTGCG

GTAGCACTTCCCAGGATGTGAACCATGCGTGTGTCTCTGCGTGTGGGGTATGGAGTTGTAAATGGTGTCC

A



>gi|71811466|gb|DR988857.1|DR988857 HDI_D_4.esd Horsegram (VZM-1) drought stressed subtracted cDNA Macrotyloma uniflorum cDNA, mRNA sequence

AAAGCTGGAGCTCACGCGGTGGCGGCGCTCTAAACTAGTGGATCCCCCGGGCTGCAGGATTCGGCACGAG

GCCTCGTGCCGAATTCGGCACGAGGGCTTTCATCGCAAGAACCCTCTACTAGTCGGAGCGATCCGAGAGA

ACCTCTGCGAACATGTTGCTTTACCAGGACCTCCTTACAGCTGACGAGCTTCTCTCTGACTCCTTCCGCT

ACACTGAAATTGAGAATGGAATGTTGTGGGAAGTTGAGGGAAAGTGGGTTGTTAAAGGAGCAGTTGATGT

AGACATTGGAGCAAACCCTTCTGCCGAAGGTGGAGATGATGAGGGTGTCGATGATCAGGCTGTTAAAGTT

GTTGATATTGTTGACACCTTTAGACTCCAGGAACAACCTGCTTATGACAAGAAACAATTTGTTACCTTTA

TGAAAAGATTTATCAAGAATTTGACTCCCAAACTTGATGCAGAGAAACAGGAGTTGTTTAAAAAACACAT

TGAGGGGGCAACCAAATTCCTGCTCCCAAAGCTTAAAGATTTTCAATTTTTTGTTGGGGAGAGCATGCAT

GATGATAGTTGCTTGGTCCTTGCTTACTACAAGGACGGTGGCCACTGATCCGACTTTCTGTACTTTGCAT

ATGCCTTGAAGGAGGTTAAGTGCTAAGTGACTCAACATAGCAGGCTATTTACCATCCTTTTTTGTCTTTT

TTCTAGGTTACTTTGGAACTTTGCCTTGGTAATCATTTATTTATAACTAGACTTTTTGTGCTTCCTTCTT

ATTCCTCTCC



>gi|71811464|gb|DR988855.1|DR988855 HDI_D_37.esd Horsegram (VZM-1) drought stressed subtracted cDNA Macrotyloma uniflorum cDNA, mRNA sequence

AAAGCTGGAGCTCACGCGGTGGCGGCGCTCTAGAACTAGTGGATCCCCCGGGCTGCAGGAATTCGGCACG

AGGCCTCGTGCCGAATTCGGCACGAGGCAATCACCAAGGACGAGTTGGATGAAATGTTATGCACATGGAG

TTGCTTCGAAACATTATACGTGTTGTCAATAGTAATTTTAAACCTACGTTATCAAGAATTAAGATATTTC

AATGTGTAGTCAGAACCTGGCTAGATGCATTTCTGATGTCTTCAACATTTGGGTTCACGTGCAACCAAAG

GAGAGTGTTCTGCTTACAATGAGAGAAAATGTGAAAAGGGACAAATAAAAACTCCTATTGGTGTATGACC

TTGTGGGCATTCATAGTAGTGTCAATATGTTAGTTCTTCACCTTCCATTTGGTGGTATTTNACTTGTAAA

CTTTTATATTCAAGAGAATCACTTATCCTTCCAGCGTTGTAACATTTCAGCGGGCAAGGTGTCCTATTGT

ATATTAGGTGTGATAATGAA



>gi|71811462|gb|DR988853.1|DR988853 HDI_D_3.esd Horsegram (VZM-1) drought stressed subtracted cDNA Macrotyloma uniflorum cDNA, mRNA sequence

CACAATTACTTGGTAACTGGTAATAGAATGGTTGTTTATCAAGAGNTACCGATGGGTGAAATGATTTGAT

ACTGTTCATTGCTGAAGTTGCCATCTGATTTGTTTGTTTGCTCTTATCTGATGTATAAGAGTCGTATGTA

TCCCTTGTTGTAGAATAGAATTCCTCTCAACCTCTACTGCCCCTGTAATGAACCCATTTCATTGTTAAAA

TAATGTAGCAAATTTTATGTATATGGCTCTGAATGGAAACCATTATCTTTTTCAAAAAAAAAAAAAAAAA

ACATNCAAGGGGGGGCCCGG



>gi|71811460|gb|DR988851.1|DR988851 HDI_D_28.esd Horsegram (VZM-1) drought stressed subtracted cDNA Macrotyloma uniflorum cDNA, mRNA sequence

AAGCTGGAGCTCACGCGGTGCCGGCCGTTCATAAACTAGTGGATCCCCCGGCGGCTGCAGTGATTCGGCA

CGAGGCCTCGTGCCGAATTCGGCACGAGGCCGCTTGCGTGGTGCTCAAGGAGGGCAAACTGACATAGATA

TTCAGGCTAATGAAATGCTGCATCACAAGGCAAATCTGAATGGATATCTCTCCTATCACACTGGCCAAAG

TTTAGAGAAGATTAACCAGGATACAGACCGTGACTTTTTTATGAGTGCAAAAGAAGCCAAGGAATATGGA

CTCATAGATGGTGTGATTATGAATCCTCTCAAAGCTCTCCAGCCTTTAAAGGCTGCAGCAGAGGGTAAAG

ATCGGGCTAGTGTTTGAACACTAGATTCTTGCATTTTAGTTACCACCAAGGTATGTAAACGAATAG



>gi|71811458|gb|DR988849.1|DR988849 HDI_D_26.esd Horsegram (VZM-1) drought stressed subtracted cDNA Macrotyloma uniflorum cDNA, mRNA sequence

AAAAGGCTGGAGCTCCACCGCGGTTGGCGGCCGCTCTAGAAACTAGTGGATCCCCCGGGGCTGCAGGAAT

TCGGCACGAAGGCGCATCGTGCCGACACTTCGTGC



>gi|71811456|gb|DR988847.1|DR988847 HDI_D_2.esd Horsegram (VZM-1) drought stressed subtracted cDNA Macrotyloma uniflorum cDNA, mRNA sequence

AGGCTGGAGCTCACCGCGGTGGCGGCCGTTCTAGAACTAGTGGATCCCCCGGGCTGCAGGATTCGGCACG

AGGTGAGTGCATCGGCGCCGCCGTCGGGAACCCGGCCGATGTCGCCATGGTCCGAATGCAGTGCCGACGG

CCGCCTCCCGCCGGCGCAGCGGGCGCACCTACCAAGGTTCCGG



>gi|71811454|gb|DR988845.1|DR988845 HDI_D_17.esd Horsegram (VZM-1) drought stressed subtracted cDNA Macrotyloma uniflorum cDNA, mRNA sequence

ATCATCAGTGAAGCAAGCGATTTGTCTGGATGAAACGAAGCTTCTAGAATATCTCTATATCTGCTGAGAG

CATACTTTCAANCGCCAATAGAAAAATCATAGCGAATTATCTATACGCACGTTCCTAGGACCATTCTTAC

TGCGAAAATGCCCTCCATGAACATGGACCAAGATGATGCTGTGCTCTATGCTCATCTAAAGTGTGTATAT

CACATGTCGTTATTATATTGCATATAAACATTAC



>gi|71811452|gb|DR988843.1|DR988843 HDI_D_15.esd Horsegram (VZM-1) drought stressed subtracted cDNA Macrotyloma uniflorum cDNA, mRNA sequence

AAAGCTGGAGCTCACGCGGTGCGCGGCGTTCTAGAACTAGTGGATCCCCCGGGCTGCAGGATTCGGCACG

AGGGCGCGTAAAACTGACAACGTTTGGAGGAGCGTGTTCCATCCTGGAAGTAACTCTGCCACTAAGACCA

TCGGTGCCCAGATGTTTGACAAACCTCTTCCCAACACTCCCACTGTCTACGATTGGATGTACAGTGGAGA

GACCAGGAGCAGGCACCGCTGAGATGTGTACGAGGGCGCGTGGGTTGCATGTAAATATTGTCGTTTNNTC

GTGAATCGCATGCCACGTTTGCATCTACTACCAATTATGTTTTCAGAAGTTTATTGGTCTGTTACTTGAC

CTGAACATCATT



>gi|71811450|gb|DR988841.1|DR988841 HDI_D_13.esd Horsegram (VZM-1) drought stressed subtracted cDNA Macrotyloma uniflorum cDNA, mRNA sequence

AGCTGGAGCTCACGCGGTGGCGGCCGNTCTAGAACTAGTGGATCCCCCGGGCTGCAGGATTCGGCACGAG

GCCTCGTGCCGAATTCGGCACGAGGAATAAACTGAACTGAAAGCTACATAGCAGCAAGTGGTGATAATTA

AGTACTGTAGACAATTATATACACCAATTAAGCTCTATCTAGCTATATATATATATCTCTCTATATATAT

AGATATATAGAGAGAGACAGAGAGAGAAAACATGTCTAGCAGAAGGTCAAGGCAACAGTCTGCATCAACA

AGGATCTCTGATGACCAAATCATCGACCTTGTTTCCAAGTTGCGTCAACTTGTTCCAGAGATTCGTGATA

GACGCTCTGATAAGGTATCAGCATCTAAGGTCCTACAAGAGACCTGTAACTATATCAGAAGCTTACACAG

AGAAGTGGATGACTTGAGTGAAAGACTGTCTCAGTTTGTTGGCCACCAACTGGATGCTGATAGTCCTGAA

GCTGCTATACATTAGGAGCCTAATTAACTAGTAATAATCAATTAATAAGCACAGTAATGATCTAATTTTC

CTATATTTTAACGGAGATAATATAAGAGTGT



>gi|71811448|gb|DR988839.1|DR988839 HDI_C_96.esd Horsegram (VZM-1) drought stressed subtracted cDNA Macrotyloma uniflorum cDNA, mRNA sequence

CTGGGCTNTTCTGTACATAGTTGTTGCATTCGCACCTCTGACCTGGGACATGCTATTGACTTGCATGTTT

AATAATCTTGTGGGTATCATAAAGTCACGGGATCTGCGTGTAGTGAAGAAAGTGTTTGATGCTTGAGAAA

TGATGACTATGATCTTTATGGTTGTACTTTTTTAGTGGGGTACTTGTATACATTACAGTGGTTTTAAATT

TTTAATGCTATGGTATAATGGTTGGCATATTTATGACAAA



>gi|71811446|gb|DR988837.1|DR988837 HDI_C_94.esd Horsegram (VZM-1) drought stressed subtracted cDNA Macrotyloma uniflorum cDNA, mRNA sequence

ACAGCTGAGCTCACCGCGGTGGCGGCGCTCTAAACTAGTGGATCCCCCGGGCTGCATGATTCGGCACGAG

GCCAAAGGGAAAATCACCTTTGTNNCCAGAGCAGCCTAAATTGGAGAGGTTATTGGGGTGC



>gi|71811444|gb|DR988835.1|DR988835 HDI_C_92.esd Horsegram (VZM-1) drought stressed subtracted cDNA Macrotyloma uniflorum cDNA, mRNA sequence

AAAGCTGGAGCTCACGCGGTTGCGCGGCCGNNTCTAGAACTAGTGGATCCCCCGGGCTGCAGGAATTCGG

CACGAGGTTTTAGTTTTAAGTTATAGGATATTTTTTGTCACAATTCCCATATTAAATATTATACACAATC

TCAAAAAAATTGGTAAGGGCAAGATAACTANTGTTAACCACCAAACGGATGCACAAAACACA



>gi|71811442|gb|DR988833.1|DR988833 HDI_C_9.esd Horsegram (VZM-1) drought stressed subtracted cDNA Macrotyloma uniflorum cDNA, mRNA sequence

ACAAGCTGGAGCTCACCGCGGTGGCGGCGCTCTAGAACTAGTGGATCCCCCGGGCTGCAGGATTCGGCAC

GAGGATTTTTCCTGTCAGAGAATTTAATTGAGGATGATTCTGGTGAATCACTTGCGCAGAAGTTTGAAAA

TTATTGTAAAGAAGTGGAGTCCACAGCCATATGGGGAGGACAACTAGAGGTTGGTGCCTTGACTCACTGT

CTAAAGAAGCATATTATGATATTTTCAGGATCCTTCCCAGATGTGGAGATGGGCAAAGAGTATAAATCTG

ATGGTGGTACTGGCTTTTCTAATTCGAGTATCATGCTTTCTTATCATAAGCATGCTTTTGGGCTAGGAGA

GCATTATAATTCTGTGGTCCCAATATGATCAAATGGGGCATATATTATCATCATAGGATGCTTGCATATT

ATGTTCTCTTGTATCATTTTTCTTTACAGCCAGGCACATTTGGCTTTAGGTCTCTTAAAACCCTAGTTGT

GATCTTTGAAATGGTTATAACTGTACATTTCACAACTGTAATTCATATTTTCTGGAAGATCAAGTTCATG

T



>gi|71811440|gb|DR988831.1|DR988831 HDI_C_82.esd Horsegram (VZM-1) drought stressed subtracted cDNA Macrotyloma uniflorum cDNA, mRNA sequence

TAAGCTGGAGCTCACGCGGNCGCGGCGTTCTAAACTAGTTGGATCCCCCGGGCTGCAGGATCGGCACGAG

GGAGAGAGAGAGTGTTGTGTTGTGAGAGAAAGAGAGAGAGTGTGTGTCNNTAGAATATTATCT



>gi|71811438|gb|DR988829.1|DR988829 HDI_C_80.esd Horsegram (VZM-1) drought stressed subtracted cDNA Macrotyloma uniflorum cDNA, mRNA sequence

TCTAGAGGTGATCTCGTCGCTGGCTCAGCAGCTTGAGGATGGGAGGACCCTTNTGATATCATTCAAGGAG

TCAACCTTTCACCTTTGTTCCTCCGATCCTCAAGGGGAGGAATGCAGATTTACGTTAAAAGACTCTTACG

GGTAAGACCATAACCCNTTAAATTTGGAAGCTTCTTAGA



>gi|71811436|gb|DR988827.1|DR988827 HDI_C_72.esd Horsegram (VZM-1) drought stressed subtracted cDNA Macrotyloma uniflorum cDNA, mRNA sequence

AAGCTGGAGCTCACGCGGTGCGCGGCGTCTCTAGAACTAGTGGATCCCCCGGGCTGCAGGATTCGGCACG

AGGCCTCGTGCCGAATTCGGCACGAGGATTTGATCAAACATCGATCGATAAAGACATTGGCAACATGTCG

ATAATTCCACGATCACGTGCTCATTTTATACTGAAAGTACCACCATTGTTTGCAGCTGAAAAGTCGAAAC

AATGATGTGAAGTTATAAAAAAGAACCAACCAAAAATGACAAATGTTATCACTTTAGAATCTGACCACGT

GTCTGTTATCACGTCCTTTCAATTTTTGCATCCGATTAGCAACCAAGGCAAATATGTGCATGTCGGTGAC

GGGCATAATAGCTCCTATCCTATTGAAGCATCGTAACATGGCTGGGTTGCTCTGTGGTTGCCATGAGAAG

TTTCGTGCCCTCCAAAATATGGTCCCATAAAAAATTAGTCTCTATCATGTGGAATGTGATCTATCCGTTC

TCCAGACTCTCGAGGGGGGGCCCGGTACCCAATTCGCCCTATAGTGAGTCGTATTACAATTCACTGGCCG

TCGTTTAACAAC



>gi|71811434|gb|DR988825.1|DR988825 HDI_C_7.esd Horsegram (VZM-1) drought stressed subtracted cDNA Macrotyloma uniflorum cDNA, mRNA sequence

AAAGCTGGAGCTCACGCGGTGGCGGCGCTCTAGAACTAGTGGATCCCCCGGGCTGCAGGAATTCGGCACG

AGGGTCTTCTAAGTCATATAGTGTTGCTGAAAGGGACAGTTACTTGAGCAAAATGTATAATGCGATATAC

AGTAGTGCTAGTAGTGGGGGACCCTGTGCTGGAGGGCTTTTTTGGCAGCTCATGGCTCAAGGAATGGATG

GTTTACGTGATGGGTATGAAGTTATCTTTCAGGACAGTCCTTCAACAACCAGAATTATAGATCAACAATC

CCACAAAATGTCAAGTATTGCTTAGAACACAACCATTAAATTCCCCTACTACCTCCCATGTATGTTACAA

ATATGTTGCTTATATATTCCCCTTACAAGCAGTAATCGTAGGAAAATATGGCGTGGTGTGAACACATTAA

GGGAGTGAAAGAATTATATATATGGGTCCAATGATATATATCAGAACAGTAATTTCTAATCTAAACAGTT

TGAAAATATTTGTTCCTTCCTTATTATATGCGAAAATAAATGTTCATTTCTT



>gi|71811432|gb|DR988823.1|DR988823 HDI_C_67.esd Horsegram (VZM-1) drought stressed subtracted cDNA Macrotyloma uniflorum cDNA, mRNA sequence

AGGAGAGGGGTAATTTAACAATTTCGTGTAATGGTGGGTTGCCATCACAAGCCTTCGAGTACATTAAATA

CAATGGTGGGCTTGAGACAGAGGAAGCNTATCCCCTACACTGCAAAAANATGGTTGTCATGCAAATATTC

CAGCTGAAAGATGTTCGCGATTCAATGTCCTTGATCTCTTGTACAATACATCACCTATGGGATGCTGAGA

GATAGACATTAAAACATTGCGGTTGCATTTAGTATCGAAACAGGTTAGTGTGGCCTNTACAGGTGGTTAT

GGGTATCCGATTACTATGAGAAAGGAAGTTTTACACCAGTTGAAATTTTGCGGGTAAACATTTTACCAAG

GATNNTTCAACATGCTTGTTNCTTGCTGTAGGGGTATGGGAGTTGAAGAATTGGTGTCCCATATGGTCAT

AAGATTCATGGGGAAAAATGGGGTNNAACAGTTGGTTATTTAAGATG



>gi|71811430|gb|DR988821.1|DR988821 HDI_C_58.esd Horsegram (VZM-1) drought stressed subtracted cDNA Macrotyloma uniflorum cDNA, mRNA sequence

CAAGCTGGAGCTCACCGCGGTGCCGGCCGTTCATAGAAACTAGTGGATCCCCCGTGCGCTGCAGTGTAAT

TCGTGCACGAGTGCCTCGTGCCGAATTCGTGCACGAGGGGACAGTGGAGAGTGCGAGTGCTGCCTGGTGT

GGAGAAGTTAGCACAGAGGAATTGGTTGGGTGAAGCTTTATGCATGAAGATAAGGAACGAGTTTGGTACC

CATGACGCGGTGCCACAGTGGATCCT



>gi|71811428|gb|DR988819.1|DR988819 HDI_C_56.esd Horsegram (VZM-1) drought stressed subtracted cDNA Macrotyloma uniflorum cDNA, mRNA sequence

TTGGATCCCCNGGCTGCAGGAATTCGGCACGAGGGAAGAAAGGAGATAGGGTGTGGCAGATAGCTTTTGG

GAGTGGGTTTAAATGTAACAGTGCTGTGTGGAAGTGCAACAAAGCTCAGACCCCCATTGATGGGGCCTTG

GGCTGATTGTATTGACCGTTATCCAGTTCACATTCCTGAGATCGTTAAGCTCTAGGAGGAGAATTGAATT

GAAGAAGGAAAAAAGACCAAAAAGAAATTGGTTATTACTTTGGTTTCAGTAATGAATTAGATGAGTTTCA

TAACCCTTCTCTGCTGTAGCCGTACCACATATCTCATTTGTCAGTCATATTGGTCATTTTACTATATCAT

ATCCCACTTTTTATATAAAGAAAAAAAAAAAAAACTTCGAGGGGGGGCCCGGTACCCATTTTTCCGC



>gi|71811426|gb|DR988817.1|DR988817 HDI_C_46.esd Horsegram (VZM-1) drought stressed subtracted cDNA Macrotyloma uniflorum cDNA, mRNA sequence

AAGCTGGAGCTCACGCGGTGCCGGCCGTTCATAGAACTAGTGGATCCCCCGGGCTGCAGGAATTCGGCAC

GAGGCCTCGTGCCGAATTCGGCACGAGGTGTTTTTTGATGCAGCAGTAAAGTTGGCCGTGAAGGTTAAGG

GAGAGAGCAAGGATCGAACTGAAGGATTTT



>gi|71811424|gb|DR988815.1|DR988815 HDI_C_44.esd Horsegram (VZM-1) drought stressed subtracted cDNA Macrotyloma uniflorum cDNA, mRNA sequence

ATATACTATGGCATCGGTGATCGAACCTAATTACTATTTCTTTTGGATATTCATCATCCTGTACTTTTGC

GTTTCTGTGTTCACTATATTAATGGATGATACATTAACTGAATTAGCTGCTGAATGTTCTGTTTGCTTTG

TTTCCCACGGGGGGGGCCCGGTACCCAATTTCGCCTATAGTGAGTCGTTATTACCATTTCCCTGGCCGGT

C



>gi|71811422|gb|DR988813.1|DR988813 HDI_C_36.esd Horsegram (VZM-1) drought stressed subtracted cDNA Macrotyloma uniflorum cDNA, mRNA sequence

GGGTGGCAAGAAACAGGACGAAATTAGCTACTACATACCGTGAGCTACAGTGTGTGGATTTCCAAGTACT

NTGACGTGGATATTCTTATACCCTTATAATCTACTATTATACCCAATACCCTTTCGGGT



>gi|71811420|gb|DR988811.1|DR988811 HDI_C_34.esd Horsegram (VZM-1) drought stressed subtracted cDNA Macrotyloma uniflorum cDNA, mRNA sequence

AAGCTGAGCTCACGCGGTGGCGGCGCTCTAGAACTAGTGGATCCCCCGGGCTGCAGGAATTCGGCACGAT

ACGGAAATAGAAAGAAGAGGGTAAGAGAGAGTGAGGTAAGGTGGTTTTCGTTGGTGCAGATCGATGGCGG

CGAGTAGAGCACTTTACCTCTCATCAAATACCTGAGAGTGCGTGTGGACGCTCTTCCCCGGCAACTCTCC

CCCTCAACCTTCGTTCCCTTTTTTCT



>gi|71811418|gb|DR988809.1|DR988809 HDI_C_32.esd Horsegram (VZM-1) drought stressed subtracted cDNA Macrotyloma uniflorum cDNA, mRNA sequence

GGAAGGTGCTGAGGGGGAGAAAAAGAAGAAGGAGAAGAAAAAAGAACATGAAGATGGCCATGGCCATGAC

AGCAGCAGCAGCAGTGACAGTGATTAGATATCACTGCTGCATCACCTATATATATTAACCGGTGTATGGA

CCATAGTAGTGATGGCTGTGACTTCTTTTTTCTTTCTTTCCTTCTTGTGATTTGCATTTATGAGTGAAAA

ACAAAAAAATGAGAGAAAAAGTGTGCGTGTATCGTGGTTGGTAATTGGAGTGTTATATGTGGTTTGGTTG

GTTGCCCACTACTTTGGTTGTAAGATTATCTTAATGAAAATAAAAGTTTTAAATTATCAGTATTAGTNAT

TA



>gi|71811416|gb|DR988807.1|DR988807 HDI_C_23.esd Horsegram (VZM-1) drought stressed subtracted cDNA Macrotyloma uniflorum cDNA, mRNA sequence

CTGGAGCTCACGCGGTGGCGGCGCTCTAGAACTAGTGGATCCCCCGGGCTGCAGGATTCGGCACGAGGGC

AAAATCTTAGAAATAAGGGAATCATTGATAAACCCGGGCACA



>gi|71811414|gb|DR988805.1|DR988805 HDI_C_21.esd Horsegram (VZM-1) drought stressed subtracted cDNA Macrotyloma uniflorum cDNA, mRNA sequence

AGCTGAGCTCACCGCGGTGGCGGCCGCTCTAGAACTAGTGGATCCCCCGGGCTGCAGGAATTCGGCACGA

GGGCAATCTACAACTGGTATCGCAATGATGACGGCAAGAGAGTACCAGCTGATTCTTTGATATTGCTTAA

CCAGTATTGGGGAAGCAATGAAGGAAAACAGAGTGCGAGTTATTGACGAAGTTGGCCATTCAATAGGAAC

TTGGAGCACTCGTAAATCCGGT



>gi|71811412|gb|DR988803.1|DR988803 HDI_C_19.esd Horsegram (VZM-1) drought stressed subtracted cDNA Macrotyloma uniflorum cDNA, mRNA sequence

AGCTCACGCGGTGGCGGCGCTCTAGAACTAGTGGATCCCCCGGGCTGCAGGATTCGGCACGAGGCCTCGT

GCCGAATTCGGCACGAGGCCTCTACAAGCATCTAAAATCAAGCAAAGGTGGACTCTTCGGGGATAATATC

AAATGGAACTTCTCCAAGTTCTTAGTTGATAAGGAAGGGAAGGTTGTTGATCGCTATGCACCCACAACTT

CTCCTCTGAGCATTGAGAAGGACTTGCTGAAGTTGTTGGATGCATGAAGCTATGAATGACTGCATCTGTC

AATAAATGAAAACATTATTATGAATAAGAAATGTATTGCAGATGTTGTTGCACTTCCCTGTCTTTCTTAC

ATGTACTGAAACAGTTGCACTTGTAGCTGATATTATGTTCATAATCATAACATTGTGTAGTAAACTTAAG

CTTCCTATTTCATCGGTTCGCATCCTGCTCTTATCACTTTGACTCAAATTTTTCATTTGGTCTATTTTCT

TGTC



>gi|71811410|gb|DR988801.1|DR988801 HDI_C_11.esd Horsegram (VZM-1) drought stressed subtracted cDNA Macrotyloma uniflorum cDNA, mRNA sequence

ACAGCTGAGCTCACCGCGGTGGCGGCCGCTCTAGAACTAGTTGGATCCCCCGGGCTGCAGGAATTCGGCA

CGAGGCTTTCTTTGCNGAGATTTCAGTACC



>gi|71811408|gb|DR988799.1|DR988799 HDI_C_89.esd Horsegram (VZM-1) drought stressed subtracted cDNA Macrotyloma uniflorum cDNA, mRNA sequence

AAGCTGGAGCTCACGCGGTGCGCGGCCGCTTCTAGAACTAGTGGATCCCCCGGGCTGCAGGAATTCGGCA

CGAGGTTTAGTTATATCAATTGTATATGAATAATATGAATAAATGGATTGTTTATTTCATTTCAGCAAAA

ACAAAAGAAGAAAAGATTCGAACCATTGCAAGTTAATTCCCAAATNGTATGAAAAAGGAAAAAAGAAGGT

TAGGGTTACCTTAAGAAAGAAGAAGAGTTAAGAATANTGTGGTCTAATAATCACGTGTGTAATAATGGAA

TTTAAAATAGAATAGATAAATAAGAATCGAGTTCTGAATTCAGAGCCAAATAANGCAAGGTAATCAAGTA

TAGAATGAAATTCAAATCANNTAAAAATAGGAGAAAAGGGTTTTTGCAATATTGAATATACATACATATG

TATTTTAATTGAATTCACAAAGTTTCCCGCATAAATTTTCTTCCATAAATT



>gi|71811406|gb|DR988797.1|DR988797 HDI_C_87.esd Horsegram (VZM-1) drought stressed subtracted cDNA Macrotyloma uniflorum cDNA, mRNA sequence

AAAGCTTGGAGCTCCACCGCGGTGCGCGGCCGCTCTAGAAACTNAGTTGGATCCCCCGGGCTGCAGGAAT

TCGGCACGAAGGCCATCGCGTCGCCGGACATTCCGTGCTGACAGATNGGATACGCCGCTGAGGAATTGAA

TTTGCGACTTTA



>gi|71811404|gb|DR988795.1|DR988795 HDI_C_85.esd Horsegram (VZM-1) drought stressed subtracted cDNA Macrotyloma uniflorum cDNA, mRNA sequence

AAGGCTGGAGCTCACCGCGGTGCNCGGCCGTTCTAGAACTAGTGGATCCCCCGGGCTGCAGGAATTCGGC

ACGAGGTGGAAAAGTTTTGGACGTACTAGGATTATTATCCTCTAAATCCTATGCTGAGGTTCTGCAGCAG

TTGAAAGATAAAGCATTGACGAGAGCAGGTCTTGGCCAAGAAACGCCCTGAAAGTT



>gi|71811402|gb|DR988793.1|DR988793 HDI_C_76.esd Horsegram (VZM-1) drought stressed subtracted cDNA Macrotyloma uniflorum cDNA, mRNA sequence

AAGCTGGAGCTCACGCGGTGCGCGGCCGNTCTAGAAACTAGTGGATCCCCCGGGCTGCAGGAATCGGCAC

GAGGCCAAACCCTAAACCAAATCCTATTTCTTTCATTCGCAGACTCGTTGAGTTGTTTCCATGGCTAGCC

TCANGCTTTGCATGCGTGCTTGCCCTGCTGTGCTTGGTGGTGATGACAGCACCCACAGCACGTGCAATCT

CGATGCGGGCAGGTCACCACTGCACCTCACTCCATGTCTCTCTTTTCTCACAAAAGGTGGGCCAAGTG



>gi|71811400|gb|DR988791.1|DR988791 HDI_C_74.esd Horsegram (VZM-1) drought stressed subtracted cDNA Macrotyloma uniflorum cDNA, mRNA sequence

AAGCTGGAGCTCACGCGGTGGCGGCCGCTCTAGAACTAGTGGATCCCCCGGGCTGCAGGAATTCGGCACG

AGGTGGACCCTGGTGTTCTTTTTGGTTCGAAAGAGTTCATCACTGATCGGATAAATGATACTGTGAGAAA

AGCAGGTAAGGGAAACATATCTTGAATCTTGGTCATGGCATTAAAGTAGGTACGCCTGAGGAGAATGTTG

CACATTTTTTAGAGAGGTTGCTAAACTCAGTCAAATACTACGCAGCTAA



>gi|71811398|gb|DR988789.1|DR988789 HDI_C_66.esd Horsegram (VZM-1) drought stressed subtracted cDNA Macrotyloma uniflorum cDNA, mRNA sequence

TGGGAAGCGCTTCGGGGGGGGTTATTTTGGGCCTATTTTCTCCTCTATGTCTTGGGACCCTTTCTTTATA

CTTCAAGTGAAGGGGCCTCTTTTTCTTNCTGTTTCTTCTTTTTATTTATAATTCTCTATGGAAATGGACA

TAGAATTGAGGGTTATTTGTGCACTAGAGATGCATGTAAATGATGATGTATTTTATGAACTCTTCAACAT

CTTACACAATGCTNAAAAAAAAAAAAACAA



>gi|71811396|gb|DR988787.1|DR988787 HDI_C_62.esd Horsegram (VZM-1) drought stressed subtracted cDNA Macrotyloma uniflorum cDNA, mRNA sequence

CAAGGCTGGAGCTCACCGCGGTGCGCGGCCGTTCTAGAACTAGTGGATCCCCCGGGCTGCAGGATTCGGC

ACGAGGCCTCGTGCCGAATTCGGCACGAGGGTGAATCGTACTGACTACCAGCTAATTGATATCTCTGAGG

ATGGATTTGTGAGTCTGCTTACTGAAAATGGGAACACTAAGGATGACCTCAAGCTCCCCACTGATGATAA

TTTGCTGGCTCAGATAAAGGATGGATTTGATCAGGGCAAGGACCTTGTTGTGTCTGTCATGTCTGCTATG

GGTGAGGAACAGATTTGTGCCCTGAAGGATATTGGGCCAAAGAACTAGCTTTTGGTGCTGGCTGTTTGTT

GTTTCTATTTAAGCAAAAATATTTTTGTAAACCTTTATATTGCTTTGTTCAAGTCCTGGCTTATAGATTC

TAGTCAGACTAGTCTTAACAATGGTGTTTGTTGTGGATGTGTTGACNGAAACTATATCACATTTTCCTGG

GCT



>gi|71811394|gb|DR988785.1|DR988785 HDI_C_6.esd Horsegram (VZM-1) drought stressed subtracted cDNA Macrotyloma uniflorum cDNA, mRNA sequence

ATAGCCTGCATGGCAATACTATGTATCCTGAAGTCCAAACTGCAGTCACAAGCTAATAAAACCACACCAC

GAGTCGCTACAGAGAGCGTTGAGAGAGAGTATCTTGAATACAATGATCCTAAAACGGTATTTCCGGGAGA

GGCTTGTGATGATCTTGGAGGATCTTTCTGTGAGTACGAGTATCAGAAGAGTGTCTTCTAGAGCAAACAA

CACTTGTATTGTCATGCCTACTGAATTTTCCATATCTACCCCTATTAATTGTAGTGTTGGTTGTACATAG

GTATATAGACTATGTGGAGTTTCATTTTCTCTATAAATTTTACAATAATTATACTCTAAATTTGAAAGCT

TCCCGTCTAGACTAGAAATGAAATAAATAGCAAAGGGAGCCATTTTCACT



>gi|71811392|gb|DR988783.1|DR988783 HDI_C_53.esd Horsegram (VZM-1) drought stressed subtracted cDNA Macrotyloma uniflorum cDNA, mRNA sequence

AAGCTGGAGCTCACGCGGTGGCGGCGTNTCTAGAACTAGTGGATCCCCCGGGCTGCAGGATTCGGCACGA

TACGCCTCGTTGCCGAAATTCGGNNAGTTATTCCATATAAACCATCTCCACACAGACCCCATTCAAAGAA

ATTTCCATTATCTCACTATTTCAAAA



>gi|71811390|gb|DR988781.1|DR988781 HDI_C_51.esd Horsegram (VZM-1) drought stressed subtracted cDNA Macrotyloma uniflorum cDNA, mRNA sequence

AAAAGCTGGAGCTCCACGCGGTGCCCGGCGTTTTAGAACTAGTGGATCCCCGGGCTGCAGGATTCGGCAC

GAGGCAAAAATGTACCTACCTGGCATCGTGATCTTTGCAGTGTCTGTGAAAACA



>gi|71811388|gb|DR988779.1|DR988779 HDI_C_49.esd Horsegram (VZM-1) drought stressed subtracted cDNA Macrotyloma uniflorum cDNA, mRNA sequence

CAAAGCTGGAGCTCCACGCGGTGGCGGCGCTCTAGAACTAGTGGATCCCCCGGGCTGCAGGATTCGGCAC

GAGGCTTGGTCTTCATTCCCTCCGTCAGCGTCACTGGTATATCCAGAGCACATGTGCCACATCTGGTGAA

GGGCTCTATGAAGGACTTGACTGGCTCTCAAACAACATTGCCAACAAGGCATAGAGATATATTAGTTAAG

GATGCGAGGAAGTTGAGTTCACTTCGGCCATCGCTCGTTTCTGATTTTCTTGCTCTTAGATTTTCATTGT

TCAGGCATGTATTATTTTTCTTCAAAAAGAACAGCGGTCTGTCACTGAAGAATAATTTGAATTTTTTTTT

CCATGTATAACCAAATAGCATTGTTTACTATTTGATTTGTGAAATCAATTGAATGATATAAAGTCTCGTA

TTATGCGCAAAAAAAAAAAAAAAAAAAAAA



>gi|71811386|gb|DR988777.1|DR988777 HDI_C_40.esd Horsegram (VZM-1) drought stressed subtracted cDNA Macrotyloma uniflorum cDNA, mRNA sequence

AAAGGCTGGAGCTCACCGCGGTGCGCGGCCGTTCTAGAACTAGTGGATCCCCCGGGCTGCAGGAATTCGG

CACGAGGGCACATGTGGAGACATGTGCTAGGACTGACCCAACACGAGAAGTAGTTCTCTCTTTACACAAC

ATATACGCTTTGTCCTTAGACCTGAGATTGTCGTGTACTCAAGAATGTGGATCGANTTACTGGGGG



>gi|71811384|gb|DR988775.1|DR988775 HDI_C_37.esd Horsegram (VZM-1) drought stressed subtracted cDNA Macrotyloma uniflorum cDNA, mRNA sequence

TTGGTGAAAACAGAATTCCGGGTTTAATGTAGTAAATATGTTTATTGTTGTAATAATGAATAAGGACTTA

TAAACTACTGTAGAAATGTTTTTTATTTTTCAAAAGCTTCAACAGTTTGGTGCCATGGACTACCTACAAC

TTCTCTGTCTTACCTTCAATCTAAAAGAAAAATAAATTATATTAGAGATCCTGTTATAGCTGTTTTTCAT

AAAAAACAAAACAAAAAAAACNCAAGGGGGGGCCCGGTTACCCATTTCGCCC



>gi|71811382|gb|DR988773.1|DR988773 HDI_C_3.esd Horsegram (VZM-1) drought stressed subtracted cDNA Macrotyloma uniflorum cDNA, mRNA sequence

AAGCTGGAGCTCACGCGGTGGCGGCGCTCTAGAACTAGTGGATCCCCCGGGCTGCAGGAATTTCATACGT

TAGCCGCATCCCTTTCACAAAAGCTGGAAATGATGGTGGCGAAAGCAGAAGCAGATGAGAGAGACCAGGT

ATGACAACCACGGAATGCATTTTTCTGGCAGCGGGCTTCATATTCTGTGTGCTTATGCTTGCCGACATGG

GACTTGTTCAATGACACCTCAGCAGGAAAACGCCCTTCGCAGCATTGCCCGTCAGGCTAATTCTGAAATC

AAAAAAAGCCAGACAGCAGTTTCCGGATAAAAACGTCGATGACATTTGCCGTAGCGTACTGAAGAAGCAC

CGCGAAACGGTAACGCTGATGGGATTCACACCGACTCATTTAAGCCTGGCAATCGGCATGTTAAACGGCG

TCTTTAAGGAACGATGAACATGAAAAGCAAAATCATCAGGGAGCTACAGGCTCCTTTTTTATTATTCGCA

TTCACCCTCAAGCGTATTAACCAACAGTTCAGGGAATTAATGAAAGATGGCAGACATCATTGATTCAGCA

TCAGAAATAGAANGAGGGGGGGCCCGGTAACCCAATTTCGCCCTATAGTGAGTCGTATTACAATTCACTG

GCCGTCGTTTACAA



>gi|71811380|gb|DR988771.1|DR988771 HDI_C_27.esd Horsegram (VZM-1) drought stressed subtracted cDNA Macrotyloma uniflorum cDNA, mRNA sequence

AGCTGAGCCACGCGGCCGCGGCGTTCATAGAACTAGTGGATCCCCCGGGCTGCAGTGAATTCGGCACGAG

GTATAAGTANCTCAAATTTTGTTTCTTTCATAGTTTATGTTCAACTGTTTTGCTACTATATATTAGAGAC

ACAATTTCTCACCATAAATATGAAGCTTTA



>gi|71811378|gb|DR988769.1|DR988769 HDI_C_2.esd Horsegram (VZM-1) drought stressed subtracted cDNA Macrotyloma uniflorum cDNA, mRNA sequence

ACAAGCTGGAGCTCACCGCGGTTGGCGGCCGTCTCTAGAACTAGTGGATCCCCCGGGCTGCAGGATTCGG

CACGAGGGCTTCTTCAATGATCTCCTCCCCAGCTATTACCACCGTTAACCGTGCCGGCGCCGGCATGGTG

GCTCCATACACCGGGCTCAAGTCCTTGGGGTTGGTTTCCCAACCAGGGAAGACCAACAATGACATTACTT

CGGTTGCAAACAACGGTGGAAGAGCACAATGCATTCAGGTGTGGCCTGACAGTTGGGAAGANGAAGTTCG

AAACTCTTTCTTACCTGCCAGACCTCACTGACGCCCAATTGGCAAAGGAAGTAGAATACCTTCTCAGGAA

TGGATGGATTCCTTGCTTGGAATTCGAGTTGGACGCACGGTTTCGTGTACCGTGAGCACAACAGGTCACC

TGGATCATATTGATGGAAGGTACTTGGACCATGATGCGANACTGCCTA



>gi|71811376|gb|DR988767.1|DR988767 HDI_C_17.esd Horsegram (VZM-1) drought stressed subtracted cDNA Macrotyloma uniflorum cDNA, mRNA sequence

AAGCTGGAGCTCACGCGGTGGCGGCGCTCTAGAACTAGTGGATCCCCCGGGCTGCAGGATTCGGCACGAG

GGAAGTAGTGGAAGCTAAGGCTGGAAAGGGTTCTGCAACTTTGTCAATGGCTTATGCTGGTGCCCTTTTC

GCTGATGCTTGTCTGAAGGGCCTCAATGGGGTCCCAGATGTTGTTGAGTGCTCATTTGTCCAATCCACTG

TCACTGAGCTTCCCTTCTTTGCTTCCAAGGTGAGGCTTGGGAAGAATGGTGTGGAGGAAGTTCTGGGCTT

GGGACCTCTCTCGGATTTTGAGAAACAAGGCCTAGAGAACCTTAAGGGTGAACTCAAATCATCAATTGAG

AAGGGAATCAAATTTGCCAACCAGTAATTGAACATGTCACATACATGTCTGGTAGGTTACTGGATATTCC

TTTAAGAACCAAATCAAATTTTGCAATCTCAGAACCATTGTTGTATTGTTGCCGGCAGTGGTGGCGGCTT

GGTTGTTTTGTTTAGAGTTAGGTATACGCCATGGTTTAATAATTCAAGTCTGAGAGCAGTTTGTTAAGGG

ATTGATTCAATCTTCAAACCAGTTTTAGGTTCTGGCCATTTCATTAATAATAGTCTCTTTTAACTGT



>gi|71811374|gb|DR988765.1|DR988765 HDI_C_15.esd Horsegram (VZM-1) drought stressed subtracted cDNA Macrotyloma uniflorum cDNA, mRNA sequence

AAGCTGGAGCTCACGCGGTGGCGGCCGCTCTAGAACTAGTGGATCCCCCGGGCTGCAGGATTCGGCACGA

GGGACAAAATGTCTCCTCCAGAGCTTGTTAAACAGTTTGAACAAGTCCTAACTGCTAAACCTGAGGTAGA

TTGTTTTGTTAAAATATTTCCGAAAGTTTCTCATGGTTGGAGTGTGAGGTACAGCAAAGAAGATGCAGAA

GCTGTTCAGGGCTGCAGAGGAGGCCCATCGAGGACTTGCTGAAGCTGGTTCTGCTAAACATCTTAAGTGA

AACAATCTTGTTCATGCTAAGAACATGTACTTGTTTGGAAAACAAAAATAAAACAATGTGGATTATCATC

TTTTTCTGCAGTGGAGGATGCAAACTTAGAAGAAAATATGTATCTTTGCTTGTTGGAATATGCTTCACAA

AAAAAAAAAAAAAA



>gi|71811372|gb|DR988763.1|DR988763 HDI_C_13.esd Horsegram (VZM-1) drought stressed subtracted cDNA Macrotyloma uniflorum cDNA, mRNA sequence

AAGCTGGAGCTCACGCGGTGGCGGCGCTCTAGAACTAGTGGATCCCCCGGGCTGCAGGATTCGGCACGAG

GGATACTTTTTATGTCACAGATGTTACTGGTAACCCAGTTGACCCCAAGATTATAGATTCAATTCGAAGA

GAGATTGGTGACAAAGTACTGAACGTAAAACATAACTCTAGTCTTTCACCAAAGGCCCCCTCAACCCACA

ACAATTTGGATTTCTCTTAGGAAATTTTTTCAAAGCTCGATCTTTTCAGAATTTTAAGTTGATCAGATCT

TATTCTTGAGTCCTCATTTGTACATTCTCAGCATTTTCATTGTGTTACTATCAAGTCAAACAGAAATTTA

TGTATGCATGTTAAATAGATTGTACATATCAATGTTCCTCCCCAGCAGAGGGGCTTGGAATTTACACCGT

ACAGTAGAAAGCTTAAACACACTCATGTACATAGCTTAGTGTAAATAGTTGTGTTCAGTAGCATTTNACC

TAGGTTGAATATGAAGCTAGTAGTCCTTTTAGCGATATCAAGT



>gi|71811370|gb|DR988761.1|DR988761 HDI_B_96.esd Horsegram (VZM-1) drought stressed subtracted cDNA Macrotyloma uniflorum cDNA, mRNA sequence

CGGCCGTTCTAGAACTAGTGGATCCCCCGGGCTGCAGGATTCGGCACGAGGCCTCGTGCCGAATTCGGCA

CGAGGGAAACAGCGAAGAAGGAGAAGAAGGTGACAAAGGTTCTGAACGCGGAGAAAAAAGAATCGCGTCC

GAAACGAAGCAGAACAGCAACCACCGCTGCTCCGAAGAGCAAGAAAACTGAGGCGGTAAAGAAAACCGAG

GCAATTAAAAAGCCCGCGAAGAAGGTTGGGCCGAAGAAGAGTAAGAAGGTGTCGACGCCGGCAAAGCCCA

AACAGCCCAAGTCCATTAGGTCTCCGGCCAAGAGGGCCCGGAAGGCTACCAGCACTGCAGCTTGATGTTT

CATATTTTCACTTGTTTAGTGAAAGTCTGTTATTCGAGATATATATATGTAGATAAGTAAAGGTGTGTGT

TGTGTAGCTTGGTGCACAAGCTTGAAGAGAGTTGTGAAGTGTAGGATAGTATTTAGGGCTATGCAGTGTT

CTGTGTCCTAAGTGATCAAATGAACATGAGTTTTTACTGTTTT



>gi|71811368|gb|DR988759.1|DR988759 HDI_B_94.esd Horsegram (VZM-1) drought stressed subtracted cDNA Macrotyloma uniflorum cDNA, mRNA sequence

AAAGCTGGAGCTCACGCGGTGGCGGCCGCTCTAGAAACTAGTGGATCCCCCGGGCTGCAGGAATTCGGCA

CGAGGAACCATTGCCACTAAGATTGGGGCTTTCAACGCCCGCAACGTAGCTTCACTCCCAGGCAAATGTG

GCGTCTCAATCCCCTACAAGATCAGCACCTCCACCAACTGTGCTACCATCAAGTTCTGAAAGATGGATGT

GGTATAAGGAAGAAAAAGGCACGAAGGGGAAATTTTCAGTAGCAGAATATTACTATATATGATGGTAACG

TAGAATTAGGTTATGAAAAATAAAGGGTGGTCGACGAGGACTCCACTTGTGCGAGTCTCTCTTGGTGTCG

GTGCCTCCCTTAGTTGTTTCAGACTTTGGTCCACTTATGTTATGTCTTCTGAAATTTGTATCAGTTGGTT

GTTGCATGTAATAATGATATATATAGTGCTGTGATTCCTTGTTTAAA



>gi|71811366|gb|DR988757.1|DR988757 HDI_B_92.esd Horsegram (VZM-1) drought stressed subtracted cDNA Macrotyloma uniflorum cDNA, mRNA sequence

CATGGACAAACAGACAGAACCTCCGACGATGAGACAAAGACCCACCAAACCACAAAGGATACTATATAGG

CGAAGTGAACGATGGCGGTCCTCGACCGGCGACGCGCTGGCTAGCGTTCGCGTTTGAGCACGTGGCGACG

GCGACGGAAGGCGTGCCCCCGGACGTGCGTGGTTACGCGCGATTGGGGAGTTGGCGAAATCGATCGGCGG

GGAAGGGCTTGTGGCGGGACAGGTGGTGGATTTAGATTCGGAGGGGGTGTCGAATGTGGGGTTGGAGAGG

TTGGAATTCATTCATGCGCACAAAACGGCGGCGTTGCTGGAAGCTGCGGTTGTCGTTAGGGGGCAATAAG



>gi|71811364|gb|DR988755.1|DR988755 HDI_B_9.esd Horsegram (VZM-1) drought stressed subtracted cDNA Macrotyloma uniflorum cDNA, mRNA sequence

TGGAGCTCACCGCGGTTGGCGGCGNTCTAGAACTAGTTGGATCCCCCGGGCTGCAGGAATTCGGCACGAG

GAGCAGGAGCTGGTGCAACCACAGCACAGGATGATGATGACGATGATGCGGTCCCTGATCTTGTCCCAGG

GGAGACTTTTGAGACAGTTGCTGAGGAGACTAAGTCTTAGAATTTTTTTACTGTGTGGTCTTTTATATAT

TTTGGATGCTTTTAAGTTTTTTTCTTTCATTTTTGCCCTTTTAAATTTGAAAGTTTTTCAGGTGTTGTTT

ATACTTTATATTGTTAAAACACACTTAATTCTGGGCCCGTCATCTAACTAGAAACTGTTACTATCTGACT

GCACGAGTTTGTGAATTGTATGTTCATTTTTGGAGGTAGAAATCAATTTTGTTTAGTTATTTTTCACCAT

CCAAAAAAAAAAAAAAAAAAAAAAA



>gi|71811362|gb|DR988753.1|DR988753 HDI_B_83.esd Horsegram (VZM-1) drought stressed subtracted cDNA Macrotyloma uniflorum cDNA, mRNA sequence

AAAGCTGGAGCTCACGCGGTGGCGGCGCTTCTAGAACTAGTGGATCCCCCGGGCTGCAGGAATTCGGCAC

GAGGCCTCGTGCCGAATTCGGCACGAGGGCTCGTTGCAGATCGTATTGATGGACTCTGGGTATCTGGTGG

TCGAGTTCTCATGAGTCGTCCTAGAGCAAGATTAGGACTTATTGGTTATTTGCTTATCTTGCATATATGG

CTACTGGGGACGATCTTGTAGAAAAGTTGTGATCAATCCTACCTGAACTGCTCAACATTGAATGTCCCGC

ATCTGCAAATTGTATTATAGATATAAAATTGTCTCCTTTTATTCATGTCTTTCTTTTAGCTTCTTAAGAA

AGCCTAACCTGTTTTTTTCTAGTCTCGTCAAATCCAATATTTGTTCTCTGCAATCACATTTTAGGGATTC

AGAGATTCTGGTCTATAGTGTTTGGGCTCATTTTTTTGACAGCTTTTTTAGAAGAAAAAAAACGAAGCCC

CTCCATTGTATTGACCTACAATTGTTTCAATATTAAAGTATGTGGTGGGTAGAACGGTAGGTAGCATGAT

TTTTTGTGCATGCCAATGTACTACACAACATGTACGAACAGATT



>gi|71811360|gb|DR988751.1|DR988751 HDI_B_81.esd Horsegram (VZM-1) drought stressed subtracted cDNA Macrotyloma uniflorum cDNA, mRNA sequence

ACGTGTANTGTGCGATGTTGTGACGATATAACTATTTGATGTGACATATCTTGGATTAGGATGCTAATGA

TACCTTCTTCATCCATCTTAAGTCCTCTTCCATTGTCCTTTGCTAACACCC



>gi|71811358|gb|DR988749.1|DR988749 HDI_B_8.esd Horsegram (VZM-1) drought stressed subtracted cDNA Macrotyloma uniflorum cDNA, mRNA sequence

AAAGGCTGGAGCTCACGCGGTGGCGGCCGCTCTAGAACTAGTGGATCCCCCGGGCTGCAGGAATTCGGCA

CGAGGCCTCGTGCCGAATTCGGCACGAGGCCGCAACAAGGGTACCAGCAACAACCGGGAATATGGGTATC

AACAAGCCCAGAAACCAAAGAAGAACAAGTTTGGAATGGGGTTGGGAGCCGGATTGCTTGGTGGTGCCTT

GGGTGGCATGTTGATTGGAGATATGGTATCTGATGCTGCGGATTATGATGCGGGCTATGATGCTGGATTT

GATGATGCTGGTGGATTTGATTTTTAATCTATTTTTGTTTAGTTAAGCGTATGTTTATGGATTCTGGATT

TCACTGTTCCTTTTTTTCGGTTGAGTACATCGATGTTCTTACTGACGTTGTNTATTCAACAACACCAAAC

AACC



>gi|71811356|gb|DR988747.1|DR988747 HDI_B_72.esd Horsegram (VZM-1) drought stressed subtracted cDNA Macrotyloma uniflorum cDNA, mRNA sequence

AAAGCTGGAGCTCACCGCGGTGCGCGGCCGTTTCTAGAACTAGTGGATCCCCCGTGGCTGCAGTGAATTC

GGCACGAGGCCTCGATGCCGAATTCGGCACGAGGCCACCGTACCATTTCCATCATCAATCACTTCCATCA

TTCACCCTCACTACATTGACTCTCCAATCAGACGGCATTAAAATGACCAGCACTGATTGATAATATATAT

ATATATATATATATATATATANATAT



>gi|71811354|gb|DR988745.1|DR988745 HDI_B_70.esd Horsegram (VZM-1) drought stressed subtracted cDNA Macrotyloma uniflorum cDNA, mRNA sequence

AGCTGGAGCTCACGCGGTGCGCGGCCGNTCTAGAACTAGTGGATCCCCCGGGCTGCAGGAATTCGGCACG

AGGAGACTTCGAGCTTAAAGATGCAAGGGATTCCGAAGAATTTATTTCAGTTGGCGAAAAGAATAGTGTG

GATGGTTGGCACATTATTCAGTTTTCTGGAGGAAAAGATGCACCGAAATTGTTTGATCTGACTCTTTACT

GGAGGTCAGGTTCAATTCATAACTCTGATTCTCCTCTTCTGAAACTTAGGACTGATGTGGACAGATTAAC

ACCAATAACCGAACGAGTCCTTATGAAGCTTCCTCGTTGGTGTTCTTTGTTTGGCAAGTCTACCTCTCCT

TATACCTTTGCTTTCTTAAGAAATCTGTATGTTAATTTTTAGGTACTTGAAACTGTGACCTTCATTCATA

GAATAGGTCTTAAAATCTTCATATAGCAGGAGCACTAATTCTTGATGCCAATTTTGTCCTATCTACTGTG

AAAACA



>gi|71811352|gb|DR988743.1|DR988743 HDI_B_68.esd Horsegram (VZM-1) drought stressed subtracted cDNA Macrotyloma uniflorum cDNA, mRNA sequence

AAGCTGGAGCTCACGCGGTGGCGGCGCTCTAGAACTAGTGGATCCCCCGGGCTGCAGGAATTCGGCACGA

GGCGGATTTGCTGTGAGACAGAATCAGGATATAGCCATGCAACCTGCTGCCGCTGCTATTCAAGGGGCTT

CCTGACGGGGTTAGCAGAACTGTGTGTTGAAGTAGGATGGATTTTATTGCTCCCCATTAAACTGTAGTAT

TATTTTTAAATTTGTAGCTGGGGGTTATACGGTATGCGAGGTTCTTAATTAAGATTGTATTAAAATGTCT

GAAATTTTGGATTGAACCCGACCATATATATGACTCGGGTATTTTTGTTTTTTTAAATCCTTAAAAACAT

ATTGGTAGCACAGACAGCTAAGATTATTTT



>gi|71811350|gb|DR988741.1|DR988741 HDI_B_60.esd Horsegram (VZM-1) drought stressed subtracted cDNA Macrotyloma uniflorum cDNA, mRNA sequence

AANAGGCTGGAGCTCACCGGCGGGCCGGCCGTTCTAGAACTAGATGGATCCCCCGGGCTGCAGTGATTCG

GCACGAGGAAGAAATATGAGGAAGGTAGGTTCGATTTCCAAGGTGCAGCTTTGTCATGGCACCATTGGTT

GTGTTACTCATAACCAACATTGTGGGTTTCTTCTGGTGGGATATGGAGAGTAGTGCATGTGAAAGATTTT

GAAGAAATGTTTGGTCAACTTTTCCTAGTCACCTATGTAACTGGTTCTCAGATTATCCACATTCTTGAAG

GCCATAATAAA



>gi|71811348|gb|DR988739.1|DR988739 HDI_B_58.esd Horsegram (VZM-1) drought stressed subtracted cDNA Macrotyloma uniflorum cDNA, mRNA sequence

CTGGAGCTCACGCGGTGGCGGCCGCTCTAGAACTAGTGGATCCCCCGGGCTGCAGGAATTCGGCACGAGG

CATTGATGAAGATGCGGCTGAGGAGCTTCAGAATCAGATGGAACAAGACTACGACATTGGGTCAACAATT

AGAGACTAG



>gi|71811346|gb|DR988737.1|DR988737 HDI_B_56.esd Horsegram (VZM-1) drought stressed subtracted cDNA Macrotyloma uniflorum cDNA, mRNA sequence

AAGCTGGAGCTCACGCGGTGGCGGCGCTCTAGAACTAGTGGATCCCCCGGGCTGCAGGATTCGGCACGAG

GTGCTAATACTAAAGCTAAAAATGAGATTGTGGAATCCACATTGTCATGGTATGTGAAGGGAGCAAGTGT

GCTGTTGCTTGGGGCATTCATGGGTTACGTCTTGGTTTCGTTACAAGTGCCAAGAAGAAGTCTGAAGAGG

CCAGGAGCAATTGGACACCAGTGAAGACCGAGGAGACTTGAACTGTATATTAATGTTTACCTCCTTTTTA

AGTAGGTCATGACGGTTCACTGAAAAGAAAAACAAAATGAGATTTCCACTCTTTTTTTGTTTAAGACAAC

AAGTTGTCACCTCATACTTGGGACTTGCTTTGTATATATGCATTTCTTTCTTTCTTACTTATGCCATCTA

AGGCGTTCCTTATAGGACCTGTGATGTTGTACAGTATTCATGGTAATGTATGATTTGTGGGATGAGACCA

TCCTATTGTAACAGAAAAAAAAGTTTTAGATGTGAGTGTACTAGTGATACAAAAACTTTTATGTCGGAAA

AC



>gi|71811344|gb|DR988735.1|DR988735 HDI_B_47.esd Horsegram (VZM-1) drought stressed subtracted cDNA Macrotyloma uniflorum cDNA, mRNA sequence

AAGCTGGAGCTCACGCGGTGCGCGGCGNTTCTAGAACTAGTGGATCCCCCGGGCTGCAGGAATTCGGCAC

GAGGAACTGACAATCACTGCCCCCCTCTCATGGGTTCTGCAACTTATAACTTCTCTCCTAATCCTGGCAA

CTACTTTGGGAATGGTTATCAGTACCAAGCTACATATCTCCACTAGTTTAGCATGCAGAGATGGTGAACA

GATGACCAAGACAACACCCCCTTTGTTTAATATTTAGTCTTTAAGTCTTAACCCTTAAGCTGTTGTATGT

TGCACTTTATCTATCTAGTTTTAAGAATCTCTGAAACAGATAAAATGTCATGTTAAAATTTAACATCTGT

ACTAGAAGAT



>gi|71811342|gb|DR988733.1|DR988733 HDI_B_44.esd Horsegram (VZM-1) drought stressed subtracted cDNA Macrotyloma uniflorum cDNA, mRNA sequence

ANGCTGGAGCTCCACCGCGGGTGNGCGGCCGCTCTAGAAACTAGGTGGATCCCCCGGGGGCTGCAGGAAT

TCGGGCACGAGGCTCTATTATTGCGGCTGAGATTCTGAACATTCGTTTCTCCCTAAGTTTAAGTCGTGAT

GGGAAAGGAAAAGGCTAACATCAAAATCGTGGTCGTTGGACATGTCTCTGAGAAATCAACTGCCACGGAG

CTTGGAAGCCCTACTGAGAAGGGTGTGACTGAAAGGTTCGAGAAGGGAGAAGGAAGTGTTTAAAACACGT

AAGATGTAAGATGTGTGAAGAAGGATGATGTAAAATAAATAAGATATAAGATGTGAAGATGGATCTATCT

TAAGATGAAGTGCTGAAGGACTTATATTCAGATAAATAAAATGAAGGATATATGTTTATCTTCTTCAAAA

AAAAAAAAAAAAAAAAAAACAAACAAAAAAAAA



>gi|71811340|gb|DR988731.1|DR988731 HDI_B_34.esd Horsegram (VZM-1) drought stressed subtracted cDNA Macrotyloma uniflorum cDNA, mRNA sequence

TAAAAAGTATATAAAATTGCCTACACAATTGATCTNTAGAAGTTAAAAAAAGTACTACCGATAGTTCAAT

GACCATAAAGAAGAGGCATAATCTCAGTAAGGATAACATAGTCTCAGACATAGGCATGGCTATATAATTG

AAATTAGTATACACATGAATTACCTTTTAAAATAGNAATTAGAAAACAATTAAATAACGTTTGAAAAACT

TATAAGTAAGGGATCTCACTTATATATGATTTTATGAA



>gi|71811338|gb|DR988729.1|DR988729 HDI_B_32.esd Horsegram (VZM-1) drought stressed subtracted cDNA Macrotyloma uniflorum cDNA, mRNA sequence

AAGGCTTGGAAGGCTCCACCGGCGGGTGCGCGGCCCGCTCTTAGGAAACTAGGTGGATCCCCCGGGGCTT

GCAGGAATTCGGGCACGAGGGAAAAATCCAAGATTTGTTTGTAACTAGCTACCTGTATAACTCATGTAAC

AACTTGTAATGAATCAGATCTGGCTATGGTTGCTATCCCTGGTGCTGTAACTTAGTACTTTAAATGTAAT

ATGTTTCGAAACCCTCCAAT



>gi|71811336|gb|DR988727.1|DR988727 HDI_B_21.esd Horsegram (VZM-1) drought stressed subtracted cDNA Macrotyloma uniflorum cDNA, mRNA sequence

TGCGCGGCCGTTCTAGAACTAGTGGATCCCCCGGGCTGCAGGAATTCGGCACGAGGGTTATCTGAACCAT

ACAAGGGAATTGGTGATTGTTTTGCTCGAACCATGAAGGATGAGGGCGTGATTGCTCTTTGGAGAGGCAA

TACTGCCAATGTTATCAGATACTTTCCTACTCAGGCTCTGAACTTTGCTTTTAAGGATTACTTCAAGAGG

CTTTTCAACTTTAAAAAGGACAAGGATGGCTACTGGAAGTGGTTTGCTGGACAATTTGGCATCTGGTGGG

GCTGCTGGTGCTTCTTCCCCCTTATTTGTGTATTCTTTGGATTATGCCCGTACTCGTTTAGCAAACGATG

CCAAGGCTGCCAAGAAGGGTGGTGAGAGACAGTTTAATGGACTGACTTGATGTGTACAGGAAAACCATTA

GTCTGAATG



>gi|71811334|gb|DR988725.1|DR988725 HDI_B_19.esd Horsegram (VZM-1) drought stressed subtracted cDNA Macrotyloma uniflorum cDNA, mRNA sequence

AAAGCTGGAGCTCACGCGGTGGCGGCGCTCTAGAAACTAGTGGATCCCCCGGGCTGCAGGAATTCGGCAC

GAGGCATGGTAGTAGCCCTCCACCCTCCCGGTCAAAGAGGTTCAGTGATAAAGATCAAATGGAGTATACT

GAGAATATTGAAGATGCGTGGCCTTCCCTTCCAAGTGACTAAATCTCAAATTGTTGAATTCTTTAAAGAT

TTCAATCTGATTGAAGACAGGGTACACATTGCATGTCGCCCTGATGGGAAAGCTACTGGAGAGGCGTATG

TGGAGTTTGTTTCTGCGGAGGAGGCTAAGAGGGCAATGTCCAAGGATAAAATGACCATAGGATCAAGGTA

TGTGGAGCTGTTTCCTTCTACTCCAGATGAAGCTAGACGGGCAGAGTCAAGATCAAGGCAGTGATCGTGA

TATTTTACCCTGTTTTAGGACTTTTACCTTGTTCCAACTGGTGCTACTATAATGTTTTAAATTTTAGAGT

ATGTACTTTATTATTAGTAGTTAAACTTGCTCTTATATTTGCATGTCGGAAATGGATGCATTAATTGATT

AAAAAAAAACAAAAAAAACACTCAAGGGGGGGCCCGGT



>gi|71811332|gb|DR988723.1|DR988723 HDI_B_11.esd Horsegram (VZM-1) drought stressed subtracted cDNA Macrotyloma uniflorum cDNA, mRNA sequence

AAGCTGGAGCTCACGCGGTGGCGGCGCTCTAGAACTAGTGGATCCCCCGGGCTGCAGGAATTCGGCACGA

GGGCAACCTTGGCTGTTGTTCAACCATCTACTGTCAAGGGCCTTGCTGGATCTTCCCTTACTGGAACTAA

GCTCTCTTTCAAGCCCTCTCAAAAGAGCTTCAGACCCAAGAATTTCAGGAATGGCGCTGTTGTAGCAAAG

TATGGTGACAAGAGTGTGTACTTTGATTTGGAGGATCTAGGCAACACTACAGGTCAGTGGGACTTGTACG

GCTCCGATGCACCTTCACCCTACAACTCTCTTCAGAGCAAGTTCTTCGAGACATTCGCTGCTCCATTCAC

AAAGAGGGGATTGTTACTCAAGTTTTTGATCCTGGGAGGTGGTTCCACCCTGGCATACTTCAGTTCCACT

GCTTCAGGTGACATTCTACCAATCAAGAAAGGCCCACAACTTCCACCAAAGCTAGGTCCTCGTGGCAAGA

TCTAATTTATTTCCGAATTCTATCTGTTTAGCTCCTTTCCCCCCTCTTCTTTTGAATTGAACGTGTATAT

TTCTGCTTGCATATAATGTGTAAGTACGCCTAAAGCCTACTTCTACTAAAACATGTTTATTTCTGGTCCA



>gi|71811330|gb|DR988721.1|DR988721 HDI_B_90.esd Horsegram (VZM-1) drought stressed subtracted cDNA Macrotyloma uniflorum cDNA, mRNA sequence

AGCTGGAGCTCACGCGGTGGCGGCCGCTCTAGAACTAGTTGGATCCCCCGGGCTGCAGGATTCGGCACGA

GGATTCAACTGGAGCATACACAATGTACGTGGATGAGGATCATGCAGATGAGATCTGCAGTGCTAAGCTT

GTGCGTAGTCCTCACCCTACATGCAAGGAAGTCACCCCTGGCCGTGACGAAGCCCTTGTTATTCTGACCC

GCTACAATGGCATTGCCTCCGACGATAGGTTCGCCAATGCCATGGGTTTCATGTCTCAGGAGGTTGCTGG

TGGCTGTGCTGAGGTCCTCAAGCAATACCAGGAGTTCGACAATGAGAACTAATTAATTAATCTCAGTATA

TTATCACTATCCACTTTCGATTTGGGTTTCCTAATGTCTATTTAATTCAATCTTGATGTGGAGTGTTATC

TCGACATTTTCATATTATGTTACATATATATATATATATGCTGATGGACCCTGCTTAATTATATCATTAC

ATCAAGACCTGTCTGGCTTGGACTTTGAGGATGTAGTCTATTTTCTGTTGGTACAATAAACTATTCTTTT

ATAAAA



>gi|71811328|gb|DR988719.1|DR988719 HDI_B_87.esd Horsegram (VZM-1) drought stressed subtracted cDNA Macrotyloma uniflorum cDNA, mRNA sequence

AAGCTGAGCTCACCGCGGTGCCGGCCGTTCTAGAACTAGTGGATCCCCCGGGCTGCAGTGATTCGGCACG

AGGCAAAAGGGAAGTAACTGAAGCCAACTTAAAGCAGGCTGGTTTCGACACATGGGAGAAGTTGATCCTC

AAGGAGCCATCTAACAGTGCACCAAATGCTCTTGAGTACAAGAAGGCTGAGAGGGCTAAGCTGGTGCAGC

AGGGAATACAACATTGTTGCAGTGGTTGGAGACCAATGGTCCGATCTGAGGGGAAAACCCAAGGGAACCA

GGAACTTTAAGCTTCCCAACCCCATGTACTCTATTGAGTAGATCCCTCTCTGCAAGGATCCCAAGCTTGC

TCCTATCTACGAAATAAGATACCTTACGTGTTCTAATGGGACGCATTAACTATGTATCTATGCATGTGAC

TTTCTGGTCTTCGGATCTTCAACAAATCACCTAAGAGACTACACTCCGTTGTTTTCTCTTTTGTGACTTT

AATAA



>gi|71811326|gb|DR988717.1|DR988717 HDI_B_78.esd Horsegram (VZM-1) drought stressed subtracted cDNA Macrotyloma uniflorum cDNA, mRNA sequence

AAAGCTGGAGCTCACGCGGTGGCGGCGCTCTAGAACTAGTGGATCCCCCGGGCTGCAGGATTCGGCACGA

GGCCTCGTGCCGAATTCGGCACGAGGGTTGAATTGGGAAAGATGACAAAGGATCTTGCGCTTCTTGTTCA

TGGACCTAAGGTTTCTAGATCCCAGTATTTGAATACTGAAGAGTTCATTGATGCTGTAGCTGAGGAGCTG

CGAACAAGATTATCTTCACAATCAAAGCTGTAAAGTTCAAGATGAATGTGTCCATTATATGATGTCCATT

TTCTTAATTTTTCTTTTTCTTTCTTGAAGGAGGGAGTTCTATTGCTTCTCTGTCAAACATTTTAGAGTAT

ATACCATTAAGATCACCAAAATTCTAATGCACCACTCGTTGCATGTTGCTTCAGCCATTAGAACTTAACA

TTTGAAGAACTCATCTTATGTATCACAAACTCCATACTTATTTGCATTCTTAAAACATATTCTGAAAACT

GAATTTTGTAAACAGTAGTGCTACTATGGAAATCCTTATGACATTATTGAAAACAGTAAAAAAATAACAA

AGGGAAAAGAACTTTCTTCGGAGAAAACAAAC



>gi|71811324|gb|DR988715.1|DR988715 HDI_B_76.esd Horsegram (VZM-1) drought stressed subtracted cDNA Macrotyloma uniflorum cDNA, mRNA sequence

ACAAGCTGGAGCTCACCGCGGTGCGCGGCCGCTTCTAGAACTAGTGGATCCCCCGGGCTGCAGGAATTCG

GCACGAGGCCTCGTGCCGAATTCGGCACGAGGATTTGTTTCTGTATTTCCGGAATATGAGTAAAGTCTAT

GATTGGTTTGAAGAACGTCTTGAAATTCAGGCGATTGCCGATGATATAACCAGTAAATACGTTCCGCCTC

ATGTGAACATATTTTATTGTTTAGGAGGAATTACACTGACTTGTTTTTTAGTCCAAGTAGCAACGGGATT

TGCTATGACTTTTTATTATCGTCCAACCGTTACTGAAGCTTTTGCTTCTGTTCAATATATAATGACTGAG

GCTAATTTTGGTTGGTTAATCCGATCAGTTCATCGATGGTCGGCAAGTATGATGGTCTTAATGATGATCC

TACACGTATTTCGTGTGTATCTTACAGGTGGTTTTAAAAAACCTCGCGAATTAACTTGGGTTACGGGTGT

AATTTTGGCTGTATTAACTGCATCTTTAGGTGTAACTGGTTATTCCTTACCTTGGGATCAAATTGGTTAT

TGGGCAGTAAAAATTGTAACAGGACGTACCCGAAGCTAATTCCTGTAATAGGATCGTCTTTGGTAGAGTT

ATTACGCGGGAAGTTCCAGTGTGGGCCAATCTAACCTTAACTCGTTTTACAAGTTTGGCAATACTTTTTG

TATTAACACTTTCTTTAACTGCCTCGTTCAACAATGGTTTCAATTGCCACCTTTTTACATGAATAACNGT

AAAGACAAAGGCATTTTCTGGACCCTTTT



>gi|71811322|gb|DR988713.1|DR988713 HDI_B_74.esd Horsegram (VZM-1) drought stressed subtracted cDNA Macrotyloma uniflorum cDNA, mRNA sequence

AGCTGGAGCTCACGCGGTNCGCGGCNTTCATAGAACTAGTGGATCCCCCGGGCTGCAGTGATTCGGCACG

AGGACGTATCACTTGCGGGTCCCTTTGCTGGTGCTGTATTATCATTTTCAATGTATATGCTGTTGGTCTT

CTTCTCTCATCGAATCCAGATACAACAGGAGATTTGGTGCAAGTTCCTAGCTTGTTGTTTCAAGGTTCCT

TGCTTCTTGGATTGATCAGTAGAGCAACCCTTGGTTATGCAGCAATGCATGCTGCAACAGTTCCAATTCA

TCCTCTTGTGATTGCAGGCTGGTGTGGAGTAACCATACAAGCTTTTAACATGCTTCCAGTGGGGTGCCTT

GATGGTGGAAGAGCAGTACAGGGTGCTTATTGGAAAAAATGCGCTAGGTTGGCTTTGGTTTAACCACTTA

TACCTTGCTTGGATTAGGAGTGCTTGGTGGGCCTCTTTCACTACCTTGAGGGGCTTTA



>gi|71811320|gb|DR988711.1|DR988711 HDI_B_66.esd Horsegram (VZM-1) drought stressed subtracted cDNA Macrotyloma uniflorum cDNA, mRNA sequence

AGCTGGAGCTCACGCGGTGGCGGCCGNTCTAGAACTAGTGGATCCCCCGGGCTGCAGGAATCGGCACGAG

GAGCAAAGCGACACCGTAAGGTTCTGAGGGATAACATTCAGGAATCACCAAGCCAGCTATTCGCCGTCTC

GCTAGACGTGGAGGTGTTAAGCGTATCAGTGGTCTCATTTATGAAGAAACCCGTGGTGTTCTCAAGATCT

TCCTTGAGAACGTTATTCGTGATGCTGTCACCTATACTGAGCACGCTCGTCGCAAGACCGTCACTGCCAT

GGATGTTGTCTATGCTCTGAAGAGGCAGGGCAGAACCCTCTACGGTTTCGGTGGTTAGAAAATTGGGAAT

TTCTTCAAGACTGCGGACAATTAGGGTTTCTTATAGTGTTAATTTCGTTGTTGATATTGCATTAGTTCTT

GAGCTTAAGCTTAGGGTTTTAGCAAGTGTTCTTGTTTGGCTTTGAGTTGTAGCTCTGAGAATTTTTTAAT

GTTAATCTGATTGAC



>gi|71811318|gb|DR988709.1|DR988709 HDI_B_62.esd Horsegram (VZM-1) drought stressed subtracted cDNA Macrotyloma uniflorum cDNA, mRNA sequence

AAAGCTGGAGCTCACCGCGGTGCNCGGCCGTTCTAGAACTAGTGGATCCCCCGGGCTGCAGTNATTCGGC

ACGAGGGTGGAAGGAAGCCAGAACAGCCTTGAGATCGATAGTTTAGCCCGCTTTGCTGTTGAAGATCACA

ACAGAAAACAGAATGCCCTTCTGGAGTTTGGAAGGGTGGTAAGTGCAAAACAGCAAGTGGTTTCTGGTAC

CTTGTACACCATCACTTTGGAGGCAAAAGATGGTGGGCAAAAGAAGGTTTATGAAGCCAAAGTCTGGGAG

AAGCCATGGTTAGAACTTCAAGGAGGTGCAGGAGTTCAAGCTTGTTGGAGATGCACCTGCATAGTGTGCT

TAGCTGGTTACTGAAGAGTGAATAACNGAGCCAGACAATGATAGAAGGTGAAAGCCTAACGGATATATGA

AAACATCCTATCTTATATATATGTATAAAATAAATGTAGC



>gi|71811316|gb|DR988707.1|DR988707 HDI_B_6.esd Horsegram (VZM-1) drought stressed subtracted cDNA Macrotyloma uniflorum cDNA, mRNA sequence

AAAGCTGGAGCTCACGCGGTGGCGGCGCTCTAGAACTAGTGGATCCCCCGGGCTGCAGGATTCGGCACGA

GGGAAGATGCTAAATTTAAGGAAGGAGAAGAGGTGTCTCTAGAGTCTCTAAAGGCGAAAGGTGTGATTAA

CCCATCGGGAAGAGAAAGGAGACTTCCTTTGAAGATTTTGGGTGAAGGAGAACTGAGCAAGAAGTTGACG

CTAAAAGCCCGTGCCTTTTCCACAGGAGCAAAGGAGAAACTTGAGAGTGTTGGATGTTCTCTCACCTTGC

TTCCTGGCCGCAAGAAATGGGTTAAGCCGTCAGTTGCTAAAAACCTTGCCCGCGCTGAGGAATACTTTGC

CAAGAAGCGTGCTGCAGCCACCACTGAGGCAGCCTCAGCTTGAACTTGAACCAATACTGTGTCATCATAT

TTTAGAAATCTTCAAGAGAGTTGCAGATTGATTTCTTTAGGAACCATCTCACATATTTGTCATCCTTCAG

ATTTCTCTCTGATTCATCTAGCTGTTGTTACTTGAGGCATTTATTCATTCTTCTTTTTGCATTCCAAA



>gi|71811314|gb|DR988705.1|DR988705 HDI_B_53.esd Horsegram (VZM-1) drought stressed subtracted cDNA Macrotyloma uniflorum cDNA, mRNA sequence

AAAGCTGGAGCTCACGCGGTGGCGGCGCTCTAGAACTAGTGGATCCCCCGGGCTGCAGGAATTCGGCACG

AGGCGACCAGTTAGTGTGGCCTTTCAGGTGGTTAATGGGTTCCGATTCTATGAGAAAGGAGTTTACACCA

GTGAAATTTGCGGTAGCACTTCCCAGGATGTGAACCATGCTGTTCTTGCTGTTGGGTATGGAGTTGAAAA

TGGTGTCCCATATTGGCTCATAAAGAATTCATGGGGAGCAAACTGGGGTGACAGTGGCTACTTCAAGATG

GAATTGGGGAAGAACATGTGTGGTGTTGCTACTTGTGCATCTTACCCGGTTGTAGCCTAAATTGCATAAC

AATATGATCCCTGGTCTACCACCTCCTTGTTATGTGCCAAAGTTTAGAGCAGCTGATGCCAGTATTAATG

ATTGATAAGTGTCGTTGTATAACGAAATTGTTGGTACTTGGGTTCACATGATGTATAAAATAAGCTAGAA

GTGTTGTAAATCGTACAAGAATATTATGAACTGAATTATGTGTATCATATACATAATAATGATCATAAAT

TTTTAGTTAC



>gi|71811312|gb|DR988703.1|DR988703 HDI_B_51.esd Horsegram (VZM-1) drought stressed subtracted cDNA Macrotyloma uniflorum cDNA, mRNA sequence

AAGCTGGAGCTCACGCGGTGGCGGCGCTCTAAACTAGTGGATCCCCCGGGCTGCAGGATTCGGCACGAGG

GCTGGGTTACGGTGCCCACTGCGCGCTAACCTAGACCCCACAAAGGGTGTTGGTCGATTAAGACAGCAGG

ACGGTGGTCATGGAAGTCGAAATCCGCTAAGGAGTGTGTAACAACTCACCTGCCGAATCAACTAGCCCCG

AAAATGGATGGCGCTAAAGCGCGCGACCTATACCCGGCCGTCGGGGCAAGGACCAGGCCCCGATGAGTAG

GAGGGCGCGGCGGTCGCTGTAAAACCCGGGGCGCGAGCCTGGGCGGAGCGGTCGTCGGTGCAGATCTTGG

TGGTAGTAGCAAATATTCAAAAAAAAAAAAAAAAAACNCNAGGGGGGGCCCGGTACCCAATTTCGCC



>gi|71811310|gb|DR988701.1|DR988701 HDI_B_5.esd Horsegram (VZM-1) drought stressed subtracted cDNA Macrotyloma uniflorum cDNA, mRNA sequence

AGCTGGAGCTCACGCGGTGGCGGCGCTCTAGAACTAGTGGATCCCCCGGGCTGCAGGAATTCGGCACGAG

GAATTTAAGTGGAAAAGTCTTACCAACTATTATTGGAGATAATGTCACTGTAGGTCATAGTGCTGTGTTA

CAAGGATGTACCGTCGAGGATGAGGCATTTATTGGCATGGGTGCAACCTTGCTTGATGGTGTATATGTTG

AGAAACATGCCATGGTTGCTGCTGGAGCCCTTGTTAGGCAGAATACAAGGATTCCTTATGGAGAAGTTTG

GGGAGGTAATCCAGCAAGGTTCTTGAGGAAGCTCACAGAAGATGAAATGACATTCTTCTCACAATCTGCC

TTAAACTATTCCAATTTGGCTCAGGCTCATGCTACTGAAAATGCAAAAGAACTTGAGAAGACTGAATTTG

TAAAAGTTCTTAACAAGAAATTTGCTCGTCCTGGTGAGGAATATGATTCAGTGCTAGGTGGTGGTCAAGA

AACGCCTGCAGAGCTTAACCTTCAGGATAATGTTTTGCTAGACAAAGCTCCCAAGGCTTAATTTTTTTTT

TCTTTCTTTTCTAGTATCTTTTATGTGCGATTTTCGGAGAATGGCTACTTTTTGTTTCCT



>gi|71811308|gb|DR988699.1|DR988699 HDI_B_41.esd Horsegram (VZM-1) drought stressed subtracted cDNA Macrotyloma uniflorum cDNA, mRNA sequence

AAGCTGGAGCTCACGCGGTGGCGGCCGCTCTAAACTAGTGGATCCCCCGGGCTGCAGGATTCGGCACGAG

GCTGCCTTCAAGTATCACAATGAAAGTGGGATTGCCAACCTCTACAGCTTGCATTCATGGCTTGGAATTG

GGGTTATTGTCCTTTATGGCATTCAGTGGATATATGGATTTGTAATCTTTTTCTACCCTGGTGGGACTCC

AGACATTAGACGTTTCTCTCTTCCTTGGCATGCACTATTTGGGCTGTTTGTATTTGTCTTGGCTGTGGGA

ACTGCTACTCTTGGATTTCTGGAGAAGCTCACTTTCCTAGAGAGTTCAGGATTGGATAAGTATGGTTCTG

AGGCATTCCTTGTGAACTTCACTGCTATCATCACAGTCTTATTTGGTGTCTTTGTTGTGTTATCTGCTTT

AGCTGATGCTCCTCCAGCAGCAGATGACTATGAAGCCATATAAACTTTGGTCTGTATCATATTTTTATTC

TTTATGAATAATCTTATGTTTGCTATTAGTTTGTTTGTATTGGGTGTCGAAAAAGGATACAGTTTGTTGT

TTGTAATTTTGGGCATAAAGCTTGTGATTGATCATCCGTATCAATTAAAAA



>gi|71811306|gb|DR988697.1|DR988697 HDI_B_4.esd Horsegram (VZM-1) drought stressed subtracted cDNA Macrotyloma uniflorum cDNA, mRNA sequence

AAGGCTGGAGCTCACGCGGTGGCGGCGCTCTAGAACTAGTGGATCCCCCGGGCTGCAGGAATTCGGCACG

AGGCCTCGTTGCCGAATTCGGCACGAGGGCAAGTTCTGTCTTACTCTCTCTTGTGTATTATCTGCATTCT

TTGGGCACCATCTCAAAGTGCAACACGATATGCTTACCGTGATGATGGGAGCGATGAATTTGATAGGGAT

GAGACTACTTTAACGCTCATAAAACCATCACCTATTTCTTCAAAAGATGTTCGAAGTGTACCAGATGCTA

GACCAGTGCAAAGCAGTAACGGGAATTCAAATGATGATTTAGAAGAAGACAAGAGAGAATGAAGTAGAAT

GTAGCCATGATTCTGTGCTTCCATTGTACCCCTCCTGAAAATTAGCTCCATGACTCTTTTCCTTTTATTC

TTTTATTTCCTTCATTAGCCTTTACTCTCACTTGATACTTTGTCAGCTCATATTTCCTTGCGCTATTCGA

TTACATGACAGAGTCCTGTGTGTTCCTGCGATGCCAATGCACAAGAAAATATCACTTTTAGTCACTTTTT

TACTTAATTACAAAAAAC



>gi|71811304|gb|DR988695.1|DR988695 HDI_B_38.esd Horsegram (VZM-1) drought stressed subtracted cDNA Macrotyloma uniflorum cDNA, mRNA sequence

AAGCTGGAGCTCACGCGGTGCGCGGCCGTTCTAGAACTAGTGGATCCCCCGGGCTGCAGGATTCGGCACG

AGGCTTGAGTGAGAATGTGTGTTTTTTAGCTTAGGTTAGAAAGAAACCCATGTTTTTAGATTATAAAAAT

TTTATTTTTTCATAATACATGTTGTTTCTTTTAAATAGATAATCAAACAACTTATCCTTGTTTTTATCTG

TTTAAAAAAAAAAAAAAAAAAAAAAAAAAAAAAAAAAAAAAAAA



>gi|71811302|gb|DR988693.1|DR988693 HDI_B_30.esd Horsegram (VZM-1) drought stressed subtracted cDNA Macrotyloma uniflorum cDNA, mRNA sequence

AAAGCTGGAGCTCACGCGGTGGCGGCCGCTCTAGAAACTAGTGGATCCCCCGGGCTGCAGGAATTCGGCA

CGAGGGGGTTTAGGATTGGTAAAACTGTTGTACAGTGCCAAGTCTACCTTTCACAGCACCAAGGCAAAGT

AAAAAAATCCACATCCACAAAGCAAAACAGGTAGTAGTAGTAGCAGCAACTACTGGTGTTTTCATCAGTG

GTTGACCCCACCATGCAATAGGTTGTTGAAGTGGAAGGAAATGTTTTGAGTTGAACTTTGGGAGGGACAT

GGGTTGTGCAGTGCATGCCTGTGCTTGTTGGGACTCACCTTCTGCCATGGTTCAATGGGGGGCATTTTCT

TGTCCATTTGTATTTTGGCCAGTTTGGAAAGGAAACGCGTGAACCGTTGCATTGGGAAGTGCCCTTTTGT

TCAGAATGGTTTTCTTTTACTATGCATTTTAATTAATCAAATGCTTGCCCCCAAATTTTGTTGATATTAG

GCCGGTGGCAGGAATGGTCCATGGAATGCATCTTTAATAGACATTGCATATGTTATGTACATAATAATGT

TGGTGGTAATTTCATTAAAACAAAAAAAAAAAAAA



>gi|71811300|gb|DR988691.1|DR988691 HDI_B_29.esd Horsegram (VZM-1) drought stressed subtracted cDNA Macrotyloma uniflorum cDNA, mRNA sequence

AAAGCTGGAGCTCACGCGGTGCGCGGCCGCTCTAGAACTAGTGGATCCCCCGGGCTGCAGGAATTCGGCA

CGAGGATTAAAAAGGACGCACCTGGTTTAACAGTTACAAAAATAGAGAACAAAATTGGACTTAGAATTGT

ACAAAATGGTGACATTGTTATGAGAAAAGTATTTGTCCCTGACGAGGACAGACTTCCTGGAGTAAACTCT

TTTCAGGACACAAACAAGGTGCTTGCTGTTTCACGTGTAATGGTTGCTTGGCAGCCCATTGGTCTATCGA

TGGGCATCTATGATATGTGTCATAGGTATCTAAAAGAGAGGAAACAATTTGGAGCTCCATTAGCAGCTTT

CCAAATCAGCCAACAGAAACTTGTCAAGATGCTCGGTAATATTCAAGCTATGATTCTTGTTGGTTGGCGC

CTCTGCAAATTGTATGAGAGTGGTAAAATGACTCCAGGTCATGCTAGCTTGGGGAAGTCATGGATCACCT

TGAGCGCAAGAGAAACAGCTGCTCTGGGGCGAGAATTGCTAGGTGGCAATGGAATTTTGGCTGATTTTCT

TGTGGCAAAGGCATTCTGTGATTTGGAACCCATCTATACCTTCGAAGGCACGTATGATATTAACACCTTG

GTTACAGGTAGAGAAGTTACTGGCTTTGCCAGCTTTAAACCAGTTGCTCAAAGAAGTCGATTGTAACACA

GGTTGAAATACATTGGTTCCCATTTGGGTGGATTCCATCAGGGCAAACTTCACATATGCCATATGGCTGT

GGACTACTGTGTGTTAGGTTATGTGTTCACATTTTGAGAAAATTGAGAAACCTTTTAATAGTTATTCAGA

TGCTTGTGT



>gi|71811298|gb|DR988689.1|DR988689 HDI_B_27.esd Horsegram (VZM-1) drought stressed subtracted cDNA Macrotyloma uniflorum cDNA, mRNA sequence

GCGGCGTTCTAGAACTAGTGGATCCCCCGGGCTGCAGGATTCGGCACGAGGCCAAGGTGTGGAGTATGAC

CGGTGGCCCCTACTGTCGTCCCAAGCACTGGAAGCGCAACACCGCCATTGCCATGCTCGGCGTCGTCCTC

GTCTGCATCCCCATCGCCATGAAATCCGCTGAACTCGAGCAACGACCACATCACCCTGTTCGCCCCATCC

CTTCTCAACTCTGGTGCAAGAACTTTGGAACTAAAGACTATAATAATCCGGAATAATTATTGAATCGCTG

GTGTGGGTGTTCACATCATGTCAAGCAAAAATTATTTGTTAAATGCTCCAAGAATATGAACAGCGTTAAA

GAGTTGCTGTTGATGTTATGCAAGACTTTGATCTAGAATCATGTAGTGACATACCTTTTTTTTGGCTATA

TTTGATGGGACATCCATTTTAGAAATGGTGAAGTTAAATAAGTGCTTATTTCTGATTTTTTATTTTATTT

GAGAGCTTGTTGAAACCGATATTACTGGTTACTATAAAATAAAATTTGGGTTAATTTTGGGTGCCTTTTT

TTAATTCCAAGGTTTTTTGTTCCCAAAAGAAA



>gi|71811296|gb|DR988687.1|DR988687 HDI_B_25.esd Horsegram (VZM-1) drought stressed subtracted cDNA Macrotyloma uniflorum cDNA, mRNA sequence

ATCGTAAAAACATTCGANAGGGTCGTAATAGGAAATAGAGCTCGAAAAAGATCGATAGCCGAGATAGGGA

TTGGTAATATTGAGATAATCGCCAAACTTCGCTTGAGGGTAGGATTGGGCAAATGGAGATTTACAAAGCT

CTCGCGATATGGG



>gi|71811294|gb|DR988685.1|DR988685 HDI_B_18.esd Horsegram (VZM-1) drought stressed subtracted cDNA Macrotyloma uniflorum cDNA, mRNA sequence

AAGCTGGAGCTCACGCGGTGGCGGCGCTCTAGAACTAGTGGATCCCCCGGGCTGCAGGAATTCGGCACGA

GGGTTGATGAGATAATATCTCGAACGCCTATAAAACGATTGGCAGAAGCACATGAAGTGTCATCCTTGGT

GACTTTCCTTTGCTTGCCAGCTGCTTCTTACATCAATGGACAAATTATTGCTGTTGATGGAGGATTTACT

GTGAATGGATTTCAACCCAGCATGAGAGTTACTTGAAGCAAAATCAACAGTTTTATTGTTGGATTATGTG

AATAAAGTGGTGCAACTTGAGTTTTGGACCACCATGATCAAATTGTTTTGATTGGTGAATGAATTATTTT

AGTCTCTTAAAAATCTAGATAATTCCATTCGGTGCCTATCATTGTAAGATTATGGGTGTACTTACCTCAA

ACCTCATTCACCATAACTAGTGCCAAGTACCATTACACTATGTATTTGAACATAATCTCAAGCCATGAAC

TGAATCAAATCAAAACTTTGTTGCCAAAAAAAAAAAAAAAAAACTCNAGGCGGGGGCCCGGTACCCATTT

CGC



>gi|71811292|gb|DR988683.1|DR988683 HDI_B_16.esd Horsegram (VZM-1) drought stressed subtracted cDNA Macrotyloma uniflorum cDNA, mRNA sequence

AAGCTGGAGCTCACGCGGTGCGCGGCNTTCTAGAACTAGTGGATCCCCCGGGCTGCAGGAATTCGGCACG

AGGCTGCGGAGGGACTAGTTGAAGGAGGAAAGAAAGAGATTTTCACACCAATGTACTTCTTCTTGGCACG

GAAGCCTGATTCAGACAGTAACTAAACTGGTTGTGTACAAATAGGTTGCTGTTGTTTATTGTGATTCTGG

TAGGATGAATCGATTAATTTTGTCCTTATTGCCGTAGTTTCGTTCTGTTTCGAGTTGCTTGTTTTTGTTT

TAGGTTACCAAAACCTTTGCTACACCTCTAGCGTATTTCTTTTTATCCTCAAAAAAAAAAAAAAAAACAA

AAACAAAAAACCCAGGGGGGGAC



>gi|71811290|gb|DR988681.1|DR988681 HDI_B_14.esd Horsegram (VZM-1) drought stressed subtracted cDNA Macrotyloma uniflorum cDNA, mRNA sequence

AATCAGCTGGAGCTCCACCGGCGGGTGCGCGGCCGTTTTAGTAACTAGTGGATCCCCGGGCTGCAGGAAT

TCGGCACGTAGGCCTGTCCGATCGGCCGAGGGGTATTGCGGTAGAGC



>gi|71811288|gb|DR988679.1|DR988679 HDI_B_1.esd Horsegram (VZM-1) drought stressed subtracted cDNA Macrotyloma uniflorum cDNA, mRNA sequence

AAGCTGGAGCTCACGCGGTGGCGGCCGTTCTAGAACTAGTGGATCCCCCGGGCTGCAGGAATTCGGCACG

AGGGCAAGAGGCTATAATAAAACTTCTTAGAAATGAGTTGGGGCTGGAAGTGTTTCCTGCCAGAACGAGA

TTAGCAGTAAGTGACACACCGAACTTAAAGCACCCCAACCTTGCAAATCTCAAGACAGAAGCACTACCAG

CAGAGAATGCATTATCCACATCAACGGGGTTTCAGACCAGGAGACCTGGAGTTCTAGTAAGGTTGAAGGT

CACCAAAAAATCGTTATACAGTTGGTTGAAGAGGAAGTAACCGTTGAATAGTTCCATTACAACTTATAGT

AATTATGTACAAATATTAGGTAACATATGCCATTTTCCCCCCTCCCAACTCTGTATATATTGCATCTGTT

ATAGCTTCGTATGCATCTGTAATCAAATAGCTGTAACATAATGAATGATACCTAGTGCCGAGGAATCAAA

TCGTGTAACTGACTATCCTC



>gi|50886895|gb|CO751631.1|CO751631 Hdi-30 Horsegram stress induced cDNA library Macrotyloma uniflorum cDNA clone Hdi-30, mRNA sequence

AAGTGAATCAGAGCTCAGGTACCTCAGTCGAATGCATCTAGATAAATAGCACACACCCCCCCCGCGTAAT

AGCTCACTGATCGAGCGCTCTAGCGCCGAAGATGAACGGGGCTAAGCGATCTGCCGAAGCTGTGGGATGT

CAAAATGAATGGTAGGGGAGCGTTACGCCTTAGAGGGAAGGACTCGCGCGAGCAGTGCTGGACGAAGCGG

AAGCGAGAATGTCGGCTAGAGTAACGCAAACGTAGGTGAGAATCCAATGCCTCTAAAACCCAAGGGTTCC

TCCGCAAGGTTCGTCACACGGGGGGTGAGTCAGGGCCTATCGCCAAGGCGTATCATGGACNCGGTG



>gi|50886893|gb|CO751629.1|CO751629 Hdi-28 Horsegram stress induced cDNA library Macrotyloma uniflorum cDNA clone Hdi-28, mRNA sequence

ATTAGAGCTCGGTACCTCGTGAATGCATCTAGATTAATTGCCACCCCCCCCCGATGAGTTGTGGTTAGGG

GTGAAATGCCACTCGAACCCAGAGCTAGCTGGTTCTCCCCGAAATGCGTTGAGGCCAGCAGTTGACTAGA

CATCTAGGGGTAAAGCACTGTTTCGGTGCGGGCCGCGAGAGCGGTACCAAATCGAGGCAAACTCTGAATA

CTAGATATGACCTCAAAATAACAAGGGTCAAGGTCGGCCAGTGAGACGATGGGGGATAAGCTTCATCGTC

GAGAGGGAAACAGCCCGGATCCCAGCTAAGGCCCCTTAAATGACCGCTCNGTTGATAAGGAGGTGGGTGC

AAGACACCA



>gi|50886891|gb|CO751627.1|CO751627 Hdi-26 Horsegram stress induced cDNA library Macrotyloma uniflorum cDNA clone Hdi-26, mRNA sequence

CGATGCGCTCCTGGCCTTAACTGGCCGGGTCGTGCCTTCGGTGCTGTTACTTTGAAGAAATTAGAGTGCT

CAAAGCAAGCCTACGCTCTGGATACATTAGCATGGGATAACACCACAGGATTCTGATCCTATTGTGTGGG

CCTTCGGGATCGGAGTAATGATTAACAGGGACAGTCGGGGGCATTCGTATTTCATAGTCAGAGGTGAAAT

TCTTGGATTTATGAAAGACGAACAACTGCGAAAGCATTTGCCAAGGATGTTTTCATTAATCAAGAACGAA

AGTTGGGGGCTCGAAGACGATCAGATACCGTCACTAGTCTCNACCATAAACGATGCCGACCAGGGATCAG

CGGATGTTGCTTTTAGGACTCCGCTGGCACCTTATGAGAAATCAAAAGTCTTTGGGTTCCGGGGGGGGTG

GCACATAATTCATGATCCCAGGGCCCGCTCCTAACCTGCCATAAGGCCTGCATCCAAGACTTGTCCCTAA

TAATGCAGTAATTAATTAACAAGCATGGCGTAAT



>gi|50886889|gb|CO751625.1|CO751625 Hdi-24 Horsegram stress induced cDNA library Macrotyloma uniflorum cDNA clone Hdi-24, mRNA sequence

AGGGCGTAGTAAGCGACGAAATGCTTCGGGGAGTTGAAAATAAGCGTAGATCCGGAGATTCCCGATATAG

GTCAACCTTTCGAACTGCTGCTGAATCCACGGGCAGGCAAGAGACAACCTGGTGAACTGAAACATCTTAG

TAGCCAGAGGAAAAAAAAAAAAAAAAGCGGCCGCTCTCTGCAATCGGATCCCGGGCCCGTCGACTGCAGA

GGCCTGCATGCAAGCTTTCCCTATAGTGAGTCGTATTAGAGCTTGGCGTAATCATGGTCATAGCTGTTTC

CTGTGTGAAATTGTTATCCGCTCACAATTCCACACAACATACGAGCCGGAAGCATAAAGTGTAAAGCCTG

GGGTGCCTAATGAGTGAGCTAACTCACATTAATTGCGTTGCGCTCACTGCCCGCTTTCCAGTCGGGAAAC

CTGTCGTGCCAGCTGCATTAATGAATCGGCCAACGCGCGGGGAGAGGCGGTTTGCGTATTGGGCGCTCTT

CCGCTTCCTCGCTCNCTGACTCCTGCGCTCGGTCCTTCGGCTGCGGCGAGCGG



>gi|50886887|gb|CO751623.1|CO751623 Hdi-22 Horsegram stress induced cDNA library Macrotyloma uniflorum cDNA clone Hdi-22, mRNA sequence

TAATTGCCACCCCCCCCCGCGCAAATTACCCAATCCTGACACGGGGAGGTAGTGACAATAAATAACAATA

CCGGGCTCATTGAGTCTGGTAATTGGAATGAGTACAATCTAAATCCCTTAACGAGGATCCATTGGAGGGC

AAGTCTGGTGCCAGCAGCCGCGGTAATTCCAGCTCCAATAGCGTATATTTAAGTTGTTGCAGTTAAAAAG

CTCGTAGTTGGACCTTGGGTTGGGTCGATCGGTCCGCCTCTGGTGTGCACCGGTCGGCTCGTCCCTTCTG

CCGGCGATGCGCTCCTGGCCTTAACTGGCCGGGTCGTGCCTTCGGTGCTGTTACTTTGAAGAAATTAGAG

TGCTCAAAGCAAGCCTACGCTCTGGATACATTAGCATGGGATAACACCACAGGATTCTGATCCTATTGTG

TTGGCCTTCGGGATCGGAGTATGATTAACAGGGACAGTCGGGGGCATTCATATTTCATAGTCAGAGGTGA

AATTCTTGGATTTATGAAAGACGAACACTGCGAAATGCATTGCGATGTTTTNTTTTTCCC



>gi|50886885|gb|CO751621.1|CO751621 Hdi-20 Horsegram stress induced cDNA library Macrotyloma uniflorum cDNA clone Hdi-20, mRNA sequence

AATTGCCACCCCCCCCCGGAACCCAAAGACTTTGATTTCTCATAAGGTGCCAGCAGAGTCCTAAAAGCAA

CATCCGCTGATCCCTGGTCGGCATCGTTTATGGTTGAGACTAGGACGGTATCTGATCGTCTTCGAGCCCC

CAACTTTCGTTNCTTGATTAATGAAAACATCCTTGGCAAATGCTTTCGCAGTTGTTCGTCTTTCATAAAT

CCAAGAATTTCACCTCTGACTATGAAATACGAATGCCCCCGACTGTCCCTGTTAATCATTACTCCGATCC

CGAAGGCCAACACAATAGGATCAGAATCCTGTGGTGTTATCCCATGCTAATGTATCCAGAGCGTAGGCTT

GCTTTGAGCACTCTAATTTCTTCAAAGTAACAGCACCGAAGGCACGACCCCGGCCAGTTAAGGCCGGAGC

GCATCGCCGGCAGAAGGGACGAGCCTGGGGGGGTGGCATTAATCGGATCCCGGGCCCCTATGCCAAGTCC

TTCTC



>gi|50886883|gb|CO751619.1|CO751619 Hdi-18 Horsegram stress induced cDNA library Macrotyloma uniflorum cDNA clone Hdi-18, mRNA sequence

AATTGCCACCCCCCCCCGCGCAAATTACCCAATCCTGACACGGGGAGGTAGTGACAATAAATAACAATAC

CGGGCTCATTGAGTCTGGTAATTGGAATGAGTACAATCTAAATCCCTTAACGAGGATCCATTGGAGGGCA

AGTCTGGTGCCAGCAGCCGCGGTAATTCCAGCTCCAATAGCGTATATTTAAGTTGTTGCAGTTAAAAAGC

TCGTAGTTGGACCTTGGGTTGGGTCGATCGGTCCGCCTCTGGTGTGCACCGGTCGGCTCGTCCCTTCTGC

CGGCGATGCGCTCCTGGCCTTAACTGGCCGGGTCGTGCCTTCGGTGCTGTTACTTTGAAGAAATTAGAGT

GCTCAAAGCAAGCCTACGCTCTGGATACATTAGCATGGGATAACCCNCAGGATTCTGATCCTATTGTGTT

GGCCTTCGGGATCGGAGTTGTTACTGGACGTCGGGGCTCTTTCTTTCGAGGTCTC



>gi|50886881|gb|CO751617.1|CO751617 Hdi-16 Horsegram stress induced cDNA library Macrotyloma uniflorum cDNA clone Hdi-16, mRNA sequence

ATTGCCACCCCCCCCCGTCAATTCCTTTGAGTTTCATTCTTGCGAACGTACTCCCCAGGCGGGATACTTA

ACGCGTTAGCTACAGCATTGCACGGGTCGATACGCACAGCGCCTAGTATCCATCGTTTACGGCTAGGACT

ACTGGGGTATCTAATCCCATTCGCTCCCCTAGCTTTCGTCTCTCAGTGTCAGTGTCGGCCCAGCAGAGTG

CTTTCGCCGTTGGTGTTCTTTCCGATCTCTACGCATTTCACCGCTCCACCGGAAATTCTCTCTGCCCCGG

GGGGGGGTGGCAATTTATCGGATCCCGGGCCCGTCGACTGCAGAGGCCTGCATGCAAGCTTTCCCTATAG

TGAGTCGTATTAGAGCTTGGCGTAATCATGGTCATAGCTGTTTTCCCTGTGTGAAATTGTTCCGCTCCNT

TTCCACCCATCGAGCCGGA



>gi|50886879|gb|CO751615.1|CO751615 Hdi-14 Horsegram stress induced cDNA library Macrotyloma uniflorum cDNA clone Hdi-14, mRNA sequence

AATTGCCACCCCCCCCCGCGCAAATTACCCAATCCTGACACGGGGAGGTAGTGACAATAAATAACAATAC

CGGGCTCATTGAGTCTGGTAATTGGAATGAGTACAATCTAAATCCCTTAACGAGGATCCATTGGAGGGCA

AGTCTGGTGCCAGCAGCCGCGGTAATTCCAGCTCCAATAGCGTATATTTAAGTTGTTGCAGTTAAAAAGC

TCGTAGTTGGACCTTGGGTTGGGTCGATCGGTCCGCCTCTGGTGTGCACCGGTCGGCTCGTCCCTTCTGC

CGGCGATGCGCTCCTGGCCTTAACTGGCCGGGTCGTGCCTTCGGTGCTGTTACTTTGAAGAAATTAGAGT

GCTCAAAGCAAGCCTACGCTCTGGATACATTAGCATGGGATAACACCACAGGATTCTGATCCTATTGTGT

TGGCCTTCGGGATCGGAGTAATGATTAACAGGNACAGTCCGGGGGG



>gi|50886877|gb|CO751613.1|CO751613 Hdi-12 Horsegram stress induced cDNA library Macrotyloma uniflorum cDNA clone Hdi-12, mRNA sequence

ATTGCCACCCCCCCCCGCGCAAATTACCCAATCCTGACACGGGGAGGTAGTGACAATAAATAACAATACC

GGGCTCATTGAGTCTGGTAATTGGAATGAGTACAATCTAAATCCCTTAACGAGGATCCATTGGAGGGCAA

GTCTGGTGCCAGCAGCCGCGGTAATTCCAGCTCCAATAGCGTATATTTAAGTTGTTGCAGTTAAAAAGCT

CGTAGTTGGACCTTGGGTTGGGTCGATCGGTCCGCCTCTGGTGTGCACCGGTCGGCTCGTCCCTTCTGCC

GGCGATGCGCTCCTGGCCTTAACTGGCCGGGTCGTGCCTTCGGTGCTGTTGCTTTGAAGAAATTAGAGTG

CTCAAAGCAAGCCTACGCTCTGGATACATTAGCATGGGATAACACCACAGGATTCTGATCCTATTGTGTT

GGCCTTCGGGATCGGAGTAATGATTAACAGGGACAGTCGGGGGGCATTCGTATTTCATAGTCAAG



>gi|50886894|gb|CO751630.1|CO751630 Hdi-29 Horsegram stress induced cDNA library Macrotyloma uniflorum cDNA clone Hdi-29, mRNA sequence

AAGAAGTAGACGGCCAGTGAATGAGCTGNGTACCTTGAATGCATACTAGATNAATANGCACACACCCCCC

CCGGAACCCAAAGACTTTGATTTNCTACATAAGGTGCCAGCGGAGTCCTAAAAGCAACATACCGCTGATC

CCTGGTCGGCATCGTTATATGGTTGAGACTAGGACGGTCTGATCGTCATATCAANGCCCCCAACTATTNC

AGTTACTATGATNTAATGAAAATACACTATGGCAATGCTTCCGCAGTTGTATACGTCATTTTACATTAAA



>gi|50886892|gb|CO751628.1|CO751628 Hdi-27 Horsegram stress induced cDNA library Macrotyloma uniflorum cDNA clone Hdi-27, mRNA sequence

ATATGCCACNCCCCCCCCGTTGGGTCGATCGGTCCGCCTCTGGTGTGCACCGGTCGGCTACGCACCTTNC

TGCCGGCGATGCGCTNCCTGGCCTTAACTGGCCGGGTCGTGCCTTACGGTGCTGTACTATTGAAGAAATT

AGAGTGCTACAAAGCAAGCCTACGCTCTGGATACATTAGNATGGGATATCACCACAGGATTCTGATCCTA

TTGTGTTGGCCTTACGGGATCGGAGTAATGATTAACAGGGAAGTCGGGGCTTCCTATTTCTATTCCAGGT

TAAATTACTGGATTTATGANAGACAACC



>gi|50886890|gb|CO751626.1|CO751626 Hdi-25 Horsegram stress induced cDNA library Macrotyloma uniflorum cDNA clone Hdi-25, mRNA sequence

GAACCCAAAGACTTTGATTTCTCATAAGGTGCCAGCGGAGTCCTAAAAGCAACATCCGCTGATCCCTGGT

CGGCATCGTTAATGGTTGAGACTAGGACGGTATCTGATCGTCNTTCGAGCCCCCAACTTTCGTTNNTTGA

TTAATGAAAACATCCTTGGCAAATGCTTTCGCAGTTGTTCGTCTTTCATAAATCCAAGAATTTCACCTCT

GACTATGAAATACGAATGCCGGGGGGGGGTGGCAATTATCGGATCCCGGGCCCGTCGACTGCGAGGCCTG

CTGCTTCCCTATATGAGTC



>gi|50886888|gb|CO751624.1|CO751624 Hdi-23 Horsegram stress induced cDNA library Macrotyloma uniflorum cDNA clone Hdi-23, mRNA sequence

TAATTGCCACCCCCCCCCGTCAAGGTCGGCCAGTGAGACGATGGGGGATAAGCTTCATCGTCGAGAGGGA

AACAGCCCGGATCACCAGCTAAGGCCCCTAAATGACCGCTCAGTGATAAAGGAGGTAGGGGTGCAGAGAC

AACCAGGAGGTTTGCCTAGAAGCAGCCACCCTTGAAAGAGTGCGTAATAGCTCACTGATCGAGCGCTCTT

GCGCCGAAGATGAACGGGGCTAAGCGATCTGCCGAAGCTGTGGGATGTCAAAATGAATCGGTAGGGGAGC

GTTCCGCCTTAGAGGGAAGGACTCGCGCGAGCAGTGCTGGACGAAGCGGAAGCGAGAATGTCGGCTTGAG

TAACGCAAACGTTGGTGAGAATCAATGCCTCGAAAACCCAAGGGTTCCTCCGCAAGGTTCGTCACACGGG

GGGGGGTGGCA



>gi|50886886|gb|CO751622.1|CO751622 Hdi-21 Horsegram stress induced cDNA library Macrotyloma uniflorum cDNA clone Hdi-21, mRNA sequence

CATTGCCACCCCCCCCCGAAGGGCGTAGTAAGCGACGAAATGCTTCGGGGAGTTGAAAATAAGCGTAGAT

CCGGAGATTCCCGATATAGGTCAACCTTTCGAACTGCTGCTGAATCCACGGGCAGGCAAGAGACAACCTG

GTGAACTGAAACATCTTAGTAGCCAGAGGAAAAAAAAAAAAAAAAGCGGCCGCTCTCTGCAATCGGATCC

CGGGCCCGTCGACTGCAGAGGCCTGCATGCAAGCTTTCCCTATAGTGAGTCGTATTAGAGCTTGGCGTAA

TCATGGTCATAGCTGTTTCCTGTGTGAAATTGTTATCCGCTCACAATTCCACACAACATACGAGCCGGAA

GCATAAAGTGTAAAGCCTGGGGTGCCTAATGAGTGAGCTAACTCACATTAATTGCGTTGCGCTCACTGCC

CGCTTTCCGTCGGGAAACCTGTCNNTGCCACTGCTTTATTGAATCGGCCCCACCCNGCGGGGA



>gi|50886884|gb|CO751620.1|CO751620 Hdi-19 Horsegram stress induced cDNA library Macrotyloma uniflorum cDNA clone Hdi-19, mRNA sequence

AATTGCCACCCCCCCCCGTGCAAATTACCCAATCCTGACACGGGGAGGTAGTGACAATAAATAACAATAC

CGGGCTCATTGAGTCTGGTAATTGGAATGAGTACAATCTAAATCCCTTAACGAGGATCCATTGGAGGGCA

AGTCTGGTGCCAGCAGCCGCGGTAATTCCAGCTCCAATAGCGTATATTTAAGTTGTTGCAGTTAAAAAGC

TCGTAGTTGGACCTTGGGTTGGGTCGATCGGTCCGCCTCTGGTGTGCACCGGTCGGCTCGTCACCTTCTG

CCGGCGATGCGCTCCTGGCCCTTAACTGGCCGGGTCGTGCCTCCGGTGCTGTTACTTTGAAGAAATTAGA

GTGCTCAAAGCAAGCCTACGCTCTG



>gi|50886882|gb|CO751618.1|CO751618 Hdi-17 Horsegram stress induced cDNA library Macrotyloma uniflorum cDNA clone Hdi-17, mRNA sequence

TAATGCCACCCCCCCCCGGAACCCAAAGACTTTGATTTCTCATAAGGTGCCAGCAGAGTCCTAAAAGCAA

CATCCGCTGATCCCTGGTCGGCATCGTTNATGGTTGAGACTAGGACGGTATCTGATCGTCATTCGAGCCC

CCAACTTTCGTTACTTGATTAATGAAAACATCCTTGGCAAATGCTTTCGCAGTTGTTCGTCATTTCATAA

AATCCAAGAATTTCACCTCTGACTATGAAATACGAATGCCCCCGACTGTCCCTGTTAATCATTACTCCGA

TCCCGAAGGCCACACAATAGGATCAGAATCCTGTGGTGTTATCCCTGCTATGTATCCAGAGCGTAGGCTT

GCTTTGACCCTCTAATTTCTTCATCCCCCGCCACCCGCCTTAGCCGACCTCCGCATGGAACACACCGTCC

CCAGCCC



>gi|50886880|gb|CO751616.1|CO751616 Hdi-15 Horsegram stress induced cDNA library Macrotyloma uniflorum cDNA clone Hdi-15, mRNA sequence

AATTGCCACCCCCCCCCGACTTTCGTTCTTGATTAATGAAAACATCCTTGGCAAATGCTTTCGCAGTTGT

TCGTCTTTCATAAATCCAAGAATTTCACCTCTGACTATGAAATACGAATGCCCCCGACTGTCCCTGTTAA

TCATTACTCCGATCCCGAAGGCCAACACAATAGGATCAGAATCCTGTGGTGTTATCCCATGCTAATGTAT

CCAGAGCGTAGGCTTGCTTTGAGCACTCTAATTTCTTCAAAGTAACAGCACCGAAGGCACGACCCGGCCA

GTTAAGGCCAGGAGCGCATCGCCGGCAGAAGGGACGAGCCGACCGGTGCACACCAGAGGCGGACCGATCG

ACCCAACCCAAGGTCCAACTACGAGCTTTTTAACTGCAACAACTTAAATATACGCTATTGGAGCTGGAAT

TACCGCGGCTGCTGGCACCAGACTTTGCCCTCCAATGGATCCCTTCGTTAAGGGATTTAGATTGTACTCT

TTCCCATTTAACCCAGACTCCACTGAGCCCCGGTATTGGTTATTTTAATTNGTTCACCCTACCCCTCCCC

CCGTTGTTTCAGGATTGGGTATTTGC



>gi|50886878|gb|CO751614.1|CO751614 Hdi-13 Horsegram stress induced cDNA library Macrotyloma uniflorum cDNA clone Hdi-13, mRNA sequence

AATTGCCACCCCCCCCCGGTGGAAACTACCAAGCTGGAGTACGGTAGGGGCAGAGGGAATTTCCGGTGGA

GCGGTGAAATGCGTAGAGATCGGAAAGAACACCAACGGCGAAAGCACTCTGCTGGGCCGACACTGACACT

GAGAGACGAAAGCTAGGGGAGCGAATGGGATTAGATACCCCAGTAGTCCTAGCCGTAAACGATGGATACT

AGGCGCTGTGCGTATCGACCCGTGCAATGCTGTAGCTAACGCGTTAAGTATCCCGCCTGGGGAGTACGTT

NCGCAAGAATGAAACTCAAAGGAATTGACGGGGGGGGGTGGCATNNTCNGATCCCGGGCCCT



>gi|50886876|gb|CO751612.1|CO751612 Hdi-11 Horsegram stress induced cDNA library Macrotyloma uniflorum cDNA clone Hdi-11, mRNA sequence

AAGTGAATTCGAGCTCGGTACCTACGCGAATGCATACTAGATTAATTGCCACTCCCCCCCGGTAATTCCA

GCTCCAATAGCGTATATTTAAGTTGTTGCAGTTAAAAAGCTCGTAGTTGGACCTTGGGTTGGGTCGATCG

GTCCGCCTCTGGTGTGCACCGGTCGGCTCGTCACCTTCTGCCGGCGATGCGCTCCTGGCCTTAACTGGCC

GGGTCGTGCCTTCGGTGCTGTTACTTTGAAGAAATTAGAGTGCTCAAAGCAAGCCTACGCTCTGGATACA

TTAGCATGGGATAACACCACAGGATTCTGATCCTATTGTGTTGGCCTTCGGGATCGGAGTAATGATTAAC

AGGGACAGTCGGGGCATTCGTATTTCATAGTCAGAGGTGAAATTCTTGGATTTATGAAAGACGAACAACT

GCGAAAGCATTTGGCCAAGGATGTTTTCATTAATC



>gi|50355582|gb|CO553849.1|CO553849 Hdi-10 Horsegram stress induced cDNA library Macrotyloma uniflorum cDNA clone Hdi-10, mRNA sequence

GTAAATTGCCTCCCCCCCCCGTCTTACCGCGGCTGCTGGCACGGAGTTAGCCGGGGCTTCTTCCTCGAGT

CCTGTCATGATCGCGCACTCGACGAAAGAGCTTTACTAGCGGCATTGCCCTTCTTCACTCCGCGATATTG

CTGGATCGGGCTTTCGCCCATTGTCCAGATTCCCCCTGCTGCCCCCCGTGGGAGTTCCGGCCTTGTTTTC

CCTCTGTTGCTGATCTCCCACTCACCCCTCT



>gi|50355580|gb|CO553847.1|CO553847 Hdi-8 Horsegram stress induced cDNA library Macrotyloma uniflorum cDNA clone Hdi-8, mRNA sequence

AATAGCACACACCCCCCCCGGAACCCAAGACTTTGATTTCTCATAAGTGCCATCAGAGTCCTAAAAGCAA

CATCCGCTGATCCCTGGTCAGCATCGTTAATGGTTGAGACTAGACGTATCTGATCGTCATTCGAGCCCAC

AACTATCGTTTTGATTAATGAAAACATCCTAGGCAAATGCTTACGCAGTTGTTCGTCATTCTAGTCCATG

AATTCTCTGACTATGAAACTACAAATGCCCCCGACTGTCCCTGTTATCATTACTCCGATGGGCCCACACA

TAGGATCGATCCTGTTGGTGTTATCCCTGCTATGTTTCCACCTAGGCTGCTGACCTCTC



>gi|50355578|gb|CO553845.1|CO553845 Hdi-6 Horsegram stress induced cDNA library Macrotyloma uniflorum cDNA clone Hdi-6, mRNA sequence

AATTGCCACCCCCCCCCGGAACCCAAAGACTTTGATTTCTCATAAGGTGCCAGCAGAGTCCTAAAAGCAA

CATCCGCTGATCCCTGGTCGGCATCGTTTATGGTTGAGACTAGGACGGTATCTGATCGTCTTCGAGCCCC

CAACTTTCGTTCTTGATTAATGAAAACATCCTTGGCAAATGCTTTCGCAGTTGTTCGTCTTTCATAAATC

CAAGAATTTCACCTCTGACTTATGAAATACAATGCCCCCGAACTGTCCCTGTTAATCATTTACTCCGATT

CCCCGAAGGCCCACCCCTTAGGATCAATCCTGTTGGTGTTATTCCCTGCCTTGTTCCAACCGTG



>gi|50355576|gb|CO553843.1|CO553843 Hdi-4 Horsegram stress induced cDNA library Macrotyloma uniflorum cDNA clone Hdi-4, mRNA sequence

AGATTAGGGCAGGCCGAATGAATAGTATCAGACTCACATTGGGCATTAATAGCCACTACCCCCCCCGCTC

AGTGATAAAGGAGGTAGGGGTGCAGAGACAACCAGGAGGTTTGCCTAGAAGCAGCCACCCTTGAAAGAGT

GCGTAATAGCTCACTGATCGAGCGCTCTTGCGCCGGAGATGAACGGGGCTAAGCGATCTGCCGAAGCTGT

GGGATGTCAAANTGATCGGTAGGGGAGCGTTCCGCCTTAGAGGGAAGGACTCCCGCAGCACTGCTCGGAC

CAACCCGAACGATATGTCGGCTTGATTTTCTTTCTTTCTTGGTTCAAATCCGTGCCT



>gi|50355581|gb|CO553848.1|CO553848 Hdi-9 Horsegram stress induced cDNA library Macrotyloma uniflorum cDNA clone Hdi-9, mRNA sequence

GTGTCACGCTGATGTTACACCATAGGCATGGCCACTCCTCCCGCCGCCTGGCCCGNATACCCCCCCCCGA

ACCCAAGACTTGATTCTCAAGGTGCCAGCGAGTCTAAAAGCACATCCGCTGATCCCTGGTCGGCATCGTT

TATGGTTGAGACTAGGACGGTATCTGATCGTCATTCGAGCCCCCAACTTTCGTTCTTGATTAATGAAAAC

ATCCTTGGCAAATGCTTTCGCTGTTGTTCGTCTTTCATAAATCCAAGAATTTCACCTCTGACTATGAAAT

ACGAATGCCCCCGACTGTCCCTGTTAATCATTACTCCGATCCCGAAGGCCAACACAATAGGATCAGAATC

CTGTGGTGTTATCCCATGCTAATGTATCCAAGAGCGTAGGCGGGGGGGGGTGGCATTC



>gi|50355579|gb|CO553846.1|CO553846 Hdi-7 Horsegram stress induced cDNA library Macrotyloma uniflorum cDNA clone Hdi-7, mRNA sequence

ATGTAACGCCAGTGATGAATAGCCCACCCCCCCCGGAACCCAAAGACTTTGATTTCTCATAAGGTGCCAG

CAGAGTCCTAAAAGCAACATCCGCTGATCCCTGGTCGGCATCTTAATGGTTGAGACTAGGACGGTATCTG

ATCGTATGCCCCCAACTTCGTTTTGATTAATGAAAACATCCTTGGATGCTTCGCAGTTGTTCGTCATTTC

TAAATCCAGAATTTCACCTCTGACTATGAAATACGAATGCCCCCGACTGTCCCCTGTTCTTCTCCGATCC

CGAGCCACCATAGGATCGAATCCTGGTGGTGTCCCTGCTATG



>gi|50355577|gb|CO553844.1|CO553844 Hdi-5 Horsegram stress induced cDNA library Macrotyloma uniflorum cDNA clone Hdi-5, mRNA sequence

GTGTAGACACGCCGTAATTGCCACCCCCCCCCGTCGTCACCGCGGCTGCTGGCACGGAGTTAGCCGGGGC

TTCCCTCAGTCCTGTCATGATCGCGCACTCGACGAAAGAGCTTACAAGCGGCATTGCCCTTCTTCACTCG

CGCGATATTGCTGGATCGGGCTTCGCCCCTTGTCCCAGAATTCCCCCCTGCTGCCCCCCGTGGGGAAGTC

CGGGCCCGTGCTCCTCC



>gi|50248890|gb|CO499852.1|CO499852 Hdi-2 Horsegram stress induced cDNA library Macrotyloma uniflorum cDNA clone Hdi-2, mRNA sequence

AATAGCACCACCCCCCCCGGAACCCAAAGACTTTGATTTCTCATAAGGTGCCAGCAGAGTCCTAAAAGCA

ACATCCGCTGATCCCTGGTCGGCATCGTTAATGGTTGAGACTAGGACGGTATCTGATCGTCATTCGAGCC

CCCAACTTTCGTTACTTGATTAATGAAAACATCCTTGGCAAATGCTTACGCAGTTGTTCGTCATTTCATA

AATCCAGAATTCACCTACTGACTATGAAATACGAATGCCCCCGACTGTCCCTGTTAATCATTACTCCGAT

CCCAAGGCCACACAATAGATCAATCCTGTGGTGTTATCCCTGCTAGTTCCAACCGTGCTGCT



>gi|50248891|gb|CO499853.1|CO499853 Hdi-3 Horsegram stress induced cDNA library Macrotyloma uniflorum cDNA clone Hdi-3, mRNA sequence

TCGCATGCTCCCGGCCGCCATGGCCGCGGGATTAATAGCCACCCCCCCCCGCTCAGTGATAAAGGAGGTA

GGGGTGCAGAGACAACCAGGAGGTTAGCCTAAAGCCACCCTAGAAAGCGTAATACACTGATCACCGCCCG

AATACGGCAACACGCCAACGTGGGATGTCAAATGATCGTAGGACTCCCCTAAGGAAGACCCCACATGCGA

CAACGGACAATGTCGCTGATACCAACTNGTAATCCATGCCAAAAGGTTCCTCAGGTTCGTCCAGGGGGGG



>gi|50248889|gb|CO499851.1|CO499851 Hdi-1 Horsegram stress induced cDNA library Macrotyloma uniflorum cDNA clone Hdi-1, mRNA sequence

TTAATTGCCACCCCCCCCCGGAACCCAAAGACTTTGATTTCTCATAAGGTGCCAGCGGAGTCCTAAAAGC

AACATCCGCTGATCCCTGGTCGGCATCGCTTATGGTTGAGACTAGGACGGTATCTGATCGTCTTCGAGCC

CCCAACTTTCGTTCTTGATTAATGAAAACATCCTTGGCAAATGCTTTCGCAGTTGTTCGTCTTTCATAAA

TCAAGAATTTCACCTCTGACTATGAAATACGAATGCCCCCGACTGTCCCTGTTAATCATTACTCCGATCC

CGAGCCAACACAATAGGATCAGAATCCTGTGGTGTTATCCCATGCTAATGTATCCAGAGCGTAGGCTTGC

TTTGAGCACTCTAATTTCTTCAGGTAACAGCACCGAAGGCCGACCCGGCCAGTTAAGGCCAGGAGCGCTC

CCGGCCGANGGACAGCGAGGGGTGGC
